# Supplementary material for: Integrative proteome-wide structural analysis and high-throughput docking identify broad-spectrum antiviral scaffolds against Zika, Yellow Fever, West Nile, Saint Louis encephalitis, and Usutu viruses
Source: Front Cell Infect Microbiol. 2026 Apr 30;16:1723132. doi: 10.3389/fcimb.2026.1723132 (PMC13171538; doi:10.3389/fcimb.2026.1723132)
Supplement: Supplementary file 3 [file DataSheet3.zip › SLEV/SLEV_E/Mol_probity_Files/SLEV_E_1FH-multi.table.pdf]

Viewing SLEV_E1FH-multi.table

When finished, you should close this window. *Hint: Use File | Save As... to save a copy of this page.*

All-Atom Contacts	Clashscore, all atoms:	1.46		99th percentile* (N=1784, all resolutions)
	Clashscore is the number of serious steric overlaps (> 0.4 Å) per 1000 atoms.			
Protein Geometry	Poor rotamers	0	0.00%	Goal: <0.3%
	Favored rotamers	407	99.51%	Goal: >98%
	Ramachandran outliers	1	0.20%	Goal: <0.05%
	Ramachandran favored	492	98.60%	Goal: >98%
	Rama distribution Z-score	0.56 ± 0.37		Goal: abs(Z score) < 2
	MolProbity score^	0.88		100th percentile* (N=27675, 0Å - 99Å)
	Cβ deviations >0.25Å	0	0.00%	Goal: 0
	Bad bonds:	7 / 3882	0.18%	Goal: 0%
	Bad angles:	9 / 5275	0.17%	Goal: <0.1%
Peptide Omegas	Cis Prolines:	0 / 17	0.00%	Expected: ≤1 per chain, or ≤5%
Low-resolution Criteria	CaBLAM outliers	9	1.8%	Goal: <1.0%
	CA Geometry outliers	4	0.80%	Goal: <0.5%
Additional validations	Chiral volume outliers	0/605		
	Waters with clashes	0/0	0.00%	See UnDowser table for details

In the two column results, the left column gives the raw count, right column gives the percentage.

* 100th percentile is the best among structures of comparable resolution; 0th percentile is the worst. For clashscore the comparative set of structures was selected in 2004, for MolProbity score in 2006.

^ MolProbity score combines the clashscore, rotamer, and Ramachandran evaluations into a single score, normalized to be on the same scale as X-ray resolution.

Key to table colors and cutoffs here:

#	Alt	Res	High B	Clash > 0.4Å	Ramachandran	Rotamer	Cβ deviation	CaBLAM	Bond lengths	Bond angles	Cis Peptides
			Avg: 1.09	Clashscore: 1.46	Outliers: 1 of 499	Poor rotamers: 0 of 409	Outliers: 0 of 449	Outliers: 10 of 497	Outliers: 7 of 501	Outliers: 9 of 501	Non-Trans: 0 of 500
A 1		PHE	1.11	0.51Å N with A 42 ASP OD2	-	Favored (23.6%) *m-10* chi angles: 295.2,343	0.12Å	-	-	-	-
A 2		ASN	1.11	0.56Å H with A 152 HIS CD2	Favored (12.25%) General / 64.6,20.8	Favored (9.5%) *m110* chi angles: 300.8,74.3	0.10Å	-	-	-	-
A 3		CYS	1.13	-	Allowed (1.16%) General / -139.3,-13.6	Favored (72%) *m* chi angles: 297.9	0.02Å	CaBLAM Disfavored (2.165%)	-	-	-
A 4		LEU	1.15	-	Favored (69.11%) General / -64.2,-27.5	Favored (96.2%) *mt* chi angles: 296.7,177.2	0.02Å	Favored (35.926%) alpha helix	-	-	-
A 5		GLY	1.17	-	Favored (82.78%) Glycine / -90.4,5.1	-	-	Favored (61.593%)	-	-	-
A 6		THR	1.17	-	Favored (43.13%) General / -99.2,123.0	Favored (87%) *m* chi angles: 296.9	0.06Å	Favored (32.736%)	-	-	-

A 7		SER	1.15	-	Favored (85.37%) General / -67.0,-38.0	Favored (65.5%) *m* chi angles: 294.3	0.06Å	Favored (15.277%)	-	-	-
A 8		ASN	1.11	-	Favored (8.81%) General / -81.9,70.5	Favored (87.9%) *m-40* chi angles: 294.8,319	0.03Å	Favored (7.419%)	-	-	-
A 9		ARG	1.07	-	Favored (37.98%) General / -133.1,129.4	Favored (59.5%) *ttt180* chi angles: 182.2,178.3,175.5,202.4	0.03Å	Favored (19.924%)	-	-	-
A 10		ASP	1.06	-	Favored (23.54%) General / -110.5,154.6	Favored (47.5%) *m-30* chi angles: 293.8,297.6	0.01Å	Favored (47.728%) beta sheet	-	-	-
A 11		PHE	1.1	-	Favored (51.25%) General / -124.7,142.6	Favored (74.9%) *m-80* chi angles: 296.5,81.3	0.08Å	Favored (68.448%) beta sheet	-	-	-
A 12		VAL	1.22	-	Favored (69.22%) Ile or Val / -127.5,127.8	Favored (67.7%) *t* chi angles: 179	0.08Å	Favored (70.529%) beta sheet	-	-	-
A 13		GLU	1.4	-	Favored (45.19%) General / -109.9,121.4	Favored (92%) *tt0* chi angles: 181.8,176.7,3.6	0.04Å	Favored (56.107%) beta sheet	-	-	-
A 14		GLY	1.63	-	Favored (50.83%) Glycine / -67.0,148.8	-	-	Favored (42.358%) beta sheet	-	-	-
A 15		ALA	1.83	-	Favored (59.71%) General / -81.9,-8.9	-	0.06Å	Favored (5.555%)	-	-	-
A 16		SER	1.91	-	Allowed (0.24%) General / 67.6,178.0	Favored (59.6%) *p* chi angles: 73.2	0.07Å	Favored (7.787%)	-	-	-
A 17		GLY	1.85	-	Favored (3.8%) Glycine / 77.8,-59.5	-	-	CaBLAM Outlier (0.213%)	-	-	-
A 18		ALA	1.66	-	Favored (46.64%) General / -59.6,144.8	-	0.04Å	Favored (18.213%)	-	-	-
A 19		THR	1.43	-	Favored (30.97%) General / -104.9,13.6	Favored (68.4%) *p* chi angles: 59	0.04Å	CaBLAM Disfavored (4.817%)	-	-	-
A 20		TRP	1.22	-	Favored (16.14%) General / -163.0,153.3	Favored (68.9%) *p-90* chi angles: 56,263.9	0.01Å	Favored (18.587%)	-	-	-
**#**	**Alt**	**Res**	**High B**	**Clash > 0.4Å**	**Ramachandran**	**Rotamer**	**Cβ deviation**	**CaBLAM**	**Bond lengths**	**Bond angles**	**Cis Peptides**
			Avg: 1.09	Clashscore: 1.46	Outliers: 1 of 499	Poor rotamers: 0 of 409	Outliers: 0 of 449	Outliers: 10 of 497	Outliers: 7 of 501	Outliers: 9 of 501	Non-Trans: 0 of 500
A 21		ILE	1.07	-	Favored (72.34%) Ile or Val / -122.6,131.9	Favored (38.9%) *mm* chi angles: 301.9,295.3	0.07Å	Favored (54.834%)	-	-	-
A 22		ASP	0.98	-	Favored (35.08%) General / -91.2,124.0	Favored (94.9%) *m-30* chi angles: 286.3,345.3	0.10Å	Favored (58.896%) beta sheet	-	-	-

A 23		LEU	0.94	-	Favored (35.78%) General / -116.0,151.3	Favored (40.1%) *mt* chi angles: 307.3,175.6	0.12Å	Favored (53.984%) beta sheet	-	-	-
A 24		VAL	0.94	-	Favored (57.33%) Ile or Val / -124.5,119.8	Favored (64.4%) *t* chi angles: 179.4	0.01Å	Favored (58.138%) beta sheet	-	-	-
A 25		LEU	0.97	-	Favored (15.22%) General / -105.2,105.3	Favored (64.1%) *mt* chi angles: 304.7,177.4	0.07Å	Favored (72.578%)	-	-	-
A 26		GLU	0.99	-	Favored (43.86%) General / -98.8,124.7	Favored (40.7%) *tt0* chi angles: 177.1,176.3,67.1	0.07Å	Favored (7.844%)	-	-	-
A 27		GLY	1.01	-	Favored (35.19%) Glycine / 55.3,-125.1	-	-	Favored (55.101%)	-	-	-
A 28		GLY	1.01	-	Favored (57.87%) Glycine / -100.9,15.8	-	-	Favored (9.641%)	-	-	-
A 29		SER	0.99	-	Favored (13.35%) General / -74.8,171.8	Favored (89.8%) *p* chi angles: 68.8	0.09Å	Favored (18.744%)	-	-	-
A 30		CYS	0.97	-	Favored (41%) General / -137.4,139.8	Favored (51.4%) *t* chi angles: 180.3	0.06Å	Favored (57.292%) beta sheet	-	-	-
A 31		VAL	0.97	-	Favored (73.18%) Ile or Val / -117.0,124.6	Favored (80.2%) *t* chi angles: 178	0.04Å	Favored (68.856%) beta sheet	-	-	-
A 32		THR	1	-	Favored (48.75%) General / -101.9,126.0	Favored (93.4%) *m* chi angles: 297.5	0.05Å	Favored (68.911%) beta sheet	-	-	-
A 33		VAL	1.08	-	Favored (68.9%) Ile or Val / -119.2,132.5	Favored (47.6%) *t* chi angles: 181.9	0.05Å	Favored (58.371%) beta sheet	-	-	-
A 34		MET	1.18	-	Favored (49.92%) General / -136.0,155.7	Favored (23.2%) *ptp* chi angles: 64.6,193.6,75	0.07Å	Favored (26.205%)	-	-	-
A 35		ALA	1.28	-	Favored (24.95%) Pre-Pro / -159.1,155.8	-	0.03Å	Favored (8.77%)	-	-	-
A 36		PRO	1.31	-	Favored (59.63%) Trans-Pro / -54.2,134.3	Favored (92.7%) *Cg_exo* chi angles: 331.2,37.8,329.7	0.07Å	Favored (33.057%)	-	-	-
A 37		GLU	1.27	-	Favored (5.72%) General / 58.4,18.7	Favored (84.4%) *mt-10* chi angles: 300,184.4,341.3	0.05Å	Favored (7.578%)	-	-	-
A 38		LYS	1.16	-	Favored (55.92%) Pre-Pro / -121.4,151.1	Favored (66.3%) *mmtt* chi angles: 300.5,295.4,187.9,192.1	0.01Å	Favored (27.866%)	-	-	-
A 39		PRO	1.02	-	Favored (43.19%) Trans-Pro / -72.5,161.8	Favored (76.7%) *Cg_endo* chi angles: 28.7,327,24.5	0.03Å	Favored (72.895%)	-	-	-

#	Alt	Res	High B	Clash > 0.4Å	Ramachandran	Rotamer	Cβ deviation	CaBLAM	Bond lengths	Bond angles	Cis Peptides
A 40		THR	0.89	-	Favored (35.33%) General / -81.3,132.4	Favored (89.2%) *m* chi angles: 301.3	0.06Å	Favored (29.963%)	-	-	-
#	Alt	Res	High B	Clash > 0.4Å	Ramachandran	Rotamer	Cβ deviation	CaBLAM	Bond lengths	Bond angles	Cis Peptides
			Avg: 1.09	Clashscore: 1.46	Outliers: 1 of 499	Poor rotamers: 0 of 409	Outliers: 0 of 449	Outliers: 10 of 497	Outliers: 7 of 501	Outliers: 9 of 501	Non-Trans: 0 of 500
A 41		LEU	0.79	-	Favored (52.72%) General / -120.0,139.3	Favored (2.2%) *mp* chi angles: 287.9,92.1	0.05Å	Favored (69.117%) beta sheet	-	-	-
A 42		ASP	0.74	0.51Å OD2 with A 1 PHE N	Favored (48.64%) General / -109.4,123.0	Favored (96.6%) *m-30* chi angles: 290.1,344.1	0.08Å	Favored (70.762%) beta sheet	-	-	-
A 43		PHE	0.73	-	Favored (56.35%) General / -112.4,128.6	Favored (77.4%) *m-80* chi angles: 299.3,83.8	0.10Å	Favored (57.563%) beta sheet	-	-	-
A 44		LYS	0.74	-	Favored (20.93%) General / -145.6,133.2	Favored (85.2%) *tttt* chi angles: 180,173.4,180.3,180.3	0.05Å	Favored (41.403%) beta sheet	-	-	-
A 45		VAL	0.76	-	Favored (38.59%) Ile or Val / -80.9,124.3	Favored (91.9%) *t* chi angles: 174.4	0.03Å	Favored (55.107%)	-	-	-
A 46		MET	0.79	-	Favored (15.79%) General / -89.3,-36.0	Favored (98.7%) *mtp* chi angles: 293.2,177.2,69.1	0.08Å	Favored (28.575%)	-	-	-
A 47		LYS	0.81	-	Favored (13.08%) General / -159.0,140.3	Favored (33.8%) *ttpt* chi angles: 181.8,171.7,63.7,167.8	0.03Å	Favored (25.641%)	-	-	-
A 48		MET	0.84	-	Favored (53.57%) General / -115.5,125.8	Favored (26.1%) *ttt* chi angles: 177.5,182.8,186.8	0.09Å	Favored (59.335%)	-	-	-
A 49		GLU	0.87	-	Favored (51.75%) General / -130.7,150.7	Favored (97.6%) *mt-10* chi angles: 292.2,181.8,346.6	0.11Å	Favored (54.998%) beta sheet	-	-	-
A 50		ALA	0.89	-	Favored (43.57%) General / -115.2,144.5	-	0.05Å	Favored (63.216%)	-	-	-
A 51		THR	0.91	-	Allowed (0.57%) General / -112.5,-86.2	Favored (62.9%) *p* chi angles: 57.8	0.07Å	CaBLAM Disfavored (1.184%)	-	-	-
A 52		GLU	0.92	-	Favored (5.52%) General / -79.0,71.8	Favored (95.7%) *mt-10* chi angles: 297,181.2,353.5	0.03Å	Favored (30.268%)	-	-	-
A 53		LEU	0.93	-	Favored (53.68%) General / -68.0,146.3	Favored (94.4%) *mt* chi angles: 295.5,175	0.06Å	Favored (16.494%)	-	-	-
A 54		ALA	0.91	-	Favored (39.73%) General / -105.8,119.7	-	0.03Å	Favored (36.559%) beta sheet	-	-	-
A 55		THR	0.88	-	Favored (53.44%)	Favored (84.7%) *m* chi angles: 301.8	0.04Å	Favored (27.023%)	-	-	-

					General / -60.1,132.3						
A 56		VAL	0.84	-	Favored (7.22%) Ile or Val / -107.4,-51.8	Favored (76.8%) *t* chi angles: 178.2	0.12Å	Favored (12.92%)	-	-	-
A 57		ARG	0.81	-	Favored (11.22%) General / -160.8,141.0	Favored (42.7%) *ttm170* chi angles: 176.9,182.7,293.9,187.1	0.06Å	Favored (16.32%)	-	-	-
A 58		GLU	0.78	-	Favored (34.83%) General / -103.2,142.0	Favored (95.4%) *mt-10* chi angles: 296,182.3,354.5	0.04Å	Favored (59.493%)	-	-	-
A 59		TYR	0.75	-	Favored (42.51%) General / -115.6,145.6	Favored (48.6%) *m-80* chi angles: 282.1,83.8	0.07Å	Favored (56.35%) beta sheet	-	-	-
A 60		CYS	0.74	-	Favored (47.43%) General / -100.8,127.0	Favored (52.1%) *t* chi angles: 180.6	0.06Å	Favored (47.222%)	-	-	-
**#**	**Alt**	**Res**	**High B**	**Clash > 0.4Å**	**Ramachandran**	**Rotamer**	**Cβ deviation**	**CaBLAM**	**Bond lengths**	**Bond angles**	**Cis Peptides**
			Avg: 1.09	Clashscore: 1.46	Outliers: 1 of 499	Poor rotamers: 0 of 409	Outliers: 0 of 449	Outliers: 10 of 497	Outliers: 7 of 501	Outliers: 9 of 501	Non-Trans: 0 of 500
A 61		TYR	0.72	-	Favored (6.33%) General / -104.3,-39.8	Favored (20%) *m-10* chi angles: 292.8,331.8	0.10Å	Favored (14.456%)	-	-	-
A 62		GLU	0.72	-	Favored (51.22%) General / -123.4,129.4	Favored (90.2%) *tt0* chi angles: 183.3,174.5,355.7	0.05Å	Favored (28.693%)	-	-	-
A 63		ALA	0.71	-	Favored (48.33%) General / -131.9,155.2	-	0.03Å	Favored (53.829%)	-	-	-
A 64		THR	0.7	-	Favored (50.94%) General / -128.3,136.4	Favored (94.6%) *m* chi angles: 299.4	0.06Å	Favored (52.129%) beta sheet	-	-	-
A 65		LEU	0.69	-	Favored (10.38%) General / -83.8,72.2	Favored (81.4%) *mt* chi angles: 302,177.6	0.05Å	Favored (25.163%)	-	-	-
A 66		ASP	0.69	-	Favored (77.01%) General / -58.5,-38.1	Favored (90.3%) *m-30* chi angles: 285.3,343.7	0.04Å	Favored (20.041%)	-	-	-
A 67		THR	0.68	-	Favored (34.35%) General / -135.3,130.0	Favored (98.8%) *m* chi angles: 300.3	0.01Å	Favored (30.915%)	-	-	-
A 68		LEU	0.67	-	Favored (42.61%) General / -122.3,150.3	Favored (90.9%) *mt* chi angles: 298.8,176.1	0.05Å	Favored (45.988%)	-	-	-
A 69		SER	0.67	-	Favored (12.59%) General / -154.5,133.8	Favored (39%) *t* chi angles: 174.9	0.04Å	Favored (40.778%) beta sheet	-	-	-
A 70		THR	0.66	-	Favored (51.84%) General / -125.8,135.3	Favored (92.9%) *m* chi angles: 299.2	0.02Å	Favored (69.738%) beta sheet	-	-	-

A 71		VAL	0.66	-	Favored (57.6%) Ile or Val / -118.8,135.9	Favored (90.6%) *t* chi angles: 174.3	0.03Å	Favored (61.645%) beta sheet	-	-	-
A 72		ALA	0.67	-	Favored (41.85%) General / -139.9,148.3	-	0.01Å	Favored (66.042%)	-	-	-
A 73		ARG	0.69	-	Favored (49.32%) General / -130.5,152.9	Favored (79.9%) *mtp85* chi angles: 297.5,183.5,73.9,84.1	0.04Å	Favored (43.826%)	-	-	-
A 74		CYS	0.7	-	Favored (87.48%) Pre-Pro / -74.4,151.2	Favored (94%) *m* chi angles: 293	0.03Å	Favored (40.717%)	-	-	-
A 75		PRO	0.71	-	Favored (3.37%) Trans-Pro / -44.4,-29.9	Favored (88.9%) *Cg_exo* chi angles: 330.8,36.7,332.3	0.06Å	Favored (11.005%)	-	-	-
A 76		THR	0.71	-	Favored (20.96%) General / -111.8,13.2	Favored (69.7%) *p* chi angles: 59.3	0.02Å	Favored (21.22%)	-	-	-
A 77		THR	0.7	-	Favored (21.98%) General / -111.1,11.7	Favored (78.5%) *p* chi angles: 60.8	0.06Å	Favored (9.037%)	-	-	-
A 78		GLY	0.68	-	Favored (38.69%) Glycine / 93.8,170.5	-	-	Favored (37.247%)	-	-	-
A 79		GLU	0.66	-	Favored (51.08%) General / -70.9,143.2	Favored (76.1%) *mt-10* chi angles: 292.4,183.9,325.3	0.03Å	Favored (9.366%)	-	-	-
A 80		ALA	0.64	-	Favored (58.29%) General / -65.5,142.1	-	0.03Å	Favored (36.448%)	-	-	-
**#**	**Alt**	**Res**	**High B**	**Clash > 0.4Å**	**Ramachandran**	**Rotamer**	**Cβ deviation**	**CaBLAM**	**Bond lengths**	**Bond angles**	**Cis Peptides**
			Avg: 1.09	Clashscore: 1.46	Outliers: 1 of 499	Poor rotamers: 0 of 409	Outliers: 0 of 449	Outliers: 10 of 497	Outliers: 7 of 501	Outliers: 9 of 501	Non-Trans: 0 of 500
A 81		HIS	0.62	-	Favored (5.99%) General / -143.9,111.5	Favored (27.1%) *t-170* chi angles: 186.7,190.7	0.01Å	Favored (38.275%) beta sheet	-	-	-
A 82		ASN	0.62	-	Favored (31.55%) General / -87.8,122.5	Favored (43.3%) *t0* chi angles: 191.6,319.7	0.03Å	Favored (50.787%)	-	-	-
A 83		THR	0.63	-	Favored (7.07%) General / -45.8,-38.4	Favored (92.6%) *m* chi angles: 299.1	0.01Å	Favored (47.449%)	-	-	-
A 84		LYS	0.66	-	Favored (59.01%) General / -59.6,-20.9	Favored (40%) *mttp* chi angles: 286.2,172.7,166.1,60.9	0.04Å	Favored (58.825%) three-ten	-	-	-
A 85		ARG	0.69	-	Favored (65.9%) General / -58.3,-29.5	Favored (66%) *ttt90* chi angles: 185.4,175.5,178.2,85.6	0.01Å	Favored (61.762%) three-ten	-	-	-
A 86		SER	0.73	-	Favored (59.42%)	Favored (87.6%) *p* chi angles: 67	0.02Å	Favored (53.883%)	-	-	-

					General / -76.5,-10.5							
A 87		ASP	0.76	-	Favored (47.01%) Pre-Pro / -90.0,111.5	Favored (21.3%) *t0* chi angles: 178.1,331.2	0.03Å	Favored (30.93%)	-	-	-
A 88		PRO	0.77	-	Favored (10.87%) Trans-Pro / -53.4,-18.3	Favored (90.9%) *Cg_exo* chi angles: 333.2,35.4,331	0.08Å	Favored (19.914%)	-	-	-
A 89		THR	0.76	-	Favored (52.64%) General / -88.5,-9.3	Favored (80%) *p* chi angles: 60.4	0.08Å	Favored (55.177%)	-	-	-
A 90		PHE	0.74	-	Favored (22.01%) General / -92.5,148.6	Favored (51.5%) *m-80* chi angles: 283.2,81.6	0.06Å	Favored (27.827%)	-	-	-
A 91		VAL	0.7	-	Favored (46.56%) Ile or Val / -102.2,116.8	Favored (54.6%) *t* chi angles: 180.7	0.12Å	Favored (60.134%)	-	-	-
A 92		CYS	0.67	-	Favored (54.09%) General / -124.5,136.6	Favored (64%) *m* chi angles: 300	0.01Å	Favored (63.056%) beta sheet	-	-	-
A 93		LYS	0.65	-	Favored (54.8%) General / -123.0,138.0	Favored (72.3%) *mmtt* chi angles: 298.9,293.2,182.5,181.8	0.01Å	Favored (70.909%) beta sheet	-	-	-
A 94		ARG	0.65	-	Favored (52.69%) General / -113.1,136.4	Favored (39.8%) *ttm170* chi angles: 178,179.2,286.9,153.7	0.03Å	Favored (40.85%) beta sheet	-	-	-
A 95		ASP	0.66	-	Favored (6.94%) General / -149.7,-179.2	Favored (10.3%) *t0* chi angles: 207.3,335.6	0.06Å	Favored (24.308%) beta sheet	-	-	-
A 96		VAL	0.69	-	Favored (60.19%) Ile or Val / -113.3,133.1	Favored (95.2%) *t* chi angles: 175.7	0.06Å	Favored (24.351%) beta sheet	-	-	-
A 97		VAL	0.73	-	Favored (34.85%) Ile or Val / -128.4,145.8	Favored (14%) *m* chi angles: 291.6	0.11Å	Favored (47.443%) beta sheet	-	-	-
A 98		ASP	0.78	0.43Å OD1 with A 110 LYS NZ	Favored (58.32%) General / -64.2,138.2	Favored (92.2%) *m-30* chi angles: 290.3,349.6	0.01Å	Favored (37.61%) beta sheet	-	-	-
A 99		ARG	0.84	-	Favored (49.49%) General / -135.6,149.7	Favored (65%) *mmm-85* chi angles: 298,293.5,294.9,272.9	0.06Å	Favored (34.272%) beta sheet	-	-	-
A 100		GLY	0.89	-	Favored (12.98%) Glycine / -158.3,-163.6	-	-	Favored (30.15%)	-	-	-
**#**	**Alt**	**Res**	**High B**	**Clash > 0.4Å**	**Ramachandran**	**Rotamer**	**Cβ deviation**	**CaBLAM**	**Bond lengths**	**Bond angles**	**Cis Peptides**
			Avg: 1.09	Clashscore: 1.46	Outliers: 1 of 499	Poor rotamers: 0 of 409	Outliers: 0 of 449	Outliers: 10 of 497	Outliers: 7 of 501	Outliers: 9 of 501	Non-Trans: 0 of 500
A 101		TRP	0.92	-	Favored (74.14%) General / -57.2,-38.7	Favored (41.6%) *m-10* chi angles: 286.1,349.3	0.02Å	Favored (17.565%)	-	-	-
A 102		GLY	0.94	-	Favored (65.39%)	-	-	Favored (61.857%)	-	-	-

					Glycine / -73.0,-5.5						
A 103		ASN	0.94	-	Favored (20.8%) General / -111.5,16.2	Favored (41.4%) *m110* chi angles: 301.5,120.3	0.08Å	Favored (49.29%)	-	-	-
A 104		GLY	0.95	-	Favored (86.25%) Glycine / 84.6,5.9	-	-	Favored (80.058%)	-	-	-
A 105		CYS	0.95	-	Favored (17.16%) General / -96.0,154.6	Favored (84.3%) *m* chi angles: 295.5	0.04Å	Favored (31.252%)	-	-	-
A 106		GLY	0.94	-	Favored (71.53%) Glycine / -86.2,-12.0	-	-	Favored (46.767%)	-	-	-
A 107		LEU	0.92	-	Favored (29.28%) General / -119.4,156.9	Favored (59.3%) *mt* chi angles: 303.9,174.5	0.07Å	Favored (18.872%)	-	-	-
A 108		PHE	0.88	-	Favored (35.48%) General / -105.0,142.1	Favored (94.1%) *m-80* chi angles: 292.5,86.4	0.05Å	CaBLAM Disfavored (1.452%)	-	-	-
A 109		GLY	0.82	-	Favored (21.38%) Glycine / 161.3,-151.2	-	-	Favored (30.885%)	-	-	-
A 110		LYS	0.75	0.43Å NZ with A 98 ASP OD1	Favored (53.22%) General / -117.2,126.7	Favored (64%) *mttm* chi angles: 294.5,184.1,183.9,295	0.01Å	Favored (6.457%)	-	-	-
A 111		GLY	0.68	-	Favored (26.35%) Glycine / -102.1,-159.7	-	-	Favored (29.132%)	-	-	-
A 112		SER	0.62	-	Favored (46.88%) General / -135.2,144.4	Favored (32.8%) *t* chi angles: 174.1	0.04Å	Favored (27.446%)	-	-	-
A 113		ILE	0.59	-	Favored (48.22%) Ile or Val / -132.0,140.3	Favored (21.2%) *tt* chi angles: 187,164.8	0.09Å	Favored (72.221%) beta sheet	-	-	-
A 114		ASP	0.59	-	Favored (11.92%) General / -130.1,110.8	Favored (41.7%) *t0* chi angles: 187.3,13.6	0.03Å	Favored (59.25%) beta sheet	-	-	-
A 115		THR	0.6	-	Favored (56.77%) General / -116.5,130.5	Favored (97.4%) *m* chi angles: 299.9	0.02Å	Favored (66.503%) beta sheet	-	-	-
A 116		CYS	0.62	-	Favored (18.62%) General / -113.7,160.4	Favored (72.4%) *m* chi angles: 297.8	0.03Å	Favored (36.154%) beta sheet	-	-	-
A 117		ALA	0.65	-	Favored (11.26%) General / -147.1,123.9	-	0.05Å	Favored (49.393%) beta sheet	-	OUTLIER(S) worst is C-N-CA: 16.0 σ	-
A 118		LYS	0.69	-	Favored (77.72%) General / -66.6,-34.4	Favored (53.4%) *tptt* chi angles: 180.8,68.7,175.5,177.9	0.05Å	CaBLAM Disfavored (4.837%) try beta sheet	-	-	-
A 119		PHE	0.72	0.95Å CD2 with A 119 PHE O	Favored (8.86%) General / 62.3,42.9	Favored (88.7%) *t80* chi angles: 178.1,82.7	0.05Å	Favored (9.387%) beta sheet	-	-	-

#	Alt	Res	High B	Clash > 0.4Å	Ramachandran	Rotamer	Cβ deviation	CaBLAM	Bond lengths	Bond angles	Cis Peptides
A 120		THR	0.75	-	Favored (30.89%) General / -59.3,127.2	Favored (84.9%) *m* chi angles: 301.7	0.07Å	Favored (23.341%) beta sheet	-	-	-
#	Alt	Res	High B	Clash > 0.4Å	Ramachandran	Rotamer	Cβ deviation	CaBLAM	Bond lengths	Bond angles	Cis Peptides
			Avg: 1.09	Clashscore: 1.46	Outliers: 1 of 499	Poor rotamers: 0 of 409	Outliers: 0 of 449	Outliers: 10 of 497	Outliers: 7 of 501	Outliers: 9 of 501	Non-Trans: 0 of 500
A 121		CYS	0.77	-	Favored (34.66%) General / -83.0,136.0	Favored (42.5%) *t* chi angles: 186.1	0.08Å	Favored (49.502%)	-	-	-
A 122		LYS	0.77	-	Favored (6.91%) General / -102.8,-39.6	Favored (72%) *mmtt* chi angles: 299.8,295.7,180.7,178.2	0.03Å	Favored (13.175%)	-	-	-
A 123		ASN	0.76	-	Favored (17.99%) General / -125.4,113.8	Favored (67.8%) *m-40* chi angles: 291.6,294.9	0.15Å	Favored (26.768%)	-	-	-
A 124		LYS	0.75	-	Favored (9.59%) General / -108.8,168.1	Favored (55%) *mtmt* chi angles: 295.9,188.7,291.9,181.1	0.05Å	Favored (19.836%)	-	-	-
A 125		ALA	0.74	-	Favored (47.25%) General / -140.0,155.0	-	0.08Å	Favored (57.968%) beta sheet	-	-	-
A 126		THR	0.73	-	Favored (47.85%) General / -129.2,134.1	Favored (82.3%) *m* chi angles: 302.2	0.04Å	Favored (63.276%) beta sheet	-	-	-
A 127		GLY	0.73	-	Favored (14.16%) Glycine / -101.2,138.9	-	-	Favored (71.493%) beta sheet	-	-	-
A 128		LYS	0.74	-	Favored (32.71%) General / -132.5,126.3	Favored (91.7%) *mttt* chi angles: 300.8,183.5,179.6,181	0.04Å	Favored (48.657%) beta sheet	-	-	-
A 129		THR	0.76	-	Favored (50.45%) General / -67.1,133.8	Favored (96.5%) *m* chi angles: 299.7	0.07Å	Favored (41.818%) beta sheet	-	-	-
A 130		ILE	0.78	-	Favored (34.98%) Ile or Val / -100.5,113.0	Favored (59.3%) *mt* chi angles: 302.8,165.5	0.09Å	Favored (67.851%) beta sheet	-	-	-
A 131		LEU	0.8	-	Favored (20.9%) General / -98.3,151.1	Favored (74.8%) *mt* chi angles: 301,173.8	0.04Å	Favored (34.06%)	-	-	-
A 132		ARG	0.81	-	Favored (77%) General / -57.0,-40.6	Favored (98.2%) *mtt180* chi angles: 289.3,175,181.9,171.2	0.06Å	Favored (63.651%)	-	-	-
A 133		GLU	0.81	-	Favored (66.41%) General / -64.5,-20.7	Favored (99.1%) *mt-10* chi angles: 291.2,180.1,356.6	0.03Å	Favored (65.719%) alpha helix	-	-	-
A 134		ASN	0.8	-	Favored (49.84%) General / -96.2,6.0	Favored (90.9%) *m-40* chi angles: 293.6,324.7	0.03Å	Favored (49.167%)	-	-	-
A 135		ILE	0.79	-	Favored (46.65%) Ile or Val / -97.5,130.1	Favored (93.5%) *mt* chi angles: 295.1,170.8	0.06Å	Favored (22.781%)	-	-	-

A 136		LYS	0.77	-	Favored (51.86%) General / -126.4,141.5	Favored (96.6%) *mttt* chi angles: 296.8,182.7,180.6,182.1	0.06Å	Favored (57.565%)	-	-	-
A 137		TYR	0.74	-	Favored (48.55%) General / -121.0,144.4	Favored (77.8%) *m-80* chi angles: 291.7,81.3	0.08Å	Favored (66.579%) beta sheet	-	-	-
A 138		GLU	0.73	-	Favored (43.29%) General / -111.9,120.8	Favored (93.3%) *mt-10* chi angles: 296.3,182.7,2.3	0.03Å	Favored (64.009%) beta sheet	-	-	-
A 139		VAL	0.72	-	Favored (68.46%) Ile or Val / -119.3,132.7	Favored (88.9%) *t* chi angles: 174.1	0.03Å	Favored (64.859%) beta sheet	-	-	-
A 140		ALA	0.71	-	Favored (37.68%) General / -108.4,143.2	-	0.04Å	Favored (66.131%) beta sheet	-	-	-
**#**	**Alt**	**Res**	**High B**	**Clash > 0.4Å**	**Ramachandran**	**Rotamer**	**Cβ deviation**	**CaBLAM**	**Bond lengths**	**Bond angles**	**Cis Peptides**
			Avg: 1.09	Clashscore: 1.46	Outliers: 1 of 499	Poor rotamers: 0 of 409	Outliers: 0 of 449	Outliers: 10 of 497	Outliers: 7 of 501	Outliers: 9 of 501	Non-Trans: 0 of 500
A 141		ILE	0.72	-	Favored (74.65%) Ile or Val / -123.6,129.3	Favored (71%) *mt* chi angles: 302,173.7	0.03Å	Favored (69.724%) beta sheet	-	-	-
A 142		PHE	0.74	-	Favored (47.09%) General / -122.1,146.6	Favored (78.1%) *m-80* chi angles: 300.6,85.8	0.07Å	Favored (63.57%) beta sheet	-	-	-
A 143		VAL	0.76	-	Favored (54.41%) Ile or Val / -107.2,132.5	Favored (46.9%) *t* chi angles: 182.1	0.06Å	Favored (55.01%) beta sheet	-	-	-
A 144		HIS	0.8	-	Favored (69.62%) General / -54.4,-49.6	Favored (27.2%) *m170* chi angles: 283.6,195	0.06Å	CaBLAM Outlier (0.498%)	-	-	-
A 145		GLY	0.84	-	Favored (27.34%) Glycine / 53.0,-124.3	-	-	Favored (5.606%)	-	-	-
A 146		SER	0.88	-	Favored (3.78%) General / -149.2,107.6	Favored (46%) *t* chi angles: 179.6	0.04Å	CA Geom Outlier (0.196%)	-	-	-
A 147		THR	0.93	-	Favored (16.24%) General / -134.5,170.1	Favored (13.6%) *p* chi angles: 75.5	0.12Å	Favored (30.606%)	-	-	-
A 148		ASP	0.98	0.42Å C with A 148 ASP OD1	Favored (4.86%) General / -89.3,-175.8	Favored (43%) *p0* chi angles: 68.2,18.8	0.04Å	Favored (50.67%)	-	-	-
A 149		SER	1.04	-	Favored (37.92%) General / -95.7,10.1	Favored (93.4%) *p* chi angles: 64.3	0.04Å	Favored (10.598%)	-	-	-
A 150		THR	1.1	-	Favored (4.18%) General / -103.4,-47.8	Favored (92.2%) *m* chi angles: 299.1	0.01Å	CaBLAM Disfavored (4.869%) try alpha helix	-	-	-
A 151		SER	1.16	-	Favored (63.01%)	Favored (87.5%) *p* chi angles: 67.1	0.03Å	Favored (51.785%)	-	-	-

					General / -65.4,-15.8			alpha helix			
A 152		HIS	1.2	0.56Å CD2 with A 2 ASN H	Favored (39.71%) General / -50.0,-38.9	Favored (52.1%) *t-90* chi angles: 176.2,271.5	0.05Å	Favored (51.568%) alpha helix	-	-	-
A 153		GLY	1.2	-	Favored (84.97%) Glycine / -88.8,4.8	-	-	Favored (54.698%)	-	-	-
A 154		ASN	1.16	-	Favored (6.34%) General / -119.8,98.9	Favored (54%) *t0* chi angles: 185.6,330	0.07Å	Favored (13.658%)	-	-	-
A 155		TYR	1.09	0.49Å CD1 with A 155 TYR N	Favored (95.06%) General / -60.8,-41.1	Allowed (0.5%) *m-10* chi angles: 293.2,38.3	0.03Å	Favored (46.068%)	-	-	-
A 156		SER	1.02	-	Favored (96.9%) General / -64.1,-40.7	Favored (62.1%) *m* chi angles: 293.8	0.01Å	Favored (85.389%) alpha helix	-	-	-
A 157		GLU	0.96	-	Favored (98.59%) General / -62.0,-42.0	Favored (90.7%) *tt0* chi angles: 183.6,175,2.4	0.05Å	Favored (92.189%) alpha helix	-	-	-
A 158		GLN	0.93	-	Favored (78.01%) General / -64.8,-34.3	Favored (84.4%) *mm-40* chi angles: 295.8,304.6,323.4	0.06Å	Favored (87.143%) alpha helix	-	-	-
A 159		ILE	0.91	-	Favored (90.97%) Ile or Val / -66.0,-45.4	Favored (96.9%) *mt* chi angles: 293.7,168.4	0.09Å	Favored (80.48%) alpha helix	-	-	-
A 160		GLY	0.9	-	Favored (65.41%) Glycine / -58.3,-31.1	-	-	Favored (88.881%) alpha helix	-	-	-
**#**	**Alt**	**Res**	**High B**	**Clash > 0.4Å**	**Ramachandran**	**Rotamer**	**Cβ deviation**	**CaBLAM**	**Bond lengths**	**Bond angles**	**Cis Peptides**
			Avg: 1.09	Clashscore: 1.46	Outliers: 1 of 499	Poor rotamers: 0 of 409	Outliers: 0 of 449	Outliers: 10 of 497	Outliers: 7 of 501	Outliers: 9 of 501	Non-Trans: 0 of 500
A 161		LYS	0.88	-	Favored (47.58%) General / -86.4,2.4	Favored (72.9%) *mmtt* chi angles: 297.1,293.5,183.9,181.2	0.03Å	Favored (50.826%)	-	-	-
A 162		ASN	0.86	-	Favored (24.86%) General / 58.9,37.3	Favored (83.4%) *m-40* chi angles: 297.5,310.1	0.04Å	Favored (14.861%)	-	-	-
A 163		GLN	0.83	-	Favored (14.32%) General / -116.8,10.8	Favored (53.9%) *mt0* chi angles: 299.3,191.9,66.5	0.03Å	Favored (21.874%)	-	-	-
A 164		ALA	0.79	-	Favored (32.97%) General / -160.1,164.6	-	0.06Å	Favored (38.191%)	-	-	-
A 165		ALA	0.77	-	Favored (37.78%) General / -153.0,155.9	-	0.06Å	Favored (61.041%)	-	-	-
A 166		ARG	0.75	-	Favored (51.18%) General / -126.5,133.3	Favored (64.1%) *ttp-170* chi angles: 183.4,176.6,67.6,184.5	0.04Å	Favored (58.281%) beta sheet	-	-	-
A 167		PHE	0.75	0.49Å CD1 with A 167 PHE N	Favored (47.08%)	Favored (53.5%) *m-80* chi angles: 305.9,89	0.13Å	Favored (52.613%) beta sheet	OUTLIER(S) worst is CB--CG: 4.8 σ	OUTLIER(S) worst is CA-CB-CG: 4.9 σ	-

#	Alt	Res	High B	Clash > 0.4Å	Ramachandran	Rotamer	Cβ deviation	CaBLAM	Bond lengths	Bond angles	Cis Peptides
					General / -129.7,154.5						
A 168		THR	0.76	-	Favored (55.24%) General / -108.4,129.8	Favored (96.8%) *m* chi angles: 299.8	0.04Å	Favored (60.729%) beta sheet	-	-	-
A 169		ILE	0.79	-	Favored (69.33%) Ile or Val / -117.3,131.7	Favored (3.6%) *mp* chi angles: 301,92.8	0.07Å	Favored (45.188%) beta sheet	-	-	-
A 170		SER	0.81	-	Favored (33.84%) Pre-Pro / -149.1,160.9	Favored (93.7%) *p* chi angles: 64.2	0.07Å	Favored (34.676%)	-	-	-
A 171		PRO	0.82	-	Favored (42.26%) Trans-Pro / -56.8,-21.9	Favored (85.6%) *Cg_exo* chi angles: 333.8,37.9,326.9	0.01Å	Favored (80.502%)	-	-	-
A 172		GLN	0.83	-	Favored (56.98%) General / -87.5,-8.0	Favored (70.1%) *mt0* chi angles: 296.7,184.2,65.3	0.04Å	Favored (33.47%)	-	-	-
A 173		ALA	0.82	-	Favored (3.6%) Pre-Pro / -158.3,68.1	-	0.03Å	Favored (11.429%)	-	-	-
A 174		PRO	0.81	-	Favored (32.4%) Trans-Pro / -70.8,-17.9	Favored (53.2%) *Cg_endo* chi angles: 25.6,325.9,27.6	0.05Å	Favored (69.563%)	-	-	-
A 175		SER	0.81	-	Favored (28.51%) General / -117.9,156.4	Favored (98.8%) *p* chi angles: 65.3	0.02Å	Favored (7.304%)	-	-	-
A 176		PHE	0.81	-	Favored (39.81%) General / -156.7,162.5	Favored (58.6%) *p90* chi angles: 62.6,90.2	0.02Å	Favored (42.354%)	-	-	-
A 177		THR	0.82	-	Favored (49.85%) General / -125.5,129.5	Favored (97.7%) *m* chi angles: 300	0.01Å	Favored (48.338%) beta sheet	-	-	-
A 178		ALA	0.85	-	Favored (29.59%) General / -93.7,140.5	-	0.04Å	Favored (49.648%) beta sheet	-	-	-
A 179		ASN	0.88	-	Favored (17.73%) General / -111.6,108.3	Favored (56.2%) *t0* chi angles: 186.7,330.2	0.06Å	Favored (53.048%) beta sheet	-	-	-
A 180		MET	0.9	-	Favored (20.27%) General / -99.5,17.5	Favored (18.8%) *mmt* chi angles: 301,299,192.8	0.05Å	CaBLAM Disfavored (1.449%)	-	-	-
**#**	**Alt**	**Res**	**High B**	**Clash > 0.4Å**	**Ramachandran**	**Rotamer**	**Cβ deviation**	**CaBLAM**	**Bond lengths**	**Bond angles**	**Cis Peptides**
			Avg: 1.09	Clashscore: 1.46	Outliers: 1 of 499	Poor rotamers: 0 of 409	Outliers: 0 of 449	Outliers: 10 of 497	Outliers: 7 of 501	Outliers: 9 of 501	Non-Trans: 0 of 500
A 181		GLY	0.91	-	Favored (3.9%) Glycine / 62.3,-111.3	-	-	Favored (18.21%)	-	-	-
A 182		GLU	0.91	-	Favored (61.19%) General / -59.0,-22.9	Favored (66.1%) *mm-30* chi angles: 293.7,295,302.8	0.03Å	Favored (15.419%)	-	-	-
A 183		TYR	0.89	-	Favored (40.76%)	Favored (84.7%) *m-80* chi angles: 301.9,100.3	0.04Å	Favored (44.765%)	-	-	-

					General / -100.4,10.8						
A 184		GLY	0.86	-	Favored (36.67%) Glycine / 83.6,-159.4	-	-	Favored (33.168%)	-	-	-
A 185		THR	0.83	-	Favored (42.67%) General / -125.5,154.1	Favored (57.5%) *p* chi angles: 64.5	0.04Å	Favored (11.37%)	-	-	-
A 186		VAL	0.81	-	Favored (57.07%) Ile or Val / -126.6,120.8	Favored (63.1%) *t* chi angles: 179.6	0.09Å	Favored (59.125%) beta sheet	-	-	-
A 187		THR	0.81	-	Favored (45.44%) General / -99.4,127.7	Favored (93%) *m* chi angles: 297.6	0.08Å	Favored (67.188%) beta sheet	-	-	-
A 188		ILE	0.83	-	Favored (66.76%) Ile or Val / -113.9,131.1	Favored (41.9%) *mm* chi angles: 306.1,298.7	0.04Å	Favored (69.746%) beta sheet	-	-	-
A 189		ASP	0.88	-	Favored (10.75%) General / -117.8,104.2	Favored (65.7%) *t0* chi angles: 184.1,355.2	0.07Å	Favored (61.982%) beta sheet	-	OUTLIER(S) worst is CA-CB-CG: 4.3 σ	-
A 190		CYS	0.97	-	Favored (35.55%) General / -108.4,144.8	Favored (68.9%) *m* chi angles: 298.9	0.06Å	Favored (48.937%) beta sheet	-	-	-
A 191		GLU	1.1	-	Favored (2.07%) General / -100.2,78.5	Favored (93.2%) *mt-10* chi angles: 298.7,182.6,357	0.03Å	Favored (26.048%) beta sheet	-	-	-
A 192		ALA	1.25	-	Favored (66.45%) General / -59.0,-28.5	-	0.03Å	Favored (42.181%)	-	-	-
A 193		ARG	1.4	-	Favored (59.3%) General / -77.6,-10.1	Favored (46.5%) *ptt180* chi angles: 65.8,185.5,181.2,181.4	0.07Å	Favored (64.299%) three-ten	-	-	-
A 194		SER	1.52	-	Favored (16.04%) General / -97.2,-23.8	Favored (70.7%) *m* chi angles: 296.2	0.03Å	Favored (37.054%)	-	-	-
A 195		GLY	1.58	-	Favored (16.74%) Glycine / -59.9,-57.9	-	-	Favored (17.945%)	-	-	-
A 196		ILE	1.57	-	Favored (75.97%) Ile or Val / -120.6,127.9	Favored (86.5%) *mt* chi angles: 298.7,170.5	0.04Å	Favored (22.487%)	-	-	-
A 197		ASN	1.48	-	Favored (5.85%) General / -79.2,72.7	Favored (59.1%) *t0* chi angles: 190.8,26.4	0.04Å	Favored (43.083%)	-	-	-
A 198		THR	1.36	-	Favored (48.73%) General / -56.9,-24.0	Favored (6.7%) *t* chi angles: 194.2	0.04Å	Favored (20.282%)	-	-	-
A 199		GLU	1.23	-	Favored (67.54%) General / -62.2,-25.0	Favored (72.7%) *mm-30* chi angles: 294,294.7,308.5	0.06Å	Favored (57.199%)	-	-	-
A 200		ASP	1.1	-	Favored (52.74%) General / -93.9,5.1	Favored (81.2%) *m-30* chi angles: 290.3,331.9	0.03Å	Favored (41.211%)	-	-	-

#	Alt	Res	High B	Clash > 0.4Å	Ramachandran	Rotamer	Cβ deviation	CaBLAM	Bond lengths	Bond angles	Cis Peptides
			Avg: 1.09	Clashscore: 1.46	Outliers: 1 of 499	Poor rotamers: 0 of 409	Outliers: 0 of 449	Outliers: 10 of 497	Outliers: 7 of 501	Outliers: 9 of 501	Non-Trans: 0 of 500
A 201		TYR	1	-	Favored (35.9%) General / -123.3,156.2	Favored (85.1%) *m-80* chi angles: 295.3,83.1	0.07Å	Favored (20.031%)	-	-	-
A 202		TYR	0.93	-	Favored (26.26%) General / -144.3,166.0	Favored (30.6%) *p90* chi angles: 74.3,98.7	0.10Å	Favored (47.808%)	OUTLIER(S) worst is CB--CG: 6.9 σ	-	-
A 203		VAL	0.9	-	Favored (34.99%) Ile or Val / -103.0,136.6	Favored (5.7%) *p* chi angles: 58.1	0.05Å	Favored (45.781%) beta sheet	-	-	-
A 204		PHE	0.92	-	Favored (52.92%) General / -110.8,134.0	Favored (35.8%) *t80* chi angles: 190.7,66	0.04Å	Favored (71.025%) beta sheet	-	-	-
A 205		THR	0.96	-	Favored (50.31%) General / -122.9,128.4	Favored (96.9%) *m* chi angles: 299.8	0.01Å	Favored (59.918%)	-	-	-
A 206		VAL	1.02	-	Favored (27.89%) Ile or Val / -129.5,113.5	Favored (59.7%) *t* chi angles: 180	0.09Å	Favored (16.993%)	-	-	-
A 207		LYS	1.07	-	Favored (23.96%) General / 49.9,46.8	Favored (98%) *mttt* chi angles: 295.8,180.6,176.5,174.4	0.05Å	Favored (34.434%)	-	-	-
A 208		GLU	1.08	-	Favored (7.28%) General / 65.5,11.3	Favored (88.4%) *mt-10* chi angles: 300.5,183.7,353.2	0.05Å	Favored (7.949%)	-	-	-
A 209		LYS	1.06	-	Favored (33.8%) General / -96.8,138.8	Favored (99.3%) *mttt* chi angles: 295.2,180.2,178.5,178.5	0.03Å	Favored (24.507%)	-	-	-
A 210		SER	1.02	-	Favored (51.12%) General / -131.7,152.1	Favored (60.5%) *m* chi angles: 298.9	0.04Å	Favored (67.669%) beta sheet	-	-	-
A 211		TRP	0.98	-	Favored (48.06%) General / -137.2,150.6	Favored (18.5%) *m-90* chi angles: 303.9,263.5	0.10Å	Favored (57.164%) beta sheet	-	-	-
A 212		LEU	0.96	-	Favored (27.39%) General / -92.8,114.8	Favored (54.7%) *tp* chi angles: 179.8,65.2	0.06Å	Favored (47.529%) beta sheet	-	-	-
A 213		VAL	0.97	-	Favored (51.84%) Ile or Val / -129.1,139.4	Favored (47.6%) *t* chi angles: 181.9	0.08Å	Favored (45.628%) beta sheet	-	-	-
A 214		ASN	1	-	Favored (58.19%) General / -62.8,137.1	Favored (20.5%) *t0* chi angles: 193,295.7	0.03Å	Favored (46.812%)	-	-	-
A 215		ARG	1.04	-	Favored (68%) General / -58.2,-32.2	Favored (81.7%) *mtp180* chi angles: 289.7,172.8,72.5,187.2	0.02Å	Favored (55.771%)	-	-	-
A 216		ASP	1.07	-	Favored (87.76%) General / -66.8,-39.7	Favored (98.7%) *m-30* chi angles: 288.2,346.3	0.02Å	Favored (76.103%) alpha helix	-	-	-

#	Alt	Res	High B	Clash > 0.4Å	Ramachandran	Rotamer	Cβ deviation	CaBLAM	Bond lengths	Bond angles	Cis Peptides
A 217		TRP	1.08	-	Favored (83.95%) General / -58.7,-47.6	Favored (70.5%) *t-100* chi angles: 183.9,259.2	0.07Å	Favored (95.825%) alpha helix	-	-	-
A 218		PHE	1.07	-	Favored (76.75%) General / -58.3,-49.5	Favored (78.6%) *t80* chi angles: 173.7,72.2	0.11Å	Favored (94.595%) alpha helix	-	OUTLIER(S) worst is CA-CB-CG: 6.9 σ	-
A 219		HIS	1.03	-	Favored (69.64%) General / -62.5,-28.1	Favored (49.9%) *m170* chi angles: 288.9,162.1	0.04Å	Favored (73.75%) alpha helix	-	-	-
A 220		ASP	0.99	-	Favored (58.83%) General / -82.8,-5.3	Favored (85.7%) *m-30* chi angles: 289.1,335.7	0.04Å	Favored (51.903%)	-	-	-
**#**	**Alt**	**Res**	**High B**	**Clash > 0.4Å**	**Ramachandran**	**Rotamer**	**Cβ deviation**	**CaBLAM**	**Bond lengths**	**Bond angles**	**Cis Peptides**
			Avg: 1.09	Clashscore: 1.46	Outliers: 1 of 499	Poor rotamers: 0 of 409	Outliers: 0 of 449	Outliers: 10 of 497	Outliers: 7 of 501	Outliers: 9 of 501	Non-Trans: 0 of 500
A 221		LEU	0.93	-	Favored (56.59%) General / -63.6,135.2	Favored (96%) *mt* chi angles: 292.7,171	0.06Å	Favored (33.209%)	-	-	-
A 222		ASN	0.87	-	Favored (36.71%) General / -92.9,9.1	Favored (39.7%) *p0* chi angles: 65.4,339.6	0.02Å	Favored (11.056%) beta sheet	-	-	-
A 223		LEU	0.84	-	Favored (81.63%) Pre-Pro / -77.0,151.2	Favored (93.5%) *mt* chi angles: 296.9,172.9	0.18Å	Favored (19.772%) beta sheet	-	OUTLIER(S) worst is C-CA-CB: 4.1 σ	-
A 224		PRO	0.82	-	Favored (89.77%) Trans-Pro / -56.5,142.1	Favored (65.1%) *Cg_exo* chi angles: 335.6,35.9,328	0.08Å	Favored (43.391%) beta sheet	-	-	-
A 225		TRP	0.85	-	Favored (43.95%) General / -145.7,157.4	Favored (74.2%) *p-90* chi angles: 59.5,265.5	0.04Å	Favored (69.625%) beta sheet	-	-	-
A 226		THR	0.93	-	Favored (47.9%) General / -128.8,132.7	Favored (94.1%) *m* chi angles: 299.3	0.07Å	Favored (56.737%) beta sheet	-	-	-
A 227		SER	1.07	-	Favored (85.76%) Pre-Pro / -75.1,156.9	Favored (85.8%) *p* chi angles: 67.8	0.10Å	Favored (45.198%)	-	-	-
A 228		PRO	1.23	-	Favored (69.99%) Trans-Pro / -61.7,-20.7	Favored (32.4%) *Cg_endo* chi angles: 21.8,325.2,33.1	0.01Å	Favored (74.62%)	-	-	-
A 229		ALA	1.37	-	Favored (59.25%) General / -83.5,-5.7	-	0.05Å	Favored (66.516%) alpha helix	-	-	-
A 230		THR	1.43	-	Favored (12.26%) General / -128.6,171.3	Favored (80.5%) *p* chi angles: 60.5	0.05Å	Favored (13.737%)	-	-	-
A 231		THR	1.38	-	Favored (11.33%) General / -116.2,-11.4	Favored (75.1%) *p* chi angles: 60	0.04Å	Favored (12.618%)	-	-	-
A 232		ASP	1.25	-	Favored (15.04%) General / -100.9,159.6	Favored (19.1%) *m-30* chi angles: 304.7,345.1	0.08Å	Favored (22.643%) beta sheet	-	-	-
A 233		TRP	1.08	-	Favored (23.5%)	Favored (35.4%) *m-90*	0.04Å	Favored (49.777%)	-	-	-

					General / -102.2,150.2	chi angles: 296,261.7					
A 234		ARG	0.93	-	Favored (15.14%) General / -92.1,-32.5	Favored (54.3%) *mmt180* chi angles: 298.6,298.2,184.2,185	0.03Å	Favored (29.647%)	-	-	-
A 235		ASN	0.82	-	Favored (2.5%) General / -128.3,46.9	Favored (68.2%) *m-40* chi angles: 291.9,279.8	0.08Å	Favored (13.58%)	-	-	-
A 236		ARG	0.76	-	Favored (68.81%) General / -57.7,-33.9	Favored (98.2%) *mtm-85* chi angles: 288.9,192.3,291.9,270.9	0.10Å	Favored (21.668%)	-	-	-
A 237		GLU	0.73	-	Favored (58.92%) General / -58.7,-23.3	Favored (26.5%) *pt0* chi angles: 66.2,182.1,354.6	0.03Å	Favored (60.355%)	-	-	-
A 238		THR	0.72	-	Favored (71.12%) General / -60.0,-32.7	Favored (92.5%) *m* chi angles: 297.7	0.09Å	Favored (52.286%) three-ten	-	-	-
A 239		LEU	0.72	-	Favored (36.78%) General / -101.7,12.7	Favored (60.8%) *mt* chi angles: 292.1,180.3	0.08Å	Favored (18.045%) alpha helix	-	-	-
A 240		VAL	0.73	-	Favored (12.16%) Ile or Val / -145.3,129.4	Favored (4.5%) *p* chi angles: 56.6	0.08Å	Favored (11.64%)	-	-	-
**#**	**Alt**	**Res**	**High B**	**Clash > 0.4Å**	**Ramachandran**	**Rotamer**	**Cβ deviation**	**CaBLAM**	**Bond lengths**	**Bond angles**	**Cis Peptides**
			Avg: 1.09	Clashscore: 1.46	Outliers: 1 of 499	Poor rotamers: 0 of 409	Outliers: 0 of 449	Outliers: 10 of 497	Outliers: 7 of 501	Outliers: 9 of 501	Non-Trans: 0 of 500
A 241		GLU	0.74	-	Favored (47.7%) General / -106.1,135.3	Favored (91.4%) *tt0* chi angles: 181,178,5.5	0.03Å	Favored (60.266%)	-	-	-
A 242		PHE	0.77	-	Favored (31.7%) General / -105.5,116.3	Favored (48.1%) *m-80* chi angles: 290.7,75.5	0.08Å	Favored (61.144%)	-	-	-
A 243		GLU	0.8	-	Favored (24.67%) General / -78.4,164.6	Favored (91.6%) *mt-10* chi angles: 296.3,184.6,347.1	0.01Å	Favored (15.605%)	-	-	-
A 244		GLU	0.85	-	Favored (46.4%) Pre-Pro / -53.1,127.4	Favored (90.9%) *tt0* chi angles: 184.7,178.6,4.7	0.06Å	Favored (26.442%)	-	-	-
A 245		PRO	0.89	-	Favored (54.96%) Trans-Pro / -72.4,155.9	Favored (76.3%) *Cg_endo* chi angles: 28.2,324.7,26.8	0.04Å	Favored (65.724%)	-	-	-
A 246		HIS	0.93	-	Favored (4.66%) General / -127.7,96.9	Favored (67.9%) *m-70* chi angles: 300.3,264.6	0.04Å	CaBLAM Outlier (0.6%)	-	-	-
A 247		ALA	0.95	-	Allowed (0.08%) General / 55.7,-98.2	-	0.05Å	CaBLAM Outlier (0.207%)	-	-	-
A 248		THR	0.94	-	Favored (26.37%) General / -103.0,-3.2	Favored (74.9%) *p* chi angles: 61.4	0.05Å	Favored (18.429%)	-	-	-
A 249		LYS	0.91	-	Favored (44.61%)	Favored (99%) *mttt* chi angles:	0.04Å	Favored (23.533%)	-	-	-

					General / -127.2,154.6	294.8,184.4,179.9,180.4					
A 250		GLN	0.87	-	Favored (18.3%) General / -148.3,133.4	Favored (58.5%) *tt0* chi angles: 184,170.9,24.6	0.04Å	Favored (44.734%)	-	-	-
A 251		THR	0.84	-	Favored (33.64%) General / -87.2,133.5	Favored (90.7%) *m* chi angles: 298.1	0.02Å	Favored (51.454%) beta sheet	-	-	-
A 252		VAL	0.81	-	Favored (67.81%) Ile or Val / -121.3,133.4	Favored (44.1%) *t* chi angles: 182.6	0.05Å	Favored (72.533%) beta sheet	-	-	-
A 253		VAL	0.8	-	Favored (63.15%) Ile or Val / -124.1,122.0	Favored (72.1%) *t* chi angles: 178.5	0.08Å	Favored (60.677%) beta sheet	-	-	-
A 254		ALA	0.81	-	Favored (54.19%) General / -69.4,139.7	-	0.04Å	Favored (39.084%) beta sheet	-	-	-
A 255		LEU	0.82	-	Favored (4.56%) General / -89.0,58.4	Favored (73.5%) *mt* chi angles: 303.2,178.6	0.07Å	Favored (5.11%)	-	-	-
A 256		GLY	0.84	-	Favored (36.89%) Glycine / 53.1,-128.0	-	-	Favored (51.508%)	-	-	-
A 257		SER	0.87	-	Favored (33.8%) General / -128.9,123.9	Favored (36.5%) *t* chi angles: 174.5	0.10Å	Favored (10.109%)	-	-	-
A 258		GLN	0.91	-	Favored (20.97%) General / -90.5,11.7	Favored (98.8%) *mm-40* chi angles: 296.1,298.9,311.8	0.11Å	Favored (9.057%)	-	-	-
A 259		GLU	0.96	-	Favored (65.38%) General / -53.0,-42.2	Favored (86.4%) *tt0* chi angles: 177.5,178.6,357.4	0.03Å	Favored (48.81%)	-	-	-
A 260		GLY	1.02	-	Favored (98.14%) Glycine / -61.3,-40.1	-	-	Favored (96.871%) alpha helix	-	-	-
**#**	**Alt**	**Res**	**High B**	**Clash > 0.4Å**	**Ramachandran**	**Rotamer**	**Cβ deviation**	**CaBLAM**	**Bond lengths**	**Bond angles**	**Cis Peptides**
			Avg: 1.09	Clashscore: 1.46	Outliers: 1 of 499	Poor rotamers: 0 of 409	Outliers: 0 of 449	Outliers: 10 of 497	Outliers: 7 of 501	Outliers: 9 of 501	Non-Trans: 0 of 500
A 261		ALA	1.08	-	Favored (87.46%) General / -62.9,-37.6	-	0.02Å	Favored (95.179%) alpha helix	-	-	-
A 262		LEU	1.14	0.60Å C with A 262 LEU HD13	Favored (68.64%) General / -64.7,-50.0	Allowed (1.1%) *tm* chi angles: 183.1,281.3	0.03Å	Favored (86.564%) alpha helix	-	-	-
A 263		HIS	1.2	-	Favored (87.8%) General / -61.1,-38.9	Favored (73.8%) *m-70* chi angles: 290.8,301.1	0.02Å	Favored (85.341%) alpha helix	-	-	-
A 264		THR	1.24	-	Favored (87.97%) General / -64.7,-45.2	Favored (96.5%) *m* chi angles: 299.7	0.03Å	Favored (84.509%) alpha helix	-	-	-

A 265		ALA	1.26	-	Favored (71.74%) General / -59.0,-34.3	-	0.04Å	Favored (77.803%) alpha helix	-	-	-
A 266		LEU	1.25	-	Favored (13.24%) General / -93.1,16.3	Favored (49.9%) *mt* chi angles: 299.2,185.3	0.04Å	Favored (29.568%) alpha helix	-	-	-
A 267		ALA	1.22	-	Favored (64.97%) General / -57.1,-31.2	-	0.02Å	Favored (48.751%) alpha helix	-	-	-
A 268		GLY	1.17	-	Favored (86.14%) Glycine / -87.4,4.3	-	-	Favored (58.142%)	-	-	-
A 269		ALA	1.1	-	Favored (14.78%) General / -90.6,162.9	-	0.04Å	Favored (32.698%)	-	-	-
A 270		ILE	1.04	-	Favored (42.52%) Pre-Pro / -97.2,126.3	Favored (49.3%) *mm* chi angles: 301.6,301.1	0.03Å	Favored (23.121%) beta sheet	-	-	-
A 271		PRO	1	-	Favored (73.17%) Trans-Pro / -56.4,146.4	Favored (86.3%) *Cg_exo* chi angles: 333.6,35.2,331.3	0.03Å	Favored (28.361%) beta sheet	-	-	-
A 272		ALA	1	-	Favored (33.25%) General / -147.5,164.5	-	0.03Å	Favored (51.884%) beta sheet	-	-	-
A 273		THR	1.03	-	Favored (53.44%) General / -124.1,134.0	Favored (93.3%) *m* chi angles: 299.2	0.01Å	Favored (53.535%) beta sheet	-	-	-
A 274		VAL	1.09	-	Favored (74.47%) Ile or Val / -117.5,129.1	Favored (84.4%) *t* chi angles: 177.1	0.03Å	Favored (70.643%)	-	-	-
A 275		SER	1.14	-	Favored (9.87%) General / -119.3,103.8	Favored (37.7%) *t* chi angles: 177.5	0.06Å	Favored (6.791%)	-	-	-
A 276		SER	1.18	-	Allowed (0.09%) General / 54.6,-99.2	Favored (8.7%) *t* chi angles: 192.2	0.08Å	CaBLAM Outlier (0.95%)	-	-	-
A 277		SER	1.18	-	Favored (21.65%) General / -106.3,-3.0	Favored (96.4%) *p* chi angles: 63.7	0.03Å	Favored (8.699%)	-	-	-
A 278		THR	1.16	-	Favored (55.04%) General / -114.9,134.6	Favored (98.8%) *m* chi angles: 300.2	0.04Å	Favored (36.368%)	-	-	-
A 279		LEU	1.14	-	Favored (51.83%) General / -105.5,126.2	Favored (35%) *tp* chi angles: 170.9,66.2	0.10Å	Favored (70.417%)	-	-	-
A 280		THR	1.14	-	Favored (54.5%) General / -107.3,128.5	Favored (98.5%) *m* chi angles: 300.6	0.01Å	Favored (69.458%) beta sheet	-	-	-
**#**	**Alt**	**Res**	**High B**	**Clash > 0.4Å**	**Ramachandran**	**Rotamer**	**Cβ deviation**	**CaBLAM**	**Bond lengths**	**Bond angles**	**Cis Peptides**
			Avg: 1.09	Clashscore: 1.46	Outliers: 1 of 499	Poor rotamers: 0 of 409	Outliers: 0 of 449	Outliers: 10 of 497	Outliers: 7 of 501	Outliers: 9 of 501	Non-Trans: 0 of 500

A 281		LEU	1.16	-	Favored (34.99%) General / -105.0,142.3	Favored (95.8%) *mt* chi angles: 296.7,176.5	0.03Å	Favored (60.433%)	-	-	-
A 282		GLN	1.19	-	Favored (43.32%) General / -95.6,-5.1	Favored (76.6%) *mt0* chi angles: 296.2,178.2,23	0.01Å	Favored (48.815%)	-	-	-
A 283		SER	1.21	-	Favored (9.74%) General / -121.6,24.6	Favored (59.7%) *p* chi angles: 57.8	0.06Å	Favored (25.809%)	-	-	-
A 284		GLY	1.2	-	Favored (51.54%) Glycine / -67.3,150.1	-	-	Favored (38.337%)	-	-	-
A 285		HIS	1.15	-	Favored (3.49%) General / -129.6,93.2	Favored (78.5%) *m90* chi angles: 297.7,82.8	0.08Å	Favored (25.054%) beta sheet	OUTLIER(S) worst is CB--CG: 4.2 σ	-	-
A 286		LEU	1.08	-	Favored (29.18%) General / -88.2,120.0	Favored (46.4%) *tp* chi angles: 183.1,65	0.01Å	Favored (53.416%) beta sheet	-	-	-
A 287		LYS	1.01	-	Favored (31.6%) General / -90.3,120.6	Favored (73.3%) *tttt* chi angles: 175.7,182,167.9,179	0.03Å	Favored (59.268%) beta sheet	-	-	-
A 288		CYS	0.97	-	Favored (47.6%) General / -131.9,136.8	Favored (69.1%) *m* chi angles: 298.8	0.04Å	Favored (56.896%) beta sheet	-	-	-
A 289		ARG	0.96	-	Favored (36.82%) General / -95.4,121.2	Favored (47.7%) *ttm170* chi angles: 177.7,167.9,289.1,181.4	0.01Å	Favored (66.359%) beta sheet	-	-	-
A 290		ALA	0.97	-	Favored (32.13%) General / -103.8,116.3	-	0.03Å	Favored (67.84%) beta sheet	-	-	-
A 291		LYS	0.98	-	Favored (29.71%) General / -84.1,-25.5	Favored (98.4%) *mttt* chi angles: 294.1,181.9,179,183.3	0.04Å	CaBLAM Disfavored (3.634%) try beta sheet	-	-	-
A 292		LEU	1	-	OUTLIER (0.01)%) General / 56.5,-20.7	Favored (92.4%) *mt* chi angles: 294.5,176.1	0.06Å	Favored (25.388%)	-	-	-
A 293		ASP	1.01	-	Allowed (0.25%) General / 44.0,-112.3	Favored (73.9%) *m-30* chi angles: 295.4,319.6	0.03Å	CaBLAM Disfavored (1.119%)	-	-	-
A 294		LYS	1.02	-	Favored (25.84%) General / -88.0,8.5	Favored (99.4%) *mttt* chi angles: 294.8,180.9,180.3,177.8	0.03Å	CaBLAM Disfavored (4.287%)	-	-	-
A 295		VAL	1.03	-	Favored (24.59%) Ile or Val / -59.3,132.8	Favored (55.9%) *t* chi angles: 170.2	0.06Å	Favored (20.161%)	-	-	-
A 296		LYS	1.06	-	Favored (37.19%) General / -128.1,158.7	Favored (98.3%) *mttt* chi angles: 295.6,182.8,180.3,180.9	0.04Å	Favored (45.749%) beta sheet	-	-	-
A 297		ILE	1.09	-	Favored (29.48%) Ile or Val / -99.5,111.6	Favored (77.4%) *mt* chi angles: 300.2,173.7	0.10Å	Favored (32.6%)	OUTLIER(S) worst is CB--CG1: 5.5 σ	-	-

A 298		LYS	1.12	-	Favored (55.02%) General / -62.4,133.4	Favored (51.3%) *tptt* chi angles: 180.6,70.9,177.3,184.2	0.02Å	Favored (6.98%)	-	-	-
A 299		GLY	1.14	-	Favored (40.81%) Glycine / 106.5,-16.6	-	-	Favored (66.588%) alpha helix	-	-	-
A 300		THR	1.13	-	Favored (14.18%) General / -82.6,7.6	Favored (43%) *p* chi angles: 55	0.11Å	CaBLAM Disfavored (4.245%) try alpha helix	-	-	-
**#**	**Alt**	**Res**	**High B**	**Clash > 0.4Å**	**Ramachandran**	**Rotamer**	**Cβ deviation**	**CaBLAM**	**Bond lengths**	**Bond angles**	**Cis Peptides**
			Avg: 1.09	Clashscore: 1.46	Outliers: 1 of 499	Poor rotamers: 0 of 409	Outliers: 0 of 449	Outliers: 10 of 497	Outliers: 7 of 501	Outliers: 9 of 501	Non-Trans: 0 of 500
A 301		THR	1.09	-	Favored (16.09%) General / -114.6,7.7	Favored (76.3%) *p* chi angles: 60.1	0.05Å	Favored (59.482%)	-	-	-
A 302		TYR	1.03	-	Favored (32.41%) General / -121.5,156.6	Favored (95.6%) *m-80* chi angles: 295.6,87	0.01Å	Favored (24.741%)	-	-	-
A 303		GLY	0.98	-	Favored (46.82%) Glycine / -84.5,-170.4	-	-	Favored (47.024%)	-	-	-
A 304		MET	0.93	-	Favored (28.75%) General / -80.7,151.8	Favored (30.5%) *mmt* chi angles: 291.8,292.7,176.6	0.04Å	Favored (19.756%)	-	-	-
A 305		CYS	0.91	-	Favored (49.68%) General / -67.0,133.3	Favored (81%) *m* chi angles: 295.9	0.06Å	Favored (37.687%)	-	-	-
A 306		ASP	0.89	-	Favored (5.18%) General / -109.9,-39.1	Favored (62.5%) *m-30* chi angles: 295.7,306.5	0.06Å	CaBLAM Disfavored (4.076%) try beta sheet	-	-	-
A 307		SER	0.88	-	Favored (21.11%) General / -64.2,159.2	Favored (63.8%) *m* chi angles: 297.5	0.06Å	Favored (6.362%) beta sheet	-	-	-
A 308		ALA	0.87	-	Favored (44.54%) General / -60.1,146.1	-	0.04Å	Favored (16.43%) beta sheet	-	-	-
A 309		PHE	0.85	-	Favored (15.43%) General / -106.8,160.5	Favored (69.1%) *m-80* chi angles: 286.6,86	0.08Å	Favored (50.921%) beta sheet	-	-	-
A 310		THR	0.83	-	Favored (42.83%) General / -141.8,151.5	Favored (8.4%) *t* chi angles: 184.2	0.04Å	Favored (38.754%) beta sheet	-	-	-
A 311		PHE	0.81	-	Favored (40.03%) General / -77.3,141.2	Favored (62.8%) *m-80* chi angles: 287.9,107.4	0.07Å	Favored (41.039%)	-	-	-
A 312		SER	0.79	-	Favored (10.44%) General / -88.0,-45.1	Favored (44.5%) *t* chi angles: 179	0.06Å	Favored (8.362%)	-	-	-
A 313		LYS	0.8	-	Favored (51.52%)	Favored (99%) *mttt* chi angles: 295.5,182.1,178,177	0.05Å	Favored (23.655%)	-	-	-

#	Alt	Res	High B	Clash > 0.4Å	Ramachandran	Rotamer	Cβ deviation	CaBLAM	Bond lengths	Bond angles	Cis Peptides
					General / -130.7,144.6						
A 314		ASN	0.83	-	Favored (98.37%) Pre-Pro / -68.0,148.6	Favored (94.3%) *m-40* chi angles: 289.9,331.7	0.03Å	Favored (27.914%)	-	-	-
A 315		PRO	0.89	-	Favored (29.13%) Trans-Pro / -49.7,131.6	Favored (77%) *Cg_exo* chi angles: 329,37.8,332.3	0.08Å	Favored (54.907%)	-	-	-
A 316		THR	0.97	-	Favored (55.06%) General / -121.2,132.8	Favored (89.1%) *m* chi angles: 298.6	0.05Å	Favored (49.845%) beta sheet	-	-	-
A 317		ASP	1.08	-	Favored (16.3%) General / -77.6,114.3	Favored (54.2%) *t0* chi angles: 188.8,355.3	0.06Å	Favored (47.615%)	-	OUTLIER(S) worst is CA-CB-CG: 4.4 σ	-
A 318		THR	1.18	-	Favored (14.72%) General / -82.1,171.1	Favored (62.1%) *p* chi angles: 63.6	0.08Å	Favored (29.613%)	-	-	-
A 319		GLY	1.24	-	Favored (51.07%) Glycine / -56.0,-27.3	-	-	Favored (45.218%)	-	-	-
A 320		HIS	1.24	-	Favored (42.09%) General / -90.0,-11.2	Favored (58.9%) *t70* chi angles: 190.3,76	0.08Å	Favored (29.135%)	-	-	-
**#**	**Alt**	**Res**	**High B**	**Clash > 0.4Å**	**Ramachandran**	**Rotamer**	**Cβ deviation**	**CaBLAM**	**Bond lengths**	**Bond angles**	**Cis Peptides**
			Avg: 1.09	Clashscore: 1.46	Outliers: 1 of 499	Poor rotamers: 0 of 409	Outliers: 0 of 449	Outliers: 10 of 497	Outliers: 7 of 501	Outliers: 9 of 501	Non-Trans: 0 of 500
A 321		GLY	1.17	-	Favored (62.63%) Glycine / 94.9,2.7	-	-	Favored (86.823%)	-	-	-
A 322		THR	1.06	-	Favored (19.17%) General / -82.2,166.9	Favored (67.5%) *p* chi angles: 62.8	0.02Å	Favored (44.128%)	-	-	-
A 323		VAL	0.94	-	Favored (60.32%) Ile or Val / -122.4,136.1	Favored (8.5%) *p* chi angles: 61.4	0.06Å	Favored (49.661%) beta sheet	-	-	-
A 324		ILE	0.84	-	Favored (57.12%) Ile or Val / -104.4,129.2	Favored (39.8%) *mm* chi angles: 308.3,303.3	0.04Å	Favored (65.301%) beta sheet	OUTLIER(S) worst is CB--CG1: 5.5 σ	-	-
A 325		VAL	0.77	-	Favored (75.91%) Ile or Val / -121.2,127.8	Favored (51.1%) *t* chi angles: 181.3	0.13Å	Favored (70.201%) beta sheet	-	-	-
A 326		GLU	0.74	-	Favored (32.84%) General / -107.5,117.0	Favored (30.2%) *tt0* chi angles: 182.3,181.9,98	0.07Å	Favored (69.311%) beta sheet	-	-	-
A 327		LEU	0.73	-	Favored (22.05%) General / -109.2,154.7	Favored (74%) *mt* chi angles: 301.6,174.6	0.10Å	Favored (35.721%) beta sheet	OUTLIER(S) worst is CB--CG: 4.4 σ	-	-
A 328		GLN	0.76	-	Favored (50.14%) General / -125.3,129.5	Favored (55%) *tt0* chi angles: 183,173.1,51.2	0.07Å	Favored (56.798%) beta sheet	-	-	-
A 329		TYR	0.8	-	Favored (28.18%)	Favored (43.8%) *t80*	0.00Å	Favored (62.872%)	-	-	-

					General / -96.2,115.9	chi angles: 181.4,59.2					
A 330		THR	0.85	-	Favored (8.91%) General / -86.0,65.0	Favored (78.9%) *p* chi angles: 60.3	0.04Å	CaBLAM Disfavored (2.152%)	-	-	-
A 331		GLY	0.88	-	Favored (40.76%) Glycine / -176.9,168.7	-	-	Favored (6.143%)	-	-	-
A 332		SER	0.9	-	Favored (10.39%) General / -119.8,24.7	Favored (60.2%) *p* chi angles: 57.8	0.02Å	Favored (5.333%)	-	-	-
A 333		ASN	0.89	-	Favored (2.55%) General / -79.1,58.7	Favored (85.5%) *m-40* chi angles: 296.4,313.7	0.08Å	CA Geom Outlier (0.47%)	-	-	-
A 334		GLY	0.86	-	Favored (23.36%) Glycine / 69.2,180.0	-	-	Favored (18.194%)	-	-	-
A 335		PRO	0.81	-	Favored (8.61%) Trans-Pro / -78.5,65.2	Favored (70.8%) *Cg_endo* chi angles: 31.1,323.7,26.7	0.04Å	CA Geom Outlier (0.483%)	-	-	-
A 336		CYS	0.76	-	Favored (29.84%) General / -67.6,159.4	Favored (36.5%) *p* chi angles: 62.5	0.08Å	Favored (7.991%)	-	-	-
A 337		ARG	0.73	-	Favored (34.51%) General / -82.2,138.9	Favored (87.6%) *mtp180* chi angles: 295.5,176.3,69.1,187.3	0.06Å	Favored (40.082%)	-	-	-
A 338		VAL	0.71	-	Favored (63.31%) Pre-Pro / -100.8,117.1	Favored (46.3%) *t* chi angles: 182.2	0.02Å	Favored (50.036%) beta sheet	-	-	-
A 339		PRO	0.69	-	Favored (2.24%) Trans-Pro / -75.9,82.1	Favored (75.8%) *Cg_endo* chi angles: 30,325.2,25	0.01Å	Favored (7.467%) beta sheet	-	-	-
A 340		ILE	0.69	-	Favored (53.84%) Ile or Val / -106.0,132.1	Favored (74%) *mt* chi angles: 301.2,169.1	0.03Å	Favored (9.295%) beta sheet	OUTLIER(S) worst is CB--CG1: 5.8 σ	-	-
**#**	**Alt**	**Res**	**High B**	**Clash > 0.4Å**	**Ramachandran**	**Rotamer**	**Cβ deviation**	**CaBLAM**	**Bond lengths**	**Bond angles**	**Cis Peptides**
			Avg: 1.09	Clashscore: 1.46	Outliers: 1 of 499	Poor rotamers: 0 of 409	Outliers: 0 of 449	Outliers: 10 of 497	Outliers: 7 of 501	Outliers: 9 of 501	Non-Trans: 0 of 500
A 341		SER	0.7	-	Favored (36.33%) General / -150.8,164.4	Favored (98%) *p* chi angles: 65.2	0.04Å	Favored (36.178%) beta sheet	-	-	-
A 342		VAL	0.72	-	Favored (68.28%) Ile or Val / -119.5,122.2	Favored (83.2%) *t* chi angles: 177.8	0.04Å	Favored (30.954%) beta sheet	-	-	-
A 343		THR	0.77	-	Favored (39.7%) General / -119.3,150.7	Favored (6.7%) *t* chi angles: 182.3	0.07Å	Favored (46.386%)	-	-	-
A 344		ALA	0.85	-	Favored (60.03%) General / -78.9,-12.5	-	0.02Å	Favored (23.576%)	-	-	-

#	Alt	Res	High B	Clash > 0.4Å	Ramachandran	Rotamer	Cβ deviation	CaBLAM	Bond lengths	Bond angles	Cis Peptides
A 345		ASN	0.95	-	Favored (9.23%) General / -154.2,128.8	Favored (22.9%) *t0* chi angles: 193.3,301.7	0.03Å	Favored (13.848%)	-	-	-
A 346		LEU	1.06	-	Favored (65.49%) General / -63.0,-20.7	Favored (88%) *mt* chi angles: 292.2,175.9	0.02Å	Favored (36.792%) alpha helix	-	-	-
A 347		MET	1.13	-	Favored (49.14%) General / -83.3,-0.3	Favored (98%) *mmm* chi angles: 296.9,302.6,292.1	0.02Å	Favored (43.458%) alpha helix	-	-	-
A 348		ASP	1.15	-	Favored (3%) General / -146.9,98.2	Favored (67.8%) *t0* chi angles: 183,355	0.04Å	Favored (10.359%)	-	-	-
A 349		LEU	1.12	-	Favored (6.16%) General / -86.4,60.3	Favored (77.1%) *mt* chi angles: 301.3,180.3	0.05Å	Favored (16.393%)	-	-	-
A 350		THR	1.05	-	Favored (55.77%) Pre-Pro / -102.8,119.4	Favored (93.3%) *m* chi angles: 297.6	0.04Å	Favored (24.187%) beta sheet	-	-	-
A 351		PRO	0.97	-	Favored (81.76%) Trans-Pro / -66.2,146.6	Favored (43.6%) *Cg_endo* chi angles: 24.3,327.4,26.9	0.02Å	Favored (24.584%) beta sheet	-	-	-
A 352		VAL	0.9	-	Favored (2.19%) Ile or Val / -122.2,-25.8	Favored (28%) *m* chi angles: 298.6	0.05Å	Favored (5.89%)	-	-	-
A 353		GLY	0.86	-	Favored (31.79%) Glycine / -98.1,-167.8	-	-	Favored (13.872%)	-	-	-
A 354		ARG	0.85	-	Favored (34.41%) General / -137.1,162.1	Favored (93.2%) *mmt-90* chi angles: 297.2,290.8,184.4,273	0.08Å	Favored (39.102%)	-	-	-
A 355		LEU	0.86	-	Favored (31.93%) General / -107.6,146.4	Favored (4.1%) *mp* chi angles: 267.3,70.5	0.08Å	Favored (57.625%) beta sheet	-	-	-
A 356		VAL	0.87	-	Favored (18.13%) Ile or Val / -83.8,-44.1	Favored (88.6%) *t* chi angles: 174	0.03Å	Favored (7.328%)	-	-	-
A 357		THR	0.88	-	Favored (21%) General / -77.2,119.6	Favored (85%) *m* chi angles: 301.7	0.08Å	Favored (25.023%)	-	-	-
A 358		VAL	0.87	-	Favored (96.05%) Ile or Val / -62.1,-42.7	Favored (60.7%) *t* chi angles: 170.9	0.06Å	Favored (20.518%)	-	-	-
A 359		ASN	0.86	-	Favored (11.27%) Pre-Pro / -147.6,75.1	Favored (65.1%) *t0* chi angles: 191.7,31.5	0.07Å	Favored (5.22%)	-	-	-
A 360		PRO	0.85	-	Favored (92.16%) Trans-Pro / -56.8,141.6	Favored (78.4%) *Cg_exo* chi angles: 334.8,35.9,329.3	0.11Å	Favored (18.068%)	-	-	-
**#**	**Alt**	**Res**	**High B**	**Clash > 0.4Å**	**Ramachandran**	**Rotamer**	**Cβ deviation**	**CaBLAM**	**Bond lengths**	**Bond angles**	**Cis Peptides**
			Avg: 1.09	Clashscore: 1.46	Outliers: 1 of 499	Poor rotamers: 0 of 409	Outliers: 0 of 449	Outliers: 10 of 497	Outliers: 7 of 501	Outliers: 9 of 501	Non-Trans: 0 of 500

A 361		PHE	0.84	-	Favored (30.32%) General / -151.2,166.6	Favored (40.1%) *p90* chi angles: 70.3,98.3	0.05Å	Favored (36.282%)	-	-	-
A 362		ILE	0.83	-	Favored (38.08%) Ile or Val / -84.8,129.2	Favored (49.9%) *mm* chi angles: 302.6,300.4	0.08Å	Favored (23.459%)	-	-	-
A 363		SER	0.83	-	Favored (65.57%) General / -62.1,-22.1	Favored (85.1%) *p* chi angles: 67.3	0.03Å	Favored (10.908%)	-	-	-
A 364		THR	0.83	-	Favored (36.11%) General / -154.7,156.3	Favored (10.4%) *t* chi angles: 186.5	0.03Å	Favored (21.335%)	-	-	-
A 365		GLY	0.84	-	Favored (2.38%) Glycine / -76.0,67.5	-	-	CaBLAM Disfavored (1.962%)	-	-	-
A 366		GLY	0.84	-	Favored (6.55%) Glycine / -150.2,138.1	-	-	Favored (36.543%)	-	-	-
A 367		ALA	0.83	-	Favored (55.19%) General / -62.1,144.5	-	0.03Å	Favored (17.929%)	-	-	-
A 368		ASN	0.82	-	Favored (7.91%) General / 69.8,15.4	Favored (76.9%) *m-40* chi angles: 301.6,321.7	0.10Å	Favored (11.19%)	-	-	-
A 369		ASN	0.8	-	Favored (55.27%) General / -67.8,137.4	Favored (18.5%) *t0* chi angles: 188.5,262.1	0.09Å	Favored (30.755%)	-	-	-
A 370		LYS	0.79	-	Favored (19.05%) General / -98.6,152.9	Favored (98.7%) *mttt* chi angles: 295.9,176.1,178.9,175.9	0.01Å	Favored (46.906%) beta sheet	-	-	-
A 371		VAL	0.77	-	Favored (26.24%) Ile or Val / -142.8,136.1	Favored (4.6%) *p* chi angles: 56.7	0.07Å	Favored (59.037%) beta sheet	-	-	-
A 372		MET	0.76	-	Favored (46.61%) General / -101.0,131.9	Favored (48.6%) *ttp* chi angles: 180.3,170.4,61.9	0.03Å	Favored (62.498%) beta sheet	-	-	-
A 373		ILE	0.76	-	Favored (73.73%) Ile or Val / -123.5,131.1	Favored (3.5%) *mp* chi angles: 304.7,98.4	0.06Å	Favored (66.75%) beta sheet	-	-	-
A 374		GLU	0.78	-	Favored (38.92%) General / -101.6,119.5	Favored (77.5%) *tt0* chi angles: 182,177.4,17.1	0.08Å	Favored (63.172%) beta sheet	-	-	-
A 375		VAL	0.82	-	Favored (25.07%) Ile or Val / -114.4,143.0	Favored (46.7%) *t* chi angles: 182.1	0.06Å	Favored (46.666%) beta sheet	-	-	-
A 376		GLU	0.88	0.49Å OE2 with A 406 LYS NZ	Favored (51.13%) Pre-Pro / -123.1,81.3	Favored (89.6%) *tt0* chi angles: 187,175.1,358.1	0.04Å	Favored (17.468%)	-	-	-
A 377		PRO	0.94	-	Favored (22.77%) Trans-Pro / -59.8,160.1	Favored (46.4%) *Cg_exo* chi angles: 338,35,326.3	0.06Å	Favored (22.79%)	-	-	-

#	Alt	Res	High B	Clash > 0.4Å	Ramachandran	Rotamer	Cβ deviation	CaBLAM	Bond lengths	Bond angles	Cis Peptides
A 378		PRO	0.98	-	Favored (32.57%) Trans-Pro / -73.3,165.4	Favored (75.6%) *Cg_endo* chi angles: 30,323.5,28.3	0.07Å	Favored (74.797%)	-	-	-
A 379		PHE	0.98	-	Favored (67.28%) General / -62.0,-24.9	Favored (28.2%) *p90* chi angles: 76,92.8	0.04Å	Favored (40.694%)	-	-	-
A 380		GLY	0.95	-	Favored (49.2%) Glycine / -87.0,-176.5	-	-	Favored (25.699%)	-	-	-
**#**	**Alt**	**Res**	**High B**	**Clash > 0.4Å**	**Ramachandran**	**Rotamer**	**Cβ deviation**	**CaBLAM**	**Bond lengths**	**Bond angles**	**Cis Peptides**
			Avg: 1.09	Clashscore: 1.46	Outliers: 1 of 499	Poor rotamers: 0 of 409	Outliers: 0 of 449	Outliers: 10 of 497	Outliers: 7 of 501	Outliers: 9 of 501	Non-Trans: 0 of 500
A 381		ASP	0.9	-	Favored (56.45%) General / -65.4,145.2	Favored (87.8%) *m-30* chi angles: 289.4,353	0.05Å	Favored (5.791%)	-	-	-
A 382		SER	0.83	-	Favored (23.42%) General / -158.1,169.9	Favored (92.1%) *p* chi angles: 66.5	0.05Å	Favored (39.71%)	-	-	-
A 383		TYR	0.79	-	Favored (53.31%) General / -121.7,139.6	Favored (87.1%) *m-80* chi angles: 299.5,87	0.09Å	Favored (39.774%) beta sheet	-	-	-
A 384		ILE	0.77	-	Favored (46.8%) Ile or Val / -97.1,120.8	Favored (76.6%) *mt* chi angles: 300.6,168.6	0.04Å	Favored (65.483%) beta sheet	-	-	-
A 385		VAL	0.8	-	Favored (71.77%) Ile or Val / -118.9,131.4	Favored (78.3%) *t* chi angles: 178.1	0.03Å	Favored (68.505%)	-	-	-
A 386		VAL	0.88	-	Favored (73.24%) Ile or Val / -121.9,125.5	Favored (59.8%) *t* chi angles: 180	0.12Å	Favored (13.479%)	-	-	-
A 387		GLY	0.99	-	Favored (43.09%) Glycine / 91.5,174.3	-	-	Favored (25.589%)	-	-	-
A 388		ARG	1.1	-	Favored (38.81%) General / -138.8,142.2	Favored (91.9%) *mmt-90* chi angles: 292,290.1,184.5,273.2	0.08Å	CA Geom Outlier (0.048%)	-	-	-
A 389		GLY	1.19	-	Favored (31.97%) Glycine / 61.3,-123.3	-	-	Favored (14.809%)	-	-	-
A 390		THR	1.22	-	Favored (3.93%) General / -44.9,-37.5	Favored (97%) *m* chi angles: 299.9	0.01Å	Favored (10.545%)	-	-	-
A 391		THR	1.17	-	Favored (11.02%) General / -112.5,25.9	Favored (43.4%) *p* chi angles: 55.1	0.11Å	Favored (24.47%)	-	-	-
A 392		GLN	1.08	-	Favored (47.61%) General / -73.1,141.2	Favored (12.3%) *mt0* chi angles: 292.9,175.8,207.3	0.14Å	Favored (31.357%)	-	-	-
A 393		ILE	0.97	-	Favored (67.44%)	Favored (47.6%) *mm* chi angles: 303.6,299.6	0.04Å	Favored (72.284%) beta sheet	-	-	-

#	Alt	Res	High B	Clash > 0.4Å	Ramachandran	Rotamer	Cβ deviation	CaBLAM	Bond lengths	Bond angles	Cis Peptides
					Ile or Val / -111.3,123.7						
A 394		ASN	0.88	-	Favored (38.28%) General / -120.0,121.1	Favored (67.7%) *m-40* chi angles: 304.2,318.9	0.07Å	Favored (68.135%) beta sheet	-	-	-
A 395		TYR	0.84	-	Favored (45.86%) General / -123.0,125.8	Favored (63.1%) *t80* chi angles: 187.3,86.8	0.05Å	Favored (57.071%) beta sheet	-	-	-
A 396		HIS	0.85	-	Favored (49.03%) General / -70.7,136.4	Favored (71.4%) *t-90* chi angles: 187,283.1	0.01Å	Favored (42.759%) beta sheet	-	-	-
A 397		TRP	0.93	-	Favored (50.93%) General / -130.3,143.7	Favored (71.6%) *t-100* chi angles: 184.6,254.8	0.02Å	Favored (69.023%) beta sheet	-	-	-
A 398		HIS	1.07	-	Favored (19.97%) General / -136.4,122.2	Favored (77.2%) *t70* chi angles: 177,82.1	0.07Å	Favored (60.366%) beta sheet	-	-	-
A 399		LYS	1.29	-	Favored (42.1%) General / -97.3,125.8	Favored (28.3%) *ttmt* chi angles: 182.6,179.4,297.1,191.9	0.02Å	Favored (64.672%) beta sheet	-	-	-
A 400		GLU	1.57	-	Favored (83.83%) General / -66.9,-37.1	Favored (99.4%) *mt-10* chi angles: 291.4,178.8,354	0.01Å	Favored (27.456%)	-	-	-
**#**	**Alt**	**Res**	**High B**	**Clash > 0.4Å**	**Ramachandran**	**Rotamer**	**Cβ deviation**	**CaBLAM**	**Bond lengths**	**Bond angles**	**Cis Peptides**
			Avg: 1.09	Clashscore: 1.46	Outliers: 1 of 499	Poor rotamers: 0 of 409	Outliers: 0 of 449	Outliers: 10 of 497	Outliers: 7 of 501	Outliers: 9 of 501	Non-Trans: 0 of 500
A 401		GLY	1.89	-	Favored (20.82%) Glycine / -88.0,148.4	-	-	Favored (15.418%)	-	-	-
A 402		SER	2.21	-	Favored (20.39%) General / -82.3,165.5	Favored (93.6%) *p* chi angles: 66.3	0.03Å	Favored (48.608%)	-	-	-
A 403		SER	2.48	-	Favored (88.4%) General / -62.7,-37.9	Favored (58.5%) *m* chi angles: 293.3	0.02Å	Favored (66.917%)	-	-	-
A 404		ILE	2.68	-	Favored (97.43%) Ile or Val / -64.2,-44.6	Favored (98.6%) *mt* chi angles: 292.5,168.3	0.01Å	Favored (84.147%) alpha helix	-	-	-
A 405		GLY	2.81	-	Favored (46.1%) Glycine / -54.6,-52.3	-	-	Favored (97.407%) alpha helix	-	-	-
A 406		LYS	2.86	0.49Å NZ with A 376 GLU OE2	Favored (79.68%) General / -56.2,-45.5	Favored (85%) *tttt* chi angles: 181.4,179.7,177.3,182.7	0.03Å	Favored (92.686%) alpha helix	-	-	-
A 407		ALA	2.83	-	Favored (87.07%) General / -60.1,-39.7	-	0.04Å	Favored (81.491%) alpha helix	-	-	-
A 408		LEU	2.72	-	Favored (69.67%) General / -71.8,-39.6	Favored (98.6%) *mt* chi angles: 292.8,172.8	0.04Å	Favored (88.438%) alpha helix	-	-	-

#	Alt	Res	High B	Clash > 0.4Å	Ramachandran	Rotamer	Cβ deviation	CaBLAM	Bond lengths	Bond angles	Cis Peptides
A 409		ALA	2.56	-	Favored (94.42%) General / -62.2,-39.9	-	0.03Å	Favored (97.335%) alpha helix	-	-	-
A 410		THR	2.37	-	Favored (78.79%) General / -67.2,-44.8	Favored (99.7%) *m* chi angles: 300.4	0.04Å	Favored (90.053%) alpha helix	-	-	-
A 411		THR	2.16	-	Favored (90.15%) General / -59.8,-46.1	Favored (89.3%) *m* chi angles: 298.7	0.03Å	Favored (96.142%) alpha helix	-	-	-
A 412		TRP	1.99	-	Favored (94.93%) General / -62.9,-44.9	Favored (31.5%) *t-100* chi angles: 175.2,235.1	0.06Å	Favored (97.449%) alpha helix	-	-	-
A 413		LYS	1.85	-	Favored (85.47%) General / -59.3,-40.2	Favored (47.3%) *tttm* chi angles: 182.4,174.3,176.9,300.7	0.05Å	Favored (93.652%) alpha helix	-	-	-
A 414		GLY	1.74	-	Favored (62.51%) Glycine / -57.7,-50.8	-	-	Favored (94.848%) alpha helix	-	-	-
A 415		ALA	1.67	-	Favored (77.79%) General / -58.8,-38.0	-	0.03Å	Favored (82.78%) alpha helix	-	-	-
A 416		GLN	1.61	-	Favored (95.39%) General / -64.5,-43.1	Favored (92.7%) *mt0* chi angles: 289.4,175.9,310.2	0.06Å	Favored (88.373%) alpha helix	-	-	-
A 417		ARG	1.58	-	Favored (96.7%) General / -62.3,-40.5	Favored (41.2%) *ttp-170* chi angles: 187.3,170.9,62.6,207.1	0.07Å	Favored (92.078%) alpha helix	-	-	-
A 418		LEU	1.56	-	Favored (96.17%) General / -64.3,-40.5	Favored (75.5%) *mt* chi angles: 287.6,170.1	0.03Å	Favored (97.584%) alpha helix	-	-	-
A 419		ALA	1.56	-	Favored (81.86%) General / -63.1,-35.9	-	0.05Å	Favored (66.361%) alpha helix	-	-	-
A 420		VAL	1.56	-	Favored (13.03%) Ile or Val / -89.7,-50.5	Favored (91.1%) *t* chi angles: 176	0.04Å	Favored (34.968%) alpha helix	-	-	-
**#**	**Alt**	**Res**	**High B**	**Clash > 0.4Å**	**Ramachandran**	**Rotamer**	**Cβ deviation**	**CaBLAM**	**Bond lengths**	**Bond angles**	**Cis Peptides**
			Avg: 1.09	Clashscore: 1.46	Outliers: 1 of 499	Poor rotamers: 0 of 409	Outliers: 0 of 449	Outliers: 10 of 497	Outliers: 7 of 501	Outliers: 9 of 501	Non-Trans: 0 of 500
A 421		LEU	1.57	-	Favored (6.31%) General / -90.7,-49.7	Favored (38.4%) *tp* chi angles: 171.2,60	0.05Å	CaBLAM Disfavored (2.513%)	-	-	-
A 422		GLY	1.57	-	Favored (19.02%) Glycine / 96.2,-148.5	-	-	Favored (14.469%)	-	-	-
A 423		ASP	1.57	-	Favored (8.23%) General / -44.8,-42.9	Favored (44.1%) *p0* chi angles: 58,356.8	0.03Å	Favored (16.673%)	-	-	-
A 424		THR	1.56	-	Favored (14.3%)	Favored (99.4%) *m* chi angles: 300.4	0.07Å	Favored (58.763%) three-ten	-	-	-

#	Alt	Res	High B	Clash > 0.4Å	Ramachandran	Rotamer	Cβ deviation	CaBLAM	Bond lengths	Bond angles	Cis Peptides
					General / -52.5,-26.9						
A 425		ALA	1.57	-	Favored (64.84%) General / -57.5,-30.1	-	0.09Å	Favored (61.323%) three-ten	-	-	-
A 426		TRP	1.6	-	Favored (51.92%) General / -71.9,-8.0	Favored (95.7%) *m100* chi angles: 288.5,104.3	0.01Å	Favored (53.978%)	-	-	-
A 427		ASP	1.66	-	Favored (59.04%) General / -86.8,-5.7	Favored (85.5%) *m-30* chi angles: 290,335	0.03Å	Favored (32.282%)	-	-	-
A 428		PHE	1.76	-	Favored (6.96%) General / -73.2,109.7	Favored (84.9%) *t80* chi angles: 182.5,78.6	0.09Å	CaBLAM Disfavored (3.534%)	-	OUTLIER(S) worst is CA-CB-CG: 5.8 σ	-
A 429		GLY	1.89	-	Favored (89.17%) Glycine / 81.3,6.6	-	-	Favored (33.709%)	-	-	-
A 430		SER	2.05	-	Favored (55.1%) General / -67.5,145.3	Favored (44.7%) *t* chi angles: 179	0.06Å	Favored (14.35%)	-	-	-
A 431		ILE	2.21	-	Favored (6.5%) Ile or Val / -119.9,17.3	Favored (40.9%) *pt* chi angles: 59.5,170.3	0.04Å	CaBLAM Disfavored (3.73%)	-	-	-
A 432		GLY	2.33	-	Favored (50.44%) Glycine / 92.0,16.6	-	-	Favored (46.008%)	-	-	-
A 433		GLY	2.36	-	Favored (51.59%) Glycine / -64.9,150.1	-	-	Favored (29.656%)	-	-	-
A 434		VAL	2.27	-	Favored (92%) Ile or Val / -62.4,-41.5	Favored (67.7%) *t* chi angles: 171.8	0.01Å	Favored (59.532%)	-	-	-
A 435		PHE	2.08	-	Favored (73.49%) General / -54.7,-47.6	Favored (93.6%) *t80* chi angles: 177.9,77.7	0.04Å	Favored (73.729%) alpha helix	-	-	-
A 436		ASN	1.84	-	Favored (81.56%) General / -63.4,-35.8	Favored (96.3%) *m-40* chi angles: 286.7,337.8	0.06Å	Favored (87.019%) alpha helix	-	-	-
A 437		SER	1.6	-	Favored (96.85%) General / -63.8,-40.5	Favored (63.3%) *m* chi angles: 294	0.01Å	Favored (88.666%) alpha helix	-	-	-
A 438		ILE	1.41	-	Favored (94.64%) Ile or Val / -65.4,-44.5	Favored (99.2%) *mt* chi angles: 292.6,168	0.01Å	Favored (74.264%) alpha helix	-	-	-
A 439		GLY	1.28	-	Favored (35.99%) Glycine / -50.3,-50.1	-	-	Favored (94.521%) alpha helix	-	-	-
A 440		LYS	1.21	-	Favored (98.36%) General / -60.9,-43.5	Favored (97.2%) *mttt* chi angles: 288.9,178,181.7,176.7	0.13Å	Favored (92.195%) alpha helix	-	-	-
**#**	**Alt**	**Res**	**High B**	**Clash > 0.4Å**	**Ramachandran**	**Rotamer**	**Cβ deviation**	**CaBLAM**	**Bond lengths**	**Bond angles**	**Cis Peptides**
			Avg: 1.09	Clashscore: 1.46	Outliers: 1 of 499	Poor rotamers: 0 of 409	Outliers: 0 of 449	Outliers: 10 of 497	Outliers: 7 of 501	Outliers: 9 of 501	Non-Trans: 0 of 500
A 441		ALA	1.2	-	Favored (92.03%)	-	0.04Å	Favored (77.811%) alpha helix	-	-	-

					General / -60.4,-40.8						
A 442		VAL	1.24	-	Favored (79.22%) Ile or Val / -69.1,-45.7	Favored (83.4%) *t* chi angles: 173.4	0.04Å	Favored (77.446%) alpha helix	-	-	-
A 443		HIS	1.31	-	Favored (87.98%) General / -59.4,-46.7	Favored (82.6%) *t70* chi angles: 182.4,79	0.08Å	Favored (85.061%) alpha helix	-	OUTLIER(S) worst is CA-CB-CG: 4.6 σ	-
A 444		GLN	1.39	-	Favored (76.41%) General / -60.0,-36.0	Favored (97.5%) *mt0* chi angles: 288,172,332.3	0.02Å	Favored (74.725%) alpha helix	-	-	-
A 445		VAL	1.47	-	Favored (97.94%) Ile or Val / -62.5,-45.8	Favored (59.4%) *t* chi angles: 170.7	0.03Å	Favored (74.824%) alpha helix	-	-	-
A 446		PHE	1.53	-	Favored (66.91%) General / -71.7,-31.6	Favored (48.5%) *m-80* chi angles: 285.7,111.2	0.02Å	Favored (72.821%) alpha helix	-	-	-
A 447		GLY	1.57	-	Favored (26.5%) Glycine / -59.2,-56.2	-	-	Favored (88.711%) alpha helix	-	-	-
A 448		GLY	1.6	-	Favored (97.09%) Glycine / -61.0,-39.5	-	-	Favored (89.277%) alpha helix	-	-	-
A 449		ALA	1.6	-	Favored (92.09%) General / -61.9,-45.8	-	0.04Å	Favored (75.292%) alpha helix	-	-	-
A 450		PHE	1.6	-	Favored (60.58%) General / -75.5,-36.5	Favored (39.7%) *m-80* chi angles: 282.4,76.2	0.12Å	Favored (74.92%) alpha helix	-	-	-
A 451		ARG	1.6	-	Favored (94.72%) General / -63.4,-39.5	Favored (96.7%) *mtt180* chi angles: 288.2,173.2,178.9,168.1	0.03Å	Favored (89.162%) alpha helix	-	-	-
A 452		THR	1.64	-	Favored (79.64%) General / -65.3,-46.6	Favored (92.6%) *m* chi angles: 299.1	0.05Å	Favored (74.567%) alpha helix	-	-	-
A 453		LEU	1.71	-	Favored (67.48%) General / -72.9,-37.1	Favored (90.9%) *mt* chi angles: 291.6,170	0.07Å	Favored (47.1%)	-	-	-
A 454		PHE	1.79	-	Favored (5.56%) General / -115.1,-32.9	Favored (69.8%) *m-80* chi angles: 304.1,104.4	0.10Å	CaBLAM Outlier (0.652%)	-	OUTLIER(S) worst is CA-CB-CG: 4.4 σ	-
A 455		GLY	1.86	-	Favored (39.87%) Glycine / 56.8,-125.8	-	-	Favored (36.679%)	-	-	-
A 456		GLY	1.9	-	Favored (78.63%) Glycine / -86.2,7.4	-	-	CaBLAM Disfavored (2.688%)	-	-	-
A 457		MET	1.88	-	Favored (14.36%) General / -49.4,137.7	Favored (65%) *ttm* chi angles: 181.7,179.4,291.1	0.09Å	Favored (18.444%)	-	-	-
A 458		SER	1.79	-	Favored (26.23%) General / -60.7,152.6	Favored (90.5%) *p* chi angles: 66.7	0.07Å	Favored (34.714%)	-	-	-

#	Alt	Res	High B	Clash > 0.4Å	Ramachandran	Rotamer	Cβ deviation	CaBLAM	Bond lengths	Bond angles	Cis Peptides
A 459		TRP	1.63	-	Favored (66.71%) General / -59.1,-28.7	Favored (60.8%) *p-90* chi angles: 70.6,266.2	0.03Å	Favored (61.063%)	-	-	-
A 460		ILE	1.43	-	Favored (79.42%) Ile or Val / -66.2,-49.0	Favored (96.9%) *mt* chi angles: 294.1,167.8	0.05Å	Favored (73.061%) alpha helix	-	-	-
#	Alt	Res	High B	Clash > 0.4Å	Ramachandran	Rotamer	Cβ deviation	CaBLAM	Bond lengths	Bond angles	Cis Peptides
			Avg: 1.09	Clashscore: 1.46	Outliers: 1 of 499	Poor rotamers: 0 of 409	Outliers: 0 of 449	Outliers: 10 of 497	Outliers: 7 of 501	Outliers: 9 of 501	Non-Trans: 0 of 500
A 461		THR	1.24	-	Favored (91.14%) General / -65.8,-42.1	Favored (88.6%) *m* chi angles: 298.6	0.08Å	Favored (80.268%) alpha helix	-	-	-
A 462		GLN	1.07	-	Favored (85.97%) General / -61.1,-38.4	Favored (77.7%) *mt0* chi angles: 289.5,182.8,54.4	0.04Å	Favored (94.929%) alpha helix	-	-	-
A 463		GLY	0.93	-	Favored (79.01%) Glycine / -59.4,-49.4	-	-	Favored (98.95%) alpha helix	-	-	-
A 464		LEU	0.82	-	Favored (94%) General / -62.8,-39.4	Favored (93.8%) *mt* chi angles: 291.7,171.5	0.05Å	Favored (91.887%) alpha helix	-	-	-
A 465		LEU	0.74	-	Favored (84.85%) General / -65.6,-36.8	Favored (93.3%) *mt* chi angles: 291.6,173	0.01Å	Favored (92.083%) alpha helix	-	-	-
A 466		GLY	0.68	-	Favored (45%) Glycine / -59.0,-53.6	-	-	Favored (91.776%) alpha helix	-	-	-
A 467		ALA	0.64	-	Favored (79.39%) General / -58.6,-39.1	-	0.04Å	Favored (81.545%) alpha helix	-	-	-
A 468		LEU	0.61	-	Favored (74.89%) General / -61.4,-49.9	Favored (57.6%) *tp* chi angles: 173.8,61.5	0.05Å	Favored (89.66%) alpha helix	-	-	-
A 469		LEU	0.58	-	Favored (78.65%) General / -65.1,-34.5	Favored (88.1%) *mt* chi angles: 292,175.4	0.07Å	Favored (80.053%) alpha helix	-	-	-
A 470		LEU	0.56	-	Favored (98.39%) General / -63.6,-42.0	Favored (85%) *mt* chi angles: 289.9,171.1	0.03Å	Favored (87.527%) alpha helix	-	-	-
A 471		TRP	0.55	-	Favored (80.68%) General / -62.9,-48.0	Favored (90.5%) *t60* chi angles: 177.8,85.6	0.02Å	Favored (91.236%) alpha helix	-	-	-
A 472		MET	0.56	-	Favored (85.33%) General / -59.7,-39.7	Favored (91.1%) *mtp* chi angles: 290,172.5,63.7	0.02Å	Favored (89.239%) alpha helix	-	-	-
A 473		GLY	0.66	-	Favored (60.49%) Glycine / -58.0,-51.1	-	-	Favored (95.82%) alpha helix	-	-	-
A 474		LEU	0.9	-	Favored (75.77%) General / -63.2,-33.6	Favored (96%) *mt* chi angles: 292.7,173.9	0.03Å	Favored (73.581%) alpha helix	-	-	-

#	Alt	Res	High B	Clash > 0.4Å	Ramachandran	Rotamer	Cβ deviation	CaBLAM	Bond lengths	Bond angles	Cis Peptides
A 475		GLN	1.46	-	Favored (41.34%) General / -79.6,-29.8	Favored (95.2%) *mm-40* chi angles: 294.7,293.7,308.6	0.03Å	Favored (31.37%)	-	-	-
A 476		ALA	2.56	-	Favored (57.53%) General / -59.3,138.7	-	0.03Å	Favored (22.65%)	-	-	-
A 477		ARG	4.16	-	Favored (20.51%) General / -82.5,-40.8	Favored (99%) *mtt180* chi angles: 292,177.4,181,174.8	0.02Å	Favored (10.991%)	-	-	-
A 478		ASP	5.51	-	Favored (40.42%) General / -70.9,130.0	Favored (20.7%) *t70* chi angles: 182.1,267.9	0.06Å	Favored (22.37%)	-	-	-
A 479		ARG	5.56	-	Favored (81.31%) General / -59.1,-39.1	Favored (72.6%) *ttt-90* chi angles: 183.1,178.3,181.9,273.5	0.02Å	Favored (50.872%)	-	-	-
A 480		SER	4.29	-	Favored (71.23%) General / -70.9,-41.5	Favored (67.8%) *m* chi angles: 294.6	0.02Å	Favored (80.246%) alpha helix	-	-	-
#	Alt	Res	High B	Clash > 0.4Å	Ramachandran	Rotamer	Cβ deviation	CaBLAM	Bond lengths	Bond angles	Cis Peptides
			Avg: 1.09	Clashscore: 1.46	Outliers: 1 of 499	Poor rotamers: 0 of 409	Outliers: 0 of 449	Outliers: 10 of 497	Outliers: 7 of 501	Outliers: 9 of 501	Non-Trans: 0 of 500
A 481		ILE	2.72	-	Favored (87.49%) Ile or Val / -67.3,-44.1	Favored (96.9%) *mt* chi angles: 292.9,169.3	0.05Å	Favored (80.55%) alpha helix	-	-	-
A 482		SER	1.61	-	Favored (75.28%) General / -58.2,-49.9	Favored (46.2%) *t* chi angles: 179.7	0.06Å	Favored (91.254%) alpha helix	-	-	-
A 483		LEU	1.04	-	Favored (88.28%) General / -64.0,-37.7	Favored (93.2%) *mt* chi angles: 291.4,172.1	0.03Å	Favored (82.266%) alpha helix	-	-	-
A 484		THR	0.78	-	Favored (84.42%) General / -63.3,-47.0	Favored (91.6%) *m* chi angles: 299	0.03Å	Favored (89.081%) alpha helix	-	-	-
A 485		LEU	0.7	-	Favored (94.47%) General / -63.5,-39.4	Favored (83.5%) *mt* chi angles: 289.7,169.7	0.06Å	Favored (88.461%) alpha helix	-	-	-
A 486		LEU	0.69	-	Favored (91.57%) General / -65.8,-40.2	Favored (90.3%) *mt* chi angles: 291.2,170.7	0.02Å	Favored (98.268%) alpha helix	-	-	-
A 487		ALA	0.72	-	Favored (97.22%) General / -63.7,-43.2	-	0.04Å	Favored (86.565%) alpha helix	-	-	-
A 488		VAL	0.75	-	Favored (83.76%) Ile or Val / -68.5,-42.6	Favored (82.8%) *t* chi angles: 173.4	0.03Å	Favored (82.673%) alpha helix	-	-	-
A 489		GLY	0.77	-	Favored (60.97%) Glycine / -55.7,-50.4	-	-	Favored (96.108%) alpha helix	-	-	-
A 490		GLY	0.8	-	Favored (46.91%)	-	-	Favored (99.734%) alpha helix	-	-	-

					Glycine / -53.8,-51.8						
A 491		ILE	0.83	-	Favored (93.8%) Ile or Val / -60.1,-43.9	Favored (94.1%) *mt* chi angles: 291.6,167.7	0.01Å	Favored (89.89%) alpha helix	-	-	-
A 492		LEU	0.85	-	Favored (83.01%) General / -62.2,-36.8	Favored (61.6%) *mt* chi angles: 287.3,174	0.04Å	Favored (84.31%) alpha helix	-	-	-
A 493		ILE	0.89	-	Favored (84.12%) Ile or Val / -68.1,-41.2	Favored (38.2%) *mm* chi angles: 296,301	0.07Å	Favored (94.643%) alpha helix	-	-	-
A 494		PHE	0.94	-	Favored (96.49%) General / -61.5,-41.0	Favored (5.5%) *m-10* chi angles: 283.2,24.6	0.04Å	Favored (87.716%) alpha helix	-	-	-
A 495		LEU	1.01	-	Favored (77.08%) General / -67.7,-44.8	Favored (88.9%) *mt* chi angles: 291,170.2	0.06Å	Favored (78.036%) alpha helix	-	-	-
A 496		ALA	1.15	-	Favored (82.94%) General / -61.0,-37.6	-	0.06Å	Favored (69.668%) alpha helix	-	-	-
A 497		THR	1.36	-	Favored (26.18%) General / -92.0,-16.6	Favored (79.2%) *p* chi angles: 60.4	0.10Å	Favored (50.601%) alpha helix	-	-	-
A 498		SER	1.69	-	Favored (10.33%) General / -101.8,-32.3	Favored (40.2%) *m* chi angles: 302	0.10Å	Favored (17.771%) alpha helix	-	-	-
A 499		VAL	2.13	-	Favored (15.07%) Ile or Val / -57.6,-21.0	Favored (4.9%) *p* chi angles: 71.8	0.02Å	Favored (48.533%)	-	-	-
A 500		GLN	2.68	-	Favored (62.83%) General / -70.6,-16.2	Favored (75.8%) *mt0* chi angles: 295.2,175.8,55.6	0.09Å	-	-	-	-
**#**	**Alt**	**Res**	**High B**	**Clash > 0.4Å**	**Ramachandran**	**Rotamer**	**Cβ deviation**	**CaBLAM**	**Bond lengths**	**Bond angles**	**Cis Peptides**
			Avg: 1.09	Clashscore: 1.46	Outliers: 1 of 499	Poor rotamers: 0 of 409	Outliers: 0 of 449	Outliers: 10 of 497	Outliers: 7 of 501	Outliers: 9 of 501	Non-Trans: 0 of 500
A 501		ALA	3.28	-	-	-	0.04Å	-	-	-	-

About MolProbity | Website for the Richardson Lab | Using ecloud x-H | Internal reference 4.5.2
