# Supplementary material for: Integrative proteome-wide structural analysis and high-throughput docking identify broad-spectrum antiviral scaffolds against Zika, Yellow Fever, West Nile, Saint Louis encephalitis, and Usutu viruses
Source: Front Cell Infect Microbiol. 2026 Apr 30;16:1723132. doi: 10.3389/fcimb.2026.1723132 (PMC13171538; doi:10.3389/fcimb.2026.1723132)
Supplement: Supplementary file 3 [file DataSheet3.zip › SLEV/SLEV_E/Mol_probity_Files/SLEV_E_1FH-rama.pdf]

MolProbity Ramachandran analysis

SLEV_E1FH.pdb, model 1

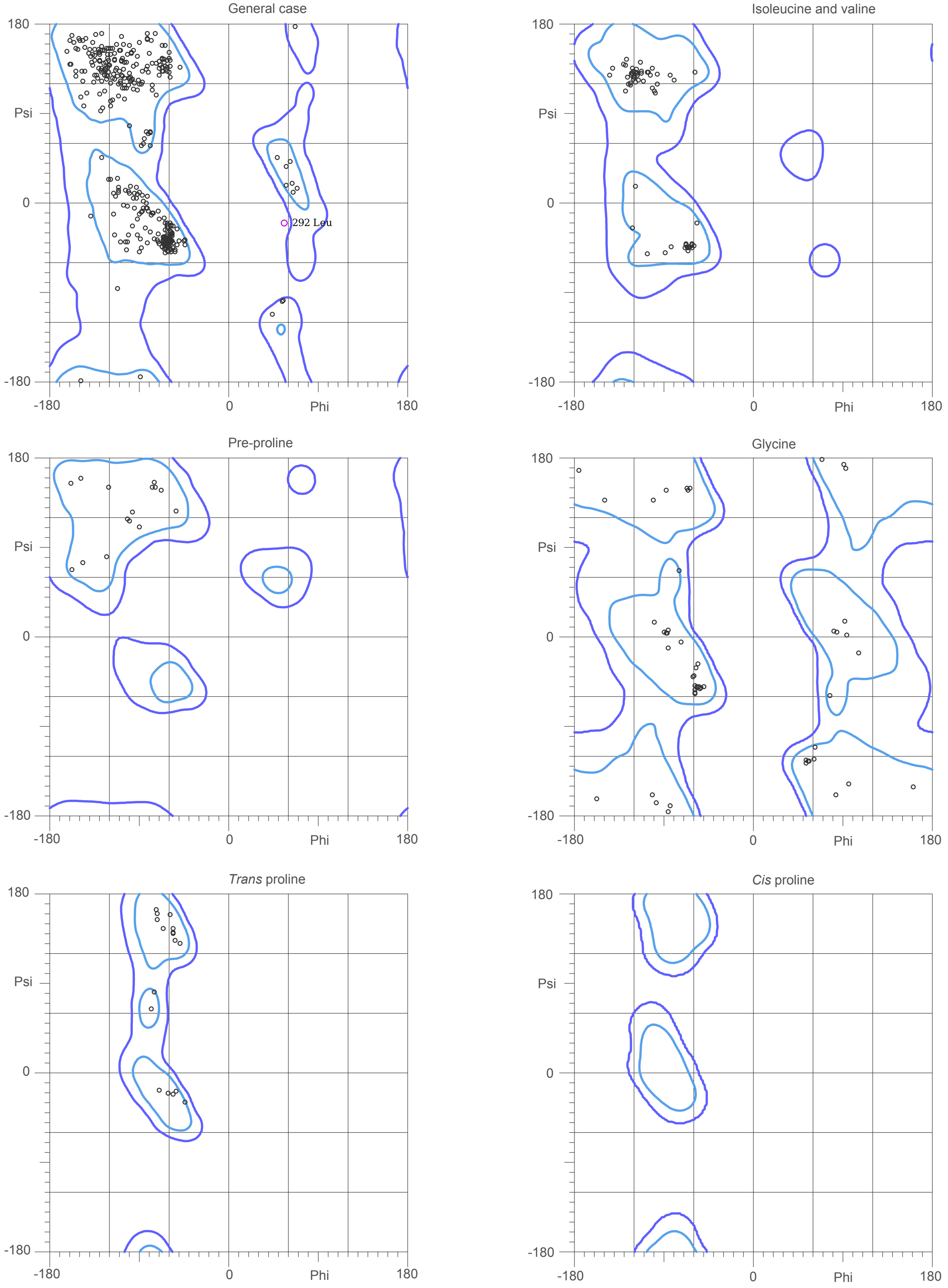

98.6% (492/499) of all residues were in favored (98%) regions.
99.8% (498/499) of all residues were in allowed (>99.8%) regions.

There were 1 outliers (phi, psi):
292 Leu (56.5, -20.7)
