# Supplementary material for: Integrative proteome-wide structural analysis and high-throughput docking identify broad-spectrum antiviral scaffolds against Zika, Yellow Fever, West Nile, Saint Louis encephalitis, and Usutu viruses
Source: Front Cell Infect Microbiol. 2026 Apr 30;16:1723132. doi: 10.3389/fcimb.2026.1723132 (PMC13171538; doi:10.3389/fcimb.2026.1723132)
Supplement: Supplementary file 3 [file DataSheet3.zip › SLEV/SLEV_M/Mol_probity_Files/SLEV_M_1FH-multi.table.pdf]

Viewing SLEV_M1FH-multi.table

When finished, you should close this window .

Hint: Use File | Save As... to save a copy of this page.

All-Atom Contacts	Clashscore, all atoms:	0.84		99th percentile* (N=1784, all resolutions)
	Clashscore is the number of serious steric overlaps (> 0.4 Å) per 1000 atoms.			
Protein Geometry	Poor rotamers	0	0.00%	Goal: <0.3%
	Favored rotamers	65	100.00%	Goal: >98%
	Ramachandran outliers	0	0.00%	Goal: <0.05%
	Ramachandran favored	72	98.63%	Goal: >98%
	Rama distribution Z-score	2.10 ± 1.01		Goal: abs(Z score) < 2
	MolProbity score^	0.76		100th percentile* (N=27675, 0Å - 99Å)
	Cβ deviations >0.25Å	0	0.00%	Goal: 0
	Bad bonds:	0 / 602	0.00%	Goal: 0%
	Bad angles:	0 / 824	0.00%	Goal: <0.1%
Peptide Omegas	Cis Prolines:	0 / 3	0.00%	Expected: ≤1 per chain, or ≤5%
Low-resolution Criteria	CaBLAM outliers	1	1.4%	Goal: <1.0%
	CA Geometry outliers	0	0.00%	Goal: <0.5%
Additional validations	Chiral volume outliers	0/100		
	Waters with clashes	0/0	0.00%	See UnDowser table for details

In the two column results, the left column gives the raw count, right column gives the percentage.

* 100th percentile is the best among structures of comparable resolution; 0th percentile is the worst. For clashscore the comparative set of structures was selected in 2004, for MolProbity score in 2006.

^ MolProbity score combines the clashscore, rotamer, and Ramachandran evaluations into a single score, normalized to be on the same scale as X-ray resolution.

Key to table colors and cutoffs here:

#	Alt	Res	High B	Clash > 0.4Å	Ramachandran	Rotamer	Cβ deviation	CaBLAM	Bond lengths	Bond angles	Cis Peptides
			Avg: 2.70	Clashscore: 0.84	Outliers: 0 of 73	Poor rotamers: 0 of 65	Outliers: 0 of 71	Outliers: 1 of 71	Outliers: 0 of 75	Outliers: 0 of 75	Non-Trans: 0 of 74
A 1		SER	6.88	-	-	Favored (71.9%) *m* chi angles: 295.3	0.02Å	-	-	-	-
A 2		ILE	6.2	-	Favored (64.02%) Ile or Val / -111.8,121.3	Favored (84.2%) *mt* chi angles: 299.3,171	0.03Å	-	-	-	-
A 3		SER	5.44	-	Favored (20.35%) General / -82.8,164.7	Favored (87.5%) *p* chi angles: 67.1	0.04Å	Favored (21.62%)	-	-	-
A 4		VAL	4.68	-	Favored (72.02%) Ile or Val / -125.0,126.9	Favored (82.9%) *t* chi angles: 177.8	0.02Å	Favored (55.476%) beta sheet	-	-	-

#	Alt	Res	High B	Clash > 0.4Å	Ramachandran	Rotamer	Cβ deviation	CaBLAM	Bond lengths	Bond angles	Cis Peptides
A 5		GLN	4.05	-	Favored (25.54%) General / -96.8,145.8	Favored (91%) *mt0* chi angles: 295.7,180.6,307.1	0.04Å	Favored (56.553%) beta sheet	-	-	-
A 6		HIS	3.61	-	Favored (14.42%) General / -108.2,-15.7	Favored (99.6%) *m-70* chi angles: 298,289.5	0.04Å	CaBLAM Disfavored (4.036%)	-	-	-
A 7		HIS	3.37	-	Favored (25.63%) General / 50.5,41.7	Favored (99.1%) *m-70* chi angles: 300.9,290	0.01Å	CaBLAM Disfavored (4.061%)	-	-	-
A 8		GLY	3.36	-	Favored (79.67%) Glycine / 75.6,18.6	-	-	Favored (11.507%)	-	-	-
A 9		ASP	3.61	-	Allowed (0.08%) General / 74.5,55.9	Favored (14.6%) *t0* chi angles: 203,27.4	0.01Å	CaBLAM Outlier (0.025%)	-	-	-
A 10		SER	4.13	-	Favored (4.04%) General / 60.9,52.7	Favored (60.2%) *m* chi angles: 298.9	0.04Å	CaBLAM Disfavored (2.258%)	-	-	-
A 11		THR	4.92	-	Favored (19.04%) General / -79.1,168.9	Favored (62.8%) *p* chi angles: 63.5	0.06Å	Favored (17.625%) beta sheet	-	-	-
A 12		LEU	5.94	-	Favored (22.76%) General / -106.3,153.0	Favored (77%) *mt* chi angles: 302.9,177.5	0.01Å	Favored (35.663%)	-	-	-
A 13		ALA	7.01	-	Favored (64.43%) General / -59.2,-26.0	-	0.04Å	Favored (34.738%)	-	-	-
A 14		THR	7.84	-	Favored (21.59%) General / -109.1,17.5	Favored (65.1%) *p* chi angles: 58.2	0.04Å	Favored (31.391%)	-	-	-
A 15		LYS	8.11	-	Favored (43.45%) General / -64.8,152.2	Favored (16.3%) *pttm* chi angles: 69.8,188.1,180.1,292.4	0.06Å	Favored (23.35%)	-	-	-
A 16		ASN	7.69	-	Favored (17.45%) General / -112.1,19.8	Favored (72%) *m-40* chi angles: 291.6,284.2	0.02Å	Favored (9.978%) beta sheet	-	-	-
A 17		THR	6.74	-	Favored (27.02%) Pre-Pro / -121.0,137.8	Favored (99.8%) *m* chi angles: 300.4	0.08Å	Favored (17.305%) beta sheet	-	-	-
A 18		PRO	5.61	-	Favored (64.34%) Trans-Pro / -53.7,141.4	Favored (96.8%) *Cg_exo* chi angles: 331.7,36.1,331.7	0.04Å	Favored (91.902%)	-	-	-
A 19		TRP	4.6	-	Favored (63.74%) General / -57.0,-29.8	Favored (62.9%) *p-90* chi angles: 71.4,270.9	0.01Å	Favored (51.294%)	-	-	-
A 20		LEU	3.81	-	Favored (19.91%) General / -115.7,111.0	Favored (58.5%) *tp* chi angles: 181.6,63.2	0.05Å	Favored (21.976%)	-	-	-
**#**	**Alt**	**Res**	**High B**	**Clash > 0.4Å**	**Ramachandran**	**Rotamer**	**Cβ deviation**	**CaBLAM**	**Bond lengths**	**Bond angles**	**Cis Peptides**

			Avg: 2.70	Clashscore: 0.84	Outliers: 0 of 73	Poor rotamers: 0 of 65	Outliers: 0 of 71	Outliers: 1 of 71	Outliers: 0 of 75	Outliers: 0 of 75	Non-Trans: 0 of 74
A 21		ASP	3.24	-	Favored (42.23%) General / -72.6,151.6	Favored (13.8%) *t70* chi angles: 193.4,84	0.06Å	Favored (32.263%)	-	-	-
A 22		THR	2.83	-	Favored (42.56%) General / -49.8,-40.8	Favored (92.5%) *m* chi angles: 299.1	0.02Å	Favored (64.828%)	-	-	-
A 23		VAL	2.55	-	Favored (80.8%) Ile or Val / -61.2,-38.9	Favored (61.5%) *t* chi angles: 171	0.01Å	Favored (75.88%) alpha helix	-	-	-
A 24		LYS	2.34	-	Favored (62.83%) General / -74.7,-35.3	Favored (71.8%) *mmtt* chi angles: 295.5,292.2,185.4,175.9	0.06Å	Favored (92.001%) alpha helix	-	-	-
A 25		THR	2.14	-	Favored (91.34%) General / -63.8,-45.1	Favored (88.6%) *m* chi angles: 298.6	0.03Å	Favored (86.473%) alpha helix	-	-	-
A 26		THR	1.95	-	Favored (90.33%) General / -62.1,-46.1	Favored (95.7%) *m* chi angles: 299.6	0.04Å	Favored (98.462%) alpha helix	-	-	-
A 27		LYS	1.78	-	Favored (99.4%) General / -63.1,-41.2	Favored (60.3%) *mttm* chi angles: 286.7,178.5,179.5,292.8	0.04Å	Favored (97.628%) alpha helix	-	-	-
A 28		TYR	1.63	-	Favored (74.21%) General / -59.9,-50.3	Favored (55.9%) *t80* chi angles: 167.3,75.7	0.05Å	Favored (86.532%) alpha helix	-	-	-
A 29		LEU	1.5	-	Favored (78.84%) General / -63.6,-34.9	Favored (86.5%) *mt* chi angles: 291.3,174.4	0.04Å	Favored (80.637%) alpha helix	-	-	-
A 30		THR	1.4	-	Favored (96.59%) General / -63.4,-43.8	Favored (96.7%) *m* chi angles: 299.8	0.02Å	Favored (84.201%) alpha helix	-	-	-
A 31		LYS	1.32	-	Favored (81.46%) General / -56.6,-44.8	Favored (84.4%) *tttt* chi angles: 179.8,180.1,177.5,180.8	0.07Å	Favored (97.04%) alpha helix	-	-	-
A 32		VAL	1.25	-	Favored (92.24%) Ile or Val / -63.9,-47.1	Favored (81.3%) *t* chi angles: 173.2	0.05Å	Favored (94.647%) alpha helix	-	-	-
A 33		GLU	1.2	-	Favored (86.25%) General / -58.8,-47.0	Favored (92.3%) *tt0* chi angles: 183.2,179.3,3.9	0.03Å	Favored (94.158%) alpha helix	-	-	-
A 34		ASN	1.18	-	Favored (85.7%) General / -63.7,-37.0	Favored (95.2%) *m-40* chi angles: 286.3,340.2	0.03Å	Favored (85.483%) alpha helix	-	-	-
A 35		TRP	1.18	-	Favored (91.88%) General / -61.4,-46.0	Favored (66.7%) *t60* chi angles: 187.5,83.8	0.05Å	Favored (86.108%) alpha helix	-	-	-

A 36		VAL	1.2	-	Favored (94.73%) Ile or Val / -65.1,-42.5	Favored (80.3%) *t* chi angles: 173.2	0.02Å	Favored (89.318%) alpha helix	-	-	-
A 37		LEU	1.22	-	Favored (88.31%) General / -62.2,-38.2	Favored (93%) *mt* chi angles: 291.6,171.4	0.03Å	Favored (79.748%) alpha helix	-	-	-
A 38		ARG	1.24	-	Favored (61.2%) General / -75.3,-34.7	Favored (95.5%) *mtt180* chi angles: 290.7,171.2,181.4,167.5	0.04Å	Favored (60.218%)	-	-	-
A 39		ASN	1.24	-	Favored (72.13%) Pre-Pro / -131.8,64.3	Favored (47.6%) *m-40* chi angles: 300.8,278	0.06Å	Favored (23.566%)	-	-	-
A 40		PRO	1.22	-	Favored (71.54%) Trans-Pro / -62.8,-20.6	Favored (40.3%) *Cg_endo* chi angles: 23.5,325.5,30.9	0.02Å	Favored (28.314%)	-	-	-
**#**	**Alt**	**Res**	**High B**	**Clash > 0.4Å**	**Ramachandran**	**Rotamer**	**Cβ deviation**	**CaBLAM**	**Bond lengths**	**Bond angles**	**Cis Peptides**
			Avg: 2.70	Clashscore: 0.84	Outliers: 0 of 73	Poor rotamers: 0 of 65	Outliers: 0 of 71	Outliers: 1 of 71	Outliers: 0 of 75	Outliers: 0 of 75	Non-Trans: 0 of 74
A 41		GLY	1.17	-	Favored (59.17%) Glycine / -56.9,-29.6	-	-	Favored (68.268%)	-	-	-
A 42		TYR	1.11	-	Favored (63.6%) General / -71.7,-28.2	Favored (51.3%) *m-80* chi angles: 286.5,111.2	0.03Å	Favored (73.409%) three-ten	-	-	-
A 43		ALA	1.04	-	Favored (89.42%) General / -66.2,-39.3	-	0.02Å	Favored (77.103%) alpha helix	-	-	-
A 44		LEU	0.96	-	Favored (91.12%) General / -65.2,-38.7	Favored (94.9%) *mt* chi angles: 293.7,174.3	0.02Å	Favored (94.105%) alpha helix	-	-	-
A 45		VAL	0.9	-	Favored (91.66%) Ile or Val / -65.4,-45.9	Favored (68%) *t* chi angles: 171.9	0.03Å	Favored (86.328%) alpha helix	-	-	-
A 46		ALA	0.84	-	Favored (82.65%) General / -60.0,-38.4	-	0.04Å	Favored (85.489%) alpha helix	-	-	-
A 47		LEU	0.79	-	Favored (94.33%) General / -63.1,-44.9	Favored (76.4%) *mt* chi angles: 288,170.7	0.05Å	Favored (88.108%) alpha helix	-	-	-
A 48		ALA	0.76	-	Favored (98.24%) General / -61.4,-42.2	-	0.03Å	Favored (79.2%) alpha helix	-	-	-
A 49		ILE	0.74	-	Favored (68.77%) Ile or Val / -69.4,-48.1	Favored (96.6%) *mt* chi angles: 294.1,168.1	0.02Å	Favored (80.07%) alpha helix	-	-	-

A 50		GLY	0.77	-	Favored (92.11%) Glycine / -59.7,-37.3	-	-	Favored (93.258%) alpha helix	-	-	-
A 51		TRP	0.89	-	Favored (93.98%) General / -65.2,-41.9	Favored (41.4%) *m-10* chi angles: 289.5,336.1	0.02Å	Favored (92.947%) alpha helix	-	-	-
A 52		MET	1.19	-	Favored (71.54%) General / -69.7,-32.7	Favored (83.4%) *mtm* chi angles: 289.6,187,285.8	0.03Å	Favored (74.658%) alpha helix	-	-	-
A 53		LEU	1.8	-	Favored (19.14%) General / -83.5,-40.5	Favored (94.5%) *mt* chi angles: 294.9,174.5	0.10Å	Favored (28.74%)	-	-	-
A 54		GLY	2.86	-	Favored (28.8%) Glycine / -77.9,144.5	-	-	Favored (15.862%)	-	-	-
A 55		SER	4.22	-	Favored (51.72%) General / -85.4,-12.6	Favored (94.9%) *p* chi angles: 64	0.04Å	Favored (14.996%)	-	-	-
A 56		ASN	5.21	-	Favored (17.33%) General / -154.7,172.8	Favored (28.2%) *p0* chi angles: 60.4,57.6	0.06Å	Favored (22.012%)	-	-	-
A 57		ASN	5.1	-	Favored (89.76%) General / -62.4,-38.5	Favored (99%) *m-40* chi angles: 287.3,340.2	0.04Å	Favored (53.803%) alpha helix	-	-	-
A 58		THR	3.99	-	Favored (75.92%) General / -64.9,-47.9	Favored (91.6%) *m* chi angles: 297.9	0.01Å	Favored (85.296%) alpha helix	-	-	-
A 59		GLN	2.67	-	Favored (93.36%) General / -61.5,-40.1	Favored (52%) *tt0* chi angles: 184,171.6,52.3	0.03Å	Favored (84.57%) alpha helix	-	-	-
A 60		ARG	1.71	-	Favored (99.66%) General / -62.8,-42.3	Favored (97%) *mtt180* chi angles: 288.7,173.4,176.3,181.4	0.03Å	Favored (92.544%) alpha helix	-	-	-
**#**	**Alt**	**Res**	**High B**	**Clash > 0.4Å**	**Ramachandran**	**Rotamer**	**Cβ deviation**	**CaBLAM**	**Bond lengths**	**Bond angles**	**Cis Peptides**
			Avg: 2.70	Clashscore: 0.84	Outliers: 0 of 73	Poor rotamers: 0 of 65	Outliers: 0 of 71	Outliers: 1 of 71	Outliers: 0 of 75	Outliers: 0 of 75	Non-Trans: 0 of 74
A 61		VAL	1.18	-	Favored (98.71%) Ile or Val / -63.4,-43.8	Favored (60%) *t* chi angles: 170.8	0.02Å	Favored (97.813%) alpha helix	-	-	-
A 62		VAL	0.93	-	Favored (94.75%) Ile or Val / -60.1,-44.3	Favored (56.2%) *t* chi angles: 170.3	0.02Å	Favored (98.037%) alpha helix	-	-	-
A 63		PHE	0.83	-	Favored (93.11%) General / -61.8,-39.8	Favored (6.2%) *m-10* chi angles: 283.2,339.7	0.03Å	Favored (94.419%) alpha helix	-	-	-

A 64		VAL	0.82	-	Favored (98.22%) Ile or Val / -63.8,-44.4	Favored (70.1%) *t* chi angles: 172.1	0.03Å	Favored (92.407%) alpha helix	-	-	-
A 65		ILE	0.85	-	Favored (96.56%) Ile or Val / -61.9,-46.4	Favored (98.5%) *mt* chi angles: 292.4,167.2	0.03Å	Favored (89.267%) alpha helix	-	-	-
A 66		MET	0.89	-	Favored (68.5%) General / -54.2,-49.8	Favored (48.9%) *ttp* chi angles: 176.1,190,71.5	0.10Å	Favored (95.716%) alpha helix	-	-	-
A 67		LEU	0.95	-	Favored (92.51%) General / -62.6,-39.0	Favored (91%) *mt* chi angles: 291.8,169.6	0.07Å	Favored (79.975%) alpha helix	-	-	-
A 68		MET	1.03	-	Favored (59.79%) General / -74.7,-26.4	Favored (91.7%) *mmm* chi angles: 295,308,290.1	0.08Å	Favored (78.605%) alpha helix	-	-	-
A 69		LEU	1.14	-	Favored (60.53%) General / -75.4,-31.5	Favored (80.5%) *mt* chi angles: 291.2,175.8	0.01Å	Favored (75.921%) alpha helix	-	-	-
A 70		ILE	1.31	-	Favored (18.56%) Ile or Val / -83.4,-46.3	Favored (94.3%) *mt* chi angles: 295.6,168	0.05Å	Favored (22.07%) alpha helix	-	-	-
A 71		ALA	1.56	-	Favored (68.32%) Pre-Pro / -50.6,-41.2	-	0.07Å	Favored (66.127%) three-ten	-	-	-
A 72		PRO	1.93	-	Favored (53.23%) Trans-Pro / -58.9,-20.6	Favored (59.7%) *Cg_exo* chi angles: 336,35.1,328.6	0.05Å	Favored (56.991%) three-ten	-	-	-
A 73		ALA	2.4	-	Favored (58.96%) General / -86.0,-3.5	-	0.03Å	Favored (52.988%)	-	-	-
A 74		TYR	2.97	0.41Å O with A 75 SER CB	Favored (71.18%) General / -55.5,-49.9	Favored (91.1%) *t80* chi angles: 177.3,80.8	0.02Å	-	-	-	-
A 75		SER	3.59	0.41Å CB with A 74 TYR O	-	Favored (41.5%) *t* chi angles: 176.2	0.08Å	-	-	-	-

About MolProbity | Website for the Richardson Lab | Using ecloud x-H | Internal reference 4.5.2
