# Supplementary material for: Integrative proteome-wide structural analysis and high-throughput docking identify broad-spectrum antiviral scaffolds against Zika, Yellow Fever, West Nile, Saint Louis encephalitis, and Usutu viruses
Source: Front Cell Infect Microbiol. 2026 Apr 30;16:1723132. doi: 10.3389/fcimb.2026.1723132 (PMC13171538; doi:10.3389/fcimb.2026.1723132)
Supplement: Supplementary file 3 [file DataSheet3.zip › SLEV/SLEV_NS1/Mol_probity_Files/SLEV_NS1_1FH-multi.table.pdf]

Viewing SLEV_NS1_1FH-multi.table

MolProbity

Duke Biochemistry
Duke University School of Medicine

When finished, you should close this window.

Hint: Use File | Save As... to save a copy of this page.

All-Atom Contacts	Clashscore, all atoms:	2.52		98th percentile* (N=1784, all resolutions)
	Clashscore is the number of serious steric overlaps (> 0.4 Å) per 1000 atoms.			
Protein Geometry	Poor rotamers	0	0.00%	Goal: <0.3%
	Favored rotamers	304	99.02%	Goal: >98%
	Ramachandran outliers	1	0.29%	Goal: <0.05%
	Ramachandran favored	340	97.14%	Goal: >98%
	Rama distribution Z-score	1.04 ± 0.45		Goal: abs(Z score) < 2
	MolProbity score^	1.19		99th percentile* (N=27675, 0Å - 99Å)
	Cβ deviations >0.25Å	0	0.00%	Goal: 0
	Bad bonds:	5 / 2891	0.17%	Goal: 0%
	Bad angles:	6 / 3919	0.15%	Goal: <0.1%
Peptide Omegas	Cis Prolines:	1 / 15	6.67%	Expected: ≤1 per chain, or ≤5%
	Twisted Peptides:	1 / 351	0.28%	Goal: 0
Low-resolution Criteria	CaBLAM outliers	13	3.7%	Goal: <1.0%
	CA Geometry outliers	3	0.86%	Goal: <0.5%
Additional validations	Chiral volume outliers	0/419		
	Waters with clashes	0/0	0.00%	See UnDowser table for details

In the two column results, the left column gives the raw count, right column gives the percentage.

* 100th percentile is the best among structures of comparable resolution; 0th percentile is the worst. For clashscore the comparative set of structures was selected in 2004, for MolProbity score in 2006.

^ MolProbity score combines the clashscore, rotamer, and Ramachandran evaluations into a single score, normalized to be on the same scale as X-ray resolution.

Key to table colors and cutoffs here:

#	Alt	Res	High B	Clash > 0.4Å	Ramachandran	Rotamer	Cβ deviation	CaBLAM	Bond lengths	Bond angles	Cis Peptides
			Avg: 1.14	Clashscore: 2.52	Outliers: 1 of 350	Poor rotamers: 0 of 307	Outliers: 0 of 323	Outliers: 15 of 348	Outliers: 4 of 352	Outliers: 6 of 352	Non-Trans: 2 of 351
A 1		ASP	3.11	-	-	Favored (12.7%) *t0* chi angles: 203.2,337.4	0.05Å	-	-	-	-
A 2		SER	3.03	-	Allowed (1.51%) General / -141.2,0.3	Favored (82.9%) *p* chi angles: 62.5	0.02Å	-	-	-	-
A 3		GLY	2.97	-	Favored (40.64%) Glycine / -83.8,-166.6	-	-	Favored (21.274%)	-	-	-
A 4		CYS	2.97	-	Favored (41.22%) General / -132.8,158.9	Favored (75.5%) *m* chi angles: 296.8	0.07Å	Favored (44.173%) beta sheet	-	-	-
A 5		ALA	3.03	-	Favored (34.79%) General / -153.1,154.4	-	0.04Å	Favored (54.154%) beta sheet	-	-	-
A 6		ILE	3.17	-	Favored (75.03%)	Favored (84.2%) *mt* chi angles: 299.3,171.2	0.04Å	Favored (54.504%) beta sheet	-	-	-

					Ile or Val / -117.8,126.8						
A 7		ASP	3.33	-	Favored (11.51%) General / -86.9,99.0	Favored (46.9%) *t0* chi angles: 179.2,342.2	0.02Å	Favored (67.2%) beta sheet	-	-	-
A 8		LEU	3.5	-	Favored (35.77%) General / -78.4,-1.9	Favored (95.2%) *mt* chi angles: 294.3,174	0.03Å	Favored (18.31%)	-	-	-
A 9		GLN	3.62	-	Favored (40.07%) General / -91.2,-10.8	Favored (90%) *mt0* chi angles: 295.3,180.3,343.3	0.02Å	Favored (44.22%) alpha helix	-	-	-
A 10		ARG	3.65	-	Favored (5.24%) General / -127.0,-15.9	Favored (82.4%) *mtt90* chi angles: 293.3,178.3,173.5,87.8	0.01Å	Favored (7.944%)	-	-	-
A 11		ARG	3.58	-	Favored (8.19%) General / 61.3,46.1	Favored (95.1%) *mtt180* chi angles: 299.9,182.4,180.9,175.4	0.01Å	Favored (20.763%)	-	-	-
A 12		GLU	3.42	-	Favored (56.37%) General / -115.8,129.2	Favored (91.7%) *tt0* chi angles: 181,176.6,355.5	0.02Å	Favored (30.644%) beta sheet	-	-	-
A 13		LEU	3.2	-	Favored (41.29%) General / -96.6,125.8	Favored (45.6%) *tp* chi angles: 173.6,66	0.06Å	Favored (64.48%) beta sheet	-	-	-
A 14		LYS	2.94	-	Favored (54.47%) General / -122.8,133.4	Favored (63%) *mttm* chi angles: 293.6,181.7,182.2,294	0.03Å	Favored (52.454%)	-	-	-
A 15		CYS	2.68	-	Favored (33.2%) General / -137.6,162.7	Favored (67.5%) *m* chi angles: 299.4	0.07Å	Favored (9.243%)	-	-	-
A 16		GLY	2.41	-	Favored (19.71%) Glycine / 96.0,-150.7	-	-	Favored (19.118%)	-	-	-
A 17		GLY	2.17	-	Allowed (1.16%) Glycine / -71.5,52.7	-	-	CaBLAM Outlier (0.455%)	-	-	-
A 18		GLY	1.97	-	Favored (7.71%) Glycine / -121.9,-148.0	-	-	Favored (39.596%)	-	-	-
A 19		ILE	1.82	-	Favored (58.79%) Ile or Val / -129.0,123.4	Favored (77.4%) *mt* chi angles: 301.1,172.3	0.05Å	Favored (5.965%) beta sheet	-	-	-
A 20		PHE	1.73	-	Favored (52.36%) General / -114.5,124.6	Favored (98.7%) *m-80* chi angles: 297.9,95.3	0.07Å	Favored (70.789%) beta sheet	-	-	-
**#**	**Alt**	**Res**	**High B**	**Clash > 0.4Å**	**Ramachandran**	**Rotamer**	**Cβ deviation**	**CaBLAM**	**Bond lengths**	**Bond angles**	**Cis Peptides**
			Avg: 1.14	Clashscore: 2.52	Outliers: 1 of 350	Poor rotamers: 0 of 307	Outliers: 0 of 323	Outliers: 15 of 348	Outliers: 4 of 352	Outliers: 6 of 352	Non-Trans: 2 of 351
A 21		VAL	1.68	-	Favored (65.61%) Ile or Val / -112.2,130.6	Favored (79.1%) *t* chi angles: 178	0.09Å	Favored (65.658%) beta sheet	-	-	-
A 22		TYR	1.67	-	Favored (47.14%)	Favored (85.6%) *m-80*	0.04Å	Favored (52.333%)	-	-	-

					General / -120.4,145.4	chi angles: 300.4,88.1		beta sheet			
A 23		ASN	1.72	-	Favored (7.74%) General / -81.6,67.9	Favored (39.1%) *t0* chi angles: 190.3,9.8	0.04Å	Favored (11.863%) beta sheet	-	-	-
A 24		ASP	1.84	-	Favored (57.51%) General / -91.1,-2.7	Favored (87.2%) *m-30* chi angles: 294.2,339.7	0.06Å	Favored (7.473%)	-	-	-
A 25		VAL	2.04	-	Favored (20.24%) Ile or Val / -58.8,-22.0	Favored (6.4%) *p* chi angles: 69	0.12Å	Favored (20.251%)	-	-	-
A 26		GLU	2.33	-	Favored (56.6%) General / -90.3,-5.1	Favored (96.5%) *mt-10* chi angles: 296.4,179.7,0.5	0.05Å	Favored (41.028%) alpha helix	-	-	-
A 27		LYS	2.64	-	Favored (2.98%) General / -130.4,-20.2	Allowed (1.2%) *mtpm* chi angles: 293.5,176.4,84.9,298.4	0.04Å	Favored (7.677%) alpha helix	-	-	-
A 28		TRP	2.88	0.57Å CD1 with A 28 TRP N	Allowed (1.98%) General / -51.1,-21.6	Allowed (1.6%) *p-90* chi angles: 61,311.1	0.08Å	Favored (8.13%)	-	-	-
A 29		LYS	2.92	-	Favored (67.43%) General / -60.6,-27.0	Favored (98%) *mttt* chi angles: 291.5,179.6,181.1,177.9	0.02Å	Favored (16.596%)	-	-	-
A 30		SER	2.71	-	Favored (8.97%) General / -115.3,-23.3	Favored (81.6%) *p* chi angles: 62	0.02Å	Favored (17.019%)	-	-	-
A 31		ASP	2.33	-	Favored (46.34%) General / -78.5,-27.2	Favored (88.9%) *m-30* chi angles: 293.3,347.7	0.07Å	Favored (72.72%) alpha helix	-	-	-
A 32		TYR	1.91	-	Favored (51.88%) General / -112.9,124.1	Favored (50.4%) *m-80* chi angles: 290.8,75.9	0.06Å	Favored (28.195%)	-	-	-
A 33		LYS	1.56	-	Favored (54.42%) General / -114.2,135.1	Favored (98.7%) *mttt* chi angles: 295.2,183.2,180.2,181.8	0.03Å	Favored (63.476%)	-	-	-
A 34		TYR	1.33	-	Favored (37.61%) General / -101.4,139.0	Favored (9.4%) *m-10* chi angles: 284.9,12.4	0.09Å	Favored (60.996%) beta sheet	-	-	-
A 35		PHE	1.2	-	Favored (47.9%) Pre-Pro / -122.0,94.8	Favored (89.8%) *m-80* chi angles: 298,85.8	0.03Å	Favored (53.83%)	-	-	-
A 36		PRO	1.14	-	Favored (43.87%) Trans-Pro / -73.5,160.8	Favored (80.8%) *Cg_endo* chi angles: 30.6,325.8,23.6	0.08Å	Favored (20.385%)	-	-	-
A 37		LEU	1.11	0.46Å O with A 38 THR C	Allowed (0.37%) General / -44.3,149.4	Allowed (1.3%) *pt* chi angles: 65.8,159.6	0.08Å	CA Geom Outlier (0.413%)	-	-	-
A 38		THR	1.08	0.46Å C with A 37 LEU O	Favored (3.73%) Pre-Pro / -43.4,126.9	Favored (71.7%) *m* chi angles: 303	0.07Å	Favored (9.973%)	-	-	-
A 39		PRO	1.04	-	Favored (14.05%) Trans-Pro / -49.0,-28.7	Favored (76.2%) *Cg_exo* chi angles: 329,37.4,332	0.03Å	Favored (67.282%)	-	-	-

#	Alt	Res	High B	Clash > 0.4Å	Ramachandran	Rotamer	Cβ deviation	CaBLAM	Bond lengths	Bond angles	Cis Peptides
A 40		THR	0.99	-	Favored (95.79%) General / -64.6,-42.0	Favored (65.4%) *m* chi angles: 303.3	0.09Å	Favored (71.305%) alpha helix	-	-	-
#	Alt	Res	High B	Clash > 0.4Å	Ramachandran	Rotamer	Cβ deviation	CaBLAM	Bond lengths	Bond angles	Cis Peptides
			Avg: 1.14	Clashscore: 2.52	Outliers: 1 of 350	Poor rotamers: 0 of 307	Outliers: 0 of 323	Outliers: 15 of 348	Outliers: 4 of 352	Outliers: 6 of 352	Non-Trans: 2 of 351
A 41		GLY	0.93	-	Favored (39.91%) Glycine / -68.9,-49.7	-	-	Favored (79.932%) alpha helix	-	-	-
A 42		LEU	0.88	-	Favored (95.99%) General / -60.1,-43.0	Favored (57.6%) *tp* chi angles: 178.9,56.7	0.09Å	Favored (88.525%) alpha helix	-	-	-
A 43		ALA	0.84	-	Favored (75.95%) General / -58.7,-37.4	-	0.06Å	Favored (79.724%) alpha helix	-	-	-
A 44		ARG	0.8	-	Favored (87.91%) General / -66.8,-40.1	Favored (41.5%) *tpt170* chi angles: 180.8,65.5,178.1,162.6	0.02Å	Favored (95.991%) alpha helix	-	-	-
A 45		VAL	0.78	-	Favored (94.98%) Ile or Val / -61.6,-42.7	Favored (62.4%) *t* chi angles: 171.1	0.05Å	Favored (97.374%) alpha helix	-	-	-
A 46		ILE	0.76	-	Favored (97.01%) Ile or Val / -64.3,-45.1	Favored (98.9%) *mt* chi angles: 292.8,167.4	0.05Å	Favored (93.497%) alpha helix	-	-	-
A 47		GLN	0.75	-	Favored (87.32%) General / -58.4,-46.0	Favored (59.9%) *tt0* chi angles: 183.1,182.3,29.7	0.06Å	Favored (86.527%) alpha helix	-	-	-
A 48		GLU	0.75	-	Favored (86.8%) General / -67.1,-41.2	Favored (70.4%) *tp30* chi angles: 182.5,66.6,20.1	0.05Å	Favored (82.888%) alpha helix	-	-	-
A 49		ALA	0.75	-	Favored (91.33%) General / -59.8,-41.5	-	0.06Å	Favored (90.598%) alpha helix	-	-	-
A 50		HIS	0.74	-	Favored (73.39%) General / -60.4,-50.5	Favored (88.8%) *t70* chi angles: 178.8,76.3	0.02Å	Favored (88.778%) alpha helix	-	-	-
A 51		ALA	0.73	-	Favored (67.71%) General / -58.0,-32.3	-	0.03Å	Favored (73.571%)	-	-	-
A 52		ASN	0.71	-	Favored (31.36%) General / -94.0,11.0	Favored (87.1%) *m-40* chi angles: 290.7,322.2	0.05Å	Favored (22.811%)	-	-	-
A 53		GLY	0.69	-	Favored (41.01%) Glycine / 100.7,11.3	-	-	Favored (66.879%)	-	-	-
A 54		ILE	0.68	-	Favored (49.15%) Ile or Val / -102.0,118.6	Favored (87.9%) *mt* chi angles: 298.2,168.7	0.04Å	Favored (18.27%)	-	-	-
A 55		CYS	0.67	-	Favored (52.51%) General / -88.9,-9.2	Favored (29.2%) *p* chi angles: 66.9	0.06Å	CaBLAM Disfavored (3.314%)	-	-	-

A 56		GLY	0.66	-	Favored (41.47%) Glycine / 175.1,174.2	-	-	Favored (39.771%)	-	-	-
A 57		ILE	0.68	0.43Å O with A 57 ILE HG23	Favored (41.38%) Ile or Val / -121.8,141.6	Favored (4.3%) *tp* chi angles: 179.6,66.2	0.06Å	Favored (37.571%)	-	-	-
A 58		ARG	0.71	-	Favored (48.49%) General / -120.0,125.5	Favored (43.3%) *ttm170* chi angles: 182.4,183.4,290,156.6	0.04Å	Favored (57.935%) beta sheet	-	-	-
A 59		SER	0.76	-	Favored (17.14%) General / -77.7,170.1	Favored (88.8%) *p* chi angles: 66.9	0.03Å	Favored (9.119%)	-	-	-
A 60		THR	0.82	-	Favored (16.29%) General / -107.6,-8.0	Favored (77.5%) *p* chi angles: 60.2	0.01Å	Favored (31.265%)	-	-	-
**#**	**Alt**	**Res**	**High B**	**Clash > 0.4Å**	**Ramachandran**	**Rotamer**	**Cβ deviation**	**CaBLAM**	**Bond lengths**	**Bond angles**	**Cis Peptides**
			Avg: 1.14	Clashscore: 2.52	Outliers: 1 of 350	Poor rotamers: 0 of 307	Outliers: 0 of 323	Outliers: 15 of 348	Outliers: 4 of 352	Outliers: 6 of 352	Non-Trans: 2 of 351
A 61		SER	0.88	-	Favored (43.04%) General / -152.9,160.3	Favored (85.3%) *p* chi angles: 67.7	0.07Å	Favored (24.598%)	-	-	-
A 62		ARG	0.94	-	Favored (81.78%) General / -60.4,-37.8	Favored (35.8%) *tpt170* chi angles: 181.3,66,179.6,180.7	0.06Å	Favored (65.454%) alpha helix	-	-	-
A 63		LEU	0.98	-	Favored (87.78%) General / -66.8,-39.1	Favored (94%) *mt* chi angles: 293.1,174.7	0.07Å	Favored (80.567%) alpha helix	-	-	-
A 64		GLU	1	-	Favored (88.87%) General / -65.2,-37.9	Favored (96%) *mt-10* chi angles: 289.6,169.8,345.9	0.02Å	Favored (90.256%) alpha helix	-	-	-
A 65		HIS	1.01	-	Favored (76.29%) General / -59.3,-49.8	Favored (82.9%) *t70* chi angles: 179.4,79.5	0.05Å	Favored (93.673%) alpha helix	OUTLIER(S) worst is CB--CG: 4.5 σ	-	-
A 66		LEU	1.02	-	Favored (80.32%) General / -62.3,-35.9	Favored (86.6%) *mt* chi angles: 290.1,172	0.04Å	Favored (77.744%) alpha helix	-	-	-
A 67		MET	1.02	0.41Å C with A 67 MET SD	Favored (78.18%) General / -57.2,-48.7	Favored (28.8%) *tmm* chi angles: 178.6,276.5,290.1	0.06Å	Favored (77.322%) alpha helix	-	-	-
A 68		TRP	1.02	-	Favored (95.54%) General / -62.6,-40.0	Favored (63.2%) *m100* chi angles: 276.6,106.1	0.04Å	Favored (81.727%) alpha helix	-	-	-
A 69		GLU	1.03	-	Favored (86.76%) General / -61.7,-47.1	Favored (92.5%) *tt0* chi angles: 182.1,178.2,357.9	0.02Å	Favored (80.633%) alpha helix	-	-	-
A 70		ASN	1.03	-	Favored (71.21%) General / -61.8,-31.0	Favored (99.2%) *m-40* chi angles: 287.7,339	0.02Å	Favored (61.254%) alpha helix	-	-	-
A 71		ILE	1.03	-	Favored (10.15%)	Favored (36.9%) *pt* chi angles: 65.3,169.2	0.04Å	Favored (41.497%) alpha helix	-	-	-

					Ile or Val / -104.9,7.0						
A 72		GLN	1.02	-	Favored (81.75%) General / -58.3,-48.1	Favored (48.7%) *tt0* chi angles: 179.5,182.9,318.4	0.05Å	Favored (44.074%) alpha helix	-	-	-
A 73		ARG	1.01	-	Favored (88.18%) General / -59.7,-40.5	Favored (50.6%) *ttm110* chi angles: 187.7,178.4,294.2,108.9	0.02Å	Favored (80.678%) alpha helix	-	-	-
A 74		GLU	1	-	Favored (70.34%) General / -70.0,-44.0	Favored (89.9%) *tt0* chi angles: 187.3,176.2,1.8	0.04Å	Favored (94.035%) alpha helix	-	-	-
A 75		LEU	0.99	-	Favored (96.89%) General / -61.6,-41.0	Favored (83.8%) *mt* chi angles: 289.7,170.9	0.08Å	Favored (96.915%) alpha helix	-	-	-
A 76		ASN	0.98	-	Favored (89.58%) General / -66.3,-40.0	Favored (95.5%) *m-40* chi angles: 286.4,340.2	0.04Å	Favored (93.841%) alpha helix	-	-	-
A 77		ALA	0.98	-	Favored (91.56%) General / -61.9,-39.3	-	0.04Å	Favored (94.506%) alpha helix	-	-	-
A 78		ILE	0.98	-	Favored (89.44%) Ile or Val / -66.5,-45.3	Favored (98.3%) *mt* chi angles: 293,168.1	0.04Å	Favored (93.371%) alpha helix	-	-	-
A 79		PHE	0.98	-	Favored (92.61%) General / -60.5,-40.7	Favored (18.3%) *m-80* chi angles: 279.9,124.5	0.04Å	Favored (94.277%) alpha helix	-	-	-
A 80		GLU	0.96	-	Favored (87.17%) General / -63.3,-46.4	Favored (91.8%) *tt0* chi angles: 181.6,176.2,358.6	0.04Å	Favored (94.69%) alpha helix	-	-	-
**#**	**Alt**	**Res**	**High B**	**Clash > 0.4Å**	**Ramachandran**	**Rotamer**	**Cβ deviation**	**CaBLAM**	**Bond lengths**	**Bond angles**	**Cis Peptides**
			Avg: 1.14	Clashscore: 2.52	Outliers: 1 of 350	Poor rotamers: 0 of 307	Outliers: 0 of 323	Outliers: 15 of 348	Outliers: 4 of 352	Outliers: 6 of 352	Non-Trans: 2 of 351
A 81		ASP	0.93	-	Favored (66.83%) General / -65.4,-23.0	Favored (94.9%) *m-30* chi angles: 285.9,347.8	0.12Å	Favored (67.432%) alpha helix	-	-	-
A 82		ASN	0.88	-	Favored (38.63%) General / -94.3,9.3	Favored (69.3%) *m-40* chi angles: 287.3,281.3	0.07Å	Favored (53.855%)	-	-	-
A 83		GLU	0.83	-	Favored (29.89%) General / 54.7,43.6	Favored (90.7%) *mt-10* chi angles: 298.9,185.1,356.7	0.04Å	Favored (34.287%)	-	-	-
A 84		ILE	0.78	-	Favored (60.4%) Ile or Val / -107.7,121.2	Favored (82.8%) *mt* chi angles: 299.2,168.1	0.07Å	Favored (32.288%) beta sheet	-	-	-
A 85		ASP	0.73	-	Favored (7.1%) General / -81.6,83.2	Favored (64%) *t0* chi angles: 183.2,343.5	0.04Å	Favored (7.354%) beta sheet	OUTLIER(S) worst is CB--CG: 4.1 σ	-	-
A 86		LEU	0.7	-	Favored (4.05%) General / -158.7,123.0	Favored (58.8%) *tp* chi angles: 181.8,63	0.02Å	CaBLAM Disfavored (4.041%) try beta sheet	-	-	-
A 87		SER	0.69	-	Favored (21.77%)	Favored (93.1%) *p* chi angles: 63.2	0.03Å	Favored (43.6%) beta sheet	-	-	-

#	Alt	Res	High B	Clash > 0.4Å	Ramachandran	Rotamer	Cβ deviation	CaBLAM	Bond lengths	Bond angles	Cis Peptides
					General / -115.2,158.8						
A 88		VAL	0.7	-	Favored (66.76%) Ile or Val / -110.5,128.4	Favored (76.7%) *t* chi angles: 178.2	0.06Å	Favored (60.104%) beta sheet	-	-	-
A 89		VAL	0.73	-	Favored (65.83%) Ile or Val / -124.5,123.2	Favored (87.5%) *t* chi angles: 176.1	0.02Å	Favored (66.524%) beta sheet	-	-	-
A 90		VAL	0.79	-	Favored (56.95%) Ile or Val / -102.7,125.7	Favored (54.2%) *t* chi angles: 180.8	0.05Å	Favored (67.145%)	-	-	-
A 91		GLN	0.87	-	Favored (7.2%) General / -90.8,174.9	Favored (77.8%) *mm-40* chi angles: 290.5,289.7,301.2	0.07Å	Favored (22.141%)	-	-	-
A 92		GLU	0.95	-	Favored (64.7%) General / -52.1,-44.9	Favored (73.8%) *tt0* chi angles: 179,178.5,339.8	0.02Å	CaBLAM Outlier (0.226%)	-	-	-
A 93		ASP	1.01	0.64Å O with A 93 ASP OD1	OUTLIER (0)%) Pre-Pro / 124.0,147.6	Favored (55.5%) *p0* chi angles: 66.9,6.1	0.18Å	CaBLAM Outlier (0.273%)	-	-	-
A 94		PRO	1.04	-	Favored (47.17%) Trans-Pro / -74.1,155.6	Favored (71.1%) *Cg_endo* chi angles: 29.3,325.4,26.2	0.05Å	Favored (38.66%)	-	-	-
A 95		LYS	1.03	-	Favored (48.85%) General / -85.5,-13.5	Favored (29%) *mmtm* chi angles: 298.9,296,181.6,284.5	0.12Å	Favored (44.573%)	-	-	-
A 96		TYR	0.98	-	Favored (15.97%) General / -131.2,115.2	Favored (2.8%) *t80* chi angles: 172.7,24.8	0.07Å	Favored (14.92%)	-	-	-
A 97		TYR	0.91	-	Favored (9.82%) General / -85.8,77.0	Favored (97.3%) *m-80* chi angles: 294.1,90	0.08Å	Favored (64.151%)	-	-	-
A 98		LYS	0.84	-	Favored (49.69%) General / -62.6,147.3	Favored (98.2%) *mttt* chi angles: 292.3,179.7,180.7,178.5	0.05Å	Favored (16.548%)	-	-	-
A 99		ARG	0.78	-	Favored (25.57%) General / -72.5,164.9	Favored (56%) *ptt90* chi angles: 67.6,177,176.3,87	0.07Å	Favored (19.046%)	-	-	-
A 100		ALA	0.74	-	Favored (53.59%) Pre-Pro / -143.0,150.6	-	0.03Å	Favored (49.583%)	-	-	-
**#**	**Alt**	**Res**	**High B**	**Clash > 0.4Å**	**Ramachandran**	**Rotamer**	**Cβ deviation**	**CaBLAM**	**Bond lengths**	**Bond angles**	**Cis Peptides**
			Avg: 1.14	Clashscore: 2.52	Outliers: 1 of 350	Poor rotamers: 0 of 307	Outliers: 0 of 323	Outliers: 15 of 348	Outliers: 4 of 352	Outliers: 6 of 352	Non-Trans: 2 of 351
A 101		PRO	0.71	-	Favored (38.49%) Trans-Pro / -69.6,-16.6	Favored (54.8%) *Cg_endo* chi angles: 25.8,325.2,28.8	0.04Å	Favored (42.376%)	-	-	-
A 102		ARG	0.71	-	Favored (10.76%) General / -104.8,165.9	Favored (93.2%) *mtt-85* chi angles: 297.1,177,185,266.4	0.04Å	Favored (13.012%)	-	-	-

A<br>103		ARG	0.73	-	Favored<br>(48.82%)<br>General /<br>-135.5,147.5	Favored (93%)<br>*mmt-90*<br>chi angles:<br>297.1,291,183,274.4	0.05Å	Favored<br>(49.998%)	-	-	-
A<br>104		LEU	0.78	-	Favored<br>(34.97%)<br>General /<br>-80.1,142.4	Favored (79.3%) *mt*<br>chi angles: 301.2,175.7	0.11Å	Favored<br>(43.905%)<br>beta sheet	-	-	-
A<br>105		LYS	0.85	-	Favored<br>(31.8%)<br>General /<br>-102.3,143.7	Favored (27.9%)<br>*mmmt*<br>chi angles:<br>299.1,290.5,288.4,186.8	0.03Å	Favored<br>(34.844%)<br>beta sheet	-	-	-
A<br>106		LYS	0.95	-	Favored<br>(38.64%)<br>General /<br>-63.1,129.1	Favored (86.1%)<br>*tttt*<br>chi angles:<br>184.3,179.6,179.3,183.2	0.07Å	Favored<br>(42.045%)<br>beta sheet	-	-	-
A<br>107		LEU	1.05	-	Favored<br>(29.05%)<br>General /<br>-113.9,153.5	Favored (76.1%) *mt*<br>chi angles: 302.6,176.7	0.02Å	Favored<br>(52.838%)<br>beta sheet	-	-	-
A<br>108		GLU	1.13	-	Favored<br>(56.78%)<br>General / -88.6,-7.2	Favored (96.8%)<br>*mt-10*<br>chi angles:<br>295.7,178.2,359.4	0.03Å	Favored<br>(20.535%)	-	-	-
A<br>109		ASP	1.19	-	Favored<br>(16.56%)<br>General /<br>-98.2,156.7	Favored (72.4%) *m-30*<br>chi angles: 294.7,317.5	0.06Å	Favored<br>(16.081%)	-	-	-
A<br>110		GLU	1.26	-	Favored<br>(47.93%)<br>General /<br>-133.8,156.5	Favored (85.2%)<br>*mt-10*<br>chi angles:<br>300.3,185.9,2	0.02Å	Favored<br>(31.212%)	-	-	-
A<br>111		LEU	1.36	-	Favored<br>(50.44%)<br>General /<br>-67.3,149.4	Favored (86.2%) *mt*<br>chi angles: 295.3,178.5	0.04Å	Favored<br>(44.582%)	-	-	-
A<br>112		ASP	1.53	-	Favored<br>(78.22%)<br>General /<br>-58.5,-38.6	Favored (98.9%) *m-30*<br>chi angles: 287.8,345.7	0.06Å	Favored<br>(27.939%)	-	-	-
A<br>113		TYR	1.82	-	Allowed<br>(0.14%)<br>General /<br>-80.9,-80.7	Favored (89.7%)<br>*t80*<br>chi angles: 178.4,75.9	0.03Å	CaBLAM<br>Outlier<br>(0.565%)	-	-	-
A<br>114		GLY	2.26	-	Favored<br>(4.25%)<br>Glycine /<br>73.7,156.6	-	-	CaBLAM<br>Outlier<br>(0.897%)	-	-	-
A<br>115		TRP	2.84	-	Favored<br>(27.53%)<br>General /<br>-89.0,142.6	Favored (95.2%)<br>*m100*<br>chi angles: 287.1,102.8	0.08Å	Favored<br>(16.811%)	-	-	-
A<br>116		LYS	3.51	-	Favored<br>(41.5%)<br>General /<br>-82.5,-18.4	Favored (98.6%)<br>*mttt*<br>chi angles:<br>293.2,179.7,179.6,178.9	0.02Å	Favored<br>(28.864%)	-	-	-
A<br>117		LYS	4.16	-	Favored<br>(6.81%)<br>General /<br>-152.3,121.8	Favored (87.1%)<br>*tttt*<br>chi angles:<br>183.2,173.9,179.6,179.2	0.03Å	Favored<br>(14.591%)	-	-	-
A<br>118		TRP	4.67	-	Favored<br>(66.61%)<br>General /<br>-60.1,-26.9	Favored (72.1%) *p-90*<br>chi angles: 68.6,268.6	0.04Å	Favored<br>(26.176%)	-	-	-
A<br>119		GLY	4.94	-	Favored<br>(37.51%)<br>Glycine /<br>-58.5,-18.5	-	-	Favored<br>(58.84%)	-	-	-

#	Alt	Res	High B	Clash > 0.4Å	Ramachandran	Rotamer	Cβ deviation	CaBLAM	Bond lengths	Bond angles	Cis Peptides
A 120		LYS	4.92	-	Favored (66.95%) General / -64.6,-22.4	Favored (71.7%) *mmtt* chi angles: 294,292.8,183.7,180.6	0.05Å	Favored (61.245%) three-ten	-	-	-
#	Alt	Res	High B	Clash > 0.4Å	Ramachandran	Rotamer	Cβ deviation	CaBLAM	Bond lengths	Bond angles	Cis Peptides
			Avg: 1.14	Clashscore: 2.52	Outliers: 1 of 350	Poor rotamers: 0 of 307	Outliers: 0 of 323	Outliers: 15 of 348	Outliers: 4 of 352	Outliers: 6 of 352	Non-Trans: 2 of 351
A 121		THR	4.56	-	Favored (56.66%) General / -78.0,-7.3	Favored (62.5%) *p* chi angles: 63.5	0.05Å	Favored (59.9%) three-ten	-	-	-
A 122		LEU	3.91	-	Favored (59.49%) General / -83.5,-7.6	Favored (94.2%) *mt* chi angles: 296.4,173.3	0.07Å	Favored (52.682%) three-ten	-	-	-
A 123		PHE	3.11	-	Favored (4.05%) General / -116.4,-36.3	Favored (81%) *m-80* chi angles: 301.7,103.6	0.07Å	Favored (36.875%) alpha helix	-	-	-
A 124		VAL	2.35	-	Favored (32.3%) Ile or Val / -120.8,111.5	Favored (63.3%) *t* chi angles: 179.6	0.03Å	Favored (28.602%)	-	-	-
A 125		GLU	1.74	-	Favored (49.06%) Pre-Pro / -96.9,124.7	Favored (31.5%) *tt0* chi angles: 181.4,174.1,276.8	0.05Å	Favored (55.741%)	-	-	-
A 126		PRO	1.32	-	Favored (42.05%) Trans-Pro / -74.7,159.7	Favored (73.9%) *Cg_endo* chi angles: 29,324.3,27.1	0.07Å	Favored (64.656%) beta sheet	-	-	-
A 127		ARG	1.06	-	Favored (20.6%) General / -83.6,163.0	Favored (87.5%) *mtm180* chi angles: 295.2,181,292.8,172.1	0.02Å	Favored (50.258%) beta sheet	-	-	-
A 128		LEU	0.9	-	Favored (57.66%) General / -87.6,-1.0	Favored (90.6%) *mt* chi angles: 294.2,177.2	0.07Å	CaBLAM Outlier (0.314%)	-	-	-
A 129		GLY	0.81	-	Favored (46.04%) Glycine / 60.9,-148.2	-	-	Favored (35.372%)	-	-	-
A 130		ASN	0.75	-	Favored (4.79%) General / -115.1,-35.2	Favored (66.1%) *m-40* chi angles: 297.7,284.1	0.04Å	CaBLAM Outlier (0.031%)	-	-	-
A 131		ASN	0.7	-	Favored (42.78%) General / -96.8,132.2	Favored (85.9%) *m-40* chi angles: 296.6,314	0.04Å	Favored (21.793%)	-	-	-
A 132		THR	0.67	-	Favored (48.71%) General / -129.8,135.7	Favored (89.6%) *m* chi angles: 298.2	0.05Å	Favored (71.471%)	-	-	-
A 133		PHE	0.64	-	Favored (27.76%) General / -96.0,115.7	Favored (78.9%) *t80* chi angles: 182.8,85.2	0.07Å	Favored (64.563%) beta sheet	-	-	-
A 134		VAL	0.64	-	Favored (40.51%) Ile or Val / -98.3,133.5	Favored (75.6%) *t* chi angles: 178.3	0.05Å	Favored (54.162%)	-	-	-
A 135		VAL	0.65	-	Favored (57.3%) Ile or Val / -107.6,119.5	Favored (66.3%) *t* chi angles: 179.2	0.07Å	Favored (11.314%)	-	-	-
A 136		ASP	0.68	-	Favored (29.24%)	Favored (76.9%) *m-30*	0.02Å	Favored (5.334%)	-	-	-

					General / 51.8,41.6	chi angles: 292.2,324.8					
A 137		GLY	0.73	-	Favored (36.61%) Glycine / -95.3,-179.9	-	-	Favored (23.427%)	-	-	-
A 138		PRO	0.79	-	Favored (63%) Trans-Pro / -63.1,155.1	Favored (49.6%) *Cg_exo* chi angles: 337.8,34.2,328.3	0.07Å	Favored (45.487%)	-	-	-
A 139		GLU	0.85	-	Favored (25.13%) General / -74.5,123.6	Favored (47.7%) *tt0* chi angles: 181.1,176.2,60.8	0.03Å	Favored (21.534%)	-	-	-
A 140		THR	0.88	-	Favored (15.57%) General / -130.9,169.6	Favored (70.5%) *p* chi angles: 62.3	0.01Å	Favored (34.452%)	-	-	-
**#**	**Alt**	**Res**	**High B**	**Clash > 0.4Å**	**Ramachandran**	**Rotamer**	**Cβ deviation**	**CaBLAM**	**Bond lengths**	**Bond angles**	**Cis Peptides**
			Avg: 1.14	Clashscore: 2.52	Outliers: 1 of 350	Poor rotamers: 0 of 307	Outliers: 0 of 323	Outliers: 15 of 348	Outliers: 4 of 352	Outliers: 6 of 352	Non-Trans: 2 of 351
A 141		LYS	0.89	-	Favored (86.35%) General / -63.6,-37.2	Favored (97.2%) *mttt* chi angles: 289.7,179.6,180,178.3	0.02Å	Favored (60.213%)	-	-	-
A 142		GLU	0.87	-	Favored (75.85%) General / -70.0,-38.2	Favored (99.9%) *mt-10* chi angles: 292,179.6,354	0.03Å	Favored (52.906%) alpha helix	-	-	-
A 143		CYS	0.83	-	Favored (31.85%) Pre-Pro / -138.4,79.0	Favored (41.6%) *t* chi angles: 186.8	0.02Å	Favored (20.233%)	-	-	-
A 144		PRO	0.77	-	Favored (34.13%) Trans-Pro / -75.0,164.0	Favored (71%) *Cg_endo* chi angles: 29.6,324.2,27.3	0.03Å	Favored (22.817%)	-	-	-
A 145		THR	0.72	-	Favored (67.07%) General / -55.6,-36.6	Favored (96.5%) *m* chi angles: 299.8	0.03Å	Favored (45.071%)	-	-	-
A 146		ALA	0.68	-	Favored (59.06%) General / -57.8,-24.8	-	0.05Å	Favored (56.462%) alpha helix	-	-	-
A 147		ASN	0.65	-	Favored (21.74%) General / -109.1,17.4	Favored (20.2%) *m110* chi angles: 289.5,108	0.06Å	Favored (37.252%)	-	-	-
A 148		ARG	0.64	-	Favored (35.99%) General / -111.4,147.1	Favored (97.3%) *mtt-85* chi angles: 291.8,180.1,179.3,276.5	0.07Å	Favored (33.446%)	-	-	-
A 149		ALA	0.64	-	Favored (16.92%) General / -92.2,157.1	-	0.02Å	Favored (41.908%)	-	-	-
A 150		TRP	0.64	-	Favored (27.41%) General / -149.1,145.9	Favored (82.3%) *t60* chi angles: 174.9,87.9	0.06Å	Favored (9.754%)	-	-	-
A 151		ASN	0.65	-	Favored (25.22%) General / 57.1,42.8	Favored (56%) *t0* chi angles: 198.8,27.6	0.01Å	Favored (11.923%)	-	OUTLIER(S) worst is CA-CB-CG: 4.5 σ	-
A 152		SER	0.67	-	Favored (57.49%) General / -91.0,0.7	Favored (68.3%) *m* chi angles: 296.7	0.04Å	Favored (11.624%)	-	-	-

A 153		PHE	0.7	-	Favored (39.06%) General / -118.7,150.9	Favored (86%) *m-80* chi angles: 290.9,85.4	0.04Å	Favored (25.975%)	-	-	-
A 154		LYS	0.74	-	Favored (42.21%) General / -136.7,139.9	Favored (87.1%) *tttt* chi angles: 182.1,176.2,180.2,180.7	0.04Å	Favored (60.26%)	-	-	-
A 155		VAL	0.82	-	Favored (93.16%) Ile or Val / -64.0,-41.6	Favored (51.6%) *t* chi angles: 169.6	0.15Å	CaBLAM Disfavored (1.92%)	-	-	-
A 156		GLU	0.95	-	Allowed (0.66%) General / 71.2,-54.9	Favored (56.6%) *tp30* chi angles: 189.2,68.7,20	0.03Å	Favored (12.437%)	-	-	-
A 157		ASP	1.14	-	Favored (21.7%) General / -154.9,170.4	Favored (13.5%) *t0* chi angles: 207.3,340.6	0.05Å	Favored (11.018%)	-	-	-
A 158		PHE	1.43	-	Favored (15.36%) General / -167.3,164.4	Favored (40.6%) *p90* chi angles: 54.6,89.3	0.03Å	Favored (59.634%)	-	-	-
A 159		GLY	1.8	-	Favored (21.59%) Glycine / -142.7,177.7	-	-	Favored (50.928%) beta sheet	-	-	-
A 160		PHE	2.22	-	Favored (12.32%) General / -112.6,-18.7	Favored (80.1%) *m-80* chi angles: 298.8,107.7	0.08Å	CaBLAM Disfavored (2.695%)	-	-	-
**#**	**Alt**	**Res**	**High B**	**Clash > 0.4Å**	**Ramachandran**	**Rotamer**	**Cβ deviation**	**CaBLAM**	**Bond lengths**	**Bond angles**	**Cis Peptides**
			Avg: 1.14	Clashscore: 2.52	Outliers: 1 of 350	Poor rotamers: 0 of 307	Outliers: 0 of 323	Outliers: 15 of 348	Outliers: 4 of 352	Outliers: 6 of 352	Non-Trans: 2 of 351
A 161		GLY	2.57	-	Favored (32.57%) Glycine / 64.1,-164.4	-	-	Favored (28.044%)	-	-	-
A 162		MET	2.74	-	Favored (10.68%) General / -99.8,-33.1	Favored (98%) *mmm* chi angles: 296.4,301.9,291.2	0.01Å	CaBLAM Outlier (0.735%)	-	-	-
A 163		VAL	2.66	-	Favored (2.97%) Ile or Val / -122.0,-56.0	Favored (77.2%) *t* chi angles: 178.2	0.01Å	CaBLAM Disfavored (3.723%)	-	-	-
A 164		PHE	2.38	-	Favored (38.16%) General / -113.0,146.9	Favored (92.8%) *m-80* chi angles: 299.4,99.1	0.10Å	Favored (17.47%)	-	-	-
A 165		THR	2	-	Favored (17.87%) General / -87.0,162.0	Favored (56.4%) *p* chi angles: 64.7	0.05Å	Favored (40.87%)	-	-	-
A 166		ARG	1.64	-	Favored (32.91%) General / -131.3,125.5	Favored (83.4%) *mtp180* chi angles: 299.2,182.2,64.1,186.5	0.07Å	Favored (46.191%) beta sheet	-	-	-
A 167		LEU	1.34	0.45Å N with A 167 LEU HD12	Favored (52.05%) General / -125.4,141.5	Favored (4.2%) *mp* chi angles: 302.7,90	0.09Å	Favored (56.386%) beta sheet	-	-	-
A 168		TRP	1.13	-	Favored (14.55%)	Favored (94.7%) *m100* chi angles: 294.1,89.2	0.07Å	Favored (27.127%) beta sheet	-	-	-

					General / -94.0,160.8						
A 169		LEU	0.98	-	Favored (51.81%) General / -130.1,147.0	Favored (80.3%) *mt* chi angles: 297.2,170.5	0.13Å	Favored (49.336%)	-	-	-
A 170		THR	0.89	-	Favored (47.9%) General / -139.3,154.1	Favored (9.4%) *t* chi angles: 185.3	0.03Å	Favored (39.406%)	-	-	-
A 171		ILE	0.84	-	Favored (33.35%) Ile or Val / -73.7,132.5	Favored (44.7%) *mm* chi angles: 299,296.8	0.08Å	Favored (38.773%)	-	-	-
A 172		ARG	0.82	-	Favored (46.81%) General / -72.5,138.3	Favored (77.2%) *ttm-80* chi angles: 189.1,181,298.7,275.7	0.03Å	Favored (40.273%) beta sheet	-	-	-
A 173		GLU	0.81	-	Favored (18.74%) General / -87.3,-34.7	Favored (95.7%) *mt-10* chi angles: 296.6,177,359.4	0.06Å	Favored (13.717%)	-	-	-
A 174		GLU	0.81	-	Favored (26.38%) General / -101.9,147.6	Favored (97.1%) *mt-10* chi angles: 295.8,180.7,356.9	0.03Å	Favored (17.626%)	-	-	-
A 175		ASN	0.81	-	Favored (17.37%) General / -85.4,107.4	Favored (44.2%) *t0* chi angles: 181.9,329	0.03Å	Favored (13.79%)	-	-	-
A 176		THR	0.81	-	Favored (8.35%) General / -139.5,176.9	Favored (11.7%) *t* chi angles: 189.8	0.02Å	Favored (28.537%) beta sheet	-	-	-
A 177		THR	0.82	-	Favored (9.21%) General / -123.2,4.6	Favored (69.4%) *p* chi angles: 59.2	0.04Å	Favored (12.121%) beta sheet	-	-	-
A 178		GLU	0.82	-	Favored (31.36%) General / -79.2,151.4	Favored (95.3%) *mt-10* chi angles: 292.8,183,345.3	0.05Å	Favored (42.21%)	-	-	-
A 179		CYS	0.84	-	Favored (22.84%) General / -77.5,166.6	Favored (70%) *m* chi angles: 298.5	0.04Å	Favored (34.129%)	-	-	-
A 180		ASP	0.86	-	Favored (17.41%) General / -83.7,109.4	Favored (62.4%) *t0* chi angles: 187.9,340.3	0.03Å	Favored (19.581%)	-	-	-
**#**	**Alt**	**Res**	**High B**	**Clash > 0.4Å**	**Ramachandran**	**Rotamer**	**Cβ deviation**	**CaBLAM**	**Bond lengths**	**Bond angles**	**Cis Peptides**
			Avg: 1.14	Clashscore: 2.52	Outliers: 1 of 350	Poor rotamers: 0 of 307	Outliers: 0 of 323	Outliers: 15 of 348	Outliers: 4 of 352	Outliers: 6 of 352	Non-Trans: 2 of 351
A 181		SER	0.9	-	Favored (63.1%) General / -60.4,-22.2	Favored (94.9%) *p* chi angles: 64.8	0.05Å	Favored (31.905%)	-	-	-
A 182		ALA	0.93	-	Favored (67.97%) General / -61.0,-26.9	-	0.05Å	Favored (40.183%)	-	-	-
A 183		ILE	0.95	-	Favored (7.05%) Ile or Val / -115.2,19.8	Favored (39.4%) *pt* chi angles: 60,168.5	0.03Å	Favored (28.214%)	-	-	-

A 184		ILE	0.98	-	Favored (62.26%) Ile or Val / -110.6,131.2	Favored (86.8%) *mt* chi angles: 298.9,172.4	0.03Å	Favored (25.873%)	-	-	-
A 185		GLY	1.01	-	Favored (19.57%) Glycine / -130.7,151.4	-	-	Favored (62.439%)	-	-	-
A 186		THR	1.05	-	Favored (33.49%) General / -140.9,137.9	Favored (87.1%) *m* chi angles: 301.4	0.04Å	Favored (51.362%) beta sheet	-	-	-
A 187		ALA	1.1	-	Favored (35.79%) General / -153.2,155.0	-	0.04Å	Favored (42.413%) beta sheet	-	-	-
A 188		ILE	1.14	-	Favored (58.52%) Ile or Val / -132.7,134.5	Favored (16.4%) *tt* chi angles: 181.7,165.7	0.02Å	Favored (59.392%)	-	-	-
A 189		LYS	1.16	-	Favored (8.08%) General / -136.7,110.4	Favored (51.5%) *mttp* chi angles: 294.8,177.8,178.6,57.7	0.02Å	Favored (12.85%)	-	-	-
A 190		GLY	1.15	-	Favored (38.37%) Glycine / 60.1,-125.2	-	-	Favored (68.18%)	-	-	-
A 191		ASP	1.11	-	Favored (35.79%) General / -92.4,9.1	Favored (47.7%) *p0* chi angles: 59.7,355	0.05Å	Favored (11.041%)	-	-	-
A 192		ARG	1.04	-	Favored (44.57%) General / -117.5,144.9	Favored (83.9%) *mtp180* chi angles: 294.9,167.5,68.3,185.9	0.04Å	Favored (31.636%)	-	-	-
A 193		ALA	0.98	-	Favored (36.08%) General / -140.0,140.0	-	0.03Å	Favored (68.266%)	-	-	-
A 194		VAL	0.92	-	Favored (34.4%) Ile or Val / -131.6,150.7	Favored (24.4%) *m* chi angles: 295.3	0.04Å	Favored (61.722%) beta sheet	-	-	-
A 195		HIS	0.89	-	Favored (42.16%) General / -137.6,144.6	Favored (44.4%) *m90* chi angles: 295.7,68.8	0.07Å	Favored (42.628%) beta sheet	-	-	-
A 196		SER	0.87	-	Favored (26.8%) General / -161.6,166.5	Favored (61.8%) *p* chi angles: 73	0.11Å	Favored (24.877%)	-	-	-
A 197		ASP	0.86	-	Favored (5.32%) General / -146.0,-176.9	Favored (6.1%) *p0* chi angles: 70.1,35.2	0.09Å	Favored (29.235%)	-	OUTLIER(S) worst is CA-CB-CG: 8.0 σ	-
A 198		LEU	0.85	-	Favored (66.83%) General / -66.2,-25.1	Favored (95.8%) *mt* chi angles: 296.7,176.5	0.14Å	Favored (23.87%)	-	-	-
A 199		SER	0.84	-	Favored (32.05%) General / -103.0,0.3	Favored (92.2%) *p* chi angles: 66.5	0.10Å	Favored (29.37%) alpha helix	-	-	-
A 200		TYR	0.83	-	Favored (17.02%) General / -154.0,138.7	Favored (86.1%) *t80* chi angles: 180.9,82.1	0.02Å	Favored (26.984%)	-	-	-

#	Alt	Res	High B	Clash > 0.4Å	Ramachandran	Rotamer	Cβ deviation	CaBLAM	Bond lengths	Bond angles	Cis Peptides
			Avg: 1.14	Clashscore: 2.52	Outliers: 1 of 350	Poor rotamers: 0 of 307	Outliers: 0 of 323	Outliers: 15 of 348	Outliers: 4 of 352	Outliers: 6 of 352	Non-Trans: 2 of 351
A 201		TRP	0.82	-	Favored (35.94%) General / -139.9,138.9	Favored (78.9%) *t60* chi angles: 175.8,89.4	0.02Å	Favored (60.316%)	-	-	-
A 202		ILE	0.81	-	Favored (33.67%) Ile or Val / -139.9,141.7	Favored (19.8%) *tt* chi angles: 183.2,167.4	0.05Å	Favored (63.089%) beta sheet	-	-	-
A 203		GLU	0.83	-	Favored (52.91%) General / -127.2,137.6	Favored (48.1%) *tt0* chi angles: 180,173.1,46.3	0.03Å	Favored (64.091%) beta sheet	-	-	-
A 204		SER	0.86	-	Favored (31.18%) General / -131.4,162.0	Favored (91.6%) *p* chi angles: 63.1	0.09Å	Favored (54.455%) beta sheet	-	-	-
A 205		LYS	0.9	-	Favored (41.5%) General / -137.7,143.8	Favored (87.9%) *tttt* chi angles: 183.5,175.4,184.8,181.1	0.04Å	Favored (43.835%) beta sheet	-	-	-
A 206		LYS	0.93	-	Favored (33.53%) General / -86.4,125.2	Favored (86.3%) *tttt* chi angles: 186.3,177,179.2,181	0.03Å	Favored (13.515%)	-	-	-
A 207		ASN	0.95	-	Allowed (0.31%) General / -126.6,-144.7	Favored (66.3%) *m-40* chi angles: 287.4,290	0.07Å	CaBLAM Outlier (0.923%)	-	-	-
A 208		GLU	0.95	-	Favored (49.37%) General / -60.8,-17.4	Favored (28.9%) *pm20* chi angles: 63.2,273.6,14.5	0.02Å	CA Geom Outlier (0.036%)	-	-	-
A 209		THR	0.92	-	Favored (43.71%) General / -150.2,160.4	Favored (12.6%) *t* chi angles: 187.7	0.04Å	Favored (20.183%)	-	-	-
A 210		TRP	0.87	-	Favored (40.71%) General / -76.0,138.1	Favored (92.3%) *m100* chi angles: 285.9,95.5	0.04Å	Favored (24.398%)	-	-	-
A 211		GLN	0.82	-	Favored (49.27%) General / -135.3,147.6	Favored (11.7%) *mm-40* chi angles: 296,285,264.5	0.09Å	Favored (46.346%) beta sheet	-	-	-
A 212		LEU	0.77	-	Favored (44.91%) General / -66.5,130.9	Favored (5.6%) *tt* chi angles: 190.6,156.4	0.08Å	Favored (41.523%)	-	-	-
A 213		GLU	0.74	-	Favored (6.28%) General / -103.5,-40.6	Favored (73.3%) *mm-30* chi angles: 295.9,289.5,330	0.02Å	Favored (19.309%)	-	-	-
A 214		ARG	0.72	-	Favored (18.01%) General / -156.3,142.2	Favored (54.2%) *ttt90* chi angles: 183.1,174.5,167,91.1	0.06Å	Favored (18.057%)	-	-	-
A 215		ALA	0.73	-	Favored (32.1%) General / -142.0,138.8	-	0.02Å	Favored (64.772%)	-	-	-
A 216		VAL	0.74	-	Favored (61.63%)	Favored (70.8%) *t* chi angles: 178.7	0.03Å	Favored (71.075%) beta sheet	-	-	-

#	Alt	Res	High B	Clash > 0.4Å	Ramachandran	Rotamer	Cβ deviation	CaBLAM	Bond lengths	Bond angles	Cis Peptides
					Ile or Val / -124.7,121.5						
A 217		MET	0.76	-	Favored (41.4%) General / -109.9,120.1	Favored (61.2%) *ttm* chi angles: 182.4,175.5,285.2	0.10Å	Favored (71.712%)	-	-	-
A 218		GLY	0.78	-	Favored (64.36%) Glycine / -71.0,-37.3	-	-	Favored (11.696%)	-	-	-
A 219		GLU	0.8	-	Favored (39.74%) General / -155.3,159.2	Favored (24.5%) *pt0* chi angles: 60.4,187.7,356	0.04Å	Favored (15.703%)	OUTLIER(S) worst is CG--CD: 5.6 σ	-	-
A 220		VAL	0.81	-	Favored (31.1%) Ile or Val / -97.8,135.8	Favored (37.4%) *t* chi angles: 184	0.08Å	Favored (41.079%)	-	-	-
**#**	**Alt**	**Res**	**High B**	**Clash > 0.4Å**	**Ramachandran**	**Rotamer**	**Cβ deviation**	**CaBLAM**	**Bond lengths**	**Bond angles**	**Cis Peptides**
			Avg: 1.14	Clashscore: 2.52	Outliers: 1 of 350	Poor rotamers: 0 of 307	Outliers: 0 of 323	Outliers: 15 of 348	Outliers: 4 of 352	Outliers: 6 of 352	Non-Trans: 2 of 351
A 221		LYS	0.8	-	Favored (47.49%) General / -138.0,151.0	Favored (56.8%) *pttt* chi angles: 61.3,187.9,180.6,182.6	0.07Å	Favored (56.885%) beta sheet	-	-	-
A 222		SER	0.79	-	Favored (56.62%) General / -84.3,-2.2	Favored (92%) *p* chi angles: 63.1	0.09Å	Favored (9.174%) beta sheet	-	-	-
A 223		CYS	0.77	-	Favored (27.13%) General / -77.0,163.0	Favored (23%) *p* chi angles: 69.3	0.07Å	Favored (33.762%) beta sheet	-	-	-
A 224		THR	0.76	-	Favored (32.57%) General / -116.0,152.9	Favored (62.7%) *p* chi angles: 63.5	0.07Å	Favored (32.376%) beta sheet	-	-	-
A 225		TRP	0.75	-	Favored (78.58%) Pre-Pro / -74.7,143.5	Favored (16%) *m-10* chi angles: 287.8,39.1	0.08Å	Favored (48.479%) beta sheet	-	-	-
A 226		PRO	0.75	-	Favored (99.53%) Trans-Pro / -59.6,144.0	Favored (73.1%) *Cg_exo* chi angles: 335.1,34.4,330.8	0.06Å	Favored (61.644%)	-	-	-
A 227		GLU	0.75	-	Favored (66.04%) General / -66.8,-21.0	Favored (96.8%) *mt-10* chi angles: 292.2,182.9,360	0.05Å	Favored (39.079%)	-	-	-
A 228		THR	0.76	-	Favored (61.54%) General / -68.1,-12.8	Favored (80.2%) *p* chi angles: 60.5	0.04Å	Favored (38.664%)	-	-	-
A 229		HIS	0.78	-	Favored (39.28%) General / -102.7,9.3	Favored (87.3%) *m-70* chi angles: 289.9,285.6	0.04Å	Favored (54.829%)	-	-	-
A 230		THR	0.8	-	Favored (38.71%) General / -97.5,135.9	Favored (77.9%) *p* chi angles: 60.9	0.08Å	Favored (33.894%)	-	-	-
A 231		LEU	0.81	-	Favored (4.18%) General / -76.9,-56.9	Favored (64.9%) *tp* chi angles: 180.5,62.2	0.05Å	Favored (17.881%)	-	-	-
A 232		TRP	0.82	-	Allowed (0.73%)	Favored (43.3%) *m100*	0.01Å	CaBLAM Disfavored	-	-	-

					General / -105.6,66.4	chi angles: 305.1,120.4		(3.212%)			
A 233		GLY	0.82	-	Favored (69.29%) Glycine / -92.1,-1.9	-	-	CaBLAM Disfavored (4.013%)	-	-	-
A 234		ASP	0.81	-	Favored (20.73%) General / -81.5,115.3	Favored (78.7%) *m-30* chi angles: 292.8,325.9	0.02Å	CaBLAM Disfavored (4.177%)	-	-	-
A 235		GLY	0.79	-	Favored (80.5%) Glycine / 85.2,9.2	-	-	Favored (42.342%)	-	-	-
A 236		VAL	0.78	-	Favored (32.79%) Ile or Val / -67.0,131.2	Favored (84%) *t* chi angles: 173.5	0.03Å	Favored (20.812%)	-	-	-
A 237		VAL	0.76	-	Favored (64.68%) Ile or Val / -110.2,122.4	Favored (80.6%) *t* chi angles: 177.9	0.05Å	Favored (63.639%)	-	-	-
A 238		GLU	0.75	-	Favored (66.16%) General / -54.0,-39.4	Favored (70.9%) *tp30* chi angles: 182.2,67.2,18.9	0.02Å	Favored (50.41%)	-	-	-
A 239		SER	0.75	-	Favored (63.25%) General / -63.8,-17.6	Favored (86.9%) *p* chi angles: 67.1	0.03Å	Favored (56.72%) alpha helix	-	-	-
A 240		GLU	0.75	-	Favored (40.48%) General / -96.8,-4.5	Favored (96.5%) *mt-10* chi angles: 296.2,180.7,357.6	0.01Å	Favored (56.498%)	-	-	-

#	Alt	Res	High B	Clash > 0.4Å	Ramachandran	Rotamer	Cβ deviation	CaBLAM	Bond lengths	Bond angles	Cis Peptides
			Avg: 1.14	Clashscore: 2.52	Outliers: 1 of 350	Poor rotamers: 0 of 307	Outliers: 0 of 323	Outliers: 15 of 348	Outliers: 4 of 352	Outliers: 6 of 352	Non-Trans: 2 of 351
A 241		MET	0.75	0.42Å O with A 262 THR HA	Favored (33.51%) General / -80.5,143.2	Favored (46.2%) *mmp* chi angles: 299.7,295.6,98.1	0.12Å	Favored (31.899%)	-	-	-
A 242		ILE	0.75	-	Favored (23.15%) Ile or Val / -78.5,-48.0	Favored (40.5%) *mm* chi angles: 299.2,304	0.02Å	Favored (13.405%)	-	-	-
A 243		ILE	0.75	-	Favored (61.29%) Pre-Pro / -97.4,120.2	Favored (87.1%) *mt* chi angles: 298.8,172.2	0.05Å	Favored (24.618%)	-	-	-
A 244		PRO	0.75	-	Favored (94.5%) Trans-Pro / -57.4,143.2	Favored (80%) *Cg_exo* chi angles: 334.7,33.7,332.5	0.10Å	Favored (66.641%)	-	-	-
A 245		VAL	0.75	-	Favored (73.85%) Ile or Val / -63.1,-36.0	Favored (66.7%) *t* chi angles: 171.7	0.10Å	Favored (42.346%)	-	-	-
A 246		THR	0.75	-	Favored (33.63%) General / -65.2,-10.6	Favored (64.4%) *p* chi angles: 58.1	0.03Å	Favored (32.782%)	-	-	-
A 247		LEU	0.74	-	Favored (26.55%) General / -108.7,11.3	Favored (90.2%) *mt* chi angles: 296.9,178.7	0.06Å	Favored (14.809%)	-	-	-
A 248		GLY	0.73	-	Favored (35.57%) Glycine / 105.6,8.1	-	-	Favored (59.685%)	-	-	-

A 249		GLY	0.71	-	Favored (28.96%) Glycine / -83.8,152.7	-	-	Favored (34.772%)	-	-	-
A 250		PRO	0.7	-	Favored (90.62%) Trans-Pro / -64.5,148.0	Favored (48.1%) *Cg_exo* chi angles: 337.9,30.5,334.4	0.12Å	Favored (63.354%)	-	-	-
A 251		LYS	0.69	-	Favored (7.37%) General / -85.6,86.4	Favored (87%) *tttt* chi angles: 185.5,177.3,182.9,182.2	0.04Å	Favored (10.381%)	-	-	-
A 252		SER	0.68	-	Favored (26.8%) General / -152.2,147.8	Favored (35.2%) *t* chi angles: 174.4	0.07Å	Favored (18.98%)	-	-	-
A 253		HIS	0.69	-	Favored (64.76%) General / -62.3,-20.9	Favored (86.3%) *m-70* chi angles: 290.8,293.4	0.05Å	Favored (34.831%)	-	-	-
A 254		HIS	0.7	-	Favored (60.68%) General / -69.8,-11.2	Favored (29.1%) *p-80* chi angles: 79.5,283	0.07Å	Favored (57.537%)	OUTLIER(S) worst is CB--CG: 6.0 σ	-	-
A 255		ASN	0.72	-	Favored (46.03%) General / -92.7,6.8	Favored (66.6%) *m-40* chi angles: 285,281.8	0.10Å	Favored (40.9%)	-	OUTLIER(S) worst is CA-CB-CG: 4.8 σ	-
A 256		LYS	0.75	-	Favored (24.15%) General / -89.4,147.3	Favored (86.9%) *tttt* chi angles: 183.3,173.7,178.3,179.8	0.05Å	Favored (26.848%)	-	-	-
A 257		ARG	0.77	-	Favored (20.39%) General / -136.9,123.0	Favored (16.7%) *tpt90* chi angles: 181.2,77,171.1,102.4	0.06Å	Favored (12.746%)	-	-	-
A 258		ASN	0.79	-	Favored (45.1%) General / -58.1,130.1	Favored (96.9%) *m-40* chi angles: 289.1,335.2	0.05Å	Favored (23.955%)	-	-	-
A 259		GLY	0.8	-	Favored (82.91%) Glycine / 89.8,-3.1	-	-	Favored (76.334%)	-	-	-
A 260		TYR	0.8	-	Favored (30.93%) General / -118.0,155.1	Favored (97.8%) *m-80* chi angles: 297.5,88.3	0.05Å	Favored (35.598%)	-	-	-
**#**	**Alt**	**Res**	**High B**	**Clash > 0.4Å**	**Ramachandran**	**Rotamer**	**Cβ deviation**	**CaBLAM**	**Bond lengths**	**Bond angles**	**Cis Peptides**
			Avg: 1.14	Clashscore: 2.52	Outliers: 1 of 350	Poor rotamers: 0 of 307	Outliers: 0 of 323	Outliers: 15 of 348	Outliers: 4 of 352	Outliers: 6 of 352	Non-Trans: 2 of 351
A 261		TYR	0.79	-	Favored (6.58%) General / -116.0,173.4	Favored (84.9%) *m-80* chi angles: 300.1,87.2	0.04Å	Favored (5.104%)	-	-	-
A 262		THR	0.77	0.42Å HA with A 241 MET O	Favored (36.56%) General / -56.3,128.6	Favored (43.2%) *m* chi angles: 304.8	0.03Å	Favored (12.778%)	-	-	-
A 263		GLN	0.75	-	Favored (8.95%) General / -83.3,66.8	Favored (78.5%) *mm-40* chi angles: 301.4,292.6,295.5	0.07Å	Favored (18.195%)	-	-	-
A 264		THR	0.75	-	Favored (61.15%) General / -73.1,-13.2	Favored (79.1%) *p* chi angles: 60.7	0.01Å	Favored (12.399%)	-	-	-

A 265		LYS	0.76	-	Favored (6.1%) General / -122.4,31.5	Favored (95.4%) *mttt* chi angles: 298,184.6,181,179.4	0.03Å	Favored (27.369%)	-	-	-
A 266		GLY	0.79	-	Favored (37.5%) Glycine / -75.1,-176.1	-	-	Favored (45.248%)	-	-	-
A 267		PRO	0.82	-	Favored (8.57%) Trans-Pro / -78.4,65.0	Favored (59.9%) *Cg_endo* chi angles: 31.9,324.8,23.8	0.07Å	CaBLAM Disfavored (1.19%)	-	-	-
A 268		TRP	0.86	-	Favored (13.09%) General / -96.2,18.7	Favored (59.7%) *m100* chi angles: 297.9,118.5	0.07Å	CaBLAM Disfavored (1.055%)	-	-	-
A 269		SER	0.89	-	Favored (21.77%) General / -96.0,-16.3	Favored (96.7%) *p* chi angles: 63.7	0.07Å	CaBLAM Outlier (0.797%)	-	OUTLIER(S) worst is O-C-N: 4.8 σ	-
A 270		GLU	0.9	-	Favored (2.13%) General / -93.8,29.2	Favored (60.4%) *mt-10* chi angles: 298.5,186.9,23.8	0.03Å	Favored (7.444%)	-	-	Twisted nonPRO omega= -143.99
A 271		GLY	0.89	-	Favored (22.05%) Glycine / 64.5,-121.3	-	-	Favored (7.495%)	-	-	-
A 272		GLU	0.86	-	Favored (18.43%) General / -72.3,120.8	Favored (89.8%) *tt0* chi angles: 183.1,177.2,353.3	0.04Å	CaBLAM Disfavored (2.626%)	-	-	-
A 273		ILE	0.82	-	Favored (65.17%) Ile or Val / -129.4,127.4	Favored (68.6%) *mt* chi angles: 301.7,166.9	0.08Å	Favored (57.629%)	-	-	-
A 274		ILE	0.79	-	Favored (72.94%) Ile or Val / -115.3,126.5	Favored (86.5%) *mt* chi angles: 298.8,171.5	0.04Å	Favored (70.639%) beta sheet	-	-	-
A 275		LEU	0.76	-	Favored (32.19%) General / -102.1,116.2	Favored (48.4%) *tp* chi angles: 172.9,60.1	0.01Å	Favored (51.877%) beta sheet	-	-	-
A 276		ASP	0.74	-	Favored (23.96%) General / -123.4,161.8	Favored (50.4%) *m-30* chi angles: 287.5,316.9	0.02Å	Favored (34.551%)	-	-	-
A 277		PHE	0.72	-	Favored (4.1%) General / -117.2,93.2	Favored (99.6%) *m-80* chi angles: 297.3,96.8	0.06Å	CaBLAM Disfavored (2.985%)	-	OUTLIER(S) worst is CA-CB-CG: 4.7 σ	-
A 278		ASP	0.7	-	Favored (3.09%) General / -171.1,179.2	Favored (11.9%) *t0* chi angles: 204.5,336.8	0.06Å	Favored (7.094%)	-	-	-
A 279		TYR	0.68	-	Favored (25.85%) General / -94.7,144.2	Favored (86.4%) *m-80* chi angles: 295,83.5	0.08Å	Favored (9.235%)	-	-	-
A 280		CYS	0.66	-	Favored (96.53%) Pre-Pro / -65.6,137.7	Favored (95.7%) *m* chi angles: 292.8	0.05Å	Favored (24.351%)	-	-	-
**#**	**Alt**	**Res**	**High B**	**Clash > 0.4Å**	**Ramachandran**	**Rotamer**	**Cβ deviation**	**CaBLAM**	**Bond lengths**	**Bond angles**	**Cis Peptides**
			Avg: 1.14	Clashscore: 2.52	Outliers: 1 of 350	Poor rotamers: 0 of 307	Outliers: 0 of 323	Outliers: 15 of 348	Outliers: 4 of 352	Outliers: 6 of 352	Non-Trans: 2 of 351

A 281		PRO	0.65	-	Favored (47.75%) Trans-Pro / -52.0,134.1	Favored (86.6%) *Cg_exo* chi angles: 330.4,37.4,330.8	0.07Å	Favored (41.12%)	-	-	-
A 282		GLY	0.64	-	Favored (86.66%) Glycine / 82.4,-1.8	-	-	Favored (71.729%)	-	-	-
A 283		THR	0.65	-	Favored (23.9%) General / -123.6,162.0	Favored (42.4%) *p* chi angles: 67.3	0.09Å	Favored (34.043%)	-	-	-
A 284		THR	0.67	-	Favored (46.93%) General / -128.8,153.9	Favored (61.6%) *p* chi angles: 63.7	0.04Å	Favored (65.914%)	-	-	-
A 285		VAL	0.7	-	Favored (70.45%) Ile or Val / -127.5,131.2	Favored (61.5%) *t* chi angles: 179.8	0.04Å	Favored (66.529%) beta sheet	-	-	-
A 286		THR	0.75	-	Favored (45.32%) General / -118.5,145.4	Favored (9.9%) *t* chi angles: 186	0.06Å	Favored (55.383%) beta sheet	-	-	-
A 287		VAL	0.81	-	Favored (43.95%) Ile or Val / -92.0,128.0	Favored (78.2%) *t* chi angles: 178.1	0.05Å	Favored (15.459%) beta sheet	-	-	-
A 288		THR	0.89	-	Favored (11.67%) General / -161.7,143.9	Favored (4.2%) *t* chi angles: 179.1	0.04Å	Favored (9.623%)	-	-	-
A 289		GLU	0.96	-	Favored (66.26%) General / -63.2,-21.5	Favored (69.1%) *mm-30* chi angles: 289.3,293.3,311.4	0.04Å	Favored (43.868%)	-	-	-
A 290		HIS	1	-	Favored (40.11%) General / -84.7,2.9	Favored (94.8%) *m-70* chi angles: 294,291.2	0.03Å	Favored (37.835%)	-	-	-
A 291		CYS	1.02	-	Favored (79.92%) General / -68.1,-36.5	Favored (21.7%) *t* chi angles: 190.7	0.05Å	CaBLAM Disfavored (3.871%)	-	-	-
A 292		GLY	0.99	-	Favored (19.48%) Glycine / 108.7,-175.6	-	-	Favored (22.376%)	-	-	-
A 293		ASN	0.94	-	Favored (8.94%) General / -91.7,171.8	Favored (14.6%) *t0* chi angles: 203.2,351.2	0.05Å	CaBLAM Disfavored (2.899%)	-	-	-
A 294		ARG	0.87	-	Favored (50.79%) General / -64.6,148.4	Favored (33.5%) *mtp-110* chi angles: 296.1,176,63.5,254	0.07Å	Favored (5.732%)	-	-	-
A 295		GLY	0.8	-	Favored (21.99%) Glycine / -160.2,-168.3	-	-	Favored (12.636%)	-	-	-
A 296		ALA	0.74	-	Favored (41.06%) General / -60.5,147.5	-	0.03Å	CaBLAM Disfavored (4.609%)	-	-	-
A 297		SER	0.69	-	Favored (47.37%) General / -56.6,140.2	Favored (23.8%) *t* chi angles: 171.2	0.04Å	Favored (30.742%)	-	-	-
A 298		LEU	0.67	-	Favored (50.45%)	Favored (71.1%) *mt* chi angles: 301.3,173.2	0.06Å	Favored (47.512%)	-	-	-

#	Alt	Res	High B	Clash > 0.4Å	Ramachandran	Rotamer	Cβ deviation	CaBLAM	Bond lengths	Bond angles	Cis Peptides
					General / -115.5,139.0			beta sheet			
A 299		ARG	0.66	-	Favored (32.68%) General / -79.0,147.9	Favored (39.2%) *ttp-170* chi angles: 197.8,169.4,70.6,179.4	0.03Å	Favored (41.358%)	-	-	-
A 300		THR	0.66	-	Favored (63.38%) General / -69.2,-15.4	Favored (59.6%) *p* chi angles: 57.3	0.10Å	Favored (49.373%)	-	-	-
**#**	**Alt**	**Res**	**High B**	**Clash > 0.4Å**	**Ramachandran**	**Rotamer**	**Cβ deviation**	**CaBLAM**	**Bond lengths**	**Bond angles**	**Cis Peptides**
			Avg: 1.14	Clashscore: 2.52	Outliers: 1 of 350	Poor rotamers: 0 of 307	Outliers: 0 of 323	Outliers: 15 of 348	Outliers: 4 of 352	Outliers: 6 of 352	Non-Trans: 2 of 351
A 301		THR	0.68	-	Favored (37.62%) General / -116.5,150.6	Favored (66.8%) *p* chi angles: 58.5	0.03Å	Favored (24.172%)	-	-	-
A 302		THR	0.71	-	Favored (18.04%) General / -79.2,169.5	Favored (78.1%) *p* chi angles: 60.3	0.07Å	CaBLAM Outlier (0.158%)	-	-	-
A 303		ALA	0.73	-	Favored (12.9%) General / -48.4,-35.5	-	0.05Å	Favored (6.949%)	-	-	-
A 304		SER	0.73	-	Favored (59.73%) General / -79.9,-12.6	Favored (74.7%) *p* chi angles: 71.3	0.09Å	Favored (34.402%)	-	-	-
A 305		GLY	0.72	-	Favored (69.57%) Glycine / 94.0,-12.2	-	-	Favored (56.646%)	-	-	-
A 306		LYS	0.7	-	Favored (57.77%) General / -60.0,135.6	Favored (87.4%) *tttt* chi angles: 183.2,176.9,178.5,179.2	0.02Å	Favored (34.052%) beta sheet	-	-	-
A 307		LEU	0.68	-	Favored (33.04%) General / -77.0,127.6	Favored (38.8%) *tp* chi angles: 183.3,67	0.05Å	Favored (45.247%) beta sheet	-	-	-
A 308		VAL	0.65	-	Favored (36.5%) Ile or Val / -86.1,120.8	Favored (71.5%) *t* chi angles: 178.6	0.15Å	Favored (52.368%) beta sheet	-	-	-
A 309		THR	0.64	-	Favored (12.41%) General / -113.2,-17.1	Favored (73.5%) *p* chi angles: 61.6	0.04Å	Favored (12.622%)	-	-	-
A 310		ASP	0.65	-	Favored (6.32%) General / -105.1,96.1	Favored (64.1%) *t0* chi angles: 185.2,354.8	0.06Å	Favored (17.389%)	-	OUTLIER(S) worst is CA-CB-CG: 5.0 σ	-
A 311		TRP	0.68	-	Favored (18.7%) General / -112.5,159.5	Favored (68.3%) *m100* chi angles: 293.6,76	0.03Å	Favored (16.568%)	-	-	-
A 312		CYS	0.73	-	Favored (23.66%) General / -143.3,166.8	Favored (16.5%) *p* chi angles: 56.7	0.08Å	Favored (64.623%) beta sheet	-	-	-
A 313		CYS	0.78	-	Favored (32%) General / -139.3,134.0	Favored (39.7%) *t* chi angles: 187.1	0.04Å	Favored (45.735%)	-	-	-
A 314		ARG	0.84	-	Favored (59.85%)	Favored (79.2%) *ttt180*	0.07Å	Favored (10.364%)	-	-	-

					General / -60.5,-53.2	chi angles: 182.7,177.2,180.1,189.3					
A 315		SER	0.87	-	Allowed (1.05%) General / -146.4,8.9	Favored (82.3%) *p* chi angles: 62.2	0.02Å	CaBLAM Outlier (0.71%)	-	-	-
A 316		CYS	0.87	-	Favored (29.49%) General / -76.3,160.6	Favored (23%) *p* chi angles: 69.3	0.06Å	Favored (35.457%)	-	-	-
A 317		SER	0.84	-	Favored (25.37%) General / -80.9,158.0	Favored (97.1%) *p* chi angles: 65.9	0.02Å	Favored (39.497%)	-	-	-
A 318		LEU	0.79	-	Favored (40.52%) Pre-Pro / -116.5,159.4	Favored (69.1%) *mt* chi angles: 303.6,179.3	0.07Å	CA Geom Outlier (0.039%)	-	-	-
A 319		PRO	0.74	-	Favored (19.31%) Cis-Pro / -57.1,141.8	Favored (76%) *Cg_exo* chi angles: 334.9,36.2,328	0.03Å	Favored (18.541%)	-	-	Cis PRO omega= 1.72
A 320		PRO	0.7	-	Favored (73.98%) Trans-Pro / -68.9,150.9	Favored (51.5%) *Cg_endo* chi angles: 25.4,328.6,24.1	0.04Å	Favored (77.199%)	-	-	-
**#**	**Alt**	**Res**	**High B**	**Clash > 0.4Å**	**Ramachandran**	**Rotamer**	**Cβ deviation**	**CaBLAM**	**Bond lengths**	**Bond angles**	**Cis Peptides**
			Avg: 1.14	Clashscore: 2.52	Outliers: 1 of 350	Poor rotamers: 0 of 307	Outliers: 0 of 323	Outliers: 15 of 348	Outliers: 4 of 352	Outliers: 6 of 352	Non-Trans: 2 of 351
A 321		LEU	0.67	-	Favored (12.73%) General / -77.1,111.5	Favored (4.4%) *tt* chi angles: 194.3,159.2	0.10Å	Favored (32.917%) beta sheet	-	-	-
A 322		ARG	0.67	-	Favored (51.05%) General / -132.4,153.2	Favored (57.4%) *ptt90* chi angles: 64.5,177.4,177.2,86.8	0.07Å	Favored (45.181%) beta sheet	-	-	-
A 323		TYR	0.68	-	Favored (47.92%) General / -119.9,143.5	Favored (98.4%) *m-80* chi angles: 295.5,91.3	0.04Å	Favored (63.908%) beta sheet	-	-	-
A 324		THR	0.7	-	Favored (55.38%) General / -117.3,128.8	Favored (97.2%) *m* chi angles: 299.9	0.04Å	Favored (52.048%)	-	-	-
A 325		THR	0.71	-	Favored (5.53%) General / -128.7,179.8	Favored (17.5%) *p* chi angles: 73.7	0.05Å	Favored (21.831%)	-	-	-
A 326		LYS	0.72	-	Favored (54.22%) General / -59.7,-20.1	Favored (14.2%) *pttp* chi angles: 71.4,181.2,181.2,70.1	0.03Å	Favored (42.931%)	-	-	-
A 327		ASP	0.71	-	Favored (45.25%) General / -97.9,8.1	Favored (86.5%) *m-30* chi angles: 294.6,342.4	0.03Å	Favored (33.095%)	-	-	-
A 328		GLY	0.69	-	Favored (13.73%) Glycine / 117.7,-170.9	-	-	Favored (28.671%)	-	-	-
A 329		CYS	0.66	-	Favored (35.75%) General / -95.5,136.6	Favored (31.1%) *t* chi angles: 189.2	0.03Å	Favored (8.481%)	-	-	-
A 330		TRP	0.64	-	Favored (39.7%)	Favored (60.6%) *m100*	0.13Å	Favored (55.553%)	-	-	-

					General / -119.0,150.6	chi angles: 294.2,73.7		beta sheet			
A 331		TYR	0.63	-	Favored (25.55%) General / -103.1,148.6	Favored (38.3%) *m-80* chi angles: 281.8,109	0.08Å	Favored (29.403%)	-	-	-
A 332		GLY	0.62	-	Favored (8.78%) Glycine / -62.6,173.7	-	-	Favored (33.154%)	-	-	-
A 333		MET	0.63	-	Favored (68.47%) General / -62.1,-26.7	Favored (91.9%) *mtp* chi angles: 291.9,183.3,68.6	0.02Å	Favored (40.53%)	-	-	-
A 334		GLU	0.65	-	Favored (59.42%) General / -82.4,-9.5	Favored (54.4%) *mp0* chi angles: 292.2,74.4,9.8	0.00Å	Favored (57.736%)	-	-	-
A 335		ILE	0.68	-	Favored (69.37%) Ile or Val / -112.4,124.7	Favored (77.8%) *mt* chi angles: 300.7,170.3	0.10Å	Favored (19.45%)	-	-	-
A 336		ARG	0.71	-	Favored (80.65%) Pre-Pro / -132.3,156.0	Favored (65.6%) *mtp180* chi angles: 296.5,181,63.5,170	0.01Å	Favored (34.6%)	-	-	-
A 337		PRO	0.76	-	Favored (94.97%) Trans-Pro / -62.1,147.5	Favored (53.3%) *Cg_exo* chi angles: 337,36.8,324.8	0.04Å	Favored (54.899%)	-	-	-
A 338		VAL	0.81	-	Favored (17.4%) Ile or Val / -85.2,-45.2	Favored (92.9%) *t* chi angles: 175.9	0.06Å	Favored (16.863%)	-	-	-
A 339		LYS	0.85	-	Favored (5.94%) General / -112.4,-34.2	Favored (94.4%) *mttt* chi angles: 297.9,178.4,180.8,173.6	0.07Å	Favored (5.547%)	-	-	-
A 340		GLU	0.88	0.55Å N with A 340 GLU OE1	Favored (58.7%) General / -63.4,138.9	Favored (45.4%) *mp0* chi angles: 295,81,344.7	0.07Å	Favored (32.111%)	-	-	-
**#**	**Alt**	**Res**	**High B**	**Clash > 0.4Å**	**Ramachandran**	**Rotamer**	**Cβ deviation**	**CaBLAM**	**Bond lengths**	**Bond angles**	**Cis Peptides**
			Avg: 1.14	Clashscore: 2.52	Outliers: 1 of 350	Poor rotamers: 0 of 307	Outliers: 0 of 323	Outliers: 15 of 348	Outliers: 4 of 352	Outliers: 6 of 352	Non-Trans: 2 of 351
A 341		GLU	0.88	0.41Å OE1 with A 344 LYS NZ	Favored (43.04%) General / -60.1,129.7	Favored (90.1%) *tt0* chi angles: 185.3,180.8,3.2	0.03Å	Favored (43.555%)	-	-	-
A 342		GLU	0.85	-	Favored (73.1%) General / -58.5,-36.0	Favored (86.9%) *tt0* chi angles: 183.9,171.3,357.6	0.05Å	Favored (48.96%)	-	-	-
A 343		ALA	0.81	-	Favored (64.34%) General / -60.0,-24.4	-	0.03Å	Favored (63.318%) alpha helix	-	-	-
A 344		LYS	0.76	0.41Å NZ with A 341 GLU OE1	Favored (46.19%) General / -99.1,7.1	Favored (52.5%) *mttp* chi angles: 294.3,179.7,171.4,67.7	0.03Å	Favored (50.543%)	-	-	-
A 345		LEU	0.74	-	Favored (17.81%) General / -91.5,156.3	Favored (75.1%) *mt* chi angles: 302.6,176.4	0.07Å	Favored (38.095%)	-	-	-

A 346		VAL	0.76	-	Favored (54.66%) Ile or Val / -101.0,126.5	Favored (86.6%) *t* chi angles: 177.4	0.08Å	Favored (52.408%)	-	-	-
A 347		LYS	0.83	-	Favored (48.01%) General / -131.4,155.0	Favored (60.8%) *pttt* chi angles: 66.4,182.3,183.8,180.6	0.02Å	Favored (41.011%)	-	-	-
A 348		SER	0.97	-	Favored (58.38%) General / -64.0,142.5	Favored (41.3%) *t* chi angles: 176.3	0.05Å	Favored (34.783%)	-	-	-
A 349		ARG	1.2	-	Favored (15.74%) General / -115.4,19.0	Favored (97%) *mtt180* chi angles: 296.6,183.4,178.7,183.8	0.04Å	Favored (12.101%)	-	-	-
A 350		VAL	1.5	-	Favored (69.18%) Ile or Val / -111.8,126.0	Favored (81%) *t* chi angles: 176.6	0.07Å	Favored (26.206%)	-	-	-
A 351		THR	1.86	-	Favored (40.97%) General / -117.2,121.4	Favored (85.1%) *m* chi angles: 301.7	0.02Å	-	-	-	-
A 352		ALA	2.23	-	-	-	0.03Å	-	-	-	-

About MolProbity | Website for the Richardson Lab | Using ecloud x-H | Internal reference 4.5.2
