# Supplementary material for: Integrative proteome-wide structural analysis and high-throughput docking identify broad-spectrum antiviral scaffolds against Zika, Yellow Fever, West Nile, Saint Louis encephalitis, and Usutu viruses
Source: Front Cell Infect Microbiol. 2026 Apr 30;16:1723132. doi: 10.3389/fcimb.2026.1723132 (PMC13171538; doi:10.3389/fcimb.2026.1723132)
Supplement: Supplementary file 3 [file DataSheet3.zip › SLEV/SLEV_NS2a/Mol_probity_Files/SLEV_NS2a_1FH-multi.table.pdf]

Viewing SLEV_NS2a1FH-multi.table

Duke Biochemistry
Duke University School of Medicine

When finished, you should close this window.

Hint: Use File | Save As... to save a copy of this page.

All-Atom Contacts	Clashscore, all atoms:	2.53		98th percentile* (N=1784, all resolutions)
	Clashscore is the number of serious steric overlaps (> 0.4 Å) per 1000 atoms.			
Protein Geometry	Poor rotamers	1	0.56%	Goal: <0.3%
	Favored rotamers	176	98.88%	Goal: >98%
	Ramachandran outliers	3	1.33%	Goal: <0.05%
	Ramachandran favored	219	97.33%	Goal: >98%
	Rama distribution Z-score	1.09 ± 0.54		Goal: abs(Z score) < 2
	MolProbity score^	1.16		99th percentile* (N=27675, 0Å - 99Å)
	Cβ deviations >0.25Å	0	0.00%	Goal: 0
	Bad bonds:	2 / 1738	0.12%	Goal: 0%
	Bad angles:	4 / 2351	0.17%	Goal: <0.1%
Peptide Omegas	Cis Prolines:	0 / 7	0.00%	Expected: ≤1 per chain, or ≤5%
Low-resolution Criteria	CaBLAM outliers	7	3.1%	Goal: <1.0%
	CA Geometry outliers	1	0.45%	Goal: <0.5%
Additional validations	Chiral volume outliers	0/289		
	Waters with clashes	0/0	0.00%	See UnDowser table for details

In the two column results, the left column gives the raw count, right column gives the percentage.

* 100th percentile is the best among structures of comparable resolution; 0th percentile is the worst. For clashscore the comparative set of structures was selected in 2004, for MolProbity score in 2006.

^ MolProbity score combines the clashscore, rotamer, and Ramachandran evaluations into a single score, normalized to be on the same scale as X-ray resolution.

Key to table colors and cutoffs here:

#	Alt	Res	High B	Clash > 0.4Å	Ramachandran	Rotamer	Cβ deviation	CaBLAM	Bond lengths	Bond angles	Cis Peptides
			Avg: 6.07	Clashscore: 2.53	Outliers: 3 of 225	Poor rotamers: 1 of 178	Outliers: 0 of 206	Outliers: 7 of 223	Outliers: 2 of 227	Outliers: 4 of 227	Non-Trans: 0 of 226
A 1		GLY	11.3	-	-	-	-	-	-	-	-
A 2		VAL	10.87	-	Favored (70.05%) Ile or Val / -124.1,125.0	Favored (74.2%) *t* chi angles: 178.4	0.03Å	-	-	-	-
A 3		ALA	10.34	-	Favored (24.71%) General / -81.7,157.3	-	0.04Å	Favored (37.108%)	-	-	-
A 4		GLY	9.75	-	Favored (55.28%) Glycine / -58.4,-24.0	-	-	Favored (58.016%)	-	-	-
A 5		GLY	9.13	-	Favored (84.71%) Glycine / -84.0,-7.9	-	-	Favored (81.516%) alpha helix	-	-	-
A 6		MET	8.54	-	Favored (12.25%) General / -94.8,-35.1	Favored (93.9%) *mtp* chi angles: 290.9,172.7,64.8	0.02Å	Favored (35.016%) alpha helix	-	-	-
A 7		GLU	7.99	-	Favored (76.58%)	Favored (95.4%) *mt-10*	0.09Å	CaBLAM Disfavored	-	-	-

					Pre-Pro / -131.7,65.8	chi angles: 292,186,355.7		(3.337%) try alpha helix			
A 8		PRO	7.48	0.42Å O with A 9 PHE HB2	Favored (2.35%) Trans-Pro / -79.3,-174.6	Favored (56.8%) *Cg_endo* chi angles: 32.3,324.8,24.2	0.07Å	CA Geom Outlier (0.269%)	-	-	-
A 9		PHE	7.03	0.42Å HB2 with A 8 PRO O	OUTLIER (0.02)%) General / 80.5,-113.1	Favored (63.3%) *m-80* chi angles: 305,106.5	0.05Å	CaBLAM Outlier (0.965%)	-	OUTLIER(S) worst is CA-CB-CG: 4.7 σ	-
A 10		GLN	6.63	-	Allowed (0.53%) General / 44.9,-116.4	Favored (92.1%) *mt0* chi angles: 293.1,182.2,331	0.07Å	CaBLAM Outlier (0.266%)	-	-	-
A 11		LEU	6.29	-	Favored (75.21%) General / -62.5,-33.6	Favored (87.7%) *mt* chi angles: 290.4,171.9	0.02Å	Favored (18.018%)	-	-	-
A 12		GLY	5.99	-	Favored (95.07%) Glycine / -65.8,-42.6	-	-	Favored (90.113%) alpha helix	-	-	-
A 13		LEU	5.73	-	Favored (96.82%) General / -64.1,-42.8	Favored (48.1%) *tp* chi angles: 179.3,55.5	0.07Å	Favored (82.96%) alpha helix	-	-	-
A 14		LEU	5.5	-	Favored (80%) General / -56.3,-46.1	Favored (60.4%) *tp* chi angles: 180.9,63.3	0.05Å	Favored (87.213%) alpha helix	-	-	-
A 15		VAL	5.33	-	Favored (99.85%) Ile or Val / -62.7,-44.9	Favored (57.9%) *t* chi angles: 170.5	0.02Å	Favored (94.352%) alpha helix	-	-	-
A 16		ALA	5.21	-	Favored (95.48%) General / -60.2,-42.5	-	0.04Å	Favored (94.242%) alpha helix	-	-	-
A 17		PHE	5.15	-	Favored (57.44%) General / -62.5,-53.2	Favored (92.1%) *t80* chi angles: 176.1,77.3	0.05Å	Favored (82.286%) alpha helix	-	-	-
A 18		ILE	5.16	-	Favored (98.12%) Ile or Val / -62.5,-45.7	Favored (98.7%) *mt* chi angles: 292.6,168.3	0.05Å	Favored (87.312%) alpha helix	-	-	-
A 19		ALA	5.24	-	Favored (85.07%) General / -58.4,-41.5	-	0.04Å	Favored (97.218%) alpha helix	-	-	-
A 20		THR	5.37	-	Favored (87.76%) General / -66.9,-41.0	Favored (99.5%) *m* chi angles: 300.4	0.06Å	Favored (82.83%) alpha helix	-	-	-
**#**	**Alt**	**Res**	**High B**	**Clash > 0.4Å**	**Ramachandran**	**Rotamer**	**Cβ deviation**	**CaBLAM**	**Bond lengths**	**Bond angles**	**Cis Peptides**
			Avg: 6.07	Clashscore: 2.53	Outliers: 3 of 225	Poor rotamers: 1 of 178	Outliers: 0 of 206	Outliers: 7 of 223	Outliers: 2 of 227	Outliers: 4 of 227	Non-Trans: 0 of 226
A 21		GLN	5.57	-	Favored (84.07%) General / -61.8,-37.3	Favored (87.9%) *mm-40* chi angles: 290.9,292.4,302.1	0.03Å	Favored (80.717%) alpha helix	-	-	-
A 22		GLU	5.84	-	Favored (84.91%) General / -66.6,-37.4	Favored (81.4%) *mm-30* chi angles: 290.9,298.3,308	0.04Å	Favored (88.221%) alpha helix	-	-	-

A<br>23		VAL	6.15	-	Favored<br>(85.89%)<br>Ile or Val /<br>-67.9,-42.6	Favored (67.6%) *t*<br>chi angles: 171.8	0.03Å	Favored<br>(77.923%)<br>alpha helix	-	-	-
A<br>24		LEU	6.53	-	Favored<br>(85.12%)<br>General /<br>-57.6,-45.6	Favored (45.8%) *tp*<br>chi angles: 183.3,65	0.08Å	Favored<br>(80.344%)<br>alpha helix	-	-	-
A<br>25		LYS	6.92	-	Favored<br>(82.92%)<br>General /<br>-65.5,-36.1	Favored (56.7%)<br>*mtmt*<br>chi angles:<br>289,185.8,287.7,183.4	0.02Å	Favored<br>(78.14%)	-	-	-
A<br>26		ARG	7.3	-	Favored<br>(10.84%)<br>General /<br>-84.5,173.5	Favored (89.1%)<br>*mtm180*<br>chi angles:<br>294.3,177.3,293,169.6	0.02Å	CaBLAM<br>Disfavored<br>(2.387%)	-	-	-
A<br>27		ARG	7.63	-	Favored<br>(21.97%)<br>General /<br>-75.0,121.4	Favored (81.5%)<br>*ttt180*<br>chi angles:<br>183.5,182.8,177.6,185.6	0.06Å	Favored<br>(16.829%)	-	-	-
A<br>28		TRP	7.89	-	Favored<br>(4.02%)<br>General /<br>-55.9,-60.0	Favored (65.2%)<br>*m100*<br>chi angles: 280.6,91.8	0.11Å	CaBLAM<br>Disfavored<br>(1.678%)	-	-	-
A<br>29		THR	8.06	-	OUTLIER<br>(0.02)%)<br>General /<br>55.0,166.3	Favored (43.3%) *p*<br>chi angles: 67.1	0.04Å	CaBLAM<br>Disfavored<br>(2.01%)	-	-	-
A<br>30		GLY	8.15	-	Favored<br>(79.44%)<br>Glycine /<br>-60.4,-33.0	-	-	Favored<br>(29.356%)	-	-	-
A<br>31		LYS	8.15	-	Favored<br>(89.09%)<br>General /<br>-66.6,-41.0	Favored (97.2%)<br>*mttt*<br>chi angles:<br>289.8,179.3,180.3,178.4	0.02Å	Favored<br>(69.76%)<br>alpha helix	-	-	-
A<br>32		LEU	8.07	-	Favored<br>(29.9%)<br>General /<br>-84.3,-24.1	Favored (95.9%) *mt*<br>chi angles: 297.1,175.9	0.06Å	Favored<br>(70.56%)<br>alpha helix	-	-	-
A<br>33		THR	7.92	-	Favored<br>(75.59%)<br>General /<br>-63.2,-49.1	Favored (93.4%) *m*<br>chi angles: 299.2	0.04Å	Favored<br>(72.696%)<br>alpha helix	-	-	-
A<br>34		LEU	7.69	-	Favored<br>(86.95%)<br>General /<br>-64.9,-37.2	Favored (92.8%) *mt*<br>chi angles: 291.2,172.4	0.03Å	Favored<br>(78.071%)<br>alpha helix	-	-	-
A<br>35		THR	7.43	-	Favored<br>(93.13%)<br>General /<br>-62.2,-45.5	Favored (88.9%) *m*<br>chi angles: 298.6	0.01Å	Favored<br>(88.289%)<br>alpha helix	-	-	-
A<br>36		SER	7.16	-	Favored<br>(96.85%)<br>General /<br>-61.4,-41.3	Favored (71.3%) *m*<br>chi angles: 295.2	0.06Å	Favored<br>(95.674%)<br>alpha helix	-	-	-
A<br>37		LEU	6.93	-	Favored<br>(93.75%)<br>General /<br>-65.0,-39.7	Favored (94.3%) *mt*<br>chi angles: 291.7,171.8	0.03Å	Favored<br>(91.3%)<br>alpha helix	-	-	-
A<br>38		ALA	6.77	-	Favored<br>(88.1%)<br>General /<br>-61.2,-38.9	-	0.03Å	Favored<br>(85.639%)<br>alpha helix	-	-	-
A<br>39		VAL	6.67	-	Favored<br>(88.37%)<br>Ile or Val /<br>-65.8,-46.5	Favored (63.8%) *t*<br>chi angles: 171.3	0.03Å	Favored<br>(83.665%)<br>alpha helix	-	-	-

#	Alt	Res	High B	Clash > 0.4Å	Ramachandran	Rotamer	Cβ deviation	CaBLAM	Bond lengths	Bond angles	Cis Peptides
A 40		CYS	6.64	-	Favored (97.31%) General / -62.6,-44.0	Favored (98.6%) *m* chi angles: 292.6	0.08Å	Favored (87.111%) alpha helix	-	-	-
#	Alt	Res	High B	Clash > 0.4Å	Ramachandran	Rotamer	Cβ deviation	CaBLAM	Bond lengths	Bond angles	Cis Peptides
			Avg: 6.07	Clashscore: 2.53	Outliers: 3 of 225	Poor rotamers: 1 of 178	Outliers: 0 of 206	Outliers: 7 of 223	Outliers: 2 of 227	Outliers: 4 of 227	Non-Trans: 0 of 226
A 41		LEU	6.67	-	Favored (82.09%) General / -67.9,-41.9	Favored (87%) *mt* chi angles: 290.3,171.5	0.01Å	Favored (88.979%) alpha helix	-	-	-
A 42		ALA	6.75	-	Favored (86.28%) General / -60.5,-39.1	-	0.04Å	Favored (83.45%) alpha helix	-	-	-
A 43		LEU	6.88	-	Favored (75.78%) General / -70.1,-38.6	Favored (87.7%) *mt* chi angles: 293.4,168.9	0.10Å	Favored (94.733%) alpha helix	-	-	-
A 44		LEU	7.02	-	Favored (77.01%) General / -65.2,-33.9	Favored (80.8%) *mt* chi angles: 289.6,167.3	0.02Å	Favored (72.352%) alpha helix	-	-	-
A 45		ILE	7.11	-	Favored (16.71%) Ile or Val / -86.2,-46.5	Favored (89.6%) *mt* chi angles: 296.5,166.8	0.02Å	Favored (47.6%) alpha helix	-	-	-
A 46		PHE	7.1	-	Favored (15.45%) General / -108.7,-8.2	Favored (78.8%) *m-80* chi angles: 301.6,105.1	0.06Å	Favored (20.102%)	-	-	-
A 47		GLY	6.93	-	Favored (12.79%) Glycine / -101.7,-142.6	-	-	Favored (17.993%)	-	-	-
A 48		ASN	6.59	-	Favored (3.5%) General / -130.4,40.6	Favored (57.3%) *t0* chi angles: 199,30	0.09Å	CaBLAM Disfavored (1.342%)	-	-	-
A 49		LEU	6.14	-	Favored (13.86%) General / -102.4,161.2	Favored (63.3%) *mt* chi angles: 302.8,183.2	0.06Å	Favored (28.186%)	-	-	-
A 50		THR	5.63	-	Favored (11.79%) General / -126.3,170.5	Favored (38.6%) *p* chi angles: 68	0.12Å	Favored (32.898%)	-	-	-
A 51		TYR	5.12	-	Favored (69.6%) General / -53.4,-46.3	Favored (87.1%) *t80* chi angles: 173.1,78.1	0.06Å	Favored (61.11%)	-	-	-
A 52		MET	4.67	-	Favored (69.64%) General / -63.5,-28.0	Favored (94.4%) *mmm* chi angles: 291.7,304.5,295.6	0.04Å	Favored (74.051%) alpha helix	-	-	-
A 53		ASP	4.31	-	Favored (87.61%) General / -64.7,-37.4	Favored (16.4%) *t70* chi angles: 195,66.8	0.12Å	Favored (75.282%) alpha helix	-	-	-
A 54		LEU	4.03	-	Favored (99.09%) General / -63.2,-41.0	Favored (58%) *tp* chi angles: 181.5,59.1	0.03Å	Favored (95.512%) alpha helix	-	-	-
A 55		VAL	3.82	-	Favored (99.09%) Ile or Val / -62.6,-45.3	Favored (83.5%) *t* chi angles: 173.5	0.04Å	Favored (96.103%) alpha helix	-	-	-

#	Alt	Res	High B	Clash > 0.4Å	Ramachandran	Rotamer	Cβ deviation	CaBLAM	Bond lengths	Bond angles	Cis Peptides
A 56		ARG	3.69	-	Favored (92.06%) General / -59.7,-42.0	Favored (46.1%) *ttm110* chi angles: 189.2,173.5,300.5,107.3	0.03Å	Favored (88.489%) alpha helix	-	-	-
A 57		TYR	3.62	-	Favored (66.96%) General / -54.3,-50.6	Favored (54.3%) *t80* chi angles: 166.9,78.2	0.02Å	Favored (88.691%) alpha helix	-	-	-
A 58		LEU	3.59	-	Favored (83.66%) General / -61.8,-37.3	Favored (96.9%) *mt* chi angles: 293.1,171.7	0.08Å	Favored (80.677%) alpha helix	-	-	-
A 59		VAL	3.62	-	Favored (85.14%) Ile or Val / -68.2,-43.2	Favored (87.4%) *t* chi angles: 173.9	0.03Å	Favored (90.293%) alpha helix	-	-	-
A 60		LEU	3.69	-	Favored (99.17%) General / -62.0,-42.4	Favored (88.2%) *mt* chi angles: 290.7,170.9	0.16Å	Favored (84.034%) alpha helix	-	-	-
**#**	**Alt**	**Res**	**High B**	**Clash > 0.4Å**	**Ramachandran**	**Rotamer**	**Cβ deviation**	**CaBLAM**	**Bond lengths**	**Bond angles**	**Cis Peptides**
			Avg: 6.07	Clashscore: 2.53	Outliers: 3 of 225	Poor rotamers: 1 of 178	Outliers: 0 of 206	Outliers: 7 of 223	Outliers: 2 of 227	Outliers: 4 of 227	Non-Trans: 0 of 226
A 61		VAL	3.8	-	Favored (62.75%) Ile or Val / -72.4,-44.5	Favored (87.3%) *t* chi angles: 173.9	0.05Å	Favored (83.861%) alpha helix	-	-	-
A 62		GLY	3.94	-	Favored (93.34%) Glycine / -59.4,-38.4	-	-	Favored (99.126%) alpha helix	-	-	-
A 63		THR	4.14	-	Favored (82.7%) General / -64.9,-46.2	Favored (99.5%) *m* chi angles: 300.4	0.05Å	Favored (87.989%) alpha helix	-	-	-
A 64		ALA	4.41	-	Favored (79.01%) General / -58.7,-38.7	-	0.07Å	Favored (79.453%) alpha helix	-	-	-
A 65		PHE	4.76	-	Favored (73.87%) General / -69.0,-43.9	Favored (58.6%) *t80* chi angles: 185.7,68.8	0.04Å	Favored (79.08%) alpha helix	-	-	-
A 66		ALA	5.2	-	Favored (72.2%) General / -57.9,-36.3	-	0.04Å	Favored (56.718%)	-	-	-
A 67		GLU	5.7	-	Favored (3.51%) General / -88.8,-55.9	Favored (51.3%) *mm-30* chi angles: 294.3,288.4,310.9	0.07Å	CaBLAM Outlier (0.42%)	-	-	-
A 68		MET	6.18	0.62Å HG3 with A 68 MET O	OUTLIER (0)%) General / 41.3,7.3	Favored (64.4%) *tpp* chi angles: 182.1,61.4,74.2	0.07Å	CaBLAM Outlier (0.856%)	-	OUTLIER(S) worst is C-N-CA: 4.7 σ	-
A 69		ASN	6.56	-	Favored (4.9%) General / -130.9,9.2	Favored (61%) *m-40* chi angles: 292.9,276.6	0.06Å	Favored (56.267%)	-	-	-
A 70		THR	6.78	-	Favored (26.34%) General / -129.7,121.1	Favored (99.4%) *m* chi angles: 300.4	0.08Å	Favored (19.643%)	-	-	-
A 71		GLY	6.8	-	Favored (79.06%) Glycine / -59.0,-34.5	-	-	Favored (42.682%)	-	-	-

A 72		GLY	6.6	-	Favored (77.82%) Glycine / -59.4,-33.8	-	-	Favored (85.731%) alpha helix	-	-	-
A 73		ASP	6.27	-	Favored (66.25%) General / -73.1,-38.9	Favored (33.9%) *t70* chi angles: 188.6,58.9	0.05Å	Favored (80.772%) alpha helix	-	-	-
A 74		VAL	5.91	-	Favored (91.12%) Ile or Val / -61.3,-41.8	Favored (75.8%) *t* chi angles: 172.7	0.03Å	Favored (95.08%) alpha helix	-	-	-
A 75		ILE	5.58	-	Favored (98.02%) Ile or Val / -62.0,-45.8	Favored (98.4%) *mt* chi angles: 292.5,167.1	0.01Å	Favored (96.38%) alpha helix	-	-	-
A 76		HIS	5.3	-	Favored (91.42%) General / -65.8,-39.9	Favored (68.7%) *m90* chi angles: 285.8,79.9	0.11Å	Favored (79.915%) alpha helix	-	-	-
A 77		LEU	5.12	-	Favored (61.25%) General / -75.0,-38.9	Favored (95.6%) *mt* chi angles: 294.7,173.4	0.06Å	Favored (84.635%) alpha helix	-	-	-
A 78		ALA	5.01	-	Favored (91.08%) General / -62.4,-38.8	-	0.03Å	Favored (95.368%) alpha helix	-	-	-
A 79		LEU	4.96	0.58Å C with A 79 LEU HD23	Favored (65.93%) General / -57.9,-52.3	Allowed (1.9%) *tt* chi angles: 180.8,165.2	0.17Å	Favored (88.561%) alpha helix	-	-	-
A 80		VAL	4.94	0.42Å HG13 with A 85 VAL HG22	Favored (89.17%) Ile or Val / -59.6,-42.7	Favored (47.7%) *t* chi angles: 169.1	0.03Å	Favored (88.982%) alpha helix	-	-	-
**#**	**Alt**	**Res**	**High B**	**Clash > 0.4Å**	**Ramachandran**	**Rotamer**	**Cβ deviation**	**CaBLAM**	**Bond lengths**	**Bond angles**	**Cis Peptides**
			Avg: 6.07	Clashscore: 2.53	Outliers: 3 of 225	Poor rotamers: 1 of 178	Outliers: 0 of 206	Outliers: 7 of 223	Outliers: 2 of 227	Outliers: 4 of 227	Non-Trans: 0 of 226
A 81		ALA	4.9	-	Favored (4.18%) General / -59.1,-60.2	-	0.06Å	Favored (67.946%) alpha helix	-	-	-
A 82		VAL	4.76	-	Favored (39.98%) Ile or Val / -66.3,-27.2	Favored (4.9%) *p* chi angles: 71.8	0.10Å	Favored (36.695%) alpha helix	-	-	-
A 83		PHE	4.53	-	Favored (17.17%) General / -108.3,21.6	Favored (51.1%) *m-80* chi angles: 299.9,118.9	0.02Å	Favored (41.628%)	-	-	-
A 84		LYS	4.22	-	Favored (31.84%) General / 54.8,41.5	Favored (47.2%) *mtpt* chi angles: 296.4,167.8,62.9,182.3	0.15Å	Favored (5.699%)	-	-	-
A 85		VAL	3.88	0.53Å O with A 85 VAL HG12	Allowed (0.15%) Ile or Val / 50.0,29.8	OUTLIER (0.2%) chi angles: 203.7	0.19Å	CaBLAM Outlier (0.813%)	-	OUTLIER(S) worst is C-CA-CB: 5.0 σ	-
A 86		GLN	3.56	0.41Å HA with A 87 PRO HD3	Favored (40.25%) Pre-Pro / -58.2,120.0	Favored (67.9%) *tt0* chi angles: 183.7,178.1,354.5	0.11Å	Favored (18.319%)	-	-	-

A 87		PRO	3.33	0.41Å HD3 with A 86 GLN HA	Favored (47.81%) Trans-Pro / -54.2,-27.5	Favored (96.7%) *Cg_exo* chi angles: 332.7,36.3,331.1	0.05Å	Favored (59.424%)	-	-	-
A 88		ALA	3.19	-	Favored (70.35%) General / -58.1,-34.5	-	0.06Å	Favored (55.716%)	-	-	-
A 89		PHE	3.14	-	Favored (27.04%) General / -79.3,-41.9	Favored (80.8%) *m-80* chi angles: 290.1,84.1	0.13Å	Favored (73.858%) alpha helix	OUTLIER(S) worst is CB--CG: 4.1 σ	-	-
A 90		LEU	3.18	-	Favored (79.23%) General / -64.6,-34.8	Favored (90.9%) *mt* chi angles: 292,174.4	0.02Å	Favored (77.806%) alpha helix	-	-	-
A 91		ALA	3.31	-	Favored (95.32%) General / -61.4,-40.7	-	0.06Å	Favored (74.235%) alpha helix	-	-	-
A 92		GLY	3.52	-	Favored (92.06%) Glycine / -59.2,-37.6	-	-	Favored (95.239%) alpha helix	-	-	-
A 93		LEU	3.84	-	Favored (43.16%) General / -76.7,-4.4	Favored (88.2%) *mt* chi angles: 294.7,178	0.05Å	Favored (45.497%) three-ten	-	-	-
A 94		PHE	4.27	-	Favored (66.37%) General / -60.5,-25.9	Favored (52.5%) *t80* chi angles: 189.6,77.2	0.05Å	Favored (56.537%) three-ten	-	-	-
A 95		LEU	4.83	-	Favored (53.77%) General / -90.7,3.6	Favored (86.2%) *mt* chi angles: 299.9,176.1	0.09Å	Favored (50.356%)	-	-	-
A 96		ARG	5.53	-	Favored (8.77%) General / -54.7,-20.6	Favored (53.7%) *ptt90* chi angles: 68.4,184.5,179.4,85.5	0.10Å	Favored (10.748%)	-	-	-
A 97		MET	6.32	-	Favored (66.3%) General / -61.2,-24.3	Favored (30.1%) *mmt* chi angles: 296.2,300.9,175.8	0.04Å	Favored (27.349%)	-	-	-
A 98		GLN	7.1	-	Favored (30.42%) General / -106.1,5.7	Favored (97%) *mm-40* chi angles: 298.3,297.2,305.5	0.03Å	Favored (32.372%)	-	-	-
A 99		TRP	7.71	-	Favored (32.14%) General / -117.0,153.8	Favored (62.3%) *m-10* chi angles: 293.3,346.1	0.06Å	Favored (29.696%)	-	-	-
A 100		SER	8.05	-	Favored (19.85%) General / -80.1,167.9	Favored (93.4%) *p* chi angles: 64.3	0.08Å	Favored (48.777%)	-	-	-
**#**	**Alt**	**Res**	**High B**	**Clash > 0.4Å**	**Ramachandran**	**Rotamer**	**Cβ deviation**	**CaBLAM**	**Bond lengths**	**Bond angles**	**Cis Peptides**
			Avg: 6.07	Clashscore: 2.53	Outliers: 3 of 225	Poor rotamers: 1 of 178	Outliers: 0 of 206	Outliers: 7 of 223	Outliers: 2 of 227	Outliers: 4 of 227	Non-Trans: 0 of 226
A 101		ASN	8.06	-	Favored (80.59%) General / -62.3,-36.0	Favored (99.1%) *m-40* chi angles: 287.5,338.2	0.04Å	Favored (64.338%)	-	-	-
A 102		GLN	7.77	-	Favored (82.7%) General / -67.7,-37.8	Favored (95.5%) *tp40* chi angles: 187.7,63.1,53.1	0.04Å	Favored (96.312%) alpha helix	-	-	-

A 103		GLU	7.27	-	Favored (83.99%) General / -65.0,-36.3	Favored (37.4%) *mt-10* chi angles: 288,167.2,309.1	0.08Å	Favored (91.108%) alpha helix	-	-	-
A 104		ASN	6.7	-	Favored (98.44%) General / -60.9,-42.7	Favored (96.4%) *m-40* chi angles: 286.6,339.9	0.05Å	Favored (89.307%) alpha helix	-	OUTLIER(S) worst is CA-CB-CG: 4.0 σ	-
A 105		ILE	6.17	-	Favored (96.83%) Ile or Val / -64.1,-45.4	Favored (95.2%) *mt* chi angles: 291.9,167.9	0.00Å	Favored (96.197%) alpha helix	-	-	-
A 106		LEU	5.71	-	Favored (86.32%) General / -62.8,-37.4	Favored (94.7%) *mt* chi angles: 291.8,171.8	0.03Å	Favored (83.641%) alpha helix	-	-	-
A 107		MET	5.36	-	Favored (99.2%) General / -62.6,-41.9	Favored (12.6%) *tpt* chi angles: 187.4,67.5,172.7	0.05Å	Favored (94.699%) alpha helix	-	-	-
A 108		VAL	5.1	-	Favored (97.09%) Ile or Val / -64.3,-43.1	Favored (72.3%) *t* chi angles: 172.4	0.11Å	Favored (97.47%) alpha helix	-	-	-
A 109		ILE	4.93	-	Favored (99.88%) Ile or Val / -62.7,-45.0	Favored (94.9%) *mt* chi angles: 291.8,167.8	0.02Å	Favored (93.052%) alpha helix	-	-	-
A 110		GLY	4.83	-	Favored (59.93%) Glycine / -55.6,-50.5	-	-	Favored (99.286%) alpha helix	-	-	-
A 111		ALA	4.81	-	Favored (75.84%) General / -58.9,-37.1	-	0.03Å	Favored (79.691%) alpha helix	-	-	-
A 112		ALA	4.85	-	Favored (97.45%) General / -61.9,-41.0	-	0.02Å	Favored (83.819%) alpha helix	-	-	-
A 113		PHE	4.95	-	Favored (91.5%) General / -65.6,-39.5	Favored (22.2%) *m-10* chi angles: 292.2,345.2	0.04Å	Favored (98.719%) alpha helix	-	-	-
A 114		LEU	5.1	-	Favored (91.79%) General / -65.5,-39.3	Favored (99.5%) *mt* chi angles: 292.4,172.5	0.02Å	Favored (97.711%) alpha helix	-	-	-
A 115		GLN	5.33	-	Favored (83.67%) General / -59.7,-47.9	Favored (48.2%) *tt0* chi angles: 179.5,187.8,21.5	0.07Å	Favored (92.723%) alpha helix	-	-	-
A 116		MET	5.64	-	Favored (86.95%) General / -59.4,-47.0	Favored (39.3%) *mtt* chi angles: 291.6,174.1,201.5	0.07Å	Favored (98.689%) alpha helix	-	-	-
A 117		ALA	6.04	-	Favored (98.82%) General / -61.6,-43.5	-	0.05Å	Favored (97.806%) alpha helix	-	-	-
A 118		ALA	6.53	-	Favored (91.11%) General / -62.3,-38.9	-	0.03Å	Favored (84.499%) alpha helix	-	-	-
A 119		ASN	7.05	-	Favored (69.8%) General / -71.1,-42.0	Favored (89.8%) *m-40* chi angles: 284.9,339.1	0.09Å	Favored (86.227%) alpha helix	-	-	-

#	Alt	Res	High B	Clash > 0.4Å	Ramachandran	Rotamer	Cβ deviation	CaBLAM	Bond lengths	Bond angles	Cis Peptides
A 120		ASP	7.46	-	Favored (76.83%) General / -62.0,-34.8	Favored (98.5%) *m-30* chi angles: 287.2,347.9	0.04Å	Favored (75.645%) alpha helix	-	-	-
#	Alt	Res	High B	Clash > 0.4Å	Ramachandran	Rotamer	Cβ deviation	CaBLAM	Bond lengths	Bond angles	Cis Peptides
			Avg: 6.07	Clashscore: 2.53	Outliers: 3 of 225	Poor rotamers: 1 of 178	Outliers: 0 of 206	Outliers: 7 of 223	Outliers: 2 of 227	Outliers: 4 of 227	Non-Trans: 0 of 226
A 121		LEU	7.6	-	Favored (56.45%) General / -91.4,2.4	Favored (94.9%) *mt* chi angles: 297.3,174.8	0.05Å	Favored (52.307%)	-	-	-
A 122		LYS	7.4	-	Favored (26.36%) General / 54.2,45.9	Favored (95.6%) *mttt* chi angles: 298.1,182,181,177.7	0.03Å	Favored (27.956%)	-	-	-
A 123		LEU	6.92	-	Favored (18.8%) General / -99.7,106.8	Favored (69.8%) *mt* chi angles: 303.9,177.4	0.05Å	Favored (43.941%) beta sheet	-	-	-
A 124		GLU	6.26	-	Favored (9.75%) General / -84.1,67.5	Favored (94.5%) *mt-10* chi angles: 297.6,182,353.2	0.03Å	Favored (10.036%)	-	-	-
A 125		VAL	5.57	-	Favored (3.75%) Ile or Val / -114.9,-24.4	Favored (28.3%) *m* chi angles: 298.5	0.08Å	CaBLAM Disfavored (2.341%)	-	-	-
A 126		LEU	4.97	0.47Å N with A 127 PRO CD	Favored (72.81%) Pre-Pro / -59.7,-39.7	Favored (83.4%) *mt* chi angles: 291.1,175	0.10Å	Favored (59.638%) three-ten	-	-	-
A 127		PRO	4.52	0.47Å CD with A 126 LEU N	Favored (59.23%) Trans-Pro / -58.7,-22.0	Favored (69.4%) *Cg_exo* chi angles: 335.4,37.2,326.2	0.03Å	Favored (54.765%) alpha helix	-	-	-
A 128		ILE	4.21	-	Favored (14.61%) Ile or Val / -88.1,-42.0	Favored (43%) *mm* chi angles: 302.6,305.1	0.05Å	Favored (61.55%) alpha helix	-	-	-
A 129		LEU	4	-	Favored (87.42%) General / -58.3,-45.7	Favored (66.7%) *tp* chi angles: 177.1,58.7	0.02Å	Favored (86.327%) alpha helix	-	-	-
A 130		ASN	3.86	-	Favored (93.2%) General / -60.8,-40.6	Favored (98.7%) *m-40* chi angles: 289.7,341.3	0.04Å	Favored (85.086%) alpha helix	-	-	-
A 131		ALA	3.78	-	Favored (91.71%) General / -59.6,-45.5	-	0.09Å	Favored (76.89%) alpha helix	-	-	-
A 132		MET	3.72	-	Favored (78.9%) General / -69.1,-39.4	Favored (84.6%) *mtm* chi angles: 290.8,187.9,290.7	0.08Å	Favored (75.649%) alpha helix	-	-	-
A 133		SER	3.71	-	Favored (93.44%) General / -64.1,-39.2	Favored (73.4%) *m* chi angles: 295.5	0.09Å	Favored (78.587%) alpha helix	-	-	-
A 134		ILE	3.73	-	Favored (99.2%) Ile or Val / -62.7,-45.3	Favored (89.1%) *mt* chi angles: 291.3,166.5	0.07Å	Favored (88.747%) alpha helix	-	-	-
A 135		ALA	3.77	-	Favored (88%) General / -61.0,-39.0	-	0.05Å	Favored (96.125%) alpha helix	-	-	-

#	Alt	Res	High B	Clash > 0.4Å	Ramachandran	Rotamer	Cβ deviation	CaBLAM	Bond lengths	Bond angles	Cis Peptides
A 136		TRP	3.82	-	Favored (87.2%) General / -66.8,-38.9	Favored (49.6%) *m-10* chi angles: 293.3,335	0.02Å	Favored (84.995%) alpha helix	-	-	-
A 137		MET	3.91	-	Favored (96.96%) General / -61.3,-41.4	Favored (7.5%) *tpt* chi angles: 187.8,62.3,248.9	0.07Å	Favored (79.78%) alpha helix	-	-	-
A 138		LEU	4.04	-	Favored (82.76%) General / -57.0,-45.6	Favored (52.4%) *tp* chi angles: 173.9,59.7	0.05Å	Favored (92.726%) alpha helix	-	-	-
A 139		ILE	4.21	0.40Å HA with A 139 ILE HD13	Favored (93.34%) Ile or Val / -62.1,-42.0	Favored (21.8%) *mm* chi angles: 296.4,307.7	0.03Å	Favored (93.826%) alpha helix	-	-	-
A 140		ARG	4.42	-	Favored (88.04%) General / -63.5,-37.6	Favored (19.5%) *tpp-160* chi angles: 179.6,62.1,70.8,202.8	0.07Å	Favored (94.152%) alpha helix	-	-	-
**#**	**Alt**	**Res**	**High B**	**Clash > 0.4Å**	**Ramachandran**	**Rotamer**	**Cβ deviation**	**CaBLAM**	**Bond lengths**	**Bond angles**	**Cis Peptides**
			Avg: 6.07	Clashscore: 2.53	Outliers: 3 of 225	Poor rotamers: 1 of 178	Outliers: 0 of 206	Outliers: 7 of 223	Outliers: 2 of 227	Outliers: 4 of 227	Non-Trans: 0 of 226
A 141		ALA	4.67	-	Favored (90.96%) General / -63.8,-38.4	-	0.03Å	Favored (96.52%) alpha helix	-	-	-
A 142		MET	4.93	-	Favored (88.83%) General / -65.9,-43.3	Favored (47.4%) *mtt* chi angles: 289.1,179.6,163.9	0.05Å	Favored (37.024%)	-	-	-
A 143		LYS	5.15	-	Favored (39.89%) General / -75.7,146.8	Favored (98.3%) *mttt* chi angles: 293,181.5,178.4,180.9	0.06Å	CaBLAM Disfavored (2.165%)	-	-	-
A 144		GLU	5.31	-	Allowed (0.59%) General / 70.2,-56.3	Favored (93.2%) *mt-10* chi angles: 292.1,181.8,337.8	0.08Å	Favored (25.953%)	-	-	-
A 145		GLY	5.36	-	Favored (68.64%) Glycine / 78.8,21.5	-	-	Favored (60.603%)	-	-	-
A 146		LYS	5.27	-	Favored (26.93%) General / -78.1,162.1	Favored (11.1%) *ptmt* chi angles: 66.3,179.3,292.7,182.6	0.05Å	Favored (26.793%)	-	-	-
A 147		VAL	5.03	-	Favored (93.29%) Ile or Val / -59.3,-45.5	Favored (62.3%) *t* chi angles: 171.1	0.04Å	Favored (65.769%)	-	-	-
A 148		ALA	4.69	-	Favored (83.52%) General / -60.3,-38.4	-	0.04Å	Favored (78.768%) alpha helix	-	-	-
A 149		MET	4.33	-	Favored (54.14%) General / -77.2,-28.6	Favored (53.1%) *mmp* chi angles: 295.6,300.7,99.9	0.08Å	Favored (82.887%) alpha helix	-	-	-
A 150		TYR	3.98	-	Favored (76.71%) General / -67.6,-45.0	Favored (82.7%) *t80* chi angles: 181.2,74.3	0.05Å	Favored (74.33%) alpha helix	-	-	-
A 151		ALA	3.66	-	Favored (77.63%) General / -59.7,-37.0	-	0.04Å	Favored (68.25%) three-ten	-	-	-

A 152		LEU	3.41	-	Favored (96.43%) Pre-Pro / -58.8,-48.6	Favored (82.8%) *mt* chi angles: 289.5,169.4	0.16Å	Favored (67.044%) three-ten	-	-	-
A 153		PRO	3.24	-	Favored (73.04%) Trans-Pro / -53.8,-33.9	Favored (89.3%) *Cg_exo* chi angles: 329.9,35.5,333.3	0.03Å	Favored (99.305%) alpha helix	-	-	-
A 154		ILE	3.14	-	Favored (78.92%) Ile or Val / -69.0,-46.3	Favored (97.2%) *mt* chi angles: 292.8,169.2	0.03Å	Favored (75.366%) alpha helix	-	-	-
A 155		LEU	3.12	-	Favored (83.46%) General / -59.8,-39.0	Favored (70.8%) *mt* chi angles: 290.3,165.8	0.11Å	Favored (82.663%) alpha helix	-	-	-
A 156		CYS	3.19	-	Favored (79.15%) General / -68.4,-42.3	Favored (40%) *t* chi angles: 187.1	0.02Å	Favored (74.825%) alpha helix	-	-	-
A 157		ALA	3.37	-	Favored (66.65%) General / -59.1,-28.6	-	0.02Å	Favored (71.496%) three-ten	-	-	-
A 158		LEU	3.65	-	Favored (53.65%) General / -78.5,-5.8	Favored (91.6%) *mt* chi angles: 296.9,172.2	0.08Å	Favored (46.459%)	-	-	-
A 159		THR	4.08	-	Favored (60.63%) Pre-Pro / -83.2,160.2	Favored (72.7%) *p* chi angles: 61.8	0.02Å	Favored (25.307%)	-	-	-
A 160		PRO	4.65	-	Favored (88.84%) Trans-Pro / -57.6,145.5	Favored (83%) *Cg_exo* chi angles: 334.4,34.4,331.5	0.03Å	Favored (39.089%)	-	-	-
**#**	**Alt**	**Res**	**High B**	**Clash > 0.4Å**	**Ramachandran**	**Rotamer**	**Cβ deviation**	**CaBLAM**	**Bond lengths**	**Bond angles**	**Cis Peptides**
			Avg: 6.07	Clashscore: 2.53	Outliers: 3 of 225	Poor rotamers: 1 of 178	Outliers: 0 of 206	Outliers: 7 of 223	Outliers: 2 of 227	Outliers: 4 of 227	Non-Trans: 0 of 226
A 161		GLY	5.31	-	Favored (50.74%) Glycine / -64.9,146.9	-	-	Favored (43.447%)	-	-	-
A 162		MET	5.99	-	Favored (7.65%) General / -80.7,74.7	Favored (29.4%) *mmt* chi angles: 299.2,292.5,178.9	0.10Å	Favored (22.21%) beta sheet	-	-	-
A 163		ARG	6.64	-	Favored (69.15%) General / -60.7,-29.1	Favored (98%) *mtt180* chi angles: 288.9,178.4,178.7,173.2	0.01Å	Favored (32.103%)	-	-	-
A 164		MET	7.15	-	Favored (75.51%) General / -61.5,-34.5	Favored (67.1%) *mtt* chi angles: 289.6,177,177	0.02Å	Favored (53.191%) alpha helix	-	-	-
A 165		ALA	7.46	-	Favored (14.79%) General / -92.6,-32.4	-	0.04Å	CaBLAM Outlier (0.919%)	-	-	-
A 166		GLY	7.56	-	Favored (3.83%) Glycine / 104.0,113.6	-	-	Favored (11.947%)	-	-	-
A 167		LEU	7.47	-	Favored (76.85%) General / -59.2,-37.2	Favored (43.5%) *tp* chi angles: 184.9,60.2	0.03Å	Favored (59.64%)	-	-	-

A 168		ASP	7.25	-	Favored (73.88%) General / -61.5,-33.5	Favored (97.3%) *m-30* chi angles: 289.4,346.7	0.04Å	Favored (76.614%) alpha helix	-	-	-
A 169		VAL	6.96	-	Favored (80.75%) Ile or Val / -69.2,-44.5	Favored (88.6%) *t* chi angles: 174	0.07Å	Favored (83.058%) alpha helix	-	-	-
A 170		ILE	6.65	-	Favored (95.51%) Ile or Val / -63.4,-46.3	Favored (97.1%) *mt* chi angles: 292.6,169.4	0.09Å	Favored (96.567%) alpha helix	OUTLIER(S) worst is CB--CG1: 4.0 σ	-	-
A 171		ARG	6.35	-	Favored (77.47%) General / -65.1,-34.0	Favored (96.4%) *mtt-85* chi angles: 289.9,183.9,183.5,280	0.16Å	Favored (82.71%) alpha helix	-	-	-
A 172		CYS	6.08	-	Favored (95.91%) General / -64.8,-40.8	Favored (94.2%) *m* chi angles: 290.7	0.01Å	Favored (86.615%) alpha helix	-	-	-
A 173		LEU	5.86	-	Favored (95.26%) General / -61.1,-45.3	Favored (68%) *tp* chi angles: 179.2,61.2	0.04Å	Favored (88.984%) alpha helix	-	-	-
A 174		LEU	5.69	-	Favored (66.89%) General / -53.6,-49.9	Favored (63.2%) *tp* chi angles: 174.8,62.9	0.12Å	Favored (88.796%) alpha helix	-	-	-
A 175		LEU	5.56	-	Favored (98.32%) General / -62.8,-40.8	Favored (80.5%) *mt* chi angles: 288.7,167.6	0.05Å	Favored (94.117%) alpha helix	-	-	-
A 176		ILE	5.46	-	Favored (83.24%) Ile or Val / -64.6,-48.8	Favored (95.7%) *mt* chi angles: 292.5,166.3	0.02Å	Favored (75.503%) alpha helix	-	-	-
A 177		ILE	5.39	-	Favored (84.12%) Ile or Val / -68.3,-41.9	Favored (43.8%) *mm* chi angles: 297.6,301.1	0.06Å	Favored (75.439%) alpha helix	-	-	-
A 178		GLY	5.37	-	Favored (35.36%) Glycine / -56.6,-54.6	-	-	Favored (94.081%) alpha helix	-	-	-
A 179		ILE	5.4	-	Favored (98.2%) Ile or Val / -61.7,-45.7	Favored (90.2%) *mt* chi angles: 291.6,166.3	0.01Å	Favored (86.569%) alpha helix	-	-	-
A 180		VAL	5.5	-	Favored (95.23%) Ile or Val / -59.9,-45.3	Favored (59.7%) *t* chi angles: 170.8	0.03Å	Favored (79.653%) alpha helix	-	-	-
**#**	**Alt**	**Res**	**High B**	**Clash > 0.4Å**	**Ramachandran**	**Rotamer**	**Cβ deviation**	**CaBLAM**	**Bond lengths**	**Bond angles**	**Cis Peptides**
			Avg: 6.07	Clashscore: 2.53	Outliers: 3 of 225	Poor rotamers: 1 of 178	Outliers: 0 of 206	Outliers: 7 of 223	Outliers: 2 of 227	Outliers: 4 of 227	Non-Trans: 0 of 226
A 181		THR	5.69	-	Favored (92.51%) General / -64.8,-43.6	Favored (92.1%) *m* chi angles: 297.8	0.05Å	Favored (76.317%) alpha helix	-	-	-
A 182		LEU	5.98	-	Favored (72.97%) General / -60.3,-33.8	Favored (17.1%) *tp* chi angles: 191.6,58	0.04Å	Favored (74.577%) alpha helix	-	-	-

A 183		LEU	6.38	-	Favored (98.41%) General / -62.3,-41.2	Favored (84.4%) *mt* chi angles: 289.8,170.8	0.02Å	Favored (82.755%) alpha helix	-	-	-
A 184		ASN	6.87	-	Favored (66.61%) General / -68.7,-28.2	Favored (95.4%) *m-40* chi angles: 287.3,334.7	0.04Å	Favored (78.591%) alpha helix	-	-	-
A 185		GLU	7.43	-	Favored (47.36%) General / -86.4,2.5	Favored (48.2%) *mm-30* chi angles: 295.3,289.3,305.4	0.06Å	Favored (45.196%) alpha helix	-	-	-
A 186		ARG	8	-	Favored (55.68%) General / -61.6,-17.5	Favored (89.3%) *mtm180* chi angles: 289,174.3,292.9,178.1	0.07Å	Favored (12.833%) three-ten	-	-	-
A 187		ARG	8.51	-	Favored (60.92%) General / -59.7,-21.7	Favored (50.8%) *ptt-90* chi angles: 68.4,188.5,184.8,276.1	0.01Å	Favored (46.762%) three-ten	-	-	-
A 188		GLU	8.88	-	Favored (39.2%) General / -119.2,121.2	Favored (90.5%) *tt0* chi angles: 179.3,177.7,355.3	0.01Å	Favored (22.992%) alpha helix	-	-	-
A 189		SER	9.03	-	Favored (79.2%) General / -59.2,-38.1	Favored (65.5%) *m* chi angles: 294.3	0.02Å	Favored (53.743%) alpha helix	-	-	-
A 190		VAL	8.92	-	Favored (91.53%) Ile or Val / -64.8,-46.6	Favored (73.9%) *t* chi angles: 172.6	0.03Å	Favored (86.431%) alpha helix	-	-	-
A 191		ALA	8.54	-	Favored (92.19%) General / -62.2,-39.3	-	0.03Å	Favored (83.352%) alpha helix	-	-	-
A 192		LYS	7.98	-	Favored (88.42%) General / -59.3,-46.5	Favored (86.8%) *tttt* chi angles: 183.3,177.6,178,182.3	0.03Å	Favored (78.24%) alpha helix	-	-	-
A 193		LYS	7.36	-	Favored (65.69%) General / -72.8,-40.8	Favored (67.5%) *mmtt* chi angles: 292.4,289.7,180,179.5	0.04Å	Favored (75.932%) alpha helix	-	-	-
A 194		LYS	6.75	-	Favored (82.45%) General / -68.1,-40.6	Favored (97%) *mttt* chi angles: 288.5,180.7,175,179.1	0.03Å	Favored (92.929%) alpha helix	-	-	-
A 195		GLY	6.24	-	Favored (93.86%) Glycine / -63.9,-36.9	-	-	Favored (90.744%) alpha helix	-	-	-
A 196		GLY	5.85	-	Favored (44.49%) Glycine / -55.9,-53.1	-	-	Favored (91.109%) alpha helix	-	-	-
A 197		TYR	5.6	-	Favored (73.12%) General / -56.2,-49.8	Favored (75.7%) *t80* chi angles: 171,79.2	0.08Å	Favored (89.43%) alpha helix	-	-	-
A 198		LEU	5.47	-	Favored (92.71%) General / -61.1,-40.1	Favored (86.6%) *mt* chi angles: 290.7,169.4	0.04Å	Favored (80.687%) alpha helix	-	-	-
A 199		LEU	5.43	-	Favored (84.86%) General / -67.6,-40.3	Favored (83.8%) *mt* chi angles: 289.6,171.9	0.03Å	Favored (88.62%) alpha helix	-	-	-

A 200		ALA	5.47	-	Favored (93.47%) General / -62.0,-39.8	-	0.04Å	Favored (93.764%) alpha helix	-	-	-
**#**	**Alt**	**Res**	**High B**	**Clash > 0.4Å**	**Ramachandran**	**Rotamer**	**Cβ deviation**	**CaBLAM**	**Bond lengths**	**Bond angles**	**Cis Peptides**
			Avg: 6.07	Clashscore: 2.53	Outliers: 3 of 225	Poor rotamers: 1 of 178	Outliers: 0 of 206	Outliers: 7 of 223	Outliers: 2 of 227	Outliers: 4 of 227	Non-Trans: 0 of 226
A 201		ALA	5.56	-	Favored (98.18%) General / -63.0,-43.6	-	0.04Å	Favored (91.58%) alpha helix	-	-	-
A 202		ALA	5.72	-	Favored (82.49%) General / -62.0,-36.8	-	0.03Å	Favored (79.774%) alpha helix	-	-	-
A 203		LEU	5.95	-	Favored (93.75%) General / -63.7,-39.2	Favored (93.7%) *mt* chi angles: 291.5,172	0.07Å	Favored (79.748%) alpha helix	-	-	-
A 204		CYS	6.24	-	Favored (73.41%) General / -69.2,-43.8	Favored (90.7%) *m* chi angles: 291.6	0.03Å	Favored (75.331%) alpha helix	-	-	-
A 205		GLN	6.57	-	Favored (71.47%) General / -65.0,-30.5	Favored (80.4%) *mt0* chi angles: 291.6,171.9,21.9	0.01Å	Favored (74.784%) alpha helix	-	-	-
A 206		ALA	6.9	-	Favored (58.22%) General / -77.7,-8.6	-	0.05Å	Favored (44.042%)	-	-	-
A 207		GLY	7.23	-	Favored (69.01%) Glycine / 74.2,26.2	-	-	Favored (79.715%)	-	-	-
A 208		VAL	7.53	-	Favored (10.24%) Ile or Val / -107.2,-4.9	Favored (26.5%) *m* chi angles: 299.6	0.08Å	Favored (10.709%)	-	-	-
A 209		CYS	7.78	-	Favored (32.99%) General / -157.9,166.3	Favored (23.8%) *p* chi angles: 68.8	0.08Å	Favored (22.192%)	-	-	-
A 210		SER	7.93	-	Favored (37.64%) Pre-Pro / -88.2,146.2	Favored (29.3%) *t* chi angles: 172.3	0.05Å	Favored (34.599%)	-	-	-
A 211		PRO	7.96	-	Favored (20.39%) Trans-Pro / -49.7,-29.7	Favored (85.9%) *Cg_exo* chi angles: 330.5,36.3,332.2	0.01Å	Favored (83.721%)	-	-	-
A 212		LEU	7.89	-	Favored (79.82%) General / -65.9,-35.1	Favored (96%) *mt* chi angles: 292.7,173.9	0.03Å	Favored (70.166%) alpha helix	-	-	-
A 213		ILE	7.75	-	Favored (70.7%) Ile or Val / -71.3,-45.4	Favored (96.4%) *mt* chi angles: 293.9,168.7	0.03Å	Favored (76.296%) alpha helix	-	-	-
A 214		MET	7.59	-	Favored (73.88%) General / -56.7,-49.9	Favored (48.6%) *ttp* chi angles: 171.1,184,71.5	0.05Å	Favored (97.316%) alpha helix	-	-	-
A 215		MET	7.45	-	Favored (89.96%) General / -64.2,-38.1	Favored (82.3%) *mtm* chi angles: 288.8,186.1,284.1	0.07Å	Favored (90.377%) alpha helix	-	-	-

A 216		GLY	7.4	-	Favored (48.07%) Glycine / -58.2,-53.1	-	-	Favored (91.523%) alpha helix	-	-	-
A 217		GLY	7.45	-	Favored (97.98%) Glycine / -60.3,-40.8	-	-	Favored (91.119%) alpha helix	-	-	-
A 218		LEU	7.65	-	Favored (91.7%) General / -64.9,-38.7	Favored (87.9%) *mt* chi angles: 291,173.4	0.01Å	Favored (90.126%) alpha helix	-	-	-
A 219		ILE	7.98	-	Favored (98.26%) Ile or Val / -63.9,-44.9	Favored (93.9%) *mt* chi angles: 291.8,167.1	0.01Å	Favored (91.648%) alpha helix	-	-	-
A 220		LEU	8.41	-	Favored (95.1%) General / -64.2,-40.0	Favored (98.8%) *mt* chi angles: 292.4,172.2	0.05Å	Favored (92.601%) alpha helix	-	-	-
**#**	**Alt**	**Res**	**High B**	**Clash > 0.4Å**	**Ramachandran**	**Rotamer**	**Cβ deviation**	**CaBLAM**	**Bond lengths**	**Bond angles**	**Cis Peptides**
			Avg: 6.07	Clashscore: 2.53	Outliers: 3 of 225	Poor rotamers: 1 of 178	Outliers: 0 of 206	Outliers: 7 of 223	Outliers: 2 of 227	Outliers: 4 of 227	Non-Trans: 0 of 226
A 221		ALA	8.92	-	Favored (77.41%) General / -63.4,-34.3	-	0.02Å	Favored (63.889%) alpha helix	-	-	-
A 222		HIS	9.48	-	Favored (81.16%) Pre-Pro / -128.7,73.1	Favored (54.9%) *m90* chi angles: 304.3,84.2	0.11Å	Favored (25.864%) alpha helix	-	-	-
A 223		PRO	10.08	-	Favored (70.91%) Trans-Pro / -61.8,-20.9	Favored (39.6%) *Cg_endo* chi angles: 23.3,325.8,30.6	0.03Å	Favored (51.729%) alpha helix	-	-	-
A 224		ASN	10.66	-	Favored (46.85%) General / -98.1,7.1	Favored (88.7%) *m-40* chi angles: 293,320.5	0.03Å	Favored (51.457%)	-	-	-
A 225		GLY	11.15	-	Favored (3.15%) Glycine / -76.5,61.7	-	-	CaBLAM Disfavored (4.295%)	-	-	-
A 226		LYS	11.53	-	Favored (56.14%) General / -59.0,133.5	Favored (87.7%) *tttt* chi angles: 183.7,176.5,179.7,179.8	0.01Å	-	-	-	-
A 227		ARG	11.79	-	-	Favored (83%) *ttt180* chi angles: 184.2,178.3,179,180.6	0.04Å	-	-	-	-
