# Supplementary material for: Integrative proteome-wide structural analysis and high-throughput docking identify broad-spectrum antiviral scaffolds against Zika, Yellow Fever, West Nile, Saint Louis encephalitis, and Usutu viruses
Source: Front Cell Infect Microbiol. 2026 Apr 30;16:1723132. doi: 10.3389/fcimb.2026.1723132 (PMC13171538; doi:10.3389/fcimb.2026.1723132)
Supplement: Supplementary file 3 [file DataSheet3.zip › SLEV/SLEV_NS2b/Mol_probity_Files/SLEV_NS2b_1FH-multi.table.pdf]

Viewing SLEV_NS2b1FH-multi.table

Duke Biochemistry
Duke University School of Medicine

When finished, you should close this window. *Hint: Use File | Save As... to save a copy of this page.*

All-Atom Contacts	Clashscore, all atoms:	0.98		99th percentile* (N=1784, all resolutions)
	Clashscore is the number of serious steric overlaps (> 0.4 Å) per 1000 atoms.			
Protein Geometry	Poor rotamers	0	0.00%	Goal: <0.3%
	Favored rotamers	105	100.00%	Goal: >98%
	Ramachandran outliers	2	1.55%	Goal: <0.05%
	Ramachandran favored	121	93.80%	Goal: >98%
	Rama distribution Z-score	-0.38 ± 0.65		Goal: abs(Z score) < 2
	MolProbity score^	1.20		99th percentile* (N=27675, 0Å - 99Å)
	Cβ deviations >0.25Å	0	0.00%	Goal: 0
	Bad bonds:	4 / 1048	0.38%	Goal: 0%
	Bad angles:	13 / 1426	0.91%	Goal: <0.1%
Peptide Omegas	Cis Prolines:	0 / 6	0.00%	Expected: ≤1 per chain, or ≤5%
Low-resolution Criteria	CaBLAM outliers	9	7.1%	Goal: <1.0%
	CA Geometry outliers	2	1.57%	Goal: <0.5%
Additional validations	Chiral volume outliers	0/158		
	Waters with clashes	0/0	0.00%	See UnDowser table for details

In the two column results, the left column gives the raw count, right column gives the percentage.

* 100th percentile is the best among structures of comparable resolution; 0th percentile is the worst. For clashscore the comparative set of structures was selected in 2004, for MolProbity score in 2006.

^ MolProbity score combines the clashscore, rotamer, and Ramachandran evaluations into a single score, normalized to be on the same scale as X-ray resolution.

Key to table colors and cutoffs here:

#	Alt	Res	High B	Clash > 0.4Å	Ramachandran	Rotamer	Cβ deviation	CaBLAM	Bond lengths	Bond angles	Cis Peptides
			Avg: 6.62	Clashscore: 0.98	Outliers: 2 of 129	Poor rotamers: 0 of 105	Outliers: 0 of 118	Outliers: 9 of 127	Outliers: 2 of 131	Outliers: 5 of 131	Non-Trans: 0 of 130
A 1		SER	8.31	-	-	Favored (64.9%) *m* chi angles: 294.2	0.04Å	-	-	-	-
A 2		TRP	8.13	-	Favored (19.88%) Pre-Pro / -48.2,-55.1	Favored (67.2%) *t-100* chi angles: 186,259.6	0.06Å	-	-	-	-
A 3		PRO	7.83	-	OUTLIER (0)%) Trans-Pro / 108.2,144.8	Favored (44%) *Cg_endo* chi angles: 33.6,323.5,29.8	0.24Å	CaBLAM Disfavored (1.021%)	OUTLIER(S) worst is N--CD: 10.6 σ	OUTLIER(S) worst is CA-N-CD: 14.7 σ	-
A 4		ALA	7.42	-	Favored (65.49%) General / -57.1,-31.7	-	0.04Å	Favored (52.868%)	-	-	-
A 5		SER	6.97	-	Favored (81.32%) General / -59.9,-38.1	Favored (63.3%) *m* chi angles: 294	0.03Å	Favored (65.402%) alpha helix	-	-	-
A 6		GLU	6.53	-	Favored (51.64%) General / -77.8,-31.4	Favored (75.6%) *mm-30* chi angles: 292.5,294.5,309.4	0.07Å	Favored (81.265%) alpha helix	-	-	-

A 7		VAL	6.15	-	Favored (89.79%)<br>Ile or Val / -63.2,-48.0	Favored (71.1%) *t*<br>chi angles: 172.2	0.12Å	Favored (76.797%)<br>alpha helix	-	-	-
A 8		LEU	5.86	-	Favored (87.22%)<br>General / -65.0,-37.3	Favored (96.6%) *mt*<br>chi angles: 292.2,171.6	0.04Å	Favored (79.022%)<br>alpha helix	-	-	-
A 9		THR	5.67	-	Favored (98.48%)<br>General / -61.4,-43.7	Favored (88.5%) *m*<br>chi angles: 298.4	0.01Å	Favored (80.04%)<br>alpha helix	-	-	-
A 10		GLY	5.54	-	Favored (53.2%)<br>Glycine / -54.4,-51.1	-	-	Favored (98.342%)<br>alpha helix	-	-	-
A 11		VAL	5.44	-	Favored (95.89%)<br>Ile or Val / -60.4,-44.3	Favored (66.7%) *t*<br>chi angles: 171.7	0.02Å	Favored (94.895%)<br>alpha helix	-	-	-
A 12		GLY	5.34	-	Favored (43.96%)<br>Glycine / -56.2,-53.3	-	-	Favored (94.439%)<br>alpha helix	-	-	-
A 13		LEU	5.23	-	Favored (97.81%)<br>General / -62.6,-40.7	Favored (97.3%) *mt*<br>chi angles: 292.2,172	0.06Å	Favored (83.149%)<br>alpha helix	-	-	-
A 14		MET	5.11	-	Favored (93.04%)<br>General / -59.5,-44.9	Favored (28.5%) *tmm*<br>chi angles: 178.7,276.3,293.1	0.04Å	Favored (95.407%)<br>alpha helix	-	-	-
A 15		CYS	4.95	-	Favored (93.63%)<br>General / -64.1,-39.2	Favored (93.7%) *m*<br>chi angles: 291.9	0.04Å	Favored (94.398%)<br>alpha helix	-	-	-
A 16		ALA	4.8	-	Favored (95.51%)<br>General / -61.1,-40.9	-	0.04Å	Favored (92.808%)<br>alpha helix	-	-	-
A 17		LEU	4.68	-	Favored (85.92%)<br>General / -67.2,-41.5	Favored (85.4%) *mt*<br>chi angles: 290.7,168.1	0.06Å	Favored (93.804%)<br>alpha helix	-	-	-
A 18		ALA	4.6	-	Favored (84.3%)<br>General / -60.7,-38.2	-	0.03Å	Favored (85.319%)<br>alpha helix	-	-	-
A 19		GLY	4.58	-	Favored (25.75%)<br>Glycine / -57.6,-56.3	-	-	Favored (90.661%)<br>alpha helix	-	-	-
A 20		GLY	4.7	-	Favored (62.82%)<br>Glycine / -55.5,-36.0	-	-	Favored (86.787%)<br>alpha helix	-	-	-
**#**	**Alt**	**Res**	**High B**	**Clash > 0.4Å**	**Ramachandran**	**Rotamer**	**Cβ deviation**	**CaBLAM**	**Bond lengths**	**Bond angles**	**Cis Peptides**
			Avg: 6.62	Clashscore: 0.98	Outliers: 2 of 129	Poor rotamers: 0 of 105	Outliers: 0 of 118	Outliers: 9 of 127	Outliers: 2 of 131	Outliers: 5 of 131	Non-Trans: 0 of 130
A 21		LEU	5.1	-	Favored (82.22%)<br>General / -63.1,-36.0	Favored (47.1%) *tp*<br>chi angles: 179.7,55.5	0.05Å	Favored (77.514%)<br>alpha helix	-	-	-
A 22		LEU	5.84	-	Favored (57.85%)<br>General / -89.2,-5.2	Favored (93.3%) *mt*<br>chi angles: 298.1,175.5	0.06Å	Favored (54.338%)	-	-	-

A 23		GLU	6.82	-	Favored (20.12%) General / -76.5,168.4	Favored (94.6%) *mt-10* chi angles: 291.2,184.3,348.1	0.09Å	Favored (20.058%)	-	-	-
A 24		PHE	7.85	-	Favored (25.27%) General / -48.0,-41.5	Favored (89.3%) *t80* chi angles: 177,81.9	0.04Å	Favored (5.19%)	-	-	-
A 25		GLU	8.63	-	Favored (37.13%) General / -61.0,149.3	Favored (66.9%) *mm-30* chi angles: 295,292.8,308.9	0.02Å	Favored (15.49%)	-	-	-
A 26		GLU	8.89	-	Favored (43.34%) General / -54.0,134.7	Favored (90.3%) *tt0* chi angles: 185,176.1,4.3	0.01Å	Favored (26.913%)	-	-	-
A 27		THR	8.56	-	Favored (46.54%) General / -55.8,131.3	Favored (66.7%) *m* chi angles: 303.2	0.02Å	Favored (7.14%)	-	-	-
A 28		SER	7.78	-	Allowed (0.71%) General / -141.4,-156.8	Favored (11%) *t* chi angles: 189.5	0.03Å	CA Geom Outlier (0.466%)	-	-	-
A 29		MET	6.87	-	Allowed (1.63%) General / -55.1,-14.5	Favored (88.1%) *mmm* chi angles: 288.1,301.3,297.6	0.05Å	CaBLAM Disfavored (4.47%)	-	-	-
A 30		VAL	6.11	-	Favored (24.54%) Ile or Val / -57.2,-26.1	Favored (7.5%) *p* chi angles: 68.4	0.04Å	Favored (45.116%)	-	-	-
A 31		VAL	5.66	0.49Å HB with A 32 PRO HD3	Favored (2.81%) Pre-Pro / -66.8,-63.6	Favored (65.6%) *t* chi angles: 171.6	0.17Å	Favored (34.607%) alpha helix	-	-	-
A 32		PRO	5.46	0.49Å HD3 with A 31 VAL HB	Favored (27.55%) Trans-Pro / -48.7,-34.5	Favored (52.9%) *Cg_exo* chi angles: 327.9,36.2,334.9	0.08Å	Favored (86.174%) alpha helix	-	-	-
A 33		PHE	5.37	-	Favored (64.53%) General / -71.1,-45.0	Favored (23.2%) *m-10* chi angles: 293.9,343.1	0.03Å	Favored (82.538%) alpha helix	-	-	-
A 34		ALA	5.29	-	Favored (84.4%) General / -60.8,-38.2	-	0.02Å	Favored (95.945%) alpha helix	-	-	-
A 35		ILE	5.25	-	Favored (92.13%) Ile or Val / -64.8,-46.4	Favored (97.7%) *mt* chi angles: 293.1,167.1	0.02Å	Favored (96.054%) alpha helix	-	-	-
A 36		ALA	5.24	-	Favored (76.71%) General / -59.7,-36.5	-	0.03Å	Favored (82.916%) alpha helix	-	-	-
A 37		GLY	5.24	-	Favored (43.05%) Glycine / -57.6,-53.7	-	-	Favored (90.982%) alpha helix	-	-	-
A 38		LEU	5.22	-	Favored (85.07%) General / -61.8,-37.6	Favored (87.9%) *mt* chi angles: 290.6,170.9	0.04Å	Favored (76.827%) alpha helix	-	-	-
A 39		MET	5.21	-	Favored (86.44%) General / -67.1,-41.4	Favored (81.7%) *mtm* chi angles: 290.6,189.4,291.2	0.03Å	Favored (88.112%) alpha helix	-	-	-

A 40		TYR	5.22	-	Favored (72.97%) General / -57.9,-50.5	Favored (87.4%) *t80* chi angles: 173,77.2	0.03Å	Favored (93.993%) alpha helix	-	-	-
**#**	**Alt**	**Res**	**High B**	**Clash > 0.4Å**	**Ramachandran**	**Rotamer**	**Cβ deviation**	**CaBLAM**	**Bond lengths**	**Bond angles**	**Cis Peptides**
			Avg: 6.62	Clashscore: 0.98	Outliers: 2 of 129	Poor rotamers: 0 of 105	Outliers: 0 of 118	Outliers: 9 of 127	Outliers: 2 of 131	Outliers: 5 of 131	Non-Trans: 0 of 130
A 41		ILE	5.24	-	Favored (90.01%) Ile or Val / -59.1,-43.7	Favored (90.4%) *mt* chi angles: 292.2,165.2	0.07Å	Favored (86.049%) alpha helix	-	-	-
A 42		THR	5.28	-	Favored (76.72%) General / -55.4,-45.4	Favored (94.3%) *m* chi angles: 299.3	0.04Å	Favored (82.745%) alpha helix	-	-	-
A 43		TYR	5.34	-	Favored (62.94%) General / -72.7,-28.9	Favored (34.8%) *m-80* chi angles: 291.7,125.6	0.06Å	Favored (77.786%) alpha helix	-	-	-
A 44		THR	5.47	0.48Å OG1 with A 45 VAL N	Favored (5.23%) General / -66.4,-58.5	Favored (68%) *p* chi angles: 62.7	0.13Å	Favored (54.694%) alpha helix	-	-	-
A 45		VAL	5.73	0.48Å N with A 44 THR OG1	Favored (86.11%) Ile or Val / -58.4,-43.2	Favored (63.6%) *t* chi angles: 171.3	0.04Å	Favored (74.125%) alpha helix	-	-	-
A 46		SER	6.18	-	Favored (91.9%) General / -61.0,-40.0	Favored (71%) *m* chi angles: 295.1	0.03Å	Favored (85.443%) alpha helix	-	-	-
A 47		GLY	6.84	-	Favored (58.63%) Glycine / -56.9,-51.2	-	-	Favored (94.458%) alpha helix	-	-	-
A 48		LYS	7.7	-	Favored (87.56%) General / -58.5,-46.0	Favored (85.4%) *tttt* chi angles: 181,178.9,177.1,182.1	0.02Å	Favored (88.105%) alpha helix	-	-	-
A 49		ALA	8.73	-	Favored (69.41%) General / -59.5,-31.5	-	0.03Å	Favored (73.791%)	-	-	-
A 50		ALA	9.87	-	Favored (28.19%) General / -80.2,1.5	-	0.03Å	Favored (20.325%)	-	-	-
A 51		GLU	10.94	-	Favored (12.9%) General / -48.7,127.0	Favored (10.9%) *tp30* chi angles: 184.7,76.5,46.2	0.06Å	Favored (17.21%)	-	-	-
A 52		MET	11.76	-	Favored (31.55%) General / -143.5,143.4	Favored (30.3%) *ttt* chi angles: 176.4,175.7,180.7	0.02Å	Favored (54.467%) beta sheet	-	-	-
A 53		TRP	12.2	-	Favored (27.56%) General / -104.7,147.5	Favored (28.6%) *m-90* chi angles: 295.9,259.1	0.07Å	Favored (57.158%) beta sheet	-	-	-
A 54		ILE	12.29	-	Favored (6.92%) Ile or Val / -105.5,-53.4	Favored (48.8%) *mm* chi angles: 303.7,301	0.04Å	CaBLAM Outlier (0.04%)	-	-	-
A 55		GLU	12.14	-	Allowed (0.09%) General / 58.0,-173.7	Favored (96.2%) *mt-10* chi angles: 296.4,181.3,356.6	0.05Å	CaBLAM Disfavored (1.817%)	-	-	-

A 56		LYS	11.9	-	Favored (31.97%) General / -127.0,121.5	Favored (36.8%) *ttpt* chi angles: 179.6,173.2,69,172.4	0.04Å	Favored (5.722%)	-	-	-
A 57		ALA	11.69	-	Favored (25.77%) General / -84.0,-31.1	-	0.03Å	Favored (26.359%)	-	-	-
A 58		ALA	11.59	-	Favored (23.72%) General / -162.3,158.2	-	0.03Å	Favored (14.925%)	-	-	-
A 59		ASP	11.69	-	Favored (23.71%) General / -86.0,115.6	Favored (45.6%) *m-30* chi angles: 290.2,307	0.01Å	Favored (34.756%)	-	-	-
A 60		ILE	11.97	-	Favored (48.37%) Ile or Val / -100.4,131.2	Favored (47.7%) *mm* chi angles: 305.6,302.2	0.02Å	Favored (55.012%) beta sheet	-	-	-
**#**	**Alt**	**Res**	**High B**	**Clash > 0.4Å**	**Ramachandran**	**Rotamer**	**Cβ deviation**	**CaBLAM**	**Bond lengths**	**Bond angles**	**Cis Peptides**
			Avg: 6.62	Clashscore: 0.98	Outliers: 2 of 129	Poor rotamers: 0 of 105	Outliers: 0 of 118	Outliers: 9 of 127	Outliers: 2 of 131	Outliers: 5 of 131	Non-Trans: 0 of 130
A 61		THR	12.36	-	Favored (32.75%) General / -125.9,159.3	Favored (59.7%) *p* chi angles: 64.1	0.03Å	Favored (53.827%) beta sheet	-	-	-
A 62		TRP	12.73	-	Favored (47.02%) General / -123.5,147.7	Favored (37.8%) *m-90* chi angles: 294.8,261.7	0.03Å	Favored (61.284%) beta sheet	-	-	-
A 63		GLU	13.04	-	Favored (46.09%) General / -98.0,7.6	Favored (94.5%) *mt-10* chi angles: 297.1,181.7,359.7	0.01Å	CaBLAM Disfavored (1.57%)	-	-	-
A 64		GLN	13.21	-	Allowed (0.07%) General / 56.3,-97.3	Favored (98.1%) *mt0* chi angles: 292.9,179.3,330.2	0.07Å	CaBLAM Outlier (0.469%)	-	-	-
A 65		ASN	13.2	-	Allowed (1.75%) General / -74.2,72.5	Favored (57.4%) *t0* chi angles: 188,28.1	0.01Å	Favored (26.803%)	-	-	-
A 66		ALA	12.99	-	Favored (4.54%) General / -78.0,74.0	-	0.02Å	Favored (43.644%)	-	-	-
A 67		GLU	12.54	-	Favored (68.15%) General / -64.5,-25.8	Favored (95.4%) *mt-10* chi angles: 292.8,183.7,1.1	0.02Å	Favored (37.799%)	-	-	-
A 68		ILE	11.83	-	Favored (57.99%) Ile or Val / -104.5,122.9	Favored (86.1%) *mt* chi angles: 298.8,170	0.02Å	Favored (19.997%)	-	-	-
A 69		THR	10.85	-	Favored (11.35%) General / -118.5,23.7	Favored (65.9%) *p* chi angles: 58.4	0.04Å	Favored (13.887%)	-	-	-
A 70		GLY	9.72	-	Favored (43.69%) Glycine / -82.1,-169.7	-	-	Favored (47.234%) beta sheet	-	-	-

A 71		THR	8.56	-	Favored (56.46%) General / -113.6,130.1	Favored (96%) *m* chi angles: 299.6	0.04Å	Favored (13.416%) beta sheet	-	-	-
A 72		SER	7.55	-	Favored (96.02%) Pre-Pro / -68.2,143.6	Favored (42.3%) *t* chi angles: 175.8	0.08Å	Favored (43.723%) beta sheet	-	-	-
A 73		PRO	6.85	-	Favored (48.51%) Trans-Pro / -71.1,160.6	Favored (65.8%) *Cg_endo* chi angles: 26.9,325.8,27.4	0.03Å	Favored (71.849%) beta sheet	-	-	-
A 74		ARG	6.53	-	Favored (36.98%) General / -134.1,130.1	Favored (52.1%) *ttt90* chi angles: 181.2,181.3,172.3,96.4	0.05Å	Favored (52.904%) beta sheet	-	-	-
A 75		LEU	6.65	-	Favored (21.78%) General / -107.0,109.8	Favored (82%) *mt* chi angles: 301.1,178.6	0.02Å	Favored (70.208%) beta sheet	-	-	-
A 76		ASP	7.19	-	Favored (23.91%) General / -86.9,114.8	Favored (51.5%) *m-30* chi angles: 292,305.3	0.04Å	Favored (58.874%) beta sheet	-	-	-
A 77		VAL	8.14	-	Favored (16.06%) Ile or Val / -124.4,169.2	Favored (27.2%) *m* chi angles: 298.9	0.05Å	Favored (28.922%) beta sheet	-	-	-
A 78		ASP	9.41	-	Favored (20.67%) General / -145.2,132.6	Favored (12.9%) *t70* chi angles: 187.6,296.1	0.04Å	Favored (53.776%) beta sheet	-	-	-
A 79		LEU	10.84	-	Favored (18.56%) General / -82.8,6.3	Favored (78.1%) *mt* chi angles: 288.5,171.7	0.04Å	CaBLAM Outlier (0.321%)	-	-	-
A 80		ASP	12.16	-	Allowed (1.37%) General / 57.2,-132.8	Favored (11.1%) *t70* chi angles: 196.2,72.6	0.11Å	CaBLAM Outlier (0.393%)	-	OUTLIER(S) worst is CA-CB-CG: 4.8 σ	-
**#**	**Alt**	**Res**	**High B**	**Clash > 0.4Å**	**Ramachandran**	**Rotamer**	**Cβ deviation**	**CaBLAM**	**Bond lengths**	**Bond angles**	**Cis Peptides**
			Avg: 6.62	Clashscore: 0.98	Outliers: 2 of 129	Poor rotamers: 0 of 105	Outliers: 0 of 118	Outliers: 9 of 127	Outliers: 2 of 131	Outliers: 5 of 131	Non-Trans: 0 of 130
A 81		SER	13.08	-	Favored (3.42%) General / -129.3,-19.9	Favored (97.2%) *p* chi angles: 63.5	0.10Å	CaBLAM Outlier (0.036%)	-	-	-
A 82		HIS	13.36	-	Favored (54.92%) General / -92.4,-3.6	Favored (79%) *m90* chi angles: 293,86.8	0.04Å	Favored (43.739%)	-	-	-
A 83		GLY	12.9	-	Favored (89.07%) Glycine / 84.8,1.2	-	-	Favored (81.785%)	-	-	-
A 84		ASN	11.84	-	Favored (35.41%) General / -88.9,130.5	Favored (88.4%) *m-40* chi angles: 293.2,320.2	0.03Å	Favored (31.331%)	-	-	-
A 85		PHE	10.5	-	Favored (38.28%) General / -78.6,141.2	Favored (49%) *m-80* chi angles: 284,106.1	0.12Å	Favored (43.518%) beta sheet	-	OUTLIER(S) worst is CA-CB-CG: 4.5 σ	-
A 86		LYS	9.13	-	Favored (42.07%) General / -119.3,149.3	Favored (97%) *mttt* chi angles: 297.5,176.8,182.9,175	0.07Å	Favored (53.847%) beta sheet	-	-	-
A 87		LEU	7.94	-	Favored (42.95%)	Favored (64.7%) *tp* chi angles: 176.7,64	0.04Å	Favored (56.398%)	-	-	-

					General / -98.2,124.8			beta sheet			
A 88		LEU	6.99	-	Favored (57.2%) General / -85.2,-9.9	Favored (74.6%) *mt* chi angles: 301.2,174.1	0.10Å	Favored (47.432%)	-	-	-
A 89		ASN	6.28	-	Favored (8.54%) General / -105.4,27.2	Favored (91.3%) *m-40* chi angles: 292.3,324.1	0.05Å	Favored (7.271%)	-	-	-
A 90		ASP	5.75	-	Favored (46.63%) Pre-Pro / -107.7,102.8	Favored (62.5%) *t0* chi angles: 182.8,342.2	0.03Å	Favored (13.903%)	-	-	-
A 91		PRO	5.34	-	Favored (7.15%) Trans-Pro / -84.8,3.9	Favored (29.5%) *Cg_endo* chi angles: 35.2,323.9,22.1	0.07Å	CaBLAM Outlier (0.846%)	-	-	-
A 92		GLY	4.97	-	Favored (53.73%) Glycine / 82.2,-175.8	-	-	Favored (47.298%)	-	-	-
A 93		ALA	4.58	-	Favored (98.21%) Pre-Pro / -56.0,-42.8	-	0.03Å	CA Geom Outlier (0.003%)	-	-	-
A 94		PRO	4.16	-	OUTLIER (0)%) Trans-Pro / 115.1,143.8	Favored (66.6%) *Cg_endo* chi angles: 31.3,323,33.4	0.23Å	CaBLAM Outlier (0.691%)	OUTLIER(S) worst is N--CD: 10.3 σ	OUTLIER(S) worst is CA-N-CD: 14.6 σ	-
A 95		VAL	3.73	-	Favored (77.83%) Ile or Val / -59.0,-39.3	Favored (65%) *t* chi angles: 171.5	0.01Å	Favored (60.637%)	-	-	-
A 96		HIS	3.32	-	Favored (63.5%) General / -55.8,-52.6	Favored (89.5%) *t70* chi angles: 178,76.1	0.04Å	Favored (73.836%) alpha helix	-	-	-
A 97		LEU	2.94	-	Favored (85.75%) General / -66.2,-37.5	Favored (88.4%) *mt* chi angles: 291.8,174.8	0.02Å	Favored (76.367%) alpha helix	-	-	-
A 98		PHE	2.6	-	Favored (71.46%) General / -56.9,-50.8	Favored (83%) *t80* chi angles: 172.5,79.7	0.10Å	Favored (84.579%) alpha helix	-	-	-
A 99		ALA	2.29	-	Favored (87.55%) General / -59.8,-40.3	-	0.04Å	Favored (87.221%) alpha helix	-	-	-
A 100		LEU	2.03	-	Favored (90%) General / -65.9,-38.9	Favored (96.3%) *mt* chi angles: 291.9,172.7	0.04Å	Favored (94.413%) alpha helix	-	-	-
**#**	**Alt**	**Res**	**High B**	**Clash > 0.4Å**	**Ramachandran**	**Rotamer**	**Cβ deviation**	**CaBLAM**	**Bond lengths**	**Bond angles**	**Cis Peptides**
			Avg: 6.62	Clashscore: 0.98	Outliers: 2 of 129	Poor rotamers: 0 of 105	Outliers: 0 of 118	Outliers: 9 of 127	Outliers: 2 of 131	Outliers: 5 of 131	Non-Trans: 0 of 130
A 101		ARG	1.84	-	Favored (87.5%) General / -66.7,-41.7	Favored (85.7%) *mtt180* chi angles: 290.6,170.4,190.5,167.5	0.09Å	Favored (86.672%) alpha helix	-	-	-
A 102		PHE	1.7	-	Favored (78.49%) General / -58.4,-49.1	Favored (88.1%) *t80* chi angles: 180,80.7	0.05Å	Favored (96.214%) alpha helix	-	-	-
A 103		ILE	1.6	-	Favored (97.34%)	Favored (90.1%) *mt* chi angles: 291.6,166.2	0.04Å	Favored (97.026%) alpha helix	-	-	-

					Ile or Val / -61.6,-46.1						
A 104		LEU	1.54	-	Favored (89.41%) General / -63.9,-38.0	Favored (86.7%) *mt* chi angles: 291.4,174.6	0.04Å	Favored (85.303%) alpha helix	-	-	-
A 105		LEU	1.57	-	Favored (73.35%) General / -70.8,-37.4	Favored (95.6%) *mt* chi angles: 292.9,174	0.04Å	Favored (93.477%) alpha helix	-	-	-
A 106		GLY	1.76	-	Favored (62.65%) Glycine / -60.6,-50.9	-	-	Favored (92.767%) alpha helix	-	-	-
A 107		LEU	2.23	-	Favored (73.6%) General / -55.7,-49.2	Favored (67.8%) *tp* chi angles: 175.5,62.3	0.06Å	Favored (97.736%) alpha helix	-	-	-
A 108		SER	3.19	-	Favored (95.12%) General / -60.3,-42.3	Favored (73.2%) *m* chi angles: 295.6	0.11Å	Favored (87.986%) alpha helix	-	-	-
A 109		ALA	4.78	-	Favored (88.16%) General / -62.4,-38.0	-	0.03Å	Favored (74.573%) alpha helix	-	-	-
A 110		ARG	6.75	-	Favored (24.79%) General / -82.5,-37.9	Favored (87.8%) *mtp180* chi angles: 291.3,175.2,65.7,192.8	0.04Å	Favored (60.908%) alpha helix	-	-	-
A 111		PHE	8.16	-	Favored (6.33%) General / -125.8,100.3	Favored (82.8%) *m-80* chi angles: 300.9,87.7	0.07Å	Favored (20.089%)	-	-	-
A 112		HIS	8.01	-	Favored (49.66%) General / -56.5,-25.0	Favored (61%) *p-80* chi angles: 66.8,282	0.01Å	Favored (35.406%)	-	-	-
A 113		TRP	6.41	-	Favored (67.82%) General / -55.4,-37.6	Favored (88.6%) *t60* chi angles: 184.3,89.7	0.06Å	Favored (61.983%)	-	-	-
A 114		PHE	4.43	-	Favored (51.63%) General / -85.0,0.3	Favored (86.7%) *m-80* chi angles: 290.2,87.6	0.07Å	Favored (46.831%) alpha helix	-	-	-
A 115		ILE	2.92	-	Favored (5.92%) Pre-Pro / -54.0,-61.0	Favored (89.7%) *mt* chi angles: 291.1,167	0.16Å	Favored (32.791%) alpha helix	-	OUTLIER(S) worst is CA-C-N: 4.1 σ	-
A 116		PRO	2.04	-	Favored (74.22%) Trans-Pro / -62.4,-22.6	Favored (37.8%) *Cg_endo* chi angles: 22.9,326.4,29.7	0.00Å	Favored (40.335%) alpha helix	-	-	-
A 117		PHE	1.61	-	Favored (20.07%) General / -79.0,-45.5	Favored (98.4%) *m-80* chi angles: 297.2,89.7	0.09Å	Favored (70.469%) alpha helix	-	-	-
A 118		GLY	1.42	-	Favored (95.98%) Glycine / -63.6,-38.2	-	-	Favored (93.343%) alpha helix	-	-	-
A 119		VAL	1.38	-	Favored (89.51%) Ile or Val / -64.9,-47.1	Favored (57.8%) *t* chi angles: 170.5	0.03Å	Favored (85.438%) alpha helix	-	-	-
A 120		LEU	1.39	-	Favored (89.29%) General / -61.8,-46.5	Favored (66.6%) *tp* chi angles: 176.7,59.6	0.04Å	Favored (84.983%) alpha helix	-	-	-

#	Alt	Res	High B	Clash > 0.4Å	Ramachandran	Rotamer	Cβ deviation	CaBLAM	Bond lengths	Bond angles	Cis Peptides
			Avg: 6.62	Clashscore: 0.98	Outliers: 2 of 129	Poor rotamers: 0 of 105	Outliers: 0 of 118	Outliers: 9 of 127	Outliers: 2 of 131	Outliers: 5 of 131	Non-Trans: 0 of 130
A 121		GLY	1.41	-	Favored (51.69%) Glycine / -54.2,-51.3	-	-	Favored (97.826%) alpha helix	-	-	-
A 122		PHE	1.46	-	Favored (73.17%) General / -54.8,-48.1	Favored (89.8%) *t80* chi angles: 178.4,81.3	0.03Å	Favored (92.626%) alpha helix	-	-	-
A 123		TRP	1.56	-	Favored (92.39%) General / -59.7,-42.1	Favored (34.6%) *m-10* chi angles: 286.5,339.3	0.07Å	Favored (91.907%) alpha helix	-	-	-
A 124		LEU	1.74	-	Favored (89.39%) General / -66.3,-39.5	Favored (97.6%) *mt* chi angles: 292.3,171.8	0.05Å	Favored (96.395%) alpha helix	-	-	-
A 125		LEU	2.02	-	Favored (75.57%) General / -64.7,-33.3	Favored (93.9%) *mt* chi angles: 292.1,170.6	0.04Å	Favored (82.395%) alpha helix	-	-	-
A 126		GLY	2.43	-	Favored (33.39%) Glycine / -59.8,-54.9	-	-	Favored (84.095%) alpha helix	-	-	-
A 127		LYS	3.01	-	Favored (70.34%) General / -60.8,-30.9	Favored (96.8%) *mttt* chi angles: 289.9,180.1,179.5,180.7	0.01Å	Favored (60.389%) alpha helix	-	-	-
A 128		HIS	3.76	-	Favored (65.86%) General / -62.5,-21.9	Favored (60%) *m170* chi angles: 291.2,170.1	0.04Å	Favored (65.955%) three-ten	-	-	-
A 129		SER	4.65	-	Favored (59.65%) General / -82.6,-7.0	Favored (89.8%) *p* chi angles: 68.9	0.03Å	Favored (55.295%)	-	-	-
A 130		LYS	5.59	-	Favored (72.96%) General / -55.6,-41.1	Favored (87%) *tttt* chi angles: 183.3,177.6,178,180.1	0.02Å	-	-	-	-
A 131		ARG	6.44	-	-	Favored (14.5%) *ptp-170* chi angles: 61.7,199.8,63.9,175.7	0.07Å	-	-	-	-
