# Supplementary material for: Integrative proteome-wide structural analysis and high-throughput docking identify broad-spectrum antiviral scaffolds against Zika, Yellow Fever, West Nile, Saint Louis encephalitis, and Usutu viruses
Source: Front Cell Infect Microbiol. 2026 Apr 30;16:1723132. doi: 10.3389/fcimb.2026.1723132 (PMC13171538; doi:10.3389/fcimb.2026.1723132)
Supplement: Supplementary file 3 [file DataSheet3.zip › SLEV/SLEV_NS3/Mol_probity_Files/SLEV_NS3_1FH-multi.table.pdf]

Viewing SLEV_NS3_1FH-multi.table

When finished, you should close this window. *Hint: Use File | Save As... to save a copy of this page.*

All-Atom Contacts	Clashscore, all atoms:	1.14		99th percentile* (N=1784, all resolutions)
	Clashscore is the number of serious steric overlaps (> 0.4 Å) per 1000 atoms.			
Protein Geometry	Poor rotamers	0	0.00%	Goal: <0.3%
	Favored rotamers	514	99.81%	Goal: >98%
	Ramachandran outliers	1	0.16%	Goal: <0.05%
	Ramachandran favored	609	98.86%	Goal: >98%
	Rama distribution Z-score	1.04 ± 0.34		Goal: abs(Z score) < 2
	MolProbity score^	0.82		100th percentile* (N=27675, 0Å - 99Å)
	Cβ deviations >0.25Å	0	0.00%	Goal: 0
	Bad bonds:	3 / 4936	0.06%	Goal: 0%
	Bad angles:	14 / 6702	0.21%	Goal: <0.1%
Peptide Omegas	Cis Prolines:	0 / 37	0.00%	Expected: ≤1 per chain, or ≤5%
Low-resolution Criteria	CaBLAM outliers	9	1.5%	Goal: <1.0%
	CA Geometry outliers	4	0.65%	Goal: <0.5%
Additional validations	Chiral volume outliers	0/734		
	Waters with clashes	0/0	0.00%	See UnDowser table for details

In the two column results, the left column gives the raw count, right column gives the percentage.

* 100th percentile is the best among structures of comparable resolution; 0th percentile is the worst. For clashscore the comparative set of structures was selected in 2004, for MolProbity score in 2006.

^ MolProbity score combines the clashscore, rotamer, and Ramachandran evaluations into a single score, normalized to be on the same scale as X-ray resolution.

Key to table colors and cutoffs here:

#	Alt	Res	High B	Clash > 0.4Å	Ramachandran	Rotamer	Cβ deviation	CaBLAM	Bond lengths	Bond angles	Cis Peptides
			Avg: 1.22	Clashscore: 1.14	Outliers: 1 of 616	Poor rotamers: 0 of 515	Outliers: 0 of 562	Outliers: 10 of 614	Outliers: 3 of 618	Outliers: 14 of 618	Non-Trans: 0 of 617
A 1		GLY	13.86	-	-	-	-	-	-	-	-
A 2		GLY	13.66	-	Favored (25.04%) Glycine / -93.8,-149.2	-	-	-	-	-	-
A 3		ALA	13.33	-	Favored (78.58%) General / -60.4,-36.6	-	0.04Å	CA Geom Outlier (0.462%)	-	-	-
A 4		LEU	12.88	-	Favored (28.02%) General / 54.0,38.0	Favored (80.8%) *mt* chi angles: 302.2,177.6	0.01Å	CaBLAM Disfavored (3.451%) try beta sheet	-	-	-
A 5		TRP	12.35	-	Favored (69.66%) General / -72.0,-35.7	Favored (32.3%) *m-10* chi angles: 289.1,328.5	0.03Å	CaBLAM Disfavored (1.204%) try beta sheet	-	-	-
A 6		ASP	11.77	-	Favored (26.54%) General / 50.4,45.1	Favored (75.5%) *m-30* chi angles: 296.4,345.8	0.05Å	Favored (5.585%) beta sheet	-	-	-
A 7		VAL	11.18	-	Favored (92.29%)	Favored (86%) *t* chi angles: 176.2	0.06Å	Favored (23.757%)	-	-	-

					Pre-Pro / -67.0,126.3						
A 8		PRO	10.59	-	Favored (85.86%) Trans-Pro / -58.0,146.7	Favored (82.7%) *Cg_exo* chi angles: 334.5,34.3,331.5	0.06Å	Favored (76.369%)	-	-	-
A 9		SER	9.94	-	Favored (98.35%) Pre-Pro / -65.8,143.0	Favored (41.9%) *t* chi angles: 175.3	0.09Å	Favored (46.57%)	-	-	-
A 10		PRO	9.2	-	Favored (21.05%) Trans-Pro / -75.0,170.2	Favored (72.8%) *Cg_endo* chi angles: 29.1,325.1,26.6	0.02Å	Favored (63.833%)	-	-	-
A 11		LYS	8.3	-	Favored (8.2%) General / -74.8,175.2	Favored (98.9%) *mttt* chi angles: 295.4,180.3,179.6,179.2	0.03Å	Favored (9.753%)	-	-	-
A 12		VAL	7.23	-	Favored (37.9%) Ile or Val / -78.2,125.6	Favored (83.9%) *t* chi angles: 176.3	0.04Å	Favored (6.561%)	-	-	-
A 13		TYR	6.06	-	Favored (71.8%) Pre-Pro / -129.3,158.8	Favored (99.4%) *m-80* chi angles: 296.6,92.6	0.01Å	Favored (28.62%)	-	-	-
A 14		PRO	4.86	-	Favored (36.05%) Trans-Pro / -53.4,147.8	Favored (94.5%) *Cg_exo* chi angles: 332.9,35.3,331.7	0.03Å	Favored (36.257%)	-	-	-
A 15		LYS	3.73	-	Favored (58.9%) General / -63.9,140.7	Favored (29.1%) *mmtm* chi angles: 293.7,291.6,189,292	0.04Å	Favored (38.171%)	-	-	-
A 16		CYS	2.78	-	Favored (44.32%) General / -67.7,130.9	Favored (49.8%) *t* chi angles: 179.8	0.10Å	Favored (47.079%) beta sheet	-	-	-
A 17		GLU	2.05	-	Favored (37.94%) General / -76.4,130.4	Favored (89.5%) *tt0* chi angles: 184.6,177.4,5.9	0.02Å	Favored (51.731%) beta sheet	-	-	-
A 18		THR	1.53	-	Favored (4.93%) General / -123.1,35.2	Favored (51.2%) *p* chi angles: 56.4	0.10Å	CaBLAM Disfavored (4.598%) try beta sheet	-	-	-
A 19		LYS	1.18	-	Favored (96.59%) Pre-Pro / -62.5,138.4	Favored (98.3%) *mttt* chi angles: 291.3,179.8,180.5,175.9	0.04Å	Favored (20.981%)	-	-	-
A 20		PRO	0.97	-	Favored (70.75%) Trans-Pro / -54.6,142.8	Favored (99.2%) *Cg_exo* chi angles: 332.6,35.7,331.2	0.04Å	CaBLAM Disfavored (4.249%)	-	-	-
**#**	**Alt**	**Res**	**High B**	**Clash > 0.4Å**	**Ramachandran**	**Rotamer**	**Cβ deviation**	**CaBLAM**	**Bond lengths**	**Bond angles**	**Cis Peptides**
			Avg: 1.22	Clashscore: 1.14	Outliers: 1 of 616	Poor rotamers: 0 of 515	Outliers: 0 of 562	Outliers: 10 of 614	Outliers: 3 of 618	Outliers: 14 of 618	Non-Trans: 0 of 617
A 21		GLY	0.85	-	Favored (29.63%) Glycine / 151.7,-177.4	-	-	Favored (48.414%)	-	-	-
A 22		ILE	0.79	-	Favored (54.97%) Ile or Val / -102.3,128.4	Favored (81.9%) *mt* chi angles: 299.8,171.1	0.05Å	Favored (10.238%) beta sheet	-	-	-

A 23		TYR	0.79	-	Favored (49.52%) General / -124.0,144.9	Favored (85.8%) *m-80* chi angles: 296.3,83.7	0.04Å	Favored (54.994%) beta sheet	-	-	-
A 24		ARG	0.83	-	Favored (30.14%) General / -82.7,144.6	Favored (82.2%) *mtt90* chi angles: 296.7,176.3,178.6,86.7	0.04Å	Favored (44.165%) beta sheet	-	-	-
A 25		ILE	0.92	-	Favored (48.34%) Ile or Val / -106.0,115.7	Favored (78%) *mt* chi angles: 300.5,170	0.05Å	Favored (63.805%) beta sheet	-	-	-
A 26		MET	1.06	-	Favored (27.91%) General / -111.7,152.2	Favored (64.4%) *mmm* chi angles: 310.5,296.4,291.4	0.04Å	Favored (43.087%) beta sheet	-	-	-
A 27		THR	1.24	-	Favored (48.31%) General / -128.5,132.6	Favored (89%) *m* chi angles: 298.3	0.05Å	Favored (59.085%) beta sheet	-	-	-
A 28		ARG	1.44	-	Favored (70.66%) General / -54.6,-49.3	Favored (55.5%) *ttp-170* chi angles: 181.5,189.2,68,198.3	0.05Å	CaBLAM Outlier (0.76%)	-	-	-
A 29		GLY	1.6	-	Favored (48.08%) Glycine / 62.1,-148.7	-	-	Favored (14.233%)	-	-	-
A 30		ILE	1.67	-	Favored (75.67%) Ile or Val / -120.8,126.8	Favored (89.7%) *mt* chi angles: 298,170.8	0.05Å	CaBLAM Outlier (0.246%)	-	-	-
A 31		LEU	1.62	-	Favored (23.91%) General / 57.6,42.5	Favored (89%) *mt* chi angles: 298.7,174.8	0.05Å	CA Geom Outlier (0.286%)	-	-	-
A 32		GLY	1.46	-	Favored (3.19%) Glycine / 125.3,128.1	-	-	Favored (28.243%)	-	-	-
A 33		THR	1.26	-	Favored (56.23%) General / -60.6,133.6	Favored (82%) *m* chi angles: 302.3	0.03Å	Favored (32.258%)	-	-	-
A 34		PHE	1.07	-	Favored (26.85%) General / -122.8,160.0	Favored (55.6%) *p90* chi angles: 67.3,90.9	0.01Å	Favored (38.994%)	-	-	-
A 35		GLN	0.92	-	Favored (27.64%) General / -85.1,121.3	Favored (50.8%) *tt0* chi angles: 182.1,176.8,310.8	0.04Å	Favored (35.79%)	-	-	-
A 36		ALA	0.82	-	Favored (18.65%) General / -88.1,-32.6	-	0.04Å	Favored (8.169%)	-	-	-
A 37		GLY	0.76	-	Favored (41.09%) Glycine / -177.7,-171.4	-	-	Favored (29.752%)	-	-	-
A 38		VAL	0.73	-	Favored (51.03%) Ile or Val / -133.6,138.4	Favored (54.9%) *t* chi angles: 180.6	0.12Å	Favored (19.1%)	-	-	-
A 39		GLY	0.73	-	Favored (13.56%) Glycine / -131.9,-174.2	-	-	Favored (48.65%) beta sheet	-	-	-

#	Alt	Res	High B	Clash > 0.4Å	Ramachandran	Rotamer	Cβ deviation	CaBLAM	Bond lengths	Bond angles	Cis Peptides
A 40		VAL	0.74	-	Favored (47.29%) Ile or Val / -136.3,138.1	Favored (8.7%) *p* chi angles: 63.4	0.08Å	Favored (38.195%) beta sheet	-	-	-
			Avg: 1.22	Clashscore: 1.14	Outliers: 1 of 616	Poor rotamers: 0 of 515	Outliers: 0 of 562	Outliers: 10 of 614	Outliers: 3 of 618	Outliers: 14 of 618	Non-Trans: 0 of 617
A 41		MET	0.77	-	Favored (41.65%) General / -95.0,130.6	Favored (38.7%) *tpp* chi angles: 188.8,75.6,68.9	0.08Å	Favored (51.998%)	-	-	-
A 42		HIS	0.81	-	Favored (12.64%) General / -146.8,125.6	Favored (78.5%) *t70* chi angles: 185.3,76.1	0.06Å	Favored (21.888%)	-	-	-
A 43		GLU	0.83	-	Favored (24.16%) General / 51.6,39.0	Favored (67.5%) *mt-10* chi angles: 302.6,189.7,359.1	0.04Å	Favored (39.006%)	-	-	-
A 44		GLY	0.84	-	Favored (89.95%) Glycine / 83.2,0.0	-	-	Favored (82.494%)	-	-	-
A 45		VAL	0.83	-	Favored (59.41%) Ile or Val / -114.7,133.9	Favored (82.6%) *t* chi angles: 177.8	0.03Å	Favored (30.562%)	-	-	-
A 46		PHE	0.81	-	Favored (36.7%) General / -90.6,129.8	Favored (66.6%) *t80* chi angles: 184.5,70.6	0.04Å	Favored (59.275%) beta sheet	-	-	-
A 47		HIS	0.78	-	Favored (54.69%) General / -122.2,134.4	Favored (47%) *m90* chi angles: 305.2,79.9	0.13Å	Favored (35.997%) beta sheet	-	-	-
A 48		THR	0.77	-	Favored (4.73%) General / -149.8,-174.6	Favored (10.4%) *t* chi angles: 188.5	0.13Å	Favored (14.955%) beta sheet	-	-	-
A 49		MET	0.78	-	Favored (28.28%) General / -93.2,141.6	Favored (91.2%) *mmm* chi angles: 303.1,301,291.3	0.03Å	Favored (12.532%)	-	-	-
A 50		TRP	0.81	-	Favored (90.03%) General / -58.9,-45.5	Favored (89.2%) *t60* chi angles: 177.4,82.5	0.11Å	Favored (54.564%)	-	-	-
A 51		HIS	0.86	-	Favored (38.18%) General / -59.2,-18.6	Favored (49.4%) *p-80* chi angles: 72.3,278.1	0.10Å	Favored (60.513%) alpha helix	-	OUTLIER(S) worst is CA-CB-CG: 4.0 σ	-
A 52		ALA	0.95	-	Favored (59.73%) General / -80.6,-10.7	-	0.06Å	Favored (36.005%) alpha helix	-	-	-
A 53		THR	1.04	-	Favored (2.86%) General / -128.7,-25.3	Favored (52.4%) *p* chi angles: 65.3	0.17Å	CaBLAM Disfavored (2.974%)	-	-	-
A 54		GLU	1.15	-	Favored (11.22%) General / 54.8,29.1	Favored (91.1%) *mt-10* chi angles: 298.6,184.5,357.9	0.04Å	Favored (5.734%)	-	-	-
A 55		GLY	1.25	-	Favored (89.93%) Glycine / 83.8,1.2	-	-	Favored (51.357%)	-	-	-

A 56		ALA	1.34	-	Favored (48.86%) General / -64.3,149.1	-	0.04Å	Favored (21.208%)	-	-	-
A 57		VAL	1.43	-	Favored (32.96%) Ile or Val / -69.7,132.6	Favored (94.9%) *t* chi angles: 174.8	0.06Å	Favored (41.63%) beta sheet	-	-	-
A 58		LEU	1.53	-	Favored (25.77%) General / -97.9,146.1	Favored (82.4%) *mt* chi angles: 300.9,178.7	0.02Å	Favored (41.479%) beta sheet	-	-	-
A 59		ARG	1.65	-	Favored (36.87%) General / -95.5,135.7	Favored (80.8%) *ttt180* chi angles: 182.4,172,177.4,175.8	0.07Å	Favored (54.504%)	-	-	-
A 60		ASN	1.77	-	Favored (3.71%) General / -133.2,96.3	Favored (56.3%) *m-40* chi angles: 295.4,276.2	0.03Å	Favored (12.177%)	-	-	-
**#**	**Alt**	**Res**	**High B**	**Clash > 0.4Å**	**Ramachandran**	**Rotamer**	**Cβ deviation**	**CaBLAM**	**Bond lengths**	**Bond angles**	**Cis Peptides**
			Avg: 1.22	Clashscore: 1.14	Outliers: 1 of 616	Poor rotamers: 0 of 515	Outliers: 0 of 562	Outliers: 10 of 614	Outliers: 3 of 618	Outliers: 14 of 618	Non-Trans: 0 of 617
A 61		GLY	1.86	-	Favored (33.48%) Glycine / 62.6,-124.6	-	-	Favored (66.356%)	-	-	-
A 62		GLU	1.92	-	Favored (57.83%) General / -88.8,-5.7	Favored (97%) *mt-10* chi angles: 295.6,178.6,359.2	0.01Å	Favored (13.111%)	-	-	-
A 63		GLY	1.91	-	Favored (21.31%) Glycine / -109.3,-178.4	-	-	Favored (17.609%)	-	-	-
A 64		ARG	1.84	-	Favored (51.33%) General / -130.8,144.3	Favored (66.7%) *mmm-85* chi angles: 299.9,295,296.9,274.6	0.03Å	Favored (56.068%)	-	-	-
A 65		LEU	1.73	-	Favored (22.86%) General / -122.8,116.0	Favored (26.2%) *tp* chi angles: 187.9,66.7	0.09Å	Favored (57.028%)	-	-	-
A 66		ASP	1.62	-	Favored (50.4%) Pre-Pro / -83.6,146.7	Favored (15.2%) *p0* chi angles: 58.9,306.8	0.03Å	Favored (37.647%)	-	-	-
A 67		PRO	1.53	-	Favored (90.81%) Trans-Pro / -57.5,139.7	Favored (84.5%) *Cg_exo* chi angles: 334.1,34.9,330.4	0.10Å	Favored (43.113%)	-	-	-
A 68		TYR	1.48	-	Favored (2.81%) General / -115.6,-47.5	Favored (89.4%) *t80* chi angles: 179.7,79.3	0.03Å	Favored (11.918%)	-	-	-
A 69		ALA	1.46	-	Favored (43.51%) General / -148.0,159.8	-	0.04Å	CA Geom Outlier (0.062%)	-	-	-
A 70		GLY	1.46	-	Favored (46.24%) Glycine / 175.4,179.9	-	-	Favored (41.288%)	-	-	-

A 71		ASP	1.43	-	Favored (3.47%) General / -147.1,102.0	Favored (62.9%) *t0* chi angles: 184,358.9	0.07Å	CaBLAM Disfavored (2.419%) try beta sheet	-	-	-
A 72		VAL	1.38	-	Favored (39.68%) Ile or Val / -61.1,-26.6	Favored (3.3%) *p* chi angles: 73.6	0.06Å	Favored (42.673%)	-	-	-
A 73		ARG	1.3	-	Favored (70.97%) General / -63.4,-29.7	Favored (80.7%) *mtt90* chi angles: 291.6,180.7,182.4,88.7	0.02Å	Favored (55.545%) alpha helix	-	-	-
A 74		ASN	1.21	-	Favored (23.6%) General / -110.0,12.5	Favored (70.6%) *m-40* chi angles: 288.3,282	0.02Å	Favored (36.414%)	-	-	-
A 75		ASP	1.12	-	Favored (29.34%) General / 51.4,42.6	Favored (41.9%) *t0* chi angles: 190.9,22.7	0.07Å	Favored (30.431%)	-	OUTLIER(S) worst is CA-CB-CG: 4.2 σ	-
A 76		LEU	1.05	-	Favored (50.06%) General / -118.6,140.3	Favored (84.8%) *mt* chi angles: 299.3,174.3	0.08Å	Favored (16.462%) beta sheet	-	-	-
A 77		ILE	1.01	-	Favored (36.79%) Ile or Val / -139.6,140.5	Favored (20.8%) *tt* chi angles: 184.5,166.9	0.06Å	Favored (59.716%) beta sheet	-	-	-
A 78		SER	1.01	-	Favored (49.1%) General / -116.9,140.7	Favored (71%) *m* chi angles: 296.2	0.07Å	Favored (70.976%)	-	-	-
A 79		TYR	1.03	-	Favored (55.45%) General / -120.4,133.3	Favored (68.5%) *m-80* chi angles: 295.5,79.9	0.07Å	Favored (12.845%)	-	-	-
A 80		GLY	1.05	-	Favored (68.03%) Glycine / 88.1,-11.2	-	-	Favored (6.819%)	-	-	-
**#**	**Alt**	**Res**	**High B**	**Clash > 0.4Å**	**Ramachandran**	**Rotamer**	**Cβ deviation**	**CaBLAM**	**Bond lengths**	**Bond angles**	**Cis Peptides**
			Avg: 1.22	Clashscore: 1.14	Outliers: 1 of 616	Poor rotamers: 0 of 515	Outliers: 0 of 562	Outliers: 10 of 614	Outliers: 3 of 618	Outliers: 14 of 618	Non-Trans: 0 of 617
A 81		GLY	1.04	-	Favored (46.43%) Glycine / 175.1,-179.3	-	-	Favored (14.31%)	-	-	-
A 82		PRO	1.01	-	Favored (61.16%) Trans-Pro / -66.8,157.5	Favored (50%) *Cg_endo* chi angles: 25.2,326.6,27.1	0.03Å	Favored (5.929%)	-	-	-
A 83		TRP	0.96	-	Favored (31.95%) General / -60.5,127.5	Favored (55.5%) *t-100* chi angles: 186.7,267.1	0.02Å	Favored (15.322%)	-	-	-
A 84		LYS	0.9	-	Favored (13.54%) General / -109.8,-18.0	Favored (70.8%) *mmtt* chi angles: 299.8,294.8,188.9,179.1	0.06Å	Favored (7.774%)	-	-	-
A 85		LEU	0.85	-	Favored (4.36%) General / -78.0,81.7	Favored (66.3%) *mt* chi angles: 304,179.6	0.03Å	Favored (12.445%)	-	-	-
A 86		SER	0.8	-	Favored (55.74%) General / -82.2,-14.4	Favored (89.8%) *p* chi angles: 68.9	0.04Å	Favored (9.986%)	-	-	-

A 87		ALA	0.78	-	Favored (29.64%) General / -79.8,152.8	-	0.02Å	Favored (19.615%)	-	-	-
A 88		THR	0.77	-	Favored (9.2%) General / -132.2,175.5	Favored (46.4%) *p* chi angles: 66.3	0.05Å	Favored (38.943%)	-	-	-
A 89		TRP	0.77	-	Favored (18.37%) General / -85.4,163.7	Favored (44.4%) *p90* chi angles: 53.3,91	0.16Å	Favored (38.784%)	-	-	-
A 90		ASP	0.75	-	Favored (10.75%) General / -120.4,1.9	Favored (56.7%) *p0* chi angles: 62.8,358.8	0.05Å	CaBLAM Disfavored (4.81%)	-	-	-
A 91		GLY	0.74	-	Favored (84.47%) Glycine / 81.2,-1.6	-	-	Favored (39.433%)	-	-	-
A 92		THR	0.71	-	Favored (2.01%) General / -132.4,-24.8	Favored (61.6%) *p* chi angles: 63.7	0.03Å	CaBLAM Disfavored (3.121%)	-	-	-
A 93		GLU	0.68	-	Favored (24.08%) General / -85.3,151.4	Favored (7.1%) *tp30* chi angles: 187.7,60.1,98.5	0.04Å	Favored (23.644%)	-	-	-
A 94		GLU	0.66	-	Favored (56.37%) General / -62.5,134.4	Favored (89.7%) *tt0* chi angles: 182.7,177.2,353	0.01Å	Favored (34.68%) beta sheet	-	-	-
A 95		VAL	0.64	-	Favored (36.96%) Ile or Val / -127.8,160.7	Favored (20.7%) *m* chi angles: 302.1	0.05Å	Favored (54.74%) beta sheet	-	-	-
A 96		GLN	0.63	-	Favored (55.69%) General / -119.3,130.9	Favored (34.8%) *tt0* chi angles: 178.1,176.2,281.9	0.04Å	Favored (59.606%) beta sheet	-	-	-
A 97		MET	0.65	-	Favored (44.02%) General / -100.7,134.2	Favored (55.5%) *ttm* chi angles: 183.3,178.3,295.5	0.03Å	Favored (66.8%) beta sheet	-	-	-
A 98		ILE	0.68	-	Favored (2.98%) Ile or Val / -114.5,94.2	Favored (3.7%) *mp* chi angles: 302.6,91.3	0.07Å	Favored (49.517%) beta sheet	-	-	-
A 99		ALA	0.74	-	Favored (34.61%) General / -93.6,135.7	-	0.04Å	Favored (32.869%) beta sheet	-	-	-
A 100		VAL	0.84	-	Favored (7.99%) Ile or Val / -114.8,99.2	Favored (55.7%) *t* chi angles: 180.5	0.03Å	Favored (58.968%)	-	-	-
**#**	**Alt**	**Res**	**High B**	**Clash > 0.4Å**	**Ramachandran**	**Rotamer**	**Cβ deviation**	**CaBLAM**	**Bond lengths**	**Bond angles**	**Cis Peptides**
			Avg: 1.22	Clashscore: 1.14	Outliers: 1 of 616	Poor rotamers: 0 of 515	Outliers: 0 of 562	Outliers: 10 of 614	Outliers: 3 of 618	Outliers: 14 of 618	Non-Trans: 0 of 617
A 101		ALA	0.95	-	Favored (69.43%) Pre-Pro / -80.6,150.2	-	0.03Å	Favored (18.454%)	-	-	-
A 102		PRO	1.05	-	Favored (46.53%) Trans-Pro / -53.6,132.0	Favored (98%) *Cg_exo* chi angles: 332,36.7,330.9	0.08Å	Favored (37.803%)	-	-	-

A 103		GLY	1.12	-	Favored (84.36%) Glycine / 80.1,-0.2	-	-	Favored (73.012%)	-	-	-
A 104		LYS	1.14	-	Favored (74.36%) Pre-Pro / -127.4,156.9	Favored (94.9%) *mttt* chi angles: 295.9,186.6,181.4,180.6	0.02Å	Favored (27.575%)	-	-	-
A 105		PRO	1.08	-	Favored (38.97%) Trans-Pro / -71.8,163.4	Favored (73.4%) *Cg_endo* chi angles: 27.6,324.7,28.3	0.01Å	Favored (21.436%)	-	-	-
A 106		ALA	0.99	-	Favored (56.53%) General / -59.0,139.6	-	0.04Å	Favored (16.552%)	-	-	-
A 107		ILE	0.88	-	Favored (64.56%) Ile or Val / -114.8,120.5	Favored (77.9%) *mt* chi angles: 300.4,169.2	0.05Å	Favored (60.74%) beta sheet	-	-	-
A 108		ASN	0.79	-	Favored (28.89%) General / -93.4,116.3	Favored (83.4%) *m-40* chi angles: 296.1,311.4	0.03Å	Favored (60.332%) beta sheet	-	-	-
A 109		VAL	0.72	-	Favored (75.95%) Ile or Val / -121.5,129.0	Favored (85.6%) *t* chi angles: 177.6	0.03Å	Favored (60.105%) beta sheet	-	-	-
A 110		GLN	0.69	-	Favored (43.31%) General / -97.3,132.1	Favored (32.2%) *mp10* chi angles: 295.8,81.4,43.2	0.02Å	Favored (27.163%) beta sheet	-	-	-
A 111		THR	0.69	-	Favored (39.71%) General / -154.2,158.0	Favored (8.5%) *t* chi angles: 184.3	0.10Å	Favored (34.298%) beta sheet	-	-	-
A 112		THR	0.71	-	Favored (45.56%) Pre-Pro / -105.6,121.6	Favored (98.5%) *m* chi angles: 300.2	0.02Å	Favored (29.989%) beta sheet	-	-	-
A 113		PRO	0.76	-	Favored (87.91%) Trans-Pro / -62.5,150.3	Favored (34.8%) *Cg_exo* chi angles: 339.3,33.8,326.8	0.07Å	Favored (47.069%)	-	-	-
A 114		GLY	0.85	-	Favored (21.86%) Glycine / -107.4,-164.6	-	-	Favored (46.55%)	-	-	-
A 115		VAL	0.97	-	Favored (68.26%) Ile or Val / -128.9,129.9	Favored (69.1%) *t* chi angles: 178.9	0.04Å	Favored (16.492%)	-	-	-
A 116		PHE	1.12	-	Favored (53.34%) General / -117.6,136.3	Favored (88.2%) *t80* chi angles: 180.2,76.7	0.04Å	Favored (59.07%) beta sheet	-	-	-
A 117		LYS	1.28	-	Favored (38.31%) General / -92.8,127.5	Favored (88.1%) *tttt* chi angles: 185.4,175.9,180.3,179.6	0.05Å	Favored (54.783%)	-	-	-
A 118		THR	1.42	-	Favored (5.74%) Pre-Pro / -116.7,170.6	Favored (19.3%) *p* chi angles: 72.9	0.12Å	Favored (22.042%)	-	-	-
A 119		PRO	1.48	-	Favored (36.14%) Trans-Pro / -58.3,-18.1	Favored (83.2%) *Cg_exo* chi angles: 334.4,35.6,329.6	0.02Å	Favored (53.08%)	-	-	-
A 120		LEU	1.46	-	Favored (13.74%)	Favored (88.6%) *mt* chi angles: 299.9,177.1	0.04Å	CaBLAM Outlier	-	-	-

#	Alt	Res	High B	Clash > 0.4Å	Ramachandran	Rotamer	Cβ deviation	CaBLAM	Bond lengths	Bond angles	Cis Peptides
					General / -96.3,-29.9			(0.839%)			
#	Alt	Res	High B	Clash > 0.4Å	Ramachandran	Rotamer	Cβ deviation	CaBLAM	Bond lengths	Bond angles	Cis Peptides
			Avg: 1.22	Clashscore: 1.14	Outliers: 1 of 616	Poor rotamers: 0 of 515	Outliers: 0 of 562	Outliers: 10 of 614	Outliers: 3 of 618	Outliers: 14 of 618	Non-Trans: 0 of 617
A 121		GLY	1.34	-	Favored (33.86%) Glycine / 155.9,-171.8	-	-	Favored (25.071%)	-	-	-
A 122		THR	1.19	-	Favored (23.15%) General / -82.4,159.0	Favored (76.9%) *p* chi angles: 60.1	0.05Å	Favored (6.785%)	-	-	-
A 123		ILE	1.04	-	Favored (21.98%) Ile or Val / -144.4,144.9	Favored (22.2%) *tt* chi angles: 190.5,166.2	0.09Å	Favored (45.772%) beta sheet	-	-	-
A 124		GLY	0.91	-	Favored (53.49%) Glycine / -69.2,155.0	-	-	Favored (44.233%) beta sheet	-	-	-
A 125		ALA	0.84	-	Favored (32.32%) General / -150.3,151.1	-	0.04Å	Favored (62.469%) beta sheet	-	-	-
A 126		VAL	0.81	-	Favored (74.71%) Ile or Val / -117.7,126.3	Favored (100%) *t* chi angles: 175.5	0.05Å	Favored (57.071%) beta sheet	-	-	-
A 127		THR	0.8	0.46Å O with A 127 THR HG22	Favored (10.11%) General / -85.8,67.7	Favored (60.5%) *m* chi angles: 303.5	0.02Å	Favored (12.498%) beta sheet	-	-	-
A 128		LEU	0.82	-	Favored (54.94%) General / -123.0,137.5	Favored (95.4%) *mt* chi angles: 294.8,173.7	0.06Å	Favored (26.038%) beta sheet	-	-	-
A 129		ASP	0.84	-	Favored (7.21%) General / -110.5,97.9	Favored (64.8%) *t0* chi angles: 184.9,354.6	0.05Å	Favored (37.004%) beta sheet	-	OUTLIER(S) worst is CA-CB-CG: 4.4 σ	-
A 130		PHE	0.85	-	Favored (42.25%) Pre-Pro / -109.5,154.6	Favored (74.6%) *m-80* chi angles: 287.7,94.1	0.03Å	Favored (27.932%)	-	-	-
A 131		PRO	0.85	-	Favored (56%) Trans-Pro / -68.1,158.8	Favored (45.9%) *Cg_endo* chi angles: 24.7,325,30.4	0.04Å	Favored (45.023%)	-	-	-
A 132		LYS	0.83	-	Favored (41.62%) General / -57.8,143.6	Favored (97.9%) *mttt* chi angles: 292,181.4,180.3,179	0.01Å	Favored (9.624%)	-	-	-
A 133		GLY	0.79	-	Favored (52.97%) Glycine / 103.6,-15.4	-	-	Favored (35.619%)	-	-	-
A 134		THR	0.75	-	Favored (57.03%) General / -89.0,-6.7	Favored (68.4%) *p* chi angles: 58.9	0.04Å	Favored (7.609%)	-	-	-
A 135		SER	0.71	-	Favored (56.99%) General / -58.6,138.2	Favored (71.1%) *m* chi angles: 295.1	0.10Å	Favored (11.532%)	-	-	-
A 136		GLY	0.68	-	Favored (72.44%)	-	-	Favored (74.701%)	-	-	-

#	Alt	Res	High B	Clash > 0.4Å	Ramachandran	Rotamer	Cβ deviation	CaBLAM	Bond lengths	Bond angles	Cis Peptides
					Glycine / 94.2,-7.0						
A 137		SER	0.66	-	Favored (87.19%) Pre-Pro / -59.0,140.0	Favored (70.3%) *m* chi angles: 295	0.07Å	Favored (29.745%)	-	-	-
A 138		PRO	0.65	-	Favored (57.69%) Trans-Pro / -71.9,152.6	Favored (69.7%) *Cg_endo* chi angles: 27.2,326.4,25.9	0.04Å	Favored (71.862%) beta sheet	-	-	-
A 139		ILE	0.66	-	Favored (75%) Ile or Val / -118.0,128.9	Favored (70.9%) *mt* chi angles: 302.1,169.8	0.07Å	Favored (68.745%) beta sheet	-	-	-
A 140		ILE	0.66	-	Favored (27.78%) Ile or Val / -123.1,158.9	Favored (46%) *pt* chi angles: 63.5,171.9	0.08Å	Favored (47.212%)	-	-	-
**#**	**Alt**	**Res**	**High B**	**Clash > 0.4Å**	**Ramachandran**	**Rotamer**	**Cβ deviation**	**CaBLAM**	**Bond lengths**	**Bond angles**	**Cis Peptides**
			Avg: 1.22	Clashscore: 1.14	Outliers: 1 of 616	Poor rotamers: 0 of 515	Outliers: 0 of 562	Outliers: 10 of 614	Outliers: 3 of 618	Outliers: 14 of 618	Non-Trans: 0 of 617
A 141		ASN	0.67	-	Favored (5.34%) General / -89.4,-177.4	Favored (47.6%) *p0* chi angles: 65.3,23	0.09Å	Favored (39.131%)	-	-	-
A 142		LYS	0.69	-	Favored (60.78%) General / -62.5,-17.9	Favored (59.5%) *pttt* chi angles: 69.3,183.4,182.5,185.2	0.02Å	Favored (35.576%)	-	-	-
A 143		LYS	0.69	-	Favored (54.41%) General / -93.5,-2.5	Favored (99%) *mttt* chi angles: 295.4,181.4,179.7,178.7	0.02Å	Favored (48.359%)	-	-	-
A 144		GLY	0.7	-	Favored (70.4%) Glycine / 88.7,8.3	-	-	Favored (89.441%)	-	-	-
A 145		GLU	0.7	-	Favored (29.77%) General / -84.1,143.4	Favored (67.5%) *mt-10* chi angles: 298,176.5,318.4	0.05Å	Favored (35.58%)	-	-	-
A 146		ILE	0.7	-	Favored (31.93%) Ile or Val / -82.1,119.6	Favored (76.7%) *mt* chi angles: 300.1,167	0.05Å	Favored (46.501%)	-	-	-
A 147		ILE	0.71	-	Favored (9.6%) Ile or Val / -104.6,-10.9	Favored (42.9%) *pt* chi angles: 63.3,169.5	0.03Å	CaBLAM Disfavored (4.475%)	-	-	-
A 148		GLY	0.73	-	Favored (39.68%) Glycine / 169.9,176.6	-	-	Favored (24.115%)	-	-	-
A 149		LEU	0.77	-	Favored (41.18%) General / -110.7,143.4	Favored (56.7%) *mt* chi angles: 305.5,179	0.10Å	Favored (14.393%)	-	-	-
A 150		TYR	0.82	-	Favored (24.16%) General / -84.6,152.0	Favored (88.8%) *t80* chi angles: 175.1,79.1	0.03Å	CaBLAM Disfavored (3.967%)	-	-	-
A 151		GLY	0.9	-	Favored (4.13%) Glycine / 122.0,-33.7	-	-	Favored (12.289%)	-	-	-
A 152		ASN	0.99	-	Favored (7.02%) General / -83.4,89.3	Favored (50.9%) *t0* chi angles: 186.6,321.2	0.12Å	Favored (6.592%)	-	-	-
A 153		GLY	1.1	-	Favored (13.73%)	-	-	Favored (41.884%)	-	-	-

					Glycine / -121.7,-169.9						
A 154		VAL	1.2	-	Favored (41.16%) Ile or Val / -134.3,142.3	Favored (6.1%) *p* chi angles: 59	0.10Å	Favored (23.343%) beta sheet	-	-	-
A 155		LEU	1.29	-	Favored (28.43%) General / -87.1,120.5	Favored (52.3%) *tp* chi angles: 182.7,63.8	0.03Å	Favored (52.711%)	-	-	-
A 156		ILE	1.35	-	Favored (64.68%) Ile or Val / -112.7,131.3	Favored (48.1%) *mm* chi angles: 301.3,298.5	0.02Å	Favored (8.577%)	-	-	-
A 157		GLY	1.36	-	Favored (6.33%) Glycine / 55.2,-116.0	-	-	CaBLAM Disfavored (3.12%)	-	-	-
A 158		GLN	1.31	-	Favored (4.63%) General / -57.7,-13.9	Favored (18%) *pm20* chi angles: 62.7,273.7,23.5	0.07Å	CaBLAM Disfavored (2.533%)	-	-	-
A 159		GLY	1.23	-	Favored (2.51%) Glycine / 113.2,31.1	-	-	Favored (12.58%)	-	-	-
A 160		GLU	1.11	-	Favored (29.67%) General / -106.1,146.9	Favored (48.7%) *mt-10* chi angles: 292.1,184.5,286.7	0.04Å	Favored (31.951%)	-	-	-
**#**	**Alt**	**Res**	**High B**	**Clash > 0.4Å**	**Ramachandran**	**Rotamer**	**Cβ deviation**	**CaBLAM**	**Bond lengths**	**Bond angles**	**Cis Peptides**
			Avg: 1.22	Clashscore: 1.14	Outliers: 1 of 616	Poor rotamers: 0 of 515	Outliers: 0 of 562	Outliers: 10 of 614	Outliers: 3 of 618	Outliers: 14 of 618	Non-Trans: 0 of 617
A 161		TYR	1	-	Favored (28.71%) General / -93.4,141.2	Favored (86.8%) *m-80* chi angles: 290.2,90.5	0.04Å	Favored (50.584%) beta sheet	-	-	-
A 162		VAL	0.9	-	Favored (56.63%) Ile or Val / -120.5,118.5	Favored (67.4%) *t* chi angles: 179.1	0.09Å	Favored (68.737%) beta sheet	-	-	-
A 163		SER	0.84	-	Favored (41.84%) General / -97.8,123.6	Favored (26%) *t* chi angles: 171.7	0.04Å	Favored (52.689%) beta sheet	-	-	-
A 164		GLY	0.82	-	Favored (49.59%) Glycine / -67.5,148.3	-	-	Favored (52.672%) beta sheet	-	-	-
A 165		ILE	0.84	-	Favored (16.31%) Ile or Val / -87.3,106.6	Favored (82.2%) *mt* chi angles: 300,172.3	0.08Å	Favored (53.728%) beta sheet	-	-	-
A 166		ILE	0.9	-	Favored (56.38%) Ile or Val / -104.3,129.6	Favored (81%) *mt* chi angles: 299.8,169.6	0.10Å	Favored (36.253%) beta sheet	-	-	-
A 167		GLN	1.01	-	Favored (28.62%) General / -145.5,141.8	Allowed (2%) *tp-100* chi angles: 190,87.4,301.7	0.13Å	Favored (31.839%)	-	-	-
A 168		GLY	1.18	-	Favored (36.3%) Glycine / -95.1,-169.2	-	-	Favored (56.241%)	-	-	-

A 169		GLU	1.42	-	Favored (23.46%) General / -97.1,148.0	Favored (97.6%) *mt-10* chi angles: 295.8,180.2,356.5	0.03Å	Favored (9.421%)	-	-	-
A 170		ARG	1.74	-	Favored (57.56%) General / -61.5,135.2	Favored (83.7%) *ttp80* chi angles: 182.6,175.3,61.8,81.8	0.02Å	Favored (36.574%)	-	-	-
A 171		THR	2.11	-	Favored (9.42%) General / -120.0,170.3	Favored (28.6%) *p* chi angles: 69.8	0.06Å	Favored (41.313%)	-	-	-
A 172		GLU	2.5	-	Favored (49.29%) General / -131.1,153.5	Favored (94.8%) *mt-10* chi angles: 296.1,182.6,357.7	0.02Å	Favored (27.326%)	-	-	-
A 173		GLU	2.82	-	Favored (88.92%) Pre-Pro / -74.4,126.4	Favored (83.7%) *tt0* chi angles: 183.1,181.7,10.3	0.04Å	Favored (38.961%)	-	-	-
A 174		PRO	2.99	-	Favored (28.84%) Trans-Pro / -76.6,165.4	Favored (70.6%) *Cg_endo* chi angles: 31.1,324.9,25	0.04Å	Favored (24.429%)	-	-	-
A 175		ILE	2.93	-	Favored (94.28%) Pre-Pro / -65.8,129.2	Favored (47.1%) *mm* chi angles: 299.5,298	0.06Å	Favored (18.279%)	-	-	-
A 176		PRO	2.69	-	Favored (74.52%) Trans-Pro / -57.5,148.2	Favored (86.2%) *Cg_exo* chi angles: 333.6,35.4,330.7	0.05Å	Favored (83.238%)	-	-	-
A 177		ASP	2.34	-	Favored (5.39%) General / -79.2,69.7	Favored (58%) *m-30* chi angles: 288.6,318.8	0.01Å	Favored (10.017%) beta sheet	-	-	-
A 178		ALA	1.99	-	Favored (48.47%) General / -82.8,-0.5	-	0.05Å	Favored (8.451%)	-	-	-
A 179		TYR	1.69	-	Favored (36.89%) General / -129.3,125.5	Favored (72.2%) *t80* chi angles: 177.7,68.2	0.09Å	Favored (19.522%)	-	-	-
A 180		ASN	1.44	-	Favored (50.48%) General / -124.9,144.2	Favored (22.9%) *t0* chi angles: 188.4,276.1	0.05Å	Favored (46.286%)	-	-	-
**#**	**Alt**	**Res**	**High B**	**Clash > 0.4Å**	**Ramachandran**	**Rotamer**	**Cβ deviation**	**CaBLAM**	**Bond lengths**	**Bond angles**	**Cis Peptides**
			Avg: 1.22	Clashscore: 1.14	Outliers: 1 of 616	Poor rotamers: 0 of 515	Outliers: 0 of 562	Outliers: 10 of 614	Outliers: 3 of 618	Outliers: 14 of 618	Non-Trans: 0 of 617
A 181		GLU	1.25	-	Favored (26.27%) General / -49.9,-36.1	Favored (90.8%) *tt0* chi angles: 183.6,175.1,3.3	0.02Å	Favored (54.485%)	-	-	-
A 182		GLU	1.11	-	Favored (65.81%) General / -61.3,-23.6	Favored (71.4%) *mm-30* chi angles: 288.5,295.4,310.8	0.03Å	Favored (61.321%) three-ten	-	-	-
A 183		MET	1	-	Favored (64.76%) General / -64.0,-18.9	Favored (67.6%) *mtp* chi angles: 288,170.7,56.5	0.06Å	Favored (46.893%) three-ten	-	-	-
A 184		LEU	0.92	-	Favored (46.75%) General / -99.0,6.2	Favored (57.4%) *mt* chi angles: 302.3,184.2	0.03Å	Favored (53.792%)	-	-	-

A 185		ARG	0.86	-	Favored (38.3%) General / -77.8,133.1	Favored (81.3%) *ttm-80* chi angles: 185.6,182.9,295.1,278.8	0.04Å	Favored (26.295%)	-	-	-
A 186		LYS	0.82	-	Favored (28.03%) General / -59.0,149.2	Favored (98.1%) *mttt* chi angles: 292.5,182.1,180.9,179.3	0.03Å	Favored (11.747%)	-	-	-
A 187		ARG	0.79	-	Favored (3.62%) General / 76.4,4.7	Favored (97.5%) *mtt-85* chi angles: 297.3,183.5,184.7,275.8	0.09Å	CaBLAM Disfavored (4.764%)	-	-	-
A 188		LYS	0.78	-	Favored (37.04%) General / -100.2,138.6	Favored (73%) *mmtt* chi angles: 301.9,297,184.2,181.8	0.02Å	Favored (22.955%)	-	-	-
A 189		LEU	0.79	-	Favored (11.56%) General / -116.8,104.7	Favored (50.1%) *mt* chi angles: 300.4,168.7	0.10Å	Favored (62.191%) beta sheet	-	-	-
A 190		THR	0.81	-	Favored (53.38%) General / -109.7,133.2	Favored (92.4%) *m* chi angles: 297.7	0.01Å	Favored (60.042%) beta sheet	-	-	-
A 191		VAL	0.86	-	Favored (65.53%) Ile or Val / -109.2,128.0	Favored (76.3%) *t* chi angles: 178.2	0.03Å	Favored (70.055%) beta sheet	-	-	-
A 192		LEU	0.93	-	Favored (6.68%) General / -107.4,96.8	Favored (83.9%) *mt* chi angles: 301.3,177.6	0.01Å	Favored (63.491%) beta sheet	-	-	-
A 193		GLU	1.04	-	Favored (12.22%) General / -92.1,99.4	Favored (86.7%) *tt0* chi angles: 181.9,182.1,351.2	0.02Å	Favored (45.277%) beta sheet	-	-	-
A 194		LEU	1.17	-	Favored (53.74%) General / -124.6,139.6	Favored (3.2%) *mp* chi angles: 285.1,82.7	0.06Å	Favored (37.154%)	-	-	-
A 195		HIS	1.31	-	Favored (43.55%) Pre-Pro / -60.7,156.7	Favored (28.1%) *p90* chi angles: 69.3,75.9	0.07Å	Favored (23.029%)	-	-	-
A 196		PRO	1.42	-	Favored (15.44%) Trans-Pro / -48.5,-30.2	Favored (87.6%) *Cg_exo* chi angles: 330.2,39,329	0.02Å	Favored (42.775%)	-	-	-
A 197		GLY	1.48	-	Favored (65.96%) Glycine / -96.8,9.1	-	-	Favored (60.525%)	-	-	-
A 198		ALA	1.49	-	Favored (41.76%) General / -82.9,1.0	-	0.05Å	CaBLAM Disfavored (3.307%)	-	-	-
A 199		GLY	1.45	-	Favored (52.84%) Glycine / 76.7,31.8	-	-	Favored (83.107%)	-	-	-
A 200		LYS	1.37	-	Favored (9.75%) General / -52.2,-57.4	Favored (26.5%) *mtmm* chi angles: 293.2,183.8,287.8,293	0.01Å	Favored (21.681%)	-	-	-
**#**	**Alt**	**Res**	**High B**	**Clash > 0.4Å**	**Ramachandran**	**Rotamer**	**Cβ deviation**	**CaBLAM**	**Bond lengths**	**Bond angles**	**Cis Peptides**
			Avg: 1.22	Clashscore: 1.14	Outliers: 1 of 616	Poor rotamers: 0 of 515	Outliers: 0 of 562	Outliers: 10 of 614	Outliers: 3 of 618	Outliers: 14 of 618	Non-Trans: 0 of 617
A 201		THR	1.28	-	Favored (63.26%)	Favored (90.8%) *m* chi angles: 298	0.06Å	Favored (36.138%)	-	-	-

					General / -73.1,-42.2						
A 202		ARG	1.18	-	Favored (59.37%) General / -76.2,-33.3	Favored (96.8%) *mtt180* chi angles: 289.2,181.9,176.1,183.5	0.06Å	Favored (29.349%) alpha helix	-	-	-
A 203		LYS	1.1	-	Favored (2.6%) General / -118.4,-46.0	Favored (72.7%) *mmtt* chi angles: 300.5,293.7,183.9,180.7	0.04Å	Favored (12.513%) alpha helix	-	-	-
A 204		VAL	1.03	-	Favored (47.95%) Ile or Val / -72.3,-48.5	Favored (97.6%) *t* chi angles: 175.2	0.03Å	Favored (78.978%) alpha helix	-	-	-
A 205		LEU	0.97	0.46Å HB3 with A 206 PRO HD3	Favored (57.35%) Pre-Pro / -53.5,-53.4	Favored (53.5%) *tp* chi angles: 175.1,65.1	0.12Å	Favored (88.356%) alpha helix	-	-	-
A 206		PRO	0.92	0.46Å HD3 with A 205 LEU HB3	Favored (51.21%) Trans-Pro / -51.0,-35.8	Favored (90.8%) *Cg_exo* chi angles: 329.6,36.2,333.6	0.02Å	Favored (96.613%) alpha helix	-	-	-
A 207		GLN	0.88	-	Favored (89.65%) General / -64.8,-44.5	Favored (68.3%) *tt0* chi angles: 181.4,179.7,2.5	0.02Å	Favored (77.093%) alpha helix	-	-	-
A 208		ILE	0.85	-	Favored (85.68%) Ile or Val / -66.5,-46.7	Favored (96.1%) *mt* chi angles: 293.5,166.9	0.09Å	Favored (78.151%) alpha helix	-	-	-
A 209		ILE	0.84	-	Favored (94%) Ile or Val / -64.7,-42.1	Favored (36.4%) *mm* chi angles: 294.9,298.8	0.01Å	Favored (91.033%) alpha helix	-	-	-
A 210		LYS	0.83	-	Favored (94.71%) General / -63.1,-39.5	Favored (96.6%) *mttt* chi angles: 288.1,177.6,180.7,178.6	0.03Å	Favored (96.298%) alpha helix	-	-	-
A 211		ASP	0.82	-	Favored (77.43%) General / -67.1,-34.4	Favored (13%) *t70* chi angles: 197.4,62.2	0.05Å	Favored (90.837%) alpha helix	-	-	-
A 212		CYS	0.82	-	Favored (94.46%) General / -65.0,-40.2	Favored (90.2%) *m* chi angles: 291.6	0.10Å	Favored (94.705%) alpha helix	-	-	-
A 213		ILE	0.82	-	Favored (88.86%) Ile or Val / -66.4,-45.7	Favored (97.6%) *mt* chi angles: 292.6,166.9	0.04Å	Favored (86.294%) alpha helix	-	-	-
A 214		GLN	0.82	-	Favored (29.49%) General / -77.4,-1.3	Favored (84.7%) *mt0* chi angles: 294.1,177.9,41.6	0.02Å	CaBLAM Outlier (0.045%) try alpha helix	-	OUTLIER(S) worst is CA-C-O: 4.8 σ	-
A 215		LYS	0.82	-	Favored (30.38%) General / -106.9,8.1	Favored (47.5%) *mttp* chi angles: 292.3,178.5,167.6,68.1	0.04Å	CaBLAM Disfavored (1.597%)	-	-	-
A 216		ARG	0.8	-	Favored (29.3%) General / 56.6,40.5	Favored (29.6%) *mmm160* chi angles: 311.6,296,294.3,165.7	0.02Å	Favored (32.613%)	-	-	-
A 217		LEU	0.78	-	Favored (28%) General / -100.0,114.6	Favored (75.1%) *mt* chi angles: 302.3,176	0.05Å	Favored (40.323%) beta sheet	-	-	-
A 218		ARG	0.75	-	Favored (28.27%) General / -61.8,126.7	Favored (14.4%) *tpt-90* chi angles: 184,81.3,181.6,266	0.03Å	Favored (38.773%) beta sheet	-	-	-

#	Alt	Res	High B	Clash > 0.4Å	Ramachandran	Rotamer	Cβ deviation	CaBLAM	Bond lengths	Bond angles	Cis Peptides
A 219		THR	0.72	-	Favored (50.62%) General / -126.7,134.9	Favored (95.6%) *m* chi angles: 300.8	0.03Å	Favored (71.694%) beta sheet	-	-	-
A 220		ALA	0.7	-	Favored (43.71%) General / -106.3,137.8	-	0.04Å	Favored (70.241%) beta sheet	-	-	-
#	Alt	Res	High B	Clash > 0.4Å	Ramachandran	Rotamer	Cβ deviation	CaBLAM	Bond lengths	Bond angles	Cis Peptides
			Avg: 1.22	Clashscore: 1.14	Outliers: 1 of 616	Poor rotamers: 0 of 515	Outliers: 0 of 562	Outliers: 10 of 614	Outliers: 3 of 618	Outliers: 14 of 618	Non-Trans: 0 of 617
A 221		VAL	0.7	-	Favored (60.21%) Ile or Val / -120.7,119.6	Favored (51%) *t* chi angles: 181.3	0.06Å	Favored (69.481%) beta sheet	-	-	-
A 222		LEU	0.71	-	Favored (39.58%) General / -108.3,141.8	Favored (19.2%) *mt* chi angles: 312.2,179.7	0.08Å	Favored (58.742%) beta sheet	-	-	-
A 223		ALA	0.73	-	Favored (52.51%) Pre-Pro / -131.6,144.2	-	0.11Å	Favored (59.828%)	-	-	-
A 224		PRO	0.77	-	Favored (56.32%) Trans-Pro / -65.6,-23.9	Favored (42.7%) *Cg_endo* chi angles: 24.1,326.1,28.4	0.05Å	Favored (30.94%)	-	-	-
A 225		THR	0.81	-	Favored (32.88%) General / -137.4,162.7	Favored (44.2%) *p* chi angles: 66.8	0.02Å	Favored (22.44%)	-	-	-
A 226		ARG	0.86	-	Favored (78.38%) General / -65.7,-34.5	Favored (95.6%) *mtm-85* chi angles: 289.1,189.8,286.3,270.4	0.12Å	Favored (62.997%) alpha helix	-	-	-
A 227		VAL	0.9	-	Favored (99.41%) Ile or Val / -62.8,-45.2	Favored (59.1%) *t* chi angles: 170.7	0.05Å	Favored (78.37%) alpha helix	-	-	-
A 228		VAL	0.94	-	Favored (94.02%) Ile or Val / -65.5,-42.8	Favored (76.4%) *t* chi angles: 172.8	0.04Å	Favored (82.472%) alpha helix	-	-	-
A 229		ALA	0.97	-	Favored (72.28%) General / -57.7,-36.7	-	0.02Å	Favored (78.556%) alpha helix	-	-	-
A 230		CYS	1	-	Favored (87.69%) General / -66.5,-38.7	Favored (99.6%) *m* chi angles: 292.5	0.02Å	Favored (94.303%) alpha helix	-	-	-
A 231		GLU	1.03	-	Favored (93.86%) General / -64.0,-39.3	Favored (61.4%) *tp30* chi angles: 187,68.7,17.9	0.03Å	Favored (98.317%) alpha helix	-	-	-
A 232		ILE	1.06	-	Favored (99.7%) Ile or Val / -62.7,-44.8	Favored (98%) *mt* chi angles: 292.3,168	0.04Å	Favored (82.442%) alpha helix	-	-	-
A 233		ALA	1.09	-	Favored (78.72%) General / -57.5,-40.5	-	0.05Å	Favored (79.226%) alpha helix	-	-	-

#	Alt	Res	High B	Clash > 0.4Å	Ramachandran	Rotamer	Cβ deviation	CaBLAM	Bond lengths	Bond angles	Cis Peptides
A 234		GLU	1.11	-	Favored (77.44%) General / -69.5,-40.0	Favored (67%) *mm-30* chi angles: 290.7,293.3,305.8	0.02Å	Favored (82.381%) alpha helix	-	-	-
A 235		ALA	1.13	-	Favored (74.71%) General / -60.2,-35.1	-	0.06Å	Favored (73.75%)	-	-	-
A 236		LEU	1.14	-	Favored (31.66%) General / -100.7,14.4	Favored (86.2%) *mt* chi angles: 299.8,178.6	0.04Å	Favored (18.258%)	-	-	-
A 237		LYS	1.12	-	Favored (36.83%) General / -53.1,131.7	Favored (29.3%) *ttmt* chi angles: 190.2,180.8,296.9,184.3	0.01Å	Favored (10.675%)	-	-	-
A 238		GLY	1.08	-	Favored (62.03%) Glycine / 98.8,-13.9	-	-	Favored (83.076%)	-	-	-
A 239		LEU	1.02	-	Favored (57.59%) Pre-Pro / -86.0,154.1	Favored (96.3%) *mt* chi angles: 297.9,177.2	0.07Å	Favored (25.978%)	-	-	-
A 240		PRO	0.97	-	Allowed (1.24%) Trans-Pro / -69.5,73.3	Favored (69.8%) *Cg_endo* chi angles: 29.4,320.8,32.6	0.04Å	CaBLAM Disfavored (4.52%) try beta sheet	-	-	-
**#**	**Alt**	**Res**	**High B**	**Clash > 0.4Å**	**Ramachandran**	**Rotamer**	**Cβ deviation**	**CaBLAM**	**Bond lengths**	**Bond angles**	**Cis Peptides**
			Avg: 1.22	Clashscore: 1.14	Outliers: 1 of 616	Poor rotamers: 0 of 515	Outliers: 0 of 562	Outliers: 10 of 614	Outliers: 3 of 618	Outliers: 14 of 618	Non-Trans: 0 of 617
A 241		ILE	0.94	0.42Å HG12 with A 258 VAL HB	Favored (70.2%) Ile or Val / -115.3,130.2	Favored (89.6%) *mt* chi angles: 297.9,168.4	0.02Å	Favored (33.557%) beta sheet	OUTLIER(S) worst is CB--CG1: 4.9 σ	-	-
A 242		ARG	0.94	-	Favored (28.42%) General / -96.6,142.7	Favored (57.3%) *ttt180* chi angles: 180,183.4,182.1,201.2	0.12Å	Favored (47.294%) beta sheet	-	-	-
A 243		TYR	1	-	Favored (55.15%) General / -113.8,133.9	Favored (75.4%) *m-80* chi angles: 300.6,85.1	0.09Å	Favored (63.437%) beta sheet	-	-	-
A 244		LEU	1.12	-	Favored (14.77%) General / -110.7,23.0	Favored (4.8%) *mp* chi angles: 286.1,71.8	0.05Å	Favored (12.291%) beta sheet	-	-	-
A 245		THR	1.31	-	Favored (56.85%) Pre-Pro / -87.0,128.1	Favored (95.4%) *m* chi angles: 299.5	0.06Å	Favored (30.303%)	-	-	-
A 246		PRO	1.58	-	Favored (19.07%) Trans-Pro / -49.4,-29.8	Favored (90%) *Cg_exo* chi angles: 329.7,36.7,332.8	0.04Å	Favored (80.594%)	-	-	-
A 247		ALA	1.94	-	Favored (56.88%) General / -82.4,-4.2	-	0.04Å	Favored (55.809%)	-	-	-
A 248		VAL	2.36	-	Favored (70.85%) Ile or Val / -120.8,132.2	Favored (63.5%) *t* chi angles: 179.5	0.08Å	Favored (32.252%)	-	-	-
A 249		ARG	2.83	-	Favored (49.37%) General / -92.3,5.8	Favored (98.4%) *mtt180* chi angles: 295.2,180.5,180.1,181.9	0.02Å	Favored (13.896%)	-	-	-

A 250		ASN	3.26	-	Favored (6.56%) General / -81.7,85.5	Favored (93.3%) *m-40* chi angles: 292.4,327.4	0.06Å	Favored (19.013%)	-	-	-
A 251		GLU	3.53	-	Favored (13.86%) General / -78.2,172.2	Favored (64.3%) *mm-30* chi angles: 285.9,292.4,333	0.04Å	CaBLAM Disfavored (2.406%)	-	-	-
A 252		HIS	3.5	-	Favored (56.99%) General / -58.1,135.2	Favored (66.4%) *t-90* chi angles: 195.9,283.4	0.07Å	Favored (10.684%)	-	-	-
A 253		GLN	3.14	-	Favored (12.71%) General / -109.6,24.7	Favored (90.8%) *mm-40* chi angles: 302.1,296.5,303.3	0.04Å	CaBLAM Disfavored (1.406%)	-	-	-
A 254		GLY	2.55	-	Favored (5.02%) Glycine / 84.9,-57.1	-	-	CaBLAM Disfavored (2.007%)	-	-	-
A 255		ASN	1.94	-	Favored (25.55%) General / -99.6,15.8	Favored (88.5%) *m-40* chi angles: 292.1,321	0.02Å	Favored (30.1%)	-	-	-
A 256		GLU	1.43	-	Favored (35.8%) General / -77.7,146.9	Favored (76.2%) *mt-10* chi angles: 291.9,177.6,319.6	0.11Å	Favored (22.118%)	-	-	-
A 257		ILE	1.08	-	Favored (3.94%) Ile or Val / -108.2,-38.6	Favored (48.6%) *mm* chi angles: 301.3,300	0.03Å	Favored (7.226%)	-	-	-
A 258		VAL	0.86	0.42Å HB with A 241 ILE HG12	Favored (63.8%) Ile or Val / -111.3,131.0	Favored (75.5%) *t* chi angles: 178.3	0.09Å	Favored (24.834%)	-	-	-
A 259		ASP	0.74	-	Favored (33.61%) General / -111.9,117.7	Favored (85.8%) *m-30* chi angles: 294.2,346.3	0.02Å	Favored (72.674%)	-	-	-
A 260		VAL	0.69	-	Favored (51.65%) Ile or Val / -101.3,129.9	Favored (98%) *t* chi angles: 175.6	0.03Å	Favored (32.263%) beta sheet	-	-	-
**#**	**Alt**	**Res**	**High B**	**Clash > 0.4Å**	**Ramachandran**	**Rotamer**	**Cβ deviation**	**CaBLAM**	**Bond lengths**	**Bond angles**	**Cis Peptides**
			Avg: 1.22	Clashscore: 1.14	Outliers: 1 of 616	Poor rotamers: 0 of 515	Outliers: 0 of 562	Outliers: 10 of 614	Outliers: 3 of 618	Outliers: 14 of 618	Non-Trans: 0 of 617
A 261		MET	0.69	-	Favored (22.5%) General / -153.7,169.8	Favored (18.9%) *ptp* chi angles: 67.4,197.1,79.1	0.08Å	Favored (26.414%) beta sheet	-	-	-
A 262		CYS	0.71	-	Favored (15.01%) General / -94.4,159.7	Favored (69.2%) *m* chi angles: 298.8	0.03Å	Favored (35.012%)	-	-	-
A 263		HIS	0.73	-	Favored (81.06%) General / -59.3,-38.7	Favored (52.5%) *m170* chi angles: 293.7,177.3	0.04Å	Favored (63.127%)	OUTLIER(S) worst is CG--CD2: 5.0 σ	-	-
A 264		ALA	0.75	-	Favored (77.84%) General / -66.9,-45.5	-	0.03Å	Favored (92.249%) alpha helix	-	-	-

#	Alt	Res	High B	Clash > 0.4Å	Ramachandran	Rotamer	Cβ deviation	CaBLAM	Bond lengths	Bond angles	Cis Peptides
A 265		THR	0.76	-	Favored (75.75%) General / -68.0,-34.0	Favored (5%) *t* chi angles: 180.4	0.10Å	Favored (87.707%) alpha helix	-	-	-
A 266		LEU	0.77	-	Favored (65.63%) General / -65.9,-50.1	Favored (61.2%) *tp* chi angles: 174.5,61.5	0.09Å	Favored (76.457%) alpha helix	-	-	-
A 267		THR	0.77	-	Favored (94.97%) General / -60.0,-43.8	Favored (94.9%) *m* chi angles: 299.4	0.04Å	Favored (86.481%) alpha helix	-	-	-
A 268		GLN	0.78	-	Favored (96.5%) General / -62.5,-40.4	Favored (93.2%) *mt0* chi angles: 289.8,174.1,349	0.03Å	Favored (99.222%) alpha helix	-	-	-
A 269		LYS	0.82	-	Favored (94.32%) General / -61.3,-40.5	Favored (54.5%) *tttp* chi angles: 184.4,174.3,176.5,65.6	0.04Å	Favored (97.012%) alpha helix	-	-	-
A 270		LEU	0.89	-	Favored (76.97%) General / -65.5,-33.9	Favored (83.1%) *mt* chi angles: 291.3,175.3	0.03Å	Favored (74.735%) alpha helix	-	-	-
A 271		LEU	1.01	-	Favored (34.39%) General / -80.2,-36.8	Favored (96.4%) *mt* chi angles: 294.8,172.6	0.10Å	Favored (23.85%)	-	-	-
A 272		THR	1.15	-	Favored (96.07%) Pre-Pro / -63.4,135.1	Favored (98.2%) *m* chi angles: 300.1	0.07Å	Favored (26.565%)	-	-	-
A 273		PRO	1.3	-	Favored (2.99%) Trans-Pro / -72.6,72.7	Favored (74.6%) *Cg_endo* chi angles: 29.9,322.5,29.3	0.02Å	Favored (7.423%)	-	-	-
A 274		THR	1.41	-	Favored (46.06%) General / -140.9,156.6	Favored (11.3%) *t* chi angles: 186.9	0.05Å	Favored (12.513%)	-	-	-
A 275		ARG	1.43	-	Favored (53.1%) General / -63.9,133.3	Favored (83.9%) *ttt180* chi angles: 184.7,175.7,180.5,178.4	0.03Å	Favored (25.896%) beta sheet	-	-	-
A 276		VAL	1.35	-	Favored (32.35%) Pre-Pro / -116.1,122.4	Favored (69.2%) *t* chi angles: 178.9	0.03Å	Favored (48.907%) beta sheet	-	-	-
A 277		PRO	1.2	-	Favored (92.35%) Trans-Pro / -57.6,144.5	Favored (72.3%) *Cg_exo* chi angles: 335.2,34.4,330.5	0.05Å	Favored (84.761%) beta sheet	-	-	-
A 278		ASN	1.02	-	Favored (27.71%) General / -93.5,115.2	Favored (81.4%) *m-40* chi angles: 294.9,310.7	0.01Å	Favored (57.01%) beta sheet	-	-	-
A 279		TYR	0.87	-	Favored (16.56%) General / -95.6,155.9	Favored (92.2%) *m-80* chi angles: 296.3,85.8	0.02Å	Favored (26.81%)	-	-	-
A 280		GLN	0.77	-	Favored (15.22%) General / -94.3,-29.1	Favored (17.6%) *mp10* chi angles: 286.1,72.5,66.4	0.04Å	Favored (21.839%)	-	-	-
**#**	**Alt**	**Res**	**High B**	**Clash > 0.4Å**	**Ramachandran**	**Rotamer**	**Cβ deviation**	**CaBLAM**	**Bond lengths**	**Bond angles**	**Cis Peptides**

			Avg: 1.22	Clashscore: 1.14	Outliers: 1 of 616	Poor rotamers: 0 of 515	Outliers: 0 of 562	Outliers: 10 of 614	Outliers: 3 of 618	Outliers: 14 of 618	Non-Trans: 0 of 617
A 281		VAL	0.7	-	Favored (64.92%) Ile or Val / -130.7,130.9	Favored (61.7%) *t* chi angles: 179.8	0.04Å	Favored (38.714%)	-	-	-
A 282		TYR	0.66	-	Favored (49.18%) General / -118.3,125.0	Favored (84.8%) *m-80* chi angles: 295.9,83.2	0.06Å	Favored (70.953%)	-	-	-
A 283		ILE	0.65	-	Favored (71.64%) Ile or Val / -115.7,124.0	Favored (66.7%) *mt* chi angles: 302.7,168.9	0.06Å	Favored (70.918%) beta sheet	-	-	-
A 284		MET	0.67	-	Favored (53.77%) General / -112.0,125.2	Favored (62.6%) *ttm* chi angles: 180.7,176.3,286.6	0.03Å	Favored (69.377%)	-	-	-
A 285		ASP	0.71	-	Favored (26.13%) General / -89.4,144.3	Favored (63.7%) *m-30* chi angles: 289.4,0.4	0.07Å	Favored (11.264%)	-	-	-
A 286		GLU	0.76	-	Favored (21.25%) General / 53.8,48.5	Favored (58.5%) *mt-10* chi angles: 300.1,177.3,127.1	0.10Å	Favored (12.613%)	-	-	-
A 287		ALA	0.8	-	Favored (61.88%) General / -60.2,-21.6	-	0.03Å	Favored (19.644%)	-	-	-
A 288		HIS	0.84	-	Favored (62.38%) General / -71.3,-15.1	Favored (52.5%) *p-80* chi angles: 61.1,275.8	0.11Å	Favored (20.091%)	-	OUTLIER(S) worst is CA-CB-CG: 4.4 σ	-
A 289		PHE	0.85	-	Favored (43.82%) General / -60.7,129.9	Favored (31%) *t80* chi angles: 187.4,57.1	0.13Å	Favored (33.48%)	-	-	-
A 290		ILE	0.84	0.49Å O with A 290 ILE HG12	Favored (16.28%) Ile or Val / -73.6,-13.9	Favored (12.8%) *tt* chi angles: 199.7,168.6	0.05Å	Favored (10.353%) beta sheet	-	-	-
A 291		ASP	0.81	-	Favored (58.25%) Pre-Pro / -53.9,132.4	Favored (9.8%) *t0* chi angles: 183.8,301.3	0.03Å	Favored (44.138%)	-	OUTLIER(S) worst is CA-CB-CG: 4.1 σ	-
A 292		PRO	0.78	-	Favored (9.46%) Trans-Pro / -44.5,-36.1	Favored (73.8%) *Cg_exo* chi angles: 328.9,37.4,332.6	0.08Å	Favored (77.277%)	-	-	-
A 293		ALA	0.73	-	Favored (81.17%) General / -66.0,-35.7	-	0.03Å	Favored (79.493%) alpha helix	-	-	-
A 294		SER	0.7	-	Favored (64.35%) General / -73.0,-41.4	Favored (69.4%) *m* chi angles: 296.5	0.08Å	Favored (78.557%) alpha helix	-	-	-
A 295		ILE	0.67	-	Favored (86.27%) Ile or Val / -65.9,-47.2	Favored (90.6%) *mt* chi angles: 294.9,166.2	0.05Å	Favored (95.287%) alpha helix	-	-	-
A 296		ALA	0.65	-	Favored (94.28%) General / -61.0,-40.7	-	0.02Å	Favored (93.162%) alpha helix	-	-	-

#	Alt	Res	High B	Clash > 0.4Å	Ramachandran	Rotamer	Cβ deviation	CaBLAM	Bond lengths	Bond angles	Cis Peptides
A 297		ALA	0.64	-	Favored (99.41%) General / -63.0,-41.1	-	0.04Å	Favored (97.277%) alpha helix	-	-	-
A 298		ARG	0.64	-	Favored (94.54%) General / -63.1,-39.4	Favored (98.9%) *mtm-85* chi angles: 286.5,192.8,293.5,276.4	0.04Å	Favored (99.11%) alpha helix	-	-	-
A 299		GLY	0.64	-	Favored (55.7%) Glycine / -62.3,-51.5	-	-	Favored (92.705%) alpha helix	-	-	-
A 300		TYR	0.65	-	Favored (70.88%) General / -54.8,-49.5	Favored (92.1%) *t80* chi angles: 178.1,79.1	0.01Å	Favored (91.597%) alpha helix	-	-	-
**#**	**Alt**	**Res**	**High B**	**Clash > 0.4Å**	**Ramachandran**	**Rotamer**	**Cβ deviation**	**CaBLAM**	**Bond lengths**	**Bond angles**	**Cis Peptides**
			Avg: 1.22	Clashscore: 1.14	Outliers: 1 of 616	Poor rotamers: 0 of 515	Outliers: 0 of 562	Outliers: 10 of 614	Outliers: 3 of 618	Outliers: 14 of 618	Non-Trans: 0 of 617
A 301		ILE	0.66	-	Favored (93.84%) Ile or Val / -60.5,-47.1	Favored (96%) *mt* chi angles: 292,168.1	0.09Å	Favored (94.931%) alpha helix	-	-	-
A 302		SER	0.68	-	Favored (87.85%) General / -59.7,-40.4	Favored (69.5%) *m* chi angles: 296.5	0.06Å	Favored (92.239%) alpha helix	-	-	-
A 303		THR	0.7	-	Favored (95.46%) General / -63.2,-44.4	Favored (84.9%) *m* chi angles: 301.7	0.09Å	Favored (96.567%) alpha helix	-	-	-
A 304		LYS	0.73	-	Favored (72.19%) General / -64.3,-31.1	Favored (63.2%) *mmtt* chi angles: 288.7,295,192.6,180.4	0.02Å	Favored (75.592%) alpha helix	-	-	-
A 305		VAL	0.75	-	Favored (46.16%) Ile or Val / -73.3,-47.5	Favored (88.1%) *t* chi angles: 174	0.05Å	Favored (70.35%) alpha helix	-	-	-
A 306		GLU	0.77	-	Favored (71.52%) General / -61.5,-31.5	Favored (97.5%) *mt-10* chi angles: 290.9,176.9,3	0.06Å	Favored (74.345%) alpha helix	-	-	-
A 307		LEU	0.78	-	Favored (60.33%) General / -74.4,-11.7	Favored (87.2%) *mt* chi angles: 290.5,170.7	0.05Å	Favored (49.88%)	-	-	-
A 308		GLY	0.78	-	Favored (58.86%) Glycine / 83.9,20.8	-	-	Favored (74.697%)	-	-	-
A 309		GLU	0.76	-	Favored (15.96%) General / -95.3,-26.3	Favored (69.2%) *mt-10* chi angles: 297.8,183.5,22.2	0.01Å	CaBLAM Disfavored (4.511%)	-	-	-
A 310		ALA	0.74	-	Favored (13.66%) General / -150.9,173.9	-	0.05Å	Favored (6.868%)	-	-	-
A 311		ALA	0.72	-	Favored (26.54%) General / -113.0,154.4	-	0.07Å	Favored (52.59%)	-	-	-
A 312		ALA	0.71	-	Favored (35.33%) General / -141.8,146.0	-	0.08Å	Favored (67.929%) beta sheet	-	-	-

A 313		ILE	0.71	-	Favored (74.12%) Ile or Val / -123.5,127.3	Favored (78%) *mt* chi angles: 300.2,168.1	0.07Å	Favored (72.092%) beta sheet	-	-	-
A 314		PHE	0.74	-	Favored (12.27%) General / -116.9,105.4	Favored (77.7%) *m-80* chi angles: 300.9,86.1	0.08Å	Favored (67.478%) beta sheet	-	-	-
A 315		MET	0.78	-	Favored (30.57%) General / -88.6,139.3	Favored (65.2%) *mtt* chi angles: 294.3,183.1,177	0.03Å	Favored (6.758%) beta sheet	-	-	-
A 316		THR	0.83	-	Favored (18.62%) General / -165.6,161.8	Favored (10.1%) *t* chi angles: 186.2	0.04Å	Favored (19.943%)	-	-	-
A 317		ALA	0.89	-	Favored (30.28%) General / -83.6,-26.1	-	0.04Å	Favored (18.954%)	-	-	-
A 318		THR	0.95	-	Favored (5.63%) Pre-Pro / -149.2,126.8	Favored (78%) *m* chi angles: 302.6	0.09Å	Favored (10.687%)	-	-	-
A 319		PRO	1	-	Favored (41.77%) Trans-Pro / -73.0,162.1	Favored (73.1%) *Cg_endo* chi angles: 29.1,324.9,26.3	0.09Å	Favored (70.005%)	-	-	-
A 320		PRO	1.02	-	Favored (66.52%) Trans-Pro / -61.2,-20.1	Favored (35%) *Cg_endo* chi angles: 22.3,325.6,31.7	0.02Å	Favored (62.343%)	-	-	-
**#**	**Alt**	**Res**	**High B**	**Clash > 0.4Å**	**Ramachandran**	**Rotamer**	**Cβ deviation**	**CaBLAM**	**Bond lengths**	**Bond angles**	**Cis Peptides**
			Avg: 1.22	Clashscore: 1.14	Outliers: 1 of 616	Poor rotamers: 0 of 515	Outliers: 0 of 562	Outliers: 10 of 614	Outliers: 3 of 618	Outliers: 14 of 618	Non-Trans: 0 of 617
A 321		GLY	1.01	-	Favored (76.32%) Glycine / -92.0,2.9	-	-	Favored (65.448%)	-	-	-
A 322		THR	0.98	-	Favored (55.96%) General / -115.7,133.5	Favored (90.8%) *m* chi angles: 297.2	0.09Å	Favored (36.435%)	-	-	-
A 323		ASN	0.95	-	Allowed (1.25%) General / -100.3,40.4	Favored (34.5%) *p0* chi angles: 62.7,337.1	0.05Å	CaBLAM Disfavored (2.85%)	-	-	-
A 324		ASP	0.94	-	Favored (38.64%) Pre-Pro / -111.4,98.1	Favored (67.8%) *t0* chi angles: 183.9,352.3	0.05Å	Favored (10.129%) beta sheet	-	-	-
A 325		PRO	0.95	-	Favored (22.21%) Trans-Pro / -74.6,-11.2	Favored (72.6%) *Cg_endo* chi angles: 29.1,326.7,23.4	0.01Å	Favored (49.6%)	-	-	-
A 326		PHE	1.01	-	Favored (78.46%) Pre-Pro / -132.6,67.5	Favored (78.4%) *m-80* chi angles: 303.4,99.3	0.08Å	Favored (11.822%)	-	OUTLIER(S) worst is CA-CB-CG: 4.3 σ	-
A 327		PRO	1.12	-	Favored (28.09%) Trans-Pro / -72.2,167.0	Favored (73.7%) *Cg_endo* chi angles: 29,322.2,31.4	0.06Å	Favored (9.34%)	-	-	-
A 328		ASP	1.26	-	Favored (22.38%) General / -59.0,151.4	Favored (87%) *m-30* chi angles: 290.7,351.9	0.05Å	Favored (15.024%)	-	-	-

#	Alt	Res	High B	Clash > 0.4Å	Ramachandran	Rotamer	Cβ deviation	CaBLAM	Bond lengths	Bond angles	Cis Peptides
A 329		SER	1.39	-	Favored (28.88%) General / -145.9,165.5	Favored (84.6%) *p* chi angles: 62.6	0.04Å	Favored (51.389%)	-	-	-
A 330		ASN	1.45	-	Favored (3.66%) General / -66.0,-59.6	Favored (18.9%) *t0* chi angles: 194.4,299.5	0.03Å	Favored (7.123%)	-	-	-
A 331		SER	1.42	-	Favored (57.34%) Pre-Pro / -85.2,151.7	Favored (51.4%) *m* chi angles: 291.7	0.03Å	Favored (8.516%)	-	-	-
A 332		PRO	1.31	-	Favored (72.09%) Trans-Pro / -69.0,149.9	Favored (51.8%) *Cg_endo* chi angles: 25.4,325.4,29	0.05Å	Favored (66.389%)	-	-	-
A 333		ILE	1.16	-	Favored (66.31%) Ile or Val / -129.9,130.3	Favored (76.8%) *mt* chi angles: 300,174.1	0.04Å	Favored (53.519%) beta sheet	-	-	-
A 334		LEU	1.01	-	Favored (23.46%) General / -88.3,113.0	Favored (73.9%) *tp* chi angles: 178.2,62.5	0.02Å	Favored (59.954%) beta sheet	-	-	-
A 335		ASP	0.89	-	Favored (26.72%) General / -88.9,115.9	Favored (58.8%) *m-30* chi angles: 293.7,307.1	0.04Å	Favored (60.886%) beta sheet	-	-	-
A 336		VAL	0.81	-	Favored (75.93%) Ile or Val / -121.5,128.8	Favored (66.1%) *t* chi angles: 179.2	0.07Å	Favored (61.038%) beta sheet	-	-	-
A 337		GLU	0.77	-	Favored (34.21%) General / -86.6,131.3	Favored (33.4%) *tt0* chi angles: 186.5,171.9,303	0.04Å	Favored (35.677%) beta sheet	-	-	-
A 338		ALA	0.75	-	Favored (44.69%) General / -151.1,159.2	-	0.04Å	Favored (34.917%) beta sheet	-	-	-
A 339		GLN	0.76	-	Favored (6.92%) General / -80.1,73.2	Favored (84.5%) *mm-40* chi angles: 298.6,294.5,295.5	0.03Å	Favored (14.208%) beta sheet	-	-	-
A 340		VAL	0.79	-	Favored (89.82%) Pre-Pro / -72.0,123.7	Favored (95.1%) *t* chi angles: 174.8	0.10Å	Favored (25.483%) beta sheet	-	-	-
**#**	**Alt**	**Res**	**High B**	**Clash > 0.4Å**	**Ramachandran**	**Rotamer**	**Cβ deviation**	**CaBLAM**	**Bond lengths**	**Bond angles**	**Cis Peptides**
			Avg: 1.22	Clashscore: 1.14	Outliers: 1 of 616	Poor rotamers: 0 of 515	Outliers: 0 of 562	Outliers: 10 of 614	Outliers: 3 of 618	Outliers: 14 of 618	Non-Trans: 0 of 617
A 341		PRO	0.84	-	Favored (65.93%) Trans-Pro / -70.3,151.6	Favored (59.5%) *Cg_endo* chi angles: 26.3,327.2,25.8	0.02Å	Favored (55.604%)	-	-	-
A 342		ASP	0.92	-	Favored (15.24%) General / -93.2,-30.3	Favored (64.5%) *m-30* chi angles: 297,305.4	0.07Å	Favored (12.558%)	-	-	-
A 343		LYS	1.05	-	Favored (13.94%) General / -104.9,161.7	Favored (98.3%) *mttt* chi angles: 295.8,181.9,180.1,179.7	0.01Å	Favored (7.764%)	-	-	-

A 344		ALA	1.2	-	Favored (36.35%) General / -62.5,151.9	-	0.07Å	Favored (15.32%)	-	-	-
A 345		TRP	1.34	-	Favored (41.35%) General / -144.5,160.3	Favored (32.9%) *p90* chi angles: 70.3,96.1	0.19Å	Favored (62.929%)	-	-	-
A 346		SER	1.45	-	Favored (2.82%) General / -137.7,8.4	Favored (81.4%) *p* chi angles: 61.9	0.02Å	Favored (25.736%)	-	-	-
A 347		THR	1.48	-	Allowed (0.61%) General / -126.2,-86.0	Favored (52.2%) *p* chi angles: 56.5	0.02Å	CaBLAM Disfavored (1.383%)	-	-	-
A 348		GLY	1.43	-	Favored (67.4%) Glycine / -58.8,-31.3	-	-	Favored (35.922%)	-	-	-
A 349		TYR	1.32	-	Favored (9.5%) General / -85.6,66.1	Favored (99.1%) *m-80* chi angles: 297.5,91.1	0.08Å	Favored (11.223%)	-	-	-
A 350		GLU	1.2	-	Favored (75.3%) General / -61.1,-34.7	Favored (98%) *mt-10* chi angles: 289.9,179.1,351.8	0.03Å	Favored (19.619%)	-	-	-
A 351		TRP	1.1	-	Favored (19.72%) General / -48.5,-38.2	Favored (75%) *t-100* chi angles: 181.8,252.8	0.04Å	Favored (49.408%)	-	-	-
A 352		ILE	1.04	-	Favored (16%) Ile or Val / -84.0,-49.7	Favored (49.9%) *mm* chi angles: 302.2,300.6	0.13Å	Favored (50.214%) alpha helix	-	-	-
A 353		THR	1.01	-	Favored (60.95%) General / -73.9,-18.0	Favored (67.8%) *p* chi angles: 62.7	0.07Å	Favored (60.139%) alpha helix	-	-	-
A 354		ASN	1	-	Favored (56.9%) General / -90.4,1.1	Favored (96.4%) *m-40* chi angles: 289.5,334.5	0.04Å	Favored (51.302%)	-	-	-
A 355		PHE	0.98	-	Favored (42.94%) General / -74.0,133.3	Favored (56.4%) *t80* chi angles: 184.9,66.5	0.09Å	Favored (26.037%)	-	-	-
A 356		THR	0.95	-	Favored (25.21%) General / -97.0,-10.9	Favored (68.6%) *p* chi angles: 62.6	0.03Å	Favored (35.942%) beta sheet	-	-	-
A 357		GLY	0.9	-	Favored (39.22%) Glycine / -93.3,-169.9	-	-	Favored (29.71%) beta sheet	-	-	-
A 358		ARG	0.83	-	Favored (34.28%) General / -86.3,131.2	Favored (97%) *mtm-85* chi angles: 293.5,184.2,293.1,267.4	0.05Å	Favored (12.425%) beta sheet	-	-	-
A 359		THR	0.77	-	Favored (54.2%) General / -125.1,138.0	Favored (90.7%) *m* chi angles: 298	0.05Å	Favored (71.212%) beta sheet	-	-	-
A 360		VAL	0.73	-	Favored (68.94%) Ile or Val / -111.9,127.6	Favored (75.7%) *t* chi angles: 178.3	0.04Å	Favored (72.741%) beta sheet	-	-	-
**#**	**Alt**	**Res**	**High B**	**Clash > 0.4Å**	**Ramachandran**	**Rotamer**	**Cβ deviation**	**CaBLAM**	**Bond lengths**	**Bond angles**	**Cis Peptides**

			Avg: 1.22	Clashscore: 1.14	Outliers: 1 of 616	Poor rotamers: 0 of 515	Outliers: 0 of 562	Outliers: 10 of 614	Outliers: 3 of 618	Outliers: 14 of 618	Non-Trans: 0 of 617
A 361		TRP	0.72	0.44Å C with A 361 TRP CD1	Favored (17.68%) General / -113.6,108.8	Favored (4.6%) *t-100* chi angles: 188.9,299.3	0.06Å	Favored (67.685%) beta sheet	-	-	-
A 362		PHE	0.73	-	Favored (33.61%) General / -87.8,124.4	Favored (94.8%) *m-80* chi angles: 293,86.7	0.03Å	Favored (60.187%) beta sheet	-	-	-
A 363		VAL	0.78	-	Favored (48.52%) Pre-Pro / -116.8,152.4	Favored (29%) *m* chi angles: 300.8	0.12Å	Favored (42.123%)	-	-	-
A 364		PRO	0.83	-	Favored (65.41%) Trans-Pro / -64.6,-20.5	Favored (35.2%) *Cg_endo* chi angles: 22.4,325.4,31.3	0.02Å	Favored (14.964%)	-	-	-
A 365		SER	0.89	-	Favored (35.43%) General / -158.1,165.2	Favored (97.2%) *p* chi angles: 65.9	0.06Å	Favored (22.898%)	-	-	-
A 366		VAL	0.94	-	Favored (92.78%) Ile or Val / -59.8,-43.9	Favored (61.2%) *t* chi angles: 171	0.06Å	Favored (66.919%) alpha helix	-	-	-
A 367		LYS	0.96	-	Favored (78.08%) General / -60.8,-49.3	Favored (52.6%) *tttm* chi angles: 181.8,179,186.2,293.9	0.01Å	Favored (88.158%) alpha helix	-	-	-
A 368		SER	0.96	-	Favored (89.5%) General / -61.3,-39.2	Favored (56.1%) *m* chi angles: 292.8	0.01Å	Favored (84.502%) alpha helix	-	-	-
A 369		GLY	0.94	-	Favored (32.53%) Glycine / -55.8,-54.8	-	-	Favored (94.836%) alpha helix	-	-	-
A 370		ASN	0.91	-	Favored (84.39%) General / -59.7,-39.4	Favored (94.7%) *m-40* chi angles: 286.3,337.9	0.04Å	Favored (82.371%) alpha helix	-	-	-
A 371		GLU	0.88	-	Favored (97.57%) General / -64.0,-41.0	Favored (99.4%) *mt-10* chi angles: 291.7,176.1,357.7	0.03Å	Favored (84.045%) alpha helix	-	-	-
A 372		ILE	0.87	-	Favored (85.17%) Ile or Val / -67.8,-45.1	Favored (90.1%) *mt* chi angles: 291.2,168.5	0.08Å	Favored (79.701%) alpha helix	-	-	-
A 373		ALA	0.86	-	Favored (87.67%) General / -59.0,-41.4	-	0.04Å	Favored (89.738%) alpha helix	-	-	-
A 374		ILE	0.87	-	Favored (94.92%) Ile or Val / -63.5,-46.5	Favored (90.7%) *mt* chi angles: 291.5,166.6	0.02Å	Favored (97.058%) alpha helix	-	-	-
A 375		CYS	0.88	-	Favored (93.24%) General / -63.6,-39.0	Favored (91.9%) *m* chi angles: 289.2	0.06Å	Favored (93.683%) alpha helix	-	-	-
A 376		LEU	0.9	-	Favored (88.62%) General / -63.9,-37.8	Favored (80.3%) *mt* chi angles: 288.8,169.9	0.05Å	Favored (98.308%) alpha helix	-	-	-

A 377		GLN	0.92	-	Favored (92.27%) General / -65.5,-39.6	Favored (95%) *mt0* chi angles: 290.5,171.1,347.1	0.02Å	Favored (93.316%) alpha helix	-	-	-
A 378		LYS	0.92	-	Favored (69.56%) General / -63.6,-27.9	Favored (96.9%) *mttt* chi angles: 288.1,179.9,176.1,178.6	0.04Å	Favored (74.87%)	-	-	-
A 379		ALA	0.9	-	Favored (38.23%) General / -78.7,-2.2	-	0.02Å	Favored (48.559%)	-	-	-
A 380		GLY	0.86	-	Favored (57.25%) Glycine / 95.0,10.7	-	-	Favored (87.472%)	-	-	-
**#**	**Alt**	**Res**	**High B**	**Clash > 0.4Å**	**Ramachandran**	**Rotamer**	**Cβ deviation**	**CaBLAM**	**Bond lengths**	**Bond angles**	**Cis Peptides**
			Avg: 1.22	Clashscore: 1.14	Outliers: 1 of 616	Poor rotamers: 0 of 515	Outliers: 0 of 562	Outliers: 10 of 614	Outliers: 3 of 618	Outliers: 14 of 618	Non-Trans: 0 of 617
A 381		LYS	0.81	-	Favored (20.58%) General / -92.5,150.8	Favored (72.5%) *mmtt* chi angles: 301.6,294.8,186.7,181.3	0.03Å	Favored (35.32%)	-	-	-
A 382		ARG	0.77	0.41Å HG2 with A 403 TRP CE3	Favored (24.51%) General / -88.8,113.6	Favored (84.4%) *ttt180* chi angles: 184.1,175.8,181,184.9	0.05Å	Favored (49.821%) beta sheet	-	-	-
A 383		VAL	0.74	-	Favored (65.03%) Ile or Val / -128.4,134.3	Favored (33%) *t* chi angles: 185.3	0.10Å	Favored (63.417%) beta sheet	-	-	-
A 384		ILE	0.74	-	Favored (35.76%) Ile or Val / -116.1,140.9	Favored (17.6%) *tt* chi angles: 184.7,163.3	0.06Å	Favored (61.798%) beta sheet	-	-	-
A 385		GLN	0.77	-	Favored (54.32%) General / -109.6,127.2	Favored (64.9%) *tp40* chi angles: 176,71.7,54	0.03Å	Favored (68.578%) beta sheet	-	-	-
A 386		LEU	0.83	-	Favored (53.73%) General / -114.8,125.5	Favored (77.2%) *mt* chi angles: 302.6,177	0.06Å	Favored (36.749%) beta sheet	-	-	-
A 387		ASN	0.91	-	Favored (7.14%) General / -148.3,-179.7	Favored (36.1%) *p0* chi angles: 59.1,41.9	0.09Å	Favored (17.339%)	-	-	-
A 388		ARG	0.99	-	Favored (68.69%) General / -58.3,-32.6	Favored (22.5%) *tpp80* chi angles: 180.2,64.1,65.3,92.3	0.02Å	Favored (32.456%)	-	-	-
A 389		LYS	1.06	-	Favored (56.47%) General / -92.8,-1.8	Favored (72.2%) *mmtt* chi angles: 303.2,295,179.8,180.5	0.07Å	Favored (23.649%) alpha helix	-	-	-
A 390		SER	1.11	-	Favored (3.25%) General / -130.3,-18.3	Favored (97.2%) *p* chi angles: 63.6	0.05Å	Favored (9.964%) alpha helix	-	-	-
A 391		PHE	1.13	-	Favored (63.24%) General / -51.9,-43.2	Favored (53%) *t80* chi angles: 186.7,67.7	0.10Å	Favored (45.62%) alpha helix	-	OUTLIER(S) worst is CA-CB-CG: 5.8 σ	-
A 392		ASP	1.13	-	Favored (66.32%) General / -59.1,-28.2	Favored (97.3%) *m-30* chi angles: 289.4,347.4	0.11Å	Favored (54.785%) alpha helix	-	-	-

A 393		THR	1.12	-	Favored (6.36%) General / -97.7,-45.5	Favored (91.2%) *m* chi angles: 298.9	0.04Å	Favored (39.23%) alpha helix	-	-	-
A 394		GLU	1.12	-	Favored (36.96%) General / -80.5,-31.8	Favored (69.8%) *mm-30* chi angles: 296.1,296.8,305.2	0.07Å	Favored (44.519%) alpha helix	-	-	-
A 395		TYR	1.13	-	Favored (85.22%) Pre-Pro / -55.5,-50.7	Favored (83.9%) *t80* chi angles: 175.6,73.4	0.24Å	Favored (58.789%) alpha helix	-	OUTLIER(S) worst is CA-CB-CG: 4.7 σ	-
A 396		PRO	1.17	-	Favored (34.72%) Trans-Pro / -53.4,-26.2	Favored (88.5%) *Cg_exo* chi angles: 333.4,36.4,329.5	0.02Å	Favored (70.908%) alpha helix	-	-	-
A 397		LYS	1.22	-	Favored (48.39%) General / -78.0,-37.0	Favored (98.9%) *mttt* chi angles: 293.2,178.3,184.2,176.5	0.05Å	Favored (74.706%) alpha helix	-	-	-
A 398		THR	1.27	-	Favored (94.06%) General / -63.0,-45.1	Favored (89.1%) *m* chi angles: 298.3	0.01Å	Favored (90.001%) alpha helix	-	-	-
A 399		LYS	1.28	-	Favored (84.65%) General / -59.0,-47.6	Favored (85%) *tttt* chi angles: 181.5,180,177.8,181.6	0.07Å	Favored (60.105%) alpha helix	-	-	-
A 400		ASN	1.24	-	Favored (13.26%) General / -95.5,-31.6	Favored (90.6%) *m-40* chi angles: 293.1,322.7	0.03Å	Favored (22.571%)	-	-	-
**#**	**Alt**	**Res**	**High B**	**Clash > 0.4Å**	**Ramachandran**	**Rotamer**	**Cβ deviation**	**CaBLAM**	**Bond lengths**	**Bond angles**	**Cis Peptides**
			Avg: 1.22	Clashscore: 1.14	Outliers: 1 of 616	Poor rotamers: 0 of 515	Outliers: 0 of 562	Outliers: 10 of 614	Outliers: 3 of 618	Outliers: 14 of 618	Non-Trans: 0 of 617
A 401		ASN	1.15	-	Favored (16.01%) General / -92.4,158.5	Favored (80.8%) *m-40* chi angles: 289.7,317.9	0.12Å	Favored (18.045%)	-	-	-
A 402		GLU	1.02	-	Favored (8.67%) General / -81.7,74.5	Favored (96.8%) *mt-10* chi angles: 296.6,179.5,352.9	0.02Å	Favored (17.682%) beta sheet	-	-	-
A 403		TRP	0.89	0.41Å CE3 with A 382 ARG HG2	Favored (29.82%) General / -81.6,147.0	Favored (94.1%) *m100* chi angles: 296.3,98.4	0.06Å	Favored (18.576%)	-	-	-
A 404		ASP	0.77	-	Favored (14.62%) General / -87.8,-39.8	Favored (57.4%) *m-30* chi angles: 295.7,302.1	0.09Å	Favored (12.437%)	-	-	-
A 405		PHE	0.69	-	Favored (51.77%) General / -131.6,145.6	Favored (88.3%) *m-80* chi angles: 292,84.3	0.07Å	Favored (28.461%)	-	-	-
A 406		VAL	0.66	-	Favored (65.59%) Ile or Val / -124.8,123.3	Favored (54.6%) *t* chi angles: 180.7	0.11Å	Favored (69.195%)	-	-	-
A 407		VAL	0.66	-	Favored (66.42%) Ile or Val / -109.3,125.1	Favored (59%) *t* chi angles: 180.1	0.05Å	Favored (65.153%) beta sheet	-	-	-

#	Alt	Res	High B	Clash > 0.4Å	Ramachandran	Rotamer	Cβ deviation	CaBLAM	Bond lengths	Bond angles	Cis Peptides
A 408		THR	0.68	-	Favored (42.96%) General / -136.7,159.1	Favored (26.4%) *p* chi angles: 70.6	0.11Å	Favored (56.886%)	-	-	-
A 409		THR	0.72	-	Favored (3.44%) General / -95.1,-175.2	Favored (34.7%) *p* chi angles: 68.7	0.09Å	Favored (24.624%)	-	-	-
A 410		ASP	0.76	0.56Å OD2 with A 431 LYS NZ	Favored (11.73%) General / -72.2,-0.3	Favored (56%) *p0* chi angles: 63,5.5	0.07Å	Favored (5.07%)	-	-	-
A 411		ILE	0.81	-	Favored (37.69%) Ile or Val / -65.4,-25.7	Favored (17.8%) *tt* chi angles: 195.9,166.3	0.07Å	Favored (44.844%)	-	-	-
A 412		SER	0.86	0.42Å O with A 460 ARG NH2	Favored (21.98%) General / -79.7,2.6	Favored (92.5%) *p* chi angles: 63.2	0.05Å	Favored (28.226%) three-ten	-	-	-
A 413		GLU	0.92	-	Favored (59.22%) General / -80.3,-7.8	Favored (96.1%) *mt-10* chi angles: 294.7,173.9,1.9	0.07Å	Favored (58.654%) three-ten	-	-	-
A 414		MET	0.98	-	Favored (50.8%) General / -124.6,143.3	Favored (41.2%) *mtm* chi angles: 300.1,167.7,290.1	0.14Å	CA Geom Outlier (0.387%)	-	-	-
A 415		GLY	1.05	-	Allowed (1.91%) Glycine / 73.3,-59.5	-	-	CaBLAM Outlier (0.144%)	-	-	-
A 416		ALA	1.11	-	Favored (38.87%) General / -53.5,136.1	-	0.04Å	Favored (13.781%)	-	-	-
A 417		ASN	1.14	-	Favored (3.05%) General / -133.0,42.5	Favored (65.9%) *t0* chi angles: 193.8,35.4	0.04Å	CaBLAM Disfavored (3.407%) try beta sheet	-	-	-
A 418		PHE	1.13	-	Favored (51.71%) General / -58.6,131.7	Favored (87.1%) *t80* chi angles: 179.8,75.7	0.04Å	Favored (23.143%) beta sheet	-	-	-
A 419		GLY	1.07	-	Favored (2.57%) Glycine / -75.0,61.8	-	-	Favored (8.696%) beta sheet	-	-	-
A 420		ALA	0.98	-	Favored (49.81%) General / -65.2,149.5	-	0.04Å	Favored (13.035%)	-	-	-
**#**	**Alt**	**Res**	**High B**	**Clash > 0.4Å**	**Ramachandran**	**Rotamer**	**Cβ deviation**	**CaBLAM**	**Bond lengths**	**Bond angles**	**Cis Peptides**
			Avg: 1.22	Clashscore: 1.14	Outliers: 1 of 616	Poor rotamers: 0 of 515	Outliers: 0 of 562	Outliers: 10 of 614	Outliers: 3 of 618	Outliers: 14 of 618	Non-Trans: 0 of 617
A 421		HIS	0.87	-	Favored (14.4%) General / -101.9,-22.0	Favored (43.3%) *m90* chi angles: 296.1,105.7	0.07Å	Favored (20.956%)	-	-	-
A 422		ARG	0.78	-	Favored (29.69%) General / -142.9,137.8	Favored (56.5%) *ttm170* chi angles: 181.2,175.9,291.5,172.2	0.03Å	Favored (38.033%)	-	-	-
A 423		VAL	0.7	-	Favored (68.3%) Ile or Val / -117.9,132.2	Favored (61.7%) *t* chi angles: 171	0.06Å	Favored (72.702%)	-	-	-

#	Alt	Res	High B	Clash > 0.4Å	Ramachandran	Rotamer	Cβ deviation	CaBLAM	Bond lengths	Bond angles	Cis Peptides
A 424		ILE	0.66	-	Favored (66.53%) Ile or Val / -110.5,128.7	Favored (36.8%) *mm* chi angles: 308.1,298.7	0.05Å	Favored (67.831%) beta sheet	-	-	-
A 425		ASP	0.64	-	Favored (8.35%) General / -136.3,110.5	Favored (61.2%) *t0* chi angles: 184.6,359.3	0.04Å	Favored (45.951%) beta sheet	-	-	-
A 426		SER	0.64	-	Favored (61.98%) General / -60.5,-21.1	Favored (86.1%) *p* chi angles: 67.2	0.12Å	Favored (7.342%) beta sheet	-	-	-
A 427		ARG	0.67	-	Favored (10.54%) General / 66.0,16.7	Favored (26%) *mmt90* chi angles: 293,287,187.4,80.2	0.09Å	Favored (5.668%) beta sheet	-	-	-
A 428		LYS	0.7	-	Favored (26.54%) General / -118.3,157.7	Favored (89.5%) *mttt* chi angles: 297.9,179.8,188.9,176.1	0.02Å	Favored (21.036%) beta sheet	-	-	-
A 429		CYS	0.75	-	Favored (43.33%) General / -149.2,157.4	Favored (22%) *p* chi angles: 58.8	0.10Å	Favored (52.951%) beta sheet	-	-	-
A 430		VAL	0.8	-	Favored (50.92%) Ile or Val / -108.9,116.0	Favored (60.2%) *t* chi angles: 180	0.05Å	Favored (34.331%) beta sheet	-	-	-
A 431		LYS	0.86	0.56Å NZ with A 410 ASP OD2	Favored (46.21%) Pre-Pro / -118.3,149.6	Favored (71.5%) *mmtt* chi angles: 300.9,292.3,186.5,184.3	0.03Å	Favored (39.271%) beta sheet	-	-	-
A 432		PRO	0.93	-	Favored (19.02%) Trans-Pro / -73.3,133.2	Favored (77.6%) *Cg_endo* chi angles: 28.6,324,27.6	0.05Å	Favored (27.959%) beta sheet	-	-	-
A 433		VAL	1.02	-	Favored (63.68%) Ile or Val / -123.5,135.3	Favored (75.3%) *t* chi angles: 178.3	0.06Å	Favored (57.007%) beta sheet	-	-	-
A 434		ILE	1.13	-	Favored (40.93%) Ile or Val / -87.1,125.6	Favored (93%) *mt* chi angles: 296.3,168.8	0.10Å	Favored (55.495%) beta sheet	-	-	-
A 435		LEU	1.25	-	Favored (12.6%) General / -99.6,101.4	Favored (6.2%) *mp* chi angles: 283,60.7	0.08Å	Favored (70.386%) beta sheet	-	-	-
A 436		GLU	1.34	-	Favored (78.13%) General / -62.5,-35.0	Favored (98.9%) *mt-10* chi angles: 290.8,177.8,352.7	0.01Å	Favored (23.438%)	-	-	-
A 437		ASP	1.37	-	Favored (52%) General / -58.1,-22.8	Favored (98.4%) *m-30* chi angles: 288.6,347.5	0.02Å	Favored (7.503%)	-	-	-
A 438		ASP	1.34	-	Favored (2.76%) General / -138.5,15.2	Favored (54.6%) *p0* chi angles: 61.6,0.5	0.09Å	Favored (21.992%)	-	-	-
A 439		ASP	1.26	-	Favored (21.43%) General / 49.3,48.1	Favored (22.2%) *t0* chi angles: 200.1,26.9	0.01Å	Favored (28.57%)	-	-	-
A 440		ARG	1.17	-	Favored (41.68%) General / -128.6,157.3	Favored (65.5%) *mtm180* chi angles: 294.9,192.3,289.7,173.8	0.09Å	Favored (30.012%)	-	-	-
#	Alt	Res	High B	Clash > 0.4Å	Ramachandran	Rotamer	Cβ deviation	CaBLAM	Bond lengths	Bond angles	Cis Peptides

			Avg: 1.22	Clashscore: 1.14	Outliers: 1 of 616	Poor rotamers: 0 of 515	Outliers: 0 of 562	Outliers: 10 of 614	Outliers: 3 of 618	Outliers: 14 of 618	Non-Trans: 0 of 617
A 441		VAL	1.1	-	Favored (47.83%) Ile or Val / -108.8,114.8	Favored (96.2%) *t* chi angles: 175.7	0.05Å	Favored (48.67%) beta sheet	-	-	-
A 442		ILE	1.07	-	Favored (55.77%) Ile or Val / -117.5,136.1	Favored (46.2%) *mm* chi angles: 303.7,298.3	0.05Å	Favored (54.229%) beta sheet	-	-	-
A 443		LEU	1.05	-	Favored (7.29%) General / -80.7,97.5	Favored (66.3%) *mt* chi angles: 290,177	0.04Å	Favored (57.02%)	-	-	-
A 444		ASN	1.04	-	Favored (2.74%) General / -80.2,57.4	Favored (65.3%) *t0* chi angles: 194.8,35.2	0.03Å	Favored (6.127%)	-	-	-
A 445		GLY	1.02	-	Favored (10.31%) Glycine / 65.1,-177.7	-	-	Favored (22.789%)	-	-	-
A 446		PRO	0.98	-	Favored (61.58%) Trans-Pro / -70.4,148.7	Favored (56.3%) *Cg_endo* chi angles: 26,326.6,26.6	0.04Å	CaBLAM Disfavored (4.682%)	-	-	-
A 447		MET	0.93	-	Favored (43.64%) General / -146.8,157.3	Favored (22.2%) *ptp* chi angles: 63.6,194.1,72.7	0.06Å	Favored (16.437%)	-	-	-
A 448		ALA	0.89	-	Favored (58.23%) General / -62.2,141.4	-	0.04Å	Favored (32.673%)	-	-	-
A 449		ILE	0.87	-	Favored (12.74%) Ile or Val / -68.9,146.5	Favored (9.8%) *tp* chi angles: 196.2,65.9	0.08Å	Favored (51.916%) beta sheet	-	-	-
A 450		THR	0.85	-	Favored (28.34%) General / -74.2,163.0	Favored (65.7%) *p* chi angles: 63.1	0.09Å	Favored (52.164%)	-	-	-
A 451		SER	0.85	-	Favored (92.37%) General / -61.8,-39.6	Favored (48.1%) *m* chi angles: 291	0.03Å	Favored (66.386%)	-	-	-
A 452		ALA	0.85	-	Favored (96.79%) General / -61.2,-41.4	-	0.02Å	Favored (78.405%) alpha helix	-	-	-
A 453		SER	0.84	-	Favored (81.26%) General / -68.4,-40.6	Favored (23%) *m* chi angles: 288	0.08Å	Favored (84.019%) alpha helix	-	-	-
A 454		ALA	0.83	-	Favored (83.42%) General / -59.5,-39.3	-	0.03Å	Favored (87.562%) alpha helix	-	-	-
A 455		ALA	0.82	-	Favored (98.38%) General / -61.5,-42.1	-	0.06Å	Favored (94.222%) alpha helix	-	-	-
A 456		GLN	0.81	-	Favored (81.21%) General / -65.0,-35.5	Favored (23.7%) *mm110* chi angles: 290.1,288.4,125.7	0.06Å	Favored (90.103%) alpha helix	-	-	-

A 457		ARG	0.82	-	Favored (82.49%) General / -67.7,-42.0	Favored (93.6%) *mtt180* chi angles: 291.8,178.4,180.4,189.5	0.04Å	Favored (89.577%) alpha helix	-	-	-
A 458		ARG	0.84	-	Favored (82.93%) General / -61.6,-37.2	Favored (65.8%) *ttt-90* chi angles: 183.6,170.4,177.6,269.1	0.06Å	Favored (63.675%) alpha helix	-	-	-
A 459		GLY	0.88	-	Favored (41.91%) Glycine / -57.5,-21.4	-	-	Favored (69.244%) three-ten	-	-	-
A 460		ARG	0.95	0.42Å NH2 with A 412 SER O	Favored (60.49%) General / -58.4,-24.6	Favored (24.2%) *tpp80* chi angles: 180.6,58.6,50.5,84.5	0.06Å	Favored (24.193%) three-ten	-	-	-
**#**	**Alt**	**Res**	**High B**	**Clash > 0.4Å**	**Ramachandran**	**Rotamer**	**Cβ deviation**	**CaBLAM**	**Bond lengths**	**Bond angles**	**Cis Peptides**
			Avg: 1.22	Clashscore: 1.14	Outliers: 1 of 616	Poor rotamers: 0 of 515	Outliers: 0 of 562	Outliers: 10 of 614	Outliers: 3 of 618	Outliers: 14 of 618	Non-Trans: 0 of 617
A 461		ILE	1.04	-	Favored (11.91%) Ile or Val / -97.7,-49.4	Favored (45.8%) *mm* chi angles: 298.8,300.5	0.07Å	Favored (16.904%) alpha helix	-	-	-
A 462		GLY	1.17	-	Favored (59.14%) Glycine / -56.4,-30.8	-	-	Favored (76.909%) three-ten	-	-	-
A 463		ARG	1.32	-	Favored (69.47%) General / -62.9,-27.6	Favored (94.1%) *mtt180* chi angles: 286.5,175.1,182.7,162.8	0.10Å	Favored (56.168%)	-	OUTLIER(S) worst is NE-CZ-NH1: 4.4 σ	-
A 464		ASN	1.51	-	Favored (16.89%) Pre-Pro / -96.7,104.1	Favored (55.1%) *t0* chi angles: 184.4,334.1	0.05Å	Favored (30.662%)	-	-	-
A 465		PRO	1.71	-	Favored (61.77%) Trans-Pro / -64.8,-18.2	Favored (53.2%) *Cg_endo* chi angles: 25.6,325.5,28.7	0.06Å	Favored (40.972%)	-	-	-
A 466		SER	1.86	-	Favored (57.21%) General / -86.6,-8.8	Favored (90.1%) *p* chi angles: 68.7	0.03Å	Favored (60.684%)	-	-	-
A 467		GLN	1.89	-	Favored (42.81%) General / -110.3,120.5	Favored (13.8%) *mt0* chi angles: 296.7,175.4,194.8	0.01Å	Favored (12.65%)	-	-	-
A 468		ILE	1.77	-	Favored (4.8%) Ile or Val / -109.9,-55.9	Favored (49.4%) *mm* chi angles: 302.9,300.4	0.06Å	CaBLAM Disfavored (3.154%)	-	-	-
A 469		GLY	1.54	-	Favored (4.38%) Glycine / -79.3,56.1	-	-	CaBLAM Disfavored (1.665%)	-	-	-
A 470		ASP	1.28	-	Favored (13.59%) General / -101.4,161.5	Favored (86.1%) *m-30* chi angles: 294.3,344.8	0.04Å	Favored (33.44%)	-	-	-
A 471		GLU	1.05	-	Favored (51.32%) General / -132.1,152.2	Favored (87.2%) *mt-10* chi angles: 300.7,182.8,2	0.03Å	Favored (66.264%) beta sheet	-	-	-
A 472		TYR	0.89	-	Favored (24.45%) General / -128.7,119.3	Favored (19%) *t80* chi angles: 177,48.4	0.05Å	Favored (64.308%) beta sheet	-	-	-

A 473		HIS	0.79	-	Favored (54.34%) General / -110.8,132.3	Favored (47.7%) *m-70* chi angles: 293.1,254	0.03Å	Favored (67.799%) beta sheet	-	-	-
A 474		TYR	0.74	-	Favored (45.04%) General / -130.3,156.2	Favored (54.3%) *p90* chi angles: 67.1,90.1	0.04Å	Favored (63.825%)	-	-	-
A 475		GLY	0.73	-	Allowed (1.52%) Glycine / -91.7,-106.8	-	-	CaBLAM Disfavored (3.172%)	-	-	-
A 476		GLY	0.75	-	Favored (52.23%) Glycine / -79.9,-179.2	-	-	Favored (17.689%)	-	-	-
A 477		ALA	0.78	-	Favored (30.22%) General / -76.8,159.2	-	0.03Å	Favored (39.55%)	-	-	-
A 478		THR	0.81	-	Favored (16.03%) General / -82.1,170.0	Favored (58.9%) *p* chi angles: 64.3	0.02Å	Favored (32.425%) beta sheet	-	-	-
A 479		ASN	0.85	-	Favored (4.9%) General / -151.2,114.9	Favored (47%) *t0* chi angles: 187.6,0.3	0.06Å	Favored (19.436%) beta sheet	-	-	-
A 480		GLU	0.9	-	Favored (55.52%) General / -88.2,0.9	Favored (13.7%) *mp0* chi angles: 294.8,76.3,49.7	0.06Å	Favored (16.448%)	-	-	-
**#**	**Alt**	**Res**	**High B**	**Clash > 0.4Å**	**Ramachandran**	**Rotamer**	**Cβ deviation**	**CaBLAM**	**Bond lengths**	**Bond angles**	**Cis Peptides**
			Avg: 1.22	Clashscore: 1.14	Outliers: 1 of 616	Poor rotamers: 0 of 515	Outliers: 0 of 562	Outliers: 10 of 614	Outliers: 3 of 618	Outliers: 14 of 618	Non-Trans: 0 of 617
A 481		ASP	0.95	-	Favored (9.71%) General / -86.8,95.1	Favored (65.7%) *t0* chi angles: 183.1,345.2	0.06Å	Favored (7.714%)	-	-	-
A 482		ASP	0.98	-	Favored (9.51%) General / -117.8,26.7	Favored (45.8%) *p0* chi angles: 58.6,356.9	0.11Å	Favored (6.678%)	-	-	-
A 483		HIS	0.98	-	Favored (66.85%) General / -67.1,-26.7	Favored (79.4%) *m90* chi angles: 292.8,80.1	0.06Å	Favored (35.04%)	-	-	-
A 484		ASP	0.95	-	Favored (55.85%) General / -92.9,3.4	Favored (74.1%) *m-30* chi angles: 294.9,320.5	0.03Å	Favored (44.226%)	-	-	-
A 485		LEU	0.9	-	Favored (52.01%) General / -70.5,141.6	Favored (61.6%) *mt* chi angles: 296.2,183.1	0.08Å	Favored (41.355%)	-	-	-
A 486		ALA	0.84	-	Favored (67.29%) General / -58.5,-30.9	-	0.06Å	Favored (39.117%)	-	-	-
A 487		ASN	0.78	-	Favored (68.38%) General / -58.9,-31.2	Favored (98.9%) *m-40* chi angles: 289.4,342.4	0.04Å	Favored (59.962%)	-	-	-
A 488		TRP	0.73	-	Favored (55.98%) General / -76.9,-37.2	Favored (77.8%) *m100* chi angles: 297.6,113.8	0.04Å	Favored (80.358%) alpha helix	-	-	-

A 489		THR	0.68	-	Favored (87.86%) General / -65.8,-43.7	Favored (91.1%) *m* chi angles: 298	0.02Å	Favored (92.28%) alpha helix	-	-	-
A 490		GLU	0.65	-	Favored (87.96%) General / -62.1,-38.2	Favored (65.8%) *mt-10* chi angles: 287.2,177.8,319.4	0.05Å	Favored (81.426%) alpha helix	-	-	-
A 491		ALA	0.63	-	Favored (92.49%) General / -60.8,-40.4	-	0.07Å	Favored (86.328%) alpha helix	-	-	-
A 492		LYS	0.61	0.58Å NZ with A 520 ASP OD1	Favored (90.98%) General / -64.7,-38.4	Favored (25.9%) *mmmt* chi angles: 292.7,291.1,285.4,189.6	0.05Å	Favored (98.089%) alpha helix	-	-	-
A 493		ILE	0.59	-	Favored (95.54%) Ile or Val / -62.4,-46.8	Favored (93.2%) *mt* chi angles: 291.5,167.7	0.01Å	Favored (90.29%) alpha helix	-	-	-
A 494		LEU	0.59	-	Favored (99.12%) General / -63.3,-42.8	Favored (87.3%) *mt* chi angles: 290.8,169.6	0.07Å	Favored (92.283%) alpha helix	-	-	-
A 495		LEU	0.6	-	Favored (99.48%) General / -63.1,-41.6	Favored (79.2%) *mt* chi angles: 288.3,168.3	0.03Å	Favored (91.861%) alpha helix	-	-	-
A 496		ASP	0.63	-	Favored (72.96%) General / -61.8,-32.7	Favored (95.3%) *m-30* chi angles: 286.4,349	0.06Å	Favored (75.34%) alpha helix	-	-	-
A 497		ASN	0.7	-	Favored (52.75%) General / -92.7,5.0	Favored (84.5%) *m-40* chi angles: 289.1,322.7	0.09Å	Favored (49.161%)	-	-	-
A 498		ILE	0.8	-	Favored (31.51%) Ile or Val / -81.1,132.5	Favored (95%) *mt* chi angles: 293.5,170.4	0.09Å	Favored (33.929%)	-	-	-
A 499		TYR	0.93	-	Favored (33.46%) General / -86.7,124.9	Favored (87.1%) *t80* chi angles: 178.5,74.5	0.06Å	Favored (53.497%) beta sheet	-	-	-
A 500		LEU	1.07	-	Favored (25.28%) Pre-Pro / -118.4,138.0	Favored (2.9%) *mp* chi angles: 284.6,84.1	0.03Å	Favored (57.894%)	-	-	-
**#**	**Alt**	**Res**	**High B**	**Clash > 0.4Å**	**Ramachandran**	**Rotamer**	**Cβ deviation**	**CaBLAM**	**Bond lengths**	**Bond angles**	**Cis Peptides**
			Avg: 1.22	Clashscore: 1.14	Outliers: 1 of 616	Poor rotamers: 0 of 515	Outliers: 0 of 562	Outliers: 10 of 614	Outliers: 3 of 618	Outliers: 14 of 618	Non-Trans: 0 of 617
A 501		PRO	1.18	-	Favored (69.07%) Trans-Pro / -63.8,-22.2	Favored (41.5%) *Cg_endo* chi angles: 23.7,326.1,29.6	0.03Å	Favored (70.497%)	-	-	-
A 502		ASN	1.21	-	Favored (56.35%) General / -92.2,2.9	Favored (49.2%) *p0* chi angles: 62.3,6.7	0.05Å	Favored (51.608%)	-	-	-
A 503		GLY	1.16	-	Favored (88.11%) Glycine / 79.9,2.1	-	-	Favored (74.775%)	-	-	-
A 504		LEU	1.05	-	Favored (32.75%) General / -86.9,124.1	Favored (62.6%) *tp* chi angles: 177.8,64.6	0.05Å	Favored (33.446%)	-	-	-
A 505		VAL	0.93	-	Favored (30.96%)	Favored (85.7%) *t* chi angles: 173.7	0.07Å	Favored (44.038%)	-	-	-

					Ile or Val / -65.6,130.5			beta sheet			
A 506		ALA	0.85	-	Favored (57.26%) General / -60.6,140.7	-	0.02Å	Favored (38.582%)	-	-	-
A 507		GLN	0.81	-	Favored (17.03%) General / -105.7,158.0	Favored (75.7%) *mt0* chi angles: 298.3,184.1,300.4	0.02Å	Favored (35.858%)	-	-	-
A 508		MET	0.82	-	Favored (39.05%) General / -73.6,155.5	Favored (99.7%) *mtp* chi angles: 292,176.4,71.7	0.04Å	Favored (33.692%)	-	-	-
A 509		TYR	0.85	-	Favored (49.06%) General / -62.4,131.4	Favored (38%) *t80* chi angles: 179.9,57.3	0.08Å	Favored (28.493%)	-	-	-
A 510		GLN	0.9	-	OUTLIER (0.04)%) Pre-Pro / -23.5,-70.0	Favored (67.1%) *tt0* chi angles: 179.3,179.4,355.8	0.17Å	Favored (33.931%)	-	-	-
A 511		PRO	0.95	-	Favored (61.82%) Trans-Pro / -61.9,-18.6	Favored (39.2%) *Cg_endo* chi angles: 23.2,324.6,32.8	0.03Å	Favored (18.533%) alpha helix	-	-	-
A 512		GLU	1	-	Favored (19.29%) General / -94.1,-21.2	Favored (36.1%) *mt-10* chi angles: 300.7,176.8,74.1	0.07Å	Favored (42.045%) alpha helix	-	-	-
A 513		ARG	1.04	-	Favored (78.19%) General / -62.4,-35.1	Favored (97.5%) *mtt180* chi angles: 290.3,179.4,184.5,179.7	0.15Å	Favored (35.434%) three-ten	-	-	-
A 514		ASP	1.06	-	Favored (57.19%) General / -60.0,-20.4	Favored (98.2%) *m-30* chi angles: 288.7,346.9	0.01Å	Favored (63.015%) three-ten	-	-	-
A 515		LYS	1.06	-	Favored (57.49%) General / -90.6,-3.5	Favored (98.4%) *mttt* chi angles: 294.3,179.9,174.5,179.2	0.05Å	Favored (59.325%)	-	-	-
A 516		VAL	1.03	0.42Å HG12 with A 518 THR H	Favored (66.11%) Ile or Val / -119.8,133.6	Favored (87.3%) *t* chi angles: 173.9	0.09Å	Favored (29.207%)	-	-	-
A 517		PHE	0.98	-	Favored (12.1%) General / -111.7,25.0	Favored (66.5%) *m-80* chi angles: 305.2,96.5	0.10Å	Favored (5.291%)	-	OUTLIER(S) worst is CA-CB-CG: 5.2 σ	-
A 518		THR	0.91	0.42Å H with A 516 VAL HG12	Favored (53.88%) General / -111.8,133.7	Favored (89%) *m* chi angles: 298.6	0.07Å	Favored (29.052%)	-	-	-
A 519		MET	0.83	-	Favored (37.26%) General / -76.7,147.7	Favored (99.2%) *mmm* chi angles: 297.7,299.6,291.8	0.04Å	Favored (38.606%)	-	-	-
A 520		ASP	0.76	0.58Å OD1 with A 492 LYS NZ	Favored (57.07%) General / -58.3,135.1	Favored (80.9%) *m-30* chi angles: 294.3,348.7	0.09Å	Favored (32.102%)	-	-	-
**#**	**Alt**	**Res**	**High B**	**Clash > 0.4Å**	**Ramachandran**	**Rotamer**	**Cβ deviation**	**CaBLAM**	**Bond lengths**	**Bond angles**	**Cis Peptides**
			Avg: 1.22	Clashscore: 1.14	Outliers: 1 of 616	Poor rotamers: 0 of 515	Outliers: 0 of 562	Outliers: 10 of 614	Outliers: 3 of 618	Outliers: 14 of 618	Non-Trans: 0 of 617

A 521		GLY	0.7	-	Favored (79.48%) Glycine / 90.7,-8.7	-	-	Favored (84.288%)	-	-	-
A 522		GLU	0.67	-	Favored (68.02%) General / -60.2,-28.5	Favored (66.8%) *mt-10* chi angles: 287.1,184.1,21.6	0.10Å	Favored (33.303%)	-	-	-
A 523		PHE	0.67	-	Favored (43.12%) General / -100.8,8.6	Favored (62.5%) *m-80* chi angles: 294.7,114.3	0.07Å	Favored (15.529%)	-	-	-
A 524		ARG	0.69	-	Favored (46.17%) General / -56.6,130.7	Favored (86.3%) *mtm180* chi angles: 287,175.5,289.6,178.9	0.04Å	Favored (21.364%)	-	-	-
A 525		LEU	0.71	-	Favored (17.12%) General / -100.3,156.6	Favored (94.7%) *mt* chi angles: 298.5,177.5	0.02Å	Favored (34.491%)	-	-	-
A 526		ARG	0.73	-	Favored (51.11%) General / -128.9,144.2	Favored (97.6%) *mtt180* chi angles: 296.1,182.3,181.1,180.8	0.04Å	CaBLAM Disfavored (4.476%)	-	-	-
A 527		GLY	0.74	-	Favored (33.35%) Glycine / 58.7,-123.4	-	-	Favored (24.133%)	-	-	-
A 528		GLU	0.74	-	Favored (67.21%) General / -59.9,-28.1	Favored (98.8%) *mt-10* chi angles: 290.8,179.1,353.4	0.01Å	Favored (15.534%)	-	-	-
A 529		GLU	0.72	-	Favored (78.88%) General / -63.3,-35.0	Favored (98.3%) *mt-10* chi angles: 291.9,173.5,340.7	0.01Å	Favored (63.463%) three-ten	-	-	-
A 530		ARG	0.7	-	Favored (51.33%) General / -78.0,-33.9	Favored (31%) *mtp-110* chi angles: 289.8,178.2,61.4,250.5	0.04Å	Favored (81.275%) alpha helix	-	-	-
A 531		LYS	0.67	-	Favored (77.64%) General / -62.0,-49.0	Favored (52.9%) *tttm* chi angles: 182,178.1,185.6,292.5	0.01Å	Favored (80.625%) alpha helix	-	-	-
A 532		ASN	0.66	-	Favored (95.58%) General / -63.0,-39.7	Favored (98.7%) *m-40* chi angles: 289,337.3	0.05Å	Favored (81.889%) alpha helix	-	-	-
A 533		PHE	0.66	-	Favored (72.12%) General / -57.4,-50.7	Favored (60.4%) *t80* chi angles: 171.6,68.2	0.08Å	Favored (82.385%) alpha helix	-	OUTLIER(S) worst is CA-CB-CG: 5.3 σ	-
A 534		VAL	0.68	-	Favored (97.81%) Ile or Val / -64.0,-44.5	Favored (69.3%) *t* chi angles: 172	0.06Å	Favored (85.445%) alpha helix	-	-	-
A 535		GLU	0.71	-	Favored (92.23%) General / -63.2,-38.7	Favored (96.9%) *mt-10* chi angles: 290,180.4,359.1	0.03Å	Favored (94.766%) alpha helix	-	-	-
A 536		LEU	0.76	-	Favored (98.64%) General / -62.3,-41.6	Favored (71.8%) *mt* chi angles: 288,166.3	0.07Å	Favored (82.165%) alpha helix	-	-	-
A 537		MET	0.82	-	Favored (71.19%) General / -71.2,-40.4	Favored (83%) *mtm* chi angles: 290.7,188,292.5	0.03Å	Favored (72.658%) alpha helix	-	-	-
A 538		ARG	0.87	-	Favored (60.82%)	Favored (96.8%) *mtt-85*	0.13Å	Favored (57.517%)	-	-	-

					General / -74.9,-40.7	chi angles: 287,181.9,179.9,275.4		alpha helix			
A 539		ASN	0.91	-	Favored (8.11%) General / -104.1,-35.3	Favored (88.8%) *m-40* chi angles: 295.2,320.8	0.04Å	Favored (28.947%) alpha helix	-	-	-
A 540		GLY	0.91	-	Favored (70.13%) Glycine / -91.7,-2.1	-	-	Favored (34.382%)	-	-	-

#	Alt	Res	High B	Clash > 0.4Å	Ramachandran	Rotamer	Cβ deviation	CaBLAM	Bond lengths	Bond angles	Cis Peptides
			Avg: 1.22	Clashscore: 1.14	Outliers: 1 of 616	Poor rotamers: 0 of 515	Outliers: 0 of 562	Outliers: 10 of 614	Outliers: 3 of 618	Outliers: 14 of 618	Non-Trans: 0 of 617
A 541		ASP	0.89	-	Favored (31.11%) General / 53.5,43.5	Favored (29.8%) *t0* chi angles: 197,27.7	0.02Å	Favored (24.518%)	-	-	-
A 542		LEU	0.85	-	Favored (38.5%) Pre-Pro / -93.4,150.2	Favored (81.7%) *mt* chi angles: 301.3,176.7	0.05Å	Favored (22.353%) beta sheet	-	-	-
A 543		PRO	0.79	-	Favored (61.31%) Trans-Pro / -68.0,157.6	Favored (45.1%) *Cg_endo* chi angles: 24.6,327.1,27.2	0.06Å	Favored (89.924%)	-	-	-
A 544		VAL	0.74	-	Favored (91.08%) Ile or Val / -58.9,-46.7	Favored (70.1%) *t* chi angles: 172.1	0.12Å	Favored (67.048%)	-	-	-
A 545		TRP	0.7	-	Favored (91.11%) General / -59.5,-45.6	Favored (93%) *t60* chi angles: 182.2,88.1	0.04Å	Favored (79.56%) alpha helix	-	-	-
A 546		LEU	0.66	-	Favored (72.33%) General / -68.7,-45.2	Favored (55.3%) *tp* chi angles: 182.3,58.8	0.01Å	Favored (77.633%) alpha helix	-	-	-
A 547		ALA	0.64	-	Favored (76.31%) General / -57.7,-39.0	-	0.05Å	Favored (88.749%) alpha helix	-	-	-
A 548		TYR	0.62	-	Favored (70.27%) General / -60.4,-51.2	Favored (89.3%) *t80* chi angles: 179.6,79.6	0.05Å	Favored (92.145%) alpha helix	-	-	-
A 549		LYS	0.61	-	Favored (82.87%) General / -61.9,-37.0	Favored (62.7%) *mmtt* chi angles: 288.6,288.9,188.7,175.3	0.07Å	Favored (78.419%) alpha helix	-	-	-
A 550		VAL	0.61	-	Favored (39.48%) Ile or Val / -67.9,-52.4	Favored (77.1%) *t* chi angles: 172.9	0.14Å	Favored (71.694%) alpha helix	-	-	-
A 551		ALA	0.61	-	Favored (86.6%) General / -60.7,-38.9	-	0.06Å	Favored (75.638%) alpha helix	-	-	-
A 552		SER	0.61	-	Favored (62.92%) General / -69.7,-14.7	Favored (71.3%) *p* chi angles: 71.9	0.03Å	Favored (61.814%) alpha helix	-	-	-
A 553		ASN	0.63	-	Favored (28.87%) General / -106.0,13.7	Favored (63.8%) *m-40* chi angles: 284.9,279.2	0.12Å	Favored (55.306%)	-	-	-
A 554		GLY	0.64	-	Favored (79.96%) Glycine / 81.3,13.8	-	-	Favored (88.064%)	-	-	-

A 555		HIS	0.67	-	Favored (29.78%) General / -90.2,118.5	Favored (93.3%) *m-70* chi angles: 292.1,287.2	0.03Å	Favored (28.257%)	-	-	-
A 556		SER	0.69	-	Favored (19.95%) General / -74.4,168.3	Favored (87.8%) *p* chi angles: 68.1	0.02Å	Favored (33.424%)	-	-	-
A 557		TYR	0.72	-	Favored (47.56%) General / -49.6,-45.3	Favored (84.2%) *t80* chi angles: 180.6,83.5	0.04Å	Favored (44.982%)	-	-	-
A 558		GLN	0.73	-	Favored (56.01%) General / -83.9,-2.2	Favored (94.1%) *mt0* chi angles: 294.8,179.8,336	0.01Å	Favored (50.366%)	-	-	-
A 559		ASP	0.73	-	Favored (9.36%) General / -82.8,79.0	Favored (66.3%) *t0* chi angles: 183.4,356.3	0.04Å	Favored (15.704%)	-	-	-
A 560		ARG	0.71	-	Favored (12.43%) General / -61.0,-12.6	Favored (51.8%) *ptt-90* chi angles: 66.8,184.6,180.5,275	0.06Å	Favored (9.812%)	-	-	-
**#**	**Alt**	**Res**	**High B**	**Clash > 0.4Å**	**Ramachandran**	**Rotamer**	**Cβ deviation**	**CaBLAM**	**Bond lengths**	**Bond angles**	**Cis Peptides**
			Avg: 1.22	Clashscore: 1.14	Outliers: 1 of 616	Poor rotamers: 0 of 515	Outliers: 0 of 562	Outliers: 10 of 614	Outliers: 3 of 618	Outliers: 14 of 618	Non-Trans: 0 of 617
A 561		SER	0.69	-	Favored (66.78%) General / -61.0,-25.4	Favored (89.2%) *p* chi angles: 69.1	0.03Å	Favored (44.459%)	-	-	-
A 562		TRP	0.66	-	Favored (62.86%) General / -69.7,-14.5	Favored (66.9%) *p-90* chi angles: 64.2,263.5	0.11Å	Favored (66.073%) three-ten	-	-	-
A 563		CYS	0.65	-	Favored (46.54%) General / -85.9,-13.8	Favored (78.9%) *m* chi angles: 296.2	0.10Å	Favored (62.976%) alpha helix	-	-	-
A 564		PHE	0.65	-	Favored (6.96%) General / -116.0,-27.4	Favored (86.3%) *m-80* chi angles: 298.3,105.2	0.03Å	Favored (16.556%) alpha helix	-	-	-
A 565		THR	0.68	-	Favored (35.54%) General / -89.0,126.8	Favored (89.6%) *m* chi angles: 298.2	0.03Å	CaBLAM Outlier (0.207%)	-	-	-
A 566		GLY	0.71	-	Favored (37.93%) Glycine / 163.9,179.0	-	-	Favored (47.44%)	-	-	-
A 567		GLN	0.75	-	Favored (38.17%) General / -73.3,156.7	Favored (17.2%) *pt0* chi angles: 67.8,181.3,349.6	0.04Å	Favored (13.246%)	-	-	-
A 568		THR	0.77	-	Favored (65.2%) General / -67.6,-19.2	Favored (70.6%) *p* chi angles: 59.5	0.06Å	Favored (48.195%)	-	-	-
A 569		ASN	0.79	-	Favored (59.1%) General / -73.2,-9.8	Favored (98.6%) *m-40* chi angles: 290.1,340.5	0.03Å	Favored (58.559%)	-	-	-
A 570		ASN	0.81	-	Favored (49.84%) General / -97.1,5.7	Favored (70.9%) *m-40* chi angles: 297,346.2	0.04Å	Favored (26.478%)	-	-	-

A 571		THR	0.85	-	Favored (52.76%) General / -58.6,132.1	Favored (37.4%) *m* chi angles: 305.4	0.04Å	Favored (40.848%)	-	-	-
A 572		ILE	0.93	-	Favored (39.39%) Ile or Val / -85.0,128.0	Favored (91.7%) *mt* chi angles: 297.7,172.4	0.01Å	Favored (53.935%) beta sheet	-	-	-
A 573		LEU	1.02	-	Favored (38.02%) General / -114.4,148.2	Favored (4.8%) *mp* chi angles: 280.9,73.9	0.02Å	Favored (50.795%)	-	-	-
A 574		GLU	1.11	-	Allowed (0.61%) General / -114.9,-83.7	Favored (90.7%) *tt0* chi angles: 180.6,178.8,352	0.05Å	CaBLAM Disfavored (1.576%)	-	-	-
A 575		ASP	1.15	-	Favored (6.44%) General / -89.7,64.1	Favored (41.3%) *t0* chi angles: 190.2,19	0.01Å	CaBLAM Disfavored (2.97%)	-	-	-
A 576		ASN	1.13	-	Favored (12.45%) General / 58.5,24.9	Favored (76.4%) *m-40* chi angles: 299.1,303.2	0.02Å	CaBLAM Disfavored (3.648%)	-	-	-
A 577		ASN	1.03	-	Favored (35.13%) General / -144.3,148.7	Favored (56.5%) *m-40* chi angles: 285.2,275.7	0.04Å	Favored (12.664%)	-	-	-
A 578		GLU	0.91	-	Favored (57.58%) General / -59.0,135.4	Favored (68.9%) *mt-10* chi angles: 297.1,184.4,322.7	0.04Å	Favored (38.692%) beta sheet	-	-	-
A 579		VAL	0.8	-	Favored (39.14%) Ile or Val / -84.3,128.2	Favored (96.2%) *t* chi angles: 175.7	0.04Å	Favored (50.437%) beta sheet	-	-	-
A 580		GLU	0.73	-	Favored (46.23%) General / -117.5,143.2	Favored (69.8%) *mt-10* chi angles: 294.1,180.7,313.9	0.03Å	Favored (67.582%) beta sheet	-	-	-
**#**	**Alt**	**Res**	**High B**	**Clash > 0.4Å**	**Ramachandran**	**Rotamer**	**Cβ deviation**	**CaBLAM**	**Bond lengths**	**Bond angles**	**Cis Peptides**
			Avg: 1.22	Clashscore: 1.14	Outliers: 1 of 616	Poor rotamers: 0 of 515	Outliers: 0 of 562	Outliers: 10 of 614	Outliers: 3 of 618	Outliers: 14 of 618	Non-Trans: 0 of 617
A 581		VAL	0.71	-	Favored (71.73%) Ile or Val / -125.8,131.6	Favored (98.5%) *t* chi angles: 175.6	0.07Å	Favored (61.281%) beta sheet	-	-	-
A 582		PHE	0.73	-	Favored (29.84%) General / -85.6,122.5	Favored (67.9%) *m-80* chi angles: 286.4,94.3	0.12Å	Favored (54.902%)	-	OUTLIER(S) worst is CA-CB-CG: 4.0 σ	-
A 583		THR	0.76	-	Favored (18.67%) General / -79.6,169.0	Favored (71.5%) *p* chi angles: 59.6	0.08Å	Favored (39.07%)	-	-	-
A 584		LYS	0.79	-	Favored (43.76%) General / -59.2,-19.3	Favored (54.6%) *mtmt* chi angles: 290.6,193.7,293.5,183.8	0.06Å	Favored (41.003%)	-	-	-
A 585		THR	0.8	-	Favored (59.54%) General / -81.0,-11.6	Favored (43.7%) *p* chi angles: 66.9	0.11Å	Favored (34.436%)	-	-	-
A 586		GLY	0.78	-	Favored (75.14%) Glycine / 93.4,-8.0	-	-	Favored (77.149%)	-	-	-

A 587		ASP	0.73	-	Favored (16.4%) General / -78.4,113.5	Favored (62.9%) *t0* chi angles: 185.9,355	0.05Å	Favored (27.602%)	-	OUTLIER(S) worst is CA-CB-CG: 4.0 σ	-
A 588		ARG	0.69	-	Favored (25.35%) General / -83.8,119.9	Favored (59.8%) *ttt90* chi angles: 184.5,180,173.6,92.9	0.05Å	Favored (55.972%) beta sheet	-	-	-
A 589		LYS	0.64	-	Favored (50.7%) General / -129.4,150.7	Favored (58.8%) *mttm* chi angles: 297.1,183.5,188.3,300.6	0.05Å	Favored (48.527%) beta sheet	-	-	-
A 590		ILE	0.61	-	Favored (34.67%) Ile or Val / -78.9,131.3	Favored (91.5%) *mt* chi angles: 297.4,168.7	0.03Å	Favored (37.558%) beta sheet	-	-	-
A 591		LEU	0.6	-	Favored (24.04%) General / -75.3,122.6	Favored (94.3%) *mt* chi angles: 292,173.3	0.06Å	Favored (24.16%) beta sheet	OUTLIER(S) worst is CB--CG: 4.0 σ	-	-
A 592		ARG	0.6	-	Favored (17.29%) Pre-Pro / -143.7,71.6	Favored (90.4%) *mtt180* chi angles: 290.1,176.3,173,160.5	0.01Å	CaBLAM Disfavored (4.942%) try beta sheet	-	-	-
A 593		PRO	0.61	-	Favored (78.72%) Trans-Pro / -60.2,150.6	Favored (55.1%) *Cg_exo* chi angles: 337.4,35.7,326.3	0.05Å	Favored (31.56%)	-	-	-
A 594		LYS	0.64	-	Favored (70.9%) General / -59.4,-33.2	Favored (95.8%) *mttt* chi angles: 288,181.4,177.5,182.2	0.04Å	Favored (24.41%)	-	-	-
A 595		TRP	0.69	-	Favored (40.14%) General / -132.2,129.8	Favored (11.7%) *m-90* chi angles: 308.9,269.9	0.11Å	Favored (27.237%)	-	-	-
A 596		MET	0.75	-	Favored (36.46%) General / -102.4,140.6	Favored (13.5%) *tpt* chi angles: 183.1,64.2,196.7	0.08Å	Favored (56.888%)	-	-	-
A 597		ASP	0.82	-	Favored (8.18%) General / -131.2,106.2	Favored (52.5%) *t0* chi angles: 181.6,338.8	0.02Å	Favored (39.957%) beta sheet	-	-	-
A 598		ALA	0.9	-	Favored (37.42%) General / -56.3,-23.6	-	0.06Å	Favored (26.853%)	-	-	-
A 599		ARG	1	-	Favored (66.24%) General / -63.0,-21.7	Favored (87.5%) *mtm180* chi angles: 289.5,174.1,292.2,181.4	0.04Å	Favored (63.832%)	-	-	-
A 600		VAL	1.12	-	Favored (84.61%) Ile or Val / -63.5,-39.2	Favored (55.5%) *t* chi angles: 170.1	0.02Å	Favored (58.754%) alpha helix	-	-	-
**#**	**Alt**	**Res**	**High B**	**Clash > 0.4Å**	**Ramachandran**	**Rotamer**	**Cβ deviation**	**CaBLAM**	**Bond lengths**	**Bond angles**	**Cis Peptides**
			Avg: 1.22	Clashscore: 1.14	Outliers: 1 of 616	Poor rotamers: 0 of 515	Outliers: 0 of 562	Outliers: 10 of 614	Outliers: 3 of 618	Outliers: 14 of 618	Non-Trans: 0 of 617
A 601		CYS	1.25	-	Favored (19.64%) General / -98.0,-16.4	Favored (29.5%) *p* chi angles: 66.7	0.05Å	Favored (28.292%) alpha helix	-	-	-
A 602		CYS	1.37	-	Favored (72.24%)	Favored (52.2%) *t* chi angles: 180.6	0.05Å	Favored (44.352%) alpha helix	-	-	-

					General / -60.3,-50.8						
A 603		ASP	1.44	-	Favored (30.75%) General / -100.1,115.9	Favored (48.1%) *t0* chi angles: 184,332.4	0.07Å	Favored (26.513%) alpha helix	-	-	-
A 604		TYR	1.45	-	Favored (5.41%) General / -44.3,-40.6	Favored (83.5%) *t80* chi angles: 172.9,79.9	0.05Å	Favored (45.415%) alpha helix	-	-	-
A 605		GLN	1.39	-	Favored (74.88%) General / -66.4,-33.1	Favored (96.4%) *mm-40* chi angles: 292.4,297.4,317.7	0.01Å	Favored (78.293%) alpha helix	-	-	-
A 606		ALA	1.26	-	Favored (84.78%) General / -67.6,-39.0	-	0.03Å	Favored (85.306%) alpha helix	-	-	-
A 607		LEU	1.11	-	Favored (77.75%) General / -64.8,-47.6	Favored (70.4%) *tp* chi angles: 177.9,60	0.04Å	Favored (95.233%) alpha helix	-	-	-
A 608		LYS	0.97	-	Favored (80.24%) General / -56.9,-47.6	Favored (54%) *tttm* chi angles: 181.6,178.4,184.7,294.3	0.01Å	Favored (97.655%) alpha helix	-	-	-
A 609		SER	0.85	-	Favored (97.42%) General / -61.9,-41.1	Favored (73.3%) *m* chi angles: 295.6	0.04Å	Favored (95.025%) alpha helix	-	-	-
A 610		PHE	0.75	-	Favored (92.21%) General / -60.5,-45.8	Favored (73.1%) *t80* chi angles: 185,83.7	0.04Å	Favored (96.472%) alpha helix	-	-	-
A 611		LYS	0.69	-	Favored (94.55%) General / -61.2,-40.6	Favored (51.6%) *mtpt* chi angles: 287.8,171.8,71.5,176.8	0.04Å	Favored (96.218%) alpha helix	-	-	-
A 612		GLU	0.65	-	Favored (94.04%) General / -65.1,-40.2	Favored (97.7%) *mt-10* chi angles: 290.9,178.2,2.2	0.01Å	Favored (97.97%) alpha helix	-	-	-
A 613		PHE	0.65	-	Favored (66.65%) General / -59.7,-52.2	Favored (77.6%) *t80* chi angles: 173.7,83.4	0.04Å	Favored (81.765%) alpha helix	-	-	-
A 614		ALA	0.69	-	Favored (80.55%) General / -59.4,-38.4	-	0.04Å	Favored (77.716%) alpha helix	-	-	-
A 615		ALA	0.79	-	Favored (68.43%) General / -62.1,-26.6	-	0.04Å	Favored (30.051%)	-	-	-
A 616		GLY	0.97	-	Favored (64.22%) Glycine / 87.0,14.7	-	-	Favored (79.593%)	-	-	-
A 617		LYS	1.23	-	Favored (77.77%) General / -66.7,-34.4	Favored (96.4%) *mttt* chi angles: 288.1,177.4,180.2,179.7	0.03Å	-	-	-	-
A 618		ARG	1.58	0.55Å HG3 with A 618 ARG OXT	-	Favored (37.5%) *ptt180* chi angles: 61.3,180.9,179.7,170.6	0.04Å	-	-	-	-
