# Supplementary material for: Integrative proteome-wide structural analysis and high-throughput docking identify broad-spectrum antiviral scaffolds against Zika, Yellow Fever, West Nile, Saint Louis encephalitis, and Usutu viruses
Source: Front Cell Infect Microbiol. 2026 Apr 30;16:1723132. doi: 10.3389/fcimb.2026.1723132 (PMC13171538; doi:10.3389/fcimb.2026.1723132)
Supplement: Supplementary file 3 [file DataSheet3.zip › SLEV/SLEV_NS4a/Mol_probity_Files/SLEV_NS4a_1FH-multi.table.pdf]

Viewing SLEV_NS4a1FH-multi.table

PROBITY

Duke Biochemistry
Duke University School of Medicine

When finished, you should close this window.

Hint: Use File | Save As... to save a copy of this page.

All-Atom Contacts	Clashscore, all atoms:	0		100th percentile* (N=1784, all resolutions)
	Clashscore is the number of serious steric overlaps (> 0.4 Å) per 1000 atoms.			
Protein Geometry	Poor rotamers	0	0.00%	Goal: <0.3%
	Favored rotamers	101	100.00%	Goal: >98%
	Ramachandran outliers	0	0.00%	Goal: <0.05%
	Ramachandran favored	123	99.19%	Goal: >98%
	Rama distribution Z-score	2.16 ± 0.63		Goal: abs(Z score) < 2
	MolProbity score^	0.50		100th percentile* (N=27675, 0Å - 99Å)
	Cβ deviations >0.25Å	0	0.00%	Goal: 0
	Bad bonds:	0 / 968	0.00%	Goal: 0%
	Bad angles:	2 / 1309	0.15%	Goal: <0.1%
Peptide Omegas	Cis Prolines:	0 / 5	0.00%	Expected: ≤1 per chain, or ≤5%
Low-resolution Criteria	CaBLAM outliers	0	0.0%	Goal: <1.0%
	CA Geometry outliers	0	0.00%	Goal: <0.5%
Additional validations	Chiral volume outliers	0/159		
	Waters with clashes	0/0	0.00%	See UnDowser table for details

In the two column results, the left column gives the raw count, right column gives the percentage.

* 100th percentile is the best among structures of comparable resolution; 0th percentile is the worst. For clashscore the comparative set of structures was selected in 2004, for MolProbity score in 2006.

^ MolProbity score combines the clashscore, rotamer, and Ramachandran evaluations into a single score, normalized to be on the same scale as X-ray resolution.

Key to table colors and cutoffs here:

#	Alt	Res	High B	Clash > 0.4Å	Ramachandran	Rotamer	Cβ deviation	CaBLAM	Bond lengths	Bond angles	Cis Peptides
			Avg: 3.35	Clashscore: 0	Outliers: 0 of 124	Poor rotamers: 0 of 101	Outliers: 0 of 115	Outliers: 0 of 122	Outliers: 0 of 126	Outliers: 2 of 126	Non-Trans: 0 of 125
A 1		SER	10.46	-	-	Favored (73%) *m* chi angles: 295.6	0.02Å	-	-	-	-
A 2		ALA	10.29	-	Favored (69.65%) General / -57.9,-34.3	-	0.03Å	-	-	-	-
A 3		LEU	10.03	-	Favored (73.29%) General / -64.5,-31.9	Favored (91.6%) *mt* chi angles: 293.1,175.7	0.01Å	Favored (74.5%)	-	-	-
A 4		GLY	9.67	-	Favored (76.39%) Glycine / -64.5,-48.1	-	-	Favored (89.833%) alpha helix	-	-	-
A 5		MET	9.24	-	Favored (79.18%) General / -56.0,-44.9	Favored (27.5%) *tmm* chi angles: 178.1,276.6,293.7	0.04Å	Favored (92.922%) alpha helix	-	-	-
A 6		MET	8.74	-	Favored (93.36%)	Favored (34%) *mtt* chi angles:	0.03Å	Favored (95.82%)	-	-	-

#	Alt	Res	High B	Clash > 0.4Å	Ramachandran	Rotamer	Cβ deviation	CaBLAM	Bond lengths	Bond angles	Cis Peptides
					General / -60.2,-45.4	292.4,175.7,204.3		alpha helix			
A 7		GLU	8.21	-	Favored (92.15%) General / -59.8,-41.9	Favored (91.8%) *tt0* chi angles: 182,176.3,358.8	0.05Å	Favored (99.062%) alpha helix	-	-	-
A 8		VAL	7.67	-	Favored (98.26%) Ile or Val / -63.8,-44.7	Favored (62.9%) *t* chi angles: 171.2	0.02Å	Favored (98.521%) alpha helix	-	-	-
A 9		MET	7.12	-	Favored (71.97%) General / -61.0,-32.4	Favored (87.7%) *mmm* chi angles: 286.7,298.6,294.1	0.03Å	Favored (74.118%) alpha helix	-	-	-
A 10		GLY	6.57	-	Favored (62.49%) Glycine / -67.4,-13.2	-	-	Favored (74.015%) three-ten	-	-	-
A 11		ARG	6	-	Favored (47.09%) General / -95.5,-3.3	Favored (95.6%) *mtt180* chi angles: 297.2,177.1,185.2,186	0.07Å	Favored (57.943%)	-	-	-
A 12		MET	5.44	-	Favored (55.69%) Pre-Pro / -86.7,153.8	Favored (97.7%) *mmm* chi angles: 299.5,299.5,289.4	0.03Å	Favored (21.865%)	-	-	-
A 13		PRO	4.87	-	Favored (68.66%) Trans-Pro / -56.2,146.9	Favored (85%) *Cg_exo* chi angles: 333.9,35.3,330.5	0.04Å	Favored (86.219%)	-	-	-
A 14		ASN	4.33	-	Favored (71.12%) General / -57.2,-36.6	Favored (96.7%) *m-40* chi angles: 286.7,340.2	0.03Å	Favored (63.705%)	-	-	-
A 15		HIS	3.82	-	Favored (71.92%) General / -60.9,-50.8	Favored (88.1%) *t70* chi angles: 181.5,74.5	0.01Å	Favored (80.468%) alpha helix	-	-	-
A 16		PHE	3.38	-	Favored (73.64%) General / -55.1,-48.4	Favored (73.7%) *t80* chi angles: 178,88.1	0.07Å	Favored (85.907%) alpha helix	-	-	-
A 17		TRP	3.01	-	Favored (77.71%) General / -60.8,-49.4	Favored (87.6%) *t60* chi angles: 184.9,89.9	0.08Å	Favored (94.721%) alpha helix	-	-	-
A 18		GLU	2.71	-	Favored (88.85%) General / -62.1,-38.4	Favored (97.8%) *mt-10* chi angles: 289.9,175.9,357.7	0.03Å	Favored (75.057%) alpha helix	-	-	-
A 19		LYS	2.46	-	Favored (59.41%) General / -76.1,-34.9	Favored (31.3%) *mmmt* chi angles: 295.9,299.8,289.5,185.1	0.01Å	Favored (90.075%) alpha helix	-	-	-
A 20		THR	2.27	-	Favored (83.45%) General / -65.8,-45.2	Favored (97.3%) *m* chi angles: 299.9	0.04Å	Favored (82.504%) alpha helix	-	-	-
**#**	**Alt**	**Res**	**High B**	**Clash > 0.4Å**	**Ramachandran**	**Rotamer**	**Cβ deviation**	**CaBLAM**	**Bond lengths**	**Bond angles**	**Cis Peptides**
			Avg: 3.35	Clashscore: 0	Outliers: 0 of 124	Poor rotamers: 0 of 101	Outliers: 0 of 115	Outliers: 0 of 122	Outliers: 0 of 126	Outliers: 2 of 126	Non-Trans: 0 of 125
A 21		VAL	2.11	-	Favored (92.79%)	Favored (68%) *t* chi angles: 171.9	0.03Å	Favored (84.57%) alpha helix	-	-	-

					Ile or Val / -65.8,-44.8						
A 22		ALA	1.95	-	Favored (83.03%) General / -59.0,-39.9	-	0.05Å	Favored (89.635%) alpha helix	-	-	-
A 23		ALA	1.81	-	Favored (99.68%) General / -63.0,-41.9	-	0.03Å	Favored (97.301%) alpha helix	-	-	-
A 24		ALA	1.68	-	Favored (94.71%) General / -62.0,-40.1	-	0.04Å	Favored (91.657%) alpha helix	-	-	-
A 25		ASP	1.55	-	Favored (75.99%) General / -70.0,-38.0	Favored (98.1%) *m-30* chi angles: 288.8,347.5	0.01Å	Favored (94.551%) alpha helix	-	-	-
A 26		THR	1.43	-	Favored (84.52%) General / -62.4,-47.4	Favored (91.7%) *m* chi angles: 299	0.08Å	Favored (85.301%) alpha helix	-	-	-
A 27		LEU	1.33	-	Favored (86.99%) General / -66.2,-38.0	Favored (96.3%) *mt* chi angles: 292.1,171.9	0.07Å	Favored (79.06%) alpha helix	-	-	-
A 28		TYR	1.25	-	Favored (70.62%) General / -56.4,-50.8	Favored (90%) *t80* chi angles: 177.2,81.7	0.03Å	Favored (81.323%) alpha helix	-	-	-
A 29		LEU	1.23	-	Favored (95.94%) General / -61.0,-41.2	Favored (79.6%) *mt* chi angles: 289.5,173.2	0.03Å	Favored (94.013%) alpha helix	-	-	-
A 30		LEU	1.36	-	Favored (76.97%) General / -65.1,-33.8	Favored (95.3%) *mt* chi angles: 292.7,174.1	0.05Å	Favored (74.703%) alpha helix	-	-	-
A 31		GLY	1.73	-	Favored (56.18%) Glycine / -81.7,-24.4	-	-	Favored (53.531%) alpha helix	-	-	-
A 32		THR	2.52	-	Favored (8.45%) General / -117.3,-22.0	Favored (74.2%) *p* chi angles: 61.5	0.01Å	Favored (18.975%)	-	-	-
A 33		SER	3.93	-	Favored (23.78%) General / -81.1,162.4	Favored (100%) *p* chi angles: 65.5	0.02Å	Favored (17.282%)	-	-	-
A 34		GLU	5.76	-	Favored (57%) General / -63.1,135.1	Favored (91%) *tt0* chi angles: 183.9,176.4,4	0.01Å	Favored (36.732%)	-	-	-
A 35		ALA	7.08	-	Favored (64.27%) General / -56.7,-31.2	-	0.04Å	Favored (37.885%)	-	-	-
A 36		ASN	6.82	-	Favored (48.96%) General / -95.3,6.5	Favored (86.5%) *m-40* chi angles: 291,321.1	0.03Å	Favored (49.47%)	-	-	-
A 37		SER	5.17	-	Favored (28.65%) General / -77.2,160.8	Favored (98.4%) *p* chi angles: 65.7	0.05Å	Favored (42.393%)	-	-	-
A 38		ARG	3.3	-	Favored (94.86%)	Favored (24.1%) *tpt-90*	0.02Å	Favored (59.229%)	-	-	-

#	Alt	Res	High B	Clash > 0.4Å	Ramachandran	Rotamer	Cβ deviation	CaBLAM	Bond lengths	Bond angles	Cis Peptides
					General / -64.4,-39.9	chi angles: 181.1,65.5,182.3,270.9					
A 39		ALA	1.98	-	Favored (84.69%) General / -59.8,-39.4	-	0.02Å	Favored (79.583%) alpha helix	-	-	-
A 40		HIS	1.27	-	Favored (71.8%) General / -62.3,-50.4	Favored (68.2%) *t70* chi angles: 187.5,79.3	0.07Å	Favored (82.633%) alpha helix	-	-	-
**#**	**Alt**	**Res**	**High B**	**Clash > 0.4Å**	**Ramachandran**	**Rotamer**	**Cβ deviation**	**CaBLAM**	**Bond lengths**	**Bond angles**	**Cis Peptides**
			Avg: 3.35	Clashscore: 0	Outliers: 0 of 124	Poor rotamers: 0 of 101	Outliers: 0 of 115	Outliers: 0 of 122	Outliers: 0 of 126	Outliers: 2 of 126	Non-Trans: 0 of 125
A 41		LYS	0.95	-	Favored (94%) General / -61.6,-40.2	Favored (97.6%) *mttt* chi angles: 290.7,176.8,183.3,178.4	0.05Å	Favored (78.783%) alpha helix	-	-	-
A 42		GLU	0.83	-	Favored (84.61%) General / -67.7,-39.3	Favored (68.8%) *mm-30* chi angles: 289.4,293.6,309.4	0.02Å	Favored (91.801%) alpha helix	-	-	-
A 43		ALA	0.83	-	Favored (93.17%) General / -62.6,-39.2	-	0.06Å	Favored (94.977%) alpha helix	-	-	-
A 44		LEU	0.9	-	Favored (95.05%) General / -64.5,-40.1	Favored (84.6%) *mt* chi angles: 290,172.8	0.06Å	Favored (94.163%) alpha helix	-	-	-
A 45		ALA	0.99	-	Favored (83.55%) General / -60.8,-37.9	-	0.03Å	Favored (73.961%) alpha helix	-	-	-
A 46		GLU	1.1	-	Favored (32.37%) General / -87.6,-17.2	Favored (78.3%) *mm-30* chi angles: 296.7,293.2,324.4	0.01Å	Favored (43.013%) alpha helix	-	-	-
A 47		LEU	1.22	-	Favored (71.97%) Pre-Pro / -48.7,-47.3	Favored (70.7%) *tp* chi angles: 177.9,63.2	0.12Å	Favored (48.367%) alpha helix	-	OUTLIER(S) worst is CA-C-N: 4.0 σ	-
A 48		PRO	1.35	-	Favored (68.02%) Trans-Pro / -57.5,-25.6	Favored (68.3%) *Cg_exo* chi angles: 335.4,36.4,327.3	0.03Å	Favored (83.412%) alpha helix	-	-	-
A 49		ASP	1.48	-	Favored (42.2%) General / -76.7,-42.2	Favored (31%) *t70* chi angles: 189.7,65.8	0.04Å	Favored (73.99%) alpha helix	-	-	-
A 50		SER	1.6	-	Favored (96.62%) General / -64.5,-41.3	Favored (68.7%) *m* chi angles: 294.8	0.05Å	Favored (95.844%) alpha helix	-	-	-
A 51		LEU	1.7	-	Favored (79.86%) General / -67.8,-43.3	Favored (94.5%) *mt* chi angles: 291.8,172.9	0.03Å	Favored (93.589%) alpha helix	-	-	-
A 52		GLU	1.8	-	Favored (87.07%) General / -58.1,-43.5	Favored (93.2%) *tt0* chi angles: 180,178.5,358.4	0.03Å	Favored (96.962%) alpha helix	-	-	-
A 53		THR	1.9	-	Favored (64.64%)	Favored (90.8%) *m* chi angles: 298	0.07Å	Favored (77.372%) alpha helix	-	-	-

#	Alt	Res	High B	Clash > 0.4Å	Ramachandran	Rotamer	Cβ deviation	CaBLAM	Bond lengths	Bond angles	Cis Peptides
					General /<br>-60.9,-52.5						
A<br>54		LEU	1.99	-	Favored<br>(85.03%)<br>General /<br>-67.2,-38.2	Favored (94.8%) *mt*<br>chi angles: 292.8,174.3	0.03Å	Favored<br>(77.177%)<br>alpha helix	-	-	-
A<br>55		LEU	2.06	-	Favored<br>(88.04%)<br>General /<br>-66.4,-38.6	Favored (88.9%) *mt*<br>chi angles: 291.8,168.6	0.07Å	Favored<br>(92.447%)<br>alpha helix	-	-	-
A<br>56		LEU	2.13	-	Favored<br>(64.57%)<br>General /<br>-73.5,-33.1	Favored (90.5%) *mt*<br>chi angles: 293.1,176.2	0.08Å	Favored<br>(94.676%)<br>alpha helix	-	-	-
A<br>57		ILE	2.18	-	Favored<br>(99.88%)<br>Ile or Val /<br>-62.9,-44.9	Favored (95%) *mt*<br>chi angles: 291.8,167.4	0.01Å	Favored<br>(78.239%)<br>alpha helix	-	-	-
A<br>58		GLY	2.22	-	Favored<br>(44.6%)<br>Glycine /<br>-53.7,-52.0	-	-	Favored<br>(97.237%)<br>alpha helix	-	-	-
A<br>59		MET	2.25	-	Favored<br>(74.16%)<br>General /<br>-54.7,-46.4	Favored (29%) *tmm*<br>chi angles:<br>180.1,276.4,293.2	0.08Å	Favored<br>(82.419%)<br>alpha helix	-	-	-
A<br>60		LEU	2.27	-	Favored<br>(90.64%)<br>General /<br>-65.0,-38.3	Favored (95.3%) *mt*<br>chi angles: 291.8,172	0.04Å	Favored<br>(95.403%)<br>alpha helix	-	-	-
**#**	**Alt**	**Res**	**High B**	**Clash > 0.4Å**	**Ramachandran**	**Rotamer**	**Cβ deviation**	**CaBLAM**	**Bond lengths**	**Bond angles**	**Cis Peptides**
			Avg:<br>3.35	Clashscore:<br>0	Outliers: 0 of<br>124	Poor rotamers: 0 of<br>101	Outliers:<br>0 of 115	Outliers: 0<br>of 122	Outliers:<br>0 of 126	Outliers: 2 of<br>126	Non-<br>Trans: 0<br>of 125
A<br>61		CYS	2.29	-	Favored<br>(87.16%)<br>General /<br>-67.1,-41.0	Favored (94.3%) *m*<br>chi angles: 292.9	0.03Å	Favored<br>(93.734%)<br>alpha helix	-	-	-
A<br>62		VAL	2.3	-	Favored<br>(96.71%)<br>Ile or Val /<br>-61.9,-46.3	Favored (50.5%) *t*<br>chi angles: 169.5	0.02Å	Favored<br>(91.105%)<br>alpha helix	-	-	-
A<br>63		MET	2.32	-	Favored<br>(84.33%)<br>General /<br>-66.4,-37.0	Favored (62.5%)<br>*tpp*<br>chi angles:<br>187,60.4,72.2	0.07Å	Favored<br>(88.715%)<br>alpha helix	-	-	-
A<br>64		SER	2.34	-	Favored<br>(78.69%)<br>General /<br>-67.1,-44.9	Favored (70.5%) *m*<br>chi angles: 296.3	0.05Å	Favored<br>(79.451%)<br>alpha helix	-	-	-
A<br>65		MET	2.38	-	Favored<br>(83.46%)<br>General /<br>-67.7,-38.3	Favored (82.3%)<br>*mtm*<br>chi angles:<br>289.3,186.9,288.6	0.04Å	Favored<br>(75.668%)<br>alpha helix	-	-	-
A<br>66		GLY	2.43	-	Favored<br>(52.88%)<br>Glycine /<br>-56.7,-52.1	-	-	Favored<br>(93.818%)<br>alpha helix	-	-	-
A<br>67		THR	2.53	-	Favored<br>(92.21%)<br>General /<br>-59.4,-45.1	Favored (92.1%) *m*<br>chi angles: 299	0.02Å	Favored<br>(94.599%)<br>alpha helix	-	-	-
A<br>68		PHE	2.67	-	Favored<br>(74.71%)	Favored (91.6%)<br>*t80*<br>chi angles: 176.1,78.2	0.02Å	Favored<br>(89.74%)<br>alpha helix	-	-	-

					General / -57.2,-49.8						
A 69		ILE	2.9	-	Favored (96.98%) Ile or Val / -60.9,-44.0	Favored (94.6%) *mt* chi angles: 292,166.8	0.04Å	Favored (85.781%) alpha helix	-	-	-
A 70		PHE	3.25	-	Favored (70.33%) General / -54.0,-48.6	Favored (86.2%) *t80* chi angles: 178,83.7	0.06Å	Favored (88.477%) alpha helix	-	-	-
A 71		LEU	3.75	-	Favored (79.1%) General / -64.6,-34.8	Favored (97.7%) *mt* chi angles: 292.5,173.3	0.03Å	Favored (77.653%) alpha helix	-	-	-
A 72		MET	4.37	-	Favored (53.19%) General / -77.7,-32.8	Favored (51.3%) *mmp* chi angles: 296,301.8,100.2	0.07Å	Favored (34.396%)	-	-	-
A 73		ASN	5.03	-	Favored (47.66%) General / -64.6,150.1	Favored (99.3%) *m-40* chi angles: 288.6,341.4	0.09Å	Favored (16.193%)	-	-	-
A 74		ARG	5.57	-	Favored (20.24%) General / -83.6,163.6	Favored (99%) *mtt180* chi angles: 294.9,179.8,181.6,176.7	0.03Å	Favored (13.745%)	-	-	-
A 75		LYS	5.83	-	Favored (40.09%) General / -54.0,131.3	Favored (86%) *tttt* chi angles: 181.5,178,178.7,180.9	0.02Å	CaBLAM Disfavored (2.354%)	-	-	-
A 76		GLY	5.72	-	Favored (28.13%) Glycine / 148.4,-159.8	-	-	Favored (29.211%)	-	-	-
A 77		VAL	5.29	-	Favored (28.89%) Ile or Val / -59.1,-25.1	Favored (5.4%) *p* chi angles: 69.6	0.05Å	Favored (6.794%)	-	-	-
A 78		GLY	4.67	-	Favored (17.57%) Glycine / -59.5,-12.1	-	-	Favored (19.708%)	-	-	-
A 79		LYS	4	-	Allowed (1.7%) General / -99.8,-59.5	Favored (69.5%) *mmtt* chi angles: 297,290.8,178.4,178.9	0.06Å	Favored (8.355%) alpha helix	-	-	-
A 80		MET	3.42	-	Favored (24.86%) General / -83.4,-35.8	Favored (49.5%) *mmp* chi angles: 294.2,298.4,96.6	0.03Å	Favored (27.235%) alpha helix	-	-	-
**#**	**Alt**	**Res**	**High B**	**Clash > 0.4Å**	**Ramachandran**	**Rotamer**	**Cβ deviation**	**CaBLAM**	**Bond lengths**	**Bond angles**	**Cis Peptides**
			Avg: 3.35	Clashscore: 0	Outliers: 0 of 124	Poor rotamers: 0 of 101	Outliers: 0 of 115	Outliers: 0 of 122	Outliers: 0 of 126	Outliers: 2 of 126	Non-Trans: 0 of 125
A 81		GLY	2.96	-	Favored (58.61%) Glycine / -64.5,-50.5	-	-	Favored (81.358%) alpha helix	-	-	-
A 82		LEU	2.6	-	Favored (77.86%) General / -57.2,-48.8	Favored (57.2%) *tp* chi angles: 178.2,65.4	0.10Å	Favored (85.298%) alpha helix	-	-	-
A 83		GLY	2.33	-	Favored (25.17%)	-	-	Favored (92.698%) alpha helix	-	-	-

					Glycine / -52.0,-54.6						
A 84		ALA	2.13	-	Favored (87.21%) General / -60.4,-39.4	-	0.04Å	Favored (78.789%) alpha helix	-	-	-
A 85		PHE	1.99	-	Favored (71.39%) General / -60.1,-51.0	Favored (91.1%) *t80* chi angles: 175.2,77.5	0.05Å	Favored (84.187%) alpha helix	-	OUTLIER(S) worst is CA-CB-CG: 4.2 σ	-
A 86		VAL	1.88	-	Favored (96.46%) Ile or Val / -63.6,-45.8	Favored (65.4%) *t* chi angles: 171.6	0.08Å	Favored (86.041%) alpha helix	-	-	-
A 87		MET	1.8	-	Favored (94.24%) General / -59.9,-44.4	Favored (65.9%) *ttp* chi angles: 181.4,178.9,70.7	0.04Å	Favored (97.625%) alpha helix	-	-	-
A 88		THR	1.73	-	Favored (92.74%) General / -61.2,-45.9	Favored (89.9%) *m* chi angles: 298.2	0.01Å	Favored (90.734%) alpha helix	-	-	-
A 89		LEU	1.68	-	Favored (95.99%) General / -64.8,-41.2	Favored (86.3%) *mt* chi angles: 290.9,173.7	0.07Å	Favored (94.719%) alpha helix	-	-	-
A 90		ALA	1.62	-	Favored (94.54%) General / -61.6,-40.3	-	0.05Å	Favored (99.042%) alpha helix	-	-	-
A 91		THR	1.58	-	Favored (87.13%) General / -65.7,-44.0	Favored (98.9%) *m* chi angles: 300.6	0.05Å	Favored (90.504%) alpha helix	-	-	-
A 92		ALA	1.54	-	Favored (82.65%) General / -59.2,-39.5	-	0.05Å	Favored (86.811%) alpha helix	-	-	-
A 93		LEU	1.52	-	Favored (79.8%) General / -68.0,-36.4	Favored (85.4%) *mt* chi angles: 290.1,170.8	0.09Å	Favored (89.929%) alpha helix	-	-	-
A 94		LEU	1.53	-	Favored (94.45%) General / -63.8,-39.5	Favored (94.6%) *mt* chi angles: 291.9,173.2	0.09Å	Favored (87.577%) alpha helix	-	-	-
A 95		TRP	1.58	-	Favored (83.27%) General / -67.6,-41.9	Favored (69%) *m100* chi angles: 282.1,114.2	0.04Å	Favored (86.053%) alpha helix	-	-	-
A 96		ALA	1.66	-	Favored (73.19%) General / -60.3,-34.1	-	0.02Å	Favored (78.691%) alpha helix	-	-	-
A 97		ALA	1.77	-	Favored (32.61%) General / -81.6,1.7	-	0.03Å	Favored (49.327%)	-	-	-
A 98		GLU	1.87	-	Favored (12.48%) General / 61.9,40.5	Favored (91.1%) *mt-10* chi angles: 299.6,184,357.6	0.05Å	Favored (19.544%)	-	-	-
A 99		VAL	1.97	-	Favored (69.96%) Pre-Pro / -77.5,132.2	Favored (94.8%) *t* chi angles: 174.8	0.08Å	Favored (21.535%) beta sheet	-	-	-

#	Alt	Res	High B	Clash > 0.4Å	Ramachandran	Rotamer	Cβ deviation	CaBLAM	Bond lengths	Bond angles	Cis Peptides
A 100		PRO	2.03	-	Favored (64.52%) Trans-Pro / -53.5,140.8	Favored (96.2%) *Cg_exo* chi angles: 331.5,35.9,331.9	0.04Å	Favored (90.887%)	-	-	-
**#**	**Alt**	**Res**	**High B**	**Clash > 0.4Å**	**Ramachandran**	**Rotamer**	**Cβ deviation**	**CaBLAM**	**Bond lengths**	**Bond angles**	**Cis Peptides**
			Avg: 3.35	Clashscore: 0	Outliers: 0 of 124	Poor rotamers: 0 of 101	Outliers: 0 of 115	Outliers: 0 of 122	Outliers: 0 of 126	Outliers: 2 of 126	Non-Trans: 0 of 125
A 101		GLY	2.04	-	Favored (57.87%) Glycine / -55.8,-31.2	-	-	Favored (59.082%)	-	-	-
A 102		THR	2.02	-	Favored (99.69%) General / -62.5,-42.7	Favored (89.4%) *m* chi angles: 298.3	0.02Å	Favored (74.873%) alpha helix	-	-	-
A 103		GLN	1.98	-	Favored (71.97%) General / -71.2,-37.8	Favored (86%) *mt0* chi angles: 293,179.5,359.4	0.01Å	Favored (83.527%) alpha helix	-	-	-
A 104		ILE	1.95	-	Favored (95.33%) Ile or Val / -64.8,-45.4	Favored (96.9%) *mt* chi angles: 292.6,169.6	0.03Å	Favored (98.558%) alpha helix	-	-	-
A 105		ALA	1.92	-	Favored (82.07%) General / -60.5,-37.8	-	0.04Å	Favored (90.291%) alpha helix	-	-	-
A 106		GLY	1.9	-	Favored (52.07%) Glycine / -57.4,-52.4	-	-	Favored (91.716%) alpha helix	-	-	-
A 107		VAL	1.92	-	Favored (93.15%) Ile or Val / -60.3,-43.2	Favored (59.2%) *t* chi angles: 170.7	0.02Å	Favored (86.924%) alpha helix	-	-	-
A 108		LEU	1.96	-	Favored (95.58%) General / -61.1,-40.9	Favored (78%) *mt* chi angles: 288.2,169.8	0.08Å	Favored (88.222%) alpha helix	-	-	-
A 109		LEU	2.03	-	Favored (78.65%) General / -65.4,-46.8	Favored (68.1%) *tp* chi angles: 178.5,59.9	0.03Å	Favored (83.746%) alpha helix	-	-	-
A 110		ILE	2.12	-	Favored (94.96%) Ile or Val / -62.0,-47.1	Favored (91.9%) *mt* chi angles: 291.8,166.4	0.03Å	Favored (93.017%) alpha helix	-	-	-
A 111		VAL	2.24	-	Favored (99.42%) Ile or Val / -62.0,-44.8	Favored (63%) *t* chi angles: 171.2	0.02Å	Favored (97.292%) alpha helix	-	-	-
A 112		PHE	2.39	-	Favored (73.76%) General / -57.6,-50.2	Favored (86.4%) *t80* chi angles: 173.5,78.8	0.03Å	Favored (96.217%) alpha helix	-	-	-
A 113		LEU	2.57	-	Favored (90.23%) General / -63.2,-38.2	Favored (92.5%) *mt* chi angles: 291.8,173.7	0.02Å	Favored (85.954%) alpha helix	-	-	-
A 114		LEU	2.76	-	Favored (92.15%) General / -64.0,-38.7	Favored (90.2%) *mt* chi angles: 290.9,171.9	0.01Å	Favored (91.904%) alpha helix	-	-	-

A 115		MET	2.97	-	Favored (85.13%) General / -58.5,-47.1	Favored (53.4%) *ttp* chi angles: 179.5,189.9,70.6	0.04Å	Favored (88.439%) alpha helix	-	-	-
A 116		ILE	3.21	-	Favored (83.07%) Ile or Val / -60.1,-40.3	Favored (35%) *mm* chi angles: 294.8,300	0.03Å	Favored (82.043%) alpha helix	-	-	-
A 117		VAL	3.49	-	Favored (97.37%) Ile or Val / -62.9,-42.7	Favored (62.2%) *t* chi angles: 171.1	0.02Å	Favored (99.428%) alpha helix	-	-	-
A 118		LEU	3.84	-	Favored (69.9%) General / -67.9,-30.5	Favored (95.5%) *mt* chi angles: 292.2,173.4	0.01Å	Favored (52.817%)	-	-	-
A 119		ILE	4.28	-	Favored (86.62%) Pre-Pro / -72.0,122.2	Favored (87.8%) *mt* chi angles: 296.8,166.4	0.09Å	Favored (32.165%)	-	-	-
A 120		PRO	4.84	-	Favored (51.56%) Trans-Pro / -73.2,154.5	Favored (76.9%) *Cg_endo* chi angles: 28.3,325.5,26.2	0.06Å	Favored (64.789%) beta sheet	-	-	-
**#**	**Alt**	**Res**	**High B**	**Clash > 0.4Å**	**Ramachandran**	**Rotamer**	**Cβ deviation**	**CaBLAM**	**Bond lengths**	**Bond angles**	**Cis Peptides**
			Avg: 3.35	Clashscore: 0	Outliers: 0 of 124	Poor rotamers: 0 of 101	Outliers: 0 of 115	Outliers: 0 of 122	Outliers: 0 of 126	Outliers: 2 of 126	Non-Trans: 0 of 125
A 121		GLU	5.55	-	Favored (79.94%) Pre-Pro / -81.4,121.3	Favored (92.8%) *tt0* chi angles: 182.4,179.6,2.4	0.05Å	Favored (40.457%)	-	-	-
A 122		PRO	6.39	-	Favored (74.8%) Trans-Pro / -62.6,-23.7	Favored (39.2%) *Cg_endo* chi angles: 23.2,327.1,28.5	0.01Å	Favored (79.76%)	-	-	-
A 123		GLU	7.27	-	Favored (72.95%) General / -65.6,-31.7	Favored (75%) *mm-30* chi angles: 290.9,295.4,308.3	0.02Å	Favored (66.591%) alpha helix	-	-	-
A 124		LYS	8.11	-	Favored (54.35%) General / -85.4,-0.3	Favored (51.2%) *mttp* chi angles: 293.2,181.6,169.5,66.7	0.07Å	Favored (47.295%)	-	-	-
A 125		GLN	8.82	-	Favored (27.04%) General / -106.8,4.1	Favored (84.6%) *mt0* chi angles: 297.4,181.5,303.7	0.04Å	-	-	-	-
A 126		ARG	9.33	-	-	Favored (73.1%) *ttp80* chi angles: 180.8,170.2,66,89.6	0.03Å	-	-	-	-

About MolProbity | Website for the Richardson Lab | Using ecloud x-H | Internal reference 4.5.2
