# Supplementary material for: Integrative proteome-wide structural analysis and high-throughput docking identify broad-spectrum antiviral scaffolds against Zika, Yellow Fever, West Nile, Saint Louis encephalitis, and Usutu viruses
Source: Front Cell Infect Microbiol. 2026 Apr 30;16:1723132. doi: 10.3389/fcimb.2026.1723132 (PMC13171538; doi:10.3389/fcimb.2026.1723132)
Supplement: Supplementary file 3 [file DataSheet3.zip › SLEV/SLEV_NS4b/Mol_probity_Files/SLEV_NS4b_1FH-multi.table.pdf]

MolProbity

Viewing SLEV_NS4b1FH-multi.table

Duke Biochemistry
Duke University School of Medicine

When finished, you should close this window.

Hint: Use File | Save As... to save a copy of this page.

All-Atom Contacts	Clashscore, all atoms:	1.79		99th percentile* (N=1784, all resolutions)
	Clashscore is the number of serious steric overlaps (> 0.4 Å) per 1000 atoms.			
Protein Geometry	Poor rotamers	0	0.00%	Goal: <0.3%
	Favored rotamers	201	99.50%	Goal: >98%
	Ramachandran outliers	5	1.95%	Goal: <0.05%
	Ramachandran favored	244	95.31%	Goal: >98%
	Rama distribution Z-score	0.86 ± 0.51		Goal: abs(Z score) < 2
	MolProbity score^	1.26		99th percentile* (N=27675, 0Å - 99Å)
	Cβ deviations >0.25Å	0	0.00%	Goal: 0
	Bad bonds:	1 / 1951	0.05%	Goal: 0%
	Bad angles:	8 / 2660	0.30%	Goal: <0.1%
Peptide Omegas	Cis Prolines:	0 / 13	0.00%	Expected: ≤1 per chain, or ≤5%
	Cis nonProlines:	1 / 244	0.41%	Goal: <0.05%
Low-resolution Criteria	CaBLAM outliers	8	3.1%	Goal: <1.0%
	CA Geometry outliers	2	0.79%	Goal: <0.5%
Additional validations	Chiral volume outliers	0/327		
	Waters with clashes	0/0	0.00%	See UnDowser table for details

In the two column results, the left column gives the raw count, right column gives the percentage.

* 100th percentile is the best among structures of comparable resolution; 0th percentile is the worst. For clashscore the comparative set of structures was selected in 2004, for MolProbity score in 2006.

^ MolProbity score combines the clashscore, rotamer, and Ramachandran evaluations into a single score, normalized to be on the same scale as X-ray resolution.

Key to table colors and cutoffs here:

#	Alt	Res	High B	Clash > 0.4Å	Ramachandran	Rotamer	Cβ deviation	CaBLAM	Bond lengths	Bond angles	Cis Peptides
			Avg: 4.48	Clashscore: 1.79	Outliers: 5 of 256	Poor rotamers: 0 of 202	Outliers: 0 of 236	Outliers: 9 of 254	Outliers: 1 of 258	Outliers: 6 of 258	Non-Trans: 1 of 257
A 1		ASN	20.05	-	-	Favored (84.4%) *m-40* chi angles: 292.3,316.7	0.04Å	-	-	-	-
A 2		GLU	20.1	0.79Å N with A 2 GLU OE1	Favored (65.19%) General / -67.8,-22.9	Favored (31.9%) *mp0* chi angles: 293.9,73.4,352.3	0.09Å	-	-	-	-
A 3		MET	20.03	-	Favored (12.22%) General / -103.5,163.6	Favored (71.2%) *mtm* chi angles: 297.3,177.8,287.5	0.03Å	Favored (11.121%)	-	-	-
A 4		GLY	19.83	-	Favored (2.09%) Glycine / -73.8,53.5	-	-	CaBLAM Disfavored (1.69%)	-	-	-
A 5		LEU	19.49	-	Favored (70.61%) General / -71.5,-35.2	Favored (95%) *mt* chi angles: 295.5,173.6	0.10Å	Favored (23.995%)	-	-	-
A 6		LEU	19.01	-	Favored (70.17%)	Favored (94.7%) *mt* chi angles: 294.1,174.3	0.07Å	Favored (87.3%)	-	-	-

					General / -71.5,-34.5			alpha helix			
A 7		GLU	18.48	-	Favored (87.3%) General / -66.9,-39.2	Favored (99.4%) *mt-10* chi angles: 292.6,175.2,352.2	0.03Å	Favored (83.643%) alpha helix	-	-	-
A 8		LYS	18	-	Favored (77.23%) General / -68.8,-42.4	Favored (53.6%) *tttm* chi angles: 182.7,175,185.6,290	0.03Å	Favored (78.486%) alpha helix	-	-	-
A 9		THR	17.66	-	Favored (99.31%) General / -62.5,-42.1	Favored (88.6%) *m* chi angles: 298.4	0.01Å	Favored (79.742%) alpha helix	-	-	-
A 10		LYS	17.44	-	Favored (67.83%) General / -54.2,-40.8	Favored (87.1%) *tttt* chi angles: 184.2,177.5,182,182.7	0.08Å	Favored (78.454%) alpha helix	-	OUTLIER(S) worst is N-CA-C: 6.5 σ	-
A 11		SER	17.29	-	OUTLIER (0.01)%) General / 85.2,-105.5	Favored (73.3%) *m* chi angles: 295.5	0.20Å	Favored (47.053%) alpha helix	-	OUTLIER(S) worst is C-N-CA: 9.9 σ	Cis nonPRO omega= 21.64
A 12		ASP	17.16	-	Favored (65.92%) General / -56.8,-32.8	Favored (92%) *m-30* chi angles: 285.3,345.7	0.10Å	Favored (74.555%) alpha helix	-	-	-
A 13		ILE	16.99	-	Favored (66.5%) Ile or Val / -67.9,-49.7	Favored (98.8%) *mt* chi angles: 292.8,167.3	0.04Å	Favored (72.474%) alpha helix	-	-	-
A 14		ALA	16.69	-	Favored (68.75%) General / -56.6,-36.0	-	0.06Å	Favored (72.532%) alpha helix	-	-	-
A 15		LYS	16.23	-	Favored (65.35%) General / -59.9,-25.8	Favored (89%) *mttt* chi angles: 289.9,175.5,186.9,170.8	0.04Å	Favored (47.81%) alpha helix	-	-	-
A 16		LEU	15.65	-	Favored (7.41%) General / -106.0,-35.0	Favored (90.6%) *mt* chi angles: 299.2,176.8	0.07Å	Favored (38.253%)	-	-	-
A 17		PHE	15.07	-	Favored (18.66%) General / -112.2,5.7	Favored (85.7%) *m-80* chi angles: 299.7,103.9	0.06Å	CaBLAM Outlier (0.786%)	-	-	-
A 18		GLY	14.59	-	Allowed (1.75%) Glycine / 138.8,119.7	-	-	Favored (7.34%)	-	-	-
A 19		SER	14.28	-	Allowed (1.36%) General / -73.3,74.3	Favored (46.2%) *t* chi angles: 179.7	0.04Å	Favored (7.171%)	-	-	-
A 20		GLN	14.16	-	Favored (7.44%) Pre-Pro / -138.0,54.4	Favored (19.4%) *pt0* chi angles: 63.4,187,55.3	0.04Å	CaBLAM Disfavored (3.287%)	-	-	-
**#**	**Alt**	**Res**	**High B**	**Clash > 0.4Å**	**Ramachandran**	**Rotamer**	**Cβ deviation**	**CaBLAM**	**Bond lengths**	**Bond angles**	**Cis Peptides**
			Avg: 4.48	Clashscore: 1.79	Outliers: 5 of 256	Poor rotamers: 0 of 202	Outliers: 0 of 236	Outliers: 9 of 254	Outliers: 1 of 258	Outliers: 6 of 258	Non-Trans: 1 of 257
A 21		PRO	14.19	-	Favored (61.77%) Trans-Pro / -65.3,-20.3	Favored (43.7%) *Cg_endo* chi angles: 24.3,325.5,29.8	0.04Å	Favored (23.642%)	-	-	-
A 22		GLY	14.35	-	Favored (69.36%)	-	-	Favored (37.638%)	-	-	-

					Glycine /<br>-95.3,10.8						
A<br>23		SER	14.59	-	Allowed<br>(0.14%)<br>General /<br>-67.8,71.2	Favored (37.8%) *t*<br>chi angles: 177.6	0.04Å	Favored<br>(6.498%)	-	-	-
A<br>24		VAL	14.81	-	Favored<br>(7.27%)<br>Ile or Val /<br>-109.3,98.6	Favored (57%) *t*<br>chi angles: 180.3	0.05Å	CaBLAM<br>Disfavored<br>(2.199%)	-	-	-
A<br>25		GLY	14.9	-	Favored<br>(89.75%)<br>Glycine / 83.6,-0.1	-	-	Favored<br>(9.548%)	-	-	-
A<br>26		PHE	14.8	-	Favored<br>(15.86%)<br>General /<br>-160.8,148.6	Favored (65.5%)<br>*t80*<br>chi angles: 183.8,68.8	0.10Å	Favored<br>(10.402%)	-	-	-
A<br>27		ALA	14.48	-	Favored<br>(73.3%)<br>General /<br>-58.5,-36.2	-	0.05Å	CaBLAM<br>Outlier<br>(0.607%)	-	-	-
A<br>28		THR	13.93	0.46Å<br>O with A 28<br>THR HG23	OUTLIER<br>(0)%)<br>General / 29.4,34.6	Favored (11.9%) *t*<br>chi angles: 190.2	0.05Å	CaBLAM<br>Outlier<br>(0.9%)<br>try beta sheet	-	-	-
A<br>29		ARG	13.13	-	Favored<br>(48.36%)<br>General /<br>-68.5,133.3	Favored (46.9%)<br>*ttm170*<br>chi angles:<br>193.3,176.1,295.7,171.7	0.05Å	Favored<br>(15.857%)<br>beta sheet	-	-	-
A<br>30		THR	12.12	-	Favored<br>(30.61%)<br>General /<br>-93.7,-11.8	Favored (22.4%) *p*<br>chi angles: 71.6	0.16Å	Favored<br>(5.859%)	-	-	-
A<br>31		THR	10.86	-	Allowed<br>(0.11%)<br>Pre-Pro /<br>61.6,154.4	Favored (79%) *p*<br>chi angles: 60.7	0.12Å	CaBLAM<br>Disfavored<br>(1.108%)	-	-	-
A<br>32		PRO	9.35	-	Favored<br>(48.46%)<br>Trans-Pro /<br>-73.4,158.2	Favored (74.5%)<br>*Cg_endo*<br>chi angles:<br>27.8,324.7,28	0.02Å	Favored<br>(21.233%)	-	-	-
A<br>33		TRP	7.67	-	Favored<br>(39.68%)<br>General /<br>-68.5,129.4	Favored (22.2%)<br>*t60*<br>chi angles: 179,40.9	0.05Å	Favored<br>(30.736%)	-	-	-
A<br>34		ASP	5.95	-	Favored<br>(14.46%)<br>General /<br>-83.0,106.0	Favored (66%) *t0*<br>chi angles: 184.2,354.4	0.04Å	Favored<br>(57.808%)<br>beta sheet	-	-	-
A<br>35		ILE	4.39	-	Favored<br>(57.98%)<br>Ile or Val /<br>-103.7,124.5	Favored (76.4%) *mt*<br>chi angles: 300.8,169.4	0.05Å	Favored<br>(55.181%)<br>beta sheet	-	-	-
A<br>36		SER	3.14	-	Favored<br>(15.83%)<br>General /<br>-141.2,123.6	Favored (46%) *t*<br>chi angles: 179.6	0.05Å	Favored<br>(41.575%)<br>beta sheet	-	-	-
A<br>37		LEU	2.24	-	Favored<br>(7.57%)<br>General /<br>-80.7,77.1	Favored (27.1%) *tp*<br>chi angles: 188.1,59	0.04Å	Favored<br>(51.049%)<br>beta sheet	-	-	-
A<br>38		ASP	1.65	-	Favored<br>(11.03%)<br>General /<br>-89.4,97.1	Favored (53.8%) *t0*<br>chi angles: 186,2.9	0.05Å	Favored<br>(44.955%)<br>beta sheet	-	OUTLIER(S)<br>worst is CA-<br>CB-CG: 4.3 σ	-
A<br>39		ILE	1.3	-	Favored<br>(60.17%)<br>Ile or Val /<br>-105.1,126.3	Favored (79%) *mt*<br>chi angles: 300.1,168.4	0.02Å	Favored<br>(55.912%)<br>beta sheet	-	-	-

#	Alt	Res	High B	Clash > 0.4Å	Ramachandran	Rotamer	Cβ deviation	CaBLAM	Bond lengths	Bond angles	Cis Peptides
A 40		LYS	1.1	-	Favored (42.56%) Pre-Pro / -121.4,85.1	Favored (86.2%) *mttt* chi angles: 300.4,179.2,189.1,177.6	0.08Å	Favored (43.482%) beta sheet	-	-	-
**#**	**Alt**	**Res**	**High B**	**Clash > 0.4Å**	**Ramachandran**	**Rotamer**	**Cβ deviation**	**CaBLAM**	**Bond lengths**	**Bond angles**	**Cis Peptides**
			Avg: 4.48	Clashscore: 1.79	Outliers: 5 of 256	Poor rotamers: 0 of 202	Outliers: 0 of 236	Outliers: 9 of 254	Outliers: 1 of 258	Outliers: 6 of 258	Non-Trans: 1 of 257
A 41		PRO	1	-	Favored (13.06%) Trans-Pro / -46.2,-34.1	Favored (91%) *Cg_exo* chi angles: 329.5,38.1,330.1	0.08Å	Favored (53.53%)	-	-	-
A 42		ALA	0.97	-	Favored (86.16%) General / -61.6,-38.0	-	0.06Å	Favored (66.671%) alpha helix	-	-	-
A 43		THR	0.97	-	Favored (22.57%) General / -85.9,-30.8	Favored (72.4%) *p* chi angles: 61.9	0.06Å	Favored (67.576%) alpha helix	-	-	-
A 44		ALA	1	-	Favored (94.2%) General / -61.7,-40.2	-	0.03Å	Favored (81.537%) alpha helix	-	-	-
A 45		TRP	1.04	-	Favored (67.79%) General / -64.9,-50.1	Favored (16%) *t60* chi angles: 187.2,13	0.07Å	Favored (95.81%) alpha helix	-	-	-
A 46		ALA	1.09	-	Favored (84.8%) General / -60.5,-38.6	-	0.10Å	Favored (85.338%) alpha helix	-	-	-
A 47		LEU	1.15	-	Favored (86.31%) General / -67.1,-41.5	Favored (85.9%) *mt* chi angles: 292.3,167.2	0.10Å	Favored (85.03%) alpha helix	-	-	-
A 48		TYR	1.22	-	Favored (61.78%) General / -59.5,-53.1	Favored (45.9%) *t80* chi angles: 168.1,84.6	0.08Å	Favored (78.022%) alpha helix	-	-	-
A 49		ALA	1.32	-	Favored (96.02%) General / -63.8,-43.5	-	0.03Å	Favored (87.776%) alpha helix	-	-	-
A 50		ALA	1.43	-	Favored (87.86%) General / -64.2,-45.5	-	0.05Å	Favored (90.42%) alpha helix	-	-	-
A 51		ALA	1.55	-	Favored (94.01%) General / -62.4,-39.7	-	0.04Å	Favored (92.564%) alpha helix	-	-	-
A 52		THR	1.67	-	Favored (89.99%) General / -64.6,-44.6	Favored (97.8%) *m* chi angles: 300	0.01Å	Favored (97.042%) alpha helix	-	-	-
A 53		MET	1.78	-	Favored (76.43%) General / -63.4,-33.9	Favored (76.6%) *mtm* chi angles: 287.6,186,281.6	0.06Å	Favored (65.903%) alpha helix	-	-	-
A 54		VAL	1.88	-	Favored (13.57%) Ile or Val / -86.6,-51.0	Favored (97.6%) *t* chi angles: 175.6	0.03Å	Favored (44.879%) alpha helix	-	-	-

#	Alt	Res	High B	Clash > 0.4Å	Ramachandran	Rotamer	Cβ deviation	CaBLAM	Bond lengths	Bond angles	Cis Peptides
A 55		MET	1.96	-	Favored (83.28%) General / -68.0,-38.9	Favored (99.4%) *mtp* chi angles: 292.3,174.4,67.8	0.03Å	Favored (47.935%) alpha helix	-	-	-
A 56		THR	2.02	-	Favored (2.54%) Pre-Pro / -48.0,-62.0	Favored (91.7%) *m* chi angles: 297.3	0.17Å	Favored (54.537%) alpha helix	-	-	-
A 57		PRO	2.08	-	Favored (76.69%) Trans-Pro / -62.2,-25.2	Favored (35.5%) *Cg_endo* chi angles: 22.4,326,30.8	0.01Å	Favored (87.012%) alpha helix	-	-	-
A 58		LEU	2.14	-	Favored (70.71%) General / -71.5,-39.7	Favored (92.2%) *mt* chi angles: 291.5,171.2	0.02Å	Favored (78.319%) alpha helix	-	-	-
A 59		ILE	2.19	-	Favored (90.23%) Ile or Val / -65.4,-46.4	Favored (97.4%) *mt* chi angles: 292.8,166.9	0.03Å	Favored (85.159%) alpha helix	-	-	-
A 60		LYS	2.24	-	Favored (83.73%) General / -62.6,-36.8	Favored (97.5%) *mttt* chi angles: 288.7,179,176.2,177.1	0.01Å	Favored (84.569%) alpha helix	-	-	-
**#**	**Alt**	**Res**	**High B**	**Clash > 0.4Å**	**Ramachandran**	**Rotamer**	**Cβ deviation**	**CaBLAM**	**Bond lengths**	**Bond angles**	**Cis Peptides**
			Avg: 4.48	Clashscore: 1.79	Outliers: 5 of 256	Poor rotamers: 0 of 202	Outliers: 0 of 236	Outliers: 9 of 254	Outliers: 1 of 258	Outliers: 6 of 258	Non-Trans: 1 of 257
A 61		HIS	2.3	-	Favored (86.51%) General / -67.2,-40.0	Favored (54.3%) *m-70* chi angles: 287.7,304.9	0.05Å	Favored (95.342%) alpha helix	-	-	-
A 62		LEU	2.36	-	Favored (75.75%) General / -66.9,-46.1	Favored (60.5%) *tp* chi angles: 180.7,59.1	0.02Å	Favored (88.182%) alpha helix	-	-	-
A 63		ILE	2.43	-	Favored (94.07%) Ile or Val / -60.1,-46.7	Favored (94.7%) *mt* chi angles: 291.8,167.9	0.03Å	Favored (95.031%) alpha helix	-	-	-
A 64		THR	2.53	-	Favored (87.49%) General / -59.3,-46.8	Favored (96.3%) *m* chi angles: 299.7	0.03Å	Favored (96.781%) alpha helix	-	-	-
A 65		THR	2.62	-	Favored (89.71%) General / -58.6,-44.4	Favored (96%) *m* chi angles: 299.6	0.01Å	Favored (89.136%) alpha helix	-	-	-
A 66		GLN	2.71	-	Favored (89.48%) General / -62.0,-38.7	Favored (96.9%) *mt0* chi angles: 290.6,171.1,341.9	0.04Å	Favored (83.863%) alpha helix	-	-	-
A 67		TYR	2.81	-	Favored (59.3%) General / -75.6,-29.2	Favored (39.6%) *m-80* chi angles: 283.7,113.2	0.17Å	Favored (88.013%) alpha helix	-	-	-
A 68		VAL	2.9	-	Favored (92.97%) Ile or Val / -65.6,-42.5	Favored (62.1%) *t* chi angles: 171.1	0.02Å	Favored (76.443%) alpha helix	-	-	-
A 69		ASN	2.97	-	Favored (90.34%) General / -59.4,-41.9	Favored (98.6%) *m-40* chi angles: 289.3,342.8	0.05Å	Favored (94.432%) alpha helix	-	-	-

A 70		PHE	3.02	-	Favored (71.85%) General / -58.7,-51.0	Favored (72.5%) *t80* chi angles: 171,81	0.09Å	Favored (91.469%) alpha helix	-	-	-
A 71		SER	3.08	-	Favored (83.8%) General / -60.4,-48.0	Favored (40.3%) *t* chi angles: 181	0.04Å	Favored (92.268%) alpha helix	-	-	-
A 72		LEU	3.16	-	Favored (82.32%) General / -63.2,-36.0	Favored (95.4%) *mt* chi angles: 292.1,173.3	0.06Å	Favored (83.541%) alpha helix	-	-	-
A 73		THR	3.3	-	Favored (89.27%) General / -63.8,-45.5	Favored (96.3%) *m* chi angles: 299.7	0.06Å	Favored (94.546%) alpha helix	-	-	-
A 74		ALA	3.58	-	Favored (76.03%) General / -61.5,-34.7	-	0.04Å	Favored (76.953%) alpha helix	-	-	-
A 75		ILE	4.05	-	Favored (21.16%) Ile or Val / -79.3,-48.5	Favored (86.6%) *mt* chi angles: 295.7,165.6	0.04Å	Favored (6.301%)	-	-	-
A 76		ALA	4.72	-	Favored (14.38%) General / -163.1,172.8	-	0.03Å	CA Geom Outlier (0.384%)	-	-	-
A 77		SER	5.6	-	Favored (3.38%) General / -59.5,-10.6	Favored (95%) *p* chi angles: 64.8	0.02Å	Favored (5.33%)	-	-	-
A 78		GLN	6.64	-	Favored (3.03%) General / -58.0,-12.1	Favored (18%) *pt0* chi angles: 72,186,322.1	0.06Å	Favored (6.834%)	-	-	-
A 79		ALA	7.7	-	Favored (53%) General / -56.2,-26.9	-	0.04Å	Favored (30.501%) three-ten	-	-	-
A 80		GLY	8.62	-	Favored (44.24%) Glycine / -59.5,-18.5	-	-	Favored (55.195%) three-ten	-	-	-
**#**	**Alt**	**Res**	**High B**	**Clash > 0.4Å**	**Ramachandran**	**Rotamer**	**Cβ deviation**	**CaBLAM**	**Bond lengths**	**Bond angles**	**Cis Peptides**
			Avg: 4.48	Clashscore: 1.79	Outliers: 5 of 256	Poor rotamers: 0 of 202	Outliers: 0 of 236	Outliers: 9 of 254	Outliers: 1 of 258	Outliers: 6 of 258	Non-Trans: 1 of 257
A 81		VAL	9.3	-	Favored (10.8%) Ile or Val / -98.4,-42.0	Favored (80.7%) *t* chi angles: 176.5	0.05Å	Favored (16.297%) alpha helix	-	-	-
A 82		LEU	9.71	0.65Å C with A 82 LEU HD12	Favored (53.33%) General / -58.3,-22.6	Allowed (1.2%) *pp* chi angles: 76.2,82.3	0.16Å	Favored (61.509%) three-ten	-	-	-
A 83		LEU	9.94	-	Favored (66.03%) General / -59.3,-27.6	Favored (96.7%) *mt* chi angles: 292,172.6	0.05Å	Favored (62.713%) three-ten	-	-	-
A 84		GLY	10.1	-	Favored (67.21%) Glycine / -67.2,-16.7	-	-	Favored (73.206%) three-ten	-	-	-
A 85		LEU	10.25	-	Favored (66.67%) General / -65.8,-23.1	Favored (97.9%) *mt* chi angles: 292.5,173.3	0.07Å	Favored (58.361%) three-ten	-	-	-

#	Alt	Res	High B	Clash > 0.4Å	Ramachandran	Rotamer	Cβ deviation	CaBLAM	Bond lengths	Bond angles	Cis Peptides
A 86		THR	10.34	-	Favored (62.71%) General / -70.5,-14.9	Favored (74.7%) *p* chi angles: 61.4	0.06Å	Favored (52.702%) three-ten	-	-	-
A 87		ASN	10.32	-	Favored (39.09%) General / -102.9,8.7	Favored (87.4%) *m-40* chi angles: 292.5,319.4	0.02Å	Favored (45.892%)	-	-	-
A 88		GLY	10.17	-	Favored (66.26%) Glycine / -96.8,11.6	-	-	CaBLAM Disfavored (3.574%)	-	-	-
A 89		MET	9.79	-	Favored (80.06%) Pre-Pro / -64.6,122.7	Favored (89.7%) *mmm* chi angles: 290.1,296.3,295.7	0.04Å	Favored (40.09%)	-	-	-
A 90		PRO	9.08	-	Favored (87.44%) Trans-Pro / -63.4,150.9	Favored (54.5%) *Cg_exo* chi angles: 337.3,34.6,328	0.06Å	Favored (53.522%) beta sheet	-	-	-
A 91		PHE	8.08	-	Favored (34.33%) General / -104.9,10.5	Favored (85.4%) *m-80* chi angles: 298.9,104.9	0.06Å	CaBLAM Disfavored (1.91%)	-	OUTLIER(S) worst is CA-CB-CG: 4.4 σ	-
A 92		THR	6.89	-	Allowed (0.77%) General / 49.8,-140.5	Favored (31.6%) *p* chi angles: 69.2	0.03Å	CaBLAM Disfavored (1.52%)	-	-	-
A 93		ALA	5.68	-	Favored (28.49%) General / -161.5,159.9	-	0.02Å	Favored (10.997%)	-	-	-
A 94		MET	4.61	-	Favored (28.9%) General / -137.9,130.0	Favored (57.4%) *ttp* chi angles: 178.5,173.8,65.6	0.04Å	Favored (22.366%) beta sheet	-	-	-
A 95		ASP	3.73	0.48Å OD1 with A 249 LYS NZ	Favored (58.8%) General / -63.0,139.7	Favored (13.8%) *t70* chi angles: 190.4,94.7	0.03Å	Favored (45.106%)	-	-	-
A 96		LEU	3.04	-	Favored (33.73%) General / -51.2,-34.7	Favored (66.4%) *tp* chi angles: 179.7,61.3	0.07Å	Favored (51.026%)	-	-	-
A 97		SER	2.54	-	Favored (66%) General / -62.8,-21.6	Favored (96.3%) *p* chi angles: 63.7	0.03Å	Favored (68.003%) alpha helix	-	-	-
A 98		VAL	2.18	-	Favored (2.48%) Pre-Pro / -72.4,-59.7	Favored (98.7%) *t* chi angles: 175.3	0.12Å	Favored (52.379%) alpha helix	-	OUTLIER(S) worst is CA-C-N: 4.1 σ	-
A 99		PRO	1.92	-	Favored (74.99%) Trans-Pro / -62.7,-24.7	Favored (31.8%) *Cg_endo* chi angles: 21.6,326.8,31.1	0.01Å	Favored (77.548%) alpha helix	-	-	-
A 100		LEU	1.73	-	Favored (74.68%) General / -70.3,-39.5	Favored (85.9%) *mt* chi angles: 290.7,173.5	0.02Å	Favored (76.667%) alpha helix	-	-	-
**#**	**Alt**	**Res**	**High B**	**Clash > 0.4Å**	**Ramachandran**	**Rotamer**	**Cβ deviation**	**CaBLAM**	**Bond lengths**	**Bond angles**	**Cis Peptides**
			Avg: 4.48	Clashscore: 1.79	Outliers: 5 of 256	Poor rotamers: 0 of 202	Outliers: 0 of 236	Outliers: 9 of 254	Outliers: 1 of 258	Outliers: 6 of 258	Non-Trans: 1 of 257
A 101		LEU	1.58	-	Favored (71.87%) General / -64.3,-49.4	Favored (63.7%) *tp* chi angles: 178.4,64.2	0.10Å	Favored (77.006%) alpha helix	-	-	-

A 102		VAL	1.49	-	Favored (98.28%) Ile or Val / -62.0,-44.0	Favored (63.9%) *t* chi angles: 171.3	0.02Å	Favored (83.659%) alpha helix	-	-	-
A 103		LEU	1.43	-	Favored (99.28%) General / -63.1,-41.1	Favored (85.8%) *mt* chi angles: 290.2,170.6	0.04Å	Favored (98.468%) alpha helix	-	-	-
A 104		GLY	1.41	-	Favored (92.53%) Glycine / -61.6,-36.4	-	-	Favored (90.939%) alpha helix	-	-	-
A 105		CYS	1.42	-	Favored (54.69%) General / -77.1,-37.2	Favored (81.6%) *m* chi angles: 294.7	0.12Å	Favored (66.646%) alpha helix	-	-	-
A 106		TRP	1.45	-	Favored (94.07%) General / -59.7,-43.0	Favored (84.8%) *t60* chi angles: 186.7,89.9	0.03Å	Favored (63.697%) alpha helix	-	-	-
A 107		ASN	1.51	-	Favored (54.81%) General / -57.8,-23.9	Favored (97.5%) *m-40* chi angles: 287,339.7	0.03Å	Favored (48.028%) alpha helix	-	-	-
A 108		GLN	1.59	-	Favored (20.45%) General / -107.0,-3.1	Favored (75.2%) *mt0* chi angles: 296.5,181.3,24	0.07Å	Favored (15.752%)	-	-	-
A 109		MET	1.66	-	Favored (56.72%) General / -66.7,137.5	Favored (63.4%) *ttp* chi angles: 184.2,181,73.8	0.01Å	Favored (24.766%)	-	-	-
A 110		THR	1.71	-	Favored (20.5%) General / -115.0,159.7	Favored (46.6%) *p* chi angles: 66.3	0.10Å	Favored (40.698%)	-	-	-
A 111		LEU	1.73	-	Favored (85.09%) Pre-Pro / -54.0,-50.3	Favored (65%) *tp* chi angles: 175.6,61.2	0.09Å	Favored (61.42%)	-	-	-
A 112		PRO	1.72	-	Favored (70.84%) Trans-Pro / -63.4,-24.9	Favored (24.4%) *Cg_endo* chi angles: 20.3,326.5,32.1	0.03Å	Favored (95.393%) alpha helix	-	-	-
A 113		SER	1.67	-	Favored (77.69%) General / -69.3,-40.6	Favored (73.2%) *m* chi angles: 295.6	0.04Å	Favored (75.585%) alpha helix	-	-	-
A 114		LEU	1.6	-	Favored (80.36%) General / -65.3,-46.4	Favored (67.4%) *tp* chi angles: 178.5,59.4	0.01Å	Favored (78.617%) alpha helix	-	-	-
A 115		ALA	1.52	-	Favored (80.54%) General / -58.4,-39.8	-	0.03Å	Favored (81.239%) alpha helix	-	-	-
A 116		VAL	1.44	-	Favored (95.56%) Ile or Val / -65.1,-44.7	Favored (60.2%) *t* chi angles: 170.8	0.03Å	Favored (86.96%) alpha helix	-	-	-
A 117		ALA	1.38	-	Favored (82.3%) General / -57.9,-41.3	-	0.08Å	Favored (85.712%) alpha helix	-	-	-
A 118		VAL	1.32	-	Favored (88.93%) Ile or Val / -66.9,-44.4	Favored (73.5%) *t* chi angles: 172.5	0.04Å	Favored (93.923%) alpha helix	-	-	-

#	Alt	Res	High B	Clash > 0.4Å	Ramachandran	Rotamer	Cβ deviation	CaBLAM	Bond lengths	Bond angles	Cis Peptides
A 119		MET	1.27	-	Favored (78.54%) General / -57.2,-48.6	Favored (50.9%) *ttp* chi angles: 177.3,189.7,70.5	0.08Å	Favored (96.893%) alpha helix	-	-	-
A 120		LEU	1.22	-	Favored (89.57%) General / -62.9,-38.1	Favored (94.3%) *mt* chi angles: 295.6,175.2	0.09Å	Favored (89.998%) alpha helix	-	-	-
**#**	**Alt**	**Res**	**High B**	**Clash > 0.4Å**	**Ramachandran**	**Rotamer**	**Cβ deviation**	**CaBLAM**	**Bond lengths**	**Bond angles**	**Cis Peptides**
			Avg: 4.48	Clashscore: 1.79	Outliers: 5 of 256	Poor rotamers: 0 of 202	Outliers: 0 of 236	Outliers: 9 of 254	Outliers: 1 of 258	Outliers: 6 of 258	Non-Trans: 1 of 257
A 121		LEU	1.19	-	Favored (88.29%) General / -65.2,-44.5	Favored (41%) *tp* chi angles: 185.4,63.1	0.04Å	Favored (96.926%) alpha helix	-	-	-
A 122		ALA	1.17	-	Favored (86.4%) General / -60.7,-38.8	-	0.04Å	Favored (93.015%) alpha helix	-	-	-
A 123		ILE	1.17	-	Favored (88.45%) Ile or Val / -66.2,-46.1	Favored (96.7%) *mt* chi angles: 293,166.8	0.02Å	Favored (89.24%) alpha helix	-	-	-
A 124		HIS	1.18	-	Favored (67.1%) General / -57.6,-52.0	Favored (90.8%) *t70* chi angles: 175.9,73.3	0.04Å	Favored (87.92%) alpha helix	-	-	-
A 125		TYR	1.21	-	Favored (93.27%) General / -62.4,-39.5	Favored (5.5%) *m-10* chi angles: 283,151	0.09Å	Favored (77.511%) alpha helix	-	-	-
A 126		ALA	1.26	-	Favored (72.03%) General / -58.2,-35.7	-	0.04Å	Favored (71.02%) alpha helix	-	-	-
A 127		PHE	1.31	-	Favored (20.87%) General / -80.8,-43.0	Favored (13.7%) *m-10* chi angles: 289.3,324.9	0.04Å	Favored (59.14%) alpha helix	-	-	-
A 128		MET	1.37	-	Favored (26.89%) General / -83.1,-32.3	Favored (52.7%) *mmp* chi angles: 296.2,299.8,100.7	0.03Å	Favored (46.647%) alpha helix	-	-	-
A 129		ILE	1.44	-	Favored (91.9%) Pre-Pro / -60.9,-48.7	Favored (84.5%) *mt* chi angles: 290.5,168.9	0.18Å	Favored (65.341%) alpha helix	-	-	-
A 130		PRO	1.5	-	Favored (51.69%) Trans-Pro / -52.1,-33.0	Favored (97.4%) *Cg_exo* chi angles: 331.8,38.1,328.2	0.03Å	Favored (95.907%) alpha helix	-	-	-
A 131		GLY	1.57	-	Favored (46.53%) Glycine / -65.4,-51.7	-	-	Favored (86.229%) alpha helix	-	-	-
A 132		TRP	1.64	-	Favored (97.6%) General / -61.6,-41.5	Favored (57.3%) *m-10* chi angles: 297.3,337.6	0.09Å	Favored (80.73%) alpha helix	-	-	-
A 133		GLN	1.73	-	Favored (81.53%) General / -68.1,-41.6	Favored (98.3%) *mt0* chi angles: 290.6,175,330.1	0.02Å	Favored (96.887%) alpha helix	-	-	-

A 134		ALA	1.83	-	Favored (98.73%) General / -61.5,-43.5	-	0.04Å	Favored (90.21%) alpha helix	-	-	-
A 135		GLU	1.95	-	Favored (82.45%) General / -68.2,-39.3	Favored (49.2%) *mm-30* chi angles: 287.3,290.8,301.1	0.11Å	Favored (89.353%) alpha helix	-	-	-
A 136		ALA	2.1	-	Favored (87.37%) General / -61.9,-38.2	-	0.06Å	Favored (91.407%) alpha helix	-	-	-
A 137		MET	2.26	-	Favored (84.62%) General / -67.4,-38.5	Favored (14.3%) *tpt* chi angles: 182.1,63.1,179.2	0.04Å	Favored (88.943%) alpha helix	-	-	-
A 138		ARG	2.42	-	Favored (96.17%) General / -62.5,-40.3	Favored (84.4%) *mtt180* chi angles: 288.7,167.5,180.1,156.5	0.08Å	Favored (89.205%) alpha helix	-	-	-
A 139		ALA	2.58	-	Favored (98.36%) General / -63.1,-40.7	-	0.06Å	Favored (95.409%) alpha helix	-	-	-
A 140		ALA	2.71	-	Favored (95.8%) General / -61.1,-41.0	-	0.03Å	Favored (96.37%) alpha helix	-	-	-
**#**	**Alt**	**Res**	**High B**	**Clash > 0.4Å**	**Ramachandran**	**Rotamer**	**Cβ deviation**	**CaBLAM**	**Bond lengths**	**Bond angles**	**Cis Peptides**
			Avg: 4.48	Clashscore: 1.79	Outliers: 5 of 256	Poor rotamers: 0 of 202	Outliers: 0 of 236	Outliers: 9 of 254	Outliers: 1 of 258	Outliers: 6 of 258	Non-Trans: 1 of 257
A 141		GLN	2.82	-	Favored (92.89%) General / -65.2,-39.6	Favored (96.8%) *mt0* chi angles: 291.1,170.2,333.9	0.04Å	Favored (96.483%) alpha helix	-	-	-
A 142		ARG	2.9	-	Favored (81.72%) General / -66.6,-44.6	Favored (95.9%) *mtt180* chi angles: 289.9,170.2,182.1,176	0.07Å	Favored (81.405%) alpha helix	-	-	-
A 143		ARG	2.99	-	Favored (89.55%) General / -66.4,-40.2	Favored (99%) *mtm-85* chi angles: 288,192.4,294.6,267.3	0.06Å	Favored (79.552%) alpha helix	-	-	-
A 144		THR	3.11	-	Favored (92.28%) General / -61.5,-45.9	Favored (93.4%) *m* chi angles: 299.2	0.01Å	Favored (85.455%) alpha helix	-	-	-
A 145		ALA	3.28	-	Favored (93.04%) General / -60.9,-45.8	-	0.05Å	Favored (95.769%) alpha helix	-	-	-
A 146		ALA	3.55	-	Favored (91.7%) General / -61.9,-39.4	-	0.04Å	Favored (94.552%) alpha helix	-	-	-
A 147		GLY	3.94	-	Favored (55.78%) Glycine / -60.9,-51.9	-	-	Favored (95.811%) alpha helix	-	-	-
A 148		ILE	4.44	-	Favored (90.59%) Ile or Val / -63.8,-40.9	Favored (91.8%) *mt* chi angles: 291.8,169.9	0.06Å	Favored (79.412%) alpha helix	-	-	-

A 149		MET	5.06	-	Favored (79.01%) General / -57.7,-40.3	Favored (90.2%) *mtp* chi angles: 289.3,167.8,68.3	0.03Å	Favored (5.185%) alpha helix	-	-	-
A 150		LYS	5.71	-	Allowed (0.5%) General / 67.4,-49.6	Favored (98.4%) *mttt* chi angles: 292.2,179,181.5,177.4	0.07Å	Favored (6.336%)	-	-	-
A 151		ASN	6.28	-	Favored (4.7%) General / -92.3,22.1	Favored (83.2%) *m-40* chi angles: 288.9,321.9	0.03Å	Favored (5.827%)	-	-	-
A 152		ALA	6.64	-	Favored (70.37%) General / -60.6,-31.2	-	0.02Å	Favored (33.09%)	-	-	-
A 153		VAL	6.7	0.51Å O with A 155 ASP N	Favored (9.86%) Ile or Val / -94.7,-52.2	Favored (94.6%) *t* chi angles: 175.8	0.04Å	CaBLAM Outlier (0.288%)	-	-	-
A 154		VAL	6.44	-	OUTLIER (0)%) Ile or Val / 38.2,-66.1	Favored (85.4%) *t* chi angles: 176.2	0.07Å	CaBLAM Outlier (0.015%)	-	-	-
A 155		ASP	5.94	0.51Å N with A 153 VAL O	Favored (56.1%) General / -93.3,-1.4	Favored (74.1%) *m-30* chi angles: 294.9,320.6	0.03Å	Favored (5.569%)	-	-	-
A 156		GLY	5.31	-	Favored (28.16%) Glycine / 105.7,11.7	-	-	Favored (31.051%)	-	-	-
A 157		ILE	4.71	-	Favored (63.91%) Ile or Val / -126.0,123.4	Favored (78.7%) *mt* chi angles: 300.6,171.3	0.05Å	Favored (13.816%)	-	-	-
A 158		VAL	4.21	-	Favored (36.2%) Ile or Val / -76.4,125.5	Favored (59.6%) *t* chi angles: 180	0.07Å	Favored (45.069%)	-	-	-
A 159		ALA	3.85	-	Favored (70.1%) General / -58.4,-33.9	-	0.04Å	Favored (52.07%)	-	-	-
A 160		THR	3.6	-	Favored (49.88%) General / -102.9,125.9	Favored (96.4%) *m* chi angles: 299.7	0.05Å	Favored (26.537%)	-	-	-
**#**	**Alt**	**Res**	**High B**	**Clash > 0.4Å**	**Ramachandran**	**Rotamer**	**Cβ deviation**	**CaBLAM**	**Bond lengths**	**Bond angles**	**Cis Peptides**
			Avg: 4.48	Clashscore: 1.79	Outliers: 5 of 256	Poor rotamers: 0 of 202	Outliers: 0 of 236	Outliers: 9 of 254	Outliers: 1 of 258	Outliers: 6 of 258	Non-Trans: 1 of 257
A 161		ASP	3.43	0.44Å O with A 162 ILE C	Favored (8.46%) General / -79.4,7.2	Favored (57.7%) *p0* chi angles: 62.6,2.1	0.06Å	CaBLAM Outlier (0.302%)	-	-	-
A 162		ILE	3.32	0.57Å O with A 162 ILE HG13	OUTLIER (0.09)%) Pre-Pro / 12.0,74.6	Favored (47.1%) *pt* chi angles: 61.6,171.9	0.05Å	CaBLAM Outlier (0.13%)	-	-	-
A 163		PRO	3.25	-	Favored (29.69%) Trans-Pro / -58.8,157.1	Favored (49%) *Cg_exo* chi angles: 337.9,34.8,327.7	0.04Å	Favored (13.115%)	-	-	-
A 164		ASP	3.2	-	Favored (21.15%) General / -62.3,156.9	Favored (96.4%) *m-30* chi angles: 288.5,349	0.05Å	Favored (28.205%)	-	-	-
A 165		LEU	3.14	-	Favored (20.43%)	Favored (90.3%) *mt* chi angles: 297.5,179.1	0.06Å	Favored (40.683%) beta sheet	-	-	-

					General / -84.3,161.6						
A 166		SER	3.04	-	Favored (51.56%) Pre-Pro / -133.6,82.2	Favored (32%) *t* chi angles: 183.4	0.05Å	Favored (11.213%)	-	-	-
A 167		PRO	2.87	-	Allowed (0.19%) Trans-Pro / -59.8,-178.9	Favored (36.8%) *Cg_exo* chi angles: 338.7,34,327.4	0.04Å	CaBLAM Disfavored (3.112%)	-	-	-
A 168		ALA	2.64	-	Favored (52.26%) General / -53.7,-32.6	-	0.03Å	CA Geom Outlier (0.094%)	-	-	-
A 169		THR	2.37	-	OUTLIER (0.06)%) Pre-Pro / 88.2,145.3	Favored (44.6%) *p* chi angles: 66.7	0.12Å	CaBLAM Disfavored (1.277%)	-	OUTLIER(S) worst is C-N-CA: 4.3 σ	-
A 170		PRO	2.08	-	Favored (71.71%) Trans-Pro / -60.4,-22.6	Favored (34%) *Cg_endo* chi angles: 22.1,325.5,32.2	0.01Å	Favored (66.452%)	-	-	-
A 171		MET	1.8	-	Favored (56.94%) General / -76.5,-37.3	Favored (52.3%) *mmp* chi angles: 295,300,98	0.03Å	Favored (74.251%) alpha helix	-	-	-
A 172		THR	1.56	-	Favored (80.12%) General / -67.9,-43.1	Favored (96.7%) *m* chi angles: 300.7	0.09Å	Favored (78.217%) alpha helix	-	-	-
A 173		GLU	1.37	-	Favored (97.33%) General / -62.9,-40.4	Favored (91.6%) *mt-10* chi angles: 293.1,167.3,1.8	0.08Å	Favored (95.986%) alpha helix	-	-	-
A 174		LYS	1.23	-	Favored (81.38%) General / -64.8,-46.6	Favored (11.8%) *ttmm* chi angles: 188.4,186.4,291.3,291	0.02Å	Favored (81.646%) alpha helix	-	-	-
A 175		LYS	1.14	-	Favored (73.5%) General / -54.5,-46.9	Favored (85%) *tttt* chi angles: 181.4,180.2,178.6,179.2	0.04Å	Favored (88.542%) alpha helix	-	-	-
A 176		MET	1.07	-	Favored (96.91%) General / -63.5,-40.4	Favored (61.4%) *tpp* chi angles: 185.8,61.1,70	0.03Å	Favored (92.943%) alpha helix	-	-	-
A 177		GLY	1.03	-	Favored (94.44%) Glycine / -61.2,-37.7	-	-	Favored (98.971%) alpha helix	-	-	-
A 178		GLN	1	-	Favored (67.18%) General / -72.2,-32.9	Favored (6.3%) *mm110* chi angles: 293.9,294.9,50.1	0.11Å	Favored (89.526%) alpha helix	-	-	-
A 179		ILE	0.99	-	Favored (95.07%) Ile or Val / -64.6,-45.6	Favored (93.7%) *mt* chi angles: 291.6,167.4	0.03Å	Favored (79.346%) alpha helix	-	-	-
A 180		LEU	1	-	Favored (87.34%) General / -63.7,-37.4	Favored (81.6%) *mt* chi angles: 290.4,174.1	0.06Å	Favored (81.973%) alpha helix	-	-	-
**#**	**Alt**	**Res**	**High B**	**Clash > 0.4Å**	**Ramachandran**	**Rotamer**	**Cβ deviation**	**CaBLAM**	**Bond lengths**	**Bond angles**	**Cis Peptides**
			Avg: 4.48	Clashscore: 1.79	Outliers: 5 of 256	Poor rotamers: 0 of 202	Outliers: 0 of 236	Outliers: 9 of 254	Outliers: 1 of 258	Outliers: 6 of 258	Non-Trans: 1 of 257

A 181		LEU	1.01	-	Favored (83.35%) General / -60.6,-48.1	Favored (65.5%) *tp* chi angles: 176,60.6	0.07Å	Favored (83.342%) alpha helix	-	-	-
A 182		ILE	1.03	-	Favored (97.8%) Ile or Val / -61.3,-44.1	Favored (94.1%) *mt* chi angles: 291.9,168.9	0.05Å	Favored (95.421%) alpha helix	-	-	-
A 183		ALA	1.06	-	Favored (84.05%) General / -57.7,-42.5	-	0.05Å	Favored (85.976%) alpha helix	-	-	-
A 184		ALA	1.1	-	Favored (95.69%) General / -64.9,-40.9	-	0.04Å	Favored (90.616%) alpha helix	-	-	-
A 185		ALA	1.16	-	Favored (99.06%) General / -62.7,-41.2	-	0.05Å	Favored (93.853%) alpha helix	-	-	-
A 186		VAL	1.25	-	Favored (90.45%) Ile or Val / -66.1,-45.5	Favored (63.9%) *t* chi angles: 171.3	0.01Å	Favored (87.914%) alpha helix	-	-	-
A 187		LEU	1.38	-	Favored (69.92%) General / -56.4,-51.0	Favored (68.5%) *tp* chi angles: 175.7,62.3	0.05Å	Favored (98.341%) alpha helix	-	-	-
A 188		ALA	1.56	-	Favored (88.19%) General / -60.1,-40.0	-	0.07Å	Favored (78.661%) alpha helix	-	-	-
A 189		VAL	1.81	-	Favored (88.41%) Ile or Val / -66.7,-41.7	Favored (69.3%) *t* chi angles: 172	0.05Å	Favored (97.851%) alpha helix	-	-	-
A 190		LEU	2.13	-	Favored (84.25%) General / -63.4,-36.6	Favored (94%) *mt* chi angles: 291.9,173.3	0.04Å	Favored (76.587%) alpha helix	-	-	-
A 191		VAL	2.5	-	Favored (29.53%) Ile or Val / -77.0,-46.2	Favored (96.8%) *t* chi angles: 175.1	0.06Å	Favored (43.085%)	-	-	-
A 192		ARG	2.83	-	Favored (63.73%) Pre-Pro / -134.5,75.3	Favored (91%) *mmt-90* chi angles: 294.3,291.2,180.6,269.3	0.08Å	Favored (11.744%)	-	-	-
A 193		PRO	3.06	-	Favored (5.72%) Trans-Pro / -77.0,57.2	Favored (60.4%) *Cg_endo* chi angles: 31.8,322.6,27.1	0.07Å	CaBLAM Disfavored (3.031%)	-	-	-
A 194		GLY	3.09	-	Favored (45.48%) Glycine / -89.7,-176.8	-	-	Favored (40.634%)	-	-	-
A 195		ILE	2.92	-	Favored (90.62%) Ile or Val / -63.9,-47.4	Favored (98.6%) *mt* chi angles: 292.7,168.2	0.01Å	Favored (39.68%)	-	-	-
A 196		CYS	2.6	-	Favored (86.84%) General / -64.7,-37.2	Favored (82.8%) *m* chi angles: 288.7	0.04Å	Favored (85.134%) alpha helix	-	-	-
A 197		SER	2.23	-	Favored (97.76%) General / -62.4,-40.8	Favored (67.7%) *m* chi angles: 294.6	0.05Å	Favored (82.861%) alpha helix	-	-	-

#	Alt	Res	High B	Clash > 0.4Å	Ramachandran	Rotamer	Cβ deviation	CaBLAM	Bond lengths	Bond angles	Cis Peptides
A 198		ILE	1.88	-	Favored (76.19%) Ile or Val / -70.7,-44.8	Favored (98.6%) *mt* chi angles: 293.3,167.5	0.11Å	Favored (81.191%) alpha helix	-	-	-
A 199		LYS	1.59	-	Favored (93.86%) General / -64.4,-39.4	Favored (96.9%) *mttt* chi angles: 288.6,180.5,176.1,179.4	0.02Å	Favored (93.915%) alpha helix	-	-	-
A 200		GLU	1.38	-	Favored (95.91%) General / -62.5,-44.6	Favored (83.5%) *tt0* chi angles: 180.8,169.9,357.9	0.09Å	Favored (91%) alpha helix	-	-	-
**#**	**Alt**	**Res**	**High B**	**Clash > 0.4Å**	**Ramachandran**	**Rotamer**	**Cβ deviation**	**CaBLAM**	**Bond lengths**	**Bond angles**	**Cis Peptides**
			Avg: 4.48	Clashscore: 1.79	Outliers: 5 of 256	Poor rotamers: 0 of 202	Outliers: 0 of 236	Outliers: 9 of 254	Outliers: 1 of 258	Outliers: 6 of 258	Non-Trans: 1 of 257
A 201		PHE	1.23	-	Favored (74.29%) General / -55.3,-48.4	Favored (74.6%) *t80* chi angles: 179.2,87.9	0.08Å	Favored (88.158%) alpha helix	-	-	-
A 202		GLY	1.12	-	Favored (32.36%) Glycine / -53.5,-54.0	-	-	Favored (92.084%) alpha helix	-	-	-
A 203		VAL	1.04	-	Favored (99.38%) Ile or Val / -63.2,-45.1	Favored (67.2%) *t* chi angles: 171.8	0.07Å	Favored (67.962%) alpha helix	-	-	-
A 204		LEU	0.98	-	Favored (58.83%) General / -75.6,-38.6	Favored (94.4%) *mt* chi angles: 296.4,173.9	0.10Å	Favored (76.208%) alpha helix	-	-	-
A 205		GLY	0.94	-	Favored (82.02%) Glycine / -61.2,-49.1	-	-	Favored (92.629%) alpha helix	-	-	-
A 206		SER	0.92	-	Favored (96.79%) General / -61.0,-41.5	Favored (72.5%) *m* chi angles: 295.3	0.04Å	Favored (90.723%) alpha helix	-	-	-
A 207		ALA	0.9	-	Favored (91.05%) General / -59.1,-42.7	-	0.06Å	Favored (84.91%) alpha helix	-	-	-
A 208		ALA	0.88	-	Favored (77.47%) General / -66.9,-45.5	-	0.06Å	Favored (83.367%) alpha helix	-	-	-
A 209		LEU	0.87	-	Favored (88.2%) General / -65.0,-37.6	Favored (89%) *mt* chi angles: 291,173.2	0.07Å	Favored (82.162%) alpha helix	-	-	-
A 210		VAL	0.87	-	Favored (95.49%) Ile or Val / -60.1,-45.7	Favored (53.9%) *t* chi angles: 169.9	0.01Å	Favored (79.001%) alpha helix	-	-	-
A 211		THR	0.89	-	Favored (83.57%) General / -64.7,-46.1	Favored (95.7%) *m* chi angles: 299.6	0.08Å	Favored (77.627%) alpha helix	-	-	-
A 212		LEU	0.94	-	Favored (71.86%) General / -65.3,-31.0	Favored (96.7%) *mt* chi angles: 294.1,173	0.07Å	Favored (62.804%) alpha helix	-	-	-

A 213		ILE	1.03	-	Favored (15.33%) Ile or Val / -88.8,-47.2	Favored (93.3%) *mt* chi angles: 296.1,168.6	0.02Å	Favored (46.811%) alpha helix	-	-	-
A 214		GLU	1.18	-	Favored (19.64%) General / -93.3,-21.4	Favored (94.2%) *mt-10* chi angles: 298.4,181.6,358.1	0.02Å	Favored (39.785%) alpha helix	-	-	-
A 215		GLY	1.4	-	Favored (88.46%) Glycine / 84.4,3.6	-	-	Favored (50.086%)	-	-	-
A 216		THR	1.67	-	Favored (3.69%) General / -143.0,-172.5	Favored (11.7%) *t* chi angles: 189.9	0.04Å	Favored (18.586%)	-	-	-
A 217		ALA	1.93	-	Favored (19.18%) General / -158.4,147.6	-	0.03Å	Favored (26.968%)	-	-	-
A 218		GLY	2.11	-	Favored (37.75%) Glycine / -94.9,-173.4	-	-	Favored (54.791%)	-	-	-
A 219		VAL	2.16	-	Favored (24.3%) Ile or Val / -60.4,-22.0	Favored (4.5%) *p* chi angles: 72.2	0.07Å	Favored (22.698%)	-	-	-
A 220		VAL	2.06	-	Favored (10.14%) Ile or Val / -104.2,-0.1	Favored (27.2%) *m* chi angles: 299.7	0.04Å	Favored (60.503%) alpha helix	-	-	-
**#**	**Alt**	**Res**	**High B**	**Clash > 0.4Å**	**Ramachandran**	**Rotamer**	**Cβ deviation**	**CaBLAM**	**Bond lengths**	**Bond angles**	**Cis Peptides**
			Avg: 4.48	Clashscore: 1.79	Outliers: 5 of 256	Poor rotamers: 0 of 202	Outliers: 0 of 236	Outliers: 9 of 254	Outliers: 1 of 258	Outliers: 6 of 258	Non-Trans: 1 of 257
A 221		TRP	1.87	-	Favored (19.54%) General / -110.7,3.7	Favored (42.9%) *m100* chi angles: 302.8,121.3	0.06Å	Favored (57.804%)	-	-	-
A 222		ASN	1.66	-	Favored (21.22%) General / -85.3,155.7	Favored (73.1%) *m-40* chi angles: 288,312.8	0.04Å	Favored (29.453%)	-	-	-
A 223		CYS	1.47	-	Favored (27.82%) General / -48.4,-40.9	Favored (36.3%) *p* chi angles: 62.7	0.06Å	Favored (56.053%)	-	-	-
A 224		THR	1.31	-	Favored (78.19%) General / -67.0,-45.2	Favored (93.8%) *m* chi angles: 299.3	0.03Å	Favored (69.125%) alpha helix	-	-	-
A 225		THR	1.22	-	Favored (18.98%) General / -79.9,-45.2	Favored (66.9%) *m* chi angles: 303.2	0.04Å	Favored (56.764%) alpha helix	-	-	-
A 226		ALA	1.16	-	Favored (86.13%) General / -59.3,-40.4	-	0.05Å	Favored (77.319%) alpha helix	-	-	-
A 227		VAL	1.13	-	Favored (70.25%) Ile or Val / -71.0,-45.9	Favored (58%) *t* chi angles: 170.5	0.06Å	Favored (76.378%) alpha helix	-	-	-
A 228		GLY	1.13	-	Favored (91.43%) Glycine / -60.6,-36.4	-	-	Favored (94.511%) alpha helix	-	-	-

A 229		LEU	1.16	-	Favored (94.85%) General / -65.0,-40.3	Favored (91.2%) *mt* chi angles: 291.1,171.8	0.04Å	Favored (96.189%) alpha helix	-	-	-
A 230		CYS	1.22	-	Favored (88.85%) General / -66.3,-42.0	Favored (98.6%) *m* chi angles: 292.6	0.11Å	Favored (86.793%) alpha helix	-	-	-
A 231		ASN	1.31	-	Favored (71.32%) General / -70.1,-33.0	Favored (90.7%) *m-40* chi angles: 285.1,339.2	0.06Å	Favored (83.523%) alpha helix	-	-	-
A 232		LEU	1.42	-	Favored (88.99%) General / -66.5,-39.6	Favored (33.9%) *tp* chi angles: 186.4,58.1	0.05Å	Favored (73.597%) alpha helix	-	-	-
A 233		MET	1.54	-	Favored (63.39%) General / -53.0,-39.7	Favored (51.9%) *ttp* chi angles: 175.7,184.3,66.1	0.04Å	Favored (78.276%)	-	-	-
A 234		ARG	1.67	-	Favored (32.95%) General / -86.9,6.2	Favored (96.4%) *mtt-85* chi angles: 291.2,179.3,177.6,273.2	0.01Å	Favored (31.468%)	-	-	-
A 235		GLY	1.76	-	Favored (58.38%) Glycine / 95.7,8.3	-	-	Favored (81.677%)	-	-	-
A 236		GLY	1.81	-	Favored (2.48%) Glycine / -75.6,65.2	-	-	Favored (11.969%)	-	-	-
A 237		TRP	1.81	-	Favored (11.39%) General / -48.1,-35.4	Favored (78.8%) *p-90* chi angles: 64.8,271.3	0.07Å	Favored (36.391%)	-	-	-
A 238		LEU	1.76	-	Favored (74.76%) General / -70.4,-39.2	Favored (39.5%) *tp* chi angles: 183,55.1	0.08Å	Favored (78.836%) alpha helix	-	-	-
A 239		ALA	1.7	-	Favored (79.7%) General / -64.9,-35.0	-	0.05Å	Favored (78.155%) alpha helix	-	-	-
A 240		GLY	1.63	-	Favored (88.96%) Glycine / -59.7,-36.2	-	-	Favored (90.276%) alpha helix	-	-	-
**#**	**Alt**	**Res**	**High B**	**Clash > 0.4Å**	**Ramachandran**	**Rotamer**	**Cβ deviation**	**CaBLAM**	**Bond lengths**	**Bond angles**	**Cis Peptides**
			Avg: 4.48	Clashscore: 1.79	Outliers: 5 of 256	Poor rotamers: 0 of 202	Outliers: 0 of 236	Outliers: 9 of 254	Outliers: 1 of 258	Outliers: 6 of 258	Non-Trans: 1 of 257
A 241		MET	1.59	-	Favored (99.53%) General / -61.2,-43.0	Favored (55.1%) *ttm* chi angles: 185.7,182,295.4	0.07Å	Favored (76.918%) alpha helix	-	-	-
A 242		SER	1.59	-	Favored (92.38%) General / -59.2,-44.5	Favored (34.5%) *t* chi angles: 182.6	0.06Å	Favored (89.75%) alpha helix	-	-	-
A 243		ILE	1.65	-	Favored (93.33%) Ile or Val / -64.2,-46.5	Favored (90.3%) *mt* chi angles: 291.7,170.4	0.07Å	Favored (88.338%) alpha helix	OUTLIER(S) worst is CB--CG1: 5.0 σ	-	-
A 244		THR	1.76	-	Favored (83.86%) General / -58.5,-47.5	Favored (91.9%) *m* chi angles: 299	0.05Å	Favored (87.942%) alpha helix	-	-	-

A 245		TRP	1.93	-	Favored (90.69%) General / -62.3,-46.0	Favored (90%) *t60* chi angles: 182.3,85.6	0.03Å	Favored (87.811%) alpha helix	-	-	-
A 246		THR	2.15	-	Favored (93.42%) General / -59.7,-45.0	Favored (88.6%) *m* chi angles: 298.4	0.04Å	Favored (95.876%) alpha helix	-	-	-
A 247		VAL	2.41	-	Favored (99.65%) Ile or Val / -62.3,-44.9	Favored (59.3%) *t* chi angles: 170.7	0.04Å	Favored (98.944%) alpha helix	-	-	-
A 248		TYR	2.71	-	Favored (92.82%) General / -60.8,-40.4	Favored (17.6%) *m-10* chi angles: 289.9,341.8	0.03Å	Favored (90.144%) alpha helix	-	-	-
A 249		LYS	3.07	0.48Å NZ with A 95 ASP OD1	Favored (81.52%) General / -56.9,-43.6	Favored (87.2%) *tttt* chi angles: 181.8,176.4,175.7,179.9	0.04Å	Favored (86.441%) alpha helix	-	-	-
A 250		ASN	3.5	-	Favored (71.66%) General / -69.5,-32.7	Favored (96.6%) *m-40* chi angles: 288,335	0.04Å	Favored (72.115%) alpha helix	-	-	-
A 251		VAL	4.08	-	Favored (81.36%) Ile or Val / -59.7,-40.1	Favored (50.3%) *t* chi angles: 169.4	0.03Å	Favored (72.531%) alpha helix	-	-	-
A 252		ASP	4.84	-	Favored (54.1%) General / -79.3,-5.3	Favored (83.7%) *m-30* chi angles: 289.4,334.3	0.04Å	Favored (49.521%)	-	-	-
A 253		LYS	5.83	-	Favored (77.32%) Pre-Pro / -78.3,150.7	Favored (97.5%) *mttt* chi angles: 291.2,182,179.4,179.7	0.03Å	Favored (32.739%)	-	-	-
A 254		PRO	7.01	-	Favored (82.33%) Trans-Pro / -66.8,151.5	Favored (41.6%) *Cg_endo* chi angles: 23.8,327.1,28.2	0.02Å	Favored (74.513%) beta sheet	-	-	-
A 255		LYS	8.28	-	Favored (28.58%) General / -107.6,11.5	Favored (72.8%) *mmtt* chi angles: 301.1,296.4,183.8,179.7	0.03Å	Favored (8.525%) beta sheet	-	-	-
A 256		GLY	9.49	-	Favored (51.35%) Glycine / -64.7,149.5	-	-	Favored (42.577%)	-	-	-
A 257		LYS	10.48	-	Favored (28.02%) General / -108.2,10.2	Favored (72.8%) *mmtt* chi angles: 300.5,295.7,182.6,180.3	0.02Å	-	-	-	-
A 258		ARG	11.22	-	-	Favored (93.2%) *mmt-90* chi angles: 296.2,290.1,183.1,273.3	0.04Å	-	-	-	-

About MolProbity | Website for the Richardson Lab | Using ecloud x-H | Internal reference 4.5.2
