# Supplementary material for: Integrative proteome-wide structural analysis and high-throughput docking identify broad-spectrum antiviral scaffolds against Zika, Yellow Fever, West Nile, Saint Louis encephalitis, and Usutu viruses
Source: Front Cell Infect Microbiol. 2026 Apr 30;16:1723132. doi: 10.3389/fcimb.2026.1723132 (PMC13171538; doi:10.3389/fcimb.2026.1723132)
Supplement: Supplementary file 3 [file DataSheet3.zip › SLEV/SLEV_NS4b/Mol_probity_Files/SLEV_NS4b_1FH-rama.pdf]

MolProbity Ramachandran analysis

SLEV_NS4b1FH.pdb, model 1

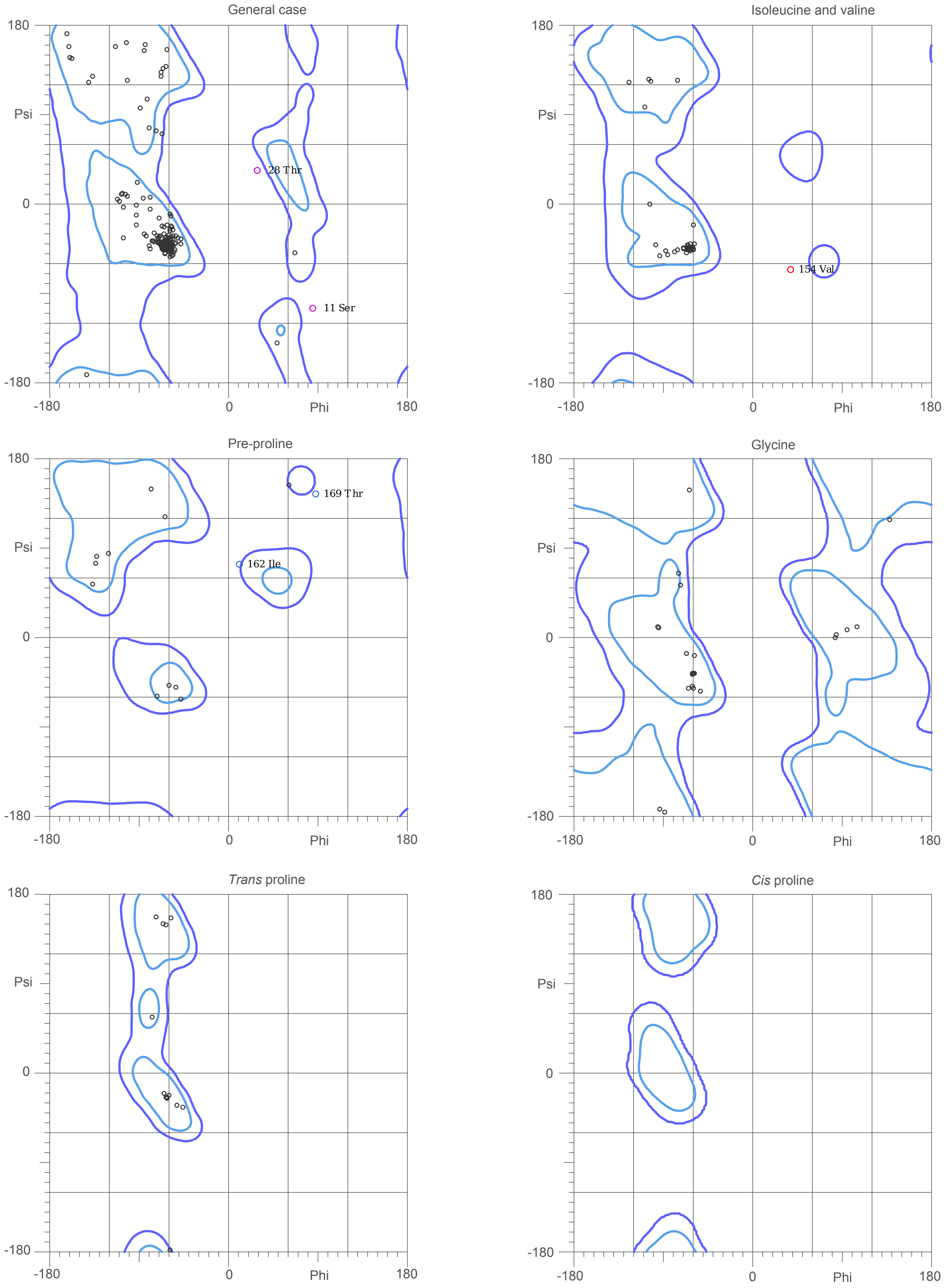

95.3% (244/256) of all residues were in favored (98%) regions.
98.0% (251/256) of all residues were in allowed (>99.8%) regions.

There were 5 outliers (phi, psi):
11 Ser (85.2, -105.5)
28 Thr (29.4, 34.6)
154 Val (38.2, -66.1)
162 Ile (12.0, 74.6)
169 Thr (88.2, 145.3)

http://kinemage.biochem.duke.edu

Lovell, Davis, et al. Proteins 50:437 (2003)
