# Supplementary material for: Integrative proteome-wide structural analysis and high-throughput docking identify broad-spectrum antiviral scaffolds against Zika, Yellow Fever, West Nile, Saint Louis encephalitis, and Usutu viruses
Source: Front Cell Infect Microbiol. 2026 Apr 30;16:1723132. doi: 10.3389/fcimb.2026.1723132 (PMC13171538; doi:10.3389/fcimb.2026.1723132)
Supplement: Supplementary file 3 [file DataSheet3.zip › SLEV/SLEV_NS5/Mol_probity_Files/SLEV_NS5_1FH-multi.table.pdf]

Viewing SLEV_NS5_1FH-multi.table

Duke Biochemistry
Duke University School of Medicine

When finished, you should close this window.

Hint: Use File | Save As... to save a copy of this page.

All-Atom Contacts	Clashscore, all atoms:	1.52		99th percentile* (N=1784, all resolutions)
	Clashscore is the number of serious steric overlaps (> 0.4 Å) per 1000 atoms.			
Protein Geometry	Poor rotamers	0	0.00%	Goal: <0.3%
	Favored rotamers	768	99.87%	Goal: >98%
	Ramachandran outliers	7	0.77%	Goal: <0.05%
	Ramachandran favored	875	96.79%	Goal: >98%
	Rama distribution Z-score	0.09 ± 0.27		Goal: abs(Z score) < 2
	MolProbity score^	1.09		100th percentile* (N=27675, 0Å - 99Å)
	Cβ deviations >0.25Å	0	0.00%	Goal: 0
	Bad bonds:	16 / 7437	0.22%	Goal: 0%
	Bad angles:	18 / 10041	0.18%	Goal: <0.1%
Peptide Omegas	Cis Prolines:	0 / 38	0.00%	Expected: ≤1 per chain, or ≤5%
Low-resolution Criteria	CaBLAM outliers	17	1.9%	Goal: <1.0%
	CA Geometry outliers	1	0.11%	Goal: <0.5%
Additional validations	Chiral volume outliers	0/1046		
	Waters with clashes	0/0	0.00%	See UnDowser table for details

In the two column results, the left column gives the raw count, right column gives the percentage.

* 100th percentile is the best among structures of comparable resolution; 0th percentile is the worst. For clashscore the comparative set of structures was selected in 2004, for MolProbity score in 2006.

^ MolProbity score combines the clashscore, rotamer, and Ramachandran evaluations into a single score, normalized to be on the same scale as X-ray resolution.

Key to table colors and cutoffs here:

#	Alt	Res	High B	Clash > 0.4Å	Ramachandran	Rotamer	Cβ deviation	CaBLAM	Bond lengths	Bond angles	Cis Peptides
			Avg: 0.98	Clashscore: 1.52	Outliers: 7 of 904	Poor rotamers: 0 of 769	Outliers: 0 of 827	Outliers: 17 of 902	Outliers: 14 of 906	Outliers: 17 of 906	Non-Trans: 0 of 905
A 1		GLY	7.68	-	-	-	-	-	-	-	-
A 2		GLY	6.77	-	Favored (33.42%) Glycine / 60.5,-123.6	-	-	-	-	-	-
A 3		GLY	5.58	-	Favored (56.18%) Glycine / -62.7,-16.6	-	-	Favored (20.683%)	-	-	-
A 4		LYS	4.31	-	Favored (56.59%) General / -90.5,-4.9	Favored (99.3%) *mttt* chi angles: 295.2,180.1,179.4,178.5	0.03Å	Favored (54.253%)	-	-	-
A 5		GLY	3.17	-	Favored (45.39%) Glycine / -69.6,147.5	-	-	Favored (46.526%)	-	-	-
A 6		ALA	2.3	-	Favored (57.5%) General / -65.4,143.8	-	0.03Å	Favored (43.877%) beta sheet	-	-	-

#	Alt	Res	High B	Clash > 0.4Å	Ramachandran	Rotamer	Cβ deviation	CaBLAM	Bond lengths	Bond angles	Cis Peptides
A 7		THR	1.71	-	Favored (14.03%) General / -91.1,164.2	Favored (74.3%) *p* chi angles: 61.5	0.07Å	Favored (43.178%)	-	-	-
A 8		LEU	1.33	-	Favored (89.28%) General / -63.0,-38.0	Favored (87.2%) *mt* chi angles: 291.3,174.1	0.03Å	Favored (67.412%)	-	-	-
A 9		GLY	1.11	-	Favored (96.57%) Glycine / -64.9,-39.5	-	-	Favored (97.665%) alpha helix	-	-	-
A 10		GLU	0.99	-	Favored (89.94%) General / -63.3,-38.1	Favored (37%) *mt-10* chi angles: 287.1,167.2,310	0.02Å	Favored (99.114%) alpha helix	-	-	-
A 11		ILE	0.94	-	Favored (93.34%) Ile or Val / -65.5,-45.3	Favored (97.7%) *mt* chi angles: 293.2,167.2	0.04Å	Favored (93.07%) alpha helix	-	-	-
A 12		TRP	0.93	-	Favored (80.24%) General / -60.4,-48.8	Favored (65.3%) *t60* chi angles: 182.3,77.9	0.03Å	Favored (95.603%) alpha helix	-	-	-
A 13		LYS	0.95	-	Favored (85.02%) General / -62.0,-37.5	Favored (23.4%) *mmmt* chi angles: 287.4,287.2,287.1,183.7	0.04Å	Favored (90.485%) alpha helix	-	-	-
A 14		SER	0.97	-	Favored (96.39%) General / -63.7,-43.5	Favored (72.7%) *m* chi angles: 295.7	0.04Å	Favored (91.169%) alpha helix	-	-	-
A 15		ARG	1.02	-	Favored (95.48%) General / -60.4,-42.3	Favored (70.4%) *mtp85* chi angles: 287.7,165.1,62.7,91.6	0.04Å	Favored (94.986%) alpha helix	-	-	-
A 16		LEU	1.08	-	Favored (95.63%) General / -62.9,-39.8	Favored (75.7%) *mt* chi angles: 288.1,172.5	0.04Å	Favored (93.894%) alpha helix	-	-	-
A 17		ASN	1.18	-	Favored (65.15%) General / -69.8,-27.8	Favored (89%) *m-40* chi angles: 285.2,335.4	0.11Å	Favored (76.902%) alpha helix	-	-	-
A 18		GLN	1.3	-	Favored (61.24%) General / -72.3,-23.7	Favored (99%) *mt0* chi angles: 291.4,173.5,337.9	0.01Å	Favored (49.241%)	-	-	-
A 19		LEU	1.44	-	Favored (45.37%) General / -69.6,151.9	Favored (94.8%) *mt* chi angles: 296.1,177.1	0.15Å	Favored (22.796%)	-	-	-
A 20		THR	1.55	-	Favored (20.41%) General / -79.9,167.5	Favored (73.1%) *p* chi angles: 61.7	0.05Å	Favored (49.179%)	-	-	-
**#**	**Alt**	**Res**	**High B**	**Clash > 0.4Å**	**Ramachandran**	**Rotamer**	**Cβ deviation**	**CaBLAM**	**Bond lengths**	**Bond angles**	**Cis Peptides**
			Avg: 0.98	Clashscore: 1.52	Outliers: 7 of 904	Poor rotamers: 0 of 769	Outliers: 0 of 827	Outliers: 17 of 902	Outliers: 14 of 906	Outliers: 17 of 906	Non-Trans: 0 of 905
A 21		ARG	1.61	-	Favored (91.25%) General / -58.9,-44.4	Favored (71%) *ttt-90* chi angles: 182.5,177.4,180.9,271	0.02Å	Favored (62.783%)	-	-	-

A 22		ALA	1.62	-	Favored (59.82%) General / -79.6,-12.8	-	0.02Å	CaBLAM Outlier (0.876%) try alpha helix	-	OUTLIER(S) worst is CA-C-O: 4.9 σ	-
A 23		GLU	1.56	-	Favored (24.08%) General / -78.6,-44.5	Favored (53.9%) *mt-10* chi angles: 291.8,168.8,313.3	0.03Å	CaBLAM Disfavored (1.427%) try alpha helix	-	-	-
A 24		PHE	1.47	-	Favored (72.43%) General / -55.7,-49.6	Favored (80.9%) *t80* chi angles: 182.6,84.4	0.04Å	Favored (82.621%) alpha helix	-	-	-
A 25		MET	1.36	-	Favored (92.21%) General / -63.2,-38.7	Favored (82.7%) *mtm* chi angles: 290.9,188.2,292.7	0.09Å	Favored (82.703%) alpha helix	-	-	-
A 26		ALA	1.27	-	Favored (88.9%) General / -62.9,-37.9	-	0.03Å	Favored (74.79%) alpha helix	-	-	-
A 27		TYR	1.2	-	Favored (48.34%) General / -78.2,-35.6	Favored (49.2%) *m-80* chi angles: 287.5,113.7	0.02Å	Favored (37.299%) alpha helix	-	-	-
A 28		ARG	1.15	-	Favored (53.12%) General / -50.2,-45.0	Favored (62.5%) *ttp-170* chi angles: 179.7,184.4,65.5,190	0.06Å	Favored (60.91%) alpha helix	-	-	-
A 29		LYS	1.09	-	Favored (58.95%) General / -87.6,-4.3	Favored (59.4%) *pttt* chi angles: 69.9,183.3,183.7,181.5	0.06Å	Favored (31.298%)	-	-	-
A 30		ASP	1.03	-	Favored (4.89%) General / -72.6,107.0	Favored (58.7%) *t0* chi angles: 181.3,343.9	0.02Å	CaBLAM Disfavored (2.097%)	-	-	-
A 31		GLY	0.96	-	Favored (87.13%) Glycine / 78.8,3.2	-	-	Favored (22.821%)	-	-	-
A 32		ILE	0.9	-	Allowed (1.59%) Ile or Val / -85.5,168.8	Favored (31%) *pt* chi angles: 67.2,168.7	0.08Å	Favored (39.943%)	-	-	-
A 33		VAL	0.85	-	Favored (50.6%) Ile or Val / -104.8,132.8	Favored (68.6%) *t* chi angles: 178.9	0.05Å	Favored (49.221%) beta sheet	-	-	-
A 34		GLU	0.81	-	Favored (31.62%) General / -112.2,149.9	Favored (96.2%) *mt-10* chi angles: 292.1,184,358.9	0.05Å	Favored (51.064%) beta sheet	-	-	-
A 35		VAL	0.78	-	Favored (44.46%) Ile or Val / -96.5,130.9	Favored (52.1%) *t* chi angles: 181.1	0.06Å	Favored (45.423%) beta sheet	-	-	-
A 36		ASP	0.78	-	Favored (61.38%) General / -51.4,-49.5	Favored (97.8%) *m-30* chi angles: 288.5,344.2	0.01Å	CaBLAM Disfavored (1.378%) try beta sheet	-	-	-
A 37		ARG	0.79	-	OUTLIER (0.04)%) General / 60.6,-12.6	Favored (73.4%) *mtm180* chi angles: 291.9,175,290.3,158.5	0.07Å	CaBLAM Disfavored (2.003%)	-	-	-
A 38		ALA	0.81	-	Favored (85.4%) Pre-Pro / -51.0,-48.2	-	0.04Å	Favored (47.637%)	-	OUTLIER(S) worst is CA-C-N: 4.1 σ	-

A 39		PRO	0.84	-	Favored (50.67%) Trans-Pro / -67.0,-20.8	Favored (43.9%) *Cg_endo* chi angles: 24.4,325.8,29.1	0.01Å	Favored (84.404%) alpha helix	-	-	-
A 40		ALA	0.88	-	Favored (28.61%) General / -79.2,-41.3	-	0.03Å	Favored (70.408%) alpha helix	-	-	-
**#**	**Alt**	**Res**	**High B**	**Clash > 0.4Å**	**Ramachandran**	**Rotamer**	**Cβ deviation**	**CaBLAM**	**Bond lengths**	**Bond angles**	**Cis Peptides**
			Avg: 0.98	Clashscore: 1.52	Outliers: 7 of 904	Poor rotamers: 0 of 769	Outliers: 0 of 827	Outliers: 17 of 902	Outliers: 14 of 906	Outliers: 17 of 906	Non-Trans: 0 of 905
A 41		ARG	0.93	-	Favored (94.47%) General / -65.3,-40.9	Favored (99.2%) *mtm-85* chi angles: 289.3,194.2,294.4,268.8	0.04Å	Favored (92.838%) alpha helix	-	-	-
A 42		LYS	1	-	Favored (86.68%) General / -66.7,-42.1	Favored (97%) *mttt* chi angles: 289.2,176.9,181.4,178.9	0.01Å	Favored (95.158%) alpha helix	-	-	-
A 43		ALA	1.08	-	Favored (92.1%) General / -59.7,-42.1	-	0.05Å	Favored (91.29%) alpha helix	-	-	-
A 44		ARG	1.18	-	Favored (73.79%) General / -63.7,-49.2	Favored (22.5%) *ttm110* chi angles: 176.3,179.8,282.8,133.5	0.04Å	Favored (86.159%) alpha helix	-	-	-
A 45		ARG	1.28	-	Favored (76.63%) General / -62.7,-34.2	Favored (97.6%) *mtt180* chi angles: 288.4,173.3,180.3,172.6	0.01Å	Favored (73.36%) alpha helix	-	-	-
A 46		GLU	1.35	-	Favored (58.53%) General / -86.4,-2.5	Favored (55%) *mt-10* chi angles: 293.7,181.8,40.6	0.03Å	Favored (55.921%)	-	-	-
A 47		GLY	1.39	-	Favored (88.52%) Glycine / 80.9,7.5	-	-	Favored (84.101%)	-	-	-
A 48		ARG	1.36	-	Favored (10.67%) General / -84.7,69.5	Favored (98.3%) *mtt-85* chi angles: 295,178.6,184.5,273	0.06Å	Favored (11.317%)	-	-	-
A 49		LEU	1.28	0.48Å C with A 49 LEU HD12	Favored (15.24%) General / -70.0,-3.2	Favored (2.1%) *pp* chi angles: 65.4,76.4	0.07Å	Favored (8.945%)	-	-	-
A 50		THR	1.16	-	Favored (49.36%) General / -89.5,-9.7	Favored (65.9%) *p* chi angles: 63	0.02Å	Favored (29.674%)	-	-	-
A 51		GLY	1.03	-	Favored (48.66%) Glycine / -61.8,143.5	-	-	Favored (54.773%)	-	-	-
A 52		GLY	0.92	-	Favored (3.65%) Glycine / -78.0,62.4	-	-	Favored (7.563%)	-	-	-
A 53		HIS	0.83	-	Favored (81.91%) Pre-Pro / -131.6,154.6	Favored (95.8%) *m-70* chi angles: 302.6,295.3	0.06Å	Favored (25.253%)	-	-	-
A 54		PRO	0.78	-	Favored (63.58%) Trans-Pro / -69.7,156.4	Favored (58.4%) *Cg_endo* chi angles: 26.2,326.7,26.5	0.03Å	Favored (77.985%)	-	-	-
A 55		VAL	0.74	-	Favored (9.83%)	Favored (19.7%) *m* chi angles: 302.5	0.05Å	Favored (18.473%)	-	-	-

#	Alt	Res	High B	Clash > 0.4Å	Ramachandran	Rotamer	Cβ deviation	CaBLAM	Bond lengths	Bond angles	Cis Peptides
					Ile or Val / -101.8,-1.6						
A 56		SER	0.72	-	Favored (12.86%) General / -163.3,150.6	Favored (40.9%) *t* chi angles: 176.5	0.05Å	Favored (13.489%)	-	-	-
A 57		ARG	0.7	-	Favored (54.35%) General / -53.4,-33.5	Favored (52.4%) *ttt90* chi angles: 187,186.3,184.8,93.2	0.10Å	Favored (49.644%) alpha helix	-	-	-
A 58		GLY	0.69	-	Favored (69.2%) Glycine / -57.8,-33.8	-	-	Favored (77.448%) alpha helix	-	-	-
A 59		SER	0.67	-	Favored (93.16%) General / -61.1,-45.8	Favored (45.9%) *t* chi angles: 179.5	0.07Å	Favored (76.637%) alpha helix	-	-	-
A 60		ALA	0.65	-	Favored (76.83%) General / -60.2,-36.0	-	0.03Å	Favored (80.152%) alpha helix	-	-	-
**#**	**Alt**	**Res**	**High B**	**Clash > 0.4Å**	**Ramachandran**	**Rotamer**	**Cβ deviation**	**CaBLAM**	**Bond lengths**	**Bond angles**	**Cis Peptides**
			Avg: 0.98	Clashscore: 1.52	Outliers: 7 of 904	Poor rotamers: 0 of 769	Outliers: 0 of 827	Outliers: 17 of 902	Outliers: 14 of 906	Outliers: 17 of 906	Non-Trans: 0 of 905
A 61		LYS	0.64	-	Favored (81.52%) General / -67.5,-43.2	Favored (92.7%) *mttt* chi angles: 291.5,179.5,187.7,178.1	0.03Å	Favored (86.995%) alpha helix	-	-	-
A 62		LEU	0.63	-	Favored (92.21%) General / -65.4,-39.5	Favored (38.7%) *tp* chi angles: 185.7,59.7	0.05Å	Favored (84.7%) alpha helix	-	-	-
A 63		ARG	0.63	-	Favored (78.55%) General / -56.7,-48.0	Favored (2.7%) *tmm160* chi angles: 181.8,267.2,312.2,185.7	0.04Å	Favored (86.032%) alpha helix	-	-	-
A 64		TRP	0.63	-	Favored (92.57%) General / -59.7,-45.3	Favored (91.3%) *t60* chi angles: 182.9,89.2	0.04Å	Favored (96.288%) alpha helix	-	-	-
A 65		ILE	0.63	-	Favored (89.36%) Ile or Val / -61.4,-41.3	Favored (97.2%) *mt* chi angles: 292.5,169.2	0.06Å	Favored (82.845%) alpha helix	-	-	-
A 66		THR	0.64	-	Favored (74.95%) General / -66.6,-46.7	Favored (87.3%) *m* chi angles: 301.4	0.06Å	Favored (83.269%) alpha helix	-	-	-
A 67		GLU	0.65	-	Favored (73.36%) General / -62.0,-32.8	Favored (90%) *mt-10* chi angles: 286,180.1,340.6	0.08Å	Favored (76.103%) alpha helix	-	-	-
A 68		ARG	0.65	-	Favored (40.36%) General / -79.4,-2.1	Favored (49.3%) *mmt180* chi angles: 293.5,288,181.2,183.1	0.02Å	Favored (54.029%)	-	-	-
A 69		GLY	0.65	-	Favored (77%) Glycine / 82.3,14.2	-	-	Favored (84.179%)	-	-	-
A 70		PHE	0.65	0.41Å HB3 with A 222 VAL HG11	Favored (23.41%) General / -84.6,-33.3	Favored (63.1%) *m-80* chi angles: 296.2,114.8	0.02Å	Favored (16.289%)	-	OUTLIER(S) worst is CA-CB-CG: 4.2 σ	-
A 71		VAL	0.65	-	Favored (69.16%)	Favored (66.6%) *t* chi angles: 179.2	0.04Å	Favored (29.726%)	-	-	-

					Ile or Val / -124.7,133.5						
A 72		LYS	0.65	-	Favored (75.6%) Pre-Pro / -128.9,78.9	Favored (96.3%) *mttt* chi angles: 297.5,179.1,180.5,178.6	0.04Å	Favored (10.649%)	-	-	-
A 73		PRO	0.65	-	Favored (89.69%) Trans-Pro / -57.8,139.2	Favored (79.7%) *Cg_exo* chi angles: 334.7,38.5,324.4	0.08Å	Favored (15.275%)	-	-	-
A 74		MET	0.64	-	Favored (42.61%) General / -152.5,158.5	Favored (17.5%) *ptt* chi angles: 62.3,184.4,179.4	0.06Å	Favored (7.312%)	-	-	-
A 75		GLY	0.63	-	Favored (38.14%) Glycine / 58.2,-125.0	-	-	Favored (38.832%)	-	-	-
A 76		LYS	0.62	-	Favored (39.82%) General / -95.9,124.2	Favored (37.8%) *ttpt* chi angles: 184.9,172.3,71.3,176.6	0.06Å	Favored (13.402%)	-	-	-
A 77		VAL	0.61	-	Favored (67.12%) Ile or Val / -112.8,130.2	Favored (61.2%) *t* chi angles: 179.8	0.08Å	Favored (68.057%)	-	-	-
A 78		VAL	0.61	-	Favored (73.34%) Ile or Val / -116.2,125.5	Favored (83.4%) *t* chi angles: 176.9	0.05Å	Favored (72.794%) beta sheet	-	-	-
A 79		ASP	0.61	-	Favored (16.58%) General / -107.1,106.2	Favored (68.6%) *t0* chi angles: 182.9,353.6	0.07Å	Favored (67.732%) beta sheet	-	-	-
A 80		LEU	0.62	-	Favored (51.67%) General / -88.0,2.5	Favored (84.3%) *mt* chi angles: 299.4,174.3	0.06Å	Favored (8.136%)	-	-	-
**#**	**Alt**	**Res**	**High B**	**Clash > 0.4Å**	**Ramachandran**	**Rotamer**	**Cβ deviation**	**CaBLAM**	**Bond lengths**	**Bond angles**	**Cis Peptides**
			Avg: 0.98	Clashscore: 1.52	Outliers: 7 of 904	Poor rotamers: 0 of 769	Outliers: 0 of 827	Outliers: 17 of 902	Outliers: 14 of 906	Outliers: 17 of 906	Non-Trans: 0 of 905
A 81		GLY	0.63	-	Allowed (0.91%) Glycine / -140.0,47.0	-	-	Favored (15.906%)	-	-	-
A 82		CYS	0.65	-	Favored (69.55%) General / -64.9,-28.5	Favored (15.4%) *p* chi angles: 72.1	0.05Å	CaBLAM Outlier (0.179%)	-	-	-
A 83		GLY	0.65	-	Favored (38.49%) Glycine / 52.8,-129.4	-	-	Favored (40.76%)	-	-	-
A 84		ARG	0.65	-	Favored (66.81%) General / -61.3,-25.0	Favored (80.6%) *mtt90* chi angles: 290.3,185.7,179.7,88.7	0.04Å	CaBLAM Disfavored (3.561%)	-	-	-
A 85		GLY	0.64	-	Favored (11.38%) Glycine / 115.1,10.9	-	-	Favored (74.485%)	-	-	-
A 86		GLY	0.62	-	Favored (8.21%) Glycine / -48.0,-57.0	-	-	Favored (20.099%)	-	-	-
A 87		TRP	0.61	-	Favored (83.85%)	Favored (38.1%) *m-10*	0.03Å	Favored (72.876%)	-	-	-

#	Alt	Res	High B	Clash > 0.4Å	Ramachandran	Rotamer	Cβ deviation	CaBLAM	Bond lengths	Bond angles	Cis Peptides
					General / -67.0,-37.3	chi angles: 284.5,12.3		alpha helix			
A 88		SER	0.6	-	Favored (99.12%) General / -63.2,-41.8	Favored (53.9%) *m* chi angles: 292.3	0.05Å	Favored (82.967%) alpha helix	-	-	-
A 89		TYR	0.59	-	Favored (64.42%) General / -74.0,-35.2	Favored (41.5%) *m-80* chi angles: 288.5,118	0.04Å	Favored (90.52%) alpha helix	-	-	-
A 90		TYR	0.59	-	Favored (74.1%) General / -60.8,-50.3	Favored (90.5%) *t80* chi angles: 177.9,75.8	0.05Å	Favored (79.531%) alpha helix	-	-	-
A 91		CYS	0.61	-	Favored (77.53%) General / -59.9,-36.8	Favored (90.8%) *m* chi angles: 291.6	0.06Å	Favored (72.605%) alpha helix	-	-	-
A 92		ALA	0.63	-	Favored (64.74%) General / -60.2,-24.3	-	0.06Å	Favored (68.595%) alpha helix	-	-	-
A 93		THR	0.66	-	Favored (58.97%) General / -86.3,-6.4	Favored (65.9%) *p* chi angles: 63	0.06Å	Favored (57.65%)	-	-	-
A 94		LEU	0.69	-	Favored (33.86%) General / -85.9,134.9	Favored (84%) *mt* chi angles: 301.2,177.3	0.04Å	Favored (35.09%)	-	-	-
A 95		LYS	0.71	-	Favored (19.18%) General / -49.3,-35.8	Favored (86.8%) *tttt* chi angles: 180.8,176.4,178.4,178.5	0.01Å	Favored (27.735%)	-	-	-
A 96		HIS	0.72	-	Favored (54.56%) General / -94.7,-0.3	Favored (99.3%) *m-70* chi angles: 298.4,291	0.04Å	Favored (53.095%)	-	-	-
A 97		VAL	0.72	-	Favored (35.04%) Ile or Val / -89.1,132.0	Favored (96.6%) *t* chi angles: 175	0.05Å	Favored (23.076%)	-	-	-
A 98		GLN	0.7	-	Favored (8.62%) General / -103.0,-35.3	Favored (90.9%) *mm-40* chi angles: 297.2,292.2,302.7	0.09Å	Favored (28.82%)	-	-	-
A 99		GLU	0.67	-	Favored (17.12%) General / -152.4,137.3	Favored (92.6%) *tt0* chi angles: 181.8,178.4,0	0.02Å	Favored (33.148%)	-	-	-
A 100		VAL	0.65	-	Favored (71.59%) Ile or Val / -123.6,125.6	Favored (63.9%) *t* chi angles: 179.5	0.07Å	Favored (68.534%)	-	-	-
**#**	**Alt**	**Res**	**High B**	**Clash > 0.4Å**	**Ramachandran**	**Rotamer**	**Cβ deviation**	**CaBLAM**	**Bond lengths**	**Bond angles**	**Cis Peptides**
			Avg: 0.98	Clashscore: 1.52	Outliers: 7 of 904	Poor rotamers: 0 of 769	Outliers: 0 of 827	Outliers: 17 of 902	Outliers: 14 of 906	Outliers: 17 of 906	Non-Trans: 0 of 905
A 101		LYS	0.64	-	Favored (47.01%) General / -114.7,142.1	Favored (95.4%) *mttt* chi angles: 296.4,185.9,180.6,183.5	0.02Å	Favored (60.899%) beta sheet	-	-	-
A 102		GLY	0.65	-	Favored (17.57%) Glycine / -137.9,145.9	-	-	Favored (70.402%) beta sheet	-	-	-
A 103		PHE	0.69	-	Favored (50.74%)	Favored (83.2%) *m-80* chi angles: 301.6,89.8	0.10Å	Favored (47.77%) beta sheet	-	-	-

					General / -132.2,144.6						
A 104		THR	0.77	-	Favored (27.29%) General / -156.2,152.0	Favored (9.2%) *t* chi angles: 185.1	0.04Å	Favored (22.293%) beta sheet	-	-	-
A 105		LYS	0.89	-	Favored (29.55%) General / -81.9,124.2	Favored (33.3%) *ttmt* chi angles: 183.6,180.9,286.8,184.4	0.02Å	Favored (43.35%) beta sheet	-	-	-
A 106		GLY	1.04	-	Favored (37.3%) Glycine / -92.2,-162.4	-	-	Favored (8.494%)	-	-	-
A 107		GLY	1.18	-	Favored (45.63%) Glycine / 89.7,176.1	-	-	Favored (11.88%)	-	-	-
A 108		PRO	1.28	-	Favored (67%) Trans-Pro / -54.0,136.6	Favored (88.9%) *Cg_exo* chi angles: 333.3,36.9,328.8	0.05Å	CaBLAM Disfavored (4.631%)	-	-	-
A 109		GLY	1.31	-	Favored (73.79%) Glycine / 91.0,-10.9	-	-	Favored (71.324%)	-	-	-
A 110		HIS	1.26	-	Favored (20.4%) General / -114.2,159.2	Favored (99.3%) *m-70* chi angles: 297.4,290.8	0.04Å	Favored (31.232%)	-	-	-
A 111		GLU	1.17	-	Favored (85.16%) General / -64.0,-36.8	Favored (93.4%) *mt-10* chi angles: 290.3,184.5,354.4	0.06Å	CaBLAM Disfavored (3.079%)	-	-	-
A 112		GLU	1.07	-	OUTLIER (0.05)%) Pre-Pro / 68.2,136.7	Favored (49.7%) *mm-30* chi angles: 293.9,289.3,5.1	0.06Å	CaBLAM Disfavored (1.099%)	-	-	-
A 113		PRO	0.99	-	Favored (71.44%) Trans-Pro / -69.3,150.6	Favored (73.2%) *Cg_endo* chi angles: 27.6,327.8,23.3	0.06Å	Favored (65.265%)	-	-	-
A 114		GLN	0.93	-	Favored (53.54%) General / -124.3,131.9	Favored (62.5%) *tt0* chi angles: 186,177.8,32.9	0.03Å	Favored (45.277%) beta sheet	-	-	-
A 115		LEU	0.88	-	Favored (8.42%) General / -85.8,81.9	Favored (76.1%) *mt* chi angles: 302.5,179.6	0.04Å	Favored (29.707%) beta sheet	-	-	-
A 116		MET	0.84	-	Favored (13.25%) General / -99.7,162.0	Favored (80%) *mmm* chi angles: 302.7,293.2,282.4	0.02Å	Favored (27.285%)	-	-	-
A 117		GLN	0.8	-	Favored (40.12%) General / -101.7,137.3	Favored (43.6%) *tt0* chi angles: 175,174.1,312.6	0.04Å	Favored (10.89%)	-	-	-
A 118		SER	0.77	-	Favored (37.23%) General / -158.6,161.7	Favored (87.3%) *p* chi angles: 68	0.07Å	CaBLAM Disfavored (4.429%)	-	-	-
A 119		TYR	0.75	-	Favored (26.37%) General / -54.2,127.3	Favored (75.7%) *t80* chi angles: 181.2,70.8	0.11Å	Favored (17.737%)	-	-	-
A 120		GLY	0.73	-	Favored (87.02%) Glycine / 84.3,5.6	-	-	Favored (73.887%)	-	-	-

#	Alt	Res	High B	Clash > 0.4Å	Ramachandran	Rotamer	Cβ deviation	CaBLAM	Bond lengths	Bond angles	Cis Peptides
			Avg: 0.98	Clashscore: 1.52	Outliers: 7 of 904	Poor rotamers: 0 of 769	Outliers: 0 of 827	Outliers: 17 of 902	Outliers: 14 of 906	Outliers: 17 of 906	Non-Trans: 0 of 905
A 121		TRP	0.73	-	Favored (37.62%) General / -54.4,-27.5	Favored (74.6%) *p-90* chi angles: 67.9,268.9	0.09Å	Favored (26.928%)	-	-	-
A 122		ASN	0.73	-	Favored (62.56%) General / -68.1,-13.9	Favored (13.9%) *p0* chi angles: 59.2,291.6	0.03Å	Favored (62.869%) three-ten	-	-	-
A 123		LEU	0.73	-	Favored (51.95%) General / -95.5,-0.7	Favored (67.5%) *mt* chi angles: 304,178.9	0.08Å	Favored (57.052%)	-	-	-
A 124		VAL	0.74	-	Favored (57.2%) Ile or Val / -105.6,120.9	Favored (79.6%) *t* chi angles: 178	0.04Å	Favored (26.833%)	-	-	-
A 125		HIS	0.76	-	Favored (9.23%) General / -123.5,104.7	Favored (93.9%) *m-70* chi angles: 304.1,285.3	0.06Å	Favored (51.787%)	-	-	-
A 126		MET	0.77	-	Favored (25.06%) General / -104.0,149.6	Favored (84.4%) *mtm* chi angles: 293.3,184.8,284.6	0.05Å	Favored (37.075%) beta sheet	-	-	-
A 127		LYS	0.79	-	Favored (33.55%) General / -130.8,125.4	Favored (87.4%) *tttt* chi angles: 181.7,175.7,181,177.9	0.07Å	Favored (62.188%) beta sheet	-	-	-
A 128		SER	0.79	-	Favored (21.68%) General / -91.2,-20.7	Favored (90.5%) *p* chi angles: 68.5	0.07Å	Favored (12.378%)	-	-	-
A 129		GLY	0.78	-	Favored (4.65%) Glycine / -85.4,52.6	-	-	CaBLAM Outlier (0.508%)	-	-	-
A 130		VAL	0.76	0.42Å O with A 130 VAL HG13	Favored (11.23%) Ile or Val / -149.6,135.1	Favored (5.8%) *p* chi angles: 58.5	0.10Å	Favored (14.251%)	-	-	-
A 131		ASP	0.74	-	Favored (16.38%) General / -103.3,106.0	Favored (51%) *t0* chi angles: 184.2,333.9	0.04Å	Favored (66.67%) beta sheet	-	-	-
A 132		VAL	0.72	-	Favored (27.2%) Ile or Val / -62.8,-21.2	Favored (30.7%) *m* chi angles: 297.9	0.06Å	Favored (41.563%)	-	-	-
A 133		PHE	0.7	-	Favored (62.88%) General / -70.6,-19.0	Favored (51%) *m-80* chi angles: 286.3,111	0.04Å	Favored (53.878%) alpha helix	-	-	-
A 134		HIS	0.7	-	Favored (18.68%) General / -113.1,10.0	Favored (98%) *m-70* chi angles: 295.8,288	0.02Å	Favored (44.338%)	-	-	-
A 135		LYS	0.69	-	Favored (78.44%) Pre-Pro / -81.5,125.5	Favored (44.5%) *tttp* chi angles: 178,175.7,164,61.2	0.02Å	Favored (31.587%)	-	-	-
A 136		PRO	0.68	-	Favored (91.29%) Trans-Pro / -57.7,139.8	Favored (87.6%) *Cg_exo* chi angles: 333.4,35.5,330.7	0.08Å	Favored (57.43%)	-	-	-

#	Alt	Res	High B	Clash > 0.4Å	Ramachandran	Rotamer	Cβ deviation	CaBLAM	Bond lengths	Bond angles	Cis Peptides
A 137		ALA	0.67	-	Favored (55.53%) General / -66.3,145.6	-	0.05Å	Favored (35.859%)	-	-	-
A 138		GLU	0.64	-	Favored (21.58%) Pre-Pro / -111.8,138.3	Favored (78.9%) *tt0* chi angles: 180.7,177.1,342.4	0.07Å	Favored (36.352%)	-	-	-
A 139		PRO	0.62	-	Favored (93.32%) Trans-Pro / -57.0,140.7	Favored (85.7%) *Cg_exo* chi angles: 333.8,35,331.4	0.03Å	Favored (31.197%)	-	-	-
A 140		ALA	0.6	-	Favored (34.41%) General / -153.0,165.8	-	0.02Å	Favored (43.688%)	-	-	-
**#**	**Alt**	**Res**	**High B**	**Clash > 0.4Å**	**Ramachandran**	**Rotamer**	**Cβ deviation**	**CaBLAM**	**Bond lengths**	**Bond angles**	**Cis Peptides**
			Avg: 0.98	Clashscore: 1.52	Outliers: 7 of 904	Poor rotamers: 0 of 769	Outliers: 0 of 827	Outliers: 17 of 902	Outliers: 14 of 906	Outliers: 17 of 906	Non-Trans: 0 of 905
A 141		ASP	0.58	-	Favored (14%) General / -91.5,-35.9	Favored (59.1%) *m-30* chi angles: 296.4,302.2	0.06Å	Favored (7.041%)	-	-	-
A 142		THR	0.58	-	Favored (44.69%) General / -131.4,132.8	Favored (74.5%) *m* chi angles: 302.8	0.06Å	Favored (40.745%)	-	-	-
A 143		VAL	0.59	-	Favored (66.77%) Ile or Val / -122.8,122.8	Favored (83.7%) *t* chi angles: 177.7	0.07Å	Favored (65.637%)	-	-	-
A 144		LEU	0.61	-	Favored (33.59%) General / -112.1,148.6	Favored (37.9%) *mt* chi angles: 307.1,174.5	0.10Å	Favored (38.155%) beta sheet	-	-	-
A 145		CYS	0.65	-	Favored (14.77%) General / -152.7,134.6	Favored (53.3%) *t* chi angles: 184	0.03Å	Favored (36.047%) beta sheet	-	-	-
A 146		ASP	0.71	-	Allowed (1.44%) General / -146.2,76.1	Favored (65.4%) *t0* chi angles: 186.8,343.9	0.05Å	CaBLAM Disfavored (3.939%)	-	-	-
A 147		ILE	0.79	-	Favored (24.38%) Ile or Val / -131.4,168.2	Favored (31%) *pt* chi angles: 64.5,178.1	0.07Å	CA Geom Outlier (0.113%)	OUTLIER(S) worst is CB--CG1: 4.9 σ	-	-
A 148		GLY	0.9	-	Favored (44.74%) Glycine / 105.2,-8.1	-	-	CaBLAM Disfavored (2.623%)	-	-	-
A 149		GLU	1.03	-	Allowed (1.82%) General / 48.8,-123.6	Favored (67.4%) *mt-10* chi angles: 299.5,180.8,316.6	0.04Å	CaBLAM Outlier (0.282%)	-	-	-
A 150		SER	1.16	-	Favored (14.9%) General / -161.3,148.4	Favored (40%) *t* chi angles: 177.9	0.05Å	CaBLAM Disfavored (2.795%)	-	-	-
A 151		ASN	1.25	-	Favored (3.74%) Pre-Pro / -152.3,123.8	Favored (31.2%) *t0* chi angles: 187.9,301.9	0.01Å	Favored (19.822%)	-	-	-
A 152		PRO	1.27	-	Favored (6.62%) Trans-Pro / -46.2,-29.4	Favored (90.7%) *Cg_exo* chi angles: 329.6,36.9,332.7	0.05Å	Favored (32.458%)	-	-	-

#	Alt	Res	High B	Clash > 0.4Å	Ramachandran	Rotamer	Cβ deviation	CaBLAM	Bond lengths	Bond angles	Cis Peptides
A 153		SER	1.23	-	Favored (21.9%) General / -85.8,113.4	Favored (39.1%) *t* chi angles: 174.9	0.04Å	Favored (34.958%)	-	-	-
A 154		CYS	1.14	-	Favored (67.24%) General / -61.5,-25.4	Favored (29.3%) *p* chi angles: 65.6	0.07Å	Favored (44.142%)	-	-	-
A 155		GLU	1.02	-	Favored (73.62%) General / -63.7,-32.2	Favored (99.7%) *mt-10* chi angles: 291.8,180.1,353.3	0.01Å	Favored (64.487%) alpha helix	-	-	-
A 156		VAL	0.91	-	Favored (33.73%) Ile or Val / -76.4,-45.8	Favored (91.6%) *t* chi angles: 174.4	0.06Å	Favored (71.44%) alpha helix	-	-	-
A 157		GLU	0.82	-	Favored (85.35%) General / -61.8,-37.7	Favored (93.5%) *mt-10* chi angles: 289.6,182.2,346.1	0.01Å	Favored (92.328%) alpha helix	-	-	-
A 158		GLU	0.75	-	Favored (77.03%) General / -61.2,-49.5	Favored (92.5%) *tt0* chi angles: 179.9,177.5,356.6	0.03Å	Favored (92.552%) alpha helix	-	-	-
A 159		ALA	0.71	-	Favored (94.5%) General / -61.5,-40.4	-	0.05Å	Favored (89.549%) alpha helix	-	-	-
A 160		ARG	0.68	-	Favored (73.45%) General / -68.5,-45.0	Favored (97.5%) *mtt180* chi angles: 289.8,178.2,182.7,180.6	0.02Å	Favored (91.326%) alpha helix	-	-	-
**#**	**Alt**	**Res**	**High B**	**Clash > 0.4Å**	**Ramachandran**	**Rotamer**	**Cβ deviation**	**CaBLAM**	**Bond lengths**	**Bond angles**	**Cis Peptides**
			Avg: 0.98	Clashscore: 1.52	Outliers: 7 of 904	Poor rotamers: 0 of 769	Outliers: 0 of 827	Outliers: 17 of 902	Outliers: 14 of 906	Outliers: 17 of 906	Non-Trans: 0 of 905
A 161		THR	0.67	-	Favored (96.93%) General / -62.4,-44.2	Favored (52.9%) *m* chi angles: 294.9	0.04Å	Favored (92.222%) alpha helix	-	-	-
A 162		ALA	0.66	-	Favored (85.75%) General / -60.2,-39.2	-	0.04Å	Favored (92.811%) alpha helix	-	-	-
A 163		ARG	0.66	-	Favored (93.11%) General / -61.3,-40.2	Favored (73.4%) *ttt180* chi angles: 187.6,180.1,180.8,191.9	0.03Å	Favored (95.756%) alpha helix	-	-	-
A 164		VAL	0.66	-	Favored (98.03%) Ile or Val / -63.9,-44.5	Favored (75.2%) *t* chi angles: 172.7	0.05Å	Favored (94.73%) alpha helix	-	-	-
A 165		LEU	0.66	-	Favored (89.15%) General / -62.3,-38.4	Favored (87.4%) *mt* chi angles: 290.9,173.5	0.07Å	Favored (86.52%) alpha helix	-	-	-
A 166		ASP	0.67	0.42Å OD1 with A 194 LYS NZ	Favored (97.82%) General / -62.9,-40.5	Favored (90.1%) *m-30* chi angles: 285,344.6	0.03Å	Favored (97.863%) alpha helix	-	-	-
A 167		MET	0.67	-	Favored (78.09%) General / -59.7,-49.3	Favored (49.6%) *ttp* chi angles: 176.3,188.5,67.1	0.07Å	Favored (88.019%) alpha helix	-	-	-

#	Alt	Res	High B	Clash > 0.4Å	Ramachandran	Rotamer	Cβ deviation	CaBLAM	Bond lengths	Bond angles	Cis Peptides
A 168		VAL	0.67	-	Favored (89.9%) Ile or Val / -63.5,-40.7	Favored (65.9%) *t* chi angles: 171.6	0.05Å	Favored (71.942%) alpha helix	-	-	-
A 169		GLU	0.67	-	Favored (65.5%) General / -53.2,-41.8	Favored (90.5%) *tt0* chi angles: 186.3,177,2.2	0.04Å	Favored (70.311%) alpha helix	-	-	-
A 170		GLU	0.67	-	Favored (84.56%) General / -57.8,-42.6	Favored (69.4%) *tt0* chi angles: 183.2,186,341.3	0.04Å	Favored (95.498%) alpha helix	-	-	-
A 171		TRP	0.67	-	Favored (87.64%) General / -64.0,-37.5	Favored (69.5%) *m100* chi angles: 283.2,88.4	0.03Å	Favored (91.165%) alpha helix	-	-	-
A 172		LEU	0.67	-	Favored (82.91%) General / -68.0,-38.5	Favored (97.7%) *mt* chi angles: 293.9,172.6	0.07Å	Favored (80.694%) alpha helix	-	-	-
A 173		LYS	0.67	-	Favored (69.15%) General / -63.2,-27.1	Favored (97%) *mttt* chi angles: 288,180.1,176.5,177.8	0.06Å	Favored (71.993%)	-	-	-
A 174		LYS	0.67	-	Favored (45.74%) General / -78.9,-3.6	Favored (73.3%) *mmtt* chi angles: 295.4,296.2,183.8,180.7	0.07Å	Favored (17.406%)	-	-	-
A 175		GLY	0.67	-	Favored (17.86%) Glycine / 116.2,4.8	-	-	Favored (62.183%) beta sheet	-	-	-
A 176		ALA	0.66	-	Favored (56.5%) General / -60.2,141.1	-	0.04Å	Favored (19.466%)	-	-	-
A 177		THR	0.66	-	Favored (3.82%) General / -98.8,-51.6	Favored (95.4%) *m* chi angles: 299.5	0.05Å	Favored (7.894%)	-	-	-
A 178		GLU	0.65	-	Favored (19.76%) General / -105.8,155.0	Favored (78.5%) *mm-30* chi angles: 301,290.6,335.9	0.01Å	CaBLAM Disfavored (2.245%)	-	-	-
A 179		PHE	0.65	-	Favored (36.69%) General / -159.0,161.4	Favored (46.4%) *p90* chi angles: 58.8,85.2	0.06Å	Favored (47.312%)	-	-	-
A 180		CYS	0.64	-	Favored (2.99%) General / -151.4,103.3	Favored (57.1%) *t* chi angles: 183.1	0.12Å	Favored (17.651%) beta sheet	-	-	-
**#**	**Alt**	**Res**	**High B**	**Clash > 0.4Å**	**Ramachandran**	**Rotamer**	**Cβ deviation**	**CaBLAM**	**Bond lengths**	**Bond angles**	**Cis Peptides**
			Avg: 0.98	Clashscore: 1.52	Outliers: 7 of 904	Poor rotamers: 0 of 769	Outliers: 0 of 827	Outliers: 17 of 902	Outliers: 14 of 906	Outliers: 17 of 906	Non-Trans: 0 of 905
A 181		ILE	0.65	-	Favored (69.95%) Ile or Val / -112.5,126.7	Favored (28.2%) *mm* chi angles: 311.3,301.3	0.11Å	Favored (54.607%) beta sheet	-	-	-
A 182		LYS	0.65	-	Favored (42.79%) General / -74.2,134.7	Favored (28.7%) *ttpt* chi angles: 189.2,171.7,79.8,183.6	0.06Å	Favored (44.26%) beta sheet	-	-	-
A 183		VAL	0.67	-	Favored (72.13%) Ile or Val / -116.5,124.0	Favored (85.5%) *t* chi angles: 177.2	0.10Å	Favored (72.396%)	-	-	-

A<br>184		LEU	0.7	-	Favored<br>(52.35%)<br>General /<br>-112.6,124.3	Favored (73.4%) *tp*<br>chi angles: 177.6,61.2	0.10Å	Favored<br>(12.26%)	-	-	-
A<br>185		CYS	0.73	-	Allowed<br>(0.36%)<br>Pre-Pro / 70.8,51.7	Favored (77.9%) *m*<br>chi angles: 296.3	0.08Å	CaBLAM<br>Disfavored<br>(4.865%)	-	-	-
A<br>186		PRO	0.77	-	Favored<br>(52.44%)<br>Trans-Pro /<br>-61.6,-17.1	Favored (39.4%)<br>*Cg_endo*<br>chi angles:<br>23.3,325.2,31.9	0.07Å	Favored<br>(35.307%)	-	-	-
A<br>187		TYR	0.81	-	Favored<br>(61.96%)<br>General /<br>-71.9,-19.3	Favored (40%) *p90*<br>chi angles: 62.6,82.5	0.03Å	Favored<br>(33.886%)	-	-	-
A<br>188		THR	0.84	-	Favored<br>(85.34%)<br>Pre-Pro /<br>-72.0,142.6	Favored (70.3%) *p*<br>chi angles: 59.4	0.11Å	Favored<br>(36.389%)	-	-	-
A<br>189		PRO	0.87	-	Favored<br>(30.48%)<br>Trans-Pro /<br>-49.7,-33.0	Favored (87.3%)<br>*Cg_exo*<br>chi angles:<br>330.2,37,331.8	0.02Å	Favored<br>(88.33%)	-	-	-
A<br>190		LYS	0.87	-	Favored<br>(64.65%)<br>General /<br>-70.0,-46.6	Favored (88.3%)<br>*tttt*<br>chi angles:<br>183.8,174.9,182.2,179.6	0.02Å	Favored<br>(72.817%)<br>alpha helix	-	-	-
A<br>191		ILE	0.86	-	Favored<br>(77.41%)<br>Ile or Val /<br>-70.3,-40.0	Favored (99.5%) *mt*<br>chi angles: 292.7,167.5	0.12Å	Favored<br>(73.302%)<br>alpha helix	-	-	-
A<br>192		ILE	0.84	-	Favored<br>(83.75%)<br>Ile or Val /<br>-58.1,-48.7	Favored (97.6%) *mt*<br>chi angles: 292.3,168.4	0.09Å	Favored<br>(86.35%)<br>alpha helix	-	-	-
A<br>193		GLU	0.81	-	Favored<br>(94.7%)<br>General /<br>-60.3,-42.1	Favored (92.8%) *tt0*<br>chi angles:<br>180.5,177.6,356.8	0.03Å	Favored<br>(90.591%)<br>alpha helix	-	-	-
A<br>194		LYS	0.78	0.42Å<br>NZ with A<br>166 ASP<br>OD1	Favored<br>(88.46%)<br>General /<br>-63.1,-46.2	Favored (54%) *tptt*<br>chi angles:<br>181.7,68.2,174.8,184.6	0.05Å	Favored<br>(88.236%)<br>alpha helix	-	-	-
A<br>195		LEU	0.76	-	Favored<br>(88.47%)<br>General /<br>-66.5,-39.0	Favored (78.4%) *mt*<br>chi angles: 288.6,172.3	0.05Å	Favored<br>(81.286%)<br>alpha helix	-	-	-
A<br>196		GLU	0.74	-	Favored<br>(92.03%)<br>General /<br>-59.1,-45.0	Favored (88.1%) *tt0*<br>chi angles:<br>178.7,179.5,352.7	0.05Å	Favored<br>(84.752%)<br>alpha helix	-	-	-
A<br>197		LYS	0.73	-	Favored<br>(98.08%)<br>General /<br>-63.7,-41.5	Favored (97.5%)<br>*mttt*<br>chi angles:<br>289.1,178,178.7,177.2	0.02Å	Favored<br>(92.757%)<br>alpha helix	-	-	-
A<br>198		LEU	0.73	-	Favored<br>(84.89%)<br>General /<br>-66.2,-37.1	Favored (93.6%) *mt*<br>chi angles: 291.9,170.8	0.02Å	Favored<br>(90.739%)<br>alpha helix	-	-	-
A<br>199		GLN	0.73	-	Favored<br>(96.29%)<br>General /<br>-64.7,-41.1	Favored (44%) *mt0*<br>chi angles:<br>290,179.9,262	0.09Å	Favored<br>(89.692%)<br>alpha helix	-	-	-
A<br>200		ARG	0.73	-	Favored<br>(78.78%)<br>General /<br>-61.7,-35.7	Favored (97%) *mtt-85*<br>chi angles:<br>288.4,178.7,181.9,276.8	0.04Å	Favored<br>(74.657%)<br>alpha helix	-	-	-

#	Alt	Res	High B	Clash > 0.4Å	Ramachandran	Rotamer	Cβ deviation	CaBLAM	Bond lengths	Bond angles	Cis Peptides
			Avg: 0.98	Clashscore: 1.52	Outliers: 7 of 904	Poor rotamers: 0 of 769	Outliers: 0 of 827	Outliers: 17 of 902	Outliers: 14 of 906	Outliers: 17 of 906	Non-Trans: 0 of 905
A 201		LYS	0.73	-	Favored (46.67%) General / -78.0,-37.7	Favored (98.4%) *mttt* chi angles: 290.5,175.4,178.6,176.3	0.06Å	Favored (59.473%) alpha helix	-	-	-
A 202		TYR	0.71	-	Favored (12.14%) General / -115.6,-8.0	Favored (63.5%) *m-80* chi angles: 302.4,112.4	0.12Å	Favored (36.667%)	-	-	-
A 203		GLY	0.7	-	Favored (88.23%) Glycine / 83.7,5.3	-	-	Favored (72.193%)	-	-	-
A 204		GLY	0.68	-	Favored (32.77%) Glycine / -94.5,-157.5	-	-	Favored (13.522%)	-	-	-
A 205		GLY	0.67	-	Favored (47.84%) Glycine / -176.5,176.5	-	-	Favored (9.249%)	-	-	-
A 206		LEU	0.67	-	Favored (33.26%) General / -91.5,136.2	Favored (78.7%) *mt* chi angles: 293.4,178.8	0.07Å	Favored (9.07%)	-	-	-
A 207		VAL	0.67	-	Favored (68.21%) Ile or Val / -128.8,129.1	Favored (65.5%) *t* chi angles: 179.3	0.09Å	Favored (62.653%) beta sheet	-	-	-
A 208		ARG	0.7	-	Favored (40.18%) General / -94.4,127.3	Favored (30.8%) *tpt170* chi angles: 177.3,74.7,175.6,180.4	0.03Å	Favored (56.176%) beta sheet	-	-	-
A 209		VAL	0.74	-	Favored (30.09%) Pre-Pro / -94.5,131.8	Favored (66.8%) *t* chi angles: 179.1	0.08Å	Favored (45.59%)	-	-	-
A 210		PRO	0.79	-	Favored (26.91%) Trans-Pro / -54.4,-22.3	Favored (98.2%) *Cg_exo* chi angles: 332.6,34.9,332.3	0.07Å	Favored (65.997%)	-	-	-
A 211		LEU	0.86	-	Favored (57.32%) General / -83.6,-3.0	Favored (90.2%) *mt* chi angles: 295.9,171.6	0.06Å	Favored (39.155%)	-	-	-
A 212		SER	0.94	-	Favored (31.72%) General / -79.0,151.2	Favored (63.6%) *m* chi angles: 294	0.03Å	Favored (35.768%)	-	-	-
A 213		ARG	0.99	-	Favored (21.44%) General / -83.7,159.9	Favored (99.4%) *mtt180* chi angles: 292.9,175.8,176.7,174.6	0.02Å	Favored (48.625%)	-	-	-
A 214		ASN	1.01	-	Favored (2.41%) General / -91.2,25.7	Favored (88.7%) *m-40* chi angles: 295.6,326.3	0.10Å	CaBLAM Outlier (0.787%)	-	OUTLIER(S) worst is CA-CB-CG: 4.0 σ	-
A 215		SER	0.98	-	Favored (12.95%) General / -111.7,-17.7	Favored (77.8%) *p* chi angles: 60.5	0.12Å	Favored (8.417%) alpha helix	-	-	-
A 216		THR	0.92	-	Favored (35.16%) General / -127.5,123.1	Favored (82.4%) *m* chi angles: 302.2	0.14Å	Favored (21.982%)	-	-	-

A 217		HIS	0.84	-	Favored (10.47%) General / -84.2,73.1	Favored (32.8%) *t-90* chi angles: 201.4,295.2	0.06Å	Favored (25.042%)	-	-	-
A 218		GLU	0.75	-	Favored (33.5%) General / -108.4,146.1	Favored (13.8%) *pt0* chi angles: 60.9,178.8,31.6	0.03Å	Favored (10.742%) beta sheet	-	-	-
A 219		MET	0.69	-	Favored (43.27%) General / -142.6,158.8	Favored (83.1%) *mtp* chi angles: 298.7,184.2,77	0.02Å	Favored (45.411%) beta sheet	-	-	-
A 220		TYR	0.65	-	Favored (37.53%) General / -106.1,141.4	Favored (92.3%) *m-80* chi angles: 291.8,87	0.04Å	Favored (45.05%) beta sheet	-	-	-
**#**	**Alt**	**Res**	**High B**	**Clash > 0.4Å**	**Ramachandran**	**Rotamer**	**Cβ deviation**	**CaBLAM**	**Bond lengths**	**Bond angles**	**Cis Peptides**
			Avg: 0.98	Clashscore: 1.52	Outliers: 7 of 904	Poor rotamers: 0 of 769	Outliers: 0 of 827	Outliers: 17 of 902	Outliers: 14 of 906	Outliers: 17 of 906	Non-Trans: 0 of 905
A 221		TRP	0.64	-	Favored (24.91%) General / -95.5,113.3	Favored (22.6%) *t60* chi angles: 179.2,44.8	0.03Å	Favored (61.004%) beta sheet	-	-	-
A 222		VAL	0.66	0.41Å HG11 with A 70 PHE HB3	Favored (58.24%) Ile or Val / -127.6,137.2	Favored (54.8%) *t* chi angles: 180.6	0.09Å	Favored (59.445%)	OUTLIER(S) worst is CB--CG1: 4.3 σ	-	-
A 223		SER	0.69	-	Favored (66.37%) General / -59.6,-27.5	Favored (69%) *p* chi angles: 58.8	0.05Å	Favored (38.007%)	-	-	-
A 224		GLY	0.74	-	Favored (79.78%) Glycine / -91.4,4.1	-	-	Favored (62.355%)	-	-	-
A 225		ALA	0.79	-	Favored (14.65%) General / -96.9,159.9	-	0.04Å	Favored (14.776%)	-	-	-
A 226		ALA	0.83	-	Favored (40.05%) General / -156.5,161.0	-	0.02Å	Favored (7.394%)	-	-	-
A 227		GLY	0.86	-	Favored (11.37%) Glycine / 128.1,163.6	-	-	Favored (18.976%) beta sheet	-	-	-
A 228		ASN	0.88	-	Favored (32.04%) General / -67.1,127.3	Favored (27.2%) *t0* chi angles: 183.4,275.8	0.04Å	Favored (7.665%)	-	-	-
A 229		ILE	0.89	-	Favored (45.09%) Ile or Val / -62.8,-28.5	Favored (20.7%) *tt* chi angles: 193.9,166.9	0.04Å	Favored (46.473%)	-	-	-
A 230		ILE	0.89	-	Favored (55.25%) Ile or Val / -73.5,-43.5	Favored (46.5%) *mm* chi angles: 299.5,300.8	0.03Å	Favored (73.362%) alpha helix	-	-	-
A 231		HIS	0.88	-	Favored (68.92%) General / -63.0,-50.9	Favored (84.7%) *t70* chi angles: 183.5,74.6	0.01Å	Favored (78.731%) alpha helix	-	-	-
A 232		ALA	0.88	-	Favored (83%) General / -58.1,-41.2	-	0.06Å	Favored (75.004%) alpha helix	-	-	-

A 233		VAL	0.88	-	Favored (69.6%) Ile or Val / -71.8,-42.9	Favored (80.2%) *t* chi angles: 173.2	0.05Å	Favored (79.427%) alpha helix	-	-	-
A 234		SER	0.88	-	Favored (99.8%) General / -62.5,-42.9	Favored (71.3%) *m* chi angles: 296.1	0.05Å	Favored (97.309%) alpha helix	-	-	-
A 235		MET	0.88	-	Favored (86.17%) General / -66.8,-38.2	Favored (50.8%) *mmp* chi angles: 294.6,301.7,98.4	0.05Å	Favored (97.247%) alpha helix	-	-	-
A 236		THR	0.88	-	Favored (93.8%) General / -62.5,-45.3	Favored (88.4%) *m* chi angles: 298.5	0.04Å	Favored (96.613%) alpha helix	-	-	-
A 237		SER	0.87	-	Favored (96.95%) General / -63.9,-40.6	Favored (62.1%) *m* chi angles: 298.4	0.05Å	Favored (89.593%) alpha helix	-	-	-
A 238		GLN	0.87	-	Favored (92.72%) General / -62.5,-39.2	Favored (92.2%) *mt0* chi angles: 289.5,177.8,345.3	0.05Å	Favored (86.035%) alpha helix	-	-	-
A 239		VAL	0.88	-	Favored (93.15%) Ile or Val / -64.8,-46.1	Favored (61.4%) *t* chi angles: 171	0.02Å	Favored (88.075%) alpha helix	-	-	-
A 240		LEU	0.88	-	Favored (92.68%) General / -64.2,-38.9	Favored (97.3%) *mt* chi angles: 293.2,173.2	0.06Å	Favored (82.86%) alpha helix	-	-	-
**#**	**Alt**	**Res**	**High B**	**Clash > 0.4Å**	**Ramachandran**	**Rotamer**	**Cβ deviation**	**CaBLAM**	**Bond lengths**	**Bond angles**	**Cis Peptides**
			Avg: 0.98	Clashscore: 1.52	Outliers: 7 of 904	Poor rotamers: 0 of 769	Outliers: 0 of 827	Outliers: 17 of 902	Outliers: 14 of 906	Outliers: 17 of 906	Non-Trans: 0 of 905
A 241		MET	0.9	-	Favored (86.49%) General / -67.1,-41.3	Favored (99.5%) *mtp* chi angles: 291.7,172.3,71.5	0.05Å	Favored (75.637%) alpha helix	-	-	-
A 242		GLY	0.96	-	Favored (86.14%) Glycine / -56.2,-41.2	-	-	Favored (92.544%) alpha helix	-	-	-
A 243		ARG	1.06	-	Favored (80.28%) General / -61.7,-36.2	Favored (98.3%) *mtt180* chi angles: 289.3,176.7,181.4,177.8	0.06Å	Favored (77.526%) alpha helix	-	-	-
A 244		MET	1.24	-	Favored (64.79%) General / -68.1,-19.1	Favored (84%) *mtp* chi angles: 292.5,187,72.7	0.10Å	Favored (69.194%) alpha helix	-	-	-
A 245		ASP	1.49	-	Favored (19.46%) General / -102.0,18.9	Favored (65.4%) *m-30* chi angles: 290.8,318.4	0.05Å	Favored (41.96%)	-	-	-
A 246		LYS	1.78	-	Allowed (0.84%) General / -77.2,14.8	Favored (71.5%) *mmtt* chi angles: 293.8,293,183.3,180.5	0.09Å	CaBLAM Outlier (0.49%)	-	-	-
A 247		GLN	2.02	-	OUTLIER (0.04)%) General / 56.5,-93.2	Favored (88.1%) *mm-40* chi angles: 304.1,301.9,299.4	0.04Å	CaBLAM Outlier (0.242%)	-	-	-

A 248		ASN	2.13	-	Favored (67.21%) General / -61.8,-24.9	Favored (99.5%) *m-40* chi angles: 287.7,340.6	0.03Å	Favored (27.958%)	-	-	-
A 249		ARG	2.04	-	Allowed (0.62%) General / 62.0,69.7	Favored (87.1%) *mtm-85* chi angles: 301.1,184,300.3,278.1	0.06Å	CaBLAM Outlier (0.674%)	-	-	-
A 250		SER	1.8	-	Favored (18.6%) General / -54.3,145.1	Favored (34.1%) *t* chi angles: 174.3	0.06Å	Favored (28.072%)	-	-	-
A 251		GLY	1.5	-	Favored (31.85%) Glycine / -74.8,-173.6	-	-	Favored (31.123%)	-	-	-
A 252		PRO	1.24	-	Favored (43.44%) Trans-Pro / -74.9,156.7	Favored (76.1%) *Cg_endo* chi angles: 28.7,326.5,24.2	0.04Å	Favored (50.694%)	-	-	-
A 253		ARG	1.04	-	Favored (17.47%) General / -91.1,157.0	Favored (76.4%) *mtm180* chi angles: 292.6,173.3,285.3,162.1	0.05Å	Favored (43.288%) beta sheet	-	-	-
A 254		TYR	0.91	-	Favored (40.11%) General / -119.6,150.6	Favored (80.9%) *m-80* chi angles: 289.5,85.7	0.12Å	Favored (57.868%) beta sheet	-	-	-
A 255		GLU	0.83	-	Favored (38.29%) General / -138.7,139.3	Favored (89.6%) *tt0* chi angles: 187,174.9,1.1	0.05Å	Favored (43.638%)	-	-	-
A 256		GLU	0.79	-	Favored (52.81%) General / -64.8,147.6	Favored (91.9%) *mt-10* chi angles: 293,182.9,8.3	0.02Å	Favored (40.791%)	-	-	-
A 257		ASP	0.76	-	Favored (35.01%) General / -60.8,149.7	Favored (2.5%) *m-30* chi angles: 287.2,260.2	0.05Å	Favored (39.813%)	OUTLIER(S) worst is CB--CG: 4.1 σ	-	-
A 258		VAL	0.75	-	Allowed (0.75%) Ile or Val / -76.6,84.0	Favored (51.7%) *t* chi angles: 181.2	0.13Å	Favored (32.971%) beta sheet	-	-	-
A 259		ASN	0.74	-	Favored (58.11%) General / -60.2,136.7	Favored (94.2%) *m-40* chi angles: 291.1,330.7	0.10Å	Favored (15.154%) beta sheet	-	-	-
A 260		LEU	0.74	-	Favored (18.2%) General / -107.3,157.1	Favored (66.5%) *mt* chi angles: 304.3,178.1	0.04Å	Favored (38.099%) beta sheet	-	-	-
**#**	**Alt**	**Res**	**High B**	**Clash > 0.4Å**	**Ramachandran**	**Rotamer**	**Cβ deviation**	**CaBLAM**	**Bond lengths**	**Bond angles**	**Cis Peptides**
			Avg: 0.98	Clashscore: 1.52	Outliers: 7 of 904	Poor rotamers: 0 of 769	Outliers: 0 of 827	Outliers: 17 of 902	Outliers: 14 of 906	Outliers: 17 of 906	Non-Trans: 0 of 905
A 261		GLY	0.73	-	Favored (41.11%) Glycine / -78.1,-173.3	-	-	Favored (37.074%)	-	-	-
A 262		SER	0.73	-	Favored (3.03%) General / -132.8,-172.9	Favored (10.1%) *t* chi angles: 190.7	0.07Å	CaBLAM Disfavored (2.957%)	-	-	-

A 263		GLY	0.75	-	Favored (33.58%) Glycine / 79.0,-150.1	-	-	Favored (31.12%)	-		-		-
A 264		THR	0.79	-	Favored (7.88%) General / -125.2,174.8	Favored (66.1%) *p* chi angles: 63	0.07Å	Favored (9.878%) beta sheet	-		-		-
A 265		ARG	0.86	-	Favored (51.22%) General / -132.9,145.8	Favored (54.6%) *mtp180* chi angles: 287.5,181.6,64.8,213.5	0.12Å	Favored (36.234%) beta sheet	-		-		-
A 266		SER	0.97	-	Favored (16.9%) General / -79.3,170.2	Favored (87.7%) *p* chi angles: 67	0.05Å	Favored (15.925%) beta sheet	-		-		-
A 267		VAL	1.11	-	Favored (19.07%) Ile or Val / -123.6,166.2	Favored (28.5%) *m* chi angles: 298.5	0.03Å	Favored (35.855%)	-		-		-
A 268		GLY	1.24	-	Favored (49.1%) Glycine / -85.4,14.8	-	-	Favored (10.902%)	-		-		-
A 269		LYS	1.34	-	Favored (41.92%) General / -74.9,146.4	Favored (73.4%) *mmtt* chi angles: 299.3,294.3,185.3,181.2	0.01Å	Favored (41.071%)	-		-		-
A 270		LEU	1.38	-	Favored (60.12%) General / -63.4,-52.8	Favored (71.4%) *tp* chi angles: 176.5,62.2	0.05Å	CaBLAM Disfavored (2.563%)	-		-		-
A 271		THR	1.33	-	OUTLIER (0.02)%) General / 63.5,121.1	Favored (81.4%) *m* chi angles: 302.4	0.09Å	CaBLAM Disfavored (1.129%)	-		-		-
A 272		GLU	1.25	-	Favored (57.93%) General / -60.4,138.6	Favored (63.2%) *mp0* chi angles: 294.5,87.4,355.5	0.01Å	Favored (43.763%)	-		-		-
A 273		LYS	1.15	-	Favored (66.9%) Pre-Pro / -82.4,128.5	Favored (87.4%) *tttt* chi angles: 184,177.4,179.5,179	0.03Å	Favored (43.289%)	-		-		-
A 274		PRO	1.07	-	Favored (56.6%) Trans-Pro / -72.0,156.0	Favored (74.4%) *Cg_endo* chi angles: 27.8,324.7,28.1	0.05Å	Favored (58.378%)	-		-		-
A 275		ASP	1.02	-	Favored (35.77%) Pre-Pro / -81.2,112.8	Favored (54.1%) *t0* chi angles: 185.1,334.9	0.03Å	Favored (34.696%)	-		-		-
A 276		PRO	0.98	-	Favored (56.92%) Trans-Pro / -65.3,-16.9	Favored (56.9%) *Cg_endo* chi angles: 26,326.2,27.4	0.03Å	Favored (63.583%)	-		-		-
A 277		ARG	0.95	-	Favored (59.91%) General / -77.1,-13.7	Favored (92%) *mtm180* chi angles: 295.7,174.1,296.7,175.3	0.04Å	Favored (55.828%) alpha helix	-		-		-
A 278		LYS	0.92	-	Favored (9.13%) General / -106.6,-30.3	Favored (60.4%) *mttm* chi angles: 293.3,177.5,181.2,287	0.02Å	Favored (46.274%) alpha helix	-		-		-
A 279		VAL	0.88	-	Favored (9.58%) Ile or Val / -111.0,-15.9	Favored (28.9%) *m* chi angles: 298.4	0.03Å	Favored (21.331%) alpha helix	-		-		-

#	Alt	Res	High B	Clash > 0.4Å	Ramachandran	Rotamer	Cβ deviation	CaBLAM	Bond lengths	Bond angles	Cis Peptides
A 280		GLY	0.84	-	Favored (86.72%) Glycine / -59.7,-35.6	-	-	Favored (62.076%) alpha helix	-	-	-
**#**	**Alt**	**Res**	**High B**	**Clash > 0.4Å**	**Ramachandran**	**Rotamer**	**Cβ deviation**	**CaBLAM**	**Bond lengths**	**Bond angles**	**Cis Peptides**
			Avg: 0.98	Clashscore: 1.52	Outliers: 7 of 904	Poor rotamers: 0 of 769	Outliers: 0 of 827	Outliers: 17 of 902	Outliers: 14 of 906	Outliers: 17 of 906	Non-Trans: 0 of 905
A 281		GLU	0.8	-	Favored (81.28%) General / -67.9,-37.0	Favored (71.7%) *mm-30* chi angles: 291,294.8,306.4	0.01Å	Favored (90.582%) alpha helix	-	-	-
A 282		ARG	0.76	-	Favored (70.88%) General / -70.8,-42.0	Favored (36.5%) *mtm180* chi angles: 282.8,178.1,278.7,153.3	0.07Å	Favored (80.592%) alpha helix	-	-	-
A 283		ILE	0.74	-	Favored (88.34%) Ile or Val / -67.2,-43.6	Favored (92.8%) *mt* chi angles: 293.6,166.1	0.11Å	Favored (86.027%) alpha helix	-	-	-
A 284		ARG	0.72	-	Favored (82.37%) General / -56.9,-45.8	Favored (83.8%) *ttp80* chi angles: 179.7,175,66.7,82.4	0.01Å	Favored (86.698%) alpha helix	-	-	-
A 285		ARG	0.72	-	Favored (90.47%) General / -61.9,-39.0	Favored (85.6%) *mtp85* chi angles: 287.6,174.3,63.4,86	0.03Å	Favored (90.74%) alpha helix	-	-	-
A 286		LEU	0.72	-	Favored (94%) General / -64.8,-39.7	Favored (93.9%) *mt* chi angles: 292.1,170.6	0.04Å	Favored (97.82%) alpha helix	-	-	-
A 287		ARG	0.72	-	Favored (97.66%) General / -64.0,-41.2	Favored (96.9%) *mtt180* chi angles: 287.7,173.3,175.8,166.4	0.04Å	Favored (88.248%) alpha helix	-	-	-
A 288		GLU	0.73	-	Favored (68.68%) General / -71.3,-42.3	Favored (98.6%) *mt-10* chi angles: 291.6,174.4,0.8	0.03Å	Favored (79.489%) alpha helix	-	-	-
A 289		GLU	0.74	-	Favored (76.84%) General / -57.7,-39.2	Favored (46%) *tp30* chi angles: 186.2,74.8,15.4	0.06Å	Favored (54.443%) alpha helix	-	-	-
A 290		TYR	0.75	-	Favored (10.26%) General / -114.9,26.3	Favored (83.9%) *m-80* chi angles: 299.7,104.7	0.06Å	Favored (14.442%) alpha helix	-	-	-
A 291		GLN	0.76	-	Favored (69.14%) General / -56.2,-37.0	Favored (92.8%) *tp40* chi angles: 187.9,66.3,56.7	0.03Å	Favored (40.126%) alpha helix	-	-	-
A 292		GLN	0.78	-	Favored (66.15%) General / -64.5,-20.3	Favored (98.3%) *mt0* chi angles: 291.5,179.5,333.3	0.02Å	Favored (44.894%) alpha helix	-	-	-
A 293		THR	0.79	-	Favored (10.99%) General / -120.9,11.2	Favored (61.7%) *p* chi angles: 57.6	0.05Å	Favored (25.687%)	-	-	-
A 294		TRP	0.81	-	Favored (32.44%) General / -84.6,138.9	Favored (34.4%) *t-100* chi angles: 191.6,272.4	0.03Å	Favored (12.051%)	-	-	-
A 295		THR	0.84	-	Favored (40.67%) General / -150.1,155.9	Favored (12.2%) *t* chi angles: 187.1	0.06Å	Favored (39.33%) beta sheet	-	-	-

A 296		TYR	0.87	-	Favored (23.83%) General / -108.5,111.8	Favored (34.9%) *t80* chi angles: 190.2,97.8	0.03Å	Favored (42.256%) beta sheet	-	-	-
A 297		ASP	0.89	-	Favored (25.15%) General / -90.8,113.1	Favored (58.3%) *t0* chi angles: 188.1,354.1	0.01Å	Favored (69.669%) beta sheet	-	-	-
A 298		HIS	0.91	-	Favored (13.7%) General / -82.0,7.2	Favored (90.8%) *m-70* chi angles: 291.3,288.5	0.01Å	Favored (13.164%)	-	-	-
A 299		ASN	0.91	-	Favored (28.4%) General / -105.3,14.8	Favored (58.3%) *m-40* chi angles: 298.9,280.4	0.09Å	Favored (7.352%)	-	-	-
A 300		ASN	0.89	-	Favored (75.43%) Pre-Pro / -57.0,127.5	Favored (25.8%) *t0* chi angles: 184.7,271.4	0.07Å	Favored (31.732%)	OUTLIER(S) worst is CB--CG: 6.8 σ	-	-
**#**	**Alt**	**Res**	**High B**	**Clash > 0.4Å**	**Ramachandran**	**Rotamer**	**Cβ deviation**	**CaBLAM**	**Bond lengths**	**Bond angles**	**Cis Peptides**
			Avg: 0.98	Clashscore: 1.52	Outliers: 7 of 904	Poor rotamers: 0 of 769	Outliers: 0 of 827	Outliers: 17 of 902	Outliers: 14 of 906	Outliers: 17 of 906	Non-Trans: 0 of 905
A 301		PRO	0.87	-	Favored (11.73%) Trans-Pro / -81.1,-2.5	Favored (43.3%) *Cg_endo* chi angles: 33.7,322.6,25.6	0.15Å	Favored (8.029%)	-	-	-
A 302		TYR	0.84	-	Favored (32.25%) General / -78.8,150.8	Favored (63.9%) *m-80* chi angles: 300.2,293.6	0.08Å	Favored (19.661%)	-	-	-
A 303		ARG	0.8	-	Allowed (0.91%) General / -118.3,-68.5	Favored (61.6%) *ttp-170* chi angles: 180.3,175.9,62.4,187.6	0.05Å	CaBLAM Disfavored (2.159%)	-	-	-
A 304		THR	0.76	-	Favored (96.95%) General / -63.7,-40.5	Favored (90.1%) *m* chi angles: 297.2	0.07Å	Favored (25.037%)	-	-	-
A 305		TRP	0.73	0.42Å CZ2 with A 601 GLN HG3	Favored (29.71%) General / -82.4,145.7	Favored (48.7%) *m100* chi angles: 292.5,69	0.08Å	Favored (17.474%)	-	-	-
A 306		ASN	0.7	-	Favored (35.1%) General / -89.6,125.1	Favored (23.6%) *t0* chi angles: 188,276.4	0.08Å	Favored (43.987%)	-	-	-
A 307		TYR	0.68	-	Favored (35.79%) General / -89.2,129.5	Favored (70.5%) *t80* chi angles: 175.2,68.8	0.07Å	Favored (50.543%)	-	-	-
A 308		HIS	0.66	-	Favored (15.51%) General / -113.5,2.9	Favored (99.2%) *m-70* chi angles: 300.2,290.1	0.10Å	CaBLAM Disfavored (2.09%)	-	-	-
A 309		GLY	0.66	-	Favored (13.54%) Glycine / 124.2,171.9	-	-	Favored (6.324%)	-	-	-
A 310		SER	0.67	-	Favored (24.79%) General / -149.4,167.9	Favored (96.6%) *p* chi angles: 65.1	0.12Å	Favored (52.835%)	-	-	-
A 311		TYR	0.71	-	Favored (43.26%) General / -145.0,155.2	Favored (51.7%) *p90* chi angles: 68.7,91.3	0.01Å	Favored (11.697%)	-	-	-

#	Alt	Res	High B	Clash > 0.4Å	Ramachandran	Rotamer	Cβ deviation	CaBLAM	Bond lengths	Bond angles	Cis Peptides
A 312		GLU	0.77	-	Favored (55.64%) General / -64.5,135.0	Favored (89.7%) *tt0* chi angles: 186.4,174.9,357	0.07Å	Favored (23.333%)	-	-	-
A 313		VAL	0.85	-	Favored (11.97%) Ile or Val / -150.4,152.1	Favored (5.2%) *p* chi angles: 69.9	0.13Å	Favored (39.816%)	-	-	-
A 314		LYS	0.96	-	Favored (96.32%) Pre-Pro / -70.0,149.3	Favored (98%) *mttt* chi angles: 292.1,181.6,179.9,179.3	0.04Å	Favored (42.358%)	-	-	-
A 315		PRO	1.09	-	Favored (81.76%) Trans-Pro / -66.9,151.7	Favored (44%) *Cg_endo* chi angles: 24.4,326.4,28.5	0.01Å	Favored (67.958%)	-	-	-
A 316		THR	1.24	-	Favored (4.74%) General / -125.0,35.6	Favored (61.2%) *p* chi angles: 57.5	0.05Å	CaBLAM Outlier (0.939%)	-	-	-
A 317		GLY	1.37	-	Favored (25.88%) Glycine / -172.9,-162.9	-	-	Favored (11.852%)	-	-	-
A 318		SER	1.46	-	Favored (28.33%) General / -148.7,146.9	Favored (41.4%) *t* chi angles: 176.2	0.09Å	Favored (18.529%)	-	-	-
A 319		ALA	1.49	-	Favored (59.02%) General / -85.8,-3.9	-	0.03Å	Favored (13.159%)	-	-	-
A 320		SER	1.44	-	Favored (19.99%) General / -156.6,171.6	Favored (89.9%) *p* chi angles: 68.8	0.02Å	Favored (21.881%)	-	-	-
**#**	**Alt**	**Res**	**High B**	**Clash > 0.4Å**	**Ramachandran**	**Rotamer**	**Cβ deviation**	**CaBLAM**	**Bond lengths**	**Bond angles**	**Cis Peptides**
			Avg: 0.98	Clashscore: 1.52	Outliers: 7 of 904	Poor rotamers: 0 of 769	Outliers: 0 of 827	Outliers: 17 of 902	Outliers: 14 of 906	Outliers: 17 of 906	Non-Trans: 0 of 905
A 321		SER	1.32	-	Favored (43.98%) General / -130.1,156.8	Favored (68.8%) *p* chi angles: 58.8	0.04Å	Favored (39.709%)	-	-	-
A 322		MET	1.18	-	Favored (98.6%) General / -61.2,-42.5	Favored (63.7%) *ttm* chi angles: 181.6,178.2,290.2	0.06Å	CaBLAM Disfavored (2.574%)	-	-	-
A 323		VAL	1.04	-	OUTLIER (0.01)%) Ile or Val / 86.8,129.2	Favored (71.8%) *t* chi angles: 178.5	0.09Å	CaBLAM Outlier (0.749%)	-	-	-
A 324		ASN	0.93	-	Favored (5.27%) General / -75.0,103.7	Favored (38.7%) *t0* chi angles: 181.3,326.2	0.07Å	Favored (25.547%)	-	-	-
A 325		GLY	0.85	-	Favored (76.2%) Glycine / -58.6,-34.4	-	-	Favored (44.738%)	-	-	-
A 326		VAL	0.81	-	Favored (84.1%) Ile or Val / -66.0,-47.7	Favored (62.5%) *t* chi angles: 171.1	0.05Å	Favored (70.917%) alpha helix	-	-	-
A 327		VAL	0.79	-	Favored (78.98%) Ile or Val / -69.8,-44.3	Favored (96.2%) *t* chi angles: 175	0.02Å	Favored (66.262%) alpha helix	-	-	-

A 328		ARG	0.8	-	Favored (68.67%) General / -53.1,-47.5	Favored (80%) *ttt180* chi angles: 179.6,178.5,183.5,177.9	0.04Å	Favored (75.941%) alpha helix	-	-	-
A 329		LEU	0.81	-	Favored (94.42%) General / -64.5,-43.3	Favored (79.3%) *mt* chi angles: 288.6,170.2	0.05Å	Favored (78.4%) alpha helix	-	-	-
A 330		LEU	0.83	-	Favored (63.04%) General / -70.8,-25.9	Favored (89.4%) *mt* chi angles: 291.1,170.3	0.06Å	Favored (75.16%)	-	-	-
A 331		SER	0.86	-	Favored (40.49%) General / -92.9,8.2	Favored (57.7%) *m* chi angles: 299.6	0.04Å	Favored (31.318%)	-	-	-
A 332		LYS	0.89	-	Allowed (0.22%) Pre-Pro / -27.1,-53.8	Favored (85.1%) *tttt* chi angles: 178.6,177.9,178.1,180.4	0.15Å	Favored (15.75%)	-	OUTLIER(S) worst is CA-C-N: 4.4 σ	-
A 333		PRO	0.93	-	Favored (45.37%) Trans-Pro / -56.1,-23.6	Favored (95.7%) *Cg_exo* chi angles: 332.8,35.9,330.8	0.09Å	Favored (66.43%) alpha helix	-	-	-
A 334		TRP	0.97	-	Favored (57.67%) General / -90.5,-3.4	Favored (42.7%) *m-90* chi angles: 289.2,266.2	0.07Å	Favored (56.272%) three-ten	-	-	-
A 335		ASP	1.03	-	Favored (61.32%) General / -71.1,-12.1	Favored (6%) *t70* chi angles: 204.5,55.9	0.01Å	Favored (56.597%) three-ten	-	-	-
A 336		MET	1.08	-	Favored (57.89%) General / -89.0,-1.0	Favored (52%) *mtt* chi angles: 300.1,176.5,193.7	0.12Å	Favored (49.741%)	-	-	-
A 337		ILE	1.11	-	Favored (69.85%) Ile or Val / -112.7,124.9	Favored (83.8%) *mt* chi angles: 299.1,169	0.06Å	Favored (33.831%)	-	-	-
A 338		THR	1.12	-	Favored (87.58%) General / -58.3,-43.0	Favored (91.8%) *m* chi angles: 299	0.03Å	Favored (46.662%)	-	-	-
A 339		ASN	1.1	-	Favored (64.37%) General / -61.2,-22.0	Favored (98.9%) *m-40* chi angles: 289.4,340.4	0.04Å	Favored (55.309%) alpha helix	-	-	-
A 340		VAL	1.07	-	Favored (12.43%) Ile or Val / -94.0,-49.8	Favored (94.4%) *t* chi angles: 174.7	0.06Å	Favored (31.942%) alpha helix	-	-	-
**#**	**Alt**	**Res**	**High B**	**Clash > 0.4Å**	**Ramachandran**	**Rotamer**	**Cβ deviation**	**CaBLAM**	**Bond lengths**	**Bond angles**	**Cis Peptides**
			Avg: 0.98	Clashscore: 1.52	Outliers: 7 of 904	Poor rotamers: 0 of 769	Outliers: 0 of 827	Outliers: 17 of 902	Outliers: 14 of 906	Outliers: 17 of 906	Non-Trans: 0 of 905
A 341		THR	1.03	-	Favored (60.27%) General / -75.5,-16.7	Favored (78%) *p* chi angles: 60.9	0.06Å	Favored (55.115%) alpha helix	-	-	-
A 342		THR	0.99	-	Favored (55.54%) General / -94.7,2.2	Favored (80.1%) *p* chi angles: 60.5	0.06Å	Favored (54.912%)	-	-	-
A 343		MET	0.97	-	Favored (37.28%) General / -79.2,137.2	Favored (15.1%) *ttt* chi angles: 185.2,159.5,185.4	0.15Å	Favored (9.669%)	-	-	-
A 344		ALA	0.97	-	Favored (37.93%)	-	0.03Å	Favored (16.484%)	-	-	-

#	Alt	Res	High B	Clash > 0.4Å	Ramachandran	Rotamer	Cβ deviation	CaBLAM	Bond lengths	Bond angles	Cis Peptides
					General / -157.8,163.5						
A 345		MET	0.96	-	Favored (54.63%) General / -59.3,141.3	Favored (58.8%) *ttp* chi angles: 184,185,71.6	0.08Å	Favored (29.158%)	-	-	-
A 346		THR	0.95	-	Favored (18.04%) General / -67.8,165.1	Favored (46.9%) *p* chi angles: 55.6	0.08Å	Favored (19.966%)	-	-	-
A 347		ASP	0.94	-	Favored (18.7%) General / -79.7,114.5	Favored (63.9%) *t0* chi angles: 187.3,340.9	0.10Å	Favored (10.128%)	-	-	-
A 348		THR	0.92	-	Favored (16.4%) General / -115.8,15.4	Favored (50.8%) *p* chi angles: 56.3	0.06Å	Favored (15.187%) beta sheet	-	-	-
A 349		THR	0.91	-	Favored (46.7%) Pre-Pro / -77.3,166.1	Favored (69.4%) *p* chi angles: 62.5	0.07Å	Favored (31.418%)	-	-	-
A 350		PRO	0.89	-	Favored (41.28%) Trans-Pro / -49.7,-36.3	Favored (86.1%) *Cg_exo* chi angles: 330.5,36.3,332.5	0.08Å	Favored (88.101%)	-	-	-
A 351		PHE	0.87	-	Favored (68.34%) General / -64.1,-50.4	Favored (90.7%) *t80* chi angles: 178.1,80.7	0.05Å	Favored (76.005%) alpha helix	-	-	-
A 352		GLY	0.87	-	Favored (44.61%) Glycine / -57.8,-53.5	-	-	Favored (91.979%) alpha helix	-	-	-
A 353		GLN	0.86	-	Favored (91.11%) General / -61.7,-39.3	Favored (85.3%) *mt0* chi angles: 291,181,36.6	0.02Å	Favored (78.358%) alpha helix	-	-	-
A 354		GLN	0.86	-	Favored (85.09%) General / -67.4,-38.7	Favored (87.2%) *tp40* chi angles: 179,60.9,55	0.04Å	Favored (97.057%) alpha helix	-	-	-
A 355		ARG	0.87	-	Favored (96.88%) General / -61.1,-44.7	Favored (77%) *ttm-80* chi angles: 182.9,174.2,289.7,278.1	0.06Å	Favored (85.128%) alpha helix	-	-	-
A 356		VAL	0.89	-	Favored (97.25%) Ile or Val / -64.2,-45.1	Favored (60.7%) *t* chi angles: 170.9	0.08Å	Favored (84.349%) alpha helix	-	-	-
A 357		PHE	0.91	-	Favored (71.73%) General / -57.5,-50.9	Favored (48.5%) *t80* chi angles: 190.6,87.9	0.08Å	Favored (74.272%) alpha helix	-	OUTLIER(S) worst is CA-CB-CG: 4.8 σ	-
A 358		LYS	0.93	-	Favored (68.58%) General / -67.2,-48.2	Favored (41.5%) *tttm* chi angles: 182.3,168.3,178.7,284.4	0.10Å	Favored (65.093%) alpha helix	-	-	-
A 359		GLU	0.95	-	Favored (82.36%) General / -66.3,-36.2	Favored (96.9%) *mt-10* chi angles: 288.7,179.4,350.3	0.06Å	Favored (48.755%) alpha helix	-	-	-
A 360		LYS	0.96	-	Favored (6.39%) General / -104.3,-39.6	Favored (30.8%) *mmmt* chi angles: 298.3,299.6,290.8,183	0.04Å	Favored (25.621%) alpha helix	-	-	-

			Avg: 0.98	Clashscore: 1.52	Outliers: 7 of 904	Poor rotamers: 0 of 769	Outliers: 0 of 827	Outliers: 17 of 902	Outliers: 14 of 906	Outliers: 17 of 906	Non-Trans: 0 of 905
A 361		VAL	0.98	-	Favored (6.4%) Ile or Val / -94.4,-35.6	Favored (53.4%) *t* chi angles: 180.9	0.06Å	Favored (57.1%)	-	-	-
A 362		ASP	0.99	-	Allowed (1.2%) General / -80.5,16.9	Favored (11.2%) *m-30* chi angles: 280.1,304.9	0.15Å	Favored (14.786%)	-	OUTLIER(S) worst is CA-CB-CG: 6.0 σ	-
A 363		THR	0.98	-	Favored (29.13%) General / -85.8,121.9	Favored (89.1%) *m* chi angles: 298.3	0.05Å	Favored (33.137%)	-	-	-
A 364		LYS	0.95	-	Favored (57.23%) General / -67.3,138.9	Favored (88.1%) *tttt* chi angles: 184.2,176,181.4,179.4	0.02Å	Favored (36.165%) beta sheet	-	-	-
A 365		ALA	0.91	-	Favored (8.94%) Pre-Pro / -97.3,101.0	-	0.07Å	Favored (44.068%) beta sheet	-	-	-
A 366		PRO	0.86	-	Favored (98.33%) Trans-Pro / -58.5,143.0	Favored (82.8%) *Cg_exo* chi angles: 334.5,34.9,330.2	0.08Å	Favored (62.832%) beta sheet	-	-	-
A 367		GLU	0.8	-	Favored (88.15%) Pre-Pro / -73.8,149.7	Favored (72.1%) *mm-30* chi angles: 295.3,294.2,311.5	0.04Å	Favored (45.632%)	-	-	-
A 368		PRO	0.74	-	Favored (63.68%) Trans-Pro / -70.0,156.1	Favored (58%) *Cg_endo* chi angles: 26.2,325.3,27.9	0.06Å	Favored (72.414%)	-	-	-
A 369		PRO	0.7	-	Favored (50.63%) Trans-Pro / -59.5,154.0	Favored (69%) *Cg_exo* chi angles: 335.4,33,332.3	0.06Å	Favored (80.505%)	-	-	-
A 370		LEU	0.67	-	Favored (65.2%) General / -52.7,-43.0	Favored (69.5%) *tp* chi angles: 178.7,61	0.04Å	Favored (65.42%)	-	-	-
A 371		GLY	0.65	-	Favored (29.82%) Glycine / -56.2,-55.4	-	-	Favored (91.011%) alpha helix	-	-	-
A 372		VAL	0.64	0.46Å HG13 with A 685 PHE CE1	Favored (62.38%) Ile or Val / -53.9,-44.8	Favored (43.1%) *t* chi angles: 168.4	0.12Å	Favored (77.322%) alpha helix	-	-	-
A 373		ALA	0.64	-	Favored (89.73%) General / -60.1,-40.5	-	0.03Å	Favored (83.687%) alpha helix	-	-	-
A 374		GLN	0.63	-	Favored (84.82%) General / -67.6,-39.6	Favored (99.3%) *mt0* chi angles: 291.4,172.7,338.3	0.01Å	Favored (97.868%) alpha helix	-	-	-
A 375		ILE	0.63	-	Favored (98.41%) Ile or Val / -63.6,-44.1	Favored (91.9%) *mt* chi angles: 294.5,166.3	0.03Å	Favored (97.042%) alpha helix	-	-	-
A 376		MET	0.63	-	Favored (88.62%) General / -64.1,-37.8	Favored (50.8%) *tpp* chi angles: 182.2,60.1,85	0.08Å	Favored (85.558%) alpha helix	-	-	-
A 377		ASP	0.64	0.46Å OD1 with A 554 LYS NZ	Favored (86.18%) General / -63.8,-37.1	Favored (25%) *t70* chi angles: 191.7,66.6	0.04Å	Favored (84.506%) alpha helix	-	-	-

#	Alt	Res	High B	Clash > 0.4Å	Ramachandran	Rotamer	Cβ deviation	CaBLAM	Bond lengths	Bond angles	Cis Peptides
A 378		VAL	0.64	-	Favored (82.62%) Ile or Val / -67.5,-46.7	Favored (66.9%) *t* chi angles: 171.7	0.02Å	Favored (78.333%) alpha helix	-	-	-
A 379		THR	0.65	-	Favored (94.26%) General / -64.7,-43.2	Favored (97.5%) *m* chi angles: 300.7	0.04Å	Favored (88.008%) alpha helix	-	-	-
A 380		THR	0.67	-	Favored (83.27%) General / -61.0,-48.1	Favored (57%) *m* chi angles: 295.3	0.10Å	Favored (96.435%) alpha helix	-	-	-
**#**	**Alt**	**Res**	**High B**	**Clash > 0.4Å**	**Ramachandran**	**Rotamer**	**Cβ deviation**	**CaBLAM**	**Bond lengths**	**Bond angles**	**Cis Peptides**
			Avg: 0.98	Clashscore: 1.52	Outliers: 7 of 904	Poor rotamers: 0 of 769	Outliers: 0 of 827	Outliers: 17 of 902	Outliers: 14 of 906	Outliers: 17 of 906	Non-Trans: 0 of 905
A 381		ASP	0.69	-	Favored (90.07%) General / -64.8,-38.2	Favored (25.9%) *t70* chi angles: 191.8,64.6	0.01Å	Favored (93.595%) alpha helix	-	-	-
A 382		TRP	0.71	-	Favored (82.66%) General / -62.6,-47.7	Favored (88.4%) *t60* chi angles: 176.8,83.1	0.06Å	Favored (94.089%) alpha helix	-	-	-
A 383		LEU	0.74	-	Favored (83.25%) General / -63.9,-36.2	Favored (22.4%) *tp* chi angles: 189.9,59.1	0.09Å	Favored (85.942%) alpha helix	-	-	-
A 384		TRP	0.78	-	Favored (99.29%) General / -62.9,-41.2	Favored (40.5%) *m100* chi angles: 281.4,120.8	0.13Å	Favored (91.653%) alpha helix	-	-	-
A 385		ASP	0.83	-	Favored (84.15%) General / -64.0,-46.5	Favored (38.4%) *t70* chi angles: 186.3,65.3	0.03Å	Favored (84.322%) alpha helix	-	-	-
A 386		PHE	0.9	-	Favored (74%) General / -55.5,-48.8	Favored (91.5%) *t80* chi angles: 179.1,77.5	0.08Å	Favored (69.47%) alpha helix	-	OUTLIER(S) worst is CA-CB-CG: 4.5 σ	-
A 387		VAL	0.98	-	Favored (26.46%) Ile or Val / -77.4,-47.4	Favored (92%) *t* chi angles: 175.9	0.05Å	Favored (52.633%) alpha helix	-	-	-
A 388		ALA	1.06	-	Favored (67.49%) General / -60.1,-28.1	-	0.03Å	Favored (54.728%)	-	-	-
A 389		ARG	1.12	-	Favored (18.65%) General / -48.6,-37.6	Favored (51.2%) *ttm170* chi angles: 185.3,178.4,300.4,171.7	0.04Å	Favored (19.952%)	-	-	-
A 390		GLU	1.16	-	Favored (8.11%) General / -108.2,-30.9	Favored (92.5%) *mt-10* chi angles: 296.1,185.2,357.6	0.05Å	Favored (18.322%)	-	-	-
A 391		LYS	1.16	-	Favored (3.16%) General / -114.6,89.7	Favored (20.7%) *mmtp* chi angles: 294.4,288.8,177.8,59.9	0.07Å	Favored (18.875%)	-	-	-
A 392		LYS	1.13	-	Favored (71.49%) Pre-Pro / -75.5,136.6	Favored (87.7%) *tttt* chi angles: 185,177.2,180.7,180.5	0.04Å	Favored (22.243%)	-	-	-
A 393		PRO	1.07	-	Favored (72.93%) Trans-Pro / -56.2,146.1	Favored (99.7%) *Cg_exo* chi angles: 332.4,35.4,332.1	0.05Å	Favored (45.667%)	-	-	-

A 394		ARG	1.01	-	Favored (35.81%) General / -147.2,163.6	Favored (53.7%) *ptt90* chi angles: 65.2,180.3,177.9,90.7	0.05Å	Favored (43.844%)	-	-	-
A 395		VAL	0.94	-	Favored (32.15%) Ile or Val / -88.4,132.9	Favored (97.4%) *t* chi angles: 175.2	0.06Å	Favored (35.586%)	-	-	-
A 396		CYS	0.89	-	Favored (25.31%) General / -91.0,144.8	Favored (73.8%) *m* chi angles: 297.3	0.05Å	Favored (40.239%) beta sheet	-	-	-
A 397		THR	0.84	-	Favored (42.35%) Pre-Pro / -89.9,159.7	Favored (78.5%) *p* chi angles: 60.8	0.10Å	Favored (47.533%)	-	-	-
A 398		PRO	0.8	-	Favored (34.27%) Trans-Pro / -50.0,-33.4	Favored (90.4%) *Cg_exo* chi angles: 329.5,37.5,331.6	0.02Å	Favored (94.708%)	-	-	-
A 399		GLU	0.77	-	Favored (67.93%) General / -71.9,-41.4	Favored (100%) *mt-10* chi angles: 292.1,179.6,355.3	0.01Å	Favored (79.329%) alpha helix	-	-	-
A 400		GLU	0.76	-	Favored (91.95%) General / -63.9,-38.7	Favored (33.6%) *mt-10* chi angles: 288.2,168.6,283.8	0.01Å	Favored (95.677%) alpha helix	-	-	-
**#**	**Alt**	**Res**	**High B**	**Clash > 0.4Å**	**Ramachandran**	**Rotamer**	**Cβ deviation**	**CaBLAM**	**Bond lengths**	**Bond angles**	**Cis Peptides**
			Avg: 0.98	Clashscore: 1.52	Outliers: 7 of 904	Poor rotamers: 0 of 769	Outliers: 0 of 827	Outliers: 17 of 902	Outliers: 14 of 906	Outliers: 17 of 906	Non-Trans: 0 of 905
A 401		PHE	0.76	-	Favored (77.79%) General / -61.7,-49.1	Favored (80.1%) *t80* chi angles: 181.4,72.8	0.03Å	Favored (87.808%) alpha helix	-	-	-
A 402		LYS	0.78	-	Favored (72.8%) General / -55.2,-42.1	Favored (87.2%) *tttt* chi angles: 181.9,174.4,179.2,178	0.01Å	Favored (85.874%) alpha helix	-	-	-
A 403		ALA	0.83	-	Favored (80.5%) General / -61.6,-36.4	-	0.02Å	Favored (91.182%) alpha helix	-	-	-
A 404		LYS	0.92	-	Favored (71.55%) General / -71.3,-39.3	Favored (50.1%) *tptt* chi angles: 187.2,69.1,174.3,182.4	0.04Å	Favored (36.323%)	-	-	-
A 405		VAL	1.03	-	Allowed (1.44%) Ile or Val / -76.5,96.6	Favored (83.8%) *t* chi angles: 177.7	0.03Å	Favored (34.548%)	-	-	-
A 406		ASN	1.15	-	Favored (28.99%) General / -56.8,145.6	Favored (20.2%) *t0* chi angles: 190.3,276.7	0.03Å	Favored (18.308%)	-	-	-
A 407		SER	1.26	-	Favored (24.9%) General / -54.6,-25.2	Favored (72.4%) *m* chi angles: 295.8	0.04Å	Favored (17.197%)	-	-	-
A 408		HIS	1.34	-	Favored (21.18%) General / -111.7,12.7	Favored (99.4%) *m-70* chi angles: 299.2,287	0.05Å	Favored (27.662%)	-	-	-

A 409		ALA	1.4	-	Favored (58.56%) General / -64.0,138.7	-	0.02Å	Favored (33.552%)	-	-	-
A 410		ALA	1.42	-	Favored (58.19%) General / -63.6,143.0	-	0.03Å	Favored (36.128%) beta sheet	-	-	-
A 411		LEU	1.41	-	Favored (29.57%) General / -112.1,115.9	Favored (70.7%) *tp* chi angles: 176.4,62.1	0.08Å	Favored (55.275%) beta sheet	-	-	-
A 412		GLY	1.38	-	Favored (37.56%) Glycine / -71.6,144.8	-	-	Favored (48.378%) beta sheet	-	-	-
A 413		ALA	1.34	-	Favored (3.72%) General / -156.2,116.7	-	0.04Å	Favored (10.18%) beta sheet	-	-	-
A 414		MET	1.29	0.56Å HB2 with A 480 TRP CZ2	Favored (6.89%) General / -125.5,-8.7	Favored (30.2%) *mtm* chi angles: 287.6,167.6,272	0.19Å	Favored (19.27%)	-	OUTLIER(S) worst is N-CA-CB: 4.6 σ	-
A 415		PHE	1.23	-	Favored (45.52%) General / -120.2,147.0	Favored (13.2%) *m-10* chi angles: 289,146.1	0.10Å	Favored (22.437%)	-	-	-
A 416		GLU	1.15	-	Favored (63.42%) General / -54.9,-34.8	Favored (68.4%) *tp30* chi angles: 184,65.4,19.6	0.03Å	Favored (57.225%)	-	-	-
A 417		GLU	1.07	-	Favored (80.68%) General / -65.2,-35.3	Favored (77.4%) *mm-30* chi angles: 294.1,297.6,306.9	0.04Å	Favored (75.95%) alpha helix	-	-	-
A 418		GLN	0.99	0.48Å HG3 with A 480 TRP CD1	Favored (51.01%) General / -84.5,-13.7	Favored (52.2%) *mm-40* chi angles: 297.1,282,316.7	0.10Å	Favored (54.566%) alpha helix	-	-	-
A 419		ASN	0.92	-	Favored (23.35%) General / -80.0,2.5	Favored (92.9%) *m-40* chi angles: 286.3,335.4	0.06Å	Favored (30.343%) three-ten	-	-	-
A 420		GLN	0.85	-	Favored (58.34%) General / -85.5,-2.6	Favored (99%) *mm-40* chi angles: 300.3,299.9,304.6	0.04Å	Favored (41.375%)	-	-	-
**#**	**Alt**	**Res**	**High B**	**Clash > 0.4Å**	**Ramachandran**	**Rotamer**	**Cβ deviation**	**CaBLAM**	**Bond lengths**	**Bond angles**	**Cis Peptides**
			Avg: 0.98	Clashscore: 1.52	Outliers: 7 of 904	Poor rotamers: 0 of 769	Outliers: 0 of 827	Outliers: 17 of 902	Outliers: 14 of 906	Outliers: 17 of 906	Non-Trans: 0 of 905
A 421		TRP	0.8	-	Favored (48.46%) General / -121.3,144.8	Favored (6%) *m-10* chi angles: 297.5,305.7	0.06Å	Favored (29.583%)	-	-	-
A 422		SER	0.76	-	Favored (43.51%) General / -86.0,-14.8	Favored (89.4%) *p* chi angles: 66.8	0.01Å	Favored (18.653%)	-	-	-
A 423		SER	0.72	-	Favored (40.1%) General / -156.3,162.0	Favored (94.2%) *p* chi angles: 66.2	0.04Å	Favored (24.23%)	-	-	-
A 424		ALA	0.7	-	Favored (85.07%) General / -60.9,-38.3	-	0.05Å	Favored (66.911%) alpha helix	-	-	-

#	Alt	Res	High B	Clash > 0.4Å	Ramachandran	Rotamer	Cβ deviation	CaBLAM	Bond lengths	Bond angles	Cis Peptides
A 425		ARG	0.69	-	Favored (90.03%) General / -60.0,-46.3	Favored (72%) *ttt180* chi angles: 175.9,178.4,167.5,182.8	0.06Å	Favored (85.506%) alpha helix	-	-	-
A 426		GLU	0.69	-	Favored (84.63%) General / -58.3,-41.6	Favored (92.7%) *tt0* chi angles: 183.2,180,1.2	0.02Å	Favored (93.543%) alpha helix	-	-	-
A 427		ALA	0.69	-	Favored (88.54%) General / -59.3,-41.3	-	0.04Å	Favored (93.833%) alpha helix	-	-	-
A 428		VAL	0.7	-	Favored (94.34%) Ile or Val / -64.7,-42.2	Favored (64.9%) *t* chi angles: 171.5	0.02Å	Favored (87.316%) alpha helix	-	-	-
A 429		GLU	0.72	-	Favored (72.41%) General / -65.8,-31.4	Favored (93.5%) *mt-10* chi angles: 290.6,184.9,353.9	0.10Å	Favored (48.058%)	-	-	-
A 430		ASP	0.73	-	Favored (35.4%) Pre-Pro / -75.7,114.3	Favored (58.5%) *t0* chi angles: 184.7,338	0.03Å	Favored (31.889%)	-	-	-
A 431		PRO	0.74	-	Favored (23.67%) Trans-Pro / -49.5,-31.2	Favored (90.4%) *Cg_exo* chi angles: 330.9,37.8,329.3	0.05Å	Favored (76.035%)	-	-	-
A 432		LYS	0.75	-	Favored (75.4%) General / -60.1,-35.5	Favored (83.9%) *tttt* chi angles: 187.1,180.1,178.6,179.9	0.05Å	Favored (63.924%) alpha helix	-	-	-
A 433		PHE	0.75	-	Favored (75.71%) General / -57.9,-49.7	Favored (78.1%) *t80* chi angles: 171.7,74.8	0.05Å	Favored (78.957%) alpha helix	-	-	-
A 434		TRP	0.74	-	Favored (73.46%) General / -66.1,-32.2	Favored (90.7%) *m100* chi angles: 289.5,109.6	0.08Å	Favored (76.365%) alpha helix	-	-	-
A 435		GLU	0.74	-	Favored (97.52%) General / -63.7,-40.7	Favored (98.6%) *mt-10* chi angles: 290.5,179.6,355.1	0.04Å	Favored (83.853%) alpha helix	-	-	-
A 436		MET	0.73	-	Favored (78.41%) General / -67.7,-35.2	Favored (83%) *mtm* chi angles: 289.8,188.8,284.6	0.08Å	Favored (84.3%) alpha helix	-	-	-
A 437		VAL	0.72	-	Favored (99.06%) Ile or Val / -61.7,-44.7	Favored (51.2%) *t* chi angles: 169.6	0.05Å	Favored (82.102%) alpha helix	-	-	-
A 438		ASP	0.72	-	Favored (92.02%) General / -60.1,-41.1	Favored (94.1%) *m-30* chi angles: 286.2,344.4	0.03Å	Favored (97.049%) alpha helix	-	-	-
A 439		GLU	0.71	-	Favored (96.29%) General / -64.2,-40.5	Favored (95.3%) *mt-10* chi angles: 289.7,182.1,354.2	0.04Å	Favored (96.811%) alpha helix	-	-	-
A 440		GLU	0.71	-	Favored (82.21%) General / -67.8,-37.7	Favored (43%) *tp30* chi angles: 189.4,69.8,29.8	0.02Å	Favored (82.831%) alpha helix	-	-	-
**#**	**Alt**	**Res**	**High B**	**Clash > 0.4Å**	**Ramachandran**	**Rotamer**	**Cβ deviation**	**CaBLAM**	**Bond lengths**	**Bond angles**	**Cis Peptides**

			Avg: 0.98	Clashscore: 1.52	Outliers: 7 of 904	Poor rotamers: 0 of 769	Outliers: 0 of 827	Outliers: 17 of 902	Outliers: 14 of 906	Outliers: 17 of 906	Non-Trans: 0 of 905
A 441		ARG	0.71	-	Favored (90.08%) General / -59.3,-45.9	Favored (50.5%) *ttm110* chi angles: 185,182.9,298.1,107.4	0.11Å	Favored (82.913%) alpha helix	-	-	-
A 442		GLU	0.71	-	Favored (91.83%) General / -62.2,-39.2	Favored (87.3%) *mt-10* chi angles: 288.8,186.9,356.6	0.06Å	Favored (88.914%) alpha helix	-	-	-
A 443		ALA	0.73	-	Favored (88.55%) General / -58.9,-42.1	-	0.06Å	Favored (88.72%) alpha helix	-	-	-
A 444		HIS	0.75	-	Favored (87.47%) General / -66.5,-42.4	Favored (39.1%) *m170* chi angles: 291.5,190.7	0.01Å	Favored (97.714%) alpha helix	-	-	-
A 445		LEU	0.78	-	Favored (68.31%) General / -65.5,-27.2	Favored (95.8%) *mt* chi angles: 292,172.9	0.05Å	Favored (74.035%) alpha helix	-	-	-
A 446		LYS	0.81	-	Favored (57.01%) General / -85.7,-1.3	Favored (98.8%) *mttt* chi angles: 293.2,174.6,182.2,175.8	0.02Å	Favored (56.244%)	-	-	-
A 447		GLY	0.83	-	Favored (54.44%) Glycine / 89.0,18.3	-	-	Favored (50.932%)	-	-	-
A 448		GLU	0.84	-	Favored (16.71%) General / -134.9,169.9	Favored (18.2%) *pt0* chi angles: 63.5,180.4,21.3	0.03Å	Favored (34.713%)	-	-	-
A 449		CYS	0.83	-	Favored (24.73%) General / -149.1,142.0	Favored (50.6%) *t* chi angles: 180.1	0.05Å	Favored (49.731%)	-	-	-
A 450		HIS	0.82	-	Favored (8.72%) General / -116.0,-23.0	Favored (98.5%) *m-70* chi angles: 299.9,292.9	0.06Å	Favored (19.082%)	-	-	-
A 451		THR	0.8	-	Favored (9.77%) General / -122.3,23.4	Favored (56.1%) *p* chi angles: 56.9	0.14Å	Favored (34.755%)	-	-	-
A 452		CYS	0.79	-	Favored (7.27%) General / -87.0,62.3	Favored (54.6%) *m* chi angles: 302.7	0.12Å	CaBLAM Disfavored (2.842%)	-	-	-
A 453		ILE	0.8	-	Favored (74.58%) Ile or Val / -123.0,130.2	Favored (3.1%) *mp* chi angles: 303.5,101.2	0.08Å	Favored (22.66%)	-	-	-
A 454		TYR	0.84	0.50Å OH with A 606 GLN NE2	Favored (29.21%) General / -103.9,146.1	Favored (88.8%) *m-80* chi angles: 294,84.1	0.14Å	Favored (48.217%) beta sheet	-	-	-
A 455		ASN	0.9	-	Favored (21.92%) General / -98.9,109.1	Favored (58.2%) *t0* chi angles: 185.5,352.1	0.04Å	Favored (56.803%) beta sheet	-	-	-
A 456		MET	0.98	-	Favored (25.08%) General / -81.6,120.4	Favored (7.8%) *tmm* chi angles: 195.2,284.5,276.1	0.06Å	Favored (43.216%) beta sheet	-	-	-

A 457		MET	1.07	-	Favored (42.77%) General / -130.7,157.4	Favored (79.5%) *mtp* chi angles: 302.2,182.1,73.1	0.04Å	Favored (36.301%) beta sheet	-	-	-
A 458		GLY	1.17	-	Favored (26.65%) Glycine / -78.9,143.5	-	-	Favored (30.376%) beta sheet	-	-	-
A 459		LYS	1.26	-	Favored (34.27%) General / -85.0,133.0	Favored (65.4%) *tttt* chi angles: 186.1,178.3,168.7,192.8	0.10Å	Favored (40.79%) beta sheet	-	-	-
A 460		ARG	1.34	-	Favored (5.56%) General / -79.5,68.4	Favored (94.4%) *mtt180* chi angles: 295.8,183.8,183.4,189.3	0.03Å	Favored (22.007%) beta sheet	-	-	-
**#**	**Alt**	**Res**	**High B**	**Clash > 0.4Å**	**Ramachandran**	**Rotamer**	**Cβ deviation**	**CaBLAM**	**Bond lengths**	**Bond angles**	**Cis Peptides**
			Avg: 0.98	Clashscore: 1.52	Outliers: 7 of 904	Poor rotamers: 0 of 769	Outliers: 0 of 827	Outliers: 17 of 902	Outliers: 14 of 906	Outliers: 17 of 906	Non-Trans: 0 of 905
A 461		GLU	1.43	-	Favored (27.03%) General / -80.4,155.6	Favored (70.3%) *mt-10* chi angles: 293,185.5,322.7	0.07Å	Favored (24.288%) beta sheet	-	-	-
A 462		LYS	1.55	-	Favored (34.68%) General / -106.5,117.7	Favored (5.7%) *mtmm* chi angles: 298.9,168.9,272.7,272.6	0.09Å	Favored (34.798%) beta sheet	-	-	-
A 463		LYS	1.71	-	Favored (48.8%) General / -134.9,146.1	Favored (97.8%) *mttt* chi angles: 296.1,184,183.7,178.3	0.06Å	Favored (42.279%) beta sheet	-	-	-
A 464		THR	1.93	-	Favored (49.19%) General / -60.7,131.0	Favored (74.8%) *m* chi angles: 302.8	0.03Å	Favored (45.055%) beta sheet	-	-	-
A 465		GLY	2.15	-	Favored (49.4%) Glycine / -76.0,178.1	-	-	Favored (29.083%)	-	-	-
A 466		GLU	2.33	-	Favored (4.47%) General / -90.3,-53.4	Favored (92.5%) *tt0* chi angles: 182.6,178.4,356.8	0.03Å	CaBLAM Disfavored (2.503%)	-	-	-
A 467		PHE	2.4	-	Favored (3.4%) General / -133.6,35.0	Favored (29.3%) *p90* chi angles: 52.9,93.4	0.04Å	CaBLAM Outlier (0.157%)	-	-	-
A 468		GLY	2.33	-	Favored (63.4%) Glycine / 96.1,-15.2	-	-	Favored (54.871%)	-	-	-
A 469		LYS	2.13	-	Favored (33.44%) General / -82.8,127.4	Favored (15.1%) *tptp* chi angles: 176.5,72.4,184.8,76.6	0.03Å	Favored (29.95%)	-	-	-
A 470		ALA	1.87	-	Favored (57.19%) General / -60.4,140.4	-	0.04Å	Favored (46.191%)	-	-	-
A 471		LYS	1.62	-	Favored (23.15%) General / -81.2,163.2	Favored (99.3%) *mttt* chi angles: 293.9,179.8,176.4,177.3	0.02Å	Favored (31.58%)	-	-	-
A 472		GLY	1.42	-	Favored (50.68%) Glycine / -65.4,147.3	-	-	Favored (30.398%)	-	-	-

A 473		SER	1.27	-	Favored (30.09%) General / -70.4,161.7	Favored (92.5%) *p* chi angles: 64.5	0.09Å	Favored (31.465%)	-	-	-
A 474		ARG	1.16	-	Favored (40.89%) General / -75.3,135.6	Favored (81.1%) *ttm-80* chi angles: 182.7,179.9,293.8,275.6	0.05Å	Favored (25.955%)	-	-	-
A 475		ALA	1.08	-	Favored (23.2%) General / -71.0,123.9	-	0.04Å	Favored (44.13%) beta sheet	-	-	-
A 476		ILE	1.01	-	Favored (70.55%) Ile or Val / -113.2,126.0	Favored (44.4%) *mm* chi angles: 304.5,298.2	0.08Å	Favored (69.437%) beta sheet	-	-	-
A 477		TRP	0.95	-	Favored (28.43%) General / -101.4,114.4	Favored (35.7%) *m-90* chi angles: 288.3,260.1	0.02Å	Favored (70.477%) beta sheet	-	-	-
A 478		TYR	0.9	-	Favored (37.08%) General / -101.4,118.5	Favored (96.2%) *m-80* chi angles: 293.2,87.4	0.06Å	Favored (69.048%)	-	-	-
A 479		MET	0.85	-	Favored (21.87%) General / -97.5,149.7	Favored (53.1%) *tpp* chi angles: 175.2,64.4,73.6	0.06Å	Favored (27.585%)	OUTLIER(S) worst is SD--CE: 4.1 σ	-	-
A 480		TRP	0.81	0.56Å CZ2 with A 414 MET HB2	Favored (32.87%) General / -58.1,146.5	Favored (18.3%) *m-90* chi angles: 290.8,281.1	0.14Å	Favored (48.274%)	OUTLIER(S) worst is CD2--CE3: 4.9 σ	-	-
**#**	**Alt**	**Res**	**High B**	**Clash > 0.4Å**	**Ramachandran**	**Rotamer**	**Cβ deviation**	**CaBLAM**	**Bond lengths**	**Bond angles**	**Cis Peptides**
			Avg: 0.98	Clashscore: 1.52	Outliers: 7 of 904	Poor rotamers: 0 of 769	Outliers: 0 of 827	Outliers: 17 of 902	Outliers: 14 of 906	Outliers: 17 of 906	Non-Trans: 0 of 905
A 481		LEU	0.77	-	Favored (68.93%) General / -60.1,-29.9	Favored (10.4%) *tp* chi angles: 198,64.8	0.05Å	Favored (57.558%)	-	-	-
A 482		GLY	0.74	-	Favored (18.36%) Glycine / -57.5,-57.7	-	-	Favored (77.807%) alpha helix	-	-	-
A 483		ALA	0.71	-	Favored (80.33%) General / -62.0,-36.1	-	0.03Å	Favored (69.661%) alpha helix	-	-	-
A 484		ARG	0.7	-	Favored (81.69%) General / -62.7,-36.1	Favored (80%) *mtp180* chi angles: 287.9,168.7,62.9,195.2	0.05Å	Favored (74.027%) alpha helix	-	-	-
A 485		PHE	0.68	-	Favored (78.94%) General / -57.8,-48.7	Favored (73.1%) *t80* chi angles: 170.6,75.3	0.14Å	Favored (82.669%) alpha helix	-	OUTLIER(S) worst is CA-CB-CG: 7.1 σ	-
A 486		LEU	0.67	-	Favored (80.73%) General / -63.8,-35.5	Favored (97.2%) *mt* chi angles: 292.4,173.3	0.09Å	Favored (84.513%) alpha helix	-	-	-
A 487		GLU	0.67	-	Favored (93.12%) General / -61.5,-40.0	Favored (97.8%) *mt-10* chi angles: 290,176.5,358.2	0.08Å	Favored (87.796%) alpha helix	-	-	-

A 488		PHE	0.68	-	Favored (68.32%) General / -67.0,-48.5	Favored (74.7%) *t80* chi angles: 184.8,79	0.03Å	Favored (78.001%) alpha helix	-	-	-
A 489		GLU	0.7	-	Favored (70.78%) General / -53.8,-45.5	Favored (92%) *tt0* chi angles: 180.8,175.9,359.8	0.04Å	Favored (62.852%) alpha helix	-	-	-
A 490		ALA	0.73	-	Favored (15.47%) General / -84.2,-43.0	-	0.08Å	Favored (35.352%) alpha helix	-	-	-
A 491		LEU	0.77	-	Favored (12.12%) General / -115.7,-7.1	Favored (78.3%) *mt* chi angles: 300.6,174.4	0.07Å	Favored (14.759%) alpha helix	-	-	-
A 492		GLY	0.82	-	Favored (58.41%) Glycine / -58.4,-25.8	-	-	Favored (38.856%) three-ten	-	-	-
A 493		PHE	0.88	-	Favored (63.29%) General / -59.8,-23.4	Favored (32.8%) *p90* chi angles: 74.1,91.4	0.02Å	Favored (61.977%) three-ten	-	-	-
A 494		LEU	0.94	-	Favored (62.51%) General / -71.0,-14.9	Favored (94.7%) *mt* chi angles: 296.1,175.4	0.04Å	Favored (37.765%) three-ten	-	-	-
A 495		ASN	0.98	-	Favored (7.02%) General / -102.1,-40.0	Favored (27.5%) *m110* chi angles: 297.4,104	0.13Å	Favored (29.728%) alpha helix	-	-	-
A 496		GLU	1	-	Favored (42.32%) General / -79.3,-34.6	Favored (72.5%) *mm-30* chi angles: 296.5,299.8,302.6	0.05Å	Favored (75.213%) alpha helix	-	-	-
A 497		ASP	1	-	Favored (56.41%) General / -85.2,-10.6	Favored (88.4%) *m-30* chi angles: 293.1,336.5	0.04Å	Favored (31.307%)	-	-	-
A 498		HIS	0.97	-	Favored (24.94%) General / 53.4,47.1	Favored (98.4%) *m-70* chi angles: 296.5,289.4	0.05Å	Favored (15.666%)	-	-	-
A 499		TRP	0.94	0.57Å CD1 with A 499 TRP H	Favored (59.43%) General / -59.4,-21.1	Favored (3%) *p-90* chi angles: 63.8,303.9	0.03Å	Favored (33.012%)	-	-	-
A 500		MET	0.9	-	Favored (41.74%) General / -88.8,5.8	Favored (72.4%) *mmm* chi angles: 299,315.2,295	0.08Å	Favored (33.441%)	-	-	-
**#**	**Alt**	**Res**	**High B**	**Clash > 0.4Å**	**Ramachandran**	**Rotamer**	**Cβ deviation**	**CaBLAM**	**Bond lengths**	**Bond angles**	**Cis Peptides**
			Avg: 0.98	Clashscore: 1.52	Outliers: 7 of 904	Poor rotamers: 0 of 769	Outliers: 0 of 827	Outliers: 17 of 902	Outliers: 14 of 906	Outliers: 17 of 906	Non-Trans: 0 of 905
A 501		SER	0.88	-	Favored (37.34%) General / -61.3,149.6	Favored (31.6%) *t* chi angles: 174	0.08Å	Favored (37.219%)	-	-	-
A 502		ARG	0.86	-	Favored (59.15%) General / -56.8,-27.1	Favored (50.9%) *ttt90* chi angles: 188.4,167.2,181.1,80.5	0.07Å	Favored (54.797%)	-	-	-
A 503		GLU	0.86	-	Favored (63.79%) General / -70.0,-25.8	Favored (99.3%) *mt-10* chi angles: 292.9,179.2,356.5	0.02Å	Favored (40.961%) alpha helix	-	-	-

A 504		ASN	0.85	-	Favored (2.96%) General / -114.1,-47.1	Favored (38.5%) *m110* chi angles: 295.7,124.9	0.03Å	Favored (14.429%) alpha helix	-	-	-
A 505		SER	0.83	-	Favored (27.68%) General / -93.5,-13.6	Favored (54.4%) *m* chi angles: 292.5	0.04Å	Favored (11.557%) alpha helix	-	-	-
A 506		TYR	0.82	-	OUTLIER (0.02)%) General / 56.1,-13.7	Favored (81.4%) *m-80* chi angles: 289,89.6	0.08Å	CaBLAM Disfavored (2.771%) try alpha helix	-	-	-
A 507		GLY	0.82	-	Favored (20.36%) Glycine / -110.7,-10.9	-	-	Favored (18.383%)	-	-	-
A 508		GLY	0.83	-	Favored (19.94%) Glycine / -96.1,151.4	-	-	Favored (8.692%)	-	-	-
A 509		VAL	0.85	-	Favored (3.47%) Ile or Val / -127.3,13.6	Favored (29.7%) *m* chi angles: 296.5	0.02Å	Favored (13.501%)	-	-	-
A 510		GLU	0.89	-	Favored (67.1%) General / -54.8,-38.3	Favored (92.1%) *tt0* chi angles: 183.7,178.7,1.3	0.04Å	Favored (46.219%)	-	-	-
A 511		GLY	0.92	-	Favored (76.36%) Glycine / -87.2,8.6	-	-	Favored (31.06%) alpha helix	-	-	-
A 512		LYS	0.93	-	Favored (4.19%) General / -102.2,-48.5	Favored (70.8%) *mmtt* chi angles: 296.4,295.3,190.1,184.7	0.08Å	CaBLAM Outlier (0.451%)	-	-	-
A 513		GLY	0.92	-	Favored (37.28%) Glycine / 93.4,164.5	-	-	Favored (39.521%)	-	-	-
A 514		LEU	0.88	-	Favored (66.6%) General / -57.3,-32.4	Favored (86.8%) *mt* chi angles: 290.5,170	0.09Å	Favored (13.554%)	-	-	-
A 515		GLN	0.83	-	Favored (53.51%) General / -86.5,0.8	Favored (28.9%) *mp10* chi angles: 295.5,83.7,35.8	0.00Å	Favored (37.406%) alpha helix	-	-	-
A 516		LYS	0.78	-	Favored (7.92%) General / -116.5,-24.2	Favored (97.2%) *mttt* chi angles: 295.6,181.3,182.3,173.7	0.05Å	Favored (11.448%) alpha helix	-	-	-
A 517		LEU	0.74	-	Favored (74.28%) General / -58.4,-36.9	Favored (82.8%) *mt* chi angles: 291,174.9	0.09Å	Favored (61.617%) alpha helix	-	-	-
A 518		GLY	0.71	-	Favored (86.03%) Glycine / -60.8,-34.2	-	-	Favored (89.586%) alpha helix	-	-	-
A 519		TYR	0.68	-	Favored (45.71%) General / -78.9,-32.4	Favored (73.6%) *m-80* chi angles: 288.9,101.2	0.06Å	Favored (81.692%) alpha helix	-	-	-
A 520		ILE	0.67	-	Favored (96.97%) Ile or Val / -64.5,-43.9	Favored (99.2%) *mt* chi angles: 292.5,167.3	0.06Å	Favored (79.98%) alpha helix	-	-	-

#	Alt	Res	High B	Clash > 0.4Å	Ramachandran	Rotamer	Cβ deviation	CaBLAM	Bond lengths	Bond angles	Cis Peptides
			Avg: 0.98	Clashscore: 1.52	Outliers: 7 of 904	Poor rotamers: 0 of 769	Outliers: 0 of 827	Outliers: 17 of 902	Outliers: 14 of 906	Outliers: 17 of 906	Non-Trans: 0 of 905
A 521		LEU	0.67	-	Favored (85.51%) General / -62.8,-37.1	Favored (96.4%) *mt* chi angles: 292.4,173.5	0.07Å	Favored (89.039%) alpha helix	-	-	-
A 522		GLN	0.67	-	Favored (98.08%) General / -63.7,-41.4	Favored (91.1%) *mt0* chi angles: 288.1,180.3,311.7	0.13Å	Favored (82.914%) alpha helix	-	-	-
A 523		GLU	0.69	-	Favored (83.44%) General / -58.2,-41.2	Favored (40%) *tt0* chi angles: 182.4,175,310.5	0.02Å	Favored (86.131%) alpha helix	-	-	-
A 524		ILE	0.72	-	Favored (96.1%) Ile or Val / -64.1,-45.6	Favored (98.1%) *mt* chi angles: 292.3,168	0.05Å	Favored (81.806%) alpha helix	-	-	-
A 525		SER	0.77	-	Favored (72.81%) General / -57.6,-37.2	Favored (34.5%) *t* chi angles: 182.2	0.04Å	Favored (74.258%) alpha helix	-	-	-
A 526		GLN	0.81	-	Favored (63.34%) General / -69.9,-17.8	Favored (98.6%) *mt0* chi angles: 292.5,173.6,339.2	0.01Å	Favored (56.691%)	-	-	-
A 527		ILE	0.84	-	Favored (75.44%) Pre-Pro / -84.1,123.5	Favored (84.8%) *mt* chi angles: 298.9,169.1	0.04Å	Favored (21.956%)	-	-	-
A 528		PRO	0.85	-	Favored (74.41%) Trans-Pro / -55.1,143.4	Favored (85.5%) *Cg_exo* chi angles: 333.8,35.4,330.9	0.05Å	Favored (10.136%)	-	-	-
A 529		GLY	0.82	-	Favored (6.16%) Glycine / 98.3,128.7	-	-	Favored (24.434%)	-	-	-
A 530		GLY	0.78	-	Favored (13.26%) Glycine / 75.1,164.8	-	-	Favored (15.52%)	-	-	-
A 531		LYS	0.74	-	Favored (22.07%) General / -84.0,156.9	Favored (50.7%) *mttp* chi angles: 294,182,184.5,71.5	0.06Å	Favored (20.684%)	-	-	-
A 532		MET	0.71	-	Favored (29.99%) General / -85.6,141.2	Favored (96%) *mtp* chi angles: 289.2,175.8,70.6	0.06Å	Favored (36.555%)	-	-	-
A 533		TYR	0.7	-	Favored (34.51%) General / -121.2,155.5	Favored (85.6%) *m-80* chi angles: 296.3,83.6	0.05Å	Favored (61.3%) beta sheet	-	-	-
A 534		ALA	0.73	-	Favored (7.39%) General / -142.7,113.3	-	0.03Å	Favored (33.362%) beta sheet	-	-	-
A 535		ASP	0.79	-	Favored (42.39%) General / -115.7,121.4	Favored (82.5%) *m-30* chi angles: 295,345.7	0.09Å	Favored (66.638%) beta sheet	-	-	-

#	Alt	Res	High B	Clash > 0.4Å	Ramachandran	Rotamer	Cβ deviation	CaBLAM	Bond lengths	Bond angles	Cis Peptides
A 536		ASP	0.85	-	Favored (17.3%) General / -107.2,106.7	Favored (66.5%) *t0* chi angles: 181.6,354.9	0.08Å	Favored (61.571%) beta sheet	-	OUTLIER(S) worst is CA-CB-CG: 4.5 σ	-
A 537		THR	0.91	-	Favored (37.94%) General / -73.0,129.4	Favored (99.6%) *m* chi angles: 300.4	0.05Å	Favored (39.712%)	-	-	-
A 538		ALA	0.94	-	Favored (79.03%) General / -60.1,-37.1	-	0.08Å	Favored (16.858%)	-	-	-
A 539		GLY	0.94	-	Allowed (1.02%) Glycine / -138.0,46.0	-	-	CaBLAM Disfavored (4.038%)	-	-	-
A 540		TRP	0.91	-	Favored (4.18%) General / -46.7,-33.2	Favored (75.3%) *p-90* chi angles: 67.7,269.5	0.04Å	Favored (19.058%)	-	-	-
**#**	**Alt**	**Res**	**High B**	**Clash > 0.4Å**	**Ramachandran**	**Rotamer**	**Cβ deviation**	**CaBLAM**	**Bond lengths**	**Bond angles**	**Cis Peptides**
			Avg: 0.98	Clashscore: 1.52	Outliers: 7 of 904	Poor rotamers: 0 of 769	Outliers: 0 of 827	Outliers: 17 of 902	Outliers: 14 of 906	Outliers: 17 of 906	Non-Trans: 0 of 905
A 541		ASP	0.86	-	Favored (56.87%) General / -87.1,-0.6	Favored (55%) *p0* chi angles: 62.3,6.6	0.07Å	Favored (37.015%) alpha helix	-	-	-
A 542		THR	0.81	-	Favored (3.66%) General / -123.0,-31.2	Favored (85.1%) *m* chi angles: 301.7	0.04Å	Favored (17.769%)	-	-	-
A 543		ARG	0.76	-	Favored (10.19%) General / -84.8,10.9	Favored (51.1%) *mmt180* chi angles: 294.7,295.1,192.2,182	0.03Å	Favored (12.101%)	-	-	-
A 544		ILE	0.73	-	Favored (34.96%) Ile or Val / -75.5,131.4	Favored (93.4%) *mt* chi angles: 296,168.9	0.06Å	Favored (28.182%)	-	-	-
A 545		THR	0.72	-	Favored (8.03%) General / -103.1,170.3	Favored (66.2%) *p* chi angles: 63	0.09Å	Favored (34.614%)	-	-	-
A 546		LYS	0.71	-	Favored (83.56%) General / -63.1,-36.4	Favored (96.8%) *mttt* chi angles: 291.5,180.2,185.1,181.1	0.04Å	Favored (58.785%)	-	-	-
A 547		GLU	0.72	-	Favored (76.9%) General / -62.0,-34.8	Favored (73.8%) *mm-30* chi angles: 289.8,295.5,309	0.04Å	Favored (77.473%) alpha helix	-	-	-
A 548		ASP	0.73	0.54Å OD2 with A 691 LYS NZ	Favored (65.24%) General / -73.6,-37.9	Favored (49.9%) *m-30* chi angles: 291.3,306.3	0.10Å	Favored (98.569%) alpha helix	-	-	-
A 549		LEU	0.76	-	Favored (81.98%) General / -65.9,-35.9	Favored (83.5%) *mt* chi angles: 291.3,175.2	0.09Å	Favored (98.284%) alpha helix	-	-	-
A 550		LYS	0.79	-	Favored (91.88%) General / -64.0,-38.7	Favored (54.8%) *mtmt* chi angles: 288.3,190.5,289.2,183.6	0.04Å	Favored (89.982%) alpha helix	-	-	-
A 551		ASN	0.82	-	Favored (81.04%) General / -64.7,-35.4	Favored (97.1%) *m-40* chi angles: 287.4,335.8	0.08Å	Favored (80.117%) alpha helix	-	-	-

A 552		GLU	0.86	-	Favored (67.11%) General / -72.9,-35.7	Favored (69.6%) *mm-30* chi angles: 291.1,297.7,345.1	0.04Å	Favored (63.433%) alpha helix	-	-	-
A 553		ALA	0.91	-	Favored (65.69%) General / -62.2,-22.2	-	0.04Å	Favored (58.446%) three-ten	-	-	-
A 554		LYS	0.96	0.46Å NZ with A 377 ASP OD1	Favored (46.02%) General / -58.1,-21.5	Favored (22.3%) *mmtp* chi angles: 293.9,293.4,181.6,65.8	0.04Å	Favored (53.803%) three-ten	-	-	-
A 555		ILE	1.01	-	Favored (35.5%) Ile or Val / -60.5,-25.7	Favored (16.4%) *tt* chi angles: 194.9,172	0.10Å	Favored (62.223%) three-ten	-	-	-
A 556		THR	1.06	-	Favored (61.2%) General / -72.8,-13.0	Favored (72.5%) *p* chi angles: 61.8	0.09Å	Favored (65.798%) three-ten	-	-	-
A 557		LYS	1.11	-	Favored (58.43%) General / -82.4,-5.1	Favored (63.1%) *mttm* chi angles: 295,180.1,179.5,294.5	0.02Å	Favored (58.011%) three-ten	-	-	-
A 558		ARG	1.16	-	Favored (31.8%) General / -105.0,4.5	Favored (10.3%) *mpt180* chi angles: 277,79.7,184.1,176.3	0.02Å	Favored (57.637%)	-	-	-
A 559		MET	1.18	-	Favored (14.67%) General / -96.0,160.0	Favored (71.8%) *mtt* chi angles: 293.6,178.3,180.3	0.02Å	Favored (29.172%)	-	-	-
A 560		GLU	1.16	-	Favored (30.47%) General / -73.1,161.6	Favored (60.9%) *mm-30* chi angles: 297.5,291.8,310	0.02Å	Favored (45.669%)	-	-	-
**#**	**Alt**	**Res**	**High B**	**Clash > 0.4Å**	**Ramachandran**	**Rotamer**	**Cβ deviation**	**CaBLAM**	**Bond lengths**	**Bond angles**	**Cis Peptides**
			Avg: 0.98	Clashscore: 1.52	Outliers: 7 of 904	Poor rotamers: 0 of 769	Outliers: 0 of 827	Outliers: 17 of 902	Outliers: 14 of 906	Outliers: 17 of 906	Non-Trans: 0 of 905
A 561		GLU	1.11	-	Favored (94.57%) General / -61.9,-40.2	Favored (50.3%) *mm-30* chi angles: 290.1,290.2,301	0.02Å	Favored (51.525%)	-	-	-
A 562		ARG	1.04	-	Favored (93.13%) General / -62.2,-45.5	Favored (68.5%) *ttt-90* chi angles: 180,182.9,179.4,277.8	0.07Å	Favored (77.819%) alpha helix	-	-	-
A 563		HIS	0.94	-	Favored (79.4%) General / -63.5,-48.0	Favored (25.2%) *t-170* chi angles: 184.7,189.3	0.06Å	Favored (80.598%) alpha helix	OUTLIER(S) worst is CB--CG: 4.4 σ	-	-
A 564		ARG	0.86	-	Favored (77.68%) General / -56.2,-47.9	Favored (82.6%) *ttp80* chi angles: 181.6,177.4,68.6,80.1	0.03Å	Favored (87.192%) alpha helix	-	-	-
A 565		LYS	0.79	-	Favored (94.91%) General / -62.9,-39.6	Favored (97.2%) *mttt* chi angles: 289.5,177.4,181.4,178.2	0.02Å	Favored (78.496%) alpha helix	-	-	-
A 566		LEU	0.73	-	Favored (68.54%) General / -69.0,-46.2	Favored (50.6%) *tp* chi angles: 183.6,60.7	0.01Å	Favored (76.863%) alpha helix	-	-	-

A 567		ALA	0.71	-	Favored (95.25%) General / -61.6,-40.6	-	0.04Å	Favored (83.559%) alpha helix	-	-	-
A 568		GLU	0.69	-	Favored (92.52%) General / -65.6,-40.3	Favored (96.7%) *mt-10* chi angles: 289.2,176.1,359.1	0.04Å	Favored (97.949%) alpha helix	-	-	-
A 569		ALA	0.69	-	Favored (94.31%) General / -61.7,-40.2	-	0.05Å	Favored (95.889%) alpha helix	-	-	-
A 570		ILE	0.7	-	Favored (81.44%) Ile or Val / -68.0,-46.6	Favored (98.1%) *mt* chi angles: 292.3,167.3	0.07Å	Favored (86.642%) alpha helix	-	-	-
A 571		ILE	0.71	-	Favored (97.77%) Ile or Val / -63.1,-42.8	Favored (97.5%) *mt* chi angles: 292.6,169.2	0.10Å	Favored (76.569%) alpha helix	-	-	-
A 572		ASP	0.72	-	Favored (69.97%) General / -71.5,-40.9	Favored (23.3%) *t70* chi angles: 193.1,63.4	0.02Å	Favored (61.96%) alpha helix	-	-	-
A 573		LEU	0.73	-	Favored (16.4%) General / -95.5,-25.0	Favored (83%) *mt* chi angles: 300.7,176.2	0.03Å	Favored (44.663%) alpha helix	-	-	-
A 574		THR	0.74	-	Favored (4.86%) General / -101.6,-46.7	Favored (86.6%) *m* chi angles: 301.5	0.03Å	Favored (31.181%) alpha helix	-	-	-
A 575		TYR	0.74	-	Favored (11.22%) General / -82.1,-48.5	Favored (21.6%) *m-80* chi angles: 291.1,69.6	0.07Å	Favored (45.643%) alpha helix	-	-	-
A 576		ARG	0.73	-	Allowed (0.79%) General / -81.8,-67.4	Favored (72.3%) *mtt180* chi angles: 291.5,175.2,189.1,151.6	0.07Å	Favored (33.198%) alpha helix	-	-	-
A 577		HIS	0.72	-	Favored (10.13%) General / -83.2,73.0	Favored (73.9%) *m-70* chi angles: 292.5,264.7	0.02Å	Favored (28.291%)	-	-	-
A 578		LYS	0.71	-	Favored (33.47%) General / -86.3,135.6	Favored (10.1%) *ttmm* chi angles: 194.2,184.9,303,299	0.04Å	Favored (18.402%)	-	-	-
A 579		VAL	0.7	-	Favored (68.62%) Ile or Val / -112.9,129.4	Favored (78.5%) *t* chi angles: 178.1	0.02Å	Favored (64.429%) beta sheet	-	-	-
A 580		VAL	0.69	-	Favored (40.88%) Ile or Val / -130.4,161.1	Favored (19.1%) *m* chi angles: 302.7	0.05Å	Favored (53.108%) beta sheet	-	-	-
**#**	**Alt**	**Res**	**High B**	**Clash > 0.4Å**	**Ramachandran**	**Rotamer**	**Cβ deviation**	**CaBLAM**	**Bond lengths**	**Bond angles**	**Cis Peptides**
			Avg: 0.98	Clashscore: 1.52	Outliers: 7 of 904	Poor rotamers: 0 of 769	Outliers: 0 of 827	Outliers: 17 of 902	Outliers: 14 of 906	Outliers: 17 of 906	Non-Trans: 0 of 905
A 581		LYS	0.71	-	Favored (55.7%) General / -114.8,133.7	Favored (46.1%) *mtpt* chi angles: 299.8,173.4,74.7,180.1	0.05Å	Favored (62.628%) beta sheet	-	-	-

A 582		VAL	0.73	-	Favored (72.48%) Ile or Val / -125.8,130.5	Favored (67.1%) *t* chi angles: 179.1	0.04Å	Favored (59.194%) beta sheet	-	-	-
A 583		MET	0.78	-	Favored (34.79%) General / -82.0,129.5	Favored (53.3%) *ttp* chi angles: 187.9,174.2,66	0.08Å	Favored (50.438%) beta sheet	-	-	-
A 584		ARG	0.86	-	Favored (26.58%) Pre-Pro / -136.6,138.5	Favored (70.2%) *ttp80* chi angles: 180.3,174.4,62.5,94.3	0.10Å	Favored (52.037%) beta sheet	-	-	-
A 585		PRO	0.94	-	Favored (44.55%) Trans-Pro / -74.0,151.0	Favored (78%) *Cg_endo* chi angles: 28.5,326.1,24.9	0.04Å	Favored (48.76%)	-	-	-
A 586		GLY	1.03	-	Favored (11.81%) Glycine / -131.5,-167.0	-	-	Favored (9.37%)	-	-	-
A 587		PRO	1.1	-	Favored (70.23%) Trans-Pro / -54.9,143.8	Favored (70.5%) *Cg_exo* chi angles: 335.3,35.8,328.3	0.01Å	Favored (7.358%)	-	-	-
A 588		ASP	1.12	-	Favored (5.11%) General / 65.1,9.0	Favored (74.5%) *m-30* chi angles: 297.1,317.9	0.04Å	CaBLAM Disfavored (1.684%)	-	-	-
A 589		GLY	1.09	-	Favored (75.79%) Glycine / 86.2,9.8	-	-	Favored (54.024%)	-	-	-
A 590		LYS	1.01	-	Favored (50.15%) General / -68.3,148.9	Favored (98.3%) *mttt* chi angles: 293,180.1,181.2,179.6	0.02Å	Favored (29.781%)	-	-	-
A 591		THR	0.93	-	Favored (54.21%) General / -113.8,135.2	Favored (98.3%) *m* chi angles: 300.1	0.07Å	Favored (56.781%) beta sheet	-	-	-
A 592		TYR	0.84	-	Favored (48.69%) General / -127.8,151.7	Favored (82.4%) *m-80* chi angles: 299.4,85.5	0.08Å	Favored (52.991%) beta sheet	-	-	-
A 593		MET	0.78	-	Favored (44.09%) General / -98.0,131.8	Favored (96%) *mmm* chi angles: 298.1,301,295.6	0.13Å	Favored (58.113%) beta sheet	-	-	-
A 594		ASP	0.74	-	Favored (34.22%) General / -101.1,141.3	Favored (74.2%) *m-30* chi angles: 291.6,323.7	0.07Å	Favored (52.808%) beta sheet	-	-	-
A 595		VAL	0.74	-	Favored (54.72%) Ile or Val / -105.9,119.1	Favored (86.9%) *t* chi angles: 177.4	0.06Å	Favored (40.634%) beta sheet	-	-	-
A 596		ILE	0.75	-	Favored (37.07%) Ile or Val / -132.8,163.7	Favored (38.2%) *pt* chi angles: 61.7,176.4	0.09Å	Favored (31.731%) beta sheet	OUTLIER(S) worst is CB--CG1: 6.3 σ	-	-
A 597		SER	0.78	-	Favored (43.65%) General / -144.6,158.7	Favored (98%) *p* chi angles: 65.8	0.02Å	Favored (63.581%) beta sheet	-	-	-
A 598		ARG	0.82	-	Favored (13.73%) General / -158.9,140.9	Favored (33%) *tpt170* chi angles: 181.2,65.9,184.4,181.1	0.06Å	Favored (44.426%)	-	-	-

#	Alt	Res	High B	Clash > 0.4Å	Ramachandran	Rotamer	Cβ deviation	CaBLAM	Bond lengths	Bond angles	Cis Peptides
A 599		GLU	0.86	-	Favored (40.86%) General / -84.2,2.2	Favored (95.1%) *mt-10* chi angles: 296,181.9,359.7	0.02Å	CaBLAM Disfavored (4.432%)	-	-	-
A 600		ASP	0.91	-	Favored (2.22%) General / -141.3,22.0	Favored (55.3%) *p0* chi angles: 61.7,1.7	0.03Å	Favored (48.295%)	-	-	-
#	Alt	Res	High B	Clash > 0.4Å	Ramachandran	Rotamer	Cβ deviation	CaBLAM	Bond lengths	Bond angles	Cis Peptides
			Avg: 0.98	Clashscore: 1.52	Outliers: 7 of 904	Poor rotamers: 0 of 769	Outliers: 0 of 827	Outliers: 17 of 902	Outliers: 14 of 906	Outliers: 17 of 906	Non-Trans: 0 of 905
A 601		GLN	0.94	0.42Å HG3 with A 305 TRP CZ2	Favored (12%) General / -105.9,164.4	Favored (76.1%) *mm-40* chi angles: 300.2,288.8,302.5	0.09Å	Favored (35.092%)	-	-	-
A 602		ARG	0.96	-	Favored (51.03%) General / -129.8,140.6	Favored (43.5%) *ttm170* chi angles: 179.9,173.6,291.8,153	0.04Å	Favored (45.549%) beta sheet	-	-	-
A 603		GLY	0.96	-	Favored (9.83%) Glycine / -86.7,130.2	-	-	Favored (43.991%)	-	-	-
A 604		SER	0.96	-	Favored (64.67%) General / -65.8,-17.5	Favored (86.7%) *p* chi angles: 67.2	0.04Å	Favored (34.567%)	-	-	-
A 605		GLY	0.94	-	Favored (72.89%) Glycine / -93.8,5.0	-	-	Favored (58.2%)	-	-	-
A 606		GLN	0.92	0.50Å NE2 with A 454 TYR OH	Favored (42.07%) General / -72.9,151.2	Favored (54.6%) *mt0* chi angles: 291,182.8,276.6	0.11Å	Favored (43.395%)	-	-	-
A 607		VAL	0.9	-	Favored (52.39%) Ile or Val / -54.4,-41.2	Favored (70%) *t* chi angles: 172.1	0.08Å	Favored (22.117%)	-	-	-
A 608		VAL	0.87	-	Favored (3.37%) Ile or Val / -110.5,25.2	Favored (23.8%) *m* chi angles: 295.1	0.05Å	Favored (21.793%) alpha helix	-	-	-
A 609		THR	0.84	-	Favored (77.17%) General / -63.1,-34.2	Favored (54%) *p* chi angles: 56.7	0.13Å	Favored (45.068%) alpha helix	-	-	-
A 610		TYR	0.81	-	Favored (60.95%) General / -75.2,-38.0	Favored (60.6%) *m-80* chi angles: 285.6,99.9	0.01Å	Favored (84.434%) alpha helix	-	-	-
A 611		ALA	0.78	-	Favored (84.91%) General / -65.7,-44.8	-	0.07Å	Favored (78.066%) alpha helix	-	-	-
A 612		LEU	0.76	-	Favored (64.6%) General / -73.9,-35.6	Favored (93.7%) *mt* chi angles: 294.6,175.2	0.05Å	Favored (78.3%) alpha helix	-	-	-
A 613		ASN	0.73	-	Favored (80.28%) General / -68.5,-41.2	Favored (58.5%) *t0* chi angles: 187.2,31	0.12Å	Favored (90.644%) alpha helix	-	-	-
A 614		THR	0.71	-	Favored (88.77%) General / -58.8,-45.9	Favored (98.9%) *m* chi angles: 300.3	0.05Å	Favored (97.4%) alpha helix	-	-	-

#	Alt	Res	High B	Clash > 0.4Å	Ramachandran	Rotamer	Cβ deviation	CaBLAM	Bond lengths	Bond angles	Cis Peptides
A 615		PHE	0.69	-	Favored (78.99%) General / -58.8,-49.1	Favored (76.9%) *t80* chi angles: 182,87.1	0.02Å	Favored (97.956%) alpha helix	-	-	-
A 616		THR	0.67	-	Favored (92.56%) General / -59.3,-44.0	Favored (49.5%) *m* chi angles: 294.5	0.05Å	Favored (96.399%) alpha helix	-	-	-
A 617		ASN	0.66	-	Favored (77.48%) General / -66.9,-34.3	Favored (67.4%) *m-40* chi angles: 286.9,280.2	0.08Å	Favored (85%) alpha helix	-	-	-
A 618		LEU	0.65	-	Favored (97.05%) General / -64.3,-41.2	Favored (66.9%) *mt* chi angles: 286.7,170.9	0.03Å	Favored (93.596%) alpha helix	-	-	-
A 619		ALA	0.64	-	Favored (96.75%) General / -62.0,-40.8	-	0.04Å	Favored (94.386%) alpha helix	-	-	-
A 620		VAL	0.63	-	Favored (91.61%) Ile or Val / -66.1,-44.9	Favored (68.5%) *t* chi angles: 171.9	0.02Å	Favored (93.696%) alpha helix	-	-	-
**#**	**Alt**	**Res**	**High B**	**Clash > 0.4Å**	**Ramachandran**	**Rotamer**	**Cβ deviation**	**CaBLAM**	**Bond lengths**	**Bond angles**	**Cis Peptides**
			Avg: 0.98	Clashscore: 1.52	Outliers: 7 of 904	Poor rotamers: 0 of 769	Outliers: 0 of 827	Outliers: 17 of 902	Outliers: 14 of 906	Outliers: 17 of 906	Non-Trans: 0 of 905
A 621		GLN	0.63	-	Favored (79.45%) General / -65.8,-35.0	Favored (27.1%) *mm110* chi angles: 297.3,289.8,103.7	0.09Å	Favored (80.91%) alpha helix	-	-	-
A 622		LEU	0.63	-	Favored (89.39%) General / -63.5,-38.0	Favored (70.2%) *mt* chi angles: 287.1,171.4	0.05Å	Favored (89.274%) alpha helix	-	-	-
A 623		ILE	0.63	-	Favored (68.47%) Ile or Val / -72.0,-41.5	Favored (90.9%) *mt* chi angles: 296.4,167	0.10Å	Favored (86.906%) alpha helix	-	-	-
A 624		ARG	0.63	-	Favored (73.25%) General / -58.3,-36.4	Favored (85.5%) *mtp180* chi angles: 290.5,175.5,69,190	0.04Å	Favored (83.883%) alpha helix	-	-	-
A 625		CYS	0.63	-	Favored (97.18%) General / -64.1,-40.9	Favored (90.5%) *m* chi angles: 291.6	0.01Å	Favored (89.282%) alpha helix	-	-	-
A 626		MET	0.63	-	Favored (76.55%) General / -64.0,-33.8	Favored (74.2%) *mtm* chi angles: 287.1,191.4,285.9	0.05Å	Favored (80.073%) alpha helix	-	-	-
A 627		GLU	0.62	-	Favored (73.22%) General / -66.2,-47.6	Favored (91.9%) *tt0* chi angles: 182,176.7,1.5	0.04Å	Favored (73.538%) alpha helix	-	-	-
A 628		ALA	0.62	-	Favored (74.97%) General / -59.1,-36.3	-	0.08Å	Favored (74.517%) alpha helix	-	-	-
A 629		GLU	0.62	-	Favored (57.08%) General / -87.1,-0.7	Favored (56.2%) *mt-10* chi angles: 294.2,179,40.5	0.05Å	Favored (57.422%)	-	-	-
A 630		GLY	0.62	-	Favored (64.48%) Glycine / 78.2,24.0	-	-	Favored (80.543%)	-	-	-

#	Alt	Res	High B	Clash > 0.4Å	Ramachandran	Rotamer	Cβ deviation	CaBLAM	Bond lengths	Bond angles	Cis Peptides
A 631		VAL	0.62	-	Favored (21.75%) Ile or Val / -80.6,-45.2	Favored (99.8%) *t* chi angles: 175.5	0.10Å	CaBLAM Disfavored (2.493%)	-	-	-
A 632		VAL	0.63	-	Favored (39.78%) Ile or Val / -127.8,156.8	Favored (30.7%) *m* chi angles: 300.3	0.06Å	Favored (12.722%)	-	-	-
A 633		ASP	0.64	-	Favored (7.02%) General / -148.2,-179.6	Favored (9.1%) *t0* chi angles: 208.1,334.3	0.08Å	Favored (6.351%)	-	OUTLIER(S) worst is CA-CB-CG: 4.9 σ	-
A 634		GLU	0.65	-	Favored (56.84%) General / -57.7,136.5	Favored (90.6%) *tt0* chi angles: 185.5,176.6,4	0.04Å	Favored (12.292%)	-	-	-
A 635		ASP	0.67	-	Favored (2.27%) General / 69.3,-1.2	Favored (71.7%) *m-30* chi angles: 293.1,318.2	0.08Å	CaBLAM Disfavored (1.241%)	-	-	-
A 636		ASP	0.69	-	Favored (21.61%) General / -85.0,-35.8	Favored (57.8%) *m-30* chi angles: 293.6,306.5	0.14Å	CaBLAM Disfavored (2.744%) try three-ten	-	OUTLIER(S) worst is CA-CB-CG: 4.0 σ	-
A 637		ILE	0.7	-	Favored (20.94%) Ile or Val / -60.9,-20.1	Favored (42.6%) *pt* chi angles: 59,172.6	0.15Å	Favored (54.403%) alpha helix	-	-	-
A 638		THR	0.72	-	Favored (8.16%) General / -90.1,-46.9	Favored (99%) *m* chi angles: 300.3	0.03Å	Favored (22.203%) alpha helix	-	-	-
A 639		ARG	0.72	-	Favored (2.62%) General / -132.3,89.1	Favored (92.2%) *mmt-90* chi angles: 294.7,290.2,182.2,269.7	0.06Å	Favored (13.446%)	-	-	-
A 640		VAL	0.72	-	Favored (38.38%) Ile or Val / -78.9,128.6	Favored (88.1%) *t* chi angles: 174	0.05Å	Favored (28.266%)	-	-	-
**#**	**Alt**	**Res**	**High B**	**Clash > 0.4Å**	**Ramachandran**	**Rotamer**	**Cβ deviation**	**CaBLAM**	**Bond lengths**	**Bond angles**	**Cis Peptides**
			Avg: 0.98	Clashscore: 1.52	Outliers: 7 of 904	Poor rotamers: 0 of 769	Outliers: 0 of 827	Outliers: 17 of 902	Outliers: 14 of 906	Outliers: 17 of 906	Non-Trans: 0 of 905
A 641		ARG	0.71	-	Favored (27.96%) General / -82.5,148.7	Favored (85.3%) *mtm180* chi angles: 293.6,170.5,285.7,170.8	0.08Å	Favored (36.902%)	-	-	-
A 642		LEU	0.7	-	Favored (72.25%) General / -61.2,-32.5	Favored (91.7%) *mt* chi angles: 292.4,175.1	0.02Å	Favored (60.655%)	-	-	-
A 643		GLY	0.68	-	Favored (69.06%) Glycine / -59.9,-30.1	-	-	Favored (85.476%) alpha helix	-	-	-
A 644		ARG	0.66	0.41Å HA with A 644 ARG NE	Favored (34.69%) General / -79.4,-38.5	Favored (13.6%) *mmp80* chi angles: 297.2,286.4,75,78	0.02Å	Favored (84.416%) alpha helix	-	-	-
A 645		LEU	0.64	-	Favored (94.38%) General / -65.0,-40.1	Favored (86.9%) *mt* chi angles: 291.4,174.6	0.04Å	Favored (94.328%) alpha helix	-	-	-
A 646		ALA	0.62	-	Favored (97.7%) General / -62.0,-41.2	-	0.03Å	Favored (97.142%) alpha helix	-	-	-

A 647		LYS	0.61	-	Favored (94.61%) General / -65.3,-41.0	Favored (81.1%) *tttt* chi angles: 190,178.2,180.3,184.5	0.03Å	Favored (93.65%) alpha helix	-	-	-
A 648		ALA	0.6	-	Favored (88.49%) General / -60.5,-39.6	-	0.01Å	Favored (93.096%) alpha helix	-	-	-
A 649		VAL	0.6	-	Favored (90.74%) Ile or Val / -64.8,-46.9	Favored (63.2%) *t* chi angles: 171.2	0.01Å	Favored (94.871%) alpha helix	-	-	-
A 650		GLU	0.6	-	Favored (93.3%) General / -59.5,-44.1	Favored (92.5%) *tt0* chi angles: 182.2,178.5,2.3	0.01Å	Favored (97.461%) alpha helix	-	-	-
A 651		TRP	0.61	-	Favored (90.55%) General / -60.9,-46.5	Favored (87.4%) *t60* chi angles: 185,90.3	0.08Å	Favored (97.693%) alpha helix	-	-	-
A 652		LEU	0.62	-	Favored (96.14%) General / -63.1,-39.9	Favored (69.5%) *mt* chi angles: 289.2,165.7	0.06Å	Favored (96.493%) alpha helix	-	-	-
A 653		ARG	0.63	-	Favored (77.63%) General / -67.1,-34.4	Favored (98.1%) *mtt180* chi angles: 289.3,175.5,179.1,170.2	0.01Å	Favored (57.983%) alpha helix	-	-	-
A 654		LYS	0.65	-	Favored (7.13%) General / -98.9,-42.5	Favored (98.8%) *mttt* chi angles: 292.7,180.5,178.2,176.3	0.01Å	Favored (28.903%) alpha helix	-	-	-
A 655		ASN	0.67	0.42Å O with A 656 GLY C	Favored (13.51%) General / -103.6,-23.1	Favored (71%) *m-40* chi angles: 293.9,287.7	0.02Å	Favored (15.687%) alpha helix	-	-	-
A 656		GLY	0.68	0.42Å C with A 655 ASN O	Allowed (0.51%) Glycine / -36.7,-66.0	-	-	Favored (54.393%) alpha helix	-	-	-
A 657		PRO	0.68	-	Favored (69.46%) Trans-Pro / -54.9,-30.6	Favored (62.4%) *Cg_exo* chi angles: 335.8,37,326.2	0.08Å	Favored (70.932%) alpha helix	-	-	-
A 658		GLU	0.68	-	Favored (55.69%) General / -76.5,-38.2	Favored (66.7%) *mm-30* chi angles: 289.3,292.2,311.7	0.02Å	Favored (82.154%) alpha helix	-	-	-
A 659		ARG	0.67	-	Favored (74.87%) General / -70.4,-39.0	Favored (93.2%) *mmt-90* chi angles: 298.5,289.6,185.1,275	0.08Å	Favored (90.426%) alpha helix	-	-	-
A 660		LEU	0.66	-	Favored (86.82%) General / -62.7,-37.5	Favored (76.5%) *mt* chi angles: 288.1,171.2	0.02Å	Favored (77.567%) alpha helix	-	-	-
**#**	**Alt**	**Res**	**High B**	**Clash > 0.4Å**	**Ramachandran**	**Rotamer**	**Cβ deviation**	**CaBLAM**	**Bond lengths**	**Bond angles**	**Cis Peptides**
			Avg: 0.98	Clashscore: 1.52	Outliers: 7 of 904	Poor rotamers: 0 of 769	Outliers: 0 of 827	Outliers: 17 of 902	Outliers: 14 of 906	Outliers: 17 of 906	Non-Trans: 0 of 905
A 661		SER	0.65	-	Favored (63.12%) General / -67.2,-14.7	Favored (55.2%) *p* chi angles: 73.8	0.03Å	Favored (66.762%) alpha helix	-	-	-

A 662		ARG	0.64	-	Favored (33.49%) General / -97.8,12.6	Favored (69.7%) *mtt90* chi angles: 288.9,163.6,171.7,77.2	0.01Å	Favored (34.912%)	-	-	-
A 663		MET	0.65	-	Favored (35.41%) General / -123.0,156.1	Favored (65.1%) *mtt* chi angles: 298.7,182.1,181	0.05Å	Favored (27.054%)	-	-	-
A 664		ALA	0.66	-	Favored (9.89%) General / -139.4,114.9	-	0.05Å	Favored (36.288%)	-	-	-
A 665		VAL	0.68	-	Favored (69.48%) Ile or Val / -127.6,132.1	Favored (49.2%) *t* chi angles: 181.6	0.02Å	Favored (50.317%)	OUTLIER(S) worst is CB--CG2: 4.2 σ	-	-
A 666		SER	0.69	-	Favored (5.23%) General / -132.5,101.2	Favored (40.2%) *t* chi angles: 178	0.07Å	Favored (7.767%)	-	-	-
A 667		GLY	0.71	-	Favored (36.45%) Glycine / 58.3,-124.5	-	-	Favored (46.133%)	-	-	-
A 668		ASP	0.71	-	Favored (26.63%) General / -80.4,2.0	Favored (54.6%) *p0* chi angles: 61.6,3.5	0.08Å	Favored (8.427%)	-	-	-
A 669		ASP	0.71	-	Favored (27.25%) General / -114.1,115.3	Favored (56.6%) *t0* chi angles: 187.6,334.2	0.06Å	Favored (26.18%)	-	-	-
A 670		CYS	0.7	-	Favored (42.2%) General / -128.9,127.6	Favored (47.9%) *t* chi angles: 178.9	0.11Å	Favored (58.171%)	-	-	-
A 671		VAL	0.7	-	Favored (46.06%) Ile or Val / -99.6,131.9	Favored (92.3%) *t* chi angles: 174.5	0.06Å	Favored (61.302%) beta sheet	-	-	-
A 672		VAL	0.71	-	Favored (56.36%) Ile or Val / -131.9,136.7	Favored (48.8%) *t* chi angles: 181.7	0.06Å	Favored (66.014%) beta sheet	-	-	-
A 673		LYS	0.72	-	Favored (37.6%) Pre-Pro / -121.1,83.0	Favored (48.9%) *tttp* chi angles: 184.3,177.1,172.2,70.8	0.06Å	Favored (25.031%) beta sheet	-	-	-
A 674		PRO	0.74	-	Favored (54.86%) Trans-Pro / -71.1,158.5	Favored (77%) *Cg_endo* chi angles: 30.1,326.2,23.3	0.08Å	Favored (28.855%)	-	-	-
A 675		ILE	0.75	-	Favored (24.02%) Ile or Val / -60.6,-21.7	Favored (6.9%) *tp* chi angles: 201.3,67.4	0.06Å	Favored (14.61%)	-	-	-
A 676		ASP	0.75	-	Favored (24.26%) General / -148.2,140.5	Favored (11.6%) *t70* chi angles: 193.5,292.1	0.04Å	Favored (18.777%)	-	-	-
A 677		ASP	0.74	-	Favored (9.47%) General / -59.7,-13.3	Favored (98.3%) *m-30* chi angles: 287,347.1	0.07Å	Favored (9.501%)	-	-	-
A 678		ARG	0.71	-	Favored (42.33%) General / -58.0,-20.9	Favored (98.9%) *mtm-85* chi angles: 285.5,190.1,292.5,273.9	0.01Å	Favored (24.132%)	-	-	-
A 679		PHE	0.69	-	Favored (17.16%)	Favored (84.2%) *t80*	0.06Å	Favored (53.179%)	-	-	-

#	Alt	Res	High B	Clash > 0.4Å	Ramachandran	Rotamer	Cβ deviation	CaBLAM	Bond lengths	Bond angles	Cis Peptides
					General / -48.4,-37.6	chi angles: 182.6,80.1		three-ten			
A 680		ALA	0.67	-	Favored (62.21%) General / -55.2,-32.5	-	0.10Å	Favored (57.87%) three-ten	-	-	-
#	Alt	Res	High B	Clash > 0.4Å	Ramachandran	Rotamer	Cβ deviation	CaBLAM	Bond lengths	Bond angles	Cis Peptides
			Avg: 0.98	Clashscore: 1.52	Outliers: 7 of 904	Poor rotamers: 0 of 769	Outliers: 0 of 827	Outliers: 17 of 902	Outliers: 14 of 906	Outliers: 17 of 906	Non-Trans: 0 of 905
A 681		THR	0.65	-	Favored (35.8%) General / -97.9,-4.8	Favored (58.8%) *p* chi angles: 64.3	0.06Å	Favored (47.091%)	-	-	-
A 682		ALA	0.65	-	Favored (5.32%) General / -81.2,62.9	-	0.05Å	Favored (5.085%)	-	-	-
A 683		LEU	0.65	-	Favored (19.6%) General / -105.3,-6.5	Favored (6.7%) *mp* chi angles: 275.6,68.7	0.03Å	Favored (11.979%)	-	-	-
A 684		HIS	0.64	-	Favored (73.13%) General / -68.8,-44.7	Favored (56.7%) *m170* chi angles: 290.9,164.2	0.01Å	Favored (23.119%)	-	-	-
A 685		PHE	0.64	0.46Å CE1 with A 372 VAL HG13	Favored (84.66%) General / -58.6,-47.3	Favored (82.4%) *t80* chi angles: 182.9,82.4	0.06Å	Favored (85.105%) alpha helix	-	-	-
A 686		LEU	0.65	-	Favored (60.34%) General / -53.3,-52.1	Favored (49.1%) *tp* chi angles: 178.6,66.7	0.14Å	Favored (85.991%) alpha helix	-	-	-
A 687		ASN	0.66	-	Favored (76.98%) General / -66.8,-34.0	Favored (98.6%) *m-40* chi angles: 287.2,340.5	0.10Å	Favored (77.494%) alpha helix	-	-	-
A 688		ASN	0.67	-	Favored (65.51%) General / -68.7,-26.9	Favored (62.3%) *m-40* chi angles: 282.5,284.7	0.09Å	Favored (75.217%) alpha helix	-	-	-
A 689		MET	0.7	-	Favored (38.72%) General / -83.9,2.4	Favored (93.4%) *mmm* chi angles: 290.2,301.1,295.9	0.06Å	Favored (53.886%)	-	-	-
A 690		SER	0.74	-	Favored (8.33%) General / 69.5,13.9	Favored (27.6%) *m* chi angles: 303.5	0.12Å	Favored (8.736%)	-	-	-
A 691		LYS	0.8	0.54Å NZ with A 548 ASP OD2	Favored (9.05%) General / -87.9,67.5	Favored (5.7%) *mptt* chi angles: 270,61.5,178.8,176.2	0.03Å	Favored (10.035%) beta sheet	-	-	-
A 692		ILE	0.89	-	Favored (36.22%) Ile or Val / -73.5,128.7	Favored (91.7%) *mt* chi angles: 297.4,169.3	0.05Å	Favored (20.348%) beta sheet	-	-	-
A 693		ARG	1.01	-	Favored (52.21%) General / -65.5,133.9	Favored (67.9%) *ttt-90* chi angles: 186.5,180.4,188.3,275	0.02Å	Favored (43.668%)	-	-	-
A 694		LYS	1.15	-	Favored (4.25%) General / -63.5,-59.7	Favored (11.8%) *ttpp* chi angles: 184.2,172.3,73.3,75.8	0.03Å	Favored (23.208%)	-	-	-
A 695		ASP	1.27	-	Favored (3.64%)	Favored (77.5%) *m-30* chi angles: 297.1,334.9	0.03Å	CaBLAM Outlier (0.153%)	-	-	-

#	Alt	Res	High B	Clash > 0.4Å	Ramachandran	Rotamer	Cβ deviation	CaBLAM	Bond lengths	Bond angles	Cis Peptides
					General / -89.5,56.6						
A 696		ILE	1.33	-	Favored (2.11%) Ile or Val / -165.3,148.4	Favored (19.9%) *tt* chi angles: 193.5,165.7	0.05Å	CaBLAM Disfavored (3.605%)	-	-	-
A 697		GLN	1.31	-	Favored (40.86%) General / -66.3,129.7	Favored (83.6%) *tp40* chi angles: 182,69.9,53.4	0.01Å	Favored (28.068%)	-	-	-
A 698		GLU	1.2	-	Allowed (1.31%) General / -40.4,-41.0	Favored (93.3%) *tt0* chi angles: 180.2,178.7,358.2	0.04Å	Favored (13.137%)	-	-	-
A 699		TRP	1.06	-	Favored (57.91%) General / -88.5,-6.0	Favored (61.5%) *p-90* chi angles: 51.7,264.8	0.01Å	Favored (53.005%)	-	-	-
A 700		LYS	0.92	-	Favored (61.98%) Pre-Pro / -80.9,147.4	Favored (97%) *mttt* chi angles: 290.7,182.6,180.2,180.4	0.01Å	Favored (29.15%)	-	-	-
**#**	**Alt**	**Res**	**High B**	**Clash > 0.4Å**	**Ramachandran**	**Rotamer**	**Cβ deviation**	**CaBLAM**	**Bond lengths**	**Bond angles**	**Cis Peptides**
			Avg: 0.98	Clashscore: 1.52	Outliers: 7 of 904	Poor rotamers: 0 of 769	Outliers: 0 of 827	Outliers: 17 of 902	Outliers: 14 of 906	Outliers: 17 of 906	Non-Trans: 0 of 905
A 701		PRO	0.81	-	Favored (76.31%) Trans-Pro / -67.4,153.6	Favored (45.9%) *Cg_endo* chi angles: 24.7,326.1,28.5	0.02Å	Favored (66.872%)	-	-	-
A 702		SER	0.74	-	Favored (53.11%) General / -67.2,147.4	Favored (68.7%) *m* chi angles: 296.7	0.03Å	Favored (46.945%)	-	-	-
A 703		THR	0.71	-	Favored (35.12%) General / -81.6,130.6	Favored (89.1%) *m* chi angles: 298.6	0.02Å	Favored (43.039%) beta sheet	-	-	-
A 704		GLY	0.7	-	Favored (15.82%) Glycine / -110.8,155.9	-	-	Favored (66.01%) beta sheet	-	-	-
A 705		TRP	0.71	-	Favored (51.06%) General / -131.3,144.1	Favored (40.2%) *m-90* chi angles: 297.8,267.4	0.04Å	Favored (68.916%)	-	-	-
A 706		HIS	0.73	-	Favored (56.85%) General / -85.4,-10.0	Favored (44.4%) *p-80* chi angles: 64.2,293.3	0.06Å	Favored (50.594%)	-	-	-
A 707		ASN	0.74	-	Favored (12.29%) General / -135.2,114.4	Favored (43.5%) *t0* chi angles: 187.9,314	0.01Å	Favored (14.111%) alpha helix	-	-	-
A 708		TRP	0.74	-	Favored (2.37%) General / -49.1,-25.8	Favored (78%) *p-90* chi angles: 65.8,271.5	0.02Å	Favored (30.33%) alpha helix	-	-	-
A 709		GLN	0.74	-	Favored (62.52%) General / -71.2,-16.5	Favored (33.8%) *mt0* chi angles: 295.3,174.2,104.2	0.02Å	Favored (62.87%) alpha helix	-	-	-
A 710		GLU	0.75	-	Favored (53.52%) General / -96.1,3.2	Favored (91.1%) *mt-10* chi angles: 297.8,184.3,0.3	0.01Å	Favored (56.761%)	-	-	-
A 711		VAL	0.78	-	Favored (65.98%)	Favored (66.6%) *t* chi angles: 179.2	0.11Å	Favored (32.139%)	-	-	-

#	Alt	Res	High B	Clash > 0.4Å	Ramachandran	Rotamer	Cβ deviation	CaBLAM	Bond lengths	Bond angles	Cis Peptides
					Pre-Pro / -96.4,117.6						
A 712		PRO	0.81	-	Favored (46.05%) Trans-Pro / -74.2,153.8	Favored (74.7%) *Cg_endo* chi angles: 28.9,327,23.6	0.01Å	Favored (31.826%)	-	-	-
A 713		PHE	0.85	-	Favored (12.08%) General / -158.2,137.9	Favored (66.7%) *t80* chi angles: 185.9,74.4	0.06Å	Favored (16.863%)	-	-	-
A 714		CYS	0.87	-	OUTLIER (0.03)%) General / 70.1,-82.4	Favored (54.5%) *t* chi angles: 181.3	0.08Å	CaBLAM Outlier (0.229%)	-	-	-
A 715		SER	0.88	-	Allowed (1.01%) General / -146.3,7.5	Favored (70.9%) *p* chi angles: 59.1	0.04Å	CaBLAM Disfavored (2.299%)	-	-	-
A 716		HIS	0.86	-	Favored (18.96%) General / -155.7,172.1	Favored (11.7%) *p-80* chi angles: 47.9,264.8	0.08Å	Favored (23.91%)	OUTLIER(S) worst is CB--CG: 5.3 σ	OUTLIER(S) worst is CA-CB-CG: 4.0 σ	-
A 717		HIS	0.83	-	Favored (12.86%) General / -103.0,162.5	Favored (70%) *m90* chi angles: 300.5,85.7	0.08Å	Favored (41.505%)	-	-	-
A 718		PHE	0.79	-	Favored (56.5%) General / -118.0,131.4	Favored (86%) *m-80* chi angles: 291.4,84.3	0.03Å	Favored (50.906%) beta sheet	-	-	-
A 719		ASN	0.76	-	Favored (29.86%) General / -107.7,148.1	Favored (81.4%) *m-40* chi angles: 290.4,317.1	0.02Å	Favored (46.383%) beta sheet	-	-	-
A 720		GLU	0.75	-	Favored (43.63%) General / -100.7,122.6	Favored (86.4%) *tt0* chi angles: 183.5,175.7,9.5	0.07Å	Favored (58.104%) beta sheet	-	-	-
**#**	**Alt**	**Res**	**High B**	**Clash > 0.4Å**	**Ramachandran**	**Rotamer**	**Cβ deviation**	**CaBLAM**	**Bond lengths**	**Bond angles**	**Cis Peptides**
			Avg: 0.98	Clashscore: 1.52	Outliers: 7 of 904	Poor rotamers: 0 of 769	Outliers: 0 of 827	Outliers: 17 of 902	Outliers: 14 of 906	Outliers: 17 of 906	Non-Trans: 0 of 905
A 721		LEU	0.77	-	Favored (27.41%) General / -109.2,150.8	Allowed (1.4%) *mm* chi angles: 286.8,302.8	0.10Å	Favored (42.433%) beta sheet	-	-	-
A 722		MET	0.8	-	Favored (52.17%) General / -109.4,134.2	Favored (59.7%) *ttm* chi angles: 181.2,174.2,283.4	0.08Å	Favored (47.787%) beta sheet	-	-	-
A 723		LEU	0.83	-	Favored (29.75%) General / -77.8,158.0	Favored (6.8%) *mp* chi angles: 279.4,67.9	0.04Å	Favored (46.825%)	-	-	-
A 724		LYS	0.86	-	Favored (66.17%) General / -61.7,-23.5	Favored (97.9%) *mttt* chi angles: 291.2,180,180.2,177.3	0.09Å	Favored (58.071%)	-	-	-
A 725		ASP	0.87	-	Favored (34.69%) General / -86.7,5.8	Favored (51.1%) *p0* chi angles: 67,14.8	0.06Å	Favored (50.488%)	-	-	-
A 726		GLY	0.86	-	Favored (71.75%) Glycine / 94.2,-10.1	-	-	Favored (70.051%)	-	-	-

A 727		ARG	0.83	-	Favored (26.54%) General / -79.9,158.9	Favored (92%) *mtm180* chi angles: 294.6,173.3,293,173.3	0.04Å	Favored (43.179%)	OUTLIER(S) worst is CZ--NH1: 4.2 σ	-	-
A 728		THR	0.79	-	Favored (22.81%) General / -115.7,158.4	Favored (58.1%) *p* chi angles: 64.4	0.08Å	Favored (56.492%) beta sheet	-	-	-
A 729		ILE	0.75	-	Favored (26.13%) Ile or Val / -142.9,142.5	Favored (22.1%) *tt* chi angles: 187.2,165.5	0.15Å	Favored (61.323%) beta sheet	-	-	-
A 730		VAL	0.73	-	Favored (67.65%) Ile or Val / -110.4,125.3	Favored (56.2%) *t* chi angles: 180.4	0.04Å	Favored (57.251%) beta sheet	-	-	-
A 731		VAL	0.71	0.55Å O with A 731 VAL HG23	Favored (73.83%) Pre-Pro / -126.4,152.2	Favored (21%) *m* chi angles: 302.1	0.23Å	Favored (44.454%) beta sheet	-	-	-
A 732		PRO	0.71	-	Favored (65.65%) Trans-Pro / -70.3,154.5	Favored (58.8%) *Cg_endo* chi angles: 26.3,325,28.3	0.06Å	Favored (63.893%) beta sheet	-	-	-
A 733		CYS	0.72	-	Favored (5.32%) General / -150.8,115.9	Favored (17.4%) *t* chi angles: 192	0.02Å	Favored (33.614%)	-	-	-
A 734		ARG	0.73	-	Favored (39.54%) General / -113.2,146.3	Favored (78.8%) *ttt180* chi angles: 183.3,170.8,182.1,177.5	0.04Å	Favored (27.866%)	-	-	-
A 735		SER	0.75	-	Favored (14.25%) General / -47.7,130.2	Favored (68.9%) *m* chi angles: 296.6	0.02Å	Favored (31.601%)	-	-	-
A 736		GLN	0.77	-	Favored (70.12%) General / -56.0,-38.2	Favored (56.8%) *tt0* chi angles: 185.3,181.1,61.3	0.04Å	Favored (55.617%)	-	-	-
A 737		ASP	0.78	-	Favored (72.95%) General / -60.5,-33.7	Favored (99%) *m-30* chi angles: 288.2,347.4	0.06Å	Favored (71.789%) alpha helix	-	-	-
A 738		GLU	0.79	-	Favored (47.92%) General / -78.2,-36.0	Favored (88.1%) *mt-10* chi angles: 294.9,180.9,14.1	0.03Å	Favored (85.825%) alpha helix	-	-	-
A 739		LEU	0.79	-	Favored (86.34%) General / -67.2,-39.7	Favored (80.9%) *mt* chi angles: 290.4,174.3	0.01Å	Favored (75.789%) alpha helix	-	-	-
A 740		ILE	0.78	-	Favored (25.36%) Ile or Val / -78.8,-43.9	Favored (92.9%) *mt* chi angles: 296,170.9	0.10Å	Favored (72.192%) alpha helix	-	-	-
**#**	**Alt**	**Res**	**High B**	**Clash > 0.4Å**	**Ramachandran**	**Rotamer**	**Cβ deviation**	**CaBLAM**	**Bond lengths**	**Bond angles**	**Cis Peptides**
			Avg: 0.98	Clashscore: 1.52	Outliers: 7 of 904	Poor rotamers: 0 of 769	Outliers: 0 of 827	Outliers: 17 of 902	Outliers: 14 of 906	Outliers: 17 of 906	Non-Trans: 0 of 905
A 741		GLY	0.79	-	Favored (55.85%) Glycine / -53.6,-49.9	-	-	Favored (94.04%) alpha helix	-	-	-

A 742		ARG	0.8	-	Favored (70.99%) General / -55.0,-49.7	Favored (52.9%) *ttt90* chi angles: 183.6,185.5,186.7,91.1	0.06Å	Favored (96.974%) alpha helix	-	-	-
A 743		ALA	0.84	-	Favored (70.28%) General / -59.8,-32.0	-	0.04Å	Favored (74.037%) alpha helix	-	-	-
A 744		ARG	0.92	-	Favored (48.55%) General / -77.3,-5.2	Favored (32%) *mtp180* chi angles: 288,176.8,55.2,160.9	0.07Å	Favored (49.572%)	-	-	-
A 745		ILE	1.05	-	Favored (67.35%) Ile or Val / -110.9,128.5	Favored (48.9%) *mm* chi angles: 302.2,299	0.05Å	Favored (35.506%)	-	-	-
A 746		SER	1.23	-	Favored (27.06%) Pre-Pro / -93.9,143.8	Favored (69%) *m* chi angles: 296.6	0.07Å	Favored (40.819%)	-	-	-
A 747		PRO	1.43	-	Favored (8%) Trans-Pro / -77.4,64.6	Favored (61.3%) *Cg_endo* chi angles: 31.7,324.1,24.9	0.04Å	CaBLAM Outlier (0.08%)	-	-	-
A 748		GLY	1.6	-	Favored (28.98%) Glycine / 151.6,-160.8	-	-	Favored (17.927%)	-	-	-
A 749		ALA	1.68	-	Favored (41.84%) General / -148.4,161.3	-	0.03Å	CaBLAM Outlier (0.812%)	-	-	-
A 750		GLY	1.62	-	Favored (68.68%) Glycine / 87.7,11.2	-	-	Favored (28.095%)	-	-	-
A 751		TRP	1.46	-	Favored (50.6%) General / -71.1,143.4	Favored (94%) *m100* chi angles: 296.4,103.9	0.18Å	Favored (31.556%)	-	-	-
A 752		ASN	1.25	-	Favored (5.07%) General / -93.2,-179.4	Favored (45.8%) *p0* chi angles: 67.5,21	0.08Å	Favored (42.36%)	-	-	-
A 753		VAL	1.06	-	Favored (94.37%) Ile or Val / -64.0,-46.3	Favored (66.9%) *t* chi angles: 171.7	0.03Å	Favored (54.466%)	-	-	-
A 754		LYS	0.91	-	Favored (78.34%) General / -56.1,-43.6	Favored (85.4%) *tttt* chi angles: 178.4,174.2,180,180.1	0.04Å	Favored (83.171%) alpha helix	-	-	-
A 755		GLU	0.8	-	Favored (96.32%) General / -63.8,-40.3	Favored (67%) *mt-10* chi angles: 290.2,169.8,321.5	0.03Å	Favored (89.073%) alpha helix	-	-	-
A 756		THR	0.74	-	Favored (66.29%) General / -72.0,-31.6	Favored (78.6%) *p* chi angles: 60.3	0.02Å	Favored (87.818%) alpha helix	-	-	-
A 757		ALA	0.7	-	Favored (94.96%) General / -64.9,-40.4	-	0.07Å	Favored (92.426%) alpha helix	-	-	-
A 758		CYS	0.67	-	Favored (90.6%) General / -65.9,-42.0	Favored (89.5%) *m* chi angles: 291.5	0.05Å	Favored (94.072%) alpha helix	-	-	-
A 759		LEU	0.65	-	Favored (96.79%)	Favored (84.2%) *mt* chi angles: 290.1,168.6	0.06Å	Favored (80.224%)	-	-	-

#	Alt	Res	High B	Clash > 0.4Å	Ramachandran	Rotamer	Cβ deviation	CaBLAM	Bond lengths	Bond angles	Cis Peptides
					General / -64.4,-41.1			alpha helix			
A 760		SER	0.64	-	Favored (91.36%) General / -59.5,-45.5	Favored (34%) *t* chi angles: 181.9	0.05Å	Favored (81.848%) alpha helix	-	-	-
#	Alt	Res	High B	Clash > 0.4Å	Ramachandran	Rotamer	Cβ deviation	CaBLAM	Bond lengths	Bond angles	Cis Peptides
			Avg: 0.98	Clashscore: 1.52	Outliers: 7 of 904	Poor rotamers: 0 of 769	Outliers: 0 of 827	Outliers: 17 of 902	Outliers: 14 of 906	Outliers: 17 of 906	Non-Trans: 0 of 905
A 761		LYS	0.64	-	Favored (94.19%) General / -59.7,-43.2	Favored (13.1%) *ttmm* chi angles: 185.8,179.8,292.8,294.9	0.06Å	Favored (91.597%) alpha helix	-	-	-
A 762		SER	0.63	-	Favored (70.32%) General / -64.7,-29.3	Favored (33%) *p* chi angles: 77	0.10Å	Favored (76.799%) alpha helix	-	-	-
A 763		TYR	0.64	-	Favored (56.6%) General / -76.1,-38.6	Favored (51.3%) *m-80* chi angles: 291.1,116.1	0.04Å	Favored (84.357%) alpha helix	-	-	-
A 764		ALA	0.64	-	Favored (97.99%) General / -63.7,-41.9	-	0.07Å	Favored (98.02%) alpha helix	-	-	-
A 765		GLN	0.65	-	Favored (77.53%) General / -69.0,-36.2	Favored (78.9%) *mt0* chi angles: 290.7,186.9,328.2	0.17Å	Favored (92.728%) alpha helix	-	-	-
A 766		MET	0.65	-	Favored (91.63%) General / -59.3,-45.3	Favored (27.8%) *tmm* chi angles: 177.6,275.1,290.6	0.03Å	Favored (87.412%) alpha helix	-	-	-
A 767		TRP	0.66	-	Favored (99.3%) General / -61.6,-42.7	Favored (30.1%) *m-10* chi angles: 284.2,343.1	0.05Å	Favored (97.263%) alpha helix	-	-	-
A 768		LEU	0.66	-	Favored (76.41%) General / -64.2,-33.7	Favored (83.5%) *mt* chi angles: 290.9,174.5	0.05Å	Favored (61.574%) alpha helix	-	-	-
A 769		LEU	0.67	-	Favored (11.36%) General / -88.6,-43.4	Favored (34.5%) *tp* chi angles: 186.7,61.2	0.03Å	Favored (45.48%) alpha helix	-	-	-
A 770		MET	0.66	-	Favored (17.1%) General / -85.2,-40.1	Favored (32%) *ttt* chi angles: 187.1,175.5,180.2	0.04Å	Favored (54.352%)	-	-	-
A 771		TYR	0.66	-	Favored (4.58%) General / -118.9,36.1	Favored (74.9%) *m-80* chi angles: 302.4,106.3	0.03Å	Favored (12.611%)	-	-	-
A 772		PHE	0.66	0.41Å C with A 772 PHE CD1	Favored (26.07%) General / -56.7,-21.2	Favored (21%) *p90* chi angles: 69.6,80.2	0.09Å	Favored (13.817%)	-	-	-
A 773		HIS	0.66	-	Favored (58.15%) General / -83.6,-10.6	Favored (25.6%) *p-80* chi angles: 64.5,300.7	0.08Å	Favored (54.178%)	-	-	-
A 774		ARG	0.66	-	Favored (36.39%) General / -90.3,128.2	Favored (26.6%) *ttm170* chi angles: 192.1,179.8,305.4,187.8	0.03Å	Favored (35.535%)	OUTLIER(S) worst is CZ--NH2: 4.8 σ	-	-

#	Alt	Res	High B	Clash > 0.4Å	Ramachandran	Rotamer	Cβ deviation	CaBLAM	Bond lengths	Bond angles	Cis Peptides
A 775		ARG	0.66	-	Favored (90.9%) General / -62.2,-38.9	Favored (98.2%) *mtt-85* chi angles: 290.1,176,183.7,275.5	0.05Å	Favored (47.628%)	-	-	-
A 776		ASP	0.66	0.43Å OD2 with A 854 SER OG	Favored (63.3%) General / -73.9,-40.2	Favored (94.6%) *m-30* chi angles: 291.4,347.2	0.10Å	Favored (89.652%) alpha helix	-	-	-
A 777		LEU	0.66	-	Favored (64.36%) General / -72.9,-30.9	Favored (82.7%) *mt* chi angles: 292.5,177.9	0.12Å	Favored (80.878%) alpha helix	-	-	-
A 778		ARG	0.66	-	Favored (98.31%) General / -61.1,-42.3	Favored (34.5%) *mmm-85* chi angles: 282.7,277,294,278.1	0.04Å	Favored (81.493%) alpha helix	-	-	-
A 779		MET	0.65	-	Favored (82.03%) General / -68.1,-41.2	Favored (97.3%) *mmm* chi angles: 292,298.3,288.1	0.03Å	Favored (76.822%) alpha helix	-	-	-
A 780		MET	0.65	-	Favored (74.11%) General / -68.2,-45.1	Favored (99.4%) *mtp* chi angles: 292.3,176.2,72.9	0.07Å	Favored (82.34%) alpha helix	-	-	-
**#**	**Alt**	**Res**	**High B**	**Clash > 0.4Å**	**Ramachandran**	**Rotamer**	**Cβ deviation**	**CaBLAM**	**Bond lengths**	**Bond angles**	**Cis Peptides**
			Avg: 0.98	Clashscore: 1.52	Outliers: 7 of 904	Poor rotamers: 0 of 769	Outliers: 0 of 827	Outliers: 17 of 902	Outliers: 14 of 906	Outliers: 17 of 906	Non-Trans: 0 of 905
A 781		ALA	0.64	-	Favored (95.77%) General / -61.9,-40.5	-	0.06Å	Favored (83.945%) alpha helix	-	-	-
A 782		ASN	0.63	-	Favored (68.83%) General / -71.7,-33.4	Favored (63%) *m-40* chi angles: 283,284.8	0.06Å	Favored (89.096%) alpha helix	-	-	-
A 783		ALA	0.63	-	Favored (99.03%) General / -63.3,-42.3	-	0.04Å	Favored (83.741%) alpha helix	-	-	-
A 784		ILE	0.62	-	Favored (90.46%) Ile or Val / -65.2,-46.6	Favored (93.7%) *mt* chi angles: 292,166.5	0.04Å	Favored (82.831%) alpha helix	-	-	-
A 785		CYS	0.63	-	Favored (71.43%) General / -59.5,-33.5	Favored (99.9%) *m* chi angles: 292.5	0.01Å	Favored (74.517%) alpha helix	-	-	-
A 786		SER	0.63	-	Favored (59.22%) General / -79.5,-13.9	Favored (55.1%) *p* chi angles: 73.8	0.09Å	Favored (69.038%) alpha helix	-	-	-
A 787		ALA	0.64	-	Favored (14.87%) General / -90.1,-36.4	-	0.04Å	Favored (17.251%)	-	-	-
A 788		VAL	0.65	-	Favored (51.76%) Pre-Pro / -93.9,125.8	Favored (61.3%) *t* chi angles: 179.8	0.05Å	Favored (17.621%)	-	-	-
A 789		PRO	0.66	-	Favored (31.67%) Trans-Pro / -50.7,142.5	Favored (54.2%) *Cg_exo* chi angles: 328,37.6,332.8	0.05Å	Favored (19.076%)	-	-	-

A 790		VAL	0.69	-	Favored (31.55%) Ile or Val / -59.8,-25.2	Favored (4.8%) *p* chi angles: 71.9	0.04Å	Favored (30.664%)	-	-	-
A 791		ASN	0.72	-	Favored (53.72%) General / -95.1,4.4	Favored (87.8%) *m-40* chi angles: 292,320.6	0.04Å	Favored (54.885%)	-	-	-
A 792		TRP	0.76	-	Favored (9.4%) General / -84.3,79.8	Favored (43.7%) *m-10* chi angles: 287.6,11.5	0.08Å	Favored (20.029%)	-	-	-
A 793		VAL	0.83	-	Favored (87.62%) Pre-Pro / -63.8,125.4	Favored (94%) *t* chi angles: 175.8	0.11Å	Favored (25.281%) beta sheet	-	-	-
A 794		PRO	0.92	-	Favored (79.92%) Trans-Pro / -55.8,143.8	Favored (95.6%) *Cg_exo* chi angles: 332.8,36.4,330.3	0.02Å	Favored (44.905%) beta sheet	-	-	-
A 795		THR	1.04	-	Favored (9.75%) General / -117.8,-17.7	Favored (64.6%) *p* chi angles: 63.2	0.05Å	Favored (17.605%)	-	-	-
A 796		GLY	1.19	-	Favored (27.88%) Glycine / -96.2,-151.2	-	-	Favored (19.164%)	-	-	-
A 797		ARG	1.35	-	Favored (33.62%) General / -134.2,128.3	Favored (93.9%) *mmt-90* chi angles: 295.2,290.9,182.7,274.2	0.04Å	Favored (9.006%)	-	-	-
A 798		THR	1.5	-	Favored (69.5%) General / -70.3,-43.9	Favored (92.4%) *m* chi angles: 301	0.09Å	Favored (18.856%)	-	-	-
A 799		THR	1.61	-	Favored (40.23%) General / -155.1,159.4	Favored (10.5%) *t* chi angles: 188.6	0.08Å	Favored (22.192%)	-	-	-
A 800		TRP	1.67	-	Favored (22.11%) General / -111.0,9.3	Favored (39.2%) *m-90* chi angles: 297.5,269.7	0.03Å	CaBLAM Disfavored (4.852%) try beta sheet	-	-	-
**#**	**Alt**	**Res**	**High B**	**Clash > 0.4Å**	**Ramachandran**	**Rotamer**	**Cβ deviation**	**CaBLAM**	**Bond lengths**	**Bond angles**	**Cis Peptides**
			Avg: 0.98	Clashscore: 1.52	Outliers: 7 of 904	Poor rotamers: 0 of 769	Outliers: 0 of 827	Outliers: 17 of 902	Outliers: 14 of 906	Outliers: 17 of 906	Non-Trans: 0 of 905
A 801		SER	1.67	-	Favored (57.31%) General / -60.4,140.3	Favored (37.1%) *t* chi angles: 174.6	0.07Å	Favored (37.973%)	-	-	-
A 802		ILE	1.61	-	Favored (20.7%) Ile or Val / -58.0,-23.2	Favored (11.1%) *tp* chi angles: 194.1,64.7	0.06Å	Favored (28.408%)	-	-	-
A 803		HIS	1.52	-	Favored (39.78%) General / -101.6,3.2	Favored (95.4%) *m-70* chi angles: 297.2,295.6	0.02Å	Favored (56.781%)	OUTLIER(S) worst is CB--CG: 5.9 σ	-	-
A 804		GLY	1.38	-	Favored (49.47%) Glycine / -79.8,-176.5	-	-	Favored (22.974%)	-	-	-
A 805		LYS	1.23	-	Favored (20.9%) General / -153.2,141.8	Favored (79.2%) *tttt* chi angles: 192.1,174.9,185.9,183	0.05Å	Favored (19.211%)	-	-	-

A 806		GLY	1.09	-	Favored (7.83%)<br>Glycine / -57.2,-12.4	-	-	Favored (19.325%)	-	-	-
A 807		GLU	0.97	-	Favored (68.89%)<br>General / -59.6,-30.7	Favored (50.7%) *mm-30*<br>chi angles: 288.9,289.8,304.8	0.03Å	Favored (46.942%)	-	-	-
A 808		TRP	0.87	-	Favored (65.65%)<br>General / -64.0,-20.1	Favored (59.1%) *m-10*<br>chi angles: 291.7,345.6	0.06Å	Favored (57.443%)<br>three-ten	-	-	-
A 809		MET	0.8	-	Favored (22.14%)<br>General / -109.6,16.5	Favored (96.5%) *mtp*<br>chi angles: 295.2,178.7,68.7	0.02Å	Favored (39.881%)	-	-	-
A 810		THR	0.76	-	Favored (59.1%)<br>General / -87.2,-4.4	Favored (68.7%) *p*<br>chi angles: 59	0.04Å	CaBLAM Disfavored (1.784%)	-	-	-
A 811		THR	0.75	-	Allowed (0.57%)<br>General / 70.2,-44.6	Favored (98.9%) *m*<br>chi angles: 300.3	0.09Å	Favored (16.94%)<br>beta sheet	-	-	-
A 812		GLU	0.75	0.47Å<br>O with A 813 ASP C	Allowed (1.33%)<br>General / -40.3,136.1	Favored (79%) *tt0*<br>chi angles: 185.2,183.4,9.9	0.10Å	Favored (7.191%)<br>beta sheet	-	-	-
A 813		ASP	0.75	0.47Å<br>C with A 812 GLU O	Favored (2.9%)<br>General / -43.3,123.3	Favored (30.3%) *t0*<br>chi angles: 186.9,321.2	0.05Å	Favored (23.298%)	OUTLIER(S)<br>worst is CB--CG: 4.5 σ	-	-
A 814		MET	0.74	-	Favored (42.57%)<br>General / -59.3,-19.0	Favored (92.7%) *mmm*<br>chi angles: 289.6,302.4,293.6	0.13Å	Favored (18.891%)	-	-	-
A 815		LEU	0.74	-	Favored (94.77%)<br>General / -64.9,-40.3	Favored (45.8%) *tp*<br>chi angles: 184.3,59.4	0.06Å	Favored (53.408%)<br>alpha helix	-	-	-
A 816		SER	0.73	-	Favored (94.91%)<br>General / -63.5,-44.2	Favored (45.7%) *t*<br>chi angles: 180.6	0.05Å	Favored (86.459%)<br>alpha helix	-	-	-
A 817		VAL	0.72	-	Favored (96.5%)<br>Ile or Val / -64.1,-42.7	Favored (80.5%) *t*<br>chi angles: 173.2	0.04Å	Favored (92.233%)<br>alpha helix	-	-	-
A 818		TRP	0.71	-	Favored (87.76%)<br>General / -58.9,-46.5	Favored (54%) *t60*<br>chi angles: 193.1,95.8	0.11Å	Favored (93.318%)<br>alpha helix	-	-	-
A 819		ASN	0.7	-	Favored (98.05%)<br>General / -61.7,-43.9	Favored (79.9%) *m-40*<br>chi angles: 282.2,338.9	0.01Å	Favored (98.177%)<br>alpha helix	-	-	-
A 820		ARG	0.71	-	Favored (75.76%)<br>General / -59.6,-50.0	Favored (50.4%) *ttm110*<br>chi angles: 183.3,175.1,300.8,112.9	0.01Å	Favored (64.93%)<br>alpha helix	-	-	-
**#**	**Alt**	**Res**	**High B**	**Clash > 0.4Å**	**Ramachandran**	**Rotamer**	**Cβ deviation**	**CaBLAM**	**Bond lengths**	**Bond angles**	**Cis Peptides**
			Avg: 0.98	Clashscore: 1.52	Outliers: 7 of 904	Poor rotamers: 0 of 769	Outliers: 0 of 827	Outliers: 17 of 902	Outliers: 14 of 906	Outliers: 17 of 906	Non-Trans: 0 of 905
A 821		VAL	0.72	-	Favored (21.62%)<br>Ile or Val / -80.3,-46.8	Favored (93.1%) *t*<br>chi angles: 174.6	0.07Å	Favored (49.41%)<br>alpha helix	-	-	-

A 822		TRP	0.74	-	Favored (34.23%) General / -82.5,-24.4	Favored (87.3%) *m100* chi angles: 286.8,110.4	0.06Å	Favored (41.817%) alpha helix	-	-	-
A 823		ILE	0.77	-	Allowed (1.97%) Ile or Val / -110.6,-63.1	Favored (48.3%) *mm* chi angles: 303.2,299.5	0.04Å	Favored (13.979%) alpha helix	-	-	-
A 824		GLU	0.8	-	Favored (93.5%) General / -65.3,-40.2	Favored (99.8%) *mt-10* chi angles: 292.2,176.8,352.9	0.02Å	Favored (48.938%) alpha helix	-	-	-
A 825		GLU	0.83	-	Favored (57.48%) General / -91.6,0.7	Favored (96.8%) *mt-10* chi angles: 295.6,179.7,1.5	0.02Å	Favored (39.308%)	-	-	-
A 826		ASN	0.85	-	Favored (9.63%) General / -86.7,94.9	Favored (58.6%) *t0* chi angles: 184.3,348.4	0.07Å	Favored (20.62%)	-	-	-
A 827		GLU	0.87	-	Favored (38.09%) General / -61.5,-15.5	Favored (11.2%) *pt0* chi angles: 69.9,180.5,312.7	0.05Å	Favored (23.407%)	-	-	-
A 828		TYR	0.89	-	Favored (44.33%) General / -100.4,6.0	Favored (89.9%) *m-80* chi angles: 292.8,100.4	0.03Å	Favored (48.537%)	-	-	-
A 829		MET	0.89	-	Favored (46.27%) General / -105.8,122.5	Favored (60.6%) *ttm* chi angles: 182.3,176.7,282.8	0.05Å	Favored (34.616%)	-	-	-
A 830		LYS	0.89	-	Favored (79.01%) General / -66.6,-34.9	Favored (97.6%) *mttt* chi angles: 290.4,179.1,180.5,177.7	0.01Å	Favored (40.308%)	-	-	-
A 831		ASP	0.88	-	Favored (4.67%) General / -118.6,95.0	Favored (27.6%) *t0* chi angles: 176.8,338.8	0.05Å	Favored (18.614%)	-	-	-
A 832		LYS	0.86	-	Favored (6.22%) General / -84.8,60.5	Favored (98.8%) *mttt* chi angles: 295.3,181.1,176.4,182.6	0.02Å	Favored (32.315%)	-	-	-
A 833		THR	0.85	-	Favored (66.19%) Pre-Pro / -86.2,126.1	Favored (78.3%) *m* chi angles: 296.3	0.02Å	Favored (32.208%) beta sheet	-	-	-
A 834		PRO	0.84	-	Favored (59.44%) Trans-Pro / -70.8,157.1	Favored (60.8%) *Cg_endo* chi angles: 26.5,325.6,27.6	0.03Å	Favored (78.382%) beta sheet	-	-	-
A 835		LEU	0.84	-	Favored (21.77%) General / -94.1,148.8	Favored (55.9%) *mt* chi angles: 301.1,184.5	0.06Å	Favored (44.518%)	-	-	-
A 836		ALA	0.84	-	Favored (16.74%) General / -91.8,-29.4	-	0.04Å	Favored (28.401%)	-	-	-
A 837		ALA	0.85	-	Favored (39.87%) General / -144.9,161.2	-	0.05Å	Favored (22.034%)	-	-	-
A 838		TRP	0.84	-	Favored (67.94%) General / -63.1,-24.5	Favored (81.9%) *m100* chi angles: 281.9,98.9	0.02Å	Favored (52.471%) alpha helix	-	-	-

#	Alt	Res	High B	Clash > 0.4Å	Ramachandran	Rotamer	Cβ deviation	CaBLAM	Bond lengths	Bond angles	Cis Peptides
A 839		ASN	0.81	-	Favored (58.29%) General / -73.2,-9.0	Favored (81.5%) *m-40* chi angles: 287,323.8	0.05Å	Favored (58.269%) three-ten	-	-	-
A 840		ASP	0.77	-	Favored (46.15%) General / -86.8,-13.1	Favored (81.9%) *m-30* chi angles: 295.2,335	0.11Å	Favored (47.163%)	-	OUTLIER(S) worst is CA-CB-CG: 4.6 σ	-
#	Alt	Res	High B	Clash > 0.4Å	Ramachandran	Rotamer	Cβ deviation	CaBLAM	Bond lengths	Bond angles	Cis Peptides
			Avg: 0.98	Clashscore: 1.52	Outliers: 7 of 904	Poor rotamers: 0 of 769	Outliers: 0 of 827	Outliers: 17 of 902	Outliers: 14 of 906	Outliers: 17 of 906	Non-Trans: 0 of 905
A 841		ILE	0.74	-	Favored (70.39%) Pre-Pro / -87.8,122.2	Favored (92.7%) *mt* chi angles: 296.5,170.6	0.08Å	Favored (30.79%)	-	-	-
A 842		PRO	0.71	-	Favored (47.14%) Trans-Pro / -72.2,160.5	Favored (74%) *Cg_endo* chi angles: 29,324.3,27.5	0.06Å	Favored (34.339%) beta sheet	-	-	-
A 843		TYR	0.7	-	Favored (37.91%) General / -121.5,153.5	Favored (94.4%) *m-80* chi angles: 298.3,88.4	0.08Å	Favored (36.058%)	-	-	-
A 844		LEU	0.71	-	Favored (55.22%) General / -68.8,140.8	Favored (17.6%) *tp* chi angles: 192.1,64.6	0.11Å	Favored (38.269%)	-	-	-
A 845		GLY	0.73	-	Favored (41.9%) Glycine / -63.7,157.5	-	-	Favored (51.809%)	-	-	-
A 846		LYS	0.76	-	Favored (77.14%) General / -55.9,-43.3	Favored (87.6%) *tttt* chi angles: 182.1,175.9,178.6,178.5	0.03Å	Favored (63.904%)	-	-	-
A 847		ARG	0.79	-	Favored (92.35%) General / -62.7,-38.9	Favored (97.9%) *mtt180* chi angles: 289.3,178.2,179.6,172.8	0.08Å	Favored (99.33%) alpha helix	-	-	-
A 848		GLU	0.82	-	Favored (83.83%) General / -67.8,-39.6	Favored (73.4%) *mt-10* chi angles: 291.4,172,320	0.10Å	Favored (96.615%) alpha helix	-	-	-
A 849		ASP	0.85	-	Favored (87.18%) General / -60.5,-39.3	Favored (68%) *m-30* chi angles: 280.1,345.3	0.07Å	Favored (97.052%) alpha helix	-	OUTLIER(S) worst is CA-CB-CG: 4.2 σ	-
A 850		ILE	0.86	-	Favored (91.25%) Ile or Val / -65.3,-41.3	Favored (93.9%) *mt* chi angles: 291.7,168.2	0.10Å	Favored (84.195%) alpha helix	-	-	-
A 851		TRP	0.87	-	Favored (70.85%) General / -60.5,-31.8	Favored (52.7%) *m-10* chi angles: 294.2,336.6	0.06Å	Favored (77.057%) alpha helix	-	-	-
A 852		CYS	0.87	-	Favored (4.89%) General / -86.3,15.9	Favored (90.5%) *m* chi angles: 291.3	0.08Å	Favored (25.627%)	-	-	-
A 853		GLY	0.87	-	Favored (84.42%) Glycine / 82.6,9.7	-	-	Favored (33.14%)	-	-	-
A 854		SER	0.88	0.43Å OG with A 776 ASP OD2	Favored (58.26%) General / -62.0,137.5	Favored (41.7%) *t* chi angles: 176.1	0.05Å	Favored (19.132%) beta sheet	-	-	-

#	Alt	Res	High B	Clash > 0.4Å	Ramachandran	Rotamer	Cβ deviation	CaBLAM	Bond lengths	Bond angles	Cis Peptides
A 855		LEU	0.91	-	Favored (62.14%) General / -62.2,-18.8	Favored (11.8%) *tp* chi angles: 196.1,60.3	0.06Å	Favored (12.664%)	-	-	-
A 856		ILE	0.95	-	Allowed (0.24%) Ile or Val / -45.2,-23.1	Favored (18.6%) *tt* chi angles: 196,167.5	0.03Å	Favored (11.196%)	-	-	-
A 857		GLY	1	-	Favored (87.24%) Glycine / -81.3,0.8	-	-	Favored (66.762%)	-	-	-
A 858		THR	1.05	-	Favored (53.91%) General / -112.6,134.9	Favored (94.7%) *m* chi angles: 299.4	0.08Å	Favored (29.756%)	-	-	-
A 859		ARG	1.08	-	Favored (67.36%) General / -59.5,-28.7	Favored (97.9%) *mtt180* chi angles: 289.3,178.6,179.3,173.2	0.04Å	Favored (44.355%)	-	-	-
A 860		THR	1.08	-	Favored (84.97%) General / -58.8,-40.8	Favored (90.7%) *m* chi angles: 298.9	0.03Å	Favored (63.497%) alpha helix	-	-	-
**#**	**Alt**	**Res**	**High B**	**Clash > 0.4Å**	**Ramachandran**	**Rotamer**	**Cβ deviation**	**CaBLAM**	**Bond lengths**	**Bond angles**	**Cis Peptides**
			Avg: 0.98	Clashscore: 1.52	Outliers: 7 of 904	Poor rotamers: 0 of 769	Outliers: 0 of 827	Outliers: 17 of 902	Outliers: 14 of 906	Outliers: 17 of 906	Non-Trans: 0 of 905
A 861		ARG	1.07	-	Favored (97.55%) General / -62.7,-43.9	Favored (4.8%) *tmt170* chi angles: 184.6,272.8,192,197.7	0.10Å	Favored (90.485%) alpha helix	-	-	-
A 862		ALA	1.04	-	Favored (95.14%) General / -60.7,-41.4	-	0.03Å	Favored (96.378%) alpha helix	-	-	-
A 863		THR	1.02	-	Favored (95.31%) General / -63.5,-44.0	Favored (89.9%) *m* chi angles: 298.7	0.02Å	Favored (98.242%) alpha helix	-	-	-
A 864		TRP	1.01	-	Favored (85.83%) General / -58.5,-46.9	Favored (45.6%) *t60* chi angles: 165.5,84.4	0.03Å	Favored (84.882%) alpha helix	-	-	-
A 865		ALA	1.02	-	Favored (82.64%) General / -58.1,-40.9	-	0.04Å	Favored (83.947%) alpha helix	-	-	-
A 866		GLU	1.03	-	Favored (81.94%) General / -62.3,-36.4	Favored (95.7%) *mt-10* chi angles: 289.6,181.6,353.2	0.05Å	Favored (74.454%) alpha helix	-	-	-
A 867		ASN	1.03	-	Favored (28%) General / -100.2,15.3	Favored (90.1%) *m-40* chi angles: 293.4,322.3	0.03Å	Favored (39.357%) alpha helix	-	-	-
A 868		ILE	1.02	-	Favored (53.38%) Ile or Val / -55.9,-38.8	Favored (81.9%) *mt* chi angles: 290,168.7	0.07Å	Favored (40.476%) alpha helix	-	-	-
A 869		TYR	0.99	-	Favored (49%) General / -49.8,-44.9	Favored (83.8%) *t80* chi angles: 174.5,81.6	0.04Å	Favored (64.654%) alpha helix	-	-	-
A 870		ALA	0.94	-	Favored (55.76%) Pre-Pro / -57.2,-54.3	-	0.08Å	Favored (70.51%) alpha helix	-	OUTLIER(S) worst is CA-C-N: 4.2 σ	-
A 871		PRO	0.9	-	Favored (59.68%)	Favored (38.2%) *Cg_endo*	0.04Å	Favored (86.412%)	-	-	-

#	Alt	Res	High B	Clash > 0.4Å	Ramachandran	Rotamer	Cβ deviation	CaBLAM	Bond lengths	Bond angles	Cis Peptides
					Trans-Pro / -65.2,-23.6	chi angles: 23,325.8,31.3		alpha helix			
A 872		ILE	0.85	-	Favored (86.9%) Ile or Val / -65.5,-47.4	Favored (94.4%) *mt* chi angles: 291.9,168.5	0.05Å	Favored (71.351%) alpha helix	-	-	-
A 873		MET	0.82	-	Favored (86.78%) General / -58.2,-46.0	Favored (50.5%) *ttp* chi angles: 177,186.8,65.9	0.04Å	Favored (87.337%) alpha helix	-	-	-
A 874		GLN	0.8	-	Favored (94.84%) General / -61.4,-40.6	Favored (96.3%) *mt0* chi angles: 291.2,171.3,346.6	0.05Å	Favored (83.182%) alpha helix	-	-	-
A 875		ILE	0.78	-	Favored (86.17%) Ile or Val / -67.7,-44.3	Favored (94.2%) *mt* chi angles: 292.6,165.9	0.05Å	Favored (84.649%) alpha helix	-	-	-
A 876		ARG	0.78	-	Favored (74.15%) General / -58.5,-36.7	Favored (46.5%) *mtm110* chi angles: 288,188.9,282.4,103.4	0.02Å	Favored (81.304%) alpha helix	-	-	-
A 877		ASN	0.78	-	Favored (85.1%) General / -63.1,-36.9	Favored (99.2%) *m-40* chi angles: 287.7,339	0.04Å	Favored (80.488%) alpha helix	-	-	-
A 878		LEU	0.78	-	Favored (71.97%) General / -61.5,-32.0	Favored (96.2%) *mt* chi angles: 292.9,173.8	0.14Å	Favored (75.859%) alpha helix	-	-	-
A 879		ILE	0.78	0.55Å CG2 with A 879 ILE O	Favored (10.73%) Ile or Val / -90.7,-7.5	Favored (86.5%) *mt* chi angles: 290.9,169.5	0.07Å	Favored (10.41%)	-	-	-
A 880		GLY	0.78	-	Favored (29.19%) Glycine / 79.8,-147.4	-	-	Favored (49.586%)	-	-	-
**#**	**Alt**	**Res**	**High B**	**Clash > 0.4Å**	**Ramachandran**	**Rotamer**	**Cβ deviation**	**CaBLAM**	**Bond lengths**	**Bond angles**	**Cis Peptides**
			Avg: 0.98	Clashscore: 1.52	Outliers: 7 of 904	Poor rotamers: 0 of 769	Outliers: 0 of 827	Outliers: 17 of 902	Outliers: 14 of 906	Outliers: 17 of 906	Non-Trans: 0 of 905
A 881		GLU	0.78	-	Favored (49.21%) General / -86.7,2.3	Favored (97.2%) *mt-10* chi angles: 295.4,178.9,359.2	0.02Å	CaBLAM Disfavored (3.933%)	-	-	-
A 882		GLU	0.77	-	Favored (55.87%) General / -58.8,133.3	Favored (18.3%) *mm-30* chi angles: 291.9,282.7,290.8	0.07Å	Favored (24.925%)	-	-	-
A 883		GLU	0.77	-	Favored (46.12%) General / -56.0,131.0	Favored (88.2%) *tt0* chi angles: 181.7,179.4,348.1	0.04Å	Favored (21.011%) beta sheet	-	-	-
A 884		TYR	0.77	-	Favored (31.53%) General / -133.6,162.6	Favored (59%) *p90* chi angles: 64.9,92.7	0.10Å	Favored (45.6%) beta sheet	-	-	-
A 885		ARG	0.78	-	Favored (48.56%) General / -122.4,145.1	Favored (30.1%) *mmt90* chi angles: 303.9,288.6,177.4,81.4	0.03Å	Favored (38.456%) beta sheet	-	-	-
A 886		ASP	0.81	-	Favored (10.51%) General / -84.0,70.5	Favored (63%) *m-30* chi angles: 289.9,319.1	0.05Å	Favored (16.775%) beta sheet	-	-	-

A 887		TYR	0.85	-	Favored (66.58%) General / -65.2,-20.8	Favored (66.5%) *m-80* chi angles: 289.6,108.1	0.05Å	Favored (18.83%)	-	-	-
A 888		MET	0.89	-	Favored (67.4%) General / -62.6,-24.2	Favored (94%) *mmm* chi angles: 291.5,305.1,295	0.02Å	Favored (61.038%)	-	-	-
A 889		VAL	0.96	-	Favored (72.55%) Ile or Val / -62.0,-36.1	Favored (68.2%) *t* chi angles: 171.9	0.05Å	Favored (53.447%) three-ten	-	-	-
A 890		ALA	1.04	-	Favored (64.27%) General / -61.6,-21.4	-	0.03Å	Favored (67.866%)	-	-	-
A 891		GLN	1.14	-	Favored (51.72%) General / -88.4,2.9	Favored (34.6%) *mm110* chi angles: 292.2,294.6,121.2	0.04Å	Favored (10.403%)	-	-	-
A 892		ASN	1.29	-	Allowed (0.19%) General / 68.3,-31.4	Favored (61.8%) *t0* chi angles: 198.1,36.4	0.04Å	CaBLAM Disfavored (3.111%)	-	-	-
A 893		ARG	1.49	-	Favored (61.96%) General / -53.2,-37.5	Favored (55.2%) *ttt90* chi angles: 186.9,178.5,187.7,87.1	0.03Å	Favored (27.64%)	-	-	-
A 894		PHE	1.78	-	Favored (36.37%) General / -103.4,11.5	Favored (97.9%) *m-80* chi angles: 294.8,94.5	0.05Å	Favored (25.264%) alpha helix	-	-	-
A 895		GLY	2.2	-	Favored (38.86%) Glycine / -63.1,-53.6	-	-	Favored (19.408%) alpha helix	-	-	-
A 896		ARG	2.76	-	Favored (91.55%) General / -59.0,-43.9	Favored (83.2%) *ttt180* chi angles: 179.8,174.5,175.1,175.9	0.01Å	Favored (84.482%) alpha helix	-	-	-
A 897		GLU	3.49	-	Favored (93.7%) General / -65.1,-39.9	Favored (95.6%) *mt-10* chi angles: 291,183.5,354.6	0.04Å	Favored (97.357%) alpha helix	-	-	-
A 898		GLU	4.39	-	Favored (92.64%) General / -64.7,-38.9	Favored (72.4%) *tp30* chi angles: 181.3,66,19.4	0.02Å	Favored (90.461%) alpha helix	-	-	-
A 899		THR	5.39	-	Favored (97.84%) General / -60.7,-43.5	Favored (95.1%) *m* chi angles: 299.5	0.04Å	Favored (89.414%) alpha helix	-	-	-
A 900		HIS	6.42	-	Favored (91%) General / -64.5,-38.4	Favored (58.6%) *m-70* chi angles: 286.6,302.5	0.04Å	Favored (91.563%) alpha helix	-	-	-
**#**	**Alt**	**Res**	**High B**	**Clash > 0.4Å**	**Ramachandran**	**Rotamer**	**Cβ deviation**	**CaBLAM**	**Bond lengths**	**Bond angles**	**Cis Peptides**
			Avg: 0.98	Clashscore: 1.52	Outliers: 7 of 904	Poor rotamers: 0 of 769	Outliers: 0 of 827	Outliers: 17 of 902	Outliers: 14 of 906	Outliers: 17 of 906	Non-Trans: 0 of 905
A 901		VAL	7.43	-	Favored (58.72%) Ile or Val / -66.2,-51.5	Favored (65.9%) *t* chi angles: 171.6	0.06Å	Favored (72.121%) alpha helix	-	-	-
A 902		VAL	8.38	-	Favored (92.12%) Ile or Val / -61.9,-41.8	Favored (63.5%) *t* chi angles: 171.3	0.04Å	Favored (70.413%) alpha helix	-	-	-

A 903		GLY	9.23	-	Favored (83.85%) Glycine / -87.0,6.1	-	-	Favored (34.264%)	-	-	-
A 904		GLY	9.94	-	Favored (26.34%) Glycine / -100.0,-152.0	-	-	Favored (27.182%)	-	-	-
A 905		VAL	10.5	-	Favored (60.12%) Ile or Val / -113.9,119.1	Favored (70.3%) *t* chi angles: 178.7	0.03Å	-	-	-	-
A 906		LEU	10.91	-	-	Favored (54.1%) *mt* chi angles: 305.4,175.9	0.06Å	-	-	-	-

About MolProbity | Website for the Richardson Lab | Using ecloud x-H | Internal reference 4.5.2
