# Supplementary material for: Integrative proteome-wide structural analysis and high-throughput docking identify broad-spectrum antiviral scaffolds against Zika, Yellow Fever, West Nile, Saint Louis encephalitis, and Usutu viruses
Source: Front Cell Infect Microbiol. 2026 Apr 30;16:1723132. doi: 10.3389/fcimb.2026.1723132 (PMC13171538; doi:10.3389/fcimb.2026.1723132)
Supplement: Supplementary file 3 [file DataSheet3.zip › SLEV/SLEV_NS5/Mol_probity_Files/SLEV_NS5_1FH-rama.pdf]

MolProbity Ramachandran analysis

SLEV_NS5_1FH.pdb, model 1

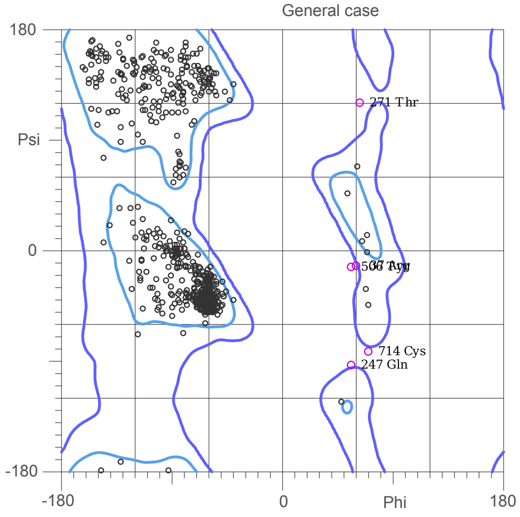

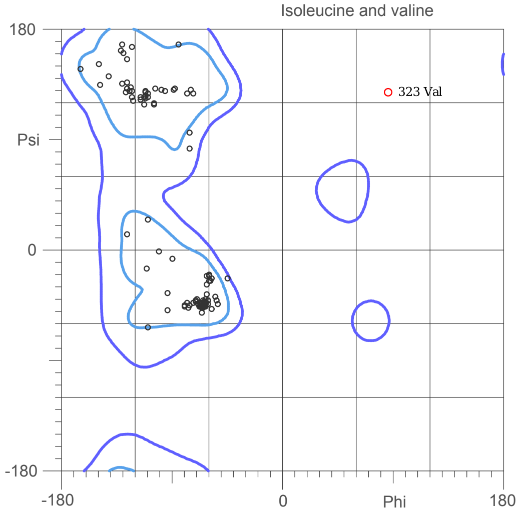

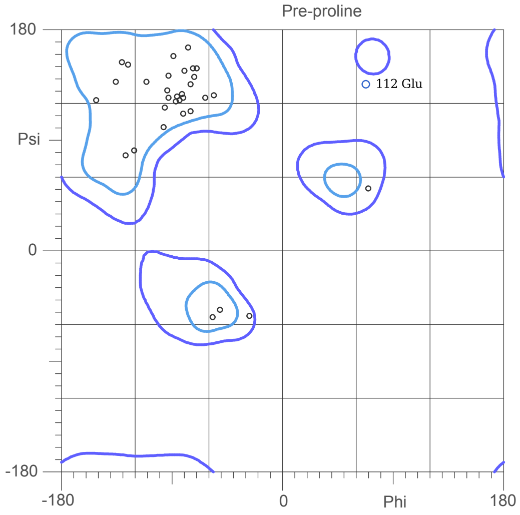

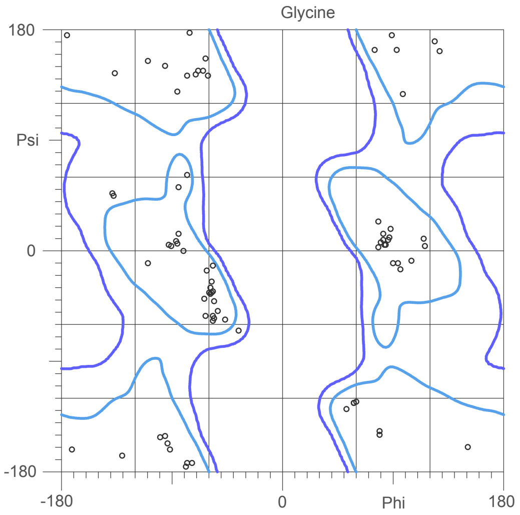

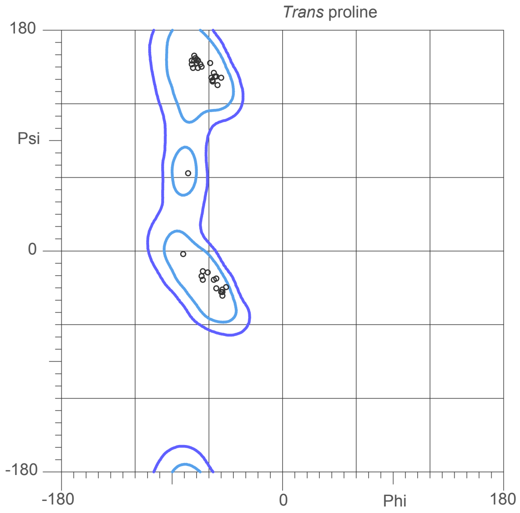

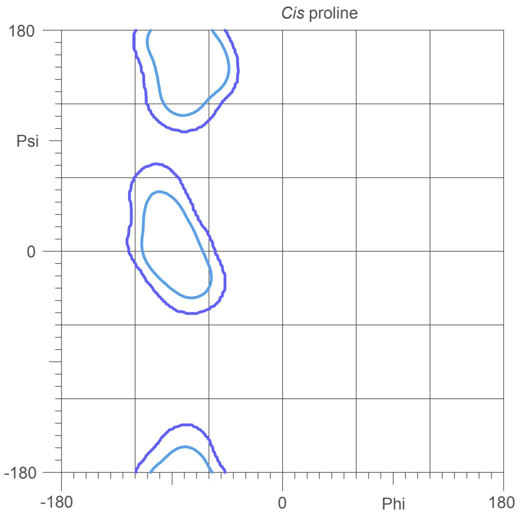

96.8% (875/904) of all residues were in favored (98%) regions.
99.2% (897/904) of all residues were in allowed (>99.8%) regions.

There were 7 outliers (phi, psi):
- 37 Arg (60.6, -12.6)
- 112 Glu (68.2, 136.7)
- 247 Gln (56.5, -93.2)
- 271 Thr (63.4, 121.1)
- 323 Val (86.8, 129.2)
- 506 Tyr (56.1, -13.7)
- 714 Cys (70.1, -82.4)
