# Supplementary figures and images for: Integrative proteome-wide structural analysis and high-throughput docking identify broad-spectrum antiviral scaffolds against Zika, Yellow Fever, West Nile, Saint Louis encephalitis, and Usutu viruses
Source: Front Cell Infect Microbiol. 2026 Apr 30;16:1723132. doi: 10.3389/fcimb.2026.1723132 (PMC13171538; doi:10.3389/fcimb.2026.1723132)

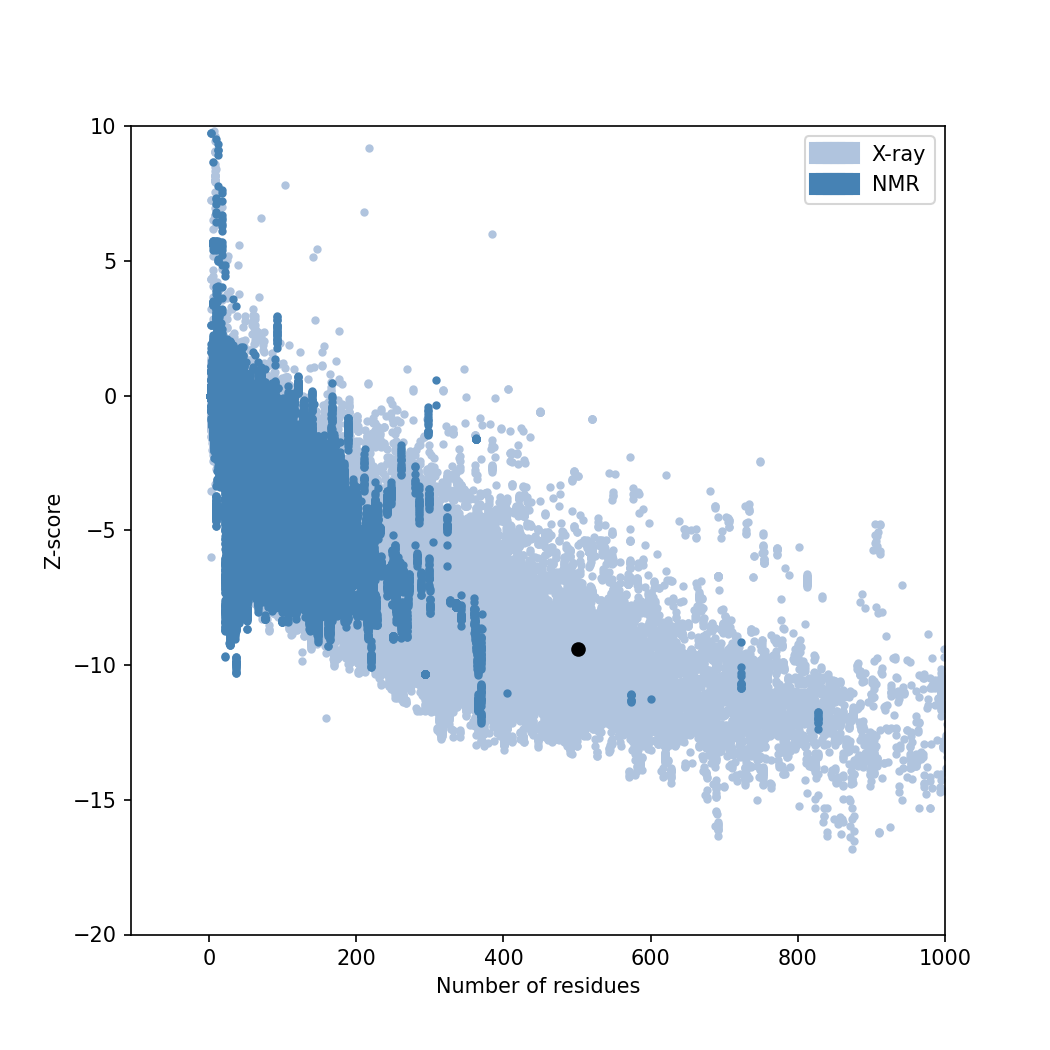

Supplement: Supplementary file 3 [file DataSheet3.zip › SLEV/SLEV_E/ProSA_global_analysis/SLEV_E_Global_z_score.png]

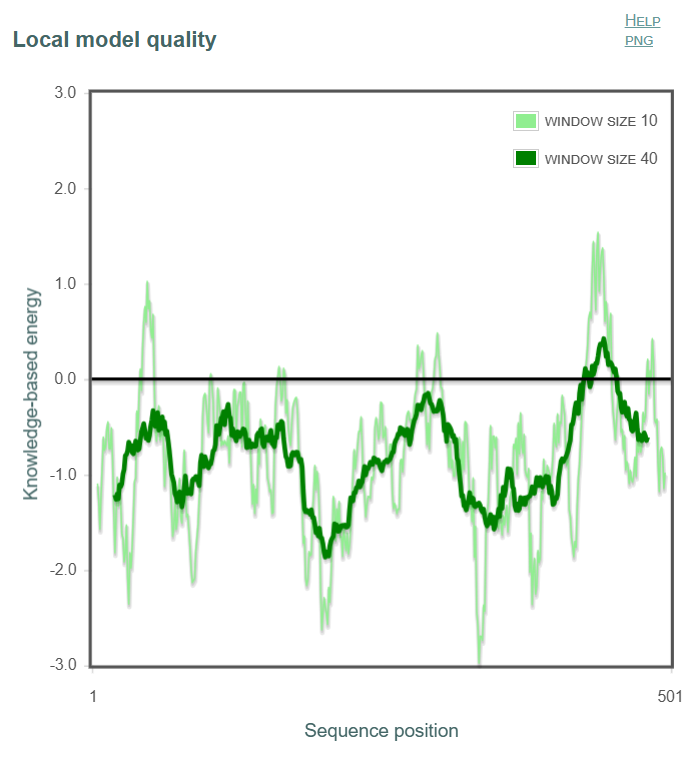

Supplement: Supplementary file 3 [file DataSheet3.zip › SLEV/SLEV_E/ProSA_global_analysis/SLEV_E_Local_model.png]

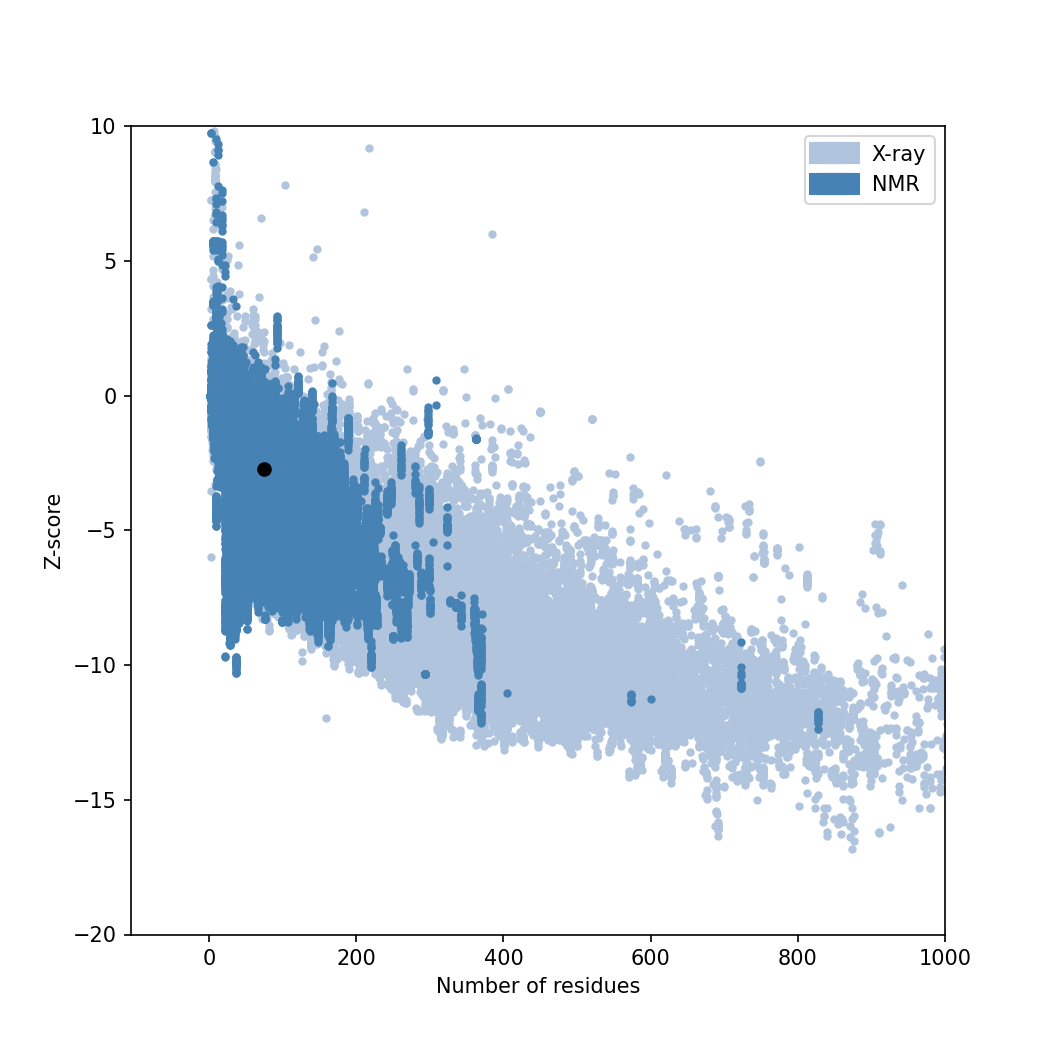

Supplement: Supplementary file 3 [file DataSheet3.zip › SLEV/SLEV_M/ProSA_global_analysis/SLEV_M_Global_z_score.png]

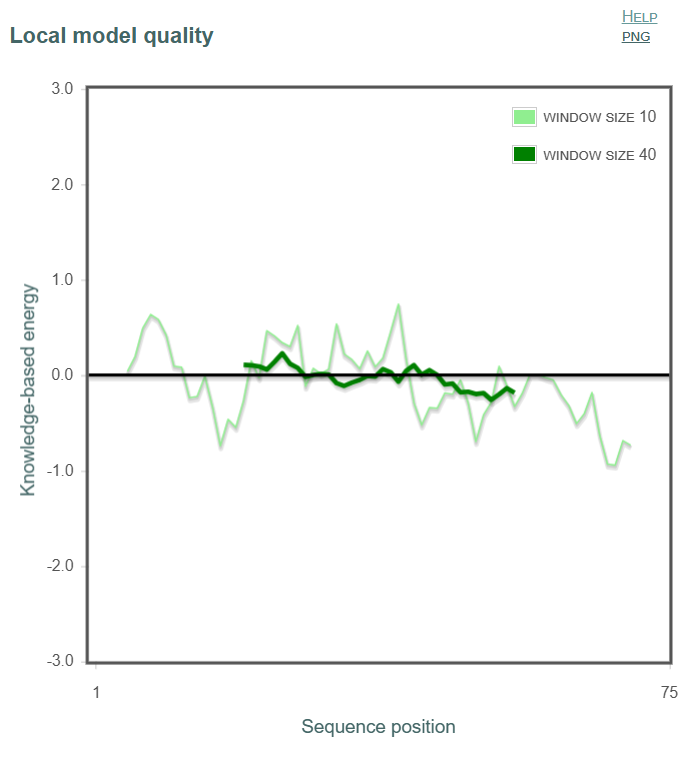

Supplement: Supplementary file 3 [file DataSheet3.zip › SLEV/SLEV_M/ProSA_global_analysis/SLEV_M_Local_model.png]

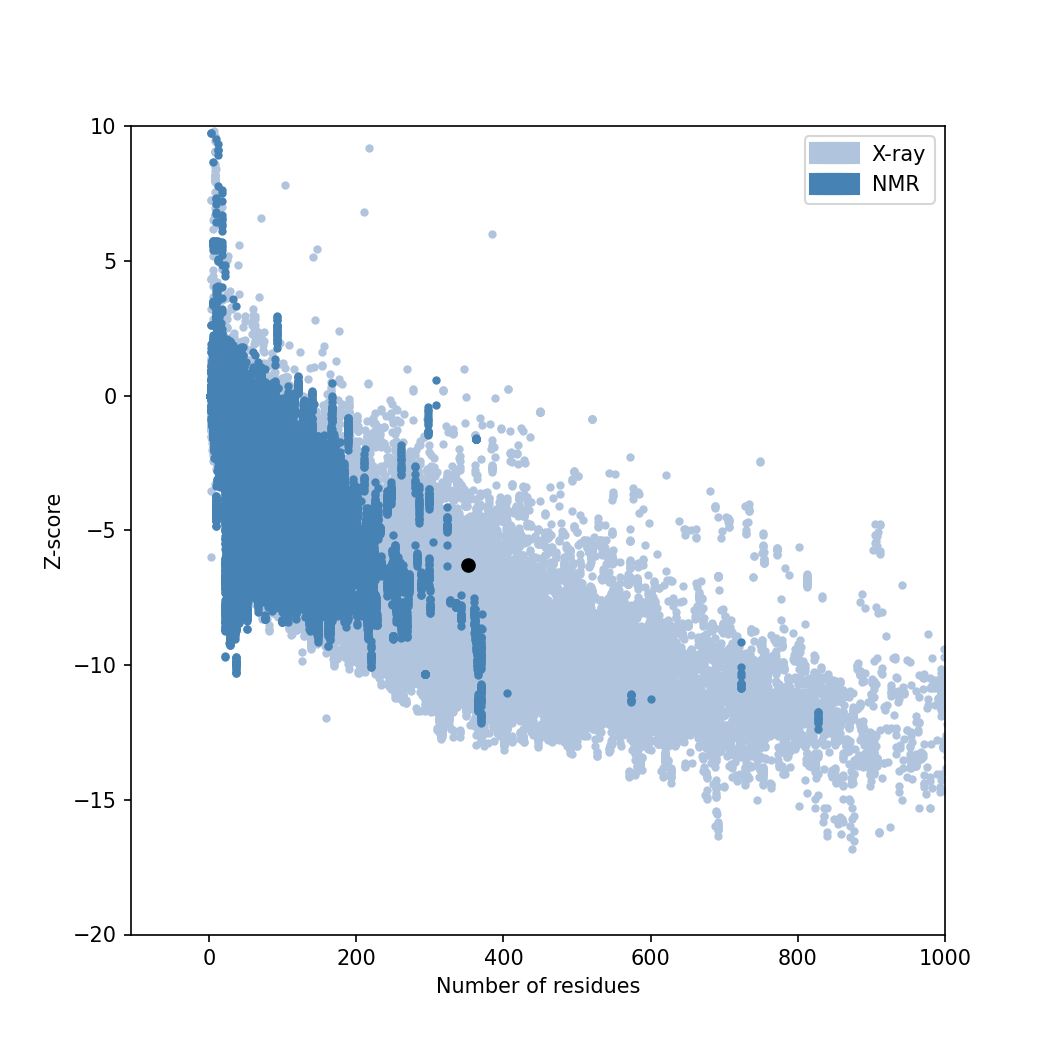

Supplement: Supplementary file 3 [file DataSheet3.zip › SLEV/SLEV_NS1/ProSA_global_analysis/SLEV_NS1_Global_z_score.png]

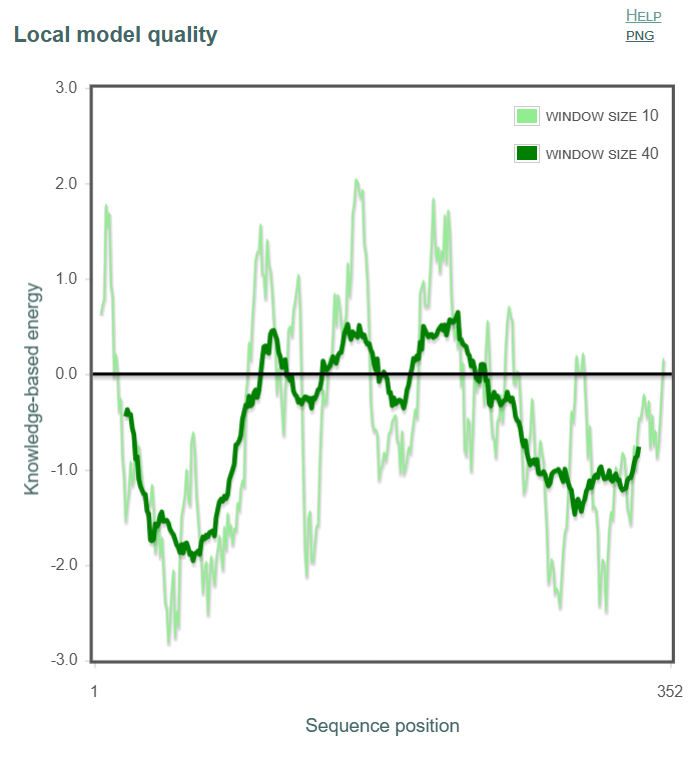

Supplement: Supplementary file 3 [file DataSheet3.zip › SLEV/SLEV_NS1/ProSA_global_analysis/SLEV_NS1_Local_model.png]

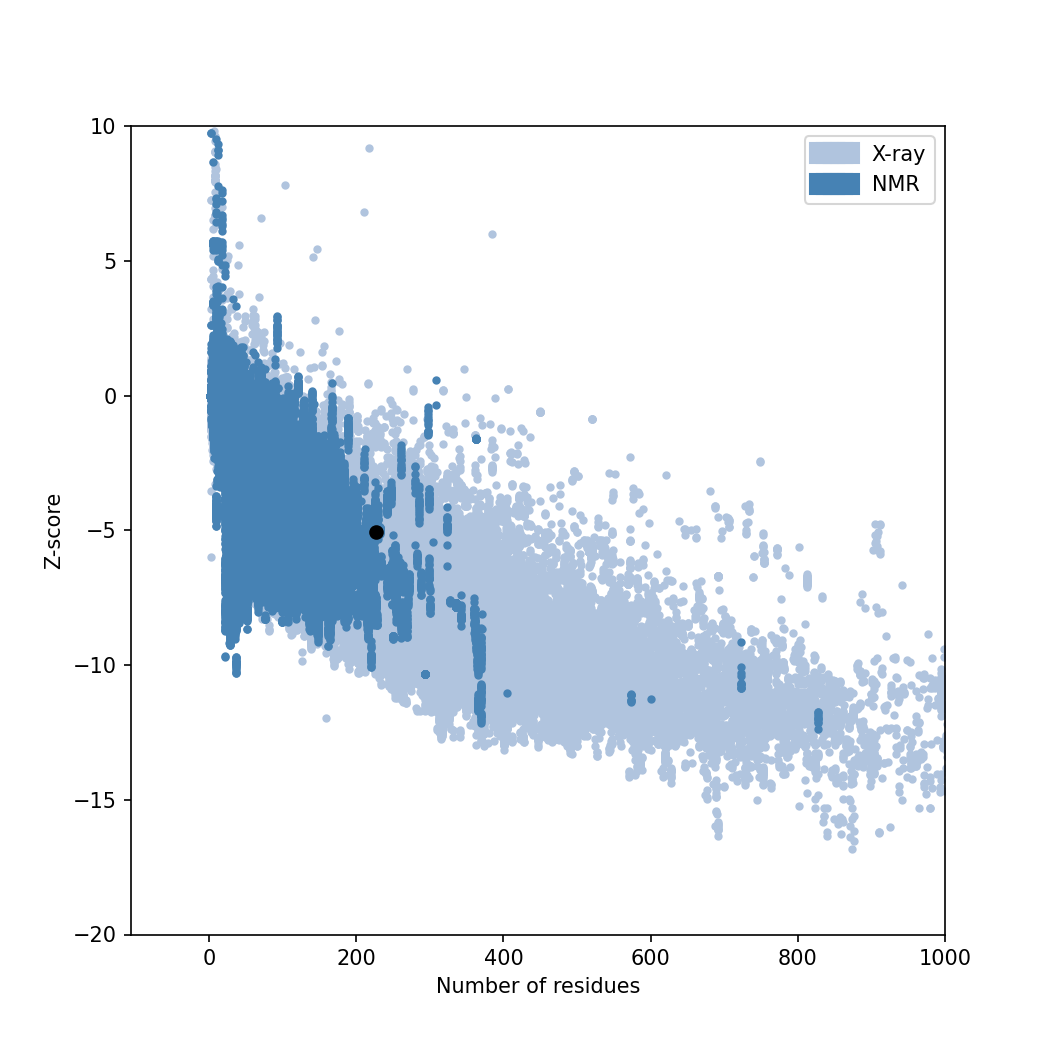

Supplement: Supplementary file 3 [file DataSheet3.zip › SLEV/SLEV_NS2a/ProSA_global_analysis/SLEV_NS2a_Global_z_score.png]

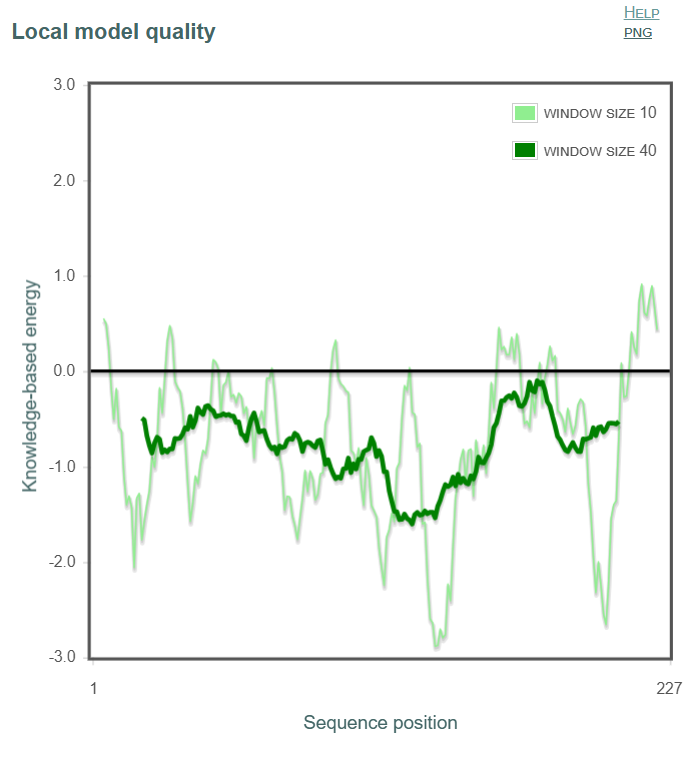

Supplement: Supplementary file 3 [file DataSheet3.zip › SLEV/SLEV_NS2a/ProSA_global_analysis/SLEV_NS2a_Local_model.png]

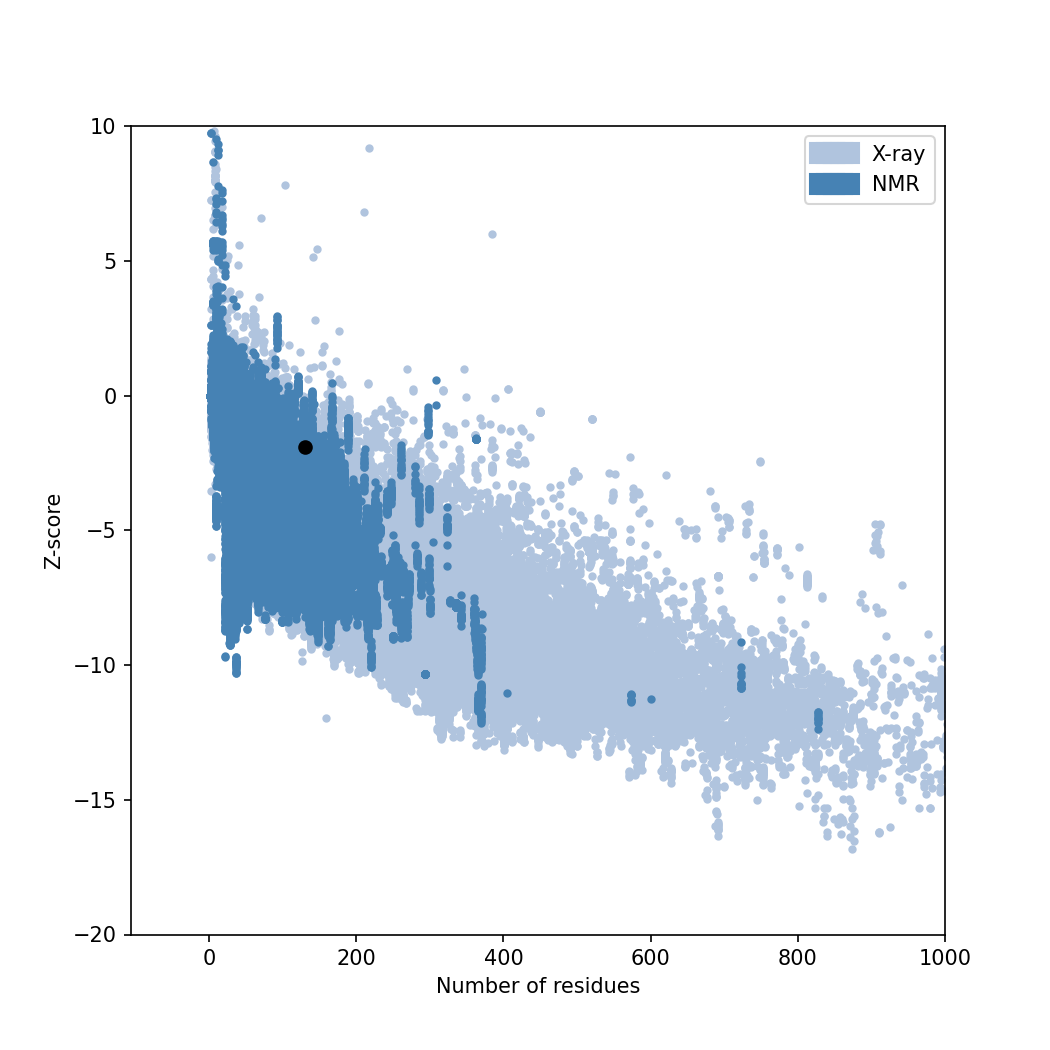

Supplement: Supplementary file 3 [file DataSheet3.zip › SLEV/SLEV_NS2b/ProSA_global_analysis/SLEV_NS2b_Global_z_score.png]

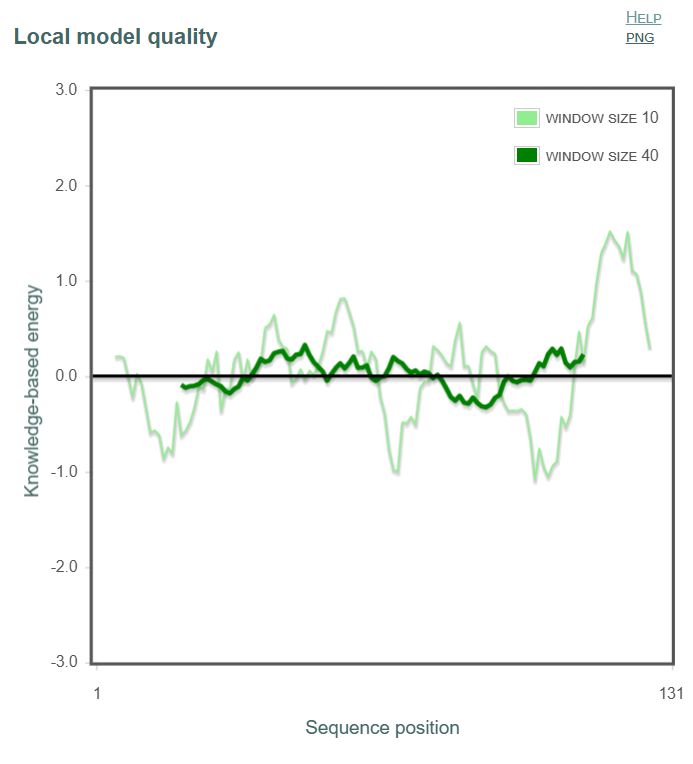

Supplement: Supplementary file 3 [file DataSheet3.zip › SLEV/SLEV_NS2b/ProSA_global_analysis/SLEV_NS2b_Local_model.png]

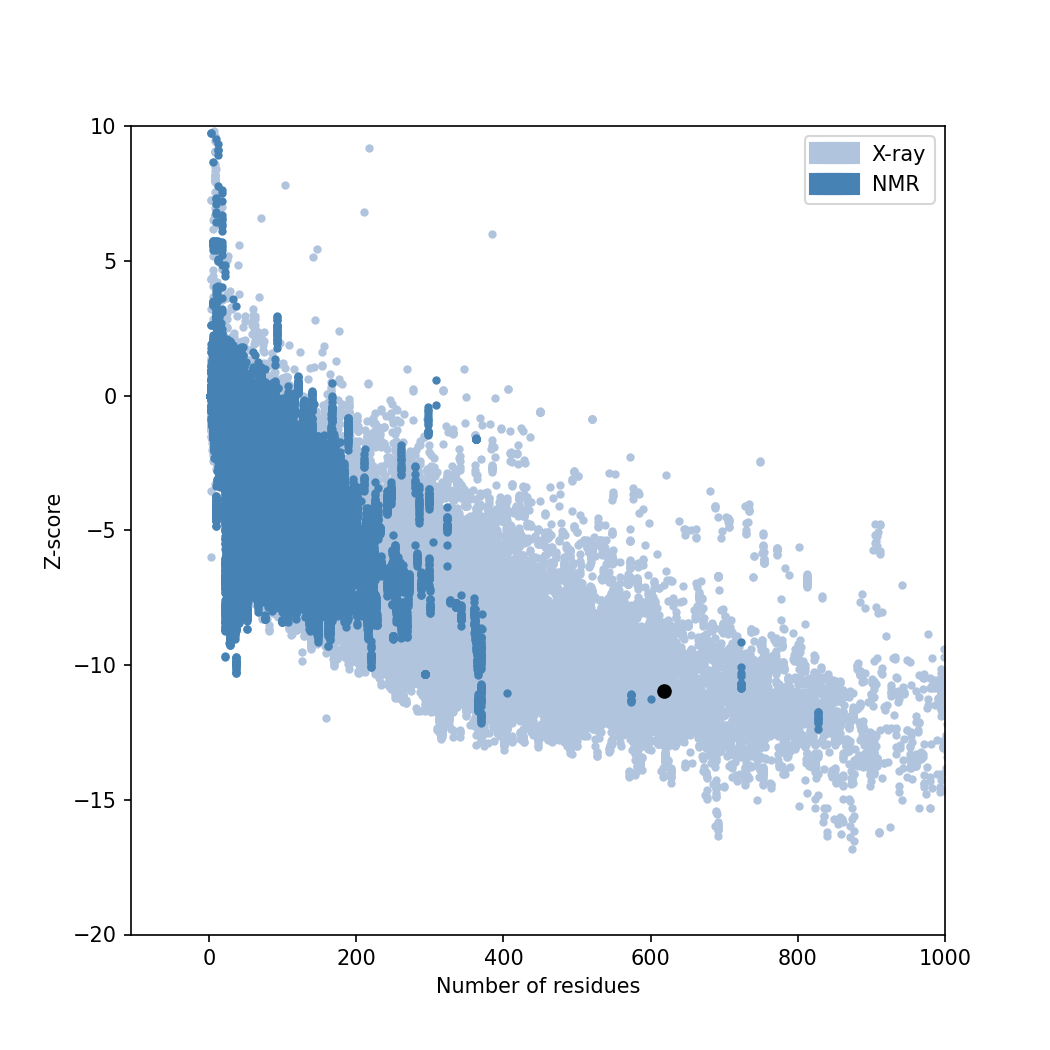

Supplement: Supplementary file 3 [file DataSheet3.zip › SLEV/SLEV_NS3/ProSA_global_analysis/SLEV_NS3_Global_z_score.png]

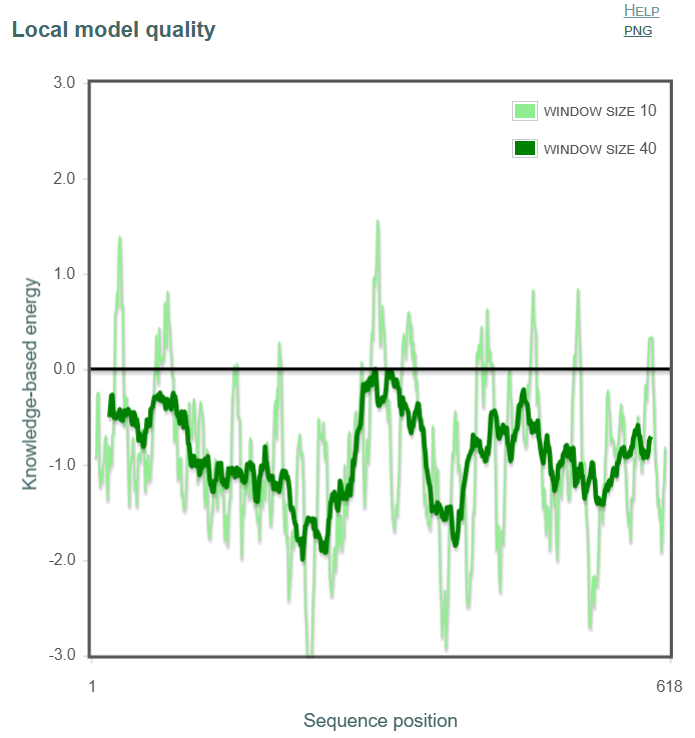

Supplement: Supplementary file 3 [file DataSheet3.zip › SLEV/SLEV_NS3/ProSA_global_analysis/SLEV_NS3_Local_model.png]

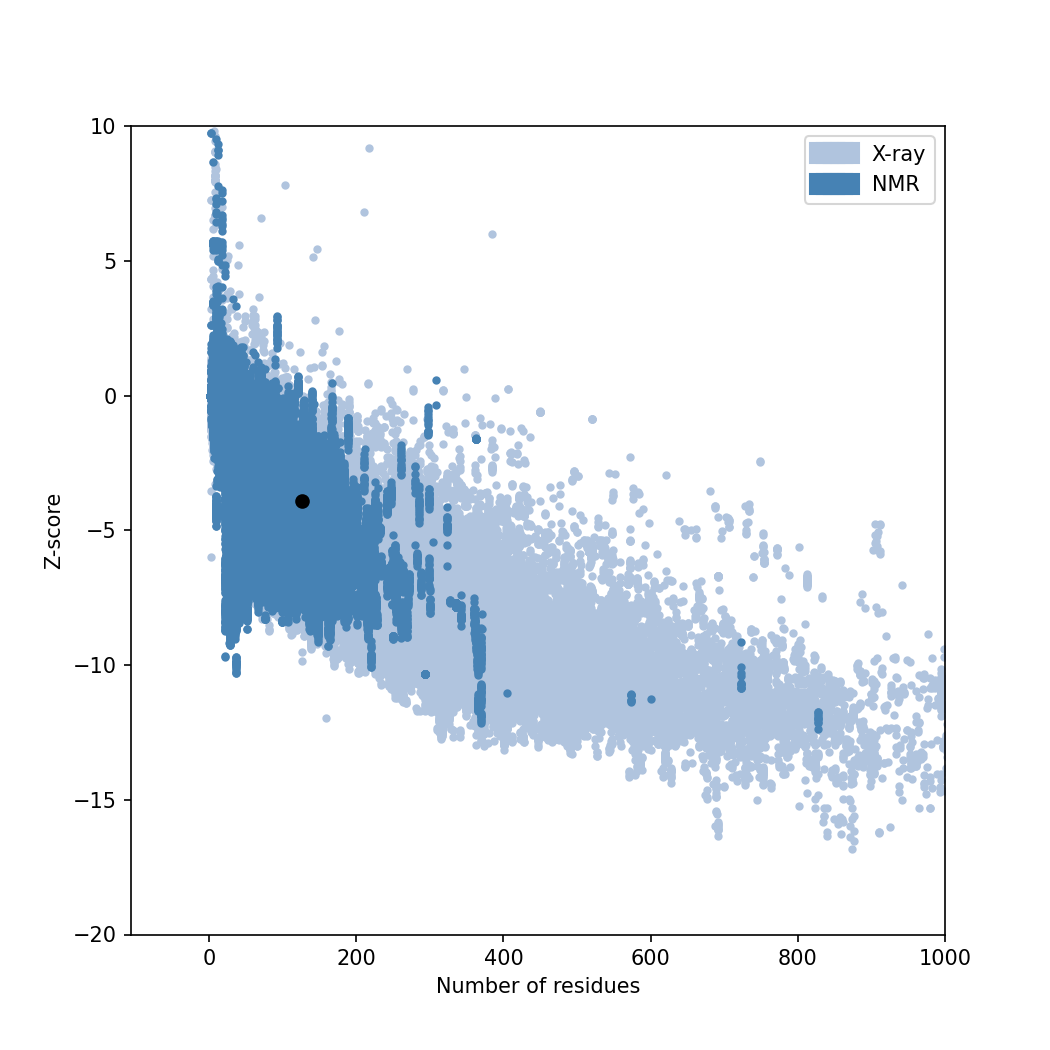

Supplement: Supplementary file 3 [file DataSheet3.zip › SLEV/SLEV_NS4a/ProSA_global_analysis/SLEV_NS4a_Global_z_score.png]

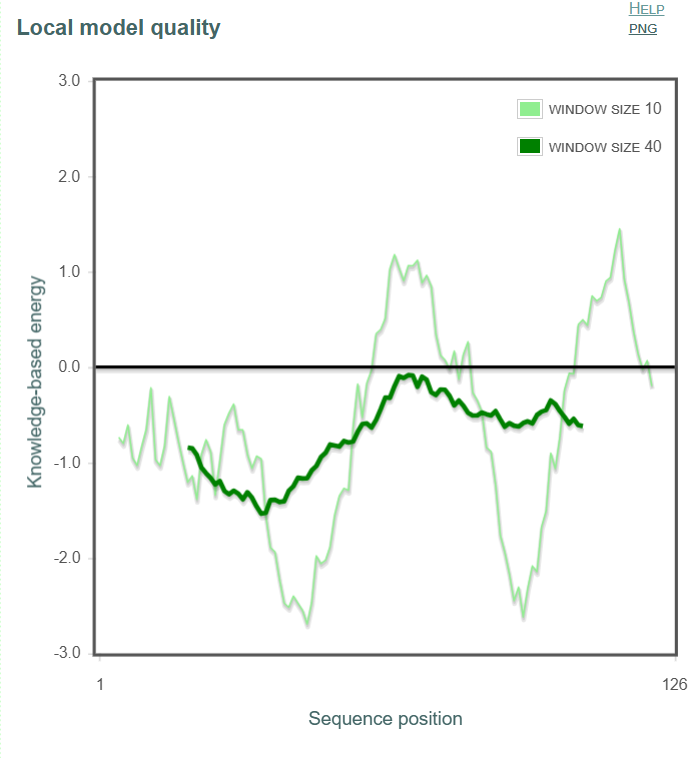

Supplement: Supplementary file 3 [file DataSheet3.zip › SLEV/SLEV_NS4a/ProSA_global_analysis/SLEV_NS4a_Local_model.png]

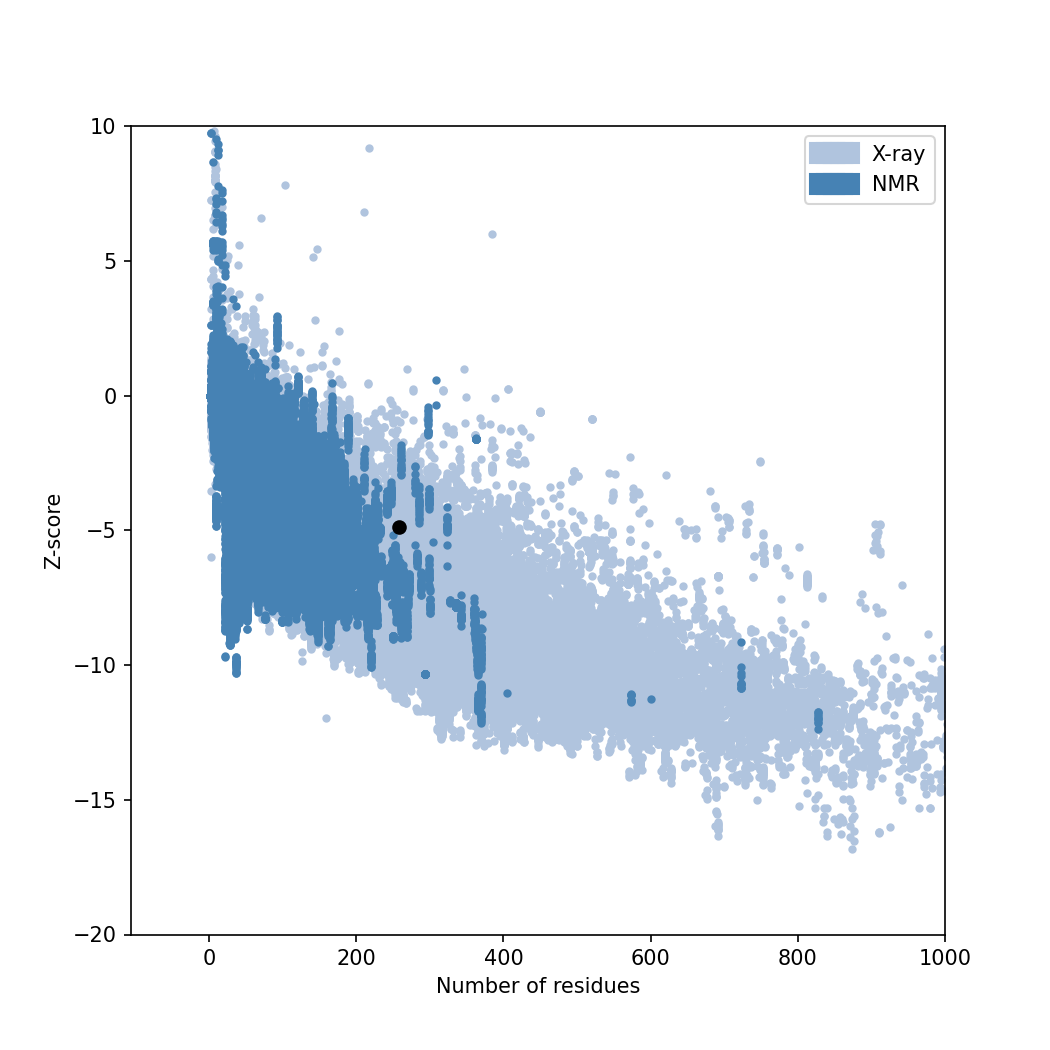

Supplement: Supplementary file 3 [file DataSheet3.zip › SLEV/SLEV_NS4b/ProSA_global_analysis/SLEV_NS4b_Global_z_score.png]

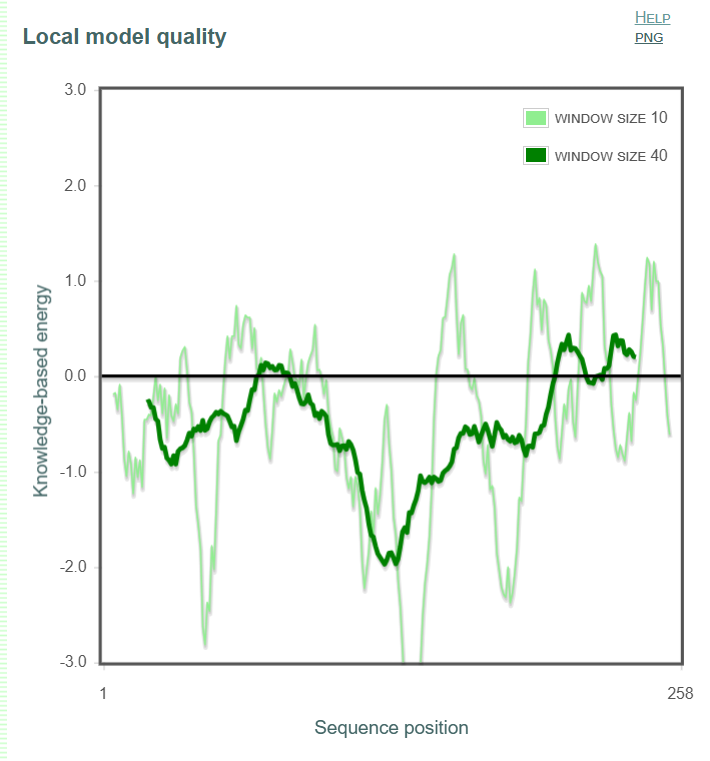

Supplement: Supplementary file 3 [file DataSheet3.zip › SLEV/SLEV_NS4b/ProSA_global_analysis/SLEV_NS4b_Local_model.png]

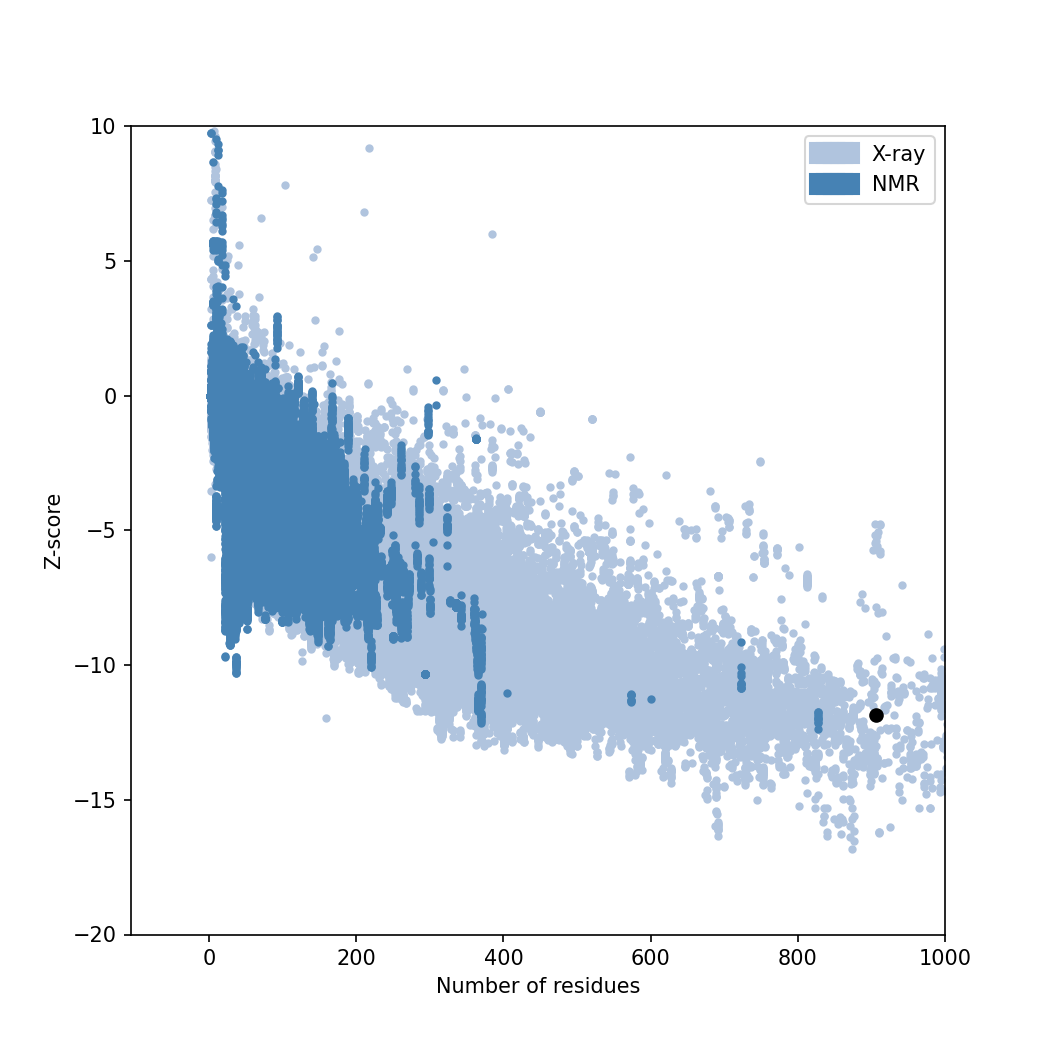

Supplement: Supplementary file 3 [file DataSheet3.zip › SLEV/SLEV_NS5/ProSA_global_analysis/SLEV_NS5_Global_z_score.png]

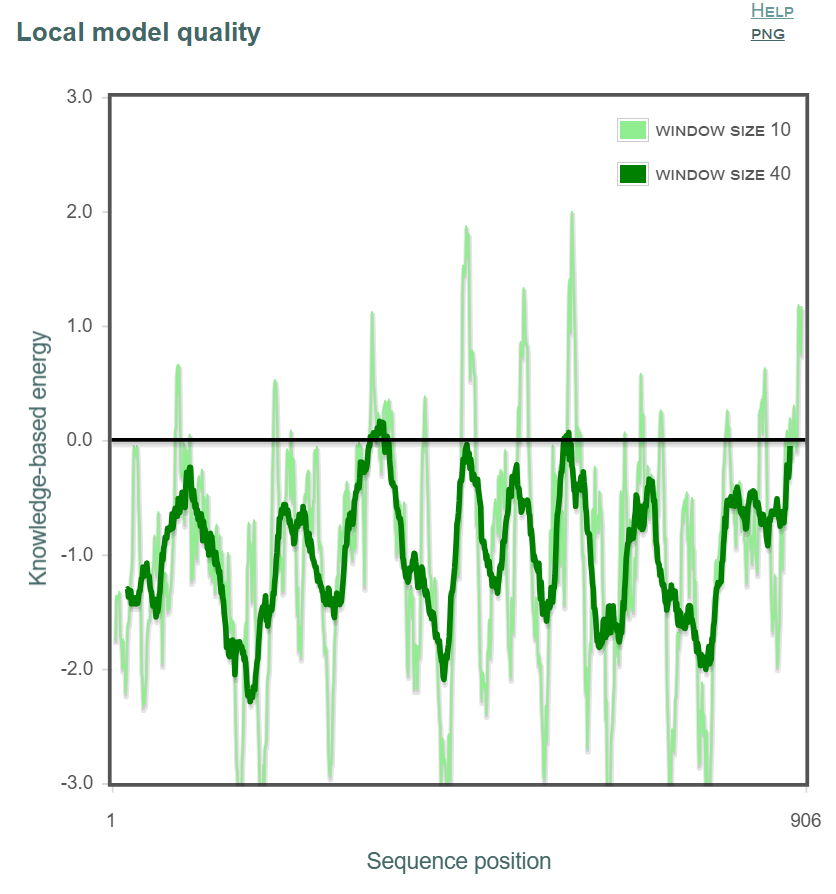

Supplement: Supplementary file 3 [file DataSheet3.zip › SLEV/SLEV_NS5/ProSA_global_analysis/SLEV_NS5_Local_model.png]

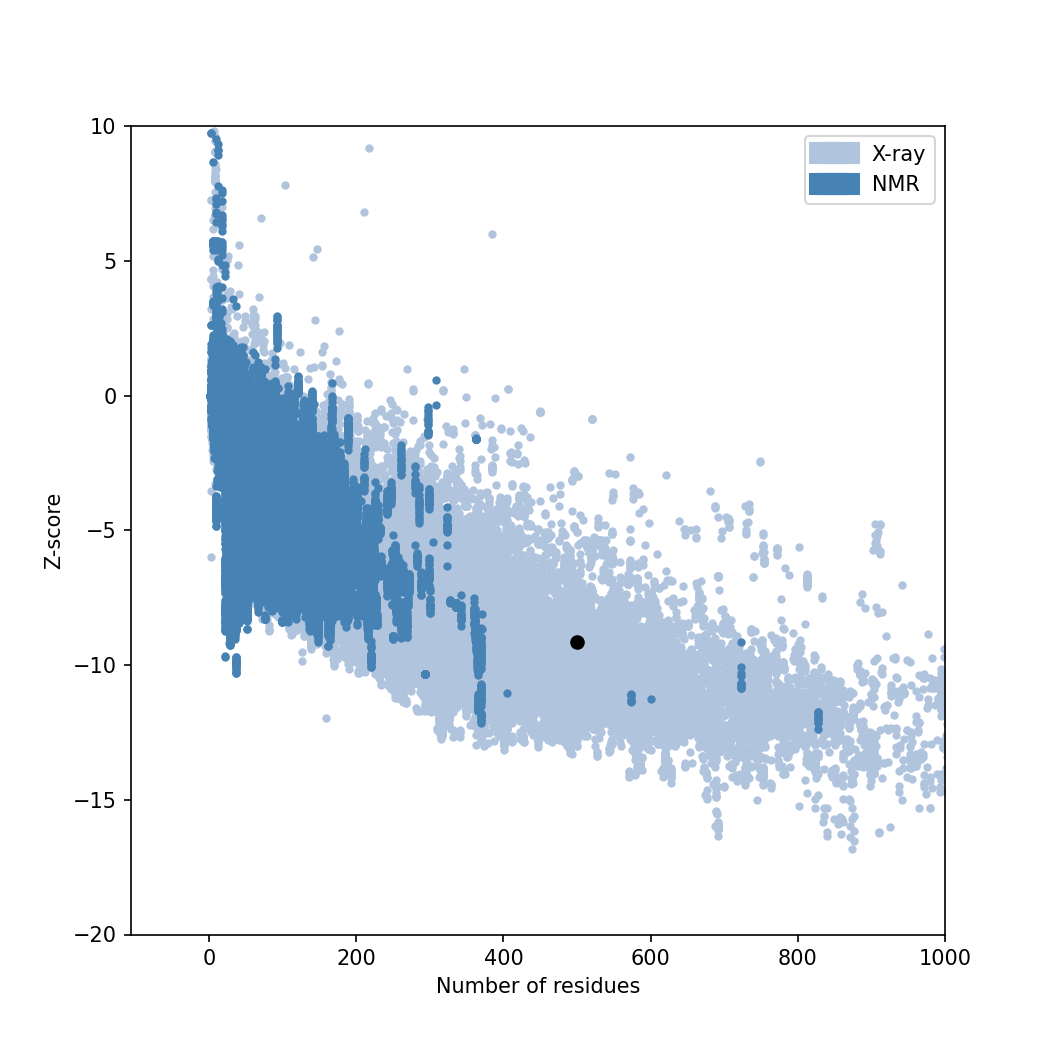

Supplement: Supplementary file 4 [file DataSheet4.zip › USUV/USU_E/ProSA_global_analysis/USU_E_Global_z_score.png]

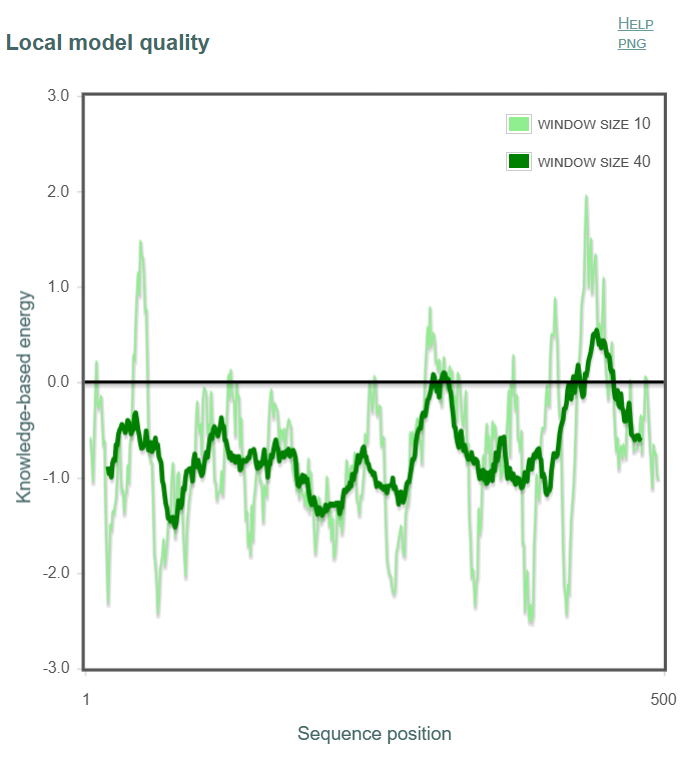

Supplement: Supplementary file 4 [file DataSheet4.zip › USUV/USU_E/ProSA_global_analysis/USU_E_Local_model.png]

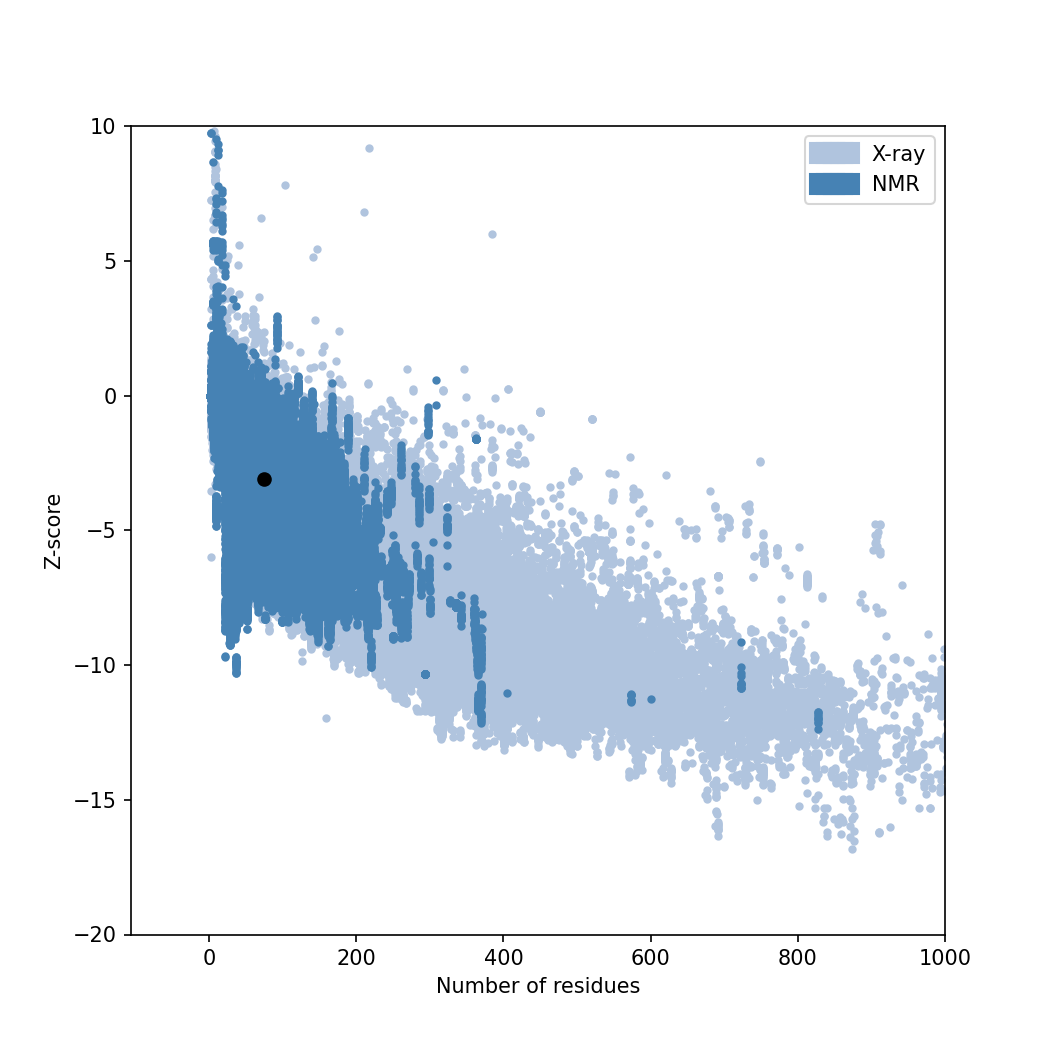

Supplement: Supplementary file 4 [file DataSheet4.zip › USUV/USU_M/ProSA_global_analysis/USU_M_Global_z_score.png]

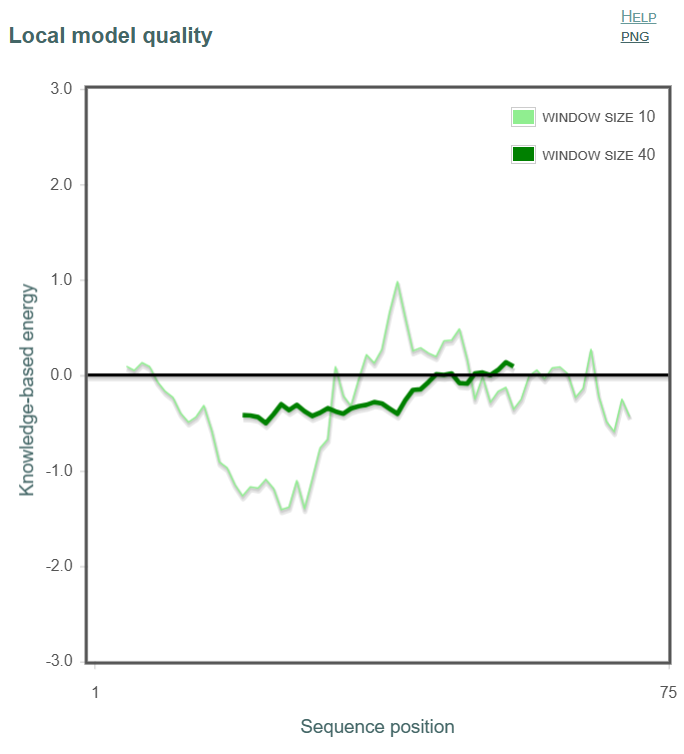

Supplement: Supplementary file 4 [file DataSheet4.zip › USUV/USU_M/ProSA_global_analysis/USU_M_Local_model.png]

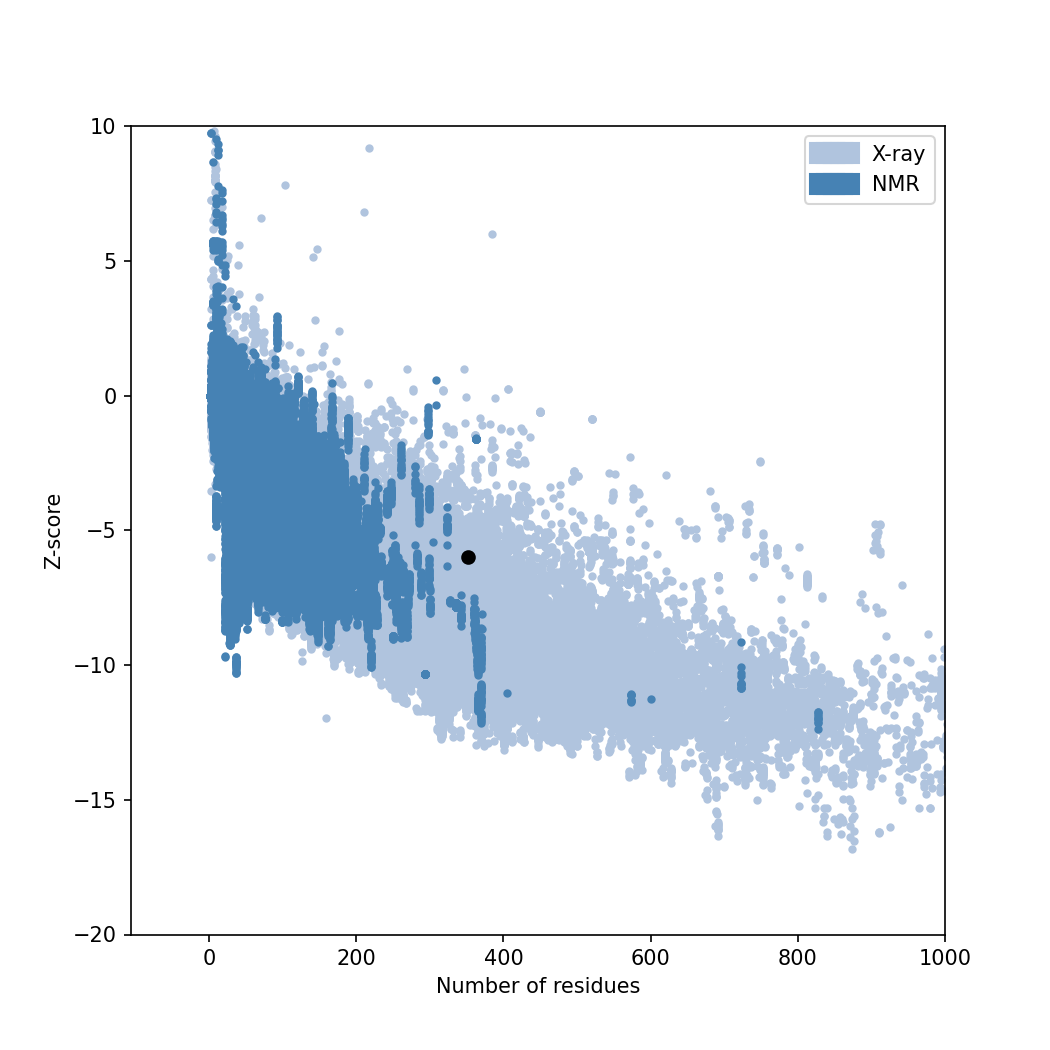

Supplement: Supplementary file 4 [file DataSheet4.zip › USUV/USU_NS1/ProSA_global_analysis/USU_NS1_Global_z_score.png]

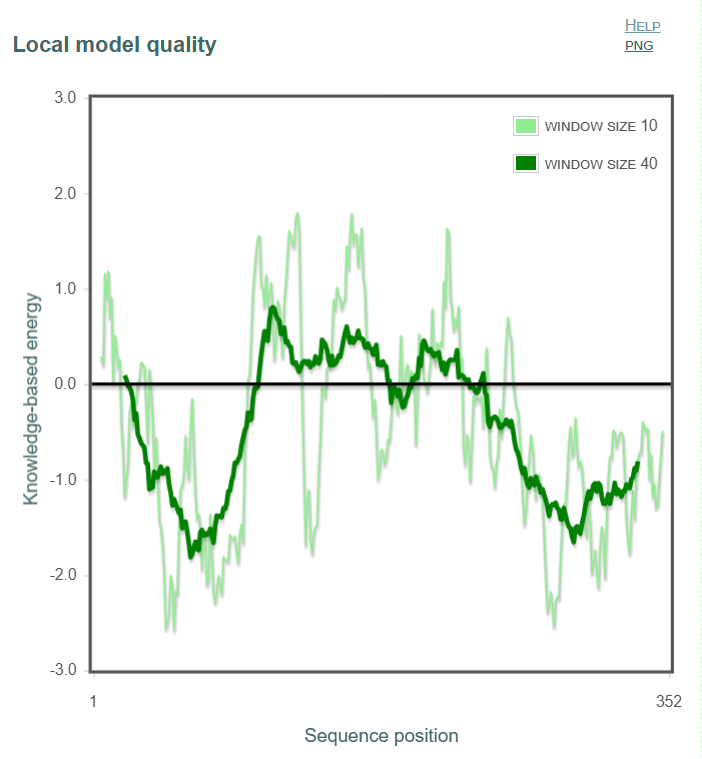

Supplement: Supplementary file 4 [file DataSheet4.zip › USUV/USU_NS1/ProSA_global_analysis/USU_NS1_Local_model.png]

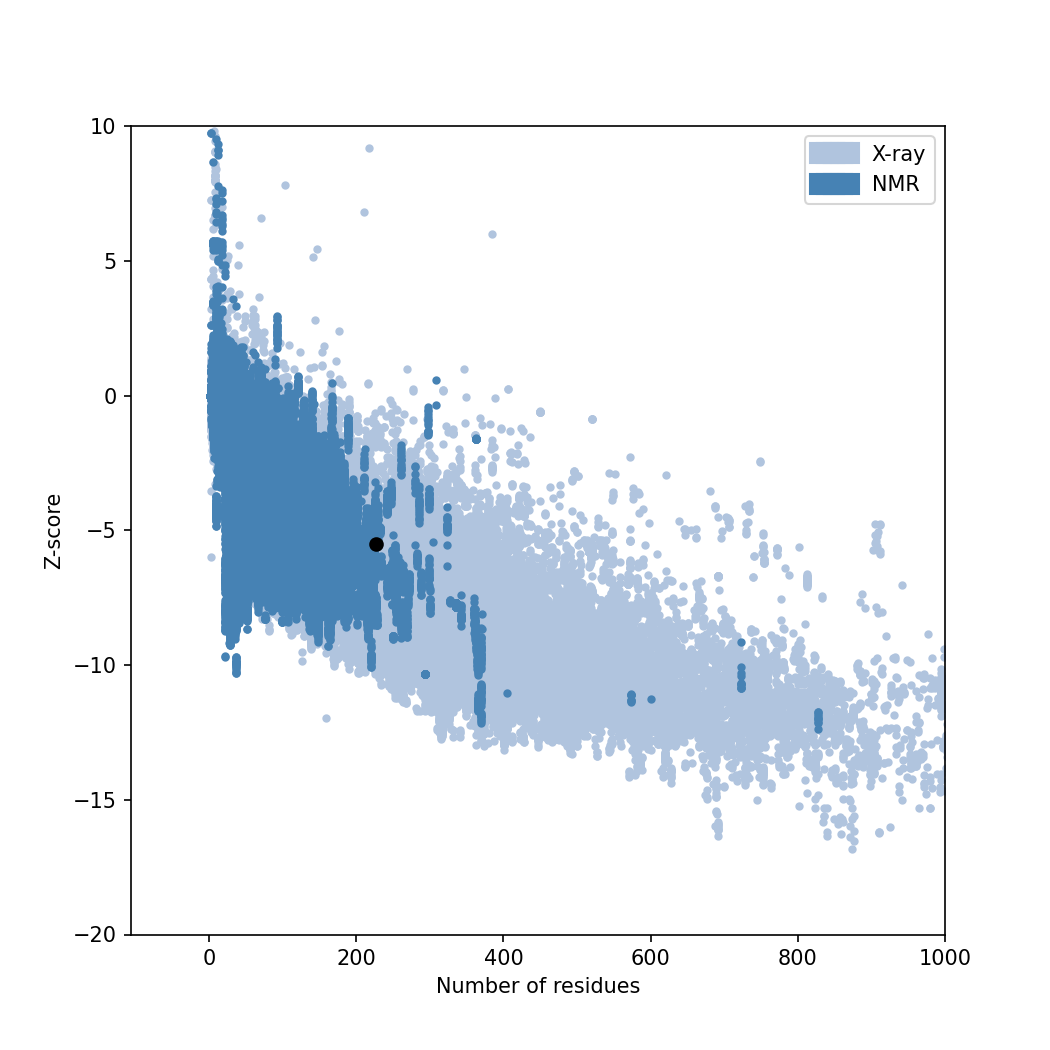

Supplement: Supplementary file 4 [file DataSheet4.zip › USUV/USU_NS2a/ProSA_global_analysis/USU_NS2a_Global_z_score.png]

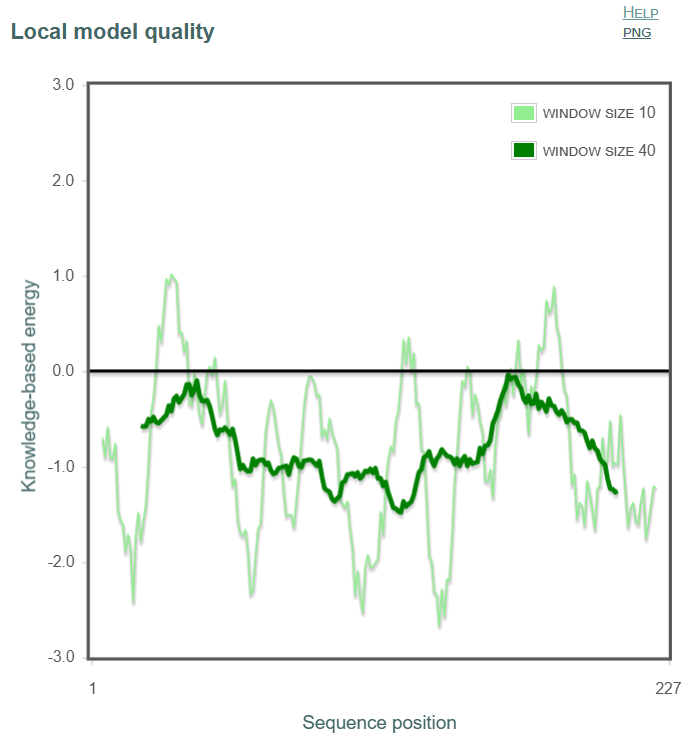

Supplement: Supplementary file 4 [file DataSheet4.zip › USUV/USU_NS2a/ProSA_global_analysis/USU_NS2a_Local_model.png]

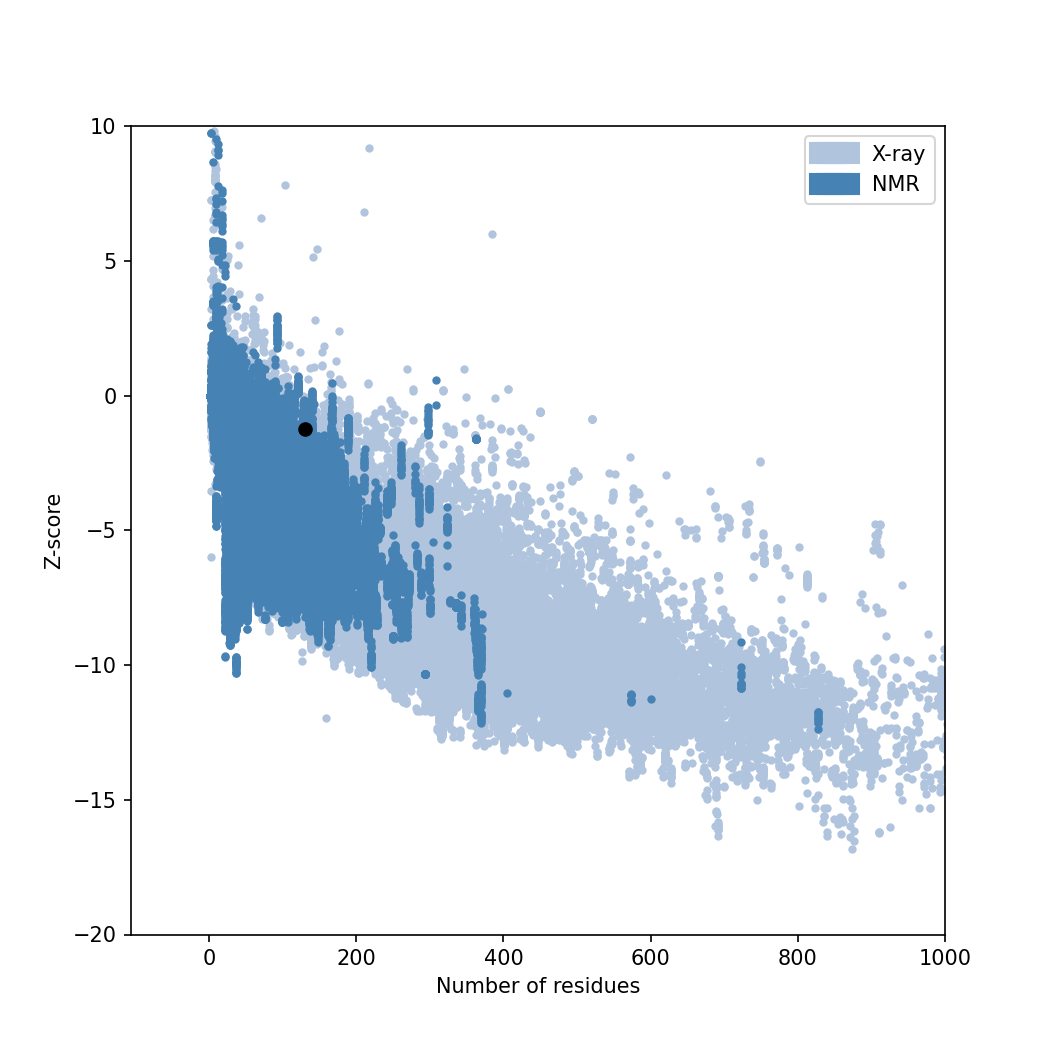

Supplement: Supplementary file 4 [file DataSheet4.zip › USUV/USU_NS2b/ProSA_global_analysis/USU_NS2b_Global_z_score.png]

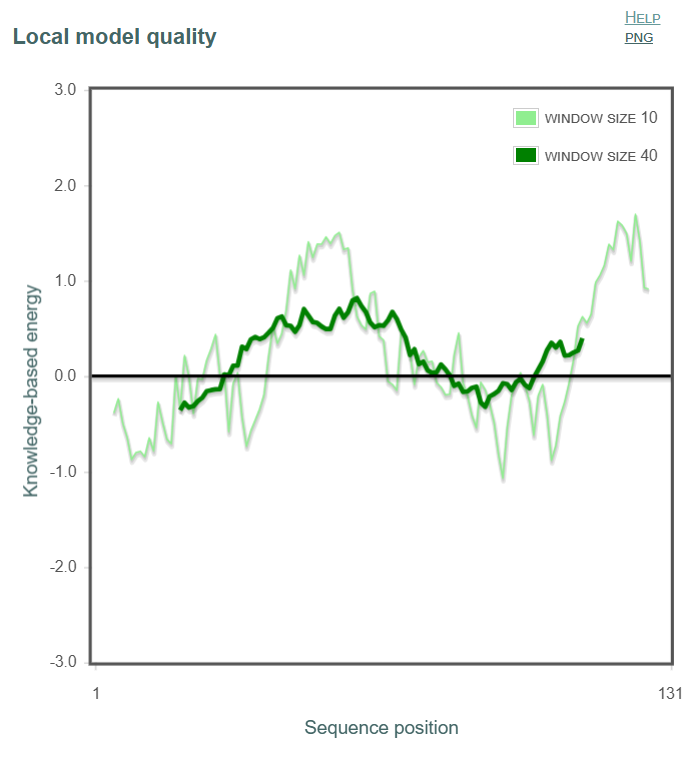

Supplement: Supplementary file 4 [file DataSheet4.zip › USUV/USU_NS2b/ProSA_global_analysis/USU_NS2b_Local_model.png]

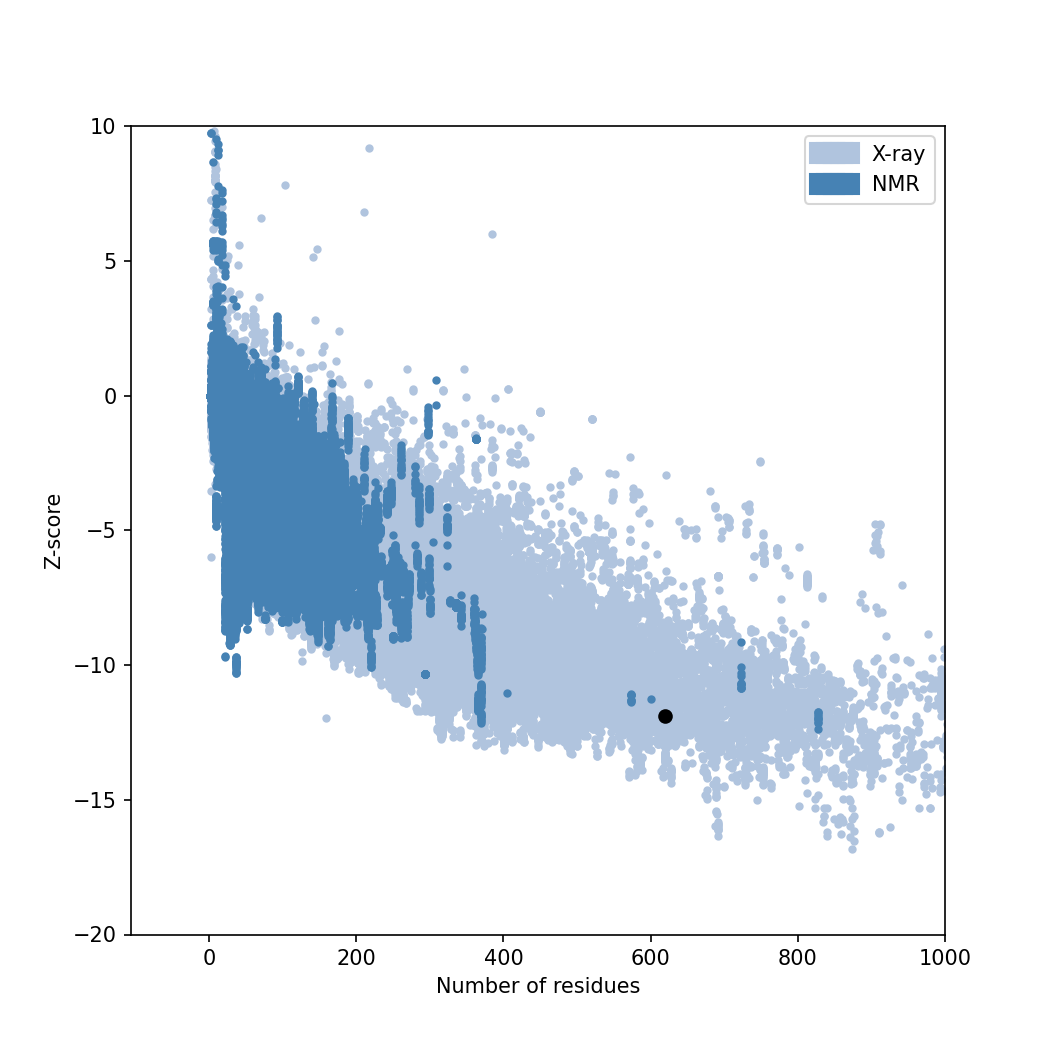

Supplement: Supplementary file 4 [file DataSheet4.zip › USUV/USU_NS3/ProSA_global_analysis/USU_NS3_Global_z_score.png]

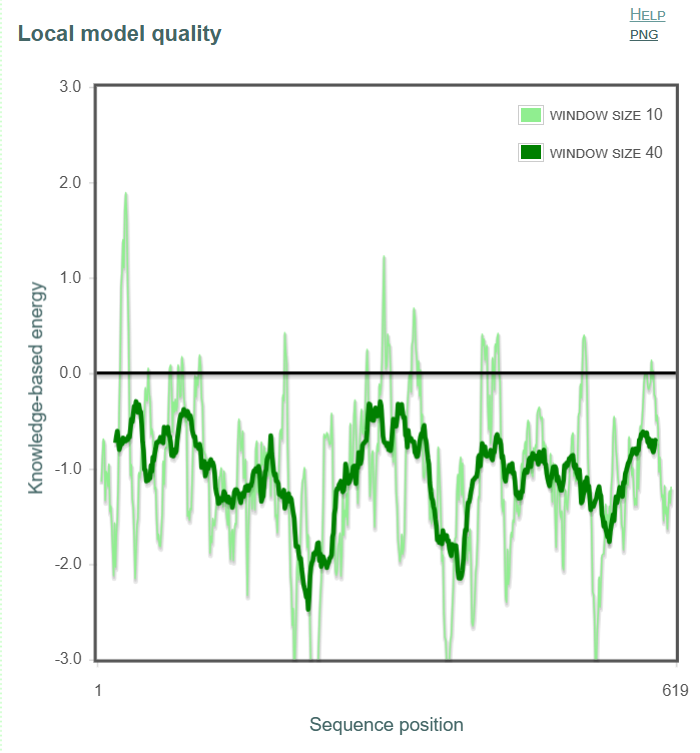

Supplement: Supplementary file 4 [file DataSheet4.zip › USUV/USU_NS3/ProSA_global_analysis/USU_NS3_Local_model.png]

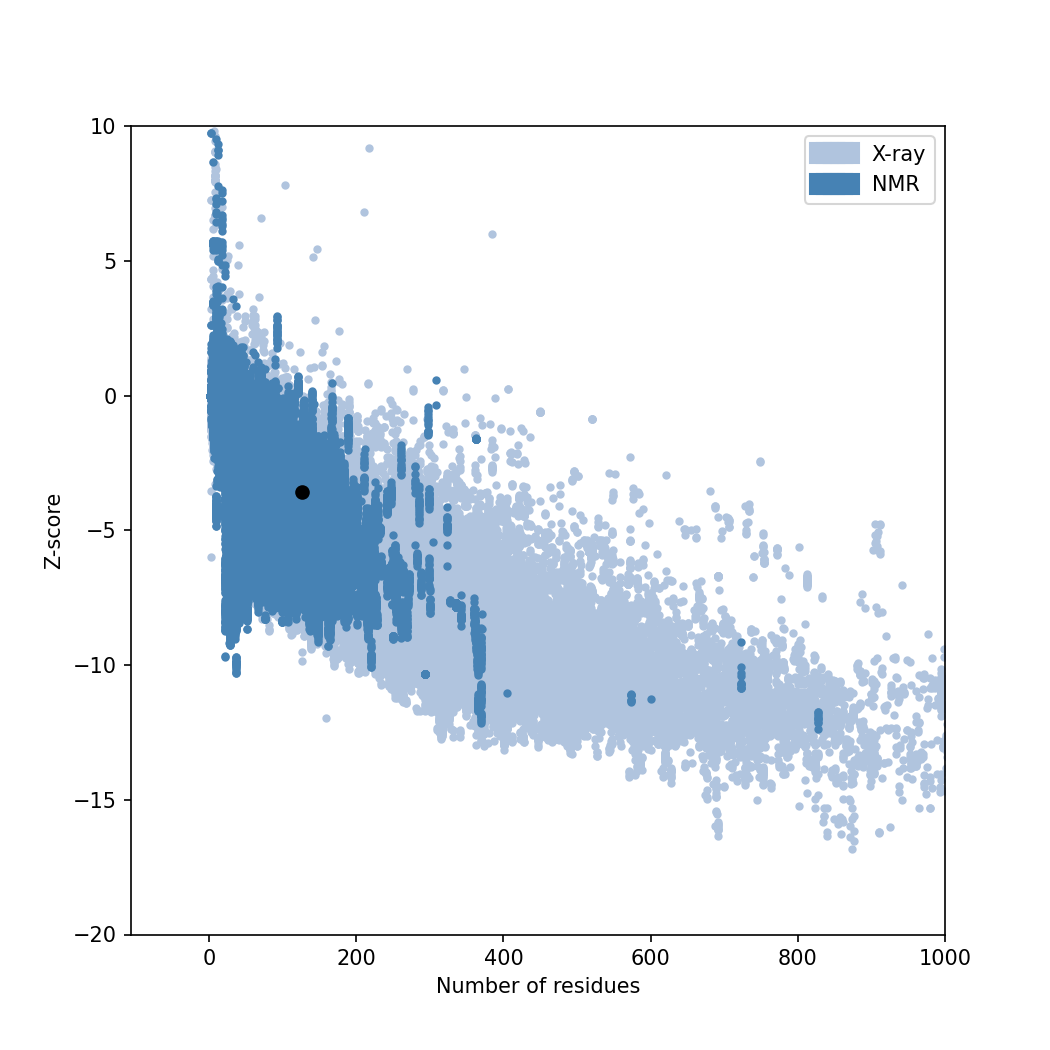

Supplement: Supplementary file 4 [file DataSheet4.zip › USUV/USU_NS4a/ProSA_global_analysis/USU_NS4a_Global_z_score.png]

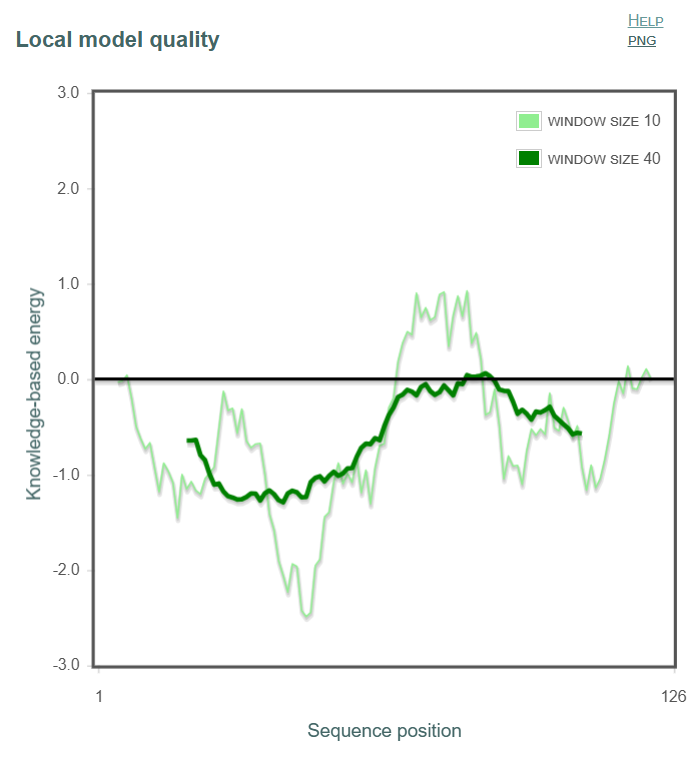

Supplement: Supplementary file 4 [file DataSheet4.zip › USUV/USU_NS4a/ProSA_global_analysis/USU_NS4a_Local_model.png]

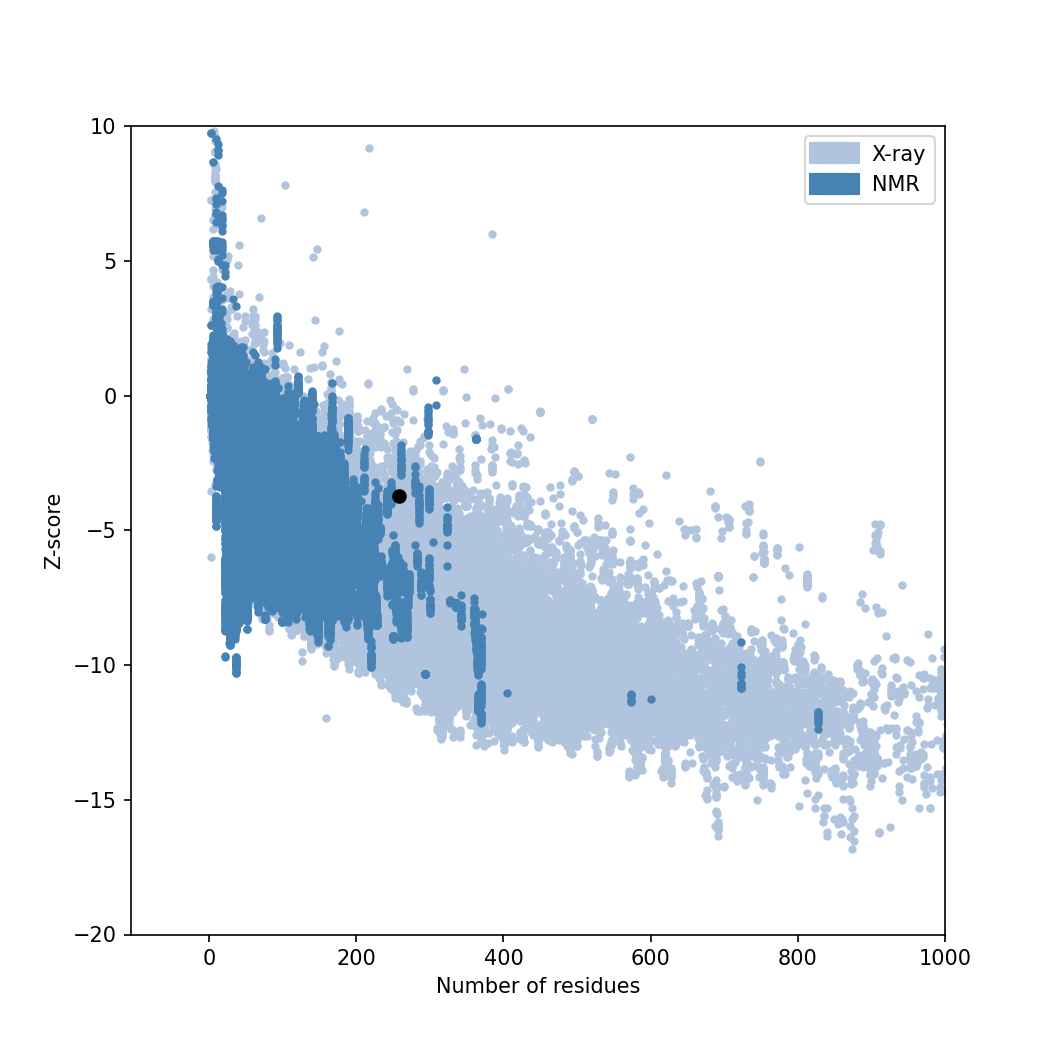

Supplement: Supplementary file 4 [file DataSheet4.zip › USUV/USU_NS4b/ProSA_global_analysis/USU_NS4b_Global_z_score.png]

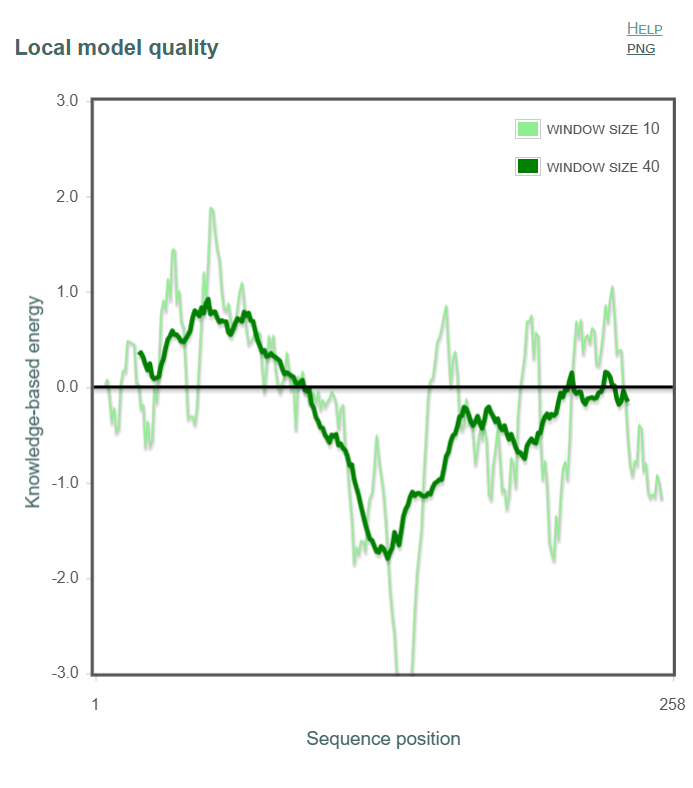

Supplement: Supplementary file 4 [file DataSheet4.zip › USUV/USU_NS4b/ProSA_global_analysis/USU_NS4b_Local_model.png]

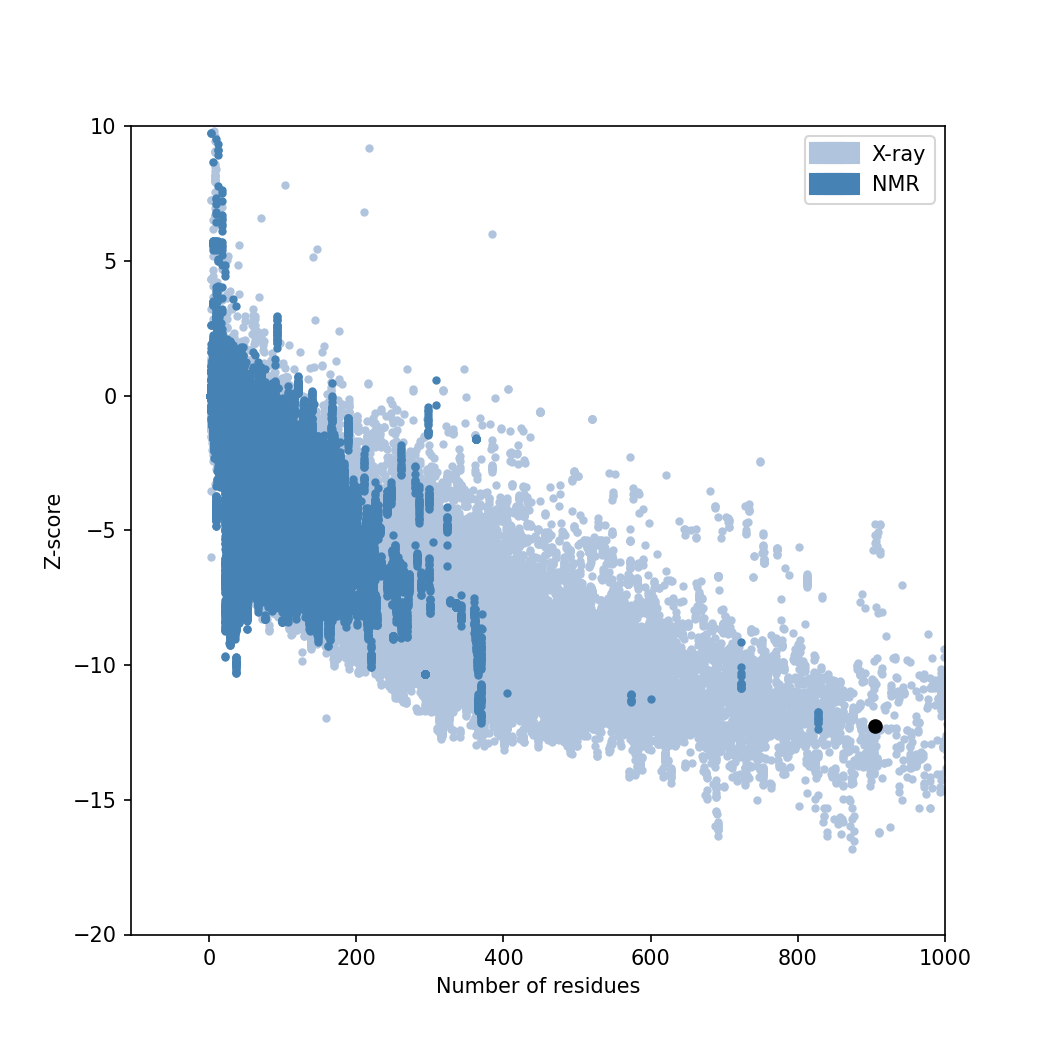

Supplement: Supplementary file 4 [file DataSheet4.zip › USUV/USU_NS5/ProSA_global_analysis/USU_NS5_Global_z_score.png]

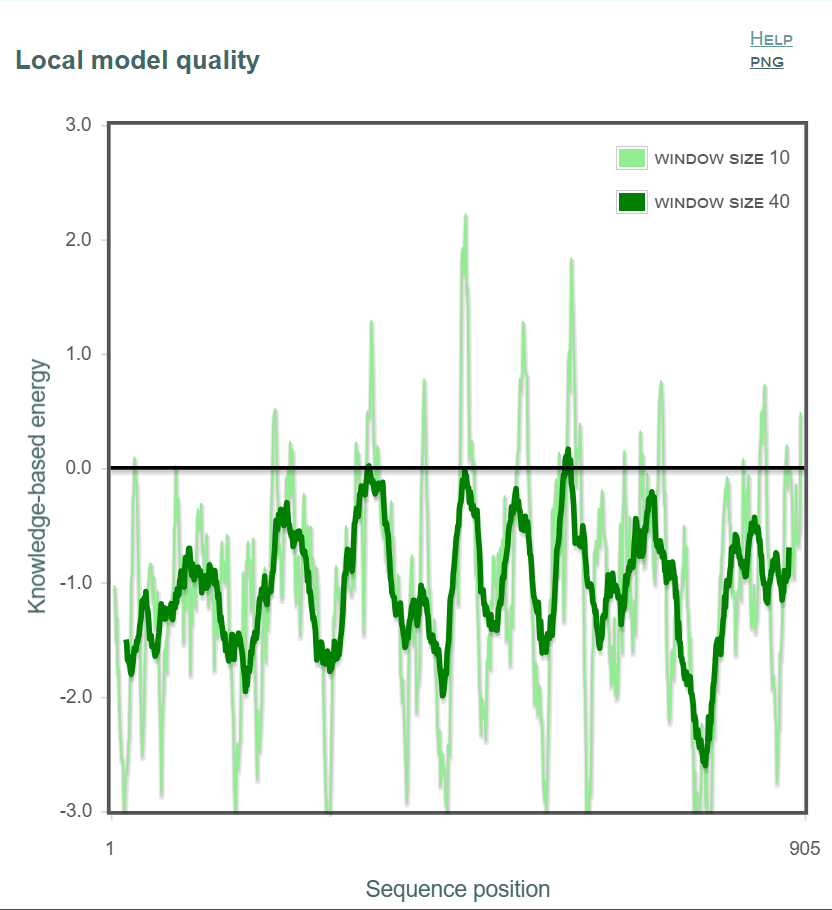

Supplement: Supplementary file 4 [file DataSheet4.zip › USUV/USU_NS5/ProSA_global_analysis/USU_NS5_Local_model.png]

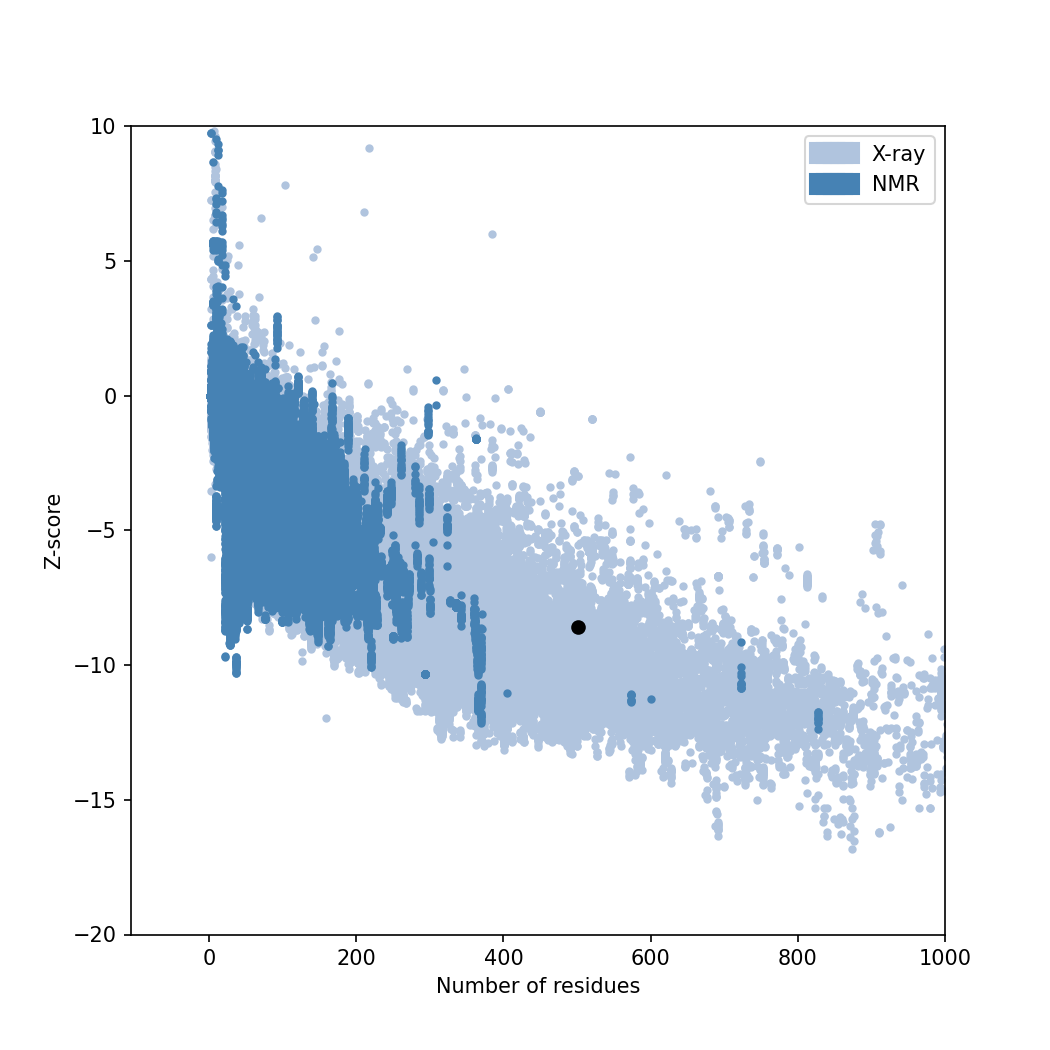

Supplement: Supplementary file 5 [file DataSheet5.zip › WNV/WNV_E/ProSA_global_analysis/WNV_E_Global_z_score.png]

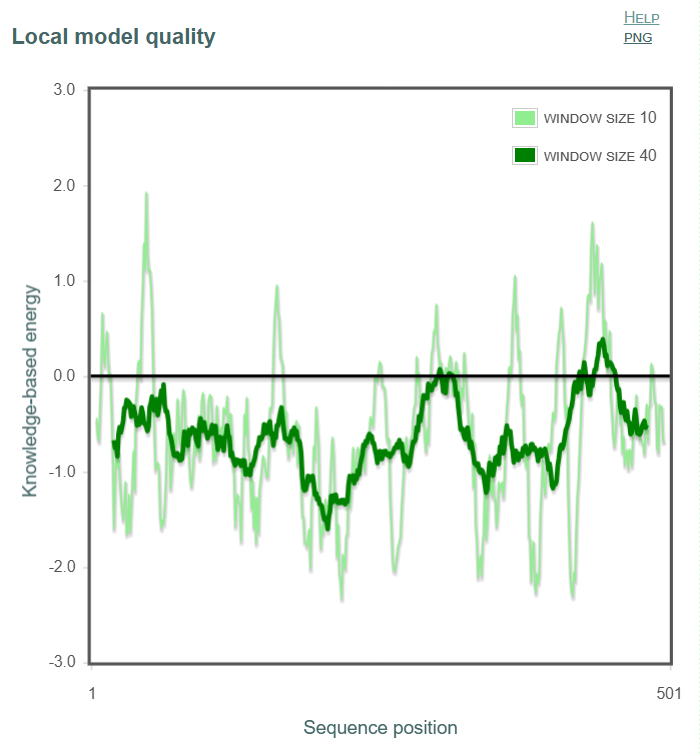

Supplement: Supplementary file 5 [file DataSheet5.zip › WNV/WNV_E/ProSA_global_analysis/WNV_E_Local_model.png]

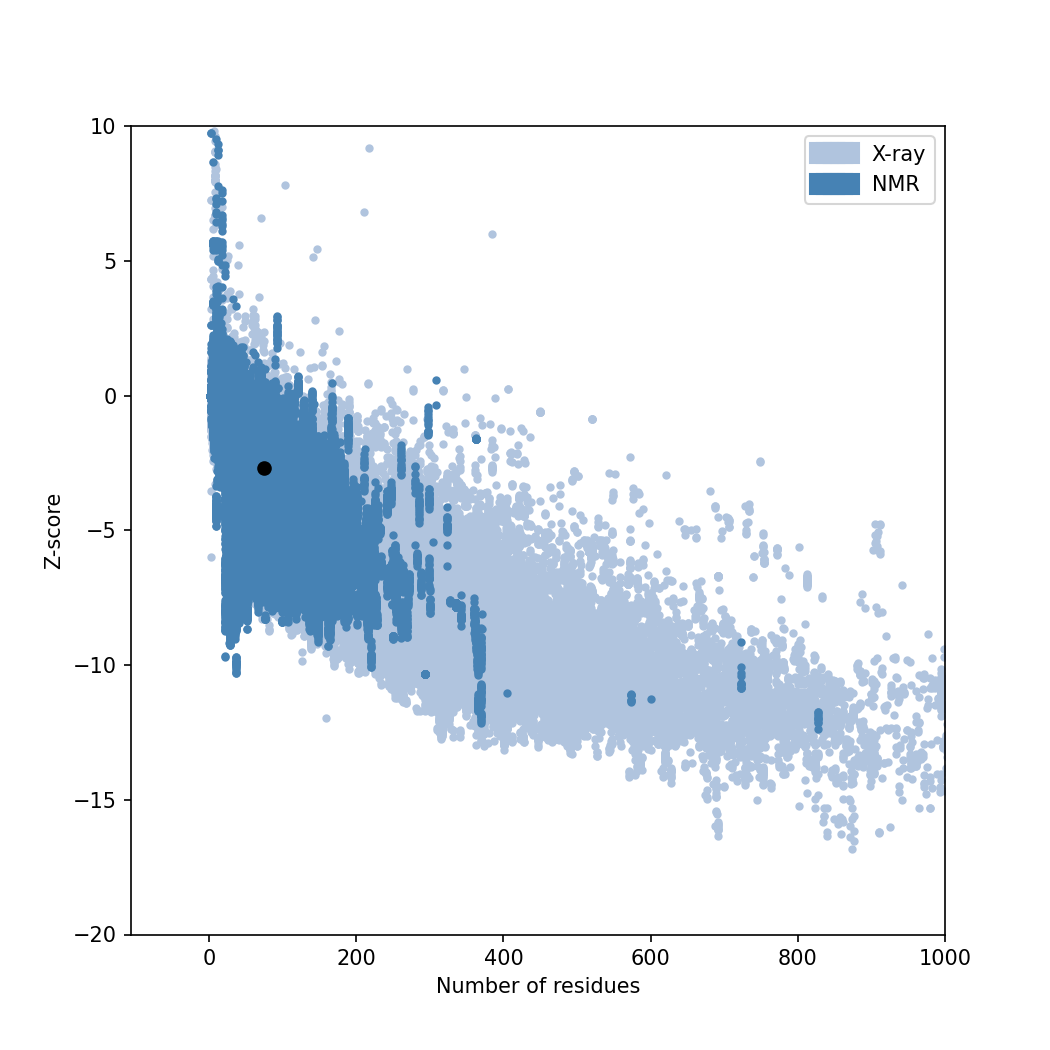

Supplement: Supplementary file 5 [file DataSheet5.zip › WNV/WNV_M/ProSA_global_analysis/WNV_M_Global_z_score.png]

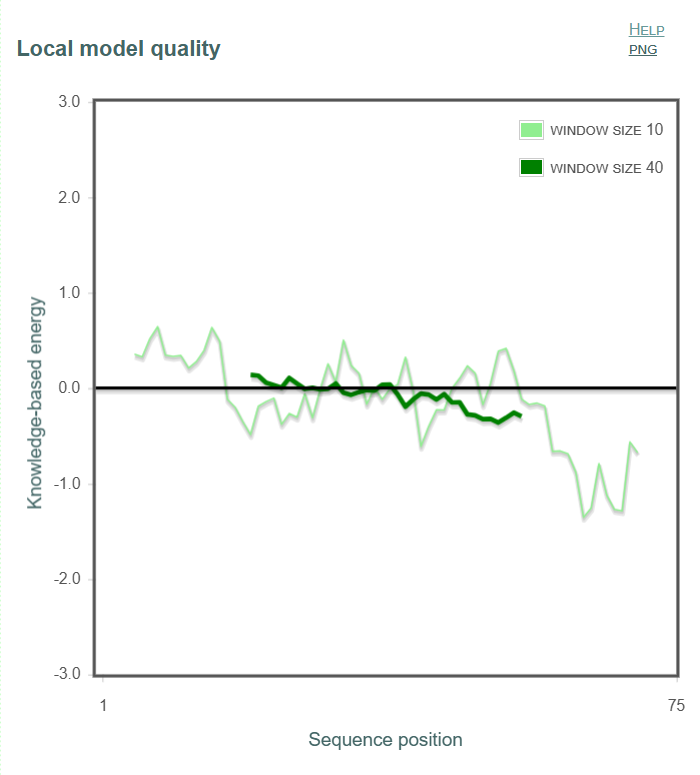

Supplement: Supplementary file 5 [file DataSheet5.zip › WNV/WNV_M/ProSA_global_analysis/WNV_M_Local_model.png]

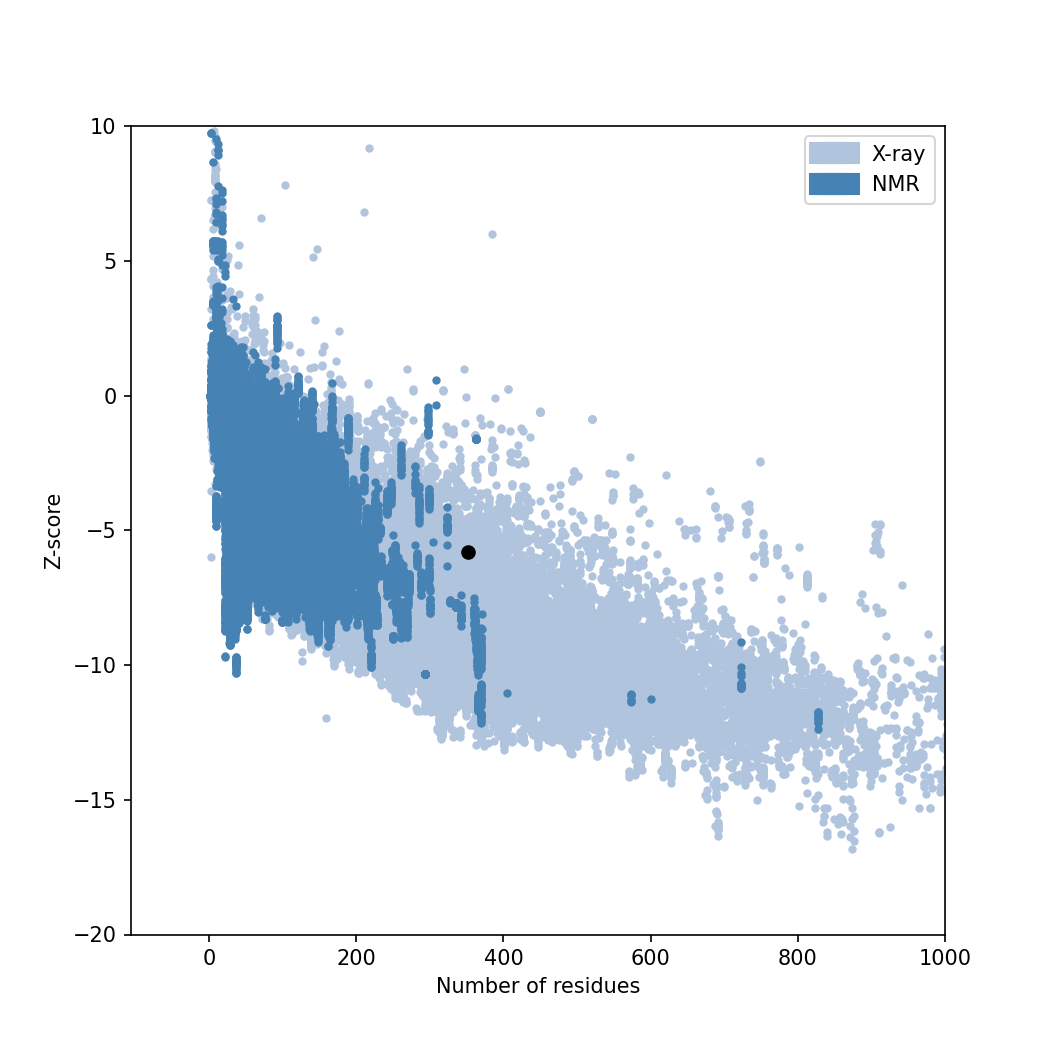

Supplement: Supplementary file 5 [file DataSheet5.zip › WNV/WNV_NS1/ProSA_global_analysis/WNV_NS1_Global_z_score.png]

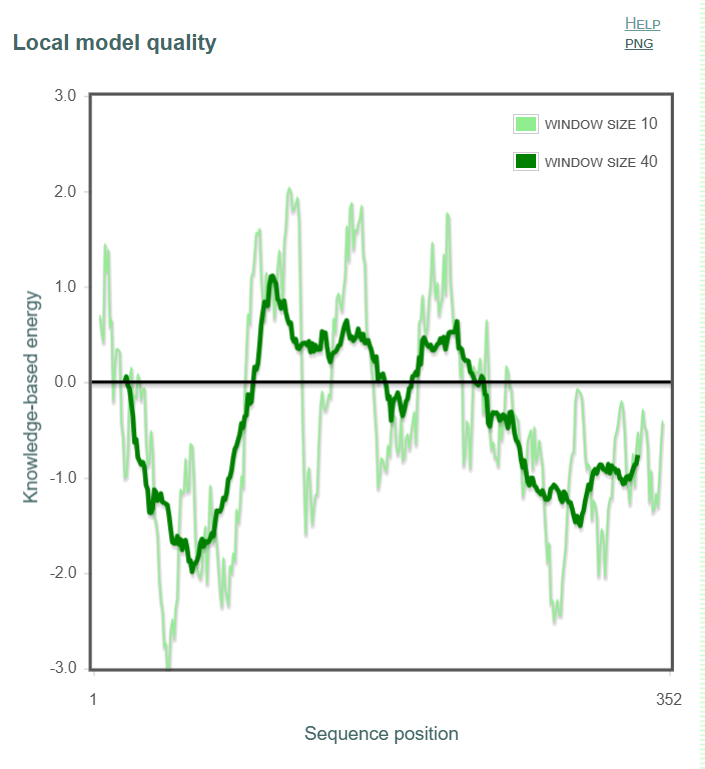

Supplement: Supplementary file 5 [file DataSheet5.zip › WNV/WNV_NS1/ProSA_global_analysis/WNV_NS1_Local_model.png]

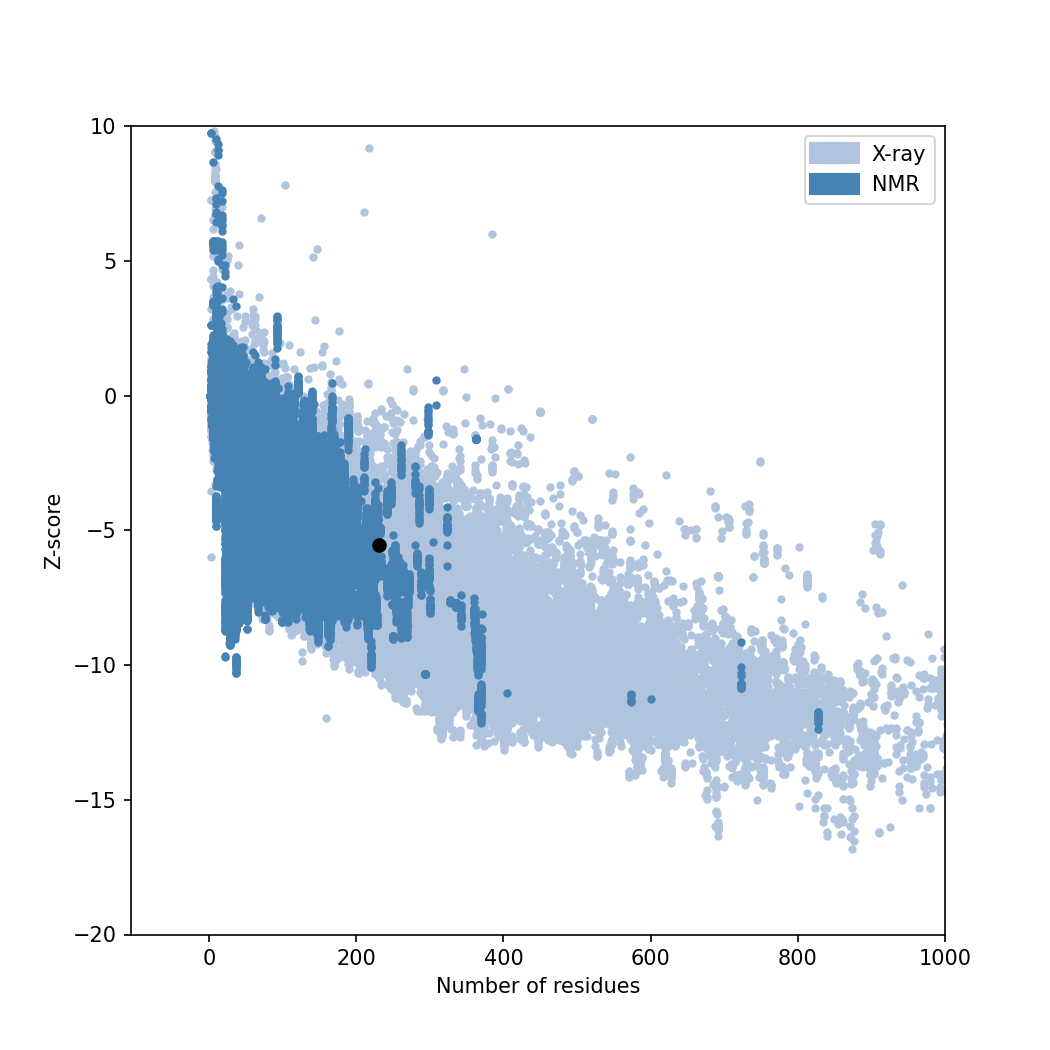

Supplement: Supplementary file 5 [file DataSheet5.zip › WNV/WNV_NS2a/ProSA_global_analysis/WNV_NS2a_Global_z_score.png]

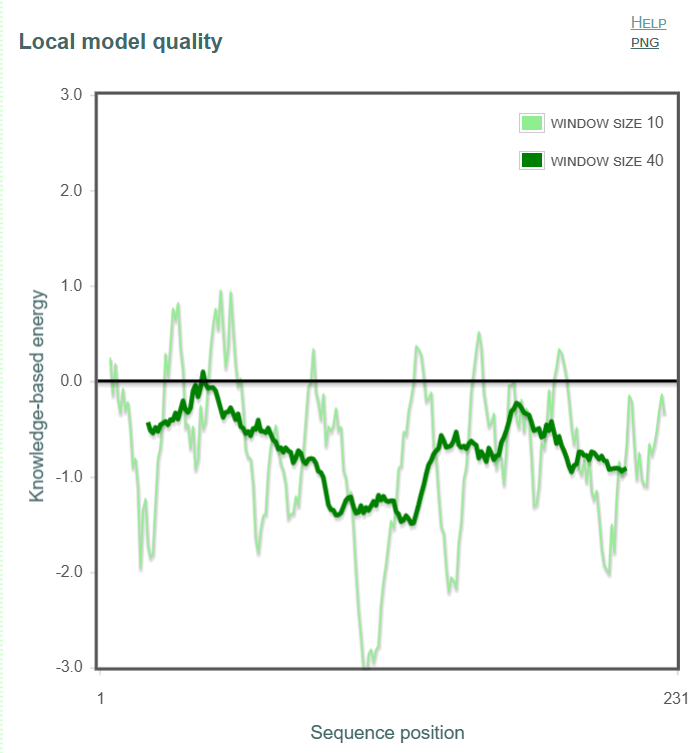

Supplement: Supplementary file 5 [file DataSheet5.zip › WNV/WNV_NS2a/ProSA_global_analysis/WNV_NS2a_Local_model.png]

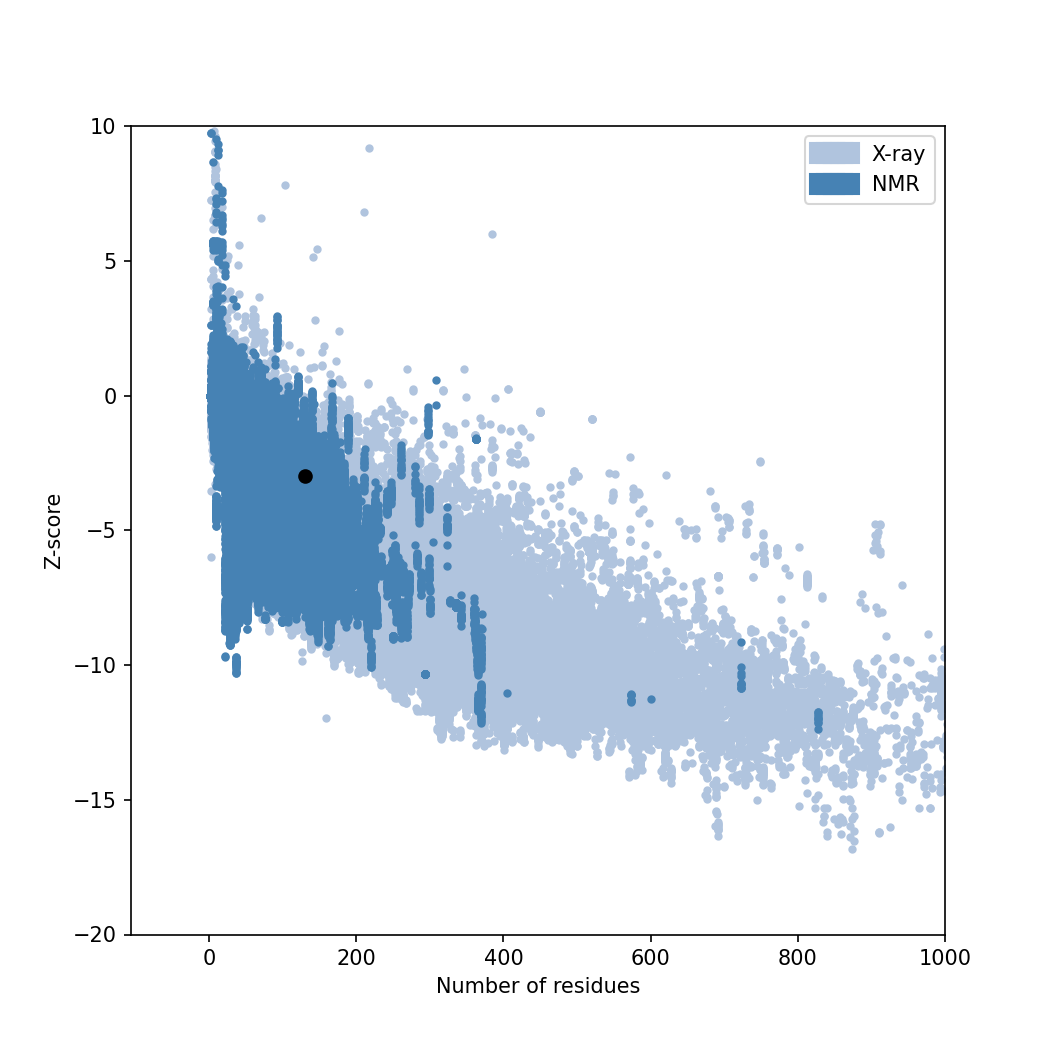

Supplement: Supplementary file 5 [file DataSheet5.zip › WNV/WNV_NS2b/ProSA_global_analysis/WNV_NS2b_Global_z_score.png]

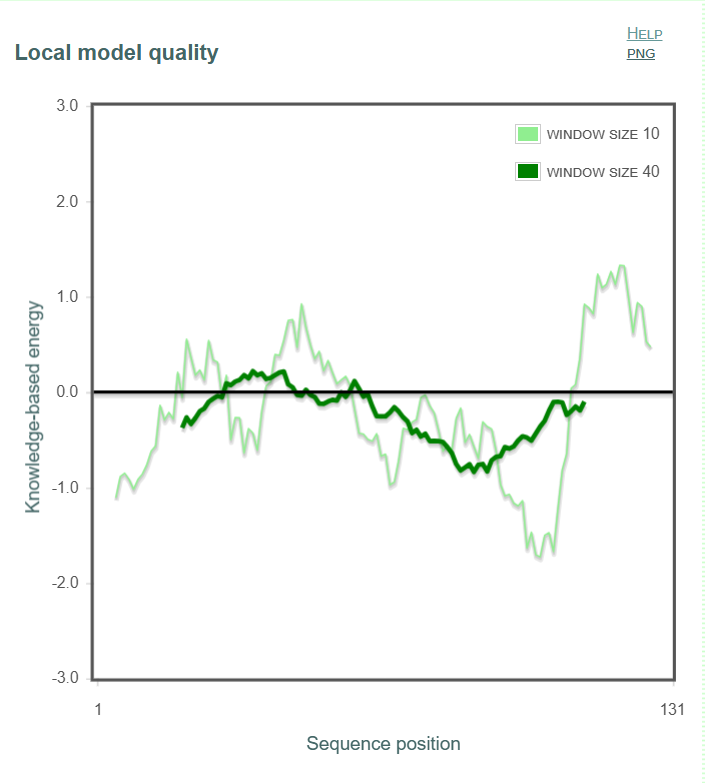

Supplement: Supplementary file 5 [file DataSheet5.zip › WNV/WNV_NS2b/ProSA_global_analysis/WNV_NS2b_Local_model.png]

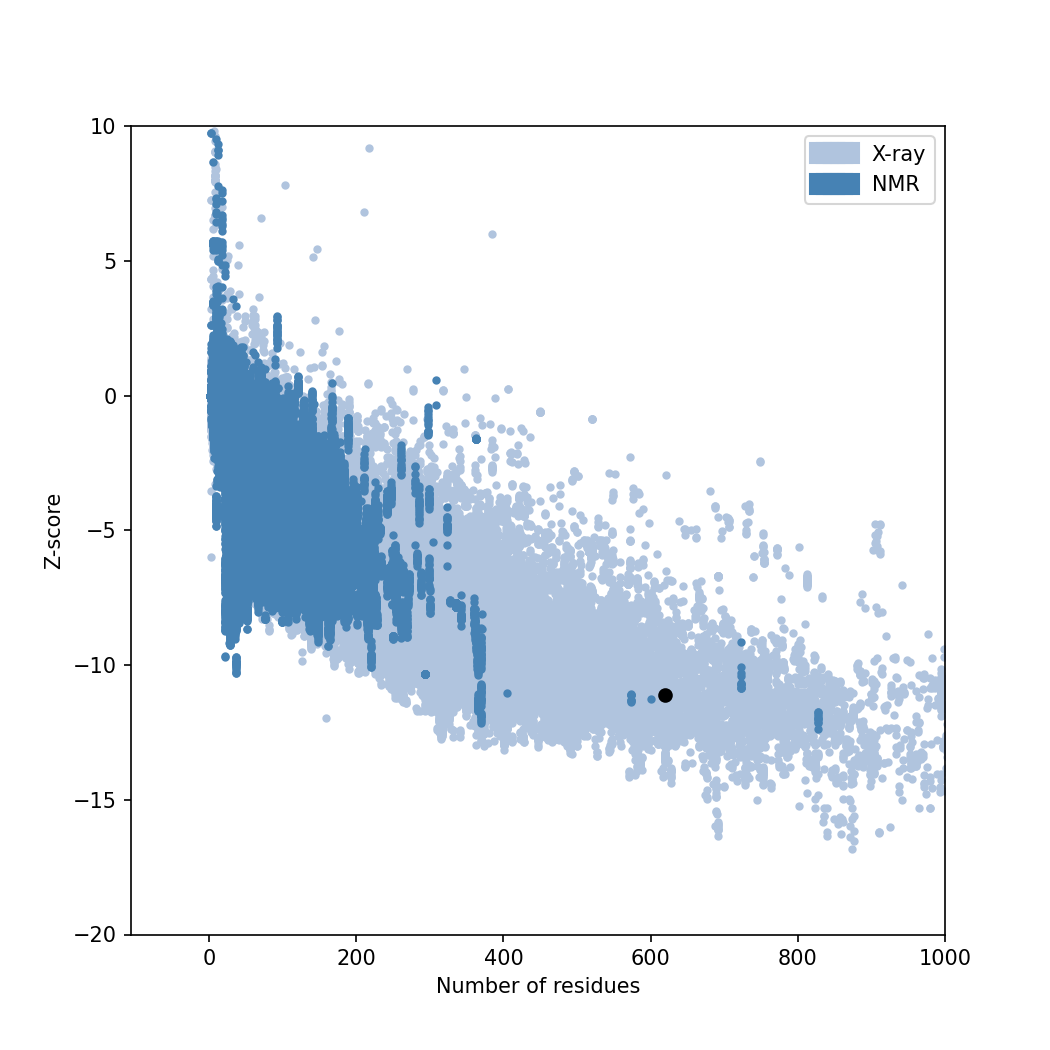

Supplement: Supplementary file 5 [file DataSheet5.zip › WNV/WNV_NS3/ProSA_global_analysis/WNV_NS3_Global_z_score.png]

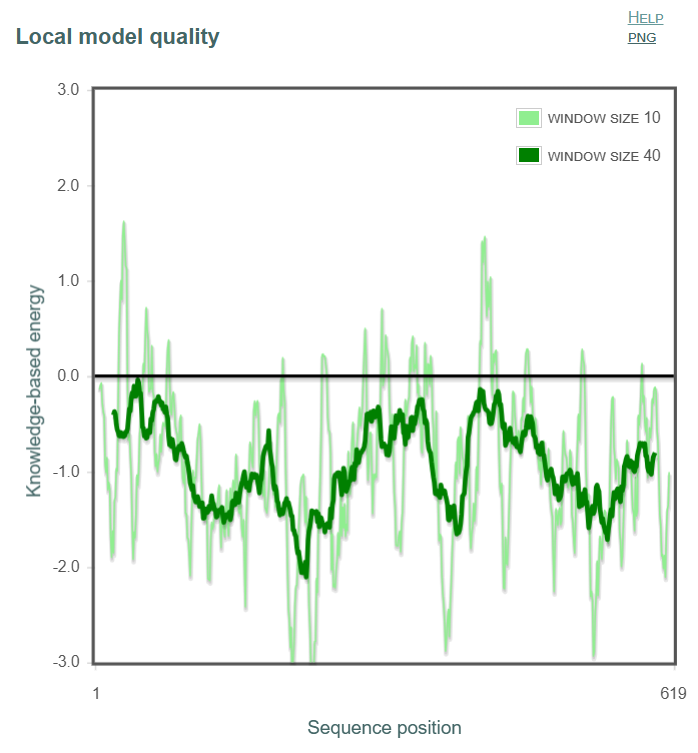

Supplement: Supplementary file 5 [file DataSheet5.zip › WNV/WNV_NS3/ProSA_global_analysis/WNV_NS3_Local_model.png]

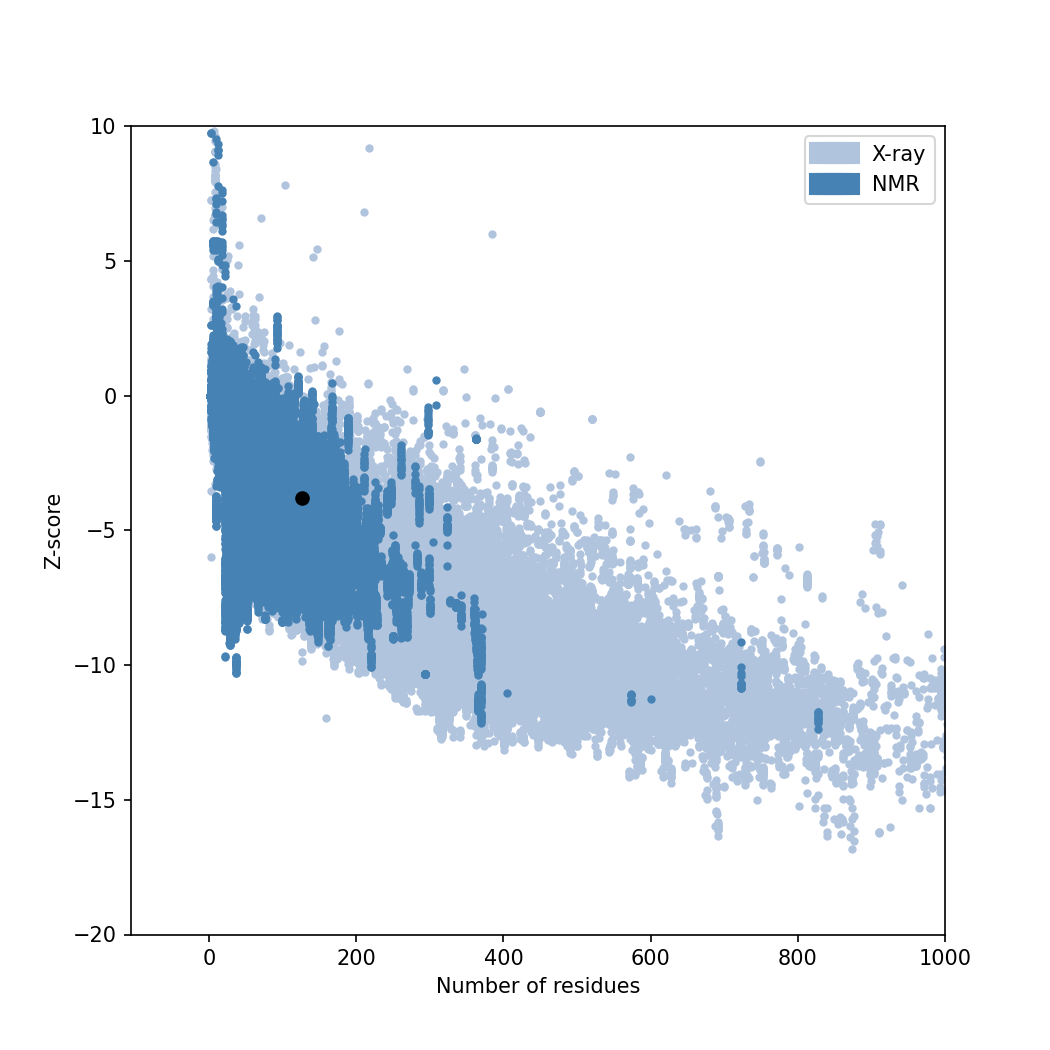

Supplement: Supplementary file 5 [file DataSheet5.zip › WNV/WNV_NS4a/ProSA_global_analysis/WNV_NS4a_Global_z_score.png]

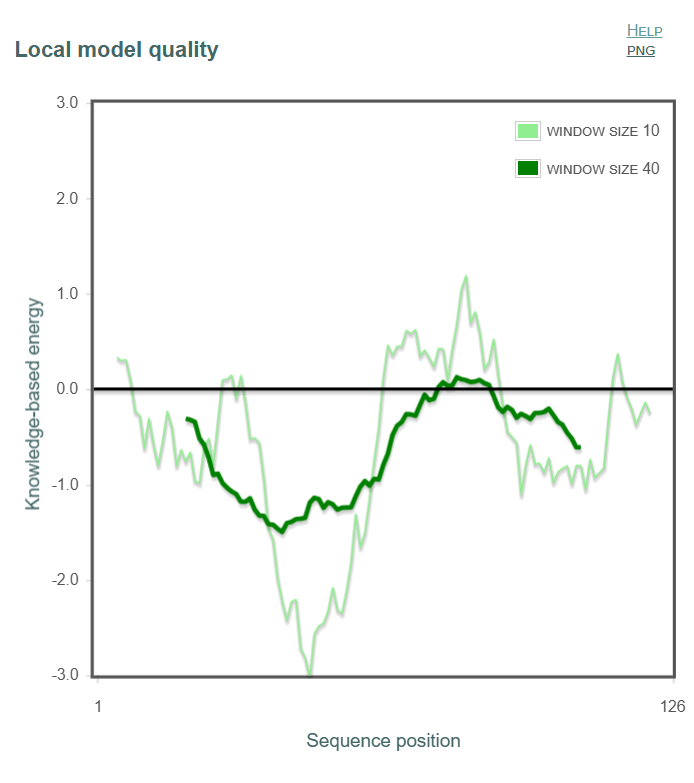

Supplement: Supplementary file 5 [file DataSheet5.zip › WNV/WNV_NS4a/ProSA_global_analysis/WNV_NS4a_Local_model.png]

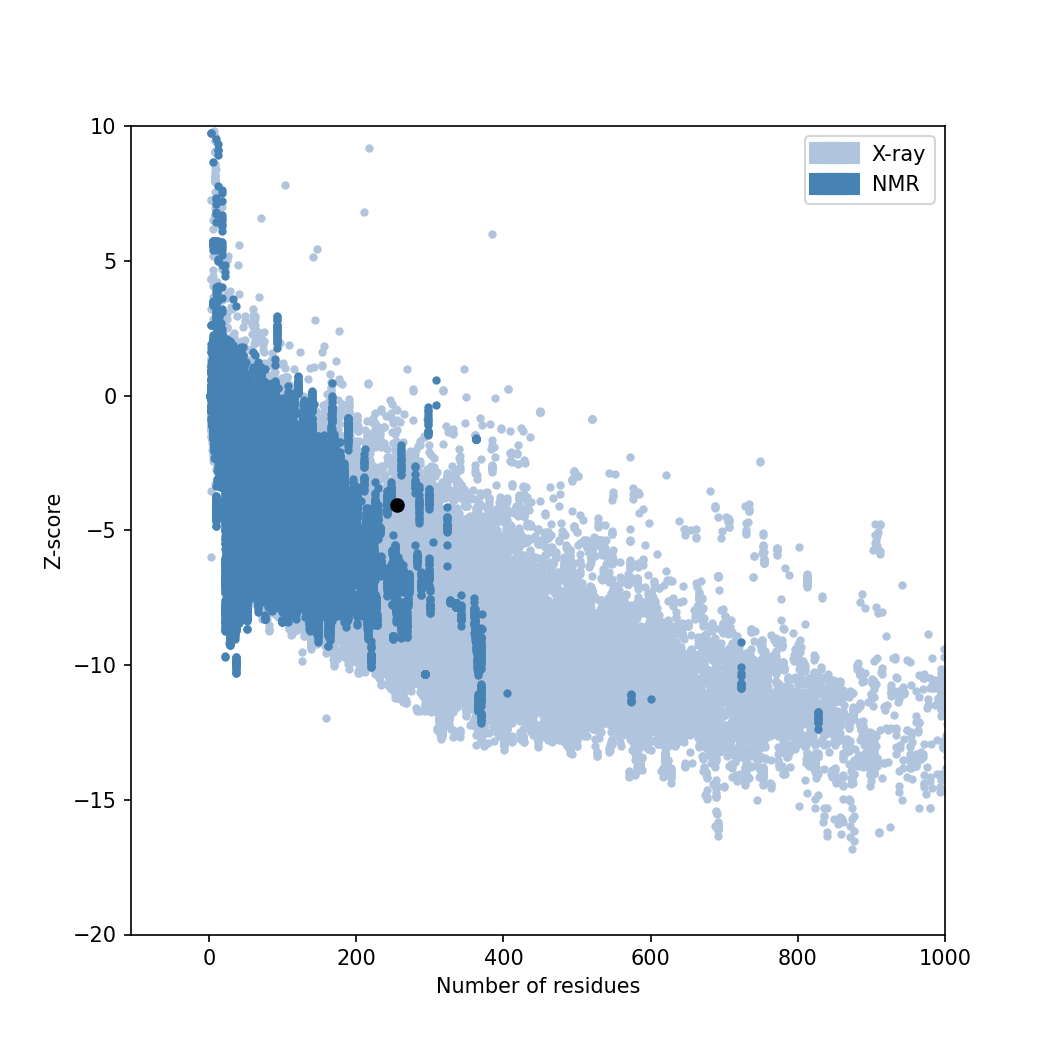

Supplement: Supplementary file 5 [file DataSheet5.zip › WNV/WNV_NS4b/ProSA_global_analysis/WNV_NS4b_Global_z_score.png]

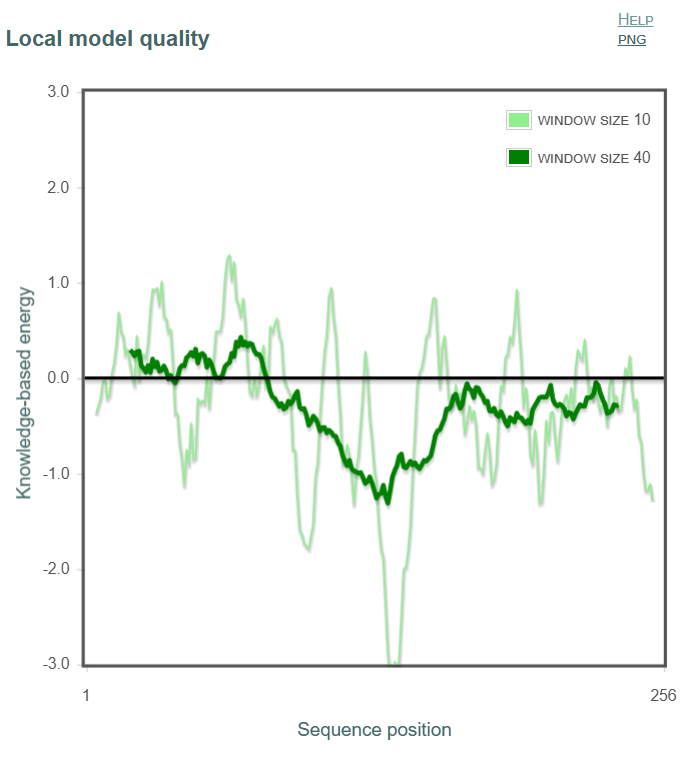

Supplement: Supplementary file 5 [file DataSheet5.zip › WNV/WNV_NS4b/ProSA_global_analysis/WNV_NS4b_Local_model.png]

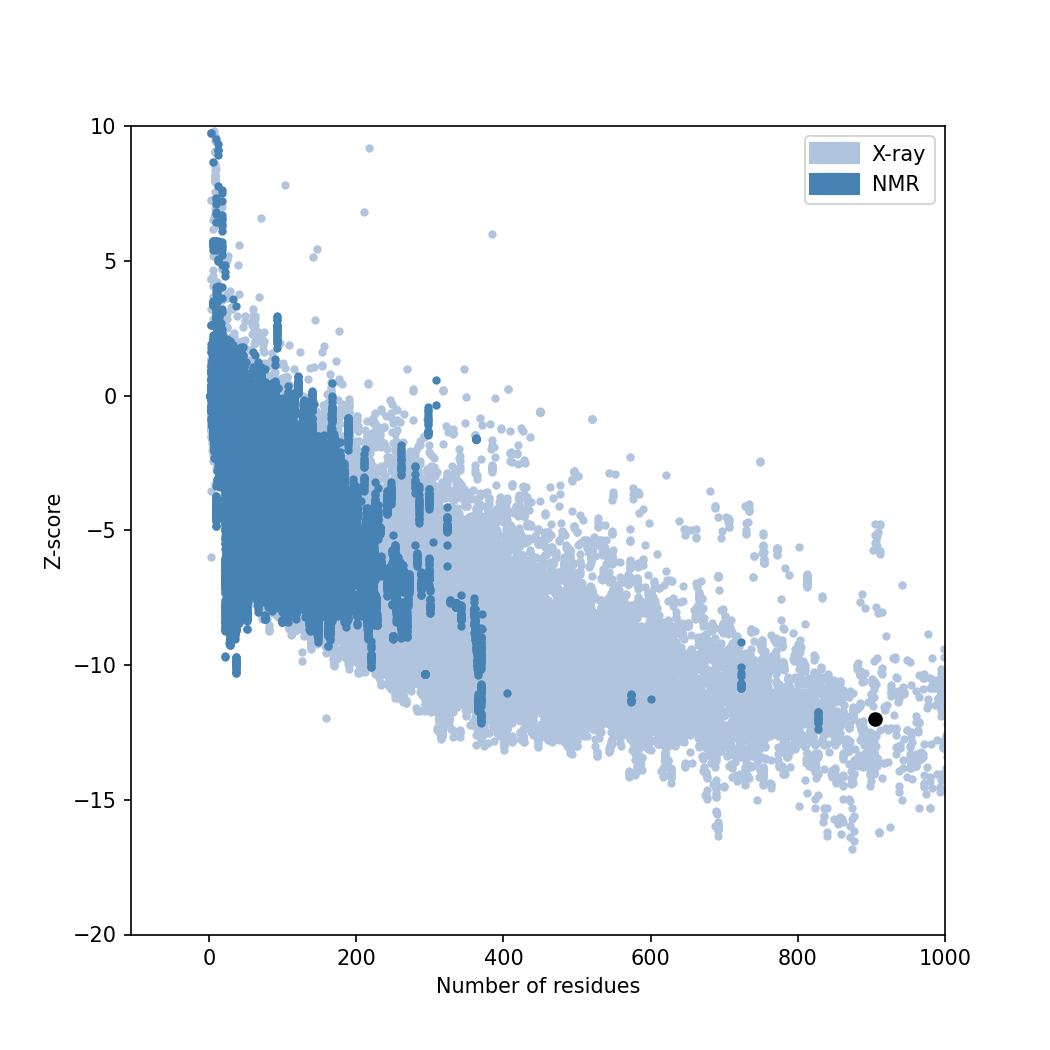

Supplement: Supplementary file 5 [file DataSheet5.zip › WNV/WNV_NS5/ProSA_global_analysis/WNV_NS5_Global_z_score.png]

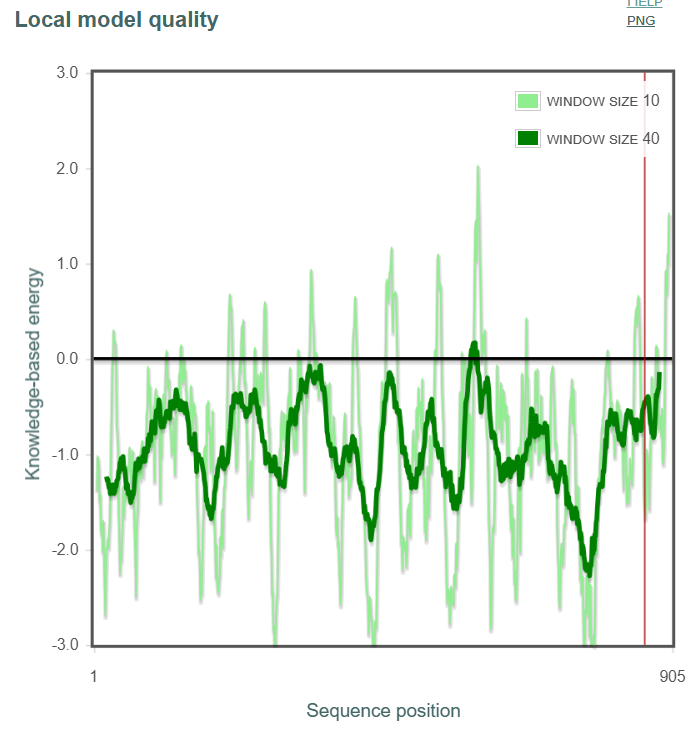

Supplement: Supplementary file 5 [file DataSheet5.zip › WNV/WNV_NS5/ProSA_global_analysis/WNV_NS5_Local_model.png]

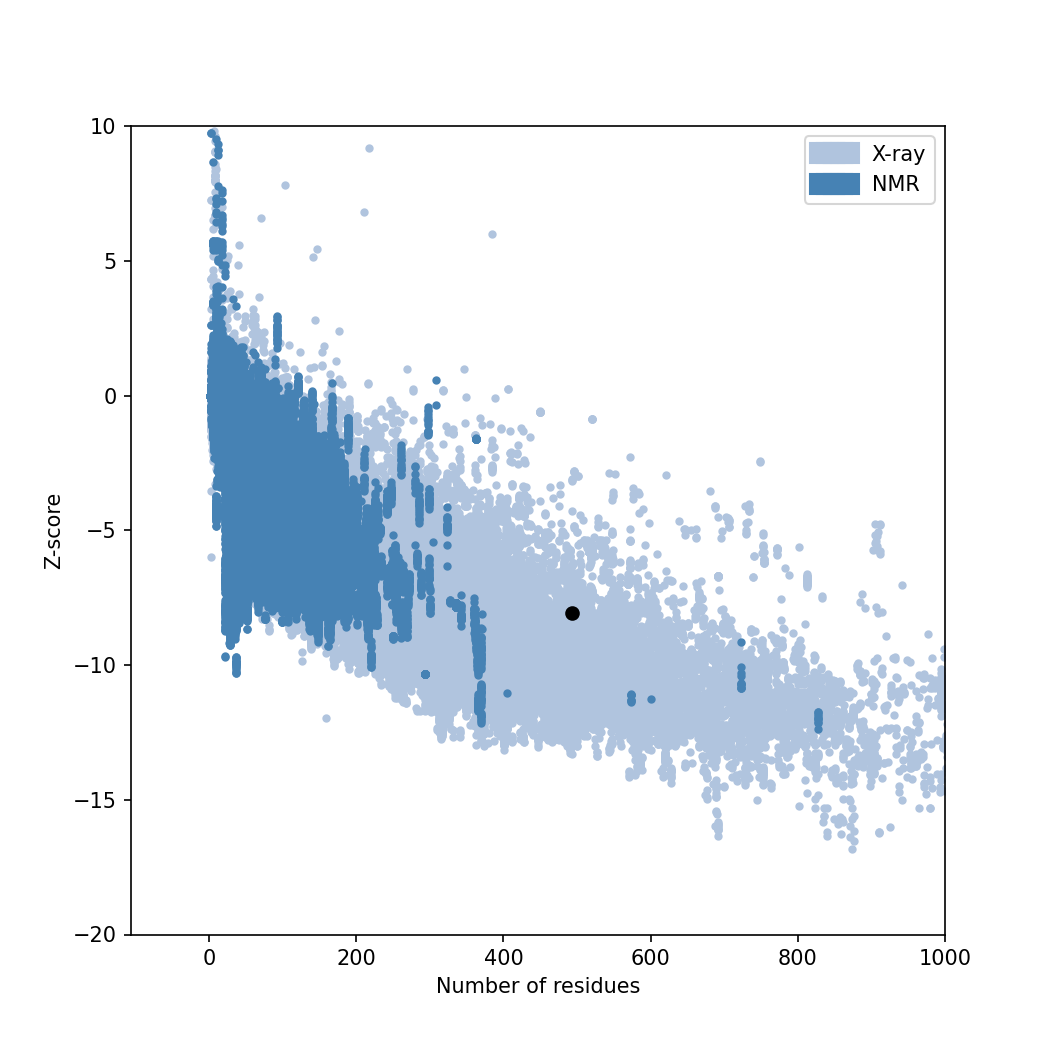

Supplement: Supplementary file 6 [file DataSheet6.zip › YFV/YF_E/ProSA_global_analysis/YF_E_Global_z_score.png]

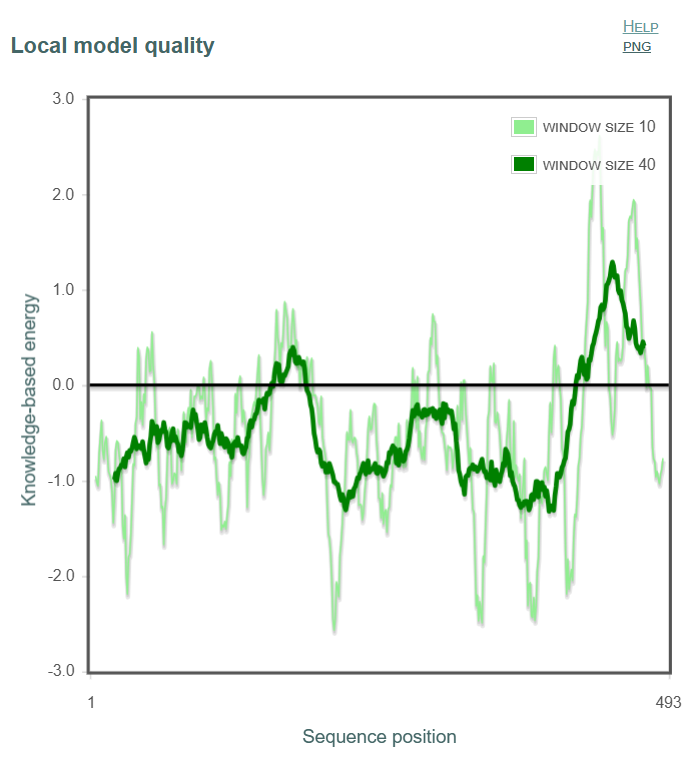

Supplement: Supplementary file 6 [file DataSheet6.zip › YFV/YF_E/ProSA_global_analysis/YF_E_Local_model.png]

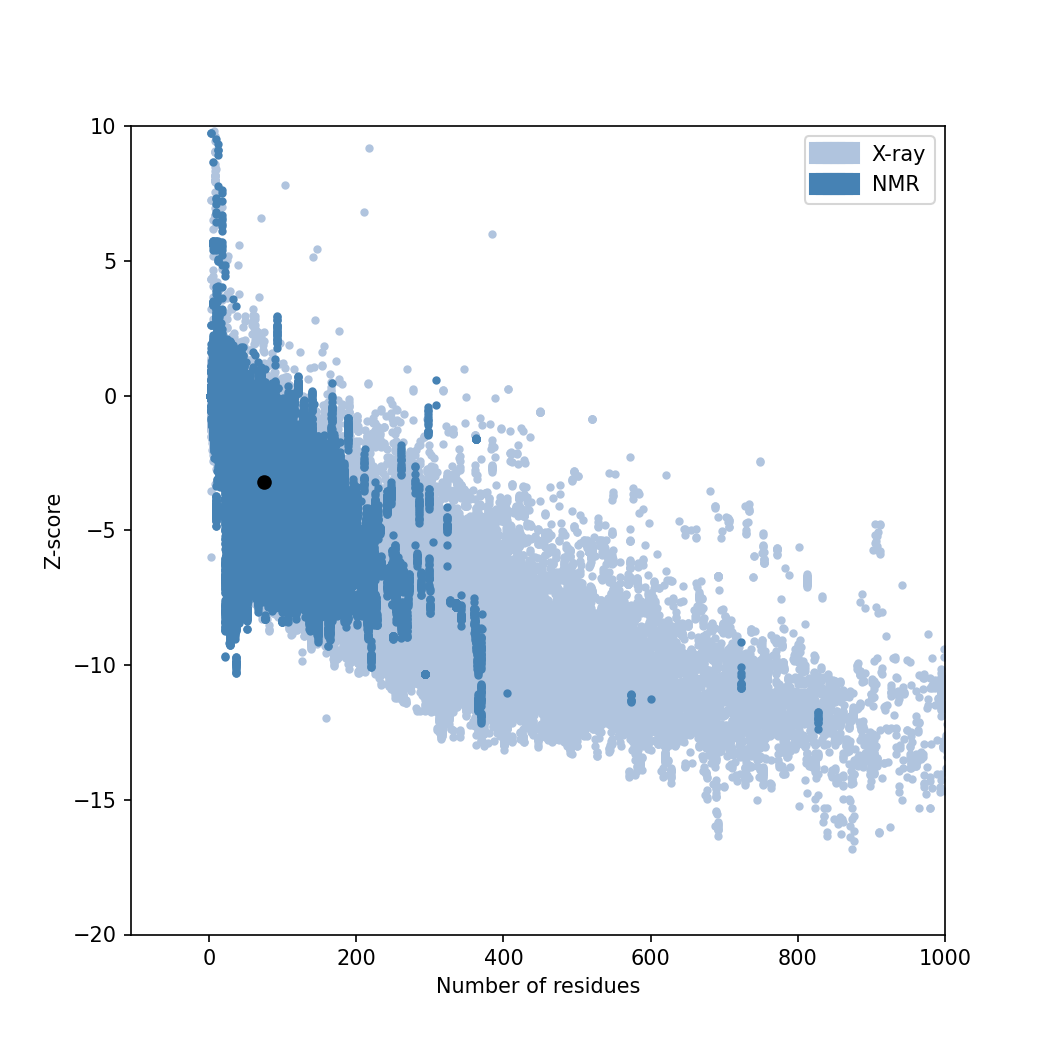

Supplement: Supplementary file 6 [file DataSheet6.zip › YFV/YF_M/ProSA_global_analysis/YF_M_Global_z_score.png]

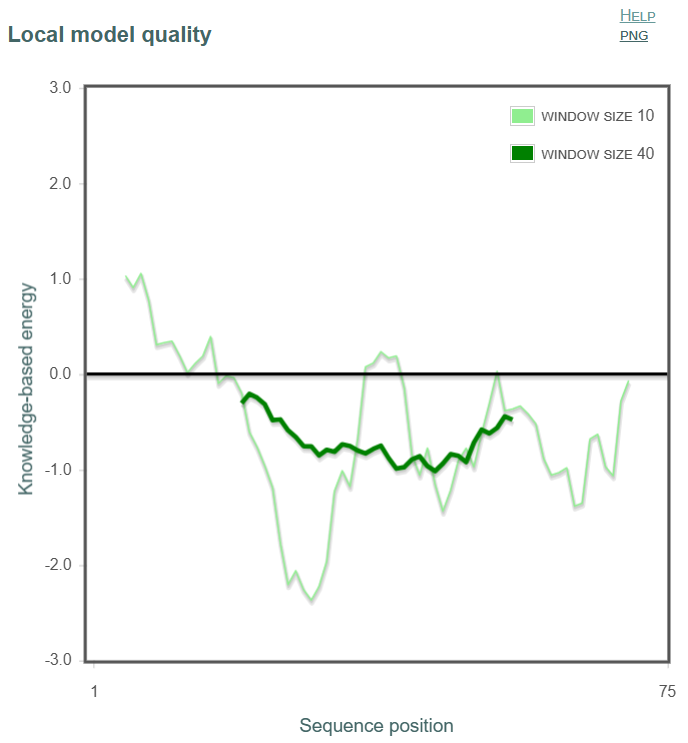

Supplement: Supplementary file 6 [file DataSheet6.zip › YFV/YF_M/ProSA_global_analysis/YF_M_Local_model.png]

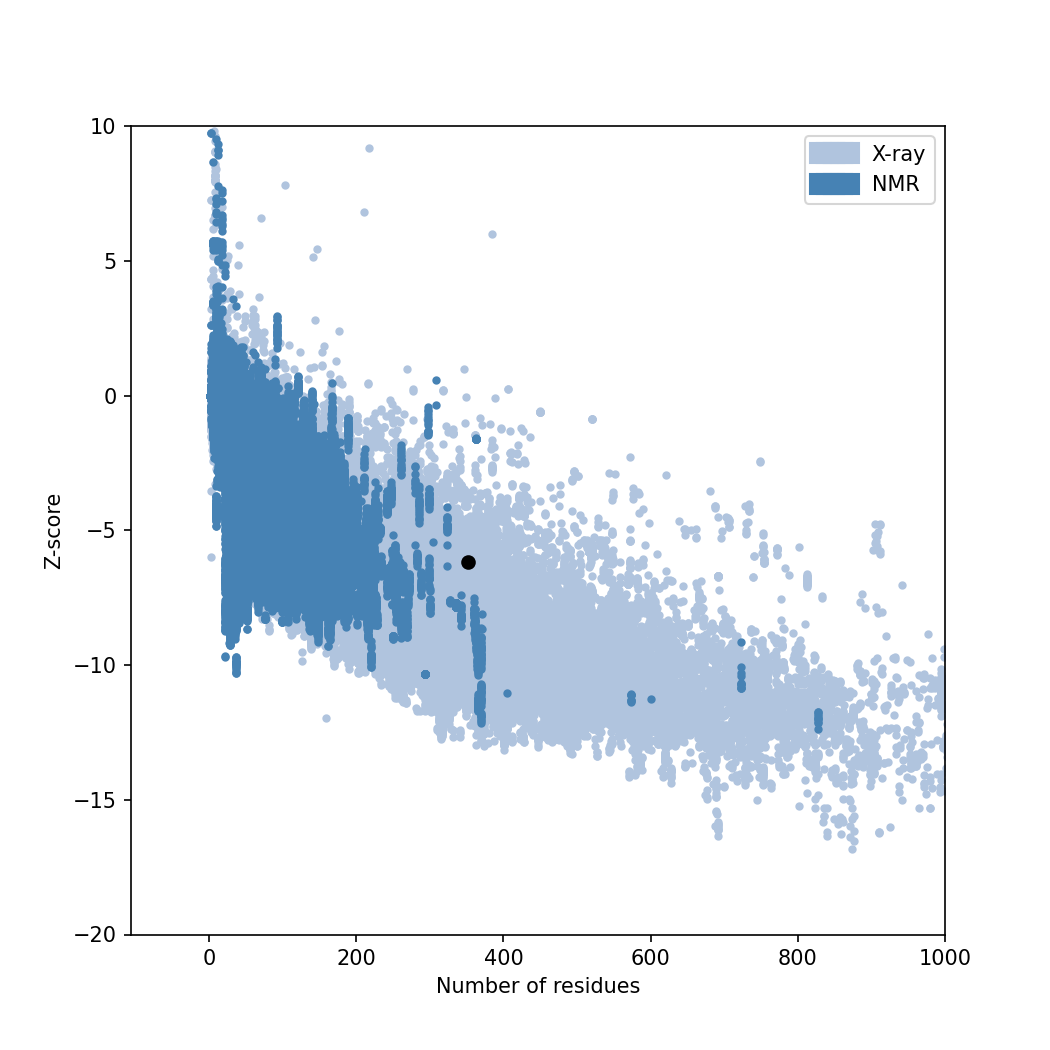

Supplement: Supplementary file 6 [file DataSheet6.zip › YFV/YF_NS1/ProSA_global_analysis/YF_NS1_Global_z_score.png]

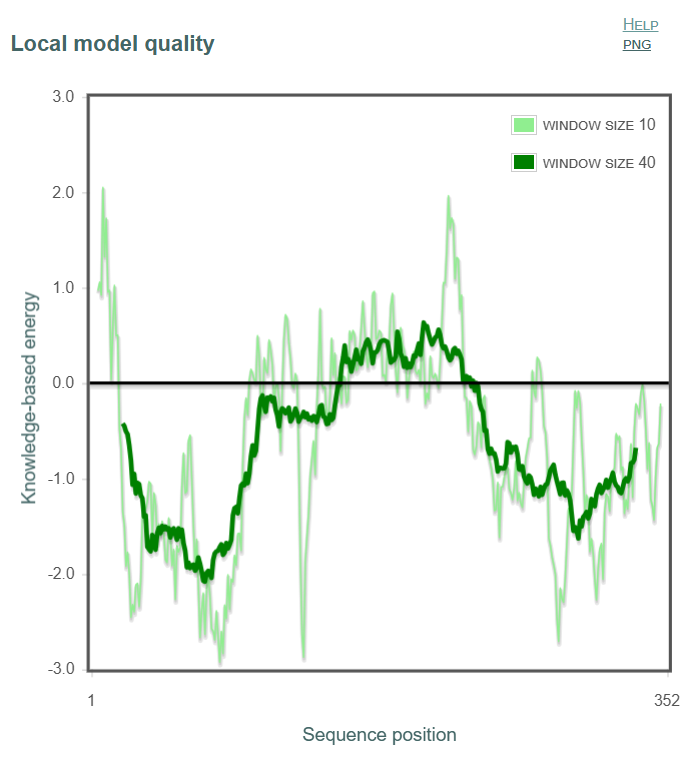

Supplement: Supplementary file 6 [file DataSheet6.zip › YFV/YF_NS1/ProSA_global_analysis/YF_NS1_Local_model.png]

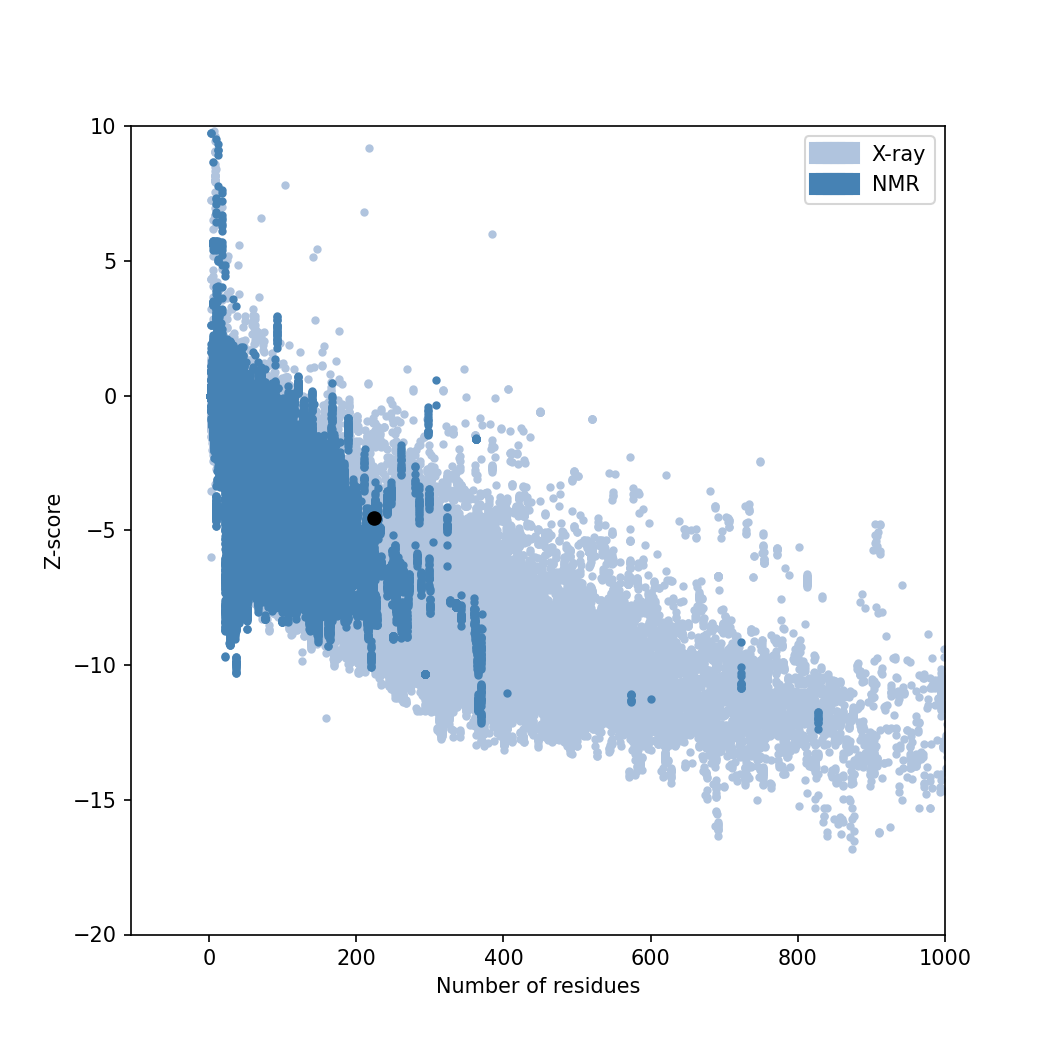

Supplement: Supplementary file 6 [file DataSheet6.zip › YFV/YF_NS2a/ProSA_global_analysis/YF_NS2a_Global_z_score.png]

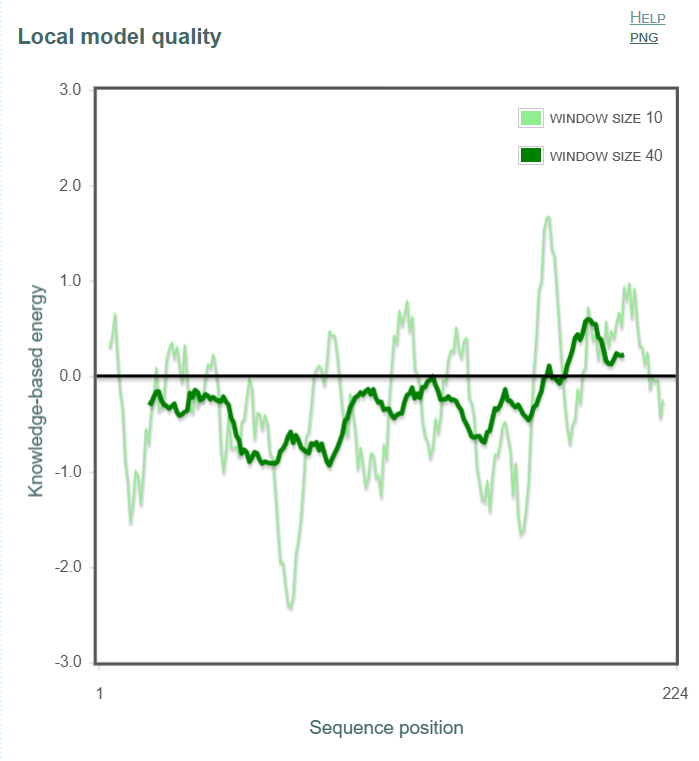

Supplement: Supplementary file 6 [file DataSheet6.zip › YFV/YF_NS2a/ProSA_global_analysis/YF_NS2a_Local_model.png]

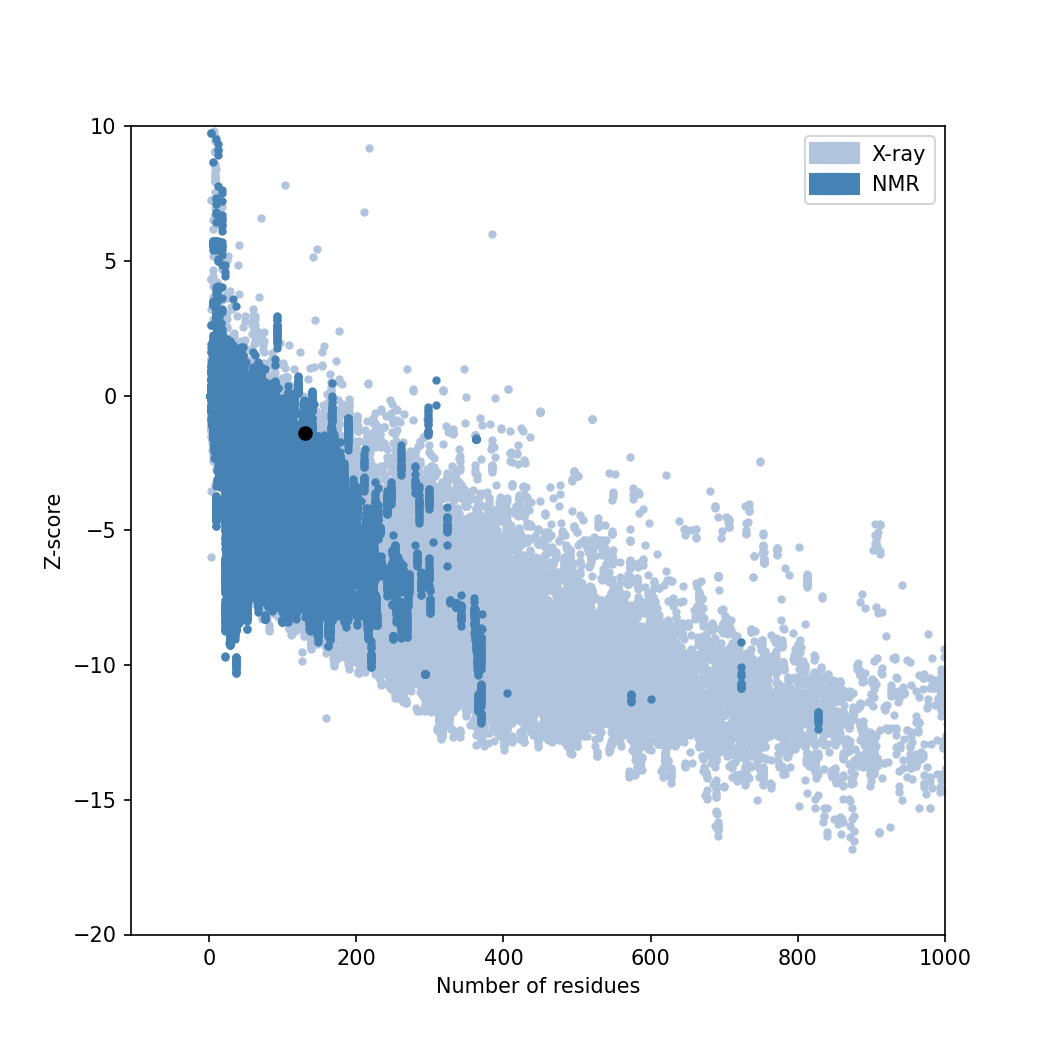

Supplement: Supplementary file 6 [file DataSheet6.zip › YFV/YF_NS2b/ProSA_global_analysis/YF_NS2b_Global_z_score.png]

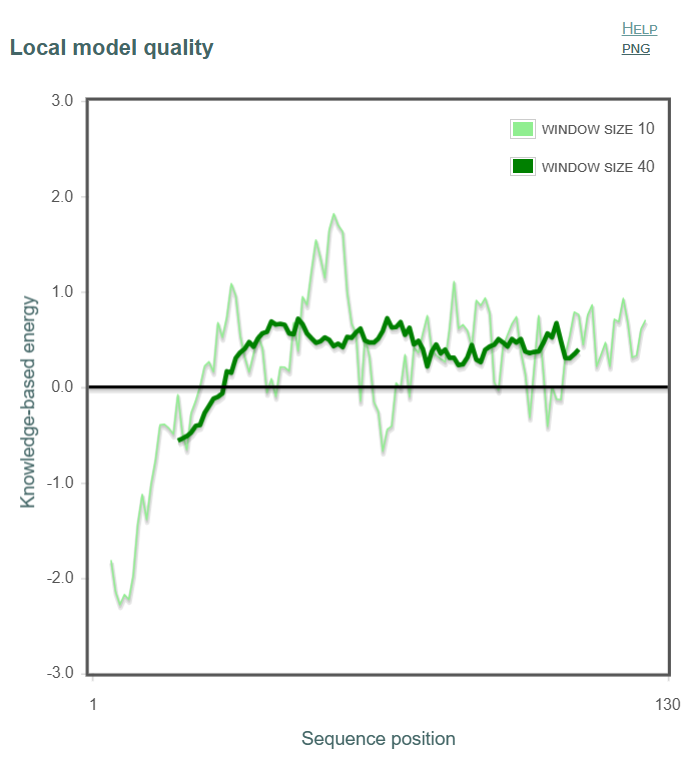

Supplement: Supplementary file 6 [file DataSheet6.zip › YFV/YF_NS2b/ProSA_global_analysis/YF_NS2b_Local_model.png]

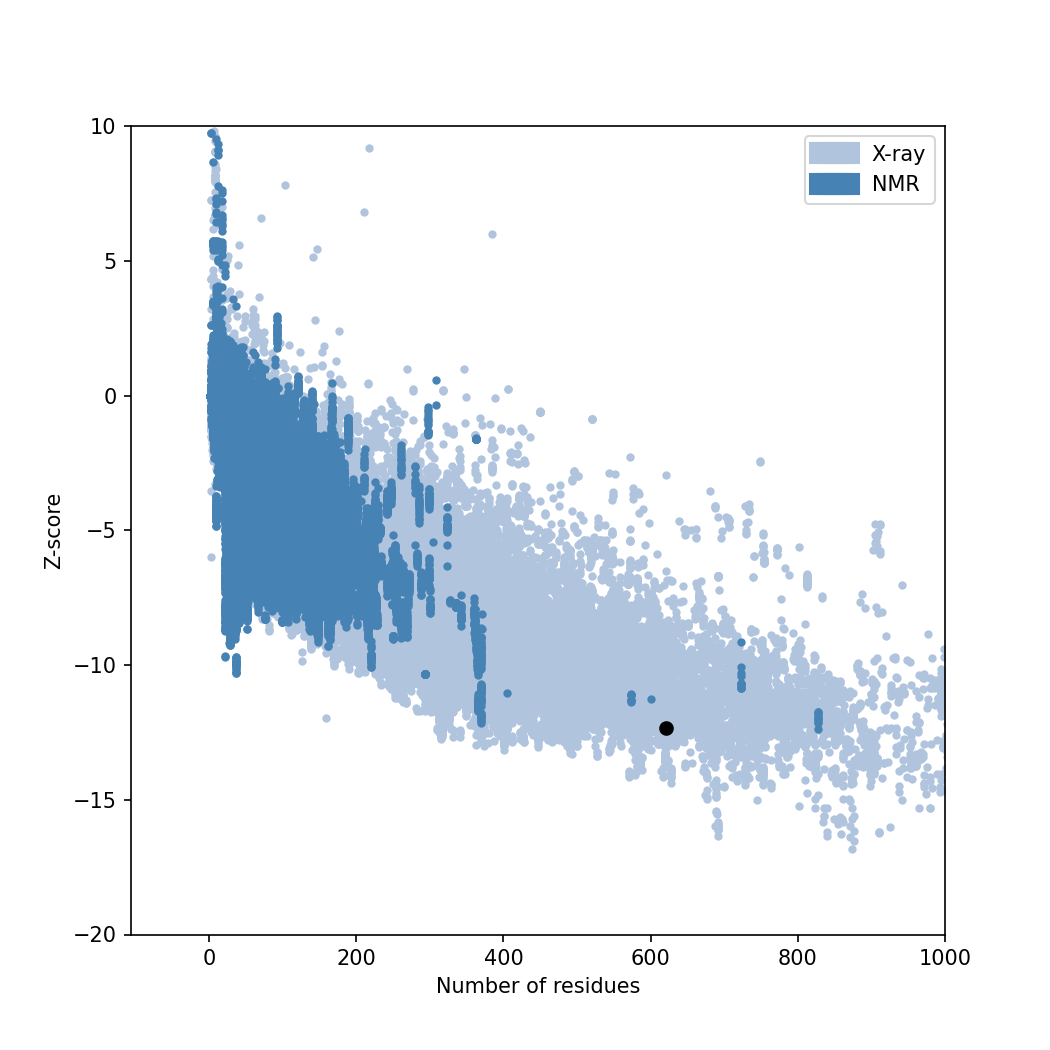

Supplement: Supplementary file 6 [file DataSheet6.zip › YFV/YF_NS3/ProSA_global_analysis/YF_NS3_Global_z_score.png]

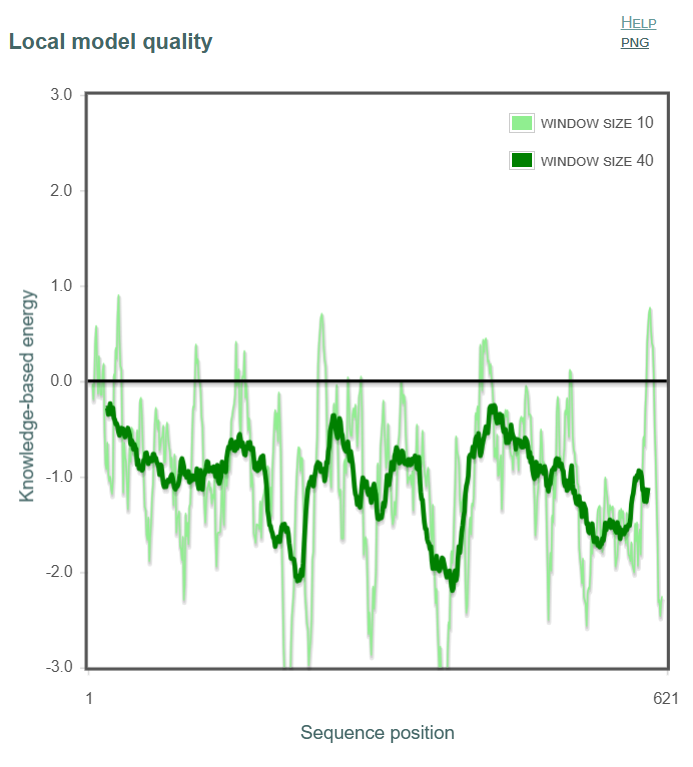

Supplement: Supplementary file 6 [file DataSheet6.zip › YFV/YF_NS3/ProSA_global_analysis/YF_NS3_Local_model.png]

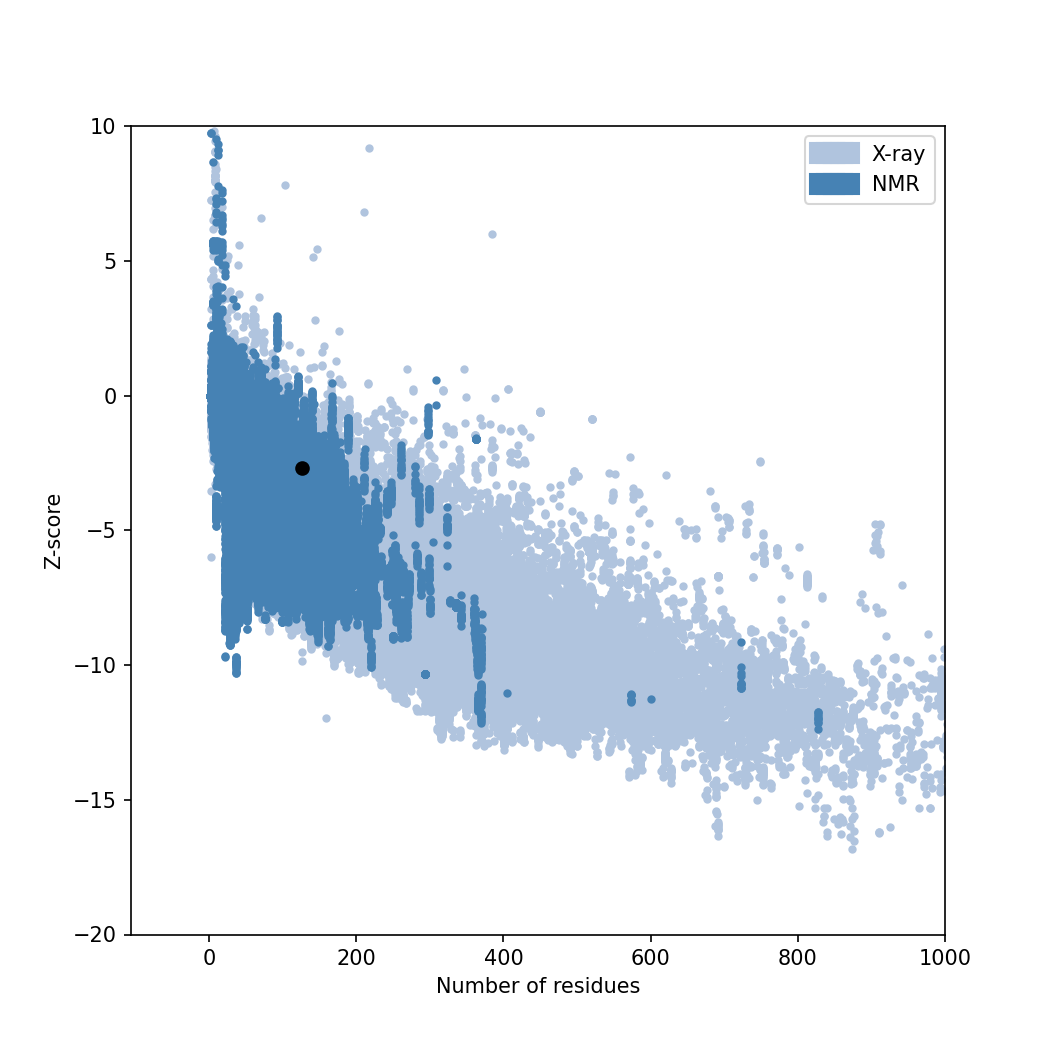

Supplement: Supplementary file 6 [file DataSheet6.zip › YFV/YF_NS4a/ProSA_global_analysis/YF_NS4a_Global_z_score.png]

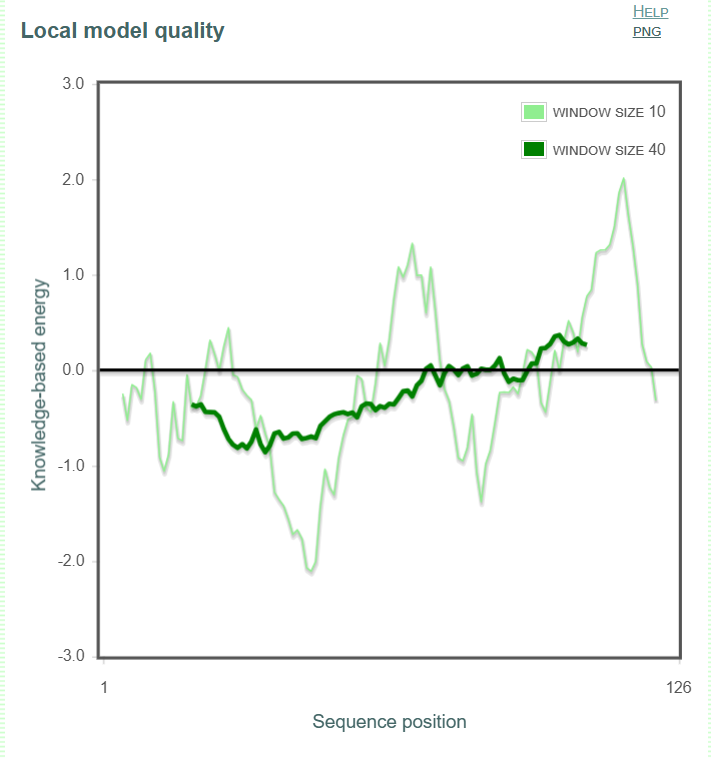

Supplement: Supplementary file 6 [file DataSheet6.zip › YFV/YF_NS4a/ProSA_global_analysis/YF_NS4a_Local_model.png]

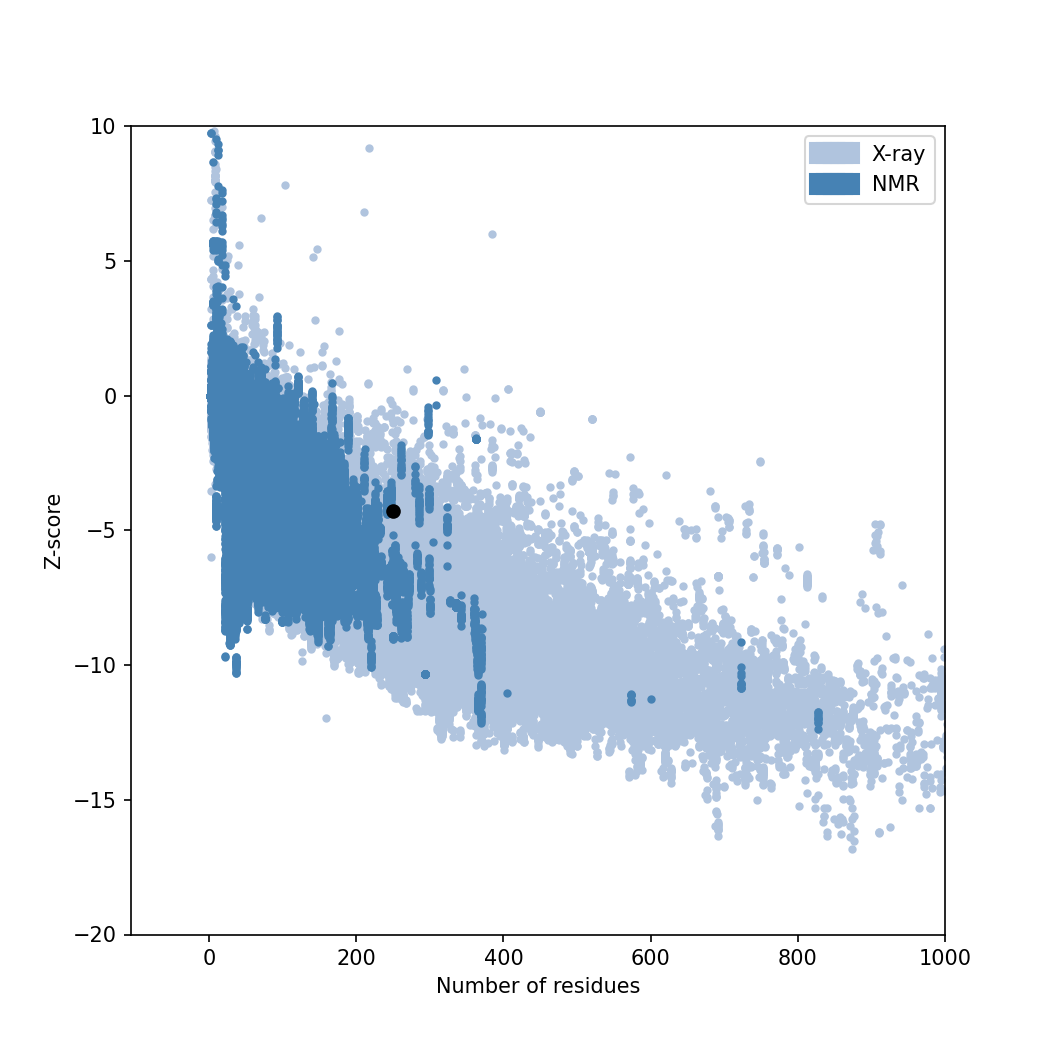

Supplement: Supplementary file 6 [file DataSheet6.zip › YFV/YF_NS4b/ProSA_global_analysis/YF_NS4b_Global_z_score.png]

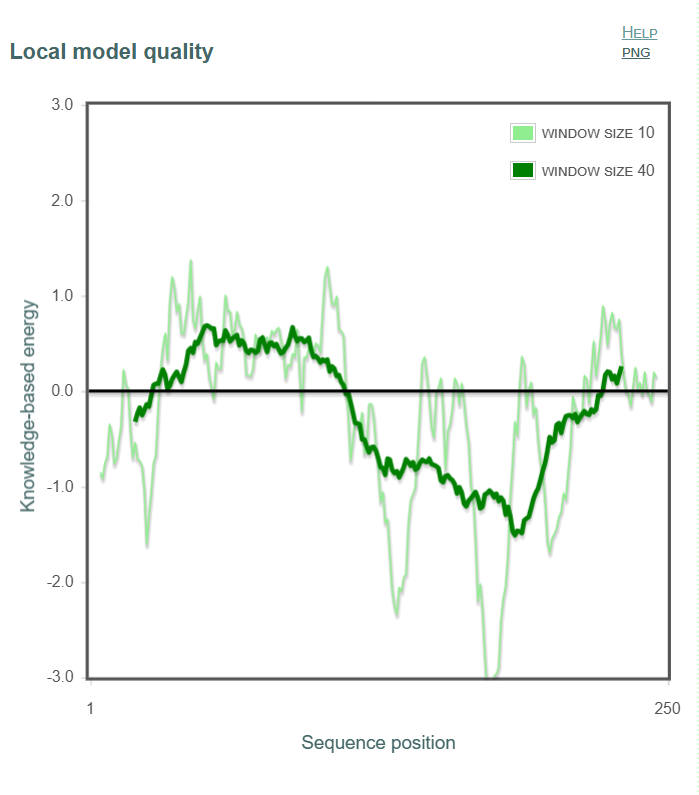

Supplement: Supplementary file 6 [file DataSheet6.zip › YFV/YF_NS4b/ProSA_global_analysis/YF_NS4b_Local_model.png]

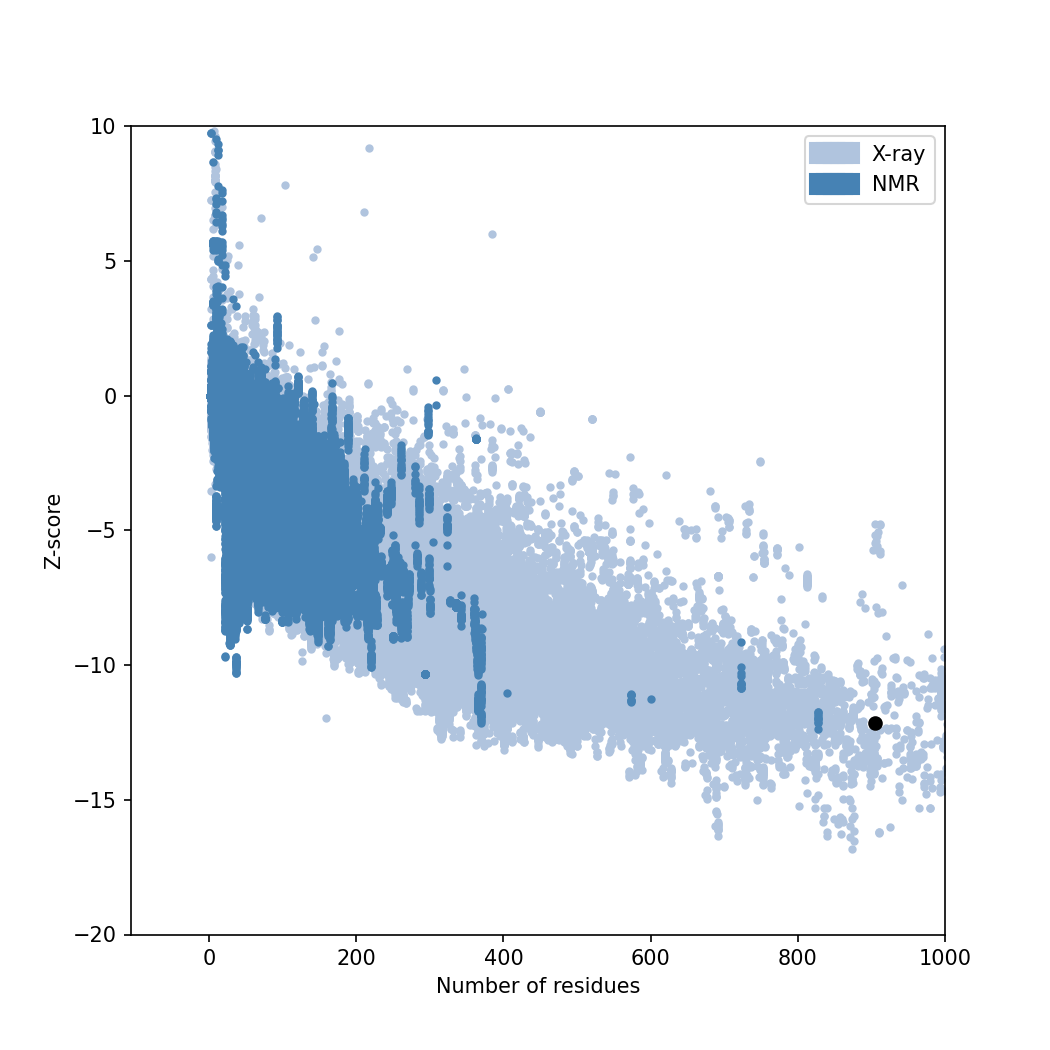

Supplement: Supplementary file 6 [file DataSheet6.zip › YFV/YF_NS5/ProSA_global_analysis/YF_NS5_Global_z_score.png]

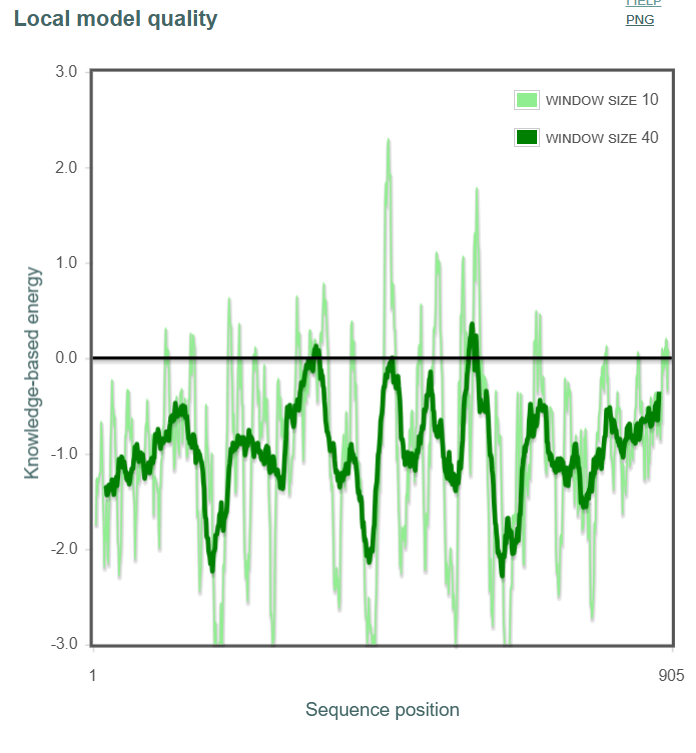

Supplement: Supplementary file 6 [file DataSheet6.zip › YFV/YF_NS5/ProSA_global_analysis/YF_NS5_Local_model.png]

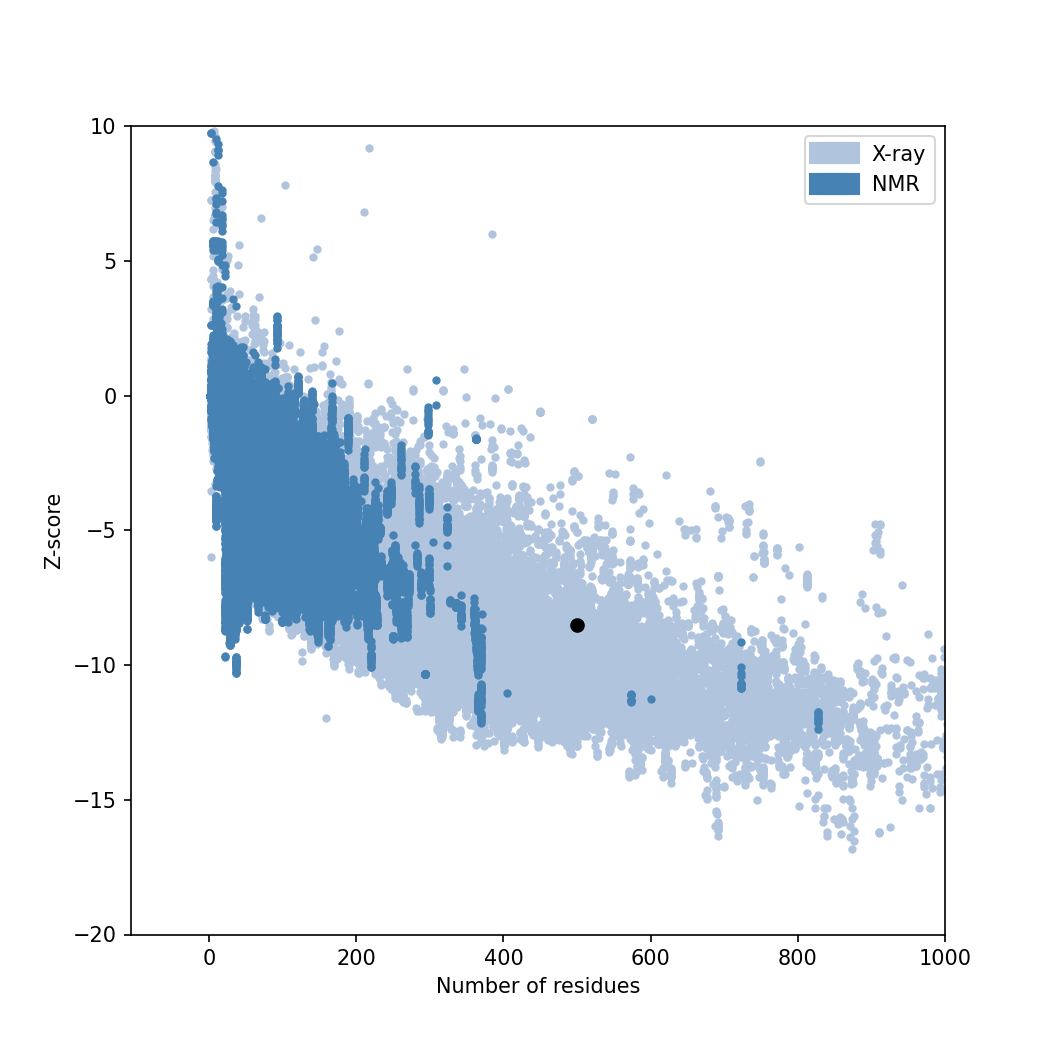

Supplement: Supplementary file 7 [file DataSheet7.zip › ZIKV/ZIKV_E/ProSA_global_analysis/ZIKV_E_Global_z_score.png]

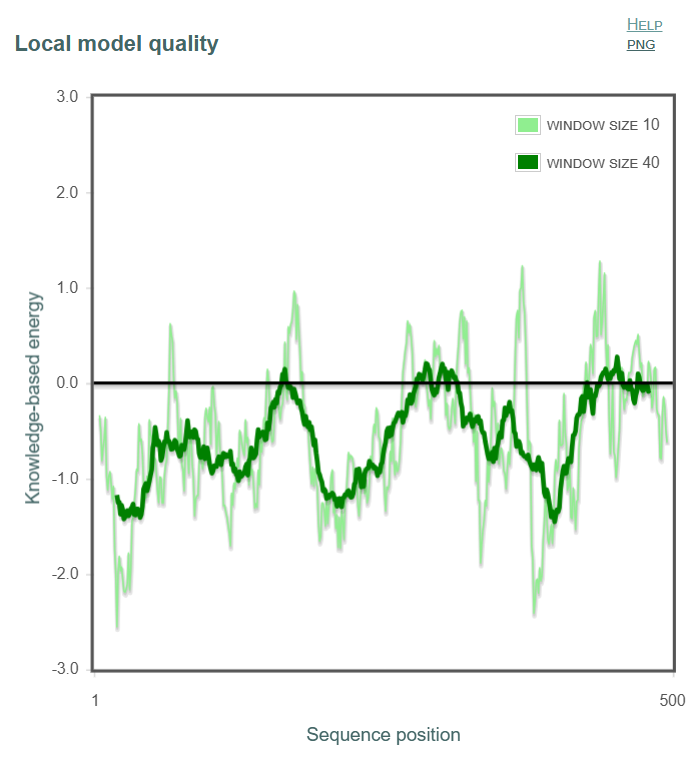

Supplement: Supplementary file 7 [file DataSheet7.zip › ZIKV/ZIKV_E/ProSA_global_analysis/ZIKV_E_Local_model.png]

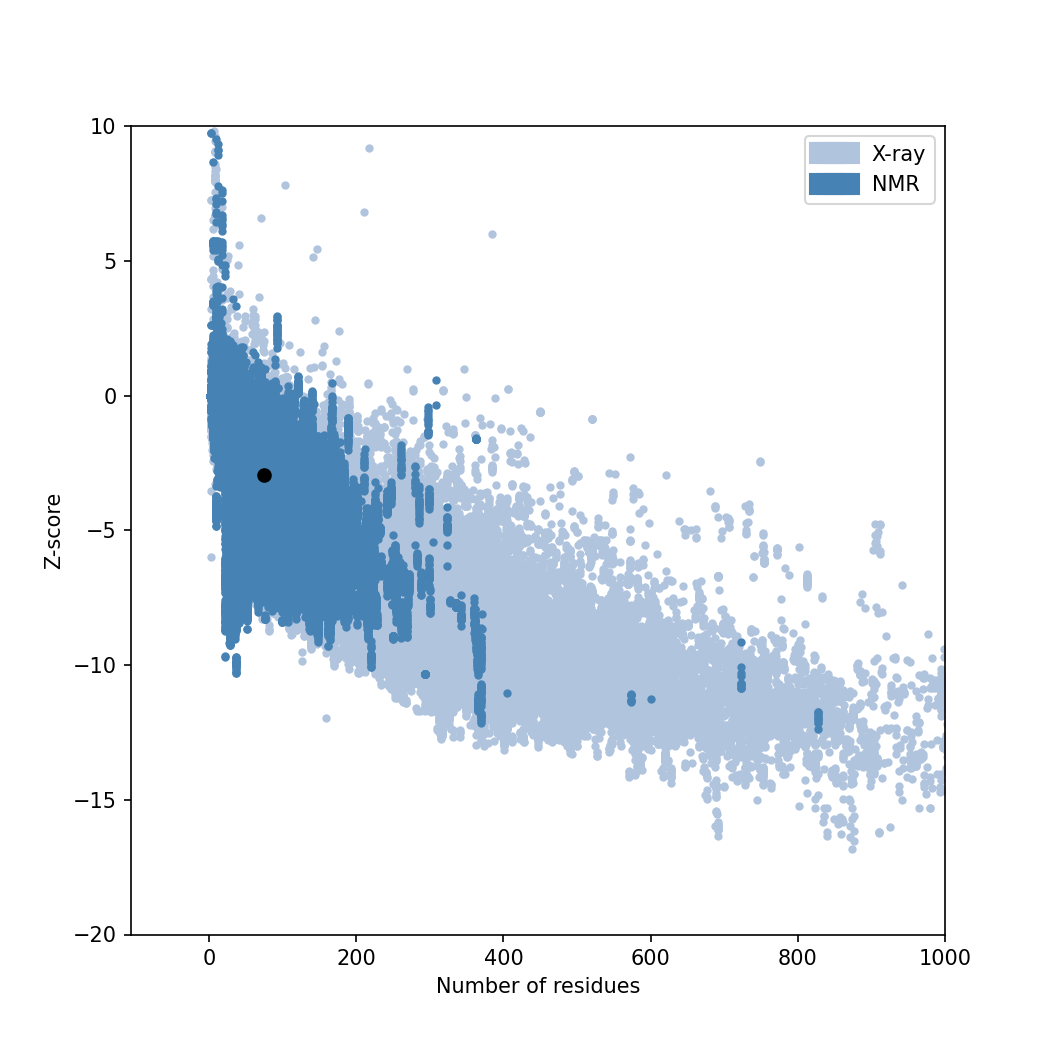

Supplement: Supplementary file 7 [file DataSheet7.zip › ZIKV/ZIKV_M/ProSA_global_analysis/ZIKV_M_Global_z_score.png]

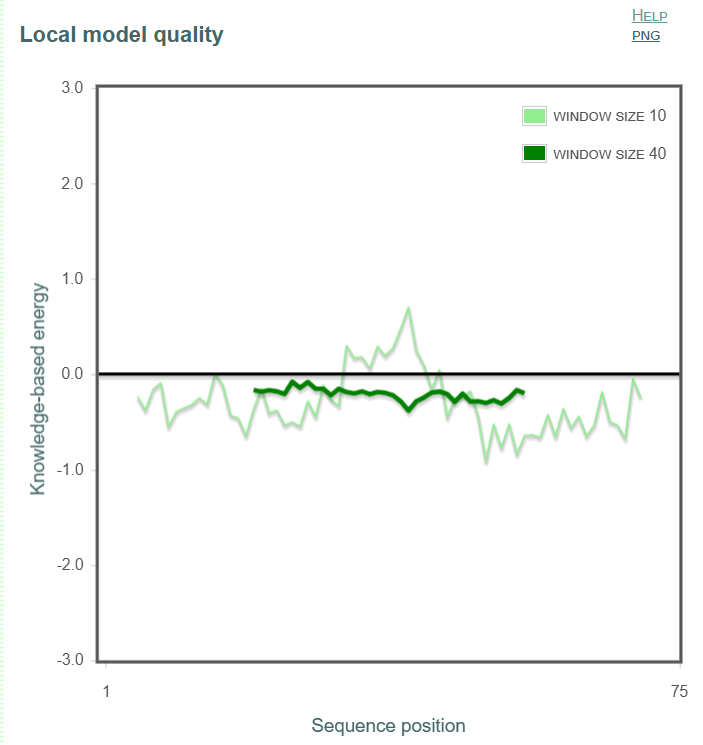

Supplement: Supplementary file 7 [file DataSheet7.zip › ZIKV/ZIKV_M/ProSA_global_analysis/ZIKV_M_Local_model.png]

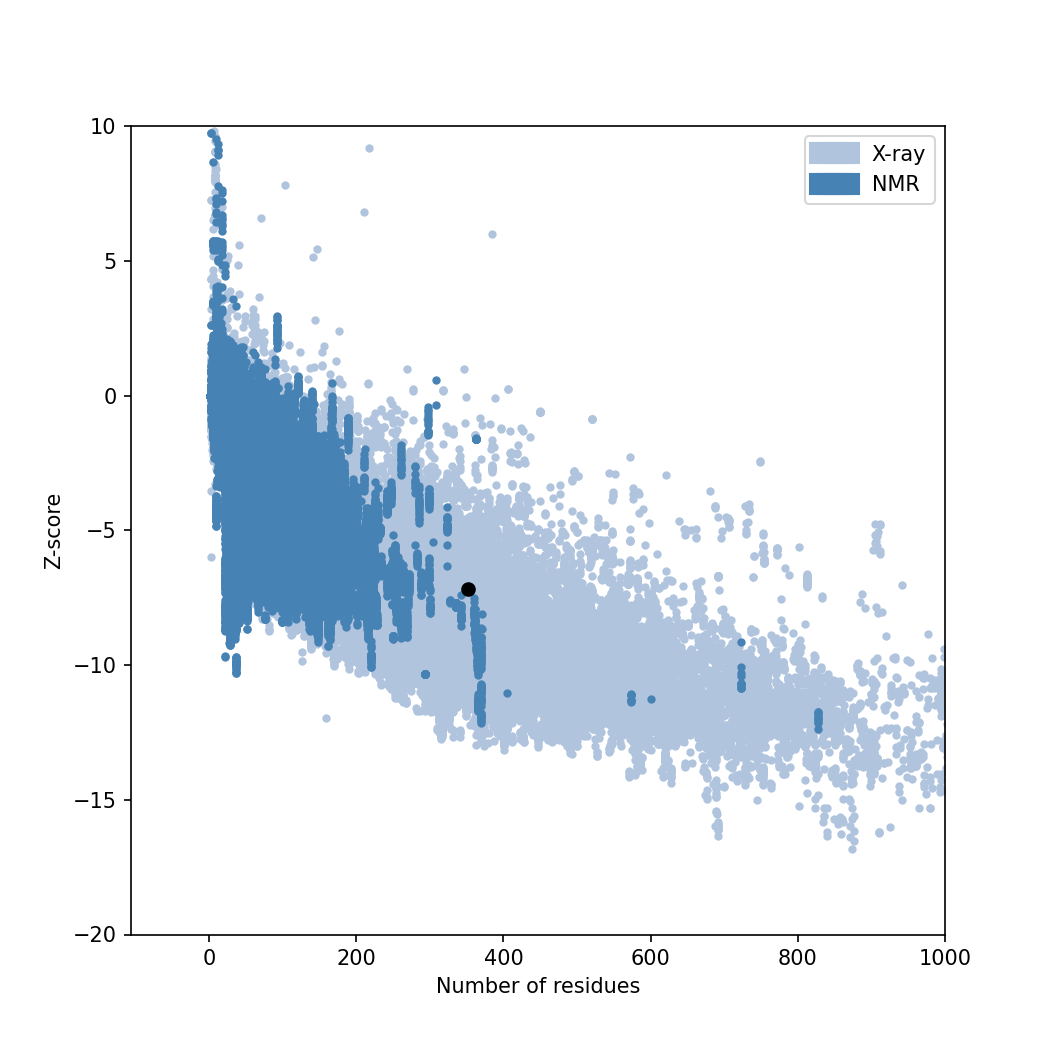

Supplement: Supplementary file 7 [file DataSheet7.zip › ZIKV/ZIKV_NS1/ProSA_global_analysis/ZIKV_NS1_Global_z_score.png]

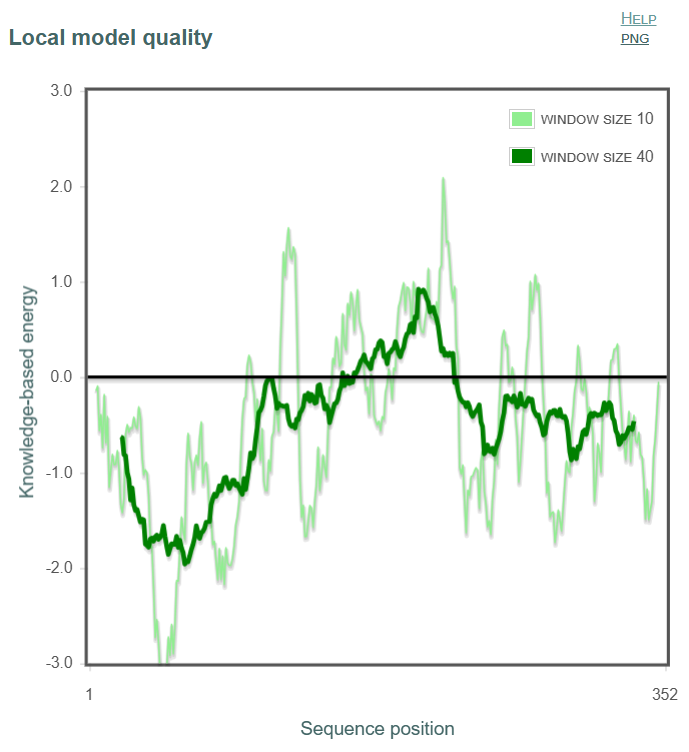

Supplement: Supplementary file 7 [file DataSheet7.zip › ZIKV/ZIKV_NS1/ProSA_global_analysis/ZIKV_NS1_Local_model.png]

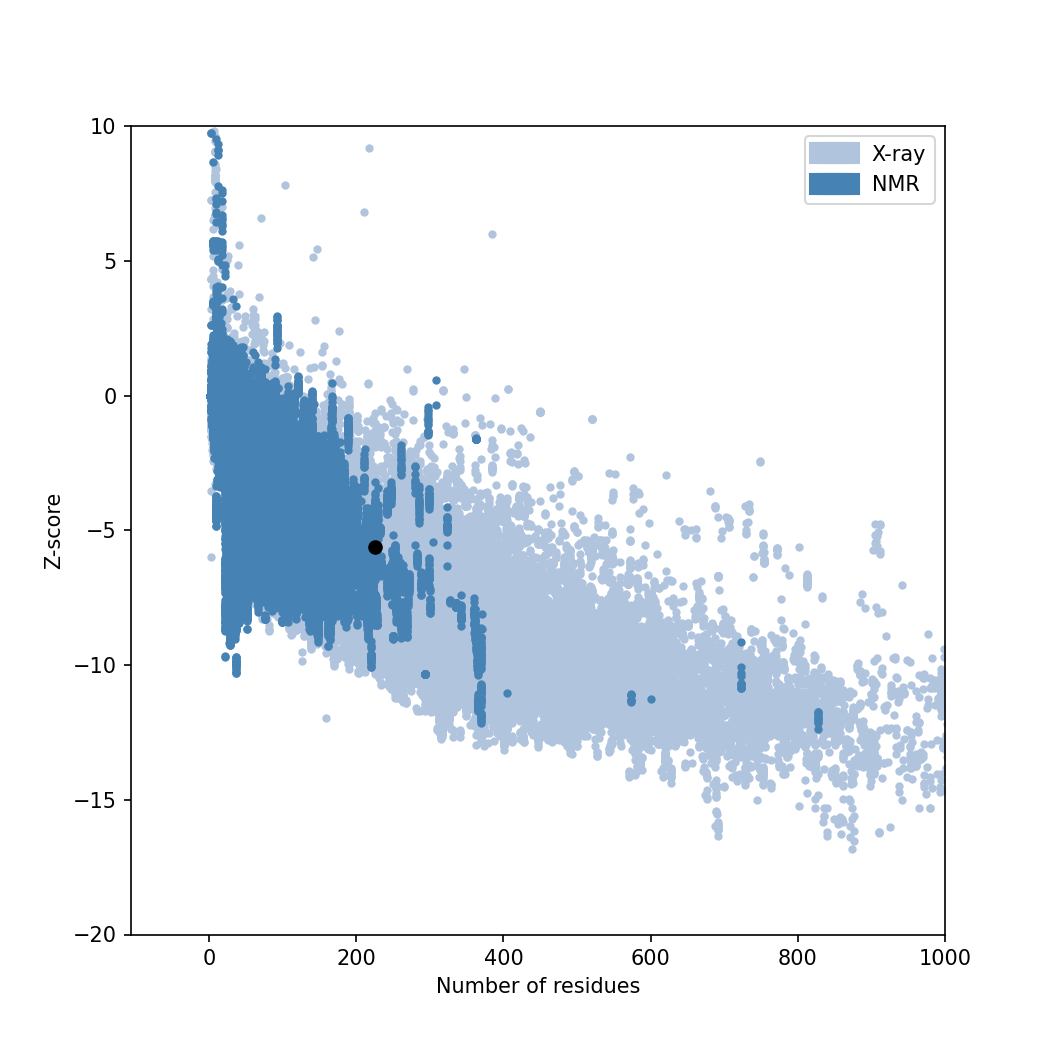

Supplement: Supplementary file 7 [file DataSheet7.zip › ZIKV/ZIKV_NS2a/ProSA_global_analysis/ZIKV_NS2a_Global_z_score.png]

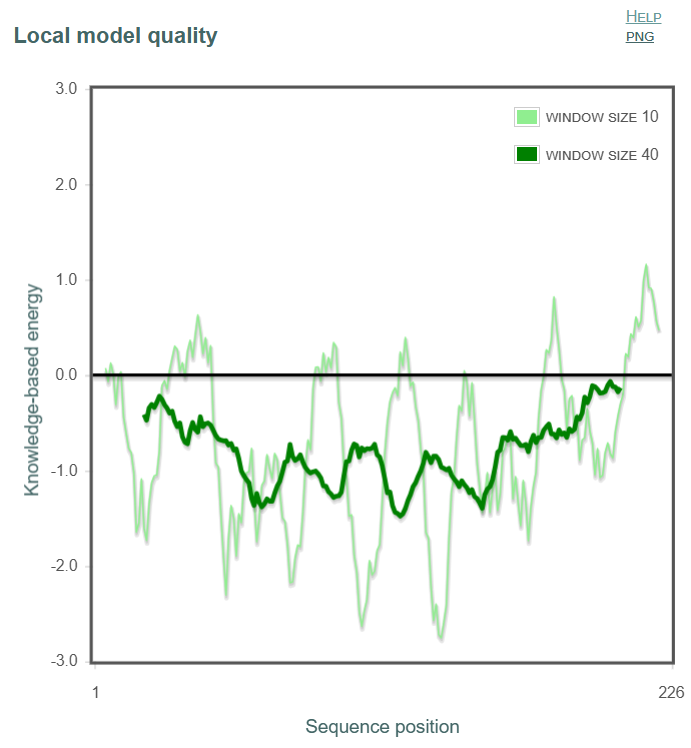

Supplement: Supplementary file 7 [file DataSheet7.zip › ZIKV/ZIKV_NS2a/ProSA_global_analysis/ZIKV_NS2a_Local_model.png]

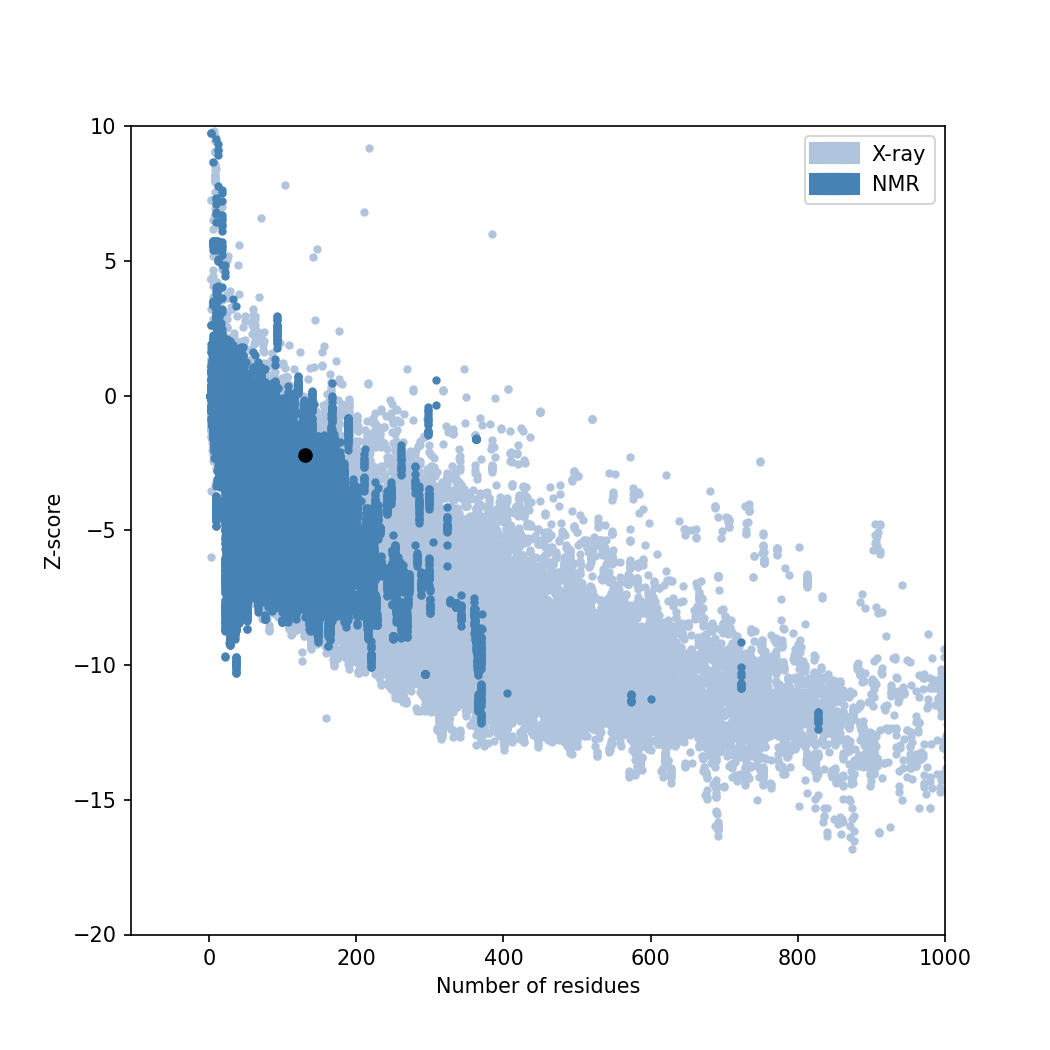

Supplement: Supplementary file 7 [file DataSheet7.zip › ZIKV/ZIKV_NS2b/ProSA_global_analysis/ZIKV_NS2b_Global_z_score.png]

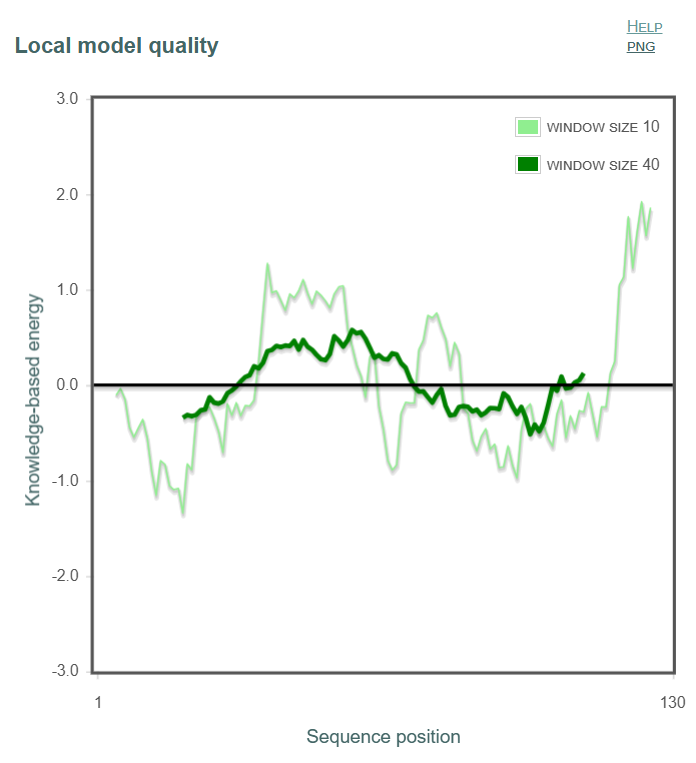

Supplement: Supplementary file 7 [file DataSheet7.zip › ZIKV/ZIKV_NS2b/ProSA_global_analysis/ZIKV_NS2b_Local_model.png]

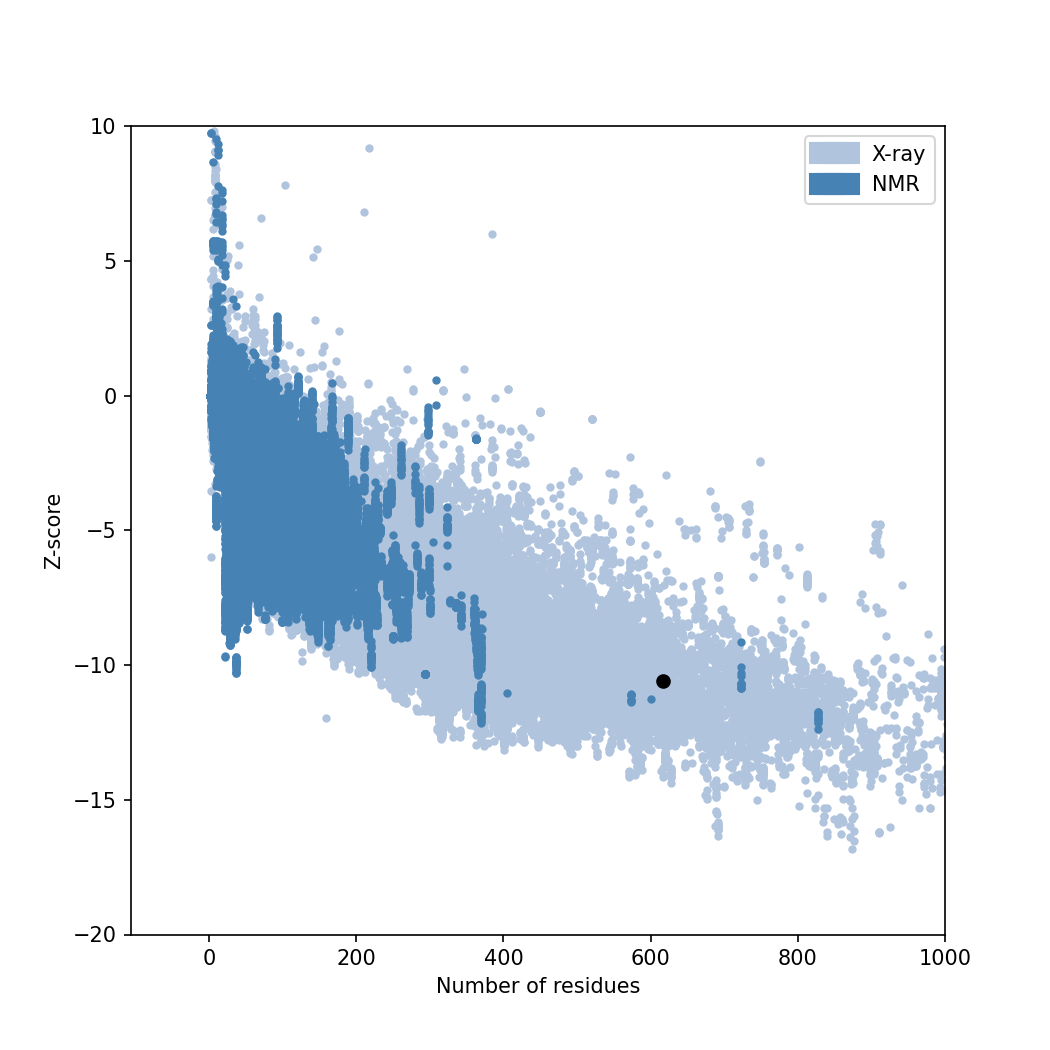

Supplement: Supplementary file 7 [file DataSheet7.zip › ZIKV/ZIKV_NS3/ProSA_global_analysis/ZIKV_NS3_Global_z_score.png]

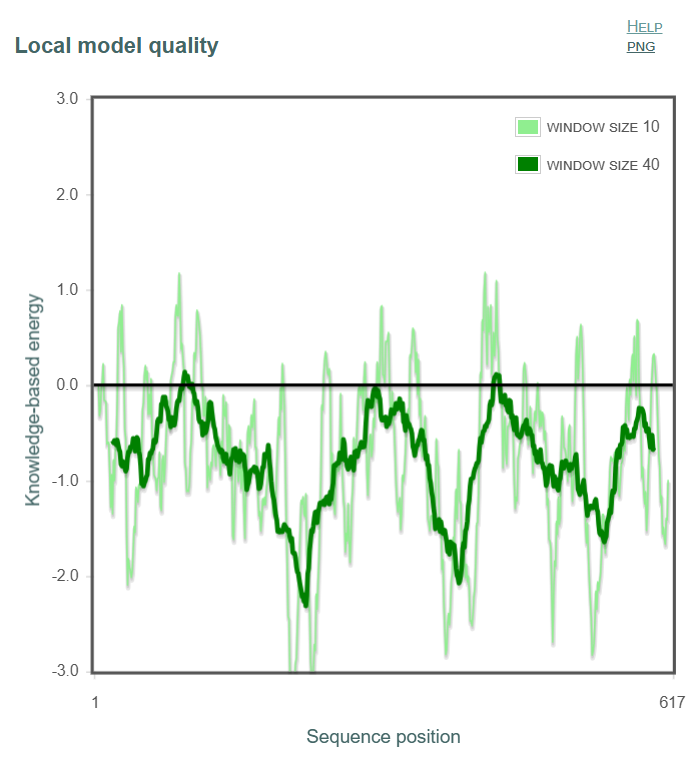

Supplement: Supplementary file 7 [file DataSheet7.zip › ZIKV/ZIKV_NS3/ProSA_global_analysis/ZIKV_NS3_Local_model.png]

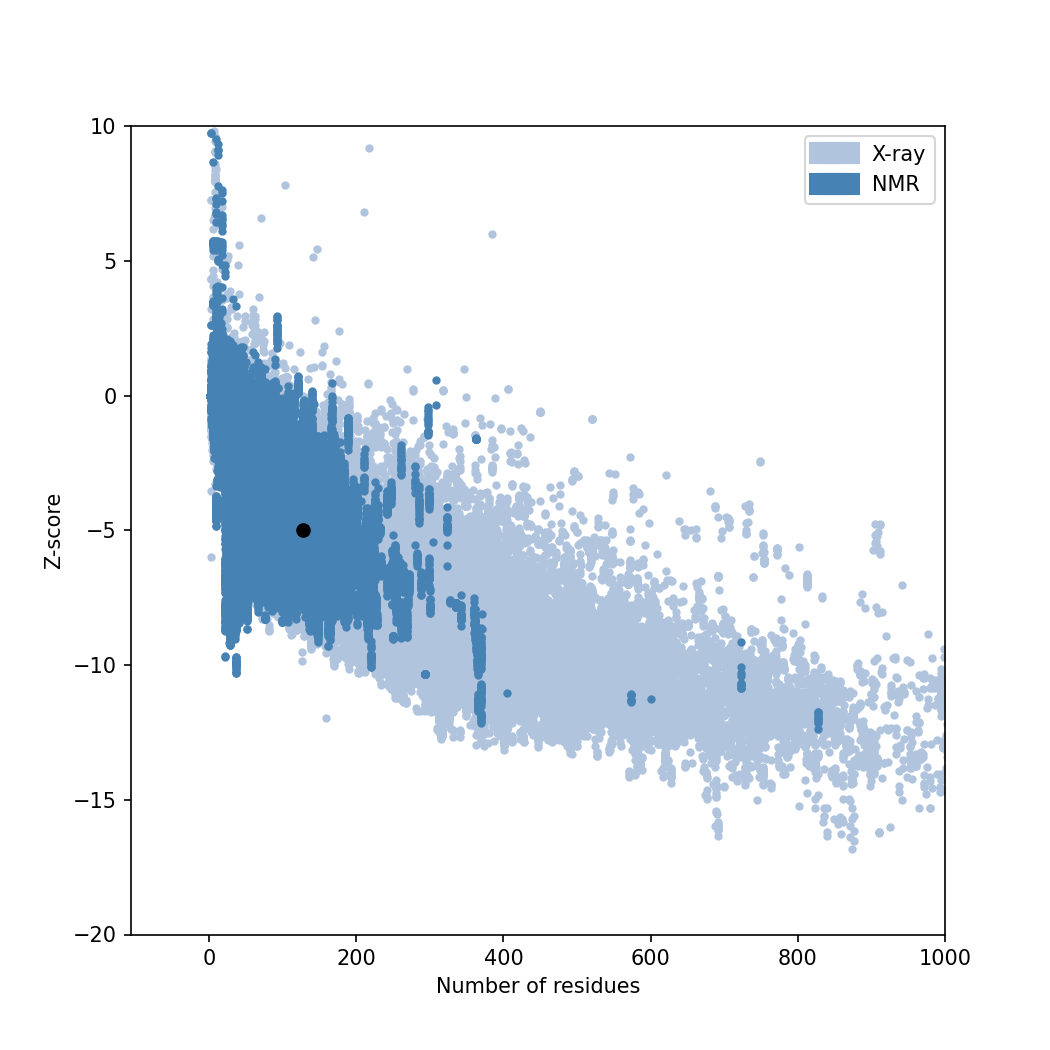

Supplement: Supplementary file 7 [file DataSheet7.zip › ZIKV/ZIKV_NS4a/ProSA_global_analysis/ZIKV_NS4a_Global_z_score.png]

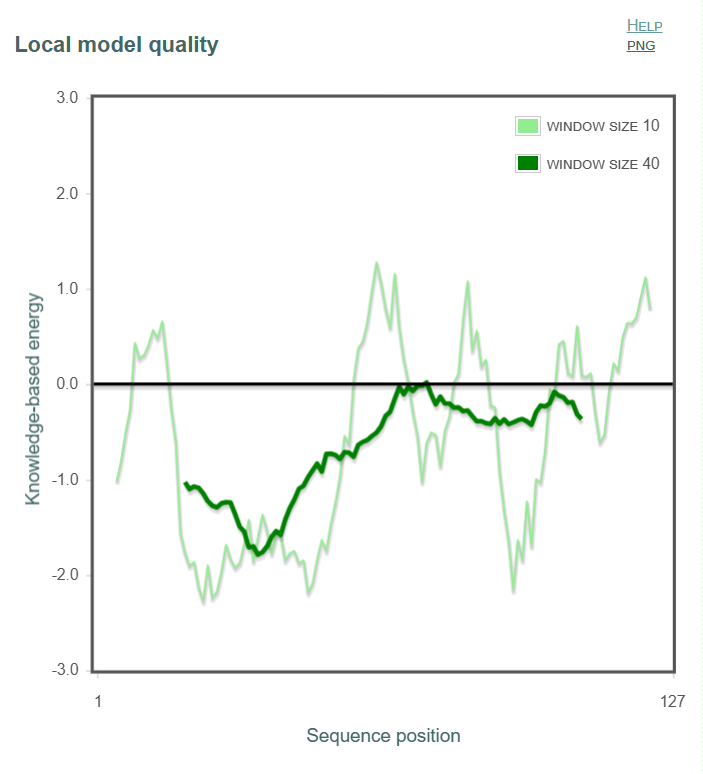

Supplement: Supplementary file 7 [file DataSheet7.zip › ZIKV/ZIKV_NS4a/ProSA_global_analysis/ZIKV_NS4a_Local_model.png]

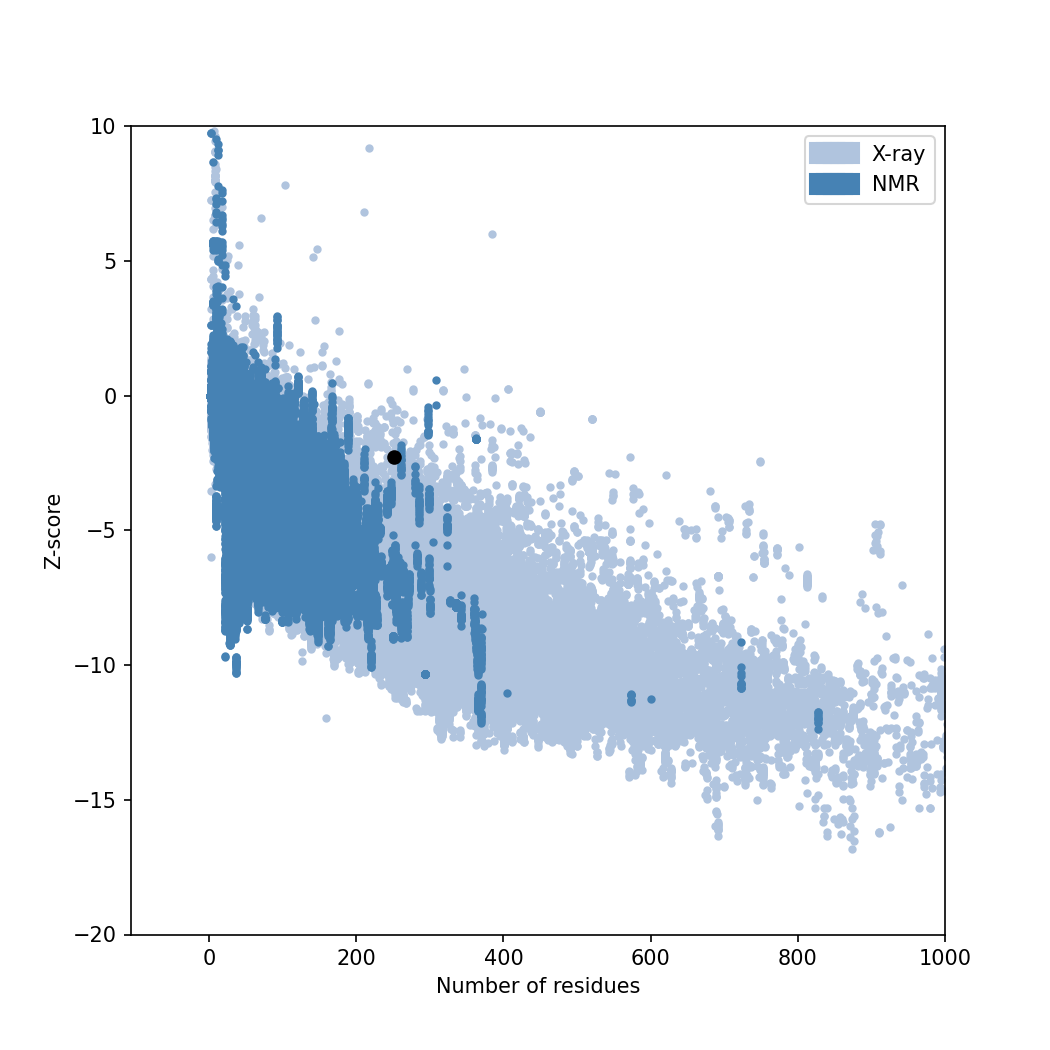

Supplement: Supplementary file 7 [file DataSheet7.zip › ZIKV/ZIKV_NS4b/ProSA_global_analysis/ZIKV_NS4b_Global_z_score.png]

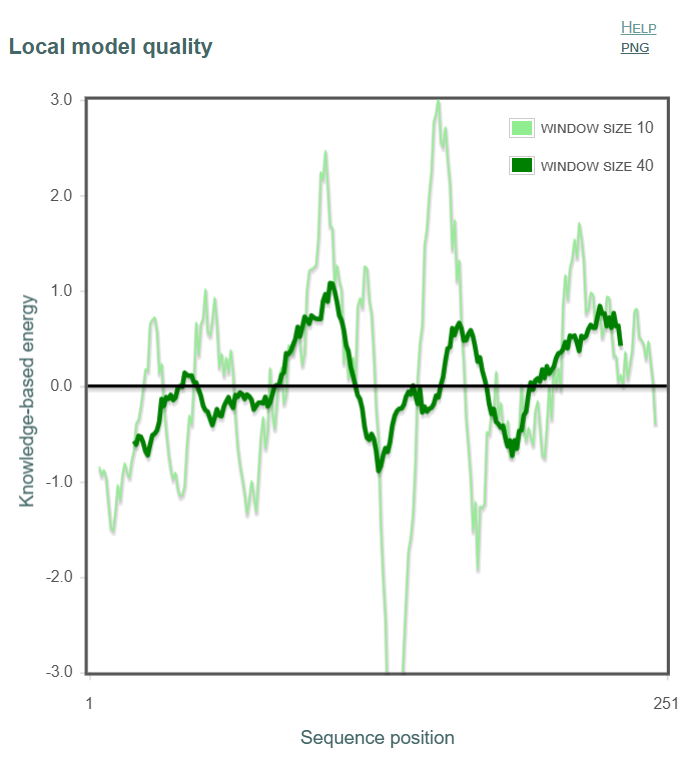

Supplement: Supplementary file 7 [file DataSheet7.zip › ZIKV/ZIKV_NS4b/ProSA_global_analysis/ZIKV_NS4b_Local_model.png]

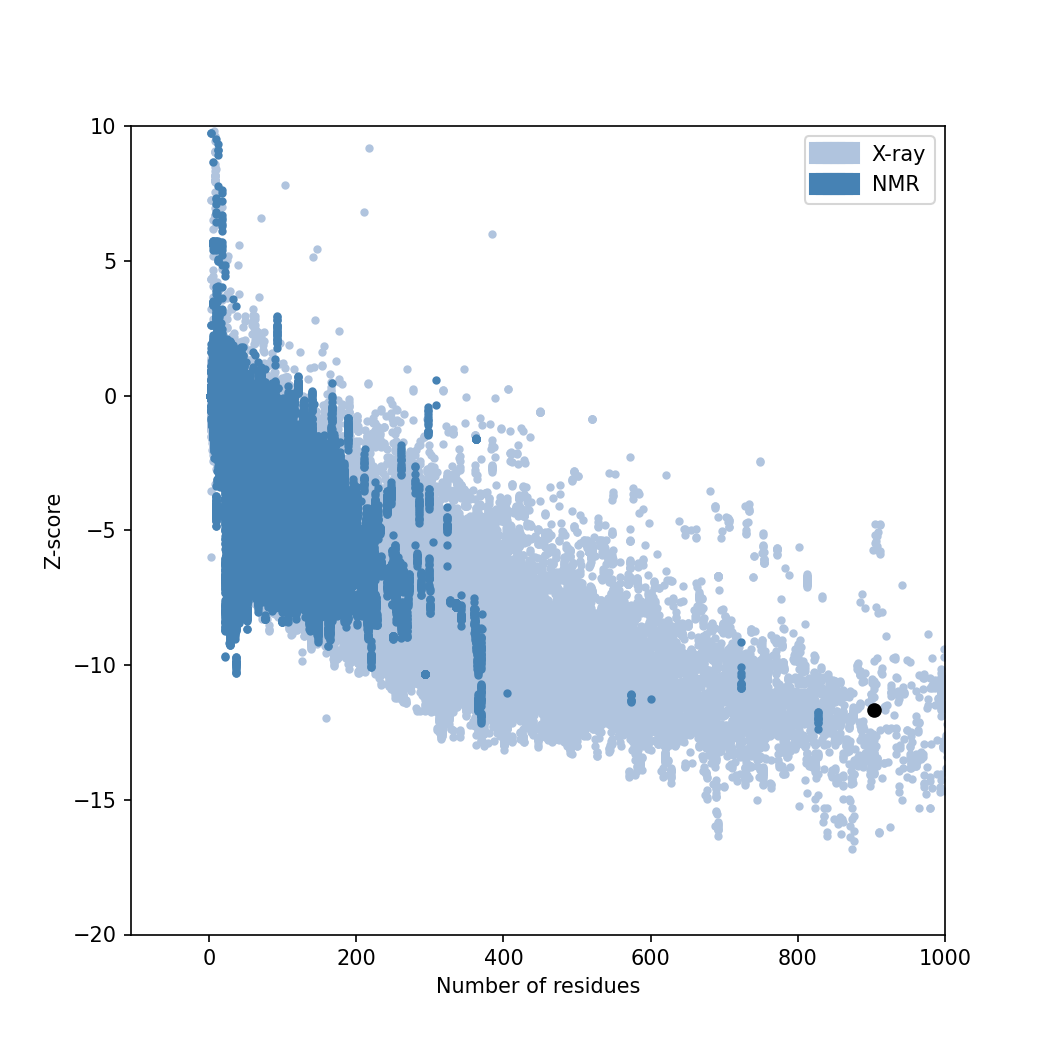

Supplement: Supplementary file 7 [file DataSheet7.zip › ZIKV/ZIKV_NS5/ProSA_global_analysis/ZIKV_NS5_Global_z_score.png]

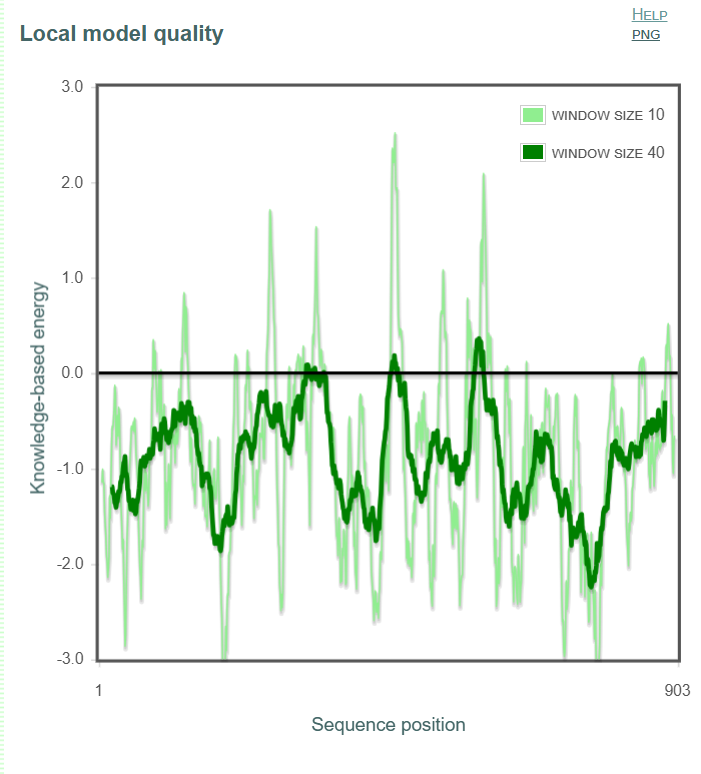

Supplement: Supplementary file 7 [file DataSheet7.zip › ZIKV/ZIKV_NS5/ProSA_global_analysis/ZIKV_NS5_Local_model.png]
