# Supplementary material for: Integrative proteome-wide structural analysis and high-throughput docking identify broad-spectrum antiviral scaffolds against Zika, Yellow Fever, West Nile, Saint Louis encephalitis, and Usutu viruses
Source: Front Cell Infect Microbiol. 2026 Apr 30;16:1723132. doi: 10.3389/fcimb.2026.1723132 (PMC13171538; doi:10.3389/fcimb.2026.1723132)
Supplement: Supplementary file 4 [file DataSheet4.zip › USUV/USU_E/Mol_probity_Files/USU_E_1FH-multi.table.pdf]

Viewing USU_E1FH-multi.table

MolProbity

Duke Biochemistry
Duke University School of Medicine

When finished, you should close this window.

Hint: Use File | Save As... to save a copy of this page.

All-Atom Contacts	Clashscore, all atoms:	1.34		99th percentile* (N=1784, all resolutions)
	Clashscore is the number of serious steric overlaps (> 0.4 Å) per 1000 atoms.			
Protein Geometry	Poor rotamers	0	0.00%	Goal: <0.3%
	Favored rotamers	404	100.00%	Goal: >98%
	Ramachandran outliers	0	0.00%	Goal: <0.05%
	Ramachandran favored	486	97.59%	Goal: >98%
	Rama distribution Z-score	0.71 ± 0.36		Goal: abs(Z score) < 2
	MolProbity score^	0.95		100th percentile* (N=27675, 0Å - 99Å)
	Cβ deviations >0.25Å	0	0.00%	Goal: 0
	Bad bonds:	8 / 3838	0.21%	Goal: 0%
	Bad angles:	10 / 5204	0.19%	Goal: <0.1%
Peptide Omegas	Cis Prolines:	1 / 18	5.56%	Expected: ≤1 per chain, or ≤5%
Low-resolution Criteria	CaBLAM outliers	10	2.0%	Goal: <1.0%
	CA Geometry outliers	6	1.21%	Goal: <0.5%
Additional validations	Chiral volume outliers	0/587		
	Waters with clashes	0/0	0.00%	See UnDowser table for details

In the two column results, the left column gives the raw count, right column gives the percentage.

* 100th percentile is the best among structures of comparable resolution; 0th percentile is the worst. For clashscore the comparative set of structures was selected in 2004, for MolProbity score in 2006.

^ MolProbity score combines the clashscore, rotamer, and Ramachandran evaluations into a single score, normalized to be on the same scale as X-ray resolution.

Key to table colors and cutoffs here:

#	Alt	Res	High B	Clash > 0.4Å	Ramachandran	Rotamer	Cβ deviation	CaBLAM	Bond lengths	Bond angles	Cis Peptides
			Avg: 1.06	Clashscore: 1.34	Outliers: 0 of 498	Poor rotamers: 0 of 404	Outliers: 0 of 446	Outliers: 12 of 496	Outliers: 7 of 500	Outliers: 10 of 500	Non-Trans: 1 of 499
A 1		PHE	1.2	0.51Å N with A 42 ASP OD2	-	Favored (84.3%) *t80* chi angles: 177.6,72.5	0.09Å	-	-	-	-
A 2		ASN	1.17	-	Favored (62.38%) General / -71.5,-17.1	Favored (16.6%) *p0* chi angles: 65.6,301.3	0.07Å	-	-	-	-
A 3		CYS	1.17	-	Favored (41.19%) General / -96.8,9.6	Favored (74.6%) *m* chi angles: 297.1	0.04Å	Favored (39.433%)	-	-	-
A 4		LEU	1.18	-	Favored (74.69%) General / -61.4,-34.0	Favored (83.3%) *mt* chi angles: 294.2,168.7	0.11Å	Favored (48.807%) alpha helix	OUTLIER(S) worst is CB--CG: 4.3 σ	-	-
A 5		GLY	1.2	-	Favored (75.43%) Glycine / -93.2,8.5	-	-	Favored (53.796%)	-	-	-
A 6		MET	1.22	-	Favored (36.36%) General / -103.8,141.3	Favored (86.1%) *mtp* chi angles: 293.1,176,60.9	0.07Å	Favored (34.003%)	-	-	-

A 7		SER	1.22	-	Favored (15.16%) General / -82.3,170.6	Favored (93.5%) *p* chi angles: 66.3	0.03Å	CaBLAM Disfavored (4.502%)	-	-	-
A 8		ASN	1.2	-	Favored (30.16%) General / 56.1,40.7	Favored (88.7%) *m-40* chi angles: 296.5,321.1	0.04Å	Favored (8.175%)	-	-	-
A 9		ARG	1.17	-	Favored (40.45%) General / -95.8,125.6	Favored (81.2%) *ttt180* chi angles: 182.5,178.4,180.8,186.6	0.04Å	Favored (32.823%)	-	-	-
A 10		ASP	1.16	-	Favored (25.42%) General / -106.4,151.1	Favored (74.2%) *m-30* chi angles: 296.6,317.9	0.02Å	Favored (45.735%) beta sheet	-	-	-
A 11		PHE	1.22	-	Favored (48.87%) General / -118.7,141.2	Favored (89.5%) *m-80* chi angles: 293.8,84.4	0.04Å	Favored (60.886%) beta sheet	-	-	-
A 12		LEU	1.36	-	Favored (20.57%) General / -141.4,128.3	Favored (74.9%) *tp* chi angles: 177.5,62.7	0.10Å	Favored (58.199%) beta sheet	-	-	-
A 13		GLU	1.58	-	Favored (35.34%) General / -101.1,140.5	Favored (98.3%) *mt-10* chi angles: 294.6,179,355.5	0.01Å	Favored (60.914%) beta sheet	-	-	-
A 14		GLY	1.84	-	Favored (36.9%) Glycine / -95.7,-177.1	-	-	Favored (39.282%) beta sheet	-	-	-
A 15		VAL	2.04	-	Favored (36.56%) Ile or Val / -131.1,151.8	Favored (27.5%) *m* chi angles: 298.8	0.03Å	Favored (33.484%)	-	-	-
A 16		SER	2.09	-	Favored (66.51%) General / -64.7,-20.8	Favored (88.7%) *p* chi angles: 66.9	0.03Å	Favored (47.514%)	-	-	-
A 17		GLY	1.96	-	Favored (87.67%) Glycine / -85.0,-3.5	-	-	Favored (60.111%) alpha helix	-	-	-
A 18		ALA	1.7	-	Favored (3.01%) General / -116.0,-43.0	-	0.06Å	CaBLAM Disfavored (1.108%)	-	-	-
A 19		THR	1.42	-	Allowed (0.65%) General / 71.1,-46.8	Favored (90.7%) *m* chi angles: 298.8	0.10Å	CaBLAM Disfavored (4.982%)	-	-	-
A 20		TRP	1.19	-	Favored (21.02%) General / -86.6,154.5	Favored (70.5%) *p-90* chi angles: 57.3,263.9	0.07Å	Favored (15.28%)	-	-	-
**#**	**Alt**	**Res**	**High B**	**Clash > 0.4Å**	**Ramachandran**	**Rotamer**	**Cβ deviation**	**CaBLAM**	**Bond lengths**	**Bond angles**	**Cis Peptides**
			Avg: 1.06	Clashscore: 1.34	Outliers: 0 of 498	Poor rotamers: 0 of 404	Outliers: 0 of 446	Outliers: 12 of 496	Outliers: 7 of 500	Outliers: 10 of 500	Non-Trans: 1 of 499
A 21		VAL	1.03	-	Favored (46.78%) Ile or Val / -133.8,140.3	Favored (7.3%) *p* chi angles: 60.5	0.10Å	Favored (53.016%)	-	-	-
A 22		ASP	0.94	-	Favored (36.34%) General / -94.4,121.9	Favored (97.4%) *m-30* chi angles: 289.2,345.6	0.10Å	Favored (58.445%) beta sheet	-	-	-

A 23		VAL	0.9	-	Favored (67.38%) Ile or Val / -110.2,125.7	Favored (83.4%) *t* chi angles: 177.8	0.11Å	Favored (68.3%) beta sheet	-	-	-
A 24		VAL	0.9	-	Favored (8.02%) Ile or Val / -109.5,99.1	Favored (68.6%) *t* chi angles: 178.9	0.18Å	Favored (64.875%) beta sheet	-	-	-
A 25		LEU	0.92	-	Favored (51.43%) General / -109.2,134.7	Favored (18.8%) *tp* chi angles: 189.9,69.5	0.12Å	Favored (40.586%)	-	-	-
A 26		GLU	0.95	-	Favored (44.26%) General / -119.8,147.8	Favored (91.8%) *tt0* chi angles: 182.6,176.8,357.9	0.03Å	Favored (23.553%)	-	-	-
A 27		GLY	0.96	-	Favored (27.89%) Glycine / -50.0,130.2	-	-	Favored (12.958%)	-	-	-
A 28		ASP	0.97	-	Favored (5.84%) General / 66.0,9.0	Favored (38.4%) *m-30* chi angles: 285.5,316	0.15Å	Favored (10.492%)	-	-	-
A 29		SER	0.95	-	Favored (13.39%) General / -87.0,168.4	Favored (89.1%) *p* chi angles: 69.2	0.10Å	Favored (36.668%)	-	-	-
A 30		CYS	0.94	-	Favored (27.24%) General / -142.6,134.6	Favored (47.7%) *t* chi angles: 178.7	0.07Å	Favored (49.264%) beta sheet	-	-	-
A 31		ILE	0.94	-	Favored (74.55%) Ile or Val / -117.8,126.0	Favored (80.3%) *mt* chi angles: 299.8,168.4	0.05Å	Favored (71.76%) beta sheet	-	-	-
A 32		THR	0.99	-	Favored (44.46%) General / -103.4,122.2	Favored (84.9%) *m* chi angles: 296.7	0.06Å	Favored (70.595%) beta sheet	-	-	-
A 33		ILE	1.07	-	Favored (64.01%) Ile or Val / -107.6,125.5	Favored (3.3%) *mp* chi angles: 301,89.9	0.05Å	Favored (63.522%) beta sheet	-	-	-
A 34		MET	1.19	-	Favored (44.95%) General / -122.8,148.8	Favored (28.3%) *mtm* chi angles: 297.3,160.4,286.7	0.09Å	Favored (24.982%)	-	-	-
A 35		ALA	1.29	-	Favored (21.89%) General / -159.7,152.8	-	0.02Å	Favored (7.795%)	-	-	-
A 36		LYS	1.34	-	Favored (40.58%) General / -56.8,129.2	Favored (50.8%) *tttm* chi angles: 182.4,175.4,182.6,286.7	0.03Å	Favored (14.972%)	-	-	-
A 37		ASP	1.3	-	Favored (7.52%) General / 65.8,11.4	Favored (71.4%) *m-30* chi angles: 298.6,316.2	0.06Å	Favored (6.655%)	-	-	-
A 38		LYS	1.2	-	Favored (46.49%) Pre-Pro / -114.3,153.6	Favored (66.6%) *mmtt* chi angles: 301.6,297.5,188,190.7	0.02Å	Favored (24.32%)	-	-	-
A 39		PRO	1.06	-	Favored (68.89%) Trans-Pro / -69.5,154.3	Favored (57.9%) *Cg_endo* chi angles: 26.1,327.1,26.3	0.04Å	Favored (69.449%)	-	-	-
A 40		THR	0.92	-	Favored (30.18%)	Favored (95.1%) *m* chi angles: 299.5	0.06Å	Favored (34.59%)	-	-	-

#	Alt	Res	High B	Clash > 0.4Å	Ramachandran	Rotamer	Cβ deviation	CaBLAM	Bond lengths	Bond angles	Cis Peptides
					General / -78.7,126.0						
			Avg: 1.06	Clashscore: 1.34	Outliers: 0 of 498	Poor rotamers: 0 of 404	Outliers: 0 of 446	Outliers: 12 of 496	Outliers: 7 of 500	Outliers: 10 of 500	Non-Trans: 1 of 499
A 41		ILE	0.82	-	Favored (66.58%) Ile or Val / -109.8,127.5	Favored (44.3%) *mm* chi angles: 306.6,300.8	0.05Å	Favored (68.659%) beta sheet	-	-	-
A 42		ASP	0.77	0.51Å OD2 with A 1 PHE N	Favored (41.48%) General / -102.4,121.0	Favored (98.1%) *m-30* chi angles: 287.4,344.2	0.07Å	Favored (71.83%) beta sheet	-	OUTLIER(S) worst is CA-CB-CG: 4.7 σ	-
A 43		ILE	0.74	-	Favored (71.09%) Ile or Val / -113.8,125.3	Favored (70.5%) *mt* chi angles: 299.9,176	0.10Å	Favored (62.588%) beta sheet	-	-	-
A 44		LYS	0.74	-	Favored (40.37%) General / -131.6,129.1	Favored (49.3%) *tttm* chi angles: 180.2,182.5,186.4,293	0.06Å	Favored (51.648%) beta sheet	-	-	-
A 45		MET	0.75	-	Favored (20.92%) General / -76.0,120.2	Favored (60.9%) *ttm* chi angles: 183.4,179.1,293.4	0.04Å	Favored (53.192%)	-	-	-
A 46		MET	0.77	-	Favored (12.14%) General / -89.6,-41.1	Favored (94.3%) *mtp* chi angles: 291.5,173.2,64.6	0.04Å	Favored (30.887%)	-	-	-
A 47		GLU	0.79	-	Favored (13.41%) General / -156.8,137.6	Favored (37.3%) *tt0* chi angles: 184.3,175.1,76.5	0.01Å	Favored (21.302%)	-	-	-
A 48		THR	0.81	-	Favored (50.9%) General / -116.8,125.0	Favored (97.1%) *m* chi angles: 300.7	0.04Å	Favored (61.731%)	-	-	-
A 49		GLU	0.84	-	Favored (32.14%) General / -140.2,135.2	Favored (42.6%) *tt0* chi angles: 183.6,173.6,65.9	0.03Å	Favored (57.329%) beta sheet	-	-	-
A 50		ALA	0.86	-	Favored (32.79%) General / -103.1,143.3	-	0.10Å	Favored (55.55%) beta sheet	-	-	-
A 51		THR	0.88	-	Allowed (0.63%) General / -116.9,-85.8	Favored (61.5%) *p* chi angles: 57.5	0.07Å	CaBLAM Disfavored (1.245%)	-	-	-
A 52		ASN	0.89	-	Favored (4.05%) General / -77.9,68.8	Favored (87.1%) *m-40* chi angles: 293.3,318.7	0.06Å	Favored (29.139%)	-	-	-
A 53		LEU	0.88	-	Favored (51.08%) General / -70.4,145.4	Favored (5.4%) *mp* chi angles: 272.8,70.9	0.07Å	Favored (17.67%)	-	-	-
A 54		ALA	0.86	-	Favored (20.73%) General / -100.8,152.3	-	0.03Å	Favored (38.828%) beta sheet	-	-	-
A 55		GLU	0.82	-	Favored (36.05%) General / -76.9,150.3	Favored (95.8%) *mt-10* chi angles: 294.1,175.6,342.2	0.12Å	CaBLAM Disfavored (3.02%)	-	OUTLIER(S) worst is CA-C-O: 5.0 σ	-

A 56		VAL	0.78	-	Allowed (1.07%) Ile or Val / -124.6,-33.0	Favored (23.3%) *m* chi angles: 295	0.03Å	CaBLAM Outlier (0.827%)	-	OUTLIER(S) worst is C-N-CA: 4.3 σ	-
A 57		ARG	0.73	-	Favored (5.3%) General / -164.9,139.8	Favored (48%) *ttm170* chi angles: 178.3,180.5,291.1,183.2	0.04Å	Favored (12.898%)	-	-	-
A 58		SER	0.7	-	Favored (46.51%) General / -103.1,134.4	Favored (41.3%) *t* chi angles: 176.3	0.03Å	Favored (64.129%)	-	-	-
A 59		TYR	0.68	-	Favored (33.03%) General / -111.4,148.4	Favored (39.8%) *m-80* chi angles: 279.9,86.2	0.05Å	Favored (53.971%) beta sheet	-	-	-
A 60		CYS	0.67	-	Favored (46.68%) General / -105.5,135.5	Favored (39.5%) *t* chi angles: 187.2	0.06Å	Favored (41.243%)	-	-	-
**#**	**Alt**	**Res**	**High B**	**Clash > 0.4Å**	**Ramachandran**	**Rotamer**	**Cβ deviation**	**CaBLAM**	**Bond lengths**	**Bond angles**	**Cis Peptides**
			Avg: 1.06	Clashscore: 1.34	Outliers: 0 of 498	Poor rotamers: 0 of 404	Outliers: 0 of 446	Outliers: 12 of 496	Outliers: 7 of 500	Outliers: 10 of 500	Non-Trans: 1 of 499
A 61		TYR	0.66	0.47Å CD1 with A 61 TYR N	Favored (3.89%) General / -113.1,-40.7	Favored (87.3%) *m-80* chi angles: 298.4,85.6	0.07Å	Favored (9.978%)	-	OUTLIER(S) worst is CA-CB-CG: 4.1 σ	-
A 62		LEU	0.66	-	Favored (20.17%) General / -124.7,115.1	Favored (63.4%) *tp* chi angles: 174.5,62.3	0.08Å	Favored (28.268%)	-	-	-
A 63		ALA	0.67	-	Favored (33.27%) General / -116.7,152.9	-	0.03Å	Favored (43.997%)	-	-	-
A 64		THR	0.67	-	Favored (53.83%) General / -117.7,135.7	Favored (88.6%) *m* chi angles: 298.6	0.05Å	Favored (56.068%) beta sheet	-	-	-
A 65		VAL	0.67	-	Favored (45.98%) Ile or Val / -94.0,123.1	Favored (85.5%) *t* chi angles: 177.2	0.09Å	Favored (27.653%)	-	-	-
A 66		SER	0.66	-	Allowed (0.58%) General / -115.9,-88.3	Favored (92.7%) *p* chi angles: 63.2	0.07Å	CaBLAM Disfavored (1.493%)	-	-	-
A 67		ASP	0.66	-	Favored (2.1%) General / -74.6,85.5	Favored (41.5%) *t0* chi angles: 184.8,17.8	0.02Å	Favored (12.216%)	-	OUTLIER(S) worst is CA-CB-CG: 4.5 σ	-
A 68		VAL	0.66	-	Favored (39.6%) Ile or Val / -93.0,131.6	Favored (82.7%) *t* chi angles: 177.8	0.02Å	Favored (26.42%)	-	-	-
A 69		SER	0.65	-	Favored (23.42%) General / -143.9,133.4	Favored (40.6%) *t* chi angles: 175.1	0.05Å	Favored (53.595%) beta sheet	-	-	-
A 70		THR	0.65	-	Favored (54.1%) General / -123.3,134.1	Favored (98.3%) *m* chi angles: 300.1	0.02Å	Favored (71.21%) beta sheet	-	-	-
A 71		VAL	0.65	-	Favored (54.99%) Ile or Val / -116.6,136.0	Favored (83.6%) *t* chi angles: 173.5	0.03Å	Favored (65.084%) beta sheet	-	-	-

A 72		SER	0.66	-	Favored (47.79%) General / -134.3,156.8	Favored (97.1%) *p* chi angles: 63.5	0.06Å	Favored (64.657%) beta sheet	-	-	-
A 73		ASN	0.67	-	Favored (49.02%) General / -136.2,150.4	Favored (4.3%) *m110* chi angles: 281.2,85.4	0.06Å	Favored (45.235%)	-	-	-
A 74		CYS	0.69	-	Favored (83.07%) Pre-Pro / -76.2,150.7	Favored (83.6%) *m* chi angles: 295.3	0.03Å	Favored (36.926%)	-	-	-
A 75		PRO	0.71	-	Favored (5.12%) Trans-Pro / -45.5,-29.6	Favored (94.2%) *Cg_exo* chi angles: 331.3,37.6,330.3	0.06Å	Favored (7.07%)	-	-	-
A 76		THR	0.73	-	Favored (13.8%) General / -118.1,18.7	Favored (61.5%) *p* chi angles: 57.5	0.05Å	Favored (14.188%)	-	-	-
A 77		THR	0.73	-	Favored (32.01%) General / -105.6,6.3	Favored (72.6%) *p* chi angles: 61.8	0.04Å	Favored (5.733%)	-	-	-
A 78		GLY	0.72	-	Favored (29.74%) Glycine / 100.3,180.0	-	-	Favored (38.032%)	-	-	-
A 79		GLU	0.7	-	Favored (30.74%) General / -80.4,148.0	Favored (95.1%) *mt-10* chi angles: 295.3,181.4,3.7	0.03Å	Favored (7.721%)	-	-	-
A 80		ALA	0.68	-	Favored (53.54%) General / -69.2,145.0	-	0.03Å	Favored (36.208%)	-	-	-

#	Alt	Res	High B	Clash > 0.4Å	Ramachandran	Rotamer	Cβ deviation	CaBLAM	Bond lengths	Bond angles	Cis Peptides
			Avg: 1.06	Clashscore: 1.34	Outliers: 0 of 498	Poor rotamers: 0 of 404	Outliers: 0 of 446	Outliers: 12 of 496	Outliers: 7 of 500	Outliers: 10 of 500	Non-Trans: 1 of 499
A 81		HIS	0.65	-	Favored (6.36%) General / -146.4,114.0	Favored (26.3%) *t-170* chi angles: 184,190.8	0.02Å	Favored (32.963%) beta sheet	-	-	-
A 82		ASN	0.63	-	Favored (61.18%) Pre-Pro / -95.2,113.3	Favored (46.1%) *t0* chi angles: 187.4,315.7	0.05Å	Favored (64.544%)	-	-	-
A 83		PRO	0.64	-	Favored (9.89%) Trans-Pro / -48.5,-27.1	Favored (88.9%) *Cg_exo* chi angles: 330.8,36.5,331.8	0.05Å	Favored (52.869%)	-	-	-
A 84		LYS	0.66	-	Favored (65.71%) General / -64.9,-19.1	Favored (59.7%) *mttm* chi angles: 291.3,176.5,184.4,284.9	0.03Å	Favored (58.005%)	-	-	-
A 85		ARG	0.69	-	Favored (64.01%) General / -59.4,-25.0	Favored (76.4%) *ttt180* chi angles: 186.4,168.1,175,175.4	0.04Å	Favored (60.547%) three-ten	-	-	-
A 86		ALA	0.73	-	Favored (56.2%) General / -80.2,-5.5	-	0.04Å	Favored (48.432%)	-	-	-
A 87		GLU	0.76	-	Favored (34.91%) General / -99.9,139.9	Favored (93.8%) *mt-10* chi angles: 295.2,183.3,1.3	0.05Å	Favored (31.185%)	-	-	-

A 88		ASP	0.78	-	Favored (60.47%) General / -60.6,-20.4	Favored (98.5%) *m-30* chi angles: 287.8,348.3	0.03Å	Favored (15.765%)	-	-	-
A 89		THR	0.77	-	Favored (51.69%) General / -97.1,3.6	Favored (67.7%) *p* chi angles: 58.8	0.08Å	Favored (52.992%)	-	-	-
A 90		TYR	0.74	-	Favored (37.39%) General / -103.6,140.5	Favored (62.2%) *m-80* chi angles: 288.7,79.2	0.03Å	Favored (35.468%)	-	-	-
A 91		VAL	0.71	-	Favored (58.55%) Ile or Val / -104.0,126.3	Favored (50.5%) *t* chi angles: 181.4	0.11Å	Favored (70.614%)	-	-	-
A 92		CYS	0.69	-	Favored (52.23%) General / -128.5,138.4	Favored (63.2%) *m* chi angles: 301.5	0.01Å	Favored (64.985%) beta sheet	-	-	-
A 93		LYS	0.67	-	Favored (44.18%) General / -127.6,155.3	Favored (71.7%) *mmtt* chi angles: 298.6,292.6,181.1,183	0.04Å	Favored (53.722%) beta sheet	-	-	-
A 94		SER	0.68	-	Favored (35.62%) General / -143.3,162.5	Favored (98.8%) *p* chi angles: 65.3	0.04Å	Favored (29.222%) beta sheet	-	-	-
A 95		GLY	0.69	-	Favored (43.22%) Glycine / -177.1,-174.1	-	-	Favored (50.967%) beta sheet	-	-	-
A 96		VAL	0.72	-	Favored (70.15%) Ile or Val / -121.0,132.6	Favored (98.3%) *t* chi angles: 175.3	0.07Å	Favored (10.738%)	-	-	-
A 97		THR	0.75	-	Favored (28.73%) General / -128.7,161.9	Favored (79.7%) *p* chi angles: 60.4	0.04Å	Favored (32.185%)	-	-	-
A 98		ASP	0.79	0.46Å OD1 with A 110 LYS NZ	Favored (58.31%) General / -61.6,139.1	Favored (89.8%) *m-30* chi angles: 291.2,350	0.05Å	Favored (21.343%)	-	-	-
A 99		ARG	0.82	-	Favored (3.75%) General / -110.8,-43.7	Favored (20.1%) *mmm160* chi angles: 296.8,292.1,291.7,147	0.03Å	CaBLAM Outlier (0.017%)	-	OUTLIER(S) worst is NE-CZ-NH1: 4.0 σ	-
A 100		GLY	0.85	-	Favored (46.05%) Glycine / 57.5,-127.9	-	-	Favored (10.585%)	-	-	-
**#**	**Alt**	**Res**	**High B**	**Clash > 0.4Å**	**Ramachandran**	**Rotamer**	**Cβ deviation**	**CaBLAM**	**Bond lengths**	**Bond angles**	**Cis Peptides**
			Avg: 1.06	Clashscore: 1.34	Outliers: 0 of 498	Poor rotamers: 0 of 404	Outliers: 0 of 446	Outliers: 12 of 496	Outliers: 7 of 500	Outliers: 10 of 500	Non-Trans: 1 of 499
A 101		TRP	0.86	-	Favored (33.18%) General / -141.8,142.9	Favored (88.7%) *t60* chi angles: 182,91.2	0.04Å	CA Geom Outlier (0.456%)	-	-	-
A 102		GLY	0.86	-	Favored (65.52%) Glycine / 96.1,-4.8	-	-	Favored (72.394%)	-	-	-
A 103		ASN	0.84	-	Favored (9.73%) General / -96.7,21.2	Favored (76.5%) *m-40* chi angles: 288,316.6	0.01Å	CA Geom Outlier (0.067%)	-	-	-

A 104		GLY	0.83	-	Favored (52.84%) Glycine / 103.8,-11.0	-	-	Favored (47.575%)	-	-	-
A 105		CYS	0.81	-	Favored (25.61%) General / -103.2,148.6	Favored (68.2%) *m* chi angles: 299.2	0.06Å	Favored (28.885%)	-	-	-
A 106		GLY	0.79	-	Favored (39.44%) Glycine / -106.3,6.7	-	-	Favored (22.677%)	-	-	-
A 107		LEU	0.77	-	Favored (40.46%) General / -125.0,123.7	Favored (54.8%) *tp* chi angles: 174.8,64.7	0.07Å	Favored (18.206%)	-	-	-
A 108		PHE	0.75	-	Favored (36.56%) General / -78.4,143.7	Favored (76.1%) *m-80* chi angles: 288,86.4	0.05Å	CaBLAM Disfavored (1.622%)	-	-	-
A 109		GLY	0.72	-	Favored (28.9%) Glycine / 147.9,-165.3	-	-	Favored (42.274%)	-	-	-
A 110		LYS	0.69	0.46Å NZ with A 98 ASP OD1	Favored (17.27%) General / -107.7,158.4	Favored (62.4%) *mttm* chi angles: 297.6,183.5,184.8,296.1	0.04Å	Favored (6.898%)	-	-	-
A 111		GLY	0.66	-	Favored (37.01%) Glycine / -161.6,169.2	-	-	Favored (26.494%)	-	-	-
A 112		SER	0.63	-	Favored (39.13%) General / -78.0,139.8	Favored (27.2%) *t* chi angles: 173.5	0.07Å	Favored (19.127%)	-	-	-
A 113		ILE	0.6	-	Favored (67.57%) Ile or Val / -126.2,134.0	Favored (78.7%) *mt* chi angles: 298.6,174.7	0.05Å	Favored (70.489%) beta sheet	-	-	-
A 114		ASP	0.59	-	Favored (14.96%) General / -124.2,110.7	Favored (44.4%) *t0* chi angles: 186.1,10.9	0.05Å	Favored (64.132%) beta sheet	-	-	-
A 115		THR	0.59	-	Favored (56.14%) General / -114.9,128.2	Favored (97%) *m* chi angles: 299.9	0.04Å	Favored (64.752%) beta sheet	-	-	-
A 116		CYS	0.6	-	Favored (30.2%) General / -115.8,154.1	Favored (82.3%) *m* chi angles: 294.9	0.03Å	Favored (49.183%) beta sheet	-	-	-
A 117		ALA	0.63	-	Favored (24.82%) General / -145.1,136.2	-	0.05Å	Favored (38.048%) beta sheet	-	-	-
A 118		ASN	0.66	-	Favored (19.05%) General / -78.8,116.3	Favored (44.7%) *t0* chi angles: 186.2,316.4	0.04Å	Favored (48.785%) beta sheet	-	-	-
A 119		PHE	0.69	-	Favored (26.52%) General / -92.8,114.1	Favored (77%) *t80* chi angles: 179.2,87.1	0.15Å	Favored (61.014%) beta sheet	-	OUTLIER(S) worst is CA-CB-CG: 4.9 σ	-
A 120		THR	0.72	-	Favored (49.56%) General / -116.1,124.1	Favored (90%) *m* chi angles: 298.8	0.01Å	Favored (58.54%) beta sheet	-	-	-

#	Alt	Res	High B	Clash > 0.4Å	Ramachandran	Rotamer	Cβ deviation	CaBLAM	Bond lengths	Bond angles	Cis Peptides
			Avg: 1.06	Clashscore: 1.34	Outliers: 0 of 498	Poor rotamers: 0 of 404	Outliers: 0 of 446	Outliers: 12 of 496	Outliers: 7 of 500	Outliers: 10 of 500	Non-Trans: 1 of 499
A 121		CYS	0.74	-	Favored (32.46%) General / -91.5,120.6	Favored (42.6%) *t* chi angles: 186.2	0.04Å	Favored (53.939%)	-	-	-
A 122		SER	0.74	-	Favored (6.98%) General / -86.6,-50.2	Favored (40.8%) *t* chi angles: 178.1	0.02Å	Favored (17.639%)	-	-	-
A 123		LEU	0.74	-	Favored (50.89%) General / -113.1,123.7	Favored (51.8%) *tp* chi angles: 175.2,65.5	0.12Å	Favored (14.837%)	-	-	-
A 124		LYS	0.73	-	Favored (37.19%) General / -130.9,159.5	Favored (60.5%) *pttt* chi angles: 63.3,182.7,183,181.2	0.03Å	Favored (38.241%)	-	-	-
A 125		ALA	0.72	-	Favored (46.06%) General / -125.3,151.4	-	0.08Å	Favored (59.362%) beta sheet	-	-	-
A 126		MET	0.74	-	Favored (54.14%) General / -125.2,138.9	Favored (72%) *mtp* chi angles: 295.6,189.4,68.6	0.04Å	Favored (62.252%) beta sheet	-	-	-
A 127		GLY	0.76	-	Favored (10.8%) Glycine / -98.1,132.0	-	-	Favored (71.487%) beta sheet	-	-	-
A 128		ARG	0.8	-	Favored (52.66%) General / -125.5,130.9	Favored (74.6%) *mtt90* chi angles: 294.6,188.1,169.8,89.8	0.04Å	Favored (57.185%) beta sheet	-	-	-
A 129		MET	0.84	-	Favored (38.07%) General / -78.5,137.6	Favored (33.2%) *ttt* chi angles: 181.6,178.2,179.9	0.04Å	Favored (41.946%) beta sheet	-	-	-
A 130		ILE	0.87	-	Favored (57.82%) Ile or Val / -103.8,123.9	Favored (86.1%) *mt* chi angles: 298.9,171.3	0.05Å	Favored (69.838%) beta sheet	-	-	-
A 131		GLN	0.88	-	Favored (35.19%) Pre-Pro / -96.4,148.9	Favored (84.6%) *mt0* chi angles: 294.2,178.3,296	0.03Å	Favored (41.549%)	-	-	-
A 132		PRO	0.87	-	Favored (31.73%) Trans-Pro / -49.1,-34.9	Favored (61.3%) *Cg_exo* chi angles: 328.5,37,333.5	0.12Å	Favored (87.674%)	-	-	-
A 133		GLU	0.84	-	Favored (66.38%) General / -66.0,-21.0	Favored (98.7%) *mt-10* chi angles: 293.8,178.9,357	0.02Å	Favored (67.638%) three-ten	-	-	-
A 134		ASN	0.81	-	Favored (48.66%) General / -95.6,6.5	Favored (91.4%) *m-40* chi angles: 292.5,323.7	0.04Å	Favored (46.478%)	-	-	-
A 135		VAL	0.78	-	Favored (60.71%) Ile or Val / -109.5,131.3	Favored (66.3%) *t* chi angles: 179.2	0.03Å	Favored (28.221%)	-	-	-
A 136		LYS	0.76	-	Favored (50.44%) General / -128.1,149.3	Favored (60.2%) *pttt* chi angles: 63.6,180.9,180.6,179	0.04Å	Favored (58.296%)	-	-	-

#	Alt	Res	High B	Clash > 0.4Å	Ramachandran	Rotamer	Cβ deviation	CaBLAM	Bond lengths	Bond angles	Cis Peptides
A 137		TYR	0.74	-	Favored (49.55%) General / -122.6,127.5	Favored (85.5%) *m-80* chi angles: 291.9,82.9	0.06Å	Favored (67.875%) beta sheet	-	-	-
A 138		GLU	0.74	-	Favored (46.17%) General / -104.3,122.8	Favored (91.6%) *tt0* chi angles: 181.2,177.6,354.7	0.05Å	Favored (66.225%) beta sheet	-	-	-
A 139		VAL	0.74	-	Favored (68.14%) Ile or Val / -126.9,133.6	Favored (70.2%) *t* chi angles: 178.7	0.07Å	Favored (61.169%) beta sheet	-	-	-
A 140		GLY	0.76	-	Favored (18.11%) Glycine / -98.3,146.3	-	-	Favored (66.418%) beta sheet	-	-	-
**#**	**Alt**	**Res**	**High B**	**Clash > 0.4Å**	**Ramachandran**	**Rotamer**	**Cβ deviation**	**CaBLAM**	**Bond lengths**	**Bond angles**	**Cis Peptides**
			Avg: 1.06	Clashscore: 1.34	Outliers: 0 of 498	Poor rotamers: 0 of 404	Outliers: 0 of 446	Outliers: 12 of 496	Outliers: 7 of 500	Outliers: 10 of 500	Non-Trans: 1 of 499
A 141		ILE	0.79	-	Favored (68.7%) Ile or Val / -123.8,124.2	Favored (67.3%) *mt* chi angles: 302.7,174.5	0.09Å	Favored (63.911%) beta sheet	-	-	-
A 142		PHE	0.83	-	Favored (49.62%) General / -126.2,147.7	Favored (73.1%) *m-80* chi angles: 301.2,85.2	0.06Å	Favored (58.097%) beta sheet	-	-	-
A 143		ILE	0.88	-	Favored (62.95%) Ile or Val / -107.9,128.7	Favored (75.5%) *mt* chi angles: 300.2,174.3	0.06Å	Favored (48.022%) beta sheet	OUTLIER(S) worst is CB--CG1: 5.2 σ	-	-
A 144		HIS	0.95	0.43Å C with A 144 HIS CD2	Favored (5.03%) General / -78.5,77.0	Favored (4.6%) *t70* chi angles: 193.4,124.5	0.04Å	Favored (11.014%) beta sheet	-	-	-
A 145		GLY	1.02	-	Favored (10.69%) Glycine / -111.5,-147.2	-	-	Favored (8.545%)	-	-	-
A 146		SER	1.1	-	Favored (2.11%) General / -74.8,79.0	Favored (43.1%) *t* chi angles: 178.5	0.05Å	CaBLAM Outlier (0.875%)	-	-	-
A 147		THR	1.17	-	Favored (45.93%) General / -127.8,154.0	Favored (69.4%) *p* chi angles: 59.2	0.09Å	Favored (29.187%)	-	-	-
A 148		SER	1.23	-	Favored (23.12%) General / -93.0,147.1	Favored (23.5%) *t* chi angles: 171.1	0.06Å	Favored (42.199%)	-	-	-
A 149		SER	1.29	-	Favored (64.43%) General / -60.5,-23.2	Favored (96.2%) *p* chi angles: 65	0.08Å	Favored (51.721%)	-	-	-
A 150		ASP	1.34	-	Favored (55.75%) General / -78.3,-6.7	Favored (85.5%) *m-30* chi angles: 289.6,335.2	0.03Å	Favored (48.16%) alpha helix	-	-	-
A 151		THR	1.37	-	Favored (15.22%) General / -112.9,0.7	Favored (72.2%) *p* chi angles: 61.9	0.03Å	Favored (48.207%) alpha helix	-	-	-
A 152		HIS	1.39	-	Favored (68.16%) General / -64.4,-25.8	Favored (29.2%) *p-80* chi angles: 79.7,281.7	0.10Å	Favored (37.801%) alpha helix	-	-	-

#	Alt	Res	High B	Clash > 0.4Å	Ramachandran	Rotamer	Cβ deviation	CaBLAM	Bond lengths	Bond angles	Cis Peptides
A 153		GLY	1.4	-	Favored (66.39%) Glycine / -94.7,1.8	-	-	Favored (53.809%)	-	-	-
A 154		ASN	1.39	-	Favored (3.94%) General / -109.9,92.2	Favored (45.9%) *t0* chi angles: 182.7,327.4	0.06Å	Favored (14.448%)	-	-	-
A 155		TYR	1.37	0.54Å CD1 with A 155 TYR N	Favored (88.72%) General / -61.9,-38.5	Favored (4.8%) *m-10* chi angles: 293.4,7.5	0.04Å	Favored (45.975%)	-	-	-
A 156		SER	1.34	-	Favored (90.72%) General / -60.2,-40.6	Favored (63.1%) *m* chi angles: 294	0.01Å	Favored (75.229%) alpha helix	-	-	-
A 157		SER	1.32	-	Favored (94.56%) General / -65.1,-40.5	Favored (58.5%) *m* chi angles: 293.3	0.02Å	Favored (96.323%) alpha helix	-	-	-
A 158		GLN	1.3	-	Favored (89.46%) General / -64.7,-38.0	Favored (74.7%) *mt0* chi angles: 292.4,175.2,58.5	0.05Å	Favored (89.241%) alpha helix	-	-	-
A 159		LEU	1.27	-	Favored (79.08%) General / -68.5,-42.0	Favored (89.1%) *mt* chi angles: 293.2,169.1	0.10Å	Favored (78.747%) alpha helix	-	-	-
A 160		GLY	1.23	-	Favored (79.71%) Glycine / -60.9,-32.4	-	-	Favored (90.077%) alpha helix	-	-	-
**#**	**Alt**	**Res**	**High B**	**Clash > 0.4Å**	**Ramachandran**	**Rotamer**	**Cβ deviation**	**CaBLAM**	**Bond lengths**	**Bond angles**	**Cis Peptides**
			Avg: 1.06	Clashscore: 1.34	Outliers: 0 of 498	Poor rotamers: 0 of 404	Outliers: 0 of 446	Outliers: 12 of 496	Outliers: 7 of 500	Outliers: 10 of 500	Non-Trans: 1 of 499
A 161		ALA	1.15	-	Favored (51.21%) General / -78.3,-5.2	-	0.06Å	Favored (35.274%)	-	-	-
A 162		SER	1.06	-	Favored (30.98%) General / 55.6,40.2	Favored (60.4%) *m* chi angles: 298.9	0.03Å	Favored (17.309%)	-	-	-
A 163		GLN	0.97	-	Favored (10.8%) General / -121.8,21.0	Favored (65.4%) *mt0* chi angles: 297.8,185.9,68.2	0.04Å	Favored (8.487%)	-	-	-
A 164		ALA	0.88	-	Favored (25.55%) General / -161.0,167.9	-	0.04Å	Favored (28.594%)	-	-	-
A 165		GLY	0.81	-	Favored (41.12%) Glycine / -165.5,172.7	-	-	Favored (55.229%)	-	-	-
A 166		ARG	0.77	-	Favored (39.39%) General / -134.6,160.0	Favored (81.8%) *mtm180* chi angles: 297.1,184.7,291.1,173.1	0.04Å	Favored (61.153%) beta sheet	-	-	-
A 167		PHE	0.75	-	Favored (44.15%) General / -144.5,156.4	Favored (59.2%) *p90* chi angles: 63.1,91.2	0.03Å	Favored (68.61%) beta sheet	-	OUTLIER(S) worst is CA-CB-CG: 6.2 σ	-
A 168		THR	0.76	-	Favored (42.66%) General / -125.9,125.4	Favored (86.9%) *m* chi angles: 301.4	0.04Å	Favored (62.181%) beta sheet	-	-	-
A 169		ILE	0.77	-	Favored (62.13%)	Favored (72.8%) *mt* chi angles: 289.9,172.9	0.18Å	Favored (47.429%)	-	-	-

					Ile or Val / -119.3,134.7			beta sheet			
A 170		THR	0.79	-	Favored (25.77%) Pre-Pro / -134.3,166.9	Favored (17.5%) *p* chi angles: 73.7	0.11Å	Favored (33.348%)	-	-	-
A 171		PRO	0.79	-	Favored (34.47%) Trans-Pro / -56.6,-20.6	Favored (84.5%) *Cg_exo* chi angles: 334.1,36,329.6	0.03Å	Favored (50.61%)	-	-	-
A 172		ASN	0.79	-	Favored (44.47%) General / -97.9,-0.7	Favored (88.9%) *m-40* chi angles: 291.1,323.5	0.02Å	Favored (53.118%) alpha helix	-	-	-
A 173		SER	0.79	-	Favored (9.48%) Pre-Pro / -148.9,68.0	Favored (51.7%) *p* chi angles: 56.6	0.03Å	Favored (19.508%) alpha helix	-	-	-
A 174		PRO	0.79	-	Favored (40.6%) Trans-Pro / -68.8,-15.5	Favored (57.4%) *Cg_endo* chi angles: 26.1,325,29	0.05Å	Favored (48.936%) alpha helix	-	-	-
A 175		ALA	0.79	-	Favored (31.42%) General / -141.7,137.0	-	0.04Å	Favored (26.885%)	-	-	-
A 176		ILE	0.81	-	Favored (55.42%) Ile or Val / -130.2,137.8	Favored (28.6%) *mm* chi angles: 302.7,292.9	0.04Å	Favored (58.656%)	-	-	-
A 177		THR	0.84	-	Favored (47.61%) General / -101.5,129.4	Favored (91.1%) *m* chi angles: 298	0.06Å	Favored (66.346%) beta sheet	-	-	-
A 178		VAL	0.9	-	Favored (69.36%) Ile or Val / -113.6,123.5	Favored (62.1%) *t* chi angles: 179.7	0.08Å	Favored (62.088%) beta sheet	-	-	-
A 179		LYS	0.96	-	Favored (35.36%) General / -81.2,132.7	Favored (62.5%) *mttm* chi angles: 294.9,182.4,180.1,292.7	0.01Å	Favored (51.403%) beta sheet	-	-	-
A 180		MET	1.02	-	Favored (5.76%) General / -110.1,31.6	Favored (85.6%) *mmm* chi angles: 305.3,300.3,290.1	0.04Å	CaBLAM Outlier (0.576%)	-	-	-
**#**	**Alt**	**Res**	**High B**	**Clash > 0.4Å**	**Ramachandran**	**Rotamer**	**Cβ deviation**	**CaBLAM**	**Bond lengths**	**Bond angles**	**Cis Peptides**
			Avg: 1.06	Clashscore: 1.34	Outliers: 0 of 498	Poor rotamers: 0 of 404	Outliers: 0 of 446	Outliers: 12 of 496	Outliers: 7 of 500	Outliers: 10 of 500	Non-Trans: 1 of 499
A 181		GLY	1.05	-	Favored (38.58%) Glycine / 61.9,-126.3	-	-	Favored (38.357%)	-	-	-
A 182		ASP	1.04	-	Favored (62.54%) General / -63.1,-17.6	Favored (91.9%) *m-30* chi angles: 291,349.1	0.04Å	Favored (14.7%)	-	-	-
A 183		TYR	1.01	-	Favored (55.84%) General / -88.6,1.0	Favored (85.4%) *m-80* chi angles: 290.3,96.5	0.06Å	Favored (21.514%)	-	-	-
A 184		GLY	0.95	-	Favored (18.89%) Glycine / 95.4,-148.2	-	-	Favored (14.953%)	-	-	-
A 185		GLU	0.89	-	Favored (51.96%)	Favored (84.4%) *mt-10*	0.06Å	Favored (10.485%)	-	-	-

					General / -130.3,149.4	chi angles: 301.8,181.3,352.7					
A 186		ILE	0.84	-	Favored (75.91%) Ile or Val / -120.2,129.1	Favored (88.7%) *mt* chi angles: 298.4,172.2	0.02Å	Favored (69.27%) beta sheet	-	-	-
A 187		SER	0.81	-	Favored (43.16%) General / -106.6,138.2	Favored (63.7%) *m* chi angles: 294.1	0.05Å	Favored (68.904%) beta sheet	-	-	-
A 188		VAL	0.81	-	Favored (71.78%) Ile or Val / -125.7,127.8	Favored (53.9%) *t* chi angles: 180.8	0.02Å	Favored (70.745%) beta sheet	-	-	-
A 189		GLU	0.83	-	Favored (22.42%) General / -114.5,112.1	Favored (93.5%) *mt-10* chi angles: 299.8,178.1,0.6	0.07Å	Favored (62.909%) beta sheet	-	-	-
A 190		CYS	0.89	-	Favored (41.72%) General / -123.9,153.0	Favored (34.9%) *m* chi angles: 305.9	0.07Å	Favored (51.867%) beta sheet	-	-	-
A 191		GLU	0.98	-	Favored (52.18%) Pre-Pro / -114.2,100.1	Favored (12%) *tp30* chi angles: 175.9,59.6,72.2	0.04Å	Favored (46.384%) beta sheet	-	-	-
A 192		PRO	1.09	-	Favored (56.61%) Trans-Pro / -66.1,-20.3	Favored (47.8%) *Cg_endo* chi angles: 24.9,326.4,27.6	0.01Å	Favored (73.991%)	-	-	-
A 193		ARG	1.22	-	Favored (37.6%) General / -83.2,-18.9	Favored (91.1%) *mtm180* chi angles: 294,172.2,292.7,177	0.01Å	Favored (57.53%) three-ten	-	-	-
A 194		ASN	1.34	-	Favored (65.45%) General / -68.5,-26.4	Favored (88.8%) *m-40* chi angles: 285.2,335.2	0.06Å	Favored (64.796%) three-ten	-	-	-
A 195		GLY	1.42	-	Favored (86.51%) Glycine / -84.5,-5.7	-	-	Favored (22.125%) alpha helix	-	-	-
A 196		LEU	1.45	-	Allowed (1.93%) General / -165.7,127.0	Favored (38.3%) *tp* chi angles: 181.4,68.1	0.08Å	CaBLAM Disfavored (4.827%)	-	-	-
A 197		ASN	1.41	-	Favored (5.31%) General / -78.8,71.6	Favored (41.7%) *t0* chi angles: 188.9,13.2	0.02Å	Favored (45.559%)	-	-	-
A 198		THR	1.32	-	Favored (31.23%) General / -48.4,-42.3	Favored (94.1%) *m* chi angles: 300.9	0.11Å	Favored (33.234%)	-	-	-
A 199		GLU	1.21	-	Favored (66.34%) General / -63.3,-21.6	Favored (95.4%) *mt-10* chi angles: 290.5,182.7,349.3	0.04Å	Favored (68.945%) three-ten	-	-	-
A 200		ALA	1.08	-	Favored (56.86%) General / -85.1,-1.4	-	0.04Å	Favored (59.244%)	-	-	-
**#**	**Alt**	**Res**	**High B**	**Clash > 0.4Å**	**Ramachandran**	**Rotamer**	**Cβ deviation**	**CaBLAM**	**Bond lengths**	**Bond angles**	**Cis Peptides**
			Avg: 1.06	Clashscore: 1.34	Outliers: 0 of 498	Poor rotamers: 0 of 404	Outliers: 0 of 446	Outliers: 12 of 496	Outliers: 7 of 500	Outliers: 10 of 500	Non-Trans: 1 of 499
A 201		TYR	0.98	-	Favored (35.9%)	Favored (84.5%) *m-80* chi angles: 294.6,82.6	0.04Å	Favored (19.574%)	-	-	-

					General / -121.3,154.8						
A 202		TYR	0.89	-	Favored (28.68%) General / -143.7,165.1	Favored (31.5%) *p90* chi angles: 74.8,97.6	0.10Å	Favored (48.675%)	OUTLIER(S) worst is CB--CG: 4.5 σ	-	-
A 203		ILE	0.84	-	Favored (57.76%) Ile or Val / -104.5,128.8	Favored (47.2%) *mm* chi angles: 303.4,298.8	0.04Å	Favored (46.173%) beta sheet	-	-	-
A 204		MET	0.82	-	Favored (45.55%) General / -102.1,134.3	Favored (45.8%) *ttm* chi angles: 178.1,175.7,275.6	0.10Å	Favored (68.199%) beta sheet	-	-	-
A 205		SER	0.82	-	Favored (37.24%) General / -116.8,120.0	Favored (67.5%) *m* chi angles: 296.9	0.10Å	Favored (47.312%)	-	-	-
A 206		VAL	0.83	0.45Å O with A 206 VAL HG13	Favored (28.49%) Ile or Val / -142.6,137.6	Favored (7%) *p* chi angles: 60.2	0.07Å	CaBLAM Disfavored (2.493%)	-	-	-
A 207		GLY	0.85	-	Favored (34.78%) Glycine / 59.2,-123.8	-	-	Favored (35.228%)	-	-	-
A 208		THR	0.86	-	Favored (13.65%) General / -118.7,16.4	Favored (66.1%) *p* chi angles: 58.4	0.02Å	Favored (5.23%)	-	-	-
A 209		LYS	0.88	-	Favored (38.08%) General / -117.7,151.0	Favored (99.1%) *mttt* chi angles: 294.8,183.1,179.5,180.3	0.01Å	Favored (35.042%)	-	-	-
A 210		HIS	0.89	-	Favored (49.48%) General / -133.9,145.3	Favored (59.6%) *m-70* chi angles: 307.9,271.9	0.03Å	Favored (72.415%)	-	-	-
A 211		PHE	0.91	-	Favored (49.62%) General / -135.2,148.5	Favored (70.2%) *m-80* chi angles: 302.4,85.9	0.12Å	Favored (59.988%) beta sheet	-	-	-
A 212		LEU	0.94	-	Favored (27.66%) General / -95.5,115.6	Favored (59.1%) *tp* chi angles: 178.1,65.1	0.01Å	Favored (49.164%) beta sheet	-	-	-
A 213		VAL	0.99	-	Favored (48.58%) Ile or Val / -131.3,140.4	Favored (42.9%) *t* chi angles: 182.9	0.09Å	Favored (45.968%) beta sheet	-	-	-
A 214		HIS	1.04	-	Favored (57.91%) General / -66.7,140.7	Favored (70.2%) *t-90* chi angles: 194.8,282.9	0.02Å	Favored (47.115%)	-	-	-
A 215		ARG	1.08	-	Favored (70.64%) General / -60.4,-31.7	Favored (82.5%) *mtp180* chi angles: 289.3,176.3,71.6,190.1	0.07Å	Favored (54.632%)	-	-	-
A 216		GLU	1.11	-	Favored (83.51%) General / -64.0,-36.3	Favored (96.5%) *mt-10* chi angles: 287.9,177.4,355.4	0.08Å	Favored (70.752%) alpha helix	-	-	-
A 217		TRP	1.1	-	Favored (85.26%) General / -57.6,-45.5	Favored (62.6%) *t60* chi angles: 177.2,94.1	0.13Å	Favored (87.161%) alpha helix	-	-	-
A 218		PHE	1.07	-	Favored (74.48%)	Favored (70.3%) *t80* chi angles: 184.2,71.8	0.12Å	Favored (80.346%) alpha helix	-	OUTLIER(S) worst is CA-CB-CG: 7.0 σ	-

#	Alt	Res	High B	Clash > 0.4Å	Ramachandran	Rotamer	Cβ deviation	CaBLAM	Bond lengths	Bond angles	Cis Peptides
					General / -66.5,-46.9						
A 219		ASN	1.02	-	Favored (69.27%) General / -60.3,-30.1	Favored (99.3%) *m-40* chi angles: 288.4,341	0.05Å	Favored (73.701%) alpha helix	-	-	-
A 220		ASP	0.95	-	Favored (50.94%) General / -89.6,3.9	Favored (85.3%) *m-30* chi angles: 288.8,335.6	0.03Å	Favored (47.353%)	-	-	-
**#**	**Alt**	**Res**	**High B**	**Clash > 0.4Å**	**Ramachandran**	**Rotamer**	**Cβ deviation**	**CaBLAM**	**Bond lengths**	**Bond angles**	**Cis Peptides**
			Avg: 1.06	Clashscore: 1.34	Outliers: 0 of 498	Poor rotamers: 0 of 404	Outliers: 0 of 446	Outliers: 12 of 496	Outliers: 7 of 500	Outliers: 10 of 500	Non-Trans: 1 of 499
A 221		LEU	0.88	-	Favored (25.29%) General / -77.6,122.5	Favored (50.3%) *tp* chi angles: 182.7,64.3	0.05Å	Favored (27.363%)	-	-	-
A 222		ALA	0.82	-	Favored (59.75%) General / -80.4,-9.4	-	0.05Å	Favored (15.325%) beta sheet	-	-	-
A 223		LEU	0.78	-	Favored (75.84%) Pre-Pro / -62.2,153.3	Favored (95.8%) *mt* chi angles: 293.2,173.9	0.09Å	Favored (14.598%) beta sheet	-	-	-
A 224		PRO	0.76	-	Favored (82.07%) Trans-Pro / -56.9,145.8	Favored (62.9%) *Cg_exo* chi angles: 335.8,34.8,329.2	0.06Å	Favored (36.555%) beta sheet	-	-	-
A 225		TRP	0.77	-	Favored (43%) General / -148.1,156.8	Favored (77%) *p-90* chi angles: 59.7,266.7	0.03Å	Favored (70.644%) beta sheet	-	-	-
A 226		THR	0.82	-	Favored (43.59%) General / -133.5,134.6	Favored (95.5%) *m* chi angles: 299.5	0.06Å	Favored (55.915%) beta sheet	-	-	-
A 227		SER	0.9	-	Favored (86.92%) Pre-Pro / -74.4,150.0	Favored (69.5%) *m* chi angles: 296.5	0.05Å	Favored (44.052%)	-	-	-
A 228		PRO	0.99	-	Favored (38.01%) Trans-Pro / -55.4,-23.3	Favored (85.3%) *Cg_exo* chi angles: 333.9,35.2,330.8	0.05Å	Favored (74.769%)	-	-	-
A 229		ALA	1.06	-	Favored (50.46%) General / -84.6,0.4	-	0.04Å	Favored (55.497%) alpha helix	-	-	-
A 230		SER	1.08	-	Favored (48.14%) General / -138.5,152.3	Favored (45.7%) *t* chi angles: 179.5	0.06Å	Favored (26.165%)	-	-	-
A 231		SER	1.05	-	Favored (53.3%) General / -91.8,-5.5	Favored (95.9%) *p* chi angles: 63.8	0.05Å	Favored (16.75%)	-	-	-
A 232		ASN	0.97	-	Favored (44.06%) General / -96.8,130.4	Favored (33.9%) *m-40* chi angles: 300.9,348.6	0.07Å	Favored (34.154%) beta sheet	-	-	-
A 233		TRP	0.88	-	Favored (34.26%) General / -85.4,132.3	Favored (99.1%) *m100* chi angles: 290.8,98.3	0.04Å	Favored (48.111%)	-	-	-
A 234		ARG	0.8	-	Favored (19.08%) General / -95.6,151.7	Favored (13.6%) *mpt180* chi angles: 275.6,70.4,171.4,171.4	0.07Å	Favored (12.018%)	-	-	-
A 235		ASN	0.74	-	Favored (30.61%)	Favored (86.8%) *m-40*	0.03Å	Favored (15.566%)	-	-	-

					General / 55.9,39.6	chi angles: 296.3,329.1						
A 236		ARG	0.7	-	Favored (70.08%) General / -60.7,-30.6	Favored (99.3%) *mtm-85* chi angles: 291.1,191.9,292.8,274.9	0.05Å	Favored (21.298%)	-	-	-
A 237		GLU	0.69	-	Favored (52.62%) General / -58.9,-20.6	Favored (26.1%) *pt0* chi angles: 67,182.5,353.5	0.04Å	Favored (52.091%)	-	-	-
A 238		ILE	0.68	-	Favored (75.66%) Ile or Val / -65.8,-35.6	Favored (40.2%) *mm* chi angles: 296.3,300.2	0.12Å	Favored (42.45%) three-ten	-	-	-
A 239		LEU	0.68	-	Favored (14.73%) General / -96.8,-27.4	Favored (86.3%) *mt* chi angles: 294.9,178.4	0.08Å	Favored (26.679%)	-	-	-
A 240		LEU	0.69	-	Favored (26.55%) General / -100.5,113.6	Favored (19.2%) *mt* chi angles: 305.2,189.8	0.10Å	Favored (29.673%)	-	-	-
**#**	**Alt**	**Res**	**High B**	**Clash > 0.4Å**	**Ramachandran**	**Rotamer**	**Cβ deviation**	**CaBLAM**	**Bond lengths**	**Bond angles**	**Cis Peptides**
			Avg: 1.06	Clashscore: 1.34	Outliers: 0 of 498	Poor rotamers: 0 of 404	Outliers: 0 of 446	Outliers: 12 of 496	Outliers: 7 of 500	Outliers: 10 of 500	Non-Trans: 1 of 499
A 241		GLU	0.71	-	Favored (36.86%) General / -90.7,129.1	Favored (90.5%) *tt0* chi angles: 182.3,179.1,6.6	0.03Å	Favored (52.567%)	-	-	-
A 242		PHE	0.75	-	Favored (41.03%) General / -98.6,121.9	Favored (25.4%) *m-80* chi angles: 287.7,69.8	0.06Å	Favored (56.597%)	-	-	-
A 243		GLU	0.79	-	Favored (28.11%) General / -77.0,161.8	Favored (92.8%) *mt-10* chi angles: 295.3,183.6,345.9	0.03Å	Favored (24.676%)	-	-	-
A 244		GLU	0.83	-	Favored (53.69%) Pre-Pro / -53.7,128.6	Favored (90.8%) *tt0* chi angles: 184.8,178.6,4.8	0.06Å	Favored (30.522%)	-	-	-
A 245		PRO	0.86	-	Favored (77.15%) Trans-Pro / -68.1,150.2	Favored (52.3%) *Cg_endo* chi angles: 25.5,327.2,26.3	0.02Å	Favored (67.674%)	-	-	-
A 246		HIS	0.88	-	Favored (6.73%) General / -113.0,172.8	Favored (99.2%) *m-70* chi angles: 300.2,289.5	0.08Å	Favored (6.732%)	-	-	-
A 247		ALA	0.89	-	Favored (48.99%) General / -55.0,135.3	-	0.02Å	CaBLAM Disfavored (3.442%)	-	-	-
A 248		THR	0.87	-	Allowed (0.51%) General / 68.0,-54.2	Favored (82.8%) *m* chi angles: 302.1	0.03Å	CaBLAM Disfavored (1.569%)	-	-	-
A 249		LYS	0.84	-	Favored (15.62%) General / -109.5,161.1	Favored (71.2%) *mmtt* chi angles: 302.3,298.7,185.2,187.2	0.07Å	Favored (9.667%)	-	-	-
A 250		GLN	0.8	-	Favored (22.31%) General / -148.7,138.8	Favored (62.4%) *tt0* chi angles: 182.5,175.4,26.2	0.05Å	Favored (56.417%)	-	-	-
A 251		SER	0.77	-	Favored (18.53%)	Favored (72.2%) *m* chi angles: 295.8	0.04Å	Favored (41.123%)	-	-	-

					General / -97.4,152.9			beta sheet			
A 252		VAL	0.76	-	Favored (68.85%) Ile or Val / -127.6,127.6	Favored (46.9%) *t* chi angles: 182.1	0.04Å	Favored (64.986%) beta sheet	-	-	-
A 253		VAL	0.75	-	Favored (67.65%) Ile or Val / -125.5,124.8	Favored (75.3%) *t* chi angles: 178.3	0.07Å	Favored (59.256%) beta sheet	-	-	-
A 254		ALA	0.76	-	Favored (55.43%) General / -68.5,138.7	-	0.03Å	Favored (41.358%) beta sheet	-	-	-
A 255		LEU	0.77	-	Favored (7.04%) General / -85.3,61.7	Favored (93.9%) *mt* chi angles: 298.6,177.6	0.06Å	Favored (6.756%)	-	-	-
A 256		GLY	0.8	-	Favored (44.47%) Glycine / 54.3,-130.3	-	-	Favored (45.572%)	-	-	-
A 257		SER	0.82	-	Favored (39.47%) General / -130.3,127.4	Favored (32.9%) *t* chi angles: 174.1	0.08Å	Favored (8.645%)	-	-	-
A 258		GLN	0.85	-	Favored (35.42%) General / -87.8,6.4	Favored (98.8%) *mm-40* chi angles: 298.2,298.6,309.2	0.08Å	Favored (10.121%)	-	-	-
A 259		GLU	0.89	-	Favored (74.1%) General / -57.4,-38.4	Favored (92.1%) *tt0* chi angles: 183.1,178.4,3.2	0.03Å	Favored (48.912%)	-	-	-
A 260		GLY	0.94	-	Favored (43.96%) Glycine / -58.0,-53.6	-	-	Favored (91.747%) alpha helix	-	-	-
**#**	**Alt**	**Res**	**High B**	**Clash > 0.4Å**	**Ramachandran**	**Rotamer**	**Cβ deviation**	**CaBLAM**	**Bond lengths**	**Bond angles**	**Cis Peptides**
			Avg: 1.06	Clashscore: 1.34	Outliers: 0 of 498	Poor rotamers: 0 of 404	Outliers: 0 of 446	Outliers: 12 of 496	Outliers: 7 of 500	Outliers: 10 of 500	Non-Trans: 1 of 499
A 261		ALA	0.99	-	Favored (76.19%) General / -59.4,-36.6	-	0.03Å	Favored (76.118%) alpha helix	-	-	-
A 262		LEU	1.06	-	Favored (77.4%) General / -59.4,-49.5	Favored (52.6%) *tp* chi angles: 175.3,65.4	0.13Å	Favored (79.577%) alpha helix	-	-	-
A 263		HIS	1.13	-	Favored (82.23%) General / -63.2,-36.0	Favored (73.1%) *m-70* chi angles: 288.2,297.9	0.05Å	Favored (80.462%) alpha helix	-	-	-
A 264		GLN	1.19	-	Favored (95.12%) General / -64.7,-42.9	Favored (98.6%) *mt0* chi angles: 291.8,173.4,342.4	0.07Å	Favored (86.347%) alpha helix	-	-	-
A 265		ALA	1.25	-	Favored (73.48%) General / -61.6,-33.2	-	0.05Å	Favored (79.706%) alpha helix	-	-	-
A 266		LEU	1.28	-	Favored (27.07%) General / -84.1,5.2	Favored (94.8%) *mt* chi angles: 292,171.3	0.04Å	Favored (37.435%) alpha helix	-	-	-
A 267		ALA	1.28	-	Favored (62.5%)	-	0.02Å	Favored (40.943%)	-	-	-

#	Alt	Res	High B	Clash > 0.4Å	Ramachandran	Rotamer	Cβ deviation	CaBLAM	Bond lengths	Bond angles	Cis Peptides
					General / -57.5,-27.3			alpha helix			
A 268		GLY	1.23	-	Favored (86.42%) Glycine / -87.1,1.1	-	-	Favored (66.275%)	-	-	-
A 269		ALA	1.16	-	Favored (19.14%) General / -85.5,161.9	-	0.04Å	Favored (35.659%)	-	-	-
A 270		VAL	1.08	-	Favored (33.48%) Pre-Pro / -98.3,128.4	Favored (82%) *t* chi angles: 176.7	0.06Å	Favored (27.737%) beta sheet	-	-	-
A 271		PRO	1.01	-	Favored (85.67%) Trans-Pro / -65.8,147.9	Favored (40.8%) *Cg_endo* chi angles: 23.6,327.8,27.1	0.01Å	Favored (58.311%) beta sheet	-	-	-
A 272		VAL	0.96	-	Favored (44.03%) Ile or Val / -132.9,141.8	Favored (95.4%) *t* chi angles: 175.7	0.07Å	Favored (63.248%) beta sheet	-	-	-
A 273		SER	0.94	-	Favored (22.66%) General / -101.6,110.6	Favored (41.4%) *t* chi angles: 176.2	0.02Å	Favored (52.648%) beta sheet	-	-	-
A 274		PHE	0.94	-	Favored (17.8%) General / -125.2,113.6	Favored (3.5%) *t80* chi angles: 182.9,18.7	0.09Å	Favored (20.218%)	-	OUTLIER(S) worst is CA-CB-CG: 6.1 σ	-
A 275		SER	0.94	-	Favored (2.69%) General / -151.8,100.6	Favored (42.5%) *t* chi angles: 180.8	0.04Å	CA Geom Outlier (0.369%)	-	-	-
A 276		GLY	0.94	-	Favored (2.59%) Glycine / 76.3,-66.6	-	-	CaBLAM Outlier (0.234%)	-	-	-
A 277		SER	0.94	-	Favored (14.35%) General / -109.7,162.5	Favored (94.5%) *p* chi angles: 64.8	0.03Å	Favored (12.373%)	-	-	-
A 278		VAL	0.96	-	Favored (36.35%) Ile or Val / -106.7,111.7	Favored (73.6%) *t* chi angles: 178.4	0.20Å	Favored (42.038%)	-	-	-
A 279		LYS	0.98	-	Favored (32.12%) General / -98.2,117.2	Favored (59.3%) *mttm* chi angles: 296.6,183.9,188.9,299.2	0.04Å	Favored (67.81%) beta sheet	-	-	-
A 280		LEU	1.02	-	Favored (25.4%) General / -89.1,145.5	Favored (84.8%) *mt* chi angles: 300.8,178	0.06Å	Favored (34.424%)	-	-	-
#	Alt	Res	High B	Clash > 0.4Å	Ramachandran	Rotamer	Cβ deviation	CaBLAM	Bond lengths	Bond angles	Cis Peptides
			Avg: 1.06	Clashscore: 1.34	Outliers: 0 of 498	Poor rotamers: 0 of 404	Outliers: 0 of 446	Outliers: 12 of 496	Outliers: 7 of 500	Outliers: 10 of 500	Non-Trans: 1 of 499
A 281		THR	1.06	-	Favored (42.34%) General / -88.0,-13.2	Favored (65.3%) *p* chi angles: 63.1	0.01Å	Favored (22.402%)	-	-	-
A 282		SER	1.08	-	Allowed (0.49%) General / -113.0,-91.8	Favored (95.9%) *p* chi angles: 63.8	0.05Å	Favored (11.552%)	-	-	-
A 283		GLY	1.08	-	Favored (17.42%)	-	-	CaBLAM Disfavored	-	-	-

					Glycine / 73.8,168.9			(1.804%)			
A 284		HIS	1.04	-	Favored (5.72%) General / -164.6,139.9	Favored (76.6%) *t70* chi angles: 187.1,70.3	0.14Å	Favored (12.185%) beta sheet	OUTLIER(S) worst is CB--CG: 5.9 σ	-	-
A 285		LEU	0.97	-	Favored (26.04%) General / -138.7,129.2	Favored (39.4%) *tp* chi angles: 182.5,67.5	0.12Å	Favored (64.885%) beta sheet	-	-	-
A 286		LYS	0.91	-	Favored (38.44%) General / -96.6,121.5	Favored (79.5%) *tttt* chi angles: 177.4,181.3,172,176.6	0.03Å	Favored (64.656%) beta sheet	-	-	-
A 287		CYS	0.87	-	Favored (53.34%) General / -126.5,137.4	Favored (70%) *m* chi angles: 298.5	0.09Å	Favored (63.471%) beta sheet	-	-	-
A 288		ARG	0.87	-	Favored (40.48%) General / -100.7,120.7	Favored (44.1%) *ttm170* chi angles: 178,164.4,288.8,182.6	0.03Å	Favored (70.209%) beta sheet	-	-	-
A 289		VAL	0.89	-	Favored (63.89%) Ile or Val / -109.3,122.4	Favored (54.4%) *t* chi angles: 180.7	0.08Å	Favored (68.644%) beta sheet	-	-	-
A 290		LYS	0.93	-	Favored (38.12%) General / -99.0,119.6	Favored (98.6%) *mttt* chi angles: 296.1,177.4,180.8,175.4	0.03Å	Favored (63.114%) beta sheet	-	-	-
A 291		MET	0.99	-	Favored (24.8%) General / -102.1,16.9	Favored (16.1%) *mmt* chi angles: 304.4,300.6,192.5	0.07Å	Favored (14.052%)	-	-	-
A 292		GLU	1.05	-	Favored (19.88%) General / -47.4,-51.3	Favored (92.3%) *tt0* chi angles: 183,178.6,0.3	0.03Å	Favored (16.874%)	-	-	-
A 293		LYS	1.09	-	Favored (49.85%) General / -94.3,-3.8	Favored (51.9%) *mtmt* chi angles: 296.5,190,293.7,189.6	0.03Å	Favored (17.692%)	-	-	-
A 294		LEU	1.13	-	Favored (36.39%) General / -62.6,128.5	Favored (31.1%) *tp* chi angles: 186.1,66.8	0.06Å	Favored (30.318%)	-	-	-
A 295		THR	1.15	-	Favored (54.93%) General / -121.7,133.0	Favored (97.9%) *m* chi angles: 300	0.11Å	Favored (54.284%) beta sheet	-	-	-
A 296		LEU	1.15	-	Favored (6.54%) General / -81.9,91.3	Favored (36.6%) *tp* chi angles: 184.2,55.4	0.09Å	Favored (50.023%)	-	-	-
A 297		LYS	1.15	-	Favored (48.26%) General / -55.0,136.0	Favored (87.8%) *tttt* chi angles: 183.2,175.6,178.7,182.2	0.05Å	CaBLAM Disfavored (4.615%)	-	-	-
A 298		GLY	1.14	-	Favored (55.17%) Glycine / 102.8,-15.9	-	-	Favored (59.659%) alpha helix	-	-	-
A 299		THR	1.11	-	Favored (19.23%) General / -84.5,7.6	Favored (61.6%) *p* chi angles: 57.6	0.07Å	CaBLAM Disfavored (4.566%) try alpha helix	-	-	-
A 300		THR	1.06	-	Favored (20.13%)	Favored (80%) *p* chi angles: 60.6	0.05Å	Favored (60.6%)	-	-	-

#	Alt	Res	High B	Clash > 0.4Å	Ramachandran	Rotamer	Cβ deviation	CaBLAM	Bond lengths	Bond angles	Cis Peptides
					General / -111.0,5.3						
			Avg: 1.06	Clashscore: 1.34	Outliers: 0 of 498	Poor rotamers: 0 of 404	Outliers: 0 of 446	Outliers: 12 of 496	Outliers: 7 of 500	Outliers: 10 of 500	Non-Trans: 1 of 499
A 301		TYR	1	-	Favored (17.14%) General / -115.3,162.4	Favored (87.9%) *m-80* chi angles: 292.6,83.1	0.02Å	Favored (22.888%)	-	-	-
A 302		GLY	0.95	-	Favored (45.77%) Glycine / -83.2,-170.2	-	-	Favored (43.601%)	-	-	-
A 303		MET	0.9	-	Favored (24.21%) General / -87.8,149.3	Favored (90.3%) *mmm* chi angles: 303.1,301.3,293.3	0.04Å	Favored (14.908%)	-	-	-
A 304		CYS	0.85	-	Favored (58.33%) General / -65.1,142.5	Favored (80.4%) *m* chi angles: 296	0.05Å	Favored (32.116%)	-	-	-
A 305		THR	0.82	-	Favored (4.95%) General / -126.0,-18.8	Favored (63.2%) *p* chi angles: 63.4	0.04Å	Favored (7.804%) beta sheet	-	-	-
A 306		GLU	0.79	-	Favored (20.04%) General / -84.9,160.9	Favored (70.5%) *mt-10* chi angles: 296.1,178.5,137.7	0.14Å	Favored (11.905%) beta sheet	-	-	-
A 307		LYS	0.77	-	Favored (57.73%) General / -60.1,135.5	Favored (88.3%) *tttt* chi angles: 185.1,175.9,178.9,180.3	0.03Å	Favored (21.575%) beta sheet	-	-	-
A 308		PHE	0.76	-	Favored (16.82%) General / -101.2,157.4	Favored (72.8%) *m-80* chi angles: 287.5,85.3	0.09Å	Favored (45.586%) beta sheet	-	-	-
A 309		SER	0.75	-	Favored (40.45%) General / -138.0,143.3	Favored (40.1%) *t* chi angles: 177.9	0.04Å	Favored (40.538%) beta sheet	-	-	-
A 310		PHE	0.75	-	Favored (49.2%) General / -72.1,140.9	Favored (8.1%) *m-10* chi angles: 286.5,13.5	0.00Å	Favored (42.529%)	-	-	-
A 311		ALA	0.76	-	Favored (11.87%) General / -90.1,-41.1	-	0.03Å	Favored (10.612%)	-	-	-
A 312		LYS	0.78	-	Favored (49.51%) General / -135.4,149.1	Favored (95.6%) *mttt* chi angles: 297.9,183.5,181.2,179.2	0.00Å	Favored (23.503%)	-	-	-
A 313		ASN	0.81	-	Favored (92.82%) Pre-Pro / -72.0,151.0	Favored (94.6%) *m-40* chi angles: 291.8,330.5	0.02Å	Favored (32.318%)	-	-	-
A 314		PRO	0.84	-	Favored (70.46%) Trans-Pro / -54.1,137.9	Favored (92%) *Cg_exo* chi angles: 331.1,37,330.4	0.07Å	Favored (58.735%)	-	-	-
A 315		ALA	0.89	-	Favored (50.58%) General / -132.1,144.2	-	0.04Å	Favored (46.897%) beta sheet	-	-	-
A 316		ASP	0.93	-	Favored (27.61%)	Favored (37.5%) *t70*	0.06Å	Favored (45.004%)	-	-	-

#	Alt	Res	High B	Clash > 0.4Å	Ramachandran	Rotamer	Cβ deviation	CaBLAM	Bond lengths	Bond angles	Cis Peptides
					General / -77.4,124.2	chi angles: 187.2,65.5					
A 317		THR	0.97	-	Favored (10.5%) General / -87.9,171.3	Favored (27.7%) *p* chi angles: 70.2	0.12Å	Favored (39.785%)	-	-	-
A 318		GLY	0.99	-	Favored (39.66%) Glycine / -74.3,3.5	-	-	Favored (9.955%)	-	-	-
A 319		HIS	0.99	-	Favored (5.41%) General / -129.9,21.6	Favored (24.7%) *p-80* chi angles: 51.4,274.8	0.09Å	Favored (42.518%)	-	-	-
A 320		GLY	0.96	-	Favored (68.85%) Glycine / 79.0,21.0	-	-	Favored (70.561%)	-	-	-
**#**	**Alt**	**Res**	**High B**	**Clash > 0.4Å**	**Ramachandran**	**Rotamer**	**Cβ deviation**	**CaBLAM**	**Bond lengths**	**Bond angles**	**Cis Peptides**
			Avg: 1.06	Clashscore: 1.34	Outliers: 0 of 498	Poor rotamers: 0 of 404	Outliers: 0 of 446	Outliers: 12 of 496	Outliers: 7 of 500	Outliers: 10 of 500	Non-Trans: 1 of 499
A 321		THR	0.91	-	Favored (7.01%) General / -114.9,172.5	Favored (75.5%) *p* chi angles: 61.3	0.04Å	Favored (35.168%)	-	-	-
A 322		VAL	0.86	-	Favored (53.61%) Ile or Val / -125.1,138.7	Favored (9.9%) *p* chi angles: 62.2	0.04Å	Favored (50.736%) beta sheet	-	-	-
A 323		VAL	0.82	-	Favored (73.41%) Ile or Val / -115.8,127.5	Favored (82.5%) *t* chi angles: 177.8	0.06Å	Favored (64.649%) beta sheet	-	-	-
A 324		LEU	0.78	-	Favored (49.13%) General / -125.9,129.3	Favored (17.1%) *tp* chi angles: 165.9,63.1	0.08Å	Favored (62.055%) beta sheet	-	-	-
A 325		GLU	0.77	-	Favored (47.68%) General / -105.8,123.3	Favored (36.1%) *tt0* chi angles: 184.2,181.7,79.5	0.03Å	Favored (70.527%) beta sheet	-	-	-
A 326		LEU	0.78	-	Favored (25.69%) General / -107.9,151.5	Favored (80.7%) *mt* chi angles: 300.2,174.5	0.10Å	Favored (45.771%) beta sheet	OUTLIER(S) worst is CB--CG: 5.2 σ	-	-
A 327		GLN	0.8	-	Favored (54.01%) General / -123.5,132.8	Favored (49.4%) *tt0* chi angles: 186.7,170.5,50.9	0.07Å	Favored (58.943%) beta sheet	-	-	-
A 328		TYR	0.86	-	Favored (21.56%) General / -99.6,109.0	Favored (39.1%) *t80* chi angles: 184.7,58.9	0.02Å	Favored (65.394%)	-	-	-
A 329		THR	0.94	-	Favored (35.72%) General / -89.0,128.1	Favored (92.4%) *m* chi angles: 299.1	0.03Å	CaBLAM Disfavored (4.378%)	-	-	-
A 330		GLY	1.05	-	Favored (26.5%) Glycine / 151.2,-156.1	-	-	Favored (14.029%)	-	-	-
A 331		SER	1.14	-	Allowed (0.93%) General / -144.5,-0.9	Favored (82.9%) *p* chi angles: 62.4	0.03Å	CaBLAM Outlier (0.665%)	-	-	-
A 332		ASP	1.2	-	Favored (14.52%)	Favored (23.6%) *t0* chi angles: 198.8,353.9	0.02Å	CaBLAM Disfavored (2.893%)	-	-	-

#	Alt	Res	High B	Clash > 0.4Å	Ramachandran	Rotamer	Cβ deviation	CaBLAM	Bond lengths	Bond angles	Cis Peptides
					General / -80.4,171.9						
A 333		GLY	1.19	-	Favored (25.81%) Glycine / 103.4,170.3	-	-	CA Geom Outlier (0.028%)	-	-	-
A 334		PRO	1.11	-	Favored (24.67%) Cis-Pro / -56.7,146.4	Favored (71%) *Cg_exo* chi angles: 335.3,35,329.5	0.12Å	CA Geom Outlier (0.121%)	-	-	Cis PRO omega= 4.85
A 335		CYS	1	-	Favored (39.1%) General / -157.3,162.6	Favored (24.1%) *p* chi angles: 59.3	0.04Å	Favored (26.567%) beta sheet	-	-	-
A 336		LYS	0.88	-	Favored (33.65%) General / -87.0,133.0	Favored (85.3%) *tttt* chi angles: 179.7,173.4,178.1,179.1	0.06Å	Favored (29.78%) beta sheet	-	-	-
A 337		ILE	0.78	-	Favored (65.86%) Pre-Pro / -93.0,121.3	Favored (63%) *mt* chi angles: 303.8,170	0.06Å	Favored (48.13%) beta sheet	-	-	-
A 338		PRO	0.71	-	Favored (2.21%) Trans-Pro / -75.8,82.1	Favored (72.9%) *Cg_endo* chi angles: 29.8,325.3,25.3	0.02Å	Favored (7.618%) beta sheet	-	-	-
A 339		ILE	0.67	-	Favored (60.21%) Ile or Val / -110.2,131.8	Favored (65.9%) *mt* chi angles: 303.7,173	0.07Å	Favored (11.586%) beta sheet	OUTLIER(S) worst is CB--CG1: 6.3 σ	-	-
A 340		SER	0.65	-	Favored (31.17%) General / -146.0,164.7	Favored (96%) *p* chi angles: 65	0.01Å	Favored (40.71%) beta sheet	-	-	-
**#**	**Alt**	**Res**	**High B**	**Clash > 0.4Å**	**Ramachandran**	**Rotamer**	**Cβ deviation**	**CaBLAM**	**Bond lengths**	**Bond angles**	**Cis Peptides**
			Avg: 1.06	Clashscore: 1.34	Outliers: 0 of 498	Poor rotamers: 0 of 404	Outliers: 0 of 446	Outliers: 12 of 496	Outliers: 7 of 500	Outliers: 10 of 500	Non-Trans: 1 of 499
A 341		ILE	0.65	-	Favored (52.57%) Ile or Val / -118.9,116.9	Favored (3.7%) *mp* chi angles: 305.2,93.8	0.09Å	Favored (38.028%) beta sheet	-	-	-
A 342		VAL	0.66	-	Favored (24.35%) Ile or Val / -120.9,156.2	Favored (27%) *m* chi angles: 299.7	0.06Å	Favored (36.391%)	-	-	-
A 343		ALA	0.69	-	Favored (77.62%) General / -61.2,-35.6	-	0.03Å	Favored (16.403%)	-	-	-
A 344		SER	0.71	-	Favored (18.63%) General / -157.5,145.1	Favored (42.3%) *t* chi angles: 178.3	0.06Å	Favored (15.396%)	-	-	-
A 345		LEU	0.74	-	Favored (67.02%) General / -60.3,-27.1	Favored (18.1%) *tp* chi angles: 191.9,61.6	0.05Å	Favored (57.576%)	-	-	-
A 346		SER	0.75	-	Favored (59.39%) General / -77.6,-10.2	Favored (83.9%) *p* chi angles: 67.5	0.01Å	Favored (42.452%) alpha helix	-	-	-
A 347		ASP	0.76	-	Allowed (1.73%) General / -154.4,94.7	Favored (67.7%) *t0* chi angles: 182,354.1	0.04Å	Favored (8.537%)	-	-	-

A 348		LEU	0.75	-	Favored (3.37%) General / -80.3,59.3	Favored (5.4%) *mp* chi angles: 278,71.9	0.05Å	Favored (23.297%)	-	-	-
A 349		THR	0.74	-	Favored (65.62%) Pre-Pro / -96.2,118.3	Favored (92.9%) *m* chi angles: 297.7	0.02Å	Favored (28.834%) beta sheet	-	-	-
A 350		PRO	0.74	-	Favored (82.93%) Trans-Pro / -55.7,139.1	Favored (98.8%) *Cg_exo* chi angles: 332.2,36.4,330.5	0.04Å	Favored (23.82%) beta sheet	-	-	-
A 351		ILE	0.74	-	Allowed (1.58%) Ile or Val / -122.8,-28.8	Favored (20.1%) *tt* chi angles: 194.2,168.4	0.05Å	Favored (7.892%)	-	-	-
A 352		GLY	0.76	-	Favored (26.36%) Glycine / -102.5,-165.0	-	-	Favored (14.413%)	-	-	-
A 353		ARG	0.78	-	Favored (47.81%) General / -134.1,156.7	Favored (92.1%) *mmt-90* chi angles: 297.9,290.9,183.3,272.3	0.05Å	Favored (30.259%)	-	-	-
A 354		MET	0.81	-	Favored (28.89%) General / -95.6,141.6	Favored (57.6%) *tpp* chi angles: 179.9,67.6,67.6	0.06Å	Favored (53.87%) beta sheet	-	-	-
A 355		VAL	0.83	-	Favored (15.36%) Ile or Val / -87.6,-48.2	Favored (93.2%) *t* chi angles: 174.6	0.01Å	Favored (6.653%)	-	-	-
A 356		THR	0.84	-	Favored (42.07%) General / -74.7,133.2	Favored (2.6%) *t* chi angles: 175.6	0.06Å	Favored (19.275%)	-	-	-
A 357		ALA	0.85	-	Favored (67.28%) General / -59.6,-28.6	-	0.05Å	Favored (12.535%)	-	-	-
A 358		ASN	0.85	-	Favored (2.4%) Pre-Pro / -157.0,92.2	Favored (51.4%) *t0* chi angles: 188.7,22.7	0.09Å	Favored (10.465%)	-	-	-
A 359		PRO	0.85	-	Favored (15.04%) Trans-Pro / -72.6,129.8	Favored (60.4%) *Cg_endo* chi angles: 26.5,330.7,19.6	0.06Å	Favored (7.816%)	-	-	-
A 360		TYR	0.87	-	Favored (42.95%) General / -150.7,161.3	Favored (36.2%) *p90* chi angles: 70,99.8	0.06Å	Favored (42.467%) beta sheet	-	-	-
**#**	**Alt**	**Res**	**High B**	**Clash > 0.4Å**	**Ramachandran**	**Rotamer**	**Cβ deviation**	**CaBLAM**	**Bond lengths**	**Bond angles**	**Cis Peptides**
			Avg: 1.06	Clashscore: 1.34	Outliers: 0 of 498	Poor rotamers: 0 of 404	Outliers: 0 of 446	Outliers: 12 of 496	Outliers: 7 of 500	Outliers: 10 of 500	Non-Trans: 1 of 499
A 361		VAL	0.89	-	Favored (32.83%) Ile or Val / -76.9,-42.9	Favored (90.7%) *t* chi angles: 176	0.10Å	CaBLAM Disfavored (2.159%)	-	-	-
A 362		ALA	0.91	-	Favored (4.63%) General / 70.2,3.9	-	0.10Å	CaBLAM Disfavored (1.186%)	-	-	-
A 363		SER	0.94	-	Favored (24.37%) General / -162.0,157.9	Favored (94.8%) *p* chi angles: 64.8	0.02Å	Favored (8.798%)	-	-	-

A 364		SER	0.95	-	Favored (6.55%) General / -85.0,60.9	Favored (27.5%) *p* chi angles: 54.4	0.03Å	Favored (6.513%)	-	-	-
A 365		GLU	0.95	-	Favored (30.33%) General / -137.0,129.7	Favored (91.7%) *tt0* chi angles: 182,176.1,358.8	0.03Å	Favored (17.161%)	-	-	-
A 366		ALA	0.94	-	Favored (61.92%) General / -59.6,-22.5	-	0.07Å	Favored (12.031%)	-	-	-
A 367		ASN	0.91	-	Favored (20.83%) General / -111.9,12.3	Favored (72.3%) *m-40* chi angles: 292.1,283.4	0.06Å	Favored (26.618%)	-	-	-
A 368		ALA	0.88	-	Favored (57.94%) General / -60.4,138.8	-	0.03Å	Favored (35.602%)	-	-	-
A 369		LYS	0.84	-	Favored (48.51%) General / -116.1,141.2	Favored (98.8%) *mttt* chi angles: 294,182.3,180.1,179.7	0.04Å	Favored (66.265%) beta sheet	-	-	-
A 370		VAL	0.81	-	Favored (62.36%) Ile or Val / -131.0,128.4	Favored (66.8%) *t* chi angles: 179.1	0.06Å	Favored (62.681%) beta sheet	-	-	-
A 371		LEU	0.78	0.44Å C with A 371 LEU HD23	Favored (37.66%) General / -91.6,129.2	Favored (7.3%) *tt* chi angles: 186.9,149.8	0.02Å	Favored (60.85%) beta sheet	-	-	-
A 372		VAL	0.76	-	Favored (75.15%) Ile or Val / -118.7,126.4	Favored (46.9%) *t* chi angles: 182.1	0.13Å	Favored (72.064%) beta sheet	-	-	-
A 373		GLU	0.74	-	Favored (55.91%) General / -111.5,128.2	Favored (88.8%) *tt0* chi angles: 182.6,177.9,8	0.08Å	Favored (61.524%) beta sheet	-	-	-
A 374		MET	0.73	-	Favored (28.87%) General / -127.5,161.4	Favored (32.5%) *mtm* chi angles: 295.4,162.5,281.5	0.07Å	Favored (42.334%) beta sheet	-	-	-
A 375		GLU	0.72	0.53Å OE2 with A 405 LYS NZ	Favored (73.47%) Pre-Pro / -128.9,80.0	Favored (89.6%) *tt0* chi angles: 187.7,176.4,355.5	0.02Å	Favored (10.171%)	-	-	-
A 376		PRO	0.71	-	Favored (16.15%) Trans-Pro / -59.1,161.4	Favored (60.4%) *Cg_exo* chi angles: 335.9,33.8,330.3	0.05Å	Favored (23.728%)	-	-	-
A 377		PRO	0.7	-	Favored (9.94%) Trans-Pro / -74.5,176.3	Favored (62.9%) *Cg_endo* chi angles: 31.5,321.7,29.6	0.07Å	Favored (63.686%)	-	-	-
A 378		PHE	0.68	-	Favored (57.64%) General / -88.7,-0.8	Favored (72.1%) *m-80* chi angles: 287.9,99	0.03Å	Favored (20.183%)	-	-	-
A 379		GLY	0.66	-	Favored (43.23%) Glycine / -90.6,179.5	-	-	Favored (25.1%)	-	-	-
A 380		ASP	0.64	-	Favored (51.18%) General / -67.9,148.4	Favored (87.8%) *m-30* chi angles: 289.9,352.5	0.03Å	Favored (9.681%) beta sheet	-	-	-

#	Alt	Res	High B	Clash > 0.4Å	Ramachandran	Rotamer	Cβ deviation	CaBLAM	Bond lengths	Bond angles	Cis Peptides
			Avg: 1.06	Clashscore: 1.34	Outliers: 0 of 498	Poor rotamers: 0 of 404	Outliers: 0 of 446	Outliers: 12 of 496	Outliers: 7 of 500	Outliers: 10 of 500	Non-Trans: 1 of 499
A 381		SER	0.62	-	Favored (29.11%) General / -154.5,167.7	Favored (98.2%) *p* chi angles: 65.7	0.05Å	Favored (45.186%) beta sheet	-	-	-
A 382		TYR	0.61	-	Favored (49.13%) General / -120.7,142.8	Favored (83.8%) *m-80* chi angles: 294.9,82.5	0.05Å	Favored (48.045%) beta sheet	-	-	-
A 383		ILE	0.62	-	Favored (56.97%) Ile or Val / -103.2,123.8	Favored (85.4%) *mt* chi angles: 298.8,169	0.02Å	Favored (66.584%) beta sheet	-	-	-
A 384		VAL	0.65	-	Favored (42.12%) Ile or Val / -121.2,141.3	Favored (23.7%) *m* chi angles: 295.1	0.04Å	Favored (66.387%)	-	-	-
A 385		VAL	0.7	-	Favored (55.56%) Ile or Val / -129.7,122.8	Favored (73.4%) *t* chi angles: 178.4	0.08Å	Favored (11.165%)	-	-	-
A 386		GLY	0.76	-	Favored (38.4%) Glycine / 94.7,175.4	-	-	Favored (23.242%)	-	-	-
A 387		ARG	0.81	-	Favored (51.56%) General / -132.6,152.8	Favored (97.2%) *mtt180* chi angles: 296.3,178.7,179.2,171.9	0.02Å	CA Geom Outlier (0.2%)	-	-	-
A 388		GLY	0.86	-	Favored (50.91%) Glycine / 69.3,-151.2	-	-	Favored (58.495%)	-	-	-
A 389		ASP	0.87	-	Favored (65.18%) General / -62.6,-20.9	Favored (94.8%) *m-30* chi angles: 290.2,348.3	0.02Å	Favored (11.008%)	-	-	-
A 390		LYS	0.85	-	Favored (58.4%) General / -88.6,-2.1	Favored (98.3%) *mttt* chi angles: 296.2,177.8,184.5,176.4	0.05Å	Favored (46.65%)	-	-	-
A 391		GLN	0.82	-	Favored (52.48%) General / -69.5,137.5	Favored (37.5%) *tt0* chi angles: 185.2,176,77.7	0.02Å	Favored (37.606%)	-	-	-
A 392		ILE	0.77	-	Favored (50.88%) Ile or Val / -102.6,131.4	Favored (45.7%) *mm* chi angles: 305.9,301.1	0.01Å	Favored (65.559%) beta sheet	-	-	-
A 393		ASN	0.73	-	Favored (51.85%) General / -120.1,139.9	Favored (36.3%) *t0* chi angles: 177.5,62.2	0.03Å	Favored (63.313%) beta sheet	-	-	-
A 394		HIS	0.7	-	Favored (17.2%) General / -141.8,125.8	Favored (60.9%) *t70* chi angles: 186.5,83.8	0.05Å	Favored (40.351%) beta sheet	-	-	-
A 395		HIS	0.7	-	Favored (51.86%) General / -69.3,136.5	Favored (65.8%) *t-90* chi angles: 186.1,284	0.04Å	Favored (43%) beta sheet	-	-	-
A 396		TRP	0.71	-	Favored (51.03%)	Favored (72.8%) *t-100* chi angles: 182.7,251.5	0.03Å	Favored (66.954%) beta sheet	-	-	-

#	Alt	Res	High B	Clash > 0.4Å	Ramachandran	Rotamer	Cβ deviation	CaBLAM	Bond lengths	Bond angles	Cis Peptides
					General / -131.6,144.4						
A 397		HIS	0.74	-	Favored (24.63%) General / -140.2,130.0	Favored (70.8%) *t70* chi angles: 179.3,84.4	0.07Å	Favored (59.055%) beta sheet	-	-	-
A 398		LYS	0.78	-	Favored (31.22%) General / -113.4,117.2	Favored (29.8%) *ttpt* chi angles: 179.8,166.9,57,172	0.02Å	Favored (56.557%)	-	-	-
A 399		ALA	0.83	-	Favored (53.5%) General / -64.9,147.3	-	0.03Å	Favored (7.517%)	-	-	-
A 400		GLY	0.89	-	Favored (12.08%) Glycine / 112.3,-164.4	-	-	Favored (8.134%)	-	-	-
**#**	**Alt**	**Res**	**High B**	**Clash > 0.4Å**	**Ramachandran**	**Rotamer**	**Cβ deviation**	**CaBLAM**	**Bond lengths**	**Bond angles**	**Cis Peptides**
			Avg: 1.06	Clashscore: 1.34	Outliers: 0 of 498	Poor rotamers: 0 of 404	Outliers: 0 of 446	Outliers: 12 of 496	Outliers: 7 of 500	Outliers: 10 of 500	Non-Trans: 1 of 499
A 401		SER	0.97	-	Favored (41.5%) General / -152.6,162.2	Favored (99.4%) *p* chi angles: 65.4	0.04Å	Favored (7.16%)	-	-	-
A 402		SER	1.07	-	Favored (87.23%) General / -60.9,-38.8	Favored (66.7%) *m* chi angles: 294.5	0.03Å	Favored (66.783%)	-	-	-
A 403		ILE	1.19	-	Favored (96.07%) Ile or Val / -64.3,-45.4	Favored (97.1%) *mt* chi angles: 293.7,168.3	0.03Å	Favored (92.438%) alpha helix	-	-	-
A 404		GLY	1.35	-	Favored (52.21%) Glycine / -56.9,-52.2	-	-	Favored (95.412%) alpha helix	-	-	-
A 405		LYS	1.53	0.53Å NZ with A 375 GLU OE2	Favored (79.88%) General / -56.8,-42.7	Favored (87.5%) *tttt* chi angles: 182.4,176.2,178.1,178.2	0.03Å	Favored (86.032%) alpha helix	-	-	-
A 406		ALA	1.73	-	Favored (93.13%) General / -60.2,-41.5	-	0.02Å	Favored (91.137%) alpha helix	-	-	-
A 407		PHE	1.92	-	Favored (74.45%) General / -61.7,-50.0	Favored (91.9%) *t80* chi angles: 176.5,78.6	0.04Å	Favored (82.56%) alpha helix	-	-	-
A 408		ILE	2.08	-	Favored (92.64%) Ile or Val / -59.8,-43.8	Favored (91.6%) *mt* chi angles: 291.8,166.4	0.05Å	Favored (92.431%) alpha helix	-	-	-
A 409		THR	2.17	-	Favored (98.39%) General / -61.8,-43.7	Favored (97.3%) *m* chi angles: 300.7	0.03Å	Favored (97.323%) alpha helix	-	-	-
A 410		THR	2.21	-	Favored (94.79%) General / -60.1,-44.5	Favored (89.4%) *m* chi angles: 298.3	0.02Å	Favored (90.552%) alpha helix	-	-	-
A 411		ILE	2.19	-	Favored (86.04%) Ile or Val / -67.9,-42.8	Favored (40.7%) *mm* chi angles: 296.9,302.1	0.03Å	Favored (90.436%) alpha helix	-	-	-

#	Alt	Res	High B	Clash > 0.4Å	Ramachandran	Rotamer	Cβ deviation	CaBLAM	Bond lengths	Bond angles	Cis Peptides
A 412		LYS	2.15	-	Favored (79.35%) General / -56.7,-42.6	Favored (32.6%) *ttpt* chi angles: 179.2,173.4,63.6,165.3	0.02Å	Favored (94.533%) alpha helix	-	-	-
A 413		GLY	2.08	-	Favored (52.35%) Glycine / -56.8,-52.2	-	-	Favored (95.001%) alpha helix	-	-	-
A 414		ALA	2.02	-	Favored (75.76%) General / -58.4,-37.6	-	0.03Å	Favored (80.121%) alpha helix	-	-	-
A 415		GLN	1.95	-	Favored (94.8%) General / -65.2,-41.3	Favored (98.1%) *mt0* chi angles: 289.6,172.8,330.6	0.01Å	Favored (93.18%) alpha helix	-	-	-
A 416		ARG	1.9	-	Favored (90.21%) General / -66.1,-39.8	Favored (87.2%) *mtp85* chi angles: 287.9,175.4,63.6,85	0.03Å	Favored (95.032%) alpha helix	-	-	-
A 417		LEU	1.86	-	Favored (87.79%) General / -65.4,-44.3	Favored (97.6%) *mt* chi angles: 293.1,172	0.09Å	Favored (86.853%) alpha helix	-	-	-
A 418		ALA	1.81	-	Favored (97.86%) General / -62.1,-41.0	-	0.07Å	Favored (65.688%) alpha helix	-	-	-
A 419		ALA	1.76	-	Favored (16.82%) General / -89.2,-34.1	-	0.04Å	Favored (52.484%) alpha helix	-	-	-
A 420		LEU	1.69	-	Favored (14.52%) General / -99.0,-25.6	Favored (55.1%) *mt* chi angles: 305.9,178.5	0.04Å	Favored (17.696%)	-	-	-
**#**	**Alt**	**Res**	**High B**	**Clash > 0.4Å**	**Ramachandran**	**Rotamer**	**Cβ deviation**	**CaBLAM**	**Bond lengths**	**Bond angles**	**Cis Peptides**
			Avg: 1.06	Clashscore: 1.34	Outliers: 0 of 498	Poor rotamers: 0 of 404	Outliers: 0 of 446	Outliers: 12 of 496	Outliers: 7 of 500	Outliers: 10 of 500	Non-Trans: 1 of 499
A 421		GLY	1.62	-	Favored (34.74%) Glycine / 86.8,-163.2	-	-	Favored (51.961%)	-	-	-
A 422		ASP	1.53	-	Favored (64.9%) General / -59.2,-26.5	Favored (42.5%) *p0* chi angles: 58,352.1	0.06Å	Favored (9.787%)	-	-	-
A 423		THR	1.45	-	Favored (36.34%) General / -54.0,-28.2	Favored (86.9%) *m* chi angles: 301.4	0.02Å	Favored (56.795%) three-ten	-	-	-
A 424		ALA	1.38	-	Favored (63.62%) General / -57.6,-28.5	-	0.06Å	Favored (63.653%) three-ten	-	-	-
A 425		TRP	1.35	-	Favored (34.95%) General / -81.0,0.6	Favored (96.7%) *m100* chi angles: 294.2,93.1	0.05Å	Favored (46.28%)	-	-	-
A 426		ASP	1.36	-	Favored (48.8%) General / -92.6,6.0	Favored (63.8%) *m-30* chi angles: 289.2,321.2	0.06Å	Favored (17.445%)	-	-	-
A 427		PHE	1.42	-	Favored (2.77%) General / -74.2,96.6	Favored (66.8%) *t80* chi angles: 186.4,76.9	0.07Å	CaBLAM Disfavored (1.999%)	-	-	-

A 428		GLY	1.54	-	Favored (2.28%) Glycine / 103.5,-40.9	-	-	CaBLAM Disfavored (1.409%)	-	-	-
A 429		SER	1.71	-	Favored (25.42%) General / 57.0,42.9	Favored (8%) *t* chi angles: 192.8	0.05Å	Favored (14.605%)	-	-	-
A 430		VAL	1.91	-	Favored (2.33%) Ile or Val / -84.4,87.7	Favored (43.2%) *t* chi angles: 182.8	0.08Å	Favored (48.913%) beta sheet	-	-	-
A 431		GLY	2.1	-	Allowed (0.97%) Glycine / -71.1,63.6	-	-	Favored (25.052%) beta sheet	-	-	-
A 432		GLY	2.2	-	Favored (51.99%) Glycine / -82.6,-177.6	-	-	Favored (32.166%)	-	-	-
A 433		ILE	2.18	-	Favored (50.24%) Ile or Val / -61.8,-31.2	Favored (12.5%) *tp* chi angles: 192,63.4	0.04Å	Favored (49.12%)	-	-	-
A 434		PHE	2.03	-	Favored (61.48%) General / -72.0,-22.0	Favored (70.3%) *m-80* chi angles: 293,110.5	0.05Å	Favored (71.986%) alpha helix	-	-	-
A 435		ASN	1.81	-	Favored (27.5%) General / -79.5,-41.4	Favored (16.6%) *t0* chi angles: 188.1,255.6	0.04Å	Favored (69.611%) alpha helix	-	-	-
A 436		SER	1.58	-	Favored (99.72%) General / -62.7,-42.5	Favored (66.7%) *m* chi angles: 294.5	0.04Å	Favored (81.309%) alpha helix	-	-	-
A 437		VAL	1.4	-	Favored (79.43%) Ile or Val / -69.5,-44.9	Favored (74%) *t* chi angles: 172.6	0.03Å	Favored (75.758%) alpha helix	-	-	-
A 438		GLY	1.27	-	Favored (34.09%) Glycine / -52.0,-52.6	-	-	Favored (94.058%) alpha helix	-	-	-
A 439		LYS	1.21	-	Favored (99.44%) General / -62.0,-43.1	Favored (97.3%) *mttt* chi angles: 287.6,176.9,177.1,177	0.07Å	Favored (84.802%) alpha helix	-	-	-
A 440		ALA	1.21	-	Favored (97.12%) General / -61.0,-41.8	-	0.05Å	Favored (82.85%) alpha helix	-	-	-
**#**	**Alt**	**Res**	**High B**	**Clash > 0.4Å**	**Ramachandran**	**Rotamer**	**Cβ deviation**	**CaBLAM**	**Bond lengths**	**Bond angles**	**Cis Peptides**
			Avg: 1.06	Clashscore: 1.34	Outliers: 0 of 498	Poor rotamers: 0 of 404	Outliers: 0 of 446	Outliers: 12 of 496	Outliers: 7 of 500	Outliers: 10 of 500	Non-Trans: 1 of 499
A 441		VAL	1.26	-	Favored (89.24%) Ile or Val / -66.6,-45.1	Favored (72.3%) *t* chi angles: 172.4	0.03Å	Favored (83.708%) alpha helix	-	-	-
A 442		HIS	1.35	-	Favored (93.78%) General / -60.9,-45.7	Favored (22.6%) *t-170* chi angles: 186.7,184.2	0.03Å	Favored (96.374%) alpha helix	-	-	-
A 443		GLN	1.45	-	Favored (90.8%) General / -61.6,-39.3	Favored (95.3%) *mt0* chi angles: 288.8,170.6,330.8	0.02Å	Favored (85.486%) alpha helix	-	-	-

A 444		VAL	1.55	-	Favored (90.1%) Ile or Val / -64.6,-47.1	Favored (60.2%) *t* chi angles: 170.8	0.01Å	Favored (78.577%) alpha helix	-	-	-
A 445		PHE	1.62	-	Favored (93.04%) General / -65.1,-39.5	Favored (10.1%) *m-10* chi angles: 286.8,329	0.01Å	Favored (76.62%) alpha helix	-	-	-
A 446		GLY	1.66	-	Favored (25.28%) Glycine / -55.6,-56.1	-	-	Favored (90.473%) alpha helix	-	-	-
A 447		GLY	1.68	-	Favored (99.23%) Glycine / -61.0,-41.1	-	-	Favored (89.919%) alpha helix	-	-	-
A 448		ALA	1.66	-	Favored (97.92%) General / -62.0,-43.9	-	0.04Å	Favored (75.423%) alpha helix	-	-	-
A 449		PHE	1.63	-	Favored (55.95%) General / -76.1,-39.2	Favored (55%) *m-80* chi angles: 283.9,97.4	0.06Å	Favored (74.563%) alpha helix	-	-	-
A 450		ARG	1.61	-	Favored (75.97%) General / -63.8,-33.6	Favored (98.5%) *mtt180* chi angles: 288.3,175.6,179.5,177.2	0.02Å	Favored (74.624%) alpha helix	-	-	-
A 451		THR	1.63	-	Favored (7.19%) General / -76.7,-54.0	Favored (97.8%) *m* chi angles: 300	0.06Å	Favored (50.243%) alpha helix	-	-	-
A 452		LEU	1.69	-	Favored (82.66%) General / -63.8,-36.1	Favored (90.5%) *mt* chi angles: 291,171.7	0.04Å	Favored (50.057%)	-	-	-
A 453		PHE	1.79	-	Favored (16.52%) General / -111.4,21.2	Favored (87%) *m-80* chi angles: 302,95.1	0.11Å	Favored (12.74%)	-	-	-
A 454		GLY	1.88	-	Favored (47.7%) Glycine / -61.5,141.2	-	-	Favored (6.179%)	-	-	-
A 455		GLY	1.95	-	Favored (75.76%) Glycine / 87.2,-8.7	-	-	Favored (84.193%)	-	-	-
A 456		MET	1.97	-	Favored (30.91%) General / -83.9,142.0	Favored (67.6%) *mtt* chi angles: 293.5,183,181.4	0.04Å	Favored (35.041%)	-	-	-
A 457		SER	1.91	-	Favored (32.21%) General / -71.4,160.7	Favored (88.1%) *p* chi angles: 67	0.05Å	Favored (51.131%)	-	-	-
A 458		TRP	1.76	-	Favored (64.33%) General / -56.5,-31.7	Favored (77.1%) *p-90* chi angles: 65.9,267.4	0.04Å	Favored (64.265%)	-	-	-
A 459		ILE	1.55	-	Favored (84.05%) Ile or Val / -67.0,-46.8	Favored (97.5%) *mt* chi angles: 293.5,168.2	0.05Å	Favored (82.003%) alpha helix	-	-	-
A 460		THR	1.34	-	Favored (99.25%) General / -62.5,-42.1	Favored (90.1%) *m* chi angles: 301.2	0.02Å	Favored (92.285%) alpha helix	-	-	-

#	Alt	Res	High B	Clash > 0.4Å	Ramachandran	Rotamer	Cβ deviation	CaBLAM	Bond lengths	Bond angles	Cis Peptides
			Avg: 1.06	Clashscore: 1.34	Outliers: 0 of 498	Poor rotamers: 0 of 404	Outliers: 0 of 446	Outliers: 12 of 496	Outliers: 7 of 500	Outliers: 10 of 500	Non-Trans: 1 of 499
A 461		GLN	1.15	-	Favored (90%) General / -62.0,-38.8	Favored (84.6%) *mt0* chi angles: 290.8,177.8,41.2	0.02Å	Favored (90.734%) alpha helix	-	-	-
A 462		GLY	1	-	Favored (98.07%) Glycine / -62.1,-39.8	-	-	Favored (97.289%) alpha helix	-	-	-
A 463		LEU	0.88	-	Favored (90.26%) General / -66.1,-39.7	Favored (98.8%) *mt* chi angles: 292.4,172.3	0.03Å	Favored (97.257%) alpha helix	-	-	-
A 464		MET	0.79	-	Favored (75.08%) General / -66.9,-33.2	Favored (88.8%) *mmm* chi angles: 288.7,302.4,297.3	0.01Å	Favored (89.115%) alpha helix	-	-	-
A 465		GLY	0.73	-	Favored (30.55%) Glycine / -60.3,-55.4	-	-	Favored (89.098%) alpha helix	-	-	-
A 466		ALA	0.68	-	Favored (78.74%) General / -58.5,-38.9	-	0.04Å	Favored (78.626%) alpha helix	-	-	-
A 467		LEU	0.64	-	Favored (68.32%) General / -61.5,-51.5	Favored (56.6%) *tp* chi angles: 173.3,61.8	0.07Å	Favored (80.732%) alpha helix	-	-	-
A 468		LEU	0.61	-	Favored (77.65%) General / -67.0,-34.4	Favored (91.1%) *mt* chi angles: 291.4,173.4	0.12Å	Favored (77.227%) alpha helix	-	-	-
A 469		LEU	0.59	-	Favored (97.64%) General / -63.9,-41.5	Favored (81.9%) *mt* chi angles: 289.3,169.4	0.04Å	Favored (89.431%) alpha helix	-	-	-
A 470		TRP	0.57	-	Favored (81.11%) General / -63.7,-47.4	Favored (92.5%) *t60* chi angles: 182.1,86.9	0.01Å	Favored (90.42%) alpha helix	-	-	-
A 471		MET	0.58	-	Favored (85.05%) General / -61.7,-37.7	Favored (98.7%) *mtp* chi angles: 291.1,174.1,70.3	0.01Å	Favored (86.607%) alpha helix	-	-	-
A 472		GLY	0.67	-	Favored (26.76%) Glycine / -58.6,-56.2	-	-	Favored (91.299%) alpha helix	-	-	-
A 473		VAL	0.91	-	Favored (31.54%) Ile or Val / -66.5,-23.5	Favored (27.4%) *m* chi angles: 298.9	0.06Å	Favored (61.703%) alpha helix	-	-	-
A 474		ASN	1.47	-	Favored (43.49%) General / -96.5,8.7	Favored (67.6%) *m-40* chi angles: 288.5,279.7	0.03Å	Favored (48.812%)	-	-	-
A 475		ALA	2.57	-	Favored (30.37%) General / -85.7,140.7	-	0.03Å	Favored (31.61%)	-	-	-
A 476		ARG	4.17	-	Favored (46.54%) General / -78.7,-34.3	Favored (98.8%) *mtt180* chi angles: 291.2,177.7,180.1,175.6	0.02Å	Favored (15.128%)	-	-	-

A 477		ASP	5.59	-	Favored (19.72%) General / -85.5,111.1	Favored (43%) *t0* chi angles: 181,335.3	0.08Å	Favored (31.717%)	-	-	-
A 478		ARG	5.76	-	Favored (45.82%) General / -53.2,-32.0	Favored (84.1%) *ttt180* chi angles: 183.8,176.9,178.5,183.3	0.05Å	Favored (42.563%)	-	-	-
A 479		SER	4.56	-	Favored (56.59%) General / -72.1,-46.5	Favored (94.1%) *p* chi angles: 64.2	0.02Å	Favored (70.789%) alpha helix	-	-	-
A 480		ILE	2.96	0.41Å HA with A 480 ILE HD12	Favored (47.8%) Ile or Val / -69.0,-30.8	Favored (9.6%) *tp* chi angles: 197.2,64.8	0.09Å	Favored (69.654%) alpha helix	-	-	-

#	Alt	Res	High B	Clash > 0.4Å	Ramachandran	Rotamer	Cβ deviation	CaBLAM	Bond lengths	Bond angles	Cis Peptides
			Avg: 1.06	Clashscore: 1.34	Outliers: 0 of 498	Poor rotamers: 0 of 404	Outliers: 0 of 446	Outliers: 12 of 496	Outliers: 7 of 500	Outliers: 10 of 500	Non-Trans: 1 of 499
A 481		ALA	1.79	-	Favored (90.99%) General / -59.6,-45.8	-	0.07Å	Favored (75.205%) alpha helix	-	-	-
A 482		LEU	1.16	-	Favored (84.91%) General / -65.0,-36.6	Favored (90.1%) *mt* chi angles: 290.8,172.3	0.05Å	Favored (82.818%) alpha helix	-	-	-
A 483		VAL	0.87	-	Favored (94%) Ile or Val / -63.3,-47.0	Favored (62.7%) *t* chi angles: 171.2	0.05Å	Favored (82.005%) alpha helix	-	-	-
A 484		MET	0.76	-	Favored (78.27%) General / -68.8,-36.4	Favored (80.4%) *mtm* chi angles: 289.7,189.9,287.1	0.05Å	Favored (81.429%) alpha helix	-	-	-
A 485		LEU	0.75	-	Favored (88.58%) General / -66.2,-42.5	Favored (85.1%) *mt* chi angles: 290.1,170	0.03Å	Favored (90.434%) alpha helix	-	-	-
A 486		ALA	0.78	-	Favored (93.85%) General / -65.2,-42.1	-	0.04Å	Favored (84.984%) alpha helix	-	-	-
A 487		THR	0.8	-	Favored (76.99%) General / -65.7,-47.0	Favored (91.6%) *m* chi angles: 301.1	0.04Å	Favored (82.565%) alpha helix	-	-	-
A 488		GLY	0.84	-	Favored (96.27%) Glycine / -62.3,-38.5	-	-	Favored (91.444%) alpha helix	-	-	-
A 489		GLY	0.88	-	Favored (35.52%) Glycine / -58.7,-54.7	-	-	Favored (91.05%) alpha helix	-	-	-
A 490		VAL	0.94	-	Favored (94.15%) Ile or Val / -60.1,-44.0	Favored (62.1%) *t* chi angles: 171.1	0.02Å	Favored (83.303%) alpha helix	-	-	-
A 491		LEU	1	-	Favored (90.33%) General / -61.8,-39.0	Favored (78.7%) *mt* chi angles: 288.5,170.6	0.07Å	Favored (88.976%) alpha helix	OUTLIER(S) worst is CB--CG: 4.9 σ	-	-
A 492		LEU	1.07	-	Favored (84.06%) General / -66.4,-44.0	Favored (58%) *tp* chi angles: 181.8,60	0.03Å	Favored (87.464%) alpha helix	-	-	-

A 493		PHE	1.18	-	Favored (98.89%) General / -61.8,-42.4	Favored (22.7%) *m-10* chi angles: 294.6,330.9	0.07Å	Favored (99.513%) alpha helix	-	-	-
A 494		LEU	1.32	-	Favored (99.44%) General / -61.5,-43.1	Favored (85.4%) *mt* chi angles: 290.1,170.6	0.09Å	Favored (93.594%) alpha helix	-	-	-
A 495		ALA	1.54	-	Favored (81.08%) General / -60.0,-38.0	-	0.07Å	Favored (73.975%) alpha helix	-	-	-
A 496		THR	1.84	-	Favored (48.92%) General / -85.2,-13.8	Favored (67.7%) *p* chi angles: 62.8	0.02Å	Favored (48.631%) alpha helix	-	-	-
A 497		ASN	2.26	-	Favored (4.97%) General / -109.6,-40.0	Favored (81.4%) *m-40* chi angles: 299.6,323.1	0.04Å	Favored (13.206%) alpha helix	-	-	-
A 498		VAL	2.77	-	Allowed (1.77%) Ile or Val / -91.2,81.3	Favored (32.5%) *t* chi angles: 185.4	0.04Å	CaBLAM Disfavored (3.106%)	-	-	-
A 499		HIS	3.35	-	Favored (30.79%) General / -158.0,156.2	Favored (33%) *p90* chi angles: 55.4,87.4	0.06Å	-	-	-	-
A 500		ALA	3.92	-	-	-	0.02Å	-	-	-	-

About MolProbity | Website for the Richardson Lab | Using ecloud x-H | Internal reference 4.5.2
