# Supplementary material for: Integrative proteome-wide structural analysis and high-throughput docking identify broad-spectrum antiviral scaffolds against Zika, Yellow Fever, West Nile, Saint Louis encephalitis, and Usutu viruses
Source: Front Cell Infect Microbiol. 2026 Apr 30;16:1723132. doi: 10.3389/fcimb.2026.1723132 (PMC13171538; doi:10.3389/fcimb.2026.1723132)
Supplement: Supplementary file 4 [file DataSheet4.zip › USUV/USU_E/Mol_probity_Files/USU_E_1FH-rama.pdf]

MolProbity Ramachandran analysis

USU_E1FH.pdb, model 1

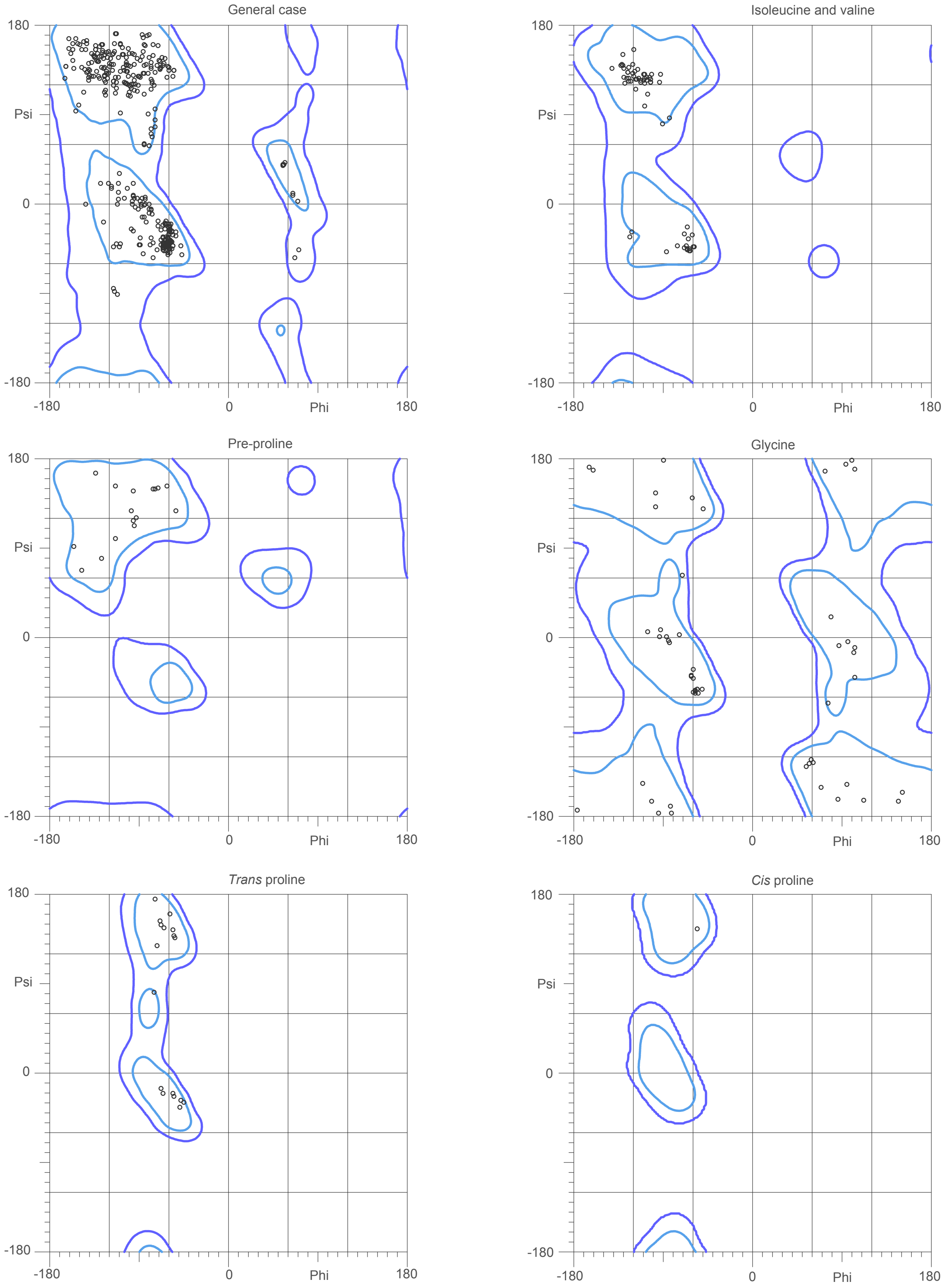

97.6% (486/498) of all residues were in favored (98%) regions.
100.0% (498/498) of all residues were in allowed (>99.8%) regions.

There were no outliers.
