# Supplementary material for: Integrative proteome-wide structural analysis and high-throughput docking identify broad-spectrum antiviral scaffolds against Zika, Yellow Fever, West Nile, Saint Louis encephalitis, and Usutu viruses
Source: Front Cell Infect Microbiol. 2026 Apr 30;16:1723132. doi: 10.3389/fcimb.2026.1723132 (PMC13171538; doi:10.3389/fcimb.2026.1723132)
Supplement: Supplementary file 4 [file DataSheet4.zip › USUV/USU_M/Mol_probity_Files/USU_M_1FH-multi.table.pdf]

Viewing USU_M1FH-multi.table

Duke Biochemistry
Duke University School of Medicine

When finished, you should close this window.

Hint: Use File | Save As... to save a copy of this page.

All-Atom Contacts	Clashscore, all atoms:	3.36		97th percentile* (N=1784, all resolutions)
	Clashscore is the number of serious steric overlaps (> 0.4 Å) per 1000 atoms.			
Protein Geometry	Poor rotamers	0	0.00%	Goal: <0.3%
	Favored rotamers	62	100.00%	Goal: >98%
	Ramachandran outliers	1	1.37%	Goal: <0.05%
	Ramachandran favored	70	95.89%	Goal: >98%
	Rama distribution Z-score	1.92 ± 1.00		Goal: abs(Z score) < 2
	MolProbity score^	1.41		97th percentile* (N=27675, 0Å - 99Å)
	Cβ deviations >0.25Å	0	0.00%	Goal: 0
	Bad bonds:	0 / 600	0.00%	Goal: 0%
	Bad angles:	1 / 816	0.12%	Goal: <0.1%
Peptide Omegas	Cis Prolines:	0 / 2	0.00%	Expected: ≤1 per chain, or ≤5%
Low-resolution Criteria	CaBLAM outliers	2	2.8%	Goal: <1.0%
	CA Geometry outliers	0	0.00%	Goal: <0.5%
Additional validations	Chiral volume outliers	0/94		
	Waters with clashes	0/0	0.00%	See UnDowser table for details

In the two column results, the left column gives the raw count, right column gives the percentage.

* 100th percentile is the best among structures of comparable resolution; 0th percentile is the worst. For clashscore the comparative set of structures was selected in 2004, for MolProbity score in 2006.

^ MolProbity score combines the clashscore, rotamer, and Ramachandran evaluations into a single score, normalized to be on the same scale as X-ray resolution.

Key to table colors and cutoffs here:

#	Alt	Res	High B	Clash > 0.4Å	Ramachandran	Rotamer	Cβ deviation	CaBLAM	Bond lengths	Bond angles	Cis Peptides
			Avg: 2.98	Clashscore: 3.36	Outliers: 1 of 73	Poor rotamers: 0 of 62	Outliers: 0 of 70	Outliers: 2 of 71	Outliers: 0 of 75	Outliers: 1 of 75	Non-Trans: 0 of 74
A 1		SER	7.26	-	-	Favored (38.8%) *t*<br>chi angles: 177.7	0.02Å	-	-	-	-
A 2		ILE	6.77	-	Favored (67.01%)<br>Ile or Val / -112.6,122.6	Favored (86.7%) *mt*<br>chi angles: 298.7,170.9	0.03Å	-	-	-	-
A 3		ALA	6.22	-	Favored (39.64%)<br>General / -77.4,141.8	-	0.03Å	Favored (38.598%)	-	-	-
A 4		VAL	5.67	-	Favored (73.99%)<br>Ile or Val / -124.2,129.6	Favored (72.4%) *t*<br>chi angles: 178.5	0.04Å	Favored (70.876%)<br>beta sheet	-	-	-
A 5		GLN	5.19	-	Favored (53.48%)<br>General / -110.2,133.2	Favored (55.9%) *tt0*<br>chi angles: 182.4,176.1,57.6	0.01Å	Favored (48.552%)<br>beta sheet	-	-	-

#	Alt	Res	High B	Clash > 0.4Å	Ramachandran	Rotamer	Cβ deviation	CaBLAM	Bond lengths	Bond angles	Cis Peptides
A 6		THR	4.81	-	Favored (50.92%) General / -60.5,144.6	Favored (4%) *t* chi angles: 178	0.05Å	Favored (45.954%)	-	-	-
A 7		HIS	4.55	-	Favored (58.42%) General / -58.5,-23.7	Favored (61%) *p-80* chi angles: 66.2,282	0.02Å	Favored (41.233%)	-	-	-
A 8		GLY	4.42	-	Favored (61.14%) Glycine / -63.2,-18.8	-	-	Favored (40.742%)	-	-	-
A 9		GLU	4.45	0.47Å OE2 with A 24 LYS NZ	Favored (5.48%) General / -109.9,-37.9	Favored (82.4%) *mt-10* chi angles: 293.6,175.7,20.7	0.06Å	Favored (22.647%) alpha helix	-	-	-
A 10		SER	4.61	-	Favored (74.02%) General / -64.6,-32.4	Favored (65.3%) *m* chi angles: 294.3	0.04Å	Favored (49.84%) three-ten	-	-	-
A 11		MET	4.94	-	Favored (8.09%) General / -56.0,-17.9	Favored (28.3%) *ptm* chi angles: 68.9,180.7,284.5	0.05Å	Favored (39.646%) three-ten	-	-	-
A 12		LEU	5.48	-	Favored (62.29%) General / -60.5,-21.3	Favored (7.1%) *mp* chi angles: 268.5,57	0.04Å	Favored (44.194%)	-	-	-
A 13		ALA	6.14	-	Favored (70.57%) General / -59.2,-33.1	-	0.04Å	Favored (15.215%)	-	-	-
A 14		ASN	6.74	-	Favored (3.51%) General / -129.8,-17.9	Favored (67.9%) *m-40* chi angles: 296.2,283.3	0.01Å	Favored (6.433%)	-	-	-
A 15		LYS	7.13	-	Favored (6.71%) General / -119.2,173.8	Favored (98.1%) *mttt* chi angles: 295.9,182.9,179.1,180.2	0.02Å	CaBLAM Disfavored (2.603%)	-	-	-
A 16		LYS	7.18	-	Favored (42.04%) General / -53.7,134.2	Favored (16.9%) *tptp* chi angles: 183.1,70.2,181.9,70	0.01Å	Favored (19.985%)	-	-	-
A 17		ASP	6.89	-	Allowed (1.06%) General / -74.2,64.1	Favored (18.7%) *p0* chi angles: 59,325.7	0.01Å	Favored (10.824%)	-	-	-
A 18		ALA	6.34	-	Favored (77.24%) General / -59.6,-36.9	-	0.04Å	CaBLAM Outlier (0.244%)	-	-	-
A 19		TRP	5.69	-	Allowed (0.09%) General / 45.1,-105.3	Favored (67.8%) *t-100* chi angles: 183.5,249.2	0.08Å	CaBLAM Outlier (0.268%)	-	-	-
A 20		LEU	5.05	0.41Å O with A 24 LYS HB3	Favored (64.92%) General / -70.6,-45.7	Favored (60.2%) *tp* chi angles: 181.7,61.7	0.07Å	CaBLAM Disfavored (1.203%)	-	-	-
**#**	**Alt**	**Res**	**High B**	**Clash > 0.4Å**	**Ramachandran**	**Rotamer**	**Cβ deviation**	**CaBLAM**	**Bond lengths**	**Bond angles**	**Cis Peptides**
			Avg: 2.98	Clashscore: 3.36	Outliers: 1 of 73	Poor rotamers: 0 of 62	Outliers: 0 of 70	Outliers: 2 of 71	Outliers: 0 of 75	Outliers: 1 of 75	Non-Trans: 0 of 74

A 21		ASP	4.49	0.43Å OD1 with A 22 SER N	OUTLIER (0.01)%) General / 53.6,165.1	Favored (46.4%) *t0* chi angles: 187.6,6.6	0.03Å	CaBLAM Disfavored (3.163%)	-	-	-
A 22		SER	4.04	0.43Å N with A 21 ASP OD1	Favored (66.47%) General / -66.3,-24.2	Favored (93.6%) *p* chi angles: 63.2	0.09Å	Favored (25.752%)	-	-	-
A 23		THR	3.66	-	Favored (71.81%) General / -65.7,-48.5	Favored (92.3%) *m* chi angles: 299.1	0.03Å	Favored (56.325%) alpha helix	-	-	-
A 24		LYS	3.3	0.47Å NZ with A 9 GLU OE2	Favored (47.33%) General / -78.0,-37.3	Favored (87.7%) *tttt* chi angles: 183.8,176.2,176.3,183.8	0.04Å	Favored (63.276%) alpha helix	-	-	-
A 25		ALA	2.96	-	Favored (87.83%) General / -58.8,-42.0	-	0.05Å	Favored (80.911%) alpha helix	-	-	-
A 26		SER	2.66	-	Favored (74.38%) General / -68.3,-44.9	Favored (71.8%) *m* chi angles: 295.9	0.06Å	Favored (81.982%) alpha helix	-	-	-
A 27		ARG	2.4	-	Favored (93.65%) General / -64.7,-39.4	Favored (98.3%) *mtt180* chi angles: 288.7,174.6,180.3,172.2	0.03Å	Favored (91.764%) alpha helix	-	-	-
A 28		TYR	2.15	-	Favored (82.4%) General / -61.2,-48.2	Favored (70.5%) *t80* chi angles: 185.9,81	0.05Å	Favored (90.248%) alpha helix	-	-	-
A 29		LEU	1.93	-	Favored (92.86%) General / -64.7,-39.0	Favored (95%) *mt* chi angles: 291.8,172.9	0.05Å	Favored (86.52%) alpha helix	-	-	-
A 30		MET	1.73	-	Favored (88.03%) General / -66.1,-38.3	Favored (82.2%) *mtm* chi angles: 289,187.6,288.5	0.01Å	Favored (97.249%) alpha helix	-	-	-
A 31		LYS	1.55	-	Favored (89.85%) General / -65.6,-38.5	Favored (96.6%) *mttt* chi angles: 288.8,178.3,180.5,179.3	0.01Å	Favored (97.743%) alpha helix	-	-	-
A 32		THR	1.4	-	Favored (85.34%) General / -63.7,-46.5	Favored (98.1%) *m* chi angles: 300.1	0.03Å	Favored (93.436%) alpha helix	-	-	-
A 33		GLU	1.28	-	Favored (94.36%) General / -63.8,-39.5	Favored (95.3%) *mt-10* chi angles: 288.9,181.3,353.2	0.04Å	Favored (93.326%) alpha helix	-	-	-
A 34		ASN	1.2	-	Favored (96.09%) General / -62.9,-39.9	Favored (99.3%) *m-40* chi angles: 287.5,339.6	0.02Å	Favored (96.594%) alpha helix	-	-	-
A 35		TRP	1.15	-	Favored (88.68%) General / -61.4,-46.8	Favored (85.6%) *t60* chi angles: 175.3,83.9	0.07Å	Favored (96.778%) alpha helix	-	-	-
A 36		ILE	1.13	-	Favored (98.19%) Ile or Val / -62.3,-43.5	Favored (98.1%) *mt* chi angles: 292.7,168.6	0.04Å	Favored (95.302%) alpha helix	-	-	-

#	Alt	Res	High B	Clash > 0.4Å	Ramachandran	Rotamer	Cβ deviation	CaBLAM	Bond lengths	Bond angles	Cis Peptides
A 37		ILE	1.12	-	Favored (98.96%) Ile or Val / -61.5,-45.3	Favored (93.1%) *mt* chi angles: 292,166.4	0.04Å	Favored (84.632%) alpha helix	-	-	-
A 38		ARG	1.13	-	Favored (75.6%) General / -68.2,-34.1	Favored (95.2%) *mtt180* chi angles: 290.1,171.2,181.7,166.7	0.04Å	Favored (59.912%)	-	-	-
A 39		ASN	1.13	-	Favored (79.53%) Pre-Pro / -130.8,66.7	Favored (52.6%) *m-40* chi angles: 300.3,279.8	0.04Å	Favored (25.182%)	-	-	-
A 40		PRO	1.1	-	Favored (69.75%) Trans-Pro / -63.1,-19.8	Favored (42.7%) *Cg_endo* chi angles: 24.1,325.6,30	0.01Å	Favored (26.898%)	-	-	-
**#**	**Alt**	**Res**	**High B**	**Clash > 0.4Å**	**Ramachandran**	**Rotamer**	**Cβ deviation**	**CaBLAM**	**Bond lengths**	**Bond angles**	**Cis Peptides**
			Avg: 2.98	Clashscore: 3.36	Outliers: 1 of 73	Poor rotamers: 0 of 62	Outliers: 0 of 70	Outliers: 2 of 71	Outliers: 0 of 75	Outliers: 1 of 75	Non-Trans: 0 of 74
A 41		GLY	1.06	-	Favored (59.18%) Glycine / -57.4,-28.4	-	-	Favored (68.061%)	-	-	-
A 42		TYR	1	-	Favored (63.31%) General / -71.7,-27.9	Favored (51%) *m-80* chi angles: 285.5,109.4	0.03Å	Favored (73.364%) alpha helix	-	-	-
A 43		ALA	0.93	-	Favored (70.82%) General / -71.3,-40.6	-	0.04Å	Favored (74.598%) alpha helix	-	-	-
A 44		PHE	0.87	-	Favored (70.73%) General / -58.0,-51.2	Favored (88.1%) *t80* chi angles: 174.2,76.7	0.03Å	Favored (81.273%) alpha helix	-	-	-
A 45		VAL	0.81	-	Favored (97.38%) Ile or Val / -64.1,-43.1	Favored (68.4%) *t* chi angles: 171.9	0.05Å	Favored (79.115%) alpha helix	-	-	-
A 46		ALA	0.77	-	Favored (78.06%) General / -58.6,-38.5	-	0.04Å	Favored (81.976%) alpha helix	-	-	-
A 47		VAL	0.73	-	Favored (93.33%) Ile or Val / -64.4,-46.3	Favored (57.1%) *t* chi angles: 170.4	0.06Å	Favored (90.81%) alpha helix	-	-	-
A 48		LEU	0.72	-	Favored (97.93%) General / -63.4,-40.7	Favored (97.5%) *mt* chi angles: 292.8,173.3	0.07Å	Favored (89.407%) alpha helix	-	-	-
A 49		LEU	0.72	-	Favored (87.77%) General / -66.3,-42.8	Favored (86.3%) *mt* chi angles: 290.2,171.2	0.05Å	Favored (90.835%) alpha helix	-	-	-
A 50		GLY	0.77	-	Favored (96.05%) Glycine / -60.3,-39.4	-	-	Favored (96.816%) alpha helix	-	-	-
A 51		TRP	0.94	-	Favored (95.99%) General / -64.5,-42.4	Favored (40.8%) *m-10* chi angles: 287.4,342	0.03Å	Favored (88.301%) alpha helix	-	-	-

#	Alt	Res	High B	Clash > 0.4Å	Ramachandran	Rotamer	Cβ deviation	CaBLAM	Bond lengths	Bond angles	Cis Peptides
A 52		MET	1.34	-	Favored (68.35%) General / -71.1,-31.9	Favored (84.2%) *mtm* chi angles: 289.9,186.9,284.6	0.06Å	Favored (77.666%) alpha helix	-	-	-
A 53		LEU	2.16	-	Favored (24.13%) General / -80.1,-42.1	Favored (91%) *mt* chi angles: 293,169.5	0.07Å	Favored (65.904%) alpha helix	-	-	-
A 54		GLY	3.65	-	Favored (25.93%) Glycine / -91.0,158.6	-	-	Favored (5.737%) alpha helix	-	-	-
A 55		SER	5.59	-	Favored (9.07%) General / -107.2,-29.9	Favored (95.9%) *p* chi angles: 63.8	0.03Å	Favored (9.715%) alpha helix	-	-	-
A 56		ASN	6.95	-	Favored (16.35%) General / -103.9,158.5	Favored (13.9%) *t0* chi angles: 192.2,270.8	0.03Å	Favored (15.706%) alpha helix	-	-	-
A 57		ASN	6.62	-	Favored (75.86%) General / -61.9,-34.3	Favored (98.5%) *m-40* chi angles: 287.2,339.6	0.03Å	Favored (65.069%) alpha helix	-	-	-
A 58		GLY	4.91	-	Favored (54.56%) Glycine / -58.9,-52.2	-	-	Favored (90.971%) alpha helix	-	-	-
A 59		GLN	3.06	-	Favored (97.18%) General / -62.1,-40.9	Favored (97.5%) *mt0* chi angles: 291.3,173.2,316.3	0.12Å	Favored (80.739%) alpha helix	-	-	-
A 60		ARG	1.82	-	Favored (98.37%) General / -63.6,-42.4	Favored (96.2%) *mtt180* chi angles: 290,171.9,177.7,166.9	0.05Å	Favored (85.947%) alpha helix	-	-	-
**#**	**Alt**	**Res**	**High B**	**Clash > 0.4Å**	**Ramachandran**	**Rotamer**	**Cβ deviation**	**CaBLAM**	**Bond lengths**	**Bond angles**	**Cis Peptides**
			Avg: 2.98	Clashscore: 3.36	Outliers: 1 of 73	Poor rotamers: 0 of 62	Outliers: 0 of 70	Outliers: 2 of 71	Outliers: 0 of 75	Outliers: 1 of 75	Non-Trans: 0 of 74
A 61		VAL	1.18	-	Favored (96.42%) Ile or Val / -64.8,-44.5	Favored (65.7%) *t* chi angles: 171.6	0.03Å	Favored (94.409%) alpha helix	-	-	-
A 62		VAL	0.89	-	Favored (94.47%) Ile or Val / -60.2,-44.0	Favored (54.2%) *t* chi angles: 170	0.03Å	Favored (97.707%) alpha helix	-	-	-
A 63		PHE	0.78	-	Favored (94.76%) General / -62.1,-40.1	Favored (11.4%) *m-10* chi angles: 287.8,328.6	0.03Å	Favored (94.726%) alpha helix	-	-	-
A 64		VAL	0.77	-	Favored (99.63%) Ile or Val / -62.8,-44.6	Favored (73%) *t* chi angles: 172.5	0.04Å	Favored (91.813%) alpha helix	-	-	-
A 65		VAL	0.8	-	Favored (95.27%) Ile or Val / -63.9,-46.0	Favored (66.4%) *t* chi angles: 171.7	0.04Å	Favored (89.821%) alpha helix	-	-	-
A 66		LEU	0.84	-	Favored (95.8%) General / -62.7,-40.0	Favored (90%) *mt* chi angles: 291.3,173.4	0.02Å	Favored (95.192%) alpha helix	-	-	-

A 67		LEU	0.9	-	Favored (98.96%) General / -62.2,-42.1	Favored (83%) *mt* chi angles: 289.7,168.2	0.05Å	Favored (92.925%) alpha helix	-	-	-
A 68		LEU	0.97	-	Favored (73.43%) General / -68.7,-33.1	Favored (84.2%) *mt* chi angles: 290.9,174.3	0.09Å	Favored (86.112%) alpha helix	-	-	-
A 69		LEU	1.06	-	Favored (69.17%) General / -69.9,-31.4	Favored (90.3%) *mt* chi angles: 290.8,172.4	0.03Å	Favored (68.477%) alpha helix	-	-	-
A 70		VAL	1.2	-	Favored (3.8%) Ile or Val / -98.7,-26.4	Favored (23.3%) *m* chi angles: 301.4	0.01Å	Favored (23.85%) alpha helix	-	-	-
A 71		ALA	1.42	0.43Å N with A 72 PRO HD2	Favored (84.75%) Pre-Pro / -54.2,-40.8	-	0.06Å	Favored (62.279%) three-ten	-	OUTLIER(S) worst is CA-C-N: 4.5 σ	-
A 72		PRO	1.73	0.43Å HD2 with A 71 ALA N	Favored (60.89%) Trans-Pro / -64.1,-17.7	Favored (35.3%) *Cg_endo* chi angles: 22.4,325.3,32.7	0.01Å	Favored (59.692%)	-	-	-
A 73		ALA	2.16	-	Favored (58.95%) General / -84.3,-4.6	-	0.03Å	Favored (53.969%)	-	-	-
A 74		TYR	2.69	-	Favored (3.94%) General / -77.9,68.3	Favored (6.7%) *m-10* chi angles: 291.5,7.1	0.01Å	-	-	-	-
A 75		SER	3.28	-	-	Favored (40.3%) *t* chi angles: 175.1	0.03Å	-	-	-	-
