# Supplementary material for: Integrative proteome-wide structural analysis and high-throughput docking identify broad-spectrum antiviral scaffolds against Zika, Yellow Fever, West Nile, Saint Louis encephalitis, and Usutu viruses
Source: Front Cell Infect Microbiol. 2026 Apr 30;16:1723132. doi: 10.3389/fcimb.2026.1723132 (PMC13171538; doi:10.3389/fcimb.2026.1723132)
Supplement: Supplementary file 4 [file DataSheet4.zip › USUV/USU_NS1/Mol_probity_Files/USU_NS1_1FH-multi.table.pdf]

Viewing USU_NS1_1FH-multi.table

Duke Biochemistry
Duke University School of Medicine

When finished, you should close this window.

Hint: Use File | Save As... to save a copy of this page.

All-Atom Contacts	Clashscore, all atoms:	1.45		99th percentile* (N=1784, all resolutions)
	Clashscore is the number of serious steric overlaps (> 0.4 Å) per 1000 atoms.			
Protein Geometry	Poor rotamers	0	0.00%	Goal: <0.3%
	Favored rotamers	307	99.68%	Goal: >98%
	Ramachandran outliers	0	0.00%	Goal: <0.05%
	Ramachandran favored	340	97.14%	Goal: >98%
	Rama distribution Z-score	0.70 ± 0.45		Goal: abs(Z score) < 2
	MolProbity score^	1.04		100th percentile* (N=27675, 0Å - 99Å)
	Cβ deviations >0.25Å	0	0.00%	Goal: 0
	Bad bonds:	2 / 2857	0.07%	Goal: 0%
	Bad angles:	7 / 3879	0.18%	Goal: <0.1%
Peptide Omegas	Cis Prolines:	1 / 16	6.25%	Expected: ≤1 per chain, or ≤5%
	Cis nonProlines:	1 / 335	0.30%	Goal: <0.05%
Low-resolution Criteria	CaBLAM outliers	5	1.4%	Goal: <1.0%
	CA Geometry outliers	7	2.01%	Goal: <0.5%
Additional validations	Chiral volume outliers	0/425		
	Waters with clashes	0/0	0.00%	See UnDowser table for details

In the two column results, the left column gives the raw count, right column gives the percentage.

* 100th percentile is the best among structures of comparable resolution; 0th percentile is the worst. For clashscore the comparative set of structures was selected in 2004, for MolProbity score in 2006.

^ MolProbity score combines the clashscore, rotamer, and Ramachandran evaluations into a single score, normalized to be on the same scale as X-ray resolution.

Key to table colors and cutoffs here:

#	Alt	Res	High B	Clash > 0.4Å	Ramachandran	Rotamer	Cβ deviation	CaBLAM	Bond lengths	Bond angles	Cis Peptides
			Avg: 1.15	Clashscore: 1.45	Outliers: 0 of 350	Poor rotamers: 0 of 308	Outliers: 0 of 327	Outliers: 11 of 348	Outliers: 2 of 352	Outliers: 7 of 352	Non-Trans: 2 of 351
A 1		ASP	2.67	-	-	Favored (13.3%) *t0* chi angles: 202.8,338.2	0.03Å	-	-	-	-
A 2		SER	2.62	-	Favored (2.72%) General / -134.1,-5.6	Favored (90.7%) *p* chi angles: 63	0.02Å	-	-	-	-
A 3		GLY	2.62	-	Favored (50.43%) Glycine / -71.9,153.8	-	-	Favored (48.964%)	-	-	-
A 4		CYS	2.71	-	Favored (16.28%) General / -97.7,157.0	Favored (91.2%) *m* chi angles: 293.2	0.05Å	Favored (47.049%) beta sheet	-	-	-
A 5		ALA	2.89	-	Favored (47.99%) General / -136.2,148.2	-	0.07Å	Favored (66.887%) beta sheet	-	-	-
A 6		ILE	3.17	-	Favored (71.45%)	Favored (86.2%) *mt* chi angles: 298.9,171.4	0.04Å	Favored (61.419%) beta sheet	-	-	-

					Ile or Val / -117.7,123.2						
A 7		ASP	3.5	-	Favored (16.53%) General / -92.9,104.3	Favored (67.5%) *t0* chi angles: 182.8,356.3	0.08Å	Favored (70.215%) beta sheet	-	-	-
A 8		VAL	3.83	-	Favored (51.03%) Ile or Val / -59.4,-33.4	Favored (73.3%) *t* chi angles: 172.5	0.06Å	Favored (50.254%)	-	-	-
A 9		GLY	4.06	-	Favored (84.84%) Glycine / -75.7,-10.1	-	-	Favored (40.374%) alpha helix	-	-	-
A 10		ARG	4.12	-	Favored (5.62%) General / -126.7,-14.9	Favored (76.4%) *mtm180* chi angles: 292.3,179.8,286.6,163.3	0.03Å	Favored (7.175%)	-	-	-
A 11		ARG	3.95	-	Favored (8.09%) General / 60.9,47.0	Favored (95.1%) *mtt180* chi angles: 299.8,182.7,180.9,175.8	0.02Å	Favored (21.297%)	-	-	-
A 12		GLU	3.61	-	Favored (54.53%) General / -116.5,134.7	Favored (96%) *mt-10* chi angles: 296.6,177.9,359.5	0.07Å	Favored (29.977%) beta sheet	-	-	-
A 13		LEU	3.17	-	Favored (43.04%) General / -101.5,122.0	Favored (50.6%) *tp* chi angles: 173.4,64.7	0.05Å	Favored (68.768%) beta sheet	-	-	-
A 14		ARG	2.75	-	Favored (45.96%) General / -124.5,126.5	Favored (93%) *mtt-85* chi angles: 291.6,175.4,177.7,266.6	0.05Å	Favored (67.288%)	-	-	-
A 15		CYS	2.38	-	Favored (25.19%) General / -134.8,165.5	Favored (41.8%) *m* chi angles: 304.4	0.10Å	CA Geom Outlier (0.006%)	-	-	-
A 16		GLY	2.09	-	Allowed (0.84%) Glycine / -152.6,24.4	-	-	CA Geom Outlier (0.321%)	-	-	Cis nonPRO omega= -3.53
A 17		GLN	1.88	-	Favored (3.61%) General / -133.1,34.1	Favored (90.3%) *mt0* chi angles: 297.6,180.7,318.1	0.02Å	Favored (19.443%)	-	-	-
A 18		GLY	1.73	-	Favored (5.47%) Glycine / -117.3,-135.6	-	-	Favored (26.775%)	-	-	-
A 19		ILE	1.64	-	Favored (69.33%) Ile or Val / -128.3,129.9	Favored (81.6%) *mt* chi angles: 300,171.9	0.04Å	Favored (7.7%) beta sheet	-	-	-
A 20		PHE	1.58	-	Favored (19.6%) General / -116.7,111.3	Favored (5.6%) *t80* chi angles: 180.5,27.4	0.01Å	Favored (67.568%) beta sheet	-	-	-
**#**	**Alt**	**Res**	**High B**	**Clash > 0.4Å**	**Ramachandran**	**Rotamer**	**Cβ deviation**	**CaBLAM**	**Bond lengths**	**Bond angles**	**Cis Peptides**
			Avg: 1.15	Clashscore: 1.45	Outliers: 0 of 350	Poor rotamers: 0 of 308	Outliers: 0 of 327	Outliers: 11 of 348	Outliers: 2 of 352	Outliers: 7 of 352	Non-Trans: 2 of 351
A 21		ILE	1.55	-	Favored (6.71%) Ile or Val / -100.0,98.2	Favored (42.3%) *mm* chi angles: 307.9,301.5	0.07Å	Favored (71.23%) beta sheet	-	-	-

A 22		HIS	1.55	-	Favored (36.01%) General / -89.5,128.5	Favored (5.2%) *t-170* chi angles: 195.9,220.3	0.02Å	Favored (43.543%) beta sheet	-	-	-
A 23		ASN	1.61	-	Favored (6.31%) General / -79.7,75.0	Favored (38.4%) *t0* chi angles: 191,10.2	0.07Å	Favored (16.062%) beta sheet	-	-	-
A 24		ASP	1.74	-	Favored (53.99%) General / -91.2,3.8	Favored (27.7%) *m-30* chi angles: 284.6,310.9	0.06Å	Favored (7.044%)	-	-	-
A 25		VAL	1.97	-	Favored (83.17%) Ile or Val / -59.3,-41.1	Favored (71.3%) *t* chi angles: 172.3	0.05Å	Favored (41.718%)	-	-	-
A 26		GLU	2.24	-	Favored (58.41%) General / -85.5,-8.3	Favored (95.7%) *mt-10* chi angles: 296.6,177.8,0.9	0.06Å	Favored (47.308%)	-	-	-
A 27		ALA	2.47	-	Favored (8.63%) General / -85.8,64.5	-	0.04Å	Favored (7.118%)	-	-	-
A 28		TRP	2.54	0.54Å CG with A 29 VAL H	Allowed (0.08%) General / 46.6,-151.4	Favored (46.8%) *t-100* chi angles: 178.3,239.5	0.16Å	CaBLAM Outlier (0.387%)	-	-	-
A 29		VAL	2.41	0.54Å H with A 28 TRP CG	Favored (7.37%) Ile or Val / -123.8,-8.3	Favored (27%) *m* chi angles: 299	0.05Å	CaBLAM Disfavored (1.326%)	-	-	-
A 30		ASP	2.1	-	Favored (59.01%) General / -82.9,-10.1	Favored (85.9%) *m-30* chi angles: 291.4,334.2	0.05Å	Favored (40.845%)	-	-	-
A 31		ARG	1.72	-	Favored (65.32%) General / -69.5,-27.6	Favored (97.1%) *mtt180* chi angles: 289.3,183.7,180,177.1	0.08Å	Favored (52.103%) three-ten	-	-	-
A 32		TYR	1.4	-	Favored (7.01%) General / -121.7,100.4	Favored (79.6%) *m-80* chi angles: 292.1,81.4	0.02Å	Favored (17.606%)	-	-	-
A 33		LYS	1.18	-	Favored (29.06%) General / -87.3,120.9	Favored (87.1%) *tttt* chi angles: 184.1,178.1,179.9,177.9	0.01Å	Favored (53.811%)	-	-	-
A 34		PHE	1.07	-	Favored (28.45%) General / -94.2,116.1	Favored (3.2%) *m-10* chi angles: 287.2,24.6	0.10Å	Favored (67.527%) beta sheet	-	-	-
A 35		MET	1.03	-	Favored (57.91%) Pre-Pro / -110.6,109.6	Favored (78.4%) *mtp* chi angles: 302.9,172.4,71.6	0.12Å	Favored (66.008%)	-	-	-
A 36		PRO	1.05	-	Favored (37.77%) Trans-Pro / -75.4,161.3	Favored (61.2%) *Cg_endo* chi angles: 31.7,325.6,22.6	0.09Å	Favored (37.303%)	-	-	-
A 37		GLU	1.07	-	Allowed (1.34%) General / -53.1,159.0	Favored (17%) *pt0* chi angles: 63.7,184.8,339.1	0.05Å	CaBLAM Disfavored (2.1%)	-	-	-
A 38		THR	1.07	-	Favored (8.96%) Pre-Pro / -47.0,129.6	Favored (55.7%) *m* chi angles: 303.8	0.07Å	Favored (8.922%)	-	-	-

#	Alt	Res	High B	Clash > 0.4Å	Ramachandran	Rotamer	Cβ deviation	CaBLAM	Bond lengths	Bond angles	Cis Peptides
A 39		PRO	1.05	-	Favored (13.3%) Trans-Pro / -48.6,-29.1	Favored (83.1%) *Cg_exo* chi angles: 329.2,37.2,332.1	0.02Å	Favored (65.115%)	-	-	-
A 40		LYS	1	-	Favored (87.52%) General / -59.3,-40.9	Favored (52.6%) *mtpt* chi angles: 288.4,168.8,64.9,176.5	0.20Å	Favored (62.855%) alpha helix	-	-	-
**#**	**Alt**	**Res**	**High B**	**Clash > 0.4Å**	**Ramachandran**	**Rotamer**	**Cβ deviation**	**CaBLAM**	**Bond lengths**	**Bond angles**	**Cis Peptides**
			Avg: 1.15	Clashscore: 1.45	Outliers: 0 of 350	Poor rotamers: 0 of 308	Outliers: 0 of 327	Outliers: 11 of 348	Outliers: 2 of 352	Outliers: 7 of 352	Non-Trans: 2 of 351
A 41		GLN	0.93	-	Favored (46.39%) General / -76.5,-41.7	Favored (92.7%) *mt0* chi angles: 294,169.3,331	0.06Å	Favored (77.164%) alpha helix	-	-	-
A 42		LEU	0.87	-	Favored (98.23%) General / -63.6,-41.3	Favored (71%) *mt* chi angles: 289.7,175.5	0.10Å	Favored (97.727%) alpha helix	-	-	-
A 43		ALA	0.81	-	Favored (93.05%) General / -62.0,-39.6	-	0.03Å	Favored (90.731%) alpha helix	-	-	-
A 44		LYS	0.77	-	Favored (78.79%) General / -69.1,-39.6	Favored (44.7%) *tptt* chi angles: 188.8,69.2,182.1,184.6	0.05Å	Favored (94.855%) alpha helix	-	-	-
A 45		VAL	0.74	-	Favored (88.89%) Ile or Val / -59.5,-42.7	Favored (56.4%) *t* chi angles: 170.3	0.03Å	Favored (97.4%) alpha helix	-	-	-
A 46		ILE	0.74	-	Favored (99.29%) Ile or Val / -62.5,-44.4	Favored (82.6%) *mt* chi angles: 290.1,168.4	0.05Å	Favored (97.816%) alpha helix	-	-	-
A 47		GLU	0.75	-	Favored (84.75%) General / -58.1,-47.0	Favored (78.8%) *tt0* chi angles: 182,186.1,5.6	0.06Å	Favored (83.199%) alpha helix	-	-	-
A 48		GLN	0.79	-	Favored (87.44%) General / -67.0,-40.2	Favored (95.1%) *mt0* chi angles: 291,169.7,328.4	0.04Å	Favored (80.783%) alpha helix	-	-	-
A 49		ALA	0.84	-	Favored (92.48%) General / -60.4,-40.8	-	0.06Å	Favored (89.053%) alpha helix	-	-	-
A 50		HIS	0.88	-	Favored (74.09%) General / -61.2,-50.2	Favored (85%) *t70* chi angles: 182.9,76.1	0.02Å	Favored (91.558%) alpha helix	-	-	-
A 51		ALA	0.91	-	Favored (68.72%) General / -58.2,-32.8	-	0.03Å	Favored (74.662%)	-	-	-
A 52		LYS	0.9	-	Favored (53.72%) General / -84.9,-0.3	Favored (98.4%) *mttt* chi angles: 292.8,178.9,180,179.4	0.02Å	Favored (38.489%)	-	-	-
A 53		GLY	0.86	-	Favored (40.37%) Glycine / 102.2,10.0	-	-	Favored (76.78%)	-	-	-
A 54		ILE	0.8	-	Favored (47.49%)	Favored (91.6%) *mt* chi angles: 297.6,171.1	0.04Å	Favored (20.559%)	-	-	-

#	Alt	Res	High B	Clash > 0.4Å	Ramachandran	Rotamer	Cβ deviation	CaBLAM	Bond lengths	Bond angles	Cis Peptides
					Ile or Val / -98.7,120.0						
A 55		CYS	0.75	-	Favored (56.16%) General / -88.5,-7.6	Favored (27.7%) *p* chi angles: 67.4	0.10Å	CaBLAM Disfavored (1.736%)	-	-	-
A 56		GLY	0.71	-	Favored (46.03%) Glycine / 175.2,-173.1	-	-	Favored (40.546%)	-	-	-
A 57		LEU	0.7	-	Favored (25.84%) General / -141.8,132.7	Favored (43%) *tp* chi angles: 173.5,66.5	0.06Å	Favored (14.404%)	-	-	-
A 58		ARG	0.73	-	Favored (55.1%) General / -110.2,131.4	Favored (78.3%) *ttm-80* chi angles: 187,182.8,297.9,278.5	0.06Å	Favored (60.852%) beta sheet	-	-	-
A 59		SER	0.77	-	Favored (15.03%) General / -80.7,171.4	Favored (59.7%) *m* chi angles: 299.1	0.09Å	Favored (9.179%)	-	-	-
A 60		VAL	0.81	-	Favored (10.08%) Ile or Val / -112.6,-1.9	Favored (32.4%) *m* chi angles: 297.5	0.06Å	Favored (33.039%)	-	-	-
**#**	**Alt**	**Res**	**High B**	**Clash > 0.4Å**	**Ramachandran**	**Rotamer**	**Cβ deviation**	**CaBLAM**	**Bond lengths**	**Bond angles**	**Cis Peptides**
			Avg: 1.15	Clashscore: 1.45	Outliers: 0 of 350	Poor rotamers: 0 of 308	Outliers: 0 of 327	Outliers: 11 of 348	Outliers: 2 of 352	Outliers: 7 of 352	Non-Trans: 2 of 351
A 61		SER	0.86	-	Favored (40.79%) General / -151.6,156.6	Favored (88.5%) *p* chi angles: 68.2	0.07Å	Favored (21.329%) alpha helix	-	-	-
A 62		ARG	0.89	-	Favored (76.28%) General / -56.2,-41.9	Favored (60.1%) *ttp-170* chi angles: 178.8,174.9,62.7,191.9	0.07Å	Favored (64.127%) alpha helix	-	-	-
A 63		LEU	0.92	-	Favored (80.17%) General / -68.5,-37.5	Favored (94%) *mt* chi angles: 293.6,174.7	0.12Å	Favored (80.103%) alpha helix	-	-	-
A 64		GLU	0.93	-	Favored (90.48%) General / -64.9,-38.3	Favored (91.5%) *mt-10* chi angles: 289.2,167.9,340.9	0.03Å	Favored (87.718%) alpha helix	-	-	-
A 65		HIS	0.93	-	Favored (80.28%) General / -59.1,-48.8	Favored (87.5%) *t70* chi angles: 180.5,76.3	0.08Å	Favored (95.406%) alpha helix	OUTLIER(S) worst is CB--CG: 4.1 σ	OUTLIER(S) worst is CA-CB-CG: 4.6 σ	-
A 66		VAL	0.92	-	Favored (83.97%) Ile or Val / -59.6,-41.1	Favored (57.9%) *t* chi angles: 170.5	0.04Å	Favored (80.93%) alpha helix	-	-	-
A 67		MET	0.93	-	Favored (75.35%) General / -55.1,-47.2	Favored (29.1%) *tmm* chi angles: 179.7,276.5,288	0.06Å	Favored (80.804%) alpha helix	-	-	-
A 68		TRP	0.93	-	Favored (96.26%) General / -64.6,-41.4	Favored (83.2%) *m100* chi angles: 284.9,110.8	0.03Å	Favored (85.57%) alpha helix	-	-	-
A 69		GLU	0.95	-	Favored (77.85%) General / -58.4,-38.7	Favored (91.1%) *tt0* chi angles: 184.9,177.2,3.1	0.03Å	Favored (80.676%) alpha helix	-	-	-
A 70		ASN	0.97	-	Favored (59.39%)	Favored (98.4%) *m-40*	0.03Å	Favored (41.991%)	-	-	-

					General / -78.5,-9.7	chi angles: 287.8,337		alpha helix			
A 71		ILE	0.98	-	Favored (10.89%) Ile or Val / -119.1,-5.0	Favored (41.4%) *pt* chi angles: 63,168.1	0.06Å	Favored (30.977%) alpha helix	-	-	-
A 72		ARG	1	-	Favored (27.76%) General / -47.9,-50.2	Favored (71.2%) *ttt180* chi angles: 181.4,184.5,180,194.2	0.10Å	Favored (55.783%) alpha helix	-	-	-
A 73		ASP	1.02	-	Favored (80.29%) General / -61.7,-36.2	Favored (98.6%) *m-30* chi angles: 288,345.6	0.03Å	Favored (77.003%) alpha helix	-	-	-
A 74		GLU	1.04	-	Favored (62.32%) General / -73.3,-42.5	Favored (91%) *tt0* chi angles: 181.5,174.1,357.2	0.05Å	Favored (81.919%) alpha helix	-	-	-
A 75		LEU	1.05	-	Favored (97.86%) General / -62.1,-41.0	Favored (90.2%) *mt* chi angles: 291.1,170.9	0.10Å	Favored (92.83%) alpha helix	-	-	-
A 76		ASN	1.07	-	Favored (76.52%) General / -69.5,-36.4	Favored (96.2%) *m-40* chi angles: 286.7,338.3	0.04Å	Favored (89.09%) alpha helix	-	-	-
A 77		THR	1.1	-	Favored (90.48%) General / -61.7,-46.2	Favored (93.7%) *m* chi angles: 297.5	0.02Å	Favored (84.426%) alpha helix	-	-	-
A 78		LEU	1.13	-	Favored (84.51%) General / -67.2,-37.9	Favored (79%) *mt* chi angles: 294.2,179.1	0.04Å	Favored (84.019%) alpha helix	-	-	-
A 79		LEU	1.15	-	Favored (95.59%) General / -64.1,-40.1	Favored (83.2%) *mt* chi angles: 289.5,172.4	0.04Å	Favored (90.008%) alpha helix	-	-	-
A 80		ARG	1.17	-	Favored (69.57%) General / -62.7,-50.8	Favored (85.2%) *ttp80* chi angles: 180.2,181.4,64.9,78.4	0.00Å	Favored (85.602%) alpha helix	-	-	-
**#**	**Alt**	**Res**	**High B**	**Clash > 0.4Å**	**Ramachandran**	**Rotamer**	**Cβ deviation**	**CaBLAM**	**Bond lengths**	**Bond angles**	**Cis Peptides**
			Avg: 1.15	Clashscore: 1.45	Outliers: 0 of 350	Poor rotamers: 0 of 308	Outliers: 0 of 327	Outliers: 11 of 348	Outliers: 2 of 352	Outliers: 7 of 352	Non-Trans: 2 of 351
A 81		GLU	1.15	-	Favored (68.76%) General / -63.7,-26.7	Favored (75.7%) *mt-10* chi angles: 288.4,179.5,324.6	0.08Å	Favored (69.758%) alpha helix	-	-	-
A 82		ASN	1.1	-	Favored (24.02%) General / -96.3,14.5	Favored (70%) *m-40* chi angles: 289.2,281.4	0.03Å	Favored (47.17%)	-	-	-
A 83		ALA	1.03	-	Favored (19.73%) General / 58.6,43.0	-	0.02Å	Favored (30.831%)	-	-	-
A 84		VAL	0.95	-	Favored (57.6%) Ile or Val / -107.7,119.6	Favored (63.9%) *t* chi angles: 179.5	0.06Å	Favored (30.523%) beta sheet	-	-	-
A 85		ASP	0.87	-	Favored (6.78%) General / -80.4,69.3	Favored (42%) *t0* chi angles: 192.3,21.5	0.04Å	Favored (7.994%) beta sheet	-	-	-
A 86		LEU	0.8	-	Favored (15.69%)	Favored (56.8%) *tp* chi angles: 178.3,65.4	0.03Å	Favored (10.977%) beta sheet	-	-	-

					General / -150.2,132.7						
A 87		SER	0.76	-	Favored (46.76%) General / -123.9,148.6	Favored (61.4%) *m* chi angles: 298.6	0.04Å	Favored (58.72%) beta sheet	-	-	-
A 88		VAL	0.75	-	Favored (44.14%) Ile or Val / -93.5,129.3	Favored (74.6%) *t* chi angles: 178.3	0.04Å	Favored (56.661%) beta sheet	-	-	-
A 89		VAL	0.77	-	Favored (75.2%) Ile or Val / -118.2,128.4	Favored (76.5%) *t* chi angles: 178.2	0.05Å	Favored (72.158%) beta sheet	-	-	-
A 90		VAL	0.81	-	Favored (72.37%) Ile or Val / -117.2,123.9	Favored (61.9%) *t* chi angles: 179.7	0.05Å	Favored (69.63%)	-	-	-
A 91		GLU	0.87	-	Favored (48.72%) General / -121.3,144.3	Favored (38.2%) *mt-10* chi angles: 296.8,182,79.5	0.04Å	Favored (39.464%)	-	-	-
A 92		LYS	0.94	-	Favored (24.54%) Pre-Pro / -50.2,131.7	Favored (49.4%) *tttm* chi angles: 181.7,180.9,183.8,289.4	0.06Å	Favored (44.267%)	-	-	-
A 93		PRO	1	-	Favored (81.72%) Trans-Pro / -66.5,147.1	Favored (42.9%) *Cg_endo* chi angles: 24.1,327.1,28.3	0.05Å	Favored (68.738%)	-	-	-
A 94		LYS	1.03	-	Favored (27.94%) General / -131.5,123.4	Favored (96.3%) *mttt* chi angles: 296.9,184.9,180.1,183.2	0.03Å	Favored (12.003%)	-	-	-
A 95		GLY	1.04	-	Favored (54.09%) Glycine / 72.8,-160.5	-	-	Favored (50.541%)	-	-	-
A 96		MET	1.02	0.40Å HB2 with A 96 MET HE3	Favored (58.76%) General / -64.5,141.5	Favored (38.3%) *ttm* chi angles: 183.9,182,303.5	0.12Å	Favored (5.803%)	-	-	-
A 97		TYR	0.98	-	Favored (5.87%) General / -79.4,78.2	Favored (94.6%) *m-80* chi angles: 295,99.3	0.09Å	Favored (42.184%)	-	-	-
A 98		LYS	0.93	-	Favored (52.42%) General / -68.7,136.2	Favored (94.6%) *mttt* chi angles: 294.6,179.6,187.6,178.3	0.04Å	Favored (24.661%) beta sheet	-	-	-
A 99		SER	0.89	-	Favored (9.13%) General / -62.9,163.7	Favored (90%) *p* chi angles: 68.4	0.02Å	CaBLAM Disfavored (3.978%) try beta sheet	-	-	-
A 100		ALA	0.85	-	Favored (54.26%) Pre-Pro / -144.5,152.8	-	0.06Å	Favored (50.568%)	-	-	-
**#**	**Alt**	**Res**	**High B**	**Clash > 0.4Å**	**Ramachandran**	**Rotamer**	**Cβ deviation**	**CaBLAM**	**Bond lengths**	**Bond angles**	**Cis Peptides**
			Avg: 1.15	Clashscore: 1.45	Outliers: 0 of 350	Poor rotamers: 0 of 308	Outliers: 0 of 327	Outliers: 11 of 348	Outliers: 2 of 352	Outliers: 7 of 352	Non-Trans: 2 of 351
A 101		PRO	0.83	-	Favored (43.7%) Trans-Pro / -68.5,-18.3	Favored (49.3%) *Cg_endo* chi angles: 25.1,324.8,29.8	0.04Å	Favored (33.376%)	-	-	-

A 102		GLN	0.81	-	Favored (14.52%) General / -99.1,160.1	Favored (93.6%) *mm-40* chi angles: 300.8,296.8,301.2	0.04Å	Favored (13.01%)	-	-	-
A 103		ARG	0.82	-	Favored (49.61%) General / -137.1,152.1	Favored (92.9%) *mmt-90* chi angles: 297.2,291.3,183.6,275.5	0.06Å	Favored (58.234%)	-	-	-
A 104		LEU	0.85	-	Favored (12.26%) General / -86.2,170.3	Favored (70%) *mt* chi angles: 303.7,178.4	0.06Å	Favored (34.286%)	-	-	-
A 105		ALA	0.91	-	Favored (39.08%) General / -147.9,162.5	-	0.03Å	Favored (23.708%)	-	-	-
A 106		LEU	0.98	-	Favored (38.01%) General / -75.4,152.1	Favored (86.8%) *mt* chi angles: 294.7,170.1	0.03Å	Favored (32.061%)	-	-	-
A 107		THR	1.05	-	Favored (38.94%) General / -144.2,151.0	Favored (8.3%) *t* chi angles: 184.1	0.03Å	Favored (65.061%) beta sheet	-	-	-
A 108		SER	1.13	-	Favored (57.61%) General / -85.1,-9.8	Favored (89.3%) *p* chi angles: 66.8	0.03Å	Favored (16.477%)	-	-	-
A 109		GLU	1.21	-	Favored (35.26%) General / -81.7,131.3	Favored (48%) *mt-10* chi angles: 294.6,179.8,59	0.02Å	Favored (22.626%)	-	-	-
A 110		GLU	1.33	-	Favored (8.22%) General / -124.5,23.3	Favored (84.7%) *mt-10* chi angles: 297.9,189,354.2	0.02Å	CaBLAM Disfavored (4.973%)	-	-	-
A 111		PHE	1.51	-	Allowed (0.06%) General / 57.8,-178.7	Favored (25.5%) *m-80* chi angles: 284.3,125	0.07Å	CaBLAM Disfavored (1.387%)	-	-	-
A 112		GLU	1.8	-	Favored (5.64%) General / -111.7,-35.8	Favored (97.2%) *mt-10* chi angles: 296.2,180.4,356.3	0.01Å	CaBLAM Outlier (0.122%)	-	-	-
A 113		ILE	2.25	-	Favored (46.36%) Ile or Val / -99.7,131.8	Favored (46.7%) *mm* chi angles: 305.8,301.7	0.03Å	CaBLAM Disfavored (3.198%)	-	-	-
A 114		GLY	2.89	-	Favored (7.9%) Glycine / -146.6,-158.7	-	-	Favored (11.166%)	-	-	-
A 115		TRP	3.68	-	Allowed (1.02%) General / -48.4,-23.7	Favored (76.3%) *p-90* chi angles: 67,268.9	0.04Å	CaBLAM Disfavored (3.839%)	-	-	-
A 116		LYS	4.52	-	Favored (65.03%) General / -60.5,-24.1	Favored (97.8%) *mttt* chi angles: 291.1,179.2,180.4,178	0.02Å	Favored (29.409%)	-	-	-
A 117		ALA	5.29	-	Favored (11.67%) General / -114.7,24.6	-	0.04Å	Favored (31.641%)	-	-	-
A 118		TRP	5.87	-	Favored (39.9%) General / -52.4,-32.8	Favored (56.1%) *p-90* chi angles: 73,271.6	0.06Å	Favored (26.395%)	-	-	-
A 119		GLY	6.19	-	Favored (54.59%)	-	-	Favored (71.557%)	-	-	-

#	Alt	Res	High B	Clash > 0.4Å	Ramachandran	Rotamer	Cβ deviation	CaBLAM	Bond lengths	Bond angles	Cis Peptides
					Glycine / -59.3,-21.9						
A 120		LYS	6.2	-	Favored (67.66%) General / -61.8,-25.7	Favored (97.1%) *mttt* chi angles: 289.6,180.7,178.5,178.8	0.03Å	Favored (63.102%) three-ten	-	-	-
#	Alt	Res	High B	Clash > 0.4Å	Ramachandran	Rotamer	Cβ deviation	CaBLAM	Bond lengths	Bond angles	Cis Peptides
			Avg: 1.15	Clashscore: 1.45	Outliers: 0 of 350	Poor rotamers: 0 of 308	Outliers: 0 of 327	Outliers: 11 of 348	Outliers: 2 of 352	Outliers: 7 of 352	Non-Trans: 2 of 351
A 121		SER	5.84	-	Favored (61.85%) General / -72.1,-14.5	Favored (90%) *p* chi angles: 68.8	0.05Å	Favored (64.676%) three-ten	-	-	-
A 122		LEU	5.13	-	Favored (15.15%) General / -90.1,-36.0	Favored (84.8%) *mt* chi angles: 299.8,175.3	0.10Å	Favored (56.742%) alpha helix	-	-	-
A 123		VAL	4.18	-	Favored (79.27%) Ile or Val / -69.7,-44.6	Favored (91%) *t* chi angles: 174.3	0.05Å	Favored (45.033%) alpha helix	-	-	-
A 124		PHE	3.19	-	Favored (12.65%) General / -140.5,119.5	Favored (74.8%) *t80* chi angles: 175.7,70	0.07Å	Favored (24.965%)	-	-	-
A 125		ALA	2.33	-	Favored (35.31%) Pre-Pro / -87.1,135.2	-	0.02Å	Favored (41.766%)	-	-	-
A 126		PRO	1.69	-	Favored (52.74%) Trans-Pro / -72.8,156.5	Favored (73.6%) *Cg_endo* chi angles: 27.6,325.3,26.7	0.03Å	Favored (86.022%)	-	-	-
A 127		GLU	1.27	-	Favored (24.85%) General / -82.1,155.8	Favored (94.7%) *mt-10* chi angles: 296.6,180.7,345.9	0.03Å	Favored (35.504%)	-	-	-
A 128		LEU	1	-	Favored (37.09%) General / -79.3,141.3	Favored (95.1%) *mt* chi angles: 296.3,176.6	0.03Å	Favored (40.489%)	-	-	-
A 129		ALA	0.84	-	Favored (28.54%) General / -75.7,162.1	-	0.06Å	Favored (42.791%)	-	-	-
A 130		ASN	0.74	-	Favored (67.16%) General / -66.7,-26.6	Favored (97.5%) *m-40* chi angles: 287.8,336	0.03Å	Favored (62.477%)	-	-	-
A 131		HIS	0.69	-	Favored (47.5%) General / -104.1,123.6	Favored (97.2%) *m-70* chi angles: 300.4,283.7	0.07Å	Favored (23.945%)	-	-	-
A 132		THR	0.65	-	Favored (21.62%) General / -127.4,164.5	Favored (55.7%) *p* chi angles: 64.8	0.05Å	Favored (46.629%)	-	-	-
A 133		PHE	0.64	-	Favored (25.55%) General / -121.0,116.7	Favored (52.8%) *t80* chi angles: 182.4,95.2	0.04Å	Favored (50.409%) beta sheet	-	-	-
A 134		VAL	0.63	-	Favored (54.16%) Ile or Val / -103.5,130.3	Favored (63.6%) *t* chi angles: 179.5	0.03Å	Favored (61.148%)	-	-	-

A 135		VAL	0.65	-	Favored (55.03%) Ile or Val / -106.2,119.1	Favored (69.3%) *t* chi angles: 178.8	0.02Å	Favored (10.401%)	-	-	-
A 136		ASP	0.67	-	Favored (7.32%) General / 56.1,23.8	Favored (59.1%) *m-30* chi angles: 293.7,307.3	0.03Å	CaBLAM Disfavored (1.169%)	-	-	-
A 137		GLY	0.71	-	Favored (46.71%) Glycine / -88.3,-179.7	-	-	Favored (31.825%)	-	-	-
A 138		PRO	0.75	-	Favored (85.34%) Trans-Pro / -61.2,150.3	Favored (51.2%) *Cg_exo* chi angles: 337.7,34.2,328.2	0.07Å	Favored (36.254%)	-	-	-
A 139		GLU	0.78	-	Favored (47.49%) General / -64.0,131.4	Favored (46.5%) *tt0* chi angles: 184.2,177.3,60	0.03Å	Favored (27.584%)	-	-	-
A 140		THR	0.8	-	Favored (14.92%) General / -131.5,170.3	Favored (49.3%) *p* chi angles: 65.8	0.04Å	Favored (40.666%)	-	-	-
**#**	**Alt**	**Res**	**High B**	**Clash > 0.4Å**	**Ramachandran**	**Rotamer**	**Cβ deviation**	**CaBLAM**	**Bond lengths**	**Bond angles**	**Cis Peptides**
			Avg: 1.15	Clashscore: 1.45	Outliers: 0 of 350	Poor rotamers: 0 of 308	Outliers: 0 of 327	Outliers: 11 of 348	Outliers: 2 of 352	Outliers: 7 of 352	Non-Trans: 2 of 351
A 141		LYS	0.81	-	Favored (91.11%) General / -64.4,-38.5	Favored (97.2%) *mttt* chi angles: 289.6,179.4,180.1,178.4	0.01Å	Favored (59.939%)	-	-	-
A 142		GLU	0.8	-	Favored (97.21%) General / -64.1,-41.8	Favored (92.9%) *tt0* chi angles: 181.6,179,359.8	0.07Å	Favored (55.81%) alpha helix	-	-	-
A 143		CYS	0.78	-	Favored (54.3%) Pre-Pro / -134.3,79.8	Favored (42.3%) *t* chi angles: 186.6	0.01Å	Favored (22.141%)	-	-	-
A 144		PRO	0.76	-	Favored (46.81%) Trans-Pro / -73.8,158.2	Favored (76%) *Cg_endo* chi angles: 28.7,325.2,26.5	0.02Å	Favored (24.267%)	-	-	-
A 145		ASP	0.73	-	Favored (64.97%) General / -60.2,-24.7	Favored (85%) *m-30* chi angles: 282.8,346.7	0.07Å	Favored (45.47%)	-	-	-
A 146		ALA	0.7	-	Favored (68.87%) General / -60.6,-29.0	-	0.04Å	Favored (50.641%) alpha helix	-	-	-
A 147		LYS	0.68	-	Favored (11.76%) General / -105.6,-26.6	Favored (98.7%) *mttt* chi angles: 293.4,182,179.7,178.1	0.03Å	Favored (14.625%)	-	-	-
A 148		ARG	0.66	-	Favored (58.66%) General / -62.8,141.4	Favored (98.5%) *mtt-85* chi angles: 291.5,181,185.3,275.2	0.13Å	Favored (30.073%)	-	-	-
A 149		ALA	0.65	-	Favored (21.28%) General / -83.9,159.4	-	0.02Å	Favored (36.909%)	-	-	-
A 150		TRP	0.65	-	Favored (30%) General / -145.5,143.4	Favored (81.4%) *t60* chi angles: 174.8,88	0.04Å	Favored (9.597%)	-	-	-

A 151		ASN	0.65	-	Favored (26.29%) General / 56.4,43.4	Favored (55.9%) *t0* chi angles: 197.1,24.7	0.01Å	Favored (12.12%)	-	-	-
A 152		SER	0.66	-	Favored (57.91%) General / -89.3,-4.9	Favored (73.4%) *m* chi angles: 295.5	0.05Å	Favored (17.529%)	-	-	-
A 153		LEU	0.68	-	Favored (28.98%) General / -111.6,151.3	Favored (79%) *mt* chi angles: 302.4,178.2	0.04Å	Favored (19.906%)	-	-	-
A 154		GLU	0.71	-	Favored (39.81%) General / -142.9,160.7	Favored (25.2%) *pt0* chi angles: 66.1,180.7,9.5	0.05Å	Favored (41.32%)	-	-	-
A 155		ILE	0.78	-	Favored (39.77%) Ile or Val / -87.9,122.6	Favored (88.3%) *mt* chi angles: 298.1,168.4	0.07Å	Favored (30.739%)	-	-	-
A 156		GLU	0.88	-	Favored (30.14%) General / -79.8,-39.8	Favored (72.3%) *tp30* chi angles: 180.1,67.8,15	0.02Å	Favored (27.201%)	-	-	-
A 157		ASP	1.04	-	Favored (18.08%) General / -157.9,172.7	Favored (11.3%) *t0* chi angles: 209.6,339.5	0.04Å	Favored (14.312%)	-	-	-
A 158		PHE	1.25	-	Favored (19.72%) General / -152.4,140.0	Favored (85.4%) *t80* chi angles: 174.4,75.1	0.03Å	Favored (13.068%)	-	-	-
A 159		GLY	1.5	-	Favored (24.08%) Glycine / -160.9,-169.4	-	-	Favored (42.129%) beta sheet	-	-	-
A 160		PHE	1.77	-	Favored (41.62%) General / -154.3,161.7	Favored (50.6%) *p90* chi angles: 58.7,91.1	0.01Å	CA Geom Outlier (0.302%)	-	-	-
**#**	**Alt**	**Res**	**High B**	**Clash > 0.4Å**	**Ramachandran**	**Rotamer**	**Cβ deviation**	**CaBLAM**	**Bond lengths**	**Bond angles**	**Cis Peptides**
			Avg: 1.15	Clashscore: 1.45	Outliers: 0 of 350	Poor rotamers: 0 of 308	Outliers: 0 of 327	Outliers: 11 of 348	Outliers: 2 of 352	Outliers: 7 of 352	Non-Trans: 2 of 351
A 161		GLY	1.96	-	Favored (7.09%) Glycine / -114.5,-139.6	-	-	Favored (35.785%)	-	-	-
A 162		ILE	2.03	-	Favored (6.58%) Ile or Val / -103.0,-54.5	Favored (49.8%) *mm* chi angles: 302.1,300.7	0.01Å	CaBLAM Outlier (0.117%)	-	-	-
A 163		MET	1.96	-	Favored (54.87%) General / -92.4,-3.7	Favored (98.1%) *mmm* chi angles: 298.3,301.8,291.3	0.02Å	Favored (7.481%)	-	-	-
A 164		SER	1.77	-	Favored (29.25%) General / -160.6,166.3	Favored (89.5%) *p* chi angles: 68.3	0.04Å	Favored (21.631%)	-	-	-
A 165		THR	1.53	-	Favored (10.77%) General / -113.8,167.3	Favored (44.6%) *p* chi angles: 66.7	0.06Å	Favored (43.114%)	-	-	-
A 166		ARG	1.31	-	Favored (45.94%) General / -131.5,134.3	Favored (98.3%) *mtt180* chi angles: 293.9,177.6,176.5,185	0.03Å	Favored (50.149%) beta sheet	-	-	-

A 167		VAL	1.12	-	Favored (65.32%) Ile or Val / -121.6,134.3	Favored (84.5%) *t* chi angles: 173.6	0.10Å	Favored (62.883%) beta sheet	-	-	-
A 168		TRP	0.99	-	Favored (53.84%) General / -110.9,132.9	Favored (35.3%) *m-90* chi angles: 288.1,259.9	0.04Å	Favored (67.169%) beta sheet	-	-	-
A 169		LEU	0.91	-	Favored (28.42%) General / -109.6,150.3	Favored (74.4%) *mt* chi angles: 301,173.7	0.09Å	Favored (36.8%)	-	-	-
A 170		LYS	0.86	-	Favored (41.61%) General / -147.3,161.1	Favored (59.6%) *pttt* chi angles: 67.2,180.1,185.6,184	0.10Å	Favored (22.319%)	-	-	-
A 171		VAL	0.85	-	Favored (32.41%) Ile or Val / -66.8,130.6	Favored (83.5%) *t* chi angles: 173.5	0.12Å	Favored (25.545%)	-	-	-
A 172		ARG	0.85	-	Favored (88.77%) General / -61.5,-38.8	Favored (75.8%) *ttm-80* chi angles: 188.2,180.7,297.9,283.1	0.05Å	CaBLAM Disfavored (2.72%) try beta sheet	-	-	-
A 173		GLU	0.85	-	Allowed (0.68%) General / 72.5,-55.0	Favored (63.4%) *tp30* chi angles: 185.1,68.7,16.4	0.04Å	Favored (8.069%) beta sheet	-	-	-
A 174		HIS	0.86	-	Favored (37.74%) General / -76.4,148.0	Favored (95.1%) *m-70* chi angles: 293.6,289.2	0.02Å	Favored (24.009%) beta sheet	-	-	-
A 175		ASN	0.85	-	Favored (14.46%) General / -91.8,101.9	Favored (47.5%) *t0* chi angles: 182.8,328.9	0.04Å	Favored (15.53%) beta sheet	-	-	-
A 176		THR	0.84	-	Favored (20.1%) General / -131.8,167.1	Favored (30.4%) *p* chi angles: 69.3	0.06Å	Favored (26.767%) beta sheet	-	-	-
A 177		THR	0.82	-	Favored (12.73%) General / -118.8,11.7	Favored (66.2%) *p* chi angles: 58.4	0.01Å	Favored (13.289%) beta sheet	-	-	-
A 178		ASP	0.8	-	Favored (29.63%) General / -79.8,152.8	Favored (87.4%) *m-30* chi angles: 293.9,345.8	0.01Å	Favored (44.249%)	-	-	-
A 179		CYS	0.79	-	Favored (19.53%) General / -83.5,164.9	Favored (68.3%) *m* chi angles: 299.1	0.06Å	Favored (31.285%)	-	-	-
A 180		ASP	0.79	-	Favored (18.88%) General / -83.2,111.4	Favored (27.8%) *t0* chi angles: 187,319.6	0.07Å	Favored (20.018%)	-	-	-
**#**	**Alt**	**Res**	**High B**	**Clash > 0.4Å**	**Ramachandran**	**Rotamer**	**Cβ deviation**	**CaBLAM**	**Bond lengths**	**Bond angles**	**Cis Peptides**
			Avg: 1.15	Clashscore: 1.45	Outliers: 0 of 350	Poor rotamers: 0 of 308	Outliers: 0 of 327	Outliers: 11 of 348	Outliers: 2 of 352	Outliers: 7 of 352	Non-Trans: 2 of 351
A 181		SER	0.81	-	Favored (62.26%) General / -60.5,-21.4	Favored (97.4%) *p* chi angles: 65.8	0.05Å	Favored (32.52%)	-	-	-
A 182		SER	0.83	-	Favored (61.1%)	Favored (89%) *p* chi angles: 69.2	0.04Å	Favored (38.834%)	-	-	-

					General / -73.2,-13.1						
A 183		ILE	0.87	-	Favored (6.56%) Ile or Val / -119.0,19.2	Favored (37.3%) *pt* chi angles: 57.7,169.8	0.08Å	Favored (31.197%)	-	-	-
A 184		ILE	0.91	-	Favored (67.72%) Ile or Val / -116.5,131.9	Favored (74.8%) *mt* chi angles: 301.3,170.3	0.04Å	Favored (18.996%)	-	-	-
A 185		GLY	0.96	-	Favored (27.52%) Glycine / -143.9,158.8	-	-	Favored (54.121%)	-	-	-
A 186		THR	1.03	-	Favored (46.33%) General / -135.7,157.7	Favored (55.9%) *p* chi angles: 64.8	0.06Å	Favored (58.336%) beta sheet	-	-	-
A 187		ALA	1.11	-	Favored (21.86%) General / -161.7,155.6	-	0.03Å	Favored (41.888%) beta sheet	-	-	-
A 188		VAL	1.19	-	Favored (59.12%) Ile or Val / -127.8,122.5	Favored (58.6%) *t* chi angles: 180.1	0.06Å	Favored (27.706%)	-	-	-
A 189		LYS	1.25	-	Favored (16.98%) General / -143.3,127.0	Favored (88.9%) *mttt* chi angles: 301.1,182.4,186.8,177.7	0.10Å	Favored (13.692%)	-	-	-
A 190		GLY	1.26	-	Favored (84.25%) Glycine / 64.3,32.5	-	-	Favored (49.528%)	-	-	-
A 191		ASP	1.22	-	Favored (8.41%) General / 69.1,12.0	Favored (68.7%) *m-30* chi angles: 292.6,316	0.07Å	Favored (10.323%)	-	-	-
A 192		ILE	1.14	-	Favored (65.91%) Ile or Val / -111.2,129.8	Favored (81.6%) *mt* chi angles: 299.5,167.9	0.04Å	Favored (24.773%)	-	-	-
A 193		ALA	1.05	-	Favored (45.25%) General / -119.2,146.3	-	0.04Å	Favored (65.373%) beta sheet	-	-	-
A 194		VAL	0.96	-	Favored (24.98%) Ile or Val / -141.6,145.1	Favored (10.1%) *p* chi angles: 62.3	0.06Å	Favored (62.872%) beta sheet	-	-	-
A 195		HIS	0.9	-	Favored (51.81%) General / -131.2,145.1	Favored (26.9%) *m90* chi angles: 307,72.6	0.16Å	Favored (45.312%) beta sheet	-	OUTLIER(S) worst is CA-CB-CG: 4.8 σ	-
A 196		SER	0.87	-	Favored (28.77%) General / -161.9,164.3	Favored (68.3%) *p* chi angles: 72.2	0.10Å	Favored (24.518%)	-	-	-
A 197		ASP	0.84	-	Allowed (1.25%) General / -140.5,-161.8	Favored (39.5%) *p0* chi angles: 66.8,21.9	0.12Å	Favored (22.912%)	-	OUTLIER(S) worst is CA-CB-CG: 7.9 σ	-
A 198		LEU	0.83	-	Favored (58.25%) General / -88.0,-1.9	Favored (89.4%) *mt* chi angles: 299.1,176	0.08Å	Favored (10.524%)	-	-	-
A 199		SER	0.82	-	Favored (6.35%) General / -122.3,-19.8	Favored (97.5%) *p* chi angles: 63.5	0.07Å	Favored (47.087%) alpha helix	-	-	-

A 200		TYR	0.81	-	Favored (14.93%) General / -143.4,125.0	Favored (15.5%) *t80* chi angles: 180.4,44.1	0.04Å	Favored (27.85%)	-	-	-
**#**	**Alt**	**Res**	**High B**	**Clash > 0.4Å**	**Ramachandran**	**Rotamer**	**Cβ deviation**	**CaBLAM**	**Bond lengths**	**Bond angles**	**Cis Peptides**
			Avg: 1.15	Clashscore: 1.45	Outliers: 0 of 350	Poor rotamers: 0 of 308	Outliers: 0 of 327	Outliers: 11 of 348	Outliers: 2 of 352	Outliers: 7 of 352	Non-Trans: 2 of 351
A 201		TRP	0.8	-	Favored (55.23%) General / -118.3,129.1	Favored (26.4%) *t-100* chi angles: 173.3,276.6	0.06Å	Favored (61.757%)	-	-	-
A 202		ILE	0.81	-	Favored (60.41%) Ile or Val / -129.8,135.8	Favored (17.5%) *tt* chi angles: 182,168.4	0.05Å	Favored (64.936%) beta sheet	-	-	-
A 203		GLU	0.83	-	Favored (40.12%) General / -135.9,136.0	Favored (89.9%) *tt0* chi angles: 182.9,174.5,355.1	0.06Å	Favored (55.756%) beta sheet	-	-	-
A 204		SER	0.88	-	Favored (15.99%) General / -134.4,170.3	Favored (94.2%) *p* chi angles: 64.2	0.10Å	Favored (38.211%) beta sheet	-	-	-
A 205		HIS	0.93	-	Favored (28.54%) General / -147.3,144.1	Favored (55.8%) *t-90* chi angles: 179.3,275.9	0.04Å	Favored (30.088%)	-	-	-
A 206		LYS	0.98	-	Favored (32.3%) General / -86.2,124.2	Favored (87.1%) *tttt* chi angles: 185,178.3,178.1,181.7	0.03Å	Favored (10.861%)	-	-	-
A 207		ASN	1	-	Allowed (0.3%) General / -125.8,-143.6	Favored (65.5%) *m-40* chi angles: 286.5,289.2	0.06Å	CA Geom Outlier (0.454%)	-	-	-
A 208		THR	0.99	-	Favored (61.91%) General / -67.3,-13.3	Favored (73.2%) *p* chi angles: 61.7	0.03Å	CA Geom Outlier (0.065%)	-	-	-
A 209		THR	0.95	-	Favored (42.42%) General / -147.8,156.1	Favored (11.4%) *t* chi angles: 186.9	0.05Å	Favored (23.088%)	-	-	-
A 210		TRP	0.89	-	Favored (37.75%) General / -78.0,134.7	Favored (90.3%) *m100* chi angles: 284.8,97.1	0.04Å	Favored (29.84%)	-	-	-
A 211		ARG	0.83	-	Favored (51.06%) General / -130.8,143.9	Favored (98.4%) *mtt180* chi angles: 294.6,179,175.3,172	0.10Å	Favored (49.455%) beta sheet	-	-	-
A 212		LEU	0.78	-	Favored (35.5%) General / -65.2,128.4	Favored (74.5%) *tp* chi angles: 177.9,62.3	0.01Å	Favored (43.312%)	-	-	-
A 213		GLU	0.75	-	Favored (5.93%) General / -103.6,-41.5	Favored (63.9%) *mm-30* chi angles: 293.7,287,329.3	0.02Å	Favored (19.533%)	-	-	-
A 214		ARG	0.73	-	Favored (23.12%) General / -153.8,145.2	Favored (54.6%) *ttt90* chi angles: 181.5,173.5,169.4,91.6	0.05Å	Favored (26.637%)	-	-	-
A 215		ALA	0.73	-	Favored (25.58%) General / -143.4,134.5	-	0.04Å	Favored (61.057%)	-	-	-

A 216		VAL	0.75	-	Favored (66.13%) Ile or Val / -124.5,123.4	Favored (82.1%) *t* chi angles: 177.8	0.06Å	Favored (71.068%) beta sheet	-	-	-
A 217		PHE	0.76	-	Favored (30.34%) General / -112.5,116.5	Favored (83.6%) *m-80* chi angles: 297.4,83.3	0.12Å	Favored (69.197%)	-	-	-
A 218		GLY	0.78	-	Favored (76.04%) Glycine / -68.7,-33.5	-	-	Favored (12.372%)	-	-	-
A 219		GLU	0.79	-	Favored (40.15%) General / -155.5,159.9	Favored (23.2%) *pt0* chi angles: 58.8,185.4,356.5	0.04Å	Favored (16.974%)	OUTLIER(S) worst is CG--CD: 6.3 σ	-	-
A 220		ILE	0.8	-	Favored (52.62%) Ile or Val / -107.8,133.4	Favored (38.4%) *mm* chi angles: 308.1,299.5	0.02Å	Favored (43.629%)	-	-	-
**#**	**Alt**	**Res**	**High B**	**Clash > 0.4Å**	**Ramachandran**	**Rotamer**	**Cβ deviation**	**CaBLAM**	**Bond lengths**	**Bond angles**	**Cis Peptides**
			Avg: 1.15	Clashscore: 1.45	Outliers: 0 of 350	Poor rotamers: 0 of 308	Outliers: 0 of 327	Outliers: 11 of 348	Outliers: 2 of 352	Outliers: 7 of 352	Non-Trans: 2 of 351
A 221		LYS	0.79	-	Favored (50.65%) General / -131.6,152.6	Favored (52.6%) *pttt* chi angles: 60.4,191.4,180.1,184.1	0.11Å	Favored (55.665%) beta sheet	-	-	-
A 222		SER	0.77	-	Favored (20.18%) General / -92.5,13.3	Favored (97.1%) *p* chi angles: 63.5	0.10Å	Favored (6.209%) beta sheet	-	-	-
A 223		CYS	0.75	-	Favored (15.85%) General / -85.7,166.8	Favored (28.9%) *p* chi angles: 67	0.07Å	Favored (40.367%) beta sheet	-	-	-
A 224		THR	0.74	-	Favored (55.3%) General / -114.1,133.8	Favored (64.8%) *m* chi angles: 295.8	0.06Å	Favored (34.292%) beta sheet	-	-	-
A 225		TRP	0.74	-	Favored (93.32%) Pre-Pro / -67.3,133.8	Favored (21.9%) *m-10* chi angles: 279.8,0.2	0.04Å	Favored (41.931%) beta sheet	-	-	-
A 226		PRO	0.74	-	Favored (87.53%) Trans-Pro / -59.2,148.1	Favored (64%) *Cg_exo* chi angles: 335.7,34,331	0.04Å	Favored (92.227%)	-	-	-
A 227		GLU	0.74	-	Favored (65.92%) General / -57.9,-30.6	Favored (89.9%) *tt0* chi angles: 186.6,175.5,1.5	0.05Å	Favored (51.721%)	-	-	-
A 228		THR	0.74	-	Favored (58.44%) General / -67.3,-11.8	Favored (79.4%) *p* chi angles: 60.7	0.05Å	Favored (53.552%) three-ten	-	-	-
A 229		HIS	0.75	-	Favored (13.53%) General / -108.5,24.1	Favored (91.6%) *m-70* chi angles: 291.5,284.7	0.07Å	Favored (17.789%)	-	-	-
A 230		THR	0.76	-	Favored (37.82%) General / -112.0,146.5	Favored (61.3%) *p* chi angles: 63.8	0.05Å	Favored (35.521%)	-	-	-
A 231		LEU	0.76	-	Favored (8.27%)	Favored (91.2%) *mt* chi angles: 291.6,170.2	0.09Å	Favored (20.354%)	-	-	-

					General / -81.2,-51.6						
A 232		TRP	0.76	0.54Å C with A 232 TRP CD1	Favored (4.3%) General / -125.7,95.0	Allowed (1.9%) *t60* chi angles: 184.8,318.4	0.01Å	CaBLAM Disfavored (4.597%)	-	-	-
A 233		SER	0.75	-	Favored (27.72%) General / -99.1,-6.8	Favored (94.4%) *p* chi angles: 64.8	0.02Å	CaBLAM Disfavored (4.795%)	-	-	-
A 234		ASP	0.74	-	Favored (4.2%) General / -75.0,100.6	Favored (64.1%) *t0* chi angles: 184,357.7	0.04Å	CaBLAM Disfavored (3.087%)	-	-	-
A 235		GLY	0.73	-	Favored (55.45%) Glycine / 96.4,10.8	-	-	Favored (36.754%)	-	-	-
A 236		VAL	0.72	-	Favored (32.39%) Ile or Val / -78.2,132.5	Favored (95.8%) *t* chi angles: 174.9	0.04Å	Favored (21.661%)	-	-	-
A 237		VAL	0.72	-	Favored (67.16%) Ile or Val / -113.7,130.8	Favored (85.6%) *t* chi angles: 177.2	0.05Å	Favored (59.688%)	-	-	-
A 238		GLU	0.72	-	Favored (59.86%) General / -51.5,-40.5	Favored (65.7%) *tp30* chi angles: 181.1,69,23.2	0.05Å	Favored (53.744%)	-	-	-
A 239		SER	0.73	-	Favored (64.43%) General / -63.3,-19.3	Favored (99.8%) *p* chi angles: 65.5	0.03Å	Favored (63.025%) alpha helix	-	-	-
A 240		ASP	0.74	0.46Å OD1 with A 261 LYS NZ	Favored (49.66%) General / -97.8,2.7	Favored (74.1%) *m-30* chi angles: 295.1,320.3	0.01Å	Favored (56.583%)	-	-	-
**#**	**Alt**	**Res**	**High B**	**Clash > 0.4Å**	**Ramachandran**	**Rotamer**	**Cβ deviation**	**CaBLAM**	**Bond lengths**	**Bond angles**	**Cis Peptides**
			Avg: 1.15	Clashscore: 1.45	Outliers: 0 of 350	Poor rotamers: 0 of 308	Outliers: 0 of 327	Outliers: 11 of 348	Outliers: 2 of 352	Outliers: 7 of 352	Non-Trans: 2 of 351
A 241		LEU	0.75	-	Favored (32.83%) General / -90.4,121.9	Favored (94.4%) *mt* chi angles: 295.9,176.6	0.08Å	Favored (34.955%)	-	-	-
A 242		VAL	0.76	-	Favored (72.68%) Ile or Val / -60.7,-51.5	Favored (62.8%) *t* chi angles: 171.2	0.05Å	Favored (40.844%)	-	-	-
A 243		VAL	0.77	-	Favored (67.92%) Pre-Pro / -89.7,122.6	Favored (89.2%) *t* chi angles: 176.1	0.09Å	Favored (23.518%)	-	-	-
A 244		PRO	0.78	-	Favored (99.24%) Trans-Pro / -59.1,144.8	Favored (58.8%) *Cg_exo* chi angles: 336,33.5,331.7	0.09Å	Favored (77.822%)	-	-	-
A 245		VAL	0.79	-	Favored (63.68%) Ile or Val / -62.7,-34.1	Favored (65.3%) *t* chi angles: 171.5	0.12Å	Favored (54.378%)	-	-	-
A 246		THR	0.8	-	Favored (57.33%) General / -67.4,-11.3	Favored (70.9%) *p* chi angles: 59.5	0.01Å	Favored (53.096%)	-	-	-
A 247		LEU	0.79	-	Favored (7.97%) General / -112.4,29.4	Favored (80.4%) *mt* chi angles: 299.6,180.3	0.06Å	Favored (24.569%)	-	-	-

A 248		ALA	0.78	-	Favored (6.52%) General / 68.2,28.3	-	0.02Å	Favored (6.256%)	-	-	-
A 249		GLY	0.76	-	Favored (31.8%) Glycine / -82.7,153.7	-	-	Favored (27.551%)	-	-	-
A 250		PRO	0.74	-	Favored (43.13%) Trans-Pro / -73.5,148.7	Favored (73.4%) *Cg_endo* chi angles: 27.6,326.1,25.9	0.04Å	Favored (52.346%)	-	-	-
A 251		LYS	0.72	-	Favored (7.38%) General / -85.0,86.4	Favored (87.6%) *tttt* chi angles: 184.9,177.3,181,181.1	0.02Å	Favored (10.722%)	-	-	-
A 252		SER	0.71	-	Favored (36.13%) General / -149.0,163.9	Favored (77.3%) *p* chi angles: 70.7	0.19Å	Favored (26.847%)	-	-	-
A 253		ASN	0.7	-	Favored (61.67%) General / -68.8,-12.6	Favored (74.6%) *m-40* chi angles: 280.9,342.6	0.14Å	Favored (11.029%)	-	OUTLIER(S) worst is CA-C-O: 4.9 σ	-
A 254		HIS	0.71	-	Favored (37.47%) General / -90.1,-12.7	Favored (77.2%) *m-70* chi angles: 291.2,267.6	0.10Å	Favored (22.754%) three-ten	-	-	-
A 255		ASN	0.72	-	Favored (41.35%) General / -100.1,10.4	Favored (38%) *t0* chi angles: 180.7,22.7	0.08Å	Favored (43.728%)	-	OUTLIER(S) worst is CA-CB-CG: 4.2 σ	-
A 256		ARG	0.74	-	Favored (17.21%) General / -99.8,156.3	Favored (84.8%) *mtp180* chi angles: 298.6,172.8,67.5,185	0.05Å	Favored (23.894%)	-	-	-
A 257		ARG	0.76	0.41Å HG2 with A 277 PHE CE2	Favored (12.87%) General / -145.2,124.3	Favored (18.8%) *tpt90* chi angles: 182.2,74.7,161.9,84.1	0.04Å	Favored (5.671%)	-	-	-
A 258		GLU	0.78	-	Favored (24.26%) General / -54.8,126.5	Favored (84.9%) *tt0* chi angles: 182.5,174.4,349.1	0.07Å	Favored (24.051%)	-	-	-
A 259		GLY	0.79	-	Favored (80.94%) Glycine / 91.3,-5.4	-	-	Favored (75.067%)	-	-	-
A 260		TYR	0.8	-	Favored (38.28%) General / -119.4,151.6	Favored (62.8%) *m-80* chi angles: 301.6,82.7	0.07Å	Favored (36.478%)	-	-	-

#	Alt	Res	High B	Clash > 0.4Å	Ramachandran	Rotamer	Cβ deviation	CaBLAM	Bond lengths	Bond angles	Cis Peptides
			Avg: 1.15	Clashscore: 1.45	Outliers: 0 of 350	Poor rotamers: 0 of 308	Outliers: 0 of 327	Outliers: 11 of 348	Outliers: 2 of 352	Outliers: 7 of 352	Non-Trans: 2 of 351
A 261		LYS	0.8	0.46Å NZ with A 240 ASP OD1	Favored (7.49%) General / -112.9,171.5	Favored (73.5%) *mmtt* chi angles: 300.2,295.7,183.2,184.2	0.05Å	Favored (5.808%)	-	-	-
A 262		VAL	0.79	-	Favored (17.54%) Ile or Val / -55.5,130.2	Favored (9.6%) *p* chi angles: 62.9	0.06Å	Favored (14.031%)	-	-	-
A 263		GLN	0.78	-	Favored (8.32%) General / -86.0,64.0	Favored (71.4%) *mm-40* chi angles: 301.7,291.1,294.1	0.07Å	Favored (16.383%)	-	-	-

A 264		SER	0.78	-	Favored (64.93%) General / -66.5,-17.7	Favored (88.3%) *p* chi angles: 68.2	0.03Å	Favored (14.681%)	-	-	-
A 265		GLN	0.79	-	Favored (5.12%) General / -121.1,34.7	Favored (81.4%) *mt0* chi angles: 300.7,181.4,311.5	0.02Å	Favored (22.054%)	-	-	-
A 266		GLY	0.81	-	Favored (45.99%) Glycine / -75.2,179.1	-	-	Favored (46.981%)	-	-	-
A 267		PRO	0.84	-	Favored (5.08%) Trans-Pro / -80.0,54.1	Favored (49.5%) *Cg_endo* chi angles: 33.1,323.4,24.7	0.10Å	CaBLAM Disfavored (1.809%)	-	-	-
A 268		TRP	0.89	-	Favored (63.11%) General / -70.1,-24.1	Favored (60.4%) *m100* chi angles: 297.9,118.3	0.12Å	Favored (24.744%)	-	-	-
A 269		ASP	0.92	-	Favored (10.9%) General / -84.8,10.6	Favored (30.9%) *m-30* chi angles: 283.1,319.3	0.11Å	Favored (25.642%)	-	-	-
A 270		GLU	0.94	-	Favored (57.42%) General / -92.5,1.1	Favored (93.3%) *mt-10* chi angles: 293.2,182.6,340.7	0.10Å	CaBLAM Disfavored (4.452%)	-	-	-
A 271		GLU	0.93	-	Allowed (0.19%) General / 66.0,-65.3	Favored (80.1%) *mt-10* chi angles: 297.8,171.3,14.3	0.10Å	CaBLAM Disfavored (2.279%)	-	-	-
A 272		ASP	0.9	0.50Å OD2 with A 326 LYS NZ	Favored (10.26%) General / -88.4,95.6	Favored (64.4%) *t0* chi angles: 183.4,343.9	0.04Å	Favored (14.994%)	-	OUTLIER(S) worst is CA-CB-CG: 4.5 σ	-
A 273		ILE	0.85	-	Favored (62.74%) Ile or Val / -129.7,126.4	Favored (69.6%) *mt* chi angles: 302.7,171.3	0.06Å	Favored (34.636%)	-	-	-
A 274		VAL	0.8	-	Favored (73.28%) Ile or Val / -115.6,126.9	Favored (85.3%) *t* chi angles: 177.6	0.03Å	Favored (69.829%) beta sheet	-	-	-
A 275		LEU	0.75	0.42Å N with A 275 LEU HD12	Favored (46.79%) General / -100.5,127.5	Favored (6.5%) *mp* chi angles: 282.8,63.1	0.12Å	Favored (56.091%) beta sheet	-	-	-
A 276		ASP	0.72	-	Favored (32.62%) General / -138.9,133.9	Favored (25.8%) *m-30* chi angles: 291.2,287.2	0.07Å	Favored (47.176%)	-	OUTLIER(S) worst is CA-CB-CG: 4.2 σ	-
A 277		PHE	0.69	0.41Å CE2 with A 257 ARG HG2	Favored (7.83%) General / -87.5,81.3	Favored (79.8%) *m-80* chi angles: 289.1,95.9	0.12Å	CaBLAM Disfavored (4.654%)	-	-	-
A 278		ASP	0.67	-	Favored (11.12%) General / -157.8,177.7	Favored (11.2%) *t0* chi angles: 205.9,336.2	0.07Å	Favored (9.778%)	-	-	-
A 279		TYR	0.65	-	Favored (24.24%) General / -94.6,146.3	Favored (82.9%) *m-80* chi angles: 294.4,82.2	0.08Å	Favored (15.818%)	-	-	-
A 280		CYS	0.64	-	Favored (88.17%) Pre-Pro / -69.6,137.4	Favored (93.7%) *m* chi angles: 293	0.05Å	Favored (25.271%)	-	-	-

#	Alt	Res	High B	Clash > 0.4Å	Ramachandran	Rotamer	Cβ deviation	CaBLAM	Bond lengths	Bond angles	Cis Peptides
			Avg: 1.15	Clashscore: 1.45	Outliers: 0 of 350	Poor rotamers: 0 of 308	Outliers: 0 of 327	Outliers: 11 of 348	Outliers: 2 of 352	Outliers: 7 of 352	Non-Trans: 2 of 351
A 281		PRO	0.64	-	Favored (57.61%) Trans-Pro / -52.9,135.4	Favored (87.9%) *Cg_exo* chi angles: 330.7,37,331.3	0.08Å	Favored (42.276%)	-	-	-
A 282		GLY	0.64	-	Favored (86.32%) Glycine / 82.4,-1.9	-	-	Favored (74.738%)	-	-	-
A 283		THR	0.64	-	Favored (16.6%) General / -120.4,164.3	Favored (42.7%) *p* chi angles: 67.2	0.06Å	Favored (33.325%)	-	-	-
A 284		THR	0.66	-	Favored (49.23%) General / -130.9,137.3	Favored (98%) *m* chi angles: 300.1	0.03Å	Favored (63.61%) beta sheet	-	-	-
A 285		VAL	0.68	-	Favored (73.85%) Ile or Val / -116.5,128.2	Favored (64%) *t* chi angles: 179.5	0.04Å	Favored (69.574%) beta sheet	-	-	-
A 286		THR	0.71	-	Favored (42.98%) General / -108.3,139.4	Favored (93.6%) *m* chi angles: 297.5	0.03Å	Favored (49.815%) beta sheet	-	-	-
A 287		ILE	0.74	-	Favored (34.32%) Ile or Val / -93.2,116.5	Favored (76.5%) *mt* chi angles: 301,170.6	0.10Å	Favored (24.964%) beta sheet	-	-	-
A 288		THR	0.79	-	Favored (18.15%) General / -148.4,133.4	Favored (90.1%) *m* chi angles: 298.8	0.21Å	Favored (16.93%)	-	-	-
A 289		GLU	0.83	-	Favored (71%) General / -63.0,-29.7	Favored (99.6%) *mt-10* chi angles: 291.6,179.1,352.9	0.02Å	Favored (49.23%)	-	-	-
A 290		ALA	0.87	-	Favored (35.72%) General / -79.8,-0.7	-	0.06Å	Favored (31.976%)	-	-	-
A 291		CYS	0.88	-	Favored (13%) General / -80.5,173.0	Favored (74.5%) *m* chi angles: 297.1	0.04Å	Favored (6.816%)	-	-	-
A 292		GLY	0.88	-	Favored (43.18%) Glycine / -61.0,150.5	-	-	Favored (19.06%)	-	-	-
A 293		LYS	0.86	-	Favored (44.69%) General / -68.9,152.9	Favored (98.3%) *mttt* chi angles: 292.7,181.8,179.9,179.2	0.04Å	Favored (25.44%)	-	-	-
A 294		ARG	0.82	-	Favored (41.68%) General / -54.6,138.1	Favored (71.6%) *ttt-90* chi angles: 185,177.4,180.5,271.1	0.03Å	CaBLAM Disfavored (2.202%)	-	-	-
A 295		GLY	0.77	-	Favored (28.38%) Glycine / -171.0,-165.5	-	-	Favored (8.483%)	-	-	-
A 296		PRO	0.72	-	Favored (61.66%) Trans-Pro / -54.3,144.2	Favored (90.9%) *Cg_exo* chi angles: 333.2,36.1,330.2	0.07Å	CaBLAM Disfavored (4.471%)	-	-	-
A 297		SER	0.68	-	Favored (44.14%)	Favored (30.1%) *t* chi angles: 172.4	0.05Å	Favored (27.256%)	-	-	-

#	Alt	Res	High B	Clash > 0.4Å	Ramachandran	Rotamer	Cβ deviation	CaBLAM	Bond lengths	Bond angles	Cis Peptides
					General / -57.1,142.0						
A 298		ILE	0.66	-	Favored (75.04%) Ile or Val / -119.8,129.7	Favored (76.6%) *mt* chi angles: 300.8,169.5	0.02Å	Favored (48.262%) beta sheet	-	-	-
A 299		ARG	0.66	-	Favored (54.54%) General / -66.0,146.6	Favored (34.6%) *ttp-170* chi angles: 199.8,169.7,71,178.7	0.04Å	Favored (38.204%)	-	-	-
A 300		THR	0.68	-	Favored (63.43%) General / -68.9,-15.2	Favored (55.1%) *p* chi angles: 56.8	0.11Å	Favored (47.509%)	-	-	-
**#**	**Alt**	**Res**	**High B**	**Clash > 0.4Å**	**Ramachandran**	**Rotamer**	**Cβ deviation**	**CaBLAM**	**Bond lengths**	**Bond angles**	**Cis Peptides**
			Avg: 1.15	Clashscore: 1.45	Outliers: 0 of 350	Poor rotamers: 0 of 308	Outliers: 0 of 327	Outliers: 11 of 348	Outliers: 2 of 352	Outliers: 7 of 352	Non-Trans: 2 of 351
A 301		THR	0.72	-	Favored (42.38%) General / -111.8,143.6	Favored (60.2%) *p* chi angles: 57.4	0.05Å	Favored (24.685%)	-	-	-
A 302		THR	0.77	-	Favored (9.13%) General / -77.5,176.2	Favored (48.5%) *p* chi angles: 65.9	0.10Å	Favored (24.437%)	-	-	-
A 303		SER	0.81	-	Favored (52.47%) General / -61.2,-17.3	Favored (89.5%) *p* chi angles: 68.3	0.02Å	Favored (39.898%)	-	-	-
A 304		SER	0.83	-	Favored (54.04%) General / -88.1,-9.2	Favored (90%) *p* chi angles: 68.4	0.08Å	Favored (36.028%)	-	-	-
A 305		GLY	0.82	-	Favored (71.38%) Glycine / 92.7,-11.8	-	-	Favored (68.657%)	-	-	-
A 306		ARG	0.79	-	Favored (54.64%) General / -69.1,142.0	Favored (87.1%) *mtm180* chi angles: 290.9,178,290.8,172.2	0.04Å	Favored (36.589%)	-	-	-
A 307		LEU	0.74	-	Favored (34.52%) General / -78.4,129.1	Favored (37.6%) *tp* chi angles: 183.7,67	0.04Å	Favored (44.474%) beta sheet	-	-	-
A 308		VAL	0.7	-	Favored (40.01%) Ile or Val / -88.5,122.5	Favored (75.4%) *t* chi angles: 178.3	0.15Å	Favored (51.839%) beta sheet	-	-	-
A 309		THR	0.66	-	Favored (10.75%) General / -113.6,-21.5	Favored (74%) *p* chi angles: 61.5	0.06Å	Favored (10.238%)	-	-	-
A 310		ASP	0.65	-	Favored (7.55%) General / -95.6,94.1	Favored (64.4%) *t0* chi angles: 184.6,343.3	0.03Å	Favored (19.129%)	-	-	-
A 311		TRP	0.66	-	Favored (29.2%) General / -112.6,152.2	Favored (76.7%) *m100* chi angles: 293,78.7	0.06Å	Favored (24.17%)	-	-	-
A 312		CYS	0.68	-	Favored (30.28%) General / -140.1,133.7	Favored (49.4%) *t* chi angles: 179.7	0.06Å	Favored (66.515%) beta sheet	-	-	-
A 313		CYS	0.71	-	Favored (50.4%)	Favored (49.2%) *t* chi angles: 184.7	0.03Å	Favored (50.673%)	-	-	-

					General /<br>-104.9,133.0						
A<br>314		ARG	0.75	-	Favored<br>(9.59%)<br>General /<br>-88.0,93.8	Favored (26.2%)<br>*mtp-110*<br>chi angles:<br>295.1,190.7,73,250.6	0.12Å	CaBLAM<br>Disfavored<br>(2.987%)	-	-	-
A<br>315		SER	0.79	-	Favored<br>(21.34%)<br>General / 60.1,34.5	Favored (52.7%) *m*<br>chi angles: 300.6	0.03Å	CaBLAM<br>Disfavored<br>(1.835%)	-	-	-
A<br>316		CYS	0.8	-	Favored<br>(13.45%)<br>General /<br>-94.5,163.3	Favored (29.4%) *p*<br>chi angles: 65.6	0.06Å	Favored<br>(30.641%)	-	-	-
A<br>317		THR	0.8	-	Favored<br>(32.12%)<br>General /<br>-91.0,120.6	Favored (91.1%) *m*<br>chi angles: 298	0.05Å	Favored<br>(10.602%)	-	-	-
A<br>318		LEU	0.77	-	Favored<br>(65.01%)<br>Pre-Pro /<br>-82.3,159.3	Favored (81.6%) *mt*<br>chi angles: 298.3,180.6	0.06Å	CA Geom<br>Outlier<br>(0.021%)	-	-	-
A<br>319		PRO	0.74	-	Favored<br>(29.2%)<br>Cis-Pro /<br>-57.5,147.1	Favored (57%)<br>*Cg_exo*<br>chi angles:<br>336.1,35.8,327.7	0.02Å	Favored<br>(18.151%)	-	-	Cis PRO<br>omega=<br>1.79
A<br>320		PRO	0.7	-	Favored<br>(46.98%)<br>Trans-Pro /<br>-73.4,159.3	Favored (73.7%)<br>*Cg_endo*<br>chi angles:<br>29.8,324.9,25.2	0.07Å	Favored<br>(57.789%)	-	-	-
**#**	**Alt**	**Res**	**High B**	**Clash > 0.4Å**	**Ramachandran**	**Rotamer**	**Cβ deviation**	**CaBLAM**	**Bond lengths**	**Bond angles**	**Cis Peptides**
			Avg:<br>1.15	Clashscore:<br>1.45	Outliers: 0 of<br>350	Poor rotamers: 0 of<br>308	Outliers:<br>0 of 327	Outliers:<br>11 of 348	Outliers: 2 of<br>352	Outliers: 7 of<br>352	Non-<br>Trans: 2<br>of 351
A<br>321		LEU	0.68	-	Favored<br>(31.97%)<br>General /<br>-81.1,126.9	Favored (56.8%) *tp*<br>chi angles: 175.5,64.7	0.11Å	Favored<br>(26.642%)	-	-	-
A<br>322		ARG	0.68	-	Favored<br>(35.63%)<br>General /<br>-130.4,159.9	Favored (57.8%)<br>*ptt90*<br>chi angles:<br>63.7,177.3,175.7,86.2	0.04Å	Favored<br>(49.124%)<br>beta sheet	-	-	-
A<br>323		TYR	0.69	-	Favored<br>(53.47%)<br>General /<br>-124.0,139.9	Favored (46.1%) *m-80*<br>chi angles: 306,85.8	0.14Å	Favored<br>(63.079%)<br>beta sheet	-	-	-
A<br>324		ARG	0.71	-	Favored<br>(46.09%)<br>General /<br>-113.0,121.9	Favored (82.2%)<br>*ttt180*<br>chi angles:<br>181.7,178.6,179.4,181.9	0.02Å	Favored<br>(45.289%)<br>beta sheet	-	-	-
A<br>325		THR	0.72	-	Favored (5.7%)<br>General /<br>-130.7,179.9	Favored (15.3%) *p*<br>chi angles: 74.6	0.05Å	Favored<br>(20.024%)	-	-	-
A<br>326		LYS	0.73	0.50Å<br>NZ with A<br>272 ASP<br>OD2	Favored<br>(61.72%)<br>General /<br>-67.7,-13.0	Favored (27.1%)<br>*mmtm*<br>chi angles:<br>290.2,295.2,191.3,291.5	0.06Å	Favored<br>(37.833%)	-	-	-
A<br>327		ASN	0.72	-	Favored<br>(40.41%)<br>General /<br>-100.5,11.0	Favored (87.9%) *m-40*<br>chi angles: 290.6,323.5	0.05Å	Favored<br>(35.804%)	-	-	-
A<br>328		GLY	0.69	-	Favored<br>(15.94%)<br>Glycine /<br>112.0,-173.9	-	-	Favored<br>(30.546%)	-	-	-
A<br>329		CYS	0.67	-	Favored<br>(33.21%)	Favored (29.9%) *t*<br>chi angles: 189.6	0.03Å	Favored<br>(8.51%)	-	-	-

					General / -91.6,136.3							
A 330		TRP	0.65	-	Favored (33.36%) General / -118.4,153.9	Favored (44.9%) *m100* chi angles: 293,68.1	0.11Å	Favored (52.651%)	-	-	-
A 331		TYR	0.63	-	Favored (26%) General / -105.5,149.7	Favored (38.7%) *m-80* chi angles: 280.1,279	0.10Å	Favored (26.016%)	-	-	-
A 332		GLY	0.63	-	Favored (7.13%) Glycine / -58.4,166.1	-	-	Favored (34.338%)	-	-	-
A 333		MET	0.63	-	Favored (67.81%) General / -61.7,-26.0	Favored (92.3%) *mtp* chi angles: 291.5,182.9,69.3	0.01Å	Favored (43.738%)	-	-	-
A 334		GLU	0.64	-	Favored (59.38%) General / -80.8,-7.2	Favored (60.4%) *mp0* chi angles: 291.9,76,11	0.01Å	Favored (60.108%)	-	-	-
A 335		ILE	0.66	-	Favored (73.53%) Ile or Val / -117.3,124.8	Favored (3.5%) *mp* chi angles: 307,97.2	0.04Å	Favored (25.334%)	-	-	-
A 336		ARG	0.7	-	Favored (55.31%) Pre-Pro / -123.7,148.1	Favored (63.5%) *mmm-85* chi angles: 299.5,292.8,301.8,272.6	0.11Å	Favored (42.976%)	-	-	-
A 337		PRO	0.77	-	Favored (98.91%) Trans-Pro / -60.0,145.2	Favored (55%) *Cg_exo* chi angles: 337.4,36.1,325.7	0.04Å	Favored (62.779%)	-	-	-
A 338		MET	0.85	-	Favored (33.36%) General / -100.3,-2.5	Favored (97%) *mmm* chi angles: 297.9,301.6,289.7	0.09Å	Favored (30.266%)	-	-	-
A 339		LYS	0.93	-	Favored (24.92%) General / -136.8,165.9	Favored (46.8%) *mttp* chi angles: 296,177.7,170.1,56.1	0.00Å	CA Geom Outlier (0.158%)	-	-	-
A 340		HIS	1	-	Allowed (0.18%) General / 69.0,81.0	Favored (99.9%) *m-70* chi angles: 297.7,287.9	0.10Å	CaBLAM Outlier (0.078%)	-	-	-
**#**	**Alt**	**Res**	**High B**	**Clash > 0.4Å**	**Ramachandran**	**Rotamer**	**Cβ deviation**	**CaBLAM**	**Bond lengths**	**Bond angles**	**Cis Peptides**
			Avg: 1.15	Clashscore: 1.45	Outliers: 0 of 350	Poor rotamers: 0 of 308	Outliers: 0 of 327	Outliers: 11 of 348	Outliers: 2 of 352	Outliers: 7 of 352	Non-Trans: 2 of 351
A 341		ASP	1.03	-	Favored (2.54%) General / -45.6,142.1	Favored (21.4%) *t70* chi angles: 185.2,266.6	0.07Å	Favored (12.412%)	-	-	-
A 342		GLU	1	-	Favored (3.94%) General / -46.6,-33.2	Favored (73.3%) *tt0* chi angles: 184.3,172.2,342.1	0.07Å	Favored (33.816%)	-	-	-
A 343		THR	0.93	-	Favored (42.27%) General / -69.0,-8.2	Favored (65.1%) *p* chi angles: 58.2	0.04Å	Favored (39.496%) alpha helix	-	-	-
A 344		THR	0.85	-	Favored (17.62%) General / -111.5,2.8	Favored (62.7%) *p* chi angles: 63.5	0.04Å	Favored (53.814%)	-	-	-
A 345		LEU	0.79	-	Favored (17.73%) General / -94.9,153.3	Favored (86.3%) *mt* chi angles: 300.7,177.4	0.02Å	Favored (35.501%)	-	-	-
A 346		VAL	0.77	-	Favored (42.65%)	Favored (77%) *t* chi angles: 178.2	0.09Å	Favored (51.972%)	-	-	-

					Ile or Val / -95.7,118.8			beta sheet			
A 347		LYS	0.8	-	Favored (41.56%) General / -120.4,149.9	Favored (49.3%) *mtmt* chi angles: 293.6,181.5,283.6,175.1	0.05Å	Favored (41.487%) beta sheet	-	-	-
A 348		SER	0.89	-	Favored (53.56%) General / -68.1,146.3	Favored (39.8%) *t* chi angles: 176.8	0.06Å	Favored (37.991%) beta sheet	-	-	-
A 349		SER	1.05	-	Favored (18.36%) General / -114.0,15.6	Favored (74.1%) *p* chi angles: 59.5	0.02Å	Favored (11.503%) beta sheet	-	-	-
A 350		VAL	1.28	-	Favored (26.77%) Ile or Val / -77.0,135.4	Favored (94.2%) *t* chi angles: 174.7	0.01Å	Favored (15.226%)	-	-	-
A 351		SER	1.57	-	Favored (4.81%) General / -131.6,17.6	Favored (78.6%) *p* chi angles: 60.8	0.03Å	-	-	-	-
A 352		ALA	1.87	-	-	-	0.04Å	-	-	-	-

About MolProbity | Website for the Richardson Lab | Using ecloud x-H | Internal reference 4.5.2
