# Supplementary material for: Integrative proteome-wide structural analysis and high-throughput docking identify broad-spectrum antiviral scaffolds against Zika, Yellow Fever, West Nile, Saint Louis encephalitis, and Usutu viruses
Source: Front Cell Infect Microbiol. 2026 Apr 30;16:1723132. doi: 10.3389/fcimb.2026.1723132 (PMC13171538; doi:10.3389/fcimb.2026.1723132)
Supplement: Supplementary file 4 [file DataSheet4.zip › USUV/USU_NS2a/Mol_probity_Files/USU_NS2a_1FH-multi.table.pdf]

Viewing USU_NS2a1FH-multi.table

When finished, you should close this window. *Hint: Use File | Save As... to save a copy of this page.*

All-Atom Contacts	Clashscore, all atoms:	3.39		97th percentile* (N=1784, all resolutions)
	Clashscore is the number of serious steric overlaps (> 0.4 Å) per 1000 atoms.			
Protein Geometry	Poor rotamers	1	0.57%	Goal: <0.3%
	Favored rotamers	173	98.30%	Goal: >98%
	Ramachandran outliers	2	0.89%	Goal: <0.05%
	Ramachandran favored	220	97.78%	Goal: >98%
	Rama distribution Z-score	0.72 ± 0.52		Goal: abs(Z score) < 2
	MolProbity score^	1.18		99th percentile* (N=27675, 0Å - 99Å)
	Cβ deviations >0.25Å	0	0.00%	Goal: 0
	Bad bonds:	4 / 1741	0.23%	Goal: 0%
	Bad angles:	5 / 2363	0.21%	Goal: <0.1%
Peptide Omegas	Cis Prolines:	0 / 8	0.00%	Expected: ≤1 per chain, or ≤5%
Low-resolution Criteria	CaBLAM outliers	3	1.3%	Goal: <1.0%
	CA Geometry outliers	1	0.45%	Goal: <0.5%
Additional validations	Chiral volume outliers	0/292		
	Waters with clashes	0/0	0.00%	See UnDowser table for details

In the two column results, the left column gives the raw count, right column gives the percentage.

* 100th percentile is the best among structures of comparable resolution; 0th percentile is the worst. For clashscore the comparative set of structures was selected in 2004, for MolProbity score in 2006.

^ MolProbity score combines the clashscore, rotamer, and Ramachandran evaluations into a single score, normalized to be on the same scale as X-ray resolution.

Key to table colors and cutoffs here:

#	Alt	Res	High B	Clash > 0.4Å	Ramachandran	Rotamer	Cβ deviation	CaBLAM	Bond lengths	Bond angles	Cis Peptides
			Avg: 5.66	Clashscore: 3.39	Outliers: 2 of 225	Poor rotamers: 1 of 176	Outliers: 0 of 209	Outliers: 3 of 223	Outliers: 3 of 227	Outliers: 5 of 227	Non-Trans: 0 of 226
A 1		HIS	9.93	-	-	Favored (68%) *t70* chi angles: 189.2,70.7	0.03Å	-	-	-	-
A 2		ARG	9.65	-	Favored (4.45%) General / -125.7,37.0	Favored (45.8%) *mmt180* chi angles: 291.6,290.5,177,181	0.10Å	-	-	-	-
A 3		SER	9.27	-	Favored (4.44%) General / -78.4,68.6	Favored (64.6%) *m* chi angles: 297.4	0.06Å	Favored (9.384%)	-	-	-
A 4		ASP	8.78	-	Favored (58.35%) General / -77.9,-8.6	Favored (84.7%) *m-30* chi angles: 290.1,334.4	0.09Å	Favored (11.834%)	-	-	-
A 5		MET	8.18	-	Favored (65.26%) General / -65.6,-18.4	Favored (94.3%) *mmm* chi angles: 291.1,303.1,295.4	0.02Å	Favored (44.691%)	-	-	-
A 6		ILE	7.52	-	Favored (81.09%) Ile or Val / -60.5,-39.4	Favored (87.1%) *mt* chi angles: 291.1,169.8	0.08Å	Favored (53.313%) three-ten	-	-	-
A 7		ASP	6.84	-	Favored (8.33%)	Favored (84.3%) *m-30*	0.23Å	Favored (65.516%)	-	-	-

					Pre-Pro /<br>-53.0,-59.7	chi angles: 282.8,346.3		three-ten			
A 8		PRO	6.16	-	Favored<br>(32.59%)<br>Trans-Pro /<br>-48.5,-37.1	Favored (76%)<br>*Cg_exo*<br>chi angles:<br>329,37.4,332.5	0.04Å	Favored<br>(96.572%)<br>alpha helix	-	-	-
A 9		PHE	5.54	0.64Å<br>C with A 9<br>PHE HD1	Favored<br>(68.89%)<br>General /<br>-64.7,-49.9	OUTLIER (0%)<br>chi angles: 163.5,338.4	0.13Å	Favored<br>(83.776%)<br>alpha helix	-	OUTLIER(S)<br>worst is CA-CB-CG: 6.5 σ	-
A 10		GLN	4.99	-	Favored<br>(76.11%)<br>General /<br>-64.4,-33.5	Favored (89.6%)<br>*mm-40*<br>chi angles:<br>297.1,291.2,306.8	0.02Å	Favored<br>(75.618%)<br>alpha helix	-	-	-
A 11		LEU	4.56	-	Favored<br>(77.29%)<br>General /<br>-61.5,-35.3	Favored (91.7%) *mt*<br>chi angles: 292.9,175.6	0.00Å	Favored<br>(72.47%)<br>alpha helix	-	-	-
A 12		GLY	4.21	-	Favored<br>(87.84%)<br>Glycine /<br>-59.6,-36.0	-	-	Favored<br>(90.91%)<br>alpha helix	-	-	-
A 13		LEU	3.95	-	Favored<br>(85.79%)<br>General /<br>-64.2,-37.0	Favored (88.3%) *mt*<br>chi angles: 295.3,170.9	0.05Å	Favored<br>(80.793%)<br>alpha helix	-	-	-
A 14		LEU	3.77	-	Favored<br>(86.25%)<br>General /<br>-60.4,-47.4	Favored (66.2%) *tp*<br>chi angles: 176.7,59.4	0.09Å	Favored<br>(87.382%)<br>alpha helix	-	-	-
A 15		VAL	3.67	-	Favored<br>(93.83%)<br>Ile or Val /<br>-60.2,-43.7	Favored (61.2%) *t*<br>chi angles: 171	0.04Å	Favored<br>(87.423%)<br>alpha helix	-	-	-
A 16		MET	3.63	-	Favored<br>(94.39%)<br>General /<br>-64.7,-39.9	Favored (79.8%)<br>*mtm*<br>chi angles:<br>291,191.2,287.7	0.09Å	Favored<br>(97.234%)<br>alpha helix	-	-	-
A 17		PHE	3.65	-	Favored<br>(65.82%)<br>General /<br>-64.7,-50.7	Favored (73.9%)<br>*t80*<br>chi angles: 185,82.8	0.01Å	Favored<br>(81.869%)<br>alpha helix	-	-	-
A 18		LEU	3.74	-	Favored<br>(95.13%)<br>General /<br>-64.2,-40.0	Favored (92.8%) *mt*<br>chi angles: 292.8,175.1	0.05Å	Favored<br>(83.577%)<br>alpha helix	-	-	-
A 19		ALA	3.88	-	Favored<br>(93.4%)<br>General /<br>-61.4,-40.1	-	0.04Å	Favored<br>(94.244%)<br>alpha helix	-	-	-
A 20		THR	4.09	-	Favored<br>(79.57%)<br>General /<br>-66.2,-45.8	Favored (90.6%) *m*<br>chi angles: 298.8	0.02Å	Favored<br>(92.332%)<br>alpha helix	-	-	-
**#**	**Alt**	**Res**	**High B**	**Clash > 0.4Å**	**Ramachandran**	**Rotamer**	**Cβ deviation**	**CaBLAM**	**Bond lengths**	**Bond angles**	**Cis Peptides**
			Avg: 5.66	Clashscore: 3.39	Outliers: 2 of 225	Poor rotamers: 1 of 176	Outliers: 0 of 209	Outliers: 3 of 223	Outliers: 3 of 227	Outliers: 5 of 227	Non-Trans: 0 of 226
A 21		GLN	4.36	-	Favored<br>(79.43%)<br>General /<br>-66.1,-35.0	Favored (97.6%)<br>*mm-40*<br>chi angles:<br>293.7,302.2,306.5	0.05Å	Favored<br>(79.794%)<br>alpha helix	-	-	-
A 22		GLU	4.68	-	Favored<br>(83.74%)<br>General /<br>-60.8,-48.0	Favored (91.1%) *tt0*<br>chi angles:<br>179,180,358.2	0.05Å	Favored<br>(77.006%)<br>alpha helix	-	-	-

A<br>23		VAL	5.04	-	Favored<br>(93.78%)<br>Ile or Val /<br>-64.2,-41.9	Favored (84.5%) *t*<br>chi angles: 173.6	0.04Å	Favored<br>(80.824%)<br>alpha helix	-	-	-
A<br>24		LEU	5.44	-	Favored<br>(77.54%)<br>General /<br>-58.7,-49.5	Favored (6.7%) *tt*<br>chi angles: 187.6,154.7	0.06Å	Favored<br>(92.368%)<br>alpha helix	-	-	-
A<br>25		ARG	5.87	-	Favored<br>(80.28%)<br>General /<br>-64.2,-35.3	Favored (96.5%)<br>*mtt180*<br>chi angles:<br>288.3,172.8,176.4,166.1	0.03Å	Favored<br>(83.927%)	-	-	-
A<br>26		LYS	6.3	-	Favored<br>(10.88%)<br>General /<br>-87.4,171.0	Favored (99.5%)<br>*mttt*<br>chi angles:<br>294.4,178.9,176.1,177.2	0.03Å	CaBLAM<br>Disfavored<br>(2.376%)	-	-	-
A<br>27		ARG	6.72	-	Favored<br>(57.68%)<br>General /<br>-63.2,136.4	Favored (83.8%)<br>*ttt180*<br>chi angles:<br>184.7,175.8,180.3,178	0.02Å	Favored<br>(15.413%)	-	-	-
A<br>28		TRP	7.11	-	Allowed<br>(0.79%)<br>General /<br>-71.5,74.9	Favored (91.3%)<br>*m100*<br>chi angles: 286.1,93	0.06Å	Favored<br>(22.554%)	-	-	-
A<br>29		THR	7.44	-	Favored<br>(31.34%)<br>General /<br>-92.6,138.6	Favored (85.3%) *m*<br>chi angles: 301.6	0.06Å	Favored<br>(27.846%)	-	-	-
A<br>30		ALA	7.69	-	Favored<br>(65.43%)<br>General /<br>-56.6,-32.7	-	0.04Å	Favored<br>(55.087%)	-	-	-
A<br>31		ARG	7.83	-	Favored<br>(66.99%)<br>General /<br>-62.7,-23.2	Favored (98.6%)<br>*mtt180*<br>chi angles:<br>291.1,178.1,179.4,174.2	0.03Å	Favored<br>(40.147%)<br>alpha helix	-	-	-
A<br>32		LEU	7.87	-	Favored<br>(6.67%)<br>General /<br>-104.3,-39.0	Favored (87.7%) *mt*<br>chi angles: 300,177	0.08Å	Favored<br>(25.773%)<br>alpha helix	-	-	-
A<br>33		THR	7.81	-	Favored<br>(75.53%)<br>General /<br>-67.2,-45.9	Favored (85.4%) *m*<br>chi angles: 301.6	0.05Å	Favored<br>(83.758%)<br>alpha helix	-	-	-
A<br>34		VAL	7.68	-	Favored<br>(54.37%)<br>Pre-Pro /<br>-62.2,-53.9	Favored (67.1%) *t*<br>chi angles: 171.8	0.12Å	Favored<br>(83.22%)<br>alpha helix	-	OUTLIER(S)<br>worst is CA-C-<br>N: 4.0 σ	-
A<br>35		PRO	7.49	-	Favored<br>(77.05%)<br>Trans-Pro /<br>-62.1,-25.3	Favored (25.2%)<br>*Cg_endo*<br>chi angles:<br>20.4,326.8,31.6	0.02Å	Favored<br>(93.571%)<br>alpha helix	-	-	-
A<br>36		ALA	7.27	-	Favored<br>(92.84%)<br>General /<br>-65.6,-40.7	-	0.02Å	Favored<br>(76.392%)<br>alpha helix	-	-	-
A<br>37		ILE	7.04	-	Favored<br>(86.3%)<br>Ile or Val /<br>-67.1,-45.6	Favored (98.2%) *mt*<br>chi angles: 292.3,167.9	0.03Å	Favored<br>(85.285%)<br>alpha helix	-	-	-
A<br>38		VAL	6.82	-	Favored<br>(96.94%)<br>Ile or Val /<br>-61.6,-43.4	Favored (67.1%) *t*<br>chi angles: 171.8	0.02Å	Favored<br>(98.829%)<br>alpha helix	-	-	-
A<br>39		GLY	6.63	-	Favored<br>(54.87%)<br>Glycine /<br>-55.6,-51.4	-	-	Favored<br>(96.579%)<br>alpha helix	-	-	-

A 40		ALA	6.47	-	Favored (80.32%) General / -59.7,-37.9	-	0.03Å	Favored (80.13%) alpha helix	-	-	-
**#**	**Alt**	**Res**	**High B**	**Clash > 0.4Å**	**Ramachandran**	**Rotamer**	**Cβ deviation**	**CaBLAM**	**Bond lengths**	**Bond angles**	**Cis Peptides**
			Avg: 5.66	Clashscore: 3.39	Outliers: 2 of 225	Poor rotamers: 1 of 176	Outliers: 0 of 209	Outliers: 3 of 223	Outliers: 3 of 227	Outliers: 5 of 227	Non-Trans: 0 of 226
A 41		LEU	6.34	-	Favored (95.9%) General / -64.4,-40.5	Favored (92.1%) *mt* chi angles: 291.2,172.1	0.02Å	Favored (91.505%) alpha helix	-	-	-
A 42		LEU	6.28	-	Favored (88.78%) General / -64.5,-37.8	Favored (91.4%) *mt* chi angles: 291,172.1	0.04Å	Favored (93.706%) alpha helix	-	-	-
A 43		VAL	6.24	-	Favored (95.8%) Ile or Val / -60.4,-46.1	Favored (45.3%) *t* chi angles: 168.7	0.11Å	Favored (92.585%) alpha helix	-	-	-
A 44		LEU	6.2	-	Favored (98.68%) General / -62.7,-40.9	Favored (94.3%) *mt* chi angles: 293.9,174.6	0.06Å	Favored (82.722%) alpha helix	-	-	-
A 45		ILE	6.09	-	Favored (83.21%) Ile or Val / -67.0,-47.1	Favored (96.7%) *mt* chi angles: 292.4,166.8	0.07Å	Favored (68.909%) alpha helix	-	-	-
A 46		LEU	5.89	-	Favored (25.98%) General / -83.1,-35.0	Favored (92.1%) *mt* chi angles: 297.7,173.5	0.07Å	Favored (59.356%) alpha helix	-	-	-
A 47		GLY	5.6	-	Favored (44.78%) Glycine / -84.3,-25.2	-	-	Favored (15.547%)	-	-	-
A 48		GLY	5.25	-	Favored (25.08%) Glycine / 74.3,45.4	-	-	Favored (22.092%)	-	-	-
A 49		ILE	4.86	-	Favored (87.35%) Ile or Val / -61.3,-40.8	Allowed (0.7%) *mp* chi angles: 274.7,49.4	0.15Å	CaBLAM Disfavored (1.415%)	-	-	-
A 50		THR	4.49	-	OUTLIER (0.03)%) General / 56.3,172.2	Favored (30.7%) *p* chi angles: 69.3	0.08Å	CaBLAM Disfavored (2.471%)	-	-	-
A 51		TYR	4.14	-	Favored (71.75%) General / -56.4,-50.4	Favored (92.6%) *t80* chi angles: 177.3,79.2	0.09Å	Favored (19.74%)	-	-	-
A 52		THR	3.83	-	Favored (97.31%) General / -61.2,-41.7	Favored (89.7%) *m* chi angles: 298.2	0.04Å	Favored (73.426%) alpha helix	-	-	-
A 53		ASP	3.58	-	Favored (41.86%) General / -79.5,-30.6	Favored (15.3%) *m-30* chi angles: 291.3,279.2	0.07Å	Favored (73.778%) alpha helix	-	-	-
A 54		LEU	3.37	-	Favored (93.86%) General / -64.7,-39.6	Favored (39.3%) *tp* chi angles: 185.4,58.4	0.02Å	Favored (79.007%) alpha helix	-	-	-
A 55		LEU	3.19	-	Favored (80.58%) General / -58.5,-48.5	Favored (66%) *tp* chi angles: 175.1,62.3	0.04Å	Favored (91.132%) alpha helix	-	-	-

A 56		ARG	3.07	0.42Å HD2 with A 56 ARG HH11	Favored (82.3%) General / -56.8,-44.8	Favored (30.5%) *ttm110* chi angles: 191.9,178.8,311.5,107.7	0.03Å	Favored (98.465%) alpha helix	-	OUTLIER(S) worst is NE-CZ-NH2: 5.7 σ	-
A 57		TYR	3	-	Favored (73.06%) General / -56.5,-50.0	Favored (36.9%) *t80* chi angles: 164.6,80.3	0.05Å	Favored (97.521%) alpha helix	-	-	-
A 58		VAL	2.98	-	Favored (94.91%) Ile or Val / -61.2,-42.8	Favored (58.3%) *t* chi angles: 170.5	0.03Å	Favored (97.195%) alpha helix	-	-	-
A 59		LEU	3.01	-	Favored (92.53%) General / -62.2,-39.4	Favored (93.8%) *mt* chi angles: 291.6,171.9	0.05Å	Favored (97.827%) alpha helix	-	-	-
A 60		LEU	3.07	-	Favored (94.03%) General / -64.5,-43.4	Favored (59%) *mt* chi angles: 285.9,171.6	0.09Å	Favored (98.301%) alpha helix	OUTLIER(S) worst is CB--CG: 6.8 σ	-	-
**#**	**Alt**	**Res**	**High B**	**Clash > 0.4Å**	**Ramachandran**	**Rotamer**	**Cβ deviation**	**CaBLAM**	**Bond lengths**	**Bond angles**	**Cis Peptides**
			Avg: 5.66	Clashscore: 3.39	Outliers: 2 of 225	Poor rotamers: 1 of 176	Outliers: 0 of 209	Outliers: 3 of 223	Outliers: 3 of 227	Outliers: 5 of 227	Non-Trans: 0 of 226
A 61		VAL	3.17	-	Favored (94.12%) Ile or Val / -65.5,-42.9	Favored (69.8%) *t* chi angles: 172.1	0.05Å	Favored (96.298%) alpha helix	-	-	-
A 62		GLY	3.32	-	Favored (39.48%) Glycine / -56.4,-54.0	-	-	Favored (92.597%) alpha helix	-	-	-
A 63		ALA	3.51	-	Favored (94.01%) General / -60.5,-41.3	-	0.06Å	Favored (82.649%) alpha helix	-	-	-
A 64		ALA	3.77	-	Favored (96.56%) General / -62.5,-40.4	-	0.04Å	Favored (91.363%) alpha helix	-	-	-
A 65		PHE	4.12	-	Favored (76.18%) General / -59.5,-49.9	Favored (90.8%) *t80* chi angles: 175.5,78.1	0.02Å	Favored (92.912%) alpha helix	-	-	-
A 66		ALA	4.52	-	Favored (84.25%) General / -59.8,-39.2	-	0.04Å	Favored (79.091%) alpha helix	-	-	-
A 67		GLU	4.95	-	Favored (66.61%) General / -72.8,-33.9	Favored (52.4%) *mm-30* chi angles: 291,300.8,349.4	0.06Å	CaBLAM Disfavored (3.189%)	-	-	-
A 68		ALA	5.32	-	Allowed (0.1%) General / 55.0,-99.4	-	0.05Å	CaBLAM Outlier (0.047%)	-	-	-
A 69		ASN	5.56	-	Favored (65.21%) General / -68.2,-25.4	Favored (98.6%) *m-40* chi angles: 288.9,337.2	0.03Å	Favored (23.123%)	-	-	-
A 70		SER	5.61	-	Favored (5.24%) General / -78.9,70.3	Favored (72.9%) *m* chi angles: 295.7	0.03Å	Favored (20.265%)	-	-	-
A 71		GLY	5.49	-	Favored (54.62%) Glycine / -53.8,-36.2	-	-	Favored (29.852%)	-	-	-

A 72		GLY	5.24	-	Favored (84.08%) Glycine / -57.1,-38.1	-	-	Favored (82.236%) alpha helix	-	-	-
A 73		ASP	4.93	-	Favored (62.61%) General / -74.7,-34.8	Favored (97.2%) *m-30* chi angles: 286.8,348.1	0.09Å	Favored (77.662%) alpha helix	-	-	-
A 74		VAL	4.64	-	Favored (91.79%) Ile or Val / -60.0,-43.1	Favored (64.4%) *t* chi angles: 171.4	0.06Å	Favored (83.834%) alpha helix	-	-	-
A 75		VAL	4.4	-	Favored (96.13%) Ile or Val / -64.9,-44.3	Favored (58.3%) *t* chi angles: 170.5	0.06Å	Favored (92.495%) alpha helix	-	-	-
A 76		HIS	4.23	-	Favored (99.86%) General / -62.6,-43.0	Favored (27.2%) *m-70* chi angles: 295.7,321	0.12Å	Favored (92.31%) alpha helix	-	-	-
A 77		LEU	4.14	-	Favored (97.82%) General / -63.8,-42.5	Favored (78.4%) *mt* chi angles: 288.3,169.7	0.10Å	Favored (86.197%) alpha helix	-	-	-
A 78		ALA	4.14	-	Favored (86.72%) General / -58.0,-43.3	-	0.13Å	Favored (80.345%) alpha helix	-	-	-
A 79		LEU	4.22	-	Favored (79.98%) General / -60.4,-37.1	Favored (22.5%) *tp* chi angles: 190,65.1	0.10Å	Favored (70.974%) alpha helix	-	-	-
A 80		ILE	4.35	-	Favored (86.45%) Ile or Val / -58.9,-48.4	Favored (99.2%) *mt* chi angles: 292.4,167.7	0.08Å	Favored (55.371%) alpha helix	-	-	-

#	Alt	Res	High B	Clash > 0.4Å	Ramachandran	Rotamer	Cβ deviation	CaBLAM	Bond lengths	Bond angles	Cis Peptides
			Avg: 5.66	Clashscore: 3.39	Outliers: 2 of 225	Poor rotamers: 1 of 176	Outliers: 0 of 209	Outliers: 3 of 223	Outliers: 3 of 227	Outliers: 5 of 227	Non-Trans: 0 of 226
A 81		ALA	4.51	-	Favored (49.35%) General / -78.2,-34.8	-	0.05Å	Favored (23.936%)	-	-	-
A 82		ALA	4.62	-	Favored (68.99%) General / -54.9,-39.5	-	0.14Å	Favored (15.02%)	-	-	-
A 83		PHE	4.62	0.50Å O with A 84 LYS C	Favored (5.34%) General / -129.2,27.7	Favored (93.2%) *m-80* chi angles: 299.8,93.7	0.08Å	CaBLAM Disfavored (3.536%)	-	-	-
A 84		LYS	4.52	0.63Å HG2 with A 84 LYS O	Favored (4.32%) General / 42.4,54.7	Favored (30%) *ttpt* chi angles: 172.4,184.9,65,173	0.12Å	CA Geom Outlier (0.052%)	-	-	-
A 85		ILE	4.33	0.52Å CG2 with A 85 ILE O	Favored (31.17%) Ile or Val / -65.6,133.1	Favored (5.5%) *tp* chi angles: 202.3,70.5	0.05Å	Favored (24.194%) beta sheet	-	-	-
A 86		GLN	4.11	-	Favored (93.13%) Pre-Pro / -67.6,130.4	Favored (29.6%) *mm-40* chi angles: 290.2,284.4,13.2	0.04Å	Favored (40.429%)	-	-	-
A 87		PRO	3.92	-	OUTLIER (0.06)%) Trans-Pro / -46.9,-10.8	Favored (90.5%) *Cg_exo* chi angles: 331,33.8,335.3	0.05Å	CaBLAM Disfavored (3.988%)	-	-	-

A 88		GLY	3.8	-	Favored (9.39%) Glycine / -47.4,-34.5	-	-	Favored (55.506%)	-	-	-
A 89		PHE	3.76	-	Favored (77.26%) General / -55.9,-47.6	Favored (50.3%) *t80* chi angles: 167.4,71.5	0.06Å	Favored (73.493%) alpha helix	-	-	-
A 90		LEU	3.82	-	Favored (73.33%) General / -64.8,-31.9	Favored (96.2%) *mt* chi angles: 293.4,173.7	0.06Å	Favored (75.103%) alpha helix	-	-	-
A 91		ALA	3.95	-	Favored (98.97%) General / -62.4,-41.9	-	0.10Å	Favored (77.741%) alpha helix	-	-	-
A 92		MET	4.14	-	Favored (90.13%) General / -65.6,-38.6	Favored (81%) *mtm* chi angles: 291.4,190.4,291.8	0.05Å	Favored (91.924%) alpha helix	-	-	-
A 93		THR	4.4	-	Favored (81.32%) General / -64.9,-46.5	Favored (93.4%) *m* chi angles: 297.5	0.05Å	Favored (86.423%) alpha helix	-	-	-
A 94		PHE	4.72	-	Favored (74.66%) General / -54.9,-46.5	Favored (63.7%) *t80* chi angles: 184.2,90.8	0.08Å	Favored (81.58%) alpha helix	-	-	-
A 95		LEU	5.11	-	Favored (49.09%) General / -83.2,-0.3	Favored (92.8%) *mt* chi angles: 297.2,172.6	0.10Å	Favored (17.961%)	-	-	-
A 96		ARG	5.55	-	Favored (3.38%) General / -134.4,32.2	Favored (93.4%) *mmt-90* chi angles: 294.9,293.7,182.6,271.4	0.03Å	Favored (29.898%)	-	-	-
A 97		GLY	6.02	-	Favored (15.03%) Glycine / -58.2,-13.3	-	-	Favored (12.623%)	-	-	-
A 98		LYS	6.46	-	Favored (58.84%) General / -86.0,-3.1	Favored (99%) *mttt* chi angles: 294.2,180.7,179.4,179	0.01Å	Favored (35.62%)	-	-	-
A 99		TRP	6.82	-	Favored (48.2%) General / -69.5,149.5	Favored (42.5%) *m-10* chi angles: 287.8,12.2	0.06Å	Favored (39.984%)	-	-	-
A 100		THR	7.04	-	Favored (17.9%) General / -76.8,169.7	Favored (70.8%) *p* chi angles: 62.2	0.07Å	Favored (42.802%)	-	-	-
**#**	**Alt**	**Res**	**High B**	**Clash > 0.4Å**	**Ramachandran**	**Rotamer**	**Cβ deviation**	**CaBLAM**	**Bond lengths**	**Bond angles**	**Cis Peptides**
			Avg: 5.66	Clashscore: 3.39	Outliers: 2 of 225	Poor rotamers: 1 of 176	Outliers: 0 of 209	Outliers: 3 of 223	Outliers: 3 of 227	Outliers: 5 of 227	Non-Trans: 0 of 226
A 101		ASN	7.11	-	Favored (86.73%) General / -58.5,-42.1	Favored (98.6%) *m-40* chi angles: 290,341.5	0.05Å	Favored (57.714%)	-	-	-
A 102		GLN	7.03	-	Favored (80.76%) General / -68.5,-38.2	Favored (95.4%) *tp40* chi angles: 188,63.1,53.6	0.04Å	Favored (96.627%) alpha helix	-	-	-
A 103		GLU	6.85	-	Favored (84.72%) General / -64.6,-36.6	Favored (46.6%) *mt-10* chi angles: 288.8,167.8,314.4	0.07Å	Favored (92.551%) alpha helix	-	-	-

A 104		ASN	6.67	-	Favored (79.01%) General / -65.5,-34.8	Favored (88.5%) *m-40* chi angles: 285.4,333.9	0.08Å	Favored (85.299%) alpha helix	-	-	-
A 105		ILE	6.5	-	Favored (95.75%) Ile or Val / -64.1,-45.7	Favored (96.3%) *mt* chi angles: 292.1,168.1	0.03Å	Favored (85.995%) alpha helix	-	-	-
A 106		LEU	6.37	-	Favored (88.1%) General / -64.2,-37.6	Favored (97.6%) *mt* chi angles: 293.5,172.9	0.06Å	Favored (92.305%) alpha helix	-	-	-
A 107		LEU	6.27	-	Favored (92.52%) General / -64.2,-44.2	Favored (45.6%) *tp* chi angles: 184.6,61.4	0.07Å	Favored (92.805%) alpha helix	-	-	-
A 108		ALA	6.21	-	Favored (98.75%) General / -61.3,-42.5	-	0.05Å	Favored (93.137%) alpha helix	-	-	-
A 109		LEU	6.17	-	Favored (92.46%) General / -65.7,-40.5	Favored (89.2%) *mt* chi angles: 290.8,171.5	0.04Å	Favored (83.881%) alpha helix	-	-	-
A 110		GLY	6.13	-	Favored (45.37%) Glycine / -55.3,-52.8	-	-	Favored (97.331%) alpha helix	-	-	-
A 111		ALA	6.12	-	Favored (82.22%) General / -59.0,-39.5	-	0.08Å	Favored (81.225%) alpha helix	-	-	-
A 112		ALA	6.13	-	Favored (99.46%) General / -61.2,-42.9	-	0.07Å	Favored (79.467%) alpha helix	-	-	-
A 113		PHE	6.18	-	Favored (60.64%) General / -75.6,-33.9	Favored (50.9%) *m-80* chi angles: 289.6,115	0.07Å	Favored (78.494%) alpha helix	-	-	-
A 114		PHE	6.27	-	Favored (68.67%) General / -59.8,-51.7	Favored (90.5%) *t80* chi angles: 177.5,81.9	0.07Å	Favored (78.682%) alpha helix	-	-	-
A 115		GLN	6.44	-	Favored (89.93%) General / -59.3,-46.0	Favored (46.4%) *tt0* chi angles: 178.6,181,307.8	0.04Å	Favored (86.652%) alpha helix	-	-	-
A 116		MET	6.68	-	Favored (95.77%) General / -64.1,-40.2	Favored (80.9%) *mtm* chi angles: 288.7,186.3,283.2	0.09Å	Favored (96.555%) alpha helix	-	-	-
A 117		ALA	7.01	-	Favored (96.87%) General / -64.1,-42.3	-	0.04Å	Favored (90.814%) alpha helix	-	-	-
A 118		ALA	7.41	-	Favored (75.75%) General / -60.6,-35.3	-	0.04Å	Favored (77.96%) alpha helix	-	-	-
A 119		THR	7.83	-	Favored (64.4%) General / -71.9,-44.1	Favored (90.7%) *m* chi angles: 301.2	0.03Å	Favored (92.341%) alpha helix	-	-	-
A 120		ASP	8.15	-	Favored (75.95%) General / -62.9,-33.8	Favored (97%) *m-30* chi angles: 286.6,347.8	0.02Å	Favored (98.728%)	-	-	-

#	Alt	Res	High B	Clash > 0.4Å	Ramachandran	Rotamer	Cβ deviation	CaBLAM	Bond lengths	Bond angles	Cis Peptides
			Avg: 5.66	Clashscore: 3.39	Outliers: 2 of 225	Poor rotamers: 1 of 176	Outliers: 0 of 209	Outliers: 3 of 223	Outliers: 3 of 227	Outliers: 5 of 227	Non-Trans: 0 of 226
A 121		LEU	8.28	-	Favored (7.02%) General / -94.6,174.6	Favored (76.4%) *mt* chi angles: 301.6,175.4	0.06Å	CaBLAM Disfavored (3.711%)	-	-	-
A 122		ASN	8.16	-	Favored (5.1%) General / -91.1,63.6	Favored (86.9%) *m-40* chi angles: 293.8,318.2	0.06Å	CaBLAM Outlier (0.674%)	-	-	-
A 123		PHE	7.78	-	Favored (54.71%) General / -109.2,131.8	Favored (81%) *m-80* chi angles: 290.2,83.9	0.08Å	Favored (16.493%)	-	-	-
A 124		SER	7.22	-	Favored (48.96%) General / -91.7,-7.8	Favored (99.2%) *p* chi angles: 65.6	0.03Å	Favored (12.391%)	-	-	-
A 125		LEU	6.57	-	Favored (74.77%) Pre-Pro / -49.8,-43.6	Favored (70.1%) *tp* chi angles: 177,60.4	0.13Å	Favored (39.768%)	-	-	-
A 126		PRO	5.96	-	Favored (57.35%) Trans-Pro / -57.5,-23.6	Favored (82.9%) *Cg_exo* chi angles: 334.5,36.6,328.2	0.02Å	Favored (62.708%) three-ten	-	-	-
A 127		GLY	5.45	-	Favored (40.56%) Glycine / -75.1,-38.4	-	-	Favored (89.893%) alpha helix	-	-	-
A 128		ILE	5.04	-	Favored (87.58%) Ile or Val / -66.7,-45.7	Favored (96.8%) *mt* chi angles: 293.4,168.9	0.03Å	Favored (80.017%) alpha helix	-	-	-
A 129		LEU	4.72	-	Favored (85.28%) General / -64.3,-36.8	Favored (96.1%) *mt* chi angles: 293.6,173.6	0.03Å	Favored (84.661%) alpha helix	-	-	-
A 130		ASN	4.49	0.43Å C with A 130 ASN OD1	Favored (29.3%) General / -58.8,-55.9	Favored (17%) *t0* chi angles: 175.8,328.8	0.01Å	Favored (77.567%) alpha helix	-	-	-
A 131		ALA	4.33	-	Favored (86.09%) General / -60.2,-39.3	-	0.04Å	Favored (79.434%) alpha helix	-	-	-
A 132		THR	4.23	-	Favored (70.93%) General / -62.5,-50.5	Favored (91.6%) *m* chi angles: 299	0.09Å	Favored (83.087%) alpha helix	-	-	-
A 133		ALA	4.18	-	Favored (77.02%) General / -62.8,-34.3	-	0.10Å	Favored (67.372%) alpha helix	-	-	-
A 134		THR	4.19	-	Favored (90.61%) General / -59.0,-42.7	Favored (92.4%) *m* chi angles: 297.4	0.10Å	Favored (68.183%) alpha helix	-	-	-
A 135		ALA	4.27	-	Favored (89.83%) General / -60.1,-40.5	-	0.03Å	Favored (88.151%) alpha helix	-	-	-
A 136		TRP	4.39	-	Favored (76%) General / -69.8,-40.4	Favored (55.2%) *m100* chi angles: 288.8,120.1	0.03Å	Favored (84.805%) alpha helix	-	-	-
A 137		MET	4.59	-	Favored (94.85%)	Favored (54.3%) *ttp* chi angles:	0.01Å	Favored (90.147%)	-	-	-

					General / -59.9,-43.7	176.3,178.8,64.7		alpha helix			
A 138		LEU	4.85	0.56Å C with A 138 LEU HD12	Favored (72.75%) General / -69.5,-33.3	Allowed (1.1%) *pp* chi angles: 77.6,85.2	0.04Å	Favored (86.42%) alpha helix	-	-	-
A 139		LEU	5.19	-	Favored (86.66%) General / -66.2,-37.9	Favored (89.9%) *mt* chi angles: 290.8,172.1	0.06Å	Favored (89.095%) alpha helix	-	-	-
A 140		ARG	5.57	-	Favored (88.58%) General / -63.4,-45.9	Favored (82.8%) *ttt180* chi angles: 184.6,177.3,180.6,186.3	0.04Å	Favored (86.855%) alpha helix	-	-	-
**#**	**Alt**	**Res**	**High B**	**Clash > 0.4Å**	**Ramachandran**	**Rotamer**	**Cβ deviation**	**CaBLAM**	**Bond lengths**	**Bond angles**	**Cis Peptides**
			Avg: 5.66	Clashscore: 3.39	Outliers: 2 of 225	Poor rotamers: 1 of 176	Outliers: 0 of 209	Outliers: 3 of 223	Outliers: 3 of 227	Outliers: 5 of 227	Non-Trans: 0 of 226
A 141		ALA	5.97	-	Favored (89.97%) General / -60.5,-40.1	-	0.04Å	Favored (80.767%) alpha helix	-	-	-
A 142		ALA	6.31	-	Favored (72.92%) General / -70.9,-38.3	-	0.04Å	Favored (61.523%) alpha helix	-	-	-
A 143		THR	6.5	-	Favored (13.96%) General / -104.3,-19.9	Favored (70%) *p* chi angles: 62.4	0.05Å	Favored (42.773%) alpha helix	-	-	-
A 144		GLN	6.49	-	Favored (76.95%) Pre-Pro / -130.1,66.0	Favored (76.1%) *mt0* chi angles: 297.2,175.3,31.5	0.11Å	Favored (13.218%)	-	-	-
A 145		PRO	6.28	-	Favored (5.45%) Trans-Pro / -76.2,57.9	Favored (54.8%) *Cg_endo* chi angles: 32.5,321.8,27.5	0.05Å	CaBLAM Disfavored (2.92%)	-	-	-
A 146		SER	5.88	-	Favored (17.78%) General / -79.0,169.7	Favored (85.2%) *p* chi angles: 67.7	0.04Å	Favored (22.316%)	-	-	-
A 147		THR	5.38	-	Favored (70.92%) General / -57.7,-51.1	Favored (93.2%) *m* chi angles: 297.6	0.02Å	Favored (33.227%)	-	-	-
A 148		SER	4.86	-	Favored (81.12%) General / -62.4,-36.0	Favored (84.9%) *p* chi angles: 67.4	0.13Å	Favored (77.04%) alpha helix	-	-	-
A 149		ALA	4.4	-	Favored (89.27%) General / -63.0,-38.0	-	0.04Å	Favored (75.105%) alpha helix	-	-	-
A 150		ILE	4	-	Favored (48.74%) Ile or Val / -74.3,-44.6	Favored (97%) *mt* chi angles: 293.9,167.4	0.05Å	Favored (73.685%) alpha helix	-	-	-
A 151		VAL	3.66	0.42Å O with A 152 MET C	Favored (94.75%) Ile or Val / -62.5,-42.2	Favored (60.3%) *t* chi angles: 170.8	0.14Å	Favored (55.869%) three-ten	-	-	-
A 152		MET	3.39	0.42Å C with A 151 VAL O	Allowed (1.28%) Pre-Pro / -43.2,-62.6	Favored (57.3%) *mtt* chi angles: 289.3,176.2,191	0.17Å	Favored (55.547%) three-ten	-	OUTLIER(S) worst is CA-C-N: 4.1 σ	-

A 153		PRO	3.18	-	Favored (78.41%) Trans-Pro / -61.5,-24.6	Favored (30.7%) *Cg_endo* chi angles: 21.4,327.3,30.8	0.02Å	Favored (71.707%) alpha helix	-	-	-
A 154		LEU	3.02	-	Favored (56.94%) General / -77.3,-19.1	Favored (94.5%) *mt* chi angles: 295.9,175.2	0.03Å	Favored (68.523%) alpha helix	-	-	-
A 155		LEU	2.92	-	Favored (10.27%) General / -91.5,-42.4	Favored (82.1%) *mt* chi angles: 297.6,171	0.18Å	Favored (33.928%) alpha helix	OUTLIER(S) worst is CB--CG: 4.5 σ	-	-
A 156		CYS	2.89	-	Favored (88.15%) General / -63.2,-37.7	Favored (99.6%) *m* chi angles: 292.5	0.05Å	Favored (74.275%) alpha helix	-	-	-
A 157		LEU	2.93	-	Favored (63.22%) General / -62.5,-19.1	Favored (3.4%) *pp* chi angles: 67.4,84.3	0.09Å	Favored (67.803%) alpha helix	-	-	-
A 158		LEU	3.04	-	Favored (53.27%) General / -88.4,-9.2	Favored (93.4%) *mt* chi angles: 297.5,173.4	0.12Å	Favored (55.612%)	-	-	-
A 159		ALA	3.26	-	Favored (85.88%) Pre-Pro / -73.3,145.7	-	0.03Å	Favored (41.715%)	-	-	-
A 160		PRO	3.59	-	Favored (40.91%) Trans-Pro / -56.2,-22.6	Favored (92.4%) *Cg_exo* chi angles: 333.1,33.5,334.1	0.01Å	Favored (42.708%)	-	-	-
**#**	**Alt**	**Res**	**High B**	**Clash > 0.4Å**	**Ramachandran**	**Rotamer**	**Cβ deviation**	**CaBLAM**	**Bond lengths**	**Bond angles**	**Cis Peptides**
			Avg: 5.66	Clashscore: 3.39	Outliers: 2 of 225	Poor rotamers: 1 of 176	Outliers: 0 of 209	Outliers: 3 of 223	Outliers: 3 of 227	Outliers: 5 of 227	Non-Trans: 0 of 226
A 161		GLY	4.02	-	Favored (67.36%) Glycine / -58.5,-31.8	-	-	Favored (67.057%)	-	-	-
A 162		MET	4.54	-	Favored (22.77%) General / -82.4,4.8	Favored (95.6%) *mmm* chi angles: 293.6,306.3,292.6	0.10Å	Favored (34.33%) three-ten	-	-	-
A 163		ARG	5.11	-	Favored (40.67%) General / -96.5,-5.0	Favored (96.2%) *mtt180* chi angles: 298,179.5,182.8,183	0.06Å	Favored (68.148%) alpha helix	-	-	-
A 164		LEU	5.65	-	Favored (8.52%) General / -102.5,-36.0	Favored (82.6%) *mt* chi angles: 301.6,177.8	0.07Å	Favored (13.289%)	-	-	-
A 165		LEU	6.05	-	Favored (20.82%) General / -81.9,165.4	Favored (4.3%) *mp* chi angles: 276,74.3	0.04Å	Favored (7.213%)	-	-	-
A 166		TYR	6.25	-	Favored (36.16%) General / -77.7,146.2	Favored (84.7%) *m-80* chi angles: 290.1,95.4	0.01Å	Favored (35.548%)	-	-	-
A 167		LEU	6.23	-	Favored (65.66%) General / -57.6,-31.0	Favored (23.6%) *tp* chi angles: 189.6,60	0.01Å	Favored (53.395%)	-	-	-
A 168		ASP	6.03	-	Favored (71.37%) General / -63.4,-30.2	Favored (14.6%) *t70* chi angles: 197.2,60.8	0.04Å	Favored (68.273%) alpha helix	-	-	-

#	Alt	Res	High B	Clash > 0.4Å	Ramachandran	Rotamer	Cβ deviation	CaBLAM	Bond lengths	Bond angles	Cis Peptides
A 169		THR	5.73	-	Favored (78.55%) General / -66.5,-45.8	Favored (89.2%) *m* chi angles: 297.1	0.02Å	Favored (75.259%) alpha helix	-	-	-
A 170		TYR	5.42	-	Favored (73.02%) General / -56.5,-50.0	Favored (38.1%) *t80* chi angles: 164.9,80.6	0.03Å	Favored (93.03%) alpha helix	-	-	-
A 171		ARG	5.13	-	Favored (49.95%) General / -57.4,-54.2	Favored (85.7%) *ttp80* chi angles: 184.2,183.4,66.7,80.6	0.10Å	Favored (85.622%) alpha helix	-	-	-
A 172		ILE	4.91	-	Favored (88.31%) Ile or Val / -58.9,-43.3	Favored (95.2%) *mt* chi angles: 291.8,167.5	0.04Å	Favored (76.378%) alpha helix	-	-	-
A 173		THR	4.76	-	Favored (49.45%) General / -51.6,-52.2	Favored (98.4%) *m* chi angles: 300.2	0.10Å	Favored (73.836%) alpha helix	-	-	-
A 174		LEU	4.65	-	Favored (71.52%) General / -71.3,-35.9	Favored (94.6%) *mt* chi angles: 292.4,174.2	0.05Å	Favored (74.327%) alpha helix	-	-	-
A 175		ILE	4.57	-	Favored (89.22%) Ile or Val / -65.2,-40.6	Favored (43.4%) *mm* chi angles: 297.2,300.3	0.07Å	Favored (85.701%) alpha helix	-	-	-
A 176		ILE	4.53	-	Favored (94.78%) Ile or Val / -62.6,-42.1	Favored (96.4%) *mt* chi angles: 292.1,168.3	0.04Å	Favored (96.872%) alpha helix	-	-	-
A 177		ILE	4.53	-	Favored (93.68%) Ile or Val / -59.5,-44.8	Favored (97.7%) *mt* chi angles: 292.8,168.8	0.10Å	Favored (94.602%) alpha helix	OUTLIER(S) worst is CB--CG1: 4.2 σ	-	-
A 178		GLY	4.6	-	Favored (35.69%) Glycine / -55.0,-54.1	-	-	Favored (93.848%) alpha helix	-	-	-
A 179		ILE	4.74	-	Favored (99.4%) Ile or Val / -61.6,-44.9	Favored (94.7%) *mt* chi angles: 291.7,167.5	0.05Å	Favored (83.269%) alpha helix	-	-	-
A 180		CYS	4.99	-	Favored (94.88%) General / -61.3,-40.6	Favored (91.3%) *m* chi angles: 291.2	0.09Å	Favored (95.705%) alpha helix	-	-	-
**#**	**Alt**	**Res**	**High B**	**Clash > 0.4Å**	**Ramachandran**	**Rotamer**	**Cβ deviation**	**CaBLAM**	**Bond lengths**	**Bond angles**	**Cis Peptides**
			Avg: 5.66	Clashscore: 3.39	Outliers: 2 of 225	Poor rotamers: 1 of 176	Outliers: 0 of 209	Outliers: 3 of 223	Outliers: 3 of 227	Outliers: 5 of 227	Non-Trans: 0 of 226
A 181		SER	5.35	-	Favored (84.03%) General / -66.8,-43.3	Favored (66.5%) *m* chi angles: 297	0.05Å	Favored (84.478%) alpha helix	-	-	-
A 182		LEU	5.86	-	Favored (84.95%) General / -61.8,-37.6	Favored (77%) *mt* chi angles: 287.8,169.2	0.02Å	Favored (75.586%) alpha helix	-	-	-
A 183		ILE	6.53	-	Favored (38.77%) Ile or Val / -70.0,-51.5	Favored (94.4%) *mt* chi angles: 294.8,167.1	0.04Å	Favored (69.397%) alpha helix	-	-	-

A 184		GLY	7.31	-	Favored (85.42%) Glycine / -61.8,-33.5	-	-	Favored (89.27%) alpha helix	-	-	-
A 185		GLU	8.16	-	Favored (19.72%) General / -94.2,14.6	Favored (69.9%) *mm-30* chi angles: 296.8,297.2,349	0.05Å	Favored (27.853%) alpha helix	-	-	-
A 186		ARG	8.97	-	Favored (58.65%) General / -58.8,-22.6	Favored (86%) *mtm180* chi angles: 288.7,171.8,290.4,170.9	0.07Å	Favored (44.409%) alpha helix	-	-	-
A 187		ARG	9.56	-	Favored (56.05%) General / -78.7,-17.4	Favored (89.4%) *mtm180* chi angles: 294.9,170.3,290.2,172.8	0.09Å	Favored (68.35%) alpha helix	-	-	-
A 188		ARG	9.77	-	Favored (4.33%) General / -133.8,99.0	Favored (65.3%) *mtm180* chi angles: 297.5,192.2,294,176.6	0.03Å	Favored (13.121%) alpha helix	-	-	-
A 189		ALA	9.56	-	Favored (68.42%) General / -56.3,-36.2	-	0.03Å	Favored (50.512%) alpha helix	-	-	-
A 190		ALA	8.95	-	Favored (77.82%) General / -60.8,-36.0	-	0.04Å	Favored (72.838%) alpha helix	-	-	-
A 191		ALA	8.08	-	Favored (23.97%) General / -84.8,-31.4	-	0.06Å	Favored (68.729%) alpha helix	-	-	-
A 192		LYS	7.16	-	Favored (92.96%) General / -59.5,-44.8	Favored (86.2%) *tttt* chi angles: 184,179.4,178.8,182.1	0.02Å	Favored (71.402%) alpha helix	-	-	-
A 193		LYS	6.31	-	Favored (71.13%) General / -55.0,-49.6	Favored (27%) *ttpt* chi angles: 179.1,189.5,68.5,171.4	0.06Å	Favored (96.599%) alpha helix	-	-	-
A 194		LYS	5.61	-	Favored (76.73%) General / -60.2,-36.0	Favored (97.2%) *mttt* chi angles: 289.8,178.2,181.9,178.3	0.02Å	Favored (79.567%) alpha helix	-	-	-
A 195		GLY	5.06	-	Favored (58.63%) Glycine / -58.3,-51.4	-	-	Favored (92.444%) alpha helix	-	-	-
A 196		ALA	4.66	-	Favored (79.39%) General / -60.2,-37.2	-	0.02Å	Favored (79.964%) alpha helix	-	-	-
A 197		VAL	4.36	-	Favored (91.91%) Ile or Val / -64.5,-46.7	Favored (68.1%) *t* chi angles: 171.9	0.00Å	Favored (84.327%) alpha helix	-	-	-
A 198		LEU	4.16	0.41Å C with A 198 LEU HD23	Favored (73.03%) General / -57.3,-50.4	Favored (6%) *tt* chi angles: 186.5,157.1	0.04Å	Favored (87.128%) alpha helix	-	-	-
A 199		LEU	4.03	0.46Å C with A 199 LEU HD23	Favored (68.8%) General / -59.3,-51.7	Favored (5.4%) *tt* chi angles: 191.3,156.4	0.04Å	Favored (89.301%) alpha helix	-	-	-
A 200		GLY	3.97	-	Favored (53.19%) Glycine / -56.0,-51.8	-	-	Favored (97.881%) alpha helix	-	-	-

#	Alt	Res	High B	Clash > 0.4Å	Ramachandran	Rotamer	Cβ deviation	CaBLAM	Bond lengths	Bond angles	Cis Peptides
			Avg: 5.66	Clashscore: 3.39	Outliers: 2 of 225	Poor rotamers: 1 of 176	Outliers: 0 of 209	Outliers: 3 of 223	Outliers: 3 of 227	Outliers: 5 of 227	Non-Trans: 0 of 226
A 201		LEU	4	-	Favored (91.15%) General / -60.1,-40.9	Favored (86.6%) *mt* chi angles: 290.5,170.2	0.05Å	Favored (82.761%) alpha helix	-	-	-
A 202		ALA	4.13	-	Favored (89.73%) General / -59.5,-41.4	-	0.05Å	Favored (82.119%) alpha helix	-	-	-
A 203		LEU	4.37	-	Favored (97.15%) General / -64.2,-41.1	Favored (94.7%) *mt* chi angles: 291.9,173.1	0.09Å	Favored (97.323%) alpha helix	-	-	-
A 204		THR	4.69	-	Favored (65.73%) General / -70.0,-28.7	Favored (67.7%) *p* chi angles: 62.8	0.02Å	Favored (73.192%) alpha helix	-	-	-
A 205		SER	5.09	-	Favored (60.84%) General / -71.4,-11.3	Favored (77.4%) *p* chi angles: 70.6	0.04Å	Favored (52.022%) alpha helix	-	-	-
A 206		THR	5.5	-	Favored (16.71%) General / -112.7,3.7	Favored (73.4%) *p* chi angles: 61.7	0.06Å	Favored (46.618%)	-	-	-
A 207		GLY	5.88	-	Favored (79.65%) Glycine / 73.6,19.0	-	-	Favored (84.216%)	-	-	-
A 208		GLN	6.19	-	Favored (45.53%) General / -98.0,-0.0	Favored (96.3%) *mm-40* chi angles: 301.3,300.9,303.5	0.02Å	Favored (20.01%)	-	-	-
A 209		PHE	6.38	-	Favored (5.17%) General / -158.4,126.2	Favored (53.6%) *t80* chi angles: 186.9,68.3	0.14Å	CaBLAM Disfavored (3.974%)	-	OUTLIER(S) worst is CA-CB-CG: 7.6 σ	-
A 210		SER	6.47	-	Favored (58.81%) General / -64.8,139.9	Favored (36.7%) *t* chi angles: 174.6	0.06Å	Favored (46.355%)	-	-	-
A 211		ALA	6.48	-	Favored (64.61%) General / -60.4,-23.7	-	0.02Å	Favored (44.191%)	-	-	-
A 212		SER	6.43	-	Favored (58.44%) General / -74.9,-24.0	Favored (94.9%) *p* chi angles: 66.2	0.03Å	Favored (70.2%) alpha helix	-	-	-
A 213		VAL	6.34	-	Favored (17.76%) Ile or Val / -84.0,-43.4	Favored (99.6%) *t* chi angles: 175.4	0.03Å	Favored (58.51%) alpha helix	-	-	-
A 214		MET	6.28	-	Favored (71.91%) General / -56.3,-50.2	Favored (45.4%) *ttp* chi angles: 177.1,191.8,68.8	0.12Å	Favored (98.628%) alpha helix	-	-	-
A 215		ALA	6.3	-	Favored (95.41%) General / -60.3,-42.4	-	0.04Å	Favored (97.423%) alpha helix	-	-	-
A 216		ALA	6.4	-	Favored (94.16%) General / -60.7,-41.0	-	0.04Å	Favored (95.853%) alpha helix	-	-	-

A 217		GLY	6.63	-	Favored (48.04%) Glycine / -63.4,-52.3	-	-	Favored (93.122%) alpha helix	-	-	-
A 218		LEU	7.01	-	Favored (85.26%) General / -65.2,-36.8	Favored (84.1%) *mt* chi angles: 294.3,178.4	0.05Å	Favored (82.58%) alpha helix	-	-	-
A 219		MET	7.52	-	Favored (90.73%) General / -65.9,-39.7	Favored (82.5%) *mtm* chi angles: 289,187.1,286.3	0.02Å	Favored (94.485%) alpha helix	-	-	-
A 220		ALA	8.13	-	Favored (81.08%) General / -61.9,-36.4	-	0.02Å	Favored (89.56%) alpha helix	-	-	-
**#**	**Alt**	**Res**	**High B**	**Clash > 0.4Å**	**Ramachandran**	**Rotamer**	**Cβ deviation**	**CaBLAM**	**Bond lengths**	**Bond angles**	**Cis Peptides**
			Avg: 5.66	Clashscore: 3.39	Outliers: 2 of 225	Poor rotamers: 1 of 176	Outliers: 0 of 209	Outliers: 3 of 223	Outliers: 3 of 227	Outliers: 5 of 227	Non-Trans: 0 of 226
A 221		CYS	8.78	-	Favored (74%) General / -68.3,-33.3	Favored (91.5%) *m* chi angles: 293.1	0.09Å	Favored (43.553%)	-	-	-
A 222		ASN	9.41	-	Favored (34.27%) Pre-Pro / -72.2,115.0	Favored (55.4%) *t0* chi angles: 186.2,330.3	0.03Å	Favored (31.298%)	-	-	-
A 223		PRO	9.96	-	Favored (48.68%) Trans-Pro / -65.3,-15.2	Favored (52.7%) *Cg_endo* chi angles: 25.5,325.9,28.3	0.02Å	Favored (58.477%)	-	-	-
A 224		ASN	10.43	-	Favored (36.64%) General / -96.4,10.9	Favored (88.5%) *m-40* chi angles: 293.1,320.3	0.02Å	Favored (31.39%)	-	-	-
A 225		LYS	10.77	-	Favored (63.01%) General / -60.4,-22.1	Favored (61%) *pttt* chi angles: 65.4,185.4,176.8,182.3	0.04Å	Favored (9.125%)	-	-	-
A 226		LYS	11	-	Favored (9.35%) General / -82.5,70.0	Favored (99.3%) *mttt* chi angles: 294.9,181.2,179.1,179.5	0.03Å	-	-	-	-
A 227		ARG	11.15	-	-	Favored (16.3%) *ptp-170* chi angles: 60.3,197.1,63.2,179.3	0.07Å	-	-	-	-

About MolProbity | Website for the Richardson Lab | Using ecloud x-H | Internal reference 4.5.2
