# Supplementary material for: Integrative proteome-wide structural analysis and high-throughput docking identify broad-spectrum antiviral scaffolds against Zika, Yellow Fever, West Nile, Saint Louis encephalitis, and Usutu viruses
Source: Front Cell Infect Microbiol. 2026 Apr 30;16:1723132. doi: 10.3389/fcimb.2026.1723132 (PMC13171538; doi:10.3389/fcimb.2026.1723132)
Supplement: Supplementary file 4 [file DataSheet4.zip › USUV/USU_NS2a/Mol_probity_Files/USU_NS2a_1FH-rama.pdf]

MolProbity Ramachandran analysis

USU_NS2a1FH.pdb, model 1

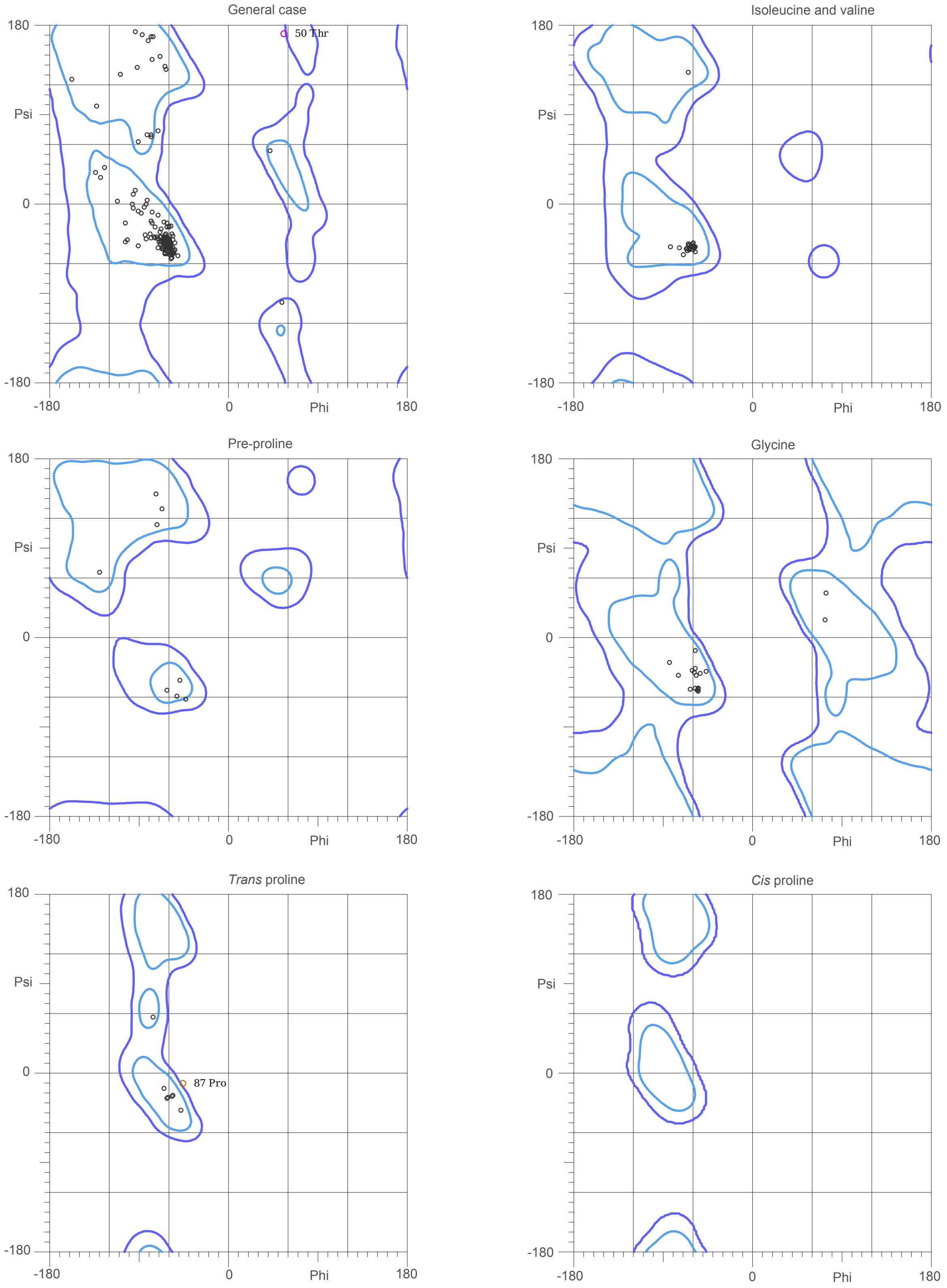

97.8% (220/225) of all residues were in favored (98%) regions.
99.1% (223/225) of all residues were in allowed (>99.8%) regions.

There were 2 outliers (phi, psi):
50 Thr (56.3, 172.2)
87 Pro (-46.9, -10.8)
