# Supplementary material for: Integrative proteome-wide structural analysis and high-throughput docking identify broad-spectrum antiviral scaffolds against Zika, Yellow Fever, West Nile, Saint Louis encephalitis, and Usutu viruses
Source: Front Cell Infect Microbiol. 2026 Apr 30;16:1723132. doi: 10.3389/fcimb.2026.1723132 (PMC13171538; doi:10.3389/fcimb.2026.1723132)
Supplement: Supplementary file 4 [file DataSheet4.zip › USUV/USU_NS2b/Mol_probity_Files/USU_NS2b_1FH-multi.table.pdf]

Viewing USU_NS2b1FH-multi.table

When finished, you should close this window.

Hint: Use File | Save As... to save a copy of this page.

All-Atom Contacts	Clashscore, all atoms:	0.5		99th percentile* (N=1784, all resolutions)
	Clashscore is the number of serious steric overlaps (> 0.4 Å) per 1000 atoms.			
Protein Geometry	Poor rotamers	0	0.00%	Goal: <0.3%
	Favored rotamers	103	100.00%	Goal: >98%
	Ramachandran outliers	5	3.88%	Goal: <0.05%
	Ramachandran favored	122	94.57%	Goal: >98%
	Rama distribution Z-score	0.40 ± 0.72		Goal: abs(Z score) < 2
	MolProbity score^	1.04		100th percentile* (N=27675, 0Å - 99Å)
	Cβ deviations >0.25Å	1	0.84%	Goal: 0
	Bad bonds:	0 / 1039	0.00%	Goal: 0%
	Bad angles:	10 / 1425	0.70%	Goal: <0.1%
Peptide Omegas	Cis Prolines:	0 / 6	0.00%	Expected: ≤1 per chain, or ≤5%
	Cis nonProlines:	2 / 124	1.61%	Goal: <0.05%
Low-resolution Criteria	CaBLAM outliers	3	2.4%	Goal: <1.0%
	CA Geometry outliers	0	0.00%	Goal: <0.5%
Additional validations	Chiral volume outliers	0/164		
	Waters with clashes	0/0	0.00%	See UnDowser table for details

In the two column results, the left column gives the raw count, right column gives the percentage.

* 100th percentile is the best among structures of comparable resolution; 0th percentile is the worst. For clashscore the comparative set of structures was selected in 2004, for MolProbity score in 2006.

^ MolProbity score combines the clashscore, rotamer, and Ramachandran evaluations into a single score, normalized to be on the same scale as X-ray resolution.

Key to table colors and cutoffs here:

#	Alt	Res	High B	Clash > 0.4Å	Ramachandran	Rotamer	Cβ deviation	CaBLAM	Bond lengths	Bond angles	Cis Peptides
			Avg: 6.24	Clashscore: 0.5	Outliers: 5 of 129	Poor rotamers: 0 of 103	Outliers: 1 of 119	Outliers: 3 of 127	Outliers: 0 of 131	Outliers: 7 of 131	Non-Trans: 2 of 130
A 1		GLY	7.09	-	-	-	-	-	-	-	-
A 2		TRP	6.79	-	OUTLIER (0.06)%) Pre-Pro / -19.6,-57.0	Favored (56.8%) *m100* chi angles: 276.3,110.1	0.20Å	-	-	-	-
A 3		PRO	6.41	-	Favored (17.47%) Trans-Pro / -48.4,-31.3	Favored (90.4%) *Cg_exo* chi angles: 329.5,37.7,331.4	0.02Å	Favored (46.332%)	-	-	-
A 4		ALA	5.96	-	Favored (88.12%) General / -61.7,-38.5	-	0.04Å	Favored (73.75%) alpha helix	-	-	-
A 5		THR	5.52	-	Favored (73.44%) General / -69.5,-43.3	Favored (90.8%) *m* chi angles: 298.9	0.02Å	Favored (81.078%) alpha helix	-	-	-
A 6		GLU	5.13	-	Favored (98.48%)	Favored (99.7%) *mt-10*	0.03Å	Favored (89.243%)	-	-	-

					General / -63.2,-40.8	chi angles: 292.4,176.8,354.4		alpha helix			
A 7		VAL	4.81	-	Favored (80.6%) Ile or Val / -67.7,-47.3	Favored (66.5%) *t* chi angles: 171.7	0.03Å	Favored (82.661%) alpha helix	-	-	-
A 8		LEU	4.57	-	Favored (82.97%) General / -62.2,-36.8	Favored (89.7%) *mt* chi angles: 290.8,171.6	0.04Å	Favored (85.031%) alpha helix	-	-	-
A 9		THR	4.42	-	Favored (80.66%) General / -64.3,-47.1	Favored (96.2%) *m* chi angles: 299.7	0.04Å	Favored (86.983%) alpha helix	-	-	-
A 10		ALA	4.34	-	Favored (89.43%) General / -60.0,-40.5	-	0.03Å	Favored (84.801%) alpha helix	-	-	-
A 11		VAL	4.29	-	Favored (80.83%) Ile or Val / -69.3,-44.0	Favored (76.5%) *t* chi angles: 172.8	0.05Å	Favored (92.068%) alpha helix	-	-	-
A 12		GLY	4.26	-	Favored (51.77%) Glycine / -57.0,-52.3	-	-	Favored (93.209%) alpha helix	-	-	-
A 13		LEU	4.24	-	Favored (97.22%) General / -62.7,-40.4	Favored (98.8%) *mt* chi angles: 292.3,172.3	0.04Å	Favored (81.647%) alpha helix	-	-	-
A 14		MET	4.22	-	Favored (86.4%) General / -58.3,-46.3	Favored (28.8%) *tmm* chi angles: 178.5,275.5,291	0.04Å	Favored (86.518%) alpha helix	-	-	-
A 15		PHE	4.19	-	Favored (89.02%) General / -64.3,-37.9	Favored (16.6%) *m-80* chi angles: 279.5,125.4	0.09Å	Favored (86.569%) alpha helix	-	-	-
A 16		ALA	4.16	-	Favored (86.06%) General / -59.9,-39.6	-	0.04Å	Favored (90.878%) alpha helix	-	-	-
A 17		ILE	4.16	-	Favored (89.12%) Ile or Val / -67.0,-42.9	Favored (97.7%) *mt* chi angles: 293.5,167.9	0.03Å	Favored (96.156%) alpha helix	-	-	-
A 18		VAL	4.16	-	Favored (87.68%) Ile or Val / -59.9,-42.0	Favored (57.8%) *t* chi angles: 170.5	0.01Å	Favored (81.346%) alpha helix	-	-	-
A 19		GLY	4.17	-	Favored (44.99%) Glycine / -53.0,-51.6	-	-	Favored (93.396%) alpha helix	-	-	-
A 20		GLY	4.24	-	Favored (31.09%) Glycine / -53.0,-54.0	-	-	Favored (99.135%) alpha helix	-	-	-
**#**	**Alt**	**Res**	**High B**	**Clash > 0.4Å**	**Ramachandran**	**Rotamer**	**Cβ deviation**	**CaBLAM**	**Bond lengths**	**Bond angles**	**Cis Peptides**
			Avg: 6.24	Clashscore: 0.5	Outliers: 5 of 129	Poor rotamers: 0 of 103	Outliers: 1 of 119	Outliers: 3 of 127	Outliers: 0 of 131	Outliers: 7 of 131	Non-Trans: 2 of 130
A 21		LEU	4.45	-	Favored (72.15%)	Favored (64.6%) *tp* chi angles: 175.5,61.1	0.06Å	Favored (77.201%) alpha helix	-	-	-

					General / -54.6,-43.4						
A 22		ALA	4.78	-	Favored (72.28%) General / -58.4,-35.5	-	0.03Å	Favored (61.64%) alpha helix	-	-	-
A 23		GLU	5.2	-	Favored (13.83%) General / -92.1,-35.5	Favored (95.8%) *mt-10* chi angles: 295.2,182.5,354.1	0.03Å	Favored (19.18%)	-	-	-
A 24		LEU	5.65	-	Favored (7.51%) General / -88.6,64.7	Favored (89.9%) *mt* chi angles: 299.5,177	0.04Å	Favored (6.582%)	-	-	-
A 25		ASP	6.04	-	Favored (18.14%) General / -81.2,5.0	Favored (57.7%) *p0* chi angles: 65.1,2.2	0.06Å	Favored (6.866%)	-	-	-
A 26		VAL	6.31	-	Favored (70.68%) Ile or Val / -71.5,-39.2	Favored (97.8%) *t* chi angles: 175.2	0.11Å	Favored (15.602%)	-	OUTLIER(S) worst is C-N-CA: 5.8 σ	-
A 27		ASP	6.42	-	Favored (8.03%) General / -146.9,178.8	Favored (11.2%) *t0* chi angles: 206.3,336.4	0.06Å	Favored (7.503%)	-	OUTLIER(S) worst is CA-CB-CG: 5.2 σ	-
A 28		SER	6.35	-	Favored (16.98%) General / -78.5,170.2	Favored (94.5%) *p* chi angles: 63.3	0.07Å	Favored (8.711%)	-	-	-
A 29		MET	6.16	-	Allowed (0.43%) General / 66.7,-7.9	Favored (96.9%) *mmm* chi angles: 291.9,301.1,295.3	0.07Å	CaBLAM Outlier (0.155%) try alpha helix	-	-	-
A 30		SER	5.94	-	Favored (96.07%) General / -63.6,-40.1	Favored (64%) *m* chi angles: 294.1	0.03Å	Favored (49.493%) alpha helix	-	-	-
A 31		ILE	5.77	0.41Å HB with A 32 PRO HD3	Favored (26.13%) Pre-Pro / -65.2,-55.4	Favored (97.5%) *mt* chi angles: 293.5,168.2	0.20Å	Favored (84.756%) alpha helix	-	-	-
A 32		PRO	5.61	0.41Å HD3 with A 31 ILE HB	Favored (35.09%) Trans-Pro / -49.4,-35.2	Favored (51.8%) *Cg_exo* chi angles: 327.9,37.3,333.5	0.04Å	Favored (95.913%) alpha helix	-	-	-
A 33		PHE	5.41	-	Favored (88.56%) General / -66.7,-40.9	Favored (15.2%) *m-10* chi angles: 290.1,330.2	0.02Å	Favored (85.162%) alpha helix	-	-	-
A 34		VAL	5.17	-	Favored (98.65%) Ile or Val / -63.1,-43.0	Favored (49.2%) *t* chi angles: 169.3	0.04Å	Favored (94.835%) alpha helix	-	-	-
A 35		LEU	4.95	-	Favored (95.93%) General / -63.0,-39.8	Favored (97.6%) *mt* chi angles: 293.3,173	0.05Å	Favored (96.058%) alpha helix	-	-	-
A 36		ALA	4.73	-	Favored (89.92%) General / -60.9,-39.6	-	0.03Å	Favored (96.284%) alpha helix	-	-	-
A 37		GLY	4.52	-	Favored (49.68%) Glycine / -60.3,-52.9	-	-	Favored (92.688%) alpha helix	-	-	-

A 38		LEU	4.32	-	Favored (89.77%) General / -61.5,-39.1	Favored (89.9%) *mt* chi angles: 291.6,169.5	0.05Å	Favored (79.85%) alpha helix	-	-	-
A 39		MET	4.13	-	Favored (84.01%) General / -67.6,-38.5	Favored (81.9%) *mtm* chi angles: 288.6,188.2,287.3	0.06Å	Favored (86.053%) alpha helix	-	-	-
A 40		ALA	3.98	-	Favored (85.92%) General / -60.6,-38.9	-	0.04Å	Favored (87.411%) alpha helix	-	-	-

#	Alt	Res	High B	Clash > 0.4Å	Ramachandran	Rotamer	Cβ deviation	CaBLAM	Bond lengths	Bond angles	Cis Peptides
			Avg: 6.24	Clashscore: 0.5	Outliers: 5 of 129	Poor rotamers: 0 of 103	Outliers: 1 of 119	Outliers: 3 of 127	Outliers: 0 of 131	Outliers: 7 of 131	Non-Trans: 2 of 130
A 41		VAL	3.88	-	Favored (83.06%) Ile or Val / -66.0,-48.0	Favored (70.2%) *t* chi angles: 172.1	0.04Å	Favored (81.566%) alpha helix	-	-	-
A 42		SER	3.83	-	Favored (97.75%) General / -61.9,-41.3	Favored (70.8%) *m* chi angles: 295.1	0.04Å	Favored (80.03%) alpha helix	-	-	-
A 43		TYR	3.87	-	Favored (67.66%) General / -72.7,-35.9	Favored (29.8%) *m-80* chi angles: 288.3,126.5	0.03Å	Favored (93.181%) alpha helix	-	-	-
A 44		THR	4.05	-	Favored (84.63%) General / -65.8,-44.8	Favored (91.1%) *m* chi angles: 301.1	0.03Å	Favored (82.589%) alpha helix	-	-	-
A 45		ILE	4.41	-	Favored (93.28%) Ile or Val / -65.7,-44.5	Favored (97.9%) *mt* chi angles: 293.1,168.3	0.02Å	Favored (83.185%) alpha helix	-	-	-
A 46		SER	4.99	-	Favored (92.29%) General / -60.0,-41.5	Favored (72.5%) *m* chi angles: 295.8	0.04Å	Favored (84.499%) alpha helix	-	-	-
A 47		GLY	5.81	-	Favored (37.75%) Glycine / -54.0,-53.3	-	-	Favored (96.48%) alpha helix	-	-	-
A 48		LYS	6.88	-	Favored (63.18%) General / -51.8,-44.0	Favored (81.8%) *tttt* chi angles: 181.6,181.7,176.8,186.5	0.05Å	Favored (80.872%) alpha helix	-	-	-
A 49		SER	8.14	-	Favored (62.11%) General / -67.9,-13.4	Favored (55.9%) *p* chi angles: 73.7	0.04Å	Favored (57.679%)	-	-	-
A 50		THR	9.46	-	Favored (35.96%) General / -104.1,10.5	Favored (74.4%) *p* chi angles: 61.5	0.02Å	Favored (45.618%)	-	-	-
A 51		ASP	10.66	-	Allowed (1.84%) General / -74.3,73.9	Favored (40.4%) *t0* chi angles: 188.8,24.5	0.08Å	Favored (14.596%)	-	-	-
A 52		LEU	11.56	-	Favored (32.13%) General / -84.4,139.6	Favored (88.4%) *mt* chi angles: 299.6,176.5	0.05Å	Favored (24.406%) beta sheet	-	-	-

A 53		TRP	12.09	-	Favored (20.59%) General / -96.9,150.6	Favored (55.6%) *m-10* chi angles: 295.2,351.9	0.04Å	Favored (41.875%) beta sheet	-	-	-
A 54		LEU	12.33	-	Favored (27.03%) General / -109.9,151.5	Favored (72.2%) *mt* chi angles: 303.4,177.2	0.03Å	Favored (57.041%) beta sheet	-	-	-
A 55		GLU	12.37	-	Favored (51.04%) General / -129.1,148.7	Favored (94.2%) *mt-10* chi angles: 297.4,181.7,0.1	0.04Å	Favored (56.435%) beta sheet	-	-	-
A 56		ARG	12.32	-	Favored (25.65%) General / -95.9,114.0	Favored (55%) *ttm170* chi angles: 179.3,176,292.1,177.6	0.05Å	Favored (58.067%)	-	-	-
A 57		ALA	12.27	-	Favored (90.02%) General / -62.2,-38.7	-	0.03Å	Favored (9.857%)	-	-	-
A 58		ALA	12.3	-	Favored (10.09%) General / -165.4,152.0	-	0.04Å	Favored (6.014%)	-	-	-
A 59		ASP	12.42	-	Favored (56.54%) General / -67.8,141.3	Favored (95.8%) *m-30* chi angles: 289.6,348.2	0.01Å	Favored (35.347%)	-	-	-
A 60		ILE	12.61	-	Favored (72.48%) Ile or Val / -125.4,131.2	Favored (77.8%) *mt* chi angles: 300.7,170.5	0.02Å	Favored (67.034%) beta sheet	-	-	-
**#**	**Alt**	**Res**	**High B**	**Clash > 0.4Å**	**Ramachandran**	**Rotamer**	**Cβ deviation**	**CaBLAM**	**Bond lengths**	**Bond angles**	**Cis Peptides**
			Avg: 6.24	Clashscore: 0.5	Outliers: 5 of 129	Poor rotamers: 0 of 103	Outliers: 1 of 119	Outliers: 3 of 127	Outliers: 0 of 131	Outliers: 7 of 131	Non-Trans: 2 of 130
A 61		THR	12.85	-	Favored (53.4%) General / -121.6,130.3	Favored (96.9%) *m* chi angles: 299.8	0.03Å	Favored (64.045%) beta sheet	-	-	-
A 62		TRP	13.08	-	Favored (22.83%) General / -102.0,-6.9	Favored (37.1%) *m-90* chi angles: 296.3,262.6	0.05Å	CaBLAM Disfavored (3.719%)	-	-	-
A 63		GLU	13.23	-	OUTLIER (0.01)%) General / 45.7,-167.2	Favored (96.5%) *mt-10* chi angles: 295,181.9,356.9	0.03Å	CaBLAM Disfavored (4.914%)	-	-	-
A 64		THR	13.3	-	Favored (7.53%) General / -121.0,-18.4	Favored (73.5%) *p* chi angles: 61.6	0.02Å	CaBLAM Outlier (0.842%)	-	-	-
A 65		ASP	13.24	-	Favored (40.18%) General / -101.6,10.4	Favored (74.3%) *m-30* chi angles: 296.6,318.6	0.03Å	Favored (39.877%) alpha helix	-	-	-
A 66		ALA	13.05	-	Favored (73.45%) General / -59.4,-35.1	-	0.04Å	Favored (34.523%) alpha helix	-	-	-
A 67		ALA	12.7	-	Favored (66.54%) General / -60.0,-27.0	-	0.04Å	Favored (54.844%) alpha helix	-	-	-

A<br>68		ILE	12.15	-	Favored<br>(9.91%)<br>Ile or Val /<br>-98.9,-41.3	Favored (48.9%)<br>*mm*<br>chi angles: 303.5,303	0.05Å	Favored<br>(25.772%)	-	-	-
A<br>69		THR	11.37	-	Favored<br>(87.72%)<br>General /<br>-59.0,-46.6	Favored (96.8%) *m*<br>chi angles: 299.8	0.05Å	Favored<br>(9.979%)	-	-	-
A<br>70		GLY	10.39	-	Favored<br>(14.62%)<br>Glycine /<br>149.5,168.1	-	-	Favored<br>(27.676%)	-	-	-
A<br>71		THR	9.33	-	Favored<br>(41.76%)<br>General /<br>-61.1,129.5	Favored (86.8%) *m*<br>chi angles: 301.5	0.03Å	Favored<br>(7.992%)	-	-	-
A<br>72		SER	8.34	-	Favored<br>(22.35%)<br>General /<br>-88.7,151.6	Favored (65.9%) *m*<br>chi angles: 294.4	0.02Å	Favored<br>(40.438%)<br>beta sheet	-	-	-
A<br>73		GLN	7.57	-	Favored<br>(10.34%)<br>General /<br>-83.6,71.8	Favored (87.5%)<br>*mm-40*<br>chi angles:<br>299.6,296.1,296	0.03Å	Favored<br>(14.858%)<br>beta sheet	-	-	-
A<br>74		ARG	7.16	-	Favored<br>(26.23%)<br>General /<br>-83.6,150.0	Favored (92.7%)<br>*mtm180*<br>chi angles:<br>295.2,174.4,292.6,174.6	0.05Å	Favored<br>(22.447%)<br>beta sheet	-	-	-
A<br>75		LEU	7.15	-	Favored<br>(48.66%)<br>General /<br>-118.2,124.8	Favored (60.4%) *tp*<br>chi angles: 179.4,64.3	0.02Å	Favored<br>(51.188%)<br>beta sheet	-	-	-
A<br>76		ASP	7.58	-	Favored<br>(16.1%)<br>General /<br>-82.6,108.5	Favored (64.4%) *t0*<br>chi angles: 185.4,354.1	0.06Å	Favored<br>(59.25%)<br>beta sheet	-	OUTLIER(S)<br>worst is CA-CB-CG: 4.9 σ	-
A<br>77		VAL	8.41	-	Favored<br>(71.95%)<br>Ile or Val /<br>-116.0,129.7	Favored (84.7%) *t*<br>chi angles: 177.7	0.04Å	Favored<br>(63.42%)<br>beta sheet	-	-	-
A<br>78		LYS	9.57	-	Favored<br>(3.62%)<br>General /<br>-117.4,91.6	Favored (51.2%)<br>*mttm*<br>chi angles:<br>297.9,186.6,187,307.3	0.06Å	Favored<br>(39.908%)<br>beta sheet	-	-	-
A<br>79		LEU	10.88	-	Favored<br>(57.78%)<br>General /<br>-61.6,135.8	Favored (93.5%) *mt*<br>chi angles: 294.8,175.6	0.07Å	Favored<br>(16.086%)	-	-	-
A<br>80		ASP	12.06	-	Favored<br>(5.66%)<br>General /<br>-88.3,-177.4	Favored (29.6%) *p0*<br>chi angles: 72.4,17	0.07Å	Favored<br>(36.428%)	-	-	-
**#**	**Alt**	**Res**	**High B**	**Clash > 0.4Å**	**Ramachandran**	**Rotamer**	**Cβ deviation**	**CaBLAM**	**Bond lengths**	**Bond angles**	**Cis Peptides**
			Avg: 6.24	Clashscore: 0.5	Outliers: 5 of 129	Poor rotamers: 0 of 103	Outliers: 1 of 119	Outliers: 3 of 127	Outliers: 0 of 131	Outliers: 7 of 131	Non-Trans: 2 of 130
A<br>81		ASP	12.85	-	Favored<br>(67.12%)<br>General /<br>-62.4,-23.9	Favored (94.8%) *m-30*<br>chi angles: 290.3,348.2	0.02Å	Favored<br>(33.718%)	-	-	-
A<br>82		ASP	13.05	-	Favored<br>(41.6%)<br>General /<br>-101.8,6.3	Favored (72.2%) *m-30*<br>chi angles: 293.1,318.7	0.01Å	Favored<br>(51.3%)	-	-	-

A 83		GLY	12.61	-	Favored (84.08%) Glycine / 85.2,6.8	-	-	Favored (86.57%)	-	-	-
A 84		ASP	11.67	-	Favored (7.58%) General / -83.2,179.9	Favored (7.1%) *t0* chi angles: 203.2,326.6	0.04Å	Favored (29.532%)	-	-	-
A 85		PHE	10.46	-	OUTLIER (0.03)%) General / -80.2,-97.1	Favored (66.8%) *m-80* chi angles: 296.8,113.7	0.21Å	CaBLAM Disfavored (4.957%) try beta sheet	-	OUTLIER(S) worst is CA-C-N: 4.6 σ	-
A 86		HIS	9.18	-	Favored (46.94%) General / -71.3,148.6	Favored (73.9%) *t-90* chi angles: 193.4,280.3	0.05Å	CaBLAM Disfavored (4.506%) try beta sheet	-	OUTLIER(S) worst is C-N-CA: 8.0 σ	Cis nonPRO omega= -10.35
A 87		LEU	7.99	-	Favored (51.67%) General / -79.5,-4.5	Favored (95%) *mt* chi angles: 296.6,175.2	0.04Å	CaBLAM Disfavored (1.628%) try beta sheet	-	-	-
A 88		ILE	6.96	-	OUTLIER (0)%) Ile or Val / 33.9,-171.9	Favored (42.7%) *pt* chi angles: 63.3,169.4	0.06Å	Favored (16.102%)	-	-	-
A 89		ASN	6.12	-	Favored (30.86%) General / 55.0,38.6	Favored (90.1%) *m-40* chi angles: 294.6,326.6	0.08Å	CaBLAM Outlier (0.018%)	-	-	-
A 90		ASP	5.46	-	Favored (4.42%) Pre-Pro / -145.6,108.0	Favored (60.5%) *t0* chi angles: 183.7,340.3	0.02Å	CaBLAM Disfavored (4.925%)	-	-	-
A 91		PRO	4.92	-	Favored (35.68%) Trans-Pro / -69.2,-13.2	Favored (67.6%) *Cg_endo* chi angles: 27.1,326.5,25.8	0.03Å	CaBLAM Disfavored (3.328%)	-	-	-
A 92		GLY	4.46	-	Favored (48.45%) Glycine / 72.6,-152.9	-	-	Favored (58.336%)	-	-	-
A 93		VAL	4.03	-	Favored (61.35%) Pre-Pro / -94.9,122.0	Favored (77.6%) *t* chi angles: 178.1	0.06Å	Favored (6.08%)	-	-	-
A 94		PRO	3.63	-	Favored (78.12%) Trans-Pro / -56.1,144.9	Favored (85.6%) *Cg_exo* chi angles: 333.8,34.6,331.5	0.04Å	Favored (84.55%)	-	-	-
A 95		TRP	3.25	-	Favored (83.66%) General / -58.0,-41.7	Favored (54.7%) *t-100* chi angles: 187.3,245.3	0.04Å	Favored (64.441%)	-	-	-
A 96		LYS	2.91	-	Favored (72.03%) General / -54.2,-45.0	Favored (86.6%) *tttt* chi angles: 180.9,176.9,178,178.9	0.00Å	Favored (76.184%) alpha helix	-	-	-
A 97		ILE	2.62	-	Favored (93.69%) Ile or Val / -65.1,-42.2	Favored (76.8%) *mt* chi angles: 290,171.5	0.10Å	Favored (88.676%) alpha helix	-	-	-
A 98		TRP	2.37	-	Favored (83.98%) General / -58.0,-47.1	Favored (89.9%) *t60* chi angles: 183.5,89.5	0.07Å	Favored (89.589%) alpha helix	-	-	-
A 99		VAL	2.16	-	Favored (99.25%) Ile or Val / -63.3,-44.8	Favored (68.5%) *t* chi angles: 171.9	0.02Å	Favored (90.014%) alpha helix	-	-	-

#	Alt	Res	High B	Clash > 0.4Å	Ramachandran	Rotamer	Cβ deviation	CaBLAM	Bond lengths	Bond angles	Cis Peptides
A 100		ILE	2	-	Favored (99.3%) Ile or Val / -62.5,-44.4	Favored (97.6%) *mt* chi angles: 292.2,167.3	0.02Å	Favored (97.755%) alpha helix	-	-	-
#	Alt	Res	High B	Clash > 0.4Å	Ramachandran	Rotamer	Cβ deviation	CaBLAM	Bond lengths	Bond angles	Cis Peptides
			Avg: 6.24	Clashscore: 0.5	Outliers: 5 of 129	Poor rotamers: 0 of 103	Outliers: 1 of 119	Outliers: 3 of 127	Outliers: 0 of 131	Outliers: 7 of 131	Non-Trans: 2 of 130
A 101		ARG	1.88	-	Favored (92.22%) General / -60.8,-46.0	Favored (97%) *mtt180* chi angles: 289.4,174.4,181.3,182.9	0.14Å	Favored (86.224%) alpha helix	-	-	-
A 102		MET	1.79	-	Favored (93.97%) General / -65.0,-39.9	Favored (92%) *mmm* chi angles: 290.1,294.5,288.5	0.04Å	Favored (87.095%) alpha helix	-	-	-
A 103		THR	1.72	-	Favored (78.9%) General / -65.6,-46.5	Favored (93.4%) *m* chi angles: 299.2	0.04Å	Favored (97.738%) alpha helix	-	-	-
A 104		ALA	1.66	-	Favored (96.55%) General / -60.8,-42.0	-	0.06Å	Favored (75.274%) alpha helix	-	-	-
A 105		LEU	1.66	-	Favored (38.14%) General / -80.1,-34.5	Favored (96.1%) *mt* chi angles: 297,176.4	0.08Å	Favored (75.605%) alpha helix	-	-	-
A 106		GLY	1.82	-	Favored (67.9%) Glycine / -61.1,-50.4	-	-	Favored (91.938%) alpha helix	-	-	-
A 107		PHE	2.24	-	Favored (74.89%) General / -55.9,-48.8	Favored (85.2%) *t80* chi angles: 180.8,75.4	0.02Å	Favored (98.067%) alpha helix	-	-	-
A 108		ALA	3.12	-	Favored (76.36%) General / -58.1,-38.4	-	0.05Å	Favored (77.807%) alpha helix	-	-	-
A 109		ALA	4.62	-	Favored (84.64%) General / -60.9,-38.1	-	0.03Å	Favored (73.613%) alpha helix	-	-	-
A 110		TRP	6.52	-	Favored (35.57%) General / -80.9,-29.3	Favored (90.3%) *m100* chi angles: 289.7,109.9	0.02Å	Favored (59.842%) alpha helix	-	-	-
A 111		THR	7.99	-	Favored (75.68%) Pre-Pro / -131.2,76.7	Favored (57.7%) *p* chi angles: 57.1	0.08Å	Favored (16.622%) alpha helix	-	-	-
A 112		PRO	8.03	-	Favored (10.22%) Trans-Pro / -46.5,-31.7	Favored (89.1%) *Cg_exo* chi angles: 329.9,37.6,331.1	0.03Å	Favored (30.146%) alpha helix	-	-	-
A 113		TRP	6.6	-	Favored (64.65%) General / -59.5,-25.7	Favored (73.5%) *p-90* chi angles: 59.9,265.2	0.01Å	Favored (62.753%) three-ten	-	-	-
A 114		ALA	4.67	-	Favored (35.06%) General / -79.3,-1.1	-	0.04Å	Favored (29.38%) three-ten	-	-	-
A 115		ILE	3.12	-	Favored (69.93%)	Favored (83.6%) *mt* chi angles: 290.2,167.1	0.03Å	Favored (54.506%)	-	-	-

					Ile or Val /<br>-58.3,-38.6			alpha helix			
A<br>116		ILE	2.2	-	Favored<br>(7.04%)<br>Pre-Pro /<br>-63.2,-61.6	Favored (96.6%) *mt*<br>chi angles: 292.2,168.4	0.16Å	Favored<br>(55.083%)<br>alpha helix	-	-	-
A<br>117		PRO	1.75	-	Favored<br>(76.09%)<br>Trans-Pro /<br>-62.4,-25.0	Favored (33.2%)<br>*Cg_endo*<br>chi angles:<br>21.9,325.9,31.7	0.02Å	Favored<br>(52.834%)<br>alpha helix	-	-	-
A<br>118		ALA	1.56	-	Favored<br>(86.09%)<br>General /<br>-67.2,-41.1	-	0.06Å	Favored<br>(74.349%)<br>alpha helix	-	-	-
A<br>119		GLY	1.53	-	Favored<br>(87.61%)<br>Glycine /<br>-61.1,-48.1	-	-	Favored<br>(93.042%)<br>alpha helix	-	-	-
A<br>120		ILE	1.56	-	Favored<br>(98.97%)<br>Ile or Val /<br>-62.7,-45.4	Favored (98.3%) *mt*<br>chi angles: 292.4,167.1	0.05Å	Favored<br>(93.865%)<br>alpha helix	-	-	-
**#**	**Alt**	**Res**	**High B**	**Clash > 0.4Å**	**Ramachandran**	**Rotamer**	**Cβ deviation**	**CaBLAM**	**Bond lengths**	**Bond angles**	**Cis Peptides**
			Avg: 6.24	Clashscore: 0.5	Outliers: 5 of 129	Poor rotamers: 0 of 103	Outliers: 1 of 119	Outliers: 3 of 127	Outliers: 0 of 131	Outliers: 7 of 131	Non-Trans: 2 of 130
A<br>121		GLY	1.61	-	Favored<br>(37.27%)<br>Glycine /<br>-56.0,-54.2	-	-	Favored<br>(92.925%)<br>alpha helix	-	-	-
A<br>122		TYR	1.68	-	Favored<br>(71.09%)<br>General /<br>-53.8,-47.1	Favored (83.1%)<br>*t80*<br>chi angles: 179,84.6	0.02Å	Favored<br>(86.171%)<br>alpha helix	-	-	-
A<br>123		TRP	1.78	-	Favored<br>(91.56%)<br>General /<br>-61.5,-39.6	Favored (47.9%) *m-10*<br>chi angles: 292.2,334.9	0.06Å	Favored<br>(74.468%)<br>alpha helix	-	-	-
A<br>124		LEU	1.9	-	Favored<br>(42.08%)<br>General /<br>-79.6,-32.5	Favored (92.9%) *mt*<br>chi angles: 292.7,175	0.02Å	Favored<br>(42.059%)<br>alpha helix	-	-	-
A<br>125		THR	2.08	-	Favored<br>(8.22%)<br>General /<br>-44.9,-42.7	Favored (90.4%) *m*<br>chi angles: 297.2	0.21Å	Favored<br>(52.134%)<br>alpha helix	-	OUTLIER(S)<br>worst is CA-C-N: 6.2 σ	-
A<br>126		VAL	2.33	-	OUTLIER<br>(0)%)<br>Ile or Val /<br>100.1,-88.5	Favored (97.8%) *t*<br>chi angles: 175.2	0.28Å	Favored<br>(24.991%)<br>alpha helix	-	OUTLIER(S)<br>worst is C-N-CA: 11.0 σ	Cis nonPRO<br>omega= 5
A<br>127		LYS	2.68	-	Favored<br>(66.32%)<br>General /<br>-54.0,-39.6	Favored (85.6%)<br>*tttt*<br>chi angles:<br>181.5,179.1,177.9,180.3	0.07Å	Favored<br>(53.312%)<br>alpha helix	-	-	-
A<br>128		TYR	3.16	-	Favored<br>(65.95%)<br>General /<br>-65.7,-19.6	Favored (34.2%) *m-80*<br>chi angles: 292.3,126.3	0.03Å	Favored<br>(62.031%)	-	-	-
A<br>129		ALA	3.75	-	Favored<br>(55.58%)<br>General / -87.3,0.3	-	0.03Å	Favored<br>(16.137%)	-	-	-
A<br>130		LYS	4.42	-	Favored<br>(23.99%)<br>General /<br>-50.2,132.2	Favored (51.3%)<br>*tttm*<br>chi angles:<br>179.2,179.9,182.4,294.8	0.05Å	-	-	-	-

A 131	ARG	5.08	-	-	Favored (98.7%) *mtt180* chi angles: 295.7,177.1,184.6,177.2	0.07Å	-	-	-	-

About MolProbity | Website for the Richardson Lab | Using ecloud x-H | Internal reference 4.5.2
