# Supplementary material for: Integrative proteome-wide structural analysis and high-throughput docking identify broad-spectrum antiviral scaffolds against Zika, Yellow Fever, West Nile, Saint Louis encephalitis, and Usutu viruses
Source: Front Cell Infect Microbiol. 2026 Apr 30;16:1723132. doi: 10.3389/fcimb.2026.1723132 (PMC13171538; doi:10.3389/fcimb.2026.1723132)
Supplement: Supplementary file 4 [file DataSheet4.zip › USUV/USU_NS2b/Mol_probity_Files/USU_NS2b_1FH-rama.pdf]

MolProbity Ramachandran analysis

USU_NS2b1FH.pdb, model 1

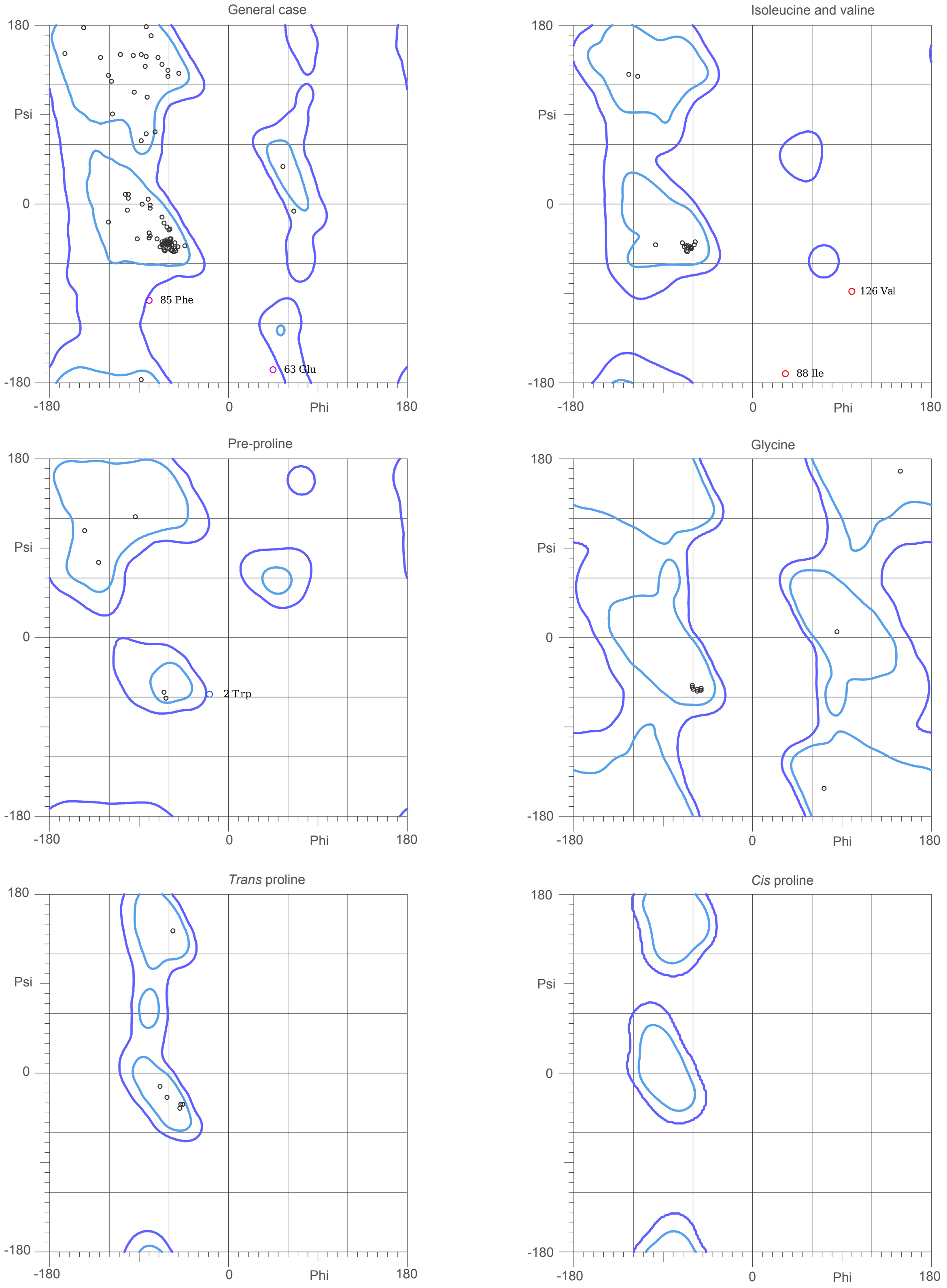

94.6% (122/129) of all residues were in favored (98%) regions.
96.1% (124/129) of all residues were in allowed (>99.8%) regions.

There were 5 outliers (phi, psi):
- 2 Trp (-19.6, -57.0)
- 63 Glu (45.7, -167.2)
- 85 Phe (-80.2, -97.1)
- 88 Ile (33.9, -171.9)
- 126 Val (100.1, -88.6)

http://kinemage.biochem.duke.edu

Lovell, Davis, et al. Proteins 50:437 (2003)
