# Supplementary material for: Integrative proteome-wide structural analysis and high-throughput docking identify broad-spectrum antiviral scaffolds against Zika, Yellow Fever, West Nile, Saint Louis encephalitis, and Usutu viruses
Source: Front Cell Infect Microbiol. 2026 Apr 30;16:1723132. doi: 10.3389/fcimb.2026.1723132 (PMC13171538; doi:10.3389/fcimb.2026.1723132)
Supplement: Supplementary file 4 [file DataSheet4.zip › USUV/USU_NS3/Mol_probity_Files/USU_NS3_1FH-multi.table.pdf]

Viewing USU_NS3_1FH-multi.table

Duke Biochemistry
Duke University School of Medicine

When finished, you should close this window.

Hint: Use File | Save As... to save a copy of this page.

All-Atom Contacts	Clashscore, all atoms:	1.45		99th percentile* (N=1784, all resolutions)
	Clashscore is the number of serious steric overlaps (> 0.4 Å) per 1000 atoms.			
Protein Geometry	Poor rotamers	1	0.20%	Goal: <0.3%
	Favored rotamers	506	99.80%	Goal: >98%
	Ramachandran outliers	1	0.16%	Goal: <0.05%
	Ramachandran favored	608	98.54%	Goal: >98%
	Rama distribution Z-score	0.64 ± 0.33		Goal: abs(Z score) < 2
	MolProbity score^	0.88		100th percentile* (N=27675, 0Å - 99Å)
	Cβ deviations >0.25Å	0	0.00%	Goal: 0
	Bad bonds:	7 / 4956	0.14%	Goal: 0%
	Bad angles:	6 / 6731	0.09%	Goal: <0.1%
Peptide Omegas	Cis Prolines:	0 / 37	0.00%	Expected: ≤1 per chain, or ≤5%
Low-resolution Criteria	CaBLAM outliers	14	2.3%	Goal: <1.0%
	CA Geometry outliers	3	0.49%	Goal: <0.5%
Additional validations	Chiral volume outliers	0/728		
	Waters with clashes	0/0	0.00%	See UnDowser table for details

In the two column results, the left column gives the raw count, right column gives the percentage.

* 100th percentile is the best among structures of comparable resolution; 0th percentile is the worst. For clashscore the comparative set of structures was selected in 2004, for MolProbity score in 2006.

^ MolProbity score combines the clashscore, rotamer, and Ramachandran evaluations into a single score, normalized to be on the same scale as X-ray resolution.

Key to table colors and cutoffs here:

#	Alt	Res	High B	Clash > 0.4Å	Ramachandran	Rotamer	Cβ deviation	CaBLAM	Bond lengths	Bond angles	Cis Peptides
			Avg: 1.13	Clashscore: 1.45	Outliers: 1 of 617	Poor rotamers: 1 of 507	Outliers: 0 of 561	Outliers: 16 of 615	Outliers: 7 of 619	Outliers: 6 of 619	Non-Trans: 0 of 618
A 1		GLY	10.92	-	-	-	-	-	-	-	-
A 2		GLY	10.18	-	Favored (66.24%) Glycine / -76.0,-0.8	-	-	-	-	-	-
A 3		VAL	9.33	-	Favored (7.64%) Ile or Val / -114.7,5.3	Favored (31.4%) *m* chi angles: 297.8	0.07Å	Favored (30.95%)	-	-	-
A 4		PHE	8.45	-	Favored (58.35%) General / -62.8,137.8	Favored (67.6%) *t80* chi angles: 186.5,79.6	0.01Å	Favored (33.412%)	-	-	-
A 5		TRP	7.64	-	Favored (57.47%) General / -66.3,138.2	Favored (96.3%) *m100* chi angles: 291.9,106	0.06Å	Favored (46.61%) beta sheet	-	-	-
A 6		ASP	6.9	-	Favored (56.93%) General / -61.3,142.4	Favored (98.4%) *m-30* chi angles: 287.5,348.7	0.04Å	Favored (47.147%) beta sheet	-	-	-

A 7		THR	6.22	-	Favored (65.93%)<br>Pre-Pro / -85.3,126.9	Favored (93.2%) *m*<br>chi angles: 299.2	0.05Å	Favored (38.843%)<br>beta sheet	-	-	-
A 8		PRO	5.58	-	Favored (75.28%)<br>Trans-Pro / -68.5,150.2	Favored (49.1%) *Cg_endo*<br>chi angles: 25.1,326.7,27.4	0.02Å	Favored (81.399%)<br>beta sheet	-	-	-
A 9		ALA	4.94	-	Favored (87.42%)<br>Pre-Pro / -74.1,149.7	-	0.03Å	Favored (51.564%)<br>beta sheet	-	-	-
A 10		PRO	4.32	-	Favored (53.99%)<br>Trans-Pro / -72.0,157.7	Favored (68.3%) *Cg_endo*<br>chi angles: 27.1,325.6,27.3	0.03Å	Favored (74.383%)<br>beta sheet	-	-	-
A 11		ARG	3.7	-	Favored (28.08%)<br>General / -81.2,151.5	Favored (98.6%) *mtt180*<br>chi angles: 294.1,180.5,181.1,178.3	0.02Å	Favored (39.832%)<br>beta sheet	-	-	-
A 12		THR	3.11	-	Favored (35.23%)<br>General / -82.4,131.1	Favored (90.5%) *m*<br>chi angles: 301.2	0.02Å	Favored (41.34%)<br>beta sheet	-	-	-
A 13		TYR	2.56	-	Favored (54.67%)<br>Pre-Pro / -127.7,87.9	Favored (98.1%) *m-80*<br>chi angles: 296.4,89.6	0.04Å	Favored (31.854%)	-	-	-
A 14		PRO	2.07	-	Favored (3%)<br>Trans-Pro / -56.7,167.3	Favored (52.2%) *Cg_exo*<br>chi angles: 336.8,34.3,329.1	0.04Å	Favored (12.739%)	-	-	-
A 15		LYS	1.67	-	Favored (33.55%)<br>General / -49.1,-40.9	Favored (88%) *tttt*<br>chi angles: 181.9,175,178.8,177.5	0.03Å	CaBLAM Outlier (0.428%)	-	-	-
A 16		GLY	1.35	-	Favored (29.98%)<br>Glycine / 99.6,-178.5	-	-	Favored (57.697%)	-	-	-
A 17		ASP	1.11	-	Favored (20.84%)<br>General / -81.6,115.4	Favored (66.3%) *t0*<br>chi angles: 186.1,348.6	0.06Å	CaBLAM Disfavored (4.406%)	-	-	-
A 18		THR	0.94	-	Favored (13%)<br>General / -115.3,22.8	Favored (55.8%) *p*<br>chi angles: 56.9	0.09Å	Favored (7.243%)	-	-	-
A 19		SER	0.83	-	Favored (93.77%)<br>Pre-Pro / -61.0,136.5	Favored (58.9%) *m*<br>chi angles: 299.3	0.10Å	Favored (23.996%)	-	-	-
A 20		PRO	0.76	-	Favored (69.62%)<br>Trans-Pro / -54.5,142.8	Favored (89.9%) *Cg_exo*<br>chi angles: 333.3,35.4,331.1	0.02Å	CaBLAM Disfavored (4.546%)	-	-	-
**#**	**Alt**	**Res**	**High B**	**Clash > 0.4Å**	**Ramachandran**	**Rotamer**	**Cβ deviation**	**CaBLAM**	**Bond lengths**	**Bond angles**	**Cis Peptides**
			Avg: 1.13	Clashscore: 1.45	Outliers: 1 of 617	Poor rotamers: 1 of 507	Outliers: 0 of 561	Outliers: 16 of 615	Outliers: 7 of 619	Outliers: 6 of 619	Non-Trans: 0 of 618
A 21		GLY	0.72	-	Favored (28.67%)<br>Glycine / 148.8,-173.7	-	-	Favored (49.337%)	-	-	-
A 22		VAL	0.7	-	Favored (63.92%)<br>Ile or Val / -107.6,126.5	Favored (58.7%) *t*<br>chi angles: 180.1	0.06Å	Favored (11.875%)<br>beta sheet	-	-	-

A 23		TYR	0.72	-	Favored (36.5%) General / -111.8,147.0	Favored (63%) *m-80* chi angles: 288.9,79.3	0.07Å	Favored (45.111%) beta sheet	-	-	-
A 24		ARG	0.77	-	Favored (27.26%) General / -87.6,143.3	Favored (81.8%) *mtt90* chi angles: 297.1,176.1,177.5,87.3	0.05Å	Favored (45.763%) beta sheet	-	-	-
A 25		ILE	0.87	-	Favored (47.49%) Ile or Val / -105.2,115.7	Favored (75.6%) *mt* chi angles: 301,169.5	0.06Å	Favored (64.682%) beta sheet	-	-	-
A 26		MET	1.04	-	Favored (17.05%) General / -108.8,159.1	Favored (60.2%) *mmm* chi angles: 311.6,297.6,290.1	0.03Å	Favored (30.126%) beta sheet	-	-	-
A 27		SER	1.29	-	Favored (26.08%) General / -139.5,130.3	Favored (39.5%) *t* chi angles: 176.9	0.02Å	Favored (54.579%) beta sheet	-	-	-
A 28		ARG	1.59	-	Favored (73.36%) General / -62.7,-32.3	Favored (97.7%) *mtt180* chi angles: 288.9,178.1,179.1,172.1	0.03Å	CaBLAM Disfavored (1.231%)	-	-	-
A 29		TYR	1.86	0.41Å CG with A 30 ILE N	Allowed (0.06%) General / 49.8,-160.9	Favored (53.7%) *t80* chi angles: 183.4,63.6	0.12Å	CaBLAM Disfavored (2.287%)	-	-	-
A 30		ILE	2.01	0.41Å N with A 29 TYR CG	Favored (12.96%) Ile or Val / -95.1,-48.1	Favored (89.9%) *mt* chi angles: 297.8,168.5	0.12Å	CaBLAM Outlier (0.258%)	-	-	-
A 31		LEU	1.96	-	Favored (46.13%) General / -99.3,6.5	Favored (85.7%) *mt* chi angles: 300.7,177.2	0.04Å	CaBLAM Outlier (0.679%)	-	-	-
A 32		GLY	1.74	-	Favored (5.26%) Glycine / 139.2,148.5	-	-	Favored (21.054%)	-	-	-
A 33		THR	1.46	-	Favored (56.29%) General / -62.0,134.2	Favored (76.2%) *m* chi angles: 302.7	0.04Å	Favored (11.175%)	-	-	-
A 34		TYR	1.19	-	Favored (34.99%) General / -134.9,161.5	Favored (37.7%) *p90* chi angles: 72.9,94.9	0.03Å	Favored (42.626%)	-	-	-
A 35		GLN	0.99	-	Favored (24.16%) General / -84.5,118.2	Favored (51.8%) *tt0* chi angles: 181.8,177.7,313.2	0.03Å	Favored (26.602%)	-	-	-
A 36		ALA	0.85	-	Favored (20.62%) General / -87.3,-30.8	-	0.04Å	Favored (7.542%)	-	-	-
A 37		GLY	0.77	-	Favored (37.26%) Glycine / -177.2,-168.6	-	-	Favored (25.375%)	-	-	-
A 38		VAL	0.72	-	Favored (56.9%) Ile or Val / -128.7,137.6	Favored (76.3%) *t* chi angles: 178.2	0.07Å	Favored (19.343%)	-	-	-
A 39		GLY	0.7	-	Favored (16.18%) Glycine / -119.9,155.3	-	-	Favored (60.459%) beta sheet	-	-	-

A 40		VAL	0.7	-	Favored (65.04%) Ile or Val / -122.9,122.2	Favored (51.6%) *t* chi angles: 181.2	0.07Å	Favored (57.796%) beta sheet	-	-	-
**#**	**Alt**	**Res**	**High B**	**Clash > 0.4Å**	**Ramachandran**	**Rotamer**	**Cβ deviation**	**CaBLAM**	**Bond lengths**	**Bond angles**	**Cis Peptides**
			Avg: 1.13	Clashscore: 1.45	Outliers: 1 of 617	Poor rotamers: 1 of 507	Outliers: 0 of 561	Outliers: 16 of 615	Outliers: 7 of 619	Outliers: 6 of 619	Non-Trans: 0 of 618
A 41		MET	0.71	-	Favored (37.66%) General / -91.8,130.2	Favored (33.3%) *tpp* chi angles: 189.4,77.5,68.2	0.06Å	Favored (41.128%)	-	-	-
A 42		TYR	0.73	-	Favored (12.07%) General / -150.9,129.3	Favored (86.8%) *t80* chi angles: 180.8,80.9	0.09Å	Favored (16.376%)	-	-	-
A 43		GLU	0.75	-	Favored (22.4%) General / 50.0,41.1	Favored (91.5%) *mt-10* chi angles: 297,185.1,358.5	0.03Å	Favored (37.58%)	-	-	-
A 44		GLY	0.75	-	Favored (90.11%) Glycine / 81.7,1.2	-	-	Favored (82.792%)	-	-	-
A 45		VAL	0.74	-	Favored (63.74%) Ile or Val / -117.3,133.5	Favored (84.6%) *t* chi angles: 177.7	0.03Å	Favored (28.341%)	-	-	-
A 46		LEU	0.73	-	Favored (37.33%) General / -96.6,120.6	Favored (62.1%) *tp* chi angles: 176.8,64.5	0.02Å	Favored (66.248%) beta sheet	-	-	-
A 47		HIS	0.73	-	Favored (53.73%) General / -107.7,127.5	Favored (33.5%) *m90* chi angles: 304.3,72	0.13Å	Favored (49.695%) beta sheet	-	-	-
A 48		THR	0.73	-	Favored (15.44%) General / -134.3,170.6	Favored (18.7%) *p* chi angles: 73.1	0.06Å	Favored (30.659%) beta sheet	-	-	-
A 49		LEU	0.75	-	Favored (25.68%) General / -88.3,145.7	Favored (88.3%) *mt* chi angles: 295.3,178.2	0.13Å	Favored (27.952%)	-	-	-
A 50		TRP	0.79	-	Favored (90.85%) General / -59.1,-45.4	Favored (88.3%) *t60* chi angles: 180.5,89.8	0.07Å	Favored (57.946%)	-	-	-
A 51		HIS	0.84	0.44Å CE1 with A 135 SER HG	Favored (23.13%) General / -62.5,-12.6	Favored (53.2%) *p-80* chi angles: 70.9,280.1	0.08Å	Favored (54.423%) alpha helix	-	-	-
A 52		THR	0.91	-	Favored (32.26%) General / -92.2,-12.6	Favored (62.5%) *p* chi angles: 63.5	0.01Å	Favored (30.04%) alpha helix	-	-	-
A 53		THR	0.99	-	Favored (2.59%) General / -130.3,-24.2	Favored (38.2%) *p* chi angles: 68.1	0.09Å	CaBLAM Disfavored (1.515%)	-	-	-
A 54		ARG	1.05	-	Favored (10.29%) General / 56.1,26.5	Favored (29.8%) *mmt90* chi angles: 306.5,299.3,178.7,92.3	0.02Å	CaBLAM Disfavored (4.018%)	-	-	-
A 55		GLY	1.1	-	Favored (72.39%) Glycine / 94.2,-7.4	-	-	Favored (53.742%)	-	-	-

A 56		ALA	1.12	-	Favored (52.86%) General / -63.3,146.7	-	0.03Å	Favored (24.612%)	-	-	-
A 57		ALA	1.13	-	Favored (43.57%) General / -64.9,152.2	-	0.03Å	Favored (39.196%) beta sheet	-	-	-
A 58		ILE	1.14	-	Favored (74.76%) Ile or Val / -117.3,127.8	Favored (91.8%) *mt* chi angles: 297.6,172.3	0.03Å	Favored (49.064%) beta sheet	-	-	-
A 59		ARG	1.15	-	Favored (34.8%) General / -83.6,134.3	Favored (72.4%) *ttt180* chi angles: 180.3,172.3,176.2,168.3	0.04Å	Favored (50.393%)	-	-	-
A 60		SER	1.17	-	Favored (8.77%) General / -132.9,108.2	Favored (38%) *t* chi angles: 177.6	0.03Å	Favored (13.029%)	-	-	-
**#**	**Alt**	**Res**	**High B**	**Clash > 0.4Å**	**Ramachandran**	**Rotamer**	**Cβ deviation**	**CaBLAM**	**Bond lengths**	**Bond angles**	**Cis Peptides**
			Avg: 1.13	Clashscore: 1.45	Outliers: 1 of 617	Poor rotamers: 1 of 507	Outliers: 0 of 561	Outliers: 16 of 615	Outliers: 7 of 619	Outliers: 6 of 619	Non-Trans: 0 of 618
A 61		GLY	1.2	-	Favored (36.33%) Glycine / 62.6,-125.9	-	-	Favored (67.423%)	-	-	-
A 62		GLU	1.22	-	Favored (57.81%) General / -90.6,-3.1	Favored (97.2%) *mt-10* chi angles: 295.6,179.2,359.1	0.03Å	Favored (11.039%)	-	-	-
A 63		GLY	1.23	-	Favored (17.29%) Glycine / -113.1,-169.3	-	-	Favored (20.47%)	-	-	-
A 64		ARG	1.22	-	Favored (22.26%) General / -143.0,131.5	Favored (54%) *ttm170* chi angles: 177.8,179,290.1,173.1	0.02Å	Favored (25.61%)	-	-	-
A 65		LEU	1.19	-	Favored (33.82%) General / -104.3,142.9	Favored (79.8%) *mt* chi angles: 302.4,177.7	0.04Å	Favored (62.18%)	-	-	-
A 66		THR	1.15	-	Favored (57.13%) Pre-Pro / -120.8,155.5	Favored (70.2%) *p* chi angles: 62.4	0.05Å	Favored (25.798%)	-	-	-
A 67		PRO	1.12	-	Favored (87.52%) Trans-Pro / -56.1,140.9	Favored (78.2%) *Cg_exo* chi angles: 334.8,35.5,329.2	0.04Å	Favored (30.785%)	-	-	-
A 68		TYR	1.1	-	Favored (3.02%) General / -112.7,-48.1	Favored (83.6%) *t80* chi angles: 182.8,79.7	0.07Å	Favored (10.187%)	-	-	-
A 69		TRP	1.1	-	Favored (10.5%) General / -147.4,175.8	Favored (30.5%) *t-100* chi angles: 199.9,251.6	0.02Å	CA Geom Outlier (0.022%)	-	-	-
A 70		GLY	1.1	-	Favored (36.99%) Glycine / 160.2,-175.6	-	-	Favored (35.335%)	-	-	-

A 71		SER	1.1	-	Favored (2.76%) General / -160.0,118.9	Favored (29.5%) *t* chi angles: 172.8	0.05Å	CaBLAM Disfavored (1.71%) try beta sheet	-	-	-
A 72		VAL	1.08	-	Favored (85.27%) Ile or Val / -67.2,-40.6	Favored (70.4%) *t* chi angles: 172.2	0.05Å	Favored (23.078%)	-	-	-
A 73		LYS	1.04	-	Favored (67.61%) General / -53.6,-42.3	Favored (87.2%) *tttt* chi angles: 182.8,176.8,179.9,180	0.03Å	Favored (67.827%) alpha helix	-	-	-
A 74		GLU	0.99	-	Favored (45.67%) General / -93.5,7.2	Favored (44.8%) *mt-10* chi angles: 293.5,168.1,308	0.03Å	Favored (41.309%)	-	-	-
A 75		ASP	0.94	-	Favored (26.29%) General / 52.5,46.6	Favored (38.3%) *t0* chi angles: 193.2,26.6	0.07Å	Favored (23.877%)	-	-	-
A 76		ARG	0.89	-	Favored (41.48%) General / -140.5,148.7	Favored (54.4%) *ptt90* chi angles: 64.6,184.3,180.2,87.3	0.08Å	Favored (18.954%)	-	-	-
A 77		ILE	0.87	-	Favored (54.75%) Ile or Val / -134.3,134.7	Favored (74.7%) *mt* chi angles: 301,168.6	0.06Å	Favored (63.697%) beta sheet	-	-	-
A 78		THR	0.88	-	Favored (47.17%) General / -114.7,141.9	Favored (66.9%) *p* chi angles: 62.9	0.07Å	Favored (69.337%)	-	-	-
A 79		TYR	0.9	-	Favored (55.11%) General / -117.0,128.4	Favored (56.1%) *m-80* chi angles: 295.6,77.8	0.07Å	Favored (12.036%)	-	-	-
A 80		GLY	0.93	-	Favored (70.06%) Glycine / 87.2,-10.1	-	-	Favored (6.525%)	-	-	-
**#**	**Alt**	**Res**	**High B**	**Clash > 0.4Å**	**Ramachandran**	**Rotamer**	**Cβ deviation**	**CaBLAM**	**Bond lengths**	**Bond angles**	**Cis Peptides**
			Avg: 1.13	Clashscore: 1.45	Outliers: 1 of 617	Poor rotamers: 1 of 507	Outliers: 0 of 561	Outliers: 16 of 615	Outliers: 7 of 619	Outliers: 6 of 619	Non-Trans: 0 of 618
A 81		GLY	0.96	-	Favored (45.45%) Glycine / 174.9,179.3	-	-	Favored (17.168%)	-	-	-
A 82		PRO	0.97	-	Favored (42.31%) Trans-Pro / -74.1,160.7	Favored (72.2%) *Cg_endo* chi angles: 29.2,325.7,24.7	0.02Å	Favored (8.434%)	-	-	-
A 83		TRP	0.96	-	Favored (33.72%) General / -58.6,127.8	Favored (55.4%) *t-100* chi angles: 187.1,267	0.04Å	Favored (16.933%)	-	-	-
A 84		LYS	0.93	-	Favored (15.92%) General / -107.8,-8.8	Favored (20.5%) *mmtp* chi angles: 300,295.3,188.2,61.6	0.04Å	Favored (12.713%)	-	-	-
A 85		PHE	0.9	-	Favored (10.2%) General / -86.0,73.9	Favored (16.8%) *m-10* chi angles: 301.4,342.5	0.04Å	Favored (10.767%)	-	-	-
A 86		ASP	0.86	-	Favored (49.71%) General / -87.0,2.4	Favored (57.3%) *p0* chi angles: 63.4,1.1	0.06Å	Favored (8.674%)	-	-	-

A 87		ARG	0.83	-	Favored (30.58%) General / -89.1,120.5	Favored (80%) *ttm-80* chi angles: 183.2,178.5,294.7,282.6	0.06Å	Favored (32.755%) beta sheet	-	-	-
A 88		LYS	0.8	-	Favored (24.21%) General / -86.3,150.7	Favored (99.4%) *mttt* chi angles: 295,179.2,180.2,178.7	0.07Å	Favored (41.224%) beta sheet	-	-	-
A 89		TRP	0.79	0.51Å CD1 with A 89 TRP H	Favored (51.12%) General / -64.0,147.8	Favored (54.9%) *p-90* chi angles: 52.4,270.7	0.14Å	Favored (36.513%) beta sheet	-	-	-
A 90		ASN	0.77	-	Favored (12.8%) General / -102.6,-27.4	Favored (89.7%) *m-40* chi angles: 294.2,322.1	0.02Å	CaBLAM Disfavored (1.292%)	-	-	-
A 91		GLY	0.75	-	Favored (89.42%) Glycine / 82.5,-0.2	-	-	Favored (45.519%)	-	-	-
A 92		LEU	0.73	-	Favored (4.99%) General / -109.5,-40.0	Favored (83.4%) *mt* chi angles: 300.7,176.4	0.11Å	CaBLAM Disfavored (2.861%)	-	-	-
A 93		ASP	0.7	-	Favored (22.68%) General / -90.7,148.3	Favored (26.7%) *m-30* chi angles: 288.6,295.2	0.07Å	Favored (15.719%)	-	-	-
A 94		ASP	0.68	-	Favored (51.95%) General / -66.0,148.3	Favored (87.5%) *m-30* chi angles: 288.6,353.7	0.06Å	Favored (36.91%)	-	-	-
A 95		VAL	0.66	-	Favored (30%) Ile or Val / -126.5,162.7	Favored (26.8%) *m* chi angles: 299.7	0.02Å	Favored (53.807%) beta sheet	-	-	-
A 96		GLN	0.65	-	Favored (50.24%) General / -121.7,127.0	Favored (84.7%) *mt0* chi angles: 294.9,181.4,351	0.01Å	Favored (54.8%) beta sheet	-	-	-
A 97		LEU	0.65	-	Favored (44.97%) General / -99.3,125.4	Favored (51.5%) *tp* chi angles: 175,65.5	0.02Å	Favored (65.104%) beta sheet	-	-	-
A 98		ILE	0.68	-	Favored (15.16%) Ile or Val / -99.8,103.7	Favored (44.1%) *mm* chi angles: 307.5,303	0.09Å	Favored (67.57%) beta sheet	-	-	-
A 99		ILE	0.74	-	Favored (52.68%) Ile or Val / -99.6,124.0	Favored (2.9%) *mp* chi angles: 297.6,92.7	0.10Å	Favored (56.848%) beta sheet	OUTLIER(S) worst is CB--CG1: 5.7 σ	-	-
A 100		VAL	0.81	-	Favored (6.31%) Ile or Val / -110.6,97.9	Favored (61.5%) *t* chi angles: 179.8	0.04Å	Favored (62.632%)	-	-	-
**#**	**Alt**	**Res**	**High B**	**Clash > 0.4Å**	**Ramachandran**	**Rotamer**	**Cβ deviation**	**CaBLAM**	**Bond lengths**	**Bond angles**	**Cis Peptides**
			Avg: 1.13	Clashscore: 1.45	Outliers: 1 of 617	Poor rotamers: 1 of 507	Outliers: 0 of 561	Outliers: 16 of 615	Outliers: 7 of 619	Outliers: 6 of 619	Non-Trans: 0 of 618
A 101		ALA	0.91	-	Favored (54.42%) Pre-Pro / -86.9,152.9	-	0.04Å	Favored (15.789%)	-	-	-
A 102		PRO	1.02	-	Favored (37.99%) Trans-Pro / -53.5,130.3	Favored (99.5%) *Cg_exo* chi angles: 332.5,36.3,330.9	0.09Å	Favored (32.161%)	-	-	-

A 103		GLY	1.11	-	Favored (81.99%) Glycine / 79.1,0.2	-	-	Favored (73.712%)	-	-	-
A 104		LYS	1.16	-	Favored (47.86%) General / -129.6,153.7	Favored (95.6%) *mttt* chi angles: 297.8,183.1,181.3,180.3	0.02Å	Favored (27.448%)	-	-	-
A 105		ALA	1.16	-	Favored (45.16%) General / -67.7,152.7	-	0.03Å	Favored (32.58%)	-	-	-
A 106		ALA	1.09	-	Favored (57.7%) General / -60.6,139.7	-	0.06Å	Favored (27.316%)	-	-	-
A 107		ILE	1	-	Favored (27.19%) Ile or Val / -123.0,147.7	Favored (45.5%) *pt* chi angles: 61.2,171.3	0.01Å	Favored (58.938%) beta sheet	-	-	-
A 108		ASN	0.9	-	Favored (18.74%) General / -107.3,107.7	Favored (47.2%) *t0* chi angles: 182,331.9	0.05Å	Favored (53.324%) beta sheet	-	-	-
A 109		ILE	0.82	-	Favored (67.44%) Ile or Val / -110.8,124.3	Favored (46.8%) *mm* chi angles: 304.1,299.6	0.06Å	Favored (60.791%) beta sheet	-	-	-
A 110		GLN	0.77	-	Favored (38.32%) General / -96.1,135.1	Favored (49.4%) *tt0* chi angles: 184.6,169.1,331.3	0.06Å	Favored (24.138%) beta sheet	-	-	-
A 111		THR	0.75	-	Favored (31.02%) General / -152.8,151.9	Favored (10%) *t* chi angles: 186.1	0.09Å	Favored (30.642%) beta sheet	-	-	-
A 112		LYS	0.76	-	Favored (48.51%) Pre-Pro / -89.0,129.4	Favored (33%) *ttmt* chi angles: 185.1,176.5,289.8,185.5	0.01Å	Favored (38.183%) beta sheet	-	-	-
A 113		PRO	0.81	-	Favored (47.45%) Trans-Pro / -73.9,153.0	Favored (73%) *Cg_endo* chi angles: 29.1,327.9,22.1	0.02Å	Favored (52.569%)	-	-	-
A 114		GLY	0.88	-	Favored (23.53%) Glycine / -105.7,-167.9	-	-	Favored (49.871%)	-	-	-
A 115		ILE	0.98	-	Favored (63.26%) Ile or Val / -126.5,123.5	Favored (73.6%) *mt* chi angles: 301.2,173.9	0.04Å	Favored (16.866%)	-	-	-
A 116		PHE	1.11	-	Favored (49.15%) General / -102.3,125.8	Favored (56.8%) *t80* chi angles: 180.2,63.6	0.06Å	Favored (59.72%) beta sheet	-	-	-
A 117		LYS	1.23	-	Favored (38.39%) General / -92.8,127.8	Favored (87.4%) *tttt* chi angles: 183.9,177.1,179.1,180	0.03Å	Favored (52.022%)	-	-	-
A 118		THR	1.33	-	Favored (7.47%) Pre-Pro / -122.3,170.7	Favored (16.4%) *p* chi angles: 74.1	0.13Å	Favored (27.559%)	-	-	-
A 119		PRO	1.38	-	Favored (35.1%) Trans-Pro / -58.1,-18.1	Favored (85.3%) *Cg_exo* chi angles: 333.9,36,329.6	0.03Å	Favored (56.021%)	-	-	-
A 120		GLN	1.35	-	Favored (45.96%)	Favored (97.4%) *mm-40*	0.03Å	Favored (36.707%)	-	-	-

					General / -99.4,3.8	chi angles: 299.1,297.3,306.7					
**#**	**Alt**	**Res**	**High B**	**Clash > 0.4Å**	**Ramachandran**	**Rotamer**	**Cβ deviation**	**CaBLAM**	**Bond lengths**	**Bond angles**	**Cis Peptides**
			Avg: 1.13	Clashscore: 1.45	Outliers: 1 of 617	Poor rotamers: 1 of 507	Outliers: 0 of 561	Outliers: 16 of 615	Outliers: 7 of 619	Outliers: 6 of 619	Non-Trans: 0 of 618
A 121		GLY	1.26	-	Favored (13.37%) Glycine / 113.3,-170.4	-	-	Favored (29.499%)	-	-	-
A 122		GLU	1.13	-	Favored (25.6%) General / -86.0,148.7	Favored (97.2%) *mt-10* chi angles: 292.9,180.8,344.6	0.03Å	Favored (6.657%)	-	-	-
A 123		ILE	1.01	-	Favored (31.02%) Ile or Val / -140.0,142.9	Favored (20.2%) *tt* chi angles: 188.3,163.7	0.06Å	Favored (45.267%) beta sheet	-	-	-
A 124		GLY	0.92	-	Favored (53.19%) Glycine / -70.4,156.0	-	-	Favored (37.616%) beta sheet	-	-	-
A 125		ALA	0.87	-	Favored (32.61%) General / -154.8,154.3	-	0.04Å	Favored (55.633%) beta sheet	-	-	-
A 126		VAL	0.86	-	Favored (40.99%) Ile or Val / -118.6,140.8	Favored (29.3%) *m* chi angles: 296.4	0.04Å	Favored (48.959%) beta sheet	-	-	-
A 127		SER	0.87	-	Favored (8.5%) General / -86.7,64.4	Favored (70.3%) *m* chi angles: 296.3	0.04Å	Favored (10.466%) beta sheet	-	-	-
A 128		LEU	0.9	-	Favored (54.06%) General / -125.3,137.8	Favored (56.7%) *mt* chi angles: 302,171.2	0.04Å	Favored (26.87%) beta sheet	-	-	-
A 129		ASP	0.93	-	Favored (27.51%) General / -112.4,114.7	Favored (7.2%) *m-30* chi angles: 281.9,285.4	0.04Å	Favored (36.42%)	-	-	-
A 130		TYR	0.95	-	Favored (58.48%) Pre-Pro / -133.5,162.3	Favored (95.1%) *m-80* chi angles: 298.5,99.3	0.03Å	Favored (17.902%)	-	-	-
A 131		PRO	0.94	-	Favored (47.17%) Trans-Pro / -56.0,150.4	Favored (85.3%) *Cg_exo* chi angles: 333.9,35.1,330.9	0.04Å	Favored (65.726%)	-	-	-
A 132		GLU	0.91	-	Favored (66.87%) General / -57.5,-32.4	Favored (98.5%) *mt-10* chi angles: 290.3,177.8,352.4	0.02Å	Favored (47.391%)	-	-	-
A 133		GLY	0.86	-	Favored (40.43%) Glycine / -62.7,-12.3	-	-	Favored (67.851%)	-	-	-
A 134		THR	0.81	-	Favored (57.22%) General / -85.1,-10.1	Favored (69.9%) *p* chi angles: 62.4	0.08Å	Favored (30.671%)	-	-	-
A 135		SER	0.76	0.44Å HG with A 51 HIS CE1	Favored (55.03%) General / -56.7,135.5	Favored (42.8%) *t* chi angles: 175.5	0.04Å	Favored (7.269%)	-	-	-
A 136		GLY	0.72	-	Favored (54.98%)	-	-	Favored (67.353%)	-	-	-

					Glycine / 101.7,-6.5						
A 137		SER	0.69	-	Favored (95.02%) Pre-Pro / -61.7,141.6	Favored (53.9%) *m* chi angles: 292.3	0.06Å	Favored (30.635%)	-	-	-
A 138		PRO	0.68	-	Favored (39.9%) Trans-Pro / -74.4,149.6	Favored (75.5%) *Cg_endo* chi angles: 28.8,329.5,20.1	0.07Å	Favored (72.713%) beta sheet	-	-	-
A 139		ILE	0.68	-	Favored (69.37%) Ile or Val / -112.5,124.7	Favored (87.5%) *mt* chi angles: 298.6,171.6	0.05Å	Favored (69.619%) beta sheet	-	-	-
A 140		LEU	0.69	-	Favored (23.67%) General / -107.8,153.0	Favored (47%) *mt* chi angles: 307,178.9	0.11Å	Favored (40.087%)	-	-	-
**#**	**Alt**	**Res**	**High B**	**Clash > 0.4Å**	**Ramachandran**	**Rotamer**	**Cβ deviation**	**CaBLAM**	**Bond lengths**	**Bond angles**	**Cis Peptides**
			Avg: 1.13	Clashscore: 1.45	Outliers: 1 of 617	Poor rotamers: 1 of 507	Outliers: 0 of 561	Outliers: 16 of 615	Outliers: 7 of 619	Outliers: 6 of 619	Non-Trans: 0 of 618
A 141		ASP	0.69	0.41Å C with A 141 ASP OD1	Favored (5.75%) General / -85.4,-175.6	Favored (50.4%) *p0* chi angles: 68.3,11	0.06Å	Favored (37.374%)	-	-	-
A 142		LYS	0.7	-	Favored (59.49%) General / -61.1,-18.7	Favored (60.6%) *pttt* chi angles: 67.1,184.9,179.6,185.2	0.03Å	Favored (32.375%)	-	-	-
A 143		ASN	0.71	-	Favored (40.89%) General / -101.8,4.9	Favored (88.5%) *m-40* chi angles: 294,320.3	0.03Å	Favored (52.37%)	-	-	-
A 144		GLY	0.72	-	Favored (84.81%) Glycine / 86.7,2.0	-	-	Favored (87.244%)	-	-	-
A 145		ASP	0.72	-	Favored (38.64%) General / -75.4,149.2	Favored (90.5%) *m-30* chi angles: 284.8,346.2	0.08Å	Favored (35.621%)	-	-	-
A 146		ILE	0.72	-	Favored (39.89%) Ile or Val / -82.0,126.3	Favored (73.5%) *mt* chi angles: 300.5,166.7	0.02Å	Favored (43.397%)	-	-	-
A 147		VAL	0.72	-	Favored (4.97%) Ile or Val / -104.2,-22.1	Favored (28.3%) *m* chi angles: 298.6	0.03Å	CaBLAM Disfavored (4.802%)	-	-	-
A 148		GLY	0.74	-	Favored (43.46%) Glycine / 169.6,-176.6	-	-	Favored (31.96%)	-	-	-
A 149		LEU	0.76	-	Favored (27.85%) General / -109.4,150.6	Favored (62%) *mt* chi angles: 304.4,180.5	0.05Å	Favored (8.272%)	-	-	-
A 150		TYR	0.81	-	Favored (35.19%) General / -82.5,133.2	Favored (85.1%) *t80* chi angles: 174.1,80.3	0.02Å	CaBLAM Disfavored (3.349%)	-	-	-
A 151		GLY	0.87	0.41Å H with A 163 SER HG	Favored (25.8%) Glycine / 138.5,-162.9	-	-	CaBLAM Outlier (0.101%)	-	-	-
A 152		ASN	0.95	-	Favored (20.21%)	Favored (65.4%) *t0* chi angles: 195,32.5	0.05Å	CaBLAM Outlier	-	-	-

					General / 51.8,49.7			(0.167%)			
A 153		GLY	1.08	-	Favored (23.93%) Glycine / -105.2,-168.9	-	-	Favored (39.687%)	-	-	-
A 154		VAL	1.28	-	Favored (37.57%) Ile or Val / -136.0,142.9	Favored (8.6%) *p* chi angles: 61.5	0.10Å	Favored (30.696%) beta sheet	-	-	-
A 155		ILE	1.55	-	Favored (38.27%) Ile or Val / -88.0,130.0	Favored (47.2%) *mm* chi angles: 299.3,297.6	0.07Å	Favored (49.209%)	-	-	-
A 156		LEU	1.86	-	Favored (11.51%) General / -89.8,169.2	Favored (58.1%) *mt* chi angles: 303.5,183.5	0.07Å	Favored (41.325%)	-	-	-
A 157		GLY	2.12	-	Favored (55.14%) Glycine / -56.6,-27.8	-	-	Favored (56.268%)	-	-	-
A 158		ASN	2.23	-	Favored (40.27%) General / -87.9,5.5	Favored (54%) *p0* chi angles: 66.5,12.1	0.04Å	Favored (54.851%)	-	-	-
A 159		GLY	2.1	-	Favored (79.47%) Glycine / 87.7,-7.9	-	-	Favored (68.708%)	-	-	-
A 160		SER	1.8	-	Favored (24.91%) General / -78.9,163.9	Favored (84.9%) *p* chi angles: 67.4	0.01Å	Favored (40.957%)	-	-	-
**#**	**Alt**	**Res**	**High B**	**Clash > 0.4Å**	**Ramachandran**	**Rotamer**	**Cβ deviation**	**CaBLAM**	**Bond lengths**	**Bond angles**	**Cis Peptides**
			Avg: 1.13	Clashscore: 1.45	Outliers: 1 of 617	Poor rotamers: 1 of 507	Outliers: 0 of 561	Outliers: 16 of 615	Outliers: 7 of 619	Outliers: 6 of 619	Non-Trans: 0 of 618
A 161		TYR	1.44	-	Favored (54.96%) General / -107.5,129.4	Favored (78.2%) *t80* chi angles: 183.6,76	0.03Å	Favored (53.697%) beta sheet	-	-	-
A 162		VAL	1.14	-	Favored (53.07%) Ile or Val / -111.9,116.6	Favored (85.3%) *t* chi angles: 177.6	0.07Å	Favored (69.197%) beta sheet	-	-	-
A 163		SER	0.93	0.41Å HG with A 151 GLY H	Favored (32.49%) General / -96.8,118.1	Favored (21.2%) *t* chi angles: 170.4	0.04Å	Favored (50.783%) beta sheet	-	-	-
A 164		ALA	0.84	-	Favored (57.36%) General / -62.0,142.7	-	0.05Å	Favored (39.608%) beta sheet	-	-	-
A 165		ILE	0.83	-	Favored (22.74%) Ile or Val / -83.9,113.4	Favored (86.6%) *mt* chi angles: 298.9,172	0.03Å	Favored (44.315%) beta sheet	-	-	-
A 166		VAL	0.88	-	Favored (27.61%) Ile or Val / -84.8,116.0	Favored (82.2%) *t* chi angles: 176.7	0.06Å	Favored (38.282%) beta sheet	-	-	-
A 167		GLN	1	-	Favored (3.53%) General / -136.4,97.3	Favored (24.9%) *tm-30* chi angles: 187.7,283.8,320.5	0.06Å	Favored (16.42%)	-	-	-
A 168		GLY	1.16	-	Favored (32.27%) Glycine / -79.0,149.0	-	-	Favored (44.405%)	-	-	-

A 169		GLU	1.37	-	Favored (55.82%) General / -60.5,133.2	Favored (17.6%) *tp30* chi angles: 189.2,65.4,52.1	0.07Å	Favored (44.873%)	-	-	-
A 170		ARG	1.6	-	Allowed (1.92%) General / -74.6,72.4	Favored (76.2%) *ttt180* chi angles: 178.1,169,178.9,185	0.06Å	Favored (22.301%) beta sheet	-	-	-
A 171		GLU	1.82	-	Favored (9.68%) General / -82.8,76.7	Favored (85.3%) *mt-10* chi angles: 295.9,180.1,331.1	0.06Å	Favored (46.213%) beta sheet	-	-	-
A 172		GLU	1.98	-	Favored (4.61%) General / -78.6,68.7	Favored (78.4%) *mt-10* chi angles: 296.4,185.4,332.7	0.08Å	Favored (68.931%) beta sheet	-	-	-
A 173		GLU	2.04	-	Favored (34.68%) Pre-Pro / -60.7,118.4	Favored (19.1%) *tp30* chi angles: 185.7,70.1,47.4	0.06Å	Favored (22.788%) beta sheet	-	-	-
A 174		PRO	2	-	Favored (37.58%) Trans-Pro / -75.9,160.0	Favored (62.1%) *Cg_endo* chi angles: 31.6,324.4,24.8	0.04Å	Favored (64.385%) beta sheet	-	-	-
A 175		VAL	1.87	-	Favored (74.24%) Pre-Pro / -80.0,128.3	Favored (84.6%) *t* chi angles: 177.7	0.07Å	Favored (20.852%) beta sheet	-	-	-
A 176		PRO	1.69	-	Favored (83.32%) Trans-Pro / -65.0,152.2	Favored (38.8%) *Cg_endo* chi angles: 23.1,327.1,28.6	0.02Å	Favored (83.058%) beta sheet	-	-	-
A 177		GLU	1.48	-	Favored (4.48%) General / -77.9,73.1	Favored (96.3%) *mt-10* chi angles: 296.1,181.1,353.3	0.01Å	Favored (10.68%) beta sheet	-	-	-
A 178		ALA	1.3	-	Favored (53.21%) General / -83.6,-1.1	-	0.07Å	Favored (7.464%)	-	-	-
A 179		TYR	1.15	-	Favored (23.38%) General / -147.6,138.3	Favored (89.8%) *t80* chi angles: 179.2,79.9	0.10Å	Favored (15.59%)	-	-	-
A 180		ASN	1.02	-	Favored (46.95%) General / -138.1,150.6	Favored (68.5%) *m-40* chi angles: 293.2,280.5	0.05Å	Favored (43.755%)	-	-	-
**#**	**Alt**	**Res**	**High B**	**Clash > 0.4Å**	**Ramachandran**	**Rotamer**	**Cβ deviation**	**CaBLAM**	**Bond lengths**	**Bond angles**	**Cis Peptides**
			Avg: 1.13	Clashscore: 1.45	Outliers: 1 of 617	Poor rotamers: 1 of 507	Outliers: 0 of 561	Outliers: 16 of 615	Outliers: 7 of 619	Outliers: 6 of 619	Non-Trans: 0 of 618
A 181		ALA	0.93	-	Favored (65.18%) General / -57.5,-30.6	-	0.03Å	Favored (46.744%)	-	-	-
A 182		ASP	0.86	-	Favored (60.54%) General / -59.8,-21.1	Favored (93.1%) *m-30* chi angles: 285.5,346.4	0.03Å	Favored (61.524%) three-ten	-	-	-
A 183		MET	0.81	-	Favored (39.94%) General / -73.5,-5.4	Favored (70.1%) *mtp* chi angles: 291.2,169.3,56.6	0.09Å	Favored (31.045%) three-ten	-	-	-
A 184		LEU	0.78	-	Favored (39.7%) General / -101.4,2.8	Favored (85.3%) *mt* chi angles: 299,179.4	0.03Å	Favored (54.248%)	-	-	-

A 185		ARG	0.76	-	Favored (43.97%) General / -73.5,135.6	Favored (96.5%) *mtt-85* chi angles: 292.7,177.3,185.4,270.8	0.03Å	Favored (34.774%)	-	-	-
A 186		LYS	0.75	-	Favored (88.07%) General / -59.3,-46.6	Favored (96.8%) *mttt* chi angles: 287.3,179.3,175.7,178.2	0.07Å	Favored (59.499%)	-	-	-
A 187		LYS	0.75	-	Favored (7.4%) General / -89.9,68.8	Favored (87.2%) *tttt* chi angles: 185.6,176.9,181.2,180.6	0.06Å	CaBLAM Disfavored (2.744%)	-	-	-
A 188		GLN	0.76	-	Favored (13.49%) General / -152.8,133.0	Favored (49.5%) *tt0* chi angles: 183.1,169.8,323.3	0.03Å	Favored (8.977%)	-	-	-
A 189		LEU	0.78	-	Favored (23.91%) General / -112.8,112.4	Favored (73.8%) *mt* chi angles: 300.5,172.5	0.08Å	Favored (69.242%) beta sheet	-	-	-
A 190		THR	0.81	-	Favored (55.47%) General / -113.8,133.3	Favored (91.7%) *m* chi angles: 299	0.01Å	Favored (63.895%) beta sheet	-	-	-
A 191		VAL	0.85	-	Favored (67.19%) Ile or Val / -111.9,129.5	Favored (67.2%) *t* chi angles: 179.1	0.04Å	Favored (69.817%) beta sheet	-	-	-
A 192		LEU	0.89	-	Favored (15.02%) General / -108.9,105.5	Favored (78.6%) *mt* chi angles: 301.7,176.2	0.01Å	Favored (66.03%) beta sheet	-	-	-
A 193		ASP	0.95	-	Favored (7.06%) General / -83.7,88.9	Favored (38%) *m-30* chi angles: 297,356.9	0.04Å	Favored (60.79%) beta sheet	-	-	-
A 194		LEU	1	-	Favored (21.2%) General / -104.7,109.3	Favored (63.2%) *mt* chi angles: 304.3,176.3	0.05Å	Favored (52.301%)	-	-	-
A 195		HIS	1.03	-	Favored (58.91%) Pre-Pro / -58.3,149.4	Favored (53.7%) *p-80* chi angles: 65.1,275.5	0.04Å	Favored (29.812%)	-	-	-
A 196		PRO	1.05	-	Favored (63.52%) Trans-Pro / -53.8,135.7	Favored (99.7%) *Cg_exo* chi angles: 332.4,37.2,329.3	0.06Å	Favored (46.181%)	-	-	-
A 197		GLY	1.06	-	Favored (86.92%) Glycine / 86.2,0.6	-	-	Favored (79.41%)	-	-	-
A 198		ALA	1.06	-	Favored (29.21%) General / -77.9,-0.9	-	0.07Å	CaBLAM Disfavored (4.693%)	-	-	-
A 199		GLY	1.05	-	Favored (49.92%) Glycine / 81.8,26.4	-	-	Favored (78.703%)	-	-	-
A 200		LYS	1.04	-	Favored (46.54%) General / -54.2,-29.5	Favored (14.4%) *pttp* chi angles: 68.1,180.9,174.4,60.7	0.05Å	Favored (12.379%)	-	-	-
**#**	**Alt**	**Res**	**High B**	**Clash > 0.4Å**	**Ramachandran**	**Rotamer**	**Cβ deviation**	**CaBLAM**	**Bond lengths**	**Bond angles**	**Cis Peptides**
			Avg: 1.13	Clashscore: 1.45	Outliers: 1 of 617	Poor rotamers: 1 of 507	Outliers: 0 of 561	Outliers: 16 of 615	Outliers: 7 of 619	Outliers: 6 of 619	Non-Trans: 0 of 618
A 201		THR	1.02	-	Favored (6.51%)	Favored (95.7%) *m* chi angles: 299.6	0.02Å	Favored (36.979%)	-	-	-

					General / -102.0,-41.3						
A 202		ARG	0.99	-	Favored (32.38%) General / -82.8,-25.8	Favored (96.7%) *mtt180* chi angles: 290.1,183.1,176.8,184.3	0.06Å	Favored (29.958%) alpha helix	-	-	-
A 203		ARG	0.95	-	Favored (2.25%) General / -120.5,-49.5	Favored (99.3%) *mtm-85* chi angles: 291,192.3,294,272	0.03Å	Favored (13.611%) alpha helix	-	-	-
A 204		ILE	0.91	-	Favored (23.15%) Ile or Val / -74.1,-51.7	Favored (91.9%) *mt* chi angles: 295.1,166.7	0.09Å	Favored (76.789%) alpha helix	-	-	-
A 205		LEU	0.86	0.47Å HB3 with A 206 PRO HD3	Favored (51.63%) Pre-Pro / -50.6,-52.9	Favored (47.7%) *tp* chi angles: 174.5,66	0.11Å	Favored (89.51%) alpha helix	-	-	-
A 206		PRO	0.82	0.47Å HD3 with A 205 LEU HB3	Favored (47.26%) Trans-Pro / -50.6,-35.6	Favored (77.2%) *Cg_exo* chi angles: 329,36.6,333.3	0.02Å	Favored (95.732%) alpha helix	-	-	-
A 207		GLN	0.79	-	Favored (78.18%) General / -69.2,-40.2	Favored (97.5%) *mt0* chi angles: 290.9,171.1,340.9	0.03Å	Favored (81.3%) alpha helix	-	-	-
A 208		ILE	0.77	-	Favored (87.53%) Ile or Val / -65.7,-47.0	Favored (98.7%) *mt* chi angles: 292.8,168	0.12Å	Favored (74.879%) alpha helix	-	-	-
A 209		ILE	0.76	-	Favored (71.23%) Ile or Val / -71.5,-44.2	Favored (45.7%) *mm* chi angles: 298.8,300.5	0.05Å	Favored (79.993%) alpha helix	-	-	-
A 210		LYS	0.76	-	Favored (83.96%) General / -57.7,-42.6	Favored (87.3%) *tttt* chi angles: 182.8,176.6,178.7,180.2	0.02Å	Favored (83.948%) alpha helix	-	-	-
A 211		ASP	0.77	-	Favored (82.98%) General / -68.0,-38.7	Favored (16.3%) *t70* chi angles: 196.1,62.7	0.01Å	Favored (92.363%) alpha helix	-	-	-
A 212		ALA	0.78	-	Favored (81.75%) General / -59.8,-38.4	-	0.03Å	Favored (93.114%) alpha helix	-	-	-
A 213		ILE	0.8	-	Favored (92.5%) Ile or Val / -65.9,-44.8	Favored (93.6%) *mt* chi angles: 291.8,167	0.05Å	Favored (93.62%) alpha helix	-	-	-
A 214		GLN	0.82	-	Favored (71.71%) General / -61.9,-31.4	Favored (92.9%) *mm-40* chi angles: 290,297.9,311.5	0.03Å	Favored (78.267%) alpha helix	-	-	-
A 215		ARG	0.84	-	Favored (42.15%) General / -88.4,5.4	Favored (98.1%) *mtt-85* chi angles: 294,176.6,185.6,273.7	0.04Å	Favored (50.113%)	-	-	-
A 216		ARG	0.85	-	Favored (26.5%) General / 56.4,43.2	Favored (14.4%) *mpt180* chi angles: 276.1,69.8,174.9,173.7	0.04Å	Favored (30.385%)	-	-	-
A 217		LEU	0.85	-	Favored (25.72%) General / -99.5,113.4	Favored (66%) *mt* chi angles: 304.3,178.5	0.06Å	Favored (42.586%) beta sheet	-	-	-
A 218		ARG	0.85	-	Favored (22.83%)	Favored (17.1%) *tpt-90*	0.05Å	Favored (35.996%) beta sheet	-	-	-

#	Alt	Res	High B	Clash > 0.4Å	Ramachandran	Rotamer	Cβ deviation	CaBLAM	Bond lengths	Bond angles	Cis Peptides
					General / -57.9,125.4	chi angles: 180.9,78.5,181.4,268.6					
A 219		THR	0.84	-	Favored (52.36%) General / -127.2,136.7	Favored (97.8%) *m* chi angles: 300	0.03Å	Favored (70.445%) beta sheet	-	-	-
A 220		ALA	0.85	-	Favored (43.9%) General / -106.4,137.7	-	0.06Å	Favored (70.824%) beta sheet	-	-	-
**#**	**Alt**	**Res**	**High B**	**Clash > 0.4Å**	**Ramachandran**	**Rotamer**	**Cβ deviation**	**CaBLAM**	**Bond lengths**	**Bond angles**	**Cis Peptides**
			Avg: 1.13	Clashscore: 1.45	Outliers: 1 of 617	Poor rotamers: 1 of 507	Outliers: 0 of 561	Outliers: 16 of 615	Outliers: 7 of 619	Outliers: 6 of 619	Non-Trans: 0 of 618
A 221		VAL	0.87	-	Favored (63.22%) Ile or Val / -120.7,120.7	Favored (46.9%) *t* chi angles: 182.1	0.03Å	Favored (69.2%) beta sheet	-	-	-
A 222		LEU	0.9	-	Favored (22.32%) General / -109.4,111.0	Favored (22.7%) *mt* chi angles: 311,175.1	0.10Å	Favored (71.181%) beta sheet	-	-	-
A 223		ALA	0.95	-	Favored (33.23%) Pre-Pro / -93.0,147.1	-	0.09Å	Favored (30.449%)	-	-	-
A 224		PRO	1.01	-	Favored (75.01%) Trans-Pro / -62.7,-25.9	Favored (28.1%) *Cg_endo* chi angles: 20.9,327,29.9	0.06Å	Favored (25.376%)	-	-	-
A 225		THR	1.07	-	Favored (23.54%) General / -139.1,166.8	Favored (30.2%) *p* chi angles: 69.4	0.04Å	Favored (20.867%)	-	-	-
A 226		ARG	1.12	-	Favored (92.74%) General / -62.9,-38.9	Favored (89.5%) *mtt180* chi angles: 290.2,170.9,179.5,159.3	0.09Å	Favored (66.839%) alpha helix	-	-	-
A 227		VAL	1.16	-	Favored (90.58%) Ile or Val / -66.4,-44.4	Favored (61.1%) *t* chi angles: 170.9	0.04Å	Favored (97.024%) alpha helix	-	-	-
A 228		VAL	1.17	-	Favored (86.35%) Ile or Val / -64.7,-39.6	Favored (70.1%) *t* chi angles: 172.1	0.10Å	Favored (84.171%) alpha helix	-	-	-
A 229		ALA	1.16	-	Favored (75.24%) General / -57.5,-38.7	-	0.03Å	Favored (80.478%) alpha helix	-	-	-
A 230		ALA	1.14	-	Favored (92.25%) General / -62.1,-39.4	-	0.02Å	Favored (93.584%) alpha helix	-	-	-
A 231		GLU	1.11	-	Favored (98.41%) General / -63.6,-41.0	Favored (60.3%) *tp30* chi angles: 184.1,70.6,18.1	0.04Å	Favored (98.053%) alpha helix	-	-	-
A 232		MET	1.08	-	Favored (98.78%) General / -62.1,-43.5	Favored (59.6%) *mtt* chi angles: 291,176.8,169.1	0.09Å	Favored (82.813%) alpha helix	-	-	-
A 233		ALA	1.05	-	Favored (78.48%) General / -59.1,-37.9	-	0.03Å	Favored (76.829%) alpha helix	-	-	-

A 234		GLU	1.02	-	Favored (73.36%) General / -70.6,-40.1	Favored (90.3%) *mt-10* chi angles: 292.9,185.4,6.8	0.03Å	Favored (82.375%) alpha helix	-	-	-
A 235		ALA	1.01	-	Favored (73.84%) General / -60.5,-34.3	-	0.04Å	Favored (74.65%) alpha helix	-	-	-
A 236		LEU	1.01	-	Favored (22.13%) General / -100.6,17.3	Favored (81.5%) *mt* chi angles: 301.1,176.4	0.07Å	Favored (33.34%) alpha helix	-	-	-
A 237		LYS	1.01	-	Favored (55.57%) General / -57.0,-24.7	Favored (17.6%) *pttm* chi angles: 64.9,188,177.2,294	0.03Å	Favored (21.479%) alpha helix	-	-	-
A 238		GLY	1.03	-	Favored (83.75%) Glycine / -89.5,3.8	-	-	Favored (66.068%)	-	-	-
A 239		LEU	1.07	-	Favored (39.9%) Pre-Pro / -112.6,157.6	Favored (71.2%) *mt* chi angles: 301.8,174.1	0.10Å	Favored (15.919%)	-	-	-
A 240		PRO	1.14	-	Allowed (0.91%) Trans-Pro / -68.7,73.5	Favored (75.7%) *Cg_endo* chi angles: 28.8,320.5,34.1	0.03Å	CaBLAM Disfavored (4.681%) try beta sheet	-	-	-

**#**	**Alt**	**Res**	**High B**	**Clash > 0.4Å**	**Ramachandran**	**Rotamer**	**Cβ deviation**	**CaBLAM**	**Bond lengths**	**Bond angles**	**Cis Peptides**
			Avg: 1.13	Clashscore: 1.45	Outliers: 1 of 617	Poor rotamers: 1 of 507	Outliers: 0 of 561	Outliers: 16 of 615	Outliers: 7 of 619	Outliers: 6 of 619	Non-Trans: 0 of 618
A 241		VAL	1.27	-	Favored (55.03%) Ile or Val / -108.9,133.1	Favored (77.8%) *t* chi angles: 178.1	0.07Å	Favored (31.331%) beta sheet	-	-	-
A 242		ARG	1.47	-	Favored (41.46%) General / -109.2,141.4	Favored (42.4%) *ttp80* chi angles: 186.6,173.3,75.8,100.7	0.10Å	Favored (54.562%) beta sheet	-	-	-
A 243		TYR	1.78	-	Favored (54.86%) General / -107.5,130.2	Favored (84.9%) *t80* chi angles: 175.3,74.2	0.06Å	Favored (63.99%) beta sheet	-	-	-
A 244		LEU	2.21	-	Favored (53.07%) General / -116.0,125.8	Favored (53.9%) *tp* chi angles: 179.8,65.3	0.01Å	Favored (40.885%) beta sheet	-	-	-
A 245		THR	2.78	-	Favored (51.11%) Pre-Pro / -144.9,152.2	Favored (3.1%) *t* chi angles: 176.6	0.05Å	Favored (31.24%)	-	-	-
A 246		PRO	3.44	-	Favored (34.53%) Trans-Pro / -56.5,-20.7	Favored (88.9%) *Cg_exo* chi angles: 333.3,35.1,331.1	0.03Å	Favored (74.03%)	-	-	-
A 247		ALA	4.11	-	Favored (52.2%) General / -80.9,-3.7	-	0.03Å	Favored (58.024%) alpha helix	-	-	-
A 248		VAL	4.65	-	Favored (41.7%) Ile or Val / -128.9,143.3	Favored (5.5%) *p* chi angles: 69.5	0.04Å	Favored (28.774%)	-	-	-
A 249		ASN	4.95	-	Favored (13.85%) General / -112.2,23.3	Favored (72.2%) *m-40* chi angles: 292.3,285.7	0.03Å	Favored (8.155%)	-	-	-

A 250		ARG	4.92	-	Favored (38.63%)<br>General / -77.9,142.1	Favored (98.5%) *mtt180*<br>chi angles: 291.7,175.5,182.8,171.9	0.02Å	Favored (35.321%)	-	-	-
A 251		GLU	4.6	-	Favored (19.03%)<br>General / -100.1,153.7	Favored (96.7%) *mt-10*<br>chi angles: 295.5,180.7,358.9	0.01Å	Favored (24.815%)	-	-	-
A 252		HIS	4.03	-	Favored (58.18%)<br>General / -60.5,137.3	Favored (59.3%) *t-90*<br>chi angles: 181.5,278.6	0.10Å	Favored (35.667%)	-	-	-
A 253		SER	3.33	-	Favored (16.99%)<br>General / -96.7,17.2	Favored (81.8%) *p*<br>chi angles: 62	0.03Å	CaBLAM Outlier (0.705%)	-	-	-
A 254		GLY	2.64	-	Favored (4.94%)<br>Glycine / 85.4,-56.8	-	-	CaBLAM Outlier (0.757%)	-	-	-
A 255		THR	2.05	-	Favored (96.65%)<br>General / -62.0,-40.7	Favored (88.4%) *m*<br>chi angles: 298.5	0.03Å	Favored (31.654%)	-	-	-
A 256		GLU	1.61	-	Favored (58.23%)<br>General / -61.0,137.4	Favored (49%) *mt-10*<br>chi angles: 292.9,172.5,288.6	0.06Å	Favored (10.979%)	-	-	-
A 257		ILE	1.3	-	Favored (4.77%)<br>Ile or Val / -107.4,-39.9	Favored (48.4%) *mm*<br>chi angles: 301.1,299.8	0.05Å	Favored (6.86%)	-	-	-
A 258		VAL	1.1	-	Favored (67.62%)<br>Ile or Val / -112.6,129.8	Favored (58.7%) *t*<br>chi angles: 180.1	0.11Å	Favored (25.17%)	-	-	-
A 259		ASP	0.99	-	Favored (35.15%)<br>General / -107.9,117.8	Favored (95.3%) *m-30*<br>chi angles: 291.5,345	0.04Å	Favored (72.693%)	-	-	-
A 260		VAL	0.95	-	Favored (55.32%)<br>Ile or Val / -102.0,127.6	Favored (82.9%) *t*<br>chi angles: 176.9	0.02Å	Favored (31.16%)<br>beta sheet	-	-	-
**#**	**Alt**	**Res**	**High B**	**Clash > 0.4Å**	**Ramachandran**	**Rotamer**	**Cβ deviation**	**CaBLAM**	**Bond lengths**	**Bond angles**	**Cis Peptides**
			Avg: 1.13	Clashscore: 1.45	Outliers: 1 of 617	Poor rotamers: 1 of 507	Outliers: 0 of 561	Outliers: 16 of 615	Outliers: 7 of 619	Outliers: 6 of 619	Non-Trans: 0 of 618
A 261		MET	0.96	-	Favored (28.02%)<br>General / -151.1,167.3	Favored (22.2%) *ptp*<br>chi angles: 65.1,194.4,77.1	0.07Å	Favored (25.019%)<br>beta sheet	-	-	-
A 262		CYS	1	-	Favored (14.68%)<br>General / -90.1,163.9	Favored (20.4%) *p*<br>chi angles: 70.6	0.06Å	Favored (33.87%)	-	-	-
A 263		HIS	1.05	-	Favored (86.93%)<br>General / -58.9,-41.3	Favored (40.9%) *m170*<br>chi angles: 295.5,181.5	0.09Å	Favored (66.877%)	OUTLIER(S)<br>worst is CG--CD2: 4.7 σ	-	-
A 264		ALA	1.09	-	Favored (97.19%)<br>General / -61.3,-41.6	-	0.02Å	Favored (91.784%)<br>alpha helix	-	-	-

#	Alt	Res	High B	Clash > 0.4Å	Ramachandran	Rotamer	Cβ deviation	CaBLAM	Bond lengths	Bond angles	Cis Peptides
A 265		THR	1.13	-	Favored (91.11%) General / -64.0,-44.9	Favored (91.5%) *m* chi angles: 297.3	0.01Å	Favored (98.23%) alpha helix	-	-	-
A 266		LEU	1.16	-	Favored (71.95%) General / -57.2,-50.7	Favored (55.3%) *tp* chi angles: 175.2,64.8	0.09Å	Favored (93.809%) alpha helix	-	-	-
A 267		THR	1.16	-	Favored (71.65%) General / -66.3,-31.1	Favored (73.4%) *p* chi angles: 59.8	0.04Å	Favored (75.366%) alpha helix	-	-	-
A 268		HIS	1.17	-	Favored (78.94%) General / -69.0,-40.0	Favored (56.2%) *m-70* chi angles: 282.7,296.7	0.04Å	Favored (93.481%) alpha helix	-	-	-
A 269		ARG	1.21	-	Favored (78.4%) General / -65.0,-34.4	Favored (48.4%) *mmt180* chi angles: 293.6,286.5,182,186.6	0.02Å	Favored (86.211%) alpha helix	-	-	-
A 270		LEU	1.31	-	Favored (62.1%) General / -75.0,-36.3	Favored (92.4%) *mt* chi angles: 293.1,175.4	0.04Å	Favored (86.523%) alpha helix	-	-	-
A 271		MET	1.48	-	Favored (63.35%) General / -74.5,-35.9	Favored (83.4%) *mtm* chi angles: 290.2,187.3,290.4	0.01Å	Favored (32.627%)	-	-	-
A 272		SER	1.68	-	Favored (86.91%) Pre-Pro / -62.2,149.4	Favored (69.7%) *m* chi angles: 296.5	0.05Å	Favored (19.462%)	-	-	-
A 273		PRO	1.88	-	Favored (6.41%) Trans-Pro / -75.6,62.9	Favored (67.4%) *Cg_endo* chi angles: 31.3,322.5,27.8	0.04Å	Favored (8.963%)	-	-	-
A 274		LEU	2	-	Favored (28.74%) General / -116.8,155.6	Favored (54.9%) *mt* chi angles: 304.9,175.1	0.09Å	Favored (24.642%)	-	-	-
A 275		ARG	1.97	-	Favored (15.38%) General / -80.0,110.5	Favored (81.4%) *ttt180* chi angles: 181.6,179.2,180.8,180.7	0.05Å	Favored (29.718%) beta sheet	-	-	-
A 276		ALA	1.78	-	Favored (93.5%) Pre-Pro / -68.8,142.3	-	0.02Å	Favored (45.315%) beta sheet	-	-	-
A 277		PRO	1.5	-	Favored (94.04%) Trans-Pro / -60.8,147.8	Favored (54.1%) *Cg_exo* chi angles: 337.2,32.6,331.4	0.04Å	Favored (86.747%) beta sheet	-	-	-
A 278		ASN	1.23	-	Favored (24.11%) General / -91.8,112.3	Favored (82.4%) *m-40* chi angles: 295,311.6	0.02Å	Favored (46.494%) beta sheet	-	-	-
A 279		TYR	1.01	-	Favored (29.58%) General / -101.2,144.8	Favored (96.8%) *m-80* chi angles: 295.5,98.2	0.04Å	Favored (46.864%)	-	-	-
A 280		ASN	0.87	-	Favored (22.35%) General / -98.8,-10.9	Favored (92.7%) *m-40* chi angles: 293.3,329.2	0.03Å	Favored (28.451%)	-	-	-

			Avg: 1.13	Clashscore: 1.45	Outliers: 1 of 617	Poor rotamers: 1 of 507	Outliers: 0 of 561	Outliers: 16 of 615	Outliers: 7 of 619	Outliers: 6 of 619	Non-Trans: 0 of 618
A 281		LEU	0.78	-	Favored (18.57%) General / -147.2,132.6	Favored (39.2%) *tp* chi angles: 174.5,67.8	0.06Å	Favored (31.806%)	-	-	-
A 282		PHE	0.74	-	Favored (56.27%) General / -116.7,129.7	Favored (90.8%) *m-80* chi angles: 293.5,84.9	0.09Å	Favored (71.788%)	-	-	-
A 283		VAL	0.73	-	Favored (58.96%) Ile or Val / -118.1,118.7	Favored (50.4%) *t* chi angles: 181.4	0.05Å	Favored (70.836%) beta sheet	-	-	-
A 284		MET	0.75	-	Favored (48.95%) General / -106.0,134.6	Favored (61.9%) *ttm* chi angles: 181.3,175.7,285.4	0.06Å	Favored (58.863%)	-	-	-
A 285		ASP	0.77	-	Favored (42.93%) General / -98.1,133.1	Favored (36.5%) *t70* chi angles: 181,71.7	0.08Å	Favored (10.073%)	-	-	-
A 286		GLU	0.8	0.41Å HA with A 286 GLU OE1	Favored (10.99%) General / 51.6,54.5	Favored (23.3%) *mm-30* chi angles: 296.3,296.4,9.5	0.07Å	Favored (8.674%)	-	-	-
A 287		ALA	0.83	0.43Å O with A 298 ARG NH1	Favored (60.75%) General / -59.6,-21.7	-	0.03Å	Favored (23.335%)	-	-	-
A 288		HIS	0.84	-	Favored (58.09%) General / -77.0,-8.8	Favored (57.9%) *p-80* chi angles: 66.8,278.6	0.08Å	Favored (31.252%)	-	-	-
A 289		PHE	0.84	-	Favored (58.42%) General / -63.6,138.2	Favored (17%) *t80* chi angles: 199.8,80.5	0.07Å	Favored (36.253%)	-	-	-
A 290		THR	0.82	-	Favored (12.75%) General / -88.2,12.9	Favored (65.5%) *p* chi angles: 58.3	0.07Å	CaBLAM Disfavored (4.093%) try beta sheet	-	-	-
A 291		ASP	0.81	-	Favored (95.14%) Pre-Pro / -63.9,132.1	Favored (12.1%) *t70* chi angles: 186,294.4	0.08Å	Favored (35.695%)	-	-	-
A 292		PRO	0.78	-	Favored (9.02%) Trans-Pro / -44.8,-35.0	Favored (90.8%) *Cg_exo* chi angles: 329.6,36.7,332.9	0.07Å	Favored (77.028%)	-	-	-
A 293		ALA	0.76	-	Favored (65.35%) General / -73.1,-33.2	-	0.03Å	Favored (89.323%) alpha helix	-	-	-
A 294		SER	0.75	-	Favored (66.93%) General / -72.4,-40.7	Favored (72.9%) *m* chi angles: 295.4	0.07Å	Favored (76.83%) alpha helix	-	-	-
A 295		ILE	0.74	-	Favored (82.52%) Ile or Val / -65.9,-48.3	Favored (85%) *mt* chi angles: 295.6,165.2	0.06Å	Favored (92.513%) alpha helix	-	-	-
A 296		ALA	0.74	-	Favored (93.55%) General / -61.3,-40.3	-	0.03Å	Favored (92.596%) alpha helix	-	-	-
A 297		ALA	0.74	-	Favored (99.21%)	-	0.03Å	Favored (97.038%)	-	-	-

#	Alt	Res	High B	Clash > 0.4Å	Ramachandran	Rotamer	Cβ deviation	CaBLAM	Bond lengths	Bond angles	Cis Peptides
					General / -62.6,-41.8			alpha helix			
A 298		ARG	0.76	0.43Å NH1 with A 287 ALA O	Favored (93.78%) General / -63.5,-39.2	Favored (99.2%) *mtm-85* chi angles: 286.8,192.6,294.9,276.6	0.05Å	Favored (96.153%) alpha helix	-	-	-
A 299		GLY	0.77	-	Favored (52.67%) Glycine / -61.5,-52.2	-	-	Favored (91.91%) alpha helix	-	-	-
A 300		TYR	0.79	-	Favored (70.65%) General / -55.3,-50.0	Favored (91.8%) *t80* chi angles: 177.1,79.6	0.04Å	Favored (90.234%) alpha helix	-	-	-
**#**	**Alt**	**Res**	**High B**	**Clash > 0.4Å**	**Ramachandran**	**Rotamer**	**Cβ deviation**	**CaBLAM**	**Bond lengths**	**Bond angles**	**Cis Peptides**
			Avg: 1.13	Clashscore: 1.45	Outliers: 1 of 617	Poor rotamers: 1 of 507	Outliers: 0 of 561	Outliers: 16 of 615	Outliers: 7 of 619	Outliers: 6 of 619	Non-Trans: 0 of 618
A 301		ILE	0.81	-	Favored (89.08%) Ile or Val / -62.5,-48.3	Favored (94.8%) *mt* chi angles: 292,166.9	0.08Å	Favored (95.845%) alpha helix	-	-	-
A 302		ALA	0.83	-	Favored (79.09%) General / -59.7,-37.5	-	0.04Å	Favored (89.001%) alpha helix	-	-	-
A 303		THR	0.84	-	Favored (99.55%) General / -62.5,-43.1	Favored (98.2%) *m* chi angles: 300.1	0.07Å	Favored (95.774%) alpha helix	-	-	-
A 304		LYS	0.85	-	Favored (67.87%) General / -67.6,-28.6	Favored (65.2%) *mmtt* chi angles: 289,296,189.3,180.2	0.01Å	Favored (75.661%) alpha helix	-	-	-
A 305		VAL	0.86	-	Favored (38.65%) Ile or Val / -75.8,-45.2	Favored (97.2%) *t* chi angles: 175.1	0.03Å	Favored (71.31%) alpha helix	-	-	-
A 306		GLU	0.87	-	Favored (25.1%) General / -78.8,0.9	Favored (98.8%) *mt-10* chi angles: 293.3,176.7,357.1	0.06Å	CaBLAM Outlier (0.069%) try alpha helix	-	OUTLIER(S) worst is CA-C-O: 4.6 σ	-
A 307		LEU	0.86	-	Favored (44.32%) General / -93.4,-7.5	Favored (96.1%) *mt* chi angles: 294.6,173.1	0.02Å	CaBLAM Outlier (0.34%)	-	-	-
A 308		GLY	0.84	-	Favored (75.85%) Glycine / 76.8,20.8	-	-	Favored (80.304%)	-	-	-
A 309		GLU	0.81	-	Favored (16.65%) General / -90.8,-31.1	Favored (79.7%) *mt-10* chi angles: 296.2,182,18.3	0.01Å	Favored (6.584%)	-	-	-
A 310		ALA	0.77	-	Favored (40.15%) General / -150.4,155.8	-	0.09Å	Favored (19.462%)	-	-	-
A 311		ALA	0.74	-	Favored (16.43%) General / -89.8,160.6	-	0.05Å	Favored (42.899%)	-	-	-
A 312		ALA	0.72	-	Favored (30.28%) General / -145.4,143.8	-	0.07Å	Favored (59.834%) beta sheet	-	-	-
A 313		ILE	0.72	-	Favored (75.55%)	Favored (75.4%) *mt* chi angles: 300.3,167	0.06Å	Favored (71.36%)	-	-	-

#	Alt	Res	High B	Clash > 0.4Å	Ramachandran	Rotamer	Cβ deviation	CaBLAM	Bond lengths	Bond angles	Cis Peptides
					Ile or Val / -122.3,128.7			beta sheet			
A 314		PHE	0.74	-	Favored (9.34%) General / -116.7,102.3	Favored (79.6%) *m-80* chi angles: 300.8,86.5	0.06Å	Favored (65.017%) beta sheet	-	-	-
A 315		MET	0.77	-	Favored (32.23%) General / -84.1,139.8	Favored (72.3%) *mtm* chi angles: 298.4,187.5,294.9	0.04Å	Favored (5.247%) beta sheet	-	-	-
A 316		THR	0.82	-	Favored (5.27%) General / -169.2,153.1	Favored (4.5%) *t* chi angles: 179.8	0.03Å	Favored (10.722%)	-	-	-
A 317		ALA	0.89	-	Favored (77.64%) General / -62.5,-34.9	-	0.06Å	Favored (17.237%)	-	-	-
A 318		THR	0.97	-	Favored (11.1%) Pre-Pro / -154.4,138.5	Favored (4%) *t* chi angles: 178	0.11Å	Favored (11.721%)	-	-	-
A 319		PRO	1.04	-	Favored (36.37%) Trans-Pro / -75.4,162.2	Favored (79%) *Cg_endo* chi angles: 30.2,324.6,25.8	0.05Å	Favored (80.392%)	-	-	-
A 320		PRO	1.1	-	Favored (64.96%) Trans-Pro / -61.8,-19.3	Favored (34.2%) *Cg_endo* chi angles: 22.2,325.9,31.5	0.01Å	Favored (64.603%)	-	-	-
**#**	**Alt**	**Res**	**High B**	**Clash > 0.4Å**	**Ramachandran**	**Rotamer**	**Cβ deviation**	**CaBLAM**	**Bond lengths**	**Bond angles**	**Cis Peptides**
			Avg: 1.13	Clashscore: 1.45	Outliers: 1 of 617	Poor rotamers: 1 of 507	Outliers: 0 of 561	Outliers: 16 of 615	Outliers: 7 of 619	Outliers: 6 of 619	Non-Trans: 0 of 618
A 321		GLY	1.12	-	Favored (83.92%) Glycine / -88.5,1.2	-	-	Favored (64.506%)	-	-	-
A 322		THR	1.1	-	Favored (52.29%) General / -110.2,134.4	Favored (86.7%) *m* chi angles: 296.9	0.10Å	Favored (35.37%)	-	-	-
A 323		HIS	1.06	-	Favored (2.9%) General / -109.1,36.4	Favored (55.4%) *m170* chi angles: 297.5,161.7	0.03Å	CaBLAM Disfavored (3.094%)	-	-	-
A 324		ASP	1.01	-	Favored (36.27%) Pre-Pro / -101.5,104.8	Favored (63.2%) *t0* chi angles: 183,0.5	0.06Å	Favored (17.582%) beta sheet	-	-	-
A 325		PRO	0.96	-	Favored (26.63%) Trans-Pro / -72.7,-12.6	Favored (72.5%) *Cg_endo* chi angles: 27.5,327.1,24.3	0.01Å	Favored (50.342%)	-	-	-
A 326		PHE	0.93	-	Favored (61.26%) Pre-Pro / -132.2,62.0	Favored (86.3%) *m-80* chi angles: 302.4,97.6	0.09Å	Favored (13.72%)	-	-	-
A 327		PRO	0.94	-	Favored (31.77%) Trans-Pro / -73.8,165.6	Favored (75.1%) *Cg_endo* chi angles: 28.8,324.2,28.5	0.03Å	Favored (6.093%)	-	-	-
A 328		ASP	0.96	-	Favored (37.35%) General / -59.2,147.0	Favored (91.6%) *m-30* chi angles: 289.5,350.8	0.03Å	Favored (13.328%)	-	-	-
A 329		THR	0.99	-	Favored (5.36%)	Favored (75.3%) *p* chi angles: 61.3	0.11Å	Favored (30.216%)	-	-	-

#	Alt	Res	High B	Clash > 0.4Å	Ramachandran	Rotamer	Cβ deviation	CaBLAM	Bond lengths	Bond angles	Cis Peptides
					General / -126.0,179.2						
A 330		ASN	1.01	-	Favored (36.93%) General / -98.2,11.7	Favored (19.8%) *t0* chi angles: 207.1,46.7	0.03Å	CaBLAM Disfavored (3.598%)	-	-	-
A 331		ALA	0.99	-	Favored (25.52%) Pre-Pro / -153.9,151.8	-	0.04Å	Favored (12.908%)	-	-	-
A 332		PRO	0.95	-	Favored (48.82%) Trans-Pro / -68.7,141.1	Favored (50.1%) *Cg_endo* chi angles: 25.2,326.9,26.7	0.03Å	Favored (36.87%)	-	-	-
A 333		VAL	0.9	-	Favored (52.57%) Ile or Val / -135.3,132.5	Favored (58.6%) *t* chi angles: 180.1	0.05Å	Favored (61.222%) beta sheet	-	-	-
A 334		THR	0.84	-	Favored (35.72%) General / -91.6,133.3	Favored (98.8%) *m* chi angles: 300.2	0.02Å	Favored (51.493%) beta sheet	-	-	-
A 335		ASP	0.81	-	Favored (33.62%) General / -104.9,117.3	Favored (18.8%) *m-30* chi angles: 289.4,284.9	0.02Å	Favored (61.107%) beta sheet	-	-	-
A 336		ILE	0.79	-	Favored (68.11%) Ile or Val / -120.5,122.4	Favored (3.7%) *mp* chi angles: 304.7,95.7	0.10Å	Favored (58.576%) beta sheet	-	-	-
A 337		GLN	0.79	-	Favored (37.02%) General / -79.0,135.8	Favored (61.5%) *tt0* chi angles: 188,176.7,36.3	0.04Å	Favored (33.792%) beta sheet	-	-	-
A 338		ALA	0.8	-	Favored (43.76%) General / -151.9,159.5	-	0.04Å	Favored (35.908%) beta sheet	-	-	-
A 339		GLU	0.82	-	Favored (5.97%) General / -79.4,75.8	Favored (96.4%) *mt-10* chi angles: 296.3,180.9,353.1	0.01Å	Favored (15.339%) beta sheet	-	-	-
A 340		VAL	0.84	-	Favored (84.91%) Pre-Pro / -78.5,125.2	Favored (88.7%) *t* chi angles: 176.1	0.07Å	Favored (31.035%) beta sheet	-	-	-
**#**	**Alt**	**Res**	**High B**	**Clash > 0.4Å**	**Ramachandran**	**Rotamer**	**Cβ deviation**	**CaBLAM**	**Bond lengths**	**Bond angles**	**Cis Peptides**
			Avg: 1.13	Clashscore: 1.45	Outliers: 1 of 617	Poor rotamers: 1 of 507	Outliers: 0 of 561	Outliers: 16 of 615	Outliers: 7 of 619	Outliers: 6 of 619	Non-Trans: 0 of 618
A 341		PRO	0.88	-	Favored (63.77%) Trans-Pro / -70.9,152.7	Favored (54.9%) *Cg_endo* chi angles: 25.8,327.6,26.5	0.03Å	Favored (52.064%)	-	-	-
A 342		ASP	0.93	-	Favored (11.33%) General / -97.8,-33.3	Favored (59.3%) *m-30* chi angles: 295.2,304.6	0.07Å	Favored (9.596%)	-	-	-
A 343		ARG	1	-	Favored (6.2%) General / -110.6,173.7	Favored (62.2%) *mmm-85* chi angles: 304.4,294.7,296.6,272.7	0.05Å	CaBLAM Disfavored (1.053%)	-	-	-
A 344		ALA	1.08	-	Favored (16.86%) General / -50.5,138.8	-	0.12Å	CaBLAM Disfavored (4.188%)	-	-	-
A 345		TRP	1.18	-	Favored (35.74%)	Favored (70.2%) *p-90*	0.14Å	Favored (36.735%)	-	-	-

					General / -148.3,151.7	chi angles: 64.5,275.5					
A 346		SER	1.26	-	Allowed (1.9%) General / -143.1,16.4	Favored (79.8%) *p* chi angles: 61.3	0.03Å	Favored (20.298%)	-	-	-
A 347		SER	1.31	-	Allowed (0.23%) General / -138.6,-88.3	Favored (75.7%) *p* chi angles: 59.9	0.02Å	CaBLAM Outlier (0.471%)	-	-	-
A 348		GLY	1.32	-	Favored (49.45%) Glycine / -54.2,-31.6	-	-	Favored (46.61%)	-	-	-
A 349		PHE	1.29	-	Favored (7.32%) General / -86.9,62.3	Favored (90.1%) *m-80* chi angles: 301,96.2	0.06Å	Favored (9.044%)	-	-	-
A 350		GLU	1.24	-	Favored (71.23%) General / -60.1,-32.7	Favored (98.6%) *mt-10* chi angles: 290.5,178.9,351.8	0.05Å	Favored (24.417%)	-	-	-
A 351		TRP	1.19	-	Favored (65.95%) General / -59.7,-26.8	Favored (82.3%) *m100* chi angles: 285.5,112	0.02Å	Favored (65.22%) alpha helix	-	-	-
A 352		ILE	1.15	-	Favored (75.8%) Ile or Val / -66.9,-49.2	Favored (84.2%) *mt* chi angles: 295,164.7	0.14Å	Favored (68.042%) alpha helix	-	-	-
A 353		THR	1.13	-	Favored (84.08%) General / -67.8,-40.8	Favored (93.9%) *m* chi angles: 299.3	0.04Å	Favored (71.729%) alpha helix	-	-	-
A 354		GLU	1.1	-	Favored (75.89%) General / -64.6,-33.4	Favored (65.3%) *mm-30* chi angles: 289.4,292.9,307.3	0.03Å	Favored (51.027%)	-	-	-
A 355		TYR	1.07	-	Favored (54.24%) General / -56.8,137.7	Favored (17.9%) *m-10* chi angles: 292.1,144.8	0.03Å	Favored (19.167%)	-	-	-
A 356		THR	1.02	-	Favored (15.79%) General / -104.8,-13.7	Favored (71.4%) *p* chi angles: 62.1	0.01Å	Favored (15.855%)	-	-	-
A 357		GLY	0.95	-	Favored (40.2%) Glycine / -84.4,164.6	-	-	Favored (25.129%) beta sheet	-	-	-
A 358		LYS	0.88	-	Favored (51.21%) General / -60.1,131.6	Favored (95.8%) *mttt* chi angles: 285.8,181.9,174.5,174.9	0.04Å	Favored (21.642%) beta sheet	-	-	-
A 359		THR	0.81	-	Favored (54.49%) General / -123.0,135.5	Favored (93%) *m* chi angles: 299.2	0.03Å	Favored (71.958%) beta sheet	-	-	-
A 360		VAL	0.76	-	Favored (63.65%) Ile or Val / -110.7,130.7	Favored (88.8%) *t* chi angles: 176.1	0.05Å	Favored (72.368%) beta sheet	-	-	-
**#**	**Alt**	**Res**	**High B**	**Clash > 0.4Å**	**Ramachandran**	**Rotamer**	**Cβ deviation**	**CaBLAM**	**Bond lengths**	**Bond angles**	**Cis Peptides**
			Avg: 1.13	Clashscore: 1.45	Outliers: 1 of 617	Poor rotamers: 1 of 507	Outliers: 0 of 561	Outliers: 16 of 615	Outliers: 7 of 619	Outliers: 6 of 619	Non-Trans: 0 of 618

A 361		TRP	0.73	-	Favored (55.39%) General / -113.3,132.9	Favored (6%) *t-100* chi angles: 187.3,296.8	0.11Å	Favored (68.492%) beta sheet	OUTLIER(S) worst is NE1--CE2: 4.5 σ	-	-
A 362		PHE	0.74	-	Favored (45.78%) General / -111.0,121.6	Favored (84.4%) *m-80* chi angles: 293.9,82.4	0.04Å	Favored (67.833%) beta sheet	-	-	-
A 363		VAL	0.76	-	Favored (8.48%) Ile or Val / -110.4,161.8	Favored (27%) *m* chi angles: 299	0.07Å	Favored (25.938%)	-	-	-
A 364		ALA	0.81	-	Favored (59.68%) General / -80.2,-12.0	-	0.04Å	Favored (8.952%)	-	-	-
A 365		SER	0.86	-	Favored (31.17%) General / -161.0,164.2	Favored (85.3%) *p* chi angles: 67.3	0.05Å	Favored (19.896%)	-	-	-
A 366		VAL	0.9	-	Favored (96.29%) Ile or Val / -61.0,-43.5	Favored (64.3%) *t* chi angles: 171.4	0.04Å	Favored (66.737%) alpha helix	-	-	-
A 367		LYS	0.92	-	Favored (95.03%) General / -64.6,-40.1	Favored (97.7%) *mttt* chi angles: 288.5,178.6,175.7,176.5	0.08Å	Favored (92.977%) alpha helix	-	-	-
A 368		MET	0.93	-	Favored (92.28%) General / -63.0,-38.7	Favored (69%) *mtt* chi angles: 288.8,179.9,180.5	0.07Å	Favored (83.665%) alpha helix	-	-	-
A 369		GLY	0.91	-	Favored (37.58%) Glycine / -55.0,-53.8	-	-	Favored (94.41%) alpha helix	-	-	-
A 370		ASN	0.89	-	Favored (81.89%) General / -58.9,-39.5	Favored (97.5%) *m-40* chi angles: 287,339.1	0.04Å	Favored (80.889%) alpha helix	-	-	-
A 371		GLU	0.87	-	Favored (99.29%) General / -63.0,-41.0	Favored (78.8%) *mt-10* chi angles: 285.3,186.6,359.1	0.03Å	Favored (79.932%) alpha helix	-	-	-
A 372		ILE	0.86	-	Favored (72.98%) Ile or Val / -71.5,-42.3	Favored (96.4%) *mt* chi angles: 293.5,169.2	0.10Å	Favored (76.22%) alpha helix	-	-	-
A 373		ALA	0.86	-	Favored (90.58%) General / -58.8,-43.6	-	0.07Å	Favored (89.301%) alpha helix	-	-	-
A 374		GLN	0.86	-	Favored (95.31%) General / -64.1,-40.0	Favored (94.8%) *mt0* chi angles: 289.7,173,347.1	0.03Å	Favored (98.061%) alpha helix	-	-	-
A 375		CYS	0.87	-	Favored (94.45%) General / -64.7,-43.2	Favored (96.4%) *m* chi angles: 292.2	0.05Å	Favored (91.959%) alpha helix	-	-	-
A 376		LEU	0.89	-	Favored (82.3%) General / -67.2,-36.7	Favored (81%) *mt* chi angles: 289.1,172.2	0.11Å	Favored (89.46%) alpha helix	-	-	-
A 377		GLN	0.9	-	Favored (89.97%) General / -65.6,-38.6	Favored (95.4%) *mt0* chi angles: 290.5,171.1,346.2	0.04Å	Favored (89.874%) alpha helix	-	-	-

#	Alt	Res	High B	Clash > 0.4Å	Ramachandran	Rotamer	Cβ deviation	CaBLAM	Bond lengths	Bond angles	Cis Peptides
A 378		ARG	0.9	-	Favored (69.84%) General / -64.3,-28.6	Favored (86.8%) *mtt180* chi angles: 288.9,169.1,185.1,159.6	0.01Å	Favored (75.368%)	-	-	-
A 379		ALA	0.88	-	Favored (38.67%) General / -78.8,-2.2	-	0.02Å	Favored (52.32%)	-	-	-
A 380		GLY	0.85	-	Favored (58.63%) Glycine / 92.3,12.4	-	-	Favored (88.087%)	-	-	-
**#**	**Alt**	**Res**	**High B**	**Clash > 0.4Å**	**Ramachandran**	**Rotamer**	**Cβ deviation**	**CaBLAM**	**Bond lengths**	**Bond angles**	**Cis Peptides**
			Avg: 1.13	Clashscore: 1.45	Outliers: 1 of 617	Poor rotamers: 1 of 507	Outliers: 0 of 561	Outliers: 16 of 615	Outliers: 7 of 619	Outliers: 6 of 619	Non-Trans: 0 of 618
A 381		LYS	0.81	-	Favored (19.97%) General / -93.6,151.0	Favored (73.2%) *mmtt* chi angles: 302.9,294.4,184.9,180.2	0.04Å	Favored (35.281%)	-	-	-
A 382		LYS	0.77	0.51Å NZ with A 402 ASP OD2	Favored (24.88%) General / -88.1,114.7	Favored (98.2%) *mttt* chi angles: 291.8,180.8,177,178.7	0.04Å	Favored (47.352%) beta sheet	-	-	-
A 383		VAL	0.75	-	Favored (72.47%) Ile or Val / -125.8,130.3	Favored (43.6%) *t* chi angles: 182.7	0.10Å	Favored (63.359%) beta sheet	-	-	-
A 384		ILE	0.75	-	Favored (62.16%) Ile or Val / -109.7,130.8	Favored (75.9%) *mt* chi angles: 300.8,168.9	0.04Å	Favored (66.262%) beta sheet	-	-	-
A 385		GLN	0.79	-	Favored (50.43%) General / -103.4,129.2	Favored (82.2%) *tp40* chi angles: 179.2,69.3,53.6	0.03Å	Favored (68.682%) beta sheet	-	-	-
A 386		LEU	0.85	-	Favored (44.89%) General / -130.5,130.9	Favored (6.1%) *tt* chi angles: 178.2,145.3	0.08Å	Favored (29.737%) beta sheet	-	-	-
A 387		ASN	0.93	-	Favored (6.96%) General / -152.2,-178.7	Favored (31.9%) *p0* chi angles: 59.9,51.9	0.02Å	Favored (12.709%)	-	-	-
A 388		ARG	1.01	-	Favored (22.89%) General / -54.0,-25.7	Favored (21.1%) *tpp80* chi angles: 182.7,64.2,64.1,95.4	0.01Å	Favored (11.33%)	-	-	-
A 389		LYS	1.09	-	Favored (26.49%) General / -106.3,2.2	Favored (72%) *mmtt* chi angles: 301.1,296.4,180.3,181.1	0.03Å	Favored (33.609%) alpha helix	-	-	-
A 390		SER	1.14	-	Favored (3.93%) General / -130.5,-12.1	Favored (80.2%) *p* chi angles: 61.4	0.03Å	Favored (13.413%) alpha helix	-	-	-
A 391		TYR	1.16	-	Favored (59.41%) General / -51.1,-50.2	Favored (85.8%) *t80* chi angles: 174.1,79.9	0.09Å	Favored (53.934%) alpha helix	OUTLIER(S) worst is CB--CG: 4.8 σ	-	-
A 392		ASP	1.16	-	Favored (64.32%) General / -63.0,-19.5	Favored (98.2%) *m-30* chi angles: 287.2,346	0.05Å	Favored (50.59%) alpha helix	-	-	-
A 393		THR	1.16	-	Favored (6.93%) General / -96.1,-45.3	Favored (94%) *m* chi angles: 299.3	0.04Å	Favored (37.247%) alpha helix	-	-	-

#	Alt	Res	High B	Clash > 0.4Å	Ramachandran	Rotamer	Cβ deviation	CaBLAM	Bond lengths	Bond angles	Cis Peptides
A 394		GLU	1.15	-	Favored (25.21%) General / -85.5,-28.2	Favored (62.8%) *mm-30* chi angles: 299.1,295,306.9	0.05Å	Favored (34.148%) alpha helix	-	-	-
A 395		TYR	1.17	-	Favored (33.46%) Pre-Pro / -48.3,-53.5	Favored (78.1%) *t80* chi angles: 175.8,85	0.08Å	Favored (52.989%) alpha helix	-	-	-
A 396		PRO	1.21	-	Favored (72.02%) Trans-Pro / -63.2,-21.0	Favored (35.2%) *Cg_endo* chi angles: 22.4,325.3,32	0.01Å	Favored (68.675%) alpha helix	-	-	-
A 397		LYS	1.29	-	Favored (53.61%) General / -76.8,-38.7	Favored (98.9%) *mttt* chi angles: 293.8,179.1,185,177.4	0.05Å	Favored (73.746%) alpha helix	-	-	-
A 398		CYS	1.38	-	Favored (77.63%) General / -69.5,-38.4	Favored (91.8%) *m* chi angles: 293.1	0.10Å	Favored (86.206%) alpha helix	-	-	-
A 399		LYS	1.44	-	Favored (79.73%) General / -61.3,-48.8	Favored (84.9%) *tttt* chi angles: 181.1,180,177.1,181.2	0.03Å	Favored (34.609%) alpha helix	-	-	-
A 400		ASN	1.43	-	Favored (13.82%) General / -107.8,-18.4	Favored (88.2%) *m-40* chi angles: 295.9,318.2	0.02Å	Favored (14.291%)	-	-	-
**#**	**Alt**	**Res**	**High B**	**Clash > 0.4Å**	**Ramachandran**	**Rotamer**	**Cβ deviation**	**CaBLAM**	**Bond lengths**	**Bond angles**	**Cis Peptides**
			Avg: 1.13	Clashscore: 1.45	Outliers: 1 of 617	Poor rotamers: 1 of 507	Outliers: 0 of 561	Outliers: 16 of 615	Outliers: 7 of 619	Outliers: 6 of 619	Non-Trans: 0 of 618
A 401		GLY	1.34	-	Favored (49.01%) Glycine / -83.5,170.9	-	-	Favored (50.01%)	-	-	-
A 402		ASP	1.19	0.51Å OD2 with A 382 LYS NZ	Favored (6.36%) General / -88.8,62.4	Favored (17.8%) *p0* chi angles: 58.1,324.7	0.03Å	CaBLAM Disfavored (4.28%) try beta sheet	-	-	-
A 403		TRP	1.02	-	Favored (20.51%) General / -86.4,155.7	Favored (47.3%) *p-90* chi angles: 50.2,270.1	0.03Å	Favored (22.965%)	-	-	-
A 404		ASP	0.86	-	Favored (9.33%) General / -96.3,-40.1	Favored (59.7%) *m-30* chi angles: 296,303.4	0.07Å	Favored (7.704%)	-	-	-
A 405		PHE	0.75	-	Favored (51.72%) General / -133.4,147.1	Favored (88.4%) *m-80* chi angles: 298.4,86	0.10Å	Favored (27.5%)	-	-	-
A 406		VAL	0.68	-	Favored (74.31%) Ile or Val / -123.9,128.6	Favored (69.5%) *t* chi angles: 178.8	0.07Å	Favored (69.035%)	-	-	-
A 407		ILE	0.67	-	Favored (67.24%) Ile or Val / -111.0,123.8	Favored (78.1%) *mt* chi angles: 300.7,171	0.04Å	Favored (62.512%) beta sheet	-	-	-
A 408		THR	0.68	-	Favored (27.48%) General / -131.9,163.8	Favored (28.6%) *p* chi angles: 69.8	0.11Å	Favored (46.64%)	-	-	-
A 409		THR	0.71	-	Favored (3.05%)	Favored (22.1%) *p* chi angles: 71.7	0.09Å	Favored (18.188%)	-	-	-

					General / -100.8,-176.0						
A 410		ASP	0.76	-	Favored (19.31%) General / -56.2,-20.7	Favored (93.6%) *m-30* chi angles: 290.8,348.4	0.03Å	Favored (8.849%)	-	-	-
A 411		ILE	0.82	-	Favored (41.25%) Ile or Val / -59.2,-29.2	Favored (20.8%) *tt* chi angles: 191.8,169.9	0.07Å	Favored (58.214%)	-	-	-
A 412		SER	0.89	0.50Å O with A 461 ARG NH2	Favored (59%) General / -75.9,-9.9	Favored (90.8%) *p* chi angles: 66.7	0.06Å	Favored (58.096%)	-	-	-
A 413		GLU	0.96	-	Favored (60.81%) General / -73.7,-19.6	Favored (99.5%) *mt-10* chi angles: 291.7,177.9,357	0.04Å	Favored (31.249%)	-	-	-
A 414		MET	1.04	-	Favored (4.33%) General / -122.2,-28.5	Favored (18.5%) *ptt* chi angles: 65.5,179.5,181.1	0.05Å	Favored (14.845%)	-	-	-
A 415		GLY	1.1	-	Favored (66.04%) Glycine / -96.8,11.8	-	-	Favored (35.894%)	-	-	-
A 416		ALA	1.14	-	Favored (39%) General / -150.1,163.1	-	0.03Å	CA Geom Outlier (0.396%)	-	OUTLIER(S) worst is C-N-CA: 4.3 σ	-
A 417		ASN	1.14	-	Allowed (1.41%) General / -147.0,76.2	Favored (66.6%) *t0* chi angles: 192.6,32.2	0.04Å	Favored (10.214%)	-	-	-
A 418		PHE	1.1	-	Favored (33.32%) General / -95.7,138.5	Favored (24.4%) *m-10* chi angles: 294.8,338.1	0.05Å	Favored (33.441%) beta sheet	-	-	-
A 419		GLY	1.03	-	Favored (2.84%) Glycine / -75.3,58.5	-	-	Favored (6.605%) beta sheet	-	-	-
A 420		ALA	0.94	-	Favored (38.05%) General / -75.8,149.0	-	0.07Å	Favored (19.259%)	-	-	-
**#**	**Alt**	**Res**	**High B**	**Clash > 0.4Å**	**Ramachandran**	**Rotamer**	**Cβ deviation**	**CaBLAM**	**Bond lengths**	**Bond angles**	**Cis Peptides**
			Avg: 1.13	Clashscore: 1.45	Outliers: 1 of 617	Poor rotamers: 1 of 507	Outliers: 0 of 561	Outliers: 16 of 615	Outliers: 7 of 619	Outliers: 6 of 619	Non-Trans: 0 of 618
A 421		SER	0.85	-	Favored (12.92%) General / -100.8,-28.2	Favored (69.4%) *m* chi angles: 296.6	0.07Å	Favored (14.343%)	-	-	-
A 422		ARG	0.77	-	Favored (47.96%) General / -135.5,146.3	Favored (83.3%) *mtt90* chi angles: 294.1,184.8,173.5,86.9	0.06Å	Favored (34.545%)	-	-	-
A 423		VAL	0.7	-	Favored (72.37%) Ile or Val / -122.4,131.8	Favored (72.2%) *t* chi angles: 172.4	0.08Å	Favored (71.812%)	-	-	-
A 424		ILE	0.66	-	Favored (29.58%) Ile or Val / -119.7,110.5	Favored (72.3%) *mt* chi angles: 301.2,174.3	0.03Å	Favored (63.79%) beta sheet	-	-	-
A 425		ASP	0.64	-	Favored (30.46%) General / -108.9,148.5	Favored (85.8%) *m-30* chi angles: 294.3,345.5	0.04Å	Favored (47.658%) beta sheet	OUTLIER(S) worst is CB--CG: 4.4 σ	-	-

A 426		CYS	0.63	-	Favored (55.29%) General / -78.1,-18.8	Favored (83%) *m* chi angles: 295.7	0.06Å	CaBLAM Outlier (0.209%) try beta sheet	-	-	-
A 427		ARG	0.65	-	Favored (2.63%) General / 42.9,41.6	Favored (79.4%) *mtt180* chi angles: 292.5,189.7,167.5,183.2	0.17Å	CaBLAM Disfavored (2.924%) try beta sheet	-	-	-
A 428		LYS	0.68	-	Favored (48.23%) General / -129.6,153.3	Favored (94.4%) *mttt* chi angles: 298,180.8,185.7,179	0.05Å	Favored (22.116%) beta sheet	-	-	-
A 429		SER	0.72	-	Favored (13.11%) General / -150.3,129.9	Favored (42%) *t* chi angles: 178.3	0.05Å	Favored (24.129%) beta sheet	-	-	-
A 430		VAL	0.76	-	Favored (2.01%) Ile or Val / -82.4,88.8	Favored (57%) *t* chi angles: 180.3	0.06Å	Favored (56.125%) beta sheet	OUTLIER(S) worst is CB--CG1: 4.2 σ	-	-
A 431		LYS	0.81	-	Favored (48.86%) Pre-Pro / -85.2,148.0	Favored (69.4%) *mmtt* chi angles: 300.9,298.3,186.3,188.5	0.04Å	Favored (20.283%) beta sheet	-	-	-
A 432		PRO	0.87	-	Favored (31.85%) Trans-Pro / -74.2,143.4	Favored (70.6%) *Cg_endo* chi angles: 29.3,323.3,28.3	0.08Å	Favored (38.707%) beta sheet	-	-	-
A 433		THR	0.93	-	Favored (23.73%) General / -131.9,165.5	Favored (65.1%) *p* chi angles: 63.1	0.06Å	Favored (46.341%) beta sheet	-	-	-
A 434		ILE	1.02	-	Favored (59.86%) Ile or Val / -105.6,123.3	Favored (90.3%) *mt* chi angles: 297.8,169.9	0.06Å	Favored (33.965%) beta sheet	-	-	-
A 435		LEU	1.12	-	Favored (7.63%) General / -81.4,81.1	Favored (25.2%) *tp* chi angles: 188.9,65.1	0.04Å	Favored (45.701%) beta sheet	-	-	-
A 436		GLU	1.22	-	Favored (66.54%) General / -63.6,-21.8	Favored (99.4%) *mt-10* chi angles: 291.5,180.1,354.6	0.03Å	Favored (22.815%)	-	-	-
A 437		GLU	1.28	-	Favored (66.54%) General / -64.1,-21.5	Favored (98.6%) *mt-10* chi angles: 293.8,179,357.5	0.02Å	CaBLAM Disfavored (4.143%)	-	-	-
A 438		GLY	1.29	-	Favored (2.57%) Glycine / 179.0,136.3	-	-	Favored (16.302%)	-	-	-
A 439		GLU	1.23	-	Allowed (0.06%) General / 60.4,-9.6	Favored (63.5%) *mm-30* chi angles: 306.3,296.5,340.7	0.04Å	CaBLAM Disfavored (4.075%)	-	-	-
A 440		GLY	1.14	-	Favored (81%) Glycine / 74.2,13.8	-	-	Favored (45.818%)	-	-	-
**#**	**Alt**	**Res**	**High B**	**Clash > 0.4Å**	**Ramachandran**	**Rotamer**	**Cβ deviation**	**CaBLAM**	**Bond lengths**	**Bond angles**	**Cis Peptides**
			Avg: 1.13	Clashscore: 1.45	Outliers: 1 of 617	Poor rotamers: 1 of 507	Outliers: 0 of 561	Outliers: 16 of 615	Outliers: 7 of 619	Outliers: 6 of 619	Non-Trans: 0 of 618
A 441		ARG	1.04	-	Favored (46.46%) General / -59.1,144.2	Favored (88.3%) *mtm180* chi angles: 288,175.9,291.7,172.8	0.04Å	Favored (8.563%) beta sheet	-	-	-

A 442		VAL	0.97	-	Favored (33.94%) Ile or Val / -91.1,117.2	Favored (99.8%) *t* chi angles: 175.5	0.08Å	Favored (50.541%) beta sheet	-	-	-
A 443		ILE	0.91	-	Favored (71.11%) Ile or Val / -126.8,131.4	Favored (78.6%) *mt* chi angles: 300.8,172.2	0.05Å	Favored (51.664%) beta sheet	-	-	-
A 444		LEU	0.88	-	Favored (49.4%) General / -65.3,132.6	Favored (60.5%) *mt* chi angles: 289.5,177.3	0.06Å	Favored (45.773%)	-	-	-
A 445		SER	0.86	-	Favored (17.54%) General / -81.0,169.4	Favored (80.6%) *p* chi angles: 61.6	0.06Å	Favored (29.129%)	-	-	-
A 446		ASN	0.84	0.46Å O with A 448 SER N	Favored (86.03%) Pre-Pro / -61.9,149.3	Favored (96.4%) *m-40* chi angles: 291.3,333.8	0.03Å	Favored (12.864%)	-	-	-
A 447		PRO	0.82	-	OUTLIER (0.01)%) Trans-Pro / -49.7,73.8	Favored (93.9%) *Cg_exo* chi angles: 333,33.8,334.2	0.05Å	Favored (5.533%)	-	-	-
A 448		SER	0.8	0.46Å N with A 446 ASN O	Favored (98.36%) Pre-Pro / -66.3,148.8	Favored (64.3%) *m* chi angles: 297.4	0.07Å	Favored (13.535%)	-	-	-
A 449		PRO	0.78	-	Favored (72.3%) Trans-Pro / -54.2,138.5	Favored (99%) *Cg_exo* chi angles: 332.2,36.4,330.7	0.05Å	Favored (80.037%)	-	-	-
A 450		ILE	0.77	-	Favored (4.01%) Ile or Val / -71.7,163.0	Favored (47%) *pt* chi angles: 62.9,173.2	0.03Å	Favored (39.027%)	-	-	-
A 451		THR	0.77	-	Favored (26.68%) General / -76.3,163.8	Favored (67.5%) *p* chi angles: 62.8	0.06Å	Favored (48.053%)	-	-	-
A 452		SER	0.77	-	Favored (72.36%) General / -61.9,-32.0	Favored (96.6%) *p* chi angles: 65.1	0.11Å	Favored (65.271%)	-	-	-
A 453		ALA	0.77	-	Favored (88.93%) General / -66.1,-42.7	-	0.03Å	Favored (78.432%) alpha helix	-	-	-
A 454		SER	0.76	-	Favored (88.48%) General / -66.7,-40.0	Favored (27.4%) *m* chi angles: 288.6	0.07Å	Favored (85.441%) alpha helix	-	-	-
A 455		ALA	0.76	-	Favored (88.3%) General / -59.9,-40.3	-	0.03Å	Favored (87.254%) alpha helix	-	-	-
A 456		ALA	0.75	-	Favored (97.57%) General / -60.9,-42.2	-	0.06Å	Favored (92.441%) alpha helix	-	-	-
A 457		GLN	0.75	-	Favored (76.65%) General / -67.7,-34.3	Favored (34.4%) *mm110* chi angles: 290,296.1,109.2	0.04Å	Favored (88.209%) alpha helix	-	-	-
A 458		ARG	0.75	-	Favored (75.19%) General / -69.6,-42.0	Favored (93.2%) *mtt180* chi angles: 292.5,181,180,190.2	0.05Å	Favored (78.149%) alpha helix	-	OUTLIER(S) worst is NE-CZ-NH2: 4.2 σ	-

A 459		ARG	0.77	-	Favored (77.39%) General / -65.2,-34.0	Favored (30.8%) *mtp-110* chi angles: 291.3,177.1,70.6,253.3	0.15Å	Favored (46.155%) alpha helix	-	-	-
A 460		GLY	0.79	0.43Å O with A 464 ARG HG3	Favored (46.58%) Glycine / -59.1,-19.7	-	-	Favored (70.857%) alpha helix	-	-	-
**#**	**Alt**	**Res**	**High B**	**Clash > 0.4Å**	**Ramachandran**	**Rotamer**	**Cβ deviation**	**CaBLAM**	**Bond lengths**	**Bond angles**	**Cis Peptides**
			Avg: 1.13	Clashscore: 1.45	Outliers: 1 of 617	Poor rotamers: 1 of 507	Outliers: 0 of 561	Outliers: 16 of 615	Outliers: 7 of 619	Outliers: 6 of 619	Non-Trans: 0 of 618
A 461		ARG	0.82	0.50Å NH2 with A 412 SER O	Favored (65.88%) General / -72.5,-41.5	Favored (25.6%) *tpp80* chi angles: 178.8,61.8,55,78.6	0.11Å	Favored (60.891%) alpha helix	-	-	-
A 462		VAL	0.87	-	Favored (11.23%) Ile or Val / -81.7,-32.0	Favored (3.2%) *p* chi angles: 74.5	0.11Å	Favored (70.535%) alpha helix	-	-	-
A 463		GLY	0.92	-	Favored (23.83%) Glycine / -85.3,19.3	-	-	Favored (8.296%) three-ten	-	-	-
A 464		ARG	0.98	0.43Å HG3 with A 460 GLY O	Favored (23.22%) General / -86.5,-28.6	Favored (97.1%) *mtt180* chi angles: 296.4,183.4,181.5,183.4	0.07Å	Favored (21.021%)	OUTLIER(S) worst is CB--CG: 4.2 σ	-	-
A 465		ASN	1.04	-	Favored (40.66%) Pre-Pro / -104.3,103.7	Favored (47.6%) *t0* chi angles: 182.4,330.1	0.02Å	Favored (26.437%)	-	-	-
A 466		PRO	1.11	-	Favored (19.12%) Trans-Pro / -49.6,-29.5	Favored (87.3%) *Cg_exo* chi angles: 330.6,37.5,330.6	0.09Å	Favored (61.107%)	-	-	-
A 467		SER	1.17	-	Favored (59.21%) General / -77.1,-16.3	Favored (85.4%) *p* chi angles: 67.3	0.07Å	Favored (53.849%)	-	-	-
A 468		GLN	1.18	-	Favored (6.98%) General / -88.0,83.9	Favored (15.9%) *mt0* chi angles: 297.3,179.2,187.7	0.09Å	Favored (16.751%)	-	-	-
A 469		ILE	1.14	-	Favored (31.82%) Ile or Val / -75.9,-47.6	Favored (48.3%) *mm* chi angles: 300.7,302.5	0.03Å	Favored (15.845%)	-	-	-
A 470		GLY	1.06	-	Favored (4.64%) Glycine / -83.7,51.9	-	-	CaBLAM Disfavored (2.091%)	-	-	-
A 471		ASP	0.96	-	Favored (15.85%) General / -93.9,158.0	Favored (95.7%) *m-30* chi angles: 287.8,350.4	0.12Å	Favored (30.061%)	-	-	-
A 472		GLU	0.86	-	Favored (49.64%) General / -135.8,150.9	Favored (58.2%) *mt-10* chi angles: 301.2,185.5,315.8	0.06Å	Favored (71.734%) beta sheet	-	-	-
A 473		TYR	0.78	-	Favored (34.37%) General / -126.6,122.2	Favored (90.3%) *t80* chi angles: 175.1,78	0.06Å	Favored (68.299%) beta sheet	-	-	-

A 474		HIS	0.72	-	Favored (48.63%) General / -114.0,140.2	Favored (54.2%) *m-70* chi angles: 293.8,257	0.07Å	Favored (52.195%) beta sheet	-	-	-
A 475		TYR	0.69	-	Favored (30.16%) General / -150.1,149.4	Favored (48.8%) *p90* chi angles: 61.4,95.8	0.10Å	Favored (48.7%)	-	-	-
A 476		GLY	0.7	-	Favored (28.86%) Glycine / -101.0,-179.7	-	-	CaBLAM Disfavored (4.985%)	-	-	-
A 477		GLY	0.72	-	Favored (42.51%) Glycine / 54.5,-128.7	-	-	Favored (15.946%)	-	-	-
A 478		GLY	0.76	-	Favored (16.52%) Glycine / -174.5,-157.6	-	-	CaBLAM Disfavored (2.239%)	-	-	-
A 479		THR	0.81	-	Favored (12.43%) General / -123.2,168.4	Favored (59.1%) *p* chi angles: 64.2	0.01Å	Favored (15.289%)	-	-	-
A 480		SER	0.87	-	Favored (12.23%) General / -145.7,124.0	Favored (41.2%) *t* chi angles: 176.3	0.12Å	Favored (33.262%) beta sheet	-	-	-
**#**	**Alt**	**Res**	**High B**	**Clash > 0.4Å**	**Ramachandran**	**Rotamer**	**Cβ deviation**	**CaBLAM**	**Bond lengths**	**Bond angles**	**Cis Peptides**
			Avg: 1.13	Clashscore: 1.45	Outliers: 1 of 617	Poor rotamers: 1 of 507	Outliers: 0 of 561	Outliers: 16 of 615	Outliers: 7 of 619	Outliers: 6 of 619	Non-Trans: 0 of 618
A 481		GLU	0.94	-	Favored (54.69%) General / -89.7,2.6	Favored (14.6%) *mp0* chi angles: 295.2,75.4,49	0.09Å	Favored (13.223%)	-	-	-
A 482		ASP	0.99	-	Favored (9.03%) General / -91.3,93.4	Favored (64.8%) *t0* chi angles: 184,344.1	0.07Å	Favored (7.769%)	-	-	-
A 483		ASP	1.02	-	Favored (9.7%) General / -114.5,27.1	Favored (44.8%) *p0* chi angles: 59.6,351.1	0.08Å	Favored (6.49%)	-	-	-
A 484		THR	1.02	-	Favored (54.71%) General / -75.3,-7.7	Favored (68%) *p* chi angles: 62.7	0.01Å	Favored (11.501%)	-	-	-
A 485		ILE	0.98	-	Favored (10.11%) Ile or Val / -104.6,-0.2	Favored (40.4%) *pt* chi angles: 64.5,170	0.03Å	Favored (41.835%)	-	-	-
A 486		ALA	0.92	-	Favored (58.65%) General / -64.4,141.8	-	0.03Å	Favored (42.942%)	-	-	-
A 487		ALA	0.86	-	Favored (65.64%) General / -61.0,-23.8	-	0.04Å	Favored (32.299%)	-	-	-
A 488		HIS	0.8	-	Favored (68.85%) General / -60.4,-29.4	Favored (60.5%) *m170* chi angles: 291.4,168.8	0.07Å	Favored (56.166%)	-	-	-
A 489		TRP	0.75	-	Favored (60.28%) General / -75.8,-35.1	Favored (70.4%) *m100* chi angles: 301.4,114.1	0.06Å	Favored (83.63%) alpha helix	-	-	-

A 490		THR	0.72	-	Favored (78.76%) General / -67.0,-45.0	Favored (91.6%) *m* chi angles: 297.3	0.01Å	Favored (85.842%) alpha helix	-	-	-
A 491		GLU	0.69	-	Favored (89.63%) General / -63.3,-38.0	Favored (69.4%) *mt-10* chi angles: 287.7,179.4,321	0.05Å	Favored (85.627%) alpha helix	-	-	-
A 492		ALA	0.67	-	Favored (97.19%) General / -61.2,-41.6	-	0.07Å	Favored (89.5%) alpha helix	-	-	-
A 493		LYS	0.65	0.44Å NZ with A 521 ASP OD1	Favored (91.61%) General / -64.0,-38.6	Favored (24.5%) *mmmt* chi angles: 292.3,289.1,286.1,190.3	0.01Å	Favored (97.727%) alpha helix	-	-	-
A 494		ILE	0.65	-	Favored (93.69%) Ile or Val / -62.6,-47.3	Favored (97.5%) *mt* chi angles: 292.1,167.5	0.01Å	Favored (89.912%) alpha helix	-	-	-
A 495		MET	0.66	-	Favored (98.5%) General / -63.5,-42.2	Favored (99.3%) *mtp* chi angles: 291.7,176.3,72	0.02Å	Favored (91.222%) alpha helix	-	-	-
A 496		LEU	0.7	-	Favored (97.89%) General / -62.0,-41.3	Favored (79.1%) *mt* chi angles: 288.2,168.2	0.05Å	Favored (90.243%) alpha helix	-	-	-
A 497		ASP	0.78	-	Favored (71.68%) General / -61.6,-31.6	Favored (96.5%) *m-30* chi angles: 286.9,349	0.05Å	Favored (75.447%) alpha helix	-	-	-
A 498		ASN	0.91	-	Favored (40.08%) General / -94.7,9.1	Favored (83.6%) *m-40* chi angles: 289.9,320.3	0.09Å	Favored (42.074%)	-	-	-
A 499		ILE	1.09	-	Favored (30.57%) Ile or Val / -82.5,132.8	Favored (92.9%) *mt* chi angles: 295.3,171.8	0.10Å	Favored (32.914%)	-	-	-
A 500		HIS	1.3	-	Favored (18.98%) General / -89.2,107.3	Favored (26.9%) *t-170* chi angles: 185.5,191	0.01Å	Favored (53.877%) beta sheet	-	-	-
**#**	**Alt**	**Res**	**High B**	**Clash > 0.4Å**	**Ramachandran**	**Rotamer**	**Cβ deviation**	**CaBLAM**	**Bond lengths**	**Bond angles**	**Cis Peptides**
			Avg: 1.13	Clashscore: 1.45	Outliers: 1 of 617	Poor rotamers: 1 of 507	Outliers: 0 of 561	Outliers: 16 of 615	Outliers: 7 of 619	Outliers: 6 of 619	Non-Trans: 0 of 618
A 501		LEU	1.51	-	Favored (21.76%) Pre-Pro / -104.0,131.6	Favored (3.9%) *mp* chi angles: 282.9,78	0.03Å	Favored (56.303%)	-	-	-
A 502		PRO	1.64	-	Favored (75.58%) Trans-Pro / -61.8,-23.0	Favored (37.4%) *Cg_endo* chi angles: 22.8,326.5,29.9	0.03Å	Favored (64.995%)	-	-	-
A 503		ASN	1.63	-	Favored (53.36%) General / -91.6,4.1	Favored (45.9%) *p0* chi angles: 62.9,1.4	0.03Å	Favored (53.798%)	-	-	-
A 504		GLY	1.49	-	Favored (89.88%) Glycine / 81.1,1.2	-	-	Favored (76.743%)	-	-	-
A 505		LEU	1.29	-	Favored (33.49%) General / -81.2,128.2	Favored (54.6%) *tp* chi angles: 179.5,65.3	0.05Å	Favored (34.336%)	-	-	-
A 506		VAL	1.11	-	Favored (36.81%)	Favored (99.9%) *t* chi angles: 175.5	0.05Å	Favored (38.744%)	-	-	-

					Ile or Val / -75.8,129.7			beta sheet			
A 507		ALA	0.98	-	Favored (53.32%) General / -60.4,143.3	-	0.03Å	Favored (36.678%)	-	-	-
A 508		GLN	0.91	-	Favored (23.15%) General / -114.5,157.3	Favored (50.5%) *mt0* chi angles: 300.8,183.3,282.4	0.06Å	Favored (34.368%)	-	-	-
A 509		MET	0.89	-	Favored (29.79%) General / -68.6,160.6	Favored (88.6%) *mtp* chi angles: 289.3,179.2,64.4	0.04Å	Favored (47.962%)	-	-	-
A 510		TYR	0.9	-	Favored (50.77%) General / -113.9,138.1	Favored (51.5%) *t80* chi angles: 174,63.8	0.04Å	CA Geom Outlier (0.259%)	-	-	-
A 511		GLY	0.94	-	Favored (50.41%) Glycine / 57.9,-130.0	-	-	Favored (22.043%)	-	-	-
A 512		PRO	0.99	-	Favored (52.1%) Trans-Pro / -65.5,-16.0	Favored (59.7%) *Cg_endo* chi angles: 26.4,326.3,27	0.03Å	CaBLAM Disfavored (3.796%)	-	-	-
A 513		GLU	1.03	-	Favored (51.5%) General / -79.8,-18.2	Favored (38.5%) *mt-10* chi angles: 296.8,177.5,75.1	0.04Å	Favored (49.733%) three-ten	-	-	-
A 514		ARG	1.06	-	Favored (73.17%) General / -62.3,-32.4	Favored (97.1%) *mtt180* chi angles: 289.2,173.4,178.6,168.8	0.05Å	Favored (59.091%) three-ten	-	-	-
A 515		ASP	1.07	-	Favored (54.82%) General / -79.4,-5.5	Favored (85.9%) *m-30* chi angles: 290.7,334.7	0.03Å	Favored (50.297%) three-ten	-	-	-
A 516		LYS	1.06	-	Favored (48.68%) General / -93.7,-5.4	Favored (60.4%) *mttm* chi angles: 297.3,178.5,185.4,289.5	0.04Å	Favored (65.972%)	-	-	-
A 517		ALA	1.02	-	Favored (42.8%) General / -115.7,145.4	-	0.02Å	Favored (27.075%)	-	-	-
A 518		PHE	0.96	-	Favored (5.14%) General / -123.1,34.4	Favored (63.9%) *m-80* chi angles: 305.5,96.4	0.10Å	CaBLAM Disfavored (3.834%)	-	OUTLIER(S) worst is CA-CB-CG: 5.3 σ	-
A 519		THR	0.88	-	Favored (53.87%) General / -125.8,138.8	Favored (97%) *m* chi angles: 300.7	0.07Å	Favored (25.849%)	-	-	-
A 520		MET	0.8	-	Favored (43.05%) General / -73.7,148.2	Favored (78.8%) *mtm* chi angles: 294.9,180.8,289.8	0.01Å	Favored (36.271%)	-	-	-
**#**	**Alt**	**Res**	**High B**	**Clash > 0.4Å**	**Ramachandran**	**Rotamer**	**Cβ deviation**	**CaBLAM**	**Bond lengths**	**Bond angles**	**Cis Peptides**
			Avg: 1.13	Clashscore: 1.45	Outliers: 1 of 617	Poor rotamers: 1 of 507	Outliers: 0 of 561	Outliers: 16 of 615	Outliers: 7 of 619	Outliers: 6 of 619	Non-Trans: 0 of 618
A 521		ASP	0.74	0.44Å OD1 with A 493 LYS NZ	Favored (55.5%) General / -61.6,133.3	Favored (51%) *m-30* chi angles: 292.3,303.9	0.06Å	Favored (28.022%)	-	-	-
A 522		GLY	0.69	-	Favored (70.06%)	-	-	Favored (83.771%)	-	-	-

				Glycine / 95.1,-10.0						
A 523	GLU	0.66	-	Favored (67.48%) General / -63.4,-23.6	Favored (37.7%) *mt-10* chi angles: 289.5,189.6,49.8	0.11Å	Favored (33.552%)	-	-	-
A 524	TYR	0.65	-	Favored (25.77%) General / -105.9,16.0	Favored (41.7%) *m-80* chi angles: 298.9,123	0.04Å	Favored (25.236%)	-	-	-
A 525	ARG	0.65	-	Favored (55.74%) General / -60.7,133.1	Favored (86.8%) *mtm180* chi angles: 287,175,289.5,178.8	0.05Å	Favored (31.222%)	-	-	-
A 526	LEU	0.66	-	Favored (19.64%) General / -100.8,153.2	Favored (94.8%) *mt* chi angles: 298,176.3	0.02Å	Favored (42.702%)	-	-	-
A 527	ARG	0.67	-	Favored (51.27%) General / -129.1,144.6	Favored (92.2%) *mtm180* chi angles: 294.7,175.2,292.4,173.3	0.05Å	Favored (6.142%)	-	-	-
A 528	GLY	0.68	-	Favored (34.01%) Glycine / 58.5,-123.7	-	-	Favored (28.64%)	-	-	-
A 529	GLU	0.69	-	Favored (68.54%) General / -61.9,-27.1	Favored (78%) *mm-30* chi angles: 295,298.1,307.5	0.02Å	Favored (16.29%)	-	-	-
A 530	GLU	0.69	-	Favored (80.03%) General / -63.9,-35.2	Favored (97.9%) *mt-10* chi angles: 292.6,173.1,342.5	0.05Å	Favored (67.096%) three-ten	-	-	-
A 531	ARG	0.69	-	Favored (62.79%) General / -74.5,-39.0	Favored (98.3%) *mtt180* chi angles: 291.8,177.5,183.3,179.3	0.12Å	Favored (84.293%) alpha helix	-	-	-
A 532	LYS	0.69	-	Favored (96.81%) General / -64.2,-42.2	Favored (97.8%) *mttt* chi angles: 290.9,178.8,183.9,176.2	0.07Å	Favored (96.814%) alpha helix	-	-	-
A 533	THR	0.69	-	Favored (83.09%) General / -64.7,-46.3	Favored (90%) *m* chi angles: 298.8	0.04Å	Favored (90.561%) alpha helix	-	-	-
A 534	PHE	0.71	-	Favored (67.8%) General / -55.3,-51.1	Favored (79.9%) *t80* chi angles: 172,75	0.07Å	Favored (90.77%) alpha helix	-	OUTLIER(S) worst is CA-CB-CG: 5.1 σ	-
A 535	LEU	0.73	-	Favored (84.37%) General / -65.5,-36.6	Favored (92.1%) *mt* chi angles: 292.7,175.3	0.08Å	Favored (82.293%) alpha helix	-	-	-
A 536	GLU	0.77	-	Favored (85.85%) General / -66.9,-38.2	Favored (58.2%) *tp30* chi angles: 188.6,66.8,16	0.03Å	Favored (95.31%) alpha helix	-	-	-
A 537	LEU	0.82	-	Favored (81.84%) General / -63.2,-35.9	Favored (80%) *mt* chi angles: 288.7,169.3	0.02Å	Favored (77.592%) alpha helix	-	-	-
A 538	LEU	0.87	-	Favored (41.51%) General / -79.3,-35.3	Favored (86.4%) *mt* chi angles: 294.8,178.3	0.04Å	Favored (67.281%) alpha helix	-	-	-
A 539	ARG	0.92	-	Favored (10.66%)	Favored (83.8%) *ttp80*	0.03Å	Favored (51.51%) alpha helix	-	-	-

#	Alt	Res	High B	Clash > 0.4Å	Ramachandran	Rotamer	Cβ deviation	CaBLAM	Bond lengths	Bond angles	Cis Peptides
					General / -84.8,-47.2	chi angles: 184.8,180.3,69.6,85.1					
A 540		THR	0.94	-	Favored (7.54%) General / -92.6,-46.6	Favored (93.5%) *m* chi angles: 301	0.02Å	Favored (29.895%) alpha helix	-	-	-
**#**	**Alt**	**Res**	**High B**	**Clash > 0.4Å**	**Ramachandran**	**Rotamer**	**Cβ deviation**	**CaBLAM**	**Bond lengths**	**Bond angles**	**Cis Peptides**
			Avg: 1.13	Clashscore: 1.45	Outliers: 1 of 617	Poor rotamers: 1 of 507	Outliers: 0 of 561	Outliers: 16 of 615	Outliers: 7 of 619	Outliers: 6 of 619	Non-Trans: 0 of 618
A 541		ALA	0.94	-	Favored (59.56%) General / -80.6,-8.3	-	0.04Å	Favored (31.14%)	-	-	-
A 542		ASP	0.91	-	Favored (28.85%) General / 54.4,44.5	Favored (28%) *t0* chi angles: 197.8,27.7	0.03Å	Favored (16.711%)	-	-	-
A 543		LEU	0.86	-	Favored (42.89%) Pre-Pro / -92.3,154.2	Favored (78.2%) *mt* chi angles: 301.4,179.7	0.02Å	Favored (20.503%) beta sheet	-	-	-
A 544		PRO	0.81	-	Favored (56.25%) Trans-Pro / -60.3,153.9	Favored (58.2%) *Cg_exo* chi angles: 336.1,33.9,330.8	0.02Å	Favored (88.641%)	-	-	-
A 545		VAL	0.76	-	Favored (92.75%) Ile or Val / -59.3,-45.8	Favored (53.2%) *t* chi angles: 169.8	0.04Å	Favored (66.174%)	-	-	-
A 546		TRP	0.71	-	Favored (94.71%) General / -60.2,-44.9	Favored (91%) *t60* chi angles: 179.7,88.1	0.01Å	Favored (87.975%) alpha helix	-	-	-
A 547		LEU	0.68	-	Favored (76.97%) General / -66.8,-45.9	Favored (51.6%) *tp* chi angles: 183.4,60.5	0.03Å	Favored (85.272%) alpha helix	-	-	-
A 548		ALA	0.65	-	Favored (81.96%) General / -58.9,-39.7	-	0.05Å	Favored (89.937%) alpha helix	-	-	-
A 549		TYR	0.63	-	Favored (73.22%) General / -59.4,-50.6	Favored (75.2%) *t80* chi angles: 170.5,78	0.04Å	Favored (93.939%) alpha helix	-	-	-
A 550		LYS	0.62	-	Favored (77.86%) General / -63.9,-34.4	Favored (65.1%) *mmtt* chi angles: 287.5,292.6,181.8,177.7	0.02Å	Favored (76.571%) alpha helix	-	-	-
A 551		VAL	0.61	-	Favored (36.79%) Ile or Val / -70.0,-51.8	Favored (89.6%) *t* chi angles: 174.1	0.12Å	Favored (64.238%) alpha helix	-	-	-
A 552		ALA	0.6	-	Favored (85.82%) General / -60.5,-38.9	-	0.05Å	Favored (74.22%) alpha helix	-	-	-
A 553		SER	0.61	-	Favored (62.13%) General / -70.4,-13.4	Favored (64.6%) *p* chi angles: 72.7	0.04Å	Favored (60.79%) alpha helix	-	-	-
A 554		ASN	0.62	-	Favored (37.46%) General / -102.8,11.5	Favored (63.6%) *m-40* chi angles: 284.4,279.6	0.12Å	Favored (57.69%)	-	-	-
A 555		GLY	0.63	-	Favored (79.98%) Glycine / 76.8,17.3	-	-	Favored (87.924%)	-	-	-

A 556		ILE	0.66	-	Favored (40.9%) Ile or Val / -87.7,124.3	Favored (76.8%) *mt* chi angles: 299.7,166.6	0.10Å	Favored (30.233%) beta sheet	-	-	-
A 557		GLN	0.68	-	Favored (27.51%) General / -79.2,159.1	Favored (90%) *mt0* chi angles: 294.6,180.9,304.2	0.02Å	Favored (46.915%)	-	-	-
A 558		TYR	0.69	-	Favored (27.17%) General / -47.7,-44.4	Favored (68.2%) *t80* chi angles: 180.2,89.9	0.05Å	Favored (46.992%)	-	-	-
A 559		THR	0.7	-	Favored (59.58%) General / -83.2,-7.7	Favored (74.2%) *p* chi angles: 59.9	0.02Å	Favored (53.463%)	-	-	-
A 560		ASP	0.7	-	Favored (9.47%) General / -83.8,79.4	Favored (66.8%) *t0* chi angles: 185.2,347	0.04Å	Favored (17.576%)	-	-	-
**#**	**Alt**	**Res**	**High B**	**Clash > 0.4Å**	**Ramachandran**	**Rotamer**	**Cβ deviation**	**CaBLAM**	**Bond lengths**	**Bond angles**	**Cis Peptides**
			Avg: 1.13	Clashscore: 1.45	Outliers: 1 of 617	Poor rotamers: 1 of 507	Outliers: 0 of 561	Outliers: 16 of 615	Outliers: 7 of 619	Outliers: 6 of 619	Non-Trans: 0 of 618
A 561		ARG	0.68	-	Favored (5.42%) General / -61.8,-8.8	Favored (50.7%) *ptt-90* chi angles: 69,183.8,182.6,275.6	0.05Å	Favored (8.073%)	-	-	-
A 562		LYS	0.66	-	Favored (68.67%) General / -60.0,-29.6	Favored (98.2%) *mttt* chi angles: 291.3,177.7,181,177	0.02Å	Favored (38.678%)	-	-	-
A 563		TRP	0.65	-	Favored (62.85%) General / -60.5,-21.7	Favored (41.3%) *p-90* chi angles: 75.4,266.4	0.09Å	Favored (66.487%) three-ten	-	-	-
A 564		CYS	0.64	-	Favored (58.75%) General / -82.6,-10.9	Favored (74.8%) *m* chi angles: 297	0.09Å	Favored (53.977%) alpha helix	-	-	-
A 565		PHE	0.65	-	Favored (7.8%) General / -123.3,-12.5	Favored (88.8%) *m-80* chi angles: 299.4,102.6	0.03Å	Favored (23.742%) alpha helix	-	-	-
A 566		ASP	0.67	-	Favored (40.13%) General / -91.0,7.5	Favored (89.3%) *m-30* chi angles: 290.3,337.2	0.08Å	Favored (11.436%)	-	-	-
A 567		GLY	0.7	-	Favored (51.87%) Glycine / -83.9,-178.8	-	-	Favored (48.354%)	-	-	-
A 568		PRO	0.73	-	Favored (58.99%) Trans-Pro / -60.1,153.4	Favored (53.7%) *Cg_exo* chi angles: 336.3,33.5,330.8	0.05Å	Favored (56.58%)	-	-	-
A 569		ARG	0.76	-	Favored (61.73%) General / -55.2,-31.9	Favored (84%) *ttt180* chi angles: 185.1,177,178.2,181.2	0.03Å	Favored (47.063%)	-	-	-
A 570		SER	0.77	-	Favored (53.79%) General / -63.4,-14.8	Favored (91.9%) *p* chi angles: 66.5	0.02Å	Favored (56.115%)	-	-	-
A 571		ASN	0.8	-	Favored (33.87%) General / -97.9,12.5	Favored (63.8%) *m-40* chi angles: 292.3,357.1	0.08Å	Favored (29.749%)	-	-	-
A 572		ILE	0.84	-	Favored (32.61%)	Favored (94.5%) *mt* chi angles: 295.1,169.2	0.06Å	Favored (36.734%)	-	-	-

#	Alt	Res	High B	Clash > 0.4Å	Ramachandran	Rotamer	Cβ deviation	CaBLAM	Bond lengths	Bond angles	Cis Peptides
					Ile or Val / -67.4,130.3						
A 573		ILE	0.92	-	Favored (35.26%) Ile or Val / -85.5,131.0	Favored (92.2%) *mt* chi angles: 294.6,172.6	0.08Å	Favored (51.781%) beta sheet	-	-	-
A 574		LEU	1	-	Favored (34.14%) General / -110.3,147.3	Favored (5.9%) *mp* chi angles: 281.2,69.7	0.04Å	Favored (51.165%)	-	-	-
A 575		GLU	1.08	-	Allowed (0.61%) General / -117.1,-80.5	Favored (89.8%) *tt0* chi angles: 181.3,173.3,355.1	0.04Å	CaBLAM Disfavored (1.656%)	-	-	-
A 576		ASP	1.12	-	Favored (7.75%) General / -88.5,65.0	Favored (41.3%) *t0* chi angles: 190.5,18.8	0.01Å	CaBLAM Disfavored (3.274%)	-	-	-
A 577		ASN	1.09	-	Favored (5.57%) General / 60.6,15.3	Favored (76.4%) *m-40* chi angles: 298.9,303.2	0.01Å	CaBLAM Disfavored (1.299%)	-	-	-
A 578		ASN	1	-	Favored (42.87%) General / -142.3,152.3	Favored (58.7%) *m-40* chi angles: 287.7,275.7	0.04Å	Favored (10.137%)	-	-	-
A 579		GLU	0.89	-	Favored (56.86%) General / -58.6,138.4	Favored (63.9%) *mp0* chi angles: 295.5,85.8,356.7	0.02Å	Favored (34.412%) beta sheet	-	-	-
A 580		VAL	0.79	-	Favored (39.54%) Ile or Val / -80.4,127.2	Favored (87.2%) *t* chi angles: 173.9	0.06Å	Favored (48.619%) beta sheet	-	-	-
**#**	**Alt**	**Res**	**High B**	**Clash > 0.4Å**	**Ramachandran**	**Rotamer**	**Cβ deviation**	**CaBLAM**	**Bond lengths**	**Bond angles**	**Cis Peptides**
			Avg: 1.13	Clashscore: 1.45	Outliers: 1 of 617	Poor rotamers: 1 of 507	Outliers: 0 of 561	Outliers: 16 of 615	Outliers: 7 of 619	Outliers: 6 of 619	Non-Trans: 0 of 618
A 581		GLU	0.73	-	Favored (51.83%) General / -117.4,138.4	Favored (94.9%) *mt-10* chi angles: 293.6,177,339.2	0.04Å	Favored (70.656%) beta sheet	-	-	-
A 582		ILE	0.72	-	Favored (71.2%) Ile or Val / -123.6,132.5	Favored (88.5%) *mt* chi angles: 298.4,172.4	0.04Å	Favored (64.422%) beta sheet	-	-	-
A 583		VAL	0.74	-	Favored (47.25%) Ile or Val / -94.9,124.0	Favored (73.5%) *t* chi angles: 178.4	0.03Å	Favored (63.207%)	-	-	-
A 584		THR	0.77	-	Favored (5.69%) General / -93.2,178.6	Favored (64.8%) *p* chi angles: 63.2	0.08Å	Favored (19.33%)	-	-	-
A 585		ARG	0.8	-	Favored (62.4%) General / -58.6,-25.3	Favored (97.8%) *mtt180* chi angles: 289.9,179.8,180.9,177.9	0.12Å	Favored (41.354%)	-	-	-
A 586		THR	0.8	-	Favored (54.38%) General / -89.3,-7.9	Favored (55.9%) *p* chi angles: 64.8	0.09Å	Favored (46.512%)	-	-	-
A 587		GLY	0.78	-	Favored (71.28%) Glycine / 93.6,-11.4	-	-	Favored (74.1%)	-	-	-
A 588		GLU	0.74	-	Favored (57.96%) General / -66.6,140.8	Favored (92.8%) *mt-10* chi angles: 291.1,181,9.6	0.02Å	Favored (37.676%)	-	-	-

A 589		ARG	0.69	-	Favored (43.15%) General / -107.0,120.8	Favored (59.7%) *ttt90* chi angles: 186,178,179.9,92.5	0.03Å	Favored (55.149%) beta sheet	-	-	-
A 590		LYS	0.65	-	Favored (50.39%) General / -129.1,150.6	Favored (95.8%) *mttt* chi angles: 297.5,181.4,181.3,181.3	0.06Å	Favored (45.646%) beta sheet	-	-	-
A 591		MET	0.62	-	Favored (43.41%) General / -75.3,141.7	Favored (61.9%) *mtt* chi angles: 290.1,183.2,173.5	0.07Å	Favored (38.462%) beta sheet	-	-	-
A 592		LEU	0.61	-	Favored (17.47%) General / -77.0,116.2	Favored (73.6%) *tp* chi angles: 178.2,62.5	0.07Å	Favored (25.789%) beta sheet	-	-	-
A 593		LYS	0.61	-	Favored (16.64%) Pre-Pro / -143.7,74.6	Favored (36.5%) *ttpt* chi angles: 181.6,173.5,64.9,171	0.09Å	Favored (7.016%) beta sheet	-	-	-
A 594		PRO	0.63	-	Favored (87.11%) Trans-Pro / -60.5,149.4	Favored (54.9%) *Cg_exo* chi angles: 337.4,36,325.9	0.02Å	Favored (35.066%)	-	-	-
A 595		ARG	0.66	-	Favored (73.32%) General / -62.6,-32.4	Favored (94%) *mtt180* chi angles: 289.8,179.6,187.2,182.8	0.02Å	Favored (26.787%)	-	-	-
A 596		TRP	0.69	-	Favored (45.97%) General / -133.2,136.8	Favored (24.7%) *m-90* chi angles: 303.6,271.5	0.12Å	Favored (30.169%)	-	-	-
A 597		LEU	0.74	-	Favored (21.56%) General / -103.5,152.5	Favored (85.5%) *mt* chi angles: 297.6,180.1	0.13Å	Favored (52.045%)	-	-	-
A 598		ASP	0.8	-	Favored (4.75%) General / -142.8,107.1	Favored (58.8%) *t0* chi angles: 183.9,338.9	0.04Å	Favored (23.179%) beta sheet	-	-	-
A 599		ALA	0.87	-	Favored (56.72%) General / -58.6,-22.7	-	0.05Å	Favored (31.428%)	-	-	-
A 600		ARG	0.96	-	Favored (66.38%) General / -65.5,-20.5	Favored (88.3%) *mtm180* chi angles: 289.6,174,292.5,180	0.03Å	Favored (65.032%)	-	-	-
**#**	**Alt**	**Res**	**High B**	**Clash > 0.4Å**	**Ramachandran**	**Rotamer**	**Cβ deviation**	**CaBLAM**	**Bond lengths**	**Bond angles**	**Cis Peptides**
			Avg: 1.13	Clashscore: 1.45	Outliers: 1 of 617	Poor rotamers: 1 of 507	Outliers: 0 of 561	Outliers: 16 of 615	Outliers: 7 of 619	Outliers: 6 of 619	Non-Trans: 0 of 618
A 601		VAL	1.07	-	Favored (71.51%) Ile or Val / -71.5,-43.7	Favored (82.3%) *t* chi angles: 173.3	0.02Å	Favored (57.076%) alpha helix	-	-	-
A 602		TYR	1.18	-	Favored (46.42%) General / -92.1,-8.2	Favored (29.5%) *p90* chi angles: 51.3,83.9	0.19Å	Favored (24.278%) alpha helix	-	-	-
A 603		ALA	1.29	-	Favored (69.59%) General / -61.0,-29.2	-	0.06Å	Favored (33.03%) alpha helix	-	-	-
A 604		ASP	1.36	-	Favored (15.06%) General / -135.3,117.1	Favored (33.5%) *t0* chi angles: 183.6,326.3	0.04Å	Favored (16.073%) alpha helix	-	-	-

A 605		HIS	1.39	-	Favored (7.3%) General / -52.9,-23.6	Favored (57%) *p-80* chi angles: 69.4,281.5	0.01Å	Favored (25.241%) alpha helix	-	-	-
A 606		GLN	1.35	-	Favored (65.78%) General / -73.4,-35.1	Favored (94.7%) *mm-40* chi angles: 292.3,293,312.3	0.03Å	Favored (75.268%) alpha helix	-	-	-
A 607		SER	1.27	-	Favored (65.57%) General / -73.3,-39.2	Favored (62.6%) *m* chi angles: 293.9	0.06Å	Favored (76.571%) alpha helix	-	-	-
A 608		LEU	1.15	-	Favored (92.94%) General / -65.6,-41.3	Favored (42.9%) *tp* chi angles: 184.6,57.5	0.04Å	Favored (87.227%) alpha helix	-	-	-
A 609		LYS	1.02	-	Favored (95.71%) General / -60.2,-42.7	Favored (17.8%) *tptp* chi angles: 180.3,67,165.4,72.4	0.04Å	Favored (89.365%) alpha helix	-	-	-
A 610		TRP	0.9	-	Favored (90.24%) General / -62.0,-38.9	Favored (70.8%) *m100* chi angles: 284.7,115.2	0.02Å	Favored (95.361%) alpha helix	-	-	-
A 611		PHE	0.81	-	Favored (79.66%) General / -68.5,-41.7	Favored (19.6%) *t80* chi angles: 185.7,49.8	0.07Å	Favored (95.626%) alpha helix	-	-	-
A 612		LYS	0.73	-	Favored (87.05%) General / -63.0,-37.4	Favored (56%) *mtmt* chi angles: 290,187.9,292.8,186.1	0.01Å	Favored (87.976%) alpha helix	-	-	-
A 613		ASP	0.68	-	Favored (73.84%) General / -70.6,-39.3	Favored (99.3%) *m-30* chi angles: 287.6,346.3	0.01Å	Favored (90.098%) alpha helix	-	-	-
A 614		PHE	0.66	-	Favored (73.64%) General / -59.1,-50.5	Favored (65.6%) *t80* chi angles: 169.1,75.6	0.03Å	Favored (84.585%) alpha helix	-	-	-
A 615		ALA	0.68	-	Favored (59.34%) General / -75.3,-10.3	-	0.07Å	CaBLAM Outlier (0.278%) try alpha helix	-	OUTLIER(S) worst is CA-C-O: 4.9 σ	-
A 616		ALA	0.74	-	Favored (54.47%) General / -84.1,-12.8	-	0.03Å	CaBLAM Outlier (0.388%)	-	-	-
A 617		GLY	0.85	-	Favored (63.48%) Glycine / 79.6,22.9	-	-	Favored (79.193%)	-	-	-
A 618		LYS	0.99	-	Favored (59.28%) General / -85.5,-5.5	Favored (99.1%) *mttt* chi angles: 293,175.7,180.9,176.6	0.02Å	-	-	-	-
A 619		ARG	1.17	0.69Å HD3 with A 619 ARG OXT	-	OUTLIER (0.2%) chi angles: 58.1,93.5,73.8,201.5	0.10Å	-	-	-	-
