# Supplementary material for: Integrative proteome-wide structural analysis and high-throughput docking identify broad-spectrum antiviral scaffolds against Zika, Yellow Fever, West Nile, Saint Louis encephalitis, and Usutu viruses
Source: Front Cell Infect Microbiol. 2026 Apr 30;16:1723132. doi: 10.3389/fcimb.2026.1723132 (PMC13171538; doi:10.3389/fcimb.2026.1723132)
Supplement: Supplementary file 4 [file DataSheet4.zip › USUV/USU_NS4a/Mol_probity_Files/USU_NS4a_1FH-multi.table.pdf]

Viewing USU_NS4a1FH-multi.table

Duke Biochemistry
Duke University School of Medicine

When finished, you should close this window. *Hint: Use File | Save As... to save a copy of this page.*

All-Atom Contacts	Clashscore, all atoms:	0.51		99th percentile* (N=1784, all resolutions)
	Clashscore is the number of serious steric overlaps (> 0.4 Å) per 1000 atoms.			
Protein Geometry	Poor rotamers	0	0.00%	Goal: <0.3%
	Favored rotamers	97	100.00%	Goal: >98%
	Ramachandran outliers	0	0.00%	Goal: <0.05%
	Ramachandran favored	122	98.39%	Goal: >98%
	Rama distribution Z-score	1.93 ± 0.69		Goal: abs(Z score) < 2
	MolProbity score^	0.68		100th percentile* (N=27675, 0Å - 99Å)
	Cβ deviations >0.25Å	0	0.00%	Goal: 0
	Bad bonds:	0 / 962	0.00%	Goal: 0%
	Bad angles:	2 / 1298	0.15%	Goal: <0.1%
Peptide Omegas	Cis Prolines:	0 / 4	0.00%	Expected: ≤1 per chain, or ≤5%
Low-resolution Criteria	CaBLAM outliers	0	0.0%	Goal: <1.0%
	CA Geometry outliers	0	0.00%	Goal: <0.5%
Additional validations	Chiral volume outliers	0/159		
	Waters with clashes	0/0	0.00%	See UnDowser table for details

In the two column results, the left column gives the raw count, right column gives the percentage.

* 100th percentile is the best among structures of comparable resolution; 0th percentile is the worst. For clashscore the comparative set of structures was selected in 2004, for MolProbity score in 2006.

^ MolProbity score combines the clashscore, rotamer, and Ramachandran evaluations into a single score, normalized to be on the same scale as X-ray resolution.

Key to table colors and cutoffs here:

#	Alt	Res	High B	Clash > 0.4Å	Ramachandran	Rotamer	Cβ deviation	CaBLAM	Bond lengths	Bond angles	Cis Peptides
			Avg: 3.33	Clashscore: 0.51	Outliers: 0 of 124	Poor rotamers: 0 of 97	Outliers: 0 of 112	Outliers: 0 of 122	Outliers: 0 of 126	Outliers: 2 of 126	Non-Trans: 0 of 125
A 1		SER	10.23	-	-	Favored (99.8%) *p* chi angles: 65.5	0.03Å	-	-	-	-
A 2		ALA	10.01	-	Favored (34.75%) General / -155.3,156.0	-	0.02Å	-	-	-	-
A 3		VAL	9.76	-	Favored (88.41%) Ile or Val / -61.5,-41.0	Favored (67.6%) *t* chi angles: 171.8	0.02Å	Favored (62.541%)	-	-	-
A 4		GLY	9.49	-	Favored (42.25%) Glycine / -53.0,-52.1	-	-	Favored (90.925%) alpha helix	-	-	-
A 5		PHE	9.2	-	Favored (78.95%) General / -56.5,-47.5	Favored (87.5%) *t80* chi angles: 175.1,75.7	0.02Å	Favored (92.252%) alpha helix	-	-	-
A 6		LEU	8.87	-	Favored (88.38%)	Favored (96.9%) *mt* chi angles: 293.1,173.4	0.04Å	Favored (85.269%)	-	-	-

					General / -62.5,-38.1			alpha helix			
A 7		GLU	8.48	-	Favored (96.87%) General / -61.1,-41.5	Favored (91.4%) *tt0* chi angles: 180.4,174.9,355.8	0.05Å	Favored (89.883%) alpha helix	-	-	-
A 8		VAL	8	-	Favored (99.13%) Ile or Val / -61.7,-44.7	Favored (59.6%) *t* chi angles: 170.7	0.01Å	Favored (97.728%) alpha helix	-	-	-
A 9		LEU	7.41	-	Favored (90.07%) General / -63.1,-38.2	Favored (85.6%) *mt* chi angles: 292.2,176.8	0.07Å	Favored (90.869%) alpha helix	-	-	-
A 10		GLY	6.68	-	Favored (97.76%) Glycine / -61.9,-39.6	-	-	Favored (89.131%) alpha helix	-	-	-
A 11		ARG	5.88	-	Favored (33.33%) General / -82.4,-25.9	Favored (98.1%) *mtt-85* chi angles: 291.6,181.4,182.7,275.8	0.04Å	Favored (38.72%) alpha helix	-	-	-
A 12		MET	5.06	-	Favored (18.43%) Pre-Pro / -44.1,-51.5	Favored (54.7%) *ttm* chi angles: 183.8,178.7,296	0.20Å	Favored (51.991%) alpha helix	-	-	-
A 13		PRO	4.28	-	Favored (64.52%) Trans-Pro / -58.8,-23.0	Favored (71.6%) *Cg_exo* chi angles: 335.2,35.9,328	0.07Å	Favored (70.283%) alpha helix	-	-	-
A 14		GLU	3.57	-	Favored (34.35%) General / -80.6,-35.4	Favored (98.6%) *mt-10* chi angles: 293.8,179.3,350.1	0.02Å	Favored (77.145%) alpha helix	-	-	-
A 15		HIS	2.98	-	Favored (64.94%) General / -73.3,-40.0	Favored (87%) *m-70* chi angles: 291,293.2	0.02Å	Favored (78.899%) alpha helix	-	-	-
A 16		PHE	2.5	-	Favored (55.02%) General / -60.0,-53.7	Favored (90%) *t80* chi angles: 177.4,82.3	0.08Å	Favored (82.638%) alpha helix	-	-	-
A 17		ALA	2.13	-	Favored (90.66%) General / -60.9,-39.8	-	0.03Å	Favored (82.364%) alpha helix	-	-	-
A 18		GLY	1.84	-	Favored (27.05%) Glycine / -57.6,-56.1	-	-	Favored (90.332%) alpha helix	-	-	-
A 19		LYS	1.64	-	Favored (83.43%) General / -61.5,-37.4	Favored (97.1%) *mttt* chi angles: 289.7,178.2,180.6,178.8	0.03Å	Favored (75.114%) alpha helix	-	-	-
A 20		THR	1.48	-	Favored (88.54%) General / -64.3,-45.3	Favored (97.8%) *m* chi angles: 300	0.04Å	Favored (92.664%) alpha helix	-	-	-
**#**	**Alt**	**Res**	**High B**	**Clash > 0.4Å**	**Ramachandran**	**Rotamer**	**Cβ deviation**	**CaBLAM**	**Bond lengths**	**Bond angles**	**Cis Peptides**
			Avg: 3.33	Clashscore: 0.51	Outliers: 0 of 124	Poor rotamers: 0 of 97	Outliers: 0 of 112	Outliers: 0 of 122	Outliers: 0 of 126	Outliers: 2 of 126	Non-Trans: 0 of 125
A 21		ARG	1.37	-	Favored (94.06%)	Favored (92.9%) *mtt180*	0.03Å	Favored (94.592%) alpha helix	-	-	-

					General / -65.1,-40.1	chi angles: 289.6,169.9,181.7,164					
A 22		GLU	1.29	-	Favored (94.28%) General / -64.1,-39.5	Favored (97.7%) *mt-10* chi angles: 289.4,178.1,355.3	0.02Å	Favored (94.977%) alpha helix	-	-	-
A 23		ALA	1.22	-	Favored (99.41%) General / -61.6,-43.1	-	0.02Å	Favored (96.711%) alpha helix	-	-	-
A 24		PHE	1.16	-	Favored (69.31%) General / -60.2,-51.5	Favored (88.1%) *t80* chi angles: 174.6,79	0.08Å	Favored (95.472%) alpha helix	-	OUTLIER(S) worst is CA-CB-CG: 4.1 σ	-
A 25		ASP	1.11	-	Favored (80.94%) General / -60.1,-37.7	Favored (97.5%) *m-30* chi angles: 286.9,346.5	0.02Å	Favored (82.154%) alpha helix	-	-	-
A 26		THR	1.07	-	Favored (79.41%) General / -64.2,-47.5	Favored (99.2%) *m* chi angles: 300.3	0.07Å	Favored (81.743%) alpha helix	-	-	-
A 27		MET	1.04	-	Favored (84.67%) General / -66.5,-37.2	Favored (78.6%) *mtm* chi angles: 290.2,187.7,294.1	0.04Å	Favored (77.532%) alpha helix	-	-	-
A 28		TYR	1.03	-	Favored (75.85%) General / -57.9,-49.7	Favored (87.8%) *t80* chi angles: 180.1,81	0.01Å	Favored (81.267%) alpha helix	-	-	-
A 29		LEU	1.07	-	Favored (93.24%) General / -62.8,-39.1	Favored (74.9%) *mt* chi angles: 289.2,173.9	0.01Å	Favored (93.422%) alpha helix	-	-	-
A 30		VAL	1.21	-	Favored (99.26%) Ile or Val / -62.3,-44.5	Favored (66.3%) *t* chi angles: 171.7	0.02Å	Favored (82.75%) alpha helix	-	-	-
A 31		ALA	1.53	-	Favored (84.04%) General / -66.5,-37.0	-	0.04Å	Favored (51.306%) alpha helix	-	-	-
A 32		THR	2.16	-	Favored (10.1%) General / -115.7,-20.5	Favored (68.9%) *p* chi angles: 62.6	0.03Å	Favored (16.706%)	-	-	-
A 33		ALA	3.22	-	Favored (48.27%) General / -69.2,149.7	-	0.02Å	Favored (28.321%)	-	-	-
A 34		GLU	4.56	-	Favored (51.68%) General / -56.2,132.8	Favored (91.5%) *tt0* chi angles: 183.1,176.7,0.6	0.02Å	Favored (42.689%)	-	-	-
A 35		LYS	5.5	-	Favored (67.9%) General / -59.3,-29.8	Favored (97%) *mttt* chi angles: 289.4,179.9,179.7,178.7	0.03Å	Favored (42.54%)	-	-	-
A 36		GLY	5.35	-	Favored (83.23%) Glycine / -88.5,0.5	-	-	Favored (66.685%)	-	-	-
A 37		GLY	4.19	-	Favored (35.69%) Glycine / -84.4,159.9	-	-	Favored (40.587%)	-	-	-

A 38		LYS	2.79	-	Favored (89.62%) General / -66.1,-39.0	Favored (97.4%) *mttt* chi angles: 290.2,179.1,180.1,178.3	0.02Å	Favored (57.871%)	-	-	-
A 39		ALA	1.73	-	Favored (78.23%) General / -60.3,-36.6	-	0.03Å	Favored (75.231%) alpha helix	-	-	-
A 40		HIS	1.12	-	Favored (52.23%) General / -65.6,-52.4	Favored (85.1%) *t70* chi angles: 183.1,75.3	0.06Å	Favored (76.716%) alpha helix	-	-	-
**#**	**Alt**	**Res**	**High B**	**Clash > 0.4Å**	**Ramachandran**	**Rotamer**	**Cβ deviation**	**CaBLAM**	**Bond lengths**	**Bond angles**	**Cis Peptides**
			Avg: 3.33	Clashscore: 0.51	Outliers: 0 of 124	Poor rotamers: 0 of 97	Outliers: 0 of 112	Outliers: 0 of 122	Outliers: 0 of 126	Outliers: 2 of 126	Non-Trans: 0 of 125
A 41		ARG	0.82	-	Favored (94.99%) General / -62.1,-40.1	Favored (99.8%) *mtm-85* chi angles: 289.9,192.4,296.3,274.9	0.02Å	Favored (79.938%) alpha helix	-	-	-
A 42		MET	0.7	-	Favored (87.35%) General / -66.9,-39.3	Favored (83.1%) *mtm* chi angles: 289.6,187.7,289.1	0.02Å	Favored (93.503%) alpha helix	-	-	-
A 43		ALA	0.68	-	Favored (89.07%) General / -61.6,-38.8	-	0.05Å	Favored (89.109%) alpha helix	-	-	-
A 44		LEU	0.71	-	Favored (91.75%) General / -64.9,-38.7	Favored (83.6%) *mt* chi angles: 290.1,173.3	0.03Å	Favored (91.224%) alpha helix	-	-	-
A 45		GLU	0.77	-	Favored (87.02%) General / -62.9,-37.5	Favored (96.6%) *mt-10* chi angles: 289.6,180.9,354.8	0.04Å	Favored (73.75%) alpha helix	-	-	-
A 46		GLU	0.84	-	Favored (43.83%) General / -89.1,-11.5	Favored (95.3%) *mt-10* chi angles: 294.7,182.6,359.4	0.04Å	Favored (42.815%) alpha helix	-	-	-
A 47		LEU	0.92	-	Favored (88.17%) Pre-Pro / -51.0,-46.4	Favored (64.1%) *tp* chi angles: 179.9,63.2	0.13Å	Favored (50.05%) alpha helix	-	-	-
A 48		PRO	1.02	-	Favored (47.93%) Trans-Pro / -52.7,-30.8	Favored (94.9%) *Cg_exo* chi angles: 331.4,38,328.7	0.03Å	Favored (78.052%) alpha helix	-	-	-
A 49		ASP	1.13	-	Favored (46.7%) General / -78.3,-36.5	Favored (84.8%) *m-30* chi angles: 294.2,347.5	0.01Å	Favored (83.779%) alpha helix	-	-	-
A 50		ALA	1.25	-	Favored (96.61%) General / -61.3,-41.2	-	0.06Å	Favored (91.404%) alpha helix	-	-	-
A 51		LEU	1.38	-	Favored (70.85%) General / -71.7,-37.0	Favored (94.1%) *mt* chi angles: 294.5,174.8	0.05Å	Favored (87.058%) alpha helix	-	-	-
A 52		GLU	1.53	-	Favored (86.06%) General / -59.6,-47.3	Favored (85.3%) *tt0* chi angles: 177.7,181.1,0.8	0.08Å	Favored (81.922%) alpha helix	-	-	-

A 53		THR	1.68	-	Favored (80.81%) General / -59.3,-48.6	Favored (88.6%) *m* chi angles: 298.4	0.05Å	Favored (84.267%) alpha helix	-	-	-
A 54		ILE	1.84	-	Favored (96.93%) Ile or Val / -63.7,-45.6	Favored (93.1%) *mt* chi angles: 292.7,165.7	0.07Å	Favored (87.062%) alpha helix	-	-	-
A 55		THR	1.99	-	Favored (99.15%) General / -61.9,-42.5	Favored (93.6%) *m* chi angles: 301	0.05Å	Favored (92.613%) alpha helix	-	-	-
A 56		LEU	2.13	-	Favored (88.32%) General / -64.7,-37.6	Favored (89.2%) *mt* chi angles: 291.6,174.2	0.02Å	Favored (94.057%) alpha helix	-	-	-
A 57		ILE	2.25	-	Favored (96.44%) Ile or Val / -61.6,-43.0	Favored (92.2%) *mt* chi angles: 291.5,168.3	0.04Å	Favored (93.525%) alpha helix	-	-	-
A 58		VAL	2.35	-	Favored (97.72%) Ile or Val / -62.4,-43.0	Favored (68.6%) *t* chi angles: 171.9	0.03Å	Favored (91.207%) alpha helix	-	-	-
A 59		ALA	2.44	-	Favored (78.77%) General / -58.1,-39.5	-	0.05Å	Favored (84.136%) alpha helix	-	-	-
A 60		LEU	2.51	-	Favored (85.06%) General / -67.5,-39.2	Favored (96.4%) *mt* chi angles: 291.9,172.2	0.03Å	Favored (90.096%) alpha helix	-	-	-
**#**	**Alt**	**Res**	**High B**	**Clash > 0.4Å**	**Ramachandran**	**Rotamer**	**Cβ deviation**	**CaBLAM**	**Bond lengths**	**Bond angles**	**Cis Peptides**
			Avg: 3.33	Clashscore: 0.51	Outliers: 0 of 124	Poor rotamers: 0 of 97	Outliers: 0 of 112	Outliers: 0 of 122	Outliers: 0 of 126	Outliers: 2 of 126	Non-Trans: 0 of 125
A 61		ALA	2.56	-	Favored (85.19%) General / -61.5,-37.9	-	0.02Å	Favored (86.898%) alpha helix	-	-	-
A 62		VAL	2.61	-	Favored (87.17%) Ile or Val / -64.0,-48.2	Favored (57.1%) *t* chi angles: 170.4	0.05Å	Favored (85.24%) alpha helix	-	-	-
A 63		MET	2.66	-	Favored (82.54%) General / -65.5,-36.0	Favored (97.5%) *mmm* chi angles: 291.7,301.4,290.6	0.02Å	Favored (84.446%) alpha helix	-	-	-
A 64		THR	2.7	-	Favored (57.17%) General / -67.4,-51.0	Favored (98%) *m* chi angles: 300.1	0.05Å	Favored (76.782%) alpha helix	-	-	-
A 65		ALA	2.75	-	Favored (80.23%) General / -61.8,-36.1	-	0.04Å	Favored (75.507%) alpha helix	-	-	-
A 66		GLY	2.82	-	Favored (41.91%) Glycine / -58.5,-53.9	-	-	Favored (91.225%) alpha helix	-	-	-
A 67		VAL	2.91	-	Favored (91.32%) Ile or Val / -60.0,-42.9	Favored (59.6%) *t* chi angles: 170.7	0.03Å	Favored (86.675%) alpha helix	-	-	-

#	Alt	Res	High B	Clash > 0.4Å	Ramachandran	Rotamer	Cβ deviation	CaBLAM	Bond lengths	Bond angles	Cis Peptides
A 68		PHE	3.03	-	Favored (62.81%) General / -55.1,-52.6	Favored (90%) *t80* chi angles: 175.4,78.5	0.02Å	Favored (83.354%) alpha helix	-	-	-
A 69		LEU	3.22	-	Favored (82.7%) General / -58.1,-41.1	Favored (56.3%) *tp* chi angles: 182.2,59.5	0.03Å	Favored (78.038%) alpha helix	-	-	-
A 70		LEU	3.53	0.43Å O with A 79 LYS NZ	Favored (67.52%) General / -61.7,-25.6	Favored (82%) *mt* chi angles: 289.3,168.8	0.01Å	Favored (66.616%) alpha helix	-	-	-
A 71		LEU	3.99	-	Favored (16.39%) General / -91.4,-30.7	Favored (95.6%) *mt* chi angles: 297.2,175.4	0.02Å	Favored (61.818%) alpha helix	-	-	-
A 72		VAL	4.57	-	Favored (9.01%) Ile or Val / -103.8,-12.8	Favored (21.4%) *m* chi angles: 301.9	0.01Å	Favored (36.25%)	-	-	-
A 73		GLN	5.21	-	Favored (51.3%) General / -70.1,145.8	Favored (13.9%) *tp-100* chi angles: 179.3,62.2,272.8	0.05Å	Favored (13.877%)	-	-	-
A 74		ARG	5.77	-	Favored (3.4%) General / -128.6,-21.7	Favored (90.8%) *mmt-90* chi angles: 296.1,291.6,179.9,269.7	0.01Å	Favored (11.979%)	-	-	-
A 75		ARG	6.07	-	Favored (30.38%) General / -139.7,133.3	Favored (93.7%) *mmt-90* chi angles: 294,289.9,184.1,274.1	0.05Å	CaBLAM Disfavored (1.843%) try alpha helix	-	-	-
A 76		GLY	5.99	-	Favored (31.25%) Glycine / 92.7,154.6	-	-	CaBLAM Disfavored (2.669%) try alpha helix	-	-	-
A 77		ILE	5.56	-	Allowed (0.18%) Ile or Val / 62.5,-56.2	Favored (92.3%) *mt* chi angles: 296.9,168.5	0.05Å	Favored (15.918%)	-	-	-
A 78		GLY	4.89	-	Favored (29.1%) Glycine / 98.7,155.0	-	-	Favored (49.707%)	-	-	-
A 79		LYS	4.17	0.43Å NZ with A 70 LEU O	Favored (81.67%) General / -62.5,-36.2	Favored (61.4%) *mttm* chi angles: 289.4,180.6,183.8,297.7	0.02Å	Favored (45.656%)	-	-	-
A 80		LEU	3.53	-	Favored (75.13%) General / -69.8,-41.4	Favored (41.8%) *tp* chi angles: 184.9,58	0.02Å	Favored (87.169%) alpha helix	-	-	-
**#**	**Alt**	**Res**	**High B**	**Clash > 0.4Å**	**Ramachandran**	**Rotamer**	**Cβ deviation**	**CaBLAM**	**Bond lengths**	**Bond angles**	**Cis Peptides**
			Avg: 3.33	Clashscore: 0.51	Outliers: 0 of 124	Poor rotamers: 0 of 97	Outliers: 0 of 112	Outliers: 0 of 122	Outliers: 0 of 126	Outliers: 2 of 126	Non-Trans: 0 of 125
A 81		GLY	3.03	-	Favored (48.62%) Glycine / -58.7,-53.1	-	-	Favored (91.98%) alpha helix	-	-	-
A 82		LEU	2.66	-	Favored (89.48%)	Favored (82.9%) *mt* chi angles: 289.7,172.8	0.06Å	Favored (76.887%) alpha helix	-	-	-

					General / -62.1,-38.6						
A 83		GLY	2.4	-	Favored (41.46%) Glycine / -55.1,-53.3	-	-	Favored (92.29%) alpha helix	-	-	-
A 84		GLY	2.21	-	Favored (92.6%) Glycine / -59.4,-37.8	-	-	Favored (90.876%) alpha helix	-	-	-
A 85		MET	2.09	-	Favored (94.12%) General / -62.7,-45.1	Favored (61.9%) *mtt* chi angles: 291.7,174.9,190.7	0.04Å	Favored (84.589%) alpha helix	-	-	-
A 86		VAL	2.02	-	Favored (90.35%) Ile or Val / -65.1,-46.7	Favored (62.6%) *t* chi angles: 171.2	0.09Å	Favored (81.538%) alpha helix	-	-	-
A 87		LEU	1.97	-	Favored (94.52%) General / -63.6,-44.2	Favored (60.8%) *tp* chi angles: 180.4,58.9	0.03Å	Favored (84.064%) alpha helix	-	-	-
A 88		GLY	1.94	-	Favored (34.16%) Glycine / -54.4,-54.1	-	-	Favored (93.068%) alpha helix	-	-	-
A 89		LEU	1.92	-	Favored (98.89%) General / -62.4,-41.8	Favored (72.8%) *mt* chi angles: 287.4,171.3	0.03Å	Favored (82.372%) alpha helix	-	-	-
A 90		ALA	1.92	-	Favored (90.97%) General / -60.9,-39.9	-	0.04Å	Favored (98.732%) alpha helix	-	-	-
A 91		THR	1.93	-	Favored (93.9%) General / -63.7,-44.4	Favored (99.2%) *m* chi angles: 300.3	0.08Å	Favored (91.924%) alpha helix	-	-	-
A 92		PHE	1.95	-	Favored (70.44%) General / -55.2,-50.1	Favored (53.5%) *t80* chi angles: 167.1,74.2	0.10Å	Favored (88.491%) alpha helix	-	OUTLIER(S) worst is CA-CB-CG: 5.6 σ	-
A 93		PHE	2	-	Favored (87.18%) General / -65.9,-37.9	Favored (52.2%) *m-80* chi angles: 289.3,114.3	0.09Å	Favored (86.3%) alpha helix	-	-	-
A 94		LEU	2.08	-	Favored (90.34%) General / -63.6,-38.2	Favored (94.4%) *mt* chi angles: 291.5,172.4	0.03Å	Favored (87.366%) alpha helix	-	-	-
A 95		TRP	2.19	-	Favored (85.09%) General / -67.5,-40.1	Favored (71.7%) *m100* chi angles: 283.9,114.5	0.05Å	Favored (96.606%) alpha helix	-	-	-
A 96		MET	2.35	-	Favored (67.21%) General / -67.0,-27.1	Favored (74.5%) *mtm* chi angles: 290.1,189.4,294.8	0.05Å	Favored (76.139%) alpha helix	-	-	-
A 97		ALA	2.56	-	Favored (31.35%) General / -80.0,0.5	-	0.03Å	Favored (45.592%)	-	-	-
A 98		ASP	2.83	-	Favored (10.95%) General / 62.4,40.6	Favored (19.2%) *t0* chi angles: 200.7,29.8	0.01Å	Favored (19.174%)	-	-	-
A 99		VAL	3.13	-	Favored (26.75%)	Favored (94.5%) *t* chi angles: 174.7	0.05Å	Favored (22.711%)	-	-	-

					Ile or Val / -80.3,134.8			beta sheet			
A 100		SER	3.36	-	Favored (34.5%) General / -54.7,140.8	Favored (29.3%) *t* chi angles: 172.8	0.08Å	Favored (42.149%)	-	-	-
**#**	**Alt**	**Res**	**High B**	**Clash > 0.4Å**	**Ramachandran**	**Rotamer**	**Cβ deviation**	**CaBLAM**	**Bond lengths**	**Bond angles**	**Cis Peptides**
			Avg: 3.33	Clashscore: 0.51	Outliers: 0 of 124	Poor rotamers: 0 of 97	Outliers: 0 of 112	Outliers: 0 of 122	Outliers: 0 of 126	Outliers: 2 of 126	Non-Trans: 0 of 125
A 101		GLY	3.44	-	Favored (48.39%) Glycine / -57.0,-23.9	-	-	Favored (51.493%)	-	-	-
A 102		THR	3.36	-	Favored (89.18%) General / -65.6,-43.5	Favored (92.7%) *m* chi angles: 297.7	0.01Å	Favored (66.248%) alpha helix	-	-	-
A 103		LYS	3.15	-	Favored (64.65%) General / -73.9,-36.4	Favored (97.2%) *mttt* chi angles: 290.1,180.7,179.3,179.5	0.01Å	Favored (77.173%) alpha helix	-	-	-
A 104		ILE	2.86	-	Favored (86.7%) Ile or Val / -67.0,-45.6	Favored (96.9%) *mt* chi angles: 292.5,169.6	0.06Å	Favored (90.927%) alpha helix	-	-	-
A 105		ALA	2.57	-	Favored (82.67%) General / -60.4,-38.0	-	0.03Å	Favored (91.92%) alpha helix	-	-	-
A 106		GLY	2.33	-	Favored (64.63%) Glycine / -57.8,-50.5	-	-	Favored (93.008%) alpha helix	-	-	-
A 107		THR	2.19	-	Favored (95.72%) General / -60.5,-44.8	Favored (90.2%) *m* chi angles: 298.1	0.03Å	Favored (89.279%) alpha helix	-	-	-
A 108		LEU	2.14	-	Favored (98.57%) General / -62.7,-43.5	Favored (81.2%) *mt* chi angles: 289.1,169.7	0.08Å	Favored (89.618%) alpha helix	-	-	-
A 109		LEU	2.15	-	Favored (64.07%) General / -66.0,-50.5	Favored (65.9%) *tp* chi angles: 176.3,60.2	0.03Å	Favored (77.634%) alpha helix	-	-	-
A 110		LEU	2.19	-	Favored (83.13%) General / -59.7,-48.1	Favored (68.2%) *tp* chi angles: 176.6,60.5	0.03Å	Favored (92.197%) alpha helix	-	-	-
A 111		ALA	2.26	-	Favored (89.43%) General / -59.6,-41.0	-	0.05Å	Favored (90.549%) alpha helix	-	-	-
A 112		LEU	2.36	-	Favored (92.53%) General / -65.2,-39.2	Favored (89.2%) *mt* chi angles: 290.9,170.8	0.03Å	Favored (97.677%) alpha helix	-	-	-
A 113		LEU	2.49	-	Favored (89.22%) General / -64.4,-37.9	Favored (94.7%) *mt* chi angles: 291.6,172.3	0.01Å	Favored (96.713%) alpha helix	-	-	-
A 114		MET	2.64	-	Favored (78.68%)	Favored (91.6%) *mmm*	0.06Å	Favored (88.419%) alpha helix	-	-	-

					General / -68.0,-35.7	chi angles: 291,306.1,296.7					
A 115		MET	2.81	-	Favored (95.72%) General / -60.2,-43.6	Favored (30.1%) *mtt* chi angles: 291.6,175.3,206.4	0.04Å	Favored (88.608%) alpha helix	-	-	-
A 116		ILE	3.04	-	Favored (96.89%) Ile or Val / -62.7,-46.2	Favored (97.5%) *mt* chi angles: 292.2,167.4	0.03Å	Favored (74.37%) alpha helix	-	-	-
A 117		VAL	3.35	-	Favored (15.16%) Ile or Val / -77.8,-27.5	Favored (22.9%) *m* chi angles: 301.5	0.05Å	Favored (73.594%) alpha helix	-	-	-
A 118		LEU	3.75	-	Favored (39.45%) General / -80.0,-33.2	Favored (95.6%) *mt* chi angles: 294.3,173.7	0.01Å	Favored (39.049%)	-	-	-
A 119		ILE	4.26	-	Favored (80.66%) Pre-Pro / -81.5,122.2	Favored (87.7%) *mt* chi angles: 298.3,168.8	0.05Å	Favored (27.268%)	-	-	-
A 120		PRO	4.88	-	Favored (70.05%) Trans-Pro / -69.6,152.1	Favored (62.1%) *Cg_endo* chi angles: 26.6,326.2,26.5	0.01Å	Favored (76.196%)	-	-	-
**#**	**Alt**	**Res**	**High B**	**Clash > 0.4Å**	**Ramachandran**	**Rotamer**	**Cβ deviation**	**CaBLAM**	**Bond lengths**	**Bond angles**	**Cis Peptides**
			Avg: 3.33	Clashscore: 0.51	Outliers: 0 of 124	Poor rotamers: 0 of 97	Outliers: 0 of 112	Outliers: 0 of 122	Outliers: 0 of 126	Outliers: 2 of 126	Non-Trans: 0 of 125
A 121		GLU	5.61	-	Favored (81.62%) Pre-Pro / -76.4,149.5	Favored (57.6%) *mt-10* chi angles: 292.4,180.2,292.8	0.03Å	Favored (49.958%)	-	-	-
A 122		PRO	6.4	-	Favored (46.26%) Trans-Pro / -70.4,161.4	Favored (64%) *Cg_endo* chi angles: 26.8,325.2,28.2	0.03Å	Favored (34.135%)	-	-	-
A 123		GLU	7.18	-	Allowed (0.69%) General / 72.5,-49.0	Favored (98.9%) *mt-10* chi angles: 293.7,179.2,355.2	0.07Å	CaBLAM Disfavored (1.036%)	-	-	-
A 124		LYS	7.9	-	Favored (70.3%) General / -63.0,-28.7	Favored (96.9%) *mttt* chi angles: 290.2,181.7,181.3,179.5	0.01Å	Favored (37.153%)	-	-	-
A 125		GLN	8.5	-	Favored (40.35%) General / -75.7,137.0	Favored (60.9%) *mt0* chi angles: 294.1,172.2,72.1	0.08Å	-	-	-	-
A 126		ARG	8.95	-	-	Favored (17.2%) *ptp-170* chi angles: 60.2,186.9,70.1,200	0.04Å	-	-	-	-

About MolProbity | Website for the Richardson Lab | Using ecloud x-H | Internal reference 4.5.2
