# Supplementary material for: Integrative proteome-wide structural analysis and high-throughput docking identify broad-spectrum antiviral scaffolds against Zika, Yellow Fever, West Nile, Saint Louis encephalitis, and Usutu viruses
Source: Front Cell Infect Microbiol. 2026 Apr 30;16:1723132. doi: 10.3389/fcimb.2026.1723132 (PMC13171538; doi:10.3389/fcimb.2026.1723132)
Supplement: Supplementary file 4 [file DataSheet4.zip › USUV/USU_NS4b/Mol_probity_Files/USU_NS4b_1FH-multi.table.pdf]

Viewing USU_NS4b1FH-multi.table

Duke Biochemistry
Duke University School of Medicine

When finished, you should close this window.

Hint: Use File | Save As... to save a copy of this page.

All-Atom Contacts	Clashscore, all atoms:	1.78		99th percentile* (N=1784, all resolutions)
	Clashscore is the number of serious steric overlaps (> 0.4 Å) per 1000 atoms.			
Protein Geometry	Poor rotamers	0	0.00%	Goal: <0.3%
	Favored rotamers	204	100.00%	Goal: >98%
	Ramachandran outliers	1	0.39%	Goal: <0.05%
	Ramachandran favored	249	97.27%	Goal: >98%
	Rama distribution Z-score	1.74 ± 0.50		Goal: abs(Z score) < 2
	MolProbity score^	1.07		100th percentile* (N=27675, 0Å - 99Å)
	Cβ deviations >0.25Å	0	0.00%	Goal: 0
	Bad bonds:	0 / 1968	0.00%	Goal: 0%
	Bad angles:	4 / 2692	0.15%	Goal: <0.1%
Peptide Omegas	Cis Prolines:	0 / 12	0.00%	Expected: ≤1 per chain, or ≤5%
Low-resolution Criteria	CaBLAM outliers	9	3.5%	Goal: <1.0%
	CA Geometry outliers	2	0.79%	Goal: <0.5%
Additional validations	Chiral volume outliers	0/334		
	Waters with clashes	0/0	0.00%	See UnDowser table for details

In the two column results, the left column gives the raw count, right column gives the percentage.

* 100th percentile is the best among structures of comparable resolution; 0th percentile is the worst. For clashscore the comparative set of structures was selected in 2004, for MolProbity score in 2006.

^ MolProbity score combines the clashscore, rotamer, and Ramachandran evaluations into a single score, normalized to be on the same scale as X-ray resolution.

Key to table colors and cutoffs here:

#	Alt	Res	High B	Clash > 0.4Å	Ramachandran	Rotamer	Cβ deviation	CaBLAM	Bond lengths	Bond angles	Cis Peptides
			Avg: 4.01	Clashscore: 1.78	Outliers: 1 of 256	Poor rotamers: 0 of 204	Outliers: 0 of 239	Outliers: 9 of 254	Outliers: 0 of 258	Outliers: 3 of 258	Non-Trans: 0 of 257
A 1		ASN	13.13	-	-	Favored (42.2%) *p0* chi angles: 66.5,24.9	0.10Å	-	-	OUTLIER(S) worst is CA-CB-CG: 4.3 σ	-
A 2		GLU	13.51	-	Favored (52.85%) General / -87.6,1.7	Favored (97.1%) *mt-10* chi angles: 295.5,179.2,359.4	0.01Å	-	-	-	-
A 3		TYR	13.88	-	Favored (42.7%) General / -126.2,154.9	Favored (86.9%) *m-80* chi angles: 300.3,88.9	0.03Å	Favored (26.793%)	-	-	-
A 4		GLY	14.18	-	Favored (47.43%) Glycine / -63.5,-12.7	-	-	Favored (21.76%)	-	-	-
A 5		MET	14.37	-	Favored (68.21%) General / -55.4,-38.0	Favored (60%) *mtt* chi angles: 292,174.4,191.7	0.11Å	Favored (55.793%)	-	-	-

A 6		LEU	14.43	-	Favored (80.28%) General / -67.0,-44.4	Favored (62.4%) *tp* chi angles: 180,59.1	0.04Å	Favored (84.787%) alpha helix	-	-	-
A 7		GLU	14.41	-	Favored (84.45%) General / -65.4,-36.6	Favored (76.2%) *mm-30* chi angles: 291.1,295.7,308.4	0.01Å	Favored (94.078%) alpha helix	-	-	-
A 8		ARG	14.41	-	Favored (89.32%) General / -66.3,-39.3	Favored (98.4%) *mtt180* chi angles: 289.3,174.9,179.4,171.2	0.02Å	Favored (95.016%) alpha helix	-	-	-
A 9		THR	14.46	-	Favored (84.19%) General / -62.5,-47.4	Favored (91.2%) *m* chi angles: 298	0.03Å	Favored (93.33%) alpha helix	-	-	-
A 10		LYS	14.54	-	Favored (91%) General / -63.0,-38.4	Favored (56.6%) *mtmt* chi angles: 289.5,188.3,291.7,184.8	0.01Å	Favored (90.886%) alpha helix	-	-	-
A 11		SER	14.65	-	Favored (97.69%) General / -63.8,-42.8	Favored (72.1%) *m* chi angles: 295.9	0.04Å	Favored (97.805%) alpha helix	-	-	-
A 12		ASP	14.79	-	Favored (89.76%) General / -65.1,-38.2	Favored (95.3%) *m-30* chi angles: 286,347.8	0.10Å	Favored (94.011%) alpha helix	-	-	-
A 13		LEU	14.95	-	Favored (91.82%) General / -62.9,-38.7	Favored (84.5%) *mt* chi angles: 289.8,171.6	0.02Å	Favored (86.476%) alpha helix	-	-	-
A 14		GLY	15.08	-	Favored (51.68%) Glycine / -56.5,-52.2	-	-	Favored (95.186%) alpha helix	-	-	-
A 15		LYS	15.16	-	Favored (87.14%) General / -62.0,-38.0	Favored (56.9%) *mtmt* chi angles: 289.1,184.9,290.5,185.7	0.01Å	Favored (82.008%) alpha helix	-	-	-
A 16		ILE	15.17	-	Favored (84.35%) Ile or Val / -65.0,-48.4	Favored (88.6%) *mt* chi angles: 291.3,166.3	0.03Å	Favored (79.657%) alpha helix	-	-	-
A 17		PHE	15.13	-	Favored (83.14%) General / -68.0,-39.1	Favored (35.2%) *m-80* chi angles: 290.1,124	0.02Å	Favored (81.474%) alpha helix	-	-	-
A 18		SER	15.08	-	Favored (94.66%) General / -64.1,-43.7	Favored (70.5%) *m* chi angles: 296.3	0.06Å	Favored (97.445%) alpha helix	-	-	-
A 19		SER	15.09	-	Favored (90.52%) General / -61.7,-39.2	Favored (70%) *m* chi angles: 295	0.04Å	Favored (85.348%) alpha helix	-	-	-
A 20		THR	15.16	-	Favored (85.77%) General / -66.3,-43.7	Favored (97.7%) *m* chi angles: 300	0.04Å	Favored (76.764%) alpha helix	-	-	-
**#**	**Alt**	**Res**	**High B**	**Clash > 0.4Å**	**Ramachandran**	**Rotamer**	**Cβ deviation**	**CaBLAM**	**Bond lengths**	**Bond angles**	**Cis Peptides**
			Avg: 4.01	Clashscore: 1.78	Outliers: 1 of 256	Poor rotamers: 0 of 204	Outliers: 0 of 239	Outliers: 9 of 254	Outliers: 0 of 258	Outliers: 3 of 258	Non-Trans: 0 of 257

A 21		ARG	15.3	-	Favored (57.46%) General / -77.0,-33.1	Favored (94.9%) *mtt180* chi angles: 291.5,170.8,183.1,166.9	0.06Å	Favored (77.284%) alpha helix	-	-	-
A 22		GLN	15.47	0.42Å N with A 23 PRO HD3	OUTLIER (0.08)%) Pre-Pro / -89.5,-3.3	Favored (92.9%) *mt0* chi angles: 295.7,174.6,340.5	0.01Å	Favored (52.725%) alpha helix	-	OUTLIER(S) worst is CA-C-N: 5.0 σ	-
A 23		PRO	15.58	0.42Å HD3 with A 22 GLN N	Favored (11.58%) Trans-Pro / -47.1,-31.2	Favored (86.8%) *Cg_exo* chi angles: 330.3,37.4,330.3	0.07Å	CA Geom Outlier (0.399%)	-	-	-
A 24		GLN	15.51	-	Favored (71.38%) General / -61.9,-31.1	Favored (93.2%) *mm-40* chi angles: 291.3,293.1,308	0.00Å	Favored (67.944%) alpha helix	-	-	-
A 25		SER	15.17	-	Favored (60.09%) General / -75.0,-11.3	Favored (93%) *p* chi angles: 66.4	0.02Å	Favored (63.594%)	-	-	-
A 26		ALA	14.57	-	Favored (53.22%) General / -94.8,-1.3	-	0.04Å	Favored (49.197%)	-	-	-
A 27		LEU	13.77	-	Favored (65.92%) Pre-Pro / -76.8,138.5	Favored (94.6%) *mt* chi angles: 295.9,174.4	0.04Å	Favored (39.58%)	-	-	-
A 28		PRO	12.9	-	Favored (8.69%) Trans-Pro / -79.7,63.0	Favored (57.6%) *Cg_endo* chi angles: 32.1,323.8,25.1	0.06Å	Favored (13.673%) beta sheet	-	-	-
A 29		LEU	12.03	-	Favored (39.12%) Pre-Pro / -63.8,-38.6	Favored (94.4%) *mt* chi angles: 294.1,174.5	0.08Å	Favored (9.109%)	-	-	-
A 30		PRO	11.2	-	Favored (79.79%) Trans-Pro / -56.4,137.3	Favored (86.3%) *Cg_exo* chi angles: 333.6,37.2,327.9	0.03Å	CA Geom Outlier (0.108%)	-	-	-
A 31		SER	10.33	-	Allowed (1.92%) General / -142.9,15.3	Favored (81.1%) *p* chi angles: 61.8	0.02Å	Favored (5.257%)	-	-	-
A 32		MET	9.28	-	Favored (35.21%) General / -82.5,131.8	Favored (15%) *tpt* chi angles: 180.9,63.9,189.3	0.04Å	Favored (22.088%) beta sheet	-	-	-
A 33		ASN	7.94	-	Favored (23.42%) General / -109.7,111.8	Favored (50.8%) *t0* chi angles: 184.1,329.7	0.06Å	Favored (67.51%)	-	-	-
A 34		ALA	6.37	-	Favored (44.05%) General / -56.4,-24.3	-	0.04Å	Favored (27.733%)	-	-	-
A 35		LEU	4.79	-	Favored (40.8%) General / -100.2,10.7	Favored (84.6%) *mt* chi angles: 299.7,179.1	0.02Å	Favored (37.327%)	-	-	-
A 36		ALA	3.42	-	Favored (50.13%) General / -61.3,145.8	-	0.06Å	Favored (34.967%)	-	-	-
A 37		LEU	2.4	-	Favored (23.96%)	Favored (94.9%) *mt* chi angles: 297.7,175.5	0.05Å	Favored (45.158%) beta sheet	-	-	-

#	Alt	Res	High B	Clash > 0.4Å	Ramachandran	Rotamer	Cβ deviation	CaBLAM	Bond lengths	Bond angles	Cis Peptides
					General / -86.2,151.0						
A 38		ASP	1.71	-	Favored (2.43%) General / -146.0,94.2	Favored (51.8%) *t0* chi angles: 185.9,4.7	0.06Å	Favored (14.957%) beta sheet	-	-	-
A 39		LEU	1.28	-	Favored (35.62%) General / -90.3,125.7	Favored (71.3%) *tp* chi angles: 176.4,62.2	0.03Å	Favored (51.09%) beta sheet	-	-	-
A 40		ARG	1.04	-	Favored (19.17%) Pre-Pro / -117.3,85.2	Favored (88.2%) *mtt180* chi angles: 300.2,167,184,173.7	0.12Å	Favored (48.113%) beta sheet	-	-	-
**#**	**Alt**	**Res**	**High B**	**Clash > 0.4Å**	**Ramachandran**	**Rotamer**	**Cβ deviation**	**CaBLAM**	**Bond lengths**	**Bond angles**	**Cis Peptides**
			Avg: 4.01	Clashscore: 1.78	Outliers: 1 of 256	Poor rotamers: 0 of 204	Outliers: 0 of 239	Outliers: 9 of 254	Outliers: 0 of 258	Outliers: 3 of 258	Non-Trans: 0 of 257
A 41		PRO	0.91	-	Favored (18.14%) Trans-Pro / -46.0,-37.8	Favored (76.2%) *Cg_exo* chi angles: 329,38.8,329.5	0.09Å	Favored (47.709%)	-	-	-
A 42		ALA	0.85	-	Favored (83.12%) General / -65.4,-36.2	-	0.02Å	Favored (79.134%) alpha helix	-	-	-
A 43		THR	0.84	-	Favored (40.56%) General / -76.1,-44.0	Favored (92.1%) *m* chi angles: 301.1	0.02Å	Favored (73.741%) alpha helix	-	-	-
A 44		ALA	0.86	-	Favored (89.23%) General / -59.8,-40.7	-	0.06Å	Favored (83.485%) alpha helix	-	-	-
A 45		TRP	0.89	0.46Å CE3 with A 45 TRP HA	Favored (66.72%) General / -71.6,-43.2	Favored (25.3%) *t-100* chi angles: 198.1,242.8	0.04Å	Favored (83.792%) alpha helix	-	-	-
A 46		ALA	0.96	-	Favored (90.89%) General / -61.3,-39.6	-	0.09Å	Favored (94.848%) alpha helix	-	-	-
A 47		LEU	1.05	-	Favored (74.55%) General / -69.1,-43.4	Favored (42.7%) *tp* chi angles: 184.7,58.2	0.12Å	Favored (95.124%) alpha helix	-	-	-
A 48		TYR	1.18	-	Favored (69.83%) General / -55.9,-50.8	Favored (32.7%) *t80* chi angles: 164.2,81.3	0.06Å	Favored (86.24%) alpha helix	-	-	-
A 49		GLY	1.34	-	Favored (29.1%) Glycine / -55.8,-55.4	-	-	Favored (98.727%) alpha helix	-	-	-
A 50		GLY	1.53	-	Favored (32.18%) Glycine / -52.9,-53.7	-	-	Favored (92.523%) alpha helix	-	-	-
A 51		SER	1.73	-	Favored (97.84%) General / -61.5,-41.9	Favored (72.7%) *m* chi angles: 295.7	0.07Å	Favored (81.522%) alpha helix	-	-	-
A 52		THR	1.92	-	Favored (90.61%)	Favored (95.8%) *m* chi angles: 299.6	0.04Å	Favored (94.842%) alpha helix	-	-	-

#	Alt	Res	High B	Clash > 0.4Å	Ramachandran	Rotamer	Cβ deviation	CaBLAM	Bond lengths	Bond angles	Cis Peptides
					General / -65.1,-43.8						
A 53		VAL	2.09	-	Favored (95.19%) Ile or Val / -61.5,-42.7	Favored (57.7%) *t* chi angles: 170.5	0.01Å	Favored (77.344%) alpha helix	-	-	-
A 54		VAL	2.23	-	Favored (72%) Ile or Val / -70.3,-46.4	Favored (85.1%) *t* chi angles: 173.6	0.05Å	Favored (64.023%) alpha helix	-	-	-
A 55		LEU	2.32	-	Favored (33.36%) General / -76.3,-45.2	Favored (59.6%) *tp* chi angles: 180.5,58.2	0.06Å	Favored (31.31%) alpha helix	-	-	-
A 56		THR	2.39	-	Allowed (1.56%) Pre-Pro / -41.0,-60.7	Favored (88.8%) *m* chi angles: 298.6	0.14Å	Favored (57.006%) alpha helix	-	-	-
A 57		PRO	2.43	-	Favored (74.03%) Trans-Pro / -61.9,-22.3	Favored (32.2%) *Cg_endo* chi angles: 21.7,326.3,31.2	0.01Å	Favored (57.532%) alpha helix	-	-	-
A 58		LEU	2.46	-	Favored (65.78%) General / -71.6,-43.7	Favored (52.6%) *tp* chi angles: 183,59	0.04Å	Favored (71.088%) alpha helix	-	-	-
A 59		ILE	2.45	-	Favored (97.56%) Ile or Val / -63.7,-45.4	Favored (99.1%) *mt* chi angles: 292.9,167.7	0.01Å	Favored (87.264%) alpha helix	-	-	-
A 60		LYS	2.44	-	Favored (83.8%) General / -61.8,-37.3	Favored (97.7%) *mttt* chi angles: 288.9,178.8,175.2,177.3	0.01Å	Favored (88.447%) alpha helix	-	-	-
**#**	**Alt**	**Res**	**High B**	**Clash > 0.4Å**	**Ramachandran**	**Rotamer**	**Cβ deviation**	**CaBLAM**	**Bond lengths**	**Bond angles**	**Cis Peptides**
			Avg: 4.01	Clashscore: 1.78	Outliers: 1 of 256	Poor rotamers: 0 of 204	Outliers: 0 of 239	Outliers: 9 of 254	Outliers: 0 of 258	Outliers: 3 of 258	Non-Trans: 0 of 257
A 61		HIS	2.42	-	Favored (84.42%) General / -67.3,-41.9	Favored (49%) *m170* chi angles: 288.4,162.3	0.07Å	Favored (92.398%) alpha helix	-	-	-
A 62		LEU	2.41	-	Favored (66.33%) General / -61.6,-52.0	Favored (60.8%) *tp* chi angles: 174.3,61.8	0.06Å	Favored (83.32%) alpha helix	-	-	-
A 63		VAL	2.42	-	Favored (98.13%) Ile or Val / -60.8,-45.3	Favored (55.7%) *t* chi angles: 170.2	0.07Å	Favored (88.114%) alpha helix	-	-	-
A 64		THR	2.46	-	Favored (94.46%) General / -62.1,-45.2	Favored (87%) *m* chi angles: 301.4	0.06Å	Favored (97.358%) alpha helix	-	-	-
A 65		SER	2.53	-	Favored (99.43%) General / -63.0,-41.1	Favored (73.5%) *m* chi angles: 295.5	0.03Å	Favored (89.56%) alpha helix	-	-	-
A 66		GLU	2.6	-	Favored (96.12%) General / -63.3,-40.0	Favored (98.1%) *mt-10* chi angles: 289.6,176.4,355.5	0.02Å	Favored (87.286%) alpha helix	-	-	-
A 67		TYR	2.68	-	Favored (60.19%) General / -75.8,-34.4	Favored (36.5%) *m-80* chi angles: 282.7,113.5	0.12Å	Favored (85.65%) alpha helix	-	-	-

A 68		ILE	2.75	-	Favored (89.67%) Ile or Val / -66.8,-43.6	Favored (92%) *mt* chi angles: 291.6,168.9	0.06Å	Favored (85.751%) alpha helix	-	-	-
A 69		THR	2.76	-	Favored (96.55%) General / -60.4,-43.6	Favored (89.8%) *m* chi angles: 301.2	0.03Å	Favored (98.425%) alpha helix	-	-	-
A 70		THR	2.74	-	Favored (92.18%) General / -59.2,-42.9	Favored (98.1%) *m* chi angles: 300.1	0.01Å	Favored (93.207%) alpha helix	-	-	-
A 71		SER	2.7	-	Favored (85.14%) General / -60.4,-47.7	Favored (34.5%) *t* chi angles: 182.2	0.06Å	Favored (91.182%) alpha helix	-	-	-
A 72		LEU	2.66	-	Favored (94.97%) General / -63.2,-39.6	Favored (98.6%) *mt* chi angles: 293.1,172.4	0.07Å	Favored (91.077%) alpha helix	-	-	-
A 73		ALA	2.68	-	Favored (80.4%) General / -62.2,-35.9	-	0.04Å	Favored (87.063%) alpha helix	-	-	-
A 74		SER	2.81	-	Favored (63.19%) General / -74.4,-34.0	Favored (65%) *m* chi angles: 297.3	0.03Å	Favored (91.487%) alpha helix	-	-	-
A 75		ILE	3.08	-	Favored (86.94%) Ile or Val / -66.7,-46.0	Favored (94.5%) *mt* chi angles: 293,166.2	0.02Å	Favored (28.632%)	-	-	-
A 76		SER	3.53	-	Favored (57.96%) General / -61.0,139.9	Favored (38.5%) *t* chi angles: 177.2	0.02Å	Favored (20.682%)	-	-	-
A 77		ALA	4.15	-	Favored (2.82%) General / -78.4,61.3	-	0.03Å	CaBLAM Disfavored (3.625%)	-	-	-
A 78		GLN	4.91	-	Favored (2.59%) General / -161.0,120.2	Favored (62.2%) *tt0* chi angles: 182.7,175.6,25.1	0.02Å	CaBLAM Disfavored (2.305%)	-	-	-
A 79		ALA	5.72	-	Favored (70.81%) General / -61.3,-31.0	-	0.03Å	Favored (37.491%)	-	-	-
A 80		GLY	6.44	-	Favored (58.21%) Glycine / -59.2,-24.0	-	-	Favored (78.061%)	-	-	-
**#**	**Alt**	**Res**	**High B**	**Clash > 0.4Å**	**Ramachandran**	**Rotamer**	**Cβ deviation**	**CaBLAM**	**Bond lengths**	**Bond angles**	**Cis Peptides**
			Avg: 4.01	Clashscore: 1.78	Outliers: 1 of 256	Poor rotamers: 0 of 204	Outliers: 0 of 239	Outliers: 9 of 254	Outliers: 0 of 258	Outliers: 3 of 258	Non-Trans: 0 of 257
A 81		SER	6.99	-	Favored (59.77%) General / -77.7,-10.9	Favored (72.4%) *m* chi angles: 295.8	0.02Å	Favored (58.303%) three-ten	-	-	-
A 82		LEU	7.31	-	Favored (51.73%) General / -74.4,-7.1	Favored (96.4%) *mt* chi angles: 294.6,172.8	0.06Å	Favored (28.452%) three-ten	-	-	-
A 83		PHE	7.45	-	Favored (28.83%)	Favored (84.4%) *m-80*	0.12Å	Favored (55.752%)	-	OUTLIER(S) worst is CA-	-

					General / -103.0,-1.7	chi angles: 301.5,101.5				CB-CG: 5.8 σ	
A 84		ASN	7.46	-	Favored (10.74%) General / -85.2,69.2	Favored (49%) *t0* chi angles: 190.9,19.5	0.03Å	Favored (5.99%)	-	-	-
A 85		LEU	7.39	-	Favored (64.59%) Pre-Pro / -125.5,70.6	Favored (73.5%) *mt* chi angles: 300.6,172.6	0.07Å	CaBLAM Disfavored (3.546%)	-	-	-
A 86		PRO	7.26	-	Favored (75.67%) Trans-Pro / -65.9,144.2	Favored (40.6%) *Cg_endo* chi angles: 23.5,327.1,28.2	0.03Å	Favored (26.888%) beta sheet	-	-	-
A 87		ARG	7.06	-	Favored (9.38%) General / -87.8,72.9	Favored (47.7%) *mtp180* chi angles: 300.7,190.2,79,200	0.02Å	Favored (29.86%) beta sheet	-	-	-
A 88		GLY	6.79	-	Favored (49.54%) Glycine / -62.9,144.6	-	-	Favored (17.506%) beta sheet	-	-	-
A 89		LEU	6.43	-	Favored (77.46%) Pre-Pro / -130.3,76.6	Favored (90%) *mt* chi angles: 296.3,171.7	0.03Å	Favored (14.695%)	-	-	-
A 90		PRO	5.96	-	Favored (86.6%) Trans-Pro / -58.3,147.1	Favored (60.1%) *Cg_exo* chi angles: 335.9,35.3,328.4	0.05Å	Favored (30.428%)	-	-	-
A 91		PHE	5.39	-	Favored (47.3%) General / -72.2,146.6	Favored (75.8%) *m-80* chi angles: 288.1,95.2	0.03Å	Favored (39.748%)	-	-	-
A 92		THR	4.75	-	Favored (10.91%) General / -103.8,165.7	Favored (58%) *p* chi angles: 64.5	0.06Å	Favored (37.669%) beta sheet	-	-	-
A 93		GLU	4.11	-	Favored (22.46%) General / -103.7,151.9	Favored (96.3%) *mt-10* chi angles: 296,181,358.4	0.01Å	Favored (48.9%) beta sheet	-	-	-
A 94		LEU	3.52	-	Favored (17.04%) General / -134.5,118.2	Favored (71%) *tp* chi angles: 178.2,63	0.04Å	Favored (31.163%) beta sheet	-	-	-
A 95		ASP	3.02	-	Favored (58.56%) General / -62.5,138.9	Favored (11.1%) *t70* chi angles: 193.4,98.8	0.03Å	Favored (37.224%)	-	-	-
A 96		PHE	2.63	-	Favored (62.84%) General / -57.8,-27.1	Favored (37.9%) *t80* chi angles: 188,62.2	0.05Å	Favored (49.445%)	-	-	-
A 97		THR	2.35	-	Favored (90.69%) General / -58.9,-43.0	Favored (97.5%) *m* chi angles: 300	0.09Å	Favored (65.523%) alpha helix	-	-	-
A 98		VAL	2.15	-	Favored (55.38%) Ile or Val / -72.8,-37.7	Favored (86.8%) *t* chi angles: 176.2	0.05Å	Favored (75.666%) alpha helix	-	-	-
A 99		VAL	2.01	-	Favored (86.59%) Ile or Val / -57.7,-45.2	Favored (63.2%) *t* chi angles: 171.2	0.09Å	Favored (77.57%) alpha helix	-	-	-

#	Alt	Res	High B	Clash > 0.4Å	Ramachandran	Rotamer	Cβ deviation	CaBLAM	Bond lengths	Bond angles	Cis Peptides
A 100		LEU	1.9	-	Favored (85.84%) General / -63.0,-37.1	Favored (89.5%) *mt* chi angles: 290.8,171.8	0.01Å	Favored (88.873%) alpha helix	-	-	-
**#**	**Alt**	**Res**	**High B**	**Clash > 0.4Å**	**Ramachandran**	**Rotamer**	**Cβ deviation**	**CaBLAM**	**Bond lengths**	**Bond angles**	**Cis Peptides**
			Avg: 4.01	Clashscore: 1.78	Outliers: 1 of 256	Poor rotamers: 0 of 204	Outliers: 0 of 239	Outliers: 9 of 254	Outliers: 0 of 258	Outliers: 3 of 258	Non-Trans: 0 of 257
A 101		VAL	1.81	-	Favored (93.12%) Ile or Val / -62.4,-47.4	Favored (61.4%) *t* chi angles: 171	0.07Å	Favored (82.045%) alpha helix	-	-	-
A 102		PHE	1.75	-	Favored (65.62%) General / -61.3,-52.2	Favored (87.1%) *t80* chi angles: 174,79	0.09Å	Favored (78.337%) alpha helix	-	-	-
A 103		LEU	1.7	-	Favored (95.07%) General / -62.3,-40.0	Favored (96.1%) *mt* chi angles: 292,171.9	0.10Å	Favored (88.447%) alpha helix	-	-	-
A 104		GLY	1.67	-	Favored (30.94%) Glycine / -59.1,-55.4	-	-	Favored (88.727%) alpha helix	-	-	-
A 105		CYS	1.65	-	Favored (63.97%) General / -69.8,-25.8	Favored (7.8%) *p* chi angles: 77.7	0.10Å	Favored (47.268%) alpha helix	-	-	-
A 106		TRP	1.64	-	Favored (83.49%) General / -59.0,-39.9	Favored (51.7%) *t-100* chi angles: 187.6,244.3	0.04Å	Favored (51.397%) alpha helix	-	-	-
A 107		GLY	1.65	-	Favored (30.62%) Glycine / -55.9,-21.5	-	-	Favored (51.823%)	-	-	-
A 108		GLN	1.66	-	Favored (8.62%) General / -122.1,26.3	Favored (66%) *mt0* chi angles: 297.4,187.7,359.9	0.09Å	Favored (24.656%)	-	-	-
A 109		VAL	1.66	-	Favored (38.44%) Ile or Val / -80.4,124.5	Favored (99.6%) *t* chi angles: 175.5	0.02Å	Favored (19.687%)	-	-	-
A 110		SER	1.63	-	Favored (12.5%) General / -95.6,165.1	Favored (89.7%) *p* chi angles: 68.9	0.05Å	Favored (25.085%)	-	-	-
A 111		LEU	1.59	-	Favored (92.88%) General / -61.3,-40.1	Favored (58.7%) *tp* chi angles: 181.3,59.3	0.04Å	Favored (65.363%)	-	-	-
A 112		THR	1.54	-	Favored (82.61%) General / -57.0,-46.0	Favored (87.7%) *m* chi angles: 301.4	0.10Å	Favored (86.865%) alpha helix	-	-	-
A 113		THR	1.48	-	Favored (92.45%) General / -65.2,-43.2	Favored (94.8%) *m* chi angles: 299.4	0.05Å	Favored (90.231%) alpha helix	-	-	-
A 114		LEU	1.42	-	Favored (87.57%) General / -62.0,-46.8	Favored (62.2%) *tp* chi angles: 180.6,60.2	0.05Å	Favored (93.13%) alpha helix	-	-	-

#	Alt	Res	High B	Clash > 0.4Å	Ramachandran	Rotamer	Cβ deviation	CaBLAM	Bond lengths	Bond angles	Cis Peptides
A 115		ILE	1.36	-	Favored (99.67%) Ile or Val / -63.0,-44.5	Favored (98.6%) *mt* chi angles: 292.4,167.9	0.01Å	Favored (90.832%) alpha helix	-	-	-
A 116		THR	1.33	-	Favored (86.83%) General / -58.8,-46.7	Favored (88.3%) *m* chi angles: 298.5	0.03Å	Favored (95.383%) alpha helix	-	-	-
A 117		ALA	1.31	-	Favored (85.5%) General / -59.9,-39.4	-	0.04Å	Favored (85.096%) alpha helix	-	-	-
A 118		ALA	1.31	-	Favored (98.81%) General / -62.2,-41.9	-	0.04Å	Favored (96.923%) alpha helix	-	-	-
A 119		ALA	1.32	-	Favored (95.86%) General / -64.7,-40.7	-	0.03Å	Favored (96.476%) alpha helix	-	-	-
A 120		LEU	1.33	-	Favored (94.28%) General / -65.0,-42.8	Favored (93.7%) *mt* chi angles: 292.3,170.1	0.09Å	Favored (97.128%) alpha helix	-	-	-
**#**	**Alt**	**Res**	**High B**	**Clash > 0.4Å**	**Ramachandran**	**Rotamer**	**Cβ deviation**	**CaBLAM**	**Bond lengths**	**Bond angles**	**Cis Peptides**
			Avg: 4.01	Clashscore: 1.78	Outliers: 1 of 256	Poor rotamers: 0 of 204	Outliers: 0 of 239	Outliers: 9 of 254	Outliers: 0 of 258	Outliers: 3 of 258	Non-Trans: 0 of 257
A 121		ALA	1.36	-	Favored (99.32%) General / -63.1,-41.3	-	0.04Å	Favored (95.23%) alpha helix	-	-	-
A 122		THR	1.39	-	Favored (92.89%) General / -61.7,-45.7	Favored (94.2%) *m* chi angles: 299.3	0.04Å	Favored (95.702%) alpha helix	-	-	-
A 123		LEU	1.42	-	Favored (99.36%) General / -63.0,-41.0	Favored (83.6%) *mt* chi angles: 289.8,169.4	0.05Å	Favored (90.996%) alpha helix	-	-	-
A 124		HIS	1.45	-	Favored (72.29%) General / -58.9,-50.9	Favored (90%) *t70* chi angles: 178.5,75.1	0.01Å	Favored (82.277%) alpha helix	-	-	-
A 125		TYR	1.49	-	Favored (90.46%) General / -64.5,-38.3	Favored (35.7%) *m-80* chi angles: 292.4,125.4	0.06Å	Favored (75.108%) alpha helix	-	-	-
A 126		GLY	1.54	-	Favored (78.34%) Glycine / -58.7,-34.7	-	-	Favored (83.207%) alpha helix	-	-	-
A 127		TYR	1.59	-	Favored (24.38%) General / -81.4,-39.9	Favored (54.6%) *m-80* chi angles: 293.2,116.3	0.06Å	Favored (58.798%) alpha helix	-	-	-
A 128		MET	1.64	-	Favored (17.07%) General / -89.2,-33.2	Favored (35.9%) *mmp* chi angles: 295.4,294.6,89.8	0.08Å	Favored (29.773%) alpha helix	-	-	-
A 129		LEU	1.7	-	Favored (80.51%) Pre-Pro / -50.4,-48.6	Favored (70.6%) *tp* chi angles: 176.5,61.8	0.08Å	Favored (60.21%) alpha helix	-	-	-

A 130		PRO	1.74	-	Favored (73.09%) Trans-Pro / -62.3,-21.7	Favored (31.4%) *Cg_endo* chi angles: 21.5,326.1,31.4	0.02Å	Favored (70.003%) alpha helix	-	-	-
A 131		GLY	1.77	-	Favored (29.52%) Glycine / -76.1,-40.5	-	-	Favored (83.162%) alpha helix	-	-	-
A 132		TRP	1.8	-	Favored (94.61%) General / -65.1,-41.9	Favored (56.6%) *m-10* chi angles: 298.1,337.9	0.06Å	Favored (91.245%) alpha helix	-	-	-
A 133		GLN	1.83	-	Favored (82.86%) General / -67.9,-41.2	Favored (96.4%) *mt0* chi angles: 290.7,174.6,315.4	0.01Å	Favored (88.472%) alpha helix	-	-	-
A 134		ALA	1.87	-	Favored (97.93%) General / -61.2,-44.0	-	0.04Å	Favored (84.154%) alpha helix	-	-	-
A 135		GLU	1.92	-	Favored (88.33%) General / -66.8,-40.8	Favored (35.4%) *mm-30* chi angles: 285.2,287.7,300.7	0.07Å	Favored (86.887%) alpha helix	-	-	-
A 136		ALA	1.99	-	Favored (89.1%) General / -60.9,-39.4	-	0.07Å	Favored (87.336%) alpha helix	-	-	-
A 137		LEU	2.07	-	Favored (69.47%) General / -67.5,-47.6	Favored (61.8%) *tp* chi angles: 176.5,58.1	0.03Å	Favored (83.286%) alpha helix	-	-	-
A 138		ARG	2.16	-	Favored (91.83%) General / -59.5,-42.3	Favored (83.8%) *mtt180* chi angles: 288.5,167.1,181.1,156.5	0.07Å	Favored (87.274%) alpha helix	-	-	-
A 139		ALA	2.23	-	Favored (98.38%) General / -63.6,-41.8	-	0.03Å	Favored (93.45%) alpha helix	-	-	-
A 140		ALA	2.29	-	Favored (92%) General / -61.3,-39.9	-	0.04Å	Favored (89.32%) alpha helix	-	-	-
**#**	**Alt**	**Res**	**High B**	**Clash > 0.4Å**	**Ramachandran**	**Rotamer**	**Cβ deviation**	**CaBLAM**	**Bond lengths**	**Bond angles**	**Cis Peptides**
			Avg: 4.01	Clashscore: 1.78	Outliers: 1 of 256	Poor rotamers: 0 of 204	Outliers: 0 of 239	Outliers: 9 of 254	Outliers: 0 of 258	Outliers: 3 of 258	Non-Trans: 0 of 257
A 141		GLN	2.32	-	Favored (92.27%) General / -65.4,-39.4	Favored (98.5%) *mt0* chi angles: 290.2,172.1,338.6	0.01Å	Favored (80.432%) alpha helix	-	-	-
A 142		ARG	2.33	-	Favored (58.58%) General / -75.8,-38.1	Favored (37.7%) *mmm160* chi angles: 294.6,299,296.6,176.4	0.04Å	Favored (79.268%) alpha helix	-	-	-
A 143		ARG	2.34	-	Favored (98.55%) General / -61.7,-42.2	Favored (73.4%) *ttt180* chi angles: 182.4,173.3,177.2,168.1	0.02Å	Favored (92.604%) alpha helix	-	-	-
A 144		THR	2.37	-	Favored (89.28%) General / -60.7,-46.7	Favored (89.2%) *m* chi angles: 298.3	0.03Å	Favored (97.74%) alpha helix	-	-	-
A 145		ALA	2.43	-	Favored (96.32%)	-	0.06Å	Favored (99.078%)	-	-	-

					General / -60.2,-43.0			alpha helix			
A 146		ALA	2.56	-	Favored (93.76%) General / -62.3,-39.7	-	0.03Å	Favored (94.137%) alpha helix	-	-	-
A 147		GLY	2.78	-	Favored (38.86%) Glycine / -64.5,-53.0	-	-	Favored (90.655%) alpha helix	-	-	-
A 148		ILE	3.07	-	Favored (89.73%) Ile or Val / -60.3,-42.2	Favored (94.4%) *mt* chi angles: 292,169.2	0.03Å	Favored (74.199%) alpha helix	-	-	-
A 149		MET	3.39	-	Favored (54.81%) General / -81.7,-3.9	Favored (83.6%) *mtm* chi angles: 293.9,186,287.3	0.09Å	CaBLAM Disfavored (3.386%)	-	-	-
A 150		LYS	3.78	-	Allowed (0.34%) General / 45.4,-140.9	Favored (91.3%) *mttt* chi angles: 301.3,184.6,180.8,178.9	0.05Å	Favored (6.915%)	-	-	-
A 151		ASN	4.23	-	Favored (12.71%) General / 47.0,42.6	Favored (88.4%) *m-40* chi angles: 294.2,320.1	0.03Å	CaBLAM Outlier (0.249%)	-	-	-
A 152		ALA	4.61	-	Favored (84.51%) General / -59.8,-39.2	-	0.03Å	Favored (24.304%)	-	-	-
A 153		VAL	4.78	-	Favored (71.72%) Ile or Val / -125.9,131.6	Favored (87.2%) *t* chi angles: 177.5	0.04Å	Favored (7.021%)	-	-	-
A 154		VAL	4.64	-	Favored (2.84%) Ile or Val / -118.0,-60.1	Favored (80.6%) *t* chi angles: 177.9	0.05Å	CaBLAM Disfavored (2.219%)	-	-	-
A 155		ASP	4.2	-	Favored (18.94%) General / -103.8,20.0	Favored (73.1%) *m-30* chi angles: 295.6,317.3	0.04Å	CaBLAM Outlier (0.638%)	-	-	-
A 156		GLY	3.62	-	Favored (69.11%) Glycine / 95.5,-10.3	-	-	Favored (32.897%)	-	-	-
A 157		LEU	3.04	-	Favored (21.91%) General / -103.0,151.9	Favored (68.3%) *mt* chi angles: 303.8,176.7	0.08Å	Favored (20.286%)	-	-	-
A 158		VAL	2.56	-	Favored (39.32%) Ile or Val / -79.9,127.3	Favored (88.9%) *t* chi angles: 176.1	0.06Å	Favored (36.842%)	-	-	-
A 159		ALA	2.23	-	Favored (81.07%) General / -61.0,-37.0	-	0.03Å	Favored (41.547%)	-	-	-
A 160		THR	2.04	-	Favored (39.19%) General / -120.7,121.9	Favored (95.7%) *m* chi angles: 299.6	0.03Å	Favored (22.278%)	-	-	-
**#**	**Alt**	**Res**	**High B**	**Clash > 0.4Å**	**Ramachandran**	**Rotamer**	**Cβ deviation**	**CaBLAM**	**Bond lengths**	**Bond angles**	**Cis Peptides**
			Avg: 4.01	Clashscore: 1.78	Outliers: 1 of 256	Poor rotamers: 0 of 204	Outliers: 0 of 239	Outliers: 9 of 254	Outliers: 0 of 258	Outliers: 3 of 258	Non-Trans: 0 of 257

A<br>161		ASP	1.98	-	Favored<br>(29.34%)<br>General /<br>-69.4,126.3	Favored (32.5%)<br>*t70*<br>chi angles: 186.5,72	0.01Å	Favored<br>(45.08%)	-	-	-
A<br>162		VAL	1.98	-	Favored<br>(42.65%)<br>Pre-Pro /<br>-126.1,93.8	Favored (49.2%) *t*<br>chi angles: 181.6	0.02Å	Favored<br>(30.093%)<br>beta sheet	-	-	-
A<br>163		PRO	2.01	-	Favored<br>(91.89%)<br>Trans-Pro /<br>-58.9,146.8	Favored (59.2%)<br>*Cg_exo*<br>chi angles:<br>336,34.7,329.3	0.06Å	Favored<br>(44.958%)	-	-	-
A<br>164		GLU	2.04	-	Favored<br>(36.59%)<br>General /<br>-76.0,153.4	Favored (94.2%)<br>*mt-10*<br>chi angles:<br>294.9,181.5,5.6	0.03Å	Favored<br>(33.613%)	-	-	-
A<br>165		LEU	2.08	-	Favored<br>(57.5%)<br>General /<br>-63.8,136.5	Favored (95.7%) *mt*<br>chi angles: 294.1,173.7	0.09Å	Favored<br>(28.99%)	-	-	-
A<br>166		GLU	2.15	-	Favored<br>(13.75%)<br>General /<br>-95.6,-30.5	Favored (94.3%)<br>*mt-10*<br>chi angles:<br>296.4,182.7,358.8	0.02Å	CaBLAM<br>Outlier<br>(0.705%)	-	-	-
A<br>167		ARG	2.22	-	Allowed<br>(0.06%)<br>General /<br>60.0,165.3	Favored (96.1%)<br>*mtt-85*<br>chi angles:<br>296.6,184.3,186.2,278.4	0.02Å	CaBLAM<br>Disfavored<br>(1.566%)	-	-	-
A<br>168		THR	2.27	-	Favored<br>(51.48%)<br>General /<br>-58.8,131.6	Favored (85%) *m*<br>chi angles: 301.7	0.03Å	Favored<br>(8.033%)	-	-	-
A<br>169		THR	2.29	-	Favored<br>(68.21%)<br>Pre-Pro /<br>-55.4,128.7	Favored (82.6%) *m*<br>chi angles: 302.2	0.06Å	Favored<br>(45.337%)	-	-	-
A<br>170		PRO	2.25	-	Favored<br>(24.39%)<br>Trans-Pro /<br>-48.8,-33.3	Favored (89%)<br>*Cg_exo*<br>chi angles:<br>329.4,36.9,332.5	0.03Å	Favored<br>(83.471%)	-	-	-
A<br>171		LEU	2.14	-	Favored<br>(94.61%)<br>General /<br>-62.1,-40.0	Favored (58.6%) *tp*<br>chi angles: 181.4,59.4	0.03Å	Favored<br>(71.717%)<br>alpha helix	-	-	-
A<br>172		MET	1.99	-	Favored<br>(94.87%)<br>General /<br>-63.9,-39.8	Favored (96.5%)<br>*mtp*<br>chi angles:<br>291.4,170.6,67.5	0.06Å	Favored<br>(96.475%)<br>alpha helix	-	-	-
A<br>173		GLN	1.81	-	Favored<br>(94.98%)<br>General /<br>-64.3,-40.0	Favored (17.7%)<br>*mt0*<br>chi angles:<br>292.2,174.7,174.5	0.04Å	Favored<br>(98.873%)<br>alpha helix	-	-	-
A<br>174		LYS	1.64	-	Favored<br>(89.86%)<br>General /<br>-66.2,-39.9	Favored (97.4%)<br>*mttt*<br>chi angles:<br>289.4,176.4,181.8,177.4	0.01Å	Favored<br>(97.113%)<br>alpha helix	-	-	-
A<br>175		LYS	1.5	-	Favored<br>(91.64%)<br>General /<br>-65.3,-39.0	Favored (94.7%)<br>*mttt*<br>chi angles:<br>288.4,182,173.8,180.3	0.06Å	Favored<br>(93.357%)<br>alpha helix	-	-	-
A<br>176		VAL	1.38	-	Favored<br>(98.93%)<br>Ile or Val /<br>-61.0,-44.8	Favored (56.3%) *t*<br>chi angles: 170.3	0.04Å	Favored<br>(95.232%)<br>alpha helix	-	-	-
A<br>177		GLY	1.28	-	Favored<br>(35.88%)	-	-	Favored<br>(95.909%)	-	-	-

					Glycine / -56.1,-54.4			alpha helix			
A 178		GLN	1.2	0.55Å HA with A 178 GLN OE1	Favored (77.17%) General / -63.0,-34.3	Favored (12.9%) *mm-40* chi angles: 293.1,300.9,12.6	0.06Å	Favored (74.875%) alpha helix	-	-	-
A 179		ILE	1.13	-	Favored (95.82%) Ile or Val / -64.5,-45.4	Favored (92.4%) *mt* chi angles: 291.9,166.4	0.05Å	Favored (86.231%) alpha helix	-	-	-
A 180		LEU	1.07	-	Favored (94.89%) General / -62.6,-39.8	Favored (83.6%) *mt* chi angles: 290.3,173.5	0.03Å	Favored (82.561%) alpha helix	-	-	-
**#**	**Alt**	**Res**	**High B**	**Clash > 0.4Å**	**Ramachandran**	**Rotamer**	**Cβ deviation**	**CaBLAM**	**Bond lengths**	**Bond angles**	**Cis Peptides**
			Avg: 4.01	Clashscore: 1.78	Outliers: 1 of 256	Poor rotamers: 0 of 204	Outliers: 0 of 239	Outliers: 9 of 254	Outliers: 0 of 258	Outliers: 3 of 258	Non-Trans: 0 of 257
A 181		LEU	1.02	-	Favored (91.12%) General / -59.0,-42.9	Favored (53%) *tp* chi angles: 176.3,56.6	0.06Å	Favored (77.075%) alpha helix	-	-	-
A 182		ILE	0.99	-	Favored (97.91%) Ile or Val / -61.5,-44.0	Favored (98.8%) *mt* chi angles: 292.6,168.2	0.05Å	Favored (94.608%) alpha helix	-	-	-
A 183		GLY	0.98	-	Favored (94.13%) Glycine / -59.5,-38.8	-	-	Favored (97.984%) alpha helix	-	-	-
A 184		VAL	0.99	-	Favored (94.04%) Ile or Val / -63.3,-47.0	Favored (68.1%) *t* chi angles: 171.9	0.14Å	Favored (91.539%) alpha helix	-	-	-
A 185		SER	1.03	-	Favored (96.99%) General / -63.4,-43.6	Favored (71.8%) *m* chi angles: 295.9	0.10Å	Favored (96.707%) alpha helix	-	-	-
A 186		ALA	1.1	-	Favored (98.13%) General / -62.0,-41.5	-	0.03Å	Favored (96.574%) alpha helix	-	-	-
A 187		ALA	1.21	-	Favored (95.96%) General / -61.8,-40.7	-	0.03Å	Favored (90.725%) alpha helix	-	-	-
A 188		ALA	1.38	-	Favored (85.7%) General / -62.2,-37.5	-	0.05Å	Favored (82.965%) alpha helix	-	-	-
A 189		LEU	1.6	-	Favored (79.14%) General / -64.5,-34.8	Favored (91.2%) *mt* chi angles: 291.2,171.4	0.04Å	Favored (82.55%) alpha helix	-	-	-
A 190		LEU	1.89	-	Favored (62.15%) General / -74.9,-34.5	Favored (97%) *mt* chi angles: 294.1,172.8	0.04Å	Favored (74.365%) alpha helix	-	-	-
A 191		VAL	2.23	-	Favored (19.63%) Ile or Val / -80.9,-48.1	Favored (82.7%) *t* chi angles: 173.4	0.02Å	Favored (31.958%)	-	-	-
A 192		ASN	2.56	-	Favored (19.14%)	Favored (42.1%) *t0* chi angles: 187.6,10.7	0.02Å	Favored (8.623%)	-	-	-

#	Alt	Res	High B	Clash > 0.4Å	Ramachandran	Rotamer	Cβ deviation	CaBLAM	Bond lengths	Bond angles	Cis Peptides
					Pre-Pro / -142.9,72.3						
A 193		PRO	2.78	-	Favored (6%) Trans-Pro / -77.2,57.8	Favored (62%) *Cg_endo* chi angles: 31.6,322.8,27	0.06Å	CaBLAM Disfavored (2.848%)	-	-	-
A 194		CYS	2.83	-	Favored (11.4%) General / -90.9,168.8	Favored (28.1%) *p* chi angles: 65.2	0.03Å	Favored (25.576%)	-	-	-
A 195		VAL	2.67	-	Favored (82.96%) Ile or Val / -58.3,-42.1	Favored (63.7%) *t* chi angles: 171.3	0.02Å	Favored (60.394%)	-	-	-
A 196		THR	2.38	-	Favored (91.19%) General / -59.7,-45.7	Favored (91%) *m* chi angles: 298	0.01Å	Favored (95.354%) alpha helix	-	-	-
A 197		THR	2.02	-	Favored (99.47%) General / -62.4,-42.5	Favored (90.1%) *m* chi angles: 298.2	0.07Å	Favored (90.198%) alpha helix	-	-	-
A 198		VAL	1.69	-	Favored (99.51%) Ile or Val / -62.9,-44.3	Favored (63.8%) *t* chi angles: 171.3	0.07Å	Favored (87.78%) alpha helix	-	-	-
A 199		ARG	1.42	-	Favored (94.14%) General / -65.1,-42.1	Favored (97%) *mtt180* chi angles: 289.6,173.8,178.7,166.9	0.05Å	Favored (81.094%) alpha helix	-	-	-
A 200		GLU	1.22	-	Favored (66.53%) General / -63.9,-51.0	Favored (86.8%) *tt0* chi angles: 178.9,174.5,354.2	0.09Å	Favored (82.427%) alpha helix	-	-	-
**#**	**Alt**	**Res**	**High B**	**Clash > 0.4Å**	**Ramachandran**	**Rotamer**	**Cβ deviation**	**CaBLAM**	**Bond lengths**	**Bond angles**	**Cis Peptides**
			Avg: 4.01	Clashscore: 1.78	Outliers: 1 of 256	Poor rotamers: 0 of 204	Outliers: 0 of 239	Outliers: 9 of 254	Outliers: 0 of 258	Outliers: 3 of 258	Non-Trans: 0 of 257
A 201		ALA	1.08	-	Favored (87.8%) General / -61.8,-38.4	-	0.04Å	Favored (79.527%) alpha helix	-	-	-
A 202		GLY	0.99	-	Favored (49.51%) Glycine / -57.7,-52.9	-	-	Favored (90.618%) alpha helix	-	-	-
A 203		ILE	0.93	-	Favored (93.7%) Ile or Val / -63.4,-47.0	Favored (92.9%) *mt* chi angles: 291.6,167.2	0.09Å	Favored (76.742%) alpha helix	-	-	-
A 204		LEU	0.89	-	Favored (90.12%) General / -66.3,-40.5	Favored (93.8%) *mt* chi angles: 292.8,170.3	0.07Å	Favored (85.93%) alpha helix	-	-	-
A 205		ILE	0.87	-	Favored (88.09%) Ile or Val / -67.1,-44.4	Favored (97.1%) *mt* chi angles: 292.2,168.2	0.03Å	Favored (87.21%) alpha helix	-	-	-
A 206		SER	0.86	-	Favored (99.55%) General / -61.5,-43.0	Favored (70.7%) *m* chi angles: 296.2	0.07Å	Favored (98.46%) alpha helix	-	-	-
A 207		ALA	0.85	-	Favored (97.49%)	-	0.07Å	Favored (96.352%) alpha helix	-	-	-

					General / -60.7,-42.5						
A 208		ALA	0.85	-	Favored (85.7%) General / -65.7,-44.6	-	0.06Å	Favored (96.875%) alpha helix	-	-	-
A 209		LEU	0.86	-	Favored (93.35%) General / -65.5,-40.7	Favored (83.9%) *mt* chi angles: 289.6,172.3	0.02Å	Favored (98.66%) alpha helix	-	-	-
A 210		LEU	0.89	-	Favored (94.75%) General / -64.2,-39.8	Favored (89.2%) *mt* chi angles: 291.1,170.4	0.05Å	Favored (99.574%) alpha helix	-	-	-
A 211		THR	0.97	-	Favored (93.42%) General / -64.0,-44.2	Favored (95.7%) *m* chi angles: 299.6	0.03Å	Favored (92.16%) alpha helix	-	-	-
A 212		LEU	1.13	-	Favored (93.39%) General / -65.2,-40.0	Favored (88.6%) *mt* chi angles: 291.2,169.6	0.06Å	Favored (88.88%) alpha helix	-	-	-
A 213		TRP	1.4	-	Favored (70.58%) General / -71.3,-40.8	Favored (46.3%) *m-10* chi angles: 290.8,336.8	0.04Å	Favored (72.656%) alpha helix	-	-	-
A 214		ASP	1.79	-	Favored (39.62%) General / -100.1,11.3	Favored (65.6%) *m-30* chi angles: 291.8,315.8	0.04Å	Favored (39.206%) alpha helix	-	-	-
A 215		ASN	2.3	-	Favored (28.03%) General / 54.8,44.7	Favored (85.1%) *m-40* chi angles: 295.9,313.8	0.02Å	CaBLAM Outlier (0.338%) try alpha helix	-	-	-
A 216		GLY	2.85	-	Favored (17.48%) Glycine / 168.5,160.9	-	-	Favored (9.312%)	-	-	-
A 217		ALA	3.26	-	Favored (55.13%) General / -77.2,-7.0	-	0.06Å	CaBLAM Outlier (0.463%)	-	-	-
A 218		ILE	3.39	0.81Å O with A 218 ILE HG22	Allowed (0.39%) Ile or Val / 45.7,48.5	Favored (87.9%) *mt* chi angles: 297.9,167.2	0.12Å	CaBLAM Outlier (0.183%)	-	-	-
A 219		ALA	3.21	-	Allowed (0.06%) General / 59.0,-95.5	-	0.06Å	CaBLAM Outlier (0.02%)	-	-	-
A 220		VAL	2.86	0.42Å O with A 220 VAL HG22	Favored (4.54%) Ile or Val / -52.6,-23.2	Favored (8.1%) *p* chi angles: 67.4	0.05Å	Favored (38.952%)	-	-	-
**#**	**Alt**	**Res**	**High B**	**Clash > 0.4Å**	**Ramachandran**	**Rotamer**	**Cβ deviation**	**CaBLAM**	**Bond lengths**	**Bond angles**	**Cis Peptides**
			Avg: 4.01	Clashscore: 1.78	Outliers: 1 of 256	Poor rotamers: 0 of 204	Outliers: 0 of 239	Outliers: 9 of 254	Outliers: 0 of 258	Outliers: 3 of 258	Non-Trans: 0 of 257
A 221		TRP	2.49	0.41Å CE3 with A 221 TRP HA	Favored (19.55%) General / -98.6,17.3	Favored (40.6%) *m100* chi angles: 300.2,122.4	0.08Å	Favored (32.501%)	-	-	-
A 222		ASN	2.16	-	Favored (10.14%)	Favored (8.6%) *t0* chi angles: 206.9,329.5	0.01Å	Favored (26.184%)	-	-	-

					General / -71.1,171.9							
A 223		SER	1.9	-	Favored (98.75%) General / -62.5,-43.5	Favored (55.8%) *m* chi angles: 292.8	0.04Å	Favored (62.944%)	-	-	-	
A 224		THR	1.7	-	Favored (86.79%) General / -62.0,-47.0	Favored (93.3%) *m* chi angles: 297.6	0.02Å	Favored (75.249%) alpha helix	-	-	-	
A 225		THR	1.56	-	Favored (26.48%) General / -74.6,-48.5	Favored (98.3%) *m* chi angles: 300.1	0.06Å	Favored (70.5%) alpha helix	-	-	-	
A 226		ALA	1.45	-	Favored (85.26%) General / -60.8,-38.4	-	0.03Å	Favored (76.309%) alpha helix	-	-	-	
A 227		THR	1.37	-	Favored (58.91%) General / -72.4,-45.6	Favored (70.5%) *m* chi angles: 303	0.08Å	Favored (78.28%) alpha helix	-	-	-	
A 228		GLY	1.31	-	Favored (95.22%) Glycine / -60.8,-38.4	-	-	Favored (95.416%) alpha helix	-	-	-	
A 229		LEU	1.29	-	Favored (96.81%) General / -63.6,-40.4	Favored (89.4%) *mt* chi angles: 290.8,171.4	0.04Å	Favored (95.392%) alpha helix	-	-	-	
A 230		CYS	1.3	-	Favored (90.62%) General / -64.2,-38.3	Favored (93.6%) *m* chi angles: 291.9	0.06Å	Favored (97.946%) alpha helix	-	-	-	
A 231		HIS	1.34	-	Favored (94.5%) General / -64.8,-40.0	Favored (41.3%) *m-70* chi angles: 279.7,299.1	0.03Å	Favored (93.059%) alpha helix	-	-	-	
A 232		VAL	1.39	-	Favored (91.08%) Ile or Val / -62.0,-41.5	Favored (60.4%) *t* chi angles: 170.8	0.06Å	Favored (87.532%) alpha helix	-	-	-	
A 233		ILE	1.46	-	Favored (68.01%) Ile or Val / -58.0,-38.6	Favored (88.8%) *mt* chi angles: 290.9,168.1	0.06Å	Favored (87.336%) alpha helix	-	-	-	
A 234		ARG	1.52	-	Favored (10.57%) General / -82.6,8.9	Favored (80%) *mtp85* chi angles: 295.2,180.1,67.3,93.5	0.05Å	Favored (41.717%)	-	-	-	
A 235		GLY	1.58	-	Favored (86.02%) Glycine / 85.2,5.1	-	-	Favored (84.404%)	-	-	-	
A 236		ASN	1.61	-	Favored (6.57%) General / -89.1,83.9	Favored (57.8%) *t0* chi angles: 184.7,336.5	0.13Å	Favored (14.953%)	-	-	-	
A 237		TRP	1.61	-	Favored (64.13%) General / -57.1,-30.1	Favored (68.8%) *p-90* chi angles: 69.6,268.8	0.06Å	Favored (36.864%)	-	-	-	
A 238		LEU	1.59	-	Favored (71.86%) General / -69.2,-44.5	Favored (54.3%) *tp* chi angles: 182.3,57.6	0.09Å	Favored (71.318%) alpha helix	-	-	-	

A 239		ALA	1.56	-	Favored (79.63%) General / -61.0,-36.5	-	0.05Å	Favored (83.824%) alpha helix	-	-	-
A 240		GLY	1.54	-	Favored (86.8%) Glycine / -59.8,-35.5	-	-	Favored (87.61%) alpha helix	-	-	-
**#**	**Alt**	**Res**	**High B**	**Clash > 0.4Å**	**Ramachandran**	**Rotamer**	**Cβ deviation**	**CaBLAM**	**Bond lengths**	**Bond angles**	**Cis Peptides**
			Avg: 4.01	Clashscore: 1.78	Outliers: 1 of 256	Poor rotamers: 0 of 204	Outliers: 0 of 239	Outliers: 9 of 254	Outliers: 0 of 258	Outliers: 3 of 258	Non-Trans: 0 of 257
A 241		ALA	1.54	-	Favored (75.5%) General / -60.3,-35.4	-	0.03Å	Favored (69.611%) alpha helix	-	-	-
A 242		SER	1.57	-	Favored (89.43%) General / -59.3,-46.1	Favored (39.7%) *t* chi angles: 181.1	0.08Å	Favored (77.248%) alpha helix	-	-	-
A 243		ILE	1.63	-	Favored (99.72%) Ile or Val / -62.7,-44.8	Favored (91%) *mt* chi angles: 291.7,169.8	0.06Å	Favored (93.884%) alpha helix	-	-	-
A 244		ALA	1.72	-	Favored (86.7%) General / -58.9,-41.3	-	0.04Å	Favored (88.654%) alpha helix	-	-	-
A 245		TRP	1.87	-	Favored (64.94%) General / -72.2,-30.4	Favored (74.4%) *m100* chi angles: 280.7,109.8	0.09Å	Favored (79.886%) alpha helix	-	-	-
A 246		THR	2.09	-	Favored (86.22%) General / -63.1,-46.6	Favored (95.3%) *m* chi angles: 299.5	0.07Å	Favored (77.879%) alpha helix	-	-	-
A 247		LEU	2.4	-	Favored (92.57%) General / -65.1,-39.1	Favored (99.5%) *mt* chi angles: 292.8,172.5	0.05Å	Favored (85.644%) alpha helix	-	-	-
A 248		ILE	2.84	-	Favored (86.51%) Ile or Val / -65.5,-47.4	Favored (95.3%) *mt* chi angles: 292.5,166.3	0.01Å	Favored (82.249%) alpha helix	-	-	-
A 249		LYS	3.44	-	Favored (71.76%) General / -55.4,-40.7	Favored (86%) *tttt* chi angles: 181.8,178,178.4,182.2	0.02Å	Favored (73.451%) alpha helix	-	-	-
A 250		ASN	4.21	-	Favored (52.4%) General / -92.5,5.0	Favored (88.4%) *m-40* chi angles: 291.4,322.3	0.03Å	Favored (43.028%) alpha helix	-	-	-
A 251		ALA	5.15	-	Favored (69.41%) General / -59.6,-31.3	-	0.03Å	Favored (46.583%) alpha helix	-	-	-
A 252		ASP	6.19	-	Favored (59.76%) General / -81.6,-8.9	Favored (86.4%) *m-30* chi angles: 291.1,334.8	0.01Å	Favored (56.715%)	-	-	-
A 253		LYS	7.25	-	Favored (67.59%) Pre-Pro / -79.0,131.2	Favored (86.6%) *tttt* chi angles: 186.1,177,181.1,179.6	0.04Å	Favored (34.407%)	-	-	-
A 254		PRO	8.2	-	Favored (18.33%)	Favored (89.6%) *Cg_exo*	0.04Å	Favored (66.845%)	-	-	-

					Trans-Pro / -48.9,-30.7	chi angles: 329.8,37.1,332.1						
A 255		ALA	8.96	-	Favored (64.23%) General / -60.2,-23.8	-	0.05Å	Favored (58.898%) alpha helix	-	-	-	
A 256		CYS	9.54	-	Favored (39.85%) General / -102.3,5.3	Favored (72.1%) *m* chi angles: 297.8	0.08Å	Favored (49.791%)	-	-	-	
A 257		LYS	9.95	-	Favored (22.07%) General / -106.2,153.5	Favored (96.1%) *mttt* chi angles: 297.3,183.1,184.3,180.3	0.02Å	-	-	-	-	
A 258		ARG	10.2	-	-	Favored (98.7%) *mtt-85* chi angles: 294.4,181.6,183.8,274.2	0.04Å	-	-	-	-	

About MolProbity | Website for the Richardson Lab | Using ecloud x-H | Internal reference 4.5.2
