# Supplementary material for: Integrative proteome-wide structural analysis and high-throughput docking identify broad-spectrum antiviral scaffolds against Zika, Yellow Fever, West Nile, Saint Louis encephalitis, and Usutu viruses
Source: Front Cell Infect Microbiol. 2026 Apr 30;16:1723132. doi: 10.3389/fcimb.2026.1723132 (PMC13171538; doi:10.3389/fcimb.2026.1723132)
Supplement: Supplementary file 4 [file DataSheet4.zip › USUV/USU_NS5/Mol_probity_Files/USU_NS5_1FH-multi.table.pdf]

Viewing USU_NS5_1FH-multi.table

Duke Biochemistry
Duke University School of Medicine

When finished, you should close this window.

Hint: Use File | Save As... to save a copy of this page.

All-Atom Contacts	Clashscore, all atoms:	1.59		99th percentile* (N=1784, all resolutions)
	Clashscore is the number of serious steric overlaps (> 0.4 Å) per 1000 atoms.			
Protein Geometry	Poor rotamers	0	0.00%	Goal: <0.3%
	Favored rotamers	771	100.00%	Goal: >98%
	Ramachandran outliers	4	0.44%	Goal: <0.05%
	Ramachandran favored	879	97.34%	Goal: >98%
	Rama distribution Z-score	0.26 ± 0.27		Goal: abs(Z score) < 2
	MolProbity score^	1.03		100th percentile* (N=27675, 0Å - 99Å)
	Cβ deviations >0.25Å	0	0.00%	Goal: 0
	Bad bonds:	16 / 7459	0.21%	Goal: 0%
	Bad angles:	19 / 10076	0.19%	Goal: <0.1%
Peptide Omegas	Cis Prolines:	0 / 35	0.00%	Expected: ≤1 per chain, or ≤5%
	Cis nonProlines:	1 / 869	0.12%	Goal: <0.05%
Low-resolution Criteria	CaBLAM outliers	21	2.3%	Goal: <1.0%
	CA Geometry outliers	1	0.11%	Goal: <0.5%
Additional validations	Chiral volume outliers	0/1047		
	Waters with clashes	0/0	0.00%	See UnDowser table for details

In the two column results, the left column gives the raw count, right column gives the percentage.

* 100th percentile is the best among structures of comparable resolution; 0th percentile is the worst. For clashscore the comparative set of structures was selected in 2004, for MolProbity score in 2006.

^ MolProbity score combines the clashscore, rotamer, and Ramachandran evaluations into a single score, normalized to be on the same scale as X-ray resolution.

Key to table colors and cutoffs here:

#	Alt	Res	High B	Clash > 0.4Å	Ramachandran	Rotamer	Cβ deviation	CaBLAM	Bond lengths	Bond angles	Cis Peptides
			Avg: 0.92	Clashscore: 1.59	Outliers: 4 of 903	Poor rotamers: 0 of 771	Outliers: 0 of 828	Outliers: 22 of 901	Outliers: 13 of 905	Outliers: 19 of 905	Non-Trans: 1 of 904
A 1		GLY	8.36	-	-	-	-	-	-	-	-
A 2		ARG	7.06	-	Favored (6.52%)<br>Pre-Pro / -40.6,-52.0	Favored (82.1%)<br>*ttt180*<br>chi angles: 179.4,178.1,177.4,181.4	0.07Å	-	-	-	-
A 3		PRO	5.52	-	Favored (65.73%)<br>Trans-Pro / -63.2,-18.6	Favored (36.9%)<br>*Cg_endo*<br>chi angles: 22.7,324.7,32.7	0.01Å	Favored (51.685%)	-	-	-
A 4		GLY	4.03	-	Favored (87.81%)<br>Glycine / -85.2,-2.5	-	-	Favored (64.526%)<br>three-ten	-	-	-
A 5		GLY	2.82	-	Favored (57.84%)<br>Glycine / -61.0,-20.5	-	-	Favored (43.663%)	-	-	-
A 6		ARG	1.97	-	Favored (82.44%)<br>General / -65.7,-36.0	Favored (42.7%)<br>*tpt170*<br>chi angles: 181.9,64.6,171.7,168.3	0.06Å	Favored (6.582%)	-	-	-

A 7		THR	1.42	-	Allowed (0.06%) General / 71.6,142.5	Favored (32.8%) *p* chi angles: 69	0.09Å	CaBLAM Disfavored (3.52%)	-	OUTLIER(S) worst is C-N-CA: 4.9 σ	-
A 8		LEU	1.1	-	Favored (80.63%) General / -61.3,-36.6	Favored (93.2%) *mt* chi angles: 291.4,172	0.11Å	Favored (62.256%)	-	-	-
A 9		GLY	0.93	-	Favored (96.97%) Glycine / -61.9,-39.0	-	-	Favored (90.745%) alpha helix	-	-	-
A 10		GLU	0.84	-	Favored (80.95%) General / -68.3,-37.9	Favored (63%) *tp30* chi angles: 186.6,64.4,18.2	0.07Å	Favored (86.617%) alpha helix	-	-	-
A 11		GLN	0.82	-	Favored (86.5%) General / -61.4,-38.3	Favored (96.3%) *mt0* chi angles: 290.8,178.8,338.5	0.06Å	Favored (86.695%) alpha helix	-	-	-
A 12		TRP	0.82	-	Favored (79.82%) General / -62.6,-48.4	Favored (47.2%) *t60* chi angles: 183.3,74.6	0.06Å	Favored (94.057%) alpha helix	-	-	-
A 13		LYS	0.83	-	Favored (84.4%) General / -61.4,-37.7	Favored (22.2%) *mmmt* chi angles: 289.3,287.4,282.2,183.2	0.05Å	Favored (88.442%) alpha helix	-	-	-
A 14		GLU	0.85	-	Favored (96.81%) General / -62.6,-44.2	Favored (90.8%) *tt0* chi angles: 182.2,174.3,357.5	0.03Å	Favored (93.845%) alpha helix	-	-	-
A 15		LYS	0.87	-	Favored (98.48%) General / -63.1,-40.7	Favored (62.4%) *mttm* chi angles: 288.7,176.2,184.4,294.5	0.03Å	Favored (96.418%) alpha helix	-	-	-
A 16		LEU	0.89	-	Favored (98.84%) General / -62.6,-41.3	Favored (77.5%) *mt* chi angles: 287.9,168.9	0.05Å	Favored (87.235%) alpha helix	-	-	-
A 17		ASN	0.9	-	Favored (68.86%) General / -70.4,-31.6	Favored (93.3%) *m-40* chi angles: 286.3,336.1	0.08Å	Favored (78.564%) alpha helix	-	-	-
A 18		GLY	0.92	-	Favored (90.16%) Glycine / -63.9,-34.5	-	-	Favored (38.109%)	-	-	-
A 19		LEU	0.94	-	Favored (55.81%) General / -66.0,145.5	Favored (79.4%) *mt* chi angles: 291.5,176.9	0.10Å	Favored (23.918%)	-	-	-
A 20		SER	0.94	-	Favored (30.72%) General / -66.3,157.8	Favored (94.8%) *p* chi angles: 66.2	0.09Å	Favored (51.555%)	-	-	-
**#**	**Alt**	**Res**	**High B**	**Clash > 0.4Å**	**Ramachandran**	**Rotamer**	**Cβ deviation**	**CaBLAM**	**Bond lengths**	**Bond angles**	**Cis Peptides**
			Avg: 0.92	Clashscore: 1.59	Outliers: 4 of 903	Poor rotamers: 0 of 771	Outliers: 0 of 828	Outliers: 22 of 901	Outliers: 13 of 905	Outliers: 19 of 905	Non-Trans: 1 of 904
A 21		LYS	0.94	-	Favored (70.82%) General / -54.9,-41.3	Favored (87.4%) *tttt* chi angles: 181.8,176,178.4,178.6	0.02Å	Favored (66.131%)	-	-	-
A 22		GLU	0.93	-	Favored (78.59%)	Favored (90.6%) *tt0* chi angles: 179.2,177.7,358	0.01Å	Favored (75.49%) alpha helix	-	-	-

					General / -60.0,-49.2						
A 23		ASP	0.91	-	Favored (69.44%) General / -71.4,-33.4	Favored (99.6%) *m-30* chi angles: 287.7,347.2	0.09Å	Favored (70.956%) alpha helix	-	-	-
A 24		PHE	0.87	-	Favored (68.08%) General / -55.7,-51.2	Favored (83.9%) *t80* chi angles: 175,82.5	0.07Å	Favored (76.76%) alpha helix	-	-	-
A 25		LEU	0.84	-	Favored (92.4%) General / -64.8,-38.9	Favored (98.5%) *mt* chi angles: 292.8,172.9	0.06Å	Favored (81.131%) alpha helix	-	-	-
A 26		LYS	0.8	-	Favored (93.35%) General / -62.7,-39.2	Favored (91.5%) *mttt* chi angles: 289,174.8,181.9,170.4	0.04Å	Favored (75.196%) alpha helix	-	-	-
A 27		TYR	0.77	-	Favored (52.58%) General / -77.5,-36.4	Favored (41.2%) *m-80* chi angles: 285.6,115.3	0.03Å	Favored (55.393%) alpha helix	-	-	-
A 28		ARG	0.75	-	Favored (67.92%) General / -54.2,-40.9	Favored (61.2%) *ttp-170* chi angles: 184.6,178.1,71.6,182.8	0.06Å	Favored (61.186%) alpha helix	-	-	-
A 29		LYS	0.75	-	Favored (23.48%) General / -106.3,17.4	Favored (5.8%) *mmmm* chi angles: 300.5,297.2,288.3,291.8	0.01Å	Favored (8.554%) alpha helix	-	-	-
A 30		GLU	0.75	-	Allowed (0.41%) General / -85.2,-74.0	Favored (81.5%) *tt0* chi angles: 180.8,183.9,8.5	0.12Å	CaBLAM Disfavored (1.176%) try alpha helix	-	-	-
A 31		ALA	0.76	-	Favored (8.45%) General / -89.0,71.6	-	0.06Å	CaBLAM Disfavored (1.096%)	-	-	-
A 32		ILE	0.78	-	Favored (20.28%) Ile or Val / -143.8,147.0	Favored (21.9%) *tt* chi angles: 191,168.9	0.14Å	Favored (28.405%)	-	-	-
A 33		THR	0.81	-	Favored (40.58%) General / -95.2,126.5	Favored (90.9%) *m* chi angles: 298.9	0.07Å	Favored (55.688%) beta sheet	-	-	-
A 34		GLU	0.85	-	Favored (52.17%) General / -107.9,133.5	Favored (85%) *tt0* chi angles: 184.9,174.3,9.9	0.08Å	Favored (51.979%) beta sheet	-	-	-
A 35		VAL	0.9	-	Favored (39.03%) Ile or Val / -83.0,123.9	Favored (70.2%) *t* chi angles: 178.7	0.11Å	Favored (43.279%) beta sheet	-	-	-
A 36		ASP	0.96	-	Favored (6.88%) General / -82.8,90.1	Favored (65.5%) *t0* chi angles: 183.4,345	0.06Å	Favored (35.811%) beta sheet	-	-	-
A 37		ARG	1.01	-	Favored (47.05%) General / -88.9,4.7	Favored (83.3%) *mtp180* chi angles: 295.2,176.1,71.2,196.5	0.10Å	Favored (10.816%)	-	-	-
A 38		SER	1.05	-	Favored (72.69%) General / -62.5,-31.9	Favored (91.5%) *p* chi angles: 66.6	0.02Å	Favored (51.803%)	-	-	-
A 39		ALA	1.09	-	Favored (92.96%)	-	0.03Å	Favored (95.144%)	-	-	-

					General / -65.5,-40.5			alpha helix			
A 40		ALA	1.1	-	Favored (87.36%) General / -67.0,-40.9	-	0.03Å	Favored (95.849%) alpha helix	-	-	-

#	Alt	Res	High B	Clash > 0.4Å	Ramachandran	Rotamer	Cβ deviation	CaBLAM	Bond lengths	Bond angles	Cis Peptides
			Avg: 0.92	Clashscore: 1.59	Outliers: 4 of 903	Poor rotamers: 0 of 771	Outliers: 0 of 828	Outliers: 22 of 901	Outliers: 13 of 905	Outliers: 19 of 905	Non-Trans: 1 of 904
A 41		ARG	1.11	-	Favored (96.42%) General / -64.5,-40.9	Favored (97.3%) *mtt180* chi angles: 288.7,174.2,177.9,167.2	0.03Å	Favored (95.763%) alpha helix	-	-	-
A 42		LYS	1.12	-	Favored (78.14%) General / -67.4,-44.8	Favored (18.3%) *tptp* chi angles: 184.1,65.2,163.7,70.7	0.01Å	Favored (86.096%) alpha helix	-	-	-
A 43		ALA	1.16	-	Favored (88.69%) General / -58.8,-42.2	-	0.05Å	Favored (83.339%) alpha helix	-	-	-
A 44		ARG	1.21	-	Favored (86.11%) General / -67.3,-40.4	Favored (98.8%) *mtm-85* chi angles: 289.6,192.7,300.9,275.8	0.04Å	Favored (90.552%) alpha helix	-	-	-
A 45		ARG	1.28	-	Favored (74.48%) General / -62.8,-33.1	Favored (96.4%) *mtt180* chi angles: 287.9,172.5,179.9,169.4	0.01Å	Favored (76.382%) alpha helix	-	-	-
A 46		ASP	1.34	-	Favored (59.1%) General / -85.2,-4.3	Favored (91.2%) *m-30* chi angles: 291.5,338	0.06Å	Favored (55.138%)	-	-	-
A 47		GLY	1.39	-	Favored (82.19%) Glycine / 79.8,13.5	-	-	Favored (85.618%)	-	-	-
A 48		ASN	1.42	-	Favored (8.3%) General / -84.5,82.7	Favored (56%) *t0* chi angles: 189.3,343.6	0.06Å	Favored (14.377%) beta sheet	-	-	-
A 49		LYS	1.41	-	Favored (23.73%) General / -73.6,-2.7	Favored (98.7%) *mttt* chi angles: 291.9,179.6,178.8,174.9	0.04Å	Favored (8.969%)	-	-	-
A 50		THR	1.35	-	Favored (22.69%) General / -109.9,5.7	Favored (67.5%) *p* chi angles: 58.7	0.03Å	Favored (57.317%)	-	-	-
A 51		GLY	1.25	-	Favored (66.03%) Glycine / -57.9,-32.3	-	-	CA Geom Outlier (0.438%)	-	-	-
A 52		GLY	1.14	-	Favored (78.07%) Glycine / 86.6,-7.7	-	-	Favored (26.83%) beta sheet	-	-	-
A 53		HIS	1.03	-	Favored (98.58%) Pre-Pro / -67.7,146.8	Favored (96.4%) *m-70* chi angles: 299.3,294.9	0.09Å	Favored (31.249%) beta sheet	-	-	-
A 54		PRO	0.93	-	Favored (52.4%) Trans-Pro / -69.5,159.7	Favored (77%) *Cg_endo* chi angles: 28.3,324.4,28.2	0.03Å	Favored (73.208%)	-	-	-
A 55		VAL	0.84	-	Favored (9.54%) Ile or Val / -87.9,-15.0	Favored (24.3%) *m* chi angles: 301.3	0.04Å	Favored (25.657%)	-	-	-

A 56		SER	0.77	-	Favored (26.49%) General / -155.4,150.3	Favored (44.5%) *t* chi angles: 179	0.05Å	Favored (22.206%)	-	-	-
A 57		ARG	0.73	-	Favored (50.75%) General / -52.8,-33.9	Favored (56.6%) *ttt90* chi angles: 187.4,182,186,87.9	0.09Å	Favored (51.47%) alpha helix	-	-	-
A 58		GLY	0.7	0.40Å O with A 87 TRP NE1	Favored (71.58%) Glycine / -57.8,-34.6	-	-	Favored (77.449%) alpha helix	-	-	-
A 59		SER	0.68	-	Favored (95.39%) General / -60.5,-45.0	Favored (45.4%) *t* chi angles: 179.3	0.06Å	Favored (77.292%) alpha helix	-	-	-
A 60		ALA	0.67	-	Favored (77.4%) General / -60.5,-36.1	-	0.04Å	Favored (79.89%) alpha helix	-	-	-
**#**	**Alt**	**Res**	**High B**	**Clash > 0.4Å**	**Ramachandran**	**Rotamer**	**Cβ deviation**	**CaBLAM**	**Bond lengths**	**Bond angles**	**Cis Peptides**
			Avg: 0.92	Clashscore: 1.59	Outliers: 4 of 903	Poor rotamers: 0 of 771	Outliers: 0 of 828	Outliers: 22 of 901	Outliers: 13 of 905	Outliers: 19 of 905	Non-Trans: 1 of 904
A 61		LYS	0.67	-	Favored (91.27%) General / -65.8,-41.8	Favored (97.9%) *mttt* chi angles: 291,179.5,183.1,176	0.03Å	Favored (86.447%) alpha helix	-	-	-
A 62		LEU	0.68	-	Favored (88.7%) General / -66.0,-43.2	Favored (57.6%) *tp* chi angles: 181.7,59.1	0.06Å	Favored (79.578%) alpha helix	-	-	-
A 63		ARG	0.7	-	Favored (73.1%) General / -54.4,-47.1	Favored (2.9%) *tmm160* chi angles: 179.9,266.2,311,184.1	0.05Å	Favored (83.515%) alpha helix	-	-	-
A 64		TRP	0.73	-	Favored (94.51%) General / -60.4,-45.2	Favored (86.5%) *t60* chi angles: 184.4,86.5	0.03Å	Favored (99.375%) alpha helix	-	-	-
A 65		MET	0.78	-	Favored (71.67%) General / -64.0,-30.6	Favored (80.4%) *mtm* chi angles: 289,187.6,282.3	0.08Å	Favored (74.259%) alpha helix	-	-	-
A 66		VAL	0.83	-	Favored (50.25%) Ile or Val / -73.9,-45.1	Favored (81.3%) *t* chi angles: 173.2	0.12Å	Favored (72.864%) alpha helix	-	-	-
A 67		GLU	0.88	-	Favored (72.48%) General / -61.2,-32.7	Favored (92.6%) *mt-10* chi angles: 286.9,178.7,342.2	0.06Å	Favored (77.05%) alpha helix	-	-	-
A 68		ARG	0.9	-	Favored (35.98%) General / -84.5,3.5	Favored (32.9%) *mmm160* chi angles: 298,285.2,292,176	0.07Å	Favored (52.949%)	-	-	-
A 69		GLN	0.9	-	Favored (16.22%) General / 60.6,40.7	Favored (54.5%) *mt0* chi angles: 298.3,186.1,76.7	0.04Å	Favored (21.476%)	-	-	-
A 70		PHE	0.87	-	Favored (11.69%) General / -95.2,-35.8	Favored (23.7%) *m-10* chi angles: 296,325.9	0.01Å	CaBLAM Disfavored (3.165%)	-	-	-
A 71		VAL	0.83	-	Favored (68.31%)	Favored (52.1%) *t* chi angles: 181.1	0.05Å	Favored (23.686%)	-	-	-

#	Alt	Res	High B	Clash > 0.4Å	Ramachandran	Rotamer	Cβ deviation	CaBLAM	Bond lengths	Bond angles	Cis Peptides
					Ile or Val / -126.3,133.6						
A 72		LYS	0.78	-	Favored (72.65%) Pre-Pro / -131.8,77.3	Favored (99.2%) *mttt* chi angles: 295,182.3,178.1,180.6	0.01Å	Favored (5.116%)	-	-	-
A 73		PRO	0.73	-	Favored (76.64%) Trans-Pro / -54.7,138.6	Favored (66.2%) *Cg_exo* chi angles: 335.6,38.1,324.3	0.04Å	Favored (14.561%)	-	-	-
A 74		ILE	0.69	-	Favored (15.18%) Ile or Val / -148.7,149.3	Favored (20.9%) *tt* chi angles: 190.3,165	0.08Å	Favored (11.856%)	-	-	-
A 75		GLY	0.66	-	Favored (33.27%) Glycine / 60.6,-123.6	-	-	Favored (25.026%)	-	-	-
A 76		LYS	0.63	-	Favored (32.33%) General / -90.7,121.1	Favored (82.9%) *tttt* chi angles: 181.5,181.3,176.9,185.8	0.05Å	Favored (13.047%)	-	-	-
A 77		VAL	0.62	-	Favored (69.27%) Ile or Val / -113.5,129.4	Favored (56.4%) *t* chi angles: 180.4	0.08Å	Favored (68.039%)	-	-	-
A 78		VAL	0.61	-	Favored (70.02%) Ile or Val / -113.4,128.9	Favored (94.1%) *t* chi angles: 175.8	0.03Å	Favored (72.586%) beta sheet	-	-	-
A 79		ASP	0.61	-	Favored (11.1%) General / -116.2,104.0	Favored (66.4%) *t0* chi angles: 181.6,355.3	0.06Å	Favored (59.9%) beta sheet	-	OUTLIER(S) worst is CA-CB-CG: 4.1 σ	-
A 80		LEU	0.62	-	Favored (44.13%) General / -85.9,2.9	Favored (95.4%) *mt* chi angles: 296.6,176	0.02Å	Favored (5.876%)	-	-	-
**#**	**Alt**	**Res**	**High B**	**Clash > 0.4Å**	**Ramachandran**	**Rotamer**	**Cβ deviation**	**CaBLAM**	**Bond lengths**	**Bond angles**	**Cis Peptides**
			Avg: 0.92	Clashscore: 1.59	Outliers: 4 of 903	Poor rotamers: 0 of 771	Outliers: 0 of 828	Outliers: 22 of 901	Outliers: 13 of 905	Outliers: 19 of 905	Non-Trans: 1 of 904
A 81		GLY	0.63	-	Allowed (0.87%) Glycine / -140.3,47.6	-	-	Favored (16.109%)	-	-	-
A 82		CYS	0.64	-	Favored (69.22%) General / -65.4,-28.4	Favored (13.9%) *p* chi angles: 73	0.04Å	CaBLAM Outlier (0.236%)	-	-	-
A 83		GLY	0.65	-	Favored (47.05%) Glycine / 57.9,-128.2	-	-	Favored (39.747%)	-	-	-
A 84		ARG	0.65	-	Favored (66.03%) General / -65.6,-19.7	Favored (97.8%) *mtt-85* chi angles: 292.4,177.2,185.8,274.1	0.02Å	CaBLAM Disfavored (2.58%)	-	-	-
A 85		GLY	0.64	-	Favored (11.14%) Glycine / 113.6,12.8	-	-	Favored (63.507%)	-	-	-
A 86		GLY	0.64	-	Favored (9.01%) Glycine / -49.4,-57.7	-	-	Favored (16.469%)	-	-	-
A 87		TRP	0.63	0.40Å NE1 with A	Favored (65.33%)	Favored (26.6%) *m-10*	0.04Å	Favored (62.884%)	-	-	-

				58 GLY O	General / -72.1,-30.6	chi angles: 286.9,23.2		alpha helix			
A 88		SER	0.63	-	Favored (93.43%) General / -65.2,-42.4	Favored (55.2%) *m* chi angles: 292.6	0.03Å	Favored (79.328%) alpha helix	-	-	-
A 89		TYR	0.63	-	Favored (67.72%) General / -72.6,-38.9	Favored (31.2%) *m-80* chi angles: 287.2,124.3	0.04Å	Favored (83.814%) alpha helix	-	-	-
A 90		TYR	0.64	-	Favored (74.8%) General / -62.9,-49.4	Favored (84.4%) *t80* chi angles: 182.6,77.4	0.06Å	Favored (83.279%) alpha helix	-	-	-
A 91		ALA	0.66	-	Favored (74.65%) General / -57.9,-37.8	-	0.04Å	Favored (74.113%) alpha helix	-	-	-
A 92		ALA	0.69	-	Favored (64.96%) General / -60.7,-23.6	-	0.05Å	Favored (67.799%) alpha helix	-	-	-
A 93		THR	0.72	-	Favored (58.17%) General / -89.8,-3.3	Favored (61.2%) *p* chi angles: 63.8	0.03Å	Favored (57.553%)	-	-	-
A 94		LEU	0.74	-	Favored (31.23%) General / -88.7,138.7	Favored (83.5%) *mt* chi angles: 301.5,177.5	0.06Å	Favored (34.504%)	-	-	-
A 95		LYS	0.74	-	Favored (67.01%) General / -60.3,-27.1	Favored (97.3%) *mttt* chi angles: 290.1,179.8,179.7,179	0.02Å	Favored (34.102%)	-	-	-
A 96		GLY	0.73	-	Favored (87.12%) Glycine / -83.7,-6.4	-	-	Favored (65.433%)	-	-	-
A 97		VAL	0.7	-	Favored (31.53%) Ile or Val / -94.7,134.6	Favored (82.3%) *t* chi angles: 176.8	0.07Å	Favored (18.81%)	-	-	-
A 98		GLN	0.66	-	Favored (8.31%) General / -107.2,-31.2	Favored (87.6%) *mm-40* chi angles: 293.9,290.9,304.7	0.05Å	Favored (27.701%)	-	-	-
A 99		GLU	0.63	-	Favored (15.7%) General / -155.4,138.7	Favored (90.4%) *tt0* chi angles: 184.5,175,1	0.02Å	Favored (32.016%)	-	-	-
A 100		VAL	0.61	-	Favored (73.35%) Ile or Val / -125.1,129.1	Favored (60.3%) *t* chi angles: 179.9	0.07Å	Favored (71.545%)	-	-	-
**#**	**Alt**	**Res**	**High B**	**Clash > 0.4Å**	**Ramachandran**	**Rotamer**	**Cβ deviation**	**CaBLAM**	**Bond lengths**	**Bond angles**	**Cis Peptides**
			Avg: 0.92	Clashscore: 1.59	Outliers: 4 of 903	Poor rotamers: 0 of 771	Outliers: 0 of 828	Outliers: 22 of 901	Outliers: 13 of 905	Outliers: 19 of 905	Non-Trans: 1 of 904
A 101		ARG	0.6	-	Favored (12.27%) General / -119.6,106.5	Favored (31.5%) *mmt90* chi angles: 303.3,292.9,186,92.1	0.08Å	Favored (65.016%) beta sheet	-	-	-
A 102		GLY	0.61	-	Favored (16.69%) Glycine / -100.5,144.3	-	-	Favored (56.289%) beta sheet	-	-	-
A 103		TYR	0.64	-	Favored (44.65%)	Favored (84.9%) *m-80* chi angles: 300.8,88.7	0.05Å	Favored (57.165%) beta sheet	-	-	-

					General / -133.9,137.1						
A 104		THR	0.7	-	Favored (24.32%) General / -147.3,139.3	Favored (83.7%) *m* chi angles: 296.6	0.19Å	Favored (26.449%) beta sheet	-	-	-
A 105		LYS	0.77	-	Favored (2.74%) General / -75.5,90.6	Favored (84.5%) *tttt* chi angles: 184.8,173.8,181.9,172.6	0.03Å	Favored (49.263%) beta sheet	-	-	-
A 106		GLY	0.87	-	Favored (46.34%) Glycine / -62.1,150.5	-	-	Favored (12.248%) beta sheet	-	-	-
A 107		GLY	0.95	-	Favored (13.38%) Glycine / 142.2,172.0	-	-	Favored (28.027%)	-	-	-
A 108		PRO	1.01	-	Favored (18.86%) Trans-Pro / -49.3,-30.1	Favored (88.1%) *Cg_exo* chi angles: 330.7,37.9,330	0.06Å	Favored (10.288%)	-	-	-
A 109		GLY	1.01	-	Favored (77.75%) Glycine / -92.2,4.7	-	-	Favored (65.703%) alpha helix	-	-	-
A 110		HIS	0.97	-	Favored (51.21%) General / -135.8,154.4	Favored (93.6%) *m-70* chi angles: 301.3,280.4	0.06Å	Favored (20.334%)	-	-	-
A 111		GLU	0.9	-	Favored (49.88%) General / -71.4,139.6	Favored (76%) *mt-10* chi angles: 291.9,183.2,323.7	0.09Å	Favored (42.004%)	-	-	-
A 112		GLU	0.85	-	Favored (48.92%) Pre-Pro / -87.6,150.6	Favored (80.1%) *mm-30* chi angles: 301.3,298.7,331.8	0.07Å	Favored (41.862%) beta sheet	-	-	-
A 113		PRO	0.81	-	Favored (75.52%) Trans-Pro / -68.4,150.1	Favored (40.6%) *Cg_endo* chi angles: 23.5,327.6,27.5	0.02Å	Favored (61.495%) beta sheet	-	-	-
A 114		MET	0.8	-	Favored (47.32%) General / -138.3,156.8	Favored (3.8%) *ppp* chi angles: 61.1,80.1,67.1	0.01Å	Favored (49.523%) beta sheet	-	-	-
A 115		LEU	0.82	-	Favored (37.74%) General / -98.5,119.7	Favored (84.8%) *mt* chi angles: 300.9,177.8	0.07Å	Favored (33.857%) beta sheet	-	-	-
A 116		MET	0.85	-	Favored (50.05%) General / -126.7,146.8	Favored (64.2%) *ttp* chi angles: 182.7,178.1,74.7	0.06Å	Favored (43.463%) beta sheet	-	-	-
A 117		GLN	0.88	-	Favored (34.75%) General / -85.5,128.5	Favored (8.6%) *tm-30* chi angles: 206.4,279.1,316.7	0.06Å	Favored (13.982%) beta sheet	-	OUTLIER(S) worst is CG-CD-NE2: 4.2 σ	-
A 118		SER	0.88	-	Favored (33.29%) General / -154.8,166.5	Favored (84%) *p* chi angles: 67.5	0.02Å	Favored (15.074%)	-	-	-
A 119		TYR	0.87	-	Allowed (0.43%) General / -69.7,76.3	Favored (73.7%) *t80* chi angles: 183.7,72.7	0.08Å	CaBLAM Disfavored (2.72%)	-	-	-
A 120		GLY	0.83	-	Favored (2.03%) Glycine / 135.7,6.5	-	-	CaBLAM Disfavored (3.406%)	-	-	-

#	Alt	Res	High B	Clash > 0.4Å	Ramachandran	Rotamer	Cβ deviation	CaBLAM	Bond lengths	Bond angles	Cis Peptides
			Avg: 0.92	Clashscore: 1.59	Outliers: 4 of 903	Poor rotamers: 0 of 771	Outliers: 0 of 828	Outliers: 22 of 901	Outliers: 13 of 905	Outliers: 19 of 905	Non-Trans: 1 of 904
A 121		TRP	0.79	-	Favored (14.56%) General / -53.0,-26.3	Favored (72.3%) *p-90* chi angles: 67.4,266.5	0.11Å	Favored (27.776%)	OUTLIER(S) worst is NE1--CE2: 4.6 σ	-	-
A 122		ASN	0.74	-	Favored (50.67%) General / -72.4,-7.6	Favored (18.6%) *p0* chi angles: 60,298.3	0.12Å	Favored (42.79%)	-	-	-
A 123		LEU	0.69	-	Favored (30.46%) General / -99.8,-4.5	Favored (6.4%) *mp* chi angles: 279.9,68.9	0.10Å	Favored (53.844%)	-	-	-
A 124		VAL	0.66	-	Favored (66.19%) Ile or Val / -110.3,123.5	Favored (60%) *t* chi angles: 180	0.02Å	Favored (24.058%)	-	-	-
A 125		THR	0.64	-	Favored (54.52%) General / -114.0,135.0	Favored (90.6%) *m* chi angles: 298.1	0.01Å	Favored (48.465%)	-	-	-
A 126		MET	0.64	-	Favored (50.49%) General / -127.9,147.7	Favored (59.2%) *mtt* chi angles: 298,184.7,184.9	0.05Å	Favored (71.222%) beta sheet	-	-	-
A 127		LYS	0.65	-	Favored (31.6%) General / -133.4,126.6	Favored (31.6%) *ttmt* chi angles: 180.1,179,286.8,187.1	0.01Å	Favored (54.62%) beta sheet	-	-	-
A 128		SER	0.67	-	Favored (15.55%) General / -93.3,-29.6	Favored (59.3%) *m* chi angles: 299.2	0.07Å	Favored (10.148%)	-	-	-
A 129		GLY	0.7	-	Favored (4.93%) Glycine / -82.1,55.6	-	-	CaBLAM Outlier (0.725%)	-	-	-
A 130		VAL	0.72	0.55Å O with A 130 VAL HG13	Favored (5.04%) Ile or Val / -149.3,130.6	Favored (5.6%) *p* chi angles: 57.6	0.13Å	Favored (9.432%)	-	-	-
A 131		ASP	0.75	-	Favored (16.89%) General / -96.8,104.8	Favored (53.8%) *t0* chi angles: 183,336.4	0.06Å	Favored (68.55%) beta sheet	-	-	-
A 132		VAL	0.76	-	Favored (30.14%) Ile or Val / -62.1,-22.9	Favored (32.4%) *m* chi angles: 297.5	0.07Å	Favored (40.534%)	-	-	-
A 133		TYR	0.76	-	Favored (70.07%) General / -61.8,-29.4	Favored (62.7%) *t80* chi angles: 186.6,72.9	0.07Å	Favored (52.916%)	-	-	-
A 134		TYR	0.76	-	Favored (25.57%) General / -109.4,9.6	Favored (88.8%) *m-80* chi angles: 299.4,102.7	0.03Å	Favored (31.388%)	-	-	-
A 135		LYS	0.75	-	Favored (84.08%) Pre-Pro / -73.9,129.9	Favored (45.4%) *tttp* chi angles: 180.7,177.7,164.3,63.4	0.01Å	Favored (34.358%)	-	-	-
A 136		PRO	0.73	-	Favored (97.73%) Trans-Pro / -58.3,142.8	Favored (91.6%) *Cg_exo* chi angles: 333.1,35.4,331	0.08Å	Favored (66.296%)	-	-	-

#	Alt	Res	High B	Clash > 0.4Å	Ramachandran	Rotamer	Cβ deviation	CaBLAM	Bond lengths	Bond angles	Cis Peptides
A 137		SER	0.7	-	Favored (35.59%) General / -66.9,156.5	Favored (98.2%) *p* chi angles: 65.7	0.09Å	Favored (23.22%)	-	-	-
A 138		GLU	0.67	-	Favored (73.02%) Pre-Pro / -133.1,149.6	Favored (94.8%) *mt-10* chi angles: 299.4,180,350.8	0.08Å	Favored (42.739%)	-	-	-
A 139		PRO	0.64	-	Favored (69.95%) Trans-Pro / -69.3,149.4	Favored (47.4%) *Cg_endo* chi angles: 24.9,327.4,26.5	0.02Å	Favored (26.296%)	-	-	-
A 140		CYS	0.62	-	Favored (20.8%) General / -158.3,171.1	Favored (29.1%) *p* chi angles: 64.1	0.02Å	Favored (41.61%)	-	-	-
**#**	**Alt**	**Res**	**High B**	**Clash > 0.4Å**	**Ramachandran**	**Rotamer**	**Cβ deviation**	**CaBLAM**	**Bond lengths**	**Bond angles**	**Cis Peptides**
			Avg: 0.92	Clashscore: 1.59	Outliers: 4 of 903	Poor rotamers: 0 of 771	Outliers: 0 of 828	Outliers: 22 of 901	Outliers: 13 of 905	Outliers: 19 of 905	Non-Trans: 1 of 904
A 141		ASP	0.6	-	Favored (11.47%) General / -95.5,-36.0	Favored (60.9%) *m-30* chi angles: 296.6,303.3	0.04Å	Favored (5.67%)	-	-	-
A 142		THR	0.59	-	Favored (46.64%) General / -129.7,132.5	Favored (85.8%) *m* chi angles: 301.5	0.03Å	Favored (39.736%)	-	-	-
A 143		LEU	0.6	-	Favored (38.37%) General / -119.4,120.9	Favored (35.4%) *tp* chi angles: 178.3,69.4	0.11Å	Favored (66.937%)	-	-	-
A 144		PHE	0.62	-	Favored (32.21%) General / -110.4,148.3	Favored (20.2%) *m-10* chi angles: 293,331.6	0.04Å	Favored (36.758%) beta sheet	-	-	-
A 145		CYS	0.65	-	Favored (13.77%) General / -149.9,130.2	Favored (57.2%) *t* chi angles: 183.1	0.01Å	Favored (30.787%) beta sheet	-	-	-
A 146		ASP	0.7	-	Favored (3.15%) General / -134.0,38.9	Favored (33.4%) *t0* chi angles: 194.9,28.5	0.04Å	Favored (9.228%) beta sheet	-	-	-
A 147		ILE	0.76	-	Favored (45.52%) Ile or Val / -93.7,127.8	Favored (48.3%) *mm* chi angles: 301.7,298.5	0.02Å	CaBLAM Outlier (0.834%)	-	-	-
A 148		GLY	0.81	-	Allowed (0.18%) Glycine / 171.8,-49.1	-	-	CaBLAM Disfavored (2.63%)	-	-	-
A 149		GLU	0.87	-	Allowed (0.74%) General / 61.1,-131.7	Favored (68.6%) *mm-30* chi angles: 295.5,293.8,308.9	0.05Å	CaBLAM Outlier (0.555%)	-	-	-
A 150		SER	0.91	-	Favored (23.32%) General / -163.8,164.9	Favored (86.3%) *p* chi angles: 67.9	0.01Å	Favored (10.504%)	-	-	-
A 151		SER	0.92	-	Favored (22.72%) General / -158.8,152.1	Favored (41.1%) *t* chi angles: 178.1	0.07Å	Favored (45.041%)	-	-	-
A 152		SER	0.92	-	Favored (65.75%)	Favored (86.4%) *p* chi angles: 67.2	0.03Å	Favored (36.366%)	-	-	-

					General / -66.5,-19.3						
A 153		SER	0.89	-	Favored (25.45%) General / -101.1,112.9	Favored (42.6%) *t* chi angles: 175.7	0.02Å	Favored (32.939%)	-	-	-
A 154		ALA	0.84	-	Favored (70.69%) General / -59.1,-33.3	-	0.03Å	Favored (49.895%)	-	-	-
A 155		GLU	0.79	-	Favored (71.95%) General / -63.6,-30.9	Favored (97.7%) *mt-10* chi angles: 290.8,181.1,352.8	0.03Å	Favored (70.779%) alpha helix	-	-	-
A 156		VAL	0.74	-	Favored (37.16%) Ile or Val / -76.1,-44.7	Favored (87.3%) *t* chi angles: 173.9	0.02Å	Favored (71.64%) alpha helix	-	-	-
A 157		GLU	0.7	-	Favored (81.3%) General / -62.5,-36.0	Favored (86.6%) *mt-10* chi angles: 289.6,188.1,358.5	0.04Å	Favored (81.51%) alpha helix	-	-	-
A 158		GLU	0.66	-	Favored (73.78%) General / -57.8,-50.3	Favored (40.7%) *tt0* chi angles: 183.1,185.7,59.9	0.09Å	Favored (80.176%) alpha helix	-	-	-
A 159		GLN	0.64	-	Favored (91.95%) General / -64.6,-38.7	Favored (83.1%) *mt0* chi angles: 290.4,177.2,39.6	0.01Å	Favored (83.616%) alpha helix	-	-	-
A 160		ARG	0.63	-	Favored (97.14%) General / -64.0,-42.3	Favored (97.7%) *mtt180* chi angles: 290,178.7,184.9,178.5	0.03Å	Favored (94.922%) alpha helix	-	-	-
**#**	**Alt**	**Res**	**High B**	**Clash > 0.4Å**	**Ramachandran**	**Rotamer**	**Cβ deviation**	**CaBLAM**	**Bond lengths**	**Bond angles**	**Cis Peptides**
			Avg: 0.92	Clashscore: 1.59	Outliers: 4 of 903	Poor rotamers: 0 of 771	Outliers: 0 of 828	Outliers: 22 of 901	Outliers: 13 of 905	Outliers: 19 of 905	Non-Trans: 1 of 904
A 161		THR	0.64	-	Favored (93.76%) General / -62.9,-45.2	Favored (60.7%) *m* chi angles: 295.6	0.02Å	Favored (96.585%) alpha helix	-	-	-
A 162		LEU	0.64	-	Favored (96.4%) General / -62.9,-40.0	Favored (89.4%) *mt* chi angles: 291.1,170.3	0.05Å	Favored (96.577%) alpha helix	-	-	-
A 163		ARG	0.65	-	Favored (96.13%) General / -62.8,-40.0	Favored (96%) *mtt180* chi angles: 289.1,172.4,184.1,169.4	0.04Å	Favored (93.465%) alpha helix	-	-	-
A 164		ILE	0.67	0.43Å HA with A 164 ILE HD13	Favored (81.44%) Ile or Val / -69.0,-40.8	Favored (32.4%) *mm* chi angles: 295,302.8	0.12Å	Favored (94.579%) alpha helix	-	-	-
A 165		LEU	0.68	-	Favored (89.11%) General / -62.5,-38.2	Favored (83.2%) *mt* chi angles: 290.3,173.5	0.06Å	Favored (90.878%) alpha helix	-	-	-
A 166		GLU	0.7	-	Favored (89.64%) General / -66.2,-39.5	Favored (98.2%) *mt-10* chi angles: 290,177.6,353.7	0.06Å	Favored (90.384%) alpha helix	-	-	-
A 167		MET	0.71	-	Favored (74.65%) General / -60.0,-50.2	Favored (49.9%) *ttp* chi angles: 177.2,190.1,70.6	0.05Å	Favored (88.829%) alpha helix	-	-	-

A 168		VAL	0.71	-	Favored (30.24%) Ile or Val / -68.8,-25.3	Favored (26.6%) *m* chi angles: 299.2	0.04Å	Favored (49.077%) alpha helix	-	-	-
A 169		SER	0.71	-	Favored (79.56%) General / -56.3,-43.9	Favored (34.3%) *t* chi angles: 182.1	0.04Å	Favored (57.886%) alpha helix	-	-	-
A 170		ASP	0.71	-	Favored (77.25%) General / -59.7,-36.9	Favored (95.8%) *m-30* chi angles: 286.2,347	0.05Å	Favored (80.412%) alpha helix	-	-	-
A 171		TRP	0.71	-	Favored (71.89%) General / -71.2,-39.5	Favored (78.9%) *m100* chi angles: 288,82.3	0.10Å	Favored (83.676%) alpha helix	-	-	-
A 172		LEU	0.71	-	Favored (81.87%) General / -65.0,-35.6	Favored (98.8%) *mt* chi angles: 292.7,172.8	0.04Å	Favored (80.519%) alpha helix	-	-	-
A 173		GLN	0.71	-	Favored (78.06%) General / -64.5,-34.4	Favored (75.4%) *mt0* chi angles: 289.7,174.2,48.4	0.04Å	Favored (73.359%)	-	-	-
A 174		ARG	0.71	-	Favored (78.43%) General / -61.8,-35.5	Favored (51.3%) *mmt180* chi angles: 292.9,295.8,181,186.7	0.04Å	Favored (11.389%)	-	-	-
A 175		GLY	0.7	-	Allowed (0.62%) Glycine / 107.4,66.2	-	-	CaBLAM Outlier (0.754%)	-	-	-
A 176		PRO	0.69	-	Favored (52.47%) Trans-Pro / -72.1,158.3	Favored (73.2%) *Cg_endo* chi angles: 27.6,326.1,26.9	0.03Å	Favored (29.379%)	-	-	-
A 177		ARG	0.67	-	Favored (38.74%) General / -78.7,-38.5	Favored (77.8%) *mtp180* chi angles: 292.8,173,65.6,174.1	0.08Å	Favored (15.151%)	-	-	-
A 178		GLU	0.66	-	Favored (47.03%) General / -137.4,157.2	Favored (80.5%) *mt-10* chi angles: 296.2,189.8,2.3	0.07Å	Favored (12.472%)	-	-	-
A 179		PHE	0.64	-	Favored (41.92%) General / -151.6,161.9	Favored (51.5%) *p90* chi angles: 61.4,86.3	0.05Å	Favored (65.551%)	-	-	-
A 180		CYS	0.63	-	Favored (3.69%) General / -150.8,108.6	Favored (56.5%) *t* chi angles: 183.4	0.04Å	Favored (21.458%) beta sheet	-	-	-
**#**	**Alt**	**Res**	**High B**	**Clash > 0.4Å**	**Ramachandran**	**Rotamer**	**Cβ deviation**	**CaBLAM**	**Bond lengths**	**Bond angles**	**Cis Peptides**
			Avg: 0.92	Clashscore: 1.59	Outliers: 4 of 903	Poor rotamers: 0 of 771	Outliers: 0 of 828	Outliers: 22 of 901	Outliers: 13 of 905	Outliers: 19 of 905	Non-Trans: 1 of 904
A 181		ILE	0.62	-	Favored (73.98%) Ile or Val / -120.5,125.3	Favored (66.5%) *mt* chi angles: 299.8,163.9	0.13Å	Favored (54.353%) beta sheet	-	-	-
A 182		LYS	0.62	-	Favored (44.39%) General / -73.1,134.3	Favored (26.1%) *ttpt* chi angles: 184.3,163.9,71.5,191.1	0.07Å	Favored (43.539%) beta sheet	-	-	-
A 183		VAL	0.63	-	Favored (75.93%)	Favored (74%) *t* chi angles: 178.4	0.13Å	Favored (70.565%)	-	-	-

					Ile or Val / -120.7,127.4						
A 184		LEU	0.65	-	Favored (38.81%) General / -105.6,140.4	Favored (67.5%) *mt* chi angles: 304,178.6	0.06Å	Favored (12.272%)	-	-	-
A 185		CYS	0.68	-	Favored (12.36%) Pre-Pro / 53.8,54.8	Favored (91.2%) *m* chi angles: 291.2	0.04Å	Favored (11.759%)	-	-	-
A 186		PRO	0.72	-	Favored (68.62%) Trans-Pro / -61.8,-20.3	Favored (37.1%) *Cg_endo* chi angles: 22.8,325.5,31.7	0.02Å	Favored (59.648%)	-	-	-
A 187		TYR	0.76	0.41Å C with A 187 TYR CD1	Favored (62.23%) General / -70.9,-23.1	Favored (26.3%) *p90* chi angles: 72.1,84.7	0.04Å	Favored (41.351%)	-	-	-
A 188		MET	0.79	-	Favored (90.21%) Pre-Pro / -67.5,124.9	Favored (86.2%) *mmm* chi angles: 292.4,288.9,284.4	0.07Å	Favored (35.355%)	-	-	-
A 189		PRO	0.82	-	Favored (20.27%) Trans-Pro / -47.0,-36.1	Favored (86.7%) *Cg_exo* chi angles: 329.4,37.6,331.7	0.03Å	Favored (69.504%)	-	-	-
A 190		ARG	0.83	-	Favored (80.5%) General / -66.0,-35.4	Favored (77.1%) *mtt90* chi angles: 292.5,187.2,178.8,96.8	0.04Å	Favored (75.469%) alpha helix	-	-	-
A 191		VAL	0.83	-	Favored (32.44%) Ile or Val / -77.0,-43.1	Favored (91.1%) *t* chi angles: 174.3	0.03Å	Favored (71.635%) alpha helix	-	-	-
A 192		MET	0.82	-	Favored (91.4%) General / -58.9,-44.5	Favored (50.8%) *ttm* chi angles: 185,172.9,280.3	0.06Å	Favored (90.889%) alpha helix	-	-	-
A 193		GLU	0.8	-	Favored (92.25%) General / -63.9,-38.8	Favored (96.9%) *mt-10* chi angles: 288.6,179.3,354.2	0.03Å	Favored (95.474%) alpha helix	-	-	-
A 194		ARG	0.78	-	Favored (95.82%) General / -64.4,-42.8	Favored (42.9%) *tpt170* chi angles: 179,63.2,179.2,168.6	0.03Å	Favored (96.074%) alpha helix	-	-	-
A 195		LEU	0.76	-	Favored (91.74%) General / -65.5,-39.3	Favored (89.9%) *mt* chi angles: 291.4,170	0.03Å	Favored (85.545%) alpha helix	-	-	-
A 196		GLU	0.76	-	Favored (95.47%) General / -60.7,-41.6	Favored (92.1%) *tt0* chi angles: 180.6,176.6,355.9	0.07Å	Favored (84.815%) alpha helix	-	-	-
A 197		VAL	0.76	-	Favored (93.38%) Ile or Val / -63.2,-47.2	Favored (61.9%) *t* chi angles: 171.1	0.05Å	Favored (79.345%) alpha helix	-	-	-
A 198		LEU	0.76	-	Favored (66.94%) General / -65.8,-24.6	Favored (97.7%) *mt* chi angles: 293.6,172.3	0.07Å	Favored (69.098%) alpha helix	-	-	-
A 199		GLN	0.77	0.65Å HG2 with A 199 GLN O	Favored (55.72%) General / -83.6,-2.3	Favored (58.9%) *tt0* chi angles: 184.5,178.4,55.9	0.07Å	Favored (46.892%) alpha helix	-	-	-
A 200		ARG	0.78	-	Favored (12.63%) General / -106.5,-23.0	Favored (97.6%) *mtt-85* chi angles: 290.4,179.8,181,275.9	0.05Å	Favored (56.169%) alpha helix	-	-	-

#	Alt	Res	High B	Clash > 0.4Å	Ramachandran	Rotamer	Cβ deviation	CaBLAM	Bond lengths	Bond angles	Cis Peptides
			Avg: 0.92	Clashscore: 1.59	Outliers: 4 of 903	Poor rotamers: 0 of 771	Outliers: 0 of 828	Outliers: 22 of 901	Outliers: 13 of 905	Outliers: 19 of 905	Non-Trans: 1 of 904
A 201		ARG	0.77	-	Favored (28.85%) General / -82.8,-30.2	Favored (97.5%) *mtt180* chi angles: 290.4,179.8,179.3,181.4	0.03Å	Favored (63.661%) alpha helix	-	-	-
A 202		TYR	0.76	-	Favored (39.69%) General / -132.1,159.2	Favored (26.3%) *m-80* chi angles: 311,95.3	0.06Å	Favored (12.978%) alpha helix	-	OUTLIER(S) worst is C-N-CA: 17.6 σ	-
A 203		GLY	0.74	-	Favored (6.12%) Glycine / 129.9,-6.2	-	-	CaBLAM Disfavored (2.27%) try alpha helix	-	-	Cis nonPRO omega= 4.12
A 204		GLY	0.72	-	Favored (8.89%) Glycine / -172.6,-152.8	-	-	Favored (10.009%)	-	-	-
A 205		GLY	0.7	-	Favored (40.97%) Glycine / -173.4,-175.2	-	-	CaBLAM Disfavored (3.254%)	-	-	-
A 206		LEU	0.68	-	Favored (35.03%) General / -96.2,137.6	Favored (94.7%) *mt* chi angles: 296.3,174.7	0.02Å	Favored (7.357%)	-	-	-
A 207		VAL	0.68	-	Favored (65.97%) Ile or Val / -128.9,127.1	Favored (70.5%) *t* chi angles: 178.7	0.08Å	Favored (63.027%) beta sheet	-	-	-
A 208		ARG	0.68	-	Favored (64.35%) General / -72.5,-42.9	Favored (40.4%) *tpt170* chi angles: 172.3,62,178.1,166.2	0.03Å	CaBLAM Disfavored (4.27%) try beta sheet	-	-	-
A 209		VAL	0.68	-	OUTLIER (0.03)%) Pre-Pro / 69.6,121.3	Favored (70.4%) *t* chi angles: 178.7	0.04Å	CaBLAM Disfavored (1.614%)	-	-	-
A 210		PRO	0.69	-	Favored (42.93%) Trans-Pro / -66.0,-14.1	Favored (52.7%) *Cg_endo* chi angles: 25.5,325.4,29.5	0.03Å	Favored (28.314%)	-	-	-
A 211		LEU	0.69	-	Favored (58.18%) General / -87.8,-6.3	Favored (85%) *mt* chi angles: 299.3,174.4	0.07Å	Favored (48.864%)	-	-	-
A 212		SER	0.68	-	Favored (51.7%) General / -69.9,145.7	Favored (63.3%) *m* chi angles: 297.8	0.07Å	Favored (38.242%)	-	-	-
A 213		ARG	0.68	-	Favored (29.14%) General / -82.6,146.4	Favored (99%) *mtt180* chi angles: 294,178.4,181.2,174.5	0.02Å	Favored (45.55%)	-	-	-
A 214		ASN	0.67	-	Favored (4.3%) General / -85.3,57.2	Favored (87.8%) *m-40* chi angles: 297.9,316.5	0.03Å	CaBLAM Disfavored (1.072%)	-	-	-
A 215		SER	0.66	-	Allowed (0.19%) General / -152.3,-10.7	Favored (88.5%) *p* chi angles: 62.9	0.06Å	CaBLAM Disfavored (3.512%)	-	-	-
A 216		ASN	0.65	-	Favored (16.28%) General / -121.1,110.5	Favored (51.1%) *t0* chi angles: 186.6,358.2	0.02Å	Favored (18.207%)	-	-	-

#	Alt	Res	High B	Clash > 0.4Å	Ramachandran	Rotamer	Cβ deviation	CaBLAM	Bond lengths	Bond angles	Cis Peptides
A 217		HIS	0.64	-	Favored (6.89%) General / -80.1,74.7	Favored (37.8%) *t-90* chi angles: 200,295.2	0.06Å	Favored (32.134%)	-	-	-
A 218		GLU	0.63	-	Favored (34.27%) General / -109.3,146.4	Favored (9.7%) *pt0* chi angles: 60.9,178,294.7	0.02Å	Favored (11.898%) beta sheet	-	-	-
A 219		MET	0.62	-	Favored (44.5%) General / -144.0,157.2	Favored (78.9%) *mtp* chi angles: 299.9,185.1,77.9	0.02Å	Favored (46.902%) beta sheet	-	-	-
A 220		TYR	0.63	-	Favored (32.81%) General / -102.5,143.0	Favored (84.5%) *m-80* chi angles: 289.7,89	0.02Å	Favored (46.446%) beta sheet	-	-	-
**#**	**Alt**	**Res**	**High B**	**Clash > 0.4Å**	**Ramachandran**	**Rotamer**	**Cβ deviation**	**CaBLAM**	**Bond lengths**	**Bond angles**	**Cis Peptides**
			Avg: 0.92	Clashscore: 1.59	Outliers: 4 of 903	Poor rotamers: 0 of 771	Outliers: 0 of 828	Outliers: 22 of 901	Outliers: 13 of 905	Outliers: 19 of 905	Non-Trans: 1 of 904
A 221		TRP	0.66	-	Favored (24.06%) General / -98.1,111.9	Favored (20.1%) *t60* chi angles: 176.4,35.3	0.07Å	Favored (60.689%) beta sheet	-	-	-
A 222		VAL	0.71	-	Favored (62.2%) Ile or Val / -124.8,136.0	Favored (39.3%) *t* chi angles: 183.5	0.05Å	Favored (53.504%)	-	-	-
A 223		SER	0.78	-	Favored (25.19%) General / -64.7,158.2	Favored (94.1%) *p* chi angles: 64.7	0.04Å	Favored (9.947%)	-	-	-
A 224		GLY	0.86	-	Favored (4.11%) Glycine / 81.3,-50.0	-	-	CaBLAM Outlier (0.399%)	-	-	-
A 225		ALA	0.93	-	Allowed (1.2%) General / -74.3,65.4	-	0.05Å	Favored (22.753%)	-	-	-
A 226		ALA	0.97	-	Favored (49.15%) General / -67.2,150.4	-	0.02Å	CaBLAM Outlier (0.416%)	-	-	-
A 227		GLY	0.97	-	Favored (11.57%) Glycine / 165.8,159.2	-	-	Favored (19.94%) beta sheet	-	-	-
A 228		ASN	0.94	-	Favored (27.57%) General / -67.0,125.9	Favored (25.4%) *t0* chi angles: 185.9,275.9	0.06Å	Favored (12.498%)	-	-	-
A 229		ILE	0.9	-	Favored (98.95%) Ile or Val / -63.4,-44.4	Favored (87.8%) *mt* chi angles: 290.7,167.3	0.04Å	Favored (47.709%)	-	-	-
A 230		VAL	0.85	-	Favored (99.34%) Ile or Val / -61.4,-44.9	Favored (84.2%) *t* chi angles: 173.5	0.06Å	Favored (84.556%) alpha helix	-	-	-
A 231		HIS	0.81	-	Favored (69.01%) General / -63.5,-50.6	Favored (83.5%) *t70* chi angles: 184.1,73.9	0.01Å	Favored (78.997%) alpha helix	-	-	-
A 232		ALA	0.78	-	Favored (76.6%) General / -57.3,-39.9	-	0.05Å	Favored (76.806%) alpha helix	-	-	-

#	Alt	Res	High B	Clash > 0.4Å	Ramachandran	Rotamer	Cβ deviation	CaBLAM	Bond lengths	Bond angles	Cis Peptides
A 233		VAL	0.75	-	Favored (76.61%) Ile or Val / -70.0,-45.4	Favored (75%) *t* chi angles: 172.7	0.04Å	Favored (84.834%) alpha helix	-	-	-
A 234		ASN	0.73	-	Favored (94.08%) General / -64.2,-39.5	Favored (94.4%) *m-40* chi angles: 286.1,339.5	0.03Å	Favored (94.024%) alpha helix	-	-	-
A 235		MET	0.72	-	Favored (80.78%) General / -68.5,-38.2	Favored (51%) *mmp* chi angles: 294.6,301.4,98.2	0.04Å	Favored (98.183%) alpha helix	-	-	-
A 236		THR	0.71	-	Favored (93.88%) General / -62.4,-45.3	Favored (90.8%) *m* chi angles: 298	0.05Å	Favored (95.475%) alpha helix	-	-	-
A 237		SER	0.71	-	Favored (96.42%) General / -63.7,-40.3	Favored (61.1%) *m* chi angles: 298.7	0.05Å	Favored (88.986%) alpha helix	-	-	-
A 238		GLN	0.71	-	Favored (94.33%) General / -62.2,-39.9	Favored (94.6%) *mt0* chi angles: 289.2,178.3,336.8	0.08Å	Favored (90.553%) alpha helix	-	-	-
A 239		VAL	0.7	-	Favored (89.53%) Ile or Val / -66.1,-45.8	Favored (62.8%) *t* chi angles: 171.2	0.01Å	Favored (85.647%) alpha helix	-	-	-
A 240		LEU	0.7	-	Favored (91.82%) General / -63.0,-38.6	Favored (94.6%) *mt* chi angles: 292,173.4	0.04Å	Favored (80.63%) alpha helix	-	-	-
**#**	**Alt**	**Res**	**High B**	**Clash > 0.4Å**	**Ramachandran**	**Rotamer**	**Cβ deviation**	**CaBLAM**	**Bond lengths**	**Bond angles**	**Cis Peptides**
			Avg: 0.92	Clashscore: 1.59	Outliers: 4 of 903	Poor rotamers: 0 of 771	Outliers: 0 of 828	Outliers: 22 of 901	Outliers: 13 of 905	Outliers: 19 of 905	Non-Trans: 1 of 904
A 241		ILE	0.71	-	Favored (84.85%) Ile or Val / -67.5,-45.7	Favored (98.5%) *mt* chi angles: 292.9,168.1	0.05Å	Favored (75.582%) alpha helix	-	-	-
A 242		GLY	0.73	-	Favored (67.85%) Glycine / -55.2,-38.7	-	-	Favored (91.545%) alpha helix	-	-	-
A 243		ARG	0.76	-	Favored (84.39%) General / -61.5,-37.6	Favored (96.8%) *mtt180* chi angles: 289.2,173.9,178.6,182.9	0.03Å	Favored (79.108%) alpha helix	-	-	-
A 244		MET	0.81	-	Favored (64.12%) General / -69.0,-19.0	Favored (86%) *mtp* chi angles: 292.8,185.9,72.8	0.07Å	Favored (66.782%) alpha helix	-	-	-
A 245		GLU	0.87	-	Favored (57.81%) General / -88.9,-0.9	Favored (96.8%) *mt-10* chi angles: 295.3,180.7,359.7	0.04Å	Favored (55.762%)	-	-	-
A 246		LYS	0.92	-	Favored (5.29%) General / -78.8,76.1	Favored (72.7%) *mmtt* chi angles: 300.9,295.9,181.8,182.1	0.03Å	Favored (17.691%)	-	-	-
A 247		ARG	0.95	-	OUTLIER (0.02)%) General / -65.5,19.0	Favored (99.1%) *mtt180* chi angles: 292,177.3,180.3,175	0.04Å	CaBLAM Outlier (0.79%)	-	-	-
A 248		THR	0.95	-	Favored (13.93%)	Favored (72.7%) *p* chi angles: 61.8	0.03Å	CaBLAM Disfavored (2.123%)	-	-	-

					General / -107.6,-18.1						
A 249		TRP	0.94	-	Favored (3.5%) General / 54.4,60.5	Favored (13.3%) *m-90* chi angles: 303.2,288.3	0.07Å	Favored (7.015%)	-	-	-
A 250		HIS	0.92	-	Favored (51.55%) General / -61.2,145.3	Favored (74.5%) *t-90* chi angles: 193.5,282.3	0.04Å	Favored (25.728%)	-	-	-
A 251		GLY	0.91	-	Favored (53%) Glycine / -71.6,166.5	-	-	Favored (54.51%)	-	-	-
A 252		PRO	0.92	-	Favored (68.51%) Trans-Pro / -69.5,154.6	Favored (48.7%) *Cg_endo* chi angles: 25,325.6,29.1	0.04Å	Favored (86.023%)	-	-	-
A 253		LYS	0.94	-	Favored (38.46%) General / -92.8,130.2	Favored (86%) *tttt* chi angles: 181.4,178.2,177.8,180.2	0.04Å	Favored (42.511%) beta sheet	-	-	-
A 254		TYR	0.97	-	Favored (25.93%) General / -98.3,146.2	Favored (79.3%) *m-80* chi angles: 288.5,89.3	0.07Å	Favored (51.981%) beta sheet	-	-	-
A 255		GLU	1	-	Favored (36.99%) General / -135.1,131.5	Favored (48.8%) *tt0* chi angles: 184.6,178.3,37.5	0.06Å	Favored (38.998%) beta sheet	-	-	-
A 256		GLU	1.02	-	Favored (56.95%) General / -57.8,136.2	Favored (85.2%) *tt0* chi angles: 187.1,176.3,9.1	0.02Å	Favored (49.581%)	-	-	-
A 257		ASP	1.03	-	Favored (7.11%) General / -62.7,164.9	Favored (21.9%) *t0* chi angles: 201.1,353.1	0.01Å	Favored (18.485%)	-	-	-
A 258		VAL	1.05	0.44Å O with A 258 VAL HG12	Allowed (1.02%) Ile or Val / -79.0,82.3	Favored (43.7%) *t* chi angles: 182.7	0.09Å	Favored (21.311%)	-	-	-
A 259		ASN	1.08	-	Favored (52.62%) General / -58.4,132.1	Favored (93.9%) *m-40* chi angles: 293.4,333.4	0.06Å	Favored (16.234%)	-	-	-
A 260		LEU	1.12	-	Favored (12.04%) General / -97.8,165.2	Favored (55.1%) *mt* chi angles: 305,175.3	0.09Å	Favored (34.785%)	-	-	-
**#**	**Alt**	**Res**	**High B**	**Clash > 0.4Å**	**Ramachandran**	**Rotamer**	**Cβ deviation**	**CaBLAM**	**Bond lengths**	**Bond angles**	**Cis Peptides**
			Avg: 0.92	Clashscore: 1.59	Outliers: 4 of 903	Poor rotamers: 0 of 771	Outliers: 0 of 828	Outliers: 22 of 901	Outliers: 13 of 905	Outliers: 19 of 905	Non-Trans: 1 of 904
A 261		GLY	1.17	-	Favored (55.27%) Glycine / -78.6,172.6	-	-	Favored (45.323%)	-	-	-
A 262		SER	1.21	-	Favored (6.08%) General / -110.2,174.0	Favored (72.5%) *m* chi angles: 295.8	0.04Å	CaBLAM Disfavored (4.043%)	-	-	-
A 263		GLY	1.24	-	Favored (22.34%) Glycine / 89.3,-151.9	-	-	Favored (23.156%)	-	-	-
A 264		THR	1.24	-	Favored (13.47%)	Favored (72.2%) *p* chi angles: 61.9	0.07Å	Favored (8.757%)	-	-	-

#	Alt	Res	High B	Clash > 0.4Å	Ramachandran	Rotamer	Cβ deviation	CaBLAM	Bond lengths	Bond angles	Cis Peptides
					General / -133.8,171.9						
A 265		ARG	1.22	-	Favored (51.72%) General / -124.0,141.8	Favored (57.2%) *mtp180* chi angles: 287.9,181.3,60.4,210.6	0.09Å	Favored (36.687%) beta sheet	-	-	-
A 266		ALA	1.21	-	Favored (30.32%) General / -78.3,155.2	-	0.03Å	Favored (36.656%) beta sheet	-	-	-
A 267		VAL	1.2	-	Favored (18.27%) Ile or Val / -122.2,165.2	Favored (28.5%) *m* chi angles: 298.5	0.04Å	Favored (41.053%)	-	-	-
A 268		GLY	1.2	-	Favored (86.6%) Glycine / -85.0,3.8	-	-	Favored (10.682%)	-	-	-
A 269		LYS	1.22	-	Favored (91.32%) Pre-Pro / -62.1,128.8	Favored (54%) *tttp* chi angles: 184.2,177.8,176.4,65.6	0.07Å	Favored (36.968%)	-	-	-
A 270		PRO	1.22	-	Favored (86.53%) Trans-Pro / -56.3,139.6	Favored (84.4%) *Cg_exo* chi angles: 334.1,34.9,331	0.03Å	Favored (79.091%)	-	-	-
A 271		GLN	1.2	-	Favored (37.96%) Pre-Pro / -100.4,152.9	Favored (81.8%) *mt0* chi angles: 295.4,179,0.8	0.07Å	Favored (34.575%)	-	-	-
A 272		PRO	1.15	-	Favored (72.33%) Trans-Pro / -68.8,153.9	Favored (46.8%) *Cg_endo* chi angles: 24.8,327,27.3	0.02Å	Favored (65.135%)	-	-	-
A 273		HIS	1.09	-	Favored (58.41%) General / -62.6,141.5	Favored (72.6%) *t-90* chi angles: 186.3,280.1	0.02Å	Favored (34.933%)	-	-	-
A 274		THR	1.02	-	Favored (52.61%) General / -125.6,132.1	Favored (95.8%) *m* chi angles: 299.6	0.03Å	Favored (51.13%) beta sheet	-	-	-
A 275		ASN	0.96	-	Favored (6.06%) General / -80.8,85.8	Favored (82.9%) *m-40* chi angles: 292.3,315.2	0.02Å	Favored (53.431%) beta sheet	-	-	-
A 276		GLN	0.92	-	Favored (70.47%) General / -59.0,-33.2	Favored (97%) *mt0* chi angles: 290.4,176.9,336.2	0.01Å	Favored (35.708%)	-	-	-
A 277		GLU	0.88	-	Favored (67.51%) General / -61.3,-26.0	Favored (79.8%) *mm-30* chi angles: 293,297.6,307.4	0.03Å	Favored (59.007%) alpha helix	-	-	-
A 278		LYS	0.85	-	Favored (47.42%) General / -93.1,-6.8	Favored (62.8%) *mttm* chi angles: 294.1,181,180.6,293.9	0.03Å	Favored (55.093%) alpha helix	-	-	-
A 279		ILE	0.82	-	Favored (7.71%) Ile or Val / -121.4,-13.9	Favored (46.2%) *pt* chi angles: 61.3,171.7	0.04Å	Favored (22.32%) alpha helix	-	-	-
A 280		LYS	0.8	-	Favored (67.63%) General / -60.1,-28.2	Favored (59.5%) *pttt* chi angles: 69.4,181.9,184.2,183.2	0.03Å	Favored (47.195%) alpha helix	-	-	-
**#**	**Alt**	**Res**	**High B**	**Clash > 0.4Å**	**Ramachandran**	**Rotamer**	**Cβ deviation**	**CaBLAM**	**Bond lengths**	**Bond angles**	**Cis Peptides**

			Avg: 0.92	Clashscore: 1.59	Outliers: 4 of 903	Poor rotamers: 0 of 771	Outliers: 0 of 828	Outliers: 22 of 901	Outliers: 13 of 905	Outliers: 19 of 905	Non-Trans: 1 of 904
A 281		ALA	0.77	-	Favored (89.32%) General / -66.5,-40.3	-	0.03Å	Favored (77.958%) alpha helix	-	-	-
A 282		ARG	0.75	-	Favored (71.85%) General / -70.9,-41.0	Favored (98.2%) *mtm-85* chi angles: 291.7,194.9,291.2,276.4	0.08Å	Favored (80.436%) alpha helix	-	-	-
A 283		ILE	0.74	-	Favored (78.56%) Ile or Val / -70.0,-44.3	Favored (93.7%) *mt* chi angles: 294.3,166.7	0.13Å	Favored (93.353%) alpha helix	-	-	-
A 284		GLN	0.73	-	Favored (96.35%) General / -60.9,-41.7	Favored (61.2%) *tp40* chi angles: 182.9,66.1,29.5	0.03Å	Favored (86.037%) alpha helix	-	-	-
A 285		ARG	0.72	-	Favored (86.32%) General / -57.7,-45.0	Favored (61.2%) *ttt90* chi angles: 186.2,183.1,173.7,87.7	0.01Å	Favored (85.386%) alpha helix	-	-	-
A 286		LEU	0.72	-	Favored (98.19%) General / -63.5,-40.9	Favored (89.2%) *mt* chi angles: 291,170.5	0.09Å	Favored (86.255%) alpha helix	-	-	-
A 287		LYS	0.72	-	Favored (92.33%) General / -59.2,-44.3	Favored (86.4%) *tttt* chi angles: 181.1,176.9,179.1,181.3	0.03Å	Favored (81.302%) alpha helix	-	-	-
A 288		GLU	0.72	-	Favored (80.62%) General / -68.5,-40.6	Favored (99%) *mt-10* chi angles: 292.1,178.6,2.2	0.02Å	Favored (77.694%) alpha helix	-	-	-
A 289		GLU	0.73	-	Favored (79.32%) General / -58.9,-38.7	Favored (54.2%) *tp30* chi angles: 183.5,72.9,13.3	0.07Å	Favored (54.159%) alpha helix	-	-	-
A 290		TYR	0.74	-	Favored (9.96%) General / -115.2,26.6	Favored (71.1%) *m-80* chi angles: 303.4,106.1	0.04Å	Favored (14.529%) alpha helix	-	-	-
A 291		ALA	0.76	-	Favored (66.39%) General / -55.0,-37.3	-	0.04Å	Favored (37.531%) alpha helix	-	-	-
A 292		ALA	0.78	-	Favored (64.79%) General / -59.5,-25.9	-	0.04Å	Favored (46.033%) alpha helix	-	-	-
A 293		THR	0.81	-	Favored (10.39%) General / -122.3,14.1	Favored (68.7%) *p* chi angles: 59	0.06Å	Favored (18.196%)	-	-	-
A 294		TRP	0.85	-	Favored (34.33%) General / -82.0,139.8	Favored (50.2%) *t-100* chi angles: 187.1,269.4	0.04Å	Favored (8.417%)	-	-	-
A 295		HIS	0.91	-	Favored (20.92%) General / -153.3,170.3	Favored (51.9%) *p-80* chi angles: 62.2,274.2	0.01Å	Favored (35.921%) beta sheet	-	-	-
A 296		HIS	0.97	-	Favored (16.78%) General / -125.5,112.9	Favored (66.9%) *m-70* chi angles: 299.8,263.8	0.02Å	Favored (19.726%) beta sheet	-	-	-

#	Alt	Res	High B	Clash > 0.4Å	Ramachandran	Rotamer	Cβ deviation	CaBLAM	Bond lengths	Bond angles	Cis Peptides
A 297		ASP	1.03	-	Favored (14.62%) General / -90.6,101.9	Favored (65.2%) *t0* chi angles: 185.5,344.2	0.01Å	Favored (71.389%) beta sheet	-	-	-
A 298		LYS	1.07	-	Favored (59.77%) General / -62.1,-18.1	Favored (14.1%) *pttp* chi angles: 72.8,179.9,183.2,69.8	0.04Å	Favored (37.597%)	-	-	-
A 299		ASP	1.08	-	Favored (16.01%) General / -101.1,20.2	Favored (86.9%) *m-30* chi angles: 294.3,344.1	0.05Å	Favored (8.19%)	-	-	-
A 300		HIS	1.05	-	Favored (3.9%) Pre-Pro / -43.7,131.9	Favored (88.1%) *t70* chi angles: 182.1,74	0.12Å	Favored (15.874%)	OUTLIER(S) worst is CB--CG: 5.5 σ	-	-
**#**	**Alt**	**Res**	**High B**	**Clash > 0.4Å**	**Ramachandran**	**Rotamer**	**Cβ deviation**	**CaBLAM**	**Bond lengths**	**Bond angles**	**Cis Peptides**
			Avg: 0.92	Clashscore: 1.59	Outliers: 4 of 903	Poor rotamers: 0 of 771	Outliers: 0 of 828	Outliers: 22 of 901	Outliers: 13 of 905	Outliers: 19 of 905	Non-Trans: 1 of 904
A 301		PRO	1.01	-	Favored (26%) Trans-Pro / -73.1,-15.2	Favored (72.9%) *Cg_endo* chi angles: 27.5,324.8,27.9	0.17Å	Favored (11.413%) beta sheet	-	-	-
A 302		TYR	0.94	-	Favored (32.88%) General / -80.1,144.7	Favored (24.3%) *m-10* chi angles: 296.6,342.7	0.05Å	Favored (26.887%)	-	-	-
A 303		ARG	0.87	-	Favored (15.21%) General / -97.2,-25.6	Favored (44.4%) *ptt180* chi angles: 69.5,182.5,179.9,184.5	0.03Å	Favored (26.637%)	-	-	-
A 304		THR	0.81	-	Favored (3.92%) General / -110.5,-43.1	Favored (91.6%) *m* chi angles: 297.9	0.03Å	Favored (10.942%)	-	-	-
A 305		TRP	0.76	-	Favored (41.68%) General / -75.2,145.9	Favored (41.1%) *m100* chi angles: 289.7,66.8	0.05Å	Favored (19.856%)	OUTLIER(S) worst is CE2--CZ2: 4.6 σ	-	-
A 306		THR	0.72	-	Favored (32.33%) General / -80.7,127.3	Favored (91.5%) *m* chi angles: 299	0.02Å	Favored (45.042%) beta sheet	-	-	-
A 307		TYR	0.69	-	Favored (40.46%) General / -94.4,131.1	Favored (72.8%) *t80* chi angles: 175.1,69.6	0.07Å	Favored (43.857%)	-	-	-
A 308		HIS	0.67	-	Favored (3.88%) General / -107.8,-46.0	Favored (99.3%) *m-70* chi angles: 298.6,286.7	0.07Å	CaBLAM Disfavored (2.457%)	-	-	-
A 309		GLY	0.67	-	Favored (41.52%) Glycine / 175.5,173.8	-	-	Favored (22.774%)	-	-	-
A 310		SER	0.7	-	Favored (24.82%) General / -138.1,166.2	Favored (52.4%) *m* chi angles: 300.7	0.10Å	Favored (58.079%)	-	-	-
A 311		TYR	0.75	-	Favored (43.66%) General / -149.1,160.1	Favored (45.2%) *p90* chi angles: 70.8,91.9	0.03Å	Favored (17.106%)	-	-	-
A 312		GLU	0.84	-	Favored (50.75%) General / -70.9,139.2	Favored (82.2%) *mt-10* chi angles: 297.6,183.8,12.6	0.02Å	Favored (25.022%)	-	-	-

A 313		VAL	0.96	-	Favored (13.56%) Ile or Val / -149.8,150.7	Favored (7.5%) *p* chi angles: 68.3	0.12Å	Favored (38.738%) beta sheet	-	-	-
A 314		LYS	1.08	-	Favored (96.25%) Pre-Pro / -68.6,144.4	Favored (97.9%) *mttt* chi angles: 291.9,181.5,180.1,179.1	0.03Å	Favored (48.315%) beta sheet	-	-	-
A 315		PRO	1.19	-	Favored (53.86%) Trans-Pro / -71.8,158.0	Favored (73%) *Cg_endo* chi angles: 27.5,324.9,27.9	0.06Å	Favored (52.566%)	-	-	-
A 316		THR	1.27	-	Favored (3.82%) General / -138.7,-174.4	Favored (12%) *t* chi angles: 190.4	0.04Å	Favored (10.283%)	-	-	-
A 317		GLY	1.3	-	Favored (49.6%) Glycine / 68.0,-161.5	-	-	Favored (27.239%)	-	-	-
A 318		SER	1.29	-	Favored (15.73%) General / -159.7,145.5	Favored (45.7%) *t* chi angles: 179.5	0.06Å	Favored (8.277%)	-	-	-
A 319		ALA	1.25	-	Favored (46.2%) General / -96.8,-1.5	-	0.03Å	Favored (40.607%)	-	-	-
A 320		SER	1.18	-	Allowed (1.42%) General / -103.5,-167.8	Favored (13.4%) *t* chi angles: 188.9	0.01Å	Favored (5.976%)	-	-	-
**#**	**Alt**	**Res**	**High B**	**Clash > 0.4Å**	**Ramachandran**	**Rotamer**	**Cβ deviation**	**CaBLAM**	**Bond lengths**	**Bond angles**	**Cis Peptides**
			Avg: 0.92	Clashscore: 1.59	Outliers: 4 of 903	Poor rotamers: 0 of 771	Outliers: 0 of 828	Outliers: 22 of 901	Outliers: 13 of 905	Outliers: 19 of 905	Non-Trans: 1 of 904
A 321		SER	1.11	-	Favored (18.76%) General / -156.4,143.2	Favored (34.8%) *t* chi angles: 182.4	0.04Å	Favored (21.353%)	-	-	-
A 322		LEU	1.02	-	Favored (22.79%) General / -96.2,148.4	Favored (93.4%) *mt* chi angles: 297.7,174.3	0.08Å	Favored (42.832%) beta sheet	-	-	-
A 323		VAL	0.93	-	Favored (52.99%) Ile or Val / -109.9,134.1	Favored (81.4%) *t* chi angles: 176.5	0.07Å	Favored (43.421%) beta sheet	-	-	-
A 324		ASN	0.86	-	Favored (4.79%) General / -75.7,100.8	Favored (27%) *t0* chi angles: 179,324.6	0.08Å	Favored (55.53%) beta sheet	-	-	-
A 325		GLY	0.8	-	Favored (76.69%) Glycine / -58.4,-34.9	-	-	Favored (43.977%)	-	-	-
A 326		VAL	0.76	-	Favored (79.74%) Ile or Val / -69.2,-45.4	Favored (63.4%) *t* chi angles: 171.3	0.04Å	Favored (75.48%) alpha helix	-	-	-
A 327		VAL	0.75	-	Favored (87.44%) Ile or Val / -67.3,-44.4	Favored (92.2%) *t* chi angles: 174.4	0.02Å	Favored (74.937%) alpha helix	-	-	-
A 328		ARG	0.76	-	Favored (79.92%) General / -56.6,-47.3	Favored (84%) *ttp80* chi angles: 176.4,183.1,64.7,79.9	0.05Å	Favored (83.994%) alpha helix	-	-	-

A 329		LEU	0.8	-	Favored (87.59%) General / -64.5,-37.5	Favored (82.4%) *mt* chi angles: 289.4,170.1	0.02Å	Favored (74.531%) alpha helix	-	-	-
A 330		MET	0.84	-	Favored (36.74%) General / -81.4,-24.8	Favored (82.7%) *mmm* chi angles: 297.5,311.2,297.3	0.02Å	Favored (76.478%)	-	-	-
A 331		SER	0.9	-	Favored (56.5%) General / -91.7,2.4	Favored (59.9%) *m* chi angles: 299	0.07Å	Favored (29.878%)	-	-	-
A 332		LYS	0.97	-	OUTLIER (0.04)%) Pre-Pro / -17.1,-57.4	Favored (86.2%) *tttt* chi angles: 180.6,177.2,179.2,179.8	0.09Å	Favored (6.625%)	-	OUTLIER(S) worst is CA-C-N: 4.4 σ	-
A 333		PRO	1.04	-	Favored (69.82%) Trans-Pro / -60.5,-21.8	Favored (35.2%) *Cg_endo* chi angles: 22.4,321.5,38.6	0.05Å	Favored (59.717%) alpha helix	-	-	-
A 334		TRP	1.11	0.41Å CZ2 with A 855 LEU HB2	Favored (59.64%) General / -80.0,-9.0	Favored (96.8%) *m100* chi angles: 287.5,101	0.04Å	Favored (57.671%) three-ten	-	-	-
A 335		ASP	1.17	-	Favored (61.96%) General / -70.3,-13.0	Favored (4.4%) *t70* chi angles: 205.9,56.7	0.01Å	Favored (53.475%) three-ten	-	-	-
A 336		ALA	1.2	-	Favored (59.3%) General / -82.8,-6.2	-	0.06Å	Favored (54.252%)	-	-	-
A 337		ILE	1.19	-	Favored (61.13%) Ile or Val / -107.8,121.7	Favored (46.2%) *mm* chi angles: 304.9,300.3	0.08Å	Favored (35.705%)	-	-	-
A 338		LEU	1.15	-	Favored (71.56%) General / -65.3,-30.7	Favored (82.4%) *mt* chi angles: 291,175	0.02Å	Favored (40.895%)	-	-	-
A 339		ASN	1.09	-	Favored (40.39%) General / -71.2,-6.5	Favored (98.2%) *m-40* chi angles: 290.5,342.5	0.05Å	Favored (16.079%) alpha helix	-	-	-
A 340		VAL	1.01	-	Favored (7.8%) Ile or Val / -106.9,-50.9	Favored (94.7%) *t* chi angles: 174.8	0.04Å	Favored (10.417%) alpha helix	-	-	-
**#**	**Alt**	**Res**	**High B**	**Clash > 0.4Å**	**Ramachandran**	**Rotamer**	**Cβ deviation**	**CaBLAM**	**Bond lengths**	**Bond angles**	**Cis Peptides**
			Avg: 0.92	Clashscore: 1.59	Outliers: 4 of 903	Poor rotamers: 0 of 771	Outliers: 0 of 828	Outliers: 22 of 901	Outliers: 13 of 905	Outliers: 19 of 905	Non-Trans: 1 of 904
A 341		THR	0.94	-	Favored (60.85%) General / -74.5,-16.0	Favored (75.6%) *p* chi angles: 60	0.05Å	Favored (52.892%) alpha helix	-	-	-
A 342		THR	0.89	-	Favored (46.88%) General / -96.7,7.3	Favored (78.3%) *p* chi angles: 60.8	0.07Å	Favored (49.628%)	-	-	-
A 343		MET	0.86	-	Favored (37.41%) General / -79.6,139.8	Favored (48%) *ttm* chi angles: 184.4,169.3,287.9	0.12Å	Favored (17.791%)	-	-	-
A 344		ALA	0.84	-	Favored (44.05%) General / -151.6,158.8	-	0.06Å	Favored (32.363%)	-	-	-
A 345		MET	0.84	-	Favored (58.54%) General / -62.7,138.6	Favored (61.4%) *ttp* chi angles: 185.4,182.2,75.3	0.05Å	Favored (38.205%)	-	-	-

A 346		THR	0.84	-	Favored (24.43%) General / -71.6,165.2	Favored (48.6%) *p* chi angles: 55.9	0.09Å	Favored (27.585%)	-	-	-
A 347		ASP	0.85	-	Favored (17.3%) General / -78.8,113.9	Favored (66.2%) *t0* chi angles: 186.9,346.7	0.08Å	Favored (11.697%)	-	-	-
A 348		THR	0.85	-	Favored (17.7%) General / -114.2,12.5	Favored (45.5%) *p* chi angles: 55.4	0.07Å	Favored (15.876%) beta sheet	-	-	-
A 349		THR	0.86	-	Favored (41.64%) Pre-Pro / -78.3,166.6	Favored (65.2%) *p* chi angles: 63.1	0.08Å	Favored (29.543%)	-	-	-
A 350		PRO	0.87	-	Favored (32.12%) Trans-Pro / -49.0,-35.5	Favored (90.9%) *Cg_exo* chi angles: 329.6,37.4,332	0.06Å	Favored (76.105%)	-	-	-
A 351		PHE	0.89	-	Favored (63.28%) General / -72.2,-44.2	Favored (55%) *t80* chi angles: 179.7,63	0.09Å	Favored (75.795%) alpha helix	-	-	-
A 352		GLY	0.91	-	Favored (50.71%) Glycine / -54.5,-51.6	-	-	Favored (96.251%) alpha helix	-	-	-
A 353		GLN	0.93	-	Favored (85.27%) General / -60.6,-38.6	Favored (84.2%) *mt0* chi angles: 290.8,177.4,40.5	0.02Å	Favored (79.574%) alpha helix	-	-	-
A 354		GLN	0.95	-	Favored (84.38%) General / -66.9,-37.4	Favored (76.9%) *mm-40* chi angles: 285.6,293.2,306.4	0.03Å	Favored (94.076%) alpha helix	-	-	-
A 355		ARG	0.98	-	Favored (95%) General / -64.4,-40.0	Favored (32.4%) *mmm160* chi angles: 288.6,293.4,295,177.2	0.04Å	Favored (88.594%) alpha helix	-	-	-
A 356		VAL	1.01	-	Favored (88.55%) Ile or Val / -66.9,-44.7	Favored (71.4%) *t* chi angles: 172.3	0.07Å	Favored (89.708%) alpha helix	-	-	-
A 357		PHE	1.04	0.51Å C with A 357 PHE CD2	Favored (68.48%) General / -58.0,-51.7	Favored (39.1%) *t80* chi angles: 185.8,98.8	0.12Å	Favored (76.613%) alpha helix	-	OUTLIER(S) worst is CA-CB-CG: 4.8 σ	-
A 358		LYS	1.07	-	Favored (78.18%) General / -66.1,-46.3	Favored (15.9%) *tptp* chi angles: 179.5,60.1,161.6,48.6	0.07Å	Favored (70.217%) alpha helix	-	-	-
A 359		GLU	1.1	-	Favored (76.04%) General / -69.0,-35.0	Favored (88.5%) *mt-10* chi angles: 289.3,182.4,338.9	0.07Å	Favored (37.456%) alpha helix	-	-	-
A 360		LYS	1.13	-	Favored (2.86%) General / -112.3,-51.2	Favored (72.5%) *mmtt* chi angles: 302.2,296.8,182.7,181.5	0.05Å	Favored (12.959%) alpha helix	-	-	-
**#**	**Alt**	**Res**	**High B**	**Clash > 0.4Å**	**Ramachandran**	**Rotamer**	**Cβ deviation**	**CaBLAM**	**Bond lengths**	**Bond angles**	**Cis Peptides**
			Avg: 0.92	Clashscore: 1.59	Outliers: 4 of 903	Poor rotamers: 0 of 771	Outliers: 0 of 828	Outliers: 22 of 901	Outliers: 13 of 905	Outliers: 19 of 905	Non-Trans: 1 of 904
A 361		VAL	1.16	-	Favored (70.68%)	Favored (68.3%) *t* chi angles: 179	0.17Å	Favored (76.121%)	-	-	-

					Ile or Val / -70.3,-37.2						
A 362		ASP	1.18	-	Favored (19.64%) General / -69.4,-4.8	Favored (3.4%) *m-30* chi angles: 267,331.2	0.20Å	Favored (38.23%)	-	OUTLIER(S) worst is CA-CB-CG: 9.3 σ	-
A 363		THR	1.18	-	Favored (26.11%) General / -60.3,126.1	Favored (98.9%) *m* chi angles: 300.3	0.01Å	Favored (33.651%)	-	-	-
A 364		LYS	1.15	-	Favored (35.15%) General / -87.2,128.3	Favored (13.9%) *tptp* chi angles: 176.5,73.2,184.2,80.4	0.08Å	Favored (47.736%) beta sheet	-	-	-
A 365		ALA	1.09	-	Favored (67.68%) Pre-Pro / -93.3,118.5	-	0.06Å	Favored (51.676%) beta sheet	-	-	-
A 366		PRO	1.01	-	Favored (92.89%) Trans-Pro / -60.8,148.2	Favored (62.7%) *Cg_exo* chi angles: 335.8,33,332.1	0.04Å	Favored (47.681%) beta sheet	-	-	-
A 367		GLU	0.91	-	Favored (81.29%) Pre-Pro / -75.2,146.0	Favored (81%) *mm-30* chi angles: 294.8,291.4,330.5	0.03Å	Favored (34.684%)	-	-	-
A 368		PRO	0.82	-	Favored (46.69%) Trans-Pro / -62.2,157.1	Favored (20.8%) *Cg_exo* chi angles: 342.2,31.9,328	0.02Å	Favored (54.539%)	-	-	-
A 369		PRO	0.74	-	Favored (44.98%) Trans-Pro / -59.6,154.9	Favored (79.5%) *Cg_exo* chi angles: 334.7,33.6,332.2	0.06Å	Favored (76.076%)	-	-	-
A 370		SER	0.69	-	Favored (81.07%) General / -56.8,-43.7	Favored (64.2%) *m* chi angles: 294.1	0.01Å	Favored (64.984%)	-	-	-
A 371		GLY	0.65	-	Favored (57.68%) Glycine / -57.6,-51.5	-	-	Favored (95.613%) alpha helix	-	-	-
A 372		VAL	0.64	-	Favored (76.34%) Ile or Val / -56.6,-42.6	Favored (65%) *t* chi angles: 171.5	0.04Å	Favored (81.26%) alpha helix	-	-	-
A 373		ARG	0.63	-	Favored (91.93%) General / -62.5,-39.0	Favored (85.2%) *mtp180* chi angles: 290.3,177.8,67.2,191.1	0.02Å	Favored (97.301%) alpha helix	-	-	-
A 374		GLU	0.63	-	Favored (93.87%) General / -65.0,-39.7	Favored (63.9%) *mt-10* chi angles: 290.3,170.9,317.9	0.03Å	Favored (98.301%) alpha helix	-	-	-
A 375		VAL	0.63	-	Favored (93.57%) Ile or Val / -65.6,-44.5	Favored (83.1%) *t* chi angles: 173.4	0.06Å	Favored (94.226%) alpha helix	-	-	-
A 376		MET	0.64	-	Favored (81.69%) General / -65.2,-35.6	Favored (12.2%) *tpt* chi angles: 193,66.2,177.7	0.04Å	Favored (90.206%) alpha helix	-	-	-
A 377		ASP	0.64	-	Favored (74.79%) General / -65.6,-47.7	Favored (25%) *t70* chi angles: 189.9,72.7	0.04Å	Favored (81.345%) alpha helix	-	-	-
A 378		GLU	0.65	-	Favored (92.18%) General / -64.3,-38.8	Favored (81.5%) *mm-30* chi angles: 291.2,291.9,331.8	0.05Å	Favored (82.807%) alpha helix	-	-	-

A 379		THR	0.65	-	Favored (78.88%) General / -67.7,-43.9	Favored (87.5%) *m* chi angles: 301.4	0.06Å	Favored (93.674%) alpha helix	-	-	-
A 380		THR	0.65	-	Favored (99.13%) General / -63.0,-43.3	Favored (88.6%) *m* chi angles: 297	0.04Å	Favored (96.546%) alpha helix	-	-	-
**#**	**Alt**	**Res**	**High B**	**Clash > 0.4Å**	**Ramachandran**	**Rotamer**	**Cβ deviation**	**CaBLAM**	**Bond lengths**	**Bond angles**	**Cis Peptides**
			Avg: 0.92	Clashscore: 1.59	Outliers: 4 of 903	Poor rotamers: 0 of 771	Outliers: 0 of 828	Outliers: 22 of 901	Outliers: 13 of 905	Outliers: 19 of 905	Non-Trans: 1 of 904
A 381		ASN	0.66	-	Favored (97.19%) General / -60.4,-43.0	Favored (98.3%) *m-40* chi angles: 287.2,339.7	0.06Å	Favored (95.78%) alpha helix	-	-	-
A 382		TRP	0.67	-	Favored (78.78%) General / -63.1,-48.4	Favored (87.3%) *t60* chi angles: 176.2,85.1	0.06Å	Favored (89.984%) alpha helix	-	-	-
A 383		LEU	0.68	-	Favored (92.74%) General / -65.6,-41.4	Favored (45.7%) *tp* chi angles: 184.4,59.5	0.11Å	Favored (85.737%) alpha helix	-	-	-
A 384		TRP	0.71	-	Favored (95.88%) General / -64.5,-40.6	Favored (46.3%) *m100* chi angles: 281.2,119.3	0.08Å	Favored (94.576%) alpha helix	-	-	-
A 385		ALA	0.74	-	Favored (87.32%) General / -61.0,-38.8	-	0.04Å	Favored (94.146%) alpha helix	-	-	-
A 386		PHE	0.79	-	Favored (66.49%) General / -61.7,-51.9	Favored (93.4%) *t80* chi angles: 177.7,78.3	0.07Å	Favored (77.993%) alpha helix	-	OUTLIER(S) worst is CA-CB-CG: 5.3 σ	-
A 387		LEU	0.86	-	Favored (78.59%) General / -63.9,-34.7	Favored (94.6%) *mt* chi angles: 295.3,174.4	0.09Å	Favored (74.818%) alpha helix	-	-	-
A 388		ALA	0.94	-	Favored (38.02%) General / -75.6,-4.1	-	0.03Å	Favored (39.382%)	-	-	-
A 389		ARG	1.01	-	Favored (71.12%) General / -66.3,-30.7	Favored (99%) *mtt180* chi angles: 289.1,175.8,179.3,174.8	0.09Å	Favored (19.677%)	-	-	-
A 390		GLU	1.06	-	Favored (6.18%) General / -107.3,-37.7	Favored (83.8%) *mm-30* chi angles: 298.8,290.1,340.2	0.03Å	Favored (30.099%)	-	-	-
A 391		LYS	1.07	-	Favored (48.07%) General / -124.6,148.1	Favored (71.7%) *mmtt* chi angles: 298.5,291.1,184.6,182.2	0.04Å	Favored (20.263%)	-	-	-
A 392		LYS	1.05	-	Favored (53.73%) Pre-Pro / -122.6,148.7	Favored (31.5%) *mmtm* chi angles: 298.6,294.6,190.8,295.5	0.02Å	Favored (39.05%)	-	-	-
A 393		PRO	1	-	Favored (80.31%) Trans-Pro / -59.6,149.8	Favored (51.5%) *Cg_exo* chi angles: 336.6,34.5,329.3	0.05Å	Favored (37.738%)	-	-	-
A 394		ARG	0.93	-	Favored (42.65%) General / -149.2,156.8	Favored (53.2%) *ptt90* chi angles: 63,183.5,175.5,89.7	0.07Å	Favored (42.267%) beta sheet	-	-	-

#	Alt	Res	High B	Clash > 0.4Å	Ramachandran	Rotamer	Cβ deviation	CaBLAM	Bond lengths	Bond angles	Cis Peptides
A 395		LEU	0.87	-	Favored (28.84%) General / -84.2,144.6	Favored (93.9%) *mt* chi angles: 295,175.2	0.04Å	Favored (39.744%) beta sheet	-	-	-
A 396		CYS	0.82	-	Favored (24.92%) General / -89.0,146.4	Favored (70.2%) *m* chi angles: 298.4	0.03Å	Favored (41.536%) beta sheet	-	-	-
A 397		THR	0.79	-	Favored (14.79%) General / -85.7,168.0	Favored (74.1%) *p* chi angles: 61.5	0.05Å	Favored (50.617%)	-	-	-
A 398		ARG	0.76	-	Favored (86.56%) General / -59.4,-40.4	Favored (79.1%) *ttt180* chi angles: 183.7,179.4,177.7,189.5	0.03Å	Favored (66.017%)	-	-	-
A 399		GLU	0.75	-	Favored (94.62%) General / -60.2,-45.0	Favored (86.2%) *tt0* chi angles: 178.9,177.8,351.1	0.02Å	Favored (88.097%) alpha helix	-	-	-
A 400		GLU	0.75	-	Favored (91.93%) General / -62.4,-39.0	Favored (35.5%) *mt-10* chi angles: 287.8,169,286.5	0.03Å	Favored (93.495%) alpha helix	-	-	-
**#**	**Alt**	**Res**	**High B**	**Clash > 0.4Å**	**Ramachandran**	**Rotamer**	**Cβ deviation**	**CaBLAM**	**Bond lengths**	**Bond angles**	**Cis Peptides**
			Avg: 0.92	Clashscore: 1.59	Outliers: 4 of 903	Poor rotamers: 0 of 771	Outliers: 0 of 828	Outliers: 22 of 901	Outliers: 13 of 905	Outliers: 19 of 905	Non-Trans: 1 of 904
A 401		PHE	0.76	-	Favored (83.43%) General / -62.3,-47.6	Favored (79.5%) *t80* chi angles: 183,74.9	0.05Å	Favored (87.254%) alpha helix	-	-	-
A 402		LYS	0.8	-	Favored (74.75%) General / -55.0,-44.6	Favored (86.7%) *tttt* chi angles: 183.3,178,177.5,181.5	0.02Å	Favored (92.666%) alpha helix	-	-	-
A 403		ARG	0.89	-	Favored (72.43%) General / -63.9,-31.3	Favored (97.3%) *mtt-85* chi angles: 288.9,179.5,182.1,276.3	0.03Å	Favored (77.989%) alpha helix	-	-	-
A 404		LYS	1.03	-	Favored (79.68%) General / -68.8,-40.3	Favored (52.4%) *tptt* chi angles: 186.8,66.7,175,180.2	0.03Å	Favored (78.234%) alpha helix	-	-	-
A 405		VAL	1.22	-	Favored (78.04%) Ile or Val / -70.6,-43.1	Favored (63.1%) *t* chi angles: 171.2	0.04Å	CaBLAM Disfavored (4.424%)	-	-	-
A 406		ASN	1.45	-	Allowed (1.7%) General / 56.8,-122.8	Favored (81%) *m-40* chi angles: 294.9,310.2	0.09Å	CaBLAM Outlier (0.65%)	-	-	-
A 407		SER	1.68	-	Favored (6.79%) General / -108.3,-34.5	Favored (70.1%) *m* chi angles: 296.4	0.02Å	CaBLAM Outlier (0.212%)	-	-	-
A 408		ASN	1.86	-	Allowed (1.33%) General / -102.6,-62.0	Favored (98.1%) *m-40* chi angles: 292,339.4	0.02Å	CaBLAM Outlier (0.292%)	-	-	-
A 409		ALA	1.95	-	Allowed (0.75%) General / 44.5,-133.0	-	0.03Å	CaBLAM Outlier (0.213%)	-	-	-
A 410		ALA	1.94	-	Favored (12.91%) General / -161.3,145.0	-	0.03Å	CaBLAM Disfavored (1.717%) try beta sheet	-	-	-

A 411		LEU	1.84	-	Favored (50.93%) General / -128.2,145.7	Favored (94.4%) *mt* chi angles: 296.8,174.1	0.08Å	Favored (64.811%) beta sheet	-	-	-
A 412		GLY	1.69	-	Favored (12.2%) Glycine / -113.5,-156.8	-	-	Favored (31.08%) beta sheet	-	-	-
A 413		ALA	1.54	-	Favored (17.06%) General / -159.0,145.7	-	0.02Å	Favored (13.049%)	-	-	-
A 414		MET	1.4	0.59Å N with A 414 MET SD	Allowed (0.91%) General / -141.1,-13.9	Favored (2.8%) *pmm* chi angles: 61.2,292.8,296.2	0.13Å	Favored (7.559%)	-	-	-
A 415		PHE	1.28	-	Favored (50.98%) General / -135.2,151.8	Favored (97%) *m-80* chi angles: 297.2,99.6	0.09Å	Favored (25.194%) alpha helix	-	-	-
A 416		GLU	1.18	-	Favored (65.42%) General / -57.4,-31.0	Favored (67.1%) *tp30* chi angles: 184,64.2,18.1	0.03Å	Favored (56.656%) alpha helix	-	-	-
A 417		GLU	1.09	-	Favored (83.19%) General / -61.1,-37.6	Favored (96.9%) *mt-10* chi angles: 289.8,171.7,341.5	0.05Å	Favored (67.328%) alpha helix	-	-	-
A 418		GLN	1.03	-	Favored (77.95%) General / -66.3,-34.4	Favored (84.4%) *tp40* chi angles: 184.4,66,62	0.06Å	Favored (69.542%) alpha helix	-	-	-
A 419		ASN	0.99	-	Favored (4.38%) General / -74.1,5.5	Favored (89.2%) *m-40* chi angles: 285.8,333.1	0.04Å	Favored (21.153%) three-ten	-	-	-
A 420		GLN	0.95	-	Favored (59.19%) General / -86.7,-4.6	Favored (97.5%) *mm-40* chi angles: 300.7,299.7,301.2	0.04Å	Favored (56.079%)	-	-	-
**#**	**Alt**	**Res**	**High B**	**Clash > 0.4Å**	**Ramachandran**	**Rotamer**	**Cβ deviation**	**CaBLAM**	**Bond lengths**	**Bond angles**	**Cis Peptides**
			Avg: 0.92	Clashscore: 1.59	Outliers: 4 of 903	Poor rotamers: 0 of 771	Outliers: 0 of 828	Outliers: 22 of 901	Outliers: 13 of 905	Outliers: 19 of 905	Non-Trans: 1 of 904
A 421		TRP	0.92	-	Favored (54.06%) General / -121.5,139.0	Favored (6.2%) *m-90* chi angles: 297.1,299.2	0.06Å	Favored (29.819%)	-	-	-
A 422		SER	0.89	-	Favored (53.06%) General / -83.7,-13.9	Favored (84.5%) *p* chi angles: 67.4	0.02Å	Favored (16.273%)	-	-	-
A 423		SER	0.86	-	Favored (36.32%) General / -157.9,159.2	Favored (95.6%) *p* chi angles: 66.1	0.04Å	Favored (20.533%)	-	-	-
A 424		ALA	0.84	-	Favored (77.74%) General / -60.2,-36.5	-	0.05Å	Favored (66.088%) alpha helix	-	-	-
A 425		ARG	0.82	-	Favored (77.95%) General / -58.5,-49.3	Favored (75.8%) *ttt180* chi angles: 177.9,173.9,170.8,174.3	0.05Å	Favored (79.47%) alpha helix	-	-	-
A 426		GLU	0.8	-	Favored (88.42%) General / -58.4,-43.2	Favored (87.8%) *tt0* chi angles: 183.6,182.3,1.4	0.03Å	Favored (94.193%) alpha helix	-	-	-

A 427		ALA	0.78	-	Favored (94.09%) General / -60.4,-41.6	-	0.04Å	Favored (95.545%) alpha helix	-	-	-
A 428		VAL	0.78	-	Favored (93.85%) Ile or Val / -65.6,-42.9	Favored (76.4%) *t* chi angles: 172.8	0.04Å	Favored (91.795%) alpha helix	-	-	-
A 429		GLU	0.77	-	Favored (86.78%) General / -65.7,-37.5	Favored (99.7%) *mt-10* chi angles: 291.6,174.9,356	0.04Å	Favored (38.826%)	-	-	-
A 430		ASP	0.76	-	Favored (24.43%) Pre-Pro / -61.8,117.0	Favored (46.2%) *t0* chi angles: 185.7,330.1	0.03Å	Favored (30.867%)	-	OUTLIER(S) worst is CA-CB-CG: 4.8 σ	-
A 431		PRO	0.76	-	Favored (35.03%) Trans-Pro / -53.1,-26.9	Favored (99.9%) *Cg_exo* chi angles: 332.5,36.1,330.5	0.06Å	Favored (78.964%)	-	-	-
A 432		ARG	0.75	-	Favored (61.03%) General / -74.5,-28.5	Favored (91.7%) *mtm180* chi angles: 294,174.9,295.8,171.3	0.07Å	Favored (73.281%) alpha helix	-	-	-
A 433		PHE	0.74	-	Favored (75.9%) General / -63.1,-49.1	Favored (86.7%) *t80* chi angles: 174.2,76	0.07Å	Favored (73.792%) alpha helix	-	-	-
A 434		TRP	0.73	0.46Å CE3 with A 434 TRP HA	Favored (90.97%) General / -59.2,-42.4	Favored (42.2%) *t-100* chi angles: 185.4,239.8	0.04Å	Favored (84.682%) alpha helix	-	-	-
A 435		GLU	0.72	-	Favored (96.82%) General / -61.3,-41.4	Favored (97.5%) *mt-10* chi angles: 289.1,177.2,354.6	0.02Å	Favored (97.408%) alpha helix	-	-	-
A 436		MET	0.71	-	Favored (93.56%) General / -64.2,-39.2	Favored (99.7%) *mtp* chi angles: 292,174.4,74.1	0.01Å	Favored (95.185%) alpha helix	-	-	-
A 437		VAL	0.7	-	Favored (98.82%) Ile or Val / -62.5,-44.0	Favored (59%) *t* chi angles: 170.6	0.04Å	Favored (98.392%) alpha helix	-	-	-
A 438		ASP	0.68	-	Favored (90.44%) General / -64.8,-38.3	Favored (24%) *t70* chi angles: 192.7,64	0.01Å	Favored (95.946%) alpha helix	-	-	-
A 439		GLU	0.67	-	Favored (97.39%) General / -64.1,-41.2	Favored (97.5%) *mt-10* chi angles: 289.3,179.2,354.7	0.03Å	Favored (93.416%) alpha helix	-	-	-
A 440		GLU	0.66	-	Favored (78.38%) General / -68.3,-35.8	Favored (29%) *mm-30* chi angles: 288.8,291.9,285.9	0.13Å	Favored (81.686%) alpha helix	-	-	-
**#**	**Alt**	**Res**	**High B**	**Clash > 0.4Å**	**Ramachandran**	**Rotamer**	**Cβ deviation**	**CaBLAM**	**Bond lengths**	**Bond angles**	**Cis Peptides**
			Avg: 0.92	Clashscore: 1.59	Outliers: 4 of 903	Poor rotamers: 0 of 771	Outliers: 0 of 828	Outliers: 22 of 901	Outliers: 13 of 905	Outliers: 19 of 905	Non-Trans: 1 of 904
A 441		ARG	0.65	-	Favored (88.79%) General / -60.1,-46.7	Favored (73.1%) *ttt-90* chi angles: 185.4,179.1,183.5,274.1	0.06Å	Favored (81.838%) alpha helix	-	-	-
A 442		GLU	0.65	-	Favored (93.43%)	Favored (92.3%) *tt0* chi angles: 180,177.8,355	0.02Å	Favored (94.055%) alpha helix	-	-	-

				General / -60.5,-45.5							
A 443	ASN	0.65	-	Favored (86.06%) General / -62.1,-37.7	Favored (99.1%) *m-40* chi angles: 288.3,339.4	0.04Å	Favored (94.206%) alpha helix	-	-	-	
A 444	HIS	0.67	-	Favored (95.54%) General / -65.0,-40.8	Favored (39.7%) *m170* chi angles: 290.6,190.6	0.06Å	Favored (87.273%) alpha helix	-	-	-	
A 445	LEU	0.7	-	Favored (69.17%) General / -62.9,-27.1	Favored (91.9%) *mt* chi angles: 291.1,172.5	0.04Å	Favored (73.306%) alpha helix	-	-	-	
A 446	LYS	0.74	-	Favored (54.39%) General / -88.0,1.3	Favored (99%) *mttt* chi angles: 293.4,180.4,178.5,177.6	0.04Å	Favored (56.161%)	-	-	-	
A 447	GLY	0.78	-	Favored (48.56%) Glycine / 84.9,23.6	-	-	Favored (60.77%)	-	-	-	
A 448	GLU	0.82	-	Favored (35.67%) General / -140.4,143.3	Favored (57.4%) *tt0* chi angles: 181.5,171.9,330.6	0.06Å	Favored (23.319%)	-	-	-	
A 449	CYS	0.85	-	Favored (33.34%) General / -139.3,134.9	Favored (43.7%) *t* chi angles: 177.2	0.03Å	Favored (61.903%) beta sheet	-	-	-	
A 450	HIS	0.86	-	Favored (12.9%) General / -104.4,-25.3	Favored (55.6%) *m170* chi angles: 297,170	0.03Å	Favored (17.326%)	-	-	-	
A 451	THR	0.86	-	Favored (29.65%) General / -106.8,6.7	Favored (74.9%) *p* chi angles: 59.9	0.03Å	Favored (47.151%)	-	-	-	
A 452	CYS	0.86	-	Favored (4.72%) General / -80.2,63.6	Favored (67.3%) *m* chi angles: 299.5	0.07Å	CaBLAM Disfavored (4.302%)	-	-	-	
A 453	ILE	0.85	-	Favored (74.67%) Ile or Val / -120.2,130.2	Favored (3%) *mp* chi angles: 301,100.5	0.05Å	Favored (26.315%)	-	-	-	
A 454	TYR	0.86	0.61Å OH with A 606 GLN NE2	Favored (29.18%) General / -100.9,145.0	Favored (96.2%) *m-80* chi angles: 293.1,87.5	0.12Å	Favored (46.378%) beta sheet	-	-	-	
A 455	ASN	0.88	-	Favored (21.95%) General / -103.6,110.0	Favored (57.7%) *t0* chi angles: 182.8,341.5	0.03Å	Favored (56.277%) beta sheet	-	-	-	
A 456	MET	0.91	-	Favored (27.79%) General / -82.2,122.3	Favored (7.4%) *tmm* chi angles: 193.5,280,271.1	0.07Å	Favored (44.247%) beta sheet	-	-	-	
A 457	MET	0.96	-	Favored (44.86%) General / -131.4,156.8	Favored (82.2%) *mtp* chi angles: 301.5,181.9,71.7	0.05Å	Favored (37.921%) beta sheet	-	-	-	
A 458	GLY	1.02	-	Favored (25.26%) Glycine / -80.3,143.8	-	-	Favored (30.048%) beta sheet	-	-	-	
A 459	LYS	1.07	-	Favored (34.27%) General / -88.4,132.9	Favored (71.3%) *tttt* chi angles: 184.8,179.3,172.8,193.1	0.07Å	Favored (40.483%) beta sheet	-	-	-	

A 460		ARG	1.11	-	Favored (3.58%) General / -77.9,66.6	Favored (96%) *mtt180* chi angles: 296.5,179,186.5,181.2	0.04Å	Favored (20.273%) beta sheet	-	-	-
**#**	**Alt**	**Res**	**High B**	**Clash > 0.4Å**	**Ramachandran**	**Rotamer**	**Cβ deviation**	**CaBLAM**	**Bond lengths**	**Bond angles**	**Cis Peptides**
			Avg: 0.92	Clashscore: 1.59	Outliers: 4 of 903	Poor rotamers: 0 of 771	Outliers: 0 of 828	Outliers: 22 of 901	Outliers: 13 of 905	Outliers: 19 of 905	Non-Trans: 1 of 904
A 461		GLU	1.14	-	Favored (26.34%) General / -81.2,155.2	Favored (68.5%) *mt-10* chi angles: 294.3,185.9,322.7	0.07Å	Favored (24.226%) beta sheet	-	-	-
A 462		LYS	1.17	-	Favored (32.42%) General / -101.5,116.3	Favored (2.3%) *mppt* chi angles: 277.4,81.3,63.5,170.4	0.06Å	Favored (35.927%) beta sheet	-	-	-
A 463		LYS	1.19	-	Favored (52.19%) General / -130.9,146.1	Favored (58.9%) *mttm* chi angles: 295.4,185.7,190.5,298.2	0.05Å	Favored (41.854%) beta sheet	-	-	-
A 464		LEU	1.2	-	Favored (38.31%) General / -63.4,129.0	Favored (31.1%) *tp* chi angles: 187.5,62.5	0.06Å	Favored (42.369%)	-	-	-
A 465		GLY	1.21	-	Favored (45.95%) Glycine / -84.3,-169.8	-	-	Favored (35.167%)	-	-	-
A 466		GLU	1.2	-	Favored (16.14%) General / -122.9,111.0	Favored (94.8%) *mt-10* chi angles: 296.2,182.2,359.2	0.01Å	CaBLAM Outlier (0.779%)	-	-	-
A 467		PHE	1.2	-	Favored (19.04%) General / 56.8,31.1	Favored (91%) *m-80* chi angles: 300.6,93.9	0.05Å	Favored (33.986%)	-	-	-
A 468		GLY	1.18	-	Favored (76.32%) Glycine / 92.9,-8.5	-	-	Favored (74.945%)	-	-	-
A 469		LYS	1.18	-	Favored (28.38%) General / -88.9,141.6	Favored (72.8%) *mmtt* chi angles: 301.8,297.9,184.5,182.2	0.01Å	Favored (30.167%)	-	-	-
A 470		ALA	1.18	-	Favored (56.38%) General / -65.4,145.3	-	0.03Å	Favored (40.265%)	-	-	-
A 471		LYS	1.18	-	Favored (13.93%) General / -90.2,165.5	Favored (97.5%) *mttt* chi angles: 292.8,181.3,174.2,178.1	0.03Å	Favored (25.352%)	-	-	-
A 472		GLY	1.18	-	Favored (49.31%) Glycine / -68.7,149.2	-	-	Favored (31.274%)	-	-	-
A 473		SER	1.18	-	Favored (29.03%) General / -71.7,162.7	Favored (94.1%) *p* chi angles: 64.2	0.09Å	Favored (36.525%)	-	-	-
A 474		ARG	1.15	-	Favored (35.33%) General / -82.2,134.8	Favored (73.1%) *ttt180* chi angles: 180.7,172.4,177.4,168.9	0.06Å	Favored (23.171%)	-	-	-
A 475		ALA	1.11	-	Favored (57.03%) General / -67.5,139.4	-	0.06Å	Favored (39.384%) beta sheet	-	-	-

A 476		ILE	1.05	-	Favored (70.46%) Ile or Val / -125.7,132.8	Favored (89.2%) *mt* chi angles: 298.2,171.8	0.10Å	Favored (66.219%) beta sheet	-	-	-
A 477		TRP	0.99	-	Favored (25.38%) General / -104.2,112.4	Favored (32.6%) *m-90* chi angles: 290.2,258.6	0.08Å	Favored (67.44%) beta sheet	-	-	-
A 478		PHE	0.92	-	Favored (39.62%) General / -97.8,121.4	Favored (88.5%) *m-80* chi angles: 291.3,85.9	0.06Å	Favored (66.097%)	-	-	-
A 479		MET	0.86	-	Favored (26.02%) General / -100.0,147.0	Favored (45%) *tpp* chi angles: 171.7,63.8,72.7	0.06Å	Favored (26.543%)	OUTLIER(S) worst is SD--CE: 4.7 σ	-	-
A 480		TRP	0.82	-	Favored (10.01%) General / -46.2,132.1	Favored (25.4%) *t60* chi angles: 177.4,61.3	0.03Å	Favored (43.081%)	-	-	-
**#**	**Alt**	**Res**	**High B**	**Clash > 0.4Å**	**Ramachandran**	**Rotamer**	**Cβ deviation**	**CaBLAM**	**Bond lengths**	**Bond angles**	**Cis Peptides**
			Avg: 0.92	Clashscore: 1.59	Outliers: 4 of 903	Poor rotamers: 0 of 771	Outliers: 0 of 828	Outliers: 22 of 901	Outliers: 13 of 905	Outliers: 19 of 905	Non-Trans: 1 of 904
A 481		LEU	0.79	-	Favored (64.39%) General / -55.6,-33.8	Favored (21.7%) *tp* chi angles: 190.4,64.8	0.05Å	Favored (50.357%)	-	-	-
A 482		GLY	0.76	-	Favored (80.7%) Glycine / -75.3,-16.3	-	-	Favored (82.05%) alpha helix	-	-	-
A 483		ALA	0.73	-	Favored (24.77%) General / -83.8,-33.8	-	0.04Å	Favored (66.486%) alpha helix	-	-	-
A 484		ARG	0.71	-	Favored (79.55%) General / -66.0,-35.1	Favored (86.5%) *mtp180* chi angles: 290.9,174,65.8,195.1	0.02Å	Favored (78.359%) alpha helix	-	-	-
A 485		PHE	0.69	0.50Å C with A 485 PHE CD2	Favored (69.62%) General / -56.3,-51.1	Favored (36.7%) *t80* chi angles: 179.9,98.1	0.11Å	Favored (76.166%) alpha helix	-	-	-
A 486		LEU	0.68	-	Favored (82.17%) General / -65.9,-36.0	Favored (97.9%) *mt* chi angles: 292.5,171.6	0.12Å	Favored (77.513%) alpha helix	-	-	-
A 487		GLU	0.67	-	Favored (96.36%) General / -61.7,-40.8	Favored (98.1%) *mt-10* chi angles: 289.7,176.6,355.9	0.11Å	Favored (81.5%) alpha helix	-	-	-
A 488		PHE	0.66	-	Favored (65.45%) General / -67.5,-49.0	Favored (81.2%) *t80* chi angles: 183.4,77.5	0.02Å	Favored (74.092%) alpha helix	-	-	-
A 489		GLU	0.67	-	Favored (55.47%) General / -50.3,-46.4	Favored (71.9%) *tt0* chi angles: 177.6,180.2,340.9	0.03Å	Favored (61.349%) alpha helix	-	-	-
A 490		ALA	0.7	-	Favored (16.15%) General / -86.7,-39.3	-	0.09Å	Favored (40.421%) alpha helix	-	-	-
A 491		LEU	0.73	-	Favored (13.01%)	Favored (71.3%) *mt* chi angles: 302.1,174.6	0.04Å	Favored (16.741%) alpha helix	-	-	-

					General / -113.3,-12.9						
A 492		GLY	0.78	-	Favored (55.11%) Glycine / -56.9,-26.5	-	-	Favored (37.27%) three-ten	-	-	-
A 493		PHE	0.84	-	Favored (61.85%) General / -57.7,-26.5	Favored (41.3%) *p90* chi angles: 72.1,92.4	0.06Å	Favored (62.823%) three-ten	-	-	-
A 494		LEU	0.9	-	Favored (80.22%) General / -62.4,-35.8	Favored (81.2%) *mt* chi angles: 289.1,171.1	0.03Å	Favored (57.688%) alpha helix	-	-	-
A 495		ASN	0.95	-	Favored (9%) General / -84.2,-49.2	Favored (97.4%) *m-40* chi angles: 292.6,340.6	0.10Å	Favored (42.997%) alpha helix	-	OUTLIER(S) worst is CA-CB-CG: 4.3 σ	-
A 496		GLU	0.97	-	Favored (76.68%) General / -66.5,-33.9	Favored (80%) *mm-30* chi angles: 293,300.3,303.6	0.06Å	Favored (67.922%) alpha helix	-	-	-
A 497		ASP	0.97	-	Favored (57.55%) General / -90.3,-0.3	Favored (84.4%) *m-30* chi angles: 294.5,336.3	0.10Å	Favored (50.279%)	-	OUTLIER(S) worst is CA-CB-CG: 6.8 σ	-
A 498		HIS	0.95	-	Favored (11.35%) General / 53.7,53.5	Favored (99.3%) *m-70* chi angles: 298.4,291.1	0.05Å	Favored (21.515%)	-	-	-
A 499		TRP	0.93	-	Favored (69.11%) General / -59.5,-31.1	Favored (14.2%) *m100* chi angles: 280.4,52.6	0.07Å	Favored (26.776%)	-	-	-
A 500		LEU	0.91	-	Favored (11.55%) General / -110.8,25.6	Favored (61.9%) *mt* chi angles: 301.2,171.3	0.15Å	Favored (12.706%)	OUTLIER(S) worst is CG--CD1: 4.1 σ	-	-
**#**	**Alt**	**Res**	**High B**	**Clash > 0.4Å**	**Ramachandran**	**Rotamer**	**Cβ deviation**	**CaBLAM**	**Bond lengths**	**Bond angles**	**Cis Peptides**
			Avg: 0.92	Clashscore: 1.59	Outliers: 4 of 903	Poor rotamers: 0 of 771	Outliers: 0 of 828	Outliers: 22 of 901	Outliers: 13 of 905	Outliers: 19 of 905	Non-Trans: 1 of 904
A 501		GLY	0.9	-	Favored (49.68%) Glycine / -84.6,173.7	-	-	Favored (31.395%)	-	-	-
A 502		ARG	0.88	-	Favored (65.74%) General / -62.3,-22.1	Favored (40.1%) *ptt180* chi angles: 67,185.1,174.7,191.5	0.05Å	Favored (38.73%)	-	-	-
A 503		LYS	0.86	-	Favored (51.72%) General / -76.8,-39.4	Favored (86.5%) *tttt* chi angles: 182.9,173.6,180.3,179.6	0.02Å	Favored (47.978%) alpha helix	-	-	-
A 504		ASN	0.84	-	Favored (14.74%) General / -96.8,-27.4	Favored (69.3%) *m-40* chi angles: 295.8,290.1	0.04Å	Favored (40.557%) alpha helix	-	-	-
A 505		SER	0.81	-	Favored (18.08%) General / -125.6,113.9	Favored (45%) *t* chi angles: 179.2	0.12Å	Favored (15.974%)	-	-	-
A 506		GLY	0.78	-	Favored (48.74%) Glycine / -60.1,138.4	-	-	Favored (11.054%)	-	-	-
A 507		GLY	0.77	-	Favored (71.05%) Glycine / 81.8,-5.2	-	-	Favored (83.081%)	-	-	-

A 508		GLY	0.78	-	Favored (31.92%) Glycine / -78.6,148.2	-	-	Favored (23.014%)	-	-	-
A 509		VAL	0.8	-	Favored (6.74%) Ile or Val / -119.6,15.1	Favored (29.8%) *m* chi angles: 296.5	0.07Å	Favored (6.065%)	-	-	-
A 510		GLU	0.83	-	Favored (53.96%) General / -59.1,132.4	Favored (87.1%) *tt0* chi angles: 183.7,174.1,8	0.01Å	Favored (17.534%)	-	-	-
A 511		GLY	0.85	-	Favored (89.73%) Glycine / 83.4,3.7	-	-	Favored (79.264%)	-	-	-
A 512		LEU	0.86	-	Favored (56.71%) General / -91.8,2.1	Favored (84.5%) *mt* chi angles: 299,179.5	0.03Å	CaBLAM Disfavored (1.779%)	-	-	-
A 513		GLY	0.86	-	Favored (50.32%) Glycine / 71.0,-152.5	-	-	Favored (29.316%)	-	-	-
A 514		VAL	0.84	-	Favored (7.17%) Ile or Val / -109.2,-47.9	Favored (84.6%) *t* chi angles: 177.1	0.13Å	CaBLAM Outlier (0.002%)	-	-	-
A 515		GLN	0.81	0.42Å CD with A 515 GLN H	Favored (61.82%) General / -57.2,-27.2	Favored (28.1%) *mp10* chi angles: 299.3,88.9,331.6	0.09Å	Favored (61.557%) alpha helix	-	-	-
A 516		LYS	0.78	-	Favored (33.84%) General / -95.0,-9.5	Favored (87.5%) *mttt* chi angles: 299.5,183.3,187.8,185.3	0.02Å	Favored (48.632%) alpha helix	-	-	-
A 517		LEU	0.75	-	Favored (74.7%) General / -65.2,-32.8	Favored (87.9%) *mt* chi angles: 295.1,178.2	0.04Å	Favored (54.439%) alpha helix	-	-	-
A 518		GLY	0.72	-	Favored (75.56%) Glycine / -60.7,-31.5	-	-	Favored (87.411%) alpha helix	-	-	-
A 519		TYR	0.7	-	Favored (42.67%) General / -79.4,-30.9	Favored (59%) *m-80* chi angles: 286,103.4	0.03Å	Favored (90.676%) alpha helix	-	-	-
A 520		ILE	0.7	-	Favored (88%) Ile or Val / -67.4,-42.9	Favored (42.7%) *mm* chi angles: 297.2,300.7	0.04Å	Favored (82.325%) alpha helix	-	-	-
**#**	**Alt**	**Res**	**High B**	**Clash > 0.4Å**	**Ramachandran**	**Rotamer**	**Cβ deviation**	**CaBLAM**	**Bond lengths**	**Bond angles**	**Cis Peptides**
			Avg: 0.92	Clashscore: 1.59	Outliers: 4 of 903	Poor rotamers: 0 of 771	Outliers: 0 of 828	Outliers: 22 of 901	Outliers: 13 of 905	Outliers: 19 of 905	Non-Trans: 1 of 904
A 521		LEU	0.7	-	Favored (94.37%) General / -65.3,-41.0	Favored (96.7%) *mt* chi angles: 293.1,173.5	0.03Å	Favored (93.136%) alpha helix	-	-	-
A 522		ARG	0.71	-	Favored (92.16%) General / -62.1,-45.7	Favored (79.1%) *ttt180* chi angles: 179.9,176,171.5,180.7	0.04Å	Favored (96.99%) alpha helix	-	-	-
A 523		GLU	0.72	-	Favored (94.03%) General / -63.6,-39.3	Favored (63.6%) *mm-30* chi angles: 288.7,293,306.8	0.02Å	Favored (92.435%) alpha helix	-	-	-
A 524		MET	0.74	-	Favored (63.76%)	Favored (28.7%) *ptm*	0.10Å	Favored (72.083%)	-	-	-

#	Alt	Res	High B	Clash > 0.4Å	Ramachandran	Rotamer	Cβ deviation	CaBLAM	Bond lengths	Bond angles	Cis Peptides
					General / -69.5,-24.3	chi angles: 67.7,181.3,283.9		alpha helix			
A 525		SER	0.75	-	Favored (61.81%) General / -70.5,-12.8	Favored (34.5%) *p* chi angles: 76.8	0.08Å	Favored (64.754%) three-ten	-	-	-
A 526		HIS	0.75	-	Favored (50.65%) General / -92.5,5.5	Favored (90.3%) *m-70* chi angles: 291.8,292.2	0.06Å	Favored (37.994%)	OUTLIER(S) worst is CB--CG: 5.6 σ	-	-
A 527		HIS	0.74	-	Favored (16.43%) General / -80.5,170.4	Favored (74.8%) *m-70* chi angles: 289.6,299.1	0.12Å	Favored (29.776%)	-	OUTLIER(S) worst is CA-CB-CG: 4.8 σ	-
A 528		SER	0.71	-	Favored (59.9%) General / -76.0,-11.2	Favored (88.4%) *p* chi angles: 67	0.06Å	Favored (6.521%)	-	-	-
A 529		GLY	0.69	-	Allowed (1.79%) Glycine / -73.1,54.2	-	-	Favored (25.484%)	-	-	-
A 530		GLY	0.67	-	Favored (26.49%) Glycine / 49.6,-132.4	-	-	Favored (17.981%)	-	-	-
A 531		LYS	0.65	-	Favored (27.48%) General / -83.6,147.8	Favored (50.4%) *mttp* chi angles: 295,175.9,185.5,61	0.06Å	CaBLAM Disfavored (4.109%)	-	-	-
A 532		MET	0.65	-	Favored (27.93%) General / -88.0,142.3	Favored (90.7%) *mtp* chi angles: 287.9,174,65.4	0.04Å	Favored (48.724%)	-	-	-
A 533		TYR	0.67	-	Favored (38.73%) General / -121.5,152.8	Favored (88.1%) *m-80* chi angles: 294.5,84	0.04Å	Favored (65.104%) beta sheet	-	-	-
A 534		ALA	0.71	-	Favored (12.68%) General / -139.4,118.6	-	0.03Å	Favored (46.9%) beta sheet	-	-	-
A 535		ASP	0.77	-	Favored (24.93%) General / -114.1,113.6	Favored (80.9%) *m-30* chi angles: 295,347.3	0.10Å	Favored (65.937%) beta sheet	-	-	-
A 536		ASP	0.83	-	Favored (14.35%) General / -107.8,104.8	Favored (66.1%) *t0* chi angles: 181.5,355.2	0.09Å	Favored (58.248%) beta sheet	-	OUTLIER(S) worst is CA-CB-CG: 4.9 σ	-
A 537		THR	0.88	-	Favored (21.39%) General / -83.8,159.5	Favored (64.7%) *p* chi angles: 58.1	0.14Å	Favored (43.758%)	-	-	-
A 538		ALA	0.91	-	Favored (7.05%) General / -80.5,69.7	-	0.05Å	CaBLAM Disfavored (1.594%)	-	-	-
A 539		GLY	0.92	-	Favored (6.01%) Glycine / 81.2,48.4	-	-	CaBLAM Disfavored (1.607%)	-	-	-
A 540		TRP	0.9	-	Favored (11.28%) General / -45.1,-46.1	Favored (46.5%) *t-100* chi angles: 188.7,269.7	0.10Å	Favored (46.068%)	-	-	-
**#**	**Alt**	**Res**	**High B**	**Clash > 0.4Å**	**Ramachandran**	**Rotamer**	**Cβ deviation**	**CaBLAM**	**Bond lengths**	**Bond angles**	**Cis Peptides**
			Avg: 0.92	Clashscore: 1.59	Outliers: 4 of 903	Poor rotamers: 0 of 771	Outliers: 0 of 828	Outliers: 22 of 901	Outliers: 13 of 905	Outliers: 19 of 905	Non-Trans: 1

											of 904
A 541		ASP	0.86	-	Favored (58.71%) General / -55.1,-29.9	Favored (94%) *m-30* chi angles: 292.1,346.6	0.06Å	Favored (61.46%) alpha helix	-	-	-
A 542		THR	0.82	-	Favored (28.67%) General / -98.2,-7.9	Favored (71.5%) *p* chi angles: 62.1	0.06Å	Favored (60.862%)	-	-	-
A 543		ARG	0.79	-	Favored (40.4%) General / -101.5,10.4	Favored (97.3%) *mtt-85* chi angles: 291.2,182,179.5,274.2	0.04Å	Favored (30.193%)	-	-	-
A 544		ILE	0.76	-	Favored (33.53%) Ile or Val / -69.9,132.1	Favored (46.6%) *mm* chi angles: 298.3,298.1	0.06Å	Favored (30.001%)	-	-	-
A 545		THR	0.74	-	Favored (9.06%) General / -101.1,169.2	Favored (71%) *p* chi angles: 62.2	0.09Å	Favored (34.479%)	-	-	-
A 546		ARG	0.73	-	Favored (73.91%) General / -65.5,-32.4	Favored (89.2%) *mtm180* chi angles: 290.6,176.5,290.2,175.3	0.02Å	Favored (53.909%)	-	-	-
A 547		ALA	0.73	-	Favored (74.51%) General / -58.9,-36.3	-	0.02Å	Favored (74.383%) alpha helix	-	-	-
A 548		ASP	0.73	-	Favored (59.18%) General / -76.1,-35.9	Favored (52.2%) *m-30* chi angles: 292.2,305.2	0.11Å	Favored (93.875%) alpha helix	-	OUTLIER(S) worst is CA-CB-CG: 4.1 σ	-
A 549		LEU	0.74	-	Favored (93.68%) General / -64.6,-39.4	Favored (96.9%) *mt* chi angles: 293.4,173.3	0.03Å	Favored (96.416%) alpha helix	-	-	-
A 550		ASP	0.76	-	Favored (85.24%) General / -65.5,-36.9	Favored (91.9%) *m-30* chi angles: 285.1,346.4	0.06Å	Favored (91.574%) alpha helix	-	-	-
A 551		ASN	0.77	-	Favored (80.78%) General / -68.5,-38.4	Favored (88.1%) *m-40* chi angles: 286.2,331.1	0.10Å	Favored (93.762%) alpha helix	-	-	-
A 552		GLU	0.79	-	Favored (71.47%) General / -69.0,-32.1	Favored (74.3%) *mm-30* chi angles: 288.7,298.9,328.5	0.06Å	Favored (62.147%) alpha helix	-	-	-
A 553		ALA	0.82	-	Favored (50.98%) General / -60.2,-18.8	-	0.03Å	Favored (54.849%) three-ten	-	-	-
A 554		LYS	0.84	-	Favored (64.15%) General / -62.7,-19.8	Favored (55.1%) *mttm* chi angles: 289.3,172.3,179.1,286.2	0.05Å	Favored (50.492%) three-ten	-	-	-
A 555		VAL	0.87	-	Favored (31.21%) Ile or Val / -59.8,-25.1	Favored (4.5%) *p* chi angles: 72.1	0.04Å	Favored (58.877%) three-ten	-	-	-
A 556		LEU	0.9	-	Favored (67.66%) General / -64.5,-24.6	Favored (94.1%) *mt* chi angles: 296.8,173.6	0.07Å	Favored (66.531%) three-ten	-	-	-
A 557		GLU	0.93	-	Favored (57.5%) General / -79.0,-6.9	Favored (97.9%) *mt-10* chi angles: 294.2,180.4,359.3	0.01Å	Favored (62.924%) three-ten	-	-	-

A 558		LEU	0.95	-	Favored (46.54%) General / -95.3,-3.8	Favored (82.5%) *mt* chi angles: 300.6,175.9	0.07Å	Favored (54.669%)	-	-	-
A 559		MET	0.95	-	Favored (14.51%) General / -102.3,160.5	Favored (65.3%) *mtt* chi angles: 298.1,177.2,186.4	0.07Å	Favored (20.326%)	-	-	-
A 560		GLU	0.93	-	Favored (46.64%) General / -129.8,155.0	Favored (94.2%) *mt-10* chi angles: 297.8,181.5,0.1	0.03Å	Favored (8.208%)	-	-	-
**#**	**Alt**	**Res**	**High B**	**Clash > 0.4Å**	**Ramachandran**	**Rotamer**	**Cβ deviation**	**CaBLAM**	**Bond lengths**	**Bond angles**	**Cis Peptides**
			Avg: 0.92	Clashscore: 1.59	Outliers: 4 of 903	Poor rotamers: 0 of 771	Outliers: 0 of 828	Outliers: 22 of 901	Outliers: 13 of 905	Outliers: 19 of 905	Non-Trans: 1 of 904
A 561		GLY	0.9	-	Favored (48.4%) Glycine / 55.1,-131.7	-	-	Favored (43.851%)	-	-	-
A 562		GLU	0.84	-	Favored (66.95%) General / -65.6,-24.0	Favored (93%) *mt-10* chi angles: 291.4,183.6,5.6	0.04Å	Favored (18.368%)	-	-	-
A 563		HIS	0.79	0.42Å C with A 563 HIS CD2	Favored (46.11%) General / -68.4,-51.2	Favored (12.3%) *t-170* chi angles: 182.4,173.5	0.04Å	Favored (50.625%) alpha helix	-	-	-
A 564		ARG	0.74	-	Favored (79.75%) General / -56.2,-46.2	Favored (49.2%) *ttm170* chi angles: 185.4,185.6,298.2,175	0.06Å	Favored (82.238%) alpha helix	-	-	-
A 565		GLN	0.7	-	Favored (92.33%) General / -63.0,-45.4	Favored (65.8%) *tt0* chi angles: 186,177,349.2	0.04Å	Favored (85.128%) alpha helix	-	-	-
A 566		LEU	0.68	-	Favored (72.12%) General / -66.3,-47.9	Favored (61.5%) *tp* chi angles: 181.1,61.2	0.04Å	Favored (78.928%) alpha helix	-	-	-
A 567		ALA	0.67	-	Favored (88.85%) General / -60.8,-39.5	-	0.04Å	Favored (87.099%) alpha helix	-	-	-
A 568		ARG	0.67	-	Favored (83.4%) General / -60.4,-48.0	Favored (83.6%) *ttt180* chi angles: 179.6,175,176.3,178.3	0.04Å	Favored (90.243%) alpha helix	-	-	-
A 569		ALA	0.67	-	Favored (81.77%) General / -59.6,-38.7	-	0.06Å	Favored (84.612%) alpha helix	-	-	-
A 570		ILE	0.68	-	Favored (88.82%) Ile or Val / -65.7,-46.6	Favored (93%) *mt* chi angles: 291.9,166.6	0.10Å	Favored (87.243%) alpha helix	-	-	-
A 571		ILE	0.7	-	Favored (90.85%) Ile or Val / -61.8,-41.5	Favored (36.7%) *mm* chi angles: 294.7,298	0.01Å	Favored (75.263%) alpha helix	-	-	-
A 572		GLU	0.71	-	Favored (63.39%) General / -74.3,-37.9	Favored (98.9%) *mt-10* chi angles: 292.6,177,359.3	0.03Å	Favored (61.892%) alpha helix	-	-	-
A 573		LEU	0.72	-	Favored (11.88%) General / -93.5,-37.5	Favored (76.9%) *mt* chi angles: 299.9,172.4	0.07Å	Favored (48.273%) alpha helix	-	-	-

#	Alt	Res	High B	Clash > 0.4Å	Ramachandran	Rotamer	Cβ deviation	CaBLAM	Bond lengths	Bond angles	Cis Peptides
A 574		THR	0.73	-	Favored (10.12%) General / -91.4,-42.8	Favored (90.1%) *m* chi angles: 301.2	0.03Å	Favored (45.622%) alpha helix	-	-	-
A 575		TYR	0.72	-	Favored (7.72%) General / -94.3,-44.9	Favored (90.8%) *m-80* chi angles: 293.2,84.8	0.02Å	Favored (37.352%) alpha helix	-	-	-
A 576		LYS	0.72	-	Favored (57.8%) General / -85.4,-9.3	Favored (96.6%) *mttt* chi angles: 292.3,181.9,174.3,184.6	0.09Å	Favored (20.05%) alpha helix	-	-	-
A 577		HIS	0.71	-	Allowed (1.74%) General / -140.4,48.8	Favored (77.4%) *m90* chi angles: 297.6,87.6	0.07Å	Favored (5.848%)	-	-	-
A 578		LYS	0.7	-	Favored (54.84%) General / -63.6,134.1	Favored (30.3%) *ttpt* chi angles: 185.4,171,69.9,162.6	0.02Å	Favored (17.32%)	-	-	-
A 579		VAL	0.7	-	Favored (68.61%) Ile or Val / -111.5,127.5	Favored (84.4%) *t* chi angles: 177.7	0.01Å	Favored (70.821%) beta sheet	-	-	-
A 580		VAL	0.7	-	Favored (57.69%) Ile or Val / -127.2,137.5	Favored (61.2%) *t* chi angles: 179.8	0.01Å	Favored (67.221%) beta sheet	-	-	-
**#**	**Alt**	**Res**	**High B**	**Clash > 0.4Å**	**Ramachandran**	**Rotamer**	**Cβ deviation**	**CaBLAM**	**Bond lengths**	**Bond angles**	**Cis Peptides**
			Avg: 0.92	Clashscore: 1.59	Outliers: 4 of 903	Poor rotamers: 0 of 771	Outliers: 0 of 828	Outliers: 22 of 901	Outliers: 13 of 905	Outliers: 19 of 905	Non-Trans: 1 of 904
A 581		LYS	0.72	-	Favored (47.81%) General / -101.9,124.6	Favored (35.8%) *mtpt* chi angles: 298.9,171.9,76.3,189.5	0.10Å	Favored (69.772%) beta sheet	-	-	-
A 582		VAL	0.76	-	Favored (69.45%) Ile or Val / -123.5,133.3	Favored (38.8%) *t* chi angles: 183.7	0.05Å	Favored (56.912%) beta sheet	-	-	-
A 583		MET	0.84	-	Favored (37.28%) General / -78.3,131.9	Favored (57%) *ttm* chi angles: 187.9,174.6,283.8	0.09Å	Favored (45.904%) beta sheet	-	-	-
A 584		ARG	0.95	-	Favored (16.68%) Pre-Pro / -142.1,138.3	Favored (67.2%) *ttp80* chi angles: 179.8,168.8,63.9,93	0.12Å	Favored (46.977%) beta sheet	-	-	-
A 585		PRO	1.11	-	Favored (89.82%) Trans-Pro / -61.2,140.6	Favored (51.5%) *Cg_exo* chi angles: 336.6,35.5,327.6	0.04Å	Favored (38.819%)	-	-	-
A 586		GLY	1.27	-	Favored (24.53%) Glycine / -104.9,-173.2	-	-	Favored (53.285%)	-	-	-
A 587		THR	1.4	-	Favored (62.36%) General / -71.3,-18.8	Favored (69.7%) *p* chi angles: 59.3	0.01Å	Favored (41.914%)	-	-	-
A 588		ASP	1.44	-	Favored (41.31%) General / -101.3,9.7	Favored (47%) *p0* chi angles: 58.8,1.6	0.05Å	Favored (56.874%)	-	-	-
A 589		GLY	1.38	-	Favored (89.07%) Glycine / 82.3,-0.3	-	-	Favored (77.373%)	-	-	-

A 590		LYS	1.24	-	Favored (13.32%) General / -88.5,167.6	Favored (55%) *mtpt* chi angles: 293.2,175,67,177.6	0.03Å	Favored (40.529%)	-	-	-
A 591		THR	1.07	-	Favored (47.28%) General / -105.2,135.1	Favored (97.7%) *m* chi angles: 300	0.05Å	Favored (38.959%) beta sheet	-	-	-
A 592		VAL	0.92	-	Favored (39.83%) Ile or Val / -128.9,160.3	Favored (30.6%) *m* chi angles: 300.3	0.08Å	Favored (46.872%) beta sheet	-	-	-
A 593		MET	0.82	-	Favored (38.27%) General / -102.4,139.2	Favored (96.4%) *mmm* chi angles: 300,297.4,292.3	0.11Å	Favored (50.355%) beta sheet	-	-	-
A 594		ASP	0.76	-	Favored (51.13%) General / -104.3,126.1	Favored (94.1%) *m-30* chi angles: 288.9,340.7	0.07Å	Favored (60.546%) beta sheet	-	-	-
A 595		VAL	0.73	-	Favored (38.33%) Ile or Val / -97.3,115.9	Favored (69.2%) *t* chi angles: 178.9	0.04Å	Favored (42.644%) beta sheet	-	-	-
A 596		ILE	0.74	-	Favored (28.92%) Ile or Val / -131.3,166.4	Favored (43.8%) *pt* chi angles: 61.5,174.1	0.10Å	Favored (26.83%) beta sheet	-	-	-
A 597		SER	0.77	-	Favored (43.75%) General / -145.4,158.9	Favored (95.9%) *p* chi angles: 65	0.03Å	Favored (61.632%) beta sheet	-	-	-
A 598		ARG	0.81	-	Favored (15.24%) General / -157.4,140.4	Favored (83.3%) *ttt180* chi angles: 181.7,174.6,174,183.5	0.04Å	Favored (48.277%)	OUTLIER(S) worst is CD--NE: 4.1 σ	-	-
A 599		GLU	0.86	-	Favored (51.6%) General / -96.3,5.2	Favored (93.7%) *mt-10* chi angles: 298.4,181.7,359.5	0.02Å	Favored (20.42%)	-	-	-
A 600		ASP	0.9	-	Allowed (1.47%) General / -138.2,65.4	Favored (16.2%) *p0* chi angles: 56.8,325.9	0.09Å	CaBLAM Disfavored (2.68%)	-	-	-
**#**	**Alt**	**Res**	**High B**	**Clash > 0.4Å**	**Ramachandran**	**Rotamer**	**Cβ deviation**	**CaBLAM**	**Bond lengths**	**Bond angles**	**Cis Peptides**
			Avg: 0.92	Clashscore: 1.59	Outliers: 4 of 903	Poor rotamers: 0 of 771	Outliers: 0 of 828	Outliers: 22 of 901	Outliers: 13 of 905	Outliers: 19 of 905	Non-Trans: 1 of 904
A 601		GLN	0.94	-	Favored (10.4%) General / -163.6,147.4	Favored (18.7%) *tt0* chi angles: 184.5,170.7,100.7	0.03Å	Favored (7.597%)	-	-	-
A 602		ARG	0.97	-	Favored (31.45%) General / -112.3,150.1	Favored (95.9%) *mtt-85* chi angles: 294.6,181.3,181.2,283	0.05Å	Favored (44.548%)	-	-	-
A 603		GLY	0.98	-	Favored (14.67%) Glycine / -93.9,139.7	-	-	Favored (35.605%)	-	-	-
A 604		SER	0.98	-	Favored (66.39%) General / -64.0,-21.1	Favored (67.5%) *m* chi angles: 296.9	0.06Å	Favored (40.103%)	-	-	-
A 605		GLY	0.97	-	Favored (70.58%) Glycine / -94.6,6.1	-	-	Favored (61.646%)	-	-	-

A 606		GLN	0.94	0.61Å NE2 with A 454 TYR OH	Favored (26.25%) General / -82.3,152.5	Favored (46.3%) *mt0* chi angles: 295,182.1,267.3	0.06Å	Favored (38.58%)	-	-	-
A 607		VAL	0.91	-	Favored (60.54%) Ile or Val / -55.6,-40.6	Favored (67.1%) *t* chi angles: 171.8	0.02Å	Favored (14.299%)	-	-	-
A 608		VAL	0.88	-	Favored (7.41%) Ile or Val / -114.7,18.8	Favored (19.7%) *m* chi angles: 293.9	0.01Å	Favored (18.073%)	-	-	-
A 609		THR	0.85	-	Favored (98.55%) General / -60.9,-42.8	Favored (55%) *m* chi angles: 295.1	0.12Å	Favored (45.175%) alpha helix	-	-	-
A 610		TYR	0.81	-	Favored (66.17%) General / -54.7,-51.2	Favored (86.2%) *t80* chi angles: 176.4,82.9	0.04Å	Favored (85.906%) alpha helix	-	-	-
A 611		ALA	0.79	-	Favored (99.21%) General / -62.7,-41.6	-	0.03Å	Favored (83.053%) alpha helix	-	-	-
A 612		LEU	0.76	-	Favored (81.89%) General / -67.8,-37.3	Favored (93.9%) *mt* chi angles: 293.8,174.8	0.08Å	Favored (97.365%) alpha helix	-	-	-
A 613		ASN	0.73	-	Favored (93.83%) General / -64.8,-39.6	Favored (92.6%) *m-40* chi angles: 286.1,345.1	0.09Å	Favored (96.07%) alpha helix	-	-	-
A 614		THR	0.71	-	Favored (87.52%) General / -62.9,-46.5	Favored (96.9%) *m* chi angles: 299.8	0.06Å	Favored (95.737%) alpha helix	-	-	-
A 615		PHE	0.69	0.47Å C with A 615 PHE CD2	Favored (71.88%) General / -58.3,-50.9	Favored (43.7%) *t80* chi angles: 180.6,96.6	0.11Å	Favored (87.092%) alpha helix	-	-	-
A 616		THR	0.68	-	Favored (91.45%) General / -60.7,-46.2	Favored (91.8%) *m* chi angles: 297.8	0.02Å	Favored (88.918%) alpha helix	-	-	-
A 617		ASN	0.67	-	Favored (89.01%) General / -59.6,-40.9	Favored (31.2%) *t0* chi angles: 194,4.5	0.10Å	Favored (83.082%) alpha helix	-	-	-
A 618		ILE	0.66	-	Favored (96.43%) Ile or Val / -61.5,-46.5	Favored (88.7%) *mt* chi angles: 291.7,165.6	0.07Å	Favored (92.832%) alpha helix	-	-	-
A 619		ALA	0.65	-	Favored (89.11%) General / -60.1,-40.3	-	0.04Å	Favored (92.355%) alpha helix	-	-	-
A 620		VAL	0.64	-	Favored (97.52%) Ile or Val / -64.2,-44.3	Favored (70%) *t* chi angles: 172.1	0.03Å	Favored (97.609%) alpha helix	-	-	-
**#**	**Alt**	**Res**	**High B**	**Clash > 0.4Å**	**Ramachandran**	**Rotamer**	**Cβ deviation**	**CaBLAM**	**Bond lengths**	**Bond angles**	**Cis Peptides**
			Avg: 0.92	Clashscore: 1.59	Outliers: 4 of 903	Poor rotamers: 0 of 771	Outliers: 0 of 828	Outliers: 22 of 901	Outliers: 13 of 905	Outliers: 19 of 905	Non-Trans: 1 of 904
A 621		GLN	0.64	-	Favored (77.85%)	Favored (25.7%) *mm110*	0.09Å	Favored (81.267%) alpha helix	-	-	-

					General / -66.8,-34.5	chi angles: 297.4,293.5,95.7					
A 622		LEU	0.64	-	Favored (89.55%) General / -62.5,-38.3	Favored (70.4%) *mt* chi angles: 287.1,170.7	0.03Å	Favored (85.846%) alpha helix	-	-	-
A 623		ILE	0.63	-	Favored (81.88%) Ile or Val / -69.1,-43.2	Favored (95.6%) *mt* chi angles: 293.6,166.9	0.14Å	Favored (93.128%) alpha helix	-	-	-
A 624		ARG	0.63	-	Favored (76.47%) General / -60.8,-35.5	Favored (85.9%) *mtp180* chi angles: 290.2,176.1,67.5,190.8	0.03Å	Favored (83.761%) alpha helix	-	-	-
A 625		LEU	0.63	-	Favored (95.1%) General / -63.9,-39.8	Favored (79.9%) *mt* chi angles: 288.7,169.3	0.03Å	Favored (83.675%) alpha helix	-	-	-
A 626		MET	0.62	-	Favored (78.86%) General / -63.6,-34.9	Favored (75.9%) *mtm* chi angles: 286.7,189.4,282.9	0.06Å	Favored (80.997%) alpha helix	-	-	-
A 627		GLU	0.62	-	Favored (69.12%) General / -65.7,-49.3	Favored (69.1%) *tt0* chi angles: 176.6,181,341.8	0.03Å	Favored (71.881%) alpha helix	-	-	-
A 628		ALA	0.62	-	Favored (77.18%) General / -58.6,-38.0	-	0.09Å	Favored (74.675%) alpha helix	-	-	-
A 629		GLU	0.62	-	Favored (57.22%) General / -86.9,-0.9	Favored (69.5%) *mt-10* chi angles: 295.4,179.2,27.5	0.03Å	Favored (56.119%)	-	-	-
A 630		GLY	0.62	-	Favored (71.95%) Glycine / 82.2,16.4	-	-	Favored (85.679%)	-	-	-
A 631		VAL	0.64	-	Favored (43.93%) Ile or Val / -74.8,-45.5	Favored (100%) *t* chi angles: 175.5	0.09Å	Favored (7.367%)	-	-	-
A 632		ILE	0.67	-	Favored (34.29%) Ile or Val / -122.8,112.5	Favored (47.3%) *mt* chi angles: 305.8,166.7	0.14Å	Favored (34.404%)	-	-	-
A 633		GLY	0.71	-	Favored (50.41%) Glycine / -84.3,174.1	-	-	Favored (44.972%)	-	-	-
A 634		GLN	0.75	-	Favored (71.31%) General / -57.7,-36.0	Favored (67.8%) *tt0* chi angles: 182.3,179.9,353	0.04Å	Favored (54.272%)	-	-	-
A 635		GLU	0.8	-	Favored (65.99%) General / -65.1,-19.4	Favored (99.5%) *mt-10* chi angles: 292.4,180,357.6	0.01Å	Favored (66.486%) three-ten	-	-	-
A 636		HIS	0.82	-	Favored (49.12%) General / -97.6,5.6	Favored (48.8%) *m-70* chi angles: 299.4,257.3	0.13Å	Favored (48.48%) three-ten	-	-	-
A 637		LEU	0.83	-	Favored (30.04%) General / -83.0,-27.8	Favored (8.4%) *mp* chi angles: 273.4,56.7	0.05Å	Favored (46.67%) three-ten	-	-	-
A 638		GLU	0.8	-	Favored (86.98%) General / -67.0,-39.1	Favored (99.3%) *mt-10* chi angles: 291.3,178.8,354	0.02Å	Favored (7.681%)	-	-	-

#	Alt	Res	High B	Clash > 0.4Å	Ramachandran	Rotamer	Cβ deviation	CaBLAM	Bond lengths	Bond angles	Cis Peptides
A 639		SER	0.76	-	Favored (10.3%) General / -161.4,140.9	Favored (45.9%) *t* chi angles: 179.6	0.06Å	Favored (7.071%)	-	-	-
A 640		LEU	0.72	-	Favored (24.86%) Pre-Pro / -94.4,134.8	Favored (94.6%) *mt* chi angles: 296.5,174.3	0.14Å	Favored (43.331%)	-	-	-
**#**	**Alt**	**Res**	**High B**	**Clash > 0.4Å**	**Ramachandran**	**Rotamer**	**Cβ deviation**	**CaBLAM**	**Bond lengths**	**Bond angles**	**Cis Peptides**
			Avg: 0.92	Clashscore: 1.59	Outliers: 4 of 903	Poor rotamers: 0 of 771	Outliers: 0 of 828	Outliers: 22 of 901	Outliers: 13 of 905	Outliers: 19 of 905	Non-Trans: 1 of 904
A 641		PRO	0.68	-	Favored (89.76%) Trans-Pro / -64.0,149.8	Favored (54.6%) *Cg_exo* chi angles: 337.3,33.1,330.1	0.02Å	Favored (32.78%)	-	-	-
A 642		ARG	0.65	-	Favored (53.31%) General / -50.4,-42.2	Favored (76.1%) *ttt180* chi angles: 181.9,180,179.1,191.2	0.04Å	Favored (64.978%)	-	-	-
A 643		LYS	0.63	-	Favored (75.94%) General / -63.0,-33.7	Favored (97.3%) *mttt* chi angles: 290,179.3,180.2,178.3	0.01Å	Favored (75.072%) alpha helix	-	-	-
A 644		THR	0.61	-	Favored (65.43%) General / -73.0,-40.2	Favored (91.7%) *m* chi angles: 301.1	0.09Å	Favored (73.574%) alpha helix	-	-	-
A 645		LYS	0.61	-	Favored (78.8%) General / -57.9,-39.8	Favored (96.8%) *mttt* chi angles: 289.1,178.8,178.5,180.2	0.04Å	Favored (81.301%) alpha helix	-	-	-
A 646		TYR	0.6	-	Favored (92.95%) General / -65.6,-40.7	Favored (18.7%) *m-10* chi angles: 290.8,159.6	0.04Å	Favored (97.549%) alpha helix	-	-	-
A 647		ALA	0.59	-	Favored (87.18%) General / -61.4,-38.4	-	0.03Å	Favored (95.406%) alpha helix	-	-	-
A 648		VAL	0.59	-	Favored (82.38%) Ile or Val / -66.3,-48.0	Favored (60.3%) *t* chi angles: 170.8	0.06Å	Favored (84.692%) alpha helix	-	-	-
A 649		ARG	0.59	-	Favored (99.18%) General / -62.5,-41.9	Favored (97.7%) *mtt180* chi angles: 288.2,176.5,178.1,170.3	0.07Å	Favored (93.812%) alpha helix	-	-	-
A 650		THR	0.59	-	Favored (86.18%) General / -63.5,-46.4	Favored (97.9%) *m* chi angles: 300	0.04Å	Favored (99.79%) alpha helix	-	-	-
A 651		TRP	0.6	-	Favored (90.43%) General / -59.5,-45.9	Favored (88.5%) *t60* chi angles: 184.1,90.3	0.08Å	Favored (98.756%) alpha helix	-	-	-
A 652		LEU	0.62	-	Favored (99.11%) General / -62.4,-43.3	Favored (74.8%) *mt* chi angles: 289.5,166.4	0.07Å	Favored (82.342%) alpha helix	-	-	-
A 653		PHE	0.63	-	Favored (65.19%) General / -72.8,-32.0	Favored (42.3%) *m-80* chi angles: 286,115.1	0.05Å	Favored (59.359%) alpha helix	-	-	-
A 654		GLU	0.64	-	Favored (13.62%)	Favored (96.2%) *mt-10*	0.02Å	Favored (40.201%) alpha helix	-	-	-

					General / -93.6,-33.6	chi angles: 295.3,181.7,351.3						
A 655		ASN	0.65	-	Favored (9.11%) General / -119.2,-16.3	Favored (69.2%) *m-40* chi angles: 293.3,281	0.04Å	Favored (12.078%) alpha helix	-	-	-
A 656		GLY	0.66	-	Favored (60.65%) Glycine / -54.6,-37.3	-	-	Favored (58.518%) alpha helix	-	-	-
A 657		GLU	0.66	-	Favored (93.12%) General / -63.5,-44.8	Favored (86%) *tt0* chi angles: 180.7,171.1,354.8	0.04Å	Favored (88.255%) alpha helix	-	-	-
A 658		GLU	0.66	-	Favored (86.35%) General / -64.3,-37.1	Favored (59.2%) *mm-30* chi angles: 286.4,292.4,308.2	0.04Å	Favored (86.033%) alpha helix	-	-	-
A 659		ARG	0.66	-	Favored (83.12%) General / -68.0,-39.8	Favored (87.1%) *mmt-90* chi angles: 299.1,292,187.5,277.8	0.08Å	Favored (87.786%) alpha helix	-	-	-
A 660		VAL	0.66	-	Favored (35.25%) Ile or Val / -68.9,-27.3	Favored (32.5%) *m* chi angles: 297.5	0.09Å	Favored (76.325%) alpha helix	-	-	-
**#**	**Alt**	**Res**	**High B**	**Clash > 0.4Å**	**Ramachandran**	**Rotamer**	**Cβ deviation**	**CaBLAM**	**Bond lengths**	**Bond angles**	**Cis Peptides**
			Avg: 0.92	Clashscore: 1.59	Outliers: 4 of 903	Poor rotamers: 0 of 771	Outliers: 0 of 828	Outliers: 22 of 901	Outliers: 13 of 905	Outliers: 19 of 905	Non-Trans: 1 of 904
A 661		THR	0.65	-	Favored (48.14%) General / -74.7,-6.3	Favored (72.4%) *p* chi angles: 61.9	0.07Å	Favored (60.794%) three-ten	-	-	-
A 662		ARG	0.65	-	Favored (21.42%) General / -103.0,18.6	Favored (64.6%) *mtt90* chi angles: 288,162.8,173.4,71.6	0.04Å	Favored (21.132%)	-	-	-
A 663		MET	0.65	-	Favored (32.15%) General / -123.9,158.4	Favored (56.4%) *mtt* chi angles: 301.4,183.3,183.9	0.08Å	Favored (29.312%)	-	-	-
A 664		ALA	0.66	-	Favored (8.91%) General / -132.0,107.8	-	0.04Å	Favored (25.699%)	-	-	-
A 665		VAL	0.67	-	Favored (71.29%) Ile or Val / -121.0,124.0	Favored (44.4%) *t* chi angles: 182.6	0.07Å	Favored (52.695%)	-	-	-
A 666		SER	0.68	-	Favored (6.24%) General / -125.7,100.0	Favored (39.4%) *t* chi angles: 176.9	0.12Å	Favored (6.281%)	-	-	-
A 667		GLY	0.69	-	Favored (42.39%) Glycine / 59.2,-126.4	-	-	Favored (61.642%)	-	-	-
A 668		ASP	0.69	-	Favored (24.86%) General / -92.5,11.9	Favored (50.7%) *p0* chi angles: 61.4,353.7	0.07Å	Favored (8.572%)	-	OUTLIER(S) worst is CA-CB-CG: 4.9 σ	-
A 669		ASP	0.68	-	Favored (23.52%) General / -110.5,111.9	Favored (68.5%) *t0* chi angles: 182.9,349.7	0.06Å	Favored (23.758%)	-	-	-
A 670		CYS	0.66	-	Favored (38.66%)	Favored (42.2%) *t* chi angles: 176.9	0.08Å	Favored (57.922%)	-	-	-

#	Alt	Res	High B	Clash > 0.4Å	Ramachandran	Rotamer	Cβ deviation	CaBLAM	Bond lengths	Bond angles	Cis Peptides
					General / -133.8,131.0						
A 671		VAL	0.65	-	Favored (47.87%) Ile or Val / -101.4,132.1	Favored (79.5%) *t* chi angles: 178	0.02Å	Favored (64.412%) beta sheet	-	-	-
A 672		VAL	0.65	-	Favored (55.02%) Ile or Val / -129.5,138.2	Favored (55.2%) *t* chi angles: 180.6	0.02Å	Favored (69.666%) beta sheet	-	-	-
A 673		LYS	0.65	-	Favored (37.32%) Pre-Pro / -121.0,83.3	Favored (42.5%) *tttp* chi angles: 187.6,173.1,173.6,75.6	0.01Å	Favored (29.567%) beta sheet	-	-	-
A 674		PRO	0.66	-	Favored (55.11%) Trans-Pro / -72.1,157.0	Favored (71.2%) *Cg_endo* chi angles: 31.1,325.1,24.3	0.11Å	Favored (32.679%)	-	-	-
A 675		LEU	0.67	0.44Å N with A 675 LEU HD22	Favored (37.65%) General / -73.0,-5.3	Favored (2.7%) *mm* chi angles: 277.1,297	0.04Å	Favored (13.185%)	-	-	-
A 676		ASP	0.68	-	Favored (8.11%) General / -150.5,122.3	Favored (44%) *t0* chi angles: 187.6,327.5	0.03Å	Favored (10.138%)	-	-	-
A 677		ASP	0.68	-	Favored (5.58%) General / -56.9,-15.8	Favored (98.9%) *m-30* chi angles: 287.7,348.2	0.06Å	Favored (12.657%)	-	-	-
A 678		ARG	0.68	-	Favored (10.99%) General / -57.9,-16.1	Favored (74.9%) *mtm180* chi angles: 287.8,181.7,288.6,164.1	0.08Å	Favored (16.383%)	-	-	-
A 679		PHE	0.68	-	Favored (9.98%) General / -49.1,-32.5	Favored (76.5%) *t80* chi angles: 184.4,78.1	0.05Å	Favored (37.849%) three-ten	-	-	-
A 680		ALA	0.68	-	Favored (61.88%) General / -54.5,-34.6	-	0.11Å	Favored (51.174%) three-ten	-	-	-
**#**	**Alt**	**Res**	**High B**	**Clash > 0.4Å**	**Ramachandran**	**Rotamer**	**Cβ deviation**	**CaBLAM**	**Bond lengths**	**Bond angles**	**Cis Peptides**
			Avg: 0.92	Clashscore: 1.59	Outliers: 4 of 903	Poor rotamers: 0 of 771	Outliers: 0 of 828	Outliers: 22 of 901	Outliers: 13 of 905	Outliers: 19 of 905	Non-Trans: 1 of 904
A 681		ASN	0.69	-	Favored (39.26%) General / -102.8,9.0	Favored (71.6%) *m-40* chi angles: 292,282	0.06Å	Favored (42.107%)	-	-	-
A 682		ALA	0.7	-	Favored (8.74%) General / -83.7,65.9	-	0.04Å	CaBLAM Disfavored (4.286%)	-	-	-
A 683		LEU	0.72	-	Favored (19.59%) General / -110.2,3.0	Favored (93.6%) *mt* chi angles: 295.3,176.1	0.03Å	Favored (9.894%)	OUTLIER(S) worst is CB--CG: 5.0 σ	-	-
A 684		HIS	0.73	-	Favored (72.08%) General / -70.8,-41.2	Favored (81.2%) *m-70* chi angles: 290.3,296.6	0.06Å	Favored (16.79%)	-	-	-
A 685		PHE	0.75	-	Favored (71.47%) General / -71.0,-34.6	Favored (20.7%) *m-80* chi angles: 277,114.5	0.07Å	Favored (75.458%) alpha helix	-	-	-
A 686		LEU	0.76	-	Favored (81.01%)	Favored (49.1%) *tp* chi angles: 182.2,65.1	0.08Å	Favored (63.097%) alpha helix	-	-	-

					General / -63.0,-47.9							
A 687		ASN	0.77	-	Favored (56.87%) General / -77.1,-34.1	Favored (67.3%) *m-40* chi angles: 287.3,279.7	0.08Å	Favored (59.871%) alpha helix	-	-	-
A 688		SER	0.79	-	Favored (68.23%) General / -65.9,-27.5	Favored (68.1%) *m* chi angles: 294.7	0.04Å	Favored (75.46%) alpha helix	-	-	-
A 689		MET	0.83	-	Favored (18.86%) General / -86.5,9.3	Favored (67%) *mtt* chi angles: 292.2,177.8,188.9	0.03Å	Favored (44.014%)	-	-	-
A 690		SER	0.88	-	Favored (12.72%) General / 64.5,30.9	Favored (44%) *m* chi angles: 301.8	0.03Å	Favored (20.744%)	-	-	-
A 691		LYS	0.95	-	Favored (42.84%) General / -110.9,142.6	Favored (22.8%) *mmtm* chi angles: 307.4,295.3,181.8,278.5	0.05Å	Favored (24.073%)	-	-	-
A 692		VAL	1.06	-	Favored (61.87%) Ile or Val / -130.8,134.4	Favored (72.3%) *t* chi angles: 178.5	0.05Å	Favored (52.704%) beta sheet	-	-	-
A 693		ARG	1.21	-	Favored (30.74%) General / -72.9,126.8	Favored (65.9%) *ttt-90* chi angles: 183,179.3,187.6,268.6	0.05Å	Favored (43.925%)	-	-	-
A 694		LYS	1.38	-	Favored (57.84%) General / -52.0,-38.7	Favored (35.5%) *ttpt* chi angles: 181.4,171.5,67,183.3	0.02Å	Favored (32.511%)	-	-	-
A 695		ASP	1.54	-	Favored (50.15%) General / -94.6,6.0	Favored (73.5%) *m-30* chi angles: 294.4,319.7	0.03Å	Favored (31.258%)	-	-	-
A 696		VAL	1.65	-	Favored (81.89%) Pre-Pro / -130.8,154.7	Favored (28.6%) *m* chi angles: 298.4	0.05Å	Favored (19.148%)	-	-	-
A 697		PRO	1.65	-	Favored (63.86%) Trans-Pro / -60.0,152.6	Favored (53.3%) *Cg_exo* chi angles: 337.6,32.5,331.1	0.01Å	Favored (33.147%)	-	-	-
A 698		GLU	1.54	-	Favored (47.52%) General / -56.9,140.7	Favored (95.7%) *mt-10* chi angles: 291.8,179.8,9,3	0.01Å	Favored (25.88%)	-	-	-
A 699		TRP	1.37	-	Favored (5.37%) General / 70.4,5.5	Favored (51.2%) *m100* chi angles: 309,106.2	0.06Å	Favored (10.477%)	-	-	-
A 700		LYS	1.17	-	Favored (33.14%) Pre-Pro / -93.0,147.1	Favored (72.2%) *mmtt* chi angles: 299.9,298.6,183.1,182.3	0.08Å	Favored (16.951%)	-	-	-
**#**	**Alt**	**Res**	**High B**	**Clash > 0.4Å**	**Ramachandran**	**Rotamer**	**Cβ deviation**	**CaBLAM**	**Bond lengths**	**Bond angles**	**Cis Peptides**
			Avg: 0.92	Clashscore: 1.59	Outliers: 4 of 903	Poor rotamers: 0 of 771	Outliers: 0 of 828	Outliers: 22 of 901	Outliers: 13 of 905	Outliers: 19 of 905	Non-Trans: 1 of 904
A 701		PRO	1	-	Favored (88.54%) Trans-Pro / -64.6,150.2	Favored (29.5%) *Cg_endo* chi angles: 21.2,327,30.4	0.04Å	Favored (23.584%) beta sheet	-	-	-
A 702		SER	0.88	-	Favored (38.98%) General / -75.9,147.6	Favored (66.6%) *m* chi angles: 297	0.03Å	Favored (49.447%) beta sheet	-	-	-

A 703		SER	0.81	-	Favored (32.61%) General / -79.1,147.8	Favored (68.7%) *m* chi angles: 296.7	0.04Å	Favored (41.377%) beta sheet	-	-	-
A 704		GLY	0.77	-	Favored (24.12%) Glycine / -136.1,158.8	-	-	Favored (70.886%) beta sheet	-	-	-
A 705		TRP	0.75	-	Favored (47.02%) General / -135.2,144.7	Favored (27%) *m-90* chi angles: 301.4,264.8	0.08Å	Favored (68.68%)	-	-	-
A 706		HIS	0.75	-	Favored (58.68%) General / -84.9,-8.7	Favored (49.9%) *p-80* chi angles: 63,291.6	0.04Å	Favored (50.823%)	-	-	-
A 707		ASP	0.75	-	Favored (10.92%) General / -129.2,109.1	Favored (59.2%) *t0* chi angles: 182.2,341.2	0.02Å	Favored (15.913%)	-	-	-
A 708		TRP	0.75	-	Favored (4.99%) General / -51.3,-24.9	Favored (78.8%) *p-90* chi angles: 64.6,268.4	0.02Å	Favored (33.559%)	-	-	-
A 709		GLN	0.75	-	Favored (62.96%) General / -70.5,-18.6	Favored (31.7%) *mt0* chi angles: 294.7,174.1,107.2	0.04Å	Favored (63.777%)	-	-	-
A 710		GLN	0.76	-	Favored (55.4%) General / -95.0,2.7	Favored (76.2%) *mt0* chi angles: 297.1,179.1,24.8	0.02Å	Favored (55.502%)	-	-	-
A 711		VAL	0.79	-	Favored (67%) Pre-Pro / -94.4,118.6	Favored (65.5%) *t* chi angles: 179.3	0.11Å	Favored (31.436%)	-	-	-
A 712		PRO	0.82	-	Favored (56.9%) Trans-Pro / -71.6,150.5	Favored (67.3%) *Cg_endo* chi angles: 27,327.6,24.5	0.02Å	Favored (31.004%)	-	-	-
A 713		PHE	0.85	-	Favored (2.16%) General / -163.3,123.0	Favored (26.4%) *t80* chi angles: 186.6,54.4	0.05Å	CaBLAM Disfavored (3.328%)	-	-	-
A 714		CYS	0.88	-	Favored (23.25%) General / 58.7,40.4	Favored (56.8%) *m* chi angles: 302.3	0.02Å	Favored (26.293%)	-	-	-
A 715		SER	0.88	-	Allowed (1.31%) General / 75.2,-9.9	Favored (6%) *p* chi angles: 84.4	0.05Å	CaBLAM Outlier (0.708%)	-	-	-
A 716		ASN	0.87	-	Favored (13.95%) General / -103.3,161.3	Favored (83%) *m-40* chi angles: 292.6,314.7	0.03Å	Favored (28.508%)	-	-	-
A 717		HIS	0.85	-	Favored (23.06%) General / -119.7,160.2	Favored (73.9%) *m90* chi angles: 294.3,77.2	0.02Å	Favored (52.977%) beta sheet	-	-	-
A 718		PHE	0.81	-	Favored (49.9%) General / -126.7,147.9	Favored (84.3%) *m-80* chi angles: 296.4,83.2	0.07Å	Favored (63.058%) beta sheet	-	-	-
A 719		GLN	0.78	-	Favored (49.74%) General / -127.1,149.8	Favored (69.6%) *mt0* chi angles: 295.4,187.1,358.4	0.05Å	Favored (55.929%) beta sheet	-	-	-
A 720		GLU	0.77	-	Favored (31.02%) General / -94.0,118.1	Favored (89.7%) *tt0* chi angles: 181,180.2,350.1	0.03Å	Favored (54.786%) beta sheet	-	-	-

#	Alt	Res	High B	Clash > 0.4Å	Ramachandran	Rotamer	Cβ deviation	CaBLAM	Bond lengths	Bond angles	Cis Peptides
			Avg: 0.92	Clashscore: 1.59	Outliers: 4 of 903	Poor rotamers: 0 of 771	Outliers: 0 of 828	Outliers: 22 of 901	Outliers: 13 of 905	Outliers: 19 of 905	Non-Trans: 1 of 904
A 721		LEU	0.77	-	Favored (27.82%) General / -109.0,150.4	Favored (3.1%) *mm* chi angles: 276.3,290.9	0.05Å	Favored (41.423%) beta sheet	-	-	-
A 722		ILE	0.78	-	Favored (70.66%) Ile or Val / -113.3,125.7	Favored (78.3%) *mt* chi angles: 300.5,170.3	0.01Å	Favored (46.619%) beta sheet	-	-	-
A 723		MET	0.79	-	Favored (28.61%) General / -73.7,163.0	Favored (92.3%) *mtp* chi angles: 295.6,181.7,69.6	0.08Å	Favored (37.107%)	-	-	-
A 724		LYS	0.79	-	Favored (64.25%) General / -65.9,-16.9	Favored (22.5%) *mmtp* chi angles: 297.3,292.9,180.9,65.9	0.01Å	Favored (41.559%)	-	-	-
A 725		ASP	0.79	-	Favored (45.23%) General / -98.8,8.2	Favored (74.1%) *m-30* chi angles: 297.4,316.3	0.16Å	Favored (55.662%)	-	-	-
A 726		GLY	0.79	0.49Å O with A 727 ARG HB2	Favored (50.33%) Glycine / 85.2,-176.7	-	-	Favored (10.565%)	-	-	-
A 727		ARG	0.77	0.49Å HB2 with A 726 GLY O	OUTLIER (0.03)%) General / 90.5,136.1	Favored (85.9%) *mtm180* chi angles: 296.4,175.8,287.2,169.6	0.10Å	Favored (6.861%)	-	OUTLIER(S) worst is NE-CZ-NH2: 6.8 σ	-
A 728		THR	0.75	-	Favored (23.9%) General / -104.5,151.2	Favored (58.9%) *p* chi angles: 64.3	0.01Å	Favored (45.82%) beta sheet	-	-	-
A 729		LEU	0.74	0.42Å C with A 729 LEU HD23	Favored (26.62%) General / -143.4,135.3	Favored (7.6%) *tt* chi angles: 184.4,152.7	0.08Å	Favored (57.613%) beta sheet	-	-	-
A 730		VAL	0.73	-	Favored (55.02%) Ile or Val / -101.6,123.9	Favored (59.5%) *t* chi angles: 180	0.05Å	Favored (55.62%) beta sheet	-	-	-
A 731		VAL	0.73	-	Favored (53.31%) Pre-Pro / -131.3,144.4	Favored (7.1%) *p* chi angles: 60.3	0.13Å	Favored (43.815%) beta sheet	-	-	-
A 732		PRO	0.75	-	Favored (20.06%) Trans-Pro / -58.3,159.1	Favored (77.3%) *Cg_exo* chi angles: 334.9,31.6,335.3	0.08Å	Favored (19.647%) beta sheet	-	-	-
A 733		CYS	0.77	-	Favored (33.01%) General / -153.2,153.6	Favored (28.7%) *p* chi angles: 61.3	0.10Å	Favored (51.014%)	-	OUTLIER(S) worst is C-N-CA: 16.7 σ	-
A 734		ARG	0.79	0.40Å O with A 735 GLY C	Favored (24.82%) General / -162.8,160.1	Favored (73.9%) *ttt180* chi angles: 179.8,169.2,176.5,171.9	0.01Å	Favored (5.713%)	-	-	-
A 735		GLY	0.82	0.40Å C with A 734 ARG O	Favored (29.21%) Glycine / -51.2,127.4	-	-	Favored (9.707%)	-	-	-
A 736		GLN	0.83	-	Favored (74.3%) General / -61.5,-33.7	Favored (15.4%) *tp40* chi angles: 182.3,64.7,349.5	0.03Å	Favored (51.899%)	-	-	-

A 737		ASP	0.84	-	Favored (78.65%) General / -61.0,-36.1	Favored (91.5%) *m-30* chi angles: 292,348.5	0.10Å	Favored (72.15%) alpha helix	-	-	-
A 738		GLU	0.83	-	Favored (40.23%) General / -79.3,-36.0	Favored (95.5%) *mt-10* chi angles: 292.1,185.1,350.6	0.03Å	Favored (82.424%) alpha helix	-	-	-
A 739		LEU	0.82	-	Favored (96.59%) General / -63.2,-40.1	Favored (87.8%) *mt* chi angles: 291.6,174.7	0.03Å	Favored (75.442%) alpha helix	-	-	-
A 740		ILE	0.8	-	Favored (37.35%) Ile or Val / -76.1,-44.5	Favored (92.4%) *mt* chi angles: 296.8,169.4	0.05Å	Favored (70.554%) alpha helix	-	-	-
**#**	**Alt**	**Res**	**High B**	**Clash > 0.4Å**	**Ramachandran**	**Rotamer**	**Cβ deviation**	**CaBLAM**	**Bond lengths**	**Bond angles**	**Cis Peptides**
			Avg: 0.92	Clashscore: 1.59	Outliers: 4 of 903	Poor rotamers: 0 of 771	Outliers: 0 of 828	Outliers: 22 of 901	Outliers: 13 of 905	Outliers: 19 of 905	Non-Trans: 1 of 904
A 741		GLY	0.79	-	Favored (45.6%) Glycine / -51.9,-50.2	-	-	Favored (95.648%) alpha helix	-	-	-
A 742		ARG	0.8	-	Favored (93.66%) General / -59.7,-44.5	Favored (85.4%) *mtp85* chi angles: 289.6,174,66.7,85.9	0.04Å	Favored (95.554%) alpha helix	-	-	-
A 743		ALA	0.84	-	Favored (71.48%) General / -60.9,-32.1	-	0.05Å	Favored (74.778%) alpha helix	-	-	-
A 744		ARG	0.93	-	Favored (34.85%) General / -80.8,0.4	Favored (38.8%) *mtp180* chi angles: 287.6,180.8,55.8,164.8	0.08Å	Favored (43.998%)	-	-	-
A 745		VAL	1.08	-	Favored (73.48%) Ile or Val / -115.9,127.5	Favored (83.3%) *t* chi angles: 176.9	0.04Å	Favored (31.459%)	-	-	-
A 746		SER	1.28	-	Favored (34.25%) Pre-Pro / -93.5,130.8	Favored (67%) *m* chi angles: 294.5	0.05Å	Favored (47.76%)	-	-	-
A 747		PRO	1.52	-	Favored (8.18%) Trans-Pro / -78.1,63.2	Favored (54.6%) *Cg_endo* chi angles: 32.5,323,25.8	0.07Å	CaBLAM Outlier (0.539%)	-	-	-
A 748		GLY	1.74	-	Favored (33.41%) Glycine / -177.4,-165.9	-	-	Favored (11.132%) alpha helix	-	-	-
A 749		SER	1.84	-	Favored (2.61%) General / -115.2,-53.7	Favored (43.7%) *t* chi angles: 178.7	0.02Å	CaBLAM Outlier (0.059%) try alpha helix	-	-	-
A 750		GLY	1.8	-	Favored (17.33%) Glycine / -92.9,144.9	-	-	CaBLAM Outlier (0.472%)	-	-	-
A 751		TRP	1.63	-	Favored (4.5%) General / -166.2,140.9	Favored (61.7%) *t-100* chi angles: 188.6,260.7	0.17Å	Favored (17.499%)	-	-	-
A 752		ASN	1.4	-	Favored (14.28%)	Favored (12.2%) *t0* chi angles: 193.1,269.8	0.01Å	Favored (38.716%)	-	-	-

					General / -104.1,161.1						
A 753		VAL	1.18	-	Favored (38.88%) Ile or Val / -59.7,-27.8	Favored (5.7%) *p* chi angles: 69.4	0.07Å	Favored (62.432%)	-	-	-
A 754		ARG	1	-	Favored (65.62%) General / -68.5,-26.7	Favored (98.6%) *mtt180* chi angles: 292.6,180.5,179.4,176	0.02Å	Favored (73.509%) alpha helix	-	-	-
A 755		ASP	0.87	-	Favored (25.39%) General / -83.5,-34.2	Favored (65.2%) *m-30* chi angles: 297.3,305.6	0.10Å	Favored (72.658%) alpha helix	-	-	-
A 756		THR	0.78	-	Favored (80.44%) General / -61.9,-48.5	Favored (89%) *m* chi angles: 298.6	0.09Å	Favored (88.209%) alpha helix	-	-	-
A 757		ALA	0.73	-	Favored (99.71%) General / -62.5,-42.7	-	0.09Å	Favored (95.57%) alpha helix	-	-	-
A 758		CYS	0.69	-	Favored (95.33%) General / -64.9,-41.8	Favored (95.3%) *m* chi angles: 290.6	0.03Å	Favored (89.632%) alpha helix	-	-	-
A 759		LEU	0.67	-	Favored (86.47%) General / -65.7,-44.3	Favored (59.9%) *tp* chi angles: 181.7,61.5	0.04Å	Favored (81.929%) alpha helix	-	-	-
A 760		ALA	0.65	-	Favored (79.39%) General / -57.7,-40.4	-	0.07Å	Favored (81.532%) alpha helix	-	-	-
**#**	**Alt**	**Res**	**High B**	**Clash > 0.4Å**	**Ramachandran**	**Rotamer**	**Cβ deviation**	**CaBLAM**	**Bond lengths**	**Bond angles**	**Cis Peptides**
			Avg: 0.92	Clashscore: 1.59	Outliers: 4 of 903	Poor rotamers: 0 of 771	Outliers: 0 of 828	Outliers: 22 of 901	Outliers: 13 of 905	Outliers: 19 of 905	Non-Trans: 1 of 904
A 761		LYS	0.65	-	Favored (92.89%) General / -64.6,-39.0	Favored (83.8%) *tttt* chi angles: 187.3,172.8,184.9,180.6	0.04Å	Favored (75.957%) alpha helix	-	-	-
A 762		ALA	0.65	-	Favored (76.76%) General / -57.2,-40.1	-	0.10Å	Favored (74.334%) alpha helix	-	-	-
A 763		TYR	0.65	-	Favored (61.88%) General / -73.8,-41.8	Favored (50.7%) *m-80* chi angles: 283,97.8	0.02Å	Favored (78.317%) alpha helix	-	-	-
A 764		ALA	0.65	-	Favored (94.13%) General / -61.5,-40.3	-	0.07Å	Favored (91.08%) alpha helix	-	-	-
A 765		GLN	0.66	-	Favored (84.27%) General / -67.1,-42.5	Favored (96.7%) *mt0* chi angles: 290.6,173.4,317.2	0.02Å	Favored (95.57%) alpha helix	-	-	-
A 766		MET	0.67	-	Favored (90.61%) General / -59.2,-45.6	Favored (57.9%) *ttp* chi angles: 177.1,184.4,68.7	0.05Å	Favored (96.605%) alpha helix	-	-	-
A 767		TRP	0.68	-	Favored (95.14%) General / -60.2,-44.2	Favored (48%) *m-10* chi angles: 288.8,344.4	0.08Å	Favored (96.709%) alpha helix	-	-	-

A 768		LEU	0.68	-	Favored (78.29%) General / -63.7,-34.6	Favored (83.1%) *mt* chi angles: 289.5,172.5	0.02Å	Favored (73.478%) alpha helix	-	-	-
A 769		LEU	0.69	-	Favored (21.03%) General / -87.7,-29.5	Favored (88.6%) *mt* chi angles: 298.3,173.1	0.10Å	Favored (60.213%) alpha helix	-	-	-
A 770		LEU	0.7	-	Favored (9.44%) General / -100.6,-35.6	Favored (77.5%) *mt* chi angles: 296,180.5	0.11Å	Favored (39.598%)	-	-	-
A 771		TYR	0.7	-	Favored (5.89%) General / -120.7,32.6	Favored (66.2%) *m-80* chi angles: 304.5,105.8	0.08Å	Favored (14.84%)	-	-	-
A 772		PHE	0.7	0.52Å C with A 772 PHE CD1	Favored (7.2%) General / -54.3,-20.5	Favored (14.5%) *p90* chi angles: 71.6,79.1	0.09Å	Favored (8.841%)	-	-	-
A 773		HIS	0.7	0.55Å CD2 with A 773 HIS H	Favored (59.65%) General / -83.1,-7.4	Favored (13.7%) *p90* chi angles: 62.7,103.5	0.08Å	Favored (49.14%)	OUTLIER(S) worst is CB--CG: 7.8 σ	-	-
A 774		ARG	0.7	-	Favored (31.95%) General / -86.4,123.7	Favored (52.4%) *ttm170* chi angles: 180.4,167.4,295.2,170	0.06Å	Favored (35.383%)	OUTLIER(S) worst is CD--NE: 5.5 σ	-	-
A 775		ARG	0.71	-	Favored (95.13%) General / -64.9,-42.0	Favored (91.1%) *mtt-85* chi angles: 290.4,171.1,180.2,270.6	0.09Å	Favored (39.94%)	-	-	-
A 776		ASP	0.7	-	Favored (66.73%) General / -72.5,-40.7	Favored (36%) *t70* chi angles: 188.1,63.3	0.02Å	Favored (77.662%) alpha helix	-	-	-
A 777		LEU	0.7	-	Favored (71.96%) General / -71.1,-39.8	Favored (15.2%) *tp* chi angles: 193.6,61.9	0.04Å	Favored (92.914%) alpha helix	OUTLIER(S) worst is CB--CG: 4.2 σ	-	-
A 778		ARG	0.69	-	Favored (88.3%) General / -58.3,-44.0	Favored (27.6%) *mmm-85* chi angles: 281.4,274.4,292.3,276.7	0.09Å	Favored (92.331%) alpha helix	-	-	-
A 779		LEU	0.67	-	Favored (80.21%) General / -67.3,-44.1	Favored (46.5%) *tp* chi angles: 181,55.6	0.08Å	Favored (88.853%) alpha helix	-	-	-
A 780		MET	0.65	-	Favored (96.88%) General / -60.5,-43.6	Favored (98.1%) *mtp* chi angles: 290.7,174.4,71.4	0.11Å	Favored (96.548%) alpha helix	-	-	-
**#**	**Alt**	**Res**	**High B**	**Clash > 0.4Å**	**Ramachandran**	**Rotamer**	**Cβ deviation**	**CaBLAM**	**Bond lengths**	**Bond angles**	**Cis Peptides**
			Avg: 0.92	Clashscore: 1.59	Outliers: 4 of 903	Poor rotamers: 0 of 771	Outliers: 0 of 828	Outliers: 22 of 901	Outliers: 13 of 905	Outliers: 19 of 905	Non-Trans: 1 of 904
A 781		ALA	0.64	-	Favored (98.21%) General / -61.8,-41.8	-	0.07Å	Favored (87.814%) alpha helix	-	-	-
A 782		ASN	0.62	-	Favored (87.7%) General / -66.4,-38.6	Favored (88.4%) *m-40* chi angles: 284.6,337.2	0.02Å	Favored (89.253%) alpha helix	-	-	-
A 783		ALA	0.61	-	Favored (93.28%) General / -62.7,-39.2	-	0.06Å	Favored (78.362%) alpha helix	-	-	-

A 784		ILE	0.6	-	Favored (57.87%) Ile or Val / -72.3,-46.2	Favored (98.5%) *mt* chi angles: 293.3,167.6	0.04Å	Favored (73.602%) alpha helix	-	-	-
A 785		CYS	0.6	-	Favored (70.35%) General / -60.2,-31.7	Favored (87.7%) *m* chi angles: 293.4	0.05Å	Favored (76.319%) alpha helix	-	-	-
A 786		SER	0.6	-	Favored (59.59%) General / -80.7,-11.7	Favored (59.6%) *m* chi angles: 299.1	0.09Å	Favored (64.917%) alpha helix	-	-	-
A 787		ALA	0.6	-	Favored (13.18%) General / -94.3,-33.5	-	0.04Å	Favored (16.458%)	-	-	-
A 788		VAL	0.61	-	Favored (47.26%) Pre-Pro / -84.0,132.7	Favored (85.4%) *t* chi angles: 177.2	0.04Å	Favored (17.991%)	-	-	-
A 789		PRO	0.62	-	Favored (64.75%) Trans-Pro / -55.8,146.9	Favored (83.7%) *Cg_exo* chi angles: 334.3,35,331	0.07Å	Favored (49.994%)	-	-	-
A 790		SER	0.63	-	Favored (34.3%) General / -66.3,156.4	Favored (95.9%) *p* chi angles: 65	0.04Å	Favored (17.131%)	-	-	-
A 791		ASN	0.66	-	Allowed (1.34%) General / 65.6,-0.1	Favored (87.3%) *m-40* chi angles: 298,320.2	0.05Å	CaBLAM Disfavored (4.076%)	-	-	-
A 792		TRP	0.7	-	Favored (38.54%) General / -78.0,142.1	Favored (38.7%) *m-10* chi angles: 288.7,13.6	0.06Å	Favored (25.676%)	-	-	-
A 793		VAL	0.76	-	Favored (24.42%) Pre-Pro / -113.7,140.2	Favored (9.7%) *p* chi angles: 64.1	0.13Å	Favored (41.829%) beta sheet	-	-	-
A 794		PRO	0.84	-	Favored (83.38%) Trans-Pro / -66.7,148.8	Favored (37.4%) *Cg_endo* chi angles: 22.8,325.2,31.2	0.05Å	Favored (53.579%) beta sheet	-	-	-
A 795		THR	0.92	-	Favored (8.23%) General / -122.2,-12.7	Favored (66.2%) *p* chi angles: 63	0.05Å	Favored (11.15%) beta sheet	-	-	-
A 796		GLY	1.01	-	Favored (36.85%) Glycine / -93.7,-164.5	-	-	Favored (30.69%) beta sheet	-	-	-
A 797		ARG	1.1	-	Favored (49.86%) General / -126.5,147.1	Favored (58.8%) *mmm-85* chi angles: 300.1,293.4,293.6,270.1	0.01Å	Favored (20.206%)	-	-	-
A 798		THR	1.17	-	Favored (12.24%) General / -107.8,-23.1	Favored (72.1%) *p* chi angles: 61.9	0.06Å	Favored (31.545%)	-	-	-
A 799		SER	1.22	-	Favored (5.28%) General / -167.0,146.4	Favored (42.3%) *t* chi angles: 178.3	0.03Å	Favored (20.999%)	-	-	-
A 800		TRP	1.25	-	Favored (25.67%) General / -109.2,10.9	Favored (41%) *m-90* chi angles: 293,270.9	0.02Å	Favored (10.093%)	-	-	-

#	Alt	Res	High B	Clash > 0.4Å	Ramachandran	Rotamer	Cβ deviation	CaBLAM	Bond lengths	Bond angles	Cis Peptides
			Avg: 0.92	Clashscore: 1.59	Outliers: 4 of 903	Poor rotamers: 0 of 771	Outliers: 0 of 828	Outliers: 22 of 901	Outliers: 13 of 905	Outliers: 19 of 905	Non-Trans: 1 of 904
A 801		SER	1.27	-	Favored (55.94%) General / -64.6,145.6	Favored (31%) *t* chi angles: 173.9	0.06Å	Favored (41.71%)	-	-	-
A 802		VAL	1.28	-	Favored (22.34%) Ile or Val / -55.8,-27.5	Favored (62%) *t* chi angles: 171.1	0.03Å	Favored (49.25%)	-	-	-
A 803		HIS	1.26	-	Favored (55.17%) General / -87.3,-9.4	Favored (75.1%) *m-70* chi angles: 289.6,269.2	0.06Å	Favored (57.797%)	OUTLIER(S) worst is CB--CG: 4.2 σ	OUTLIER(S) worst is CA-CB-CG: 4.3 σ	-
A 804		ALA	1.21	-	Favored (31.17%) General / -75.7,159.4	-	0.03Å	Favored (17.427%)	-	-	-
A 805		THR	1.13	-	Favored (3.83%) General / -134.5,-175.3	Favored (11.7%) *t* chi angles: 189.9	0.05Å	Favored (35.834%)	-	-	-
A 806		GLY	1.04	-	Favored (27.86%) Glycine / -111.6,5.0	-	-	Favored (12.601%)	-	-	-
A 807		GLU	0.95	-	Favored (73.27%) General / -62.0,-32.7	Favored (57.5%) *mm-30* chi angles: 295.8,292.4,303.8	0.10Å	Favored (53.939%)	-	-	-
A 808		TRP	0.86	-	Favored (84.46%) General / -67.6,-38.8	Favored (18.5%) *m-10* chi angles: 310.5,336.8	0.17Å	Favored (38.484%) three-ten	-	-	-
A 809		MET	0.8	-	Favored (10.37%) General / -85.1,75.2	Favored (94%) *mtp* chi angles: 293,182.2,72.8	0.05Å	CaBLAM Disfavored (3.198%)	-	-	-
A 810		THR	0.75	-	Favored (3.14%) General / -142.0,-170.4	Favored (10.7%) *t* chi angles: 188.8	0.10Å	Favored (33.394%)	-	-	-
A 811		THR	0.72	-	Favored (8.39%) General / -122.4,-10.9	Favored (76.2%) *p* chi angles: 60.1	0.04Å	CaBLAM Outlier (0.744%) try beta sheet	-	-	-
A 812		ASP	0.71	-	Favored (44.14%) General / -62.1,148.5	Favored (84.1%) *m-30* chi angles: 294.3,335	0.09Å	Favored (20.551%) beta sheet	-	-	-
A 813		ASP	0.71	-	Favored (51.71%) General / -58.8,131.7	Favored (13.3%) *t0* chi angles: 191.9,305.9	0.01Å	Favored (38.01%)	-	-	-
A 814		MET	0.7	0.41Å HA with A 814 MET HE2	Favored (63.46%) General / -58.0,-27.4	Favored (41.7%) *mmp* chi angles: 290.1,293.8,96.2	0.08Å	Favored (41.36%)	-	-	-
A 815		LEU	0.7	-	Favored (69.91%) General / -71.0,-32.9	Favored (68.5%) *mt* chi angles: 289.2,175.1	0.07Å	Favored (79.785%) alpha helix	-	-	-
A 816		GLU	0.69	-	Favored (80.41%) General / -68.8,-39.0	Favored (97.5%) *mt-10* chi angles: 291.2,171.5,340.7	0.04Å	Favored (83.702%) alpha helix	-	-	-

A 817		VAL	0.68	-	Favored (78%) Ile or Val / -70.6,-43.1	Favored (89.5%) *t* chi angles: 176	0.04Å	Favored (84.666%) alpha helix	-	-	-
A 818		TRP	0.67	-	Favored (92.56%) General / -59.8,-45.4	Favored (56.2%) *t60* chi angles: 193.5,93.5	0.09Å	Favored (96.073%) alpha helix	-	-	-
A 819		ASN	0.67	-	Favored (93.91%) General / -60.4,-41.6	Favored (76.7%) *m-40* chi angles: 281.8,336	0.03Å	Favored (89.274%) alpha helix	-	-	-
A 820		LYS	0.67	-	Favored (46.41%) General / -64.2,-53.6	Favored (52%) *tttm* chi angles: 180.4,178.3,182.1,295.9	0.03Å	Favored (65.628%) alpha helix	-	-	-
**#**	**Alt**	**Res**	**High B**	**Clash > 0.4Å**	**Ramachandran**	**Rotamer**	**Cβ deviation**	**CaBLAM**	**Bond lengths**	**Bond angles**	**Cis Peptides**
			Avg: 0.92	Clashscore: 1.59	Outliers: 4 of 903	Poor rotamers: 0 of 771	Outliers: 0 of 828	Outliers: 22 of 901	Outliers: 13 of 905	Outliers: 19 of 905	Non-Trans: 1 of 904
A 821		VAL	0.69	-	Favored (88.69%) Ile or Val / -66.7,-45.3	Favored (74.8%) *t* chi angles: 172.7	0.04Å	Favored (55.065%) alpha helix	-	-	-
A 822		TRP	0.71	-	Favored (17.8%) General / -93.4,-25.2	Favored (94.1%) *m100* chi angles: 288.1,107.5	0.04Å	Favored (39.384%) alpha helix	-	-	-
A 823		ILE	0.75	-	Favored (5.06%) Ile or Val / -110.7,-55.0	Favored (49.6%) *mm* chi angles: 302.4,299.7	0.03Å	Favored (15.779%) alpha helix	-	-	-
A 824		GLN	0.79	-	Favored (29.58%) General / -79.1,-41.1	Favored (93%) *mm-40* chi angles: 299.1,305.4,316.4	0.02Å	Favored (49.901%) alpha helix	-	-	-
A 825		ASP	0.84	-	Favored (59.08%) General / -83.1,-9.7	Favored (90.5%) *m-30* chi angles: 292.6,348.4	0.06Å	Favored (44.65%)	-	-	-
A 826		ASN	0.89	-	Favored (9.57%) General / -85.3,96.6	Favored (58.2%) *t0* chi angles: 183.5,353.1	0.04Å	Favored (28.718%)	-	-	-
A 827		GLU	0.92	-	Favored (17.43%) General / -62.2,-11.9	Favored (16.5%) *pm20* chi angles: 64.8,268,3.8	0.08Å	Favored (22.209%)	-	-	-
A 828		TRP	0.93	-	Favored (47.56%) General / -93.6,-6.0	Favored (81.1%) *m100* chi angles: 301.8,101.8	0.04Å	Favored (54.948%)	-	-	-
A 829		MET	0.92	-	Favored (30.21%) General / -82.3,124.7	Favored (62.7%) *tpp* chi angles: 178.9,61.8,76.5	0.08Å	Favored (32.542%)	-	-	-
A 830		LEU	0.88	-	Favored (59.07%) General / -87.2,-4.0	Favored (93.7%) *mt* chi angles: 298.6,177.2	0.02Å	Favored (14.942%)	-	-	-
A 831		ASP	0.83	-	Favored (2.28%) General / -149.5,95.0	Favored (62.9%) *t0* chi angles: 180.1,355.8	0.03Å	Favored (11.103%)	-	-	-
A 832		LYS	0.78	-	Favored (8.93%) General / -86.7,65.3	Favored (96.3%) *mttt* chi angles: 295.8,183.2,175.3,185.9	0.04Å	Favored (33.911%)	-	-	-
A 833		THR	0.74	-	Favored (66.11%)	Favored (88.4%) *m* chi angles: 297	0.04Å	Favored (35.64%) beta sheet	-	-	-

#	Alt	Res	High B	Clash > 0.4Å	Ramachandran	Rotamer	Cβ deviation	CaBLAM	Bond lengths	Bond angles	Cis Peptides
					Pre-Pro / -94.5,119.6						
A 834		PRO	0.72	-	Favored (97.24%) Trans-Pro / -60.1,146.0	Favored (66.9%) *Cg_exo* chi angles: 335.5,34,331.1	0.09Å	Favored (82.258%) beta sheet	-	-	-
A 835		VAL	0.71	-	Favored (24.11%) Ile or Val / -75.7,136.8	Favored (56.7%) *t* chi angles: 170.3	0.03Å	Favored (42.31%)	-	-	-
A 836		GLN	0.71	-	Favored (13.3%) General / -104.4,-22.6	Favored (91.5%) *mm-40* chi angles: 304.2,300,303.7	0.05Å	Favored (23.911%)	-	-	-
A 837		SER	0.72	-	Favored (33.36%) General / -147.0,149.5	Favored (29.6%) *t* chi angles: 172.8	0.07Å	Favored (23.413%)	-	-	-
A 838		TRP	0.73	-	Favored (52.09%) General / -58.4,-22.1	Favored (59.3%) *m100* chi angles: 275.8,107	0.09Å	Favored (51.147%) alpha helix	-	-	-
A 839		THR	0.73	-	Favored (58.81%) General / -75.1,-9.7	Favored (71.4%) *p* chi angles: 59.6	0.10Å	Favored (63.811%) three-ten	-	-	-
A 840		ASP	0.74	-	Favored (34.09%) General / -83.9,-19.8	Favored (75.2%) *m-30* chi angles: 297.8,334.5	0.16Å	Favored (39.533%)	-	-	-
**#**	**Alt**	**Res**	**High B**	**Clash > 0.4Å**	**Ramachandran**	**Rotamer**	**Cβ deviation**	**CaBLAM**	**Bond lengths**	**Bond angles**	**Cis Peptides**
			Avg: 0.92	Clashscore: 1.59	Outliers: 4 of 903	Poor rotamers: 0 of 771	Outliers: 0 of 828	Outliers: 22 of 901	Outliers: 13 of 905	Outliers: 19 of 905	Non-Trans: 1 of 904
A 841		ILE	0.75	-	Favored (67.65%) Pre-Pro / -90.5,117.0	Favored (92.5%) *mt* chi angles: 296.7,171	0.11Å	Favored (28.867%)	-	-	-
A 842		PRO	0.78	-	Favored (48.65%) Trans-Pro / -71.5,160.3	Favored (72.5%) *Cg_endo* chi angles: 29.1,324.6,27	0.05Å	Favored (15.189%) beta sheet	-	-	-
A 843		TYR	0.83	-	Favored (20.49%) General / -115.6,160.1	Favored (99.2%) *m-80* chi angles: 296.7,91.9	0.07Å	Favored (39.198%)	-	-	-
A 844		THR	0.89	-	Favored (6.72%) General / -86.2,-179.5	Favored (10.2%) *t* chi angles: 191.1	0.05Å	Favored (18.399%)	-	-	-
A 845		GLY	0.95	-	Favored (44%) Glycine / -70.9,148.0	-	-	Favored (19.364%)	-	-	-
A 846		LYS	1.01	-	Favored (83.98%) General / -58.8,-40.5	Favored (88.4%) *tttt* chi angles: 183.2,175.1,178.4,177.9	0.06Å	Favored (57.068%)	-	-	-
A 847		ARG	1.05	-	Favored (98.15%) General / -62.6,-40.8	Favored (98%) *mtt180* chi angles: 289.4,177.3,179.5,172.2	0.06Å	Favored (88.426%) alpha helix	-	-	-
A 848		GLU	1.08	-	Favored (78.33%) General / -68.9,-36.6	Favored (99.8%) *mt-10* chi angles: 292.3,176.5,354.9	0.04Å	Favored (83.59%) alpha helix	-	-	-
A 849		ASP	1.1	-	Favored (95.01%)	Favored (8.4%) *t70* chi angles: 197.3,76.3	0.10Å	Favored (80.298%) alpha helix	-	OUTLIER(S) worst is CA-CB-CG: 6.1 σ	-

					General / -61.9,-45.1						
A 850		ILE	1.12	-	Favored (91.31%) Ile or Val / -66.4,-43.4	Favored (96.9%) *mt* chi angles: 292.3,168.7	0.04Å	Favored (78.243%) alpha helix	-	-	-
A 851		TRP	1.12	-	Favored (69.79%) General / -59.5,-31.8	Favored (43.4%) *m-10* chi angles: 290.3,335.9	0.00Å	Favored (74.527%)	-	-	-
A 852		CYS	1.12	-	Favored (16.45%) General / -81.3,5.7	Favored (75.6%) *m* chi angles: 296.8	0.06Å	Favored (36.397%)	-	-	-
A 853		GLY	1.11	-	Favored (57.82%) Glycine / 97.3,6.0	-	-	Favored (69.198%)	-	-	-
A 854		SER	1.1	-	Favored (58.57%) General / -63.1,141.9	Favored (40.3%) *t* chi angles: 176.7	0.06Å	Favored (25.062%) beta sheet	-	-	-
A 855		LEU	1.09	0.41Å HB2 with A 334 TRP CZ2	Favored (50.06%) General / -78.0,-5.0	Favored (17.9%) *tp* chi angles: 191.7,60.2	0.05Å	Favored (10.591%)	-	-	-
A 856		ILE	1.09	-	Favored (9.57%) Ile or Val / -52.0,-27.6	Favored (20.6%) *tt* chi angles: 194.3,167.7	0.04Å	Favored (16.696%)	-	-	-
A 857		GLY	1.11	-	Favored (3.84%) Glycine / -79.8,50.3	-	-	CaBLAM Outlier (0.304%)	-	-	-
A 858		THR	1.14	-	Favored (10.23%) General / -166.5,154.7	Favored (4.2%) *t* chi angles: 179.1	0.14Å	Favored (8.225%)	-	-	-
A 859		ARG	1.16	-	Favored (68.97%) General / -63.4,-26.9	Favored (98%) *mtt180* chi angles: 290,177.6,180,172.4	0.04Å	Favored (54.898%)	-	-	-
A 860		THR	1.16	-	Favored (79.73%) General / -61.7,-48.7	Favored (87%) *m* chi angles: 296.9	0.05Å	Favored (57.007%) alpha helix	-	-	-
**#**	**Alt**	**Res**	**High B**	**Clash > 0.4Å**	**Ramachandran**	**Rotamer**	**Cβ deviation**	**CaBLAM**	**Bond lengths**	**Bond angles**	**Cis Peptides**
			Avg: 0.92	Clashscore: 1.59	Outliers: 4 of 903	Poor rotamers: 0 of 771	Outliers: 0 of 828	Outliers: 22 of 901	Outliers: 13 of 905	Outliers: 19 of 905	Non-Trans: 1 of 904
A 861		ARG	1.15	-	Favored (81.88%) General / -66.3,-45.0	Favored (4.6%) *tmt170* chi angles: 190.5,268.4,182.5,177.2	0.04Å	Favored (75.761%) alpha helix	-	-	-
A 862		ALA	1.13	-	Favored (89.29%) General / -59.9,-40.6	-	0.04Å	Favored (86.09%) alpha helix	-	-	-
A 863		THR	1.1	-	Favored (93.36%) General / -61.9,-45.5	Favored (94.2%) *m* chi angles: 299.3	0.04Å	Favored (97.846%) alpha helix	-	-	-
A 864		TRP	1.08	-	Favored (85.54%) General / -58.9,-47.3	Favored (40.2%) *t60* chi angles: 164.1,81.8	0.04Å	Favored (85.62%) alpha helix	-	-	-
A 865		ALA	1.06	-	Favored (80.69%) General / -57.5,-41.4	-	0.04Å	Favored (82.796%) alpha helix	-	-	-

A 866		GLU	1.04	-	Favored (77.45%) General / -63.2,-34.4	Favored (98%) *mt-10* chi angles: 290.2,180.2,354	0.03Å	Favored (77.155%) alpha helix	-	-	-
A 867		ASN	1.01	-	Favored (35.72%) General / -95.5,10.7	Favored (88.2%) *m-40* chi angles: 291.1,322.6	0.02Å	Favored (37.979%) alpha helix	-	-	-
A 868		ILE	0.98	-	Favored (91.61%) Ile or Val / -60.9,-42.2	Favored (84.5%) *mt* chi angles: 290.5,168.9	0.07Å	Favored (39.742%) alpha helix	OUTLIER(S) worst is CB--CG1: 5.2 σ	-	-
A 869		TYR	0.94	-	Favored (67.93%) General / -53.0,-47.9	Favored (88.8%) *t80* chi angles: 180.4,78.2	0.07Å	Favored (76.257%) alpha helix	-	-	-
A 870		ALA	0.9	-	Favored (98.76%) General / -62.0,-42.1	-	0.03Å	Favored (94.152%) alpha helix	-	-	-
A 871		ALA	0.86	-	Favored (84.3%) General / -61.8,-37.4	-	0.01Å	Favored (87.488%) alpha helix	-	-	-
A 872		ILE	0.82	-	Favored (92.53%) Ile or Val / -63.8,-47.1	Favored (92.7%) *mt* chi angles: 291.6,168.5	0.04Å	Favored (90.299%) alpha helix	-	-	-
A 873		ASN	0.79	-	Favored (79.47%) General / -61.7,-36.0	Favored (96.7%) *m-40* chi angles: 286.8,339.1	0.07Å	Favored (81.281%) alpha helix	-	-	-
A 874		GLN	0.77	-	Favored (94.59%) General / -64.5,-43.3	Favored (98.2%) *mt0* chi angles: 290.8,171.9,342	0.05Å	Favored (86.824%) alpha helix	-	-	-
A 875		VAL	0.75	-	Favored (85.18%) Ile or Val / -67.7,-41.1	Favored (71.8%) *t* chi angles: 172.3	0.07Å	Favored (85.906%) alpha helix	-	-	-
A 876		ARG	0.75	-	Favored (89.28%) General / -59.5,-41.2	Favored (97.4%) *mtt180* chi angles: 288.8,179.6,175,171.8	0.07Å	Favored (85.699%) alpha helix	-	-	-
A 877		ALA	0.75	-	Favored (82.75%) General / -60.4,-38.0	-	0.03Å	Favored (83.241%) alpha helix	-	-	-
A 878		ILE	0.77	-	Favored (82.32%) Ile or Val / -68.6,-44.9	Favored (98.5%) *mt* chi angles: 293.1,167.9	0.00Å	Favored (90.21%) alpha helix	-	-	-
A 879		ILE	0.79	-	Favored (75.83%) Ile or Val / -69.6,-46.2	Favored (95.3%) *mt* chi angles: 294.8,168.4	0.04Å	Favored (5.543%)	-	-	-
A 880		GLY	0.8	-	Favored (35.79%) Glycine / 159.1,-172.1	-	-	Favored (21.014%)	-	-	-
**#**	**Alt**	**Res**	**High B**	**Clash > 0.4Å**	**Ramachandran**	**Rotamer**	**Cβ deviation**	**CaBLAM**	**Bond lengths**	**Bond angles**	**Cis Peptides**
			Avg: 0.92	Clashscore: 1.59	Outliers: 4 of 903	Poor rotamers: 0 of 771	Outliers: 0 of 828	Outliers: 22 of 901	Outliers: 13 of 905	Outliers: 19 of 905	Non-Trans: 1 of 904
A 881		GLN	0.8	-	Favored (42.84%) General / -94.0,8.0	Favored (97.9%) *mm-40*	0.01Å	CaBLAM Outlier (0.895%)	-	-	-

						chi angles: 299.2,297.7,307.7					
A 882		GLU	0.79	-	Favored (48.51%) General / -63.3,148.4	Favored (15%) *mm-30* chi angles: 296.3,281.4,297	0.10Å	Favored (20.137%)	-	-	-
A 883		LYS	0.77	-	Favored (56.3%) General / -61.6,134.0	Favored (86.9%) *tttt* chi angles: 182.9,177.4,178.5,180.6	0.01Å	Favored (31.927%) beta sheet	-	-	-
A 884		TYR	0.75	-	Favored (35.52%) General / -120.2,154.0	Favored (80.6%) *m-80* chi angles: 302.1,89.4	0.09Å	Favored (52.732%) beta sheet	-	-	-
A 885		ARG	0.75	-	Favored (51.1%) General / -110.3,135.4	Favored (72.1%) *ttt180* chi angles: 176,171.1,169.7,185.9	0.05Å	Favored (45.652%) beta sheet	-	-	-
A 886		ASP	0.75	-	Favored (9.9%) General / -82.8,72.9	Favored (73.7%) *m-30* chi angles: 293.3,320.6	0.01Å	Favored (24.987%) beta sheet	-	-	-
A 887		TYR	0.77	-	Favored (65.76%) General / -66.6,-19.4	Favored (68.7%) *m-80* chi angles: 287.2,98.8	0.07Å	Favored (19.132%)	-	-	-
A 888		MET	0.81	-	Favored (63.9%) General / -59.3,-25.1	Favored (89.1%) *mmm* chi angles: 289.2,292.8,286.4	0.12Å	Favored (61.507%)	-	-	-
A 889		LEU	0.87	-	Favored (52.94%) General / -86.1,0.7	Favored (91.8%) *mt* chi angles: 298.8,176.5	0.04Å	Favored (47.999%) three-ten	-	-	-
A 890		SER	0.94	-	Favored (59.2%) General / -82.9,-9.7	Favored (89.2%) *p* chi angles: 69.1	0.03Å	Favored (58.536%)	-	-	-
A 891		LEU	1.04	-	Favored (49.21%) General / -106.4,124.2	Favored (60.8%) *tp* chi angles: 177.2,64.9	0.04Å	Favored (24.317%)	-	-	-
A 892		ARG	1.15	-	Favored (33.92%) General / -54.8,-26.4	Favored (42.2%) *ptt180* chi angles: 66.1,183,181.9,172.2	0.03Å	Favored (31.207%)	-	-	-
A 893		ARG	1.3	-	Favored (50.72%) General / -52.9,-33.6	Favored (62.3%) *ttt90* chi angles: 182.5,175.1,171,85.7	0.01Å	Favored (35.451%)	-	-	-
A 894		TYR	1.49	-	Favored (25.78%) General / -108.7,6.5	Favored (97.6%) *m-80* chi angles: 295.2,97.1	0.09Å	Favored (37.173%) alpha helix	-	-	-
A 895		GLU	1.73	-	Favored (82.56%) General / -62.8,-36.2	Favored (96.8%) *mt-10* chi angles: 287.9,176,354.6	0.05Å	Favored (37.746%) alpha helix	-	-	-
A 896		GLU	2.05	-	Favored (75.22%) General / -59.1,-36.5	Favored (72%) *tp30* chi angles: 180.3,67.3,21.7	0.03Å	Favored (73.722%) alpha helix	-	-	-
A 897		VAL	2.46	-	Favored (91.84%) Ile or Val / -64.8,-46.5	Favored (74.9%) *t* chi angles: 172.7	0.04Å	Favored (80.883%) alpha helix	-	-	-
A 898		ASN	2.99	-	Favored (85.63%) General / -59.7,-39.7	Favored (43.6%) *t0* chi angles: 188.1,65.2	0.03Å	Favored (92.146%) alpha helix	-	-	-

#	Alt	Res	High B	Clash > 0.4Å	Ramachandran	Rotamer	Cβ deviation	CaBLAM	Bond lengths	Bond angles	Cis Peptides
A 899		VAL	3.63	-	Favored (95.72%) Ile or Val / -62.6,-46.7	Favored (61.3%) *t* chi angles: 171	0.03Å	Favored (91.685%) alpha helix	-	-	-
A 900		GLN	4.35	-	Favored (93.09%) General / -64.7,-39.1	Favored (79.7%) *mt0* chi angles: 290.5,178.9,24.4	0.03Å	Favored (89.924%) alpha helix	-	-	-
**#**	**Alt**	**Res**	**High B**	**Clash > 0.4Å**	**Ramachandran**	**Rotamer**	**Cβ deviation**	**CaBLAM**	**Bond lengths**	**Bond angles**	**Cis Peptides**
			Avg: 0.92	Clashscore: 1.59	Outliers: 4 of 903	Poor rotamers: 0 of 771	Outliers: 0 of 828	Outliers: 22 of 901	Outliers: 13 of 905	Outliers: 19 of 905	Non-Trans: 1 of 904
A 901		GLU	5.09	-	Favored (85.88%) General / -64.5,-37.0	Favored (61.6%) *mm-30* chi angles: 289.8,291.9,305.6	0.04Å	Favored (82.895%) alpha helix	-	-	-
A 902		ASP	5.85	-	Favored (77.29%) General / -68.9,-35.9	Favored (93.9%) *m-30* chi angles: 285.7,348.2	0.05Å	Favored (97.601%) alpha helix	-	-	-
A 903		ARG	6.57	-	Favored (71.18%) General / -70.5,-33.4	Favored (97.7%) *mtt180* chi angles: 288.7,176,178.2,169.1	0.02Å	Favored (46.022%)	-	-	-
A 904		VAL	7.21	-	Favored (35.69%) Ile or Val / -83.0,130.5	Favored (94%) *t* chi angles: 175.8	0.04Å	-	-	-	-
A 905		LEU	7.76	-	-	Favored (95.1%) *mt* chi angles: 296.3,176.2	0.10Å	-	-	-	-
