# Supplementary material for: Integrative proteome-wide structural analysis and high-throughput docking identify broad-spectrum antiviral scaffolds against Zika, Yellow Fever, West Nile, Saint Louis encephalitis, and Usutu viruses
Source: Front Cell Infect Microbiol. 2026 Apr 30;16:1723132. doi: 10.3389/fcimb.2026.1723132 (PMC13171538; doi:10.3389/fcimb.2026.1723132)
Supplement: Supplementary file 4 [file DataSheet4.zip › USUV/USU_NS5/Mol_probity_Files/USU_NS5_1FH-rama.pdf]

# MolProbity Ramachandran analysis

USU_NS5_1FH.pdb, model 1

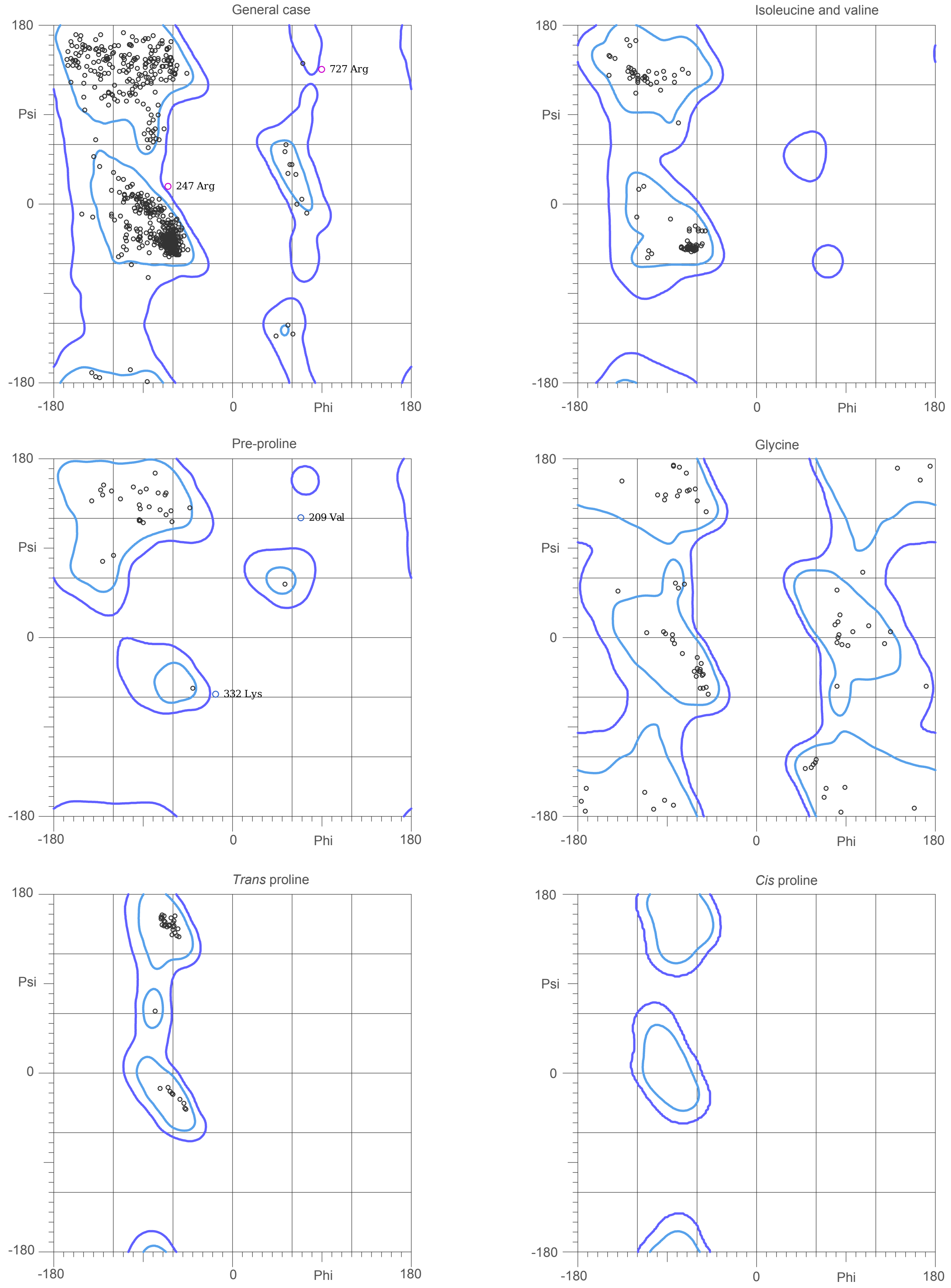


97.3% (879/903) of all residues were in favored (98%) regions.
99.6% (899/903) of all residues were in allowed (>99.8%) regions.

There were 4 outliers (phi, psi):
209 Val (69.6, 121.3)
247 Arg (-65.5, 19.0)
332 Lys (-17.2, -57.4)
727 Arg (90.5, 136.0)
