# Supplementary material for: Integrative proteome-wide structural analysis and high-throughput docking identify broad-spectrum antiviral scaffolds against Zika, Yellow Fever, West Nile, Saint Louis encephalitis, and Usutu viruses
Source: Front Cell Infect Microbiol. 2026 Apr 30;16:1723132. doi: 10.3389/fcimb.2026.1723132 (PMC13171538; doi:10.3389/fcimb.2026.1723132)
Supplement: Supplementary file 5 [file DataSheet5.zip › WNV/WNV_E/Mol_probity_Files/WNV_E_1FH-multi.table.pdf]

Viewing WNV_E1FH-multi.table

Duke Biochemistry
Duke University School of Medicine

When finished, you should close this window.

Hint: Use File | Save As... to save a copy of this page.

All-Atom Contacts	Clashscore, all atoms:	1.47		99th percentile* (N=1784, all resolutions)
	Clashscore is the number of serious steric overlaps (> 0.4 Å) per 1000 atoms.			
Protein Geometry	Poor rotamers	0	0.00%	Goal: <0.3%
	Favored rotamers	402	99.75%	Goal: >98%
	Ramachandran outliers	1	0.20%	Goal: <0.05%
	Ramachandran favored	487	97.60%	Goal: >98%
	Rama distribution Z-score	0.95 ± 0.36		Goal: abs(Z score) < 2
	MolProbity score^	0.97		100th percentile* (N=27675, 0Å - 99Å)
	Cβ deviations >0.25Å	0	0.00%	Goal: 0
	Bad bonds:	6 / 3850	0.16%	Goal: 0%
	Bad angles:	4 / 5223	0.08%	Goal: <0.1%
Peptide Omegas	Cis Prolines:	1 / 17	5.88%	Expected: ≤1 per chain, or ≤5%
Low-resolution Criteria	CaBLAM outliers	7	1.4%	Goal: <1.0%
	CA Geometry outliers	1	0.20%	Goal: <0.5%
Additional validations	Chiral volume outliers	0/591		
	Waters with clashes	0/0	0.00%	See UnDowser table for details

In the two column results, the left column gives the raw count, right column gives the percentage.

* 100th percentile is the best among structures of comparable resolution; 0th percentile is the worst. For clashscore the comparative set of structures was selected in 2004, for MolProbity score in 2006.

^ MolProbity score combines the clashscore, rotamer, and Ramachandran evaluations into a single score, normalized to be on the same scale as X-ray resolution.

Key to table colors and cutoffs here:

#	Alt	Res	High B	Clash > 0.4Å	Ramachandran	Rotamer	Cβ deviation	CaBLAM	Bond lengths	Bond angles	Cis Peptides
			Avg: 1.11	Clashscore: 1.47	Outliers: 1 of 499	Poor rotamers: 0 of 403	Outliers: 0 of 447	Outliers: 8 of 497	Outliers: 6 of 501	Outliers: 4 of 501	Non-Trans: 1 of 500
A 1		PHE	1.4	0.42Å N with A 42 ASP OD2	-	Favored (27.3%) *t80* chi angles: 177,53.2	0.12Å	-	-	-	-
A 2		ASN	1.32	-	Favored (67.39%) General / -63.2,-23.3	Favored (18.4%) *p0* chi angles: 64.1,306.8	0.05Å	-	-	-	-
A 3		CYS	1.27	-	Favored (56.3%) General / -85.6,-0.9	Favored (75.9%) *m* chi angles: 296.7	0.03Å	Favored (47.764%)	-	-	-
A 4		LEU	1.23	-	Favored (72.82%) General / -62.9,-31.7	Favored (97.1%) *mt* chi angles: 294.1,172.4	0.07Å	Favored (48.335%) alpha helix	-	-	-
A 5		GLY	1.22	-	Favored (69.53%) Glycine / -95.1,7.2	-	-	Favored (53.872%)	-	-	-
A 6		MET	1.22	-	Favored (25.6%) General / -103.6,148.8	Favored (92.8%) *mmm* chi angles: 301.1,297.9,293.5	0.03Å	Favored (34.439%)	-	-	-

A 7		SER	1.21	-	Favored (16.26%) General / -85.2,166.8	Favored (93.8%) *p* chi angles: 66.3	0.03Å	Favored (7.566%)	-	-	-
A 8		ASN	1.21	-	Favored (15.66%) General / 54.4,50.7	Favored (61.8%) *t0* chi angles: 198.2,33	0.08Å	Favored (8.69%)	-	-	-
A 9		ARG	1.2	-	Favored (43.45%) General / -97.3,132.0	Favored (56.3%) *ttt180* chi angles: 181.4,180.1,171.8,201.8	0.05Å	Favored (37.439%)	OUTLIER(S) worst is CD--NE: 4.2 σ	-	-
A 10		ASP	1.21	-	Favored (23.9%) General / -104.0,150.8	Favored (60.8%) *m-30* chi angles: 289.9,317.5	0.02Å	Favored (50.023%) beta sheet	-	-	-
A 11		PHE	1.28	-	Favored (51.19%) General / -120.0,140.3	Favored (86.6%) *m-80* chi angles: 293.7,83.1	0.06Å	Favored (67.219%) beta sheet	-	-	-
A 12		LEU	1.44	-	Favored (14.13%) General / -138.0,118.8	Favored (68.1%) *tp* chi angles: 176.6,60.5	0.09Å	Favored (52.22%) beta sheet	-	-	-
A 13		GLU	1.69	-	Favored (43.54%) General / -95.5,-5.2	Favored (96.7%) *mt-10* chi angles: 296.1,178.7,359.2	0.01Å	Favored (6.955%)	-	-	-
A 14		GLY	2.03	-	Favored (51.21%) Glycine / 69.0,-162.7	-	-	Favored (32.34%)	-	-	-
A 15		VAL	2.4	-	Favored (3.5%) Ile or Val / -127.5,2.8	Favored (28.8%) *m* chi angles: 298.4	0.03Å	CaBLAM Outlier (0.732%)	-	-	-
A 16		SER	2.66	-	Allowed (0.25%) General / 69.4,178.9	Favored (58.7%) *p* chi angles: 73.3	0.07Å	Favored (8.863%)	-	-	-
A 17		GLY	2.69	-	Favored (2.99%) Glycine / 76.6,-64.0	-	-	CaBLAM Outlier (0.132%)	-	-	-
A 18		ALA	2.48	-	Favored (51.12%) General / -62.1,146.2	-	0.03Å	Favored (15.753%)	-	-	-
A 19		THR	2.12	-	Favored (53.3%) General / -95.6,0.8	Favored (72%) *p* chi angles: 61.9	0.03Å	Favored (38.42%)	-	-	-
A 20		TRP	1.74	-	Favored (45.55%) General / -141.1,157.2	Favored (75%) *p-90* chi angles: 58.4,270.1	0.12Å	Favored (26.815%)	-	-	-
**#**	**Alt**	**Res**	**High B**	**Clash > 0.4Å**	**Ramachandran**	**Rotamer**	**Cβ deviation**	**CaBLAM**	**Bond lengths**	**Bond angles**	**Cis Peptides**
			Avg: 1.11	Clashscore: 1.47	Outliers: 1 of 499	Poor rotamers: 0 of 403	Outliers: 0 of 447	Outliers: 8 of 497	Outliers: 6 of 501	Outliers: 4 of 501	Non-Trans: 1 of 500
A 21		VAL	1.43	-	Favored (34.28%) Ile or Val / -131.9,150.9	Favored (31.3%) *m* chi angles: 297.8	0.15Å	Favored (56.553%)	-	-	-
A 22		ASP	1.21	-	Favored (26.71%) General / -95.8,143.8	Favored (96.3%) *m-30* chi angles: 290.9,342.9	0.06Å	Favored (46.505%) beta sheet	-	-	-
A 23		LEU	1.09	0.47Å C with A 23	Favored (38.37%)	Favored (3.7%) *pp* chi angles: 61,81.5	0.04Å	Favored (47.407%)	-	-	-

				LEU HD12	General / -145.8,162.1			beta sheet			
A 24		VAL	1.04	-	Favored (33.8%) Ile or Val / -123.2,144.2	Favored (26%) *m* chi angles: 299.6	0.06Å	Favored (62.356%) beta sheet	-	-	-
A 25		LEU	1.04	-	Favored (47.58%) General / -113.4,141.0	Favored (69.5%) *mt* chi angles: 302.1,174	0.10Å	Favored (63.251%)	-	-	-
A 26		GLU	1.07	-	Favored (20.75%) General / -147.5,134.9	Favored (89.7%) *tt0* chi angles: 187.7,175.8,1.1	0.10Å	Favored (9.786%)	-	-	-
A 27		GLY	1.09	-	Favored (30.19%) Glycine / 55.5,-123.4	-	-	Favored (57.784%)	-	-	-
A 28		ASP	1.1	-	Favored (33.88%) General / -102.4,13.7	Favored (72.6%) *m-30* chi angles: 295.5,316.8	0.07Å	Favored (5.665%)	-	-	-
A 29		SER	1.09	-	Favored (13.75%) General / -73.4,171.1	Favored (81.7%) *p* chi angles: 70.1	0.12Å	Favored (11.507%)	-	-	-
A 30		CYS	1.08	-	Favored (28.02%) General / -147.0,142.9	Favored (49.3%) *t* chi angles: 179.6	0.06Å	Favored (56.84%) beta sheet	-	-	-
A 31		VAL	1.08	-	Favored (72.83%) Ile or Val / -115.3,126.2	Favored (81.8%) *t* chi angles: 177.9	0.04Å	Favored (67.855%) beta sheet	-	-	-
A 32		THR	1.12	-	Favored (42.63%) General / -102.4,121.6	Favored (92.8%) *m* chi angles: 297.4	0.04Å	Favored (71.322%) beta sheet	-	-	-
A 33		ILE	1.21	-	Favored (65.51%) Ile or Val / -108.6,124.9	Favored (3.6%) *mp* chi angles: 303.6,90.9	0.03Å	Favored (64.326%) beta sheet	-	-	-
A 34		MET	1.32	-	Favored (40.39%) General / -123.7,153.7	Favored (30.8%) *mtm* chi angles: 297.5,161.2,284.1	0.09Å	Favored (23.612%) beta sheet	-	-	-
A 35		SER	1.42	-	Favored (14.64%) General / -160.1,144.7	Favored (46.9%) *t* chi angles: 180.2	0.04Å	Favored (10.862%)	-	-	-
A 36		LYS	1.45	-	Favored (5.23%) General / -42.9,-46.3	Favored (9.7%) *ttpp* chi angles: 181.4,181.9,72.6,78.4	0.03Å	Favored (15.508%)	-	-	-
A 37		ASP	1.4	-	Favored (23.79%) General / -107.8,16.3	Favored (74.7%) *m-30* chi angles: 297.6,318.8	0.02Å	Favored (8.23%)	-	-	-
A 38		LYS	1.27	-	Favored (72.35%) Pre-Pro / -125.4,154.0	Favored (66.1%) *mmtt* chi angles: 300.7,296,185.8,193.7	0.01Å	Favored (17.865%)	-	-	-
A 39		PRO	1.11	-	Favored (75.42%) Trans-Pro / -68.6,151.0	Favored (56.8%) *Cg_endo* chi angles: 26,327.9,25.1	0.02Å	Favored (67.191%) beta sheet	-	-	-
A 40		THR	0.96	-	Favored (36.24%)	Favored (84.3%) *m* chi angles: 301.8	0.04Å	Favored (38.122%) beta sheet	-	-	-

#	Alt	Res	High B	Clash > 0.4Å	Ramachandran	Rotamer	Cβ deviation	CaBLAM	Bond lengths	Bond angles	Cis Peptides
					General / -79.8,133.1						
#	Alt	Res	High B	Clash > 0.4Å	Ramachandran	Rotamer	Cβ deviation	CaBLAM	Bond lengths	Bond angles	Cis Peptides
			Avg: 1.11	Clashscore: 1.47	Outliers: 1 of 499	Poor rotamers: 0 of 403	Outliers: 0 of 447	Outliers: 8 of 497	Outliers: 6 of 501	Outliers: 4 of 501	Non-Trans: 1 of 500
A 41		ILE	0.86	-	Favored (69.65%) Ile or Val / -122.4,133.1	Favored (55.3%) *mt* chi angles: 296.6,179.5	0.09Å	Favored (68.732%) beta sheet	-	-	-
A 42		ASP	0.79	0.42Å OD2 with A 1 PHE N	Favored (43.19%) General / -98.6,124.0	Favored (96.8%) *m-30* chi angles: 289.8,345.8	0.08Å	Favored (69.096%) beta sheet	-	OUTLIER(S) worst is CA-CB-CG: 4.4 σ	-
A 43		VAL	0.77	-	Favored (69%) Ile or Val / -113.7,123.1	Favored (60.6%) *t* chi angles: 179.9	0.05Å	Favored (61.554%) beta sheet	-	-	-
A 44		LYS	0.76	-	Favored (37.3%) General / -137.5,136.0	Favored (50%) *tttm* chi angles: 179,174.1,185.3,300	0.03Å	Favored (51.647%) beta sheet	-	-	-
A 45		MET	0.77	-	Favored (34.41%) General / -75.9,128.1	Favored (47.4%) *ttm* chi angles: 185.9,178,299	0.02Å	Favored (52.24%)	-	-	-
A 46		MET	0.79	-	Favored (11.01%) General / -95.8,-36.8	Favored (90.1%) *mmm* chi angles: 300.2,299.2,282.6	0.10Å	Favored (25.423%)	-	-	-
A 47		ASN	0.8	-	Favored (3.38%) General / -165.9,135.7	Favored (24.8%) *t0* chi angles: 189.5,72.1	0.02Å	Favored (8.828%)	-	-	-
A 48		MET	0.8	-	Favored (53.35%) General / -112.2,135.1	Favored (53.8%) *ttp* chi angles: 181.5,176.5,80.3	0.09Å	Favored (56.362%) beta sheet	-	-	-
A 49		GLU	0.8	-	Favored (41.74%) General / -145.5,154.0	Favored (10.5%) *pt0* chi angles: 65,184,54.6	0.04Å	Favored (47.515%) beta sheet	-	-	-
A 50		ALA	0.8	-	Favored (30.9%) General / -104.0,144.9	-	0.03Å	Favored (63.033%)	-	-	-
A 51		ALA	0.8	-	Favored (50.86%) General / -135.8,152.2	-	0.04Å	Favored (14.001%)	-	-	-
A 52		ASN	0.8	-	Favored (20.52%) General / 57.2,45.5	Favored (82%) *m-40* chi angles: 297.2,308.9	0.03Å	Favored (8.565%)	-	-	-
A 53		LEU	0.8	-	Favored (58.4%) General / -64.8,142.5	Favored (3.8%) *mp* chi angles: 283.6,78.7	0.11Å	Favored (18.011%)	-	-	-
A 54		ALA	0.8	-	Favored (31.3%) General / -91.5,138.8	-	0.02Å	Favored (39.872%) beta sheet	-	-	-
A 55		ASP	0.79	-	Favored (18.95%) General / -78.6,116.5	Favored (67.3%) *t0* chi angles: 184.2,351.8	0.04Å	Favored (37.849%)	-	-	-
A 56		VAL	0.77	-	Favored (7.71%)	Favored (81.5%) *t* chi angles: 176.6	0.04Å	Favored (9.486%)	-	-	-

#	Alt	Res	High B	Clash > 0.4Å	Ramachandran	Rotamer	Cβ deviation	CaBLAM	Bond lengths	Bond angles	Cis Peptides
					Ile or Val / -105.1,-52.3						
A 57		ARG	0.75	-	Favored (15.51%) General / -157.5,140.9	Favored (41.9%) *ttm170* chi angles: 177.8,183.9,293.8,187.2	0.04Å	Favored (17.568%)	-	-	-
A 58		SER	0.73	-	Favored (31.21%) General / -108.5,147.8	Favored (72.9%) *m* chi angles: 295.4	0.03Å	Favored (58.881%)	-	-	-
A 59		TYR	0.72	-	Favored (42.94%) General / -116.9,146.4	Favored (53.8%) *m-80* chi angles: 283.2,87.2	0.09Å	Favored (57.724%) beta sheet	-	-	-
A 60		CYS	0.72	-	Favored (41.83%) General / -107.3,139.4	Favored (36%) *t* chi angles: 187.8	0.05Å	Favored (49.888%)	-	-	-
#	Alt	Res	High B	Clash > 0.4Å	Ramachandran	Rotamer	Cβ deviation	CaBLAM	Bond lengths	Bond angles	Cis Peptides
			Avg: 1.11	Clashscore: 1.47	Outliers: 1 of 499	Poor rotamers: 0 of 403	Outliers: 0 of 447	Outliers: 8 of 497	Outliers: 6 of 501	Outliers: 4 of 501	Non-Trans: 1 of 500
A 61		TYR	0.71	-	Favored (5.48%) General / -110.6,-37.4	Favored (73%) *m-80* chi angles: 301.9,108.1	0.09Å	Favored (15.969%)	OUTLIER(S) worst is CB--CG: 5.8 σ	-	-
A 62		LEU	0.72	-	Favored (31.92%) General / -135.2,128.4	Favored (56.3%) *tp* chi angles: 178.4,65.5	0.07Å	Favored (21.486%)	-	-	-
A 63		ALA	0.72	-	Favored (44.63%) General / -141.2,152.6	-	0.06Å	Favored (49.937%)	-	-	-
A 64		SER	0.72	-	Favored (12.46%) General / -100.1,163.4	Favored (93.4%) *p* chi angles: 64.3	0.04Å	Favored (39.61%) beta sheet	-	-	-
A 65		VAL	0.72	-	Favored (70.05%) Ile or Val / -115.9,130.7	Favored (78.4%) *t* chi angles: 178.1	0.06Å	Favored (32.621%)	-	-	-
A 66		SER	0.71	-	Favored (26.97%) General / -144.1,165.7	Favored (97.9%) *p* chi angles: 65.8	0.03Å	Favored (8.298%)	-	-	-
A 67		ASP	0.7	-	Favored (26.32%) General / 55.1,45.3	Favored (25.5%) *t0* chi angles: 198.5,28.4	0.02Å	Favored (7.018%)	-	-	-
A 68		LEU	0.68	-	Favored (38.13%) General / -66.7,128.9	Favored (49.3%) *tp* chi angles: 181.6,65.4	0.01Å	Favored (18.733%)	-	-	-
A 69		SER	0.67	-	Favored (16.72%) General / -141.3,124.8	Favored (37.6%) *t* chi angles: 174.7	0.05Å	Favored (54.165%) beta sheet	-	-	-
A 70		THR	0.65	-	Favored (55.3%) General / -121.0,134.3	Favored (89.7%) *m* chi angles: 298.7	0.01Å	Favored (60.487%) beta sheet	-	-	-
A 71		ARG	0.64	-	Favored (39.18%) General / -124.7,155.4	Favored (98%) *mtt180* chi angles: 295.7,182.7,181.9,179.2	0.03Å	Favored (49.473%) beta sheet	-	-	-
A 72		ALA	0.65	-	Favored (44.32%)	-	0.01Å	Favored (68.031%)	-	-	-

#	Alt	Res	High B	Clash > 0.4Å	Ramachandran	Rotamer	Cβ deviation	CaBLAM	Bond lengths	Bond angles	Cis Peptides
					General / -148.9,159.3			beta sheet			
A 73		ALA	0.66	-	Favored (50.17%) General / -134.7,147.5	-	0.04Å	Favored (36.708%)	-	-	-
A 74		CYS	0.68	-	Favored (96.44%) Pre-Pro / -67.6,152.2	Favored (90.5%) *m* chi angles: 291.6	0.04Å	Favored (42.721%)	-	-	-
A 75		PRO	0.7	-	Favored (2.95%) Trans-Pro / -44.2,-29.6	Favored (87%) *Cg_exo* chi angles: 330.3,35.6,334.5	0.07Å	Favored (15.527%)	-	-	-
A 76		THR	0.71	-	Favored (22.64%) General / -107.8,1.2	Favored (71.4%) *p* chi angles: 62.1	0.01Å	Favored (30.515%)	-	-	-
A 77		MET	0.72	-	Favored (56.88%) General / -91.8,-2.5	Favored (68.7%) *mtt* chi angles: 293.4,178.1,187.1	0.03Å	Favored (11.859%)	-	-	-
A 78		GLY	0.71	-	Favored (28.15%) Glycine / 93.1,-165.8	-	-	Favored (40.109%)	-	-	-
A 79		GLU	0.69	-	Favored (40.2%) General / -75.8,146.1	Favored (48.6%) *mt-10* chi angles: 293.3,183.3,51.6	0.05Å	Favored (6.72%)	-	-	-
A 80		ALA	0.66	-	Favored (41.98%) General / -75.6,144.1	-	0.02Å	Favored (43.767%)	-	-	-
**#**	**Alt**	**Res**	**High B**	**Clash > 0.4Å**	**Ramachandran**	**Rotamer**	**Cβ deviation**	**CaBLAM**	**Bond lengths**	**Bond angles**	**Cis Peptides**
			Avg: 1.11	Clashscore: 1.47	Outliers: 1 of 499	Poor rotamers: 0 of 403	Outliers: 0 of 447	Outliers: 8 of 497	Outliers: 6 of 501	Outliers: 4 of 501	Non-Trans: 1 of 500
A 81		HIS	0.64	-	Favored (21.57%) General / -137.6,124.8	Favored (92.7%) *m-70* chi angles: 299.2,278	0.04Å	Favored (59.947%) beta sheet	-	-	-
A 82		ASN	0.62	-	Favored (35.62%) General / -104.5,118.0	Favored (41.4%) *t0* chi angles: 193.1,322.1	0.10Å	Favored (68.695%) beta sheet	-	-	-
A 83		GLU	0.62	-	Favored (62.73%) General / -58.2,-26.3	Favored (51.6%) *mt-10* chi angles: 288.1,172.8,307.8	0.02Å	Favored (38.358%)	-	-	-
A 84		LYS	0.63	-	Favored (64.86%) General / -64.2,-18.9	Favored (60.8%) *mttm* chi angles: 290.4,179.4,183.6,289.2	0.03Å	Favored (57.612%)	-	-	-
A 85		ARG	0.66	-	Favored (66.03%) General / -59.8,-26.7	Favored (68.7%) *ttt180* chi angles: 188.8,165.7,176,173.6	0.08Å	Favored (59.799%) three-ten	-	-	-
A 86		ALA	0.68	-	Favored (59.84%) General / -79.8,-11.3	-	0.05Å	Favored (57.277%)	-	-	-
A 87		ASP	0.71	-	Favored (59.1%) Pre-Pro / -92.1,124.7	Favored (77.9%) *m-30* chi angles: 285.9,335.7	0.06Å	Favored (29.298%)	-	-	-

A 88		PRO	0.72	-	Favored (34.39%) Trans-Pro / -56.0,152.9	Favored (63.2%) *Cg_exo* chi angles: 335.7,33.1,332	0.02Å	Favored (14.182%)	-	-	-
A 89		ALA	0.72	-	Favored (2.62%) General / 77.9,-1.3	-	0.05Å	CaBLAM Disfavored (1.165%)	-	-	-
A 90		PHE	0.71	-	Favored (31.86%) General / -103.0,144.0	Favored (61.2%) *m-80* chi angles: 286.2,81.3	0.06Å	Favored (30.518%)	-	-	-
A 91		VAL	0.69	-	Favored (72.56%) Ile or Val / -117.3,130.2	Favored (97%) *t* chi angles: 175.1	0.04Å	Favored (71.738%) beta sheet	-	-	-
A 92		CYS	0.67	-	Favored (52.13%) General / -129.0,139.4	Favored (44.4%) *m* chi angles: 304.1	0.04Å	Favored (70.466%) beta sheet	-	-	-
A 93		LYS	0.65	-	Favored (46.17%) General / -118.8,144.7	Favored (72.6%) *mmtt* chi angles: 295.5,292.9,183.8,181.2	0.08Å	Favored (70.851%) beta sheet	-	-	-
A 94		GLN	0.65	-	Favored (51.53%) General / -129.5,139.6	Favored (67.7%) *tt0* chi angles: 184,180,3.9	0.02Å	Favored (26.342%) beta sheet	-	-	-
A 95		GLY	0.66	-	Favored (15.91%) Glycine / -161.2,-164.3	-	-	Favored (37.296%) beta sheet	-	-	-
A 96		VAL	0.68	-	Favored (59.21%) Ile or Val / -115.5,134.2	Favored (88.8%) *t* chi angles: 176.1	0.07Å	Favored (12.949%) beta sheet	-	-	-
A 97		VAL	0.72	-	Favored (47.88%) Ile or Val / -132.2,140.4	Favored (8.8%) *p* chi angles: 61.6	0.09Å	Favored (45.783%) beta sheet	-	-	-
A 98		ASP	0.76	0.49Å OD1 with A 110 LYS NZ	Favored (58.44%) General / -62.1,139.8	Favored (89.4%) *m-30* chi angles: 291.1,350.3	0.04Å	Favored (35.597%) beta sheet	-	-	-
A 99		ARG	0.81	-	Favored (52.53%) General / -130.8,148.0	Favored (61%) *mmm-85* chi angles: 302.2,294.4,292.5,274.5	0.07Å	Favored (30.232%) beta sheet	-	-	-
A 100		GLY	0.85	-	Favored (27.32%) Glycine / -166.7,-167.4	-	-	Favored (28.114%)	-	-	-
**#**	**Alt**	**Res**	**High B**	**Clash > 0.4Å**	**Ramachandran**	**Rotamer**	**Cβ deviation**	**CaBLAM**	**Bond lengths**	**Bond angles**	**Cis Peptides**
			Avg: 1.11	Clashscore: 1.47	Outliers: 1 of 499	Poor rotamers: 0 of 403	Outliers: 0 of 447	Outliers: 8 of 497	Outliers: 6 of 501	Outliers: 4 of 501	Non-Trans: 1 of 500
A 101		TRP	0.89	-	Favored (6.09%) General / -51.2,-26.0	Favored (78.5%) *p-90* chi angles: 64.8,268	0.01Å	CaBLAM Disfavored (4.644%)	-	-	-
A 102		GLY	0.91	-	Favored (78.75%) Glycine / -90.4,0.9	-	-	Favored (67.317%)	-	-	-
A 103		ASN	0.91	-	Favored (11.91%) General / -106.8,164.7	Favored (21.9%) *m110* chi angles: 305,133.8	0.03Å	Favored (14.438%)	-	-	-

#	Alt	Res	High B	Clash > 0.4Å	Ramachandran	Rotamer	Cβ deviation	CaBLAM	Bond lengths	Bond angles	Cis Peptides
A 104		GLY	0.9	-	Allowed (1.8%) Glycine / -73.8,65.6	-	-	CaBLAM Outlier (0.524%)	-	-	-
A 105		CYS	0.87	-	Favored (7.26%) General / -162.4,137.9	Favored (42.4%) *t* chi angles: 186	0.04Å	Favored (6.257%)	-	-	-
A 106		GLY	0.84	-	Favored (83.79%) Glycine / -87.1,6.2	-	-	Favored (19.536%)	-	-	-
A 107		LEU	0.79	-	Favored (47.71%) General / -120.6,145.1	Favored (3.7%) *mp* chi angles: 293.6,81.7	0.09Å	Favored (30.687%)	-	-	-
A 108		PHE	0.74	-	Favored (46.7%) General / -100.6,130.8	Favored (86.9%) *t80* chi angles: 178,74.1	0.02Å	CaBLAM Disfavored (3.007%)	-	-	-
A 109		GLY	0.69	-	Favored (24.31%) Glycine / 155.0,-152.8	-	-	Favored (26.673%)	-	-	-
A 110		LYS	0.65	0.49Å NZ with A 98 ASP OD1	Favored (56.7%) General / -116.0,130.2	Favored (63.8%) *mttm* chi angles: 294.8,184,182.5,295	0.02Å	Favored (7.51%)	-	-	-
A 111		GLY	0.61	-	Favored (19.99%) Glycine / -109.8,-167.3	-	-	Favored (39.183%)	-	-	-
A 112		SER	0.59	-	Favored (46.82%) General / -118.4,143.2	Favored (26.6%) *t* chi angles: 171.8	0.04Å	Favored (26.964%)	-	-	-
A 113		ILE	0.57	-	Favored (49.36%) Ile or Val / -130.8,140.2	Favored (20.7%) *tt* chi angles: 185.8,165.5	0.07Å	Favored (71.825%) beta sheet	-	-	-
A 114		ASP	0.57	-	Favored (10.1%) General / -133.4,110.5	Favored (68%) *t0* chi angles: 183.4,348.9	0.03Å	Favored (57.487%) beta sheet	-	OUTLIER(S) worst is CA-CB-CG: 4.1 σ	-
A 115		THR	0.58	-	Favored (48.8%) General / -116.1,123.8	Favored (98%) *m* chi angles: 300.1	0.04Å	Favored (64.961%) beta sheet	-	-	-
A 116		CYS	0.6	-	Favored (35.28%) General / -115.2,151.0	Favored (77.1%) *m* chi angles: 296.4	0.05Å	Favored (38.149%) beta sheet	-	-	-
A 117		ALA	0.63	-	Favored (29.4%) General / -150.6,149.1	-	0.06Å	Favored (46.604%) beta sheet	-	-	-
A 118		LYS	0.67	-	Favored (38.79%) General / -93.0,128.2	Favored (86.2%) *tttt* chi angles: 186.5,176.5,179.8,181.1	0.03Å	Favored (51.642%) beta sheet	-	-	-
A 119		PHE	0.71	-	Favored (31.06%) General / -104.1,115.8	Favored (20.9%) *m-10* chi angles: 294.8,346.9	0.04Å	Favored (68.666%) beta sheet	-	-	-
A 120		ALA	0.74	-	Favored (20.5%) General / -110.5,110.0	-	0.02Å	Favored (60.494%) beta sheet	-	-	-
**#**	**Alt**	**Res**	**High B**	**Clash > 0.4Å**	**Ramachandran**	**Rotamer**	**Cβ deviation**	**CaBLAM**	**Bond lengths**	**Bond angles**	**Cis Peptides**

			Avg: 1.11	Clashscore: 1.47	Outliers: 1 of 499	Poor rotamers: 0 of 403	Outliers: 0 of 447	Outliers: 8 of 497	Outliers: 6 of 501	Outliers: 4 of 501	Non-Trans: 1 of 500
A 121		CYS	0.77	-	Favored (49.06%) General / -67.1,133.0	Favored (42.2%) *t* chi angles: 185.8	0.03Å	Favored (25.322%)	-	-	-
A 122		THR	0.78	-	Favored (4.58%) General / -101.4,-47.8	Favored (90.5%) *m* chi angles: 298.1	0.01Å	Favored (11.553%)	-	-	-
A 123		THR	0.79	-	Favored (52.34%) General / -120.1,127.2	Favored (95.9%) *m* chi angles: 299.6	0.04Å	Favored (13.191%)	-	-	-
A 124		LYS	0.78	-	Favored (25.88%) General / -139.6,165.9	Favored (59.9%) *pttt* chi angles: 62.2,182.9,182.4,180.5	0.02Å	Favored (37.1%)	-	-	-
A 125		ALA	0.77	-	Favored (44.52%) General / -125.9,153.2	-	0.12Å	Favored (55.143%) beta sheet	-	-	-
A 126		THR	0.77	-	Favored (54.71%) General / -122.2,133.0	Favored (88.5%) *m* chi angles: 297	0.07Å	Favored (62.792%) beta sheet	-	-	-
A 127		GLY	0.77	-	Favored (13.64%) Glycine / -98.6,138.1	-	-	Favored (68.446%) beta sheet	-	-	-
A 128		TRP	0.78	-	Favored (45.31%) General / -126.8,153.6	Favored (40.8%) *m-90* chi angles: 295.1,263.9	0.13Å	Favored (54.855%) beta sheet	-	-	-
A 129		ILE	0.8	-	Favored (40.07%) Ile or Val / -108.5,137.5	Favored (19.8%) *tt* chi angles: 186.9,163.5	0.09Å	Favored (56.524%) beta sheet	-	-	-
A 130		ILE	0.82	-	Favored (9.11%) Ile or Val / -98.5,-8.2	Favored (44.9%) *pt* chi angles: 62.4,174.3	0.12Å	CaBLAM Disfavored (2.804%)	-	-	-
A 131		GLN	0.84	-	Allowed (0.87%) General / 46.7,-135.5	Favored (31%) *mm-40* chi angles: 300.4,297.9,0.8	0.03Å	CaBLAM Disfavored (1.637%)	-	-	-
A 132		LYS	0.84	-	Allowed (1.59%) General / -127.2,-44.5	Favored (99.6%) *mttt* chi angles: 294.7,179.7,179.9,176.4	0.03Å	CaBLAM Outlier (0.001%)	-	-	-
A 133		GLU	0.83	-	Favored (63.97%) General / -62.1,-20.4	Favored (19.8%) *pm20* chi angles: 59.1,276.8,30.5	0.05Å	Favored (65.188%) alpha helix	-	-	-
A 134		ASN	0.81	-	Favored (48.19%) General / -93.5,6.4	Favored (85.8%) *m-40* chi angles: 288.9,324.3	0.03Å	Favored (39.349%)	-	-	-
A 135		ILE	0.79	-	Favored (48.57%) Ile or Val / -96.0,126.4	Favored (43.7%) *mm* chi angles: 306.6,300.5	0.05Å	Favored (29.957%)	-	-	-
A 136		LYS	0.76	-	Favored (55.12%) General / -120.0,131.5	Favored (36.9%) *ttpt* chi angles: 180.3,169.4,67.7,174.3	0.04Å	Favored (68.49%) beta sheet	-	-	-
A 137		TYR	0.75	-	Favored (40.67%)	Favored (56.7%) *m-80*	0.14Å	Favored (69.891%)	-	-	-

					General / -117.2,121.2	chi angles: 298.2,78.9		beta sheet			
A 138		GLU	0.74	-	Favored (38.23%) General / -94.9,123.2	Favored (91.3%) *tt0* chi angles: 181.4,177.4,354.5	0.05Å	Favored (65.085%) beta sheet	-	-	-
A 139		VAL	0.75	-	Favored (67.69%) Ile or Val / -120.9,133.4	Favored (85.1%) *t* chi angles: 176.3	0.03Å	Favored (65.214%) beta sheet	-	-	-
A 140		ALA	0.77	-	Favored (45.41%) General / -114.2,143.1	-	0.05Å	Favored (68.424%) beta sheet	-	-	-

#	Alt	Res	High B	Clash > 0.4Å	Ramachandran	Rotamer	Cβ deviation	CaBLAM	Bond lengths	Bond angles	Cis Peptides
			Avg: 1.11	Clashscore: 1.47	Outliers: 1 of 499	Poor rotamers: 0 of 403	Outliers: 0 of 447	Outliers: 8 of 497	Outliers: 6 of 501	Outliers: 4 of 501	Non-Trans: 1 of 500
A 141		ILE	0.81	-	Favored (68.16%) Ile or Val / -124.3,124.3	Favored (55.2%) *mt* chi angles: 300,179.4	0.10Å	Favored (63.832%) beta sheet	-	-	-
A 142		PHE	0.86	-	Favored (49.93%) General / -124.9,145.2	Favored (60.8%) *m-80* chi angles: 303.3,84.7	0.13Å	Favored (57.455%) beta sheet	-	-	-
A 143		VAL	0.93	-	Favored (28.26%) Ile or Val / -100.4,137.3	Favored (53.9%) *t* chi angles: 180.8	0.03Å	Favored (40.537%) beta sheet	-	-	-
A 144		HIS	1.01	-	Allowed (0.41%) General / -70.6,68.9	Favored (45.9%) *m-70* chi angles: 286,258.7	0.09Å	Favored (21.108%)	-	OUTLIER(S) worst is CA-CB-CG: 4.0 σ	-
A 145		GLY	1.08	-	Favored (52.03%) Glycine / -84.0,177.8	-	-	Favored (30.236%)	-	-	-
A 146		PRO	1.15	0.43Å O with A 147 THR C	Favored (80.81%) Trans-Pro / -60.7,-25.4	Favored (21.9%) *Cg_endo* chi angles: 19.8,328.5,29.2	0.03Å	CaBLAM Outlier (0.87%)	-	-	-
A 147		THR	1.21	0.43Å C with A 146 PRO O	OUTLIER (0)%) General / 16.7,-124.5	Favored (65.8%) *p* chi angles: 58.3	0.14Å	CaBLAM Disfavored (2.814%)	-	-	-
A 148		THR	1.24	-	Allowed (1.24%) General / -174.3,148.1	Favored (6.3%) *t* chi angles: 181.8	0.03Å	CaBLAM Outlier (0.034%)	-	-	-
A 149		VAL	1.27	-	Favored (75.23%) Ile or Val / -58.1,-40.0	Favored (63.6%) *t* chi angles: 171.3	0.01Å	Favored (60.194%)	-	-	-
A 150		GLU	1.3	-	Favored (65.23%) General / -59.9,-25.7	Favored (99.5%) *mt-10* chi angles: 292.1,179.2,359.2	0.02Å	Favored (61.779%) alpha helix	-	-	-
A 151		SER	1.32	-	Favored (47.29%) General / -96.7,-1.1	Favored (71.6%) *m* chi angles: 295.2	0.02Å	Favored (55.392%) alpha helix	-	-	-
A 152		HIS	1.33	-	Favored (68.29%) General / -64.6,-26.2	Favored (24.1%) *p-80* chi angles: 81,282.9	0.06Å	Favored (50.118%) alpha helix	-	-	-
A 153		GLY	1.34	-	Favored (82.31%)	-	-	Favored (55.767%)	-	-	-

					Glycine / -90.1,3.2						
A 154		ASN	1.32	-	Favored (3.94%) General / -119.1,93.0	Favored (47.3%) *t0* chi angles: 183.1,327.9	0.06Å	Favored (10.373%)	-	-	-
A 155		TYR	1.3	-	Favored (66.26%) General / -66.6,-23.8	Favored (46%) *p90* chi angles: 65.8,85.8	0.07Å	Favored (39.085%)	-	-	-
A 156		SER	1.27	-	Favored (76.65%) General / -68.7,-43.2	Favored (62.2%) *m* chi angles: 293.9	0.02Å	Favored (72.446%) alpha helix	-	-	-
A 157		THR	1.25	-	Favored (92.29%) General / -64.3,-44.2	Favored (92.9%) *m* chi angles: 297.4	0.02Å	Favored (84.434%) alpha helix	-	-	-
A 158		GLN	1.24	-	Favored (94.37%) General / -64.1,-39.6	Favored (69.3%) *mt0* chi angles: 291.9,173.2,61.3	0.05Å	Favored (96.75%) alpha helix	-	-	-
A 159		THR	1.24	-	Favored (99.55%) General / -62.1,-43.1	Favored (99.6%) *m* chi angles: 300.4	0.05Å	Favored (97.558%) alpha helix	-	-	-
A 160		GLY	1.23	-	Favored (69.23%) Glycine / -73.3,-6.2	-	-	Favored (57.626%)	-	-	-
**#**	**Alt**	**Res**	**High B**	**Clash > 0.4Å**	**Ramachandran**	**Rotamer**	**Cβ deviation**	**CaBLAM**	**Bond lengths**	**Bond angles**	**Cis Peptides**
			Avg: 1.11	Clashscore: 1.47	Outliers: 1 of 499	Poor rotamers: 0 of 403	Outliers: 0 of 447	Outliers: 8 of 497	Outliers: 6 of 501	Outliers: 4 of 501	Non-Trans: 1 of 500
A 161		ALA	1.19	-	Favored (58.45%) General / -85.4,-2.8	-	0.04Å	Favored (13.761%)	-	-	-
A 162		THR	1.12	-	Allowed (0.16%) General / 78.3,-33.2	Favored (99.1%) *m* chi angles: 300.6	0.11Å	CaBLAM Disfavored (1.607%)	-	-	-
A 163		GLN	1.03	-	Favored (2.89%) General / -77.4,9.9	Favored (76.4%) *mt0* chi angles: 297.7,175.8,51	0.09Å	CaBLAM Outlier (0.148%)	-	-	-
A 164		ALA	0.93	-	Favored (41.06%) General / -155.3,161.4	-	0.06Å	Favored (36.192%)	-	-	-
A 165		GLY	0.84	-	Favored (33.57%) Glycine / -158.6,164.5	-	-	Favored (55.662%)	-	-	-
A 166		ARG	0.78	-	Favored (51.6%) General / -130.1,150.0	Favored (54.4%) *mtm110* chi angles: 293.4,181.1,288.6,110	0.02Å	Favored (60.831%) beta sheet	-	-	-
A 167		PHE	0.75	-	Favored (22.16%) General / -150.2,140.3	Favored (78.2%) *t80* chi angles: 183.5,83.9	0.02Å	Favored (51.611%) beta sheet	-	-	-
A 168		SER	0.74	-	Favored (26.59%) General / -94.0,114.4	Favored (68.8%) *m* chi angles: 294.8	0.07Å	Favored (62.432%) beta sheet	-	-	-
A 169		ILE	0.74	-	Favored (52.51%) Ile or Val / -100.5,128.5	Favored (58.2%) *mt* chi angles: 303.1,165.6	0.14Å	Favored (39.921%) beta sheet	-	-	-

A<br>170		THR	0.75	-	Favored<br>(56.42%)<br>Pre-Pro /<br>-132.8,162.6	Favored (43.5%) *p*<br>chi angles: 67	0.07Å	Favored<br>(39.274%)	-	-	-
A<br>171		PRO	0.76	-	Favored<br>(44.82%)<br>Trans-Pro /<br>-55.4,-24.7	Favored (93.1%)<br>*Cg_exo*<br>chi angles:<br>333,36.3,330.1	0.02Å	Favored<br>(93.244%)	-	-	-
A<br>172		SER	0.76	-	Favored<br>(59.12%)<br>General / -83.5,-9.2	Favored (89.2%) *p*<br>chi angles: 69.1	0.02Å	Favored<br>(45.084%)	-	-	-
A<br>173		ALA	0.75	-	Favored<br>(6.45%)<br>Pre-Pro /<br>-153.2,67.9	-	0.04Å	Favored<br>(18.745%)	-	-	-
A<br>174		PRO	0.74	-	Favored<br>(27.6%)<br>Trans-Pro /<br>-72.5,-15.9	Favored (71%)<br>*Cg_endo*<br>chi angles:<br>27.3,325,27.6	0.04Å	Favored<br>(64.955%)	-	-	-
A<br>175		SER	0.74	-	Favored<br>(45.55%)<br>General /<br>-127.9,154.4	Favored (92.8%) *p*<br>chi angles: 64.4	0.02Å	Favored<br>(7.175%)	-	-	-
A<br>176		TYR	0.75	-	Favored<br>(33.38%)<br>General /<br>-160.3,163.4	Favored (36.7%)<br>*p90*<br>chi angles: 61.4,99.4	0.06Å	Favored<br>(38.383%)	-	-	-
A<br>177		THR	0.77	-	Favored<br>(50.87%)<br>General /<br>-126.9,132.4	Favored (97.2%) *m*<br>chi angles: 299.9	0.02Å	Favored<br>(55.038%)<br>beta sheet	-	-	-
A<br>178		LEU	0.81	-	Favored<br>(45.41%)<br>General /<br>-110.7,140.1	Favored (87.1%) *mt*<br>chi angles: 299.8,176.3	0.05Å	Favored<br>(59.332%)<br>beta sheet	-	-	-
A<br>179		LYS	0.85	-	Favored<br>(41.36%)<br>General /<br>-96.2,132.6	Favored (62.3%)<br>*mttm*<br>chi angles:<br>294.7,180.9,180.4,292	0.01Å	Favored<br>(59.006%)<br>beta sheet	-	-	-
A<br>180		LEU	0.89	-	Favored<br>(6.62%)<br>General /<br>-117.3,31.6	Favored (54.1%) *mt*<br>chi angles: 299.6,168.5	0.10Å	CaBLAM<br>Disfavored<br>(1.223%)	-	-	-
**#**	**Alt**	**Res**	**High B**	**Clash > 0.4Å**	**Ramachandran**	**Rotamer**	**Cβ deviation**	**CaBLAM**	**Bond lengths**	**Bond angles**	**Cis Peptides**
			Avg: 1.11	Clashscore: 1.47	Outliers: 1 of 499	Poor rotamers: 0 of 403	Outliers: 0 of 447	Outliers: 8 of 497	Outliers: 6 of 501	Outliers: 4 of 501	Non-Trans: 1 of 500
A<br>181		GLY	0.92	-	Favored<br>(30.59%)<br>Glycine /<br>62.3,-123.3	-	-	Favored<br>(38.284%)	-	-	-
A<br>182		GLU	0.93	-	Favored<br>(37.59%)<br>General / -72.1,-5.8	Favored (4.8%)<br>*mp0*<br>chi angles:<br>300.2,85.6,50.4	0.01Å	Favored<br>(10.686%)	-	-	-
A<br>183		TYR	0.92	-	Favored<br>(56.86%)<br>General / -92.4,2.1	Favored (67.2%) *m-80*<br>chi angles: 293.3,112.1	0.08Å	Favored<br>(19.011%)	-	-	-
A<br>184		GLY	0.9	-	Favored<br>(20.95%)<br>Glycine /<br>87.5,-148.0	-	-	Favored<br>(17.716%)	-	-	-
A<br>185		GLU	0.87	-	Favored<br>(48.03%)<br>General /<br>-131.1,154.7	Favored (89.5%)<br>*mt-10*<br>chi angles:<br>300.3,183.9,353.6	0.05Å	Favored<br>(10.113%)	-	-	-

A 186		VAL	0.84	-	Favored (73.99%) Ile or Val / -123.7,130.5	Favored (84.1%) *t* chi angles: 176.3	0.03Å	Favored (65.786%) beta sheet	-	-	-
A 187		THR	0.82	-	Favored (55.13%) General / -112.4,132.2	Favored (90.3%) *m* chi angles: 298.1	0.07Å	Favored (71.892%) beta sheet	-	-	-
A 188		VAL	0.82	-	Favored (64.99%) Ile or Val / -125.0,135.2	Favored (49.7%) *t* chi angles: 181.5	0.07Å	Favored (70.463%) beta sheet	-	-	-
A 189		ASP	0.85	-	Favored (8.2%) General / -122.0,102.5	Favored (65.9%) *t0* chi angles: 184.4,354.3	0.06Å	Favored (52.135%) beta sheet	-	-	-
A 190		CYS	0.9	-	Favored (22.8%) General / -107.8,153.6	Favored (71.2%) *m* chi angles: 298.1	0.05Å	Favored (33.751%) beta sheet	-	-	-
A 191		GLU	0.99	-	Favored (34.31%) Pre-Pro / -116.9,91.0	Favored (11.5%) *tp30* chi angles: 177.4,58.4,74.3	0.06Å	Favored (18.118%) beta sheet	-	-	-
A 192		PRO	1.11	-	Favored (58.58%) Trans-Pro / -65.4,-17.8	Favored (46.2%) *Cg_endo* chi angles: 24.7,326.3,27.8	0.03Å	Favored (54.374%)	-	-	-
A 193		ARG	1.25	-	Favored (53.59%) General / -86.8,-10.4	Favored (89.3%) *mtm180* chi angles: 297.3,172.7,293.5,178.5	0.04Å	Favored (69.318%) three-ten	-	-	-
A 194		SER	1.36	-	Favored (20.2%) General / -91.5,-22.8	Favored (93.6%) *p* chi angles: 64.3	0.03Å	Favored (60.662%)	-	-	-
A 195		GLY	1.42	-	Favored (6.65%) Glycine / -64.7,-60.7	-	-	Favored (6.353%)	-	-	-
A 196		ILE	1.42	-	Favored (72.79%) Ile or Val / -125.5,129.8	Favored (89.5%) *mt* chi angles: 298,172.7	0.05Å	Favored (20.6%)	-	-	-
A 197		ASP	1.36	-	Favored (5.1%) General / -79.5,90.6	Favored (58.4%) *t0* chi angles: 182.1,341	0.07Å	Favored (50.506%)	-	-	-
A 198		THR	1.26	-	Favored (51.53%) General / -65.3,-12.4	Favored (35.6%) *p* chi angles: 54	0.13Å	Favored (23.064%)	-	-	-
A 199		SER	1.14	-	Favored (59.86%) General / -77.9,-11.1	Favored (88.5%) *p* chi angles: 69.4	0.04Å	Favored (66.093%) three-ten	-	-	-
A 200		ALA	1.03	-	Favored (57.55%) General / -91.0,0.3	-	0.04Å	Favored (59.431%)	-	-	-
**#**	**Alt**	**Res**	**High B**	**Clash > 0.4Å**	**Ramachandran**	**Rotamer**	**Cβ deviation**	**CaBLAM**	**Bond lengths**	**Bond angles**	**Cis Peptides**
			Avg: 1.11	Clashscore: 1.47	Outliers: 1 of 499	Poor rotamers: 0 of 403	Outliers: 0 of 447	Outliers: 8 of 497	Outliers: 6 of 501	Outliers: 4 of 501	Non-Trans: 1 of 500
A 201		TYR	0.94	-	Favored (48.8%) General / -125.5,148.7	Favored (83.7%) *m-80* chi angles: 296.2,82.9	0.01Å	Favored (29.429%)	-	-	-
A 202		TYR	0.89	-	Favored (28.4%)	Favored (40.1%) *m-80*	0.14Å	Favored (51.816%)	-	-	-

					General / -115.5,154.9	chi angles: 293.3,123.1					
A 203		VAL	0.87	-	Favored (57.87%) Ile or Val / -104.6,128.8	Favored (97.9%) *t* chi angles: 175.2	0.03Å	Favored (60.827%) beta sheet	-	-	-
A 204		MET	0.9	-	Favored (38.51%) General / -109.2,143.5	Favored (88.4%) *mtp* chi angles: 294.6,183.9,69.4	0.10Å	Favored (66.457%) beta sheet	-	-	-
A 205		SER	0.95	-	Favored (25.76%) General / -121.3,116.9	Favored (61.2%) *m* chi angles: 293.7	0.06Å	Favored (59.652%)	-	-	-
A 206		VAL	1.01	-	Favored (17.31%) Ile or Val / -117.1,105.0	Favored (47.9%) *t* chi angles: 181.9	0.14Å	Favored (7.517%)	-	-	-
A 207		GLY	1.06	-	Favored (43.5%) Glycine / 58.7,-126.8	-	-	Favored (67.585%)	-	-	-
A 208		ALA	1.07	-	Favored (57.86%) General / -85.6,-2.1	-	0.04Å	Favored (12.779%)	-	-	-
A 209		LYS	1.04	-	Favored (43.57%) General / -117.2,145.9	Favored (99%) *mttt* chi angles: 295.1,182.5,179.7,180.6	0.02Å	Favored (31.906%)	-	-	-
A 210		SER	0.99	-	Favored (45.27%) General / -122.3,148.3	Favored (62.5%) *m* chi angles: 298.2	0.01Å	Favored (66.146%)	-	-	-
A 211		PHE	0.95	-	Favored (46.39%) General / -138.1,149.9	Favored (97.3%) *m-80* chi angles: 296.1,88.1	0.04Å	Favored (57.52%) beta sheet	-	-	-
A 212		LEU	0.93	-	Favored (28.53%) General / -95.0,116.3	Favored (47.3%) *tp* chi angles: 183,64.9	0.01Å	Favored (53.346%) beta sheet	-	-	-
A 213		VAL	0.94	-	Favored (67.12%) Ile or Val / -123.6,134.2	Favored (54.5%) *t* chi angles: 180.7	0.09Å	Favored (47.844%) beta sheet	-	-	-
A 214		HIS	0.99	-	Favored (55.45%) General / -59.3,141.0	Favored (77.3%) *t-90* chi angles: 191.4,282.1	0.03Å	Favored (48.659%)	-	-	-
A 215		ARG	1.05	-	Favored (74.4%) General / -58.4,-37.0	Favored (85%) *mtp180* chi angles: 290.5,177.6,69.5,185.1	0.02Å	Favored (57.416%)	-	-	-
A 216		GLU	1.1	-	Favored (87.63%) General / -66.9,-39.8	Favored (97.9%) *mt-10* chi angles: 289.8,177.8,356.7	0.06Å	Favored (88.227%) alpha helix	-	-	-
A 217		TRP	1.13	-	Favored (92.96%) General / -61.2,-45.8	Favored (72.8%) *t-100* chi angles: 183.6,252.4	0.06Å	Favored (99.384%) alpha helix	-	-	-
A 218		PHE	1.12	-	Favored (69.11%) General / -55.6,-50.8	Favored (83.8%) *t80* chi angles: 175.8,83.1	0.09Å	Favored (97.717%) alpha helix	-	-	-
A 219		MET	1.08	-	Favored (68.62%) General / -64.9,-27.1	Favored (84%) *mtm* chi angles: 289.9,187.6,286.8	0.06Å	Favored (71.809%)	-	-	-

#	Alt	Res	High B	Clash > 0.4Å	Ramachandran	Rotamer	Cβ deviation	CaBLAM	Bond lengths	Bond angles	Cis Peptides
A 220		ASP	1.01	-	Favored (57.83%) General / -81.5,-5.3	Favored (85.7%) *m-30* chi angles: 289.5,335.4	0.04Å	Favored (49.243%)	-	-	-
**#**	**Alt**	**Res**	**High B**	**Clash > 0.4Å**	**Ramachandran**	**Rotamer**	**Cβ deviation**	**CaBLAM**	**Bond lengths**	**Bond angles**	**Cis Peptides**
			Avg: 1.11	Clashscore: 1.47	Outliers: 1 of 499	Poor rotamers: 0 of 403	Outliers: 0 of 447	Outliers: 8 of 497	Outliers: 6 of 501	Outliers: 4 of 501	Non-Trans: 1 of 500
A 221		LEU	0.93	-	Favored (58.3%) General / -62.7,137.6	Favored (94.5%) *mt* chi angles: 293.4,171.2	0.05Å	Favored (35.569%)	-	-	-
A 222		ASN	0.86	-	Favored (29.3%) General / -93.6,11.3	Favored (39.6%) *p0* chi angles: 65.6,338.8	0.04Å	Favored (8.906%) beta sheet	-	-	-
A 223		LEU	0.8	-	Favored (76.97%) Pre-Pro / -79.3,154.9	Favored (95.2%) *mt* chi angles: 297.8,176	0.09Å	Favored (18.685%) beta sheet	-	-	-
A 224		PRO	0.77	-	Favored (87.97%) Trans-Pro / -57.9,146.1	Favored (55.6%) *Cg_exo* chi angles: 336.2,35.1,327.9	0.07Å	Favored (42.946%) beta sheet	-	-	-
A 225		TRP	0.76	-	Favored (41.74%) General / -144.6,160.1	Favored (76.7%) *p-90* chi angles: 59.2,269.5	0.07Å	Favored (69.404%) beta sheet	-	-	-
A 226		SER	0.78	-	Favored (15.23%) General / -137.9,119.7	Favored (44.2%) *t* chi angles: 178.9	0.07Å	Favored (32.96%)	-	-	-
A 227		SER	0.82	-	Favored (58.05%) General / -61.5,140.9	Favored (42.4%) *t* chi angles: 175.8	0.03Å	Favored (40.022%)	-	-	-
A 228		ALA	0.86	-	Favored (53.58%) General / -57.4,139.2	-	0.03Å	Favored (31.737%)	-	-	-
A 229		GLY	0.89	-	Favored (66.84%) Glycine / 94.7,-13.6	-	-	Favored (79.427%)	-	-	-
A 230		SER	0.9	-	Favored (19.5%) General / -89.1,155.4	Favored (44.6%) *t* chi angles: 179	0.06Å	Favored (32.397%)	-	-	-
A 231		THR	0.89	-	Favored (27.4%) General / -107.1,4.9	Favored (77.2%) *p* chi angles: 60.2	0.03Å	Favored (36.763%)	-	-	-
A 232		THR	0.85	-	Favored (17.1%) General / -121.0,164.2	Favored (62.7%) *p* chi angles: 63.5	0.01Å	Favored (24.886%)	-	-	-
A 233		TRP	0.8	-	Favored (39.93%) General / -115.4,147.9	Favored (93.1%) *m100* chi angles: 293.4,86.3	0.03Å	Favored (53.566%)	-	-	-
A 234		ARG	0.76	-	Favored (24.6%) General / -108.6,152.6	Favored (61.4%) *mmm-85* chi angles: 303.4,290.6,297.2,276.3	0.10Å	Favored (10.459%)	-	-	-
A 235		ASN	0.72	-	Favored (31.49%) General / 54.0,40.4	Favored (92.5%) *m-40* chi angles: 293.6,330.4	0.07Å	Favored (14.745%)	-	-	-
A 236		ARG	0.7	-	Favored (70.64%)	Favored (99.6%) *mtm-85*	0.11Å	Favored (27.965%)	-	-	-

					General / -59.5,-32.8	chi angles: 292.7,192,296.9,275.4					
A 237		GLU	0.69	-	Favored (68.16%) General / -60.8,-27.7	Favored (79.4%) *mt-10* chi angles: 293.9,178.9,22.8	0.09Å	Favored (55.966%) three-ten	-	-	-
A 238		THR	0.68	-	Favored (64.94%) General / -56.6,-32.0	Favored (88.4%) *m* chi angles: 297	0.10Å	Favored (35.626%) three-ten	-	-	-
A 239		LEU	0.68	-	Favored (53.79%) General / -95.2,0.1	Favored (91.7%) *mt* chi angles: 296.5,178.2	0.07Å	Favored (47.816%) alpha helix	-	-	-
A 240		MET	0.68	-	Favored (51.75%) General / -133.8,152.0	Favored (8.7%) *mmt* chi angles: 308.7,294,198.4	0.06Å	Favored (26.431%)	OUTLIER(S) worst is CG-- SD: 4.5 σ	-	-
**#**	**Alt**	**Res**	**High B**	**Clash > 0.4Å**	**Ramachandran**	**Rotamer**	**Cβ deviation**	**CaBLAM**	**Bond lengths**	**Bond angles**	**Cis Peptides**
			Avg: 1.11	Clashscore: 1.47	Outliers: 1 of 499	Poor rotamers: 0 of 403	Outliers: 0 of 447	Outliers: 8 of 497	Outliers: 6 of 501	Outliers: 4 of 501	Non-Trans: 1 of 500
A 241		GLU	0.7	-	Favored (44.95%) General / -123.8,150.0	Favored (26%) *pt0* chi angles: 64.3,181.3,6.2	0.02Å	Favored (58.105%)	-	-	-
A 242		PHE	0.72	-	Favored (43.98%) General / -104.1,136.3	Favored (39.6%) *m-80* chi angles: 287,73.6	0.07Å	Favored (48.136%)	-	-	-
A 243		GLU	0.75	-	Favored (20.85%) General / -83.6,162.5	Favored (92.4%) *mt-10* chi angles: 294.3,184,344.5	0.06Å	Favored (30.534%)	-	-	-
A 244		GLU	0.78	-	Favored (88.89%) Pre-Pro / -59.7,132.5	Favored (90.8%) *tt0* chi angles: 184.4,178.8,5	0.06Å	Favored (34.496%)	-	-	-
A 245		PRO	0.81	-	Favored (96.06%) Trans-Pro / -58.7,140.7	Favored (89%) *Cg_exo* chi angles: 333.3,34.9,332	0.11Å	Favored (59.03%)	-	-	-
A 246		HIS	0.84	-	Favored (25.43%) General / -138.2,128.1	Favored (95.3%) *m-70* chi angles: 298.7,280.3	0.03Å	Favored (10.71%)	-	-	-
A 247		ALA	0.85	-	Allowed (0.17%) General / 55.7,-103.3	-	0.03Å	CaBLAM Disfavored (2.922%)	-	-	-
A 248		THR	0.85	-	Favored (9.52%) General / -122.7,4.1	Favored (77.1%) *p* chi angles: 60.2	0.04Å	Favored (8.811%)	-	-	-
A 249		LYS	0.84	-	Favored (35.64%) General / -125.7,158.1	Favored (99%) *mttt* chi angles: 294.7,184.3,180.3,179.8	0.05Å	Favored (21.698%)	-	-	-
A 250		GLN	0.81	-	Favored (18.24%) General / -152.3,138.4	Favored (62.9%) *tt0* chi angles: 181.7,175,31.3	0.04Å	Favored (44.638%)	-	-	-
A 251		SER	0.79	-	Favored (19.24%) General / -108.3,156.6	Favored (66.9%) *m* chi angles: 297	0.04Å	Favored (42.309%) beta sheet	-	-	-
A 252		VAL	0.78	-	Favored (75.24%)	Favored (59.5%) *t* chi angles: 180	0.03Å	Favored (66.272%)	-	-	-

#	Alt	Res	High B	Clash > 0.4Å	Ramachandran	Rotamer	Cβ deviation	CaBLAM	Bond lengths	Bond angles	Cis Peptides
					Ile or Val / -123.0,129.2			beta sheet			
A 253		VAL	0.77	-	Favored (74.56%) Ile or Val / -120.9,130.5	Favored (78.9%) *t* chi angles: 178.1	0.08Å	Favored (57.869%) beta sheet	-	-	-
A 254		ALA	0.78	-	Favored (43.59%) General / -73.1,148.9	-	0.03Å	Favored (40.364%)	-	-	-
A 255		LEU	0.8	-	Favored (14.23%) General / -91.0,163.7	Favored (79.7%) *mt* chi angles: 302.4,177.8	0.04Å	Favored (35.341%)	-	-	-
A 256		GLY	0.83	-	Favored (44.6%) Glycine / -71.3,172.5	-	-	Favored (27.584%)	-	-	-
A 257		SER	0.86	-	Favored (58.17%) General / -60.4,137.3	Favored (41.9%) *t* chi angles: 176	0.04Å	Favored (13.77%)	-	-	-
A 258		GLN	0.9	-	Favored (49.5%) General / -92.2,5.7	Favored (98.3%) *mm-40* chi angles: 298.4,299.2,302.3	0.06Å	Favored (11.696%)	-	-	-
A 259		GLU	0.94	-	Favored (75.21%) General / -57.1,-39.6	Favored (90.6%) *tt0* chi angles: 179.8,173.7,356.7	0.02Å	Favored (50.622%)	-	-	-
A 260		GLY	0.99	-	Favored (49.6%) Glycine / -58.3,-53.0	-	-	Favored (92.165%) alpha helix	-	-	-
**#**	**Alt**	**Res**	**High B**	**Clash > 0.4Å**	**Ramachandran**	**Rotamer**	**Cβ deviation**	**CaBLAM**	**Bond lengths**	**Bond angles**	**Cis Peptides**
			Avg: 1.11	Clashscore: 1.47	Outliers: 1 of 499	Poor rotamers: 0 of 403	Outliers: 0 of 447	Outliers: 8 of 497	Outliers: 6 of 501	Outliers: 4 of 501	Non-Trans: 1 of 500
A 261		ALA	1.04	-	Favored (73.4%) General / -59.3,-35.3	-	0.03Å	Favored (76.493%) alpha helix	-	-	-
A 262		LEU	1.09	0.69Å C with A 262 LEU HD13	Favored (71.33%) General / -63.8,-49.9	Allowed (1.3%) *tm* chi angles: 185.8,285.3	0.05Å	Favored (80.71%) alpha helix	-	-	-
A 263		HIS	1.14	-	Favored (82.96%) General / -61.5,-37.2	Favored (70.2%) *m-70* chi angles: 290.3,302.1	0.03Å	Favored (84.721%) alpha helix	-	-	-
A 264		GLN	1.17	-	Favored (94.02%) General / -65.1,-40.1	Favored (97.9%) *mt0* chi angles: 291.6,173.2,344.3	0.05Å	Favored (88.062%) alpha helix	-	-	-
A 265		ALA	1.19	-	Favored (74.14%) General / -59.8,-35.1	-	0.03Å	Favored (80.873%) alpha helix	-	-	-
A 266		LEU	1.2	-	Favored (8.68%) General / -97.2,22.4	Favored (8.2%) *mp* chi angles: 271.1,61.1	0.07Å	Favored (23.251%) alpha helix	-	-	-
A 267		ALA	1.18	-	Favored (67.07%) General / -58.1,-31.4	-	0.01Å	Favored (45.504%) alpha helix	-	-	-

A 268		GLY	1.14	-	Favored (83.54%) Glycine / -89.7,4.2	-	-	Favored (61.541%)	-	-	-
A 269		ALA	1.09	-	Favored (21.89%) General / -83.3,159.3	-	0.03Å	Favored (37.952%)	-	-	-
A 270		ILE	1.03	-	Favored (61.62%) Pre-Pro / -93.4,123.0	Favored (49.2%) *mm* chi angles: 301.5,301	0.03Å	Favored (31.652%) beta sheet	-	-	-
A 271		PRO	0.99	-	Favored (33.6%) Trans-Pro / -67.9,134.9	Favored (44.2%) *Cg_endo* chi angles: 24.5,328.2,25.6	0.02Å	Favored (42.245%) beta sheet	-	-	-
A 272		VAL	0.97	-	Favored (16.17%) Ile or Val / -132.4,172.6	Favored (31.6%) *m* chi angles: 300.5	0.07Å	Favored (30.35%) beta sheet	-	-	-
A 273		GLU	0.98	-	Favored (43.09%) General / -119.3,123.0	Favored (92.7%) *tt0* chi angles: 181.9,178.4,356.5	0.01Å	Favored (28.403%) beta sheet	-	-	-
A 274		PHE	1	-	Favored (50.8%) General / -111.1,136.0	Favored (64%) *t80* chi angles: 173.4,67.6	0.04Å	Favored (47.427%)	-	-	-
A 275		SER	1.01	-	Favored (11.02%) General / -146.9,123.3	Favored (41.3%) *t* chi angles: 178.2	0.05Å	Favored (7.846%)	-	-	-
A 276		SER	1.01	-	Allowed (0.08%) General / 55.5,-98.2	Favored (9.1%) *t* chi angles: 191.8	0.07Å	CaBLAM Disfavored (2.189%)	-	-	-
A 277		ASN	0.99	-	Favored (9.47%) General / -123.7,15.2	Favored (71.6%) *m-40* chi angles: 293.3,286.9	0.02Å	Favored (12.677%)	-	-	-
A 278		THR	0.96	-	Favored (50.19%) General / -130.3,137.8	Favored (90.2%) *m* chi angles: 301.2	0.02Å	Favored (33.481%)	-	-	-
A 279		VAL	0.95	-	Favored (71.51%) Ile or Val / -119.9,123.8	Favored (71.5%) *t* chi angles: 178.6	0.08Å	Favored (64.125%)	-	-	-
A 280		LYS	0.96	-	Favored (28.27%) General / -84.8,121.8	Favored (37%) *ttpt* chi angles: 182.7,174.1,72.8,179.8	0.05Å	Favored (59.283%) beta sheet	-	-	-
**#**	**Alt**	**Res**	**High B**	**Clash > 0.4Å**	**Ramachandran**	**Rotamer**	**Cβ deviation**	**CaBLAM**	**Bond lengths**	**Bond angles**	**Cis Peptides**
			Avg: 1.11	Clashscore: 1.47	Outliers: 1 of 499	Poor rotamers: 0 of 403	Outliers: 0 of 447	Outliers: 8 of 497	Outliers: 6 of 501	Outliers: 4 of 501	Non-Trans: 1 of 500
A 281		LEU	0.99	-	Favored (33.96%) General / -102.0,142.0	Favored (76.7%) *mt* chi angles: 300.9,174.3	0.15Å	Favored (59.186%) beta sheet	OUTLIER(S) worst is CB--CG: 5.6 σ	-	-
A 282		THR	1.04	-	Favored (15.56%) General / -106.1,-13.1	Favored (55.1%) *p* chi angles: 64.9	0.04Å	Favored (50.566%)	-	-	-
A 283		SER	1.09	-	Favored (10.12%) General / -112.8,26.9	Favored (69%) *m* chi angles: 296.6	0.03Å	Favored (17.129%)	-	-	-

A 284		GLY	1.1	-	Favored (36.29%) Glycine / -60.9,154.3	-	-	Favored (22.454%)	-	-	-
A 285		HIS	1.08	-	Favored (23.43%) General / -147.8,138.9	Favored (45.5%) *t-90* chi angles: 181.3,284	0.01Å	Favored (65.603%) beta sheet	-	-	-
A 286		LEU	1.04	-	Favored (20.85%) General / -141.2,128.3	Favored (41.8%) *tp* chi angles: 182.1,67	0.08Å	Favored (58.349%) beta sheet	OUTLIER(S) worst is CB--CG: 4.0 σ	-	-
A 287		LYS	1.01	-	Favored (36.22%) General / -91.6,125.1	Favored (79.7%) *tttt* chi angles: 177.7,180.4,171.8,177.2	0.03Å	Favored (61.022%) beta sheet	-	-	-
A 288		CYS	0.99	-	Favored (48.79%) General / -131.6,138.4	Favored (67.9%) *m* chi angles: 299.3	0.05Å	Favored (63.426%) beta sheet	-	-	-
A 289		ARG	1	-	Favored (41.4%) General / -97.1,125.1	Favored (47.9%) *ttm170* chi angles: 178.4,165.7,290.7,179.4	0.03Å	Favored (69.075%) beta sheet	-	-	-
A 290		VAL	1.04	-	Favored (67.68%) Ile or Val / -115.0,121.7	Favored (62.2%) *t* chi angles: 179.7	0.03Å	Favored (71.628%) beta sheet	-	-	-
A 291		LYS	1.09	-	Favored (43.72%) General / -97.3,131.8	Favored (97.2%) *mttt* chi angles: 296.6,180,180.4,180.6	0.05Å	Favored (64.613%) beta sheet	-	-	-
A 292		MET	1.13	-	Favored (20.1%) General / -106.3,19.7	Favored (17%) *mmt* chi angles: 301.5,302.1,193.1	0.10Å	Favored (5.109%)	-	-	-
A 293		GLU	1.17	-	Favored (56.8%) General / -59.7,134.2	Favored (91.7%) *mt-10* chi angles: 291,182.1,8.6	0.03Å	Favored (15.598%)	-	-	-
A 294		LYS	1.19	-	Favored (7.14%) General / 67.7,9.6	Favored (40.8%) *mtmt* chi angles: 305.3,187.6,298.3,182.4	0.07Å	Favored (13.728%)	-	-	-
A 295		LEU	1.2	-	Favored (41.62%) General / -74.9,134.3	Favored (16.4%) *tp* chi angles: 191.4,69.7	0.04Å	Favored (30.263%)	-	-	-
A 296		GLN	1.23	-	Favored (56.05%) General / -115.3,128.3	Favored (14.8%) *tt0* chi angles: 179,174,240.2	0.04Å	Favored (56.862%) beta sheet	-	-	-
A 297		LEU	1.26	-	Favored (6.53%) General / -82.2,87.2	Favored (45.9%) *tp* chi angles: 182.4,55.9	0.05Å	Favored (62.918%) beta sheet	-	-	-
A 298		LYS	1.29	-	Favored (42.16%) General / -50.7,-37.6	Favored (86.3%) *tttt* chi angles: 181.9,173.9,178.1,181.9	0.05Å	Favored (43.196%)	-	-	-
A 299		GLY	1.3	-	Favored (59.11%) Glycine / -57.9,-27.3	-	-	Favored (81.597%) three-ten	-	-	-
A 300		THR	1.28	-	Favored (47.8%) General / -85.3,1.6	Favored (70.1%) *p* chi angles: 59.4	0.05Å	Favored (48.984%) three-ten	-	-	-

#	Alt	Res	High B	Clash > 0.4Å	Ramachandran	Rotamer	Cβ deviation	CaBLAM	Bond lengths	Bond angles	Cis Peptides
			Avg: 1.11	Clashscore: 1.47	Outliers: 1 of 499	Poor rotamers: 0 of 403	Outliers: 0 of 447	Outliers: 8 of 497	Outliers: 6 of 501	Outliers: 4 of 501	Non-Trans: 1 of 500
A 301		THR	1.23	-	Favored (55.86%) General / -93.8,-0.4	Favored (52.4%) *p* chi angles: 65.3	0.05Å	Favored (66.761%)	-	-	-
A 302		TYR	1.16	-	Favored (26.84%) General / -124.8,161.3	Favored (88.4%) *m-80* chi angles: 301.5,97.9	0.02Å	Favored (20.509%)	-	-	-
A 303		GLY	1.1	-	Favored (43.25%) Glycine / -82.0,-169.5	-	-	Favored (46.162%)	-	-	-
A 304		VAL	1.05	-	Favored (28.98%) Ile or Val / -93.4,135.1	Favored (82.1%) *t* chi angles: 176.7	0.04Å	Favored (7.832%)	-	-	-
A 305		CYS	1.01	-	Favored (18.7%) General / -61.4,123.8	Favored (83.5%) *m* chi angles: 295.3	0.10Å	Favored (40.586%)	-	-	-
A 306		SER	0.99	-	Favored (38.99%) General / -93.7,-9.1	Favored (73.1%) *m* chi angles: 295.4	0.02Å	Favored (28.19%) beta sheet	-	-	-
A 307		LYS	0.96	-	Favored (10.5%) General / -99.4,167.6	Favored (30.7%) *mmtm* chi angles: 304.3,297,193.1,293.7	0.04Å	Favored (10.941%) beta sheet	-	-	-
A 308		ALA	0.94	-	Favored (33.31%) General / -60.8,150.2	-	0.08Å	Favored (17.701%) beta sheet	-	-	-
A 309		PHE	0.91	-	Favored (35.5%) General / -116.7,151.9	Favored (84.3%) *m-80* chi angles: 291,84.1	0.09Å	Favored (61.799%) beta sheet	-	-	-
A 310		LYS	0.88	-	Favored (35.91%) General / -119.8,153.3	Favored (98.5%) *mttt* chi angles: 293.1,180.9,178.9,179.8	0.03Å	Favored (40.369%) beta sheet	-	-	-
A 311		PHE	0.87	-	Favored (37.42%) General / -79.5,138.1	Favored (57.3%) *m-80* chi angles: 286.3,107	0.04Å	Favored (43.307%)	-	-	-
A 312		ALA	0.87	-	Favored (59.02%) General / -85.0,-4.1	-	0.04Å	CaBLAM Disfavored (2.733%)	-	-	-
A 313		GLY	0.89	-	Favored (37.84%) Glycine / 170.1,173.9	-	-	Favored (14.029%)	-	-	-
A 314		ASN	0.93	-	Favored (83.67%) Pre-Pro / -73.4,144.1	Favored (90.7%) *m-40* chi angles: 291.5,325.2	0.03Å	Favored (10.666%)	-	-	-
A 315		PRO	0.99	-	Favored (78.78%) Trans-Pro / -54.9,139.6	Favored (99.8%) *Cg_exo* chi angles: 332.5,36.7,330.1	0.04Å	Favored (84.868%)	-	-	-
A 316		ALA	1.07	-	Favored (41.2%) General / -104.0,138.0	-	0.03Å	Favored (44.332%) beta sheet	-	-	-
A 317		ASP	1.16	-	Favored (19.49%)	Favored (55.8%) *t0* chi angles: 187.5,358.8	0.06Å	Favored (58.309%)	-	-	-

					General / -86.9,109.7						
A 318		THR	1.24	-	Favored (15.01%) General / -82.0,170.9	Favored (33.9%) *p* chi angles: 68.8	0.12Å	Favored (31.798%)	-	-	-
A 319		GLY	1.28	-	Favored (48.55%) Glycine / -70.7,-3.2	-	-	Favored (19.224%)	-	-	-
A 320		HIS	1.27	-	Favored (15.76%) General / -115.9,12.4	Favored (79.1%) *m90* chi angles: 297.2,83.6	0.06Å	Favored (53.632%)	-	OUTLIER(S) worst is CA-CB-CG: 5.8 σ	-

#	Alt	Res	High B	Clash > 0.4Å	Ramachandran	Rotamer	Cβ deviation	CaBLAM	Bond lengths	Bond angles	Cis Peptides
			Avg: 1.11	Clashscore: 1.47	Outliers: 1 of 499	Poor rotamers: 0 of 403	Outliers: 0 of 447	Outliers: 8 of 497	Outliers: 6 of 501	Outliers: 4 of 501	Non-Trans: 1 of 500
A 321		GLY	1.2	-	Favored (77.39%) Glycine / 89.2,-9.3	-	-	Favored (66.969%)	-	-	-
A 322		THR	1.1	-	Favored (21.52%) General / -81.1,165.5	Favored (61.3%) *p* chi angles: 63.8	0.02Å	Favored (41.077%)	-	-	-
A 323		VAL	0.99	-	Favored (52.97%) Ile or Val / -124.8,138.8	Favored (5.8%) *p* chi angles: 58.4	0.07Å	Favored (55.355%) beta sheet	-	-	-
A 324		VAL	0.9	-	Favored (65.02%) Ile or Val / -108.3,126.8	Favored (83.6%) *t* chi angles: 177.7	0.01Å	Favored (63.963%) beta sheet	-	-	-
A 325		LEU	0.83	-	Favored (49.14%) General / -120.8,142.9	Favored (3.9%) *mp* chi angles: 305.3,92.9	0.12Å	Favored (60.976%) beta sheet	-	-	-
A 326		GLU	0.79	-	Favored (31.02%) General / -119.3,118.3	Favored (39.7%) *tt0* chi angles: 184,176.7,75	0.04Å	Favored (65.165%) beta sheet	-	-	-
A 327		LEU	0.76	-	Favored (29.86%) General / -106.6,147.1	Favored (2.1%) *mm* chi angles: 278.9,299.6	0.07Å	Favored (48.209%) beta sheet	-	-	-
A 328		GLN	0.76	-	Favored (56.43%) General / -113.1,128.2	Favored (56.3%) *tt0* chi angles: 182,177,331.6	0.02Å	Favored (55.453%) beta sheet	-	-	-
A 329		TYR	0.76	-	Favored (32.16%) General / -85.2,124.7	Favored (14.3%) *t80* chi angles: 186.5,46.3	0.01Å	Favored (58.596%) beta sheet	-	-	-
A 330		THR	0.78	-	Favored (55.08%) General / -90.7,-6.0	Favored (51.4%) *p* chi angles: 65.5	0.03Å	Favored (19.261%)	-	-	-
A 331		GLY	0.8	-	Favored (48.2%) Glycine / -86.8,177.7	-	-	Favored (24.641%)	-	-	-
A 332		THR	0.83	-	Favored (9.6%) General / -123.4,20.4	Favored (59.1%) *p* chi angles: 57.3	0.05Å	Favored (7.969%)	-	-	-
A 333		ASP	0.86	-	Favored (17.02%) General / -93.9,155.7	Favored (72.6%) *m-30* chi angles: 298.1,315.9	0.12Å	CA Geom Outlier (0.413%)	-	-	-

A 334		GLY	0.88	-	Favored (21.37%) Glycine / 143.3,-178.6	-	-	Favored (8.947%)	-	-	-
A 335		PRO	0.88	-	Favored (38.36%) Cis-Pro / -58.5,151.6	Favored (50.5%) *Cg_exo* chi angles: 337.8,34.3,328.2	0.02Å	Favored (6.983%)	-	-	Cis PRO omega= -2.79
A 336		CYS	0.88	-	Favored (27.37%) General / -157.5,153.7	Favored (19.4%) *p* chi angles: 57.9	0.07Å	Favored (24.172%)	-	-	-
A 337		LYS	0.87	-	Favored (54.63%) General / -66.6,135.8	Favored (83.2%) *tttt* chi angles: 178.7,179.4,178.3,185.5	0.04Å	Favored (43.787%) beta sheet	-	-	-
A 338		VAL	0.85	-	Favored (66.84%) Pre-Pro / -94.3,116.8	Favored (69.3%) *t* chi angles: 178.8	0.04Å	Favored (52.77%) beta sheet	-	-	-
A 339		PRO	0.82	-	Favored (5.39%) Trans-Pro / -78.5,74.5	Favored (64.5%) *Cg_endo* chi angles: 31.4,324.9,24.2	0.01Å	Favored (7.337%) beta sheet	-	-	-
A 340		ILE	0.79	0.46Å O with A 340 ILE HG23	Favored (38.23%) Ile or Val / -108.9,138.0	Favored (17.7%) *tt* chi angles: 184.1,164	0.04Å	Favored (6.977%) beta sheet	-	-	-
**#**	**Alt**	**Res**	**High B**	**Clash > 0.4Å**	**Ramachandran**	**Rotamer**	**Cβ deviation**	**CaBLAM**	**Bond lengths**	**Bond angles**	**Cis Peptides**
			Avg: 1.11	Clashscore: 1.47	Outliers: 1 of 499	Poor rotamers: 0 of 403	Outliers: 0 of 447	Outliers: 8 of 497	Outliers: 6 of 501	Outliers: 4 of 501	Non-Trans: 1 of 500
A 341		SER	0.76	-	Favored (37.85%) General / -156.3,164.8	Favored (88.9%) *p* chi angles: 66.9	0.02Å	Favored (33.657%) beta sheet	-	-	-
A 342		SER	0.74	-	Favored (43.93%) General / -114.0,121.5	Favored (27.9%) *t* chi angles: 173.2	0.04Å	Favored (35.469%) beta sheet	-	-	-
A 343		VAL	0.73	-	Favored (22.63%) Ile or Val / -120.0,151.6	Favored (18.7%) *m* chi angles: 302.9	0.13Å	Favored (44.5%)	-	-	-
A 344		ALA	0.73	-	Favored (80.83%) General / -63.4,-35.6	-	0.04Å	Favored (23.359%)	-	-	-
A 345		SER	0.74	-	Favored (20.29%) General / -154.1,142.1	Favored (42%) *t* chi angles: 175.9	0.06Å	Favored (19.13%)	-	-	-
A 346		LEU	0.75	-	Favored (66.21%) General / -66.4,-21.3	Favored (84.2%) *mt* chi angles: 295,178.7	0.03Å	Favored (41.064%) alpha helix	-	-	-
A 347		ASN	0.76	-	Favored (64.79%) General / -70.1,-27.6	Favored (95.7%) *m-40* chi angles: 287.2,335.3	0.03Å	Favored (46.835%) alpha helix	-	-	-
A 348		ASP	0.77	-	Favored (7.14%) General / -104.8,-37.2	Favored (66%) *m-30* chi angles: 298.6,307.5	0.08Å	CaBLAM Disfavored (2.591%)	-	-	-
A 349		LEU	0.77	-	Favored (16.53%) General / 58.0,28.6	Favored (96.3%) *mt* chi angles: 298,177.5	0.08Å	Favored (7.094%)	-	-	-

A 350		THR	0.76	-	Favored (71.87%) Pre-Pro / -86.6,122.0	Favored (93.4%) *m* chi angles: 299.2	0.04Å	Favored (34.171%) beta sheet	-	-	-
A 351		PRO	0.77	-	Favored (58.8%) Trans-Pro / -67.5,142.3	Favored (47.7%) *Cg_endo* chi angles: 24.9,326.9,27.3	0.03Å	Favored (29.579%) beta sheet	-	-	-
A 352		VAL	0.78	-	Allowed (1.9%) Ile or Val / -127.1,-22.0	Favored (32.5%) *m* chi angles: 297.5	0.06Å	Favored (5.784%)	-	-	-
A 353		GLY	0.8	-	Favored (36.07%) Glycine / -94.8,-166.8	-	-	Favored (17.653%)	-	-	-
A 354		ARG	0.84	-	Favored (50.06%) General / -137.5,153.4	Favored (93.2%) *mmt-90* chi angles: 297.5,291,184.3,273.5	0.05Å	Favored (30.088%)	-	-	-
A 355		LEU	0.88	-	Favored (35.79%) General / -97.1,119.5	Favored (77.8%) *mt* chi angles: 301.6,179.8	0.05Å	Favored (52.328%) beta sheet	-	-	-
A 356		VAL	0.91	-	Favored (83.96%) Ile or Val / -64.0,-39.0	Favored (69.6%) *t* chi angles: 172.1	0.01Å	Favored (39.232%)	-	-	-
A 357		THR	0.94	-	Favored (25.69%) General / -76.3,-1.2	Favored (72.6%) *p* chi angles: 61.8	0.02Å	CaBLAM Disfavored (2.568%)	-	-	-
A 358		VAL	0.95	-	Allowed (0.16%) Ile or Val / 67.3,-68.5	Favored (79.7%) *t* chi angles: 173.1	0.09Å	CaBLAM Disfavored (3.82%)	-	-	-
A 359		ASN	0.95	-	Favored (7.66%) Pre-Pro / -146.5,84.6	Favored (48%) *t0* chi angles: 184,326.1	0.04Å	Favored (11.964%)	-	-	-
A 360		PRO	0.95	-	Favored (58.91%) Trans-Pro / -71.8,153.4	Favored (73.7%) *Cg_endo* chi angles: 27.7,327.2,23.8	0.02Å	Favored (24.251%)	-	-	-
**#**	**Alt**	**Res**	**High B**	**Clash > 0.4Å**	**Ramachandran**	**Rotamer**	**Cβ deviation**	**CaBLAM**	**Bond lengths**	**Bond angles**	**Cis Peptides**
			Avg: 1.11	Clashscore: 1.47	Outliers: 1 of 499	Poor rotamers: 0 of 403	Outliers: 0 of 447	Outliers: 8 of 497	Outliers: 6 of 501	Outliers: 4 of 501	Non-Trans: 1 of 500
A 361		PHE	0.95	-	Favored (42.24%) General / -153.3,161.5	Favored (55.3%) *p90* chi angles: 61.6,93.6	0.05Å	Favored (45.496%) beta sheet	-	-	-
A 362		VAL	0.94	-	Favored (39.03%) Ile or Val / -84.8,123.3	Favored (84%) *t* chi angles: 177	0.13Å	Favored (39.465%)	-	-	-
A 363		SER	0.92	-	Favored (24.79%) General / -89.5,-19.7	Favored (96.5%) *p* chi angles: 63.4	0.05Å	Favored (43.34%)	-	-	-
A 364		VAL	0.91	-	Favored (70.78%) Ile or Val / -127.1,131.5	Favored (66%) *t* chi angles: 179.2	0.01Å	Favored (21.488%)	-	-	-
A 365		ALA	0.89	-	Favored (2.4%) General / -75.4,71.9	-	0.03Å	Favored (9.074%)	-	-	-
A 366		THR	0.88	-	Favored (37.57%)	Favored (4.6%) *t* chi angles: 180	0.05Å	Favored (20.557%)	-	-	-

					General / -144.4,150.4						
A 367		ALA	0.86	-	Favored (52.4%) General / -71.6,-8.2	-	0.03Å	Favored (8.748%)	-	-	-
A 368		ASN	0.84	-	Favored (22.03%) General / -110.6,15.5	Favored (72%) *m-40* chi angles: 292.9,284.8	0.06Å	Favored (45.788%)	-	-	-
A 369		SER	0.83	-	Favored (39.5%) General / -76.5,137.3	Favored (40.1%) *t* chi angles: 176.7	0.05Å	Favored (29.255%)	-	-	-
A 370		LYS	0.83	-	Favored (35.86%) General / -110.7,146.6	Favored (99.1%) *mttt* chi angles: 294,181.4,178.7,177.6	0.04Å	Favored (62.563%) beta sheet	-	-	-
A 371		VAL	0.82	-	Favored (55.3%) Ile or Val / -128.8,121.7	Favored (54.6%) *t* chi angles: 180.7	0.06Å	Favored (58.863%) beta sheet	-	-	-
A 372		LEU	0.81	0.48Å C with A 372 LEU HD23	Favored (32.98%) General / -88.3,123.5	Favored (6.6%) *tt* chi angles: 186.3,155.2	0.06Å	Favored (61.317%) beta sheet	-	-	-
A 373		ILE	0.8	-	Favored (74.72%) Ile or Val / -123.2,127.8	Favored (67.4%) *mt* chi angles: 303.1,171	0.04Å	Favored (68.526%) beta sheet	-	-	-
A 374		GLU	0.8	-	Favored (48.98%) General / -105.4,124.1	Favored (76.1%) *tt0* chi angles: 183.6,182,15	0.07Å	Favored (65.597%) beta sheet	-	-	-
A 375		LEU	0.8	-	Favored (53.44%) General / -124.6,139.9	Favored (6.3%) *tt* chi angles: 177.6,149.8	0.11Å	Favored (56.346%) beta sheet	-	-	-
A 376		GLU	0.8	0.48Å OE2 with A 406 LYS NZ	Favored (60.11%) Pre-Pro / -105.0,108.0	Favored (90.2%) *tt0* chi angles: 183.7,177.4,5.5	0.02Å	Favored (61.276%)	-	-	-
A 377		PRO	0.8	-	Favored (22.72%) Trans-Pro / -79.7,165.3	Favored (47.6%) *Cg_endo* chi angles: 33.3,325.5,21.7	0.06Å	Favored (34.773%)	-	-	-
A 378		PRO	0.79	-	Favored (30.04%) Trans-Pro / -75.4,165.8	Favored (79.6%) *Cg_endo* chi angles: 30.3,323.3,28.1	0.07Å	Favored (41.403%)	-	-	-
A 379		PHE	0.77	-	Favored (39.05%) General / -76.0,147.1	Favored (59.6%) *m-80* chi angles: 286,103	0.05Å	Favored (13.801%)	-	-	-
A 380		GLY	0.74	-	Favored (32.04%) Glycine / 87.9,-161.6	-	-	Favored (40.67%)	-	-	-
**#**	**Alt**	**Res**	**High B**	**Clash > 0.4Å**	**Ramachandran**	**Rotamer**	**Cβ deviation**	**CaBLAM**	**Bond lengths**	**Bond angles**	**Cis Peptides**
			Avg: 1.11	Clashscore: 1.47	Outliers: 1 of 499	Poor rotamers: 0 of 403	Outliers: 0 of 447	Outliers: 8 of 497	Outliers: 6 of 501	Outliers: 4 of 501	Non-Trans: 1 of 500
A 381		ASP	0.71	-	Favored (53.49%) General / -69.0,145.6	Favored (85.2%) *m-30* chi angles: 290.1,353.2	0.06Å	CaBLAM Disfavored (1.074%)	-	-	-
A 382		SER	0.68	-	Favored (25.4%)	Favored (86.9%) *p* chi angles: 68	0.03Å	Favored (27.258%)	-	-	-

					General / -161.3,167.7						
A 383		TYR	0.67	-	Favored (49.31%) General / -121.0,142.9	Favored (91.7%) *m-80* chi angles: 296.9,85.7	0.06Å	Favored (44.138%) beta sheet	-	-	-
A 384		ILE	0.67	-	Favored (56.75%) Ile or Val / -104.4,121.9	Favored (75.8%) *mt* chi angles: 300.9,169.6	0.11Å	Favored (66.682%) beta sheet	-	-	-
A 385		VAL	0.7	-	Favored (66.79%) Ile or Val / -109.8,124.7	Favored (60.7%) *t* chi angles: 179.9	0.16Å	Favored (63.833%)	-	-	-
A 386		VAL	0.74	-	Favored (68.13%) Ile or Val / -126.5,125.9	Favored (72.9%) *t* chi angles: 178.5	0.02Å	Favored (14.368%)	-	-	-
A 387		GLY	0.8	-	Favored (40.94%) Glycine / 92.6,178.7	-	-	Favored (12.897%)	-	-	-
A 388		ARG	0.86	-	Favored (41.59%) General / -151.2,162.1	Favored (47.7%) *ptt180* chi angles: 64.7,185,182.2,185.2	0.06Å	CaBLAM Disfavored (4.743%)	-	-	-
A 389		GLY	0.89	-	Favored (49.6%) Glycine / 73.5,-155.0	-	-	Favored (56.025%)	-	-	-
A 390		GLU	0.9	-	Favored (67.9%) General / -62.4,-25.3	Favored (99.7%) *mt-10* chi angles: 291.7,179,353.6	0.01Å	Favored (9.664%)	-	-	-
A 391		GLN	0.89	-	Favored (55.93%) General / -90.7,2.5	Favored (68.1%) *mt0* chi angles: 293.8,181,71.7	0.02Å	Favored (34.099%)	-	-	-
A 392		GLN	0.85	-	Favored (58.12%) General / -59.9,136.8	Favored (35.3%) *tt0* chi angles: 185.9,177.2,81	0.03Å	Favored (33.498%)	-	-	-
A 393		ILE	0.8	0.43Å N with A 393 ILE HD12	Favored (61.4%) Ile or Val / -105.9,125.1	Favored (3.3%) *mp* chi angles: 299.7,92	0.07Å	Favored (67.12%) beta sheet	-	-	-
A 394		ASN	0.76	-	Favored (25.44%) General / -124.8,118.0	Favored (70.7%) *m-40* chi angles: 296.5,296.9	0.05Å	Favored (66.767%) beta sheet	-	-	-
A 395		HIS	0.74	-	Favored (54.04%) General / -116.6,127.0	Favored (84.8%) *t70* chi angles: 182.1,77.8	0.05Å	Favored (57.23%) beta sheet	-	-	-
A 396		HIS	0.75	-	Favored (47.52%) General / -71.9,137.7	Favored (66.8%) *t-90* chi angles: 185,281.6	0.01Å	Favored (44.392%) beta sheet	-	-	-
A 397		TRP	0.79	-	Favored (47.73%) General / -132.1,155.7	Favored (73.1%) *t-100* chi angles: 182.8,251.7	0.02Å	Favored (59.339%) beta sheet	-	-	-
A 398		HIS	0.86	-	Favored (15.88%) General / -146.9,129.4	Favored (70.8%) *t70* chi angles: 181.2,84.2	0.09Å	Favored (57.93%) beta sheet	-	-	-
A 399		LYS	0.96	-	Favored (32.99%)	Favored (43.2%) *tptt*	0.08Å	Favored (72.008%) beta sheet	-	-	-

#	Alt	Res	High B	Clash > 0.4Å	Ramachandran	Rotamer	Cβ deviation	CaBLAM	Bond lengths	Bond angles	Cis Peptides
					General / -110.9,117.3	chi angles: 181.5,67,188.3,180.4					
A 400		SER	1.09	-	Favored (54.11%) General / -77.8,-6.4	Favored (93.6%) *p* chi angles: 66.3	0.02Å	Favored (19.071%)	-	-	-
**#**	**Alt**	**Res**	**High B**	**Clash > 0.4Å**	**Ramachandran**	**Rotamer**	**Cβ deviation**	**CaBLAM**	**Bond lengths**	**Bond angles**	**Cis Peptides**
			Avg: 1.11	Clashscore: 1.47	Outliers: 1 of 499	Poor rotamers: 0 of 403	Outliers: 0 of 447	Outliers: 8 of 497	Outliers: 6 of 501	Outliers: 4 of 501	Non-Trans: 1 of 500
A 401		GLY	1.23	-	Favored (31.54%) Glycine / -88.2,-157.0	-	-	Favored (11.299%)	-	-	-
A 402		SER	1.38	-	Favored (43.46%) General / -151.8,158.6	Favored (98.8%) *p* chi angles: 65.3	0.05Å	Favored (18.293%)	-	-	-
A 403		SER	1.53	-	Favored (66.9%) General / -65.2,-22.3	Favored (88.9%) *p* chi angles: 66.9	0.04Å	Favored (59.388%)	-	-	-
A 404		ILE	1.69	-	Favored (38.16%) Ile or Val / -74.0,-48.5	Favored (95%) *mt* chi angles: 294.9,168.7	0.02Å	Favored (67.326%) alpha helix	-	-	-
A 405		GLY	1.85	-	Favored (95.25%) Glycine / -61.5,-38.1	-	-	Favored (94.195%) alpha helix	-	-	-
A 406		LYS	2.03	0.48Å NZ with A 376 GLU OE2	Favored (77.78%) General / -63.5,-48.4	Favored (88.1%) *tttt* chi angles: 182.8,175.3,180.3,177.8	0.03Å	Favored (89.527%) alpha helix	-	-	-
A 407		ALA	2.21	-	Favored (94.23%) General / -61.1,-40.5	-	0.05Å	Favored (89.657%) alpha helix	-	-	-
A 408		PHE	2.38	-	Favored (73.06%) General / -62.6,-50.0	Favored (88.3%) *t80* chi angles: 177.5,74.4	0.04Å	Favored (83.548%) alpha helix	-	-	-
A 409		THR	2.5	-	Favored (94.44%) General / -59.9,-44.3	Favored (96.1%) *m* chi angles: 299.7	0.05Å	Favored (92.315%) alpha helix	-	-	-
A 410		THR	2.55	-	Favored (97.38%) General / -61.0,-44.5	Favored (96.2%) *m* chi angles: 299.7	0.04Å	Favored (96.316%) alpha helix	-	-	-
A 411		THR	2.56	-	Favored (92.61%) General / -59.6,-45.2	Favored (90.4%) *m* chi angles: 298.8	0.03Å	Favored (97.107%) alpha helix	-	-	-
A 412		LEU	2.52	-	Favored (92.32%) General / -64.3,-38.8	Favored (88.7%) *mt* chi angles: 291.6,174.3	0.01Å	Favored (93.954%) alpha helix	-	-	-
A 413		ARG	2.46	-	Favored (87.45%) General / -64.1,-37.4	Favored (67.4%) *mtp180* chi angles: 287,168.5,61.8,204.1	0.03Å	Favored (93.913%) alpha helix	-	-	-
A 414		GLY	2.38	-	Favored (61.56%) Glycine / -59.3,-51.0	-	-	Favored (92.421%) alpha helix	-	-	-
A 415		ALA	2.29	-	Favored (79.94%)	-	0.02Å	Favored (84.388%)	-	-	-

#	Alt	Res	High B	Clash > 0.4Å	Ramachandran	Rotamer	Cβ deviation	CaBLAM	Bond lengths	Bond angles	Cis Peptides
					General / -58.8,-39.0			alpha helix			
A 416		GLN	2.21	-	Favored (83.05%) General / -61.5,-48.0	Favored (61.1%) *tt0* chi angles: 179.7,182.8,49.1	0.04Å	Favored (89.243%) alpha helix	-	-	-
A 417		ARG	2.13	-	Favored (85.37%) General / -58.3,-46.8	Favored (24.6%) *ttm170* chi angles: 189.7,189.3,292.5,143.6	0.01Å	Favored (93.275%) alpha helix	-	-	-
A 418		LEU	2.04	-	Favored (97.52%) General / -62.7,-43.9	Favored (70.1%) *mt* chi angles: 286.6,167.7	0.03Å	Favored (92.297%) alpha helix	-	-	-
A 419		ALA	1.95	-	Favored (89.25%) General / -61.2,-39.1	-	0.06Å	Favored (70.408%) alpha helix	-	-	-
A 420		ALA	1.86	-	Favored (23.22%) General / -84.4,-35.4	-	0.05Å	Favored (56.359%) alpha helix	-	-	-
**#**	**Alt**	**Res**	**High B**	**Clash > 0.4Å**	**Ramachandran**	**Rotamer**	**Cβ deviation**	**CaBLAM**	**Bond lengths**	**Bond angles**	**Cis Peptides**
			Avg: 1.11	Clashscore: 1.47	Outliers: 1 of 499	Poor rotamers: 0 of 403	Outliers: 0 of 447	Outliers: 8 of 497	Outliers: 6 of 501	Outliers: 4 of 501	Non-Trans: 1 of 500
A 421		LEU	1.77	-	Favored (4.83%) General / -103.6,-45.4	Favored (73.7%) *tp* chi angles: 178.2,62.5	0.07Å	CaBLAM Disfavored (3.655%)	OUTLIER(S) worst is CB--CG: 4.1 σ	-	-
A 422		GLY	1.69	-	Favored (19.83%) Glycine / 99.5,-159.2	-	-	Favored (25.026%)	-	-	-
A 423		ASP	1.63	-	Favored (13.18%) General / -46.1,-42.1	Favored (44%) *p0* chi angles: 58.1,355.9	0.03Å	Favored (14.613%)	-	-	-
A 424		THR	1.59	-	Favored (3.64%) General / -50.7,-24.7	Favored (91.4%) *m* chi angles: 301.1	0.08Å	Favored (43.524%) three-ten	-	-	-
A 425		ALA	1.6	-	Favored (64.23%) General / -55.8,-33.2	-	0.12Å	Favored (58.948%) three-ten	-	-	-
A 426		TRP	1.68	-	Favored (56.16%) General / -73.3,-8.5	Favored (87.6%) *m100* chi angles: 284.2,96.3	0.08Å	Favored (54.975%)	-	-	-
A 427		ASP	1.83	-	Favored (59.75%) General / -80.5,-10.1	Favored (83.5%) *m-30* chi angles: 288.7,334.6	0.03Å	Favored (23.495%)	-	-	-
A 428		PHE	2.04	-	Favored (14.74%) General / -66.0,121.3	Favored (82.3%) *t80* chi angles: 181.1,74	0.12Å	Favored (8.61%)	-	-	-
A 429		GLY	2.31	-	Favored (90.36%) Glycine / 80.8,4.0	-	-	Favored (51.913%)	-	-	-
A 430		SER	2.59	-	Favored (58.38%) General / -64.2,142.5	Favored (39.5%) *t* chi angles: 176.9	0.06Å	Favored (9.829%)	-	-	-
A 431		VAL	2.84	-	Favored (3.57%)	Favored (29.6%) *m* chi angles: 296.4	0.07Å	CaBLAM Disfavored (3.2%)	-	-	-

					Ile or Val / -127.0,16.0						
A 432		GLY	2.98	-	Favored (63.91%) Glycine / 86.7,15.2	-	-	Favored (56.126%)	-	-	-
A 433		GLY	2.95	-	Favored (51.79%) Glycine / -65.5,150.7	-	-	Favored (29.954%)	-	-	-
A 434		VAL	2.74	-	Favored (96.83%) Ile or Val / -64.5,-43.7	Favored (70.9%) *t* chi angles: 172.2	0.01Å	Favored (57.922%)	-	-	-
A 435		PHE	2.44	-	Favored (71.37%) General / -54.2,-48.1	Favored (90.7%) *t80* chi angles: 175.4,78.1	0.03Å	Favored (78.618%) alpha helix	-	-	-
A 436		THR	2.1	-	Favored (92.51%) General / -59.9,-45.4	Favored (89.6%) *m* chi angles: 298.2	0.04Å	Favored (96.569%) alpha helix	-	-	-
A 437		SER	1.8	-	Favored (91.35%) General / -60.7,-40.2	Favored (64.8%) *m* chi angles: 294.2	0.02Å	Favored (85.025%) alpha helix	-	-	-
A 438		VAL	1.57	-	Favored (92.31%) Ile or Val / -66.0,-44.2	Favored (69.7%) *t* chi angles: 172.1	0.01Å	Favored (84.613%) alpha helix	-	-	-
A 439		GLY	1.41	-	Favored (91.51%) Glycine / -57.5,-40.0	-	-	Favored (96.83%) alpha helix	-	-	-
A 440		LYS	1.33	-	Favored (91.54%) General / -64.3,-44.4	Favored (52.9%) *mtpt* chi angles: 286.6,173.4,70.2,175.7	0.05Å	Favored (92.103%) alpha helix	-	-	-
**#**	**Alt**	**Res**	**High B**	**Clash > 0.4Å**	**Ramachandran**	**Rotamer**	**Cβ deviation**	**CaBLAM**	**Bond lengths**	**Bond angles**	**Cis Peptides**
			Avg: 1.11	Clashscore: 1.47	Outliers: 1 of 499	Poor rotamers: 0 of 403	Outliers: 0 of 447	Outliers: 8 of 497	Outliers: 6 of 501	Outliers: 4 of 501	Non-Trans: 1 of 500
A 441		ALA	1.31	-	Favored (91.25%) General / -60.5,-40.4	-	0.04Å	Favored (86.653%) alpha helix	-	-	-
A 442		ILE	1.34	-	Favored (87.13%) Ile or Val / -65.0,-47.7	Favored (96.9%) *mt* chi angles: 293.5,167.2	0.03Å	Favored (85.595%) alpha helix	-	-	-
A 443		HIS	1.42	-	Favored (93.84%) General / -59.8,-44.5	Favored (74.2%) *t70* chi angles: 186.4,77.6	0.07Å	Favored (83.125%) alpha helix	-	-	-
A 444		GLN	1.51	-	Favored (79.86%) General / -59.5,-38.0	Favored (96.7%) *mt0* chi angles: 288,171.6,332.5	0.03Å	Favored (75.723%) alpha helix	-	-	-
A 445		VAL	1.58	-	Favored (97.08%) Ile or Val / -62.2,-46.2	Favored (57.6%) *t* chi angles: 170.4	0.04Å	Favored (73.72%) alpha helix	-	-	-
A 446		PHE	1.64	-	Favored (63.75%) General / -74.0,-33.4	Favored (54%) *m-80* chi angles: 288.3,112.5	0.03Å	Favored (68.659%) alpha helix	-	-	-
A 447		GLY	1.68	-	Favored (52.33%)	-	-	Favored (91.196%)	-	-	-

					Glycine / -57.9,-52.4			alpha helix			
A 448		GLY	1.68	-	Favored (59.63%) Glycine / -56.4,-50.9	-	-	Favored (95.446%) alpha helix	-	-	-
A 449		ALA	1.66	-	Favored (89.1%) General / -59.3,-41.5	-	0.05Å	Favored (78.851%) alpha helix	-	-	-
A 450		PHE	1.62	-	Favored (62.81%) General / -74.3,-39.7	Favored (37.5%) *m-80* chi angles: 279.5,90.3	0.08Å	Favored (80.557%) alpha helix	-	-	-
A 451		ARG	1.59	-	Favored (82.12%) General / -67.8,-37.6	Favored (97.7%) *mtt180* chi angles: 288.9,178.6,177,180.1	0.03Å	Favored (86.41%) alpha helix	-	-	-
A 452		SER	1.58	-	Favored (89.71%) General / -61.5,-39.1	Favored (72.2%) *m* chi angles: 295.3	0.05Å	Favored (65.061%) alpha helix	-	-	-
A 453		LEU	1.59	-	Favored (15.07%) General / -89.4,-37.1	Favored (94.2%) *mt* chi angles: 296.6,173.6	0.07Å	Favored (43.167%) alpha helix	-	-	-
A 454		PHE	1.62	-	Favored (10.06%) General / -120.1,-4.3	Favored (56.8%) *m-80* chi angles: 306.7,101.5	0.07Å	Favored (16.87%) alpha helix	-	-	-
A 455		GLY	1.63	-	Favored (60.85%) Glycine / -57.1,-30.6	-	-	Favored (51.847%) alpha helix	-	-	-
A 456		GLY	1.63	-	Favored (82.95%) Glycine / -89.1,1.6	-	-	Favored (65.204%)	-	-	-
A 457		MET	1.6	-	Favored (23.63%) General / -90.5,147.1	Favored (69.6%) *mtt* chi angles: 295.3,179.6,184	0.05Å	Favored (28.845%)	-	-	-
A 458		SER	1.53	-	Favored (32.41%) General / -65.6,156.5	Favored (86.4%) *p* chi angles: 67.9	0.06Å	Favored (49.638%)	-	-	-
A 459		TRP	1.43	-	Favored (67.11%) General / -58.3,-31.0	Favored (70.4%) *p-90* chi angles: 68.8,267.3	0.03Å	Favored (65.426%)	-	-	-
A 460		ILE	1.29	-	Favored (87.48%) Ile or Val / -66.4,-46.1	Favored (97%) *mt* chi angles: 293.7,168.2	0.04Å	Favored (81.946%) alpha helix	-	-	-
**#**	**Alt**	**Res**	**High B**	**Clash > 0.4Å**	**Ramachandran**	**Rotamer**	**Cβ deviation**	**CaBLAM**	**Bond lengths**	**Bond angles**	**Cis Peptides**
			Avg: 1.11	Clashscore: 1.47	Outliers: 1 of 499	Poor rotamers: 0 of 403	Outliers: 0 of 447	Outliers: 8 of 497	Outliers: 6 of 501	Outliers: 4 of 501	Non-Trans: 1 of 500
A 461		THR	1.16	-	Favored (98.08%) General / -61.6,-43.9	Favored (95.5%) *m* chi angles: 299.5	0.05Å	Favored (97.299%) alpha helix	-	-	-
A 462		GLN	1.04	-	Favored (91.73%) General / -61.5,-39.7	Favored (83.7%) *mt0* chi angles: 291.4,175.7,37	0.07Å	Favored (95.763%) alpha helix	-	-	-
A 463		GLY	0.93	-	Favored (78.33%)	-	-	Favored (99.414%)	-	-	-

					Glycine / -59.9,-49.5			alpha helix			
A 464		LEU	0.83	-	Favored (97.02%) General / -62.8,-40.4	Favored (95.2%) *mt* chi angles: 292,171.4	0.05Å	Favored (95.564%) alpha helix	-	-	-
A 465		LEU	0.76	-	Favored (87.25%) General / -65.3,-37.5	Favored (95.7%) *mt* chi angles: 291.8,172.2	0.03Å	Favored (92.092%) alpha helix	-	-	-
A 466		GLY	0.71	-	Favored (46.37%) Glycine / -58.1,-53.3	-	-	Favored (92.816%) alpha helix	-	-	-
A 467		ALA	0.67	-	Favored (83.16%) General / -59.0,-39.9	-	0.04Å	Favored (84.011%) alpha helix	-	-	-
A 468		LEU	0.63	-	Favored (98.73%) General / -63.4,-42.1	Favored (87.4%) *mt* chi angles: 290.5,171.1	0.02Å	Favored (88.792%) alpha helix	-	-	-
A 469		LEU	0.61	-	Favored (87.05%) General / -66.9,-38.8	Favored (74.2%) *mt* chi angles: 287.4,166.9	0.05Å	Favored (90.814%) alpha helix	-	-	-
A 470		LEU	0.59	-	Favored (94.12%) General / -64.1,-39.5	Favored (87.2%) *mt* chi angles: 290.6,170.4	0.03Å	Favored (91.813%) alpha helix	-	-	-
A 471		TRP	0.57	-	Favored (69.5%) General / -70.2,-44.1	Favored (51.1%) *m-10* chi angles: 293.6,335.8	0.04Å	Favored (82.175%) alpha helix	-	-	-
A 472		MET	0.59	-	Favored (83.72%) General / -66.7,-36.9	Favored (79.3%) *mtm* chi angles: 289.3,188.2,293.1	0.04Å	Favored (90.076%) alpha helix	-	-	-
A 473		GLY	0.69	-	Favored (29.39%) Glycine / -61.7,-55.3	-	-	Favored (91.677%) alpha helix	-	-	-
A 474		ILE	0.94	-	Favored (46.56%) Ile or Val / -62.6,-29.3	Favored (9.7%) *tp* chi angles: 196.7,65.3	0.07Å	Favored (65.235%) alpha helix	-	-	-
A 475		ASN	1.52	-	Favored (45.53%) General / -96.9,7.9	Favored (87.6%) *m-40* chi angles: 291.6,321.2	0.02Å	Favored (47.577%)	-	-	-
A 476		ALA	2.64	-	Favored (39%) General / -76.6,145.4	-	0.02Å	Favored (35.275%)	-	-	-
A 477		ARG	4.27	-	Favored (53.2%) General / -76.8,-38.8	Favored (98.6%) *mtt180* chi angles: 291,177.8,180.4,174.3	0.03Å	Favored (14.571%)	-	-	-
A 478		ASP	5.66	-	Favored (34.6%) General / -80.4,129.6	Favored (26.8%) *t70* chi angles: 183.7,81.9	0.06Å	Favored (22.585%)	-	-	-
A 479		ARG	5.78	-	Favored (73.35%) General / -64.7,-31.9	Favored (98.2%) *mtt180* chi angles: 289.7,178.2,179.7,173.6	0.02Å	Favored (51.756%)	-	-	-
A 480		SER	4.55	-	Favored (67.85%) General / -71.9,-41.6	Favored (67.1%) *m* chi angles: 294.5	0.02Å	Favored (76.423%) alpha helix	-	-	-

#	Alt	Res	High B	Clash > 0.4Å	Ramachandran	Rotamer	Cβ deviation	CaBLAM	Bond lengths	Bond angles	Cis Peptides
			Avg: 1.11	Clashscore: 1.47	Outliers: 1 of 499	Poor rotamers: 0 of 403	Outliers: 0 of 447	Outliers: 8 of 497	Outliers: 6 of 501	Outliers: 4 of 501	Non-Trans: 1 of 500
A 481		ILE	2.95	-	Favored (88.76%) Ile or Val / -67.0,-44.1	Favored (96.9%) *mt* chi angles: 292.7,169.5	0.04Å	Favored (79.686%) alpha helix	-	-	-
A 482		ALA	1.79	-	Favored (86.08%) General / -58.0,-45.8	-	0.07Å	Favored (86.76%) alpha helix	-	-	-
A 483		MET	1.17	-	Favored (81.6%) General / -67.6,-36.8	Favored (82.9%) *mtm* chi angles: 289.2,186.9,285.3	0.04Å	Favored (83.671%) alpha helix	-	-	-
A 484		THR	0.89	-	Favored (68.45%) General / -64.7,-50.1	Favored (90.1%) *m* chi angles: 298.8	0.04Å	Favored (80.321%) alpha helix	-	-	-
A 485		PHE	0.79	-	Favored (95.69%) General / -62.6,-40.0	Favored (3.4%) *m-10* chi angles: 279,345.1	0.04Å	Favored (81.631%) alpha helix	-	-	-
A 486		LEU	0.79	-	Favored (94.38%) General / -65.3,-41.1	Favored (83.3%) *mt* chi angles: 289.7,169.8	0.02Å	Favored (98.173%) alpha helix	-	-	-
A 487		ALA	0.83	-	Favored (96.83%) General / -64.1,-42.8	-	0.04Å	Favored (82.904%) alpha helix	-	-	-
A 488		VAL	0.86	-	Favored (78.21%) Ile or Val / -70.2,-44.1	Favored (91.9%) *t* chi angles: 174.4	0.01Å	Favored (81.184%) alpha helix	-	-	-
A 489		GLY	0.9	-	Favored (93.08%) Glycine / -59.8,-37.8	-	-	Favored (94.932%) alpha helix	-	-	-
A 490		GLY	0.94	-	Favored (52.56%) Glycine / -59.5,-52.5	-	-	Favored (93.148%) alpha helix	-	-	-
A 491		VAL	0.98	-	Favored (96.91%) Ile or Val / -61.4,-43.5	Favored (64.6%) *t* chi angles: 171.4	0.02Å	Favored (89.51%) alpha helix	-	-	-
A 492		LEU	1.02	-	Favored (85.04%) General / -62.5,-37.2	Favored (71.6%) *mt* chi angles: 289.8,175.5	0.04Å	Favored (88.66%) alpha helix	-	-	-
A 493		LEU	1.06	-	Favored (91.99%) General / -63.4,-45.3	Favored (64.8%) *tp* chi angles: 179.5,59.8	0.04Å	Favored (92.564%) alpha helix	-	-	-
A 494		PHE	1.13	-	Favored (93.38%) General / -61.5,-40.1	Favored (23.7%) *m-80* chi angles: 276.9,106.3	0.05Å	Favored (87.702%) alpha helix	-	-	-
A 495		LEU	1.23	-	Favored (89.38%) General / -66.4,-40.2	Favored (93.3%) *mt* chi angles: 291.7,171.2	0.03Å	Favored (85.75%) alpha helix	-	-	-
A 496		SER	1.38	-	Favored (93.92%)	Favored (46.6%) *t* chi angles: 180	0.07Å	Favored (74.029%) alpha helix	-	-	-

#	Alt	Res	High B	Clash > 0.4Å	Ramachandran	Rotamer	Cβ deviation	CaBLAM	Bond lengths	Bond angles	Cis Peptides
					General / -59.9,-45.0						
A 497		VAL	1.61	-	Favored (36.27%) Ile or Val / -75.0,-47.5	Favored (88.7%) *t* chi angles: 174	0.03Å	Favored (58.384%) alpha helix	-	-	-
A 498		ASN	1.93	-	Favored (44.04%) General / -95.5,-4.8	Favored (86.9%) *m-40* chi angles: 293.1,318.6	0.06Å	Favored (26.782%) alpha helix	-	-	-
A 499		VAL	2.32	-	Favored (31.53%) Ile or Val / -59.9,-25.1	Favored (7.2%) *p* chi angles: 68.6	0.04Å	Favored (43.54%)	-	-	-
A 500		HIS	2.79	-	Favored (62.79%) General / -69.9,-14.6	Favored (39.1%) *p-80* chi angles: 76.5,280.9	0.12Å	-	-	-	-
**#**	**Alt**	**Res**	**High B**	**Clash > 0.4Å**	**Ramachandran**	**Rotamer**	**Cβ deviation**	**CaBLAM**	**Bond lengths**	**Bond angles**	**Cis Peptides**
			Avg: 1.11	Clashscore: 1.47	Outliers: 1 of 499	Poor rotamers: 0 of 403	Outliers: 0 of 447	Outliers: 8 of 497	Outliers: 6 of 501	Outliers: 4 of 501	Non-Trans: 1 of 500
A 501		ALA	3.27	-	-	-	0.04Å	-	-	-	-

About MolProbity | Website for the Richardson Lab | Using ecloud x-H | Internal reference 4.5.2
