# Supplementary material for: Integrative proteome-wide structural analysis and high-throughput docking identify broad-spectrum antiviral scaffolds against Zika, Yellow Fever, West Nile, Saint Louis encephalitis, and Usutu viruses
Source: Front Cell Infect Microbiol. 2026 Apr 30;16:1723132. doi: 10.3389/fcimb.2026.1723132 (PMC13171538; doi:10.3389/fcimb.2026.1723132)
Supplement: Supplementary file 5 [file DataSheet5.zip › WNV/WNV_M/Mol_probity_Files/WNV_M_1FH-multi.table.pdf]

Viewing WNV_M1FH-multi.table

Duke Biochemistry
Duke University School of Medicine

When finished, you should close this window.

Hint: Use File | Save As... to save a copy of this page.

All-Atom Contacts	Clashscore, all atoms:	0		100th percentile* (N=1784, all resolutions)
	Clashscore is the number of serious steric overlaps (> 0.4 Å) per 1000 atoms.			
Protein Geometry	Poor rotamers	0	0.00%	Goal: <0.3%
	Favored rotamers	61	100.00%	Goal: >98%
	Ramachandran outliers	0	0.00%	Goal: <0.05%
	Ramachandran favored	73	100.00%	Goal: >98%
	Rama distribution Z-score	2.08 ± 0.90		Goal: abs(Z score) < 2
	MolProbity score^	0.50		100th percentile* (N=27675, 0Å - 99Å)
	Cβ deviations >0.25Å	0	0.00%	Goal: 0
	Bad bonds:	0 / 590	0.00%	Goal: 0%
	Bad angles:	2 / 805	0.25%	Goal: <0.1%
Peptide Omegas	Cis Prolines:	0 / 2	0.00%	Expected: ≤1 per chain, or ≤5%
Low-resolution Criteria	CaBLAM outliers	0	0.0%	Goal: <1.0%
	CA Geometry outliers	0	0.00%	Goal: <0.5%
Additional validations	Chiral volume outliers	0/98		
	Waters with clashes	0/0	0.00%	See UnDowser table for details

In the two column results, the left column gives the raw count, right column gives the percentage.

* 100th percentile is the best among structures of comparable resolution; 0th percentile is the worst. For clashscore the comparative set of structures was selected in 2004, for MolProbity score in 2006.

^ MolProbity score combines the clashscore, rotamer, and Ramachandran evaluations into a single score, normalized to be on the same scale as X-ray resolution.

Key to table colors and cutoffs here:

#	Alt	Res	High B	Clash > 0.4Å	Ramachandran	Rotamer	Cβ deviation	CaBLAM	Bond lengths	Bond angles	Cis Peptides
			Avg: 2.75	Clashscore: 0	Outliers: 0 of 73	Poor rotamers: 0 of 61	Outliers: 0 of 70	Outliers: 0 of 71	Outliers: 0 of 75	Outliers: 2 of 75	Non-Trans: 0 of 74
A 1		SER	7.27	-	-	Favored (42.3%) *t* chi angles: 175.4	0.03Å	-	-	-	-
A 2		LEU	6.72	-	Favored (27%) General / -84.1,148.1	Favored (91.6%) *mt* chi angles: 299.1,177	0.03Å	-	-	-	-
A 3		THR	6.1	-	Favored (17.61%) General / -128.3,167.0	Favored (56.3%) *p* chi angles: 64.7	0.03Å	Favored (49.855%)	-	-	-
A 4		VAL	5.48	-	Favored (74.72%) Ile or Val / -123.1,129.9	Favored (81.6%) *t* chi angles: 177.9	0.03Å	Favored (55.565%) beta sheet	-	-	-
A 5		GLN	4.93	-	Favored (49.39%) General / -102.8,131.9	Favored (62.8%) *tt0* chi angles: 183.2,177.9,43.2	0.02Å	Favored (42.507%) beta sheet	-	-	-
A 6		THR	4.52	-	Favored (57.89%)	Favored (94.6%) *m* chi angles: 300.9	0.07Å	Favored (49.221%)	-	-	-

					General / -60.6,135.9						
A 7		HIS	4.3	-	Favored (27.51%) General / -54.3,-26.1	Favored (61.1%) *p-80* chi angles: 64.4,282	0.06Å	Favored (37.081%)	-	-	-
A 8		GLY	4.27	-	Favored (68.33%) Glycine / -80.1,-20.0	-	-	Favored (92.743%) alpha helix	-	-	-
A 9		GLU	4.43	-	Favored (20.17%) General / -90.7,-25.2	Favored (95.7%) *mt-10* chi angles: 295,174.6,345.3	0.02Å	Favored (19.282%)	-	-	-
A 10		SER	4.77	-	Favored (44.19%) General / -58.4,143.8	Favored (34.9%) *t* chi angles: 174.3	0.03Å	Favored (17.636%)	-	-	-
A 11		THR	5.25	-	Favored (29.98%) General / -104.3,14.6	Favored (68.7%) *p* chi angles: 59	0.06Å	Favored (8.965%)	-	-	-
A 12		LEU	5.8	-	Favored (59.25%) General / -50.9,-41.7	Favored (64.3%) *tp* chi angles: 180.4,62.8	0.05Å	Favored (8.284%)	-	-	-
A 13		ALA	6.3	-	Favored (12.17%) General / -149.0,127.3	-	0.03Å	Favored (9.92%)	-	-	-
A 14		ASN	6.62	-	Favored (8.56%) General / -82.3,67.9	Favored (88%) *m-40* chi angles: 294.9,319.1	0.05Å	Favored (13.901%)	-	-	-
A 15		LYS	6.67	-	Favored (41.46%) General / -82.5,-18.4	Favored (99%) *mttt* chi angles: 293.9,179.7,179.2,178.9	0.04Å	Favored (7.706%)	-	-	-
A 16		LYS	6.47	-	Favored (12.59%) General / -154.7,134.1	Favored (82.9%) *tttt* chi angles: 188.5,177.4,182.9,185.2	0.03Å	Favored (17.962%)	-	-	-
A 17		GLY	6.06	-	Favored (84.04%) Glycine / -79.1,-12.4	-	-	Favored (17.394%)	-	-	-
A 18		ALA	5.54	-	Favored (8.46%) General / -81.4,72.5	-	0.05Å	Favored (19.553%)	-	-	-
A 19		TRP	4.97	-	Favored (48.04%) General / -51.6,-37.0	Favored (63.2%) *p-90* chi angles: 71.3,271.9	0.03Å	Favored (29.002%)	-	-	-
A 20		LEU	4.43	-	Favored (17.8%) General / -115.7,109.5	Favored (68.9%) *tp* chi angles: 176.9,60.3	0.02Å	Favored (25.107%)	-	-	-
**#**	**Alt**	**Res**	**High B**	**Clash > 0.4Å**	**Ramachandran**	**Rotamer**	**Cβ deviation**	**CaBLAM**	**Bond lengths**	**Bond angles**	**Cis Peptides**
			Avg: 2.75	Clashscore: 0	Outliers: 0 of 73	Poor rotamers: 0 of 61	Outliers: 0 of 70	Outliers: 0 of 71	Outliers: 0 of 75	Outliers: 2 of 75	Non-Trans: 0 of 74
A 21		ASP	3.95	-	Favored (40.86%)	Favored (11.6%) *t70*	0.03Å	Favored (32.084%)	-	-	-

					General / -76.4,143.2	chi angles: 193.3,284					
A 22		SER	3.52	-	Favored (62.31%) General / -60.4,-21.5	Favored (88.8%) *p* chi angles: 66.9	0.02Å	Favored (49.817%)	-	-	-
A 23		THR	3.17	-	Favored (91.2%) General / -64.7,-44.2	Favored (98.7%) *m* chi angles: 300.2	0.07Å	Favored (67.358%) alpha helix	-	-	-
A 24		LYS	2.87	-	Favored (64.11%) General / -71.8,-44.4	Favored (48.9%) *tttm* chi angles: 181.2,172.7,183.2,287.1	0.02Å	Favored (76.216%) alpha helix	-	-	-
A 25		ALA	2.62	-	Favored (88.98%) General / -59.7,-40.8	-	0.05Å	Favored (93.19%) alpha helix	-	-	-
A 26		THR	2.39	-	Favored (80.29%) General / -64.7,-46.9	Favored (90.4%) *m* chi angles: 298.8	0.02Å	Favored (91.786%) alpha helix	-	-	-
A 27		ARG	2.19	-	Favored (86.27%) General / -61.3,-38.3	Favored (97.3%) *mtt180* chi angles: 287.6,173.6,179.8,170.8	0.02Å	Favored (82.636%) alpha helix	-	-	-
A 28		TYR	2.02	-	Favored (76.28%) General / -58.5,-49.7	Favored (84.2%) *t80* chi angles: 182.2,82.6	0.07Å	Favored (84.746%) alpha helix	-	-	-
A 29		LEU	1.86	-	Favored (91.78%) General / -64.4,-38.6	Favored (97.9%) *mt* chi angles: 292.2,172.3	0.06Å	Favored (82.733%) alpha helix	-	-	-
A 30		VAL	1.72	-	Favored (95.19%) Ile or Val / -64.6,-42.5	Favored (60.8%) *t* chi angles: 170.9	0.03Å	Favored (96.269%) alpha helix	-	-	-
A 31		LYS	1.6	-	Favored (91.68%) General / -63.0,-38.6	Favored (96.7%) *mttt* chi angles: 288.9,178.4,180.5,179.2	0.01Å	Favored (99.42%) alpha helix	-	-	-
A 32		THR	1.51	-	Favored (83.92%) General / -64.2,-46.4	Favored (97.9%) *m* chi angles: 300.1	0.02Å	Favored (93.299%) alpha helix	-	-	-
A 33		GLU	1.46	-	Favored (96.22%) General / -60.2,-42.9	Favored (91.4%) *tt0* chi angles: 183.6,178.3,4.5	0.03Å	Favored (95.916%) alpha helix	-	-	-
A 34		SER	1.44	-	Favored (98.6%) General / -61.8,-42.1	Favored (69.7%) *m* chi angles: 296.5	0.06Å	Favored (96.697%) alpha helix	-	-	-
A 35		TRP	1.46	-	Favored (88.24%) General / -60.2,-46.9	Favored (76.1%) *t60* chi angles: 171.1,82.5	0.08Å	Favored (97.683%) alpha helix	-	-	-
A 36		ILE	1.48	-	Favored (98.25%) Ile or Val / -61.8,-44.1	Favored (98.2%) *mt* chi angles: 292.4,168.1	0.05Å	Favored (97.904%) alpha helix	-	-	-
A 37		LEU	1.5	-	Favored (88.64%)	Favored (91.7%) *mt* chi angles: 291.2,171.7	0.04Å	Favored (80.808%) alpha helix	-	-	-

					General / -63.2,-37.8						
A 38		ARG	1.5	-	Favored (65.15%) General / -73.4,-33.8	Favored (97.1%) *mtt180* chi angles: 290.1,172.9,180.5,170.6	0.03Å	Favored (61.163%)	-	-	-
A 39		ASN	1.44	-	Favored (77.86%) Pre-Pro / -131.0,65.9	Favored (52.5%) *m-40* chi angles: 300.1,279.6	0.04Å	Favored (25.085%)	-	-	-
A 40		PRO	1.34	-	Favored (70.13%) Trans-Pro / -62.9,-19.9	Favored (39.7%) *Cg_endo* chi angles: 23.3,325.6,30.8	0.02Å	Favored (27.977%)	-	-	-
**#**	**Alt**	**Res**	**High B**	**Clash > 0.4Å**	**Ramachandran**	**Rotamer**	**Cβ deviation**	**CaBLAM**	**Bond lengths**	**Bond angles**	**Cis Peptides**
			Avg: 2.75	Clashscore: 0	Outliers: 0 of 73	Poor rotamers: 0 of 61	Outliers: 0 of 70	Outliers: 0 of 71	Outliers: 0 of 75	Outliers: 2 of 75	Non-Trans: 0 of 74
A 41		GLY	1.22	-	Favored (59.02%) Glycine / -56.9,-29.4	-	-	Favored (68.016%)	-	-	-
A 42		TYR	1.09	-	Favored (63.73%) General / -71.4,-28.1	Favored (50.7%) *m-80* chi angles: 285.7,110.1	0.04Å	Favored (73.129%) three-ten	-	-	-
A 43		ALA	0.99	-	Favored (86.67%) General / -66.3,-38.0	-	0.03Å	Favored (80.596%) alpha helix	-	-	-
A 44		LEU	0.91	-	Favored (73.59%) General / -70.4,-40.9	Favored (95.6%) *mt* chi angles: 294.2,173.7	0.04Å	Favored (90.848%) alpha helix	-	-	-
A 45		VAL	0.86	-	Favored (95.39%) Ile or Val / -64.5,-45.5	Favored (64.8%) *t* chi angles: 171.5	0.01Å	Favored (94.153%) alpha helix	-	-	-
A 46		ALA	0.82	-	Favored (82.77%) General / -60.4,-38.0	-	0.05Å	Favored (85.22%) alpha helix	-	-	-
A 47		ALA	0.79	-	Favored (97.16%) General / -62.1,-44.2	-	0.03Å	Favored (85.379%) alpha helix	-	-	-
A 48		VAL	0.77	-	Favored (92.29%) Ile or Val / -66.0,-43.9	Favored (73%) *t* chi angles: 172.5	0.03Å	Favored (89.02%) alpha helix	-	-	-
A 49		ILE	0.77	-	Favored (97.34%) Ile or Val / -62.9,-46.0	Favored (99.5%) *mt* chi angles: 292.7,167.5	0.02Å	Favored (93.07%) alpha helix	-	-	-
A 50		GLY	0.8	-	Favored (92.57%) Glycine / -59.1,-38.0	-	-	Favored (94.254%) alpha helix	-	-	-
A 51		TRP	0.93	-	Favored (94.93%) General / -62.5,-39.9	Favored (51%) *m-10* chi angles: 291.7,338.4	0.06Å	Favored (82.549%) alpha helix	-	-	-
A 52		MET	1.22	-	Favored (55.77%)	Favored (52.5%) *mmp*	0.01Å	Favored (76.399%)	-	-	-

					General / -76.7,-28.3	chi angles: 294.9,300.6,98.9		alpha helix			
A 53		LEU	1.73	-	Favored (19.29%) General / -82.0,-42.5	Favored (96.6%) *mt* chi angles: 293.7,173.4	0.08Å	Favored (58.436%) alpha helix	-	-	-
A 54		GLY	2.55	-	Favored (31.56%) Glycine / -88.6,162.3	-	-	Favored (6.307%) alpha helix	-	-	-
A 55		SER	3.46	-	Favored (7.26%) General / -110.6,-31.4	Favored (88.3%) *p* chi angles: 62.9	0.03Å	Favored (7.394%) alpha helix	-	-	-
A 56		ASN	4.02	-	Favored (17.31%) General / -102.5,156.9	Favored (87.4%) *m-40* chi angles: 292.3,319.8	0.06Å	Favored (15.798%) alpha helix	-	-	-
A 57		THR	3.82	-	Favored (75.93%) General / -55.3,-47.2	Favored (90.9%) *m* chi angles: 298	0.02Å	Favored (60.935%) alpha helix	-	-	-
A 58		MET	3.02	-	Favored (66.6%) General / -68.8,-28.3	Favored (83.4%) *mmm* chi angles: 290.2,309.5,299.7	0.12Å	Favored (75.746%) alpha helix	-	-	-
A 59		GLN	2.12	-	Favored (94.43%) General / -65.3,-41.1	Favored (96.5%) *mt0* chi angles: 291,174.9,314.3	0.10Å	Favored (80.369%) alpha helix	-	-	-
A 60		ARG	1.44	-	Favored (93.31%) General / -65.3,-40.0	Favored (97.3%) *mtt180* chi angles: 291.5,172.9,175.8,168.1	0.08Å	Favored (89.941%) alpha helix	-	-	-
**#**	**Alt**	**Res**	**High B**	**Clash > 0.4Å**	**Ramachandran**	**Rotamer**	**Cβ deviation**	**CaBLAM**	**Bond lengths**	**Bond angles**	**Cis Peptides**
			Avg: 2.75	Clashscore: 0	Outliers: 0 of 73	Poor rotamers: 0 of 61	Outliers: 0 of 70	Outliers: 0 of 71	Outliers: 0 of 75	Outliers: 2 of 75	Non-Trans: 0 of 74
A 61		VAL	1.07	-	Favored (85.14%) Ile or Val / -66.0,-47.4	Favored (64.5%) *t* chi angles: 171.4	0.04Å	Favored (88.918%) alpha helix	-	-	-
A 62		VAL	0.89	-	Favored (97.28%) Ile or Val / -61.0,-44.1	Favored (58.1%) *t* chi angles: 170.5	0.03Å	Favored (95.35%) alpha helix	-	-	-
A 63		PHE	0.82	-	Favored (98.31%) General / -62.2,-41.4	Favored (10.6%) *m-10* chi angles: 287.3,326.7	0.06Å	Favored (96.166%) alpha helix	-	-	-
A 64		ALA	0.81	-	Favored (89.68%) General / -60.2,-40.3	-	0.03Å	Favored (90.647%) alpha helix	-	-	-
A 65		ILE	0.84	-	Favored (85.39%) Ile or Val / -66.3,-47.1	Favored (99.4%) *mt* chi angles: 292.6,167.4	0.03Å	Favored (90.934%) alpha helix	-	-	-
A 66		LEU	0.88	-	Favored (92.64%) General / -62.0,-39.6	Favored (92.9%) *mt* chi angles: 291.5,172.9	0.03Å	Favored (92.113%) alpha helix	-	-	-
A 67		LEU	0.93	-	Favored (99.58%)	Favored (84.5%) *mt* chi angles: 290.3,168.1	0.07Å	Favored (94.222%)	-	-	-

					General / -62.9,-41.8			alpha helix			
A 68		LEU	0.99	-	Favored (82.54%) General / -66.4,-36.3	Favored (92.4%) *mt* chi angles: 291.3,172.8	0.05Å	Favored (89.671%) alpha helix	-	-	-
A 69		LEU	1.08	-	Favored (77.18%) General / -66.2,-34.1	Favored (86.9%) *mt* chi angles: 290.2,171.8	0.02Å	Favored (67.428%) alpha helix	-	-	-
A 70		VAL	1.23	-	Favored (5.13%) Ile or Val / -98.1,-21.7	Favored (24.7%) *m* chi angles: 301.3	0.01Å	Favored (28.252%) alpha helix	-	-	-
A 71		ALA	1.45	-	Favored (90.55%) Pre-Pro / -53.8,-42.0	-	0.05Å	Favored (46.279%) alpha helix	-	OUTLIER(S) worst is CA-C-N: 4.1 σ	-
A 72		PRO	1.76	-	Favored (9.42%) Trans-Pro / -82.0,1.1	Favored (52.3%) *Cg_endo* chi angles: 32.8,323.6,24.9	0.02Å	Favored (17.496%)	-	-	-
A 73		ALA	2.18	-	Favored (11.85%) General / -112.9,25.0	-	0.04Å	Favored (16.87%)	-	-	-
A 74		TYR	2.68	-	Favored (61.53%) General / -58.9,-24.0	Favored (57.4%) *p90* chi angles: 66.1,91.4	0.05Å	-	-	-	-
A 75		SER	3.22	-	-	Favored (82.1%) *p* chi angles: 70.1	0.10Å	-	-	OUTLIER(S) worst is C-N-CA: 4.1 σ	-

About MolProbity | Website for the Richardson Lab | Using ecloud x-H | Internal reference 4.5.2
