# Supplementary material for: Integrative proteome-wide structural analysis and high-throughput docking identify broad-spectrum antiviral scaffolds against Zika, Yellow Fever, West Nile, Saint Louis encephalitis, and Usutu viruses
Source: Front Cell Infect Microbiol. 2026 Apr 30;16:1723132. doi: 10.3389/fcimb.2026.1723132 (PMC13171538; doi:10.3389/fcimb.2026.1723132)
Supplement: Supplementary file 5 [file DataSheet5.zip › WNV/WNV_NS1/Mol_probity_Files/WNV_NS1_1FH-multi.table.pdf]

Viewing WNV_NS1_1FH-multi.table

When finished, you should close this window.

Hint: Use File | Save As... to save a copy of this page.

All-Atom Contacts	Clashscore, all atoms:	1.47		99th percentile* (N=1784, all resolutions)
	Clashscore is the number of serious steric overlaps (> 0.4 Å) per 1000 atoms.			
Protein Geometry	Poor rotamers	0	0.00%	Goal: <0.3%
	Favored rotamers	299	99.34%	Goal: >98%
	Ramachandran outliers	1	0.29%	Goal: <0.05%
	Ramachandran favored	340	97.14%	Goal: >98%
	Rama distribution Z-score	0.72 ± 0.45		Goal: abs(Z score) < 2
	MolProbity score^	1.04		100th percentile* (N=27675, 0Å - 99Å)
	Cβ deviations >0.25Å	0	0.00%	Goal: 0
	Bad bonds:	2 / 2838	0.07%	Goal: 0%
	Bad angles:	9 / 3852	0.23%	Goal: <0.1%
Peptide Omegas	Cis Prolines:	1 / 16	6.25%	Expected: ≤1 per chain, or ≤5%
Low-resolution Criteria	CaBLAM outliers	5	1.4%	Goal: <1.0%
	CA Geometry outliers	6	1.72%	Goal: <0.5%
Additional validations	Chiral volume outliers	0/418		
	Waters with clashes	0/0	0.00%	See UnDowser table for details

In the two column results, the left column gives the raw count, right column gives the percentage.

* 100th percentile is the best among structures of comparable resolution; 0th percentile is the worst. For clashscore the comparative set of structures was selected in 2004, for MolProbity score in 2006.

^ MolProbity score combines the clashscore, rotamer, and Ramachandran evaluations into a single score, normalized to be on the same scale as X-ray resolution.

Key to table colors and cutoffs here:

#	Alt	Res	High B	Clash > 0.4Å	Ramachandran	Rotamer	Cβ deviation	CaBLAM	Bond lengths	Bond angles	Cis Peptides
			Avg: 1.18	Clashscore: 1.47	Outliers: 1 of 350	Poor rotamers: 0 of 301	Outliers: 0 of 323	Outliers: 10 of 348	Outliers: 2 of 352	Outliers: 9 of 352	Non-Trans: 1 of 351
A 1		ASP	3.19	-	-	Favored (80.9%) *m-30* chi angles: 296,337.2	0.11Å	-	-	OUTLIER(S) worst is CA-CB-CG: 5.3 σ	-
A 2		THR	3.09	-	Favored (44.17%) General / -132.2,133.5	Favored (83.8%) *m* chi angles: 301.9	0.02Å	-	-	-	-
A 3		GLY	3.03	-	Favored (32.68%) Glycine / 161.1,-161.1	-	-	Favored (23.113%)	-	-	-
A 4		CYS	3.02	-	Favored (28.27%) General / -132.4,163.7	Favored (63.3%) *m* chi angles: 301.4	0.05Å	Favored (13.796%) beta sheet	-	-	-
A 5		ALA	3.09	-	Favored (37.77%) General / -148.4,152.9	-	0.03Å	Favored (69.804%) beta sheet	-	-	-

A 6		ILE	3.23	-	Favored (6.14%) Ile or Val / -111.0,-46.5	Favored (84.9%) *mt* chi angles: 299.1,170.8	0.06Å	CaBLAM Outlier (0.154%) try beta sheet	-	-	-
A 7		ASP	3.43	-	Allowed (0.19%) General / 76.5,157.8	Favored (44%) *t0* chi angles: 191.1,356	0.06Å	Favored (5.046%)	-	-	-
A 8		ILE	3.62	-	Favored (7.37%) Ile or Val / -120.2,-15.8	Favored (45.2%) *pt* chi angles: 61.9,170.2	0.06Å	Favored (7.616%)	-	-	-
A 9		GLY	3.74	-	Favored (55.92%) Glycine / -80.2,-26.1	-	-	Favored (38.106%) alpha helix	-	-	-
A 10		ARG	3.74	-	Favored (9.17%) General / -118.7,-17.8	Favored (88.3%) *mtp85* chi angles: 295.8,181.8,64.8,84.5	0.02Å	Favored (9.802%)	-	-	-
A 11		GLN	3.59	-	Favored (20.53%) General / 54.5,48.5	Favored (68.4%) *mm-40* chi angles: 298.1,286.7,302.1	0.07Å	Favored (13.638%)	-	-	-
A 12		GLU	3.35	-	Favored (7.81%) General / -119.4,101.2	Favored (92%) *mt-10* chi angles: 287.9,179.9,340	0.05Å	Favored (18.484%) beta sheet	-	-	-
A 13		LEU	3.05	-	Favored (35.39%) General / -64.1,128.3	Favored (51.3%) *tp* chi angles: 178.4,66.4	0.05Å	Favored (22.616%) beta sheet	-	-	-
A 14		ARG	2.77	-	Favored (37.82%) General / -136.1,134.0	Favored (93.4%) *mmt-90* chi angles: 294.7,290,183.2,272.4	0.03Å	Favored (54.629%) beta sheet	-	-	-
A 15		CYS	2.51	-	Favored (43.42%) General / -127.6,155.7	Favored (64.5%) *m* chi angles: 300	0.01Å	Favored (5.588%) beta sheet	-	-	-
A 16		GLY	2.28	-	Favored (28.48%) Glycine / 160.8,-157.0	-	-	Favored (40.94%)	-	-	-
A 17		SER	2.08	-	Favored (31.23%) General / -103.5,14.4	Favored (80.2%) *p* chi angles: 61.4	0.02Å	CaBLAM Disfavored (1.257%)	-	-	-
A 18		GLY	1.9	-	Favored (31.38%) Glycine / -83.4,-161.5	-	-	Favored (36.729%)	-	-	-
A 19		VAL	1.76	-	Favored (41.96%) Ile or Val / -96.3,131.9	Favored (83.9%) *t* chi angles: 177	0.06Å	Favored (11.074%) beta sheet	-	-	-
A 20		PHE	1.65	-	Favored (12.7%) General / -124.0,108.8	Favored (75.1%) *m-80* chi angles: 302,86.8	0.03Å	Favored (60.137%) beta sheet	-	-	-
**#**	**Alt**	**Res**	**High B**	**Clash > 0.4Å**	**Ramachandran**	**Rotamer**	**Cβ deviation**	**CaBLAM**	**Bond lengths**	**Bond angles**	**Cis Peptides**
			Avg: 1.18	Clashscore: 1.47	Outliers: 1 of 350	Poor rotamers: 0 of 301	Outliers: 0 of 323	Outliers: 10 of 348	Outliers: 2 of 352	Outliers: 9 of 352	Non-Trans: 1 of 351
A 21		ILE	1.56	-	Favored (58.41%)	Favored (42.7%) *mm* chi angles: 306.9,300.2	0.05Å	Favored (53.867%) beta sheet	-	-	-

					Ile or Val / -106.9,130.7						
A 22		HIS	1.5	-	Favored (51.12%) General / -128.1,144.6	Favored (88.3%) *m-70* chi angles: 298.1,273.1	0.03Å	Favored (55.685%) beta sheet	-	-	-
A 23		ASN	1.49	-	Favored (8.59%) General / -81.6,71.5	Favored (38.3%) *t0* chi angles: 191.1,10	0.02Å	Favored (14.167%) beta sheet	-	-	-
A 24		ASP	1.55	-	Favored (59.64%) General / -82.3,-8.3	Favored (84.3%) *m-30* chi angles: 290,334.2	0.02Å	Favored (10.346%)	-	-	-
A 25		VAL	1.68	-	Favored (83.31%) Ile or Val / -59.9,-40.6	Favored (76.1%) *t* chi angles: 172.8	0.07Å	Favored (47.634%)	-	-	-
A 26		GLU	1.86	-	Favored (58.75%) General / -84.6,-4.0	Favored (96.1%) *mt-10* chi angles: 296.4,180.5,0.2	0.05Å	Favored (51.472%)	-	-	-
A 27		ALA	2.04	-	Favored (8.74%) General / -82.3,68.6	-	0.04Å	CA Geom Outlier (0.178%)	-	-	-
A 28		TRP	2.15	-	Favored (3.39%) General / 54.2,22.0	Favored (8.4%) *m-90* chi angles: 312,282.5	0.09Å	CA Geom Outlier (0.032%)	-	-	-
A 29		MET	2.12	-	Favored (5.19%) General / 48.9,34.2	Favored (24.6%) *mmt* chi angles: 299.5,295.9,161.8	0.03Å	CaBLAM Disfavored (1.075%)	-	-	-
A 30		ASP	1.96	0.40Å OD1 with A 33 LYS NZ	Allowed (1.22%) General / -144.8,38.9	Favored (32.6%) *t0* chi angles: 195.5,27.9	0.05Å	CA Geom Outlier (0.459%)	-	OUTLIER(S) worst is CA-CB-CG: 4.8 σ	-
A 31		ARG	1.71	-	Favored (36.84%) General / -100.6,-0.9	Favored (94%) *mtt180* chi angles: 290.8,181.6,172.3,171.1	0.08Å	Favored (45.726%)	-	-	-
A 32		TYR	1.46	-	Favored (50.91%) General / -128.8,142.7	Favored (80.2%) *m-80* chi angles: 294.4,81.7	0.06Å	Favored (28.555%)	-	-	-
A 33		LYS	1.27	0.40Å NZ with A 30 ASP OD1	Favored (53.34%) General / -126.1,136.8	Favored (71.8%) *mmtt* chi angles: 302,292.5,183,185.4	0.06Å	Favored (65.28%)	-	-	-
A 34		PHE	1.16	-	Favored (37.2%) General / -102.7,140.2	Favored (10.1%) *m-10* chi angles: 285.2,10.4	0.11Å	Favored (62.509%) beta sheet	-	-	-
A 35		TYR	1.11	-	Favored (35.83%) Pre-Pro / -119.5,112.2	Favored (82%) *m-80* chi angles: 299.3,85.2	0.07Å	Favored (67.471%)	-	-	-
A 36		PRO	1.11	-	Favored (13.43%) Trans-Pro / -76.7,174.1	Favored (53.9%) *Cg_endo* chi angles: 32.6,324,24.4	0.07Å	Favored (17.931%)	-	-	-
A 37		GLU	1.12	0.51Å O with A 38 THR C	Favored (7.05%) General / -57.7,157.6	Favored (98.7%) *mt-10* chi angles: 293.5,180.5,355.5	0.12Å	CA Geom Outlier (0.384%)	-	-	-
A 38		THR	1.12	0.51Å C with A 37 GLU O	Favored (5.53%) Pre-Pro / -45.1,130.2	Favored (59%) *m* chi angles: 303.6	0.07Å	Favored (15.338%)	-	-	-

#	Alt	Res	High B	Clash > 0.4Å	Ramachandran	Rotamer	Cβ deviation	CaBLAM	Bond lengths	Bond angles	Cis Peptides
A 39		PRO	1.11	-	Favored (7.44%) Trans-Pro / -47.4,-27.6	Favored (76.3%) *Cg_exo* chi angles: 329,37.1,332.3	0.08Å	Favored (50.399%)	-	-	-
A 40		GLN	1.07	-	Favored (87.9%) General / -58.2,-43.9	Favored (55.2%) *tt0* chi angles: 183.2,185.6,59.7	0.09Å	Favored (56.1%) alpha helix	-	-	-
**#**	**Alt**	**Res**	**High B**	**Clash > 0.4Å**	**Ramachandran**	**Rotamer**	**Cβ deviation**	**CaBLAM**	**Bond lengths**	**Bond angles**	**Cis Peptides**
			Avg: 1.18	Clashscore: 1.47	Outliers: 1 of 350	Poor rotamers: 0 of 301	Outliers: 0 of 323	Outliers: 10 of 348	Outliers: 2 of 352	Outliers: 9 of 352	Non-Trans: 1 of 351
A 41		GLY	1.02	-	Favored (94.9%) Glycine / -64.6,-44.5	-	-	Favored (94.206%) alpha helix	-	-	-
A 42		LEU	0.96	-	Favored (91.36%) General / -63.5,-38.5	Favored (76.1%) *mt* chi angles: 291.9,178.1	0.07Å	Favored (88.582%) alpha helix	-	-	-
A 43		ALA	0.91	-	Favored (87.04%) General / -61.4,-38.4	-	0.03Å	Favored (90.771%) alpha helix	-	-	-
A 44		ARG	0.87	-	Favored (83.83%) General / -67.8,-39.5	Favored (41.6%) *tpt170* chi angles: 185.5,64.5,181.5,165.1	0.04Å	Favored (94.361%) alpha helix	-	-	-
A 45		ILE	0.84	-	Favored (91.18%) Ile or Val / -59.9,-43.0	Favored (26.5%) *mm* chi angles: 293.9,292.2	0.08Å	Favored (90.978%) alpha helix	-	-	-
A 46		ILE	0.82	-	Favored (89.31%) Ile or Val / -66.5,-45.2	Favored (86.2%) *mt* chi angles: 290.9,169.7	0.07Å	Favored (91.509%) alpha helix	-	-	-
A 47		GLN	0.81	-	Favored (88.35%) General / -59.2,-46.5	Favored (62.9%) *tt0* chi angles: 181.9,181.6,8.2	0.02Å	Favored (84.061%) alpha helix	-	-	-
A 48		LYS	0.81	-	Favored (91.58%) General / -64.4,-44.4	Favored (88.2%) *tttt* chi angles: 182.6,175.1,178.7,177.8	0.03Å	Favored (80.113%) alpha helix	-	-	-
A 49		ALA	0.81	-	Favored (81.46%) General / -57.8,-41.1	-	0.08Å	Favored (84.959%) alpha helix	-	-	-
A 50		HIS	0.8	-	Favored (76.24%) General / -61.9,-49.4	Favored (78.1%) *t70* chi angles: 185.6,75.6	0.03Å	Favored (89.431%) alpha helix	-	-	-
A 51		ALA	0.79	-	Favored (69.32%) General / -58.6,-32.8	-	0.02Å	Favored (74.637%)	-	-	-
A 52		GLU	0.77	-	Favored (47.33%) General / -83.5,0.2	Favored (97.5%) *mt-10* chi angles: 291.3,182.1,356.1	0.06Å	Favored (41.96%)	-	-	-
A 53		GLY	0.75	-	Favored (42.36%) Glycine / 99.7,11.9	-	-	Favored (76.458%)	-	-	-
A 54		VAL	0.72	-	Favored (51.33%) Ile or Val / -98.0,125.2	Favored (94.1%) *t* chi angles: 175.8	0.06Å	Favored (20.449%)	-	-	-

A 55		CYS	0.71	-	Favored (45.25%) General / -92.8,-7.9	Favored (28.4%) *p* chi angles: 67.1	0.07Å	CaBLAM Disfavored (3.84%)	-	-	-
A 56		GLY	0.71	-	Favored (48.36%) Glycine / 178.7,178.9	-	-	Favored (43.302%)	-	-	-
A 57		LEU	0.73	-	Favored (29.51%) General / -137.6,130.1	Favored (44.5%) *tp* chi angles: 173.3,66	0.05Å	Favored (23.412%)	-	-	-
A 58		ARG	0.77	0.54Å HH21 with A 98 LYS H	Favored (45.38%) General / -102.8,122.9	Favored (87.3%) *mtp85* chi angles: 295.3,178.2,66.1,80.9	0.08Å	Favored (61.239%) beta sheet	-	-	-
A 59		SER	0.82	-	Favored (17.11%) General / -78.3,170.1	Favored (85%) *p* chi angles: 67.7	0.03Å	Favored (10.107%)	-	-	-
A 60		VAL	0.88	-	Favored (8.04%) Ile or Val / -107.3,-46.8	Favored (88.1%) *t* chi angles: 174	0.06Å	CaBLAM Disfavored (3.109%)	-	-	-
**#**	**Alt**	**Res**	**High B**	**Clash > 0.4Å**	**Ramachandran**	**Rotamer**	**Cβ deviation**	**CaBLAM**	**Bond lengths**	**Bond angles**	**Cis Peptides**
			Avg: 1.18	Clashscore: 1.47	Outliers: 1 of 350	Poor rotamers: 0 of 301	Outliers: 0 of 323	Outliers: 10 of 348	Outliers: 2 of 352	Outliers: 9 of 352	Non-Trans: 1 of 351
A 61		SER	0.94	-	Favored (15.14%) General / -101.8,159.7	Favored (95.6%) *p* chi angles: 64.9	0.05Å	Favored (12.581%) alpha helix	-	-	-
A 62		ARG	0.99	-	Favored (67.98%) General / -63.9,-25.0	Favored (51.7%) *ptt90* chi angles: 67.8,183.2,172,83.2	0.03Å	Favored (60.964%) alpha helix	-	-	-
A 63		LEU	1.01	-	Favored (58.24%) General / -76.1,-37.2	Allowed (1.9%) *mm* chi angles: 264.6,296.5	0.04Å	Favored (78.422%) alpha helix	-	-	-
A 64		GLU	1.02	-	Favored (91.56%) General / -64.1,-38.6	Favored (84.6%) *mt-10* chi angles: 289.2,164.4,339.2	0.04Å	Favored (92.77%) alpha helix	-	-	-
A 65		HIS	1.02	-	Favored (70.76%) General / -59.5,-51.2	Favored (88%) *t70* chi angles: 179.8,76.3	0.05Å	Favored (90.911%) alpha helix	-	-	-
A 66		GLN	1.01	-	Favored (81.4%) General / -60.8,-37.3	Favored (92.3%) *mt0* chi angles: 289.6,173.4,350.4	0.02Å	Favored (79.393%) alpha helix	-	-	-
A 67		MET	1.01	-	Favored (78.22%) General / -59.1,-49.4	Favored (28.7%) *tmm* chi angles: 180.8,277.7,290.5	0.04Å	Favored (81.103%) alpha helix	-	-	-
A 68		TRP	1.01	-	Favored (92.28%) General / -63.1,-38.7	Favored (73.6%) *m100* chi angles: 284.3,114.2	0.04Å	Favored (80.746%) alpha helix	-	-	-
A 69		GLU	1.02	-	Favored (84.16%) General / -61.9,-47.6	Favored (92.4%) *tt0* chi angles: 182.8,178.8,1	0.03Å	Favored (77.548%) alpha helix	-	-	-
A 70		ALA	1.03	-	Favored (71.68%) General / -58.8,-34.6	-	0.03Å	Favored (62.705%) alpha helix	-	-	-

A 71		ILE	1.03	-	Favored (10.61%) Ile or Val / -102.2,3.9	Favored (36.1%) *pt* chi angles: 65.6,169.2	0.03Å	Favored (43.899%) alpha helix	-	-	-
A 72		LYS	1.04	-	Favored (73.47%) General / -54.6,-47.4	Favored (2.5%) *tmmt* chi angles: 180.8,260.8,292.5,174.7	0.02Å	Favored (53.541%) alpha helix	-	-	-
A 73		ASP	1.05	-	Favored (91.24%) General / -62.5,-38.8	Favored (97.8%) *m-30* chi angles: 288.7,345.1	0.05Å	Favored (81.15%) alpha helix	-	-	-
A 74		GLU	1.06	-	Favored (80.26%) General / -68.7,-39.7	Favored (74.6%) *mt-10* chi angles: 286.2,183.1,333.1	0.11Å	Favored (87.923%) alpha helix	-	-	-
A 75		LEU	1.07	-	Favored (97.85%) General / -62.8,-40.6	Favored (93.2%) *mt* chi angles: 291.6,171.5	0.07Å	Favored (92.174%) alpha helix	-	-	-
A 76		ASN	1.09	-	Favored (78.53%) General / -68.8,-36.6	Favored (95.2%) *m-40* chi angles: 286.3,339.5	0.03Å	Favored (90.441%) alpha helix	-	-	-
A 77		THR	1.13	-	Favored (92.79%) General / -62.8,-45.4	Favored (90.1%) *m* chi angles: 298.8	0.03Å	Favored (86.211%) alpha helix	-	-	-
A 78		LEU	1.16	0.40Å HB2 with A 115 TRP CZ2	Favored (84.29%) General / -65.9,-44.7	Favored (61.7%) *tp* chi angles: 179.9,58.4	0.05Å	Favored (92.776%) alpha helix	OUTLIER(S) worst is CB--CG: 4.8 σ	-	-
A 79		LEU	1.19	-	Favored (92.84%) General / -60.8,-40.5	Favored (81.7%) *mt* chi angles: 289.5,172.8	0.05Å	Favored (97.786%) alpha helix	-	-	-
A 80		LYS	1.2	-	Favored (83.4%) General / -61.1,-48.1	Favored (48.1%) *tttp* chi angles: 179.7,178.6,170.7,68.2	0.01Å	Favored (94.779%) alpha helix	-	-	-
**#**	**Alt**	**Res**	**High B**	**Clash > 0.4Å**	**Ramachandran**	**Rotamer**	**Cβ deviation**	**CaBLAM**	**Bond lengths**	**Bond angles**	**Cis Peptides**
			Avg: 1.18	Clashscore: 1.47	Outliers: 1 of 350	Poor rotamers: 0 of 301	Outliers: 0 of 323	Outliers: 10 of 348	Outliers: 2 of 352	Outliers: 9 of 352	Non-Trans: 1 of 351
A 81		GLU	1.18	-	Favored (68.05%) General / -64.3,-25.4	Favored (56.3%) *mt-10* chi angles: 288.6,185.5,315.3	0.10Å	Favored (70.714%) alpha helix	-	-	-
A 82		ASN	1.11	-	Favored (50.53%) General / -89.3,3.9	Favored (75.7%) *m-40* chi angles: 288.3,315.1	0.05Å	Favored (58.319%)	-	-	-
A 83		GLY	1.02	-	Favored (70.38%) Glycine / 78.2,21.5	-	-	Favored (87.957%)	-	-	-
A 84		VAL	0.93	-	Favored (42.97%) Ile or Val / -99.0,116.8	Favored (53.3%) *t* chi angles: 180.9	0.08Å	Favored (29.599%)	-	-	-
A 85		ASP	0.84	-	Favored (6.8%) General / -80.2,70.1	Favored (42%) *t0* chi angles: 191.7,21.6	0.05Å	Favored (8.744%) beta sheet	-	-	-
A 86		LEU	0.77	-	Favored (14.45%) General / -150.0,131.1	Favored (60.9%) *tp* chi angles: 177.7,65	0.04Å	Favored (10.049%) beta sheet	-	-	-
A 87		SER	0.73	-	Favored (35.1%)	Favored (62.4%) *m* chi angles: 298.2	0.04Å	Favored (54.329%)	-	-	-

					General / -121.5,155.3			beta sheet			
A 88		VAL	0.73	-	Favored (52.63%) Ile or Val / -101.7,129.6	Favored (70.7%) *t* chi angles: 178.7	0.06Å	Favored (58.448%) beta sheet	-	-	-
A 89		VAL	0.76	-	Favored (72.3%) Ile or Val / -121.7,124.8	Favored (71%) *t* chi angles: 178.6	0.07Å	Favored (70.68%) beta sheet	-	-	-
A 90		VAL	0.82	-	Favored (59.08%) Ile or Val / -110.0,119.4	Favored (58.9%) *t* chi angles: 180.1	0.02Å	Favored (72.578%)	-	-	-
A 91		GLU	0.91	-	Favored (17.11%) General / -92.9,156.2	Favored (68.8%) *mm-30* chi angles: 296.3,288.3,330.1	0.05Å	Favored (21.922%)	-	-	-
A 92		LYS	1	-	Favored (55.88%) General / -59.5,133.2	Favored (86.4%) *tttt* chi angles: 182.5,178,177.3,181.1	0.02Å	Favored (36.711%)	-	-	-
A 93		GLN	1.09	-	Favored (58.89%) General / -63.3,140.6	Favored (13%) *pm20* chi angles: 70.3,273.9,47	0.04Å	Favored (38.469%)	-	-	-
A 94		ASN	1.14	-	Favored (20.26%) General / -135.7,121.9	Favored (71.7%) *m-40* chi angles: 290.2,282.9	0.05Å	Favored (9.253%)	-	-	-
A 95		GLY	1.15	-	Allowed (0.7%) Glycine / 69.6,-99.8	-	-	CaBLAM Disfavored (1.063%)	-	-	-
A 96		MET	1.12	-	Favored (54.23%) General / -125.0,138.4	Favored (29.6%) *ttm* chi angles: 182.4,182.6,307.3	0.06Å	Favored (18.415%)	-	-	-
A 97		TYR	1.08	-	Favored (8.97%) General / -82.1,77.0	Favored (89.7%) *m-80* chi angles: 293.1,100.8	0.03Å	Favored (52.829%)	-	-	-
A 98		LYS	1.02	0.54Å H with A 58 ARG HH21	Favored (54.66%) General / -69.2,140.5	Favored (97.9%) *mttt* chi angles: 292.3,182,182.7,178.6	0.05Å	Favored (23.462%)	-	-	-
A 99		ALA	0.97	-	Favored (37.4%) General / -62.3,151.1	-	0.04Å	Favored (18.556%)	-	-	-
A 100		ALA	0.93	-	Favored (70.5%) Pre-Pro / -136.8,150.6	-	0.05Å	Favored (52.557%)	-	-	-
**#**	**Alt**	**Res**	**High B**	**Clash > 0.4Å**	**Ramachandran**	**Rotamer**	**Cβ deviation**	**CaBLAM**	**Bond lengths**	**Bond angles**	**Cis Peptides**
			Avg: 1.18	Clashscore: 1.47	Outliers: 1 of 350	Poor rotamers: 0 of 301	Outliers: 0 of 323	Outliers: 10 of 348	Outliers: 2 of 352	Outliers: 9 of 352	Non-Trans: 1 of 351
A 101		PRO	0.91	-	Favored (47.41%) Trans-Pro / -67.8,-18.2	Favored (48.1%) *Cg_endo* chi angles: 25,325.4,29.2	0.03Å	Favored (30.425%)	-	-	-
A 102		LYS	0.91	-	Favored (13.62%) General / -99.2,161.4	Favored (99.1%) *mttt* chi angles: 295.4,180.7,176.7,177.9	0.15Å	Favored (13.724%)	-	-	-
A 103		ARG	0.94	-	Favored (43.54%)	Favored (91.6%) *mmt-90*	0.07Å	Favored (55.592%)	-	-	-

					General / -136.6,143.3	chi angles: 297.7,290.8,183.3,276.2					
A 104		LEU	1	-	Favored (20.23%) General / -99.8,108.0	Favored (55.7%) *mt* chi angles: 305.2,181.4	0.03Å	Favored (47.324%)	-	-	-
A 105		ALA	1.1	-	Favored (53.67%) General / -64.9,147.3	-	0.03Å	Favored (40.834%)	-	-	-
A 106		ALA	1.24	-	Favored (57.05%) General / -59.3,139.4	-	0.03Å	Favored (34.738%)	-	-	-
A 107		THR	1.38	-	Favored (53.06%) General / -124.7,133.7	Favored (95%) *m* chi angles: 299.4	0.09Å	Favored (72.126%) beta sheet	-	-	-
A 108		THR	1.53	-	Favored (48.21%) General / -96.8,-0.4	Favored (76%) *p* chi angles: 61.2	0.04Å	Favored (16.839%) beta sheet	-	-	-
A 109		GLU	1.68	-	Favored (30.85%) General / -79.9,149.6	Favored (93.8%) *mt-10* chi angles: 296.9,178.4,344.1	0.06Å	Favored (31.568%) beta sheet	-	-	-
A 110		LYS	1.85	-	Favored (35.55%) General / -123.3,156.3	Favored (99.2%) *mttt* chi angles: 295,182.6,180.2,179.8	0.02Å	Favored (44.318%) beta sheet	-	-	-
A 111		LEU	2.07	-	Favored (23.01%) General / -86.9,151.8	Favored (83.2%) *mt* chi angles: 298.1,171.6	0.07Å	Favored (45.17%)	-	-	-
A 112		GLU	2.38	-	Favored (96.7%) General / -63.8,-40.4	Favored (98.9%) *mt-10* chi angles: 290.7,177.6,354.1	0.00Å	Favored (57.604%)	-	-	-
A 113		MET	2.82	-	Favored (28.92%) General / -91.3,141.3	Favored (15.6%) *tpt* chi angles: 183.1,66.3,188.2	0.10Å	Favored (8.302%)	-	-	-
A 114		GLY	3.39	-	Favored (3.74%) Glycine / -130.3,29.0	-	-	Favored (7.596%)	-	-	-
A 115		TRP	4.03	0.40Å CZ2 with A 78 LEU HB2	Allowed (0.06%) General / 60.1,-8.3	Favored (28.8%) *m-90* chi angles: 300.9,274.3	0.13Å	Favored (41.399%)	-	-	-
A 116		LYS	4.61	-	OUTLIER (0.04)%) General / 58.4,-61.3	Favored (97.8%) *mttt* chi angles: 291.4,180,179.7,179	0.06Å	CaBLAM Disfavored (1.493%)	-	-	-
A 117		ALA	5.04	-	Favored (13.37%) General / -99.7,-28.1	-	0.04Å	Favored (8.102%)	-	-	-
A 118		TRP	5.27	-	Allowed (0.07%) General / 67.9,151.1	Favored (32.3%) *m-90* chi angles: 284.6,261.5	0.12Å	Favored (39.699%)	-	-	-
A 119		GLY	5.27	-	Favored (89.01%) Glycine / 85.0,-1.2	-	-	Favored (20.514%)	-	-	-
A 120		LYS	5.05	-	Favored (69.36%) General / -57.1,-35.4	Favored (88.9%) *tttt* chi angles: 184.9,174.9,182.1,178.4	0.01Å	Favored (39.759%)	-	-	-

#	Alt	Res	High B	Clash > 0.4Å	Ramachandran	Rotamer	Cβ deviation	CaBLAM	Bond lengths	Bond angles	Cis Peptides
			Avg: 1.18	Clashscore: 1.47	Outliers: 1 of 350	Poor rotamers: 0 of 301	Outliers: 0 of 323	Outliers: 10 of 348	Outliers: 2 of 352	Outliers: 9 of 352	Non-Trans: 1 of 351
A 121		SER	4.6	-	Favored (63.76%) General / -68.9,-16.4	Favored (76.5%) *p* chi angles: 70.9	0.10Å	Favored (63.071%) alpha helix	-	-	-
A 122		ILE	3.95	-	Favored (18.91%) Ile or Val / -82.6,-43.4	Favored (93%) *mt* chi angles: 296.3,169.1	0.04Å	Favored (36.292%) alpha helix	-	-	-
A 123		ILE	3.2	-	Favored (99.39%) Ile or Val / -62.4,-44.6	Favored (98.7%) *mt* chi angles: 292.4,168.1	0.05Å	Favored (37.128%) alpha helix	-	-	-
A 124		PHE	2.48	-	Favored (7.11%) General / -141.4,112.1	Favored (90.7%) *t80* chi angles: 177.5,81.8	0.09Å	Favored (13.822%)	-	-	-
A 125		ALA	1.87	-	Favored (28.2%) Pre-Pro / -89.8,141.2	-	0.02Å	Favored (36.512%)	-	-	-
A 126		PRO	1.43	-	Favored (39.23%) Trans-Pro / -75.6,159.3	Favored (71%) *Cg_endo* chi angles: 29.6,323.6,27.7	0.06Å	Favored (81.557%)	-	-	-
A 127		GLU	1.13	-	Favored (28.94%) General / -76.3,161.2	Favored (26%) *pm20* chi angles: 71.8,278,2	0.06Å	Favored (41.674%)	-	-	-
A 128		LEU	0.94	-	Favored (29.4%) General / -86.0,141.5	Favored (94.9%) *mt* chi angles: 296.2,176.2	0.04Å	Favored (33.551%)	-	-	-
A 129		ALA	0.82	-	Favored (29.79%) General / -75.7,160.7	-	0.05Å	Favored (40.059%)	-	-	-
A 130		ASN	0.75	-	Favored (70.55%) General / -67.2,-30.6	Favored (97.1%) *m-40* chi angles: 287.7,335.6	0.04Å	Favored (32.851%)	-	-	-
A 131		ASN	0.7	-	Favored (29.96%) General / -92.2,117.5	Favored (80.9%) *m-40* chi angles: 300.2,311	0.08Å	Favored (22.277%)	-	-	-
A 132		THR	0.67	-	Favored (32.73%) General / -126.4,159.6	Favored (77.7%) *p* chi angles: 60.9	0.04Å	Favored (50.351%)	-	-	-
A 133		PHE	0.65	-	Favored (26.33%) General / -118.9,116.4	Favored (56.8%) *t80* chi angles: 183.4,93.8	0.05Å	Favored (55.276%) beta sheet	-	-	-
A 134		VAL	0.65	-	Favored (50.6%) Ile or Val / -101.1,130.4	Favored (76.1%) *t* chi angles: 178.2	0.04Å	Favored (59.864%)	-	-	-
A 135		ILE	0.67	-	Favored (61.89%) Ile or Val / -107.8,122.3	Favored (81.7%) *mt* chi angles: 299.3,173.4	0.10Å	Favored (11.466%)	-	-	-
A 136		ASP	0.71	-	Favored (23.92%) General / 51.7,38.9	Favored (76.1%) *m-30* chi angles: 295.1,324.2	0.05Å	Favored (5.672%)	-	-	-

A 137		GLY	0.76	-	Favored (34.11%) Glycine / -94.9,175.8	-	-	Favored (24.845%)	-	-	-
A 138		PRO	0.82	-	Favored (86.58%) Trans-Pro / -62.0,150.4	Favored (54.8%) *Cg_exo* chi angles: 337.4,34,329.1	0.09Å	Favored (48.943%)	-	-	-
A 139		GLU	0.86	-	Favored (45.68%) General / -64.6,131.0	Favored (45.6%) *tt0* chi angles: 182.3,175.1,61.7	0.03Å	Favored (27.914%)	-	-	-
A 140		THR	0.89	-	Favored (18.11%) General / -132.0,168.3	Favored (70.6%) *p* chi angles: 62.3	0.02Å	Favored (42.345%)	-	-	-
**#**	**Alt**	**Res**	**High B**	**Clash > 0.4Å**	**Ramachandran**	**Rotamer**	**Cβ deviation**	**CaBLAM**	**Bond lengths**	**Bond angles**	**Cis Peptides**
			Avg: 1.18	Clashscore: 1.47	Outliers: 1 of 350	Poor rotamers: 0 of 301	Outliers: 0 of 323	Outliers: 10 of 348	Outliers: 2 of 352	Outliers: 9 of 352	Non-Trans: 1 of 351
A 141		GLU	0.88	-	Favored (89.36%) General / -63.4,-38.0	Favored (99%) *mt-10* chi angles: 290.9,177.5,352.9	0.03Å	Favored (56.901%)	-	-	-
A 142		GLU	0.86	-	Favored (75.5%) General / -70.0,-37.3	Favored (99.9%) *mt-10* chi angles: 291.9,179.6,354.4	0.02Å	Favored (57.443%) alpha helix	-	-	-
A 143		CYS	0.82	-	Favored (46.24%) Pre-Pro / -135.8,79.4	Favored (42.5%) *t* chi angles: 186.1	0.02Å	Favored (22.071%)	-	-	-
A 144		PRO	0.77	-	Favored (44.58%) Trans-Pro / -72.8,161.2	Favored (76%) *Cg_endo* chi angles: 28.7,324.9,27	0.03Å	Favored (27.111%)	-	-	-
A 145		THR	0.73	-	Favored (63.77%) General / -53.6,-38.2	Favored (87.5%) *m* chi angles: 301.4	0.03Å	Favored (47.121%)	-	-	-
A 146		ALA	0.7	-	Favored (61.75%) General / -56.9,-28.1	-	0.04Å	Favored (59.002%) alpha helix	-	-	-
A 147		ASN	0.68	-	Favored (25.24%) General / -105.7,16.5	Favored (81.2%) *m-40* chi angles: 289,319.6	0.10Å	Favored (32.798%)	-	-	-
A 148		ARG	0.67	-	Favored (32.67%) General / -108.2,146.5	Favored (97.9%) *mtt-85* chi angles: 293.9,178.6,182.8,273.2	0.04Å	Favored (33.88%)	-	-	-
A 149		ALA	0.67	-	Favored (13.59%) General / -93.8,163.3	-	0.03Å	Favored (35.742%)	-	-	-
A 150		TRP	0.67	-	Favored (25.77%) General / -151.3,146.1	Favored (85.8%) *t60* chi angles: 175.8,87.6	0.04Å	Favored (8.064%)	-	-	-
A 151		ASN	0.68	-	Favored (18.96%) General / 56.8,47.0	Favored (61.5%) *t0* chi angles: 197.8,30.4	0.01Å	Favored (9.578%)	-	OUTLIER(S) worst is CA-CB-CG: 4.3 σ	-
A 152		SER	0.7	-	Favored (55.15%) General / -93.3,-2.2	Favored (68%) *m* chi angles: 296.8	0.02Å	Favored (21.993%)	-	-	-

A 153		MET	0.73	-	Favored (27.48%) General / -122.8,159.7	Favored (54.8%) *mtp* chi angles: 302,190.5,81.2	0.04Å	Favored (19.378%)	-	-	-
A 154		GLU	0.77	-	Favored (26.04%) General / -147.8,142.0	Favored (43.8%) *tt0* chi angles: 184.6,174.8,46.4	0.08Å	Favored (41.124%)	-	-	-
A 155		VAL	0.85	-	Favored (35.15%) Ile or Val / -76.9,124.3	Favored (90.4%) *t* chi angles: 174.2	0.05Å	Favored (45.303%)	-	-	-
A 156		GLU	0.98	-	Favored (12.81%) General / -89.2,-40.5	Favored (28.9%) *tt0* chi angles: 186.5,167.1,60.6	0.02Å	Favored (26.17%)	-	-	-
A 157		ASP	1.18	-	Favored (14.42%) General / -155.3,175.0	Favored (11.9%) *t0* chi angles: 207.9,338.3	0.05Å	Favored (8.312%)	-	-	-
A 158		PHE	1.47	-	Favored (36.01%) General / -158.6,164.4	Favored (50.1%) *p90* chi angles: 58.4,90.8	0.08Å	CA Geom Outlier (0.363%)	-	-	-
A 159		GLY	1.83	-	Favored (22.2%) Glycine / -167.6,-163.8	-	-	Favored (51.196%) beta sheet	-	-	-
A 160		PHE	2.19	-	Favored (39.06%) General / -156.4,159.8	Favored (48.8%) *p90* chi angles: 57.9,91.1	0.01Å	Favored (44.003%)	-	-	-
**#**	**Alt**	**Res**	**High B**	**Clash > 0.4Å**	**Ramachandran**	**Rotamer**	**Cβ deviation**	**CaBLAM**	**Bond lengths**	**Bond angles**	**Cis Peptides**
			Avg: 1.18	Clashscore: 1.47	Outliers: 1 of 350	Poor rotamers: 0 of 301	Outliers: 0 of 323	Outliers: 10 of 348	Outliers: 2 of 352	Outliers: 9 of 352	Non-Trans: 1 of 351
A 161		GLY	2.45	-	Favored (12.15%) Glycine / -110.7,-150.1	-	-	Favored (48.122%)	-	-	-
A 162		LEU	2.52	-	Favored (14.16%) General / -100.0,-25.9	Favored (88.7%) *mt* chi angles: 299.5,178.1	0.01Å	Favored (6.585%)	-	-	-
A 163		THR	2.38	-	Favored (12.86%) General / -113.5,-12.7	Favored (79%) *p* chi angles: 60.7	0.03Å	Favored (23.106%)	-	-	-
A 164		SER	2.09	-	Favored (34.91%) General / -159.3,164.3	Favored (89%) *p* chi angles: 68.3	0.03Å	Favored (21.989%)	-	-	-
A 165		THR	1.78	-	Favored (10.09%) General / -102.1,167.3	Favored (62.8%) *p* chi angles: 63.5	0.06Å	Favored (45.779%)	-	-	-
A 166		ARG	1.5	-	Favored (49.06%) General / -130.2,136.3	Favored (82.1%) *mtp180* chi angles: 300.6,179.5,66.2,183.1	0.08Å	Favored (55.517%) beta sheet	-	-	-
A 167		MET	1.28	-	Favored (50.83%) General / -129.0,146.9	Favored (35.1%) *mtt* chi angles: 294.7,188.1,162.7	0.05Å	Favored (61.503%) beta sheet	-	-	-
A 168		PHE	1.13	-	Favored (54.7%)	Favored (69.4%) *m-80* chi angles: 301.7,84.7	0.07Å	Favored (61.65%) beta sheet	-	OUTLIER(S) worst is CA-CB-CG: 4.8 σ	-

					General / -116.7,127.8						
A 169		LEU	1.03	-	Favored (16.07%) General / -95.9,157.1	Favored (67.3%) *mt* chi angles: 298.3,169.3	0.07Å	Favored (24.569%)	-	-	-
A 170		ARG	0.98	-	Favored (37.51%) General / -140.9,161.6	Favored (39.3%) *ptt180* chi angles: 66.2,182,182.2,192.9	0.05Å	Favored (41.408%)	-	-	-
A 171		ILE	0.95	-	Favored (32.37%) Ile or Val / -84.5,132.1	Favored (48.2%) *mm* chi angles: 303,299	0.02Å	Favored (24.846%)	-	-	-
A 172		ARG	0.94	-	Favored (49.67%) General / -70.6,147.1	Favored (95.1%) *mtt-85* chi angles: 289.5,178.1,179.6,270.8	0.03Å	Favored (32.398%) beta sheet	-	-	-
A 173		GLU	0.96	-	Favored (25.67%) General / -106.6,2.0	Favored (96.9%) *mt-10* chi angles: 296.5,180.1,358.2	0.01Å	Favored (29.373%)	-	-	-
A 174		THR	0.97	-	Favored (54.41%) General / -122.8,132.9	Favored (91.7%) *m* chi angles: 299	0.00Å	Favored (23.9%)	-	-	-
A 175		ASN	0.98	-	Favored (11.01%) General / -82.2,102.2	Favored (55.3%) *t0* chi angles: 182.9,336.1	0.03Å	Favored (16.569%)	-	-	-
A 176		THR	0.99	-	Favored (7.8%) General / -140.7,177.8	Favored (12%) *t* chi angles: 190.4	0.02Å	Favored (27.761%) beta sheet	-	-	-
A 177		THR	0.98	-	Favored (8.36%) General / -124.5,3.3	Favored (71.4%) *p* chi angles: 59.6	0.02Å	Favored (13.746%) beta sheet	-	-	-
A 178		GLU	0.96	-	Favored (27.08%) General / -81.8,152.2	Favored (95.2%) *mt-10* chi angles: 292,183.6,344.8	0.05Å	Favored (38.878%)	-	-	-
A 179		CYS	0.94	-	Favored (18.47%) General / -83.2,166.6	Favored (84.2%) *m* chi angles: 295.5	0.03Å	Favored (34.76%)	-	-	-
A 180		ASP	0.93	-	Favored (11.71%) General / -79.4,106.8	Favored (63.9%) *t0* chi angles: 182.7,343.6	0.08Å	Favored (20.052%)	-	-	-
**#**	**Alt**	**Res**	**High B**	**Clash > 0.4Å**	**Ramachandran**	**Rotamer**	**Cβ deviation**	**CaBLAM**	**Bond lengths**	**Bond angles**	**Cis Peptides**
			Avg: 1.18	Clashscore: 1.47	Outliers: 1 of 350	Poor rotamers: 0 of 301	Outliers: 0 of 323	Outliers: 10 of 348	Outliers: 2 of 352	Outliers: 9 of 352	Non-Trans: 1 of 351
A 181		SER	0.93	-	Favored (61.38%) General / -62.0,-18.7	Favored (94.4%) *p* chi angles: 64.8	0.03Å	Favored (38.579%)	-	-	-
A 182		LYS	0.95	-	Favored (58.99%) General / -81.4,-6.5	Favored (73.7%) *mmtt* chi angles: 295.5,294.3,185.1,180.8	0.04Å	Favored (49.512%) alpha helix	-	-	-
A 183		ILE	0.97	-	Favored (7.24%) Ile or Val / -117.1,10.2	Favored (38.5%) *pt* chi angles: 64.6,168.9	0.01Å	Favored (48.023%)	-	-	-
A 184		ILE	1	-	Favored (63.39%)	Favored (85.2%) *mt* chi angles: 299,170.2	0.02Å	Favored (25.64%)	-	-	-

					Ile or Val / -113.0,131.9							
A 185		GLY	1.06	-	Favored (21.28%) Glycine / -134.2,154.2	-	-	Favored (59.235%)	-	-	-	
A 186		THR	1.13	-	Favored (33.21%) General / -141.6,139.8	Favored (62.5%) *m* chi angles: 303.4	0.05Å	Favored (54.082%) beta sheet	-	-	-	
A 187		ALA	1.21	-	Favored (42.84%) General / -151.7,158.0	-	0.03Å	Favored (46.36%) beta sheet	-	-	-	
A 188		VAL	1.28	-	Favored (52.83%) Ile or Val / -133.8,127.6	Favored (6.1%) *p* chi angles: 58.9	0.07Å	Favored (31.767%)	-	-	-	
A 189		LYS	1.32	-	Favored (20.74%) General / -148.8,136.7	Favored (88.4%) *mttt* chi angles: 301.1,183,186.9,179.6	0.08Å	Favored (12.311%)	-	-	-	
A 190		ASN	1.3	-	Favored (30.02%) General / 51.8,42.5	Favored (86.5%) *m-40* chi angles: 298.2,315.6	0.03Å	Favored (27.03%)	-	-	-	
A 191		ASN	1.24	-	Favored (9.22%) General / 65.8,13.9	Favored (88.7%) *m-40* chi angles: 296.8,320.6	0.07Å	Favored (9.1%)	-	-	-	
A 192		MET	1.14	-	Favored (28.44%) General / -112.7,152.9	Favored (90.8%) *mmm* chi angles: 300.9,295.8,293.3	0.03Å	Favored (30.603%)	-	-	-	
A 193		ALA	1.04	-	Favored (45.52%) General / -137.8,148.4	-	0.04Å	Favored (71.643%) beta sheet	-	-	-	
A 194		VAL	0.96	-	Favored (29.52%) Ile or Val / -140.8,142.9	Favored (8.8%) *p* chi angles: 61.6	0.06Å	Favored (66.741%) beta sheet	-	-	-	
A 195		HIS	0.91	-	Favored (51.94%) General / -130.9,145.2	Favored (33.4%) *m90* chi angles: 302.4,70.1	0.11Å	Favored (44.441%) beta sheet	-	OUTLIER(S) worst is CA-CB-CG: 5.6 σ	-	
A 196		SER	0.89	-	Favored (31.93%) General / -160.7,164.3	Favored (86.1%) *p* chi angles: 69.7	0.07Å	Favored (25.804%)	-	-	-	
A 197		ASP	0.87	-	Favored (4.96%) General / -148.7,-175.5	Favored (4.8%) *p0* chi angles: 71.2,35.4	0.08Å	Favored (25.556%)	-	OUTLIER(S) worst is CA-CB-CG: 8.1 σ	-	
A 198		LEU	0.86	-	Favored (68.8%) General / -61.4,-27.8	Favored (94.5%) *mt* chi angles: 296.1,172.7	0.10Å	Favored (14.598%)	-	-	-	
A 199		SER	0.85	-	Favored (18.8%) General / -109.8,0.6	Favored (99%) *p* chi angles: 65.4	0.10Å	Favored (26.017%) alpha helix	-	-	-	
A 200		TYR	0.84	-	Favored (18.79%) General / -154.0,140.5	Favored (61.7%) *t80* chi angles: 171.3,69.1	0.04Å	Favored (26.771%)	-	-	-	
**#**	**Alt**	**Res**	**High B**	**Clash > 0.4Å**	**Ramachandran**	**Rotamer**	**Cβ deviation**	**CaBLAM**	**Bond lengths**	**Bond angles**	**Cis Peptides**	
			Avg: 1.18	Clashscore: 1.47	Outliers: 1 of 350	Poor rotamers: 0 of 301	Outliers: 0 of 323	Outliers: 10 of 348	Outliers: 2 of 352	Outliers: 9 of 352	Non-Trans: 1	

											of 351
A 201		TRP	0.83	-	Favored (33.45%) General / -141.2,138.6	Favored (83.8%) *t60* chi angles: 178.2,90.1	0.08Å	Favored (60.226%)	-	-	-
A 202		ILE	0.84	-	Favored (48.94%) Ile or Val / -136.4,136.2	Favored (16.1%) *tt* chi angles: 180.9,167.6	0.09Å	Favored (65.699%) beta sheet	-	-	-
A 203		GLU	0.87	-	Favored (46.93%) General / -129.5,132.8	Favored (46.8%) *tt0* chi angles: 180.6,175.2,62.1	0.05Å	Favored (53.273%) beta sheet	-	-	-
A 204		SER	0.93	-	Favored (14.31%) General / -135.9,171.6	Favored (16.6%) *m* chi angles: 305.9	0.12Å	Favored (20.425%)	-	-	-
A 205		GLY	1.02	-	Favored (39.25%) Glycine / -163.3,172.3	-	-	Favored (33.717%)	-	-	-
A 206		LEU	1.09	-	Favored (55.48%) General / -109.2,130.8	Favored (8.1%) *tt* chi angles: 182.3,147.3	0.06Å	Favored (9.189%)	-	-	-
A 207		ASN	1.14	-	Allowed (0.91%) General / -101.2,-66.5	Favored (46.8%) *t0* chi angles: 185.2,65.5	0.04Å	CaBLAM Disfavored (2.057%)	-	-	-
A 208		ASP	1.13	-	Allowed (1.42%) General / -140.0,-3.3	Favored (16.3%) *p0* chi angles: 60.9,312	0.02Å	CaBLAM Outlier (0.341%)	-	-	-
A 209		THR	1.07	-	Favored (33.14%) General / -156.2,155.9	Favored (9.8%) *t* chi angles: 185.8	0.07Å	Favored (18.401%)	-	-	-
A 210		TRP	0.98	-	Favored (37.11%) General / -79.6,137.5	Favored (98%) *m100* chi angles: 289.2,99.6	0.03Å	Favored (23.288%)	-	-	-
A 211		LYS	0.88	-	Favored (36.75%) General / -147.1,151.4	Favored (60.3%) *pttt* chi angles: 63.8,181.5,180.4,178.8	0.08Å	Favored (37.002%) beta sheet	-	-	-
A 212		LEU	0.8	-	Favored (43.78%) General / -64.7,130.5	Favored (73.2%) *tp* chi angles: 177.4,63	0.03Å	Favored (33.645%)	-	-	-
A 213		GLU	0.74	-	Favored (5.6%) General / -107.2,-39.8	Favored (88.8%) *mt-10* chi angles: 294.9,188.4,359.2	0.03Å	Favored (15.538%)	-	-	-
A 214		ARG	0.72	-	Favored (18.28%) General / -157.6,144.7	Favored (54.2%) *ttt90* chi angles: 181,172.9,166.4,86.5	0.06Å	Favored (20.254%)	-	-	-
A 215		ALA	0.72	-	Favored (33.38%) General / -145.1,148.2	-	0.03Å	Favored (66.112%)	-	-	-
A 216		VAL	0.74	-	Favored (57.41%) Ile or Val / -129.1,122.9	Favored (74.9%) *t* chi angles: 178.3	0.04Å	Favored (67.535%) beta sheet	-	-	-
A 217		LEU	0.76	-	Favored (21.87%) General / -106.0,109.8	Favored (4.6%) *mp* chi angles: 283.4,74.9	0.02Å	Favored (71.632%)	-	-	-

#	Alt	Res	High B	Clash > 0.4Å	Ramachandran	Rotamer	Cβ deviation	CaBLAM	Bond lengths	Bond angles	Cis Peptides
A 218		GLY	0.79	-	Favored (71.25%) Glycine / -62.2,-49.8	-	-	Favored (13.491%)	-	-	-
A 219		GLU	0.82	-	Favored (45.81%) General / -139.6,151.2	Favored (91%) *mt-10* chi angles: 297.6,184.6,0.1	0.08Å	Favored (17.846%)	-	-	-
A 220		VAL	0.84	-	Favored (19.92%) Ile or Val / -100.3,140.2	Favored (39.9%) *t* chi angles: 183.4	0.05Å	Favored (49.209%)	-	-	-
**#**	**Alt**	**Res**	**High B**	**Clash > 0.4Å**	**Ramachandran**	**Rotamer**	**Cβ deviation**	**CaBLAM**	**Bond lengths**	**Bond angles**	**Cis Peptides**
			Avg: 1.18	Clashscore: 1.47	Outliers: 1 of 350	Poor rotamers: 0 of 301	Outliers: 0 of 323	Outliers: 10 of 348	Outliers: 2 of 352	Outliers: 9 of 352	Non-Trans: 1 of 351
A 221		LYS	0.84	-	Favored (49.92%) General / -135.4,155.8	Favored (49.3%) *pttt* chi angles: 60.5,190.1,179.2,188.6	0.09Å	Favored (56.177%) beta sheet	-	-	-
A 222		SER	0.83	-	Favored (56.32%) General / -79.0,-6.5	Favored (52.3%) *m* chi angles: 291.9	0.10Å	Favored (8.674%) beta sheet	-	-	-
A 223		CYS	0.81	-	Favored (56.44%) General / -65.6,136.5	Favored (52.4%) *t* chi angles: 184.2	0.07Å	Favored (38.605%) beta sheet	-	-	-
A 224		THR	0.8	-	Favored (30.72%) General / -102.5,144.6	Favored (68.6%) *p* chi angles: 59	0.09Å	Favored (39.508%) beta sheet	-	-	-
A 225		TRP	0.78	-	Favored (68.73%) Pre-Pro / -76.4,140.4	Favored (21.9%) *m-10* chi angles: 283.3,30.9	0.06Å	Favored (46.824%) beta sheet	-	-	-
A 226		PRO	0.77	-	Favored (97.19%) Trans-Pro / -60.1,146.0	Favored (61.5%) *Cg_exo* chi angles: 335.9,33.5,331.4	0.05Å	Favored (73.753%)	-	-	-
A 227		GLU	0.77	-	Favored (67.54%) General / -63.9,-24.0	Favored (98.2%) *mt-10* chi angles: 290,178.4,354.7	0.03Å	Favored (38.254%)	-	-	-
A 228		THR	0.78	-	Favored (60.01%) General / -67.9,-12.1	Favored (79%) *p* chi angles: 60.4	0.05Å	Favored (31.611%)	-	-	-
A 229		HIS	0.81	-	Favored (35.12%) General / -104.3,6.3	Favored (97.2%) *m-70* chi angles: 295.1,287.9	0.04Å	Favored (57.245%)	OUTLIER(S) worst is CB--CG: 4.9 σ	-	-
A 230		THR	0.86	-	Favored (26.26%) General / -88.2,144.8	Favored (77.5%) *p* chi angles: 61	0.08Å	Favored (33.028%)	-	-	-
A 231		LEU	0.93	-	Favored (5.76%) General / -76.7,-55.2	Favored (60.6%) *tp* chi angles: 180.8,59.5	0.05Å	Favored (16.745%)	-	-	-
A 232		TRP	0.98	-	Favored (3.46%) General / -129.2,92.9	Favored (19.1%) *t60* chi angles: 184.1,20.4	0.04Å	CaBLAM Disfavored (2.518%)	-	-	-
A 233		GLY	1.02	-	Favored (51.69%) Glycine / -101.9,0.6	-	-	CaBLAM Disfavored (4.346%)	-	-	-

A 234		ASP	1.03	-	Favored (22.04%) General / -77.7,120.0	Favored (78.4%) *m-30* chi angles: 293.2,325.8	0.02Å	Favored (5.949%)	-	-	-
A 235		GLY	0.99	-	Favored (87.13%) Glycine / 83.3,7.0	-	-	Favored (56.256%)	-	-	-
A 236		VAL	0.93	-	Favored (36.15%) Ile or Val / -74.1,130.0	Favored (90.5%) *t* chi angles: 174.2	0.03Å	Favored (23.747%)	-	-	-
A 237		LEU	0.86	-	Favored (43.6%) General / -98.5,125.0	Favored (53.9%) *tp* chi angles: 175.3,65.1	0.02Å	Favored (59.677%)	-	-	-
A 238		GLU	0.8	-	Favored (73.93%) General / -58.4,-36.8	Favored (98.7%) *mt-10* chi angles: 290.4,176.2,354.1	0.06Å	Favored (48.617%)	-	-	-
A 239		SER	0.75	-	Favored (64.18%) General / -61.1,-21.9	Favored (64.1%) *m* chi angles: 294.1	0.03Å	Favored (62.569%) alpha helix	-	-	-
A 240		ASP	0.73	-	Favored (44.74%) General / -99.0,8.7	Favored (83.4%) *m-30* chi angles: 295,344.1	0.04Å	Favored (50.575%)	-	-	-
**#**	**Alt**	**Res**	**High B**	**Clash > 0.4Å**	**Ramachandran**	**Rotamer**	**Cβ deviation**	**CaBLAM**	**Bond lengths**	**Bond angles**	**Cis Peptides**
			Avg: 1.18	Clashscore: 1.47	Outliers: 1 of 350	Poor rotamers: 0 of 301	Outliers: 0 of 323	Outliers: 10 of 348	Outliers: 2 of 352	Outliers: 9 of 352	Non-Trans: 1 of 351
A 241		LEU	0.72	-	Favored (29.88%) General / -91.1,117.8	Favored (81.3%) *mt* chi angles: 297.3,180.8	0.06Å	Favored (32.877%)	-	-	-
A 242		ILE	0.73	0.42Å CD1 with A 242 ILE N	Favored (92.86%) Ile or Val / -63.6,-41.5	Allowed (0.4%) *mp* chi angles: 277.7,44.6	0.05Å	Favored (50.746%)	-	-	-
A 243		ILE	0.76	-	Favored (64.9%) Pre-Pro / -96.1,119.2	Favored (71.5%) *mt* chi angles: 301.7,168.6	0.10Å	Favored (24.393%)	-	-	-
A 244		PRO	0.79	-	Favored (92.55%) Trans-Pro / -58.5,146.1	Favored (68.6%) *Cg_exo* chi angles: 335.4,34.6,330.5	0.07Å	Favored (70.81%)	-	-	-
A 245		ILE	0.83	0.41Å HA with A 245 ILE HD13	Favored (80.19%) Ile or Val / -62.8,-37.8	Favored (41%) *mm* chi angles: 297.1,302.3	0.02Å	Favored (54.34%)	-	-	-
A 246		THR	0.85	-	Favored (29.26%) General / -70.5,-5.5	Favored (77.2%) *p* chi angles: 60.2	0.09Å	Favored (33.282%)	-	-	-
A 247		LEU	0.86	-	Favored (8.38%) General / -117.2,28.6	Favored (97.1%) *mt* chi angles: 297.1,177.4	0.10Å	Favored (31.428%)	-	-	-
A 248		ALA	0.85	-	Favored (3.92%) General / 69.7,30.2	-	0.02Å	Favored (6.158%)	-	-	-
A 249		GLY	0.83	-	Favored (34.39%) Glycine / -81.9,154.9	-	-	Favored (28.056%)	-	-	-
A 250		PRO	0.81	-	Favored (31.96%)	Favored (79.4%) *Cg_endo*	0.03Å	Favored (53.323%)	-	-	-

					Trans-Pro / -77.1,153.3	chi angles: 30.3,325.1,25.1					
A 251		ARG	0.79	-	Favored (10.06%) General / -83.7,75.5	Favored (98.5%) *mtt-85* chi angles: 295,176.4,182.1,274.1	0.02Å	Favored (9.91%)	-	-	-
A 252		SER	0.78	-	Favored (34.92%) General / -147.0,150.3	Favored (40.5%) *t* chi angles: 181	0.06Å	Favored (23.659%)	-	-	-
A 253		ASN	0.77	-	Favored (61.41%) General / -59.8,-21.8	Favored (66.1%) *m-40* chi angles: 284.4,282.3	0.03Å	Favored (41.858%)	-	-	-
A 254		HIS	0.76	-	Favored (61.26%) General / -71.5,-12.2	Favored (78.7%) *m90* chi angles: 292.3,86.8	0.06Å	Favored (52.159%)	-	-	-
A 255		ASN	0.76	-	Favored (40.51%) General / -99.7,10.9	Favored (36.5%) *t0* chi angles: 179.8,21.8	0.12Å	Favored (42.68%)	-	OUTLIER(S) worst is CA-CB-CG: 4.1 σ	-
A 256		ARG	0.75	-	Favored (18.79%) General / -100.0,153.9	Favored (68.4%) *mtp180* chi angles: 299.2,175.3,77.1,197.7	0.05Å	Favored (23.271%)	-	-	-
A 257		ARG	0.74	-	Favored (6.47%) Pre-Pro / -145.0,122.8	Favored (11.9%) *tpt90* chi angles: 180.9,77.8,158.8,87.9	0.09Å	Favored (7.349%)	-	-	-
A 258		PRO	0.72	-	Favored (70.7%) Trans-Pro / -54.1,137.8	Favored (96%) *Cg_exo* chi angles: 331.5,36.7,330.9	0.03Å	Favored (30.534%)	-	-	-
A 259		GLY	0.71	-	Favored (75.59%) Glycine / 90.7,-10.2	-	-	Favored (70.158%)	-	-	-
A 260		TYR	0.71	-	Favored (44.53%) General / -122.3,148.8	Favored (92.5%) *m-80* chi angles: 294,85.7	0.03Å	Favored (36.841%)	-	-	-
**#**	**Alt**	**Res**	**High B**	**Clash > 0.4Å**	**Ramachandran**	**Rotamer**	**Cβ deviation**	**CaBLAM**	**Bond lengths**	**Bond angles**	**Cis Peptides**
			Avg: 1.18	Clashscore: 1.47	Outliers: 1 of 350	Poor rotamers: 0 of 301	Outliers: 0 of 323	Outliers: 10 of 348	Outliers: 2 of 352	Outliers: 9 of 352	Non-Trans: 1 of 351
A 261		LYS	0.71	-	Favored (6.51%) General / -112.4,173.1	Favored (71.4%) *mmtt* chi angles: 301,297.5,185,187.5	0.02Å	Favored (7.293%)	-	-	-
A 262		THR	0.71	-	Favored (29.99%) General / -57.9,127.0	Favored (91.4%) *m* chi angles: 301.1	0.03Å	Favored (13.051%)	-	-	-
A 263		GLN	0.73	-	Favored (8.95%) General / -83.5,66.5	Favored (72%) *mm-40* chi angles: 302,290.2,297.1	0.09Å	Favored (20.896%)	-	-	-
A 264		ASN	0.75	-	Favored (67.83%) General / -62.9,-24.5	Favored (83.6%) *m-40* chi angles: 283.9,334	0.03Å	Favored (16.744%)	-	-	-
A 265		GLN	0.78	-	Favored (4.51%) General / -119.1,36.3	Favored (89.8%) *mt0* chi angles: 298.9,177.4,318.4	0.04Å	Favored (17.518%)	-	-	-

#	Alt	Res	High B	Clash > 0.4Å	Ramachandran	Rotamer	Cβ deviation	CaBLAM	Bond lengths	Bond angles	Cis Peptides
A 266		GLY	0.82	-	Favored (42.31%) Glycine / -75.5,-178.1	-	-	Favored (45.834%)	-	-	-
A 267		PRO	0.87	-	Favored (7.66%) Trans-Pro / -79.3,60.1	Favored (56.3%) *Cg_endo* chi angles: 32.3,324.1,24	0.06Å	CaBLAM Disfavored (1.495%)	-	-	-
A 268		TRP	0.92	-	Favored (58.9%) General / -87.8,-4.2	Favored (62%) *m100* chi angles: 301.9,116.7	0.04Å	Favored (12.174%)	-	-	-
A 269		ASP	0.97	-	Favored (52.37%) General / -95.1,5.2	Favored (46.6%) *m-30* chi angles: 287.9,313.8	0.08Å	Favored (55.086%)	-	OUTLIER(S) worst is CA-CB-CG: 5.8 σ	-
A 270		GLU	0.99	-	Favored (30.24%) General / -98.8,14.1	Favored (68%) *mt-10* chi angles: 297,187,326	0.09Å	CaBLAM Disfavored (2.047%)	-	-	-
A 271		GLY	0.97	-	Favored (6.1%) Glycine / 73.5,-118.3	-	-	CaBLAM Disfavored (2.555%)	-	-	-
A 272		ARG	0.93	-	Favored (19.62%) General / -64.4,123.6	Favored (59%) *ttt180* chi angles: 182.2,171.4,173.1,161.8	0.04Å	CaBLAM Disfavored (2.229%)	-	-	-
A 273		VAL	0.87	-	Favored (44.66%) Ile or Val / -130.4,118.8	Favored (66.7%) *t* chi angles: 179.2	0.06Å	Favored (50.83%)	-	-	-
A 274		GLU	0.8	-	Favored (30.25%) General / -100.8,144.0	Favored (88.7%) *mt-10* chi angles: 299.3,184.7,2.1	0.07Å	Favored (54.192%) beta sheet	-	-	-
A 275		ILE	0.75	-	Favored (72.29%) Ile or Val / -123.9,126.2	Favored (63%) *mt* chi angles: 304.6,172.5	0.09Å	Favored (45.66%) beta sheet	-	-	-
A 276		ASP	0.7	-	Favored (43.25%) General / -139.6,159.0	Favored (27.7%) *m-30* chi angles: 293.7,285.2	0.09Å	Favored (40.259%)	-	-	-
A 277		PHE	0.67	-	Favored (6.73%) General / -108.3,97.0	Favored (97.1%) *m-80* chi angles: 293.1,91.2	0.09Å	CaBLAM Disfavored (4.47%)	-	-	-
A 278		ASP	0.65	-	Favored (3.36%) General / -171.1,178.5	Favored (11.9%) *t0* chi angles: 205.8,337.2	0.06Å	Favored (8.469%)	-	-	-
A 279		TYR	0.64	-	Favored (26.12%) General / -97.8,145.7	Favored (87.4%) *m-80* chi angles: 295.2,83.9	0.06Å	Favored (10.546%)	-	-	-
A 280		CYS	0.63	-	Favored (91.63%) Pre-Pro / -68.1,136.7	Favored (98%) *m* chi angles: 292.3	0.05Å	Favored (24.128%)	-	-	-
**#**	**Alt**	**Res**	**High B**	**Clash > 0.4Å**	**Ramachandran**	**Rotamer**	**Cβ deviation**	**CaBLAM**	**Bond lengths**	**Bond angles**	**Cis Peptides**
			Avg: 1.18	Clashscore: 1.47	Outliers: 1 of 350	Poor rotamers: 0 of 301	Outliers: 0 of 323	Outliers: 10 of 348	Outliers: 2 of 352	Outliers: 9 of 352	Non-Trans: 1 of 351
A 281		PRO	0.63	-	Favored (54.21%) Trans-Pro / -52.5,135.1	Favored (88%) *Cg_exo* chi angles: 330.7,36.8,331.5	0.07Å	Favored (41.459%)	-	-	-
A 282		GLY	0.64	-	Favored (87.05%)	-	-	Favored (74.937%)	-	-	-

					Glycine / 82.4,-1.6						
A 283		THR	0.65	-	Favored (19.98%) General / -121.6,162.8	Favored (44.4%) *p* chi angles: 66.7	0.06Å	Favored (34.197%)	-	-	-
A 284		THR	0.66	-	Favored (52.2%) General / -127.1,136.5	Favored (95.5%) *m* chi angles: 299.5	0.02Å	Favored (66.66%) beta sheet	-	-	-
A 285		VAL	0.69	-	Favored (73.85%) Ile or Val / -116.5,128.6	Favored (79%) *t* chi angles: 178	0.04Å	Favored (65.608%) beta sheet	-	-	-
A 286		THR	0.72	-	Favored (10.7%) General / -116.0,167.8	Favored (63.6%) *p* chi angles: 63.4	0.07Å	Favored (27.707%) beta sheet	-	-	-
A 287		ILE	0.76	-	Favored (68.86%) Ile or Val / -111.5,126.5	Favored (66.4%) *mt* chi angles: 303.2,170.3	0.06Å	Favored (21.369%) beta sheet	-	-	-
A 288		SER	0.8	-	Favored (18.51%) General / -156.6,143.2	Favored (42.9%) *t* chi angles: 178.4	0.06Å	Favored (20.789%)	-	-	-
A 289		ASP	0.83	-	Favored (66.22%) General / -62.6,-22.1	Favored (94.9%) *m-30* chi angles: 290.2,348.2	0.04Å	Favored (42.97%)	-	-	-
A 290		SER	0.84	-	Favored (59.35%) General / -83.3,-9.0	Favored (83.9%) *p* chi angles: 67.5	0.03Å	Favored (46.12%)	-	-	-
A 291		CYS	0.84	-	Favored (58.86%) General / -64.0,140.3	Favored (50.8%) *t* chi angles: 184.5	0.03Å	Favored (31.507%)	-	-	-
A 292		GLY	0.82	-	Favored (28.24%) Glycine / -57.0,150.7	-	-	Favored (40.864%)	-	-	-
A 293		HIS	0.79	-	Favored (46.84%) General / -60.1,145.4	Favored (73.5%) *t-90* chi angles: 193.7,281.4	0.01Å	Favored (17.174%)	-	-	-
A 294		ARG	0.75	-	Favored (13.96%) General / -49.8,139.0	Favored (70.6%) *ttt-90* chi angles: 183.7,178.6,179.9,270.8	0.05Å	CaBLAM Disfavored (2.105%)	-	-	-
A 295		GLY	0.72	-	Favored (29.67%) Glycine / -170.5,-166.6	-	-	Favored (8.296%)	-	-	-
A 296		PRO	0.69	-	Favored (56.96%) Trans-Pro / -53.8,143.8	Favored (95.8%) *Cg_exo* chi angles: 332.8,36.7,329.9	0.07Å	CaBLAM Disfavored (4.809%)	-	-	-
A 297		ALA	0.67	-	Favored (31.06%) General / -58.9,148.2	-	0.03Å	Favored (23.74%)	-	-	-
A 298		ALA	0.66	-	Favored (51.7%) General / -127.9,140.9	-	0.05Å	Favored (47.726%) beta sheet	-	-	-
A 299		ARG	0.67	-	Favored (38.15%) General / -76.5,146.9	Favored (34.6%) *ttp-170* chi angles: 200.3,169.1,73.3,185.2	0.03Å	Favored (42.405%)	-	-	-

#	Alt	Res	High B	Clash > 0.4Å	Ramachandran	Rotamer	Cβ deviation	CaBLAM	Bond lengths	Bond angles	Cis Peptides
A 300		THR	0.69	-	Favored (66.11%) General / -66.2,-20.4	Favored (54.1%) *p* chi angles: 56.7	0.10Å	Favored (56.451%)	-	-	-
#	Alt	Res	High B	Clash > 0.4Å	Ramachandran	Rotamer	Cβ deviation	CaBLAM	Bond lengths	Bond angles	Cis Peptides
			Avg: 1.18	Clashscore: 1.47	Outliers: 1 of 350	Poor rotamers: 0 of 301	Outliers: 0 of 323	Outliers: 10 of 348	Outliers: 2 of 352	Outliers: 9 of 352	Non-Trans: 1 of 351
A 301		THR	0.72	-	Favored (40.13%) General / -112.7,145.6	Favored (63.8%) *p* chi angles: 58	0.02Å	Favored (24.276%)	-	-	-
A 302		THR	0.76	-	Favored (10.93%) General / -77.7,174.5	Favored (71.5%) *p* chi angles: 62.1	0.07Å	Favored (27.895%)	-	-	-
A 303		GLU	0.8	-	Favored (66.86%) General / -59.3,-28.6	Favored (93%) *mt-10* chi angles: 289.1,183,350.1	0.03Å	Favored (54.998%)	-	-	-
A 304		SER	0.81	-	Favored (59.82%) General / -79.3,-10.5	Favored (71.2%) *p* chi angles: 71.9	0.12Å	Favored (51.54%)	-	-	-
A 305		GLY	0.81	-	Favored (68.76%) Glycine / 94.2,-12.7	-	-	Favored (65.505%)	-	-	-
A 306		LYS	0.78	-	Favored (55.81%) General / -60.1,133.1	Favored (86.3%) *tttt* chi angles: 182.3,178.2,178.3,180.3	0.03Å	Favored (37.272%)	-	-	-
A 307		LEU	0.74	-	Favored (34.54%) General / -80.8,129.5	Favored (43.9%) *tp* chi angles: 182.1,66.5	0.05Å	Favored (46.381%) beta sheet	-	-	-
A 308		ILE	0.71	-	Favored (41.82%) Ile or Val / -91.6,121.1	Favored (81.5%) *mt* chi angles: 299.7,169.5	0.11Å	Favored (57.94%) beta sheet	-	-	-
A 309		THR	0.67	-	Favored (14.55%) General / -106.7,-16.2	Favored (72.2%) *p* chi angles: 61.9	0.05Å	Favored (14.126%)	-	-	-
A 310		ASP	0.66	-	Favored (10.24%) General / -109.5,101.3	Favored (62.6%) *t0* chi angles: 184.8,341.4	0.10Å	Favored (16.387%)	-	OUTLIER(S) worst is CA-CB-CG: 5.8 σ	-
A 311		TRP	0.66	-	Favored (24.54%) General / -117.9,158.6	Favored (69.1%) *m100* chi angles: 293.2,76.1	0.02Å	Favored (17.29%)	-	-	-
A 312		CYS	0.68	-	Favored (19.32%) General / -143.3,168.6	Favored (28.7%) *p* chi angles: 61.3	0.17Å	Favored (62.531%) beta sheet	-	-	-
A 313		CYS	0.71	-	Favored (28.78%) General / -142.8,164.9	Favored (18.4%) *p* chi angles: 71.1	0.03Å	Favored (44.436%)	-	-	-
A 314		ARG	0.75	-	Favored (9%) General / -105.7,99.4	Favored (84.6%) *mtp85* chi angles: 296.1,185,67.1,81.8	0.06Å	CaBLAM Disfavored (1.678%)	-	-	-
A 315		SER	0.78	-	Favored (23.47%) General / 53.9,36.2	Favored (58.5%) *m* chi angles: 299.4	0.02Å	CaBLAM Disfavored (1.52%)	-	-	-

A 316		CYS	0.79	-	Favored (13.66%) General / -86.4,168.6	Favored (11.1%) *p* chi angles: 74.9	0.09Å	Favored (32.79%)	-	-	-
A 317		THR	0.78	-	Favored (19.85%) General / -104.4,154.4	Favored (74.6%) *p* chi angles: 61.4	0.05Å	Favored (12.324%)	-	-	-
A 318		LEU	0.75	0.40Å HA with A 319 PRO C	Favored (28.44%) Pre-Pro / -109.9,160.3	Favored (80.1%) *mt* chi angles: 302.1,177.1	0.09Å	CA Geom Outlier (0.48%)	-	-	-
A 319		PRO	0.71	0.40Å C with A 318 LEU HA	Favored (29.47%) Cis-Pro / -58.3,145.2	Favored (59.4%) *Cg_exo* chi angles: 336,36,327.6	0.05Å	Favored (46.508%)	-	-	Cis PRO omega= -7.79
A 320		PRO	0.68	-	Favored (52.54%) Trans-Pro / -72.9,156.3	Favored (73.3%) *Cg_endo* chi angles: 29,326.1,24.4	0.05Å	Favored (55.941%)	-	-	-
**#**	**Alt**	**Res**	**High B**	**Clash > 0.4Å**	**Ramachandran**	**Rotamer**	**Cβ deviation**	**CaBLAM**	**Bond lengths**	**Bond angles**	**Cis Peptides**
			Avg: 1.18	Clashscore: 1.47	Outliers: 1 of 350	Poor rotamers: 0 of 301	Outliers: 0 of 323	Outliers: 10 of 348	Outliers: 2 of 352	Outliers: 9 of 352	Non-Trans: 1 of 351
A 321		LEU	0.66	-	Favored (27.7%) General / -69.0,125.8	Favored (63.8%) *tp* chi angles: 178.1,64.3	0.06Å	Favored (30%)	-	-	-
A 322		ARG	0.67	-	Favored (49.62%) General / -135.5,156.0	Favored (49.7%) *ptt90* chi angles: 69,179.9,184.5,89.1	0.06Å	Favored (51.244%) beta sheet	-	-	-
A 323		PHE	0.69	-	Favored (49.66%) General / -122.6,143.6	Favored (88.7%) *m-80* chi angles: 297.3,84.8	0.07Å	Favored (66.397%) beta sheet	-	-	-
A 324		GLN	0.72	-	Favored (35.47%) General / -126.7,122.7	Favored (54.4%) *tt0* chi angles: 182.2,173.9,326.8	0.06Å	Favored (39.027%)	-	-	-
A 325		THR	0.74	-	Favored (4.46%) General / -129.0,-178.0	Favored (17.2%) *p* chi angles: 73.8	0.05Å	Favored (14.704%)	-	-	-
A 326		GLU	0.75	-	Favored (64.87%) General / -63.0,-20.0	Favored (98%) *mt-10* chi angles: 292,181.5,0.7	0.01Å	Favored (43.038%)	-	-	-
A 327		ASN	0.73	-	Favored (40.05%) General / -97.7,10.4	Favored (87.6%) *m-40* chi angles: 290.6,323.1	0.04Å	Favored (41.717%)	-	-	-
A 328		GLY	0.7	-	Favored (14.28%) Glycine / 112.7,-171.9	-	-	Favored (30.819%)	-	-	-
A 329		CYS	0.66	-	Favored (37.78%) General / -93.8,132.8	Favored (31%) *t* chi angles: 189.4	0.04Å	Favored (8.364%)	-	-	-
A 330		TRP	0.63	-	Favored (38.12%) General / -117.1,150.8	Favored (46.7%) *m100* chi angles: 292.7,68.5	0.12Å	Favored (52.415%) beta sheet	-	-	-
A 331		TYR	0.61	-	Favored (23.86%)	Favored (32.5%) *m-80* chi angles: 280.1,109.1	0.10Å	Favored (33.358%)	-	-	-

					General / -97.5,147.8						
A 332		GLY	0.6	-	Favored (26.52%) Glycine / -67.9,175.8	-	-	Favored (44.247%)	-	-	-
A 333		MET	0.6	-	Favored (68.91%) General / -63.0,-26.6	Favored (93.6%) *mtp* chi angles: 292.5,182.5,71.4	0.01Å	Favored (41.973%)	-	-	-
A 334		GLU	0.61	0.41Å N with A 334 GLU OE1	Favored (59.46%) General / -82.5,-6.7	Favored (58%) *mp0* chi angles: 290.9,76.7,2.8	0.03Å	Favored (59.864%)	-	-	-
A 335		ILE	0.65	-	Favored (70.36%) Ile or Val / -116.6,122.7	Favored (2.7%) *mp* chi angles: 303.8,103.8	0.01Å	Favored (22.122%)	-	-	-
A 336		ARG	0.72	-	Favored (71.24%) Pre-Pro / -126.2,157.0	Favored (36.4%) *mmm160* chi angles: 303.4,291.2,296.1,176.2	0.03Å	Favored (31.533%)	-	-	-
A 337		PRO	0.82	-	Favored (65.76%) Trans-Pro / -62.6,154.3	Favored (46.1%) *Cg_exo* chi angles: 338,34.9,326.9	0.08Å	Favored (59.153%)	-	-	-
A 338		THR	0.94	-	Allowed (0.53%) General / -105.0,-81.7	Favored (55.9%) *p* chi angles: 56.9	0.04Å	CaBLAM Outlier (0.541%)	-	-	-
A 339		ARG	1.06	-	Favored (6.66%) General / -80.3,69.3	Favored (72.8%) *mtm180* chi angles: 293.4,181.3,289,160.9	0.03Å	CaBLAM Outlier (0.632%)	-	-	-
A 340		HIS	1.14	-	Favored (15.6%) General / -159.1,143.6	Favored (50.1%) *t70* chi angles: 192,76.3	0.07Å	Favored (7.129%)	-	-	-
**#**	**Alt**	**Res**	**High B**	**Clash > 0.4Å**	**Ramachandran**	**Rotamer**	**Cβ deviation**	**CaBLAM**	**Bond lengths**	**Bond angles**	**Cis Peptides**
			Avg: 1.18	Clashscore: 1.47	Outliers: 1 of 350	Poor rotamers: 0 of 301	Outliers: 0 of 323	Outliers: 10 of 348	Outliers: 2 of 352	Outliers: 9 of 352	Non-Trans: 1 of 351
A 341		ASP	1.15	-	Favored (15.73%) General / -82.0,108.8	Favored (65.1%) *t0* chi angles: 184.8,344.3	0.01Å	Favored (47.708%) beta sheet	-	-	-
A 342		GLU	1.08	-	Favored (62.34%) General / -60.0,-22.3	Favored (80.7%) *mt-10* chi angles: 288.7,180.7,329.3	0.03Å	Favored (22.665%)	-	-	-
A 343		LYS	0.97	-	Favored (62.97%) General / -63.0,-18.1	Favored (6.7%) *ptpp* chi angles: 70.5,179.7,62.1,58.4	0.09Å	Favored (47.626%)	-	-	-
A 344		THR	0.86	-	Favored (38.87%) General / -102.0,3.5	Favored (74.3%) *p* chi angles: 61.5	0.04Å	Favored (52.107%)	-	-	-
A 345		LEU	0.79	-	Favored (33.85%) General / -86.1,126.0	Favored (45.8%) *tp* chi angles: 181.6,66.3	0.07Å	Favored (32.702%)	-	-	-
A 346		VAL	0.78	-	Favored (32.55%) Ile or Val / -76.6,123.2	Favored (95.2%) *t* chi angles: 175.7	0.07Å	Favored (50.714%) beta sheet	-	-	-
A 347		GLN	0.84	-	Favored (51.72%)	Favored (58.1%) *mt0*	0.04Å	Favored (46.603%)	-	-	-

					General / -131.9,151.4	chi angles: 298.2,175,77.3		beta sheet			
A 348		SER	0.97	-	Favored (46.03%) General / -73.6,144.2	Favored (39.9%) *t* chi angles: 177.9	0.03Å	Favored (38.435%) beta sheet	-	-	-
A 349		ARG	1.21	-	Favored (12%) General / -119.2,10.4	Favored (96.4%) *mtt180* chi angles: 297.8,181.5,180,180.7	0.02Å	Favored (9.065%) beta sheet	-	-	-
A 350		VAL	1.55	-	Favored (18.57%) Ile or Val / -64.7,141.2	Favored (5.1%) *p* chi angles: 70	0.04Å	Favored (22.023%)	-	-	-
A 351		ASN	1.99	-	Allowed (1.78%) General / -137.9,55.2	Favored (68.2%) *m-40* chi angles: 294,280.9	0.09Å	-	-	-	-
A 352		ALA	2.47	-	-	-	0.03Å	-	-	-	-

About MolProbity | Website for the Richardson Lab | Using ecloud x-H | Internal reference 4.5.2
