# Supplementary material for: Integrative proteome-wide structural analysis and high-throughput docking identify broad-spectrum antiviral scaffolds against Zika, Yellow Fever, West Nile, Saint Louis encephalitis, and Usutu viruses
Source: Front Cell Infect Microbiol. 2026 Apr 30;16:1723132. doi: 10.3389/fcimb.2026.1723132 (PMC13171538; doi:10.3389/fcimb.2026.1723132)
Supplement: Supplementary file 5 [file DataSheet5.zip › WNV/WNV_NS2a/Mol_probity_Files/WNV_NS2a_1FH-multi.table.pdf]

Viewing WNV_NS2a1FH-multi.table

Duke Biochemistry
Duke University School of Medicine

When finished, you should close this window.

Hint: Use File | Save As... to save a copy of this page.

All-Atom Contacts	Clashscore, all atoms:	1.91		99th percentile* (N=1784, all resolutions)
	Clashscore is the number of serious steric overlaps (> 0.4 Å) per 1000 atoms.			
Protein Geometry	Poor rotamers	0	0.00%	Goal: <0.3%
	Favored rotamers	190	100.00%	Goal: >98%
	Ramachandran outliers	4	1.75%	Goal: <0.05%
	Ramachandran favored	214	93.45%	Goal: >98%
	Rama distribution Z-score	0.53 ± 0.52		Goal: abs(Z score) < 2
	MolProbity score^	1.38		97th percentile* (N=27675, 0Å - 99Å)
	Cβ deviations >0.25Å	1	0.46%	Goal: 0
	Bad bonds:	2 / 1804	0.11%	Goal: 0%
	Bad angles:	11 / 2453	0.45%	Goal: <0.1%
Peptide Omegas	Cis Prolines:	0 / 8	0.00%	Expected: ≤1 per chain, or ≤5%
Low-resolution Criteria	CaBLAM outliers	8	3.5%	Goal: <1.0%
	CA Geometry outliers	4	1.76%	Goal: <0.5%
Additional validations	Chiral volume outliers	0/304		
	Waters with clashes	0/0	0.00%	See UnDowser table for details

In the two column results, the left column gives the raw count, right column gives the percentage.

* 100th percentile is the best among structures of comparable resolution; 0th percentile is the worst. For clashscore the comparative set of structures was selected in 2004, for MolProbity score in 2006.

^ MolProbity score combines the clashscore, rotamer, and Ramachandran evaluations into a single score, normalized to be on the same scale as X-ray resolution.

Key to table colors and cutoffs here:

#	Alt	Res	High B	Clash > 0.4Å	Ramachandran	Rotamer	Cβ deviation	CaBLAM	Bond lengths	Bond angles	Cis Peptides
			Avg: 6.62	Clashscore: 1.91	Outliers: 4 of 229	Poor rotamers: 0 of 190	Outliers: 1 of 219	Outliers: 9 of 227	Outliers: 2 of 231	Outliers: 9 of 231	Non-Trans: 0 of 230
A 1		TYR	8.6	-	-	Favored (62.5%) *t80* chi angles: 185.3,70.1	0.06Å	-	-	-	-
A 2		ASN	8.59	-	Allowed (1.39%) General / -84.5,-63.1	Favored (17.4%) *t0* chi angles: 187.5,255.5	0.04Å	-	-	-	-
A 3		ALA	8.49	-	Allowed (1.34%) General / 47.4,-132.1	-	0.04Å	CA Geom Outlier (0.443%)	-	-	-
A 4		ASP	8.28	-	Favored (45.64%) General / -99.0,7.8	Favored (74.9%) *m-30* chi angles: 297.5,341.3	0.07Å	Favored (5.909%)	-	-	-
A 5		MET	7.96	-	Favored (40.74%) General / -76.8,140.5	Favored (62.6%) *ttp* chi angles: 186,179.7,71.5	0.01Å	Favored (37.324%)	-	-	-
A 6		ILE	7.56	-	Allowed (1.23%) Ile or Val / -79.5,85.8	Favored (70.5%) *mt* chi angles: 302.4,173.5	0.06Å	Favored (43.407%) beta sheet	-	-	-

A 7		ASP	7.11	-	Favored (87.42%) Pre-Pro / -62.7,125.9	Favored (45.9%) *t0* chi angles: 191.8,334.2	0.07Å	Favored (20.328%)	-	OUTLIER(S) worst is C-N-CA: 4.1 σ	-
A 8		PRO	6.67	-	Favored (4.58%) Trans-Pro / -46.2,-27.4	Favored (89.6%) *Cg_exo* chi angles: 329.8,36.1,333.2	0.02Å	Favored (50.005%)	-	-	-
A 9		PHE	6.27	-	Favored (61.44%) General / -51.5,-49.7	Favored (73.9%) *t80* chi angles: 171.6,72.8	0.17Å	Favored (33.665%)	-	OUTLIER(S) worst is CA-CB-CG: 10.9 σ	-
A 10		GLN	5.92	-	Favored (26.16%) General / -85.2,-27.4	Favored (67.3%) *mm-40* chi angles: 296.1,294.3,289	0.03Å	Favored (56.322%) alpha helix	-	-	-
A 11		LEU	5.65	0.41Å C with A 11 LEU HD23	Favored (73.28%) General / -59.8,-50.6	Favored (6.5%) *tt* chi angles: 186.8,155.3	0.03Å	Favored (68.016%) alpha helix	-	-	-
A 12		GLY	5.46	-	Favored (40.35%) Glycine / -54.9,-53.3	-	-	Favored (93.048%) alpha helix	-	-	-
A 13		LEU	5.34	-	Favored (66.18%) General / -52.4,-46.6	Favored (62.2%) *tp* chi angles: 180.4,63.2	0.04Å	Favored (78.172%) alpha helix	-	-	-
A 14		LEU	5.3	-	Favored (73.96%) General / -59.9,-50.4	Favored (59.7%) *tp* chi angles: 174.7,63.6	0.14Å	Favored (85.98%) alpha helix	-	-	-
A 15		VAL	5.33	-	Favored (96.13%) Ile or Val / -62.8,-42.4	Favored (74.1%) *t* chi angles: 172.6	0.04Å	Favored (86.245%) alpha helix	-	-	-
A 16		VAL	5.43	-	Favored (98.87%) Ile or Val / -62.8,-43.6	Favored (70.8%) *t* chi angles: 172.2	0.04Å	Favored (96.896%) alpha helix	-	-	-
A 17		PHE	5.58	-	Favored (70.93%) General / -56.8,-50.9	Favored (86.6%) *t80* chi angles: 172.8,76.9	0.01Å	Favored (85.719%) alpha helix	-	-	-
A 18		LEU	5.79	-	Favored (85.2%) General / -64.8,-36.7	Favored (90.2%) *mt* chi angles: 293.2,176.4	0.04Å	Favored (79.166%) alpha helix	-	-	-
A 19		ALA	6.03	-	Favored (86.83%) General / -60.7,-39.0	-	0.03Å	Favored (88.872%) alpha helix	-	-	-
A 20		THR	6.29	-	Favored (92.38%) General / -65.8,-41.0	Favored (94.8%) *m* chi angles: 300.9	0.03Å	Favored (80.709%) alpha helix	-	-	-
**#**	**Alt**	**Res**	**High B**	**Clash > 0.4Å**	**Ramachandran**	**Rotamer**	**Cβ deviation**	**CaBLAM**	**Bond lengths**	**Bond angles**	**Cis Peptides**
			Avg: 6.62	Clashscore: 1.91	Outliers: 4 of 229	Poor rotamers: 0 of 190	Outliers: 1 of 219	Outliers: 9 of 227	Outliers: 2 of 231	Outliers: 9 of 231	Non-Trans: 0 of 230
A 21		GLN	6.58	-	Favored (81.71%) General / -62.0,-36.5	Favored (97.2%) *mm-40* chi angles: 296.5,301.8,301.5	0.07Å	Favored (75.544%) alpha helix	-	-	-

A<br>22		GLU	6.89	-	Favored<br>(94.78%)<br>General /<br>-60.1,-42.5	Favored (92%) *tt0*<br>chi angles:<br>183.3,178.1,2.6	0.07Å	Favored<br>(85.315%)<br>alpha helix	-	-	-
A<br>23		VAL	7.24	-	Favored<br>(96.99%)<br>Ile or Val /<br>-61.1,-46.3	Favored (66.9%) *t*<br>chi angles: 171.7	0.09Å	Favored<br>(94.241%)<br>alpha helix	-	-	-
A<br>24		LEU	7.64	-	Favored<br>(93.55%)<br>General /<br>-65.2,-39.8	Favored (93.7%) *mt*<br>chi angles: 294,174.9	0.06Å	Favored<br>(88.056%)<br>alpha helix	-	-	-
A<br>25		ARG	8.09	-	Favored<br>(72.67%)<br>General /<br>-64.0,-31.5	Favored (44.5%)<br>*mmt180*<br>chi angles:<br>290.9,297.3,183.7,195.7	0.03Å	Favored<br>(74.918%)<br>alpha helix	-	-	-
A<br>26		LYS	8.54	-	Favored<br>(16.74%)<br>General /<br>-100.8,19.6	Favored (98.5%)<br>*mttt*<br>chi angles:<br>295.9,177.2,179,177.5	0.10Å	Favored<br>(6.967%)	-	-	-
A<br>27		ARG	8.94	-	OUTLIER<br>(0.03)%)<br>General /<br>72.7,-83.3	Favored (51.5%)<br>*ttp-170*<br>chi angles:<br>181.5,196,65.8,190.3	0.06Å	CaBLAM<br>Disfavored<br>(1.083%)	-	-	-
A<br>28		TRP	9.25	-	Allowed (1.1%)<br>General /<br>-173.9,145.1	Favored (86.5%)<br>*t60*<br>chi angles: 185.5,88.1	0.08Å	CA Geom<br>Outlier<br>(0.074%)	-	-	-
A<br>29		THR	9.42	-	Favored<br>(9.83%)<br>General /<br>-163.7,146.4	Favored (3.2%) *t*<br>chi angles: 176.8	0.06Å	Favored<br>(10.013%)	-	-	-
A<br>30		ALA	9.46	-	Favored<br>(68.33%)<br>General /<br>-59.4,-30.2	-	0.03Å	Favored<br>(47.944%)	-	-	-
A<br>31		LYS	9.39	-	Favored<br>(67.07%)<br>General /<br>-63.7,-22.8	Favored (97.9%)<br>*mttt*<br>chi angles:<br>291.4,179,179.8,178.7	0.03Å	Favored<br>(26.506%)<br>alpha helix	-	-	-
A<br>32		ILE	9.28	-	Favored<br>(6.01%)<br>Ile or Val /<br>-109.7,-44.2	Favored (82.1%) *mt*<br>chi angles: 299.6,170	0.06Å	Favored<br>(13.836%)<br>alpha helix	-	-	-
A<br>33		SER	9.12	-	Favored<br>(93.37%)<br>General /<br>-59.7,-45.0	Favored (34%) *t*<br>chi angles: 181.9	0.06Å	Favored<br>(74.953%)<br>alpha helix	-	-	-
A<br>34		ILE	8.95	-	Favored<br>(28.99%)<br>Pre-Pro /<br>-59.9,-57.1	Favored (97.5%) *mt*<br>chi angles: 292.2,167.9	0.13Å	Favored<br>(75.359%)<br>alpha helix	-	-	-
A<br>35		PRO	8.74	-	Favored<br>(74.95%)<br>Trans-Pro /<br>-62.7,-25.5	Favored (26.2%)<br>*Cg_endo*<br>chi angles:<br>20.6,327.1,31.1	0.04Å	Favored<br>(85.067%)<br>alpha helix	-	-	-
A<br>36		ALA	8.51	-	Favored<br>(92.08%)<br>General /<br>-65.8,-40.5	-	0.01Å	Favored<br>(75.746%)<br>alpha helix	-	-	-
A<br>37		ILE	8.26	-	Favored<br>(81.91%)<br>Ile or Val /<br>-68.6,-45.2	Favored (97.2%) *mt*<br>chi angles: 292.4,166.9	0.03Å	Favored<br>(78.135%)<br>alpha helix	-	-	-
A<br>38		MET	8.02	-	Favored<br>(87.18%)<br>General /<br>-59.0,-41.2	Favored (97.4%)<br>*mtp*<br>chi angles:<br>291,171.2,69.6	0.03Å	Favored<br>(90.618%)<br>alpha helix	-	-	-

A 39		LEU	7.84	0.48Å C with A 39 LEU HD23	Favored (71.71%) General / -58.3,-51.0	Favored (6.1%) *tt* chi angles: 187.7,156.7	0.02Å	Favored (92.104%) alpha helix	-	-	-
A 40		ALA	7.73	-	Favored (77.23%) General / -58.4,-38.4	-	0.03Å	Favored (82.846%) alpha helix	-	-	-
**#**	**Alt**	**Res**	**High B**	**Clash > 0.4Å**	**Ramachandran**	**Rotamer**	**Cβ deviation**	**CaBLAM**	**Bond lengths**	**Bond angles**	**Cis Peptides**
			Avg: 6.62	Clashscore: 1.91	Outliers: 4 of 229	Poor rotamers: 0 of 190	Outliers: 1 of 219	Outliers: 9 of 227	Outliers: 2 of 231	Outliers: 9 of 231	Non-Trans: 0 of 230
A 41		LEU	7.69	-	Favored (93.18%) General / -65.1,-39.5	Favored (96.2%) *mt* chi angles: 292,172.8	0.01Å	Favored (91.709%) alpha helix	-	-	-
A 42		LEU	7.74	-	Favored (86.37%) General / -64.5,-37.1	Favored (91.6%) *mt* chi angles: 291.2,171.8	0.04Å	Favored (93.224%) alpha helix	-	-	-
A 43		VAL	7.83	-	Favored (98.78%) Ile or Val / -63.3,-43.7	Favored (58%) *t* chi angles: 170.5	0.07Å	Favored (94.106%) alpha helix	-	-	-
A 44		LEU	7.91	-	Favored (83.54%) General / -63.5,-36.4	Favored (93.5%) *mt* chi angles: 291.5,171.8	0.02Å	Favored (87.663%) alpha helix	-	-	-
A 45		VAL	7.9	-	Favored (83.48%) Ile or Val / -68.7,-42.9	Favored (68.3%) *t* chi angles: 171.9	0.03Å	Favored (52.477%) alpha helix	-	-	-
A 46		PHE	7.76	-	Favored (12.33%) General / -104.2,-26.9	Favored (82.5%) *m-80* chi angles: 297,107.5	0.04Å	Favored (18.331%)	-	-	-
A 47		GLY	7.47	-	Favored (27.25%) Glycine / -87.5,155.6	-	-	Favored (16.009%)	-	-	-
A 48		GLY	7.05	-	Favored (4.65%) Glycine / -80.2,56.8	-	-	CaBLAM Disfavored (2.394%)	-	-	-
A 49		ILE	6.58	-	Favored (33.38%) Ile or Val / -68.7,131.9	Favored (49.3%) *mm* chi angles: 302.5,299.2	0.04Å	Favored (16.032%)	-	-	-
A 50		THR	6.14	-	Favored (11.26%) General / -113.0,166.6	Favored (55.8%) *p* chi angles: 64.8	0.08Å	Favored (29.559%)	-	-	-
A 51		TYR	5.74	-	Favored (63.85%) General / -51.6,-47.2	Favored (86.7%) *t80* chi angles: 175.3,80.9	0.09Å	Favored (64.414%)	-	-	-
A 52		THR	5.41	-	Favored (95.02%) General / -60.0,-42.9	Favored (90.4%) *m* chi angles: 297.2	0.04Å	Favored (94.245%) alpha helix	-	-	-
A 53		ASP	5.16	-	Favored (74.9%) General / -65.0,-32.9	Favored (2.1%) *t70* chi angles: 206.3,65.7	0.03Å	Favored (92.735%) alpha helix	-	-	-

#	Alt	Res	High B	Clash > 0.4Å	Ramachandran	Rotamer	Cβ deviation	CaBLAM	Bond lengths	Bond angles	Cis Peptides
A 54		VAL	4.97	-	Favored (91.17%) Ile or Val / -66.5,-43.0	Favored (71%) *t* chi angles: 172.2	0.01Å	Favored (88.301%) alpha helix	-	-	-
A 55		LEU	4.8	-	Favored (82.57%) General / -56.9,-45.5	Favored (71.6%) *tp* chi angles: 178.6,62.6	0.03Å	Favored (91.562%) alpha helix	-	-	-
A 56		ARG	4.66	-	Favored (94.78%) General / -60.8,-41.1	Favored (66.8%) *mtp180* chi angles: 291.1,180.8,72.7,204.3	0.06Å	Favored (91.669%) alpha helix	-	-	-
A 57		TYR	4.56	-	Favored (72.37%) General / -56.1,-50.0	Favored (75.3%) *t80* chi angles: 170.6,76.8	0.08Å	Favored (93.93%) alpha helix	-	-	-
A 58		VAL	4.49	-	Favored (95.73%) Ile or Val / -60.1,-44.7	Favored (64.2%) *t* chi angles: 171.4	0.05Å	Favored (80.362%) alpha helix	-	-	-
A 59		ILE	4.46	-	Favored (96.77%) Ile or Val / -64.6,-44.6	Favored (98.3%) *mt* chi angles: 293.4,167.5	0.01Å	Favored (86.15%) alpha helix	-	-	-
A 60		LEU	4.49	-	Favored (91.53%) General / -59.7,-45.6	Favored (44.2%) *mt* chi angles: 286.5,162.4	0.10Å	Favored (81.115%) alpha helix	-	-	-
**#**	**Alt**	**Res**	**High B**	**Clash > 0.4Å**	**Ramachandran**	**Rotamer**	**Cβ deviation**	**CaBLAM**	**Bond lengths**	**Bond angles**	**Cis Peptides**
			Avg: 6.62	Clashscore: 1.91	Outliers: 4 of 229	Poor rotamers: 0 of 190	Outliers: 1 of 219	Outliers: 9 of 227	Outliers: 2 of 231	Outliers: 9 of 231	Non-Trans: 0 of 230
A 61		VAL	4.57	-	Favored (71.33%) Ile or Val / -71.6,-43.3	Favored (79.5%) *t* chi angles: 173.1	0.05Å	Favored (79.755%) alpha helix	-	-	-
A 62		GLY	4.7	-	Favored (38.4%) Glycine / -54.0,-53.2	-	-	Favored (92.211%) alpha helix	-	-	-
A 63		ALA	4.9	-	Favored (99.23%) General / -61.1,-43.2	-	0.07Å	Favored (78.943%) alpha helix	-	-	-
A 64		ALA	5.18	-	Favored (85.39%) General / -59.5,-39.9	-	0.06Å	Favored (76.974%) alpha helix	-	-	-
A 65		PHE	5.56	-	Favored (68.41%) General / -64.4,-50.2	Favored (87.5%) *t80* chi angles: 176.9,74.4	0.02Å	Favored (77.98%) alpha helix	-	-	-
A 66		ALA	6.03	-	Favored (74.23%) General / -58.0,-37.5	-	0.05Å	Favored (70.871%) alpha helix	-	-	-
A 67		GLU	6.57	-	Favored (25.56%) General / -100.8,16.3	Favored (73.9%) *mm-30* chi angles: 295.3,294,314.6	0.04Å	Favored (31.464%) alpha helix	-	-	-
A 68		ALA	7.07	-	Favored (63.78%) General / -60.0,-23.6	-	0.03Å	Favored (35.501%) alpha helix	-	-	-

A 69		ASN	7.45	-	Favored (59.24%) General / -76.2,-10.2	Favored (96.9%) *m-40* chi angles: 290.1,334.9	0.04Å	Favored (30.004%) alpha helix	-	-	-
A 70		SER	7.66	-	Favored (2.75%) General / -114.2,-51.8	Favored (43.5%) *t* chi angles: 178.6	0.03Å	CaBLAM Outlier (0.921%)	-	-	-
A 71		GLY	7.67	-	Favored (42.16%) Glycine / 91.5,-20.3	-	-	CaBLAM Disfavored (4.827%)	-	-	-
A 72		GLY	7.5	-	Favored (88.27%) Glycine / -59.4,-36.2	-	-	Favored (56.55%)	-	-	-
A 73		ASP	7.21	-	Favored (74.72%) General / -63.0,-33.1	Favored (34.3%) *t70* chi angles: 188.8,63.1	0.04Å	Favored (71.695%) alpha helix	-	-	-
A 74		VAL	6.91	-	Favored (94.85%) Ile or Val / -63.5,-42.1	Favored (76.8%) *t* chi angles: 172.8	0.02Å	Favored (73.425%) alpha helix	-	-	-
A 75		VAL	6.64	-	Favored (91.44%) Ile or Val / -66.3,-42.8	Favored (82.3%) *t* chi angles: 173.3	0.06Å	Favored (90.089%) alpha helix	-	-	-
A 76		HIS	6.44	-	Favored (91.51%) General / -60.6,-40.4	Favored (66%) *m-70* chi angles: 286.8,299.2	0.02Å	Favored (96.776%) alpha helix	-	-	-
A 77		LEU	6.31	-	Favored (96.98%) General / -64.3,-41.2	Favored (97.4%) *mt* chi angles: 292.8,171.7	0.10Å	Favored (97.599%) alpha helix	-	-	-
A 78		ALA	6.25	-	Favored (89.46%) General / -62.7,-38.2	-	0.02Å	Favored (85.281%) alpha helix	-	-	-
A 79		LEU	6.26	-	Favored (99.49%) General / -62.9,-41.7	Favored (83.8%) *mt* chi angles: 289.7,170.4	0.05Å	Favored (80.169%) alpha helix	-	-	-
A 80		MET	6.33	-	Favored (72.77%) General / -71.0,-39.1	Favored (84%) *mtm* chi angles: 290.4,187.8,290.4	0.03Å	Favored (81.12%) alpha helix	-	-	-
**#**	**Alt**	**Res**	**High B**	**Clash > 0.4Å**	**Ramachandran**	**Rotamer**	**Cβ deviation**	**CaBLAM**	**Bond lengths**	**Bond angles**	**Cis Peptides**
			Avg: 6.62	Clashscore: 1.91	Outliers: 4 of 229	Poor rotamers: 0 of 190	Outliers: 1 of 219	Outliers: 9 of 227	Outliers: 2 of 231	Outliers: 9 of 231	Non-Trans: 0 of 230
A 81		ALA	6.43	-	Favored (78.65%) General / -63.0,-35.0	-	0.04Å	Favored (46.744%)	-	-	-
A 82		THR	6.51	-	Favored (33.19%) General / -79.2,128.4	Favored (65.7%) *m* chi angles: 303.3	0.11Å	CaBLAM Disfavored (2.566%)	-	-	-
A 83		PHE	6.54	-	OUTLIER (0)%) General / 38.7,1.2	Favored (16.5%) *m-10* chi angles: 296.7,349.3	0.09Å	CA Geom Outlier (0.169%)	-	OUTLIER(S) worst is N-CA-C: 4.6 σ	-
A 84		LYS	6.51	-	Allowed (1.1%) General / 70.2,41.0	Favored (44.3%) *mtmt* chi angles: 301.7,179.5,284.4,181.8	0.05Å	CA Geom Outlier (0.023%)	-	-	-

#	Alt	Res	High B	Clash > 0.4Å	Ramachandran	Rotamer	Cβ deviation	CaBLAM	Bond lengths	Bond angles	Cis Peptides
A 85		ILE	6.39	0.51Å O with A 85 ILE HG13	OUTLIER (0)%) Ile or Val / -21.6,-17.1	Favored (8.6%) *tp* chi angles: 198.6,65.5	0.08Å	CaBLAM Disfavored (3.56%)	-	OUTLIER(S) worst is C-N-CA: 6.7 σ	-
A 86		GLN	6.2	0.66Å HB3 with A 87 PRO CD	OUTLIER (0.01)%) Pre-Pro / 59.2,-154.9	Favored (60.7%) *mt0* chi angles: 299.3,191.2,331.3	0.28Å	CaBLAM Outlier (0.921%)	OUTLIER(S) worst is CA--C: 4.2 σ	OUTLIER(S) worst is C-CA-CB: 5.2 σ	-
A 87		PRO	5.99	0.66Å CD with A 86 GLN HB3	Allowed (0.57%) Trans-Pro / -84.7,-168.9	Favored (23.2%) *Cg_endo* chi angles: 36.4,320.8,25.6	0.09Å	CaBLAM Disfavored (2.952%)	-	-	-
A 88		VAL	5.79	-	Allowed (0.28%) Ile or Val / 74.2,-58.7	Favored (89.5%) *t* chi angles: 174.1	0.04Å	CaBLAM Outlier (0.39%) try alpha helix	-	-	-
A 89		PHE	5.66	-	Favored (44.27%) General / -79.1,-31.4	Favored (98.5%) *m-80* chi angles: 295.1,92.2	0.12Å	Favored (89.302%) alpha helix	-	OUTLIER(S) worst is CA-CB-CG: 6.3 σ	-
A 90		LEU	5.61	-	Favored (58.27%) General / -76.2,-31.3	Favored (8.6%) *mp* chi angles: 274.3,62.8	0.07Å	Favored (84.889%) alpha helix	-	-	-
A 91		VAL	5.64	-	Favored (30.62%) Ile or Val / -77.2,-44.7	Favored (84%) *t* chi angles: 177	0.07Å	Favored (78.18%) alpha helix	-	-	-
A 92		ALA	5.76	-	Favored (79.8%) General / -59.7,-37.8	-	0.05Å	Favored (85.651%) alpha helix	-	-	-
A 93		SER	5.97	-	Favored (87.93%) General / -62.6,-46.5	Favored (70.2%) *m* chi angles: 295	0.03Å	Favored (81.116%) alpha helix	-	-	-
A 94		PHE	6.28	-	Favored (68.68%) General / -59.4,-51.7	Favored (83.7%) *t80* chi angles: 173.8,80.7	0.09Å	Favored (85.724%) alpha helix	-	-	-
A 95		LEU	6.68	-	Favored (93.52%) General / -64.3,-39.2	Favored (95.4%) *mt* chi angles: 291.8,172.2	0.02Å	Favored (60.513%) alpha helix	-	-	-
A 96		LYS	7.17	-	Favored (10.52%) General / -84.5,-47.5	Favored (50.6%) *tttp* chi angles: 185.4,178.1,178.1,68.2	0.03Å	CaBLAM Disfavored (3.085%)	-	-	-
A 97		ALA	7.7	-	Favored (3.75%) General / 55.3,20.8	-	0.02Å	CaBLAM Outlier (0.183%)	-	-	-
A 98		ARG	8.22	-	Favored (2.67%) General / -137.3,30.6	Favored (92.7%) *mmt-90* chi angles: 294.3,292.4,182.7,271.4	0.03Å	Favored (5.011%)	-	-	-
A 99		TRP	8.65	-	Favored (30.7%) General / -80.9,146.8	Favored (98.6%) *m100* chi angles: 291.3,99.8	0.08Å	Favored (37.148%)	-	-	-
A 100		THR	8.9	-	Favored (22.57%) General / -78.1,166.6	Favored (76.7%) *p* chi angles: 61.1	0.04Å	Favored (52.235%)	-	-	-
			Avg: 6.62	Clashscore: 1.91	Outliers: 4 of 229	Poor rotamers: 0 of 190	Outliers: 1 of 219	Outliers: 9 of 227	Outliers: 2 of 231	Outliers: 9 of 231	Non-Trans: 0

											of 230
A 101		ASN	8.92	-	Favored (88.82%) General / -62.7,-38.0	Favored (97.3%) *m-40* chi angles: 286.8,340.7	0.04Å	Favored (66.788%)	-	-	-
A 102		GLN	8.73	-	Favored (86.88%) General / -62.1,-46.9	Favored (65%) *tt0* chi angles: 181,180.6,8.8	0.06Å	Favored (86.505%) alpha helix	-	-	-
A 103		GLU	8.41	-	Favored (89.95%) General / -64.0,-38.1	Favored (44.2%) *mt-10* chi angles: 289.2,166.8,314.6	0.02Å	Favored (83.711%) alpha helix	-	-	-
A 104		SER	8.03	-	Favored (99.33%) General / -62.9,-41.2	Favored (71.6%) *m* chi angles: 296	0.08Å	Favored (87.299%) alpha helix	-	-	-
A 105		ILE	7.66	-	Favored (99.31%) Ile or Val / -63.2,-44.5	Favored (97.6%) *mt* chi angles: 292.4,167	0.04Å	Favored (94.476%) alpha helix	-	-	-
A 106		LEU	7.33	-	Favored (81.86%) General / -63.5,-35.9	Favored (97.4%) *mt* chi angles: 292.2,172.1	0.04Å	Favored (84.113%) alpha helix	-	-	-
A 107		LEU	7.08	-	Favored (96.95%) General / -63.2,-40.2	Favored (88.4%) *mt* chi angles: 291.2,173.8	0.09Å	Favored (89.052%) alpha helix	-	-	-
A 108		MET	6.9	-	Favored (84.62%) General / -61.7,-47.6	Favored (29.8%) *tmm* chi angles: 179.6,275.8,291.7	0.03Å	Favored (93.002%) alpha helix	-	-	-
A 109		LEU	6.79	-	Favored (95.51%) General / -64.8,-41.9	Favored (97.2%) *mt* chi angles: 292.6,171.4	0.06Å	Favored (92.797%) alpha helix	-	-	-
A 110		ALA	6.74	-	Favored (94.53%) General / -61.3,-40.5	-	0.05Å	Favored (88.509%) alpha helix	-	-	-
A 111		ALA	6.73	-	Favored (85.81%) General / -60.7,-38.7	-	0.05Å	Favored (77.017%) alpha helix	-	-	-
A 112		ALA	6.75	-	Favored (90.96%) General / -60.4,-40.5	-	0.04Å	Favored (81.353%) alpha helix	-	-	-
A 113		PHE	6.78	-	Favored (65.37%) General / -73.6,-36.2	Favored (62.3%) *m-80* chi angles: 286.8,103.8	0.04Å	Favored (88.646%) alpha helix	-	-	-
A 114		PHE	6.8	-	Favored (82.03%) General / -68.3,-39.6	Favored (22.9%) *m-80* chi angles: 275.5,99.6	0.08Å	Favored (89.352%) alpha helix	-	-	-
A 115		GLN	6.83	-	Favored (89.53%) General / -60.5,-39.9	Favored (63.6%) *tt0* chi angles: 183.4,181.4,349.5	0.09Å	Favored (89.583%) alpha helix	-	-	-
A 116		MET	6.89	-	Favored (95.13%) General / -63.4,-39.7	Favored (83.1%) *mtm* chi angles: 289,187.6,285.4	0.08Å	Favored (98.136%) alpha helix	-	-	-
A 117		ALA	6.97	-	Favored (95.32%) General / -62.6,-39.9	-	0.03Å	Favored (98.19%) alpha helix	-	-	-

A 118		TYR	7.1	-	Favored (93.32%) General / -65.5,-40.5	Favored (24.5%) *m-10* chi angles: 295.5,341	0.04Å	Favored (90.084%) alpha helix	-	-	-
A 119		TYR	7.3	-	Favored (72.37%) General / -58.8,-50.9	Favored (84.6%) *t80* chi angles: 172.3,78.2	0.04Å	Favored (88.545%) alpha helix	-	-	-
A 120		ASP	7.56	-	Favored (92.53%) General / -60.8,-40.4	Favored (95.2%) *m-30* chi angles: 286.2,346.1	0.02Å	Favored (86.595%) alpha helix	-	-	-
**#**	**Alt**	**Res**	**High B**	**Clash > 0.4Å**	**Ramachandran**	**Rotamer**	**Cβ deviation**	**CaBLAM**	**Bond lengths**	**Bond angles**	**Cis Peptides**
			Avg: 6.62	Clashscore: 1.91	Outliers: 4 of 229	Poor rotamers: 0 of 190	Outliers: 1 of 219	Outliers: 9 of 227	Outliers: 2 of 231	Outliers: 9 of 231	Non-Trans: 0 of 230
A 121		ALA	7.88	-	Favored (80.25%) General / -60.4,-37.3	-	0.02Å	Favored (79.236%) alpha helix	-	-	-
A 122		LYS	8.21	-	Favored (78.23%) General / -66.0,-46.4	Favored (87.8%) *tttt* chi angles: 183.8,174.6,183.3,182.1	0.04Å	Favored (85.567%) alpha helix	-	-	-
A 123		ASN	8.5	-	Favored (72.25%) General / -61.7,-32.0	Favored (81.9%) *m-40* chi angles: 282.9,345.3	0.07Å	Favored (60.317%) alpha helix	-	-	-
A 124		VAL	8.69	-	Favored (14.89%) Ile or Val / -89.9,-47.2	Favored (96.8%) *t* chi angles: 175.7	0.03Å	Favored (44.213%) alpha helix	-	-	-
A 125		LEU	8.7	-	Favored (28.06%) General / -102.4,-2.9	Favored (64.9%) *mt* chi angles: 303.2,174.6	0.08Å	Favored (34.461%)	-	-	-
A 126		SER	8.53	-	Favored (27.11%) General / 57.2,41.3	Favored (59.3%) *m* chi angles: 299.2	0.04Å	Favored (26.061%)	-	-	-
A 127		TRP	8.19	-	Favored (29.49%) General / -88.8,119.7	Favored (50.8%) *m-10* chi angles: 288.8,350	0.07Å	Favored (24.73%) beta sheet	-	-	-
A 128		GLU	7.74	-	Favored (56.79%) General / -93.2,-0.4	Favored (97.1%) *mt-10* chi angles: 296,179.9,358.9	0.02Å	Favored (16.031%)	-	-	-
A 129		VAL	7.26	-	Favored (99.69%) Pre-Pro / -56.2,-44.4	Favored (62.2%) *t* chi angles: 171.1	0.12Å	Favored (45.54%)	-	-	-
A 130		PRO	6.79	-	Favored (51.05%) Trans-Pro / -57.9,-21.8	Favored (67%) *Cg_exo* chi angles: 335.5,36.6,327.1	0.03Å	Favored (61.654%) alpha helix	-	-	-
A 131		ASP	6.38	-	Favored (20.96%) General / -86.8,-31.1	Favored (65.9%) *m-30* chi angles: 296.6,307.6	0.02Å	Favored (80.434%) alpha helix	-	-	-
A 132		VAL	6.04	-	Favored (90.69%) Ile or Val / -65.1,-46.6	Favored (88.8%) *t* chi angles: 174	0.14Å	Favored (75.103%) alpha helix	-	-	-
A 133		LEU	5.76	-	Favored (80.22%) General / -66.1,-35.3	Favored (93.3%) *mt* chi angles: 293.9,175.2	0.05Å	Favored (74.265%) alpha helix	-	-	-

A 134		ASN	5.52	-	Favored (81.58%) General / -57.4,-47.5	Favored (50.2%) *t0* chi angles: 181.5,62.6	0.07Å	Favored (74.675%) alpha helix	-	-	-
A 135		SER	5.32	-	Favored (89.76%) General / -58.6,-45.2	Favored (46.9%) *t* chi angles: 180.2	0.04Å	Favored (93.033%) alpha helix	-	-	-
A 136		LEU	5.14	-	Favored (91.73%) General / -63.6,-38.6	Favored (91.7%) *mt* chi angles: 291.3,171.4	0.05Å	Favored (86.204%) alpha helix	-	-	-
A 137		SER	4.99	-	Favored (86.92%) General / -62.2,-46.9	Favored (22.9%) *t* chi angles: 185.1	0.07Å	Favored (81.068%) alpha helix	-	-	-
A 138		VAL	4.89	-	Favored (37.27%) Ile or Val / -71.2,-29.7	Favored (28.4%) *m* chi angles: 298.5	0.09Å	Favored (76.599%) alpha helix	-	-	-
A 139		ALA	4.84	-	Favored (84.92%) General / -61.9,-37.5	-	0.02Å	Favored (76.554%) alpha helix	-	-	-
A 140		TRP	4.83	-	Favored (84.42%) General / -66.5,-37.1	Favored (68.9%) *m100* chi angles: 280.1,111.6	0.04Å	Favored (85.833%) alpha helix	-	-	-

#	Alt	Res	High B	Clash > 0.4Å	Ramachandran	Rotamer	Cβ deviation	CaBLAM	Bond lengths	Bond angles	Cis Peptides
			Avg: 6.62	Clashscore: 1.91	Outliers: 4 of 229	Poor rotamers: 0 of 190	Outliers: 1 of 219	Outliers: 9 of 227	Outliers: 2 of 231	Outliers: 9 of 231	Non-Trans: 0 of 230
A 141		MET	4.87	-	Favored (83.06%) General / -64.1,-36.1	Favored (76.6%) *mtm* chi angles: 288.6,191.1,287.1	0.07Å	Favored (81.371%) alpha helix	-	-	-
A 142		ILE	4.97	-	Favored (35.19%) Ile or Val / -76.5,-41.3	Favored (47%) *mm* chi angles: 303.6,298.8	0.05Å	Favored (73.178%) alpha helix	-	-	-
A 143		LEU	5.12	-	Favored (63.11%) General / -51.4,-47.0	Favored (7.5%) *tt* chi angles: 183.3,153.2	0.03Å	Favored (78.908%) alpha helix	-	-	-
A 144		ARG	5.31	-	Favored (93.77%) General / -61.5,-45.6	Favored (29%) *tpt170* chi angles: 181.8,71.5,185.9,178.4	0.13Å	Favored (86.048%) alpha helix	-	-	-
A 145		ALA	5.54	-	Favored (85.61%) General / -59.6,-39.9	-	0.07Å	Favored (75.774%) alpha helix	-	-	-
A 146		ILE	5.77	-	Favored (72.99%) Ile or Val / -70.7,-45.7	Favored (94.8%) *mt* chi angles: 294,166.8	0.04Å	Favored (80.069%) alpha helix	-	-	-
A 147		SER	5.96	-	Favored (93.22%) General / -63.5,-39.0	Favored (69.9%) *m* chi angles: 296.4	0.06Å	Favored (55.979%) alpha helix	-	-	-
A 148		PHE	6.08	-	Allowed (1.43%) General / -127.5,59.8	Favored (78.6%) *m-80* chi angles: 303.5,96.6	0.06Å	Favored (11.933%)	-	-	-

#	Alt	Res	High B	Clash > 0.4Å	Ramachandran	Rotamer	Cβ deviation	CaBLAM	Bond lengths	Bond angles	Cis Peptides
A 149		THR	6.09	-	Favored (58.82%) General / -64.4,139.9	Favored (34%) *p* chi angles: 53.8	0.01Å	CaBLAM Outlier (0.532%)	-	-	-
A 150		ASN	5.98	-	Favored (2.64%) General / -166.1,-173.5	Favored (29.1%) *p0* chi angles: 61.8,53.2	0.06Å	Favored (9.541%)	-	-	-
A 151		THR	5.75	-	Favored (78.82%) General / -56.0,-46.0	Favored (93.5%) *m* chi angles: 297.5	0.03Å	Favored (14.996%)	-	-	-
A 152		SER	5.45	-	Favored (63.97%) General / -65.5,-50.8	Favored (37.9%) *t* chi angles: 177.4	0.05Å	Favored (72.709%) alpha helix	-	-	-
A 153		ASN	5.14	-	Favored (66.59%) General / -67.1,-26.2	Favored (84.8%) *m-40* chi angles: 283.4,339.3	0.18Å	Favored (58.939%) alpha helix	-	-	-
A 154		VAL	4.84	-	Favored (30.2%) Ile or Val / -77.3,-41.6	Favored (68.8%) *t* chi angles: 172	0.08Å	Favored (71.024%) alpha helix	-	-	-
A 155		VAL	4.58	-	Favored (84.89%) Ile or Val / -61.0,-40.2	Favored (59.6%) *t* chi angles: 170.7	0.08Å	Favored (59.397%) alpha helix	-	-	-
A 156		VAL	4.38	-	Allowed (1.53%) Pre-Pro / -45.0,-62.6	Favored (49.8%) *t* chi angles: 169.4	0.17Å	Favored (57.862%) alpha helix	-	OUTLIER(S) worst is CA-C-N: 4.5 σ	-
A 157		PRO	4.24	-	Favored (77.42%) Trans-Pro / -62.2,-26.2	Favored (29.7%) *Cg_endo* chi angles: 21.2,327.4,31.7	0.05Å	Favored (76.993%) alpha helix	OUTLIER(S) worst is N--CD: 5.2 σ	-	-
A 158		LEU	4.16	-	Favored (83.13%) General / -67.4,-37.4	Favored (94.2%) *mt* chi angles: 293.1,174.6	0.03Å	Favored (62.529%) alpha helix	-	-	-
A 159		LEU	4.14	-	Favored (23.79%) General / -83.0,-37.5	Favored (79.8%) *mt* chi angles: 288.8,171.9	0.02Å	Favored (40.197%) alpha helix	-	-	-
A 160		ALA	4.18	-	Favored (75.79%) General / -60.2,-35.6	-	0.02Å	Favored (72.783%) alpha helix	-	-	-
**#**	**Alt**	**Res**	**High B**	**Clash > 0.4Å**	**Ramachandran**	**Rotamer**	**Cβ deviation**	**CaBLAM**	**Bond lengths**	**Bond angles**	**Cis Peptides**
			Avg: 6.62	Clashscore: 1.91	Outliers: 4 of 229	Poor rotamers: 0 of 190	Outliers: 1 of 219	Outliers: 9 of 227	Outliers: 2 of 231	Outliers: 9 of 231	Non-Trans: 0 of 230
A 161		LEU	4.3	-	Favored (51.76%) General / -76.0,-6.6	Favored (94.7%) *mt* chi angles: 294.9,174.3	0.03Å	Favored (52.519%) alpha helix	-	-	-
A 162		LEU	4.49	-	Favored (42.51%) General / -100.4,2.6	Favored (92.1%) *mt* chi angles: 298.9,177.7	0.03Å	Favored (56.787%)	-	-	-
A 163		THR	4.76	-	Favored (69.02%) Pre-Pro / -90.4,118.8	Favored (88.3%) *m* chi angles: 298.5	0.05Å	Favored (32.087%)	-	-	-
A 164		PRO	5.11	-	Favored (39.35%) Trans-Pro / -52.0,-30.4	Favored (97.4%) *Cg_exo* chi angles: 331.8,36.3,331.2	0.06Å	Favored (66.989%)	-	-	-

#	Alt	Res	High B	Clash > 0.4Å	Ramachandran	Rotamer	Cβ deviation	CaBLAM	Bond lengths	Bond angles	Cis Peptides
A 165		GLY	5.49	-	Favored (61.66%) Glycine / -60.7,-24.0	-	-	Favored (71.135%)	-	-	-
A 166		LEU	5.89	-	Favored (66.53%) General / -56.7,-33.6	Favored (65.1%) *tp* chi angles: 178.3,57.8	0.04Å	Favored (62.364%)	-	-	-
A 167		LYS	6.24	-	Favored (7.95%) General / -85.4,12.6	Favored (72.1%) *mmtt* chi angles: 301,297.9,186.1,181.6	0.02Å	Favored (18.216%)	-	-	-
A 168		CYS	6.5	-	Allowed (0.49%) General / -59.8,174.3	Favored (21%) *p* chi angles: 70.3	0.05Å	CaBLAM Disfavored (1.984%)	-	-	-
A 169		LEU	6.62	-	Allowed (1.05%) General / -40.2,-40.2	Favored (66.4%) *tp* chi angles: 179.8,62.8	0.10Å	Favored (26.866%)	-	-	-
A 170		ASN	6.6	-	Favored (7.67%) General / -100.5,25.5	Favored (62.5%) *m-40* chi angles: 284.1,278.9	0.16Å	Favored (17.974%) alpha helix	-	-	-
A 171		LEU	6.46	-	Favored (43.09%) General / -51.5,-36.1	Favored (63.8%) *tp* chi angles: 180.9,62.3	0.06Å	Favored (40.061%) alpha helix	-	-	-
A 172		ASP	6.23	-	Favored (71.13%) General / -61.0,-31.6	Favored (28%) *t70* chi angles: 191.3,63.5	0.02Å	Favored (71.141%) alpha helix	-	-	-
A 173		VAL	5.96	-	Favored (82.2%) Ile or Val / -68.7,-44.6	Favored (86.8%) *t* chi angles: 173.8	0.05Å	Favored (78.167%) alpha helix	-	-	-
A 174		TYR	5.69	-	Favored (69.45%) General / -55.7,-50.8	Favored (80.7%) *t80* chi angles: 174.4,83	0.06Å	Favored (91.95%) alpha helix	-	-	-
A 175		ARG	5.44	-	Favored (58.22%) General / -52.8,-52.0	Favored (84.6%) *ttp80* chi angles: 179.3,185,65.8,80.3	0.08Å	Favored (87.778%) alpha helix	-	-	-
A 176		ILE	5.23	-	Favored (93.67%) Ile or Val / -61.4,-47.4	Favored (96.5%) *mt* chi angles: 292.3,166.8	0.04Å	Favored (89.113%) alpha helix	-	-	-
A 177		LEU	5.07	-	Favored (94.94%) General / -63.8,-39.7	Favored (90.2%) *mt* chi angles: 291.3,173.4	0.04Å	Favored (91.681%) alpha helix	-	-	-
A 178		LEU	4.97	-	Favored (97.17%) General / -64.2,-40.9	Favored (6.6%) *mp* chi angles: 267.1,60.6	0.06Å	Favored (94.099%) alpha helix	-	-	-
A 179		LEU	4.9	-	Favored (82.02%) General / -68.1,-38.2	Favored (97.4%) *mt* chi angles: 293.9,172.8	0.08Å	Favored (96.75%) alpha helix	-	-	-
A 180		MET	4.88	-	Favored (94.41%) General / -62.0,-45.2	Favored (93.8%) *mtp* chi angles: 289.4,170,67.8	0.05Å	Favored (86.716%) alpha helix	-	-	-

			Avg: 6.62	Clashscore: 1.91	Outliers: 4 of 229	Poor rotamers: 0 of 190	Outliers: 1 of 219	Outliers: 9 of 227	Outliers: 2 of 231	Outliers: 9 of 231	Non-Trans: 0 of 230
A 181		VAL	4.9	-	Favored (87.16%) Ile or Val / -67.6,-43.0	Favored (88.6%) *t* chi angles: 174	0.03Å	Favored (79.731%) alpha helix	-	-	-
A 182		GLY	4.96	-	Favored (29.41%) Glycine / -53.7,-54.7	-	-	Favored (91.266%) alpha helix	-	-	-
A 183		VAL	5.09	-	Favored (96.84%) Ile or Val / -64.5,-44.3	Favored (70.5%) *t* chi angles: 172.2	0.04Å	Favored (78.712%) alpha helix	-	-	-
A 184		GLY	5.28	-	Favored (87.42%) Glycine / -58.5,-37.1	-	-	Favored (96.186%) alpha helix	-	-	-
A 185		SER	5.54	-	Favored (90.19%) General / -66.3,-40.7	Favored (67.1%) *m* chi angles: 296.9	0.04Å	Favored (87.611%) alpha helix	-	-	-
A 186		LEU	5.9	-	Favored (80.61%) General / -61.6,-36.4	Favored (73.2%) *mt* chi angles: 289.8,175.1	0.09Å	Favored (82.379%) alpha helix	-	-	-
A 187		ILE	6.38	-	Favored (81.62%) Ile or Val / -67.4,-47.3	Favored (98.3%) *mt* chi angles: 293.3,167.7	0.04Å	Favored (81.005%) alpha helix	-	-	-
A 188		LYS	6.97	-	Favored (76.57%) General / -60.9,-35.4	Favored (97.2%) *mttt* chi angles: 289.7,178,181.7,178.4	0.04Å	Favored (75.821%) alpha helix	-	-	-
A 189		GLU	7.63	-	Favored (15.6%) General / -96.5,17.7	Favored (84.1%) *mm-30* chi angles: 295.2,296.5,330.4	0.04Å	Favored (26.21%) alpha helix	-	-	-
A 190		LYS	8.31	-	Favored (58.82%) General / -58.9,-21.9	Favored (60.1%) *pttt* chi angles: 68.2,184.1,181.3,181.3	0.01Å	Favored (40.022%) alpha helix	-	-	-
A 191		ARG	8.92	-	Favored (58.83%) General / -78.3,-8.8	Favored (89.1%) *mtm180* chi angles: 294.7,172,290.4,171.1	0.08Å	Favored (40.923%) alpha helix	-	OUTLIER(S) worst is C-N-CA: 4.3 σ	-
A 192		SER	9.35	-	Favored (13.58%) General / -135.6,172.0	Favored (75%) *p* chi angles: 71.3	0.06Å	Favored (16.523%) alpha helix	-	-	-
A 193		SER	9.51	-	Favored (4.34%) General / -108.6,-43.2	Favored (70.7%) *m* chi angles: 296.2	0.03Å	CaBLAM Disfavored (3.579%) try alpha helix	-	-	-
A 194		ALA	9.35	-	Favored (83.07%) General / -64.3,-46.5	-	0.03Å	Favored (85.023%) alpha helix	-	-	-
A 195		ALA	8.92	-	Favored (83.79%) General / -64.5,-36.3	-	0.04Å	Favored (79.144%) alpha helix	-	-	-
A 196		LYS	8.32	-	Favored (89.45%) General / -60.2,-46.5	Favored (30.1%) *ttmt* chi angles: 181.6,183.4,285.4,174.5	0.03Å	Favored (84.529%) alpha helix	-	-	-

#	Alt	Res	High B	Clash > 0.4Å	Ramachandran	Rotamer	Cβ deviation	CaBLAM	Bond lengths	Bond angles	Cis Peptides
A 197		LYS	7.64	-	Favored (96.69%) General / -63.8,-40.4	Favored (97.1%) *mttt* chi angles: 289.3,176.7,181.3,178.5	0.03Å	Favored (96.316%) alpha helix	-	-	-
A 198		LYS	6.96	-	Favored (89.79%) General / -65.6,-38.5	Favored (97.7%) *mttt* chi angles: 290.6,176.6,184.3,177.4	0.05Å	Favored (97.371%) alpha helix	-	-	-
A 199		GLY	6.33	-	Favored (55.01%) Glycine / -59.0,-52.2	-	-	Favored (92.767%) alpha helix	-	-	-
A 200		ALA	5.8	-	Favored (79.5%) General / -59.5,-37.9	-	0.04Å	Favored (79.915%) alpha helix	-	-	-
**#**	**Alt**	**Res**	**High B**	**Clash > 0.4Å**	**Ramachandran**	**Rotamer**	**Cβ deviation**	**CaBLAM**	**Bond lengths**	**Bond angles**	**Cis Peptides**
			Avg: 6.62	Clashscore: 1.91	Outliers: 4 of 229	Poor rotamers: 0 of 190	Outliers: 1 of 219	Outliers: 9 of 227	Outliers: 2 of 231	Outliers: 9 of 231	Non-Trans: 0 of 230
A 201		CYS	5.36	-	Favored (95.62%) General / -64.8,-41.8	Favored (89.5%) *m* chi angles: 291.5	0.02Å	Favored (90.204%) alpha helix	-	-	-
A 202		LEU	5.02	-	Favored (71.87%) General / -69.6,-32.9	Favored (91.5%) *mt* chi angles: 291.5,173.4	0.11Å	Favored (85.813%) alpha helix	-	-	-
A 203		ILE	4.77	-	Favored (93.37%) Ile or Val / -64.4,-46.3	Favored (91.6%) *mt* chi angles: 291.3,167.8	0.07Å	Favored (79.969%) alpha helix	-	-	-
A 204		CYS	4.6	-	Favored (92.8%) General / -63.1,-38.8	Favored (98.5%) *m* chi angles: 289.7	0.04Å	Favored (87.465%) alpha helix	-	-	-
A 205		LEU	4.51	-	Favored (97.42%) General / -61.2,-44.4	Favored (59.7%) *tp* chi angles: 181.5,60.7	0.05Å	Favored (92.175%) alpha helix	-	-	-
A 206		ALA	4.51	-	Favored (96.92%) General / -60.6,-42.5	-	0.05Å	Favored (98.442%) alpha helix	-	-	-
A 207		LEU	4.57	-	Favored (83.87%) General / -65.9,-36.6	Favored (92.9%) *mt* chi angles: 291.6,173	0.04Å	Favored (76.333%) alpha helix	-	-	-
A 208		ALA	4.69	-	Favored (10.62%) General / -72.2,-54.1	-	0.07Å	Favored (60.742%) alpha helix	-	-	-
A 209		SER	4.86	-	Favored (65.82%) General / -67.2,-21.2	Favored (93%) *p* chi angles: 64.4	0.07Å	Favored (53.725%) alpha helix	-	-	-
A 210		THR	5.07	-	Favored (45.17%) General / -100.0,5.0	Favored (78%) *p* chi angles: 60.9	0.07Å	Favored (56.413%)	-	-	-
A 211		GLY	5.32	-	Favored (62.42%) Glycine / 74.6,28.9	-	-	Favored (85.327%)	-	-	-
A 212		VAL	5.57	-	Favored (16.23%) Ile or Val / -85.0,-48.8	Favored (85.3%) *t* chi angles: 177.6	0.05Å	CaBLAM Disfavored (2.908%)	-	-	-

#	Alt	Res	High B	Clash > 0.4Å	Ramachandran	Rotamer	Cβ deviation	CaBLAM	Bond lengths	Bond angles	Cis Peptides
A 213		PHE	5.8	-	Favored (47.87%) General / -126.4,151.3	Favored (96.7%) *m-80* chi angles: 293.6,88.4	0.10Å	Favored (19.996%)	-	OUTLIER(S) worst is CA-CB-CG: 4.7 σ	-
A 214		ASN	5.99	-	Favored (85.07%) Pre-Pro / -60.7,126.4	Favored (33.1%) *t0* chi angles: 186.9,304.4	0.01Å	Favored (42.566%)	-	-	-
A 215		PRO	6.12	-	Favored (12.9%) Trans-Pro / -47.9,-30.1	Favored (89.2%) *Cg_exo* chi angles: 329.4,36.5,333.3	0.05Å	Favored (70.37%)	-	-	-
A 216		MET	6.18	-	Favored (59.79%) General / -74.5,-26.2	Favored (50.7%) *mmp* chi angles: 293.9,299.5,96.4	0.06Å	Favored (74.024%) alpha helix	-	-	-
A 217		ILE	6.21	-	Favored (62.58%) Ile or Val / -71.3,-46.9	Favored (96.8%) *mt* chi angles: 294.1,167.4	0.14Å	Favored (63.367%) alpha helix	-	-	-
A 218		LEU	6.22	-	Favored (97.02%) General / -64.2,-41.7	Favored (93.9%) *mt* chi angles: 293.8,171.2	0.12Å	Favored (88.659%) alpha helix	-	-	-
A 219		ALA	6.28	-	Favored (99.76%) General / -63.0,-42.6	-	0.04Å	Favored (93.803%) alpha helix	-	-	-
A 220		ALA	6.43	-	Favored (87.58%) General / -60.5,-39.4	-	0.03Å	Favored (91.357%) alpha helix	-	-	-
**#**	**Alt**	**Res**	**High B**	**Clash > 0.4Å**	**Ramachandran**	**Rotamer**	**Cβ deviation**	**CaBLAM**	**Bond lengths**	**Bond angles**	**Cis Peptides**
			Avg: 6.62	Clashscore: 1.91	Outliers: 4 of 229	Poor rotamers: 0 of 190	Outliers: 1 of 219	Outliers: 9 of 227	Outliers: 2 of 231	Outliers: 9 of 231	Non-Trans: 0 of 230
A 221		GLY	6.71	-	Favored (40.7%) Glycine / -60.4,-54.0	-	-	Favored (91.394%) alpha helix	-	-	-
A 222		LEU	7.14	-	Favored (90.06%) General / -62.1,-38.8	Favored (91.3%) *mt* chi angles: 292,174.4	0.06Å	Favored (77.542%) alpha helix	-	-	-
A 223		MET	7.69	-	Favored (83.95%) General / -67.8,-40.2	Favored (83.5%) *mtm* chi angles: 289.6,187.9,287.5	0.04Å	Favored (93.333%) alpha helix	-	-	-
A 224		ALA	8.34	-	Favored (77.15%) General / -60.7,-35.8	-	0.04Å	Favored (86.172%) alpha helix	-	-	-
A 225		CYS	9.03	-	Favored (59.47%) General / -80.0,-8.6	Favored (74.6%) *m* chi angles: 297.1	0.07Å	Favored (37.89%) alpha helix	-	-	-
A 226		ASP	9.66	-	Favored (8.99%) Pre-Pro / -150.5,73.5	Favored (41.7%) *t0* chi angles: 190.2,21.2	0.04Å	Favored (7.996%)	-	-	-
A 227		PRO	10.19	-	Favored (4.28%) Trans-Pro / -46.9,-26.0	Favored (89.7%) *Cg_exo* chi angles: 330.9,37.1,330.7	0.03Å	Favored (20.569%)	-	-	-
A 228		ASN	10.58	-	Favored (65.49%) General / -60.8,-24.1	Favored (99.3%) *m-40* chi angles: 288.3,340.7	0.04Å	Favored (62.354%)	-	-	-

A 229		ARG	10.82	-	Favored (58.99%) General / -86.7,-3.0	Favored (94.4%) *mtt-85* chi angles: 295,174.9,185.7,265.8	0.09Å	Favored (56.948%)	-	-	-
A 230		LYS	10.95	-	Favored (47.08%) General / -86.7,-12.9	Favored (54.4%) *mtpt* chi angles: 293.5,174.5,72.7,176.1	0.01Å	-	-	-	-
A 231		ARG	11.01	-	-	Favored (93.6%) *mmt-90* chi angles: 294.6,290.1,183.3,273.3	0.02Å	-	-	-	-

About MolProbity | Website for the Richardson Lab | Using ecloud x-H | Internal reference 4.5.2
