# Supplementary material for: Integrative proteome-wide structural analysis and high-throughput docking identify broad-spectrum antiviral scaffolds against Zika, Yellow Fever, West Nile, Saint Louis encephalitis, and Usutu viruses
Source: Front Cell Infect Microbiol. 2026 Apr 30;16:1723132. doi: 10.3389/fcimb.2026.1723132 (PMC13171538; doi:10.3389/fcimb.2026.1723132)
Supplement: Supplementary file 5 [file DataSheet5.zip › WNV/WNV_NS2a/Mol_probity_Files/WNV_NS2a_1FH-rama.pdf]

MolProbity Ramachandran analysis

WNV_NS2a1FH.pdb, model 1

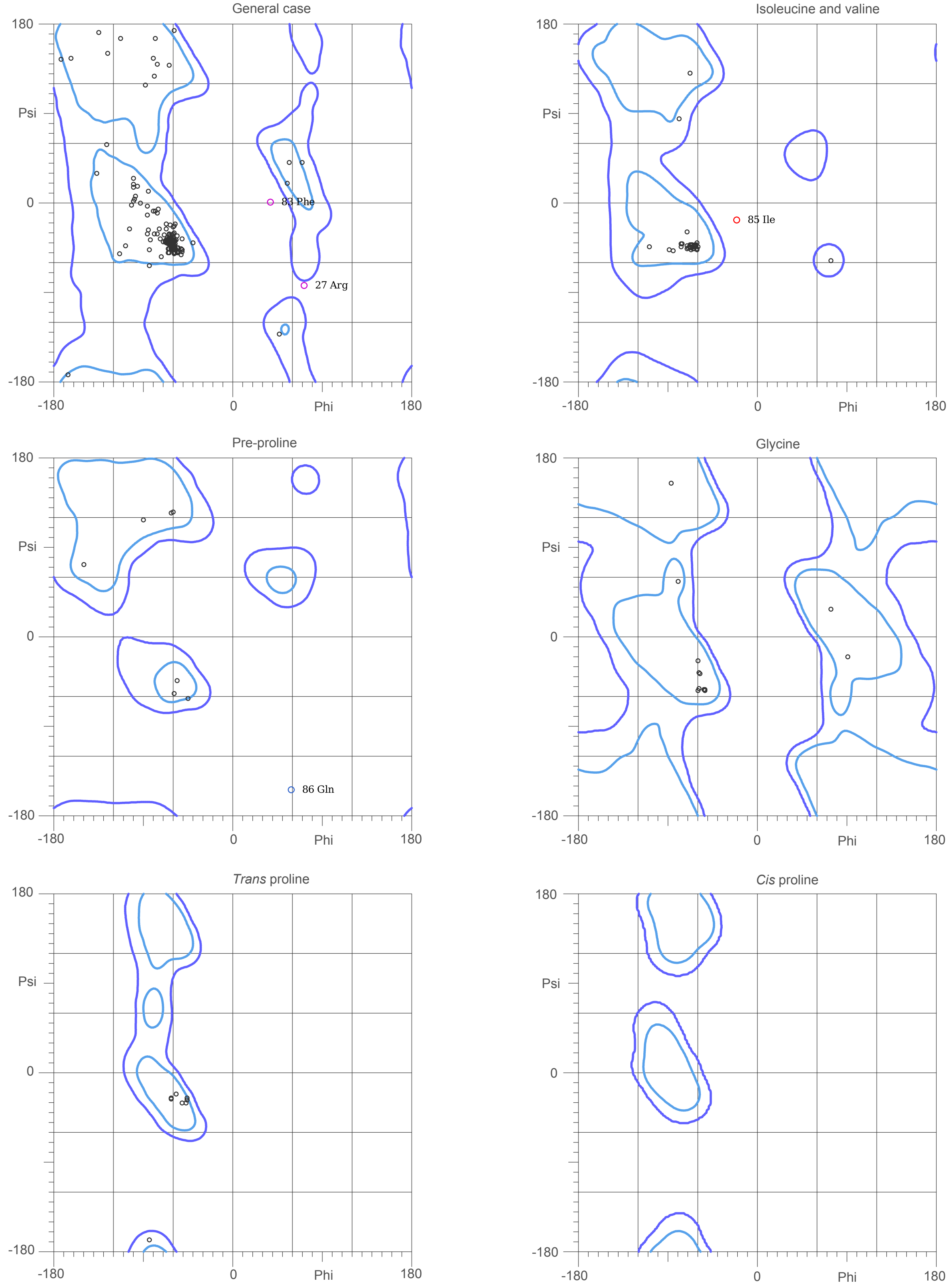

93.4% (214/229) of all residues were in favored (98%) regions.
98.3% (225/229) of all residues were in allowed (>99.8%) regions.

There were 4 outliers (phi, psi):
27 Arg (72.6, -83.3)
83 Phe (38.7, 1.2)
85 Ile (-21.6, -17.1)
86 Gln (59.2, -154.9)

http://kinemage.biochem.duke.edu

Lovell, Davis, et al. Proteins 50:437 (2003)
