# Supplementary material for: Integrative proteome-wide structural analysis and high-throughput docking identify broad-spectrum antiviral scaffolds against Zika, Yellow Fever, West Nile, Saint Louis encephalitis, and Usutu viruses
Source: Front Cell Infect Microbiol. 2026 Apr 30;16:1723132. doi: 10.3389/fcimb.2026.1723132 (PMC13171538; doi:10.3389/fcimb.2026.1723132)
Supplement: Supplementary file 5 [file DataSheet5.zip › WNV/WNV_NS2b/Mol_probity_Files/WNV_NS2b_1FH-multi.table.pdf]

Viewing WNV_NS2b1FH-multi.table

Duke Biochemistry
Duke University School of Medicine

When finished, you should close this window.

Hint: Use File | Save As... to save a copy of this page.

All-Atom Contacts	Clashscore, all atoms:	0.49		99th percentile* (N=1784, all resolutions)
	Clashscore is the number of serious steric overlaps (> 0.4 Å) per 1000 atoms.			
Protein Geometry	Poor rotamers	0	0.00%	Goal: <0.3%
	Favored rotamers	107	100.00%	Goal: >98%
	Ramachandran outliers	1	0.78%	Goal: <0.05%
	Ramachandran favored	127	98.45%	Goal: >98%
	Rama distribution Z-score	0.93 ± 0.65		Goal: abs(Z score) < 2
	MolProbity score^	0.67		100th percentile* (N=27675, 0Å - 99Å)
	Cβ deviations >0.25Å	0	0.00%	Goal: 0
	Bad bonds:	0 / 1043	0.00%	Goal: 0%
	Bad angles:	0 / 1424	0.00%	Goal: <0.1%
Peptide Omegas	Cis Prolines:	0 / 6	0.00%	Expected: ≤1 per chain, or ≤5%
Low-resolution Criteria	CaBLAM outliers	1	0.8%	Goal: <1.0%
	CA Geometry outliers	0	0.00%	Goal: <0.5%
Additional validations	Chiral volume outliers	0/163		
	Waters with clashes	0/0	0.00%	See UnDowser table for details

In the two column results, the left column gives the raw count, right column gives the percentage.

* 100th percentile is the best among structures of comparable resolution; 0th percentile is the worst. For clashscore the comparative set of structures was selected in 2004, for MolProbity score in 2006.

^ MolProbity score combines the clashscore, rotamer, and Ramachandran evaluations into a single score, normalized to be on the same scale as X-ray resolution.

Key to table colors and cutoffs here:

#	Alt	Res	High B	Clash > 0.4Å	Ramachandran	Rotamer	Cβ deviation	CaBLAM	Bond lengths	Bond angles	Cis Peptides
			Avg: 6.68	Clashscore: 0.49	Outliers: 1 of 129	Poor rotamers: 0 of 107	Outliers: 0 of 121	Outliers: 1 of 127	Outliers: 0 of 131	Outliers: 0 of 131	Non-Trans: 0 of 130
A 1		GLY	8.16	-	-	-	-	-	-	-	-
A 2		TRP	7.88	-	OUTLIER (0.02)%) Pre-Pro / 95.2,84.8	Favored (94.3%) *m100* chi angles: 294.1,107.7	0.11Å	-	-	-	-
A 3		PRO	7.49	-	Favored (29.9%) Trans-Pro / -59.7,158.1	Favored (61.1%) *Cg_exo* chi angles: 335.9,34.3,330	0.04Å	Favored (47.586%)	-	-	-
A 4		ALA	7	-	Favored (61.67%) General / -56.3,-29.7	-	0.04Å	Favored (50.458%)	-	-	-
A 5		THR	6.47	-	Favored (82.87%) General / -57.5,-42.5	Favored (89.1%) *m* chi angles: 298.3	0.03Å	Favored (66.311%) alpha helix	-	-	-
A 6		GLU	5.97	-	Favored (70.04%)	Favored (84.4%) *mm-30*	0.02Å	Favored (92.254%)	-	-	-

					General / -71.2,-33.7	chi angles: 292.9,298.5,309.3		alpha helix			
A 7		VAL	5.55	-	Favored (84.79%) Ile or Val / -64.2,-48.6	Favored (65.4%) *t* chi angles: 171.5	0.08Å	Favored (75.169%) alpha helix	-	-	-
A 8		MET	5.2	-	Favored (85.4%) General / -66.5,-37.6	Favored (84.3%) *mtm* chi angles: 290.4,186.4,285.8	0.06Å	Favored (78.634%) alpha helix	-	-	-
A 9		THR	4.94	-	Favored (93.44%) General / -64.1,-44.1	Favored (90.1%) *m* chi angles: 298.8	0.02Å	Favored (85.455%) alpha helix	-	-	-
A 10		ALA	4.77	-	Favored (86.14%) General / -59.5,-40.1	-	0.04Å	Favored (90.793%) alpha helix	-	-	-
A 11		VAL	4.66	-	Favored (96.2%) Ile or Val / -63.2,-46.3	Favored (67.9%) *t* chi angles: 171.9	0.04Å	Favored (92.707%) alpha helix	-	-	-
A 12		GLY	4.59	-	Favored (47.94%) Glycine / -57.3,-53.0	-	-	Favored (95.34%) alpha helix	-	-	-
A 13		LEU	4.57	-	Favored (96.9%) General / -62.9,-40.2	Favored (95.1%) *mt* chi angles: 291.8,172.8	0.07Å	Favored (83.68%) alpha helix	-	-	-
A 14		MET	4.58	-	Favored (78.82%) General / -68.9,-37.2	Favored (51%) *mmp* chi angles: 294.5,301.4,101.4	0.03Å	Favored (94.507%) alpha helix	-	-	-
A 15		PHE	4.6	-	Favored (85.01%) General / -66.9,-37.8	Favored (7%) *m-10* chi angles: 284.2,329.8	0.06Å	Favored (82.244%) alpha helix	-	-	-
A 16		ALA	4.64	-	Favored (81.39%) General / -60.0,-38.0	-	0.03Å	Favored (79.756%) alpha helix	-	-	-
A 17		ILE	4.7	-	Favored (91.36%) Ile or Val / -64.4,-47.0	Favored (98.5%) *mt* chi angles: 292.4,167.9	0.02Å	Favored (84.76%) alpha helix	-	-	-
A 18		VAL	4.77	-	Favored (98.73%) Ile or Val / -61.6,-44.5	Favored (63.4%) *t* chi angles: 171.3	0.06Å	Favored (92.3%) alpha helix	-	-	-
A 19		GLY	4.86	-	Favored (35.93%) Glycine / -57.3,-54.6	-	-	Favored (96.324%) alpha helix	-	-	-
A 20		GLY	4.97	-	Favored (88.99%) Glycine / -57.8,-38.6	-	-	Favored (90.603%) alpha helix	-	-	-
**#**	**Alt**	**Res**	**High B**	**Clash > 0.4Å**	**Ramachandran**	**Rotamer**	**Cβ deviation**	**CaBLAM**	**Bond lengths**	**Bond angles**	**Cis Peptides**
			Avg: 6.68	Clashscore: 0.49	Outliers: 1 of 129	Poor rotamers: 0 of 107	Outliers: 0 of 121	Outliers: 1 of 127	Outliers: 0 of 131	Outliers: 0 of 131	Non-Trans: 0 of 130

A 21		LEU	5.14	-	Favored (85%) General / -67.0,-42.6	Favored (81.8%) *mt* chi angles: 289.3,170.4	0.06Å	Favored (77.094%) alpha helix	-	-	-
A 22		ALA	5.42	-	Favored (75.3%) General / -59.6,-35.9	-	0.03Å	Favored (73.872%) alpha helix	-	-	-
A 23		GLU	5.79	-	Favored (69.35%) General / -60.4,-30.0	Favored (96%) *mt-10* chi angles: 289.2,181,353.9	0.05Å	Favored (63.01%)	-	-	-
A 24		LEU	6.21	-	Favored (2.51%) General / -87.2,52.4	Favored (70.8%) *mt* chi angles: 302.7,175.5	0.14Å	CaBLAM Outlier (0.493%)	-	-	-
A 25		ASP	6.56	-	Favored (28.74%) General / 51.0,43.9	Favored (74.3%) *m-30* chi angles: 294.4,321.1	0.03Å	Favored (5.059%)	-	-	-
A 26		ILE	6.77	-	Favored (36.02%) Ile or Val / -121.0,112.5	Favored (83.2%) *mt* chi angles: 299.1,173.2	0.05Å	Favored (8.999%)	-	-	-
A 27		ASP	6.8	-	Favored (30.98%) General / -106.1,6.5	Favored (69.8%) *m-30* chi angles: 295,314.4	0.04Å	Favored (16.14%)	-	-	-
A 28		SER	6.64	-	Favored (2.81%) General / -115.2,-48.6	Favored (73.1%) *m* chi angles: 295.6	0.02Å	Favored (9.211%) alpha helix	-	-	-
A 29		MET	6.34	-	Favored (69.02%) General / -65.1,-27.8	Favored (99.2%) *mtp* chi angles: 291.5,178.5,68.3	0.12Å	Favored (56.727%) alpha helix	-	-	-
A 30		ALA	6	-	Favored (63.41%) General / -56.6,-30.6	-	0.05Å	Favored (64.842%) alpha helix	-	-	-
A 31		ILE	5.71	-	Allowed (1.5%) Pre-Pro / -68.5,-65.0	Favored (96.4%) *mt* chi angles: 293.4,167	0.15Å	Favored (34.373%) alpha helix	-	-	-
A 32		PRO	5.5	-	Favored (73.63%) Trans-Pro / -63.1,-23.2	Favored (35.6%) *Cg_endo* chi angles: 22.5,326.2,31.4	0.02Å	Favored (34.308%) alpha helix	-	-	-
A 33		MET	5.37	-	Favored (60.77%) General / -75.5,-33.6	Favored (96.3%) *mmm* chi angles: 290.2,299.9,292.5	0.07Å	Favored (79.317%) alpha helix	-	-	-
A 34		THR	5.26	-	Favored (80.31%) General / -67.5,-43.7	Favored (95.4%) *m* chi angles: 299.5	0.04Å	Favored (76.308%) alpha helix	-	-	-
A 35		ILE	5.14	-	Favored (77.44%) Ile or Val / -64.9,-49.9	Favored (97.3%) *mt* chi angles: 292.9,167	0.07Å	Favored (79.969%) alpha helix	-	-	-
A 36		ALA	5.03	-	Favored (81.36%) General / -60.5,-37.6	-	0.05Å	Favored (81.299%) alpha helix	-	-	-
A 37		GLY	4.92	-	Favored (45.82%) Glycine / -58.9,-53.5	-	-	Favored (92.494%) alpha helix	-	-	-

#	Alt	Res	High B	Clash > 0.4Å	Ramachandran	Rotamer	Cβ deviation	CaBLAM	Bond lengths	Bond angles	Cis Peptides
A 38		LEU	4.81	-	Favored (73.96%) General / -64.4,-32.4	Favored (88.4%) *mt* chi angles: 291.6,174.4	0.12Å	Favored (74.831%) alpha helix	-	-	-
A 39		MET	4.72	-	Favored (98.53%) General / -62.1,-41.8	Favored (96.7%) *mtp* chi angles: 290.8,170.8,69.5	0.02Å	Favored (78.812%) alpha helix	-	-	-
A 40		PHE	4.65	-	Favored (84.87%) General / -58.8,-47.4	Favored (84.1%) *t80* chi angles: 174.3,74.5	0.07Å	Favored (97.598%) alpha helix	-	-	-
**#**	**Alt**	**Res**	**High B**	**Clash > 0.4Å**	**Ramachandran**	**Rotamer**	**Cβ deviation**	**CaBLAM**	**Bond lengths**	**Bond angles**	**Cis Peptides**
			Avg: 6.68	Clashscore: 0.49	Outliers: 1 of 129	Poor rotamers: 0 of 107	Outliers: 0 of 121	Outliers: 1 of 127	Outliers: 0 of 131	Outliers: 0 of 131	Non-Trans: 0 of 130
A 41		VAL	4.61	-	Favored (94.77%) Ile or Val / -60.5,-43.6	Favored (72.6%) *t* chi angles: 172.4	0.07Å	Favored (96.75%) alpha helix	-	-	-
A 42		ALA	4.61	-	Favored (78.09%) General / -59.3,-37.5	-	0.02Å	Favored (82.203%) alpha helix	-	-	-
A 43		PHE	4.7	-	Favored (76.36%) General / -59.0,-49.8	Favored (67.9%) *t80* chi angles: 169.5,78.9	0.02Å	Favored (81.768%) alpha helix	-	-	-
A 44		VAL	4.93	-	Favored (95.12%) Ile or Val / -60.5,-43.8	Favored (63.9%) *t* chi angles: 171.4	0.06Å	Favored (87.956%) alpha helix	-	-	-
A 45		ILE	5.3	-	Favored (98.24%) Ile or Val / -63.9,-44.9	Favored (98.9%) *mt* chi angles: 292.9,167.8	0.04Å	Favored (97.756%) alpha helix	-	-	-
A 46		SER	5.85	-	Favored (96.27%) General / -60.5,-42.5	Favored (71.1%) *m* chi angles: 296.1	0.07Å	Favored (90.594%) alpha helix	-	-	-
A 47		GLY	6.6	-	Favored (41.63%) Glycine / -54.9,-53.1	-	-	Favored (94.291%) alpha helix	-	-	-
A 48		LYS	7.53	-	Favored (61.27%) General / -51.2,-43.1	Favored (53.4%) *tttm* chi angles: 180.3,175.8,181.9,294.3	0.02Å	Favored (75.214%) alpha helix	-	-	-
A 49		SER	8.6	-	Favored (62.32%) General / -71.2,-19.9	Favored (65.1%) *p* chi angles: 72.6	0.02Å	Favored (55.502%)	-	-	-
A 50		THR	9.72	-	Favored (7.25%) General / -81.5,66.9	Favored (48.9%) *p* chi angles: 56	0.05Å	Favored (17.599%)	-	-	-
A 51		ASP	10.74	-	Favored (4.13%) General / -77.6,72.9	Favored (40.1%) *t0* chi angles: 192.6,25.5	0.07Å	Favored (40.832%)	-	-	-
A 52		MET	11.56	-	Favored (34.34%) General / -84.2,135.5	Favored (45.1%) *mmp* chi angles: 300,295.9,102.8	0.04Å	Favored (20.584%) beta sheet	-	-	-

#	Alt	Res	High B	Clash > 0.4Å	Ramachandran	Rotamer	Cβ deviation	CaBLAM	Bond lengths	Bond angles	Cis Peptides
A 53		TRP	12.12	-	Favored (48.54%) General / -122.4,145.2	Favored (92%) *m100* chi angles: 291.8,85.5	0.03Å	Favored (69.128%) beta sheet	-	-	-
A 54		ILE	12.44	-	Favored (73.06%) Ile or Val / -123.4,126.4	Favored (82.5%) *mt* chi angles: 299.8,171.6	0.01Å	Favored (71.254%) beta sheet	-	-	-
A 55		GLU	12.58	-	Favored (27.43%) General / -104.2,147.5	Favored (97.1%) *mt-10* chi angles: 295.6,180.8,356.7	0.01Å	Favored (43.904%) beta sheet	-	-	-
A 56		ARG	12.6	-	Favored (38.89%) General / -93.0,129.6	Favored (77.8%) *ttp80* chi angles: 182.2,170.1,64.9,86.8	0.01Å	Favored (54.399%)	-	-	-
A 57		THR	12.58	-	Favored (16.3%) General / -110.6,-2.7	Favored (76%) *p* chi angles: 61.2	0.04Å	Favored (21.746%)	-	-	-
A 58		ALA	12.6	-	Favored (30%) General / -157.6,155.4	-	0.03Å	Favored (19.306%)	-	-	-
A 59		ASP	12.72	-	Favored (41.15%) General / -74.2,149.0	Favored (95.6%) *m-30* chi angles: 290.8,346.2	0.01Å	Favored (40.368%)	-	-	-
A 60		ILE	12.96	-	Favored (74.18%) Ile or Val / -123.4,130.5	Favored (72.6%) *mt* chi angles: 301.7,169.9	0.02Å	Favored (59.482%) beta sheet	-	-	-
#	Alt	Res	High B	Clash > 0.4Å	Ramachandran	Rotamer	Cβ deviation	CaBLAM	Bond lengths	Bond angles	Cis Peptides
			Avg: 6.68	Clashscore: 0.49	Outliers: 1 of 129	Poor rotamers: 0 of 107	Outliers: 0 of 121	Outliers: 1 of 127	Outliers: 0 of 131	Outliers: 0 of 131	Non-Trans: 0 of 130
A 61		THR	13.27	-	Favored (13.93%) General / -105.8,-18.8	Favored (76.1%) *p* chi angles: 61.2	0.04Å	CaBLAM Disfavored (3.866%) try beta sheet	-	-	-
A 62		TRP	13.59	-	Favored (31.71%) General / 53.7,42.8	Favored (30.9%) *m-90* chi angles: 296.8,276	0.04Å	Favored (8.926%) beta sheet	-	-	-
A 63		GLU	13.86	-	Favored (38.28%) General / -54.5,130.0	Favored (89.8%) *tt0* chi angles: 185.5,177.1,5	0.03Å	Favored (19.213%)	-	-	-
A 64		SER	14	-	Favored (27.5%) General / -75.4,163.4	Favored (89.9%) *p* chi angles: 68.8	0.04Å	Favored (10.882%)	-	-	-
A 65		ASP	13.92	-	Favored (26.11%) General / 56.5,35.5	Favored (52.1%) *m-30* chi angles: 303.5,304.7	0.07Å	Favored (11.216%)	-	-	-
A 66		ALA	13.58	-	Favored (3.68%) General / -80.3,60.1	-	0.03Å	Favored (5.799%)	-	-	-
A 67		GLU	13	-	Favored (2.56%) General / -130.3,-24.5	Favored (95.9%) *mt-10* chi angles: 298.4,179.4,356.9	0.05Å	CaBLAM Disfavored (1.142%)	-	-	-
A 68		ILE	12.2	-	Favored (96.59%)	Favored (97.8%) *mt* chi angles: 293.5,167.9	0.04Å	Favored (34.419%)	-	-	-

					Ile or Val / -64.7,-43.4			three-ten			
A 69		THR	11.24	-	Favored (42.25%) General / -124.2,153.0	Favored (67.9%) *p* chi angles: 62.7	0.04Å	Favored (21.097%)	-	-	-
A 70		GLY	10.2	-	Favored (23.95%) Glycine / -112.2,18.0	-	-	Favored (16.434%)	-	-	-
A 71		SER	9.19	-	Favored (59.57%) General / -78.1,-10.3	Favored (83.8%) *p* chi angles: 67.5	0.02Å	Favored (8.586%)	-	-	-
A 72		SER	8.29	-	Favored (58.5%) General / -62.3,138.8	Favored (37.6%) *t* chi angles: 177.5	0.03Å	Favored (43.436%)	-	-	-
A 73		GLU	7.63	-	Favored (37.74%) General / -78.8,137.5	Favored (88.7%) *tt0* chi angles: 186.1,177.3,6.2	0.02Å	Favored (44.672%) beta sheet	-	-	-
A 74		ARG	7.3	-	Favored (43.54%) General / -130.5,130.0	Favored (53%) *ttt90* chi angles: 182.9,183.5,176.3,97.4	0.06Å	Favored (69.465%) beta sheet	-	-	-
A 75		VAL	7.35	-	Favored (56%) Ile or Val / -119.1,118.0	Favored (57.9%) *t* chi angles: 180.2	0.06Å	Favored (67.021%) beta sheet	-	-	-
A 76		ASP	7.79	-	Favored (28.9%) General / -91.3,116.5	Favored (72.6%) *m-30* chi angles: 290,325.5	0.01Å	Favored (51.246%) beta sheet	-	-	-
A 77		VAL	8.65	-	Favored (22.83%) Ile or Val / -125.2,165.3	Favored (28.1%) *m* chi angles: 299.9	0.04Å	Favored (31.276%) beta sheet	-	-	-
A 78		ARG	9.83	-	Favored (49.98%) General / -130.2,152.1	Favored (41.8%) *ptt180* chi angles: 66.2,181.1,183.3,189.7	0.04Å	Favored (50.89%) beta sheet	-	-	-
A 79		LEU	11.17	-	Favored (49.07%) General / -103.5,124.6	Favored (50.5%) *tp* chi angles: 180.6,65.7	0.08Å	Favored (56.791%)	-	-	-
A 80		ASP	12.41	0.41Å C with A 80 ASP OD1	Favored (6.08%) General / -87.1,-177.7	Favored (36.3%) *p0* chi angles: 71.3,15.1	0.06Å	Favored (32.059%)	-	-	-
**#**	**Alt**	**Res**	**High B**	**Clash > 0.4Å**	**Ramachandran**	**Rotamer**	**Cβ deviation**	**CaBLAM**	**Bond lengths**	**Bond angles**	**Cis Peptides**
			Avg: 6.68	Clashscore: 0.49	Outliers: 1 of 129	Poor rotamers: 0 of 107	Outliers: 0 of 121	Outliers: 1 of 127	Outliers: 0 of 131	Outliers: 0 of 131	Non-Trans: 0 of 130
A 81		ASP	13.28	-	Favored (66.24%) General / -63.9,-21.0	Favored (94.7%) *m-30* chi angles: 290.5,348.1	0.01Å	Favored (35.588%)	-	-	-
A 82		ASP	13.53	-	Favored (42.82%) General / -101.3,7.4	Favored (71.7%) *m-30* chi angles: 292.9,318.3	0.02Å	Favored (55.236%)	-	-	-
A 83		GLY	13.07	-	Favored (76.66%) Glycine / 85.7,10.3	-	-	Favored (88.306%)	-	-	-

A<br>84		ASN	12.04	-	Favored<br>(19.1%)<br>General /<br>-91.0,154.9	Favored (80.7%) *m-40*<br>chi angles: 298.6,308.2	0.08Å	Favored<br>(36.658%)	-	-	-
A<br>85		PHE	10.73	-	Favored<br>(30.01%)<br>General /<br>-105.4,146.0	Favored (71.7%) *m-80*<br>chi angles: 286.8,89.6	0.09Å	Favored<br>(55.657%)<br>beta sheet	-	-	-
A<br>86		GLN	9.4	-	Favored<br>(52.12%)<br>General /<br>-130.2,149.0	Favored (83.2%)<br>*mt0*<br>chi angles:<br>298,183.5,322.8	0.05Å	Favored<br>(53.038%)<br>beta sheet	-	-	-
A<br>87		LEU	8.18	-	Favored<br>(27.28%)<br>General /<br>-88.5,117.2	Favored (75.5%) *tp*<br>chi angles: 177.5,62	0.01Å	Favored<br>(51.797%)	-	-	-
A<br>88		ILE	7.16	-	Favored<br>(33.24%)<br>Ile or Val /<br>-64.8,-23.4	Favored (7.6%) *tp*<br>chi angles: 200.1,65.8	0.01Å	Favored<br>(49.945%)	-	-	-
A<br>89		ASN	6.33	-	Favored<br>(41.11%)<br>General / -94.6,8.8	Favored (90.6%) *m-40*<br>chi angles: 292.2,323.1	0.02Å	Favored<br>(33.059%)	-	-	-
A<br>90		ASP	5.69	-	Favored<br>(19.02%)<br>Pre-Pro /<br>-92.2,106.8	Favored (63.3%) *t0*<br>chi angles: 184.2,342.3	0.03Å	Favored<br>(21.542%)	-	-	-
A<br>91		PRO	5.19	-	Favored<br>(19.04%)<br>Trans-Pro /<br>-76.3,-12.3	Favored (66.3%)<br>*Cg_endo*<br>chi angles:<br>31.3,324.9,24.2	0.08Å	CaBLAM<br>Disfavored<br>(2.684%)	-	-	-
A<br>92		GLY	4.75	-	Favored<br>(45.24%)<br>Glycine /<br>77.5,176.6	-	-	Favored<br>(54.467%)	-	-	-
A<br>93		ALA	4.34	-	Favored<br>(96.62%)<br>Pre-Pro /<br>-69.5,146.9	-	0.02Å	Favored<br>(6.163%)	-	-	-
A<br>94		PRO	3.98	-	Favored<br>(90.96%)<br>Trans-Pro /<br>-57.9,145.5	Favored (73.6%)<br>*Cg_exo*<br>chi angles:<br>335.1,34.3,331	0.05Å	Favored<br>(89.873%)	-	-	-
A<br>95		TRP	3.67	-	Favored<br>(89.93%)<br>General /<br>-58.8,-42.8	Favored (62.1%) *t-100*<br>chi angles: 184.4,246.8	0.04Å	Favored<br>(63.506%)	-	-	-
A<br>96		LYS	3.41	-	Favored<br>(79.73%)<br>General /<br>-56.5,-43.6	Favored (86.8%) *tttt*<br>chi angles:<br>181.2,176.5,178.9,178.8	0.02Å	Favored<br>(86.833%)<br>alpha helix	-	-	-
A<br>97		ILE	3.18	-	Favored<br>(95.09%)<br>Ile or Val /<br>-63.5,-42.1	Favored (30.6%)<br>*mm*<br>chi angles: 292.8,297.4	0.05Å	Favored<br>(97.427%)<br>alpha helix	-	-	-
A<br>98		TRP	2.95	-	Favored<br>(84.65%)<br>General /<br>-59.8,-47.7	Favored (92.3%)<br>*t60*<br>chi angles: 180.4,87.1	0.05Å	Favored<br>(91.359%)<br>alpha helix	-	-	-
A<br>99		MET	2.74	-	Favored<br>(92.42%)<br>General /<br>-63.9,-38.8	Favored (56.3%)<br>*tpp*<br>chi angles:<br>190.1,63,72.1	0.02Å	Favored<br>(81.921%)<br>alpha helix	-	-	-

#	Alt	Res	High B	Clash > 0.4Å	Ramachandran	Rotamer	Cβ deviation	CaBLAM	Bond lengths	Bond angles	Cis Peptides
A 100		LEU	2.55	-	Favored (93.85%) General / -64.0,-39.3	Favored (86%) *mt* chi angles: 290.2,170.8	0.04Å	Favored (95.161%) alpha helix	-	-	-
#	Alt	Res	High B	Clash > 0.4Å	Ramachandran	Rotamer	Cβ deviation	CaBLAM	Bond lengths	Bond angles	Cis Peptides
			Avg: 6.68	Clashscore: 0.49	Outliers: 1 of 129	Poor rotamers: 0 of 107	Outliers: 0 of 121	Outliers: 1 of 127	Outliers: 0 of 131	Outliers: 0 of 131	Non-Trans: 0 of 130
A 101		ARG	2.4	-	Favored (85.75%) General / -67.3,-38.8	Favored (95.9%) *mtt180* chi angles: 291,173.9,186.1,170.3	0.03Å	Favored (98.081%) alpha helix	-	-	-
A 102		MET	2.28	-	Favored (86.76%) General / -66.2,-38.0	Favored (49.5%) *mmp* chi angles: 295.5,303.5,100.1	0.07Å	Favored (96.495%) alpha helix	-	-	-
A 103		ALA	2.18	-	Favored (95.26%) General / -62.2,-40.2	-	0.03Å	Favored (89.955%) alpha helix	-	-	-
A 104		CYS	2.12	-	Favored (97.03%) General / -62.3,-40.7	Favored (89.9%) *m* chi angles: 291.4	0.08Å	Favored (84.146%) alpha helix	-	-	-
A 105		LEU	2.15	-	Favored (69.99%) General / -70.9,-42.3	Favored (93.1%) *mt* chi angles: 293.9,171.1	0.07Å	Favored (84.063%) alpha helix	-	-	-
A 106		ALA	2.36	-	Favored (94.16%) General / -62.9,-39.4	-	0.04Å	Favored (90.644%) alpha helix	-	-	-
A 107		ILE	2.87	-	Favored (89.42%) Ile or Val / -65.1,-47.0	Favored (91.9%) *mt* chi angles: 291.8,166.4	0.06Å	Favored (82.304%) alpha helix	-	-	-
A 108		SER	3.88	-	Favored (77.12%) General / -58.4,-38.3	Favored (69%) *m* chi angles: 294.8	0.08Å	Favored (73.712%) alpha helix	-	-	-
A 109		ALA	5.51	-	Favored (68.3%) General / -59.6,-29.9	-	0.04Å	Favored (43.329%) alpha helix	-	-	-
A 110		TYR	7.49	-	Favored (9.49%) General / -114.9,-22.6	Favored (59.2%) *m-80* chi angles: 303.3,113.1	0.03Å	Favored (33.993%) alpha helix	-	-	-
A 111		THR	8.98	-	Favored (80.83%) Pre-Pro / -129.9,74.2	Favored (50.6%) *p* chi angles: 56.3	0.04Å	Favored (15.987%) alpha helix	-	-	-
A 112		PRO	9.04	-	Favored (13.32%) Trans-Pro / -47.6,-31.1	Favored (86%) *Cg_exo* chi angles: 330.5,37.6,330.4	0.05Å	Favored (43.033%) alpha helix	-	-	-
A 113		TRP	7.61	-	Favored (64.29%) General / -66.9,-16.4	Favored (48.2%) *p90* chi angles: 54.6,86.7	0.05Å	Favored (67.77%) three-ten	-	-	-
A 114		ALA	5.62	-	Favored (57.1%) General / -86.2,-1.1	-	0.04Å	Favored (46.272%) three-ten	-	-	-

A 115		ILE	3.96	-	Favored (93.67%) Ile or Val / -60.0,-46.8	Favored (86.6%) *mt* chi angles: 290.6,167.1	0.08Å	Favored (51.882%) alpha helix	-	-	-
A 116		LEU	2.93	-	Favored (73.08%) Pre-Pro / -58.4,-52.6	Favored (60.1%) *tp* chi angles: 174.9,60.7	0.13Å	Favored (77.53%) alpha helix	-	-	-
A 117		PRO	2.41	-	Favored (71.17%) Trans-Pro / -63.4,-24.3	Favored (28.2%) *Cg_endo* chi angles: 21,326.8,31.3	0.04Å	Favored (91.151%) alpha helix	-	-	-
A 118		SER	2.2	-	Favored (73.99%) General / -70.3,-40.9	Favored (71%) *m* chi angles: 295.1	0.04Å	Favored (76.607%) alpha helix	-	-	-
A 119		ILE	2.16	-	Favored (97.89%) Ile or Val / -63.9,-43.8	Favored (87%) *mt* chi angles: 290.7,168.3	0.01Å	Favored (95.538%) alpha helix	-	-	-
A 120		ILE	2.18	-	Favored (99.75%) Ile or Val / -62.6,-44.9	Favored (98.2%) *mt* chi angles: 292.8,167.1	0.05Å	Favored (95.853%) alpha helix	-	-	-
**#**	**Alt**	**Res**	**High B**	**Clash > 0.4Å**	**Ramachandran**	**Rotamer**	**Cβ deviation**	**CaBLAM**	**Bond lengths**	**Bond angles**	**Cis Peptides**
			Avg: 6.68	Clashscore: 0.49	Outliers: 1 of 129	Poor rotamers: 0 of 107	Outliers: 0 of 121	Outliers: 1 of 127	Outliers: 0 of 131	Outliers: 0 of 131	Non-Trans: 0 of 130
A 121		GLY	2.19	-	Favored (58.56%) Glycine / -54.6,-50.1	-	-	Favored (98.383%) alpha helix	-	-	-
A 122		PHE	2.21	-	Favored (76.27%) General / -56.1,-48.3	Favored (83.5%) *t80* chi angles: 182.6,82.2	0.03Å	Favored (97.476%) alpha helix	-	-	-
A 123		TRP	2.24	-	Favored (80.1%) General / -57.6,-40.8	Favored (37.9%) *m-10* chi angles: 288.1,337.2	0.05Å	Favored (83.22%) alpha helix	-	-	-
A 124		ILE	2.29	-	Favored (93.11%) Ile or Val / -61.3,-42.3	Favored (94.3%) *mt* chi angles: 291.7,167.3	0.08Å	Favored (93.34%) alpha helix	-	-	-
A 125		THR	2.38	-	Favored (61.79%) General / -72.5,-15.6	Favored (52%) *p* chi angles: 65.4	0.05Å	Favored (65.419%) alpha helix	-	-	-
A 126		LEU	2.52	-	Favored (35.67%) General / -77.3,-42.7	Favored (94.8%) *mt* chi angles: 296.2,175.9	0.08Å	Favored (36.498%) alpha helix	-	-	-
A 127		GLN	2.74	-	Favored (74.29%) General / -63.7,-32.7	Favored (35.8%) *mm110* chi angles: 292.7,295.2,118.3	0.01Å	Favored (70.003%) alpha helix	-	-	-
A 128		TYR	3.04	-	Favored (62.72%) General / -63.6,-17.3	Favored (10.3%) *m-10* chi angles: 285.6,342.2	0.06Å	Favored (55.595%) alpha helix	-	-	-
A 129		THR	3.41	-	Favored (28.39%) General / -105.6,2.7	Favored (73.8%) *p* chi angles: 61.6	0.03Å	Favored (50.467%)	-	-	-

A 130		LYS	3.83	-	Favored (50.04%) General / -92.2,-6.8	Favored (99%) *mttt* chi angles: 293.9,181.4,180.4,178	0.03Å	-	-	-	-
A 131		ARG	4.25	-	-	Favored (93.9%) *mmt-90* chi angles: 294.9,290.1,183.3,273.5	0.02Å	-	-	-	-

About MolProbity | Website for the Richardson Lab | Using ecloud x-H | Internal reference 4.5.2
