# Supplementary material for: Integrative proteome-wide structural analysis and high-throughput docking identify broad-spectrum antiviral scaffolds against Zika, Yellow Fever, West Nile, Saint Louis encephalitis, and Usutu viruses
Source: Front Cell Infect Microbiol. 2026 Apr 30;16:1723132. doi: 10.3389/fcimb.2026.1723132 (PMC13171538; doi:10.3389/fcimb.2026.1723132)
Supplement: Supplementary file 5 [file DataSheet5.zip › WNV/WNV_NS3/Mol_probity_Files/WNV_NS3_1FH-multi.table.pdf]

Viewing WNV_NS3_1FH-multi.table

When finished, you should close this window.

Hint: Use File | Save As... to save a copy of this page.

All-Atom Contacts	Clashscore, all atoms:	1.76		99th percentile* (N=1784, all resolutions)
	Clashscore is the number of serious steric overlaps (> 0.4 Å) per 1000 atoms.			
Protein Geometry	Poor rotamers	1	0.19%	Goal: <0.3%
	Favored rotamers	511	99.42%	Goal: >98%
	Ramachandran outliers	4	0.65%	Goal: <0.05%
	Ramachandran favored	599	97.08%	Goal: >98%
	Rama distribution Z-score	0.36 ± 0.33		Goal: abs(Z score) < 2
	MolProbity score^	1.09		100th percentile* (N=27675, 0Å - 99Å)
	Cβ deviations >0.25Å	0	0.00%	Goal: 0
	Bad bonds:	6 / 4970	0.12%	Goal: 0%
	Bad angles:	12 / 6740	0.18%	Goal: <0.1%
Peptide Omegas	Cis Prolines:	1 / 37	2.70%	Expected: ≤1 per chain, or ≤5%
	Cis nonProlines:	3 / 581	0.52%	Goal: <0.05%
Low-resolution Criteria	CaBLAM outliers	16	2.6%	Goal: <1.0%
	CA Geometry outliers	5	0.81%	Goal: <0.5%
Additional validations	Chiral volume outliers	0/723		
	Waters with clashes	0/0	0.00%	See UnDowser table for details

In the two column results, the left column gives the raw count, right column gives the percentage.

* 100th percentile is the best among structures of comparable resolution; 0th percentile is the worst. For clashscore the comparative set of structures was selected in 2004, for MolProbity score in 2006.

^ MolProbity score combines the clashscore, rotamer, and Ramachandran evaluations into a single score, normalized to be on the same scale as X-ray resolution.

Key to table colors and cutoffs here:

#	Alt	Res	High B	Clash > 0.4Å	Ramachandran	Rotamer	Cβ deviation	CaBLAM	Bond lengths	Bond angles	Cis Peptides
			Avg: 1.12	Clashscore: 1.76	Outliers: 4 of 617	Poor rotamers: 1 of 514	Outliers: 0 of 563	Outliers: 19 of 615	Outliers: 6 of 619	Outliers: 10 of 619	Non-Trans: 4 of 618
A 1		GLY	8.42	-	-	-	-	-	-	-	-
A 2		GLY	7.85	-	Favored (64.75%) Glycine / 77.0,25.2	-	-	-	-	-	-
A 3		VAL	7.24	-	Favored (23.9%) Ile or Val / -79.2,-45.5	Favored (93.9%) *t* chi angles: 174.7	0.06Å	Favored (10.476%)	-	-	-
A 4		LEU	6.67	-	Favored (12.04%) General / -103.0,102.0	Favored (81.7%) *mt* chi angles: 302,177.5	0.02Å	Favored (5.858%)	-	-	-
A 5		TRP	6.2	-	Favored (11.52%) General / -117.5,-2.3	Favored (70.9%) *m100* chi angles: 299.5,114.9	0.04Å	Favored (21.887%)	-	-	-
A 6		ASP	5.86	-	Favored (7.62%) General / -117.3,29.9	Favored (46.1%) *p0* chi angles: 59.1,354.6	0.11Å	Favored (10.835%)	-	-	-

A 7		THR	5.61	-	Favored (68.97%) Pre-Pro / -90.4,118.7	Favored (88.7%) *m* chi angles: 298.6	0.02Å	Favored (27.508%)	-	-	-
A 8		PRO	5.39	-	Favored (39.07%) Trans-Pro / -75.3,160.5	Favored (76%) *Cg_endo* chi angles: 30.8,324,26.2	0.04Å	Favored (33.413%)	-	-	-
A 9		SER	5.11	-	Favored (87.64%) Pre-Pro / -59.2,132.2	Favored (41.5%) *t* chi angles: 176.2	0.08Å	Favored (29.428%)	-	-	-
A 10		PRO	4.74	-	Favored (52.15%) Trans-Pro / -72.5,157.8	Favored (75.3%) *Cg_endo* chi angles: 28,325.1,27.1	0.04Å	Favored (83.67%)	-	-	-
A 11		ARG	4.25	-	Favored (25.22%) General / -78.5,163.8	Favored (46.7%) *mmt180* chi angles: 297.5,284.3,183.2,175.3	0.01Å	Favored (34.359%)	-	-	-
A 12		GLU	3.7	-	Favored (35.73%) General / -77.6,129.5	Favored (65.6%) *tp30* chi angles: 183.8,68.2,13.8	0.03Å	Favored (17.876%)	-	-	-
A 13		TYR	3.13	-	Favored (48.97%) General / -129.9,152.8	Favored (93.8%) *m-80* chi angles: 296.7,86.4	0.05Å	Favored (39.07%)	-	-	-
A 14		LYS	2.61	-	Favored (57.99%) General / -58.9,136.8	Favored (37.3%) *ttpt* chi angles: 183.2,171,72.1,176.3	0.02Å	Favored (34.639%)	-	-	-
A 15		LYS	2.16	-	Favored (57.03%) General / -58.7,134.7	Favored (87.8%) *tttt* chi angles: 183.5,176.1,177.9,180.2	0.04Å	Favored (47.896%)	-	-	-
A 16		GLY	1.77	-	Favored (46.64%) Glycine / -68.2,165.0	-	-	Favored (45.775%)	-	-	-
A 17		ASP	1.46	-	Favored (57.52%) General / -63.9,136.6	Favored (11.9%) *t70* chi angles: 192.2,94.5	0.05Å	Favored (19.214%)	-	-	-
A 18		THR	1.21	-	Favored (9.88%) General / -120.4,25.3	Favored (52%) *p* chi angles: 56.5	0.09Å	CaBLAM Disfavored (4.307%) try beta sheet	-	-	-
A 19		THR	1.03	-	Favored (48.58%) General / -59.1,130.8	Favored (79.7%) *m* chi angles: 302.6	0.01Å	Favored (26.021%)	-	-	-
A 20		THR	0.91	-	Favored (51.92%) General / -56.2,132.9	Favored (88.7%) *m* chi angles: 301.3	0.03Å	CaBLAM Disfavored (2.109%)	-	-	-
**#**	**Alt**	**Res**	**High B**	**Clash > 0.4Å**	**Ramachandran**	**Rotamer**	**Cβ deviation**	**CaBLAM**	**Bond lengths**	**Bond angles**	**Cis Peptides**
			Avg: 1.12	Clashscore: 1.76	Outliers: 4 of 617	Poor rotamers: 1 of 514	Outliers: 0 of 563	Outliers: 19 of 615	Outliers: 6 of 619	Outliers: 10 of 619	Non-Trans: 4 of 618
A 21		GLY	0.83	-	Favored (34.08%) Glycine / 156.2,-173.2	-	-	Favored (48.593%)	-	-	-
A 22		VAL	0.79	-	Favored (59.85%)	Favored (67%) *t* chi angles: 179.1	0.05Å	Favored (10.166%) beta sheet	-	-	-

					Ile or Val / -107.4,130.3						
A 23		TYR	0.79	-	Favored (40.2%) General / -124.3,154.4	Favored (86.5%) *m-80* chi angles: 296.6,84	0.05Å	Favored (47.601%) beta sheet	-	-	-
A 24		ARG	0.83	-	Favored (20.27%) General / -92.8,151.0	Favored (42.5%) *ptt180* chi angles: 66.9,182.1,184.7,172.6	0.05Å	Favored (44.509%) beta sheet	-	-	-
A 25		ILE	0.92	-	Favored (59.42%) Ile or Val / -111.2,119.3	Favored (81.9%) *mt* chi angles: 299.8,170.6	0.03Å	Favored (60.323%) beta sheet	-	-	-
A 26		MET	1.07	-	Favored (23.92%) General / -105.1,151.5	Favored (79.4%) *mmm* chi angles: 305.5,295.4,288.4	0.02Å	Favored (39.294%) beta sheet	-	-	-
A 27		THR	1.27	-	Favored (48.76%) General / -131.9,139.6	Favored (89.3%) *m* chi angles: 298.3	0.07Å	Favored (68.494%) beta sheet	-	-	-
A 28		ARG	1.5	-	Favored (19.1%) General / -76.6,-48.5	Favored (53.3%) *ttp-170* chi angles: 183.9,190.6,69.7,199.9	0.10Å	CaBLAM Disfavored (1.222%)	-	-	-
A 29		GLY	1.69	-	Favored (31.77%) Glycine / 66.3,-129.9	-	-	Favored (5.241%)	-	-	-
A 30		LEU	1.77	-	Favored (4.99%) General / -104.7,-43.9	Favored (93.6%) *mt* chi angles: 298.6,177.1	0.02Å	CaBLAM Outlier (0.077%)	-	-	-
A 31		LEU	1.71	-	Favored (52.65%) General / -119.2,138.9	Favored (71.6%) *mt* chi angles: 302.2,174.8	0.06Å	CA Geom Outlier (0.449%)	-	-	-
A 32		GLY	1.53	-	Favored (47.87%) Glycine / 57.5,-128.6	-	-	Favored (16.551%)	-	-	-
A 33		SER	1.3	-	Favored (25.52%) General / -163.2,163.4	Favored (85.7%) *p* chi angles: 67.3	0.02Å	Favored (7.991%)	-	-	-
A 34		TYR	1.08	-	Favored (39.92%) General / -147.2,161.9	Favored (56.9%) *p90* chi angles: 64.6,93.7	0.01Å	Favored (31.53%)	-	-	-
A 35		GLN	0.92	-	Favored (31%) General / -87.4,122.3	Favored (50.3%) *tt0* chi angles: 183.2,174.8,310	0.06Å	Favored (30.144%)	-	-	-
A 36		ALA	0.81	-	Favored (18.2%) General / -89.4,-30.7	-	0.04Å	Favored (7.536%)	-	-	-
A 37		GLY	0.74	-	Favored (25.06%) Glycine / -175.9,-161.4	-	-	Favored (12.396%)	-	-	-
A 38		ALA	0.71	-	Favored (14.08%) General / -150.2,173.3	-	0.03Å	Favored (39.217%)	-	-	-
A 39		GLY	0.7	-	Favored (41.37%) Glycine / -173.2,-175.9	-	-	Favored (47.026%) beta sheet	-	-	-

A 40		VAL	0.72	-	Favored (42.2%) Ile or Val / -133.0,142.4	Favored (8.7%) *p* chi angles: 65.6	0.07Å	Favored (28.861%) beta sheet	-	-	-
**#**	**Alt**	**Res**	**High B**	**Clash > 0.4Å**	**Ramachandran**	**Rotamer**	**Cβ deviation**	**CaBLAM**	**Bond lengths**	**Bond angles**	**Cis Peptides**
			Avg: 1.12	Clashscore: 1.76	Outliers: 4 of 617	Poor rotamers: 1 of 514	Outliers: 0 of 563	Outliers: 19 of 615	Outliers: 6 of 619	Outliers: 10 of 619	Non-Trans: 4 of 618
A 41		MET	0.74	0.44Å HB2 with A 41 MET HE2	Favored (34.64%) General / -103.1,117.5	Favored (36.4%) *tpp* chi angles: 187.1,76.8,68.2	0.11Å	Favored (56.403%)	-	-	-
A 42		VAL	0.77	-	Favored (61.38%) Ile or Val / -129.3,125.1	Favored (80.6%) *t* chi angles: 177.9	0.06Å	Favored (20.186%)	-	-	-
A 43		GLU	0.79	-	Favored (20.82%) General / 51.0,38.5	Favored (67.6%) *mt-10* chi angles: 297.3,185.6,21.5	0.05Å	Favored (39.144%)	-	-	-
A 44		GLY	0.79	-	Favored (90.12%) Glycine / 83.6,1.2	-	-	Favored (83.351%)	-	-	-
A 45		VAL	0.78	-	Favored (64.26%) Ile or Val / -113.7,131.9	Favored (79.3%) *t* chi angles: 178	0.02Å	Favored (29.766%)	-	-	-
A 46		PHE	0.77	-	Favored (35.71%) General / -89.2,130.0	Favored (70.5%) *t80* chi angles: 183.6,70.9	0.04Å	Favored (58.744%) beta sheet	-	-	-
A 47		HIS	0.75	-	Favored (54.71%) General / -122.2,133.4	Favored (47.1%) *m90* chi angles: 305,79.2	0.14Å	Favored (39.603%) beta sheet	OUTLIER(S) worst is CB--CG: 4.4 σ	-	-
A 48		THR	0.75	-	Favored (4.04%) General / -146.3,-172.9	Favored (11.7%) *t* chi angles: 189.7	0.07Å	Favored (14.054%) beta sheet	-	-	-
A 49		LEU	0.75	-	Favored (31.31%) General / -101.0,143.3	Favored (86.6%) *mt* chi angles: 294.9,178.3	0.12Å	Favored (12.294%)	-	-	-
A 50		TRP	0.78	-	Favored (91.98%) General / -59.1,-44.7	Favored (72.5%) *t60* chi angles: 190,93.4	0.03Å	Favored (56.873%)	-	-	-
A 51		HIS	0.83	-	Favored (17.42%) General / -63.3,-10.7	Favored (47.1%) *p-80* chi angles: 73.7,280	0.09Å	Favored (47.012%) alpha helix	-	OUTLIER(S) worst is CA-CB-CG: 4.0 σ	-
A 52		THR	0.89	-	Favored (27.91%) General / -93.5,-13.3	Favored (56.9%) *p* chi angles: 64.6	0.03Å	Favored (30.506%) alpha helix	-	-	-
A 53		THR	0.97	-	Favored (2.51%) General / -130.7,-23.9	Favored (43.3%) *p* chi angles: 67.1	0.09Å	CaBLAM Disfavored (1.55%)	-	-	-
A 54		LYS	1.06	-	Favored (12.65%) General / 55.9,28.6	Favored (54.4%) *mttp* chi angles: 298.7,180.8,175.6,64.7	0.05Å	CaBLAM Disfavored (4.607%)	-	-	-
A 55		GLY	1.14	-	Favored (72.02%) Glycine / 94.4,-8.3	-	-	Favored (53.82%)	-	-	-

A 56		ALA	1.22	-	Favored (56.74%) General / -63.4,144.7	-	0.03Å	Favored (27.169%)	-	-	-
A 57		ALA	1.31	-	Favored (52.8%) General / -64.3,147.3	-	0.03Å	Favored (42.782%) beta sheet	-	-	-
A 58		LEU	1.39	-	Favored (26.37%) General / -110.2,152.2	Favored (71.9%) *mt* chi angles: 303.5,178.2	0.04Å	Favored (41.492%) beta sheet	-	-	-
A 59		MET	1.48	-	Favored (22.21%) General / -97.0,149.2	Favored (91.9%) *mmm* chi angles: 301.7,298.3,288.8	0.05Å	Favored (52.322%)	-	-	-
A 60		SER	1.56	-	Favored (6.89%) General / -148.3,117.6	Favored (43.9%) *t* chi angles: 178.7	0.02Å	Favored (10.681%)	-	-	-

#	Alt	Res	High B	Clash > 0.4Å	Ramachandran	Rotamer	Cβ deviation	CaBLAM	Bond lengths	Bond angles	Cis Peptides
			Avg: 1.12	Clashscore: 1.76	Outliers: 4 of 617	Poor rotamers: 1 of 514	Outliers: 0 of 563	Outliers: 19 of 615	Outliers: 6 of 619	Outliers: 10 of 619	Non-Trans: 4 of 618
A 61		GLY	1.62	-	Favored (37.77%) Glycine / 61.5,-125.6	-	-	Favored (66.987%)	-	-	-
A 62		GLU	1.63	-	Favored (57.54%) General / -91.7,-0.1	Favored (97.4%) *mt-10* chi angles: 295.2,178.6,358.6	0.02Å	CaBLAM Disfavored (2.838%)	-	-	-
A 63		GLY	1.61	-	Favored (10.65%) Glycine / -134.7,-164.8	-	-	Favored (10.864%)	-	-	-
A 64		ARG	1.56	-	Favored (51.16%) General / -120.8,140.9	Favored (73.3%) *ttm-80* chi angles: 187.8,177.1,293.4,269.7	0.01Å	Favored (26.324%)	-	-	-
A 65		LEU	1.49	-	Favored (12.23%) General / -115.9,105.0	Favored (79.5%) *mt* chi angles: 301.5,176.2	0.01Å	Favored (58.879%)	-	-	-
A 66		ASP	1.43	-	Favored (81.11%) Pre-Pro / -77.1,150.8	Favored (96.2%) *m-30* chi angles: 290.3,345.8	0.02Å	Favored (29.358%)	-	-	-
A 67		PRO	1.39	-	Favored (73.64%) Trans-Pro / -65.8,143.4	Favored (43%) *Cg_endo* chi angles: 24.2,328,26.5	0.01Å	Favored (40.877%)	-	-	-
A 68		TYR	1.37	-	Favored (2.88%) General / -116.0,-44.9	Favored (90.1%) *t80* chi angles: 178.3,81.1	0.05Å	Favored (13.88%)	-	-	-
A 69		TRP	1.38	-	Favored (26.44%) General / -154.3,149.0	Favored (68.9%) *t-100* chi angles: 184.6,259.6	0.04Å	CA Geom Outlier (0.028%)	-	-	-
A 70		GLY	1.39	-	Favored (44.2%) Glycine / 178.9,-172.0	-	-	Favored (43.989%)	-	-	-
A 71		SER	1.38	-	Favored (2.89%)	Favored (42.7%) *t* chi angles: 178.4	0.04Å	CaBLAM Disfavored	-	-	-

#	Alt	Res	High B	Clash > 0.4Å	Ramachandran	Rotamer	Cβ deviation	CaBLAM	Bond lengths	Bond angles	Cis Peptides
					General / -157.8,114.3			(1.106%) try beta sheet			
A 72		VAL	1.35	-	Favored (86.31%) Ile or Val / -61.0,-40.7	Favored (80%) *t* chi angles: 173.1	0.08Å	Favored (37.225%)	-	-	-
A 73		LYS	1.29	-	Favored (58.64%) General / -52.9,-36.1	Favored (86.9%) *tttt* chi angles: 181.7,176.7,178.7,179.3	0.01Å	Favored (59.779%) alpha helix	-	-	-
A 74		GLU	1.21	-	Favored (33.73%) General / -105.2,10.4	Favored (95.3%) *mt-10* chi angles: 296.5,179.5,345.6	0.03Å	Favored (42.517%)	-	-	-
A 75		ASP	1.13	-	Favored (27.02%) General / 53.9,45.8	Favored (37.9%) *t0* chi angles: 193.4,26.6	0.05Å	Favored (28.38%)	-	-	-
A 76		ARG	1.05	-	Favored (49.79%) General / -135.5,150.5	Favored (54.9%) *ptt90* chi angles: 60.6,182.1,175.2,84.3	0.04Å	Favored (18.698%)	-	-	-
A 77		LEU	0.99	-	Favored (39.11%) General / -138.6,142.7	Favored (86.7%) *mt* chi angles: 291.2,168.2	0.05Å	Favored (63.647%) beta sheet	-	-	-
A 78		CYS	0.97	-	Favored (53.92%) General / -118.0,135.9	Favored (35.5%) *m* chi angles: 305.8	0.15Å	Favored (72.129%)	OUTLIER(S) worst is CB--SG: 4.4 σ	-	-
A 79		TYR	0.96	-	Favored (53.84%) General / -120.8,130.0	Favored (78.4%) *m-80* chi angles: 296.2,81.8	0.05Å	Favored (12.908%)	-	-	-
A 80		GLY	0.97	-	Favored (71.43%) Glycine / 88.9,-10.8	-	-	Favored (7.082%)	-	-	-
**#**	**Alt**	**Res**	**High B**	**Clash > 0.4Å**	**Ramachandran**	**Rotamer**	**Cβ deviation**	**CaBLAM**	**Bond lengths**	**Bond angles**	**Cis Peptides**
			Avg: 1.12	Clashscore: 1.76	Outliers: 4 of 617	Poor rotamers: 1 of 514	Outliers: 0 of 563	Outliers: 19 of 615	Outliers: 6 of 619	Outliers: 10 of 619	Non-Trans: 4 of 618
A 81		GLY	0.98	-	Favored (45.72%) Glycine / 173.8,-178.4	-	-	Favored (15.712%)	-	-	-
A 82		PRO	0.99	-	Favored (61.18%) Trans-Pro / -70.0,157.1	Favored (61.7%) *Cg_endo* chi angles: 26.6,326,26.9	0.02Å	Favored (6.031%)	-	-	-
A 83		TRP	0.99	-	Favored (29.33%) General / -57.6,126.9	Favored (51.9%) *t-100* chi angles: 187.2,268.6	0.03Å	Favored (15.468%)	-	-	-
A 84		LYS	0.99	-	Favored (15.09%) General / -109.0,-9.2	Favored (66%) *mmtt* chi angles: 299.2,291,186.3,171.5	0.08Å	Favored (10.221%)	-	-	-
A 85		LEU	0.99	-	Favored (10.73%) General / -84.6,71.8	Favored (31.4%) *mt* chi angles: 309.5,182	0.03Å	Favored (11.747%)	-	-	-
A 86		GLN	0.99	-	Favored (76.37%) General / -64.4,-33.6	Favored (56.6%) *tt0* chi angles: 185.8,173.1,334.8	0.01Å	Favored (9.344%)	-	-	-
A 87		HIS	1	-	Favored (44.72%)	Favored (94.9%) *m-70*	0.03Å	Favored (20.147%)	-	-	-

#	Alt	Res	High B	Clash > 0.4Å	Ramachandran	Rotamer	Cβ deviation	CaBLAM	Bond lengths	Bond angles	Cis Peptides
					General / -71.9,149.8	chi angles: 293.9,290.7					
A 88		LYS	1.01	-	Favored (18.3%) General / -118.2,162.4	Favored (97.1%) *mttt* chi angles: 296.9,178.9,181.6,178.6	0.04Å	Favored (39.034%)	-	-	-
A 89		TRP	1.01	0.46Å CD1 with A 89 TRP H	Favored (22.1%) General / -84.3,156.2	Favored (57.2%) *p-90* chi angles: 50.7,266.9	0.14Å	Favored (41.329%) beta sheet	-	-	-
A 90		ASN	0.99	-	Favored (8.1%) General / -124.9,2.6	Favored (45.3%) *p0* chi angles: 65,358.7	0.02Å	Favored (5.652%)	-	-	-
A 91		GLY	0.95	-	Favored (81.2%) Glycine / 79.7,-0.6	-	-	Favored (39.994%)	-	-	-
A 92		HIS	0.9	-	Allowed (1.91%) General / -132.1,-26.6	Favored (74.4%) *m90* chi angles: 298,80.5	0.03Å	CaBLAM Disfavored (3.164%)	-	-	-
A 93		ASP	0.84	-	Favored (18.29%) General / -78.5,169.4	Favored (19.4%) *t0* chi angles: 204.8,354	0.03Å	Favored (11.992%)	-	-	-
A 94		GLU	0.78	-	Favored (33.91%) General / -86.3,132.7	Favored (90.6%) *tt0* chi angles: 183.5,177.3,354.5	0.02Å	Favored (29.671%) beta sheet	-	-	-
A 95		VAL	0.73	-	Favored (35.34%) Ile or Val / -127.0,160.6	Favored (21.6%) *m* chi angles: 301.9	0.03Å	Favored (53.868%) beta sheet	-	-	-
A 96		GLN	0.69	-	Favored (55.1%) General / -119.7,130.6	Favored (32.7%) *tt0* chi angles: 176.9,177.3,280	0.02Å	Favored (58.679%) beta sheet	-	-	-
A 97		MET	0.67	-	Favored (42.52%) General / -99.2,134.1	Favored (51.8%) *ttm* chi angles: 186.6,180,298	0.04Å	Favored (60.168%) beta sheet	-	-	-
A 98		ILE	0.68	-	Favored (3.1%) Ile or Val / -107.3,94.4	Favored (65.3%) *mt* chi angles: 303.9,171.8	0.11Å	Favored (55.254%) beta sheet	-	-	-
A 99		VAL	0.71	-	Favored (41.95%) Ile or Val / -88.9,126.5	Favored (72.3%) *t* chi angles: 178.5	0.06Å	Favored (40.412%) beta sheet	-	-	-
A 100		VAL	0.79	-	Favored (11.3%) Ile or Val / -107.2,100.7	Favored (42.9%) *t* chi angles: 182.9	0.14Å	Favored (69.496%) beta sheet	-	-	-
**#**	**Alt**	**Res**	**High B**	**Clash > 0.4Å**	**Ramachandran**	**Rotamer**	**Cβ deviation**	**CaBLAM**	**Bond lengths**	**Bond angles**	**Cis Peptides**
			Avg: 1.12	Clashscore: 1.76	Outliers: 4 of 617	Poor rotamers: 1 of 514	Outliers: 0 of 563	Outliers: 19 of 615	Outliers: 6 of 619	Outliers: 10 of 619	Non-Trans: 4 of 618
A 101		GLU	0.89	-	Favored (37.65%) Pre-Pro / -90.2,147.8	Favored (91.3%) *mt-10* chi angles: 295.5,185.5,1.5	0.04Å	Favored (30.267%)	-	-	-
A 102		PRO	0.99	-	Favored (17.98%) Trans-Pro / -47.6,-33.3	Favored (90%) *Cg_exo* chi angles: 329.5,36.8,333.1	0.06Å	Favored (19.741%)	-	-	-
A 103		GLY	1.07	-	Favored (19.62%) Glycine / -115.6,9.1	-	-	Favored (24.811%)	-	-	-

A 104		LYS	1.11	-	Favored (21.24%) General / -137.5,167.6	Favored (95.9%) *mttt* chi angles: 297.7,182.4,180.7,179.2	0.05Å	Favored (16.525%)	-	-	-
A 105		ASN	1.08	-	Favored (2.76%) General / -70.2,178.5	Favored (8.2%) *t0* chi angles: 207,328	0.03Å	Favored (10.434%)	-	-	-
A 106		VAL	1	-	Favored (33.5%) Ile or Val / -83.5,131.6	Favored (67.2%) *t* chi angles: 179.1	0.09Å	Favored (15.866%)	-	-	-
A 107		LYS	0.9	-	Favored (48.62%) General / -126.6,150.9	Favored (61.4%) *pttt* chi angles: 64.5,183.1,184.4,182.1	0.02Å	Favored (57.137%) beta sheet	-	-	-
A 108		ASN	0.82	-	Favored (43.04%) General / -99.3,122.9	Favored (88.7%) *m-40* chi angles: 296.6,321.7	0.04Å	Favored (55.792%) beta sheet	-	-	-
A 109		VAL	0.77	-	Favored (74.8%) Ile or Val / -123.2,128.1	Favored (67.9%) *t* chi angles: 179	0.05Å	Favored (61.257%) beta sheet	-	-	-
A 110		GLN	0.75	-	Favored (43.02%) General / -98.2,133.2	Favored (31.9%) *mp10* chi angles: 295,81.7,41.9	0.02Å	Favored (16.488%) beta sheet	-	-	-
A 111		THR	0.77	-	Favored (38.46%) General / -156.3,159.1	Favored (8.5%) *t* chi angles: 184.4	0.11Å	Favored (26.671%) beta sheet	-	-	-
A 112		LYS	0.8	-	Favored (20.35%) Pre-Pro / -101.9,139.5	Favored (98.7%) *mttt* chi angles: 293.8,182.5,177.8,180.9	0.03Å	Favored (31.246%) beta sheet	-	-	-
A 113		PRO	0.86	-	Favored (80.6%) Trans-Pro / -65.7,152.8	Favored (23.6%) *Cg_exo* chi angles: 341.3,33.4,325.3	0.04Å	Favored (59.716%)	-	-	-
A 114		GLY	0.94	-	Favored (19.48%) Glycine / -109.8,-163.1	-	-	Favored (46.248%)	-	-	-
A 115		VAL	1.05	-	Favored (68.38%) Ile or Val / -128.1,132.6	Favored (75.4%) *t* chi angles: 178.3	0.04Å	Favored (18.927%)	-	-	-
A 116		PHE	1.19	-	Favored (36.37%) General / -117.9,120.0	Favored (41.1%) *t80* chi angles: 187.1,98	0.03Å	Favored (64.776%) beta sheet	-	-	-
A 117		LYS	1.33	-	Favored (34.98%) General / -86.7,128.0	Favored (87.3%) *tttt* chi angles: 183.7,177.2,178.9,179.9	0.03Å	Favored (49.65%)	-	-	-
A 118		THR	1.44	-	Favored (7.05%) Pre-Pro / -120.0,170.4	Favored (19%) *p* chi angles: 73	0.13Å	Favored (27.571%)	-	-	-
A 119		PRO	1.49	-	Favored (38.51%) Trans-Pro / -58.1,-18.8	Favored (84.5%) *Cg_exo* chi angles: 334.1,36.2,329.2	0.03Å	Favored (65.728%)	-	-	-
A 120		GLU	1.45	-	Favored (51.53%) General / -97.0,2.4	Favored (69.2%) *mm-30* chi angles: 298.8,293.8,314.7	0.03Å	Favored (30.448%)	-	-	-

#	Alt	Res	High B	Clash > 0.4Å	Ramachandran	Rotamer	Cβ deviation	CaBLAM	Bond lengths	Bond angles	Cis Peptides
			Avg: 1.12	Clashscore: 1.76	Outliers: 4 of 617	Poor rotamers: 1 of 514	Outliers: 0 of 563	Outliers: 19 of 615	Outliers: 6 of 619	Outliers: 10 of 619	Non-Trans: 4 of 618
A 121		GLY	1.34	-	Favored (16.93%) Glycine / 116.0,-177.2	-	-	Favored (30.568%)	-	-	-
A 122		GLU	1.19	-	Favored (34.82%) General / -83.5,134.8	Favored (98.3%) *mt-10* chi angles: 293.7,180.2,359	0.03Å	Favored (7.942%)	-	-	-
A 123		ILE	1.04	-	Favored (70.41%) Ile or Val / -125.8,132.8	Favored (74.4%) *mt* chi angles: 301.8,172.2	0.03Å	Favored (53.219%) beta sheet	-	-	-
A 124		GLY	0.93	-	Favored (50.5%) Glycine / -63.8,149.5	-	-	Favored (42.692%) beta sheet	-	-	-
A 125		ALA	0.87	-	Favored (35.35%) General / -149.5,152.3	-	0.04Å	Favored (59.026%) beta sheet	-	-	-
A 126		VAL	0.84	-	Favored (68.49%) Ile or Val / -112.3,123.9	Favored (90.3%) *t* chi angles: 176	0.06Å	Favored (55.292%) beta sheet	-	-	-
A 127		THR	0.83	-	Favored (5.51%) General / -82.7,60.9	Favored (28.4%) *p* chi angles: 52.7	0.03Å	Favored (11.52%) beta sheet	-	-	-
A 128		LEU	0.84	-	Favored (54.23%) General / -123.8,139.2	Favored (94%) *mt* chi angles: 296.6,173.2	0.03Å	Favored (25.318%) beta sheet	-	-	-
A 129		ASP	0.86	-	Favored (31.71%) General / -104.2,116.2	Favored (84.5%) *m-30* chi angles: 294.6,345.3	0.09Å	Favored (49.485%)	-	-	-
A 130		TYR	0.86	-	Favored (73.03%) Pre-Pro / -126.2,155.9	Favored (88%) *m-80* chi angles: 293.9,102.5	0.09Å	Favored (35.976%)	-	-	-
A 131		PRO	0.84	-	Favored (39.26%) Trans-Pro / -57.2,153.6	Favored (72.7%) *Cg_exo* chi angles: 335.1,34.2,331.2	0.06Å	Favored (60.138%)	-	-	-
A 132		THR	0.8	-	Favored (24.82%) General / -65.8,159.7	Favored (75.6%) *p* chi angles: 61.3	0.02Å	Favored (7.526%)	-	-	-
A 133		GLY	0.76	-	Favored (63.21%) Glycine / 93.9,-15.4	-	-	Favored (23.678%)	-	-	-
A 134		THR	0.73	-	Favored (51.37%) General / -86.2,-11.8	Favored (72.6%) *p* chi angles: 59.7	0.01Å	Favored (7.479%)	-	-	-
A 135		SER	0.7	-	Favored (57.29%) General / -58.7,137.9	Favored (61.2%) *m* chi angles: 293.7	0.09Å	Favored (7.809%)	-	-	-
A 136		GLY	0.68	-	Favored (62.37%) Glycine / 98.9,-11.1	-	-	Favored (73.051%)	-	-	-

A 137		SER	0.68	-	Favored (84.38%) Pre-Pro / -58.1,137.4	Favored (64.4%) *m* chi angles: 294.2	0.08Å	Favored (33.129%)	-	-	-
A 138		PRO	0.7	-	Favored (58.57%) Trans-Pro / -71.9,153.2	Favored (62.3%) *Cg_endo* chi angles: 26.6,327.5,25	0.03Å	Favored (73.969%) beta sheet	-	-	-
A 139		ILE	0.73	-	Favored (72.83%) Ile or Val / -115.5,128.2	Favored (79%) *mt* chi angles: 300.4,170.1	0.05Å	Favored (66.203%) beta sheet	-	-	-
A 140		VAL	0.77	-	Favored (25.27%) Ile or Val / -122.5,160.0	Favored (30.8%) *m* chi angles: 300.3	0.07Å	Favored (43.569%)	-	-	-
**#**	**Alt**	**Res**	**High B**	**Clash > 0.4Å**	**Ramachandran**	**Rotamer**	**Cβ deviation**	**CaBLAM**	**Bond lengths**	**Bond angles**	**Cis Peptides**
			Avg: 1.12	Clashscore: 1.76	Outliers: 4 of 617	Poor rotamers: 1 of 514	Outliers: 0 of 563	Outliers: 19 of 615	Outliers: 6 of 619	Outliers: 10 of 619	Non-Trans: 4 of 618
A 141		ASP	0.8	0.41Å C with A 141 ASP OD1	Favored (5.69%) General / -84.8,-175.3	Favored (47.1%) *p0* chi angles: 69.3,11	0.02Å	Favored (30.765%)	-	-	-
A 142		LYS	0.82	-	Favored (58.53%) General / -61.7,-18.2	Favored (7.9%) *ptpp* chi angles: 67.5,174.7,66.8,66.9	0.06Å	Favored (33.519%)	-	-	-
A 143		ASN	0.83	-	Favored (49.67%) General / -97.9,3.4	Favored (88.9%) *m-40* chi angles: 293.3,320.7	0.03Å	Favored (53.212%)	-	-	-
A 144		GLY	0.82	-	Favored (79.5%) Glycine / 87.0,6.3	-	-	Favored (89.052%)	-	-	-
A 145		ASP	0.8	-	Favored (35.31%) General / -81.8,137.2	Favored (68.9%) *m-30* chi angles: 285,333.3	0.06Å	Favored (35.147%)	-	-	-
A 146		VAL	0.77	-	Favored (37.22%) Ile or Val / -75.4,129.0	Favored (77.7%) *t* chi angles: 178.1	0.03Å	Favored (45.723%)	-	-	-
A 147		ILE	0.76	-	Favored (11.42%) Ile or Val / -109.6,-9.7	Favored (39.7%) *pt* chi angles: 64,168.6	0.03Å	CaBLAM Disfavored (4.196%)	-	-	-
A 148		GLY	0.76	-	Favored (38.17%) Glycine / 161.6,-175.0	-	-	Favored (25.157%)	-	-	-
A 149		LEU	0.78	-	Favored (32.58%) General / -112.6,149.6	Favored (47.6%) *mt* chi angles: 306.8,176.9	0.06Å	Favored (8.29%)	-	-	-
A 150		TYR	0.82	-	Favored (34.57%) General / -84.1,135.0	Favored (88.7%) *t80* chi angles: 174,77.1	0.04Å	CaBLAM Disfavored (2.916%)	-	-	-
A 151		GLY	0.89	-	Favored (25.13%) Glycine / 136.5,-164.5	-	-	CaBLAM Outlier (0.154%)	-	-	-
A 152		ASN	0.99	-	Favored (17.38%) General / 52.7,50.8	Favored (64%) *t0* chi angles: 195.3,30.7	0.06Å	CaBLAM Outlier (0.256%)	-	-	-
A 153		GLY	1.1	-	Favored (25.95%)	-	-	Favored (35.509%)	-	-	-

#	Alt	Res	High B	Clash > 0.4Å	Ramachandran	Rotamer	Cβ deviation	CaBLAM	Bond lengths	Bond angles	Cis Peptides
					Glycine / -103.6,-177.6						
A 154		VAL	1.24	-	Favored (54.78%) Ile or Val / -130.4,138.0	Favored (70.5%) *t* chi angles: 178.7	0.05Å	Favored (33.605%) beta sheet	-	-	-
A 155		ILE	1.37	-	Favored (39.82%) Ile or Val / -82.9,127.3	Favored (89%) *mt* chi angles: 298.1,169.8	0.05Å	Favored (45.886%)	-	-	-
A 156		MET	1.46	0.41Å HA with A 156 MET HE2	Favored (62.13%) Pre-Pro / -83.2,159.4	Favored (42.3%) *mmp* chi angles: 294.4,292,96.2	0.09Å	Favored (44.014%)	-	-	-
A 157		PRO	1.5	-	Favored (18.19%) Trans-Pro / -49.2,-30.0	Favored (88.2%) *Cg_exo* chi angles: 330.1,37.2,331.6	0.03Å	Favored (74.833%)	-	-	-
A 158		ASN	1.46	-	Favored (39.99%) General / -88.7,6.1	Favored (53.5%) *p0* chi angles: 65.5,10.9	0.05Å	Favored (55.263%)	-	-	-
A 159		GLY	1.37	-	Favored (74.21%) Glycine / 90.7,-10.6	-	-	Favored (64.754%)	-	-	-
A 160		SER	1.23	-	Favored (18.29%) General / -80.1,169.1	Favored (85.3%) *p* chi angles: 67.7	0.01Å	Favored (37.353%)	-	-	-
#	Alt	Res	High B	Clash > 0.4Å	Ramachandran	Rotamer	Cβ deviation	CaBLAM	Bond lengths	Bond angles	Cis Peptides
			Avg: 1.12	Clashscore: 1.76	Outliers: 4 of 617	Poor rotamers: 1 of 514	Outliers: 0 of 563	Outliers: 19 of 615	Outliers: 6 of 619	Outliers: 10 of 619	Non-Trans: 4 of 618
A 161		TYR	1.09	-	Favored (26.12%) General / -109.5,151.9	Favored (62%) *m-80* chi angles: 292,78	0.07Å	Favored (51.197%) beta sheet	-	-	-
A 162		ILE	0.98	0.47Å O with A 162 ILE HG23	Favored (63.31%) Ile or Val / -127.7,124.5	Favored (17.4%) *tt* chi angles: 182.5,165.4	0.07Å	Favored (57.928%) beta sheet	-	-	-
A 163		SER	0.91	-	Favored (40.11%) General / -99.6,120.7	Favored (6.1%) *t* chi angles: 163.3	0.05Å	Favored (51.857%) beta sheet	-	-	-
A 164		ALA	0.89	-	Favored (58.44%) General / -62.1,139.7	-	0.06Å	Favored (40.867%) beta sheet	-	-	-
A 165		ILE	0.92	-	Favored (27.75%) Ile or Val / -82.8,116.9	Favored (87.6%) *mt* chi angles: 298.5,170.9	0.01Å	Favored (46.195%) beta sheet	-	-	-
A 166		VAL	1.01	-	Favored (33.09%) Ile or Val / -87.1,118.4	Favored (84.8%) *t* chi angles: 176.3	0.07Å	Favored (38.398%) beta sheet	-	-	-
A 167		GLN	1.15	-	Favored (3.54%) General / -142.2,99.6	Favored (25%) *tm-30* chi angles: 191.9,283,321.9	0.05Å	Favored (13.657%)	-	-	-
A 168		GLY	1.35	-	Favored (38.81%) Glycine / -82.3,159.2	-	-	Favored (42.018%)	-	-	-
A 169		GLU	1.63	-	Favored (41.53%)	Favored (9.5%) *tp30*	0.05Å	Favored (36.428%)	-	-	-

#	Alt	Res	High B	Clash > 0.4Å	Ramachandran	Rotamer	Cβ deviation	CaBLAM	Bond lengths	Bond angles	Cis Peptides
					General / -68.9,154.7	chi angles: 191.8,60.3,71.5					
A 170		ARG	2	-	Favored (2.22%) General / -75.0,79.3	Favored (77.6%) *ttt180* chi angles: 178.9,177.9,170.7,184.5	0.06Å	Favored (17.941%)	-	-	-
A 171		MET	2.46	-	Favored (5.52%) General / -79.0,75.9	Favored (95.3%) *mmm* chi angles: 299.3,297.9,287.4	0.03Å	Favored (60.019%) beta sheet	-	-	-
A 172		GLU	3.01	-	Favored (6.31%) General / -81.0,65.8	Favored (7.2%) *tp30* chi angles: 187.1,74,65.3	0.07Å	Favored (23.009%)	-	-	-
A 173		GLU	3.59	-	Favored (3.92%) Pre-Pro / -164.0,173.7	Favored (9.6%) *pt0* chi angles: 64.3,182.3,72.6	0.06Å	CA Geom Outlier (0%)	-	-	-
A 174		PRO	4.04	-	Allowed (0.48%) Cis-Pro / -91.0,58.3	Favored (39.6%) *Cg_endo* chi angles: 34,324.6,21.6	0.07Å	CaBLAM Outlier (0.465%)	-	-	Cis PRO omega= -16.37
A 175		ALA	4.17	-	Favored (84.78%) Pre-Pro / -60.8,126.2	-	0.03Å	Favored (34.762%)	-	-	-
A 176		PRO	3.87	-	Favored (32.65%) Trans-Pro / -77.5,156.8	Favored (62.7%) *Cg_endo* chi angles: 31.5,322.9,26.7	0.08Å	Favored (61.726%) beta sheet	-	-	-
A 177		ALA	3.26	-	Favored (9.97%) General / -84.2,68.0	-	0.02Å	Favored (10.657%)	-	-	-
A 178		GLY	2.56	-	Favored (78.68%) Glycine / -58.9,-34.5	-	-	Favored (7.071%)	-	-	-
A 179		PHE	1.92	-	Favored (27.08%) General / -158.6,154.8	Favored (41.3%) *p90* chi angles: 55.6,91.9	0.05Å	Favored (29.084%)	-	-	-
A 180		GLU	1.45	-	Favored (57.95%) Pre-Pro / -139.9,149.2	Favored (95.6%) *mt-10* chi angles: 296,181.3,0.3	0.04Å	Favored (51.049%)	-	-	-
**#**	**Alt**	**Res**	**High B**	**Clash > 0.4Å**	**Ramachandran**	**Rotamer**	**Cβ deviation**	**CaBLAM**	**Bond lengths**	**Bond angles**	**Cis Peptides**
			Avg: 1.12	Clashscore: 1.76	Outliers: 4 of 617	Poor rotamers: 1 of 514	Outliers: 0 of 563	Outliers: 19 of 615	Outliers: 6 of 619	Outliers: 10 of 619	Non-Trans: 4 of 618
A 181		PRO	1.13	-	Favored (76.63%) Trans-Pro / -61.3,-23.2	Favored (34%) *Cg_endo* chi angles: 22.1,325.9,31.3	0.03Å	Favored (67.165%)	-	-	-
A 182		GLU	0.94	-	Favored (65.83%) General / -67.2,-21.2	Favored (94.3%) *mt-10* chi angles: 291.3,184.2,0.4	0.04Å	Favored (62.2%) three-ten	-	-	-
A 183		MET	0.83	-	Favored (59.57%) General / -71.6,-10.3	Favored (60.3%) *mtt* chi angles: 295.2,180.1,191.9	0.03Å	Favored (50.487%)	-	-	-
A 184		LEU	0.78	-	Favored (49.53%) General / -97.8,2.5	Favored (84.7%) *mt* chi angles: 300.7,178.1	0.07Å	Favored (52.669%)	-	-	-
A 185		ARG	0.75	-	Favored (38.43%)	Favored (78%) *mtm180*	0.01Å	Favored (35.159%)	-	-	-

					General / -78.8,138.9	chi angles: 295,180.7,300,185.2						
A 186		LYS	0.73	-	Favored (92.13%) General / -60.6,-45.9	Favored (96.8%) *mttt* chi angles: 288.2,180.1,178.1,177.9	0.07Å	Favored (60.781%)	-	-	-
A 187		LYS	0.73	-	Favored (7.87%) General / -88.9,66.2	Favored (98.9%) *mttt* chi angles: 293.9,180.6,177.1,180.2	0.09Å	CaBLAM Disfavored (2.001%)	-	-	-
A 188		GLN	0.74	-	Favored (14.51%) General / -154.7,136.5	Favored (45.4%) *tt0* chi angles: 181,167.4,323.7	0.02Å	Favored (11.018%)	-	-	-
A 189		ILE	0.76	-	Favored (70.01%) Ile or Val / -117.7,122.5	Favored (77.2%) *mt* chi angles: 301.2,172.4	0.08Å	Favored (70.611%)	-	-	-
A 190		THR	0.81	-	Favored (55.49%) General / -115.7,134.0	Favored (81.4%) *m* chi angles: 296.4	0.03Å	Favored (67.341%) beta sheet	-	-	-
A 191		VAL	0.88	-	Favored (67.49%) Ile or Val / -110.2,126.8	Favored (59.3%) *t* chi angles: 180.1	0.04Å	Favored (68.631%) beta sheet	-	-	-
A 192		LEU	0.97	-	Favored (22.84%) General / -106.1,110.5	Favored (83.1%) *mt* chi angles: 301.6,177.5	0.04Å	Favored (65.501%) beta sheet	-	-	-
A 193		ASP	1.08	-	Favored (6.75%) General / -82.8,88.3	Favored (22.1%) *m-30* chi angles: 300,356.5	0.06Å	Favored (55.544%) beta sheet	-	-	-
A 194		LEU	1.2	-	Favored (7.15%) General / -111.2,97.9	Favored (61%) *mt* chi angles: 302.8,172.9	0.06Å	Favored (43.268%)	-	-	-
A 195		HIS	1.31	-	Favored (35.64%) Pre-Pro / -60.2,157.1	Favored (38.9%) *p90* chi angles: 65.8,78.4	0.02Å	Favored (11.955%)	-	-	-
A 196		PRO	1.4	-	Favored (62.28%) Trans-Pro / -53.6,135.7	Favored (98.4%) *Cg_exo* chi angles: 332.1,37.9,328.9	0.06Å	Favored (45.629%)	-	-	-
A 197		GLY	1.44	-	Favored (88.03%) Glycine / 85.5,0.8	-	-	Favored (77.516%)	-	-	-
A 198		ALA	1.46	-	Favored (48.56%) General / -79.9,-3.5	-	0.05Å	Favored (6.312%)	-	-	-
A 199		GLY	1.47	-	Favored (76.4%) Glycine / 85.5,10.6	-	-	Favored (85.902%)	-	-	-
A 200		LYS	1.46	-	Allowed (0.53%) General / -34.7,-57.0	Allowed (0.3%) *ttpm* chi angles: 184.7,177,91.7,267.9	0.04Å	Favored (10.502%)	-	-	-
**#**	**Alt**	**Res**	**High B**	**Clash > 0.4Å**	**Ramachandran**	**Rotamer**	**Cβ deviation**	**CaBLAM**	**Bond lengths**	**Bond angles**	**Cis Peptides**
			Avg: 1.12	Clashscore: 1.76	Outliers: 4 of 617	Poor rotamers: 1 of 514	Outliers: 0 of 563	Outliers: 19 of 615	Outliers: 6 of 619	Outliers: 10 of 619	Non-Trans: 4 of 618
A 201		THR	1.42	-	Favored (14.15%) General / -87.7,-40.5	Favored (58.4%) *m* chi angles: 303.7	0.04Å	Favored (29.279%)	-	-	-

A 202		ARG	1.36	-	Favored (26.25%) General / -85.6,-26.2	Favored (52.6%) *mtp180* chi angles: 293.1,186.2,69.4,212.7	0.05Å	Favored (36.926%) alpha helix	-	-	-
A 203		LYS	1.29	-	Favored (2.61%) General / -116.8,-50.4	Favored (73.1%) *mmtt* chi angles: 300.5,295.1,182.7,180.6	0.04Å	Favored (15.286%) alpha helix	-	-	-
A 204		ILE	1.2	-	Favored (31.09%) Ile or Val / -72.7,-50.8	Favored (95%) *mt* chi angles: 295.1,168.2	0.09Å	Favored (77.832%) alpha helix	-	-	-
A 205		LEU	1.11	0.47Å HB3 with A 206 PRO HD3	Favored (37.77%) Pre-Pro / -52.5,-54.9	Favored (45.8%) *tp* chi angles: 172.5,65.2	0.13Å	Favored (96.957%) alpha helix	-	OUTLIER(S) worst is CA-C-N: 4.0 σ	-
A 206		PRO	1.02	0.47Å HD3 with A 205 LEU HB3	Favored (56.42%) Trans-Pro / -52.0,-34.3	Favored (89.5%) *Cg_exo* chi angles: 329.8,36.9,332.1	0.01Å	Favored (95.666%) alpha helix	-	-	-
A 207		GLN	0.93	-	Favored (61.99%) General / -75.0,-36.9	Favored (89%) *mm-40* chi angles: 289.7,294,312.6	0.02Å	Favored (83.863%) alpha helix	-	-	-
A 208		ILE	0.87	-	Favored (87.71%) Ile or Val / -67.0,-45.1	Favored (97.7%) *mt* chi angles: 292.7,167	0.02Å	Favored (80.466%) alpha helix	-	-	-
A 209		ILE	0.82	-	Favored (95.03%) Ile or Val / -64.5,-42.4	Favored (32.7%) *mm* chi angles: 293.5,297.4	0.01Å	Favored (90.628%) alpha helix	-	-	-
A 210		LYS	0.79	-	Favored (85.78%) General / -58.5,-46.9	Favored (86.7%) *tttt* chi angles: 182,177.3,178.2,179.8	0.02Å	Favored (86.029%) alpha helix	-	-	-
A 211		GLU	0.77	-	Favored (99.52%) General / -62.7,-42.2	Favored (90.3%) *tt0* chi angles: 184.2,174.6,356.2	0.04Å	Favored (86.865%) alpha helix	-	-	-
A 212		ALA	0.76	-	Favored (82%) General / -59.6,-38.7	-	0.03Å	Favored (89.702%) alpha helix	-	-	-
A 213		ILE	0.77	-	Favored (92.17%) Ile or Val / -66.0,-44.5	Favored (94.5%) *mt* chi angles: 292.1,166.7	0.03Å	Favored (88.064%) alpha helix	-	-	-
A 214		ASN	0.77	-	Favored (70.99%) General / -58.9,-33.9	Favored (99.3%) *m-40* chi angles: 287.4,340.4	0.02Å	Favored (77.659%) alpha helix	-	-	-
A 215		ARG	0.77	-	Favored (42.06%) General / -88.1,5.3	Favored (98.2%) *mtt-85* chi angles: 293.1,180.9,184.7,273.3	0.01Å	Favored (50.38%)	-	-	-
A 216		ARG	0.77	-	Favored (25.79%) General / 57.0,42.7	Favored (14.4%) *mpt180* chi angles: 276,69.5,174,173.9	0.03Å	Favored (30.537%)	-	-	-
A 217		LEU	0.75	-	Favored (22.44%) General / -98.8,109.7	Favored (64.5%) *mt* chi angles: 304.6,178.1	0.07Å	Favored (42.599%) beta sheet	-	-	-
A 218		ARG	0.73	-	Favored (25.67%) General / -58.0,126.0	Favored (16.1%) *tpt-90* chi angles: 181,79.8,181.5,268.3	0.05Å	Favored (34.136%) beta sheet	-	-	-

#	Alt	Res	High B	Clash > 0.4Å	Ramachandran	Rotamer	Cβ deviation	CaBLAM	Bond lengths	Bond angles	Cis Peptides
A 219		THR	0.71	-	Favored (52.92%) General / -125.9,136.3	Favored (98.1%) *m* chi angles: 300.6	0.01Å	Favored (71.149%) beta sheet	-	-	-
A 220		ALA	0.7	-	Favored (49.51%) General / -108.5,135.6	-	0.05Å	Favored (71.93%) beta sheet	-	-	-
**#**	**Alt**	**Res**	**High B**	**Clash > 0.4Å**	**Ramachandran**	**Rotamer**	**Cβ deviation**	**CaBLAM**	**Bond lengths**	**Bond angles**	**Cis Peptides**
			Avg: 1.12	Clashscore: 1.76	Outliers: 4 of 617	Poor rotamers: 1 of 514	Outliers: 0 of 563	Outliers: 19 of 615	Outliers: 6 of 619	Outliers: 10 of 619	Non-Trans: 4 of 618
A 221		VAL	0.7	-	Favored (67.4%) Ile or Val / -119.0,121.7	Favored (49.2%) *t* chi angles: 181.6	0.04Å	Favored (71.033%) beta sheet	-	-	-
A 222		LEU	0.72	-	Favored (40.44%) General / -110.3,119.8	Favored (21.4%) *mt* chi angles: 311.8,176.6	0.08Å	Favored (71.785%) beta sheet	-	-	-
A 223		ALA	0.77	-	Favored (30.87%) Pre-Pro / -99.9,145.8	-	0.09Å	Favored (42.935%)	-	-	-
A 224		PRO	0.82	-	Favored (67.58%) Trans-Pro / -63.9,-24.6	Favored (39.5%) *Cg_endo* chi angles: 23.3,326.3,29.2	0.04Å	Favored (27.518%)	-	-	-
A 225		THR	0.88	-	Favored (33.5%) General / -137.7,162.6	Favored (40.1%) *p* chi angles: 67.8	0.02Å	Favored (23.232%)	-	-	-
A 226		ARG	0.94	-	Favored (78.93%) General / -63.0,-35.0	Favored (81.9%) *mtt180* chi angles: 289.4,169.6,177.9,153.5	0.05Å	Favored (66.031%) alpha helix	-	-	-
A 227		VAL	0.99	-	Favored (96.08%) Ile or Val / -64.0,-45.7	Favored (56.7%) *t* chi angles: 170.3	0.06Å	Favored (83.224%) alpha helix	-	-	-
A 228		VAL	1.02	-	Favored (84.9%) Ile or Val / -65.1,-39.2	Favored (66.8%) *t* chi angles: 171.7	0.13Å	Favored (82.358%) alpha helix	-	-	-
A 229		ALA	1.03	-	Favored (72.97%) General / -57.3,-37.8	-	0.03Å	Favored (77.886%) alpha helix	-	-	-
A 230		ALA	1.05	-	Favored (97.72%) General / -63.1,-40.5	-	0.02Å	Favored (96.676%) alpha helix	-	-	-
A 231		GLU	1.06	-	Favored (87.53%) General / -65.4,-37.6	Favored (61.5%) *mm-30* chi angles: 291.7,290.1,310.7	0.03Å	Favored (99.554%) alpha helix	-	-	-
A 232		MET	1.07	-	Favored (97.45%) General / -63.9,-42.2	Favored (60.4%) *mtt* chi angles: 290.3,176,170.1	0.11Å	Favored (80.504%) alpha helix	OUTLIER(S) worst is SD--CE: 4.0 σ	-	-
A 233		SER	1.08	-	Favored (83.47%) General / -59.5,-39.3	Favored (71%) *m* chi angles: 296.1	0.05Å	Favored (79.505%) alpha helix	-	-	-
A 234		GLU	1.1	-	Favored (77.32%)	Favored (44.1%) *tp30*	0.04Å	Favored (81.491%) alpha helix	-	-	-

					General / -68.0,-44.2	chi angles: 184.6,67.1,35.7					
A 235		ALA	1.11	-	Favored (71.22%) General / -59.7,-33.1	-	0.04Å	Favored (74.414%)	-	-	-
A 236		LEU	1.1	-	Favored (35.05%) General / -95.5,10.8	Favored (95.9%) *mt* chi angles: 297.5,175.5	0.11Å	Favored (29.847%)	-	-	-
A 237		ARG	1.07	-	Favored (53.4%) General / -56.5,133.8	Favored (78.8%) *ttm-80* chi angles: 182.6,180.6,292,276.6	0.03Å	Favored (10.703%)	-	-	-
A 238		GLY	1.03	-	Favored (59.51%) Glycine / 100.3,-14.6	-	-	Favored (74.914%)	-	-	-
A 239		LEU	0.97	-	Favored (39.92%) Pre-Pro / -92.3,157.9	Favored (86.2%) *mt* chi angles: 300.2,176.7	0.06Å	Favored (25.306%)	-	-	-
A 240		PRO	0.92	-	Favored (3.11%) Trans-Pro / -72.3,71.1	Favored (70.2%) *Cg_endo* chi angles: 29.4,321.1,32.3	0.03Å	Favored (5.77%)	-	-	-
**#**	**Alt**	**Res**	**High B**	**Clash > 0.4Å**	**Ramachandran**	**Rotamer**	**Cβ deviation**	**CaBLAM**	**Bond lengths**	**Bond angles**	**Cis Peptides**
			Avg: 1.12	Clashscore: 1.76	Outliers: 4 of 617	Poor rotamers: 1 of 514	Outliers: 0 of 563	Outliers: 19 of 615	Outliers: 6 of 619	Outliers: 10 of 619	Non-Trans: 4 of 618
A 241		ILE	0.9	-	Favored (32.75%) Ile or Val / -113.0,140.5	Favored (21.8%) *tt* chi angles: 186.6,165.7	0.02Å	Favored (25.911%)	-	-	-
A 242		ARG	0.93	-	Favored (55.2%) General / -121.2,133.8	Favored (68.7%) *ttp80* chi angles: 180.7,175.5,70.7,93.4	0.12Å	Favored (61.158%) beta sheet	-	-	-
A 243		TYR	1.03	-	Favored (38.46%) General / -92.5,129.2	Favored (70.4%) *t80* chi angles: 174.6,69.1	0.10Å	Favored (59.741%) beta sheet	-	-	-
A 244		GLN	1.23	-	Favored (25.21%) General / -121.3,116.7	Favored (69.6%) *tp40* chi angles: 174.6,64.9,59.9	0.05Å	Favored (27.142%) beta sheet	-	-	-
A 245		THR	1.54	-	Favored (3.79%) General / -142.8,-173.1	Favored (11.4%) *t* chi angles: 189.3	0.09Å	Favored (10.933%)	-	-	-
A 246		SER	1.98	-	Favored (66.51%) General / -65.7,-21.2	Favored (86.1%) *p* chi angles: 67.8	0.02Å	Favored (33.902%)	-	-	-
A 247		ALA	2.53	-	Favored (58.93%) General / -86.3,-3.1	-	0.04Å	Favored (52.811%)	-	-	-
A 248		VAL	3.1	-	Favored (51.29%) Pre-Pro / -113.0,110.5	Favored (66.7%) *t* chi angles: 179.1	0.07Å	Favored (21.778%)	-	-	-
A 249		PRO	3.58	-	Favored (7.11%) Trans-Pro / -79.4,58.8	Favored (57.2%) *Cg_endo* chi angles: 32.2,323.8,24.8	0.03Å	CaBLAM Outlier (0.129%)	-	-	-
A 250		ARG	3.84	-	Favored (21.84%)	Favored (87.5%) *mtm-85*	0.03Å	CaBLAM Disfavored	-	-	-

					General / 52.4,48.8	chi angles: 299.3,180.9,294.8,276.8		(3.114%)			
A 251		GLU	3.77	-	Favored (4.82%) General / -79.2,67.0	Favored (82.3%) *mt-10* chi angles: 297.1,179.3,329.9	0.04Å	Favored (51.395%) beta sheet	-	-	-
A 252		HIS	3.41	-	Favored (34.35%) General / -85.2,127.7	Favored (16%) *t-170* chi angles: 186.7,229.5	0.04Å	Favored (26.346%) beta sheet	-	-	-
A 253		SER	2.84	-	Favored (35.25%) General / -89.5,7.6	Favored (63%) *m* chi angles: 297.9	0.02Å	CaBLAM Disfavored (2.685%)	-	-	-
A 254		GLY	2.23	-	Favored (4.89%) Glycine / 84.7,-59.7	-	-	CaBLAM Disfavored (1.278%)	-	-	-
A 255		ASN	1.69	-	Favored (65.28%) General / -67.8,-24.6	Favored (96.7%) *m-40* chi angles: 287.1,336.5	0.02Å	Favored (50.222%)	-	-	-
A 256		GLU	1.28	-	Favored (57.82%) General / -62.6,136.3	Favored (58.6%) *mt-10* chi angles: 289.6,169.1,318.9	0.06Å	Favored (22.084%)	-	-	-
A 257		ILE	1	-	Favored (4.95%) Ile or Val / -104.1,-38.4	Favored (49.4%) *mm* chi angles: 301.7,301	0.05Å	Favored (9.677%)	-	-	-
A 258		VAL	0.83	-	Favored (68.87%) Ile or Val / -111.5,125.7	Favored (51.5%) *t* chi angles: 181.2	0.08Å	Favored (26.272%)	-	-	-
A 259		ASP	0.74	-	Favored (54.66%) General / -107.2,128.7	Favored (96.1%) *m-30* chi angles: 291.2,342.9	0.01Å	Favored (70.015%)	-	-	-
A 260		VAL	0.7	-	Favored (63.13%) Ile or Val / -116.6,133.5	Favored (3.9%) *p* chi angles: 56	0.06Å	Favored (37.367%) beta sheet	-	-	-
**#**	**Alt**	**Res**	**High B**	**Clash > 0.4Å**	**Ramachandran**	**Rotamer**	**Cβ deviation**	**CaBLAM**	**Bond lengths**	**Bond angles**	**Cis Peptides**
			Avg: 1.12	Clashscore: 1.76	Outliers: 4 of 617	Poor rotamers: 1 of 514	Outliers: 0 of 563	Outliers: 19 of 615	Outliers: 6 of 619	Outliers: 10 of 619	Non-Trans: 4 of 618
A 261		MET	0.69	-	Favored (22.8%) General / -154.6,169.9	Favored (21.2%) *ptp* chi angles: 64.6,195.3,77.4	0.07Å	Favored (21.813%) beta sheet	-	-	-
A 262		CYS	0.7	-	Favored (14.92%) General / -90.4,162.9	Favored (27.9%) *p* chi angles: 67.4	0.04Å	Favored (30.102%)	-	-	-
A 263		HIS	0.73	-	Favored (82.23%) General / -58.9,-39.7	Favored (41.4%) *m170* chi angles: 295,182.1	0.07Å	Favored (64.599%)	OUTLIER(S) worst is CG--CD2: 4.8 σ	-	-
A 264		ALA	0.75	-	Favored (98.77%) General / -63.4,-42.2	-	0.03Å	Favored (90.701%) alpha helix	-	-	-
A 265		THR	0.77	-	Favored (84.65%) General / -65.8,-44.8	Favored (77.1%) *m* chi angles: 296.3	0.04Å	Favored (96.141%) alpha helix	-	-	-
A 266		LEU	0.79	-	Favored (79.08%)	Favored (60.5%) *tp* chi angles: 174.6,63.3	0.09Å	Favored (89.053%)	-	-	-

					General / -59.6,-49.1			alpha helix			
A 267		THR	0.81	-	Favored (92.42%) General / -59.2,-43.5	Favored (81.8%) *m* chi angles: 302.3	0.08Å	Favored (83.889%) alpha helix	-	-	-
A 268		HIS	0.83	-	Favored (98.17%) General / -63.7,-41.3	Favored (49.2%) *m-70* chi angles: 280.6,295.4	0.02Å	Favored (97.9%) alpha helix	-	-	-
A 269		ARG	0.88	-	Favored (92.64%) General / -60.1,-41.4	Favored (64.1%) *ttp-170* chi angles: 183.5,175.8,67,183.7	0.04Å	Favored (97.54%) alpha helix	-	-	-
A 270		LEU	0.97	-	Favored (88.3%) General / -64.8,-37.6	Favored (85.9%) *mt* chi angles: 291.6,175.2	0.04Å	Favored (78.731%) alpha helix	-	-	-
A 271		MET	1.1	-	Favored (62.42%) General / -74.7,-33.2	Favored (84.4%) *mtm* chi angles: 291.6,188.8,291.2	0.02Å	Favored (38.096%)	-	-	-
A 272		SER	1.25	-	Favored (94.41%) Pre-Pro / -64.4,149.8	Favored (66.7%) *m* chi angles: 297	0.05Å	Favored (20.67%)	-	-	-
A 273		PRO	1.41	-	Favored (6.08%) Trans-Pro / -75.6,61.1	Favored (64.9%) *Cg_endo* chi angles: 31.4,322.5,27.6	0.03Å	Favored (9.385%)	-	-	-
A 274		HIS	1.52	-	Favored (31.61%) General / -116.7,153.9	Favored (70.5%) *t-90* chi angles: 188.8,272.9	0.04Å	Favored (29.841%)	-	-	-
A 275		ARG	1.53	-	Favored (61.75%) General / -51.8,-41.5	Favored (62.1%) *ttp-170* chi angles: 179.1,184.4,64.8,194.4	0.05Å	CaBLAM Disfavored (2.173%)	-	-	-
A 276		VAL	1.44	-	OUTLIER (0.03)%) Pre-Pro / 67.9,119.7	Favored (82.9%) *t* chi angles: 176.8	0.03Å	CaBLAM Outlier (0.559%)	-	-	-
A 277		PRO	1.26	-	Favored (83.89%) Trans-Pro / -57.8,146.8	Favored (82.1%) *Cg_exo* chi angles: 334.5,34.3,331.3	0.04Å	Favored (87.207%)	-	-	-
A 278		ASN	1.06	-	Favored (30.06%) General / -90.6,118.5	Favored (81.6%) *m-40* chi angles: 295,310.8	0.02Å	Favored (46.633%) beta sheet	-	-	-
A 279		TYR	0.9	-	Favored (28.15%) General / -107.6,149.3	Favored (98.9%) *m-80* chi angles: 296.8,91	0.06Å	Favored (47.824%)	-	-	-
A 280		ASN	0.77	-	Favored (20.67%) General / -98.8,-12.6	Favored (93.9%) *m-40* chi angles: 292.6,328.4	0.03Å	Favored (29.554%)	-	-	-
**#**	**Alt**	**Res**	**High B**	**Clash > 0.4Å**	**Ramachandran**	**Rotamer**	**Cβ deviation**	**CaBLAM**	**Bond lengths**	**Bond angles**	**Cis Peptides**
			Avg: 1.12	Clashscore: 1.76	Outliers: 4 of 617	Poor rotamers: 1 of 514	Outliers: 0 of 563	Outliers: 19 of 615	Outliers: 6 of 619	Outliers: 10 of 619	Non-Trans: 4 of 618
A 281		LEU	0.7	-	Favored (17.89%) General / -146.2,130.9	Favored (40.8%) *tp* chi angles: 174.8,67.6	0.06Å	Favored (31.771%)	-	-	-

A 282		PHE	0.66	-	Favored (56.82%) General / -114.6,130.6	Favored (55.2%) *m-80* chi angles: 283.8,83.5	0.06Å	Favored (71.868%)	-	-	-
A 283		ILE	0.65	-	Favored (71.94%) Ile or Val / -117.5,123.6	Favored (71.4%) *mt* chi angles: 301.2,174.6	0.06Å	Favored (71.781%) beta sheet	-	-	-
A 284		MET	0.67	-	Favored (51.22%) General / -111.3,123.9	Favored (61.8%) *ttm* chi angles: 183.1,176.6,286.2	0.06Å	Favored (67.441%)	-	-	-
A 285		ASP	0.7	-	Favored (34.13%) General / -87.7,132.4	Favored (14%) *t70* chi angles: 190.1,94.3	0.12Å	Favored (10.255%)	-	OUTLIER(S) worst is CA-CB-CG: 8.1 σ	-
A 286		GLU	0.73	-	Favored (13.45%) General / 55.2,51.3	Favored (57.2%) *mm-30* chi angles: 304.3,293.9,353.3	0.03Å	Favored (8.736%)	-	-	-
A 287		ALA	0.77	0.41Å O with A 298 ARG NH1	Favored (53.3%) General / -59.8,-19.9	-	0.04Å	Favored (19.274%)	-	-	-
A 288		HIS	0.79	-	Favored (58.6%) General / -77.1,-9.2	Favored (41.5%) *p-80* chi angles: 69.1,291.5	0.06Å	Favored (23.365%)	OUTLIER(S) worst is CB--CG: 4.8 σ	-	-
A 289		PHE	0.79	-	Favored (57.5%) General / -61.7,141.9	Favored (29.1%) *m-80* chi angles: 278.6,286.1	0.17Å	Favored (29.482%)	-	OUTLIER(S) worst is CA-CB-CG: 5.2 σ	-
A 290		THR	0.77	-	Favored (29.4%) General / -93.7,11.4	Favored (65.7%) *p* chi angles: 58.3	0.08Å	CaBLAM Disfavored (4.758%) try beta sheet	-	-	-
A 291		ASP	0.75	-	Favored (92.05%) Pre-Pro / -60.7,134.5	Favored (13.5%) *t70* chi angles: 185.2,284.3	0.07Å	Favored (36.684%)	-	-	-
A 292		PRO	0.72	-	Favored (12.83%) Trans-Pro / -45.9,-34.7	Favored (72.2%) *Cg_exo* chi angles: 328.9,37.4,332.5	0.10Å	Favored (75.797%)	-	-	-
A 293		ALA	0.69	-	Favored (56.02%) General / -76.8,-29.6	-	0.04Å	Favored (96.135%) alpha helix	-	-	-
A 294		SER	0.67	-	Favored (68.7%) General / -71.9,-40.8	Favored (72%) *m* chi angles: 295.3	0.09Å	Favored (77.827%) alpha helix	-	-	-
A 295		ILE	0.65	-	Favored (78.9%) Ile or Val / -66.9,-48.7	Favored (87.5%) *mt* chi angles: 294.8,165.4	0.07Å	Favored (89.746%) alpha helix	-	-	-
A 296		ALA	0.64	-	Favored (94.05%) General / -61.0,-40.6	-	0.02Å	Favored (93.586%) alpha helix	-	-	-
A 297		ALA	0.63	-	Favored (99.45%) General / -62.9,-41.6	-	0.03Å	Favored (98.354%) alpha helix	-	-	-
A 298		ARG	0.63	0.41Å NH1 with A 287 ALA O	Favored (92.79%) General / -64.2,-38.9	Favored (99.6%) *mtm-85* chi angles: 286.3,193,297.4,275.2	0.07Å	Favored (95.99%) alpha helix	-	-	-

#	Alt	Res	High B	Clash > 0.4Å	Ramachandran	Rotamer	Cβ deviation	CaBLAM	Bond lengths	Bond angles	Cis Peptides
A 299		GLY	0.63	-	Favored (76.74%) Glycine / -61.3,-49.5	-	-	Favored (92.855%) alpha helix	-	-	-
A 300		TYR	0.64	-	Favored (67.94%) General / -54.5,-50.4	Favored (91.5%) *t80* chi angles: 175.6,77.7	0.01Å	Favored (88.319%) alpha helix	-	-	-
**#**	**Alt**	**Res**	**High B**	**Clash > 0.4Å**	**Ramachandran**	**Rotamer**	**Cβ deviation**	**CaBLAM**	**Bond lengths**	**Bond angles**	**Cis Peptides**
			Avg: 1.12	Clashscore: 1.76	Outliers: 4 of 617	Poor rotamers: 1 of 514	Outliers: 0 of 563	Outliers: 19 of 615	Outliers: 6 of 619	Outliers: 10 of 619	Non-Trans: 4 of 618
A 301		ILE	0.65	-	Favored (95.31%) Ile or Val / -62.8,-46.9	Favored (96.4%) *mt* chi angles: 293,166.8	0.04Å	Favored (91.896%) alpha helix	-	-	-
A 302		ALA	0.67	-	Favored (80.6%) General / -59.7,-38.1	-	0.04Å	Favored (90.902%) alpha helix	-	-	-
A 303		THR	0.69	-	Favored (86.17%) General / -66.1,-43.9	Favored (82.4%) *m* chi angles: 302.2	0.09Å	Favored (84.627%) alpha helix	-	-	-
A 304		LYS	0.71	-	Favored (73.48%) General / -58.8,-35.8	Favored (11.4%) *ttmm* chi angles: 187.2,177.5,288.1,286.3	0.04Å	Favored (75.793%) alpha helix	-	-	-
A 305		VAL	0.73	-	Favored (81.6%) Ile or Val / -68.1,-46.4	Favored (80%) *t* chi angles: 173.1	0.02Å	Favored (75.835%) alpha helix	-	-	-
A 306		GLU	0.75	-	Favored (72.32%) General / -60.3,-33.4	Favored (93.8%) *mt-10* chi angles: 289.3,182.8,356.3	0.04Å	Favored (77.61%) alpha helix	-	-	-
A 307		LEU	0.75	-	Favored (59.9%) General / -76.1,-11.2	Favored (91.5%) *mt* chi angles: 294.6,171.2	0.06Å	Favored (49.82%)	-	-	-
A 308		GLY	0.74	-	Favored (68.78%) Glycine / 78.0,22.4	-	-	Favored (80.102%)	-	-	-
A 309		GLU	0.72	-	Favored (15.93%) General / -89.4,-35.6	Favored (97.2%) *mt-10* chi angles: 294.9,178.4,0.2	0.03Å	CaBLAM Disfavored (3.381%)	-	-	-
A 310		ALA	0.7	-	Favored (37.06%) General / -153.7,156.0	-	0.10Å	Favored (18.782%)	-	-	-
A 311		ALA	0.68	-	Favored (20.16%) General / -85.0,159.6	-	0.06Å	Favored (42.724%)	-	-	-
A 312		ALA	0.67	-	Favored (33.81%) General / -141.4,141.5	-	0.07Å	Favored (60.204%) beta sheet	-	-	-
A 313		ILE	0.68	-	Favored (64.22%) Ile or Val / -123.8,122.2	Favored (77.5%) *mt* chi angles: 300.7,170.1	0.03Å	Favored (70.163%) beta sheet	-	-	-
A 314		PHE	0.71	-	Favored (11.28%) General / -107.3,102.0	Favored (20%) *m-10* chi angles: 290.9,344.3	0.05Å	Favored (70.817%) beta sheet	-	-	-

A 315		MET	0.76	-	Favored (31.42%) General / -89.5,138.5	Favored (66.9%) *mtt* chi angles: 296.1,179.3,175.4	0.06Å	Favored (7.673%) beta sheet	-	-	-
A 316		THR	0.82	-	Favored (8.34%) General / -167.9,155.2	Favored (3.7%) *t* chi angles: 177.6	0.03Å	Favored (14.833%)	-	-	-
A 317		ALA	0.87	-	Favored (75.67%) General / -60.7,-35.2	-	0.04Å	Favored (13.854%)	-	-	-
A 318		THR	0.92	-	Favored (14.39%) Pre-Pro / -152.8,141.9	Favored (11.6%) *t* chi angles: 189.6	0.10Å	Favored (11.131%)	-	-	-
A 319		PRO	0.96	-	Favored (51.09%) Trans-Pro / -58.8,153.3	Favored (56.7%) *Cg_exo* chi angles: 336.2,31.5,334.2	0.02Å	Favored (89.61%)	-	-	-
A 320		PRO	0.98	-	Favored (70.71%) Trans-Pro / -62.0,-20.7	Favored (35%) *Cg_endo* chi angles: 22.3,326.1,31.2	0.04Å	Favored (59.811%)	-	-	-
**#**	**Alt**	**Res**	**High B**	**Clash > 0.4Å**	**Ramachandran**	**Rotamer**	**Cβ deviation**	**CaBLAM**	**Bond lengths**	**Bond angles**	**Cis Peptides**
			Avg: 1.12	Clashscore: 1.76	Outliers: 4 of 617	Poor rotamers: 1 of 514	Outliers: 0 of 563	Outliers: 19 of 615	Outliers: 6 of 619	Outliers: 10 of 619	Non-Trans: 4 of 618
A 321		GLY	0.97	-	Favored (82.08%) Glycine / -89.2,0.9	-	-	Favored (63.713%)	-	-	-
A 322		THR	0.96	-	Favored (15.68%) General / -84.9,167.7	Favored (75.6%) *p* chi angles: 61.3	0.04Å	Favored (19.67%)	-	-	-
A 323		SER	0.94	-	Favored (3.32%) General / -135.9,23.2	Favored (74.7%) *p* chi angles: 59.7	0.02Å	Favored (15.09%) beta sheet	-	-	-
A 324		ASP	0.92	-	Favored (38.21%) Pre-Pro / -103.1,104.1	Favored (64.2%) *t0* chi angles: 184.5,356.4	0.07Å	Favored (17.054%) beta sheet	-	-	-
A 325		PRO	0.92	-	Favored (31.07%) Trans-Pro / -71.1,-13.1	Favored (63.1%) *Cg_endo* chi angles: 26.7,327.1,24.9	0.02Å	Favored (53.899%)	-	-	-
A 326		PHE	0.95	-	Favored (70.96%) Pre-Pro / -129.9,64.1	Favored (71%) *m-80* chi angles: 304.5,99.1	0.07Å	Favored (13.011%)	-	-	-
A 327		PRO	0.99	-	Favored (31.87%) Trans-Pro / -72.7,165.7	Favored (78%) *Cg_endo* chi angles: 28.5,324.2,28.6	0.01Å	Favored (5.908%)	-	-	-
A 328		GLU	1.06	-	Favored (4.27%) General / -55.3,156.5	Favored (27%) *pt0* chi angles: 65.3,181.8,356.1	0.02Å	CaBLAM Disfavored (4.121%)	-	-	-
A 329		SER	1.11	-	Favored (10.16%) General / -145.2,175.6	Favored (81.8%) *p* chi angles: 62	0.05Å	Favored (44.523%)	-	-	-
A 330		ASN	1.14	-	Favored (33.1%) General / -98.1,12.8	Favored (51.8%) *t0* chi angles: 200.4,41.7	0.03Å	CaBLAM Disfavored (3.555%)	-	-	-

#	Alt	Res	High B	Clash > 0.4Å	Ramachandran	Rotamer	Cβ deviation	CaBLAM	Bond lengths	Bond angles	Cis Peptides
A 331		ALA	1.12	-	Favored (17.79%) Pre-Pro / -156.8,149.2	-	0.04Å	Favored (9.709%)	-	-	-
A 332		PRO	1.06	-	Favored (93.56%) Trans-Pro / -58.7,140.1	Favored (81%) *Cg_exo* chi angles: 334.6,35.7,329.5	0.05Å	Favored (53.594%)	-	-	-
A 333		ILE	0.98	-	Favored (55.96%) Ile or Val / -132.9,127.9	Favored (77.8%) *mt* chi angles: 301.1,172.4	0.05Å	Favored (59.164%) beta sheet	-	-	-
A 334		SER	0.9	-	Favored (19.12%) General / -90.4,107.4	Favored (42.1%) *t* chi angles: 178.3	0.02Å	Favored (64.334%) beta sheet	-	-	-
A 335		ASP	0.83	-	Favored (26.13%) General / -89.0,114.9	Favored (46.8%) *m-30* chi angles: 290.9,305.5	0.05Å	Favored (51.754%) beta sheet	-	-	-
A 336		MET	0.79	-	Favored (47.56%) General / -132.5,138.5	Favored (44.8%) *mtm* chi angles: 297.2,167.5,285.8	0.09Å	Favored (48.759%) beta sheet	-	-	-
A 337		GLN	0.76	-	Favored (33.56%) General / -86.7,134.8	Favored (65.5%) *tt0* chi angles: 185.6,174.9,349.4	0.04Å	Favored (29.047%) beta sheet	-	-	-
A 338		THR	0.76	-	Favored (39.41%) General / -154.3,157.9	Favored (12%) *t* chi angles: 187.1	0.03Å	Favored (25.476%) beta sheet	-	-	-
A 339		GLU	0.77	-	Favored (3.34%) General / -76.7,85.0	Favored (94%) *mt-10* chi angles: 295.5,181.8,4.6	0.02Å	Favored (20.715%) beta sheet	-	-	-
A 340		ILE	0.8	-	Favored (81.95%) Pre-Pro / -78.9,126.6	Favored (91.9%) *mt* chi angles: 297.3,169.7	0.07Å	Favored (32.161%) beta sheet	-	-	-
**#**	**Alt**	**Res**	**High B**	**Clash > 0.4Å**	**Ramachandran**	**Rotamer**	**Cβ deviation**	**CaBLAM**	**Bond lengths**	**Bond angles**	**Cis Peptides**
			Avg: 1.12	Clashscore: 1.76	Outliers: 4 of 617	Poor rotamers: 1 of 514	Outliers: 0 of 563	Outliers: 19 of 615	Outliers: 6 of 619	Outliers: 10 of 619	Non-Trans: 4 of 618
A 341		PRO	0.86	-	Favored (71.52%) Trans-Pro / -69.3,152.6	Favored (53.2%) *Cg_endo* chi angles: 25.6,328.7,24.1	0.01Å	Favored (58.987%)	-	-	-
A 342		ASP	0.95	-	Favored (13.75%) General / -96.4,-29.8	Favored (64.2%) *m-30* chi angles: 299,306.5	0.08Å	Favored (21.117%)	-	-	-
A 343		ARG	1.1	-	Allowed (1.88%) General / -114.7,-171.2	Favored (62.4%) *mmm-85* chi angles: 304.3,297,294.9,274.2	0.05Å	CaBLAM Disfavored (1.333%)	-	-	-
A 344		ALA	1.31	-	Favored (61.4%) General / -53.0,-37.2	-	0.08Å	CaBLAM Outlier (0.013%)	-	-	-
A 345		TRP	1.56	0.61Å HE1 with A 349 TYR H	Allowed (0.06%) General / 67.1,-75.0	Favored (17.6%) *t60* chi angles: 159.2,77.5	0.18Å	Favored (29.836%)	-	-	-
A 346		ASN	1.81	-	Allowed (0.28%)	Favored (80.2%) *m-40* chi angles: 300.7,310.7	0.03Å	Favored (9.927%)	-	-	-

					General / 71.8,-18.9						
A 347		THR	2	-	Favored (5.58%) General / -168.4,151.6	Favored (7.7%) *t* chi angles: 183.2	0.03Å	CA Geom Outlier (0.159%)	-	-	-
A 348		GLY	2.06	-	Favored (2.76%) Glycine / 115.7,-39.4	-	-	CaBLAM Disfavored (3.327%)	-	-	-
A 349		TYR	2.01	0.61Å H with A 345 TRP HE1	Allowed (0.93%) General / -102.2,64.6	Favored (61.7%) *m-80* chi angles: 300.6,114.3	0.00Å	Favored (7.063%)	-	-	-
A 350		GLU	1.85	-	Favored (69.37%) General / -61.7,-28.4	Favored (68.6%) *mm-30* chi angles: 294.8,294.5,306.9	0.04Å	Favored (16.979%)	-	-	-
A 351		TRP	1.67	-	Favored (4.95%) General / -48.1,-30.9	Favored (88.8%) *m100* chi angles: 288.2,110.4	0.09Å	Favored (54.154%)	-	-	-
A 352		ILE	1.49	-	Favored (97.52%) Ile or Val / -62.1,-43.2	Favored (95.3%) *mt* chi angles: 291.9,167.9	0.05Å	Favored (56.559%) alpha helix	-	-	-
A 353		THR	1.35	-	Favored (32.93%) General / -95.3,-9.5	Favored (75.1%) *p* chi angles: 60	0.10Å	Favored (29.848%)	-	-	-
A 354		GLU	1.24	-	Favored (59.35%) General / -84.5,-6.2	Favored (97.2%) *mt-10* chi angles: 293.1,181.7,360	0.06Å	Favored (48.428%)	-	-	-
A 355		TYR	1.15	-	Favored (49.52%) General / -70.0,147.9	Favored (16.4%) *m-10* chi angles: 290.9,149.4	0.04Å	Favored (26.941%)	-	-	-
A 356		VAL	1.06	-	Favored (12.15%) Ile or Val / -116.5,-8.3	Favored (28%) *m* chi angles: 298.6	0.03Å	Favored (26.347%) beta sheet	-	-	-
A 357		GLY	0.98	-	Favored (30.61%) Glycine / -99.2,-169.7	-	-	Favored (22.564%) beta sheet	-	-	-
A 358		LYS	0.9	-	Favored (32.32%) General / -83.5,126.0	Favored (78.4%) *tttt* chi angles: 185.2,169.2,179,171.6	0.11Å	Favored (11.029%) beta sheet	-	-	-
A 359		THR	0.83	-	Favored (54.73%) General / -117.6,134.6	Favored (98%) *m* chi angles: 300.1	0.03Å	Favored (69.565%) beta sheet	-	-	-
A 360		VAL	0.79	-	Favored (64.39%) Ile or Val / -108.8,123.1	Favored (79.9%) *t* chi angles: 178	0.02Å	Favored (72.414%) beta sheet	-	-	-
**#**	**Alt**	**Res**	**High B**	**Clash > 0.4Å**	**Ramachandran**	**Rotamer**	**Cβ deviation**	**CaBLAM**	**Bond lengths**	**Bond angles**	**Cis Peptides**
			Avg: 1.12	Clashscore: 1.76	Outliers: 4 of 617	Poor rotamers: 1 of 514	Outliers: 0 of 563	Outliers: 19 of 615	Outliers: 6 of 619	Outliers: 10 of 619	Non-Trans: 4 of 618
A 361		TRP	0.76	-	Favored (21.52%) General / -113.0,111.0	Favored (7%) *t60* chi angles: 188.9,346.3	0.04Å	Favored (68.144%) beta sheet	-	-	-
A 362		PHE	0.76	-	Favored (25.48%)	Favored (77%) *m-80*	0.02Å	Favored (61.913%)	-	-	-

				General / -86.6,117.6	chi angles: 288.8,84.8		beta sheet			
A 363	VAL	0.78	-	Favored (22.49%) Pre-Pro / -114.7,136.6	Favored (67.9%) *t* chi angles: 179	0.10Å	Favored (51.497%)	-	-	-
A 364	PRO	0.82	-	Favored (26.55%) Trans-Pro / -54.9,-21.5	Favored (86.4%) *Cg_exo* chi angles: 333.6,35.1,331.7	0.04Å	Favored (7.1%)	-	-	-
A 365	SER	0.86	-	Favored (37.85%) General / -156.8,164.7	Favored (93.8%) *p* chi angles: 66.3	0.07Å	Favored (21.964%)	-	-	-
A 366	VAL	0.89	-	Favored (93.73%) Ile or Val / -61.6,-42.3	Favored (65.8%) *t* chi angles: 171.6	0.06Å	Favored (66.969%) alpha helix	-	-	-
A 367	LYS	0.92	-	Favored (94.95%) General / -64.7,-42.9	Favored (59.5%) *mttm* chi angles: 289.2,176.8,181.3,287.5	0.05Å	Favored (85.594%) alpha helix	-	-	-
A 368	MET	0.93	-	Favored (84.39%) General / -66.6,-37.2	Favored (98.5%) *mtp* chi angles: 291.8,171.2,69.4	0.05Å	Favored (77.609%) alpha helix	-	-	-
A 369	GLY	0.92	-	Favored (43.26%) Glycine / -56.6,-53.5	-	-	Favored (92.96%) alpha helix	-	-	-
A 370	ASN	0.91	-	Favored (86.39%) General / -59.9,-39.7	Favored (96.2%) *m-40* chi angles: 286.7,338.2	0.04Å	Favored (84.301%) alpha helix	-	-	-
A 371	GLU	0.91	-	Favored (97.07%) General / -61.5,-44.4	Favored (75.5%) *tt0* chi angles: 180.9,172.6,342.7	0.06Å	Favored (85.782%) alpha helix	-	-	-
A 372	ILE	0.91	-	Favored (94.27%) Ile or Val / -65.6,-43.0	Favored (86.4%) *mt* chi angles: 290.6,168.2	0.07Å	Favored (82.254%) alpha helix	-	-	-
A 373	ALA	0.92	-	Favored (90.84%) General / -59.3,-42.2	-	0.03Å	Favored (88.194%) alpha helix	-	-	-
A 374	LEU	0.95	-	Favored (95.47%) General / -63.9,-40.0	Favored (88.5%) *mt* chi angles: 290.7,171.3	0.03Å	Favored (89.02%) alpha helix	-	-	-
A 375	CYS	0.98	-	Favored (69.54%) General / -55.2,-50.4	Favored (13.7%) *t* chi angles: 170.7	0.09Å	Favored (79.312%) alpha helix	-	-	-
A 376	LEU	1.02	-	Favored (77.22%) General / -69.4,-36.9	Favored (95.9%) *mt* chi angles: 293.1,171.3	0.10Å	Favored (76.959%) alpha helix	-	-	-
A 377	GLN	1.05	-	Favored (86.46%) General / -63.0,-37.3	Favored (95.5%) *mt0* chi angles: 289.3,172.6,344.7	0.03Å	Favored (84.497%) alpha helix	-	-	-
A 378	ARG	1.06	-	Favored (73.66%) General / -62.5,-32.7	Favored (97.5%) *mtt180* chi angles: 288,173.3,180.9,173	0.02Å	Favored (21.594%)	-	OUTLIER(S) worst is CA-C-O: 4.6 σ	-
A 379	ALA	1.03	-	Favored (47.65%) General / -83.6,0.2	-	0.03Å	Favored (7.788%)	-	-	-

#	Alt	Res	High B	Clash > 0.4Å	Ramachandran	Rotamer	Cβ deviation	CaBLAM	Bond lengths	Bond angles	Cis Peptides
A 380		GLY	0.98	-	Favored (52.77%) Glycine / 97.4,11.1	-	-	Favored (83.458%)	-	-	-
#	Alt	Res	High B	Clash > 0.4Å	Ramachandran	Rotamer	Cβ deviation	CaBLAM	Bond lengths	Bond angles	Cis Peptides
			Avg: 1.12	Clashscore: 1.76	Outliers: 4 of 617	Poor rotamers: 1 of 514	Outliers: 0 of 563	Outliers: 19 of 615	Outliers: 6 of 619	Outliers: 10 of 619	Non-Trans: 4 of 618
A 381		LYS	0.91	-	Favored (18.68%) General / -95.7,152.2	Favored (71.5%) *mmtt* chi angles: 304.8,296.5,187.4,181.8	0.05Å	Favored (33.665%)	-	-	-
A 382		LYS	0.84	0.41Å HG2 with A 403 TRP CE3	Favored (25.59%) General / -91.9,113.3	Favored (87.4%) *tttt* chi angles: 185.1,173.5,177.5,176.6	0.08Å	Favored (51.76%) beta sheet	-	-	-
A 383		VAL	0.8	-	Favored (67.89%) Ile or Val / -128.2,132.9	Favored (35.5%) *t* chi angles: 184.4	0.11Å	Favored (65.183%) beta sheet	-	-	-
A 384		ILE	0.78	-	Favored (36.13%) Ile or Val / -115.6,140.7	Favored (16.7%) *tt* chi angles: 183.8,163.1	0.09Å	Favored (61.167%) beta sheet	-	-	-
A 385		GLN	0.8	-	Favored (33.94%) General / -115.6,118.7	Favored (69.7%) *tp40* chi angles: 176.9,70.6,59	0.04Å	Favored (66.987%) beta sheet	-	-	-
A 386		LEU	0.85	0.49Å N with A 386 LEU HD12	Favored (56.31%) General / -112.3,129.4	Favored (3.9%) *mp* chi angles: 293.3,85.8	0.02Å	Favored (26.825%) beta sheet	-	-	-
A 387		ASN	0.92	-	Favored (6.09%) General / -150.0,-177.8	Favored (30.2%) *p0* chi angles: 60.8,53.1	0.05Å	Favored (13.142%)	-	-	-
A 388		ARG	1	-	Favored (45.08%) General / -56.1,-25.1	Favored (22%) *tpp80* chi angles: 184.4,63,62.3,94.2	0.04Å	Favored (15.288%)	-	-	-
A 389		LYS	1.07	-	Favored (27.54%) General / -105.5,1.3	Favored (70.5%) *mmtt* chi angles: 298.6,294.2,189.2,181.1	0.02Å	Favored (30.008%) alpha helix	-	-	-
A 390		SER	1.11	-	Favored (3.62%) General / -128.5,-20.5	Favored (88.5%) *p* chi angles: 68.2	0.05Å	Favored (8.519%) alpha helix	-	-	-
A 391		TYR	1.14	-	Favored (51.68%) General / -49.9,-46.8	Favored (89.1%) *t80* chi angles: 175.9,80	0.09Å	Favored (52.222%) alpha helix	-	-	-
A 392		GLU	1.15	-	Favored (66.7%) General / -61.4,-24.7	Favored (97%) *mt-10* chi angles: 293.5,175.2,3.8	0.05Å	Favored (52.791%) alpha helix	-	-	-
A 393		THR	1.15	-	Favored (7.61%) General / -97.1,-42.9	Favored (93.9%) *m* chi angles: 299.3	0.04Å	Favored (41.184%) alpha helix	-	-	-
A 394		GLU	1.15	-	Favored (36.6%) General / -80.7,-29.4	Favored (75.5%) *mm-30* chi angles: 297.9,300.8,306	0.10Å	Favored (43.774%) alpha helix	-	-	-
A 395		TYR	1.16	-	Favored (25.9%) Pre-Pro / -50.7,-55.6	Favored (81%) *t80* chi angles: 174.2,82.7	0.09Å	Favored (49.246%) alpha helix	-	-	-

A 396		PRO	1.2	-	Favored (69.28%) Trans-Pro / -63.8,-22.2	Favored (38.2%) *Cg_endo* chi angles: 23,325.7,30.8	0.01Å	Favored (77.737%) alpha helix	-	-	-
A 397		LYS	1.25	-	Favored (65.14%) General / -73.6,-38.5	Favored (98.5%) *mttt* chi angles: 292.3,179,184.6,176.1	0.02Å	Favored (75.479%) alpha helix	-	-	-
A 398		CYS	1.3	-	Favored (90.76%) General / -65.1,-38.5	Favored (96.3%) *m* chi angles: 290.3	0.06Å	Favored (95.715%) alpha helix	-	-	-
A 399		LYS	1.32	-	Favored (95.67%) General / -64.4,-40.4	Favored (42.5%) *mtpt* chi angles: 283.9,172.6,57.7,173	0.05Å	Favored (57.335%) alpha helix	-	-	-
A 400		ASN	1.28	-	Favored (13.34%) General / -104.2,-22.9	Favored (69.9%) *m-40* chi angles: 294.7,283.9	0.03Å	Favored (22.436%)	-	-	-
**#**	**Alt**	**Res**	**High B**	**Clash > 0.4Å**	**Ramachandran**	**Rotamer**	**Cβ deviation**	**CaBLAM**	**Bond lengths**	**Bond angles**	**Cis Peptides**
			Avg: 1.12	Clashscore: 1.76	Outliers: 4 of 617	Poor rotamers: 1 of 514	Outliers: 0 of 563	Outliers: 19 of 615	Outliers: 6 of 619	Outliers: 10 of 619	Non-Trans: 4 of 618
A 401		ASP	1.19	-	Favored (10.54%) General / -97.7,168.1	Favored (15.4%) *t0* chi angles: 201.8,339.7	0.07Å	Favored (15.459%)	-	OUTLIER(S) worst is CA-CB-CG: 4.9 σ	-
A 402		ASP	1.07	-	Favored (5.18%) General / -92.5,74.5	Favored (40.7%) *t0* chi angles: 189.6,13.7	0.02Å	Favored (10.221%)	-	-	-
A 403		TRP	0.93	0.41Å CE3 with A 382 LYS HG2	Favored (27.33%) General / -83.9,147.8	Favored (95.1%) *m100* chi angles: 295.6,97.2	0.08Å	Favored (19.112%)	-	-	-
A 404		ASP	0.81	-	Favored (15.51%) General / -85.8,-40.9	Favored (51.9%) *m-30* chi angles: 294.8,299.3	0.08Å	Favored (12.578%)	-	-	-
A 405		PHE	0.73	-	Favored (50.94%) General / -127.4,145.1	Favored (76.2%) *m-80* chi angles: 288.5,85	0.11Å	Favored (25.759%)	-	OUTLIER(S) worst is CA-CB-CG: 4.4 σ	-
A 406		VAL	0.7	-	Favored (57.51%) Ile or Val / -123.3,119.4	Favored (58.8%) *t* chi angles: 180.1	0.10Å	Favored (67.577%)	-	-	-
A 407		ILE	0.7	-	Favored (60.5%) Ile or Val / -105.9,128.1	Favored (34.9%) *mm* chi angles: 309.4,300.5	0.05Å	Favored (61.108%) beta sheet	-	-	-
A 408		THR	0.72	-	Favored (33.91%) General / -135.5,162.0	Favored (29%) *p* chi angles: 69.7	0.06Å	Favored (54.131%)	-	-	-
A 409		THR	0.77	-	Favored (2.56%) General / -98.9,-173.6	Favored (28.7%) *p* chi angles: 69.8	0.08Å	Favored (20.847%)	-	-	-
A 410		ASP	0.83	0.53Å OD2 with A 431 LYS NZ	Favored (14.18%) General / -70.0,-3.0	Favored (56.1%) *p0* chi angles: 62.1,3.7	0.03Å	Favored (5.416%)	-	-	-
A 411		ILE	0.89	-	Favored (45.54%) Ile or Val / -63.4,-28.7	Favored (20.4%) *tt* chi angles: 194.2,168	0.08Å	Favored (52.871%)	-	-	-

#	Alt	Res	High B	Clash > 0.4Å	Ramachandran	Rotamer	Cβ deviation	CaBLAM	Bond lengths	Bond angles	Cis Peptides
A 412		SER	0.96	-	Favored (61.52%) General / -73.0,-14.8	Favored (94.5%) *p* chi angles: 64.1	0.08Å	Favored (63.9%) three-ten	-	-	-
A 413		GLU	1.03	-	Favored (63.85%) General / -69.3,-19.5	Favored (82.9%) *mt-10* chi angles: 295.9,174.5,17.1	0.07Å	Favored (60.391%)	-	-	-
A 414		MET	1.09	-	Favored (24.35%) General / -81.7,3.8	Favored (68%) *mtt* chi angles: 294.8,177.8,176.3	0.03Å	CaBLAM Outlier (0.497%)	-	-	-
A 415		GLY	1.14	-	Allowed (0.73%) Glycine / 135.3,20.4	-	-	CaBLAM Outlier (0.627%)	-	-	-
A 416		ALA	1.17	-	Allowed (1.93%) General / -74.5,80.1	-	0.05Å	Favored (32.888%)	-	-	-
A 417		ASN	1.16	-	Allowed (1.64%) General / -74.4,69.7	Favored (83%) *m-40* chi angles: 289.8,319.9	0.06Å	Favored (52.883%) beta sheet	-	-	-
A 418		PHE	1.12	-	Favored (26.99%) General / -92.6,142.8	Favored (26.1%) *m-10* chi angles: 296.7,339.5	0.08Å	Favored (25.516%) beta sheet	-	-	-
A 419		LYS	1.04	-	Favored (8.11%) General / -81.1,74.6	Favored (54.4%) *tttm* chi angles: 185.9,175.4,187.3,294.6	0.01Å	Favored (19.677%) beta sheet	-	-	-
A 420		ALA	0.95	-	Favored (23.63%) General / -85.0,152.2	-	0.04Å	Favored (16.969%)	-	-	-
#	Alt	Res	High B	Clash > 0.4Å	Ramachandran	Rotamer	Cβ deviation	CaBLAM	Bond lengths	Bond angles	Cis Peptides
			Avg: 1.12	Clashscore: 1.76	Outliers: 4 of 617	Poor rotamers: 1 of 514	Outliers: 0 of 563	Outliers: 19 of 615	Outliers: 6 of 619	Outliers: 10 of 619	Non-Trans: 4 of 618
A 421		SER	0.86	-	Favored (13.36%) General / -106.6,-20.5	Favored (87.7%) *p* chi angles: 62.8	0.07Å	Favored (18.265%)	-	-	-
A 422		ARG	0.78	-	Favored (35.43%) General / -140.4,141.9	Favored (80.8%) *mtt90* chi angles: 292.6,184.6,171.8,84.5	0.07Å	Favored (38.285%)	-	-	-
A 423		VAL	0.72	-	Favored (73.83%) Ile or Val / -124.1,128.1	Favored (96.9%) *t* chi angles: 175.7	0.04Å	Favored (71.69%)	-	-	-
A 424		ILE	0.68	-	Favored (51.25%) Ile or Val / -107.3,116.6	Favored (81.5%) *mt* chi angles: 299.9,171	0.05Å	Favored (71.287%) beta sheet	-	-	-
A 425		ASP	0.66	-	Favored (14.24%) General / -117.0,107.2	Favored (63.3%) *t0* chi angles: 181.3,359.7	0.05Å	Favored (65.909%) beta sheet	-	-	-
A 426		SER	0.66	-	Favored (61.08%) General / -61.0,-19.2	Favored (89.4%) *p* chi angles: 68.3	0.12Å	Favored (7.45%) beta sheet	-	-	-
A 427		ARG	0.68	-	Favored (9.33%) General / 68.0,14.3	Favored (26.6%) *mmt90* chi angles: 290.4,287.4,179.5,88.9	0.13Å	CaBLAM Disfavored (4.356%)	-	-	-

A 428		LYS	0.7	-	Favored (48.58%) General / -126.4,150.6	Favored (44.8%) *mtmt* chi angles: 295,174.1,291.4,190	0.08Å	Favored (25.475%)	-	-	-
A 429		SER	0.73	-	Favored (18.24%) General / -151.4,137.4	Favored (46.9%) *t* chi angles: 180.3	0.04Å	Favored (33.3%)	-	-	-
A 430		VAL	0.76	-	Favored (32.52%) Ile or Val / -89.1,117.5	Favored (64%) *t* chi angles: 179.5	0.07Å	Favored (52.191%) beta sheet	-	-	-
A 431		LYS	0.79	0.53Å NZ with A 410 ASP OD2	Favored (38.89%) Pre-Pro / -116.9,146.9	Favored (72.6%) *mmtt* chi angles: 301.8,293.2,185.7,184.6	0.07Å	Favored (44.177%) beta sheet	-	-	-
A 432		PRO	0.84	-	Favored (46.15%) Trans-Pro / -69.7,141.7	Favored (51.9%) *Cg_endo* chi angles: 25.4,326.5,26.6	0.04Å	Favored (46.388%) beta sheet	-	-	-
A 433		THR	0.93	-	Favored (44.24%) General / -128.1,155.5	Favored (69.9%) *p* chi angles: 62.4	0.05Å	Favored (45.695%) beta sheet	-	-	-
A 434		ILE	1.07	-	Favored (42.8%) Ile or Val / -89.9,125.2	Favored (48.2%) *mm* chi angles: 304.9,302	0.05Å	Favored (44.914%) beta sheet	-	-	-
A 435		ILE	1.26	-	Favored (60%) Ile or Val / -105.8,128.5	Favored (44.8%) *mm* chi angles: 306.8,301.3	0.02Å	Favored (69.76%) beta sheet	-	-	-
A 436		GLU	1.46	-	Favored (6.66%) General / -109.7,30.4	Favored (91.5%) *mt-10* chi angles: 296.8,186.1,356.7	0.02Å	CaBLAM Disfavored (3.716%) try beta sheet	-	-	-
A 437		GLU	1.6	-	Favored (3.56%) General / -149.0,105.7	Favored (91.1%) *tt0* chi angles: 183.4,175.8,358.3	0.02Å	CA Geom Outlier (0.234%)	-	-	-
A 438		GLY	1.6	-	Allowed (0.96%) Glycine / -62.4,-70.7	-	-	CaBLAM Outlier (0.186%)	-	-	Cis nonPRO omega= -3.47
A 439		ASP	1.48	-	Allowed (0.37%) General / -142.5,-67.7	Favored (63.6%) *m-30* chi angles: 279.7,343.8	0.24Å	Favored (7.101%)	-	OUTLIER(S) worst is C-N-CA: 7.1 σ	Cis nonPRO omega= -16.69
A 440		GLY	1.29	-	Favored (33.36%) Glycine / -96.9,178.3	-	-	CaBLAM Outlier (0.112%)	-	-	Cis nonPRO omega= -2.2
**#**	**Alt**	**Res**	**High B**	**Clash > 0.4Å**	**Ramachandran**	**Rotamer**	**Cβ deviation**	**CaBLAM**	**Bond lengths**	**Bond angles**	**Cis Peptides**
			Avg: 1.12	Clashscore: 1.76	Outliers: 4 of 617	Poor rotamers: 1 of 514	Outliers: 0 of 563	Outliers: 19 of 615	Outliers: 6 of 619	Outliers: 10 of 619	Non-Trans: 4 of 618
A 441		ARG	1.12	-	Favored (33.34%) General / -160.0,160.4	Favored (44.2%) *ptt-90* chi angles: 65.4,183.3,193.3,274.3	0.04Å	Favored (28.466%)	-	-	-
A 442		VAL	0.99	-	Favored (63.47%) Ile or Val / -115.5,120.0	Favored (74.4%) *t* chi angles: 178.3	0.06Å	Favored (39.686%) beta sheet	-	-	-
A 443		ILE	0.92	-	Favored (62.18%) Ile or Val / -117.5,134.1	Favored (48.1%) *mm* chi angles: 302.5,297.5	0.04Å	Favored (48.969%) beta sheet	-	-	-

A 444		LEU	0.9	-	Favored (15.87%) General / -62.9,122.4	Favored (96.2%) *mt* chi angles: 294.5,173	0.09Å	Favored (44.53%)	-	-	-
A 445		GLY	0.88	-	Favored (4.51%) Glycine / -81.0,52.6	-	-	CaBLAM Disfavored (3.607%)	-	-	-
A 446		GLU	0.87	0.41Å HB3 with A 447 PRO HD2	OUTLIER (0.03)%) Pre-Pro / 51.0,172.7	Favored (96.9%) *mt-10* chi angles: 290.5,181.3,357	0.09Å	CaBLAM Outlier (0.224%)	-	-	-
A 447		PRO	0.84	0.41Å HD2 with A 446 GLU HB3	Favored (5.85%) Trans-Pro / -75.4,69.3	Favored (70.5%) *Cg_endo* chi angles: 29.4,322.2,30.1	0.07Å	CaBLAM Outlier (0.307%)	-	-	-
A 448		SER	0.81	-	Favored (43.36%) General / -65.7,152.8	Favored (53.4%) *m* chi angles: 292.2	0.02Å	Favored (10.722%)	-	-	-
A 449		ALA	0.78	-	Favored (56.46%) General / -60.6,141.6	-	0.04Å	Favored (39.514%)	-	-	-
A 450		ILE	0.77	0.57Å C with A 450 ILE HD12	Favored (5.94%) Ile or Val / -75.8,156.1	Allowed (1.5%) *pp* chi angles: 59.4,84.6	0.04Å	Favored (49.257%)	-	-	-
A 451		THR	0.77	-	Favored (36.28%) General / -73.6,157.6	Favored (62.3%) *p* chi angles: 63.6	0.14Å	Favored (50.028%)	-	-	-
A 452		ALA	0.78	-	Favored (72.02%) General / -57.6,-36.6	-	0.06Å	Favored (63.078%)	-	-	-
A 453		ALA	0.79	-	Favored (95.87%) General / -61.9,-40.5	-	0.03Å	Favored (75.331%) alpha helix	-	-	-
A 454		SER	0.79	-	Favored (92.9%) General / -65.1,-39.2	Favored (32.8%) *m* chi angles: 289	0.07Å	Favored (90.291%) alpha helix	-	-	-
A 455		ALA	0.8	-	Favored (85.98%) General / -58.9,-40.9	-	0.04Å	Favored (88.539%) alpha helix	-	-	-
A 456		ALA	0.81	-	Favored (96.84%) General / -61.8,-40.9	-	0.05Å	Favored (93.759%) alpha helix	-	-	-
A 457		GLN	0.82	-	Favored (82.18%) General / -66.2,-36.1	Favored (35.2%) *mm110* chi angles: 293.7,295.1,119.8	0.03Å	Favored (85.897%) alpha helix	-	-	-
A 458		ARG	0.84	-	Favored (93.98%) General / -63.9,-39.4	Favored (72.5%) *mtt90* chi angles: 292.3,186.3,179.8,101.1	0.03Å	Favored (87.064%) alpha helix	-	-	-
A 459		ARG	0.88	-	Favored (65.31%) General / -67.6,-22.7	Favored (30.3%) *mtp-110* chi angles: 292.7,175.9,73.4,253.7	0.03Å	Favored (62.033%) three-ten	-	-	-
A 460		GLY	0.93	-	Favored (62.08%) Glycine / -67.0,-13.5	-	-	Favored (74.998%) three-ten	-	-	-

#	Alt	Res	High B	Clash > 0.4Å	Ramachandran	Rotamer	Cβ deviation	CaBLAM	Bond lengths	Bond angles	Cis Peptides
			Avg: 1.12	Clashscore: 1.76	Outliers: 4 of 617	Poor rotamers: 1 of 514	Outliers: 0 of 563	Outliers: 19 of 615	Outliers: 6 of 619	Outliers: 10 of 619	Non-Trans: 4 of 618
A 461		ARG	1	-	Favored (59.47%) General / -81.2,-11.3	Favored (12.5%) *mmp80* chi angles: 300.5,281.4,74.6,75.9	0.08Å	Favored (46.888%) three-ten	-	-	-
A 462		ILE	1.09	0.41Å HD13 with A 462 ILE HG21	Allowed (1.51%) Ile or Val / -121.9,-30.4	Favored (21.6%) *tt* chi angles: 192.4,168.9	0.18Å	Favored (14.939%) alpha helix	OUTLIER(S) worst is CB--CG1: 5.8 σ	-	-
A 463		GLY	1.2	-	Favored (85.4%) Glycine / -80.2,-0.1	-	-	Favored (51.203%) three-ten	-	-	-
A 464		ARG	1.34	-	Favored (49.28%) General / -89.5,-9.6	Favored (13.2%) *ptm-80* chi angles: 65.7,177.9,293.4,274.6	0.05Å	Favored (47.108%)	-	-	-
A 465		ASN	1.49	-	Favored (14.43%) Pre-Pro / -107.0,96.2	Favored (75.7%) *m-40* chi angles: 300.5,303	0.05Å	Favored (18.802%)	-	-	-
A 466		PRO	1.64	-	Favored (60.52%) Trans-Pro / -65.3,-18.4	Favored (43.5%) *Cg_endo* chi angles: 24.3,326.2,28.8	0.04Å	Favored (66.832%)	-	-	-
A 467		SER	1.74	-	Favored (45.79%) General / -82.3,-0.4	Favored (95.8%) *p* chi angles: 63.8	0.03Å	Favored (41.976%)	-	-	-
A 468		GLN	1.73	-	Favored (49.17%) General / -124.7,146.4	Favored (17.3%) *mt0* chi angles: 297.7,177.6,179.5	0.04Å	Favored (12.373%)	-	-	-
A 469		VAL	1.6	-	Favored (2.28%) Ile or Val / -124.7,-53.2	Favored (88%) *t* chi angles: 176.1	0.04Å	CaBLAM Disfavored (1.494%)	-	-	-
A 470		GLY	1.4	-	Favored (4.14%) Glycine / -78.7,54.8	-	-	CaBLAM Disfavored (1.555%)	-	-	-
A 471		ASP	1.18	-	Favored (23.37%) General / -99.4,149.3	Favored (53.3%) *m-30* chi angles: 293.1,359.5	0.04Å	Favored (28.364%)	-	-	-
A 472		GLU	0.99	-	Favored (50.68%) General / -130.8,143.4	Favored (92.6%) *mt-10* chi angles: 299.6,175.7,348.8	0.05Å	Favored (69.965%) beta sheet	-	-	-
A 473		TYR	0.85	-	Favored (28.73%) General / -123.2,118.9	Favored (83.8%) *t80* chi angles: 172.5,75.8	0.03Å	Favored (69.395%) beta sheet	-	-	-
A 474		CYS	0.76	-	Favored (41.15%) General / -110.9,143.7	Favored (75.2%) *m* chi angles: 296.9	0.07Å	Favored (60.375%) beta sheet	-	-	-
A 475		TYR	0.71	-	Favored (44.26%) General / -141.3,152.2	Favored (55.2%) *p90* chi angles: 60.5,92	0.10Å	Favored (66.787%)	-	-	-
A 476		GLY	0.7	-	Favored (86.37%) Glycine / -86.5,-0.3	-	-	Favored (36.29%)	-	-	-
A 477		GLY	0.71	-	Favored (31.93%)	-	-	Favored (15.599%)	-	-	-

#	Alt	Res	High B	Clash > 0.4Å	Ramachandran	Rotamer	Cβ deviation	CaBLAM	Bond lengths	Bond angles	Cis Peptides
					Glycine / 154.1,-166.1						
A 478		HIS	0.73	-	Favored (12.42%) General / -72.4,171.3	Favored (40.3%) *p90* chi angles: 68.1,82.9	0.02Å	CaBLAM Disfavored (2.13%)	-	-	-
A 479		THR	0.76	-	Favored (5.56%) General / -107.0,175.3	Favored (40.1%) *p* chi angles: 67.8	0.04Å	Favored (42.179%)	-	-	-
A 480		ASN	0.79	-	Favored (3.66%) General / -151.3,109.1	Favored (46.8%) *t0* chi angles: 186.7,1.7	0.05Å	Favored (11.045%) beta sheet	-	-	-
**#**	**Alt**	**Res**	**High B**	**Clash > 0.4Å**	**Ramachandran**	**Rotamer**	**Cβ deviation**	**CaBLAM**	**Bond lengths**	**Bond angles**	**Cis Peptides**
			Avg: 1.12	Clashscore: 1.76	Outliers: 4 of 617	Poor rotamers: 1 of 514	Outliers: 0 of 563	Outliers: 19 of 615	Outliers: 6 of 619	Outliers: 10 of 619	Non-Trans: 4 of 618
A 481		GLU	0.83	-	Favored (58.94%) General / -87.3,-2.9	Favored (13%) *mp0* chi angles: 290.7,76.4,51.8	0.05Å	Favored (16.482%)	-	-	-
A 482		ASP	0.86	-	Favored (7.28%) General / -87.9,87.2	Favored (65.6%) *t0* chi angles: 184.9,345.1	0.06Å	Favored (10.585%)	-	-	-
A 483		ASP	0.88	-	Favored (8.75%) General / -100.0,24.3	Favored (33.1%) *p0* chi angles: 60.2,340.8	0.07Å	Favored (5.368%)	-	-	-
A 484		SER	0.88	-	Favored (33.69%) General / -66.3,156.7	Favored (94.1%) *p* chi angles: 66.3	0.03Å	Favored (13.021%)	-	-	-
A 485		ASN	0.86	-	Favored (5.97%) General / 67.1,8.2	Favored (86.3%) *m-40* chi angles: 298.7,318.4	0.06Å	Favored (6.047%)	-	-	-
A 486		PHE	0.82	-	Favored (45.44%) General / -72.6,147.8	Favored (6.1%) *m-10* chi angles: 287.6,16	0.08Å	Favored (38.29%)	-	-	-
A 487		ALA	0.77	-	Favored (68.58%) General / -59.5,-30.5	-	0.04Å	Favored (54.102%)	-	-	-
A 488		HIS	0.72	-	Favored (72.06%) General / -61.6,-32.0	Favored (58.7%) *m170* chi angles: 290.5,170.1	0.07Å	Favored (65.321%) alpha helix	-	-	-
A 489		TRP	0.68	-	Favored (61.1%) General / -75.3,-36.3	Favored (85.1%) *m100* chi angles: 287.8,112.4	0.07Å	Favored (87.18%) alpha helix	-	-	-
A 490		THR	0.65	-	Favored (79.89%) General / -66.7,-45.1	Favored (90.2%) *m* chi angles: 298.1	0.03Å	Favored (85.491%) alpha helix	-	-	-
A 491		GLU	0.63	-	Favored (90.46%) General / -63.1,-38.3	Favored (66%) *mt-10* chi angles: 288.2,180.9,318.6	0.04Å	Favored (84.318%) alpha helix	-	-	-
A 492		ALA	0.61	-	Favored (99.16%) General / -62.0,-43.3	-	0.09Å	Favored (88.545%) alpha helix	-	-	-
A 493		ARG	0.6	0.53Å NH1 with A	Favored (94.17%)	Favored (21.9%) *tpp-160*	0.01Å	Favored (94.942%) alpha helix	-	-	-

				521 ASP OD1	General / -61.6,-40.2	chi angles: 177.8,68.5,74.4,192.9						
A 494		ILE	0.59	-	Favored (95.37%) Ile or Val / -61.7,-47.0	Favored (89.6%) *mt* chi angles: 291,167.3	0.02Å	Favored (89.385%) alpha helix	-	-	-
A 495		MET	0.59	-	Favored (80.33%) General / -67.2,-35.8	Favored (77.8%) *mtm* chi angles: 289.4,190.9,289	0.11Å	Favored (86.327%) alpha helix	-	-	-
A 496		LEU	0.61	-	Favored (98.35%) General / -62.1,-41.5	Favored (76.5%) *mt* chi angles: 288,170.6	0.09Å	Favored (87.246%) alpha helix	-	-	-
A 497		ASP	0.64	-	Favored (71.51%) General / -62.3,-31.0	Favored (94.6%) *m-30* chi angles: 286.3,349.4	0.05Å	Favored (75.664%) alpha helix	-	-	-
A 498		ASN	0.7	-	Favored (31.49%) General / -94.3,11.1	Favored (83.1%) *m-40* chi angles: 289.4,320.8	0.09Å	Favored (38.622%)	-	-	-
A 499		ILE	0.79	-	Favored (32.84%) Ile or Val / -79.2,132.1	Favored (93.8%) *mt* chi angles: 293.5,171.5	0.10Å	Favored (31.787%)	-	-	-
A 500		ASN	0.91	-	Favored (6.38%) General / -80.1,69.1	Favored (80%) *m-40* chi angles: 290.9,314.7	0.04Å	Favored (32.531%)	-	-	-
**#**	**Alt**	**Res**	**High B**	**Clash > 0.4Å**	**Ramachandran**	**Rotamer**	**Cβ deviation**	**CaBLAM**	**Bond lengths**	**Bond angles**	**Cis Peptides**
			Avg: 1.12	Clashscore: 1.76	Outliers: 4 of 617	Poor rotamers: 1 of 514	Outliers: 0 of 563	Outliers: 19 of 615	Outliers: 6 of 619	Outliers: 10 of 619	Non-Trans: 4 of 618
A 501		MET	1.03	-	Favored (80.47%) Pre-Pro / -77.5,151.3	Favored (88.3%) *mtp* chi angles: 292.3,185,71.3	0.05Å	Favored (18.505%)	-	-	-
A 502		PRO	1.14	-	Favored (87.42%) Trans-Pro / -64.8,150.9	Favored (36.3%) *Cg_endo* chi angles: 22.6,326.7,29.9	0.02Å	Favored (35.545%)	-	-	-
A 503		ASN	1.19	-	Favored (5.63%) General / 64.0,10.9	Favored (87.6%) *m-40* chi angles: 297.9,320.1	0.04Å	CaBLAM Disfavored (1.563%)	-	-	-
A 504		GLY	1.17	-	Favored (80.89%) Glycine / 80.9,13.7	-	-	Favored (50.502%)	-	-	-
A 505		LEU	1.1	-	Favored (52.22%) General / -66.0,148.2	Favored (92.7%) *mt* chi angles: 295.4,171.9	0.06Å	Favored (22.473%) beta sheet	-	-	-
A 506		VAL	1.01	-	Favored (66.68%) Ile or Val / -109.9,124.5	Favored (72.9%) *t* chi angles: 178.5	0.02Å	Favored (37.299%) beta sheet	-	-	-
A 507		ALA	0.93	-	Favored (56.39%) General / -61.9,143.5	-	0.03Å	Favored (29.888%)	-	-	-
A 508		GLN	0.89	-	Favored (34.08%) General / -118.0,153.2	Favored (51.2%) *mt0* chi angles: 300.9,182.8,282.9	0.07Å	Favored (31.164%)	-	-	-
A 509		LEU	0.88	-	Favored (51.58%) General / -64.0,147.7	Favored (83.3%) *mt* chi angles: 289.6,170.1	0.03Å	Favored (36.539%)	-	-	-

#	Alt	Res	High B	Clash > 0.4Å	Ramachandran	Rotamer	Cβ deviation	CaBLAM	Bond lengths	Bond angles	Cis Peptides
A 510		TYR	0.9	-	Favored (57.31%) General / -62.8,135.4	Favored (34.8%) *t80* chi angles: 177.2,56.3	0.10Å	Favored (31.436%)	-	-	-
A 511		GLN	0.93	-	OUTLIER (0.03)%) Pre-Pro / -20.0,-71.3	Favored (66.5%) *tt0* chi angles: 179.1,179.7,358.2	0.16Å	Favored (28.49%)	-	-	-
A 512		PRO	0.96	-	Favored (49.72%) Trans-Pro / -62.9,-15.8	Favored (41.9%) *Cg_endo* chi angles: 23.9,324.3,32.6	0.04Å	Favored (14.711%) alpha helix	-	-	-
A 513		GLU	0.97	-	Favored (17.97%) General / -95.7,-21.8	Favored (38.5%) *mt-10* chi angles: 302.3,180.2,57.6	0.08Å	Favored (40.188%) alpha helix	-	-	-
A 514		ARG	0.97	-	Favored (76.41%) General / -63.4,-33.8	Favored (98.1%) *mtt180* chi angles: 291.2,178,184.4,173	0.12Å	Favored (33.49%) three-ten	-	-	-
A 515		GLU	0.96	-	Favored (63.83%) General / -62.8,-19.3	Favored (98.7%) *mt-10* chi angles: 292.8,180.9,356.6	0.01Å	Favored (61.346%) three-ten	-	-	-
A 516		LYS	0.93	-	Favored (51.33%) General / -93.0,-5.1	Favored (20.8%) *mmtp* chi angles: 299.8,291.7,181.5,58.4	0.04Å	Favored (62.665%)	-	-	-
A 517		VAL	0.91	-	Favored (72.93%) Ile or Val / -122.9,131.6	Favored (80.9%) *t* chi angles: 176.6	0.11Å	Favored (28.561%)	-	-	-
A 518		TYR	0.87	-	Favored (29.06%) General / -104.4,15.0	Favored (90.7%) *m-80* chi angles: 298.7,102.7	0.05Å	Favored (9.565%)	-	-	-
A 519		THR	0.84	-	Favored (49.65%) General / -105.3,133.7	Favored (92.9%) *m* chi angles: 299.2	0.05Å	Favored (28.716%)	-	-	-
A 520		MET	0.79	-	Favored (40.19%) General / -74.1,151.2	Favored (78.5%) *mtm* chi angles: 295.1,180.7,290.1	0.01Å	Favored (38.108%)	-	-	-
**#**	**Alt**	**Res**	**High B**	**Clash > 0.4Å**	**Ramachandran**	**Rotamer**	**Cβ deviation**	**CaBLAM**	**Bond lengths**	**Bond angles**	**Cis Peptides**
			Avg: 1.12	Clashscore: 1.76	Outliers: 4 of 617	Poor rotamers: 1 of 514	Outliers: 0 of 563	Outliers: 19 of 615	Outliers: 6 of 619	Outliers: 10 of 619	Non-Trans: 4 of 618
A 521		ASP	0.75	0.53Å OD1 with A 493 ARG NH1	Favored (40.34%) General / -58.2,129.1	Favored (53.6%) *m-30* chi angles: 296.7,297.2	0.10Å	Favored (29.479%)	-	-	-
A 522		GLY	0.72	-	Favored (74.49%) Glycine / 93.5,-9.0	-	-	Favored (84.19%)	-	-	-
A 523		GLU	0.7	-	Favored (67.53%) General / -62.9,-23.9	Favored (36%) *mt-10* chi angles: 289.1,190.2,49.7	0.12Å	Favored (31.251%)	-	-	-
A 524		TYR	0.69	-	Favored (37.38%) General / -103.3,10.6	Favored (42.1%) *m-80* chi angles: 292.4,121.7	0.05Å	Favored (15.582%)	-	-	-
A 525		ARG	0.69	-	Favored (46.72%) General / -56.1,131.1	Favored (87.9%) *mtm180* chi angles: 287.5,174.8,289.7,177.2	0.04Å	Favored (22.975%)	-	-	-

A 526		LEU	0.69	-	Favored (17.51%) General / -98.6,155.0	Favored (95.6%) *mt* chi angles: 298,177	0.02Å	Favored (36.36%)	-	-	-
A 527		ARG	0.69	-	Favored (51.13%) General / -128.8,144.3	Favored (91%) *mtm180* chi angles: 294.9,176.2,292.5,172	0.06Å	Favored (5.215%)	-	-	-
A 528		GLY	0.69	-	Favored (33.17%) Glycine / 58.1,-123.5	-	-	Favored (26.744%)	-	-	-
A 529		GLU	0.69	-	Favored (69.14%) General / -61.7,-28.1	Favored (75.1%) *mm-30* chi angles: 295.2,297.7,306.3	0.03Å	Favored (15.997%)	-	-	-
A 530		GLU	0.68	-	Favored (79.01%) General / -62.7,-35.2	Favored (98.5%) *mt-10* chi angles: 292.1,171.5,344.1	0.04Å	Favored (65.905%) three-ten	-	-	-
A 531		ARG	0.67	-	Favored (65.76%) General / -73.4,-38.0	Favored (97.8%) *mtt180* chi angles: 292.1,178,184,182.5	0.10Å	Favored (88.04%) alpha helix	-	-	-
A 532		LYS	0.66	-	Favored (93.18%) General / -65.4,-41.8	Favored (98%) *mttt* chi angles: 291.2,178.9,183.9,175.8	0.06Å	Favored (96.84%) alpha helix	-	-	-
A 533		ASN	0.66	-	Favored (88.74%) General / -66.5,-41.4	Favored (96.8%) *m-40* chi angles: 288.9,335.1	0.05Å	Favored (89.478%) alpha helix	-	-	-
A 534		PHE	0.67	-	Favored (69.46%) General / -57.3,-51.4	Favored (77.4%) *t80* chi angles: 172.7,72.3	0.06Å	Favored (87.428%) alpha helix	-	OUTLIER(S) worst is CA-CB-CG: 4.5 σ	-
A 535		LEU	0.69	-	Favored (81.07%) General / -65.6,-35.5	Favored (91.1%) *mt* chi angles: 292.4,175.5	0.06Å	Favored (77.458%) alpha helix	-	-	-
A 536		GLU	0.73	-	Favored (80.81%) General / -68.5,-38.2	Favored (49.4%) *tp30* chi angles: 191.1,65.9,16.8	0.02Å	Favored (89.364%) alpha helix	-	-	-
A 537		PHE	0.77	-	Favored (86.99%) General / -61.4,-38.4	Favored (16.7%) *m-80* chi angles: 279.1,124.6	0.04Å	Favored (77.67%) alpha helix	-	-	-
A 538		LEU	0.82	-	Favored (48.89%) General / -77.6,-38.0	Favored (91.1%) *mt* chi angles: 294.6,177.5	0.06Å	Favored (66.666%) alpha helix	-	-	-
A 539		ARG	0.86	-	Favored (20.14%) General / -89.1,-28.4	Favored (89.3%) *mtm180* chi angles: 295.1,177.7,296.8,171.4	0.03Å	Favored (55.281%) alpha helix	-	-	-
A 540		THR	0.88	-	Favored (3.53%) General / -107.0,-48.2	Favored (99.8%) *m* chi angles: 300.5	0.03Å	Favored (26.753%) alpha helix	-	-	-
**#**	**Alt**	**Res**	**High B**	**Clash > 0.4Å**	**Ramachandran**	**Rotamer**	**Cβ deviation**	**CaBLAM**	**Bond lengths**	**Bond angles**	**Cis Peptides**
			Avg: 1.12	Clashscore: 1.76	Outliers: 4 of 617	Poor rotamers: 1 of 514	Outliers: 0 of 563	Outliers: 19 of 615	Outliers: 6 of 619	Outliers: 10 of 619	Non-Trans: 4 of 618
A 541		ALA	0.88	-	Favored (59.37%) General / -80.3,-8.2	-	0.03Å	Favored (33.828%)	-	-	-

A 542		ASP	0.85	-	Favored (30.03%) General / 54.7,43.5	Favored (22.6%) *t0* chi angles: 198.9,31	0.02Å	Favored (17.586%)	-	-	-
A 543		LEU	0.8	-	Favored (51.09%) Pre-Pro / -88.5,154.7	Favored (79.9%) *mt* chi angles: 300.7,179.7	0.01Å	Favored (22.271%) beta sheet	-	-	-
A 544		PRO	0.75	-	Favored (58.11%) Trans-Pro / -68.8,158.4	Favored (45.8%) *Cg_endo* chi angles: 24.7,326.9,27.3	0.06Å	Favored (85.48%)	-	-	-
A 545		VAL	0.71	-	Favored (95.93%) Ile or Val / -60.4,-44.4	Favored (57.6%) *t* chi angles: 170.4	0.04Å	Favored (66.849%)	-	-	-
A 546		TRP	0.68	-	Favored (94.04%) General / -60.8,-45.5	Favored (90.8%) *t60* chi angles: 183.2,89.1	0.01Å	Favored (81.453%) alpha helix	-	-	-
A 547		LEU	0.65	-	Favored (71.78%) General / -67.5,-46.9	Favored (59.3%) *tp* chi angles: 181.4,60	0.01Å	Favored (77.585%) alpha helix	-	-	-
A 548		ALA	0.63	-	Favored (77.15%) General / -58.1,-38.8	-	0.07Å	Favored (87.838%) alpha helix	-	-	-
A 549		TYR	0.61	-	Favored (69.44%) General / -60.4,-51.5	Favored (91.3%) *t80* chi angles: 178.7,78.7	0.06Å	Favored (89.589%) alpha helix	-	-	-
A 550		LYS	0.6	-	Favored (80.62%) General / -65.4,-35.3	Favored (66.6%) *mmtt* chi angles: 289.2,292.2,182.5,178.5	0.03Å	Favored (78.773%) alpha helix	-	-	-
A 551		VAL	0.6	-	Favored (70.35%) Ile or Val / -69.4,-47.9	Favored (97.2%) *t* chi angles: 175.1	0.09Å	Favored (74.343%) alpha helix	-	-	-
A 552		ALA	0.6	-	Favored (99.05%) General / -62.8,-41.0	-	0.05Å	Favored (77.505%) alpha helix	-	-	-
A 553		ALA	0.6	-	Favored (71.61%) General / -61.3,-31.8	-	0.02Å	Favored (73.961%) alpha helix	-	-	-
A 554		ALA	0.61	-	Favored (40.2%) General / -78.8,-2.6	-	0.04Å	Favored (55.056%)	-	-	-
A 555		GLY	0.62	-	Favored (65.28%) Glycine / 83.4,18.0	-	-	Favored (86.414%)	-	-	-
A 556		ILE	0.63	-	Favored (41.43%) Ile or Val / -87.9,125.1	Favored (85.2%) *mt* chi angles: 298.9,169.2	0.07Å	Favored (29.877%)	-	-	-
A 557		SER	0.65	-	Favored (26.89%) General / -75.5,163.9	Favored (88.4%) *p* chi angles: 67	0.02Å	Favored (46.019%)	-	-	-
A 558		TYR	0.66	-	Favored (60.93%) General / -51.0,-44.6	Favored (82.3%) *t80* chi angles: 182.7,83.2	0.07Å	Favored (43.848%)	-	-	-
A 559		HIS	0.67	-	Favored (58.3%) General / -86.3,-2.3	Favored (85.3%) *m-70* chi angles: 289.8,290.4	0.01Å	Favored (49.167%)	-	-	-

#	Alt	Res	High B	Clash > 0.4Å	Ramachandran	Rotamer	Cβ deviation	CaBLAM	Bond lengths	Bond angles	Cis Peptides
A 560		ASP	0.67	-	Favored (9.6%) General / -85.4,96.5	Favored (57.3%) *t0* chi angles: 180.9,344	0.04Å	Favored (17.786%)	-	-	-
#	Alt	Res	High B	Clash > 0.4Å	Ramachandran	Rotamer	Cβ deviation	CaBLAM	Bond lengths	Bond angles	Cis Peptides
			Avg: 1.12	Clashscore: 1.76	Outliers: 4 of 617	Poor rotamers: 1 of 514	Outliers: 0 of 563	Outliers: 19 of 615	Outliers: 6 of 619	Outliers: 10 of 619	Non-Trans: 4 of 618
A 561		ARG	0.66	-	Favored (45.02%) General / -92.2,6.9	Favored (72.7%) *mtt90* chi angles: 292.3,166,173,87.2	0.05Å	Favored (12.626%)	-	-	-
A 562		LYS	0.65	-	Favored (75.79%) General / -61.6,-34.5	Favored (97.9%) *mttt* chi angles: 290.6,177.5,181.8,177.1	0.02Å	Favored (28.323%)	-	-	-
A 563		TRP	0.64	-	Favored (43.49%) General / -57.6,-22.2	Favored (75.8%) *p-90* chi angles: 67.5,268.6	0.06Å	Favored (63.631%) three-ten	-	-	-
A 564		CYS	0.63	-	Favored (54.75%) General / -79.0,-17.9	Favored (81.3%) *m* chi angles: 294.4	0.10Å	Favored (52.044%) alpha helix	-	-	-
A 565		PHE	0.64	-	Favored (6.04%) General / -119.2,-25.9	Favored (82.5%) *m-80* chi angles: 299.9,105.3	0.05Å	Favored (22.171%)	-	-	-
A 566		ASP	0.67	-	Favored (7.29%) General / -99.5,25.2	Favored (72.4%) *m-30* chi angles: 292.7,319.2	0.04Å	Favored (5.684%)	-	-	-
A 567		GLY	0.7	-	Favored (47.05%) Glycine / -88.7,-175.9	-	-	Favored (34.646%)	-	-	-
A 568		PRO	0.73	-	Favored (78.24%) Trans-Pro / -59.0,149.6	Favored (51.3%) *Cg_exo* chi angles: 336.5,34,329.7	0.04Å	Favored (26.655%)	-	-	-
A 569		ARG	0.75	-	Favored (68.42%) General / -62.2,-26.5	Favored (98.6%) *mtt180* chi angles: 289.9,177.9,181.1,175	0.02Å	Favored (53.058%)	-	-	-
A 570		THR	0.77	-	Favored (61.26%) General / -71.2,-12.0	Favored (77.4%) *p* chi angles: 61	0.04Å	Favored (57.667%)	-	-	-
A 571		ASN	0.79	-	Favored (45.33%) General / -96.4,7.9	Favored (98.8%) *m-40* chi angles: 289.7,341	0.07Å	Favored (24.344%)	-	-	-
A 572		THR	0.82	-	Favored (45.97%) General / -55.0,132.0	Favored (76.7%) *m* chi angles: 302.7	0.06Å	Favored (37.438%)	-	-	-
A 573		ILE	0.88	-	Favored (41.49%) Ile or Val / -88.1,126.9	Favored (84.4%) *mt* chi angles: 299.2,170.1	0.04Å	Favored (58.424%) beta sheet	-	-	-
A 574		LEU	0.96	-	Favored (46.63%) General / -121.1,146.5	Favored (2.4%) *mp* chi angles: 292.1,97.3	0.06Å	Favored (68.203%)	-	-	-
A 575		GLU	1.02	-	Allowed (1.24%) General / -113.0,-167.8	Favored (24.8%) *pt0* chi angles: 61.8,183.4,6.4	0.08Å	CaBLAM Disfavored (1.022%)	-	-	-

#	Alt	Res	High B	Clash > 0.4Å	Ramachandran	Rotamer	Cβ deviation	CaBLAM	Bond lengths	Bond angles	Cis Peptides
A 576		ASP	1.05	-	OUTLIER (0.04)% General / -59.6,96.8	Favored (61.7%) *t0* chi angles: 185.9,356.3	0.07Å	CaBLAM Outlier (0.164%)	-	-	-
A 577		ASN	1.02	-	Favored (20.68%) General / 58.2,31.4	Favored (75%) *m-40* chi angles: 292.2,306.9	0.06Å	CaBLAM Disfavored (1.611%)	-	OUTLIER(S) worst is C-N-CA: 4.1 σ	-
A 578		ASN	0.94	-	Favored (28.61%) General / -147.1,143.9	Favored (56.2%) *m-40* chi angles: 287,275.1	0.03Å	Favored (14.645%)	-	-	-
A 579		GLU	0.84	-	Favored (48.2%) General / -72.3,144.8	Favored (55.4%) *mt-10* chi angles: 293.8,180.2,291.5	0.03Å	Favored (41.155%) beta sheet	-	-	-
A 580		VAL	0.76	-	Favored (37.47%) Ile or Val / -79.4,124.3	Favored (92.6%) *t* chi angles: 175.9	0.01Å	Favored (42.058%) beta sheet	-	-	-
**#**	**Alt**	**Res**	**High B**	**Clash > 0.4Å**	**Ramachandran**	**Rotamer**	**Cβ deviation**	**CaBLAM**	**Bond lengths**	**Bond angles**	**Cis Peptides**
			Avg: 1.12	Clashscore: 1.76	Outliers: 4 of 617	Poor rotamers: 1 of 514	Outliers: 0 of 563	Outliers: 19 of 615	Outliers: 6 of 619	Outliers: 10 of 619	Non-Trans: 4 of 618
A 581		GLU	0.72	-	Favored (43.75%) General / -117.4,145.9	Favored (58.6%) *mt-10* chi angles: 294.2,181.3,299.4	0.03Å	Favored (61.526%) beta sheet	-	-	-
A 582		VAL	0.73	-	Favored (71.04%) Ile or Val / -122.6,132.5	Favored (99.3%) *t* chi angles: 175.4	0.09Å	Favored (63.978%) beta sheet	-	-	-
A 583		ILE	0.8	-	Favored (45.84%) Ile or Val / -98.0,119.3	Favored (82.4%) *mt* chi angles: 299.7,171.2	0.05Å	Favored (58.39%)	-	-	-
A 584		THR	0.91	-	Favored (19.1%) General / -80.3,168.4	Favored (71.2%) *p* chi angles: 62.1	0.07Å	Favored (37.133%)	-	-	-
A 585		LYS	1.01	-	Favored (20.48%) General / -56.9,-20.0	Favored (59.6%) *pttt* chi angles: 68.1,181,181.9,180	0.03Å	Favored (35.15%)	-	-	-
A 586		LEU	1.07	-	Favored (57.75%) General / -91.1,-1.6	Favored (90.1%) *mt* chi angles: 299.5,177.8	0.03Å	Favored (50.079%)	-	-	-
A 587		GLY	1.05	-	Favored (79.08%) Glycine / 91.4,-8.5	-	-	Favored (74.113%)	-	-	-
A 588		GLU	0.96	-	Favored (34.41%) General / -80.9,129.2	Favored (87.3%) *tt0* chi angles: 183.3,176.7,350.4	0.04Å	Favored (30.443%)	-	-	-
A 589		ARG	0.85	-	Favored (35.61%) General / -90.3,125.8	Favored (58.6%) *ttt90* chi angles: 187.4,175.7,179.1,93.6	0.01Å	Favored (55.939%) beta sheet	-	-	-
A 590		LYS	0.74	-	Favored (52.57%) General / -131.8,149.1	Favored (62.2%) *mttm* chi angles: 294.9,181.6,180.4,291.7	0.04Å	Favored (52.449%) beta sheet	-	-	-
A 591		ILE	0.66	-	Favored (34.1%) Ile or Val / -83.6,131.3	Favored (90.5%) *mt* chi angles: 297.7,168.4	0.01Å	Favored (36.698%) beta sheet	-	-	-
A 592		LEU	0.62	-	Favored (20.62%)	Favored (83.5%) *mt* chi angles: 290,173.1	0.04Å	Favored (21.31%)	-	-	-

#	Alt	Res	High B	Clash > 0.4Å	Ramachandran	Rotamer	Cβ deviation	CaBLAM	Bond lengths	Bond angles	Cis Peptides
					General / -75.4,120.2			beta sheet			
A 593		ARG	0.61	-	Favored (20.25%) Pre-Pro / -142.6,73.1	Favored (91.5%) *mtt180* chi angles: 291.4,173.9,175.3,158.8	0.03Å	Favored (6.264%) beta sheet	-	-	-
A 594		PRO	0.63	-	Favored (83.42%) Trans-Pro / -66.8,150.0	Favored (43.1%) *Cg_endo* chi angles: 24.2,327,27.7	0.02Å	Favored (42.341%)	-	-	-
A 595		ARG	0.66	-	Favored (72.13%) General / -60.8,-32.7	Favored (97.2%) *mtt180* chi angles: 287.9,179.1,182.4,178.9	0.04Å	Favored (25.402%)	-	-	-
A 596		TRP	0.7	-	Favored (44.91%) General / -130.6,131.3	Favored (23.8%) *m-90* chi angles: 303.7,267.4	0.12Å	Favored (30.833%)	-	-	-
A 597		ALA	0.74	-	Favored (26%) General / -104.8,149.0	-	0.03Å	Favored (56.688%)	-	-	-
A 598		ASP	0.8	-	Favored (5.06%) General / -140.2,105.5	Favored (63.1%) *t0* chi angles: 183.7,342.3	0.04Å	Favored (30.187%) beta sheet	-	-	-
A 599		ALA	0.86	-	Favored (60.1%) General / -58.9,-22.5	-	0.08Å	Favored (29.269%)	-	-	-
A 600		ARG	0.95	-	Favored (66.17%) General / -56.8,-32.9	Favored (83.3%) *ttt180* chi angles: 184.3,173.9,175.4,174.8	0.02Å	Favored (57.924%)	-	-	-
**#**	**Alt**	**Res**	**High B**	**Clash > 0.4Å**	**Ramachandran**	**Rotamer**	**Cβ deviation**	**CaBLAM**	**Bond lengths**	**Bond angles**	**Cis Peptides**
			Avg: 1.12	Clashscore: 1.76	Outliers: 4 of 617	Poor rotamers: 1 of 514	Outliers: 0 of 563	Outliers: 19 of 615	Outliers: 6 of 619	Outliers: 10 of 619	Non-Trans: 4 of 618
A 601		VAL	1.05	-	Favored (62.75%) Ile or Val / -60.5,-35.4	Favored (59.1%) *t* chi angles: 170.7	0.04Å	Favored (71.381%) alpha helix	-	-	-
A 602		TYR	1.17	-	Favored (25.88%) General / -87.3,-21.2	Favored (80.5%) *t80* chi angles: 183.5,77.8	0.06Å	Favored (41.978%) alpha helix	-	-	-
A 603		SER	1.28	-	Favored (62.26%) General / -61.6,-19.4	Favored (72.3%) *p* chi angles: 71.7	0.09Å	Favored (18.719%) alpha helix	-	-	-
A 604		ASP	1.35	-	Favored (12.63%) General / -140.4,119.3	Favored (41.2%) *t0* chi angles: 185.5,328	0.03Å	Favored (14.889%) alpha helix	-	-	-
A 605		HIS	1.36	-	Favored (19.76%) General / -54.7,-23.9	Favored (57.5%) *p-80* chi angles: 69.2,281.8	0.01Å	Favored (33.092%) alpha helix	-	-	-
A 606		GLN	1.31	-	Favored (65.78%) General / -72.6,-32.1	Favored (93.9%) *mm-40* chi angles: 291.6,293.5,312.6	0.02Å	Favored (75.39%) alpha helix	-	-	-
A 607		ALA	1.2	-	Favored (54.04%) General / -77.1,-37.6	-	0.02Å	Favored (76.878%) alpha helix	-	-	-
A 608		LEU	1.07	-	Favored (86.85%)	Favored (63%) *tp* chi angles: 180.1,59.8	0.05Å	Favored (95.365%)	-	-	-

					General / -65.2,-44.9			alpha helix			
A 609		LYS	0.94	-	Favored (95.31%) General / -60.1,-43.8	Favored (18.6%) *tptp* chi angles: 178.8,66.9,165.5,70.2	0.04Å	Favored (99.367%) alpha helix	-	-	-
A 610		SER	0.82	-	Favored (97.25%) General / -61.5,-41.4	Favored (72.8%) *m* chi angles: 295.7	0.04Å	Favored (99.207%) alpha helix	-	-	-
A 611		PHE	0.74	-	Favored (87.64%) General / -61.1,-47.1	Favored (76.8%) *t80* chi angles: 184.3,80.4	0.01Å	Favored (99.036%) alpha helix	-	-	-
A 612		LYS	0.68	-	Favored (97.97%) General / -61.7,-41.7	Favored (51.6%) *mtpt* chi angles: 287.9,174,72.1,178.7	0.05Å	Favored (98.292%) alpha helix	-	-	-
A 613		ASP	0.64	-	Favored (95.43%) General / -64.2,-40.1	Favored (97.6%) *m-30* chi angles: 287,345.9	0.03Å	Favored (97.714%) alpha helix	-	-	-
A 614		PHE	0.64	-	Favored (62.28%) General / -59.2,-53.1	Favored (72%) *t80* chi angles: 170.2,75.9	0.05Å	Favored (77.489%) alpha helix	-	-	-
A 615		ALA	0.67	-	Favored (70.88%) General / -58.9,-33.8	-	0.04Å	Favored (72.841%) alpha helix	-	-	-
A 616		SER	0.73	-	Favored (61.51%) General / -72.9,-14.7	Favored (89.2%) *p* chi angles: 69.1	0.05Å	Favored (45.363%)	-	-	-
A 617		GLY	0.84	-	Favored (52.34%) Glycine / 87.2,20.6	-	-	Favored (80.185%)	-	-	-
A 618		LYS	0.98	-	Favored (58.65%) General / -85.7,-7.7	Favored (97.3%) *mttt* chi angles: 291.8,174.2,183.1,176.8	0.03Å	-	-	-	-
A 619		ARG	1.15	0.49Å HD3 with A 619 ARG OXT	-	OUTLIER (0.3%) chi angles: 58.4,93.1,71.5,198.4	0.11Å	-	-	-	-
