# Supplementary material for: Integrative proteome-wide structural analysis and high-throughput docking identify broad-spectrum antiviral scaffolds against Zika, Yellow Fever, West Nile, Saint Louis encephalitis, and Usutu viruses
Source: Front Cell Infect Microbiol. 2026 Apr 30;16:1723132. doi: 10.3389/fcimb.2026.1723132 (PMC13171538; doi:10.3389/fcimb.2026.1723132)
Supplement: Supplementary file 5 [file DataSheet5.zip › WNV/WNV_NS3/Mol_probity_Files/WNV_NS3_1FH-rama.pdf]

# MolProbity Ramachandran analysis

WNV_NS3_1FH.pdb, model 1

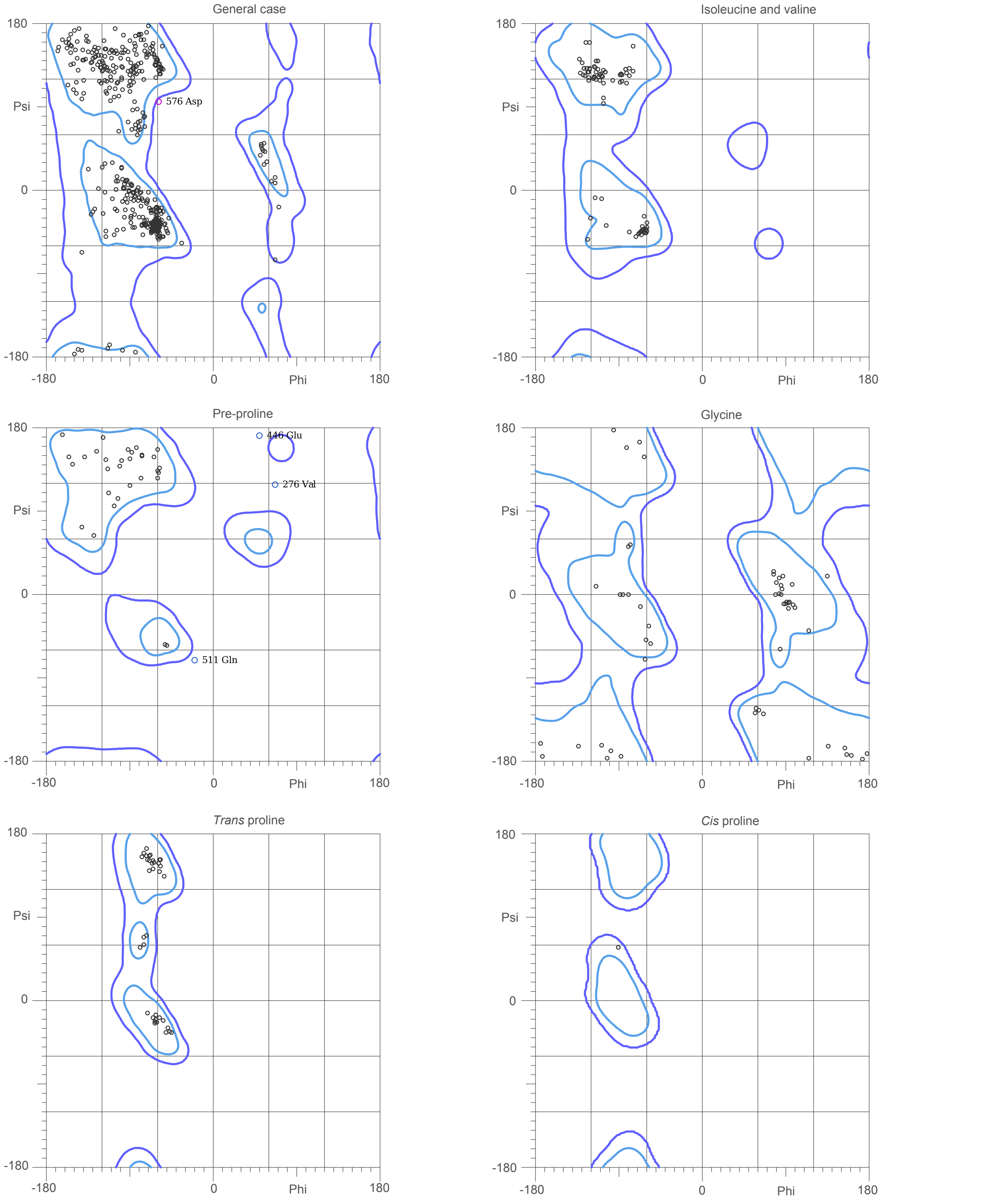


97.1% (599/617) of all residues were in favored (98%) regions.
99.4% (613/617) of all residues were in allowed (>99.8%) regions.

There were 4 outliers (phi, psi):
- 276 Val (67.9, 119.7)
- 446 Glu (51.0, 172.7)
- 511 Gln (-20.0, -71.3)
- 576 Asp (-59.6, 96.8)



Lovell, Davis, et al. Proteins 50:437 (2003)
