# Supplementary material for: Integrative proteome-wide structural analysis and high-throughput docking identify broad-spectrum antiviral scaffolds against Zika, Yellow Fever, West Nile, Saint Louis encephalitis, and Usutu viruses
Source: Front Cell Infect Microbiol. 2026 Apr 30;16:1723132. doi: 10.3389/fcimb.2026.1723132 (PMC13171538; doi:10.3389/fcimb.2026.1723132)
Supplement: Supplementary file 5 [file DataSheet5.zip › WNV/WNV_NS4a/Mol_probity_Files/WNV_NS4a_1FH-multi.table.pdf]

Viewing WNV_NS4a1FH-multi.table

Duke Biochemistry
Duke University School of Medicine

When finished, you should close this window.

Hint: Use File | Save As... to save a copy of this page.

All-Atom Contacts	Clashscore, all atoms:	1.52		99th percentile* (N=1784, all resolutions)
	Clashscore is the number of serious steric overlaps (> 0.4 Å) per 1000 atoms.			
Protein Geometry	Poor rotamers	0	0.00%	Goal: <0.3%
	Favored rotamers	100	100.00%	Goal: >98%
	Ramachandran outliers	1	0.81%	Goal: <0.05%
	Ramachandran favored	122	98.39%	Goal: >98%
	Rama distribution Z-score	2.26 ± 0.68		Goal: abs(Z score) < 2
	MolProbity score^	0.89		100th percentile* (N=27675, 0Å - 99Å)
	Cβ deviations >0.25Å	0	0.00%	Goal: 0
	Bad bonds:	0 / 972	0.00%	Goal: 0%
	Bad angles:	2 / 1311	0.15%	Goal: <0.1%
Peptide Omegas	Cis Prolines:	0 / 5	0.00%	Expected: ≤1 per chain, or ≤5%
Low-resolution Criteria	CaBLAM outliers	1	0.8%	Goal: <1.0%
	CA Geometry outliers	0	0.00%	Goal: <0.5%
Additional validations	Chiral volume outliers	0/157		
	Waters with clashes	0/0	0.00%	See UnDowser table for details

In the two column results, the left column gives the raw count, right column gives the percentage.

* 100th percentile is the best among structures of comparable resolution; 0th percentile is the worst. For clashscore the comparative set of structures was selected in 2004, for MolProbity score in 2006.

^ MolProbity score combines the clashscore, rotamer, and Ramachandran evaluations into a single score, normalized to be on the same scale as X-ray resolution.

Key to table colors and cutoffs here:

#	Alt	Res	High B	Clash > 0.4Å	Ramachandran	Rotamer	Cβ deviation	CaBLAM	Bond lengths	Bond angles	Cis Peptides
			Avg: 3.28	Clashscore: 1.52	Outliers: 1 of 124	Poor rotamers: 0 of 100	Outliers: 0 of 112	Outliers: 1 of 122	Outliers: 0 of 126	Outliers: 2 of 126	Non-Trans: 0 of 125
A 1		SER	11.15	-	-	Favored (73.4%) *m* chi angles: 295.5	0.02Å	-	-	-	-
A 2		GLN	10.98	-	Favored (75.29%) General / -61.6,-34.2	Favored (93.2%) *tp40* chi angles: 188.2,64.8,50.5	0.04Å	-	-	-	-
A 3		ILE	10.8	-	Favored (97.56%) Ile or Val / -61.3,-44.0	Favored (96.1%) *mt* chi angles: 292.1,168.2	0.01Å	Favored (78.258%)	-	-	-
A 4		GLY	10.6	-	Favored (98.57%) Glycine / -61.2,-40.4	-	-	Favored (96.971%) alpha helix	-	-	-
A 5		LEU	10.34	-	Favored (89.71%) General / -63.9,-38.1	Favored (96.6%) *mt* chi angles: 292.5,173.7	0.03Å	Favored (91.732%) alpha helix	-	-	-
A 6		VAL	9.95	-	Favored (96.81%)	Favored (62.4%) *t* chi angles: 171.1	0.04Å	Favored (93.923%)	-	-	-

#	Alt	Res	High B	Clash > 0.4Å	Ramachandran	Rotamer	Cβ deviation	CaBLAM	Bond lengths	Bond angles	Cis Peptides
					Ile or Val / -64.5,-43.9			alpha helix			
A 7		GLU	9.39	-	Favored (85.93%) General / -58.4,-41.9	Favored (91.5%) *tt0* chi angles: 181.1,175.2,357.1	0.04Å	Favored (97.875%) alpha helix	-	-	-
A 8		VAL	8.66	-	Favored (96.74%) Ile or Val / -61.8,-46.3	Favored (59.8%) *t* chi angles: 170.8	0.02Å	Favored (95.029%) alpha helix	-	-	-
A 9		LEU	7.76	-	Favored (86.82%) General / -63.6,-37.3	Favored (92.1%) *mt* chi angles: 291.5,171.2	0.04Å	Favored (85.489%) alpha helix	-	-	-
A 10		GLY	6.77	-	Favored (99.74%) Glycine / -63.1,-40.9	-	-	Favored (89.644%) alpha helix	-	-	-
A 11		ARG	5.77	-	Favored (35.26%) General / -81.3,-27.2	Favored (98%) *mtt-85* chi angles: 291.5,181.5,183.1,276.4	0.05Å	Favored (38.191%) alpha helix	-	-	-
A 12		MET	4.85	-	Favored (16.36%) Pre-Pro / -44.0,-52.0	Favored (53.8%) *ttm* chi angles: 183.5,178.9,296.6	0.20Å	Favored (52.709%) alpha helix	-	-	-
A 13		PRO	4.04	-	Favored (63.72%) Trans-Pro / -58.1,-23.9	Favored (74.1%) *Cg_exo* chi angles: 335.1,35.9,328.2	0.07Å	Favored (72.669%) alpha helix	-	-	-
A 14		GLU	3.37	-	Favored (44.21%) General / -78.3,-37.7	Favored (98.8%) *mt-10* chi angles: 293.4,179.2,349.4	0.02Å	Favored (74.327%) alpha helix	-	-	-
A 15		HIS	2.82	-	Favored (65.72%) General / -73.1,-39.5	Favored (88.8%) *m-70* chi angles: 291.6,293.1	0.03Å	Favored (77.878%) alpha helix	-	-	-
A 16		PHE	2.39	-	Favored (63.73%) General / -59.6,-52.9	Favored (85.4%) *t80* chi angles: 179.8,83.3	0.06Å	Favored (77.165%) alpha helix	-	-	-
A 17		MET	2.06	-	Favored (68.63%) General / -69.9,-31.1	Favored (78.7%) *mmm* chi angles: 289.9,309.6,301.5	0.07Å	Favored (73.694%) alpha helix	-	-	-
A 18		GLY	1.79	-	Favored (31.98%) Glycine / -58.4,-55.2	-	-	Favored (87.994%) alpha helix	-	-	-
A 19		LYS	1.59	-	Favored (85.64%) General / -61.8,-37.8	Favored (97.2%) *mttt* chi angles: 289.7,178.1,180.9,178.8	0.03Å	Favored (75.509%) alpha helix	-	-	-
A 20		THR	1.44	-	Favored (89.01%) General / -62.1,-46.5	Favored (88.4%) *m* chi angles: 298.5	0.04Å	Favored (88.643%) alpha helix	-	-	-
**#**	**Alt**	**Res**	**High B**	**Clash > 0.4Å**	**Ramachandran**	**Rotamer**	**Cβ deviation**	**CaBLAM**	**Bond lengths**	**Bond angles**	**Cis Peptides**
			Avg: 3.28	Clashscore: 1.52	Outliers: 1 of 124	Poor rotamers: 0 of 100	Outliers: 0 of 112	Outliers: 1 of 122	Outliers: 0 of 126	Outliers: 2 of 126	Non-Trans: 0 of 125
A 21		TRP	1.32	-	Favored (98.51%)	Favored (27.9%) *m-10* chi angles: 286.6,329.5	0.04Å	Favored (93.004%) alpha helix	-	-	-

					General / -63.5,-41.0						
A 22		GLU	1.23	-	Favored (93.93%) General / -62.9,-39.3	Favored (97.6%) *mt-10* chi angles: 289.3,178.6,355	0.02Å	Favored (95.38%) alpha helix	-	-	-
A 23		ALA	1.16	-	Favored (99.61%) General / -62.1,-43.1	-	0.03Å	Favored (96.609%) alpha helix	-	-	-
A 24		LEU	1.11	-	Favored (92.23%) General / -65.8,-40.7	Favored (93.4%) *mt* chi angles: 291.7,171.3	0.03Å	Favored (91.237%) alpha helix	-	-	-
A 25		ASP	1.05	-	Favored (83.82%) General / -67.7,-38.6	Favored (98.8%) *m-30* chi angles: 287.8,348.1	0.03Å	Favored (94.626%) alpha helix	-	-	-
A 26		THR	1	-	Favored (86.33%) General / -62.0,-47.1	Favored (94.6%) *m* chi angles: 299.4	0.05Å	Favored (87.321%) alpha helix	-	-	-
A 27		MET	0.96	-	Favored (86.23%) General / -66.1,-37.6	Favored (80.1%) *mtm* chi angles: 290.3,187.3,293.4	0.05Å	Favored (81.453%) alpha helix	-	-	-
A 28		TYR	0.92	-	Favored (76.35%) General / -58.1,-49.6	Favored (90.7%) *t80* chi angles: 178.2,80.6	0.02Å	Favored (85.042%) alpha helix	-	-	-
A 29		VAL	0.92	-	Favored (96.99%) Ile or Val / -62.2,-42.9	Favored (66%) *t* chi angles: 171.6	0.04Å	Favored (95.67%) alpha helix	-	-	-
A 30		VAL	1.04	-	Favored (87.76%) Ile or Val / -58.8,-47.9	Favored (62.6%) *t* chi angles: 171.2	0.08Å	Favored (82.598%) alpha helix	-	-	-
A 31		ALA	1.36	-	Favored (84.92%) General / -64.4,-36.7	-	0.03Å	Favored (48.452%) alpha helix	-	-	-
A 32		THR	2.06	-	Favored (12.03%) General / -114.3,-16.3	Favored (71.3%) *p* chi angles: 62.1	0.02Å	Favored (20.693%)	-	-	-
A 33		ALA	3.36	-	Favored (72.26%) General / -60.2,-33.4	-	0.06Å	CaBLAM Disfavored (3.363%)	-	-	-
A 34		GLU	5.17	-	OUTLIER (0.01)%) General / 105.2,129.7	Favored (4.9%) *tm-30* chi angles: 191.9,272.7,307.1	0.09Å	CaBLAM Disfavored (2.915%)	-	-	-
A 35		LYS	6.62	-	Favored (68.71%) General / -57.5,-34.1	Favored (96.9%) *mttt* chi angles: 289.2,179.5,180.1,178.5	0.03Å	Favored (39.594%)	-	-	-
A 36		GLY	6.58	-	Favored (89.6%) Glycine / -83.9,-2.6	-	-	Favored (66.922%)	-	-	-
A 37		GLY	5.07	-	Favored (28.65%) Glycine / -83.0,151.4	-	-	Favored (31.879%)	-	-	-

A 38		ARG	3.22	-	Favored (93.5%) General / -60.5,-41.0	Favored (72.7%) *ttt-90* chi angles: 182.6,176.2,181.8,272.5	0.04Å	Favored (57.682%)	-	-	-
A 39		ALA	1.89	-	Favored (75.78%) General / -59.9,-35.9	-	0.03Å	Favored (74.078%) alpha helix	-	-	-
A 40		HIS	1.18	-	Favored (65.84%) General / -62.5,-51.9	Favored (88.8%) *t70* chi angles: 177.5,77	0.06Å	Favored (75.708%) alpha helix	-	-	-
**#**	**Alt**	**Res**	**High B**	**Clash > 0.4Å**	**Ramachandran**	**Rotamer**	**Cβ deviation**	**CaBLAM**	**Bond lengths**	**Bond angles**	**Cis Peptides**
			Avg: 3.28	Clashscore: 1.52	Outliers: 1 of 124	Poor rotamers: 0 of 100	Outliers: 0 of 112	Outliers: 1 of 122	Outliers: 0 of 126	Outliers: 2 of 126	Non-Trans: 0 of 125
A 41		ARG	0.85	-	Favored (93.6%) General / -61.7,-40.0	Favored (99.8%) *mtm-85* chi angles: 290.3,192.4,294.8,274.3	0.02Å	Favored (77.521%) alpha helix	-	-	-
A 42		MET	0.73	-	Favored (77.48%) General / -69.6,-38.7	Favored (52%) *mmp* chi angles: 294.5,300.2,97.6	0.03Å	Favored (93.802%) alpha helix	-	-	-
A 43		ALA	0.71	-	Favored (85.95%) General / -62.2,-37.6	-	0.07Å	Favored (86.802%) alpha helix	-	-	-
A 44		LEU	0.74	-	Favored (88.43%) General / -64.0,-37.7	Favored (75.3%) *mt* chi angles: 289.6,174.3	0.04Å	Favored (84.283%) alpha helix	-	-	-
A 45		GLU	0.79	-	Favored (85.49%) General / -62.5,-37.3	Favored (97.6%) *mt-10* chi angles: 290.4,180.8,355.4	0.04Å	Favored (73.317%) alpha helix	-	-	-
A 46		GLU	0.85	-	Favored (41.81%) General / -88.8,-12.5	Favored (95.4%) *mt-10* chi angles: 294.8,182.5,359.3	0.03Å	Favored (41.838%) alpha helix	-	-	-
A 47		LEU	0.92	-	Favored (88.2%) Pre-Pro / -51.0,-46.0	Favored (64.3%) *tp* chi angles: 180.3,62.9	0.13Å	Favored (49.555%) alpha helix	-	-	-
A 48		PRO	1	-	Favored (44.88%) Trans-Pro / -52.6,-30.3	Favored (97.3%) *Cg_exo* chi angles: 331.8,37.5,329	0.02Å	Favored (76.717%) alpha helix	-	-	-
A 49		ASP	1.07	-	Favored (49.12%) General / -77.9,-36.8	Favored (87.6%) *m-30* chi angles: 293.7,347.1	0.02Å	Favored (83.369%) alpha helix	-	-	-
A 50		ALA	1.15	-	Favored (98.6%) General / -61.7,-42.2	-	0.06Å	Favored (88.826%) alpha helix	-	-	-
A 51		LEU	1.23	-	Favored (72.73%) General / -71.0,-38.2	Favored (95%) *mt* chi angles: 294.5,174.1	0.05Å	Favored (90.398%) alpha helix	-	-	-
A 52		GLN	1.32	-	Favored (91.93%) General / -60.1,-45.8	Favored (59.1%) *tt0* chi angles: 180.6,182.7,57.8	0.08Å	Favored (86.852%) alpha helix	-	-	-

A 53		THR	1.42	-	Favored (84.61%) General / -58.0,-46.9	Favored (89.1%) *m* chi angles: 298.3	0.06Å	Favored (96.686%) alpha helix	-	-	-
A 54		ILE	1.52	-	Favored (97.65%) Ile or Val / -62.7,-45.9	Favored (91.9%) *mt* chi angles: 292.1,165.9	0.07Å	Favored (96.984%) alpha helix	-	-	-
A 55		ALA	1.62	-	Favored (86.79%) General / -60.7,-39.0	-	0.03Å	Favored (92.557%) alpha helix	-	-	-
A 56		LEU	1.72	-	Favored (76.6%) General / -69.7,-37.3	Favored (93.2%) *mt* chi angles: 291.9,173.5	0.06Å	Favored (98.863%) alpha helix	-	-	-
A 57		ILE	1.81	-	Favored (97.6%) Ile or Val / -64.1,-44.0	Favored (93.9%) *mt* chi angles: 291.8,168.6	0.01Å	Favored (94.819%) alpha helix	-	-	-
A 58		ALA	1.89	-	Favored (86.45%) General / -59.7,-39.9	-	0.04Å	Favored (91.552%) alpha helix	-	-	-
A 59		LEU	1.97	0.51Å C with A 59 LEU HD23	Favored (81.79%) General / -58.6,-48.2	Favored (7.2%) *tt* chi angles: 186.9,153.2	0.06Å	Favored (94.1%) alpha helix	-	-	-
A 60		LEU	2.05	-	Favored (83.98%) General / -61.5,-37.5	Favored (97.9%) *mt* chi angles: 292.4,173.1	0.04Å	Favored (91.064%) alpha helix	-	-	-
**#**	**Alt**	**Res**	**High B**	**Clash > 0.4Å**	**Ramachandran**	**Rotamer**	**Cβ deviation**	**CaBLAM**	**Bond lengths**	**Bond angles**	**Cis Peptides**
			Avg: 3.28	Clashscore: 1.52	Outliers: 1 of 124	Poor rotamers: 0 of 100	Outliers: 0 of 112	Outliers: 1 of 122	Outliers: 0 of 126	Outliers: 2 of 126	Non-Trans: 0 of 125
A 61		SER	2.14	-	Favored (97.22%) General / -64.2,-41.1	Favored (71.5%) *m* chi angles: 295.2	0.02Å	Favored (94.058%) alpha helix	-	-	-
A 62		VAL	2.23	-	Favored (95.62%) Ile or Val / -61.4,-46.9	Favored (47.9%) *t* chi angles: 169.1	0.10Å	Favored (88.901%) alpha helix	-	-	-
A 63		MET	2.32	-	Favored (72.31%) General / -69.8,-33.4	Favored (95.7%) *mmm* chi angles: 293.2,305.4,293.4	0.06Å	Favored (86.197%) alpha helix	-	-	-
A 64		SER	2.42	-	Favored (74.24%) General / -67.5,-45.9	Favored (71.1%) *m* chi angles: 296.1	0.06Å	Favored (77.926%) alpha helix	-	-	-
A 65		LEU	2.51	-	Favored (81.5%) General / -66.5,-36.0	Favored (81.1%) *mt* chi angles: 289.1,171.9	0.01Å	Favored (72.411%) alpha helix	-	-	-
A 66		GLY	2.61	-	Favored (30.83%) Glycine / -56.1,-55.1	-	-	Favored (90.067%) alpha helix	-	-	-
A 67		VAL	2.73	-	Favored (88.67%) Ile or Val / -59.6,-42.5	Favored (60.9%) *t* chi angles: 170.9	0.03Å	Favored (79.689%) alpha helix	-	-	-

A 68		PHE	2.87	-	Favored (66.71%) General / -53.6,-49.9	Favored (54.2%) *t80* chi angles: 166.9,76	0.06Å	Favored (76.945%) alpha helix	-	OUTLIER(S) worst is CA-CB-CG: 4.6 σ	-
A 69		PHE	3.09	-	Favored (69.67%) General / -71.3,-33.3	Favored (48%) *m-80* chi angles: 286.7,113.3	0.00Å	Favored (75.148%) alpha helix	-	-	-
A 70		LEU	3.42	0.48Å O with A 79 LYS NZ	Favored (81.72%) General / -63.4,-35.8	Favored (78.7%) *mt* chi angles: 288.3,169.2	0.01Å	Favored (68.658%) alpha helix	-	-	-
A 71		LEU	3.89	-	Favored (13.32%) General / -85.2,-44.0	Favored (94.6%) *mt* chi angles: 296.4,174.5	0.06Å	Favored (50.685%) alpha helix	-	-	-
A 72		MET	4.46	-	Favored (69.72%) General / -68.2,-46.8	Favored (58.5%) *ttp* chi angles: 179,180.3,64.8	0.04Å	Favored (30.784%)	-	-	-
A 73		GLN	5.06	-	Favored (15.79%) General / -53.2,124.9	Favored (16.2%) *tp-100* chi angles: 177.9,65.9,252.9	0.04Å	Favored (14.998%)	-	-	-
A 74		ARG	5.54	-	Favored (14%) General / -93.7,-32.6	Favored (98.7%) *mtt180* chi angles: 293.8,179.1,180,178.4	0.03Å	Favored (32.67%)	-	-	-
A 75		LYS	5.75	-	Favored (6.35%) General / -159.8,131.8	Favored (87.2%) *tttt* chi angles: 185.5,174,180.4,179.3	0.02Å	CaBLAM Outlier (0.5%)	-	-	-
A 76		GLY	5.62	-	Favored (21.6%) Glycine / 106.5,157.5	-	-	CaBLAM Disfavored (4.692%) try alpha helix	-	-	-
A 77		ILE	5.17	-	Allowed (0.27%) Ile or Val / 68.4,-58.1	Favored (91.6%) *mt* chi angles: 297.4,168.7	0.03Å	Favored (16.696%)	-	-	-
A 78		GLY	4.54	-	Favored (25.78%) Glycine / 97.7,149.9	-	-	Favored (37.347%)	-	-	-
A 79		LYS	3.88	0.48Å NZ with A 70 LEU O	Favored (76.95%) General / -63.6,-34.0	Favored (61.8%) *mttm* chi angles: 290.3,179,186.4,295.6	0.04Å	Favored (56.408%)	-	-	-
A 80		ILE	3.31	-	Favored (88.5%) Ile or Val / -67.2,-42.8	Favored (95.6%) *mt* chi angles: 293,170.2	0.03Å	Favored (87.067%) alpha helix	-	-	-
**#**	**Alt**	**Res**	**High B**	**Clash > 0.4Å**	**Ramachandran**	**Rotamer**	**Cβ deviation**	**CaBLAM**	**Bond lengths**	**Bond angles**	**Cis Peptides**
			Avg: 3.28	Clashscore: 1.52	Outliers: 1 of 124	Poor rotamers: 0 of 100	Outliers: 0 of 112	Outliers: 1 of 122	Outliers: 0 of 126	Outliers: 2 of 126	Non-Trans: 0 of 125
A 81		GLY	2.86	-	Favored (50.32%) Glycine / -57.5,-52.7	-	-	Favored (92.86%) alpha helix	-	-	-
A 82		LEU	2.52	-	Favored (94.23%) General / -60.5,-41.5	Favored (80%) *mt* chi angles: 288.8,170.6	0.07Å	Favored (79.318%) alpha helix	-	-	-

A 83		GLY	2.27	-	Favored (39.75%) Glycine / -54.8,-53.4	-	-	Favored (94.912%) alpha helix	-	-	-
A 84		GLY	2.09	-	Favored (86.69%) Glycine / -57.9,-38.0	-	-	Favored (91.807%) alpha helix	-	-	-
A 85		VAL	1.97	-	Favored (90.43%) Ile or Val / -66.4,-45.0	Favored (71.7%) *t* chi angles: 172.3	0.03Å	Favored (84.425%) alpha helix	-	-	-
A 86		ILE	1.89	-	Favored (79.46%) Ile or Val / -64.0,-49.9	Favored (82.3%) *mt* chi angles: 293.9,163.5	0.14Å	Favored (80.402%) alpha helix	-	-	-
A 87		LEU	1.83	-	Favored (88.99%) General / -64.3,-37.9	Favored (4.7%) *mp* chi angles: 270.6,48.1	0.08Å	Favored (82.219%) alpha helix	-	-	-
A 88		GLY	1.78	-	Favored (35.74%) Glycine / -59.2,-54.7	-	-	Favored (90.663%) alpha helix	-	-	-
A 89		ALA	1.73	-	Favored (89.37%) General / -60.5,-39.9	-	0.04Å	Favored (81.509%) alpha helix	-	-	-
A 90		ALA	1.69	-	Favored (91.33%) General / -62.6,-38.8	-	0.03Å	Favored (96.346%) alpha helix	-	-	-
A 91		THR	1.66	-	Favored (92.77%) General / -63.2,-45.2	Favored (92.6%) *m* chi angles: 299.1	0.05Å	Favored (92.17%) alpha helix	-	-	-
A 92		PHE	1.64	-	Favored (71.62%) General / -55.9,-50.1	Favored (56.8%) *t80* chi angles: 167.6,74.8	0.09Å	Favored (92.637%) alpha helix	-	OUTLIER(S) worst is CA-CB-CG: 5.5 σ	-
A 93		PHE	1.63	-	Favored (81.4%) General / -65.8,-35.7	Favored (41.7%) *m-80* chi angles: 284.8,113.8	0.07Å	Favored (82.144%) alpha helix	-	-	-
A 94		CYS	1.65	-	Favored (98%) General / -62.8,-40.7	Favored (96%) *m* chi angles: 290.4	0.12Å	Favored (85.341%) alpha helix	-	-	-
A 95		TRP	1.71	-	Favored (87.33%) General / -67.0,-40.2	Favored (75.1%) *m100* chi angles: 284.9,114	0.03Å	Favored (97.457%) alpha helix	-	-	-
A 96		MET	1.81	-	Favored (67.57%) General / -67.3,-28.0	Favored (73.8%) *mtm* chi angles: 290.5,189.6,295.2	0.05Å	Favored (77.956%) alpha helix	-	-	-
A 97		ALA	1.96	-	Favored (29.69%) General / -81.8,2.5	-	0.02Å	Favored (44.855%)	-	-	-
A 98		GLU	2.18	-	Favored (17.9%) General / 60.4,39.9	Favored (91.9%) *mt-10* chi angles: 299.1,183.6,357.3	0.04Å	Favored (22.775%)	-	-	-
A 99		VAL	2.43	-	Favored (66.04%) Pre-Pro / -78.5,132.3	Favored (97%) *t* chi angles: 175.7	0.07Å	Favored (21.852%) beta sheet	-	-	-

#	Alt	Res	High B	Clash > 0.4Å	Ramachandran	Rotamer	Cβ deviation	CaBLAM	Bond lengths	Bond angles	Cis Peptides
A 100		PRO	2.65	-	Favored (69.98%) Trans-Pro / -54.4,142.3	Favored (99.1%) *Cg_exo* chi angles: 332.3,35.8,331.4	0.03Å	Favored (91.846%)	-	-	-
#	Alt	Res	High B	Clash > 0.4Å	Ramachandran	Rotamer	Cβ deviation	CaBLAM	Bond lengths	Bond angles	Cis Peptides
			Avg: 3.28	Clashscore: 1.52	Outliers: 1 of 124	Poor rotamers: 0 of 100	Outliers: 0 of 112	Outliers: 1 of 122	Outliers: 0 of 126	Outliers: 2 of 126	Non-Trans: 0 of 125
A 101		GLY	2.79	-	Favored (62.24%) Glycine / -56.6,-33.1	-	-	Favored (60.853%)	-	-	-
A 102		THR	2.81	-	Favored (88.31%) General / -63.7,-45.8	Favored (89.1%) *m* chi angles: 298.3	0.00Å	Favored (77.329%) alpha helix	-	-	-
A 103		LYS	2.72	-	Favored (95.11%) General / -63.7,-39.8	Favored (84.7%) *tttt* chi angles: 183.2,171.8,178.1,179.9	0.05Å	Favored (90.675%) alpha helix	-	-	-
A 104		ILE	2.57	-	Favored (97.29%) Ile or Val / -63.9,-45.3	Favored (95.8%) *mt* chi angles: 292.9,170.2	0.01Å	Favored (98.274%) alpha helix	-	-	-
A 105		ALA	2.4	-	Favored (82.01%) General / -59.8,-38.5	-	0.04Å	Favored (98.383%) alpha helix	-	-	-
A 106		GLY	2.27	-	Favored (50.05%) Glycine / -60.8,-52.8	-	-	Favored (91.223%) alpha helix	-	-	-
A 107		MET	2.2	-	Favored (74.26%) General / -55.0,-47.8	Favored (29.2%) *tmm* chi angles: 179.5,277.4,292.1	0.08Å	Favored (88.261%) alpha helix	-	-	-
A 108		LEU	2.2	-	Favored (74.76%) General / -65.3,-48.0	Favored (64.9%) *tp* chi angles: 179.3,59.5	0.03Å	Favored (90.455%) alpha helix	-	-	-
A 109		LEU	2.24	-	Favored (76.59%) General / -59.1,-49.8	Favored (62.5%) *tp* chi angles: 174.6,61.8	0.07Å	Favored (88.24%) alpha helix	-	-	-
A 110		LEU	2.31	-	Favored (88.57%) General / -63.3,-37.8	Favored (95.4%) *mt* chi angles: 292.1,173.2	0.04Å	Favored (85.422%) alpha helix	-	-	-
A 111		SER	2.39	-	Favored (96.61%) General / -64.4,-41.6	Favored (72.8%) *m* chi angles: 295.7	0.04Å	Favored (97.434%) alpha helix	-	-	-
A 112		LEU	2.5	-	Favored (93.52%) General / -65.3,-40.1	Favored (86.4%) *mt* chi angles: 290.5,169.8	0.02Å	Favored (97.411%) alpha helix	-	-	-
A 113		LEU	2.63	-	Favored (93.86%) General / -64.8,-39.6	Favored (95.8%) *mt* chi angles: 291.8,172.4	0.02Å	Favored (98.179%) alpha helix	-	-	-
A 114		LEU	2.77	-	Favored (92.94%) General / -63.8,-44.5	Favored (63.8%) *tp* chi angles: 179.6,59.2	0.03Å	Favored (88.106%) alpha helix	-	-	-

#	Alt	Res	High B	Clash > 0.4Å	Ramachandran	Rotamer	Cβ deviation	CaBLAM	Bond lengths	Bond angles	Cis Peptides
A 115		MET	2.93	-	Favored (71.78%) General / -54.0,-47.3	Favored (52.7%) *ttp* chi angles: 178.3,189.7,72.2	0.08Å	Favored (91.854%) alpha helix	-	-	-
A 116		ILE	3.12	-	Favored (96.96%) Ile or Val / -60.6,-44.5	Favored (92.2%) *mt* chi angles: 291.6,166.9	0.05Å	Favored (90.106%) alpha helix	-	-	-
A 117		VAL	3.38	-	Favored (97.67%) Ile or Val / -64.0,-43.7	Favored (61.2%) *t* chi angles: 171	0.03Å	Favored (96.205%) alpha helix	-	-	-
A 118		LEU	3.72	-	Favored (77.54%) General / -67.3,-34.5	Favored (91%) *mt* chi angles: 292.1,174.7	0.03Å	Favored (49.393%)	-	-	-
A 119		ILE	4.16	-	Favored (84.77%) Pre-Pro / -79.2,123.2	Favored (88.8%) *mt* chi angles: 298.1,169.5	0.06Å	Favored (28.193%)	-	-	-
A 120		PRO	4.72	-	Favored (66.41%) Trans-Pro / -70.3,152.5	Favored (59.2%) *Cg_endo* chi angles: 26.3,326.1,27.2	0.04Å	Favored (75.1%)	-	-	-
**#**	**Alt**	**Res**	**High B**	**Clash > 0.4Å**	**Ramachandran**	**Rotamer**	**Cβ deviation**	**CaBLAM**	**Bond lengths**	**Bond angles**	**Cis Peptides**
			Avg: 3.28	Clashscore: 1.52	Outliers: 1 of 124	Poor rotamers: 0 of 100	Outliers: 0 of 112	Outliers: 1 of 122	Outliers: 0 of 126	Outliers: 2 of 126	Non-Trans: 0 of 125
A 121		GLU	5.41	0.52Å OE1 with A 124 LYS NZ	Favored (79.22%) Pre-Pro / -81.6,125.1	Favored (89.4%) *tt0* chi angles: 184.1,180.4,6.2	0.06Å	Favored (38.023%)	-	-	-
A 122		PRO	6.21	-	Favored (30.99%) Trans-Pro / -50.8,-30.8	Favored (86.9%) *Cg_exo* chi angles: 330.3,37.2,331.1	0.05Å	Favored (76.453%)	-	-	-
A 123		GLU	7.05	-	Favored (72.75%) General / -64.1,-31.5	Favored (76.5%) *mm-30* chi angles: 292.1,295.6,307.4	0.01Å	Favored (68.639%) alpha helix	-	-	-
A 124		LYS	7.84	0.52Å NZ with A 121 GLU OE1	Favored (55.63%) General / -83.9,-2.0	Favored (54.5%) *mttp* chi angles: 293.9,181.8,171.9,64.6	0.04Å	Favored (51.665%)	-	-	-
A 125		GLN	8.53	-	Favored (45.65%) General / -99.5,3.6	Favored (90.5%) *mt0* chi angles: 297.4,180.6,328.5	0.02Å	-	-	-	-
A 126		ARG	9.05	-	-	Favored (92.2%) *mmt-90* chi angles: 296.5,290.2,182.4,271.8	0.03Å	-	-	-	-

About MolProbity | Website for the Richardson Lab | Using ecloud x-H | Internal reference 4.5.2
