# Supplementary material for: Integrative proteome-wide structural analysis and high-throughput docking identify broad-spectrum antiviral scaffolds against Zika, Yellow Fever, West Nile, Saint Louis encephalitis, and Usutu viruses
Source: Front Cell Infect Microbiol. 2026 Apr 30;16:1723132. doi: 10.3389/fcimb.2026.1723132 (PMC13171538; doi:10.3389/fcimb.2026.1723132)
Supplement: Supplementary file 5 [file DataSheet5.zip › WNV/WNV_NS4b/Mol_probity_Files/WNV_NS4b_1FH-multi.table.pdf]

Viewing WNV_NS4b1FH-multi.table

Duke Biochemistry
Duke University School of Medicine

When finished, you should close this window.

Hint: Use File | Save As... to save a copy of this page.

All-Atom Contacts	Clashscore, all atoms:	1.02		99th percentile* (N=1784, all resolutions)
	Clashscore is the number of serious steric overlaps (> 0.4 Å) per 1000 atoms.			
Protein Geometry	Poor rotamers	0	0.00%	Goal: <0.3%
	Favored rotamers	207	100.00%	Goal: >98%
	Ramachandran outliers	1	0.39%	Goal: <0.05%
	Ramachandran favored	245	96.46%	Goal: >98%
	Rama distribution Z-score	1.71 ± 0.54		Goal: abs(Z score) < 2
	MolProbity score^	1.03		100th percentile* (N=27675, 0Å - 99Å)
	Cβ deviations >0.25Å	0	0.00%	Goal: 0
	Bad bonds:	3 / 1970	0.15%	Goal: 0%
	Bad angles:	5 / 2691	0.19%	Goal: <0.1%
Peptide Omegas	Cis Prolines:	0 / 9	0.00%	Expected: ≤1 per chain, or ≤5%
Low-resolution Criteria	CaBLAM outliers	6	2.4%	Goal: <1.0%
	CA Geometry outliers	2	0.79%	Goal: <0.5%
Additional validations	Chiral volume outliers	0/334		
	Waters with clashes	0/0	0.00%	See UnDowser table for details

In the two column results, the left column gives the raw count, right column gives the percentage.

* 100th percentile is the best among structures of comparable resolution; 0th percentile is the worst. For clashscore the comparative set of structures was selected in 2004, for MolProbity score in 2006.

^ MolProbity score combines the clashscore, rotamer, and Ramachandran evaluations into a single score, normalized to be on the same scale as X-ray resolution.

Key to table colors and cutoffs here:

#	Alt	Res	High B	Clash > 0.4Å	Ramachandran	Rotamer	Cβ deviation	CaBLAM	Bond lengths	Bond angles	Cis Peptides
			Avg: 4.25	Clashscore: 1.02	Outliers: 1 of 254	Poor rotamers: 0 of 207	Outliers: 0 of 238	Outliers: 8 of 252	Outliers: 3 of 256	Outliers: 1 of 256	Non-Trans: 0 of 255
A 1		ASN	18.33	-	-	Favored (95.8%) *m-40* chi angles: 290.8,333	0.03Å	-	-	-	-
A 2		GLU	18.2	-	Favored (73.78%) General / -55.4,-42.4	Favored (89.1%) *tt0* chi angles: 185.6,181.3,0.5	0.03Å	-	-	-	-
A 3		MET	17.92	-	Favored (68.69%) General / -68.2,-29.8	Favored (50.1%) *mmp* chi angles: 293.3,299.4,95.7	0.04Å	Favored (82.205%)	-	-	-
A 4		GLY	17.5	-	Favored (65.8%) Glycine / -63.8,-49.8	-	-	Favored (87.731%) alpha helix	-	-	-
A 5		TRP	16.99	-	Favored (96.1%) General / -62.3,-40.4	Favored (54.5%) *m-10* chi angles: 295.2,337.1	0.05Å	Favored (83.499%) alpha helix	-	-	-
A 6		LEU	16.47	-	Favored (76.24%) General / -63.8,-48.6	Favored (68.8%) *tp* chi angles: 178.4,60	0.01Å	Favored (91.954%) alpha helix	-	-	-

A 7		ASP	16.04	-	Favored (86.21%) General / -61.4,-38.2	Favored (87.9%) *m-30* chi angles: 284.3,344.9	0.07Å	Favored (93.161%) alpha helix	-	-	-
A 8		LYS	15.74	-	Favored (96.79%) General / -63.6,-43.5	Favored (18.7%) *tptp* chi angles: 181,66.2,165.9,70.6	0.03Å	Favored (93.742%) alpha helix	-	-	-
A 9		THR	15.57	-	Favored (87.32%) General / -60.1,-47.1	Favored (88.5%) *m* chi angles: 298.5	0.05Å	Favored (96.87%) alpha helix	-	-	-
A 10		LYS	15.43	-	Favored (92.99%) General / -62.7,-39.1	Favored (55.4%) *mtmt* chi angles: 288.7,185.8,294.6,187.3	0.03Å	Favored (90.051%) alpha helix	-	-	-
A 11		ASN	15.28	-	Favored (81.34%) General / -67.7,-36.8	Favored (99.2%) *m-40* chi angles: 288.3,339.6	0.01Å	Favored (92.312%) alpha helix	-	-	-
A 12		ASP	15.12	-	Favored (66.25%) General / -72.3,-41.8	Favored (97.6%) *m-30* chi angles: 288.8,347.9	0.01Å	Favored (78.996%) alpha helix	-	-	-
A 13		ILE	14.94	-	Favored (93.08%) Ile or Val / -65.7,-45.0	Favored (99%) *mt* chi angles: 292.6,168.1	0.01Å	Favored (81.688%) alpha helix	-	-	-
A 14		GLY	14.68	-	Favored (36.75%) Glycine / -53.9,-53.4	-	-	Favored (97.35%) alpha helix	-	-	-
A 15		SER	14.35	-	Favored (78.77%) General / -56.7,-42.2	Favored (72.1%) *m* chi angles: 295.9	0.06Å	Favored (85.48%) alpha helix	-	-	-
A 16		LEU	13.96	-	Favored (72.37%) General / -62.7,-31.4	Favored (90.2%) *mt* chi angles: 290.8,172.2	0.01Å	Favored (74.271%) alpha helix	-	-	-
A 17		LEU	13.52	-	Favored (53.39%) General / -94.1,4.8	Favored (93.4%) *mt* chi angles: 298.7,177.1	0.03Å	Favored (45.136%)	-	-	-
A 18		GLY	13.11	-	Favored (82.58%) Glycine / 86.1,5.9	-	-	Favored (77.126%)	-	-	-
A 19		HIS	12.78	-	Allowed (0.8%) General / -111.6,73.1	Favored (99.6%) *m-70* chi angles: 298.3,286.9	0.04Å	CaBLAM Disfavored (4.979%)	-	-	-
A 20		LYS	12.58	-	Allowed (0.26%) Pre-Pro / -45.2,-23.8	Favored (18%) *pttm* chi angles: 66.9,182.3,182.4,292.2	0.00Å	CA Geom Outlier (0.139%)	-	-	-
**#**	**Alt**	**Res**	**High B**	**Clash > 0.4Å**	**Ramachandran**	**Rotamer**	**Cβ deviation**	**CaBLAM**	**Bond lengths**	**Bond angles**	**Cis Peptides**
			Avg: 4.25	Clashscore: 1.02	Outliers: 1 of 254	Poor rotamers: 0 of 207	Outliers: 0 of 238	Outliers: 8 of 252	Outliers: 3 of 256	Outliers: 1 of 256	Non-Trans: 0 of 255
A 21		PRO	12.54	-	OUTLIER (0)%) Trans-Pro / 64.5,151.5	Favored (3.8%) *Cg_endo* chi angles: 40.6,333.8,6.4	0.18Å	CaBLAM Outlier (0.449%) try beta sheet	OUTLIER(S) worst is N--CD: 9.4 σ	OUTLIER(S) worst is CA-N-CD: 14.9 σ	-
A 22		GLU	12.71	-	Favored (3.12%) General / -107.5,35.3	Favored (91.8%) *mt-10* chi angles: 296.1,186.6,356.1	0.03Å	CaBLAM Disfavored (2.919%) try beta sheet	-	-	-
A 23		THR	13.05	-	Favored (50.65%)	Favored (97.6%) *m* chi angles: 300.7	0.01Å	Favored (6.559%)	-	-	-

					General / -59.1,131.3			beta sheet			
A 24		ARG	13.45	-	Favored (3%) General / -133.7,-2.2	Favored (91.5%) *mmt-90* chi angles: 294.5,292.6,180.8,269.9	0.02Å	Favored (5.904%)	-	-	-
A 25		GLU	13.78	-	Favored (57.44%) General / -58.1,-24.1	Favored (15.9%) *mp0* chi angles: 287,81,332.1	0.01Å	Favored (29.387%)	-	-	-
A 26		THR	13.93	-	Favored (52.83%) General / -92.0,-5.5	Favored (60.3%) *p* chi angles: 64	0.04Å	Favored (57.148%)	-	-	-
A 27		THR	13.76	-	Favored (2.9%) General / -78.2,62.3	Favored (49.8%) *p* chi angles: 56.1	0.02Å	Favored (7.135%)	-	-	-
A 28		LEU	13.21	-	Favored (80.33%) General / -65.4,-35.2	Favored (95.7%) *mt* chi angles: 293,174	0.04Å	CA Geom Outlier (0.421%)	-	-	-
A 29		GLY	12.22	-	Allowed (0.42%) Glycine / -157.8,24.9	-	-	CaBLAM Disfavored (4.209%)	-	-	-
A 30		VAL	10.76	-	Favored (75.55%) Ile or Val / -121.2,126.8	Favored (85.4%) *t* chi angles: 177.6	0.03Å	Favored (23.452%)	-	-	-
A 31		GLU	8.95	-	Favored (18.75%) General / -95.0,106.7	Favored (91.8%) *tt0* chi angles: 181.8,178.8,354.4	0.01Å	Favored (67.372%) beta sheet	-	-	-
A 32		ASN	6.96	-	Favored (9.52%) General / -83.0,78.7	Favored (79.8%) *m-40* chi angles: 288.8,318.5	0.04Å	Favored (66.858%) beta sheet	-	-	-
A 33		PHE	5.12	-	Favored (46.11%) General / -56.4,130.7	Favored (89.7%) *t80* chi angles: 176.6,80.7	0.06Å	Favored (15.185%) beta sheet	-	-	-
A 34		LEU	3.62	-	Favored (41.66%) General / -117.4,148.5	Favored (64.6%) *mt* chi angles: 304,176.1	0.07Å	Favored (44.51%) beta sheet	-	-	-
A 35		LEU	2.54	-	Favored (54.59%) General / -68.8,143.4	Favored (93.7%) *mt* chi angles: 293.7,174.9	0.03Å	Favored (39.034%) beta sheet	-	-	-
A 36		ASP	1.82	-	Favored (2.56%) General / -147.1,95.4	Favored (53.8%) *t0* chi angles: 186.7,1.6	0.05Å	Favored (18.477%) beta sheet	-	-	-
A 37		LEU	1.37	-	Favored (35.95%) General / -94.1,121.8	Favored (72.1%) *tp* chi angles: 176.8,61.8	0.04Å	Favored (55.236%) beta sheet	-	-	-
A 38		ARG	1.11	-	Favored (18.46%) Pre-Pro / -116.9,85.9	Favored (94.8%) *mtt180* chi angles: 300.6,179.2,184.4,179.8	0.10Å	Favored (49.895%) beta sheet	-	-	-
A 39		PRO	0.96	-	Favored (77.18%) Trans-Pro / -60.0,-24.6	Favored (42.9%) *Cg_endo* chi angles: 24.1,327,28.1	0.01Å	Favored (38.352%)	-	-	-
A 40		ALA	0.9	-	Favored (83.52%) General / -67.4,-37.6	-	0.03Å	Favored (73.763%) alpha helix	-	-	-

#	Alt	Res	High B	Clash > 0.4Å	Ramachandran	Rotamer	Cβ deviation	CaBLAM	Bond lengths	Bond angles	Cis Peptides
			Avg: 4.25	Clashscore: 1.02	Outliers: 1 of 254	Poor rotamers: 0 of 207	Outliers: 0 of 238	Outliers: 8 of 252	Outliers: 3 of 256	Outliers: 1 of 256	Non-Trans: 0 of 255
A 41		THR	0.87	-	Favored (42.72%) General / -76.0,-43.9	Favored (88%) *m* chi angles: 301.4	0.02Å	Favored (74.472%) alpha helix	-	-	-
A 42		ALA	0.88	-	Favored (80.17%) General / -59.0,-38.8	-	0.04Å	Favored (91.029%) alpha helix	-	-	-
A 43		TRP	0.91	0.47Å CE3 with A 43 TRP HA	Favored (74.88%) General / -69.9,-35.9	Favored (19.3%) *t-100* chi angles: 199,240.6	0.04Å	Favored (89.259%) alpha helix	-	-	-
A 44		SER	0.96	-	Favored (70.83%) General / -66.8,-30.6	Favored (52.6%) *p* chi angles: 74.1	0.10Å	Favored (83.235%) alpha helix	-	-	-
A 45		LEU	1.03	-	Favored (62.48%) General / -73.9,-41.2	Favored (93.6%) *mt* chi angles: 294,175	0.06Å	Favored (79.23%) alpha helix	-	-	-
A 46		TYR	1.12	-	Favored (65.25%) General / -59.2,-52.5	Favored (78.5%) *t80* chi angles: 172.7,82.3	0.05Å	Favored (84.814%) alpha helix	-	-	-
A 47		ALA	1.24	-	Favored (93.15%) General / -60.3,-41.4	-	0.04Å	Favored (73.514%) alpha helix	-	-	-
A 48		VAL	1.39	-	Favored (31.88%) Ile or Val / -76.5,-46.3	Favored (95.2%) *t* chi angles: 174.9	0.04Å	Favored (72.693%) alpha helix	-	-	-
A 49		ALA	1.56	-	Favored (76.67%) General / -59.5,-36.8	-	0.04Å	Favored (89.781%) alpha helix	-	-	-
A 50		THR	1.75	-	Favored (88.31%) General / -64.6,-45.1	Favored (91.6%) *m* chi angles: 299	0.01Å	Favored (86.023%) alpha helix	-	-	-
A 51		ALA	1.95	-	Favored (73.3%) General / -58.9,-35.6	-	0.04Å	Favored (69.075%) alpha helix	-	-	-
A 52		VAL	2.13	-	Favored (16.09%) Ile or Val / -83.6,-49.8	Favored (81.7%) *t* chi angles: 176.7	0.05Å	Favored (49.772%) alpha helix	-	-	-
A 53		LEU	2.29	-	Favored (75.44%) General / -69.6,-41.6	Favored (46.2%) *tp* chi angles: 182.7,56.2	0.08Å	Favored (45.402%) alpha helix	-	-	-
A 54		THR	2.41	-	Allowed (0.5%) Pre-Pro / -43.5,-66.0	Favored (92.6%) *m* chi angles: 297.4	0.17Å	Favored (50.721%) alpha helix	-	-	-
A 55		PRO	2.51	-	Favored (76.26%) Trans-Pro / -62.2,-24.6	Favored (33.5%) *Cg_endo* chi angles: 22,325.4,32.1	0.04Å	Favored (58.433%) alpha helix	-	-	-
A 56		LEU	2.57	-	Favored (64.27%) General / -66.5,-50.1	Favored (59.2%) *tp* chi angles: 175.4,59.4	0.02Å	Favored (67.657%) alpha helix	-	-	-

A 57		LEU	2.61	-	Favored (94.54%) General / -63.4,-39.4	Favored (90%) *mt* chi angles: 291,172.9	0.03Å	Favored (79.186%) alpha helix	-	-	-
A 58		LYS	2.65	-	Favored (88.01%) General / -62.1,-38.2	Favored (95.6%) *mttt* chi angles: 289.3,177.8,182.3,173.5	0.01Å	Favored (80.741%) alpha helix	-	-	-
A 59		HIS	2.68	-	Favored (74.99%) General / -69.6,-42.1	Favored (41.1%) *m170* chi angles: 292.3,189	0.06Å	Favored (85.914%) alpha helix	-	-	-
A 60		LEU	2.72	-	Favored (87.6%) General / -66.9,-41.1	Favored (98.3%) *mt* chi angles: 292.6,171.8	0.05Å	Favored (93.993%) alpha helix	-	-	-
**#**	**Alt**	**Res**	**High B**	**Clash > 0.4Å**	**Ramachandran**	**Rotamer**	**Cβ deviation**	**CaBLAM**	**Bond lengths**	**Bond angles**	**Cis Peptides**
			Avg: 4.25	Clashscore: 1.02	Outliers: 1 of 254	Poor rotamers: 0 of 207	Outliers: 0 of 238	Outliers: 8 of 252	Outliers: 3 of 256	Outliers: 1 of 256	Non-Trans: 0 of 255
A 61		ILE	2.76	-	Favored (86.34%) Ile or Val / -65.2,-47.8	Favored (95.9%) *mt* chi angles: 292.4,166.6	0.05Å	Favored (87.125%) alpha helix	-	-	-
A 62		THR	2.83	-	Favored (90.52%) General / -59.0,-45.5	Favored (99.5%) *m* chi angles: 300.4	0.03Å	Favored (96.14%) alpha helix	-	-	-
A 63		SER	2.91	-	Favored (98.34%) General / -61.1,-42.3	Favored (68.3%) *m* chi angles: 296.7	0.05Å	Favored (86.75%) alpha helix	-	-	-
A 64		ASP	3.01	-	Favored (86.77%) General / -62.8,-37.4	Favored (96.2%) *m-30* chi angles: 286.3,347.9	0.02Å	Favored (93.347%) alpha helix	-	-	-
A 65		TYR	3.13	-	Favored (61.13%) General / -74.9,-30.1	Favored (36%) *m-80* chi angles: 282.2,112.7	0.17Å	Favored (85.172%) alpha helix	-	-	-
A 66		ILE	3.26	-	Favored (94.21%) Ile or Val / -64.5,-46.0	Favored (99.2%) *mt* chi angles: 292.6,167.9	0.05Å	Favored (78.693%) alpha helix	-	-	-
A 67		ASN	3.38	-	Favored (81.51%) General / -61.5,-36.8	Favored (91%) *m-40* chi angles: 285.2,338.9	0.05Å	Favored (89.136%) alpha helix	-	-	-
A 68		THR	3.5	-	Favored (95.09%) General / -63.3,-44.4	Favored (90.6%) *m* chi angles: 298.8	0.04Å	Favored (92.265%) alpha helix	-	-	-
A 69		SER	3.65	-	Favored (88.79%) General / -58.8,-45.8	Favored (36.3%) *t* chi angles: 181.3	0.13Å	Favored (88.636%) alpha helix	-	-	-
A 70		LEU	3.82	-	Favored (76.81%) General / -69.6,-37.4	Favored (89.6%) *mt* chi angles: 292,174.8	0.05Å	Favored (75.247%) alpha helix	-	-	-
A 71		THR	4.04	-	Favored (79.31%) General / -57.4,-40.9	Favored (94.9%) *m* chi angles: 299.4	0.03Å	Favored (71.807%) alpha helix	-	-	-
A 72		SER	4.35	-	Favored (49.36%) General / -70.8,-8.0	Favored (70.5%) *m* chi angles: 296.3	0.04Å	Favored (39.461%) alpha helix	-	-	-

A 73		ILE	4.76	-	Favored (9.68%) Ile or Val / -105.7,8.5	Favored (41.1%) *pt* chi angles: 64.8,171.2	0.06Å	Favored (6.282%)	-	-	-
A 74		ASN	5.26	-	Allowed (0.93%) General / 55.0,-115.2	Favored (85.6%) *m-40* chi angles: 297.2,312.7	0.07Å	CaBLAM Outlier (0.422%)	-	-	-
A 75		VAL	5.85	-	Favored (60.59%) Ile or Val / -54.2,-43.5	Favored (63.9%) *t* chi angles: 171.3	0.03Å	Favored (23.217%)	-	-	-
A 76		GLN	6.51	-	Favored (67.82%) General / -62.9,-24.4	Favored (92.7%) *mt0* chi angles: 290.7,181.5,334.8	0.04Å	Favored (61.793%) three-ten	-	-	-
A 77		ALA	7.17	-	Favored (68.74%) General / -60.6,-28.7	-	0.05Å	Favored (57.597%) three-ten	-	-	-
A 78		SER	7.8	-	Favored (62.98%) General / -60.8,-21.4	Favored (93.8%) *p* chi angles: 64.7	0.03Å	Favored (26.216%) three-ten	-	-	-
A 79		ALA	8.37	-	Favored (6.83%) General / -101.4,-41.0	-	0.04Å	Favored (22.241%) alpha helix	-	-	-
A 80		LEU	8.87	-	Favored (60.44%) General / -75.4,-14.2	Favored (94.1%) *mt* chi angles: 296.4,173	0.06Å	Favored (54.47%) three-ten	-	-	-
**#**	**Alt**	**Res**	**High B**	**Clash > 0.4Å**	**Ramachandran**	**Rotamer**	**Cβ deviation**	**CaBLAM**	**Bond lengths**	**Bond angles**	**Cis Peptides**
			Avg: 4.25	Clashscore: 1.02	Outliers: 1 of 254	Poor rotamers: 0 of 207	Outliers: 0 of 238	Outliers: 8 of 252	Outliers: 3 of 256	Outliers: 1 of 256	Non-Trans: 0 of 255
A 81		PHE	9.26	-	Favored (67.07%) General / -64.0,-22.8	Favored (19.7%) *m-80* chi angles: 281.4,125.5	0.10Å	Favored (58.597%) three-ten	-	-	-
A 82		THR	9.54	-	Favored (60.57%) General / -72.3,-11.1	Favored (72.3%) *p* chi angles: 61.9	0.05Å	Favored (63.66%) three-ten	-	-	-
A 83		LEU	9.68	-	Favored (46.21%) General / -88.6,-11.2	Favored (84.6%) *mt* chi angles: 299.9,175.4	0.07Å	Favored (68.199%)	-	-	-
A 84		ALA	9.66	-	Favored (58.98%) General / -85.7,-7.2	-	0.04Å	Favored (51.286%)	-	-	-
A 85		ARG	9.45	-	Favored (8.33%) General / -81.6,69.9	Favored (98.2%) *mtt180* chi angles: 296,178,181.5,179	0.03Å	Favored (8.148%)	-	-	-
A 86		GLY	9.05	-	Favored (42.15%) Glycine / -89.9,-167.2	-	-	Favored (30.39%)	-	-	-
A 87		PHE	8.44	-	Favored (47.64%) Pre-Pro / -119.9,148.9	Favored (95.8%) *m-80* chi angles: 293.6,87.2	0.03Å	Favored (27.947%) beta sheet	-	-	-
A 88		PRO	7.6	-	Favored (3.68%) Trans-Pro / -85.2,10.6	Favored (32.9%) *Cg_endo* chi angles: 34.7,323.3,23.6	0.05Å	CaBLAM Disfavored (3.915%) try beta sheet	-	-	-

#	Alt	Res	High B	Clash > 0.4Å	Ramachandran	Rotamer	Cβ deviation	CaBLAM	Bond lengths	Bond angles	Cis Peptides
A 89		PHE	6.59	-	Favored (50.84%) General / -71.1,141.7	Favored (5.2%) *m-10* chi angles: 291.9,9.5	0.09Å	Favored (32.942%) beta sheet	-	-	-
A 90		VAL	5.51	-	Favored (73.18%) Ile or Val / -125.0,128.5	Favored (80.9%) *t* chi angles: 176.5	0.02Å	Favored (58.051%) beta sheet	-	-	-
A 91		ASP	4.48	-	Favored (8.23%) General / -81.5,78.8	Favored (44.8%) *t0* chi angles: 187.8,8.3	0.02Å	Favored (45.121%) beta sheet	-	-	-
A 92		VAL	3.6	-	Favored (86.76%) Ile or Val / -64.2,-39.8	Favored (87.2%) *t* chi angles: 173.9	0.02Å	CaBLAM Disfavored (4.136%)	-	-	-
A 93		GLY	2.91	-	Favored (24.8%) Glycine / 94.0,-161.9	-	-	Favored (13.43%)	-	-	-
A 94		VAL	2.4	-	Favored (6.24%) Ile or Val / -109.1,-53.0	Favored (86.2%) *t* chi angles: 176.2	0.04Å	CaBLAM Outlier (0.007%)	-	-	-
A 95		SER	2.04	-	Favored (67.98%) General / -60.9,-27.2	Favored (96.6%) *p* chi angles: 65.9	0.03Å	Favored (68.1%) alpha helix	-	-	-
A 96		ALA	1.8	-	Favored (84.71%) General / -63.6,-36.7	-	0.04Å	Favored (71.365%) alpha helix	-	-	-
A 97		LEU	1.64	-	Favored (16.59%) General / -91.0,-30.9	Favored (91%) *mt* chi angles: 299.4,177.3	0.11Å	Favored (73.692%) alpha helix	-	-	-
A 98		LEU	1.52	-	Favored (94.17%) General / -64.3,-39.5	Favored (86.3%) *mt* chi angles: 290.4,173	0.08Å	Favored (80.633%) alpha helix	-	-	-
A 99		LEU	1.45	-	Favored (63.41%) General / -63.8,-51.7	Favored (68.1%) *tp* chi angles: 179.3,61.5	0.04Å	Favored (82.65%) alpha helix	-	-	-
A 100		ALA	1.41	-	Favored (82.93%) General / -57.8,-41.7	-	0.07Å	Favored (84.195%) alpha helix	-	-	-
**#**	**Alt**	**Res**	**High B**	**Clash > 0.4Å**	**Ramachandran**	**Rotamer**	**Cβ deviation**	**CaBLAM**	**Bond lengths**	**Bond angles**	**Cis Peptides**
			Avg: 4.25	Clashscore: 1.02	Outliers: 1 of 254	Poor rotamers: 0 of 207	Outliers: 0 of 238	Outliers: 8 of 252	Outliers: 3 of 256	Outliers: 1 of 256	Non-Trans: 0 of 255
A 101		ALA	1.41	-	Favored (75.59%) General / -59.6,-36.0	-	0.04Å	Favored (75.774%) alpha helix	-	-	-
A 102		GLY	1.45	-	Favored (87.2%) Glycine / -86.6,1.4	-	-	Favored (47.871%) alpha helix	-	-	-
A 103		CYS	1.5	-	Favored (5.77%) General / -116.5,-30.8	Favored (59.6%) *m* chi angles: 302	0.04Å	Favored (9.277%) alpha helix	-	-	-
A 104		TRP	1.58	-	Favored (62.33%) General / -53.1,-38.1	Favored (18.7%) *m-10* chi angles: 282.1,333.5	0.03Å	Favored (62.561%) alpha helix	-	-	-

#	Alt	Res	High B	Clash > 0.4Å	Ramachandran	Rotamer	Cβ deviation	CaBLAM	Bond lengths	Bond angles	Cis Peptides
A 105		GLY	1.67	-	Favored (53.05%) Glycine / -60.5,-19.2	-	-	Favored (62.35%) alpha helix	-	-	-
A 106		GLN	1.76	-	Favored (15.85%) General / -115.1,19.2	Favored (65.4%) *mt0* chi angles: 295.5,185.9,68.8	0.06Å	Favored (37.201%)	-	-	-
A 107		VAL	1.82	-	Favored (39.79%) Ile or Val / -81.3,126.4	Favored (99.6%) *t* chi angles: 175.5	0.03Å	Favored (18.646%)	-	-	-
A 108		THR	1.84	-	Favored (7.11%) General / -104.1,171.9	Favored (35.9%) *p* chi angles: 68.5	0.05Å	Favored (21.94%)	-	-	-
A 109		LEU	1.83	-	Favored (89.89%) General / -59.1,-45.8	Favored (73.2%) *tp* chi angles: 177.7,61.2	0.03Å	Favored (53.896%)	-	-	-
A 110		THR	1.78	-	Favored (81.55%) General / -56.6,-45.1	Favored (72.8%) *m* chi angles: 302.9	0.08Å	Favored (86.271%) alpha helix	-	-	-
A 111		VAL	1.72	-	Favored (98.21%) Ile or Val / -63.7,-44.1	Favored (55.3%) *t* chi angles: 170.1	0.05Å	Favored (95.581%) alpha helix	-	-	-
A 112		THR	1.64	-	Favored (86.47%) General / -60.6,-47.4	Favored (88.8%) *m* chi angles: 298.4	0.07Å	Favored (88.803%) alpha helix	-	-	-
A 113		VAL	1.57	-	Favored (94.72%) Ile or Val / -65.4,-43.5	Favored (71.2%) *t* chi angles: 172.3	0.02Å	Favored (88.835%) alpha helix	-	-	-
A 114		THR	1.51	-	Favored (89.42%) General / -60.4,-46.6	Favored (88.6%) *m* chi angles: 298.6	0.03Å	Favored (96.998%) alpha helix	-	-	-
A 115		ALA	1.47	-	Favored (90.66%) General / -60.1,-40.8	-	0.04Å	Favored (93.255%) alpha helix	-	-	-
A 116		ALA	1.47	-	Favored (98.47%) General / -62.0,-41.8	-	0.04Å	Favored (98.375%) alpha helix	-	-	-
A 117		ALA	1.48	-	Favored (96.89%) General / -63.3,-40.3	-	0.04Å	Favored (97.343%) alpha helix	-	-	-
A 118		LEU	1.51	-	Favored (95.08%) General / -64.7,-42.7	Favored (88.5%) *mt* chi angles: 291.1,169.8	0.09Å	Favored (98.338%) alpha helix	-	-	-
A 119		LEU	1.56	-	Favored (92.85%) General / -65.6,-41.5	Favored (79.2%) *mt* chi angles: 291,166.7	0.09Å	Favored (97.744%) alpha helix	-	-	-
A 120		PHE	1.62	-	Favored (73.17%) General / -58.0,-50.5	Favored (82.1%) *t80* chi angles: 171.9,78.5	0.07Å	Favored (91.395%) alpha helix	-	-	-
**#**	**Alt**	**Res**	**High B**	**Clash > 0.4Å**	**Ramachandran**	**Rotamer**	**Cβ deviation**	**CaBLAM**	**Bond lengths**	**Bond angles**	**Cis Peptides**

			Avg: 4.25	Clashscore: 1.02	Outliers: 1 of 254	Poor rotamers: 0 of 207	Outliers: 0 of 238	Outliers: 8 of 252	Outliers: 3 of 256	Outliers: 1 of 256	Non-Trans: 0 of 255
A 121		CYS	1.68	-	Favored (88.63%) General / -63.1,-37.8	Favored (94.9%) *m* chi angles: 292.9	0.06Å	Favored (80.762%) alpha helix	-	-	-
A 122		HIS	1.74	-	Favored (71.36%) General / -63.7,-49.9	Favored (90.6%) *t70* chi angles: 180.3,72.1	0.02Å	Favored (73.109%) alpha helix	-	-	-
A 123		TYR	1.79	-	Favored (60.05%) General / -75.5,-30.7	Favored (54.4%) *m-80* chi angles: 290.6,114.6	0.08Å	Favored (63.953%) alpha helix	-	-	-
A 124		ALA	1.82	-	Favored (81.03%) General / -60.1,-37.8	-	0.03Å	Favored (72.327%) alpha helix	-	-	-
A 125		TYR	1.85	-	Favored (11.93%) General / -71.7,-54.0	Favored (91.7%) *t80* chi angles: 178.4,76.9	0.01Å	Favored (53.493%) alpha helix	-	-	-
A 126		MET	1.87	-	Favored (56.93%) General / -76.7,-30.9	Favored (23.8%) *mmt* chi angles: 291.4,301.1,188.9	0.06Å	Favored (40.708%) alpha helix	-	-	-
A 127		VAL	1.88	-	Favored (95.72%) Pre-Pro / -59.6,-48.3	Favored (46.5%) *t* chi angles: 168.9	0.18Å	Favored (62.296%) alpha helix	-	-	-
A 128		PRO	1.9	-	Favored (53.5%) Trans-Pro / -52.6,-32.4	Favored (86.1%) *Cg_exo* chi angles: 330.5,38.4,329.1	0.06Å	Favored (95.86%) alpha helix	-	-	-
A 129		GLY	1.92	-	Favored (53.27%) Glycine / -65.2,-51.1	-	-	Favored (87.032%) alpha helix	-	-	-
A 130		TRP	1.96	-	Favored (98.07%) General / -62.3,-41.0	Favored (60.5%) *m-10* chi angles: 298.3,345.1	0.04Å	Favored (82.329%) alpha helix	-	-	-
A 131		GLN	2.01	-	Favored (87.3%) General / -67.0,-40.5	Favored (98.5%) *mt0* chi angles: 290.4,173.4,330.2	0.02Å	Favored (93.45%) alpha helix	-	-	-
A 132		ALA	2.09	-	Favored (90.42%) General / -62.3,-46.1	-	0.03Å	Favored (84.097%) alpha helix	-	-	-
A 133		GLU	2.19	-	Favored (82.27%) General / -67.7,-37.5	Favored (54.6%) *mm-30* chi angles: 284.7,293.2,304.2	0.12Å	Favored (83.403%) alpha helix	-	-	-
A 134		ALA	2.31	-	Favored (91.18%) General / -63.3,-38.5	-	0.07Å	Favored (89.286%) alpha helix	-	-	-
A 135		MET	2.45	-	Favored (68.56%) General / -72.2,-39.5	Favored (15%) *tpt* chi angles: 184.6,62.3,183.3	0.06Å	Favored (87.876%) alpha helix	-	-	-
A 136		ARG	2.59	-	Favored (93.52%) General / -63.2,-39.0	Favored (86.1%) *mtt180* chi angles: 288.8,167.2,180.7,158.5	0.06Å	Favored (97.319%) alpha helix	-	-	-

#	Alt	Res	High B	Clash > 0.4Å	Ramachandran	Rotamer	Cβ deviation	CaBLAM	Bond lengths	Bond angles	Cis Peptides
A 137		SER	2.73	-	Favored (92.54%) General / -64.7,-43.8	Favored (66%) *m* chi angles: 297.1	0.08Å	Favored (87.016%) alpha helix	-	-	-
A 138		ALA	2.86	-	Favored (91.79%) General / -60.0,-41.3	-	0.05Å	Favored (89.16%) alpha helix	-	-	-
A 139		GLN	2.97	-	Favored (96.1%) General / -64.8,-40.9	Favored (95.4%) *mt0* chi angles: 290.5,169.8,340.5	0.04Å	Favored (93.915%) alpha helix	-	-	-
A 140		ARG	3.07	-	Favored (89.27%) General / -66.4,-39.9	Favored (99.6%) *mtm-85* chi angles: 290.3,194.3,295.3,270.9	0.02Å	Favored (95.064%) alpha helix	-	-	-
**#**	**Alt**	**Res**	**High B**	**Clash > 0.4Å**	**Ramachandran**	**Rotamer**	**Cβ deviation**	**CaBLAM**	**Bond lengths**	**Bond angles**	**Cis Peptides**
			Avg: 4.25	Clashscore: 1.02	Outliers: 1 of 254	Poor rotamers: 0 of 207	Outliers: 0 of 238	Outliers: 8 of 252	Outliers: 3 of 256	Outliers: 1 of 256	Non-Trans: 0 of 255
A 141		ARG	3.19	-	Favored (79.82%) General / -68.6,-37.5	Favored (99%) *mtt180* chi angles: 290.7,175,181.5,173.9	0.04Å	Favored (94.769%) alpha helix	-	-	-
A 142		THR	3.35	-	Favored (87.78%) General / -61.8,-46.9	Favored (52.5%) *m* chi angles: 294.8	0.02Å	Favored (90.303%) alpha helix	-	-	-
A 143		ALA	3.58	-	Favored (93.75%) General / -60.6,-41.0	-	0.04Å	Favored (92.8%) alpha helix	-	-	-
A 144		ALA	3.92	-	Favored (85.77%) General / -60.1,-39.3	-	0.03Å	Favored (85.823%) alpha helix	-	-	-
A 145		GLY	4.39	-	Favored (40.39%) Glycine / -63.7,-53.1	-	-	Favored (89.008%) alpha helix	-	-	-
A 146		ILE	4.96	-	Favored (85.12%) Ile or Val / -59.6,-41.5	Favored (92.1%) *mt* chi angles: 291.5,168.6	0.02Å	Favored (75.879%) alpha helix	-	-	-
A 147		MET	5.63	-	Favored (66.24%) General / -61.1,-24.5	Favored (95.7%) *mtp* chi angles: 288.4,173.7,69.1	0.03Å	Favored (6.946%)	-	-	-
A 148		LYS	6.28	-	Allowed (0.41%) General / 66.5,-54.9	Favored (98.3%) *mttt* chi angles: 292.6,179.9,180.9,178.9	0.06Å	Favored (6.074%)	-	-	-
A 149		ASN	6.75	-	Favored (6.32%) General / -81.5,64.7	Favored (87.2%) *m-40* chi angles: 294,318.4	0.02Å	Favored (14.451%)	-	-	-
A 150		ALA	6.95	0.42Å O with A 157 ALA N	Favored (75.57%) General / -61.4,-34.5	-	0.03Å	Favored (18.984%)	-	-	-
A 151		VAL	6.88	-	Favored (46.24%) Ile or Val / -128.5,141.6	Favored (8.4%) *p* chi angles: 61.3	0.07Å	Favored (8.299%)	-	-	-

A 152		VAL	6.58	-	Favored (2.17%) Ile or Val / -119.5,-62.1	Favored (70.9%) *t* chi angles: 178.7	0.04Å	CaBLAM Disfavored (2.809%)	-	-	-
A 153		ASP	6.1	-	Favored (17.41%) General / -104.8,21.2	Favored (72.7%) *m-30* chi angles: 295.1,317.4	0.03Å	CaBLAM Outlier (0.539%)	-	-	-
A 154		GLY	5.51	-	Favored (70.13%) Glycine / 95.1,-9.6	-	-	Favored (37.037%)	-	-	-
A 155		ILE	4.9	-	Favored (54.31%) Ile or Val / -100.6,126.0	Favored (80.2%) *mt* chi angles: 299.8,168.5	0.09Å	Favored (31.453%)	-	-	-
A 156		VAL	4.34	-	Favored (24.25%) Ile or Val / -81.2,115.7	Favored (62.5%) *t* chi angles: 179.7	0.07Å	Favored (50.614%)	-	-	-
A 157		ALA	3.87	0.42Å N with A 150 ALA O	Favored (72.26%) General / -60.8,-32.9	-	0.04Å	Favored (36.956%)	-	-	-
A 158		THR	3.5	-	Favored (10.57%) General / -106.3,25.9	Favored (69.5%) *p* chi angles: 59.2	0.06Å	Favored (11.061%)	-	-	-
A 159		ASP	3.23	-	Favored (19.04%) General / -49.1,130.6	Favored (25.1%) *t70* chi angles: 187.4,80.3	0.03Å	Favored (19.911%)	-	-	-
A 160		VAL	3.06	-	Favored (60.61%) Pre-Pro / -107.1,115.1	Favored (71.4%) *t* chi angles: 178.6	0.07Å	Favored (46.756%)	-	-	-
**#**	**Alt**	**Res**	**High B**	**Clash > 0.4Å**	**Ramachandran**	**Rotamer**	**Cβ deviation**	**CaBLAM**	**Bond lengths**	**Bond angles**	**Cis Peptides**
			Avg: 4.25	Clashscore: 1.02	Outliers: 1 of 254	Poor rotamers: 0 of 207	Outliers: 0 of 238	Outliers: 8 of 252	Outliers: 3 of 256	Outliers: 1 of 256	Non-Trans: 0 of 255
A 161		PRO	2.99	-	Favored (85.23%) Trans-Pro / -56.0,142.2	Favored (98.5%) *Cg_exo* chi angles: 332.1,35.1,332.5	0.03Å	Favored (61.387%)	-	-	-
A 162		GLU	2.97	-	Favored (58.45%) General / -62.1,139.3	Favored (89.1%) *tt0* chi angles: 186,172.7,2.4	0.05Å	Favored (31.315%)	-	-	-
A 163		LEU	2.98	-	Favored (11.81%) General / -47.9,127.3	Favored (4.4%) *tt* chi angles: 194.7,156.3	0.14Å	Favored (42.435%)	-	-	-
A 164		GLU	2.96	-	Favored (9.5%) General / -83.0,78.8	Favored (91.7%) *mt-10* chi angles: 296,186.3,353.6	0.08Å	Favored (45.178%)	-	-	-
A 165		ARG	2.89	-	Favored (51.31%) General / -59.6,143.1	Favored (83.6%) *mtt90* chi angles: 295.2,181.4,180.5,87.6	0.03Å	Favored (16.066%)	-	-	-
A 166		THR	2.77	-	Favored (50.49%) General / -56.6,132.0	Favored (88.7%) *m* chi angles: 301.3	0.04Å	Favored (43.513%)	-	-	-
A 167		THR	2.6	-	Favored (94.7%) Pre-Pro / -64.7,130.3	Favored (84.4%) *m* chi angles: 301.8	0.06Å	Favored (46.434%)	-	-	-

A 168		PRO	2.4	-	Favored (17.09%) Trans-Pro / -48.1,-31.8	Favored (90.8%) *Cg_exo* chi angles: 329.6,36.7,332.7	0.03Å	Favored (79.01%)	-	-	-
A 169		VAL	2.17	-	Favored (90.12%) Ile or Val / -62.2,-41.1	Favored (68.7%) *t* chi angles: 171.9	0.01Å	Favored (70.921%) alpha helix	-	-	-
A 170		MET	1.96	-	Favored (95.11%) General / -65.0,-40.5	Favored (94.4%) *mtp* chi angles: 291.6,170.7,65.7	0.09Å	Favored (98.592%) alpha helix	-	-	-
A 171		GLN	1.77	-	Favored (96.88%) General / -64.0,-40.6	Favored (17%) *mt0* chi angles: 292.1,172.3,173.1	0.04Å	Favored (98.371%) alpha helix	-	-	-
A 172		LYS	1.61	-	Favored (89.87%) General / -65.5,-43.5	Favored (36.5%) *ttpt* chi angles: 182.8,166.5,68.7,174.5	0.07Å	Favored (94.091%) alpha helix	-	-	-
A 173		LYS	1.48	-	Favored (96.38%) General / -61.8,-40.8	Favored (96.8%) *mttt* chi angles: 287.7,178.6,177.3,178.3	0.06Å	Favored (92.066%) alpha helix	-	-	-
A 174		VAL	1.37	-	Favored (99.03%) Ile or Val / -62.2,-44.4	Favored (61.1%) *t* chi angles: 170.9	0.04Å	Favored (96.42%) alpha helix	-	-	-
A 175		GLY	1.29	-	Favored (68.53%) Glycine / -59.8,-50.4	-	-	Favored (95.569%) alpha helix	-	-	-
A 176		GLN	1.22	0.56Å HA with A 176 GLN OE1	Favored (75.11%) General / -63.0,-33.3	Favored (13.5%) *mm-40* chi angles: 292,299.6,14.5	0.08Å	Favored (75.31%) alpha helix	-	-	-
A 177		ILE	1.18	-	Favored (97.95%) Ile or Val / -62.9,-45.8	Favored (94.2%) *mt* chi angles: 292,166.7	0.04Å	Favored (82.291%) alpha helix	-	-	-
A 178		MET	1.14	-	Favored (95.81%) General / -60.2,-42.8	Favored (69%) *mtt* chi angles: 290.2,176.6,180.9	0.04Å	Favored (78.398%) alpha helix	-	-	-
A 179		LEU	1.12	-	Favored (78.34%) General / -56.5,-42.4	Favored (57.6%) *tp* chi angles: 177.7,56.4	0.06Å	Favored (75.776%) alpha helix	-	-	-
A 180		ILE	1.1	-	Favored (99.57%) Ile or Val / -62.9,-44.4	Favored (97.2%) *mt* chi angles: 292.9,169	0.04Å	Favored (94.646%) alpha helix	-	-	-
**#**	**Alt**	**Res**	**High B**	**Clash > 0.4Å**	**Ramachandran**	**Rotamer**	**Cβ deviation**	**CaBLAM**	**Bond lengths**	**Bond angles**	**Cis Peptides**
			Avg: 4.25	Clashscore: 1.02	Outliers: 1 of 254	Poor rotamers: 0 of 207	Outliers: 0 of 238	Outliers: 8 of 252	Outliers: 3 of 256	Outliers: 1 of 256	Non-Trans: 0 of 255
A 181		LEU	1.11	-	Favored (90.89%) General / -62.1,-39.0	Favored (97.4%) *mt* chi angles: 293.4,172.1	0.05Å	Favored (88.055%) alpha helix	-	-	-
A 182		VAL	1.14	-	Favored (90.05%) Ile or Val / -66.6,-44.1	Favored (68.6%) *t* chi angles: 171.9	0.05Å	Favored (90.594%) alpha helix	-	-	-

A 183		SER	1.19	-	Favored (99.73%) General / -62.2,-42.9	Favored (69.4%) *m* chi angles: 296.5	0.08Å	Favored (93.299%) alpha helix	-	-	-
A 184		MET	1.29	-	Favored (88.66%) General / -66.7,-40.4	Favored (79.9%) *mtm* chi angles: 290.3,189.2,292.7	0.02Å	Favored (93.425%) alpha helix	-	-	-
A 185		ALA	1.42	-	Favored (85.9%) General / -60.8,-38.6	-	0.04Å	Favored (79.745%) alpha helix	-	-	-
A 186		ALA	1.6	-	Favored (93.26%) General / -60.8,-40.6	-	0.07Å	Favored (79.614%) alpha helix	-	-	-
A 187		VAL	1.83	-	Favored (91.68%) Ile or Val / -64.1,-41.2	Favored (69.7%) *t* chi angles: 172.1	0.06Å	Favored (96.085%) alpha helix	-	-	-
A 188		VAL	2.1	-	Favored (97.89%) Ile or Val / -63.9,-44.2	Favored (69.7%) *t* chi angles: 172.1	0.04Å	Favored (85.85%) alpha helix	-	-	-
A 189		VAL	2.39	-	Favored (89.7%) Ile or Val / -66.4,-45.3	Favored (64.5%) *t* chi angles: 171.4	0.02Å	Favored (34.147%)	-	-	-
A 190		ASN	2.62	-	Favored (13.18%) Pre-Pro / -144.3,79.0	Favored (44.2%) *t0* chi angles: 185.9,9.7	0.02Å	Favored (7.664%)	-	-	-
A 191		PRO	2.74	-	Favored (5.47%) Trans-Pro / -77.0,56.7	Favored (59.9%) *Cg_endo* chi angles: 31.9,322.8,26.8	0.07Å	CaBLAM Disfavored (3.872%)	-	-	-
A 192		SER	2.71	-	Favored (18.51%) General / -82.1,167.7	Favored (79.9%) *p* chi angles: 70.3	0.02Å	Favored (30.093%)	-	-	-
A 193		VAL	2.53	-	Favored (97.39%) Ile or Val / -60.9,-44.2	Favored (64.2%) *t* chi angles: 171.4	0.03Å	Favored (59.039%)	-	-	-
A 194		ARG	2.24	-	Favored (90.84%) General / -63.6,-38.4	Favored (95.8%) *mtt-85* chi angles: 288,181.5,179.6,278.1	0.02Å	Favored (89.921%) alpha helix	-	-	-
A 195		THR	1.92	-	Favored (96.78%) General / -62.8,-44.2	Favored (96.5%) *m* chi angles: 299.8	0.09Å	Favored (90.479%) alpha helix	-	-	-
A 196		VAL	1.62	-	Favored (96.46%) Ile or Val / -64.8,-44.0	Favored (72.7%) *t* chi angles: 172.4	0.06Å	Favored (92.896%) alpha helix	-	-	-
A 197		ARG	1.38	-	Favored (96.7%) General / -62.6,-40.4	Favored (97.8%) *mtt180* chi angles: 289.1,174.8,179,168.7	0.03Å	Favored (97.676%) alpha helix	-	-	-
A 198		GLU	1.19	-	Favored (89.01%) General / -62.5,-46.3	Favored (81.1%) *tt0* chi angles: 181.5,174.2,345.9	0.07Å	Favored (96.82%) alpha helix	-	-	-
A 199		ALA	1.06	-	Favored (82.31%) General / -59.8,-38.5	-	0.04Å	Favored (87.825%) alpha helix	-	-	-

#	Alt	Res	High B	Clash > 0.4Å	Ramachandran	Rotamer	Cβ deviation	CaBLAM	Bond lengths	Bond angles	Cis Peptides
A 200		GLY	0.97	-	Favored (81.61%) Glycine / -60.9,-49.2	-	-	Favored (96.508%) alpha helix	-	-	-
#	Alt	Res	High B	Clash > 0.4Å	Ramachandran	Rotamer	Cβ deviation	CaBLAM	Bond lengths	Bond angles	Cis Peptides
			Avg: 4.25	Clashscore: 1.02	Outliers: 1 of 254	Poor rotamers: 0 of 207	Outliers: 0 of 238	Outliers: 8 of 252	Outliers: 3 of 256	Outliers: 1 of 256	Non-Trans: 0 of 255
A 201		ILE	0.91	-	Favored (97.78%) Ile or Val / -61.9,-45.9	Favored (96.7%) *mt* chi angles: 292,167.5	0.07Å	Favored (79.564%) alpha helix	-	-	-
A 202		LEU	0.87	-	Favored (78.4%) General / -69.0,-40.7	Favored (80.3%) *mt* chi angles: 294.4,168.1	0.10Å	Favored (83.807%) alpha helix	-	-	-
A 203		THR	0.85	-	Favored (92.2%) General / -64.8,-43.7	Favored (93%) *m* chi angles: 299.2	0.02Å	Favored (98.133%) alpha helix	-	-	-
A 204		THR	0.84	-	Favored (90.18%) General / -60.6,-46.5	Favored (90.9%) *m* chi angles: 298	0.05Å	Favored (97.951%) alpha helix	-	-	-
A 205		ALA	0.83	-	Favored (89.75%) General / -58.6,-43.8	-	0.09Å	Favored (92.489%) alpha helix	-	-	-
A 206		ALA	0.83	-	Favored (92.79%) General / -65.2,-43.1	-	0.07Å	Favored (94.879%) alpha helix	-	-	-
A 207		ALA	0.83	-	Favored (98.12%) General / -63.5,-43.1	-	0.04Å	Favored (98.113%) alpha helix	-	-	-
A 208		VAL	0.84	-	Favored (98.06%) Ile or Val / -63.2,-45.6	Favored (61%) *t* chi angles: 170.9	0.03Å	Favored (95.917%) alpha helix	-	-	-
A 209		THR	0.88	-	Favored (96.01%) General / -62.6,-44.6	Favored (99%) *m* chi angles: 300.3	0.06Å	Favored (96.383%) alpha helix	-	-	-
A 210		LEU	0.96	-	Favored (82.5%) General / -63.1,-36.1	Favored (88.8%) *mt* chi angles: 290.9,170.7	0.04Å	Favored (77.969%) alpha helix	-	-	-
A 211		TRP	1.1	-	Favored (65.34%) General / -73.0,-40.1	Favored (48.9%) *m-10* chi angles: 292.6,334.9	0.09Å	Favored (57.271%) alpha helix	-	-	-
A 212		GLU	1.3	-	Favored (12.31%) General / -104.0,-27.2	Favored (96.3%) *mt-10* chi angles: 297.4,180.1,352.5	0.02Å	Favored (11.221%) alpha helix	-	-	-
A 213		ASN	1.58	-	Allowed (0.16%) General / 68.2,-29.5	Favored (85.8%) *m-40* chi angles: 299,317.5	0.04Å	CaBLAM Disfavored (3.807%)	-	-	-
A 214		GLY	1.92	-	Favored (19.96%) Glycine / -91.2,-27.0	-	-	CaBLAM Outlier (0.735%)	-	-	-

#	Alt	Res	High B	Clash > 0.4Å	Ramachandran	Rotamer	Cβ deviation	CaBLAM	Bond lengths	Bond angles	Cis Peptides
A 215		ALA	2.25	-	Allowed (1.49%) General / 47.8,-131.6	-	0.02Å	CaBLAM Outlier (0.918%)	-	-	-
A 216		SER	2.46	-	Favored (23.45%) General / -161.9,168.2	Favored (94.7%) *p* chi angles: 66.2	0.01Å	CaBLAM Disfavored (3.277%)	-	-	-
A 217		SER	2.47	-	Favored (64.43%) General / -65.7,-17.2	Favored (89.7%) *p* chi angles: 68.9	0.01Å	Favored (45.033%)	-	-	-
A 218		VAL	2.31	-	Favored (16.84%) Ile or Val / -86.2,-45.0	Favored (94.8%) *t* chi angles: 174.8	0.06Å	Favored (50.815%) alpha helix	-	-	-
A 219		TRP	2.04	-	Favored (9.94%) General / -101.0,24.0	Favored (53.6%) *m100* chi angles: 290.4,120.5	0.11Å	Favored (12.711%)	-	-	-
A 220		ASN	1.77	0.52Å C with A 220 ASN OD1	Favored (7.37%) General / -85.3,178.8	Favored (45.5%) *p0* chi angles: 67.1,22.1	0.04Å	Favored (33.328%)	-	-	-
#	Alt	Res	High B	Clash > 0.4Å	Ramachandran	Rotamer	Cβ deviation	CaBLAM	Bond lengths	Bond angles	Cis Peptides
			Avg: 4.25	Clashscore: 1.02	Outliers: 1 of 254	Poor rotamers: 0 of 207	Outliers: 0 of 238	Outliers: 8 of 252	Outliers: 3 of 256	Outliers: 1 of 256	Non-Trans: 0 of 255
A 221		ALA	1.53	-	Favored (90.53%) General / -65.7,-43.0	-	0.03Å	Favored (58.614%)	-	-	-
A 222		THR	1.35	-	Favored (92.16%) General / -63.1,-45.4	Favored (90.6%) *m* chi angles: 298.1	0.03Å	Favored (87.381%) alpha helix	-	-	-
A 223		THR	1.22	-	Favored (90.72%) General / -64.9,-44.1	Favored (91.4%) *m* chi angles: 297.9	0.11Å	Favored (85.009%) alpha helix	-	-	-
A 224		ALA	1.14	-	Favored (82.5%) General / -59.2,-39.3	-	0.06Å	Favored (83.283%) alpha helix	-	-	-
A 225		ILE	1.09	-	Favored (81.1%) Ile or Val / -66.1,-48.5	Favored (94.5%) *mt* chi angles: 292.5,166	0.06Å	Favored (88.064%) alpha helix	-	-	-
A 226		GLY	1.07	-	Favored (89.58%) Glycine / -62.8,-34.4	-	-	Favored (91.646%) alpha helix	-	-	-
A 227		LEU	1.08	-	Favored (94.83%) General / -64.0,-39.7	Favored (88%) *mt* chi angles: 290.4,172.3	0.05Å	Favored (89.198%) alpha helix	-	-	-
A 228		CYS	1.13	-	Favored (89.91%) General / -65.7,-38.7	Favored (95.8%) *m* chi angles: 292.1	0.12Å	Favored (93.722%) alpha helix	-	-	-
A 229		HIS	1.22	-	Favored (65.5%) General / -73.5,-38.4	Favored (35.4%) *m170* chi angles: 286.5,156.5	0.14Å	Favored (95.273%) alpha helix	OUTLIER(S) worst is CB--CG: 4.0 σ	-	-

A 230		ILE	1.36	-	Favored (56.81%) Ile or Val / -65.5,-32.1	Favored (11.2%) *tp* chi angles: 194.6,63.8	0.07Å	Favored (75.074%) alpha helix	-	-	-
A 231		MET	1.52	-	Favored (62.78%) General / -57.8,-27.1	Favored (65.2%) *ttp* chi angles: 180.4,181.9,70	0.03Å	Favored (68.832%) alpha helix	-	-	-
A 232		ARG	1.69	-	Favored (36.94%) General / -103.9,7.9	Favored (86.6%) *mtp85* chi angles: 296.1,172.9,63.7,85.8	0.06Å	Favored (37.594%)	-	-	-
A 233		GLY	1.83	-	Favored (83.91%) Glycine / 85.1,7.1	-	-	Favored (86.965%)	-	-	-
A 234		GLY	1.9	-	Favored (27.71%) Glycine / -79.1,144.8	-	-	Favored (13.262%)	-	-	-
A 235		TRP	1.9	-	Favored (5.69%) General / -118.1,-29.1	Favored (79.9%) *p-90* chi angles: 62,271.8	0.06Å	Favored (6.826%)	-	-	-
A 236		LEU	1.84	-	Favored (79.13%) General / -69.0,-39.7	Favored (40.3%) *tp* chi angles: 182.2,54.8	0.17Å	Favored (69.988%) alpha helix	-	-	-
A 237		SER	1.75	-	Favored (78.35%) General / -58.3,-39.0	Favored (45.5%) *t* chi angles: 180.6	0.09Å	Favored (95.285%) alpha helix	-	-	-
A 238		CYS	1.65	-	Favored (57.02%) General / -76.7,-20.0	Favored (63.1%) *m* chi angles: 300.2	0.05Å	Favored (67.025%) alpha helix	-	-	-
A 239		LEU	1.58	-	Favored (91.16%) General / -65.8,-39.8	Favored (48.4%) *tp* chi angles: 183.7,58.8	0.05Å	Favored (47.039%) alpha helix	-	-	-
A 240		SER	1.53	-	Favored (92.29%) General / -59.2,-44.2	Favored (42.9%) *t* chi angles: 180.8	0.05Å	Favored (90.868%) alpha helix	-	-	-
**#**	**Alt**	**Res**	**High B**	**Clash > 0.4Å**	**Ramachandran**	**Rotamer**	**Cβ deviation**	**CaBLAM**	**Bond lengths**	**Bond angles**	**Cis Peptides**
			Avg: 4.25	Clashscore: 1.02	Outliers: 1 of 254	Poor rotamers: 0 of 207	Outliers: 0 of 238	Outliers: 8 of 252	Outliers: 3 of 256	Outliers: 1 of 256	Non-Trans: 0 of 255
A 241		ILE	1.53	-	Favored (98.15%) Ile or Val / -62.6,-45.7	Favored (92.1%) *mt* chi angles: 292,170.8	0.13Å	Favored (88.29%) alpha helix	OUTLIER(S) worst is CB--CG1: 4.2 σ	-	-
A 242		THR	1.56	-	Favored (88.32%) General / -59.0,-46.4	Favored (91.2%) *m* chi angles: 298.9	0.04Å	Favored (99.043%) alpha helix	-	-	-
A 243		TRP	1.63	-	Favored (89.74%) General / -59.6,-46.2	Favored (90.7%) *t60* chi angles: 183.6,88.3	0.04Å	Favored (98.507%) alpha helix	-	-	-
A 244		THR	1.74	-	Favored (88.46%) General / -61.7,-46.7	Favored (89.3%) *m* chi angles: 298.7	0.03Å	Favored (96.915%) alpha helix	-	-	-
A 245		LEU	1.9	-	Favored (89.91%) General / -63.8,-38.1	Favored (90.6%) *mt* chi angles: 292.2,175	0.02Å	Favored (91.795%) alpha helix	-	-	-

A 246		ILE	2.14	-	Favored (98.48%) Ile or Val / -63.2,-45.4	Favored (93.1%) *mt* chi angles: 291.4,167.5	0.03Å	Favored (95.261%) alpha helix	-	-	-
A 247		LYS	2.47	-	Favored (72.1%) General / -66.3,-31.4	Favored (68.8%) *mmtt* chi angles: 292.6,300.5,181.2,184.5	0.05Å	Favored (77.662%) alpha helix	-	-	-
A 248		ASN	2.94	-	Favored (78.88%) General / -68.5,-42.2	Favored (93.3%) *m-40* chi angles: 285.8,340.1	0.03Å	Favored (76.667%) alpha helix	-	-	-
A 249		MET	3.58	-	Favored (66.61%) General / -66.4,-25.1	Favored (81.8%) *mtm* chi angles: 288.7,187.2,287.6	0.07Å	Favored (60.415%) three-ten	-	-	-
A 250		GLU	4.42	-	Favored (67.75%) General / -60.7,-27.2	Favored (92.6%) *mt-10* chi angles: 287.7,182,352.3	0.07Å	Favored (46.773%)	-	-	-
A 251		LYS	5.46	-	Favored (87.06%) Pre-Pro / -77.2,122.9	Favored (87.4%) *tttt* chi angles: 183.8,177.1,180.4,179.5	0.04Å	Favored (29.912%)	-	-	-
A 252		PRO	6.62	-	Favored (20.71%) Trans-Pro / -47.6,-34.6	Favored (68.8%) *Cg_exo* chi angles: 328.8,38.1,331.5	0.07Å	Favored (61.734%)	-	-	-
A 253		GLY	7.78	-	Favored (64.29%) Glycine / -62.4,-23.1	-	-	Favored (71.773%) alpha helix	-	-	-
A 254		LEU	8.79	-	Favored (27.43%) General / -108.2,11.4	Favored (92%) *mt* chi angles: 298.8,176.6	0.05Å	Favored (36.342%)	-	-	-
A 255		LYS	9.54	-	Favored (44.61%) General / -83.1,-17.0	Favored (97.5%) *mttt* chi angles: 291.7,181.4,182.5,179.6	0.05Å	-	-	-	-
A 256		ARG	10	-	-	Favored (93.8%) *mmt-90* chi angles: 294.5,290.4,183.5,274	0.02Å	-	-	-	-

About MolProbity | Website for the Richardson Lab | Using ecloud x-H | Internal reference 4.5.2
