# Supplementary material for: Integrative proteome-wide structural analysis and high-throughput docking identify broad-spectrum antiviral scaffolds against Zika, Yellow Fever, West Nile, Saint Louis encephalitis, and Usutu viruses
Source: Front Cell Infect Microbiol. 2026 Apr 30;16:1723132. doi: 10.3389/fcimb.2026.1723132 (PMC13171538; doi:10.3389/fcimb.2026.1723132)
Supplement: Supplementary file 5 [file DataSheet5.zip › WNV/WNV_NS5/Mol_probity_Files/WNV_NS5_1FH-multi.table.pdf]

Viewing WNV_NS5_1FH-multi.table

Duke Biochemistry
Duke University School of Medicine

When finished, you should close this window. *Hint: Use File | Save As... to save a copy of this page.*

All-Atom Contacts	Clashscore, all atoms:	1.46		99th percentile* (N=1784, all resolutions)
	Clashscore is the number of serious steric overlaps (> 0.4 Å) per 1000 atoms.			
Protein Geometry	Poor rotamers	0	0.00%	Goal: <0.3%
	Favored rotamers	770	99.48%	Goal: >98%
	Ramachandran outliers	4	0.44%	Goal: <0.05%
	Ramachandran favored	875	96.90%	Goal: >98%
	Rama distribution Z-score	-0.01 ± 0.26		Goal: abs(Z score) < 2
	MolProbity score^	1.07		100th percentile* (N=27675, 0Å - 99Å)
	Cβ deviations >0.25Å	0	0.00%	Goal: 0
	Bad bonds:	17 / 7432	0.23%	Goal: 0%
	Bad angles:	10 / 10043	0.10%	Goal: <0.1%
Peptide Omegas	Cis Prolines:	0 / 34	0.00%	Expected: ≤1 per chain, or ≤5%
	Twisted Peptides:	1 / 904	0.11%	Goal: 0
Low-resolution Criteria	CaBLAM outliers	24	2.7%	Goal: <1.0%
	CA Geometry outliers	4	0.44%	Goal: <0.5%
Additional validations	Chiral volume outliers	0/1057		
	Waters with clashes	0/0	0.00%	See UnDowser table for details

In the two column results, the left column gives the raw count, right column gives the percentage.

* 100th percentile is the best among structures of comparable resolution; 0th percentile is the worst. For clashscore the comparative set of structures was selected in 2004, for MolProbity score in 2006.

^ MolProbity score combines the clashscore, rotamer, and Ramachandran evaluations into a single score, normalized to be on the same scale as X-ray resolution.

Key to table colors and cutoffs here:

#	Alt	Res	High B	Clash > 0.4Å	Ramachandran	Rotamer	Cβ deviation	CaBLAM	Bond lengths	Bond angles	Cis Peptides
			Avg: 0.93	Clashscore: 1.46	Outliers: 4 of 903	Poor rotamers: 0 of 774	Outliers: 0 of 826	Outliers: 27 of 901	Outliers: 15 of 905	Outliers: 10 of 905	Non-Trans: 1 of 904
A 1		GLY	7.39	-	-	-	-	-	-	-	-
A 2		GLY	6.45	-	Favored (39.65%) Glycine / -91.2,-164.9	-	-	-	-	-	-
A 3		ALA	5.2	-	Favored (67.58%) General / -58.7,-30.7	-	0.04Å	Favored (24.174%)	-	-	-
A 4		LYS	3.88	-	Favored (41.95%) General / -97.0,9.5	Favored (99.2%) *mttt* chi angles: 294.7,182.2,179.3,179.2	0.02Å	Favored (37.95%)	-	-	-
A 5		GLY	2.74	-	Favored (50.79%) Glycine / -63.9,148.7	-	-	Favored (55.405%)	-	-	-
A 6		ARG	1.91	-	Favored (22.39%) General / -88.0,152.0	Favored (86.8%) *mtm180* chi angles: 295.1,183.1,293,173.3	0.04Å	Favored (47.431%) beta sheet	-	-	-

A 7		THR	1.37	-	Favored (37.09%) General / -79.9,139.6	Favored (94.4%) *m* chi angles: 300.9	0.06Å	Favored (41.809%)	-	-	-
A 8		LEU	1.05	-	Favored (68.33%) General / -57.0,-34.6	Favored (87.7%) *mt* chi angles: 290.7,173.1	0.06Å	Favored (57.745%)	-	-	-
A 9		GLY	0.87	-	Favored (70.94%) Glycine / -61.2,-29.3	-	-	Favored (88.819%) alpha helix	-	-	-
A 10		GLU	0.79	-	Favored (63.94%) General / -72.1,-44.0	Favored (92.7%) *tt0* chi angles: 183.6,180,0.1	0.06Å	Favored (74.928%) alpha helix	-	-	-
A 11		VAL	0.76	-	Favored (94.04%) Ile or Val / -61.6,-42.4	Favored (70.2%) *t* chi angles: 172.1	0.07Å	Favored (95.028%) alpha helix	-	-	-
A 12		TRP	0.77	-	Favored (77.05%) General / -61.0,-49.5	Favored (20.9%) *t60* chi angles: 182.9,60.1	0.01Å	Favored (93.769%) alpha helix	OUTLIER(S) worst is NE1--CE2: 4.4 σ	-	-
A 13		LYS	0.79	-	Favored (81.26%) General / -59.6,-38.4	Favored (21.3%) *mmmt* chi angles: 288.5,284.9,283.2,183.2	0.09Å	Favored (85.449%) alpha helix	-	-	-
A 14		GLU	0.82	-	Favored (85.47%) General / -62.5,-47.1	Favored (47.8%) *tt0* chi angles: 182.3,176.9,59.1	0.00Å	Favored (86.001%) alpha helix	-	-	-
A 15		ARG	0.84	-	Favored (90.13%) General / -58.9,-42.7	Favored (62.4%) *ttt90* chi angles: 184.6,172.4,180.2,83.9	0.03Å	Favored (94.508%) alpha helix	-	-	-
A 16		LEU	0.88	-	Favored (97.68%) General / -60.8,-42.5	Favored (76.8%) *mt* chi angles: 287.6,168.8	0.10Å	Favored (92.9%) alpha helix	-	-	-
A 17		ASN	0.91	-	Favored (66.3%) General / -66.3,-22.9	Favored (92.2%) *m-40* chi angles: 285.4,341.6	0.10Å	Favored (70.994%) alpha helix	-	-	-
A 18		HIS	0.94	-	Favored (48.41%) General / -90.7,5.4	Favored (59.9%) *m170* chi angles: 293.5,172.8	0.02Å	Favored (46.337%)	-	-	-
A 19		MET	0.97	-	Favored (23.68%) General / -83.3,155.2	Favored (44%) *mmp* chi angles: 294.3,292.5,102.2	0.10Å	Favored (37.038%)	-	-	-
A 20		THR	0.99	-	Favored (22.28%) General / -78.8,166.6	Favored (79.5%) *p* chi angles: 60.4	0.04Å	Favored (49.883%)	-	-	-
**#**	**Alt**	**Res**	**High B**	**Clash > 0.4Å**	**Ramachandran**	**Rotamer**	**Cβ deviation**	**CaBLAM**	**Bond lengths**	**Bond angles**	**Cis Peptides**
			Avg: 0.93	Clashscore: 1.46	Outliers: 4 of 903	Poor rotamers: 0 of 774	Outliers: 0 of 826	Outliers: 27 of 901	Outliers: 15 of 905	Outliers: 10 of 905	Non-Trans: 1 of 904
A 21		LYS	0.99	-	Favored (75.98%) General / -61.5,-34.8	Favored (97.1%) *mttt* chi angles: 289.5,179.8,180.1,178.4	0.02Å	Favored (62.773%)	-	-	-
A 22		GLU	0.98	-	Favored (64.49%) General / -68.2,-48.8	Favored (91.7%) *tt0* chi angles: 182.1,176.4,1.4	0.00Å	Favored (75.491%) alpha helix	-	-	-

A 23		GLU	0.95	-	Favored (94.94%) General / -64.8,-40.2	Favored (42.1%) *mt-10* chi angles: 289.1,165.8,315.7	0.05Å	Favored (80.423%) alpha helix	-	-	-
A 24		PHE	0.92	-	Favored (79.18%) General / -58.7,-49.0	Favored (84.8%) *t80* chi angles: 182.5,77.4	0.06Å	Favored (90.483%) alpha helix	-	-	-
A 25		THR	0.88	-	Favored (96.57%) General / -62.0,-44.5	Favored (99.4%) *m* chi angles: 300.4	0.03Å	Favored (94.165%) alpha helix	-	-	-
A 26		ARG	0.85	-	Favored (86.47%) General / -60.9,-38.7	Favored (95.3%) *mtt-85* chi angles: 288.5,179.3,187.5,276.9	0.02Å	Favored (82.148%) alpha helix	-	-	-
A 27		TYR	0.84	-	Favored (63.85%) General / -74.3,-36.9	Favored (43.2%) *m-80* chi angles: 288,116.6	0.04Å	Favored (77.065%) alpha helix	-	-	-
A 28		ARG	0.83	-	Favored (70.73%) General / -56.9,-37.0	Favored (66%) *mtm180* chi angles: 282.6,185.9,287.7,170.4	0.10Å	Favored (70.333%) alpha helix	-	-	-
A 29		LYS	0.83	-	Favored (38.91%) General / -101.0,11.8	Favored (96.6%) *mttt* chi angles: 296.7,182.8,180.3,183.5	0.06Å	Favored (13.329%) alpha helix	-	-	-
A 30		GLU	0.84	-	Allowed (0.33%) General / -90.5,-80.3	Favored (63.9%) *tt0* chi angles: 184.5,186.3,16.5	0.12Å	CaBLAM Disfavored (1.038%) try alpha helix	-	-	-
A 31		ALA	0.85	-	Favored (8.41%) General / -89.0,72.4	-	0.06Å	CaBLAM Outlier (0.763%)	-	-	-
A 32		ILE	0.86	-	Favored (15.9%) Ile or Val / -148.7,148.4	Favored (22.8%) *tt* chi angles: 189.4,166.4	0.07Å	Favored (21.515%)	-	-	-
A 33		THR	0.87	-	Favored (8.14%) General / -78.6,-52.7	Favored (93.2%) *m* chi angles: 297.6	0.10Å	CaBLAM Outlier (0.221%)	-	-	-
A 34		GLU	0.9	-	OUTLIER (0.02)%) General / 54.4,170.7	Favored (36.3%) *mt-10* chi angles: 291.6,191.2,289.2	0.11Å	CaBLAM Disfavored (3.84%)	-	-	-
A 35		VAL	0.94	-	Favored (69.88%) Ile or Val / -126.2,133.0	Favored (62.8%) *t* chi angles: 179.6	0.04Å	Favored (13.894%)	-	-	-
A 36		ASP	0.98	-	Favored (7.27%) General / -82.6,93.2	Favored (63.8%) *t0* chi angles: 182.4,344.1	0.07Å	Favored (36.428%) beta sheet	-	-	-
A 37		ARG	1.03	-	Favored (41.01%) General / -93.4,8.3	Favored (77.7%) *mtp180* chi angles: 293.5,173.4,67.2,202.3	0.09Å	Favored (12.457%)	-	-	-
A 38		SER	1.08	-	Favored (72%) General / -61.0,-32.5	Favored (93.4%) *p* chi angles: 64.3	0.02Å	Favored (49.523%)	-	-	-
A 39		ALA	1.12	-	Favored (97.8%) General / -63.8,-40.9	-	0.03Å	Favored (91.694%) alpha helix	-	-	-

#	Alt	Res	High B	Clash > 0.4Å	Ramachandran	Rotamer	Cβ deviation	CaBLAM	Bond lengths	Bond angles	Cis Peptides
A 40		ALA	1.16	-	Favored (93.98%) General / -64.8,-39.7	-	0.04Å	Favored (91.108%) alpha helix	-	-	-
**#**	**Alt**	**Res**	**High B**	**Clash > 0.4Å**	**Ramachandran**	**Rotamer**	**Cβ deviation**	**CaBLAM**	**Bond lengths**	**Bond angles**	**Cis Peptides**
			Avg: 0.93	Clashscore: 1.46	Outliers: 4 of 903	Poor rotamers: 0 of 774	Outliers: 0 of 826	Outliers: 27 of 901	Outliers: 15 of 905	Outliers: 10 of 905	Non-Trans: 1 of 904
A 41		LYS	1.2	-	Favored (93.98%) General / -64.2,-39.4	Favored (96.9%) *mttt* chi angles: 289.6,178.9,181,179.2	0.01Å	Favored (84.546%) alpha helix	-	-	-
A 42		HIS	1.25	-	Favored (76.99%) General / -69.7,-39.3	Favored (74.6%) *m-70* chi angles: 289.3,298.7	0.01Å	Favored (76.874%) alpha helix	-	-	-
A 43		ALA	1.32	-	Favored (45.12%) General / -76.1,-5.1	-	0.05Å	CaBLAM Outlier (0.037%) try alpha helix	-	OUTLIER(S) worst is CA-C-O: 4.9 σ	-
A 44		ARG	1.4	-	Favored (8%) General / -96.9,-42.3	Favored (8.6%) *mpt180* chi angles: 274.9,72.4,181.6,157.1	0.02Å	CaBLAM Outlier (0.375%) try alpha helix	-	-	-
A 45		ARG	1.49	-	Favored (75.92%) General / -61.2,-34.9	Favored (95.7%) *mtt180* chi angles: 287.9,171.8,182,169.3	0.01Å	Favored (73.137%) alpha helix	-	-	-
A 46		GLU	1.55	-	Favored (57.58%) General / -86.2,-1.6	Favored (96.8%) *mt-10* chi angles: 293.9,181.9,358.7	0.02Å	Favored (55.045%)	-	-	-
A 47		GLY	1.58	-	Favored (89.13%) Glycine / 82.0,0.1	-	-	Favored (73.911%)	-	-	-
A 48		ASN	1.57	0.46Å C with A 48 ASN OD1	Favored (9.24%) General / -82.3,74.6	Favored (60.6%) *t0* chi angles: 187.7,343.8	0.05Å	Favored (10.865%)	-	-	-
A 49		ILE	1.52	-	Allowed (0.42%) Ile or Val / -80.1,14.1	Favored (38.8%) *pt* chi angles: 64.4,168.8	0.07Å	CaBLAM Disfavored (1.22%)	-	-	-
A 50		THR	1.42	-	Favored (30.86%) General / -101.5,-2.4	Favored (28.4%) *p* chi angles: 69.9	0.03Å	Favored (29.982%)	-	-	-
A 51		GLY	1.29	-	Favored (62.78%) Glycine / -57.5,-31.0	-	-	Favored (5.242%)	-	-	-
A 52		GLY	1.16	-	Favored (78.81%) Glycine / 90.8,-9.1	-	-	Favored (44.691%) beta sheet	-	-	-
A 53		HIS	1.04	-	Favored (98.1%) Pre-Pro / -67.8,145.7	Favored (99.3%) *m-70* chi angles: 297.4,290.6	0.08Å	Favored (22.673%) beta sheet	-	-	-
A 54		PRO	0.93	-	Favored (47.82%) Trans-Pro / -56.4,150.9	Favored (97.7%) *Cg_exo* chi angles: 331.9,33.5,334.6	0.10Å	Favored (46.13%)	-	-	-
A 55		VAL	0.85	-	Favored (62.72%)	Favored (76.9%) *t* chi angles: 172.8	0.06Å	Favored (23.853%)	-	-	-

					Ile or Val / -72.7,-41.8						
A 56		SER	0.78	-	Favored (43.96%) General / -145.7,157.3	Favored (42.1%) *t* chi angles: 175.9	0.02Å	Favored (20.004%)	-	-	-
A 57		ARG	0.74	-	Favored (58.16%) General / -56.7,-27.0	Favored (48.7%) *ttm170* chi angles: 187.1,166.2,293.5,171.7	0.03Å	Favored (38.719%)	-	-	-
A 58		GLY	0.71	-	Favored (64.19%) Glycine / -57.0,-33.6	-	-	Favored (72.221%)	-	-	-
A 59		THR	0.7	-	Favored (84.3%) General / -60.7,-47.9	Favored (52.3%) *m* chi angles: 294.8	0.12Å	Favored (78.249%) alpha helix	-	-	-
A 60		ALA	0.69	-	Favored (80.89%) General / -61.5,-36.6	-	0.04Å	Favored (83.096%) alpha helix	-	-	-
**#**	**Alt**	**Res**	**High B**	**Clash > 0.4Å**	**Ramachandran**	**Rotamer**	**Cβ deviation**	**CaBLAM**	**Bond lengths**	**Bond angles**	**Cis Peptides**
			Avg: 0.93	Clashscore: 1.46	Outliers: 4 of 903	Poor rotamers: 0 of 774	Outliers: 0 of 826	Outliers: 27 of 901	Outliers: 15 of 905	Outliers: 10 of 905	Non-Trans: 1 of 904
A 61		LYS	0.69	-	Favored (92.1%) General / -65.6,-39.9	Favored (91.6%) *mttt* chi angles: 290.8,179.5,188.1,178.1	0.02Å	Favored (91.005%) alpha helix	-	-	-
A 62		LEU	0.69	-	Favored (86.42%) General / -67.0,-38.8	Favored (30.8%) *tp* chi angles: 187,58.3	0.06Å	Favored (79.964%) alpha helix	-	-	-
A 63		ARG	0.71	-	Favored (84.63%) General / -57.8,-42.8	Favored (30.6%) *tpt170* chi angles: 174.9,65.8,180.3,186.2	0.04Å	Favored (80.738%) alpha helix	-	-	-
A 64		TRP	0.73	-	Favored (92.76%) General / -60.4,-45.6	Favored (90.1%) *t60* chi angles: 177.7,84.7	0.02Å	Favored (98.674%) alpha helix	-	-	-
A 65		LEU	0.76	-	Favored (74.27%) General / -61.2,-33.9	Favored (88.5%) *mt* chi angles: 290.5,171.9	0.02Å	Favored (74.14%) alpha helix	-	-	-
A 66		VAL	0.79	-	Favored (30.51%) Ile or Val / -77.3,-44.2	Favored (89.3%) *t* chi angles: 174.1	0.12Å	Favored (73.176%) alpha helix	-	-	-
A 67		GLU	0.81	-	Favored (74.9%) General / -60.8,-34.7	Favored (95.4%) *mt-10* chi angles: 288.5,180.9,355.9	0.07Å	Favored (79.94%) alpha helix	-	-	-
A 68		ARG	0.82	-	Favored (28.3%) General / -81.6,2.6	Favored (64.3%) *mmm-85* chi angles: 293.3,293.4,299.7,271.8	0.03Å	Favored (47.703%)	-	-	-
A 69		ARG	0.81	-	Favored (19.74%) General / 59.1,41.9	Favored (59.3%) *mmm-85* chi angles: 305,289.9,296,277.3	0.03Å	Favored (23.245%)	-	-	-
A 70		PHE	0.79	-	Favored (8.78%) General / -92.4,-44.3	Favored (44.4%) *m-80* chi angles: 296.9,122.1	0.04Å	CaBLAM Disfavored (1.042%)	-	-	-

A 71		LEU	0.76	-	Favored (43.61%) General / -111.9,143.0	Favored (84%) *mt* chi angles: 298.9,172.5	0.06Å	Favored (7.463%)	-	-	-
A 72		GLU	0.72	-	Favored (44.03%) Pre-Pro / -138.1,74.1	Favored (96.3%) *mt-10* chi angles: 295.1,182.2,355.4	0.04Å	CaBLAM Disfavored (2.495%)	-	-	-
A 73		PRO	0.69	-	Favored (56%) Trans-Pro / -52.4,138.5	Favored (98.8%) *Cg_exo* chi angles: 332.6,37.1,329	0.06Å	Favored (13.234%)	-	-	-
A 74		VAL	0.67	-	Favored (15.98%) Ile or Val / -148.0,148.7	Favored (7.3%) *p* chi angles: 60.5	0.09Å	Favored (10.458%)	-	-	-
A 75		GLY	0.64	-	Favored (32.46%) Glycine / 61.2,-123.4	-	-	Favored (31.524%)	-	-	-
A 76		LYS	0.62	-	Favored (39.84%) General / -95.2,125.5	Favored (38.6%) *ttpt* chi angles: 184.7,174.3,72.1,175.7	0.06Å	Favored (12.815%)	-	-	-
A 77		VAL	0.61	0.44Å HG11 with A 91 MET SD	Favored (65.1%) Ile or Val / -117.8,133.3	Favored (37.7%) *t* chi angles: 183.9	0.08Å	Favored (68.609%)	-	-	-
A 78		VAL	0.6	-	Favored (55.76%) Ile or Val / -108.9,132.9	Favored (97.9%) *t* chi angles: 175.6	0.04Å	Favored (71.628%) beta sheet	-	-	-
A 79		ASP	0.6	-	Favored (10.9%) General / -122.1,106.2	Favored (64.1%) *t0* chi angles: 184.6,356.4	0.03Å	Favored (62.532%)	-	-	-
A 80		LEU	0.61	-	Favored (17.62%) General / -94.1,105.6	Favored (69.3%) *mt* chi angles: 303.6,176.8	0.08Å	Favored (13.317%)	-	-	-
**#**	**Alt**	**Res**	**High B**	**Clash > 0.4Å**	**Ramachandran**	**Rotamer**	**Cβ deviation**	**CaBLAM**	**Bond lengths**	**Bond angles**	**Cis Peptides**
			Avg: 0.93	Clashscore: 1.46	Outliers: 4 of 903	Poor rotamers: 0 of 774	Outliers: 0 of 826	Outliers: 27 of 901	Outliers: 15 of 905	Outliers: 10 of 905	Non-Trans: 1 of 904
A 81		GLY	0.62	-	Favored (3.21%) Glycine / 107.8,32.9	-	-	CaBLAM Disfavored (4.761%)	-	-	-
A 82		CYS	0.63	-	Favored (70.84%) General / -58.2,-34.9	Favored (41.1%) *t* chi angles: 186.8	0.01Å	CaBLAM Outlier (0.453%)	-	-	-
A 83		GLY	0.64	-	Favored (46.76%) Glycine / 59.4,-128.3	-	-	Favored (47.026%)	-	-	-
A 84		ARG	0.64	-	Favored (65.41%) General / -67.4,-19.3	Favored (94.4%) *mtt-85* chi angles: 291.9,174.3,184.3,267.8	0.04Å	CaBLAM Disfavored (2.219%)	-	-	-
A 85		GLY	0.64	-	Favored (15.08%) Glycine / 118.5,2.4	-	-	Favored (76.998%)	-	-	-
A 86		GLY	0.63	-	Favored (7.84%) Glycine / -49.8,-58.9	-	-	Favored (17.04%)	-	-	-

A 87		TRP	0.62	-	Favored (79.61%) General / -68.8,-38.2	Favored (29.3%) *m-10* chi angles: 280.9,5.3	0.03Å	Favored (51.514%) alpha helix	-	-	-
A 88		CYS	0.62	-	Favored (98.79%) General / -62.3,-41.8	Favored (93.6%) *m* chi angles: 290.8	0.05Å	Favored (75.406%) alpha helix	-	-	-
A 89		TYR	0.63	-	Favored (61.59%) General / -75.1,-33.4	Favored (37%) *m-80* chi angles: 286.6,118.8	0.04Å	Favored (77.11%) alpha helix	-	-	-
A 90		TYR	0.65	-	Favored (70.35%) General / -58.1,-51.3	Favored (90.6%) *t80* chi angles: 178.8,79.4	0.14Å	Favored (76.729%) alpha helix	-	-	-
A 91		MET	0.69	0.44Å SD with A 77 VAL HG11	Favored (73.26%) General / -64.2,-31.9	Favored (84.2%) *mtm* chi angles: 290.2,188,288.8	0.06Å	Favored (63.643%) alpha helix	OUTLIER(S) worst is CG--SD: 8.1 σ	-	-
A 92		ALA	0.72	-	Favored (62.7%) General / -60.9,-20.9	-	0.06Å	Favored (65.998%) alpha helix	-	-	-
A 93		THR	0.76	-	Favored (57.97%) General / -89.7,-4.0	Favored (62.9%) *p* chi angles: 63.5	0.09Å	Favored (57.705%)	-	-	-
A 94		GLN	0.79	-	Favored (33.54%) General / -87.0,133.9	Favored (72.3%) *mm-40* chi angles: 301.8,294.7,291.3	0.09Å	Favored (34.684%)	-	-	-
A 95		LYS	0.79	-	Favored (66.69%) General / -60.9,-25.5	Favored (97.5%) *mttt* chi angles: 290.7,179.7,180.1,178.8	0.01Å	Favored (36.328%)	-	-	-
A 96		ARG	0.77	-	Favored (57.65%) General / -91.3,-1.5	Favored (84.7%) *mtp180* chi angles: 295.7,179.8,69.4,193.3	0.04Å	Favored (53.965%)	-	-	-
A 97		VAL	0.74	-	Favored (38.94%) Ile or Val / -97.0,133.3	Favored (89.3%) *t* chi angles: 176	0.06Å	Favored (22.366%)	-	-	-
A 98		GLN	0.69	-	Favored (7.96%) General / -103.1,-36.9	Favored (87.6%) *mm-40* chi angles: 294.9,291,304.3	0.09Å	Favored (28.224%)	-	-	-
A 99		GLU	0.64	-	Favored (14.69%) General / -153.6,135.6	Favored (92.2%) *tt0* chi angles: 181,176.5,358.2	0.03Å	Favored (30.384%)	-	-	-
A 100		VAL	0.61	-	Favored (73.9%) Ile or Val / -120.6,125.2	Favored (67.6%) *t* chi angles: 179	0.05Å	Favored (71.96%)	-	-	-
**#**	**Alt**	**Res**	**High B**	**Clash > 0.4Å**	**Ramachandran**	**Rotamer**	**Cβ deviation**	**CaBLAM**	**Bond lengths**	**Bond angles**	**Cis Peptides**
			Avg: 0.93	Clashscore: 1.46	Outliers: 4 of 903	Poor rotamers: 0 of 774	Outliers: 0 of 826	Outliers: 27 of 901	Outliers: 15 of 905	Outliers: 10 of 905	Non-Trans: 1 of 904
A 101		LYS	0.59	-	Favored (12.66%) General / -114.0,105.0	Favored (96.6%) *mttt* chi angles: 295.8,185.5,180.9,184.4	0.04Å	Favored (67.93%) beta sheet	-	-	-
A 102		GLY	0.59	-	Favored (17.35%) Glycine / -95.8,144.6	-	-	Favored (53.144%) beta sheet	-	-	-

A 103		TYR	0.61	-	Favored (47.65%) General / -129.5,134.2	Favored (95.6%) *m-80* chi angles: 298,87.8	0.01Å	Favored (59.215%) beta sheet	-	-	-
A 104		THR	0.65	-	Favored (48.1%) General / -131.9,155.3	Favored (47.2%) *p* chi angles: 66.2	0.10Å	Favored (51.52%) beta sheet	-	-	-
A 105		LYS	0.71	-	Favored (12.33%) General / -87.8,-42.8	Favored (74.7%) *tttt* chi angles: 182.1,167.7,178.9,170.4	0.08Å	CaBLAM Disfavored (1.14%) try beta sheet	-	-	-
A 106		GLY	0.78	-	Favored (53.35%) Glycine / 70.5,-162.8	-	-	Favored (13.591%) beta sheet	-	-	-
A 107		GLY	0.84	-	Favored (49.17%) Glycine / 87.0,176.2	-	-	Favored (6.609%)	-	-	-
A 108		PRO	0.88	-	Favored (30.04%) Trans-Pro / -50.9,-30.4	Favored (89.1%) *Cg_exo* chi angles: 330.8,38,329.6	0.05Å	Favored (10.014%)	-	-	-
A 109		GLY	0.89	-	Favored (62.76%) Glycine / -96.4,2.1	-	-	Favored (64.953%) alpha helix	-	-	-
A 110		HIS	0.88	-	Favored (30.2%) General / -138.8,164.2	Favored (98.9%) *m-70* chi angles: 299.6,286.3	0.03Å	Favored (18.021%)	-	-	-
A 111		GLU	0.85	-	Favored (35.95%) General / -77.2,148.4	Favored (74.7%) *mt-10* chi angles: 294.9,182.7,324.1	0.03Å	Favored (41.229%)	-	-	-
A 112		GLU	0.83	-	Favored (37.89%) Pre-Pro / -98.6,153.5	Favored (79%) *mm-30* chi angles: 300.6,289.7,349	0.08Å	Favored (30.867%)	-	-	-
A 113		PRO	0.82	-	Favored (75.11%) Trans-Pro / -68.5,149.9	Favored (50.1%) *Cg_endo* chi angles: 25.2,327.1,26.6	0.02Å	Favored (71.864%)	-	-	-
A 114		GLN	0.83	-	Favored (46.16%) General / -123.9,149.2	Favored (71%) *mt0* chi angles: 299.7,182.6,295.3	0.05Å	Favored (47.469%) beta sheet	-	-	-
A 115		LEU	0.85	-	Favored (20.22%) General / -96.4,107.8	Favored (67.7%) *mt* chi angles: 303.9,176.8	0.06Å	Favored (39.098%) beta sheet	-	-	-
A 116		VAL	0.87	-	Favored (64.67%) Ile or Val / -124.4,135.2	Favored (98.6%) *t* chi angles: 175.3	0.06Å	Favored (48.884%) beta sheet	-	-	-
A 117		GLN	0.89	-	Favored (8.57%) General / -87.0,64.6	Favored (34.2%) *tt0* chi angles: 185.7,183.6,291.9	0.05Å	Favored (12.789%)	-	-	-
A 118		SER	0.88	-	Favored (17.74%) General / -85.9,163.9	Favored (89%) *p* chi angles: 69.2	0.06Å	Favored (6.674%)	-	-	-
A 119		TYR	0.85	-	Favored (42.15%) General / -56.7,129.7	Favored (87%) *t80* chi angles: 178.3,74.3	0.04Å	Favored (22.12%)	-	-	-

#	Alt	Res	High B	Clash > 0.4Å	Ramachandran	Rotamer	Cβ deviation	CaBLAM	Bond lengths	Bond angles	Cis Peptides
A 120		GLY	0.81	-	Favored (85.79%) Glycine / 84.5,6.4	-	-	Favored (73.738%)	-	-	-
#	Alt	Res	High B	Clash > 0.4Å	Ramachandran	Rotamer	Cβ deviation	CaBLAM	Bond lengths	Bond angles	Cis Peptides
			Avg: 0.93	Clashscore: 1.46	Outliers: 4 of 903	Poor rotamers: 0 of 774	Outliers: 0 of 826	Outliers: 27 of 901	Outliers: 15 of 905	Outliers: 10 of 905	Non-Trans: 1 of 904
A 121		TRP	0.76	-	Favored (10.08%) General / -50.8,-28.8	Favored (77.7%) *p-90* chi angles: 65.6,268.1	0.04Å	Favored (23.618%)	-	-	-
A 122		ASN	0.71	-	Favored (64.71%) General / -67.6,-17.6	Favored (13.9%) *p0* chi angles: 61.6,291.3	0.02Å	Favored (62.396%) three-ten	-	-	-
A 123		ILE	0.66	-	Favored (6.28%) Ile or Val / -100.8,12.8	Favored (37.8%) *pt* chi angles: 64.5,168.4	0.11Å	Favored (40.046%)	-	-	-
A 124		VAL	0.63	-	Favored (66.82%) Ile or Val / -111.4,123.0	Favored (70.7%) *t* chi angles: 178.7	0.07Å	Favored (27.192%)	-	-	-
A 125		THR	0.61	-	Favored (56.97%) General / -116.4,131.3	Favored (90.7%) *m* chi angles: 298	0.01Å	Favored (59.838%)	-	-	-
A 126		MET	0.6	-	Favored (52.45%) General / -127.8,137.5	Favored (68.6%) *mtm* chi angles: 293.8,174.9,284.9	0.01Å	Favored (71.567%) beta sheet	-	-	-
A 127		LYS	0.6	-	Favored (19.03%) General / -109.1,157.1	Favored (73.3%) *mmtt* chi angles: 302.9,296.3,184.8,181.4	0.02Å	Favored (50.67%)	-	-	-
A 128		SER	0.61	-	Favored (44.67%) General / -138.5,158.2	Favored (96.9%) *p* chi angles: 63.6	0.04Å	Favored (8.321%)	-	-	-
A 129		GLY	0.62	-	Favored (70.7%) Glycine / 78.9,20.4	-	-	Favored (37.191%)	-	-	-
A 130		VAL	0.64	-	Favored (35.71%) Ile or Val / -109.0,111.3	Favored (68.2%) *t* chi angles: 179	0.07Å	Favored (22.669%)	-	-	-
A 131		ASP	0.66	-	Favored (9.98%) General / -85.2,97.5	Favored (59.7%) *t0* chi angles: 183.8,339.7	0.06Å	Favored (70.772%) beta sheet	-	-	-
A 132		VAL	0.68	-	Favored (5.19%) Ile or Val / -55.5,-19.1	Favored (30.1%) *m* chi angles: 296.6	0.03Å	Favored (30.359%)	-	-	-
A 133		PHE	0.69	-	Favored (63.58%) General / -69.7,-20.1	Favored (53.7%) *m-80* chi angles: 285.4,107.4	0.02Å	Favored (45.105%)	-	-	-
A 134		TYR	0.7	-	Favored (14.96%) General / -115.7,8.4	Favored (84.3%) *m-80* chi angles: 302.3,99.8	0.07Å	Favored (36.121%)	-	-	-
A 135		ARG	0.69	-	Favored (66.65%) Pre-Pro / -76.7,137.6	Favored (47.7%) *ttt-90* chi angles: 188.6,191.5,183.1,285	0.07Å	Favored (35.511%)	-	-	-
A 136		PRO	0.68	-	Favored (70.07%)	Favored (73.3%) *Cg_exo*	0.04Å	Favored (79.86%)	-	-	-

					Trans-Pro / -58.5,150.2	chi angles: 335.1,33.7,331.8		beta sheet			
A 137		SER	0.66	-	Favored (25.76%) General / -73.4,165.1	Favored (85.6%) *p* chi angles: 67.8	0.06Å	Favored (29.015%) beta sheet	-	-	-
A 138		GLU	0.63	-	Favored (24.19%) General / -144.0,134.0	Favored (75%) *tt0* chi angles: 180.7,174.3,341.3	0.02Å	Favored (18.992%) beta sheet	-	-	-
A 139		ALA	0.61	-	Favored (57.84%) General / -61.6,141.4	-	0.02Å	Favored (26.607%) beta sheet	-	-	-
A 140		SER	0.59	-	Favored (31.3%) General / -152.0,166.5	Favored (94%) *p* chi angles: 64.7	0.04Å	Favored (44.463%)	-	-	-
**#**	**Alt**	**Res**	**High B**	**Clash > 0.4Å**	**Ramachandran**	**Rotamer**	**Cβ deviation**	**CaBLAM**	**Bond lengths**	**Bond angles**	**Cis Peptides**
			Avg: 0.93	Clashscore: 1.46	Outliers: 4 of 903	Poor rotamers: 0 of 774	Outliers: 0 of 826	Outliers: 27 of 901	Outliers: 15 of 905	Outliers: 10 of 905	Non-Trans: 1 of 904
A 141		ASP	0.58	-	Favored (13.7%) General / -89.2,-39.2	Favored (51%) *m-30* chi angles: 295.6,297.1	0.06Å	Favored (6.15%)	-	-	-
A 142		THR	0.58	-	Favored (49.04%) General / -129.1,135.5	Favored (86.6%) *m* chi angles: 301.5	0.01Å	Favored (35.738%)	-	-	-
A 143		LEU	0.59	-	Favored (27.77%) General / -123.6,118.6	Favored (50.7%) *tp* chi angles: 176.6,66.4	0.09Å	Favored (61.756%)	-	-	-
A 144		LEU	0.62	-	Favored (31.87%) General / -109.5,148.0	Favored (68.1%) *mt* chi angles: 303.2,181.4	0.03Å	Favored (44.606%) beta sheet	-	-	-
A 145		CYS	0.68	-	Favored (23.67%) General / -143.4,133.1	Favored (57.8%) *t* chi angles: 182.2	0.03Å	Favored (42.015%) beta sheet	-	-	-
A 146		ASP	0.75	-	Favored (2.38%) General / -137.1,43.0	Favored (41.8%) *t0* chi angles: 191.7,23	0.04Å	Favored (7.003%) beta sheet	-	-	-
A 147		ILE	0.82	-	Favored (29.99%) Ile or Val / -92.2,134.6	Favored (47.2%) *mm* chi angles: 300,297.5	0.04Å	CaBLAM Disfavored (1.635%)	-	-	-
A 148		GLY	0.89	-	Favored (4.42%) Glycine / 114.7,127.8	-	-	Favored (32.67%)	-	-	-
A 149		GLU	0.93	-	Favored (16.31%) General / -100.3,157.8	Favored (76.7%) *mm-30* chi angles: 301.1,291.7,330.2	0.03Å	Favored (37.503%)	-	-	-
A 150		SER	0.94	-	Favored (23.79%) General / -77.6,165.8	Favored (88.1%) *p* chi angles: 67	0.03Å	Favored (17.605%) beta sheet	-	-	-
A 151		SER	0.92	-	Favored (20.99%) General / -160.7,153.4	Favored (39.5%) *t* chi angles: 177.8	0.05Å	Favored (43.609%)	-	-	-

A 152		SER	0.88	-	Favored (66.45%) General / -65.1,-20.4	Favored (90.3%) *p* chi angles: 66.7	0.02Å	Favored (19.589%)	-	-	-
A 153		SER	0.83	-	Favored (23.64%) General / -93.5,111.9	Favored (20.5%) *t* chi angles: 170.2	0.03Å	Favored (34.725%)	-	-	-
A 154		ALA	0.79	-	Favored (77.54%) General / -60.7,-35.9	-	0.03Å	Favored (51.21%)	-	-	-
A 155		GLU	0.75	-	Favored (96.76%) General / -61.2,-44.7	Favored (89.3%) *tt0* chi angles: 178.9,176.8,358.3	0.04Å	Favored (79.159%) alpha helix	-	-	-
A 156		VAL	0.71	-	Favored (98.9%) Ile or Val / -62.4,-44.1	Favored (61.1%) *t* chi angles: 170.9	0.03Å	Favored (95.66%) alpha helix	-	-	-
A 157		GLU	0.68	-	Favored (81.93%) General / -63.1,-35.9	Favored (86.4%) *mt-10* chi angles: 288.9,187.4,357.6	0.07Å	Favored (81.649%) alpha helix	-	-	-
A 158		GLU	0.65	-	Favored (87.31%) General / -58.3,-45.9	Favored (50.6%) *tt0* chi angles: 183.7,179.9,37.5	0.06Å	Favored (80.353%) alpha helix	-	-	-
A 159		HIS	0.64	-	Favored (79.39%) General / -68.7,-41.2	Favored (81.9%) *m-70* chi angles: 291.5,298.3	0.06Å	Favored (85.656%) alpha helix	-	-	-
A 160		ARG	0.63	-	Favored (96.62%) General / -63.8,-43.4	Favored (98%) *mtt180* chi angles: 288.8,174.8,178.2,169.5	0.03Å	Favored (96.718%) alpha helix	-	-	-
**#**	**Alt**	**Res**	**High B**	**Clash > 0.4Å**	**Ramachandran**	**Rotamer**	**Cβ deviation**	**CaBLAM**	**Bond lengths**	**Bond angles**	**Cis Peptides**
			Avg: 0.93	Clashscore: 1.46	Outliers: 4 of 903	Poor rotamers: 0 of 774	Outliers: 0 of 826	Outliers: 27 of 901	Outliers: 15 of 905	Outliers: 10 of 905	Non-Trans: 1 of 904
A 161		THR	0.63	-	Favored (88.62%) General / -64.1,-45.5	Favored (52%) *m* chi angles: 294.8	0.03Å	Favored (94.348%) alpha helix	-	-	-
A 162		VAL	0.63	-	Favored (97.39%) Ile or Val / -64.1,-43.0	Favored (72.1%) *t* chi angles: 172.4	0.07Å	Favored (91.577%) alpha helix	-	-	-
A 163		ARG	0.64	-	Favored (73.27%) General / -55.0,-43.2	Favored (62.9%) *ttp-170* chi angles: 184.4,182.2,62.7,190.4	0.03Å	Favored (86.367%) alpha helix	-	-	-
A 164		VAL	0.65	-	Favored (96.93%) Ile or Val / -64.5,-43.9	Favored (62.1%) *t* chi angles: 171.1	0.03Å	Favored (95.319%) alpha helix	-	-	-
A 165		LEU	0.66	-	Favored (90.54%) General / -62.0,-38.9	Favored (88%) *mt* chi angles: 290.4,172.2	0.03Å	Favored (94.22%) alpha helix	-	-	-
A 166		GLU	0.67	-	Favored (91.96%) General / -65.8,-40.3	Favored (98.7%) *mt-10* chi angles: 290.5,176.8,354.6	0.02Å	Favored (92.111%) alpha helix	-	-	-
A 167		MET	0.68	-	Favored (76.9%)	Favored (48.6%) *ttp* chi angles: 174.8,186.5,66.3	0.06Å	Favored (86.517%) alpha helix	-	-	-

					General / -56.6,-48.7						
A 168		VAL	0.69	-	Favored (86.68%) Ile or Val / -66.0,-40.2	Favored (61.4%) *t* chi angles: 171	0.08Å	Favored (57.382%) alpha helix	-	-	-
A 169		GLU	0.69	-	Favored (46.02%) General / -49.6,-42.9	Favored (79.3%) *tt0* chi angles: 181.5,179.6,341.2	0.03Å	Favored (65.083%) alpha helix	-	-	-
A 170		ASP	0.69	-	Favored (66.57%) General / -58.9,-28.8	Favored (89.4%) *m-30* chi angles: 284,347.3	0.09Å	Favored (69.431%) alpha helix	-	-	-
A 171		TRP	0.69	-	Favored (29.42%) General / -82.2,-33.6	Favored (57.6%) *m100* chi angles: 290.2,72.8	0.12Å	Favored (62.877%) alpha helix	-	-	-
A 172		LEU	0.68	-	Favored (69.07%) General / -66.2,-28.8	Favored (94.3%) *mt* chi angles: 294.1,174.6	0.04Å	Favored (70.816%) three-ten	-	-	-
A 173		HIS	0.68	-	Favored (22.67%) General / -76.2,-0.6	Favored (76%) *m-70* chi angles: 287.4,293.3	0.07Å	Favored (44.653%)	-	-	-
A 174		ARG	0.67	-	Favored (49.07%) General / -94.0,-4.7	Favored (50.5%) *mmt180* chi angles: 300.1,297.1,183.6,191.8	0.02Å	Favored (19.538%)	-	-	-
A 175		GLY	0.67	-	Favored (4.29%) Glycine / 85.5,49.7	-	-	Favored (25.034%)	-	-	-
A 176		PRO	0.66	-	Favored (70.94%) Trans-Pro / -68.8,154.5	Favored (53.8%) *Cg_endo* chi angles: 25.6,327.9,25.7	0.01Å	Favored (29.281%)	-	-	-
A 177		LYS	0.65	-	Favored (27.85%) General / -85.6,-22.6	Favored (56.3%) *mttp* chi angles: 294.6,177.3,175.4,63.7	0.10Å	Favored (30.88%)	-	-	-
A 178		GLU	0.64	-	Favored (23.92%) General / -153.5,145.9	Favored (27%) *tt0* chi angles: 173.7,176.6,290.2	0.01Å	Favored (27.897%)	OUTLIER(S) worst is CG--CD: 4.0 σ	-	-
A 179		PHE	0.63	-	Favored (43.51%) General / -150.6,160.9	Favored (43.8%) *p90* chi angles: 56.4,85.6	0.05Å	Favored (59.695%)	-	-	-
A 180		CYS	0.62	-	Favored (4.82%) General / -151.8,115.4	Favored (48.1%) *t* chi angles: 179	0.12Å	Favored (28.132%) beta sheet	-	-	-
**#**	**Alt**	**Res**	**High B**	**Clash > 0.4Å**	**Ramachandran**	**Rotamer**	**Cβ deviation**	**CaBLAM**	**Bond lengths**	**Bond angles**	**Cis Peptides**
			Avg: 0.93	Clashscore: 1.46	Outliers: 4 of 903	Poor rotamers: 0 of 774	Outliers: 0 of 826	Outliers: 27 of 901	Outliers: 15 of 905	Outliers: 10 of 905	Non-Trans: 1 of 904
A 181		ILE	0.62	-	Favored (73.76%) Ile or Val / -120.6,125.1	Favored (3.6%) *mp* chi angles: 301.5,96.2	0.07Å	Favored (56.831%) beta sheet	-	-	-
A 182		LYS	0.62	-	Favored (46.97%) General / -67.9,132.2	Favored (34.2%) *ttpt* chi angles: 184,174.7,73.9,186.5	0.04Å	Favored (43.343%) beta sheet	-	-	-
A 183		VAL	0.62	-	Favored (74.17%)	Favored (83.4%) *t* chi angles: 177.8	0.11Å	Favored (72.266%)	-	-	-

					Ile or Val / -118.2,125.3						
A 184		LEU	0.64	-	Favored (39.59%) General / -104.6,139.3	Favored (79.9%) *mt* chi angles: 302.2,178	0.02Å	Favored (13.173%)	-	-	-
A 185		CYS	0.66	-	Favored (9.04%) Pre-Pro / 56.4,56.2	Favored (65.1%) *m* chi angles: 286.4	0.09Å	Favored (9.044%)	-	-	-
A 186		PRO	0.69	-	Favored (33.65%) Trans-Pro / -66.6,-11.9	Favored (56.3%) *Cg_endo* chi angles: 26,323.9,31.2	0.05Å	Favored (45.873%)	-	-	-
A 187		TYR	0.71	0.43Å C with A 187 TYR CD1	Favored (42.62%) General / -87.6,-13.5	Favored (32%) *p90* chi angles: 71.6,86.3	0.07Å	Favored (47.792%)	-	-	-
A 188		MET	0.74	-	Favored (74.91%) Pre-Pro / -74.5,136.4	Favored (89.2%) *mmm* chi angles: 292.9,290.3,287.3	0.05Å	Favored (34.755%)	-	-	-
A 189		PRO	0.76	-	Favored (31.05%) Trans-Pro / -49.7,-33.2	Favored (86.2%) *Cg_exo* chi angles: 330.5,37.4,330.7	0.03Å	Favored (75.33%)	-	-	-
A 190		ARG	0.77	-	Favored (56.63%) General / -76.3,-38.1	Favored (69.2%) *ttt180* chi angles: 189.4,181,175.6,195	0.04Å	Favored (82.32%) alpha helix	-	-	-
A 191		VAL	0.77	-	Favored (83.94%) Ile or Val / -68.2,-41.6	Favored (93.6%) *t* chi angles: 174.6	0.03Å	Favored (79.993%) alpha helix	-	-	-
A 192		ILE	0.76	-	Favored (92.39%) Ile or Val / -60.5,-47.5	Favored (95.9%) *mt* chi angles: 292.1,168.3	0.07Å	Favored (94.373%) alpha helix	-	-	-
A 193		GLU	0.74	-	Favored (83.53%) General / -62.2,-37.0	Favored (96.6%) *mt-10* chi angles: 288.4,179.7,352.1	0.03Å	Favored (83.164%) alpha helix	-	-	-
A 194		LYS	0.72	-	Favored (75.36%) General / -69.1,-43.0	Favored (53.9%) *tptt* chi angles: 184,66.2,177.3,179.1	0.01Å	Favored (86.091%) alpha helix	-	-	-
A 195		MET	0.71	-	Favored (93.42%) General / -65.4,-40.3	Favored (76.7%) *mtm* chi angles: 290.3,187.7,295	0.06Å	Favored (95.042%) alpha helix	-	-	-
A 196		GLU	0.7	-	Favored (98.23%) General / -62.3,-43.7	Favored (92.1%) *tt0* chi angles: 179.8,177.3,356.6	0.05Å	Favored (98.483%) alpha helix	-	-	-
A 197		THR	0.69	-	Favored (84.72%) General / -59.8,-47.7	Favored (89.5%) *m* chi angles: 298.7	0.06Å	Favored (88.403%) alpha helix	-	-	-
A 198		LEU	0.69	0.45Å HB3 with A 221 TRP CH2	Favored (87.08%) General / -66.2,-38.1	Favored (97.1%) *mt* chi angles: 293.1,173.3	0.03Å	Favored (82.394%) alpha helix	-	-	-
A 199		GLN	0.69	-	Favored (83.05%) General / -59.6,-48.1	Favored (60%) *tt0* chi angles: 178.1,179.4,51.1	0.04Å	Favored (87.315%) alpha helix	-	-	-
A 200		ARG	0.69	-	Favored (77.14%) General / -61.1,-35.5	Favored (97.2%) *mtt-85* chi angles: 288.6,179.8,181.7,276.3	0.02Å	Favored (73.241%) alpha helix	-	-	-

#	Alt	Res	High B	Clash > 0.4Å	Ramachandran	Rotamer	Cβ deviation	CaBLAM	Bond lengths	Bond angles	Cis Peptides
			Avg: 0.93	Clashscore: 1.46	Outliers: 4 of 903	Poor rotamers: 0 of 774	Outliers: 0 of 826	Outliers: 27 of 901	Outliers: 15 of 905	Outliers: 10 of 905	Non-Trans: 1 of 904
A 201		ARG	0.68	-	Favored (36.7%) General / -80.4,-34.1	Favored (99.1%) *mtt180* chi angles: 290.7,175.9,180,175.3	0.03Å	Favored (61.17%) alpha helix	-	-	-
A 202		TYR	0.67	-	Favored (12.84%) General / -113.2,-14.7	Favored (71.9%) *m-80* chi angles: 300.5,109.9	0.13Å	Favored (24.456%)	-	-	-
A 203		GLY	0.66	-	Favored (6.7%) Glycine / 84.4,44.1	-	-	Favored (40.006%)	-	-	-
A 204		GLY	0.64	-	Favored (9.67%) Glycine / -124.8,-157.3	-	-	Favored (28.244%)	-	-	-
A 205		GLY	0.63	-	Favored (46.2%) Glycine / -175.5,173.4	-	-	Favored (10.853%)	-	-	-
A 206		LEU	0.63	-	Favored (32.37%) General / -96.8,139.8	Favored (94.3%) *mt* chi angles: 297,174	0.03Å	Favored (13.607%)	OUTLIER(S) worst is CB--CG: 4.1 σ	-	-
A 207		VAL	0.64	-	Favored (37.48%) Ile or Val / -130.4,152.4	Favored (30.3%) *m* chi angles: 300.3	0.07Å	Favored (58.013%) beta sheet	-	-	-
A 208		ARG	0.66	-	Favored (24.93%) General / -117.7,115.4	Favored (25.5%) *tpt170* chi angles: 172.9,75.6,178.8,182.6	0.05Å	Favored (46.555%) beta sheet	-	-	-
A 209		ASN	0.67	-	Favored (86.26%) Pre-Pro / -77.7,122.8	Favored (32.6%) *t0* chi angles: 190.7,307.4	0.06Å	Favored (39.433%)	-	-	-
A 210		PRO	0.69	-	Favored (13.89%) Trans-Pro / -54.9,-17.4	Favored (91.3%) *Cg_exo* chi angles: 333.2,34.8,332	0.06Å	Favored (39.681%)	-	-	-
A 211		LEU	0.71	-	Favored (58.92%) General / -88.0,-3.0	Favored (93.6%) *mt* chi angles: 297.9,175.2	0.06Å	Favored (48.564%)	-	-	-
A 212		SER	0.72	-	Favored (36.97%) General / -78.9,133.5	Favored (64.3%) *m* chi angles: 294.1	0.02Å	Favored (28.539%)	-	-	-
A 213		ARG	0.73	-	Favored (57.82%) General / -59.0,137.7	Favored (98.1%) *mtt180* chi angles: 288.7,178.7,177.4,173.7	0.03Å	Favored (47.36%)	-	-	-
A 214		ASN	0.74	-	Favored (4.51%) General / -82.8,58.8	Favored (85.4%) *m-40* chi angles: 293.8,316.3	0.04Å	CaBLAM Disfavored (1.326%)	-	-	-
A 215		SER	0.74	-	Allowed (0.17%) General / -152.7,-12.5	Favored (78.5%) *p* chi angles: 60.7	0.07Å	CaBLAM Disfavored (2.637%)	-	-	-
A 216		THR	0.73	-	Favored (38.74%) General / -116.5,120.4	Favored (98.3%) *m* chi angles: 300.6	0.01Å	Favored (23.163%)	-	-	-
A 217		HIS	0.71	-	Favored (10.17%)	Favored (2.8%) *t-90*	0.04Å	Favored (29.106%)	-	-	-

					General / -84.4,76.2	chi angles: 199.7,319.6					
A 218		GLU	0.68	-	Favored (36.78%) General / -111.5,146.7	Favored (20.5%) *pt0* chi angles: 64.2,179.5,16.6	0.04Å	Favored (10.905%) beta sheet	-	-	-
A 219		MET	0.66	-	Favored (39.47%) General / -142.9,160.9	Favored (81%) *mtp* chi angles: 298.1,184,78.5	0.03Å	Favored (45.864%) beta sheet	-	-	-
A 220		TYR	0.64	-	Favored (41.87%) General / -107.7,139.7	Favored (96.6%) *m-80* chi angles: 292.8,94.1	0.02Å	Favored (46.731%) beta sheet	-	-	-
**#**	**Alt**	**Res**	**High B**	**Clash > 0.4Å**	**Ramachandran**	**Rotamer**	**Cβ deviation**	**CaBLAM**	**Bond lengths**	**Bond angles**	**Cis Peptides**
			Avg: 0.93	Clashscore: 1.46	Outliers: 4 of 903	Poor rotamers: 0 of 774	Outliers: 0 of 826	Outliers: 27 of 901	Outliers: 15 of 905	Outliers: 10 of 905	Non-Trans: 1 of 904
A 221		TRP	0.63	0.45Å CH2 with A 198 LEU HB3	Favored (25.67%) General / -95.4,114.0	Favored (22.7%) *t60* chi angles: 180.4,46.1	0.03Å	Favored (62.762%) beta sheet	-	-	-
A 222		VAL	0.65	-	Favored (69.56%) Ile or Val / -125.6,133.3	Favored (47.4%) *t* chi angles: 182	0.08Å	Favored (54.574%)	-	-	-
A 223		SER	0.67	-	Favored (39.14%) General / -65.0,153.9	Favored (96.5%) *p* chi angles: 65	0.04Å	Favored (13.634%)	-	-	-
A 224		HIS	0.7	-	Favored (4.07%) General / 68.5,4.0	Favored (89.6%) *m-70* chi angles: 306.3,294.9	0.05Å	CaBLAM Disfavored (4.667%)	-	-	-
A 225		ALA	0.74	-	Favored (12.37%) General / -92.1,167.0	-	0.03Å	Favored (27.505%)	-	-	-
A 226		SER	0.77	-	Favored (26.35%) General / -156.6,168.8	Favored (85.5%) *p* chi angles: 67.3	0.02Å	CA Geom Outlier (0.394%)	-	-	-
A 227		GLY	0.79	-	Favored (10.77%) Glycine / 124.4,160.4	-	-	Favored (17.031%) beta sheet	-	-	-
A 228		ASN	0.8	-	Favored (33.8%) General / -67.6,127.7	Favored (26.4%) *t0* chi angles: 184.3,274.8	0.04Å	Favored (8.315%)	-	-	-
A 229		ILE	0.81	-	Favored (95.26%) Ile or Val / -63.9,-46.1	Favored (77.3%) *mt* chi angles: 289.4,168.9	0.07Å	Favored (45.862%)	-	-	-
A 230		VAL	0.81	-	Favored (98.88%) Ile or Val / -62.7,-43.7	Favored (69.3%) *t* chi angles: 172	0.04Å	Favored (89.939%) alpha helix	-	-	-
A 231		HIS	0.8	-	Favored (75.53%) General / -62.0,-49.6	Favored (85.6%) *t70* chi angles: 183.2,74.5	0.01Å	Favored (82.123%) alpha helix	-	-	-
A 232		SER	0.79	-	Favored (85.04%) General / -58.6,-41.1	Favored (72.3%) *m* chi angles: 295.8	0.06Å	Favored (79.146%) alpha helix	-	-	-
A 233		VAL	0.78	-	Favored (88.89%)	Favored (69.2%) *t* chi angles: 172	0.03Å	Favored (90.179%)	-	-	-

					Ile or Val / -66.3,-45.7			alpha helix			
A 234		ASN	0.76	-	Favored (88.92%) General / -62.7,-38.1	Favored (90.8%) *m-40* chi angles: 285.1,339	0.04Å	Favored (91.412%) alpha helix	-	-	-
A 235		MET	0.75	-	Favored (78.44%) General / -69.0,-36.9	Favored (50.5%) *mmp* chi angles: 294.7,301.5,97.8	0.02Å	Favored (98.75%) alpha helix	-	-	-
A 236		THR	0.75	-	Favored (95.88%) General / -63.0,-44.5	Favored (91.1%) *m* chi angles: 298.9	0.05Å	Favored (96.891%) alpha helix	-	-	-
A 237		SER	0.75	-	Favored (94.43%) General / -63.0,-39.3	Favored (71.5%) *m* chi angles: 295.2	0.03Å	Favored (84.737%) alpha helix	-	-	-
A 238		GLN	0.75	-	Favored (92.06%) General / -62.0,-39.4	Favored (93.3%) *mt0* chi angles: 289.3,179.3,340.5	0.07Å	Favored (84.958%) alpha helix	-	-	-
A 239		VAL	0.75	-	Favored (82.61%) Ile or Val / -66.4,-47.9	Favored (66.8%) *t* chi angles: 171.7	0.03Å	Favored (79.444%) alpha helix	-	-	-
A 240		LEU	0.76	-	Favored (87.57%) General / -63.0,-37.5	Favored (98.3%) *mt* chi angles: 292.4,171.9	0.05Å	Favored (83.774%) alpha helix	-	-	-
**#**	**Alt**	**Res**	**High B**	**Clash > 0.4Å**	**Ramachandran**	**Rotamer**	**Cβ deviation**	**CaBLAM**	**Bond lengths**	**Bond angles**	**Cis Peptides**
			Avg: 0.93	Clashscore: 1.46	Outliers: 4 of 903	Poor rotamers: 0 of 774	Outliers: 0 of 826	Outliers: 27 of 901	Outliers: 15 of 905	Outliers: 10 of 905	Non-Trans: 1 of 904
A 241		LEU	0.77	-	Favored (76.77%) General / -68.0,-44.4	Favored (42.6%) *tp* chi angles: 185,60.1	0.02Å	Favored (81.542%) alpha helix	-	-	-
A 242		GLY	0.78	-	Favored (73.36%) Glycine / -57.5,-35.7	-	-	Favored (89.62%) alpha helix	-	-	-
A 243		ARG	0.81	-	Favored (70.1%) General / -62.3,-29.0	Favored (98.1%) *mtt180* chi angles: 290.4,175.1,181.1,181	0.03Å	Favored (73.467%) alpha helix	-	-	-
A 244		MET	0.85	-	Favored (58.19%) General / -85.6,-2.5	Favored (96.4%) *mmm* chi angles: 295.1,299.1,295	0.09Å	Favored (49.351%) alpha helix	-	-	-
A 245		GLU	0.9	-	Favored (12.38%) General / -106.0,-24.5	Favored (97.4%) *mt-10* chi angles: 295.2,180.4,351.2	0.04Å	Favored (5.353%)	-	-	-
A 246		LYS	0.93	-	Favored (51.7%) General / -55.7,135.0	Favored (96.9%) *mttt* chi angles: 291.4,183.4,180,183.4	0.02Å	Favored (24.191%)	-	-	-
A 247		LYS	0.95	-	Favored (29.44%) General / -90.8,-15.9	Favored (99.3%) *mttt* chi angles: 295.2,178.6,180.4,178.1	0.02Å	Favored (49.876%)	-	-	-
A 248		THR	0.96	-	Favored (30.61%) General / -118.3,118.0	Favored (97.7%) *m* chi angles: 300	0.01Å	Favored (17.627%)	-	-	-

A 249		TRP	0.95	-	Favored (43.93%) General / -71.5,131.9	Favored (60.9%) *t60* chi angles: 187.4,82.2	0.04Å	Favored (37.668%)	-	-	-
A 250		LYS	0.95	-	Favored (44.36%) General / -101.4,134.5	Favored (86.5%) *tttt* chi angles: 181.8,177.7,178,178.3	0.02Å	Favored (48.898%)	-	-	-
A 251		GLY	0.96	-	Favored (52.8%) Glycine / -76.5,163.4	-	-	Favored (54.57%)	-	-	-
A 252		PRO	0.99	-	Favored (78.33%) Trans-Pro / -66.7,146.2	Favored (43.7%) *Cg_endo* chi angles: 24.3,326.6,28.2	0.02Å	Favored (76.166%)	-	-	-
A 253		GLN	1.02	-	Allowed (1.88%) General / -99.3,73.5	Favored (80.7%) *mm-40* chi angles: 302.3,294.9,296.2	0.06Å	Favored (8.854%) beta sheet	-	-	-
A 254		PHE	1.05	-	Favored (56.21%) General / -59.1,133.6	Favored (57.5%) *m-80* chi angles: 286.2,106.2	0.04Å	Favored (9.155%) beta sheet	-	-	-
A 255		GLU	1.07	-	Favored (35.26%) General / -94.7,136.2	Favored (84.7%) *tt0* chi angles: 179.2,176.8,348.4	0.04Å	Favored (35.957%)	-	-	-
A 256		GLU	1.07	-	Favored (57.15%) General / -62.2,134.9	Favored (90.8%) *tt0* chi angles: 184.7,176.4,3.1	0.01Å	Favored (34.034%)	-	-	-
A 257		ASP	1.05	-	Favored (49.3%) General / -55.2,133.4	Favored (27.9%) *t0* chi angles: 196,32.1	0.08Å	Favored (45.247%)	-	-	-
A 258		VAL	1.03	0.48Å O with A 258 VAL HG12	Allowed (0.2%) Ile or Val / -69.8,84.4	Favored (69%) *t* chi angles: 178.9	0.11Å	Favored (35.38%) beta sheet	-	-	-
A 259		ASN	1.01	-	Favored (5.54%) General / -51.9,120.7	Favored (51.8%) *t0* chi angles: 190.3,330.2	0.04Å	Favored (16.046%) beta sheet	-	-	-
A 260		LEU	1.01	-	Favored (53.72%) General / -92.2,-4.7	Favored (93.7%) *mt* chi angles: 298,178.1	0.06Å	Favored (7.66%)	-	-	-
**#**	**Alt**	**Res**	**High B**	**Clash > 0.4Å**	**Ramachandran**	**Rotamer**	**Cβ deviation**	**CaBLAM**	**Bond lengths**	**Bond angles**	**Cis Peptides**
			Avg: 0.93	Clashscore: 1.46	Outliers: 4 of 903	Poor rotamers: 0 of 774	Outliers: 0 of 826	Outliers: 27 of 901	Outliers: 15 of 905	Outliers: 10 of 905	Non-Trans: 1 of 904
A 261		GLY	1.02	-	Favored (45.8%) Glycine / 71.9,-172.8	-	-	Favored (29.252%)	-	-	-
A 262		SER	1.02	-	Favored (2.66%) General / -134.8,-170.9	Favored (9.7%) *t* chi angles: 191.3	0.04Å	CA Geom Outlier (0.089%)	-	-	-
A 263		GLY	1.03	-	Favored (32.34%) Glycine / 89.6,-165.5	-	-	Favored (28.237%)	-	-	-
A 264		THR	1.05	-	Favored (13.01%) General / -121.1,167.1	Favored (78.6%) *p* chi angles: 60.8	0.05Å	Favored (10.151%) beta sheet	-	-	-

A 265		ARG	1.09	-	Favored (40.12%) General / -121.9,122.7	Favored (34.7%) *tpt170* chi angles: 181.7,70.9,182.9,166.4	0.01Å	Favored (39.298%) beta sheet	-	-	-
A 266		ALA	1.16	-	Favored (42.41%) General / -70.2,154.0	-	0.04Å	Favored (22.554%) beta sheet	-	-	-
A 267		VAL	1.25	-	Favored (16.45%) Ile or Val / -122.8,167.4	Favored (29.7%) *m* chi angles: 298.2	0.06Å	Favored (40.484%)	-	-	-
A 268		GLY	1.35	-	Favored (25.6%) Glycine / -89.1,-152.2	-	-	Favored (59.145%)	-	-	-
A 269		LYS	1.44	-	Allowed (0.12%) Pre-Pro / 80.2,165.7	Favored (73.2%) *mmtt* chi angles: 299.9,294,184.2,181.7	0.03Å	CA Geom Outlier (0.405%)	-	-	-
A 270		PRO	1.48	-	Favored (61.39%) Trans-Pro / -53.1,136.5	Favored (94.8%) *Cg_exo* chi angles: 332.9,36.9,329.2	0.03Å	Favored (8.34%) beta sheet	-	-	-
A 271		LEU	1.44	-	Favored (49.31%) General / -98.0,5.0	Favored (84.8%) *mt* chi angles: 300.8,178	0.03Å	Favored (5.651%) beta sheet	-	-	-
A 272		LEU	1.34	-	OUTLIER (0.02)%) General / 61.9,142.2	Favored (92.5%) *mt* chi angles: 298.3,175.6	0.04Å	CaBLAM Outlier (0.712%) try beta sheet	-	-	-
A 273		ASN	1.2	-	Favored (14.53%) General / -91.9,162.1	Favored (86.5%) *m-40* chi angles: 294.2,317.2	0.02Å	Favored (38.801%)	-	-	-
A 274		SER	1.07	-	Favored (42.99%) General / -152.9,159.8	Favored (90.1%) *p* chi angles: 66.8	0.02Å	Favored (19.985%)	-	-	-
A 275		ASP	0.97	-	Favored (5.13%) General / -79.6,88.4	Favored (65.1%) *t0* chi angles: 185.8,344	0.06Å	Favored (18.56%)	-	-	-
A 276		THR	0.89	-	Favored (63.06%) General / -52.8,-40.4	Favored (97.8%) *m* chi angles: 300	0.02Å	Favored (44.054%)	-	-	-
A 277		SER	0.83	-	Favored (65.63%) General / -65.6,-18.9	Favored (74.3%) *p* chi angles: 71.5	0.04Å	Favored (60.889%) alpha helix	-	-	-
A 278		LYS	0.79	-	Favored (17.95%) General / -94.8,-22.6	Favored (56%) *mttp* chi angles: 292.8,181.3,177.4,64.7	0.06Å	Favored (70.336%) alpha helix	-	-	-
A 279		ILE	0.77	-	Favored (8.77%) Ile or Val / -102.6,-12.7	Favored (48.5%) *pt* chi angles: 62.1,172.8	0.03Å	Favored (37.462%) alpha helix	-	-	-
A 280		LYS	0.75	-	Favored (71.43%) General / -54.8,-49.2	Favored (85.6%) *tttt* chi angles: 180,178,178.1,180	0.02Å	Favored (54.327%) alpha helix	-	-	-
**#**	**Alt**	**Res**	**High B**	**Clash > 0.4Å**	**Ramachandran**	**Rotamer**	**Cβ deviation**	**CaBLAM**	**Bond lengths**	**Bond angles**	**Cis Peptides**
			Avg: 0.93	Clashscore: 1.46	Outliers: 4 of 903	Poor rotamers: 0 of 774	Outliers: 0 of 826	Outliers: 27 of 901	Outliers: 15 of 905	Outliers: 10 of 905	Non-Trans: 1 of 904

A 281		ASN	0.74	-	Favored (76.3%)<br>General / -63.2,-33.8	Favored (97.8%) *m-40*<br>chi angles: 287.1,338.9	0.02Å	Favored (78.862%)<br>alpha helix	-	-	-
A 282		ARG	0.73	-	Favored (82.19%)<br>General / -68.2,-40.5	Favored (97.6%) *mtm-85*<br>chi angles: 292.3,194.7,288.2,275.8	0.07Å	Favored (82.237%)<br>alpha helix	-	-	-
A 283		ILE	0.73	-	Favored (59.26%)<br>Ile or Val / -73.0,-42.6	Favored (96.1%) *mt*<br>chi angles: 294.6,167.7	0.09Å	Favored (80.009%)<br>alpha helix	-	-	-
A 284		GLU	0.73	-	Favored (81.95%)<br>General / -62.9,-36.0	Favored (68.9%) *mm-30*<br>chi angles: 290.8,293.3,307.3	0.01Å	Favored (77.482%)<br>alpha helix	-	-	-
A 285		ARG	0.74	-	Favored (90.25%)<br>General / -58.8,-45.2	Favored (65.5%) *ttt90*<br>chi angles: 183.9,176.5,174.8,87.2	0.01Å	Favored (79.973%)<br>alpha helix	-	-	-
A 286		LEU	0.76	-	Favored (90.6%)<br>General / -63.6,-38.3	Favored (85.5%) *mt*<br>chi angles: 291.2,174.5	0.03Å	Favored (86.031%)<br>alpha helix	-	-	-
A 287		LYS	0.78	-	Favored (86.34%)<br>General / -61.2,-38.3	Favored (97.2%) *mttt*<br>chi angles: 289.2,179.3,180.7,177.2	0.07Å	Favored (81.437%)<br>alpha helix	-	-	-
A 288		LYS	0.81	-	Favored (74.62%)<br>General / -69.3,-42.9	Favored (97.2%) *mttt*<br>chi angles: 289.7,178,181.5,178.4	0.02Å	Favored (80.425%)<br>alpha helix	-	-	-
A 289		GLU	0.84	-	Favored (79.31%)<br>General / -60.6,-36.7	Favored (14.9%) *mm-30*<br>chi angles: 288,279,304.2	0.08Å	Favored (57.026%)<br>alpha helix	-	-	-
A 290		TYR	0.87	-	Favored (9.49%)<br>General / -116.5,27.0	Favored (64.4%) *m-80*<br>chi angles: 304.8,106.2	0.07Å	Favored (17.033%)<br>alpha helix	-	-	-
A 291		SER	0.89	-	Favored (67.62%)<br>General / -54.2,-40.4	Favored (39%) *m*<br>chi angles: 289.6	0.05Å	Favored (39.692%)<br>alpha helix	-	-	-
A 292		SER	0.92	-	Favored (61.77%)<br>General / -64.3,-16.2	Favored (94.2%) *p*<br>chi angles: 66.3	0.03Å	Favored (35.869%)<br>alpha helix	-	-	-
A 293		THR	0.95	-	Favored (9.69%)<br>General / -122.3,8.9	Favored (53.1%) *p*<br>chi angles: 56.6	0.04Å	Favored (20.517%)	-	-	-
A 294		TRP	1.01	-	Favored (35.73%)<br>General / -81.0,139.6	Favored (25.9%) *t-100*<br>chi angles: 198.5,268	0.05Å	Favored (5.154%)	-	-	-
A 295		HIS	1.09	-	Favored (22.65%)<br>General / -156.6,170.3	Favored (54.6%) *p-80*<br>chi angles: 64.5,275.9	0.03Å	Favored (28.887%)	-	-	-
A 296		GLN	1.18	-	Favored (40.77%)<br>General / -116.5,148.4	Favored (85.8%) *mt0*<br>chi angles: 293.5,183.7,305.1	0.05Å	Favored (49.551%)<br>beta sheet	-	-	-
A 297		ASP	1.26	-	Favored (5.96%)<br>General / -137.8,106.7	Favored (60.2%) *t0*<br>chi angles: 185.4,358.8	0.03Å	Favored (43.034%)<br>beta sheet	-	-	-

A 298		ALA	1.31	-	Favored (55.32%) General / -61.4,-17.7	-	0.03Å	Favored (36.176%)	-	-	-
A 299		ASN	1.31	-	Favored (38.62%) General / -90.7,7.6	Favored (81.3%) *m-40* chi angles: 289.9,318	0.04Å	Favored (12.693%)	-	-	-
A 300		HIS	1.25	-	Favored (37.62%) Pre-Pro / -51.8,132.3	Favored (82.5%) *t70* chi angles: 183.5,66.4	0.06Å	Favored (37.302%)	OUTLIER(S) worst is CB--CG: 4.4 σ	-	-
**#**	**Alt**	**Res**	**High B**	**Clash > 0.4Å**	**Ramachandran**	**Rotamer**	**Cβ deviation**	**CaBLAM**	**Bond lengths**	**Bond angles**	**Cis Peptides**
			Avg: 0.93	Clashscore: 1.46	Outliers: 4 of 903	Poor rotamers: 0 of 774	Outliers: 0 of 826	Outliers: 27 of 901	Outliers: 15 of 905	Outliers: 10 of 905	Non-Trans: 1 of 904
A 301		PRO	1.16	-	Favored (6.47%) Trans-Pro / -75.8,68.0	Favored (59.3%) *Cg_endo* chi angles: 31.9,322.5,28.1	0.14Å	Favored (9.803%)	-	-	-
A 302		TYR	1.05	-	Favored (26.08%) General / -137.2,165.4	Favored (36.7%) *p90* chi angles: 72.7,97.7	0.07Å	CA Geom Outlier (0.177%)	-	-	-
A 303		ARG	0.96	-	Allowed (1.5%) General / -127.8,-44.2	Favored (33.9%) *ttm170* chi angles: 179.4,170.2,286.6,149.4	0.03Å	CaBLAM Disfavored (1.749%)	-	OUTLIER(S) worst is CA-C-O: 4.1 σ	-
A 304		THR	0.88	-	Favored (3.07%) General / -110.9,-49.0	Favored (99.7%) *m* chi angles: 300.4	0.04Å	Favored (33.339%)	-	-	Twisted nonPRO omega= -145.21
A 305		TRP	0.82	-	Favored (38.93%) General / -77.0,144.2	Favored (45.1%) *m100* chi angles: 285.6,72.3	0.07Å	Favored (17.905%)	-	-	-
A 306		ASN	0.77	-	Favored (20.16%) General / -81.6,114.2	Favored (47.3%) *t0* chi angles: 186.7,1.2	0.03Å	Favored (42.835%)	-	-	-
A 307		TYR	0.74	-	Favored (34.9%) General / -82.5,129.4	Favored (82.2%) *t80* chi angles: 172.8,74.6	0.03Å	Favored (39.022%)	-	-	-
A 308		HIS	0.73	-	Favored (4.9%) General / -111.3,-38.8	Favored (99.3%) *m-70* chi angles: 298.7,286.6	0.05Å	CaBLAM Disfavored (4.785%)	-	-	-
A 309		GLY	0.74	-	Favored (39.4%) Glycine / 175.9,171.1	-	-	Favored (28.424%)	-	-	-
A 310		SER	0.78	-	Favored (26.3%) General / -138.0,165.5	Favored (31.2%) *m* chi angles: 302.8	0.11Å	Favored (58.71%)	-	-	-
A 311		TYR	0.83	-	Favored (44.48%) General / -150.3,159.4	Favored (45.3%) *p90* chi angles: 70.9,92.2	0.05Å	Favored (19.583%)	-	-	-
A 312		GLU	0.9	-	Favored (46.22%) General / -72.7,137.9	Favored (52.4%) *mt-10* chi angles: 293.9,179.7,49.4	0.03Å	Favored (31.233%)	-	-	-
A 313		VAL	0.99	-	Favored (14.52%) Ile or Val / -148.7,150.0	Favored (7.9%) *p* chi angles: 67.7	0.13Å	Favored (39.963%) beta sheet	-	-	-

A 314		LYS	1.09	-	Favored (93.93%) Pre-Pro / -62.1,132.8	Favored (86.3%) *tttt* chi angles: 182.6,178.5,178.6,179.4	0.06Å	Favored (42.839%) beta sheet	-	-	-
A 315		PRO	1.2	-	Favored (78.85%) Trans-Pro / -67.8,150.5	Favored (42.2%) *Cg_endo* chi angles: 23.9,326.8,28.5	0.04Å	Favored (68.221%)	-	-	-
A 316		THR	1.32	-	Favored (29.17%) General / -125.7,160.6	Favored (69.6%) *p* chi angles: 59.3	0.02Å	Favored (12.351%)	-	-	-
A 317		GLY	1.46	-	Favored (48.75%) Glycine / 84.3,-172.2	-	-	Favored (18.628%)	-	-	-
A 318		SER	1.57	-	Favored (18.41%) General / -159.5,149.1	Favored (34.4%) *t* chi angles: 181.4	0.05Å	Favored (18.397%)	-	-	-
A 319		ALA	1.63	-	Favored (53.71%) General / -94.8,-0.8	-	0.02Å	Favored (15.86%) beta sheet	-	-	-
A 320		SER	1.62	-	Favored (23.56%) General / -78.9,165.4	Favored (95.5%) *p* chi angles: 66.1	0.03Å	Favored (20.697%) beta sheet	-	-	-
**#**	**Alt**	**Res**	**High B**	**Clash > 0.4Å**	**Ramachandran**	**Rotamer**	**Cβ deviation**	**CaBLAM**	**Bond lengths**	**Bond angles**	**Cis Peptides**
			Avg: 0.93	Clashscore: 1.46	Outliers: 4 of 903	Poor rotamers: 0 of 774	Outliers: 0 of 826	Outliers: 27 of 901	Outliers: 15 of 905	Outliers: 10 of 905	Non-Trans: 1 of 904
A 321		SER	1.52	-	Favored (9.89%) General / -149.6,124.5	Favored (44%) *t* chi angles: 180.7	0.07Å	Favored (40.276%) beta sheet	-	-	-
A 322		LEU	1.35	-	Favored (22.52%) General / -101.9,151.1	Favored (8.7%) *mp* chi angles: 276.9,61.4	0.01Å	Favored (37.698%) beta sheet	-	-	-
A 323		VAL	1.17	-	Favored (39.57%) Ile or Val / -102.5,135.4	Favored (82%) *t* chi angles: 176.7	0.08Å	Favored (38.997%) beta sheet	-	-	-
A 324		ASN	1.01	-	Favored (4.17%) General / -74.1,102.2	Favored (32.1%) *t0* chi angles: 180,325.5	0.08Å	Favored (54.719%)	-	-	-
A 325		GLY	0.89	-	Favored (77.65%) Glycine / -58.9,-34.3	-	-	Favored (44.924%)	-	-	-
A 326		VAL	0.8	-	Favored (78.8%) Ile or Val / -68.7,-47.0	Favored (65.6%) *t* chi angles: 171.6	0.04Å	Favored (75.541%) alpha helix	-	-	-
A 327		VAL	0.76	-	Favored (88.52%) Ile or Val / -67.2,-43.1	Favored (98.5%) *t* chi angles: 175.3	0.02Å	Favored (77.525%) alpha helix	-	-	-
A 328		ARG	0.74	-	Favored (77.38%) General / -56.4,-48.2	Favored (83.1%) *ttp80* chi angles: 176.8,182.3,63.5,79.4	0.04Å	Favored (95.851%) alpha helix	-	-	-
A 329		LEU	0.75	-	Favored (72.06%) General / -62.1,-31.6	Favored (81.4%) *mt* chi angles: 289.1,172	0.02Å	Favored (70.257%) alpha helix	-	-	-

A 330		LEU	0.76	-	Favored (56.41%) General / -93.9,1.0	Favored (91.3%) *mt* chi angles: 298.1,174.2	0.07Å	Favored (45.16%)	-	-	-
A 331		SER	0.79	-	Favored (5.44%) General / -124.2,32.8	Favored (51.6%) *m* chi angles: 300.9	0.09Å	Favored (20.55%)	-	-	-
A 332		LYS	0.83	-	Favored (81.56%) Pre-Pro / -50.7,-43.4	Favored (96.2%) *mttt* chi angles: 287,178.5,179,178.7	0.14Å	Favored (28.466%)	-	OUTLIER(S) worst is CA-C-N: 4.0 σ	-
A 333		PRO	0.88	-	Favored (49.84%) Trans-Pro / -56.9,-23.2	Favored (99.1%) *Cg_exo* chi angles: 332.3,35,332.1	0.08Å	Favored (66.902%) alpha helix	-	-	-
A 334		TRP	0.95	-	Favored (47.23%) General / -95.8,-2.7	Favored (41.7%) *m-90* chi angles: 295.7,265.7	0.07Å	Favored (53.238%) three-ten	-	-	-
A 335		ASP	1.04	-	Favored (60.18%) General / -71.8,-10.7	Favored (5.9%) *t70* chi angles: 204.9,54.9	0.03Å	Favored (48.806%) three-ten	-	-	-
A 336		THR	1.11	-	Favored (46.8%) General / -97.3,-0.3	Favored (39.8%) *p* chi angles: 67.8	0.06Å	Favored (55.767%)	-	-	-
A 337		ILE	1.16	-	Favored (72.65%) Ile or Val / -117.5,130.3	Favored (84.9%) *mt* chi angles: 298.9,172.9	0.11Å	Favored (28.556%)	-	-	-
A 338		THR	1.19	-	Favored (27.83%) General / -72.6,163.6	Favored (80.4%) *p* chi angles: 60.5	0.06Å	CaBLAM Disfavored (4.636%)	-	-	-
A 339		ASN	1.18	-	Favored (3.2%) General / 69.3,1.3	Favored (88.1%) *m-40* chi angles: 297.7,320.6	0.09Å	CaBLAM Outlier (0.047%) try alpha helix	-	-	-
A 340		VAL	1.16	-	Favored (3.92%) Ile or Val / -115.8,-56.9	Favored (83.7%) *t* chi angles: 177	0.08Å	CaBLAM Outlier (0.951%) try alpha helix	-	-	-
**#**	**Alt**	**Res**	**High B**	**Clash > 0.4Å**	**Ramachandran**	**Rotamer**	**Cβ deviation**	**CaBLAM**	**Bond lengths**	**Bond angles**	**Cis Peptides**
			Avg: 0.93	Clashscore: 1.46	Outliers: 4 of 903	Poor rotamers: 0 of 774	Outliers: 0 of 826	Outliers: 27 of 901	Outliers: 15 of 905	Outliers: 10 of 905	Non-Trans: 1 of 904
A 341		THR	1.12	-	Favored (58.58%) General / -79.0,-8.1	Favored (65.2%) *p* chi angles: 58.2	0.05Å	Favored (14.014%) alpha helix	-	-	-
A 342		THR	1.08	-	Favored (14.87%) General / -115.8,8.6	Favored (61.5%) *p* chi angles: 63.7	0.03Å	Favored (53.216%)	-	-	-
A 343		MET	1.06	-	Favored (33.53%) General / -82.5,140.1	Favored (24.2%) *ttt* chi angles: 186.7,164.9,180.7	0.11Å	Favored (17.023%)	-	-	-
A 344		ALA	1.03	-	Favored (37.15%) General / -153.1,164.9	-	0.04Å	Favored (28.814%)	-	-	-
A 345		MET	1	-	Favored (57.78%) General / -66.0,142.7	Favored (78.8%) *mmm* chi angles: 301.7,308.1,302.2	0.13Å	Favored (34.024%)	-	-	-
A 346		THR	0.97	-	Favored (27.34%)	Favored (51.8%) *p* chi angles: 56.5	0.10Å	Favored (33.576%)	-	-	-

#	Alt	Res	High B	Clash > 0.4Å	Ramachandran	Rotamer	Cβ deviation	CaBLAM	Bond lengths	Bond angles	Cis Peptides
					General / -73.1,164.0						
A 347		ASP	0.93	-	Favored (18.31%) General / -80.3,113.2	Favored (66.3%) *t0* chi angles: 184.8,346.1	0.07Å	Favored (13.958%)	-	-	-
A 348		THR	0.9	-	Favored (25.5%) General / -108.8,12.5	Favored (47.9%) *p* chi angles: 55.8	0.05Å	Favored (17.318%) beta sheet	-	-	-
A 349		THR	0.87	-	Favored (53.4%) Pre-Pro / -76.2,165.4	Favored (70.9%) *p* chi angles: 62.2	0.10Å	Favored (31.76%)	-	-	-
A 350		PRO	0.86	-	Favored (32.33%) Trans-Pro / -48.7,-36.3	Favored (88.7%) *Cg_exo* chi angles: 330,38,330.7	0.05Å	Favored (88.61%)	-	-	-
A 351		PHE	0.85	-	Favored (66.61%) General / -62.3,-51.7	Favored (88.9%) *t80* chi angles: 176.5,81.2	0.05Å	Favored (75.039%) alpha helix	-	OUTLIER(S) worst is CA-CB-CG: 4.3 σ	-
A 352		GLY	0.85	-	Favored (95.46%) Glycine / -60.4,-38.9	-	-	Favored (91.419%) alpha helix	-	-	-
A 353		GLN	0.85	-	Favored (90.44%) General / -66.0,-39.4	Favored (86.5%) *mt0* chi angles: 291.3,178.6,40.5	0.05Å	Favored (88.556%) alpha helix	-	-	-
A 354		GLN	0.86	-	Favored (66.45%) General / -72.3,-32.4	Favored (85.3%) *mm-40* chi angles: 287,298.1,309.8	0.08Å	Favored (77.785%) alpha helix	-	-	-
A 355		ARG	0.87	-	Favored (62.41%) General / -59.3,-53.1	Favored (83.4%) *ttt180* chi angles: 180,176.6,177.5,184.3	0.02Å	Favored (68.034%) alpha helix	-	-	-
A 356		VAL	0.89	-	Favored (65.89%) Ile or Val / -66.4,-33.8	Favored (5.3%) *p* chi angles: 69.7	0.12Å	Favored (65.027%) alpha helix	-	-	-
A 357		PHE	0.91	-	Favored (24.9%) General / -56.5,-56.1	Favored (82.9%) *t80* chi angles: 172.9,74.9	0.05Å	Favored (51.53%) alpha helix	-	-	-
A 358		LYS	0.93	-	Favored (73.25%) General / -69.9,-42.4	Favored (37%) *tttm* chi angles: 180.9,166.1,173.3,287.5	0.12Å	Favored (54.711%) alpha helix	-	-	-
A 359		GLU	0.95	-	Favored (89.71%) General / -63.9,-38.1	Favored (97.9%) *mt-10* chi angles: 289.6,178,355.7	0.05Å	Favored (21.004%) alpha helix	-	-	-
A 360		LYS	0.96	-	Favored (2.8%) General / -117.3,-43.9	Favored (72.3%) *mmtt* chi angles: 302.3,296,181.6,180.3	0.06Å	Favored (7.464%) alpha helix	-	-	-
**#**	**Alt**	**Res**	**High B**	**Clash > 0.4Å**	**Ramachandran**	**Rotamer**	**Cβ deviation**	**CaBLAM**	**Bond lengths**	**Bond angles**	**Cis Peptides**
			Avg: 0.93	Clashscore: 1.46	Outliers: 4 of 903	Poor rotamers: 0 of 774	Outliers: 0 of 826	Outliers: 27 of 901	Outliers: 15 of 905	Outliers: 10 of 905	Non-Trans: 1 of 904
A 361		VAL	0.97	-	Favored (75.98%) Ile or Val / -70.4,-39.1	Favored (84%) *t* chi angles: 176.3	0.16Å	Favored (77.144%)	-	-	-

A 362		ASP	0.98	-	Allowed (1.16%) General / -79.1,15.4	Favored (30.4%) *m-30* chi angles: 274.9,341.3	0.18Å	Favored (23.147%)	-	OUTLIER(S) worst is CA-CB-CG: 6.6 σ	-
A 363		THR	0.98	-	Favored (39.26%) General / -60.7,129.0	Favored (92.2%) *m* chi angles: 301.1	0.04Å	Favored (37.512%)	-	-	-
A 364		LYS	0.97	-	Favored (36.02%) General / -80.7,139.7	Favored (88.3%) *tttt* chi angles: 184.5,176,176.7,180	0.05Å	Favored (46.584%) beta sheet	-	-	-
A 365		ALA	0.94	-	Favored (11.34%) Pre-Pro / -117.7,77.7	-	0.06Å	Favored (25.241%)	-	-	-
A 366		PRO	0.89	-	Favored (87.77%) Trans-Pro / -65.4,149.8	Favored (39.8%) *Cg_endo* chi angles: 23.3,328.6,26.4	0.02Å	Favored (31.658%)	-	-	-
A 367		GLU	0.84	-	Favored (98.15%) Pre-Pro / -68.3,149.2	Favored (79.1%) *mm-30* chi angles: 298.3,300.6,308.6	0.02Å	Favored (46.616%)	-	-	-
A 368		PRO	0.78	-	Favored (51.62%) Trans-Pro / -70.6,159.8	Favored (53.5%) *Cg_endo* chi angles: 25.6,325.3,28.6	0.05Å	Favored (59.851%)	-	-	-
A 369		PRO	0.73	-	Favored (33.12%) Trans-Pro / -58.8,156.4	Favored (67.4%) *Cg_exo* chi angles: 335.5,32.9,332.3	0.06Å	Favored (72.344%)	-	-	-
A 370		GLU	0.7	-	Favored (83.92%) General / -59.6,-39.4	Favored (97.1%) *mt-10* chi angles: 289.1,174.7,345.3	0.03Å	Favored (65.835%)	-	-	-
A 371		GLY	0.68	-	Favored (37.38%) Glycine / -57.3,-54.4	-	-	Favored (90.637%) alpha helix	-	-	-
A 372		VAL	0.66	-	Favored (82.53%) Ile or Val / -57.0,-44.4	Favored (67.4%) *t* chi angles: 171.8	0.14Å	Favored (76.788%) alpha helix	OUTLIER(S) worst is CB--CG2: 4.2 σ	-	-
A 373		LYS	0.66	-	Favored (93.59%) General / -59.8,-42.5	Favored (85.8%) *tttt* chi angles: 186.8,173.9,184.3,178.6	0.05Å	Favored (84.046%) alpha helix	-	-	-
A 374		TYR	0.66	-	Favored (82.82%) General / -68.1,-39.7	Favored (31.7%) *m-80* chi angles: 292.8,128.1	0.01Å	Favored (87.642%) alpha helix	-	-	-
A 375		VAL	0.65	-	Favored (95.31%) Ile or Val / -65.2,-43.7	Favored (70.7%) *t* chi angles: 172.2	0.03Å	Favored (94.386%) alpha helix	-	-	-
A 376		LEU	0.65	-	Favored (88.24%) General / -66.7,-39.8	Favored (28.1%) *tp* chi angles: 187.8,59.2	0.04Å	Favored (90.44%) alpha helix	-	-	-
A 377		ASN	0.65	-	Favored (93.17%) General / -60.5,-45.6	Favored (94.1%) *m-40* chi angles: 286.4,344.5	0.09Å	Favored (88.449%) alpha helix	-	OUTLIER(S) worst is CA-CB-CG: 4.3 σ	-
A 378		GLU	0.65	-	Favored (91.02%) General / -64.6,-38.4	Favored (93.2%) *mt-10* chi angles: 289.2,180.6,341	0.06Å	Favored (87.517%) alpha helix	-	-	-

#	Alt	Res	High B	Clash > 0.4Å	Ramachandran	Rotamer	Cβ deviation	CaBLAM	Bond lengths	Bond angles	Cis Peptides
A 379		THR	0.65	-	Favored (88.82%) General / -65.8,-43.3	Favored (88.7%) *m* chi angles: 301.3	0.06Å	Favored (96.025%) alpha helix	-	-	-
A 380		THR	0.65	-	Favored (88.11%) General / -60.7,-47.0	Favored (74.4%) *m* chi angles: 296.2	0.08Å	Favored (98.529%) alpha helix	-	-	-
#	Alt	Res	High B	Clash > 0.4Å	Ramachandran	Rotamer	Cβ deviation	CaBLAM	Bond lengths	Bond angles	Cis Peptides
			Avg: 0.93	Clashscore: 1.46	Outliers: 4 of 903	Poor rotamers: 0 of 774	Outliers: 0 of 826	Outliers: 27 of 901	Outliers: 15 of 905	Outliers: 10 of 905	Non-Trans: 1 of 904
A 381		ASN	0.65	-	Favored (93.43%) General / -63.7,-39.1	Favored (98.8%) *m-40* chi angles: 289.5,339.1	0.04Å	Favored (97.655%) alpha helix	-	-	-
A 382		TRP	0.66	-	Favored (81.77%) General / -61.9,-48.2	Favored (85.6%) *t60* chi angles: 175.4,85.5	0.06Å	Favored (89.032%) alpha helix	-	-	-
A 383		LEU	0.68	-	Favored (89.33%) General / -65.8,-38.6	Favored (28.1%) *tp* chi angles: 187.5,58.3	0.10Å	Favored (84.762%) alpha helix	-	-	-
A 384		TRP	0.71	-	Favored (92.69%) General / -64.1,-44.3	Favored (45.2%) *m100* chi angles: 281.4,119.7	0.14Å	Favored (87.682%) alpha helix	-	-	-
A 385		ALA	0.75	-	Favored (87.15%) General / -62.0,-38.0	-	0.04Å	Favored (86.555%) alpha helix	-	-	-
A 386		PHE	0.8	-	Favored (36.62%) General / -62.7,-54.6	Favored (88.8%) *t80* chi angles: 179.1,81.5	0.06Å	Favored (72.06%) alpha helix	-	-	-
A 387		LEU	0.87	-	Favored (80.25%) General / -65.4,-35.2	Favored (92.4%) *mt* chi angles: 294.9,176.8	0.04Å	Favored (66.494%) alpha helix	-	-	-
A 388		ALA	0.94	-	Favored (63.29%) General / -63.4,-18.1	-	0.03Å	Favored (59.426%) three-ten	-	-	-
A 389		ARG	1.02	-	Favored (62.94%) General / -70.5,-25.0	Favored (34.6%) *ptt180* chi angles: 76.5,190.8,180.7,179.6	0.09Å	Favored (48.188%) three-ten	-	-	-
A 390		ASP	1.06	-	Favored (8.45%) General / -102.5,-36.3	Favored (62.6%) *m-30* chi angles: 296.8,304.4	0.02Å	Favored (43.737%) alpha helix	-	-	-
A 391		LYS	1.08	-	Favored (52.57%) General / -130.8,148.8	Favored (71.5%) *mmtt* chi angles: 297.3,290.7,183.8,183.5	0.03Å	Favored (27.026%)	-	-	-
A 392		LYS	1.05	-	Favored (33.23%) Pre-Pro / -126.8,138.8	Favored (85.7%) *tttt* chi angles: 179.6,177.8,176.7,179.2	0.03Å	Favored (39.78%)	-	-	-
A 393		PRO	0.99	-	Favored (67.51%) Trans-Pro / -53.7,139.5	Favored (97.7%) *Cg_exo* chi angles: 331.9,35.4,333.3	0.05Å	Favored (45.496%)	-	-	-
A 394		ARG	0.92	-	Favored (4.49%)	Favored (42.2%) *mtm180*	0.03Å	CaBLAM Outlier	-	-	-

					General / -122.2,-27.5	chi angles: 294.7,187.8,288.6,148.1		(0.511%) try beta sheet			
A 395		MET	0.86	-	OUTLIER (0.04)% General / 89.2,134.5	Favored (31.4%) *tpp* chi angles: 187.3,72.2,89.7	0.12Å	CaBLAM Disfavored (3.405%) try beta sheet	-	-	-
A 396		CYS	0.81	-	Favored (18.27%) General / -98.7,154.0	Favored (68.4%) *m* chi angles: 299.1	0.06Å	Favored (41.718%) beta sheet	-	-	-
A 397		SER	0.77	-	Favored (12.44%) General / -92.4,166.7	Favored (95.9%) *p* chi angles: 65	0.05Å	Favored (47.188%)	-	-	-
A 398		ARG	0.75	-	Favored (92.6%) General / -63.9,-38.9	Favored (97.8%) *mtt180* chi angles: 289.8,181.4,179.4,176.3	0.05Å	Favored (63.238%)	-	-	-
A 399		GLU	0.74	-	Favored (94.09%) General / -63.8,-39.4	Favored (98.6%) *mt-10* chi angles: 290.5,178.4,354	0.01Å	Favored (82.164%) alpha helix	-	-	-
A 400		GLU	0.75	-	Favored (90.95%) General / -63.5,-38.4	Favored (34.4%) *mt-10* chi angles: 287.1,167.3,305.1	0.06Å	Favored (96.622%) alpha helix	-	-	-

#	Alt	Res	High B	Clash > 0.4Å	Ramachandran	Rotamer	Cβ deviation	CaBLAM	Bond lengths	Bond angles	Cis Peptides
			Avg: 0.93	Clashscore: 1.46	Outliers: 4 of 903	Poor rotamers: 0 of 774	Outliers: 0 of 826	Outliers: 27 of 901	Outliers: 15 of 905	Outliers: 10 of 905	Non-Trans: 1 of 904
A 401		PHE	0.77	-	Favored (82.49%) General / -65.2,-46.0	Favored (69.2%) *t80* chi angles: 184.5,71.9	0.04Å	Favored (89.432%) alpha helix	-	-	-
A 402		ILE	0.82	-	Favored (89.18%) Ile or Val / -59.3,-43.1	Favored (89.2%) *mt* chi angles: 291.4,166.4	0.05Å	Favored (87.394%) alpha helix	-	-	-
A 403		GLY	0.91	-	Favored (49.38%) Glycine / -59.9,-53.0	-	-	Favored (87.139%) alpha helix	-	-	-
A 404		LYS	1.04	-	Favored (71.45%) General / -55.2,-41.0	Favored (53.2%) *tttp* chi angles: 183.5,175.8,179.6,64.9	0.01Å	Favored (73.837%) alpha helix	-	-	-
A 405		VAL	1.2	-	Favored (19.88%) Ile or Val / -68.1,-15.6	Favored (26.8%) *m* chi angles: 299.1	0.07Å	CaBLAM Disfavored (3.617%)	-	-	-
A 406		ASN	1.38	-	Allowed (1.88%) General / 51.4,-121.9	Favored (82.1%) *m-40* chi angles: 296.6,309.6	0.03Å	CaBLAM Outlier (0.514%)	-	-	-
A 407		SER	1.54	-	Allowed (1.38%) General / -141.9,0.1	Favored (83.1%) *p* chi angles: 62.5	0.05Å	CaBLAM Disfavored (2.166%)	-	-	-
A 408		ASN	1.64	-	Favored (13.96%) General / -106.9,-18.3	Favored (88.8%) *m-40* chi angles: 295.8,321.2	0.01Å	Favored (15.277%)	-	-	-
A 409		ALA	1.67	-	Favored (58.28%) General / -61.5,138.4	-	0.04Å	Favored (43.127%)	-	-	-

A 410		ALA	1.63	-	Favored (29.32%) General / -80.7,150.6	-	0.05Å	Favored (38.585%) beta sheet	-	-	-
A 411		LEU	1.54	-	Favored (43.16%) General / -116.0,145.2	Favored (90%) *mt* chi angles: 299,176.1	0.05Å	Favored (68.626%) beta sheet	-	-	-
A 412		GLY	1.43	-	Favored (23.12%) Glycine / -105.6,-161.2	-	-	Favored (28.169%) beta sheet	-	-	-
A 413		ALA	1.32	-	Favored (6.11%) General / -160.2,132.0	-	0.02Å	CaBLAM Disfavored (4.73%)	-	-	-
A 414		MET	1.23	0.64Å N with A 414 MET SD	Allowed (1.63%) General / -133.9,-25.2	Favored (2.9%) *pmm* chi angles: 62.3,293,295.4	0.10Å	CaBLAM Disfavored (2.73%)	-	-	-
A 415		PHE	1.15	-	Favored (37.02%) General / -121.6,154.2	Favored (63.2%) *m-80* chi angles: 299.2,114.4	0.06Å	Favored (22.215%) alpha helix	-	-	-
A 416		GLU	1.08	-	Favored (67.24%) General / -55.6,-36.7	Favored (69.8%) *tp30* chi angles: 180.8,67,23.4	0.01Å	Favored (62.406%) alpha helix	-	-	-
A 417		GLU	1.02	-	Favored (70.63%) General / -63.7,-29.4	Favored (86.4%) *mm-30* chi angles: 291.2,299.4,313.1	0.02Å	Favored (68.697%) three-ten	-	-	-
A 418		GLN	0.97	-	Favored (70.28%) General / -64.7,-29.3	Favored (85.4%) *tp40* chi angles: 190.3,65.7,59.8	0.05Å	Favored (67.704%) three-ten	-	-	-
A 419		ASN	0.94	-	Favored (17.3%) General / -83.5,7.4	Favored (79.6%) *m-40* chi angles: 286.6,323	0.02Å	Favored (29.655%) three-ten	-	-	-
A 420		GLN	0.92	-	Favored (58.03%) General / -89.5,-4.2	Favored (95.1%) *mm-40* chi angles: 301.4,298.6,304.2	0.03Å	Favored (57.187%)	-	-	-
**#**	**Alt**	**Res**	**High B**	**Clash > 0.4Å**	**Ramachandran**	**Rotamer**	**Cβ deviation**	**CaBLAM**	**Bond lengths**	**Bond angles**	**Cis Peptides**
			Avg: 0.93	Clashscore: 1.46	Outliers: 4 of 903	Poor rotamers: 0 of 774	Outliers: 0 of 826	Outliers: 27 of 901	Outliers: 15 of 905	Outliers: 10 of 905	Non-Trans: 1 of 904
A 421		TRP	0.9	-	Favored (54.56%) General / -122.6,138.5	Favored (6.1%) *m-90* chi angles: 298.2,300.1	0.03Å	Favored (29.281%)	-	-	-
A 422		LYS	0.88	-	Favored (74.34%) General / -70.4,-37.2	Favored (97.8%) *mttt* chi angles: 290.5,178.6,180,177.6	0.02Å	Favored (16.897%)	-	-	-
A 423		ASN	0.86	-	Favored (17.58%) General / -155.6,172.8	Favored (25.5%) *p0* chi angles: 61,60	0.05Å	Favored (15.915%)	-	-	-
A 424		ALA	0.84	-	Favored (67.67%) General / -61.6,-25.9	-	0.04Å	Favored (45.26%)	-	-	-
A 425		ARG	0.82	-	Favored (39.25%) General / -74.1,-47.1	Favored (80.3%) *ttt180* chi angles: 180.6,174.8,172.5,175.2	0.04Å	Favored (67.309%) alpha helix	-	-	-

A 426		GLU	0.8	-	Favored (85.43%) General / -58.5,-41.5	Favored (45.2%) *tt0* chi angles: 183.3,174.6,56.2	0.02Å	Favored (87.034%) alpha helix	-	-	-
A 427		ALA	0.79	-	Favored (95.26%) General / -60.2,-42.5	-	0.05Å	Favored (79.552%) alpha helix	-	-	-
A 428		VAL	0.78	-	Favored (13.02%) Ile or Val / -79.0,-18.7	Favored (31.3%) *m* chi angles: 300.4	0.06Å	Favored (66.12%) alpha helix	-	-	-
A 429		GLU	0.78	-	Favored (40.96%) General / -80.1,-23.7	Favored (99.2%) *mt-10* chi angles: 291.5,178.3,358.2	0.10Å	Favored (35.566%)	-	-	-
A 430		ASP	0.78	-	Favored (32.74%) Pre-Pro / -78.1,113.4	Favored (69%) *t0* chi angles: 182.8,351.1	0.06Å	Favored (33.718%)	-	-	-
A 431		PRO	0.77	-	Favored (33.18%) Trans-Pro / -51.0,-31.0	Favored (96.7%) *Cg_exo* chi angles: 331.7,37.3,329.4	0.05Å	Favored (80.32%)	-	-	-
A 432		LYS	0.76	-	Favored (81.86%) General / -60.3,-37.9	Favored (86.4%) *tttt* chi angles: 185.1,179.2,184,182.6	0.07Å	Favored (63.84%) alpha helix	-	-	-
A 433		PHE	0.75	-	Favored (77.82%) General / -58.9,-49.4	Favored (83.6%) *t80* chi angles: 172.7,75.4	0.06Å	Favored (84.975%) alpha helix	-	-	-
A 434		TRP	0.73	-	Favored (80.6%) General / -64.6,-35.3	Favored (69.9%) *m100* chi angles: 290,116.9	0.07Å	Favored (78.404%) alpha helix	-	-	-
A 435		GLU	0.72	-	Favored (99.23%) General / -63.1,-41.1	Favored (98%) *mt-10* chi angles: 289.9,179.7,355.3	0.04Å	Favored (88.962%) alpha helix	-	-	-
A 436		MET	0.71	-	Favored (88.77%) General / -65.3,-38.0	Favored (99%) *mtp* chi angles: 291.5,178.1,71.7	0.06Å	Favored (85.705%) alpha helix	-	-	-
A 437		VAL	0.7	-	Favored (95.67%) Ile or Val / -60.4,-44.2	Favored (46.1%) *t* chi angles: 168.9	0.04Å	Favored (82.655%) alpha helix	-	-	-
A 438		ASP	0.68	-	Favored (91.38%) General / -62.3,-39.0	Favored (98.1%) *m-30* chi angles: 288.3,345.1	0.06Å	Favored (98.034%) alpha helix	-	-	-
A 439		GLU	0.67	-	Favored (94.65%) General / -64.3,-39.8	Favored (96%) *mt-10* chi angles: 290,181.8,354.1	0.03Å	Favored (99.061%) alpha helix	-	-	-
A 440		GLU	0.66	-	Favored (90%) General / -64.9,-38.1	Favored (97.9%) *mt-10* chi angles: 290.2,173.6,344.5	0.03Å	Favored (85.622%) alpha helix	-	-	-
**#**	**Alt**	**Res**	**High B**	**Clash > 0.4Å**	**Ramachandran**	**Rotamer**	**Cβ deviation**	**CaBLAM**	**Bond lengths**	**Bond angles**	**Cis Peptides**
			Avg: 0.93	Clashscore: 1.46	Outliers: 4 of 903	Poor rotamers: 0 of 774	Outliers: 0 of 826	Outliers: 27 of 901	Outliers: 15 of 905	Outliers: 10 of 905	Non-Trans: 1 of 904
A 441		ARG	0.66	-	Favored (97.34%)	Favored (62.4%) *ttt-90*	0.03Å	Favored (85.799%) alpha helix	-	-	-

					General / -62.9,-44.0	chi angles: 190,174.1,188.8,272.8					
A 442		GLU	0.65	-	Favored (97.18%) General / -61.2,-44.5	Favored (91.7%) *tt0* chi angles: 181,175.6,356.7	0.03Å	Favored (93.549%) alpha helix	-	-	-
A 443		ALA	0.66	-	Favored (75.01%) General / -60.3,-35.2	-	0.02Å	Favored (76.032%) alpha helix	-	-	-
A 444		HIS	0.67	-	Favored (68.78%) General / -70.1,-44.5	Favored (57.7%) *m90* chi angles: 282.3,78	0.08Å	Favored (73.609%) alpha helix	-	-	-
A 445		LEU	0.68	-	Favored (70.87%) General / -63.7,-29.7	Favored (97.9%) *mt* chi angles: 292.9,173.1	0.05Å	Favored (73.158%) alpha helix	-	-	-
A 446		ARG	0.7	-	Favored (55.63%) General / -86.0,-0.5	Favored (97.8%) *mtt-85* chi angles: 291,182,181,274.7	0.05Å	Favored (57.413%)	-	-	-
A 447		GLY	0.71	-	Favored (66.25%) Glycine / 82.4,18.6	-	-	Favored (60.097%)	-	-	-
A 448		GLU	0.73	-	Favored (5.25%) General / -108.7,-39.9	Favored (96.1%) *mt-10* chi angles: 296.6,176.9,351.5	0.06Å	CaBLAM Outlier (0.053%)	-	-	-
A 449		CYS	0.74	0.53Å O with A 449 CYS SG	Allowed (0.72%) General / 39.4,65.2	Favored (52.4%) *t* chi angles: 180.7	0.11Å	CaBLAM Disfavored (3.464%)	-	-	-
A 450		ASN	0.74	-	Favored (2.17%) General / -52.5,-19.3	Favored (96.3%) *m-40* chi angles: 286.6,339.7	0.05Å	Favored (11.249%)	-	-	-
A 451		THR	0.74	-	Favored (20.38%) General / -112.3,13.3	Favored (68.5%) *p* chi angles: 59	0.04Å	Favored (41.11%)	-	-	-
A 452		CYS	0.75	-	Allowed (1.29%) General / -74.5,65.6	Favored (82.1%) *m* chi angles: 294.9	0.15Å	CaBLAM Disfavored (4.574%)	-	-	-
A 453		ILE	0.76	-	Favored (65.66%) Ile or Val / -111.6,130.2	Favored (47.2%) *mm* chi angles: 299.8,298.9	0.06Å	Favored (28.205%)	-	-	-
A 454		TYR	0.78	0.48Å OH with A 606 GLN NE2	Favored (28.53%) General / -105.0,146.8	Favored (92.4%) *m-80* chi angles: 291.6,88.4	0.10Å	Favored (50.359%) beta sheet	-	-	-
A 455		ASN	0.82	-	Favored (22.86%) General / -106.8,110.6	Favored (58.4%) *t0* chi angles: 183.6,345.3	0.07Å	Favored (57.009%) beta sheet	-	-	-
A 456		MET	0.87	-	Favored (26.51%) General / -84.5,120.6	Favored (18.5%) *tmm* chi angles: 189,279.4,281	0.04Å	Favored (41.945%) beta sheet	-	-	-
A 457		MET	0.94	-	Favored (51.82%) General / -133.6,153.2	Favored (67%) *mtm* chi angles: 297.2,175.6,283.7	0.05Å	Favored (37.276%) beta sheet	-	-	-
A 458		GLY	1.02	-	Favored (26.66%) Glycine / -79.3,144.1	-	-	Favored (35.019%) beta sheet	-	-	-

#	Alt	Res	High B	Clash > 0.4Å	Ramachandran	Rotamer	Cβ deviation	CaBLAM	Bond lengths	Bond angles	Cis Peptides
A 459		LYS	1.1	-	Favored (30.68%) General / -85.3,123.3	Favored (52%) *tptt* chi angles: 181.1,69.7,176.7,177.1	0.07Å	Favored (43.469%) beta sheet	-	-	-
A 460		ARG	1.19	-	Favored (8.62%) General / -82.4,80.2	Favored (37.2%) *ttm110* chi angles: 198,174,297.1,110.1	0.05Å	Favored (49.493%) beta sheet	-	-	-
#	Alt	Res	High B	Clash > 0.4Å	Ramachandran	Rotamer	Cβ deviation	CaBLAM	Bond lengths	Bond angles	Cis Peptides
			Avg: 0.93	Clashscore: 1.46	Outliers: 4 of 903	Poor rotamers: 0 of 774	Outliers: 0 of 826	Outliers: 27 of 901	Outliers: 15 of 905	Outliers: 10 of 905	Non-Trans: 1 of 904
A 461		GLU	1.28	-	Favored (25.15%) General / -80.9,159.1	Favored (75%) *mt-10* chi angles: 295.5,179.6,320.8	0.02Å	Favored (19.384%) beta sheet	-	-	-
A 462		LYS	1.37	0.43Å NZ with A 745 ILE O	Favored (23.18%) General / -103.2,111.0	Favored (98.9%) *mttt* chi angles: 295.2,176,176.1,183	0.04Å	Favored (16.568%) beta sheet	-	-	-
A 463		LYS	1.47	-	Favored (71.75%) Pre-Pro / -137.6,151.9	Favored (99.6%) *mttt* chi angles: 294.4,184.5,176.5,175.5	0.02Å	Favored (27.626%) beta sheet	-	-	-
A 464		PRO	1.56	-	Favored (62.62%) Trans-Pro / -53.2,136.6	Favored (97.3%) *Cg_exo* chi angles: 331.8,36.6,330.7	0.05Å	Favored (66.171%) beta sheet	-	-	-
A 465		GLY	1.62	-	Favored (87.2%) Glycine / -81.8,1.1	-	-	Favored (10.189%)	-	-	-
A 466		GLU	1.66	-	Allowed (0.33%) General / 65.6,-56.5	Favored (99.4%) *mt-10* chi angles: 292.7,180.2,353.8	0.06Å	Favored (6.974%)	-	-	-
A 467		PHE	1.64	-	Favored (4.81%) General / -127.0,-17.7	Favored (52.7%) *p90* chi angles: 67.7,89.8	0.08Å	CaBLAM Outlier (0.01%)	-	-	-
A 468		GLY	1.57	-	Favored (25.65%) Glycine / 143.8,-170.9	-	-	CaBLAM Outlier (0.077%)	-	-	-
A 469		LYS	1.47	-	Allowed (1.78%) General / 51.7,-121.2	Favored (63.1%) *mttm* chi angles: 294.4,182.4,181.7,296	0.02Å	CaBLAM Disfavored (1.185%)	-	-	-
A 470		ALA	1.36	-	Favored (26.75%) General / -159.5,155.6	-	0.02Å	CaBLAM Disfavored (2.7%)	-	-	-
A 471		LYS	1.25	-	Favored (26.67%) General / -79.7,159.5	Favored (23.2%) *mtpp* chi angles: 290.6,176.2,67.7,61.7	0.01Å	Favored (33.247%)	-	-	-
A 472		GLY	1.16	-	Favored (50.41%) Glycine / -68.1,149.8	-	-	Favored (37.632%)	-	-	-
A 473		SER	1.08	-	Favored (22.09%) General / -71.4,166.2	Favored (79.5%) *p* chi angles: 61.2	0.07Å	Favored (30.654%)	-	-	-
A 474		ARG	1.02	-	Favored (36.12%) General / -90.3,127.3	Favored (74.1%) *ttt180* chi angles: 179.8,182.1,180,191.4	0.05Å	Favored (17.895%)	-	-	-

A 475		ALA	0.98	-	Favored (42.44%) General / -64.3,130.1	-	0.07Å	Favored (42.107%) beta sheet	-	-	-
A 476		ILE	0.93	-	Favored (60.68%) Ile or Val / -124.2,136.4	Favored (40.4%) *mm* chi angles: 303.3,296	0.04Å	Favored (62.423%) beta sheet	-	-	-
A 477		TRP	0.89	-	Favored (36.2%) General / -105.9,118.3	Favored (36.2%) *m-90* chi angles: 291.8,259.8	0.06Å	Favored (66.687%) beta sheet	-	-	-
A 478		PHE	0.85	-	Favored (52.03%) General / -106.5,126.3	Favored (87.2%) *m-80* chi angles: 293.1,83	0.07Å	Favored (66.48%)	-	-	-
A 479		MET	0.81	-	Favored (24.25%) General / -99.1,148.2	Favored (44.3%) *tpp* chi angles: 172.4,64,68.9	0.09Å	Favored (25.304%)	OUTLIER(S) worst is SD--CE: 4.1 σ	-	-
A 480		TRP	0.78	-	Favored (16.86%) General / -49.6,136.4	Favored (41.6%) *t60* chi angles: 194.2,87.4	0.06Å	Favored (42.974%)	-	OUTLIER(S) worst is CB-CG-CD2: 4.4 σ	-
**#**	**Alt**	**Res**	**High B**	**Clash > 0.4Å**	**Ramachandran**	**Rotamer**	**Cβ deviation**	**CaBLAM**	**Bond lengths**	**Bond angles**	**Cis Peptides**
			Avg: 0.93	Clashscore: 1.46	Outliers: 4 of 903	Poor rotamers: 0 of 774	Outliers: 0 of 826	Outliers: 27 of 901	Outliers: 15 of 905	Outliers: 10 of 905	Non-Trans: 1 of 904
A 481		LEU	0.76	-	Favored (62.36%) General / -54.1,-35.5	Favored (24.9%) *tp* chi angles: 189,65.4	0.07Å	Favored (52.588%)	-	-	-
A 482		GLY	0.73	-	Favored (64.6%) Glycine / -62.2,-50.5	-	-	Favored (93.388%) alpha helix	-	-	-
A 483		ALA	0.71	-	Favored (76.11%) General / -60.3,-35.7	-	0.07Å	Favored (85.74%) alpha helix	-	-	-
A 484		ARG	0.7	-	Favored (87.99%) General / -63.4,-37.6	Favored (86.7%) *mtp180* chi angles: 290.1,173.8,65.5,192.2	0.04Å	Favored (82.742%) alpha helix	-	-	-
A 485		PHE	0.68	-	Favored (82.47%) General / -58.1,-47.7	Favored (61.7%) *t80* chi angles: 169.3,72.3	0.09Å	Favored (91.621%) alpha helix	-	-	-
A 486		LEU	0.67	-	Favored (77.79%) General / -65.7,-34.2	Favored (98.2%) *mt* chi angles: 293.5,172.6	0.12Å	Favored (85.183%) alpha helix	-	-	-
A 487		GLU	0.67	-	Favored (93.83%) General / -61.0,-40.6	Favored (98%) *mt-10* chi angles: 289.5,176.2,354.4	0.10Å	Favored (84.94%) alpha helix	-	-	-
A 488		PHE	0.67	-	Favored (70.85%) General / -65.4,-49.0	Favored (87.8%) *t80* chi angles: 179.1,75.4	0.07Å	Favored (84.273%) alpha helix	-	-	-
A 489		GLU	0.69	-	Favored (77.08%) General / -56.4,-42.0	Favored (82.5%) *mt-10* chi angles: 286.7,186.6,351	0.19Å	Favored (70.187%) alpha helix	-	-	-
A 490		ALA	0.72	-	Favored (6.81%)	-	0.09Å	Favored (36.044%) alpha helix	-	-	-

					General / -80.8,-53.1						
A 491		LEU	0.76	-	Favored (17.23%) General / -108.9,-3.9	Favored (92.4%) *mt* chi angles: 298.9,177.1	0.05Å	Favored (10.972%) alpha helix	-	-	-
A 492		GLY	0.81	-	Favored (60.98%) Glycine / -57.9,-28.6	-	-	Favored (43.284%) three-ten	-	-	-
A 493		PHE	0.86	-	Favored (62.94%) General / -58.7,-25.6	Favored (31.9%) *p90* chi angles: 74,90.5	0.07Å	Favored (65.785%) three-ten	-	-	-
A 494		LEU	0.91	-	Favored (96.74%) General / -63.5,-40.3	Favored (98.3%) *mt* chi angles: 292.6,171.9	0.11Å	Favored (55.917%) alpha helix	-	-	-
A 495		ASN	0.95	-	Favored (16.61%) General / -78.5,-47.8	Favored (98.4%) *m-40* chi angles: 290.3,342.1	0.08Å	Favored (53.18%) alpha helix	-	-	-
A 496		GLU	0.98	-	Favored (80.37%) General / -65.4,-35.2	Favored (77.8%) *mm-30* chi angles: 295.3,299.3,305.7	0.02Å	Favored (68.644%) alpha helix	-	-	-
A 497		ASP	0.98	-	Favored (56.98%) General / -91.7,1.7	Favored (91.5%) *m-30* chi angles: 292.4,337.6	0.06Å	Favored (41.913%)	-	-	-
A 498		HIS	0.97	-	Favored (14.78%) General / 51.2,52.5	Favored (96.3%) *m-70* chi angles: 294.9,290.5	0.06Å	Favored (21.167%)	-	-	-
A 499		TRP	0.94	0.60Å CD1 with A 499 TRP N	Favored (33.29%) General / -57.9,-20.0	Allowed (1%) *p-90* chi angles: 60.4,318.4	0.03Å	Favored (20.512%)	-	-	-
A 500		LEU	0.91	-	Favored (23.2%) General / -109.3,15.4	Favored (80.8%) *mt* chi angles: 300.1,174.3	0.07Å	Favored (43.48%)	-	-	-
**#**	**Alt**	**Res**	**High B**	**Clash > 0.4Å**	**Ramachandran**	**Rotamer**	**Cβ deviation**	**CaBLAM**	**Bond lengths**	**Bond angles**	**Cis Peptides**
			Avg: 0.93	Clashscore: 1.46	Outliers: 4 of 903	Poor rotamers: 0 of 774	Outliers: 0 of 826	Outliers: 27 of 901	Outliers: 15 of 905	Outliers: 10 of 905	Non-Trans: 1 of 904
A 501		GLY	0.88	-	Favored (54.54%) Glycine / -74.1,163.3	-	-	Favored (45.557%)	-	-	-
A 502		ARG	0.84	-	Favored (57.99%) General / -58.8,-22.0	Favored (12.4%) *ptm-80* chi angles: 70,186.3,296.6,271.8	0.04Å	Favored (37.436%)	-	-	-
A 503		LYS	0.81	-	Favored (49.69%) General / -80.0,-18.7	Favored (98.9%) *mttt* chi angles: 293.5,179.3,180.2,178	0.01Å	Favored (55.21%) alpha helix	-	-	-
A 504		ASN	0.77	-	Favored (4.58%) General / -110.1,-40.9	Favored (88.4%) *m-40* chi angles: 295.5,319.7	0.03Å	Favored (25.536%) alpha helix	-	-	-
A 505		SER	0.75	-	Favored (8.8%) General / -128.2,106.1	Favored (40.3%) *t* chi angles: 181	0.13Å	Favored (23.081%) alpha helix	-	-	-
A 506		GLY	0.74	-	Favored (52.58%) Glycine / -55.6,-28.9	-	-	Favored (34.263%) alpha helix	-	-	-

A 507		GLY	0.75	-	Favored (73.89%) Glycine / -89.5,-4.2	-	-	Favored (63.769%)	-	-	-
A 508		GLY	0.78	-	Favored (19.32%) Glycine / -99.0,150.6	-	-	Favored (9.781%)	-	-	-
A 509		VAL	0.82	-	Favored (5.88%) Ile or Val / -120.2,20.4	Favored (23.8%) *m* chi angles: 295.1	0.06Å	Favored (15.56%)	-	-	-
A 510		GLU	0.87	-	Favored (63.64%) General / -55.4,-33.6	Favored (80.3%) *tt0* chi angles: 178.7,170.7,354.3	0.04Å	Favored (41.441%)	-	-	-
A 511		GLY	0.92	-	Favored (87.9%) Glycine / -85.9,1.5	-	-	Favored (60.321%)	-	-	-
A 512		LEU	0.94	-	Favored (29.59%) General / -110.3,149.8	Allowed (1%) *mm* chi angles: 292.7,313	0.10Å	Favored (30.738%)	-	-	-
A 513		GLY	0.95	-	Favored (38.35%) Glycine / -94.5,-178.4	-	-	Favored (49.292%)	-	-	-
A 514		LEU	0.92	-	Favored (72.21%) General / -60.5,-33.1	Favored (91.4%) *mt* chi angles: 291.2,172.8	0.06Å	Favored (51.591%)	-	-	-
A 515		GLN	0.88	0.44Å N with A 515 GLN OE1	Favored (59.48%) General / -77.7,-10.3	Favored (23.8%) *mp10* chi angles: 299.8,84.2,345.6	0.04Å	Favored (55.663%) alpha helix	-	-	-
A 516		LYS	0.84	-	Favored (11.64%) General / -110.4,-22.5	Favored (98.5%) *mttt* chi angles: 295.9,181.8,181.1,176.6	0.02Å	Favored (18.559%) alpha helix	-	-	-
A 517		LEU	0.8	-	Favored (72.89%) General / -60.4,-33.7	Favored (74.3%) *mt* chi angles: 294.1,179.7	0.13Å	Favored (60.733%) three-ten	-	-	-
A 518		GLY	0.76	-	Favored (69.89%) Glycine / -58.9,-32.3	-	-	Favored (84.992%) three-ten	-	-	-
A 519		TYR	0.73	-	Favored (42.54%) General / -79.4,-33.4	Favored (89.3%) *m-80* chi angles: 290.8,93	0.05Å	Favored (89.268%) alpha helix	-	-	-
A 520		ILE	0.71	-	Favored (89.74%) Ile or Val / -66.8,-42.8	Favored (44.9%) *mm* chi angles: 298.2,300.7	0.10Å	Favored (90.671%) alpha helix	-	-	-
**#**	**Alt**	**Res**	**High B**	**Clash > 0.4Å**	**Ramachandran**	**Rotamer**	**Cβ deviation**	**CaBLAM**	**Bond lengths**	**Bond angles**	**Cis Peptides**
			Avg: 0.93	Clashscore: 1.46	Outliers: 4 of 903	Poor rotamers: 0 of 774	Outliers: 0 of 826	Outliers: 27 of 901	Outliers: 15 of 905	Outliers: 10 of 905	Non-Trans: 1 of 904
A 521		LEU	0.7	-	Favored (91.92%) General / -64.7,-38.7	Favored (92.6%) *mt* chi angles: 293.1,170.1	0.06Å	Favored (93.102%) alpha helix	-	-	-
A 522		LYS	0.69	-	Favored (94.74%) General / -61.1,-45.4	Favored (86.5%) *tttt* chi angles: 180.9,176.6,179.4,180.8	0.04Å	Favored (87.869%) alpha helix	-	-	-

A 523		GLU	0.69	-	Favored (94.7%) General / -60.4,-41.8	Favored (97.6%) *mt-10* chi angles: 289.2,177.4,355.5	0.03Å	Favored (94.881%) alpha helix	-	-	-
A 524		VAL	0.69	-	Favored (94.63%) Ile or Val / -60.7,-43.3	Favored (52.4%) *t* chi angles: 169.7	0.03Å	Favored (72.987%) alpha helix	-	-	-
A 525		GLY	0.7	-	Favored (38.64%) Glycine / -60.5,-15.2	-	-	Favored (60.102%) alpha helix	-	-	-
A 526		THR	0.7	-	Favored (26.47%) General / -105.4,-0.1	Favored (75%) *p* chi angles: 61.4	0.02Å	Favored (56.223%)	-	-	-
A 527		LYS	0.7	-	Favored (72.1%) Pre-Pro / -77.5,145.5	Favored (71.6%) *mmtt* chi angles: 299.3,291.6,185.3,180.5	0.07Å	Favored (35.84%)	-	-	-
A 528		PRO	0.69	-	Favored (53.16%) Trans-Pro / -72.9,154.1	Favored (65%) *Cg_endo* chi angles: 26.9,326.2,26.2	0.02Å	CaBLAM Disfavored (4.297%)	-	-	-
A 529		GLY	0.68	-	Favored (2.37%) Glycine / 133.8,-34.7	-	-	Favored (15.814%)	-	-	-
A 530		GLY	0.68	-	Favored (36.17%) Glycine / 172.8,-164.2	-	-	Favored (10.996%)	-	-	-
A 531		LYS	0.67	-	Favored (33.41%) General / -75.4,158.0	Favored (52.3%) *mtpt* chi angles: 292.6,175,75.1,173.2	0.01Å	Favored (7.042%)	-	-	-
A 532		ILE	0.68	-	Favored (52.99%) Ile or Val / -99.9,127.2	Favored (54.4%) *mt* chi angles: 303.8,165.6	0.10Å	Favored (46.699%)	-	-	-
A 533		TYR	0.7	-	Favored (23.77%) General / -114.6,156.9	Favored (86.8%) *m-80* chi angles: 299,86.3	0.07Å	Favored (46.888%) beta sheet	-	-	-
A 534		ALA	0.74	-	Favored (8.61%) General / -142.6,115.2	-	0.05Å	Favored (27.25%) beta sheet	-	-	-
A 535		ASP	0.79	-	Favored (28.65%) General / -114.9,154.4	Favored (81.3%) *m-30* chi angles: 295.5,344.2	0.07Å	Favored (29.022%) beta sheet	-	-	-
A 536		ASP	0.84	-	Favored (4.58%) General / -145.7,108.7	Favored (63%) *t0* chi angles: 184.8,357.4	0.02Å	Favored (13.261%) beta sheet	-	-	-
A 537		THR	0.87	-	Favored (53.09%) General / -66.0,134.5	Favored (72.7%) *m* chi angles: 296.1	0.12Å	Favored (45.193%)	-	-	-
A 538		ALA	0.89	-	Allowed (1.26%) General / -73.3,71.8	-	0.06Å	CaBLAM Disfavored (3.68%)	-	-	-
A 539		GLY	0.88	-	Favored (3.01%) Glycine / 91.8,49.0	-	-	CaBLAM Outlier (0.401%)	-	-	-

A 540		TRP	0.86	-	Favored (15.22%) General / -45.9,-49.5	Favored (50.8%) *t-100* chi angles: 183.8,270.6	0.09Å	Favored (45.884%)	-	-	-
**#**	**Alt**	**Res**	**High B**	**Clash > 0.4Å**	**Ramachandran**	**Rotamer**	**Cβ deviation**	**CaBLAM**	**Bond lengths**	**Bond angles**	**Cis Peptides**
			Avg: 0.93	Clashscore: 1.46	Outliers: 4 of 903	Poor rotamers: 0 of 774	Outliers: 0 of 826	Outliers: 27 of 901	Outliers: 15 of 905	Outliers: 10 of 905	Non-Trans: 1 of 904
A 541		ASP	0.82	-	Favored (50.3%) General / -54.3,-30.2	Favored (95.4%) *m-30* chi angles: 286.7,344	0.04Å	Favored (60.459%) alpha helix	-	-	-
A 542		THR	0.78	-	Favored (26.61%) General / -98.3,-8.8	Favored (73.1%) *p* chi angles: 61.7	0.06Å	Favored (60.513%)	-	-	-
A 543		ARG	0.74	-	Favored (40.27%) General / -100.0,11.1	Favored (96.6%) *mtt-85* chi angles: 288.4,181.1,178.3,274.4	0.03Å	Favored (27.144%)	-	-	-
A 544		ILE	0.72	-	Favored (35.25%) Ile or Val / -71.8,130.3	Favored (46.9%) *mm* chi angles: 299.5,299.2	0.07Å	Favored (28.02%)	-	-	-
A 545		THR	0.71	-	Favored (8.17%) General / -99.6,171.1	Favored (56.6%) *p* chi angles: 64.7	0.07Å	Favored (33.111%)	-	-	-
A 546		LYS	0.71	-	Favored (87.24%) General / -63.9,-37.4	Favored (96.9%) *mttt* chi angles: 290.5,179.5,181.8,180.6	0.02Å	Favored (40.901%)	-	-	-
A 547		ALA	0.71	-	Favored (69.2%) General / -57.3,-35.0	-	0.02Å	Favored (73.196%) alpha helix	-	-	-
A 548		ASP	0.73	-	Favored (55.89%) General / -77.1,-36.0	Favored (49.8%) *m-30* chi angles: 293.3,300.6	0.10Å	Favored (98.04%) alpha helix	-	-	-
A 549		LEU	0.75	-	Favored (77.79%) General / -68.0,-35.2	Favored (89.1%) *mt* chi angles: 291.4,173.8	0.08Å	Favored (97.482%) alpha helix	-	-	-
A 550		GLU	0.78	-	Favored (93.44%) General / -63.5,-39.1	Favored (97.4%) *mt-10* chi angles: 288.4,174.6,353	0.03Å	Favored (83.346%) alpha helix	-	-	-
A 551		ASN	0.81	-	Favored (81.95%) General / -63.3,-35.9	Favored (94.2%) *m-40* chi angles: 286.6,336	0.08Å	Favored (78.513%) alpha helix	-	-	-
A 552		GLU	0.85	-	Favored (64.55%) General / -73.6,-33.2	Favored (74.2%) *mm-30* chi angles: 290.8,297,341.9	0.06Å	Favored (64.099%) alpha helix	-	-	-
A 553		ALA	0.88	-	Favored (66.75%) General / -61.0,-25.3	-	0.04Å	Favored (60.253%) three-ten	-	-	-
A 554		LYS	0.91	-	Favored (57.78%) General / -60.5,-19.6	Favored (26.1%) *mmmt* chi angles: 294,293.5,280.7,183.2	0.06Å	Favored (57.858%) three-ten	-	-	-
A 555		VAL	0.94	-	Favored (32.88%) Ile or Val / -59.4,-26.1	Favored (3.8%) *p* chi angles: 72.9	0.14Å	Favored (53.225%) three-ten	-	-	-

#	Alt	Res	High B	Clash > 0.4Å	Ramachandran	Rotamer	Cβ deviation	CaBLAM	Bond lengths	Bond angles	Cis Peptides
A 556		LEU	0.96	-	Favored (68.31%) General / -61.2,-27.2	Favored (95.1%) *mt* chi angles: 295,172.3	0.07Å	Favored (65.03%) three-ten	-	-	-
A 557		GLU	0.97	-	Favored (63.19%) General / -70.1,-17.4	Favored (93.9%) *mt-10* chi angles: 293.9,184.3,1	0.04Å	Favored (68.023%) three-ten	-	-	-
A 558		LEU	0.96	-	Favored (53.65%) General / -92.9,-4.0	Favored (82%) *mt* chi angles: 300.6,175.7	0.06Å	Favored (54.661%)	-	-	-
A 559		LEU	0.95	-	Favored (25.55%) General / -104.9,149.6	Favored (5.9%) *mp* chi angles: 279.9,70.5	0.06Å	Favored (28.577%)	-	-	-
A 560		ASP	0.91	-	Favored (22.47%) General / -109.6,154.6	Favored (73.9%) *m-30* chi angles: 297.4,316	0.07Å	CaBLAM Disfavored (3.001%)	-	-	-
**#**	**Alt**	**Res**	**High B**	**Clash > 0.4Å**	**Ramachandran**	**Rotamer**	**Cβ deviation**	**CaBLAM**	**Bond lengths**	**Bond angles**	**Cis Peptides**
			Avg: 0.93	Clashscore: 1.46	Outliers: 4 of 903	Poor rotamers: 0 of 774	Outliers: 0 of 826	Outliers: 27 of 901	Outliers: 15 of 905	Outliers: 10 of 905	Non-Trans: 1 of 904
A 561		GLY	0.87	-	Favored (35.06%) Glycine / 51.6,-130.6	-	-	Favored (30.121%)	-	-	-
A 562		GLU	0.82	-	Favored (65.75%) General / -67.2,-22.2	Favored (92.1%) *mt-10* chi angles: 290.6,185.2,0	0.07Å	Favored (18.278%)	-	-	-
A 563		HIS	0.77	-	Favored (54.56%) General / -66.5,-51.7	Favored (27.5%) *t-170* chi angles: 187.9,192	0.03Å	Favored (39.815%) alpha helix	OUTLIER(S) worst is CB--CG: 4.6 σ	-	-
A 564		ARG	0.73	-	Favored (78.11%) General / -57.6,-48.9	Favored (45.1%) *ttm170* chi angles: 186,186.6,300.1,172.3	0.08Å	Favored (80.818%) alpha helix	-	-	-
A 565		ARG	0.7	-	Favored (97.75%) General / -61.5,-44.1	Favored (81%) *ttt180* chi angles: 184.1,173.8,173.4,174.7	0.01Å	Favored (87.734%) alpha helix	-	-	-
A 566		LEU	0.68	-	Favored (77.1%) General / -64.7,-47.8	Favored (62.3%) *tp* chi angles: 181.1,61.8	0.02Å	Favored (82.472%) alpha helix	-	-	-
A 567		ALA	0.68	-	Favored (84.14%) General / -60.2,-38.7	-	0.04Å	Favored (83.009%) alpha helix	-	-	-
A 568		ARG	0.68	-	Favored (79.6%) General / -60.3,-48.9	Favored (83.3%) *ttt180* chi angles: 179.5,174.7,176.8,179.3	0.04Å	Favored (86.278%) alpha helix	-	-	-
A 569		SER	0.68	-	Favored (88.28%) General / -61.9,-38.4	Favored (59.5%) *m* chi angles: 293.5	0.04Å	Favored (81.656%) alpha helix	-	-	-
A 570		ILE	0.69	-	Favored (85.93%) Ile or Val / -64.8,-48.1	Favored (91.9%) *mt* chi angles: 292.4,165.4	0.08Å	Favored (79.209%) alpha helix	-	-	-
A 571		ILE	0.71	-	Favored (98.99%) Ile or Val / -62.6,-45.4	Favored (96.8%) *mt* chi angles: 293.4,168.9	0.10Å	Favored (74.882%) alpha helix	-	-	-

A 572		GLU	0.72	-	Favored (78.81%) General / -68.4,-42.5	Favored (98.7%) *mt-10* chi angles: 291.1,175.4,358.4	0.01Å	Favored (60.945%) alpha helix	-	-	-
A 573		LEU	0.73	-	Favored (13.4%) General / -93.7,-34.0	Favored (75.4%) *mt* chi angles: 301.4,174.7	0.07Å	Favored (42.248%) alpha helix	-	-	-
A 574		THR	0.73	-	Favored (13.71%) General / -87.3,-41.3	Favored (82.6%) *m* chi angles: 302.2	0.03Å	Favored (49.504%) alpha helix	-	-	-
A 575		TYR	0.73	-	Favored (6.67%) General / -97.8,-44.6	Favored (90.6%) *m-80* chi angles: 293,84.7	0.02Å	Favored (29.931%) alpha helix	-	-	-
A 576		ARG	0.73	-	Favored (57.52%) General / -91.9,-0.5	Favored (85.3%) *mtp180* chi angles: 296.5,173.3,69.9,187.8	0.04Å	Favored (13.5%) alpha helix	-	-	-
A 577		HIS	0.72	-	Allowed (1.39%) General / -143.4,58.2	Favored (99%) *m-70* chi angles: 300.3,291.5	0.08Å	Favored (6.855%)	-	-	-
A 578		LYS	0.71	-	Favored (38.03%) General / -77.9,133.8	Favored (12.3%) *ttmm* chi angles: 187.6,181.5,287.4,294.5	0.03Å	Favored (17.03%)	-	-	-
A 579		VAL	0.7	-	Favored (74.79%) Ile or Val / -117.3,127.5	Favored (65.6%) *t* chi angles: 179.3	0.01Å	Favored (70.4%) beta sheet	-	-	-
A 580		VAL	0.71	-	Favored (57.53%) Ile or Val / -128.3,137.4	Favored (85.6%) *t* chi angles: 177.2	0.01Å	Favored (64.524%) beta sheet	-	-	-
**#**	**Alt**	**Res**	**High B**	**Clash > 0.4Å**	**Ramachandran**	**Rotamer**	**Cβ deviation**	**CaBLAM**	**Bond lengths**	**Bond angles**	**Cis Peptides**
			Avg: 0.93	Clashscore: 1.46	Outliers: 4 of 903	Poor rotamers: 0 of 774	Outliers: 0 of 826	Outliers: 27 of 901	Outliers: 15 of 905	Outliers: 10 of 905	Non-Trans: 1 of 904
A 581		LYS	0.74	-	Favored (42.25%) General / -97.7,125.1	Favored (33.6%) *mtpt* chi angles: 299.3,171.7,77.5,189.9	0.11Å	Favored (66.301%) beta sheet	-	-	-
A 582		VAL	0.78	-	Favored (24.01%) Ile or Val / -120.8,152.6	Favored (29.8%) *m* chi angles: 300.2	0.02Å	Favored (52%) beta sheet	-	-	-
A 583		MET	0.86	-	Favored (43.67%) General / -100.8,134.5	Favored (60.2%) *ttm* chi angles: 185.6,176.1,283.9	0.06Å	Favored (49.684%) beta sheet	-	-	-
A 584		ARG	0.97	-	Favored (30.67%) Pre-Pro / -137.3,140.4	Favored (54.4%) *ttp80* chi angles: 179.2,170.2,64.1,98.9	0.08Å	Favored (49.519%) beta sheet	OUTLIER(S) worst is CD--NE: 4.1 σ	-	-
A 585		PRO	1.09	-	Favored (93.99%) Trans-Pro / -62.0,142.8	Favored (52.3%) *Cg_exo* chi angles: 337.7,33.3,330.2	0.08Å	Favored (53.456%) beta sheet	-	-	-
A 586		ALA	1.2	-	Favored (15.68%) General / -103.3,159.3	-	0.03Å	Favored (39.891%)	-	-	-
A 587		ALA	1.27	-	Favored (68.12%)	-	0.02Å	Favored (62.531%)	-	-	-

					General / -59.2,-30.4						
A 588		ASP	1.27	-	Favored (24.89%) General / -84.4,6.0	Favored (54.8%) *p0* chi angles: 65.4,7.3	0.05Å	Favored (50.151%)	-	-	-
A 589		GLY	1.22	-	Favored (76.81%) Glycine / 92.6,-8.4	-	-	Favored (68.185%)	-	-	-
A 590		LYS	1.13	-	Favored (10.41%) General / -84.3,174.1	Favored (99.1%) *mttt* chi angles: 295.5,178.8,180.1,176.7	0.06Å	Favored (28.944%)	-	-	-
A 591		THR	1.02	-	Favored (52.88%) General / -116.8,136.5	Favored (92.5%) *m* chi angles: 299.1	0.05Å	Favored (43.289%) beta sheet	-	-	-
A 592		VAL	0.92	-	Favored (39.57%) Ile or Val / -127.8,158.1	Favored (28.1%) *m* chi angles: 298.6	0.07Å	Favored (51.277%) beta sheet	-	-	-
A 593		MET	0.84	-	Favored (29.47%) General / -99.9,144.0	Favored (98.6%) *mmm* chi angles: 299.1,299,292.1	0.10Å	Favored (55.494%) beta sheet	-	-	-
A 594		ASP	0.79	-	Favored (50.89%) General / -121.1,126.9	Favored (63%) *m-30* chi angles: 301.6,311.4	0.08Å	Favored (61.852%) beta sheet	-	-	-
A 595		VAL	0.77	-	Favored (47.56%) Ile or Val / -96.4,122.1	Favored (83.2%) *t* chi angles: 177.8	0.04Å	Favored (45.357%) beta sheet	-	-	-
A 596		ILE	0.76	-	Favored (39.17%) Ile or Val / -130.8,162.2	Favored (43.2%) *pt* chi angles: 60.9,173.9	0.12Å	Favored (31.279%) beta sheet	-	-	-
A 597		SER	0.77	-	Favored (43.26%) General / -145.8,159.4	Favored (86.3%) *p* chi angles: 67.9	0.04Å	Favored (59.506%) beta sheet	-	-	-
A 598		ARG	0.78	-	Favored (37.18%) General / -157.5,164.6	Favored (13.5%) *ptm160* chi angles: 69.9,186.9,291.5,188.2	0.12Å	Favored (48.695%)	-	-	-
A 599		GLU	0.8	-	Favored (21.32%) General / -111.6,13.0	Favored (92.3%) *mt-10* chi angles: 298.9,183.7,355.7	0.04Å	Favored (19.746%)	-	-	-
A 600		ASP	0.82	-	Allowed (1.56%) General / -140.7,57.7	Favored (14.6%) *p0* chi angles: 55.4,329.2	0.12Å	CaBLAM Disfavored (4.653%)	-	-	-
**#**	**Alt**	**Res**	**High B**	**Clash > 0.4Å**	**Ramachandran**	**Rotamer**	**Cβ deviation**	**CaBLAM**	**Bond lengths**	**Bond angles**	**Cis Peptides**
			Avg: 0.93	Clashscore: 1.46	Outliers: 4 of 903	Poor rotamers: 0 of 774	Outliers: 0 of 826	Outliers: 27 of 901	Outliers: 15 of 905	Outliers: 10 of 905	Non-Trans: 1 of 904
A 601		GLN	0.85	-	Favored (16.75%) General / -159.3,146.0	Favored (48.3%) *tt0* chi angles: 183.9,168.8,327.7	0.07Å	Favored (10.139%)	-	-	-
A 602		ARG	0.87	-	Favored (27.28%) General / -107.5,150.0	Favored (94.6%) *mtt180* chi angles: 294.5,172.2,187.1,172	0.04Å	Favored (43.056%)	-	-	-
A 603		GLY	0.88	-	Favored (16.36%)	-	-	Favored (35.844%)	-	-	-

					Glycine / -93.1,143.2						
A 604		SER	0.89	-	Favored (58.67%) General / -75.7,-9.5	Favored (63.1%) *m* chi angles: 297.9	0.06Å	Favored (21.769%)	-	-	-
A 605		GLY	0.9	-	Favored (34.75%) Glycine / -108.9,11.1	-	-	Favored (62.073%)	-	-	-
A 606		GLN	0.9	0.48Å NE2 with A 454 TYR OH	Favored (25.52%) General / -82.5,153.6	Favored (32.5%) *mt0* chi angles: 297.8,187.3,251.8	0.05Å	Favored (37.745%)	-	-	-
A 607		VAL	0.89	-	Favored (48.61%) Ile or Val / -54.4,-40.4	Favored (64.6%) *t* chi angles: 171.4	0.01Å	Favored (13.073%)	-	-	-
A 608		VAL	0.88	-	Favored (8.21%) Ile or Val / -114.0,14.4	Favored (28%) *m* chi angles: 296.1	0.04Å	Favored (18.314%)	-	-	-
A 609		THR	0.86	-	Favored (96.78%) General / -60.4,-42.9	Favored (77%) *m* chi angles: 296.3	0.12Å	Favored (48.041%) alpha helix	-	-	-
A 610		TYR	0.84	-	Favored (66.11%) General / -54.0,-50.6	Favored (83.5%) *t80* chi angles: 173.2,80.2	0.04Å	Favored (83.194%) alpha helix	-	-	-
A 611		ALA	0.81	-	Favored (98.76%) General / -63.4,-41.2	-	0.05Å	Favored (82.722%) alpha helix	-	-	-
A 612		LEU	0.78	-	Favored (88.41%) General / -65.6,-38.1	Favored (93.5%) *mt* chi angles: 293.8,175	0.05Å	Favored (98.865%) alpha helix	-	-	-
A 613		ASN	0.75	-	Favored (92.27%) General / -65.7,-41.5	Favored (94.1%) *m-40* chi angles: 287.4,347.5	0.06Å	Favored (92.503%) alpha helix	-	-	-
A 614		THR	0.72	-	Favored (89.71%) General / -61.6,-46.5	Favored (99.3%) *m* chi angles: 300.4	0.08Å	Favored (95.506%) alpha helix	-	-	-
A 615		PHE	0.7	-	Favored (71.82%) General / -59.2,-51.0	Favored (74.2%) *t80* chi angles: 176.7,87.2	0.08Å	Favored (89.296%) alpha helix	-	-	-
A 616		THR	0.67	-	Favored (97.64%) General / -63.1,-40.5	Favored (89.9%) *m* chi angles: 298.7	0.14Å	Favored (88.396%) alpha helix	-	-	-
A 617		ASN	0.66	-	Favored (79.28%) General / -62.4,-35.5	Favored (47.5%) *m-40* chi angles: 281.1,274.9	0.05Å	Favored (86.188%) alpha helix	-	OUTLIER(S) worst is CA-CB-CG: 4.5 σ	-
A 618		LEU	0.64	-	Favored (97.67%) General / -63.9,-42.0	Favored (78.6%) *mt* chi angles: 288,168.1	0.03Å	Favored (92.757%) alpha helix	-	-	-
A 619		ALA	0.63	-	Favored (99.86%) General / -62.7,-42.8	-	0.02Å	Favored (94.171%) alpha helix	-	-	-
A 620		VAL	0.63	-	Favored (93.1%) Ile or Val / -65.8,-44.3	Favored (64.1%) *t* chi angles: 171.4	0.03Å	Favored (94.123%) alpha helix	-	-	-

#	Alt	Res	High B	Clash > 0.4Å	Ramachandran	Rotamer	Cβ deviation	CaBLAM	Bond lengths	Bond angles	Cis Peptides
			Avg: 0.93	Clashscore: 1.46	Outliers: 4 of 903	Poor rotamers: 0 of 774	Outliers: 0 of 826	Outliers: 27 of 901	Outliers: 15 of 905	Outliers: 10 of 905	Non-Trans: 1 of 904
A 621		GLN	0.62	-	Favored (82.07%) General / -66.3,-36.1	Favored (29.4%) *mm110* chi angles: 295.8,289.8,107.9	0.09Å	Favored (88.873%) alpha helix	-	-	-
A 622		LEU	0.62	-	Favored (91.84%) General / -62.9,-38.6	Favored (79.5%) *mt* chi angles: 288.6,170.1	0.03Å	Favored (93.253%) alpha helix	-	-	-
A 623		VAL	0.61	-	Favored (94.95%) Ile or Val / -65.4,-43.1	Favored (75.9%) *t* chi angles: 172.7	0.05Å	Favored (99.039%) alpha helix	OUTLIER(S) worst is CB--CG1: 4.8 σ	-	-
A 624		ARG	0.61	-	Favored (79.65%) General / -61.8,-35.9	Favored (84.7%) *mtp180* chi angles: 288.8,177.6,66.7,191.7	0.03Å	Favored (82.799%) alpha helix	-	-	-
A 625		MET	0.61	-	Favored (94.73%) General / -62.5,-39.8	Favored (78.8%) *mtp* chi angles: 292.6,170,81.7	0.10Å	Favored (76.472%) alpha helix	-	-	-
A 626		MET	0.62	-	Favored (78.33%) General / -63.0,-34.8	Favored (77.6%) *mtm* chi angles: 288.1,190.4,285.6	0.05Å	Favored (75.699%) alpha helix	-	-	-
A 627		GLU	0.62	-	Favored (68.05%) General / -66.9,-48.7	Favored (72.6%) *tt0* chi angles: 177.9,178.6,341.2	0.04Å	Favored (71.707%) alpha helix	-	-	-
A 628		GLY	0.62	-	Favored (77.82%) Glycine / -59.4,-33.9	-	-	Favored (88.786%) alpha helix	-	-	-
A 629		GLU	0.63	-	Favored (58.58%) General / -82.5,-5.2	Favored (78.4%) *mt-10* chi angles: 292.9,168.3,17.9	0.04Å	Favored (57.499%)	-	-	-
A 630		GLY	0.64	-	Favored (71.12%) Glycine / 81.2,17.7	-	-	Favored (84.219%)	-	-	-
A 631		VAL	0.67	-	Favored (28.56%) Ile or Val / -77.9,-44.4	Favored (96%) *t* chi angles: 175.7	0.03Å	Favored (6.456%)	-	-	-
A 632		ILE	0.69	-	Favored (70.19%) Ile or Val / -122.2,124.0	Favored (62.8%) *mt* chi angles: 303.8,169.9	0.10Å	Favored (34.952%)	-	-	-
A 633		GLY	0.72	-	Favored (41.42%) Glycine / -89.5,174.3	-	-	Favored (48.338%)	-	-	-
A 634		PRO	0.74	-	Favored (73.38%) Trans-Pro / -62.9,-21.8	Favored (38.5%) *Cg_endo* chi angles: 23.1,325.8,30.4	0.03Å	Favored (80.086%)	-	-	-
A 635		ASP	0.76	-	Favored (32.17%) General / -82.8,-26.0	Favored (46.9%) *m-30* chi angles: 295.3,295.1	0.05Å	Favored (88.108%) alpha helix	-	-	-
A 636		ASP	0.76	-	Favored (25.42%) General / -85.6,-27.6	Favored (61.1%) *m-30* chi angles: 295.7,305.2	0.03Å	Favored (63.32%) alpha helix	-	-	-

A 637		VAL	0.76	-	Favored (97.38%) Ile or Val / -60.7,-44.5	Favored (64.7%) *t* chi angles: 171.5	0.08Å	Favored (59.306%) alpha helix	-	-	-
A 638		GLU	0.76	-	Favored (31.38%) General / -74.6,-47.6	Favored (97.5%) *mt-10* chi angles: 291,181.6,356.4	0.03Å	Favored (36.952%) alpha helix	-	-	-
A 639		LYS	0.77	-	Favored (2.79%) General / -134.0,91.3	Favored (70.2%) *mmtt* chi angles: 294.5,290,183.2,178.1	0.03Å	Favored (11.209%)	-	-	-
A 640		LEU	0.77	-	Favored (28.57%) General / -62.7,126.8	Favored (30%) *tp* chi angles: 186.6,66.5	0.04Å	Favored (26.925%)	-	-	-
**#**	**Alt**	**Res**	**High B**	**Clash > 0.4Å**	**Ramachandran**	**Rotamer**	**Cβ deviation**	**CaBLAM**	**Bond lengths**	**Bond angles**	**Cis Peptides**
			Avg: 0.93	Clashscore: 1.46	Outliers: 4 of 903	Poor rotamers: 0 of 774	Outliers: 0 of 826	Outliers: 27 of 901	Outliers: 15 of 905	Outliers: 10 of 905	Non-Trans: 1 of 904
A 641		GLY	0.79	-	Favored (48.06%) Glycine / -82.4,168.3	-	-	Favored (62.331%)	-	-	-
A 642		LYS	0.8	-	Favored (75.38%) General / -61.5,-34.3	Favored (97.2%) *mttt* chi angles: 289.7,179.4,180.2,178.3	0.02Å	Favored (59.913%)	-	-	-
A 643		GLY	0.79	-	Favored (86.59%) Glycine / -87.4,3.3	-	-	Favored (48.874%) alpha helix	-	-	-
A 644		LYS	0.78	-	Favored (6.85%) General / -107.0,-35.5	Favored (21.7%) *mmtp* chi angles: 300.9,289.6,178.7,65.8	0.04Å	Favored (5.682%) three-ten	-	-	-
A 645		GLY	0.74	-	Allowed (0.48%) Glycine / -38.0,-67.7	-	-	Favored (59.279%) three-ten	-	-	-
A 646		PRO	0.7	-	Favored (69.27%) Trans-Pro / -63.1,-19.6	Favored (38.3%) *Cg_endo* chi angles: 23,325.9,30.5	0.01Å	Favored (23.375%) alpha helix	-	-	-
A 647		LYS	0.67	-	Favored (42.38%) General / -74.2,-46.3	Favored (85.9%) *tttt* chi angles: 183.7,179.3,181.3,183.9	0.04Å	Favored (44.932%) alpha helix	-	-	-
A 648		VAL	0.63	-	Favored (96.54%) Ile or Val / -61.7,-46.4	Favored (63%) *t* chi angles: 171.2	0.08Å	Favored (85.498%) alpha helix	-	-	-
A 649		ARG	0.61	-	Favored (98.31%) General / -63.6,-41.8	Favored (99.6%) *mtm-85* chi angles: 290.8,192.9,296.5,272.9	0.02Å	Favored (97.426%) alpha helix	-	-	-
A 650		THR	0.61	-	Favored (88.67%) General / -62.1,-46.5	Favored (92.1%) *m* chi angles: 297.8	0.02Å	Favored (97.171%) alpha helix	-	-	-
A 651		TRP	0.61	-	Favored (91.57%) General / -60.4,-40.6	Favored (32.2%) *m100* chi angles: 273,94.2	0.05Å	Favored (87.027%) alpha helix	-	-	-
A 652		LEU	0.62	-	Favored (99.55%) General / -63.0,-41.7	Favored (81.3%) *mt* chi angles: 289.1,168.4	0.04Å	Favored (87.014%) alpha helix	-	-	-

A 653		PHE	0.63	-	Favored (65.07%) General / -72.4,-30.9	Favored (45.2%) *m-80* chi angles: 286.6,114.4	0.04Å	Favored (61.059%) alpha helix	-	-	-
A 654		GLU	0.64	-	Favored (13.63%) General / -92.8,-35.0	Favored (96.8%) *mt-10* chi angles: 294.3,181.7,350.5	0.03Å	Favored (45.622%) alpha helix	-	-	-
A 655		ASN	0.64	-	Favored (10.62%) General / -117.4,-11.0	Favored (62.9%) *m-40* chi angles: 292.7,277.1	0.04Å	Favored (17.369%) alpha helix	-	-	-
A 656		GLY	0.65	-	Favored (22.23%) Glycine / -50.0,-53.5	-	-	Favored (54.727%) alpha helix	-	-	-
A 657		GLU	0.65	-	Favored (84.4%) General / -60.8,-38.1	Favored (75%) *tt0* chi angles: 183.8,167.6,0.1	0.07Å	Favored (82.474%) alpha helix	-	-	-
A 658		GLU	0.64	-	Favored (85.98%) General / -66.8,-38.2	Favored (73.5%) *mm-30* chi angles: 288.6,296.2,311.2	0.02Å	Favored (97.959%) alpha helix	-	-	-
A 659		ARG	0.63	-	Favored (92.44%) General / -65.7,-41.2	Favored (74.7%) *mtt180* chi angles: 288.9,173,178.5,149	0.09Å	Favored (86.566%) alpha helix	OUTLIER(S) worst is CD--NE: 4.5 σ	-	-
A 660		LEU	0.63	-	Favored (89.76%) General / -61.0,-39.4	Favored (44.6%) *tp* chi angles: 184.7,60.3	0.09Å	Favored (80.023%) alpha helix	-	-	-
**#**	**Alt**	**Res**	**High B**	**Clash > 0.4Å**	**Ramachandran**	**Rotamer**	**Cβ deviation**	**CaBLAM**	**Bond lengths**	**Bond angles**	**Cis Peptides**
			Avg: 0.93	Clashscore: 1.46	Outliers: 4 of 903	Poor rotamers: 0 of 774	Outliers: 0 of 826	Outliers: 27 of 901	Outliers: 15 of 905	Outliers: 10 of 905	Non-Trans: 1 of 904
A 661		SER	0.62	-	Favored (58.2%) General / -70.5,-10.0	Favored (52.5%) *p* chi angles: 74.1	0.04Å	Favored (55.381%) three-ten	-	-	-
A 662		ARG	0.62	-	Favored (35.26%) General / -100.2,13.1	Favored (67.2%) *mtt90* chi angles: 286.5,163.6,171.1,74.2	0.04Å	Favored (37.585%)	-	-	-
A 663		MET	0.62	-	Favored (25.46%) General / -124.9,162.0	Favored (71.3%) *mtm* chi angles: 298.6,192,285	0.04Å	Favored (24.584%)	-	-	-
A 664		ALA	0.63	-	Favored (8.59%) General / -140.2,113.7	-	0.02Å	Favored (29.366%)	-	-	-
A 665		VAL	0.65	-	Favored (74.19%) Ile or Val / -124.0,129.5	Favored (55.1%) *t* chi angles: 180.6	0.03Å	Favored (53.806%)	-	-	-
A 666		SER	0.67	-	Favored (6.73%) General / -129.2,103.0	Favored (38.8%) *t* chi angles: 177.1	0.07Å	Favored (9.111%)	-	-	-
A 667		GLY	0.69	-	Favored (31.36%) Glycine / 60.7,-123.0	-	-	Favored (64.937%)	-	-	-
A 668		ASP	0.69	-	Favored (58.98%) General / -87.1,-5.5	Favored (50.4%) *m-30* chi angles: 301.7,338	0.14Å	Favored (13.676%)	-	OUTLIER(S) worst is CA-CB-CG: 5.2 σ	-

A 669		ASP	0.69	-	Favored (32.06%) General / -98.8,116.9	Favored (56.7%) *t0* chi angles: 187.7,334.4	0.09Å	Favored (27.955%)	OUTLIER(S) worst is CG--OD2: 4.1 σ	-	-
A 670		CYS	0.68	-	Favored (33.65%) General / -134.7,128.8	Favored (48.5%) *t* chi angles: 179.2	0.09Å	Favored (58.445%)	-	-	-
A 671		VAL	0.67	-	Favored (48.08%) Ile or Val / -101.3,131.9	Favored (91.8%) *t* chi angles: 174.4	0.06Å	Favored (61.358%) beta sheet	-	-	-
A 672		VAL	0.67	-	Favored (56.34%) Ile or Val / -132.3,136.5	Favored (45%) *t* chi angles: 182.5	0.07Å	Favored (66.091%) beta sheet	-	-	-
A 673		LYS	0.68	-	Favored (51.01%) Pre-Pro / -123.1,84.0	Favored (46.1%) *tttp* chi angles: 187.8,174.6,179.6,71	0.04Å	Favored (16.138%) beta sheet	-	-	-
A 674		PRO	0.69	-	Favored (35.3%) Trans-Pro / -59.2,156.3	Favored (38.9%) *Cg_exo* chi angles: 338.4,35,326.2	0.10Å	Favored (8.761%)	-	-	-
A 675		LEU	0.71	-	Favored (69.93%) General / -63.1,-28.2	Favored (87%) *mt* chi angles: 291.4,174.5	0.02Å	Favored (23.398%)	-	-	-
A 676		ASP	0.71	-	Favored (29.34%) General / -142.2,135.9	Favored (11.3%) *m-30* chi angles: 290.4,276.9	0.09Å	Favored (18.991%)	-	-	-
A 677		ASP	0.72	-	Favored (5.77%) General / -60.7,163.2	Favored (90.5%) *m-30* chi angles: 285,345.4	0.10Å	CaBLAM Disfavored (1.703%)	-	-	-
A 678		ARG	0.72	-	OUTLIER (0.04)%) General / 99.3,-15.3	Favored (89.5%) *mtm180* chi angles: 292.8,170.3,295.5,179	0.12Å	CaBLAM Outlier (0.004%)	-	-	-
A 679		PHE	0.72	-	Favored (9.15%) General / -46.5,-38.2	Favored (89.5%) *t80* chi angles: 178.5,81.6	0.07Å	Favored (28.273%)	-	-	-
A 680		ALA	0.73	-	Favored (43.98%) General / -52.5,-33.2	-	0.13Å	Favored (61.363%)	-	-	-
**#**	**Alt**	**Res**	**High B**	**Clash > 0.4Å**	**Ramachandran**	**Rotamer**	**Cβ deviation**	**CaBLAM**	**Bond lengths**	**Bond angles**	**Cis Peptides**
			Avg: 0.93	Clashscore: 1.46	Outliers: 4 of 903	Poor rotamers: 0 of 774	Outliers: 0 of 826	Outliers: 27 of 901	Outliers: 15 of 905	Outliers: 10 of 905	Non-Trans: 1 of 904
A 681		THR	0.74	-	Favored (55.28%) General / -94.9,1.5	Favored (54.8%) *p* chi angles: 65	0.05Å	Favored (50.562%)	-	-	-
A 682		SER	0.76	-	Allowed (0.62%) General / -73.8,58.1	Favored (93.8%) *p* chi angles: 64.7	0.13Å	CaBLAM Disfavored (4.226%)	-	-	-
A 683		LEU	0.78	-	Favored (34.24%) General / -101.9,-0.2	Favored (87.3%) *mt* chi angles: 300.1,177.9	0.05Å	Favored (15.342%)	-	-	-
A 684		HIS	0.79	-	Favored (52.5%) General / -72.6,-46.8	Favored (80.9%) *m90* chi angles: 294.4,82.8	0.02Å	Favored (10.847%)	-	-	-

#	Alt	Res	High B	Clash > 0.4Å	Ramachandran	Rotamer	Cβ deviation	CaBLAM	Bond lengths	Bond angles	Cis Peptides
A 685		PHE	0.8	-	Favored (94.29%) General / -64.2,-39.6	Favored (10.9%) *m-80* chi angles: 275,123.7	0.08Å	Favored (74.693%) alpha helix	-	-	-
A 686		LEU	0.8	-	Favored (84.31%) General / -62.2,-47.5	Favored (46.4%) *tp* chi angles: 182.2,65.8	0.10Å	Favored (71.007%) alpha helix	-	-	-
A 687		ASN	0.81	-	Favored (60.57%) General / -75.5,-37.0	Favored (97.5%) *m-40* chi angles: 292.4,341.5	0.07Å	Favored (61.357%) alpha helix	-	-	-
A 688		ALA	0.82	-	Favored (71.65%) General / -60.6,-32.5	-	0.03Å	Favored (74.565%) alpha helix	-	-	-
A 689		MET	0.85	-	Favored (27.32%) General / -85.2,6.1	Favored (62.5%) *mtt* chi angles: 293.3,176.5,190.6	0.04Å	Favored (46.937%)	-	-	-
A 690		SER	0.91	-	Favored (17.33%) General / 61.0,38.3	Favored (51%) *m* chi angles: 301	0.05Å	Favored (25.523%)	-	-	-
A 691		LYS	1	-	Favored (2.76%) General / -117.6,41.8	Favored (24.1%) *mmtm* chi angles: 301.9,299.1,186,278.6	0.01Å	CaBLAM Disfavored (1.157%)	-	-	-
A 692		VAL	1.13	-	Favored (10.82%) Ile or Val / -50.6,131.6	Favored (82.4%) *t* chi angles: 173.3	0.09Å	Favored (9.032%)	-	-	-
A 693		ARG	1.3	-	Favored (52.23%) General / -69.5,137.1	Favored (63%) *ttt-90* chi angles: 185.3,186.7,186.4,280.2	0.01Å	Favored (36.43%)	-	-	-
A 694		LYS	1.48	-	Favored (51.95%) General / -56.7,132.4	Favored (97.5%) *mttt* chi angles: 290.9,177.4,183,174.4	0.10Å	Favored (9.685%)	-	-	-
A 695		ASP	1.65	-	Favored (2.14%) General / 61.6,8.0	Favored (74.5%) *m-30* chi angles: 297.1,317.4	0.02Å	CaBLAM Disfavored (1.571%)	-	-	-
A 696		ILE	1.75	0.63Å O with A 696 ILE HG23	Favored (4.84%) Ile or Val / -106.9,20.2	Favored (11.7%) *tt* chi angles: 199.8,165	0.05Å	CaBLAM Disfavored (1.863%)	-	-	-
A 697		GLN	1.76	-	Allowed (1.82%) General / 55.1,-132.3	Favored (93.4%) *mt0* chi angles: 294,181.2,332.4	0.03Å	CaBLAM Outlier (0.112%)	-	-	-
A 698		GLU	1.67	-	Favored (2.22%) General / -123.8,-41.7	Favored (96.3%) *mt-10* chi angles: 297.1,180.7,356.7	0.01Å	CaBLAM Outlier (0.006%)	-	-	-
A 699		TRP	1.51	-	Favored (31.5%) General / -93.2,-11.9	Favored (70%) *p-90* chi angles: 55.3,268.4	0.03Å	Favored (46.456%)	-	-	-
A 700		LYS	1.33	-	Favored (78.21%) Pre-Pro / -73.3,138.3	Favored (97.4%) *mttt* chi angles: 289.8,181.2,175.3,179.7	0.05Å	Favored (32.32%)	-	-	-
**#**	**Alt**	**Res**	**High B**	**Clash > 0.4Å**	**Ramachandran**	**Rotamer**	**Cβ deviation**	**CaBLAM**	**Bond lengths**	**Bond angles**	**Cis Peptides**
			Avg: 0.93	Clashscore: 1.46	Outliers: 4 of 903	Poor rotamers: 0 of 774	Outliers: 0 of 826	Outliers: 27 of 901	Outliers: 15 of 905	Outliers: 10 of 905	Non-Trans: 1 of 904

A 701		PRO	1.17	-	Favored (83.91%) Trans-Pro / -66.2,147.8	Favored (43.5%) *Cg_endo* chi angles: 24.3,326.9,27.7	0.02Å	Favored (76.95%)	-	-	-
A 702		SER	1.04	-	Favored (57.09%) General / -64.2,144.5	Favored (67.9%) *m* chi angles: 296.8	0.03Å	Favored (48.278%)	-	-	-
A 703		THR	0.94	-	Favored (36.59%) General / -79.0,132.3	Favored (93.8%) *m* chi angles: 299.3	0.01Å	Favored (42.665%) beta sheet	-	-	-
A 704		GLY	0.88	-	Favored (15.4%) Glycine / -120.5,159.1	-	-	Favored (68.931%) beta sheet	-	-	-
A 705		TRP	0.84	-	Favored (46.16%) General / -131.5,156.1	Favored (35.3%) *m-90* chi angles: 298.8,265.7	0.04Å	Favored (60.409%)	-	-	-
A 706		TYR	0.82	-	Favored (41.65%) General / -100.7,10.1	Favored (86.6%) *m-80* chi angles: 293.6,102.9	0.05Å	Favored (27.18%)	-	-	-
A 707		ASP	0.81	-	Favored (5.17%) General / -146.5,111.0	Favored (61.5%) *t0* chi angles: 183.2,341.4	0.03Å	Favored (6.689%) alpha helix	-	OUTLIER(S) worst is CA-CB-CG: 4.2 σ	-
A 708		TRP	0.8	-	Favored (5.49%) General / -51.5,-24.9	Favored (80.8%) *p-90* chi angles: 62.7,268	0.04Å	Favored (34.695%) alpha helix	-	-	-
A 709		GLN	0.8	-	Favored (62.56%) General / -71.0,-19.2	Favored (32.5%) *mt0* chi angles: 293.7,173.2,106.5	0.02Å	Favored (65.568%) alpha helix	-	-	-
A 710		GLN	0.81	-	Favored (55.77%) General / -94.4,2.3	Favored (76%) *mt0* chi angles: 296.6,178.4,22.9	0.02Å	Favored (55.526%)	-	-	-
A 711		VAL	0.83	-	Favored (59.45%) Pre-Pro / -98.9,120.1	Favored (59.3%) *t* chi angles: 180.1	0.12Å	Favored (31.359%)	-	-	-
A 712		PRO	0.86	-	Favored (55.13%) Trans-Pro / -72.2,151.8	Favored (71.6%) *Cg_endo* chi angles: 27.4,327.8,24	0.02Å	Favored (36.59%)	-	-	-
A 713		PHE	0.9	-	Favored (6.66%) General / -156.2,126.7	Favored (41.5%) *t80* chi angles: 186.3,61.7	0.04Å	Favored (9.017%)	-	-	-
A 714		CYS	0.93	-	Allowed (0.21%) General / 67.4,-123.7	Favored (70.6%) *m* chi angles: 298.3	0.07Å	CaBLAM Outlier (0.569%)	-	-	-
A 715		SER	0.95	-	Favored (44.39%) General / -98.0,8.7	Favored (83.7%) *p* chi angles: 62.5	0.02Å	CaBLAM Disfavored (1.143%)	-	-	-
A 716		ASN	0.94	-	Favored (9.38%) General / -154.9,179.2	Favored (27.2%) *p0* chi angles: 54.2,51.8	0.09Å	Favored (21.865%)	-	-	-
A 717		HIS	0.92	-	Favored (16.48%) General / -113.9,162.3	Favored (69%) *m90* chi angles: 298,77.3	0.08Å	Favored (37.076%)	-	-	-
A 718		PHE	0.88	-	Favored (43.87%)	Favored (85%) *m-80*	0.08Å	Favored (53.812%)	-	-	-

#	Alt	Res	High B	Clash > 0.4Å	Ramachandran	Rotamer	Cβ deviation	CaBLAM	Bond lengths	Bond angles	Cis Peptides
					General / -117.3,145.6	chi angles: 291.4,83.6		beta sheet			
A 719		THR	0.84	-	Favored (53.33%) General / -126.5,137.2	Favored (98.9%) *m* chi angles: 300.3	0.04Å	Favored (60.085%) beta sheet	-	-	-
A 720		GLU	0.81	-	Favored (35.53%) General / -91.8,123.9	Favored (32.1%) *tt0* chi angles: 183.8,173.2,108.4	0.06Å	Favored (59.665%) beta sheet	-	-	-
**#**	**Alt**	**Res**	**High B**	**Clash > 0.4Å**	**Ramachandran**	**Rotamer**	**Cβ deviation**	**CaBLAM**	**Bond lengths**	**Bond angles**	**Cis Peptides**
			Avg: 0.93	Clashscore: 1.46	Outliers: 4 of 903	Poor rotamers: 0 of 774	Outliers: 0 of 826	Outliers: 27 of 901	Outliers: 15 of 905	Outliers: 10 of 905	Non-Trans: 1 of 904
A 721		LEU	0.8	-	Favored (26.36%) General / -107.9,151.0	Allowed (1.5%) *mm* chi angles: 282.3,304.1	0.10Å	Favored (42.953%) beta sheet	-	-	-
A 722		ILE	0.81	-	Favored (69.01%) Ile or Val / -113.5,123.3	Favored (72.6%) *mt* chi angles: 301.5,168.8	0.02Å	Favored (47.518%)	-	-	-
A 723		MET	0.82	-	Favored (29.07%) General / -71.4,162.7	Favored (82.5%) *mtp* chi angles: 295.8,186.4,72.2	0.04Å	Favored (31.557%)	-	-	-
A 724		LYS	0.83	-	Favored (36.05%) General / -49.4,-40.3	Favored (35.3%) *ttpt* chi angles: 179.6,181.4,70.9,175.4	0.04Å	Favored (59.61%)	-	-	-
A 725		ASP	0.83	-	Favored (27.19%) General / -85.6,6.5	Favored (50.8%) *p0* chi angles: 66.5,15.3	0.07Å	Favored (45.73%)	-	-	-
A 726		GLY	0.83	-	Favored (72.53%) Glycine / 93.9,-10.0	-	-	Favored (71.11%)	-	-	-
A 727		ARG	0.82	-	Favored (26.8%) General / -76.5,163.6	Favored (88.5%) *mtm180* chi angles: 292.7,174.1,291.9,168.5	0.05Å	Favored (40.508%)	-	-	-
A 728		THR	0.8	-	Favored (36.9%) General / -120.3,153.1	Favored (78.8%) *p* chi angles: 60.3	0.01Å	Favored (57.047%) beta sheet	-	-	-
A 729		LEU	0.78	-	Favored (52.3%) General / -130.4,148.8	Allowed (1.6%) *mp* chi angles: 308.1,104.3	0.11Å	Favored (64.16%) beta sheet	-	-	-
A 730		VAL	0.77	-	Favored (56.86%) Ile or Val / -110.1,118.4	Favored (64.7%) *t* chi angles: 179.4	0.04Å	Favored (51.925%) beta sheet	-	-	-
A 731		VAL	0.77	0.47Å O with A 731 VAL HG23	Favored (70.96%) Pre-Pro / -124.9,153.5	Favored (19.6%) *m* chi angles: 302.5	0.23Å	Favored (36.636%) beta sheet	-	-	-
A 732		PRO	0.78	-	Favored (58.72%) Trans-Pro / -71.5,156.0	Favored (57.8%) *Cg_endo* chi angles: 26.1,326.4,26.5	0.03Å	Favored (66.308%) beta sheet	-	-	-
A 733		CYS	0.8	-	Favored (15.25%) General / -144.9,126.7	Favored (30.9%) *t* chi angles: 189.5	0.05Å	Favored (47.378%)	-	-	-
A 734		ARG	0.82	0.42Å O with A 735	Favored (52.71%)	Favored (82%) *ttt180*	0.05Å	Favored (25.286%)	-	-	-

#	Alt	Res	High B	Clash > 0.4Å	Ramachandran	Rotamer	Cβ deviation	CaBLAM	Bond lengths	Bond angles	Cis Peptides
				GLY C	General / -131.0,148.1	chi angles: 181.7,173.9,181.7,180.6					
A 735		GLY	0.83	0.42Å C with A 734 ARG O	Favored (6.57%) Glycine / -45.4,124.3	-	-	Favored (27.474%)	-	-	-
A 736		GLN	0.84	-	Favored (72.66%) General / -56.9,-38.2	Favored (61.3%) *tt0* chi angles: 180.5,177.2,49.9	0.06Å	Favored (54.817%)	-	-	-
A 737		ASP	0.84	-	Favored (73.8%) General / -60.2,-34.5	Favored (98.2%) *m-30* chi angles: 287.9,344.1	0.05Å	Favored (73.432%) alpha helix	-	-	-
A 738		GLU	0.83	-	Favored (59.3%) General / -75.8,-37.4	Favored (90.5%) *mt-10* chi angles: 294.3,181.5,11.5	0.01Å	Favored (91.53%) alpha helix	-	-	-
A 739		LEU	0.82	-	Favored (87.95%) General / -66.8,-41.2	Favored (84.2%) *mt* chi angles: 289.9,172.8	0.01Å	Favored (74.833%) alpha helix	-	-	-
A 740		ILE	0.81	-	Favored (32.44%) Ile or Val / -76.8,-45.1	Favored (93.1%) *mt* chi angles: 295.8,170.7	0.05Å	Favored (71.055%) alpha helix	-	-	-
**#**	**Alt**	**Res**	**High B**	**Clash > 0.4Å**	**Ramachandran**	**Rotamer**	**Cβ deviation**	**CaBLAM**	**Bond lengths**	**Bond angles**	**Cis Peptides**
			Avg: 0.93	Clashscore: 1.46	Outliers: 4 of 903	Poor rotamers: 0 of 774	Outliers: 0 of 826	Outliers: 27 of 901	Outliers: 15 of 905	Outliers: 10 of 905	Non-Trans: 1 of 904
A 741		GLY	0.81	-	Favored (53.28%) Glycine / -53.8,-50.6	-	-	Favored (96.246%) alpha helix	-	-	-
A 742		ARG	0.83	-	Favored (70.95%) General / -54.9,-49.6	Favored (61.3%) *ttt90* chi angles: 185.3,182.4,182.9,87.3	0.05Å	Favored (98.04%) alpha helix	-	-	-
A 743		ALA	0.89	-	Favored (70.52%) General / -59.8,-32.3	-	0.04Å	Favored (73.753%) alpha helix	-	-	-
A 744		ARG	1	-	Favored (55.91%) General / -77.4,-7.3	Favored (36.5%) *mtp180* chi angles: 287.8,179.7,56.1,163.2	0.08Å	Favored (51.85%)	-	-	-
A 745		ILE	1.18	0.43Å O with A 462 LYS NZ	Favored (62.09%) Ile or Val / -109.1,130.4	Favored (41.5%) *mm* chi angles: 305.6,297.7	0.04Å	Favored (34.371%)	-	-	-
A 746		SER	1.44	-	Favored (47.36%) Pre-Pro / -92.6,127.7	Favored (65.5%) *m* chi angles: 294.3	0.03Å	Favored (48.525%)	-	-	-
A 747		PRO	1.75	-	Favored (7.47%) Trans-Pro / -77.4,61.9	Favored (56.9%) *Cg_endo* chi angles: 32.2,322.9,26.5	0.02Å	CaBLAM Outlier (0.119%)	-	-	-
A 748		GLY	2.04	-	Favored (31.04%) Glycine / 152.9,-176.4	-	-	Favored (8.568%)	-	-	-
A 749		ALA	2.18	-	Favored (10.48%) General / -87.4,171.5	-	0.02Å	Favored (11.909%)	-	-	-

#	Alt	Res	High B	Clash > 0.4Å	Ramachandran	Rotamer	Cβ deviation	CaBLAM	Bond lengths	Bond angles	Cis Peptides
A 750		GLY	2.1	-	Favored (4.12%) Glycine / 74.3,-113.5	-	-	Favored (5.279%)	-	-	-
A 751		TRP	1.84	-	Allowed (1.15%) General / 60.2,64.9	Favored (88.3%) *m100* chi angles: 284.8,94.9	0.12Å	CaBLAM Outlier (0.036%)	-	-	-
A 752		ASN	1.52	-	Favored (7.62%) General / -52.2,147.5	Favored (26.4%) *t0* chi angles: 184.5,275.9	0.06Å	Favored (33.807%)	-	-	-
A 753		VAL	1.23	-	Favored (4.55%) Ile or Val / -48.4,-33.5	Favored (9%) *p* chi angles: 66.6	0.03Å	Favored (54.575%)	-	-	-
A 754		ARG	1.01	-	Favored (73.76%) General / -65.2,-32.2	Favored (88.5%) *mtt180* chi angles: 291.2,178.2,177.7,158.1	0.05Å	Favored (73.495%) alpha helix	-	-	-
A 755		ASP	0.88	-	Favored (58.7%) General / -76.3,-35.9	Favored (93.4%) *m-30* chi angles: 292.7,344.7	0.03Å	Favored (81.897%) alpha helix	-	-	-
A 756		THR	0.8	-	Favored (65.11%) General / -70.7,-29.0	Favored (51.9%) *p* chi angles: 65.4	0.04Å	Favored (77.376%) alpha helix	-	-	-
A 757		ALA	0.76	-	Favored (99.59%) General / -63.1,-42.4	-	0.06Å	Favored (77.828%) alpha helix	-	-	-
A 758		CYS	0.73	-	Favored (95.77%) General / -64.7,-42.0	Favored (90.8%) *m* chi angles: 291.6	0.05Å	Favored (91.269%) alpha helix	-	-	-
A 759		LEU	0.71	-	Favored (90.77%) General / -64.4,-44.6	Favored (61.1%) *tp* chi angles: 181.5,62	0.03Å	Favored (90.102%) alpha helix	-	-	-
A 760		ALA	0.7	-	Favored (87.12%) General / -59.0,-41.3	-	0.07Å	Favored (87.181%) alpha helix	-	-	-
**#**	**Alt**	**Res**	**High B**	**Clash > 0.4Å**	**Ramachandran**	**Rotamer**	**Cβ deviation**	**CaBLAM**	**Bond lengths**	**Bond angles**	**Cis Peptides**
			Avg: 0.93	Clashscore: 1.46	Outliers: 4 of 903	Poor rotamers: 0 of 774	Outliers: 0 of 826	Outliers: 27 of 901	Outliers: 15 of 905	Outliers: 10 of 905	Non-Trans: 1 of 904
A 761		LYS	0.69	-	Favored (99.56%) General / -61.6,-42.9	Favored (50.9%) *tttp* chi angles: 183.3,173.8,172.7,68.2	0.06Å	Favored (82.927%) alpha helix	-	-	-
A 762		SER	0.68	0.66Å OG with A 798 THR OG1	Favored (89.43%) General / -58.9,-45.7	Favored (22.2%) *t* chi angles: 185.5	0.07Å	Favored (83.138%) alpha helix	-	-	-
A 763		TYR	0.68	-	Favored (95.77%) General / -64.7,-41.7	Favored (30.3%) *m-80* chi angles: 278,100.8	0.03Å	Favored (90.647%) alpha helix	-	-	-
A 764		ALA	0.68	-	Favored (99.8%) General / -62.6,-43.0	-	0.09Å	Favored (97.297%) alpha helix	-	-	-
A 765		GLN	0.69	-	Favored (86.11%) General / -67.2,-39.2	Favored (94.9%) *mt0* chi angles: 291,172.1,311.2	0.06Å	Favored (96.671%) alpha helix	-	-	-

A 766		MET	0.69	-	Favored (89.67%) General / -61.4,-46.5	Favored (61.4%) *ttp* chi angles: 181,184.7,70.1	0.04Å	Favored (83.372%) alpha helix	-	-	-
A 767		TRP	0.7	-	Favored (80.01%) General / -57.6,-40.8	Favored (49.6%) *m-10* chi angles: 288.5,346.9	0.07Å	Favored (77.385%) alpha helix	-	-	-
A 768		LEU	0.7	-	Favored (64.53%) General / -65.0,-17.5	Favored (85.4%) *mt* chi angles: 289.9,172.4	0.08Å	Favored (48.271%) alpha helix	-	-	-
A 769		LEU	0.7	-	Favored (12.3%) General / -99.9,-30.0	Favored (82.1%) *mt* chi angles: 298.2,180.5	0.05Å	Favored (44.601%) alpha helix	-	-	-
A 770		LEU	0.69	-	Favored (6.61%) General / -99.4,-43.3	Favored (91.6%) *mt* chi angles: 298.3,178.3	0.06Å	Favored (34.163%) alpha helix	-	-	-
A 771		TYR	0.68	-	Favored (7.5%) General / -118.4,29.9	Favored (42.5%) *m-80* chi angles: 309.4,107	0.04Å	Favored (11.423%) alpha helix	-	-	-
A 772		PHE	0.67	-	Favored (50.85%) General / -58.5,-21.2	Favored (28.7%) *p90* chi angles: 66.9,81	0.07Å	Favored (23.287%) alpha helix	-	-	-
A 773		HIS	0.66	-	Favored (59.84%) General / -79.8,-10.9	Favored (20.8%) *p90* chi angles: 63.9,100.2	0.05Å	Favored (47.798%)	-	-	-
A 774		ARG	0.65	-	Favored (35.22%) General / -89.2,125.8	Favored (46%) *ttm170* chi angles: 177,174,291.4,155.4	0.04Å	Favored (35.773%)	OUTLIER(S) worst is CZ--NH2: 6.4 σ	-	-
A 775		ARG	0.65	-	Favored (83.96%) General / -61.6,-37.5	Favored (87.4%) *mtp180* chi angles: 291.5,175.5,67.4,184.5	0.04Å	Favored (49.363%)	-	-	-
A 776		ASP	0.65	0.41Å OD2 with A 854 SER OG	Favored (63.34%) General / -74.2,-39.3	Favored (88.6%) *m-30* chi angles: 293.6,345.9	0.06Å	Favored (80.546%) alpha helix	-	-	-
A 777		LEU	0.64	-	Favored (48.96%) General / -78.3,-31.3	Favored (60.4%) *mt* chi angles: 291.4,179.7	0.12Å	Favored (80.688%) alpha helix	-	-	-
A 778		ARG	0.64	-	Favored (97.82%) General / -60.6,-43.1	Favored (64.6%) *ttp-170* chi angles: 184,177.1,65.3,191.9	0.01Å	Favored (78.61%) alpha helix	-	-	-
A 779		LEU	0.63	-	Favored (71.2%) General / -66.8,-47.7	Favored (62.2%) *tp* chi angles: 179,57.4	0.05Å	Favored (77.173%) alpha helix	-	-	-
A 780		MET	0.62	-	Favored (98.16%) General / -60.7,-42.9	Favored (97.7%) *mtp* chi angles: 290.4,174.8,71	0.10Å	Favored (90.101%) alpha helix	-	-	-
**#**	**Alt**	**Res**	**High B**	**Clash > 0.4Å**	**Ramachandran**	**Rotamer**	**Cβ deviation**	**CaBLAM**	**Bond lengths**	**Bond angles**	**Cis Peptides**
			Avg: 0.93	Clashscore: 1.46	Outliers: 4 of 903	Poor rotamers: 0 of 774	Outliers: 0 of 826	Outliers: 27 of 901	Outliers: 15 of 905	Outliers: 10 of 905	Non-Trans: 1 of 904
A 781		ALA	0.62	-	Favored (90.07%) General / -60.7,-39.8	-	0.07Å	Favored (79.653%) alpha helix	-	-	-

A 782		ASN	0.61	-	Favored (65.67%) General / -73.1,-33.6	Favored (65.4%) *m-40* chi angles: 283.9,282.3	0.09Å	Favored (88.694%) alpha helix	-	-	-
A 783		ALA	0.61	-	Favored (94.31%) General / -63.0,-39.3	-	0.03Å	Favored (84.198%) alpha helix	-	-	-
A 784		ILE	0.6	-	Favored (67.66%) Ile or Val / -70.5,-47.0	Favored (96.1%) *mt* chi angles: 293.4,166.9	0.03Å	Favored (75.528%) alpha helix	-	-	-
A 785		CYS	0.6	-	Favored (77%) General / -59.1,-37.4	Favored (94.3%) *m* chi angles: 292	0.04Å	Favored (91.14%) alpha helix	-	-	-
A 786		SER	0.6	-	Favored (60.2%) General / -75.0,-11.8	Favored (62.2%) *m* chi angles: 298.4	0.09Å	Favored (57.407%) alpha helix	-	-	-
A 787		ALA	0.61	-	Favored (26.89%) General / -101.5,-4.3	-	0.05Å	Favored (62.12%)	-	-	-
A 788		VAL	0.62	-	Favored (23.25%) Pre-Pro / -116.8,132.7	Favored (71.8%) *t* chi angles: 178.5	0.04Å	Favored (14.614%)	-	-	-
A 789		PRO	0.64	-	Favored (53.82%) Trans-Pro / -52.7,141.3	Favored (96.2%) *Cg_exo* chi angles: 332.8,37.8,328.2	0.04Å	Favored (57.983%)	-	-	-
A 790		VAL	0.67	-	Favored (38.04%) Ile or Val / -59.8,-27.4	Favored (5.4%) *p* chi angles: 69.6	0.05Å	Favored (32.131%)	-	-	-
A 791		ASN	0.72	-	Favored (54.48%) General / -95.0,3.8	Favored (89.5%) *m-40* chi angles: 292.2,321.9	0.02Å	Favored (54.247%)	-	-	-
A 792		TRP	0.79	-	Favored (9.94%) General / -83.8,77.1	Favored (38.5%) *m-10* chi angles: 288.7,13.7	0.06Å	Favored (18.503%)	-	-	-
A 793		VAL	0.9	0.53Å O with A 793 VAL HG13	Favored (78.07%) Pre-Pro / -56.7,135.6	Favored (8.7%) *p* chi angles: 66.8	0.15Å	Favored (21.648%)	-	-	-
A 794		PRO	1.03	-	Favored (83.71%) Trans-Pro / -66.7,149.0	Favored (39.2%) *Cg_endo* chi angles: 23.2,325.5,30.7	0.04Å	Favored (50.169%)	-	-	-
A 795		THR	1.19	-	Favored (6.81%) General / -121.6,-19.5	Favored (64.5%) *p* chi angles: 63.2	0.05Å	Favored (7.19%) beta sheet	-	-	-
A 796		GLY	1.37	-	Favored (30.23%) Glycine / -84.2,154.5	-	-	Favored (27.084%) beta sheet	-	-	-
A 797		ARG	1.55	-	Favored (39.6%) General / -76.1,146.0	Favored (64.7%) *mmm-85* chi angles: 294.9,294.6,296.9,270.1	0.02Å	Favored (38.256%)	-	-	-
A 798		THR	1.69	0.66Å OG1 with A 762 SER OG	Favored (6.74%) General / -100.7,-41.9	Favored (15.5%) *m* chi angles: 310.2	0.19Å	Favored (11.257%)	-	-	-
A 799		THR	1.78	-	Favored (27.96%)	Favored (5.9%) *t* chi angles: 181.3	0.03Å	Favored (27.64%)	-	-	-

					General / -155.2,151.3						
A 800		TRP	1.81	-	Favored (52.4%) General / -94.0,-2.9	Favored (64.4%) *p-90* chi angles: 52.5,263.6	0.03Å	CaBLAM Disfavored (4.932%)	-	-	-
**#**	**Alt**	**Res**	**High B**	**Clash > 0.4Å**	**Ramachandran**	**Rotamer**	**Cβ deviation**	**CaBLAM**	**Bond lengths**	**Bond angles**	**Cis Peptides**
			Avg: 0.93	Clashscore: 1.46	Outliers: 4 of 903	Poor rotamers: 0 of 774	Outliers: 0 of 826	Outliers: 27 of 901	Outliers: 15 of 905	Outliers: 10 of 905	Non-Trans: 1 of 904
A 801		SER	1.8	-	Favored (55.94%) General / -57.9,138.2	Favored (32.5%) *t* chi angles: 174.1	0.07Å	Favored (34.43%)	-	-	-
A 802		ILE	1.77	-	Favored (20.9%) Ile or Val / -58.6,-22.5	Favored (9.5%) *tp* chi angles: 196.4,67.3	0.06Å	Favored (25.902%)	-	-	-
A 803		HIS	1.7	-	Favored (27.05%) General / -100.1,-6.0	Favored (99.2%) *m-70* chi angles: 297.9,292.2	0.08Å	Favored (53.844%)	OUTLIER(S) worst is CB--CG: 5.3 σ	-	-
A 804		ALA	1.59	-	Favored (9.1%) General / -88.2,72.6	-	0.07Å	Favored (15.453%)	-	-	-
A 805		LYS	1.45	-	Favored (11.69%) General / -53.4,123.3	Favored (79.4%) *tttt* chi angles: 183,182.2,180.2,188.2	0.10Å	Favored (22.615%) beta sheet	-	-	-
A 806		GLY	1.3	-	Favored (2.24%) Glycine / -77.3,44.9	-	-	Favored (10.43%)	-	-	-
A 807		GLU	1.16	-	Favored (74.37%) General / -65.3,-32.6	Favored (47.6%) *tp30* chi angles: 191.2,69.2,16.8	0.04Å	Favored (30.275%)	-	-	-
A 808		TRP	1.03	-	Favored (65.39%) General / -66.6,-18.5	Favored (25%) *p90* chi angles: 69.4,85.6	0.06Å	Favored (53.412%) three-ten	-	-	-
A 809		MET	0.93	-	Favored (8.14%) General / -81.1,74.7	Favored (87.5%) *mtp* chi angles: 294.7,184.5,74	0.06Å	CaBLAM Disfavored (2.68%)	-	-	-
A 810		THR	0.85	-	Favored (3.48%) General / -144.7,-170.8	Favored (11%) *t* chi angles: 189	0.08Å	Favored (29.649%)	-	-	-
A 811		THR	0.8	-	Favored (8%) General / -123.0,-12.0	Favored (75.5%) *p* chi angles: 60	0.06Å	CaBLAM Outlier (0.349%)	-	-	-
A 812		GLU	0.77	-	Favored (58.05%) General / -60.2,137.8	Favored (77.2%) *tt0* chi angles: 189.1,180.1,12.7	0.03Å	Favored (24.273%)	-	-	-
A 813		ASP	0.76	-	Favored (31.68%) General / -51.8,133.1	Favored (15.3%) *t70* chi angles: 188.2,279.3	0.06Å	Favored (39.427%)	-	-	-
A 814		MET	0.74	-	Favored (66.16%) General / -61.5,-23.8	Favored (89.6%) *mmm* chi angles: 289.3,303.9,296.4	0.10Å	Favored (31.902%)	-	-	-
A 815		LEU	0.72	-	Favored (79.3%) General / -67.5,-35.5	Favored (81.3%) *mt* chi angles: 289.1,170.6	0.05Å	Favored (71.347%) alpha helix	-	-	-

A 816		ALA	0.71	-	Favored (88.4%) General / -65.8,-38.2	-	0.04Å	Favored (94.376%) alpha helix	-	-	-
A 817		VAL	0.7	-	Favored (82.64%) Ile or Val / -68.8,-43.5	Favored (81.6%) *t* chi angles: 176.4	0.05Å	Favored (88.581%) alpha helix	-	-	-
A 818		TRP	0.69	-	Favored (90.78%) General / -59.0,-45.4	Favored (73.8%) *t60* chi angles: 190.1,91.8	0.08Å	Favored (93.906%) alpha helix	-	-	-
A 819		ASN	0.69	-	Favored (94.1%) General / -59.8,-43.9	Favored (76.8%) *m-40* chi angles: 281.7,336.3	0.03Å	Favored (95.734%) alpha helix	-	-	-
A 820		ARG	0.7	-	Favored (77.21%) General / -61.8,-49.2	Favored (48.4%) *ttm110* chi angles: 185,173.8,303.6,112.3	0.01Å	Favored (72.68%) alpha helix	-	-	-
**#**	**Alt**	**Res**	**High B**	**Clash > 0.4Å**	**Ramachandran**	**Rotamer**	**Cβ deviation**	**CaBLAM**	**Bond lengths**	**Bond angles**	**Cis Peptides**
			Avg: 0.93	Clashscore: 1.46	Outliers: 4 of 903	Poor rotamers: 0 of 774	Outliers: 0 of 826	Outliers: 27 of 901	Outliers: 15 of 905	Outliers: 10 of 905	Non-Trans: 1 of 904
A 821		VAL	0.71	-	Favored (68.5%) Ile or Val / -71.0,-46.3	Favored (80.6%) *t* chi angles: 173.2	0.05Å	Favored (58.488%) alpha helix	-	-	-
A 822		TRP	0.74	-	Favored (18.57%) General / -92.3,-25.4	Favored (91.6%) *m100* chi angles: 285.7,104.8	0.04Å	Favored (40.514%) alpha helix	-	-	-
A 823		ILE	0.78	-	Favored (6.2%) Ile or Val / -109.5,-52.8	Favored (49.9%) *mm* chi angles: 302.3,300.4	0.07Å	Favored (19.239%) alpha helix	-	-	-
A 824		GLU	0.83	-	Favored (14.8%) General / -78.6,-48.6	Favored (98.2%) *mt-10* chi angles: 294.4,177.4,355.7	0.01Å	Favored (53.182%) alpha helix	-	-	-
A 825		GLU	0.88	-	Favored (58.94%) General / -87.8,-3.3	Favored (93.8%) *mt-10* chi angles: 294.5,184.1,0.3	0.04Å	Favored (36.485%)	-	-	-
A 826		ASN	0.93	-	Favored (21.95%) General / -84.0,114.8	Favored (44.9%) *t0* chi angles: 185.9,317.2	0.11Å	Favored (33.024%)	-	-	-
A 827		GLU	0.98	-	Favored (63.86%) General / -62.5,-19.7	Favored (95.5%) *mt-10* chi angles: 294,177.9,6.6	0.01Å	Favored (29.009%)	-	-	-
A 828		TRP	1.01	-	Favored (48.5%) General / -98.2,2.6	Favored (45.3%) *m-90* chi angles: 291.5,265.3	0.04Å	Favored (55.748%)	-	-	-
A 829		MET	1.01	-	Favored (6.7%) General / -82.6,88.0	Favored (19.9%) *mmt* chi angles: 295.9,291.9,157	0.07Å	Favored (18.915%)	-	-	-
A 830		GLU	0.98	-	Favored (70.6%) General / -58.6,-34.0	Favored (98.8%) *mt-10* chi angles: 290.5,177,350.8	0.03Å	Favored (28.156%)	-	-	-
A 831		ASP	0.93	-	Favored (5.01%) General / -118.5,95.8	Favored (22.2%) *t0* chi angles: 176.2,337.2	0.05Å	Favored (17.92%)	-	-	-

A 832		LYS	0.87	-	Favored (6.47%) General / -86.6,60.9	Favored (96.4%) *mttt* chi angles: 296.2,183.9,177.2,185.5	0.04Å	Favored (29.651%)	-	-	-
A 833		THR	0.82	-	Favored (64.5%) Pre-Pro / -86.7,126.2	Favored (86.6%) *m* chi angles: 296.8	0.03Å	Favored (32.814%) beta sheet	-	-	-
A 834		PRO	0.78	-	Favored (50.58%) Trans-Pro / -72.9,158.1	Favored (75.9%) *Cg_endo* chi angles: 28.1,324.7,27.5	0.02Å	Favored (79.777%) beta sheet	-	-	-
A 835		VAL	0.76	-	Favored (16.04%) Ile or Val / -89.0,140.3	Favored (38.8%) *t* chi angles: 167.3	0.06Å	Favored (47.11%)	-	-	-
A 836		GLU	0.75	-	Favored (8.85%) General / -106.8,-30.6	Favored (96.2%) *mt-10* chi angles: 296.2,181.4,355.4	0.02Å	Favored (18.29%)	-	-	-
A 837		ARG	0.75	-	Favored (36.71%) General / -136.2,161.1	Favored (93.5%) *mmt-90* chi angles: 296.3,290.8,184.4,272.4	0.02Å	Favored (21.305%)	-	-	-
A 838		TRP	0.75	-	Favored (65.74%) General / -67.4,-25.0	Favored (74.1%) *m100* chi angles: 281.3,111.2	0.10Å	Favored (45.74%) alpha helix	-	-	-
A 839		SER	0.74	-	Favored (59.15%) General / -64.1,-15.2	Favored (98.3%) *p* chi angles: 65.3	0.06Å	Favored (59.524%) three-ten	-	-	-
A 840		ASP	0.73	-	Favored (33.89%) General / -82.8,-23.6	Favored (71.4%) *m-30* chi angles: 298.5,334.2	0.15Å	Favored (30.098%)	-	-	-

#	Alt	Res	High B	Clash > 0.4Å	Ramachandran	Rotamer	Cβ deviation	CaBLAM	Bond lengths	Bond angles	Cis Peptides
			Avg: 0.93	Clashscore: 1.46	Outliers: 4 of 903	Poor rotamers: 0 of 774	Outliers: 0 of 826	Outliers: 27 of 901	Outliers: 15 of 905	Outliers: 10 of 905	Non-Trans: 1 of 904
A 841		VAL	0.72	-	Favored (75.84%) Pre-Pro / -84.1,122.9	Favored (81.5%) *t* chi angles: 176.6	0.07Å	Favored (28.229%)	-	-	-
A 842		PRO	0.72	-	Favored (48.63%) Trans-Pro / -70.3,160.7	Favored (72.6%) *Cg_endo* chi angles: 27.5,325.7,26.7	0.04Å	Favored (23.241%) beta sheet	-	-	-
A 843		TYR	0.74	-	Favored (43.53%) General / -132.1,157.7	Favored (60.2%) *p90* chi angles: 62.7,92.1	0.08Å	Favored (38.19%)	-	-	-
A 844		SER	0.78	-	Favored (28.93%) General / -77.9,159.4	Favored (60%) *m* chi angles: 293.6	0.08Å	Favored (33.034%)	-	-	-
A 845		GLY	0.82	-	Favored (50.44%) Glycine / -64.5,151.7	-	-	Favored (38.02%)	-	-	-
A 846		LYS	0.87	-	Favored (62.96%) General / -52.3,-41.5	Favored (87.5%) *tttt* chi angles: 183.5,176.8,178.6,179.7	0.02Å	Favored (65.492%)	-	-	-
A 847		ARG	0.92	-	Favored (80.53%)	Favored (97.8%) *mtt180*	0.04Å	Favored (79.815%) alpha helix	-	-	-

					General / -62.2,-36.0	chi angles: 289.5,178.1,179.8,172.3					
A 848		GLU	0.97	-	Favored (75.04%) General / -70.1,-40.1	Favored (60.2%) *mt-10* chi angles: 291.2,174.2,308.3	0.04Å	Favored (95.8%) alpha helix	-	-	-
A 849		ASP	1	-	Favored (92.65%) General / -62.0,-39.6	Favored (74.7%) *m-30* chi angles: 281,346.2	0.07Å	Favored (98.366%) alpha helix	-	-	-
A 850		ILE	1.02	-	Favored (90.99%) Ile or Val / -66.0,-42.1	Favored (93.5%) *mt* chi angles: 291.7,168.2	0.08Å	Favored (80.511%) alpha helix	-	-	-
A 851		TRP	1.01	-	Favored (71.54%) General / -58.2,-35.4	Favored (45.9%) *m-10* chi angles: 291.1,335.8	0.05Å	Favored (76.345%) alpha helix	-	-	-
A 852		CYS	0.98	-	Favored (5.08%) General / -90.1,19.3	Favored (95.1%) *m* chi angles: 292	0.08Å	Favored (20.288%)	-	-	-
A 853		GLY	0.94	-	Favored (82.67%) Glycine / 84.1,9.2	-	-	Favored (57.695%)	-	-	-
A 854		SER	0.92	0.41Å OG with A 776 ASP OD2	Favored (55.53%) General / -62.9,145.3	Favored (33.5%) *t* chi angles: 174.2	0.05Å	Favored (21.212%) beta sheet	-	-	-
A 855		LEU	0.9	-	Favored (11.05%) General / -84.8,10.6	Favored (39.8%) *mt* chi angles: 292.2,183.5	0.15Å	Favored (7.358%)	-	-	-
A 856		ILE	0.91	-	Favored (13.42%) Ile or Val / -51.2,-33.2	Favored (11%) *tp* chi angles: 190.5,65.6	0.07Å	Favored (26.348%)	-	-	-
A 857		GLY	0.93	-	Favored (4.23%) Glycine / -82.7,49.7	-	-	CaBLAM Outlier (0.322%)	-	-	-
A 858		THR	0.95	-	Favored (12.67%) General / -164.3,152.4	Favored (4.7%) *t* chi angles: 180	0.16Å	Favored (7.469%)	-	-	-
A 859		ARG	0.97	-	Favored (68.86%) General / -65.1,-27.6	Favored (98%) *mtt180* chi angles: 290.2,177.5,180.2,172.4	0.04Å	Favored (54.949%)	-	-	-
A 860		THR	0.97	-	Favored (88.58%) General / -64.1,-45.4	Favored (92.6%) *m* chi angles: 299.1	0.01Å	Favored (66.123%) alpha helix	-	-	-
**#**	**Alt**	**Res**	**High B**	**Clash > 0.4Å**	**Ramachandran**	**Rotamer**	**Cβ deviation**	**CaBLAM**	**Bond lengths**	**Bond angles**	**Cis Peptides**
			Avg: 0.93	Clashscore: 1.46	Outliers: 4 of 903	Poor rotamers: 0 of 774	Outliers: 0 of 826	Outliers: 27 of 901	Outliers: 15 of 905	Outliers: 10 of 905	Non-Trans: 1 of 904
A 861		ARG	0.95	-	Favored (85.95%) General / -64.8,-45.5	Favored (4.6%) *tmt170* chi angles: 186.5,272.9,190.2,199.8	0.07Å	Favored (80.873%) alpha helix	-	-	-
A 862		ALA	0.93	-	Favored (90.35%) General / -59.7,-41.2	-	0.04Å	Favored (91.577%) alpha helix	-	-	-
A 863		THR	0.92	-	Favored (93.25%)	Favored (95.7%) *m* chi angles: 299.6	0.04Å	Favored (99.117%)	-	-	-

					General / -62.9,-45.3			alpha helix			
A 864		TRP	0.91	-	Favored (85.67%) General / -58.5,-46.9	Favored (29.5%) *t60* chi angles: 162.6,84.1	0.02Å	Favored (87.421%) alpha helix	-	-	-
A 865		ALA	0.91	-	Favored (84.11%) General / -58.2,-41.4	-	0.03Å	Favored (83.569%) alpha helix	-	-	-
A 866		GLU	0.92	-	Favored (77.62%) General / -63.5,-34.4	Favored (95.8%) *mt-10* chi angles: 290,181.9,354	0.03Å	Favored (78.618%) alpha helix	-	-	-
A 867		ASN	0.91	-	Favored (32.41%) General / -95.7,11.7	Favored (89.7%) *m-40* chi angles: 291.5,323.7	0.02Å	Favored (35.636%) alpha helix	-	-	-
A 868		ILE	0.9	-	Favored (88.66%) Ile or Val / -59.9,-42.2	Favored (86.4%) *mt* chi angles: 290.8,169.1	0.12Å	Favored (44.566%) alpha helix	OUTLIER(S) worst is CB--CG1: 4.2 σ	-	-
A 869		HIS	0.87	-	Favored (74.24%) General / -59.0,-36.0	Favored (36.9%) *m170* chi angles: 283.7,169.2	0.04Å	Favored (76.268%) alpha helix	-	-	-
A 870		VAL	0.84	-	Favored (87.02%) Ile or Val / -66.2,-46.7	Favored (79.4%) *t* chi angles: 173.1	0.04Å	Favored (82.365%) alpha helix	-	-	-
A 871		ALA	0.8	-	Favored (80.46%) General / -60.1,-37.6	-	0.04Å	Favored (83.369%) alpha helix	-	-	-
A 872		ILE	0.77	-	Favored (94.51%) Ile or Val / -63.4,-46.8	Favored (92.6%) *mt* chi angles: 291.5,168.2	0.01Å	Favored (91.596%) alpha helix	-	-	-
A 873		ASN	0.74	-	Favored (78.05%) General / -62.7,-34.9	Favored (98.7%) *m-40* chi angles: 287.4,338.1	0.06Å	Favored (81.741%) alpha helix	-	-	-
A 874		GLN	0.72	-	Favored (95.3%) General / -64.7,-42.5	Favored (98%) *mt0* chi angles: 290.6,172.6,341.6	0.02Å	Favored (84.282%) alpha helix	-	-	-
A 875		VAL	0.71	-	Favored (89.71%) Ile or Val / -66.9,-43.0	Favored (67.8%) *t* chi angles: 171.8	0.05Å	Favored (87.369%) alpha helix	-	-	-
A 876		ARG	0.71	-	Favored (86.83%) General / -60.1,-39.6	Favored (97.3%) *mtt180* chi angles: 289.1,179.7,175.3,171.5	0.06Å	Favored (89.343%) alpha helix	-	-	-
A 877		SER	0.71	-	Favored (95.07%) General / -62.0,-40.2	Favored (72.6%) *m* chi angles: 295.7	0.04Å	Favored (89.084%) alpha helix	-	-	-
A 878		VAL	0.72	-	Favored (90.98%) Ile or Val / -66.5,-43.4	Favored (66.2%) *t* chi angles: 171.7	0.03Å	Favored (84.129%) alpha helix	-	-	-
A 879		ILE	0.73	-	Favored (48.68%) Ile or Val / -72.7,-47.9	Favored (96%) *mt* chi angles: 294.2,168.7	0.02Å	CaBLAM Disfavored (4.272%)	-	-	-
A 880		GLY	0.75	-	Favored (31.42%) Glycine / 152.6,-174.5	-	-	Favored (17.695%)	-	-	-

#	Alt	Res	High B	Clash > 0.4Å	Ramachandran	Rotamer	Cβ deviation	CaBLAM	Bond lengths	Bond angles	Cis Peptides
			Avg: 0.93	Clashscore: 1.46	Outliers: 4 of 903	Poor rotamers: 0 of 774	Outliers: 0 of 826	Outliers: 27 of 901	Outliers: 15 of 905	Outliers: 10 of 905	Non-Trans: 1 of 904
A 881		GLU	0.76	-	Favored (50.94%) General / -88.1,2.9	Favored (97.2%) *mt-10* chi angles: 295.4,178.9,359.1	0.01Å	CaBLAM Outlier (0.893%)	-	-	-
A 882		GLU	0.78	-	Favored (56.07%) General / -61.1,143.1	Favored (12.8%) *mm-30* chi angles: 292.7,279.1,293.6	0.10Å	Favored (17.69%)	-	-	-
A 883		LYS	0.79	-	Favored (46.95%) General / -55.8,131.5	Favored (86.4%) *tttt* chi angles: 180.7,177,177.9,179.8	0.03Å	Favored (22.076%) beta sheet	-	-	-
A 884		TYR	0.79	-	Favored (31.84%) General / -121.1,156.6	Favored (82.2%) *m-80* chi angles: 302.1,90.2	0.11Å	Favored (50.779%) beta sheet	-	-	-
A 885		VAL	0.79	-	Favored (64.31%) Ile or Val / -118.0,133.6	Favored (90.9%) *t* chi angles: 174.3	0.04Å	Favored (44.49%) beta sheet	-	-	-
A 886		ASP	0.79	-	Favored (8.92%) General / -81.9,72.1	Favored (74.1%) *m-30* chi angles: 294.3,320.8	0.02Å	Favored (25.738%) beta sheet	-	-	-
A 887		TYR	0.8	-	Favored (66.06%) General / -66.0,-20.1	Favored (73.6%) *m-80* chi angles: 292.9,108.8	0.06Å	Favored (16.689%)	-	-	-
A 888		MET	0.82	-	Favored (64.32%) General / -58.0,-28.2	Favored (90.7%) *mmm* chi angles: 290.2,292.1,285.8	0.13Å	Favored (58.041%)	-	-	-
A 889		SER	0.85	-	Favored (63.94%) General / -69.2,-19.4	Favored (84.6%) *p* chi angles: 67.4	0.02Å	Favored (66.446%) three-ten	-	-	-
A 890		SER	0.9	-	Favored (58.24%) General / -80.2,-6.7	Favored (62.8%) *m* chi angles: 293.9	0.03Å	Favored (60.394%)	-	-	-
A 891		LEU	0.97	-	Favored (55.67%) General / -90.9,-5.3	Favored (94.7%) *mt* chi angles: 297.3,174.6	0.09Å	Favored (22.365%)	-	-	-
A 892		ARG	1.07	-	Allowed (0.3%) General / 69.2,-35.3	Favored (49.7%) *mmt180* chi angles: 298.8,290,187.6,178.9	0.07Å	CaBLAM Disfavored (3.542%)	-	-	-
A 893		ARG	1.21	-	Favored (46.73%) General / -51.8,-36.2	Favored (54.3%) *ttt90* chi angles: 177.7,176.5,165.6,85.2	0.02Å	Favored (26.07%)	-	-	-
A 894		TYR	1.42	-	Favored (35.6%) General / -103.8,5.0	Favored (98.5%) *m-80* chi angles: 295.8,95.6	0.07Å	Favored (35.097%) alpha helix	-	-	-
A 895		GLU	1.7	-	Favored (81.15%) General / -65.0,-46.5	Favored (91.2%) *tt0* chi angles: 183.2,175.9,1.9	0.03Å	Favored (34.871%) alpha helix	-	-	-
A 896		ASP	2.11	-	Favored (81.59%) General / -60.6,-37.5	Favored (98.9%) *m-30* chi angles: 287.8,345.8	0.04Å	Favored (80.726%) alpha helix	-	-	-

A 897		THR	2.64	-	Favored (87.54%) General / -62.4,-46.7	Favored (88.8%) *m* chi angles: 297	0.02Å	Favored (86.05%) alpha helix	-	-	-
A 898		ILE	3.32	-	Favored (96.99%) Ile or Val / -64.4,-43.7	Favored (99%) *mt* chi angles: 292.5,167.3	0.11Å	Favored (90.104%) alpha helix	-	-	-
A 899		VAL	4.11	-	Favored (94.04%) Ile or Val / -59.7,-44.6	Favored (56.9%) *t* chi angles: 170.4	0.03Å	Favored (93.779%) alpha helix	-	-	-
A 900		VAL	4.92	-	Favored (94.93%) Ile or Val / -64.1,-46.0	Favored (63.9%) *t* chi angles: 171.3	0.02Å	Favored (92.73%) alpha helix	-	-	-
**#**	**Alt**	**Res**	**High B**	**Clash > 0.4Å**	**Ramachandran**	**Rotamer**	**Cβ deviation**	**CaBLAM**	**Bond lengths**	**Bond angles**	**Cis Peptides**
			Avg: 0.93	Clashscore: 1.46	Outliers: 4 of 903	Poor rotamers: 0 of 774	Outliers: 0 of 826	Outliers: 27 of 901	Outliers: 15 of 905	Outliers: 10 of 905	Non-Trans: 1 of 904
A 901		GLU	5.65	-	Favored (93.5%) General / -61.8,-40.0	Favored (97.3%) *mt-10* chi angles: 288.9,178,354.2	0.03Å	Favored (96.303%) alpha helix	-	-	-
A 902		ASP	6.21	-	Favored (80.3%) General / -63.8,-35.3	Favored (98.4%) *m-30* chi angles: 287.2,347.9	0.03Å	Favored (89.471%) alpha helix	-	-	-
A 903		THR	6.58	-	Favored (85.92%) General / -66.5,-43.2	Favored (95.6%) *m* chi angles: 299.6	0.04Å	Favored (72.598%)	-	-	-
A 904		VAL	6.77	-	Favored (26.26%) Ile or Val / -78.5,-43.8	Favored (87.2%) *t* chi angles: 173.9	0.03Å	-	-	-	-
A 905		LEU	6.85	-	-	Favored (67.4%) *mt* chi angles: 302.9,174.9	0.07Å	-	-	-	-

About MolProbity | Website for the Richardson Lab | Using ecloud x-H | Internal reference 4.5.2
