# Supplementary material for: Integrative proteome-wide structural analysis and high-throughput docking identify broad-spectrum antiviral scaffolds against Zika, Yellow Fever, West Nile, Saint Louis encephalitis, and Usutu viruses
Source: Front Cell Infect Microbiol. 2026 Apr 30;16:1723132. doi: 10.3389/fcimb.2026.1723132 (PMC13171538; doi:10.3389/fcimb.2026.1723132)
Supplement: Supplementary file 5 [file DataSheet5.zip › WNV/WNV_NS5/Mol_probity_Files/WNV_NS5_1FH-rama.pdf]

MolProbity Ramachandran analysis

WNV_NS5_1FH.pdb, model 1

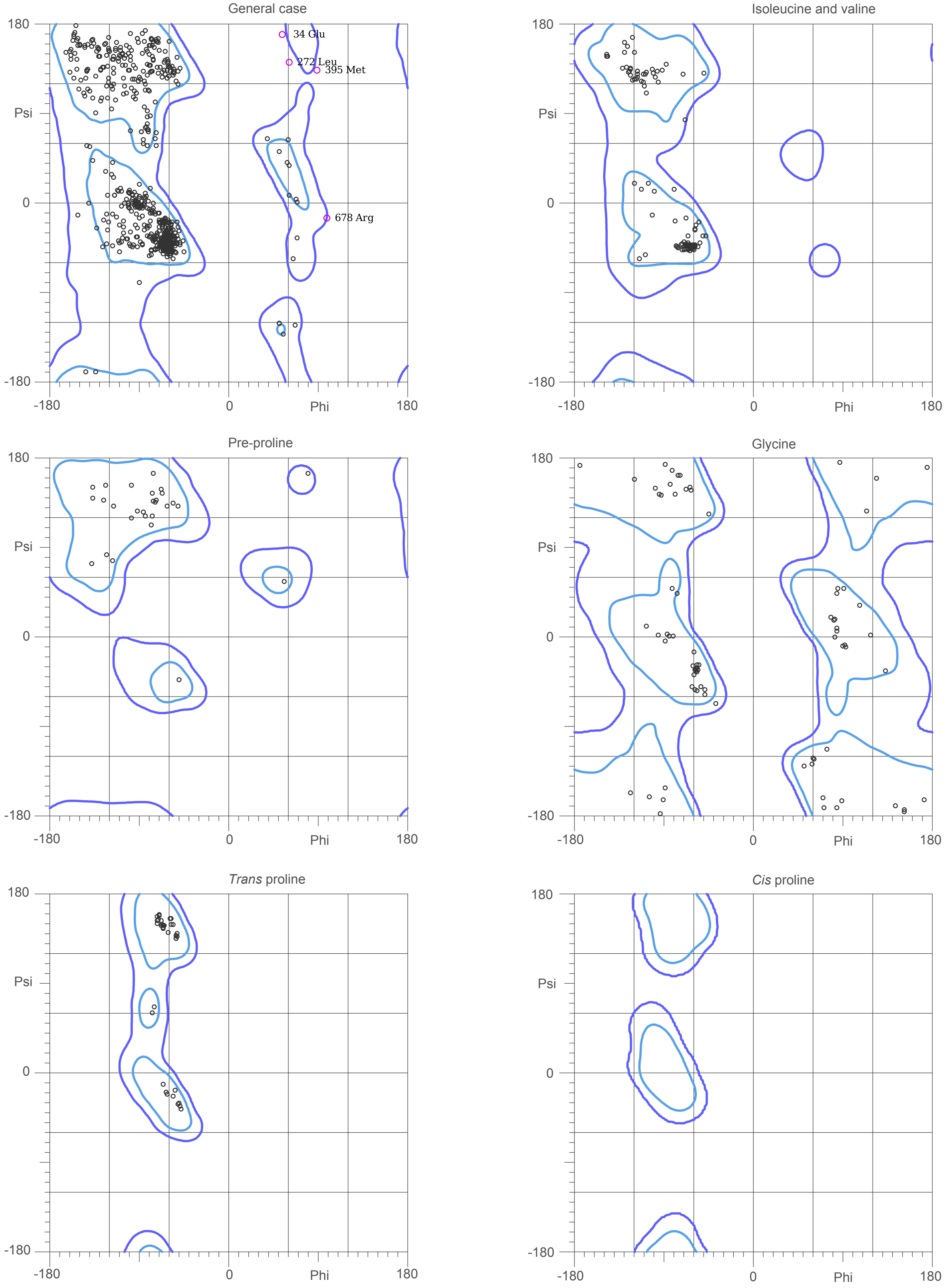

96.9% (875/903) of all residues were in favored (98%) regions.
99.6% (899/903) of all residues were in allowed (>99.8%) regions.

There were 4 outliers (phi, psi):

- 34 Glu (54.4, 170.7)
- 272 Leu (61.9, 142.2)
- 395 Met (89.2, 134.5)
- 678 Arg (99.3, -15.4)
