# Supplementary material for: Integrative proteome-wide structural analysis and high-throughput docking identify broad-spectrum antiviral scaffolds against Zika, Yellow Fever, West Nile, Saint Louis encephalitis, and Usutu viruses
Source: Front Cell Infect Microbiol. 2026 Apr 30;16:1723132. doi: 10.3389/fcimb.2026.1723132 (PMC13171538; doi:10.3389/fcimb.2026.1723132)
Supplement: Supplementary file 6 [file DataSheet6.zip › YFV/YF_E/Mol_probity_Files/YF_E_1FH-multi.table.pdf]

Viewing YF_E1FH-multi.table

Duke Biochemistry
Duke University School of Medicine

When finished, you should close this window.

Hint: Use File | Save As... to save a copy of this page.

All-Atom Contacts	Clashscore, all atoms:	0.81		99th percentile* (N=1784, all resolutions)
	Clashscore is the number of serious steric overlaps (> 0.4 Å) per 1000 atoms.			
Protein Geometry	Poor rotamers	0	0.00%	Goal: <0.3%
	Favored rotamers	400	99.50%	Goal: >98%
	Ramachandran outliers	1	0.20%	Goal: <0.05%
	Ramachandran favored	479	97.56%	Goal: >98%
	Rama distribution Z-score	1.75 ± 0.38		Goal: abs(Z score) < 2
	MolProbity score^	0.84		100th percentile* (N=27675, 0Å - 99Å)
	Cβ deviations >0.25Å	0	0.00%	Goal: 0
	Bad bonds:	5 / 3802	0.13%	Goal: 0%
	Bad angles:	10 / 5148	0.19%	Goal: <0.1%
Peptide Omegas	Cis Prolines:	1 / 14	7.14%	Expected: ≤1 per chain, or ≤5%
	Cis nonProlines:	1 / 478	0.21%	Goal: <0.05%
Low-resolution Criteria	CaBLAM outliers	13	2.7%	Goal: <1.0%
	CA Geometry outliers	5	1.02%	Goal: <0.5%
Additional validations	Chiral volume outliers	0/586		
	Waters with clashes	0/0	0.00%	See UnDowser table for details

In the two column results, the left column gives the raw count, right column gives the percentage.

* 100th percentile is the best among structures of comparable resolution; 0th percentile is the worst. For clashscore the comparative set of structures was selected in 2004, for MolProbity score in 2006.

^ MolProbity score combines the clashscore, rotamer, and Ramachandran evaluations into a single score, normalized to be on the same scale as X-ray resolution.

Key to table colors and cutoffs here:

#	Alt	Res	High B	Clash > 0.4Å	Ramachandran	Rotamer	Cβ deviation	CaBLAM	Bond lengths	Bond angles	Cis Peptides
			Avg: 1.15	Clashscore: 0.81	Outliers: 1 of 491	Poor rotamers: 0 of 402	Outliers: 0 of 442	Outliers: 17 of 489	Outliers: 5 of 493	Outliers: 10 of 493	Non-Trans: 2 of 492
A 1		ALA	1.67	-	-	-	0.02Å	-	-	-	-
A 2		HIS	1.6	-	Favored (63.86%) General / -51.9,-44.3	Favored (74.4%) *t70* chi angles: 171.5,66.4	0.03Å	-	-	-	-
A 3		CYS	1.54	-	Favored (7.42%) General / -74.3,3.4	Favored (81.4%) *m* chi angles: 294.4	0.06Å	Favored (23.034%)	-	-	-
A 4		ILE	1.49	-	Favored (23.88%) Ile or Val / -67.7,-18.5	Allowed (1.3%) *pp* chi angles: 64.5,96.2	0.09Å	Favored (50.157%) three-ten	-	-	-
A 5		GLY	1.44	-	Favored (85.77%) Glycine / -88.0,3.4	-	-	Favored (63.82%)	-	-	-
A 6		ILE	1.39	-	Favored (64.14%) Ile or Val / -111.7,121.4	Favored (85.6%) *mt* chi angles: 298.9,170.7	0.04Å	Favored (33.867%)	-	-	-
A 7		THR	1.32	-	Favored (76.86%) General / -66.3,-46.5	Favored (93.7%) *m* chi angles: 299.3	0.03Å	Favored (22.407%)	-	-	-

A 8		ASP	1.25	-	Favored (10.17%) General / -83.6,69.7	Favored (45.3%) *m-30* chi angles: 285.7,319.5	0.07Å	Favored (9.396%)	-	-	-
A 9		ARG	1.2	-	Favored (37.26%) General / -111.6,119.0	Favored (77.8%) *ttt180* chi angles: 184.6,177.5,181.6,191	0.04Å	Favored (27.399%)	-	-	-
A 10		ASP	1.19	-	Favored (44.3%) General / -101.2,122.8	Favored (90.3%) *m-30* chi angles: 293.5,344.2	0.06Å	Favored (60.403%) beta sheet	-	-	-
A 11		PHE	1.27	-	Favored (33.95%) General / -96.8,138.7	Favored (90.7%) *m-80* chi angles: 291.4,95.1	0.02Å	Favored (55.21%) beta sheet	-	-	-
A 12		ILE	1.43	-	Favored (68.2%) Ile or Val / -125.5,125.1	Favored (72.9%) *mt* chi angles: 299.5,175.6	0.07Å	Favored (60.341%) beta sheet	-	-	-
A 13		GLU	1.7	-	Favored (39.25%) General / -76.6,136.8	Favored (84.2%) *mt-10* chi angles: 295.2,184.2,12.6	0.02Å	Favored (34.85%)	-	-	-
A 14		GLY	2.01	-	Favored (2.19%) Glycine / -75.2,66.9	-	-	CaBLAM Disfavored (4.704%)	-	-	-
A 15		VAL	2.29	-	Allowed (0.13%) Ile or Val / 61.2,-50.4	Favored (79.7%) *t* chi angles: 173.1	0.15Å	Favored (16.584%)	-	-	-
A 16		HIS	2.4	-	Allowed (0.19%) General / 72.4,-24.5	Favored (93.9%) *m-70* chi angles: 304.9,289.2	0.07Å	CaBLAM Disfavored (2.008%)	-	-	-
A 17		GLY	2.29	-	Favored (47.04%) Glycine / -103.2,19.1	-	-	Favored (7.132%)	-	-	-
A 18		GLY	2.01	-	Favored (33.32%) Glycine / -174.4,-167.0	-	-	Favored (13.973%)	-	-	-
A 19		THR	1.68	-	Favored (16.57%) General / -113.3,4.8	Favored (73.1%) *p* chi angles: 59.8	0.02Å	Favored (43.209%)	-	-	-
A 20		TRP	1.38	-	Favored (50.16%) General / -137.3,154.2	Favored (74.2%) *p-90* chi angles: 58.3,265.6	0.06Å	Favored (27.413%)	-	-	-
**#**	**Alt**	**Res**	**High B**	**Clash > 0.4Å**	**Ramachandran**	**Rotamer**	**Cβ deviation**	**CaBLAM**	**Bond lengths**	**Bond angles**	**Cis Peptides**
			Avg: 1.15	Clashscore: 0.81	Outliers: 1 of 491	Poor rotamers: 0 of 402	Outliers: 0 of 442	Outliers: 17 of 489	Outliers: 5 of 493	Outliers: 10 of 493	Non-Trans: 2 of 492
A 21		VAL	1.16	-	Favored (66.44%) Ile or Val / -129.8,131.5	Favored (51.6%) *t* chi angles: 181.2	0.07Å	Favored (57.66%)	-	-	-
A 22		SER	1.02	-	Favored (23.04%) General / -83.6,155.9	Favored (92.9%) *p* chi angles: 64.4	0.04Å	Favored (21.616%) beta sheet	-	-	-
A 23		ALA	0.94	-	Favored (25.68%) General / -162.7,160.4	-	0.07Å	Favored (36.439%) beta sheet	-	-	-

A 24		THR	0.91	-	Favored (48.13%) General / -122.4,145.6	Favored (77.6%) *p* chi angles: 60.9	0.09Å	Favored (61.129%) beta sheet	-	-	-
A 25		LEU	0.91	-	Favored (38.06%) General / -120.2,152.2	Favored (46.7%) *mt* chi angles: 305.2,173.3	0.03Å	Favored (64.594%) beta sheet	-	-	-
A 26		GLU	0.92	0.48Å OE2 with A 29 LYS NZ	Favored (38.86%) General / -138.0,138.4	Favored (34.1%) *tt0* chi angles: 179.7,177,284.4	0.04Å	Favored (44.086%)	-	-	-
A 27		GLN	0.93	-	Favored (4.96%) General / -43.6,-42.1	Favored (30.4%) *tt0* chi angles: 178.9,184.5,282.1	0.07Å	Favored (23.82%)	-	-	-
A 28		ASP	0.94	-	Favored (42.24%) General / -95.4,8.8	Favored (85%) *m-30* chi angles: 294.9,340.7	0.04Å	Favored (21.57%)	-	-	-
A 29		LYS	0.94	0.48Å NZ with A 26 GLU OE2	Favored (6.16%) General / -120.1,175.0	Favored (42.7%) *mttp* chi angles: 297.7,190.1,171.8,73.1	0.03Å	Favored (15.716%)	-	-	-
A 30		CYS	0.95	-	Favored (32.89%) General / -142.0,142.4	Favored (47.9%) *t* chi angles: 178.9	0.06Å	Favored (57.731%)	-	-	-
A 31		VAL	0.96	-	Favored (66.41%) Ile or Val / -115.5,131.9	Favored (100%) *t* chi angles: 175.5	0.01Å	Favored (70.253%) beta sheet	-	-	-
A 32		THR	0.99	-	Favored (55.17%) General / -114.5,126.6	Favored (91.9%) *m* chi angles: 297.3	0.06Å	Favored (71.782%) beta sheet	-	-	-
A 33		VAL	1.05	-	Favored (48.1%) Ile or Val / -106.3,115.5	Favored (83.9%) *t* chi angles: 177.7	0.02Å	Favored (71.828%) beta sheet	-	-	-
A 34		MET	1.12	-	Allowed (1.19%) General / -102.7,71.8	Favored (44.2%) *mtm* chi angles: 300.9,169,287.9	0.11Å	Favored (50.591%)	-	-	-
A 35		ALA	1.19	-	Favored (81.27%) Pre-Pro / -74.7,145.1	-	0.05Å	Favored (20.469%)	-	-	-
A 36		PRO	1.21	-	Favored (67.88%) Trans-Pro / -65.6,141.7	Favored (43%) *Cg_endo* chi angles: 24.2,326.5,28.6	0.04Å	Favored (32.517%)	-	-	-
A 37		ASP	1.19	-	Favored (8.59%) General / 59.3,20.4	Favored (76.1%) *m-30* chi angles: 296.6,325.5	0.04Å	Favored (7.28%)	-	-	-
A 38		LYS	1.12	-	Favored (78.59%) Pre-Pro / -130.9,151.2	Favored (55.1%) *mttm* chi angles: 299.8,183,191.1,291.5	0.07Å	Favored (18.236%)	-	-	-
A 39		PRO	1.03	-	Favored (98.09%) Trans-Pro / -61.5,145.2	Favored (31.1%) *Cg_exo* chi angles: 339.9,34.2,325.4	0.07Å	Favored (53.044%)	-	-	-
A 40		SER	0.92	-	Favored (58.42%) General / -63.7,142.4	Favored (56.1%) *m* chi angles: 299.9	0.04Å	Favored (39.271%)	-	-	-
**#**	**Alt**	**Res**	**High B**	**Clash > 0.4Å**	**Ramachandran**	**Rotamer**	**Cβ deviation**	**CaBLAM**	**Bond lengths**	**Bond angles**	**Cis Peptides**

			Avg: 1.15	Clashscore: 0.81	Outliers: 1 of 491	Poor rotamers: 0 of 402	Outliers: 0 of 442	Outliers: 17 of 489	Outliers: 5 of 493	Outliers: 10 of 493	Non-Trans: 2 of 492
A 41		LEU	0.84	-	Favored (42.76%) General / -116.8,121.9	Favored (57.9%) *tp* chi angles: 177.7,65.5	0.05Å	Favored (64.399%) beta sheet	-	-	-
A 42		ASP	0.79	-	Favored (36.97%) General / -93.3,123.9	Favored (99%) *m-30* chi angles: 288,347.6	0.12Å	Favored (64.537%) beta sheet	-	OUTLIER(S) worst is CA-CB-CG: 6.6 σ	-
A 43		ILE	0.76	-	Favored (74.59%) Ile or Val / -122.9,130.2	Favored (77.3%) *mt* chi angles: 301.1,172.2	0.02Å	Favored (51.454%) beta sheet	-	-	-
A 44		SER	0.74	-	Favored (43.51%) General / -143.8,154.5	Favored (95.9%) *p* chi angles: 63.8	0.02Å	Favored (45.568%) beta sheet	-	-	-
A 45		LEU	0.74	-	Favored (23.96%) General / -94.0,112.2	Favored (65.2%) *tp* chi angles: 176.8,58.9	0.08Å	Favored (42.286%)	-	-	-
A 46		GLU	0.75	-	Favored (11.93%) General / -84.7,-46.0	Favored (91%) *tt0* chi angles: 182.2,174.5,0.6	0.01Å	Favored (34.544%)	-	-	-
A 47		THR	0.77	-	Favored (27.11%) General / -144.7,138.2	Favored (99.8%) *m* chi angles: 300.5	0.13Å	Favored (36.128%)	-	-	-
A 48		VAL	0.8	-	Favored (74.55%) Ile or Val / -117.1,128.2	Favored (58.8%) *t* chi angles: 180.1	0.07Å	Favored (57.537%)	-	-	-
A 49		ALA	0.83	-	Favored (44.02%) General / -149.1,159.7	-	0.03Å	Favored (49.191%) beta sheet	-	-	-
A 50		ILE	0.86	-	Favored (67.42%) Ile or Val / -116.7,121.3	Favored (3.8%) *mp* chi angles: 304.1,93.6	0.09Å	Favored (51.773%)	-	-	-
A 51		ASP	0.89	-	Favored (60.3%) General / -60.7,-20.2	Favored (92.9%) *m-30* chi angles: 290.9,348.7	0.04Å	CaBLAM Disfavored (1.063%)	-	-	-
A 52		GLY	0.91	-	Allowed (1.64%) Glycine / 149.6,128.4	-	-	Favored (8.412%)	-	-	-
A 53		PRO	0.92	-	Favored (9.75%) Trans-Pro / -83.5,173.3	Favored (29.9%) *Cg_endo* chi angles: 35.2,321.6,24.9	0.08Å	Favored (58.795%)	-	-	-
A 54		ALA	0.91	-	Favored (41.82%) General / -102.9,121.1	-	0.03Å	Favored (12.837%)	-	-	-
A 55		GLU	0.89	-	Favored (31.29%) General / -60.1,127.3	Favored (55.4%) *tt0* chi angles: 193.3,170.6,13.2	0.06Å	Favored (32.032%)	-	-	-
A 56		ALA	0.86	-	Favored (4.48%) General / -107.3,-43.9	-	0.05Å	Favored (18.518%)	-	-	-

A<br>57		ARG	0.83	-	Favored<br>(28.37%)<br>General /<br>-147.9,144.8	Favored (51.6%)<br>*ttm170*<br>chi angles:<br>179,180,292.1,179.2	0.01Å	Favored<br>(32.96%)	-	-	-
A<br>58		LYS	0.8	-	Favored<br>(54.25%)<br>General /<br>-112.0,133.4	Favored (97.9%)<br>*mttt*<br>chi angles:<br>296.4,176.8,181.1,178	0.04Å	Favored<br>(69.366%)	-	-	-
A<br>59		VAL	0.78	-	Favored<br>(74.11%)<br>Ile or Val /<br>-120.3,130.7	Favored (70.1%) *t*<br>chi angles: 178.7	0.04Å	Favored<br>(59.756%)<br>beta sheet	-	-	-
A<br>60		CYS	0.77	-	Favored<br>(34.49%)<br>General /<br>-84.5,132.9	Favored (42.4%) *t*<br>chi angles: 186	0.03Å	Favored<br>(42.137%)	-	-	-

#	Alt	Res	High B	Clash > 0.4Å	Ramachandran	Rotamer	Cβ deviation	CaBLAM	Bond lengths	Bond angles	Cis Peptides
			Avg: 1.15	Clashscore: 0.81	Outliers: 1 of 491	Poor rotamers: 0 of 402	Outliers: 0 of 442	Outliers: 17 of 489	Outliers: 5 of 493	Outliers: 10 of 493	Non-Trans: 2 of 492
A<br>61		TYR	0.76	-	Favored<br>(6.41%)<br>General /<br>-112.2,-32.8	Favored (96.5%) *m-80*<br>chi angles: 293,88.2	0.14Å	Favored<br>(17.511%)	-	OUTLIER(S)<br>worst is CA-CB-CG: 4.2 σ	-
A<br>62		SER	0.75	-	Favored<br>(10.14%)<br>General /<br>-136.2,112.9	Favored (42.5%) *t*<br>chi angles: 180.8	0.04Å	Favored<br>(24.719%)	-	-	-
A<br>63		ALA	0.74	-	Favored<br>(22.45%)<br>General /<br>-104.8,152.6	-	0.03Å	Favored<br>(33.642%)	-	-	-
A<br>64		VAL	0.72	-	Favored<br>(71.37%)<br>Ile or Val /<br>-114.2,128.1	Favored (66.5%) *t*<br>chi angles: 179.2	0.08Å	Favored<br>(55.656%)<br>beta sheet	-	-	-
A<br>65		LEU	0.71	-	Favored<br>(33.61%)<br>General /<br>-87.6,124.5	Favored (5%) *tt*<br>chi angles: 192.8,157.4	0.06Å	Favored<br>(43.059%)	-	-	-
A<br>66		THR	0.69	-	Allowed<br>(0.53%)<br>General /<br>-107.4,-83.7	Favored (63.9%) *p*<br>chi angles: 58	0.08Å	CaBLAM<br>Disfavored<br>(2.493%)	-	-	-
A<br>67		ASN	0.67	-	Favored<br>(21.86%)<br>General /<br>-88.5,152.5	Favored (84.6%) *m-40*<br>chi angles: 292.1,317.4	0.04Å	CaBLAM<br>Disfavored<br>(3.248%)	-	-	-
A<br>68		VAL	0.66	-	Favored<br>(32.89%)<br>Ile or Val /<br>-139.7,142.3	Favored (10.5%) *p*<br>chi angles: 62.5	0.04Å	Favored<br>(64.635%)	-	-	-
A<br>69		LYS	0.65	-	Favored<br>(22.61%)<br>General /<br>-147.3,136.7	Favored (84.3%)<br>*tttt*<br>chi angles:<br>187.6,173.3,179.8,176.6	0.04Å	Favored<br>(56.102%)<br>beta sheet	-	-	-
A<br>70		ILE	0.65	-	Favored<br>(65.55%)<br>Ile or Val /<br>-123.3,134.7	Favored (75.3%) *mt*<br>chi angles: 301.3,170.9	0.04Å	Favored<br>(71.741%)<br>beta sheet	-	-	-
A<br>71		ASN	0.65	-	Favored<br>(19.59%)<br>General /<br>-126.0,115.3	Favored (25.2%) *t0*<br>chi angles: 183.1,294.2	0.05Å	Favored<br>(67.131%)<br>beta sheet	-	-	-
A<br>72		ASP	0.66	-	Favored<br>(20.73%)	Favored (9.5%) *p0*<br>chi angles: 65.3,299.9	0.04Å	Favored<br>(41.361%)<br>beta sheet	-	-	-

#	Alt	Res	High B	Clash > 0.4Å	Ramachandran	Rotamer	Cβ deviation	CaBLAM	Bond lengths	Bond angles	Cis Peptides
					General / -106.1,154.4						
A 73		LYS	0.67	-	Favored (47.95%) General / -135.1,145.5	Favored (70.3%) *mmtt* chi angles: 296.4,288.6,184.3,181.8	0.06Å	Favored (47.877%)	-	-	-
A 74		CYS	0.69	-	Favored (91.46%) Pre-Pro / -71.9,147.6	Favored (82.3%) *m* chi angles: 294.2	0.01Å	Favored (35.56%)	-	-	-
A 75		PRO	0.69	-	Favored (89.03%) Trans-Pro / -61.3,149.5	Favored (22.9%) *Cg_endo* chi angles: 20,327.7,30.6	0.06Å	Favored (30.605%)	-	-	-
A 76		SER	0.69	-	Allowed (1.77%) General / 77.1,-7.9	Favored (4.7%) *p* chi angles: 85.6	0.07Å	CaBLAM Outlier (0.698%)	-	-	-
A 77		THR	0.69	-	Favored (45.51%) General / -97.9,-0.1	Favored (43%) *p* chi angles: 67.2	0.03Å	CaBLAM Disfavored (3.854%)	-	-	-
A 78		GLY	0.67	-	Favored (23.87%) Glycine / 105.8,176.9	-	-	Favored (33.57%)	-	-	-
A 79		GLU	0.65	-	Favored (25.67%) General / -82.2,153.9	Favored (93%) *mt-10* chi angles: 293,184.9,345	0.04Å	Favored (10.253%)	-	-	-
A 80		ALA	0.64	-	Favored (36.68%) General / -76.6,148.8	-	0.04Å	Favored (37.384%)	-	-	-
**#**	**Alt**	**Res**	**High B**	**Clash > 0.4Å**	**Ramachandran**	**Rotamer**	**Cβ deviation**	**CaBLAM**	**Bond lengths**	**Bond angles**	**Cis Peptides**
			Avg: 1.15	Clashscore: 0.81	Outliers: 1 of 491	Poor rotamers: 0 of 402	Outliers: 0 of 442	Outliers: 17 of 489	Outliers: 5 of 493	Outliers: 10 of 493	Non-Trans: 2 of 492
A 81		HIS	0.63	-	Favored (33.71%) General / -142.8,145.9	Favored (87.8%) *m-70* chi angles: 297.4,272	0.08Å	Favored (62.993%) beta sheet	-	-	-
A 82		LEU	0.63	-	Favored (47.19%) General / -119.7,144.4	Allowed (1.9%) *mp* chi angles: 282.5,88.2	0.09Å	Favored (58.258%)	-	-	-
A 83		GLU	0.65	-	Favored (66.62%) General / -62.2,-23.4	Favored (27.2%) *pm20* chi angles: 63.8,276.4,9.5	0.04Å	Favored (42.655%)	-	-	-
A 84		GLU	0.67	-	Favored (66.56%) General / -65.7,-21.4	Favored (95.8%) *mt-10* chi angles: 291.5,184,357.6	0.04Å	Favored (57.239%) three-ten	-	-	-
A 85		GLU	0.7	-	Favored (66.88%) General / -63.6,-22.4	Favored (52%) *mt-10* chi angles: 292.1,179.3,52.9	0.01Å	Favored (53.122%) three-ten	-	-	-
A 86		ASN	0.73	-	Favored (44.43%) General / -99.5,8.6	Favored (88.2%) *m-40* chi angles: 292.8,320.1	0.03Å	Favored (43.601%)	-	-	-
A 87		GLU	0.75	-	Favored (58.16%) General / -89.7,-3.6	Favored (96.8%) *mt-10* chi angles: 291.6,180.6,342.2	0.07Å	CaBLAM Disfavored (3.538%)	-	-	-
A 88		GLY	0.75	-	Favored (4.45%) Glycine / 79.9,-60.1	-	-	CaBLAM Disfavored (1.327%)	-	-	-

A 89		ASP	0.73	-	Favored (63.04%) General / -62.4,-19.1	Favored (99.5%) *m-30* chi angles: 287.5,347.8	0.05Å	Favored (42.266%)	-	-	-
A 90		ASN	0.7	-	Favored (22.6%) General / -91.1,148.1	Favored (85.4%) *m-40* chi angles: 299,316.5	0.05Å	Favored (24.983%)	-	-	-
A 91		ALA	0.66	-	Favored (53.02%) General / -104.9,128.3	-	0.04Å	Favored (66.261%)	-	-	-
A 92		CYS	0.63	-	Favored (46.54%) General / -126.7,152.6	Favored (66.3%) *m* chi angles: 299.7	0.01Å	Favored (63.712%) beta sheet	-	-	-
A 93		LYS	0.62	-	Favored (40.26%) General / -136.5,137.4	Favored (85.6%) *tttt* chi angles: 181.1,177.8,183.3,182.4	0.05Å	Favored (69.954%) beta sheet	-	-	-
A 94		ARG	0.62	-	Favored (56.37%) General / -117.5,130.3	Favored (46.7%) *ttm110* chi angles: 179.4,175.1,288.9,106.7	0.05Å	Favored (39.234%) beta sheet	-	-	-
A 95		THR	0.63	-	Favored (2.88%) General / -141.1,-169.7	Favored (11.2%) *t* chi angles: 189.2	0.06Å	Favored (31.392%) beta sheet	-	-	-
A 96		TYR	0.67	-	Favored (44.76%) General / -129.9,156.2	Favored (74.2%) *m-80* chi angles: 303.5,90.6	0.07Å	Favored (41.257%)	-	-	-
A 97		SER	0.73	-	Favored (41.62%) General / -149.5,161.7	Favored (90.9%) *p* chi angles: 66.7	0.03Å	Favored (34.93%)	-	-	-
A 98		ASP	0.81	-	Favored (22.26%) General / -84.7,114.8	Favored (55.2%) *m-30* chi angles: 292.4,306.6	0.02Å	Favored (32.944%)	-	-	-
A 99		ARG	0.9	-	Favored (7.35%) General / -109.6,171.6	Favored (42.1%) *mmm-85* chi angles: 307.2,302.4,291.1,269.3	0.09Å	CA Geom Outlier (0.043%)	-	-	-
A 100		GLY	0.99	-	Allowed (1.86%) Glycine / -139.1,-121.1	-	-	CA Geom Outlier (0.102%)	-	OUTLIER(S) worst is C-N-CA: 4.4 σ	Cis nonPRO omega= 2.95
**#**	**Alt**	**Res**	**High B**	**Clash > 0.4Å**	**Ramachandran**	**Rotamer**	**Cβ deviation**	**CaBLAM**	**Bond lengths**	**Bond angles**	**Cis Peptides**
			Avg: 1.15	Clashscore: 0.81	Outliers: 1 of 491	Poor rotamers: 0 of 402	Outliers: 0 of 442	Outliers: 17 of 489	Outliers: 5 of 493	Outliers: 10 of 493	Non-Trans: 2 of 492
A 101		TRP	1.06	-	OUTLIER (0.02)%) General / 58.0,-80.7	Favored (70%) *t-100* chi angles: 185.6,255.7	0.03Å	CaBLAM Outlier (0.013%) try alpha helix	-	-	-
A 102		GLY	1.09	-	Favored (68.5%) Glycine / -61.5,-27.8	-	-	Favored (83.61%) alpha helix	-	-	-
A 103		ASN	1.07	-	Favored (32.83%) General / -101.5,14.2	Favored (70%) *m-40* chi angles: 292.7,281	0.08Å	Favored (42.295%)	-	-	-
A 104		GLY	1.02	-	Favored (79.75%)	-	-	Favored (57.844%)	-	-	-

#	Alt	Res	High B	Clash > 0.4Å	Ramachandran	Rotamer	Cβ deviation	CaBLAM	Bond lengths	Bond angles	Cis Peptides
					Glycine / 88.5,-8.2						
A 105		CYS	0.95	-	Favored (19.19%) General / -95.6,151.6	Favored (67.3%) *m* chi angles: 299.5	0.05Å	Favored (32.786%)	-	-	-
A 106		GLY	0.89	-	Favored (86.99%) Glycine / -85.9,-1.4	-	-	Favored (35.288%)	-	-	-
A 107		LEU	0.84	-	Favored (29.53%) General / -112.5,151.8	Favored (72%) *mt* chi angles: 302.1,174.9	0.06Å	Favored (26.661%)	-	-	-
A 108		PHE	0.79	-	Favored (11.5%) General / -113.8,25.1	Favored (99.4%) *m-80* chi angles: 296.9,95.4	0.05Å	Favored (5.074%)	-	-	-
A 109		GLY	0.75	-	Favored (38.81%) Glycine / -92.8,-166.7	-	-	Favored (45.776%) beta sheet	-	-	-
A 110		LYS	0.72	-	Favored (35.33%) General / -95.6,120.2	Favored (63.4%) *mttm* chi angles: 294.7,183.4,184.8,292.6	0.05Å	Favored (11.462%)	-	-	-
A 111		GLY	0.68	-	Favored (33.92%) Glycine / -94.8,-160.1	-	-	Favored (22.957%)	-	-	-
A 112		SER	0.65	-	Favored (42.13%) General / -134.6,135.3	Favored (39.3%) *t* chi angles: 175	0.07Å	Favored (32.103%)	-	OUTLIER(S) worst is CA-C-O: 4.4 σ	-
A 113		ILE	0.62	-	Favored (63.92%) Ile or Val / -122.2,134.9	Favored (57.7%) *mt* chi angles: 304.2,167.5	0.15Å	Favored (33.993%) beta sheet	-	-	-
A 114		VAL	0.61	-	Favored (67.31%) Ile or Val / -115.6,131.7	Favored (83.7%) *t* chi angles: 177	0.07Å	Favored (70.141%) beta sheet	-	-	-
A 115		ALA	0.61	-	Favored (52.57%) General / -120.5,139.7	-	0.05Å	Favored (67.927%) beta sheet	-	-	-
A 116		CYS	0.63	-	Favored (45.37%) General / -126.8,153.6	Favored (83.5%) *m* chi angles: 295.3	0.03Å	Favored (59.433%) beta sheet	-	-	-
A 117		ALA	0.65	-	Favored (28.18%) General / -146.4,142.4	-	0.04Å	Favored (48.765%) beta sheet	-	-	-
A 118		LYS	0.69	-	Favored (37.73%) General / -92.3,127.3	Favored (88.3%) *tttt* chi angles: 185.5,174.8,179.6,178.2	0.06Å	Favored (56.872%) beta sheet	-	-	-
A 119		PHE	0.73	-	Favored (34.01%) General / -114.7,151.2	Favored (92.1%) *m-80* chi angles: 291.6,87.3	0.03Å	Favored (47.634%) beta sheet	-	-	-
A 120		THR	0.78	-	Favored (44.3%) General / -140.4,150.7	Favored (9.5%) *t* chi angles: 185.4	0.06Å	Favored (48.524%) beta sheet	-	-	-
**#**	**Alt**	**Res**	**High B**	**Clash > 0.4Å**	**Ramachandran**	**Rotamer**	**Cβ deviation**	**CaBLAM**	**Bond lengths**	**Bond angles**	**Cis Peptides**
			Avg: 1.15	Clashscore: 0.81	Outliers: 1 of 491	Poor rotamers: 0 of 402	Outliers: 0 of 442	Outliers: 17 of 489	Outliers: 5 of 493	Outliers: 10 of 493	Non-Trans: 2

											of 492
A 121		CYS	0.83	-	Favored (30.33%) General / -101.8,144.6	Favored (83.6%) *m* chi angles: 295.3	0.11Å	Favored (47.161%)	-	-	-
A 122		ALA	0.86	-	Favored (12.75%) General / -88.6,-41.2	-	0.04Å	Favored (17.005%)	-	-	-
A 123		LYS	0.87	-	Favored (47.06%) General / -133.8,140.7	Favored (58.6%) *mttm* chi angles: 295.8,186.3,180.5,301.4	0.04Å	Favored (11.209%)	-	-	-
A 124		SER	0.86	-	Favored (41.22%) General / -148.5,161.6	Favored (93.4%) *p* chi angles: 64.3	0.04Å	Favored (44.36%)	-	-	-
A 125		MET	0.84	-	Favored (51.03%) General / -113.1,137.7	Favored (58.7%) *ttp* chi angles: 184.1,176.7,77.4	0.04Å	Favored (47.983%) beta sheet	-	-	-
A 126		SER	0.83	-	Favored (46.33%) General / -100.4,131.4	Favored (51.6%) *m* chi angles: 291.7	0.06Å	Favored (61.886%) beta sheet	-	-	-
A 127		LEU	0.82	0.41Å N with A 127 LEU HD12	Favored (24.45%) General / -111.2,112.6	Favored (3.2%) *mp* chi angles: 293.4,75.4	0.09Å	Favored (70.903%) beta sheet	-	-	-
A 128		PHE	0.84	-	Favored (53.39%) General / -115.0,125.3	Favored (64.7%) *m-80* chi angles: 302.1,83.8	0.16Å	Favored (57.781%) beta sheet	-	OUTLIER(S) worst is CA-CB-CG: 6.2 σ	-
A 129		GLU	0.87	-	Favored (52.57%) General / -68.1,135.7	Favored (89.9%) *tt0* chi angles: 182.8,172.8,4.4	0.03Å	Favored (41.642%) beta sheet	-	-	-
A 130		VAL	0.92	-	Favored (43.45%) Ile or Val / -98.4,117.5	Favored (73.1%) *t* chi angles: 178.4	0.02Å	Favored (60.166%) beta sheet	-	-	-
A 131		ASP	0.96	-	Favored (23.99%) General / -86.3,115.6	Favored (47.9%) *t0* chi angles: 181.8,335.6	0.05Å	Favored (58.671%)	-	-	-
A 132		GLN	0.98	-	Favored (61.42%) General / -59.3,-22.6	Favored (88%) *mt0* chi angles: 288.2,181.6,309.6	0.09Å	Favored (39.346%)	-	-	-
A 133		THR	0.98	-	Favored (54.61%) General / -75.2,-7.7	Favored (73.3%) *p* chi angles: 61.7	0.10Å	Favored (61.793%) alpha helix	-	-	-
A 134		LYS	0.95	-	Favored (38.62%) General / -102.9,9.8	Favored (99%) *mttt* chi angles: 295.1,184.8,180.1,180.1	0.02Å	Favored (47.745%)	-	-	-
A 135		ILE	0.91	-	Favored (40.88%) Ile or Val / -100.7,134.3	Favored (79.3%) *mt* chi angles: 299.8,173.5	0.06Å	Favored (24.008%)	-	-	-
A 136		GLN	0.85	-	Favored (51.39%) General / -133.8,149.2	Favored (19.7%) *pt0* chi angles: 59.8,175.3,287.7	0.06Å	Favored (60.675%) beta sheet	-	-	-
A 137		TYR	0.8	-	Favored (55.75%) General / -118.4,130.0	Favored (46.3%) *m-80* chi angles: 287,75.4	0.05Å	Favored (68.766%) beta sheet	-	-	-

#	Alt	Res	High B	Clash > 0.4Å	Ramachandran	Rotamer	Cβ deviation	CaBLAM	Bond lengths	Bond angles	Cis Peptides
A 138		VAL	0.77	-	Favored (54.07%) Ile or Val / -111.4,117.0	Favored (63.4%) *t* chi angles: 179.5	0.04Å	Favored (69.334%) beta sheet	-	-	-
A 139		ILE	0.75	-	Favored (68.87%) Ile or Val / -116.9,131.7	Favored (92.2%) *mt* chi angles: 297.2,170.6	0.05Å	Favored (62.481%) beta sheet	-	-	-
A 140		ARG	0.76	-	Favored (26.58%) General / -98.2,145.4	Favored (80%) *mtp85* chi angles: 296.9,176.9,74.5,86.6	0.05Å	Favored (59.157%) beta sheet	-	-	-
**#**	**Alt**	**Res**	**High B**	**Clash > 0.4Å**	**Ramachandran**	**Rotamer**	**Cβ deviation**	**CaBLAM**	**Bond lengths**	**Bond angles**	**Cis Peptides**
			Avg: 1.15	Clashscore: 0.81	Outliers: 1 of 491	Poor rotamers: 0 of 402	Outliers: 0 of 442	Outliers: 17 of 489	Outliers: 5 of 493	Outliers: 10 of 493	Non-Trans: 2 of 492
A 141		ALA	0.8	-	Favored (36.08%) General / -138.1,135.8	-	0.06Å	Favored (62.285%) beta sheet	-	-	-
A 142		GLN	0.89	-	Favored (39.18%) General / -137.4,137.8	Favored (57.1%) *tt0* chi angles: 186.1,184.4,43	0.15Å	Favored (56.219%) beta sheet	-	-	-
A 143		LEU	1.05	-	Favored (33.62%) General / -88.2,135.1	Favored (96.8%) *mt* chi angles: 297.2,176.8	0.04Å	Favored (41.866%) beta sheet	-	-	-
A 144		HIS	1.27	-	Favored (6.33%) General / -81.8,87.6	Favored (4.9%) *t70* chi angles: 191.2,123.8	0.05Å	Favored (16.227%) beta sheet	-	-	-
A 145		VAL	1.58	-	Favored (12.07%) Ile or Val / -120.7,169.3	Favored (30.4%) *m* chi angles: 300.6	0.12Å	CaBLAM Disfavored (4.204%)	-	-	-
A 146		GLY	1.97	-	Favored (48.14%) Glycine / -55.0,131.9	-	-	CaBLAM Disfavored (4.909%)	-	-	-
A 147		ALA	2.39	-	Favored (20.2%) General / -161.3,153.7	-	0.03Å	Favored (22.467%)	-	-	-
A 148		LYS	2.76	-	Favored (57.42%) General / -92.3,1.1	Favored (99%) *mttt* chi angles: 293.7,179.8,178.2,178.5	0.09Å	CaBLAM Disfavored (3.212%)	-	-	-
A 149		GLN	3	-	Favored (7.17%) General / 69.6,19.9	Favored (52.7%) *mt0* chi angles: 303.7,190.6,58	0.06Å	Favored (7.439%)	-	-	-
A 150		GLU	3.02	-	Favored (53.84%) General / -120.6,138.6	Favored (96.9%) *mt-10* chi angles: 296.2,180.6,355.4	0.05Å	CaBLAM Disfavored (2.025%)	-	-	-
A 151		ASN	2.82	-	Favored (13.3%) General / 58.9,25.2	Favored (84.8%) *m-40* chi angles: 298.7,322.2	0.02Å	Favored (15.674%)	-	-	-
A 152		TRP	2.44	0.64Å CD1 with A 152 TRP H	Favored (66.88%) General / -63.3,-22.4	Favored (7.3%) *p-90* chi angles: 58,292.7	0.10Å	Favored (19.001%)	-	-	-
A 153		ASN	2	-	Favored (65.91%) General / -63.7,-20.7	Favored (88%) *m-40* chi angles: 284.9,335.5	0.06Å	Favored (52.16%)	-	-	-

A 154		THR	1.58	-	Favored (67.84%) General / -55.4,-37.5	Favored (99%) *m* chi angles: 300.3	0.06Å	Favored (41.45%) three-ten	-	-	-
A 155		ASP	1.25	-	Favored (32.43%) General / -102.1,14.3	Favored (71.5%) *m-30* chi angles: 296.5,314.1	0.06Å	Favored (22.911%)	-	-	-
A 156		ILE	1.03	-	Favored (71.96%) Ile or Val / -114.9,128.7	Favored (80.1%) *mt* chi angles: 298.8,174.2	0.06Å	Favored (30.5%)	OUTLIER(S) worst is CB--CG1: 4.5 σ	-	-
A 157		LYS	0.89	-	Favored (29.66%) General / -110.2,149.7	Favored (29%) *mmmt* chi angles: 302.9,298.7,287.7,187.1	0.02Å	Favored (52.869%)	-	-	-
A 158		THR	0.82	-	Favored (56.78%) General / -116.1,132.0	Favored (99.9%) *m* chi angles: 300.5	0.03Å	Favored (66.566%) beta sheet	-	-	-
A 159		LEU	0.8	-	Favored (46.09%) General / -119.3,145.5	Favored (4.2%) *mp* chi angles: 298.4,87.4	0.07Å	Favored (63.127%) beta sheet	-	-	-
A 160		LYS	0.82	-	Favored (37.02%) General / -126.3,123.0	Favored (32.3%) *ttmt* chi angles: 183.1,181.8,291.2,181.3	0.04Å	Favored (62.957%) beta sheet	-	-	-
**#**	**Alt**	**Res**	**High B**	**Clash > 0.4Å**	**Ramachandran**	**Rotamer**	**Cβ deviation**	**CaBLAM**	**Bond lengths**	**Bond angles**	**Cis Peptides**
			Avg: 1.15	Clashscore: 0.81	Outliers: 1 of 491	Poor rotamers: 0 of 402	Outliers: 0 of 442	Outliers: 17 of 489	Outliers: 5 of 493	Outliers: 10 of 493	Non-Trans: 2 of 492
A 161		PHE	0.87	-	Favored (9.88%) General / -120.6,104.3	Favored (72.5%) *m-80* chi angles: 301.2,85	0.15Å	Favored (60.737%)	-	-	-
A 162		ASP	0.93	-	Favored (7.45%) General / -95.2,173.7	Favored (51%) *p0* chi angles: 63.7,16.2	0.04Å	Favored (20.301%)	-	-	-
A 163		ALA	0.98	-	Favored (65.56%) General / -59.6,-26.5	-	0.03Å	Favored (37.395%)	-	-	-
A 164		LEU	1.01	-	Favored (42.77%) General / -95.4,-5.6	Favored (94%) *mt* chi angles: 298.4,177.8	0.01Å	Favored (65.747%) alpha helix	-	-	-
A 165		SER	1.01	-	Favored (5.48%) General / -107.8,-39.7	Favored (63.9%) *m* chi angles: 297.5	0.04Å	CaBLAM Disfavored (2.771%) try alpha helix	-	-	-
A 166		GLY	0.99	-	Favored (2.76%) Glycine / 75.9,-51.5	-	-	CaBLAM Disfavored (4.984%) try alpha helix	-	-	-
A 167		SER	0.96	-	Favored (41.59%) General / -138.5,159.6	Favored (95.1%) *p* chi angles: 64.9	0.05Å	Favored (17.223%)	-	-	-
A 168		GLN	0.93	-	Favored (17.19%) General / -154.1,139.0	Favored (67.8%) *tt0* chi angles: 180.4,173.7,358.3	0.04Å	Favored (43.072%)	-	-	-
A 169		GLU	0.91	-	Favored (36.78%)	Favored (88.8%) *tt0* chi angles:	0.05Å	Favored (54.257%)	-	-	-

#	Alt	Res	High B	Clash > 0.4Å	Ramachandran	Rotamer	Cβ deviation	CaBLAM	Bond lengths	Bond angles	Cis Peptides
					General / -92.5,132.5	185.4,177.5,6.4		beta sheet			
A 170		ALA	0.91	-	Favored (30.38%) General / -107.5,147.6	-	0.04Å	Favored (60.888%) beta sheet	-	-	-
A 171		GLU	0.93	-	Favored (45.92%) General / -129.9,131.1	Favored (41.1%) *tt0* chi angles: 180.9,175.5,73	0.03Å	Favored (70.322%) beta sheet	-	-	-
A 172		PHE	0.95	-	Favored (20.99%) General / -116.3,112.0	Favored (72.7%) *m-80* chi angles: 302.7,87.1	0.09Å	Favored (47.42%)	-	-	-
A 173		THR	0.96	-	Favored (27.98%) General / -57.7,126.6	Favored (84.4%) *m* chi angles: 301.8	0.02Å	Favored (17.94%)	-	-	-
A 174		GLY	0.96	-	Favored (75.76%) Glycine / 87.6,-8.9	-	-	Favored (50.71%)	-	-	-
A 175		TYR	0.94	-	Favored (2.76%) General / -120.5,-39.4	Favored (78%) *m-80* chi angles: 303.5,94.2	0.02Å	CaBLAM Outlier (0.241%)	-	-	-
A 176		GLY	0.91	-	Favored (24.21%) Glycine / 136.0,-159.2	-	-	Favored (11.069%)	-	-	-
A 177		ARG	0.88	-	Favored (37.03%) General / -126.2,157.9	Favored (93%) *mtt180* chi angles: 297.1,187.9,180.3,181.1	0.04Å	Favored (10.051%)	-	-	-
A 178		ALA	0.86	-	Favored (33.82%) General / -142.7,145.9	-	0.03Å	Favored (63.27%) beta sheet	-	-	-
A 179		THR	0.85	-	Favored (56.5%) General / -117.8,131.8	Favored (99.3%) *m* chi angles: 300.4	0.03Å	Favored (65.91%) beta sheet	-	-	-
A 180		LEU	0.88	-	Favored (53.53%) General / -108.0,132.4	Favored (4.5%) *mp* chi angles: 275.9,73.8	0.01Å	Favored (69.419%) beta sheet	-	-	-
**#**	**Alt**	**Res**	**High B**	**Clash > 0.4Å**	**Ramachandran**	**Rotamer**	**Cβ deviation**	**CaBLAM**	**Bond lengths**	**Bond angles**	**Cis Peptides**
			Avg: 1.15	Clashscore: 0.81	Outliers: 1 of 491	Poor rotamers: 0 of 402	Outliers: 0 of 442	Outliers: 17 of 489	Outliers: 5 of 493	Outliers: 10 of 493	Non-Trans: 2 of 492
A 181		GLU	0.95	-	Favored (33.08%) General / -118.9,119.0	Favored (96.1%) *mt-10* chi angles: 295.9,181.2,358.6	0.03Å	Favored (69.142%) beta sheet	-	-	-
A 182		CYS	1.07	-	Favored (53.83%) General / -125.8,139.0	Favored (83.2%) *m* chi angles: 295.2	0.02Å	Favored (65.223%) beta sheet	-	-	-
A 183		GLN	1.23	-	Favored (16.62%) General / -97.3,104.6	Favored (95.5%) *mm-40* chi angles: 296.8,297.1,300.9	0.04Å	Favored (58.493%) beta sheet	-	-	-
A 184		VAL	1.41	-	Allowed (1.34%) Ile or Val / -102.0,24.8	Favored (29.7%) *m* chi angles: 296.5	0.08Å	Favored (6.749%) beta sheet	-	-	-
A 185		GLN	1.56	-	Favored (67.94%)	Favored (86.9%) *mt0*	0.09Å	CA Geom Outlier	-	-	-

					General / -57.5,-33.4	chi angles: 291.9,178.4,37		(0.256%)			
A 186		THR	1.63	-	Allowed (0.07%) General / 71.5,146.1	Favored (33.2%) *m* chi angles: 306.3	0.07Å	CaBLAM Disfavored (2.094%)	-	-	-
A 187		ALA	1.6	-	Favored (63.47%) General / -57.0,-29.6	-	0.04Å	Favored (11.086%)	-	-	-
A 188		VAL	1.49	-	Favored (37.48%) Ile or Val / -80.5,123.7	Favored (87.8%) *t* chi angles: 176.1	0.15Å	Favored (28.89%)	-	-	-
A 189		ASP	1.34	-	Favored (6.28%) General / -80.2,95.4	Favored (60.5%) *t0* chi angles: 180.9,346.6	0.06Å	Favored (58.492%)	-	-	-
A 190		PHE	1.2	-	Favored (66.07%) General / -66.7,-20.9	Favored (34.3%) *m-80* chi angles: 282.2,114.3	0.11Å	Favored (40.344%)	-	-	-
A 191		SER	1.08	-	Favored (61.62%) General / -72.8,-15.4	Favored (88.4%) *p* chi angles: 69.5	0.05Å	Favored (59.025%) alpha helix	-	-	-
A 192		ASN	0.99	-	Favored (14.76%) General / -116.9,18.3	Favored (69.2%) *m-40* chi angles: 288.6,287.8	0.05Å	Favored (16.717%)	-	-	-
A 193		SER	0.92	-	Favored (33.16%) General / -133.3,161.9	Favored (22.3%) *m* chi angles: 304.3	0.05Å	Favored (26.551%)	-	-	-
A 194		TYR	0.88	-	Favored (26.95%) General / -143.9,165.7	Favored (39.6%) *p90* chi angles: 72.3,94.8	0.07Å	Favored (47.796%)	OUTLIER(S) worst is CB--CG: 4.0 σ	-	-
A 195		ILE	0.88	-	Favored (54.36%) Ile or Val / -100.9,124.2	Favored (85.2%) *mt* chi angles: 297.5,166.2	0.03Å	Favored (42.586%) beta sheet	-	-	-
A 196		ALA	0.91	-	Favored (25.72%) General / -94.5,144.3	-	0.02Å	Favored (57.233%) beta sheet	-	-	-
A 197		GLU	0.96	-	Favored (29.84%) General / -137.6,130.2	Favored (81.7%) *tt0* chi angles: 177.3,175.9,352.9	0.04Å	Favored (33.729%)	-	-	-
A 198		MET	1.03	-	Favored (13.5%) General / -153.7,134.1	Favored (67%) *ttp* chi angles: 180,180.6,69	0.05Å	Favored (7.606%)	-	-	-
A 199		GLU	1.08	-	Favored (20.52%) General / 48.6,43.5	Favored (40.5%) *mm-30* chi angles: 296.9,285.5,317.6	0.01Å	Favored (34.673%)	-	-	-
A 200		LYS	1.1	-	Favored (9.68%) General / 66.5,14.3	Favored (89.6%) *mttt* chi angles: 302.9,183.2,181.8,178	0.04Å	Favored (8.98%)	-	-	-
**#**	**Alt**	**Res**	**High B**	**Clash > 0.4Å**	**Ramachandran**	**Rotamer**	**Cβ deviation**	**CaBLAM**	**Bond lengths**	**Bond angles**	**Cis Peptides**
			Avg: 1.15	Clashscore: 0.81	Outliers: 1 of 491	Poor rotamers: 0 of 402	Outliers: 0 of 442	Outliers: 17 of 489	Outliers: 5 of 493	Outliers: 10 of 493	Non-Trans: 2 of 492

A 201		GLU	1.09	-	Favored (34.48%) General / -111.1,147.6	Favored (37.5%) *mt-10* chi angles: 295.4,183.8,79	0.06Å	Favored (29.632%)	-	-	-
A 202		SER	1.05	-	Favored (51.73%) General / -133.8,153.4	Favored (59.9%) *m* chi angles: 299	0.06Å	Favored (71.332%) beta sheet	-	-	-
A 203		TRP	1	-	Favored (45.34%) General / -138.6,149.5	Favored (17.4%) *m-90* chi angles: 304.5,263.7	0.09Å	Favored (56.943%) beta sheet	-	-	-
A 204		ILE	0.98	-	Favored (34.17%) Ile or Val / -92.8,116.6	Favored (48.8%) *mm* chi angles: 303.3,300.4	0.10Å	Favored (48.095%) beta sheet	OUTLIER(S) worst is CB--CG1: 4.7 σ	-	-
A 205		VAL	0.98	-	Favored (52.67%) Ile or Val / -128.6,139.2	Favored (53.1%) *t* chi angles: 180.9	0.08Å	Favored (45.835%) beta sheet	-	-	-
A 206		ASP	1.01	-	Favored (55.79%) General / -60.1,133.1	Favored (13.6%) *t0* chi angles: 190.5,306	0.05Å	Favored (46.865%)	-	-	-
A 207		LYS	1.05	-	Favored (62.95%) General / -56.8,-29.7	Favored (92.8%) *mttt* chi angles: 285.8,176.1,178.4,171.9	0.04Å	Favored (53.652%)	-	-	-
A 208		GLN	1.09	-	Favored (79.36%) General / -63.8,-35.1	Favored (91.5%) *tp40* chi angles: 185,64.5,49.9	0.03Å	Favored (67.959%) alpha helix	-	-	-
A 209		TRP	1.11	-	Favored (92.95%) General / -61.1,-45.9	Favored (85.5%) *t60* chi angles: 181.8,92.6	0.12Å	Favored (83.878%) alpha helix	-	-	-
A 210		ALA	1.12	-	Favored (93.01%) General / -61.5,-40.0	-	0.04Å	Favored (91.831%) alpha helix	-	-	-
A 211		GLN	1.1	-	Favored (69.96%) General / -66.1,-29.6	Favored (73.3%) *mm-40* chi angles: 294.6,306.7,326.2	0.05Å	Favored (76.194%) alpha helix	-	-	-
A 212		ASP	1.06	-	Favored (41.28%) General / -92.5,7.9	Favored (64.3%) *m-30* chi angles: 290,319.7	0.06Å	Favored (38.065%)	-	-	-
A 213		LEU	1	-	Favored (57.26%) General / -63.5,144.1	Favored (77.4%) *mt* chi angles: 288.3,171.5	0.04Å	Favored (34.401%)	-	-	-
A 214		THR	0.94	-	Favored (7.58%) General / -109.3,29.4	Favored (50.1%) *p* chi angles: 56.2	0.02Å	CaBLAM Disfavored (2.088%) try beta sheet	-	-	-
A 215		LEU	0.91	-	Favored (50.84%) Pre-Pro / -118.5,152.3	Favored (94.6%) *mt* chi angles: 296,173.7	0.14Å	Favored (12.306%) beta sheet	-	-	-
A 216		PRO	0.91	-	Favored (91.58%) Trans-Pro / -57.1,143.9	Favored (51.3%) *Cg_exo* chi angles: 336.5,37.4,324.7	0.06Å	Favored (29.122%) beta sheet	-	-	-
A 217		TRP	0.98	-	Favored (43.92%) General / -146.7,158.5	Favored (77%) *p-90* chi angles: 61.9,273.3	0.03Å	Favored (63.84%)	-	-	-

#	Alt	Res	High B	Clash > 0.4Å	Ramachandran	Rotamer	Cβ deviation	CaBLAM	Bond lengths	Bond angles	Cis Peptides
A 218		GLN	1.09	-	Favored (47.73%) General / -138.3,156.4	Favored (26.1%) *pt0* chi angles: 65.8,177.6,307	0.06Å	Favored (29.052%)	-	-	-
A 219		SER	1.24	-	Favored (42.86%) General / -61.8,148.5	Favored (63.3%) *m* chi angles: 297.7	0.04Å	Favored (44.246%)	-	-	-
A 220		GLY	1.36	-	Favored (53.41%) Glycine / -55.3,-29.7	-	-	Favored (60.24%)	-	-	-
**#**	**Alt**	**Res**	**High B**	**Clash > 0.4Å**	**Ramachandran**	**Rotamer**	**Cβ deviation**	**CaBLAM**	**Bond lengths**	**Bond angles**	**Cis Peptides**
			Avg: 1.15	Clashscore: 0.81	Outliers: 1 of 491	Poor rotamers: 0 of 402	Outliers: 0 of 442	Outliers: 17 of 489	Outliers: 5 of 493	Outliers: 10 of 493	Non-Trans: 2 of 492
A 221		SER	1.4	-	Favored (60.27%) General / -74.8,-11.9	Favored (86%) *p* chi angles: 67.2	0.04Å	Favored (32.761%)	-	-	-
A 222		GLY	1.34	-	Favored (10.05%) Glycine / 101.7,138.2	-	-	CaBLAM Disfavored (3.237%)	-	-	-
A 223		GLY	1.2	-	Favored (43.71%) Glycine / 175.4,-170.6	-	-	Favored (10.887%)	-	-	-
A 224		VAL	1.04	-	Favored (22.83%) Ile or Val / -66.9,124.8	Favored (86%) *t* chi angles: 173.7	0.10Å	Favored (5.152%)	-	OUTLIER(S) worst is CA-C-O: 4.5 σ	-
A 225		TRP	0.91	-	Favored (9.89%) General / -53.7,122.5	Favored (92.9%) *m100* chi angles: 289.9,108.5	0.10Å	Favored (50.522%)	-	-	-
A 226		ARG	0.81	-	Favored (27.7%) General / -102.4,146.7	Favored (53.9%) *mmt180* chi angles: 303.2,295,183.4,182.7	0.08Å	Favored (11.576%)	-	-	-
A 227		GLU	0.76	-	Favored (25.7%) General / 58.0,40.1	Favored (31%) *mt-10* chi angles: 303.9,184.8,56.9	0.01Å	Favored (16.07%)	-	-	-
A 228		MET	0.74	-	Favored (62.93%) General / -61.1,-20.7	Favored (59.1%) *tpp* chi angles: 183.5,57.9,73.2	0.15Å	Favored (14.99%)	-	-	-
A 229		HIS	0.73	-	Favored (61.73%) General / -59.2,-22.9	Favored (77.9%) *m90* chi angles: 291.4,79.6	0.04Å	Favored (49.616%)	-	-	-
A 230		HIS	0.72	-	Favored (61.43%) General / -72.8,-14.0	Favored (85.7%) *m-70* chi angles: 297.2,301	0.05Å	Favored (45.719%) three-ten	-	-	-
A 231		LEU	0.71	-	Favored (19.48%) General / -111.8,17.9	Favored (78.4%) *mt* chi angles: 291.2,176.4	0.10Å	Favored (30.71%)	-	-	-
A 232		VAL	0.7	-	Favored (36.16%) Ile or Val / -140.5,138.6	Favored (3.1%) *p* chi angles: 55.1	0.07Å	Favored (21.952%)	-	-	-
A 233		GLU	0.7	-	Favored (52.97%)	Favored (90.4%) *tt0* chi angles: 179.2,177.2,2.7	0.03Å	Favored (69.404%)	-	-	-

					General / -111.3,134.4						
A 234		PHE	0.73	-	Favored (33.81%) General / -112.0,117.8	Favored (82.3%) *m-80* chi angles: 294.8,82.2	0.09Å	Favored (63.634%)	-	-	-
A 235		GLU	0.77	-	Favored (72.66%) Pre-Pro / -78.5,160.3	Favored (94.2%) *mt-10* chi angles: 293.4,185.3,349.3	0.04Å	Favored (19.369%)	-	-	-
A 236		PRO	0.83	-	Favored (27.58%) Trans-Pro / -51.3,145.2	Favored (86.8%) *Cg_exo* chi angles: 330.6,37.2,331	0.05Å	Favored (55.229%)	-	-	-
A 237		PRO	0.91	0.40Å O with A 238 HIS C	Favored (35.98%) Trans-Pro / -69.9,-14.6	Favored (60.5%) *Cg_endo* chi angles: 26.5,324.3,29.2	0.11Å	CaBLAM Outlier (0.996%)	-	-	-
A 238		HIS	0.98	0.40Å C with A 237 PRO O	Favored (2.32%) General / 54.3,-127.2	Favored (99.4%) *m-70* chi angles: 299.9,288.6	0.06Å	CaBLAM Outlier (0.496%)	-	-	-
A 239		ALA	1.01	-	Favored (8.35%) General / -98.5,-40.3	-	0.04Å	CaBLAM Outlier (0.083%)	-	-	-
A 240		ALA	1	-	Favored (2.09%) General / -142.1,17.2	-	0.03Å	Favored (16.443%)	-	-	-
**#**	**Alt**	**Res**	**High B**	**Clash > 0.4Å**	**Ramachandran**	**Rotamer**	**Cβ deviation**	**CaBLAM**	**Bond lengths**	**Bond angles**	**Cis Peptides**
			Avg: 1.15	Clashscore: 0.81	Outliers: 1 of 491	Poor rotamers: 0 of 402	Outliers: 0 of 442	Outliers: 17 of 489	Outliers: 5 of 493	Outliers: 10 of 493	Non-Trans: 2 of 492
A 241		THR	0.95	-	Favored (14.06%) General / -150.9,131.7	Favored (70.1%) *m* chi angles: 303	0.05Å	Favored (12.143%)	-	-	-
A 242		ILE	0.89	-	Favored (58.91%) Ile or Val / -109.4,119.5	Favored (86.5%) *mt* chi angles: 298.6,169.8	0.10Å	Favored (66.54%)	-	-	-
A 243		LYS	0.83	-	Favored (35.09%) General / -83.0,133.8	Favored (86.5%) *tttt* chi angles: 185.6,178,180.3,181.7	0.02Å	Favored (48.037%) beta sheet	-	-	-
A 244		VAL	0.8	-	Favored (75.26%) Ile or Val / -118.2,128.2	Favored (38.1%) *t* chi angles: 183.8	0.07Å	Favored (68.048%) beta sheet	-	-	-
A 245		LEU	0.8	-	Favored (51.55%) General / -126.3,142.4	Favored (3.5%) *mp* chi angles: 304.9,88.4	0.11Å	Favored (55.158%) beta sheet	-	-	-
A 246		ALA	0.82	-	Favored (38.59%) General / -78.5,139.9	-	0.03Å	Favored (42.283%) beta sheet	-	-	-
A 247		LEU	0.86	-	Favored (5.61%) General / -90.3,63.1	Favored (82.6%) *mt* chi angles: 300.8,178.7	0.09Å	Favored (7.598%)	-	-	-
A 248		GLY	0.9	-	Favored (32.63%) Glycine / 57.5,-123.4	-	-	Favored (49.902%)	-	-	-

#	Alt	Res	High B	Clash > 0.4Å	Ramachandran	Rotamer	Cβ deviation	CaBLAM	Bond lengths	Bond angles	Cis Peptides
A 249		ASN	0.94	-	Favored (5.89%) General / -142.2,110.2	Favored (50.4%) *t0* chi angles: 183.8,4	0.04Å	CaBLAM Disfavored (2.394%)	-	-	-
A 250		GLN	0.98	-	Favored (23.9%) General / -86.2,7.7	Favored (99.2%) *mm-40* chi angles: 298.9,299.3,311.5	0.04Å	Favored (10.412%)	-	-	-
A 251		GLU	1.01	-	Favored (63.21%) General / -52.8,-40.3	Favored (86%) *tt0* chi angles: 177.6,177.5,356.9	0.06Å	Favored (48.587%)	-	-	-
A 252		GLY	1.04	-	Favored (99.7%) Glycine / -61.9,-42.0	-	-	Favored (95.523%) alpha helix	-	-	-
A 253		SER	1.07	-	Favored (89.3%) General / -66.5,-40.7	Favored (66%) *m* chi angles: 294.4	0.02Å	Favored (92.958%) alpha helix	-	-	-
A 254		LEU	1.12	-	Favored (56.66%) General / -60.4,-53.5	Favored (46.6%) *tp* chi angles: 171.5,63.2	0.12Å	Favored (92.674%) alpha helix	-	-	-
A 255		LYS	1.16	-	Favored (79.2%) General / -59.9,-37.4	Favored (25.1%) *ttmt* chi angles: 187.1,169.4,283.6,188.9	0.12Å	Favored (83.369%) alpha helix	-	-	-
A 256		THR	1.2	-	Favored (89.16%) General / -62.8,-46.2	Favored (96.4%) *m* chi angles: 299.7	0.05Å	Favored (81.671%) alpha helix	-	-	-
A 257		ALA	1.22	-	Favored (68.84%) General / -59.6,-30.7	-	0.01Å	Favored (72.05%)	-	-	-
A 258		LEU	1.21	-	Favored (42.2%) General / -78.9,-2.9	Favored (84.5%) *mt* chi angles: 290.3,168.4	0.02Å	Favored (32.728%)	-	-	-
A 259		THR	1.18	-	Favored (45.47%) General / -56.7,130.4	Favored (88.2%) *m* chi angles: 301.3	0.02Å	Favored (14.986%)	-	-	-
A 260		GLY	1.13	-	Favored (60.77%) Glycine / 99.3,-14.9	-	-	Favored (84.113%)	-	-	-
**#**	**Alt**	**Res**	**High B**	**Clash > 0.4Å**	**Ramachandran**	**Rotamer**	**Cβ deviation**	**CaBLAM**	**Bond lengths**	**Bond angles**	**Cis Peptides**
			Avg: 1.15	Clashscore: 0.81	Outliers: 1 of 491	Poor rotamers: 0 of 402	Outliers: 0 of 442	Outliers: 17 of 489	Outliers: 5 of 493	Outliers: 10 of 493	Non-Trans: 2 of 492
A 261		ALA	1.06	-	Favored (47.61%) General / -67.0,151.5	-	0.04Å	Favored (34.93%)	-	-	-
A 262		MET	1	-	Favored (34.29%) General / -80.2,142.8	Favored (67.1%) *mtt* chi angles: 293.6,180.5,172.8	0.05Å	Favored (38.388%) beta sheet	-	-	-
A 263		ARG	0.97	-	Favored (36.59%) General / -79.4,135.0	Favored (81.1%) *ttm-80* chi angles: 184.4,179.3,293.3,277.7	0.06Å	Favored (43.963%) beta sheet	-	-	-
A 264		VAL	0.98	-	Favored (57.96%) Ile or Val / -122.2,136.8	Favored (74.4%) *t* chi angles: 178.3	0.06Å	Favored (68.695%) beta sheet	-	-	-

A 265		THR	1.03	-	Favored (55.19%) General / -115.3,134.4	Favored (93.2%) *m* chi angles: 297.4	0.03Å	Favored (56.499%) beta sheet	-	-	-
A 266		LYS	1.13	-	Favored (33.86%) General / -87.1,132.3	Favored (48.3%) *mtpt* chi angles: 291.7,176.4,70.1,169.2	0.06Å	Favored (35.407%) beta sheet	-	-	-
A 267		ASP	1.26	-	Favored (57.41%) General / -60.1,139.6	Favored (11.9%) *t70* chi angles: 192.4,91.4	0.03Å	Favored (35.307%)	-	-	-
A 268		THR	1.38	-	Favored (58.44%) General / -86.5,-7.4	Favored (74.7%) *p* chi angles: 61.4	0.06Å	Favored (27.778%)	-	-	-
A 269		ASN	1.46	-	Favored (9.15%) General / -101.0,-36.1	Favored (89.4%) *m-40* chi angles: 294.5,321.7	0.02Å	Favored (26.231%) alpha helix	-	-	-
A 270		ASN	1.45	-	Favored (3.51%) General / -135.1,96.5	Favored (52.6%) *t0* chi angles: 183.4,333.2	0.05Å	Favored (19.53%) alpha helix	-	-	-
A 271		SER	1.37	-	Favored (6.57%) General / -61.0,-10.9	Favored (99.9%) *p* chi angles: 65.5	0.04Å	Favored (10.025%) alpha helix	-	-	-
A 272		LYS	1.25	-	Favored (49.27%) General / -85.1,-13.8	Favored (99.2%) *mttt* chi angles: 294.3,179.6,179.6,178.4	0.02Å	Favored (42.368%)	-	-	-
A 273		LEU	1.13	-	Favored (34.13%) General / -92.5,121.6	Favored (47.4%) *tp* chi angles: 180.3,66.4	0.04Å	Favored (33.464%)	-	-	-
A 274		TYR	1.06	-	Favored (49.9%) General / -104.9,133.3	Favored (68.4%) *m-80* chi angles: 289.3,80.6	0.06Å	Favored (64.173%)	-	-	-
A 275		LYS	1.02	-	Favored (55.08%) General / -110.1,127.8	Favored (98.5%) *mttt* chi angles: 295.2,176.7,182.1,174.1	0.07Å	Favored (62.129%) beta sheet	-	-	-
A 276		LEU	1.02	-	Favored (24.06%) General / -100.2,111.8	Favored (62.1%) *tp* chi angles: 176.1,59.2	0.03Å	Favored (66.444%) beta sheet	-	-	-
A 277		HIS	1.04	-	Favored (59.71%) General / -75.1,-20.1	Favored (94.9%) *m-70* chi angles: 293.1,286.3	0.05Å	Favored (43.397%)	-	-	-
A 278		GLY	1.06	-	Favored (70.44%) Glycine / -94.9,9.7	-	-	Favored (46.34%)	-	-	-
A 279		GLY	1.07	-	Favored (35.69%) Glycine / -75.5,147.5	-	-	Favored (23.484%)	-	-	-
A 280		HIS	1.07	-	Favored (50.63%) General / -136.7,154.3	Favored (57.7%) *m90* chi angles: 302.1,77.7	0.15Å	Favored (61.296%) beta sheet	OUTLIER(S) worst is CB--CG: 6.8 σ	-	-
**#**	**Alt**	**Res**	**High B**	**Clash > 0.4Å**	**Ramachandran**	**Rotamer**	**Cβ deviation**	**CaBLAM**	**Bond lengths**	**Bond angles**	**Cis Peptides**
			Avg: 1.15	Clashscore: 0.81	Outliers: 1 of 491	Poor rotamers: 0 of 402	Outliers: 0 of 442	Outliers: 17 of 489	Outliers: 5 of 493	Outliers: 10 of 493	Non-Trans: 2 of 492

A 281		VAL	1.04	-	Favored (25.72%) Ile or Val / -143.5,141.6	Favored (8.3%) *p* chi angles: 61.3	0.08Å	Favored (60.283%) beta sheet	-	-	-
A 282		ALA	1.01	-	Favored (46.06%) General / -100.0,129.8	-	0.04Å	Favored (58.559%) beta sheet	-	-	-
A 283		CYS	0.99	-	Favored (37.16%) General / -129.1,159.1	Favored (69%) *m* chi angles: 298.9	0.03Å	Favored (51.228%) beta sheet	-	-	-
A 284		ARG	0.98	-	Favored (51.7%) General / -125.9,135.3	Favored (42.2%) *ttm170* chi angles: 178.5,175.8,291.6,189.8	0.02Å	Favored (67.637%) beta sheet	-	-	-
A 285		VAL	1	-	Favored (62.62%) Ile or Val / -117.1,133.8	Favored (7.5%) *p* chi angles: 60.7	0.02Å	Favored (67.89%) beta sheet	-	-	-
A 286		LYS	1.02	-	Favored (48.17%) General / -101.6,125.0	Favored (97.9%) *mttt* chi angles: 296.6,176.5,181,174.9	0.04Å	Favored (63.77%) beta sheet	-	-	-
A 287		LEU	1.05	-	Favored (15.73%) General / -112.9,21.2	Favored (80.5%) *mt* chi angles: 300.3,174.5	0.09Å	Favored (15.6%)	-	-	-
A 288		SER	1.07	-	Favored (62.18%) General / -60.4,-21.4	Favored (84.8%) *p* chi angles: 67.4	0.06Å	Favored (13.717%)	-	-	-
A 289		ALA	1.1	-	Favored (14.26%) General / -117.6,18.1	-	0.03Å	Favored (40.739%)	-	-	-
A 290		LEU	1.12	-	Favored (44.7%) General / -102.9,122.5	Favored (62.4%) *tp* chi angles: 177.9,64.6	0.03Å	Favored (28.514%)	-	-	-
A 291		THR	1.14	-	Favored (53.31%) General / -113.8,136.0	Favored (89%) *m* chi angles: 298.6	0.01Å	Favored (51.037%) beta sheet	-	-	-
A 292		LEU	1.15	-	Favored (3.2%) General / -76.3,90.1	Favored (56.8%) *tp* chi angles: 181.9,59.1	0.06Å	Favored (46.68%)	-	-	-
A 293		LYS	1.15	-	Favored (31.45%) General / -52.7,130.1	Favored (84.8%) *tttt* chi angles: 180.7,179.7,181.2,179.2	0.10Å	Favored (7.941%)	-	-	-
A 294		GLY	1.14	-	Favored (59.38%) Glycine / 98.0,-1.4	-	-	Favored (75.628%)	-	-	-
A 295		THR	1.1	-	Favored (67.06%) General / -62.6,-23.4	Favored (47.5%) *p* chi angles: 55.7	0.05Å	Favored (46.753%) alpha helix	-	-	-
A 296		SER	1.05	-	Favored (59.03%) General / -85.9,-3.9	Favored (92.1%) *p* chi angles: 66.5	0.02Å	Favored (54.866%)	-	-	-
A 297		TYR	1	-	Favored (27.34%) General / -81.5,152.2	Favored (23%) *m-10* chi angles: 294,163.5	0.07Å	Favored (33.374%)	-	-	-
A 298		LYS	0.98	-	Favored (36.05%) General / -71.5,158.5	Favored (98.8%) *mttt* chi angles: 294,181.3,179.7,179.4	0.01Å	Favored (45.615%)	-	-	-

A 299		MET	0.97	-	Favored (31.83%) General / -80.4,145.9	Favored (69.4%) *mtt* chi angles: 292.2,180.7,174.7	0.02Å	Favored (34.189%)	-	-	-
A 300		CYS	0.98	-	Favored (22.88%) General / -79.6,119.7	Favored (54%) *m* chi angles: 302.8	0.05Å	Favored (22.695%) beta sheet	-	-	-
**#**	**Alt**	**Res**	**High B**	**Clash > 0.4Å**	**Ramachandran**	**Rotamer**	**Cβ deviation**	**CaBLAM**	**Bond lengths**	**Bond angles**	**Cis Peptides**
			Avg: 1.15	Clashscore: 0.81	Outliers: 1 of 491	Poor rotamers: 0 of 402	Outliers: 0 of 442	Outliers: 17 of 489	Outliers: 5 of 493	Outliers: 10 of 493	Non-Trans: 2 of 492
A 301		THR	0.99	-	Favored (8.94%) General / -123.8,10.2	Favored (72%) *p* chi angles: 59.6	0.03Å	Favored (11.388%) beta sheet	-	-	-
A 302		ASP	0.98	-	Favored (4.53%) General / -85.1,-173.1	Favored (52.7%) *p0* chi angles: 65.1,12.6	0.09Å	CaBLAM Disfavored (4.663%) try beta sheet	-	-	-
A 303		LYS	0.96	-	Favored (7.64%) General / -80.7,72.1	Favored (99.1%) *mttt* chi angles: 294.6,180.4,179.5,180	0.03Å	Favored (7.414%) beta sheet	-	-	-
A 304		MET	0.91	-	Favored (37.62%) General / -58.2,145.3	Favored (3.1%) *mpp* chi angles: 277.6,80.9,74.4	0.07Å	Favored (12.008%) beta sheet	-	-	-
A 305		SER	0.86	-	Favored (48.26%) General / -124.9,148.5	Favored (71.8%) *m* chi angles: 296	0.02Å	Favored (47.551%) beta sheet	-	-	-
A 306		PHE	0.81	-	Favored (37.13%) General / -78.9,142.1	Favored (39.5%) *m-80* chi angles: 283.9,113.8	0.08Å	Favored (46.399%)	-	-	-
A 307		VAL	0.79	-	Favored (12.07%) Ile or Val / -98.7,-48.3	Favored (87.1%) *t* chi angles: 177.5	0.10Å	Favored (9.425%)	-	-	-
A 308		LYS	0.78	-	Favored (46.4%) General / -132.2,135.6	Favored (88.6%) *mttt* chi angles: 298.2,179.6,185.4,170.2	0.03Å	Favored (21.293%)	-	-	-
A 309		ASN	0.81	-	Favored (91.19%) Pre-Pro / -72.9,153.1	Favored (89.1%) *m-40* chi angles: 288,328.4	0.04Å	Favored (27.659%)	-	-	-
A 310		PRO	0.85	-	Favored (36.3%) Trans-Pro / -49.9,136.8	Favored (57.1%) *Cg_exo* chi angles: 328.2,37.5,333	0.06Å	Favored (36.431%)	-	-	-
A 311		THR	0.92	-	Favored (46.83%) General / -137.9,150.1	Favored (11.2%) *t* chi angles: 186.8	0.05Å	Favored (45.394%) beta sheet	-	-	-
A 312		ASP	1.01	-	Favored (21.86%) General / -79.5,118.9	Favored (60.1%) *t0* chi angles: 187.1,336.9	0.07Å	Favored (40.533%)	-	-	-
A 313		THR	1.09	-	Favored (60.53%) General / -74.0,-12.1	Favored (70.6%) *p* chi angles: 62.3	0.11Å	Favored (5.008%)	-	-	-
A 314		GLY	1.14	-	Favored (56.38%)	-	-	Favored (31.259%)	-	-	-

#	Alt	Res	High B	Clash > 0.4Å	Ramachandran	Rotamer	Cβ deviation	CaBLAM	Bond lengths	Bond angles	Cis Peptides
					Glycine / 100.8,-17.4						
A 315		HIS	1.15	0.43Å CD2 with A 315 HIS O	Favored (40.28%) General / -99.7,11.0	Favored (55.4%) *t70* chi angles: 190.3,78.9	0.09Å	Favored (5.928%)	-	-	-
A 316		GLY	1.1	-	Favored (74.36%) Glycine / 88.4,-9.7	-	-	Favored (64.688%)	-	-	-
A 317		THR	1.03	-	Favored (21.97%) General / -82.5,162.6	Favored (62.6%) *p* chi angles: 63.5	0.02Å	Favored (44.592%)	-	-	-
A 318		ALA	0.95	-	Favored (55.25%) General / -113.6,133.5	-	0.04Å	Favored (59.137%) beta sheet	-	-	-
A 319		VAL	0.88	-	Favored (68.22%) Ile or Val / -120.2,133.0	Favored (91.3%) *t* chi angles: 174.3	0.04Å	Favored (56.647%) beta sheet	-	-	-
A 320		MET	0.84	-	Favored (42.98%) General / -139.5,159.1	Favored (13.9%) *ptt* chi angles: 61.2,186.8,171.3	0.07Å	Favored (45.587%) beta sheet	-	-	-
**#**	**Alt**	**Res**	**High B**	**Clash > 0.4Å**	**Ramachandran**	**Rotamer**	**Cβ deviation**	**CaBLAM**	**Bond lengths**	**Bond angles**	**Cis Peptides**
			Avg: 1.15	Clashscore: 0.81	Outliers: 1 of 491	Poor rotamers: 0 of 402	Outliers: 0 of 442	Outliers: 17 of 489	Outliers: 5 of 493	Outliers: 10 of 493	Non-Trans: 2 of 492
A 321		GLN	0.82	-	Favored (42.9%) General / -122.7,124.2	Favored (24%) *mt0* chi angles: 290.8,180.5,232.5	0.10Å	Favored (52.692%) beta sheet	-	-	-
A 322		VAL	0.83	-	Favored (67.39%) Ile or Val / -119.8,133.2	Favored (99.7%) *t* chi angles: 175.5	0.04Å	Favored (64.357%) beta sheet	-	-	-
A 323		LYS	0.87	-	Favored (38.53%) General / -94.5,133.1	Favored (83.5%) *tttt* chi angles: 179.8,180.7,174.5,182.5	0.03Å	Favored (56.81%) beta sheet	-	-	-
A 324		VAL	0.94	-	Favored (54.77%) Pre-Pro / -112.8,102.6	Favored (65.6%) *t* chi angles: 179.3	0.04Å	Favored (56.411%)	-	-	-
A 325		PRO	1.02	-	Favored (12.97%) Trans-Pro / -75.4,-23.6	Favored (70.5%) *Cg_endo* chi angles: 29.4,325.3,24.7	0.06Å	Favored (12.521%)	-	-	-
A 326		LYS	1.1	-	Allowed (1.68%) General / -124.9,-52.2	Favored (98.5%) *mttt* chi angles: 293.7,184.6,178.3,180.8	0.05Å	Favored (6.492%)	-	-	-
A 327		GLY	1.14	-	Favored (10.07%) Glycine / -81.2,131.0	-	-	Favored (7.304%)	-	-	-
A 328		ALA	1.13	-	Favored (5.09%) Pre-Pro / -166.0,145.3	-	0.04Å	Favored (5.699%)	-	-	-
A 329		PRO	1.06	-	Favored (34.29%) Cis-Pro / -58.6,147.0	Favored (70.7%) *Cg_exo* chi angles: 335.3,36.5,327.4	0.05Å	Favored (44.618%)	-	-	Cis PRO omega= -12.18
A 330		CYS	0.96	-	Favored (16.35%)	Favored (56.8%) *t* chi angles: 183.2	0.11Å	Favored (36.359%)	-	-	-

					General / -154.8,138.7						
A 331		ARG	0.85	-	Favored (34.49%) General / -81.2,141.1	Favored (84%) *mtp180* chi angles: 296.5,168.5,67,185.2	0.12Å	Favored (45.379%) beta sheet	-	-	-
A 332		ILE	0.77	-	Favored (52.32%) Pre-Pro / -116.1,99.0	Favored (70.1%) *mt* chi angles: 302.9,172.6	0.10Å	Favored (43.253%) beta sheet	-	-	-
A 333		PRO	0.71	-	Favored (84.57%) Trans-Pro / -58.3,138.2	Favored (86.2%) *Cg_exo* chi angles: 333.6,35.8,330	0.08Å	Favored (38.295%) beta sheet	-	-	-
A 334		VAL	0.67	-	Favored (53.22%) Ile or Val / -134.5,136.0	Favored (51.7%) *t* chi angles: 181.2	0.03Å	Favored (52.15%) beta sheet	-	-	-
A 335		MET	0.66	-	Favored (36.97%) General / -146.4,163.0	Favored (27.6%) *ptm* chi angles: 64.6,177.9,289.7	0.03Å	Favored (43.3%) beta sheet	-	-	-
A 336		VAL	0.66	-	Favored (65.84%) Ile or Val / -119.6,133.6	Favored (65.5%) *t* chi angles: 179.3	0.02Å	Favored (57.348%) beta sheet	-	-	-
A 337		ALA	0.68	-	Favored (43%) General / -130.2,157.2	-	0.04Å	Favored (56.801%)	-	-	-
A 338		ASP	0.72	-	Favored (58.44%) General / -80.9,-14.0	Favored (85.3%) *m-30* chi angles: 293.8,335.2	0.09Å	Favored (23.963%)	-	-	-
A 339		ASP	0.79	-	Favored (15.04%) General / -156.6,139.3	Favored (12.9%) *t70* chi angles: 189.8,293.5	0.04Å	Favored (11.491%)	-	-	-
A 340		LEU	0.88	-	Favored (59.93%) General / -58.8,-23.3	Favored (83.9%) *mt* chi angles: 291.6,175.7	0.01Å	Favored (22.037%) alpha helix	-	-	-
**#**	**Alt**	**Res**	**High B**	**Clash > 0.4Å**	**Ramachandran**	**Rotamer**	**Cβ deviation**	**CaBLAM**	**Bond lengths**	**Bond angles**	**Cis Peptides**
			Avg: 1.15	Clashscore: 0.81	Outliers: 1 of 491	Poor rotamers: 0 of 402	Outliers: 0 of 442	Outliers: 17 of 489	Outliers: 5 of 493	Outliers: 10 of 493	Non-Trans: 2 of 492
A 341		THR	0.96	-	Favored (32.93%) General / -104.1,3.2	Favored (73.6%) *p* chi angles: 61.6	0.04Å	Favored (50.779%)	-	-	-
A 342		ALA	1.03	-	Favored (80.41%) General / -61.1,-36.7	-	0.07Å	CaBLAM Disfavored (2.591%)	-	-	-
A 343		ALA	1.05	-	Favored (14.81%) General / 59.3,26.1	-	0.01Å	CaBLAM Disfavored (3.928%)	-	-	-
A 344		VAL	1.03	-	Favored (73.34%) Ile or Val / -124.0,127.0	Favored (86.8%) *t* chi angles: 177.4	0.02Å	Favored (21.159%) beta sheet	-	-	-
A 345		ASN	0.99	-	Favored (20.91%) General / -83.4,113.7	Favored (41.4%) *t0* chi angles: 188.7,3.1	0.04Å	Favored (50.004%) beta sheet	-	-	-
A 346		LYS	0.95	-	Favored (31.29%)	Favored (5.4%) *mmmm*	0.02Å	Favored (20.32%)	-	-	-

#	Alt	Res	High B	Clash > 0.4Å	Ramachandran	Rotamer	Cβ deviation	CaBLAM	Bond lengths	Bond angles	Cis Peptides
					General / -105.8,5.9	chi angles: 298.8,297.7,290,285.7					
A 347		GLY	0.92	-	Favored (14.32%) Glycine / -121.3,-172.1	-	-	Favored (13.587%)	-	-	-
A 348		ILE	0.92	-	Favored (72.97%) Ile or Val / -125.2,128.6	Favored (86.8%) *mt* chi angles: 298.7,170.8	0.04Å	Favored (21.781%)	-	-	-
A 349		LEU	0.93	-	Favored (32.75%) General / -82.0,142.1	Favored (81.7%) *mt* chi angles: 298.2,180.6	0.07Å	Favored (47.97%) beta sheet	-	-	-
A 350		VAL	0.95	-	Favored (15.08%) Ile or Val / -88.8,-43.3	Favored (84.3%) *t* chi angles: 173.5	0.04Å	Favored (7.153%)	-	-	-
A 351		THR	0.96	-	Favored (24.52%) General / -81.6,120.0	Favored (92.2%) *m* chi angles: 301.1	0.12Å	Favored (23.983%)	-	-	-
A 352		VAL	0.96	-	Favored (33.89%) Ile or Val / -78.4,122.6	Favored (97.8%) *t* chi angles: 175.2	0.03Å	CaBLAM Disfavored (4.189%)	-	-	-
A 353		ASN	0.96	-	Favored (4.37%) Pre-Pro / 44.4,67.0	Favored (65.5%) *t0* chi angles: 194.5,34.9	0.03Å	CaBLAM Outlier (0.878%)	-	-	-
A 354		PRO	0.98	-	Favored (78.36%) Trans-Pro / -55.1,138.2	Favored (77.6%) *Cg_exo* chi angles: 334.8,35.5,329.8	0.12Å	Favored (31.732%) beta sheet	-	-	-
A 355		ILE	1.01	-	Favored (36.9%) Ile or Val / -131.9,152.4	Favored (46%) *pt* chi angles: 60.9,172	0.06Å	Favored (65.037%) beta sheet	-	-	-
A 356		ALA	1.05	-	Favored (32.52%) General / -103.6,143.6	-	0.04Å	Favored (63.183%)	-	-	-
A 357		SER	1.1	-	Favored (14.97%) General / -104.3,-17.1	Favored (95%) *p* chi angles: 64.8	0.02Å	Favored (27.814%)	-	-	-
A 358		THR	1.14	-	Favored (44.17%) General / -151.3,159.7	Favored (9.7%) *t* chi angles: 185.6	0.05Å	Favored (14.928%)	-	-	-
A 359		ASN	1.16	-	Favored (56.84%) General / -59.0,134.3	Favored (7.2%) *m110* chi angles: 290.6,69.5	0.03Å	Favored (20.33%)	-	-	-
A 360		ASP	1.15	-	Favored (7.15%) General / 70.3,16.9	Favored (67.6%) *m-30* chi angles: 293.2,314.5	0.07Å	Favored (14.744%)	-	-	-
**#**	**Alt**	**Res**	**High B**	**Clash > 0.4Å**	**Ramachandran**	**Rotamer**	**Cβ deviation**	**CaBLAM**	**Bond lengths**	**Bond angles**	**Cis Peptides**
			Avg: 1.15	Clashscore: 0.81	Outliers: 1 of 491	Poor rotamers: 0 of 402	Outliers: 0 of 442	Outliers: 17 of 489	Outliers: 5 of 493	Outliers: 10 of 493	Non-Trans: 2 of 492
A 361		ASP	1.1	-	Favored (21.38%) General / -70.6,166.1	Favored (22.8%) *t0* chi angles: 200.9,354.3	0.10Å	Favored (26.336%)	-	-	-
A 362		GLU	1.03	-	Favored (52.57%)	Favored (92.3%) *mt-10*	0.03Å	Favored (58.177%) beta sheet	-	-	-

					General /<br>-125.0,135.2	chi angles:<br>299.2,182.2,0.2					
A<br>363		VAL	0.95	-	Favored<br>(75.56%)<br>Ile or Val /<br>-118.5,127.1	Favored (62.7%) *t*<br>chi angles: 179.6	0.08Å	Favored<br>(63.227%)<br>beta sheet	-	-	-
A<br>364		LEU	0.88	-	Favored<br>(30.41%)<br>General /<br>-87.7,121.7	Favored (6.4%) *tt*<br>chi angles: 188.6,154.7	0.01Å	Favored<br>(61.854%)<br>beta sheet	-	-	-
A<br>365		ILE	0.83	-	Favored<br>(74.71%)<br>Ile or Val /<br>-117.0,127.1	Favored (69.8%) *mt*<br>chi angles: 300.1,165.1	0.10Å	Favored<br>(71.106%)<br>beta sheet	-	-	-
A<br>366		GLU	0.79	-	Favored<br>(56.27%)<br>General /<br>-112.6,128.1	Favored (87.4%) *tt0*<br>chi angles:<br>183.7,173.6,7.3	0.07Å	Favored<br>(60.582%)<br>beta sheet	-	-	-
A<br>367		VAL	0.78	-	Favored<br>(43.29%)<br>Ile or Val /<br>-132.1,142.3	Favored (4.9%) *p*<br>chi angles: 56.9	0.08Å	Favored<br>(52.42%)<br>beta sheet	-	-	-
A<br>368		ASN	0.78	-	Favored<br>(22.12%)<br>Pre-Pro /<br>-108.6,97.2	Favored (43.9%) *t0*<br>chi angles: 187.5,3.4	0.03Å	Favored<br>(54.819%)	-	-	-
A<br>369		PRO	0.79	-	Favored<br>(36.6%)<br>Trans-Pro /<br>-72.6,164.1	Favored (72%)<br>*Cg_endo*<br>chi angles:<br>27.4,325.9,25.8	0.06Å	Favored<br>(32.135%)	-	-	-
A<br>370		PRO	0.79	-	Favored<br>(8.39%)<br>Trans-Pro /<br>-75.1,177.6	Favored (74.3%)<br>*Cg_endo*<br>chi angles:<br>30.9,324.4,25.8	0.03Å	Favored<br>(32.08%)	-	-	-
A<br>371		PHE	0.78	-	Favored<br>(34.99%)<br>General /<br>-77.9,147.4	Favored (91.5%) *m-80*<br>chi angles: 291.7,97.3	0.04Å	Favored<br>(10.782%)	-	OUTLIER(S)<br>worst is CA-CB-CG: 4.0 σ	-
A<br>372		GLY	0.76	-	Favored<br>(39.98%)<br>Glycine /<br>78.6,-154.4	-	-	Favored<br>(44.415%)	-	-	-
A<br>373		ASP	0.73	-	Favored<br>(32.9%)<br>General /<br>-75.2,127.3	Favored (35.8%) *m-30*<br>chi angles: 288.8,303.9	0.05Å	CaBLAM<br>Outlier<br>(0.714%)	-	-	-
A<br>374		SER	0.69	-	Favored<br>(28.98%)<br>General /<br>-153.0,167.5	Favored (92.4%) *p*<br>chi angles: 66.5	0.02Å	Favored<br>(28.486%)	-	-	-
A<br>375		TYR	0.67	-	Favored<br>(49.4%)<br>General /<br>-120.4,142.2	Favored (87.1%) *m-80*<br>chi angles: 300.1,88.4	0.06Å	Favored<br>(45.237%)<br>beta sheet	-	-	-
A<br>376		ILE	0.67	-	Favored<br>(58.29%)<br>Ile or Val /<br>-103.7,124.8	Favored (79.9%) *mt*<br>chi angles: 300,169.1	0.04Å	Favored<br>(66.808%)<br>beta sheet	-	-	-
A<br>377		ILE	0.7	-	Favored<br>(71.5%)<br>Ile or Val /<br>-118.5,131.4	Favored (73.9%) *mt*<br>chi angles: 301.4,169.7	0.04Å	Favored<br>(70.668%)	-	-	-
A<br>378		VAL	0.76	-	Favored<br>(65.94%)<br>Ile or Val /<br>-120.5,121.6	Favored (59.5%) *t*<br>chi angles: 180	0.05Å	Favored<br>(13.055%)	-	-	-
A<br>379		GLY	0.84	-	Favored<br>(46.3%)	-	-	Favored<br>(22.578%)	-	-	-

					Glycine / 89.2,173.8						
A 380		THR	0.92	-	Favored (36.41%) General / -129.6,159.4	Favored (59.1%) *p* chi angles: 64.2	0.03Å	CA Geom Outlier (0.465%)	-	-	-
**#**	**Alt**	**Res**	**High B**	**Clash > 0.4Å**	**Ramachandran**	**Rotamer**	**Cβ deviation**	**CaBLAM**	**Bond lengths**	**Bond angles**	**Cis Peptides**
			Avg: 1.15	Clashscore: 0.81	Outliers: 1 of 491	Poor rotamers: 0 of 402	Outliers: 0 of 442	Outliers: 17 of 489	Outliers: 5 of 493	Outliers: 10 of 493	Non-Trans: 2 of 492
A 381		GLY	0.98	-	Favored (53.57%) Glycine / 69.2,-157.0	-	-	Favored (40.566%)	-	-	-
A 382		ASP	0.99	-	Favored (51.27%) General / -61.2,145.4	Favored (90.8%) *m-30* chi angles: 289.6,351.2	0.01Å	CaBLAM Outlier (0.8%)	-	-	-
A 383		SER	0.96	-	Favored (6.57%) General / 70.7,8.4	Favored (27.7%) *m* chi angles: 303.5	0.04Å	Favored (7.998%)	-	-	-
A 384		ARG	0.89	-	Favored (43.9%) General / -63.7,150.6	Favored (42.8%) *ptt-90* chi angles: 69.7,191.7,187.8,283.5	0.03Å	Favored (28.311%)	-	-	-
A 385		LEU	0.81	-	Favored (28.91%) General / -86.3,141.9	Favored (95.3%) *mt* chi angles: 292.5,170.5	0.11Å	Favored (45.236%) beta sheet	OUTLIER(S) worst is CB--CG: 4.1 σ	-	-
A 386		THR	0.74	-	Favored (51.15%) General / -131.4,151.9	Favored (51.8%) *p* chi angles: 65.4	0.06Å	Favored (60.042%) beta sheet	-	-	-
A 387		TYR	0.69	-	Favored (18.67%) General / -150.1,136.2	Favored (78.7%) *t80* chi angles: 183.4,83.7	0.06Å	Favored (31.799%) beta sheet	-	-	-
A 388		GLN	0.69	-	Favored (31.87%) General / -86.0,123.9	Favored (61.8%) *tt0* chi angles: 183.3,174.1,343.4	0.07Å	Favored (53.011%) beta sheet	-	-	-
A 389		TRP	0.73	-	Favored (33.08%) General / -107.7,145.7	Favored (71.9%) *t-100* chi angles: 178.8,259.1	0.01Å	Favored (50.044%) beta sheet	-	-	-
A 390		HIS	0.82	-	Favored (10.58%) General / -137.2,114.1	Favored (9%) *t70* chi angles: 187.4,109.8	0.01Å	Favored (43.288%) beta sheet	-	-	-
A 391		LYS	0.97	-	Favored (32.84%) General / -91.8,120.8	Favored (29.5%) *ttmt* chi angles: 182,185.4,288.9,194.2	0.05Å	Favored (68.834%) beta sheet	-	-	-
A 392		GLU	1.19	-	Favored (52.86%) General / -85.5,0.3	Favored (97.8%) *mt-10* chi angles: 294.6,178.8,358.8	0.01Å	Favored (15.021%)	-	-	-
A 393		GLY	1.48	-	Favored (32.74%) Glycine / -92.8,-156.4	-	-	Favored (16.482%)	-	-	-
A 394		SER	1.82	-	Favored (44.49%) General / -149.3,159.2	Favored (98.7%) *p* chi angles: 65.3	0.05Å	Favored (18.363%)	-	-	-
A 395		SER	2.18	-	Favored (85.08%)	Favored (64.2%) *m* chi angles: 294.1	0.02Å	Favored (65.302%)	-	-	-

					General / -60.6,-38.6						
A 396		ILE	2.52	-	Favored (89.9%) Ile or Val / -65.8,-46.0	Favored (98.2%) *mt* chi angles: 293,168.2	0.03Å	Favored (95.033%) alpha helix	-	-	-
A 397		GLY	2.79	-	Favored (96.35%) Glycine / -60.4,-39.6	-	-	Favored (97.704%) alpha helix	-	-	-
A 398		LYS	2.94	-	Favored (64.66%) General / -61.0,-52.5	Favored (30.2%) *ttpt* chi angles: 178.8,185.9,70.8,169.9	0.06Å	Favored (81.685%) alpha helix	-	-	-
A 399		LEU	2.99	-	Favored (93.19%) General / -64.7,-39.2	Favored (98.5%) *mt* chi angles: 292.2,172.5	0.05Å	Favored (79.214%) alpha helix	-	-	-
A 400		PHE	2.93	-	Favored (77.9%) General / -61.9,-49.0	Favored (91.8%) *t80* chi angles: 177.9,76.5	0.05Å	Favored (84.456%) alpha helix	-	-	-
**#**	**Alt**	**Res**	**High B**	**Clash > 0.4Å**	**Ramachandran**	**Rotamer**	**Cβ deviation**	**CaBLAM**	**Bond lengths**	**Bond angles**	**Cis Peptides**
			Avg: 1.15	Clashscore: 0.81	Outliers: 1 of 491	Poor rotamers: 0 of 402	Outliers: 0 of 442	Outliers: 17 of 489	Outliers: 5 of 493	Outliers: 10 of 493	Non-Trans: 2 of 492
A 401		THR	2.8	-	Favored (85.54%) General / -57.9,-46.1	Favored (94.1%) *m* chi angles: 299.3	0.03Å	Favored (95.655%) alpha helix	-	-	-
A 402		GLN	2.61	-	Favored (88.84%) General / -62.6,-38.1	Favored (96.7%) *mt0* chi angles: 289.9,175.2,338.6	0.01Å	Favored (91.804%) alpha helix	-	-	-
A 403		THR	2.41	-	Favored (95.53%) General / -62.4,-44.8	Favored (96.7%) *m* chi angles: 299.8	0.03Å	Favored (93.629%) alpha helix	-	-	-
A 404		MET	2.23	-	Favored (79.2%) General / -67.5,-35.5	Favored (50.4%) *mmp* chi angles: 292.7,298.5,96.9	0.02Å	Favored (89.519%) alpha helix	-	-	-
A 405		LYS	2.08	-	Favored (76.44%) General / -69.4,-36.1	Favored (65.5%) *mmtt* chi angles: 293.1,286.9,177,175.7	0.03Å	Favored (95.253%) alpha helix	-	-	-
A 406		GLY	1.96	-	Favored (57.7%) Glycine / -59.9,-51.6	-	-	Favored (91.817%) alpha helix	-	-	-
A 407		ALA	1.87	-	Favored (79.06%) General / -58.2,-39.5	-	0.03Å	Favored (80.651%) alpha helix	-	-	-
A 408		GLU	1.79	-	Favored (95.63%) General / -64.5,-40.4	Favored (98.1%) *mt-10* chi angles: 290,179.1,354.4	0.03Å	Favored (97.45%) alpha helix	-	-	-
A 409		ARG	1.71	-	Favored (97.65%) General / -63.3,-43.5	Favored (40.9%) *ttm110* chi angles: 192.7,186.1,299.5,110.1	0.02Å	Favored (93.803%) alpha helix	-	-	-
A 410		LEU	1.65	-	Favored (94.72%) General / -63.0,-39.4	Favored (81%) *mt* chi angles: 288.9,167.8	0.07Å	Favored (88.689%) alpha helix	-	-	-

A 411		ALA	1.59	-	Favored (88.43%) General / -64.4,-37.7	-	0.08Å	Favored (53.184%) alpha helix	-	-	-
A 412		VAL	1.54	-	Favored (10.38%) Ile or Val / -102.6,-49.5	Favored (86.5%) *t* chi angles: 177.5	0.04Å	Favored (33.866%) alpha helix	-	-	-
A 413		MET	1.49	-	Favored (46.65%) General / -78.7,-33.7	Favored (27.8%) *ttt* chi angles: 187.3,176.4,188.1	0.03Å	Favored (11.442%)	-	-	-
A 414		GLY	1.45	-	Favored (20.71%) Glycine / 91.4,-150.9	-	-	Favored (26.858%)	-	-	-
A 415		ASP	1.42	-	Favored (19.38%) General / -47.0,-43.2	Favored (30.6%) *p0* chi angles: 54.8,355.3	0.02Å	Favored (17.523%)	-	-	-
A 416		ALA	1.38	-	Favored (47.97%) General / -59.4,-19.6	-	0.04Å	Favored (52.959%) three-ten	-	-	-
A 417		ALA	1.37	-	Favored (65.79%) General / -56.8,-32.5	-	0.11Å	Favored (55.428%) three-ten	-	-	-
A 418		TRP	1.39	-	Favored (66.34%) General / -65.8,-20.7	Favored (88.6%) *m100* chi angles: 283.9,101.2	0.01Å	Favored (61.398%) three-ten	-	-	-
A 419		ASP	1.46	-	Favored (67.7%) General / -61.7,-25.9	Favored (97.8%) *m-30* chi angles: 286.8,347.4	0.03Å	Favored (48.48%)	-	-	-
A 420		PHE	1.6	-	Favored (68.06%) General / -53.5,-49.2	Favored (85.7%) *t80* chi angles: 172.8,78.6	0.06Å	Favored (24.414%)	-	OUTLIER(S) worst is CA-CB-CG: 4.1 σ	-
**#**	**Alt**	**Res**	**High B**	**Clash > 0.4Å**	**Ramachandran**	**Rotamer**	**Cβ deviation**	**CaBLAM**	**Bond lengths**	**Bond angles**	**Cis Peptides**
			Avg: 1.15	Clashscore: 0.81	Outliers: 1 of 491	Poor rotamers: 0 of 402	Outliers: 0 of 442	Outliers: 17 of 489	Outliers: 5 of 493	Outliers: 10 of 493	Non-Trans: 2 of 492
A 421		GLY	1.81	-	Favored (9.89%) Glycine / -116.2,24.5	-	-	Favored (6.479%)	-	-	-
A 422		SER	2.09	-	Allowed (0.54%) General / -70.4,-66.9	Favored (38.4%) *t* chi angles: 174.8	0.09Å	CaBLAM Outlier (0.045%)	-	-	-
A 423		ALA	2.41	-	Favored (2.83%) General / 54.9,19.8	-	0.03Å	CA Geom Outlier (0.148%)	-	-	-
A 424		GLY	2.68	-	Favored (52.44%) Glycine / 82.9,178.7	-	-	CaBLAM Disfavored (4.488%)	-	-	-
A 425		GLY	2.81	-	Favored (12.72%) Glycine / 110.3,150.7	-	-	Favored (7.828%)	-	-	-
A 426		PHE	2.73	-	Favored (70.77%) General / -57.7,-51.1	Favored (92.2%) *t80* chi angles: 176.5,78.2	0.01Å	Favored (45.143%)	-	-	-

A 427		PHE	2.48	-	Favored (73.3%) General / -54.7,-47.7	Favored (87.5%) *t80* chi angles: 174.4,79.1	0.07Å	Favored (82.551%) alpha helix	-	-	-
A 428		THR	2.14	-	Favored (98.5%) General / -61.2,-42.4	Favored (96%) *m* chi angles: 299.6	0.02Å	Favored (96.015%) alpha helix	-	-	-
A 429		SER	1.81	-	Favored (92.8%) General / -60.8,-40.5	Favored (67.5%) *m* chi angles: 294.6	0.02Å	Favored (95.177%) alpha helix	-	-	-
A 430		VAL	1.54	-	Favored (97.93%) Ile or Val / -63.7,-43.2	Favored (63.2%) *t* chi angles: 171.2	0.02Å	Favored (87.183%) alpha helix	-	-	-
A 431		GLY	1.36	-	Favored (54.31%) Glycine / -55.1,-51.4	-	-	Favored (97.385%) alpha helix	-	-	-
A 432		LYS	1.26	-	Favored (89.83%) General / -61.5,-39.1	Favored (91.3%) *mttt* chi angles: 286.8,180.9,172.2,181.8	0.05Å	Favored (88.128%) alpha helix	-	-	-
A 433		GLY	1.23	-	Favored (40.45%) Glycine / -58.9,-54.1	-	-	Favored (90.831%) alpha helix	-	-	-
A 434		ILE	1.25	-	Favored (99.26%) Ile or Val / -62.4,-45.2	Favored (99.4%) *mt* chi angles: 292.7,167.7	0.03Å	Favored (76.604%) alpha helix	-	-	-
A 435		HIS	1.3	-	Favored (95.5%) General / -64.1,-43.4	Favored (72.6%) *t70* chi angles: 187.6,64.3	0.05Å	Favored (88.122%) alpha helix	-	-	-
A 436		THR	1.36	-	Favored (83.08%) General / -57.0,-44.7	Favored (89.7%) *m* chi angles: 298.2	0.02Å	Favored (85.87%) alpha helix	-	-	-
A 437		VAL	1.4	-	Favored (92.77%) Ile or Val / -65.0,-46.0	Favored (64.1%) *t* chi angles: 171.4	0.02Å	Favored (86.276%) alpha helix	-	-	-
A 438		PHE	1.43	-	Favored (97.93%) General / -62.2,-41.0	Favored (4.9%) *m-10* chi angles: 282.3,329.5	0.05Å	Favored (87.35%) alpha helix	-	-	-
A 439		GLY	1.45	-	Favored (53.73%) Glycine / -63.0,-51.7	-	-	Favored (92.143%) alpha helix	-	-	-
A 440		SER	1.46	-	Favored (98.89%) General / -61.0,-42.7	Favored (68.9%) *m* chi angles: 296.6	0.08Å	Favored (90.264%) alpha helix	-	-	-
**#**	**Alt**	**Res**	**High B**	**Clash > 0.4Å**	**Ramachandran**	**Rotamer**	**Cβ deviation**	**CaBLAM**	**Bond lengths**	**Bond angles**	**Cis Peptides**
			Avg: 1.15	Clashscore: 0.81	Outliers: 1 of 491	Poor rotamers: 0 of 402	Outliers: 0 of 442	Outliers: 17 of 489	Outliers: 5 of 493	Outliers: 10 of 493	Non-Trans: 2 of 492
A 441		ALA	1.46	-	Favored (98.06%) General / -62.7,-43.7	-	0.03Å	Favored (97.588%) alpha helix	-	-	-
A 442		PHE	1.46	-	Favored (80.12%)	Favored (90.7%) *t80* chi angles: 177.7,75.8	0.09Å	Favored (95.612%) alpha helix	-	OUTLIER(S) worst is CA-CB-CG: 6.3 σ	-

				General / -59.5,-48.8						
A 443	GLN	1.48	-	Favored (99.54%) General / -63.1,-42.2	Favored (97.2%) *mt0* chi angles: 290.8,172.8,344.3	0.04Å	Favored (92.321%) alpha helix	-	-	-
A 444	GLY	1.54	-	Favored (97.17%) Glycine / -64.1,-39.4	-	-	Favored (88.613%) alpha helix	-	-	-
A 445	LEU	1.63	-	Favored (31.93%) General / -80.9,-36.0	Favored (97.1%) *mt* chi angles: 292.8,171.6	0.07Å	Favored (49.465%)	-	-	-
A 446	PHE	1.74	-	Favored (3.91%) General / -113.7,-40.0	Favored (75.8%) *m-80* chi angles: 301.5,107.1	0.10Å	CaBLAM Outlier (0.68%)	-	OUTLIER(S) worst is CA-CB-CG: 4.6 σ	-
A 447	GLY	1.84	-	Favored (52.58%) Glycine / 57.6,-132.5	-	-	Favored (15.712%)	-	-	-
A 448	GLY	1.89	-	Favored (2.94%) Glycine / -75.6,56.6	-	-	CaBLAM Outlier (0.306%)	-	-	-
A 449	LEU	1.88	-	Favored (21.07%) General / -103.8,153.1	Favored (79.5%) *mt* chi angles: 301.6,176.3	0.04Å	Favored (22.11%)	-	-	-
A 450	SER	1.78	-	Favored (36.56%) General / -65.1,154.9	Favored (86%) *p* chi angles: 67.8	0.06Å	Favored (47.316%)	-	-	-
A 451	TRP	1.61	-	Favored (67.64%) General / -58.6,-31.1	Favored (67.5%) *p-90* chi angles: 69.9,267.5	0.02Å	Favored (65.207%)	-	-	-
A 452	ILE	1.4	-	Favored (89.61%) Ile or Val / -65.9,-46.0	Favored (96.1%) *mt* chi angles: 293.8,169.1	0.02Å	Favored (80.515%) alpha helix	-	-	-
A 453	THR	1.2	-	Favored (95.73%) General / -60.2,-43.7	Favored (97.8%) *m* chi angles: 300	0.06Å	Favored (97.261%) alpha helix	-	-	-
A 454	LYS	1.04	-	Favored (89.03%) General / -64.1,-37.9	Favored (88.3%) *mttt* chi angles: 290.4,171.3,172.8,173.6	0.08Å	Favored (89.616%) alpha helix	-	-	-
A 455	VAL	0.91	-	Favored (97.07%) Ile or Val / -64.4,-43.3	Favored (63.5%) *t* chi angles: 171.3	0.08Å	Favored (89.258%) alpha helix	-	-	-
A 456	ILE	0.81	-	Favored (93.81%) Ile or Val / -59.9,-46.5	Favored (92.9%) *mt* chi angles: 291.6,167.2	0.02Å	Favored (97.782%) alpha helix	-	-	-
A 457	MET	0.73	-	Favored (80.67%) General / -61.2,-36.7	Favored (78.6%) *mmm* chi angles: 284.1,299.1,299.6	0.03Å	Favored (86.76%) alpha helix	-	-	-
A 458	GLY	0.68	-	Favored (43.83%) Glycine / -57.4,-53.6	-	-	Favored (90.439%) alpha helix	-	-	-
A 459	VAL	0.64	-	Favored (95.75%)	Favored (62%) *t* chi angles: 171.1	0.06Å	Favored (77.502%) alpha helix	-	-	-

#	Alt	Res	High B	Clash > 0.4Å	Ramachandran	Rotamer	Cβ deviation	CaBLAM	Bond lengths	Bond angles	Cis Peptides
					Ile or Val / -61.5,-42.9						
A 460		VAL	0.61	-	Favored (99.37%) Ile or Val / -62.9,-44.1	Favored (57.4%) *t* chi angles: 170.4	0.03Å	Favored (96.894%) alpha helix	-	-	-
**#**	**Alt**	**Res**	**High B**	**Clash > 0.4Å**	**Ramachandran**	**Rotamer**	**Cβ deviation**	**CaBLAM**	**Bond lengths**	**Bond angles**	**Cis Peptides**
			Avg: 1.15	Clashscore: 0.81	Outliers: 1 of 491	Poor rotamers: 0 of 402	Outliers: 0 of 442	Outliers: 17 of 489	Outliers: 5 of 493	Outliers: 10 of 493	Non-Trans: 2 of 492
A 461		LEU	0.59	-	Favored (84.36%) General / -62.7,-36.9	Favored (98.3%) *mt* chi angles: 293,172.1	0.04Å	Favored (97.149%) alpha helix	-	-	-
A 462		ILE	0.57	-	Favored (95.76%) Ile or Val / -62.5,-46.7	Favored (99.3%) *mt* chi angles: 292.7,167.7	0.02Å	Favored (93.03%) alpha helix	-	-	-
A 463		TRP	0.56	-	Favored (95.03%) General / -60.6,-41.6	Favored (38.4%) *m-10* chi angles: 287.9,338.5	0.07Å	Favored (91.619%) alpha helix	-	-	-
A 464		VAL	0.58	-	Favored (97.82%) Ile or Val / -63.9,-43.6	Favored (71%) *t* chi angles: 172.2	0.02Å	Favored (98.872%) alpha helix	-	-	-
A 465		GLY	0.68	-	Favored (98.66%) Glycine / -60.3,-42.1	-	-	Favored (95.632%) alpha helix	-	-	-
A 466		ILE	0.93	-	Favored (79.89%) Ile or Val / -59.2,-40.0	Favored (87%) *mt* chi angles: 290.6,167.9	0.04Å	Favored (76.392%) alpha helix	-	-	-
A 467		ASN	1.5	-	Favored (38.05%) General / -90.3,7.5	Favored (86.2%) *m-40* chi angles: 290.6,321.6	0.04Å	Favored (40.381%)	-	-	-
A 468		THR	2.6	-	Favored (35.96%) General / -90.9,126.2	Favored (83.7%) *m* chi angles: 296.6	0.05Å	Favored (33.054%)	-	-	-
A 469		ARG	4.19	-	Favored (69.07%) General / -68.2,-30.1	Favored (98.4%) *mtt180* chi angles: 290.3,178,179.8,173.8	0.02Å	Favored (19.773%)	-	-	-
A 470		ASN	5.53	-	Favored (21.09%) General / -81.1,116.6	Favored (26.9%) *t0* chi angles: 182,281.3	0.04Å	Favored (32.609%)	-	-	-
A 471		MET	5.56	-	Favored (73.94%) General / -66.4,-32.5	Favored (52.2%) *mmp* chi angles: 293.3,300,97.2	0.08Å	Favored (53.728%)	-	-	-
A 472		THR	4.28	-	Favored (82.38%) General / -64.8,-46.3	Favored (89.6%) *m* chi angles: 298.2	0.01Å	Favored (75.883%) alpha helix	-	-	-
A 473		MET	2.72	-	Favored (76.68%) General / -68.9,-35.3	Favored (80.8%) *mtm* chi angles: 290.2,189.9,289.3	0.05Å	Favored (75.076%) alpha helix	-	-	-
A 474		SER	1.61	-	Favored (78.29%) General / -59.3,-49.3	Favored (46.3%) *t* chi angles: 179.8	0.07Å	Favored (80.034%) alpha helix	-	-	-
A 475		MET	1.03	-	Favored (86.63%)	Favored (82.8%) *mtm*	0.05Å	Favored (79.393%)	-	-	-

					General / -64.8,-37.1	chi angles: 289.2,186.6,285.4		alpha helix			
A 476		SER	0.78	-	Favored (84.08%) General / -66.3,-44.2	Favored (71.6%) *m* chi angles: 296	0.05Å	Favored (91.012%) alpha helix	-	-	-
A 477		MET	0.69	-	Favored (92.96%) General / -64.8,-39.1	Favored (91%) *mmm* chi angles: 289.4,295.5,288.6	0.04Å	Favored (89.989%) alpha helix	-	-	-
A 478		ILE	0.68	-	Favored (83.08%) Ile or Val / -68.4,-44.8	Favored (97.8%) *mt* chi angles: 293.2,168.3	0.09Å	Favored (89.538%) alpha helix	-	-	-
A 479		LEU	0.7	-	Favored (73.75%) General / -57.2,-50.2	Favored (68.2%) *tp* chi angles: 175.6,62.4	0.07Å	Favored (87.262%) alpha helix	-	-	-
A 480		VAL	0.73	-	Favored (97.85%) Ile or Val / -62.0,-43.6	Favored (68.9%) *t* chi angles: 172	0.02Å	Favored (86.741%) alpha helix	-	-	-
**#**	**Alt**	**Res**	**High B**	**Clash > 0.4Å**	**Ramachandran**	**Rotamer**	**Cβ deviation**	**CaBLAM**	**Bond lengths**	**Bond angles**	**Cis Peptides**
			Avg: 1.15	Clashscore: 0.81	Outliers: 1 of 491	Poor rotamers: 0 of 402	Outliers: 0 of 442	Outliers: 17 of 489	Outliers: 5 of 493	Outliers: 10 of 493	Non-Trans: 2 of 492
A 481		GLY	0.76	-	Favored (66.59%) Glycine / -56.5,-49.8	-	-	Favored (98.781%) alpha helix	-	-	-
A 482		VAL	0.8	-	Favored (90.64%) Ile or Val / -61.3,-41.7	Favored (58.8%) *t* chi angles: 170.6	0.02Å	Favored (88.216%) alpha helix	-	-	-
A 483		ILE	0.85	-	Favored (96.29%) Ile or Val / -61.9,-46.5	Favored (90.5%) *mt* chi angles: 291.1,167.7	0.03Å	Favored (87.238%) alpha helix	-	-	-
A 484		MET	0.9	-	Favored (98.11%) General / -62.0,-41.4	Favored (91%) *mtp* chi angles: 292,183.8,69.8	0.10Å	Favored (91.62%) alpha helix	-	-	-
A 485		MET	0.97	-	Favored (69.99%) General / -60.2,-51.3	Favored (55.6%) *ttp* chi angles: 179,188.8,71.6	0.07Å	Favored (88.343%) alpha helix	-	-	-
A 486		PHE	1.05	-	Favored (95.89%) General / -63.7,-40.1	Favored (3.5%) *m-10* chi angles: 279.1,325.3	0.02Å	Favored (82.047%) alpha helix	-	-	-
A 487		LEU	1.17	-	Favored (96.96%) General / -64.3,-41.2	Favored (94.7%) *mt* chi angles: 292.1,173.5	0.05Å	Favored (91.689%) alpha helix	-	-	-
A 488		SER	1.32	-	Favored (78.48%) General / -68.8,-41.7	Favored (61.9%) *m* chi angles: 298.5	0.10Å	Favored (90.63%) alpha helix	-	-	-
A 489		LEU	1.56	-	Favored (86.04%) General / -65.8,-37.3	Favored (92%) *mt* chi angles: 291.3,171.7	0.02Å	Favored (88.31%) alpha helix	-	-	-
A 490		GLY	1.89	-	Favored (99.31%) Glycine / -63.2,-40.7	-	-	Favored (94.971%) alpha helix	-	-	-

A 491		VAL	2.33	-	Favored (86.14%) Ile or Val / -67.6,-41.8	Favored (66%) *t* chi angles: 171.6	0.07Å	Favored (36.432%)	-	-	-
A 492		GLY	2.88	-	Favored (2.92%) Glycine / -75.6,59.7	-	-	-	-	-	-
A 493		ALA	3.5	-	-	-	0.04Å	-	-	-	-

About MolProbity | Website for the Richardson Lab | Using ecloud x-H | Internal reference 4.5.2
