# Supplementary material for: Integrative proteome-wide structural analysis and high-throughput docking identify broad-spectrum antiviral scaffolds against Zika, Yellow Fever, West Nile, Saint Louis encephalitis, and Usutu viruses
Source: Front Cell Infect Microbiol. 2026 Apr 30;16:1723132. doi: 10.3389/fcimb.2026.1723132 (PMC13171538; doi:10.3389/fcimb.2026.1723132)
Supplement: Supplementary file 6 [file DataSheet6.zip › YFV/YF_M/Mol_probity_Files/YF_M_1FH-multi.table.pdf]

Viewing YF_M1FH-multi.table

Duke Biochemistry
Duke University School of Medicine

When finished, you should close this window.

Hint: Use File | Save As... to save a copy of this page.

All-Atom Contacts	Clashscore, all atoms:	1.65		99th percentile* (N=1784, all resolutions)
	Clashscore is the number of serious steric overlaps (> 0.4 Å) per 1000 atoms.			
Protein Geometry	Poor rotamers	0	0.00%	Goal: <0.3%
	Favored rotamers	61	100.00%	Goal: >98%
	Ramachandran outliers	1	1.37%	Goal: <0.05%
	Ramachandran favored	68	93.15%	Goal: >98%
	Rama distribution Z-score	1.02 ± 0.93		Goal: abs(Z score) < 2
	MolProbity score^	1.36		98th percentile* (N=27675, 0Å - 99Å)
	Cβ deviations >0.25Å	0	0.00%	Goal: 0
	Bad bonds:	1 / 607	0.16%	Goal: 0%
	Bad angles:	6 / 823	0.73%	Goal: <0.1%
Peptide Omegas	Cis Prolines:	0 / 3	0.00%	Expected: ≤1 per chain, or ≤5%
Low-resolution Criteria	CaBLAM outliers	2	2.8%	Goal: <1.0%
	CA Geometry outliers	2	2.82%	Goal: <0.5%
Additional validations	Chiral volume outliers	0/94		
	Waters with clashes	0/0	0.00%	See UnDowser table for details

In the two column results, the left column gives the raw count, right column gives the percentage.

* 100th percentile is the best among structures of comparable resolution; 0th percentile is the worst. For clashscore the comparative set of structures was selected in 2004, for MolProbity score in 2006.

^ MolProbity score combines the clashscore, rotamer, and Ramachandran evaluations into a single score, normalized to be on the same scale as X-ray resolution.

Key to table colors and cutoffs here:

#	Alt	Res	High B	Clash > 0.4Å	Ramachandran	Rotamer	Cβ deviation	CaBLAM	Bond lengths	Bond angles	Cis Peptides
			Avg: 2.95	Clashscore: 1.65	Outliers: 1 of 73	Poor rotamers: 0 of 61	Outliers: 0 of 70	Outliers: 3 of 71	Outliers: 1 of 75	Outliers: 2 of 75	Non-Trans: 0 of 74
A 1		ALA	5.53	-	-	-	0.03Å	-	-	-	-
A 2		ILE	5.45	-	Favored (74.58%) Ile or Val / -118.1,129.4	Favored (82.8%) *mt* chi angles: 299.5,170.3	0.03Å	-	-	-	-
A 3		ASP	5.28	-	Favored (6.05%) General / -79.4,74.1	Favored (41.4%) *t0* chi angles: 190.3,19.2	0.01Å	Favored (24.156%)	-	-	-
A 4		LEU	5.05	-	Favored (16.03%) Pre-Pro / -59.4,-33.3	Favored (93%) *mt* chi angles: 291.8,173.5	0.03Å	CA Geom Outlier (0.038%)	-	-	-
A 5		PRO	4.83	-	OUTLIER (0)%) Trans-Pro / 69.3,157.4	Favored (6.1%) *Cg_endo* chi angles: 40,331.5,10.7	0.20Å	CaBLAM Outlier (0.343%) try beta sheet	OUTLIER(S) worst is N--CD: 9.6 σ	OUTLIER(S) worst is CA-N-CD: 14.7 σ	-
A 6		THR	4.7	-	Favored (26.05%) General / -73.2,164.9	Favored (71.3%) *p* chi angles: 62.1	0.04Å	Favored (47.889%)	-	-	-
A 7		HIS	4.71	-	Favored (68.82%)	Favored (90.9%) *t70*	0.04Å	Favored (64.817%)	-	-	-

					General / -53.2,-47.6	chi angles: 176.6,73.9					
A 8		GLU	4.9	0.40Å CD with A 8 GLU H	Favored (64.8%) General / -61.3,-22.3	Favored (55%) *mp0* chi angles: 298.2,81.2,2.3	0.09Å	Favored (51.836%) alpha helix	-	-	-
A 9		ASN	5.27	-	Favored (16.21%) General / -116.0,15.6	Favored (67.6%) *m-40* chi angles: 290.6,279.5	0.07Å	Favored (32.382%)	-	-	-
A 10		HIS	5.8	-	Allowed (0.76%) General / -86.9,-162.2	Favored (40.2%) *t-90* chi angles: 201.1,284.1	0.05Å	CaBLAM Disfavored (1.826%)	-	-	-
A 11		GLY	6.4	-	Favored (7.34%) Glycine / -77.8,16.0	-	-	CaBLAM Disfavored (1.305%)	-	-	-
A 12		LEU	6.95	-	Favored (41.57%) General / -58.5,129.3	Favored (56.2%) *tp* chi angles: 177.4,65.8	0.08Å	Favored (12.279%)	-	-	-
A 13		LYS	7.29	-	Favored (6.02%) General / -123.0,-19.6	Favored (97.6%) *mttt* chi angles: 296.4,180.8,180.7,178.6	0.02Å	Favored (10.478%)	-	-	-
A 14		THR	7.31	-	Favored (14.13%) General / -117.3,19.3	Favored (64.1%) *p* chi angles: 58	0.05Å	Favored (31.677%)	-	-	-
A 15		ARG	7.02	-	Favored (48.11%) General / -125.6,149.8	Favored (85.3%) *mtp85* chi angles: 296.2,177.1,70.6,85	0.05Å	Favored (28.187%)	-	-	-
A 16		GLN	6.53	-	Favored (58.39%) General / -70.8,-9.8	Favored (20.5%) *pm20* chi angles: 70.2,275.6,31	0.01Å	Favored (27.488%)	-	-	-
A 17		GLU	5.96	-	Favored (58.67%) General / -86.7,-2.6	Favored (69.5%) *mt-10* chi angles: 293.5,181.4,314.3	0.02Å	Favored (16.306%)	-	-	-
A 18		LYS	5.42	-	Favored (42.5%) General / -57.5,142.9	Favored (47.4%) *tttp* chi angles: 188.3,177.9,178,68.4	0.06Å	Favored (8.595%)	-	-	-
A 19		TRP	4.96	-	Allowed (0.15%) General / 81.2,-64.6	Favored (77.7%) *m100* chi angles: 300.9,111.6	0.08Å	CA Geom Outlier (0.298%)	-	-	-
A 20		MET	4.59	-	Favored (14.49%) General / -160.1,144.4	Favored (30.3%) *ttt* chi angles: 189,173.8,180.8	0.07Å	Favored (16.707%)	-	-	-
**#**	**Alt**	**Res**	**High B**	**Clash > 0.4Å**	**Ramachandran**	**Rotamer**	**Cβ deviation**	**CaBLAM**	**Bond lengths**	**Bond angles**	**Cis Peptides**
			Avg: 2.95	Clashscore: 1.65	Outliers: 1 of 73	Poor rotamers: 0 of 61	Outliers: 0 of 70	Outliers: 3 of 71	Outliers: 1 of 75	Outliers: 2 of 75	Non-Trans: 0 of 74
A 21		THR	4.28	-	Favored (13.73%) General / -119.0,165.8	Favored (47.1%) *p* chi angles: 66.2	0.03Å	Favored (37.522%)	-	-	-
A 22		GLY	3.99	-	Favored (63.13%) Glycine / -56.6,-33.7	-	-	Favored (61.468%)	-	-	-

A 23		ARG	3.72	-	Favored (77.19%) General / -65.5,-34.0	Favored (98.5%) *mtt180* chi angles: 289.5,178.4,179,174.7	0.02Å	Favored (74.614%) alpha helix	-	-	-
A 24		MET	3.43	-	Favored (78.62%) General / -69.2,-39.7	Favored (98.2%) *mtp* chi angles: 292.1,170.9,67.9	0.05Å	Favored (78.269%) alpha helix	-	-	-
A 25		GLY	3.12	-	Favored (42.7%) Glycine / -57.3,-53.7	-	-	Favored (94.314%) alpha helix	-	-	-
A 26		GLU	2.83	-	Favored (96.07%) General / -61.2,-41.0	Favored (72.9%) *tp30* chi angles: 179.7,67.3,20.7	0.02Å	Favored (83.229%) alpha helix	-	-	-
A 27		ARG	2.56	-	Favored (92.66%) General / -63.8,-38.9	Favored (96%) *mtt180* chi angles: 287.9,171.8,180,168.8	0.03Å	Favored (93.366%) alpha helix	-	-	-
A 28		GLN	2.31	-	Favored (93.85%) General / -65.2,-40.2	Favored (91.5%) *mt0* chi angles: 290.8,173.7,354	0.02Å	Favored (98.942%) alpha helix	-	-	-
A 29		LEU	2.09	-	Favored (95.85%) General / -64.8,-40.9	Favored (91.7%) *mt* chi angles: 291.1,172.1	0.03Å	Favored (99.324%) alpha helix	-	-	-
A 30		GLN	1.91	-	Favored (87.49%) General / -65.6,-37.7	Favored (76.3%) *mt0* chi angles: 289.8,180.3,20.5	0.06Å	Favored (85.505%) alpha helix	-	-	-
A 31		LYS	1.75	-	Favored (84.78%) General / -57.5,-45.4	Favored (87.6%) *tttt* chi angles: 180.9,175.6,177.3,175.5	0.03Å	Favored (82.666%) alpha helix	-	-	-
A 32		ILE	1.62	-	Favored (84.34%) Ile or Val / -67.3,-46.2	Favored (97.7%) *mt* chi angles: 293.8,167.5	0.03Å	Favored (85.379%) alpha helix	-	-	-
A 33		GLU	1.52	-	Favored (94.31%) General / -63.0,-39.3	Favored (97.5%) *mt-10* chi angles: 289.3,179,353.9	0.02Å	Favored (98.283%) alpha helix	-	-	-
A 34		ARG	1.46	-	Favored (99.45%) General / -63.1,-42.1	Favored (94.9%) *mtt180* chi angles: 289.5,171.7,185.5,170.1	0.04Å	Favored (96.764%) alpha helix	-	-	-
A 35		TRP	1.41	-	Favored (69.05%) General / -55.6,-50.8	Favored (48.7%) *t-100* chi angles: 169.8,259.7	0.05Å	Favored (97.324%) alpha helix	-	-	-
A 36		LEU	1.37	-	Favored (81.05%) General / -62.2,-36.1	Favored (97.7%) *mt* chi angles: 292.5,173.3	0.05Å	Favored (73.953%) alpha helix	-	-	-
A 37		VAL	1.32	-	Favored (64.39%) Ile or Val / -72.2,-44.3	Favored (75.4%) *t* chi angles: 172.7	0.03Å	Favored (75.751%) alpha helix	-	-	-
A 38		ARG	1.27	-	Favored (73.06%) General / -69.4,-33.5	Favored (96.3%) *mtt180* chi angles: 290.4,172.4,181.4,168.5	0.03Å	Favored (59.247%)	-	-	-
A 39		ASN	1.2	-	Favored (66.91%) Pre-Pro / -131.6,62.9	Favored (50.7%) *m-40* chi angles: 300.2,278.5	0.09Å	Favored (23.382%)	-	-	-

#	Alt	Res	High B	Clash > 0.4Å	Ramachandran	Rotamer	Cβ deviation	CaBLAM	Bond lengths	Bond angles	Cis Peptides
A 40		PRO	1.13	-	Favored (64.6%) Trans-Pro / -56.6,-26.5	Favored (61.8%) *Cg_exo* chi angles: 335.8,35.8,327.8	0.08Å	Favored (56.081%)	-	-	-
**#**	**Alt**	**Res**	**High B**	**Clash > 0.4Å**	**Ramachandran**	**Rotamer**	**Cβ deviation**	**CaBLAM**	**Bond lengths**	**Bond angles**	**Cis Peptides**
			Avg: 2.95	Clashscore: 1.65	Outliers: 1 of 73	Poor rotamers: 0 of 61	Outliers: 0 of 70	Outliers: 3 of 71	Outliers: 1 of 75	Outliers: 2 of 75	Non-Trans: 0 of 74
A 41		PHE	1.06	-	Favored (67.63%) General / -64.0,-24.2	Favored (54.8%) *p90* chi angles: 67.2,90.4	0.03Å	Favored (67.108%) alpha helix	-	-	-
A 42		PHE	0.99	-	Favored (68.44%) General / -71.3,-42.5	Favored (42.4%) *t80* chi angles: 191.3,74	0.06Å	Favored (73.663%) alpha helix	-	-	-
A 43		ALA	0.93	-	Favored (95.46%) General / -61.6,-40.6	-	0.03Å	Favored (98.482%) alpha helix	-	-	-
A 44		ALA	0.88	-	Favored (95.57%) General / -61.9,-40.5	-	0.03Å	Favored (96.579%) alpha helix	-	-	-
A 45		THR	0.84	-	Favored (84.83%) General / -66.1,-44.3	Favored (94%) *m* chi angles: 299.3	0.02Å	Favored (85.883%) alpha helix	-	-	-
A 46		ALA	0.8	-	Favored (83.8%) General / -59.0,-40.1	-	0.04Å	Favored (81.052%) alpha helix	-	-	-
A 47		LEU	0.77	-	Favored (84.01%) General / -67.3,-42.2	Favored (83.1%) *mt* chi angles: 289.6,170.3	0.02Å	Favored (86.768%) alpha helix	-	-	-
A 48		ALA	0.74	-	Favored (92.97%) General / -60.8,-40.5	-	0.03Å	Favored (94.276%) alpha helix	-	-	-
A 49		ILE	0.72	-	Favored (91.86%) Ile or Val / -63.0,-47.6	Favored (96.2%) *mt* chi angles: 292.3,166.7	0.05Å	Favored (88.13%) alpha helix	-	-	-
A 50		ALA	0.73	-	Favored (78.46%) General / -58.9,-38.3	-	0.07Å	Favored (80.964%) alpha helix	-	-	-
A 51		TYR	0.84	-	Favored (65.04%) General / -70.3,-28.3	Favored (42.3%) *m-80* chi angles: 283.9,111.6	0.05Å	Favored (76.022%) alpha helix	-	-	-
A 52		LEU	1.12	-	Favored (35.3%) General / -81.8,-25.4	Favored (96%) *mt* chi angles: 295,172.7	0.03Å	Favored (63.917%) alpha helix	-	-	-
A 53		VAL	1.75	-	Favored (10.85%) Ile or Val / -102.1,-49.1	Favored (94.4%) *t* chi angles: 175.8	0.02Å	Favored (11.455%)	-	-	-
A 54		GLY	2.92	-	Favored (43.78%) Glycine / -69.4,146.3	-	-	Favored (17.249%)	-	-	-
A 55		SER	4.57	-	Favored (34.41%)	Favored (96%) *p* chi angles: 63.8	0.03Å	Favored (18.472%)	-	-	-

#	Alt	Res	High B	Clash > 0.4Å	Ramachandran	Rotamer	Cβ deviation	CaBLAM	Bond lengths	Bond angles	Cis Peptides
					General / -89.2,-15.1						
A 56		ASN	5.93	-	Favored (15.87%) General / -153.1,173.1	Favored (28.1%) *p0* chi angles: 60.6,57.3	0.05Å	Favored (22.487%)	-	-	-
A 57		MET	5.97	-	Favored (68.07%) General / -69.3,-30.1	Favored (86.9%) *mmm* chi angles: 290.7,308.6,299.6	0.10Å	Favored (56.664%) alpha helix	-	-	-
A 58		THR	4.67	-	Favored (76.45%) General / -65.2,-47.5	Favored (91.1%) *m* chi angles: 298	0.01Å	Favored (77.614%) alpha helix	-	-	-
A 59		GLN	3.03	-	Favored (97.16%) General / -61.8,-41.0	Favored (50.1%) *tt0* chi angles: 183.7,170.6,51.7	0.03Å	Favored (81.868%) alpha helix	-	-	-
A 60		ARG	1.85	-	Favored (93.56%) General / -63.5,-39.1	Favored (96.8%) *mtt180* chi angles: 289.2,171,178.6,173.9	0.05Å	Favored (83.038%) alpha helix	-	-	-
**#**	**Alt**	**Res**	**High B**	**Clash > 0.4Å**	**Ramachandran**	**Rotamer**	**Cβ deviation**	**CaBLAM**	**Bond lengths**	**Bond angles**	**Cis Peptides**
			Avg: 2.95	Clashscore: 1.65	Outliers: 1 of 73	Poor rotamers: 0 of 61	Outliers: 0 of 70	Outliers: 3 of 71	Outliers: 1 of 75	Outliers: 2 of 75	Non-Trans: 0 of 74
A 61		VAL	1.21	-	Favored (90.44%) Ile or Val / -66.4,-44.9	Favored (64.9%) *t* chi angles: 171.5	0.02Å	Favored (85.133%) alpha helix	-	-	-
A 62		VAL	0.91	-	Favored (97.55%) Ile or Val / -61.5,-43.8	Favored (60.2%) *t* chi angles: 170.8	0.03Å	Favored (97.613%) alpha helix	-	-	-
A 63		ILE	0.8	-	Favored (95.61%) Ile or Val / -61.3,-46.9	Favored (92.8%) *mt* chi angles: 291.8,166.7	0.05Å	Favored (97.779%) alpha helix	-	-	-
A 64		ALA	0.79	-	Favored (89.73%) General / -59.5,-41.5	-	0.05Å	Favored (96.831%) alpha helix	-	-	-
A 65		LEU	0.81	-	Favored (91.23%) General / -65.6,-39.3	Favored (92.4%) *mt* chi angles: 291.2,172.6	0.01Å	Favored (94.818%) alpha helix	-	-	-
A 66		LEU	0.84	-	Favored (93.95%) General / -64.7,-39.6	Favored (90.6%) *mt* chi angles: 291,171.6	0.02Å	Favored (98.394%) alpha helix	-	-	-
A 67		VAL	0.88	-	Favored (98.81%) Ile or Val / -63.4,-44.1	Favored (60.7%) *t* chi angles: 170.9	0.03Å	Favored (97.647%) alpha helix	-	-	-
A 68		LEU	0.95	0.42Å HA with A 68 LEU HD23	Favored (80.91%) General / -66.9,-35.9	Favored (91.6%) *mt* chi angles: 294.6,177.1	0.08Å	Favored (81.099%) alpha helix	-	-	-
A 69		ALA	1.05	-	Favored (82.39%) General / -61.7,-36.9	-	0.02Å	Favored (71.811%) alpha helix	-	-	-
A 70		VAL	1.21	-	Favored (8.82%) Ile or Val / -91.1,-14.1	Favored (22.2%) *m* chi angles: 301.6	0.03Å	Favored (40.154%) alpha helix	-	-	-

A 71		GLY	1.47	-	Allowed (0.71%) Glycine / -37.0,-64.0	-	-	Favored (30.742%) alpha helix	-	-	-
A 72		PRO	1.84	-	Favored (4.02%) Trans-Pro / -83.1,9.6	Favored (35.5%) *Cg_endo* chi angles: 34.4,323.8,22.7	0.04Å	CaBLAM Disfavored (1.054%)	-	-	-
A 73		ALA	2.35	-	Allowed (1.52%) General / -141.2,58.9	-	0.04Å	Favored (10.553%)	-	-	-
A 74		TYR	2.98	-	Favored (64.76%) General / -69.1,-26.1	Favored (57.7%) *m-80* chi angles: 289.3,112.1	0.04Å	-	-	-	-
A 75		SER	3.68	-	-	Favored (74.9%) *p* chi angles: 71.3	0.11Å	-	-	OUTLIER(S) worst is C-N-CA: 4.3 σ	-

About MolProbity | Website for the Richardson Lab | Using ecloud x-H | Internal reference 4.5.2
