# Supplementary material for: Integrative proteome-wide structural analysis and high-throughput docking identify broad-spectrum antiviral scaffolds against Zika, Yellow Fever, West Nile, Saint Louis encephalitis, and Usutu viruses
Source: Front Cell Infect Microbiol. 2026 Apr 30;16:1723132. doi: 10.3389/fcimb.2026.1723132 (PMC13171538; doi:10.3389/fcimb.2026.1723132)
Supplement: Supplementary file 6 [file DataSheet6.zip › YFV/YF_NS1/Mol_probity_Files/YF_NS1_1FH-multi.table.pdf]

Viewing YF_NS1_1FH-multi.table

Duke Biochemistry
Duke University School of Medicine

When finished, you should close this window.

Hint: Use File | Save As... to save a copy of this page.

All-Atom Contacts	Clashscore, all atoms:	0.37		99th percentile* (N=1784, all resolutions)
	Clashscore is the number of serious steric overlaps (> 0.4 Å) per 1000 atoms.			
Protein Geometry	Poor rotamers	0	0.00%	Goal: <0.3%
	Favored rotamers	306	99.67%	Goal: >98%
	Ramachandran outliers	0	0.00%	Goal: <0.05%
	Ramachandran favored	340	97.14%	Goal: >98%
	Rama distribution Z-score	0.54 ± 0.47		Goal: abs(Z score) < 2
	MolProbity score^	0.79		100th percentile* (N=27675, 0Å - 99Å)
	Cβ deviations >0.25Å	0	0.00%	Goal: 0
	Bad bonds:	2 / 2855	0.07%	Goal: 0%
	Bad angles:	6 / 3867	0.16%	Goal: <0.1%
Peptide Omegas	Cis Prolines:	2 / 18	11.11%	Expected: ≤1 per chain, or ≤5%
	Twisted Peptides:	1 / 351	0.28%	Goal: 0
Low-resolution Criteria	CaBLAM outliers	7	2.0%	Goal: <1.0%
	CA Geometry outliers	1	0.29%	Goal: <0.5%
Additional validations	Chiral volume outliers	0/398		
	Waters with clashes	0/0	0.00%	See UnDowser table for details

In the two column results, the left column gives the raw count, right column gives the percentage.

* 100th percentile is the best among structures of comparable resolution; 0th percentile is the worst. For clashscore the comparative set of structures was selected in 2004, for MolProbity score in 2006.

^ MolProbity score combines the clashscore, rotamer, and Ramachandran evaluations into a single score, normalized to be on the same scale as X-ray resolution.

Key to table colors and cutoffs here:

#	Alt	Res	High B	Clash > 0.4Å	Ramachandran	Rotamer	Cβ deviation	CaBLAM	Bond lengths	Bond angles	Cis Peptides
			Avg: 1.21	Clashscore: 0.37	Outliers: 0 of 350	Poor rotamers: 0 of 307	Outliers: 0 of 318	Outliers: 8 of 348	Outliers: 2 of 352	Outliers: 6 of 352	Non-Trans: 3 of 351
A 1		ASP	2.79	-	-	Favored (23.5%) *t0* chi angles: 202.2,356.4	0.05Å	-	-	-	-
A 2		GLN	2.75	-	Favored (9.04%) General / -158.8,134.9	Favored (37.4%) *tt0* chi angles: 185.2,174.4,76.9	0.03Å	-	-	-	-
A 3		GLY	2.76	-	Favored (34.91%) Glycine / 160.5,-165.2	-	-	Favored (27.783%)	-	-	-
A 4		CYS	2.87	-	Favored (44.72%) General / -130.3,156.4	Favored (80.9%) *m* chi angles: 295.9	0.04Å	Favored (9.789%) beta sheet	-	-	-
A 5		ALA	3.07	-	Favored (34.5%) General / -153.2,154.3	-	0.03Å	Favored (50.25%) beta sheet	-	-	-
A 6		ILE	3.37	-	Favored (72.14%)	Favored (87.3%) *mt* chi angles: 298.5,170.3	0.04Å	Favored (58.405%) beta sheet	-	-	-

#	Alt	Res	High B	Clash > 0.4Å	Ramachandran	Rotamer	Cβ deviation	CaBLAM	Bond lengths	Bond angles	Cis Peptides
					Ile or Val / -115.9,129.5						
A 7		ASN	3.74	-	Favored (13.75%) General / -95.2,102.0	Favored (56.5%) *t0* chi angles: 184.3,335.7	0.04Å	Favored (68.239%) beta sheet	-	-	-
A 8		PHE	4.1	-	Favored (47.08%) General / -78.0,-4.5	Favored (75.2%) *m-80* chi angles: 288.7,99.2	0.04Å	Favored (24.209%)	-	-	-
A 9		GLY	4.34	-	Favored (80.02%) Glycine / -85.8,-8.2	-	-	Favored (43.155%)	-	-	-
A 10		LYS	4.38	-	Favored (3.71%) General / -126.7,-23.6	Favored (51.2%) *mtpt* chi angles: 296.7,177.3,68.6,177.8	0.03Å	CaBLAM Disfavored (2.016%)	-	-	-
A 11		ARG	4.17	-	Favored (3.13%) General / 64.6,46.6	Favored (94.9%) *mtt180* chi angles: 299.9,183.2,180.9,176	0.03Å	Favored (16.065%)	-	-	-
A 12		GLU	3.77	-	Favored (46.51%) General / -118.5,143.8	Favored (94.2%) *mt-10* chi angles: 294,184.9,357.9	0.05Å	Favored (30.741%) beta sheet	-	-	-
A 13		LEU	3.28	-	Favored (44.04%) General / -99.4,123.8	Favored (51.8%) *tp* chi angles: 175.8,65.9	0.03Å	Favored (62.575%) beta sheet	-	-	-
A 14		LYS	2.81	-	Favored (43.81%) General / -110.0,120.9	Favored (84.1%) *tttt* chi angles: 179.5,180.6,177.1,180.1	0.06Å	Favored (59.935%) beta sheet	-	-	-
A 15		CYS	2.43	-	Favored (51.56%) General / -129.8,145.0	Favored (82%) *m* chi angles: 294.3	0.03Å	Favored (6.172%) beta sheet	-	-	-
A 16		GLY	2.14	-	Favored (29.6%) Glycine / 155.3,-160.1	-	-	Favored (40.272%)	-	-	-
A 17		ASP	1.93	-	Favored (26.25%) General / -100.3,15.9	Favored (79.4%) *m-30* chi angles: 296.1,343.6	0.02Å	CaBLAM Disfavored (1.084%)	-	-	-
A 18		GLY	1.78	-	Favored (49.26%) Glycine / -81.2,-174.7	-	-	Favored (50.569%)	-	-	-
A 19		ILE	1.69	-	Favored (43.95%) Ile or Val / -91.6,127.4	Favored (89.5%) *mt* chi angles: 298,169.7	0.01Å	Favored (13.745%) beta sheet	-	-	-
A 20		PHE	1.64	-	Favored (40.27%) General / -116.3,148.7	Favored (91.5%) *m-80* chi angles: 294.8,85.4	0.04Å	Favored (58.291%) beta sheet	-	-	-
**#**	**Alt**	**Res**	**High B**	**Clash > 0.4Å**	**Ramachandran**	**Rotamer**	**Cβ deviation**	**CaBLAM**	**Bond lengths**	**Bond angles**	**Cis Peptides**
			Avg: 1.21	Clashscore: 0.37	Outliers: 0 of 350	Poor rotamers: 0 of 307	Outliers: 0 of 318	Outliers: 8 of 348	Outliers: 2 of 352	Outliers: 6 of 352	Non-Trans: 3 of 351
A 21		VAL	1.63	-	Favored (29.98%) Ile or Val / -125.4,149.2	Favored (29.1%) *m* chi angles: 296.3	0.02Å	Favored (71.959%) beta sheet	-	-	-
A 22		PHE	1.66	-	Favored (50.99%)	Favored (87.2%) *m-80* chi angles: 301.6,92.2	0.06Å	Favored (67.083%) beta sheet	-	-	-

					General / -131.7,152.3						
A 23		ARG	1.77	-	Allowed (0.93%) General / -88.2,-66.3	Favored (74.2%) *ttt180* chi angles: 181.4,172.6,174.2,169.9	0.05Å	CaBLAM Outlier (0.07%) try beta sheet	-	-	-
A 24		ASP	1.98	-	Allowed (1.63%) General / 54.1,18.1	Favored (68.7%) *m-30* chi angles: 290.6,321.7	0.04Å	CaBLAM Outlier (0.032%)	-	-	-
A 25		SER	2.31	-	Favored (68.53%) General / -62.4,-26.5	Favored (98.1%) *p* chi angles: 65.2	0.02Å	Favored (20.927%)	-	-	-
A 26		ASP	2.73	-	Favored (46.67%) General / -93.9,7.0	Favored (55.1%) *p0* chi angles: 62.2,357.1	0.03Å	Favored (11.444%) alpha helix	-	-	-
A 27		ASP	3.11	-	Allowed (1.08%) General / -140.2,-12.1	Favored (38.3%) *t0* chi angles: 189.2,27.7	0.04Å	Favored (6.111%) alpha helix	-	-	-
A 28		TRP	3.3	-	Favored (28.89%) General / -47.6,-47.7	Favored (6.9%) *t-100* chi angles: 197.1,286.1	0.10Å	Favored (46.093%) alpha helix	-	-	-
A 29		LEU	3.2	-	Favored (64.07%) General / -60.7,-22.6	Favored (94.1%) *mt* chi angles: 295.4,175.4	0.04Å	Favored (39.361%) alpha helix	-	-	-
A 30		ASN	2.83	-	Favored (12.25%) General / -106.4,-24.4	Favored (69.1%) *m-40* chi angles: 295.9,286.5	0.04Å	Favored (47.079%) alpha helix	-	-	-
A 31		LYS	2.34	-	Favored (23.74%) General / -83.8,-36.1	Favored (77.6%) *tttt* chi angles: 182.8,168,181.2,172.4	0.08Å	Favored (94.555%) alpha helix	-	-	-
A 32		TYR	1.9	-	Favored (34.96%) General / -111.0,118.0	Favored (48.6%) *m-80* chi angles: 296.1,76.7	0.09Å	Favored (28.333%)	-	-	-
A 33		SER	1.57	-	Favored (42.14%) General / -117.3,147.9	Favored (71%) *m* chi angles: 295.1	0.02Å	Favored (53.569%)	-	-	-
A 34		TYR	1.37	-	Favored (32.08%) General / -100.6,142.4	Favored (8.6%) *m-10* chi angles: 287.2,11.9	0.07Å	Favored (58.081%) beta sheet	-	-	-
A 35		TYR	1.26	-	Favored (44.09%) Pre-Pro / -126.1,93.2	Favored (88.3%) *m-80* chi angles: 299.7,87.7	0.04Å	Favored (46.314%)	-	-	-
A 36		PRO	1.22	-	Favored (34.93%) Trans-Pro / -72.2,164.7	Favored (71.4%) *Cg_endo* chi angles: 29.7,328.1,21.3	0.02Å	Favored (19.99%)	-	-	-
A 37		GLU	1.19	-	Favored (7.62%) General / -58.8,158.8	Favored (7.2%) *tp30* chi angles: 181,59.9,278.9	0.04Å	CA Geom Outlier (0.33%)	-	-	-
A 38		ASP	1.16	-	Allowed (1.11%) Pre-Pro / -38.6,129.4	Favored (14.1%) *t0* chi angles: 185.7,308.7	0.07Å	Favored (10.406%)	-	-	-
A 39		PRO	1.12	-	Favored (53.05%) Trans-Pro / -60.2,-18.6	Favored (43.7%) *Cg_endo* chi angles: 24.3,324.9,30.8	0.06Å	Favored (30.689%)	-	-	-

#	Alt	Res	High B	Clash > 0.4Å	Ramachandran	Rotamer	Cβ deviation	CaBLAM	Bond lengths	Bond angles	Cis Peptides
A 40		VAL	1.06	-	Favored (47.63%) Ile or Val / -66.0,-29.7	Allowed (1.8%) *p* chi angles: 77.1	0.05Å	Favored (52.896%) alpha helix	-	-	-
#	Alt	Res	High B	Clash > 0.4Å	Ramachandran	Rotamer	Cβ deviation	CaBLAM	Bond lengths	Bond angles	Cis Peptides
			Avg: 1.21	Clashscore: 0.37	Outliers: 0 of 350	Poor rotamers: 0 of 307	Outliers: 0 of 318	Outliers: 8 of 348	Outliers: 2 of 352	Outliers: 6 of 352	Non-Trans: 3 of 351
A 41		LYS	1	-	Favored (18.93%) General / -86.0,-37.5	Favored (73%) *mmtt* chi angles: 297.8,293.3,185.6,184.2	0.08Å	Favored (61.85%) alpha helix	-	-	-
A 42		LEU	0.95	-	Favored (94.51%) General / -65.3,-41.0	Favored (93.6%) *mt* chi angles: 298,175.6	0.11Å	Favored (93.963%) alpha helix	-	-	-
A 43		ALA	0.91	-	Favored (99.08%) General / -62.8,-41.0	-	0.06Å	Favored (97.657%) alpha helix	-	-	-
A 44		SER	0.88	-	Favored (98.44%) General / -62.0,-41.8	Favored (47.1%) *t* chi angles: 180.3	0.04Å	Favored (93.597%) alpha helix	-	-	-
A 45		ILE	0.87	-	Favored (98.1%) Ile or Val / -60.6,-45.1	Favored (89.4%) *mt* chi angles: 290.9,167.8	0.03Å	Favored (91.898%) alpha helix	-	-	-
A 46		VAL	0.87	-	Favored (95.54%) Ile or Val / -62.8,-46.8	Favored (51.4%) *t* chi angles: 169.6	0.04Å	Favored (89.372%) alpha helix	-	-	-
A 47		LYS	0.88	-	Favored (67.65%) General / -58.5,-52.0	Favored (48.4%) *tttm* chi angles: 179.9,180.9,185.7,289.3	0.04Å	Favored (88.439%) alpha helix	-	-	-
A 48		ALA	0.9	-	Favored (96.05%) General / -61.0,-41.1	-	0.04Å	Favored (86.822%) alpha helix	-	-	-
A 49		SER	0.92	-	Favored (99.79%) General / -62.8,-42.6	Favored (73.4%) *m* chi angles: 295.5	0.06Å	Favored (97.612%) alpha helix	-	-	-
A 50		PHE	0.93	-	Favored (78.39%) General / -58.4,-49.1	Favored (83.2%) *t80* chi angles: 181,83.9	0.04Å	Favored (96.879%) alpha helix	-	-	-
A 51		GLU	0.93	-	Favored (72.16%) General / -60.8,-32.8	Favored (98.5%) *mt-10* chi angles: 290.6,172.2,357.8	0.04Å	Favored (75.013%) alpha helix	-	-	-
A 52		GLU	0.92	-	Favored (41.76%) General / -82.2,0.3	Favored (90.3%) *mt-10* chi angles: 292.6,179.7,16	0.06Å	Favored (51.216%)	-	-	-
A 53		GLY	0.89	-	Favored (58.41%) Glycine / 96.0,7.8	-	-	Favored (87.678%)	-	-	-
A 54		LYS	0.85	-	Favored (35.37%) General / -90.3,132.8	Favored (5.2%) *mptt* chi angles: 284.2,78.9,171.8,176.8	0.05Å	Favored (20.768%)	-	-	-
A 55		CYS	0.82	-	Favored (30.71%) General / -97.1,-8.2	Favored (29.8%) *p* chi angles: 66.1	0.06Å	CaBLAM Disfavored (4.605%)	-	-	-

#	Alt	Res	High B	Clash > 0.4Å	Ramachandran	Rotamer	Cβ deviation	CaBLAM	Bond lengths	Bond angles	Cis Peptides
A 56		GLY	0.8	-	Favored (48.85%) Glycine / -179.4,179.6	-	-	Favored (40.135%)	-	-	-
A 57		LEU	0.8	-	Favored (31.21%) General / -135.8,128.7	Favored (36.3%) *tp* chi angles: 171.4,66.5	0.06Å	Favored (22.832%)	-	-	-
A 58		ASN	0.82	-	Favored (53.71%) General / -105.7,130.4	Favored (24.2%) *m-40* chi angles: 292,7.8	0.11Å	Favored (59.867%) beta sheet	-	-	-
A 59		SER	0.85	-	Favored (17.52%) General / -79.7,169.8	Favored (86.7%) *p* chi angles: 67.9	0.06Å	Favored (12.081%)	-	-	-
A 60		VAL	0.9	-	Favored (11.19%) Ile or Val / -112.6,-4.5	Favored (32.2%) *m* chi angles: 297.4	0.07Å	Favored (30.233%)	-	-	-
**#**	**Alt**	**Res**	**High B**	**Clash > 0.4Å**	**Ramachandran**	**Rotamer**	**Cβ deviation**	**CaBLAM**	**Bond lengths**	**Bond angles**	**Cis Peptides**
			Avg: 1.21	Clashscore: 0.37	Outliers: 0 of 350	Poor rotamers: 0 of 307	Outliers: 0 of 318	Outliers: 8 of 348	Outliers: 2 of 352	Outliers: 6 of 352	Non-Trans: 3 of 351
A 61		ASP	0.94	-	Favored (17.38%) General / -154.5,172.7	Favored (3.9%) *p0* chi angles: 64.8,56	0.05Å	Favored (24.362%)	-	-	-
A 62		SER	0.98	-	Favored (65.93%) General / -66.8,-20.3	Favored (88.4%) *p* chi angles: 69.4	0.07Å	Favored (37.36%) alpha helix	-	-	-
A 63		LEU	1.02	-	Favored (14.13%) General / -88.2,-39.8	Favored (81.1%) *mt* chi angles: 295.7,179.6	0.05Å	Favored (69.035%) alpha helix	-	-	-
A 64		GLU	1.04	-	Favored (91.41%) General / -60.2,-40.9	Favored (87.9%) *tt0* chi angles: 181.5,182.8,0.6	0.01Å	Favored (97.576%) alpha helix	-	-	-
A 65		HIS	1.05	-	Favored (61.61%) General / -58.9,-53.1	Favored (87.5%) *t70* chi angles: 179.5,77.1	0.06Å	Favored (80.719%) alpha helix	-	-	-
A 66		GLU	1.05	-	Favored (78.94%) General / -62.6,-35.2	Favored (54.3%) *mm-30* chi angles: 288.2,290.9,305	0.03Å	Favored (73.981%) alpha helix	-	-	-
A 67		MET	1.06	-	Favored (78.25%) General / -58.3,-49.1	Favored (55.1%) *ttp* chi angles: 180.4,189.7,73.2	0.11Å	Favored (76.532%) alpha helix	-	-	-
A 68		TRP	1.07	-	Favored (93.41%) General / -64.0,-39.1	Favored (70.5%) *m100* chi angles: 288.3,116.7	0.01Å	Favored (85.88%) alpha helix	-	-	-
A 69		ARG	1.07	-	Favored (77.86%) General / -60.3,-36.5	Favored (76.5%) *mtp180* chi angles: 291.5,181.1,71.6,197.9	0.07Å	Favored (80.823%) alpha helix	-	-	-
A 70		SER	1.07	-	Favored (59.06%) General / -80.3,-7.5	Favored (66.3%) *p* chi angles: 72.5	0.04Å	Favored (33.193%) alpha helix	-	-	-
A 71		ARG	1.06	-	Favored (7.69%) General / -123.9,-11.2	Favored (90.4%) *mmt-90* chi angles: 294.4,291.2,179.6,268.9	0.04Å	Favored (21.556%) alpha helix	-	-	-

A 72		ALA	1.05	-	Favored (68.24%) General / -55.5,-37.8	-	0.06Å	Favored (54.557%) alpha helix	-	-	-
A 73		ASP	1.04	-	Favored (81.83%) General / -61.5,-37.0	Favored (97.4%) *m-30* chi angles: 289.1,345.4	0.13Å	Favored (77.267%) alpha helix	-	OUTLIER(S) worst is CA-CB-CG: 5.2 σ	-
A 74		GLU	1.02	-	Favored (61.33%) General / -73.8,-42.2	Favored (98.9%) *mt-10* chi angles: 293.2,179.5,358.3	0.12Å	Favored (78.355%) alpha helix	-	-	-
A 75		ILE	1	-	Favored (99.32%) Ile or Val / -62.6,-45.2	Favored (99.2%) *mt* chi angles: 292.8,167.7	0.07Å	Favored (97.857%) alpha helix	-	-	-
A 76		ASN	1	-	Favored (96.25%) General / -63.4,-40.1	Favored (98.9%) *m-40* chi angles: 288.2,337.7	0.05Å	Favored (92.844%) alpha helix	-	-	-
A 77		ALA	1	-	Favored (92.15%) General / -60.7,-40.4	-	0.05Å	Favored (93.712%) alpha helix	-	-	-
A 78		ILE	1	-	Favored (83.1%) Ile or Val / -68.5,-44.2	Favored (95.2%) *mt* chi angles: 293.5,170.2	0.10Å	Favored (95.307%) alpha helix	-	-	-
A 79		LEU	1.01	-	Favored (81.5%) General / -60.6,-37.5	Favored (85.3%) *mt* chi angles: 289.9,172.1	0.06Å	Favored (95.405%) alpha helix	-	-	-
A 80		GLU	1.02	-	Favored (80.55%) General / -67.9,-42.8	Favored (63.8%) *tp30* chi angles: 186.8,66.5,20.4	0.02Å	Favored (93.201%) alpha helix	-	-	-
**#**	**Alt**	**Res**	**High B**	**Clash > 0.4Å**	**Ramachandran**	**Rotamer**	**Cβ deviation**	**CaBLAM**	**Bond lengths**	**Bond angles**	**Cis Peptides**
			Avg: 1.21	Clashscore: 0.37	Outliers: 0 of 350	Poor rotamers: 0 of 307	Outliers: 0 of 318	Outliers: 8 of 348	Outliers: 2 of 352	Outliers: 6 of 352	Non-Trans: 3 of 351
A 81		GLU	1.02	-	Favored (69.79%) General / -63.1,-28.0	Favored (86.4%) *mt-10* chi angles: 287.5,182.1,339.4	0.08Å	Favored (74.037%) alpha helix	-	-	-
A 82		ASN	1	-	Favored (28.25%) General / -94.3,12.0	Favored (68.2%) *m-40* chi angles: 289,280	0.05Å	Favored (47.056%)	-	-	-
A 83		GLU	0.97	-	Favored (24.93%) General / 55.8,45.1	Favored (90.9%) *mt-10* chi angles: 299,184.9,356.7	0.04Å	Favored (31.992%)	-	-	-
A 84		VAL	0.92	-	Favored (46.97%) Ile or Val / -110.7,114.4	Favored (54.1%) *t* chi angles: 180.8	0.11Å	Favored (26.427%) beta sheet	-	-	-
A 85		ASP	0.87	-	Favored (4.25%) General / -82.7,58.4	Favored (66.4%) *m-30* chi angles: 291,318.8	0.01Å	Favored (7.522%) beta sheet	-	-	-
A 86		ILE	0.82	-	Favored (67.02%) Ile or Val / -129.5,131.6	Favored (73.9%) *mt* chi angles: 301.4,169.8	0.08Å	Favored (20.767%) beta sheet	-	-	-
A 87		SER	0.79	-	Favored (46.57%)	Favored (69.9%) *m* chi angles: 296.4	0.03Å	Favored (58.63%) beta sheet	-	-	-

#	Alt	Res	High B	Clash > 0.4Å	Ramachandran	Rotamer	Cβ deviation	CaBLAM	Bond lengths	Bond angles	Cis Peptides
					General / -117.2,142.8						
A 88		VAL	0.78	-	Favored (44.2%) Ile or Val / -91.5,125.3	Favored (83.3%) *t* chi angles: 176.9	0.01Å	Favored (58.365%) beta sheet	-	-	-
A 89		VAL	0.81	-	Favored (60.35%) Ile or Val / -116.6,118.9	Favored (71.2%) *t* chi angles: 178.6	0.10Å	Favored (71.704%) beta sheet	-	-	-
A 90		VAL	0.87	-	Favored (57.15%) Ile or Val / -106.6,120.1	Favored (51.6%) *t* chi angles: 181.2	0.03Å	Favored (71.775%)	-	-	-
A 91		GLN	0.96	-	Favored (16.76%) General / -95.5,155.4	Favored (69.9%) *mt0* chi angles: 296.9,184.2,7.5	0.06Å	Favored (24.882%)	-	-	-
A 92		ASP	1.07	-	Favored (53.09%) General / -60.3,143.3	Favored (92%) *m-30* chi angles: 289.9,350.1	0.06Å	Favored (33.658%)	-	-	-
A 93		SER	1.15	-	Favored (54.62%) General / -59.8,141.8	Favored (22.9%) *t* chi angles: 171	0.05Å	Favored (35.001%)	-	-	-
A 94		LYS	1.19	-	Favored (14.13%) General / -107.1,23.6	Favored (72.8%) *mmtt* chi angles: 299.3,296,182.5,180.6	0.11Å	CaBLAM Disfavored (1.051%)	-	-	-
A 95		ASN	1.17	-	Allowed (0.43%) General / 80.4,-19.5	Favored (25%) *m-40* chi angles: 308.3,328.1	0.02Å	CaBLAM Disfavored (1.639%)	-	-	-
A 96		ILE	1.11	-	Favored (58.98%) Ile or Val / -123.2,119.9	Favored (74.3%) *mt* chi angles: 301.9,172.4	0.08Å	Favored (17.995%)	-	-	-
A 97		TYR	1.03	-	Favored (9.14%) General / -85.1,80.7	Favored (65.5%) *m-80* chi angles: 296.4,114	0.04Å	Favored (68.648%)	-	-	-
A 98		GLN	0.96	-	Favored (55.67%) General / -68.1,143.0	Favored (82.9%) *mt0* chi angles: 293.6,183,298.6	0.01Å	Favored (23.141%)	-	-	-
A 99		ARG	0.92	-	Favored (50.55%) General / -66.5,149.4	Favored (43.1%) *ptt-90* chi angles: 71.5,191.2,186.7,283.7	0.03Å	Favored (27.32%)	-	-	-
A 100		GLY	0.92	-	Favored (28.24%) Glycine / -100.2,-159.9	-	-	Favored (28.723%)	-	-	-
**#**	**Alt**	**Res**	**High B**	**Clash > 0.4Å**	**Ramachandran**	**Rotamer**	**Cβ deviation**	**CaBLAM**	**Bond lengths**	**Bond angles**	**Cis Peptides**
			Avg: 1.21	Clashscore: 0.37	Outliers: 0 of 350	Poor rotamers: 0 of 307	Outliers: 0 of 318	Outliers: 8 of 348	Outliers: 2 of 352	Outliers: 6 of 352	Non-Trans: 3 of 351
A 101		THR	0.95	-	Favored (7.21%) General / -124.9,-8.6	Favored (65.9%) *p* chi angles: 63	0.07Å	Favored (15.875%)	-	-	-
A 102		HIS	1.01	-	Favored (61.63%) Pre-Pro / -122.1,154.6	Favored (99.2%) *m-70* chi angles: 301.3,287.6	0.08Å	Favored (16.585%)	-	-	-

A 103		PRO	1.1	-	Allowed (1.18%) Trans-Pro / -88.9,-175.4	Favored (25.9%) *Cg_endo* chi angles: 35.9,322.9,22.9	0.07Å	Favored (26.873%)	-	-	-
A 104		PHE	1.23	-	Favored (22.22%) General / -99.5,150.4	Favored (2.1%) *m-10* chi angles: 276.9,329.7	0.05Å	Favored (47.08%)	-	OUTLIER(S) worst is CA-C-O: 4.1 σ	-
A 105		SER	1.38	-	Favored (53.75%) General / -63.1,146.2	Favored (71%) *m* chi angles: 295.1	0.05Å	Favored (23.876%) beta sheet	-	-	-
A 106		ARG	1.55	-	Favored (35.16%) General / -88.2,126.5	Favored (39%) *tpt170* chi angles: 180.4,70,174.2,166.5	0.02Å	Favored (42.286%) beta sheet	-	-	-
A 107		ILE	1.68	-	Favored (44.79%) Ile or Val / -94.6,129.5	Favored (49.6%) *mm* chi angles: 301.7,301.2	0.03Å	Favored (54.437%)	-	-	-
A 108		ARG	1.76	-	Favored (2.26%) General / -90.6,-59.1	Favored (55.1%) *ttp-170* chi angles: 183,186.2,68,202.1	0.06Å	Favored (9.551%)	-	-	-
A 109		ASP	1.76	-	Favored (12.75%) General / -80.9,-48.2	Favored (70.6%) *m-30* chi angles: 297.4,346.3	0.05Å	Favored (9.225%)	-	-	-
A 110		GLY	1.73	-	Favored (45.29%) Glycine / 89.9,176.4	-	-	Favored (41.124%)	-	-	-
A 111		LEU	1.72	-	Favored (24.56%) General / -81.6,158.0	Favored (78.4%) *mt* chi angles: 300.8,180.2	0.02Å	Favored (12.384%)	-	-	-
A 112		GLN	1.76	-	Favored (82.58%) General / -58.2,-40.8	Favored (64.6%) *tt0* chi angles: 182.2,177.9,10.9	0.03Å	Favored (30.169%)	-	-	-
A 113		TYR	1.88	-	Favored (36.56%) General / -116.1,150.9	Favored (94.1%) *m-80* chi angles: 299.5,96.3	0.07Å	CaBLAM Disfavored (1.278%)	-	-	-
A 114		GLY	2.08	-	Favored (37.33%) Glycine / -173.1,165.2	-	-	Favored (38.424%)	-	-	-
A 115		TRP	2.37	-	Favored (34.82%) General / -82.2,137.8	Favored (94.8%) *m100* chi angles: 293.3,107.8	0.11Å	Favored (23.326%)	-	-	-
A 116		LYS	2.72	-	Favored (23.56%) General / -88.1,-23.9	Favored (98.6%) *mttt* chi angles: 293,180.7,179.2,178.5	0.00Å	Favored (28.454%)	-	-	-
A 117		THR	3.09	-	Favored (38.84%) General / -131.8,128.5	Favored (98.2%) *m* chi angles: 300.1	0.08Å	Favored (24.633%) alpha helix	-	-	-
A 118		TRP	3.45	-	Favored (66.8%) General / -60.0,-27.4	Favored (74%) *p-90* chi angles: 68.1,268.9	0.01Å	Favored (41.986%) alpha helix	-	-	-
A 119		GLY	3.73	-	Favored (61.6%) Glycine / -67.0,-13.3	-	-	Favored (74.128%) three-ten	-	-	-

#	Alt	Res	High B	Clash > 0.4Å	Ramachandran	Rotamer	Cβ deviation	CaBLAM	Bond lengths	Bond angles	Cis Peptides
A 120		LYS	3.84	-	Favored (51.86%) General / -82.2,-2.3	Favored (73.2%) *mmtt* chi angles: 299.1,293.9,184.6,181.2	0.03Å	Favored (37.625%) three-ten	-	-	-
#	Alt	Res	High B	Clash > 0.4Å	Ramachandran	Rotamer	Cβ deviation	CaBLAM	Bond lengths	Bond angles	Cis Peptides
			Avg: 1.21	Clashscore: 0.37	Outliers: 0 of 350	Poor rotamers: 0 of 307	Outliers: 0 of 318	Outliers: 8 of 348	Outliers: 2 of 352	Outliers: 6 of 352	Non-Trans: 3 of 351
A 121		ASN	3.72	-	Favored (64.41%) General / -60.8,-22.8	Favored (53.7%) *t0* chi angles: 188.2,55.3	0.04Å	Favored (46.638%) three-ten	-	-	-
A 122		LEU	3.35	-	Favored (50.76%) General / -80.1,-3.9	Favored (94.4%) *mt* chi angles: 296.8,174.1	0.07Å	Favored (51.84%)	-	-	-
A 123		VAL	2.8	-	Favored (67.46%) Ile or Val / -70.4,-47.2	Favored (84.9%) *t* chi angles: 173.6	0.02Å	CaBLAM Disfavored (1.896%)	-	-	-
A 124		PHE	2.21	-	Favored (38.35%) General / -157.9,162.6	Favored (51.9%) *p90* chi angles: 68.5,90.9	0.09Å	Favored (17.827%) beta sheet	-	-	-
A 125		SER	1.71	-	Favored (30.18%) Pre-Pro / -125.0,137.7	Favored (63.2%) *m* chi angles: 297.8	0.04Å	Favored (44.618%) beta sheet	-	-	-
A 126		PRO	1.34	-	Favored (31.81%) Trans-Pro / -76.9,162.9	Favored (80.9%) *Cg_endo* chi angles: 30.6,323.8,26.4	0.05Å	Favored (62.988%)	-	-	-
A 127		GLY	1.1	-	Favored (40.79%) Glycine / -91.6,-167.8	-	-	Favored (55.5%)	-	-	-
A 128		ARG	0.95	-	Favored (45%) General / -103.8,135.6	Favored (81%) *ttt180* chi angles: 180.1,172.4,176.5,177.7	0.05Å	Favored (17.979%)	-	-	-
A 129		LYS	0.86	-	Favored (9.15%) General / -98.2,170.1	Favored (20.9%) *mmtp* chi angles: 295,288.5,180.6,60.4	0.07Å	Favored (33.168%)	-	-	-
A 130		ASN	0.8	-	Favored (90.27%) General / -62.5,-38.5	Favored (99.4%) *m-40* chi angles: 287.6,339.9	0.02Å	Favored (55.135%)	-	-	-
A 131		GLY	0.76	-	Favored (28.66%) Glycine / -86.4,155.8	-	-	CaBLAM Disfavored (3.829%)	-	-	-
A 132		SER	0.73	-	Favored (41.82%) General / -153.8,161.7	Favored (86.6%) *p* chi angles: 67.2	0.02Å	Favored (62.958%)	-	-	-
A 133		PHE	0.72	-	Favored (36.12%) General / -131.2,126.6	Favored (65.5%) *t80* chi angles: 187.2,82	0.10Å	Favored (56.366%) beta sheet	-	-	-
A 134		ILE	0.73	-	Favored (55.45%) Ile or Val / -106.8,131.9	Favored (92.1%) *mt* chi angles: 297.1,169.8	0.04Å	Favored (61.298%)	-	-	-
A 135		ILE	0.78	-	Favored (65.13%) Ile or Val / -109.4,123.2	Favored (76.8%) *mt* chi angles: 298.8,175	0.13Å	Favored (11.45%)	-	-	-

A 136		ASP	0.86	-	Favored (4.18%) General / 60.9,13.0	Favored (76.9%) *m-30* chi angles: 291.9,325.2	0.07Å	CaBLAM Outlier (0.819%)	-	-	-
A 137		GLY	0.98	-	Favored (3.75%) Glycine / -77.5,55.9	-	-	Favored (40.582%)	-	-	-
A 138		LYS	1.12	-	Allowed (1.71%) General / 49.7,-122.0	Favored (88.7%) *mttt* chi angles: 299.9,187.4,179.2,180.9	0.05Å	CaBLAM Outlier (0.18%)	-	-	-
A 139		SER	1.22	-	Favored (5.23%) General / -154.8,120.9	Favored (39.8%) *t* chi angles: 176.8	0.04Å	CaBLAM Outlier (0.337%)	-	-	-
A 140		ARG	1.27	-	Favored (33.65%) General / -133.5,161.8	Favored (81.4%) *mtt90* chi angles: 292.2,185.1,173.4,89.1	0.06Å	Favored (41.324%)	-	-	-
**#**	**Alt**	**Res**	**High B**	**Clash > 0.4Å**	**Ramachandran**	**Rotamer**	**Cβ deviation**	**CaBLAM**	**Bond lengths**	**Bond angles**	**Cis Peptides**
			Avg: 1.21	Clashscore: 0.37	Outliers: 0 of 350	Poor rotamers: 0 of 307	Outliers: 0 of 318	Outliers: 8 of 348	Outliers: 2 of 352	Outliers: 6 of 352	Non-Trans: 3 of 351
A 141		LYS	1.24	-	Favored (66.82%) General / -56.7,-33.8	Favored (96.9%) *mttt* chi angles: 289.2,179.6,180.2,178.6	0.01Å	Favored (63.993%)	-	-	-
A 142		GLU	1.15	-	Favored (78.93%) General / -64.3,-34.8	Favored (99.3%) *mt-10* chi angles: 291.1,176.9,349.8	0.06Å	Favored (32.691%) alpha helix	-	-	-
A 143		CYS	1.04	-	Favored (10.22%) Pre-Pro / -148.8,74.6	Favored (52.1%) *t* chi angles: 184.2	0.01Å	Favored (8.992%)	-	-	-
A 144		PRO	0.93	-	Favored (70.81%) Trans-Pro / -60.7,152.2	Favored (36.8%) *Cg_exo* chi angles: 338.7,33.8,327.7	0.06Å	Favored (37.08%)	-	-	-
A 145		PHE	0.84	-	Favored (42.7%) General / -54.5,-28.1	Favored (26.3%) *p90* chi angles: 76.7,94.5	0.04Å	Favored (51.788%)	-	-	-
A 146		SER	0.78	-	Favored (62.47%) General / -69.8,-13.8	Favored (89.4%) *p* chi angles: 69	0.04Å	Favored (46.531%) alpha helix	-	-	-
A 147		ASN	0.75	-	Favored (20.75%) General / -111.1,17.0	Favored (91.5%) *m-40* chi angles: 292.3,324.3	0.06Å	Favored (45.227%)	-	-	-
A 148		ARG	0.74	-	Favored (39.58%) General / -111.4,145.0	Favored (81.5%) *mtt-85* chi angles: 293.5,175,182.4,259.8	0.04Å	Favored (32.564%)	-	-	-
A 149		VAL	0.73	-	Favored (14.02%) Ile or Val / -89.5,141.7	Favored (95.1%) *t* chi angles: 174.8	0.06Å	Favored (46.533%)	-	-	-
A 150		TRP	0.74	-	Favored (35.89%) General / -140.2,141.6	Favored (81.7%) *t60* chi angles: 175.2,88.2	0.07Å	Favored (13.265%)	-	-	-
A 151		ASN	0.76	-	Favored (27.64%) General / 56.4,42.4	Favored (56.1%) *t0* chi angles: 196.7,24.6	0.04Å	Favored (11.81%)	-	-	-

A 152		SER	0.77	-	Favored (59.51%) General / -83.6,-7.5	Favored (92.2%) *p* chi angles: 66.5	0.06Å	Favored (16.345%)	-	-	-
A 153		PHE	0.8	-	Favored (49.85%) General / -118.9,140.5	Favored (35%) *m-80* chi angles: 278.7,82.4	0.16Å	Favored (23.917%)	-	-	-
A 154		GLN	0.84	-	Favored (37.8%) General / -132.9,160.2	Favored (21.7%) *pt0* chi angles: 66.8,182.3,22.4	0.06Å	Favored (42.436%)	-	-	-
A 155		ILE	0.9	-	Favored (37.3%) Ile or Val / -84.4,129.7	Favored (87.4%) *mt* chi angles: 298.3,168.8	0.08Å	Favored (30.179%)	-	-	-
A 156		GLU	1.01	-	Favored (7.74%) General / -99.6,-40.6	Favored (84.3%) *mm-30* chi angles: 297.9,297.4,331.2	0.04Å	Favored (21.983%)	-	-	-
A 157		GLU	1.18	-	Favored (48%) General / -136.9,150.2	Favored (86.3%) *mt-10* chi angles: 300.6,183.8,2	0.04Å	Favored (25.262%)	-	-	-
A 158		PHE	1.43	-	Favored (11.31%) General / -143.7,120.6	Favored (24.6%) *t80* chi angles: 180.3,50.9	0.03Å	Favored (25.681%)	-	-	-
A 159		GLY	1.78	-	Favored (13.84%) Glycine / -127.7,-174.7	-	-	Favored (31.465%) beta sheet	-	-	-
A 160		THR	2.17	-	Favored (52.49%) General / -125.3,135.5	Favored (99.7%) *m* chi angles: 300.4	0.06Å	Favored (33.513%) beta sheet	-	-	-
**#**	**Alt**	**Res**	**High B**	**Clash > 0.4Å**	**Ramachandran**	**Rotamer**	**Cβ deviation**	**CaBLAM**	**Bond lengths**	**Bond angles**	**Cis Peptides**
			Avg: 1.21	Clashscore: 0.37	Outliers: 0 of 350	Poor rotamers: 0 of 307	Outliers: 0 of 318	Outliers: 8 of 348	Outliers: 2 of 352	Outliers: 6 of 352	Non-Trans: 3 of 351
A 161		GLY	2.52	-	Favored (2.12%) Glycine / -117.7,41.2	-	-	CaBLAM Disfavored (3.114%)	-	-	-
A 162		VAL	2.7	-	Allowed (0.21%) Ile or Val / 64.3,-59.0	Favored (95%) *t* chi angles: 174.8	0.03Å	CaBLAM Disfavored (1.514%)	-	-	-
A 163		PHE	2.65	-	Favored (13.36%) General / -103.1,-25.6	Favored (78.1%) *m-80* chi angles: 299.4,108	0.07Å	Favored (38.337%)	-	-	-
A 164		THR	2.39	-	Favored (13.07%) General / -131.7,171.8	Favored (48%) *p* chi angles: 66	0.01Å	Favored (8.303%)	-	-	-
A 165		THR	2.04	-	Favored (19.55%) General / -126.2,165.1	Favored (49.9%) *p* chi angles: 65.7	0.04Å	Favored (45.426%)	-	-	-
A 166		ARG	1.69	-	Favored (30.76%) General / -132.7,125.7	Favored (94%) *mtt180* chi angles: 299.5,185.7,181.1,176.7	0.04Å	Favored (44.016%) beta sheet	-	-	-
A 167		VAL	1.41	-	Favored (69.8%) Ile or Val / -112.4,126.8	Favored (81.6%) *t* chi angles: 176.6	0.14Å	Favored (66.385%) beta sheet	-	-	-

A 168		TYR	1.21	-	Favored (50.6%) General / -107.9,134.6	Favored (83.4%) *m-80* chi angles: 295.4,82.5	0.05Å	Favored (67.092%) beta sheet	-	-	-
A 169		MET	1.08	-	Favored (15.39%) General / -109.1,161.2	Favored (48.2%) *mmm* chi angles: 313.7,295.5,288.4	0.09Å	Favored (27.636%)	-	-	-
A 170		ASP	1.01	-	Favored (42.2%) General / -151.9,161.7	Favored (23.2%) *t0* chi angles: 198.2,352.7	0.06Å	Favored (24.397%)	-	-	-
A 171		ALA	0.97	-	Favored (35.62%) General / -76.6,153.1	-	0.03Å	Favored (30.462%)	-	-	-
A 172		VAL	0.96	-	Favored (71.04%) Ile or Val / -126.3,127.9	Favored (60.2%) *t* chi angles: 179.9	0.07Å	Favored (67.809%) beta sheet	-	-	-
A 173		PHE	0.95	-	Favored (50.12%) General / -93.2,-5.5	Favored (36%) *p90* chi angles: 54,83.7	0.08Å	Favored (27.017%) beta sheet	-	-	-
A 174		GLU	0.95	-	Favored (32.21%) General / -103.0,143.7	Favored (96%) *mt-10* chi angles: 295.8,182,355.2	0.03Å	Favored (25.553%) beta sheet	-	-	-
A 175		TYR	0.95	-	Favored (26.22%) General / -81.2,121.5	Favored (69%) *t80* chi angles: 186.1,79.5	0.03Å	Favored (49.539%) beta sheet	-	-	-
A 176		THR	0.95	-	Favored (39.49%) General / -125.5,123.6	Favored (97.8%) *m* chi angles: 300	0.07Å	Favored (66.561%) beta sheet	-	-	-
A 177		MET	0.95	-	Favored (55.36%) General / -88.2,-8.4	Favored (98%) *mmm* chi angles: 293.2,299,293.4	0.06Å	Favored (15.518%) beta sheet	-	-	-
A 178		ASP	0.95	-	Favored (31.27%) General / -78.8,152.5	Favored (90.9%) *m-30* chi angles: 293.1,346.2	0.07Å	Favored (33.187%)	-	-	-
A 179		CYS	0.95	-	Favored (18.04%) General / -88.4,159.3	Favored (70.8%) *m* chi angles: 298.3	0.02Å	Favored (34.422%)	-	-	-
A 180		ASP	0.96	-	Favored (7.93%) General / -80.9,98.6	Favored (55%) *t0* chi angles: 182.2,338.4	0.04Å	Favored (25.95%)	-	-	-
**#**	**Alt**	**Res**	**High B**	**Clash > 0.4Å**	**Ramachandran**	**Rotamer**	**Cβ deviation**	**CaBLAM**	**Bond lengths**	**Bond angles**	**Cis Peptides**
			Avg: 1.21	Clashscore: 0.37	Outliers: 0 of 350	Poor rotamers: 0 of 307	Outliers: 0 of 318	Outliers: 8 of 348	Outliers: 2 of 352	Outliers: 6 of 352	Non-Trans: 3 of 351
A 181		GLY	0.98	-	Favored (5.65%) Glycine / -52.0,-19.8	-	-	Favored (20.238%)	-	-	-
A 182		SER	0.99	-	Favored (63.55%) General / -68.8,-15.6	Favored (88.4%) *p* chi angles: 69.4	0.04Å	Favored (30.172%)	-	-	-
A 183		ILE	1	-	Favored (7.08%) Ile or Val / -118.5,15.9	Favored (32.2%) *pt* chi angles: 56.3,169.5	0.04Å	Favored (23.985%)	-	-	-

A 184		LEU	1.02	-	Favored (24.65%) General / -112.5,155.2	Favored (50%) *mt* chi angles: 306.5,179.4	0.06Å	Favored (18.841%)	-	-	-
A 185		GLY	1.07	-	Favored (36.59%) Glycine / -163.1,165.6	-	-	Favored (46.487%)	-	-	-
A 186		ALA	1.14	-	Favored (17.41%) General / -159.0,146.2	-	0.07Å	Favored (52.541%) beta sheet	-	-	-
A 187		ALA	1.24	-	Favored (37.8%) General / -154.7,157.2	-	0.04Å	Favored (51.878%) beta sheet	-	-	-
A 188		VAL	1.33	-	Favored (69.79%) Ile or Val / -125.7,133.2	Favored (56.4%) *t* chi angles: 180.4	0.00Å	Favored (62.609%)	-	-	-
A 189		ASN	1.39	-	Favored (3.4%) General / -128.5,92.3	Favored (66.8%) *m-40* chi angles: 297,282.8	0.02Å	Favored (9.28%)	-	-	-
A 190		GLY	1.4	-	Favored (14.88%) Glycine / 64.9,-119.4	-	-	Favored (54.984%)	-	-	-
A 191		LYS	1.35	-	Favored (54.57%) General / -94.1,4.0	Favored (72.5%) *mmtt* chi angles: 301.1,298.2,183.9,182.5	0.02Å	Favored (13.217%)	-	-	-
A 192		LYS	1.26	-	Favored (47.91%) General / -113.5,140.7	Favored (85.5%) *tttt* chi angles: 179.9,177.6,179.4,181.1	0.04Å	Favored (28.146%)	-	-	-
A 193		SER	1.15	0.61Å OG with A 204 SER OG	Favored (48.34%) General / -136.6,156.6	Favored (95%) *p* chi angles: 64.8	0.08Å	Favored (57.056%)	-	-	-
A 194		ALA	1.06	-	Favored (16.25%) General / -159.6,173.3	-	0.02Å	Favored (45.472%) beta sheet	-	-	-
A 195		HIS	1	-	Favored (2.32%) General / -169.7,141.6	Favored (76.5%) *t-90* chi angles: 189,277	0.10Å	Favored (8.688%) beta sheet	OUTLIER(S) worst is CB--CG: 7.1 σ	-	Twisted nonPRO omega= -147.14
A 196		GLY	0.96	-	Favored (44.63%) Glycine / 175.3,177.8	-	-	Favored (26.062%)	-	-	-
A 197		SER	0.93	-	Favored (7.34%) Pre-Pro / -155.6,171.4	Favored (50.1%) *p* chi angles: 74.4	0.19Å	Favored (10.514%)	-	-	-
A 198		PRO	0.92	-	Favored (12.39%) Trans-Pro / -54.5,-17.3	Favored (86.1%) *Cg_exo* chi angles: 333.7,35.2,331.1	0.07Å	Favored (6.021%)	-	-	-
A 199		THR	0.9	-	Favored (10.29%) General / -119.4,-6.3	Favored (68.9%) *p* chi angles: 59.1	0.04Å	Favored (48.047%) alpha helix	-	-	-
A 200		PHE	0.89	-	Favored (16.36%) General / -150.0,133.2	Favored (64%) *t80* chi angles: 173.9,87.4	0.09Å	Favored (24.17%)	-	-	-

#	Alt	Res	High B	Clash > 0.4Å	Ramachandran	Rotamer	Cβ deviation	CaBLAM	Bond lengths	Bond angles	Cis Peptides
			Avg: 1.21	Clashscore: 0.37	Outliers: 0 of 350	Poor rotamers: 0 of 307	Outliers: 0 of 318	Outliers: 8 of 348	Outliers: 2 of 352	Outliers: 6 of 352	Non-Trans: 3 of 351
A 201		TRP	0.89	-	Favored (49.93%) General / -135.0,148.4	Favored (25.2%) *m-90* chi angles: 300.8,262.3	0.02Å	Favored (49.308%)	-	-	-
A 202		MET	0.9	-	Favored (26.86%) General / -147.3,142.2	Favored (63.9%) *ttp* chi angles: 181.5,175.9,70.7	0.05Å	Favored (62.95%) beta sheet	-	-	-
A 203		GLY	0.94	-	Favored (15.68%) Glycine / -128.1,145.0	-	-	Favored (52.795%) beta sheet	-	-	-
A 204		SER	1.01	0.61Å OG with A 193 SER OG	Favored (43.52%) General / -147.8,159.7	Favored (96.2%) *p* chi angles: 65	0.10Å	Favored (48.3%)	-	-	-
A 205		HIS	1.1	-	Favored (50.38%) General / -136.4,155.2	Favored (73.1%) *m90* chi angles: 297.7,79.2	0.06Å	Favored (39.301%)	-	-	-
A 206		GLU	1.21	-	Favored (24.21%) General / -82.6,119.4	Favored (30.7%) *tt0* chi angles: 183,182.6,106.8	0.01Å	Favored (39.837%)	-	-	-
A 207		VAL	1.31	-	Favored (50.9%) Ile or Val / -120.8,116.8	Favored (61.9%) *t* chi angles: 179.7	0.07Å	Favored (21.683%)	-	-	-
A 208		ASN	1.38	-	Favored (27.86%) General / 52.7,39.1	Favored (88.6%) *m-40* chi angles: 296.8,322	0.02Å	Favored (40.958%)	-	-	-
A 209		GLY	1.38	-	Favored (88.91%) Glycine / 83.2,-1.3	-	-	Favored (81.747%)	-	-	-
A 210		THR	1.33	-	Favored (56.69%) General / -114.3,131.0	Favored (88.3%) *m* chi angles: 298.5	0.03Å	Favored (30.299%)	-	-	-
A 211		TRP	1.25	-	Favored (37.43%) General / -78.3,132.2	Favored (98%) *m100* chi angles: 287.4,97.4	0.04Å	Favored (49.384%) beta sheet	-	-	-
A 212		MET	1.14	-	Favored (45.54%) General / -133.7,137.9	Favored (64.7%) *ttp* chi angles: 180.8,176.8,75.3	0.04Å	Favored (51.075%) beta sheet	-	-	-
A 213		ILE	1.04	-	Favored (24.14%) Ile or Val / -67.1,125.3	Favored (92.5%) *mt* chi angles: 296.6,171.7	0.17Å	Favored (45.701%)	-	-	-
A 214		HIS	0.96	-	Favored (13.72%) General / -99.7,-27.3	Favored (38.3%) *m170* chi angles: 291.2,191.3	0.08Å	Favored (30.47%)	-	-	-
A 215		THR	0.9	-	Favored (26.12%) General / -150.9,146.2	Favored (9.1%) *t* chi angles: 185	0.04Å	Favored (31.06%)	-	-	-
A 216		LEU	0.86	-	Favored (29.38%) General / -142.7,136.9	Favored (7.3%) *tt* chi angles: 187.1,150	0.04Å	Favored (61.497%)	-	-	-

#	Alt	Res	High B	Clash > 0.4Å	Ramachandran	Rotamer	Cβ deviation	CaBLAM	Bond lengths	Bond angles	Cis Peptides
A 217		GLU	0.84	-	Favored (42.82%) General / -133.7,134.2	Favored (87.4%) *tt0* chi angles: 183.4,178,9.4	0.07Å	Favored (66.188%) beta sheet	-	-	-
A 218		THR	0.84	-	Favored (47%) General / -130.6,134.4	Favored (64.3%) *m* chi angles: 303.3	0.10Å	Favored (60.072%)	-	-	-
A 219		LEU	0.84	-	Favored (13.35%) General / -96.7,-30.3	Favored (92.8%) *mt* chi angles: 296.3,178	0.08Å	Favored (30.932%)	-	-	-
A 220		ASP	0.84	-	Favored (33.14%) General / -148.5,150.3	Favored (4.9%) *m-30* chi angles: 289.9,86	0.07Å	Favored (23.722%)	-	OUTLIER(S) worst is CA-CB-CG: 4.6 σ	-
**#**	**Alt**	**Res**	**High B**	**Clash > 0.4Å**	**Ramachandran**	**Rotamer**	**Cβ deviation**	**CaBLAM**	**Bond lengths**	**Bond angles**	**Cis Peptides**
			Avg: 1.21	Clashscore: 0.37	Outliers: 0 of 350	Poor rotamers: 0 of 307	Outliers: 0 of 318	Outliers: 8 of 348	Outliers: 2 of 352	Outliers: 6 of 352	Non-Trans: 3 of 351
A 221		TYR	0.85	-	Favored (18.74%) General / -102.8,154.9	Favored (70.5%) *m-80* chi angles: 301.5,109.7	0.08Å	Favored (50.987%)	OUTLIER(S) worst is CB--CG: 4.7 σ	-	-
A 222		LYS	0.85	-	Favored (22.6%) General / -153.2,143.8	Favored (59%) *pttt* chi angles: 59.7,184.9,182.2,181.7	0.07Å	Favored (41.48%) beta sheet	-	-	-
A 223		GLU	0.84	-	Favored (10%) General / -87.3,70.5	Favored (94.1%) *mt-10* chi angles: 296,183.7,354.1	0.07Å	Favored (9.904%) beta sheet	-	-	-
A 224		CYS	0.83	-	Favored (23.08%) General / -144.2,133.5	Favored (56.6%) *t* chi angles: 181.9	0.09Å	Favored (19.041%) beta sheet	-	-	-
A 225		GLU	0.82	-	Favored (29.35%) General / -99.2,143.5	Favored (95.5%) *mt-10* chi angles: 296.3,181.7,358.1	0.04Å	Favored (39.244%) beta sheet	-	-	-
A 226		TRP	0.82	-	Favored (70.26%) Pre-Pro / -76.0,140.7	Favored (45.9%) *m100* chi angles: 289.2,68.9	0.11Å	Favored (47.501%) beta sheet	-	-	-
A 227		PRO	0.82	-	Favored (98.36%) Trans-Pro / -60.6,145.7	Favored (64.7%) *Cg_exo* chi angles: 335.7,34.2,330.4	0.07Å	Favored (63.257%)	-	-	-
A 228		LEU	0.83	-	Favored (67%) General / -64.3,-22.6	Favored (88.8%) *mt* chi angles: 292,175.2	0.05Å	Favored (46.215%)	-	-	-
A 229		THR	0.87	-	Favored (62.61%) General / -67.5,-14.1	Favored (64.8%) *p* chi angles: 58.1	0.05Å	Favored (46.218%) alpha helix	-	-	-
A 230		HIS	0.96	-	Favored (15.14%) General / -114.4,20.8	Favored (79.7%) *m90* chi angles: 295.4,81.9	0.07Å	Favored (31.373%)	-	-	-
A 231		THR	1.09	-	Favored (7.51%) General / -106.2,171.1	Favored (57.1%) *p* chi angles: 64.6	0.06Å	Favored (24.171%)	-	-	-
A 232		ILE	1.24	-	Favored (7.18%) Ile or Val / -108.6,-50.8	Favored (90.3%) *mt* chi angles: 298,172.3	0.07Å	CaBLAM Disfavored (2.758%)	-	-	-

A 233		GLY	1.38	-	Favored (51.42%) Glycine / -82.1,-176.7	-	-	CaBLAM Disfavored (3.756%) try alpha helix	-	-	-
A 234		THR	1.46	-	Allowed (0.6%) General / -126.4,-86.8	Favored (51.3%) *p* chi angles: 56.4	0.02Å	CaBLAM Outlier (0.188%)	-	-	-
A 235		SER	1.44	-	Favored (10.8%) General / -82.3,101.8	Favored (43.7%) *t* chi angles: 178.7	0.04Å	CaBLAM Disfavored (2.963%)	-	-	-
A 236		VAL	1.33	-	Favored (36.19%) Ile or Val / -128.0,152.5	Favored (30.3%) *m* chi angles: 298	0.06Å	Favored (34.515%)	-	-	-
A 237		GLU	1.18	-	Favored (33.91%) General / -87.8,133.3	Favored (91.1%) *tt0* chi angles: 186.7,178.4,355.9	0.00Å	Favored (43.344%)	-	-	-
A 238		GLU	1.02	-	Favored (66.79%) General / -55.5,-36.5	Favored (72.2%) *tp30* chi angles: 179.4,67.8,16.1	0.02Å	Favored (42.847%)	-	-	-
A 239		SER	0.9	-	Favored (43.94%) General / -56.9,-23.4	Favored (63.8%) *m* chi angles: 294.1	0.03Å	Favored (53.091%) alpha helix	-	-	-
A 240		ASP	0.83	-	Favored (50.87%) General / -97.4,2.7	Favored (68.5%) *m-30* chi angles: 291.2,320	0.04Å	Favored (55.285%)	-	-	-
**#**	**Alt**	**Res**	**High B**	**Clash > 0.4Å**	**Ramachandran**	**Rotamer**	**Cβ deviation**	**CaBLAM**	**Bond lengths**	**Bond angles**	**Cis Peptides**
			Avg: 1.21	Clashscore: 0.37	Outliers: 0 of 350	Poor rotamers: 0 of 307	Outliers: 0 of 318	Outliers: 8 of 348	Outliers: 2 of 352	Outliers: 6 of 352	Non-Trans: 3 of 351
A 241		MET	0.8	-	Favored (19.09%) General / -83.2,111.6	Favored (46.7%) *mtp* chi angles: 288.3,175.6,51.3	0.05Å	Favored (30.895%)	-	-	-
A 242		PHE	0.8	-	Favored (72.49%) General / -54.2,-46.5	Favored (73.1%) *t80* chi angles: 185.2,80.1	0.03Å	Favored (33.219%)	-	-	-
A 243		MET	0.81	-	Favored (58%) Pre-Pro / -107.1,116.6	Favored (32%) *ttt* chi angles: 179.7,173.5,181.9	0.02Å	Favored (24.697%)	-	-	-
A 244		PRO	0.82	-	Favored (91.49%) Trans-Pro / -62.7,149.3	Favored (20.3%) *Cg_endo* chi angles: 19.4,331.1,25.9	0.03Å	Favored (62.478%)	-	-	-
A 245		ARG	0.84	-	Favored (68.19%) General / -62.3,-26.1	Favored (21%) *ptp-170* chi angles: 72.9,181.2,70,189.5	0.04Å	Favored (56.406%)	-	-	-
A 246		SER	0.85	-	Favored (58.8%) General / -73.8,-9.6	Favored (92.6%) *p* chi angles: 64.5	0.03Å	Favored (34.077%)	-	-	-
A 247		ILE	0.86	-	Favored (9.64%) Ile or Val / -110.2,-0.6	Favored (8.3%) *tp* chi angles: 199,66.4	0.06Å	Favored (7.388%)	-	-	-
A 248		GLY	0.86	-	Favored (18.09%) Glycine / 112.7,9.9	-	-	Favored (47.959%)	-	-	-
A 249		GLY	0.87	-	Favored (29.4%)	-	-	Favored (30.735%)	-	-	-

#	Alt	Res	High B	Clash > 0.4Å	Ramachandran	Rotamer	Cβ deviation	CaBLAM	Bond lengths	Bond angles	Cis Peptides
					Glycine / -84.1,153.4						
A 250		PRO	0.88	-	Favored (34.12%) Trans-Pro / -75.5,149.0	Favored (76.7%) *Cg_endo* chi angles: 28.7,324.7,26.5	0.05Å	Favored (38.104%)	-	-	-
A 251		VAL	0.89	-	Favored (2.8%) Ile or Val / -85.2,89.8	Favored (46.8%) *t* chi angles: 182.1	0.04Å	Favored (9.331%)	-	-	-
A 252		SER	0.91	-	Favored (25.5%) General / -154.9,148.6	Favored (42.9%) *t* chi angles: 178.4	0.08Å	Favored (19.373%)	-	-	-
A 253		SER	0.92	-	Favored (63.16%) General / -64.1,-17.1	Favored (96.2%) *p* chi angles: 66	0.03Å	Favored (46.832%)	-	-	-
A 254		HIS	0.93	-	Favored (58.72%) General / -77.3,-9.3	Favored (37.9%) *m-70* chi angles: 294.9,250.4	0.07Å	Favored (61.337%) three-ten	-	-	-
A 255		ASN	0.93	-	Favored (31.15%) General / -99.2,14.0	Favored (60.1%) *t0* chi angles: 197.9,41.2	0.03Å	Favored (29.57%)	-	OUTLIER(S) worst is CA-CB-CG: 4.8 σ	-
A 256		HIS	0.91	-	Favored (25.26%) General / -104.7,149.8	Favored (28.3%) *m170* chi angles: 295.3,192.1	0.10Å	Favored (28.187%)	-	-	-
A 257		ILE	0.89	-	Favored (16.53%) Pre-Pro / -131.8,118.2	Favored (66.4%) *mt* chi angles: 303.1,173.9	0.03Å	Favored (12.593%)	-	-	-
A 258		PRO	0.86	-	Favored (57.06%) Trans-Pro / -53.1,134.7	Favored (92.8%) *Cg_exo* chi angles: 331.2,37.3,330.3	0.03Å	Favored (33.174%)	-	-	-
A 259		GLY	0.83	-	Favored (80.31%) Glycine / 90.9,-8.1	-	-	Favored (74.985%)	-	-	-
A 260		TYR	0.81	-	Favored (44.55%) General / -120.5,147.9	Favored (79.7%) *m-80* chi angles: 298.4,83.4	0.04Å	Favored (37.11%)	-	-	-
**#**	**Alt**	**Res**	**High B**	**Clash > 0.4Å**	**Ramachandran**	**Rotamer**	**Cβ deviation**	**CaBLAM**	**Bond lengths**	**Bond angles**	**Cis Peptides**
			Avg: 1.21	Clashscore: 0.37	Outliers: 0 of 350	Poor rotamers: 0 of 307	Outliers: 0 of 318	Outliers: 8 of 348	Outliers: 2 of 352	Outliers: 6 of 352	Non-Trans: 3 of 351
A 261		LYS	0.8	-	Favored (4.26%) General / -108.8,179.2	Favored (70.1%) *mmtt* chi angles: 299.9,291.8,180,177.4	0.04Å	CaBLAM Disfavored (4.987%)	-	-	-
A 262		VAL	0.78	-	Favored (18.18%) Ile or Val / -60.1,126.6	Favored (83%) *t* chi angles: 176.9	0.14Å	Favored (10.545%)	-	-	-
A 263		GLN	0.78	-	Favored (7.68%) General / -80.7,73.7	Favored (98.5%) *mm-40* chi angles: 299.7,299.4,301	0.03Å	Favored (28.536%) beta sheet	-	-	-
A 264		THR	0.78	-	Favored (62.11%) General / -70.5,-13.4	Favored (68.5%) *p* chi angles: 59	0.02Å	Favored (13.708%)	-	-	-
A 265		ASN	0.78	-	Favored (5.19%) General / -124.0,33.9	Favored (69.5%) *m-40* chi angles: 293.3,281.3	0.04Å	Favored (23.423%)	-	-	-

A 266		GLY	0.8	-	Favored (54.31%) Glycine / -77.9,175.7	-	-	Favored (46.556%)	-	-	-
A 267		PRO	0.82	-	Favored (7.09%) Trans-Pro / -80.6,58.6	Favored (51%) *Cg_endo* chi angles: 32.9,324.7,23.1	0.06Å	CaBLAM Disfavored (3.534%)	-	-	-
A 268		TRP	0.85	-	Allowed (1.9%) General / -93.6,29.9	Favored (58%) *m100* chi angles: 304,116.8	0.09Å	CaBLAM Outlier (0.143%)	-	-	-
A 269		MET	0.87	-	Favored (6.37%) General / -127.8,9.3	Favored (14.8%) *ptt* chi angles: 59.6,185.5,173.8	0.07Å	Favored (60.298%)	-	-	-
A 270		GLN	0.89	-	Favored (37.04%) General / -134.2,130.3	Favored (22.1%) *tt0* chi angles: 183,170.1,94.2	0.02Å	Favored (15.962%)	-	-	-
A 271		VAL	0.89	-	Favored (67.9%) Pre-Pro / -133.0,160.6	Favored (27.3%) *m* chi angles: 299.8	0.08Å	Favored (5.595%)	-	-	-
A 272		PRO	0.87	-	Favored (46.95%) Cis-Pro / -60.5,148.6	Favored (53.3%) *Cg_exo* chi angles: 337.6,34.5,327.7	0.02Å	Favored (68.84%)	-	-	Cis PRO omega= -11.75
A 273		LEU	0.85	-	Favored (40.73%) General / -126.7,124.8	Favored (61.6%) *tp* chi angles: 175.4,63.7	0.03Å	Favored (65.725%)	-	-	-
A 274		GLU	0.81	-	Favored (52.31%) General / -114.0,137.0	Favored (91.8%) *tt0* chi angles: 184.9,178.4,356.6	0.02Å	Favored (65.626%) beta sheet	-	-	-
A 275		VAL	0.78	-	Favored (68.9%) Ile or Val / -112.0,128.0	Favored (54.7%) *t* chi angles: 180.7	0.03Å	Favored (61.3%) beta sheet	-	-	-
A 276		LYS	0.74	-	Favored (52.6%) General / -131.7,148.8	Favored (37.4%) *mtpt* chi angles: 301.7,181.4,76.8,183.5	0.06Å	Favored (51.282%)	-	-	-
A 277		ARG	0.71	-	Favored (9.44%) General / -87.9,71.3	Favored (62.3%) *ttt90* chi angles: 187,175.2,182.9,86.5	0.03Å	CaBLAM Disfavored (4.795%)	-	-	-
A 278		GLU	0.69	-	Favored (28.69%) General / -157.5,154.5	Favored (24.8%) *pt0* chi angles: 65.8,185.8,8.1	0.01Å	Favored (9.424%)	-	-	-
A 279		ALA	0.66	-	Favored (50.23%) General / -66.2,149.5	-	0.02Å	Favored (37.755%)	-	-	-
A 280		CYS	0.64	-	Favored (95.07%) Pre-Pro / -66.5,136.9	Favored (89.4%) *m* chi angles: 293.3	0.06Å	Favored (29.472%)	-	-	-
**#**	**Alt**	**Res**	**High B**	**Clash > 0.4Å**	**Ramachandran**	**Rotamer**	**Cβ deviation**	**CaBLAM**	**Bond lengths**	**Bond angles**	**Cis Peptides**
			Avg: 1.21	Clashscore: 0.37	Outliers: 0 of 350	Poor rotamers: 0 of 307	Outliers: 0 of 318	Outliers: 8 of 348	Outliers: 2 of 352	Outliers: 6 of 352	Non-Trans: 3 of 351
A 281		PRO	0.62	-	Favored (63.57%) Trans-Pro / -53.2,137.1	Favored (87.8%) *Cg_exo* chi angles: 330.7,36.8,331.5	0.08Å	Favored (40.879%)	-	-	-

A 282		GLY	0.61	-	Favored (88.5%) Glycine / 84.4,-1.8	-	-	Favored (73.434%)	-	-	-
A 283		THR	0.61	-	Favored (44.02%) General / -127.0,154.9	Favored (38.7%) *p* chi angles: 68	0.11Å	Favored (36.506%)	-	-	-
A 284		SER	0.62	-	Favored (33.69%) General / -114.7,151.4	Favored (87.8%) *p* chi angles: 62.8	0.07Å	Favored (62.839%)	-	-	-
A 285		VAL	0.64	-	Favored (69.66%) Ile or Val / -126.8,132.8	Favored (62.7%) *t* chi angles: 179.6	0.04Å	Favored (65.705%) beta sheet	-	-	-
A 286		VAL	0.66	-	Favored (22.47%) Ile or Val / -121.7,161.2	Favored (26.5%) *m* chi angles: 301.1	0.09Å	Favored (45.703%) beta sheet	-	-	-
A 287		VAL	0.7	-	Favored (59.05%) Ile or Val / -108.4,120.0	Favored (51.6%) *t* chi angles: 181.2	0.04Å	Favored (26.046%) beta sheet	-	-	-
A 288		ASP	0.75	0.50Å OD2 with A 338 LYS NZ	Favored (3.8%) General / -151.8,110.5	Favored (64.3%) *t0* chi angles: 182.3,344.8	0.09Å	Favored (11.28%) beta sheet	-	OUTLIER(S) worst is CA-CB-CG: 4.8 σ	-
A 289		GLY	0.8	-	Favored (36.1%) Glycine / -60.5,-14.8	-	-	Favored (26.35%)	-	-	-
A 290		GLY	0.84	-	Favored (90.56%) Glycine / -82.3,-2.2	-	-	Favored (54.282%)	-	-	-
A 291		CYS	0.86	-	Favored (15.98%) General / -81.5,170.3	Favored (90.2%) *m* chi angles: 293.2	0.01Å	Favored (17.296%)	-	-	-
A 292		ASP	0.86	-	Favored (17.69%) General / -71.4,168.3	Favored (22.1%) *t0* chi angles: 199.7,351.9	0.05Å	Favored (28.632%)	-	-	-
A 293		GLY	0.84	-	Favored (43.42%) Glycine / -85.1,-167.6	-	-	Favored (17.578%)	-	-	-
A 294		ARG	0.81	-	Favored (57.93%) General / -61.8,136.3	Favored (84.2%) *mtt90* chi angles: 293.1,174.8,177.7,85.7	0.03Å	CaBLAM Disfavored (2.145%)	-	-	-
A 295		GLY	0.76	-	Favored (4.76%) Glycine / -121.9,-135.6	-	-	Favored (18.508%)	-	-	-
A 296		LYS	0.72	-	Favored (35.11%) General / -116.3,151.8	Favored (97.4%) *mttt* chi angles: 296.5,181.1,180.5,179.2	0.05Å	CaBLAM Disfavored (1.76%)	-	-	-
A 297		SER	0.68	-	Favored (49.28%) General / -55.2,135.8	Favored (28.3%) *t* chi angles: 173.7	0.06Å	Favored (24.402%)	-	-	-
A 298		THR	0.66	-	Favored (53.37%) General / -124.6,133.1	Favored (81.3%) *m* chi angles: 302.4	0.05Å	Favored (49.666%) beta sheet	-	-	-
A 299		ARG	0.66	-	Favored (55.14%)	Favored (33.6%) *ttp-170*	0.04Å	Favored (41.54%)	-	-	-

#	Alt	Res	High B	Clash > 0.4Å	Ramachandran	Rotamer	Cβ deviation	CaBLAM	Bond lengths	Bond angles	Cis Peptides
					General / -67.0,145.7	chi angles: 201.1,169.5,71.5,186					
A 300		SER	0.68	-	Favored (56.04%) General / -61.7,-17.5	Favored (91%) *p* chi angles: 63	0.05Å	Favored (41.554%)	-	-	-
**#**	**Alt**	**Res**	**High B**	**Clash > 0.4Å**	**Ramachandran**	**Rotamer**	**Cβ deviation**	**CaBLAM**	**Bond lengths**	**Bond angles**	**Cis Peptides**
			Avg: 1.21	Clashscore: 0.37	Outliers: 0 of 350	Poor rotamers: 0 of 307	Outliers: 0 of 318	Outliers: 8 of 348	Outliers: 2 of 352	Outliers: 6 of 352	Non-Trans: 3 of 351
A 301		THR	0.72	-	Favored (44.47%) General / -121.2,148.2	Favored (64.4%) *p* chi angles: 58.1	0.04Å	Favored (25.081%)	-	-	-
A 302		THR	0.76	-	Favored (8.65%) General / -81.7,177.9	Favored (42%) *p* chi angles: 67.4	0.09Å	Favored (29.467%)	-	-	-
A 303		ASP	0.79	-	Favored (45.24%) General / -57.4,-22.8	Favored (89.5%) *m-30* chi angles: 284.5,345.9	0.06Å	Favored (42.025%)	-	-	-
A 304		SER	0.8	-	Favored (58.12%) General / -83.8,-10.4	Favored (88.7%) *p* chi angles: 69.3	0.08Å	Favored (38.537%)	-	-	-
A 305		GLY	0.79	-	Favored (68.01%) Glycine / 93.9,-13.2	-	-	Favored (65.615%)	-	-	-
A 306		LYS	0.76	-	Favored (52.03%) General / -61.4,132.0	Favored (86.6%) *tttt* chi angles: 182.5,177.7,178.9,179.7	0.01Å	Favored (38.633%)	-	-	-
A 307		ILE	0.72	-	Favored (34.67%) Ile or Val / -77.7,131.4	Favored (92.8%) *mt* chi angles: 296.4,169.8	0.02Å	Favored (43.936%) beta sheet	-	-	-
A 308		ILE	0.68	-	Favored (21.66%) Pre-Pro / -83.4,110.0	Favored (82%) *mt* chi angles: 299.4,168.3	0.16Å	Favored (60.946%)	-	-	-
A 309		PRO	0.66	-	Favored (21.28%) Trans-Pro / -74.5,-16.9	Favored (69.3%) *Cg_endo* chi angles: 29.5,325.2,25.1	0.07Å	Favored (55.208%)	-	-	-
A 310		GLU	0.65	-	Favored (11.43%) General / -141.3,118.7	Favored (90.7%) *tt0* chi angles: 182.8,174.4,357.4	0.02Å	Favored (17.139%)	-	-	-
A 311		TRP	0.65	-	Favored (29.64%) General / -119.2,156.6	Favored (55%) *m100* chi angles: 295.6,72.8	0.08Å	Favored (26.5%)	-	-	-
A 312		CYS	0.67	-	Favored (29.69%) General / -147.4,146.6	Favored (53.2%) *t* chi angles: 180.9	0.08Å	Favored (70.822%) beta sheet	-	-	-
A 313		CYS	0.7	-	Favored (24.15%) General / -136.8,166.2	Favored (24.2%) *p* chi angles: 68.6	0.06Å	Favored (52.39%)	-	-	-
A 314		ARG	0.74	-	Favored (16.18%) General / -112.4,107.4	Favored (24.4%) *mtp-110* chi angles: 302,184.4,77.9,251.9	0.04Å	CaBLAM Disfavored (2.586%)	-	-	-

A 315		SER	0.77	-	Favored (17.77%) General / 57.5,29.9	Favored (52%) *m* chi angles: 300.8	0.02Å	CaBLAM Disfavored (2.202%)	-	-	-
A 316		CYS	0.78	-	Favored (22.45%) General / -83.2,158.2	Favored (15.2%) *p* chi angles: 72.1	0.01Å	Favored (29.817%)	-	-	-
A 317		THR	0.78	-	Favored (20.99%) General / -89.5,153.2	Favored (74%) *p* chi angles: 61.5	0.06Å	Favored (17.617%)	-	-	-
A 318		MET	0.75	-	Favored (39.65%) Pre-Pro / -95.2,152.8	Favored (38.8%) *mmp* chi angles: 297.1,289.4,99.5	0.03Å	Favored (12.019%)	-	-	-
A 319		PRO	0.72	-	Favored (50.64%) Cis-Pro / -60.3,153.2	Favored (62.6%) *Cg_exo* chi angles: 335.8,34.5,329.5	0.05Å	Favored (66.006%)	-	-	Cis PRO omega= -11.43
A 320		PRO	0.69	-	Favored (70.57%) Trans-Pro / -69.3,153.7	Favored (63.2%) *Cg_endo* chi angles: 26.7,325.7,27.1	0.07Å	Favored (86.183%)	-	-	-
**#**	**Alt**	**Res**	**High B**	**Clash > 0.4Å**	**Ramachandran**	**Rotamer**	**Cβ deviation**	**CaBLAM**	**Bond lengths**	**Bond angles**	**Cis Peptides**
			Avg: 1.21	Clashscore: 0.37	Outliers: 0 of 350	Poor rotamers: 0 of 307	Outliers: 0 of 318	Outliers: 8 of 348	Outliers: 2 of 352	Outliers: 6 of 352	Non-Trans: 3 of 351
A 321		VAL	0.68	-	Favored (22.71%) Ile or Val / -88.3,136.7	Favored (81.2%) *t* chi angles: 177.9	0.05Å	Favored (47.328%) beta sheet	-	-	-
A 322		SER	0.69	-	Favored (40.87%) General / -139.2,160.0	Favored (95%) *p* chi angles: 64.8	0.04Å	Favored (55.954%) beta sheet	-	-	-
A 323		PHE	0.73	-	Favored (44.59%) General / -110.4,140.5	Favored (95.1%) *m-80* chi angles: 292.2,94.2	0.16Å	Favored (58.881%) beta sheet	-	OUTLIER(S) worst is CA-CB-CG: 5.9 σ	-
A 324		HIS	0.77	-	Favored (16.6%) General / -123.3,111.6	Favored (56.8%) *m-70* chi angles: 299.4,260	0.04Å	Favored (35.184%)	-	-	-
A 325		GLY	0.82	-	Favored (15.42%) Glycine / -117.7,-171.3	-	-	Favored (23.27%)	-	-	-
A 326		SER	0.84	-	Favored (66.08%) General / -66.7,-21.6	Favored (90.2%) *p* chi angles: 68.7	0.03Å	Favored (40.655%)	-	-	-
A 327		ASP	0.82	-	Favored (41.39%) General / -97.3,9.8	Favored (77.2%) *m-30* chi angles: 291.4,326.7	0.05Å	Favored (45.458%)	-	-	-
A 328		GLY	0.79	-	Favored (16.04%) Glycine / 110.3,-173.0	-	-	Favored (32.048%)	-	-	-
A 329		CYS	0.73	-	Favored (35.31%) General / -81.9,136.3	Favored (39.5%) *t* chi angles: 187.2	0.03Å	Favored (7.13%)	-	-	-
A 330		TRP	0.68	-	Favored (39.46%) General / -121.3,151.9	Favored (60.2%) *m100* chi angles: 293.2,73	0.12Å	Favored (56.827%) beta sheet	-	-	-

A 331		TYR	0.64	-	Favored (39.58%) Pre-Pro / -107.7,150.0	Favored (50.9%) *m-80* chi angles: 283.3,99.2	0.01Å	Favored (33.627%)	-	-	-
A 332		PRO	0.62	-	Favored (38.04%) Trans-Pro / -66.0,161.8	Favored (42.9%) *Cg_endo* chi angles: 24.1,325.8,30.8	0.01Å	Favored (61.682%)	-	-	-
A 333		MET	0.61	-	Favored (39.44%) General / -57.0,-22.6	Favored (17.2%) *ptt* chi angles: 68.5,182.5,175.9	0.02Å	Favored (29.869%)	-	-	-
A 334		GLU	0.62	-	Favored (60.26%) General / -74.3,-20.1	Favored (54.7%) *mp0* chi angles: 285.2,84,3.8	0.07Å	Favored (66.64%)	-	-	-
A 335		ILE	0.64	-	Favored (51.24%) Ile or Val / -113.5,116.0	Favored (79.9%) *mt* chi angles: 300.1,169.7	0.07Å	Favored (17.662%)	-	-	-
A 336		ARG	0.68	-	Favored (77.96%) Pre-Pro / -127.8,154.1	Favored (79.7%) *mtp180* chi angles: 294.7,180.1,67.3,175.4	0.05Å	Favored (33.084%)	-	-	-
A 337		PRO	0.74	-	Favored (87.05%) Trans-Pro / -56.5,139.4	Favored (83.5%) *Cg_exo* chi angles: 334.3,38.5,325.3	0.06Å	Favored (57.265%)	-	-	-
A 338		LYS	0.83	0.50Å NZ with A 288 ASP OD2	Favored (18.69%) General / -87.4,-34.5	Favored (79.2%) *tttt* chi angles: 188.6,169.4,182.8,176.5	0.05Å	Favored (25.961%)	-	-	-
A 339		LYS	0.92	-	Favored (13.52%) General / -107.8,-19.5	Favored (94.8%) *mttt* chi angles: 296,178.1,177.6,172.1	0.04Å	Favored (22.739%)	-	-	-
A 340		THR	0.99	-	Favored (40.06%) General / -95.8,125.0	Favored (92%) *m* chi angles: 299	0.05Å	Favored (28.971%)	-	-	-
**#**	**Alt**	**Res**	**High B**	**Clash > 0.4Å**	**Ramachandran**	**Rotamer**	**Cβ deviation**	**CaBLAM**	**Bond lengths**	**Bond angles**	**Cis Peptides**
			Avg: 1.21	Clashscore: 0.37	Outliers: 0 of 350	Poor rotamers: 0 of 307	Outliers: 0 of 318	Outliers: 8 of 348	Outliers: 2 of 352	Outliers: 6 of 352	Non-Trans: 3 of 351
A 341		HIS	1.02	-	Favored (56.45%) General / -63.2,145.0	Favored (76.8%) *t-90* chi angles: 190.7,279.7	0.02Å	Favored (42.856%)	-	-	-
A 342		ASP	1	-	Favored (38.42%) General / -52.5,-32.1	Favored (91%) *m-30* chi angles: 284.9,346.3	0.04Å	Favored (44.985%)	-	-	-
A 343		SER	0.94	-	Favored (61.71%) General / -64.9,-15.1	Favored (82.3%) *p* chi angles: 62.2	0.05Å	Favored (48.236%) alpha helix	-	-	-
A 344		HIS	0.86	-	Favored (22.23%) General / -111.0,9.1	Favored (98.2%) *m-70* chi angles: 296,287.2	0.05Å	Favored (52.009%)	-	-	-
A 345		LEU	0.81	-	Favored (33.75%) General / -102.7,142.5	Favored (82.6%) *mt* chi angles: 300.6,178.9	0.03Å	Favored (33.617%)	-	-	-
A 346		VAL	0.81	-	Favored (30.74%) Ile or Val / -88.9,116.6	Favored (79.8%) *t* chi angles: 178	0.08Å	Favored (57.395%) beta sheet	-	-	-

A 347		ARG	0.86	-	Favored (51.33%) General / -129.5,146.2	Favored (87.8%) *mtm180* chi angles: 293.1,174.3,290.2,168.1	0.05Å	Favored (47.059%) beta sheet	-	-	-
A 348		SER	0.98	-	Favored (57.3%) General / -66.0,143.6	Favored (40.4%) *t* chi angles: 176.6	0.04Å	Favored (34.475%) beta sheet	-	-	-
A 349		TRP	1.17	-	Favored (15.48%) General / -114.1,20.7	Favored (46.8%) *p90* chi angles: 53.6,86.7	0.07Å	Favored (8.476%) beta sheet	-	-	-
A 350		VAL	1.44	-	Favored (34.8%) Ile or Val / -70.7,130.3	Favored (89.4%) *t* chi angles: 174.1	0.03Å	Favored (14.765%)	-	-	-
A 351		THR	1.75	-	Favored (8.6%) General / -124.8,14.3	Favored (70.9%) *p* chi angles: 59.5	0.01Å	-	-	-	-
A 352		ALA	2.08	-	-	-	0.02Å	-	-	-	-

About MolProbity | Website for the Richardson Lab | Using ecloud x-H | Internal reference 4.5.2
