# Supplementary material for: Integrative proteome-wide structural analysis and high-throughput docking identify broad-spectrum antiviral scaffolds against Zika, Yellow Fever, West Nile, Saint Louis encephalitis, and Usutu viruses
Source: Front Cell Infect Microbiol. 2026 Apr 30;16:1723132. doi: 10.3389/fcimb.2026.1723132 (PMC13171538; doi:10.3389/fcimb.2026.1723132)
Supplement: Supplementary file 6 [file DataSheet6.zip › YFV/YF_NS2a/Mol_probity_Files/YF_NS2a_1FH-multi.table.pdf]

Viewing YF_NS2a1FH-multi.table

Duke Biochemistry
Duke University School of Medicine

When finished, you should close this window.

Hint: Use File | Save As... to save a copy of this page.

All-Atom Contacts	Clashscore, all atoms:	2.85		98th percentile* (N=1784, all resolutions)
	Clashscore is the number of serious steric overlaps (> 0.4 Å) per 1000 atoms.			
Protein Geometry	Poor rotamers	0	0.00%	Goal: <0.3%
	Favored rotamers	181	100.00%	Goal: >98%
	Ramachandran outliers	1	0.45%	Goal: <0.05%
	Ramachandran favored	215	96.85%	Goal: >98%
	Rama distribution Z-score	0.20 ± 0.52		Goal: abs(Z score) < 2
	MolProbity score^	1.27		99th percentile* (N=27675, 0Å - 99Å)
	Cβ deviations >0.25Å	0	0.00%	Goal: 0
	Bad bonds:	2 / 1709	0.12%	Goal: 0%
	Bad angles:	8 / 2314	0.35%	Goal: <0.1%
Peptide Omegas	Cis Prolines:	1 / 8	12.50%	Expected: ≤1 per chain, or ≤5%
	Cis nonProlines:	1 / 215	0.47%	Goal: <0.05%
Low-resolution Criteria	CaBLAM outliers	6	2.7%	Goal: <1.0%
	CA Geometry outliers	3	1.36%	Goal: <0.5%
Additional validations	Chiral volume outliers	0/291		
	Waters with clashes	0/0	0.00%	See UnDowser table for details

In the two column results, the left column gives the raw count, right column gives the percentage.

* 100th percentile is the best among structures of comparable resolution; 0th percentile is the worst. For clashscore the comparative set of structures was selected in 2004, for MolProbity score in 2006.

^ MolProbity score combines the clashscore, rotamer, and Ramachandran evaluations into a single score, normalized to be on the same scale as X-ray resolution.

Key to table colors and cutoffs here:

#	Alt	Res	High B	Clash > 0.4Å	Ramachandran	Rotamer	Cβ deviation	CaBLAM	Bond lengths	Bond angles	Cis Peptides
			Avg: 4.77	Clashscore: 2.85	Outliers: 1 of 222	Poor rotamers: 0 of 181	Outliers: 0 of 202	Outliers: 8 of 220	Outliers: 2 of 224	Outliers: 7 of 224	Non-Trans: 2 of 223
A 1		GLY	7.89	-	-	-	-	-	-	-	-
A 2		GLU	7.65	-	Favored (11.94%) General / -114.0,24.6	Favored (24%) *pt0* chi angles: 62.9,177.5,359.4	0.06Å	-	-	-	-
A 3		ILE	7.3	-	Favored (2.3%) Ile or Val / -84.9,86.5	Favored (42.7%) *mm* chi angles: 307.5,303.5	0.06Å	Favored (18.55%)	-	-	-
A 4		HIS	6.87	-	Favored (8.74%) General / -81.8,76.8	Favored (94.5%) *m-70* chi angles: 296.4,295.2	0.04Å	Favored (62.694%) beta sheet	-	-	-
A 5		ALA	6.38	-	Favored (30.9%) General / -51.7,132.3	-	0.04Å	Favored (17.711%) beta sheet	-	-	-
A 6		ILE	5.89	-	OUTLIER (0)%) Pre-Pro / -6.2,-79.5	Favored (82%) *mt* chi angles: 299.8,171.1	0.20Å	CaBLAM Disfavored (2.482%) try beta sheet	OUTLIER(S) worst is CB--CG2: 4.1 σ	-	-

A 7		PRO	5.45	-	Favored<br>(90.85%)<br>Cis-Pro /<br>-78.4,160.8	Favored (76.4%)<br>*Cg_endo*<br>chi angles: 30,322.6,29	0.06Å	Favored<br>(12.699%)	-	-	Cis PRO<br>omega=<br>-11.63
A 8		PHE	5.07	-	Favored<br>(25.32%)<br>General /<br>-49.0,-38.9	Favored (86.3%)<br>*t80*<br>chi angles: 181.7,78.6	0.06Å	Favored<br>(35.474%)	-	-	-
A 9		GLY	4.78	-	Favored<br>(17.19%)<br>Glycine /<br>-52.4,-24.5	-	-	Favored<br>(70.203%)	-	-	-
A<br>10		LEU	4.57	-	Favored<br>(91.12%)<br>General /<br>-61.5,-39.5	Favored (63.8%) *mt*<br>chi angles: 287.6,165	0.08Å	Favored<br>(57.825%)<br>three-ten	-	-	-
A<br>11		VAL	4.45	0.42Å<br>HB with A 80<br>PHE CE1	Favored<br>(15.8%)<br>Ile or Val /<br>-78.4,-31.1	Favored (3.2%) *p*<br>chi angles: 74.5	0.10Å	Favored<br>(66.035%)<br>alpha helix	-	-	-
A<br>12		SER	4.38	-	Favored<br>(84.57%)<br>General /<br>-59.6,-39.5	Favored (68.7%) *m*<br>chi angles: 294.8	0.02Å	Favored<br>(73.468%)<br>alpha helix	-	-	-
A<br>13		MET	4.35	-	Favored<br>(96.56%)<br>General /<br>-63.9,-43.3	Favored (62.1%)<br>*mtm*<br>chi angles:<br>291.9,192.6,299.1	0.14Å	Favored<br>(96.009%)<br>alpha helix	-	-	-
A<br>14		MET	4.36	-	Favored<br>(92.8%)<br>General /<br>-60.3,-45.6	Favored (28.3%)<br>*tmm*<br>chi angles:<br>181.6,277.4,290.5	0.04Å	Favored<br>(99.227%)<br>alpha helix	-	-	-
A<br>15		ILE	4.4	-	Favored<br>(98.16%)<br>Ile or Val /<br>-62.2,-43.7	Favored (93.8%) *mt*<br>chi angles: 291.9,166.8	0.05Å	Favored<br>(92.008%)<br>alpha helix	-	-	-
A<br>16		ALA	4.44	-	Favored<br>(78.8%)<br>General /<br>-57.8,-39.9	-	0.04Å	Favored<br>(87.835%)<br>alpha helix	-	-	-
A<br>17		MET	4.52	-	Favored<br>(75.71%)<br>General /<br>-69.7,-36.1	Favored (49%)<br>*mmp*<br>chi angles:<br>293.1,298.3,95.8	0.05Å	Favored<br>(83.287%)<br>alpha helix	-	-	-
A<br>18		GLU	4.63	-	Favored<br>(95.63%)<br>General /<br>-60.1,-43.4	Favored (89.3%) *tt0*<br>chi angles:<br>178.8,178,355.7	0.05Å	Favored<br>(83.939%)<br>alpha helix	-	-	-
A<br>19		VAL	4.79	-	Favored<br>(96.58%)<br>Ile or Val /<br>-64.1,-42.8	Favored (87.3%) *t*<br>chi angles: 173.9	0.03Å	Favored<br>(96.849%)<br>alpha helix	-	-	-
A<br>20		VAL	5.04	-	Favored<br>(84.98%)<br>Ile or Val /<br>-60.0,-41.1	Favored (58.7%) *t*<br>chi angles: 170.6	0.05Å	Favored<br>(90.817%)<br>alpha helix	-	-	-
**#**	**Alt**	**Res**	**High B**	**Clash > 0.4Å**	**Ramachandran**	**Rotamer**	**Cβ deviation**	**CaBLAM**	**Bond lengths**	**Bond angles**	**Cis Peptides**
			Avg: 4.77	Clashscore: 2.85	Outliers: 1 of 222	Poor rotamers: 0 of 181	Outliers: 0 of 202	Outliers: 8 of 220	Outliers: 2 of 224	Outliers: 7 of 224	Non-Trans: 2 of 223
A<br>21		LEU	5.37	-	Favored<br>(90.26%)<br>General /<br>-63.6,-38.2	Favored (72.5%) *mt*<br>chi angles: 289.8,175.3	0.14Å	Favored<br>(44.206%)	-	-	-
A<br>22		ARG	5.77	-	Favored<br>(49.8%)	Favored (96.9%)<br>*mtt-85*	0.05Å	CA Geom<br>Outlier<br>(0.393%)	-	-	-

					General /<br>-70.5,137.1	chi angles:<br>288.7,182.6,179.8,275.8					
A<br>23		LYS	6.17	-	Allowed<br>(1.52%)<br>General /<br>57.0,-121.6	Favored (92.6%)<br>*mttt*<br>chi angles:<br>300.4,183.2,181,178.4	0.04Å	CA Geom<br>Outlier<br>(0.072%)	-	-	-
A<br>24		ARG	6.48	-	Favored<br>(2.86%)<br>General /<br>-130.4,-21.6	Favored (91.3%)<br>*mmt-90*<br>chi angles:<br>295.9,291.3,180.3,270.4	0.03Å	CaBLAM<br>Outlier<br>(0.001%)	-	-	-
A<br>25		GLN	6.63	-	Allowed<br>(1.21%)<br>General /<br>45.5,-126.0	Favored (98.2%)<br>*mm-40*<br>chi angles:<br>298.7,298.1,312.3	0.02Å	CaBLAM<br>Disfavored<br>(1.374%)	-	-	-
A<br>26		GLY	6.54	-	Favored<br>(41.01%)<br>Glycine /<br>173.0,175.7	-	-	Favored<br>(8.583%)	-	-	-
A<br>27		PRO	6.25	-	Favored<br>(34.89%)<br>Trans-Pro /<br>-51.0,-31.5	Favored (89.1%)<br>*Cg_exo*<br>chi angles:<br>329.9,38.5,329.7	0.07Å	Favored<br>(16.675%)	-	-	-
A<br>28		LYS	5.84	-	Favored<br>(61.7%)<br>General /<br>-63.7,-52.2	Favored (84.9%)<br>*tttt*<br>chi angles:<br>179.4,178.8,178.4,180.4	0.02Å	Favored<br>(63.121%)<br>alpha helix	-	-	-
A<br>29		GLN	5.39	-	Favored<br>(78.67%)<br>General /<br>-67.2,-35.0	Favored (94%) *tp40*<br>chi angles:<br>185.1,64.5,52.5	0.04Å	Favored<br>(72.744%)<br>alpha helix	-	-	-
A<br>30		ILE	4.96	-	Favored<br>(99.18%)<br>Ile or Val /<br>-62.5,-44.3	Favored (97.7%) *mt*<br>chi angles: 292.3,167.3	0.06Å	Favored<br>(82.339%)<br>alpha helix	-	-	-
A<br>31		LEU	4.61	-	Favored<br>(80.83%)<br>General /<br>-59.3,-48.6	Favored (67.2%) *tp*<br>chi angles: 175.8,61.6	0.04Å	Favored<br>(90.06%)<br>alpha helix	-	-	-
A<br>32		VAL	4.34	-	Favored<br>(81.9%)<br>Ile or Val /<br>-57.3,-43.3	Favored (55%) *t*<br>chi angles: 170.1	0.04Å	Favored<br>(85.331%)<br>alpha helix	-	-	-
A<br>33		GLY	4.15	-	Favored<br>(20.89%)<br>Glycine /<br>-52.7,-55.9	-	-	Favored<br>(93.66%)<br>alpha helix	-	-	-
A<br>34		GLY	4.02	-	Favored<br>(91.76%)<br>Glycine /<br>-59.2,-37.5	-	-	Favored<br>(90.304%)<br>alpha helix	-	-	-
A<br>35		MET	3.93	-	Favored<br>(76.73%)<br>General /<br>-68.0,-34.5	Favored (52.4%)<br>*mmp*<br>chi angles:<br>293.8,300.8,98.6	0.02Å	Favored<br>(85.304%)<br>alpha helix	-	-	-
A<br>36		VAL	3.86	-	Favored<br>(86.01%)<br>Ile or Val /<br>-67.7,-44.4	Favored (68.3%) *t*<br>chi angles: 171.9	0.01Å	Favored<br>(87.284%)<br>alpha helix	-	-	-
A<br>37		LEU	3.82	-	Favored<br>(97.55%)<br>General /<br>-63.8,-42.9	Favored (6.9%) *mp*<br>chi angles: 270.1,54.2	0.04Å	Favored<br>(82%)<br>alpha helix	-	-	-
A<br>38		LEU	3.83	-	Favored<br>(81.54%)<br>General /<br>-67.9,-42.1	Favored (88.6%) *mt*<br>chi angles: 290.5,172.1	0.04Å	Favored<br>(80.257%)<br>alpha helix	-	-	-
A<br>39		GLY	3.89	-	Favored<br>(40.02%)	-	-	Favored<br>(98.406%)<br>alpha helix	-	-	-

#	Alt	Res	High B	Clash > 0.4Å	Ramachandran	Rotamer	Cβ deviation	CaBLAM	Bond lengths	Bond angles	Cis Peptides
					Glycine / -56.9,-54.0						
A 40		ALA	4.02	-	Favored (72.35%) General / -58.5,-35.6	-	0.07Å	Favored (70.207%) alpha helix	-	-	-
#	Alt	Res	High B	Clash > 0.4Å	Ramachandran	Rotamer	Cβ deviation	CaBLAM	Bond lengths	Bond angles	Cis Peptides
			Avg: 4.77	Clashscore: 2.85	Outliers: 1 of 222	Poor rotamers: 0 of 181	Outliers: 0 of 202	Outliers: 8 of 220	Outliers: 2 of 224	Outliers: 7 of 224	Non-Trans: 2 of 223
A 41		MET	4.19	-	Favored (62.99%) General / -73.9,-40.5	Favored (83.1%) *mtm* chi angles: 289.2,187.7,286.7	0.03Å	Favored (72.148%) alpha helix	-	-	-
A 42		LEU	4.37	-	Favored (76.66%) General / -63.9,-33.9	Favored (84.5%) *mt* chi angles: 290.1,169.2	0.04Å	Favored (74.817%) alpha helix	-	-	-
A 43		VAL	4.54	-	Favored (7.23%) Ile or Val / -78.4,-4.9	Favored (27.5%) *m* chi angles: 299.8	0.04Å	Favored (55.664%)	-	-	-
A 44		GLY	4.63	-	Favored (83.15%) Glycine / 90.1,-5.2	-	-	Favored (69.77%)	-	-	-
A 45		GLN	4.65	-	Favored (79.25%) General / -68.4,-36.5	Favored (81.6%) *mt0* chi angles: 288.9,179.2,37.3	0.03Å	Favored (42.066%)	-	-	-
A 46		VAL	4.59	-	Favored (69.19%) Ile or Val / -116.6,131.4	Favored (71.8%) *t* chi angles: 178.5	0.08Å	Favored (27.073%)	-	-	-
A 47		THR	4.46	-	Favored (8.19%) General / -96.6,172.0	Favored (47.1%) *p* chi angles: 66.2	0.05Å	Favored (29.275%)	-	-	-
A 48		ILE	4.3	-	Favored (92.99%) Ile or Val / -59.8,-44.0	Favored (95.8%) *mt* chi angles: 292,168.1	0.01Å	Favored (63.6%)	-	-	-
A 49		LEU	4.14	-	Favored (73.8%) General / -63.6,-32.4	Favored (93%) *mt* chi angles: 292.5,175	0.01Å	Favored (78.165%) alpha helix	-	-	-
A 50		ASP	3.98	-	Favored (65.4%) General / -73.5,-38.3	Favored (62.2%) *m-30* chi angles: 293.7,309.7	0.12Å	Favored (91.898%) alpha helix	-	-	-
A 51		LEU	3.82	-	Favored (83.28%) General / -68.0,-39.8	Favored (39%) *tp* chi angles: 184.6,56.6	0.08Å	Favored (95.091%) alpha helix	-	-	-
A 52		LEU	3.65	-	Favored (97.63%) General / -63.3,-40.5	Favored (88.9%) *mt* chi angles: 290.6,172.2	0.03Å	Favored (97.112%) alpha helix	-	-	-
A 53		LYS	3.5	-	Favored (96.34%) General / -63.5,-40.2	Favored (97.3%) *mttt* chi angles: 287.9,175.4,177.3,174.4	0.02Å	Favored (96.917%) alpha helix	-	-	-
A 54		LEU	3.39	-	Favored (90.94%) General / -64.2,-44.8	Favored (65.6%) *tp* chi angles: 178.1,57.9	0.05Å	Favored (76.861%) alpha helix	-	-	-
A 55		THR	3.31	-	Favored (70.32%)	Favored (98%) *m* chi angles: 300.1	0.05Å	Favored (76.946%)	-	-	-

					General / -53.7,-45.4			alpha helix			
A 56		VAL	3.27	-	Favored (96.72%) Ile or Val / -62.1,-46.3	Favored (66.7%) *t* chi angles: 171.7	0.04Å	Favored (93.477%) alpha helix	-	-	-
A 57		ALA	3.29	-	Favored (76.4%) General / -61.1,-35.2	-	0.05Å	Favored (76.67%) alpha helix	-	-	-
A 58		VAL	3.37	-	Favored (96.6%) Ile or Val / -62.4,-42.7	Favored (59.7%) *t* chi angles: 170.7	0.08Å	Favored (81.542%) alpha helix	-	-	-
A 59		GLY	3.51	-	Favored (61.22%) Glycine / -59.4,-51.1	-	-	Favored (90.455%) alpha helix	-	-	-
A 60		LEU	3.7	-	Favored (88.97%) General / -62.0,-38.5	Favored (85.2%) *mt* chi angles: 294.6,169.6	0.11Å	Favored (81.4%) alpha helix	-	-	-

#	Alt	Res	High B	Clash > 0.4Å	Ramachandran	Rotamer	Cβ deviation	CaBLAM	Bond lengths	Bond angles	Cis Peptides
			Avg: 4.77	Clashscore: 2.85	Outliers: 1 of 222	Poor rotamers: 0 of 181	Outliers: 0 of 202	Outliers: 8 of 220	Outliers: 2 of 224	Outliers: 7 of 224	Non-Trans: 2 of 223
A 61		HIS	3.96	-	Favored (79.96%) General / -60.4,-48.9	Favored (5.5%) *t-170* chi angles: 177.8,166.5	0.02Å	Favored (82.454%) alpha helix	-	-	-
A 62		PHE	4.29	-	Favored (72.3%) General / -55.6,-49.6	Favored (88.2%) *t80* chi angles: 179.2,75.8	0.05Å	Favored (96.081%) alpha helix	-	-	-
A 63		HIS	4.7	-	Favored (89.84%) General / -61.2,-39.3	Favored (74.6%) *m-70* chi angles: 289.8,299.5	0.02Å	Favored (71.816%) alpha helix	-	-	-
A 64		GLU	5.14	-	Favored (51.88%) General / -91.8,4.8	Favored (80.2%) *mm-30* chi angles: 290.8,294.9,331.5	0.09Å	Favored (37.442%) alpha helix	-	-	-
A 65		MET	5.54	-	Favored (67.44%) General / -61.2,-26.1	Favored (56.6%) *ttm* chi angles: 184.2,174.8,282.3	0.01Å	Favored (35.915%) three-ten	-	-	-
A 66		ASN	5.79	-	Favored (67.63%) General / -65.3,-25.4	Favored (98.6%) *m-40* chi angles: 287.5,337.2	0.03Å	Favored (51.577%) three-ten	-	-	-
A 67		ASN	5.86	-	Favored (5.59%) General / -106.3,-40.5	Favored (88.3%) *m-40* chi angles: 294.5,319.8	0.04Å	CaBLAM Disfavored (2.013%) try alpha helix	-	-	-
A 68		GLY	5.71	-	Favored (46.28%) Glycine / 79.8,174.2	-	-	Favored (17.363%) alpha helix	-	-	-
A 69		GLY	5.39	-	Allowed (1.88%) Glycine / 74.2,-66.6	-	-	CaBLAM Outlier (0.657%) try alpha helix	-	-	-
A 70		ASP	4.96	-	Favored (89.81%)	Favored (89.7%) *m-30* chi angles: 291.1,350.1	0.05Å	Favored (81.087%) alpha helix	-	-	-

					General / -64.4,-38.1						
A 71		ALA	4.53	-	Favored (91.84%) General / -60.1,-41.1	-	0.09Å	Favored (91.248%) alpha helix	-	-	-
A 72		MET	4.16	0.40Å HB3 with A 72 MET HE3	Favored (82.92%) General / -64.4,-46.5	Favored (26.6%) *tmm* chi angles: 181.2,277.3,294.8	0.03Å	Favored (93.555%) alpha helix	-	-	-
A 73		TYR	3.86	-	Favored (92.64%) General / -61.7,-39.8	Favored (31%) *m-80* chi angles: 284.1,120.3	0.05Å	Favored (94.498%) alpha helix	-	-	-
A 74		MET	3.63	-	Favored (94.62%) General / -64.9,-40.1	Favored (84.3%) *mtm* chi angles: 290.2,188.1,287.5	0.05Å	Favored (96.115%) alpha helix	-	-	-
A 75		ALA	3.48	-	Favored (97.99%) General / -61.8,-41.6	-	0.06Å	Favored (79.562%) alpha helix	-	-	-
A 76		LEU	3.41	-	Favored (53.99%) General / -77.4,-35.8	Favored (2.9%) *mm* chi angles: 268.2,288.6	0.04Å	Favored (82.182%) alpha helix	-	-	-
A 77		ILE	3.43	-	Favored (79.17%) Ile or Val / -63.4,-50.3	Favored (92%) *mt* chi angles: 291.7,166.6	0.08Å	Favored (70.884%) alpha helix	-	-	-
A 78		ALA	3.52	-	Favored (92.09%) General / -65.7,-40.3	-	0.08Å	Favored (59.8%) alpha helix	-	-	-
A 79		ALA	3.68	-	Allowed (1.05%) General / -72.1,-63.9	-	0.07Å	Favored (24.175%) alpha helix	-	-	-
A 80		PHE	3.84	0.42Å CE1 with A 11 VAL HB	Favored (23.1%) General / -84.5,-35.3	Favored (3%) *m-10* chi angles: 278.3,347.3	0.06Å	Favored (9.137%)	-	OUTLIER(S) worst is CA-CB-CG: 4.7 σ	-
**#**	**Alt**	**Res**	**High B**	**Clash > 0.4Å**	**Ramachandran**	**Rotamer**	**Cβ deviation**	**CaBLAM**	**Bond lengths**	**Bond angles**	**Cis Peptides**
			Avg: 4.77	Clashscore: 2.85	Outliers: 1 of 222	Poor rotamers: 0 of 181	Outliers: 0 of 202	Outliers: 8 of 220	Outliers: 2 of 224	Outliers: 7 of 224	Non-Trans: 2 of 223
A 81		SER	3.95	-	Favored (29.76%) General / 56.8,39.1	Favored (70.5%) *m* chi angles: 296.3	0.03Å	CA Geom Outlier (0.435%)	-	-	-
A 82		ILE	3.99	0.47Å HA with A 82 ILE HD12	Favored (11.49%) Ile or Val / -62.9,147.9	Favored (7.2%) *tp* chi angles: 200.5,63.6	0.03Å	Favored (6.072%)	-	-	-
A 83		ARG	3.91	0.46Å C with A 82 ILE O	Favored (3%) Pre-Pro / -42.8,123.1	Favored (50.5%) *mtm110* chi angles: 283.5,182.2,295.5,107.6	0.13Å	Favored (17.525%)	-	-	-
A 84		PRO	3.74	-	Favored (28.16%) Trans-Pro / -56.2,-19.6	Favored (99.5%) *Cg_exo* chi angles: 332.4,35.1,332.8	0.04Å	Favored (22.846%)	-	-	-
A 85		GLY	3.54	-	Favored (33.5%) Glycine / -55.5,-23.0	-	-	Favored (62.114%)	-	-	-
A 86		LEU	3.36	0.42Å CD1 with A	Favored (64.72%)	Favored (5.8%) *mp* chi angles: 274.8,50	0.07Å	Favored (58.586%)	-	-	-

#	Alt	Res	High B	Clash > 0.4Å	Ramachandran	Rotamer	Cβ deviation	CaBLAM	Bond lengths	Bond angles	Cis Peptides
				86 LEU N	General / -68.3,-20.5			three-ten			
A 87		LEU	3.24	-	Favored (51.39%) General / -71.7,-8.0	Favored (89.4%) *mt* chi angles: 294.8,170.8	0.09Å	Favored (27.443%) three-ten	-	-	-
A 88		ILE	3.2	-	Favored (33.97%) Ile or Val / -58.9,-26.9	Favored (11.7%) *tp* chi angles: 193.8,63.6	0.03Å	Favored (42.004%) three-ten	-	-	-
A 89		GLY	3.24	-	Favored (65.16%) Glycine / -64.7,-19.5	-	-	Favored (78.827%) three-ten	-	-	-
A 90		PHE	3.37	-	Favored (27.76%) General / -71.5,-50.8	Favored (69.8%) *t80* chi angles: 185.4,85.7	0.10Å	Favored (28.074%) alpha helix	-	-	-
A 91		GLY	3.58	-	Favored (71.84%) Glycine / -59.9,-31.3	-	-	Favored (83.467%) alpha helix	-	-	-
A 92		LEU	3.89	-	Favored (14.28%) General / -103.2,22.3	Favored (89.1%) *mt* chi angles: 298.6,174.6	0.03Å	Favored (23.53%) alpha helix	-	-	-
A 93		ARG	4.29	-	Favored (38.24%) General / -75.5,150.3	Favored (41.6%) *ptt180* chi angles: 67.9,189.5,178.5,182	0.07Å	Favored (6.35%) alpha helix	-	-	-
A 94		THR	4.77	1.05Å O with A 94 THR HG22	Allowed (0.81%) General / 80.1,15.3	Favored (97.7%) *m* chi angles: 300.7	0.15Å	Favored (5.11%) alpha helix	-	OUTLIER(S) worst is CA-C-N: 5.4 σ	-
A 95		LEU	5.3	-	Allowed (1.41%) General / 46.3,-125.8	Favored (75.7%) *mt* chi angles: 301.7,175.3	0.20Å	CaBLAM Outlier (0.047%) try alpha helix	-	OUTLIER(S) worst is C-N-CA: 8.4 σ	Cis nonPRO omega= 5.99
A 96		TRP	5.78	0.48Å O with A 101 ARG NH1	Favored (55.91%) General / -66.9,145.0	Favored (23.8%) *m-10* chi angles: 297.3,8.3	0.03Å	CaBLAM Disfavored (2.007%)	-	-	-
A 97		SER	6.13	-	Favored (59.35%) Pre-Pro / -84.8,158.3	Favored (94.7%) *p* chi angles: 64.1	0.09Å	Favored (44.268%)	-	-	-
A 98		PRO	6.29	-	Favored (46.48%) Trans-Pro / -51.3,-33.7	Favored (86.2%) *Cg_exo* chi angles: 330.5,36.9,331.4	0.07Å	Favored (94.195%)	-	-	-
A 99		ARG	6.26	-	Favored (59.61%) General / -72.1,-45.7	Favored (41.9%) *tpt170* chi angles: 179.9,63.3,180,172.8	0.03Å	Favored (77.138%) alpha helix	-	-	-
A 100		GLU	6.05	-	Favored (88.17%) General / -63.9,-37.6	Favored (38%) *mt-10* chi angles: 288.6,167.1,309	0.01Å	Favored (84.383%) alpha helix	-	-	-
**#**	**Alt**	**Res**	**High B**	**Clash > 0.4Å**	**Ramachandran**	**Rotamer**	**Cβ deviation**	**CaBLAM**	**Bond lengths**	**Bond angles**	**Cis Peptides**
			Avg: 4.77	Clashscore: 2.85	Outliers: 1 of 222	Poor rotamers: 0 of 181	Outliers: 0 of 202	Outliers: 8 of 220	Outliers: 2 of 224	Outliers: 7 of 224	Non-Trans: 2 of 223
A 101		ARG	5.75	0.48Å NH1 with A 96 TRP O	Favored (98.46%) General / -62.4,-41.2	Favored (99.8%) *mtm-85* chi angles: 290.5,194.3,296.3,274.6	0.04Å	Favored (82.807%) alpha helix	-	-	-
A 102		LEU	5.43	-	Favored (69.16%)	Favored (66.2%) *tp* chi angles: 175.6,63	0.07Å	Favored (77.35%)	-	-	-

					General / -63.8,-50.4			alpha helix			
A 103		VAL	5.14	-	Favored (99.12%) Ile or Val / -63.4,-44.9	Favored (66.1%) *t* chi angles: 171.6	0.05Å	Favored (83.363%) alpha helix	-	-	-
A 104		LEU	4.91	-	Favored (88.9%) General / -65.2,-38.0	Favored (96.8%) *mt* chi angles: 292.4,171.3	0.06Å	Favored (95.198%) alpha helix	-	-	-
A 105		THR	4.74	-	Favored (85.45%) General / -64.5,-45.9	Favored (93.2%) *m* chi angles: 299.2	0.02Å	Favored (82.974%) alpha helix	-	-	-
A 106		LEU	4.64	-	Favored (79.65%) General / -68.9,-38.6	Favored (93.8%) *mt* chi angles: 292.8,174.7	0.01Å	Favored (83.222%) alpha helix	-	-	-
A 107		GLY	4.57	-	Favored (43.22%) Glycine / -59.1,-53.8	-	-	Favored (91.508%) alpha helix	-	-	-
A 108		ALA	4.56	-	Favored (74.31%) General / -59.1,-36.0	-	0.06Å	Favored (73.116%) alpha helix	-	-	-
A 109		ALA	4.6	-	Favored (89.7%) General / -60.8,-39.6	-	0.04Å	Favored (75.918%) alpha helix	-	-	-
A 110		MET	4.69	-	Favored (63.27%) General / -72.2,-28.6	Favored (50.6%) *mmp* chi angles: 293.7,300,95.8	0.13Å	Favored (82.37%) alpha helix	-	-	-
A 111		VAL	4.81	-	Favored (89.09%) Ile or Val / -65.0,-40.5	Favored (76.2%) *t* chi angles: 172.8	0.04Å	Favored (68.301%) alpha helix	-	-	-
A 112		GLU	4.99	-	Favored (67.23%) General / -52.8,-45.8	Favored (49.1%) *tt0* chi angles: 180,180.5,317.4	0.06Å	Favored (78.958%) alpha helix	-	-	-
A 113		ILE	5.21	-	Favored (97.54%) Ile or Val / -64.1,-43.6	Favored (38.4%) *mm* chi angles: 295.3,297.2	0.05Å	Favored (93.184%) alpha helix	-	-	-
A 114		ALA	5.45	-	Favored (83.94%) General / -61.9,-37.2	-	0.04Å	Favored (48.623%)	-	-	-
A 115		LEU	5.66	-	Favored (4.69%) General / -105.8,-44.2	Favored (89%) *mt* chi angles: 299.5,178	0.04Å	CaBLAM Outlier (0.317%)	-	-	-
A 116		GLY	5.82	-	Favored (34.63%) Glycine / 55.0,-125.1	-	-	Favored (14.358%)	-	-	-
A 117		GLY	5.83	-	Favored (47.27%) Glycine / -61.9,-15.0	-	-	Favored (11.056%)	-	-	-
A 118		MET	5.67	-	Favored (70.43%) General / -62.1,-29.6	Favored (97.1%) *mtp* chi angles: 289.6,175,68.4	0.03Å	Favored (27.458%)	-	-	-
A 119		MET	5.35	-	Favored (9.67%)	Favored (95%) *mmm*	0.11Å	CaBLAM Outlier (0.331%)	-	-	-

					General / -96.6,-39.2	chi angles: 295.3,295.9,287.1					
A 120		GLY	4.94	-	Favored (29.23%) Glycine / 54.5,-123.8	-	-	Favored (11.276%)	-	-	-

#	Alt	Res	High B	Clash > 0.4Å	Ramachandran	Rotamer	Cβ deviation	CaBLAM	Bond lengths	Bond angles	Cis Peptides
			Avg: 4.77	Clashscore: 2.85	Outliers: 1 of 222	Poor rotamers: 0 of 181	Outliers: 0 of 202	Outliers: 8 of 220	Outliers: 2 of 224	Outliers: 7 of 224	Non-Trans: 2 of 223
A 121		GLY	4.49	-	Favored (58.51%) Glycine / -59.8,-23.1	-	-	Favored (18.586%)	-	-	-
A 122		LEU	4.06	0.48Å C with A 122 LEU HD23	Favored (65.93%) General / -59.6,-52.4	Favored (5.8%) *tt* chi angles: 186.3,157.4	0.05Å	Favored (37.07%) three-ten	-	-	-
A 123		TRP	3.68	-	Favored (90.52%) General / -65.8,-39.0	Favored (11.4%) *m-10* chi angles: 278.1,341	0.04Å	Favored (67.701%) alpha helix	-	-	-
A 124		LYS	3.36	-	Favored (88.24%) General / -59.9,-40.3	Favored (97.1%) *mttt* chi angles: 290.6,180,182.2,179.7	0.02Å	Favored (74.816%) alpha helix	-	-	-
A 125		TYR	3.11	-	Favored (72.62%) General / -58.9,-50.8	Favored (88.1%) *t80* chi angles: 176.4,81.9	0.05Å	Favored (84.891%) alpha helix	-	-	-
A 126		LEU	2.91	-	Favored (81.45%) General / -63.1,-35.8	Favored (92.8%) *mt* chi angles: 292.5,174.9	0.03Å	Favored (78.218%) alpha helix	-	-	-
A 127		ASN	2.76	-	Favored (92.64%) General / -64.9,-43.5	Favored (98.6%) *m-40* chi angles: 290.3,339.3	0.12Å	Favored (87.186%) alpha helix	-	-	-
A 128		ALA	2.67	-	Favored (69.99%) General / -60.0,-51.4	-	0.07Å	Favored (84.404%) alpha helix	-	-	-
A 129		VAL	2.62	-	Favored (95.61%) Ile or Val / -62.8,-42.2	Favored (73.7%) *t* chi angles: 172.5	0.06Å	Favored (79.826%) alpha helix	-	-	-
A 130		SER	2.62	-	Favored (82.33%) General / -62.1,-36.7	Favored (65.7%) *m* chi angles: 297.2	0.10Å	Favored (75.957%) alpha helix	-	-	-
A 131		LEU	2.68	-	Favored (88.7%) General / -61.7,-38.7	Favored (81.2%) *mt* chi angles: 289.1,171.9	0.05Å	Favored (84.165%) alpha helix	-	-	-
A 132		CYS	2.8	-	Favored (80.05%) General / -68.8,-39.6	Favored (49.7%) *t* chi angles: 184.6	0.12Å	Favored (94.797%) alpha helix	-	-	-
A 133		ILE	2.96	-	Favored (99.26%) Ile or Val / -61.0,-45.1	Favored (90.6%) *mt* chi angles: 291.2,168	0.06Å	Favored (92.985%) alpha helix	-	-	-
A 134		LEU	3.15	-	Favored (74.97%) General / -56.3,-49.2	Favored (45.1%) *tp* chi angles: 180.9,66.7	0.08Å	Favored (95.693%) alpha helix	-	-	-

A 135		THR	3.38	-	Favored (75.21%) General / -59.1,-50.1	Favored (80%) *m* chi angles: 296.4	0.07Å	Favored (88.223%) alpha helix	-	-	-
A 136		ILE	3.65	-	Favored (79.55%) Ile or Val / -69.1,-39.5	Favored (40.7%) *mm* chi angles: 296.9,301.8	0.11Å	Favored (84.228%) alpha helix	-	-	-
A 137		ASN	3.95	-	Favored (86.73%) General / -61.1,-38.5	Favored (93.7%) *m-40* chi angles: 285.9,339.7	0.06Å	Favored (96.393%) alpha helix	-	-	-
A 138		ALA	4.26	-	Favored (94.15%) General / -62.8,-39.4	-	0.05Å	Favored (96.341%) alpha helix	-	-	-
A 139		VAL	4.52	-	Favored (90.56%) Ile or Val / -61.9,-41.4	Favored (57.6%) *t* chi angles: 170.5	0.01Å	Favored (87.386%) alpha helix	-	-	-
A 140		ALA	4.68	-	Favored (76.29%) General / -61.1,-35.1	-	0.03Å	Favored (53.228%)	-	-	-
**#**	**Alt**	**Res**	**High B**	**Clash > 0.4Å**	**Ramachandran**	**Rotamer**	**Cβ deviation**	**CaBLAM**	**Bond lengths**	**Bond angles**	**Cis Peptides**
			Avg: 4.77	Clashscore: 2.85	Outliers: 1 of 222	Poor rotamers: 0 of 181	Outliers: 0 of 202	Outliers: 8 of 220	Outliers: 2 of 224	Outliers: 7 of 224	Non-Trans: 2 of 223
A 141		SER	4.71	-	Favored (14.84%) General / -78.3,171.6	Favored (70.8%) *p* chi angles: 71.9	0.02Å	CaBLAM Disfavored (3.505%)	-	-	-
A 142		ARG	4.6	-	Favored (4.25%) General / -129.5,35.2	Favored (90.7%) *mmt-90* chi angles: 293.2,291.7,181.7,270.1	0.04Å	Favored (5.402%)	-	-	-
A 143		LYS	4.38	-	Favored (48.53%) General / -123.0,145.4	Favored (97.8%) *mttt* chi angles: 296.2,181.6,180.2,180	0.02Å	Favored (28.073%)	-	-	-
A 144		ALA	4.11	-	Favored (70.64%) General / -58.5,-34.2	-	0.03Å	Favored (56.571%)	-	-	-
A 145		SER	3.84	-	Favored (61.64%) General / -69.5,-12.4	Favored (92.8%) *p* chi angles: 64.4	0.05Å	Favored (45.517%) alpha helix	-	-	-
A 146		ASN	3.62	-	Favored (9.41%) General / -103.9,-32.2	Favored (36.8%) *m110* chi angles: 294.9,122.7	0.05Å	Favored (20.558%) alpha helix	-	-	-
A 147		VAL	3.45	-	Favored (77.54%) Ile or Val / -59.7,-38.8	Favored (58.1%) *t* chi angles: 170.5	0.02Å	Favored (66.242%) three-ten	-	-	-
A 148		ILE	3.32	-	Favored (29.28%) Ile or Val / -58.8,-25.6	Favored (10.9%) *tp* chi angles: 195.1,63.7	0.09Å	Favored (61.163%) three-ten	-	-	-
A 149		LEU	3.22	-	Favored (90.97%) Pre-Pro / -60.3,-49.4	Favored (86.8%) *mt* chi angles: 290.8,169.2	0.05Å	Favored (48.11%) three-ten	-	-	-
A 150		PRO	3.15	-	Favored (68.64%)	Favored (19.7%) *Cg_endo*	0.04Å	Favored (95.336%) alpha helix	-	-	-

					Trans-Pro / -63.7,-25.2	chi angles: 19.2,327,34.1					
A 151		LEU	3.13	-	Favored (70.43%) General / -71.7,-38.4	Favored (93.7%) *mt* chi angles: 294.2,175	0.07Å	Favored (75.701%) alpha helix	-	OUTLIER(S) worst is C-N-CA: 4.0 σ	-
A 152		MET	3.16	-	Favored (77.49%) General / -67.8,-34.8	Favored (98.7%) *mtp* chi angles: 292.8,176,75.5	0.12Å	Favored (76.204%) alpha helix	-	-	-
A 153		ALA	3.26	-	Favored (73.38%) General / -60.1,-34.3	-	0.04Å	Favored (73.935%) alpha helix	-	-	-
A 154		LEU	3.41	-	Favored (34.61%) General / -81.1,-30.6	Favored (95.3%) *mt* chi angles: 294.8,173.6	0.06Å	Favored (81.794%) alpha helix	-	-	-
A 155		LEU	3.61	-	Favored (69.12%) General / -68.1,-30.0	Favored (86.4%) *mt* chi angles: 291.4,174.7	0.06Å	Favored (32.961%)	-	-	-
A 156		THR	3.84	-	Favored (49.23%) Pre-Pro / -53.1,135.7	Favored (98.9%) *m* chi angles: 300.6	0.09Å	Favored (32.907%)	-	-	-
A 157		PRO	4.02	-	Favored (36.98%) Trans-Pro / -60.7,-15.4	Favored (36.6%) *Cg_endo* chi angles: 22.7,324.5,33.5	0.02Å	Favored (6.154%)	-	-	-
A 158		VAL	4.11	-	Favored (20.01%) Ile or Val / -57.5,130.0	Favored (72.8%) *t* chi angles: 172.4	0.05Å	Favored (31.422%)	-	-	-
A 159		THR	4.08	-	Favored (22.31%) General / -55.0,144.7	Favored (4.2%) *t* chi angles: 179.1	0.03Å	Favored (24.865%)	-	-	-
A 160		MET	3.96	-	Favored (65.45%) General / -60.3,-25.0	Favored (52.5%) *mmp* chi angles: 294.4,300.8,98.9	0.03Å	Favored (45.706%)	-	-	-
**#**	**Alt**	**Res**	**High B**	**Clash > 0.4Å**	**Ramachandran**	**Rotamer**	**Cβ deviation**	**CaBLAM**	**Bond lengths**	**Bond angles**	**Cis Peptides**
			Avg: 4.77	Clashscore: 2.85	Outliers: 1 of 222	Poor rotamers: 0 of 181	Outliers: 0 of 202	Outliers: 8 of 220	Outliers: 2 of 224	Outliers: 7 of 224	Non-Trans: 2 of 223
A 161		ALA	3.77	-	Favored (75.51%) General / -60.3,-35.4	-	0.03Å	Favored (57.348%) alpha helix	-	-	-
A 162		GLU	3.55	-	Favored (35.84%) General / -80.8,-31.3	Favored (70.2%) *mt-10* chi angles: 290.4,175.7,27.8	0.05Å	Favored (79.654%) alpha helix	-	-	-
A 163		VAL	3.35	-	Favored (90.96%) Ile or Val / -66.4,-44.2	Favored (86.7%) *t* chi angles: 173.8	0.05Å	Favored (84.495%) alpha helix	-	-	-
A 164		ARG	3.22	-	Favored (99.36%) General / -62.5,-42.2	Favored (99.6%) *mtm-85* chi angles: 287.6,193.4,295.1,271.1	0.02Å	Favored (94.057%) alpha helix	-	-	-
A 165		LEU	3.15	-	Favored (76.73%) General / -57.2,-49.2	Favored (60.6%) *tp* chi angles: 175.2,63.8	0.09Å	Favored (93.943%) alpha helix	-	-	-

A 166		ALA	3.15	-	Favored (81.12%) General / -59.2,-38.9	-	0.12Å	Favored (76.661%) alpha helix	-	-	-
A 167		THR	3.23	-	Favored (96.42%) General / -63.9,-43.3	Favored (98.3%) *m* chi angles: 300.1	0.09Å	Favored (87.719%) alpha helix	-	-	-
A 168		MET	3.37	-	Favored (79.15%) General / -60.4,-49.0	Favored (50.4%) *ttp* chi angles: 181.1,191.3,72.3	0.08Å	Favored (84.535%) alpha helix	-	-	-
A 169		LEU	3.57	-	Favored (99.16%) General / -63.2,-43.1	Favored (94.1%) *mt* chi angles: 291.8,171.3	0.12Å	Favored (82.364%) alpha helix	-	-	-
A 170		PHE	3.82	-	Favored (71.01%) General / -68.5,-46.0	Favored (69%) *t80* chi angles: 185.3,74.1	0.04Å	Favored (85.619%) alpha helix	-	-	-
A 171		CYS	4.11	-	Favored (78.99%) General / -59.2,-38.0	Favored (98.3%) *m* chi angles: 289.8	0.01Å	Favored (84.349%) alpha helix	-	-	-
A 172		THR	4.42	-	Favored (92.25%) General / -59.6,-45.3	Favored (92.4%) *m* chi angles: 299.1	0.03Å	Favored (91.025%) alpha helix	-	-	-
A 173		VAL	4.75	-	Favored (96.9%) Ile or Val / -62.5,-42.7	Favored (61.2%) *t* chi angles: 171	0.02Å	Favored (95.142%) alpha helix	-	-	-
A 174		VAL	5.09	-	Favored (93.52%) Ile or Val / -60.2,-43.5	Favored (58.7%) *t* chi angles: 170.6	0.01Å	Favored (96.443%) alpha helix	-	-	-
A 175		ILE	5.41	-	Favored (97.14%) Ile or Val / -61.3,-46.2	Favored (87.6%) *mt* chi angles: 290.7,168	0.05Å	Favored (97.291%) alpha helix	-	-	-
A 176		ILE	5.72	-	Favored (94.98%) Ile or Val / -60.4,-46.6	Favored (96.8%) *mt* chi angles: 292.7,166.7	0.04Å	Favored (96.242%) alpha helix	-	-	-
A 177		GLY	6.02	-	Favored (26.53%) Glycine / -54.7,-55.6	-	-	Favored (93.847%) alpha helix	-	-	-
A 178		VAL	6.31	-	Favored (87.31%) Ile or Val / -60.8,-41.1	Favored (55%) *t* chi angles: 170.1	0.04Å	Favored (73.672%) alpha helix	-	-	-
A 179		LEU	6.6	-	Favored (72.5%) General / -59.3,-34.6	Favored (76.4%) *mt* chi angles: 289.8,174.3	0.03Å	Favored (60.901%) alpha helix	-	-	-
A 180		HIS	6.92	-	Favored (10.16%) General / -84.8,-47.6	Favored (91%) *m-70* chi angles: 292.8,293.9	0.05Å	Favored (37.884%) alpha helix	-	-	-
**#**	**Alt**	**Res**	**High B**	**Clash > 0.4Å**	**Ramachandran**	**Rotamer**	**Cβ deviation**	**CaBLAM**	**Bond lengths**	**Bond angles**	**Cis Peptides**
			Avg: 4.77	Clashscore: 2.85	Outliers: 1 of 222	Poor rotamers: 0 of 181	Outliers: 0 of 202	Outliers: 8 of 220	Outliers: 2 of 224	Outliers: 7 of 224	Non-Trans: 2 of 223
A 181		GLN	7.29	-	Favored (81.11%)	Favored (98.5%) *mt0*	0.02Å	Favored (61.996%) alpha helix	-	-	-

					General / -63.3,-35.7	chi angles: 290,172.1,335.7					
A 182		ASN	7.68	-	Favored (11.75%) General / -108.7,25.4	Favored (71.7%) *m-40* chi angles: 290.1,282.8	0.06Å	Favored (25.539%) alpha helix	-	-	-
A 183		SER	8.01	-	Favored (44.96%) General / -60.9,-16.7	Favored (95.4%) *p* chi angles: 63.9	0.05Å	Favored (20.241%) alpha helix	-	-	-
A 184		LYS	8.2	-	Favored (59.27%) General / -85.4,-5.7	Favored (99%) *mttt* chi angles: 294.2,180.7,179.4,178.9	0.01Å	Favored (67.935%) alpha helix	-	-	-
A 185		ASP	8.21	-	Favored (5.58%) General / -132.9,102.3	Favored (38.3%) *t0* chi angles: 178,341	0.01Å	Favored (13.383%) alpha helix	-	-	-
A 186		THR	8	-	Favored (73.02%) General / -57.9,-36.9	Favored (90.1%) *m* chi angles: 298.2	0.02Å	Favored (47.208%) alpha helix	-	-	-
A 187		SER	7.6	-	Favored (62.39%) General / -74.7,-32.9	Favored (88.3%) *p* chi angles: 68.2	0.01Å	Favored (76.144%) alpha helix	-	-	-
A 188		MET	7.12	-	Favored (57.11%) General / -75.6,-40.1	Favored (64.7%) *tpp* chi angles: 184.8,60.9,75.7	0.02Å	Favored (66.328%) alpha helix	-	-	-
A 189		GLN	6.64	-	Favored (77.25%) General / -59.4,-37.2	Favored (60.5%) *tt0* chi angles: 186.5,178,47.2	0.03Å	Favored (69.995%) alpha helix	-	-	-
A 190		LYS	6.22	-	Favored (16.81%) General / -81.4,-44.9	Favored (86.4%) *tttt* chi angles: 182.4,177.3,179.4,184.1	0.01Å	Favored (48.875%) alpha helix	-	-	-
A 191		THR	5.9	-	Favored (94.74%) General / -62.2,-45.1	Favored (90.5%) *m* chi angles: 298.1	0.05Å	Favored (77.389%) alpha helix	-	-	-
A 192		ILE	5.67	-	Favored (28.15%) Pre-Pro / -67.3,-52.6	Favored (44.2%) *mm* chi angles: 297.5,300.5	0.20Å	Favored (86.379%) alpha helix	-	OUTLIER(S) worst is C-N-CA: 4.3 σ	-
A 193		PRO	5.5	-	Favored (67.13%) Trans-Pro / -52.8,-34.8	Favored (96.4%) *Cg_exo* chi angles: 331.6,38.5,327.7	0.02Å	Favored (94.872%) alpha helix	-	-	-
A 194		LEU	5.37	-	Favored (77.54%) General / -59.3,-49.5	Favored (63.5%) *tp* chi angles: 175.1,61.4	0.02Å	Favored (72.818%) alpha helix	-	-	-
A 195		VAL	5.28	-	Favored (88%) Ile or Val / -66.1,-46.4	Favored (74.9%) *t* chi angles: 172.7	0.05Å	Favored (73.334%) alpha helix	-	-	-
A 196		ALA	5.22	-	Favored (88.31%) General / -61.3,-38.9	-	0.05Å	Favored (77.209%) alpha helix	-	-	-
A 197		LEU	5.18	-	Favored (54.8%) General / -77.0,-37.4	Favored (86%) *mt* chi angles: 295.2,170.3	0.06Å	Favored (79.247%) alpha helix	-	-	-
A 198		THR	5.16	-	Favored (93.94%) General / -62.8,-45.2	Favored (91.7%) *m* chi angles: 297.9	0.01Å	Favored (88.124%) alpha helix	-	-	-

A 199		LEU	5.15	-	Favored (66.45%) General / -57.6,-52.2	Favored (61.2%) *tp* chi angles: 175,63.5	0.10Å	Favored (81.722%) alpha helix	-	-	-
A 200		THR	5.14	-	Favored (60.49%) General / -74.9,-18.0	Favored (68.5%) *p* chi angles: 59	0.15Å	Favored (57%) alpha helix	-	-	-
**#**	**Alt**	**Res**	**High B**	**Clash > 0.4Å**	**Ramachandran**	**Rotamer**	**Cβ deviation**	**CaBLAM**	**Bond lengths**	**Bond angles**	**Cis Peptides**
			Avg: 4.77	Clashscore: 2.85	Outliers: 1 of 222	Poor rotamers: 0 of 181	Outliers: 0 of 202	Outliers: 8 of 220	Outliers: 2 of 224	Outliers: 7 of 224	Non-Trans: 2 of 223
A 201		SER	5.11	-	Favored (55.03%) General / -73.6,-45.1	Favored (73.2%) *m* chi angles: 295.6	0.04Å	Favored (51.627%) alpha helix	-	-	-
A 202		TYR	5.06	-	Favored (86.94%) General / -61.4,-38.4	Favored (8.4%) *m-10* chi angles: 283.5,346.1	0.06Å	Favored (80.12%) alpha helix	-	-	-
A 203		LEU	4.99	-	Favored (53.51%) General / -78.6,-5.7	Favored (96.1%) *mt* chi angles: 294.3,173.3	0.05Å	Favored (55.678%)	-	-	-
A 204		GLY	4.94	-	Favored (61.62%) Glycine / 74.7,29.3	-	-	Favored (72.503%)	-	-	-
A 205		LEU	4.93	-	Favored (6.09%) General / -99.3,-44.8	Favored (3.9%) *mp* chi angles: 273.5,45.7	0.16Å	CaBLAM Disfavored (2.935%)	OUTLIER(S) worst is CB--CG: 5.4 σ	-	-
A 206		THR	4.98	-	Favored (41.08%) General / -94.3,128.8	Favored (91.4%) *m* chi angles: 298.9	0.06Å	Favored (20.591%)	-	-	-
A 207		GLN	5.06	-	Favored (89.2%) Pre-Pro / -59.6,134.4	Favored (62.7%) *tt0* chi angles: 184.6,180.8,10.6	0.07Å	Favored (47.972%)	-	-	-
A 208		PRO	5.15	-	Favored (19.93%) Trans-Pro / -47.5,-34.4	Favored (82.9%) *Cg_exo* chi angles: 329.2,37.2,332.3	0.04Å	Favored (90.868%)	-	-	-
A 209		PHE	5.25	-	Favored (71.03%) General / -57.1,-51.0	Favored (86.4%) *t80* chi angles: 172.9,78.2	0.05Å	Favored (73.593%) alpha helix	-	-	-
A 210		LEU	5.3	-	Favored (72.83%) General / -70.7,-35.6	Favored (96.9%) *mt* chi angles: 294.1,172.9	0.08Å	Favored (74.858%) alpha helix	-	-	-
A 211		GLY	5.28	-	Favored (32.06%) Glycine / -58.3,-55.2	-	-	Favored (87.568%) alpha helix	-	-	-
A 212		LEU	5.2	-	Favored (91.75%) General / -65.6,-39.6	Favored (91%) *mt* chi angles: 291.1,171.6	0.03Å	Favored (74.935%) alpha helix	-	-	-
A 213		CYS	5.1	-	Favored (97.46%) General / -63.6,-43.3	Favored (96.2%) *m* chi angles: 290.4	0.03Å	Favored (82.469%) alpha helix	-	-	-
A 214		ALA	5.02	-	Favored (94.7%) General / -65.1,-41.8	-	0.04Å	Favored (79.065%) alpha helix	-	-	-

A 215		PHE	5.01	-	Favored (65.36%) General / -67.5,-49.0	Favored (81.2%) *t80* chi angles: 178.6,71.8	0.09Å	Favored (81.523%) alpha helix	-	OUTLIER(S) worst is CA-CB-CG: 4.2 σ	-
A 216		MET	5.09	-	Favored (99.22%) General / -63.1,-41.1	Favored (81.8%) *mtm* chi angles: 290.2,187.8,292.7	0.05Å	Favored (93.589%) alpha helix	-	-	-
A 217		ALA	5.31	-	Favored (90.2%) General / -61.3,-39.4	-	0.04Å	Favored (94.397%) alpha helix	-	-	-
A 218		THR	5.7	-	Favored (80.52%) General / -64.5,-47.0	Favored (91.8%) *m* chi angles: 299	0.04Å	Favored (86.75%) alpha helix	-	-	-
A 219		ARG	6.31	-	Favored (85.67%) General / -58.9,-47.2	Favored (62.2%) *ttp-170* chi angles: 180,184.1,66,192.9	0.03Å	Favored (93.616%) alpha helix	-	-	-
A 220		ILE	7.09	-	Favored (96.81%) Ile or Val / -64.0,-45.4	Favored (91.8%) *mt* chi angles: 292,166.1	0.05Å	Favored (69.744%) alpha helix	-	-	-
**#**	**Alt**	**Res**	**High B**	**Clash > 0.4Å**	**Ramachandran**	**Rotamer**	**Cβ deviation**	**CaBLAM**	**Bond lengths**	**Bond angles**	**Cis Peptides**
			Avg: 4.77	Clashscore: 2.85	Outliers: 1 of 222	Poor rotamers: 0 of 181	Outliers: 0 of 202	Outliers: 8 of 220	Outliers: 2 of 224	Outliers: 7 of 224	Non-Trans: 2 of 223
A 221		PHE	7.96	-	Favored (46.4%) General / -94.4,-5.4	Favored (78.3%) *m-80* chi angles: 293.9,107.3	0.12Å	Favored (30.912%)	-	OUTLIER(S) worst is CA-CB-CG: 5.7 σ	-
A 222		GLY	8.79	-	Favored (60.31%) Glycine / -57.2,-29.7	-	-	Favored (20.086%)	-	-	-
A 223		ARG	9.47	-	Favored (5.01%) General / -127.9,32.0	Favored (96.3%) *mtt180* chi angles: 298.1,180.6,179.5,180.8	0.01Å	-	-	-	-
A 224		ARG	9.94	0.59Å HG2 with A 224 ARG OXT	-	Favored (77.7%) *ttt180* chi angles: 180.6,177.7,171.5,177.1	0.02Å	-	-	-	-

About MolProbity | Website for the Richardson Lab | Using ecloud x-H | Internal reference 4.5.2
