# Supplementary material for: Integrative proteome-wide structural analysis and high-throughput docking identify broad-spectrum antiviral scaffolds against Zika, Yellow Fever, West Nile, Saint Louis encephalitis, and Usutu viruses
Source: Front Cell Infect Microbiol. 2026 Apr 30;16:1723132. doi: 10.3389/fcimb.2026.1723132 (PMC13171538; doi:10.3389/fcimb.2026.1723132)
Supplement: Supplementary file 6 [file DataSheet6.zip › YFV/YF_NS2b/Mol_probity_Files/YF_NS2b_1FH-multi.table.pdf]

Viewing YF_NS2b1FH-multi.table

Duke Biochemistry
Duke University School of Medicine

When finished, you should close this window.

Hint: Use File | Save As... to save a copy of this page.

All-Atom Contacts	Clashscore, all atoms:	0.51		99th percentile* (N=1784, all resolutions)
	Clashscore is the number of serious steric overlaps (> 0.4 Å) per 1000 atoms.			
Protein Geometry	Poor rotamers	0	0.00%	Goal: <0.3%
	Favored rotamers	100	100.00%	Goal: >98%
	Ramachandran outliers	1	0.78%	Goal: <0.05%
	Ramachandran favored	126	98.44%	Goal: >98%
	Rama distribution Z-score	0.83 ± 0.72		Goal: abs(Z score) < 2
	MolProbity score^	0.68		100th percentile* (N=27675, 0Å - 99Å)
	Cβ deviations >0.25Å	0	0.00%	Goal: 0
	Bad bonds:	0 / 990	0.00%	Goal: 0%
	Bad angles:	3 / 1343	0.22%	Goal: <0.1%
Peptide Omegas	Cis Prolines:	0 / 4	0.00%	Expected: ≤1 per chain, or ≤5%
	Cis nonProlines:	2 / 125	1.60%	Goal: <0.05%
Low-resolution Criteria	CaBLAM outliers	2	1.6%	Goal: <1.0%
	CA Geometry outliers	2	1.59%	Goal: <0.5%
Additional validations	Chiral volume outliers	0/158		
	Waters with clashes	0/0	0.00%	See UnDowser table for details

In the two column results, the left column gives the raw count, right column gives the percentage.

* 100th percentile is the best among structures of comparable resolution; 0th percentile is the worst. For clashscore the comparative set of structures was selected in 2004, for MolProbity score in 2006.

^ MolProbity score combines the clashscore, rotamer, and Ramachandran evaluations into a single score, normalized to be on the same scale as X-ray resolution.

Key to table colors and cutoffs here:

#	Alt	Res	High B	Clash > 0.4Å	Ramachandran	Rotamer	Cβ deviation	CaBLAM	Bond lengths	Bond angles	Cis Peptides
			Avg: 7.71	Clashscore: 0.51	Outliers: 1 of 128	Poor rotamers: 0 of 100	Outliers: 0 of 116	Outliers: 3 of 126	Outliers: 0 of 130	Outliers: 2 of 130	Non-Trans: 2 of 129
A 1		SER	7.46	-	-	Favored (33%) *t*<br>chi angles: 183.1	0.03Å	-	-	-	-
A 2		ILE	6.77	-	Favored (71.74%)<br>Pre-Pro / -85.8,119.2	Favored (51%) *mm*<br>chi angles: 302.7,302.1	0.05Å	-	-	-	-
A 3		PRO	6.13	-	Favored (76.9%)<br>Trans-Pro / -61.0,-23.2	Favored (36.7%) *Cg_endo*<br>chi angles: 22.7,326.7,29.6	0.02Å	Favored (69.963%)	-	-	-
A 4		VAL	5.62	-	Favored (47.57%)<br>Ile or Val / -74.7,-42.4	Favored (88.9%) *t*<br>chi angles: 174.1	0.03Å	Favored (75.394%)<br>alpha helix	-	-	-
A 5		ASN	5.29	-	Favored (71.69%)<br>General / -71.3,-36.5	Favored (97.5%) *m-40*<br>chi angles: 289,335.9	0.04Å	Favored (78.638%)<br>alpha helix	-	-	-

A 6		GLU	5.12	-	Favored (91.39%) General / -64.8,-38.6	Favored (99.5%) *mt-10* chi angles: 291.4,176.2,354.8	0.05Å	Favored (84.037%) alpha helix	-	-	-
A 7		ALA	5.07	-	Favored (93.52%) General / -59.5,-43.1	-	0.08Å	Favored (84.773%) alpha helix	-	-	-
A 8		LEU	5.1	-	Favored (82.83%) General / -68.1,-39.3	Favored (97.7%) *mt* chi angles: 292.1,172.4	0.07Å	Favored (97.598%) alpha helix	-	-	-
A 9		ALA	5.17	-	Favored (99.71%) General / -62.8,-42.5	-	0.04Å	Favored (96.535%) alpha helix	-	-	-
A 10		ALA	5.2	-	Favored (99.08%) General / -63.1,-40.9	-	0.04Å	Favored (87.508%) alpha helix	-	-	-
A 11		ALA	5.2	-	Favored (90.05%) General / -60.8,-39.8	-	0.04Å	Favored (82.328%) alpha helix	-	-	-
A 12		GLY	5.15	-	Favored (28.99%) Glycine / -59.7,-55.7	-	-	Favored (90.426%) alpha helix	-	-	-
A 13		LEU	5.09	-	Favored (75.17%) General / -60.3,-50.0	Favored (61.1%) *tp* chi angles: 175.1,60.6	0.05Å	Favored (76.243%) alpha helix	-	-	-
A 14		VAL	5.05	-	Favored (53.95%) Ile or Val / -65.5,-31.5	Favored (5.3%) *p* chi angles: 69.7	0.05Å	Favored (77.957%) alpha helix	-	-	-
A 15		GLY	5.01	-	Favored (56.53%) Glycine / -59.7,-51.9	-	-	Favored (89.822%) alpha helix	-	-	-
A 16		VAL	4.99	-	Favored (95.04%) Ile or Val / -60.1,-44.5	Favored (56.1%) *t* chi angles: 170.2	0.02Å	Favored (84.618%) alpha helix	-	-	-
A 17		LEU	5	-	Favored (97.61%) General / -64.0,-41.3	Favored (98.2%) *mt* chi angles: 292.4,173	0.05Å	Favored (90.182%) alpha helix	-	-	-
A 18		ALA	5.07	-	Favored (95.01%) General / -63.8,-39.8	-	0.04Å	Favored (81.605%) alpha helix	-	-	-
A 19		GLY	5.26	-	Favored (64.34%) Glycine / -58.2,-50.6	-	-	Favored (94.912%) alpha helix	-	-	-
A 20		LEU	5.65	-	Favored (98.42%) General / -62.3,-41.3	Favored (89.9%) *mt* chi angles: 291,170.9	0.05Å	Favored (81.281%) alpha helix	-	-	-
**#**	**Alt**	**Res**	**High B**	**Clash > 0.4Å**	**Ramachandran**	**Rotamer**	**Cβ deviation**	**CaBLAM**	**Bond lengths**	**Bond angles**	**Cis Peptides**
			Avg: 7.71	Clashscore: 0.51	Outliers: 1 of 128	Poor rotamers: 0 of 100	Outliers: 0 of 116	Outliers: 3 of 126	Outliers: 0 of 130	Outliers: 2 of 130	Non-Trans: 2 of 129

A<br>21		ALA	6.39	-	Favored<br>(74.99%)<br>General /<br>-59.0,-36.5	-	0.04Å	Favored<br>(54.893%)<br>alpha helix	-	-	-
A<br>22		PHE	7.53	-	Favored<br>(11.87%)<br>General /<br>-115.6,24.0	Favored (80.8%) *m-80*<br>chi angles: 300.7,105.2	0.05Å	Favored<br>(16.823%)<br>alpha helix	-	-	-
A<br>23		GLN	8.99	-	Favored<br>(49.32%)<br>General /<br>-60.9,-17.2	Favored (23.9%)<br>*pt0*<br>chi angles:<br>68.1,187.6,314.6	0.07Å	Favored<br>(16.028%)<br>alpha helix	-	-	-
A<br>24		GLU	10.47	-	Favored<br>(58.7%)<br>General / -84.7,-3.8	Favored (97.8%)<br>*mt-10*<br>chi angles:<br>294.6,179.6,359.5	0.01Å	Favored<br>(52.829%)	-	-	-
A<br>25		MET	11.51	-	Favored<br>(35.37%)<br>General /<br>-101.3,140.6	Favored (86.1%)<br>*mtp*<br>chi angles:<br>294.4,176.4,61.9	0.09Å	Favored<br>(33.52%)	-	-	-
A<br>26		GLU	11.67	-	Favored<br>(68.13%)<br>General /<br>-64.7,-25.8	Favored (99.9%)<br>*mt-10*<br>chi angles:<br>292.1,179.9,354	0.01Å	Favored<br>(52.461%)	-	-	-
A<br>27		ASN	10.92	-	Favored<br>(13.19%)<br>General /<br>-100.0,21.1	Favored (88.5%) *m-40*<br>chi angles: 292.8,320.4	0.02Å	Favored<br>(25.928%)	-	-	-
A<br>28		PHE	9.63	-	Favored<br>(31.06%)<br>General /<br>-54.7,-26.0	Favored (25.6%)<br>*p90*<br>chi angles: 76.9,94.4	0.05Å	Favored<br>(26.379%)	-	-	-
A<br>29		LEU	8.24	-	Favored<br>(68.04%)<br>General /<br>-62.8,-25.0	Favored (86.7%) *mt*<br>chi angles: 290.6,173.1	0.03Å	Favored<br>(63.588%)	-	-	-
A<br>30		GLY	7.06	-	Allowed<br>(0.84%)<br>Glycine /<br>-55.4,-70.8	-	-	Favored<br>(43.791%)<br>three-ten	-	-	-
A<br>31		PRO	6.19	-	Favored<br>(24.55%)<br>Trans-Pro /<br>-48.9,-33.1	Favored (88.8%)<br>*Cg_exo*<br>chi angles:<br>329.4,37.5,331.9	0.02Å	Favored<br>(42.336%)<br>alpha helix	-	-	-
A<br>32		VAL	5.6	-	Favored<br>(80.93%)<br>Ile or Val /<br>-64.3,-49.5	Favored (61.5%) *t*<br>chi angles: 171	0.05Å	Favored<br>(71.258%)<br>alpha helix	-	-	-
A<br>33		ALA	5.17	-	Favored<br>(97.77%)<br>General /<br>-63.1,-40.5	-	0.04Å	Favored<br>(78.993%)<br>alpha helix	-	-	-
A<br>34		VAL	4.83	-	Favored<br>(88.2%)<br>Ile or Val /<br>-66.4,-45.8	Favored (73.7%) *t*<br>chi angles: 172.5	0.01Å	Favored<br>(97.182%)<br>alpha helix	-	-	-
A<br>35		GLY	4.56	-	Favored<br>(54.68%)<br>Glycine /<br>-56.3,-51.6	-	-	Favored<br>(94.036%)<br>alpha helix	-	-	-
A<br>36		GLY	4.35	-	Favored<br>(23.3%)<br>Glycine /<br>-51.6,-54.8	-	-	Favored<br>(97.716%)<br>alpha helix	-	-	-
A<br>37		ILE	4.19	-	Favored<br>(99.47%)	Favored (94.9%) *mt*<br>chi angles: 292,167	0.02Å	Favored<br>(87.171%)	-	-	-

#	Alt	Res	High B	Clash > 0.4Å	Ramachandran	Rotamer	Cβ deviation	CaBLAM	Bond lengths	Bond angles	Cis Peptides
					Ile or Val / -61.2,-45.0			alpha helix			
A 38		LEU	4.08	-	Favored (94.44%) General / -61.8,-40.2	Favored (93.6%) *mt* chi angles: 291.8,173.2	0.03Å	Favored (87.184%) alpha helix	-	-	-
A 39		MET	4.02	-	Favored (69.08%) General / -70.2,-31.6	Favored (51.4%) *mmp* chi angles: 292.1,300.5,96.6	0.09Å	Favored (87.836%) alpha helix	-	-	-
A 40		MET	3.99	-	Favored (81.81%) General / -67.8,-37.4	Favored (83.5%) *mtm* chi angles: 289.6,188.4,285	0.03Å	Favored (89.161%) alpha helix	-	-	-
**#**	**Alt**	**Res**	**High B**	**Clash > 0.4Å**	**Ramachandran**	**Rotamer**	**Cβ deviation**	**CaBLAM**	**Bond lengths**	**Bond angles**	**Cis Peptides**
			Avg: 7.71	Clashscore: 0.51	Outliers: 1 of 128	Poor rotamers: 0 of 100	Outliers: 0 of 116	Outliers: 3 of 126	Outliers: 0 of 130	Outliers: 2 of 130	Non-Trans: 2 of 129
A 41		LEU	3.98	-	Favored (98.59%) General / -63.5,-41.2	Favored (91.7%) *mt* chi angles: 291.3,171.4	0.04Å	Favored (85.834%) alpha helix	-	-	-
A 42		VAL	4.01	-	Favored (78.33%) Ile or Val / -66.8,-49.0	Favored (68.5%) *t* chi angles: 171.9	0.07Å	Favored (75.041%) alpha helix	-	-	-
A 43		SER	4.08	-	Favored (78.79%) General / -68.0,-43.4	Favored (72.7%) *m* chi angles: 295.7	0.05Å	Favored (78.55%) alpha helix	-	-	-
A 44		VAL	4.2	-	Favored (88.39%) Ile or Val / -67.2,-42.9	Favored (76.9%) *t* chi angles: 172.8	0.03Å	Favored (93.075%) alpha helix	-	-	-
A 45		ALA	4.42	-	Favored (70.42%) General / -60.0,-32.0	-	0.02Å	Favored (24.146%)	-	-	-
A 46		GLY	4.72	-	Favored (57.44%) Glycine / 93.5,12.1	-	-	Favored (76.865%)	-	-	-
A 47		ARG	5.13	-	Favored (68.03%) General / -64.4,-25.4	Favored (96.9%) *mtt180* chi angles: 289.8,173.2,180.7,169.3	0.04Å	Favored (21.338%)	-	-	-
A 48		VAL	5.66	-	Favored (45.55%) Ile or Val / -60.3,-30.1	Favored (8%) *p* chi angles: 67.5	0.04Å	CaBLAM Disfavored (1.986%)	-	-	-
A 49		ASP	6.31	-	Favored (6.81%) General / -168.5,174.4	Favored (14.4%) *t0* chi angles: 207.4,342.1	0.02Å	Favored (9.353%)	-	-	-
A 50		GLY	7.07	-	Favored (3.04%) Glycine / -77.7,66.3	-	-	CaBLAM Disfavored (2.222%)	-	-	-
A 51		LEU	7.91	-	Favored (31.89%) General / -81.3,144.0	Favored (86%) *mt* chi angles: 300.7,177.7	0.06Å	Favored (11.641%) beta sheet	-	-	-
A 52		GLU	8.77	-	Favored (17%) General / -152.0,136.6	Favored (91.5%) *tt0* chi angles: 183.2,176.7,358.2	0.02Å	Favored (35.175%) beta sheet	-	-	-

A 53		LEU	9.56	-	Favored (53.02%) General / -117.0,136.3	Favored (89.2%) *mt* chi angles: 298.3,173.7	0.03Å	Favored (61.335%) beta sheet	-	-	-
A 54		ARG	10.24	-	Favored (51.75%) General / -135.6,153.3	Favored (95.1%) *mtt180* chi angles: 297.2,186.1,180.2,184.1	0.06Å	Favored (52.839%) beta sheet	-	-	-
A 55		LYS	10.74	-	Favored (11.95%) General / -99.7,100.9	Favored (32.6%) *ttpt* chi angles: 179.6,170,62.4,168.6	0.03Å	Favored (43.273%)	-	-	-
A 56		LEU	10.99	-	Favored (7.77%) General / -89.0,66.1	Favored (74.9%) *mt* chi angles: 302.7,179.3	0.05Å	Favored (9.014%)	-	-	-
A 57		GLY	11.04	-	Favored (43.18%) Glycine / 89.5,168.2	-	-	Favored (18.086%)	-	-	-
A 58		GLU	11.01	-	Favored (24.32%) General / -84.2,152.2	Favored (79.8%) *mm-30* chi angles: 298.9,291.4,330	0.03Å	Favored (11.929%)	-	-	-
A 59		VAL	11.06	-	Favored (72.34%) Ile or Val / -125.0,131.6	Favored (77%) *t* chi angles: 178.2	0.03Å	Favored (51.132%) beta sheet	-	-	-
A 60		SER	11.26	-	Favored (11.78%) General / -146.7,124.3	Favored (44.6%) *t* chi angles: 179	0.03Å	Favored (26.432%) beta sheet	-	-	-
**#**	**Alt**	**Res**	**High B**	**Clash > 0.4Å**	**Ramachandran**	**Rotamer**	**Cβ deviation**	**CaBLAM**	**Bond lengths**	**Bond angles**	**Cis Peptides**
			Avg: 7.71	Clashscore: 0.51	Outliers: 1 of 128	Poor rotamers: 0 of 100	Outliers: 0 of 116	Outliers: 3 of 126	Outliers: 0 of 130	Outliers: 2 of 130	Non-Trans: 2 of 129
A 61		TRP	11.67	-	Favored (46.78%) General / -72.7,138.6	Favored (87.9%) *t60* chi angles: 178.2,82.3	0.06Å	Favored (38.797%) beta sheet	-	-	-
A 62		GLU	12.25	-	Favored (55.32%) General / -108.7,129.0	Favored (91.5%) *tt0* chi angles: 180.2,177.2,5.5	0.02Å	Favored (57.466%)	-	-	-
A 63		GLU	12.85	-	Favored (72.15%) General / -59.0,-34.7	Favored (98.5%) *mt-10* chi angles: 290,175.6,352.3	0.02Å	Favored (39.37%)	-	-	-
A 64		GLU	13.25	-	Favored (58.08%) General / -90.1,-2.3	Favored (96.2%) *mt-10* chi angles: 295.2,181.3,359.9	0.04Å	Favored (54.448%)	-	-	-
A 65		ALA	13.31	-	Favored (8.95%) General / -82.7,68.0	-	0.03Å	Favored (10.384%)	-	-	-
A 66		GLU	12.96	-	Favored (95.44%) General / -62.5,-40.0	Favored (98.5%) *mt-10* chi angles: 290.2,177.2,353	0.04Å	Favored (5.84%)	-	-	-
A 67		ILE	12.23	-	Favored (3.51%) Ile or Val / -128.2,-10.6	Favored (45.3%) *pt* chi angles: 62.7,170.1	0.03Å	CaBLAM Disfavored (2.292%)	-	-	-

A 68		SER	11.26	-	Favored (6.33%) General / -83.4,61.7	Favored (63.4%) *m* chi angles: 297.7	0.02Å	Favored (5.714%)	-	-	-
A 69		GLY	10.17	-	Favored (88.85%) Glycine / -79.6,-3.1	-	-	Favored (11.134%)	-	-	-
A 70		SER	9.12	-	Favored (9.77%) General / -123.3,15.8	Favored (72%) *p* chi angles: 59.2	0.02Å	Favored (16.31%)	-	-	-
A 71		SER	8.22	-	Favored (56.4%) General / -57.3,134.9	Favored (39.5%) *t* chi angles: 176.9	0.04Å	Favored (31.648%)	-	-	-
A 72		ALA	7.55	-	Favored (53.48%) General / -67.8,146.7	-	0.02Å	Favored (44.548%) beta sheet	-	-	-
A 73		ARG	7.21	-	Favored (38.33%) General / -95.8,134.8	Favored (78.6%) *ttp80* chi angles: 178.8,171.7,62.2,85.8	0.05Å	Favored (54.255%) beta sheet	-	-	-
A 74		TYR	7.23	-	Favored (49.61%) General / -128.7,135.7	Favored (84.2%) *m-80* chi angles: 300.2,87	0.06Å	Favored (61.002%) beta sheet	-	-	-
A 75		ASP	7.63	-	Favored (26.04%) General / -95.1,114.3	Favored (89.6%) *m-30* chi angles: 293.3,338.3	0.04Å	Favored (57.548%) beta sheet	-	-	-
A 76		VAL	8.42	-	Favored (62.66%) Ile or Val / -125.8,135.8	Favored (83.6%) *t* chi angles: 177.7	0.05Å	Favored (58.329%) beta sheet	-	-	-
A 77		THR	9.5	-	Favored (38.82%) General / -113.3,146.8	Favored (8.5%) *t* chi angles: 184.4	0.05Å	Favored (53.43%) beta sheet	-	-	-
A 78		LEU	10.69	-	Favored (44.84%) General / -103.1,122.5	Favored (71%) *tp* chi angles: 176.2,62.5	0.02Å	Favored (56.907%)	-	-	-
A 79		SER	11.75	-	Favored (18.36%) General / -81.3,168.5	Favored (96.8%) *p* chi angles: 65.9	0.03Å	Favored (38.347%)	-	-	-
A 80		GLU	12.44	-	Favored (66.57%) General / -62.8,-22.4	Favored (99.9%) *mt-10* chi angles: 292.1,179,355	0.01Å	Favored (47.636%)	-	-	-
**#**	**Alt**	**Res**	**High B**	**Clash > 0.4Å**	**Ramachandran**	**Rotamer**	**Cβ deviation**	**CaBLAM**	**Bond lengths**	**Bond angles**	**Cis Peptides**
			Avg: 7.71	Clashscore: 0.51	Outliers: 1 of 128	Poor rotamers: 0 of 100	Outliers: 0 of 116	Outliers: 3 of 126	Outliers: 0 of 130	Outliers: 2 of 130	Non-Trans: 2 of 129
A 81		GLN	12.59	-	Favored (56.98%) General / -89.2,0.1	Favored (98.2%) *mm-40* chi angles: 297.6,297.9,309.6	0.01Å	Favored (53.15%)	-	-	-
A 82		GLY	12.17	-	Favored (77.24%) Glycine / 92.5,-8.3	-	-	Favored (77.248%)	-	-	-
A 83		GLU	11.32	-	Favored (34.48%)	Favored (87.9%) *tt0* chi angles: 184.5,176,352.3	0.03Å	Favored (31.085%)	-	-	-

					General / -83.6,135.8						
A 84		PHE	10.27	-	Favored (29.8%) General / -93.7,140.3	Favored (87.1%) *m-80* chi angles: 290.4,87.2	0.06Å	Favored (53.993%) beta sheet	-	-	-
A 85		LYS	9.2	-	Favored (49.33%) General / -124.1,145.3	Favored (71.6%) *mmtt* chi angles: 300.7,291.5,184,182.4	0.03Å	Favored (53.701%) beta sheet	-	-	-
A 86		LEU	8.3	-	Favored (32.57%) General / -87.5,123.6	Favored (66.6%) *tp* chi angles: 178.8,63.4	0.04Å	Favored (53.357%)	-	-	-
A 87		LEU	7.68	-	Favored (53.87%) General / -87.1,-10.1	Favored (87.3%) *mt* chi angles: 298.7,173.9	0.08Å	Favored (22.385%)	-	-	-
A 88		SER	7.38	-	Favored (8%) General / -149.7,121.3	Favored (42.4%) *t* chi angles: 178.3	0.02Å	Favored (9.778%)	-	-	-
A 89		GLU	7.4	-	Favored (23%) General / -147.1,136.9	Favored (90.9%) *tt0* chi angles: 185.5,180.3,4	0.06Å	Favored (32.763%)	-	-	-
A 90		GLU	7.65	-	Favored (53.04%) General / -64.3,133.6	Favored (91.3%) *tt0* chi angles: 184.3,177.2,2	0.01Å	Favored (40.584%) beta sheet	-	-	-
A 91		LYS	7.99	-	Favored (42.76%) General / -124.4,152.9	Favored (71.2%) *mmtt* chi angles: 299.3,291.4,183.2,180.3	0.03Å	Favored (48.519%) beta sheet	-	-	-
A 92		VAL	8.2	-	Favored (69.98%) Pre-Pro / -87.1,123.6	Favored (85.2%) *t* chi angles: 177.2	0.07Å	Favored (32.992%) beta sheet	-	-	-
A 93		PRO	8.12	-	Favored (86.9%) Trans-Pro / -57.0,145.2	Favored (84.3%) *Cg_exo* chi angles: 334.1,34.3,331.7	0.04Å	Favored (88.699%)	-	-	-
A 94		TRP	7.73	-	Favored (65.57%) General / -53.2,-41.7	Favored (65.8%) *t-100* chi angles: 181.3,247.2	0.05Å	Favored (63.511%)	-	-	-
A 95		ASP	7.12	-	Favored (68.06%) General / -57.8,-33.0	Favored (98.3%) *m-30* chi angles: 288.5,346.4	0.03Å	Favored (71.66%) alpha helix	-	-	-
A 96		GLN	6.42	-	Favored (49.78%) General / -78.0,-29.6	Favored (80%) *mt0* chi angles: 293.5,182.3,0.3	0.02Å	Favored (88.246%) alpha helix	-	-	-
A 97		VAL	5.77	-	Favored (95.1%) Ile or Val / -64.8,-45.4	Favored (75.8%) *t* chi angles: 172.7	0.06Å	Favored (78.52%) alpha helix	-	-	-
A 98		VAL	5.25	-	Favored (83.54%) Ile or Val / -67.2,-46.7	Favored (76.7%) *t* chi angles: 172.8	0.02Å	Favored (83.228%) alpha helix	-	-	-
A 99		MET	4.88	-	Favored (87.83%) General / -63.2,-37.6	Favored (81.2%) *mtm* chi angles: 288.4,186.6,287.1	0.02Å	Favored (92.053%) alpha helix	-	-	-
A 100		THR	4.69	-	Favored (88.75%)	Favored (97.1%) *m* chi angles: 299.9	0.04Å	Favored (91.534%)	-	-	-

					General / -65.1,-44.5			alpha helix			

#	Alt	Res	High B	Clash > 0.4Å	Ramachandran	Rotamer	Cβ deviation	CaBLAM	Bond lengths	Bond angles	Cis Peptides
			Avg: 7.71	Clashscore: 0.51	Outliers: 1 of 128	Poor rotamers: 0 of 100	Outliers: 0 of 116	Outliers: 3 of 126	Outliers: 0 of 130	Outliers: 2 of 130	Non-Trans: 2 of 129
A 101		SER	4.62	-	Favored (97.58%) General / -61.0,-42.1	Favored (71.5%) *m* chi angles: 296	0.06Å	Favored (94.622%) alpha helix	-	-	-
A 102		LEU	4.59	-	Favored (93.18%) General / -64.7,-39.2	Favored (93.7%) *mt* chi angles: 291.5,172	0.03Å	Favored (96.331%) alpha helix	-	-	-
A 103		ALA	4.61	-	Favored (94.37%) General / -61.6,-40.3	-	0.04Å	Favored (91.643%) alpha helix	-	-	-
A 104		LEU	4.78	-	Favored (82.01%) General / -65.8,-35.9	Favored (89.3%) *mt* chi angles: 290.8,171.3	0.03Å	Favored (82.663%) alpha helix	-	-	-
A 105		VAL	5.24	-	Favored (76.46%) Ile or Val / -68.2,-47.8	Favored (73.5%) *t* chi angles: 172.5	0.03Å	Favored (74.108%) alpha helix	-	-	-
A 106		GLY	6.14	-	Favored (91.49%) Glycine / -57.2,-46.6	-	-	Favored (94.291%) alpha helix	-	-	-
A 107		ALA	7.62	-	Favored (63.23%) General / -59.3,-24.4	-	0.04Å	Favored (64.197%) alpha helix	-	-	-
A 108		ALA	9.69	-	Favored (59.63%) General / -82.9,-7.8	-	0.04Å	Favored (67.683%) alpha helix	-	-	-
A 109		ILE	12.03	-	Favored (3.28%) Ile or Val / -122.5,27.1	Favored (37.4%) *pt* chi angles: 57.8,169.7	0.08Å	Favored (18.168%) alpha helix	-	-	-
A 110		HIS	13.91	0.44Å HB3 with A 111 PRO HD3	OUTLIER (0.01)%) Pre-Pro / -156.6,-79.0	Favored (47.7%) *t-90* chi angles: 179.4,280.2	0.10Å	CA Geom Outlier (0.001%)	-	OUTLIER(S) worst is C-N-CA: 4.6 σ	Cis nonPRO omega= 0.62
A 111		PRO	14.5	0.44Å HD3 with A 110 HIS HB3	Favored (73.34%) Trans-Pro / -63.1,-25.5	Favored (43.5%) *Cg_endo* chi angles: 24.3,325.5,29.5	0.08Å	Favored (8.547%) alpha helix	-	-	-
A 112		PHE	13.37	-	Favored (8.72%) General / -78.8,-52.1	Favored (80.5%) *t80* chi angles: 179.5,71.9	0.03Å	Favored (52.809%) alpha helix	-	-	-
A 113		ALA	11.09	-	Favored (79.57%) General / -60.2,-37.2	-	0.05Å	Favored (72.718%) alpha helix	-	-	-
A 114		LEU	8.72	-	Favored (73.63%) General / -57.5,-50.3	Favored (68.7%) *tp* chi angles: 175.7,62.5	0.05Å	Favored (75.285%) alpha helix	-	-	-
A 115		LEU	6.85	-	Favored (84.7%) General / -64.2,-36.6	Favored (94.2%) *mt* chi angles: 292.1,173.6	0.04Å	Favored (81.31%) alpha helix	-	-	-

A 116		LEU	5.56	-	Favored (94.49%) General / -64.8,-40.0	Favored (94.6%) *mt* chi angles: 292.5,174.3	0.06Å	Favored (96.731%) alpha helix	-	-	-
A 117		VAL	4.78	-	Favored (88.42%) Ile or Val / -66.7,-45.4	Favored (50.3%) *t* chi angles: 169.4	0.04Å	Favored (95.822%) alpha helix	-	-	-
A 118		LEU	4.39	-	Favored (98.03%) General / -63.7,-42.5	Favored (94.7%) *mt* chi angles: 291.9,171.4	0.05Å	Favored (97.535%) alpha helix	-	-	-
A 119		ALA	4.28	-	Favored (98.5%) General / -63.1,-40.8	-	0.03Å	Favored (88.73%) alpha helix	-	-	-
A 120		GLY	4.41	-	Favored (48.37%) Glycine / -56.4,-52.7	-	-	Favored (96.687%) alpha helix	-	-	-
**#**	**Alt**	**Res**	**High B**	**Clash > 0.4Å**	**Ramachandran**	**Rotamer**	**Cβ deviation**	**CaBLAM**	**Bond lengths**	**Bond angles**	**Cis Peptides**
			Avg: 7.71	Clashscore: 0.51	Outliers: 1 of 128	Poor rotamers: 0 of 100	Outliers: 0 of 116	Outliers: 3 of 126	Outliers: 0 of 130	Outliers: 2 of 130	Non-Trans: 2 of 129
A 121		TRP	4.77	-	Favored (86.95%) General / -58.8,-41.5	Favored (35.6%) *m-10* chi angles: 287.5,337	0.03Å	Favored (87.064%) alpha helix	-	-	-
A 122		LEU	5.33	-	Favored (83.93%) General / -62.4,-36.9	Favored (86.9%) *mt* chi angles: 290.2,172.1	0.02Å	Favored (85.195%) alpha helix	-	-	-
A 123		PHE	6.03	-	Favored (65.49%) General / -70.2,-28.7	Favored (45%) *m-80* chi angles: 286.2,114.1	0.03Å	Favored (82.89%) alpha helix	-	-	-
A 124		HIS	6.81	-	Favored (61.95%) General / -74.9,-32.7	Favored (77.9%) *m-70* chi angles: 288.7,295.3	0.03Å	Favored (84.529%) alpha helix	-	-	-
A 125		VAL	7.63	-	Favored (28.18%) Ile or Val / -78.0,-42.6	Favored (77.2%) *t* chi angles: 172.9	0.01Å	Favored (73.441%) alpha helix	-	-	-
A 126		LYS	8.49	-	Favored (9.24%) General / -108.2,27.5	Favored (95.4%) *mttt* chi angles: 298.2,180,181.1,178.8	0.06Å	CA Geom Outlier (0.024%)	-	-	-
A 127		GLY	9.35	-	Favored (21.53%) Glycine / 86.0,-147.0	-	-	CaBLAM Outlier (0.128%) try alpha helix	-	OUTLIER(S) worst is C-N-CA: 4.8 σ	Cis nonPRO omega= 4.45
A 128		ALA	10.21	-	Favored (70.19%) General / -59.5,-32.4	-	0.06Å	Favored (29.111%)	-	-	-
A 129		ARG	11.02	-	Favored (56.93%) General / -58.8,138.8	Favored (96.5%) *mtt180* chi angles: 288.4,179.5,180.5,183.9	0.04Å	-	-	-	-
A 130		ARG	11.73	-	-	Favored (91.8%) *mmt-90* chi angles: 297.1,290.1,181.8,272.7	0.04Å	-	-	-	-

About MolProbity | Website for the Richardson Lab | Using ecloud x-H | Internal reference 4.5.2
