# Supplementary material for: Integrative proteome-wide structural analysis and high-throughput docking identify broad-spectrum antiviral scaffolds against Zika, Yellow Fever, West Nile, Saint Louis encephalitis, and Usutu viruses
Source: Front Cell Infect Microbiol. 2026 Apr 30;16:1723132. doi: 10.3389/fcimb.2026.1723132 (PMC13171538; doi:10.3389/fcimb.2026.1723132)
Supplement: Supplementary file 6 [file DataSheet6.zip › YFV/YF_NS3/Mol_probity_Files/YF_NS3_1FH-multi.table.pdf]

Viewing YF_NS3_1FH-multi.table

When finished, you should close this window.

Hint: Use File | Save As... to save a copy of this page.

All-Atom Contacts	Clashscore, all atoms:	0.94		99th percentile* (N=1784, all resolutions)
	Clashscore is the number of serious steric overlaps (> 0.4 Å) per 1000 atoms.			
Protein Geometry	Poor rotamers	0	0.00%	Goal: <0.3%
	Favored rotamers	510	99.61%	Goal: >98%
	Ramachandran outliers	2	0.32%	Goal: <0.05%
	Ramachandran favored	603	97.42%	Goal: >98%
	Rama distribution Z-score	0.78 ± 0.33		Goal: abs(Z score) < 2
	MolProbity score^	0.90		100th percentile* (N=27675, 0Å - 99Å)
	Cβ deviations >0.25Å	1	0.18%	Goal: 0
	Bad bonds:	7 / 4935	0.14%	Goal: 0%
	Bad angles:	8 / 6687	0.12%	Goal: <0.1%
Peptide Omegas	Cis Prolines:	0 / 34	0.00%	Expected: ≤1 per chain, or ≤5%
Low-resolution Criteria	CaBLAM outliers	14	2.3%	Goal: <1.0%
	CA Geometry outliers	2	0.32%	Goal: <0.5%
Additional validations	Chiral volume outliers	0/728		
	Waters with clashes	0/0	0.00%	See UnDowser table for details

In the two column results, the left column gives the raw count, right column gives the percentage.

* 100th percentile is the best among structures of comparable resolution; 0th percentile is the worst. For clashscore the comparative set of structures was selected in 2004, for MolProbity score in 2006.

^ MolProbity score combines the clashscore, rotamer, and Ramachandran evaluations into a single score, normalized to be on the same scale as X-ray resolution.

Key to table colors and cutoffs here:

#	Alt	Res	High B	Clash > 0.4Å	Ramachandran	Rotamer	Cβ deviation	CaBLAM	Bond lengths	Bond angles	Cis Peptides
			Avg: 1.18	Clashscore: 0.94	Outliers: 2 of 619	Poor rotamers: 0 of 512	Outliers: 1 of 567	Outliers: 15 of 617	Outliers: 7 of 621	Outliers: 8 of 621	Non-Trans: 0 of 620
A 1		SER	2.99	-	-	Favored (80.8%) *p* chi angles: 61.7	0.06Å	-	-	-	-
A 2		GLY	3.1	-	Favored (42.69%) Glycine / -85.8,-166.9	-	-	-	-	-	-
A 3		ASP	3.35	-	Favored (8.74%) General / -82.2,68.9	Favored (71%) *m-30* chi angles: 292.5,317.9	0.07Å	Favored (5.544%)	-	-	-
A 4		VAL	3.77	-	Favored (2.16%) Ile or Val / -79.2,96.2	Favored (60.9%) *t* chi angles: 179.9	0.11Å	Favored (25.164%) beta sheet	-	-	-
A 5		LEU	4.4	0.40Å O with A 6 TRP C	Favored (19.32%) General / -109.1,156.9	Favored (65.6%) *mt* chi angles: 303.5,175.6	0.07Å	Favored (22.071%)	-	-	-
A 6		TRP	5.22	0.40Å C with A 5 LEU O	Favored (4.84%) General / -44.0,-40.4	Favored (59.3%) *t60* chi angles: 191.6,88.1	0.01Å	Favored (17.209%)	-	-	-

A 7		ASP	6.17	-	Favored (20.83%) General / -90.0,-25.4	Favored (44.9%) *m-30* chi angles: 295.7,293.3	0.08Å	Favored (44.169%)	-	-	-
A 8		ILE	7.12	-	Favored (27.63%) Pre-Pro / -122.8,120.7	Favored (90%) *mt* chi angles: 298.1,172	0.03Å	Favored (18.944%)	-	-	-
A 9		PRO	7.93	-	Allowed (0.88%) Trans-Pro / -98.5,10.6	Favored (15.9%) *Cg_endo* chi angles: 37.6,325.4,16.7	0.08Å	CaBLAM Outlier (0.358%)	-	-	-
A 10		THR	8.5	-	Favored (84.88%) Pre-Pro / -60.6,126.4	Favored (91.7%) *m* chi angles: 301.1	0.04Å	Favored (19.845%) beta sheet	-	-	-
A 11		PRO	8.76	-	Favored (26.02%) Trans-Pro / -79.1,162.1	Favored (73.8%) *Cg_endo* chi angles: 31,324.3,25.6	0.07Å	Favored (40.868%) beta sheet	-	-	-
A 12		LYS	8.76	-	Favored (14.29%) General / -93.0,161.8	Favored (29.2%) *mmtm* chi angles: 301,291.5,189,288.9	0.01Å	Favored (42.799%) beta sheet	-	-	-
A 13		ILE	8.53	-	Favored (47%) Ile or Val / -95.9,128.5	Favored (89.8%) *mt* chi angles: 297.9,169.5	0.02Å	Favored (44.14%) beta sheet	-	-	-
A 14		ILE	8.07	-	Favored (50.72%) Ile or Val / -105.3,117.3	Favored (61.2%) *mt* chi angles: 304.5,171.1	0.08Å	Favored (57.192%)	-	-	-
A 15		GLU	7.3	-	Favored (13.22%) General / -82.3,-46.7	Favored (99.1%) *mt-10* chi angles: 293.3,178.5,356.3	0.01Å	Favored (24.473%)	-	-	-
A 16		GLU	6.2	-	Favored (4.73%) General / -120.1,95.4	Favored (95.5%) *mt-10* chi angles: 296.8,181.1,359	0.01Å	Favored (23.37%)	-	-	-
A 17		CYS	4.9	-	Allowed (0.41%) General / -74.0,14.5	Favored (90.8%) *m* chi angles: 291.6	0.06Å	CaBLAM Disfavored (1.468%)	-	-	-
A 18		GLU	3.64	-	Favored (74.74%) General / -62.2,-33.5	Favored (55.8%) *mm-30* chi angles: 289.6,290.2,307.4	0.05Å	Favored (8.522%)	-	-	-
A 19		TYR	2.61	-	Favored (50.58%) General / -130.2,142.4	Favored (87.7%) *m-80* chi angles: 299.3,87	0.04Å	Favored (22.82%)	-	-	-
A 20		LEU	1.85	-	Favored (14.52%) General / -99.4,160.1	Favored (57.3%) *mt* chi angles: 304.6,175.2	0.04Å	Favored (40.396%)	-	-	-
**#**	**Alt**	**Res**	**High B**	**Clash > 0.4Å**	**Ramachandran**	**Rotamer**	**Cβ deviation**	**CaBLAM**	**Bond lengths**	**Bond angles**	**Cis Peptides**
			Avg: 1.18	Clashscore: 0.94	Outliers: 2 of 619	Poor rotamers: 0 of 512	Outliers: 1 of 567	Outliers: 15 of 617	Outliers: 7 of 621	Outliers: 8 of 621	Non-Trans: 0 of 620
A 21		GLU	1.36	-	Favored (19.42%) General / -91.8,153.7	Favored (50.6%) *mm-30* chi angles: 298.8,290.8,307.1	0.04Å	Favored (18.723%)	-	-	-
A 22		ASP	1.07	-	Favored (55.72%) General / -61.4,143.5	Favored (91.2%) *m-30* chi angles: 290.6,349.8	0.07Å	CaBLAM Disfavored (1.551%)	-	-	-

A 23		GLY	0.9	-	Favored (24.46%) Glycine / 160.6,169.9	-	-	Favored (32.023%)	-	-	-
A 24		ILE	0.83	-	Favored (53.29%) Ile or Val / -99.8,126.0	Favored (73.8%) *mt* chi angles: 301.3,169.1	0.04Å	Favored (13.519%) beta sheet	-	-	-
A 25		TYR	0.82	-	Favored (28.71%) General / -131.7,123.9	Favored (85.2%) *t80* chi angles: 173.4,75.7	0.03Å	Favored (55.164%) beta sheet	-	-	-
A 26		GLY	0.87	-	Favored (44.65%) Glycine / -67.5,144.8	-	-	Favored (47.044%) beta sheet	-	-	-
A 27		ILE	0.97	-	Favored (57.85%) Ile or Val / -108.0,119.6	Favored (83.2%) *mt* chi angles: 299.3,168.9	0.02Å	Favored (62.274%) beta sheet	-	-	-
A 28		PHE	1.16	-	Favored (33.49%) General / -114.6,151.4	Favored (88.3%) *m-80* chi angles: 292.7,83.4	0.03Å	Favored (43.933%) beta sheet	-	-	-
A 29		GLN	1.42	-	Favored (38.15%) General / -125.0,122.7	Favored (66.6%) *tp40* chi angles: 174,68.7,59.8	0.03Å	Favored (52.495%) beta sheet	-	-	-
A 30		SER	1.73	-	Favored (48.96%) General / -70.8,136.6	Favored (38.8%) *t* chi angles: 177.1	0.03Å	Favored (44.719%)	-	-	-
A 31		THR	2.01	-	Favored (6%) General / -128.7,178.9	Favored (20.7%) *p* chi angles: 72.3	0.05Å	Favored (28.13%)	-	-	-
A 32		PHE	2.16	-	Favored (71.13%) General / -53.9,-45.5	Favored (93.7%) *t80* chi angles: 177.7,78	0.00Å	Favored (18.624%)	-	-	-
A 33		LEU	2.09	-	Favored (23.68%) General / -85.5,-30.1	Favored (96.9%) *mt* chi angles: 297.4,176.5	0.02Å	CaBLAM Disfavored (1.065%)	-	-	-
A 34		GLY	1.85	-	Favored (23%) Glycine / 158.7,170.2	-	-	Favored (20.434%)	-	-	-
A 35		ALA	1.54	-	Favored (56.09%) General / -63.5,145.2	-	0.02Å	Favored (6.253%)	-	-	-
A 36		SER	1.25	-	Favored (38.91%) General / -138.7,142.4	Favored (44.6%) *t* chi angles: 179	0.04Å	Favored (49.178%) beta sheet	-	-	-
A 37		GLN	1.03	-	Favored (30.72%) General / -79.3,126.4	Favored (52.2%) *tt0* chi angles: 186.5,175.5,315.1	0.03Å	Favored (46.932%)	-	-	-
A 38		ARG	0.9	-	Favored (13.42%) General / -94.5,-32.6	Favored (80.7%) *mtm180* chi angles: 292.2,182.1,289,181.1	0.10Å	Favored (8.025%)	-	-	-
A 39		GLY	0.82	-	Favored (28.33%) Glycine / -176.4,-163.1	-	-	Favored (12.73%)	-	-	-
A 40		VAL	0.8	-	Favored (15.21%)	Favored (10%) *p* chi angles: 62.2	0.08Å	Favored (26.625%)	-	-	-

#	Alt	Res	High B	Clash > 0.4Å	Ramachandran	Rotamer	Cβ deviation	CaBLAM	Bond lengths	Bond angles	Cis Peptides
					Ile or Val / -149.1,149.1						
			Avg: 1.18	Clashscore: 0.94	Outliers: 2 of 619	Poor rotamers: 0 of 512	Outliers: 1 of 567	Outliers: 15 of 617	Outliers: 7 of 621	Outliers: 8 of 621	Non-Trans: 0 of 620
A 41		GLY	0.81	-	Favored (12.38%) Glycine / -135.4,-171.4	-	-	Favored (41.891%) beta sheet	-	-	-
A 42		VAL	0.85	-	Favored (32.17%) Ile or Val / -132.1,148.4	Favored (26.7%) *m* chi angles: 299.1	0.10Å	Favored (39.238%) beta sheet	-	-	-
A 43		ALA	0.91	-	Favored (47.66%) General / -101.4,131.1	-	0.06Å	Favored (46.075%)	-	-	-
A 44		GLN	0.96	-	Favored (10.12%) General / -147.4,122.4	Favored (79.8%) *tp40* chi angles: 182.9,70.6,57.2	0.01Å	Favored (8.524%)	-	-	-
A 45		GLY	0.99	-	Favored (80.36%) Glycine / 76.6,16.9	-	-	Favored (32.725%)	-	-	-
A 46		GLY	0.98	-	Favored (89.44%) Glycine / 80.3,6.0	-	-	Favored (68.061%)	-	-	-
A 47		VAL	0.95	-	Favored (50.05%) Ile or Val / -107.4,134.1	Favored (84.4%) *t* chi angles: 177.1	0.04Å	Favored (32.755%)	-	-	-
A 48		PHE	0.89	-	Favored (35.15%) General / -88.4,130.6	Favored (78.3%) *t80* chi angles: 182.8,73.3	0.06Å	Favored (56.137%) beta sheet	-	-	-
A 49		HIS	0.83	-	Favored (53.06%) General / -124.4,135.2	Favored (39.8%) *m90* chi angles: 307.2,82.6	0.15Å	Favored (38.521%) beta sheet	-	-	-
A 50		THR	0.79	-	Favored (3.05%) General / -146.8,-168.5	Favored (10.4%) *t* chi angles: 188.5	0.09Å	Favored (11.805%) beta sheet	-	-	-
A 51		MET	0.77	-	Favored (30.39%) General / -102.8,145.0	Favored (75.2%) *mmm* chi angles: 306.5,294.3,286.8	0.05Å	Favored (12.002%)	-	-	-
A 52		TRP	0.78	-	Favored (96.24%) General / -61.6,-44.8	Favored (91.6%) *t60* chi angles: 180.9,88.3	0.08Å	Favored (51.019%)	-	-	-
A 53		HIS	0.82	-	Favored (56.2%) General / -60.8,-18.5	Favored (54%) *p-80* chi angles: 70.5,280.1	0.10Å	Favored (65.012%) alpha helix	-	-	-
A 54		VAL	0.88	-	Favored (22.16%) Ile or Val / -80.3,-45.4	Favored (84.2%) *t* chi angles: 173.5	0.03Å	Favored (23.005%) alpha helix	-	-	-
A 55		THR	0.97	-	Favored (13.05%) General / -105.4,-22.3	Favored (44.7%) *p* chi angles: 66.7	0.07Å	Favored (7.086%)	-	-	-
A 56		ARG	1.06	-	Favored (8.63%) General / 58.1,22.1	Favored (29.8%) *mmt90*	0.02Å	CaBLAM Disfavored (3.529%)	-	-	-

#	Alt	Res	High B	Clash > 0.4Å	Ramachandran	Rotamer	Cβ deviation	CaBLAM	Bond lengths	Bond angles	Cis Peptides
						chi angles: 306.5,298.5,179,93.4					
A 57		GLY	1.17	-	Favored (77.2%) Glycine / 89.3,2.2	-	-	Favored (49.652%)	-	-	-
A 58		ALA	1.27	-	Favored (50.03%) General / -62.8,147.3	-	0.03Å	Favored (22.76%)	-	-	-
A 59		PHE	1.38	-	Favored (52.88%) General / -69.5,145.3	Favored (4.4%) *m-10* chi angles: 292.7,9.7	0.03Å	Favored (40.74%) beta sheet	-	-	-
A 60		LEU	1.49	-	Favored (24.74%) General / -112.1,154.9	Favored (82%) *mt* chi angles: 301.7,177.2	0.01Å	Favored (44.25%) beta sheet	-	-	-
**#**	**Alt**	**Res**	**High B**	**Clash > 0.4Å**	**Ramachandran**	**Rotamer**	**Cβ deviation**	**CaBLAM**	**Bond lengths**	**Bond angles**	**Cis Peptides**
			Avg: 1.18	Clashscore: 0.94	Outliers: 2 of 619	Poor rotamers: 0 of 512	Outliers: 1 of 567	Outliers: 15 of 617	Outliers: 7 of 621	Outliers: 8 of 621	Non-Trans: 0 of 620
A 61		VAL	1.61	-	Favored (75.28%) Ile or Val / -118.1,127.3	Favored (81.2%) *t* chi angles: 177.9	0.05Å	Favored (59.174%)	-	-	-
A 62		ARG	1.71	-	Favored (10.4%) General / -118.1,104.0	Favored (66%) *mmm-85* chi angles: 297.7,296,298.8,273.4	0.05Å	Favored (14.36%)	-	-	-
A 63		ASN	1.79	-	Favored (31.47%) General / 53.0,40.7	Favored (54.6%) *m-40* chi angles: 299.7,344	0.05Å	Favored (17.135%)	-	-	-
A 64		GLY	1.83	-	Favored (65.63%) Glycine / 94.8,-14.3	-	-	Favored (68.315%)	-	-	-
A 65		LYS	1.82	-	Favored (31.65%) General / -89.1,121.7	Favored (44.1%) *tptt* chi angles: 170.6,70.3,179.4,181.6	0.06Å	Favored (29.773%)	-	-	-
A 66		LYS	1.76	-	Favored (31.69%) General / -83.7,125.3	Favored (36.8%) *ttpt* chi angles: 184.5,167.5,66,176.6	0.04Å	Favored (53.081%) beta sheet	-	-	-
A 67		LEU	1.67	-	Favored (41.06%) General / -106.3,139.3	Favored (79.6%) *mt* chi angles: 302.5,177.4	0.05Å	Favored (58.987%) beta sheet	-	-	-
A 68		VAL	1.59	-	Favored (20.09%) Pre-Pro / -107.4,134.1	Favored (69.1%) *t* chi angles: 178.9	0.09Å	Favored (36.553%) beta sheet	-	-	-
A 69		PRO	1.52	-	Favored (76.7%) Trans-Pro / -55.4,137.2	Favored (90.4%) *Cg_exo* chi angles: 333.2,34.6,332	0.12Å	Favored (53.414%)	-	-	-
A 70		SER	1.48	-	Favored (9.25%) General / -109.9,-27.4	Favored (67.3%) *m* chi angles: 294.6	0.05Å	Favored (22.809%)	-	-	-
A 71		TRP	1.45	-	Favored (29.35%) General / -145.6,142.7	Favored (61.8%) *t60* chi angles: 173.8,91.7	0.05Å	Favored (12.462%)	-	-	-
A 72		ALA	1.42	-	Favored (30.87%)	-	0.04Å	Favored (34.473%)	-	-	-

					General / -160.0,158.5						
A 73		SER	1.37	-	Favored (5.85%) General / -149.1,115.5	Favored (37.7%) *t* chi angles: 174.7	0.04Å	Favored (24.508%) beta sheet	-	-	-
A 74		VAL	1.29	-	Favored (77.89%) Ile or Val / -69.9,-39.6	Favored (99%) *t* chi angles: 175.4	0.02Å	Favored (27.735%)	-	-	-
A 75		LYS	1.2	-	Favored (80.45%) General / -58.3,-39.8	Favored (98.3%) *mttt* chi angles: 289,175.2,177.4,175.4	0.05Å	Favored (62.627%) alpha helix	-	-	-
A 76		GLU	1.11	-	Favored (45.67%) General / -94.6,-5.5	Favored (82.5%) *mm-30* chi angles: 299.7,296.2,328.4	0.03Å	Favored (40.853%)	-	-	-
A 77		ASP	1.04	-	Favored (14.53%) General / 60.5,42.4	Favored (35.9%) *t0* chi angles: 194.4,26.5	0.03Å	Favored (30.754%)	-	-	-
A 78		LEU	1	-	Favored (51.15%) General / -125.4,143.1	Favored (82.4%) *mt* chi angles: 298.3,171.8	0.07Å	Favored (21.116%) beta sheet	-	-	-
A 79		VAL	0.99	-	Favored (57.87%) Ile or Val / -133.4,133.1	Favored (44.3%) *t* chi angles: 182.6	0.03Å	Favored (68.086%) beta sheet	-	-	-
A 80		ALA	1.03	-	Favored (51.2%) General / -115.7,138.3	-	0.03Å	Favored (71.607%)	-	-	-
**#**	**Alt**	**Res**	**High B**	**Clash > 0.4Å**	**Ramachandran**	**Rotamer**	**Cβ deviation**	**CaBLAM**	**Bond lengths**	**Bond angles**	**Cis Peptides**
			Avg: 1.18	Clashscore: 0.94	Outliers: 2 of 619	Poor rotamers: 0 of 512	Outliers: 1 of 567	Outliers: 15 of 617	Outliers: 7 of 621	Outliers: 8 of 621	Non-Trans: 0 of 620
A 81		TYR	1.09	-	Favored (54.5%) General / -119.1,135.8	Favored (56.2%) *m-80* chi angles: 294.9,77.6	0.10Å	Favored (13.63%)	-	-	-
A 82		GLY	1.15	-	Favored (90.29%) Glycine / 81.9,1.5	-	-	Favored (7.249%)	-	-	-
A 83		GLY	1.19	-	Favored (39.8%) Glycine / 168.9,177.5	-	-	Favored (12.855%)	-	-	-
A 84		SER	1.19	-	Favored (27.1%) General / -70.6,163.4	Favored (88.4%) *p* chi angles: 69.4	0.10Å	CaBLAM Disfavored (4.321%)	-	-	-
A 85		TRP	1.15	-	Favored (41.77%) General / -58.1,129.4	Favored (62.2%) *t-100* chi angles: 183.5,264.8	0.07Å	Favored (15.897%)	-	-	-
A 86		LYS	1.09	-	Favored (15.43%) General / -113.7,3.1	Favored (73.3%) *mmtt* chi angles: 299.7,294.7,184.9,177.5	0.04Å	Favored (13.071%)	-	-	-
A 87		LEU	1.02	-	Favored (9.3%) General / -93.7,95.6	Favored (45.1%) *mt* chi angles: 307.4,177.9	0.04Å	Favored (11.869%)	-	-	-
A 88		ASP	0.96	-	Favored (33.18%) General / -83.1,-23.4	Favored (17.7%) *m-30* chi angles: 285.2,296	0.15Å	Favored (9.286%)	-	-	-

A 89		GLY	0.91	-	Favored (39.67%) Glycine / -61.9,154.5	-	-	Favored (24.795%) beta sheet	-	-	-
A 90		ARG	0.89	-	Favored (30.25%) General / -144.2,142.0	Favored (51.4%) *ttm170* chi angles: 185.6,183.9,292.7,164.7	0.02Å	Favored (42.25%) beta sheet	-	-	-
A 91		TRP	0.87	0.41Å CD1 with A 91 TRP H	Favored (30.76%) General / -60.9,151.2	Favored (50.3%) *p-90* chi angles: 50,268.7	0.13Å	Favored (25.243%) beta sheet	-	-	-
A 92		ASP	0.86	-	Favored (7.88%) General / -125.1,4.9	Favored (56.7%) *p0* chi angles: 64.5,359.4	0.04Å	CaBLAM Disfavored (4.793%)	-	-	-
A 93		GLY	0.84	-	Favored (75.77%) Glycine / 86.5,-8.2	-	-	Favored (31.757%)	-	-	-
A 94		GLU	0.81	-	Favored (2.46%) General / -125.2,-36.9	Favored (96%) *mt-10* chi angles: 296.9,180.9,357.5	0.03Å	CaBLAM Disfavored (1.985%)	-	-	-
A 95		GLU	0.79	-	Favored (58.18%) General / -63.9,137.8	Favored (89.4%) *tt0* chi angles: 186.1,177,5.3	0.04Å	Favored (28.712%)	-	-	-
A 96		GLU	0.77	-	Favored (56.91%) General / -66.1,137.3	Favored (81.9%) *mt-10* chi angles: 292.6,177.2,324.4	0.06Å	Favored (42.301%) beta sheet	-	-	-
A 97		VAL	0.75	-	Favored (26.79%) Ile or Val / -123.1,159.8	Favored (30.1%) *m* chi angles: 300.2	0.02Å	Favored (52.16%) beta sheet	-	-	-
A 98		GLN	0.75	-	Favored (53.74%) General / -121.5,130.7	Favored (60.8%) *tt0* chi angles: 184.5,181.8,50.7	0.03Å	Favored (57.724%) beta sheet	-	-	-
A 99		LEU	0.77	-	Favored (41.57%) General / -97.2,125.1	Favored (42.3%) *tp* chi angles: 174,66.9	0.03Å	Favored (63.76%) beta sheet	-	-	-
A 100		ILE	0.82	-	Allowed (1.04%) Ile or Val / -98.5,82.8	Favored (35.4%) *mm* chi angles: 309.3,300.4	0.21Å	Favored (57.542%) beta sheet	-	-	-
**#**	**Alt**	**Res**	**High B**	**Clash > 0.4Å**	**Ramachandran**	**Rotamer**	**Cβ deviation**	**CaBLAM**	**Bond lengths**	**Bond angles**	**Cis Peptides**
			Avg: 1.18	Clashscore: 0.94	Outliers: 2 of 619	Poor rotamers: 0 of 512	Outliers: 1 of 567	Outliers: 15 of 617	Outliers: 7 of 621	Outliers: 8 of 621	Non-Trans: 0 of 620
A 101		ALA	0.91	-	Favored (21.89%) General / -87.8,111.9	-	0.03Å	Favored (55.201%) beta sheet	-	-	-
A 102		ALA	1.03	-	Favored (10.51%) General / -86.3,70.4	-	0.03Å	Favored (65.031%)	-	-	-
A 103		ALA	1.18	-	Favored (80.95%) Pre-Pro / -57.8,141.0	-	0.06Å	Favored (17.095%)	-	-	-
A 104		PRO	1.32	-	Favored (44.7%) Trans-Pro / -62.3,133.7	Favored (36.9%) *Cg_endo* chi angles: 22.7,327,29.1	0.04Å	Favored (38.53%)	-	-	-

A 105		GLY	1.43	-	Favored (83.27%) Glycine / 79.7,-0.0	-	-	Favored (64.555%)	-	-	-
A 106		LYS	1.45	-	Favored (8.98%) General / -126.8,174.2	Favored (94.1%) *mttt* chi angles: 299,181.7,180.7,180.4	0.03Å	Favored (20.778%)	-	-	-
A 107		ASN	1.39	-	Favored (9.79%) General / -89.7,171.3	Favored (20.1%) *t0* chi angles: 199.7,349.4	0.04Å	Favored (9.087%)	-	-	-
A 108		VAL	1.26	-	Favored (28.79%) Ile or Val / -63.1,131.9	Favored (70.6%) *t* chi angles: 172.2	0.04Å	Favored (14.714%)	-	-	-
A 109		VAL	1.1	-	Favored (58.7%) Ile or Val / -110.0,119.3	Favored (70.5%) *t* chi angles: 178.7	0.09Å	Favored (64.887%) beta sheet	-	-	-
A 110		ASN	0.96	-	Favored (38.26%) General / -92.7,127.6	Favored (64.1%) *m-40* chi angles: 295.3,351.4	0.06Å	Favored (54.405%) beta sheet	-	-	-
A 111		VAL	0.86	-	Favored (51.09%) Ile or Val / -131.5,139.3	Favored (6.2%) *p* chi angles: 59.1	0.06Å	Favored (55.351%) beta sheet	-	-	-
A 112		GLN	0.8	-	Favored (53.67%) General / -112.2,134.6	Favored (62.6%) *tt0* chi angles: 184.9,174.4,13	0.04Å	Favored (23.963%) beta sheet	-	-	-
A 113		THR	0.78	-	Favored (27.33%) General / -156.8,152.8	Favored (9.2%) *t* chi angles: 185.2	0.11Å	Favored (20.583%) beta sheet	-	-	-
A 114		LYS	0.81	-	Favored (71.68%) Pre-Pro / -79.0,148.4	Favored (97.4%) *mttt* chi angles: 291.4,184.4,177.2,181.8	0.03Å	Favored (45.743%) beta sheet	-	-	-
A 115		PRO	0.87	-	Favored (37.17%) Trans-Pro / -76.3,156.1	Favored (71.2%) *Cg_endo* chi angles: 29.6,326.5,23.5	0.02Å	Favored (57.105%) beta sheet	-	-	-
A 116		SER	0.99	-	Favored (8.4%) General / -106.4,169.6	Favored (84.5%) *p* chi angles: 67.4	0.03Å	Favored (39.555%) beta sheet	-	-	-
A 117		LEU	1.19	-	Favored (55.35%) General / -115.3,127.3	Favored (86.6%) *mt* chi angles: 298.6,173.2	0.03Å	Favored (41.942%) beta sheet	-	-	-
A 118		PHE	1.47	-	Favored (54.73%) General / -107.3,130.7	Favored (12.1%) *m-10* chi angles: 286.2,344.1	0.02Å	Favored (63.529%) beta sheet	-	-	-
A 119		LYS	1.83	-	Favored (21.31%) General / -87.2,153.7	Favored (70.8%) *mmtt* chi angles: 303.5,297.1,188.7,184.4	0.06Å	Favored (33.6%) beta sheet	-	-	-
A 120		VAL	2.18	-	Favored (17.48%) Ile or Val / -121.7,165.4	Favored (31.6%) *m* chi angles: 300.5	0.02Å	Favored (37.148%)	-	-	-
**#**	**Alt**	**Res**	**High B**	**Clash > 0.4Å**	**Ramachandran**	**Rotamer**	**Cβ deviation**	**CaBLAM**	**Bond lengths**	**Bond angles**	**Cis Peptides**
			Avg: 1.18	Clashscore: 0.94	Outliers: 2 of 619	Poor rotamers: 0 of 512	Outliers: 1 of 567	Outliers: 15 of 617	Outliers: 7 of 621	Outliers: 8 of 621	Non-Trans: 0 of 620

A 121		LYS	2.42	-	Favored (54.44%) General / -60.1,-19.6	Favored (59.9%) *pttt* chi angles: 68,182.1,181.8,181.1	0.01Å	Favored (40.847%)	-	-	-
A 122		ASN	2.44	-	Favored (47.87%) General / -92.9,6.4	Favored (90.2%) *m-40* chi angles: 292.3,322.3	0.03Å	Favored (49.233%)	-	-	-
A 123		GLY	2.22	-	Favored (88.52%) Glycine / 84.9,2.2	-	-	Favored (76.636%)	-	-	-
A 124		GLY	1.86	-	Favored (43.42%) Glycine / -89.8,-168.8	-	-	Favored (50.469%)	-	-	-
A 125		GLU	1.5	-	Favored (22.27%) General / -146.9,135.8	Favored (44.6%) *tt0* chi angles: 182,174.6,64.5	0.03Å	Favored (35.043%) beta sheet	-	-	-
A 126		ILE	1.2	-	Favored (71.99%) Ile or Val / -126.3,130.7	Favored (78.2%) *mt* chi angles: 301,172.4	0.05Å	Favored (58.575%) beta sheet	-	-	-
A 127		GLY	1.01	-	Favored (45.85%) Glycine / -72.5,150.9	-	-	Favored (44.989%) beta sheet	-	-	-
A 128		ALA	0.9	-	Favored (37.67%) General / -149.9,154.0	-	0.06Å	Favored (58.173%) beta sheet	-	-	-
A 129		VAL	0.85	-	Favored (47.69%) Ile or Val / -118.8,139.0	Favored (23.5%) *m* chi angles: 295	0.05Å	Favored (47.591%) beta sheet	-	-	-
A 130		ALA	0.85	-	Favored (7.83%) General / -85.3,63.0	-	0.02Å	Favored (11.25%) beta sheet	-	-	-
A 131		LEU	0.88	-	Favored (9%) General / -116.8,102.0	Favored (70.6%) *mt* chi angles: 302.3,174.6	0.08Å	Favored (14.928%) beta sheet	-	-	-
A 132		ASP	0.9	-	Favored (7.65%) General / -87.2,88.2	Favored (62.4%) *t0* chi angles: 188.3,343.1	0.07Å	Favored (48.969%) beta sheet	-	-	-
A 133		TYR	0.92	-	Favored (41.86%) Pre-Pro / -109.3,151.0	Favored (50%) *m-80* chi angles: 282.5,83.3	0.06Å	Favored (27.642%)	-	-	-
A 134		PRO	0.91	-	Favored (32.95%) Trans-Pro / -71.6,165.3	Favored (74.4%) *Cg_endo* chi angles: 27.8,323.5,29.7	0.05Å	Favored (45.975%)	-	-	-
A 135		SER	0.87	-	Favored (12.74%) General / -60.6,158.2	Favored (85.8%) *p* chi angles: 67.8	0.04Å	Favored (5.421%)	-	-	-
A 136		GLY	0.82	-	Favored (57.36%) Glycine / 98.7,-18.1	-	-	Favored (12.092%)	-	-	-
A 137		THR	0.77	-	Favored (17.54%) General / -101.9,-13.7	Favored (79.3%) *p* chi angles: 60.7	0.04Å	Favored (9.601%)	-	-	-
A 138		SER	0.73	-	Favored (56.66%)	Favored (60.9%) *m* chi angles: 293.7	0.05Å	Favored (9.221%)	-	-	-

#	Alt	Res	High B	Clash > 0.4Å	Ramachandran	Rotamer	Cβ deviation	CaBLAM	Bond lengths	Bond angles	Cis Peptides
					General / -58.5,138.5						
A 139		GLY	0.71	-	Favored (71.7%) Glycine / 94.4,-7.3	-	-	Favored (73.985%)	-	-	-
A 140		SER	0.71	-	Favored (86.73%) Pre-Pro / -58.9,139.8	Favored (55.2%) *m* chi angles: 292.6	0.07Å	Favored (31.654%)	-	-	-
**#**	**Alt**	**Res**	**High B**	**Clash > 0.4Å**	**Ramachandran**	**Rotamer**	**Cβ deviation**	**CaBLAM**	**Bond lengths**	**Bond angles**	**Cis Peptides**
			Avg: 1.18	Clashscore: 0.94	Outliers: 2 of 619	Poor rotamers: 0 of 512	Outliers: 1 of 567	Outliers: 15 of 617	Outliers: 7 of 621	Outliers: 8 of 621	Non-Trans: 0 of 620
A 141		PRO	0.74	-	Favored (57.52%) Trans-Pro / -71.5,150.4	Favored (68.3%) *Cg_endo* chi angles: 27.1,328.1,23.6	0.02Å	Favored (74.62%) beta sheet	-	-	-
A 142		ILE	0.79	-	Favored (68.61%) Ile or Val / -112.2,128.7	Favored (86.5%) *mt* chi angles: 298.7,170.6	0.06Å	Favored (69.19%) beta sheet	-	-	-
A 143		VAL	0.85	-	Favored (23.69%) Ile or Val / -121.1,158.6	Favored (25.8%) *m* chi angles: 299.5	0.04Å	Favored (46.57%)	-	-	-
A 144		ASN	0.92	-	Favored (4.57%) General / -86.3,-173.5	Favored (49.9%) *p0* chi angles: 64.9,21.4	0.09Å	Favored (34.442%)	-	-	-
A 145		ARG	0.98	-	Favored (56.73%) General / -72.7,-8.7	Favored (91.5%) *mtm180* chi angles: 292.8,176.6,294.8,173.3	0.04Å	Favored (29.891%)	-	-	-
A 146		ASN	1.01	-	Favored (44.93%) General / -100.1,6.6	Favored (87.9%) *m-40* chi angles: 292.1,320.4	0.02Å	Favored (56.712%)	-	-	-
A 147		GLY	1.01	-	Favored (88.21%) Glycine / 82.4,7.0	-	-	Favored (87.527%)	-	-	-
A 148		GLU	0.96	-	Favored (31.36%) General / -82.1,143.7	Favored (69.9%) *mt-10* chi angles: 298.1,177.2,320.3	0.06Å	Favored (35.652%)	-	-	-
A 149		VAL	0.9	-	Favored (38.75%) Ile or Val / -79.7,128.2	Favored (86.1%) *t* chi angles: 177.3	0.06Å	Favored (46.766%)	-	-	-
A 150		ILE	0.84	-	Favored (7.45%) Ile or Val / -112.8,18.5	Favored (40.3%) *pt* chi angles: 61.3,167.6	0.08Å	CaBLAM Disfavored (1.093%)	-	-	-
A 151		GLY	0.8	-	Favored (12.47%) Glycine / 131.5,169.8	-	-	Favored (14.068%)	-	-	-
A 152		LEU	0.79	-	Favored (31.61%) General / -110.0,148.4	Favored (62.9%) *mt* chi angles: 304.3,180.3	0.05Å	Favored (9.069%)	-	-	-
A 153		TYR	0.81	-	Favored (50.39%) General / -71.4,141.7	Favored (86.9%) *t80* chi angles: 181.2,79.4	0.05Å	CA Geom Outlier (0.427%)	-	-	-
A 154		GLY	0.87	-	Favored (26.68%)	-	-	CA Geom Outlier (0.283%)	-	-	-

#	Alt	Res	High B	Clash > 0.4Å	Ramachandran	Rotamer	Cβ deviation	CaBLAM	Bond lengths	Bond angles	Cis Peptides
					Glycine / 144.8,-169.1						
A 155		ASN	0.96	-	Favored (11.39%) General / 52.5,54.0	Favored (64.8%) *t0* chi angles: 194.6,31.3	0.07Å	CaBLAM Outlier (0.098%)	-	-	-
A 156		GLY	1.09	-	Favored (31.45%) Glycine / -76.0,144.7	-	-	Favored (32.817%)	-	-	-
A 157		ILE	1.24	-	Favored (70.95%) Ile or Val / -114.0,128.6	Favored (92.2%) *mt* chi angles: 297.1,170.7	0.02Å	Favored (50.622%) beta sheet	-	-	-
A 158		LEU	1.41	-	Favored (29.55%) General / -79.5,125.4	Favored (59.1%) *tp* chi angles: 181.1,63.4	0.04Å	Favored (50.252%)	-	-	-
A 159		VAL	1.57	-	Favored (66.07%) Ile or Val / -111.7,122.4	Favored (70.2%) *t* chi angles: 178.7	0.09Å	Favored (16.436%)	-	-	-
A 160		GLY	1.66	-	Favored (48.68%) Glycine / 72.4,-152.9	-	-	Favored (18.644%)	-	-	-
**#**	**Alt**	**Res**	**High B**	**Clash > 0.4Å**	**Ramachandran**	**Rotamer**	**Cβ deviation**	**CaBLAM**	**Bond lengths**	**Bond angles**	**Cis Peptides**
			Avg: 1.18	Clashscore: 0.94	Outliers: 2 of 619	Poor rotamers: 0 of 512	Outliers: 1 of 567	Outliers: 15 of 617	Outliers: 7 of 621	Outliers: 8 of 621	Non-Trans: 0 of 620
A 161		ASP	1.67	-	Favored (16.18%) General / -90.2,-33.4	Favored (62.9%) *m-30* chi angles: 297.1,303.9	0.03Å	CaBLAM Disfavored (1.336%)	-	-	-
A 162		THR	1.56	0.40Å O with A 163 SER CB	Favored (37.38%) General / -136.3,133.9	Favored (88.9%) *m* chi angles: 301.3	0.01Å	CaBLAM Outlier (0.688%)	-	-	-
A 163		SER	1.38	0.40Å CB with A 162 THR O	OUTLIER (0)%) General / 125.3,88.8	Favored (72.5%) *m* chi angles: 295.3	0.13Å	Favored (12.492%)	-	-	-
A 164		PHE	1.18	-	Favored (34.6%) General / -82.2,138.9	Favored (61.9%) *m-80* chi angles: 285,91	0.03Å	Favored (41.808%)	-	-	-
A 165		VAL	1	-	Favored (65.77%) Ile or Val / -115.4,120.8	Favored (54%) *t* chi angles: 180.8	0.08Å	Favored (58.337%) beta sheet	-	-	-
A 166		SER	0.87	-	Favored (34.59%) General / -93.2,121.4	Favored (7.2%) *t* chi angles: 164.2	0.07Å	Favored (44.565%) beta sheet	-	-	-
A 167		ALA	0.81	-	Favored (57.99%) General / -61.5,140.9	-	0.04Å	Favored (36.819%) beta sheet	-	-	-
A 168		ILE	0.8	-	Favored (23.52%) Ile or Val / -91.6,111.2	Favored (77.8%) *mt* chi angles: 300.9,171.8	0.05Å	Favored (55.09%) beta sheet	-	-	-
A 169		SER	0.86	-	Favored (35.31%) General / -93.4,122.0	Favored (46.2%) *m* chi angles: 290.7	0.07Å	Favored (34.673%) beta sheet	-	-	-
A 170		GLN	0.99	-	Favored (24.07%)	Favored (18.1%) *tm-30*	0.07Å	Favored (23.725%)	-	-	-

					General / -148.7,140.9	chi angles: 189.4,263.8,330.9					
A 171		THR	1.2	-	Favored (53.81%) General / -125.9,138.7	Favored (89.1%) *m* chi angles: 298.6	0.06Å	Favored (43.373%)	-	-	-
A 172		GLU	1.53	-	Favored (57.9%) General / -60.7,139.3	Favored (97.6%) *mt-10* chi angles: 293.6,181.5,357.3	0.08Å	Favored (51.801%)	-	-	-
A 173		VAL	2.02	-	Favored (39.38%) Ile or Val / -82.4,127.7	Favored (82.8%) *t* chi angles: 176.8	0.08Å	Favored (47.065%)	-	-	-
A 174		LYS	2.66	-	Favored (19.99%) General / -107.2,155.3	Favored (72.8%) *mmtt* chi angles: 304.2,297,183.5,182.7	0.03Å	Favored (35.457%)	-	-	-
A 175		GLU	3.42	-	Favored (7.56%) General / -81.7,82.0	Favored (8.7%) *tp30* chi angles: 181.8,62.4,83.4	0.08Å	Favored (8.232%)	-	-	-
A 176		GLU	4.14	-	Favored (55.1%) General / -91.3,3.4	Favored (95.6%) *mt-10* chi angles: 295.9,181.3,0.5	0.02Å	Favored (8.3%)	-	-	-
A 177		GLY	4.63	-	Favored (29.22%) Glycine / -60.4,-13.4	-	-	Favored (14.449%)	-	-	-
A 178		LYS	4.75	-	Favored (59.77%) General / -59.5,-21.2	Favored (99%) *mttt* chi angles: 293.9,177.9,182.3,178.4	0.07Å	Favored (31.447%)	-	-	-
A 179		GLU	4.44	-	Favored (64.01%) General / -61.7,-20.9	Favored (20.7%) *pt0* chi angles: 68.6,177,347.7	0.06Å	Favored (17.883%)	-	-	-
A 180		GLU	3.86	-	Favored (56.36%) General / -57.3,134.9	Favored (89.1%) *tt0* chi angles: 186.2,176.6,5.4	0.04Å	Favored (35.44%)	-	-	-
**#**	**Alt**	**Res**	**High B**	**Clash > 0.4Å**	**Ramachandran**	**Rotamer**	**Cβ deviation**	**CaBLAM**	**Bond lengths**	**Bond angles**	**Cis Peptides**
			Avg: 1.18	Clashscore: 0.94	Outliers: 2 of 619	Poor rotamers: 0 of 512	Outliers: 1 of 567	Outliers: 15 of 617	Outliers: 7 of 621	Outliers: 8 of 621	Non-Trans: 0 of 620
A 181		LEU	3.19	-	Favored (55.45%) General / -59.3,132.9	Favored (35.9%) *tp* chi angles: 185.8,64.8	0.04Å	Favored (49.79%)	-	-	-
A 182		GLN	2.54	-	Allowed (1.57%) General / -54.9,161.6	Favored (17.8%) *pt0* chi angles: 69.1,182,76.5	0.04Å	CaBLAM Disfavored (4.751%)	-	-	-
A 183		GLU	2	-	Favored (34.4%) General / -52.4,132.4	Favored (93.8%) *mt-10* chi angles: 289.6,183.3,355.8	0.03Å	Favored (12.03%)	-	-	-
A 184		ILE	1.57	0.41Å N with A 185 PRO CD	Favored (3.13%) Pre-Pro / -74.1,-37.3	Favored (95.3%) *mt* chi angles: 295,167.9	0.15Å	Favored (18.131%)	-	-	-
A 185		PRO	1.25	0.41Å CD with A 184 ILE N	Favored (24.01%) Trans-Pro / -56.4,155.8	Favored (84.4%) *Cg_exo* chi angles: 334.1,35.6,329.8	0.05Å	CaBLAM Outlier (0.538%)	-	-	-

A 186		THR	1.03	-	Allowed (0.53%) General / 77.3,-49.5	Favored (98.7%) *m* chi angles: 300.2	0.10Å	CaBLAM Outlier (0.779%) try alpha helix	-	-	-
A 187		MET	0.89	-	Favored (64.96%) General / -57.7,-29.7	Favored (82.2%) *mtp* chi angles: 288.9,173.8,59.8	0.05Å	Favored (55.324%) alpha helix	-	-	-
A 188		LEU	0.8	-	Favored (45.04%) General / -98.2,8.3	Favored (73.5%) *mt* chi angles: 301.7,181.5	0.04Å	Favored (46.748%)	-	-	-
A 189		LYS	0.75	-	Favored (34.05%) General / -85.7,126.5	Favored (85.9%) *tttt* chi angles: 182,178.8,177.8,180.3	0.02Å	Favored (28.136%)	-	-	-
A 190		LYS	0.73	-	Favored (55.82%) General / -57.6,137.9	Favored (53.4%) *mttp* chi angles: 294.3,186,177.4,70	0.03Å	Favored (14.56%)	-	-	-
A 191		GLY	0.73	-	Favored (60.98%) Glycine / 98.8,-15.2	-	-	Favored (77.192%)	-	-	-
A 192		MET	0.74	-	Favored (23.22%) General / -94.8,147.6	Favored (92.3%) *mmm* chi angles: 302.2,298.4,291.5	0.04Å	Favored (29.188%)	-	-	-
A 193		THR	0.76	-	Favored (46.18%) General / -123.0,126.0	Favored (96.3%) *m* chi angles: 300.8	0.05Å	Favored (66.902%) beta sheet	-	-	-
A 194		THR	0.79	-	Favored (55.3%) General / -120.8,134.1	Favored (89.7%) *m* chi angles: 297.1	0.01Å	Favored (66.752%) beta sheet	-	-	-
A 195		ILE	0.83	-	Favored (58.93%) Ile or Val / -112.3,118.9	Favored (76.3%) *mt* chi angles: 301.3,172.7	0.02Å	Favored (69.333%) beta sheet	-	-	-
A 196		LEU	0.89	-	Favored (5.82%) General / -96.5,91.5	Favored (86.5%) *mt* chi angles: 299.9,176.4	0.04Å	Favored (71.012%) beta sheet	-	-	-
A 197		ASP	0.95	-	Favored (10.42%) General / -88.7,95.8	Favored (64.7%) *t0* chi angles: 182.5,344.4	0.05Å	Favored (42.523%) beta sheet	-	OUTLIER(S) worst is CA-CB-CG: 4.0 σ	-
A 198		PHE	1.03	-	Favored (50.21%) General / -131.0,141.0	Favored (93.3%) *m-80* chi angles: 296.5,86.2	0.04Å	Favored (31.009%)	-	-	-
A 199		HIS	1.12	-	Favored (40.36%) Pre-Pro / -58.1,152.4	Favored (58.6%) *p-80* chi angles: 65,279	0.04Å	Favored (9.203%)	-	-	-
A 200		PRO	1.19	-	Favored (92.76%) Trans-Pro / -57.4,140.3	Favored (80.3%) *Cg_exo* chi angles: 334.7,37,327	0.06Å	Favored (27.489%)	-	-	-
**#**	**Alt**	**Res**	**High B**	**Clash > 0.4Å**	**Ramachandran**	**Rotamer**	**Cβ deviation**	**CaBLAM**	**Bond lengths**	**Bond angles**	**Cis Peptides**
			Avg: 1.18	Clashscore: 0.94	Outliers: 2 of 619	Poor rotamers: 0 of 512	Outliers: 1 of 567	Outliers: 15 of 617	Outliers: 7 of 621	Outliers: 8 of 621	Non-Trans: 0 of 620
A 201		GLY	1.24	-	Favored (83.17%) Glycine / 88.4,-0.4	-	-	Favored (67.314%)	-	-	-

A 202		ALA	1.27	-	Favored (42.3%) General / -80.1,-2.0	-	0.05Å	Favored (5.64%)	-	-	-
A 203		GLY	1.29	-	Favored (58.16%) Glycine / 74.8,31.5	-	-	Favored (83.718%)	-	-	-
A 204		LYS	1.28	-	Favored (11.34%) General / -55.9,-57.6	Favored (26.6%) *mtmm* chi angles: 296.3,185.8,290.4,291	0.05Å	Favored (16.809%)	-	-	-
A 205		THR	1.25	-	Favored (97.26%) General / -64.2,-41.1	Favored (89.3%) *m* chi angles: 298.3	0.01Å	Favored (45.609%)	-	-	-
A 206		ARG	1.2	-	Favored (64.17%) General / -74.0,-34.4	Favored (98%) *mtt180* chi angles: 290.1,178.4,177.5,172.6	0.04Å	Favored (35.217%) alpha helix	-	-	-
A 207		ARG	1.15	-	Favored (2.61%) General / -115.7,-53.0	Favored (60.6%) *ttp-170* chi angles: 180.6,175.6,62.8,194.4	0.02Å	Favored (13.834%) alpha helix	-	-	-
A 208		PHE	1.1	-	Favored (61.71%) General / -72.6,-44.6	Favored (22.8%) *m-10* chi angles: 294.9,329.3	0.07Å	Favored (79.863%) alpha helix	-	-	-
A 209		LEU	1.04	0.51Å HB3 with A 210 PRO HD3	Favored (54.93%) Pre-Pro / -56.9,-54.4	Favored (63.8%) *tp* chi angles: 176.5,64	0.16Å	Favored (89.772%) alpha helix	OUTLIER(S) worst is CB--CG: 5.5 σ	-	-
A 210		PRO	0.99	0.51Å HD3 with A 209 LEU HB3	Favored (52.38%) Trans-Pro / -51.3,-35.0	Favored (87.3%) *Cg_exo* chi angles: 329.4,36.9,332.4	0.01Å	Favored (96.403%) alpha helix	-	-	-
A 211		GLN	0.94	-	Favored (81.03%) General / -68.3,-41.4	Favored (97.1%) *mt0* chi angles: 290.7,171.1,341.5	0.00Å	Favored (79.803%) alpha helix	-	-	-
A 212		ILE	0.92	-	Favored (85.35%) Ile or Val / -66.8,-46.5	Favored (92.6%) *mt* chi angles: 294.4,166.5	0.04Å	Favored (78.549%) alpha helix	-	-	-
A 213		LEU	0.91	-	Favored (89.4%) General / -65.1,-38.0	Favored (95.1%) *mt* chi angles: 291.7,172.4	0.06Å	Favored (80.931%) alpha helix	OUTLIER(S) worst is CB--CG: 4.3 σ	-	-
A 214		ALA	0.92	-	Favored (93.18%) General / -62.2,-39.6	-	0.03Å	Favored (87.151%) alpha helix	-	-	-
A 215		GLU	0.93	-	Favored (83.3%) General / -68.0,-38.9	Favored (62.7%) *tp30* chi angles: 185.4,65.8,14.7	0.01Å	Favored (93.788%) alpha helix	-	-	-
A 216		CYS	0.95	-	Favored (99.12%) General / -62.6,-41.6	Favored (98.2%) *m* chi angles: 292.3	0.11Å	Favored (97.841%) alpha helix	-	-	-
A 217		ALA	0.96	-	Favored (98.21%) General / -63.6,-42.0	-	0.03Å	Favored (94.266%) alpha helix	-	-	-
A 218		ARG	0.95	-	Favored (80.47%) General / -61.4,-36.5	Favored (94.2%) *mtm-85* chi angles: 284.8,190.7,285.3,269.2	0.02Å	Favored (79.905%) alpha helix	-	-	-
A 219		ARG	0.92	-	Favored (48.69%)	Favored (90%) *mtt-85*	0.03Å	Favored (49.926%)	-	-	-

					General / -87.3,3.0	chi angles: 291.4,173.2,181.5,264.7					
A 220		ARG	0.88	-	Favored (31.87%) General / 55.2,40.9	Favored (14.2%) *mpt180* chi angles: 276.6,71.1,174,174.2	0.02Å	Favored (29.617%)	-	-	-
**#**	**Alt**	**Res**	**High B**	**Clash > 0.4Å**	**Ramachandran**	**Rotamer**	**Cβ deviation**	**CaBLAM**	**Bond lengths**	**Bond angles**	**Cis Peptides**
			Avg: 1.18	Clashscore: 0.94	Outliers: 2 of 619	Poor rotamers: 0 of 512	Outliers: 1 of 567	Outliers: 15 of 617	Outliers: 7 of 621	Outliers: 8 of 621	Non-Trans: 0 of 620
A 221		LEU	0.83	-	Favored (27.36%) General / -91.5,142.7	Favored (77.4%) *mt* chi angles: 301.8,175.9	0.07Å	Favored (26.071%) beta sheet	-	-	-
A 222		ARG	0.78	-	Favored (23.77%) General / -82.8,119.0	Favored (20.3%) *tpt-90* chi angles: 181.4,72.6,178.6,267.2	0.05Å	Favored (52.91%) beta sheet	-	-	-
A 223		THR	0.74	-	Favored (54.74%) General / -122.3,135.5	Favored (86%) *m* chi angles: 301.5	0.01Å	Favored (67.549%) beta sheet	-	-	-
A 224		LEU	0.71	-	Favored (53.52%) General / -106.9,127.6	Favored (49.7%) *tp* chi angles: 176.1,66.4	0.08Å	Favored (72.044%) beta sheet	-	-	-
A 225		VAL	0.71	-	Favored (68.99%) Ile or Val / -118.1,122.2	Favored (48.3%) *t* chi angles: 181.8	0.07Å	Favored (71.464%) beta sheet	-	-	-
A 226		LEU	0.72	-	Favored (26.83%) General / -106.8,150.0	Favored (77.7%) *mt* chi angles: 301.6,175.8	0.07Å	Favored (46.225%) beta sheet	-	-	-
A 227		ALA	0.76	-	Favored (45.16%) Pre-Pro / -140.4,146.4	-	0.09Å	Favored (56.996%)	-	-	-
A 228		PRO	0.81	-	Favored (44.19%) Trans-Pro / -67.0,-25.8	Favored (43.8%) *Cg_endo* chi angles: 24.4,325.4,29.4	0.06Å	Favored (26.376%)	-	-	-
A 229		THR	0.85	-	Favored (31.82%) General / -140.6,163.8	Favored (41.5%) *p* chi angles: 67.5	0.04Å	Favored (22.47%)	-	-	-
A 230		ARG	0.9	-	Favored (74.13%) General / -61.0,-34.0	Favored (77.9%) *ttt180* chi angles: 188.4,173.4,181.9,178.7	0.03Å	Favored (65.877%) alpha helix	-	-	-
A 231		VAL	0.93	-	Favored (96.84%) Ile or Val / -63.1,-46.1	Favored (54.1%) *t* chi angles: 170	0.02Å	Favored (76.732%) alpha helix	-	-	-
A 232		VAL	0.95	-	Favored (85.35%) Ile or Val / -67.6,-41.2	Favored (84.7%) *t* chi angles: 173.6	0.08Å	Favored (80.045%) alpha helix	-	-	-
A 233		LEU	0.97	-	Favored (74.96%) General / -57.9,-38.0	Favored (40.1%) *tp* chi angles: 185.5,60.4	0.02Å	Favored (81.771%) alpha helix	-	-	-
A 234		SER	0.98	-	Favored (96.46%) General / -64.2,-40.6	Favored (70.9%) *m* chi angles: 296.2	0.02Å	Favored (96.707%) alpha helix	-	-	-

A 235		GLU	1	-	Favored (98.85%) General / -62.3,-41.8	Favored (46.6%) *tp30* chi angles: 187.8,73.7,19.6	0.09Å	Favored (93.319%) alpha helix	-	-	-
A 236		MET	1.02	-	Favored (92.88%) General / -59.4,-44.5	Favored (60.8%) *ttm* chi angles: 183.7,180.7,293.6	0.06Å	Favored (79.566%) alpha helix	OUTLIER(S) worst is CB--CG: 4.2 σ	-	-
A 237		LYS	1.05	-	Favored (84.97%) General / -57.5,-44.5	Favored (86.9%) *tttt* chi angles: 182.1,174.2,180,180.4	0.04Å	Favored (78.142%) alpha helix	-	-	-
A 238		GLU	1.08	-	Favored (99.89%) General / -62.8,-43.0	Favored (90.5%) *tt0* chi angles: 182.2,173.6,357.1	0.05Å	Favored (83.588%) alpha helix	-	-	-
A 239		ALA	1.11	-	Favored (69.28%) General / -58.7,-32.5	-	0.05Å	Favored (74.345%)	-	-	-
A 240		PHE	1.13	-	Favored (26.88%) General / -84.0,5.2	Favored (10.9%) *m-10* chi angles: 289,1.2	0.05Å	Favored (22.438%)	-	-	-
**#**	**Alt**	**Res**	**High B**	**Clash > 0.4Å**	**Ramachandran**	**Rotamer**	**Cβ deviation**	**CaBLAM**	**Bond lengths**	**Bond angles**	**Cis Peptides**
			Avg: 1.18	Clashscore: 0.94	Outliers: 2 of 619	Poor rotamers: 0 of 512	Outliers: 1 of 567	Outliers: 15 of 617	Outliers: 7 of 621	Outliers: 8 of 621	Non-Trans: 0 of 620
A 241		HIS	1.13	-	Favored (29.1%) General / -52.7,129.3	Favored (77.8%) *t70* chi angles: 186.6,70.5	0.01Å	Favored (13.97%)	-	-	-
A 242		GLY	1.11	-	Favored (61.56%) Glycine / 99.5,-12.8	-	-	Favored (80.794%)	-	-	-
A 243		LEU	1.06	-	Favored (20.88%) General / -96.4,150.2	Favored (92.1%) *mt* chi angles: 297.5,172.6	0.03Å	Favored (29.932%)	-	-	-
A 244		ASP	1.01	-	Favored (8.45%) General / -81.4,72.3	Favored (66.9%) *m-30* chi angles: 291,319.2	0.03Å	Favored (14.914%) beta sheet	-	-	-
A 245		VAL	0.98	-	Favored (65.81%) Ile or Val / -113.8,131.4	Favored (47.1%) *t* chi angles: 182	0.11Å	Favored (30.986%) beta sheet	-	-	-
A 246		LYS	0.98	-	Favored (42.05%) General / -96.2,132.1	Favored (49.7%) *tptt* chi angles: 174.1,69.8,178.4,178	0.06Å	Favored (56.878%) beta sheet	-	-	-
A 247		PHE	1.04	-	Favored (39.97%) General / -103.7,138.7	Favored (88.3%) *m-80* chi angles: 293.9,83.9	0.04Å	Favored (63.684%) beta sheet	-	OUTLIER(S) worst is CA-CB-CG: 4.2 σ	-
A 248		HIS	1.19	-	Favored (51.65%) General / -130.0,145.3	Favored (76.2%) *m-70* chi angles: 303.1,271.3	0.17Å	Favored (45.226%)	-	-	-
A 249		THR	1.44	-	Favored (5.68%) General / -145.3,-177.7	Favored (11.9%) *t* chi angles: 190.3	0.09Å	Favored (31.512%)	-	-	-
A 250		GLN	1.83	-	Favored (73.83%) General / -63.8,-32.4	Favored (82.4%) *mt0* chi angles: 291.1,180.1,56.3	0.02Å	Favored (51.832%)	-	-	-

A 251		ALA	2.33	-	Favored (58.41%) General / -79.9,-6.9	-	0.04Å	Favored (47.197%)	-	-	-
A 252		PHE	2.9	-	Favored (4.19%) General / -108.4,-44.1	Favored (22%) *m-10* chi angles: 297.6,344.5	0.06Å	CaBLAM Outlier (0.233%)	-	-	-
A 253		SER	3.42	-	Allowed (0.64%) General / 47.5,-139.4	Favored (16.4%) *t* chi angles: 187.3	0.03Å	CaBLAM Disfavored (3.727%)	-	-	-
A 254		ALA	3.76	-	Favored (2.35%) General / 51.5,-128.7	-	0.02Å	CaBLAM Disfavored (1.233%) try beta sheet	-	-	-
A 255		HIS	3.82	-	Favored (16.9%) General / -161.9,171.9	Favored (43%) *p90* chi angles: 59.1,81.5	0.02Å	Favored (6.982%)	-	-	-
A 256		GLY	3.57	-	Favored (31.63%) Glycine / -97.4,-162.4	-	-	Favored (37.976%)	-	-	-
A 257		SER	3.07	-	Favored (21.58%) General / -91.3,149.8	Favored (22.8%) *t* chi angles: 170.9	0.04Å	Favored (13.118%)	-	-	-
A 258		GLY	2.47	-	Favored (50.66%) Glycine / -61.6,-16.4	-	-	Favored (47.263%)	-	-	-
A 259		LYS	1.9	-	Favored (57.4%) General / -89.0,-6.2	Favored (99.1%) *mttt* chi angles: 294.3,181.1,179,178.9	0.01Å	Favored (55.73%)	-	-	-
A 260		GLU	1.43	-	Favored (19.95%) General / -93.6,151.0	Favored (51.8%) *mt-10* chi angles: 294.9,175.7,293.6	0.04Å	Favored (18.467%)	-	-	-
**#**	**Alt**	**Res**	**High B**	**Clash > 0.4Å**	**Ramachandran**	**Rotamer**	**Cβ deviation**	**CaBLAM**	**Bond lengths**	**Bond angles**	**Cis Peptides**
			Avg: 1.18	Clashscore: 0.94	Outliers: 2 of 619	Poor rotamers: 0 of 512	Outliers: 1 of 567	Outliers: 15 of 617	Outliers: 7 of 621	Outliers: 8 of 621	Non-Trans: 0 of 620
A 261		VAL	1.1	-	Favored (9.04%) Ile or Val / -118.9,-14.6	Favored (23.6%) *m* chi angles: 295.1	0.10Å	Favored (17.467%)	-	-	-
A 262		ILE	0.88	-	Favored (70.82%) Ile or Val / -122.3,132.6	Favored (56.9%) *mt* chi angles: 298.9,161	0.07Å	Favored (28.871%)	-	-	-
A 263		ASP	0.76	-	Favored (32.53%) General / -113.4,117.7	Favored (40.1%) *m-30* chi angles: 293.1,293.5	0.09Å	Favored (72.422%)	-	-	-
A 264		ALA	0.7	-	Favored (50.82%) General / -103.6,128.6	-	0.05Å	Favored (49.882%) beta sheet	-	-	-
A 265		MET	0.68	-	Favored (33.93%) General / -148.5,150.8	Favored (58.4%) *ttm* chi angles: 182.6,180.7,294.8	0.14Å	Favored (38.886%) beta sheet	-	-	-
A 266		CYS	0.69	-	Favored (29.11%) General / -79.0,154.9	Favored (84%) *m* chi angles: 294	0.07Å	Favored (45.656%)	-	-	-

A 267		HIS	0.72	-	Favored (78.49%) General / -57.8,-39.8	Favored (50.7%) *m-70* chi angles: 288.6,306.8	0.04Å	Favored (61.872%)	OUTLIER(S) worst is CB--CG: 4.1 σ	-	-
A 268		ALA	0.74	-	Favored (94.93%) General / -63.0,-39.5	-	0.04Å	Favored (97.921%) alpha helix	-	-	-
A 269		THR	0.76	-	Favored (84.22%) General / -66.8,-43.3	Favored (95.8%) *m* chi angles: 299.6	0.01Å	Favored (96.974%) alpha helix	-	-	-
A 270		LEU	0.79	0.59Å C with A 270 LEU HD13	Favored (71.64%) General / -63.1,-50.1	Allowed (1.2%) *tm* chi angles: 184,281.6	0.03Å	Favored (95.021%) alpha helix	-	-	-
A 271		THR	0.81	-	Favored (81.7%) General / -56.8,-46.2	Favored (92%) *m* chi angles: 297.8	0.04Å	Favored (97.306%) alpha helix	-	-	-
A 272		TYR	0.83	-	Favored (97.73%) General / -62.5,-40.7	Favored (23.7%) *m-10* chi angles: 294.5,162.1	0.05Å	Favored (93.874%) alpha helix	-	-	-
A 273		ARG	0.88	-	Favored (81.82%) General / -61.3,-37.0	Favored (82.7%) *mtp85* chi angles: 286.3,176.4,62.7,88.1	0.09Å	Favored (83.375%) alpha helix	-	-	-
A 274		MET	0.97	-	Favored (54.44%) General / -77.0,-27.4	Favored (53.3%) *mmp* chi angles: 295.2,300.6,99.8	0.07Å	Favored (84.168%) alpha helix	-	-	-
A 275		LEU	1.09	-	Favored (69.65%) General / -72.0,-38.5	Favored (94.7%) *mt* chi angles: 293.4,174.3	0.10Å	Favored (24.412%)	-	-	-
A 276		GLU	1.24	-	Favored (90.27%) Pre-Pro / -73.1,155.3	Favored (55.9%) *mm-30* chi angles: 292.9,284.7,329.2	0.06Å	Favored (12.604%)	-	-	-
A 277		PRO	1.39	-	Favored (6.88%) Trans-Pro / -75.9,64.8	Favored (74.1%) *Cg_endo* chi angles: 30.9,322.8,27.9	0.03Å	Favored (8.37%)	-	-	-
A 278		THR	1.49	-	Favored (51.97%) General / -110.7,135.0	Favored (89.7%) *m* chi angles: 298.7	0.04Å	Favored (22.269%)	-	-	-
A 279		ARG	1.5	-	Favored (26.37%) General / -53.1,128.1	Favored (83.5%) *ttt180* chi angles: 183.4,175.9,178.7,177	0.04Å	Favored (45.079%) beta sheet	-	-	-
A 280		VAL	1.4	-	Favored (40.7%) Ile or Val / -86.3,125.8	Favored (81.5%) *t* chi angles: 176.6	0.04Å	Favored (41.9%) beta sheet	-	-	-
**#**	**Alt**	**Res**	**High B**	**Clash > 0.4Å**	**Ramachandran**	**Rotamer**	**Cβ deviation**	**CaBLAM**	**Bond lengths**	**Bond angles**	**Cis Peptides**
			Avg: 1.18	Clashscore: 0.94	Outliers: 2 of 619	Poor rotamers: 0 of 512	Outliers: 1 of 567	Outliers: 15 of 617	Outliers: 7 of 621	Outliers: 8 of 621	Non-Trans: 0 of 620
A 281		VAL	1.22	-	Favored (31.61%) Ile or Val / -71.7,133.5	Favored (82.4%) *t* chi angles: 173.4	0.04Å	Favored (42.632%) beta sheet	-	-	-
A 282		ASN	1.03	-	Favored (5.46%)	Favored (88.2%) *m-40* chi angles: 294.4,319.7	0.02Å	Favored (36.166%) beta sheet	-	-	-

					General / -79.5,68.0						
A 283		TRP	0.87	-	Favored (58.84%) General / -63.7,140.1	Favored (16.8%) *m-90* chi angles: 291.1,282.1	0.06Å	Favored (16.038%)	-	-	-
A 284		GLU	0.75	-	Favored (14.7%) General / -94.2,-30.2	Favored (41.5%) *mt-10* chi angles: 298.1,178.4,98.3	0.05Å	Favored (25.28%)	-	-	-
A 285		VAL	0.68	-	Favored (60.9%) Ile or Val / -132.2,131.9	Favored (57.9%) *t* chi angles: 180.2	0.03Å	Favored (39.119%)	-	-	-
A 286		ILE	0.64	-	Favored (74.25%) Ile or Val / -120.3,125.5	Favored (81.7%) *mt* chi angles: 299.5,168.1	0.03Å	Favored (71.287%)	-	-	-
A 287		ILE	0.63	-	Favored (72.71%) Ile or Val / -116.2,124.7	Favored (67.6%) *mt* chi angles: 303.4,172.2	0.03Å	Favored (71.505%) beta sheet	-	-	-
A 288		MET	0.65	-	Favored (16.35%) General / -113.1,107.7	Favored (74.4%) *mtp* chi angles: 301.4,184.2,67	0.06Å	Favored (65.524%)	-	-	-
A 289		ASP	0.68	-	Favored (43.13%) General / -73.1,149.4	Favored (82.8%) *m-30* chi angles: 288.1,355.8	0.13Å	Favored (9.821%)	-	-	-
A 290		GLU	0.72	-	Favored (15.25%) General / 48.5,51.5	Favored (86.1%) *mm-30* chi angles: 298.9,293.2,337.3	0.07Å	Favored (14.373%)	-	-	-
A 291		ALA	0.76	-	Favored (61.98%) General / -60.4,-21.4	-	0.03Å	Favored (20.665%)	-	-	-
A 292		HIS	0.8	-	Favored (47.43%) General / -80.8,-2.5	Favored (53.6%) *p-80* chi angles: 59.4,284.6	0.13Å	Favored (29.166%)	-	-	-
A 293		PHE	0.83	-	Favored (41.5%) General / -59.7,129.3	Favored (16.1%) *t80* chi angles: 186.5,48	0.05Å	Favored (34.297%)	-	OUTLIER(S) worst is CA-CB-CG: 5.2 σ	-
A 294		LEU	0.83	-	Favored (33.23%) General / -93.7,10.3	Favored (67.6%) *mt* chi angles: 296.7,182.7	0.11Å	Favored (7.283%) beta sheet	-	-	-
A 295		ASP	0.81	-	Favored (85.05%) Pre-Pro / -58.6,130.3	Favored (8.1%) *t0* chi angles: 181.7,301.2	0.06Å	Favored (38.437%)	-	-	-
A 296		PRO	0.78	-	Favored (13.53%) Trans-Pro / -46.4,-34.0	Favored (70.7%) *Cg_exo* chi angles: 328.8,37.8,331.9	0.09Å	Favored (75.844%)	-	-	-
A 297		ALA	0.74	-	Favored (83.03%) General / -64.9,-36.0	-	0.03Å	Favored (74.724%) alpha helix	-	-	-
A 298		SER	0.7	-	Favored (39.31%) General / -79.4,-36.2	Favored (69.9%) *m* chi angles: 296.4	0.04Å	Favored (76.613%) alpha helix	-	-	-
A 299		ILE	0.67	-	Favored (93.32%) Ile or Val / -65.2,-45.6	Favored (97.3%) *mt* chi angles: 293.9,167.8	0.04Å	Favored (88.982%) alpha helix	-	-	-

#	Alt	Res	High B	Clash > 0.4Å	Ramachandran	Rotamer	Cβ deviation	CaBLAM	Bond lengths	Bond angles	Cis Peptides
A 300		ALA	0.66	-	Favored (97.63%) General / -61.9,-41.2	-	0.03Å	Favored (90.59%) alpha helix	-	-	-
**#**	**Alt**	**Res**	**High B**	**Clash > 0.4Å**	**Ramachandran**	**Rotamer**	**Cβ deviation**	**CaBLAM**	**Bond lengths**	**Bond angles**	**Cis Peptides**
			Avg: 1.18	Clashscore: 0.94	Outliers: 2 of 619	Poor rotamers: 0 of 512	Outliers: 1 of 567	Outliers: 15 of 617	Outliers: 7 of 621	Outliers: 8 of 621	Non-Trans: 0 of 620
A 301		ALA	0.65	-	Favored (84.56%) General / -61.3,-37.8	-	0.04Å	Favored (85.884%) alpha helix	-	-	-
A 302		ARG	0.65	-	Favored (83.25%) General / -68.0,-39.0	Favored (98.5%) *mtm-85* chi angles: 287.6,192.2,296.4,277.6	0.05Å	Favored (92.806%) alpha helix	-	-	-
A 303		GLY	0.66	-	Favored (62.13%) Glycine / -58.0,-50.9	-	-	Favored (92.137%) alpha helix	-	-	-
A 304		TRP	0.67	-	Favored (73.15%) General / -59.6,-50.6	Favored (78.1%) *t60* chi angles: 171.9,82	0.08Å	Favored (84.495%) alpha helix	-	-	-
A 305		ALA	0.69	-	Favored (96.96%) General / -61.6,-41.1	-	0.05Å	Favored (80.45%) alpha helix	-	-	-
A 306		ALA	0.72	-	Favored (83.87%) General / -67.8,-39.8	-	0.04Å	Favored (84.051%) alpha helix	-	-	-
A 307		HIS	0.75	-	Favored (79.65%) General / -60.1,-48.9	Favored (87.1%) *t70* chi angles: 181.8,75.6	0.04Å	Favored (90.708%) alpha helix	-	-	-
A 308		ARG	0.78	-	Favored (83.39%) General / -58.5,-40.7	Favored (82.8%) *mtp85* chi angles: 287.9,173.9,62.3,88	0.06Å	Favored (88.971%) alpha helix	-	-	-
A 309		ALA	0.81	-	Favored (98.25%) General / -63.6,-42.1	-	0.02Å	Favored (91.091%) alpha helix	-	-	-
A 310		ARG	0.83	-	Favored (71.92%) General / -63.9,-30.9	Favored (98.9%) *mtt180* chi angles: 288.4,175.7,177,176.1	0.04Å	Favored (76.309%) alpha helix	-	-	-
A 311		ALA	0.83	-	Favored (49.61%) General / -81.8,-2.0	-	0.04Å	Favored (53.162%)	-	-	-
A 312		ASN	0.81	-	Favored (24%) General / 59.1,36.3	Favored (84.9%) *m-40* chi angles: 298.5,313.8	0.04Å	Favored (23.917%)	-	-	-
A 313		GLU	0.77	-	Favored (17.85%) General / -92.6,-26.4	Favored (95.7%) *mt-10* chi angles: 297.1,177.9,359.4	0.03Å	CaBLAM Disfavored (3.86%)	-	-	-
A 314		SER	0.73	-	Favored (29.98%) General / -157.8,167.4	Favored (96.6%) *p* chi angles: 65.9	0.04Å	Favored (14.95%)	-	-	-
A 315		ALA	0.69	-	Favored (30.11%) General / -99.7,143.3	-	0.05Å	Favored (41.698%)	-	-	-

#	Alt	Res	High B	Clash > 0.4Å	Ramachandran	Rotamer	Cβ deviation	CaBLAM	Bond lengths	Bond angles	Cis Peptides
A 316		THR	0.67	-	Favored (51.97%) General / -124.3,130.1	Favored (94%) *m* chi angles: 299.3	0.03Å	Favored (66.742%) beta sheet	-	-	-
A 317		ILE	0.67	-	Favored (71.13%) Ile or Val / -117.5,123.0	Favored (79%) *mt* chi angles: 300,167.6	0.05Å	Favored (68.844%) beta sheet	-	-	-
A 318		LEU	0.7	-	Favored (49.96%) General / -107.1,134.6	Favored (77.2%) *mt* chi angles: 302.3,179.2	0.06Å	Favored (66.221%) beta sheet	-	-	-
A 319		MET	0.74	-	Favored (55.67%) General / -110.6,130.0	Favored (61.6%) *mtt* chi angles: 290.9,183.7,172.7	0.06Å	Favored (18.725%) beta sheet	-	-	-
A 320		THR	0.79	-	Favored (3.61%) General / -154.4,-170.7	Favored (10.9%) *t* chi angles: 188.3	0.06Å	Favored (27.92%)	-	-	-
**#**	**Alt**	**Res**	**High B**	**Clash > 0.4Å**	**Ramachandran**	**Rotamer**	**Cβ deviation**	**CaBLAM**	**Bond lengths**	**Bond angles**	**Cis Peptides**
			Avg: 1.18	Clashscore: 0.94	Outliers: 2 of 619	Poor rotamers: 0 of 512	Outliers: 1 of 567	Outliers: 15 of 617	Outliers: 7 of 621	Outliers: 8 of 621	Non-Trans: 0 of 620
A 321		ALA	0.85	-	Allowed (1.51%) General / -137.4,-14.1	-	0.06Å	Favored (8.723%)	-	-	-
A 322		THR	0.91	0.54Å O with A 322 THR HG23	Favored (5.33%) Pre-Pro / -154.8,76.8	Favored (4.3%) *t* chi angles: 179.7	0.07Å	CaBLAM Disfavored (2.797%)	-	-	-
A 323		PRO	0.96	-	Favored (63.51%) Trans-Pro / -55.7,146.8	Favored (80.8%) *Cg_exo* chi angles: 334.6,33.5,332.8	0.10Å	Favored (26.671%)	-	-	-
A 324		PRO	0.99	-	Favored (64.27%) Trans-Pro / -53.3,137.7	Favored (93.5%) *Cg_exo* chi angles: 331.2,37.5,329.6	0.06Å	Favored (38.819%)	-	-	-
A 325		GLY	0.99	-	Favored (70.52%) Glycine / 94.7,-10.8	-	-	Favored (76.109%)	-	-	-
A 326		THR	0.97	-	Favored (99.77%) General / -62.1,-43.0	Favored (91.7%) *m* chi angles: 297.9	0.01Å	CaBLAM Disfavored (1.285%)	-	-	-
A 327		SER	0.95	-	Favored (26.32%) General / 57.5,35.9	Favored (32%) *m* chi angles: 302.6	0.02Å	CaBLAM Disfavored (3.158%) try beta sheet	-	-	-
A 328		ASP	0.95	-	Favored (17.61%) General / -102.2,106.7	Favored (63.7%) *t0* chi angles: 183.7,358.6	0.08Å	Favored (22.353%)	-	-	-
A 329		GLU	0.96	-	Favored (72.15%) General / -63.9,-31.1	Favored (83.3%) *mt-10* chi angles: 289.5,179.5,328.7	0.05Å	Favored (22.623%)	-	-	-
A 330		PHE	1.01	-	Favored (64.51%) Pre-Pro / -135.0,65.0	Favored (63.6%) *m-80* chi angles: 305.6,97.8	0.07Å	Favored (15.39%)	-	-	-
A 331		PRO	1.09	-	Favored (89.76%)	Favored (53.7%) *Cg_exo*	0.06Å	Favored (22.597%)	-	-	-

#	Alt	Res	High B	Clash > 0.4Å	Ramachandran	Rotamer	Cβ deviation	CaBLAM	Bond lengths	Bond angles	Cis Peptides
					Trans-Pro / -61.1,149.3	chi angles: 337.1,35,327.4					
A 332		HIS	1.18	-	Favored (56.73%) General / -67.7,140.3	Favored (72.4%) *t70* chi angles: 188.2,71.6	0.10Å	Favored (35.091%)	-	OUTLIER(S) worst is CA-CB-CG: 4.4 σ	-
A 333		SER	1.26	-	Favored (3.92%) General / -142.0,101.3	Favored (38.7%) *t* chi angles: 177.7	0.06Å	CaBLAM Disfavored (1.602%)	-	-	-
A 334		ASN	1.27	-	Allowed (0.06%) General / 44.5,-103.0	Favored (96.9%) *m-40* chi angles: 292.9,337.9	0.25Å	CaBLAM Outlier (0.057%)	-	-	-
A 335		GLY	1.22	-	Favored (43.35%) Glycine / -76.9,-176.4	-	-	Favored (18.209%)	-	-	-
A 336		GLU	1.11	-	Favored (36.69%) General / -78.7,131.7	Favored (90%) *tt0* chi angles: 182,178.7,351.8	0.05Å	Favored (12.581%)	-	-	-
A 337		ILE	0.98	-	Favored (60.53%) Ile or Val / -126.8,122.4	Favored (76.8%) *mt* chi angles: 300.6,173.4	0.06Å	Favored (60.796%) beta sheet	-	-	-
A 338		GLU	0.86	-	Favored (27.18%) General / -94.3,115.0	Favored (90.3%) *tt0* chi angles: 182.9,180.8,353.8	0.01Å	Favored (63.755%) beta sheet	-	-	-
A 339		ASP	0.77	-	Favored (25.84%) General / -89.1,114.6	Favored (49.9%) *m-30* chi angles: 291.5,305.7	0.05Å	Favored (54.518%) beta sheet	-	-	-
A 340		VAL	0.72	-	Favored (69.03%) Ile or Val / -128.2,131.7	Favored (61.5%) *t* chi angles: 179.8	0.07Å	Favored (51.704%) beta sheet	-	-	-
**#**	**Alt**	**Res**	**High B**	**Clash > 0.4Å**	**Ramachandran**	**Rotamer**	**Cβ deviation**	**CaBLAM**	**Bond lengths**	**Bond angles**	**Cis Peptides**
			Avg: 1.18	Clashscore: 0.94	Outliers: 2 of 619	Poor rotamers: 0 of 512	Outliers: 1 of 567	Outliers: 15 of 617	Outliers: 7 of 621	Outliers: 8 of 621	Non-Trans: 0 of 620
A 341		GLN	0.69	-	Favored (34.01%) General / -87.3,132.2	Favored (62.6%) *tt0* chi angles: 186.4,173.4,11.5	0.02Å	Favored (21.41%)	-	-	-
A 342		THR	0.69	-	Favored (9.77%) General / -151.8,178.1	Favored (9.6%) *t* chi angles: 192.6	0.06Å	Favored (21.6%)	-	-	-
A 343		ASP	0.72	-	Favored (10.4%) General / -83.9,72.7	Favored (41.3%) *t0* chi angles: 190,18.9	0.01Å	Favored (6.689%)	-	-	-
A 344		ILE	0.76	-	Favored (80.09%) Pre-Pro / -81.9,123.4	Favored (78.5%) *mt* chi angles: 300.5,170.3	0.08Å	Favored (38.889%) beta sheet	-	-	-
A 345		PRO	0.82	-	Favored (53.73%) Trans-Pro / -70.5,159.2	Favored (65.5%) *Cg_endo* chi angles: 26.9,325.8,27.6	0.02Å	Favored (24.987%) beta sheet	-	-	-
A 346		SER	0.91	-	Favored (8.72%) General / -124.7,17.3	Favored (71.8%) *p* chi angles: 59.2	0.05Å	Favored (42.705%) beta sheet	-	-	-

A 347		GLU	1.03	-	Favored (62.71%) Pre-Pro / -140.7,159.4	Favored (25.5%) *pt0* chi angles: 61.7,185.5,3.4	0.13Å	Favored (11.825%) beta sheet	-	-	-
A 348		PRO	1.17	-	Favored (79.36%) Trans-Pro / -66.3,153.1	Favored (42.3%) *Cg_endo* chi angles: 24,326.1,29.5	0.02Å	Favored (31.285%) beta sheet	-	-	-
A 349		TRP	1.31	-	Favored (29.85%) General / -150.7,166.6	Favored (34.9%) *p90* chi angles: 69.3,96.8	0.13Å	Favored (54.567%)	-	-	-
A 350		ASN	1.42	-	Favored (2.55%) General / -112.2,-55.1	Favored (43.6%) *p0* chi angles: 60.8,6.6	0.10Å	CaBLAM Outlier (0.527%)	-	OUTLIER(S) worst is CA-CB-CG: 4.2 σ	-
A 351		THR	1.47	-	Favored (6.38%) General / -124.8,-15.8	Favored (79%) *p* chi angles: 60.7	0.07Å	CaBLAM Disfavored (1.411%)	-	-	-
A 352		GLY	1.44	-	Favored (59.47%) Glycine / -56.4,-31.0	-	-	Favored (24.729%)	-	-	-
A 353		HIS	1.35	-	Allowed (1.89%) General / -126.9,51.2	Favored (99.9%) *m-70* chi angles: 298.2,287.6	0.11Å	Favored (13.044%)	-	-	-
A 354		ASP	1.23	-	Favored (66.1%) General / -58.7,-28.8	Favored (29.5%) *t70* chi angles: 190.5,64.5	0.02Å	Favored (17.336%)	-	-	-
A 355		TRP	1.11	-	Favored (15.7%) General / -54.0,-24.0	Favored (76.1%) *p-90* chi angles: 59.8,266.3	0.02Å	Favored (49.087%)	-	-	-
A 356		ILE	1.02	-	Favored (18.29%) Ile or Val / -77.8,-51.3	Favored (92.6%) *mt* chi angles: 296.6,167.7	0.14Å	Favored (36.034%) alpha helix	OUTLIER(S) worst is CB--CG1: 4.1 σ	-	-
A 357		LEU	0.96	-	Favored (65.48%) General / -68.1,-25.7	Favored (7.9%) *mp* chi angles: 274.2,55.5	0.08Å	Favored (63.004%) alpha helix	-	-	-
A 358		ALA	0.93	-	Favored (59.95%) General / -77.7,-13.2	-	0.05Å	Favored (53.643%)	-	-	-
A 359		ASP	0.9	-	Favored (25.65%) General / -89.1,114.4	Favored (47.5%) *t0* chi angles: 190.2,332	0.09Å	Favored (35.561%)	-	-	-
A 360		LYS	0.87	-	Favored (58.94%) General / -77.0,-9.8	Favored (99.1%) *mttt* chi angles: 294.2,178.8,179.8,178.8	0.03Å	Favored (15.447%)	-	-	-
**#**	**Alt**	**Res**	**High B**	**Clash > 0.4Å**	**Ramachandran**	**Rotamer**	**Cβ deviation**	**CaBLAM**	**Bond lengths**	**Bond angles**	**Cis Peptides**
			Avg: 1.18	Clashscore: 0.94	Outliers: 2 of 619	Poor rotamers: 0 of 512	Outliers: 1 of 567	Outliers: 15 of 617	Outliers: 7 of 621	Outliers: 8 of 621	Non-Trans: 0 of 620
A 361		ARG	0.83	-	Favored (52.67%) Pre-Pro / -83.9,147.9	Favored (90.5%) *mtm180* chi angles: 296.4,177.9,295.3,175.8	0.06Å	Favored (16.499%) beta sheet	-	-	-
A 362		PRO	0.79	-	Favored (25.25%)	Favored (86%) *Cg_exo*	0.05Å	Favored (53.818%) beta sheet	-	-	-

					Trans-Pro / -52.1,127.9	chi angles: 330.5,37.2,330.8					
A 363		THR	0.75	-	Favored (51.33%) General / -127.5,136.1	Favored (96.6%) *m* chi angles: 299.8	0.06Å	Favored (70.03%) beta sheet	-	-	-
A 364		ALA	0.72	-	Favored (32.43%) General / -108.5,146.9	-	0.07Å	Favored (65.841%) beta sheet	-	-	-
A 365		TRP	0.71	-	Favored (16.71%) General / -133.6,117.1	Favored (6.8%) *t60* chi angles: 195.6,340.3	0.11Å	Favored (51.032%) beta sheet	-	-	-
A 366		PHE	0.72	-	Favored (27.88%) General / -92.0,115.2	Favored (91.7%) *m-80* chi angles: 292.5,85.1	0.10Å	Favored (65.629%) beta sheet	-	-	-
A 367		LEU	0.76	-	Favored (39.87%) Pre-Pro / -106.5,150.7	Favored (93%) *mt* chi angles: 297.8,174.6	0.11Å	Favored (32.804%)	-	-	-
A 368		PRO	0.81	-	Favored (66.57%) Trans-Pro / -64.1,-19.7	Favored (44%) *Cg_endo* chi angles: 24.4,324.6,30.8	0.02Å	Favored (10.334%)	-	-	-
A 369		SER	0.87	-	Favored (32.01%) General / -160.9,163.2	Favored (94.6%) *p* chi angles: 66.2	0.07Å	Favored (18.596%)	-	-	-
A 370		ILE	0.91	-	Favored (53.01%) Ile or Val / -62.4,-31.8	Favored (11.9%) *tp* chi angles: 188.6,63	0.07Å	Favored (64.36%) alpha helix	-	-	-
A 371		ARG	0.93	-	Favored (89.41%) General / -64.9,-44.5	Favored (98.4%) *mtt180* chi angles: 290.4,178.5,183,175.3	0.07Å	Favored (75.363%) alpha helix	-	-	-
A 372		ALA	0.93	-	Favored (95.36%) General / -61.8,-40.5	-	0.01Å	Favored (93.677%) alpha helix	-	-	-
A 373		ALA	0.91	-	Favored (98.29%) General / -62.7,-40.8	-	0.04Å	Favored (84.25%) alpha helix	-	-	-
A 374		ASN	0.89	-	Favored (72.91%) General / -70.9,-37.0	Favored (93.9%) *m-40* chi angles: 286.2,336.9	0.06Å	Favored (95.526%) alpha helix	-	-	-
A 375		VAL	0.86	-	Favored (85.68%) Ile or Val / -67.7,-45.0	Favored (71%) *t* chi angles: 172.2	0.05Å	Favored (87.874%) alpha helix	-	-	-
A 376		MET	0.84	-	Favored (78.11%) General / -66.9,-34.6	Favored (60.3%) *mtt* chi angles: 290.4,185.7,176.2	0.06Å	Favored (76.847%) alpha helix	-	-	-
A 377		ALA	0.83	-	Favored (91.31%) General / -59.6,-41.9	-	0.04Å	Favored (77.337%) alpha helix	-	-	-
A 378		ALA	0.83	-	Favored (98.4%) General / -62.1,-41.7	-	0.03Å	Favored (98.77%) alpha helix	-	-	-
A 379		SER	0.83	-	Favored (99.56%)	Favored (67.4%) *m* chi angles: 294.6	0.03Å	Favored (79.114%) alpha helix	-	-	-

					General / -63.0,-41.7						
A 380		LEU	0.84	-	Favored (71.12%) General / -71.3,-40.0	Favored (80.7%) *mt* chi angles: 288.9,168.8	0.06Å	Favored (78.767%) alpha helix	-	-	-
**#**	**Alt**	**Res**	**High B**	**Clash > 0.4Å**	**Ramachandran**	**Rotamer**	**Cβ deviation**	**CaBLAM**	**Bond lengths**	**Bond angles**	**Cis Peptides**
			Avg: 1.18	Clashscore: 0.94	Outliers: 2 of 619	Poor rotamers: 0 of 512	Outliers: 1 of 567	Outliers: 15 of 617	Outliers: 7 of 621	Outliers: 8 of 621	Non-Trans: 0 of 620
A 381		ARG	0.85	-	Favored (84.78%) General / -64.0,-36.7	Favored (87.3%) *mtt180* chi angles: 288.4,171.3,181.2,157.5	0.05Å	Favored (79.203%) alpha helix	-	-	-
A 382		LYS	0.86	-	Favored (66.76%) General / -65.1,-21.2	Favored (96.9%) *mttt* chi angles: 289.3,176.9,180.9,179.6	0.03Å	Favored (70.642%)	-	-	-
A 383		ALA	0.85	-	Favored (55.19%) General / -86.0,-0.3	-	0.02Å	Favored (52.289%)	-	-	-
A 384		GLY	0.82	-	Favored (63.26%) Glycine / 86.9,15.3	-	-	Favored (88.522%)	-	-	-
A 385		LYS	0.79	-	Favored (21.54%) General / -92.3,149.2	Favored (68.6%) *mmtt* chi angles: 308.1,296.9,180.6,181.2	0.06Å	Favored (36.128%)	-	-	-
A 386		SER	0.75	-	Favored (24.81%) General / -87.2,115.8	Favored (42.4%) *t* chi angles: 175.4	0.03Å	Favored (51.665%) beta sheet	-	-	-
A 387		VAL	0.73	-	Favored (72.45%) Ile or Val / -124.0,131.7	Favored (46.9%) *t* chi angles: 182.1	0.07Å	Favored (68.089%) beta sheet	-	-	-
A 388		VAL	0.74	-	Favored (45.8%) Ile or Val / -110.5,136.7	Favored (83.5%) *t* chi angles: 173.4	0.01Å	Favored (61.842%) beta sheet	-	-	-
A 389		VAL	0.78	-	Favored (61.38%) Ile or Val / -113.1,119.7	Favored (72.5%) *t* chi angles: 178.5	0.02Å	Favored (65.969%) beta sheet	-	-	-
A 390		LEU	0.85	-	Favored (39.11%) General / -106.6,119.4	Favored (86%) *mt* chi angles: 300.4,176.9	0.04Å	Favored (27.778%) beta sheet	-	-	-
A 391		ASN	0.93	-	Favored (24.73%) General / -147.9,167.5	Favored (47.2%) *t0* chi angles: 198.6,22.1	0.04Å	Favored (17.7%)	-	-	-
A 392		ARG	1.03	-	Favored (2.83%) General / -42.9,-40.1	Favored (78.3%) *ttm-80* chi angles: 185,185.1,296,272.1	0.09Å	Favored (35.52%)	-	-	-
A 393		LYS	1.11	-	Favored (57.94%) General / -90.1,-1.3	Favored (71.7%) *mmtt* chi angles: 300.2,293.6,186.8,180.6	0.04Å	Favored (29.359%) alpha helix	-	-	-
A 394		THR	1.15	-	Favored (7.23%) General / -123.1,-15.7	Favored (32%) *p* chi angles: 69.1	0.04Å	Favored (14.976%) alpha helix	-	-	-
A 395		PHE	1.16	-	Favored (66.9%)	Favored (31.1%) *m-80* chi angles: 279.6,109	0.11Å	Favored (44.272%) alpha helix	-	-	-

					General / -54.7,-38.3						
A 396		GLU	1.15	-	Favored (66.4%) General / -62.3,-22.9	Favored (87%) *mt-10* chi angles: 291.2,182.1,332.7	0.01Å	Favored (37.644%) alpha helix	-	-	-
A 397		LYS	1.14	-	Favored (5.12%) General / -109.1,-40.0	Favored (72.2%) *mmtt* chi angles: 301.5,297.8,183.1,180.8	0.06Å	Favored (27.188%) alpha helix	-	-	-
A 398		GLU	1.13	-	Favored (51.43%) General / -78.0,-33.4	Favored (70.5%) *mm-30* chi angles: 294.2,300.1,344	0.05Å	Favored (75.402%) alpha helix	-	-	-
A 399		TYR	1.14	0.48Å HB3 with A 400 PRO HD3	Favored (35.99%) Pre-Pro / -59.7,-56.4	Favored (87.4%) *t80* chi angles: 176,81.6	0.17Å	Favored (72.655%) alpha helix	-	-	-
A 400		PRO	1.17	0.48Å HD3 with A 399 TYR HB3	Favored (58.93%) Trans-Pro / -51.5,-36.3	Favored (88.9%) *Cg_exo* chi angles: 329.9,37.9,330.3	0.07Å	Favored (95.085%) alpha helix	-	-	-

#	Alt	Res	High B	Clash > 0.4Å	Ramachandran	Rotamer	Cβ deviation	CaBLAM	Bond lengths	Bond angles	Cis Peptides
			Avg: 1.18	Clashscore: 0.94	Outliers: 2 of 619	Poor rotamers: 0 of 512	Outliers: 1 of 567	Outliers: 15 of 617	Outliers: 7 of 621	Outliers: 8 of 621	Non-Trans: 0 of 620
A 401		THR	1.21	-	Favored (93.47%) General / -62.2,-45.4	Favored (91.6%) *m* chi angles: 297.9	0.02Å	Favored (80.382%) alpha helix	-	-	-
A 402		ILE	1.24	-	Favored (89.06%) Ile or Val / -60.4,-41.9	Favored (97.5%) *mt* chi angles: 292.3,168.3	0.02Å	Favored (88.857%) alpha helix	-	-	-
A 403		LYS	1.24	-	Favored (74.08%) General / -69.0,-33.7	Favored (72.2%) *mmtt* chi angles: 293.5,295.3,182.7,183.1	0.02Å	Favored (74.973%) alpha helix	-	-	-
A 404		GLN	1.18	-	Favored (29.52%) General / -82.1,-34.0	Favored (96.3%) *mt0* chi angles: 293,180.6,331.2	0.02Å	Favored (59.205%) alpha helix	-	-	-
A 405		LYS	1.08	-	Favored (9.66%) General / -135.3,111.5	Favored (86%) *tttt* chi angles: 181.7,178.4,177.9,179.7	0.06Å	Favored (13.38%)	-	-	-
A 406		LYS	0.97	-	Favored (82.36%) Pre-Pro / -73.9,130.9	Favored (98.9%) *mttt* chi angles: 293.3,179.7,181.8,176.9	0.04Å	Favored (36.636%)	-	-	-
A 407		PRO	0.85	-	Favored (16.57%) Trans-Pro / -80.3,170.1	Favored (54.9%) *Cg_endo* chi angles: 32.5,323.7,25.1	0.06Å	Favored (45.646%)	-	-	-
A 408		ASP	0.75	-	Favored (14.49%) General / -90.0,-37.2	Favored (63%) *m-30* chi angles: 297.6,303.3	0.07Å	Favored (5.19%)	-	-	-
A 409		PHE	0.68	-	Favored (49.95%) General / -132.0,143.3	Favored (94.7%) *m-80* chi angles: 296.4,86.7	0.06Å	Favored (34.863%)	-	-	-
A 410		ILE	0.65	-	Favored (48.81%) Ile or Val / -122.3,116.3	Favored (73.2%) *mt* chi angles: 301.6,170.1	0.06Å	Favored (66.101%)	-	-	-

#	Alt	Res	High B	Clash > 0.4Å	Ramachandran	Rotamer	Cβ deviation	CaBLAM	Bond lengths	Bond angles	Cis Peptides
A 411		LEU	0.66	-	Favored (30.23%) General / -99.5,143.0	Favored (71.5%) *mt* chi angles: 302.8,181.1	0.03Å	Favored (46.14%) beta sheet	-	-	-
A 412		ALA	0.69	-	Favored (41.68%) General / -149.4,156.2	-	0.08Å	Favored (54.704%)	-	-	-
A 413		THR	0.73	-	Favored (5.63%) General / -93.9,178.6	Favored (22.3%) *p* chi angles: 71.7	0.06Å	Favored (31.338%)	-	-	-
A 414		ASP	0.79	-	Favored (61.25%) General / -59.0,-23.0	Favored (79.1%) *m-30* chi angles: 294.5,349	0.13Å	Favored (21.967%)	-	-	-
A 415		ILE	0.84	-	Favored (78.05%) Ile or Val / -61.2,-37.7	Favored (46.5%) *mm* chi angles: 298.9,298.9	0.01Å	Favored (54.845%)	-	-	-
A 416		ALA	0.91	-	Favored (65.1%) General / -60.6,-23.9	-	0.04Å	Favored (64.142%)	-	-	-
A 417		GLU	0.97	-	Favored (72.8%) General / -63.3,-31.6	Favored (95.8%) *mt-10* chi angles: 295.3,173.1,0.2	0.11Å	Favored (40.022%)	-	-	-
A 418		MET	1.03	-	Favored (8.85%) General / -109.2,-28.8	Favored (82.3%) *mmm* chi angles: 301.5,299.7,281	0.14Å	Favored (19.362%)	-	-	-
A 419		GLY	1.08	-	Favored (74.56%) Glycine / -91.8,1.4	-	-	Favored (55.833%) alpha helix	-	-	-
A 420		ALA	1.11	-	Favored (9.26%) General / -153.5,128.0	-	0.03Å	Favored (8.593%)	-	-	-
**#**	**Alt**	**Res**	**High B**	**Clash > 0.4Å**	**Ramachandran**	**Rotamer**	**Cβ deviation**	**CaBLAM**	**Bond lengths**	**Bond angles**	**Cis Peptides**
			Avg: 1.18	Clashscore: 0.94	Outliers: 2 of 619	Poor rotamers: 0 of 512	Outliers: 1 of 567	Outliers: 15 of 617	Outliers: 7 of 621	Outliers: 8 of 621	Non-Trans: 0 of 620
A 421		ASN	1.12	-	Favored (9.95%) General / -87.4,70.2	Favored (87.8%) *m-40* chi angles: 296.9,316.1	0.04Å	Favored (32.884%)	-	-	-
A 422		LEU	1.09	-	Favored (22.87%) General / -100.0,150.0	Favored (82.9%) *mt* chi angles: 301.3,177.1	0.07Å	Favored (25.292%) beta sheet	-	-	-
A 423		CYS	1.02	-	Favored (10.48%) General / -112.1,102.2	Favored (76%) *m* chi angles: 296.7	0.04Å	Favored (38.367%) beta sheet	-	-	-
A 424		VAL	0.93	-	Favored (61.83%) Ile or Val / -121.0,135.2	Favored (82.3%) *t* chi angles: 176.8	0.09Å	Favored (50.655%)	-	-	-
A 425		GLU	0.84	-	Favored (47.68%) General / -78.4,-30.3	Favored (87.5%) *mt-10* chi angles: 295.6,176.8,14.2	0.10Å	Favored (29.254%)	-	-	-
A 426		ARG	0.75	-	Favored (37.26%) General / -134.6,130.9	Favored (73.1%) *ttt180* chi angles: 180.8,172.7,181.3,169.5	0.03Å	Favored (39.041%)	-	-	-

A 427		VAL	0.69	-	Favored (75.93%) Ile or Val / -119.8,128.8	Favored (92.3%) *t* chi angles: 175.9	0.05Å	Favored (72.149%)	-	-	-
A 428		LEU	0.64	-	Favored (34.43%) General / -103.1,142.3	Favored (65.6%) *mt* chi angles: 303.9,180.4	0.04Å	Favored (63.351%) beta sheet	-	-	-
A 429		ASP	0.62	-	Favored (7.14%) General / -138.3,110.1	Favored (62.1%) *t0* chi angles: 182.5,2.6	0.06Å	Favored (45.707%) beta sheet	-	-	-
A 430		CYS	0.63	-	Favored (64.27%) General / -64.5,-17.8	Favored (86.7%) *m* chi angles: 293.5	0.03Å	Favored (6.66%) beta sheet	-	-	-
A 431		ARG	0.65	-	Favored (5.29%) General / 65.2,9.1	Favored (23.6%) *mmt90* chi angles: 290.1,282.7,182.3,88.9	0.09Å	CaBLAM Outlier (0.865%)	-	-	-
A 432		THR	0.69	-	Favored (44.85%) General / -128.2,155.3	Favored (72.2%) *p* chi angles: 61.9	0.06Å	Favored (31.26%)	-	-	-
A 433		ALA	0.73	-	Favored (34.38%) General / -151.7,153.5	-	0.04Å	Favored (44.319%)	-	-	-
A 434		PHE	0.76	-	Favored (9.19%) General / -82.1,71.8	Favored (57.7%) *m-80* chi angles: 284.5,97	0.04Å	Favored (15.504%) beta sheet	-	-	-
A 435		LYS	0.8	-	Favored (63.02%) Pre-Pro / -78.2,142.0	Favored (67%) *mmtt* chi angles: 300.4,296.3,189.5,189.5	0.06Å	Favored (20.554%) beta sheet	-	-	-
A 436		PRO	0.84	-	Favored (27.99%) Trans-Pro / -72.1,136.9	Favored (75.1%) *Cg_endo* chi angles: 27.9,323.8,28.2	0.07Å	Favored (35.804%) beta sheet	-	-	-
A 437		VAL	0.89	-	Favored (63.16%) Ile or Val / -121.4,134.9	Favored (59.5%) *t* chi angles: 180	0.07Å	Favored (59.084%) beta sheet	-	-	-
A 438		LEU	0.95	-	Favored (71.52%) General / -57.0,-37.4	Favored (52.3%) *tp* chi angles: 183.1,59.7	0.03Å	CaBLAM Disfavored (1.505%)	-	-	-
A 439		VAL	1.01	-	Allowed (0.22%) Ile or Val / 64.9,-58.6	Favored (94.9%) *t* chi angles: 174.8	0.04Å	Favored (11.071%)	-	-	-
A 440		ASP	1.06	-	Allowed (1.91%) General / -140.3,-165.4	Favored (47.7%) *p0* chi angles: 62.2,19.2	0.02Å	CaBLAM Outlier (0.69%)	-	-	-
**#**	**Alt**	**Res**	**High B**	**Clash > 0.4Å**	**Ramachandran**	**Rotamer**	**Cβ deviation**	**CaBLAM**	**Bond lengths**	**Bond angles**	**Cis Peptides**
			Avg: 1.18	Clashscore: 0.94	Outliers: 2 of 619	Poor rotamers: 0 of 512	Outliers: 1 of 567	Outliers: 15 of 617	Outliers: 7 of 621	Outliers: 8 of 621	Non-Trans: 0 of 620
A 441		GLU	1.08	-	Favored (3%) General / -78.5,61.9	Favored (76.1%) *mt-10* chi angles: 298,174.5,327.5	0.11Å	CaBLAM Disfavored (1.122%)	-	-	-
A 442		GLY	1.07	-	Favored (66.49%) Glycine / 73.2,28.3	-	-	Favored (42.278%)	-	-	-

A<br>443		ARG	1.03	-	Favored<br>(5.56%)<br>General /<br>-110.6,-37.1	Favored (83%)<br>*mtt90*<br>chi angles:<br>293.3,177.7,175.8,89.3	0.01Å	CaBLAM<br>Disfavored<br>(1.382%)	-	-	-
A<br>444		LYS	0.98	-	Favored<br>(12.99%)<br>General /<br>-150.6,130.0	Favored (83.6%)<br>*tttt*<br>chi angles:<br>182,171.2,179,179.5	0.04Å	Favored<br>(25.685%)	-	-	-
A<br>445		VAL	0.94	-	Favored (47%)<br>Ile or Val /<br>-97.2,129.7	Favored (93.3%) *t*<br>chi angles: 174.6	0.03Å	Favored<br>(67.39%)	-	-	-
A<br>446		ALA	0.91	-	Favored<br>(36.62%)<br>General /<br>-122.8,155.4	-	0.02Å	Favored<br>(57.302%)<br>beta sheet	-	-	-
A<br>447		ILE	0.9	-	Favored<br>(9.42%)<br>Ile or Val /<br>-103.4,-4.7	Favored (48.3%) *pt*<br>chi angles: 62.9,172.6	0.08Å	CaBLAM<br>Disfavored<br>(2.704%)	-	-	-
A<br>448		LYS	0.88	-	Allowed<br>(0.72%)<br>General /<br>45.2,-134.9	Favored (83.6%)<br>*mttt*<br>chi angles:<br>301.1,189.7,176,182.4	0.05Å	CaBLAM<br>Disfavored<br>(1.164%)	-	-	-
A<br>449		GLY	0.85	-	Favored<br>(29.08%)<br>Glycine /<br>-99.3,176.4	-	-	CaBLAM<br>Disfavored<br>(1.509%)	-	-	-
A<br>450		PRO	0.82	-	Favored<br>(74.58%)<br>Trans-Pro /<br>-56.3,136.3	Favored (70.9%)<br>*Cg_exo*<br>chi angles:<br>335.3,35.7,328.9	0.06Å	Favored<br>(23.711%)	-	-	-
A<br>451		LEU	0.79	-	Favored<br>(39.26%)<br>General /<br>-114.6,147.6	Favored (58.5%) *mt*<br>chi angles: 305,176.4	0.07Å	Favored<br>(24.691%)	-	-	-
A<br>452		ARG	0.76	-	Favored<br>(58.24%)<br>General /<br>-60.9,137.0	Favored (98.2%)<br>*mtt180*<br>chi angles:<br>288,174.8,177,179.4	0.07Å	Favored<br>(41.851%)	-	-	-
A<br>453		ILE	0.75	-	Favored<br>(16.07%)<br>Ile or Val /<br>-80.1,140.9	Favored (93.1%) *mt*<br>chi angles: 295.8,170.8	0.06Å	Favored<br>(43.75%)	-	-	-
A<br>454		SER	0.75	-	Favored<br>(19.71%)<br>General /<br>-62.5,157.7	Favored (84.6%) *p*<br>chi angles: 67.4	0.11Å	Favored<br>(34.446%)	-	-	-
A<br>455		ALA	0.76	-	Favored<br>(79.17%)<br>General /<br>-61.0,-36.3	-	0.04Å	Favored<br>(64.507%)	-	-	-
A<br>456		SER	0.77	-	Favored<br>(98.75%)<br>General /<br>-63.4,-42.3	Favored (50.9%) *m*<br>chi angles: 291.5	0.03Å	Favored<br>(76.419%)<br>alpha helix	-	-	-
A<br>457		SER	0.77	-	Favored<br>(89.93%)<br>General /<br>-66.2,-39.6	Favored (50%) *m*<br>chi angles: 291.4	0.07Å	Favored<br>(85.379%)<br>alpha helix	-	-	-
A<br>458		ALA	0.77	-	Favored<br>(84.42%)<br>General /<br>-59.6,-39.5	-	0.02Å	Favored<br>(82.26%)<br>alpha helix	-	-	-
A<br>459		ALA	0.77	-	Favored<br>(95.55%)<br>General /<br>-60.9,-41.2	-	0.06Å	Favored<br>(88.9%)<br>alpha helix	-	-	-
A<br>460		GLN	0.77	-	Favored<br>(80.78%)	Favored (32.7%)<br>*mm110*	0.03Å	Favored<br>(91.476%)	-	-	-

#	Alt	Res	High B	Clash > 0.4Å	Ramachandran	Rotamer	Cβ deviation	CaBLAM	Bond lengths	Bond angles	Cis Peptides
					General / -67.4,-36.2	chi angles: 290.4,294.4,122.4		alpha helix			
			Avg: 1.18	Clashscore: 0.94	Outliers: 2 of 619	Poor rotamers: 0 of 512	Outliers: 1 of 567	Outliers: 15 of 617	Outliers: 7 of 621	Outliers: 8 of 621	Non-Trans: 0 of 620
A 461		ARG	0.77	-	Favored (93.73%) General / -64.8,-43.3	Favored (97.2%) *mtm-85* chi angles: 287.4,190.5,289,272.1	0.04Å	Favored (86.884%) alpha helix	OUTLIER(S) worst is CD--NE: 4.1 σ	-	-
A 462		ARG	0.78	-	Favored (78.42%) General / -64.1,-34.6	Favored (28.3%) *mtp-110* chi angles: 290.7,176.1,71.3,256.8	0.07Å	Favored (68.449%) alpha helix	-	-	-
A 463		GLY	0.81	-	Favored (61.53%) Glycine / -57.4,-30.4	-	-	Favored (76.5%) three-ten	-	-	-
A 464		ARG	0.85	-	Favored (65.59%) General / -60.7,-24.4	Favored (31.6%) *ttm170* chi angles: 181.8,168,287,194.8	0.07Å	Favored (53.449%) three-ten	-	-	-
A 465		ILE	0.91	-	Favored (9.29%) Ile or Val / -91.0,-53.2	Favored (87.6%) *mt* chi angles: 298.4,169.4	0.14Å	Favored (25.327%) alpha helix	OUTLIER(S) worst is CB--CG1: 5.6 σ	-	-
A 466		GLY	1	-	Favored (59.12%) Glycine / -55.6,-33.4	-	-	Favored (61.211%) three-ten	-	-	-
A 467		ARG	1.1	-	Favored (3.42%) General / -44.7,-37.0	Favored (80.6%) *ttm-80* chi angles: 183.8,181.2,296.7,277.5	0.05Å	Favored (40.498%) three-ten	-	-	-
A 468		ASN	1.22	-	Favored (51.32%) Pre-Pro / -112.0,101.3	Favored (46.6%) *t0* chi angles: 189.3,356.3	0.04Å	Favored (20.829%)	-	-	-
A 469		PRO	1.33	-	Favored (22.93%) Trans-Pro / -73.6,-9.6	Favored (72%) *Cg_endo* chi angles: 29.2,325.3,25.7	0.01Å	Favored (22.542%)	-	-	-
A 470		ASN	1.4	-	Allowed (1.34%) General / -86.7,25.0	Favored (65.5%) *m-40* chi angles: 294.2,279.2	0.09Å	CaBLAM Outlier (0.057%)	-	-	-
A 471		ARG	1.38	-	Favored (4.75%) General / -128.4,-14.5	Favored (92.6%) *mmt-90* chi angles: 295,293.4,181.5,270.3	0.05Å	CaBLAM Disfavored (1.946%) try alpha helix	-	-	-
A 472		ASP	1.29	-	OUTLIER (0.04)%) General / 46.7,-99.5	Favored (85.5%) *m-30* chi angles: 283.1,346.5	0.17Å	CaBLAM Outlier (0.387%)	-	-	-
A 473		GLY	1.15	-	Allowed (1.19%) Glycine / -71.8,64.1	-	-	CaBLAM Disfavored (1.44%)	-	-	-
A 474		ASP	1	-	Favored (14.05%) General / -91.4,163.8	Favored (89.4%) *m-30* chi angles: 292.6,336.1	0.04Å	Favored (35.544%)	-	-	-
A 475		SER	0.86	-	Favored (47.98%) General / -138.5,152.1	Favored (45%) *m* chi angles: 301.7	0.07Å	Favored (66.47%) beta sheet	-	-	-

#	Alt	Res	High B	Clash > 0.4Å	Ramachandran	Rotamer	Cβ deviation	CaBLAM	Bond lengths	Bond angles	Cis Peptides
A 476		TYR	0.76	-	Favored (35.42%) General / -127.2,123.0	Favored (86.8%) *t80* chi angles: 175.8,74.8	0.05Å	Favored (70.213%) beta sheet	-	-	-
A 477		TYR	0.69	-	Favored (45.44%) General / -113.5,142.8	Favored (65.7%) *m-80* chi angles: 287.6,81.2	0.04Å	Favored (56.028%) beta sheet	-	-	-
A 478		TYR	0.65	-	Favored (44.54%) General / -143.2,157.4	Favored (58.7%) *p90* chi angles: 64.4,93	0.05Å	Favored (56.207%)	-	-	-
A 479		SER	0.64	-	Favored (9.62%) General / -102.7,-33.0	Favored (58%) *m* chi angles: 293.2	0.04Å	Favored (20.802%)	-	-	-
A 480		GLU	0.64	-	Favored (79.2%) Pre-Pro / -134.9,154.4	Favored (70.6%) *mt-10* chi angles: 299,185.5,18	0.02Å	Favored (18.709%)	-	-	-
**#**	**Alt**	**Res**	**High B**	**Clash > 0.4Å**	**Ramachandran**	**Rotamer**	**Cβ deviation**	**CaBLAM**	**Bond lengths**	**Bond angles**	**Cis Peptides**
			Avg: 1.18	Clashscore: 0.94	Outliers: 2 of 619	Poor rotamers: 0 of 512	Outliers: 1 of 567	Outliers: 15 of 617	Outliers: 7 of 621	Outliers: 8 of 621	Non-Trans: 0 of 620
A 481		PRO	0.66	-	Favored (58.94%) Trans-Pro / -57.8,151.0	Favored (82%) *Cg_exo* chi angles: 334.6,33.8,332.2	0.06Å	Favored (85.206%)	-	-	-
A 482		THR	0.69	-	Favored (16.17%) General / -79.1,170.8	Favored (75.2%) *p* chi angles: 61.3	0.03Å	Favored (28.879%) beta sheet	-	-	-
A 483		SER	0.72	-	Favored (21.98%) General / -136.0,123.4	Favored (37.8%) *t* chi angles: 177.4	0.11Å	Favored (28.681%) beta sheet	-	-	-
A 484		GLU	0.76	-	Favored (54.28%) General / -87.5,1.2	Favored (36.7%) *mm-30* chi angles: 296,289.9,296.4	0.09Å	Favored (13.192%)	-	-	-
A 485		ASP	0.79	-	Favored (17.02%) General / -95.5,105.1	Favored (67.2%) *t0* chi angles: 183.4,354.7	0.10Å	Favored (7.042%)	-	-	-
A 486		ASN	0.81	-	Favored (5.41%) General / -130.5,16.7	Favored (41.9%) *p0* chi angles: 59.8,11	0.10Å	Favored (6.7%)	-	-	-
A 487		ALA	0.81	-	Favored (72.5%) General / -61.0,-32.9	-	0.02Å	Favored (48.772%)	-	-	-
A 488		HIS	0.79	-	Favored (55.19%) General / -79.2,-5.8	Favored (39.4%) *p-80* chi angles: 55.3,287.1	0.08Å	Favored (52.788%)	-	-	-
A 489		HIS	0.76	-	Favored (43.64%) General / -73.5,133.3	Favored (57.4%) *m170* chi angles: 294.3,173.3	0.07Å	Favored (37.902%)	-	OUTLIER(S) worst is CA-CB-CG: 4.1 σ	-
A 490		VAL	0.73	-	Favored (26.09%) Ile or Val / -61.8,-21.5	Allowed (1.6%) *p* chi angles: 77.7	0.06Å	Favored (23.523%)	-	-	-
A 491		CYS	0.69	-	Favored (51.89%) General / -56.5,-25.6	Favored (28.3%) *p* chi angles: 67.2	0.09Å	Favored (47.046%)	-	-	-

A 492		TRP	0.67	-	Favored (59.89%) General / -75.5,-30.9	Favored (70.9%) *m100* chi angles: 298.5,115.3	0.07Å	Favored (82.489%) alpha helix	-	-	-
A 493		LEU	0.65	-	Favored (27.61%) General / -78.8,-42.6	Favored (30.4%) *tp* chi angles: 187.5,63.2	0.03Å	Favored (73.877%) alpha helix	-	-	-
A 494		GLU	0.63	-	Favored (91.91%) General / -62.2,-39.2	Favored (65.2%) *mt-10* chi angles: 287.7,179.6,317.7	0.01Å	Favored (90.193%) alpha helix	-	-	-
A 495		ALA	0.62	-	Favored (97.19%) General / -61.4,-41.4	-	0.09Å	Favored (96.788%) alpha helix	-	-	-
A 496		SER	0.61	-	Favored (96.68%) General / -64.3,-40.8	Favored (70%) *m* chi angles: 296.4	0.05Å	Favored (97.659%) alpha helix	-	-	-
A 497		MET	0.6	-	Favored (93.51%) General / -60.1,-45.3	Favored (64.3%) *mtt* chi angles: 290.1,175.3,172.9	0.04Å	Favored (97.607%) alpha helix	-	-	-
A 498		LEU	0.61	-	Favored (99.45%) General / -62.9,-41.5	Favored (83.1%) *mt* chi angles: 289.7,168.7	0.06Å	Favored (92.824%) alpha helix	-	-	-
A 499		LEU	0.63	-	Favored (87.8%) General / -66.8,-41.4	Favored (82.7%) *mt* chi angles: 289.5,169.7	0.04Å	Favored (90.011%) alpha helix	-	-	-
A 500		ASP	0.67	-	Favored (75.33%) General / -62.0,-34.0	Favored (95.4%) *m-30* chi angles: 286.8,349.9	0.04Å	Favored (74.629%) alpha helix	-	-	-
**#**	**Alt**	**Res**	**High B**	**Clash > 0.4Å**	**Ramachandran**	**Rotamer**	**Cβ deviation**	**CaBLAM**	**Bond lengths**	**Bond angles**	**Cis Peptides**
			Avg: 1.18	Clashscore: 0.94	Outliers: 2 of 619	Poor rotamers: 0 of 512	Outliers: 1 of 567	Outliers: 15 of 617	Outliers: 7 of 621	Outliers: 8 of 621	Non-Trans: 0 of 620
A 501		ASN	0.75	-	Favored (33.67%) General / -97.8,12.5	Favored (84.8%) *m-40* chi angles: 291.5,318.6	0.08Å	Favored (36.468%)	-	OUTLIER(S) worst is CA-CB-CG: 4.6 σ	-
A 502		MET	0.89	-	Favored (29.91%) General / -91.1,140.3	Favored (58.3%) *ttm* chi angles: 184.9,174.8,289.5	0.03Å	Favored (31.556%)	-	-	-
A 503		GLU	1.07	-	Favored (24.87%) General / -84.5,119.2	Favored (87.1%) *tt0* chi angles: 181.6,176,350.1	0.07Å	Favored (48.353%) beta sheet	-	-	-
A 504		VAL	1.27	-	Favored (74.04%) Ile or Val / -120.8,131.0	Favored (85.3%) *t* chi angles: 177.2	0.06Å	Favored (47.281%)	-	-	-
A 505		ARG	1.44	-	Favored (57.23%) General / -59.1,139.1	Favored (97.5%) *mtt-85* chi angles: 291.4,184.1,179.1,275.4	0.02Å	Favored (18.104%)	-	-	-
A 506		GLY	1.51	-	Favored (71.26%) Glycine / 93.9,-11.2	-	-	Favored (17.494%)	-	-	-
A 507		GLY	1.45	-	Favored (84.49%) Glycine / 83.8,8.4	-	-	Favored (79.626%)	-	-	-

A 508		MET	1.3	-	Favored (36.88%) General / -75.2,155.4	Favored (79.4%) *mtm* chi angles: 295.5,180.7,286	0.07Å	Favored (34.933%)	-	-	-
A 509		VAL	1.12	-	Favored (61.78%) Ile or Val / -112.9,120.0	Favored (67.6%) *t* chi angles: 179	0.01Å	Favored (30.778%) beta sheet	-	-	-
A 510		ALA	0.98	-	Favored (91.23%) Pre-Pro / -68.6,132.0	-	0.06Å	Favored (38.31%)	-	-	-
A 511		PRO	0.89	-	Favored (15.09%) Trans-Pro / -81.2,170.4	Favored (43%) *Cg_endo* chi angles: 33.7,322.8,25.5	0.05Å	Favored (38.359%)	-	-	-
A 512		LEU	0.85	-	Favored (36.3%) General / -80.7,137.9	Favored (5.2%) *mp* chi angles: 277.3,72.6	0.02Å	Favored (11.002%)	-	-	-
A 513		TYR	0.84	-	Favored (39.75%) General / -71.3,129.8	Favored (71.2%) *t80* chi angles: 177.9,67.9	0.02Å	CaBLAM Disfavored (2.735%)	-	-	-
A 514		GLY	0.86	-	Favored (46.64%) Glycine / 55.7,-129.2	-	-	Favored (19.199%)	-	-	-
A 515		ILE	0.88	-	Favored (35.45%) Ile or Val / -59.8,-26.5	Favored (10.1%) *tp* chi angles: 195.5,65.9	0.03Å	Favored (17.859%)	-	-	-
A 516		GLU	0.89	-	Favored (72.78%) General / -67.3,-32.1	Favored (39.4%) *mt-10* chi angles: 294.3,182.3,77.4	0.04Å	Favored (57.974%) three-ten	-	-	-
A 517		GLY	0.89	-	Favored (87.37%) Glycine / -62.6,-33.7	-	-	Favored (88.293%) three-ten	-	-	-
A 518		THR	0.89	-	Favored (59.24%) General / -73.9,-10.0	Favored (30.6%) *p* chi angles: 69.3	0.05Å	Favored (59.841%) three-ten	-	-	-
A 519		LYS	0.88	-	Favored (55.78%) General / -88.5,-7.9	Favored (71.3%) *mmtt* chi angles: 301.5,299.7,184,186.4	0.02Å	Favored (61.305%)	-	-	-
A 520		THR	0.87	-	Favored (46.29%) Pre-Pro / -113.3,116.8	Favored (96.1%) *m* chi angles: 299.7	0.03Å	Favored (29.833%)	-	-	-
**#**	**Alt**	**Res**	**High B**	**Clash > 0.4Å**	**Ramachandran**	**Rotamer**	**Cβ deviation**	**CaBLAM**	**Bond lengths**	**Bond angles**	**Cis Peptides**
			Avg: 1.18	Clashscore: 0.94	Outliers: 2 of 619	Poor rotamers: 0 of 512	Outliers: 1 of 567	Outliers: 15 of 617	Outliers: 7 of 621	Outliers: 8 of 621	Non-Trans: 0 of 620
A 521		PRO	0.85	-	Favored (22.59%) Trans-Pro / -74.4,-14.4	Favored (75.2%) *Cg_endo* chi angles: 28.8,326,24.6	0.02Å	Favored (16.558%)	-	-	-
A 522		VAL	0.83	-	Favored (41.23%) Ile or Val / -87.6,126.7	Favored (86.3%) *t* chi angles: 177.4	0.10Å	Favored (30.986%)	-	-	-
A 523		SER	0.8	-	Favored (83.67%)	Favored (96.2%) *p* chi angles: 66	0.04Å	Favored (30.974%)	-	-	-

					Pre-Pro / -76.6,155.1						
A 524		PRO	0.77	-	Favored (76.19%) Trans-Pro / -55.0,137.3	Favored (88.1%) *Cg_exo* chi angles: 333.4,35.9,330.2	0.04Å	Favored (47.945%)	-	-	-
A 525		GLY	0.74	-	Favored (66.58%) Glycine / 92.7,-13.8	-	-	Favored (75.822%)	-	-	-
A 526		GLU	0.71	-	Favored (69.63%) General / -62.7,-28.0	Favored (62%) *mt-10* chi angles: 289.1,183.8,28.6	0.08Å	Favored (40.127%)	-	-	-
A 527		MET	0.69	-	Favored (21.4%) General / -104.9,18.9	Favored (81.7%) *mtm* chi angles: 294.4,185.2,291.1	0.10Å	Favored (25.48%)	-	-	-
A 528		ARG	0.69	-	Favored (57.87%) General / -60.0,136.0	Favored (85.9%) *mtm180* chi angles: 286.1,175.3,289.3,179	0.04Å	Favored (31.711%)	-	-	-
A 529		LEU	0.69	-	Favored (18.12%) General / -99.9,154.8	Favored (95.2%) *mt* chi angles: 297.6,175.3	0.03Å	Favored (37.25%)	-	-	-
A 530		ARG	0.7	-	Favored (25.48%) General / -75.9,165.1	Favored (98.8%) *mtt180* chi angles: 294.4,179.6,180.6,178.2	0.02Å	Favored (40.673%)	-	-	-
A 531		ASP	0.7	-	Favored (73.52%) General / -56.6,-39.4	Favored (94.2%) *m-30* chi angles: 286.2,344.5	0.07Å	Favored (46.561%)	-	-	-
A 532		ASP	0.7	-	Favored (82.13%) General / -68.1,-38.2	Favored (38.4%) *t70* chi angles: 185.1,62	0.06Å	Favored (89.567%) alpha helix	-	-	-
A 533		GLN	0.69	-	Favored (86.91%) General / -66.1,-37.9	Favored (98.4%) *mt0* chi angles: 292,175.3,315.8	0.04Å	Favored (96.293%) alpha helix	-	-	-
A 534		ARG	0.68	-	Favored (93.5%) General / -64.1,-39.2	Favored (50.5%) *mtp180* chi angles: 288.7,174.1,68.1,215.7	0.06Å	Favored (87.125%) alpha helix	-	-	-
A 535		ARG	0.67	-	Favored (76.33%) General / -59.1,-49.9	Favored (85.5%) *ttp80* chi angles: 182.4,185.2,65.8,78.7	0.03Å	Favored (82.685%) alpha helix	-	-	-
A 536		VAL	0.66	-	Favored (93.03%) Ile or Val / -59.3,-45.7	Favored (52.3%) *t* chi angles: 169.7	0.03Å	Favored (89.098%) alpha helix	-	-	-
A 537		PHE	0.67	-	Favored (66.96%) General / -55.7,-51.5	Favored (73.5%) *t80* chi angles: 170.6,75.4	0.06Å	Favored (95.226%) alpha helix	-	OUTLIER(S) worst is CA-CB-CG: 4.7 σ	-
A 538		ARG	0.69	-	Favored (98.25%) General / -62.2,-41.3	Favored (98.2%) *mtm-85* chi angles: 291.6,193.5,303.5,270.9	0.04Å	Favored (85.367%) alpha helix	-	-	-
A 539		GLU	0.72	-	Favored (90%) General / -66.0,-39.1	Favored (89.6%) *mt-10* chi angles: 288.5,184.8,354.8	0.03Å	Favored (97.721%) alpha helix	-	-	-
A 540		LEU	0.77	-	Favored (90.06%)	Favored (91.8%) *mt* chi angles: 291.1,172.3	0.01Å	Favored (81.804%) alpha helix	-	-	-

#	Alt	Res	High B	Clash > 0.4Å	Ramachandran	Rotamer	Cβ deviation	CaBLAM	Bond lengths	Bond angles	Cis Peptides
					General /<br>-64.7,-38.2						
			Avg: 1.18	Clashscore: 0.94	Outliers: 2 of 619	Poor rotamers: 0 of 512	Outliers: 1 of 567	Outliers: 15 of 617	Outliers: 7 of 621	Outliers: 8 of 621	Non-Trans: 0 of 620
A 541		VAL	0.82	-	Favored (65.68%)<br>Ile or Val /<br>-72.0,-44.8	Favored (76%) *t*<br>chi angles: 172.8	0.02Å	Favored (62.557%)<br>alpha helix	-	-	-
A 542		ARG	0.87	-	Favored (16.98%)<br>General /<br>-88.5,-35.4	Favored (78.6%) *mtp180*<br>chi angles:<br>294.6,179.5,68.8,200.4	0.04Å	Favored (51.98%)<br>alpha helix	-	-	-
A 543		ASN	0.9	-	Favored (8.7%)<br>General /<br>-101.1,-37.2	Favored (88.6%) *m-40*<br>chi angles: 296.2,323.8	0.04Å	Favored (37.344%)<br>alpha helix	-	-	-
A 544		CYS	0.9	-	Favored (52.52%)<br>General / -90.2,-7.9	Favored (61.3%) *m*<br>chi angles: 300.6	0.04Å	Favored (39.058%)	-	-	-
A 545		ASP	0.88	-	Favored (30.29%)<br>General / 54.9,43.2	Favored (20.3%) *t0*<br>chi angles: 200,30.7	0.02Å	Favored (19.481%)	-	-	-
A 546		LEU	0.83	-	Favored (37.2%)<br>Pre-Pro /<br>-95.4,150.2	Favored (88.4%) *mt*<br>chi angles: 299.4,176.1	0.06Å	Favored (19.726%)<br>beta sheet	-	-	-
A 547		PRO	0.78	-	Favored (64.1%)<br>Trans-Pro /<br>-58.4,151.0	Favored (60.1%) *Cg_exo*<br>chi angles:<br>335.9,33.8,330.8	0.02Å	Favored (86.173%)	-	-	-
A 548		VAL	0.73	-	Favored (85.97%)<br>Ile or Val /<br>-58.2,-43.5	Favored (54.7%) *t*<br>chi angles: 170	0.06Å	Favored (66.213%)	-	-	-
A 549		TRP	0.69	-	Favored (85.95%)<br>General /<br>-59.0,-47.2	Favored (89.4%) *t60*<br>chi angles: 179.6,88.7	0.02Å	Favored (79.083%)<br>alpha helix	-	-	-
A 550		LEU	0.66	-	Favored (82.26%)<br>General /<br>-66.2,-45.1	Favored (61.3%) *tp*<br>chi angles: 180.7,59.9	0.01Å	Favored (77.409%)<br>alpha helix	-	-	-
A 551		SER	0.64	-	Favored (86.49%)<br>General /<br>-58.6,-41.8	Favored (70.9%) *m*<br>chi angles: 296.2	0.10Å	Favored (85.668%)<br>alpha helix	-	-	-
A 552		TRP	0.63	-	Favored (94%)<br>General /<br>-63.4,-44.7	Favored (84.1%) *t60*<br>chi angles: 187,89.6	0.04Å	Favored (98.236%)<br>alpha helix	-	-	-
A 553		GLN	0.62	-	Favored (86.63%)<br>General /<br>-63.9,-37.2	Favored (88.7%) *mm-40*<br>chi angles:<br>294.8,300.6,327	0.07Å	Favored (88.514%)<br>alpha helix	-	-	-
A 554		VAL	0.62	-	Favored (80.61%)<br>Ile or Val /<br>-68.2,-46.8	Favored (79.4%) *t*<br>chi angles: 173.1	0.08Å	Favored (80.246%)<br>alpha helix	-	-	-
A 555		ALA	0.62	-	Favored (97.12%)<br>General /<br>-63.1,-40.3	-	0.06Å	Favored (87.422%)<br>alpha helix	-	-	-
A 556		LYS	0.62	-	Favored (86.76%)<br>General /<br>-63.8,-37.3	Favored (52.4%) *tptt*<br>chi angles:<br>184.8,67.2,172.5,175.8	0.00Å	Favored (74.547%)	-	-	-

#	Alt	Res	High B	Clash > 0.4Å	Ramachandran	Rotamer	Cβ deviation	CaBLAM	Bond lengths	Bond angles	Cis Peptides
A 557		ALA	0.64	-	Favored (61.95%) General / -62.6,-18.4	-	0.03Å	Favored (44.216%)	-	-	-
A 558		GLY	0.66	-	Favored (58.11%) Glycine / 91.1,14.0	-	-	Favored (85.696%)	-	-	-
A 559		LEU	0.69	-	Favored (33.03%) General / -78.5,149.5	Favored (78.5%) *mt* chi angles: 297.5,170.3	0.07Å	Favored (34.176%) beta sheet	-	-	-
A 560		LYS	0.72	-	Favored (13.2%) General / -96.0,163.3	Favored (28.6%) *mmtm* chi angles: 302.5,293.5,186.7,283.5	0.01Å	Favored (41.583%)	-	-	-
**#**	**Alt**	**Res**	**High B**	**Clash > 0.4Å**	**Ramachandran**	**Rotamer**	**Cβ deviation**	**CaBLAM**	**Bond lengths**	**Bond angles**	**Cis Peptides**
			Avg: 1.18	Clashscore: 0.94	Outliers: 2 of 619	Poor rotamers: 0 of 512	Outliers: 1 of 567	Outliers: 15 of 617	Outliers: 7 of 621	Outliers: 8 of 621	Non-Trans: 0 of 620
A 561		THR	0.74	-	Favored (64.45%) General / -68.3,-18.1	Favored (69.5%) *p* chi angles: 59.2	0.06Å	Favored (49.245%)	-	-	-
A 562		ASN	0.76	-	Favored (54.3%) General / -92.8,4.2	Favored (61.9%) *m-40* chi angles: 283.3,314.1	0.11Å	Favored (51.591%)	-	-	-
A 563		ASP	0.76	-	Favored (16.24%) General / -95.5,104.3	Favored (64.9%) *t0* chi angles: 183.1,344.4	0.01Å	Favored (18.25%)	-	-	-
A 564		ARG	0.74	-	Favored (51.98%) General / -97.0,3.5	Favored (13.1%) *mpt180* chi angles: 274.1,75.5,175.4,184.4	0.07Å	Favored (13.257%)	-	-	-
A 565		LYS	0.72	-	Favored (82.84%) General / -62.6,-36.5	Favored (97.1%) *mttt* chi angles: 289.6,179.7,179.6,178.8	0.02Å	Favored (33.02%)	-	-	-
A 566		TRP	0.69	-	Favored (40.05%) General / -58.7,-19.5	Favored (74%) *p-90* chi angles: 66.4,266.7	0.07Å	Favored (60.372%) three-ten	-	-	-
A 567		CYS	0.67	-	Favored (46.06%) General / -80.6,-19.1	Favored (70.9%) *m* chi angles: 298.2	0.11Å	Favored (57.213%) alpha helix	-	-	-
A 568		PHE	0.65	-	Favored (6.74%) General / -115.2,-29.1	Favored (96.3%) *m-80* chi angles: 296.6,99.5	0.05Å	Favored (14.775%) alpha helix	-	-	-
A 569		GLU	0.65	-	Favored (14.34%) General / -87.7,102.1	Favored (93.3%) *mt-10* chi angles: 295.2,183.3,3.3	0.04Å	CaBLAM Outlier (0.594%)	-	-	-
A 570		GLY	0.66	-	Favored (48.07%) Glycine / -179.8,177.3	-	-	Favored (36.956%)	-	-	-
A 571		PRO	0.68	-	Favored (81.06%) Trans-Pro / -61.1,151.1	Favored (53.3%) *Cg_exo* chi angles: 337,34.4,328.6	0.08Å	Favored (14.278%)	-	-	-
A 572		GLU	0.69	-	Favored (69.73%) General / -61.4,-29.2	Favored (99.8%) *mt-10* chi angles: 291.8,178,351.5	0.04Å	Favored (44.808%)	-	-	-

#	Alt	Res	High B	Clash > 0.4Å	Ramachandran	Rotamer	Cβ deviation	CaBLAM	Bond lengths	Bond angles	Cis Peptides
A 573		GLU	0.71	-	Favored (64.41%) General / -63.0,-19.5	Favored (99.7%) *mt-10* chi angles: 292.4,179.4,356.2	0.02Å	Favored (60.341%)	-	-	-
A 574		HIS	0.74	-	Favored (42.95%) General / -99.1,9.7	Favored (39.8%) *m170* chi angles: 292.5,189.8	0.03Å	Favored (31.852%)	-	-	-
A 575		GLU	0.79	-	Favored (57.48%) General / -59.0,135.0	Favored (68.7%) *mt-10* chi angles: 283.1,188,175.8	0.09Å	Favored (42.963%)	-	-	-
A 576		ILE	0.86	-	Favored (41.07%) Ile or Val / -88.6,128.1	Favored (49%) *mm* chi angles: 303.4,300.7	0.05Å	Favored (53.886%) beta sheet	-	-	-
A 577		LEU	0.94	-	Favored (30.22%) General / -109.9,149.2	Favored (8%) *mp* chi angles: 274.8,55.8	0.01Å	Favored (48.072%)	-	-	-
A 578		ASN	1	0.45Å C with A 578 ASN OD1	Favored (4.75%) General / -85.7,-173.7	Favored (50.5%) *p0* chi angles: 68,14.8	0.03Å	Favored (32.734%)	-	-	-
A 579		ASP	1.04	-	Favored (65.23%) General / -65.5,-18.3	Favored (82.8%) *m-30* chi angles: 293.1,350.4	0.04Å	Favored (34.33%)	-	-	-
A 580		SER	1.02	-	Favored (56.47%) General / -92.7,-1.9	Favored (70.2%) *m* chi angles: 296.4	0.03Å	Favored (50.414%)	-	-	-
**#**	**Alt**	**Res**	**High B**	**Clash > 0.4Å**	**Ramachandran**	**Rotamer**	**Cβ deviation**	**CaBLAM**	**Bond lengths**	**Bond angles**	**Cis Peptides**
			Avg: 1.18	Clashscore: 0.94	Outliers: 2 of 619	Poor rotamers: 0 of 512	Outliers: 1 of 567	Outliers: 15 of 617	Outliers: 7 of 621	Outliers: 8 of 621	Non-Trans: 0 of 620
A 581		GLY	0.95	-	Favored (77.86%) Glycine / 83.5,12.6	-	-	Favored (88.789%)	-	-	-
A 582		GLU	0.86	-	Favored (24.45%) General / -93.0,145.3	Favored (61.3%) *mt-10* chi angles: 298.6,171.6,318.5	0.08Å	Favored (25.435%)	-	-	-
A 583		THR	0.77	-	Favored (52.34%) General / -63.6,132.8	Favored (99.2%) *m* chi angles: 300.3	0.04Å	Favored (47.128%) beta sheet	-	-	-
A 584		VAL	0.71	-	Favored (37.69%) Ile or Val / -77.3,126.2	Favored (92.8%) *t* chi angles: 174.5	0.09Å	Favored (50.4%) beta sheet	-	-	-
A 585		LYS	0.7	-	Favored (39.91%) General / -114.0,146.6	Favored (99.2%) *mttt* chi angles: 294.9,182.8,179.3,180.2	0.03Å	Favored (60.1%) beta sheet	-	-	-
A 586		CYS	0.74	-	Favored (52.06%) General / -135.2,153.1	Favored (51.7%) *m* chi angles: 303.3	0.09Å	Favored (46.302%) beta sheet	-	-	-
A 587		ARG	0.83	-	Favored (37.75%) General / -78.2,136.1	Favored (83.6%) *mtp180* chi angles: 293,180.5,72.2,185.9	0.04Å	Favored (37.071%)	-	-	-
A 588		ALA	0.94	-	Favored (70.8%) Pre-Pro / -70.1,162.2	-	0.07Å	Favored (42.666%)	-	-	-
A 589		PRO	1.05	-	Favored (16.01%)	Favored (90.9%) *Cg_exo*	0.05Å	Favored (87.688%)	-	-	-

#	Alt	Res	High B	Clash > 0.4Å	Ramachandran	Rotamer	Cβ deviation	CaBLAM	Bond lengths	Bond angles	Cis Peptides
					Trans-Pro / -48.3,-30.9	chi angles: 329.6,36.4,333.6					
A 590		GLY	1.1	-	Favored (89.13%) Glycine / -84.6,-1.6	-	-	Favored (45.782%)	-	-	-
A 591		GLY	1.08	-	Favored (63.49%) Glycine / 93.3,-15.2	-	-	Favored (49.809%)	-	-	-
A 592		ALA	0.98	-	Favored (57.66%) General / -64.0,143.7	-	0.03Å	Favored (39.045%)	-	-	-
A 593		LYS	0.86	-	Favored (28.33%) General / -85.4,121.6	Favored (98.3%) *mttt* chi angles: 292.7,180.5,180,179.2	0.03Å	Favored (35.915%) beta sheet	-	-	-
A 594		LYS	0.74	-	Favored (79.59%) Pre-Pro / -132.0,152.1	Favored (96%) *mttt* chi angles: 297.1,184.4,182,182.6	0.06Å	Favored (39.584%) beta sheet	-	-	-
A 595		PRO	0.66	-	Favored (78.64%) Trans-Pro / -67.0,146.9	Favored (43.1%) *Cg_endo* chi angles: 24.2,327,27.8	0.03Å	Favored (61.834%) beta sheet	-	-	-
A 596		LEU	0.63	-	Favored (22.59%) General / -83.9,116.1	Favored (51.1%) *tp* chi angles: 182.6,64.3	0.11Å	Favored (27.586%) beta sheet	-	-	-
A 597		ARG	0.62	-	Favored (56%) Pre-Pro / -136.7,72.8	Favored (98.5%) *mtt180* chi angles: 292.9,180.3,182.3,174.3	0.13Å	Favored (7.586%) beta sheet	-	-	-
A 598		PRO	0.63	-	Favored (91.84%) Trans-Pro / -58.5,146.1	Favored (52.1%) *Cg_exo* chi angles: 336.7,36.5,326	0.03Å	Favored (37.868%)	-	-	-
A 599		ARG	0.67	-	Favored (69.75%) General / -62.0,-28.7	Favored (97.8%) *mtt180* chi angles: 288.4,178.5,182.6,176.8	0.04Å	Favored (23.603%)	-	-	-
A 600		TRP	0.7	-	Favored (47.93%) General / -133.7,142.3	Favored (14.7%) *m-90* chi angles: 306.8,267.4	0.13Å	Favored (31.305%)	-	-	-
**#**	**Alt**	**Res**	**High B**	**Clash > 0.4Å**	**Ramachandran**	**Rotamer**	**Cβ deviation**	**CaBLAM**	**Bond lengths**	**Bond angles**	**Cis Peptides**
			Avg: 1.18	Clashscore: 0.94	Outliers: 2 of 619	Poor rotamers: 0 of 512	Outliers: 1 of 567	Outliers: 15 of 617	Outliers: 7 of 621	Outliers: 8 of 621	Non-Trans: 0 of 620
A 601		CYS	0.74	-	Favored (32.05%) General / -121.5,156.8	Favored (78.4%) *m* chi angles: 296.3	0.07Å	Favored (58.884%)	-	-	-
A 602		ASP	0.79	-	Favored (4.46%) General / -145.1,107.8	Favored (59.9%) *t0* chi angles: 183,340.4	0.02Å	Favored (23.628%) beta sheet	-	-	-
A 603		GLU	0.85	-	Favored (64.21%) General / -60.6,-22.9	Favored (93.2%) *mt-10* chi angles: 289.9,181.7,342.6	0.09Å	Favored (33.102%)	-	-	-
A 604		ARG	0.93	-	Favored (66.28%) General / -60.5,-25.7	Favored (82.3%) *mtm180* chi angles: 287.7,177.4,290,183.1	0.07Å	Favored (63.418%)	-	-	-
A 605		VAL	1.04	-	Favored (82.77%)	Favored (70.1%) *t* chi angles: 172.1	0.01Å	Favored (66.977%)	-	-	-

					Ile or Val / -63.6,-38.6			alpha helix			
A 606		SER	1.16	-	Favored (28.14%) General / -84.7,-25.7	Favored (87.7%) *p* chi angles: 68.1	0.04Å	Favored (53.774%) alpha helix	-	-	-
A 607		SER	1.28	-	Favored (75.58%) General / -63.8,-48.7	Favored (46.8%) *t* chi angles: 180.2	0.03Å	Favored (62.334%) alpha helix	-	-	-
A 608		ASP	1.36	-	Favored (27.85%) General / -93.0,115.2	Favored (49.6%) *t0* chi angles: 183.9,333.3	0.11Å	Favored (26.446%) alpha helix	-	-	-
A 609		GLN	1.39	-	Favored (62.37%) General / -55.5,-31.9	Favored (96.7%) *mt0* chi angles: 291.6,177.8,342.1	0.10Å	Favored (43.292%) alpha helix	-	-	-
A 610		SER	1.35	-	Favored (69.93%) General / -71.1,-42.0	Favored (65.4%) *m* chi angles: 294.3	0.02Å	Favored (80.711%) alpha helix	-	-	-
A 611		ALA	1.25	-	Favored (93.8%) General / -63.1,-39.1	-	0.03Å	Favored (97.679%) alpha helix	-	-	-
A 612		LEU	1.11	-	Favored (80.84%) General / -64.1,-47.3	Favored (62.5%) *tp* chi angles: 179.5,58.2	0.03Å	Favored (90.844%) alpha helix	-	-	-
A 613		ALA	0.98	-	Favored (84.44%) General / -58.6,-40.8	-	0.04Å	Favored (87.034%) alpha helix	-	-	-
A 614		ASP	0.87	-	Favored (74.69%) General / -69.5,-34.8	Favored (96.5%) *m-30* chi angles: 286.4,347.3	0.14Å	Favored (92.263%) alpha helix	-	-	-
A 615		PHE	0.78	-	Favored (94.21%) General / -65.0,-42.8	Favored (9.4%) *t80* chi angles: 183.2,35.9	0.05Å	Favored (93.908%) alpha helix	-	-	-
A 616		ILE	0.72	-	Favored (98.56%) Ile or Val / -60.8,-45.1	Favored (91.2%) *mt* chi angles: 291.3,167.1	0.03Å	Favored (94.44%) alpha helix	-	-	-
A 617		LYS	0.69	-	Favored (84.8%) General / -59.5,-39.7	Favored (77.5%) *mttt* chi angles: 289.9,172.7,191.1,169.4	0.07Å	Favored (87.179%) alpha helix	-	-	-
A 618		PHE	0.68	-	Favored (72.21%) General / -60.6,-50.8	Favored (90.3%) *t80* chi angles: 174.7,77.6	0.03Å	Favored (84.329%) alpha helix	-	-	-
A 619		ALA	0.69	-	Favored (77.73%) General / -60.2,-36.4	-	0.04Å	Favored (49.644%)	-	-	-
A 620		GLU	0.72	-	Favored (10.17%) General / -83.2,71.6	Favored (32%) *mt-10* chi angles: 296.6,192.5,293.7	0.07Å	-	-	-	-
**#**	**Alt**	**Res**	**High B**	**Clash > 0.4Å**	**Ramachandran**	**Rotamer**	**Cβ deviation**	**CaBLAM**	**Bond lengths**	**Bond angles**	**Cis Peptides**
			Avg: 1.18	Clashscore: 0.94	Outliers: 2 of 619	Poor rotamers: 0 of 512	Outliers: 1 of 567	Outliers: 15 of 617	Outliers: 7 of 621	Outliers: 8 of 621	Non-Trans: 0 of 620

A 621	GLY	0.75	-	-	-	-	-	-	-	-

About MolProbity | Website for the Richardson Lab | Using ecloud x-H | Internal reference 4.5.2
