# Supplementary material for: Integrative proteome-wide structural analysis and high-throughput docking identify broad-spectrum antiviral scaffolds against Zika, Yellow Fever, West Nile, Saint Louis encephalitis, and Usutu viruses
Source: Front Cell Infect Microbiol. 2026 Apr 30;16:1723132. doi: 10.3389/fcimb.2026.1723132 (PMC13171538; doi:10.3389/fcimb.2026.1723132)
Supplement: Supplementary file 6 [file DataSheet6.zip › YFV/YF_NS4a/Mol_probity_Files/YF_NS4a_1FH-multi.table.pdf]

Viewing YF_NS4a1FH-multi.table

Duke Biochemistry
Duke University School of Medicine

When finished, you should close this window.

Hint: Use File | Save As... to save a copy of this page.

All-Atom Contacts	Clashscore, all atoms:	1.56		99th percentile* (N=1784, all resolutions)
	Clashscore is the number of serious steric overlaps (> 0.4 Å) per 1000 atoms.			
Protein Geometry	Poor rotamers	0	0.00%	Goal: <0.3%
	Favored rotamers	103	100.00%	Goal: >98%
	Ramachandran outliers	0	0.00%	Goal: <0.05%
	Ramachandran favored	122	98.39%	Goal: >98%
	Rama distribution Z-score	1.93 ± 0.68		Goal: abs(Z score) < 2
	MolProbity score^	0.90		100th percentile* (N=27675, 0Å - 99Å)
	Cβ deviations >0.25Å	0	0.00%	Goal: 0
	Bad bonds:	0 / 959	0.00%	Goal: 0%
	Bad angles:	2 / 1292	0.15%	Goal: <0.1%
Peptide Omegas	Cis Prolines:	0 / 6	0.00%	Expected: ≤1 per chain, or ≤5%
Low-resolution Criteria	CaBLAM outliers	1	0.8%	Goal: <1.0%
	CA Geometry outliers	0	0.00%	Goal: <0.5%
Additional validations	Chiral volume outliers	0/153		
	Waters with clashes	0/0	0.00%	See UnDowser table for details

In the two column results, the left column gives the raw count, right column gives the percentage.

* 100th percentile is the best among structures of comparable resolution; 0th percentile is the worst. For clashscore the comparative set of structures was selected in 2004, for MolProbity score in 2006.

^ MolProbity score combines the clashscore, rotamer, and Ramachandran evaluations into a single score, normalized to be on the same scale as X-ray resolution.

Key to table colors and cutoffs here:

#	Alt	Res	High B	Clash > 0.4Å	Ramachandran	Rotamer	Cβ deviation	CaBLAM	Bond lengths	Bond angles	Cis Peptides
			Avg: 3.52	Clashscore: 1.56	Outliers: 0 of 124	Poor rotamers: 0 of 103	Outliers: 0 of 113	Outliers: 1 of 122	Outliers: 0 of 126	Outliers: 2 of 126	Non-Trans: 0 of 125
A 1		GLY	7.91	-	-	-	-	-	-	-	-
A 2		ALA	7.85	-	Favored (68.49%) General / -57.9,-33.2	-	0.03Å	-	-	-	-
A 3		ALA	7.76	-	Favored (77.35%) General / -59.8,-36.7	-	0.04Å	Favored (70.071%)	-	-	-
A 4		GLU	7.65	-	Favored (62.73%) General / -74.5,-38.6	Favored (98.4%) *mt-10* chi angles: 293.1,181,357.3	0.00Å	Favored (78.849%) alpha helix	-	-	-
A 5		MET	7.55	-	Favored (79.06%) General / -57.8,-48.6	Favored (48%) *ttp* chi angles: 176.9,190.5,69.1	0.07Å	Favored (92.778%) alpha helix	-	-	-
A 6		LEU	7.44	-	Favored (87.28%)	Favored (92.1%) *mt* chi angles: 291.5,171.1	0.06Å	Favored (80.994%) alpha helix	-	-	-

					General / -62.9,-37.5						
A 7		VAL	7.33	-	Favored (96.78%) Ile or Val / -64.0,-45.4	Favored (60.6%) *t* chi angles: 170.9	0.02Å	Favored (84.29%) alpha helix	-	-	-
A 8		VAL	7.22	-	Favored (95.8%) Ile or Val / -61.6,-42.9	Favored (58.7%) *t* chi angles: 170.6	0.02Å	Favored (89.732%) alpha helix	-	-	-
A 9		LEU	7.08	-	Favored (97.35%) General / -63.4,-40.5	Favored (92%) *mt* chi angles: 291.2,171.9	0.04Å	Favored (97.617%) alpha helix	-	-	-
A 10		SER	6.89	-	Favored (83.84%) General / -63.2,-36.5	Favored (71.9%) *m* chi angles: 295.9	0.04Å	Favored (74.431%)	-	-	-
A 11		GLU	6.61	-	Favored (6.28%) General / -105.5,29.8	Favored (90.6%) *mt-10* chi angles: 296.4,187.1,357.2	0.02Å	Favored (17.077%)	-	-	-
A 12		LEU	6.21	-	Favored (60.1%) Pre-Pro / -57.1,146.7	Favored (86.5%) *mt* chi angles: 291,173.8	0.02Å	Favored (8.271%)	-	-	-
A 13		PRO	5.73	-	Favored (37.4%) Trans-Pro / -50.2,134.3	Favored (89.9%) *Cg_exo* chi angles: 329.7,38.2,330.5	0.07Å	Favored (35.86%)	-	-	-
A 14		ASP	5.18	-	Favored (73.4%) General / -59.9,-34.6	Favored (98.5%) *m-30* chi angles: 288.1,345.6	0.05Å	Favored (52.103%)	-	-	-
A 15		PHE	4.62	-	Favored (67.85%) General / -58.8,-51.9	Favored (93.3%) *t80* chi angles: 177.1,78.1	0.03Å	Favored (58.995%) alpha helix	-	-	-
A 16		LEU	4.09	-	Favored (61.75%) General / -75.1,-34.0	Favored (88.7%) *mt* chi angles: 293.6,177.4	0.02Å	Favored (64.225%) alpha helix	-	-	-
A 17		ALA	3.61	-	Favored (95.88%) General / -61.3,-40.9	-	0.04Å	Favored (83.531%) alpha helix	-	-	-
A 18		LYS	3.2	-	Favored (74.15%) General / -67.2,-46.3	Favored (87.3%) *tttt* chi angles: 180.5,175.3,177.3,178.3	0.02Å	Favored (85.02%) alpha helix	-	-	-
A 19		LYS	2.87	-	Favored (72.28%) General / -67.7,-32.0	Favored (68.3%) *mmtt* chi angles: 292.3,294.2,175.8,179	0.05Å	Favored (81.382%) alpha helix	-	-	-
A 20		GLY	2.6	-	Favored (45.92%) Glycine / -60.4,-53.4	-	-	Favored (87.254%) alpha helix	-	-	-
**#**	**Alt**	**Res**	**High B**	**Clash > 0.4Å**	**Ramachandran**	**Rotamer**	**Cβ deviation**	**CaBLAM**	**Bond lengths**	**Bond angles**	**Cis Peptides**
			Avg: 3.52	Clashscore: 1.56	Outliers: 0 of 124	Poor rotamers: 0 of 103	Outliers: 0 of 113	Outliers: 1 of 122	Outliers: 0 of 126	Outliers: 2 of 126	Non-Trans: 0 of 125
A 21		GLY	2.37	-	Favored (48%) Glycine / -54.3,-51.9	-	-	Favored (95.792%) alpha helix	-	-	-

A 22		GLU	2.17	-	Favored (83.73%) General / -61.5,-37.5	Favored (94.8%) *mt-10* chi angles: 288.6,181.1,348	0.04Å	Favored (80.994%) alpha helix	-	-	-
A 23		ALA	1.99	-	Favored (97.21%) General / -61.3,-41.6	-	0.03Å	Favored (87.237%) alpha helix	-	-	-
A 24		VAL	1.83	-	Favored (94.63%) Ile or Val / -65.3,-44.6	Favored (69.8%) *t* chi angles: 172.1	0.05Å	Favored (91.297%) alpha helix	-	-	-
A 25		ASP	1.68	-	Favored (94.21%) General / -63.2,-39.3	Favored (99.6%) *m-30* chi angles: 287.5,346.7	0.03Å	Favored (95.147%) alpha helix	-	-	-
A 26		THR	1.56	-	Favored (90.44%) General / -61.3,-46.4	Favored (93.5%) *m* chi angles: 299.2	0.07Å	Favored (87.922%) alpha helix	-	-	-
A 27		ILE	1.49	-	Favored (94.25%) Ile or Val / -64.3,-46.0	Favored (91.4%) *mt* chi angles: 293,165.4	0.07Å	Favored (82.987%) alpha helix	-	-	-
A 28		SER	1.48	-	Favored (90.51%) General / -59.5,-41.6	Favored (67.7%) *m* chi angles: 296.8	0.05Å	Favored (82.685%) alpha helix	-	-	-
A 29		VAL	1.56	-	Favored (99.76%) Ile or Val / -63.0,-44.7	Favored (59.1%) *t* chi angles: 170.7	0.03Å	Favored (97.378%) alpha helix	-	-	-
A 30		LEU	1.78	-	Favored (75.28%) General / -63.5,-33.3	Favored (96.1%) *mt* chi angles: 291.9,172.7	0.04Å	Favored (76.076%) alpha helix	-	-	-
A 31		LEU	2.24	-	Favored (59.84%) General / -75.9,-33.4	Favored (95.7%) *mt* chi angles: 292.8,174	0.06Å	Favored (55.062%) alpha helix	-	-	-
A 32		HIS	3.05	-	Favored (11.2%) General / -115.5,-17.5	Favored (98.9%) *m-70* chi angles: 300.7,291.8	0.02Å	Favored (21.027%)	-	-	-
A 33		SER	4.19	-	Favored (22.72%) General / -78.5,166.3	Favored (93.3%) *p* chi angles: 66.4	0.03Å	Favored (17.668%)	-	-	-
A 34		GLU	5.27	-	Favored (36.44%) General / -77.8,130.2	Favored (90.5%) *tt0* chi angles: 184.4,177.6,4.9	0.01Å	Favored (16.784%)	-	-	-
A 35		GLU	5.62	-	Favored (53.54%) General / -57.0,132.7	Favored (91.5%) *tt0* chi angles: 184.2,177.6,1.8	0.01Å	Favored (23.956%)	-	-	-
A 36		GLY	4.93	-	Favored (74.91%) Glycine / 92.3,-10.1	-	-	Favored (84.031%)	-	-	-
A 37		SER	3.65	-	Favored (27.17%) General / -69.0,162.3	Favored (97.8%) *p* chi angles: 65.2	0.03Å	Favored (30.997%)	-	-	-
A 38		ARG	2.42	-	Favored (92.01%)	Favored (97.9%) *mtt180*	0.02Å	Favored (66.036%)	-	-	-

#	Alt	Res	High B	Clash > 0.4Å	Ramachandran	Rotamer	Cβ deviation	CaBLAM	Bond lengths	Bond angles	Cis Peptides
					General / -61.7,-39.6	chi angles: 289.3,177.5,180,172.2					
A 39		ALA	1.57	-	Favored (99.21%) General / -62.6,-41.9	-	0.03Å	Favored (98.587%) alpha helix	-	-	-
A 40		TYR	1.1	-	Favored (70.36%) General / -59.2,-51.4	Favored (88.4%) *t80* chi angles: 178.7,82.6	0.04Å	Favored (90.656%) alpha helix	-	-	-
**#**	**Alt**	**Res**	**High B**	**Clash > 0.4Å**	**Ramachandran**	**Rotamer**	**Cβ deviation**	**CaBLAM**	**Bond lengths**	**Bond angles**	**Cis Peptides**
			Avg: 3.52	Clashscore: 1.56	Outliers: 0 of 124	Poor rotamers: 0 of 103	Outliers: 0 of 113	Outliers: 1 of 122	Outliers: 0 of 126	Outliers: 2 of 126	Non-Trans: 0 of 125
A 41		ARG	0.87	-	Favored (86.43%) General / -61.5,-38.2	Favored (97.1%) *mtt-85* chi angles: 289.2,175.6,184.2,272.8	0.05Å	Favored (77.982%) alpha helix	-	-	-
A 42		ASN	0.81	-	Favored (74.23%) General / -67.7,-45.7	Favored (99.4%) *m-40* chi angles: 287.6,339.8	0.04Å	Favored (82.223%) alpha helix	-	-	-
A 43		ALA	0.84	-	Favored (88.64%) General / -62.4,-38.2	-	0.05Å	Favored (90.941%) alpha helix	-	-	-
A 44		LEU	0.94	-	Favored (88.73%) General / -66.4,-41.7	Favored (35.3%) *tp* chi angles: 186.3,59.2	0.02Å	Favored (97.594%) alpha helix	-	-	-
A 45		SER	1.09	-	Favored (96.22%) General / -62.6,-40.2	Favored (73%) *m* chi angles: 295.6	0.04Å	Favored (83.793%) alpha helix	-	-	-
A 46		MET	1.28	-	Favored (62.64%) General / -72.8,-28.6	Favored (83.7%) *mtm* chi angles: 291,188.4,292.1	0.03Å	Favored (43.019%) alpha helix	-	-	-
A 47		MET	1.52	-	Favored (5.14%) Pre-Pro / -39.5,-49.9	Favored (6.2%) *tpt* chi angles: 190.1,65,158.4	0.18Å	Favored (32.817%) alpha helix	-	OUTLIER(S) worst is CA-C-N: 4.2 σ	-
A 48		PRO	1.79	-	Favored (58.82%) Trans-Pro / -56.2,-26.0	Favored (76.7%) *Cg_exo* chi angles: 334.9,37,326.9	0.04Å	Favored (73.132%) alpha helix	-	-	-
A 49		GLU	2.06	-	Favored (32.72%) General / -78.5,-41.4	Favored (91.1%) *tt0* chi angles: 184.3,176.5,359.6	0.01Å	Favored (72.932%) alpha helix	-	-	-
A 50		ALA	2.33	-	Favored (92.01%) General / -60.4,-40.7	-	0.05Å	Favored (87.574%) alpha helix	-	-	-
A 51		MET	2.58	0.41Å HA with A 51 MET HE2	Favored (60.06%) General / -75.8,-35.4	Favored (47.9%) *mmp* chi angles: 293.2,298.2,94.7	0.06Å	Favored (87.635%) alpha helix	-	-	-
A 52		THR	2.82	-	Favored (94.1%) General / -64.8,-43.1	Favored (93.6%) *m* chi angles: 299.3	0.01Å	Favored (84.299%) alpha helix	-	-	-
A 53		THR	3.07	-	Favored (82.75%)	Favored (94.6%) *m* chi angles: 299.4	0.06Å	Favored (90.697%) alpha helix	-	-	-

#	Alt	Res	High B	Clash > 0.4Å	Ramachandran	Rotamer	Cβ deviation	CaBLAM	Bond lengths	Bond angles	Cis Peptides
					General / -58.6,-47.9						
A 54		VAL	3.32	-	Favored (99.85%) Ile or Val / -62.8,-44.9	Favored (62.1%) *t* chi angles: 171.1	0.02Å	Favored (89.758%) alpha helix	-	-	-
A 55		MET	3.56	-	Favored (85.42%) General / -57.6,-44.5	Favored (29.2%) *tmm* chi angles: 180.4,276.9,290.2	0.03Å	Favored (92.724%) alpha helix	-	-	-
A 56		LEU	3.77	-	Favored (82.39%) General / -65.2,-35.9	Favored (89.4%) *mt* chi angles: 290.9,173	0.05Å	Favored (88.718%) alpha helix	-	-	-
A 57		PHE	3.93	-	Favored (97.41%) General / -62.6,-40.6	Favored (12.7%) *m-10* chi angles: 287,342.7	0.02Å	Favored (88.894%) alpha helix	-	-	-
A 58		VAL	4.05	-	Favored (91.89%) Ile or Val / -66.1,-44.6	Favored (63.4%) *t* chi angles: 171.3	0.01Å	Favored (87.406%) alpha helix	-	-	-
A 59		LEU	4.13	0.52Å C with A 59 LEU HD23	Favored (65.34%) General / -58.3,-52.5	Favored (4.6%) *tt* chi angles: 189.2,161.6	0.07Å	Favored (92.173%) alpha helix	-	-	-
A 60		ALA	4.15	-	Favored (79.79%) General / -59.8,-37.7	-	0.04Å	Favored (82.061%) alpha helix	-	-	-
**#**	**Alt**	**Res**	**High B**	**Clash > 0.4Å**	**Ramachandran**	**Rotamer**	**Cβ deviation**	**CaBLAM**	**Bond lengths**	**Bond angles**	**Cis Peptides**
			Avg: 3.52	Clashscore: 1.56	Outliers: 0 of 124	Poor rotamers: 0 of 103	Outliers: 0 of 113	Outliers: 1 of 122	Outliers: 0 of 126	Outliers: 2 of 126	Non-Trans: 0 of 125
A 61		GLY	4.13	-	Favored (51.27%) Glycine / -59.8,-52.7	-	-	Favored (90.539%) alpha helix	-	-	-
A 62		LEU	4.09	-	Favored (86.19%) General / -65.0,-37.0	Favored (97.3%) *mt* chi angles: 292.1,172.4	0.03Å	Favored (77.712%) alpha helix	-	-	-
A 63		LEU	4.03	-	Favored (74.34%) General / -63.6,-49.2	Favored (69.8%) *tp* chi angles: 177.2,59.9	0.04Å	Favored (76.64%) alpha helix	-	-	-
A 64		THR	3.95	-	Favored (80.82%) General / -63.8,-47.5	Favored (97.9%) *m* chi angles: 300	0.06Å	Favored (78.108%) alpha helix	-	-	-
A 65		SER	3.87	-	Favored (93.74%) General / -60.8,-40.8	Favored (71.4%) *m* chi angles: 296.1	0.06Å	Favored (85.767%) alpha helix	-	-	-
A 66		GLY	3.78	-	Favored (50.66%) Glycine / -56.5,-52.3	-	-	Favored (94.762%) alpha helix	-	-	-
A 67		MET	3.72	-	Favored (69.72%) General / -67.4,-30.0	Favored (93.5%) *mmm* chi angles: 292,307.8,294.9	0.10Å	Favored (76.235%) alpha helix	-	-	-
A 68		VAL	3.68	-	Favored (92.8%)	Favored (62.3%) *t* chi angles: 171.1	0.02Å	Favored (76.632%) alpha helix	-	-	-

					Ile or Val / -65.0,-46.0					
A 69		ILE	3.69	-	Favored (99.24%) Ile or Val / -61.1,-44.9	Favored (90.6%) *mt* chi angles: 291.5,166.6	0.02Å	Favored (78.617%) alpha helix	-	-	-
A 70		PHE	3.79	-	Favored (17.7%) General / -51.1,-55.4	Favored (63.9%) *t80* chi angles: 172.9,86.6	0.13Å	Favored (75.85%) alpha helix	-	OUTLIER(S) worst is CA-CB-CG: 7.4 σ	-
A 71		PHE	3.99	-	Favored (67.72%) General / -69.2,-29.7	Favored (43.9%) *m-80* chi angles: 285.9,114.2	0.03Å	Favored (54.316%) alpha helix	-	-	-
A 72		MET	4.26	-	Favored (53.74%) General / -91.7,-5.4	Favored (70.8%) *mtt* chi angles: 294.8,178.5,180.7	0.01Å	Favored (46.093%)	-	-	-
A 73		SER	4.55	-	Favored (98.42%) Pre-Pro / -66.3,148.7	Favored (51.6%) *m* chi angles: 291.7	0.05Å	Favored (34.454%)	-	-	-
A 74		PRO	4.75	-	Favored (72.65%) Trans-Pro / -68.5,154.0	Favored (46.6%) *Cg_endo* chi angles: 24.8,326.9,27.4	0.02Å	Favored (77.77%)	-	-	-
A 75		LYS	4.79	-	Favored (65.01%) General / -59.7,-25.6	Favored (97.4%) *mttt* chi angles: 290.1,179.6,179.9,178.4	0.01Å	Favored (26.328%)	-	-	-
A 76		GLY	4.62	-	Favored (48.37%) Glycine / -58.3,-21.7	-	-	Favored (34.004%)	-	-	-
A 77		ILE	4.27	-	Favored (6.5%) Ile or Val / -119.7,17.9	Favored (44%) *pt* chi angles: 60,171.9	0.06Å	CaBLAM Disfavored (3.349%)	-	-	-
A 78		SER	3.85	0.41Å OG with A 79 ARG N	Allowed (0.51%) General / 51.1,-145.2	Favored (20.1%) *t* chi angles: 186	0.04Å	CaBLAM Disfavored (1.479%)	-	-	-
A 79		ARG	3.43	0.41Å N with A 78 SER OG	Allowed (0.88%) General / -134.3,-37.0	Favored (88.3%) *mmt-90* chi angles: 292,287.9,180.8,269.6	0.10Å	CaBLAM Outlier (0.002%)	-	-	-
A 80		MET	3.09	-	Favored (83.8%) General / -67.4,-38.0	Favored (61.6%) *tpp* chi angles: 186.9,61.5,77.7	0.06Å	Favored (67.408%) alpha helix	-	-	-
**#**	**Alt**	**Res**	**High B**	**Clash > 0.4Å**	**Ramachandran**	**Rotamer**	**Cβ deviation**	**CaBLAM**	**Bond lengths**	**Bond angles**	**Cis Peptides**
			Avg: 3.52	Clashscore: 1.56	Outliers: 0 of 124	Poor rotamers: 0 of 103	Outliers: 0 of 113	Outliers: 1 of 122	Outliers: 0 of 126	Outliers: 2 of 126	Non-Trans: 0 of 125
A 81		SER	2.85	-	Favored (92.86%) General / -62.3,-39.4	Favored (60.3%) *m* chi angles: 293.6	0.04Å	Favored (83.87%) alpha helix	-	-	-
A 82		MET	2.7	-	Favored (76.15%) General / -70.0,-39.0	Favored (52%) *mmp* chi angles: 294.8,300.7,98.4	0.07Å	Favored (96.171%) alpha helix	-	-	-
A 83		ALA	2.62	-	Favored (92.22%) General / -64.8,-43.7	-	0.07Å	Favored (97.011%) alpha helix	-	-	-

A 84		MET	2.58	-	Favored (72.59%) General / -69.5,-33.2	Favored (50.9%) *mmp* chi angles: 293.9,300.2,96.3	0.06Å	Favored (85.598%) alpha helix	-	-	-
A 85		GLY	2.57	-	Favored (64.7%) Glycine / -61.5,-50.6	-	-	Favored (90.108%) alpha helix	-	-	-
A 86		THR	2.57	-	Favored (87.25%) General / -58.2,-45.7	Favored (99.5%) *m* chi angles: 300.5	0.07Å	Favored (91.943%) alpha helix	-	-	-
A 87		MET	2.57	-	Favored (76.96%) General / -59.6,-49.6	Favored (56.3%) *ttp* chi angles: 180.7,188.4,68.3	0.10Å	Favored (97.637%) alpha helix	-	-	-
A 88		ALA	2.58	-	Favored (89.3%) General / -61.2,-39.2	-	0.04Å	Favored (91.268%) alpha helix	-	-	-
A 89		GLY	2.59	-	Favored (50.6%) Glycine / -59.5,-52.9	-	-	Favored (91.418%) alpha helix	-	-	-
A 90		CYS	2.63	-	Favored (85.15%) General / -63.9,-36.8	Favored (94.8%) *m* chi angles: 292.9	0.04Å	Favored (75.226%) alpha helix	-	-	-
A 91		GLY	2.68	-	Favored (58.66%) Glycine / -56.7,-51.1	-	-	Favored (92.182%) alpha helix	-	-	-
A 92		TYR	2.79	-	Favored (76.05%) General / -56.2,-48.6	Favored (87.2%) *t80* chi angles: 174.3,79.2	0.06Å	Favored (97.109%) alpha helix	-	-	-
A 93		LEU	2.96	-	Favored (83.98%) General / -62.7,-36.7	Favored (94%) *mt* chi angles: 292,173.6	0.07Å	Favored (82.977%) alpha helix	-	-	-
A 94		MET	3.17	-	Favored (99.06%) General / -60.9,-43.1	Favored (94.9%) *mtp* chi angles: 289.6,170.8,70.3	0.04Å	Favored (90.919%) alpha helix	-	-	-
A 95		PHE	3.41	-	Favored (87.26%) General / -60.7,-47.2	Favored (85.3%) *t80* chi angles: 181.9,76.8	0.01Å	Favored (86.896%) alpha helix	-	-	-
A 96		LEU	3.63	-	Favored (65.06%) General / -55.2,-35.5	Favored (42.3%) *tp* chi angles: 185.4,62.1	0.06Å	Favored (75.798%)	-	-	-
A 97		GLY	3.72	-	Favored (77.73%) Glycine / -78.7,0.3	-	-	Favored (41.862%)	-	-	-
A 98		GLY	3.62	-	Favored (57.77%) Glycine / 94.4,10.9	-	-	Favored (85.271%)	-	-	-
A 99		VAL	3.36	-	Favored (34.48%) Ile or Val / -81.5,131.2	Favored (85.3%) *t* chi angles: 177.6	0.03Å	Favored (32.868%)	-	-	-
A 100		LYS	2.99	-	Favored (98.69%) Pre-Pro / -65.0,142.6	Favored (98%) *mttt* chi angles: 292,181.8,180.2,178.8	0.02Å	Favored (49.875%)	-	-	-

#	Alt	Res	High B	Clash > 0.4Å	Ramachandran	Rotamer	Cβ deviation	CaBLAM	Bond lengths	Bond angles	Cis Peptides
			Avg: 3.52	Clashscore: 1.56	Outliers: 0 of 124	Poor rotamers: 0 of 103	Outliers: 0 of 113	Outliers: 1 of 122	Outliers: 0 of 126	Outliers: 2 of 126	Non-Trans: 0 of 125
A 101		PRO	2.6	-	Favored (16.29%) Trans-Pro / -48.5,-30.7	Favored (85.9%) *Cg_exo* chi angles: 329.3,36.7,332.9	0.04Å	Favored (63.36%)	-	-	-
A 102		THR	2.27	-	Favored (93.25%) General / -64.6,-43.6	Favored (89.1%) *m* chi angles: 298.6	0.01Å	Favored (69.293%) alpha helix	-	-	-
A 103		HIS	2.04	-	Favored (66.54%) General / -71.9,-42.7	Favored (77.8%) *t70* chi angles: 186.5,67.4	0.05Å	Favored (74.281%) alpha helix	-	-	-
A 104		ILE	1.89	-	Favored (98.77%) Ile or Val / -61.3,-45.4	Favored (99%) *mt* chi angles: 292.4,167.7	0.06Å	Favored (96.95%) alpha helix	-	-	-
A 105		SER	1.81	-	Favored (98.09%) General / -60.9,-42.5	Favored (72.7%) *m* chi angles: 295.7	0.06Å	Favored (93.677%) alpha helix	-	-	-
A 106		TYR	1.79	-	Favored (69.62%) General / -56.0,-50.9	Favored (83.2%) *t80* chi angles: 172,78.1	0.07Å	Favored (83.612%) alpha helix	-	-	-
A 107		ILE	1.8	-	Favored (90.72%) Ile or Val / -65.7,-41.6	Favored (37.9%) *mm* chi angles: 295.9,300.9	0.03Å	Favored (81.852%) alpha helix	-	-	-
A 108		MET	1.82	-	Favored (96.53%) General / -63.0,-40.1	Favored (98.6%) *mtp* chi angles: 291,174,70.4	0.05Å	Favored (96.324%) alpha helix	-	-	-
A 109		LEU	1.85	-	Favored (93.1%) General / -64.7,-39.1	Favored (86.1%) *mt* chi angles: 290.3,170.6	0.01Å	Favored (98.69%) alpha helix	-	-	-
A 110		ILE	1.89	-	Favored (96.79%) Ile or Val / -63.3,-46.0	Favored (94.3%) *mt* chi angles: 291.9,167	0.01Å	Favored (94.049%) alpha helix	-	-	-
A 111		PHE	1.95	-	Favored (79%) General / -57.1,-48.3	Favored (88%) *t80* chi angles: 180.1,80.6	0.02Å	Favored (98.036%) alpha helix	-	-	-
A 112		PHE	2.01	-	Favored (73.09%) General / -55.0,-48.7	Favored (81.8%) *t80* chi angles: 172.6,74.6	0.06Å	Favored (95.677%) alpha helix	-	-	-
A 113		VAL	2.08	-	Favored (86.05%) Ile or Val / -59.4,-42.0	Favored (58.5%) *t* chi angles: 170.6	0.03Å	Favored (95.091%) alpha helix	-	-	-
A 114		LEU	2.16	-	Favored (84.1%) General / -61.8,-37.3	Favored (97.3%) *mt* chi angles: 292.2,172.8	0.03Å	Favored (90.501%) alpha helix	-	-	-
A 115		MET	2.25	-	Favored (71.94%) General / -66.8,-31.4	Favored (50.3%) *mmp* chi angles: 292.4,298.4,96.8	0.04Å	Favored (79.998%) alpha helix	-	-	-

A 116		VAL	2.39	-	Favored (94.59%) Ile or Val / -63.9,-42.1	Favored (66.3%) *t* chi angles: 171.7	0.03Å	Favored (80.435%) alpha helix	-	-	-
A 117		VAL	2.61	-	Favored (89.73%) Ile or Val / -66.7,-44.3	Favored (77.1%) *t* chi angles: 172.9	0.05Å	Favored (74.661%) alpha helix	-	-	-
A 118		VAL	2.94	-	Favored (64.29%) Ile or Val / -72.4,-43.2	Favored (77.1%) *t* chi angles: 172.9	0.04Å	Favored (37.109%)	-	-	-
A 119		VAL	3.38	-	Favored (70.19%) Pre-Pro / -80.3,118.0	Favored (82.7%) *t* chi angles: 176.8	0.07Å	Favored (28.242%)	-	-	-
A 120		PRO	3.96	-	Favored (37.01%) Trans-Pro / -76.3,156.0	Favored (81.4%) *Cg_endo* chi angles: 30.4,325.3,24.5	0.06Å	Favored (54.22%)	-	-	-
**#**	**Alt**	**Res**	**High B**	**Clash > 0.4Å**	**Ramachandran**	**Rotamer**	**Cβ deviation**	**CaBLAM**	**Bond lengths**	**Bond angles**	**Cis Peptides**
			Avg: 3.52	Clashscore: 1.56	Outliers: 0 of 124	Poor rotamers: 0 of 103	Outliers: 0 of 113	Outliers: 1 of 122	Outliers: 0 of 126	Outliers: 2 of 126	Non-Trans: 0 of 125
A 121		GLU	4.66	-	Favored (65.08%) Pre-Pro / -86.8,126.0	Favored (89.8%) *tt0* chi angles: 182.5,181.1,4.9	0.07Å	Favored (39.826%)	-	-	-
A 122		PRO	5.45	-	Favored (34.74%) Trans-Pro / -50.0,-33.5	Favored (88.3%) *Cg_exo* chi angles: 330,37.3,331.4	0.05Å	Favored (80.968%)	-	-	-
A 123		GLY	6.22	-	Favored (72.29%) Glycine / -60.9,-30.1	-	-	Favored (84.421%) three-ten	-	-	-
A 124		GLN	6.9	-	Favored (55.56%) General / -83.7,-2.1	Favored (84.6%) *mt0* chi angles: 294.8,178.3,44.8	0.02Å	Favored (52.726%)	-	-	-
A 125		GLN	7.44	-	Favored (57.34%) General / -92.5,-0.4	Favored (94%) *mt0* chi angles: 295.9,179.6,327.6	0.01Å	-	-	-	-
A 126		ARG	7.83	-	-	Favored (98.2%) *mtt180* chi angles: 295.3,181.4,179.7,183.5	0.02Å	-	-	-	-

About MolProbity | Website for the Richardson Lab | Using ecloud x-H | Internal reference 4.5.2
