# Supplementary material for: Integrative proteome-wide structural analysis and high-throughput docking identify broad-spectrum antiviral scaffolds against Zika, Yellow Fever, West Nile, Saint Louis encephalitis, and Usutu viruses
Source: Front Cell Infect Microbiol. 2026 Apr 30;16:1723132. doi: 10.3389/fcimb.2026.1723132 (PMC13171538; doi:10.3389/fcimb.2026.1723132)
Supplement: Supplementary file 6 [file DataSheet6.zip › YFV/YF_NS4a/Mol_probity_Files/YF_NS4a_1FH-rama.pdf]

MolProbity Ramachandran analysis

YF_NS4a1FH.pdb, model 1

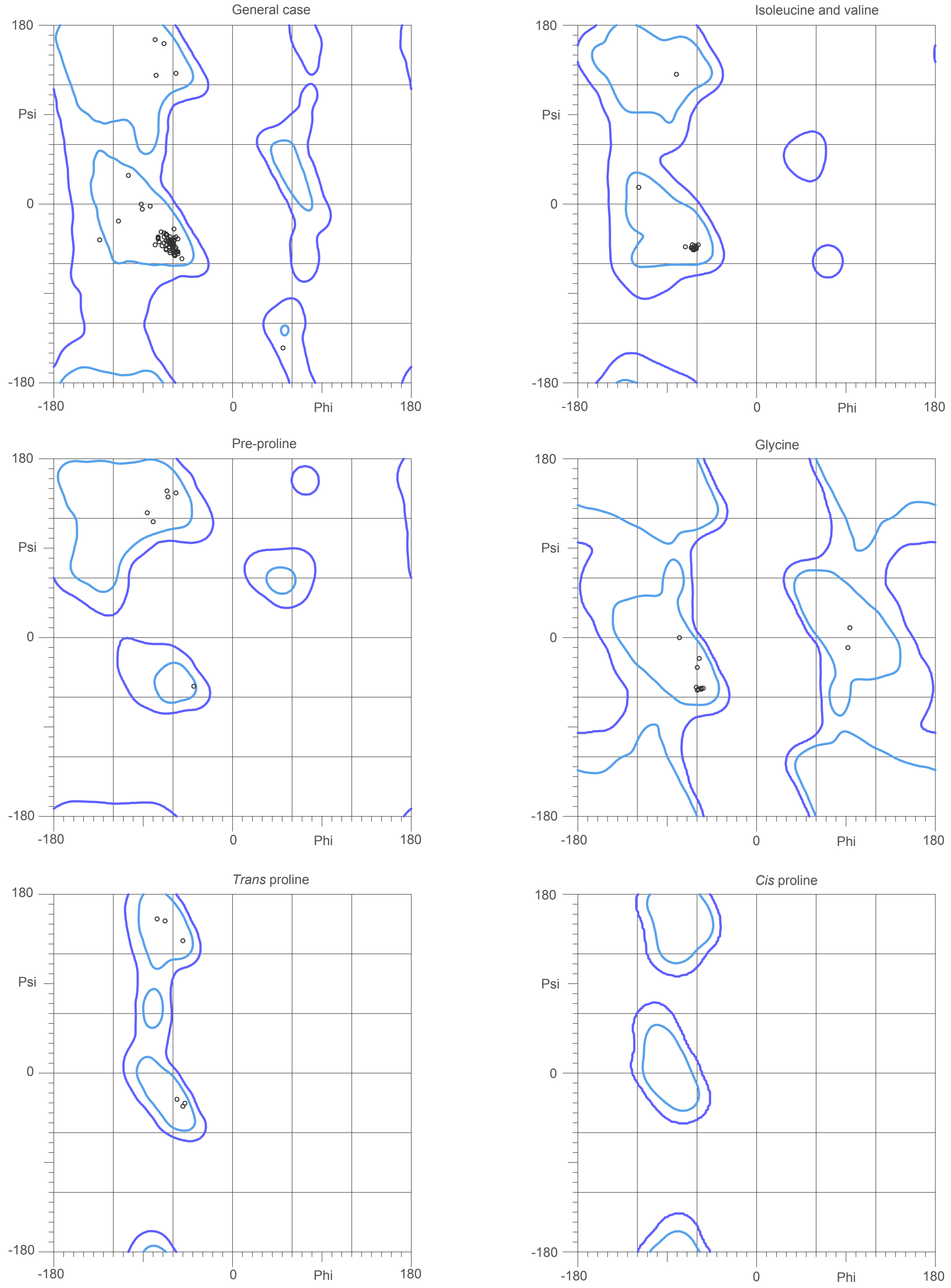

98.4% (122/124) of all residues were in favored (98%) regions.
100.0% (124/124) of all residues were in allowed (>99.8%) regions.

There were no outliers.
