# Supplementary material for: Integrative proteome-wide structural analysis and high-throughput docking identify broad-spectrum antiviral scaffolds against Zika, Yellow Fever, West Nile, Saint Louis encephalitis, and Usutu viruses
Source: Front Cell Infect Microbiol. 2026 Apr 30;16:1723132. doi: 10.3389/fcimb.2026.1723132 (PMC13171538; doi:10.3389/fcimb.2026.1723132)
Supplement: Supplementary file 6 [file DataSheet6.zip › YFV/YF_NS4b/Mol_probity_Files/YF_NS4b_1FH-multi.table.pdf]

Viewing YF_NS4b1FH-multi.table

Duke Biochemistry
Duke University School of Medicine

When finished, you should close this window.   *Hint: Use File | Save As... to save a copy of this page.*

All-Atom Contacts	Clashscore, all atoms:	1.54		99th percentile* (N=1784, all resolutions)
	Clashscore is the number of serious steric overlaps (> 0.4 Å) per 1000 atoms.			
Protein Geometry	Poor rotamers	0	0.00%	Goal: <0.3%
	Favored rotamers	206	99.52%	Goal: >98%
	Ramachandran outliers	5	2.02%	Goal: <0.05%
	Ramachandran favored	239	96.37%	Goal: >98%
	Rama distribution Z-score	0.85 ± 0.54		Goal: abs(Z score) < 2
	MolProbity score^	1.14		99th percentile* (N=27675, 0Å - 99Å)
	Cβ deviations >0.25Å	1	0.44%	Goal: 0
	Bad bonds:	1 / 1963	0.05%	Goal: 0%
	Bad angles:	2 / 2668	0.07%	Goal: <0.1%
Peptide Omegas	Cis Prolines:	0 / 15	0.00%	Expected: ≤1 per chain, or ≤5%
Low-resolution Criteria	CaBLAM outliers	2	0.8%	Goal: <1.0%
	CA Geometry outliers	0	0.00%	Goal: <0.5%
Additional validations	Chiral volume outliers	0/306		
	Waters with clashes	0/0	0.00%	See UnDowser table for details

In the two column results, the left column gives the raw count, right column gives the percentage.

* 100th percentile is the best among structures of comparable resolution; 0th percentile is the worst. For clashscore the comparative set of structures was selected in 2004, for MolProbity score in 2006.

^ MolProbity score combines the clashscore, rotamer, and Ramachandran evaluations into a single score, normalized to be on the same scale as X-ray resolution.

Key to table colors and cutoffs here:

#	Alt	Res	High B	Clash > 0.4Å	Ramachandran	Rotamer	Cβ deviation	CaBLAM	Bond lengths	Bond angles	Cis Peptides
			Avg: 3.90	Clashscore: 1.54	Outliers: 5 of 248	Poor rotamers: 0 of 207	Outliers: 1 of 229	Outliers: 2 of 246	Outliers: 1 of 250	Outliers: 2 of 250	Non-Trans: 0 of 249
A 1		ASN	16.47	-	-	Favored (85.7%) *m-40* chi angles: 291.4,319.7	0.06Å	-	-	-	-
A 2		GLU	16.17	-	Favored (46.4%) General / -63.4,149.2	Favored (97%) *mt-10* chi angles: 293.2,182.6,357.4	0.04Å	-	-	-	-
A 3		LEU	15.84	-	Favored (57.55%) General / -51.0,-40.2	Favored (58.8%) *tp* chi angles: 181.6,60.1	0.04Å	Favored (63.13%)	-	-	-
A 4		GLY	15.54	-	Favored (78.37%) Glycine / -59.4,-33.9	-	-	Favored (90.434%) alpha helix	-	-	-
A 5		MET	15.35	-	Favored (76.54%) General / -69.2,-42.0	Favored (95.1%) *mtp* chi angles: 292.3,170.8,66.1	0.06Å	Favored (85.984%) alpha helix	-	-	-
A 6		LEU	15.3	-	Favored (86.64%) General / -67.2,-40.1	Favored (97.2%) *mt* chi angles: 292.8,171.6	0.06Å	Favored (99.333%) alpha helix	-	-	-

A 7		GLU	15.38	-	Favored (92.06%) General / -64.3,-44.3	Favored (91.5%) *tt0* chi angles: 183.2,176.7,357.7	0.05Å	Favored (94.703%) alpha helix	-	-	-
A 8		LYS	15.53	-	Favored (97.26%) General / -62.3,-40.7	Favored (18.4%) *tptp* chi angles: 180.6,66,164.4,70.8	0.03Å	Favored (89.844%) alpha helix	-	-	-
A 9		THR	15.73	-	Favored (88.01%) General / -58.9,-46.4	Favored (88.7%) *m* chi angles: 298.4	0.03Å	Favored (90.579%) alpha helix	-	-	-
A 10		LYS	15.89	-	Favored (94.97%) General / -62.6,-39.8	Favored (97.4%) *mttt* chi angles: 289.1,177.5,181.4,176.8	0.03Å	Favored (87.161%) alpha helix	-	-	-
A 11		GLU	15.94	-	Favored (86.68%) General / -60.1,-39.6	Favored (91.4%) *tt0* chi angles: 182.4,175.8,359.5	0.04Å	Favored (80.931%) alpha helix	-	-	-
A 12		ASP	15.86	-	Favored (62.65%) General / -73.2,-29.2	Favored (96.5%) *m-30* chi angles: 290,347	0.04Å	Favored (66.786%) alpha helix	-	-	-
A 13		LEU	15.7	-	Favored (12.36%) General / -97.2,-31.6	Favored (95.2%) *mt* chi angles: 298.2,177.1	0.04Å	Favored (55.001%)	-	-	-
A 14		PHE	15.58	-	Favored (18.09%) General / -126.8,166.1	Favored (70.4%) *m-80* chi angles: 304.6,96.4	0.05Å	Favored (7.12%)	-	-	-
A 15		GLY	15.53	-	Favored (45.47%) Glycine / -60.3,147.1	-	-	Favored (28.23%)	-	-	-
A 16		LYS	15.55	-	Favored (64.32%) General / -61.0,-22.3	Favored (97.2%) *mttt* chi angles: 290.2,180.5,178.5,179.8	0.05Å	Favored (23.379%)	-	-	-
A 17		LYS	15.58	-	Favored (59.59%) General / -59.6,-21.0	Favored (60.4%) *pttt* chi angles: 67.4,183.3,181.4,181.9	0.02Å	Favored (44.021%)	-	-	-
A 18		ASN	15.58	-	Favored (41.9%) General / -99.6,10.1	Favored (88.1%) *m-40* chi angles: 291.9,321	0.02Å	Favored (49.545%)	-	-	-
A 19		LEU	15.53	-	Favored (9.69%) General / -83.2,68.7	Favored (89.5%) *mt* chi angles: 299.6,177	0.02Å	Favored (6.169%)	-	-	-
A 20		ILE	15.4	-	Favored (61.08%) Pre-Pro / -103.9,108.9	Favored (46.8%) *mm* chi angles: 305.7,301.7	0.04Å	Favored (21.506%)	-	-	-

#	Alt	Res	High B	Clash > 0.4Å	Ramachandran	Rotamer	Cβ deviation	CaBLAM	Bond lengths	Bond angles	Cis Peptides
			Avg: 3.90	Clashscore: 1.54	Outliers: 5 of 248	Poor rotamers: 0 of 207	Outliers: 1 of 229	Outliers: 2 of 246	Outliers: 1 of 250	Outliers: 2 of 250	Non-Trans: 0 of 249
A 21		PRO	15.13	-	Favored (28.34%) Trans-Pro / -71.4,-19.8	Favored (63.2%) *Cg_endo* chi angles: 26.7,326.3,26.1	0.04Å	CaBLAM Disfavored (3.971%) try beta sheet	-	-	-
A 22		SER	14.71	-	Favored (13.33%) General / 46.2,47.3	Favored (59.5%) *m* chi angles: 299.1	0.01Å	Favored (10.621%) beta sheet	-	-	-

A 23		SER	14.16	-	Allowed (0.6%) General / -74.0,56.5	Favored (78.4%) *p* chi angles: 60.7	0.03Å	Favored (39.271%) beta sheet	-	-	-
A 24		ALA	13.41	-	Favored (7.04%) General / -82.2,64.8	-	0.03Å	Favored (17.622%) beta sheet	-	-	-
A 25		ALA	12.41	-	Favored (92.84%) Pre-Pro / -70.1,144.6	-	0.03Å	Favored (18.832%) beta sheet	-	-	-
A 26		PRO	11.13	-	Favored (42.25%) Trans-Pro / -75.2,156.0	Favored (71.3%) *Cg_endo* chi angles: 29.7,324.8,26.1	0.04Å	Favored (68.039%) beta sheet	-	-	-
A 27		TRP	9.6	-	Favored (76.43%) General / -65.5,-47.3	Favored (74.2%) *t-100* chi angles: 182.9,252.8	0.01Å	CaBLAM Outlier (0.544%)	-	-	-
A 28		SER	7.94	-	Allowed (0.19%) General / 64.6,-170.1	Favored (8.2%) *t* chi angles: 192.6	0.05Å	CaBLAM Outlier (0.855%)	-	-	-
A 29		TRP	6.27	-	Favored (58.46%) Pre-Pro / -79.2,141.9	Favored (98.6%) *m100* chi angles: 289.7,98.5	0.03Å	Favored (7.325%)	-	-	-
A 30		PRO	4.75	-	Favored (66.98%) Trans-Pro / -60.0,152.1	Favored (70.5%) *Cg_exo* chi angles: 335.3,33.3,332.2	0.07Å	Favored (60.649%)	-	-	-
A 31		ASP	3.49	-	Favored (58.83%) General / -63.8,140.1	Favored (97.7%) *m-30* chi angles: 288.2,348.4	0.06Å	Favored (28.682%)	-	-	-
A 32		PHE	2.55	-	Favored (30.28%) General / -129.9,123.2	Favored (87.2%) *m-80* chi angles: 298.6,85.9	0.05Å	Favored (42.377%) beta sheet	-	-	-
A 33		ASP	1.9	-	Favored (2.86%) General / -147.9,97.8	Favored (59.1%) *t0* chi angles: 184.9,0.5	0.05Å	Favored (8.049%) beta sheet	-	-	-
A 34		LEU	1.48	-	Favored (4.84%) General / -79.2,89.0	Favored (55.8%) *tp* chi angles: 181.9,58	0.10Å	Favored (64.199%) beta sheet	-	-	-
A 35		LYS	1.22	-	Favored (40.97%) Pre-Pro / -84.9,112.2	Favored (55.8%) *mttp* chi angles: 295.4,178,174.1,64.1	0.08Å	Favored (56.541%)	-	-	-
A 36		PRO	1.08	-	Favored (68.04%) Trans-Pro / -53.8,-32.7	Favored (96.2%) *Cg_exo* chi angles: 331.5,37.5,329	0.09Å	Favored (90.148%)	-	-	-
A 37		GLY	1.01	-	Favored (25.48%) Glycine / -61.6,-56.0	-	-	Favored (79.489%) alpha helix	-	-	-
A 38		ALA	0.99	-	Favored (97.19%) General / -62.9,-40.3	-	0.02Å	Favored (73.195%) alpha helix	-	-	-
A 39		ALA	0.99	-	Favored (98.98%) General / -62.4,-41.9	-	0.04Å	Favored (86.029%) alpha helix	-	-	-
A 40		TRP	1.02	-	Favored (90.8%)	Favored (91.8%) *t60*	0.06Å	Favored (85.397%)	-	-	-

					General / -64.2,-44.8	chi angles: 180.9,88.2		alpha helix			

#	Alt	Res	High B	Clash > 0.4Å	Ramachandran	Rotamer	Cβ deviation	CaBLAM	Bond lengths	Bond angles	Cis Peptides
			Avg: 3.90	Clashscore: 1.54	Outliers: 5 of 248	Poor rotamers: 0 of 207	Outliers: 1 of 229	Outliers: 2 of 246	Outliers: 1 of 250	Outliers: 2 of 250	Non-Trans: 0 of 249
A 41		THR	1.05	-	Favored (99.74%) General / -62.9,-42.2	Favored (71.2%) *m* chi angles: 303	0.10Å	Favored (91.487%) alpha helix	-	-	-
A 42		VAL	1.1	-	Favored (99.05%) Ile or Val / -61.6,-44.7	Favored (50.5%) *t* chi angles: 169.5	0.03Å	Favored (86.358%) alpha helix	-	-	-
A 43		TYR	1.15	-	Favored (62.85%) General / -53.6,-51.5	Favored (54%) *t80* chi angles: 168,81.8	0.05Å	Favored (84.968%) alpha helix	-	-	-
A 44		VAL	1.21	-	Favored (96.22%) Ile or Val / -64.4,-42.8	Favored (62.7%) *t* chi angles: 171.2	0.02Å	Favored (80.466%) alpha helix	-	-	-
A 45		GLY	1.3	-	Favored (37%) Glycine / -55.1,-54.0	-	-	Favored (87.704%) alpha helix	-	-	-
A 46		ILE	1.41	-	Favored (79.78%) Ile or Val / -64.3,-49.7	Favored (96.6%) *mt* chi angles: 293.5,167.1	0.10Å	Favored (66.855%) alpha helix	-	-	-
A 47		VAL	1.54	-	Favored (86.23%) Ile or Val / -65.9,-47.2	Favored (64.7%) *t* chi angles: 171.5	0.02Å	Favored (76.758%) alpha helix	-	-	-
A 48		THR	1.72	-	Favored (80.92%) General / -68.3,-41.4	Favored (82.4%) *m* chi angles: 302.2	0.08Å	Favored (80.011%) alpha helix	-	-	-
A 49		MET	1.93	-	Favored (89.29%) General / -65.7,-38.5	Favored (77.8%) *mtm* chi angles: 288.6,187.6,281.1	0.03Å	Favored (72.206%) alpha helix	-	-	-
A 50		LEU	2.15	-	Favored (43.59%) General / -77.2,-40.9	Favored (56.1%) *tp* chi angles: 181.7,57.9	0.04Å	Favored (27.667%) alpha helix	-	-	-
A 51		SER	2.36	-	Allowed (1.1%) Pre-Pro / -40.1,-61.6	Favored (38.4%) *t* chi angles: 177.7	0.15Å	Favored (46.947%) alpha helix	-	-	-
A 52		PRO	2.54	-	Favored (72.84%) Trans-Pro / -63.2,-23.5	Favored (44%) *Cg_endo* chi angles: 24.4,326.4,28.5	0.01Å	Favored (81.548%) alpha helix	-	-	-
A 53		MET	2.65	-	Favored (65.61%) General / -68.5,-48.0	Favored (94.2%) *mtp* chi angles: 291.3,174.9,65	0.10Å	Favored (66.56%) alpha helix	-	-	-
A 54		LEU	2.68	-	Favored (83.53%) General / -67.1,-37.2	Favored (84.8%) *mt* chi angles: 291.3,174.8	0.02Å	Favored (75.635%) alpha helix	-	-	-
A 55		HIS	2.65	-	Favored (72.18%) General / -70.2,-42.6	Favored (75.3%) *m-70* chi angles: 287.9,295.7	0.02Å	Favored (74.279%) alpha helix	-	-	-
A 56		HIS	2.58	-	Favored (64.21%)	Favored (71.7%) *m-70* chi angles: 285,284.7	0.10Å	Favored (68.017%) alpha helix	-	-	-

					General / -73.8,-39.2						
A 57		TRP	2.5	-	Favored (62.88%) General / -51.5,-48.4	Allowed (1.4%) *t60* chi angles: 175.8,337	0.08Å	Favored (78.55%) alpha helix	-	-	-
A 58		ILE	2.43	-	Favored (96.73%) Ile or Val / -62.2,-46.3	Favored (97.4%) *mt* chi angles: 292.2,167.8	0.05Å	Favored (94.13%) alpha helix	-	-	-
A 59		LYS	2.39	-	Favored (79.94%) General / -60.5,-37.1	Favored (96.9%) *mttt* chi angles: 289.1,177.6,179.9,179.6	0.00Å	Favored (83.517%) alpha helix	-	-	-
A 60		VAL	2.37	-	Favored (85.96%) Ile or Val / -67.0,-45.9	Favored (65.3%) *t* chi angles: 171.5	0.02Å	Favored (92.093%) alpha helix	-	-	-
**#**	**Alt**	**Res**	**High B**	**Clash > 0.4Å**	**Ramachandran**	**Rotamer**	**Cβ deviation**	**CaBLAM**	**Bond lengths**	**Bond angles**	**Cis Peptides**
			Avg: 3.90	Clashscore: 1.54	Outliers: 5 of 248	Poor rotamers: 0 of 207	Outliers: 1 of 229	Outliers: 2 of 246	Outliers: 1 of 250	Outliers: 2 of 250	Non-Trans: 0 of 249
A 61		GLU	2.38	-	Favored (95.87%) General / -62.7,-40.0	Favored (94.4%) *mt-10* chi angles: 289.2,180.1,341.5	0.03Å	Favored (83.585%) alpha helix	-	-	-
A 62		TYR	2.43	-	Favored (61.05%) General / -74.5,-28.5	Favored (31.6%) *m-80* chi angles: 279.8,109.3	0.13Å	Favored (78.291%) alpha helix	-	-	-
A 63		GLY	2.51	-	Favored (26.65%) Glycine / -61.5,-55.8	-	-	Favored (71.972%) alpha helix	-	-	-
A 64		ASN	2.59	-	Favored (78.3%) General / -61.7,-35.5	Favored (98.8%) *m-40* chi angles: 287.4,338.9	0.05Å	Favored (72.941%) alpha helix	-	-	-
A 65		LEU	2.69	-	Favored (74.52%) General / -58.9,-50.3	Favored (63.7%) *tp* chi angles: 178.8,64	0.13Å	Favored (78.138%) alpha helix	-	-	-
A 66		SER	2.82	-	Favored (66.74%) General / -67.2,-26.6	Favored (81%) *p* chi angles: 70.2	0.04Å	Favored (72.547%) alpha helix	-	-	-
A 67		LEU	2.98	-	Favored (67.78%) General / -72.7,-38.0	Favored (99.7%) *mt* chi angles: 292.5,172.4	0.04Å	Favored (75.609%) alpha helix	-	-	-
A 68		SER	3.21	-	Favored (64.86%) General / -72.5,-42.5	Favored (70.7%) *m* chi angles: 296.2	0.06Å	Favored (33.84%) alpha helix	-	-	-
A 69		GLY	3.56	-	Favored (36.99%) Glycine / -60.5,-14.9	-	-	Favored (51.922%) three-ten	-	-	-
A 70		ILE	4.06	-	Favored (73.8%) Ile or Val / -63.2,-35.9	Favored (99%) *mt* chi angles: 292.7,167.9	0.08Å	Favored (18.697%)	-	-	-
A 71		ALA	4.72	-	Favored (36.54%) General / -61.6,150.3	-	0.03Å	Favored (20.011%)	-	-	-

A 72		GLN	5.5	-	Favored (65.23%) General / -61.5,-22.6	Favored (20.6%) *pm20* chi angles: 65.8,275.9,30.7	0.03Å	Favored (22.984%)	-	-	-
A 73		SER	6.27	-	Favored (42.89%) General / -96.9,127.4	Favored (35.6%) *t* chi angles: 174.4	0.10Å	Favored (33.857%)	-	-	-
A 74		ALA	6.9	-	Favored (63.67%) General / -62.7,-19.2	-	0.02Å	Favored (26.488%)	-	-	-
A 75		SER	7.28	-	Favored (65.24%) General / -63.3,-20.2	Favored (94.3%) *p* chi angles: 66.2	0.01Å	Favored (57.872%)	-	-	-
A 76		VAL	7.42	-	Favored (27.39%) Ile or Val / -68.1,-22.1	Favored (18.2%) *m* chi angles: 303.1	0.08Å	Favored (65.252%) three-ten	-	-	-
A 77		LEU	7.42	-	Favored (63.54%) General / -69.7,-24.0	Favored (92.9%) *mt* chi angles: 297.3,172.9	0.10Å	Favored (62.526%) three-ten	-	-	-
A 78		SER	7.37	-	Favored (53.72%) General / -77.2,-6.6	Favored (88.6%) *p* chi angles: 69.4	0.03Å	Favored (51.075%) three-ten	-	-	-
A 79		PHE	7.35	-	Favored (28.69%) General / -103.9,15.4	Favored (99.6%) *m-80* chi angles: 297.3,96	0.03Å	Favored (30.521%)	-	-	-
A 80		MET	7.4	-	Favored (61.29%) General / -72.2,-23.9	Favored (26.6%) *ptm* chi angles: 70.8,179.2,285.1	0.09Å	Favored (7.535%)	-	-	-
**#**	**Alt**	**Res**	**High B**	**Clash > 0.4Å**	**Ramachandran**	**Rotamer**	**Cβ deviation**	**CaBLAM**	**Bond lengths**	**Bond angles**	**Cis Peptides**
			Avg: 3.90	Clashscore: 1.54	Outliers: 5 of 248	Poor rotamers: 0 of 207	Outliers: 1 of 229	Outliers: 2 of 246	Outliers: 1 of 250	Outliers: 2 of 250	Non-Trans: 0 of 249
A 81		ASP	7.47	-	Favored (28.02%) General / -75.7,162.7	Favored (7.1%) *p0* chi angles: 63.4,281.3	0.14Å	CaBLAM Disfavored (3.168%)	-	-	-
A 82		LYS	7.49	-	Favored (2.42%) General / 70.1,34.5	Favored (48.1%) *mttp* chi angles: 300.1,181.3,183.7,69.7	0.04Å	Favored (13.344%)	-	OUTLIER(S) worst is C-N-CA: 4.9 σ	-
A 83		GLY	7.44	-	Favored (5.17%) Glycine / -84.6,120.1	-	-	Favored (23.237%)	-	-	-
A 84		ILE	7.24	-	Favored (32.19%) Pre-Pro / -121.1,114.4	Favored (67.8%) *mt* chi angles: 303.5,172.5	0.04Å	Favored (67.146%) beta sheet	-	-	-
A 85		PRO	6.83	-	Favored (13.63%) Trans-Pro / -77.8,-17.9	Favored (73.9%) *Cg_endo* chi angles: 29.8,325.3,24.1	0.04Å	CaBLAM Disfavored (3.051%) try beta sheet	-	-	-
A 86		PHE	6.19	-	OUTLIER (0.02)%) General / 57.6,-11.4	Favored (95.5%) *m-80* chi angles: 297.8,100.7	0.08Å	Favored (33.086%)	-	-	-
A 87		MET	5.36	-	OUTLIER (0.03)%) General / 38.8,-143.6	Favored (95.3%) *mmm* chi angles: 295.8,300.7,297.3	0.14Å	CaBLAM Disfavored (1.547%)	-	-	-

#	Alt	Res	High B	Clash > 0.4Å	Ramachandran	Rotamer	Cβ deviation	CaBLAM	Bond lengths	Bond angles	Cis Peptides
A 88		LYS	4.48	-	Favored (22.03%) General / -153.2,143.0	Favored (88.2%) *tttt* chi angles: 184.2,175.8,179.5,180.3	0.05Å	Favored (5.636%)	-	-	-
A 89		MET	3.66	-	Favored (10.06%) General / -91.8,170.2	Favored (94.7%) *mtp* chi angles: 292.5,178.7,65.6	0.05Å	Favored (26.502%)	-	-	-
A 90		ASN	2.99	-	Favored (8.62%) General / -145.5,177.8	Favored (27.1%) *p0* chi angles: 62.4,55.7	0.02Å	Favored (28.921%)	-	-	-
A 91		ILE	2.48	-	Favored (99.25%) Ile or Val / -63.2,-44.5	Favored (94.4%) *mt* chi angles: 291.9,168.7	0.03Å	Favored (54.846%)	-	-	-
A 92		SER	2.13	-	Favored (87.25%) General / -61.8,-38.2	Favored (60.8%) *m* chi angles: 293.7	0.05Å	Favored (92.554%) alpha helix	-	-	-
A 93		VAL	1.92	-	Favored (96.76%) Ile or Val / -63.7,-45.7	Favored (69.8%) *t* chi angles: 172.1	0.07Å	Favored (91.987%) alpha helix	-	-	-
A 94		ILE	1.83	-	Favored (99.12%) Ile or Val / -63.3,-44.6	Favored (97%) *mt* chi angles: 292.3,167.1	0.02Å	Favored (96.609%) alpha helix	-	-	-
A 95		ILE	1.82	-	Favored (97.24%) Ile or Val / -62.3,-46.1	Favored (96.5%) *mt* chi angles: 292.9,166.7	0.06Å	Favored (85.964%) alpha helix	-	-	-
A 96		LEU	1.88	-	Favored (97.43%) General / -64.0,-41.4	Favored (89.1%) *mt* chi angles: 291,173.2	0.04Å	Favored (78.784%) alpha helix	-	-	-
A 97		LEU	2	-	Favored (79.15%) General / -56.5,-47.4	Favored (69.5%) *tp* chi angles: 179.1,61.8	0.09Å	Favored (80.265%) alpha helix	-	-	-
A 98		VAL	2.15	-	Favored (97.12%) Ile or Val / -64.3,-43.6	Favored (66.3%) *t* chi angles: 171.7	0.01Å	Favored (89.036%) alpha helix	-	-	-
A 99		SER	2.31	-	Favored (69.81%) General / -62.4,-28.5	Favored (70.8%) *p* chi angles: 71.9	0.03Å	Favored (79.004%) alpha helix	-	-	-
A 100		GLY	2.44	-	Favored (29.32%) Glycine / -66.5,-53.3	-	-	Favored (61.924%) alpha helix	-	-	-
**#**	**Alt**	**Res**	**High B**	**Clash > 0.4Å**	**Ramachandran**	**Rotamer**	**Cβ deviation**	**CaBLAM**	**Bond lengths**	**Bond angles**	**Cis Peptides**
			Avg: 3.90	Clashscore: 1.54	Outliers: 5 of 248	Poor rotamers: 0 of 207	Outliers: 1 of 229	Outliers: 2 of 246	Outliers: 1 of 250	Outliers: 2 of 250	Non-Trans: 0 of 249
A 101		TRP	2.5	-	Favored (61.42%) General / -52.8,-38.6	Favored (31%) *m-10* chi angles: 285.4,339.4	0.01Å	Favored (65.679%) three-ten	-	-	-
A 102		ASN	2.49	-	Favored (50.47%) General / -57.6,-23.3	Favored (96.6%) *m-40* chi angles: 288.5,344.8	0.02Å	Favored (30.104%)	-	-	-
A 103		SER	2.4	-	Favored (5.2%) General / -128.1,30.7	Favored (66.3%) *m* chi angles: 297.1	0.04Å	Favored (10.309%)	-	-	-

A 104		ILE	2.24	-	Favored (4.95%) Ile or Val / -82.6,98.7	Favored (50.6%) *mm* chi angles: 302.8,301.6	0.12Å	Favored (13.503%)	OUTLIER(S) worst is CB--CG1: 4.7 σ	-	-
A 105		THR	2.05	-	Favored (28.98%) General / -86.3,141.8	Favored (86.5%) *m* chi angles: 296.8	0.07Å	Favored (24.801%)	-	-	-
A 106		VAL	1.83	-	Favored (74.38%) Ile or Val / -55.2,-44.3	Favored (62.5%) *t* chi angles: 171.1	0.04Å	Favored (59.235%)	-	-	-
A 107		MET	1.63	0.57Å HB3 with A 108 PRO HD3	OUTLIER (0.06)%) Pre-Pro / -38.9,-74.8	Favored (15.1%) *tpt* chi angles: 181.5,66.3,182.8	0.28Å	Favored (65.792%) alpha helix	-	OUTLIER(S) worst is C-CA-CB: 4.3 σ	-
A 108		PRO	1.45	0.57Å HD3 with A 107 MET HB3	Favored (23.83%) Trans-Pro / -48.3,-34.0	Favored (67%) *Cg_exo* chi angles: 328.7,36,333.9	0.12Å	Favored (58.751%) alpha helix	-	-	-
A 109		LEU	1.3	-	Favored (64.1%) General / -62.8,-52.2	Favored (74.5%) *tp* chi angles: 177.7,61.9	0.08Å	Favored (65.315%) alpha helix	-	-	-
A 110		LEU	1.19	-	Favored (83.41%) General / -66.5,-36.7	Favored (91.5%) *mt* chi angles: 293.5,175.9	0.03Å	Favored (73.537%) alpha helix	-	-	-
A 111		CYS	1.11	-	Favored (86.98%) General / -67.1,-40.3	Favored (97.6%) *m* chi angles: 292.7	0.04Å	Favored (97.828%) alpha helix	-	-	-
A 112		GLY	1.05	-	Favored (98.28%) Glycine / -63.4,-43.4	-	-	Favored (97.36%) alpha helix	-	-	-
A 113		ILE	1.02	-	Favored (93.99%) Ile or Val / -63.9,-46.5	Favored (90.8%) *mt* chi angles: 292.9,165.2	0.04Å	Favored (89.368%) alpha helix	-	-	-
A 114		GLY	1	-	Favored (50.33%) Glycine / -54.5,-51.7	-	-	Favored (97.795%) alpha helix	-	-	-
A 115		CYS	1	-	Favored (86.88%) General / -59.9,-39.9	Favored (96.9%) *m* chi angles: 290.2	0.05Å	Favored (82.012%) alpha helix	-	-	-
A 116		ALA	1.01	-	Favored (93%) General / -61.8,-39.8	-	0.02Å	Favored (86.874%) alpha helix	-	-	-
A 117		MET	1.04	-	Favored (68.77%) General / -72.1,-34.5	Favored (51.1%) *mmp* chi angles: 295.2,302,100.4	0.04Å	Favored (94.453%) alpha helix	-	-	-
A 118		LEU	1.06	-	Favored (93.37%) General / -64.9,-39.3	Favored (92.9%) *mt* chi angles: 291.3,172.4	0.04Å	Favored (89.263%) alpha helix	-	-	-
A 119		HIS	1.1	-	Favored (64.89%) General / -59.6,-52.6	Favored (89%) *t70* chi angles: 178.6,76.2	0.04Å	Favored (80.479%) alpha helix	-	-	-
A 120		TRP	1.14	-	Favored (86.64%) General / -64.8,-37.1	Favored (47.4%) *m100* chi angles: 290.5,121.8	0.02Å	Favored (77.306%) alpha helix	-	-	-
**#**	**Alt**	**Res**	**High B**	**Clash > 0.4Å**	**Ramachandran**	**Rotamer**	**Cβ deviation**	**CaBLAM**	**Bond lengths**	**Bond angles**	**Cis Peptides**

			Avg: 3.90	Clashscore: 1.54	Outliers: 5 of 248	Poor rotamers: 0 of 207	Outliers: 1 of 229	Outliers: 2 of 246	Outliers: 1 of 250	Outliers: 2 of 250	Non-Trans: 0 of 249
A 121		THR	1.19	-	Favored (91.61%) General / -59.0,-44.3	Favored (88.8%) *m* chi angles: 298.4	0.03Å	Favored (82.879%) alpha helix	-	-	-
A 122		LEU	1.24	-	Favored (73.47%) General / -69.2,-33.5	Favored (95.2%) *mt* chi angles: 291.9,172.9	0.01Å	Favored (57.364%) alpha helix	-	-	-
A 123		ILE	1.3	-	Favored (4.18%) Ile or Val / -108.4,-24.1	Favored (45.1%) *pt* chi angles: 63.6,171.5	0.06Å	Favored (20.539%) alpha helix	-	-	-
A 124		LEU	1.36	-	Favored (90.68%) Pre-Pro / -58.5,-41.7	Favored (88.9%) *mt* chi angles: 291.7,174.5	0.11Å	Favored (59.748%) alpha helix	-	-	-
A 125		PRO	1.43	-	Favored (75.34%) Trans-Pro / -59.7,-24.4	Favored (57.2%) *Cg_exo* chi angles: 336.1,36,327.5	0.05Å	Favored (93.919%) alpha helix	-	-	-
A 126		GLY	1.49	-	Favored (24.52%) Glycine / -78.8,-38.1	-	-	Favored (89.247%) alpha helix	-	-	-
A 127		ILE	1.55	-	Favored (63.81%) Ile or Val / -72.4,-43.6	Favored (45.6%) *mm* chi angles: 298.9,301	0.04Å	Favored (80.979%) alpha helix	-	-	-
A 128		LYS	1.6	-	Favored (98.7%) General / -63.4,-42.0	Favored (96.9%) *mttt* chi angles: 289.5,178.1,180.5,179.5	0.01Å	Favored (97.811%) alpha helix	-	-	-
A 129		ALA	1.66	-	Favored (95.5%) General / -62.2,-40.3	-	0.04Å	Favored (95.959%) alpha helix	-	-	-
A 130		GLN	1.73	-	Favored (93.48%) General / -65.5,-40.8	Favored (91%) *mt0* chi angles: 290.6,171.8,354.7	0.04Å	Favored (92.01%) alpha helix	-	-	-
A 131		GLN	1.82	-	Favored (64.88%) General / -72.8,-31.4	Favored (99.4%) *mm-40* chi angles: 293.7,300.1,314.3	0.08Å	Favored (80.615%) alpha helix	-	-	-
A 132		SER	1.93	-	Favored (81.62%) General / -62.8,-47.9	Favored (46.7%) *t* chi angles: 180.1	0.04Å	Favored (77.277%) alpha helix	-	-	-
A 133		LYS	2.05	-	Favored (83.28%) General / -57.8,-42.0	Favored (80.9%) *tttt* chi angles: 180.2,171.2,176.6,172.9	0.10Å	Favored (89.348%) alpha helix	-	-	-
A 134		LEU	2.19	-	Favored (90.79%) General / -65.1,-38.4	Favored (98.7%) *mt* chi angles: 292.6,172.9	0.02Å	Favored (96.733%) alpha helix	-	-	-
A 135		ALA	2.32	-	Favored (94.86%) General / -62.2,-40.1	-	0.03Å	Favored (93.343%) alpha helix	-	-	-
A 136		GLN	2.44	-	Favored (95.29%) General / -63.8,-39.9	Favored (77.5%) *mt0* chi angles: 291.1,172.6,46.1	0.03Å	Favored (94.241%) alpha helix	-	-	-

#	Alt	Res	High B	Clash > 0.4Å	Ramachandran	Rotamer	Cβ deviation	CaBLAM	Bond lengths	Bond angles	Cis Peptides
A 137		ARG	2.55	-	Favored (91.2%) General / -61.1,-46.3	Favored (76.1%) *ttt180* chi angles: 177.3,169.7,180.6,183.9	0.03Å	Favored (97.12%) alpha helix	-	-	-
A 138		ARG	2.67	-	Favored (91.52%) General / -62.3,-39.0	Favored (98.8%) *mtm-85* chi angles: 287.3,192.5,293.7,269.7	0.03Å	Favored (96.627%) alpha helix	-	-	-
A 139		VAL	2.81	-	Favored (97.86%) Ile or Val / -63.5,-45.4	Favored (63.1%) *t* chi angles: 171.2	0.05Å	Favored (83.985%) alpha helix	-	-	-
A 140		PHE	2.99	-	Favored (66.4%) General / -53.6,-50.0	Favored (61.9%) *t80* chi angles: 168.4,78.7	0.05Å	Favored (81.693%) alpha helix	-	-	-
**#**	**Alt**	**Res**	**High B**	**Clash > 0.4Å**	**Ramachandran**	**Rotamer**	**Cβ deviation**	**CaBLAM**	**Bond lengths**	**Bond angles**	**Cis Peptides**
			Avg: 3.90	Clashscore: 1.54	Outliers: 5 of 248	Poor rotamers: 0 of 207	Outliers: 1 of 229	Outliers: 2 of 246	Outliers: 1 of 250	Outliers: 2 of 250	Non-Trans: 0 of 249
A 141		HIS	3.24	-	Favored (92.03%) General / -65.8,-40.7	Favored (65%) *m-70* chi angles: 291.7,305.2	0.00Å	Favored (83.922%) alpha helix	-	-	-
A 142		GLY	3.59	-	Favored (88.48%) Glycine / -60.5,-35.2	-	-	Favored (84.426%) alpha helix	-	-	-
A 143		VAL	4.06	-	Favored (20.79%) Ile or Val / -81.3,-45.3	Favored (88.6%) *t* chi angles: 174	0.03Å	Favored (58.504%) alpha helix	-	-	-
A 144		ALA	4.68	-	Favored (77.52%) General / -60.2,-36.4	-	0.03Å	Favored (7.748%) alpha helix	-	-	-
A 145		LYS	5.44	-	Allowed (0.58%) General / 68.9,-51.7	Favored (31.1%) *mmtm* chi angles: 298.6,296.7,188.1,298.2	0.07Å	Favored (9.057%)	-	-	-
A 146		ASN	6.28	-	Favored (71.84%) Pre-Pro / -128.2,66.2	Favored (56.7%) *m-40* chi angles: 299.5,280.7	0.02Å	Favored (12.327%)	-	-	-
A 147		PRO	7.1	-	Favored (26.88%) Trans-Pro / -72.5,-12.3	Favored (75.3%) *Cg_endo* chi angles: 28,324,28.7	0.08Å	Favored (35.568%)	-	-	-
A 148		VAL	7.67	0.40Å O with A 148 VAL HG13	Favored (56.31%) Ile or Val / -126.4,137.9	Favored (6.1%) *p* chi angles: 58.9	0.13Å	Favored (27.749%)	-	-	-
A 149		VAL	7.75	-	Favored (61.36%) Ile or Val / -122.8,120.7	Favored (84.9%) *t* chi angles: 177.7	0.04Å	Favored (16.939%)	-	-	-
A 150		ASP	7.29	-	Favored (22.16%) General / 52.2,37.6	Favored (68.6%) *m-30* chi angles: 297.8,345.5	0.01Å	Favored (35.37%)	-	-	-
A 151		GLY	6.42	-	Favored (75.29%) Glycine / 89.3,-9.9	-	-	Favored (78.807%)	-	-	-
A 152		ASN	5.42	-	Favored (63.22%) Pre-Pro / -98.8,112.6	Favored (80%) *m-40* chi angles: 294.4,309.7	0.03Å	Favored (32.227%)	-	-	-

#	Alt	Res	High B	Clash > 0.4Å	Ramachandran	Rotamer	Cβ deviation	CaBLAM	Bond lengths	Bond angles	Cis Peptides
A 153		PRO	4.49	-	Favored (82.14%) Trans-Pro / -55.3,140.6	Favored (86.1%) *Cg_exo* chi angles: 333.6,35.8,329.9	0.05Å	Favored (49.134%) beta sheet	-	-	-
A 154		THR	3.74	-	Favored (16.7%) General / -102.3,-15.2	Favored (71.6%) *p* chi angles: 62	0.04Å	Favored (42.896%)	-	-	-
A 155		VAL	3.2	-	Favored (70.66%) Ile or Val / -126.5,127.8	Favored (69.1%) *t* chi angles: 178.9	0.04Å	Favored (20.85%)	-	-	-
A 156		ASP	2.81	-	Favored (4.86%) General / -78.3,73.4	Favored (41.6%) *t0* chi angles: 190.6,20.1	0.03Å	Favored (40.207%)	-	-	-
A 157		ILE	2.55	-	Favored (27.74%) Ile or Val / -89.6,114.6	Favored (45.1%) *mm* chi angles: 305.1,299.7	0.04Å	Favored (48.953%) beta sheet	-	-	-
A 158		GLU	2.37	-	Favored (36.11%) General / -80.3,134.6	Favored (43.5%) *tt0* chi angles: 186.7,176.5,65.4	0.03Å	Favored (38.306%) beta sheet	-	-	-
A 159		GLU	2.24	-	Favored (50.67%) General / -70.6,138.3	Favored (86.5%) *tt0* chi angles: 188.7,174.5,3.6	0.02Å	Favored (45.926%) beta sheet	-	-	-
A 160		ALA	2.16	-	Favored (98.63%) Pre-Pro / -67.5,148.5	-	0.07Å	Favored (51.274%) beta sheet	-	-	-
**#**	**Alt**	**Res**	**High B**	**Clash > 0.4Å**	**Ramachandran**	**Rotamer**	**Cβ deviation**	**CaBLAM**	**Bond lengths**	**Bond angles**	**Cis Peptides**
			Avg: 3.90	Clashscore: 1.54	Outliers: 5 of 248	Poor rotamers: 0 of 207	Outliers: 1 of 229	Outliers: 2 of 246	Outliers: 1 of 250	Outliers: 2 of 250	Non-Trans: 0 of 249
A 161		PRO	2.1	-	Favored (44.2%) Trans-Pro / -72.8,161.3	Favored (74.9%) *Cg_endo* chi angles: 27.9,325.5,27	0.04Å	Favored (84.401%) beta sheet	-	-	-
A 162		GLU	2.07	-	Favored (50.28%) General / -119.0,125.8	Favored (92.3%) *tt0* chi angles: 181.7,177.3,358	0.02Å	Favored (17.249%) beta sheet	-	-	-
A 163		MET	2.03	-	Favored (20.49%) Pre-Pro / -56.2,118.3	Favored (64.7%) *ttm* chi angles: 184.8,180.1,292	0.08Å	Favored (49.722%) beta sheet	-	-	-
A 164		PRO	1.98	-	Favored (99.43%) Trans-Pro / -59.9,144.7	Favored (53.6%) *Cg_exo* chi angles: 336.3,34.4,329.4	0.05Å	Favored (84.49%)	-	-	-
A 165		ALA	1.9	-	Favored (70.23%) General / -58.7,-33.5	-	0.03Å	Favored (54.644%)	-	-	-
A 166		LEU	1.78	-	Favored (71.74%) General / -57.2,-37.1	Favored (58.4%) *tp* chi angles: 181.6,59.6	0.03Å	Favored (68.341%) alpha helix	-	-	-
A 167		TYR	1.64	-	Favored (78.57%) General / -61.9,-48.9	Favored (87.1%) *t80* chi angles: 181.2,77.1	0.03Å	Favored (78.015%) alpha helix	-	-	-

A 168		GLU	1.51	-	Favored (90.09%) General / -62.6,-38.4	Favored (99.1%) *mt-10* chi angles: 292.5,180.7,354.2	0.02Å	Favored (83.351%) alpha helix	-	-	-
A 169		LYS	1.38	-	Favored (92.23%) General / -65.6,-40.1	Favored (96.9%) *mttt* chi angles: 289.5,177.4,181.7,179.2	0.01Å	Favored (92.258%) alpha helix	-	-	-
A 170		LYS	1.28	-	Favored (87.92%) General / -64.1,-37.6	Favored (95.1%) *mttt* chi angles: 287.6,182.9,174.4,181.8	0.07Å	Favored (91.772%) alpha helix	-	-	-
A 171		LEU	1.19	-	Favored (97.03%) General / -63.8,-40.6	Favored (85.1%) *mt* chi angles: 292.4,177.5	0.06Å	Favored (95.761%) alpha helix	-	-	-
A 172		ALA	1.12	-	Favored (88.56%) General / -61.2,-39.0	-	0.04Å	Favored (88.832%) alpha helix	-	-	-
A 173		LEU	1.07	-	Favored (61.58%) General / -75.0,-32.6	Favored (85.6%) *mt* chi angles: 293.9,178	0.08Å	Favored (84.336%) alpha helix	-	-	-
A 174		TYR	1.02	-	Favored (96.68%) General / -63.2,-40.1	Favored (21.1%) *m-80* chi angles: 279.8,121.8	0.03Å	Favored (83.753%) alpha helix	-	-	-
A 175		LEU	0.99	-	Favored (89.86%) General / -66.3,-40.5	Favored (87.4%) *mt* chi angles: 291.2,168.7	0.08Å	Favored (95.542%) alpha helix	-	-	-
A 176		LEU	0.96	-	Favored (91.09%) General / -60.5,-46.2	Favored (70.7%) *tp* chi angles: 178.1,60.6	0.02Å	Favored (97.272%) alpha helix	-	-	-
A 177		LEU	0.96	-	Favored (85.56%) General / -60.8,-47.6	Favored (67.3%) *tp* chi angles: 179.5,61.4	0.04Å	Favored (97.113%) alpha helix	-	-	-
A 178		ALA	0.97	-	Favored (90.01%) General / -60.0,-40.7	-	0.04Å	Favored (92.306%) alpha helix	-	-	-
A 179		LEU	1	-	Favored (95.54%) General / -64.3,-40.3	Favored (81.2%) *mt* chi angles: 289,167.9	0.05Å	Favored (98.036%) alpha helix	-	-	-
A 180		SER	1.05	-	Favored (92.93%) General / -64.8,-43.5	Favored (71.2%) *m* chi angles: 296.1	0.07Å	Favored (95.256%) alpha helix	-	-	-
**#**	**Alt**	**Res**	**High B**	**Clash > 0.4Å**	**Ramachandran**	**Rotamer**	**Cβ deviation**	**CaBLAM**	**Bond lengths**	**Bond angles**	**Cis Peptides**
			Avg: 3.90	Clashscore: 1.54	Outliers: 5 of 248	Poor rotamers: 0 of 207	Outliers: 1 of 229	Outliers: 2 of 246	Outliers: 1 of 250	Outliers: 2 of 250	Non-Trans: 0 of 249
A 181		LEU	1.11	-	Favored (88.17%) General / -66.6,-41.5	Favored (94.3%) *mt* chi angles: 293,170.6	0.08Å	Favored (93.748%) alpha helix	-	-	-
A 182		ALA	1.21	-	Favored (98.61%) General / -63.5,-41.9	-	0.04Å	Favored (93.841%) alpha helix	-	-	-

A 183		SER	1.35	-	Favored (84.48%) General / -61.0,-47.9	Favored (44.8%) *t* chi angles: 179.1	0.09Å	Favored (93.45%) alpha helix	-	-	-
A 184		VAL	1.54	-	Favored (96.69%) Ile or Val / -61.9,-43.0	Favored (58.7%) *t* chi angles: 170.6	0.04Å	Favored (92.988%) alpha helix	-	-	-
A 185		ALA	1.79	-	Favored (97.58%) General / -61.9,-41.1	-	0.05Å	Favored (73.407%) alpha helix	-	-	-
A 186		MET	2.08	-	Favored (21.23%) General / -79.2,-44.8	Favored (83.4%) *mtm* chi angles: 290,187,288.3	0.04Å	Favored (58.286%) alpha helix	-	-	-
A 187		CYS	2.34	-	Favored (2.68%) General / -113.4,87.8	Favored (62.1%) *m* chi angles: 301.7	0.09Å	Favored (20.382%)	-	-	-
A 188		ARG	2.51	-	Favored (10.54%) General / -84.4,73.0	Favored (93.4%) *mtt180* chi angles: 297.3,178.6,184.4,188.7	0.04Å	Favored (22.638%)	-	-	-
A 189		THR	2.51	-	Favored (67.54%) Pre-Pro / -124.8,157.1	Favored (30.8%) *p* chi angles: 69.3	0.10Å	Favored (26.91%)	-	-	-
A 190		PRO	2.34	-	Favored (35.85%) Trans-Pro / -50.3,-33.2	Favored (83.1%) *Cg_exo* chi angles: 329.2,37.9,331.4	0.04Å	Favored (91.059%)	-	-	-
A 191		PHE	2.06	-	Favored (47.43%) General / -77.9,-37.8	Favored (59.3%) *m-80* chi angles: 284.5,91.2	0.03Å	Favored (83.321%) alpha helix	-	-	-
A 192		SER	1.77	-	Favored (96.28%) General / -63.9,-40.4	Favored (69.2%) *m* chi angles: 296.6	0.10Å	Favored (96.983%) alpha helix	-	-	-
A 193		LEU	1.51	-	Favored (83.82%) General / -65.0,-45.9	Favored (81.4%) *mt* chi angles: 289.1,168.6	0.06Å	Favored (88.676%) alpha helix	-	-	-
A 194		ALA	1.31	-	Favored (97.95%) General / -61.8,-41.6	-	0.04Å	Favored (86.21%) alpha helix	-	-	-
A 195		GLU	1.16	-	Favored (76.52%) General / -66.9,-45.9	Favored (73.4%) *tt0* chi angles: 182.1,166.8,2.6	0.04Å	Favored (86.489%) alpha helix	-	-	-
A 196		GLY	1.06	-	Favored (96.85%) Glycine / -60.8,-39.4	-	-	Favored (95.957%) alpha helix	-	-	-
A 197		ILE	1	-	Favored (87.7%) Ile or Val / -65.7,-46.9	Favored (93.5%) *mt* chi angles: 292.2,166.3	0.03Å	Favored (90.464%) alpha helix	-	-	-
A 198		VAL	0.97	-	Favored (98.51%) Ile or Val / -61.9,-44.2	Favored (53.9%) *t* chi angles: 169.9	0.03Å	Favored (73.95%) alpha helix	-	-	-
A 199		LEU	0.95	-	Favored (37.47%) General / -79.3,-37.5	Favored (95.7%) *mt* chi angles: 296.8,176.2	0.11Å	Favored (75.583%) alpha helix	-	-	-

A 200		ALA	0.95	-	Favored (94.48%) General / -63.1,-39.4	-	0.04Å	Favored (95.602%) alpha helix	-	-	-

#	Alt	Res	High B	Clash > 0.4Å	Ramachandran	Rotamer	Cβ deviation	CaBLAM	Bond lengths	Bond angles	Cis Peptides
			Avg: 3.90	Clashscore: 1.54	Outliers: 5 of 248	Poor rotamers: 0 of 207	Outliers: 1 of 229	Outliers: 2 of 246	Outliers: 1 of 250	Outliers: 2 of 250	Non-Trans: 0 of 249
A 201		SER	0.95	-	Favored (95.86%) General / -63.9,-43.5	Favored (73%) *m* chi angles: 295.6	0.06Å	Favored (98.516%) alpha helix	-	-	-
A 202		ALA	0.96	-	Favored (84.66%) General / -60.8,-38.3	-	0.05Å	Favored (80.224%) alpha helix	-	-	-
A 203		ALA	0.97	-	Favored (49.86%) General / -71.2,-48.7	-	0.05Å	Favored (73.331%) alpha helix	-	-	-
A 204		LEU	0.98	0.40Å O with A 205 GLY C	Favored (87.29%) General / -62.5,-37.8	Favored (84.8%) *mt* chi angles: 289.9,170.9	0.07Å	Favored (67.4%) alpha helix	-	-	-
A 205		GLY	1	0.40Å C with A 204 LEU O	OUTLIER (0.05)%) Glycine / -44.4,-79.9	-	-	Favored (46.325%) alpha helix	-	-	-
A 206		PRO	1.03	-	Favored (56%) Trans-Pro / -52.5,-33.0	Favored (88.2%) *Cg_exo* chi angles: 333.4,37.6,327.4	0.07Å	Favored (20.927%) alpha helix	-	-	-
A 207		LEU	1.08	-	Favored (71.53%) General / -64.3,-30.5	Favored (89.8%) *mt* chi angles: 290.7,172.5	0.02Å	Favored (57.412%) alpha helix	-	-	-
A 208		ILE	1.16	-	Favored (13.5%) Ile or Val / -94.6,-45.4	Favored (92.9%) *mt* chi angles: 296.4,168.9	0.05Å	Favored (38.671%) alpha helix	-	-	-
A 209		GLU	1.28	-	Favored (18.98%) General / -90.8,-27.3	Favored (96.9%) *mt-10* chi angles: 296.4,179,352.9	0.03Å	Favored (37.783%)	-	-	-
A 210		GLY	1.44	-	Favored (87.82%) Glycine / 83.3,-2.0	-	-	Favored (58.75%)	-	-	-
A 211		ASN	1.63	-	Favored (25.59%) General / -109.5,152.3	Favored (87.3%) *m-40* chi angles: 294.6,318	0.02Å	Favored (33.665%) beta sheet	-	-	-
A 212		THR	1.82	-	Favored (47.32%) General / -127.3,129.2	Favored (91.3%) *m* chi angles: 301.1	0.04Å	Favored (58.956%) beta sheet	-	-	-
A 213		SER	1.96	-	Favored (19.11%) General / -81.6,167.6	Favored (94.2%) *p* chi angles: 66.3	0.04Å	Favored (21.801%)	-	-	-
A 214		LEU	2.01	-	Favored (54.21%) General / -93.9,-1.9	Favored (92.7%) *mt* chi angles: 298.6,176.6	0.02Å	Favored (31.795%)	-	-	-
A 215		LEU	1.97	-	Favored (6.3%) General / -109.4,-35.2	Favored (90%) *mt* chi angles: 299.7,177.4	0.01Å	Favored (40.747%) alpha helix	-	-	-
A 216		TRP	1.86	-	Favored (9.12%)	Favored (54.5%) *m100*	0.08Å	Favored (20.43%)	-	-	-

#	Alt	Res	High B	Clash > 0.4Å	Ramachandran	Rotamer	Cβ deviation	CaBLAM	Bond lengths	Bond angles	Cis Peptides
					General / -113.5,28.0	chi angles: 282,84.9					
A 217		ASN	1.7	0.43Å C with A 217 ASN OD1	Favored (6.87%) General / -83.4,-177.9	Favored (48.6%) *p0* chi angles: 67.2,19	0.03Å	Favored (12.57%)	-	-	-
A 218		GLY	1.54	-	OUTLIER (0.06)%) Glycine / -39.0,-78.1	-	-	Favored (12.52%)	-	-	-
A 219		PRO	1.4	-	Favored (79.57%) Trans-Pro / -61.4,-25.0	Favored (32.8%) *Cg_endo* chi angles: 21.9,327.1,29.7	0.03Å	Favored (18.694%) alpha helix	-	-	-
A 220		MET	1.31	-	Favored (18.32%) General / -75.5,-49.6	Favored (27%) *mmt* chi angles: 292.1,293.8,165.3	0.04Å	Favored (58.954%) alpha helix	-	-	-
**#**	**Alt**	**Res**	**High B**	**Clash > 0.4Å**	**Ramachandran**	**Rotamer**	**Cβ deviation**	**CaBLAM**	**Bond lengths**	**Bond angles**	**Cis Peptides**
			Avg: 3.90	Clashscore: 1.54	Outliers: 5 of 248	Poor rotamers: 0 of 207	Outliers: 1 of 229	Outliers: 2 of 246	Outliers: 1 of 250	Outliers: 2 of 250	Non-Trans: 0 of 249
A 221		ALA	1.25	-	Favored (89.7%) General / -61.9,-38.8	-	0.07Å	Favored (77.551%) alpha helix	-	-	-
A 222		VAL	1.22	-	Favored (94.32%) Ile or Val / -65.5,-44.2	Favored (55.9%) *t* chi angles: 170.2	0.10Å	Favored (93.316%) alpha helix	-	-	-
A 223		SER	1.23	-	Favored (80.62%) General / -61.3,-36.6	Favored (72.5%) *m* chi angles: 295.8	0.05Å	Favored (76.611%) alpha helix	-	-	-
A 224		MET	1.27	-	Favored (79.68%) General / -56.3,-44.2	Favored (34.4%) *mtt* chi angles: 291.5,175,203.8	0.11Å	Favored (75.522%) alpha helix	-	-	-
A 225		THR	1.34	-	Favored (58.21%) General / -75.8,-27.9	Favored (68%) *p* chi angles: 62.7	0.05Å	Favored (75.651%) alpha helix	-	-	-
A 226		GLY	1.43	-	Favored (51.2%) Glycine / -60.9,-52.6	-	-	Favored (86.243%) alpha helix	-	-	-
A 227		VAL	1.55	-	Favored (95.16%) Ile or Val / -62.9,-42.1	Favored (77.2%) *t* chi angles: 172.9	0.04Å	Favored (73.141%) alpha helix	-	-	-
A 228		MET	1.7	-	Favored (60.49%) General / -51.5,-40.9	Favored (57.2%) *ttp* chi angles: 176.9,184.9,69.6	0.06Å	Favored (73.192%) alpha helix	-	-	-
A 229		ARG	1.88	-	Favored (27.68%) General / -86.9,7.4	Favored (96.9%) *mtt-85* chi angles: 290.2,184.7,178.8,276.1	0.04Å	Favored (37.416%)	-	-	-
A 230		GLY	2.07	-	Favored (61.86%) Glycine / 97.9,-5.7	-	-	Favored (74.874%)	-	-	-
A 231		ASN	2.23	-	Favored (10.12%) General / -83.1,71.8	Favored (41.1%) *t0* chi angles: 188.6,10.7	0.05Å	Favored (12.438%)	-	-	-
A 232		TYR	2.34	-	Favored (52.39%)	Favored (90.5%) *t80*	0.10Å	Favored (26.5%)	-	-	-

#	Alt	Res	High B	Clash > 0.4Å	Ramachandran	Rotamer	Cβ deviation	CaBLAM	Bond lengths	Bond angles	Cis Peptides
					General / -50.4,-41.3	chi angles: 178.5,80.2					
A 233		TYR	2.37	-	Favored (3.74%) General / -48.7,-28.7	Favored (70.6%) *t80* chi angles: 185.9,81.5	0.06Å	Favored (59.034%)	-	-	-
A 234		ALA	2.32	-	Favored (65.87%) General / -58.1,-30.0	-	0.05Å	Favored (58.086%) three-ten	-	-	-
A 235		PHE	2.21	-	Favored (66.27%) General / -60.8,-25.1	Favored (35%) *t80* chi angles: 191.9,68.9	0.02Å	Favored (66.123%) three-ten	-	-	-
A 236		VAL	2.08	-	Favored (84.02%) Ile or Val / -62.9,-39.2	Favored (76%) *t* chi angles: 172.8	0.04Å	Favored (61.053%) three-ten	-	-	-
A 237		GLY	1.95	-	Favored (94.58%) Glycine / -65.2,-38.0	-	-	Favored (96.975%) alpha helix	-	-	-
A 238		VAL	1.86	-	Favored (81.08%) Ile or Val / -65.1,-49.1	Favored (81.8%) *t* chi angles: 173.3	0.07Å	Favored (80.308%) alpha helix	-	-	-
A 239		MET	1.82	0.49Å HA with A 239 MET HE2	Favored (91.29%) General / -65.3,-43.4	Favored (43.3%) *mmp* chi angles: 291.7,295.3,93.2	0.04Å	Favored (84.185%) alpha helix	-	-	-
A 240		TYR	1.82	-	Favored (71.03%) General / -60.2,-51.1	Favored (91.8%) *t80* chi angles: 178.8,77.9	0.04Å	Favored (79.187%) alpha helix	-	-	-
**#**	**Alt**	**Res**	**High B**	**Clash > 0.4Å**	**Ramachandran**	**Rotamer**	**Cβ deviation**	**CaBLAM**	**Bond lengths**	**Bond angles**	**Cis Peptides**
			Avg: 3.90	Clashscore: 1.54	Outliers: 5 of 248	Poor rotamers: 0 of 207	Outliers: 1 of 229	Outliers: 2 of 246	Outliers: 1 of 250	Outliers: 2 of 250	Non-Trans: 0 of 249
A 241		ASN	1.87	-	Favored (86.94%) General / -67.0,-39.4	Favored (86.8%) *m-40* chi angles: 284.8,334.3	0.04Å	Favored (76.107%) alpha helix	-	-	-
A 242		LEU	1.98	-	Favored (78.49%) General / -68.8,-36.5	Favored (79.1%) *mt* chi angles: 291.4,176.7	0.06Å	Favored (98.481%) alpha helix	-	-	-
A 243		TRP	2.15	-	Favored (97.25%) General / -64.2,-41.0	Favored (43.4%) *m-10* chi angles: 289.7,337.6	0.02Å	Favored (94.59%) alpha helix	-	-	-
A 244		LYS	2.42	-	Favored (89.29%) General / -58.6,-42.9	Favored (86.5%) *tttt* chi angles: 183,178.5,177.4,179.9	0.01Å	Favored (91.221%) alpha helix	-	-	-
A 245		MET	2.82	-	Favored (69.93%) General / -70.3,-32.2	Favored (50.5%) *mmp* chi angles: 292.9,298.7,96.8	0.01Å	Favored (75.743%) alpha helix	-	-	-
A 246		LYS	3.39	-	Favored (30.56%) General / -82.2,-31.3	Favored (97.5%) *mttt* chi angles: 291.1,179,180.5,179.8	0.01Å	Favored (36.587%)	-	-	-
A 247		THR	4.12	-	Favored (38.64%) General / -76.7,131.2	Favored (81.9%) *m* chi angles: 302.3	0.05Å	Favored (25.475%)	-	-	-

A 248		GLY	5.01	-	Favored (51.24%) Glycine / -64.7,148.3	-	-	Favored (58.559%)	-	-	-
A 249		ARG	5.96	-	Favored (70.62%) General / -53.7,-46.3	Favored (50.4%) *ttp-170* chi angles: 181.8,195.5,68.8,197.9	0.08Å	-	-	-	-
A 250		ARG	6.84	-	-	Favored (98.6%) *mtt-85* chi angles: 295,179.2,184.3,273.7	0.04Å	-	-	-	-
