# Supplementary material for: Integrative proteome-wide structural analysis and high-throughput docking identify broad-spectrum antiviral scaffolds against Zika, Yellow Fever, West Nile, Saint Louis encephalitis, and Usutu viruses
Source: Front Cell Infect Microbiol. 2026 Apr 30;16:1723132. doi: 10.3389/fcimb.2026.1723132 (PMC13171538; doi:10.3389/fcimb.2026.1723132)
Supplement: Supplementary file 6 [file DataSheet6.zip › YFV/YF_NS4b/Mol_probity_Files/YF_NS4b_1FH-rama.pdf]

# MolProbity Ramachandran analysis

YF_NS4b1FH.pdb, model 1

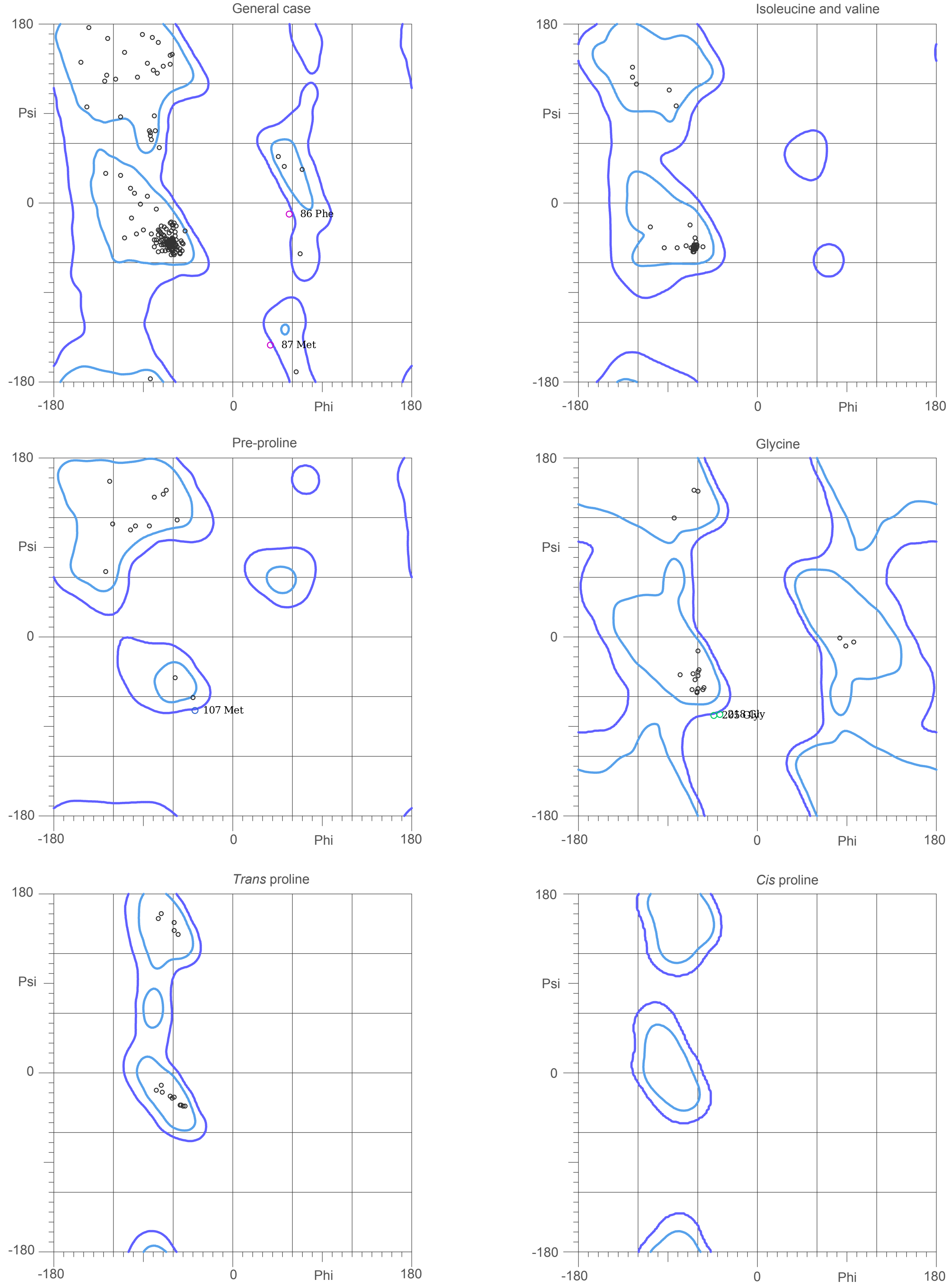


96.4% (239/248) of all residues were in favored (98%) regions.
98.0% (243/248) of all residues were in allowed (>99.8%) regions.

There were 5 outliers (phi, psi):
- 86 Phe (57.6, -11.4)
- 87 Met (38.8, -143.6)
- 107 Met (-38.9, -74.7)
- 205 Gly (-44.4, -79.9)
- 218 Gly (-39.0, -78.1)

http://kinemage.biochem.duke.edu

Lovell, Davis, et al. Proteins 50:437 (2003)
