# Supplementary material for: Integrative proteome-wide structural analysis and high-throughput docking identify broad-spectrum antiviral scaffolds against Zika, Yellow Fever, West Nile, Saint Louis encephalitis, and Usutu viruses
Source: Front Cell Infect Microbiol. 2026 Apr 30;16:1723132. doi: 10.3389/fcimb.2026.1723132 (PMC13171538; doi:10.3389/fcimb.2026.1723132)
Supplement: Supplementary file 6 [file DataSheet6.zip › YFV/YF_NS5/Mol_probity_Files/YF_NS5_1FH-multi.table.pdf]

Viewing YF_NS5_1FH-multi.table

Duke Biochemistry
Duke University School of Medicine

When finished, you should close this window.

Hint: Use File | Save As... to save a copy of this page.

All-Atom Contacts	Clashscore, all atoms:	1.51		99th percentile* (N=1784, all resolutions)
	Clashscore is the number of serious steric overlaps (> 0.4 Å) per 1000 atoms.			
Protein Geometry	Poor rotamers	0	0.00%	Goal: <0.3%
	Favored rotamers	776	99.49%	Goal: >98%
	Ramachandran outliers	4	0.44%	Goal: <0.05%
	Ramachandran favored	882	97.67%	Goal: >98%
	Rama distribution Z-score	-0.01 ± 0.27		Goal: abs(Z score) < 2
	MolProbity score^	0.96		100th percentile* (N=27675, 0Å - 99Å)
	Cβ deviations >0.25Å	0	0.00%	Goal: 0
	Bad bonds:	13 / 7458	0.17%	Goal: 0%
	Bad angles:	16 / 10076	0.16%	Goal: <0.1%
Peptide Omegas	Cis Prolines:	0 / 29	0.00%	Expected: ≤1 per chain, or ≤5%
Low-resolution Criteria	CaBLAM outliers	17	1.9%	Goal: <1.0%
	CA Geometry outliers	3	0.33%	Goal: <0.5%
Additional validations	Chiral volume outliers	0/1080		
	Waters with clashes	0/0	0.00%	See UnDowser table for details

In the two column results, the left column gives the raw count, right column gives the percentage.

* 100th percentile is the best among structures of comparable resolution; 0th percentile is the worst. For clashscore the comparative set of structures was selected in 2004, for MolProbity score in 2006.

^ MolProbity score combines the clashscore, rotamer, and Ramachandran evaluations into a single score, normalized to be on the same scale as X-ray resolution.

Key to table colors and cutoffs here:

#	Alt	Res	High B	Clash > 0.4Å	Ramachandran	Rotamer	Cβ deviation	CaBLAM	Bond lengths	Bond angles	Cis Peptides
			Avg: 0.92	Clashscore: 1.51	Outliers: 4 of 903	Poor rotamers: 0 of 780	Outliers: 0 of 837	Outliers: 18 of 901	Outliers: 11 of 905	Outliers: 16 of 905	Non-Trans: 0 of 904
A 1		GLY	7.82	-	-	-	-	-	-	-	-
A 2		ARG	6.62	-	Favored (23.67%) General / -156.3,148.9	Favored (45.3%) *ptt-90* chi angles: 62.6,182.9,185.8,269.3	0.07Å	-	-	-	-
A 3		ALA	5.17	-	Favored (85.91%) General / -64.9,-36.9	-	0.04Å	Favored (16.467%)	-	-	-
A 4		ASN	3.76	-	Favored (10.32%) General / -83.5,70.9	Favored (88.2%) *m-40* chi angles: 295.1,319.5	0.03Å	Favored (18.219%)	-	-	-
A 5		GLY	2.62	-	Favored (69.19%) Glycine / -56.8,-35.8	-	-	Favored (31.394%)	-	-	-
A 6		LYS	1.83	-	Favored (36.93%) General / -104.7,141.2	Favored (72.3%) *mmtt* chi angles: 300.7,296.9,186.9,184.7	0.03Å	Favored (21.213%)	-	-	-

A 7		THR	1.33	-	Favored (13.42%) General / -91.2,165.6	Favored (56.5%) *p* chi angles: 64.7	0.02Å	Favored (42.284%)	-	-	-
A 8		LEU	1.04	-	Favored (91.25%) General / -61.9,-39.2	Favored (94.7%) *mt* chi angles: 291.6,172.6	0.08Å	Favored (67.777%)	-	-	-
A 9		GLY	0.88	-	Favored (99.77%) Glycine / -63.0,-42.0	-	-	Favored (96.937%) alpha helix	-	-	-
A 10		GLU	0.81	-	Favored (92.46%) General / -62.4,-39.2	Favored (86.5%) *tt0* chi angles: 183.8,174.3,351.7	0.07Å	Favored (99.312%) alpha helix	-	-	-
A 11		VAL	0.79	-	Favored (97.86%) Ile or Val / -61.6,-43.9	Favored (60.5%) *t* chi angles: 170.9	0.03Å	Favored (96.441%) alpha helix	-	-	-
A 12		TRP	0.79	-	Favored (91.31%) General / -59.7,-45.7	Favored (89.5%) *t60* chi angles: 178.5,84.4	0.03Å	Favored (94.561%) alpha helix	-	-	-
A 13		LYS	0.8	-	Favored (77.07%) General / -59.5,-36.9	Favored (20.2%) *mmmt* chi angles: 287.5,286.1,281.8,180.9	0.04Å	Favored (84.206%) alpha helix	-	-	-
A 14		ARG	0.82	-	Favored (85.56%) General / -65.7,-44.6	Favored (44.7%) *tpt170* chi angles: 178.9,63.1,175.6,168.8	0.01Å	Favored (86.204%) alpha helix	-	-	-
A 15		GLU	0.85	-	Favored (94.29%) General / -62.0,-40.0	Favored (98%) *mt-10* chi angles: 289.9,179.4,353.8	0.01Å	Favored (94.52%) alpha helix	-	-	-
A 16		LEU	0.88	-	Favored (77.3%) General / -58.4,-49.4	Favored (51.2%) *tp* chi angles: 175.9,66	0.07Å	Favored (95.494%) alpha helix	-	-	-
A 17		ASN	0.93	-	Favored (68.86%) General / -66.2,-28.6	Favored (95.3%) *m-40* chi angles: 286.4,338.3	0.04Å	Favored (73.359%) alpha helix	-	-	-
A 18		LEU	0.98	-	Favored (61.35%) General / -72.1,-23.7	Favored (95.9%) *mt* chi angles: 291.8,172.3	0.04Å	Favored (49.676%)	-	-	-
A 19		LEU	1.03	-	Favored (56.3%) General / -62.6,144.3	Favored (95.5%) *mt* chi angles: 295.2,173	0.13Å	Favored (30.263%)	-	-	-
A 20		ASP	1.08	-	Favored (7.99%) General / -81.2,179.1	Favored (31.1%) *p0* chi angles: 68.6,23.1	0.05Å	Favored (40.523%)	-	-	-
**#**	**Alt**	**Res**	**High B**	**Clash > 0.4Å**	**Ramachandran**	**Rotamer**	**Cβ deviation**	**CaBLAM**	**Bond lengths**	**Bond angles**	**Cis Peptides**
			Avg: 0.92	Clashscore: 1.51	Outliers: 4 of 903	Poor rotamers: 0 of 780	Outliers: 0 of 837	Outliers: 18 of 901	Outliers: 11 of 905	Outliers: 16 of 905	Non-Trans: 0 of 904
A 21		LYS	1.11	-	Favored (90.57%) General / -60.9,-46.5	Favored (86.9%) *tttt* chi angles: 181.8,176.8,179,179.6	0.05Å	Favored (45.327%)	-	-	-

A 22		GLN	1.13	-	Favored (89.89%) General / -66.3,-40.1	Favored (97.5%) *mt0* chi angles: 290.7,177.7,333.8	0.02Å	Favored (80.957%) alpha helix	-	-	-
A 23		GLN	1.12	-	Favored (85.49%) General / -67.4,-39.3	Favored (74.5%) *mt0* chi angles: 292.4,177.1,62.4	0.02Å	Favored (77.811%) alpha helix	-	-	-
A 24		PHE	1.09	-	Favored (74.53%) General / -56.3,-49.3	Favored (74.2%) *t80* chi angles: 182.9,87.8	0.04Å	Favored (80.707%) alpha helix	-	-	-
A 25		GLU	1.04	-	Favored (90.72%) General / -65.4,-38.7	Favored (66.9%) *mm-30* chi angles: 290.4,293.5,305.9	0.01Å	Favored (87.789%) alpha helix	-	-	-
A 26		LEU	1	-	Favored (95.12%) General / -63.5,-39.7	Favored (78.6%) *mt* chi angles: 288.6,171.8	0.05Å	Favored (80.739%) alpha helix	-	-	-
A 27		TYR	0.96	-	Favored (60.52%) General / -75.6,-35.5	Favored (33.6%) *m-80* chi angles: 283.5,117	0.05Å	Favored (64.667%) alpha helix	-	-	-
A 28		LYS	0.92	-	Favored (65.99%) General / -54.1,-39.1	Favored (36.9%) *ttpt* chi angles: 181.9,175.2,68.8,180.8	0.03Å	Favored (66.599%) alpha helix	-	-	-
A 29		ARG	0.89	-	Favored (43.76%) General / -98.1,9.1	Favored (69.6%) *mtp180* chi angles: 301,180.9,73.9,198.8	0.04Å	Favored (32.757%)	-	-	-
A 30		THR	0.85	-	Favored (27.64%) General / -77.4,124.3	Favored (36%) *m* chi angles: 293.4	0.02Å	CaBLAM Disfavored (1.967%)	-	-	-
A 31		ASP	0.82	-	Favored (27.72%) General / 50.6,43.1	Favored (77%) *m-30* chi angles: 294.3,324.9	0.06Å	CaBLAM Disfavored (4.098%)	-	-	-
A 32		ILE	0.78	-	Favored (6.28%) Ile or Val / -117.6,171.2	Favored (40.8%) *pt* chi angles: 63.6,168.7	0.09Å	Favored (31.383%)	-	-	-
A 33		VAL	0.76	-	Favored (49.25%) Ile or Val / -106.4,134.0	Favored (54.3%) *t* chi angles: 180.7	0.06Å	Favored (45.999%) beta sheet	-	-	-
A 34		GLU	0.74	-	Favored (38.02%) General / -121.8,153.7	Favored (99.7%) *mt-10* chi angles: 291.7,177.5,349.2	0.05Å	Favored (53.985%) beta sheet	-	-	-
A 35		VAL	0.73	-	Favored (51.71%) Ile or Val / -101.9,130.4	Favored (75.7%) *t* chi angles: 178.3	0.04Å	Favored (41.274%) beta sheet	-	-	-
A 36		ASP	0.74	-	Favored (5.79%) General / -80.8,89.5	Favored (65.7%) *t0* chi angles: 183.8,345.2	0.05Å	Favored (35.093%) beta sheet	-	-	-
A 37		ARG	0.77	-	Favored (40.02%) General / -90.7,7.3	Favored (77.7%) *mtp180* chi angles: 293.9,179.9,67.6,201.5	0.08Å	Favored (9.936%)	-	-	-
A 38		ASP	0.8	-	Favored (75.94%) General / -62.6,-33.9	Favored (11.6%) *t70* chi angles: 197,66.1	0.01Å	Favored (50.467%)	-	-	-

A 39		THR	0.84	-	Favored (84.6%) General / -64.2,-46.3	Favored (92.4%) *m* chi angles: 297.7	0.01Å	Favored (90.558%) alpha helix	-	-	-
A 40		ALA	0.89	-	Favored (98.36%) General / -61.9,-41.9	-	0.07Å	Favored (94.336%) alpha helix	-	-	-
**#**	**Alt**	**Res**	**High B**	**Clash > 0.4Å**	**Ramachandran**	**Rotamer**	**Cβ deviation**	**CaBLAM**	**Bond lengths**	**Bond angles**	**Cis Peptides**
			Avg: 0.92	Clashscore: 1.51	Outliers: 4 of 903	Poor rotamers: 0 of 780	Outliers: 0 of 837	Outliers: 18 of 901	Outliers: 11 of 905	Outliers: 16 of 905	Non-Trans: 0 of 904
A 41		ARG	0.93	-	Favored (93.55%) General / -64.6,-43.5	Favored (98.8%) *mtm-85* chi angles: 287.6,193.3,294.1,267.5	0.04Å	Favored (92.246%) alpha helix	-	-	-
A 42		ARG	0.98	-	Favored (81.11%) General / -68.2,-41.5	Favored (96.6%) *mtt-85* chi angles: 287.9,178,181.4,273.3	0.02Å	Favored (92.204%) alpha helix	-	-	-
A 43		HIS	1.04	-	Favored (86.64%) General / -67.2,-40.6	Favored (49.4%) *m170* chi angles: 286.7,169.6	0.02Å	Favored (80.483%) alpha helix	-	-	-
A 44		LEU	1.11	-	Favored (72.83%) General / -70.8,-39.8	Favored (95.1%) *mt* chi angles: 292.1,171.1	0.10Å	Favored (78.453%) alpha helix	-	-	-
A 45		ALA	1.18	-	Favored (77.44%) General / -60.7,-35.9	-	0.04Å	Favored (78.094%) alpha helix	-	-	-
A 46		GLU	1.24	-	Favored (54.53%) General / -85.3,-0.3	Favored (97.7%) *mt-10* chi angles: 294,176.9,0.2	0.01Å	Favored (56.537%)	-	-	-
A 47		GLY	1.28	-	Favored (87.13%) Glycine / 83.6,6.6	-	-	Favored (87.435%)	-	-	-
A 48		LYS	1.28	-	Favored (10.01%) General / -84.0,68.4	Favored (90.9%) *mttt* chi angles: 295.3,172.5,177.9,170.8	0.03Å	Favored (7.923%)	-	-	-
A 49		VAL	1.24	-	Favored (84.33%) Ile or Val / -68.0,-41.2	Favored (66.3%) *t* chi angles: 171.7	0.07Å	Favored (11.754%)	-	-	-
A 50		ASP	1.17	-	Favored (4%) General / -78.5,66.4	Favored (17.6%) *p0* chi angles: 57.9,324.9	0.08Å	Favored (5.325%)	-	-	-
A 51		THR	1.07	-	Favored (6.86%) General / -135.4,178.7	Favored (11.5%) *t* chi angles: 190.7	0.03Å	Favored (25.228%)	-	-	-
A 52		GLY	0.98	-	Favored (13.24%) Glycine / -97.6,28.7	-	-	Favored (7.104%)	-	-	-
A 53		VAL	0.9	-	Favored (64.21%) Ile or Val / -120.1,134.3	Favored (47.4%) *t* chi angles: 182	0.09Å	Favored (30.595%)	-	-	-
A 54		ALA	0.83	-	Favored (25.41%) General / -59.7,151.3	-	0.05Å	Favored (15.472%)	-	-	-

#	Alt	Res	High B	Clash > 0.4Å	Ramachandran	Rotamer	Cβ deviation	CaBLAM	Bond lengths	Bond angles	Cis Peptides
A 55		VAL	0.77	-	Favored (21.06%) Ile or Val / -79.7,-40.5	Favored (90.1%) *t* chi angles: 176	0.04Å	Favored (25.938%)	-	-	-
A 56		SER	0.73	-	Favored (49.11%) General / -138.0,155.2	Favored (42.8%) *t* chi angles: 175.6	0.04Å	Favored (20.779%)	-	-	-
A 57		ARG	0.7	-	Favored (53.98%) General / -54.6,-30.1	Favored (43.4%) *ttm170* chi angles: 186.8,178.6,298.6,184.7	0.08Å	Favored (46.977%) alpha helix	-	-	-
A 58		GLY	0.67	-	Favored (66.28%) Glycine / -57.2,-34.2	-	-	Favored (74.599%) alpha helix	-	-	-
A 59		THR	0.65	-	Favored (92.24%) General / -60.5,-45.9	Favored (70.6%) *m* chi angles: 296	0.09Å	Favored (79.761%) alpha helix	-	-	-
A 60		ALA	0.64	-	Favored (83.09%) General / -61.7,-37.2	-	0.03Å	Favored (84.733%) alpha helix	-	-	-
**#**	**Alt**	**Res**	**High B**	**Clash > 0.4Å**	**Ramachandran**	**Rotamer**	**Cβ deviation**	**CaBLAM**	**Bond lengths**	**Bond angles**	**Cis Peptides**
			Avg: 0.92	Clashscore: 1.51	Outliers: 4 of 903	Poor rotamers: 0 of 780	Outliers: 0 of 837	Outliers: 18 of 901	Outliers: 11 of 905	Outliers: 16 of 905	Non-Trans: 0 of 904
A 61		LYS	0.62	-	Favored (88.48%) General / -66.6,-41.4	Favored (94.3%) *mttt* chi angles: 290.5,179.8,186.5,178.2	0.02Å	Favored (91.498%) alpha helix	-	-	-
A 62		LEU	0.62	-	Favored (86.03%) General / -67.3,-40.3	Favored (38.3%) *tp* chi angles: 185.6,58.6	0.05Å	Favored (81.436%) alpha helix	-	-	-
A 63		ARG	0.62	-	Favored (64.53%) General / -52.7,-49.8	Favored (78.8%) *ttt180* chi angles: 178.3,181.2,178,187	0.05Å	Favored (83.16%) alpha helix	-	-	-
A 64		TRP	0.63	-	Favored (91.43%) General / -59.9,-45.8	Favored (92.9%) *t60* chi angles: 182.5,88.4	0.03Å	Favored (95.806%) alpha helix	-	-	-
A 65		PHE	0.64	-	Favored (84.65%) General / -62.5,-37.1	Favored (24.1%) *m-80* chi angles: 279.9,118.6	0.02Å	Favored (79.078%) alpha helix	-	-	-
A 66		HIS	0.65	-	Favored (41.17%) General / -69.8,-50.6	Favored (76.9%) *t70* chi angles: 184.6,63.6	0.05Å	Favored (73.171%) alpha helix	-	-	-
A 67		GLU	0.67	-	Favored (73.32%) General / -62.1,-32.7	Favored (97.3%) *mt-10* chi angles: 289.1,179.6,353.8	0.04Å	Favored (73.796%) alpha helix	-	-	-
A 68		ARG	0.68	-	Favored (49.11%) General / -84.1,0.2	Favored (49.5%) *mmt180* chi angles: 294.7,289.4,177.4,183	0.02Å	Favored (57.202%)	-	-	-
A 69		GLY	0.69	-	Favored (84.34%) Glycine / 83.2,9.2	-	-	Favored (82.075%)	-	-	-
A 70		TYR	0.69	-	Favored (35.73%) General / -80.4,-35.1	Favored (53.1%) *m-80* chi angles: 290.1,114.7	0.02Å	Favored (17.973%)	-	-	-

A 71		VAL	0.69	-	Favored (13.05%) Ile or Val / -130.4,106.5	Favored (53.5%) *t* chi angles: 180.9	0.07Å	Favored (22.991%)	-	-	-
A 72		LYS	0.69	-	Favored (34.67%) General / -85.8,127.9	Favored (53.4%) *tttp* chi angles: 183.6,174.6,178,66	0.05Å	Favored (49.525%)	-	-	-
A 73		LEU	0.68	-	Favored (22.01%) General / -102.7,110.2	Favored (64.2%) *mt* chi angles: 303.5,175	0.09Å	Favored (43.239%)	-	-	-
A 74		GLU	0.67	-	Favored (31.56%) General / -143.7,143.8	Favored (91.6%) *tt0* chi angles: 183.5,177.2,1.8	0.03Å	CaBLAM Disfavored (1.787%)	-	-	-
A 75		GLY	0.65	-	Favored (30.01%) Glycine / 63.4,-123.7	-	-	Favored (23.628%)	-	-	-
A 76		ARG	0.63	-	Favored (38.63%) General / -94.7,124.3	Favored (52.2%) *ttm170* chi angles: 179,170.6,290.5,177.6	0.04Å	Favored (14.845%)	-	-	-
A 77		VAL	0.62	-	Favored (66.7%) Ile or Val / -112.8,130.4	Favored (58.9%) *t* chi angles: 180.1	0.09Å	Favored (68.845%)	-	-	-
A 78		THR	0.61	-	Favored (50.94%) General / -108.5,134.7	Favored (91.6%) *m* chi angles: 297.9	0.07Å	Favored (71.002%) beta sheet	-	-	-
A 79		ASP	0.6	-	Favored (9.98%) General / -126.5,107.1	Favored (32.9%) *t0* chi angles: 186.2,323.4	0.09Å	Favored (61.405%)	-	OUTLIER(S) worst is CA-CB-CG: 4.7 σ	-
A 80		LEU	0.6	-	Favored (34.16%) General / -93.5,136.1	Favored (70.4%) *mt* chi angles: 302.8,181.7	0.04Å	Favored (12.456%)	-	-	-
**#**	**Alt**	**Res**	**High B**	**Clash > 0.4Å**	**Ramachandran**	**Rotamer**	**Cβ deviation**	**CaBLAM**	**Bond lengths**	**Bond angles**	**Cis Peptides**
			Avg: 0.92	Clashscore: 1.51	Outliers: 4 of 903	Poor rotamers: 0 of 780	Outliers: 0 of 837	Outliers: 18 of 901	Outliers: 11 of 905	Outliers: 16 of 905	Non-Trans: 0 of 904
A 81		GLY	0.61	-	Favored (45.6%) Glycine / 76.3,34.4	-	-	Favored (26.206%)	-	-	-
A 82		CYS	0.62	-	Favored (3.38%) General / -77.1,69.5	Favored (31.5%) *t* chi angles: 188.9	0.04Å	Favored (56.515%)	-	-	-
A 83		GLY	0.63	-	Favored (40.05%) Glycine / -64.2,135.7	-	-	Favored (6.272%)	-	-	-
A 84		ARG	0.63	-	Allowed (0.21%) General / 55.8,6.5	Favored (94.7%) *mtt-85* chi angles: 299.6,182.3,186.2,274.6	0.06Å	CaBLAM Disfavored (2.102%)	-	-	-
A 85		GLY	0.62	-	Favored (89.58%) Glycine / 79.9,4.6	-	-	Favored (67.287%)	-	-	-
A 86		GLY	0.62	-	Favored (6.82%) Glycine / -49.2,-59.6	-	-	Favored (13.438%)	-	-	-

A 87		TRP	0.6	-	Favored (63.19%) General / -72.8,-29.4	Favored (26.3%) *m-10* chi angles: 279.4,8.5	0.02Å	Favored (40.805%) alpha helix	-	-	-
A 88		CYS	0.6	-	Favored (94.4%) General / -65.1,-40.4	Favored (91.7%) *m* chi angles: 291.7	0.04Å	Favored (74.718%) alpha helix	-	-	-
A 89		TYR	0.59	-	Favored (70.47%) General / -71.7,-39.0	Favored (37.6%) *m-80* chi angles: 289.3,121.7	0.03Å	Favored (92.94%) alpha helix	-	-	-
A 90		TYR	0.6	-	Favored (68.71%) General / -61.0,-51.6	Favored (77.6%) *t80* chi angles: 184.2,77.9	0.11Å	Favored (79.875%) alpha helix	-	-	-
A 91		ALA	0.61	-	Favored (96.64%) General / -61.3,-41.2	-	0.04Å	Favored (79.775%) alpha helix	-	-	-
A 92		ALA	0.62	-	Favored (71.61%) General / -61.0,-32.0	-	0.06Å	Favored (73.641%) alpha helix	-	-	-
A 93		ALA	0.65	-	Favored (56.51%) General / -77.2,-7.8	-	0.04Å	Favored (55.479%)	-	-	-
A 94		GLN	0.67	-	Favored (26.82%) General / -86.2,145.8	Favored (86%) *mm-40* chi angles: 303.8,307,301.8	0.03Å	Favored (37.14%)	-	-	-
A 95		ARG	0.7	-	Favored (69.34%) General / -62.1,-28.0	Favored (98.2%) *mtt180* chi angles: 289.7,177.6,180.2,173.3	0.06Å	Favored (43.694%)	-	-	-
A 96		GLU	0.72	-	Favored (58.5%) General / -89.2,-2.5	Favored (68.8%) *mt-10* chi angles: 294.4,181,313.5	0.03Å	Favored (54.436%)	-	-	-
A 97		VAL	0.72	-	Favored (52.14%) Ile or Val / -98.9,126.4	Favored (95.3%) *t* chi angles: 175.7	0.07Å	Favored (30.869%)	-	-	-
A 98		SER	0.71	-	Favored (28.96%) General / -92.2,-14.4	Favored (98.2%) *p* chi angles: 65.7	0.05Å	Favored (8.043%)	-	-	-
A 99		GLY	0.68	-	Favored (9.19%) Glycine / 168.2,155.1	-	-	Favored (14.833%)	-	-	-
A 100		VAL	0.66	-	Favored (63.93%) Ile or Val / -130.2,133.6	Favored (60.7%) *t* chi angles: 179.9	0.09Å	Favored (61.71%) beta sheet	-	-	-
**#**	**Alt**	**Res**	**High B**	**Clash > 0.4Å**	**Ramachandran**	**Rotamer**	**Cβ deviation**	**CaBLAM**	**Bond lengths**	**Bond angles**	**Cis Peptides**
			Avg: 0.92	Clashscore: 1.51	Outliers: 4 of 903	Poor rotamers: 0 of 780	Outliers: 0 of 837	Outliers: 18 of 901	Outliers: 11 of 905	Outliers: 16 of 905	Non-Trans: 0 of 904
A 101		ARG	0.65	-	Favored (16.85%) General / -119.6,110.4	Favored (80.4%) *ttt180* chi angles: 187,174.5,183.7,176.4	0.03Å	Favored (64.5%) beta sheet	-	-	-
A 102		GLY	0.66	-	Favored (16.01%) Glycine / -96.9,142.1	-	-	Favored (58.465%) beta sheet	-	-	-

A 103		PHE	0.69	-	Favored (23.55%) General / -126.9,117.9	Favored (88.9%) *m-80* chi angles: 297.1,84.9	0.04Å	Favored (53.58%) beta sheet	-	-	-
A 104		THR	0.76	-	Favored (45.79%) General / -132.9,135.8	Favored (68.9%) *m* chi angles: 303.1	0.04Å	Favored (49.058%) beta sheet	-	-	-
A 105		LEU	0.87	-	Favored (6.37%) General / -79.7,72.5	Favored (29.3%) *tp* chi angles: 188,63	0.07Å	Favored (42.957%) beta sheet	-	-	-
A 106		GLY	0.98	0.50Å H with A 129 LYS H	Favored (3.25%) Glycine / -76.5,54.4	-	-	Favored (9.529%) beta sheet	-	-	-
A 107		LYS	1.08	-	Favored (6.56%) General / -92.9,176.1	Favored (98.6%) *mttt* chi angles: 293.7,184.5,174.8,179.1	0.11Å	Favored (18.256%)	-	-	-
A 108		GLU	1.15	-	Favored (69.17%) General / -58.0,-33.6	Favored (98.6%) *mt-10* chi angles: 290.4,178.1,352.6	0.00Å	Favored (37.833%)	-	-	-
A 109		GLY	1.15	-	Favored (80.68%) Glycine / -91.4,6.1	-	-	Favored (50.085%)	-	-	-
A 110		HIS	1.12	-	Favored (24.69%) General / -134.6,165.7	Favored (98.4%) *m-70* chi angles: 301,285.9	0.03Å	Favored (11.414%)	-	-	-
A 111		GLU	1.07	-	Favored (58.01%) General / -65.3,143.1	Favored (65%) *mt-10* chi angles: 288.3,181.1,318	0.04Å	Favored (29.61%)	-	-	-
A 112		LYS	1.02	-	Favored (39.43%) Pre-Pro / -109.9,156.7	Favored (47.9%) *mttp* chi angles: 300.3,174,182.3,66.6	0.10Å	Favored (32.025%)	-	-	-
A 113		PRO	0.99	-	Favored (75.08%) Trans-Pro / -62.1,152.6	Favored (52%) *Cg_exo* chi angles: 336.7,35.7,326.7	0.07Å	Favored (75.949%)	-	-	-
A 114		MET	0.98	-	Favored (54.74%) General / -118.1,134.7	Favored (94.1%) *mtp* chi angles: 293.3,179.6,65.9	0.02Å	Favored (42.903%) beta sheet	-	-	-
A 115		ASN	0.97	-	Favored (7.36%) General / -86.1,85.9	Favored (78.8%) *m-40* chi angles: 290.7,313.8	0.10Å	Favored (33.045%) beta sheet	-	-	-
A 116		VAL	0.95	-	Favored (48.33%) Ile or Val / -113.0,136.7	Favored (81.9%) *t* chi angles: 176.7	0.06Å	Favored (38.286%) beta sheet	-	-	-
A 117		GLN	0.93	0.64Å HE22 with A 262 GLY H	Favored (35.35%) General / -87.9,127.4	Favored (7.4%) *tm-30* chi angles: 198.6,266.3,312.2	0.02Å	Favored (7.048%) beta sheet	-	-	-
A 118		SER	0.9	-	Favored (23.01%) General / -162.2,168.1	Favored (88.3%) *p* chi angles: 69.5	0.03Å	CaBLAM Disfavored (3.313%)	-	-	-
A 119		LEU	0.86	-	Favored (43.75%) General / -55.2,130.9	Favored (34.6%) *tp* chi angles: 183.6,68.1	0.05Å	Favored (12.087%)	-	-	-
A 120		GLY	0.83	-	Favored (89.07%)	-	-	Favored (82.371%)	-	-	-

#	Alt	Res	High B	Clash > 0.4Å	Ramachandran	Rotamer	Cβ deviation	CaBLAM	Bond lengths	Bond angles	Cis Peptides
					Glycine / 84.9,0.2						
			Avg: 0.92	Clashscore: 1.51	Outliers: 4 of 903	Poor rotamers: 0 of 780	Outliers: 0 of 837	Outliers: 18 of 901	Outliers: 11 of 905	Outliers: 16 of 905	Non-Trans: 0 of 904
A 121		TRP	0.81	-	Favored (54.2%) General / -53.6,-33.3	Favored (61.3%) *p-90* chi angles: 71.9,272.3	0.06Å	Favored (38.826%) alpha helix	-	-	-
A 122		ASN	0.79	-	Favored (52.99%) General / -74.5,-7.4	Favored (16.3%) *p0* chi angles: 63.7,295.4	0.04Å	Favored (50.72%) three-ten	-	-	-
A 123		ILE	0.78	-	Favored (9.24%) Ile or Val / -107.4,10.0	Favored (34.4%) *pt* chi angles: 66,168.8	0.09Å	Favored (53.845%)	-	-	-
A 124		ILE	0.77	-	Favored (60.86%) Ile or Val / -106.5,128.6	Favored (67.8%) *mt* chi angles: 302.7,169.5	0.10Å	Favored (33.76%)	-	-	-
A 125		THR	0.77	-	Favored (53.12%) General / -111.6,134.5	Favored (93.6%) *m* chi angles: 297.5	0.01Å	Favored (66.915%)	-	-	-
A 126		PHE	0.76	-	Favored (44.7%) General / -120.0,147.5	Favored (96.4%) *m-80* chi angles: 292.9,94.7	0.08Å	Favored (70.235%) beta sheet	-	OUTLIER(S) worst is CA-CB-CG: 4.5 σ	-
A 127		LYS	0.76	-	Favored (31.99%) General / -123.8,120.2	Favored (85.8%) *tttt* chi angles: 183.6,180,182.5,178.4	0.01Å	Favored (65.909%)	-	-	-
A 128		ASP	0.76	-	Favored (19.62%) General / -101.8,153.6	Favored (3.9%) *m-30* chi angles: 304.7,359.3	0.07Å	Favored (12.225%)	-	-	-
A 129		LYS	0.75	0.50Å H with A 106 GLY H	Favored (21.79%) General / 59.6,39.1	Favored (47.3%) *mtpt* chi angles: 297.8,172.9,76.5,177.1	0.05Å	Favored (10.68%)	-	-	-
A 130		THR	0.74	-	Favored (49.38%) General / -112.8,138.6	Favored (6.5%) *t* chi angles: 182	0.05Å	Favored (32.343%)	-	-	-
A 131		ASP	0.72	-	Favored (18.33%) General / -88.9,106.3	Favored (60%) *t0* chi angles: 182.8,340.6	0.09Å	Favored (59.713%) beta sheet	-	-	-
A 132		VAL	0.71	-	Favored (9.11%) Ile or Val / -60.2,-15.0	Favored (31%) *m* chi angles: 296.9	0.05Å	Favored (27.522%)	-	-	-
A 133		HIS	0.7	-	Favored (54.91%) General / -92.0,-4.2	Favored (96.1%) *m-70* chi angles: 298.6,295.3	0.06Å	Favored (67.041%) alpha helix	-	-	-
A 134		ARG	0.69	-	Favored (14.55%) General / -115.7,7.0	Favored (90.7%) *mtt180* chi angles: 299.5,184.8,181.9,190.2	0.04Å	Favored (49.248%)	-	-	-
A 135		LEU	0.68	-	Favored (42.97%) General / -75.4,143.0	Favored (95.3%) *mt* chi angles: 295.3,172.4	0.10Å	Favored (39.866%)	-	-	-
A 136		GLU	0.67	-	Favored (86.07%) Pre-Pro / -70.8,134.0	Favored (90.8%) *tt0* chi angles: 185.3,176.8,3.5	0.05Å	Favored (31.426%)	-	-	-

A 137		PRO	0.66	-	Favored (76.23%) Trans-Pro / -54.8,138.2	Favored (96.6%) *Cg_exo* chi angles: 331.7,37.1,330.2	0.04Å	Favored (69.577%)	-	-	-
A 138		ILE	0.64	-	Favored (74.86%) Ile or Val / -122.6,127.1	Favored (77.3%) *mt* chi angles: 301,171.7	0.04Å	Favored (43.664%) beta sheet	-	-	-
A 139		LYS	0.63	-	Favored (55.14%) General / -61.2,132.9	Favored (88.7%) *tttt* chi angles: 184.7,175.3,180.7,180.1	0.02Å	Favored (19.423%) beta sheet	-	-	-
A 140		CYS	0.61	-	Favored (17.27%) General / -148.9,170.9	Favored (27.4%) *p* chi angles: 65	0.01Å	Favored (38.756%)	-	-	-
**#**	**Alt**	**Res**	**High B**	**Clash > 0.4Å**	**Ramachandran**	**Rotamer**	**Cβ deviation**	**CaBLAM**	**Bond lengths**	**Bond angles**	**Cis Peptides**
			Avg: 0.92	Clashscore: 1.51	Outliers: 4 of 903	Poor rotamers: 0 of 780	Outliers: 0 of 837	Outliers: 18 of 901	Outliers: 11 of 905	Outliers: 16 of 905	Non-Trans: 0 of 904
A 141		ASP	0.6	-	Favored (12.32%) General / -91.1,-39.2	Favored (59.4%) *m-30* chi angles: 295.9,303.4	0.05Å	Favored (5.729%)	-	-	-
A 142		THR	0.59	-	Favored (51.61%) General / -126.1,133.4	Favored (97.9%) *m* chi angles: 300	0.02Å	Favored (36.179%)	-	-	-
A 143		LEU	0.6	-	Favored (29.8%) General / -119.1,117.8	Favored (50.9%) *tp* chi angles: 177,66.5	0.08Å	Favored (65.84%)	-	-	-
A 144		LEU	0.62	-	Favored (47.05%) General / -110.6,138.4	Favored (53.7%) *mt* chi angles: 305.7,176.4	0.08Å	Favored (54.1%) beta sheet	-	-	-
A 145		CYS	0.67	-	Favored (37.4%) General / -135.5,132.4	Favored (61.6%) *m* chi angles: 300.7	0.01Å	Favored (43.578%) beta sheet	-	-	-
A 146		ASP	0.73	-	Favored (2.83%) General / -135.8,38.5	Favored (39.9%) *t0* chi angles: 192.8,25.3	0.05Å	Favored (7.838%) beta sheet	-	-	-
A 147		ILE	0.79	-	Allowed (0.6%) Ile or Val / -91.2,68.7	Favored (41.3%) *pt* chi angles: 58.5,172.8	0.07Å	CaBLAM Disfavored (1.09%)	-	-	-
A 148		GLY	0.84	-	Favored (19.84%) Glycine / -175.9,155.6	-	-	CaBLAM Disfavored (3.012%)	-	-	-
A 149		GLU	0.87	-	Favored (52.51%) General / -131.8,147.9	Favored (95.1%) *mt-10* chi angles: 298.5,180,1.2	0.03Å	Favored (36.695%)	-	-	-
A 150		SER	0.88	-	Favored (23.9%) General / -74.0,166.2	Favored (95.4%) *p* chi angles: 66.1	0.04Å	Favored (6.307%) beta sheet	-	-	-
A 151		SER	0.87	-	Favored (11.08%) Pre-Pro / -163.0,150.4	Favored (42.1%) *t* chi angles: 175.9	0.02Å	Favored (38.863%)	-	-	-
A 152		PRO	0.84	-	Favored (53.26%) Trans-Pro / -57.0,-23.6	Favored (86.6%) *Cg_exo* chi angles: 333.5,34.9,331.6	0.05Å	Favored (14.156%)	-	-	-

A 153		SER	0.8	-	Favored (21.33%) General / -89.3,110.5	Favored (24.5%) *t* chi angles: 171.4	0.04Å	Favored (35.085%)	-	-	-
A 154		SER	0.76	-	Favored (66.75%) General / -65.5,-22.9	Favored (95.4%) *p* chi angles: 64.9	0.05Å	Favored (50.559%)	-	-	-
A 155		VAL	0.72	-	Favored (49.99%) Ile or Val / -73.2,-46.7	Favored (87.3%) *t* chi angles: 173.9	0.04Å	Favored (71.833%) alpha helix	-	-	-
A 156		THR	0.69	-	Favored (96.88%) General / -63.6,-43.5	Favored (92.9%) *m* chi angles: 297.6	0.04Å	Favored (89.871%) alpha helix	-	-	-
A 157		GLU	0.66	-	Favored (86.19%) General / -61.7,-38.0	Favored (84%) *mt-10* chi angles: 287.9,185.5,344.2	0.04Å	Favored (97.902%) alpha helix	-	-	-
A 158		GLY	0.64	-	Favored (46.87%) Glycine / -59.7,-53.3	-	-	Favored (94.342%) alpha helix	-	-	-
A 159		GLU	0.63	-	Favored (91.62%) General / -61.8,-39.4	Favored (97.7%) *mt-10* chi angles: 289.4,178,353	0.02Å	Favored (85.168%) alpha helix	-	-	-
A 160		ARG	0.63	-	Favored (97.41%) General / -64.0,-42.0	Favored (97.8%) *mtt180* chi angles: 288.6,174.7,179.5,169.6	0.04Å	Favored (92.156%) alpha helix	-	-	-
**#**	**Alt**	**Res**	**High B**	**Clash > 0.4Å**	**Ramachandran**	**Rotamer**	**Cβ deviation**	**CaBLAM**	**Bond lengths**	**Bond angles**	**Cis Peptides**
			Avg: 0.92	Clashscore: 1.51	Outliers: 4 of 903	Poor rotamers: 0 of 780	Outliers: 0 of 837	Outliers: 18 of 901	Outliers: 11 of 905	Outliers: 16 of 905	Non-Trans: 0 of 904
A 161		THR	0.63	-	Favored (99.66%) General / -62.0,-42.9	Favored (87.8%) *m* chi angles: 297	0.03Å	Favored (85.328%) alpha helix	-	-	-
A 162		MET	0.64	-	Favored (82.75%) General / -65.6,-36.1	Favored (78.3%) *mtm* chi angles: 288.9,187.9,281.1	0.09Å	Favored (87.826%) alpha helix	-	-	-
A 163		ARG	0.65	-	Favored (89.25%) General / -59.1,-41.9	Favored (35.9%) *ttp-170* chi angles: 186.8,177.7,69.4,213.7	0.04Å	Favored (87.527%) alpha helix	-	-	-
A 164		VAL	0.66	-	Favored (84.25%) Ile or Val / -65.6,-48.0	Favored (64.8%) *t* chi angles: 171.5	0.05Å	Favored (86.939%) alpha helix	-	-	-
A 165		LEU	0.66	-	Favored (89.52%) General / -63.5,-38.0	Favored (85.2%) *mt* chi angles: 291.9,176	0.06Å	Favored (85.398%) alpha helix	-	-	-
A 166		ASP	0.67	-	Favored (90.17%) General / -65.3,-38.4	Favored (98.4%) *m-30* chi angles: 287,347.5	0.03Å	Favored (97.661%) alpha helix	-	-	-
A 167		THR	0.67	-	Favored (90.45%) General / -61.6,-46.3	Favored (95.9%) *m* chi angles: 299.6	0.06Å	Favored (89.887%) alpha helix	-	-	-

A 168		VAL	0.67	-	Favored (96.52%) Ile or Val / -61.4,-43.4	Favored (46.9%) *t* chi angles: 169	0.04Å	Favored (80.71%) alpha helix	-	-	-
A 169		GLU	0.67	-	Favored (74.2%) General / -55.0,-44.1	Favored (56.3%) *tt0* chi angles: 180,176.8,326	0.04Å	Favored (79.209%) alpha helix	-	-	-
A 170		LYS	0.68	-	Favored (90.48%) General / -61.8,-39.1	Favored (97.4%) *mttt* chi angles: 290.4,178,181.9,178.6	0.04Å	Favored (80.358%) alpha helix	-	-	-
A 171		TRP	0.68	-	Favored (64.74%) General / -73.7,-38.9	Favored (28%) *m100* chi angles: 281,71.6	0.02Å	Favored (82.614%) alpha helix	-	-	-
A 172		LEU	0.69	-	Favored (74.52%) General / -65.3,-32.7	Favored (80.9%) *mt* chi angles: 292.5,178.1	0.03Å	Favored (65.532%) three-ten	-	-	-
A 173		SER	0.69	-	Favored (44.73%) General / -62.9,-14.3	Favored (60.7%) *p* chi angles: 73.1	0.04Å	Favored (53.616%)	-	-	-
A 174		CYS	0.7	-	Favored (51.14%) General / -77.8,-5.6	Favored (79.8%) *m* chi angles: 296.1	0.06Å	CaBLAM Disfavored (4.222%)	-	-	-
A 175		GLY	0.7	-	Favored (3.06%) Glycine / 106.2,34.9	-	-	Favored (5.297%) beta sheet	-	-	-
A 176		VAL	0.69	-	Favored (37.93%) Ile or Val / -78.5,129.1	Favored (86.2%) *t* chi angles: 177.3	0.06Å	Favored (22.12%)	-	-	-
A 177		GLU	0.68	-	Favored (60.94%) General / -74.9,-40.2	Favored (67%) *tp30* chi angles: 184.6,67.2,18.4	0.02Å	Favored (27.272%)	-	-	-
A 178		SER	0.66	-	Favored (39.87%) General / -141.4,160.6	Favored (95.4%) *p* chi angles: 63.9	0.01Å	Favored (9.3%)	-	-	-
A 179		PHE	0.65	-	Favored (31.24%) General / -161.1,163.9	Favored (36.4%) *p90* chi angles: 53.8,84.3	0.03Å	Favored (57.215%)	-	-	-
A 180		CYS	0.64	-	Favored (4.32%) General / -150.8,111.8	Favored (47.4%) *t* chi angles: 184.9	0.12Å	Favored (19.523%) beta sheet	-	-	-
**#**	**Alt**	**Res**	**High B**	**Clash > 0.4Å**	**Ramachandran**	**Rotamer**	**Cβ deviation**	**CaBLAM**	**Bond lengths**	**Bond angles**	**Cis Peptides**
			Avg: 0.92	Clashscore: 1.51	Outliers: 4 of 903	Poor rotamers: 0 of 780	Outliers: 0 of 837	Outliers: 18 of 901	Outliers: 11 of 905	Outliers: 16 of 905	Non-Trans: 0 of 904
A 181		VAL	0.63	-	Favored (70.09%) Ile or Val / -120.8,123.4	Favored (65.9%) *t* chi angles: 179.2	0.11Å	Favored (56.744%) beta sheet	-	-	-
A 182		LYS	0.63	-	Favored (45.8%) General / -69.5,132.2	Favored (31.5%) *ttpt* chi angles: 186.6,172.7,74.1,187.5	0.04Å	Favored (41.6%) beta sheet	-	-	-
A 183		VAL	0.63	-	Favored (72.12%) Ile or Val / -114.6,126.3	Favored (57.2%) *t* chi angles: 180.3	0.14Å	Favored (72.557%)	-	-	-

A 184		LEU	0.64	-	Favored (20.62%) General / -86.9,-31.7	Favored (73.4%) *mt* chi angles: 297.9,169.9	0.10Å	Favored (27.497%)	-	-	-
A 185		ALA	0.67	-	Favored (16.24%) Pre-Pro / -143.9,68.7	-	0.03Å	Favored (14.837%)	-	-	-
A 186		PRO	0.7	-	Favored (52.06%) Trans-Pro / -65.8,-16.1	Favored (48.9%) *Cg_endo* chi angles: 25.1,325.9,28.2	0.05Å	Favored (37.763%)	-	-	-
A 187		TYR	0.73	-	Favored (58.78%) General / -84.7,-8.7	Favored (53.8%) *p90* chi angles: 60.3,88.1	0.07Å	Favored (41.688%)	-	-	-
A 188		MET	0.76	-	Favored (76.59%) Pre-Pro / -77.8,129.4	Favored (94.8%) *mmm* chi angles: 296.5,295.8,287.9	0.03Å	Favored (32.173%)	-	-	-
A 189		PRO	0.79	-	Favored (19.51%) Trans-Pro / -48.3,-32.3	Favored (88%) *Cg_exo* chi angles: 330.1,36.9,332.1	0.05Å	Favored (71.657%)	-	-	-
A 190		ASP	0.8	-	Favored (59.48%) General / -76.1,-33.5	Favored (81.6%) *m-30* chi angles: 295,346.2	0.03Å	Favored (82.355%) alpha helix	-	-	-
A 191		VAL	0.8	-	Favored (43.01%) Ile or Val / -75.4,-43.1	Favored (88%) *t* chi angles: 174	0.05Å	Favored (66.59%) alpha helix	-	-	-
A 192		LEU	0.79	-	Favored (77.65%) General / -57.0,-48.8	Favored (65.4%) *tp* chi angles: 180.3,61.9	0.03Å	Favored (87.257%) alpha helix	-	-	-
A 193		GLU	0.77	-	Favored (92.44%) General / -59.3,-43.6	Favored (92.5%) *tt0* chi angles: 182.4,178.7,3.5	0.04Å	Favored (97.846%) alpha helix	-	-	-
A 194		LYS	0.75	-	Favored (98.32%) General / -61.9,-41.8	Favored (9.9%) *ttmm* chi angles: 189.4,168.3,292.2,288.8	0.07Å	Favored (97.364%) alpha helix	-	-	-
A 195		LEU	0.73	-	Favored (96.7%) General / -62.5,-40.4	Favored (85.1%) *mt* chi angles: 290.7,167.8	0.04Å	Favored (96.094%) alpha helix	-	-	-
A 196		GLU	0.71	-	Favored (97.65%) General / -61.0,-44.2	Favored (83%) *tt0* chi angles: 178.6,178.1,347.7	0.03Å	Favored (97.375%) alpha helix	-	-	-
A 197		LEU	0.7	-	Favored (93.06%) General / -64.3,-39.0	Favored (95.6%) *mt* chi angles: 292,171.8	0.03Å	Favored (95.386%) alpha helix	-	-	-
A 198		LEU	0.69	-	Favored (92.36%) General / -65.2,-39.1	Favored (91.8%) *mt* chi angles: 291.1,172.7	0.06Å	Favored (97.346%) alpha helix	-	-	-
A 199		GLN	0.69	-	Favored (86.12%) General / -61.8,-47.2	Favored (46.8%) *tt0* chi angles: 177.9,175,63.8	0.02Å	Favored (88.408%) alpha helix	-	-	-
A 200		ARG	0.68	-	Favored (72.43%) General / -59.8,-34.0	Favored (97.3%) *mtt-85* chi angles: 288.4,178.5,181,275.5	0.03Å	Favored (64.17%) alpha helix	-	-	-
**#**	**Alt**	**Res**	**High B**	**Clash > 0.4Å**	**Ramachandran**	**Rotamer**	**Cβ deviation**	**CaBLAM**	**Bond lengths**	**Bond angles**	**Cis Peptides**

			Avg: 0.92	Clashscore: 1.51	Outliers: 4 of 903	Poor rotamers: 0 of 780	Outliers: 0 of 837	Outliers: 18 of 901	Outliers: 11 of 905	Outliers: 16 of 905	Non-Trans: 0 of 904
A 201		ARG	0.67	-	Favored (15.84%) General / -92.6,-30.1	Favored (82.5%) *mtm180* chi angles: 293.7,176.5,291.7,162.9	0.04Å	Favored (56.492%) alpha helix	-	-	-
A 202		PHE	0.66	-	Favored (11.95%) General / -115.0,-12.9	Favored (79%) *m-80* chi angles: 300.7,106.2	0.06Å	Favored (36.987%)	-	-	-
A 203		GLY	0.65	-	Favored (70.61%) Glycine / 79.5,19.8	-	-	Favored (78.689%)	-	-	-
A 204		GLY	0.63	-	Favored (29.32%) Glycine / -99.6,-164.4	-	-	Favored (42.449%)	-	-	-
A 205		THR	0.62	-	Favored (5.8%) General / -145.9,-177.8	Favored (11.9%) *t* chi angles: 190.3	0.07Å	Favored (24.899%)	-	-	-
A 206		VAL	0.62	-	Favored (38.58%) Ile or Val / -119.0,112.6	Favored (55.1%) *t* chi angles: 180.6	0.12Å	Favored (13.026%)	-	-	-
A 207		ILE	0.63	-	Favored (70.03%) Ile or Val / -117.0,131.3	Favored (42.2%) *mm* chi angles: 303.5,296.6	0.06Å	Favored (57.74%) beta sheet	-	-	-
A 208		ARG	0.64	-	Favored (34.98%) General / -91.6,123.4	Favored (56.8%) *ttt90* chi angles: 183.6,170.6,170.7,87.2	0.07Å	Favored (48.681%) beta sheet	-	-	-
A 209		ASN	0.66	-	Favored (82.68%) Pre-Pro / -72.4,134.1	Favored (19.7%) *t0* chi angles: 193.6,286.2	0.03Å	Favored (38.943%)	-	-	-
A 210		PRO	0.68	-	Favored (28.84%) Trans-Pro / -54.7,-22.4	Favored (99.9%) *Cg_exo* chi angles: 332.5,35.1,332.2	0.04Å	Favored (74.678%)	-	-	-
A 211		LEU	0.69	-	Favored (59.66%) General / -82.2,-9.0	Favored (95.6%) *mt* chi angles: 294.9,173.3	0.06Å	Favored (49.566%)	-	-	-
A 212		SER	0.71	-	Favored (57.39%) General / -67.2,140.7	Favored (69.1%) *m* chi angles: 294.8	0.04Å	Favored (42.505%)	-	-	-
A 213		ARG	0.72	-	Favored (34.3%) General / -75.3,157.4	Favored (37.9%) *ptt180* chi angles: 66.3,189.1,183.6,193	0.06Å	Favored (45.77%)	-	OUTLIER(S) worst is NE-CZ-NH2: 5.3 σ	-
A 214		ASN	0.72	-	Favored (19.25%) General / -89.2,11.3	Favored (90.6%) *m-40* chi angles: 294.1,325.6	0.07Å	CaBLAM Disfavored (4.646%)	-	-	-
A 215		SER	0.72	-	Favored (40.68%) General / -89.5,-12.2	Favored (87.6%) *p* chi angles: 67.1	0.09Å	Favored (56.472%)	-	-	-
A 216		THR	0.71	-	Favored (31.96%) General / -133.6,126.9	Favored (62.6%) *m* chi angles: 303.4	0.11Å	Favored (19.609%)	-	-	-
A 217		HIS	0.69	-	Favored (9.31%) General / -85.5,65.7	Favored (40.5%) *t-90* chi angles: 195.1,297	0.13Å	Favored (20.355%)	-	-	-

#	Alt	Res	High B	Clash > 0.4Å	Ramachandran	Rotamer	Cβ deviation	CaBLAM	Bond lengths	Bond angles	Cis Peptides
A 218		GLU	0.66	-	Favored (22.85%) General / -96.9,148.5	Favored (13.3%) *pt0* chi angles: 62.2,177.1,31.7	0.03Å	Favored (13.531%) beta sheet	-	-	-
A 219		MET	0.65	-	Favored (36.82%) General / -143.9,162.2	Favored (73.8%) *mtp* chi angles: 302.1,184.4,77.5	0.03Å	Favored (48.975%) beta sheet	-	-	-
A 220		TYR	0.64	-	Favored (55%) General / -115.0,134.7	Favored (73%) *m-80* chi angles: 291.6,80.3	0.04Å	Favored (40.837%) beta sheet	-	-	-
**#**	**Alt**	**Res**	**High B**	**Clash > 0.4Å**	**Ramachandran**	**Rotamer**	**Cβ deviation**	**CaBLAM**	**Bond lengths**	**Bond angles**	**Cis Peptides**
			Avg: 0.92	Clashscore: 1.51	Outliers: 4 of 903	Poor rotamers: 0 of 780	Outliers: 0 of 837	Outliers: 18 of 901	Outliers: 11 of 905	Outliers: 16 of 905	Non-Trans: 0 of 904
A 221		TYR	0.65	-	Favored (24.97%) General / -87.5,115.5	Favored (51.6%) *t80* chi angles: 175,63.3	0.13Å	Favored (58.428%) beta sheet	-	-	-
A 222		VAL	0.67	-	Favored (31.34%) Ile or Val / -126.5,147.5	Favored (27.9%) *m* chi angles: 296.1	0.04Å	Favored (58.583%)	-	-	-
A 223		SER	0.7	-	Favored (56.73%) General / -83.1,-12.9	Favored (98.4%) *p* chi angles: 65.7	0.04Å	Favored (34.687%)	-	-	-
A 224		GLY	0.73	-	Favored (82.05%) Glycine / -87.1,7.1	-	-	Favored (22.92%)	-	-	-
A 225		ALA	0.76	-	Favored (35.61%) General / -151.8,154.1	-	0.04Å	Favored (29.496%)	-	-	-
A 226		ARG	0.77	-	Favored (46.01%) General / -132.9,136.2	Favored (97.7%) *mtt180* chi angles: 294.2,178.2,183.5,169.9	0.01Å	Favored (23.055%)	-	-	-
A 227		SER	0.78	-	Favored (26.42%) General / -162.6,165.0	Favored (89.5%) *p* chi angles: 66.8	0.03Å	Favored (10.278%) beta sheet	-	-	-
A 228		ASN	0.78	-	Favored (16.9%) General / -68.5,121.7	Favored (26.9%) *t0* chi angles: 183.8,276.3	0.04Å	Favored (14.545%)	-	-	-
A 229		ILE	0.77	-	Favored (90.09%) Ile or Val / -66.5,-44.8	Favored (96%) *mt* chi angles: 292.6,170.2	0.13Å	Favored (45.233%)	-	-	-
A 230		THR	0.76	-	Favored (85.61%) General / -58.2,-46.6	Favored (88.2%) *m* chi angles: 298.5	0.01Å	Favored (87.81%) alpha helix	-	-	-
A 231		PHE	0.75	-	Favored (75.9%) General / -59.6,-49.9	Favored (91.5%) *t80* chi angles: 177,79.8	0.01Å	Favored (84.205%) alpha helix	-	-	-
A 232		THR	0.74	-	Favored (89.14%) General / -58.4,-44.4	Favored (91.1%) *m* chi angles: 301.1	0.09Å	Favored (82.673%) alpha helix	-	-	-
A 233		VAL	0.73	-	Favored (94.65%) Ile or Val / -65.4,-44.3	Favored (70.8%) *t* chi angles: 172.2	0.04Å	Favored (95.743%) alpha helix	-	-	-

#	Alt	Res	High B	Clash > 0.4Å	Ramachandran	Rotamer	Cβ deviation	CaBLAM	Bond lengths	Bond angles	Cis Peptides
A 234		ASN	0.73	-	Favored (93.28%) General / -64.1,-39.1	Favored (92.1%) *m-40* chi angles: 285.4,340	0.04Å	Favored (98.786%) alpha helix	-	-	-
A 235		GLN	0.73	-	Favored (83.73%) General / -67.0,-37.2	Favored (94.7%) *mm-40* chi angles: 290.8,297.6,312.2	0.01Å	Favored (99.167%) alpha helix	-	-	-
A 236		THR	0.73	-	Favored (97.23%) General / -63.5,-43.4	Favored (90.8%) *m* chi angles: 298.9	0.04Å	Favored (95.354%) alpha helix	-	-	-
A 237		SER	0.74	-	Favored (96.88%) General / -61.5,-41.1	Favored (63.2%) *m* chi angles: 297.8	0.06Å	Favored (93.539%) alpha helix	-	-	-
A 238		ARG	0.75	-	Favored (94.62%) General / -63.2,-39.4	Favored (97.4%) *mtt-85* chi angles: 289.3,181.9,184.7,277.6	0.03Å	Favored (94.509%) alpha helix	-	-	-
A 239		LEU	0.75	-	Favored (93.58%) General / -64.8,-39.4	Favored (94%) *mt* chi angles: 291.8,173.2	0.01Å	Favored (98.234%) alpha helix	-	-	-
A 240		LEU	0.76	-	Favored (87.19%) General / -65.4,-37.5	Favored (90%) *mt* chi angles: 290.8,172.2	0.07Å	Favored (92.487%) alpha helix	-	-	-
**#**	**Alt**	**Res**	**High B**	**Clash > 0.4Å**	**Ramachandran**	**Rotamer**	**Cβ deviation**	**CaBLAM**	**Bond lengths**	**Bond angles**	**Cis Peptides**
			Avg: 0.92	Clashscore: 1.51	Outliers: 4 of 903	Poor rotamers: 0 of 780	Outliers: 0 of 837	Outliers: 18 of 901	Outliers: 11 of 905	Outliers: 16 of 905	Non-Trans: 0 of 904
A 241		MET	0.78	-	Favored (90.42%) General / -63.4,-38.2	Favored (68.6%) *mtt* chi angles: 289.5,177.6,179.3	0.05Å	Favored (85.478%) alpha helix	-	-	-
A 242		ARG	0.82	-	Favored (92.73%) General / -62.5,-39.2	Favored (97.7%) *mtt180* chi angles: 289.4,177.5,182.8,172.2	0.03Å	Favored (86.196%) alpha helix	-	-	-
A 243		ARG	0.88	-	Favored (78.11%) General / -63.7,-34.6	Favored (98%) *mtt180* chi angles: 291,178.7,184.4,178.3	0.05Å	Favored (74.191%) alpha helix	-	-	-
A 244		MET	0.98	-	Favored (55.82%) General / -65.7,-12.7	Favored (21.4%) *mmt* chi angles: 289.9,295.8,160	0.11Å	Favored (52.681%) alpha helix	-	-	-
A 245		ARG	1.11	-	Favored (33.47%) General / -104.0,3.4	Favored (95.6%) *mtt180* chi angles: 298.7,173.9,184,178.4	0.07Å	Favored (67.603%) alpha helix	-	-	-
A 246		ARG	1.26	-	Favored (63.51%) Pre-Pro / -136.0,68.2	Favored (92.4%) *mmt-90* chi angles: 295.6,290.7,182.4,270.4	0.06Å	Favored (6.763%) alpha helix	-	-	-
A 247		PRO	1.38	-	Favored (43.28%) Trans-Pro / -61.6,-15.7	Favored (39%) *Cg_endo* chi angles: 23.2,326.4,29.6	0.04Å	Favored (8.552%)	-	-	-
A 248		THR	1.43	-	Favored (26.24%) General / -109.0,10.6	Favored (74.5%) *p* chi angles: 59.9	0.07Å	Favored (21.925%)	-	-	-

A 249		GLY	1.38	-	Favored (45.29%) Glycine / -56.5,128.0	-	-	Favored (36.847%)	-	-	-
A 250		LYS	1.25	-	Favored (56.64%) General / -58.7,134.3	Favored (87.4%) *tttt* chi angles: 182.7,176.3,179,180	0.01Å	Favored (34.626%) beta sheet	-	-	-
A 251		VAL	1.1	-	Favored (33.07%) Ile or Val / -81.6,131.8	Favored (81%) *t* chi angles: 173.2	0.04Å	Favored (46.741%) beta sheet	-	-	-
A 252		THR	0.95	-	Favored (40.98%) General / -96.0,132.7	Favored (83.6%) *m* chi angles: 296.6	0.04Å	Favored (50.772%) beta sheet	-	-	-
A 253		LEU	0.85	-	Favored (31.45%) General / -106.1,145.4	Favored (89.5%) *mt* chi angles: 299.5,176.9	0.03Å	Favored (59.33%) beta sheet	-	-	-
A 254		GLU	0.78	-	Favored (48.73%) General / -121.2,144.2	Favored (98.8%) *mt-10* chi angles: 293,180.2,358.8	0.03Å	Favored (49.272%)	-	-	-
A 255		ALA	0.74	-	Favored (48.26%) General / -70.4,148.5	-	0.03Å	Favored (31.826%)	-	-	-
A 256		ASP	0.72	-	Favored (36.36%) General / -54.0,138.6	Favored (19%) *t70* chi angles: 191.1,80	0.05Å	Favored (28.717%)	-	-	-
A 257		VAL	0.72	-	Favored (19.38%) Ile or Val / -59.3,127.9	Favored (93.6%) *t* chi angles: 174.6	0.03Å	Favored (36.109%)	-	-	-
A 258		ILE	0.73	-	Favored (45.81%) Ile or Val / -93.4,124.2	Favored (50.6%) *mm* chi angles: 302.7,301.5	0.05Å	Favored (61.367%) beta sheet	-	-	-
A 259		LEU	0.74	-	Favored (29.8%) Pre-Pro / -108.5,144.6	Favored (80.5%) *mt* chi angles: 301,175.8	0.05Å	Favored (43.413%) beta sheet	-	-	-
A 260		PRO	0.76	-	Favored (26.66%) Trans-Pro / -63.3,162.5	Favored (34.4%) *Cg_exo* chi angles: 339.5,31.7,330.6	0.05Å	Favored (76.928%)	-	-	-
**#**	**Alt**	**Res**	**High B**	**Clash > 0.4Å**	**Ramachandran**	**Rotamer**	**Cβ deviation**	**CaBLAM**	**Bond lengths**	**Bond angles**	**Cis Peptides**
			Avg: 0.92	Clashscore: 1.51	Outliers: 4 of 903	Poor rotamers: 0 of 780	Outliers: 0 of 837	Outliers: 18 of 901	Outliers: 11 of 905	Outliers: 16 of 905	Non-Trans: 0 of 904
A 261		ILE	0.77	-	Favored (3.07%) Ile or Val / -117.0,175.6	Favored (45.7%) *pt* chi angles: 63.1,170.9	0.08Å	CaBLAM Disfavored (2.394%)	-	-	-
A 262		GLY	0.8	0.64Å H with A 117 GLN HE22	Favored (16.04%) Glycine / 103.9,-145.1	-	-	Favored (34.835%)	-	-	-
A 263		THR	0.85	-	Favored (16.42%) General / -128.8,168.1	Favored (70.8%) *p* chi angles: 62.2	0.09Å	Favored (7.273%) beta sheet	-	-	-

A 264		ARG	0.93	-	Favored (46.39%) General / -129.6,131.0	Favored (17.3%) *tpp-160* chi angles: 178,72.8,78.3,193.7	0.02Å	Favored (34.826%) beta sheet	-	-	-
A 265		SER	1.04	-	Favored (30.15%) General / -77.6,157.6	Favored (85%) *p* chi angles: 67.7	0.06Å	Favored (31.351%) beta sheet	-	-	-
A 266		VAL	1.18	-	Favored (59.56%) Ile or Val / -132.3,129.5	Favored (42.5%) *t* chi angles: 182.9	0.03Å	Favored (60.76%) beta sheet	-	-	-
A 267		GLU	1.33	-	Favored (78.67%) General / -65.3,-34.6	Favored (99.4%) *mt-10* chi angles: 291.4,178.4,352.5	0.00Å	CaBLAM Disfavored (1.316%)	-	-	-
A 268		THR	1.46	0.44Å OG1 with A 269 ASP N	OUTLIER (0.03)%) General / 28.4,-113.5	Favored (72.5%) *p* chi angles: 61.8	0.06Å	CA Geom Outlier (0.232%)	-	-	-
A 269		ASP	1.54	0.44Å N with A 268 THR OG1	Allowed (1.8%) General / -156.5,98.2	Favored (66%) *t0* chi angles: 184.6,353.7	0.06Å	CaBLAM Outlier (0.172%)	-	OUTLIER(S) worst is CA-CB-CG: 4.2 σ	-
A 270		LYS	1.53	-	Favored (19.6%) General / -87.0,156.8	Favored (58.5%) *mttm* chi angles: 298.7,184.5,187.7,291	0.02Å	Favored (23.178%)	-	-	-
A 271		GLY	1.47	-	Favored (52.51%) Glycine / -82.3,-178.7	-	-	Favored (50.861%)	-	-	-
A 272		PRO	1.37	-	Favored (58.99%) Trans-Pro / -71.5,155.9	Favored (66.2%) *Cg_endo* chi angles: 26.9,325.9,26.9	0.02Å	Favored (45.825%)	-	-	-
A 273		LEU	1.27	-	Favored (18.49%) General / -134.7,119.6	Favored (60.8%) *tp* chi angles: 179.7,64	0.06Å	Favored (34.347%) beta sheet	-	-	-
A 274		ASP	1.18	-	Favored (9.3%) General / -83.2,79.7	Favored (50.4%) *t0* chi angles: 187.3,3.4	0.02Å	Favored (57.752%) beta sheet	-	-	-
A 275		ARG	1.11	-	Favored (71.22%) General / -57.2,-36.7	Favored (97.6%) *mtt-85* chi angles: 288.5,180.5,182.8,275.8	0.04Å	Favored (42.452%)	-	-	-
A 276		ALA	1.04	-	Favored (68.75%) General / -59.3,-31.1	-	0.03Å	Favored (69.563%) alpha helix	-	-	-
A 277		ALA	0.97	-	Favored (55.35%) General / -82.1,-14.7	-	0.04Å	Favored (58.088%) alpha helix	-	-	-
A 278		ILE	0.91	-	Favored (11.51%) Ile or Val / -118.7,-9.9	Favored (39.7%) *pt* chi angles: 63.5,167.9	0.07Å	Favored (27.802%) alpha helix	-	-	-
A 279		GLU	0.85	-	Favored (68.31%) General / -53.1,-45.6	Favored (92.7%) *tt0* chi angles: 181.7,178.7,1.9	0.02Å	Favored (56.019%) alpha helix	-	-	-
A 280		GLU	0.8	-	Favored (83.86%) General / -58.6,-40.6	Favored (76.4%) *tt0* chi angles: 186.3,174.7,15.8	0.02Å	Favored (77.998%) alpha helix	-	-	-
**#**	**Alt**	**Res**	**High B**	**Clash > 0.4Å**	**Ramachandran**	**Rotamer**	**Cβ deviation**	**CaBLAM**	**Bond lengths**	**Bond angles**	**Cis Peptides**

			Avg: 0.92	Clashscore: 1.51	Outliers: 4 of 903	Poor rotamers: 0 of 780	Outliers: 0 of 837	Outliers: 18 of 901	Outliers: 11 of 905	Outliers: 16 of 905	Non-Trans: 0 of 904
A 281		ARG	0.77	-	Favored (72.04%) General / -71.0,-40.3	Favored (97.3%) *mtm-85* chi angles: 291.8,192.6,291,277.7	0.06Å	Favored (89.257%) alpha helix	-	-	-
A 282		VAL	0.74	-	Favored (91.25%) Ile or Val / -66.0,-42.2	Favored (73.4%) *t* chi angles: 172.5	0.06Å	Favored (96.698%) alpha helix	-	-	-
A 283		GLU	0.72	-	Favored (81.56%) General / -64.0,-35.7	Favored (62.1%) *mm-30* chi angles: 289.9,292.2,305.1	0.03Å	Favored (83.95%) alpha helix	-	-	-
A 284		ARG	0.72	-	Favored (99.68%) General / -63.1,-42.6	Favored (25.5%) *tpt-90* chi angles: 177.6,64.6,182.2,274.4	0.06Å	Favored (84.352%) alpha helix	-	-	-
A 285		ILE	0.71	-	Favored (91.08%) Ile or Val / -64.5,-47.0	Favored (94.8%) *mt* chi angles: 294.1,166.9	0.05Å	Favored (93.288%) alpha helix	-	-	-
A 286		LYS	0.71	0.43Å O with A 290 THR N	Favored (78.32%) General / -56.4,-42.6	Favored (37%) *ttpt* chi angles: 175.7,176.5,66,173.9	0.05Å	Favored (88.975%) alpha helix	-	-	-
A 287		SER	0.72	-	Favored (98.84%) General / -61.4,-43.5	Favored (72%) *m* chi angles: 295.9	0.05Å	Favored (90.869%) alpha helix	-	-	-
A 288		GLU	0.73	-	Favored (80.42%) General / -61.7,-36.3	Favored (34.2%) *mm-30* chi angles: 292.1,285.2,305.1	0.07Å	Favored (56.924%) alpha helix	-	-	-
A 289		TYR	0.74	-	Favored (17%) General / -114.0,18.8	Favored (44.6%) *m-80* chi angles: 301.1,121.3	0.04Å	Favored (16.512%) alpha helix	-	-	-
A 290		THR	0.76	0.43Å N with A 286 LYS O	Favored (11.94%) General / -45.4,-44.3	Favored (89.2%) *m* chi angles: 297.1	0.03Å	Favored (33.313%) alpha helix	-	-	-
A 291		ALA	0.79	-	Favored (63.28%) General / -56.8,-29.9	-	0.04Å	Favored (41.057%) alpha helix	-	-	-
A 292		THR	0.81	-	Favored (9.17%) General / -123.8,21.2	Favored (47.5%) *p* chi angles: 55.7	0.08Å	Favored (15.866%)	-	-	-
A 293		TRP	0.85	-	Favored (35.22%) General / -82.4,132.1	Favored (71.4%) *t-100* chi angles: 181.8,259.8	0.03Å	Favored (7.92%)	-	-	-
A 294		PHE	0.89	-	Favored (19.63%) General / -149.8,170.1	Favored (41%) *p90* chi angles: 67.1,99.4	0.03Å	Favored (36.702%) beta sheet	-	-	-
A 295		HIS	0.93	-	Favored (26.97%) General / -119.6,116.9	Favored (56.3%) *t70* chi angles: 187.8,84.1	0.06Å	Favored (30.917%) beta sheet	-	-	-
A 296		ASP	0.96	-	Favored (20.42%) General / -96.5,107.9	Favored (57%) *t0* chi angles: 188,335.2	0.02Å	Favored (72.075%) beta sheet	-	-	-
A 297		SER	0.97	-	Favored (58.16%)	Favored (98.7%) *p* chi angles: 65.3	0.03Å	Favored (34.549%)	-	-	-

#	Alt	Res	High B	Clash > 0.4Å	Ramachandran	Rotamer	Cβ deviation	CaBLAM	Bond lengths	Bond angles	Cis Peptides
					General / -63.5,-15.5						
A 298		ASP	0.97	-	Favored (8.7%) General / -104.0,26.5	Favored (19.2%) *m-30* chi angles: 281.7,314.2	0.09Å	Favored (8.929%)	-	-	-
A 299		ASN	0.95	-	Favored (11.92%) Pre-Pro / -48.1,134.0	Favored (24.2%) *t0* chi angles: 176.3,271.4	0.11Å	Favored (25.334%)	-	-	-
A 300		PRO	0.92	-	Favored (35.86%) Trans-Pro / -68.4,-12.8	Favored (42.9%) *Cg_endo* chi angles: 24.1,325,30.7	0.15Å	Favored (7.205%) beta sheet	-	-	-
**#**	**Alt**	**Res**	**High B**	**Clash > 0.4Å**	**Ramachandran**	**Rotamer**	**Cβ deviation**	**CaBLAM**	**Bond lengths**	**Bond angles**	**Cis Peptides**
			Avg: 0.92	Clashscore: 1.51	Outliers: 4 of 903	Poor rotamers: 0 of 780	Outliers: 0 of 837	Outliers: 18 of 901	Outliers: 11 of 905	Outliers: 16 of 905	Non-Trans: 0 of 904
A 301		TYR	0.88	-	Favored (29.48%) General / -80.6,150.6	Favored (36.8%) *m-80* chi angles: 301.6,304.9	0.09Å	Favored (27.041%)	-	-	-
A 302		ARG	0.84	-	Allowed (0.63%) General / -90.7,-71.3	Favored (73.5%) *mtm180* chi angles: 291.5,180.8,289.5,161.4	0.03Å	CaBLAM Disfavored (2.646%)	-	-	-
A 303		THR	0.81	0.47Å O with A 303 THR HG22	Favored (54.44%) General / -77.3,-31.3	Favored (67.5%) *m* chi angles: 303.2	0.07Å	Favored (15.328%)	-	-	-
A 304		TRP	0.78	-	Favored (44.45%) General / -73.7,146.4	Favored (41%) *m100* chi angles: 289.2,66.9	0.04Å	Favored (22.885%)	-	-	-
A 305		HIS	0.76	-	Favored (27.74%) General / -84.3,146.4	Favored (80.1%) *m-70* chi angles: 289.5,273.7	0.02Å	Favored (44.439%) beta sheet	-	-	-
A 306		TYR	0.74	-	Favored (50.29%) General / -111.2,136.4	Favored (70.1%) *t80* chi angles: 170.7,73.1	0.10Å	Favored (51.39%)	-	-	-
A 307		CYS	0.73	-	Favored (4.27%) General / -110.3,-41.9	Favored (54.1%) *m* chi angles: 302.8	0.10Å	CaBLAM Disfavored (3.286%)	-	-	-
A 308		GLY	0.73	-	Favored (37.57%) Glycine / 171.3,172.6	-	-	Favored (26.364%)	-	-	-
A 309		SER	0.75	-	Favored (26.53%) General / -137.2,165.3	Favored (53.7%) *m* chi angles: 300.4	0.03Å	Favored (51.421%)	-	-	-
A 310		TYR	0.79	-	Favored (32.61%) General / -139.2,134.3	Favored (27.4%) *t80* chi angles: 162.1,73.3	0.06Å	Favored (31.018%) beta sheet	-	-	-
A 311		VAL	0.85	0.41Å O with A 311 VAL HG13	Favored (24.42%) Ile or Val / -63.9,137.6	Favored (7.9%) *p* chi angles: 67.6	0.02Å	Favored (27.733%)	-	-	-
A 312		THR	0.93	-	Favored (2.78%) General / -142.9,-168.4	Favored (11.4%) *t* chi angles: 189.3	0.07Å	Favored (11.299%)	-	-	-
A 313		ARG	1.01	-	Favored (38.6%)	Favored (83.6%) *ttt180*	0.03Å	Favored (7.454%)	-	-	-

					General / -96.4,135.2	chi angles: 181.4,176.1,178.1,182.2					
A 314		THR	1.08	-	Favored (54.33%) General / -62.5,133.0	Favored (98.2%) *m* chi angles: 300.1	0.02Å	Favored (41.987%)	-	-	-
A 315		SER	1.14	-	Favored (25.38%) General / -147.6,141.0	Favored (39.7%) *t* chi angles: 176.8	0.05Å	Favored (5.391%)	-	-	-
A 316		GLY	1.19	-	Favored (16.36%) Glycine / 120.5,-177.2	-	-	Favored (7.599%)	-	-	-
A 317		SER	1.2	-	Favored (27.23%) General / -149.7,146.5	Favored (45%) *t* chi angles: 179.2	0.08Å	Favored (20.209%)	-	-	-
A 318		ALA	1.19	-	Favored (35.37%) General / -104.5,7.9	-	0.03Å	Favored (10.385%)	-	-	-
A 319		ALA	1.14	-	Allowed (0.91%) General / -137.3,-28.8	-	0.04Å	CaBLAM Disfavored (1.28%)	-	-	-
A 320		SER	1.08	0.43Å OG with A 745 SER O	Allowed (1.64%) General / 54.6,-133.9	Favored (9.8%) *t* chi angles: 191.1	0.03Å	CaBLAM Outlier (0.499%)	-	-	-
**#**	**Alt**	**Res**	**High B**	**Clash > 0.4Å**	**Ramachandran**	**Rotamer**	**Cβ deviation**	**CaBLAM**	**Bond lengths**	**Bond angles**	**Cis Peptides**
			Avg: 0.92	Clashscore: 1.51	Outliers: 4 of 903	Poor rotamers: 0 of 780	Outliers: 0 of 837	Outliers: 18 of 901	Outliers: 11 of 905	Outliers: 16 of 905	Non-Trans: 0 of 904
A 321		MET	1	-	Favored (23.17%) General / -155.4,147.1	Favored (49.5%) *tpp* chi angles: 182.6,58.4,63.7	0.04Å	CaBLAM Disfavored (2.51%)	-	-	-
A 322		ILE	0.91	-	Favored (33.29%) Ile or Val / -89.8,132.9	Favored (49.8%) *mm* chi angles: 303.5,301.8	0.07Å	Favored (34.39%)	-	-	-
A 323		ASN	0.83	-	Favored (4.16%) General / -68.6,110.3	Favored (20.7%) *t0* chi angles: 177.5,326.9	0.06Å	Favored (45.333%)	-	-	-
A 324		GLY	0.77	-	Favored (38.46%) Glycine / -54.9,-53.7	-	-	Favored (39.848%)	-	-	-
A 325		VAL	0.73	-	Favored (95.57%) Ile or Val / -61.0,-47.0	Favored (64.6%) *t* chi angles: 171.4	0.06Å	Favored (74.75%) alpha helix	-	-	-
A 326		ILE	0.7	-	Favored (76.92%) Ile or Val / -71.0,-42.2	Favored (96.1%) *mt* chi angles: 292.9,169.9	0.20Å	Favored (74.849%) alpha helix	-	-	-
A 327		LYS	0.69	-	Favored (66.47%) General / -52.4,-47.0	Favored (84.1%) *tttt* chi angles: 177.9,179.2,178.2,181.1	0.04Å	Favored (85.498%) alpha helix	-	-	-
A 328		ILE	0.7	-	Favored (96.07%) Ile or Val / -60.6,-44.1	Favored (96.2%) *mt* chi angles: 293.2,166.8	0.06Å	Favored (83.295%) alpha helix	-	-	-

A 329		LEU	0.71	-	Favored (27.08%) General / -79.1,0.7	Favored (78.3%) *mt* chi angles: 288.2,169.3	0.05Å	Favored (27.988%)	OUTLIER(S) worst is CB--CG: 4.0 σ	-	-
A 330		THR	0.73	-	Favored (5.11%) General / -120.2,34.7	Favored (38.6%) *p* chi angles: 54.4	0.10Å	Favored (14.561%)	-	-	-
A 331		TYR	0.77	-	Favored (28.8%) Pre-Pro / -45.5,-51.0	Favored (81.4%) *t80* chi angles: 172.3,80.1	0.13Å	Favored (32.974%)	-	-	-
A 332		PRO	0.81	-	Favored (42.71%) Trans-Pro / -56.2,-22.9	Favored (83.8%) *Cg_exo* chi angles: 334.3,35.9,329.2	0.08Å	Favored (41.305%) alpha helix	-	-	-
A 333		TRP	0.86	-	Favored (17.04%) General / -97.0,-22.1	Favored (38.8%) *m-90* chi angles: 293.3,272.1	0.06Å	Favored (37.725%) alpha helix	-	-	-
A 334		ASP	0.91	-	Favored (70.22%) General / -62.3,-29.1	Favored (20.3%) *t70* chi angles: 194.4,63.1	0.02Å	Favored (57.988%) three-ten	-	-	-
A 335		ARG	0.95	-	Favored (57.41%) General / -70.9,-9.3	Favored (84.3%) *mtp85* chi angles: 293.8,178.4,64.2,90.5	0.07Å	Favored (52.308%)	-	-	-
A 336		ILE	0.97	-	Favored (65.46%) Ile or Val / -109.2,124.0	Favored (79.6%) *mt* chi angles: 300.3,170.7	0.05Å	Favored (32.94%)	-	-	-
A 337		GLU	0.97	-	Favored (79.1%) General / -60.8,-36.5	Favored (40.3%) *mm-30* chi angles: 288,287.7,303.8	0.04Å	Favored (50.013%)	-	-	-
A 338		GLU	0.95	-	Favored (68.07%) General / -63.9,-25.3	Favored (77.9%) *mm-30* chi angles: 294.1,297.5,307.5	0.05Å	Favored (67.494%) alpha helix	-	-	-
A 339		VAL	0.91	-	Favored (19.17%) Ile or Val / -81.6,-48.0	Favored (91.9%) *t* chi angles: 174.4	0.10Å	Favored (52.468%) alpha helix	-	-	-
A 340		THR	0.86	-	Favored (60.72%) General / -74.8,-16.8	Favored (61.8%) *p* chi angles: 63.7	0.05Å	Favored (56.231%) alpha helix	-	-	-

#	Alt	Res	High B	Clash > 0.4Å	Ramachandran	Rotamer	Cβ deviation	CaBLAM	Bond lengths	Bond angles	Cis Peptides
			Avg: 0.92	Clashscore: 1.51	Outliers: 4 of 903	Poor rotamers: 0 of 780	Outliers: 0 of 837	Outliers: 18 of 901	Outliers: 11 of 905	Outliers: 16 of 905	Non-Trans: 0 of 904
A 341		ARG	0.82	-	Favored (42.55%) General / -84.6,2.2	Favored (96.7%) *mtt180* chi angles: 290.8,173.7,183.8,182.3	0.04Å	Favored (31.695%)	-	-	-
A 342		MET	0.8	-	Favored (8.6%) General / -61.3,-58.4	Favored (57.5%) *tpp* chi angles: 187.6,66.5,67.8	0.04Å	CaBLAM Outlier (0.118%)	-	-	-
A 343		ALA	0.79	-	Favored (9.61%) General / 45.2,46.0	-	0.07Å	CaBLAM Outlier (0.122%)	-	-	-
A 344		MET	0.79	-	Allowed (0.83%) General / 46.6,-118.4	Favored (15.6%) *mmt* chi angles: 287.2,295.2,155.1	0.14Å	CaBLAM Outlier (0.037%)	-	-	-

A 345		THR	0.8	-	Favored (13.13%) General / -167.1,159.6	Favored (10%) *t* chi angles: 186.1	0.06Å	CaBLAM Outlier (0.672%)	-	-	-
A 346		ASP	0.81	-	Favored (19.25%) General / -82.2,112.5	Favored (59.6%) *t0* chi angles: 185.5,338.1	0.08Å	Favored (8.992%)	-	-	-
A 347		THR	0.83	-	Favored (22.05%) General / -111.0,13.3	Favored (48.8%) *p* chi angles: 56	0.06Å	Favored (16.409%) beta sheet	-	-	-
A 348		THR	0.84	-	Favored (31.17%) Pre-Pro / -78.6,167.9	Favored (52.3%) *p* chi angles: 65.3	0.10Å	Favored (27.393%)	-	-	-
A 349		PRO	0.85	-	Favored (42.18%) Trans-Pro / -50.2,-35.2	Favored (87.9%) *Cg_exo* chi angles: 330.7,36.9,331	0.09Å	Favored (81.669%)	-	-	-
A 350		PHE	0.87	-	Favored (58.5%) General / -65.0,-52.0	Favored (91.1%) *t80* chi angles: 176.5,79.3	0.05Å	Favored (73.445%) alpha helix	-	-	-
A 351		GLY	0.88	-	Favored (95.12%) Glycine / -60.1,-38.9	-	-	Favored (92.032%) alpha helix	-	-	-
A 352		GLN	0.88	-	Favored (82.15%) General / -68.2,-41.1	Favored (87.1%) *mt0* chi angles: 291.3,180.5,39.1	0.07Å	Favored (84.806%) alpha helix	-	-	-
A 353		GLN	0.89	-	Favored (76.54%) General / -69.3,-36.1	Favored (76.7%) *mm-40* chi angles: 285.2,294.3,313.3	0.07Å	Favored (74.774%) alpha helix	-	-	-
A 354		ARG	0.91	-	Favored (62.21%) General / -58.4,-53.1	Favored (83.4%) *ttt180* chi angles: 180,177.5,176.4,184.9	0.02Å	Favored (73.067%) alpha helix	-	-	-
A 355		VAL	0.93	-	Favored (68.96%) Ile or Val / -67.8,-34.7	Favored (8.7%) *p* chi angles: 66.9	0.05Å	Favored (72.168%) alpha helix	-	-	-
A 356		PHE	0.94	-	Favored (68.47%) General / -62.6,-51.1	Favored (34.1%) *t80* chi angles: 190.9,97.2	0.04Å	Favored (65.108%) alpha helix	-	-	-
A 357		LYS	0.96	-	Favored (79.31%) General / -61.0,-49.0	Favored (2.4%) *tmmt* chi angles: 183.7,265,298.3,188	0.04Å	Favored (71.229%) alpha helix	-	-	-
A 358		GLU	0.97	-	Favored (86.17%) General / -65.1,-37.0	Favored (97.6%) *mt-10* chi angles: 289.2,176.7,356.4	0.05Å	Favored (30.975%) alpha helix	-	-	-
A 359		LYS	0.97	-	Favored (2.61%) General / -111.2,-54.4	Favored (71.9%) *mmtt* chi angles: 303.1,297.7,181.3,182.2	0.06Å	Favored (13.626%) alpha helix	-	-	-
A 360		VAL	0.96	-	Favored (76.31%) Ile or Val / -70.3,-39.1	Favored (81.7%) *t* chi angles: 176.7	0.07Å	Favored (74.242%)	-	-	-
**#**	**Alt**	**Res**	**High B**	**Clash > 0.4Å**	**Ramachandran**	**Rotamer**	**Cβ deviation**	**CaBLAM**	**Bond lengths**	**Bond angles**	**Cis Peptides**

			Avg: 0.92	Clashscore: 1.51	Outliers: 4 of 903	Poor rotamers: 0 of 780	Outliers: 0 of 837	Outliers: 18 of 901	Outliers: 11 of 905	Outliers: 16 of 905	Non-Trans: 0 of 904
A 361		ASP	0.95	-	Favored (35.8%) General / -65.5,-10.6	Favored (42.2%) *m-30* chi angles: 276.8,341.7	0.14Å	Favored (44.687%)	-	OUTLIER(S) worst is CA-CB-CG: 4.3 σ	-
A 362		THR	0.93	-	Favored (55.78%) General / -60.2,133.1	Favored (99.8%) *m* chi angles: 300.4	0.04Å	Favored (40.88%)	-	-	-
A 363		ARG	0.89	-	Favored (29.46%) General / -83.6,123.1	Favored (76.2%) *ttt180* chi angles: 182.2,169.1,176.1,173.4	0.06Å	Favored (46.038%) beta sheet	-	-	-
A 364		ALA	0.84	-	Favored (7.23%) General / -80.5,77.7	-	0.04Å	Favored (54.547%) beta sheet	-	-	-
A 365		LYS	0.79	-	Favored (28.71%) General / -51.5,130.9	Favored (86.6%) *tttt* chi angles: 182,177.2,180,180	0.01Å	Favored (33.151%) beta sheet	-	-	-
A 366		ASP	0.73	-	Favored (46.06%) Pre-Pro / -62.2,119.1	Favored (55.1%) *t0* chi angles: 190.2,344.8	0.05Å	Favored (44.544%)	-	-	-
A 367		PRO	0.68	-	Favored (15.33%) Trans-Pro / -56.2,158.4	Favored (68.9%) *Cg_exo* chi angles: 335.4,36.1,327.9	0.04Å	Favored (19.286%)	-	-	-
A 368		PRO	0.64	-	Favored (49.72%) Trans-Pro / -59.2,153.9	Favored (68%) *Cg_exo* chi angles: 335.5,34.1,330.8	0.07Å	Favored (62.648%)	-	-	-
A 369		ALA	0.61	-	Favored (73.83%) General / -57.7,-37.7	-	0.03Å	Favored (63.984%)	-	-	-
A 370		GLY	0.59	-	Favored (28.46%) Glycine / -58.6,-55.9	-	-	Favored (88.54%) alpha helix	-	-	-
A 371		THR	0.58	-	Favored (62.56%) General / -51.4,-45.4	Favored (79.4%) *m* chi angles: 302.6	0.06Å	Favored (76.206%) alpha helix	-	-	-
A 372		ARG	0.57	-	Favored (94.66%) General / -61.8,-40.3	Favored (99%) *mtm-85* chi angles: 287.8,194.2,294.4,267.7	0.06Å	Favored (86.416%) alpha helix	-	-	-
A 373		LYS	0.57	-	Favored (89.05%) General / -65.9,-38.6	Favored (97.1%) *mttt* chi angles: 290.7,179,184.3,179	0.02Å	Favored (96.751%) alpha helix	-	-	-
A 374		ILE	0.57	-	Favored (97.62%) Ile or Val / -63.6,-45.4	Favored (91.6%) *mt* chi angles: 292,166	0.08Å	Favored (97.8%) alpha helix	-	-	-
A 375		MET	0.57	-	Favored (83.87%) General / -62.8,-36.7	Favored (79.3%) *mtm* chi angles: 288.9,189.5,290.7	0.06Å	Favored (90.417%) alpha helix	-	-	-
A 376		LYS	0.57	-	Favored (87.39%) General / -67.0,-40.1	Favored (16.1%) *tppt* chi angles: 184.7,62.6,76.6,183.1	0.02Å	Favored (91.846%) alpha helix	-	-	-

A 377		VAL	0.57	-	Favored (92.97%) Ile or Val / -64.7,-46.2	Favored (64.8%) *t* chi angles: 171.5	0.03Å	Favored (79.562%) alpha helix	-	-	-
A 378		VAL	0.58	-	Favored (91.12%) Ile or Val / -66.4,-43.5	Favored (78.2%) *t* chi angles: 173	0.03Å	Favored (84.101%) alpha helix	-	-	-
A 379		ASN	0.58	-	Favored (92.85%) General / -64.4,-44.0	Favored (41.3%) *t0* chi angles: 190.6,62.5	0.04Å	Favored (99.497%) alpha helix	-	-	-
A 380		ARG	0.59	-	Favored (90.2%) General / -63.2,-38.2	Favored (96.7%) *mtt180* chi angles: 287,177,176.1,166.9	0.03Å	Favored (99.434%) alpha helix	-	-	-
**#**	**Alt**	**Res**	**High B**	**Clash > 0.4Å**	**Ramachandran**	**Rotamer**	**Cβ deviation**	**CaBLAM**	**Bond lengths**	**Bond angles**	**Cis Peptides**
			Avg: 0.92	Clashscore: 1.51	Outliers: 4 of 903	Poor rotamers: 0 of 780	Outliers: 0 of 837	Outliers: 18 of 901	Outliers: 11 of 905	Outliers: 16 of 905	Non-Trans: 0 of 904
A 381		TRP	0.61	-	Favored (83.7%) General / -62.8,-47.4	Favored (90.3%) *t60* chi angles: 178.9,85.5	0.03Å	Favored (85.66%) alpha helix	-	-	-
A 382		LEU	0.63	-	Favored (91.05%) General / -62.2,-46.0	Favored (63.4%) *tp* chi angles: 179.4,58.6	0.06Å	Favored (92.779%) alpha helix	-	-	-
A 383		PHE	0.65	-	Favored (89.08%) General / -63.3,-37.9	Favored (16.5%) *m-80* chi angles: 278.1,302.8	0.11Å	Favored (90.037%) alpha helix	-	-	-
A 384		ARG	0.68	-	Favored (82.64%) General / -63.3,-36.1	Favored (79.1%) *mtm180* chi angles: 286,178.2,288.6,165.4	0.01Å	Favored (96.106%) alpha helix	-	-	-
A 385		HIS	0.73	-	Favored (63.78%) General / -63.5,-51.9	Favored (89.5%) *t70* chi angles: 175.4,73.9	0.02Å	Favored (74.22%) alpha helix	-	-	-
A 386		LEU	0.78	-	Favored (93.62%) General / -62.5,-39.5	Favored (94.8%) *mt* chi angles: 294,171.7	0.11Å	Favored (82.746%) alpha helix	-	-	-
A 387		ALA	0.83	-	Favored (42.63%) General / -79.4,-2.6	-	0.04Å	Favored (37.509%)	-	-	-
A 388		ARG	0.87	-	Favored (81.43%) General / -62.5,-36.1	Favored (84.9%) *ttt180* chi angles: 184.5,175.2,177.3,181	0.06Å	Favored (13.735%)	-	-	-
A 389		GLU	0.89	-	Favored (7.1%) General / -112.9,-30.3	Favored (92.7%) *mt-10* chi angles: 296.5,184.8,357	0.05Å	Favored (13.558%)	-	-	-
A 390		LYS	0.9	-	Favored (40.95%) General / -111.2,144.1	Favored (60.3%) *mttm* chi angles: 293.1,180.3,183.5,285.7	0.07Å	Favored (23.299%)	-	-	-
A 391		ASN	0.88	-	Favored (38.8%) Pre-Pro / -116.9,115.3	Favored (69%) *m-40* chi angles: 296.2,292.3	0.03Å	Favored (48.426%)	-	-	-
A 392		PRO	0.85	-	Favored (73.27%) Trans-Pro / -55.4,144.3	Favored (96.5%) *Cg_exo* chi angles: 332.8,36.3,330	0.08Å	Favored (25.345%) beta sheet	-	-	-

A 393		ARG	0.81	-	Favored (43.74%) General / -149.4,157.9	Favored (51.8%) *ptt90* chi angles: 64.8,185.3,179.1,90.8	0.08Å	Favored (41.606%) beta sheet	-	-	-
A 394		LEU	0.78	-	Favored (35.73%) General / -79.5,142.5	Favored (95.5%) *mt* chi angles: 295.2,173.1	0.01Å	Favored (37.91%) beta sheet	-	-	-
A 395		CYS	0.76	-	Favored (25.06%) General / -85.8,149.7	Favored (70.7%) *m* chi angles: 298.3	0.04Å	Favored (43.071%) beta sheet	-	-	-
A 396		THR	0.74	-	Favored (14.77%) General / -86.9,166.9	Favored (72%) *p* chi angles: 62	0.05Å	Favored (51.304%)	-	-	-
A 397		LYS	0.73	-	Favored (88.36%) General / -58.4,-43.3	Favored (79.2%) *tttt* chi angles: 183.7,181.8,179.9,188.7	0.03Å	Favored (64.953%)	-	-	-
A 398		GLU	0.72	-	Favored (95.23%) General / -63.2,-39.6	Favored (98.5%) *mt-10* chi angles: 290.3,177.9,353.9	0.02Å	Favored (84.223%) alpha helix	-	-	-
A 399		GLU	0.72	-	Favored (93.64%) General / -64.6,-39.4	Favored (31.4%) *mt-10* chi angles: 287.4,165.5,308.8	0.04Å	Favored (97.565%) alpha helix	-	-	-
A 400		PHE	0.73	-	Favored (81.35%) General / -60.6,-48.5	Favored (91.1%) *t80* chi angles: 179,77.2	0.04Å	Favored (80.377%) alpha helix	-	-	-
**#**	**Alt**	**Res**	**High B**	**Clash > 0.4Å**	**Ramachandran**	**Rotamer**	**Cβ deviation**	**CaBLAM**	**Bond lengths**	**Bond angles**	**Cis Peptides**
			Avg: 0.92	Clashscore: 1.51	Outliers: 4 of 903	Poor rotamers: 0 of 780	Outliers: 0 of 837	Outliers: 18 of 901	Outliers: 11 of 905	Outliers: 16 of 905	Non-Trans: 0 of 904
A 401		ILE	0.76	-	Favored (62.42%) Ile or Val / -55.4,-41.4	Favored (86.9%) *mt* chi angles: 290.8,166.8	0.01Å	Favored (75.893%) alpha helix	-	-	-
A 402		ALA	0.82	-	Favored (67.57%) General / -58.6,-30.9	-	0.02Å	Favored (72.24%) alpha helix	-	-	-
A 403		LYS	0.91	-	Favored (26.74%) General / -89.0,-18.8	Favored (70.5%) *mmtt* chi angles: 301.7,302.5,181.5,183.9	0.05Å	Favored (35.871%)	-	-	-
A 404		VAL	1.02	0.54Å O with A 404 VAL HG12	Allowed (0.51%) Ile or Val / -73.3,88.7	Favored (81.3%) *t* chi angles: 176.6	0.05Å	Favored (32.723%)	-	-	-
A 405		ARG	1.14	-	Favored (46.88%) General / -73.5,141.2	Favored (98.9%) *mtt180* chi angles: 293.3,178.9,180.3,176	0.03Å	Favored (22.711%)	-	-	-
A 406		SER	1.24	-	Favored (63.88%) General / -64.2,-17.8	Favored (86.9%) *p* chi angles: 67.1	0.02Å	Favored (38.463%)	-	-	-
A 407		HIS	1.28	-	Favored (46.74%) General / -96.8,7.4	Favored (98.6%) *m-70* chi angles: 296.2,287.6	0.06Å	Favored (32.13%)	-	-	-
A 408		ALA	1.27	-	Favored (56.37%) General / -61.7,143.3	-	0.03Å	Favored (42.729%)	-	-	-

A 409		ALA	1.23	-	Favored (72.25%) General / -60.7,-33.0	-	0.06Å	CaBLAM Disfavored (3.836%) try beta sheet	-	-	-
A 410		ILE	1.18	-	OUTLIER (0.03)%) Ile or Val / 64.3,111.7	Favored (92.3%) *mt* chi angles: 297.3,172	0.03Å	CaBLAM Outlier (0.219%) try beta sheet	-	-	-
A 411		GLY	1.14	-	Favored (48.32%) Glycine / -65.5,145.2	-	-	Favored (25.029%) beta sheet	-	-	-
A 412		ALA	1.12	0.47Å H with A 454 ASN HD22	Allowed (0.57%) General / -142.8,-155.2	-	0.07Å	CaBLAM Outlier (0.542%)	-	-	-
A 413		PHE	1.13	-	Allowed (1.22%) General / 78.5,15.8	Favored (3.8%) *m-10* chi angles: 309.1,342.4	0.06Å	CaBLAM Outlier (0.013%)	-	-	-
A 414		LEU	1.14	-	Favored (47.19%) General / -124.9,127.5	Favored (53.1%) *tp* chi angles: 177.2,66.2	0.05Å	Favored (13.406%)	-	-	-
A 415		GLU	1.13	-	Favored (62.84%) General / -53.5,-37.6	Favored (91.4%) *tt0* chi angles: 183.2,176.3,0.6	0.04Å	Favored (56.078%)	-	-	-
A 416		GLU	1.1	-	Favored (66.33%) General / -63.3,-21.6	Favored (85.4%) *mt-10* chi angles: 289.1,177.6,330.7	0.05Å	Favored (52.317%) alpha helix	-	-	-
A 417		GLN	1.05	0.41Å OE1 with A 480 LEU N	Favored (11.81%) General / -101.3,-29.9	Favored (77.6%) *mm-40* chi angles: 306.7,296.8,298.9	0.07Å	Favored (24.803%) alpha helix	-	OUTLIER(S) worst is CB-CG-CD: 4.6 σ	-
A 418		GLU	0.99	-	Favored (73.92%) General / -61.8,-33.3	Favored (48.8%) *mm-30* chi angles: 287.5,285.2,331.4	0.07Å	Favored (62.706%) three-ten	-	-	-
A 419		GLN	0.93	-	Favored (60.21%) General / -60.4,-20.6	Favored (22.7%) *pt0* chi angles: 70.6,181.9,316	0.04Å	Favored (49.655%)	-	-	-
A 420		TRP	0.87	-	Favored (28.63%) General / -90.5,141.6	Favored (22.8%) *m-10* chi angles: 289.3,322.4	0.07Å	Favored (22.144%)	-	-	-
**#**	**Alt**	**Res**	**High B**	**Clash > 0.4Å**	**Ramachandran**	**Rotamer**	**Cβ deviation**	**CaBLAM**	**Bond lengths**	**Bond angles**	**Cis Peptides**
			Avg: 0.92	Clashscore: 1.51	Outliers: 4 of 903	Poor rotamers: 0 of 780	Outliers: 0 of 837	Outliers: 18 of 901	Outliers: 11 of 905	Outliers: 16 of 905	Non-Trans: 0 of 904
A 421		LYS	0.82	-	Favored (10.97%) General / -84.0,-47.5	Favored (87.2%) *tttt* chi angles: 183.5,177.2,178.9,179.9	0.02Å	Favored (17.593%)	-	-	-
A 422		THR	0.78	-	Favored (40.31%) General / -128.0,157.5	Favored (62.3%) *p* chi angles: 63.6	0.03Å	Favored (14.794%)	-	-	-
A 423		ALA	0.74	-	Favored (71.6%) General / -61.3,-31.8	-	0.07Å	Favored (64.998%) alpha helix	-	-	-
A 424		ASN	0.72	-	Favored (80.48%) General / -66.0,-45.7	Favored (52.7%) *t0* chi angles: 184.6,62.3	0.04Å	Favored (76.084%) alpha helix	-	-	-

A 425		GLU	0.71	-	Favored (76.33%) General / -56.5,-41.3	Favored (48.3%) *tt0* chi angles: 180.6,175.5,55.9	0.04Å	Favored (96.015%) alpha helix	-	-	-
A 426		ALA	0.7	-	Favored (70.14%) General / -63.3,-50.4	-	0.08Å	Favored (91.091%) alpha helix	-	-	-
A 427		VAL	0.71	-	Favored (42.48%) Ile or Val / -63.4,-27.1	Favored (3.3%) *p* chi angles: 74.2	0.10Å	Favored (70.365%) alpha helix	-	-	-
A 428		GLN	0.72	-	Favored (59.24%) General / -86.5,-4.8	Favored (97.1%) *mm-40* chi angles: 298.1,296.5,309	0.04Å	Favored (56.181%)	-	-	-
A 429		ASP	0.73	-	Favored (66.04%) Pre-Pro / -88.1,116.4	Favored (68.1%) *t0* chi angles: 183.5,350.4	0.03Å	Favored (34.025%)	-	-	-
A 430		PRO	0.73	-	Favored (15.97%) Trans-Pro / -48.8,-30.0	Favored (86.7%) *Cg_exo* chi angles: 330.4,37.3,330.9	0.04Å	Favored (84.516%)	-	-	-
A 431		LYS	0.74	-	Favored (87.34%) General / -64.6,-37.4	Favored (54%) *tptt* chi angles: 184.5,67.5,176,181.4	0.03Å	Favored (69.165%) alpha helix	-	-	-
A 432		PHE	0.73	-	Favored (76.85%) General / -58.8,-49.7	Favored (60%) *t80* chi angles: 169,81.6	0.09Å	Favored (83.382%) alpha helix	-	-	-
A 433		TRP	0.73	-	Favored (97.19%) General / -61.2,-41.6	Favored (43.2%) *t-100* chi angles: 190.7,243.7	0.04Å	Favored (87.339%) alpha helix	-	-	-
A 434		GLU	0.72	-	Favored (84.16%) General / -57.5,-46.1	Favored (37.6%) *tt0* chi angles: 183,183.8,75.2	0.07Å	Favored (95.374%) alpha helix	-	-	-
A 435		LEU	0.72	-	Favored (92.6%) General / -64.1,-38.9	Favored (93.9%) *mt* chi angles: 291.9,171.2	0.05Å	Favored (91.495%) alpha helix	-	-	-
A 436		VAL	0.71	-	Favored (97.9%) Ile or Val / -61.8,-43.9	Favored (49%) *t* chi angles: 169.2	0.04Å	Favored (95.935%) alpha helix	-	-	-
A 437		ASP	0.71	-	Favored (84.24%) General / -67.2,-42.4	Favored (26.1%) *t70* chi angles: 191.5,65.8	0.02Å	Favored (98.136%) alpha helix	-	-	-
A 438		GLU	0.71	-	Favored (95.62%) General / -62.4,-44.8	Favored (87.7%) *tt0* chi angles: 186,174.9,6.4	0.04Å	Favored (89.002%) alpha helix	-	-	-
A 439		GLU	0.71	-	Favored (80.4%) General / -66.3,-35.5	Favored (60.8%) *mm-30* chi angles: 291.8,292.3,301.2	0.07Å	Favored (79.207%) alpha helix	-	-	-
A 440		ARG	0.71	-	Favored (87.8%) General / -59.4,-46.8	Favored (51.7%) *ttm110* chi angles: 184.5,180.7,296.7,109.4	0.11Å	Favored (82.581%) alpha helix	-	-	-
**#**	**Alt**	**Res**	**High B**	**Clash > 0.4Å**	**Ramachandran**	**Rotamer**	**Cβ deviation**	**CaBLAM**	**Bond lengths**	**Bond angles**	**Cis Peptides**

			Avg: 0.92	Clashscore: 1.51	Outliers: 4 of 903	Poor rotamers: 0 of 780	Outliers: 0 of 837	Outliers: 18 of 901	Outliers: 11 of 905	Outliers: 16 of 905	Non-Trans: 0 of 904
A 441		ARG	0.72	-	Favored (96.6%) General / -63.0,-40.0	Favored (98.2%) *mtt-85* chi angles: 289.8,175.9,183.7,274.8	0.06Å	Favored (93.237%) alpha helix	-	-	-
A 442		LEU	0.73	-	Favored (93.73%) General / -63.7,-39.2	Favored (89.9%) *mt* chi angles: 290.8,172.1	0.01Å	Favored (94.293%) alpha helix	-	-	-
A 443		HIS	0.75	-	Favored (96.41%) General / -63.9,-40.4	Favored (45.5%) *m90* chi angles: 280,76.9	0.04Å	Favored (90.465%) alpha helix	-	-	-
A 444		GLN	0.77	-	Favored (71.11%) General / -61.5,-31.1	Favored (95.6%) *mt0* chi angles: 291.4,169.8,343.1	0.06Å	Favored (75.62%) alpha helix	-	-	-
A 445		GLN	0.79	-	Favored (58.48%) General / -86.3,-2.5	Favored (91.9%) *mt0* chi angles: 295.4,174.2,344.5	0.08Å	Favored (52.977%)	-	-	-
A 446		GLY	0.8	-	Favored (61.07%) Glycine / 83.9,19.6	-	-	Favored (70.797%)	-	-	-
A 447		ARG	0.8	-	Favored (33.52%) General / -125.7,158.9	Favored (74.4%) *mtm180* chi angles: 297.4,187.8,292.1,171.9	0.02Å	Favored (22.599%)	-	-	-
A 448		CYS	0.79	-	Favored (5.91%) General / -161.4,133.7	Favored (51.1%) *t* chi angles: 180.3	0.04Å	Favored (35.047%) beta sheet	-	-	-
A 449		ARG	0.78	-	Favored (11.17%) General / -104.9,-28.3	Favored (88.6%) *mtt180* chi angles: 294.9,170.4,183.1,159.1	0.06Å	Favored (14.748%)	-	-	-
A 450		THR	0.77	-	Favored (37.59%) General / -103.6,8.0	Favored (74.9%) *p* chi angles: 59.9	0.04Å	Favored (49.039%)	-	-	-
A 451		CYS	0.77	-	Favored (4.52%) General / -80.0,63.5	Favored (66.9%) *m* chi angles: 299.6	0.06Å	CaBLAM Disfavored (4.231%)	-	-	-
A 452		VAL	0.78	-	Favored (66.75%) Ile or Val / -124.4,134.4	Favored (80.9%) *t* chi angles: 176.5	0.03Å	Favored (26.797%)	-	-	-
A 453		TYR	0.79	0.50Å CZ with A 478 MET HE2	Favored (28.96%) General / -98.3,143.3	Favored (84%) *m-80* chi angles: 290,86.2	0.03Å	Favored (46.925%) beta sheet	-	-	-
A 454		ASN	0.82	0.47Å HD22 with A 412 ALA H	Favored (14.21%) General / -101.5,103.3	Favored (22%) *t0* chi angles: 174,353.9	0.05Å	Favored (62.423%) beta sheet	-	-	-
A 455		MET	0.85	-	Favored (35.12%) General / -87.1,127.3	Favored (5.2%) *tmm* chi angles: 186.3,273.2,262.5	0.08Å	Favored (38.756%) beta sheet	-	-	-
A 456		MET	0.89	-	Favored (51.71%) General / -133.5,150.1	Favored (68.7%) *mtm* chi angles: 300.9,182.8,281.7	0.02Å	Favored (40.132%) beta sheet	-	-	-

#	Alt	Res	High B	Clash > 0.4Å	Ramachandran	Rotamer	Cβ deviation	CaBLAM	Bond lengths	Bond angles	Cis Peptides
A 457		GLY	0.93	-	Favored (30.51%) Glycine / -77.2,145.3	-	-	Favored (40.813%) beta sheet	-	-	-
A 458		LYS	0.97	-	Favored (32.2%) General / -86.8,123.7	Favored (30.8%) *ttmt* chi angles: 181.9,183.2,281.1,185.1	0.06Å	Favored (43.983%) beta sheet	-	-	-
A 459		ARG	1.01	-	Favored (10.13%) General / -84.2,76.1	Favored (39.5%) *ttm110* chi angles: 193.3,170,298.7,112.9	0.10Å	Favored (43.129%) beta sheet	-	-	-
A 460		GLU	1.05	-	Favored (27.14%) General / -78.2,161.7	Favored (60.3%) *mt-10* chi angles: 295.9,179.7,301.7	0.10Å	Favored (16.401%) beta sheet	-	-	-
**#**	**Alt**	**Res**	**High B**	**Clash > 0.4Å**	**Ramachandran**	**Rotamer**	**Cβ deviation**	**CaBLAM**	**Bond lengths**	**Bond angles**	**Cis Peptides**
			Avg: 0.92	Clashscore: 1.51	Outliers: 4 of 903	Poor rotamers: 0 of 780	Outliers: 0 of 837	Outliers: 18 of 901	Outliers: 11 of 905	Outliers: 16 of 905	Non-Trans: 0 of 904
A 461		LYS	1.1	-	Favored (12.9%) General / -105.2,103.4	Favored (10.5%) *mtmm* chi angles: 297,178.9,278.5,276.2	0.03Å	Favored (11.561%) beta sheet	-	-	-
A 462		LYS	1.18	-	Favored (49.57%) General / -132.4,143.1	Favored (99.5%) *mttt* chi angles: 294.8,183.8,178.7,177.1	0.02Å	Favored (29.597%) beta sheet	-	-	-
A 463		LEU	1.29	-	Favored (52.37%) General / -59.5,131.9	Favored (96.5%) *mt* chi angles: 292.5,171	0.06Å	Favored (36.606%) beta sheet	-	-	-
A 464		SER	1.43	-	Favored (59.9%) General / -76.8,-14.6	Favored (84.8%) *p* chi angles: 67.4	0.09Å	CaBLAM Disfavored (3.472%)	-	-	-
A 465		GLU	1.58	-	Allowed (0.49%) General / 67.8,-54.7	Favored (85.7%) *tt0* chi angles: 188.9,175.1,2.7	0.07Å	Favored (10.013%)	-	-	-
A 466		PHE	1.71	-	Favored (16.07%) General / -115.1,10.1	Favored (62.3%) *m-80* chi angles: 305.3,106	0.07Å	Favored (5.912%)	-	-	-
A 467		GLY	1.77	-	Favored (57.73%) Glycine / 98.4,-18.0	-	-	Favored (37.412%)	-	-	-
A 468		LYS	1.76	-	Favored (30.1%) General / -94.3,117.4	Favored (13.9%) *tptp* chi angles: 172.2,70.7,186.7,76	0.04Å	Favored (30.578%)	-	-	-
A 469		ALA	1.66	-	Favored (52.24%) General / -57.0,139.3	-	0.04Å	Favored (29.953%)	-	-	-
A 470		LYS	1.51	-	Favored (25.54%) General / -80.8,158.0	Favored (62.8%) *mttm* chi angles: 295,182.1,181,292.8	0.02Å	Favored (23.824%)	-	-	-
A 471		GLY	1.36	-	Favored (47.61%) Glycine / -61.4,140.8	-	-	Favored (27.438%)	-	-	-

#	Alt	Res	High B	Clash > 0.4Å	Ramachandran	Rotamer	Cβ deviation	CaBLAM	Bond lengths	Bond angles	Cis Peptides
A 472		SER	1.22	-	Favored (29.43%) General / -69.4,161.4	Favored (96.4%) *p* chi angles: 63.7	0.09Å	Favored (33.289%)	-	-	-
A 473		ARG	1.11	-	Favored (39.12%) General / -76.8,134.5	Favored (68.8%) *ttt180* chi angles: 180.4,172.5,173.2,166.9	0.07Å	Favored (26.442%)	-	-	-
A 474		ALA	1.03	-	Favored (14.06%) General / -78.0,111.7	-	0.07Å	Favored (52.589%) beta sheet	-	-	-
A 475		ILE	0.97	-	Favored (64.68%) Ile or Val / -108.4,124.3	Favored (43.4%) *mm* chi angles: 306.1,299.8	0.06Å	Favored (67.883%) beta sheet	-	-	-
A 476		TRP	0.92	-	Favored (20.56%) General / -100.3,108.5	Favored (20.2%) *m-10* chi angles: 297.6,10.8	0.10Å	Favored (70.711%) beta sheet	OUTLIER(S) worst is NE1--CE2: 7.7 σ	-	-
A 477		TYR	0.87	-	Favored (39.59%) General / -99.7,136.5	Favored (99.3%) *m-80* chi angles: 296.5,92.2	0.04Å	Favored (46.581%)	-	-	-
A 478		MET	0.83	0.50Å HE2 with A 453 TYR CZ	Favored (22.95%) General / -91.3,147.6	Favored (52%) *tpp* chi angles: 176.4,61.7,80.4	0.12Å	Favored (35.174%)	OUTLIER(S) worst is SD--CE: 5.3 σ	-	-
A 479		TRP	0.8	-	Favored (22.92%) General / -54.1,143.2	Favored (52.5%) *m-10* chi angles: 292.2,355.9	0.14Å	Favored (47.16%)	-	-	-
A 480		LEU	0.76	0.41Å N with A 417 GLN OE1	Favored (82.77%) General / -62.8,-36.3	Favored (97.8%) *mt* chi angles: 292.9,173.2	0.01Å	Favored (64.696%)	-	-	-
**#**	**Alt**	**Res**	**High B**	**Clash > 0.4Å**	**Ramachandran**	**Rotamer**	**Cβ deviation**	**CaBLAM**	**Bond lengths**	**Bond angles**	**Cis Peptides**
			Avg: 0.92	Clashscore: 1.51	Outliers: 4 of 903	Poor rotamers: 0 of 780	Outliers: 0 of 837	Outliers: 18 of 901	Outliers: 11 of 905	Outliers: 16 of 905	Non-Trans: 0 of 904
A 481		GLY	0.74	-	Favored (13.67%) Glycine / -55.6,-58.5	-	-	Favored (84.518%) alpha helix	-	-	-
A 482		ALA	0.71	-	Favored (79.52%) General / -62.7,-35.4	-	0.03Å	Favored (61.07%) alpha helix	-	-	-
A 483		ARG	0.69	-	Favored (82.67%) General / -58.8,-40.0	Favored (63%) *ttt90* chi angles: 185.6,179,180.5,83.2	0.05Å	Favored (71.053%) alpha helix	-	-	-
A 484		TYR	0.68	0.50Å C with A 484 TYR CD2	Favored (72.3%) General / -55.0,-49.1	Favored (53.9%) *t80* chi angles: 178.5,93.1	0.11Å	Favored (85.304%) alpha helix	-	-	-
A 485		LEU	0.66	-	Favored (79.9%) General / -65.6,-35.1	Favored (96.6%) *mt* chi angles: 293.3,171.8	0.11Å	Favored (78.932%) alpha helix	-	-	-
A 486		GLU	0.66	-	Favored (98.29%) General / -62.6,-40.9	Favored (97.4%) *mt-10* chi angles: 289.6,180.3,356.1	0.13Å	Favored (84.362%) alpha helix	-	-	-

#	Alt	Res	High B	Clash > 0.4Å	Ramachandran	Rotamer	Cβ deviation	CaBLAM	Bond lengths	Bond angles	Cis Peptides
A 487		PHE	0.66	-	Favored (66.45%) General / -64.4,-50.8	Favored (86.4%) *t80* chi angles: 180.6,76	0.06Å	Favored (77.508%) alpha helix	-	-	-
A 488		GLU	0.67	-	Favored (66.03%) General / -52.5,-45.2	Favored (93.1%) *tt0* chi angles: 181.6,179.3,0.2	0.01Å	Favored (62.741%) alpha helix	-	-	-
A 489		ALA	0.68	-	Favored (15.36%) General / -85.8,-41.1	-	0.09Å	Favored (38.374%) alpha helix	-	-	-
A 490		LEU	0.71	-	Favored (12.23%) General / -112.0,-19.7	Favored (78.3%) *mt* chi angles: 301.5,175.8	0.04Å	Favored (11.772%) alpha helix	-	-	-
A 491		GLY	0.74	-	Favored (31.23%) Glycine / -56.5,-20.6	-	-	Favored (32.384%) three-ten	-	-	-
A 492		PHE	0.77	-	Favored (63.27%) General / -58.7,-25.9	Favored (38.7%) *p90* chi angles: 72.7,92.1	0.06Å	Favored (59.095%) three-ten	-	-	-
A 493		LEU	0.81	-	Favored (78.55%) General / -62.1,-35.4	Favored (82.9%) *mt* chi angles: 289.5,170.8	0.03Å	Favored (57.06%) three-ten	-	-	-
A 494		ASN	0.83	-	Favored (10%) General / -84.8,-47.8	Favored (96.3%) *m-40* chi angles: 292.9,339.4	0.09Å	Favored (44.46%) alpha helix	-	-	-
A 495		GLU	0.85	-	Favored (85.54%) General / -65.7,-37.1	Favored (63.6%) *tp30* chi angles: 183.3,60.9,14.8	0.05Å	Favored (70.376%) alpha helix	-	-	-
A 496		ASP	0.86	-	Favored (57.97%) General / -88.9,-1.2	Favored (86%) *m-30* chi angles: 293.5,334.9	0.08Å	Favored (51.511%)	-	OUTLIER(S) worst is CA-CB-CG: 7.0 σ	-
A 497		HIS	0.85	-	Favored (6.9%) General / 57.6,53.8	Favored (98.9%) *m-70* chi angles: 301.3,290.3	0.06Å	Favored (18.729%)	-	-	-
A 498		TRP	0.84	-	Favored (67.87%) General / -64.3,-25.1	Favored (24.6%) *m100* chi angles: 281.6,63.2	0.05Å	Favored (35.43%)	-	-	-
A 499		ALA	0.83	-	Favored (9%) General / -103.8,26.1	-	0.08Å	Favored (16.01%)	-	-	-
A 500		SER	0.82	-	Favored (21.77%) General / -79.8,166.4	Favored (91.5%) *p* chi angles: 63.1	0.09Å	Favored (40.025%)	-	-	-
**#**	**Alt**	**Res**	**High B**	**Clash > 0.4Å**	**Ramachandran**	**Rotamer**	**Cβ deviation**	**CaBLAM**	**Bond lengths**	**Bond angles**	**Cis Peptides**
			Avg: 0.92	Clashscore: 1.51	Outliers: 4 of 903	Poor rotamers: 0 of 780	Outliers: 0 of 837	Outliers: 18 of 901	Outliers: 11 of 905	Outliers: 16 of 905	Non-Trans: 0 of 904
A 501		ARG	0.81	-	Favored (83.07%) General / -63.7,-36.2	Favored (83%) *mtp180* chi angles: 290.5,176.5,69.6,195.5	0.08Å	Favored (67.465%)	OUTLIER(S) worst is NE--CZ: 4.1 σ	-	-
A 502		GLU	0.8	-	Favored (89.07%) General / -65.6,-38.3	Favored (98.9%) *mt-10* chi angles: 290.9,179.1,353.9	0.01Å	Favored (50.281%) alpha helix	-	-	-
A 503		ASN	0.78	-	Favored (4.24%)	Favored (33.2%) *t0* chi angles: 193.9,61.1	0.03Å	Favored (28.099%) alpha helix	-	-	-

					General / -96.0,-51.9						
A 504		SER	0.76	-	Favored (32.86%) General / -88.1,-16.7	Favored (86.8%) *p* chi angles: 67.9	0.06Å	Favored (12.887%) alpha helix	-	-	-
A 505		GLY	0.74	-	Favored (4.62%) Glycine / 81.1,-61.1	-	-	CaBLAM Disfavored (3.458%) try alpha helix	-	-	-
A 506		GLY	0.75	-	Favored (61.45%) Glycine / -81.8,-21.5	-	-	Favored (54.36%)	-	-	-
A 507		GLY	0.77	-	Favored (34.48%) Glycine / -83.1,156.8	-	-	Favored (16.7%)	-	-	-
A 508		VAL	0.81	-	Favored (2.4%) Ile or Val / -121.0,30.3	Favored (17.3%) *m* chi angles: 293.1	0.05Å	Favored (8.374%)	-	-	-
A 509		GLU	0.85	-	Favored (67.06%) General / -55.4,-36.9	Favored (45.8%) *tt0* chi angles: 179,173,51.9	0.01Å	Favored (29.642%)	-	-	-
A 510		GLY	0.9	-	Favored (77.92%) Glycine / -91.8,8.8	-	-	Favored (49.682%)	-	-	-
A 511		ILE	0.92	-	Favored (64%) Ile or Val / -109.3,129.5	Favored (36.4%) *mm* chi angles: 308.2,298.5	0.11Å	Favored (33.3%)	-	-	-
A 512		GLY	0.93	-	Favored (53.41%) Glycine / -82.3,174.3	-	-	Favored (57.434%)	-	-	-
A 513		LEU	0.9	-	Favored (79.2%) General / -59.4,-37.9	Favored (92.9%) *mt* chi angles: 292.2,174.4	0.10Å	Favored (59.202%)	-	-	-
A 514		GLN	0.86	-	Favored (63.95%) General / -66.7,-16.1	Favored (19.3%) *pm20* chi angles: 59.7,271.7,29.8	0.04Å	Favored (52.845%) alpha helix	-	-	-
A 515		TYR	0.81	-	Favored (13.72%) General / -110.5,-16.3	Favored (23.8%) *m-10* chi angles: 302.5,333.1	0.04Å	Favored (27.73%) alpha helix	-	-	-
A 516		LEU	0.76	-	Favored (71.61%) General / -60.5,-32.7	Favored (80.6%) *mt* chi angles: 292.3,178	0.07Å	Favored (57.901%) alpha helix	-	-	-
A 517		GLY	0.72	-	Favored (76.35%) Glycine / -60.3,-32.3	-	-	Favored (87.512%) alpha helix	-	-	-
A 518		TYR	0.69	-	Favored (40.65%) General / -79.7,-29.7	Favored (78.6%) *m-80* chi angles: 289.5,84.1	0.04Å	Favored (80.13%) alpha helix	-	-	-
A 519		VAL	0.68	-	Favored (92.12%) Ile or Val / -64.6,-46.6	Favored (91.8%) *t* chi angles: 174.4	0.10Å	Favored (76.483%) alpha helix	-	-	-
A 520		ILE	0.67	-	Favored (95.28%) Ile or Val / -63.9,-42.3	Favored (96.5%) *mt* chi angles: 292.1,168.3	0.13Å	Favored (85.15%) alpha helix	-	-	-

#	Alt	Res	High B	Clash > 0.4Å	Ramachandran	Rotamer	Cβ deviation	CaBLAM	Bond lengths	Bond angles	Cis Peptides
			Avg: 0.92	Clashscore: 1.51	Outliers: 4 of 903	Poor rotamers: 0 of 780	Outliers: 0 of 837	Outliers: 18 of 901	Outliers: 11 of 905	Outliers: 16 of 905	Non-Trans: 0 of 904
A 521		ARG	0.68	-	Favored (89.44%) General / -59.2,-41.7	Favored (23.4%) *tpt-90* chi angles: 179.8,69.7,181.9,276	0.05Å	Favored (90.778%) alpha helix	-	-	-
A 522		ASP	0.7	-	Favored (79.67%) General / -62.4,-35.6	Favored (92.4%) *m-30* chi angles: 285,347.4	0.07Å	Favored (90.631%) alpha helix	-	-	-
A 523		LEU	0.73	-	Favored (89.74%) General / -65.3,-38.3	Favored (79.8%) *mt* chi angles: 294.7,179.2	0.12Å	Favored (77.782%) alpha helix	-	-	-
A 524		ALA	0.77	-	Favored (74.22%) General / -60.8,-34.2	-	0.03Å	Favored (71.498%) three-ten	-	-	-
A 525		ALA	0.82	-	Favored (65.16%) General / -63.4,-20.0	-	0.03Å	Favored (51.992%)	-	-	-
A 526		LEU	0.86	-	Favored (34.31%) General / -80.9,141.7	Favored (85.6%) *mt* chi angles: 300.6,177	0.07Å	Favored (28.95%)	-	-	-
A 527		GLU	0.88	-	Favored (51.45%) General / -60.9,131.7	Favored (92.1%) *tt0* chi angles: 183.3,178.5,1.8	0.04Å	CaBLAM Disfavored (1.467%)	-	-	-
A 528		GLY	0.88	-	Favored (44.21%) Glycine / 170.4,-172.8	-	-	Favored (43.985%)	-	-	-
A 529		GLY	0.84	0.43Å O with A 672 ARG NE	Favored (33.42%) Glycine / -86.1,-160.1	-	-	Favored (11.351%)	-	-	-
A 530		GLY	0.8	-	Favored (54.74%) Glycine / -74.0,166.2	-	-	Favored (9.45%)	-	-	-
A 531		PHE	0.75	-	Favored (27.33%) General / -83.7,148.1	Favored (35.6%) *m-80* chi angles: 279.1,94.9	0.15Å	Favored (37.004%)	-	OUTLIER(S) worst is CA-CB-CG: 6.3 σ	-
A 532		TYR	0.73	-	Favored (41.69%) General / -125.0,154.1	Favored (91.5%) *m-80* chi angles: 295.3,85.5	0.07Å	Favored (70.288%) beta sheet	-	-	-
A 533		ALA	0.72	-	Favored (6.05%) General / -144.1,111.6	-	0.03Å	Favored (33.517%) beta sheet	-	-	-
A 534		ASP	0.74	-	Favored (32.5%) General / -117.3,118.5	Favored (75.7%) *m-30* chi angles: 296.1,347.4	0.06Å	Favored (66.578%) beta sheet	-	-	-
A 535		ASP	0.77	-	Favored (16.88%) General / -101.0,105.6	Favored (62.7%) *t0* chi angles: 182.3,1.8	0.07Å	Favored (67.375%) beta sheet	-	OUTLIER(S) worst is CA-CB-CG: 4.6 σ	-

#	Alt	Res	High B	Clash > 0.4Å	Ramachandran	Rotamer	Cβ deviation	CaBLAM	Bond lengths	Bond angles	Cis Peptides
A 536		THR	0.8	-	Favored (34.62%) General / -84.6,131.3	Favored (95.5%) *m* chi angles: 299.5	0.08Å	Favored (39.78%)	-	-	-
A 537		ALA	0.81	-	Favored (30.49%) General / -81.6,125.4	-	0.09Å	Favored (6.663%)	-	-	-
A 538		GLY	0.81	-	Favored (73.61%) Glycine / 69.0,32.8	-	-	Favored (28.953%)	-	-	-
A 539		TRP	0.8	-	Favored (29.16%) General / -47.7,-48.9	Favored (45%) *t-100* chi angles: 192.9,265.8	0.04Å	Favored (40.595%)	-	-	-
A 540		ASP	0.78	-	Favored (59.96%) General / -56.9,-27.0	Favored (99.3%) *m-30* chi angles: 287.5,346.2	0.02Å	Favored (63.035%) alpha helix	-	-	-
**#**	**Alt**	**Res**	**High B**	**Clash > 0.4Å**	**Ramachandran**	**Rotamer**	**Cβ deviation**	**CaBLAM**	**Bond lengths**	**Bond angles**	**Cis Peptides**
			Avg: 0.92	Clashscore: 1.51	Outliers: 4 of 903	Poor rotamers: 0 of 780	Outliers: 0 of 837	Outliers: 18 of 901	Outliers: 11 of 905	Outliers: 16 of 905	Non-Trans: 0 of 904
A 541		THR	0.75	-	Favored (32.24%) General / -95.0,-10.0	Favored (44.6%) *p* chi angles: 66.7	0.03Å	Favored (72.99%)	-	-	-
A 542		ARG	0.73	-	Favored (33.76%) General / -103.9,12.9	Favored (97.3%) *mtt-85* chi angles: 292.5,179.7,181.9,278.2	0.03Å	Favored (35.01%)	-	-	-
A 543		ILE	0.71	-	Favored (37.52%) Ile or Val / -80.4,123.7	Favored (46.9%) *mm* chi angles: 299.7,300.3	0.06Å	Favored (23.503%)	-	-	-
A 544		THR	0.71	-	Favored (12.19%) General / -100.8,163.7	Favored (50.5%) *p* chi angles: 65.6	0.14Å	Favored (30.267%)	-	-	-
A 545		GLU	0.71	-	Favored (61.4%) General / -53.6,-35.9	Favored (91.9%) *tt0* chi angles: 182.9,177.6,3.2	0.06Å	Favored (57.985%)	-	-	-
A 546		ALA	0.71	-	Favored (68.91%) General / -58.9,-31.8	-	0.01Å	Favored (69.549%) alpha helix	-	-	-
A 547		ASP	0.72	-	Favored (27.74%) General / -82.3,-35.7	Favored (41.2%) *m-30* chi angles: 292.7,294.8	0.07Å	Favored (79.2%) alpha helix	-	-	-
A 548		LEU	0.73	-	Favored (86.64%) General / -65.7,-37.5	Favored (94.6%) *mt* chi angles: 291.8,173	0.05Å	Favored (93.061%) alpha helix	-	-	-
A 549		ASP	0.74	-	Favored (93.04%) General / -62.0,-45.6	Favored (39.2%) *t70* chi angles: 185.1,64.3	0.02Å	Favored (97.738%) alpha helix	-	-	-
A 550		ASP	0.74	-	Favored (93.64%) General / -61.9,-39.9	Favored (87.2%) *m-30* chi angles: 284,345.4	0.01Å	Favored (96.252%) alpha helix	-	-	-
A 551		GLU	0.74	0.40Å OE2 with A 601 ARG NH2	Favored (79.78%) General / -63.1,-35.3	Favored (98.8%) *mt-10* chi angles: 290.9,172.6,357.4	0.05Å	Favored (69.885%) alpha helix	OUTLIER(S) worst is CG--CD: 4.8 σ	-	-

#	Alt	Res	High B	Clash > 0.4Å	Ramachandran	Rotamer	Cβ deviation	CaBLAM	Bond lengths	Bond angles	Cis Peptides
A 552		GLN	0.75	-	Favored (38.96%) General / -62.0,-14.8	Favored (88.3%) *mt0* chi angles: 287.3,177.2,306.9	0.09Å	Favored (49.361%) three-ten	-	-	-
A 553		GLU	0.75	-	Favored (66.5%) General / -61.2,-24.6	Favored (74.1%) *mm-30* chi angles: 290.7,291.8,322.8	0.04Å	Favored (50.495%) three-ten	-	-	-
A 554		ILE	0.75	-	Favored (28.24%) Ile or Val / -60.0,-23.8	Favored (12.3%) *tp* chi angles: 192.9,63.5	0.13Å	Favored (63.949%) three-ten	-	-	-
A 555		LEU	0.76	-	Favored (65.83%) General / -66.4,-19.5	Favored (6.2%) *mp* chi angles: 273.7,50.6	0.01Å	Favored (63.19%) three-ten	-	-	-
A 556		ASN	0.78	-	Favored (57.89%) General / -73.7,-8.9	Favored (96.9%) *m-40* chi angles: 290,334.9	0.03Å	Favored (50.832%) three-ten	-	-	-
A 557		TYR	0.81	-	Favored (27.84%) General / -107.3,5.8	Favored (87.9%) *m-80* chi angles: 301,91.3	0.09Å	Favored (55.449%)	-	-	-
A 558		MET	0.84	-	Favored (19.67%) General / -97.8,151.9	Favored (69.3%) *mtt* chi angles: 295,176.9,182.1	0.07Å	Favored (26.741%)	-	-	-
A 559		SER	0.86	-	Favored (93.27%) Pre-Pro / -69.8,155.6	Favored (85.9%) *p* chi angles: 67.8	0.07Å	Favored (44.884%)	-	-	-
A 560		PRO	0.86	-	Favored (36.11%) Trans-Pro / -49.5,-35.2	Favored (78.4%) *Cg_exo* chi angles: 329.1,38.4,330.9	0.05Å	Favored (73.584%)	-	-	-
**#**	**Alt**	**Res**	**High B**	**Clash > 0.4Å**	**Ramachandran**	**Rotamer**	**Cβ deviation**	**CaBLAM**	**Bond lengths**	**Bond angles**	**Cis Peptides**
			Avg: 0.92	Clashscore: 1.51	Outliers: 4 of 903	Poor rotamers: 0 of 780	Outliers: 0 of 837	Outliers: 18 of 901	Outliers: 11 of 905	Outliers: 16 of 905	Non-Trans: 0 of 904
A 561		HIS	0.85	-	Favored (64.44%) General / -67.8,-49.1	Favored (79.1%) *t70* chi angles: 186,68.3	0.03Å	Favored (73.542%) alpha helix	-	-	-
A 562		HIS	0.82	-	Favored (98.12%) General / -61.5,-43.9	Favored (16.8%) *t-170* chi angles: 183.6,180.2	0.07Å	Favored (75.915%) alpha helix	-	-	-
A 563		ARG	0.78	-	Favored (83.06%) General / -58.7,-40.3	Favored (92.2%) *mtt180* chi angles: 289.8,179.8,188.9,181	0.15Å	Favored (76.154%) alpha helix	-	-	-
A 564		LYS	0.75	-	Favored (93.51%) General / -65.5,-41.1	Favored (96.8%) *mttt* chi angles: 288.6,177.1,181.4,178.5	0.00Å	Favored (80.103%) alpha helix	-	-	-
A 565		LEU	0.72	-	Favored (62.04%) General / -70.8,-46.9	Favored (55.8%) *tp* chi angles: 182.7,61.3	0.02Å	Favored (73.375%) alpha helix	-	-	-
A 566		ALA	0.71	-	Favored (85.42%) General / -60.9,-38.4	-	0.03Å	Favored (83.477%) alpha helix	-	-	-
A 567		LEU	0.71	-	Favored (79.89%) General / -64.4,-47.3	Favored (65.7%) *tp* chi angles: 179.5,60.3	0.02Å	Favored (86.525%) alpha helix	-	-	-

A 568		ALA	0.72	-	Favored (79.59%) General / -58.7,-39.1	-	0.07Å	Favored (82.894%) alpha helix	-	-	-
A 569		VAL	0.74	-	Favored (81.03%) Ile or Val / -67.3,-47.5	Favored (66.5%) *t* chi angles: 171.7	0.03Å	Favored (82.395%) alpha helix	-	-	-
A 570		MET	0.76	-	Favored (87.56%) General / -63.7,-37.5	Favored (93.7%) *mmm* chi angles: 289,298.8,291.9	0.04Å	Favored (75.431%) alpha helix	-	-	-
A 571		GLU	0.77	-	Favored (55.88%) General / -75.7,-40.6	Favored (71.6%) *mm-30* chi angles: 291.5,293.6,308	0.04Å	Favored (61.793%) alpha helix	-	-	-
A 572		MET	0.79	-	Favored (16.18%) General / -95.0,-26.3	Favored (86.7%) *mtm* chi angles: 292.1,187.3,286.7	0.03Å	Favored (45.808%) alpha helix	-	-	-
A 573		THR	0.8	-	Favored (4.6%) General / -102.7,-46.9	Favored (83.3%) *m* chi angles: 302	0.06Å	Favored (34.538%) alpha helix	-	-	-
A 574		TYR	0.81	-	Favored (25.87%) General / -80.1,-41.2	Favored (21.8%) *m-80* chi angles: 290,69.4	0.11Å	Favored (52.901%) alpha helix	-	-	-
A 575		LYS	0.81	-	Allowed (1.06%) General / -92.4,-64.8	Favored (86.6%) *mttt* chi angles: 291.7,179.1,191.5,175.4	0.03Å	Favored (20.253%) alpha helix	-	-	-
A 576		ASN	0.8	-	Favored (10.04%) General / -84.2,68.1	Favored (87.2%) *m-40* chi angles: 295.5,316.9	0.07Å	Favored (25.171%)	-	-	-
A 577		LYS	0.79	-	Favored (33.92%) General / -86.8,132.2	Allowed (0.4%) *ttmp* chi angles: 182.1,186.4,266.7,70.2	0.05Å	Favored (18.032%)	-	-	-
A 578		VAL	0.79	-	Favored (67.94%) Ile or Val / -110.7,127.2	Favored (74.4%) *t* chi angles: 178.3	0.02Å	Favored (71.985%) beta sheet	-	-	-
A 579		VAL	0.8	-	Favored (60.59%) Ile or Val / -125.9,136.5	Favored (40%) *t* chi angles: 183.4	0.06Å	Favored (67.118%) beta sheet	-	-	-
A 580		LYS	0.83	-	Favored (40.53%) General / -103.6,120.2	Favored (22.6%) *mtpp* chi angles: 291,179.8,67.9,62.8	0.02Å	Favored (66.421%) beta sheet	-	-	-
**#**	**Alt**	**Res**	**High B**	**Clash > 0.4Å**	**Ramachandran**	**Rotamer**	**Cβ deviation**	**CaBLAM**	**Bond lengths**	**Bond angles**	**Cis Peptides**
			Avg: 0.92	Clashscore: 1.51	Outliers: 4 of 903	Poor rotamers: 0 of 780	Outliers: 0 of 837	Outliers: 18 of 901	Outliers: 11 of 905	Outliers: 16 of 905	Non-Trans: 0 of 904
A 581		VAL	0.91	-	Favored (71.54%) Ile or Val / -126.8,130.8	Favored (62.1%) *t* chi angles: 179.7	0.03Å	Favored (57.252%) beta sheet	-	-	-
A 582		LEU	1.04	-	Favored (48.5%) General / -67.8,133.0	Favored (25%) *tp* chi angles: 188.4,66.7	0.07Å	Favored (36.904%) beta sheet	-	-	-
A 583		ARG	1.23	-	Favored (59.44%) Pre-Pro / -142.8,158.4	Favored (42.7%) *ptt90* chi angles: 69.6,169.7,171.5,91.9	0.14Å	Favored (45.326%) beta sheet	-	-	-

#	Alt	Res	High B	Clash > 0.4Å	Ramachandran	Rotamer	Cβ deviation	CaBLAM	Bond lengths	Bond angles	Cis Peptides
A 584		PRO	1.48	-	Favored (48.41%) Trans-Pro / -72.5,159.7	Favored (77.2%) *Cg_endo* chi angles: 28.3,324.4,27.9	0.01Å	Favored (46.271%)	-	-	-
A 585		ALA	1.74	-	Favored (19.6%) Pre-Pro / -161.2,163.9	-	0.09Å	Favored (8.516%)	-	-	-
A 586		PRO	1.93	-	Favored (45.71%) Trans-Pro / -51.1,137.1	Favored (86.8%) *Cg_exo* chi angles: 330.3,37.9,330.1	0.03Å	Favored (21.908%)	-	-	-
A 587		GLY	1.98	-	Favored (69.25%) Glycine / 94.0,-12.4	-	-	Favored (16.748%)	-	-	-
A 588		GLY	1.85	-	Favored (88.65%) Glycine / 84.8,2.2	-	-	Favored (82.833%)	-	-	-
A 589		LYS	1.6	-	Favored (34.12%) General / -80.2,142.9	Favored (98.7%) *mttt* chi angles: 293.6,181.1,180.7,178.5	0.04Å	Favored (37.585%)	-	-	-
A 590		ALA	1.32	-	Favored (23.54%) General / -88.0,150.2	-	0.04Å	Favored (45.819%) beta sheet	-	-	-
A 591		TYR	1.08	-	Favored (43.96%) General / -127.2,155.1	Favored (66.8%) *m-80* chi angles: 290.1,79.7	0.04Å	Favored (55.144%) beta sheet	-	-	-
A 592		MET	0.92	-	Favored (50.61%) General / -103.5,131.5	Favored (29.1%) *mmt* chi angles: 295.7,291.8,180.7	0.08Å	Favored (57.519%) beta sheet	-	-	-
A 593		ASP	0.82	-	Favored (49.19%) General / -102.8,132.2	Favored (96.9%) *m-30* chi angles: 289.7,345.7	0.07Å	Favored (58.988%) beta sheet	-	-	-
A 594		VAL	0.78	-	Favored (43.34%) Ile or Val / -100.8,116.0	Favored (65.3%) *t* chi angles: 179.3	0.06Å	Favored (46.352%) beta sheet	-	-	-
A 595		ILE	0.78	-	Favored (26.61%) Ile or Val / -129.3,166.6	Favored (39.2%) *pt* chi angles: 60.1,174.9	0.09Å	Favored (26.641%) beta sheet	-	-	-
A 596		SER	0.81	-	Favored (26.81%) General / -151.4,147.4	Favored (40.4%) *t* chi angles: 181	0.03Å	Favored (48.112%) beta sheet	-	-	-
A 597		ARG	0.84	-	Favored (17.01%) General / -156.7,141.5	Favored (29.5%) *ttp-170* chi angles: 188.2,169.1,72.1,161.7	0.04Å	Favored (26.926%)	OUTLIER(S) worst is CZ--NH1: 4.1 σ	OUTLIER(S) worst is NE-CZ-NH2: 6.3 σ	-
A 598		ARG	0.88	-	Favored (35.18%) General / -81.1,-28.8	Favored (29.6%) *mtp-110* chi angles: 297.3,183.5,73.9,253.3	0.03Å	Favored (40.037%)	-	-	-
A 599		ASP	0.92	-	Favored (3.35%) General / -94.6,67.6	Favored (71.6%) *m-30* chi angles: 292.6,318.4	0.08Å	CaBLAM Disfavored (2.257%)	-	OUTLIER(S) worst is CA-CB-CG: 4.7 σ	-
A 600		GLN	0.95	-	Favored (2.62%) General / -166.6,134.1	Favored (48.5%) *tp40* chi angles: 176.7,76.9,50.5	0.07Å	CaBLAM Disfavored (4.072%)	-	-	-

			Avg: 0.92	Clashscore: 1.51	Outliers: 4 of 903	Poor rotamers: 0 of 780	Outliers: 0 of 837	Outliers: 18 of 901	Outliers: 11 of 905	Outliers: 16 of 905	Non-Trans: 0 of 904
A 601		ARG	0.97	0.40Å NH2 with A 551 GLU OE2	Favored (32.7%) General / -102.5,143.1	Favored (49.8%) *ttm110* chi angles: 186.3,176.5,300.6,108.1	0.09Å	Favored (46.008%)	OUTLIER(S) worst is CD--NE: 5.1 σ	-	-
A 602		GLY	0.98	-	Favored (20.55%) Glycine / -85.1,143.7	-	-	Favored (37.307%)	-	-	-
A 603		SER	0.98	-	Favored (59.16%) General / -77.3,-10.0	Favored (67.8%) *m* chi angles: 294.6	0.06Å	Favored (22.733%)	-	-	-
A 604		GLY	0.98	-	Favored (60.58%) Glycine / -99.1,7.9	-	-	Favored (53.064%)	-	-	-
A 605		GLN	0.97	0.40Å NE2 with A 453 TYR OH	Favored (46.62%) General / -70.1,150.4	Favored (33.5%) *mt0* chi angles: 294.3,188.6,253.8	0.12Å	Favored (41.63%)	-	-	-
A 606		VAL	0.95	-	Favored (61.61%) Ile or Val / -55.4,-41.2	Favored (64.1%) *t* chi angles: 171.4	0.01Å	Favored (21.51%)	-	-	-
A 607		VAL	0.91	-	Allowed (1.04%) Ile or Val / -111.3,32.8	Favored (17.8%) *m* chi angles: 293.3	0.05Å	Favored (12.551%) alpha helix	-	-	-
A 608		THR	0.87	-	Favored (2.19%) General / -59.2,-62.0	Favored (25%) *p* chi angles: 51.9	0.10Å	Favored (11.373%) alpha helix	-	-	-
A 609		TYR	0.83	-	Favored (69.59%) General / -53.9,-48.9	Favored (91%) *t80* chi angles: 179.2,78.2	0.04Å	Favored (73.252%) alpha helix	-	-	-
A 610		ALA	0.79	-	Favored (99.07%) General / -61.3,-42.7	-	0.04Å	Favored (82.225%) alpha helix	-	-	-
A 611		LEU	0.75	-	Favored (75.31%) General / -70.0,-36.6	Favored (88.5%) *mt* chi angles: 291.1,173.5	0.03Å	Favored (83.307%) alpha helix	-	-	-
A 612		ASN	0.71	-	Favored (99.08%) General / -63.3,-42.0	Favored (96.3%) *m-40* chi angles: 286.5,342.6	0.03Å	Favored (91.547%) alpha helix	-	-	-
A 613		THR	0.69	-	Favored (96.94%) General / -62.1,-44.3	Favored (86.3%) *m* chi angles: 301.5	0.05Å	Favored (98.424%) alpha helix	-	-	-
A 614		ILE	0.66	-	Favored (99.45%) Ile or Val / -61.5,-44.9	Favored (97.4%) *mt* chi angles: 292.3,167.1	0.06Å	Favored (94.044%) alpha helix	-	-	-
A 615		THR	0.65	-	Favored (67.04%) General / -68.7,-28.7	Favored (63.2%) *p* chi angles: 57.8	0.05Å	Favored (81.127%) alpha helix	-	-	-
A 616		ASN	0.63	-	Favored (65.39%) General / -73.6,-35.6	Favored (52.5%) *m-40* chi angles: 281.7,276	0.07Å	Favored (88.129%) alpha helix	-	-	-

A 617		LEU	0.62	-	Favored (98.72%) General / -63.3,-40.9	Favored (79.8%) *mt* chi angles: 288.6,168.7	0.03Å	Favored (93.944%) alpha helix	-	-	-
A 618		LYS	0.61	-	Favored (68%) General / -58.3,-51.9	Favored (31.4%) *ttpt* chi angles: 179.9,183.3,76.5,182.5	0.05Å	Favored (86.683%) alpha helix	-	-	-
A 619		VAL	0.61	-	Favored (95.13%) Ile or Val / -60.3,-44.2	Favored (46.4%) *t* chi angles: 168.9	0.07Å	Favored (81.287%) alpha helix	-	-	-
A 620		GLN	0.6	-	Favored (81.53%) General / -67.0,-36.2	Favored (39.2%) *mm-40* chi angles: 292.4,294,359.1	0.04Å	Favored (92.786%) alpha helix	-	-	-
**#**	**Alt**	**Res**	**High B**	**Clash > 0.4Å**	**Ramachandran**	**Rotamer**	**Cβ deviation**	**CaBLAM**	**Bond lengths**	**Bond angles**	**Cis Peptides**
			Avg: 0.92	Clashscore: 1.51	Outliers: 4 of 903	Poor rotamers: 0 of 780	Outliers: 0 of 837	Outliers: 18 of 901	Outliers: 11 of 905	Outliers: 16 of 905	Non-Trans: 0 of 904
A 621		LEU	0.6	-	Favored (89.92%) General / -63.8,-38.1	Favored (80.5%) *mt* chi angles: 288.9,170.5	0.01Å	Favored (95.853%) alpha helix	-	-	-
A 622		ILE	0.6	-	Favored (92.22%) Ile or Val / -65.0,-46.2	Favored (99.5%) *mt* chi angles: 292.7,167.5	0.03Å	Favored (96.55%) alpha helix	-	-	-
A 623		ARG	0.6	-	Favored (86.83%) General / -62.0,-38.0	Favored (86.3%) *mtp180* chi angles: 289.5,178.2,65.4,188.2	0.02Å	Favored (89.805%) alpha helix	-	-	-
A 624		MET	0.6	-	Favored (89.25%) General / -65.9,-38.6	Favored (74.9%) *mtm* chi angles: 287.5,187,280.4	0.12Å	Favored (86.625%) alpha helix	-	-	-
A 625		ALA	0.6	-	Favored (77.08%) General / -61.0,-35.5	-	0.03Å	Favored (78.848%) alpha helix	-	-	-
A 626		GLU	0.6	-	Favored (72.16%) General / -65.5,-48.5	Favored (63.6%) *tt0* chi angles: 180.5,179,329.9	0.04Å	Favored (74.464%) alpha helix	-	-	-
A 627		ALA	0.6	-	Favored (74.63%) General / -59.1,-36.1	-	0.08Å	Favored (74.897%) alpha helix	-	-	-
A 628		GLU	0.6	-	Favored (40.3%) General / -88.9,6.2	Favored (53.5%) *mt-10* chi angles: 294.8,178.9,42.8	0.05Å	Favored (51.442%)	-	-	-
A 629		MET	0.61	-	Favored (12.36%) General / 60.8,43.1	Favored (81.6%) *mtm* chi angles: 294.2,181.5,285	0.07Å	Favored (18.894%)	-	-	-
A 630		VAL	0.62	-	Favored (13.87%) Ile or Val / -87.5,-50.3	Favored (84.5%) *t* chi angles: 176.3	0.11Å	CaBLAM Outlier (0.945%)	-	-	-
A 631		ILE	0.63	-	Favored (68.21%) Ile or Val / -115.4,131.3	Favored (41.5%) *mm* chi angles: 303.8,296.6	0.06Å	Favored (33.59%)	-	-	-

#	Alt	Res	High B	Clash > 0.4Å	Ramachandran	Rotamer	Cβ deviation	CaBLAM	Bond lengths	Bond angles	Cis Peptides
A 632		HIS	0.64	-	Favored (9.03%) General / -106.5,168.6	Favored (78.5%) *m-70* chi angles: 294.7,266.6	0.04Å	Favored (29.836%)	-	-	-
A 633		HIS	0.65	-	Favored (55.01%) General / -50.3,-46.2	Favored (91.3%) *t70* chi angles: 179.4,73	0.04Å	Favored (59.993%)	-	-	-
A 634		GLN	0.65	-	Favored (61.79%) General / -60.5,-21.0	Favored (20.9%) *pm20* chi angles: 63.9,271.9,29.6	0.02Å	Favored (67.684%) alpha helix	-	-	-
A 635		HIS	0.66	-	Favored (29.69%) General / -87.3,-18.6	Favored (25.8%) *m90* chi angles: 297,129.4	0.04Å	Favored (73.55%) alpha helix	-	-	-
A 636		VAL	0.65	-	Favored (85.99%) Ile or Val / -67.7,-42.0	Favored (93.4%) *t* chi angles: 174.6	0.10Å	Favored (63.561%) alpha helix	-	-	-
A 637		GLN	0.64	-	Favored (59.26%) General / -71.5,-46.6	Favored (96%) *mt0* chi angles: 293.5,176.6,339.5	0.06Å	Favored (24.828%)	-	-	-
A 638		ASP	0.63	-	Favored (2.96%) General / -148.1,98.7	Favored (59.2%) *t0* chi angles: 186.6,357.5	0.05Å	Favored (7.234%)	-	-	-
A 639		CYS	0.62	-	Favored (17.63%) General / -90.3,105.2	Favored (20.3%) *t* chi angles: 191.1	0.07Å	Favored (56.431%)	-	-	-
A 640		ASP	0.6	-	Favored (17.78%) General / -57.4,150.8	Favored (15.5%) *t70* chi angles: 190.8,87.4	0.06Å	Favored (18.306%)	-	-	-
**#**	**Alt**	**Res**	**High B**	**Clash > 0.4Å**	**Ramachandran**	**Rotamer**	**Cβ deviation**	**CaBLAM**	**Bond lengths**	**Bond angles**	**Cis Peptides**
			Avg: 0.92	Clashscore: 1.51	Outliers: 4 of 903	Poor rotamers: 0 of 780	Outliers: 0 of 837	Outliers: 18 of 901	Outliers: 11 of 905	Outliers: 16 of 905	Non-Trans: 0 of 904
A 641		ASP	0.59	-	Favored (63.27%) General / -55.9,-32.0	Favored (98.2%) *m-30* chi angles: 288.5,346	0.07Å	Favored (60.64%)	-	-	-
A 642		THR	0.58	-	Favored (84.61%) General / -62.6,-47.2	Favored (97.2%) *m* chi angles: 300.7	0.09Å	Favored (74.304%) alpha helix	-	-	-
A 643		ALA	0.57	-	Favored (93.76%) General / -64.8,-39.5	-	0.04Å	Favored (87.43%) alpha helix	-	-	-
A 644		LEU	0.56	-	Favored (86.1%) General / -65.8,-37.4	Favored (97.6%) *mt* chi angles: 292.2,172.7	0.01Å	Favored (96.665%) alpha helix	-	-	-
A 645		THR	0.55	-	Favored (84.96%) General / -65.3,-45.2	Favored (95.8%) *m* chi angles: 299.6	0.02Å	Favored (92.074%) alpha helix	-	-	-
A 646		LYS	0.55	-	Favored (92.41%) General / -60.1,-41.3	Favored (82.9%) *tttt* chi angles: 188.3,178.8,180.9,183.2	0.01Å	Favored (97.541%) alpha helix	-	-	-

A 647		LEU	0.55	-	Favored (80.65%) General / -60.6,-48.7	Favored (75.5%) *tp* chi angles: 177.3,62.5	0.02Å	Favored (98.708%) alpha helix	-	-	-
A 648		GLU	0.55	-	Favored (92.05%) General / -59.5,-42.3	Favored (67.1%) *tp30* chi angles: 179.2,69.5,20.7	0.03Å	Favored (96.008%) alpha helix	-	-	-
A 649		ALA	0.56	-	Favored (98.36%) General / -63.6,-42.1	-	0.03Å	Favored (94.588%) alpha helix	-	-	-
A 650		TRP	0.58	-	Favored (91.08%) General / -61.7,-46.1	Favored (88.9%) *t60* chi angles: 179.5,84.3	0.04Å	Favored (95.449%) alpha helix	-	-	-
A 651		LEU	0.59	-	Favored (99.53%) General / -63.1,-43.0	Favored (71.5%) *mt* chi angles: 290.7,165.8	0.09Å	Favored (92.384%) alpha helix	-	-	-
A 652		ALA	0.61	-	Favored (85.06%) General / -61.3,-38.0	-	0.03Å	Favored (61.398%) alpha helix	-	-	-
A 653		GLU	0.63	-	Favored (13.31%) General / -91.5,-37.2	Favored (97.4%) *mt-10* chi angles: 294.2,181.1,351.3	0.03Å	Favored (35.294%) alpha helix	-	-	-
A 654		HIS	0.64	-	Favored (10.45%) General / -117.0,-17.1	Favored (99.1%) *m-70* chi angles: 300.7,289.5	0.06Å	Favored (13.276%) alpha helix	-	-	-
A 655		GLY	0.65	-	Favored (19.29%) Glycine / -47.7,-50.6	-	-	Favored (55.841%) alpha helix	-	-	-
A 656		CYS	0.66	-	Favored (86.04%) General / -64.7,-37.0	Favored (95.6%) *m* chi angles: 292.1	0.01Å	Favored (84.137%) alpha helix	-	-	-
A 657		ASP	0.66	-	Favored (73.68%) General / -65.3,-32.2	Favored (12.4%) *t70* chi angles: 195.9,70.9	0.05Å	Favored (85.612%) alpha helix	-	-	-
A 658		ARG	0.66	-	Favored (58.77%) General / -75.6,-38.8	Favored (87.8%) *mmt-90* chi angles: 298.1,295.6,177.5,271.1	0.08Å	Favored (75.468%) alpha helix	-	-	-
A 659		LEU	0.66	-	Favored (95.68%) General / -61.4,-40.8	Favored (79%) *mt* chi angles: 288.7,172	0.04Å	Favored (81.098%) alpha helix	-	-	-
A 660		LYS	0.65	-	Favored (61.77%) General / -68.5,-12.8	Favored (47.1%) *pttt* chi angles: 73,183.6,186.4,191.9	0.05Å	Favored (57.714%) three-ten	-	-	-
**#**	**Alt**	**Res**	**High B**	**Clash > 0.4Å**	**Ramachandran**	**Rotamer**	**Cβ deviation**	**CaBLAM**	**Bond lengths**	**Bond angles**	**Cis Peptides**
			Avg: 0.92	Clashscore: 1.51	Outliers: 4 of 903	Poor rotamers: 0 of 780	Outliers: 0 of 837	Outliers: 18 of 901	Outliers: 11 of 905	Outliers: 16 of 905	Non-Trans: 0 of 904
A 661		ARG	0.64	-	Favored (44.79%) General / -90.8,6.3	Favored (91.8%) *mtt-85* chi angles: 290.4,171,181.9,271.4	0.04Å	Favored (43.634%)	-	-	-

A 662		MET	0.64	-	Favored (42.32%) General / -123.1,151.2	Favored (71%) *mtm* chi angles: 298.8,186.8,279.7	0.04Å	Favored (28.596%)	-	-	-
A 663		ALA	0.65	-	Favored (9.9%) General / -122.7,105.2	-	0.03Å	Favored (40.158%)	-	-	-
A 664		VAL	0.66	-	Favored (73.22%) Ile or Val / -117.5,124.6	Favored (51.4%) *t* chi angles: 181.2	0.06Å	Favored (50.105%)	-	-	-
A 665		SER	0.67	-	Favored (6.93%) General / -126.5,102.0	Favored (37.6%) *t* chi angles: 177.5	0.07Å	Favored (7.746%)	-	-	-
A 666		GLY	0.68	-	Favored (32.81%) Glycine / 59.8,-123.3	-	-	Favored (60.629%)	-	-	-
A 667		ASP	0.68	-	Favored (56.77%) General / -89.2,0.4	Favored (44.4%) *m-30* chi angles: 302.9,336.7	0.13Å	Favored (12.522%)	-	OUTLIER(S) worst is CA-CB-CG: 4.6 σ	-
A 668		ASP	0.68	-	Favored (30.13%) General / -107.3,115.4	Favored (57.2%) *t0* chi angles: 187,335	0.04Å	Favored (27.589%)	-	-	-
A 669		CYS	0.67	-	Favored (39.77%) General / -132.7,130.3	Favored (45.5%) *t* chi angles: 177.6	0.08Å	Favored (59.561%)	-	-	-
A 670		VAL	0.67	-	Favored (49.59%) Ile or Val / -99.0,129.3	Favored (95.6%) *t* chi angles: 174.9	0.04Å	Favored (66.039%) beta sheet	-	-	-
A 671		VAL	0.67	-	Favored (61.96%) Ile or Val / -126.8,136.0	Favored (46.3%) *t* chi angles: 182.2	0.05Å	Favored (69.798%) beta sheet	-	-	-
A 672		ARG	0.68	0.43Å NE with A 529 GLY O	Favored (18.66%) Pre-Pro / -119.3,78.1	Favored (19.8%) *tpt-90* chi angles: 176.4,73.9,187,277.5	0.09Å	Favored (24.501%) beta sheet	-	OUTLIER(S) worst is NE-CZ-NH1: 4.7 σ	-
A 673		PRO	0.7	-	Favored (46.34%) Trans-Pro / -73.1,160.1	Favored (77.2%) *Cg_endo* chi angles: 30.8,326.4,22.5	0.07Å	Favored (23.788%)	-	-	-
A 674		ILE	0.72	-	Favored (27.99%) Ile or Val / -62.1,-22.1	Favored (9.5%) *tp* chi angles: 197,65.9	0.06Å	Favored (16.62%)	-	-	-
A 675		ASP	0.73	-	Favored (27.34%) General / -149.3,146.0	Favored (10%) *t70* chi angles: 195.5,291	0.02Å	Favored (21.771%)	-	-	-
A 676		ASP	0.73	-	Favored (43.41%) General / -59.5,-18.8	Favored (97.5%) *m-30* chi angles: 286.8,347	0.03Å	Favored (15.194%)	-	-	-
A 677		ARG	0.72	-	Favored (36.6%) General / -58.3,-19.7	Favored (99.4%) *mtm-85* chi angles: 286.2,190.5,293.6,274.2	0.02Å	Favored (24.179%)	-	-	-
A 678		PHE	0.71	-	Favored (32.23%) General / -48.6,-41.8	Favored (85.8%) *t80* chi angles: 179.1,83.5	0.06Å	Favored (53.948%) three-ten	-	-	-
A 679		GLY	0.69	-	Favored (59.55%)	-	-	Favored (79.328%) alpha helix	-	-	-

					Glycine / -60.0,-23.6						
A 680		LEU	0.68	-	Favored (42.22%) General / -91.0,-10.4	Favored (72.7%) *mt* chi angles: 301.7,174.5	0.12Å	Favored (48.955%)	-	-	-
**#**	**Alt**	**Res**	**High B**	**Clash > 0.4Å**	**Ramachandran**	**Rotamer**	**Cβ deviation**	**CaBLAM**	**Bond lengths**	**Bond angles**	**Cis Peptides**
			Avg: 0.92	Clashscore: 1.51	Outliers: 4 of 903	Poor rotamers: 0 of 780	Outliers: 0 of 837	Outliers: 18 of 901	Outliers: 11 of 905	Outliers: 16 of 905	Non-Trans: 0 of 904
A 681		ALA	0.68	-	Favored (6.14%) General / -80.5,66.8	-	0.05Å	CaBLAM Disfavored (4.623%)	-	-	-
A 682		LEU	0.68	-	Favored (15.18%) General / -113.4,1.7	Favored (94.7%) *mt* chi angles: 296.3,174.7	0.04Å	Favored (10.088%)	-	-	-
A 683		SER	0.68	-	Favored (78.61%) General / -64.9,-47.2	Favored (34.5%) *t* chi angles: 181.4	0.03Å	Favored (15.749%)	-	-	-
A 684		HIS	0.68	-	Favored (77.22%) General / -69.6,-38.2	Favored (55.6%) *m90* chi angles: 284.6,87.8	0.07Å	Favored (70.75%) alpha helix	OUTLIER(S) worst is CB--CG: 4.8 σ	-	-
A 685		LEU	0.68	-	Favored (79.04%) General / -57.0,-48.2	Favored (50.3%) *tp* chi angles: 181.3,65.3	0.07Å	Favored (71.995%) alpha helix	-	-	-
A 686		ASN	0.69	-	Favored (63.18%) General / -74.4,-33.9	Favored (71.2%) *m-40* chi angles: 289.1,282.2	0.06Å	Favored (69.025%) alpha helix	-	-	-
A 687		ALA	0.7	-	Favored (72.13%) General / -60.4,-33.2	-	0.04Å	Favored (74.711%) alpha helix	-	-	-
A 688		MET	0.72	-	Favored (15.01%) General / -82.5,7.2	Favored (84.2%) *mtm* chi angles: 291,184.3,284.4	0.04Å	Favored (42.535%)	-	-	-
A 689		SER	0.75	-	Favored (16.94%) General / 62.5,31.9	Favored (62.9%) *m* chi angles: 298	0.03Å	Favored (26.021%)	-	-	-
A 690		LYS	0.8	-	Favored (53.64%) General / -109.0,132.9	Favored (21.7%) *mmtm* chi angles: 307.1,296.1,183.3,276.5	0.06Å	Favored (24.796%)	-	-	-
A 691		VAL	0.87	-	Favored (61.76%) Ile or Val / -129.4,125.4	Favored (77.7%) *t* chi angles: 178.1	0.03Å	Favored (50.76%) beta sheet	-	-	-
A 692		ARG	0.95	-	Favored (53.93%) General / -61.7,132.7	Favored (71.2%) *ttt-90* chi angles: 184.5,180.5,186.6,274.3	0.03Å	Favored (40.253%)	-	-	-
A 693		LYS	1.04	-	Favored (13.56%) General / -60.7,-57.2	Favored (12.1%) *ttpp* chi angles: 183.6,170.6,73.3,74.7	0.02Å	Favored (29.615%)	-	-	-
A 694		ASP	1.13	-	Favored (2.37%) General / -90.5,53.1	Favored (72.3%) *m-30* chi angles: 298.4,330.6	0.04Å	CaBLAM Outlier (0.091%)	-	-	-
A 695		ILE	1.18	-	Allowed (1.88%)	Favored (21%) *tt* chi angles: 192.4,165.6	0.08Å	Favored (5.676%)	OUTLIER(S) worst is CB--CG1: 4.5 σ	-	-

#	Alt	Res	High B	Clash > 0.4Å	Ramachandran	Rotamer	Cβ deviation	CaBLAM	Bond lengths	Bond angles	Cis Peptides
					Ile or Val / -166.0,151.3						
A 696		SER	1.18	-	Favored (40.17%) General / -74.7,148.9	Favored (37.8%) *t* chi angles: 174.7	0.06Å	Favored (17.598%)	-	-	-
A 697		GLU	1.13	-	Favored (38.27%) General / -54.5,139.4	Favored (92.9%) *mt-10* chi angles: 291.4,179.5,12.5	0.02Å	Favored (25.053%)	-	-	-
A 698		TRP	1.05	-	Favored (4.91%) General / 71.5,4.1	Favored (55.8%) *m100* chi angles: 308.3,108.3	0.04Å	Favored (9.859%)	-	-	-
A 699		GLN	0.96	-	Favored (47.62%) Pre-Pro / -84.7,146.9	Favored (81.3%) *mt0* chi angles: 293.4,183.9,298.5	0.08Å	Favored (21.995%)	-	-	-
A 700		PRO	0.88	-	Favored (85.42%) Trans-Pro / -66.0,150.3	Favored (41.3%) *Cg_endo* chi angles: 23.7,326.6,28.5	0.02Å	Favored (72.796%) beta sheet	-	-	-
**#**	**Alt**	**Res**	**High B**	**Clash > 0.4Å**	**Ramachandran**	**Rotamer**	**Cβ deviation**	**CaBLAM**	**Bond lengths**	**Bond angles**	**Cis Peptides**
			Avg: 0.92	Clashscore: 1.51	Outliers: 4 of 903	Poor rotamers: 0 of 780	Outliers: 0 of 837	Outliers: 18 of 901	Outliers: 11 of 905	Outliers: 16 of 905	Non-Trans: 0 of 904
A 701		SER	0.82	-	Favored (47.17%) General / -71.7,147.6	Favored (63.7%) *m* chi angles: 297.6	0.01Å	Favored (50.916%) beta sheet	-	-	-
A 702		LYS	0.78	-	Favored (34.3%) General / -85.2,132.2	Favored (87.3%) *tttt* chi angles: 181.8,176.1,179.4,177.7	0.04Å	Favored (48.072%) beta sheet	-	-	-
A 703		GLY	0.77	-	Favored (15.73%) Glycine / -119.3,175.3	-	-	Favored (50.395%) beta sheet	-	-	-
A 704		TRP	0.76	-	Favored (44.04%) General / -137.9,147.0	Favored (27.1%) *m-90* chi angles: 301.5,265.1	0.07Å	Favored (63.121%)	-	-	-
A 705		ASP	0.76	-	Favored (27.94%) General / -101.4,15.7	Favored (72.6%) *m-30* chi angles: 294.8,317.6	0.04Å	Favored (11.951%)	-	-	-
A 706		ASP	0.75	-	Favored (4.75%) General / -147.7,110.6	Favored (60.3%) *t0* chi angles: 183.1,340.7	0.03Å	CaBLAM Disfavored (4.93%) try alpha helix	-	-	-
A 707		TRP	0.74	-	Favored (11.59%) General / -51.7,-27.4	Favored (76.2%) *p-90* chi angles: 67.5,270.4	0.01Å	Favored (36.308%) alpha helix	-	-	-
A 708		GLU	0.73	-	Favored (65.03%) General / -68.0,-21.5	Favored (73.4%) *mt-10* chi angles: 292,193,357.5	0.09Å	Favored (65.585%) alpha helix	-	-	-
A 709		SER	0.73	-	Favored (56.67%) General / -92.3,2.4	Favored (73.1%) *m* chi angles: 295.6	0.03Å	Favored (54.078%)	-	-	-
A 710		VAL	0.75	-	Favored (67.64%) Pre-Pro / -92.3,117.4	Favored (65%) *t* chi angles: 179.3	0.12Å	Favored (32.19%)	-	-	-
A 711		PRO	0.77	-	Favored (46.05%)	Favored (74.4%) *Cg_endo*	0.03Å	Favored (35.859%)	-	-	-

					Trans-Pro / -74.1,152.9	chi angles: 28.9,326.8,23.9					
A 712		PHE	0.8	-	Favored (9%) General / -156.5,131.3	Favored (38.9%) *t80* chi angles: 185.6,59.7	0.08Å	Favored (12.914%)	-	-	-
A 713		CYS	0.82	-	Allowed (0.21%) General / 70.8,-68.5	Favored (76.3%) *m* chi angles: 296.6	0.07Å	CaBLAM Outlier (0.446%)	-	-	-
A 714		SER	0.82	-	Allowed (0.37%) General / -150.8,-1.5	Favored (82.7%) *p* chi angles: 62.4	0.02Å	CaBLAM Outlier (0.252%)	-	-	-
A 715		HIS	0.81	-	Favored (18.92%) General / -151.3,170.7	Allowed (0.4%) *p-80* chi angles: 50,219.1	0.06Å	Favored (22.274%)	-	-	-
A 716		HIS	0.79	-	Favored (13.61%) General / -103.6,161.8	Favored (70.1%) *m90* chi angles: 300.4,86	0.08Å	Favored (43.624%)	-	-	-
A 717		PHE	0.77	-	Favored (46.09%) General / -119.6,145.8	Favored (93.1%) *m-80* chi angles: 293.9,86	0.02Å	Favored (58.593%) beta sheet	-	-	-
A 718		HIS	0.74	-	Favored (51.39%) General / -130.4,151.0	Favored (96.8%) *m-70* chi angles: 296.2,283.1	0.07Å	Favored (53.967%) beta sheet	-	-	-
A 719		GLU	0.74	-	Favored (35.63%) General / -92.6,123.1	Favored (50.5%) *tt0* chi angles: 181,179.8,62.9	0.05Å	Favored (53.42%) beta sheet	-	-	-
A 720		LEU	0.74	0.47Å N with A 720 LEU HD22	Favored (30.75%) General / -107.7,147.4	Allowed (1.2%) *mm* chi angles: 289,300.6	0.11Å	Favored (48.205%) beta sheet	-	-	-
**#**	**Alt**	**Res**	**High B**	**Clash > 0.4Å**	**Ramachandran**	**Rotamer**	**Cβ deviation**	**CaBLAM**	**Bond lengths**	**Bond angles**	**Cis Peptides**
			Avg: 0.92	Clashscore: 1.51	Outliers: 4 of 903	Poor rotamers: 0 of 780	Outliers: 0 of 837	Outliers: 18 of 901	Outliers: 11 of 905	Outliers: 16 of 905	Non-Trans: 0 of 904
A 721		GLN	0.76	-	Favored (51.51%) General / -108.8,125.0	Favored (54.3%) *tt0* chi angles: 180.5,178.5,321.1	0.00Å	Favored (52.589%)	-	-	-
A 722		LEU	0.78	-	Favored (25.03%) General / -78.8,163.8	Favored (7.7%) *mp* chi angles: 280,62.3	0.04Å	Favored (40.883%)	-	-	-
A 723		LYS	0.8	-	Favored (66.59%) General / -61.5,-24.4	Favored (22.8%) *mmtp* chi angles: 296.1,295,176.2,66.7	0.06Å	Favored (51.875%)	-	-	-
A 724		ASP	0.8	-	Favored (36.28%) General / -92.0,8.7	Favored (57.1%) *p0* chi angles: 65.4,0.8	0.01Å	Favored (53.875%)	-	-	-
A 725		GLY	0.8	-	Favored (79.92%) Glycine / 91.7,-7.2	-	-	Favored (75.265%)	-	-	-
A 726		ARG	0.78	-	Favored (34.04%) General / -76.1,156.2	Favored (52.7%) *mtm110* chi angles: 290.5,178.9,292.5,110.6	0.04Å	Favored (42.748%)	OUTLIER(S) worst is CD--NE: 4.1 σ	-	-
A 727		ARG	0.76	-	Favored (47.75%) General / -112.8,140.2	Favored (13.4%) *mpt180* chi angles: 273.8,69.9,166.1,174.2	0.01Å	Favored (56.011%) beta sheet	-	-	-

A 728		ILE	0.74	-	Favored (62.4%) Ile or Val / -129.7,135.0	Favored (68.4%) *mt* chi angles: 296.3,177.1	0.11Å	Favored (59.631%) beta sheet	-	-	-
A 729		VAL	0.72	-	Favored (43.78%) Ile or Val / -96.0,119.2	Favored (63.3%) *t* chi angles: 179.6	0.03Å	Favored (60.843%) beta sheet	-	-	-
A 730		VAL	0.71	-	Favored (28.29%) Pre-Pro / -122.7,137.7	Favored (39.9%) *t* chi angles: 183.4	0.12Å	Favored (50.717%) beta sheet	-	-	-
A 731		PRO	0.72	-	Favored (74.51%) Trans-Pro / -66.8,154.4	Favored (31.6%) *Cg_endo* chi angles: 21.6,328.4,27.9	0.04Å	Favored (54.787%) beta sheet	-	-	-
A 732		CYS	0.73	-	Favored (9.5%) General / -149.2,123.3	Favored (25.1%) *t* chi angles: 190.2	0.05Å	Favored (45.125%)	-	-	-
A 733		ARG	0.75	-	Favored (39.92%) General / -117.6,149.9	Favored (71.7%) *ttt180* chi angles: 184.2,168.7,184.7,171.1	0.05Å	Favored (30.474%)	-	-	-
A 734		ASP	0.76	-	Favored (5.8%) General / -53.7,120.1	Favored (46.4%) *t0* chi angles: 186.3,329.7	0.01Å	Favored (33.903%)	-	-	-
A 735		GLN	0.77	-	Favored (64.89%) General / -56.3,-32.7	Favored (11.5%) *tt0* chi angles: 180.6,167.7,236.8	0.06Å	Favored (42.926%)	-	-	-
A 736		ASP	0.77	-	Favored (72.6%) General / -62.5,-31.8	Favored (87.4%) *m-30* chi angles: 284.1,345	0.07Å	Favored (67.068%) alpha helix	-	-	-
A 737		GLU	0.76	-	Favored (31.86%) General / -79.9,-38.8	Favored (98.5%) *mt-10* chi angles: 291.8,181.4,350	0.04Å	Favored (73.445%) alpha helix	-	-	-
A 738		LEU	0.75	-	Favored (98.79%) General / -63.4,-41.6	Favored (88.9%) *mt* chi angles: 290.7,172.7	0.07Å	Favored (76.226%) alpha helix	-	-	-
A 739		VAL	0.73	-	Favored (47.09%) Ile or Val / -74.8,-43.2	Favored (90.7%) *t* chi angles: 174.3	0.08Å	Favored (74.007%) alpha helix	-	-	-
A 740		GLY	0.72	-	Favored (84.57%) Glycine / -56.6,-38.9	-	-	Favored (97.307%) alpha helix	-	-	-
**#**	**Alt**	**Res**	**High B**	**Clash > 0.4Å**	**Ramachandran**	**Rotamer**	**Cβ deviation**	**CaBLAM**	**Bond lengths**	**Bond angles**	**Cis Peptides**
			Avg: 0.92	Clashscore: 1.51	Outliers: 4 of 903	Poor rotamers: 0 of 780	Outliers: 0 of 837	Outliers: 18 of 901	Outliers: 11 of 905	Outliers: 16 of 905	Non-Trans: 0 of 904
A 741		ARG	0.73	-	Favored (94.98%) General / -64.7,-42.8	Favored (78.5%) *mtp85* chi angles: 286.3,168.1,64.3,86.9	0.02Å	Favored (82.519%) alpha helix	-	-	-
A 742		GLY	0.76	-	Favored (70.05%) Glycine / -59.2,-31.7	-	-	Favored (87.381%) alpha helix	-	-	-
A 743		ARG	0.82	-	Favored (35.79%) General / -81.1,0.5	Favored (23.8%) *mtp180* chi angles: 288.8,178.1,56.4,156.7	0.05Å	Favored (41.165%)	-	-	-

A 744		VAL	0.92	-	Favored (73.92%) Ile or Val / -121.2,131.1	Favored (38.7%) *t* chi angles: 183.7	0.06Å	Favored (34.709%)	-	-	-
A 745		SER	1.07	0.43Å O with A 320 SER OG	Favored (20.31%) Pre-Pro / -100.6,134.9	Favored (37.6%) *t* chi angles: 177.5	0.04Å	Favored (48.796%)	-	-	-
A 746		PRO	1.24	-	Favored (7.53%) Trans-Pro / -77.6,61.7	Favored (58.9%) *Cg_endo* chi angles: 32,322.5,26.8	0.03Å	CaBLAM Outlier (0.48%)	-	-	-
A 747		GLY	1.4	-	Favored (29.72%) Glycine / 169.7,167.1	-	-	Favored (6.067%) alpha helix	-	-	-
A 748		ASN	1.48	-	Favored (60.59%) General / -75.3,-31.3	Favored (98%) *m-40* chi angles: 288.5,336.5	0.03Å	Favored (61.056%)	-	-	-
A 749		GLY	1.46	-	Favored (40.72%) Glycine / -92.8,-178.5	-	-	CaBLAM Disfavored (1.768%)	-	-	-
A 750		TRP	1.35	0.48Å CG with A 751 MET H	OUTLIER (0.02)%) General / -167.2,-88.2	Favored (12.3%) *p-90* chi angles: 59.7,290.3	0.14Å	Favored (15.844%)	-	-	-
A 751		MET	1.19	0.48Å H with A 750 TRP CG	Allowed (0.05%) General / 85.3,135.4	Favored (62.3%) *tpp* chi angles: 185.8,62.1,72.2	0.05Å	CaBLAM Disfavored (3.79%)	-	-	-
A 752		ILE	1.02	-	Favored (37.97%) Ile or Val / -59.6,-27.6	Favored (18.2%) *tt* chi angles: 195.5,168.8	0.07Å	Favored (54.526%)	-	-	-
A 753		LYS	0.89	-	Favored (79.44%) General / -64.7,-34.9	Favored (98.3%) *mttt* chi angles: 292.1,181.7,177.6,178.3	0.04Å	Favored (70.992%) alpha helix	-	-	-
A 754		GLU	0.79	-	Favored (58.26%) General / -75.3,-40.6	Favored (66.5%) *tp30* chi angles: 181.9,65.1,26.5	0.05Å	Favored (75.446%) alpha helix	-	-	-
A 755		THR	0.73	-	Favored (67.51%) General / -68.9,-29.3	Favored (71.1%) *p* chi angles: 59.6	0.04Å	Favored (78.713%) alpha helix	-	-	-
A 756		ALA	0.68	-	Favored (97.43%) General / -63.9,-42.1	-	0.06Å	Favored (79.312%) alpha helix	-	-	-
A 757		CYS	0.66	-	Favored (90.7%) General / -64.7,-44.3	Favored (91%) *m* chi angles: 291.7	0.06Å	Favored (82.921%) alpha helix	-	-	-
A 758		LEU	0.64	-	Favored (97.8%) General / -63.8,-42.6	Favored (69.8%) *tp* chi angles: 177.5,59.2	0.02Å	Favored (82.721%) alpha helix	-	-	-
A 759		SER	0.62	-	Favored (96.15%) General / -61.0,-41.4	Favored (68.8%) *m* chi angles: 294.8	0.10Å	Favored (82.903%) alpha helix	-	-	-
A 760		LYS	0.62	-	Favored (99.6%) General / -62.3,-42.7	Favored (88.2%) *tttt* chi angles: 184.7,175.8,183.7,182	0.05Å	Favored (92.989%) alpha helix	-	-	-

#	Alt	Res	High B	Clash > 0.4Å	Ramachandran	Rotamer	Cβ deviation	CaBLAM	Bond lengths	Bond angles	Cis Peptides
			Avg: 0.92	Clashscore: 1.51	Outliers: 4 of 903	Poor rotamers: 0 of 780	Outliers: 0 of 837	Outliers: 18 of 901	Outliers: 11 of 905	Outliers: 16 of 905	Non-Trans: 0 of 904
A 761		ALA	0.62	-	Favored (93.47%) General / -59.9,-42.4	-	0.07Å	Favored (92.819%) alpha helix	-	-	-
A 762		TYR	0.62	-	Favored (94.02%) General / -65.2,-41.8	Favored (22.4%) *m-80* chi angles: 283.9,126.6	0.09Å	Favored (91.422%) alpha helix	-	-	-
A 763		ALA	0.63	-	Favored (98.45%) General / -62.4,-41.2	-	0.06Å	Favored (82.849%) alpha helix	-	-	-
A 764		ASN	0.64	-	Favored (67.94%) General / -72.7,-36.6	Favored (99.6%) *m-40* chi angles: 288.2,342.4	0.09Å	Favored (89.625%) alpha helix	-	-	-
A 765		MET	0.66	-	Favored (86.76%) General / -60.7,-47.3	Favored (63.4%) *ttp* chi angles: 180.3,181.2,66.9	0.06Å	Favored (85.322%) alpha helix	-	-	-
A 766		TRP	0.67	-	Favored (94.72%) General / -64.9,-42.6	Favored (45.9%) *m-10* chi angles: 291.8,334.2	0.10Å	Favored (87.343%) alpha helix	-	-	-
A 767		SER	0.68	-	Favored (83.38%) General / -61.5,-37.4	Favored (70.8%) *m* chi angles: 295.1	0.08Å	Favored (73.846%) alpha helix	-	-	-
A 768		LEU	0.69	-	Favored (17.83%) General / -79.8,-45.9	Favored (27.3%) *tp* chi angles: 188.7,63	0.07Å	Favored (57.003%) alpha helix	-	-	-
A 769		MET	0.7	-	Favored (22.86%) General / -83.8,-37.2	Favored (51.8%) *mmp* chi angles: 294.5,298.6,98.9	0.06Å	Favored (52.48%)	-	-	-
A 770		TYR	0.7	-	Favored (4.48%) General / -119.8,36.5	Favored (65.2%) *m-80* chi angles: 304.5,106.9	0.03Å	Favored (12.966%)	-	-	-
A 771		PHE	0.69	-	Favored (32.97%) General / -55.5,-24.7	Favored (21.6%) *p90* chi angles: 69,80	0.10Å	Favored (19.941%)	-	OUTLIER(S) worst is CA-CB-CG: 4.5 σ	-
A 772		HIS	0.69	-	Favored (59.66%) General / -83.2,-7.2	Allowed (1.4%) *p-80* chi angles: 66.3,322.9	0.08Å	Favored (45.18%)	-	-	-
A 773		LYS	0.69	-	Favored (35.55%) General / -88.5,128.0	Favored (43.7%) *mtpt* chi angles: 298.2,181.8,75.5,179.6	0.02Å	Favored (35.182%)	-	-	-
A 774		ARG	0.69	-	Favored (91.44%) General / -60.6,-40.4	Favored (87.6%) *mtp85* chi angles: 290.2,178.6,64.9,85.3	0.05Å	Favored (44.253%)	-	-	-
A 775		ASP	0.68	-	Favored (64.44%) General / -73.5,-39.8	Favored (95.7%) *m-30* chi angles: 290.8,346.1	0.11Å	Favored (84.305%) alpha helix	-	OUTLIER(S) worst is CA-CB-CG: 4.5 σ	-
A 776		MET	0.68	-	Favored (67.2%) General / -71.9,-32.2	Favored (95.9%) *mmm* chi angles: 293.5,304.8,293.7	0.03Å	Favored (92.591%) alpha helix	-	-	-

A 777		ARG	0.67	-	Favored (78.95%) General / -65.1,-47.0	Favored (37%) *ttm170* chi angles: 186.9,183.6,304,182.7	0.05Å	Favored (79.427%) alpha helix	-	-	-
A 778		LEU	0.67	-	Favored (87.08%) General / -61.4,-47.1	Favored (65.2%) *tp* chi angles: 177.2,57.6	0.03Å	Favored (81.803%) alpha helix	-	-	-
A 779		LEU	0.66	-	Favored (88.45%) General / -59.9,-46.7	Favored (70.4%) *tp* chi angles: 177.1,60.5	0.01Å	Favored (93.873%) alpha helix	-	-	-
A 780		SER	0.65	-	Favored (97.87%) General / -61.0,-42.1	Favored (70.7%) *m* chi angles: 296.2	0.10Å	Favored (87.51%) alpha helix	-	-	-
**#**	**Alt**	**Res**	**High B**	**Clash > 0.4Å**	**Ramachandran**	**Rotamer**	**Cβ deviation**	**CaBLAM**	**Bond lengths**	**Bond angles**	**Cis Peptides**
			Avg: 0.92	Clashscore: 1.51	Outliers: 4 of 903	Poor rotamers: 0 of 780	Outliers: 0 of 837	Outliers: 18 of 901	Outliers: 11 of 905	Outliers: 16 of 905	Non-Trans: 0 of 904
A 781		LEU	0.64	-	Favored (92.85%) General / -65.3,-39.6	Favored (92.8%) *mt* chi angles: 291.3,172	0.03Å	Favored (95.807%) alpha helix	-	-	-
A 782		ALA	0.63	-	Favored (94.35%) General / -62.8,-45.1	-	0.04Å	Favored (90.705%) alpha helix	-	-	-
A 783		VAL	0.63	-	Favored (97.77%) Ile or Val / -64.0,-44.0	Favored (69.6%) *t* chi angles: 172.1	0.04Å	Favored (90.628%) alpha helix	-	-	-
A 784		SER	0.63	-	Favored (73.82%) General / -58.8,-36.0	Favored (65.2%) *m* chi angles: 297.3	0.04Å	Favored (76.959%) alpha helix	-	-	-
A 785		SER	0.64	-	Favored (59.86%) General / -76.9,-11.8	Favored (72.6%) *p* chi angles: 71.7	0.03Å	Favored (63.327%) alpha helix	-	-	-
A 786		ALA	0.66	-	Favored (12.11%) General / -90.2,-40.6	-	0.05Å	Favored (10.596%)	-	-	-
A 787		VAL	0.68	-	Favored (51.25%) Pre-Pro / -84.1,131.4	Favored (66.8%) *t* chi angles: 179.1	0.05Å	Favored (15.288%)	-	-	-
A 788		PRO	0.71	-	Favored (30.53%) Trans-Pro / -49.7,139.9	Favored (90.5%) *Cg_exo* chi angles: 329.6,38.1,331.1	0.04Å	Favored (54.292%)	-	-	-
A 789		THR	0.74	-	Favored (87.19%) General / -58.0,-44.1	Favored (92.1%) *m* chi angles: 299	0.01Å	Favored (52.84%)	-	-	-
A 790		SER	0.77	-	Favored (57.71%) General / -80.5,-6.2	Favored (91.6%) *p* chi angles: 66.6	0.02Å	Favored (50.617%)	-	-	-
A 791		TRP	0.81	-	Favored (46.1%) General / -72.7,137.7	Favored (42.5%) *m-10* chi angles: 289.9,9.4	0.01Å	Favored (35.486%)	-	-	-
A 792		VAL	0.85	-	Favored (61.68%) Pre-Pro / -99.5,118.8	Favored (72.2%) *t* chi angles: 178.5	0.09Å	Favored (43.547%) beta sheet	-	-	-

A 793		PRO	0.9	-	Favored (83.11%) Trans-Pro / -55.7,139.1	Favored (87.3%) *Cg_exo* chi angles: 333.5,36.1,330.3	0.06Å	Favored (35.389%) beta sheet	-	-	-
A 794		GLN	0.96	-	Favored (5.89%) General / -115.1,-31.9	Favored (94.5%) *mm-40* chi angles: 300.5,296.1,304.9	0.02Å	CaBLAM Disfavored (4.596%) try beta sheet	-	-	-
A 795		GLY	1.04	-	Favored (35.59%) Glycine / -75.3,147.2	-	-	Favored (34.864%) beta sheet	-	-	-
A 796		ARG	1.14	-	Favored (38.71%) General / -77.2,134.1	Favored (77.9%) *ttm-80* chi angles: 181.7,178.3,290.9,276.6	0.01Å	Favored (43.028%)	-	-	-
A 797		THR	1.23	-	Favored (12.88%) General / -97.5,-30.4	Favored (70.1%) *p* chi angles: 59.4	0.10Å	Favored (25.416%)	-	-	-
A 798		THR	1.31	-	Favored (36.72%) General / -152.2,154.9	Favored (10.1%) *t* chi angles: 186.2	0.02Å	Favored (33.758%)	-	-	-
A 799		TRP	1.36	-	Favored (29.1%) General / -105.3,2.6	Favored (32.3%) *m-90* chi angles: 297.8,261.9	0.08Å	Favored (5.115%)	-	-	-
A 800		SER	1.38	-	Favored (56.59%) General / -59.2,139.8	Favored (42.2%) *t* chi angles: 175.9	0.05Å	Favored (32.456%)	-	-	-
**#**	**Alt**	**Res**	**High B**	**Clash > 0.4Å**	**Ramachandran**	**Rotamer**	**Cβ deviation**	**CaBLAM**	**Bond lengths**	**Bond angles**	**Cis Peptides**
			Avg: 0.92	Clashscore: 1.51	Outliers: 4 of 903	Poor rotamers: 0 of 780	Outliers: 0 of 837	Outliers: 18 of 901	Outliers: 11 of 905	Outliers: 16 of 905	Non-Trans: 0 of 904
A 801		VAL	1.37	-	Favored (18.5%) Ile or Val / -66.5,-14.9	Favored (25.8%) *m* chi angles: 299.5	0.09Å	Favored (27.617%)	-	-	-
A 802		HIS	1.31	-	Favored (39.59%) General / -102.8,6.8	Favored (98.9%) *m-70* chi angles: 296.9,289.6	0.07Å	Favored (50.097%)	OUTLIER(S) worst is CB--CG: 6.2 σ	-	-
A 803		GLY	1.22	-	Favored (29.45%) Glycine / -82.7,151.7	-	-	Favored (18.805%)	-	-	-
A 804		LYS	1.11	-	Favored (8.16%) General / -112.5,-27.6	Favored (30.1%) *mmmt* chi angles: 300.8,298.9,286.2,184.8	0.02Å	CaBLAM Outlier (0.755%) try beta sheet	-	-	-
A 805		GLY	1.01	-	Favored (86.97%) Glycine / 83.4,7.1	-	-	Favored (73.504%)	-	-	-
A 806		GLU	0.92	-	Favored (65.51%) General / -60.2,-25.4	Favored (48.3%) *mm-30* chi angles: 287.1,288.8,311.9	0.04Å	Favored (30.627%)	-	-	-
A 807		TRP	0.85	-	Favored (59.13%) General / -81.8,-11.1	Favored (45.4%) *p90* chi angles: 53.5,90.6	0.05Å	Favored (56.333%) three-ten	-	-	-
A 808		MET	0.8	-	Favored (7.34%) General / -90.0,69.6	Favored (90.2%) *mtp* chi angles: 294.8,183,70.3	0.08Å	CaBLAM Disfavored (3.245%)	-	-	-

#	Alt	Res	High B	Clash > 0.4Å	Ramachandran	Rotamer	Cβ deviation	CaBLAM	Bond lengths	Bond angles	Cis Peptides
A 809		THR	0.77	-	Favored (3.77%) General / -145.4,-171.9	Favored (11.2%) *t* chi angles: 189.2	0.07Å	Favored (27.664%)	-	-	-
A 810		THR	0.75	-	Favored (9.07%) General / -121.5,-8.9	Favored (63.4%) *p* chi angles: 57.9	0.04Å	CaBLAM Disfavored (1.246%)	-	-	-
A 811		GLU	0.75	-	Favored (58.51%) General / -63.5,142.1	Favored (78.8%) *mt-10* chi angles: 291,178.3,322.9	0.03Å	Favored (26.772%)	-	-	-
A 812		ASP	0.75	-	Favored (51.34%) General / -56.5,132.4	Favored (12.5%) *t70* chi angles: 190.7,296	0.04Å	Favored (42.954%)	-	-	-
A 813		MET	0.74	-	Favored (65.31%) General / -60.9,-23.6	Favored (85.8%) *mmm* chi angles: 287.5,304.4,295.9	0.11Å	Favored (37.907%)	-	-	-
A 814		LEU	0.73	-	Favored (75.04%) General / -66.7,-33.2	Favored (71.2%) *mt* chi angles: 289.7,175.4	0.07Å	Favored (71.609%) alpha helix	-	-	-
A 815		GLU	0.71	-	Favored (69.44%) General / -71.9,-39.7	Favored (73.7%) *tp30* chi angles: 179.7,63,19.1	0.04Å	Favored (81.706%) alpha helix	-	-	-
A 816		VAL	0.7	-	Favored (97.79%) Ile or Val / -63.8,-43.2	Favored (88.1%) *t* chi angles: 174	0.02Å	Favored (92.794%) alpha helix	-	-	-
A 817		TRP	0.7	-	Favored (87.26%) General / -58.7,-46.4	Favored (61.9%) *t60* chi angles: 191.8,94.9	0.10Å	Favored (98.228%) alpha helix	-	-	-
A 818		ASN	0.69	-	Favored (97.93%) General / -61.7,-41.6	Favored (78.7%) *m-40* chi angles: 282.3,335.3	0.03Å	Favored (97.918%) alpha helix	-	-	-
A 819		ARG	0.7	-	Favored (92.96%) General / -65.4,-42.2	Favored (98.6%) *mtt180* chi angles: 289.8,175.2,184.3,176.7	0.03Å	Favored (71.224%) alpha helix	-	-	-
A 820		VAL	0.71	-	Favored (20.87%) Ile or Val / -78.8,-49.2	Favored (82.8%) *t* chi angles: 173.4	0.08Å	Favored (56.865%) alpha helix	-	-	-
**#**	**Alt**	**Res**	**High B**	**Clash > 0.4Å**	**Ramachandran**	**Rotamer**	**Cβ deviation**	**CaBLAM**	**Bond lengths**	**Bond angles**	**Cis Peptides**
			Avg: 0.92	Clashscore: 1.51	Outliers: 4 of 903	Poor rotamers: 0 of 780	Outliers: 0 of 837	Outliers: 18 of 901	Outliers: 11 of 905	Outliers: 16 of 905	Non-Trans: 0 of 904
A 821		TRP	0.73	-	Favored (24.96%) General / -86.1,-26.9	Favored (94%) *m100* chi angles: 287.8,107.3	0.04Å	Favored (38.052%) alpha helix	-	-	-
A 822		ILE	0.75	-	Favored (3.31%) Ile or Val / -111.7,-59.2	Favored (49%) *mm* chi angles: 303,299.9	0.06Å	Favored (16.67%) alpha helix	-	-	-
A 823		ILE	0.78	-	Favored (58.24%) Ile or Val / -69.0,-49.9	Favored (92.3%) *mt* chi angles: 295.4,166.9	0.07Å	Favored (59.302%) alpha helix	-	-	-
A 824		ASN	0.81	-	Favored (48.93%) General / -96.7,6.3	Favored (88.5%) *m-40* chi angles: 291.8,321.6	0.02Å	Favored (24.975%)	-	-	-

A 825		ASN	0.83	-	Favored (53.21%) Pre-Pro / -82.0,114.8	Favored (47.1%) *t0* chi angles: 185.4,320.3	0.05Å	Favored (29.073%)	-	-	-
A 826		PRO	0.85	-	Favored (51.58%) Trans-Pro / -64.6,-15.8	Favored (49.2%) *Cg_endo* chi angles: 25.1,326.3,27.8	0.01Å	Favored (39.707%)	-	-	-
A 827		HIS	0.87	-	Favored (27.78%) General / -102.1,-3.3	Favored (95.8%) *m-70* chi angles: 294.2,285.5	0.06Å	Favored (56.25%)	-	-	-
A 828		MET	0.87	-	Favored (7.56%) General / -85.2,89.1	Favored (96.1%) *mmm* chi angles: 296.2,297.5,288.3	0.10Å	Favored (21.574%)	-	-	-
A 829		GLN	0.86	-	Favored (73.78%) General / -59.8,-35.0	Favored (97.6%) *mt0* chi angles: 290.8,176.7,335.5	0.03Å	Favored (38.168%)	-	-	-
A 830		ASP	0.84	-	Favored (4.2%) General / -108.2,92.8	Favored (51.4%) *t0* chi angles: 180.2,342.3	0.03Å	Favored (20.777%)	-	-	-
A 831		LYS	0.81	-	Favored (8.34%) General / -85.1,63.9	Favored (98.6%) *mttt* chi angles: 295.4,184.6,175.2,182.9	0.03Å	Favored (38.195%)	-	-	-
A 832		THR	0.79	-	Favored (35.83%) General / -89.2,129.1	Favored (83.8%) *m* chi angles: 296.6	0.09Å	Favored (30.437%) beta sheet	-	-	-
A 833		THR	0.78	-	Favored (19.37%) General / -82.8,166.0	Favored (71%) *p* chi angles: 59.5	0.06Å	Favored (38.219%) beta sheet	-	-	-
A 834		VAL	0.77	-	Favored (23.37%) Ile or Val / -95.8,137.6	Favored (46.1%) *t* chi angles: 168.8	0.05Å	Favored (44.958%)	-	-	-
A 835		LYS	0.77	-	Favored (6.72%) General / -107.5,-35.5	Favored (99.4%) *mttt* chi angles: 294.9,181.7,179.9,178.3	0.02Å	Favored (17.232%)	-	-	-
A 836		GLU	0.77	-	Favored (48.27%) General / -133.7,156.2	Favored (51.2%) *mt-10* chi angles: 294.3,183.8,293.6	0.02Å	Favored (23.359%)	-	-	-
A 837		TRP	0.77	-	Favored (66.76%) General / -61.5,-24.7	Favored (96.4%) *m100* chi angles: 287,99.1	0.02Å	Favored (49.015%) alpha helix	-	-	-
A 838		ARG	0.76	-	Favored (64.05%) General / -65.4,-16.9	Favored (11.4%) *ptm-80* chi angles: 70.6,190.9,299.7,272.9	0.02Å	Favored (64.299%) three-ten	-	-	-
A 839		ASP	0.74	-	Favored (44.19%) General / -80.5,-20.2	Favored (79%) *m-30* chi angles: 296.4,334.6	0.14Å	Favored (46.018%)	-	-	-
A 840		ILE	0.72	-	Favored (68.91%) Pre-Pro / -90.1,121.3	Favored (87.4%) *mt* chi angles: 298.7,172.3	0.06Å	Favored (33.098%)	-	-	-
**#**	**Alt**	**Res**	**High B**	**Clash > 0.4Å**	**Ramachandran**	**Rotamer**	**Cβ deviation**	**CaBLAM**	**Bond lengths**	**Bond angles**	**Cis Peptides**
			Avg: 0.92	Clashscore: 1.51	Outliers: 4 of 903	Poor rotamers: 0 of 780	Outliers: 0 of 837	Outliers: 18 of 901	Outliers: 11 of 905	Outliers: 16 of 905	Non-Trans: 0 of 904

A 841		PRO	0.71	-	Favored (8.57%) Trans-Pro / -76.3,177.6	Favored (50%) *Cg_endo* chi angles: 33,321.1,28.8	0.10Å	Favored (10.889%)	-	-	-
A 842		TYR	0.71	-	Favored (46.52%) General / -137.4,157.5	Favored (56.4%) *p90* chi angles: 64.8,93.9	0.07Å	Favored (16.652%)	-	-	-
A 843		LEU	0.73	-	Favored (57.06%) General / -65.9,137.3	Favored (24.4%) *tp* chi angles: 189.4,64.7	0.12Å	Favored (31.638%)	-	-	-
A 844		THR	0.75	-	Favored (34.25%) General / -53.5,138.1	Favored (99.3%) *m* chi angles: 300.6	0.04Å	Favored (43.512%)	-	-	-
A 845		LYS	0.77	-	Favored (41.06%) General / -51.5,-35.7	Favored (97.2%) *mttt* chi angles: 288.9,179.1,181.3,176.4	0.01Å	Favored (57.426%)	-	-	-
A 846		ARG	0.79	-	Favored (90.89%) General / -61.6,-39.3	Favored (99.6%) *mtm-85* chi angles: 290,191.8,295.9,272.4	0.07Å	Favored (83.702%) alpha helix	-	-	-
A 847		GLN	0.8	-	Favored (73.27%) General / -70.6,-40.3	Favored (87.3%) *mt0* chi angles: 291,170.1,309.3	0.05Å	Favored (82.15%) alpha helix	-	-	-
A 848		ASP	0.81	-	Favored (76.79%) General / -58.5,-38.0	Favored (46.5%) *m-30* chi angles: 276.2,346.2	0.11Å	Favored (80.952%) alpha helix	-	OUTLIER(S) worst is CA-CB-CG: 6.2 σ	-
A 849		LYS	0.81	-	Favored (84.68%) General / -66.3,-37.2	Favored (96.6%) *mttt* chi angles: 289.5,179.5,180.7,180.3	0.07Å	Favored (88.418%) alpha helix	-	-	-
A 850		LEU	0.8	-	Favored (72.22%) General / -63.2,-31.1	Favored (92.9%) *mt* chi angles: 292.2,174.2	0.02Å	Favored (83.618%)	-	-	-
A 851		CYS	0.78	-	Favored (4.57%) General / -91.0,20.9	Favored (100%) *m* chi angles: 292.5	0.08Å	Favored (12.816%)	-	-	-
A 852		GLY	0.77	-	Favored (73.48%) Glycine / 84.8,12.9	-	-	Favored (62.287%)	-	-	-
A 853		SER	0.77	-	Favored (51.11%) General / -65.9,148.9	Favored (41.4%) *t* chi angles: 176.2	0.11Å	Favored (18.7%) beta sheet	-	-	-
A 854		LEU	0.78	-	Favored (43.7%) General / -99.2,9.3	Favored (86.7%) *mt* chi angles: 298.1,179.6	0.05Å	Favored (5.688%)	-	-	-
A 855		ILE	0.82	-	Favored (23.91%) Ile or Val / -58.8,133.7	Favored (8.3%) *tp* chi angles: 192.7,70	0.07Å	Favored (13.178%)	-	-	-
A 856		GLY	0.87	-	Favored (64.64%) Glycine / 96.5,-14.1	-	-	Favored (83.512%)	-	-	-
A 857		MET	0.93	-	Favored (36.02%) General / -80.3,134.0	Favored (64.7%) *ttp* chi angles: 183,179,73.1	0.03Å	Favored (31.348%)	-	-	-
A 858		THR	0.99	-	Favored (68.59%)	Favored (89.8%) *m* chi angles: 298.7	0.02Å	Favored (54.581%)	-	-	-

#	Alt	Res	High B	Clash > 0.4Å	Ramachandran	Rotamer	Cβ deviation	CaBLAM	Bond lengths	Bond angles	Cis Peptides
					General / -53.5,-43.7						
A 859		ASN	1.02	-	Favored (68.74%) General / -65.9,-28.2	Favored (90.5%) *m-40* chi angles: 286.5,332.2	0.10Å	Favored (74.122%) alpha helix	-	-	-
A 860		ARG	1.04	-	Favored (63.59%) General / -68.7,-48.5	Favored (4.6%) *tmt170* chi angles: 189,269,190.2,198.8	0.06Å	Favored (69.36%) alpha helix	-	-	-
**#**	**Alt**	**Res**	**High B**	**Clash > 0.4Å**	**Ramachandran**	**Rotamer**	**Cβ deviation**	**CaBLAM**	**Bond lengths**	**Bond angles**	**Cis Peptides**
			Avg: 0.92	Clashscore: 1.51	Outliers: 4 of 903	Poor rotamers: 0 of 780	Outliers: 0 of 837	Outliers: 18 of 901	Outliers: 11 of 905	Outliers: 16 of 905	Non-Trans: 0 of 904
A 861		ALA	1.03	-	Favored (91.39%) General / -61.9,-39.3	-	0.04Å	Favored (85.055%) alpha helix	-	-	-
A 862		THR	1.02	-	Favored (77.47%) General / -64.4,-47.9	Favored (97.1%) *m* chi angles: 299.9	0.05Å	Favored (84.258%) alpha helix	-	-	-
A 863		TRP	1.02	-	Favored (84.17%) General / -59.2,-47.8	Favored (45.7%) *t60* chi angles: 165.3,80	0.02Å	Favored (89.821%) alpha helix	-	-	-
A 864		ALA	1.02	-	Favored (82.04%) General / -58.3,-40.5	-	0.04Å	Favored (88.061%) alpha helix	-	-	-
A 865		SER	1.02	-	Favored (85.41%) General / -62.3,-37.4	Favored (71.1%) *m* chi angles: 296.1	0.04Å	Favored (75.375%) alpha helix	-	-	-
A 866		HIS	1.01	-	Favored (30.3%) General / -98.2,13.7	Favored (94.7%) *m-70* chi angles: 293.1,288.7	0.01Å	Favored (37.652%) alpha helix	-	-	-
A 867		ILE	0.99	-	Favored (83.85%) Ile or Val / -60.2,-40.5	Favored (81.4%) *mt* chi angles: 289.9,168.5	0.07Å	Favored (42.381%) alpha helix	-	-	-
A 868		HIS	0.95	-	Favored (76.48%) General / -57.9,-38.9	Favored (79.9%) *m-70* chi angles: 287.1,283.2	0.04Å	Favored (74.658%) alpha helix	-	-	-
A 869		LEU	0.91	-	Favored (88.44%) General / -62.0,-46.6	Favored (65.8%) *tp* chi angles: 176.2,60.3	0.03Å	Favored (89.544%) alpha helix	-	-	-
A 870		VAL	0.87	-	Favored (95.37%) Ile or Val / -63.2,-42.1	Favored (80.6%) *t* chi angles: 173.2	0.04Å	Favored (94.353%) alpha helix	-	-	-
A 871		ILE	0.83	-	Favored (92.05%) Ile or Val / -61.9,-41.8	Favored (33.7%) *mm* chi angles: 294.2,299	0.02Å	Favored (91.825%) alpha helix	-	-	-
A 872		HIS	0.8	-	Favored (83.98%) General / -59.2,-47.8	Favored (90.3%) *t70* chi angles: 177.4,69.1	0.03Å	Favored (94.781%) alpha helix	-	-	-
A 873		ARG	0.77	-	Favored (98.3%) General / -61.5,-42.1	Favored (98.1%) *mtt180* chi angles: 288.8,174.1,175.7,170.3	0.04Å	Favored (83.979%) alpha helix	-	-	-

A 874		ILE	0.74	-	Favored (80.99%) Ile or Val / -68.7,-45.5	Favored (96.7%) *mt* chi angles: 293.9,168.2	0.07Å	Favored (85.179%) alpha helix	-	-	-
A 875		ARG	0.72	-	Favored (79.7%) General / -56.5,-43.6	Favored (3.6%) *tmt170* chi angles: 186,262,181.9,166.8	0.02Å	Favored (88.487%) alpha helix	-	-	-
A 876		THR	0.71	-	Favored (77.49%) General / -55.6,-44.8	Favored (98.5%) *m* chi angles: 300.2	0.03Å	Favored (87.419%) alpha helix	-	-	-
A 877		LEU	0.7	-	Favored (68.01%) General / -61.9,-26.2	Favored (96.2%) *mt* chi angles: 292.4,173.6	0.06Å	Favored (71.491%) alpha helix	-	-	-
A 878		ILE	0.71	0.62Å O with A 878 ILE HG22	Favored (10.57%) Ile or Val / -90.1,-9.2	Favored (96.6%) *mt* chi angles: 293.8,168.6	0.01Å	Favored (46.542%)	-	-	-
A 879		GLY	0.72	-	Favored (15.24%) Glycine / 115.7,-174.3	-	-	Favored (21.628%)	-	-	-
A 880		LYS	0.73	-	Favored (37.35%) General / -88.1,6.2	Favored (99%) *mttt* chi angles: 294.2,181.4,179.8,178.8	0.01Å	CaBLAM Disfavored (1.037%)	-	-	-
**#**	**Alt**	**Res**	**High B**	**Clash > 0.4Å**	**Ramachandran**	**Rotamer**	**Cβ deviation**	**CaBLAM**	**Bond lengths**	**Bond angles**	**Cis Peptides**
			Avg: 0.92	Clashscore: 1.51	Outliers: 4 of 903	Poor rotamers: 0 of 780	Outliers: 0 of 837	Outliers: 18 of 901	Outliers: 11 of 905	Outliers: 16 of 905	Non-Trans: 0 of 904
A 881		GLU	0.75	-	Favored (55.99%) General / -61.5,143.4	Favored (15.4%) *mm-30* chi angles: 294.7,281.2,294.8	0.08Å	Favored (22.035%)	-	-	-
A 882		ARG	0.76	-	Favored (58.26%) General / -62.2,137.5	Favored (83.9%) *ttt180* chi angles: 183.4,176.1,177.8,179.2	0.05Å	Favored (29.302%) beta sheet	-	-	-
A 883		TYR	0.78	-	Favored (38.98%) General / -135.5,160.3	Favored (43.4%) *p90* chi angles: 70.5,96.3	0.05Å	Favored (54.229%) beta sheet	-	-	-
A 884		THR	0.79	-	Favored (55.09%) General / -109.5,131.4	Favored (91.5%) *m* chi angles: 297.9	0.03Å	Favored (38.87%) beta sheet	-	-	-
A 885		ASP	0.81	-	Favored (6.91%) General / -83.2,88.4	Favored (64%) *t0* chi angles: 184.9,342.6	0.06Å	Favored (42.608%) beta sheet	-	-	-
A 886		TYR	0.82	-	Favored (3.47%) General / -78.7,10.2	Favored (56.4%) *m-80* chi angles: 287.8,110.6	0.05Å	CaBLAM Disfavored (2.124%)	-	-	-
A 887		LEU	0.84	-	Favored (25.63%) General / -86.9,-22.4	Favored (97%) *mt* chi angles: 294.2,172.4	0.05Å	Favored (7.645%)	-	-	-
A 888		THR	0.87	-	Favored (54.12%) General / -88.8,2.1	Favored (64.2%) *p* chi angles: 63.3	0.04Å	Favored (45.267%) three-ten	-	-	-
A 889		VAL	0.9	-	Favored (10.33%) Ile or Val / -95.4,-4.7	Favored (21.8%) *m* chi angles: 301.8	0.07Å	Favored (58.543%)	-	-	-

A 890		MET	0.96	-	Favored (51.13%) General / -103.7,130.2	Favored (31.7%) *ttt* chi angles: 181,180.4,182.9	0.10Å	Favored (26.159%)	-	-	-
A 891		ASP	1.07	-	Favored (64.03%) General / -55.2,-34.6	Favored (97.4%) *m-30* chi angles: 286.7,347.7	0.05Å	Favored (38.345%)	-	-	-
A 892		ARG	1.25	-	Favored (5.94%) General / -45.7,-37.5	Favored (63.1%) *ttt90* chi angles: 180.8,175.9,173.6,83.2	0.03Å	Favored (26.881%)	-	-	-
A 893		TYR	1.53	-	Favored (17.44%) General / -111.9,3.3	Favored (96.6%) *m-80* chi angles: 297.3,100.1	0.02Å	Favored (28.162%) alpha helix	-	-	-
A 894		SER	1.96	-	Favored (78.75%) General / -63.0,-35.0	Favored (94.4%) *p* chi angles: 64.1	0.05Å	Favored (52.476%) alpha helix	-	-	-
A 895		VAL	2.56	-	Favored (24.16%) Ile or Val / -70.4,-23.0	Favored (26.5%) *m* chi angles: 299.2	0.05Å	Favored (68.817%) alpha helix	-	-	-
A 896		ASP	3.34	-	Favored (57.03%) General / -91.0,-3.7	Favored (89.8%) *m-30* chi angles: 292.7,336.6	0.01Å	Favored (55.607%) alpha helix	-	-	-
A 897		ALA	4.3	-	Favored (2.66%) General / -109.2,-53.3	-	0.05Å	Favored (12.071%) alpha helix	-	-	-
A 898		ASP	5.34	-	Favored (69.39%) General / -63.9,-27.8	Favored (97.6%) *m-30* chi angles: 288.4,348.3	0.02Å	Favored (61.206%) alpha helix	-	-	-
A 899		LEU	6.39	-	Favored (35.9%) General / -100.8,-1.0	Favored (79.6%) *mt* chi angles: 302,176.9	0.04Å	CaBLAM Disfavored (3.977%) try alpha helix	-	-	-
A 900		GLN	7.37	0.44Å HB2 with A 901 PRO HD3	OUTLIER (0.02)%) Pre-Pro / -149.5,-74.1	Favored (81.8%) *mt0* chi angles: 288.9,184.5,331.4	0.17Å	CA Geom Outlier (0.069%)	-	-	-
**#**	**Alt**	**Res**	**High B**	**Clash > 0.4Å**	**Ramachandran**	**Rotamer**	**Cβ deviation**	**CaBLAM**	**Bond lengths**	**Bond angles**	**Cis Peptides**
			Avg: 0.92	Clashscore: 1.51	Outliers: 4 of 903	Poor rotamers: 0 of 780	Outliers: 0 of 837	Outliers: 18 of 901	Outliers: 11 of 905	Outliers: 16 of 905	Non-Trans: 0 of 904
A 901		PRO	8.29	0.44Å HD3 with A 900 GLN HB2	Favored (5.06%) Trans-Pro / -74.0,65.4	Favored (58.7%) *Cg_endo* chi angles: 32,320.9,30.3	0.07Å	CA Geom Outlier (0.055%)	-	-	-
A 902		GLY	9.11	-	Favored (52.92%) Glycine / -58.9,-21.9	-	-	Favored (19.535%)	-	-	-
A 903		GLU	9.77	-	Favored (68.05%) General / -61.1,-27.0	Favored (99.9%) *mt-10* chi angles: 291.9,178,352.7	0.05Å	Favored (53.989%)	-	-	-
A 904		LEU	10.25	-	Favored (39.26%) General / -102.1,10.7	Favored (84.1%) *mt* chi angles: 300.2,178.8	0.01Å	-	-	-	-
A 905		ILE	10.57	0.42Å O with A 905 ILE HG23	-	Favored (23.8%) *tt* chi angles: 188.9,167.7	0.03Å	-	-	-	-
