# Supplementary material for: Integrative proteome-wide structural analysis and high-throughput docking identify broad-spectrum antiviral scaffolds against Zika, Yellow Fever, West Nile, Saint Louis encephalitis, and Usutu viruses
Source: Front Cell Infect Microbiol. 2026 Apr 30;16:1723132. doi: 10.3389/fcimb.2026.1723132 (PMC13171538; doi:10.3389/fcimb.2026.1723132)
Supplement: Supplementary file 6 [file DataSheet6.zip › YFV/YF_NS5/Mol_probity_Files/YF_NS5_1FH-rama.pdf]

MolProbity Ramachandran analysis

YF_NS5_1FH.pdb, model 1

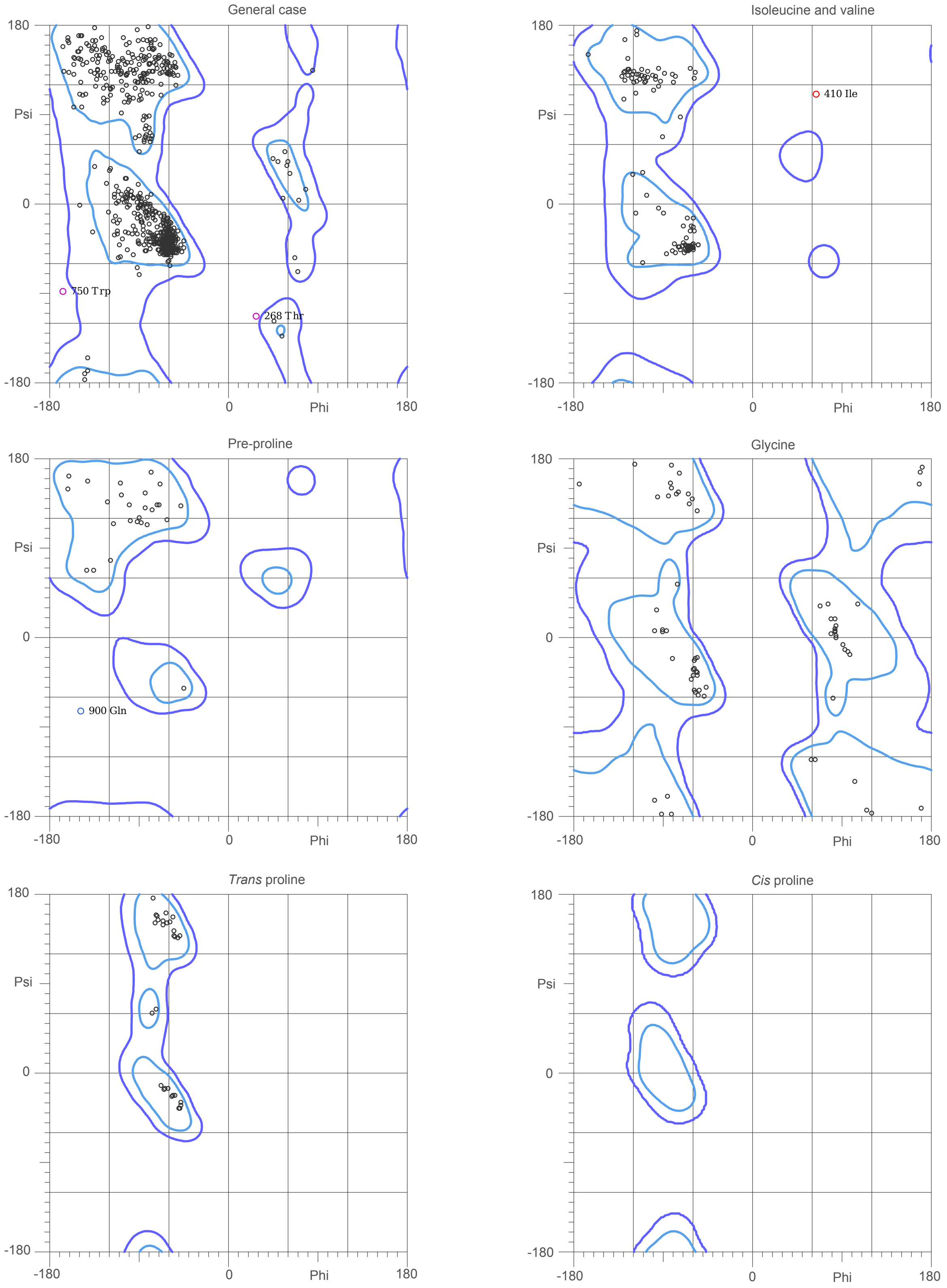

97.7% (882/903) of all residues were in favored (98%) regions.
99.6% (899/903) of all residues were in allowed (>99.8%) regions.

There were 4 outliers (phi, psi):
- 268 Thr (28.4, -113.5)
- 410 Ile (64.3, 111.7)
- 750 Trp (-167.2, -88.2)
- 900 Gln (-149.5, -74.1)
