# Supplementary material for: Integrative proteome-wide structural analysis and high-throughput docking identify broad-spectrum antiviral scaffolds against Zika, Yellow Fever, West Nile, Saint Louis encephalitis, and Usutu viruses
Source: Front Cell Infect Microbiol. 2026 Apr 30;16:1723132. doi: 10.3389/fcimb.2026.1723132 (PMC13171538; doi:10.3389/fcimb.2026.1723132)
Supplement: Supplementary file 7 [file DataSheet7.zip › ZIKV/ZIKV_E/Mol_probity_Files/ZIKV_E_1FH-multi.table.pdf]

Viewing ZIKV_E1FH-multi.table

Duke Biochemistry
Duke University School of Medicine

When finished, you should close this window.

Hint: Use File | Save As... to save a copy of this page.

All-Atom Contacts	Clashscore, all atoms:	1.46		99th percentile* (N=1784, all resolutions)
	Clashscore is the number of serious steric overlaps (> 0.4 Å) per 1000 atoms.			
Protein Geometry	Poor rotamers	1	0.24%	Goal: <0.3%
	Favored rotamers	404	98.78%	Goal: >98%
	Ramachandran outliers	6	1.20%	Goal: <0.05%
	Ramachandran favored	472	94.78%	Goal: >98%
	Rama distribution Z-score	-0.25 ± 0.37		Goal: abs(Z score) < 2
	MolProbity score^	1.24		99th percentile* (N=27675, 0Å - 99Å)
	Cβ deviations >0.25Å	0	0.00%	Goal: 0
	Bad bonds:	3 / 3872	0.08%	Goal: 0%
	Bad angles:	25 / 5243	0.48%	Goal: <0.1%
Peptide Omegas	Cis Prolines:	1 / 16	6.25%	Expected: ≤1 per chain, or ≤5%
	Cis nonProlines:	7 / 483	1.45%	Goal: <0.05%
Low-resolution Criteria	CaBLAM outliers	24	4.8%	Goal: <1.0%
	CA Geometry outliers	2	0.40%	Goal: <0.5%
Additional validations	Chiral volume outliers	0/591		
	Waters with clashes	0/0	0.00%	See UnDowser table for details

In the two column results, the left column gives the raw count, right column gives the percentage.

* 100th percentile is the best among structures of comparable resolution; 0th percentile is the worst. For clashscore the comparative set of structures was selected in 2004, for MolProbity score in 2006.

^ MolProbity score combines the clashscore, rotamer, and Ramachandran evaluations into a single score, normalized to be on the same scale as X-ray resolution.

Key to table colors and cutoffs here:

#	Alt	Res	High B	Clash > 0.4Å	Ramachandran	Rotamer	Cβ deviation	CaBLAM	Bond lengths	Bond angles	Cis Peptides
			Avg: 7.04	Clashscore: 1.46	Outliers: 6 of 498	Poor rotamers: 1 of 409	Outliers: 0 of 445	Outliers: 25 of 496	Outliers: 3 of 500	Outliers: 20 of 500	Non-Trans: 8 of 499
A 1		ILE	7.04	-	-	Favored (47.4%) *mm* chi angles: 300.1,299.1	0.05Å	-	-	-	-
A 2		ARG	7.04	0.42Å C with A 2 ARG HD3	Favored (68.4%) General / -56.7,-35.3	Favored (4.2%) *tmt-80* chi angles: 182.5,268.8,179.2,283.5	0.11Å	-	-	-	-
A 3		CYS	7.04	-	Favored (23.19%) General / -82.4,4.7	Favored (83.5%) *m* chi angles: 295.3	0.06Å	Favored (30.998%)	-	-	-
A 4		ILE	7.04	0.44Å HA with A 4 ILE HD12	Favored (37.79%) Ile or Val / -64.0,-25.3	Favored (8.2%) *tp* chi angles: 199.3,63.6	0.04Å	Favored (56.192%) alpha helix	-	-	-
A 5		GLY	7.04	-	Favored (78.03%) Glycine / -92.0,8.4	-	-	Favored (56.987%)	-	-	-
A 6		VAL	7.04	-	Favored (61.4%) Ile or Val / -107.2,122.4	Favored (81.8%) *t* chi angles: 176.7	0.05Å	Favored (32.814%)	-	-	-

A 7		SER	7.04	-	Favored (87.49%) General / -63.5,-46.1	Favored (45.6%) *t* chi angles: 179.4	0.03Å	Favored (33.429%)	-	-	-
A 8		ASN	7.04	-	Favored (7.99%) General / -88.5,65.7	Favored (86.8%) *m-40* chi angles: 296.1,315.6	0.06Å	Favored (8.739%)	-	-	-
A 9		ARG	7.04	-	Favored (50.39%) General / -109.6,124.0	Favored (73.5%) *ttt180* chi angles: 189,179.5,184.8,189.3	0.07Å	Favored (22.852%)	-	-	-
A 10		ASP	7.04	-	Favored (21.25%) General / -107.2,154.4	Favored (63.7%) *m-30* chi angles: 290.6,317.8	0.02Å	Favored (42.796%) beta sheet	-	-	-
A 11		PHE	7.04	-	Favored (47.66%) General / -123.8,147.3	Favored (87.3%) *m-80* chi angles: 296.8,84.3	0.05Å	Favored (66.318%) beta sheet	-	-	-
A 12		VAL	7.04	-	Favored (75.46%) Ile or Val / -122.7,128.9	Favored (68.8%) *t* chi angles: 178.9	0.03Å	Favored (39.847%) beta sheet	-	-	-
A 13		GLU	7.04	-	Favored (25.94%) General / -151.0,168.0	Favored (10.2%) *pt0* chi angles: 63.8,178.9,294.2	0.09Å	CaBLAM Disfavored (4.058%)	-	-	-
A 14		GLY	7.04	-	Favored (71.28%) Glycine / 94.5,-9.9	-	-	Favored (17.508%)	-	-	-
A 15		MET	7.04	-	Favored (6.04%) General / -80.2,67.6	Favored (69%) *mtt* chi angles: 296.8,180,183.5	0.06Å	Favored (9.606%)	-	-	-
A 16		SER	7.04	-	Favored (67.44%) General / -64.6,-24.0	Favored (86.4%) *p* chi angles: 67.9	0.04Å	Favored (13.022%)	-	-	-
A 17		GLY	7.04	-	Favored (22.13%) Glycine / -113.9,13.8	-	-	Favored (27.78%)	-	-	-
A 18		GLY	7.04	-	Favored (38.69%) Glycine / -75.0,148.9	-	-	Favored (26.11%)	-	-	-
A 19		THR	7.04	-	Favored (11.25%) General / -119.2,5.7	Favored (75.2%) *p* chi angles: 60	0.04Å	Favored (11.964%)	-	-	-
A 20		TRP	7.04	-	Favored (8.46%) General / -170.5,163.4	Favored (74%) *p-90* chi angles: 57.7,265.7	0.08Å	Favored (25.632%)	-	-	-
**#**	**Alt**	**Res**	**High B**	**Clash > 0.4Å**	**Ramachandran**	**Rotamer**	**Cβ deviation**	**CaBLAM**	**Bond lengths**	**Bond angles**	**Cis Peptides**
			Avg: 7.04	Clashscore: 1.46	Outliers: 6 of 498	Poor rotamers: 1 of 409	Outliers: 0 of 445	Outliers: 25 of 496	Outliers: 3 of 500	Outliers: 20 of 500	Non-Trans: 8 of 499
A 21		VAL	7.04	0.55Å O with A 21 VAL HG13	Favored (26.33%) Ile or Val / -143.2,137.5	Favored (6.4%) *p* chi angles: 59.3	0.12Å	Favored (30.933%)	-	-	-
A 22		ASP	7.04	-	Favored (36.84%) General / -94.8,121.8	Favored (99.2%) *m-30* chi angles: 287.8,346.6	0.11Å	Favored (55.664%) beta sheet	-	-	-

A 23		VAL	7.04	-	Favored (65.37%) Ile or Val / -122.1,134.4	Favored (96.7%) *t* chi angles: 175	0.08Å	Favored (57.794%) beta sheet	-	-	-
A 24		VAL	7.04	-	Favored (55.67%) Ile or Val / -103.4,122.2	Favored (53.1%) *t* chi angles: 180.9	0.01Å	Favored (63.794%) beta sheet	-	-	-
A 25		LEU	7.04	-	Favored (25.31%) General / -96.4,145.8	Favored (64.8%) *mt* chi angles: 304,176.2	0.07Å	Favored (46.751%) beta sheet	-	-	-
A 26		GLU	7.04	-	Favored (23.5%) General / -148.3,139.8	Favored (91.7%) *tt0* chi angles: 181.8,176,357.8	0.05Å	Favored (6.979%)	-	OUTLIER(S) worst is CA-C-O: 4.9 σ	-
A 27		HIS	7.04	-	Favored (67.6%) General / -67.4,-28.0	Favored (61.5%) *m90* chi angles: 284.8,75	0.09Å	CaBLAM Disfavored (2.601%)	OUTLIER(S) worst is CB--CG: 4.6 σ	-	-
A 28		GLY	7.04	-	Favored (87.82%) Glycine / -85.7,-0.5	-	-	Favored (52.445%) alpha helix	-	-	-
A 29		GLY	7.04	-	Favored (9.52%) Glycine / -134.5,-161.4	-	-	Favored (6.388%)	-	-	-
A 30		CYS	7.04	-	Favored (22.74%) General / -156.4,148.1	Favored (51.6%) *t* chi angles: 180.4	0.07Å	Favored (33.441%)	-	-	-
A 31		VAL	7.04	-	Favored (66.18%) Ile or Val / -117.5,132.8	Favored (68.5%) *t* chi angles: 178.9	0.02Å	Favored (63.882%) beta sheet	-	-	-
A 32		THR	7.04	-	Favored (52.75%) General / -110.3,125.1	Favored (92.6%) *m* chi angles: 297.7	0.08Å	Favored (72.781%) beta sheet	-	-	-
A 33		VAL	7.04	-	Favored (64.29%) Ile or Val / -114.4,132.2	Favored (64.1%) *t* chi angles: 179.5	0.04Å	Favored (56.487%) beta sheet	-	-	-
A 34		MET	7.04	-	Favored (46.3%) General / -140.6,156.3	Favored (22.2%) *ptp* chi angles: 64.6,194.4,73.4	0.05Å	Favored (29.893%)	-	-	-
A 35		ALA	7.04	-	Favored (37.33%) General / -155.4,157.6	-	0.02Å	Favored (29.257%)	-	-	-
A 36		GLN	7.04	-	Favored (9.08%) General / -44.2,-49.6	Favored (33.7%) *tt0* chi angles: 177,180.9,283.2	0.04Å	Favored (14.819%)	-	-	-
A 37		ASP	7.04	-	Favored (21.15%) General / -108.3,18.4	Favored (61.3%) *m-30* chi angles: 294.3,308	0.05Å	Favored (5.777%)	-	-	-
A 38		LYS	7.04	-	Favored (68.39%) Pre-Pro / -125.8,150.3	Favored (54.7%) *mttp* chi angles: 293.1,178.2,173,64.7	0.03Å	Favored (19.948%)	-	-	-
A 39		PRO	7.04	-	Favored (63.54%) Trans-Pro / -70.9,154.1	Favored (73.3%) *Cg_endo* chi angles: 27.6,325.1,27.9	0.05Å	Favored (68.041%)	-	-	-

#	Alt	Res	High B	Clash > 0.4Å	Ramachandran	Rotamer	Cβ deviation	CaBLAM	Bond lengths	Bond angles	Cis Peptides
A 40		THR	7.04	-	Favored (40.44%) General / -75.9,132.7	Favored (96.7%) *m* chi angles: 299.8	0.05Å	Favored (35.666%)	-	-	-
#	Alt	Res	High B	Clash > 0.4Å	Ramachandran	Rotamer	Cβ deviation	CaBLAM	Bond lengths	Bond angles	Cis Peptides
			Avg: 7.04	Clashscore: 1.46	Outliers: 6 of 498	Poor rotamers: 1 of 409	Outliers: 0 of 445	Outliers: 25 of 496	Outliers: 3 of 500	Outliers: 20 of 500	Non-Trans: 8 of 499
A 41		VAL	7.04	-	Favored (73.79%) Ile or Val / -123.1,131.2	Favored (68.1%) *t* chi angles: 179	0.02Å	Favored (67.063%) beta sheet	-	-	-
A 42		ASP	7.04	-	Favored (31.18%) General / -88.0,138.7	Favored (97.5%) *m-30* chi angles: 288.7,343.6	0.10Å	Favored (57.732%) beta sheet	-	OUTLIER(S) worst is CA-CB-CG: 5.7 σ	-
A 43		ILE	7.04	-	Favored (63.37%) Ile or Val / -130.9,129.1	Favored (17.2%) *tt* chi angles: 181.6,167.9	0.05Å	Favored (59.859%) beta sheet	-	-	-
A 44		GLU	7.04	-	Favored (37.42%) General / -135.4,132.4	Favored (47.9%) *tt0* chi angles: 178.5,178.9,62	0.07Å	Favored (54.48%) beta sheet	-	-	-
A 45		LEU	7.04	-	Favored (20.1%) General / -85.0,111.9	Favored (65.1%) *tp* chi angles: 179.2,59.2	0.05Å	Favored (57.081%)	-	-	-
A 46		VAL	7.04	-	Favored (17.35%) Ile or Val / -83.2,-48.7	Favored (97.6%) *t* chi angles: 175.6	0.06Å	Favored (24.359%)	-	-	-
A 47		THR	7.04	-	Favored (23.89%) General / -159.1,153.4	Favored (10%) *t* chi angles: 186	0.04Å	Favored (21.1%)	-	-	-
A 48		THR	7.04	-	Favored (56.84%) General / -115.7,131.8	Favored (93.2%) *m* chi angles: 299.2	0.06Å	Favored (62.955%)	-	-	-
A 49		THR	7.04	-	Favored (52.62%) General / -113.2,136.6	Favored (89.2%) *m* chi angles: 298.6	0.04Å	Favored (67.648%) beta sheet	-	-	-
A 50		VAL	7.04	-	Favored (59.04%) Ile or Val / -120.5,136.0	Favored (7%) *p* chi angles: 60.1	0.08Å	Favored (56.218%)	-	-	-
A 51		SER	7.04	-	Favored (37.41%) General / -143.2,161.8	Favored (98.7%) *p* chi angles: 65.3	0.11Å	Favored (9.132%)	-	-	-
A 52		ASN	7.04	-	Favored (31.92%) General / 53.9,40.8	Favored (87.9%) *m-40* chi angles: 295.7,317.7	0.03Å	Favored (11.733%)	-	-	-
A 53		MET	7.04	-	Favored (47.86%) General / -71.4,147.5	Favored (78.2%) *mtm* chi angles: 293,179.2,282.5	0.04Å	Favored (19.418%)	-	-	-
A 54		ALA	7.04	-	Favored (31.09%) General / -90.4,139.0	-	0.03Å	Favored (38.746%) beta sheet	-	-	-
A 55		GLU	7.04	-	Favored (38.5%) General / -77.4,132.4	Favored (98.5%) *mt-10* chi angles: 293.9,179,357.8	0.04Å	Favored (43.851%)	-	-	-

#	Alt	Res	High B	Clash > 0.4Å	Ramachandran	Rotamer	Cβ deviation	CaBLAM	Bond lengths	Bond angles	Cis Peptides
A 56		VAL	7.04	-	Favored (5.21%) Ile or Val / -108.1,-55.5	Favored (83.9%) *t* chi angles: 177	0.08Å	Favored (9.917%)	-	-	-
A 57		ARG	7.04	-	Favored (15.26%) General / -156.6,139.5	Favored (42.4%) *ttm170* chi angles: 175.5,186.1,293.8,184.9	0.03Å	Favored (21.451%)	-	-	-
A 58		SER	7.04	-	Favored (33.19%) General / -110.6,147.9	Favored (58.6%) *m* chi angles: 293.3	0.04Å	Favored (60.976%) beta sheet	-	-	-
A 59		TYR	7.04	-	Favored (48.97%) General / -122.4,144.6	Favored (70.1%) *m-80* chi angles: 286.4,87.9	0.06Å	Favored (55.976%) beta sheet	-	OUTLIER(S) worst is CB-CG-CD2: 4.2 σ	-
A 60		CYS	7.04	-	Favored (30.41%) General / -95.2,117.5	Favored (45.3%) *t* chi angles: 185.1	0.06Å	Favored (46.662%)	-	-	-
**#**	**Alt**	**Res**	**High B**	**Clash > 0.4Å**	**Ramachandran**	**Rotamer**	**Cβ deviation**	**CaBLAM**	**Bond lengths**	**Bond angles**	**Cis Peptides**
			Avg: 7.04	Clashscore: 1.46	Outliers: 6 of 498	Poor rotamers: 1 of 409	Outliers: 0 of 445	Outliers: 25 of 496	Outliers: 3 of 500	Outliers: 20 of 500	Non-Trans: 8 of 499
A 61		TYR	7.04	-	Favored (13.06%) General / -95.2,-32.5	Favored (8.9%) *m-10* chi angles: 288.5,9.1	0.05Å	Favored (26.696%)	-	-	-
A 62		GLU	7.04	-	Favored (31.47%) General / -140.5,135.0	Favored (86.1%) *tt0* chi angles: 181.8,175.1,349.7	0.02Å	Favored (22.349%)	-	-	-
A 63		ALA	7.04	-	Favored (14.02%) General / -139.2,171.6	-	0.05Å	Favored (33.903%)	-	-	-
A 64		SER	7.04	-	Favored (39.17%) General / -136.8,160.3	Favored (91.5%) *p* chi angles: 63.1	0.05Å	Favored (48.899%) beta sheet	-	-	-
A 65		ILE	7.04	-	Favored (71.1%) Ile or Val / -115.0,129.5	Favored (66%) *mt* chi angles: 303.4,173.5	0.02Å	Favored (26.117%)	-	-	-
A 66		SER	7.04	-	Allowed (0.49%) General / -109.3,-88.4	Favored (89.1%) *p* chi angles: 62.9	0.06Å	CaBLAM Disfavored (1.645%)	-	-	-
A 67		ASP	7.04	-	Favored (13.97%) General / -76.6,113.2	Favored (63.4%) *t0* chi angles: 181.6,346.5	0.01Å	CaBLAM Disfavored (4.103%)	-	OUTLIER(S) worst is CA-CB-CG: 4.1 σ	-
A 68		MET	7.04	-	Favored (40.27%) General / -121.6,151.5	Favored (83.8%) *mtp* chi angles: 297.6,184.6,68.7	0.04Å	Favored (17.278%)	-	-	-
A 69		ALA	7.04	-	Favored (30.33%) General / -156.6,154.5	-	0.03Å	Favored (29.932%) beta sheet	-	-	-
A 70		SER	7.04	-	Favored (15.75%) General / -139.5,170.5	Favored (95.5%) *p* chi angles: 64.9	0.07Å	Favored (40.397%) beta sheet	-	-	-
A 71		ASP	7.04	-	Favored (29.22%)	Favored (38.7%) *t70* chi angles: 185.5,62.8	0.02Å	Favored (69.052%) beta sheet	-	-	-

					General / -139.8,132.7							
A 72		SER	7.04	-	Favored (46.9%) General / -127.8,153.2	Favored (93%) *p* chi angles: 64.4	0.07Å	Favored (67.534%) beta sheet	-	-	-
A 73		ARG	7.04	-	Favored (46.54%) General / -130.9,155.6	Favored (29.5%) *mmt90* chi angles: 303.3,292.1,174.1,94.2	0.05Å	Favored (46.81%)	-	-	-
A 74		CYS	7.04	-	Favored (76.49%) Pre-Pro / -78.6,150.7	Favored (67.5%) *m* chi angles: 299.4	0.01Å	Favored (36.618%)	-	-	-
A 75		PRO	7.04	-	Favored (2.86%) Trans-Pro / -44.9,-28.1	Favored (86.5%) *Cg_exo* chi angles: 330.4,36.6,332.6	0.03Å	Favored (20.576%)	-	-	-
A 76		THR	7.04	-	Favored (33.55%) General / -105.2,7.2	Favored (76.9%) *p* chi angles: 61.1	0.04Å	Favored (34.487%)	-	-	-
A 77		GLN	7.04	-	Favored (40.14%) General / -101.6,10.5	Favored (94.9%) *mt0* chi angles: 294.6,177.9,314.6	0.06Å	Favored (8.705%)	-	-	-
A 78		GLY	7.04	-	Favored (42.58%) Glycine / 85.0,-168.0	-	-	Favored (45.998%)	-	-	-
A 79		GLU	7.04	-	Favored (28.77%) General / -90.1,141.4	Favored (94.8%) *mt-10* chi angles: 294.1,179.6,339.5	0.01Å	CaBLAM Disfavored (4.448%)	-	-	-
A 80		ALA	7.04	-	Favored (54.52%) General / -62.2,145.1	-	0.03Å	Favored (25.207%)	-	-	-
**#**	**Alt**	**Res**	**High B**	**Clash > 0.4Å**	**Ramachandran**	**Rotamer**	**Cβ deviation**	**CaBLAM**	**Bond lengths**	**Bond angles**	**Cis Peptides**
			Avg: 7.04	Clashscore: 1.46	Outliers: 6 of 498	Poor rotamers: 1 of 409	Outliers: 0 of 445	Outliers: 25 of 496	Outliers: 3 of 500	Outliers: 20 of 500	Non-Trans: 8 of 499
A 81		TYR	7.04	-	Favored (17.52%) General / -154.4,139.6	Favored (85.5%) *t80* chi angles: 180.9,82.7	0.03Å	Favored (54.076%) beta sheet	-	-	-
A 82		LEU	7.04	-	Favored (48.74%) General / -120.7,143.4	Favored (2.5%) *mp* chi angles: 288.5,91.5	0.04Å	Favored (61.093%)	-	-	-
A 83		ASP	7.04	-	Favored (67.98%) General / -60.3,-28.3	Favored (97.4%) *m-30* chi angles: 289.3,347.6	0.04Å	Favored (39.924%)	-	-	-
A 84		LYS	7.04	-	Favored (62.68%) General / -61.4,-20.1	Favored (98.1%) *mttt* chi angles: 291.2,176.2,177,179.7	0.08Å	Favored (58.112%)	-	-	-
A 85		GLN	7.04	-	Favored (65.74%) General / -66.8,-19.4	Favored (41.9%) *mt0* chi angles: 290.1,181.1,260.1	0.03Å	Favored (59.247%) three-ten	-	-	-
A 86		SER	7.04	-	Favored (58.47%) General / -87.3,-6.6	Favored (88.7%) *p* chi angles: 66.9	0.02Å	Favored (57.721%)	-	-	-
A 87		ASP	7.04	-	Favored (28.18%)	Favored (45.6%) *t0* chi angles: 184,331.3	0.10Å	Favored (35.606%)	-	-	-

					General / -96.6,115.9						
A 88		THR	7.04	-	Favored (19.37%) General / -63.7,-10.5	Favored (69.1%) *p* chi angles: 59.1	0.03Å	Favored (16.567%)	-	-	-
A 89		GLN	7.04	-	Favored (58.52%) General / -88.2,-2.3	Favored (78%) *mt0* chi angles: 294.7,178.6,59.5	0.05Å	Favored (63.299%)	-	-	-
A 90		TYR	7.04	-	Favored (30.82%) General / -111.9,150.2	Favored (49.3%) *m-80* chi angles: 291.2,75.7	0.12Å	Favored (26.257%)	-	-	-
A 91		VAL	7.04	-	Favored (60.55%) Ile or Val / -108.7,131.0	Favored (90.8%) *t* chi angles: 174.3	0.07Å	Favored (66.414%)	-	-	-
A 92		CYS	7.04	-	Favored (52.55%) General / -126.9,140.2	Favored (69.8%) *m* chi angles: 298.5	0.02Å	Favored (69.945%) beta sheet	-	-	-
A 93		LYS	7.04	-	Favored (49.37%) General / -124.2,145.3	Favored (72%) *mmtt* chi angles: 297.4,291.6,184.6,180.9	0.01Å	Favored (67.624%) beta sheet	-	-	-
A 94		ARG	7.04	-	Favored (55.1%) General / -117.2,128.4	Favored (48%) *ttm170* chi angles: 178.6,173.6,286.6,160.5	0.03Å	Favored (37.313%) beta sheet	-	-	-
A 95		THR	7.04	-	Favored (3.93%) General / -140.2,-174.4	Favored (11.6%) *t* chi angles: 189.6	0.06Å	Favored (18.713%) beta sheet	-	-	-
A 96		LEU	7.04	-	Favored (44.16%) General / -115.6,144.3	Favored (84.5%) *mt* chi angles: 299.6,174.8	0.02Å	Favored (21.624%) beta sheet	-	-	-
A 97		VAL	7.04	-	Favored (41.34%) Ile or Val / -132.8,155.5	Favored (23.3%) *m* chi angles: 295	0.06Å	Favored (56.675%) beta sheet	-	-	-
A 98		ASP	7.04	-	Favored (55.75%) General / -88.3,0.8	Favored (74.1%) *m-30* chi angles: 294.8,320.5	0.02Å	CaBLAM Outlier (0.108%) try beta sheet	-	-	-
A 99		ARG	7.04	-	Allowed (0.29%) General / 42.2,-114.9	Favored (25.8%) *mmm160* chi angles: 300.8,295.7,287.4,155.5	0.06Å	CaBLAM Disfavored (4.49%)	-	-	-
A 100		GLY	7.04	-	Favored (45.99%) Glycine / 87.6,170.1	-	-	Favored (10.291%)	-	-	-
**#**	**Alt**	**Res**	**High B**	**Clash > 0.4Å**	**Ramachandran**	**Rotamer**	**Cβ deviation**	**CaBLAM**	**Bond lengths**	**Bond angles**	**Cis Peptides**
			Avg: 7.04	Clashscore: 1.46	Outliers: 6 of 498	Poor rotamers: 1 of 409	Outliers: 0 of 445	Outliers: 25 of 496	Outliers: 3 of 500	Outliers: 20 of 500	Non-Trans: 8 of 499
A 101		TRP	7.04	-	Favored (58.82%) General / -63.5,139.9	Favored (44.6%) *m-10* chi angles: 291,4	0.05Å	CaBLAM Disfavored (2.81%)	-	-	-
A 102		GLY	7.04	-	Favored (63.85%) Glycine / 94.9,-15.2	-	-	Favored (51.783%)	-	-	-
A 103		ASN	7.04	-	Favored (22.98%)	Favored (81.2%) *m-40*	0.08Å	CaBLAM Disfavored	-	-	-

					General / -105.5,18.0	chi angles: 289.8,318.2		(2.476%)			
A 104		GLY	7.04	-	Favored (64.32%) Glycine / 97.8,-10.5	-	-	Favored (33.589%)	-	-	-
A 105		CYS	7.04	-	Favored (22.01%) General / -107.2,153.9	Favored (71.6%) *m* chi angles: 298	0.08Å	Favored (33.251%)	-	-	-
A 106		GLY	7.04	-	Favored (74.68%) Glycine / -91.2,0.1	-	-	Favored (31.687%)	-	-	-
A 107		LEU	7.04	-	Favored (37.8%) General / -114.3,148.3	Favored (73%) *mt* chi angles: 302.2,175.2	0.12Å	Favored (29.608%)	-	-	-
A 108		PHE	7.04	-	Favored (31.55%) General / -110.6,148.8	Favored (92.2%) *m-80* chi angles: 294.2,85.6	0.09Å	CaBLAM Disfavored (3.338%)	-	-	-
A 109		GLY	7.04	-	Favored (11.09%) Glycine / 128.9,162.8	-	-	Favored (24.531%)	-	-	-
A 110		LYS	7.04	-	Favored (47.3%) General / -66.8,132.1	Favored (59.7%) *mttm* chi angles: 294.3,185.3,189.3,299	0.07Å	CaBLAM Disfavored (2.388%)	-	-	-
A 111		GLY	7.04	-	Favored (12.86%) Glycine / -127.9,-170.6	-	-	Favored (25.636%)	-	-	-
A 112		SER	7.04	-	Favored (21.21%) General / -111.0,156.4	Favored (86.1%) *p* chi angles: 62.7	0.03Å	Favored (18.987%)	-	-	-
A 113		LEU	7.04	-	Favored (24.73%) General / -139.3,129.1	Favored (46.2%) *tp* chi angles: 177.1,67.3	0.07Å	Favored (65.098%) beta sheet	-	-	-
A 114		VAL	7.04	-	Favored (73.35%) Ile or Val / -118.5,130.4	Favored (81.6%) *t* chi angles: 176.6	0.08Å	Favored (71.753%) beta sheet	-	-	-
A 115		THR	7.04	-	Favored (55.26%) General / -120.7,134.9	Favored (96%) *m* chi angles: 300.8	0.04Å	Favored (68.064%) beta sheet	-	-	-
A 116		CYS	7.04	-	Favored (44.57%) General / -124.7,151.8	Favored (82.4%) *m* chi angles: 295	0.04Å	Favored (60.299%) beta sheet	-	-	-
A 117		ALA	7.04	-	Favored (16.04%) General / -151.3,134.5	-	0.06Å	Favored (42.201%) beta sheet	-	-	-
A 118		LYS	7.04	-	Favored (36.15%) General / -89.8,129.9	Favored (86.1%) *tttt* chi angles: 186.9,176,179.6,181.8	0.09Å	Favored (54.678%) beta sheet	-	-	-
A 119		PHE	7.04	-	Favored (32.41%) General / -111.8,148.9	Favored (21.4%) *m-80* chi angles: 303.1,319.6	0.05Å	Favored (58.593%) beta sheet	-	OUTLIER(S) worst is CA-CB-CG: 4.1 σ	-
A 120		THR	7.04	-	Favored (47.95%) General / -128.7,131.9	Favored (90.9%) *m* chi angles: 298.9	0.03Å	Favored (59.107%) beta sheet	-	-	-

#	Alt	Res	High B	Clash > 0.4Å	Ramachandran	Rotamer	Cβ deviation	CaBLAM	Bond lengths	Bond angles	Cis Peptides
			Avg: 7.04	Clashscore: 1.46	Outliers: 6 of 498	Poor rotamers: 1 of 409	Outliers: 0 of 445	Outliers: 25 of 496	Outliers: 3 of 500	Outliers: 20 of 500	Non-Trans: 8 of 499
A 121		CYS	7.04	-	Favored (32.52%) General / -89.2,137.2	Favored (90%) *m* chi angles: 293.3	0.09Å	Favored (51.342%)	-	-	-
A 122		SER	7.04	-	Favored (12.21%) General / -95.8,-33.6	Favored (72.7%) *m* chi angles: 295.4	0.03Å	Favored (20.639%)	-	-	-
A 123		LYS	7.04	-	Favored (33.15%) General / -136.0,130.2	Favored (48.5%) *mttp* chi angles: 299.6,186.8,180.8,68.8	0.05Å	Favored (34.13%)	-	-	-
A 124		LYS	7.04	-	Favored (45.34%) General / -126.8,153.7	Favored (41.3%) *mtpt* chi angles: 298.9,169.4,70.1,167	0.08Å	Favored (54.5%)	-	-	-
A 125		MET	7.04	-	Favored (49.97%) General / -117.0,140.0	Favored (20.1%) *tmm* chi angles: 179.3,274.7,277.4	0.03Å	Favored (65.281%) beta sheet	-	-	-
A 126		THR	7.04	-	Favored (51.63%) General / -123.0,129.6	Favored (89.3%) *m* chi angles: 298.3	0.04Å	Favored (69.222%) beta sheet	-	-	-
A 127		GLY	7.04	-	Favored (12.54%) Glycine / -97.1,136.0	-	-	Favored (70.861%) beta sheet	-	-	-
A 128		LYS	7.04	-	Favored (50.69%) General / -125.0,143.8	Favored (93.6%) *mttt* chi angles: 299.5,181.3,181,179.8	0.05Å	Favored (53.672%) beta sheet	-	-	-
A 129		SER	7.04	-	Favored (33.42%) General / -86.7,135.4	Favored (27.4%) *t* chi angles: 173.3	0.05Å	Favored (47.059%) beta sheet	-	-	-
A 130		ILE	7.04	-	Favored (48.55%) Ile or Val / -105.8,115.9	Favored (73.6%) *mt* chi angles: 299.5,175.3	0.08Å	Favored (70.67%) beta sheet	-	-	-
A 131		GLN	7.04	-	Favored (41.64%) Pre-Pro / -92.5,151.8	Favored (92.2%) *mm-40* chi angles: 298,292.9,303.9	0.07Å	Favored (31.008%)	-	-	-
A 132		PRO	7.04	-	Favored (29.77%) Trans-Pro / -49.4,-33.6	Favored (89.6%) *Cg_exo* chi angles: 329.8,37.2,331.9	0.06Å	Favored (70.819%)	-	-	-
A 133		GLU	7.04	-	Favored (66.08%) General / -63.4,-21.1	Favored (99.2%) *mt-10* chi angles: 293.2,179.4,356.8	0.01Å	Favored (64.509%) three-ten	-	-	-
A 134		ASN	7.04	-	Favored (56.09%) General / -89.4,1.2	Favored (98.4%) *m-40* chi angles: 289.2,336.9	0.03Å	Favored (47.548%)	-	-	-
A 135		LEU	7.04	-	Favored (33.19%) General / -105.6,143.9	Favored (84.3%) *mt* chi angles: 299.7,174.8	0.04Å	Favored (28.862%)	-	-	-
A 136		GLU	7.04	-	Favored (51.26%) General / -128.8,141.6	Favored (47.4%) *mt-10* chi angles: 301.2,180.5,295	0.06Å	Favored (65.181%)	-	-	-

A 137		TYR	7.04	-	Favored (52.7%) General / -118.6,138.2	Favored (51.7%) *m-80* chi angles: 291.9,76.1	0.06Å	Favored (66.55%) beta sheet	-	-	-
A 138		ARG	7.04	-	Favored (45.4%) General / -113.2,121.8	Favored (94%) *mtt180* chi angles: 284.4,172.3,171.4,174.2	0.04Å	Favored (67.211%) beta sheet	-	-	-
A 139		ILE	7.04	-	Favored (75.71%) Ile or Val / -118.9,128.9	Favored (3.6%) *mp* chi angles: 305.5,95.7	0.07Å	Favored (63.07%) beta sheet	-	-	-
A 140		MET	7.04	-	Favored (47.36%) General / -102.1,133.2	Favored (23.4%) *tmm* chi angles: 188,279.7,290.9	0.06Å	Favored (65.806%) beta sheet	-	-	-
**#**	**Alt**	**Res**	**High B**	**Clash > 0.4Å**	**Ramachandran**	**Rotamer**	**Cβ deviation**	**CaBLAM**	**Bond lengths**	**Bond angles**	**Cis Peptides**
			Avg: 7.04	Clashscore: 1.46	Outliers: 6 of 498	Poor rotamers: 1 of 409	Outliers: 0 of 445	Outliers: 25 of 496	Outliers: 3 of 500	Outliers: 20 of 500	Non-Trans: 8 of 499
A 141		LEU	7.04	-	Favored (44.46%) General / -111.9,142.3	Favored (81.3%) *mt* chi angles: 299.5,173.2	0.08Å	Favored (62.592%) beta sheet	-	-	-
A 142		SER	7.04	-	Favored (13.07%) General / -145.3,124.7	Favored (47%) *t* chi angles: 180.3	0.05Å	Favored (39.513%) beta sheet	-	-	-
A 143		VAL	7.04	-	Favored (29.21%) Ile or Val / -84.6,133.5	Favored (70%) *t* chi angles: 178.8	0.04Å	Favored (44%) beta sheet	-	-	-
A 144		HIS	7.04	-	Favored (7.77%) General / -59.9,-58.6	Favored (31.8%) *m170* chi angles: 288.1,196.4	0.04Å	CaBLAM Outlier (0.403%) try beta sheet	-	-	-
A 145		GLY	7.04	-	Favored (40.93%) Glycine / 53.3,-130.0	-	-	Favored (5.239%)	-	-	-
A 146		SER	7.04	-	Favored (4.3%) General / -136.4,100.2	Favored (69.8%) *m* chi angles: 296.5	0.08Å	CA Geom Outlier (0.256%)	-	-	-
A 147		GLN	7.04	0.41Å O with A 148 HIS HB3	Favored (5.47%) General / -113.0,-35.3	Favored (93.7%) *mm-40* chi angles: 294.8,294.3,303.8	0.04Å	CaBLAM Outlier (0.115%)	-	-	-
A 148		HIS	7.04	0.41Å HB3 with A 147 GLN O	OUTLIER (0.03)%) General / 92.8,159.5	Favored (73%) *t-90* chi angles: 187.6,273.8	0.03Å	CaBLAM Outlier (0.782%)	-	-	-
A 149		SER	7.04	-	Favored (48.95%) General / -78.6,-21.3	Favored (89.5%) *p* chi angles: 69	0.05Å	Favored (35.376%)	-	-	-
A 150		GLY	7.04	-	Favored (83.01%) Glycine / -63.8,-32.1	-	-	Favored (73.88%) three-ten	-	-	-
A 151		MET	7.04	-	Favored (42.18%) General / -77.0,-4.1	Favored (95.3%) *mmm* chi angles: 294.2,304.8,294.6	0.08Å	Favored (42.839%) three-ten	-	-	-
A 152		ILE	7.04	-	Favored (23.23%) Ile or Val / -68.4,-18.4	Allowed (1.2%) *pp* chi angles: 68.7,93	0.08Å	Favored (48.608%) three-ten	-	-	-

#	Alt	Res	High B	Clash > 0.4Å	Ramachandran	Rotamer	Cβ deviation	CaBLAM	Bond lengths	Bond angles	Cis Peptides
A 153		GLY	7.04	-	Favored (83.93%) Glycine / -86.1,5.5	-	-	Favored (60.739%)	-	-	-
A 154		TYR	7.04	-	Favored (38.64%) General / -114.3,147.7	Favored (62.2%) *m-80* chi angles: 293.9,78.4	0.07Å	Favored (24.521%)	-	-	-
A 155		GLU	7.04	-	Favored (7.39%) General / -112.8,-29.5	Favored (97.9%) *mt-10* chi angles: 295.2,179.6,354.4	0.05Å	Favored (14.135%)	-	-	-
A 156		THR	7.04	-	Favored (20.85%) General / -107.6,-1.8	Favored (79.8%) *p* chi angles: 60.6	0.04Å	Favored (51.321%)	-	-	-
A 157		ASP	7.04	-	Favored (28.4%) General / -80.5,153.1	Favored (26.8%) *m-30* chi angles: 288.8,294.4	0.07Å	CaBLAM Disfavored (4.804%)	-	-	-
A 158		GLU	7.04	-	Favored (56.68%) General / -63.2,144.7	Favored (75.6%) *mt-10* chi angles: 291.6,181.5,320.9	0.04Å	Favored (5.557%)	-	-	-
A 159		ASP	7.04	-	Allowed (1%) General / -98.4,46.6	Favored (67.7%) *m-30* chi angles: 300,319.7	0.06Å	CaBLAM Outlier (0.103%)	-	-	Cis nonPRO omega= 5.15
A 160		ARG	7.04	-	Favored (11.66%) General / -108.3,165.2	Favored (67.2%) *mtt90* chi angles: 301.5,166,176.5,76.4	0.12Å	Favored (12.71%)	-	-	-
**#**	**Alt**	**Res**	**High B**	**Clash > 0.4Å**	**Ramachandran**	**Rotamer**	**Cβ deviation**	**CaBLAM**	**Bond lengths**	**Bond angles**	**Cis Peptides**
			Avg: 7.04	Clashscore: 1.46	Outliers: 6 of 498	Poor rotamers: 1 of 409	Outliers: 0 of 445	Outliers: 25 of 496	Outliers: 3 of 500	Outliers: 20 of 500	Non-Trans: 8 of 499
A 161		ALA	7.04	-	Favored (26.18%) General / -154.2,148.7	-	0.04Å	Favored (47.933%)	-	-	-
A 162		LYS	7.04	-	Favored (54.59%) General / -108.6,128.2	Favored (30%) *ttmt* chi angles: 182.9,172.4,286.5,183.5	0.07Å	Favored (64.171%) beta sheet	-	-	-
A 163		VAL	7.04	-	Favored (74.65%) Ile or Val / -117.5,128.8	Favored (65.9%) *t* chi angles: 179.2	0.10Å	Favored (68.576%) beta sheet	-	-	-
A 164		GLU	7.04	-	Favored (38.02%) General / -99.3,137.3	Favored (95%) *mt-10* chi angles: 291.3,178.4,337.5	0.03Å	Favored (61.227%) beta sheet	-	-	-
A 165		VAL	7.04	-	Favored (60.48%) Ile or Val / -132.0,133.8	Favored (60.5%) *t* chi angles: 179.9	0.03Å	Favored (56.869%) beta sheet	-	-	-
A 166		THR	7.04	-	Favored (50.51%) Pre-Pro / -133.1,163.5	Favored (21.1%) *p* chi angles: 72.1	0.05Å	Favored (43.806%)	-	-	-
A 167		PRO	7.04	-	Favored (32.27%) Trans-Pro / -51.4,-29.8	Favored (98.2%) *Cg_exo* chi angles: 332,35.4,332.7	0.06Å	Favored (76.449%)	-	-	-
A 168		ASN	7.04	-	Favored (56.03%) General / -92.8,-2.4	Favored (90.9%) *m-40* chi angles: 291.4,325.6	0.04Å	Favored (34.577%)	-	-	-

A 169		SER	7.04	-	Favored (7.04%) Pre-Pro / -151.4,66.5	Favored (74.9%) *p* chi angles: 59.7	0.05Å	Favored (17.504%)	-	-	-
A 170		PRO	7.04	-	Favored (66.72%) Trans-Pro / -64.3,-22.1	Favored (39.5%) *Cg_endo* chi angles: 23.3,326,30.3	0.03Å	Favored (39.533%)	-	-	-
A 171		ARG	7.04	-	Favored (35.32%) General / -100.8,140.2	Favored (99.2%) *mtt180* chi angles: 293,174.6,178.1,174	0.02Å	Favored (7.938%)	-	-	-
A 172		ALA	7.04	-	Favored (42.66%) General / -152.7,161.4	-	0.05Å	Favored (33.911%)	-	-	-
A 173		GLU	7.04	-	Favored (31.59%) General / -141.1,136.0	Favored (91.4%) *tt0* chi angles: 183,176.2,357.4	0.02Å	Favored (57.591%) beta sheet	-	-	-
A 174		ALA	7.04	-	Favored (43.54%) General / -113.0,143.9	-	0.08Å	Favored (58.386%) beta sheet	-	-	-
A 175		THR	7.04	-	Favored (33.73%) General / -96.4,118.8	Favored (93.8%) *m* chi angles: 299.3	0.01Å	Favored (60.605%)	-	-	-
A 176		LEU	7.04	-	Allowed (1.4%) General / -99.7,66.1	Favored (86.6%) *mt* chi angles: 300.4,177.9	0.11Å	CaBLAM Disfavored (1.916%)	-	-	-
A 177		GLY	7.04	-	Favored (77.24%) Glycine / 70.3,23.7	-	-	Favored (13.11%)	-	-	-
A 178		GLY	7.04	-	Favored (71.32%) Glycine / 94.3,-10.5	-	-	Favored (79.981%)	-	-	-
A 179		PHE	7.04	-	Favored (4.17%) General / -83.0,58.0	Favored (79.6%) *m-80* chi angles: 289.1,96.7	0.10Å	CaBLAM Disfavored (3.889%)	-	OUTLIER(S) worst is CA-CB-CG: 4.3 σ	-
A 180		GLY	7.04	-	Favored (51.05%) Glycine / 59.2,-136.3	-	-	Favored (26.099%)	-	-	-
**#**	**Alt**	**Res**	**High B**	**Clash > 0.4Å**	**Ramachandran**	**Rotamer**	**Cβ deviation**	**CaBLAM**	**Bond lengths**	**Bond angles**	**Cis Peptides**
			Avg: 7.04	Clashscore: 1.46	Outliers: 6 of 498	Poor rotamers: 1 of 409	Outliers: 0 of 445	Outliers: 25 of 496	Outliers: 3 of 500	Outliers: 20 of 500	Non-Trans: 8 of 499
A 181		SER	7.04	-	Favored (31.53%) General / -147.8,165.1	Favored (93%) *p* chi angles: 64.4	0.01Å	Favored (11.134%)	-	-	-
A 182		LEU	7.04	-	Favored (27.94%) General / -131.9,123.7	Favored (6.6%) *tt* chi angles: 189.1,151.1	0.04Å	Favored (37.627%) beta sheet	-	-	-
A 183		GLY	7.04	-	Favored (17.93%) Glycine / -87.8,142.7	-	-	Favored (63.436%) beta sheet	-	-	-
A 184		LEU	7.04	-	Favored (30.98%) General / -134.7,127.4	Favored (38.1%) *tp* chi angles: 183.6,67	0.11Å	Favored (67.227%) beta sheet	-	-	-

#	Alt	Res	High B	Clash > 0.4Å	Ramachandran	Rotamer	Cβ deviation	CaBLAM	Bond lengths	Bond angles	Cis Peptides
A 185		ASP	7.04	-	Favored (9.36%) General / -127.0,106.5	Favored (63.1%) *t0* chi angles: 184.5,341.9	0.03Å	Favored (52.898%) beta sheet	-	-	-
A 186		CYS	7.04	-	Favored (29.64%) General / -117.0,155.2	Favored (33.6%) *m* chi angles: 306.3	0.06Å	Favored (41.218%) beta sheet	-	-	-
A 187		GLU	7.04	-	Favored (52%) Pre-Pro / -110.5,102.5	Favored (11.5%) *tp30* chi angles: 174.3,59.8,72.2	0.03Å	Favored (57.387%) beta sheet	-	-	-
A 188		PRO	7.04	-	Favored (32.61%) Trans-Pro / -69.4,-12.0	Favored (64.5%) *Cg_endo* chi angles: 26.8,326.3,26	0.03Å	Favored (42.734%)	-	-	-
A 189		ARG	7.04	-	Favored (42.89%) General / -100.2,9.4	Favored (90.4%) *mtm180* chi angles: 296.2,170.5,294.1,177.4	0.06Å	Favored (47.145%)	-	-	-
A 190		THR	7.04	-	Favored (69.62%) General / -66.5,-29.5	OUTLIER (0.1%) chi angles: 212.8	0.01Å	Favored (12.023%)	-	-	-
A 191		GLY	7.04	-	Favored (21.72%) Glycine / -131.7,170.1	-	-	CaBLAM Disfavored (1.472%)	-	-	-
A 192		LEU	7.04	0.43Å O with A 194 PHE N	Allowed (1.64%) General / 48.5,64.5	Favored (96.4%) *mt* chi angles: 296.8,177.5	0.07Å	CaBLAM Outlier (0.901%)	-	-	-
A 193		ASP	7.04	-	Allowed (0.34%) General / -62.9,102.3	Favored (53.5%) *t0* chi angles: 183.3,336	0.05Å	Favored (51.096%)	-	-	-
A 194		PHE	7.04	0.43Å N with A 192 LEU O	Favored (65.04%) General / -64.1,-19.1	Favored (46.6%) *m-80* chi angles: 283,103.1	0.07Å	Favored (23.684%)	-	-	-
A 195		SER	7.04	-	Favored (65.85%) General / -64.4,-20.0	Favored (85.4%) *p* chi angles: 67.3	0.06Å	Favored (39.861%)	-	-	-
A 196		ASP	7.04	-	Favored (35.21%) General / -104.4,6.8	Favored (85.8%) *m-30* chi angles: 290.5,334.8	0.05Å	Favored (40.192%)	-	-	-
A 197		LEU	7.04	-	Favored (46.44%) General / -115.9,142.7	Favored (53.7%) *mt* chi angles: 305.5,175.9	0.04Å	Favored (34.839%)	-	-	-
A 198		TYR	7.04	-	Favored (32.61%) General / -116.6,153.2	Favored (21.8%) *m-10* chi angles: 294,166.1	0.04Å	Favored (50.676%)	-	-	-
A 199		TYR	7.04	-	Favored (31.36%) General / -103.7,115.9	Favored (32.2%) *t80* chi angles: 194.9,79.6	0.11Å	Favored (60.728%) beta sheet	-	-	-
A 200		LEU	7.04	-	Favored (39.61%) General / -104.5,139.3	Favored (16.5%) *mt* chi angles: 309.9,187.1	0.07Å	Favored (65.951%) beta sheet	-	-	-
**#**	**Alt**	**Res**	**High B**	**Clash > 0.4Å**	**Ramachandran**	**Rotamer**	**Cβ deviation**	**CaBLAM**	**Bond lengths**	**Bond angles**	**Cis Peptides**
			Avg: 7.04	Clashscore: 1.46	Outliers: 6 of 498	Poor rotamers: 1 of 409	Outliers: 0 of 445	Outliers: 25 of 496	Outliers: 3 of 500	Outliers: 20 of 500	Non-Trans: 8 of 499

A 201		THR	7.04	-	Favored (51.7%) General / -122.6,129.3	Favored (93.8%) *m* chi angles: 299.3	0.04Å	Favored (51.776%)	-	-	-
A 202		MET	7.04	-	Favored (10.45%) General / -145.0,120.6	Favored (14.4%) *tpt* chi angles: 183.5,68.9,193.1	0.07Å	Favored (10.013%)	-	-	-
A 203		ASN	7.04	-	Favored (26.14%) General / 50.2,43.7	Favored (88.5%) *m-40* chi angles: 290.4,324.6	0.07Å	Favored (41.111%)	-	-	-
A 204		ASN	7.04	-	Favored (6.67%) General / 67.6,8.9	Favored (80.8%) *m-40* chi angles: 300.6,319.7	0.05Å	Favored (8.806%)	-	-	-
A 205		LYS	7.04	-	Favored (23.3%) General / -96.2,147.9	Favored (98.4%) *mttt* chi angles: 295.7,180.5,181,179.6	0.05Å	Favored (24.964%)	-	-	-
A 206		HIS	7.04	-	Favored (48.42%) General / -135.6,146.9	Favored (59.3%) *m-70* chi angles: 307.6,271	0.06Å	Favored (70.445%) beta sheet	-	-	-
A 207		TRP	7.04	-	Favored (47.76%) General / -136.2,147.8	Favored (23.7%) *m-90* chi angles: 301.8,263.3	0.08Å	Favored (59.632%) beta sheet	-	-	-
A 208		LEU	7.04	-	Favored (28.87%) General / -97.8,115.9	Favored (52.2%) *tp* chi angles: 181.2,64.9	0.04Å	Favored (53.417%) beta sheet	-	-	-
A 209		VAL	7.04	0.41Å HG11 with A 213 TRP CE3	Favored (58.53%) Ile or Val / -127.7,137.1	Favored (70.4%) *t* chi angles: 178.7	0.09Å	Favored (46.928%) beta sheet	-	-	-
A 210		HIS	7.04	-	Favored (58.27%) General / -61.4,138.5	Favored (71.2%) *t-90* chi angles: 194.4,283.3	0.02Å	Favored (48.853%)	-	-	-
A 211		LYS	7.04	-	Favored (65.21%) General / -58.7,-27.9	Favored (57.8%) *pttt* chi angles: 68.5,181.9,178.7,186.3	0.02Å	Favored (48.749%)	-	-	-
A 212		GLU	7.04	-	Favored (68.62%) General / -72.4,-37.7	Favored (97.8%) *mt-10* chi angles: 289.9,178.9,357.3	0.08Å	Favored (77.57%) alpha helix	-	-	-
A 213		TRP	7.04	0.41Å CE3 with A 209 VAL HG11	Favored (88.55%) General / -58.9,-46.1	Favored (63.8%) *t60* chi angles: 178.5,94.5	0.10Å	Favored (89.947%) alpha helix	-	-	-
A 214		PHE	7.04	-	Favored (77.19%) General / -62.2,-49.1	Favored (90.5%) *t80* chi angles: 177.2,81.1	0.06Å	Favored (82.551%) alpha helix	-	-	-
A 215		HIS	7.04	-	Favored (69.15%) General / -58.8,-32.3	Favored (54.8%) *m170* chi angles: 289.7,165.2	0.07Å	Favored (74.075%) alpha helix	-	-	-
A 216		ASP	7.04	-	Favored (48.88%) General / -83.9,0.1	Favored (84.6%) *m-30* chi angles: 288.3,335.5	0.05Å	Favored (42.31%)	-	-	-
A 217		ILE	7.04	-	Favored (90.93%) Pre-Pro / -68.0,125.0	Favored (93.5%) *mt* chi angles: 295.9,169.1	0.07Å	Favored (29.008%)	-	-	-
A 218		PRO	7.04	-	Favored (19.48%)	Favored (80.3%) *Cg_endo*	0.06Å	Favored (12.286%) beta sheet	-	-	-

					Trans-Pro / -76.1,-10.4	chi angles: 30.3,323.9,26.6					
A 219		LEU	7.04	-	Favored (84.56%) Pre-Pro / -66.6,157.0	Favored (87.1%) *mt* chi angles: 297.4,179.7	0.08Å	Favored (13.284%) beta sheet	-	-	-
A 220		PRO	7.04	-	Favored (88.32%) Trans-Pro / -56.2,141.5	Favored (71%) *Cg_exo* chi angles: 335.3,37.1,326.2	0.09Å	Favored (33.645%) beta sheet	-	-	-
**#**	**Alt**	**Res**	**High B**	**Clash > 0.4Å**	**Ramachandran**	**Rotamer**	**Cβ deviation**	**CaBLAM**	**Bond lengths**	**Bond angles**	**Cis Peptides**
			Avg: 7.04	Clashscore: 1.46	Outliers: 6 of 498	Poor rotamers: 1 of 409	Outliers: 0 of 445	Outliers: 25 of 496	Outliers: 3 of 500	Outliers: 20 of 500	Non-Trans: 8 of 499
A 221		TRP	7.04	-	Favored (39.12%) General / -143.1,161.1	Favored (76%) *p-90* chi angles: 59.9,271.9	0.03Å	Favored (61.427%)	-	-	-
A 222		HIS	7.04	-	Favored (43.8%) General / -141.6,158.5	Favored (39.1%) *p90* chi angles: 67.5,80.4	0.08Å	Favored (20.144%)	-	OUTLIER(S) worst is CA-CB-CG: 5.3 σ	-
A 223		ALA	7.04	-	Favored (58.05%) General / -59.2,136.7	-	0.03Å	Favored (35.538%)	-	-	-
A 224		GLY	7.04	-	Favored (15.9%) Glycine / -57.0,-58.2	-	-	Favored (33.802%)	-	-	-
A 225		ALA	7.04	-	Favored (2.89%) General / -80.5,57.4	-	0.03Å	CaBLAM Outlier (0.589%)	-	-	-
A 226		ASP	7.04	-	Allowed (0.7%) General / -164.9,95.6	Favored (63.8%) *t0* chi angles: 186.3,353.2	0.06Å	CaBLAM Disfavored (4.152%)	-	-	-
A 227		THR	7.04	-	Favored (17.41%) General / -112.9,5.5	Favored (73.9%) *p* chi angles: 59.8	0.07Å	Favored (8.708%)	-	-	-
A 228		GLY	7.04	-	Favored (30.6%) Glycine / -63.5,-54.6	-	-	CaBLAM Disfavored (4.264%)	-	-	-
A 229		THR	7.04	-	Favored (24.6%) Pre-Pro / -120.8,131.0	Favored (92.7%) *m* chi angles: 299.1	0.05Å	Favored (22.294%)	-	-	-
A 230		PRO	7.04	-	Favored (7.61%) Trans-Pro / -76.8,66.0	Favored (75.4%) *Cg_endo* chi angles: 30.9,323.9,26.1	0.04Å	Favored (11.997%)	-	-	-
A 231		HIS	7.04	-	Favored (5.46%) General / -79.0,78.0	Favored (15%) *t-170* chi angles: 190.5,167.7	0.03Å	Favored (55.22%) beta sheet	-	-	-
A 232		TRP	7.04	-	Favored (35.46%) General / -76.9,128.9	Favored (97.3%) *m100* chi angles: 292.4,104.2	0.07Å	Favored (28.79%)	-	-	-
A 233		ASN	7.04	-	Favored (33.99%) General / -81.2,-31.7	Favored (59.1%) *m-40* chi angles: 285.9,276.3	0.04Å	Favored (26.184%)	-	-	-
A 234		ASN	7.04	-	Favored (2.77%)	Favored (41.4%) *t0* chi angles: 203.9,37.1	0.06Å	Favored (14.498%)	-	-	-

#	Alt	Res	High B	Clash > 0.4Å	Ramachandran	Rotamer	Cβ deviation	CaBLAM	Bond lengths	Bond angles	Cis Peptides
					General / -131.7,45.5						
A 235		LYS	7.04	-	Favored (46.41%) General / -53.9,-30.4	Favored (20.8%) *mtmm* chi angles: 290.7,184.6,284.4,286.1	0.07Å	Favored (21.702%)	-	-	-
A 236		GLU	7.04	-	Favored (63.51%) General / -59.9,-23.4	Favored (27.3%) *pt0* chi angles: 66.7,181.8,355.9	0.03Å	Favored (57.206%)	-	-	-
A 237		ALA	7.04	-	Favored (67.77%) General / -58.9,-30.4	-	0.03Å	Favored (31.069%) three-ten	-	-	-
A 238		LEU	7.04	-	Favored (28.23%) General / -105.3,1.6	Favored (93.4%) *mt* chi angles: 295.5,177.4	0.11Å	Favored (35.638%) alpha helix	-	-	-
A 239		VAL	7.04	-	Allowed (0.46%) Ile or Val / -134.4,46.4	Favored (17.4%) *m* chi angles: 293.1	0.04Å	CaBLAM Disfavored (1.393%)	-	-	-
A 240		GLU	7.04	-	Favored (7.05%) General / -44.7,129.7	Favored (80.3%) *tt0* chi angles: 187.9,179.6,13.6	0.06Å	Favored (6.757%)	-	-	-
**#**	**Alt**	**Res**	**High B**	**Clash > 0.4Å**	**Ramachandran**	**Rotamer**	**Cβ deviation**	**CaBLAM**	**Bond lengths**	**Bond angles**	**Cis Peptides**
			Avg: 7.04	Clashscore: 1.46	Outliers: 6 of 498	Poor rotamers: 1 of 409	Outliers: 0 of 445	Outliers: 25 of 496	Outliers: 3 of 500	Outliers: 20 of 500	Non-Trans: 8 of 499
A 241		PHE	7.04	-	Favored (27.32%) General / -83.8,147.9	Favored (2.1%) *m-80* chi angles: 287.4,51.9	0.02Å	Favored (40.245%)	-	-	-
A 242		LYS	7.04	-	Favored (9.42%) General / -87.1,173.2	Favored (72.5%) *mmtt* chi angles: 301.3,297.3,183.5,180.8	0.09Å	Favored (25.593%)	-	-	-
A 243		ASP	7.04	-	Favored (2.05%) General / -76.0,65.6	Favored (17.1%) *p0* chi angles: 57.2,326.8	0.10Å	CaBLAM Disfavored (3.731%)	-	-	-
A 244		ALA	7.04	-	Allowed (0.45%) General / 59.2,-109.5	-	0.03Å	CaBLAM Outlier (0.094%)	-	-	-
A 245		HIS	7.04	-	OUTLIER (0.01)%) General / 86.0,71.0	Favored (98.4%) *m-70* chi angles: 302.7,289.5	0.08Å	CaBLAM Outlier (0.475%)	-	-	-
A 246		ALA	7.04	-	Allowed (1.19%) General / 56.4,-118.4	-	0.03Å	CaBLAM Outlier (0.276%)	-	-	-
A 247		LYS	7.04	-	Favored (49.28%) General / -77.0,-5.6	Favored (60.5%) *pttt* chi angles: 67.5,184.9,179.7,182.1	0.11Å	Favored (6.939%)	-	-	-
A 248		ARG	7.04	-	Favored (23.78%) General / -133.0,165.8	Favored (92.4%) *mmt-90* chi angles: 296.2,290.3,183.6,269.9	0.07Å	Favored (15.622%)	-	-	-
A 249		GLN	7.04	-	Favored (17.47%) General / -157.1,142.7	Favored (60.1%) *tt0* chi angles: 184.4,171.9,19.8	0.03Å	Favored (35.495%)	-	-	-
A 250		THR	7.04	-	Favored (43.93%)	Favored (90.6%) *m* chi angles: 298.8	0.05Å	Favored (57.736%)	-	-	-

#	Alt	Res	High B	Clash > 0.4Å	Ramachandran	Rotamer	Cβ deviation	CaBLAM	Bond lengths	Bond angles	Cis Peptides
					General / -106.0,137.4			beta sheet			
A 251		VAL	7.04	-	Favored (71.68%) Ile or Val / -114.1,127.1	Favored (53.3%) *t* chi angles: 180.9	0.06Å	Favored (72.455%) beta sheet	-	-	-
A 252		VAL	7.04	-	Favored (75.78%) Ile or Val / -121.5,128.1	Favored (75.5%) *t* chi angles: 178.3	0.05Å	Favored (58.678%) beta sheet	-	-	-
A 253		VAL	7.04	-	Favored (34.81%) Ile or Val / -72.2,131.4	Favored (84.3%) *t* chi angles: 176.3	0.06Å	Favored (41.42%) beta sheet	-	-	-
A 254		LEU	7.04	-	Favored (5.51%) General / -83.1,60.5	Favored (70.1%) *mt* chi angles: 303.8,178.1	0.07Å	Favored (8.952%)	-	-	-
A 255		GLY	7.04	-	Favored (42.09%) Glycine / 56.0,-127.0	-	-	Favored (43.088%)	-	-	-
A 256		SER	7.04	-	Favored (34.52%) General / -134.4,128.9	Favored (27.5%) *t* chi angles: 173.6	0.09Å	Favored (7.833%)	-	-	-
A 257		GLN	7.04	-	Favored (49.22%) General / -92.0,5.7	Favored (97.5%) *mm-40* chi angles: 297.4,297.3,311.4	0.09Å	Favored (12.336%)	-	-	-
A 258		GLU	7.04	-	Favored (83.85%) General / -57.3,-45.7	Favored (91.5%) *tt0* chi angles: 182.3,176,0.7	0.01Å	Favored (49.176%)	-	-	-
A 259		GLY	7.04	-	Favored (93.65%) Glycine / -62.0,-37.0	-	-	Favored (91.946%) alpha helix	-	-	-
A 260		ALA	7.04	-	Favored (98%) General / -63.5,-40.8	-	0.02Å	Favored (82.444%) alpha helix	-	-	-
**#**	**Alt**	**Res**	**High B**	**Clash > 0.4Å**	**Ramachandran**	**Rotamer**	**Cβ deviation**	**CaBLAM**	**Bond lengths**	**Bond angles**	**Cis Peptides**
			Avg: 7.04	Clashscore: 1.46	Outliers: 6 of 498	Poor rotamers: 1 of 409	Outliers: 0 of 445	Outliers: 25 of 496	Outliers: 3 of 500	Outliers: 20 of 500	Non-Trans: 8 of 499
A 261		VAL	7.04	-	Favored (57.04%) Ile or Val / -73.4,-42.6	Favored (94.7%) *t* chi angles: 174.8	0.05Å	Favored (80.932%) alpha helix	-	-	-
A 262		HIS	7.04	-	Favored (81.59%) General / -62.8,-36.0	Favored (44.9%) *m170* chi angles: 289.1,186.5	0.08Å	Favored (85.814%) alpha helix	-	-	-
A 263		THR	7.04	-	Favored (88.67%) General / -63.3,-46.0	Favored (93.8%) *m* chi angles: 299.3	0.05Å	Favored (78.818%) alpha helix	-	-	-
A 264		ALA	7.04	-	Favored (67.83%) General / -59.6,-29.2	-	0.04Å	Favored (70.387%)	-	-	-
A 265		LEU	7.04	-	Favored (38.45%) General / -78.7,-2.3	Favored (76.1%) *mt* chi angles: 287.8,170.3	0.07Å	Favored (30.41%)	-	-	-
A 266		ALA	7.04	-	Favored (44.31%)	-	0.03Å	Favored (13.639%)	-	-	-

					General / -54.2,133.0						
A 267		GLY	7.04	-	Favored (67.14%) Glycine / 96.0,-12.4	-	-	Favored (81.582%)	-	-	-
A 268		ALA	7.04	-	Favored (40.62%) General / -72.5,154.9	-	0.04Å	Favored (30.841%)	-	-	-
A 269		LEU	7.04	-	Favored (26.5%) General / -84.5,120.6	Favored (53.7%) *tp* chi angles: 173.7,64.2	0.02Å	Favored (27.1%) beta sheet	-	-	-
A 270		GLU	7.04	-	Favored (54.31%) General / -67.2,146.4	Favored (95.6%) *mt-10* chi angles: 293.2,181.8,4.8	0.01Å	Favored (23.146%) beta sheet	-	-	-
A 271		ALA	7.04	-	Favored (26.35%) General / -144.0,165.9	-	0.04Å	Favored (53.149%) beta sheet	-	-	-
A 272		GLU	7.04	-	Favored (51.57%) General / -124.8,142.2	Favored (98.7%) *mt-10* chi angles: 294.1,179.5,355.5	0.01Å	Favored (35.434%) beta sheet	-	-	-
A 273		MET	7.04	-	Favored (9.73%) General / -162.8,143.6	Favored (25.7%) *ttt* chi angles: 178.8,173,189.6	0.12Å	Favored (16.002%)	-	-	-
A 274		ASP	7.04	-	Allowed (0.37%) General / -110.5,-137.8	Favored (10.7%) *t70* chi angles: 194.4,287.8	0.10Å	CaBLAM Outlier (0.613%)	-	-	-
A 275		GLY	7.04	-	Favored (26.99%) Glycine / -75.5,-169.8	-	-	CaBLAM Disfavored (1.167%)	-	OUTLIER(S) worst is N-CA-C: 4.0 σ	-
A 276		ALA	7.04	-	Favored (49.91%) General / -60.5,145.1	-	0.02Å	CaBLAM Disfavored (1.391%)	-	-	-
A 277		LYS	7.04	-	Favored (14.07%) General / -100.6,160.8	Favored (93%) *mttt* chi angles: 299.5,179.8,180.5,174.1	0.08Å	Favored (11.853%)	-	-	Cis nonPRO omega= 2.66
A 278		GLY	7.04	-	Favored (35.2%) Glycine / -95.9,179.0	-	-	Favored (30.979%)	-	-	-
A 279		ARG	7.04	-	Favored (25.34%) General / -141.8,132.3	Favored (65.7%) *ttt180* chi angles: 186.3,175.3,185.3,197.8	0.04Å	Favored (44.494%) beta sheet	-	-	-
A 280		LEU	7.04	-	Favored (37.07%) General / -107.4,142.5	Favored (85.2%) *mt* chi angles: 299.2,174.3	0.05Å	Favored (62.389%)	-	-	-
**#**	**Alt**	**Res**	**High B**	**Clash > 0.4Å**	**Ramachandran**	**Rotamer**	**Cβ deviation**	**CaBLAM**	**Bond lengths**	**Bond angles**	**Cis Peptides**
			Avg: 7.04	Clashscore: 1.46	Outliers: 6 of 498	Poor rotamers: 1 of 409	Outliers: 0 of 445	Outliers: 25 of 496	Outliers: 3 of 500	Outliers: 20 of 500	Non-Trans: 8 of 499
A 281		PHE	7.04	-	Favored (11.61%) General / -117.0,-3.2	Favored (88.9%) *m-80* chi angles: 301.4,95.2	0.07Å	Favored (30.083%)	-	-	-

A 282		SER	7.04	-	Favored (3.22%) General / -136.1,27.4	Favored (53.3%) *m* chi angles: 300.5	0.07Å	Favored (45.685%)	-	-	-
A 283		GLY	7.04	-	Favored (50.38%) Glycine / -84.1,-174.5	-	-	Favored (51.741%)	-	-	-
A 284		HIS	7.04	-	Favored (52%) General / -135.1,153.5	Favored (39.8%) *m90* chi angles: 306.9,80.9	0.06Å	Favored (59.492%) beta sheet	-	-	-
A 285		LEU	7.04	-	Favored (19.75%) General / -139.4,125.3	Favored (48.5%) *tp* chi angles: 180.7,66	0.08Å	Favored (52.386%) beta sheet	-	-	-
A 286		LYS	7.04	-	Favored (40.18%) General / -95.5,125.5	Favored (34.3%) *ttpt* chi angles: 179.4,168.5,63.9,170.7	0.09Å	Favored (62.902%) beta sheet	-	-	-
A 287		CYS	7.04	-	Favored (53.12%) General / -126.7,139.5	Favored (61.2%) *m* chi angles: 300.5	0.08Å	Favored (63.449%) beta sheet	-	-	-
A 288		ARG	7.04	-	Favored (39.55%) General / -95.8,123.8	Favored (48%) *ttm170* chi angles: 179.2,166.7,291.7,180.5	0.01Å	Favored (66.95%) beta sheet	-	-	-
A 289		LEU	7.04	-	Favored (47.8%) General / -101.5,128.4	Favored (4.3%) *mp* chi angles: 287.7,62.6	0.08Å	Favored (70.031%) beta sheet	-	-	-
A 290		LYS	7.04	-	Favored (14.84%) General / -83.6,-44.2	Favored (97.5%) *mttt* chi angles: 291.2,182.1,178.9,179.9	0.05Å	CaBLAM Disfavored (1.485%) try beta sheet	-	-	-
A 291		MET	7.04	-	Favored (5.69%) General / 58.4,18.6	Favored (78.1%) *mtm* chi angles: 297,183.6,290.3	0.04Å	CaBLAM Disfavored (1.898%)	-	-	-
A 292		ASP	7.04	-	Favored (33.06%) General / -69.1,127.4	Favored (38.2%) *t70* chi angles: 184.4,69.5	0.02Å	CaBLAM Disfavored (4.135%)	-	-	-
A 293		LYS	7.04	-	Favored (9.45%) General / 67.5,21.8	Favored (27.4%) *mmtm* chi angles: 310.3,297.2,185.4,287.1	0.05Å	Favored (15.197%)	-	-	-
A 294		LEU	7.04	-	Favored (33.86%) General / -79.4,128.8	Favored (42.4%) *tp* chi angles: 182,66.9	0.06Å	Favored (23.483%)	-	-	-
A 295		ARG	7.04	-	Favored (34.11%) General / -118.9,153.7	Favored (96%) *mtt-85* chi angles: 296.6,176.7,183.8,271.3	0.02Å	Favored (52.575%) beta sheet	-	-	-
A 296		LEU	7.04	-	Favored (59.7%) General / -80.1,-9.5	Favored (79.9%) *mt* chi angles: 288.7,169.6	0.03Å	CaBLAM Disfavored (2.645%)	-	-	-
A 297		LYS	7.04	-	Allowed (0.28%) General / 65.6,-59.6	Favored (31%) *ttmt* chi angles: 186.2,183.8,286.8,191.1	0.05Å	CaBLAM Disfavored (4.072%)	-	-	-
A 298		GLY	7.04	-	Favored (42.44%) Glycine / -106.0,16.8	-	-	Favored (40.801%) alpha helix	-	-	-
A 299		VAL	7.04	-	Favored (31.82%)	Favored (7.9%) *p* chi angles: 67.7	0.05Å	Favored (38.301%)	-	-	-

#	Alt	Res	High B	Clash > 0.4Å	Ramachandran	Rotamer	Cβ deviation	CaBLAM	Bond lengths	Bond angles	Cis Peptides
					Ile or Val / -59.1,-26.0			alpha helix			
A 300		SER	7.04	-	Favored (58.1%) General / -80.2,-6.6	Favored (85.9%) *p* chi angles: 67.3	0.02Å	Favored (53.118%)	-	-	-
**#**	**Alt**	**Res**	**High B**	**Clash > 0.4Å**	**Ramachandran**	**Rotamer**	**Cβ deviation**	**CaBLAM**	**Bond lengths**	**Bond angles**	**Cis Peptides**
			Avg: 7.04	Clashscore: 1.46	Outliers: 6 of 498	Poor rotamers: 1 of 409	Outliers: 0 of 445	Outliers: 25 of 496	Outliers: 3 of 500	Outliers: 20 of 500	Non-Trans: 8 of 499
A 301		TYR	7.04	-	Favored (24.1%) General / -84.0,153.0	Favored (72.3%) *m-80* chi angles: 287.1,92.3	0.06Å	Favored (38.114%)	-	-	-
A 302		SER	7.04	-	Favored (14.08%) General / -77.1,171.9	Favored (90.1%) *p* chi angles: 68.7	0.05Å	Favored (26.151%)	-	-	-
A 303		LEU	7.04	-	Favored (51.27%) General / -69.9,146.4	Favored (92.1%) *mt* chi angles: 292.6,175.3	0.07Å	Favored (26.138%)	-	-	-
A 304		CYS	7.04	-	Favored (58.79%) General / -63.3,139.6	Favored (80%) *m* chi angles: 296.1	0.08Å	Favored (38.031%)	-	-	-
A 305		THR	7.04	-	Favored (13.37%) General / -111.0,-17.1	Favored (78%) *p* chi angles: 60.9	0.06Å	Favored (13.529%) beta sheet	-	-	-
A 306		ALA	7.04	-	Favored (28.24%) General / -78.1,160.2	-	0.04Å	Favored (16.403%) beta sheet	-	-	-
A 307		ALA	7.04	-	Favored (36.46%) General / -61.1,149.7	-	0.05Å	Favored (20.498%) beta sheet	-	-	-
A 308		PHE	7.04	-	Favored (20.45%) General / -111.3,157.2	Favored (76.8%) *m-80* chi angles: 288.3,86.1	0.06Å	Favored (58.826%) beta sheet	-	-	-
A 309		THR	7.04	-	Favored (47.19%) General / -136.7,148.1	Favored (5.9%) *t* chi angles: 181.2	0.09Å	Favored (46.884%) beta sheet	-	-	-
A 310		PHE	7.04	-	Favored (35.91%) General / -76.9,150.9	Favored (39.9%) *m-80* chi angles: 289,119.8	0.02Å	Favored (39.201%)	-	-	-
A 311		THR	7.04	-	Favored (7.26%) General / -118.1,-23.7	Favored (70.5%) *p* chi angles: 62.3	0.03Å	Favored (10.088%)	-	-	-
A 312		LYS	7.04	-	Favored (19.08%) General / -138.9,124.1	Favored (31.8%) *ttmt* chi angles: 182.2,178,285.6,187.6	0.04Å	Favored (11.731%)	-	-	-
A 313		VAL	7.04	0.43Å O with A 313 VAL HG13	Favored (81%) Pre-Pro / -57.6,139.6	Favored (8.9%) *p* chi angles: 66.7	0.13Å	Favored (38.941%)	-	-	-
A 314		PRO	7.04	-	Favored (53.11%) Trans-Pro / -52.0,137.8	Favored (86.6%) *Cg_exo* chi angles: 330.4,36.3,332.7	0.05Å	Favored (57.869%)	-	-	-
A 315		ALA	7.04	-	Favored (50.13%)	-	0.04Å	Favored (50.122%) beta sheet	-	-	-

#	Alt	Res	High B	Clash > 0.4Å	Ramachandran	Rotamer	Cβ deviation	CaBLAM	Bond lengths	Bond angles	Cis Peptides
					General / -132.2,143.7						
A 316		GLU	7.04	-	Favored (34.98%) General / -83.2,134.5	Favored (74.6%) *tt0* chi angles: 190.8,178,8.3	0.03Å	Favored (44.838%)	-	-	-
A 317		THR	7.04	-	Favored (4.54%) General / -99.1,179.7	Favored (33.7%) *p* chi angles: 68.8	0.10Å	Favored (25.022%)	-	-	-
A 318		LEU	7.04	-	Favored (12.41%) General / -75.5,2.1	Favored (94.8%) *mt* chi angles: 295.6,173.7	0.05Å	Favored (5.301%)	-	-	-
A 319		HIS	7.04	-	Favored (13.92%) General / -112.6,-6.8	Favored (99.5%) *m-70* chi angles: 300,287.5	0.09Å	Favored (10.309%)	OUTLIER(S) worst is CB--CG: 5.7 σ	OUTLIER(S) worst is CA-CB-CG: 5.3 σ	-
A 320		GLY	7.04	-	Favored (68.35%) Glycine / 93.6,-13.1	-	-	Favored (75.743%)	-	-	-
**#**	**Alt**	**Res**	**High B**	**Clash > 0.4Å**	**Ramachandran**	**Rotamer**	**Cβ deviation**	**CaBLAM**	**Bond lengths**	**Bond angles**	**Cis Peptides**
			Avg: 7.04	Clashscore: 1.46	Outliers: 6 of 498	Poor rotamers: 1 of 409	Outliers: 0 of 445	Outliers: 25 of 496	Outliers: 3 of 500	Outliers: 20 of 500	Non-Trans: 8 of 499
A 321		THR	7.04	-	Favored (58.28%) General / -62.6,137.5	Favored (99.3%) *m* chi angles: 300.3	0.03Å	Favored (40.719%)	-	-	-
A 322		VAL	7.04	-	Favored (23.16%) Ile or Val / -105.7,140.6	Favored (9.6%) *p* chi angles: 64.1	0.04Å	Favored (58.788%) beta sheet	-	-	-
A 323		THR	7.04	-	Favored (55.43%) General / -115.5,134.1	Favored (98.8%) *m* chi angles: 300.2	0.04Å	Favored (55.247%) beta sheet	-	-	-
A 324		VAL	7.04	-	Favored (22.01%) Ile or Val / -144.4,144.9	Favored (9.2%) *p* chi angles: 63.1	0.07Å	Favored (46.564%) beta sheet	-	-	-
A 325		GLU	7.04	-	Favored (44.68%) General / -109.0,138.9	Favored (78.9%) *mt-10* chi angles: 293,175.1,323	0.04Å	Favored (63.669%) beta sheet	-	-	-
A 326		VAL	7.04	-	Favored (40.51%) Ile or Val / -130.6,143.6	Favored (8.6%) *p* chi angles: 66.9	0.07Å	Favored (66.761%) beta sheet	-	-	-
A 327		GLN	7.04	-	Favored (53.91%) General / -112.6,134.9	Favored (40.3%) *tt0* chi angles: 176.9,181.3,297.2	0.04Å	Favored (54.188%) beta sheet	-	-	-
A 328		TYR	7.04	-	Favored (34.89%) General / -86.7,129.4	Favored (36.8%) *t80* chi angles: 185.8,58.8	0.06Å	Favored (54.904%) beta sheet	-	-	-
A 329		ALA	7.04	-	Favored (57.61%) General / -83.2,-3.7	-	0.03Å	Favored (38.198%)	-	-	-
A 330		GLY	7.04	-	Favored (20.87%) Glycine / -108.9,-170.6	-	-	Favored (16.731%)	-	-	-
A 331		THR	7.04	-	Favored (10.49%)	Favored (70.1%) *p* chi angles: 59.4	0.02Å	Favored (6.7%)	-	-	-

					General / -119.7,-2.0						
A 332		ASP	7.04	-	Favored (51.08%) General / -66.4,149.0	Favored (86.9%) *m-30* chi angles: 292.2,350.4	0.03Å	Favored (17.197%)	-	-	-
A 333		GLY	7.04	-	Favored (22.18%) Glycine / 151.2,174.5	-	-	CA Geom Outlier (0.049%)	-	-	-
A 334		PRO	7.04	-	Favored (34.48%) Cis-Pro / -58.4,147.8	Favored (57.9%) *Cg_exo* chi angles: 336.1,34.6,329.1	0.06Å	CaBLAM Outlier (0.319%)	-	-	Cis PRO omega= -2.38
A 335		CYS	7.04	-	Favored (30.44%) General / -157.6,155.7	Favored (23.7%) *p* chi angles: 59.2	0.05Å	Favored (21.055%)	-	-	-
A 336		LYS	7.04	-	Favored (31.65%) General / -82.1,143.2	Favored (94.9%) *mttt* chi angles: 292.5,176.3,182.9,171.9	0.03Å	Favored (40.896%) beta sheet	-	-	-
A 337		ILE	7.04	-	Favored (64.76%) Pre-Pro / -94.9,120.3	Favored (72%) *mt* chi angles: 301.7,169.2	0.03Å	Favored (46.132%) beta sheet	-	-	-
A 338		PRO	7.04	-	Favored (5%) Trans-Pro / -75.1,71.6	Favored (70.8%) *Cg_endo* chi angles: 29.6,323.5,27.4	0.04Å	Favored (10.411%) beta sheet	-	-	-
A 339		VAL	7.04	-	Favored (50.92%) Ile or Val / -99.5,122.1	Favored (41.9%) *t* chi angles: 183.1	0.12Å	Favored (12.998%) beta sheet	-	-	-
A 340		GLN	7.04	-	Favored (52.08%) General / -135.1,153.2	Favored (78.9%) *mt0* chi angles: 296.9,185.3,44.7	0.01Å	Favored (40.281%) beta sheet	-	-	-
**#**	**Alt**	**Res**	**High B**	**Clash > 0.4Å**	**Ramachandran**	**Rotamer**	**Cβ deviation**	**CaBLAM**	**Bond lengths**	**Bond angles**	**Cis Peptides**
			Avg: 7.04	Clashscore: 1.46	Outliers: 6 of 498	Poor rotamers: 1 of 409	Outliers: 0 of 445	Outliers: 25 of 496	Outliers: 3 of 500	Outliers: 20 of 500	Non-Trans: 8 of 499
A 341		MET	7.04	-	Favored (54.97%) General / -122.2,136.9	Favored (58.7%) *mtm* chi angles: 295.7,172.2,287.1	0.03Å	Favored (43.108%) beta sheet	OUTLIER(S) worst is CG-- SD: 4.5 σ	-	-
A 342		ALA	7.04	-	Favored (23.37%) General / -144.2,167.0	-	0.04Å	Favored (39.832%)	-	-	-
A 343		VAL	7.04	-	Favored (9.95%) Ile or Val / -106.4,-1.1	Favored (26.9%) *m* chi angles: 299	0.07Å	Favored (32.829%)	-	-	-
A 344		ASP	7.04	-	Favored (3.23%) General / -150.4,104.5	Favored (66.8%) *t0* chi angles: 184.5,352.6	0.06Å	Favored (9.789%)	-	-	-
A 345		MET	7.04	-	Favored (36.75%) General / -56.8,-22.5	Favored (39%) *ttm* chi angles: 190.6,179.1,301.8	0.02Å	Favored (23.361%)	-	-	-
A 346		GLN	7.04	-	Favored (67.03%) General / -64.9,-22.6	Favored (22.6%) *pt0* chi angles: 72.3,183,301.5	0.07Å	Favored (40.321%)	-	-	-

A 347		THR	7.04	-	Favored (20.17%) General / -133.8,120.2	Favored (95.4%) *m* chi angles: 300.8	0.01Å	Favored (13.697%)	-	-	-
A 348		LEU	7.04	-	Favored (9.88%) General / -83.4,77.0	Favored (23.6%) *tp* chi angles: 189.5,59.3	0.05Å	Favored (25.857%)	-	-	-
A 349		THR	7.04	-	Favored (62.93%) Pre-Pro / -102.2,116.5	Favored (92.7%) *m* chi angles: 297.7	0.05Å	Favored (36.428%) beta sheet	-	-	-
A 350		PRO	7.04	-	Favored (54.59%) Trans-Pro / -67.0,140.4	Favored (48.2%) *Cg_endo* chi angles: 25,327.8,25.7	0.01Å	Favored (24.337%) beta sheet	-	-	-
A 351		VAL	7.04	-	Favored (2.54%) Ile or Val / -120.8,-45.7	Favored (82.9%) *t* chi angles: 176.8	0.04Å	CaBLAM Disfavored (3.474%)	-	-	-
A 352		GLY	7.04	-	Favored (41.78%) Glycine / -89.2,-166.2	-	-	Favored (12.019%)	-	-	-
A 353		ARG	7.04	-	Favored (45.15%) General / -140.2,157.7	Favored (45.5%) *ptt180* chi angles: 65.8,180,183.7,182.3	0.11Å	Favored (31.165%)	-	-	-
A 354		LEU	7.04	-	Favored (54.4%) General / -106.6,130.2	Allowed (1.2%) *mp* chi angles: 273.4,83.9	0.07Å	Favored (53.483%) beta sheet	-	-	-
A 355		ILE	7.04	-	Favored (80.77%) Ile or Val / -66.5,-38.0	Favored (35.3%) *mm* chi angles: 295.2,300.8	0.05Å	Favored (31.478%)	-	-	-
A 356		THR	7.04	-	Favored (4.37%) General / -70.6,2.2	Favored (48.9%) *p* chi angles: 56	0.04Å	CaBLAM Outlier (0.728%)	-	-	-
A 357		ALA	7.04	-	OUTLIER (0.05)%) General / 64.2,-96.0	-	0.06Å	CaBLAM Outlier (0.201%)	-	-	-
A 358		ASN	7.04	-	Favored (76.98%) Pre-Pro / -127.1,73.2	Favored (60.9%) *m-40* chi angles: 298.7,281.8	0.04Å	Favored (11.034%)	-	-	-
A 359		PRO	7.04	-	Favored (40.7%) Trans-Pro / -53.0,131.0	Favored (87.1%) *Cg_exo* chi angles: 330.6,37.7,330.4	0.10Å	Favored (32.834%)	-	-	-
A 360		VAL	7.04	-	Favored (64.74%) Ile or Val / -130.7,130.7	Favored (84.1%) *t* chi angles: 177.7	0.05Å	Favored (59.44%) beta sheet	-	-	-
**#**	**Alt**	**Res**	**High B**	**Clash > 0.4Å**	**Ramachandran**	**Rotamer**	**Cβ deviation**	**CaBLAM**	**Bond lengths**	**Bond angles**	**Cis Peptides**
			Avg: 7.04	Clashscore: 1.46	Outliers: 6 of 498	Poor rotamers: 1 of 409	Outliers: 0 of 445	Outliers: 25 of 496	Outliers: 3 of 500	Outliers: 20 of 500	Non-Trans: 8 of 499
A 361		ILE	7.04	-	Favored (28.6%) Ile or Val / -57.0,-28.3	Favored (18.2%) *tt* chi angles: 195.1,169.5	0.02Å	CaBLAM Outlier (0.754%) try beta sheet	-	-	-
A 362		THR	7.04	-	Allowed (0.25%) General / 64.7,-58.1	Favored (85.1%) *m* chi angles: 301.7	0.06Å	Favored (5.61%)	-	-	-

A 363		GLU	7.04	-	Favored (31.11%) General / -95.2,118.0	Favored (91.2%) *tt0* chi angles: 184.2,180.8,356	0.02Å	Favored (26.853%)	-	-	-
A 364		SER	7.04	-	Allowed (1.6%) General / -75.7,63.6	Favored (56.3%) *p* chi angles: 57.3	0.04Å	Favored (7.153%)	-	-	-
A 365		THR	7.04	-	Favored (35.96%) General / -150.4,153.5	Favored (10.7%) *t* chi angles: 190.9	0.10Å	Favored (13.203%)	-	-	-
A 366		GLU	7.04	-	Favored (32.81%) General / -88.5,136.8	Favored (92.3%) *tt0* chi angles: 182.3,180.3,3.6	0.10Å	Favored (17.316%)	-	-	-
A 367		ASN	7.04	-	Favored (2.65%) General / -96.0,67.6	Favored (75.4%) *m-40* chi angles: 288.6,314	0.09Å	Favored (9.316%)	-	OUTLIER(S) worst is C-N-CA: 5.2 σ	Cis nonPRO omega= -4.9
A 368		SER	7.04	-	Favored (57.69%) General / -58.5,137.0	Favored (34.8%) *t* chi angles: 174.3	0.02Å	Favored (24.34%)	-	-	-
A 369		LYS	7.04	-	Favored (43.54%) General / -118.7,147.7	Favored (98.4%) *mttt* chi angles: 296.1,179,181.7,176.1	0.01Å	Favored (66.345%) beta sheet	-	-	-
A 370		MET	7.04	-	Favored (30.8%) General / -142.5,138.2	Allowed (1.4%) *tmt* chi angles: 187.7,271.9,161.7	0.09Å	Favored (59.739%) beta sheet	-	-	-
A 371		MET	7.04	-	Favored (43.78%) General / -98.2,126.8	Favored (60.9%) *ttp* chi angles: 181.7,177.8,77.7	0.08Å	Favored (62.226%) beta sheet	-	-	-
A 372		LEU	7.04	-	Favored (32.4%) General / -103.9,143.8	Favored (55.3%) *mt* chi angles: 298.3,167.4	0.09Å	Favored (53.26%) beta sheet	-	-	-
A 373		GLU	7.04	-	Favored (21.21%) General / -125.5,116.1	Favored (41.5%) *mt-10* chi angles: 304,177.7,30.5	0.04Å	Favored (48.563%) beta sheet	-	-	-
A 374		LEU	7.04	-	Favored (47.83%) General / -127.8,152.4	Allowed (1.2%) *mp* chi angles: 313,86.2	0.15Å	Favored (48.369%) beta sheet	-	-	-
A 375		ASP	7.04	-	Favored (37.46%) Pre-Pro / -114.3,94.9	Favored (93.9%) *m-30* chi angles: 285.6,347.1	0.07Å	Favored (37.968%)	-	-	-
A 376		PRO	7.04	-	Favored (36.98%) Trans-Pro / -63.8,160.4	Favored (28.9%) *Cg_exo* chi angles: 340.2,30.1,332	0.05Å	Favored (30.11%)	-	-	-
A 377		PRO	7.04	-	Favored (27.94%) Trans-Pro / -72.0,167.0	Favored (73.4%) *Cg_endo* chi angles: 27.6,325.3,27.5	0.03Å	Favored (46.676%)	-	-	-
A 378		PHE	7.04	-	Favored (42.53%) General / -70.2,153.9	Favored (79.9%) *m-80* chi angles: 289.1,96.3	0.05Å	Favored (24.088%)	-	-	-
A 379		GLY	7.04	-	Favored (48.45%) Glycine / 73.2,-153.7	-	-	Favored (42.765%)	-	-	-
A 380		ASP	7.04	-	Favored (54.74%)	Favored (87.9%) *m-30*	0.07Å	CaBLAM Disfavored	-	-	-

#	Alt	Res	High B	Clash > 0.4Å	Ramachandran	Rotamer	Cβ deviation	CaBLAM	Bond lengths	Bond angles	Cis Peptides
					General / -68.4,144.5	chi angles: 289.6,352.7		(1.245%)			
#	Alt	Res	High B	Clash > 0.4Å	Ramachandran	Rotamer	Cβ deviation	CaBLAM	Bond lengths	Bond angles	Cis Peptides
			Avg: 7.04	Clashscore: 1.46	Outliers: 6 of 498	Poor rotamers: 1 of 409	Outliers: 0 of 445	Outliers: 25 of 496	Outliers: 3 of 500	Outliers: 20 of 500	Non-Trans: 8 of 499
A 381		SER	7.04	-	Favored (17.74%) General / -161.9,171.4	Favored (89.5%) *p* chi angles: 69	0.02Å	Favored (28.673%)	-	-	-
A 382		TYR	7.04	-	Favored (48.9%) General / -121.3,143.9	Favored (90.3%) *m-80* chi angles: 300.6,92.3	0.06Å	Favored (45.454%) beta sheet	-	-	-
A 383		ILE	7.04	-	Favored (55.59%) Ile or Val / -105.0,120.5	Favored (80.8%) *mt* chi angles: 299.7,168.7	0.01Å	Favored (65.926%) beta sheet	-	-	-
A 384		VAL	7.04	-	Favored (74.83%) Ile or Val / -123.3,128.4	Favored (70%) *t* chi angles: 178.8	0.06Å	Favored (68.181%)	-	-	-
A 385		ILE	7.04	-	Favored (42.47%) Ile or Val / -119.9,114.0	Favored (55.5%) *mt* chi angles: 303,178.4	0.07Å	Favored (6.542%)	-	-	-
A 386		GLY	7.04	-	Favored (12.5%) Glycine / 109.0,147.9	-	-	Favored (12.526%)	-	-	-
A 387		VAL	7.04	-	Favored (9.27%) Ile or Val / -105.3,-47.5	Favored (85.2%) *t* chi angles: 177.2	0.05Å	CaBLAM Disfavored (3.039%)	-	-	-
A 388		GLY	7.04	-	Favored (36.9%) Glycine / -88.2,-161.3	-	-	Favored (15.953%)	-	-	-
A 389		ASP	7.04	-	Favored (27.16%) General / -47.5,-46.6	Favored (23%) *p0* chi angles: 53.1,355	0.04Å	Favored (9.016%)	-	-	-
A 390		LYS	7.04	-	Favored (4.07%) General / -77.5,74.9	Favored (5.6%) *mptt* chi angles: 275.2,72.2,175.6,185.5	0.03Å	CaBLAM Disfavored (3.709%)	-	-	-
A 391		LYS	7.04	-	Favored (33.06%) General / -143.9,163.6	Favored (58.2%) *pttt* chi angles: 64.7,183.2,173.1,184	0.04Å	Favored (31.892%)	-	-	-
A 392		ILE	7.04	-	Favored (53.26%) Ile or Val / -125.9,138.8	Favored (20.9%) *tt* chi angles: 185.8,165.7	0.01Å	Favored (60.688%) beta sheet	-	-	-
A 393		THR	7.04	-	Favored (41.62%) General / -126.3,155.7	Favored (52.9%) *p* chi angles: 65.2	0.08Å	Favored (60.928%) beta sheet	-	-	-
A 394		HIS	7.04	-	Favored (15.75%) General / -148.6,131.0	Favored (75.8%) *t70* chi angles: 184.9,79	0.05Å	Favored (36.343%) beta sheet	-	-	-
A 395		HIS	7.04	-	Favored (44.32%) General / -73.4,135.7	Favored (58.6%) *t-90* chi angles: 183.3,282.7	0.01Å	Favored (47.891%) beta sheet	-	-	-

A 396		TRP	7.04	-	Favored (47.02%) General / -132.2,156.2	Favored (72.8%) *t-100* chi angles: 182.3,251.3	0.03Å	Favored (58.939%) beta sheet	-	-	-
A 397		HIS	7.04	-	Favored (14.11%) General / -144.8,125.4	Favored (73.1%) *t70* chi angles: 184.3,81.3	0.07Å	Favored (44.53%) beta sheet	-	-	-
A 398		ARG	7.04	0.40Å O with A 399 SER C	Favored (29.72%) General / -100.6,-3.9	Favored (81.6%) *mtp180* chi angles: 290.6,182.6,61.2,186.2	0.10Å	CaBLAM Disfavored (2.684%) try beta sheet	-	-	-
A 399		SER	7.04	0.40Å C with A 398 ARG O	Allowed (0.42%) General / 45.9,-140.3	Favored (17.5%) *t* chi angles: 186.7	0.07Å	Favored (5.107%) beta sheet	-	-	-
A 400		GLY	7.04	-	Favored (34.64%) Glycine / 61.4,-156.4	-	-	Favored (11.735%) beta sheet	-	-	-
**#**	**Alt**	**Res**	**High B**	**Clash > 0.4Å**	**Ramachandran**	**Rotamer**	**Cβ deviation**	**CaBLAM**	**Bond lengths**	**Bond angles**	**Cis Peptides**
			Avg: 7.04	Clashscore: 1.46	Outliers: 6 of 498	Poor rotamers: 1 of 409	Outliers: 0 of 445	Outliers: 25 of 496	Outliers: 3 of 500	Outliers: 20 of 500	Non-Trans: 8 of 499
A 401		SER	7.04	-	Favored (21.6%) General / -164.5,162.0	Favored (97.3%) *p* chi angles: 65.8	0.01Å	CaBLAM Disfavored (3.662%)	-	-	-
A 402		THR	7.04	-	Favored (99.3%) General / -61.1,-43.2	Favored (88.3%) *m* chi angles: 298.5	0.01Å	Favored (63.313%)	-	-	-
A 403		ILE	7.04	-	Favored (99.09%) Ile or Val / -63.5,-45.0	Favored (98.7%) *mt* chi angles: 292.8,168.1	0.03Å	Favored (88.755%) alpha helix	-	-	-
A 404		GLY	7.04	-	Favored (37.93%) Glycine / -55.0,-53.8	-	-	Favored (92.788%) alpha helix	-	-	-
A 405		LYS	7.04	-	Favored (95.52%) General / -60.7,-41.7	Favored (49.9%) *tttp* chi angles: 181.7,174.4,171.2,68.3	0.06Å	Favored (78.652%) alpha helix	-	-	-
A 406		ALA	7.04	-	Favored (73.73%) General / -58.0,-37.2	-	0.04Å	Favored (76.35%) alpha helix	-	-	-
A 407		PHE	7.04	-	Favored (62.82%) General / -70.6,-16.3	Favored (13.1%) *m-10* chi angles: 287.6,357.8	0.02Å	Favored (60.09%) alpha helix	-	-	-
A 408		GLU	7.04	-	Favored (10.3%) General / -92.7,-41.2	Favored (69.2%) *tp30* chi angles: 182.1,66,22.2	0.02Å	Favored (25.509%) alpha helix	-	-	-
A 409		ALA	7.04	-	Favored (79.04%) General / -56.4,-43.1	-	0.03Å	Favored (76.041%) alpha helix	-	-	-
A 410		THR	7.04	-	Favored (17.32%) General / -130.6,115.8	Favored (83.2%) *m* chi angles: 302	0.07Å	CaBLAM Disfavored (1.027%) try alpha helix	-	OUTLIER(S) worst is N-CA-C: 5.1 σ	-
A 411		VAL	7.04	0.43Å O with A 411	Favored (22.95%)	Favored (5.2%) *p* chi angles: 69.8	0.03Å	CaBLAM Outlier	-	OUTLIER(S) worst is C-N-	Cis nonPRO

				VAL HG22	Ile or Val / -56.3,-26.9			(0.418%) try alpha helix		CA: 6.3 σ	omega= 15.47
A 412		ARG	7.04	-	Favored (74.54%) General / -56.4,-40.3	Favored (95%) *mtt180* chi angles: 288.7,173.5,186.4,178	0.05Å	Favored (79.305%) alpha helix	-	-	-
A 413		GLY	7.04	-	Favored (82.36%) Glycine / -59.1,-35.1	-	-	Favored (90.993%) alpha helix	-	-	-
A 414		ALA	7.04	-	Favored (84.89%) General / -66.9,-37.7	-	0.02Å	Favored (89.054%) alpha helix	-	-	-
A 415		LYS	7.04	-	Favored (88.25%) General / -66.8,-40.5	Favored (97.3%) *mttt* chi angles: 290,179.2,179.9,178.4	0.01Å	Favored (87.793%) alpha helix	-	-	-
A 416		ARG	7.04	-	Favored (92.95%) General / -59.5,-44.8	Favored (48.8%) *ttp-170* chi angles: 184,174.2,67.1,205.7	0.01Å	Favored (95.312%) alpha helix	-	-	-
A 417		MET	7.04	-	Favored (92.95%) General / -59.7,-45.2	Favored (97.6%) *mtp* chi angles: 289.9,172.4,69.1	0.06Å	Favored (98.312%) alpha helix	-	-	-
A 418		ALA	7.04	-	Favored (81.33%) General / -60.0,-38.0	-	0.06Å	Favored (65.923%) alpha helix	-	-	-
A 419		VAL	7.04	-	Favored (16.82%) Ile or Val / -86.1,-46.2	Favored (90.2%) *t* chi angles: 176	0.07Å	Favored (49.444%) alpha helix	-	-	-
A 420		LEU	7.04	-	Favored (58.16%) General / -68.5,-49.7	Favored (57.5%) *tp* chi angles: 174.8,59.9	0.02Å	Favored (11.708%)	-	-	-
**#**	**Alt**	**Res**	**High B**	**Clash > 0.4Å**	**Ramachandran**	**Rotamer**	**Cβ deviation**	**CaBLAM**	**Bond lengths**	**Bond angles**	**Cis Peptides**
			Avg: 7.04	Clashscore: 1.46	Outliers: 6 of 498	Poor rotamers: 1 of 409	Outliers: 0 of 445	Outliers: 25 of 496	Outliers: 3 of 500	Outliers: 20 of 500	Non-Trans: 8 of 499
A 421		GLY	7.04	-	Favored (19.74%) Glycine / 99.7,-159.1	-	-	Favored (27.615%)	-	-	-
A 422		ASP	7.04	-	Favored (63.6%) General / -59.3,-24.8	Favored (42.4%) *p0* chi angles: 59,349.7	0.06Å	Favored (12.718%)	-	-	-
A 423		THR	7.04	-	Favored (12.85%) General / -53.1,-25.4	Favored (87.9%) *m* chi angles: 297	0.03Å	Favored (19.463%)	-	-	-
A 424		ALA	7.04	-	Favored (14.21%) General / -57.0,151.9	-	0.03Å	CaBLAM Disfavored (3.56%)	-	-	-
A 425		TRP	7.04	-	Favored (2.69%) General / 78.2,0.5	Favored (63.5%) *m100* chi angles: 306.1,111.3	0.07Å	CaBLAM Outlier (0.698%)	-	-	-
A 426		ASP	7.04	-	Favored (54.3%) General / -91.7,3.8	Favored (96.5%) *m-30* chi angles: 290.3,343.1	0.09Å	Favored (8.937%)	-	-	-
A 427		PHE	7.04	0.52Å C with A 427	Favored (22.65%)	Favored (33%) *t80* chi angles: 185.4,100.8	0.10Å	CaBLAM Disfavored	-	-	-

#	Alt	Res	High B	Clash > 0.4Å	Ramachandran	Rotamer	Cβ deviation	CaBLAM	Bond lengths	Bond angles	Cis Peptides
				PHE CD2	General / -85.9,114.2			(3.069%)			
A 428		GLY	7.04	-	Favored (60.59%) Glycine / 76.6,27.7	-	-	Favored (21.997%)	-	-	-
A 429		SER	7.04	-	Favored (23.78%) General / -95.0,147.1	Favored (42.4%) *t* chi angles: 175.8	0.03Å	CaBLAM Disfavored (4.962%)	-	-	-
A 430		VAL	7.04	-	Allowed (0.11%) Ile or Val / -147.6,-15.8	Favored (5.6%) *p* chi angles: 69.4	0.06Å	CaBLAM Disfavored (2.235%)	-	-	-
A 431		GLY	7.04	-	Favored (33.65%) Glycine / -97.8,-175.2	-	-	Favored (18.996%)	-	-	-
A 432		GLY	7.04	-	Favored (6.45%) Glycine / -126.8,16.9	-	-	Favored (32.852%)	-	-	-
A 433		VAL	7.04	-	Favored (4.37%) Ile or Val / -115.5,-55.1	Favored (80.6%) *t* chi angles: 176.5	0.04Å	CaBLAM Disfavored (1.785%)	-	-	-
A 434		PHE	7.04	-	Favored (44.89%) General / -74.2,144.3	Favored (89.1%) *m-80* chi angles: 291,94.7	0.11Å	CaBLAM Disfavored (3.134%)	-	OUTLIER(S) worst is CA-CB-CG: 5.8 σ	-
A 435		ASN	7.04	-	Favored (6.12%) General / 70.9,7.1	Favored (83.1%) *m-40* chi angles: 299.9,319	0.06Å	Favored (11.723%)	-	-	-
A 436		SER	7.04	0.40Å C with A 438 GLY H	Favored (36.08%) General / -67.2,156.5	Favored (92.5%) *p* chi angles: 64.5	0.03Å	Favored (17.788%)	-	-	-
A 437		LEU	7.04	-	OUTLIER (0.05)%) General / 62.1,-23.2	Favored (94.2%) *mt* chi angles: 297.9,175.6	0.02Å	CaBLAM Outlier (0.269%)	-	-	-
A 438		GLY	7.04	0.40Å H with A 436 SER C	Favored (35.69%) Glycine / -65.0,-53.1	-	-	Favored (10.288%) alpha helix	-	-	-
A 439		LYS	7.04	-	Favored (77.45%) General / -60.1,-36.5	Favored (51.9%) *mtpt* chi angles: 287.1,175.6,63.5,178	0.03Å	Favored (74.798%) alpha helix	-	-	-
A 440		GLY	7.04	-	Favored (43.1%) Glycine / -58.7,-53.8	-	-	Favored (89.942%) alpha helix	-	-	-
**#**	**Alt**	**Res**	**High B**	**Clash > 0.4Å**	**Ramachandran**	**Rotamer**	**Cβ deviation**	**CaBLAM**	**Bond lengths**	**Bond angles**	**Cis Peptides**
			Avg: 7.04	Clashscore: 1.46	Outliers: 6 of 498	Poor rotamers: 1 of 409	Outliers: 0 of 445	Outliers: 25 of 496	Outliers: 3 of 500	Outliers: 20 of 500	Non-Trans: 8 of 499
A 441		ILE	7.04	-	Favored (89.62%) Ile or Val / -61.6,-41.3	Favored (90.7%) *mt* chi angles: 291.2,168	0.04Å	Favored (77.723%) alpha helix	-	-	-
A 442		HIS	7.04	-	Favored (87.15%) General / -58.2,-45.7	Favored (24.8%) *t-170* chi angles: 188.2,187.2	0.02Å	Favored (84.919%) alpha helix	-	-	-
A 443		GLN	7.04	-	Favored (84.82%)	Favored (93.3%) *mt0*	0.05Å	Favored (91.212%) alpha helix	-	-	-

					General / -61.8,-37.6	chi angles: 287.9,178.4,321.5					
A 444		ILE	7.04	-	Favored (94.11%) Ile or Val / -63.1,-47.1	Favored (94.7%) *mt* chi angles: 291.8,167.9	0.01Å	Favored (83.155%) alpha helix	-	-	-
A 445		PHE	7.04	-	Favored (93.7%) General / -65.0,-39.7	Favored (9.6%) *m-10* chi angles: 286.4,329.4	0.03Å	Favored (81.092%) alpha helix	-	-	-
A 446		GLY	7.04	-	Favored (30.53%) Glycine / -55.9,-55.2	-	-	Favored (91%) alpha helix	-	-	-
A 447		ALA	7.04	-	Favored (88.38%) General / -60.5,-39.7	-	0.05Å	Favored (76.902%) alpha helix	-	-	-
A 448		ALA	7.04	-	Favored (86.58%) General / -64.2,-45.8	-	0.04Å	Favored (83.199%) alpha helix	-	-	-
A 449		PHE	7.04	-	Favored (75.01%) General / -70.3,-39.1	Favored (20.1%) *m-80* chi angles: 281.7,68.1	0.07Å	Favored (82.928%) alpha helix	-	-	-
A 450		LYS	7.04	-	Favored (94.72%) General / -64.2,-39.8	Favored (96.8%) *mttt* chi angles: 289,178.4,180.1,179.1	0.03Å	Favored (96.862%) alpha helix	-	-	-
A 451		SER	7.04	-	Favored (93.31%) General / -63.0,-39.0	Favored (72.8%) *m* chi angles: 295.7	0.04Å	Favored (71.505%) alpha helix	-	-	-
A 452		LEU	7.04	-	Favored (20.15%) General / -83.8,-39.4	Favored (95%) *mt* chi angles: 293.9,171.7	0.09Å	Favored (49.053%)	-	-	-
A 453		PHE	7.04	-	Favored (8.3%) General / -117.1,-22.6	Favored (41.7%) *m-80* chi angles: 308.4,113.5	0.10Å	CaBLAM Disfavored (1.051%)	-	OUTLIER(S) worst is CA-CB-CG: 4.1 σ	-
A 454		GLY	7.04	-	Favored (40.42%) Glycine / 54.3,-128.0	-	-	Favored (40.793%)	-	-	-
A 455		GLY	7.04	-	Favored (48.09%) Glycine / -97.6,21.1	-	-	Favored (6.537%)	-	-	-
A 456		MET	7.04	-	Allowed (0.59%) General / -71.3,70.4	Favored (98.7%) *mtp* chi angles: 291.2,177.1,70	0.07Å	Favored (23.975%)	-	-	-
A 457		SER	7.04	-	OUTLIER (0.03)%) General / 44.0,-153.6	Favored (23.5%) *t* chi angles: 184.8	0.03Å	CaBLAM Outlier (0.886%)	-	-	-
A 458		TRP	7.04	-	Favored (4.4%) General / -122.6,-27.2	Favored (71.1%) *p-90* chi angles: 59.9,273.7	0.09Å	CaBLAM Outlier (0.011%)	-	-	-
A 459		PHE	7.04	-	Favored (37.78%) General / -72.2,-49.1	Favored (72.5%) *t80* chi angles: 184,72.6	0.05Å	Favored (73.328%) alpha helix	-	-	-
A 460		SER	7.04	-	Favored (79.91%) General / -61.8,-36.0	Favored (59.8%) *m* chi angles: 293.5	0.05Å	Favored (76.688%) alpha helix	-	-	-

#	Alt	Res	High B	Clash > 0.4Å	Ramachandran	Rotamer	Cβ deviation	CaBLAM	Bond lengths	Bond angles	Cis Peptides
			Avg: 7.04	Clashscore: 1.46	Outliers: 6 of 498	Poor rotamers: 1 of 409	Outliers: 0 of 445	Outliers: 25 of 496	Outliers: 3 of 500	Outliers: 20 of 500	Non-Trans: 8 of 499
A 461		GLN	7.04	-	Favored (76.55%) General / -63.5,-33.9	Favored (46.6%) *mt0* chi angles: 290.5,188.7,79.1	0.08Å	Favored (74.244%) alpha helix	-	-	-
A 462		ILE	7.04	-	Favored (75.39%) Ile or Val / -59.2,-38.6	Favored (88.5%) *mt* chi angles: 291.2,169.3	0.11Å	Favored (84.644%) alpha helix	-	-	-
A 463		LEU	7.04	-	Favored (18.29%) General / -84.0,7.5	Favored (92.5%) *mt* chi angles: 298.5,176.3	0.05Å	Favored (13.332%) alpha helix	-	-	-
A 464		ILE	7.04	-	Favored (8.51%) Ile or Val / -97.6,-39.3	Favored (91.6%) *mt* chi angles: 292.5,172.7	0.15Å	CaBLAM Disfavored (1.037%) try alpha helix	-	OUTLIER(S) worst is CA-C-N: 4.3 σ	-
A 465		GLY	7.04	-	Favored (4.19%) Glycine / 89.2,-113.8	-	-	Favored (82.617%) alpha helix	-	OUTLIER(S) worst is N-CA-C: 5.5 σ	Cis nonPRO omega= 29.04
A 466		THR	7.04	-	Favored (19.04%) General / -112.5,109.5	Favored (97%) *m* chi angles: 299.9	0.11Å	CaBLAM Disfavored (1.464%) try alpha helix	-	-	-
A 467		LEU	7.04	-	Favored (14.3%) General / -45.8,-45.0	Favored (66.8%) *tp* chi angles: 179.6,62.9	0.07Å	CaBLAM Outlier (0.289%) try alpha helix	-	OUTLIER(S) worst is C-N-CA: 8.4 σ	Cis nonPRO omega= 11.7
A 468		LEU	7.04	-	Favored (60.47%) General / -54.2,-34.4	Favored (71.4%) *mt* chi angles: 287.3,171.5	0.08Å	Favored (63.636%) alpha helix	-	-	-
A 469		VAL	7.04	-	Favored (99.5%) Ile or Val / -63.1,-44.6	Favored (66.7%) *t* chi angles: 171.7	0.02Å	Favored (84.176%) alpha helix	-	-	-
A 470		TRP	7.04	-	Favored (96.17%) General / -63.4,-40.0	Favored (33.8%) *m-10* chi angles: 285.7,341.3	0.04Å	Favored (85.733%) alpha helix	-	-	-
A 471		LEU	7.04	-	Favored (68.82%) General / -57.9,-51.6	Favored (71.8%) *tp* chi angles: 177.4,63.2	0.09Å	Favored (78.47%) alpha helix	-	-	-
A 472		GLY	7.04	-	Favored (45.09%) Glycine / -51.3,-49.3	-	-	Favored (93.529%) alpha helix	-	-	-
A 473		LEU	7.04	-	Favored (66.85%) General / -60.3,-26.8	Favored (96.6%) *mt* chi angles: 292.1,171.9	0.04Å	Favored (66.078%) alpha helix	-	-	-
A 474		ASN	7.04	-	Favored (28.15%) General / -104.6,15.4	Favored (68.8%) *m-40* chi angles: 291.6,280.2	0.04Å	Favored (33.25%)	-	-	-
A 475		THR	7.04	-	Favored (59.1%) General / -85.8,-6.4	Favored (51.3%) *p* chi angles: 56.4	0.07Å	CaBLAM Disfavored (1.079%)	-	-	-
A 476		LYS	7.04	-	Allowed (1.95%)	Favored (91.5%) *mttt*	0.02Å	CaBLAM Disfavored	-	-	-

					General / 51.0,-132.1	chi angles: 301.3,183.4,181.6,178.2		(2.685%)			
A 477		ASN	7.04	-	Allowed (0.58%) General / 45.6,-116.3	Favored (83.3%) *m-40* chi angles: 296.7,336.1	0.07Å	CaBLAM Outlier (0.382%)	-	-	-
A 478		GLY	7.04	-	OUTLIER (0.08)%) Glycine / 179.6,-64.9	-	-	Favored (31.381%)	-	-	-
A 479		SER	7.04	-	Favored (95.25%) General / -62.2,-40.2	Favored (65.3%) *m* chi angles: 294.3	0.03Å	Favored (80.034%) alpha helix	-	-	-
A 480		ILE	7.04	-	Favored (35.85%) Ile or Val / -60.8,-25.6	Favored (11.8%) *tp* chi angles: 192,64.8	0.07Å	Favored (68.162%) alpha helix	-	-	-
**#**	**Alt**	**Res**	**High B**	**Clash > 0.4Å**	**Ramachandran**	**Rotamer**	**Cβ deviation**	**CaBLAM**	**Bond lengths**	**Bond angles**	**Cis Peptides**
			Avg: 7.04	Clashscore: 1.46	Outliers: 6 of 498	Poor rotamers: 1 of 409	Outliers: 0 of 445	Outliers: 25 of 496	Outliers: 3 of 500	Outliers: 20 of 500	Non-Trans: 8 of 499
A 481		SER	7.04	-	Favored (59.33%) General / -78.4,-9.6	Favored (72.6%) *p* chi angles: 71.7	0.05Å	Favored (64.111%) alpha helix	-	-	-
A 482		LEU	7.04	-	Favored (8.78%) General / -96.2,-41.3	Favored (83.7%) *mt* chi angles: 300.3,175.9	0.06Å	Favored (27.12%) alpha helix	-	-	-
A 483		THR	7.04	-	Favored (74.8%) General / -57.5,-49.9	Favored (90.6%) *m* chi angles: 297.2	0.05Å	Favored (84.525%) alpha helix	-	-	-
A 484		CYS	7.04	-	Favored (44.83%) General / -124.2,125.7	Favored (66.1%) *m* chi angles: 299.7	0.13Å	CaBLAM Disfavored (1.982%) try alpha helix	-	OUTLIER(S) worst is N-CA-C: 5.1 σ	-
A 485		LEU	7.04	-	Favored (39.53%) General / -74.5,-4.9	Favored (4%) *pp* chi angles: 63.4,85.9	0.05Å	CaBLAM Outlier (0.372%) try alpha helix	-	OUTLIER(S) worst is N-CA-C: 4.4 σ	Cis nonPRO omega= 11.26
A 486		ALA	7.04	-	Favored (88.85%) General / -59.8,-40.5	-	0.04Å	Favored (77.96%) alpha helix	-	-	-
A 487		LEU	7.04	-	Favored (80.91%) General / -62.1,-48.3	Favored (68.5%) *tp* chi angles: 177.5,58.7	0.04Å	Favored (84.43%) alpha helix	-	-	-
A 488		GLY	7.04	-	Favored (96.36%) Glycine / -61.5,-38.8	-	-	Favored (91.934%) alpha helix	-	-	-
A 489		GLY	7.04	-	Favored (59.45%) Glycine / -58.7,-51.4	-	-	Favored (94.207%) alpha helix	-	-	-
A 490		VAL	7.04	-	Favored (94.78%) Ile or Val / -63.5,-42.0	Favored (77.6%) *t* chi angles: 172.9	0.03Å	Favored (81.503%) alpha helix	-	-	-
A 491		MET	7.04	-	Favored (83.4%) General / -57.8,-42.0	Favored (82.5%) *mmm* chi angles: 286,294,284.3	0.06Å	Favored (79.985%) alpha helix	-	-	-

A 492		ILE	7.04	-	Favored (80.58%) Ile or Val / -68.7,-45.9	Favored (95.8%) *mt* chi angles: 294.2,167.2	0.05Å	Favored (83.346%) alpha helix	-	-	-
A 493		PHE	7.04	-	Favored (78.78%) General / -59.5,-49.2	Favored (91.9%) *t80* chi angles: 178.6,78.1	0.09Å	Favored (93.058%) alpha helix	-	OUTLIER(S) worst is CA-CB-CG: 5.5 σ	-
A 494		LEU	7.04	-	Favored (91.64%) General / -61.4,-39.7	Favored (90.7%) *mt* chi angles: 291.7,173.9	0.10Å	Favored (92.578%) alpha helix	-	-	-
A 495		SER	7.04	-	Favored (93.37%) General / -65.2,-39.8	Favored (71.1%) *m* chi angles: 296.1	0.09Å	Favored (96.984%) alpha helix	-	-	-
A 496		THR	7.04	-	Favored (78.25%) General / -65.5,-46.8	Favored (95.5%) *m* chi angles: 299.5	0.06Å	Favored (78.472%) alpha helix	-	-	-
A 497		ALA	7.04	-	Favored (77.92%) General / -67.6,-44.4	-	0.05Å	Favored (72.71%)	-	-	-
A 498		VAL	7.04	-	Allowed (1.04%) Ile or Val / -94.1,77.0	Favored (34.8%) *t* chi angles: 184.6	0.04Å	Favored (8.908%)	-	-	-
A 499		SER	7.04	-	Favored (59.26%) General / -83.3,-9.2	Favored (84.8%) *p* chi angles: 67.6	0.02Å	-	-	-	-
A 500		ALA	7.04	-	-	-	0.06Å	-	-	-	-

About MolProbity | Website for the Richardson Lab | Using ecloud x-H | Internal reference 4.5.2
