# Supplementary material for: Integrative proteome-wide structural analysis and high-throughput docking identify broad-spectrum antiviral scaffolds against Zika, Yellow Fever, West Nile, Saint Louis encephalitis, and Usutu viruses
Source: Front Cell Infect Microbiol. 2026 Apr 30;16:1723132. doi: 10.3389/fcimb.2026.1723132 (PMC13171538; doi:10.3389/fcimb.2026.1723132)
Supplement: Supplementary file 7 [file DataSheet7.zip › ZIKV/ZIKV_E/Mol_probity_Files/ZIKV_E_1FH-rama.pdf]

MolProbity Ramachandran analysis

ZIKV_E1FH.pdb, model 1

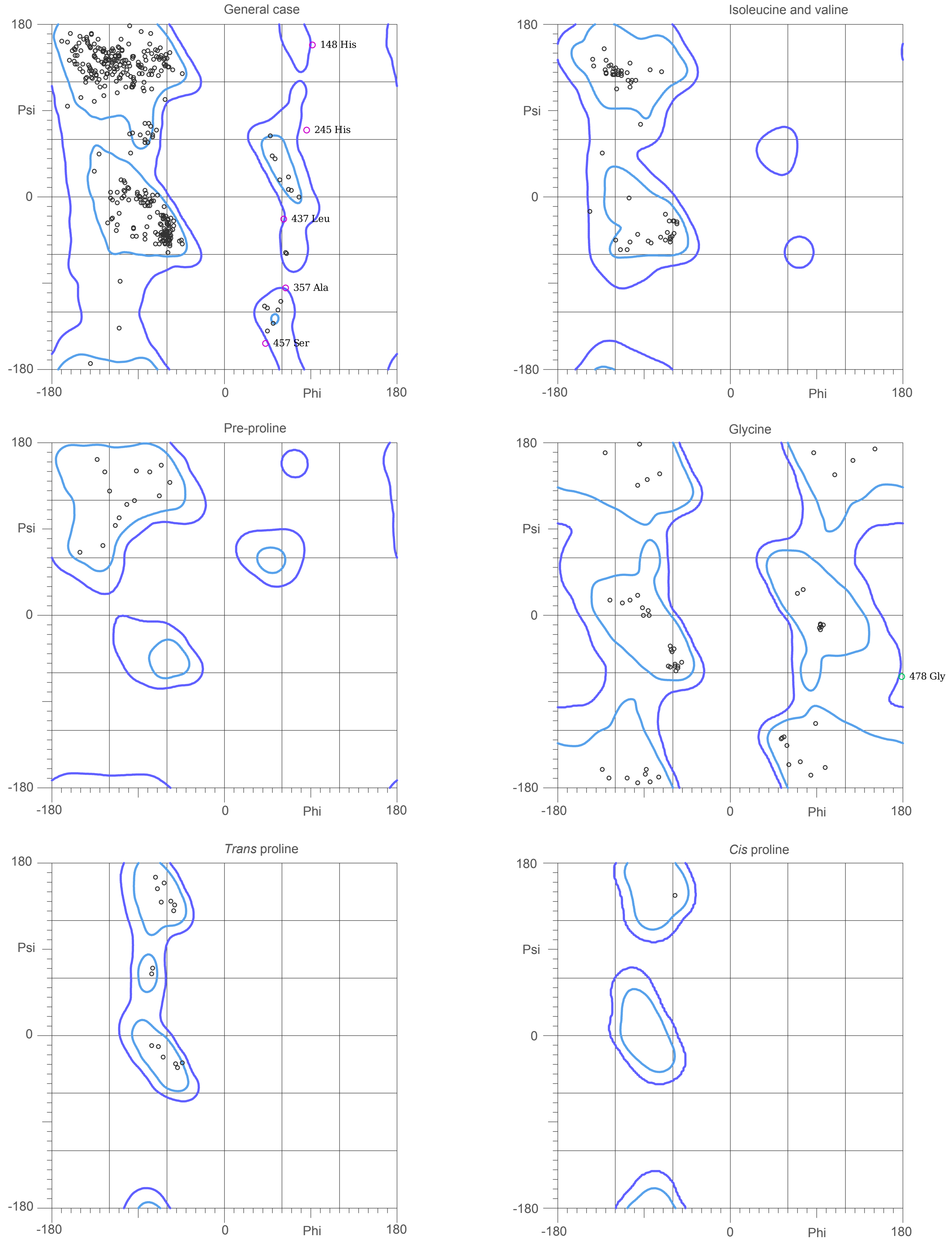

94.8% (472/498) of all residues were in favored (98%) regions.
98.8% (492/498) of all residues were in allowed (>99.8%) regions.

There were 6 outliers (phi, psi):
- 148 His (92.8, 159.5)
- 245 His (86.0, 71.0)
- 357 Ala (64.2, -96.0)
- 437 Leu (62.1, -23.2)
- 457 Ser (44.0, -153.6)
- 478 Gly (179.6, -64.9)
