# Supplementary material for: Integrative proteome-wide structural analysis and high-throughput docking identify broad-spectrum antiviral scaffolds against Zika, Yellow Fever, West Nile, Saint Louis encephalitis, and Usutu viruses
Source: Front Cell Infect Microbiol. 2026 Apr 30;16:1723132. doi: 10.3389/fcimb.2026.1723132 (PMC13171538; doi:10.3389/fcimb.2026.1723132)
Supplement: Supplementary file 7 [file DataSheet7.zip › ZIKV/ZIKV_M/Mol_probity_Files/ZIKV_M_1FH-multi.table.pdf]

Viewing ZIKV_M1FH-multi.table

Duke Biochemistry
Duke University School of Medicine

When finished, you should close this window.

Hint: Use File | Save As... to save a copy of this page.

All-Atom Contacts	Clashscore, all atoms:	0		100th percentile* (N=1784, all resolutions)
	Clashscore is the number of serious steric overlaps (> 0.4 Å) per 1000 atoms.			
Protein Geometry	Poor rotamers	0	0.00%	Goal: <0.3%
	Favored rotamers	66	100.00%	Goal: >98%
	Ramachandran outliers	1	1.37%	Goal: <0.05%
	Ramachandran favored	69	94.52%	Goal: >98%
	Rama distribution Z-score	0.51 ± 0.95		Goal: abs(Z score) < 2
	MolProbity score^	0.87		100th percentile* (N=27675, 0Å - 99Å)
	Cβ deviations >0.25Å	0	0.00%	Goal: 0
	Bad bonds:	0 / 618	0.00%	Goal: 0%
	Bad angles:	1 / 843	0.12%	Goal: <0.1%
Peptide Omegas	Cis Prolines:	0 / 3	0.00%	Expected: ≤1 per chain, or ≤5%
Low-resolution Criteria	CaBLAM outliers	6	8.5%	Goal: <1.0%
	CA Geometry outliers	0	0.00%	Goal: <0.5%
Additional validations	Chiral volume outliers	0/101		
	Waters with clashes	0/0	0.00%	See UnDowser table for details

In the two column results, the left column gives the raw count, right column gives the percentage.

* 100th percentile is the best among structures of comparable resolution; 0th percentile is the worst. For clashscore the comparative set of structures was selected in 2004, for MolProbity score in 2006.

^ MolProbity score combines the clashscore, rotamer, and Ramachandran evaluations into a single score, normalized to be on the same scale as X-ray resolution.

Key to table colors and cutoffs here:

#	Alt	Res	High B	Clash > 0.4Å	Ramachandran	Rotamer	Cβ deviation	CaBLAM	Bond lengths	Bond angles	Cis Peptides
			Avg: 2.92	Clashscore: 0	Outliers: 1 of 73	Poor rotamers: 0 of 66	Outliers: 0 of 73	Outliers: 6 of 71	Outliers: 0 of 75	Outliers: 1 of 75	Non-Trans: 0 of 74
A 1		ALA	5.61	-	-	-	0.03Å	-	-	-	-
A 2		VAL	5.37	-	Favored (71.91%) Ile or Val / -116.6,123.7	Favored (76.4%) *t* chi angles: 178.2	0.03Å	-	-	-	-
A 3		THR	5.07	-	Favored (24.07%) General / -131.5,165.2	Favored (52%) *p* chi angles: 65.4	0.04Å	Favored (38.482%)	-	-	-
A 4		LEU	4.75	-	Favored (31.21%) Pre-Pro / -103.5,145.8	Favored (82%) *mt* chi angles: 301.8,177.7	0.06Å	Favored (29.256%) beta sheet	-	-	-
A 5		PRO	4.45	-	Favored (5.8%) Trans-Pro / -77.5,57.0	Favored (58.2%) *Cg_endo* chi angles: 32.1,322.7,26.4	0.04Å	CaBLAM Outlier (0.017%)	-	-	-

A 6		SER	4.19	-	OUTLIER (0.04)%) General / 45.0,-152.1	Favored (20.7%) *t* chi angles: 185.8	0.07Å	CaBLAM Outlier (0.962%)	-	-	-
A 7		HIS	4.03	-	Favored (4.73%) General / -126.4,-19.0	Favored (98.9%) *m-70* chi angles: 300.8,291.7	0.05Å	CaBLAM Outlier (0.014%)	-	-	-
A 8		SER	4.05	-	Favored (59.79%) General / -81.0,-8.5	Favored (95%) *p* chi angles: 66.1	0.02Å	Favored (42.625%) alpha helix	-	-	-
A 9		THR	4.39	-	Favored (10.3%) General / -122.6,20.5	Favored (47.8%) *p* chi angles: 55.8	0.18Å	Favored (35.007%)	-	-	-
A 10		ARG	5.1	-	Favored (53.1%) General / -125.8,139.9	Favored (85.2%) *mtp180* chi angles: 294.7,169.2,66.5,191.8	0.08Å	Favored (34.603%)	-	-	-
A 11		LYS	6.11	-	Favored (38.21%) General / -89.5,-13.1	Favored (99.3%) *mttt* chi angles: 294.2,180.1,179.6,177.2	0.04Å	CaBLAM Disfavored (4.835%)	-	-	-
A 12		LEU	7.38	-	Allowed (0.43%) General / 59.1,-150.8	Favored (43.9%) *mt* chi angles: 307.1,176.4	0.06Å	CaBLAM Outlier (0.682%)	-	-	-
A 13		GLN	8.73	-	Favored (9%) General / -111.1,-26.9	Favored (94.7%) *mm-40* chi angles: 299.3,295.2,304.4	0.01Å	CaBLAM Disfavored (1.105%)	-	-	-
A 14		THR	9.66	-	Favored (7.99%) General / -125.0,3.0	Favored (80.3%) *p* chi angles: 60.5	0.02Å	Favored (15.58%)	-	-	-
A 15		ARG	9.8	-	Favored (22.17%) General / -85.8,113.7	Favored (42%) *ttp-170* chi angles: 188.9,166.4,70.1,199.5	0.07Å	Favored (22.775%)	-	-	-
A 16		SER	9.14	-	Favored (63.58%) General / -68.4,-15.5	Favored (89.2%) *p* chi angles: 68.3	0.02Å	Favored (42.224%)	-	-	-
A 17		GLN	8.01	-	Favored (56.82%) General / -86.3,-0.9	Favored (95.7%) *mt0* chi angles: 294.9,175.7,332.6	0.01Å	Favored (5.06%)	-	-	-
A 18		THR	6.81	-	Allowed (1.7%) General / 56.4,-131.2	Favored (65.8%) *p* chi angles: 63	0.09Å	CaBLAM Outlier (0.371%)	-	-	-
A 19		TRP	5.82	-	Allowed (0.53%) General / -106.2,-82.7	Favored (34.9%) *p-90* chi angles: 59.1,281.8	0.14Å	CaBLAM Outlier (0.009%)	-	-	-
A 20		LEU	5.05	-	Favored (7.59%) General / -87.0,88.0	Favored (34.2%) *tp* chi angles: 186.7,63.2	0.06Å	Favored (33.092%)	-	-	-
**#**	**Alt**	**Res**	**High B**	**Clash > 0.4Å**	**Ramachandran**	**Rotamer**	**Cβ deviation**	**CaBLAM**	**Bond lengths**	**Bond angles**	**Cis Peptides**
			Avg: 2.92	Clashscore: 0	Outliers: 1 of 73	Poor rotamers: 0 of 66	Outliers: 0 of 73	Outliers: 6 of 71	Outliers: 0 of 75	Outliers: 1 of 75	Non-Trans: 0 of 74

A 21		GLU	4.38	-	Favored (24.15%) General / -59.7,151.9	Favored (10.9%) *pt0* chi angles: 64.3,181.7,300.7	0.03Å	Favored (17.432%)	-	-	-
A 22		SER	3.8	-	Favored (61.33%) General / -59.6,-22.1	Favored (90.8%) *p* chi angles: 66.7	0.02Å	Favored (37.805%)	-	-	-
A 23		ARG	3.34	-	Favored (69.26%) General / -71.9,-34.6	Favored (80.3%) *mtm180* chi angles: 291,185.5,292.8,178.3	0.02Å	Favored (73.433%) alpha helix	-	-	-
A 24		GLU	2.95	-	Favored (43.46%) General / -79.2,-34.1	Favored (80.5%) *mm-30* chi angles: 293.6,297.3,309.3	0.05Å	Favored (76.873%) alpha helix	-	-	-
A 25		TYR	2.58	-	Favored (74.17%) General / -57.2,-50.0	Favored (87.3%) *t80* chi angles: 173.7,78.5	0.04Å	Favored (79.139%) alpha helix	-	-	-
A 26		THR	2.27	-	Favored (95.21%) General / -61.3,-45.3	Favored (94.7%) *m* chi angles: 299.4	0.03Å	Favored (86.238%) alpha helix	-	-	-
A 27		LYS	2.02	-	Favored (79.72%) General / -58.5,-39.3	Favored (97%) *mttt* chi angles: 287.8,176.3,184.2,175.3	0.05Å	Favored (79.335%) alpha helix	-	-	-
A 28		HIS	1.8	-	Favored (73.7%) General / -59.7,-50.5	Favored (89.4%) *t70* chi angles: 176.2,74.9	0.06Å	Favored (88.175%) alpha helix	-	-	-
A 29		LEU	1.63	-	Favored (83.33%) General / -62.5,-36.7	Favored (93.1%) *mt* chi angles: 292.2,174.1	0.06Å	Favored (80.857%) alpha helix	-	-	-
A 30		ILE	1.48	-	Favored (99.24%) Ile or Val / -62.7,-44.1	Favored (94.4%) *mt* chi angles: 291.8,168	0.03Å	Favored (89.329%) alpha helix	-	-	-
A 31		LYS	1.36	-	Favored (81.24%) General / -61.2,-36.9	Favored (96.7%) *mttt* chi angles: 288.7,178.3,180.9,178.9	0.01Å	Favored (89.398%) alpha helix	-	-	-
A 32		VAL	1.27	-	Favored (92.12%) Ile or Val / -64.7,-46.5	Favored (73.5%) *t* chi angles: 172.5	0.04Å	Favored (86.823%) alpha helix	-	-	-
A 33		GLU	1.22	-	Favored (94.3%) General / -63.5,-39.4	Favored (92.5%) *mt-10* chi angles: 288.5,182.8,354.3	0.04Å	Favored (91.505%) alpha helix	-	-	-
A 34		ASN	1.21	-	Favored (95.79%) General / -63.2,-39.9	Favored (92%) *m-40* chi angles: 285.3,342.8	0.03Å	Favored (94.052%) alpha helix	-	-	-
A 35		TRP	1.22	-	Favored (91.73%) General / -61.0,-46.2	Favored (88.3%) *t60* chi angles: 180.1,84.1	0.04Å	Favored (95.711%) alpha helix	-	-	-
A 36		ILE	1.24	-	Favored (99.16%) Ile or Val / -63.2,-44.3	Favored (98.5%) *mt* chi angles: 292.7,168.3	0.03Å	Favored (94.124%) alpha helix	-	-	-

#	Alt	Res	High B	Clash > 0.4Å	Ramachandran	Rotamer	Cβ deviation	CaBLAM	Bond lengths	Bond angles	Cis Peptides
A 37		PHE	1.27	-	Favored (91.44%) General / -63.3,-38.5	Favored (29.4%) *m-80* chi angles: 285.8,124	0.01Å	Favored (85.553%) alpha helix	-	-	-
A 38		ARG	1.27	-	Favored (69.25%) General / -71.7,-34.1	Favored (95.5%) *mtt180* chi angles: 289.9,171.3,181.4,168	0.03Å	Favored (60.282%)	-	-	-
A 39		ASN	1.23	-	Favored (75.8%) Pre-Pro / -131.1,65.0	Favored (48.1%) *m-40* chi angles: 300.8,278.2	0.06Å	Favored (24.477%)	-	-	-
A 40		PRO	1.15	-	Favored (69.8%) Trans-Pro / -63.1,-19.9	Favored (41.7%) *Cg_endo* chi angles: 23.8,325.6,30.3	0.03Å	Favored (27.983%)	-	-	-
**#**	**Alt**	**Res**	**High B**	**Clash > 0.4Å**	**Ramachandran**	**Rotamer**	**Cβ deviation**	**CaBLAM**	**Bond lengths**	**Bond angles**	**Cis Peptides**
			Avg: 2.92	Clashscore: 0	Outliers: 1 of 73	Poor rotamers: 0 of 66	Outliers: 0 of 73	Outliers: 6 of 71	Outliers: 0 of 75	Outliers: 1 of 75	Non-Trans: 0 of 74
A 41		GLY	1.05	-	Favored (61.16%) Glycine / -57.4,-29.9	-	-	Favored (68.533%)	-	-	-
A 42		PHE	0.95	-	Favored (63.75%) General / -70.4,-26.6	Favored (47.3%) *m-80* chi angles: 284.3,108.9	0.04Å	Favored (72.817%) three-ten	-	-	-
A 43		ALA	0.86	-	Favored (67.1%) General / -72.7,-39.4	-	0.03Å	Favored (75.102%) alpha helix	-	-	-
A 44		LEU	0.78	-	Favored (95.49%) General / -64.2,-40.2	Favored (96.3%) *mt* chi angles: 293.3,173.7	0.04Å	Favored (86.065%) alpha helix	-	-	-
A 45		VAL	0.72	-	Favored (85.85%) Ile or Val / -67.6,-44.9	Favored (69.4%) *t* chi angles: 172	0.02Å	Favored (78.466%) alpha helix	-	-	-
A 46		ALA	0.68	-	Favored (77.71%) General / -58.4,-38.6	-	0.04Å	Favored (79.925%) alpha helix	-	-	-
A 47		VAL	0.65	-	Favored (90.4%) Ile or Val / -64.8,-47.0	Favored (60.3%) *t* chi angles: 170.8	0.02Å	Favored (90.204%) alpha helix	-	-	-
A 48		ALA	0.63	-	Favored (88.54%) General / -60.2,-39.9	-	0.03Å	Favored (89.557%) alpha helix	-	-	-
A 49		ILE	0.63	-	Favored (88.38%) Ile or Val / -65.7,-46.8	Favored (99.1%) *mt* chi angles: 292.8,167.4	0.05Å	Favored (87.831%) alpha helix	-	-	-
A 50		ALA	0.66	-	Favored (77.72%) General / -58.2,-39.0	-	0.05Å	Favored (84.956%) alpha helix	-	-	-
A 51		TRP	0.78	-	Favored (95.96%) General / -62.8,-40.0	Favored (34%) *m-10* chi angles: 285.4,342.4	0.04Å	Favored (86.706%) alpha helix	-	-	-

A 52		LEU	1.08	-	Favored (63.41%) General / -72.9,-29.8	Favored (98.9%) *mt* chi angles: 293.1,172.5	0.03Å	Favored (76.182%) alpha helix	-	-	-
A 53		LEU	1.73	-	Favored (13.89%) General / -83.9,-44.8	Favored (95.2%) *mt* chi angles: 295.1,173.6	0.09Å	Favored (70.487%) alpha helix	-	-	-
A 54		GLY	2.95	-	Favored (32.83%) Glycine / -89.8,166.7	-	-	Favored (5.391%) alpha helix	-	-	-
A 55		SER	4.63	-	Favored (6.06%) General / -114.1,-32.3	Favored (97.3%) *p* chi angles: 63.6	0.03Å	Favored (5.101%) alpha helix	-	-	-
A 56		SER	5.95	-	Favored (13.26%) General / -101.6,161.9	Favored (81.6%) *p* chi angles: 62	0.08Å	Favored (12.07%) alpha helix	-	-	-
A 57		THR	5.85	-	Favored (94.9%) General / -60.0,-43.6	Favored (90.6%) *m* chi angles: 298.1	0.01Å	Favored (65.404%) alpha helix	-	-	-
A 58		SER	4.43	-	Favored (96.91%) General / -60.5,-42.7	Favored (62.6%) *m* chi angles: 293.9	0.02Å	Favored (87.22%) alpha helix	-	-	-
A 59		GLN	2.78	-	Favored (95.54%) General / -63.5,-39.9	Favored (74%) *mt0* chi angles: 288.8,167.6,309.6	0.04Å	Favored (96.265%) alpha helix	-	-	-
A 60		LYS	1.64	-	Favored (93.53%) General / -64.7,-39.4	Favored (96%) *mttt* chi angles: 290,173.5,181.7,175.5	0.08Å	Favored (86.214%) alpha helix	-	-	-
**#**	**Alt**	**Res**	**High B**	**Clash > 0.4Å**	**Ramachandran**	**Rotamer**	**Cβ deviation**	**CaBLAM**	**Bond lengths**	**Bond angles**	**Cis Peptides**
			Avg: 2.92	Clashscore: 0	Outliers: 1 of 73	Poor rotamers: 0 of 66	Outliers: 0 of 73	Outliers: 6 of 71	Outliers: 0 of 75	Outliers: 1 of 75	Non-Trans: 0 of 74
A 61		VAL	1.05	-	Favored (81.59%) Ile or Val / -69.0,-43.8	Favored (76.4%) *t* chi angles: 172.8	0.02Å	Favored (85.714%) alpha helix	-	-	-
A 62		ILE	0.79	-	Favored (93.73%) Ile or Val / -60.1,-47.0	Favored (88.4%) *mt* chi angles: 291.3,166.3	0.03Å	Favored (96.835%) alpha helix	-	-	-
A 63		TYR	0.69	-	Favored (96.21%) General / -62.5,-40.3	Favored (10.2%) *m-10* chi angles: 286,141.5	0.02Å	Favored (89.031%) alpha helix	-	-	-
A 64		LEU	0.68	-	Favored (79.51%) General / -57.6,-48.4	Favored (67.6%) *tp* chi angles: 176.8,59.8	0.04Å	Favored (83.291%) alpha helix	-	-	-
A 65		VAL	0.71	-	Favored (96.73%) Ile or Val / -64.4,-45.1	Favored (69.2%) *t* chi angles: 172	0.04Å	Favored (85.237%) alpha helix	-	-	-
A 66		MET	0.74	-	Favored (93.39%) General / -59.6,-44.3	Favored (94.8%) *mtp* chi angles: 289.9,170.8,67.2	0.05Å	Favored (98.705%) alpha helix	-	-	-

A 67		ILE	0.77	-	Favored (92.09%) Ile or Val / -62.1,-47.7	Favored (94%) *mt* chi angles: 292.7,165.9	0.05Å	Favored (91.203%) alpha helix	-	-	-
A 68		LEU	0.82	-	Favored (76.89%) General / -68.1,-34.7	Favored (81.3%) *mt* chi angles: 290.8,174.8	0.11Å	Favored (77.919%) alpha helix	-	-	-
A 69		LEU	0.89	-	Favored (78.95%) General / -68.3,-36.2	Favored (86.1%) *mt* chi angles: 290.3,172.9	0.03Å	Favored (73.948%) alpha helix	-	-	-
A 70		ILE	1	-	Favored (21.5%) Ile or Val / -80.6,-43.8	Favored (95%) *mt* chi angles: 295.1,168	0.01Å	Favored (24.57%) alpha helix	-	-	-
A 71		ALA	1.19	-	Favored (36.87%) Pre-Pro / -46.5,-43.5	-	0.03Å	Favored (57.837%) three-ten	-	OUTLIER(S) worst is CA-C-N: 4.5 σ	-
A 72		PRO	1.45	-	Favored (51.37%) Trans-Pro / -66.0,-16.1	Favored (36%) *Cg_endo* chi angles: 22.6,325.2,32.4	0.02Å	Favored (7.125%)	-	-	-
A 73		ALA	1.81	-	Favored (4.84%) General / -78.8,68.5	-	0.03Å	Favored (17.906%)	-	-	-
A 74		TYR	2.25	-	Favored (27.92%) General / -84.1,146.4	Favored (83.1%) *m-80* chi angles: 289.7,95.1	0.01Å	-	-	-	-
A 75		SER	2.72	-	-	Favored (42.7%) *t* chi angles: 178.4	0.02Å	-	-	-	-

About MolProbity | Website for the Richardson Lab | Using ecloud x-H | Internal reference 4.5.2
