# Supplementary material for: Integrative proteome-wide structural analysis and high-throughput docking identify broad-spectrum antiviral scaffolds against Zika, Yellow Fever, West Nile, Saint Louis encephalitis, and Usutu viruses
Source: Front Cell Infect Microbiol. 2026 Apr 30;16:1723132. doi: 10.3389/fcimb.2026.1723132 (PMC13171538; doi:10.3389/fcimb.2026.1723132)
Supplement: Supplementary file 7 [file DataSheet7.zip › ZIKV/ZIKV_M/Mol_probity_Files/ZIKV_M_1FH-rama.pdf]

MolProbity Ramachandran analysis

ZIKV_M1FH.pdb, model 1

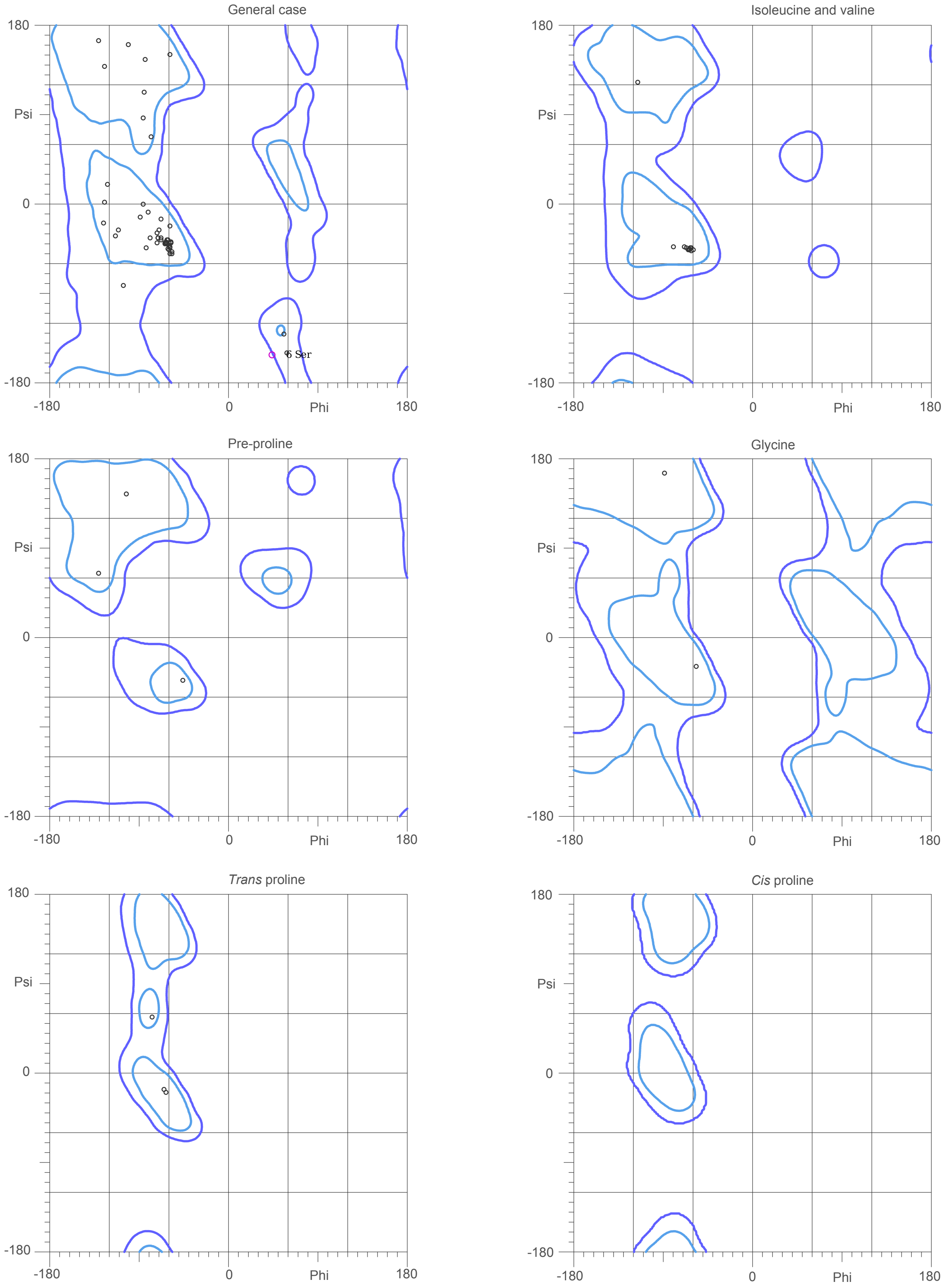

94.5% (69/73) of all residues were in favored (98%) regions.
98.6% (72/73) of all residues were in allowed (>99.8%) regions.

There were 1 outliers (phi, psi):
6 Ser (45.0, -152.0)
