# Supplementary material for: Integrative proteome-wide structural analysis and high-throughput docking identify broad-spectrum antiviral scaffolds against Zika, Yellow Fever, West Nile, Saint Louis encephalitis, and Usutu viruses
Source: Front Cell Infect Microbiol. 2026 Apr 30;16:1723132. doi: 10.3389/fcimb.2026.1723132 (PMC13171538; doi:10.3389/fcimb.2026.1723132)
Supplement: Supplementary file 7 [file DataSheet7.zip › ZIKV/ZIKV_NS1/Mol_probity_Files/ZIKV_NS1_1FH-multi.table.pdf]

Viewing ZIKV_NS1_1FH-multi.table

Duke Biochemistry
Duke University School of Medicine

When finished, you should close this window.

Hint: Use File | Save As... to save a copy of this page.

All-Atom Contacts	Clashscore, all atoms:	1.63		99th percentile* (N=1784, all resolutions)
	Clashscore is the number of serious steric overlaps (> 0.4 Å) per 1000 atoms.			
Protein Geometry	Poor rotamers	0	0.00%	Goal: <0.3%
	Favored rotamers	305	99.67%	Goal: >98%
	Ramachandran outliers	1	0.29%	Goal: <0.05%
	Ramachandran favored	339	96.86%	Goal: >98%
	Rama distribution Z-score	1.11 ± 0.49		Goal: abs(Z score) < 2
	MolProbity score^	1.10		100th percentile* (N=27675, 0Å - 99Å)
	Cβ deviations >0.25Å	0	0.00%	Goal: 0
	Bad bonds:	2 / 2895	0.07%	Goal: 0%
	Bad angles:	10 / 3928	0.25%	Goal: <0.1%
Peptide Omegas	Cis Prolines:	2 / 19	10.53%	Expected: ≤1 per chain, or ≤5%
	Cis nonProlines:	1 / 332	0.30%	Goal: <0.05%
Low-resolution Criteria	CaBLAM outliers	5	1.4%	Goal: <1.0%
	CA Geometry outliers	1	0.29%	Goal: <0.5%
Additional validations	Chiral volume outliers	0/410		
	Waters with clashes	0/0	0.00%	See UnDowser table for details

In the two column results, the left column gives the raw count, right column gives the percentage.

* 100th percentile is the best among structures of comparable resolution; 0th percentile is the worst. For clashscore the comparative set of structures was selected in 2004, for MolProbity score in 2006.

^ MolProbity score combines the clashscore, rotamer, and Ramachandran evaluations into a single score, normalized to be on the same scale as X-ray resolution.

Key to table colors and cutoffs here:

#	Alt	Res	High B	Clash > 0.4Å	Ramachandran	Rotamer	Cβ deviation	CaBLAM	Bond lengths	Bond angles	Cis Peptides
			Avg: 1.14	Clashscore: 1.63	Outliers: 1 of 350	Poor rotamers: 0 of 306	Outliers: 0 of 325	Outliers: 5 of 348	Outliers: 2 of 352	Outliers: 9 of 352	Non-Trans: 3 of 351
A 1		ASP	3.4	-	-	Allowed (2%) *t0* chi angles: 210.6,301.3	0.04Å	-	-	-	-
A 2		VAL	3.3	-	Allowed (1.06%) Ile or Val / -134.7,-8.1	Favored (32.1%) *m* chi angles: 297.6	0.05Å	-	-	-	-
A 3		GLY	3.26	-	Favored (43.83%) Glycine / -87.2,-167.6	-	-	Favored (13.344%)	-	-	-
A 4		CYS	3.31	-	Favored (24.27%) General / -137.6,166.3	Favored (44.6%) *m* chi angles: 304.1	0.06Å	Favored (59.623%) beta sheet	-	-	-
A 5		SER	3.47	-	Favored (38.71%) General / -155.8,164.3	Favored (90.2%) *p* chi angles: 66.7	0.03Å	Favored (57.284%) beta sheet	-	-	-
A 6		VAL	3.73	-	Favored (75.25%)	Favored (66.3%) *t* chi angles: 179.2	0.02Å	Favored (51.637%) beta sheet	-	-	-

					Ile or Val / -121.2,126.6						
A 7		ASP	4.04	-	Favored (13.41%) General / -95.3,101.7	Favored (61.1%) *t0* chi angles: 182.6,341.4	0.05Å	Favored (68.5%) beta sheet	-	-	-
A 8		PHE	4.34	-	Favored (49.91%) General / -77.1,-5.7	Favored (78.4%) *m-80* chi angles: 289.4,98.9	0.04Å	Favored (24.335%)	-	-	-
A 9		SER	4.53	-	Favored (47.4%) General / -87.2,-12.2	Favored (88%) *p* chi angles: 69.5	0.04Å	Favored (50.63%) alpha helix	-	-	-
A 10		LYS	4.57	-	Favored (7.14%) General / -120.3,-20.9	Favored (49%) *mttp* chi angles: 300,178.2,183.1,67.9	0.04Å	Favored (6.77%)	-	-	-
A 11		LYS	4.42	-	Favored (3.8%) General / 64.0,46.3	Favored (31.3%) *mmtm* chi angles: 303.5,294.6,188.6,298.2	0.06Å	Favored (17.02%)	-	-	-
A 12		GLU	4.11	-	Favored (46.5%) General / -117.8,143.2	Favored (96.4%) *mt-10* chi angles: 294.4,182.1,350.4	0.06Å	Favored (28.331%) beta sheet	-	-	-
A 13		THR	3.7	-	Favored (56.3%) General / -113.3,127.9	Favored (99.4%) *m* chi angles: 300.4	0.04Å	Favored (63.985%) beta sheet	-	-	-
A 14		ARG	3.28	-	Favored (52.53%) General / -130.9,147.4	Favored (91.9%) *mtt-85* chi angles: 292.7,176.2,179.9,264.6	0.01Å	Favored (56.883%) beta sheet	-	-	-
A 15		CYS	2.91	-	Favored (38.99%) General / -128.1,158.1	Favored (67.9%) *m* chi angles: 299.3	0.02Å	Favored (13.909%) beta sheet	-	-	-
A 16		GLY	2.58	-	Favored (43.24%) Glycine / 177.8,-170.8	-	-	Favored (51.534%)	-	-	-
A 17		THR	2.31	-	Favored (15.54%) General / -116.4,17.4	Favored (63.3%) *p* chi angles: 57.9	0.04Å	Favored (6.529%)	-	-	-
A 18		GLY	2.12	-	Favored (4.21%) Glycine / -88.6,-132.4	-	-	CaBLAM Disfavored (4.767%)	-	-	-
A 19		VAL	1.98	-	Favored (73.75%) Ile or Val / -124.3,128.4	Favored (83.9%) *t* chi angles: 177.7	0.02Å	Favored (7.639%) beta sheet	-	-	-
A 20		PHE	1.89	-	Favored (54.58%) General / -109.9,127.2	Favored (86.7%) *m-80* chi angles: 291.4,84.6	0.04Å	Favored (72.481%) beta sheet	-	-	-
**#**	**Alt**	**Res**	**High B**	**Clash > 0.4Å**	**Ramachandran**	**Rotamer**	**Cβ deviation**	**CaBLAM**	**Bond lengths**	**Bond angles**	**Cis Peptides**
			Avg: 1.14	Clashscore: 1.63	Outliers: 1 of 350	Poor rotamers: 0 of 306	Outliers: 0 of 325	Outliers: 5 of 348	Outliers: 2 of 352	Outliers: 9 of 352	Non-Trans: 3 of 351
A 21		ILE	1.85	-	Favored (60.66%) Ile or Val / -107.7,130.1	Favored (45.4%) *mm* chi angles: 305,299.7	0.03Å	Favored (66.933%) beta sheet	-	-	-
A 22		TYR	1.84	-	Favored (49.68%)	Favored (71.8%) *m-80*	0.06Å	Favored (54.014%)	-	-	-

					General / -125.3,146.2	chi angles: 303.2,88.2		beta sheet			
A 23		ASN	1.9	-	Favored (10.51%) General / -83.9,71.2	Favored (38.1%) *t0* chi angles: 191.7,10.8	0.06Å	Favored (11.594%) beta sheet	-	-	-
A 24		ASP	2.05	-	Favored (58.89%) General / -77.1,-9.7	Favored (80.7%) *m-30* chi angles: 287.7,333.8	0.02Å	Favored (11.824%)	-	-	-
A 25		VAL	2.3	-	Favored (53.66%) Ile or Val / -62.9,-31.8	Favored (70.3%) *t* chi angles: 178.7	0.08Å	Favored (56.709%)	-	-	-
A 26		GLU	2.61	-	Favored (56.12%) General / -82.9,-2.9	Favored (98.5%) *mt-10* chi angles: 292.6,180.6,0.1	0.04Å	Favored (54.956%)	-	-	-
A 27		ALA	2.86	-	Favored (6.05%) General / -79.4,75.3	-	0.03Å	Favored (18.619%)	-	-	-
A 28		TRP	2.93	-	Allowed (0.48%) General / -46.9,-23.7	Favored (79.1%) *p-90* chi angles: 64.2,268.6	0.04Å	Favored (17.513%)	-	-	-
A 29		ARG	2.76	-	Favored (57.84%) General / -88.5,-1.0	Favored (56.3%) *ptt90* chi angles: 68.1,180.3,175.4,84.4	0.02Å	Favored (37.98%)	-	-	-
A 30		ASP	2.39	-	Allowed (0.73%) General / 58.7,69.5	Favored (69.1%) *m-30* chi angles: 291.3,320.2	0.13Å	CaBLAM Disfavored (3.633%)	-	-	-
A 31		ARG	1.95	-	Favored (20.65%) General / -48.2,-39.4	Favored (61.4%) *ttt90* chi angles: 181.7,183.8,174.5,87.6	0.08Å	Favored (27.439%)	-	-	-
A 32		TYR	1.57	-	Favored (31.46%) General / -87.2,122.8	Favored (60.8%) *m-80* chi angles: 289.6,78.4	0.12Å	Favored (21.273%)	-	-	-
A 33		LYS	1.3	-	Favored (44%) General / -111.9,142.7	Favored (73.3%) *mmtt* chi angles: 301.6,294.4,184.2,181.3	0.05Å	Favored (56.621%)	-	-	-
A 34		TYR	1.14	-	Favored (45.66%) General / -102.6,134.6	Favored (10%) *m-10* chi angles: 281.3,10.7	0.05Å	Favored (62.48%) beta sheet	-	-	-
A 35		HIS	1.08	-	Favored (41.88%) Pre-Pro / -120.3,105.5	Favored (58.6%) *m-70* chi angles: 299.8,260.9	0.08Å	Favored (62.395%)	-	-	-
A 36		PRO	1.05	-	Favored (39.79%) Trans-Pro / -72.5,163.0	Favored (75.4%) *Cg_endo* chi angles: 30,329.1,19.7	0.07Å	Favored (31.468%)	-	-	-
A 37		ASP	1.03	-	Favored (2.02%) General / -60.3,168.5	Favored (25%) *t0* chi angles: 197.1,353	0.02Å	CA Geom Outlier (0.255%)	-	-	-
A 38		SER	1.01	-	Favored (7.66%) Pre-Pro / -47.0,137.9	Favored (28.2%) *t* chi angles: 173.6	0.09Å	CaBLAM Disfavored (4.601%)	-	-	-
A 39		PRO	0.96	-	Favored (15.12%) Trans-Pro / -50.0,-26.8	Favored (82.4%) *Cg_exo* chi angles: 329.2,37,332.3	0.03Å	Favored (58.787%)	-	-	-

A 40		ARG	0.9	-	Favored (87.16%) General / -58.7,-41.8	Favored (46.6%) *ttp-170* chi angles: 188.8,179.2,73.6,202.9	0.10Å	Favored (45.358%) alpha helix	-	-	-
**#**	**Alt**	**Res**	**High B**	**Clash > 0.4Å**	**Ramachandran**	**Rotamer**	**Cβ deviation**	**CaBLAM**	**Bond lengths**	**Bond angles**	**Cis Peptides**
			Avg: 1.14	Clashscore: 1.63	Outliers: 1 of 350	Poor rotamers: 0 of 306	Outliers: 0 of 325	Outliers: 5 of 348	Outliers: 2 of 352	Outliers: 9 of 352	Non-Trans: 3 of 351
A 41		ARG	0.84	-	Favored (19.61%) General / -82.9,-40.9	Favored (93.9%) *mtt180* chi angles: 287.1,175.5,170.6,167.1	0.04Å	Favored (52.935%) alpha helix	-	-	-
A 42		LEU	0.78	-	Favored (78.14%) General / -66.2,-46.2	Favored (51.3%) *tp* chi angles: 178.6,55.8	0.10Å	Favored (89.566%) alpha helix	-	-	-
A 43		ALA	0.73	-	Favored (83.88%) General / -59.1,-40.0	-	0.04Å	Favored (91.354%) alpha helix	-	-	-
A 44		ALA	0.7	-	Favored (93.71%) General / -62.4,-39.6	-	0.04Å	Favored (97.004%) alpha helix	-	-	-
A 45		ALA	0.68	-	Favored (99.05%) General / -62.1,-42.2	-	0.06Å	Favored (96.218%) alpha helix	-	-	-
A 46		VAL	0.67	-	Favored (89.05%) Ile or Val / -65.5,-46.7	Favored (58.8%) *t* chi angles: 170.6	0.15Å	Favored (94.627%) alpha helix	-	-	-
A 47		LYS	0.67	-	Favored (78.45%) General / -57.3,-48.6	Favored (50.8%) *tttm* chi angles: 180.1,180.7,183.4,293.1	0.02Å	Favored (96.315%) alpha helix	-	-	-
A 48		GLN	0.67	-	Favored (91.1%) General / -62.1,-39.0	Favored (60.1%) *tp40* chi angles: 183.9,65,29.1	0.04Å	Favored (79.275%) alpha helix	-	-	-
A 49		ALA	0.69	-	Favored (86.81%) General / -59.2,-40.7	-	0.07Å	Favored (77.728%) alpha helix	-	-	-
A 50		TRP	0.7	-	Favored (73.02%) General / -58.1,-50.6	Favored (67.7%) *t-100* chi angles: 183.2,261.6	0.08Å	Favored (89.941%) alpha helix	-	-	-
A 51		GLU	0.71	-	Favored (70.4%) General / -61.6,-30.0	Favored (91.8%) *mt-10* chi angles: 292.1,181.7,12	0.04Å	Favored (72.144%)	-	-	-
A 52		GLU	0.72	-	Favored (54.18%) General / -86.4,0.4	Favored (84.7%) *mt-10* chi angles: 295.1,188.9,3.4	0.06Å	Favored (33.345%)	-	-	-
A 53		GLY	0.73	-	Favored (29.43%) Glycine / 103.6,12.9	-	-	Favored (66.757%)	-	-	-
A 54		ILE	0.73	-	Favored (54.66%) Ile or Val / -100.8,124.8	Favored (91.9%) *mt* chi angles: 297.1,168.2	0.06Å	Favored (18.49%)	-	-	-
A 55		CYS	0.74	-	Favored (36.36%) General / -96.0,-7.4	Favored (30.1%) *p* chi angles: 66.4	0.07Å	Favored (7.189%)	-	-	-

A 56		GLY	0.75	-	Favored (45.11%) Glycine / -179.2,173.0	-	-	Favored (42.71%)	-	-	-
A 57		ILE	0.77	-	Favored (51.62%) Ile or Val / -130.6,121.9	Favored (62.6%) *mt* chi angles: 303.4,168.3	0.11Å	Favored (22.708%)	-	-	-
A 58		SER	0.81	-	Favored (32.26%) General / -99.4,141.4	Favored (40.1%) *t* chi angles: 178	0.07Å	Favored (48.677%) beta sheet	-	-	-
A 59		SER	0.86	-	Favored (12.85%) General / -86.5,169.4	Favored (98.8%) *p* chi angles: 65.7	0.06Å	Favored (25.74%)	-	-	-
A 60		VAL	0.9	-	Favored (12.57%) Ile or Val / -90.9,-40.4	Favored (92.1%) *t* chi angles: 174.4	0.04Å	Favored (10.166%)	-	-	-
**#**	**Alt**	**Res**	**High B**	**Clash > 0.4Å**	**Ramachandran**	**Rotamer**	**Cβ deviation**	**CaBLAM**	**Bond lengths**	**Bond angles**	**Cis Peptides**
			Avg: 1.14	Clashscore: 1.63	Outliers: 1 of 350	Poor rotamers: 0 of 306	Outliers: 0 of 325	Outliers: 5 of 348	Outliers: 2 of 352	Outliers: 9 of 352	Non-Trans: 3 of 351
A 61		SER	0.94	-	Favored (50.81%) General / -129.7,150.9	Favored (30.7%) *t* chi angles: 173.9	0.07Å	Favored (23.422%)	-	-	-
A 62		ARG	0.97	-	Favored (37.25%) General / -51.2,-35.7	Favored (85.6%) *ttp80* chi angles: 181.8,178.6,64.6,82.3	0.03Å	Favored (58.779%) alpha helix	-	-	-
A 63		MET	0.99	-	Favored (71.49%) General / -64.3,-30.4	Favored (37.1%) *tpp* chi angles: 198,61.7,73.2	0.06Å	Favored (69.798%) alpha helix	-	-	-
A 64		GLU	1	-	Favored (64.31%) General / -74.1,-36.4	Favored (96.5%) *mt-10* chi angles: 290.6,178.6,341.4	0.02Å	Favored (78.753%) alpha helix	-	-	-
A 65		ASN	0.99	-	Favored (85.1%) General / -57.6,-43.7	Favored (29.2%) *t0* chi angles: 181.6,262.4	0.03Å	Favored (85.118%) alpha helix	-	-	-
A 66		ILE	0.98	-	Favored (99.59%) Ile or Val / -62.9,-44.5	Favored (92.9%) *mt* chi angles: 291.5,167.8	0.04Å	Favored (84.474%) alpha helix	-	-	-
A 67		MET	0.98	-	Favored (65.23%) General / -53.1,-50.1	Favored (58.9%) *ttp* chi angles: 181.4,186.5,69.8	0.12Å	Favored (83.744%) alpha helix	-	-	-
A 68		TRP	0.98	-	Favored (99.2%) General / -62.7,-43.3	Favored (54.1%) *m100* chi angles: 274.8,103.2	0.06Å	Favored (87.229%) alpha helix	-	-	-
A 69		LYS	0.98	-	Favored (79.78%) General / -62.9,-35.3	Favored (40.6%) *tptt* chi angles: 192.2,66.3,178.3,173	0.01Å	Favored (78.919%) alpha helix	-	-	-
A 70		SER	0.99	-	Favored (60.98%) General / -73.3,-12.9	Favored (58.3%) *p* chi angles: 73.4	0.04Å	Favored (48.91%) alpha helix	-	-	-
A 71		VAL	0.99	-	Favored (11.14%)	Favored (30.7%) *m* chi angles: 298	0.06Å	Favored (32.225%) alpha helix	-	-	-

					Ile or Val / -118.7,-5.4						
A 72		GLU	0.98	-	Favored (22.43%) General / -47.0,-47.7	Favored (60.3%) *tt0* chi angles: 176.4,187.1,343.7	0.11Å	Favored (55.233%) alpha helix	-	-	-
A 73		GLY	0.98	-	Favored (44.57%) Glycine / -56.0,-53.1	-	-	Favored (91.961%) alpha helix	-	-	-
A 74		GLU	0.97	-	Favored (84.8%) General / -66.8,-37.6	Favored (81.7%) *mm-30* chi angles: 291.3,294.8,330	0.04Å	Favored (77.444%) alpha helix	-	-	-
A 75		LEU	0.96	-	Favored (96.86%) General / -64.3,-40.9	Favored (88.1%) *mt* chi angles: 291,173.4	0.07Å	Favored (84.449%) alpha helix	-	-	-
A 76		ASN	0.95	-	Favored (69.68%) General / -72.0,-35.8	Favored (96.2%) *m-40* chi angles: 287.9,334.7	0.06Å	Favored (83.903%) alpha helix	-	-	-
A 77		ALA	0.95	-	Favored (98.53%) General / -61.2,-43.7	-	0.03Å	Favored (83.707%) alpha helix	-	-	-
A 78		ILE	0.94	-	Favored (93.37%) Ile or Val / -64.4,-46.3	Favored (95.3%) *mt* chi angles: 291.9,168.1	0.03Å	Favored (95.599%) alpha helix	-	-	-
A 79		LEU	0.94	-	Favored (99.12%) General / -61.1,-42.8	Favored (85.6%) *mt* chi angles: 290.1,170.9	0.05Å	Favored (90.645%) alpha helix	-	-	-
A 80		GLU	0.92	-	Favored (95.04%) General / -64.5,-43.1	Favored (31.4%) *tt0* chi angles: 183.1,170.9,295.9	0.04Å	Favored (87.254%) alpha helix	-	-	-
**#**	**Alt**	**Res**	**High B**	**Clash > 0.4Å**	**Ramachandran**	**Rotamer**	**Cβ deviation**	**CaBLAM**	**Bond lengths**	**Bond angles**	**Cis Peptides**
			Avg: 1.14	Clashscore: 1.63	Outliers: 1 of 350	Poor rotamers: 0 of 306	Outliers: 0 of 325	Outliers: 5 of 348	Outliers: 2 of 352	Outliers: 9 of 352	Non-Trans: 3 of 351
A 81		GLU	0.9	-	Favored (68.01%) General / -63.5,-24.9	Favored (84.8%) *mt-10* chi angles: 289.7,170.6,331	0.03Å	Favored (69.767%) alpha helix	-	-	-
A 82		ASN	0.86	-	Favored (50.9%) General / -93.0,5.6	Favored (70.4%) *m-40* chi angles: 289.3,286	0.07Å	Favored (58.31%)	-	-	-
A 83		GLY	0.82	-	Favored (79.36%) Glycine / 76.5,18.1	-	-	Favored (88.75%)	-	-	-
A 84		VAL	0.78	-	Favored (36.56%) Ile or Val / -95.5,116.2	Favored (59.8%) *t* chi angles: 180	0.08Å	Favored (29.252%)	-	-	-
A 85		GLN	0.74	-	Favored (8.97%) General / -84.0,66.0	Favored (87.1%) *mm-40* chi angles: 297.8,296,296	0.02Å	Favored (9.44%) beta sheet	-	-	-
A 86		LEU	0.72	-	Favored (47.5%) General / -134.7,144.0	Favored (84.5%) *mt* chi angles: 297.7,171.4	0.04Å	Favored (21.536%) beta sheet	-	-	-
A 87		THR	0.71	-	Favored (43.51%)	Favored (81.5%) *m* chi angles: 302.4	0.04Å	Favored (60.709%) beta sheet	-	-	-

					General / -132.2,132.9						
A 88		VAL	0.72	-	Favored (38.52%) Ile or Val / -85.8,129.2	Favored (87.6%) *t* chi angles: 176.1	0.06Å	Favored (50.536%) beta sheet	-	-	-
A 89		VAL	0.74	-	Favored (11.1%) Ile or Val / -102.2,-47.7	Favored (83.3%) *t* chi angles: 176.4	0.04Å	CaBLAM Outlier (0.457%) try beta sheet	-	-	-
A 90		VAL	0.79	-	OUTLIER (0.03)%) Ile or Val / 59.9,108.0	Favored (51%) *t* chi angles: 181.3	0.05Å	CaBLAM Outlier (0.47%)	-	-	-
A 91		GLY	0.84	-	Favored (34.99%) Glycine / -96.5,-172.3	-	-	Favored (47.31%)	-	-	-
A 92		SER	0.9	-	Favored (17.12%) General / -80.6,169.8	Favored (90%) *p* chi angles: 68.8	0.02Å	Favored (19.389%)	-	-	-
A 93		VAL	0.94	-	Favored (40.2%) Ile or Val / -87.0,127.8	Favored (99.9%) *t* chi angles: 175.5	0.04Å	Favored (26.299%)	-	-	-
A 94		LYS	0.98	-	Favored (28.57%) General / -109.3,150.0	Favored (20%) *mmtp* chi angles: 299.1,295.1,181.7,76.4	0.07Å	Favored (30.023%)	-	-	-
A 95		ASN	1.02	-	Favored (24.13%) Pre-Pro / -109.3,129.2	Favored (64.8%) *m-40* chi angles: 293.5,278.2	0.10Å	Favored (7.112%)	-	-	-
A 96		PRO	1.05	-	Favored (99.66%) Cis-Pro / -73.9,156.5	Favored (77.5%) *Cg_endo* chi angles: 28.6,325.8,25.7	0.03Å	Favored (89.757%)	-	-	Cis PRO omega= -6.19
A 97		MET	1.1	-	Favored (3.28%) General / -76.6,85.5	Favored (93.2%) *mtp* chi angles: 298.3,177.5,73.6	0.08Å	Favored (57.538%)	-	-	-
A 98		TRP	1.14	-	Favored (25.53%) General / -67.8,125.2	Favored (95.7%) *m100* chi angles: 288.1,104	0.10Å	Favored (32.038%) beta sheet	-	-	-
A 99		ARG	1.16	-	Favored (52.42%) General / -68.5,147.2	Favored (49.2%) *ptt90* chi angles: 68.8,185.6,178.3,90.7	0.08Å	Favored (25.669%) beta sheet	-	-	-
A 100		GLY	1.16	-	Favored (29.7%) Glycine / -94.7,170.6	-	-	Favored (41.656%)	-	-	-
**#**	**Alt**	**Res**	**High B**	**Clash > 0.4Å**	**Ramachandran**	**Rotamer**	**Cβ deviation**	**CaBLAM**	**Bond lengths**	**Bond angles**	**Cis Peptides**
			Avg: 1.14	Clashscore: 1.63	Outliers: 1 of 350	Poor rotamers: 0 of 306	Outliers: 0 of 325	Outliers: 5 of 348	Outliers: 2 of 352	Outliers: 9 of 352	Non-Trans: 3 of 351
A 101		PRO	1.12	-	Favored (6.28%) Trans-Pro / -88.3,-6.2	Favored (31.2%) *Cg_endo* chi angles: 35,326.1,18.5	0.05Å	Favored (53.219%)	-	-	-
A 102		GLN	1.04	-	Favored (5.87%) General / -109.9,174.4	Favored (59.5%) *mt0* chi angles: 300.4,191.9,43.8	0.04Å	Favored (14.293%)	-	-	-

A 103		ARG	0.97	-	Favored (46.66%) General / -128.8,154.2	Favored (56.8%) *ptt90* chi angles: 64.3,180.8,175.5,87.2	0.07Å	Favored (28.596%)	-	-	-
A 104		LEU	0.9	-	Favored (88.27%) Pre-Pro / -75.7,123.2	Favored (39.4%) *tp* chi angles: 184.9,64.8	0.11Å	Favored (34.393%) beta sheet	-	-	-
A 105		PRO	0.87	-	Favored (91.82%) Trans-Pro / -61.7,149.0	Favored (48.6%) *Cg_exo* chi angles: 337.9,33.7,329.2	0.05Å	Favored (81.185%) beta sheet	-	-	-
A 106		VAL	0.86	-	Favored (84.45%) Pre-Pro / -72.7,131.3	Favored (83.2%) *t* chi angles: 173.4	0.07Å	Favored (37.938%) beta sheet	-	-	-
A 107		PRO	0.87	-	Favored (23.21%) Trans-Pro / -47.0,-37.8	Favored (59.9%) *Cg_exo* chi angles: 328.4,37.1,333.6	0.14Å	CaBLAM Disfavored (1.572%) try beta sheet	-	-	-
A 108		VAL	0.88	-	Allowed (0.17%) Ile or Val / 64.6,-65.4	Favored (84.5%) *t* chi angles: 173.6	0.07Å	CaBLAM Disfavored (4.388%)	-	-	-
A 109		ASN	0.89	-	Favored (14.99%) General / -93.0,160.1	Favored (87.5%) *m-40* chi angles: 295,318	0.03Å	Favored (6.986%)	-	-	-
A 110		GLU	0.93	-	Favored (43.39%) General / -129.0,156.7	Favored (61%) *mt-10* chi angles: 305.5,182.2,6.9	0.05Å	Favored (33.737%)	-	-	-
A 111		LEU	1.02	-	Favored (72.26%) Pre-Pro / -77.8,146.4	Favored (94.6%) *mt* chi angles: 295.8,174.2	0.08Å	Favored (45.977%)	-	-	-
A 112		PRO	1.19	-	Favored (64.89%) Trans-Pro / -64.6,-22.5	Favored (36.9%) *Cg_endo* chi angles: 22.7,325.8,31	0.01Å	Favored (22.065%)	-	-	-
A 113		HIS	1.47	-	Favored (7.8%) General / -86.7,82.6	Favored (69.5%) *m-70* chi angles: 295.6,306.1	0.06Å	Favored (24.539%)	-	-	-
A 114		GLY	1.88	-	Favored (45.39%) Glycine / -83.2,-169.9	-	-	Favored (20.358%)	-	-	-
A 115		TRP	2.43	-	Allowed (1.12%) General / -48.3,-24.1	Favored (78.9%) *p-90* chi angles: 64.4,267.9	0.00Å	CaBLAM Disfavored (4.497%)	-	-	-
A 116		LYS	3.03	-	Favored (67.46%) General / -62.5,-24.3	Favored (97.7%) *mttt* chi angles: 291,179.4,180,178.7	0.02Å	Favored (59.427%)	-	-	-
A 117		ALA	3.58	-	Favored (59.16%) General / -84.0,-5.2	-	0.04Å	Favored (40.951%)	-	-	-
A 118		TRP	3.96	-	Favored (58.2%) General / -61.1,138.8	Favored (88.6%) *t60* chi angles: 183.9,90.6	0.04Å	Favored (6.683%)	-	-	-
A 119		GLY	4.11	-	Favored (39.72%) Glycine / 106.8,-16.7	-	-	Favored (67.72%) alpha helix	-	-	-
A 120		LYS	4.03	-	Favored (67.17%)	Favored (82.6%) *tttt*	0.07Å	Favored (30.419%)	-	-	-

#	Alt	Res	High B	Clash > 0.4Å	Ramachandran	Rotamer	Cβ deviation	CaBLAM	Bond lengths	Bond angles	Cis Peptides
					General / -57.2,-33.1	chi angles: 182.1,170.1,178.3,174.7		alpha helix			
			Avg: 1.14	Clashscore: 1.63	Outliers: 1 of 350	Poor rotamers: 0 of 306	Outliers: 0 of 325	Outliers: 5 of 348	Outliers: 2 of 352	Outliers: 9 of 352	Non-Trans: 3 of 351
A 121		SER	3.75	-	Favored (42.85%) General / -64.8,-11.9	Favored (83.9%) *p* chi angles: 67.5	0.05Å	Favored (51.532%) alpha helix	-	-	-
A 122		TYR	3.29	-	Favored (59.29%) General / -84.0,-7.9	Favored (62.6%) *m-80* chi angles: 293.1,113.7	0.04Å	Favored (48.266%) alpha helix	-	-	-
A 123		PHE	2.74	-	Favored (2.64%) General / -122.2,-39.0	Favored (72.3%) *m-80* chi angles: 303.8,103.2	0.11Å	Favored (23.587%) alpha helix	-	-	-
A 124		VAL	2.19	-	Favored (67.44%) Ile or Val / -129.4,130.4	Favored (71.7%) *t* chi angles: 178.6	0.05Å	Favored (35.959%)	-	-	-
A 125		ARG	1.72	-	Favored (42.79%) General / -116.6,146.3	Favored (32%) *mmt90* chi angles: 298,291.2,179.7,89.2	0.05Å	Favored (49.754%)	-	-	-
A 126		ALA	1.35	-	Favored (40.51%) General / -73.2,153.8	-	0.06Å	Favored (49.53%)	-	-	-
A 127		ALA	1.1	-	Favored (50.3%) General / -69.0,148.3	-	0.04Å	Favored (49.127%)	-	-	-
A 128		LYS	0.93	-	Favored (32.18%) General / -86.8,123.6	Favored (86.5%) *tttt* chi angles: 181.8,177.3,178.3,180.7	0.03Å	Favored (48.675%) beta sheet	-	-	-
A 129		THR	0.82	-	Favored (10.76%) General / -97.5,167.8	Favored (64.6%) *p* chi angles: 63.2	0.05Å	Favored (25.995%)	-	-	-
A 130		ASN	0.75	-	Favored (67.18%) General / -70.2,-30.1	Favored (97%) *m-40* chi angles: 287.9,335.5	0.02Å	Favored (16.159%)	-	-	-
A 131		ASN	0.71	-	Favored (8.23%) General / -84.2,64.4	Favored (86.1%) *m-40* chi angles: 298.7,318.7	0.07Å	Favored (16.217%)	-	-	-
A 132		SER	0.68	-	Favored (20.13%) General / -84.9,160.5	Favored (98.6%) *p* chi angles: 65.3	0.05Å	Favored (24.929%)	-	-	-
A 133		PHE	0.68	-	Favored (45.96%) General / -111.7,140.9	Favored (95.2%) *m-80* chi angles: 294.2,86.9	0.17Å	Favored (55.092%) beta sheet	OUTLIER(S) worst is CB--CG: 5.3 σ	OUTLIER(S) worst is CA-CB-CG: 4.4 σ	-
A 134		VAL	0.71	-	Favored (57.03%) Ile or Val / -107.6,131.7	Favored (86.2%) *t* chi angles: 177.3	0.08Å	Favored (64.691%)	-	-	-
A 135		VAL	0.78	-	Favored (68.71%) Ile or Val / -112.2,124.3	Favored (81.3%) *t* chi angles: 177.9	0.10Å	Favored (7.669%)	-	-	-
A 136		ASP	0.91	-	Allowed (1.99%)	Favored (74.6%) *m-30*	0.06Å	CaBLAM Outlier	-	-	-

					General / 49.4,-129.8	chi angles: 296.6,320.8		(0.628%)			
A 137		GLY	1.1	-	Favored (48.39%) Glycine / 68.8,-165.4	-	-	Favored (5.9%)	-	-	-
A 138		ASP	1.32	-	Favored (32.18%) General / -77.2,155.5	Favored (95.2%) *m-30* chi angles: 291.2,346	0.06Å	Favored (6.764%)	-	-	-
A 139		THR	1.51	-	Favored (37.18%) General / -76.1,129.7	Favored (85.3%) *m* chi angles: 301.6	0.01Å	Favored (36.575%)	-	-	-
A 140		LEU	1.59	0.45Å HB2 with A 143 CYS H	Favored (8.01%) General / -108.5,170.5	Favored (64.8%) *mt* chi angles: 303.7,175.7	0.23Å	Favored (32.353%)	-	OUTLIER(S) worst is C-CA-CB: 4.9 σ	-

#	Alt	Res	High B	Clash > 0.4Å	Ramachandran	Rotamer	Cβ deviation	CaBLAM	Bond lengths	Bond angles	Cis Peptides
			Avg: 1.14	Clashscore: 1.63	Outliers: 1 of 350	Poor rotamers: 0 of 306	Outliers: 0 of 325	Outliers: 5 of 348	Outliers: 2 of 352	Outliers: 9 of 352	Non-Trans: 3 of 351
A 141		LYS	1.54	-	Allowed (0.07%) General / 85.6,-46.8	Favored (98.9%) *mttt* chi angles: 293.8,178.6,181.2,178.4	0.16Å	CaBLAM Disfavored (3.469%)	-	OUTLIER(S) worst is C-N-CA: 8.6 σ	Cis nonPRO omega= 12.6
A 142		GLU	1.39	0.43Å H with A 140 LEU HB3	Allowed (1.88%) General / -129.8,-32.5	Favored (94.5%) *mt-10* chi angles: 299.6,177.9,356.7	0.10Å	Favored (9.707%) alpha helix	-	-	-
A 143		CYS	1.2	0.45Å H with A 140 LEU HB2	Favored (12.89%) Pre-Pro / -144.8,78.4	Favored (51.4%) *t* chi angles: 184.4	0.02Å	Favored (17.098%)	-	-	-
A 144		PRO	1.03	-	Favored (48.58%) Trans-Pro / -60.3,155.0	Favored (48.8%) *Cg_exo* chi angles: 337.9,34.6,327.6	0.06Å	Favored (36.661%)	-	-	-
A 145		LEU	0.9	-	Favored (66.61%) General / -59.8,-27.5	Favored (70.8%) *mt* chi angles: 291.3,178.2	0.08Å	Favored (47.292%)	-	-	-
A 146		GLU	0.82	-	Favored (66.94%) General / -61.7,-24.7	Favored (99.5%) *mt-10* chi angles: 291.5,178.8,352.9	0.01Å	Favored (60.082%) alpha helix	-	-	-
A 147		HIS	0.78	-	Favored (26%) General / -105.1,16.4	Favored (86.4%) *m-70* chi angles: 290.3,278.4	0.02Å	Favored (30.252%)	-	-	-
A 148		ARG	0.76	-	Favored (41.08%) General / -113.8,145.6	Favored (92.9%) *mtt180* chi angles: 296.2,184.2,189.1,183.9	0.06Å	Favored (31.521%)	-	-	-
A 149		ALA	0.74	-	Favored (23.93%) General / -81.8,159.1	-	0.03Å	Favored (27.951%)	-	-	-
A 150		TRP	0.73	-	Favored (20.96%) General / -158.1,149.1	Favored (91.4%) *t60* chi angles: 182.5,86.4	0.09Å	Favored (6.61%)	-	-	-
A 151		ASN	0.73	-	Favored (28.51%) General / 54.2,44.8	Favored (52.2%) *t0* chi angles: 196.6,22.4	0.11Å	Favored (9.504%)	-	-	-
A 152		SER	0.74	-	Favored (58.42%) General / -89.6,-2.9	Favored (62.5%) *m* chi angles: 293.9	0.05Å	Favored (13.74%)	-	-	-

A 153		PHE	0.75	-	Favored (49.79%) General / -125.3,145.9	Favored (95.4%) *m-80* chi angles: 295.4,87	0.04Å	Favored (28.155%)	-	-	-
A 154		LEU	0.79	-	Favored (50.44%) General / -127.8,148.9	Favored (83.7%) *mt* chi angles: 295.6,170.1	0.07Å	Favored (49.419%)	-	-	-
A 155		VAL	0.86	-	Favored (39.69%) Ile or Val / -81.4,125.9	Favored (90.7%) *t* chi angles: 174.3	0.07Å	Favored (44.285%)	-	-	-
A 156		GLU	0.99	-	Favored (12.35%) General / -89.8,-40.6	Favored (72.2%) *tp30* chi angles: 180.7,65.5,17.6	0.01Å	Favored (28.875%)	-	-	-
A 157		ASP	1.18	-	Favored (35.78%) General / -155.2,165.7	Favored (20.2%) *t0* chi angles: 204.1,354.1	0.02Å	Favored (22.933%)	-	-	-
A 158		HIS	1.44	-	Favored (7.46%) General / -146.2,116.5	Favored (26.6%) *t-170* chi angles: 183.8,191.2	0.04Å	Favored (20.515%)	-	-	-
A 159		GLY	1.76	-	Favored (14.88%) Glycine / -117.1,173.8	-	-	Favored (39.816%) beta sheet	-	-	-
A 160		PHE	2.08	-	Favored (50.39%) General / -134.0,146.4	Favored (85.3%) *m-80* chi angles: 300,87.1	0.06Å	Favored (47.42%)	-	-	-
**#**	**Alt**	**Res**	**High B**	**Clash > 0.4Å**	**Ramachandran**	**Rotamer**	**Cβ deviation**	**CaBLAM**	**Bond lengths**	**Bond angles**	**Cis Peptides**
			Avg: 1.14	Clashscore: 1.63	Outliers: 1 of 350	Poor rotamers: 0 of 306	Outliers: 0 of 325	Outliers: 5 of 348	Outliers: 2 of 352	Outliers: 9 of 352	Non-Trans: 3 of 351
A 161		GLY	2.3	-	Favored (9.89%) Glycine / -114.5,-149.4	-	-	Favored (32.867%)	-	-	-
A 162		VAL	2.36	-	Favored (10.85%) Ile or Val / -98.6,-50.7	Favored (81.3%) *t* chi angles: 176.5	0.03Å	CaBLAM Outlier (0.302%)	-	-	-
A 163		PHE	2.24	-	Favored (16.56%) General / -105.1,-11.5	Favored (79.5%) *m-80* chi angles: 300.5,106.1	0.05Å	Favored (15.095%)	-	-	-
A 164		HIS	1.98	-	Favored (29.99%) General / -137.8,164.0	Favored (73.2%) *m90* chi angles: 299.4,85.2	0.06Å	Favored (20.034%)	-	-	-
A 165		THR	1.67	-	Favored (43.48%) General / -140.6,150.3	Favored (8.5%) *t* chi angles: 184.5	0.10Å	Favored (57.505%)	-	-	-
A 166		SER	1.39	-	Favored (52.52%) General / -122.8,130.1	Favored (53.7%) *m* chi angles: 292.3	0.03Å	Favored (62.008%) beta sheet	-	-	-
A 167		VAL	1.17	-	Favored (70.98%) Ile or Val / -116.3,130.5	Favored (69.2%) *t* chi angles: 178.9	0.11Å	Favored (65.508%) beta sheet	-	-	-
A 168		TRP	1.01	-	Favored (45.71%)	Favored (42.7%) *m-90* chi angles: 290,262.6	0.05Å	Favored (63.707%) beta sheet	-	-	-

					General / -107.3,137.3						
A 169		LEU	0.91	-	Favored (45.99%) General / -121.2,147.2	Favored (3.2%) *mp* chi angles: 290.5,89.7	0.03Å	Favored (62.896%)	-	-	-
A 170		LYS	0.86	-	Favored (29.05%) General / -128.9,161.8	Favored (60.8%) *pttt* chi angles: 63.8,182.9,180.8,184.1	0.07Å	Favored (33.081%)	-	-	-
A 171		VAL	0.85	-	Favored (33.98%) Ile or Val / -77.6,131.8	Favored (81.7%) *t* chi angles: 176.7	0.06Å	Favored (29.454%)	-	-	-
A 172		ARG	0.86	-	Favored (34.77%) General / -83.0,135.8	Favored (54.5%) *ttt90* chi angles: 186.1,173.1,171.7,95.2	0.01Å	Favored (45.474%)	-	-	-
A 173		GLU	0.88	-	Favored (59.04%) General / -84.1,-8.7	Favored (98.2%) *mt-10* chi angles: 293.5,177.6,0.3	0.04Å	Favored (39.914%)	-	-	-
A 174		ASP	0.89	-	Favored (32.19%) General / -135.0,128.3	Favored (31.2%) *m-30* chi angles: 292.6,288.4	0.09Å	Favored (15.699%)	-	-	-
A 175		TYR	0.9	-	Favored (57.94%) General / -63.7,137.1	Favored (76.4%) *t80* chi angles: 184.4,78.5	0.04Å	Favored (33.551%)	-	-	-
A 176		SER	0.89	-	Favored (31.35%) General / -151.3,151.1	Favored (43.2%) *t* chi angles: 178.5	0.06Å	Favored (47.256%) beta sheet	-	-	-
A 177		LEU	0.88	-	Favored (48.08%) General / -98.5,3.4	Favored (76.9%) *mt* chi angles: 301,180.6	0.01Å	Favored (15.081%) beta sheet	-	-	-
A 178		GLU	0.87	-	Favored (23.36%) General / -98.1,148.6	Favored (93.9%) *mt-10* chi angles: 294,184.5,0.4	0.07Å	Favored (30.62%)	-	-	-
A 179		CYS	0.87	-	Favored (32.24%) General / -79.1,149.0	Favored (70.1%) *m* chi angles: 298.5	0.01Å	Favored (35.405%)	-	-	-
A 180		ASP	0.89	-	Favored (15.07%) Pre-Pro / -64.5,114.6	Favored (52.1%) *t0* chi angles: 188.6,332.5	0.04Å	Favored (28.712%)	-	-	-
**#**	**Alt**	**Res**	**High B**	**Clash > 0.4Å**	**Ramachandran**	**Rotamer**	**Cβ deviation**	**CaBLAM**	**Bond lengths**	**Bond angles**	**Cis Peptides**
			Avg: 1.14	Clashscore: 1.63	Outliers: 1 of 350	Poor rotamers: 0 of 306	Outliers: 0 of 325	Outliers: 5 of 348	Outliers: 2 of 352	Outliers: 9 of 352	Non-Trans: 3 of 351
A 181		PRO	0.92	-	Favored (63.32%) Trans-Pro / -64.9,-18.8	Favored (53.3%) *Cg_endo* chi angles: 25.6,326,27.9	0.03Å	Favored (36.395%)	-	-	-
A 182		ALA	0.95	-	Favored (67.4%) General / -60.1,-28.0	-	0.07Å	Favored (28.997%)	-	-	-
A 183		VAL	0.97	-	Favored (5.82%) Ile or Val / -117.1,22.4	Favored (23.2%) *m* chi angles: 294.9	0.07Å	Favored (14.089%)	-	-	-
A 184		ILE	1	-	Favored (58.48%)	Favored (64.6%) *mt* chi angles: 303.6,170.6	0.05Å	Favored (12.693%)	-	-	-

					Ile or Val / -123.8,137.0						
A 185		GLY	1.04	-	Favored (34.46%) Glycine / -157.1,171.0	-	-	Favored (36.741%)	-	-	-
A 186		THR	1.09	-	Favored (32.8%) General / -155.9,155.4	Favored (10.9%) *t* chi angles: 186.7	0.08Å	Favored (65.832%) beta sheet	-	-	-
A 187		ALA	1.15	-	Favored (34.99%) General / -156.0,156.7	-	0.06Å	Favored (59.432%) beta sheet	-	-	-
A 188		VAL	1.21	-	Favored (66.78%) Ile or Val / -125.2,124.1	Favored (62.7%) *t* chi angles: 179.6	0.08Å	Favored (28.875%)	-	-	-
A 189		LYS	1.24	-	Favored (14.79%) General / -147.5,128.8	Favored (99.3%) *mttt* chi angles: 295.1,182.1,178.8,174.7	0.03Å	Favored (11.37%)	-	-	-
A 190		GLY	1.23	-	Favored (77.84%) Glycine / 65.9,30.8	-	-	Favored (48.942%)	-	-	-
A 191		ARG	1.18	-	Favored (6.64%) General / 70.0,8.4	Favored (93.8%) *mtt180* chi angles: 301.2,184.3,180.6,177.3	0.06Å	Favored (8.14%)	-	-	-
A 192		GLU	1.1	-	Favored (20.03%) General / -108.0,155.7	Favored (93.9%) *mt-10* chi angles: 295.9,182.8,0.8	0.04Å	Favored (27.652%)	-	-	-
A 193		ALA	1.02	-	Favored (42.57%) General / -148.0,156.4	-	0.04Å	Favored (67.757%) beta sheet	-	-	-
A 194		ALA	0.95	-	Favored (41.15%) General / -151.3,156.6	-	0.02Å	Favored (67.49%) beta sheet	-	-	-
A 195		HIS	0.91	-	Favored (51.77%) General / -132.3,151.6	Favored (37.4%) *m90* chi angles: 305.6,75.3	0.16Å	Favored (57.757%) beta sheet	-	OUTLIER(S) worst is CA-CB-CG: 7.0 σ	-
A 196		SER	0.9	-	Favored (28.77%) General / -161.6,165.2	Favored (76.4%) *p* chi angles: 70.9	0.08Å	Favored (36.668%)	-	-	-
A 197		ASP	0.89	-	Favored (5.39%) General / -146.4,-177.0	Favored (4.2%) *p0* chi angles: 71.7,35.7	0.07Å	Favored (25.713%)	-	OUTLIER(S) worst is CA-CB-CG: 7.4 σ	-
A 198		LEU	0.89	-	Favored (68.18%) General / -63.3,-25.1	Favored (88%) *mt* chi angles: 293.6,177.6	0.03Å	Favored (11.248%)	-	-	-
A 199		GLY	0.88	-	Favored (16.65%) Glycine / -116.7,9.4	-	-	Favored (27.14%)	-	-	-
A 200		TYR	0.86	-	Favored (9.39%) General / -153.6,128.2	Favored (10.5%) *t80* chi angles: 173.9,41.8	0.04Å	Favored (15.756%)	-	-	-
**#**	**Alt**	**Res**	**High B**	**Clash > 0.4Å**	**Ramachandran**	**Rotamer**	**Cβ deviation**	**CaBLAM**	**Bond lengths**	**Bond angles**	**Cis Peptides**
			Avg: 1.14	Clashscore: 1.63	Outliers: 1 of 350	Poor rotamers: 0 of 306	Outliers: 0 of 325	Outliers: 5 of 348	Outliers: 2 of 352	Outliers: 9 of 352	Non-Trans: 3

											of 351
A 201		TRP	0.85	-	Favored (46.73%) General / -133.6,139.8	Favored (75%) *t60* chi angles: 175.8,90.4	0.05Å	Favored (51.577%)	-	-	-
A 202		ILE	0.84	-	Favored (38.77%) Ile or Val / -139.1,140.1	Favored (18%) *tt* chi angles: 183.8,164.7	0.13Å	Favored (67.804%) beta sheet	-	-	-
A 203		GLU	0.86	-	Favored (49.25%) General / -130.8,137.0	Favored (40.5%) *tt0* chi angles: 181.7,178.4,76.6	0.02Å	Favored (60.683%) beta sheet	-	-	-
A 204		SER	0.89	-	Favored (19.88%) General / -131.9,167.3	Favored (96.6%) *p* chi angles: 63.7	0.10Å	Favored (39.278%) beta sheet	-	-	-
A 205		GLU	0.94	-	Favored (27.16%) General / -146.7,141.8	Favored (90.8%) *tt0* chi angles: 183.6,175.2,359.1	0.02Å	Favored (32.502%) beta sheet	-	-	-
A 206		LYS	0.98	-	Favored (35.11%) General / -87.0,127.0	Favored (86.7%) *tttt* chi angles: 186,176.8,179.2,181.1	0.03Å	Favored (44.393%)	-	-	-
A 207		ASN	0.99	-	Allowed (1.77%) General / -130.5,56.5	Favored (62.7%) *m-40* chi angles: 296.9,279.5	0.02Å	CaBLAM Disfavored (2.177%)	-	-	-
A 208		ASP	0.98	-	Favored (5.34%) General / 69.2,5.9	Favored (73.6%) *m-30* chi angles: 298,317.4	0.04Å	CaBLAM Disfavored (1.665%)	-	-	-
A 209		THR	0.93	-	Favored (8.83%) General / -165.7,150.9	Favored (7.4%) *t* chi angles: 182.9	0.05Å	CaBLAM Disfavored (3.724%)	-	-	-
A 210		TRP	0.88	-	Favored (33.3%) General / -83.6,138.5	Favored (98.5%) *m100* chi angles: 288.9,98.1	0.01Å	Favored (34.145%)	-	-	-
A 211		ARG	0.82	-	Favored (40.23%) General / -138.4,144.6	Favored (94.5%) *mmt-90* chi angles: 294.6,289.6,184.5,274.8	0.09Å	Favored (43.895%) beta sheet	-	-	-
A 212		LEU	0.77	-	Favored (47.51%) General / -65.3,131.8	Favored (67.6%) *tp* chi angles: 179.5,62.8	0.03Å	Favored (39.189%)	-	-	-
A 213		LYS	0.75	-	Favored (4.83%) General / -107.0,-42.8	Favored (54.1%) *mtpt* chi angles: 289.2,173.3,65.4,174.1	0.07Å	Favored (14.399%)	-	-	-
A 214		ARG	0.74	-	Favored (19.12%) General / -156.0,143.1	Favored (48.6%) *ttt-90* chi angles: 183,172.2,179,256.9	0.01Å	Favored (25.538%)	-	-	-
A 215		ALA	0.75	-	Favored (44.47%) General / -137.8,147.3	-	0.03Å	Favored (69.576%)	-	-	-
A 216		HIS	0.77	-	Favored (21.49%) General / -133.8,121.2	Favored (18.6%) *t-170* chi angles: 183.3,222.3	0.04Å	Favored (61.171%) beta sheet	-	-	-
A 217		LEU	0.8	-	Favored (45.46%) General / -115.1,122.2	Favored (17.7%) *mt* chi angles: 295.4,158.5	0.17Å	Favored (58.45%)	-	-	-

A 218		ILE	0.82	0.42Å HA with A 218 ILE HD12	Favored (6.55%) Ile or Val / -92.9,-20.2	Favored (5.2%) *tp* chi angles: 204.5,66.8	0.04Å	Favored (34.169%)	-	-	-
A 219		GLU	0.83	-	Favored (36.11%) General / -150.9,153.8	Favored (26.1%) *pt0* chi angles: 64.2,185.8,0.2	0.12Å	Favored (21.549%)	-	-	-
A 220		MET	0.84	-	Favored (44.27%) General / -105.5,136.9	Favored (67.2%) *mmm* chi angles: 303.7,307.2,304.4	0.03Å	Favored (56.248%)	-	-	-
**#**	**Alt**	**Res**	**High B**	**Clash > 0.4Å**	**Ramachandran**	**Rotamer**	**Cβ deviation**	**CaBLAM**	**Bond lengths**	**Bond angles**	**Cis Peptides**
			Avg: 1.14	Clashscore: 1.63	Outliers: 1 of 350	Poor rotamers: 0 of 306	Outliers: 0 of 325	Outliers: 5 of 348	Outliers: 2 of 352	Outliers: 9 of 352	Non-Trans: 3 of 351
A 221		LYS	0.82	-	Favored (46.58%) General / -125.7,151.4	Favored (42.4%) *mtmt* chi angles: 302.8,178,283.5,185.3	0.11Å	Favored (59.396%) beta sheet	-	-	-
A 222		THR	0.8	-	Favored (4.5%) General / -95.6,25.5	Favored (66.5%) *p* chi angles: 58.5	0.09Å	CaBLAM Disfavored (3.914%) try beta sheet	-	-	-
A 223		CYS	0.77	-	Favored (35.78%) General / -98.6,138.4	Favored (53.5%) *t* chi angles: 181	0.09Å	Favored (32.552%) beta sheet	-	-	-
A 224		GLU	0.75	0.46Å OE1 with A 245 LYS NZ	Favored (30.01%) General / -99.3,143.0	Favored (93.6%) *mt-10* chi angles: 298.1,182.1,359.3	0.06Å	Favored (39.734%) beta sheet	-	-	-
A 225		TRP	0.72	-	Favored (81.13%) Pre-Pro / -72.9,140.8	Favored (21.6%) *m-10* chi angles: 279.5,1.6	0.03Å	Favored (43.763%) beta sheet	-	-	-
A 226		PRO	0.71	-	Favored (97.28%) Trans-Pro / -59.6,145.7	Favored (51.2%) *Cg_exo* chi angles: 336.5,33.4,331.1	0.04Å	Favored (84.674%)	-	-	-
A 227		LYS	0.7	-	Favored (68.08%) General / -57.9,-32.8	Favored (87.1%) *tttt* chi angles: 184.7,178.1,179.1,181.8	0.04Å	Favored (43.646%)	-	-	-
A 228		SER	0.7	-	Favored (42.7%) General / -61.7,-15.8	Favored (97.1%) *p* chi angles: 65.9	0.03Å	Favored (42.759%) alpha helix	-	-	-
A 229		HIS	0.71	-	Favored (20.7%) General / -107.4,19.1	Favored (96.1%) *m-70* chi angles: 294.3,286.1	0.04Å	Favored (33.496%)	-	-	-
A 230		THR	0.72	-	Favored (34.72%) General / -109.6,146.4	Favored (71.6%) *p* chi angles: 62	0.08Å	Favored (36.907%)	-	-	-
A 231		LEU	0.74	-	Favored (2.83%) General / -84.8,-58.1	Favored (93.8%) *mt* chi angles: 294.1,171.4	0.04Å	Favored (18.221%)	-	-	-
A 232		TRP	0.75	-	Favored (7.31%) General / -118.4,100.2	Favored (41.6%) *m100* chi angles: 286.7,68.6	0.05Å	Favored (6.824%)	-	-	-
A 233		THR	0.75	-	Favored (14.82%)	Favored (66.6%) *p* chi angles: 62.9	0.13Å	Favored (5.656%)	-	-	-

					General / -102.5,-19.6						
A 234		ASP	0.75	-	Favored (24.52%) General / -75.9,122.7	Favored (57%) *m-30* chi angles: 297.7,350.4	0.08Å	Favored (8.262%)	-	OUTLIER(S) worst is CA-CB-CG: 4.6 σ	-
A 235		GLY	0.74	-	Favored (76.8%) Glycine / 88.8,3.8	-	-	Favored (67.692%)	-	-	-
A 236		VAL	0.74	-	Favored (40.12%) Ile or Val / -83.2,126.7	Favored (94.2%) *t* chi angles: 175.8	0.07Å	Favored (20.263%)	-	-	-
A 237		GLU	0.73	-	Favored (32.81%) General / -106.0,144.4	Favored (93.5%) *mt-10* chi angles: 295.4,184.1,359.3	0.02Å	Favored (52.705%)	-	-	-
A 238		GLU	0.72	-	Favored (74.39%) General / -56.9,-39.4	Favored (71%) *tp30* chi angles: 181.8,67.7,20.5	0.03Å	Favored (59.44%)	-	-	-
A 239		SER	0.71	-	Favored (64.7%) General / -65.0,-17.8	Favored (96.3%) *p* chi angles: 66	0.04Å	Favored (59.843%) alpha helix	-	-	-
A 240		ASP	0.7	-	Favored (50.44%) General / -93.2,-5.2	Favored (88.6%) *m-30* chi angles: 293.6,338	0.03Å	Favored (57.428%)	-	-	-
**#**	**Alt**	**Res**	**High B**	**Clash > 0.4Å**	**Ramachandran**	**Rotamer**	**Cβ deviation**	**CaBLAM**	**Bond lengths**	**Bond angles**	**Cis Peptides**
			Avg: 1.14	Clashscore: 1.63	Outliers: 1 of 350	Poor rotamers: 0 of 306	Outliers: 0 of 325	Outliers: 5 of 348	Outliers: 2 of 352	Outliers: 9 of 352	Non-Trans: 3 of 351
A 241		LEU	0.69	0.40Å O with A 262 THR HA	Favored (37.23%) General / -78.5,142.6	Favored (94.5%) *mt* chi angles: 295.8,174.9	0.09Å	Favored (30.314%)	-	-	-
A 242		ILE	0.69	-	Favored (19.95%) Ile or Val / -79.0,-49.5	Favored (46.6%) *mm* chi angles: 299.5,300.7	0.03Å	Favored (11.374%)	-	-	-
A 243		ILE	0.69	-	Favored (65.54%) Pre-Pro / -93.8,120.9	Favored (76.7%) *mt* chi angles: 300.5,168.3	0.10Å	Favored (24.169%)	-	-	-
A 244		PRO	0.71	-	Favored (80.92%) Trans-Pro / -57.6,147.2	Favored (55.8%) *Cg_exo* chi angles: 336.2,32.6,332.7	0.07Å	Favored (70.785%)	-	-	-
A 245		LYS	0.72	0.46Å NZ with A 224 GLU OE1	Favored (73.54%) General / -57.8,-37.3	Favored (45.8%) *tttm* chi angles: 182.1,172.2,182.5,283.9	0.05Å	Favored (52.131%)	-	-	-
A 246		SER	0.74	-	Favored (47.4%) General / -63.3,-14.2	Favored (97.2%) *p* chi angles: 63.6	0.02Å	Favored (49.979%)	-	-	-
A 247		LEU	0.74	-	Favored (9.33%) General / -113.9,27.7	Favored (90.1%) *mt* chi angles: 291.7,174.1	0.10Å	Favored (30.559%)	-	-	-
A 248		ALA	0.74	-	Favored (7.08%) General / 68.7,23.3	-	0.02Å	Favored (6.961%)	-	-	-
A 249		GLY	0.73	-	Favored (41.24%) Glycine / -77.8,154.2	-	-	Favored (29.958%)	-	-	-

A 250		PRO	0.71	-	Favored (42.86%) Trans-Pro / -74.1,150.4	Favored (75.4%) *Cg_endo* chi angles: 28,325.7,26.3	0.06Å	Favored (60.418%)	-	-	-
A 251		LEU	0.69	0.72Å O with A 251 LEU HD23	Favored (8.93%) General / -82.2,78.2	Favored (5.6%) *tt* chi angles: 187.8,158.3	0.01Å	Favored (10.581%)	-	-	-
A 252		SER	0.68	-	Favored (36.58%) General / -148.1,152.0	Favored (45.5%) *t* chi angles: 179.4	0.06Å	Favored (24.311%)	-	-	-
A 253		HIS	0.68	-	Favored (64.42%) General / -61.8,-21.2	Favored (79.3%) *m-70* chi angles: 287,285.6	0.04Å	Favored (44.64%)	-	-	-
A 254		HIS	0.68	-	Favored (62.8%) General / -70.4,-15.0	Favored (16.3%) *m170* chi angles: 293.5,204.5	0.05Å	Favored (57.455%)	-	-	-
A 255		ASN	0.69	-	Favored (40.14%) General / -99.8,11.1	Favored (33.7%) *t0* chi angles: 177.9,20.9	0.08Å	Favored (43.062%)	-	OUTLIER(S) worst is CA-CB-CG: 4.6 σ	-
A 256		THR	0.7	-	Favored (9.79%) General / -104.4,167.4	Favored (39.3%) *p* chi angles: 67.9	0.06Å	Favored (19.301%)	-	-	-
A 257		ARG	0.71	-	Favored (8.01%) General / -153.5,125.8	Favored (9.5%) *tpt170* chi angles: 186.4,88.7,175.6,195.1	0.05Å	CaBLAM Disfavored (3.086%)	-	-	-
A 258		GLU	0.71	-	Favored (47.31%) General / -56.9,130.8	Favored (84.9%) *tt0* chi angles: 183.8,176.4,348	0.04Å	Favored (22.777%)	-	-	-
A 259		GLY	0.71	-	Favored (73.57%) Glycine / 93.8,-6.5	-	-	Favored (75.984%)	-	-	-
A 260		TYR	0.7	-	Favored (45.36%) General / -121.1,147.6	Favored (75%) *m-80* chi angles: 300.5,84.7	0.05Å	Favored (36.998%)	-	-	-
**#**	**Alt**	**Res**	**High B**	**Clash > 0.4Å**	**Ramachandran**	**Rotamer**	**Cβ deviation**	**CaBLAM**	**Bond lengths**	**Bond angles**	**Cis Peptides**
			Avg: 1.14	Clashscore: 1.63	Outliers: 1 of 350	Poor rotamers: 0 of 306	Outliers: 0 of 325	Outliers: 5 of 348	Outliers: 2 of 352	Outliers: 9 of 352	Non-Trans: 3 of 351
A 261		ARG	0.7	-	Favored (8.21%) General / -108.0,170.1	Favored (85.3%) *mtt90* chi angles: 295.5,179.6,175.9,84.2	0.03Å	Favored (7.748%)	-	-	-
A 262		THR	0.69	0.40Å HA with A 241 LEU O	Favored (40.09%) General / -56.1,129.4	Favored (55.2%) *m* chi angles: 303.9	0.04Å	Favored (15.959%)	-	-	-
A 263		GLN	0.69	-	Favored (9.64%) General / -84.8,66.4	Favored (79.8%) *mm-40* chi angles: 301.9,292.8,297	0.09Å	Favored (18.484%)	-	-	-
A 264		VAL	0.7	-	Favored (30.78%) Ile or Val / -65.5,-22.3	Favored (30.8%) *m* chi angles: 297.9	0.06Å	Favored (15.659%)	-	-	-
A 265		LYS	0.71	-	Favored (4.5%) General / -119.0,36.3	Favored (99.5%) *mttt* chi angles: 295.1,179.5,181.5,177.2	0.05Å	Favored (16.681%)	-	-	-

A 266		GLY	0.74	-	Favored (34.05%) Glycine / -72.5,-179.0	-	-	Favored (39.381%)	-	-	-
A 267		PRO	0.79	-	Favored (8.71%) Trans-Pro / -79.4,63.3	Favored (60.8%) *Cg_endo* chi angles: 31.8,324.7,23.8	0.06Å	CaBLAM Disfavored (1.499%)	-	-	-
A 268		TRP	0.85	-	Favored (59.07%) General / -83.4,-5.2	Favored (9.7%) *m100* chi angles: 299.1,134.4	0.04Å	Favored (11.314%)	-	-	-
A 269		HIS	0.9	-	Favored (58.24%) General / -84.2,-9.8	Favored (54%) *p-80* chi angles: 60.1,286.9	0.02Å	Favored (45.285%)	-	-	-
A 270		SER	0.94	-	Favored (23.27%) General / -79.6,165.1	Favored (97.7%) *p* chi angles: 65.8	0.05Å	Favored (19.259%)	-	-	-
A 271		GLU	0.94	-	Allowed (0.76%) General / -91.6,-69.0	Favored (91.8%) *tt0* chi angles: 182.4,176.8,0.5	0.04Å	CaBLAM Disfavored (1.392%)	-	-	-
A 272		GLU	0.91	-	Favored (33.96%) General / -85.7,126.5	Favored (91.8%) *tt0* chi angles: 182.1,177.9,355.2	0.01Å	CaBLAM Disfavored (4.493%)	-	-	-
A 273		LEU	0.85	0.40Å HD11 with A 323 PHE HB3	Favored (12.59%) General / -142.8,121.5	Favored (64.5%) *tp* chi angles: 180.7,62.4	0.09Å	Favored (40.004%)	OUTLIER(S) worst is CB--CG: 4.6 σ	-	-
A 274		GLU	0.79	-	Favored (56.43%) General / -113.5,128.4	Favored (87.2%) *tt0* chi angles: 183.2,181.7,6.2	0.03Å	Favored (67.9%) beta sheet	-	-	-
A 275		ILE	0.73	-	Favored (61.36%) Ile or Val / -107.4,122.3	Favored (70.1%) *mt* chi angles: 302.6,171.3	0.05Å	Favored (53.543%) beta sheet	-	-	-
A 276		ARG	0.68	-	Favored (49.67%) General / -135.1,147.9	Favored (76.3%) *mtp85* chi angles: 299.2,176.3,71.5,89.9	0.09Å	Favored (46.22%)	-	-	-
A 277		PHE	0.65	-	Favored (4.59%) General / -92.8,68.3	Favored (93.8%) *m-80* chi angles: 292.1,95.4	0.14Å	Favored (7.259%)	-	OUTLIER(S) worst is CA-CB-CG: 6.0 σ	-
A 278		GLU	0.63	-	Favored (40.77%) General / -143.9,152.2	Favored (58.2%) *mt-10* chi angles: 293.6,186.6,307.2	0.06Å	Favored (15.966%)	-	-	-
A 279		GLU	0.62	-	Favored (50.43%) General / -69.3,147.9	Favored (53.4%) *mt-10* chi angles: 292,174,293.6	0.04Å	Favored (45.469%)	-	-	-
A 280		CYS	0.62	-	Favored (84.35%) Pre-Pro / -71.2,137.6	Favored (91.9%) *m* chi angles: 291.7	0.04Å	Favored (26.063%)	-	-	-
**#**	**Alt**	**Res**	**High B**	**Clash > 0.4Å**	**Ramachandran**	**Rotamer**	**Cβ deviation**	**CaBLAM**	**Bond lengths**	**Bond angles**	**Cis Peptides**
			Avg: 1.14	Clashscore: 1.63	Outliers: 1 of 350	Poor rotamers: 0 of 306	Outliers: 0 of 325	Outliers: 5 of 348	Outliers: 2 of 352	Outliers: 9 of 352	Non-Trans: 3 of 351
A 281		PRO	0.61	-	Favored (62.44%) Trans-Pro / -53.1,137.3	Favored (95.6%) *Cg_exo* chi angles: 331.5,36.5,331.3	0.07Å	Favored (40.602%)	-	-	-

A 282		GLY	0.61	-	Favored (87.29%) Glycine / 85.0,-3.4	-	-	Favored (70.057%)	-	-	-
A 283		THR	0.61	-	Favored (22.63%) General / -126.8,163.9	Favored (42%) *p* chi angles: 67.4	0.08Å	Favored (33.541%)	-	-	-
A 284		LYS	0.62	-	Favored (51.02%) General / -128.8,142.3	Favored (97.4%) *mttt* chi angles: 296.4,182.2,181,179.8	0.04Å	Favored (62.892%)	-	-	-
A 285		VAL	0.63	-	Favored (68.84%) Ile or Val / -117.4,131.9	Favored (73.4%) *t* chi angles: 178.4	0.03Å	Favored (66.42%) beta sheet	-	-	-
A 286		TYR	0.65	-	Favored (36.33%) General / -118.7,152.3	Favored (76.6%) *m-80* chi angles: 300.4,85.1	0.08Å	Favored (50.163%) beta sheet	-	-	-
A 287		VAL	0.68	-	Favored (58.76%) Ile or Val / -105.5,122.3	Favored (63.7%) *t* chi angles: 179.5	0.08Å	Favored (28.758%) beta sheet	-	-	-
A 288		GLU	0.72	-	Favored (18.48%) General / -150.9,137.2	Favored (90.3%) *tt0* chi angles: 184.6,176.7,355	0.04Å	Favored (26.163%)	-	-	-
A 289		GLU	0.76	-	Favored (65.84%) General / -64.5,-19.8	Favored (99.8%) *mt-10* chi angles: 292.2,179.7,356.4	0.01Å	Favored (30.23%)	-	-	-
A 290		THR	0.79	-	Favored (55.37%) General / -88.2,-8.4	Favored (43%) *p* chi angles: 67.1	0.05Å	Favored (49.13%)	-	-	-
A 291		CYS	0.81	-	Favored (57.06%) General / -61.9,143.0	Favored (47.3%) *t* chi angles: 184.9	0.01Å	Favored (33.14%)	-	-	-
A 292		GLY	0.8	-	Favored (17.04%) Glycine / -61.2,165.1	-	-	Favored (44.121%)	-	-	-
A 293		THR	0.78	-	Favored (20.04%) General / -77.9,168.4	Favored (65%) *p* chi angles: 63.2	0.03Å	Favored (13.61%)	-	-	-
A 294		ARG	0.74	-	Favored (23.69%) General / -60.5,153.2	Favored (11.9%) *ptm-80* chi angles: 69,190.5,299.8,274.1	0.01Å	CaBLAM Disfavored (2.297%)	-	-	-
A 295		GLY	0.7	-	Favored (21.75%) Glycine / -169.6,-162.4	-	-	Favored (9.266%)	-	-	-
A 296		PRO	0.67	-	Favored (87.43%) Trans-Pro / -64.7,150.9	Favored (37.7%) *Cg_endo* chi angles: 22.9,328.4,26.6	0.05Å	Favored (5.648%)	-	-	-
A 297		SER	0.64	-	Favored (54.48%) General / -57.6,139.1	Favored (26.4%) *t* chi angles: 171.8	0.05Å	Favored (29.437%)	-	-	-
A 298		LEU	0.62	-	Favored (49.16%) General / -116.1,140.6	Favored (64.8%) *mt* chi angles: 303,174.3	0.07Å	Favored (46.395%) beta sheet	-	-	-
A 299		ARG	0.62	-	Favored (33.9%)	Favored (40.9%) *ttp-170*	0.05Å	Favored (45.415%)	-	-	-

					General / -78.4,147.7	chi angles: 196.9,171.3,69.7,175.1					
A 300		SER	0.63	-	Favored (48.23%) General / -61.8,-16.2	Favored (93%) *p* chi angles: 64.4	0.05Å	Favored (37.975%)	-	-	-
**#**	**Alt**	**Res**	**High B**	**Clash > 0.4Å**	**Ramachandran**	**Rotamer**	**Cβ deviation**	**CaBLAM**	**Bond lengths**	**Bond angles**	**Cis Peptides**
			Avg: 1.14	Clashscore: 1.63	Outliers: 1 of 350	Poor rotamers: 0 of 306	Outliers: 0 of 325	Outliers: 5 of 348	Outliers: 2 of 352	Outliers: 9 of 352	Non-Trans: 3 of 351
A 301		THR	0.65	-	Favored (42.99%) General / -119.5,148.7	Favored (64.9%) *p* chi angles: 58.2	0.02Å	Favored (24.029%)	-	-	-
A 302		THR	0.68	-	Favored (11.84%) General / -77.6,173.6	Favored (72.2%) *p* chi angles: 59.7	0.04Å	Favored (25.258%)	-	-	-
A 303		ALA	0.7	-	Favored (63.17%) General / -57.3,-28.6	-	0.04Å	Favored (53.264%)	-	-	-
A 304		SER	0.71	-	Favored (59.88%) General / -78.8,-10.7	Favored (76.6%) *p* chi angles: 70.8	0.09Å	Favored (50.289%)	-	-	-
A 305		GLY	0.71	-	Favored (73.56%) Glycine / 93.1,-10.5	-	-	Favored (72.164%)	-	-	-
A 306		ARG	0.7	-	Favored (45.44%) General / -74.1,143.5	Favored (87.9%) *mtm180* chi angles: 292.4,177.5,292,170.5	0.02Å	Favored (36.036%)	-	-	-
A 307		VAL	0.68	-	Favored (31.59%) Ile or Val / -74.3,133.4	Favored (95.5%) *t* chi angles: 174.9	0.01Å	Favored (46.18%) beta sheet	-	-	-
A 308		ILE	0.65	-	Favored (9.94%) Ile or Val / -88.6,100.0	Favored (73.4%) *mt* chi angles: 301.3,168.8	0.12Å	Favored (61.13%) beta sheet	-	-	-
A 309		GLU	0.63	-	Favored (80.42%) General / -66.5,-35.5	Favored (99.8%) *mt-10* chi angles: 291.8,178.4,353.5	0.02Å	Favored (51.844%)	-	-	-
A 310		GLU	0.62	-	Favored (23.91%) General / -117.6,114.8	Favored (39.3%) *mt-10* chi angles: 297.3,179.6,78.6	0.01Å	Favored (16.001%)	-	-	-
A 311		TRP	0.63	-	Favored (25.37%) General / -118.8,158.6	Favored (62.3%) *m100* chi angles: 294.1,74.3	0.05Å	Favored (19.617%)	-	-	-
A 312		CYS	0.65	-	Favored (23.05%) General / -144.0,167.2	Favored (28.2%) *p* chi angles: 60	0.10Å	Favored (67.918%) beta sheet	-	-	-
A 313		CYS	0.67	-	Favored (30.67%) General / -145.2,144.2	Favored (55.7%) *t* chi angles: 183.6	0.05Å	Favored (64.926%)	-	-	-
A 314		ARG	0.7	-	Favored (18.49%) General / -83.0,-41.7	Favored (86.7%) *mtp85* chi angles: 291.7,180.3,64.6,82.5	0.08Å	Favored (21.84%)	-	-	-

A 315		GLU	0.73	-	Favored (6.96%) General / -127.6,13.4	Favored (59.3%) *mt-10* chi angles: 296.8,181.3,302.6	0.01Å	CaBLAM Disfavored (4.287%)	-	-	-
A 316		CYS	0.75	-	Favored (31.09%) General / -77.2,157.1	Favored (81.1%) *m* chi angles: 295.9	0.16Å	Favored (33.335%)	-	-	-
A 317		THR	0.76	-	Favored (35.18%) General / -105.1,142.3	Favored (75.4%) *p* chi angles: 61.3	0.03Å	Favored (8.269%)	-	-	-
A 318		MET	0.74	-	Favored (46.46%) Pre-Pro / -89.5,151.8	Favored (42.8%) *mmp* chi angles: 295.4,291.1,99.3	0.08Å	Favored (6.497%)	-	-	-
A 319		PRO	0.72	-	Favored (92.21%) Cis-Pro / -69.6,156.8	Favored (52.9%) *Cg_endo* chi angles: 25.5,324.9,29.4	0.04Å	Favored (52.376%)	-	-	Cis PRO omega= -0.39
A 320		PRO	0.69	-	Favored (55.38%) Trans-Pro / -71.4,158.0	Favored (77.3%) *Cg_endo* chi angles: 28.4,324.8,26.9	0.10Å	Favored (61.713%)	-	-	-
**#**	**Alt**	**Res**	**High B**	**Clash > 0.4Å**	**Ramachandran**	**Rotamer**	**Cβ deviation**	**CaBLAM**	**Bond lengths**	**Bond angles**	**Cis Peptides**
			Avg: 1.14	Clashscore: 1.63	Outliers: 1 of 350	Poor rotamers: 0 of 306	Outliers: 0 of 325	Outliers: 5 of 348	Outliers: 2 of 352	Outliers: 9 of 352	Non-Trans: 3 of 351
A 321		LEU	0.68	-	Favored (26.54%) General / -82.9,121.1	Favored (52.7%) *tp* chi angles: 172.9,63.7	0.09Å	Favored (31.848%) beta sheet	-	-	-
A 322		SER	0.68	-	Favored (49.73%) General / -132.8,155.0	Favored (99.3%) *p* chi angles: 65.6	0.05Å	Favored (51.772%) beta sheet	-	-	-
A 323		PHE	0.7	0.40Å HB3 with A 273 LEU HD11	Favored (27.86%) General / -119.3,117.2	Favored (86.4%) *m-80* chi angles: 291.9,83.4	0.13Å	Favored (62.539%) beta sheet	-	OUTLIER(S) worst is CA-CB-CG: 4.2 σ	-
A 324		ARG	0.73	-	Favored (43.26%) General / -102.5,135.9	Favored (98.7%) *mtt180* chi angles: 295.1,179.9,182.7,179.5	0.05Å	Favored (42.347%) beta sheet	-	-	-
A 325		ALA	0.75	-	Favored (22.24%) General / -146.2,167.9	-	0.05Å	Favored (33.12%)	-	-	-
A 326		LYS	0.75	-	Favored (46.77%) General / -58.3,-20.9	Favored (14.8%) *pttp* chi angles: 71.1,179.7,180.1,65.9	0.03Å	Favored (52.087%)	-	-	-
A 327		ASP	0.73	-	Favored (56.91%) General / -91.8,1.8	Favored (76.4%) *m-30* chi angles: 297.5,335	0.05Å	Favored (18.02%)	-	-	-
A 328		GLY	0.7	-	Favored (15.22%) Glycine / 121.5,176.2	-	-	Favored (25.564%)	-	-	-
A 329		CYS	0.66	-	Favored (40.52%) General / -93.9,129.1	Favored (48.1%) *t* chi angles: 184.8	0.05Å	Favored (10.604%)	-	-	-
A 330		TRP	0.63	-	Favored (31.25%) General / -114.0,152.2	Favored (55.2%) *m100* chi angles: 294.1,71.9	0.10Å	Favored (48.04%) beta sheet	-	-	-

A 331		TYR	0.6	-	Favored (26.41%) General / -101.7,147.4	Favored (45.1%) *m-80* chi angles: 282.1,100	0.06Å	Favored (30.34%)	-	-	-
A 332		GLY	0.59	-	Favored (15.3%) Glycine / -64.2,173.3	-	-	Favored (39.195%)	-	-	-
A 333		MET	0.59	-	Favored (66.62%) General / -62.1,-23.6	Favored (92%) *mtp* chi angles: 291.4,183,71.1	0.03Å	Favored (36.63%)	-	-	-
A 334		GLU	0.6	-	Favored (57.38%) General / -88.1,-7.2	Favored (85.3%) *mm-30* chi angles: 298.5,294.2,336.9	0.06Å	Favored (58.084%)	-	-	-
A 335		ILE	0.61	-	Favored (71.61%) Ile or Val / -114.5,125.0	Favored (75.7%) *mt* chi angles: 300.9,169.5	0.09Å	Favored (20.314%)	-	-	-
A 336		ARG	0.64	-	Favored (78.66%) Pre-Pro / -131.2,157.2	Favored (85.3%) *mtp85* chi angles: 297.1,181.8,65,87.5	0.03Å	Favored (34.554%)	-	-	-
A 337		PRO	0.69	-	Favored (96.03%) Trans-Pro / -60.8,147.1	Favored (61%) *Cg_exo* chi angles: 335.9,36.7,326.1	0.05Å	Favored (62.991%)	-	-	-
A 338		ARG	0.75	-	Favored (22.64%) General / -88.6,-25.2	Favored (54.2%) *mtp180* chi angles: 296.4,174.6,78.5,206.4	0.07Å	Favored (28.055%)	-	-	-
A 339		LYS	0.81	-	Favored (2.12%) General / -125.5,-40.0	Favored (97.1%) *mttt* chi angles: 295.8,177.3,178.5,173.6	0.03Å	CaBLAM Disfavored (2.786%)	-	-	-
A 340		GLU	0.85	-	Favored (89.28%) Pre-Pro / -65.5,125.3	Favored (13.2%) *tp30* chi angles: 189.5,72.3,49.9	0.04Å	Favored (32.834%)	-	-	-
**#**	**Alt**	**Res**	**High B**	**Clash > 0.4Å**	**Ramachandran**	**Rotamer**	**Cβ deviation**	**CaBLAM**	**Bond lengths**	**Bond angles**	**Cis Peptides**
			Avg: 1.14	Clashscore: 1.63	Outliers: 1 of 350	Poor rotamers: 0 of 306	Outliers: 0 of 325	Outliers: 5 of 348	Outliers: 2 of 352	Outliers: 9 of 352	Non-Trans: 3 of 351
A 341		PRO	0.87	-	Favored (82.25%) Trans-Pro / -55.4,141.4	Favored (96.1%) *Cg_exo* chi angles: 332.8,35.6,331	0.05Å	Favored (91.053%)	-	-	-
A 342		GLU	0.86	-	Favored (68.12%) General / -57.3,-33.8	Favored (41.4%) *tt0* chi angles: 185,171.8,58.2	0.07Å	Favored (53.916%)	-	-	-
A 343		SER	0.81	-	Favored (62.82%) General / -63.0,-18.0	Favored (87.9%) *p* chi angles: 67	0.02Å	Favored (56.736%) alpha helix	-	-	-
A 344		ASN	0.77	-	Favored (47.79%) General / -98.1,1.6	Favored (83.8%) *m-40* chi angles: 296.4,337.4	0.06Å	Favored (57.321%)	-	-	-
A 345		LEU	0.73	-	Favored (19.42%) General / -93.4,152.0	Favored (66.5%) *mt* chi angles: 304.1,179	0.07Å	Favored (37.798%)	-	-	-
A 346		VAL	0.73	-	Favored (44.45%) Ile or Val / -94.5,129.6	Favored (87.8%) *t* chi angles: 176.1	0.08Å	Favored (54.412%) beta sheet	-	-	-

A 347		ARG	0.76	-	Favored (45.7%) General / -141.7,155.4	Favored (45.4%) *ptt180* chi angles: 63.7,183,182.9,178.9	0.04Å	Favored (49.603%) beta sheet	-	-	-
A 348		SER	0.86	-	Favored (28.06%) General / -90.5,116.0	Favored (29.1%) *t* chi angles: 173.7	0.11Å	Favored (38.253%) beta sheet	-	-	-
A 349		MET	1	-	Favored (18.54%) General / -104.5,20.4	Favored (95.6%) *mmm* chi angles: 300.6,300.3,288.2	0.05Å	Favored (13.436%) beta sheet	-	-	-
A 350		VAL	1.21	-	Favored (46.46%) Ile or Val / -102.0,116.9	Favored (77.8%) *t* chi angles: 178.1	0.04Å	Favored (23.415%)	-	-	-
A 351		THR	1.45	-	Favored (48.94%) General / -102.4,129.6	Favored (98.6%) *m* chi angles: 300.2	0.02Å	-	-	-	-
A 352		ALA	1.7	-	-	-	0.03Å	-	-	-	-
