# Supplementary material for: Integrative proteome-wide structural analysis and high-throughput docking identify broad-spectrum antiviral scaffolds against Zika, Yellow Fever, West Nile, Saint Louis encephalitis, and Usutu viruses
Source: Front Cell Infect Microbiol. 2026 Apr 30;16:1723132. doi: 10.3389/fcimb.2026.1723132 (PMC13171538; doi:10.3389/fcimb.2026.1723132)
Supplement: Supplementary file 7 [file DataSheet7.zip › ZIKV/ZIKV_NS2a/Mol_probity_Files/ZIKV_NS2a_1FH-multi.table.pdf]

Viewing ZIKV_NS2a1FH-multi.table

Duke Biochemistry
Duke University School of Medicine

When finished, you should close this window.

Hint: Use File | Save As... to save a copy of this page.

All-Atom Contacts	Clashscore, all atoms:	2.28		99th percentile* (N=1784, all resolutions)
	Clashscore is the number of serious steric overlaps (> 0.4 Å) per 1000 atoms.			
Protein Geometry	Poor rotamers	0	0.00%	Goal: <0.3%
	Favored rotamers	179	100.00%	Goal: >98%
	Ramachandran outliers	3	1.34%	Goal: <0.05%
	Ramachandran favored	215	95.98%	Goal: >98%
	Rama distribution Z-score	0.53 ± 0.52		Goal: abs(Z score) < 2
	MolProbity score^	1.28		99th percentile* (N=27675, 0Å - 99Å)
	Cβ deviations >0.25Å	0	0.00%	Goal: 0
	Bad bonds:	2 / 1697	0.12%	Goal: 0%
	Bad angles:	4 / 2299	0.17%	Goal: <0.1%
Peptide Omegas	Cis Prolines:	0 / 7	0.00%	Expected: ≤1 per chain, or ≤5%
Low-resolution Criteria	CaBLAM outliers	7	3.2%	Goal: <1.0%
	CA Geometry outliers	3	1.35%	Goal: <0.5%
Additional validations	Chiral volume outliers	0/296		
	Waters with clashes	0/0	0.00%	See UnDowser table for details

In the two column results, the left column gives the raw count, right column gives the percentage.

* 100th percentile is the best among structures of comparable resolution; 0th percentile is the worst. For clashscore the comparative set of structures was selected in 2004, for MolProbity score in 2006.

^ MolProbity score combines the clashscore, rotamer, and Ramachandran evaluations into a single score, normalized to be on the same scale as X-ray resolution.

Key to table colors and cutoffs here:

#	Alt	Res	High B	Clash > 0.4Å	Ramachandran	Rotamer	Cβ deviation	CaBLAM	Bond lengths	Bond angles	Cis Peptides
			Avg: 5.35	Clashscore: 2.28	Outliers: 3 of 224	Poor rotamers: 0 of 179	Outliers: 0 of 207	Outliers: 7 of 222	Outliers: 2 of 226	Outliers: 4 of 226	Non-Trans: 0 of 225
A 1		GLY	8.57	-	-	-	-	-	-	-	-
A 2		SER	8.11	-	Favored (65.34%) General / -61.0,-23.4	Favored (91%) *p* chi angles: 66.7	0.03Å	-	-	-	-
A 3		THR	7.53	-	Favored (95.37%) General / -63.9,-40.0	Favored (97.1%) *m* chi angles: 299.9	0.01Å	Favored (57.037%)	-	-	-
A 4		ASP	6.89	-	Favored (16%) General / -90.1,-34.1	Favored (61.7%) *m-30* chi angles: 299.8,305.2	0.07Å	Favored (38.183%) alpha helix	-	-	-
A 5		HIS	6.24	-	Favored (77.88%) General / -56.0,-47.3	Favored (77.6%) *t70* chi angles: 177.1,82	0.10Å	Favored (66.679%) three-ten	-	-	-
A 6		MET	5.62	-	Favored (64.33%) General / -60.4,-23.4	Favored (37.2%) *ttm* chi angles: 195.5,172.5,292	0.10Å	Favored (65.227%) three-ten	-	-	-
A 7		ASP	5.06	-	Favored (66.69%)	Favored (17.8%) *t70*	0.07Å	Favored (68.245%)	-	-	-

					General / -73.1,-37.8	chi angles: 195.2,63.6		alpha helix			
A 8		HIS	4.58	-	Favored (75.27%) General / -57.0,-49.6	Favored (81.7%) *t70* chi angles: 172.1,76.3	0.06Å	Favored (83.556%) alpha helix	-	-	-
A 9		PHE	4.18	-	Favored (66.53%) General / -54.3,-50.8	Favored (23.6%) *t80* chi angles: 164.1,85.3	0.11Å	Favored (81.619%) alpha helix	-	-	-
A 10		SER	3.87	0.41Å HB3 with A 83 PHE CD2	Favored (90.55%) General / -59.1,-42.5	Favored (66.7%) *m* chi angles: 297	0.11Å	Favored (74.182%) alpha helix	-	-	-
A 11		LEU	3.63	-	Favored (94.51%) General / -64.9,-42.6	Favored (93.3%) *mt* chi angles: 291.9,170.8	0.05Å	Favored (98.14%) alpha helix	-	-	-
A 12		GLY	3.46	-	Favored (90.53%) Glycine / -63.7,-34.7	-	-	Favored (91.895%) alpha helix	-	-	-
A 13		VAL	3.36	-	Favored (96.97%) Ile or Val / -64.5,-44.1	Favored (63.7%) *t* chi angles: 171.3	0.12Å	Favored (78.218%) alpha helix	-	-	-
A 14		LEU	3.31	-	Favored (81.02%) General / -57.4,-41.8	Favored (62.8%) *tp* chi angles: 180.7,61.1	0.06Å	Favored (87.478%) alpha helix	-	-	-
A 15		VAL	3.31	-	Favored (91.34%) Ile or Val / -59.5,-43.7	Favored (63.3%) *t* chi angles: 171.3	0.03Å	Favored (95.67%) alpha helix	-	-	-
A 16		ILE	3.35	-	Favored (97.78%) Ile or Val / -62.0,-45.9	Favored (80.9%) *mt* chi angles: 290,169	0.08Å	Favored (96.384%) alpha helix	-	-	-
A 17		LEU	3.42	-	Favored (79.86%) General / -65.6,-35.1	Favored (94%) *mt* chi angles: 294.2,174.8	0.09Å	Favored (83.3%) alpha helix	-	-	-
A 18		LEU	3.52	-	Favored (90.09%) General / -64.5,-38.2	Favored (83.2%) *mt* chi angles: 289.6,170	0.03Å	Favored (90.699%) alpha helix	-	-	-
A 19		MET	3.63	-	Favored (98.26%) General / -62.3,-41.2	Favored (81.6%) *mtm* chi angles: 289.4,189.3,285	0.13Å	Favored (95.179%) alpha helix	-	-	-
A 20		VAL	3.76	-	Favored (87.79%) Ile or Val / -66.0,-46.6	Favored (67.7%) *t* chi angles: 171.8	0.05Å	Favored (92.706%) alpha helix	-	-	-
**#**	**Alt**	**Res**	**High B**	**Clash > 0.4Å**	**Ramachandran**	**Rotamer**	**Cβ deviation**	**CaBLAM**	**Bond lengths**	**Bond angles**	**Cis Peptides**
			Avg: 5.35	Clashscore: 2.28	Outliers: 3 of 224	Poor rotamers: 0 of 179	Outliers: 0 of 207	Outliers: 7 of 222	Outliers: 2 of 226	Outliers: 4 of 226	Non-Trans: 0 of 225
A 21		GLN	3.91	-	Favored (98.6%) General / -63.5,-41.7	Favored (71.5%) *mt0* chi angles: 289.6,168.6,23.2	0.05Å	Favored (93.105%) alpha helix	-	-	-
A 22		GLU	4.09	-	Favored (79.92%) General / -67.0,-35.5	Favored (85.4%) *mm-30* chi angles: 291.2,299.5,308.4	0.04Å	Favored (93.93%) alpha helix	-	-	-

A 23		GLY	4.32	-	Favored (53.34%) Glycine / -63.5,-51.6	-	-	Favored (89.441%) alpha helix	-	-	-
A 24		LEU	4.64	-	Favored (78.02%) General / -61.9,-35.3	Favored (90.1%) *mt* chi angles: 291.3,173.4	0.04Å	Favored (50.821%)	-	-	-
A 25		LYS	5.03	-	Favored (41.74%) General / -70.3,154.6	Favored (57%) *mtmt* chi angles: 291.1,185,288.4,182.5	0.02Å	CaBLAM Disfavored (1.796%)	-	-	-
A 26		LYS	5.5	-	Allowed (0.86%) General / 59.9,-118.2	Favored (93%) *mttt* chi angles: 300,182.6,180.6,178.5	0.04Å	CA Geom Outlier (0.159%)	-	-	-
A 27		ARG	6	-	Favored (43.19%) General / -136.8,143.5	Favored (93.9%) *mmt-90* chi angles: 295.6,289.9,184.2,272.9	0.06Å	Favored (15.838%)	-	-	-
A 28		MET	6.46	-	Favored (17.9%) General / -103.6,156.3	Favored (67.4%) *mtp* chi angles: 298.9,182.7,84.9	0.05Å	Favored (48.514%)	-	-	-
A 29		THR	6.82	-	Favored (12.41%) General / -126.0,169.8	Favored (46%) *p* chi angles: 66.4	0.06Å	Favored (37.034%)	-	-	-
A 30		THR	7.01	-	Favored (87.21%) General / -58.1,-44.0	Favored (92.3%) *m* chi angles: 297.8	0.01Å	Favored (61.82%)	-	-	-
A 31		LYS	7.01	-	Favored (88.54%) General / -62.8,-37.9	Favored (97.1%) *mttt* chi angles: 289.6,179.3,180.1,178.6	0.03Å	Favored (84.511%) alpha helix	-	-	-
A 32		ILE	6.84	-	Favored (94.08%) Ile or Val / -65.5,-44.0	Favored (96.3%) *mt* chi angles: 292.1,168.3	0.01Å	Favored (89.27%) alpha helix	-	-	-
A 33		ILE	6.54	-	Favored (87.82%) Ile or Val / -63.8,-48.1	Favored (95.4%) *mt* chi angles: 292.4,166.5	0.08Å	Favored (79.24%) alpha helix	-	-	-
A 34		MET	6.17	-	Favored (87.31%) General / -66.4,-42.7	Favored (98.3%) *mtp* chi angles: 290.8,176.4,68	0.03Å	Favored (82.924%) alpha helix	-	-	-
A 35		SER	5.79	-	Favored (98.7%) General / -61.7,-42.3	Favored (71.7%) *m* chi angles: 295.2	0.05Å	Favored (83.997%) alpha helix	-	-	-
A 36		THR	5.43	-	Favored (76.27%) General / -67.0,-45.9	Favored (90.6%) *m* chi angles: 301.2	0.05Å	Favored (79.705%) alpha helix	-	-	-
A 37		SER	5.12	-	Favored (99.44%) General / -61.4,-42.9	Favored (70.9%) *m* chi angles: 296.2	0.06Å	Favored (86.668%) alpha helix	-	-	-
A 38		MET	4.89	-	Favored (87.58%) General / -66.9,-39.6	Favored (82.9%) *mtm* chi angles: 289.5,187,287.2	0.02Å	Favored (91.513%) alpha helix	-	-	-
A 39		ALA	4.75	-	Favored (90.09%) General / -61.1,-39.5	-	0.04Å	Favored (92.307%) alpha helix	-	-	-

#	Alt	Res	High B	Clash > 0.4Å	Ramachandran	Rotamer	Cβ deviation	CaBLAM	Bond lengths	Bond angles	Cis Peptides
A 40		VAL	4.72	-	Favored (97.59%) Ile or Val / -64.1,-44.3	Favored (63%) *t* chi angles: 171.2	0.02Å	Favored (97.501%) alpha helix	-	-	-
#	Alt	Res	High B	Clash > 0.4Å	Ramachandran	Rotamer	Cβ deviation	CaBLAM	Bond lengths	Bond angles	Cis Peptides
			Avg: 5.35	Clashscore: 2.28	Outliers: 3 of 224	Poor rotamers: 0 of 179	Outliers: 0 of 207	Outliers: 7 of 222	Outliers: 2 of 226	Outliers: 4 of 226	Non-Trans: 0 of 225
A 41		LEU	4.8	-	Favored (91.57%) General / -62.6,-38.8	Favored (92.7%) *mt* chi angles: 291.4,172.8	0.03Å	Favored (88.902%) alpha helix	-	-	-
A 42		VAL	5.03	-	Favored (94.15%) Ile or Val / -60.6,-43.1	Favored (56.8%) *t* chi angles: 170.3	0.02Å	Favored (92.663%) alpha helix	-	-	-
A 43		VAL	5.36	-	Favored (96.4%) Ile or Val / -62.1,-42.8	Favored (70.4%) *t* chi angles: 172.2	0.02Å	Favored (98.311%) alpha helix	-	-	-
A 44		MET	5.74	-	Favored (81.67%) General / -62.6,-36.1	Favored (47%) *mmp* chi angles: 296.3,303.1,102.3	0.11Å	Favored (84.232%) alpha helix	-	-	-
A 45		ILE	6.1	-	Favored (69.57%) Ile or Val / -71.7,-44.4	Favored (95.1%) *mt* chi angles: 293,166.4	0.03Å	Favored (62.662%) alpha helix	-	-	-
A 46		LEU	6.36	-	Favored (16.79%) General / -93.9,-26.6	Favored (88.3%) *mt* chi angles: 300.2,177.4	0.03Å	Favored (38.661%)	-	-	-
A 47		GLY	6.45	-	Favored (37.88%) Glycine / -92.2,-163.3	-	-	Favored (21.958%)	-	-	-
A 48		GLY	6.39	-	Favored (36.32%) Glycine / -100.9,23.2	-	-	Favored (6.322%)	-	-	-
A 49		PHE	6.22	0.45Å CD1 with A 49 PHE N	Favored (25.04%) General / -85.1,119.1	Favored (15.7%) *m-80* chi angles: 295,69.4	0.17Å	Favored (25.229%)	-	-	-
A 50		SER	6	-	Favored (15.36%) General / -85.8,167.2	Favored (86%) *p* chi angles: 67.8	0.04Å	Favored (28.061%)	-	-	-
A 51		MET	5.78	-	Favored (63.77%) General / -69.9,-25.6	Favored (51.4%) *mmp* chi angles: 293.2,299.7,96.7	0.09Å	Favored (62.676%)	-	-	-
A 52		SER	5.6	-	Favored (87.56%) General / -64.4,-37.5	Favored (63.7%) *m* chi angles: 294.1	0.03Å	Favored (74.406%) alpha helix	-	-	-
A 53		ASP	5.44	-	Favored (46.28%) General / -77.9,-38.1	Favored (33.6%) *m-30* chi angles: 290.8,294.1	0.15Å	Favored (75.164%) alpha helix	-	OUTLIER(S) worst is CA-CB-CG: 4.8 σ	-
A 54		LEU	5.3	-	Favored (93.03%) General / -60.0,-45.4	Favored (66.6%) *tp* chi angles: 178.8,59.6	0.04Å	Favored (80.41%) alpha helix	-	-	-
A 55		ALA	5.19	-	Favored (77.76%)	-	0.05Å	Favored (82.972%) alpha helix	-	-	-

#	Alt	Res	High B	Clash > 0.4Å	Ramachandran	Rotamer	Cβ deviation	CaBLAM	Bond lengths	Bond angles	Cis Peptides
					General / -57.2,-40.5						
A 56		LYS	5.11	-	Favored (74.05%) General / -69.5,-34.3	Favored (55%) *mttp* chi angles: 290.5,184.2,177.2,64	0.05Å	Favored (91.5%) alpha helix	-	-	-
A 57		LEU	5.07	-	Favored (96.29%) General / -64.3,-40.6	Favored (88.5%) *mt* chi angles: 294.9,178	0.12Å	Favored (85.208%) alpha helix	-	-	-
A 58		VAL	5.08	-	Favored (91.03%) Ile or Val / -63.9,-41.0	Favored (64.2%) *t* chi angles: 171.4	0.08Å	Favored (83.472%) alpha helix	-	-	-
A 59		ILE	5.14	-	Favored (92.05%) Ile or Val / -59.1,-46.3	Favored (90.9%) *mt* chi angles: 291.2,167.9	0.06Å	Favored (82.824%) alpha helix	-	-	-
A 60		LEU	5.24	-	Favored (76.93%) General / -68.3,-34.9	Favored (96.3%) *mt* chi angles: 292.6,171	0.17Å	Favored (79.433%) alpha helix	-	-	-
**#**	**Alt**	**Res**	**High B**	**Clash > 0.4Å**	**Ramachandran**	**Rotamer**	**Cβ deviation**	**CaBLAM**	**Bond lengths**	**Bond angles**	**Cis Peptides**
			Avg: 5.35	Clashscore: 2.28	Outliers: 3 of 224	Poor rotamers: 0 of 179	Outliers: 0 of 207	Outliers: 7 of 222	Outliers: 2 of 226	Outliers: 4 of 226	Non-Trans: 0 of 225
A 61		MET	5.39	-	Favored (98.8%) General / -62.1,-42.0	Favored (51.8%) *mtt* chi angles: 290.5,175.6,194.8	0.08Å	Favored (85.039%) alpha helix	-	-	-
A 62		GLY	5.57	-	Favored (49.81%) Glycine / -58.4,-52.9	-	-	Favored (91.86%) alpha helix	-	-	-
A 63		ALA	5.78	-	Favored (75.34%) General / -59.8,-35.7	-	0.04Å	Favored (74.049%) alpha helix	-	-	-
A 64		THR	6.01	-	Favored (92.48%) General / -60.4,-45.8	Favored (90%) *m* chi angles: 298.2	0.02Å	Favored (74.354%) alpha helix	-	-	-
A 65		PHE	6.27	-	Favored (65.58%) General / -60.8,-52.3	Favored (81.8%) *t80* chi angles: 174.4,73.2	0.04Å	Favored (74.341%) alpha helix	-	-	-
A 66		ALA	6.55	-	Favored (75.15%) General / -58.5,-37.2	-	0.05Å	Favored (62.617%) alpha helix	-	-	-
A 67		GLU	6.81	-	Favored (31.04%) General / -103.1,14.8	Favored (80.7%) *mm-30* chi angles: 300.6,297.9,327.1	0.01Å	Favored (33.448%)	-	-	-
A 68		MET	7.03	-	Favored (9.31%) General / -51.8,-26.3	Favored (58.3%) *tpp* chi angles: 188.4,63.8,78.7	0.04Å	Favored (17.827%)	-	-	-
A 69		ASN	7.16	-	Favored (67.52%) General / -63.5,-23.7	Favored (99.2%) *m-40* chi angles: 287.6,338.9	0.02Å	Favored (65.296%)	-	-	-
A 70		THR	7.18	-	Favored (19.14%) General / -111.2,109.2	Favored (95.7%) *m* chi angles: 299.6	0.02Å	Favored (25.401%) alpha helix	-	-	-

A 71		GLY	7.06	-	Favored (90.16%) Glycine / -58.7,-37.6	-	-	Favored (45.708%) alpha helix	-	-	-
A 72		GLY	6.83	-	Favored (93.44%) Glycine / -62.1,-36.8	-	-	Favored (91.633%) alpha helix	-	-	-
A 73		ASP	6.53	-	Favored (60.63%) General / -75.5,-36.4	Favored (97.6%) *m-30* chi angles: 289.1,346.3	0.07Å	Favored (85.922%) alpha helix	-	-	-
A 74		VAL	6.22	-	Favored (98.49%) Ile or Val / -61.2,-45.5	Favored (56.6%) *t* chi angles: 170.3	0.07Å	Favored (89.238%) alpha helix	-	-	-
A 75		ALA	5.94	-	Favored (85.47%) General / -64.4,-36.9	-	0.08Å	Favored (88.906%) alpha helix	-	-	-
A 76		HIS	5.71	-	Favored (89.97%) General / -66.4,-40.9	Favored (56.6%) *m90* chi angles: 282.1,78.3	0.16Å	Favored (96.277%) alpha helix	-	OUTLIER(S) worst is CA-CB-CG: 7.0 σ	-
A 77		LEU	5.55	-	Favored (99.89%) General / -62.8,-43.0	Favored (82.7%) *mt* chi angles: 290.2,167.4	0.07Å	Favored (97.157%) alpha helix	-	-	-
A 78		ALA	5.45	-	Favored (93.09%) General / -63.6,-39.0	-	0.02Å	Favored (92.763%) alpha helix	-	-	-
A 79		LEU	5.41	-	Favored (77.1%) General / -63.0,-48.8	Favored (44.8%) *tp* chi angles: 181.2,66.7	0.12Å	Favored (78.332%) alpha helix	-	-	-
A 80		VAL	5.42	-	Favored (90.85%) Ile or Val / -66.4,-44.4	Favored (70.8%) *t* chi angles: 172.2	0.02Å	Favored (76.374%) alpha helix	-	-	-

#	Alt	Res	High B	Clash > 0.4Å	Ramachandran	Rotamer	Cβ deviation	CaBLAM	Bond lengths	Bond angles	Cis Peptides
			Avg: 5.35	Clashscore: 2.28	Outliers: 3 of 224	Poor rotamers: 0 of 179	Outliers: 0 of 207	Outliers: 7 of 222	Outliers: 2 of 226	Outliers: 4 of 226	Non-Trans: 0 of 225
A 81		ALA	5.47	-	Favored (98.41%) General / -61.9,-43.6	-	0.11Å	Favored (52.3%) alpha helix	-	-	-
A 82		ALA	5.52	-	Favored (2.12%) General / -95.8,-58.4	-	0.06Å	Favored (16.686%) alpha helix	-	-	-
A 83		PHE	5.55	0.41Å CD2 with A 10 SER HB3	Favored (34.98%) General / -98.3,-4.5	Favored (80.6%) *m-80* chi angles: 299.6,106.6	0.06Å	Favored (33.974%)	-	OUTLIER(S) worst is CA-CB-CG: 7.9 σ	-
A 84		LYS	5.53	-	Favored (8.89%) General / 63.2,40.3	Favored (60%) *mmtt* chi angles: 310.8,296.9,193.8,182.8	0.02Å	CaBLAM Disfavored (2.143%)	-	-	-
A 85		VAL	5.45	-	OUTLIER (0.03)%) Ile or Val / 81.4,110.9	Favored (83.3%) *t* chi angles: 176.9	0.10Å	CaBLAM Outlier (0.001%)	-	-	-
A 86		ARG	5.3	-	Favored (20.81%) Pre-Pro / -134.8,133.1	Favored (60.1%) *ttt90* chi angles: 187,171.5,180,84.1	0.04Å	Favored (62.195%)	OUTLIER(S) worst is CG--CD: 4.2 σ	-	-

A 87		PRO	5.12	0.42Å CD with A 133 ALA HB2	Favored (25.01%) Trans-Pro / -72.7,-18.7	Favored (69.6%) *Cg_endo* chi angles: 29.5,328.1,21	0.06Å	Favored (50.94%)	-	-	-
A 88		ALA	4.94	-	Favored (69.63%) General / -58.9,-32.5	-	0.05Å	Favored (36.101%)	-	-	-
A 89		LEU	4.8	-	Favored (5.24%) General / -96.7,-49.1	Favored (48.2%) *tp* chi angles: 184.2,62.8	0.04Å	Favored (28.942%) alpha helix	-	-	-
A 90		LEU	4.74	-	Favored (88.9%) General / -65.8,-38.4	Favored (67.2%) *mt* chi angles: 291.6,178.9	0.09Å	Favored (76.186%) alpha helix	-	-	-
A 91		VAL	4.8	-	Favored (81.47%) Ile or Val / -68.5,-40.0	Favored (80.8%) *t* chi angles: 173.2	0.05Å	Favored (83.906%) alpha helix	-	-	-
A 92		SER	4.99	-	Favored (81.38%) General / -57.5,-41.6	Favored (69.6%) *m* chi angles: 294.9	0.04Å	Favored (93.334%) alpha helix	-	-	-
A 93		PHE	5.35	-	Favored (93.32%) General / -64.0,-39.1	Favored (7.7%) *m-10* chi angles: 283.8,342.9	0.09Å	Favored (77.589%) alpha helix	-	-	-
A 94		ILE	5.85	-	Favored (66.47%) Ile or Val / -57.2,-39.3	Favored (36.6%) *mm* chi angles: 294.7,298.2	0.07Å	Favored (67.757%) alpha helix	-	-	-
A 95		PHE	6.43	-	Favored (7.89%) General / -107.4,28.7	Favored (80.3%) *m-80* chi angles: 301,105	0.07Å	Favored (7.029%)	-	-	-
A 96		ARG	6.96	-	Allowed (0.36%) General / -79.5,-73.0	Favored (33.9%) *ttt-90* chi angles: 167.3,181.5,162.9,272.7	0.11Å	CaBLAM Outlier (0.003%)	-	-	-
A 97		ALA	7.33	-	OUTLIER (0.01)%) General / 56.1,-18.8	-	0.02Å	CaBLAM Disfavored (3.942%)	-	-	-
A 98		ASN	7.43	-	Favored (3.92%) General / -133.3,7.5	Favored (57%) *m-40* chi angles: 295.7,276.5	0.10Å	Favored (46.032%)	-	-	-
A 99		TRP	7.26	-	Favored (22.89%) General / -91.4,147.6	Favored (39.8%) *m-10* chi angles: 293.2,329	0.04Å	Favored (36.533%)	-	-	-
A 100		THR	6.86	-	Favored (57.81%) Pre-Pro / -81.4,162.7	Favored (76.2%) *p* chi angles: 61.2	0.11Å	Favored (46.087%)	-	-	-
**#**	**Alt**	**Res**	**High B**	**Clash > 0.4Å**	**Ramachandran**	**Rotamer**	**Cβ deviation**	**CaBLAM**	**Bond lengths**	**Bond angles**	**Cis Peptides**
			Avg: 5.35	Clashscore: 2.28	Outliers: 3 of 224	Poor rotamers: 0 of 179	Outliers: 0 of 207	Outliers: 7 of 222	Outliers: 2 of 226	Outliers: 4 of 226	Non-Trans: 0 of 225
A 101		PRO	6.33	-	Favored (49.9%) Trans-Pro / -52.1,-32.6	Favored (97%) *Cg_exo* chi angles: 331.8,35.9,332.1	0.04Å	Favored (93.726%)	-	-	-
A 102		ARG	5.78	-	Favored (54.4%)	Favored (43.9%) *tpt170*	0.02Å	Favored (71.197%) alpha helix	-	-	-

					General / -72.3,-46.8	chi angles: 178.9,62.1,176.9,169.1					
A 103		GLU	5.3	-	Favored (92.12%) General / -64.2,-38.7	Favored (37.7%) *mt-10* chi angles: 289.1,165.5,312.8	0.04Å	Favored (80.151%) alpha helix	-	-	-
A 104		SER	4.91	-	Favored (99.34%) General / -63.0,-43.2	Favored (70.9%) *m* chi angles: 296.2	0.08Å	Favored (78.755%) alpha helix	-	-	-
A 105		MET	4.6	-	Favored (64.45%) General / -72.7,-30.7	Favored (86.9%) *mmm* chi angles: 291.6,309.5,297.9	0.06Å	Favored (79.716%) alpha helix	-	-	-
A 106		LEU	4.37	-	Favored (83.34%) General / -66.6,-36.7	Favored (85.8%) *mt* chi angles: 290,172	0.04Å	Favored (83.109%) alpha helix	-	-	-
A 107		LEU	4.21	-	Favored (93.77%) General / -64.6,-39.5	Favored (85.2%) *mt* chi angles: 290,170.6	0.02Å	Favored (88.826%) alpha helix	-	-	-
A 108		ALA	4.13	-	Favored (90.87%) General / -60.9,-39.9	-	0.01Å	Favored (90.12%) alpha helix	-	-	-
A 109		LEU	4.11	-	Favored (63.06%) General / -65.8,-50.9	Favored (66.2%) *tp* chi angles: 176.2,60.6	0.01Å	Favored (79.763%) alpha helix	-	-	-
A 110		ALA	4.17	-	Favored (79.09%) General / -59.0,-38.4	-	0.04Å	Favored (83.703%) alpha helix	-	-	-
A 111		SER	4.28	-	Favored (99.87%) General / -63.0,-42.6	Favored (71.8%) *m* chi angles: 295.2	0.07Å	Favored (83.843%) alpha helix	-	-	-
A 112		CYS	4.45	-	Favored (61.76%) General / -72.3,-25.8	Favored (14.6%) *p* chi angles: 72.4	0.07Å	Favored (76.044%) alpha helix	-	-	-
A 113		LEU	4.68	-	Favored (86.39%) General / -66.5,-38.0	Favored (98.9%) *mt* chi angles: 293,172.6	0.10Å	Favored (77.715%) alpha helix	-	-	-
A 114		LEU	4.95	-	Favored (86.6%) General / -65.2,-45.0	Favored (67.3%) *tp* chi angles: 178.5,59.5	0.01Å	Favored (80.252%) alpha helix	-	-	-
A 115		GLN	5.26	-	Favored (78.72%) General / -57.2,-41.0	Favored (35.4%) *tt0* chi angles: 181.9,164,293	0.03Å	Favored (79.767%) alpha helix	-	-	-
A 116		THR	5.59	-	Favored (96.77%) General / -62.6,-44.2	Favored (90.3%) *m* chi angles: 298.1	0.02Å	Favored (89.004%) alpha helix	-	-	-
A 117		ALA	5.97	-	Favored (70.55%) General / -57.5,-35.7	-	0.03Å	Favored (74.1%) alpha helix	-	-	-
A 118		ILE	6.34	-	Favored (31.59%) Ile or Val / -76.7,-39.3	Favored (96.8%) *mt* chi angles: 293.8,168.4	0.09Å	Favored (68.068%) alpha helix	-	-	-
A 119		SER	6.63	-	Favored (65.29%)	Favored (75.6%) *p* chi angles: 71.1	0.07Å	Favored (65.571%) three-ten	-	-	-

#	Alt	Res	High B	Clash > 0.4Å	Ramachandran	Rotamer	Cβ deviation	CaBLAM	Bond lengths	Bond angles	Cis Peptides
					General / -61.9,-22.1						
A 120		ALA	6.8	-	Favored (64.56%) General / -66.6,-17.0	-	0.03Å	Favored (48.846%)	-	-	-
**#**	**Alt**	**Res**	**High B**	**Clash > 0.4Å**	**Ramachandran**	**Rotamer**	**Cβ deviation**	**CaBLAM**	**Bond lengths**	**Bond angles**	**Cis Peptides**
			Avg: 5.35	Clashscore: 2.28	Outliers: 3 of 224	Poor rotamers: 0 of 179	Outliers: 0 of 207	Outliers: 7 of 222	Outliers: 2 of 226	Outliers: 4 of 226	Non-Trans: 0 of 225
A 121		LEU	6.75	-	Favored (20.62%) General / -85.2,157.6	Favored (76.9%) *mt* chi angles: 301.9,175.9	0.05Å	Favored (23.828%)	-	-	-
A 122		GLU	6.48	-	Favored (54.44%) General / -85.6,-0.2	Favored (97.7%) *mt-10* chi angles: 294.6,178.8,359.2	0.01Å	Favored (11.72%)	-	-	-
A 123		GLY	6.01	-	Favored (45.23%) Glycine / -89.2,-170.8	-	-	Favored (42.761%)	-	-	-
A 124		ASP	5.43	-	Favored (22.34%) General / -104.4,18.3	Favored (71.1%) *m-30* chi angles: 294.3,316.5	0.06Å	Favored (7.767%)	-	-	-
A 125		LEU	4.85	-	Favored (81.91%) General / -58.8,-39.7	Favored (89.3%) *mt* chi angles: 290.9,172.9	0.06Å	Favored (45.97%)	-	-	-
A 126		MET	4.32	-	Favored (67.5%) General / -65.1,-24.7	Favored (25%) *ptm* chi angles: 68.7,186.8,288.1	0.08Å	Favored (71.102%) alpha helix	-	-	-
A 127		VAL	3.88	-	Favored (21.55%) Ile or Val / -80.8,-44.9	Favored (94.7%) *t* chi angles: 174.8	0.03Å	Favored (64.818%) alpha helix	-	-	-
A 128		LEU	3.55	-	Favored (95.7%) General / -61.7,-40.6	Favored (93.5%) *mt* chi angles: 292.2,170.3	0.10Å	Favored (90.946%) alpha helix	-	-	-
A 129		ILE	3.29	-	Favored (82.08%) Ile or Val / -66.7,-47.7	Favored (91.6%) *mt* chi angles: 291.3,167.8	0.09Å	Favored (90.608%) alpha helix	-	-	-
A 130		ASN	3.09	-	Favored (71.71%) General / -65.3,-30.8	Favored (91%) *m-40* chi angles: 290.9,347.3	0.05Å	Favored (76.86%) alpha helix	-	-	-
A 131		GLY	2.94	-	Favored (42.9%) Glycine / -59.4,-53.8	-	-	Favored (89.365%) alpha helix	-	-	-
A 132		PHE	2.83	-	Favored (69.59%) General / -53.3,-46.8	Favored (81.8%) *t80* chi angles: 173.6,73.8	0.07Å	Favored (86.709%) alpha helix	-	-	-
A 133		ALA	2.77	0.42Å HB2 with A 87 PRO CD	Favored (92.35%) General / -60.1,-41.4	-	0.13Å	Favored (95.864%) alpha helix	-	-	-
A 134		LEU	2.77	-	Favored (89.91%) General / -64.4,-38.1	Favored (50.8%) *tp* chi angles: 182,56.7	0.13Å	Favored (82.143%) alpha helix	-	-	-

A 135		ALA	2.82	-	Favored (91.86%) General / -59.3,-42.7	-	0.07Å	Favored (81.566%) alpha helix	-	-	-
A 136		TRP	2.94	-	Favored (69.27%) General / -70.6,-32.0	Favored (35.6%) *m100* chi angles: 272.4,111.7	0.06Å	Favored (80.39%) alpha helix	-	-	-
A 137		LEU	3.12	-	Favored (80.05%) General / -62.5,-48.3	Favored (61.2%) *tp* chi angles: 180.8,59.9	0.05Å	Favored (76.385%) alpha helix	-	-	-
A 138		ALA	3.36	-	Favored (96.93%) General / -62.3,-40.6	-	0.03Å	Favored (84.334%) alpha helix	-	-	-
A 139		ILE	3.67	-	Favored (99.59%) Ile or Val / -62.5,-44.7	Favored (86.5%) *mt* chi angles: 290.6,167.1	0.04Å	Favored (81.969%) alpha helix	-	-	-
A 140		ARG	4.06	-	Favored (83.72%) General / -67.4,-42.1	Favored (41.9%) *tpt170* chi angles: 184.4,64.2,177.4,162	0.03Å	Favored (78.819%) alpha helix	-	-	-
**#**	**Alt**	**Res**	**High B**	**Clash > 0.4Å**	**Ramachandran**	**Rotamer**	**Cβ deviation**	**CaBLAM**	**Bond lengths**	**Bond angles**	**Cis Peptides**
			Avg: 5.35	Clashscore: 2.28	Outliers: 3 of 224	Poor rotamers: 0 of 179	Outliers: 0 of 207	Outliers: 7 of 222	Outliers: 2 of 226	Outliers: 4 of 226	Non-Trans: 0 of 225
A 141		ALA	4.54	-	Favored (81.72%) General / -59.0,-39.4	-	0.05Å	Favored (75.875%) alpha helix	-	-	-
A 142		MET	5.11	-	Favored (66.82%) General / -72.9,-38.8	Favored (83.1%) *mtm* chi angles: 289.9,186.7,288.2	0.02Å	Favored (81.542%) alpha helix	-	-	-
A 143		ALA	5.77	-	Favored (97.98%) General / -63.7,-42.4	-	0.06Å	Favored (35.718%) alpha helix	-	-	-
A 144		VAL	6.47	0.55Å O with A 144 VAL HG23	Favored (53.8%) Pre-Pro / -137.4,70.9	Favored (8%) *m* chi angles: 289.2	0.09Å	Favored (9.366%)	-	-	-
A 145		PRO	7.1	-	Favored (52.17%) Trans-Pro / -56.3,132.5	Favored (87.1%) *Cg_exo* chi angles: 333.5,37.3,327.8	0.10Å	Favored (19.695%)	-	-	-
A 146		ARG	7.52	-	Favored (2.05%) General / -140.0,30.1	Favored (56%) *mtm110* chi angles: 291.4,185.4,291.3,109.9	0.02Å	CaBLAM Disfavored (2.71%)	-	-	-
A 147		THR	7.65	-	Allowed (0.24%) General / 65.0,-60.3	Favored (95.9%) *m* chi angles: 299.6	0.05Å	CaBLAM Disfavored (1.201%)	-	-	-
A 148		ASP	7.43	-	Favored (72.67%) General / -61.6,-32.5	Favored (31.6%) *t70* chi angles: 189.5,65.7	0.03Å	Favored (71.237%) alpha helix	-	-	-
A 149		ASN	6.97	-	Favored (18.95%) General / -82.9,6.3	Favored (46.2%) *p0* chi angles: 63.7,1.1	0.09Å	Favored (29.574%) alpha helix	-	-	-
A 150		ILE	6.39	-	Favored (12.68%) Ile or Val / -96.3,-43.7	Favored (45.8%) *mm* chi angles: 304.4,303.6	0.06Å	Favored (8.489%) alpha helix	-	-	-

A 151		ALA	5.82	-	Favored (80.39%) General / -60.3,-37.4	-	0.03Å	Favored (70.739%) alpha helix	-	-	-
A 152		LEU	5.34	-	Favored (45.36%) Pre-Pro / -55.4,-55.0	Favored (78.5%) *mt* chi angles: 288.4,170.6	0.03Å	Favored (64.356%) alpha helix	-	-	-
A 153		PRO	4.99	-	Favored (44.14%) Trans-Pro / -67.5,-22.9	Favored (38.8%) *Cg_endo* chi angles: 23.1,326.4,30.6	0.03Å	Favored (74.168%) alpha helix	-	-	-
A 154		ILE	4.77	-	Favored (73.96%) Ile or Val / -69.0,-47.7	Favored (99.2%) *mt* chi angles: 292.4,167.7	0.03Å	Favored (68.962%) alpha helix	-	-	-
A 155		LEU	4.67	-	Favored (91.49%) General / -63.7,-38.5	Favored (85.1%) *mt* chi angles: 290.3,169.1	0.09Å	Favored (82.215%) alpha helix	-	-	-
A 156		ALA	4.65	-	Favored (96.31%) General / -61.7,-40.8	-	0.04Å	Favored (98.324%) alpha helix	-	-	-
A 157		ALA	4.69	-	Favored (77.28%) General / -64.7,-34.0	-	0.02Å	Favored (79.655%) alpha helix	-	-	-
A 158		LEU	4.75	-	Favored (63.98%) General / -72.5,-29.8	Favored (93.2%) *mt* chi angles: 291.9,173.5	0.03Å	Favored (39.279%)	-	-	-
A 159		THR	4.77	-	Favored (68.52%) Pre-Pro / -55.1,132.2	Favored (89.6%) *m* chi angles: 298.2	0.09Å	Favored (36.97%)	-	-	-
A 160		PRO	4.72	-	Favored (9.51%) Trans-Pro / -45.5,-33.6	Favored (91.1%) *Cg_exo* chi angles: 329.5,36.5,333.5	0.01Å	Favored (61.567%)	-	-	-
**#**	**Alt**	**Res**	**High B**	**Clash > 0.4Å**	**Ramachandran**	**Rotamer**	**Cβ deviation**	**CaBLAM**	**Bond lengths**	**Bond angles**	**Cis Peptides**
			Avg: 5.35	Clashscore: 2.28	Outliers: 3 of 224	Poor rotamers: 0 of 179	Outliers: 0 of 207	Outliers: 7 of 222	Outliers: 2 of 226	Outliers: 4 of 226	Non-Trans: 0 of 225
A 161		LEU	4.55	-	Favored (49.26%) General / -79.6,-3.9	Favored (96.4%) *mt* chi angles: 297.3,177.8	0.04Å	Favored (50.734%) three-ten	-	-	-
A 162		ALA	4.29	0.40Å O with A 163 ARG C	Favored (70.19%) General / -61.1,-30.2	-	0.07Å	Favored (40.312%) three-ten	-	-	-
A 163		ARG	3.97	0.40Å C with A 162 ALA O	Favored (92.43%) General / -59.5,-45.2	Favored (97.8%) *mtt180* chi angles: 289,177,179.3,171.1	0.01Å	Favored (41.371%) three-ten	-	OUTLIER(S) worst is C-N-CA: 4.3 σ	-
A 164		GLY	3.63	-	Favored (25.98%) Glycine / -69.7,-51.3	-	-	Favored (31.634%) alpha helix	-	-	-
A 165		THR	3.32	-	Favored (96.16%) General / -63.8,-43.5	Favored (77.7%) *m* chi angles: 302.7	0.05Å	Favored (83.945%) alpha helix	-	-	-
A 166		LEU	3.06	-	Favored (85.21%) General / -66.6,-43.4	Favored (50.2%) *tp* chi angles: 183.4,59.1	0.03Å	Favored (97.895%) alpha helix	-	-	-

A 167		LEU	2.88	-	Favored (94.7%) General / -60.1,-44.7	Favored (63%) *tp* chi angles: 176.6,58.3	0.04Å	Favored (94.233%) alpha helix	-	-	-
A 168		VAL	2.78	-	Favored (88.94%) Ile or Val / -60.5,-41.8	Favored (62.7%) *t* chi angles: 171.2	0.03Å	Favored (89.59%) alpha helix	-	-	-
A 169		ALA	2.73	-	Favored (79.19%) General / -60.2,-37.1	-	0.07Å	Favored (78.962%) alpha helix	-	-	-
A 170		TRP	2.75	-	Favored (90.6%) General / -65.9,-42.2	Favored (35.4%) *m-10* chi angles: 287.6,336	0.04Å	Favored (81.401%) alpha helix	-	-	-
A 171		ARG	2.81	-	Favored (75.81%) General / -66.3,-46.8	Favored (88.5%) *mtt180* chi angles: 290.3,169.7,169.5,182.6	0.11Å	Favored (77.39%) alpha helix	-	-	-
A 172		ALA	2.91	-	Favored (92.1%) General / -64.5,-38.8	-	0.04Å	Favored (79.251%) alpha helix	-	-	-
A 173		GLY	3.05	-	Favored (51.05%) Glycine / -59.6,-52.8	-	-	Favored (92.522%) alpha helix	-	-	-
A 174		LEU	3.21	-	Favored (99.22%) General / -61.6,-42.7	Favored (86.2%) *mt* chi angles: 290.7,169	0.10Å	Favored (84.502%) alpha helix	-	-	-
A 175		ALA	3.38	-	Favored (97.62%) General / -63.6,-40.7	-	0.03Å	Favored (92.905%) alpha helix	-	-	-
A 176		THR	3.56	-	Favored (80.12%) General / -66.2,-45.7	Favored (93%) *m* chi angles: 299.2	0.03Å	Favored (93.414%) alpha helix	-	-	-
A 177		CYS	3.77	-	Favored (91.01%) General / -62.7,-38.6	Favored (89.8%) *m* chi angles: 291.4	0.03Å	Favored (84.963%) alpha helix	-	-	-
A 178		GLY	3.99	-	Favored (42.9%) Glycine / -57.5,-53.7	-	-	Favored (91.442%) alpha helix	-	-	-
A 179		GLY	4.26	-	Favored (93.58%) Glycine / -60.2,-37.8	-	-	Favored (90.7%) alpha helix	-	-	-
A 180		ILE	4.59	-	Favored (88.17%) Ile or Val / -64.2,-47.9	Favored (94.8%) *mt* chi angles: 291.7,167.4	0.06Å	Favored (82.142%) alpha helix	-	-	-
**#**	**Alt**	**Res**	**High B**	**Clash > 0.4Å**	**Ramachandran**	**Rotamer**	**Cβ deviation**	**CaBLAM**	**Bond lengths**	**Bond angles**	**Cis Peptides**
			Avg: 5.35	Clashscore: 2.28	Outliers: 3 of 224	Poor rotamers: 0 of 179	Outliers: 0 of 207	Outliers: 7 of 222	Outliers: 2 of 226	Outliers: 4 of 226	Non-Trans: 0 of 225
A 181		MET	4.98	-	Favored (91.2%) General / -62.3,-45.9	Favored (95.7%) *mtp* chi angles: 289.9,173.4,66.7	0.02Å	Favored (82.731%) alpha helix	-	-	-
A 182		LEU	5.45	0.43Å C with A 182 LEU HD23	Favored (75.5%)	Favored (7.4%) *tt* chi angles: 186.8,152.3	0.03Å	Favored (84.996%) alpha helix	-	-	-

				General / -58.0,-49.8						
A 183	LEU	6	-	Favored (78.26%) General / -65.2,-34.4	Favored (96.4%) *mt* chi angles: 292.2,173.1	0.05Å	Favored (75.399%) alpha helix	-	-	-
A 184	SER	6.64	-	Favored (74.44%) General / -70.4,-37.7	Favored (68.8%) *m* chi angles: 296.6	0.07Å	Favored (76.484%) alpha helix	-	-	-
A 185	LEU	7.28	-	Favored (3.93%) General / -75.3,-57.4	Favored (64.3%) *tp* chi angles: 178.3,64.1	0.16Å	CaBLAM Disfavored (2.013%) try alpha helix	OUTLIER(S) worst is CB--CG: 7.0 σ	-	-
A 186	LYS	7.84	-	Allowed (0.09%) General / 41.8,-107.8	Favored (98.2%) *mttt* chi angles: 291.8,181.1,178,178	0.12Å	CaBLAM Outlier (0.336%) try alpha helix	-	-	-
A 187	GLY	8.21	-	Favored (6.97%) Glycine / -54.3,-16.7	-	-	Favored (20.829%)	-	-	-
A 188	LYS	8.32	-	Favored (67.78%) General / -63.0,-24.2	Favored (96.6%) *mttt* chi angles: 289.7,185,179.6,179	0.09Å	CaBLAM Disfavored (2.424%)	-	-	-
A 189	GLY	8.15	-	Allowed (1.54%) Glycine / 72.9,-64.7	-	-	CaBLAM Disfavored (1.028%)	-	-	-
A 190	SER	7.75	-	Favored (95.5%) General / -60.1,-42.9	Favored (68%) *m* chi angles: 294.7	0.03Å	Favored (58.696%) alpha helix	-	-	-
A 191	VAL	7.24	-	Favored (84.64%) Ile or Val / -68.3,-42.9	Favored (88.6%) *t* chi angles: 174	0.02Å	Favored (75.749%) alpha helix	-	-	-
A 192	LYS	6.71	-	Favored (76.55%) General / -57.0,-40.5	Favored (85.5%) *tttt* chi angles: 180.4,178.2,175.6,183	0.03Å	Favored (75.71%) alpha helix	-	-	-
A 193	LYS	6.24	-	Favored (81.2%) General / -61.8,-36.5	Favored (96.9%) *mttt* chi angles: 288.8,176.7,183,177.9	0.02Å	Favored (62.775%) alpha helix	-	-	-
A 194	ASN	5.84	-	Favored (17.14%) General / -96.6,-22.2	Favored (88.1%) *m-40* chi angles: 293,319.9	0.04Å	Favored (25.838%) alpha helix	-	-	-
A 195	LEU	5.52	-	Favored (88.92%) Pre-Pro / -53.3,-49.3	Favored (54.8%) *tp* chi angles: 172.7,62	0.14Å	Favored (56.046%) alpha helix	-	-	-
A 196	PRO	5.26	-	Favored (68.82%) Trans-Pro / -59.0,-23.5	Favored (69.7%) *Cg_exo* chi angles: 335.3,35.8,328	0.03Å	Favored (85.786%) alpha helix	-	-	-
A 197	PHE	5.05	-	Favored (22.93%) General / -79.2,-44.0	Favored (50.8%) *t80* chi angles: 181.4,61.6	0.08Å	Favored (65.961%) alpha helix	-	-	-
A 198	VAL	4.87	-	Favored (94.75%) Ile or Val / -60.6,-43.5	Favored (63.5%) *t* chi angles: 171.3	0.07Å	Favored (97.439%) alpha helix	-	-	-

#	Alt	Res	High B	Clash > 0.4Å	Ramachandran	Rotamer	Cβ deviation	CaBLAM	Bond lengths	Bond angles	Cis Peptides
A 199		MET	4.74	-	Favored (72.06%) General / -57.1,-50.7	Favored (47.7%) *ttp* chi angles: 177.7,191.4,71	0.10Å	Favored (89.187%) alpha helix	-	-	-
A 200		ALA	4.65	-	Favored (75.33%) General / -58.8,-37.0	-	0.06Å	Favored (75.629%) alpha helix	-	-	-
**#**	**Alt**	**Res**	**High B**	**Clash > 0.4Å**	**Ramachandran**	**Rotamer**	**Cβ deviation**	**CaBLAM**	**Bond lengths**	**Bond angles**	**Cis Peptides**
			Avg: 5.35	Clashscore: 2.28	Outliers: 3 of 224	Poor rotamers: 0 of 179	Outliers: 0 of 207	Outliers: 7 of 222	Outliers: 2 of 226	Outliers: 4 of 226	Non-Trans: 0 of 225
A 201		LEU	4.63	-	Favored (92.15%) General / -65.2,-39.0	Favored (85.9%) *mt* chi angles: 290.5,173.3	0.03Å	Favored (92.065%) alpha helix	-	-	-
A 202		GLY	4.66	-	Favored (92.35%) Glycine / -61.2,-47.2	-	-	Favored (93.647%) alpha helix	-	-	-
A 203		LEU	4.78	-	Favored (80.63%) General / -62.5,-35.9	Favored (97.5%) *mt* chi angles: 292.6,173.4	0.07Å	Favored (83.62%) alpha helix	-	-	-
A 204		THR	4.96	-	Favored (92.42%) General / -63.0,-45.5	Favored (93.5%) *m* chi angles: 301	0.06Å	Favored (76.701%) alpha helix	-	-	-
A 205		ALA	5.2	-	Favored (72.37%) General / -58.5,-35.5	-	0.02Å	Favored (73.744%) alpha helix	-	-	-
A 206		VAL	5.51	-	Favored (3.08%) Ile or Val / -94.3,13.0	Favored (25.9%) *m* chi angles: 299.4	0.02Å	Favored (42.556%)	-	-	-
A 207		ARG	5.85	-	Favored (9.59%) General / 58.7,49.8	Favored (14.5%) *mpt180* chi angles: 275.9,68.7,172.8,174.6	0.05Å	Favored (30.965%)	-	-	-
A 208		VAL	6.21	-	Favored (34.94%) Ile or Val / -129.1,146.0	Favored (9%) *p* chi angles: 66.6	0.04Å	CaBLAM Disfavored (1.835%)	-	-	-
A 209		VAL	6.53	-	OUTLIER (0)%) Ile or Val / 88.9,-113.9	Favored (64.8%) *t* chi angles: 179.4	0.11Å	CA Geom Outlier (0.368%)	-	-	-
A 210		ASP	6.71	-	Allowed (0.99%) Pre-Pro / -165.6,121.4	Favored (37.5%) *t0* chi angles: 187,324.9	0.04Å	CaBLAM Outlier (0.062%)	-	-	-
A 211		PRO	6.69	-	Favored (13.92%) Trans-Pro / -47.5,-31.5	Favored (87.6%) *Cg_exo* chi angles: 330.2,36.5,332.3	0.03Å	Favored (79.032%)	-	-	-
A 212		ILE	6.49	-	Favored (45.94%) Ile or Val / -69.1,-30.3	Favored (7.7%) *tp* chi angles: 199.9,63.7	0.09Å	Favored (75.14%) alpha helix	-	-	-
A 213		ASN	6.16	-	Favored (63.37%) General / -72.7,-29.5	Favored (98.9%) *m-40* chi angles: 288.4,337.7	0.03Å	Favored (85.199%) alpha helix	-	-	-
A 214		VAL	5.77	-	Favored (90.38%)	Favored (86%) *t* chi angles: 173.7	0.10Å	Favored (80.428%) alpha helix	-	-	-

#	Alt	Res	High B	Clash > 0.4Å	Ramachandran	Rotamer	Cβ deviation	CaBLAM	Bond lengths	Bond angles	Cis Peptides
					Ile or Val / -66.6,-43.9						
A 215		VAL	5.41	-	Favored (99.64%) Ile or Val / -63.0,-44.5	Favored (64.8%) *t* chi angles: 171.5	0.04Å	Favored (80.601%) alpha helix	-	-	-
A 216		GLY	5.14	-	Favored (38.51%) Glycine / -53.1,-52.7	-	-	Favored (96.996%) alpha helix	-	-	-
A 217		LEU	5.03	0.56Å C with A 217 LEU HD23	Favored (68.9%) General / -53.5,-48.4	Favored (5.5%) *tt* chi angles: 186.1,158.4	0.07Å	Favored (93.201%) alpha helix	-	-	-
A 218		LEU	5.09	-	Favored (83.13%) General / -57.0,-45.3	Favored (63.7%) *tp* chi angles: 180.2,60.3	0.06Å	Favored (91.057%) alpha helix	-	-	-
A 219		LEU	5.32	-	Favored (75.51%) General / -65.9,-33.3	Favored (96.2%) *mt* chi angles: 293.4,173.6	0.04Å	Favored (80.335%) alpha helix	-	-	-
A 220		LEU	5.68	-	Favored (61.89%) General / -74.8,-31.9	Favored (98.4%) *mt* chi angles: 292.5,171.8	0.02Å	Favored (77.178%) alpha helix	-	-	-
**#**	**Alt**	**Res**	**High B**	**Clash > 0.4Å**	**Ramachandran**	**Rotamer**	**Cβ deviation**	**CaBLAM**	**Bond lengths**	**Bond angles**	**Cis Peptides**
			Avg: 5.35	Clashscore: 2.28	Outliers: 3 of 224	Poor rotamers: 0 of 179	Outliers: 0 of 207	Outliers: 7 of 222	Outliers: 2 of 226	Outliers: 4 of 226	Non-Trans: 0 of 225
A 221		THR	6.18	-	Favored (3.94%) General / -90.7,-174.0	Favored (11.7%) *t* chi angles: 190.6	0.09Å	CA Geom Outlier (0.14%)	-	-	-
A 222		ARG	6.8	-	Favored (16.22%) General / 52.5,51.4	Favored (97.6%) *mtt180* chi angles: 296.1,178.6,181.3,172.3	0.02Å	CaBLAM Disfavored (2.965%)	-	-	-
A 223		SER	7.53	-	Favored (23.75%) General / -60.2,-15.4	Favored (99.5%) *p* chi angles: 65.6	0.02Å	Favored (18.198%)	-	-	-
A 224		GLY	8.29	-	Favored (89.32%) Glycine / -79.5,-5.3	-	-	Favored (74.94%)	-	-	-
A 225		LYS	9	-	Favored (25.96%) General / -118.8,158.2	Favored (98.7%) *mttt* chi angles: 294.2,184.2,179,181.1	0.02Å	-	-	-	-
A 226		ARG	9.61	-	-	Favored (93.9%) *mmt-90* chi angles: 295.7,290.3,183.1,274.7	0.04Å	-	-	-	-

About MolProbity | Website for the Richardson Lab | Using ecloud x-H | Internal reference 4.5.2
