# Supplementary material for: Integrative proteome-wide structural analysis and high-throughput docking identify broad-spectrum antiviral scaffolds against Zika, Yellow Fever, West Nile, Saint Louis encephalitis, and Usutu viruses
Source: Front Cell Infect Microbiol. 2026 Apr 30;16:1723132. doi: 10.3389/fcimb.2026.1723132 (PMC13171538; doi:10.3389/fcimb.2026.1723132)
Supplement: Supplementary file 7 [file DataSheet7.zip › ZIKV/ZIKV_NS2a/Mol_probity_Files/ZIKV_NS2a_1FH-rama.pdf]

MolProbity Ramachandran analysis

ZIKV_NS2a1FH.pdb, model 1

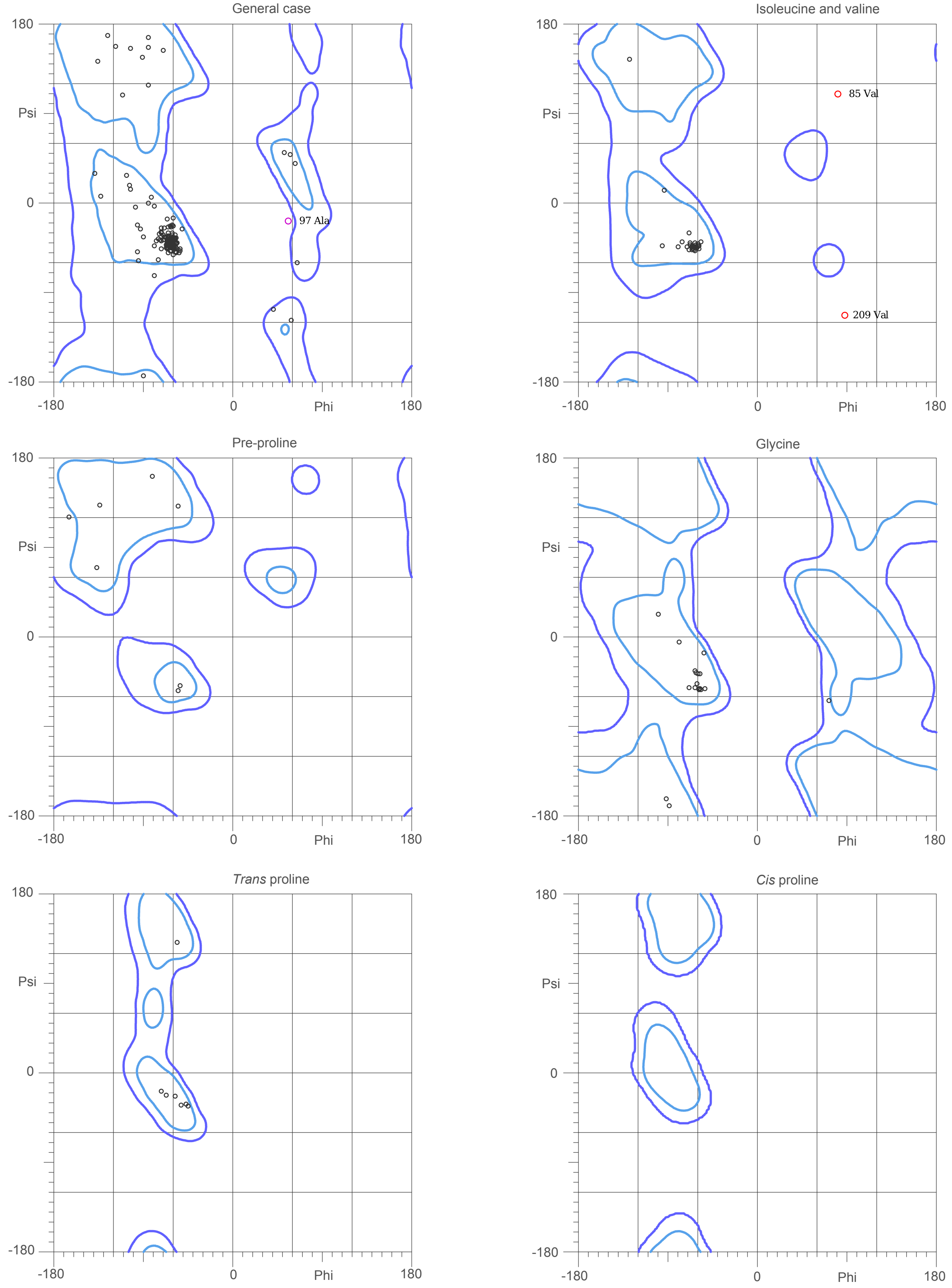

96.0% (215/224) of all residues were in favored (98%) regions.
98.7% (221/224) of all residues were in allowed (>99.8%) regions.

There were 3 outliers (phi, psi):
85 Val (81.4, 110.9)
97 Ala (56.1, -18.8)
209 Val (88.9, -113.9)
