# Supplementary material for: Integrative proteome-wide structural analysis and high-throughput docking identify broad-spectrum antiviral scaffolds against Zika, Yellow Fever, West Nile, Saint Louis encephalitis, and Usutu viruses
Source: Front Cell Infect Microbiol. 2026 Apr 30;16:1723132. doi: 10.3389/fcimb.2026.1723132 (PMC13171538; doi:10.3389/fcimb.2026.1723132)
Supplement: Supplementary file 7 [file DataSheet7.zip › ZIKV/ZIKV_NS2b/Mol_probity_Files/ZIKV_NS2b_1FH-multi.table.pdf]

Viewing ZIKV_NS2b1FH-multi.table

Duke Biochemistry
Duke University School of Medicine

When finished, you should close this window.

Hint: Use File | Save As... to save a copy of this page.

All-Atom Contacts	Clashscore, all atoms:	0.52		99th percentile* (N=1784, all resolutions)
	Clashscore is the number of serious steric overlaps (> 0.4 Å) per 1000 atoms.			
Protein Geometry	Poor rotamers	0	0.00%	Goal: <0.3%
	Favored rotamers	100	99.01%	Goal: >98%
	Ramachandran outliers	0	0.00%	Goal: <0.05%
	Ramachandran favored	120	93.75%	Goal: >98%
	Rama distribution Z-score	0.63 ± 0.73		Goal: abs(Z score) < 2
	MolProbity score^	1.09		100th percentile* (N=27675, 0Å - 99Å)
	Cβ deviations >0.25Å	0	0.00%	Goal: 0
	Bad bonds:	0 / 985	0.00%	Goal: 0%
	Bad angles:	4 / 1341	0.30%	Goal: <0.1%
Peptide Omegas	Cis Prolines:	1 / 8	12.50%	Expected: ≤1 per chain, or ≤5%
	Cis nonProlines:	2 / 121	1.65%	Goal: <0.05%
Low-resolution Criteria	CaBLAM outliers	4	3.2%	Goal: <1.0%
	CA Geometry outliers	2	1.59%	Goal: <0.5%
Additional validations	Chiral volume outliers	0/155		
	Waters with clashes	0/0	0.00%	See UnDowser table for details

In the two column results, the left column gives the raw count, right column gives the percentage.

* 100th percentile is the best among structures of comparable resolution; 0th percentile is the worst. For clashscore the comparative set of structures was selected in 2004, for MolProbity score in 2006.

^ MolProbity score combines the clashscore, rotamer, and Ramachandran evaluations into a single score, normalized to be on the same scale as X-ray resolution.

Key to table colors and cutoffs here:

#	Alt	Res	High B	Clash > 0.4Å	Ramachandran	Rotamer	Cβ deviation	CaBLAM	Bond lengths	Bond angles	Cis Peptides
			Avg: 6.84	Clashscore: 0.52	Outliers: 0 of 128	Poor rotamers: 0 of 101	Outliers: 0 of 117	Outliers: 6 of 126	Outliers: 0 of 130	Outliers: 4 of 130	Non-Trans: 3 of 129
A 1		SER	7.53	-	-	Favored (64.8%) *m*<br>chi angles: 294.2	0.03Å	-	-	-	-
A 2		TRP	7.26	-	Favored (93.9%)<br>Pre-Pro / -52.2,-45.5	Favored (69.1%) *t-100*<br>chi angles: 175.5,250.9	0.17Å	-	-	-	-
A 3		PRO	6.95	-	Favored (92.32%)<br>Cis-Pro / -76.6,162.2	Favored (64.3%) *Cg_endo*<br>chi angles: 31.4,323.5,26.4	0.06Å	Favored (5.206%)	-	-	Cis PRO<br>omega= 2.37
A 4		PRO	6.64	-	Favored (21.02%)<br>Trans-Pro / -49.4,-30.7	Favored (87.2%) *Cg_exo*<br>chi angles: 329.4,37.6,331.7	0.05Å	Favored (75.548%)	-	-	-
A 5		SER	6.39	-	Favored (93.82%)<br>General / -62.7,-39.4	Favored (65.3%) *m*<br>chi angles: 294.3	0.04Å	Favored (65.282%)<br>alpha helix	-	-	-

A 6		GLU	6.23	-	Favored (58.55%) General / -76.2,-31.7	Favored (75.9%) *mm-30* chi angles: 291.6,294.7,309.2	0.10Å	Favored (79.599%) alpha helix	-	-	-
A 7		VAL	6.17	-	Favored (96.12%) Ile or Val / -63.7,-45.9	Favored (65.3%) *t* chi angles: 171.5	0.11Å	Favored (79.978%) alpha helix	-	-	-
A 8		LEU	6.17	-	Favored (79.64%) General / -63.0,-35.3	Favored (91.5%) *mt* chi angles: 291,172.6	0.05Å	Favored (82.163%) alpha helix	-	-	-
A 9		THR	6.21	-	Favored (93.15%) General / -61.6,-45.7	Favored (93.2%) *m* chi angles: 297.6	0.02Å	Favored (77.788%) alpha helix	-	-	-
A 10		ALA	6.25	-	Favored (74.78%) General / -58.8,-36.6	-	0.02Å	Favored (79.902%) alpha helix	-	-	-
A 11		VAL	6.27	-	Favored (91.49%) Ile or Val / -65.7,-45.6	Favored (76.6%) *t* chi angles: 172.8	0.01Å	Favored (83.687%) alpha helix	-	-	-
A 12		GLY	6.27	-	Favored (85.06%) Glycine / -58.8,-36.1	-	-	Favored (88.726%) alpha helix	-	-	-
A 13		LEU	6.29	-	Favored (66.47%) General / -65.4,-20.7	Favored (93.7%) *mt* chi angles: 292.6,174.7	0.03Å	Favored (64.087%) alpha helix	-	-	-
A 14		ILE	6.32	-	Favored (10.15%) Ile or Val / -105.8,5.9	Favored (36.6%) *pt* chi angles: 63.2,166.3	0.03Å	Favored (45.94%) alpha helix	-	-	-
A 15		CYS	6.36	-	Favored (5.91%) General / -127.7,26.4	Favored (67.1%) *m* chi angles: 299.5	0.10Å	CaBLAM Outlier (0.632%) try alpha helix	-	OUTLIER(S) worst is CA-C-N: 5.5 σ	-
A 16		ALA	6.39	-	Allowed (0.79%) General / 60.9,-119.0	-	0.16Å	CaBLAM Outlier (0.584%) try alpha helix	-	OUTLIER(S) worst is C-N-CA: 9.9 σ	Cis nonPRO omega= 6.37
A 17		LEU	6.39	-	Favored (87.16%) General / -58.0,-45.3	Favored (86.6%) *mt* chi angles: 290.6,169.6	0.06Å	Favored (53.547%) alpha helix	-	-	-
A 18		ALA	6.34	-	Favored (86.36%) General / -63.2,-37.2	-	0.02Å	Favored (80.905%) alpha helix	-	-	-
A 19		GLY	6.23	-	Favored (53.05%) Glycine / -62.6,-51.9	-	-	Favored (95.547%) alpha helix	-	-	-
A 20		GLY	6.18	-	Favored (55.16%) Glycine / -56.8,-51.7	-	-	Favored (96.928%) alpha helix	-	-	-
**#**	**Alt**	**Res**	**High B**	**Clash > 0.4Å**	**Ramachandran**	**Rotamer**	**Cβ deviation**	**CaBLAM**	**Bond lengths**	**Bond angles**	**Cis Peptides**
			Avg: 6.84	Clashscore: 0.52	Outliers: 0 of 128	Poor rotamers: 0 of 101	Outliers: 0 of 117	Outliers: 6 of 126	Outliers: 0 of 130	Outliers: 4 of 130	Non-Trans: 3

											of 129
A 21		PHE	6.3	-	Favored (81.87%) General / -61.0,-37.3	Allowed (1.6%) *m-10* chi angles: 288.6,34.2	0.03Å	Favored (73.897%) alpha helix	-	-	-
A 22		ALA	6.55	-	Favored (62.86%) General / -60.9,-21.1	-	0.02Å	Favored (66.111%) three-ten	-	-	-
A 23		LYS	6.9	-	Favored (56.16%) General / -85.6,-10.2	Favored (99.3%) *mttt* chi angles: 294.5,178.2,181.2,177.8	0.04Å	Favored (56.971%)	-	-	-
A 24		ALA	7.29	-	Favored (8.28%) General / -95.8,172.0	-	0.16Å	CA Geom Outlier (0.481%)	-	OUTLIER(S) worst is C-CA-CB: 4.2 σ	-
A 25		ASP	7.62	-	Favored (5.81%) General / 70.9,19.2	Favored (64.6%) *m-30* chi angles: 300.3,325	0.13Å	Favored (6.096%)	-	OUTLIER(S) worst is C-N-CA: 7.3 σ	Cis nonPRO omega= 11.33
A 26		ILE	7.79	-	Allowed (0.36%) Ile or Val / -136.8,-34.9	Favored (17.4%) *tt* chi angles: 196.3,168.6	0.05Å	CaBLAM Outlier (0.006%)	-	-	-
A 27		GLU	7.8	-	Allowed (0.1%) General / 65.0,-14.8	Favored (91.9%) *mt-10* chi angles: 299,183.7,354	0.05Å	CaBLAM Disfavored (1.201%)	-	-	-
A 28		MET	7.68	-	Favored (16.9%) General / -76.1,1.0	Favored (97.9%) *mmm* chi angles: 294.1,302.3,292.8	0.03Å	CaBLAM Disfavored (4.022%)	-	-	-
A 29		ALA	7.47	-	Favored (69.77%) General / -58.6,-33.2	-	0.03Å	Favored (49.628%)	-	-	-
A 30		GLY	7.27	-	Allowed (0.67%) Glycine / -59.3,-72.1	-	-	Favored (54.58%) alpha helix	-	-	-
A 31		PRO	7.13	-	Favored (27.78%) Trans-Pro / -48.2,-35.9	Favored (85.2%) *Cg_exo* chi angles: 329.3,37.4,332	0.03Å	Favored (49.914%) alpha helix	-	-	-
A 32		MET	7.03	0.60Å HE3 with A 32 MET O	Favored (98.18%) General / -63.6,-43.0	Favored (7%) *tpt* chi angles: 185.6,62.3,252.4	0.06Å	Favored (73.881%) alpha helix	-	-	-
A 33		ALA	6.86	-	Favored (94.34%) General / -61.1,-40.6	-	0.03Å	Favored (97.361%) alpha helix	-	-	-
A 34		ALA	6.68	-	Favored (99.96%) General / -63.0,-42.9	-	0.03Å	Favored (96.619%) alpha helix	-	-	-
A 35		VAL	6.53	-	Favored (93.07%) Ile or Val / -64.6,-41.7	Favored (57.8%) *t* chi angles: 170.5	0.05Å	Favored (82.415%) alpha helix	-	-	-
A 36		GLY	6.39	-	Favored (48.49%) Glycine / -53.4,-51.3	-	-	Favored (94.795%) alpha helix	-	-	-

#	Alt	Res	High B	Clash > 0.4Å	Ramachandran	Rotamer	Cβ deviation	CaBLAM	Bond lengths	Bond angles	Cis Peptides
A 37		LEU	6.26	-	Favored (83.43%) General / -62.2,-36.9	Favored (97.6%) *mt* chi angles: 292.1,172.4	0.05Å	Favored (81.597%) alpha helix	-	-	-
A 38		LEU	6.12	-	Favored (95.37%) General / -64.2,-40.1	Favored (89.6%) *mt* chi angles: 290.7,171.9	0.02Å	Favored (88.758%) alpha helix	-	-	-
A 39		ILE	6	-	Favored (93.36%) Ile or Val / -61.4,-47.5	Favored (88.8%) *mt* chi angles: 291.1,166.8	0.04Å	Favored (94.059%) alpha helix	-	-	-
A 40		VAL	5.86	-	Favored (90.97%) Ile or Val / -62.1,-41.4	Favored (65.6%) *t* chi angles: 171.6	0.08Å	Favored (86.466%) alpha helix	-	-	-
**#**	**Alt**	**Res**	**High B**	**Clash > 0.4Å**	**Ramachandran**	**Rotamer**	**Cβ deviation**	**CaBLAM**	**Bond lengths**	**Bond angles**	**Cis Peptides**
			Avg: 6.84	Clashscore: 0.52	Outliers: 0 of 128	Poor rotamers: 0 of 101	Outliers: 0 of 117	Outliers: 6 of 126	Outliers: 0 of 130	Outliers: 4 of 130	Non-Trans: 3 of 129
A 41		SER	5.74	-	Favored (84.06%) General / -60.2,-38.7	Favored (72%) *m* chi angles: 295.9	0.03Å	Favored (83.31%) alpha helix	-	-	-
A 42		TYR	5.65	-	Favored (64.57%) General / -69.8,-27.1	Favored (46.3%) *m-80* chi angles: 287.1,114.5	0.04Å	Favored (75.142%) alpha helix	-	-	-
A 43		VAL	5.63	-	Favored (18.32%) Ile or Val / -77.8,-51.3	Favored (84.6%) *t* chi angles: 173.6	0.09Å	Favored (44.765%) alpha helix	-	-	-
A 44		VAL	5.72	-	Favored (95.63%) Ile or Val / -60.7,-43.8	Favored (60.5%) *t* chi angles: 170.9	0.07Å	Favored (77.923%) alpha helix	-	-	-
A 45		SER	5.96	-	Favored (61.65%) General / -72.3,-14.0	Favored (97.1%) *p* chi angles: 65.1	0.10Å	Favored (52.624%)	-	-	-
A 46		GLY	6.39	-	Favored (88.28%) Glycine / 81.8,-0.5	-	-	Favored (71.175%)	-	-	-
A 47		LYS	7.02	-	Favored (66.71%) General / -60.7,-25.8	Favored (96.5%) *mttt* chi angles: 288.4,178.9,178.7,179.8	0.04Å	Favored (23.655%)	-	-	-
A 48		SER	7.84	-	Favored (71.31%) General / -56.1,-38.9	Favored (68%) *m* chi angles: 294.7	0.06Å	Favored (6.09%)	-	-	-
A 49		VAL	8.8	-	Favored (4.67%) Ile or Val / -161.3,146.0	Favored (8.2%) *p* chi angles: 67.3	0.02Å	Favored (11.442%)	-	-	-
A 50		ASP	9.84	-	Favored (6.3%) General / -119.2,98.7	Favored (42.1%) *t0* chi angles: 187,12.5	0.04Å	Favored (34.407%)	-	-	-
A 51		MET	10.81	-	Favored (36.06%) General / -108.8,144.9	Favored (68.4%) *mtm* chi angles: 299.1,193.1,286.8	0.02Å	Favored (39.384%) beta sheet	-	-	-
A 52		TYR	11.6	-	Favored (47.95%)	Favored (81.6%) *m-80* chi angles: 297.1,82.6	0.05Å	Favored (64.907%) beta sheet	-	-	-

#	Alt	Res	High B	Clash > 0.4Å	Ramachandran	Rotamer	Cβ deviation	CaBLAM	Bond lengths	Bond angles	Cis Peptides
					General / -125.5,149.8						
A 53		ILE	12.1	-	Favored (75.27%) Ile or Val / -120.6,129.7	Favored (80.5%) *mt* chi angles: 300.2,171.2	0.04Å	Favored (62.673%) beta sheet	-	-	-
A 54		GLU	12.34	-	Favored (48.92%) General / -124.2,146.1	Favored (91.2%) *mt-10* chi angles: 299.6,182.5,1.9	0.02Å	Favored (51.325%) beta sheet	-	-	-
A 55		ARG	12.38	-	Favored (27.12%) General / -86.3,145.0	Favored (83.9%) *mtp180* chi angles: 292.1,184.1,67.6,186.8	0.02Å	Favored (43.069%)	-	-	-
A 56		ALA	12.31	-	Favored (18.28%) General / -94.6,-22.3	-	0.04Å	CaBLAM Disfavored (2.04%)	-	-	-
A 57		GLY	12.23	-	Favored (17%) Glycine / 110.1,158.5	-	-	Favored (40.671%)	-	-	-
A 58		ASP	12.18	-	Favored (51.24%) General / -60.7,144.8	Favored (92.2%) *m-30* chi angles: 289.5,350.5	0.04Å	Favored (7.651%)	-	-	-
A 59		ILE	12.22	-	Favored (68.51%) Ile or Val / -116.1,131.5	Favored (80.2%) *mt* chi angles: 300.1,170.5	0.04Å	Favored (66.319%) beta sheet	-	-	-
A 60		THR	12.34	-	Favored (31.43%) General / -123.5,158.4	Favored (63.8%) *p* chi angles: 63.3	0.03Å	Favored (59.595%) beta sheet	-	-	-
**#**	**Alt**	**Res**	**High B**	**Clash > 0.4Å**	**Ramachandran**	**Rotamer**	**Cβ deviation**	**CaBLAM**	**Bond lengths**	**Bond angles**	**Cis Peptides**
			Avg: 6.84	Clashscore: 0.52	Outliers: 0 of 128	Poor rotamers: 0 of 101	Outliers: 0 of 117	Outliers: 6 of 126	Outliers: 0 of 130	Outliers: 4 of 130	Non-Trans: 3 of 129
A 61		TRP	12.53	-	Favored (52.78%) General / -131.0,148.8	Favored (34.8%) *m-90* chi angles: 297.6,262.8	0.05Å	Favored (63.824%) beta sheet	-	-	-
A 62		GLU	12.74	-	Favored (56.68%) General / -91.8,2.2	Favored (95.1%) *mt-10* chi angles: 297.3,180.5,1.9	0.01Å	CaBLAM Disfavored (2.092%)	-	-	-
A 63		LYS	12.89	-	Allowed (0.43%) General / 66.8,-55.8	Favored (98.6%) *mttt* chi angles: 293,179.9,180.6,178.3	0.04Å	CaBLAM Disfavored (3.556%)	-	-	-
A 64		ASP	12.92	-	Favored (19.75%) General / -103.4,19.4	Favored (73%) *m-30* chi angles: 294.6,318.6	0.01Å	Favored (23.755%)	-	-	-
A 65		ALA	12.79	-	Favored (56.55%) General / -60.0,140.8	-	0.05Å	Favored (28.471%)	-	-	-
A 66		GLU	12.45	-	Favored (55.06%) General / -91.1,-5.6	Favored (97%) *mt-10* chi angles: 295.6,178.4,359.1	0.03Å	Favored (40.917%)	-	-	-
A 67		VAL	11.89	-	Favored (75.19%) Ile or Val / -122.5,129.5	Favored (87%) *t* chi angles: 177.5	0.04Å	Favored (19.815%)	-	-	-

A 68		THR	11.12	-	Favored (15.67%) General / -111.4,-2.3	Favored (79.9%) *p* chi angles: 60.6	0.04Å	Favored (9.746%)	-	-	-
A 69		GLY	10.21	-	Favored (2.43%) Glycine / -76.1,67.5	-	-	CA Geom Outlier (0.48%)	-	-	-
A 70		ASN	9.27	-	Favored (56.59%) General / -58.3,134.5	Favored (94.4%) *m-40* chi angles: 291,331.1	0.03Å	Favored (18.105%) beta sheet	-	-	-
A 71		SER	8.43	-	Favored (97.56%) Pre-Pro / -68.0,144.9	Favored (42.2%) *t* chi angles: 175.8	0.07Å	Favored (49.597%) beta sheet	-	-	-
A 72		PRO	7.82	-	Favored (43.87%) Trans-Pro / -70.9,162.1	Favored (58.8%) *Cg_endo* chi angles: 26.3,326,27.8	0.03Å	Favored (84.797%) beta sheet	-	-	-
A 73		ARG	7.55	-	Favored (44.22%) General / -128.6,128.4	Favored (65.9%) *ttt180* chi angles: 177.3,174.4,169.5,167.6	0.03Å	Favored (46.816%) beta sheet	-	-	-
A 74		LEU	7.67	-	Favored (19.8%) General / -114.5,110.4	Favored (84.9%) *mt* chi angles: 299.6,175	0.07Å	Favored (67.048%) beta sheet	-	-	-
A 75		ASP	8.19	-	Favored (24.02%) General / -86.5,115.5	Favored (47.9%) *m-30* chi angles: 290.4,308	0.02Å	Favored (61.409%) beta sheet	-	-	-
A 76		VAL	9.11	-	Favored (47.41%) Ile or Val / -94.8,125.7	Favored (57%) *t* chi angles: 180.3	0.05Å	Favored (63.668%) beta sheet	-	-	-
A 77		ALA	10.34	-	Favored (47.96%) General / -78.2,-26.9	-	0.03Å	CaBLAM Disfavored (3.847%) try beta sheet	-	-	-
A 78		LEU	11.69	-	Allowed (0.05%) General / 57.2,-179.7	Favored (84.6%) *mt* chi angles: 299.7,175	0.02Å	Favored (5.411%)	-	-	-
A 79		ASP	12.89	-	Favored (18.89%) General / -159.8,171.8	Favored (12.7%) *t0* chi angles: 206.5,338.8	0.03Å	Favored (8.726%)	-	-	-
A 80		GLU	13.69	-	Favored (72.61%) General / -61.7,-32.4	Favored (99.5%) *mt-10* chi angles: 291.5,179.8,355	0.01Å	Favored (56.074%)	-	-	-
**#**	**Alt**	**Res**	**High B**	**Clash > 0.4Å**	**Ramachandran**	**Rotamer**	**Cβ deviation**	**CaBLAM**	**Bond lengths**	**Bond angles**	**Cis Peptides**
			Avg: 6.84	Clashscore: 0.52	Outliers: 0 of 128	Poor rotamers: 0 of 101	Outliers: 0 of 117	Outliers: 6 of 126	Outliers: 0 of 130	Outliers: 4 of 130	Non-Trans: 3 of 129
A 81		SER	13.84	-	Favored (59.71%) General / -79.7,-9.9	Favored (85.4%) *p* chi angles: 67.7	0.09Å	Favored (52.356%)	-	-	-
A 82		GLY	13.31	-	Favored (70.26%) Glycine / 94.9,-10.4	-	-	Favored (77.439%)	-	-	-

A 83		ASP	12.27	-	Favored (15.04%) General / -74.2,170.7	Favored (78.4%) *m-30* chi angles: 292.1,326.3	0.07Å	Favored (18.555%)	-	-	-
A 84		PHE	10.97	-	Favored (40.1%) General / -150.9,162.8	Favored (57.7%) *p90* chi angles: 65.5,91.4	0.05Å	Favored (61.465%) beta sheet	-	-	-
A 85		SER	9.68	-	Favored (45.49%) General / -120.5,147.2	Favored (30.3%) *t* chi angles: 172.5	0.04Å	Favored (58.733%) beta sheet	-	-	-
A 86		LEU	8.55	-	Favored (33.34%) General / -94.3,10.7	Favored (86.1%) *mt* chi angles: 296.6,179.3	0.04Å	CaBLAM Disfavored (1.256%) try beta sheet	-	-	-
A 87		VAL	7.65	-	Allowed (0.22%) Ile or Val / 69.1,-50.5	Favored (90%) *t* chi angles: 174.2	0.08Å	Favored (42.502%) beta sheet	-	-	-
A 88		GLU	6.93	-	Allowed (1.4%) General / 60.4,63.3	Favored (89.2%) *mt-10* chi angles: 299.9,185.9,354.6	0.05Å	CaBLAM Outlier (0.427%) try beta sheet	-	-	-
A 89		GLU	6.35	-	Favored (35.6%) General / -53.0,136.1	Favored (84%) *tt0* chi angles: 185.7,179.5,12.1	0.03Å	Favored (32.855%) beta sheet	-	-	-
A 90		ASP	5.87	-	Favored (13.35%) General / -59.4,156.1	Favored (97.1%) *m-30* chi angles: 288.2,348.8	0.03Å	Favored (20.993%)	-	-	-
A 91		GLY	5.38	-	Favored (26.44%) Glycine / -76.0,-168.7	-	-	Favored (18.188%)	-	-	-
A 92		PRO	4.86	-	Favored (40.27%) Trans-Pro / -55.9,151.7	Favored (69.5%) *Cg_exo* chi angles: 335.4,35.5,329	0.05Å	Favored (6.987%)	-	-	-
A 93		PRO	4.33	-	Favored (59.99%) Trans-Pro / -69.4,157.8	Favored (52.7%) *Cg_endo* chi angles: 25.5,326.4,27.5	0.01Å	Favored (83.128%)	-	-	-
A 94		MET	3.83	-	Favored (74.48%) General / -56.9,-39.5	Favored (92%) *mtp* chi angles: 289.6,173.2,64.6	0.02Å	Favored (62.146%)	-	-	-
A 95		ARG	3.39	-	Favored (93.47%) General / -59.6,-44.3	Favored (71.1%) *ttt-90* chi angles: 182.4,176.1,183.7,269.7	0.04Å	Favored (76.819%) alpha helix	-	-	-
A 96		GLU	3	-	Favored (90.13%) General / -66.1,-39.5	Favored (97.8%) *mt-10* chi angles: 289.8,178.7,356.7	0.03Å	Favored (82.605%) alpha helix	-	-	-
A 97		ILE	2.65	-	Favored (83.39%) Ile or Val / -66.2,-47.8	Favored (98%) *mt* chi angles: 293,167.2	0.04Å	Favored (85.648%) alpha helix	-	-	-
A 98		ILE	2.33	-	Favored (95.63%) Ile or Val / -63.1,-46.6	Favored (93.4%) *mt* chi angles: 291.8,166.9	0.05Å	Favored (90.849%) alpha helix	-	-	-
A 99		LEU	2.07	-	Favored (91%) General /	Favored (93.4%) *mt* chi angles: 291.6,173	0.03Å	Favored (91.826%)	-	-	-

					-63.6,-38.4			alpha helix			
A 100		LYS	1.85	-	Favored (90.75%) General / -59.6,-45.8	Favored (86.5%) *tttt* chi angles: 182.2,177.7,178.2,179.8	0.03Å	Favored (92.579%) alpha helix	-	-	-

#	Alt	Res	High B	Clash > 0.4Å	Ramachandran	Rotamer	Cβ deviation	CaBLAM	Bond lengths	Bond angles	Cis Peptides
			Avg: 6.84	Clashscore: 0.52	Outliers: 0 of 128	Poor rotamers: 0 of 101	Outliers: 0 of 117	Outliers: 6 of 126	Outliers: 0 of 130	Outliers: 4 of 130	Non-Trans: 3 of 129
A 101		VAL	1.68	-	Favored (93.06%) Ile or Val / -61.1,-42.4	Favored (59.3%) *t* chi angles: 170.7	0.04Å	Favored (91.414%) alpha helix	-	-	-
A 102		VAL	1.56	-	Favored (98.29%) Ile or Val / -60.8,-45.1	Favored (58%) *t* chi angles: 170.5	0.04Å	Favored (96.734%) alpha helix	-	-	-
A 103		LEU	1.48	-	Favored (90.14%) General / -63.5,-38.2	Favored (92.2%) *mt* chi angles: 292.2,174.5	0.04Å	Favored (90.793%) alpha helix	-	-	-
A 104		MET	1.5	-	Favored (99.28%) General / -63.2,-42.6	Favored (95.3%) *mtp* chi angles: 290.6,170.2,70	0.03Å	Favored (91.918%) alpha helix	-	-	-
A 105		ALA	1.67	-	Favored (91.96%) General / -60.4,-40.7	-	0.03Å	Favored (96.89%) alpha helix	-	-	-
A 106		ILE	2.1	-	Favored (96.93%) Ile or Val / -63.9,-45.5	Favored (96%) *mt* chi angles: 291.9,167.5	0.04Å	Favored (96.209%) alpha helix	-	-	-
A 107		CYS	2.95	-	Favored (88.47%) General / -59.2,-41.4	Favored (89.5%) *m* chi angles: 291.5	0.12Å	Favored (88.978%) alpha helix	-	-	-
A 108		GLY	4.36	-	Favored (42.95%) Glycine / -63.8,-52.7	-	-	Favored (90.847%) alpha helix	-	-	-
A 109		MET	6.13	-	Favored (84.36%) General / -64.9,-36.4	Favored (82.6%) *mtm* chi angles: 289.4,186.7,287.4	0.03Å	Favored (37.446%) alpha helix	-	-	-
A 110		ASN	7.46	-	Favored (8.95%) Pre-Pro / -145.3,83.8	Favored (38.3%) *t0* chi angles: 190.4,7.8	0.02Å	Favored (10.73%)	-	-	-
A 111		PRO	7.44	-	Favored (8.21%) Trans-Pro / -47.3,-28.6	Favored (88.3%) *Cg_exo* chi angles: 330,37.3,331.3	0.01Å	Favored (31.96%)	-	-	-
A 112		ILE	6.08	-	Favored (27.94%) Ile or Val / -60.7,-23.1	Favored (17.3%) *tt* chi angles: 196.7,168.1	0.03Å	Favored (66.085%)	-	-	-
A 113		ALA	4.3	-	Favored (58.84%) General / -79.8,-7.7	-	0.06Å	Favored (53.326%) three-ten	-	-	-
A 114		ILE	2.87	-	Favored (51.78%) Pre-Pro / -51.7,-53.3	Favored (83%) *mt* chi angles: 290.3,166.4	0.12Å	Favored (51.254%) alpha helix	-	-	-

#	Alt	Res	High B	Clash > 0.4Å	Ramachandran	Rotamer	Cβ deviation	CaBLAM	Bond lengths	Bond angles	Cis Peptides
A 115		PRO	2.01	-	Favored (64.46%) Trans-Pro / -64.9,-20.5	Favored (39.8%) *Cg_endo* chi angles: 23.3,326,29.9	0.00Å	Favored (72.767%) alpha helix	-	-	-
A 116		PHE	1.57	-	Favored (16.77%) General / -82.4,-43.9	Favored (97.4%) *m-80* chi angles: 294.4,90	0.07Å	Favored (65.593%) alpha helix	-	-	-
A 117		ALA	1.39	-	Favored (96.61%) General / -63.2,-40.1	-	0.06Å	Favored (92.603%) alpha helix	-	-	-
A 118		ALA	1.35	-	Favored (98.1%) General / -61.5,-42.0	-	0.03Å	Favored (88.505%) alpha helix	-	-	-
A 119		GLY	1.39	-	Favored (53.31%) Glycine / -56.9,-52.1	-	-	Favored (97.279%) alpha helix	-	-	-
A 120		ALA	1.45	-	Favored (78.78%) General / -57.5,-40.4	-	0.04Å	Favored (87.211%) alpha helix	-	-	-
**#**	**Alt**	**Res**	**High B**	**Clash > 0.4Å**	**Ramachandran**	**Rotamer**	**Cβ deviation**	**CaBLAM**	**Bond lengths**	**Bond angles**	**Cis Peptides**
			Avg: 6.84	Clashscore: 0.52	Outliers: 0 of 128	Poor rotamers: 0 of 101	Outliers: 0 of 117	Outliers: 6 of 126	Outliers: 0 of 130	Outliers: 4 of 130	Non-Trans: 3 of 129
A 121		TRP	1.54	-	Favored (88.65%) General / -62.6,-46.4	Favored (86.6%) *t60* chi angles: 183.5,85.6	0.04Å	Favored (97.017%) alpha helix	-	-	-
A 122		TYR	1.65	-	Favored (90.66%) General / -60.4,-40.4	Favored (34.2%) *m-80* chi angles: 284.3,117.6	0.01Å	Favored (91.637%) alpha helix	-	-	-
A 123		VAL	1.78	-	Favored (95.86%) Ile or Val / -65.1,-43.7	Favored (62.7%) *t* chi angles: 171.2	0.01Å	Favored (86.892%) alpha helix	-	-	-
A 124		TYR	1.96	-	Favored (82.83%) General / -58.6,-40.3	Favored (16.1%) *m-10* chi angles: 290.1,154.4	0.03Å	Favored (74.186%) alpha helix	-	-	-
A 125		VAL	2.21	-	Favored (37.12%) Ile or Val / -75.4,-46.8	Favored (90.4%) *t* chi angles: 174.2	0.06Å	Favored (66.865%) alpha helix	-	-	-
A 126		LYS	2.57	-	Favored (76.28%) General / -69.0,-35.1	Favored (96.8%) *mttt* chi angles: 289.6,178.4,180.9,180.2	0.02Å	Favored (66.862%) alpha helix	-	-	-
A 127		THR	3.06	-	Favored (42.3%) General / -100.9,3.9	Favored (62%) *p* chi angles: 63.6	0.01Å	Favored (35.951%)	-	-	-
A 128		GLY	3.68	-	Favored (48.27%) Glycine / -84.1,-171.8	-	-	Favored (43.323%)	-	-	-
A 129		LYS	4.38	-	Favored (27.27%) General / -82.2,150.7	Favored (98.9%) *mttt* chi angles: 294.9,184.9,181.4,180.6	0.04Å	-	-	-	-

A 130		ARG	5.09	-	-	Favored (47.4%) *mmt180* chi angles: 298.6,291.5,190.1,175.1	0.04Å	-	-	-	-

About MolProbity | Website for the Richardson Lab | Using ecloud x-H | Internal reference 4.5.2
