# Supplementary material for: Integrative proteome-wide structural analysis and high-throughput docking identify broad-spectrum antiviral scaffolds against Zika, Yellow Fever, West Nile, Saint Louis encephalitis, and Usutu viruses
Source: Front Cell Infect Microbiol. 2026 Apr 30;16:1723132. doi: 10.3389/fcimb.2026.1723132 (PMC13171538; doi:10.3389/fcimb.2026.1723132)
Supplement: Supplementary file 7 [file DataSheet7.zip › ZIKV/ZIKV_NS3/Mol_probity_Files/ZIKV_NS3_1FH-multi.table.pdf]

Viewing ZIKV_NS3_1FH-multi.table

MolProbity | Duke Biochemistry | Duke University School of Medicine

When finished, you should close this window.

Hint: Use File | Save As... to save a copy of this page.

All-Atom Contacts	Clashscore, all atoms:	0.83		99th percentile* (N=1784, all resolutions)
	Clashscore is the number of serious steric overlaps (> 0.4 Å) per 1000 atoms.			
Protein Geometry	Poor rotamers	0	0.00%	Goal: <0.3%
	Favored rotamers	510	100.00%	Goal: >98%
	Ramachandran outliers	4	0.65%	Goal: <0.05%
	Ramachandran favored	604	98.21%	Goal: >98%
	Rama distribution Z-score	0.52 ± 0.33		Goal: abs(Z score) < 2
	MolProbity score^	0.76		100th percentile* (N=27675, 0Å - 99Å)
	Cβ deviations >0.25Å	0	0.00%	Goal: 0
	Bad bonds:	5 / 4913	0.10%	Goal: 0%
	Bad angles:	10 / 6660	0.15%	Goal: <0.1%
Peptide Omegas	Cis Prolines:	0 / 33	0.00%	Expected: ≤1 per chain, or ≤5%
	Cis nonProlines:	1 / 583	0.17%	Goal: <0.05%
Low-resolution Criteria	CaBLAM outliers	12	2.0%	Goal: <1.0%
	CA Geometry outliers	5	0.82%	Goal: <0.5%
Additional validations	Chiral volume outliers	0/734		
	Waters with clashes	0/0	0.00%	See UnDowser table for details

In the two column results, the left column gives the raw count, right column gives the percentage.

* 100th percentile is the best among structures of comparable resolution; 0th percentile is the worst. For clashscore the comparative set of structures was selected in 2004, for MolProbity score in 2006.

^ MolProbity score combines the clashscore, rotamer, and Ramachandran evaluations into a single score, normalized to be on the same scale as X-ray resolution.

Key to table colors and cutoffs here:

#	Alt	Res	High B	Clash > 0.4Å	Ramachandran	Rotamer	Cβ deviation	CaBLAM	Bond lengths	Bond angles	Cis Peptides
			Avg: 1.07	Clashscore: 0.83	Outliers: 4 of 615	Poor rotamers: 0 of 510	Outliers: 0 of 564	Outliers: 16 of 613	Outliers: 5 of 617	Outliers: 10 of 617	Non-Trans: 1 of 616
A 1		SER	6.05	-	-	Favored (41.3%) *t* chi angles: 176.3	0.02Å	-	-	-	-
A 2		GLY	5.81	-	Favored (40.83%) Glycine / -178.3,-170.8	-	-	-	-	-	-
A 3		ALA	5.55	-	Favored (39.39%) General / -152.8,156.6	-	0.04Å	Favored (26.71%)	-	-	-
A 4		LEU	5.3	-	Favored (18.49%) General / -98.8,17.8	Favored (92.3%) *mt* chi angles: 298.8,176.8	0.07Å	Favored (6.813%)	-	-	-
A 5		TRP	5.06	-	Favored (56.85%) General / -58.5,-23.0	Favored (45.4%) *m100* chi angles: 276.4,92.4	0.03Å	Favored (40.262%)	-	-	-
A 6		ASP	4.85	-	Favored (46.44%) General / -86.1,2.5	Favored (4.1%) *t70* chi angles: 206.3,56.5	0.03Å	Favored (27.597%)	-	-	-

#	Alt	Res	High B	Clash > 0.4Å	Ramachandran	Rotamer	Cβ deviation	CaBLAM	Bond lengths	Bond angles	Cis Peptides
A 7		VAL	4.66	-	Favored (86.5%) Pre-Pro / -58.7,135.0	Favored (52.6%) *t* chi angles: 169.7	0.09Å	Favored (19.635%)	-	-	-
A 8		PRO	4.47	-	Favored (19.59%) Trans-Pro / -48.9,142.5	Favored (89.6%) *Cg_exo* chi angles: 329.8,36.6,332.6	0.06Å	Favored (24.393%) beta sheet	-	-	-
A 9		ALA	4.28	-	Favored (57.07%) Pre-Pro / -125.0,69.0	-	0.14Å	Favored (7.16%)	-	-	-
A 10		PRO	4.06	-	Favored (27.01%) Trans-Pro / -72.7,-12.9	Favored (60.9%) *Cg_endo* chi angles: 26.5,325.5,27.7	0.08Å	Favored (7.71%)	-	-	-
A 11		LYS	3.79	-	Favored (8.59%) General / -159.4,135.1	Favored (84.7%) *tttt* chi angles: 187.9,176.4,179.2,179.3	0.03Å	Favored (12.139%)	-	-	-
A 12		GLU	3.46	0.42Å O with A 13 VAL C	Favored (75.22%) General / -57.8,-38.3	Favored (90.9%) *mt-10* chi angles: 287.1,177.4,338.7	0.09Å	CaBLAM Outlier (0.983%)	-	-	-
A 13		VAL	3.05	0.42Å C with A 12 GLU O	OUTLIER (0.01)%) Ile or Val / 36.7,-115.2	Favored (58.3%) *t* chi angles: 180.2	0.10Å	CaBLAM Outlier (0.188%)	-	-	-
A 14		LYS	2.6	-	Favored (7.07%) General / -164.6,143.6	Favored (84.9%) *tttt* chi angles: 183.7,172.1,180.1,174.4	0.03Å	CaBLAM Outlier (0.303%)	-	-	-
A 15		LYS	2.14	-	Favored (52.2%) General / -62.1,132.2	Favored (87.2%) *tttt* chi angles: 183.5,177.2,179,180	0.02Å	Favored (38.838%)	-	-	-
A 16		GLY	1.72	-	Favored (51.77%) Glycine / -65.9,152.7	-	-	Favored (65.141%) beta sheet	-	-	-
A 17		GLU	1.37	-	Favored (33.94%) General / -86.6,132.3	Favored (91.2%) *tt0* chi angles: 183.1,177.5,355	0.01Å	Favored (42.434%) beta sheet	-	-	-
A 18		THR	1.11	-	Favored (26.51%) General / -131.0,122.2	Favored (69.3%) *p* chi angles: 59.2	0.04Å	Favored (40.457%)	-	-	-
A 19		THR	0.93	-	Favored (49.59%) General / -129.3,151.8	Favored (62%) *p* chi angles: 63.6	0.03Å	Favored (29.03%)	-	-	-
A 20		ASP	0.81	-	Favored (53.4%) General / -63.9,146.8	Favored (73.8%) *m-30* chi angles: 291.6,355.8	0.14Å	CA Geom Outlier (0.27%)	-	-	-
**#**	**Alt**	**Res**	**High B**	**Clash > 0.4Å**	**Ramachandran**	**Rotamer**	**Cβ deviation**	**CaBLAM**	**Bond lengths**	**Bond angles**	**Cis Peptides**
			Avg: 1.07	Clashscore: 0.83	Outliers: 4 of 615	Poor rotamers: 0 of 510	Outliers: 0 of 564	Outliers: 16 of 613	Outliers: 5 of 617	Outliers: 10 of 617	Non-Trans: 1 of 616
A 21		GLY	0.75	-	Favored (27.02%) Glycine / 145.3,-169.2	-	-	Favored (46.272%)	-	-	-
A 22		VAL	0.72	-	Favored (65.69%)	Favored (65.7%) *t* chi angles: 179.3	0.05Å	Favored (10.776%) beta sheet	-	-	-

					Ile or Val /<br>-110.3,129.2						
A<br>23		TYR	0.72	-	Favored<br>(50.01%)<br>General /<br>-126.8,147.2	Favored (85.6%) *m-80*<br>chi angles: 298.3,85	0.03Å	Favored<br>(53.854%)<br>beta sheet	-	-	-
A<br>24		ARG	0.76	-	Favored<br>(30.99%)<br>General /<br>-81.4,145.1	Favored (79.6%)<br>*mtt90*<br>chi angles:<br>297.8,174.7,178.9,87.5	0.06Å	Favored<br>(44.362%)<br>beta sheet	-	-	-
A<br>25		VAL	0.85	-	Favored<br>(59.74%)<br>Ile or Val /<br>-107.1,121.3	Favored (62.3%) *t*<br>chi angles: 179.7	0.04Å	Favored<br>(65.574%)<br>beta sheet	-	-	-
A<br>26		MET	0.98	-	Favored<br>(25.32%)<br>General /<br>-112.8,155.1	Favored (71.3%)<br>*mmm*<br>chi angles:<br>308.2,294.2,290.2	0.06Å	Favored<br>(42.35%)<br>beta sheet	-	-	-
A<br>27		THR	1.15	-	Favored<br>(48.62%)<br>General /<br>-129.0,135.2	Favored (91.2%) *m*<br>chi angles: 298	0.05Å	Favored<br>(49.671%)<br>beta sheet	-	-	-
A<br>28		ARG	1.34	-	Favored<br>(32.69%)<br>General /<br>-83.3,126.4	Favored (83%)<br>*ttt180*<br>chi angles:<br>182.1,177,179.5,181.5	0.03Å	Favored<br>(52.587%)	-	-	-
A<br>29		ARG	1.5	-	Favored<br>(9.44%)<br>General /<br>-118.6,169.9	Favored (91.2%)<br>*mtt-85*<br>chi angles:<br>298.8,180.3,187.8,270.8	0.03Å	Favored<br>(30.241%)	-	-	-
A<br>30		LEU	1.57	-	Favored<br>(67.37%)<br>General /<br>-61.1,-26.0	Favored (95.4%) *mt*<br>chi angles: 292.7,174.1	0.03Å	Favored<br>(42.646%)	-	-	-
A<br>31		LEU	1.5	-	Favored<br>(42.99%)<br>General /<br>-100.1,9.4	Favored (86.4%) *mt*<br>chi angles: 299.8,178.5	0.02Å	Favored<br>(6.695%)	-	-	-
A<br>32		GLY	1.34	-	Favored<br>(13.2%)<br>Glycine /<br>123.2,169.1	-	-	Favored<br>(23.287%)	-	-	-
A<br>33		SER	1.14	-	Favored<br>(30.43%)<br>General /<br>-78.3,155.0	Favored (96.2%) *p*<br>chi angles: 66	0.03Å	Favored<br>(7.99%)	-	-	-
A<br>34		THR	0.96	-	Favored<br>(36.71%)<br>General /<br>-134.2,160.8	Favored (47.4%) *p*<br>chi angles: 66.1	0.03Å	Favored<br>(44.66%)	-	-	-
A<br>35		GLN	0.83	-	Favored<br>(33.95%)<br>General /<br>-86.2,132.7	Favored (51.9%) *tt0*<br>chi angles:<br>182,176.7,313.6	0.04Å	Favored<br>(35.373%)	-	-	-
A<br>36		VAL	0.74	-	Favored<br>(11.04%)<br>Ile or Val /<br>-114.5,-14.0	Favored (32.4%) *m*<br>chi angles: 297.4	0.03Å	Favored<br>(10.449%)	-	-	-
A<br>37		GLY	0.7	-	Favored<br>(45.63%)<br>Glycine /<br>178.7,-173.3	-	-	Favored<br>(42.24%)	-	-	-
A<br>38		VAL	0.68	-	Favored<br>(47.58%)<br>Ile or Val /<br>-135.0,139.3	Favored (49.6%) *t*<br>chi angles: 181.5	0.11Å	Favored<br>(24.947%)	-	-	-
A<br>39		GLY	0.68	-	Favored<br>(12.94%)	-	-	Favored<br>(45.373%)<br>beta sheet	-	-	-

					Glycine / -128.3,-171.1						
A 40		VAL	0.7	-	Favored (31.56%) Ile or Val / -140.6,141.9	Favored (10.4%) *p* chi angles: 62.4	0.07Å	Favored (42.621%) beta sheet	-	-	-
**#**	**Alt**	**Res**	**High B**	**Clash > 0.4Å**	**Ramachandran**	**Rotamer**	**Cβ deviation**	**CaBLAM**	**Bond lengths**	**Bond angles**	**Cis Peptides**
			Avg: 1.07	Clashscore: 0.83	Outliers: 4 of 615	Poor rotamers: 0 of 510	Outliers: 0 of 564	Outliers: 16 of 613	Outliers: 5 of 617	Outliers: 10 of 617	Non-Trans: 1 of 616
A 41		MET	0.72	-	Favored (46.32%) General / -106.2,136.2	Favored (67.5%) *mtt* chi angles: 296.1,180.4,186.4	0.07Å	Favored (52.362%)	-	-	-
A 42		GLN	0.74	-	Favored (16.58%) General / -143.7,126.9	Favored (48.7%) *tt0* chi angles: 176.9,178.7,323.9	0.03Å	Favored (18.396%)	-	-	-
A 43		GLU	0.76	-	Favored (20.43%) General / 57.8,31.4	Favored (43.8%) *mt-10* chi angles: 296.6,174.9,51.7	0.01Å	Favored (33.505%)	-	-	-
A 44		GLY	0.76	-	Favored (88.52%) Glycine / 80.8,0.7	-	-	Favored (75.857%)	-	-	-
A 45		VAL	0.74	-	Favored (54.03%) Ile or Val / -100.5,126.8	Favored (55%) *t* chi angles: 180.6	0.08Å	Favored (28.675%)	-	-	-
A 46		PHE	0.72	-	Favored (34.45%) General / -86.7,130.5	Favored (84.6%) *t80* chi angles: 180,74.4	0.05Å	Favored (58.641%) beta sheet	-	-	-
A 47		HIS	0.7	-	Favored (54.3%) General / -124.6,138.6	Favored (38.1%) *m90* chi angles: 307.4,81.7	0.16Å	Favored (38.402%) beta sheet	-	-	-
A 48		THR	0.7	-	Favored (4.27%) General / -150.0,-172.9	Favored (11.3%) *t* chi angles: 188.2	0.14Å	Favored (16%) beta sheet	-	-	-
A 49		MET	0.7	-	Favored (29.86%) General / -94.6,140.4	Favored (86.3%) *mmm* chi angles: 303.5,296.8,289.6	0.06Å	Favored (12.729%)	-	-	-
A 50		TRP	0.72	-	Favored (76.19%) General / -57.6,-49.5	Favored (35.4%) *t-100* chi angles: 188.5,274.9	0.09Å	Favored (45.965%)	-	-	-
A 51		HIS	0.76	-	Favored (38.11%) General / -57.3,-21.9	Favored (47.9%) *p-80* chi angles: 72,276.6	0.12Å	Favored (61.705%) alpha helix	-	OUTLIER(S) worst is CA-CB-CG: 4.5 σ	-
A 52		VAL	0.81	-	Favored (35.67%) Ile or Val / -76.4,-43.9	Favored (63%) *t* chi angles: 171.2	0.01Å	Favored (25.75%) alpha helix	-	-	-
A 53		THR	0.87	-	Favored (14.92%) General / -102.5,-19.3	Favored (48.1%) *p* chi angles: 66	0.10Å	Favored (16.462%)	-	-	-
A 54		LYS	0.94	-	Favored (12.82%) General / 59.4,24.2	Favored (90.8%) *mttt* chi angles: 301.9,182.9,182.1,178	0.04Å	Favored (5.235%)	-	-	-
A 55		GLY	1	-	Favored (89.58%)	-	-	Favored (56.675%)	-	-	-

#	Alt	Res	High B	Clash > 0.4Å	Ramachandran	Rotamer	Cβ deviation	CaBLAM	Bond lengths	Bond angles	Cis Peptides
					Glycine / 84.0,2.3						
A 56		ALA	1.05	-	Favored (51.07%) General / -67.8,148.6	-	0.04Å	Favored (23.934%)	-	-	-
A 57		ALA	1.11	-	Favored (55.13%) General / -63.8,145.8	-	0.03Å	Favored (41.011%) beta sheet	-	-	-
A 58		LEU	1.18	-	Favored (25.74%) General / -110.2,152.8	Favored (68.6%) *mt* chi angles: 304,177.8	0.04Å	Favored (41.539%) beta sheet	-	-	-
A 59		ARG	1.25	-	Favored (22.82%) General / -98.0,149.1	Favored (63.8%) *mmm-85* chi angles: 298.8,296.5,295.7,272.2	0.05Å	Favored (52.844%)	-	-	-
A 60		SER	1.33	-	Favored (6.71%) General / -150.1,119.1	Favored (45.9%) *t* chi angles: 179.6	0.04Å	Favored (8.979%)	-	-	-
**#**	**Alt**	**Res**	**High B**	**Clash > 0.4Å**	**Ramachandran**	**Rotamer**	**Cβ deviation**	**CaBLAM**	**Bond lengths**	**Bond angles**	**Cis Peptides**
			Avg: 1.07	Clashscore: 0.83	Outliers: 4 of 615	Poor rotamers: 0 of 510	Outliers: 0 of 564	Outliers: 16 of 613	Outliers: 5 of 617	Outliers: 10 of 617	Non-Trans: 1 of 616
A 61		GLY	1.39	-	Favored (35.21%) Glycine / 62.7,-125.5	-	-	Favored (62.662%)	-	-	-
A 62		GLU	1.43	-	Favored (38.02%) General / -89.4,-13.3	Favored (96.9%) *mt-10* chi angles: 295.6,176.5,357.4	0.04Å	Favored (11.554%)	-	-	-
A 63		GLY	1.44	-	Favored (13.02%) Glycine / -114.7,-160.4	-	-	Favored (18.567%)	-	-	-
A 64		ARG	1.42	-	Favored (45.98%) General / -133.3,137.6	Favored (71.8%) *ttp80* chi angles: 180.9,167.5,59,81	0.04Å	Favored (29.185%)	-	-	-
A 65		LEU	1.37	-	Favored (16.9%) General / -112.5,108.0	Favored (75.8%) *mt* chi angles: 302.4,176.3	0.06Å	Favored (66.843%)	-	-	-
A 66		ASP	1.31	-	Favored (73.19%) Pre-Pro / -80.4,152.6	Favored (98.4%) *m-30* chi angles: 287.2,346.1	0.06Å	Favored (27.824%)	-	OUTLIER(S) worst is CA-CB-CG: 4.8 σ	-
A 67		PRO	1.26	-	Favored (68.03%) Trans-Pro / -68.2,146.0	Favored (63.7%) *Cg_endo* chi angles: 26.7,326.4,26	0.04Å	Favored (46.646%)	-	-	-
A 68		TYR	1.23	-	Favored (7.94%) General / -116.8,-23.9	Favored (85.3%) *m-80* chi angles: 289.9,88.3	0.08Å	Favored (23.155%)	-	-	-
A 69		TRP	1.2	-	Favored (28.26%) General / -150.2,147.9	Favored (72.6%) *t60* chi angles: 176.3,91.6	0.03Å	Favored (13.66%)	-	-	-
A 70		GLY	1.17	-	Favored (37%) Glycine / -165.2,164.2	-	-	Favored (45.852%)	-	-	-
A 71		ASP	1.13	-	Favored (3%) General / -150.5,101.9	Favored (47.3%) *t0* chi angles: 180.7,338.5	0.07Å	Favored (18.983%) beta sheet	-	-	-

#	Alt	Res	High B	Clash > 0.4Å	Ramachandran	Rotamer	Cβ deviation	CaBLAM	Bond lengths	Bond angles	Cis Peptides
A 72		VAL	1.08	-	Favored (12.89%) Ile or Val / -74.4,-9.4	Favored (27.3%) *m* chi angles: 299.8	0.06Å	Favored (28.84%)	-	-	-
A 73		LYS	1.02	-	Favored (14.75%) General / -85.1,-42.5	Favored (55%) *tttp* chi angles: 183.4,173.8,174.8,65.1	0.05Å	Favored (25.759%) alpha helix	-	-	-
A 74		GLN	0.96	-	Favored (58.13%) General / -87.8,-6.5	Favored (87.5%) *mm-40* chi angles: 295.3,290.7,306	0.05Å	Favored (44.09%)	-	-	-
A 75		ASP	0.9	-	Favored (11.69%) General / 61.4,42.4	Favored (41.5%) *t0* chi angles: 192.7,21	0.04Å	Favored (31.754%)	-	-	-
A 76		LEU	0.86	-	Favored (45.42%) General / -123.0,148.5	Favored (65.4%) *mt* chi angles: 301.8,172.4	0.04Å	Favored (15.086%) beta sheet	-	-	-
A 77		VAL	0.84	-	Favored (20.1%) Ile or Val / -147.3,142.7	Favored (6.6%) *p* chi angles: 59.6	0.07Å	Favored (51.492%) beta sheet	-	-	-
A 78		SER	0.85	-	Favored (41.25%) General / -116.5,148.0	Favored (95.6%) *p* chi angles: 66.1	0.06Å	Favored (71.241%)	-	-	-
A 79		TYR	0.87	-	Favored (13.99%) General / -117.7,12.6	Favored (90.5%) *m-80* chi angles: 291.1,90.9	0.07Å	Favored (8.126%)	-	-	-
A 80		CYS	0.9	-	Allowed (0.48%) General / -160.5,72.6	Favored (31.4%) *t* chi angles: 189	0.04Å	CaBLAM Outlier (0.453%)	-	-	-
**#**	**Alt**	**Res**	**High B**	**Clash > 0.4Å**	**Ramachandran**	**Rotamer**	**Cβ deviation**	**CaBLAM**	**Bond lengths**	**Bond angles**	**Cis Peptides**
			Avg: 1.07	Clashscore: 0.83	Outliers: 4 of 615	Poor rotamers: 0 of 510	Outliers: 0 of 564	Outliers: 16 of 613	Outliers: 5 of 617	Outliers: 10 of 617	Non-Trans: 1 of 616
A 81		GLY	0.92	-	Favored (40.14%) Glycine / 92.5,-178.9	-	-	Favored (56.554%)	-	-	-
A 82		PRO	0.92	-	Favored (60.45%) Trans-Pro / -60.1,153.2	Favored (52.8%) *Cg_exo* chi angles: 336.9,34.4,328.8	0.12Å	CaBLAM Disfavored (3.905%)	-	-	-
A 83		TRP	0.91	-	Favored (27.29%) General / -57.3,126.5	Favored (62.1%) *t-100* chi angles: 183.9,264.6	0.09Å	Favored (21.137%)	-	-	-
A 84		LYS	0.89	-	Favored (15.78%) General / -112.7,1.7	Favored (21.5%) *mmtp* chi angles: 297.1,291.9,185.3,61.7	0.04Å	Favored (12.329%)	-	-	-
A 85		LEU	0.87	-	Favored (8.58%) General / -85.7,81.5	Favored (43.8%) *mt* chi angles: 306.5,183	0.04Å	Favored (9.304%)	-	-	-
A 86		ASP	0.85	-	Favored (37.27%) General / -81.3,-24.4	Favored (87.3%) *m-30* chi angles: 292.5,334.2	0.04Å	Favored (9.841%)	-	-	-
A 87		ALA	0.84	-	Favored (29.8%) General / -75.4,160.9	-	0.04Å	Favored (13.499%)	-	-	-

A 88		ALA	0.83	-	Favored (43.84%) General / -147.3,158.1	-	0.02Å	Favored (32.12%)	-	-	-
A 89		TRP	0.82	-	Favored (36.52%) General / -62.2,151.3	Favored (77.2%) *p-90* chi angles: 61.2,272.8	0.15Å	Favored (22.77%)	-	-	-
A 90		ASP	0.81	-	Favored (6.33%) General / -127.2,-3.0	Favored (55.8%) *p0* chi angles: 61.9,1.1	0.02Å	CaBLAM Disfavored (2.961%)	-	-	-
A 91		GLY	0.78	-	Favored (78.63%) Glycine / 78.8,-0.2	-	-	Favored (44.478%)	-	-	-
A 92		LEU	0.75	-	Favored (3.22%) General / -115.3,-42.0	Favored (88%) *mt* chi angles: 299.5,176.2	0.07Å	CaBLAM Disfavored (2.725%)	-	-	-
A 93		SER	0.71	-	Favored (24.98%) General / -80.1,162.1	Favored (96.7%) *p* chi angles: 63.7	0.05Å	Favored (11.349%)	-	-	-
A 94		GLU	0.68	-	Favored (22.1%) General / -90.4,149.9	Favored (96.4%) *mt-10* chi angles: 294,182.3,349.3	0.06Å	Favored (42.146%)	-	-	-
A 95		VAL	0.65	-	Favored (26.2%) Ile or Val / -127.7,165.7	Favored (21%) *m* chi angles: 302.1	0.06Å	Favored (45.948%) beta sheet	-	-	-
A 96		GLN	0.63	-	Favored (47.83%) General / -121.7,125.9	Favored (85.1%) *mt0* chi angles: 294.7,181.3,351	0.01Å	Favored (48.681%) beta sheet	-	-	-
A 97		LEU	0.63	-	Favored (34.67%) General / -96.6,119.2	Favored (48.8%) *tp* chi angles: 173.6,65.2	0.04Å	Favored (66.415%) beta sheet	-	-	-
A 98		LEU	0.65	-	Favored (5.51%) General / -90.9,64.5	Favored (74.7%) *mt* chi angles: 301.8,180.9	0.02Å	Favored (44.028%) beta sheet	-	-	-
A 99		ALA	0.68	-	Favored (55.94%) General / -61.9,133.8	-	0.02Å	Favored (14.392%) beta sheet	-	-	-
A 100		VAL	0.72	-	Favored (73.56%) Pre-Pro / -128.2,79.7	Favored (17.8%) *t* chi angles: 187.4	0.09Å	Favored (18.832%)	-	-	-
**#**	**Alt**	**Res**	**High B**	**Clash > 0.4Å**	**Ramachandran**	**Rotamer**	**Cβ deviation**	**CaBLAM**	**Bond lengths**	**Bond angles**	**Cis Peptides**
			Avg: 1.07	Clashscore: 0.83	Outliers: 4 of 615	Poor rotamers: 0 of 510	Outliers: 0 of 564	Outliers: 16 of 613	Outliers: 5 of 617	Outliers: 10 of 617	Non-Trans: 1 of 616
A 101		PRO	0.77	-	Favored (59.58%) Trans-Pro / -71.0,156.7	Favored (53.6%) *Cg_endo* chi angles: 25.6,328.6,23.9	0.04Å	Favored (16.662%)	-	-	-
A 102		PRO	0.81	-	Favored (60%) Trans-Pro / -54.1,134.4	Favored (98.8%) *Cg_exo* chi angles: 332.2,36.6,330.5	0.09Å	Favored (48.441%)	-	-	-
A 103		GLY	0.84	-	Favored (86.71%) Glycine / 81.2,-0.9	-	-	Favored (71.789%)	-	-	-

#	Alt	Res	High B	Clash > 0.4Å	Ramachandran	Rotamer	Cβ deviation	CaBLAM	Bond lengths	Bond angles	Cis Peptides
A 104		GLU	0.84	-	Favored (26.06%) General / -128.2,162.8	Favored (85.9%) *mt-10* chi angles: 300.4,184.4,3.6	0.03Å	Favored (24.425%)	-	-	-
A 105		ARG	0.83	-	Favored (28.25%) General / -78.1,160.1	Favored (98.7%) *mtt180* chi angles: 293.6,180.1,180.6,176.9	0.03Å	Favored (22.777%)	-	-	-
A 106		ALA	0.79	-	Favored (56.59%) General / -60.5,141.3	-	0.03Å	Favored (22.586%)	-	-	-
A 107		ARG	0.75	-	Favored (38.82%) General / -117.8,150.6	Favored (97.7%) *mtt-85* chi angles: 295.5,178.8,182.1,273.3	0.05Å	Favored (54.699%) beta sheet	-	-	-
A 108		ASN	0.72	-	Favored (16.37%) General / -113.4,107.8	Favored (44.1%) *t0* chi angles: 182.3,327.2	0.07Å	Favored (47.489%) beta sheet	-	-	-
A 109		ILE	0.69	-	Favored (59.39%) Ile or Val / -112.6,119.0	Favored (3.7%) *mp* chi angles: 302,91.7	0.05Å	Favored (63.788%) beta sheet	-	-	-
A 110		GLN	0.68	-	Favored (33.92%) General / -88.0,133.5	Favored (47.5%) *tt0* chi angles: 185.8,167.9,332.8	0.10Å	Favored (21.43%) beta sheet	-	-	-
A 111		THR	0.68	-	Favored (36.98%) General / -153.6,155.9	Favored (8.3%) *t* chi angles: 183.9	0.09Å	Favored (36.294%) beta sheet	-	-	-
A 112		LEU	0.71	-	Favored (62.84%) Pre-Pro / -98.7,118.6	Favored (62.1%) *tp* chi angles: 176.6,64.3	0.09Å	Favored (33.95%) beta sheet	-	-	-
A 113		PRO	0.76	-	Favored (92.23%) Trans-Pro / -63.6,148.7	Favored (42.5%) *Cg_exo* chi angles: 338.2,34.1,327.3	0.06Å	Favored (50.48%)	-	-	-
A 114		GLY	0.84	-	Favored (23.11%) Glycine / -106.1,-166.2	-	-	Favored (48.684%)	-	-	-
A 115		ILE	0.95	-	Favored (70.53%) Ile or Val / -125.8,126.7	Favored (81.2%) *mt* chi angles: 299.1,173.7	0.10Å	Favored (16.49%)	-	-	-
A 116		PHE	1.1	-	Favored (40.06%) General / -100.0,120.6	Favored (51.6%) *t80* chi angles: 178.2,61.8	0.12Å	Favored (64.042%) beta sheet	-	OUTLIER(S) worst is CA-CB-CG: 6.0 σ	-
A 117		LYS	1.25	-	Favored (41.74%) General / -97.8,123.3	Favored (12.8%) *ttmm* chi angles: 186.9,177.5,293.1,294.1	0.01Å	Favored (48.15%)	-	-	-
A 118		THR	1.36	-	Favored (5.1%) General / -126.3,179.8	Favored (19.5%) *p* chi angles: 72.8	0.10Å	Favored (18.909%)	-	-	-
A 119		LYS	1.41	-	Favored (64.77%) General / -63.4,-19.6	Favored (97.9%) *mttt* chi angles: 291.4,179.8,179.6,178.8	0.01Å	Favored (44.778%)	-	-	-
A 120		ASP	1.37	-	Favored (41.9%) General / -101.1,9.5	Favored (73.6%) *m-30* chi angles: 295.8,317.9	0.05Å	Favored (48.83%)	-	-	-

			Avg: 1.07	Clashscore: 0.83	Outliers: 4 of 615	Poor rotamers: 0 of 510	Outliers: 0 of 564	Outliers: 16 of 613	Outliers: 5 of 617	Outliers: 10 of 617	Non-Trans: 1 of 616
A 121		GLY	1.26	-	Favored (19.52%) Glycine / 106.5,-173.4	-	-	Favored (33.966%)	-	-	-
A 122		ASP	1.13	-	Favored (34.49%) General / -82.4,128.7	Favored (88.7%) *m-30* chi angles: 292,349.8	0.03Å	Favored (7.471%)	-	-	-
A 123		ILE	1	-	Favored (71.3%) Ile or Val / -124.4,132.5	Favored (72.1%) *mt* chi angles: 301.9,170	0.04Å	Favored (54.02%) beta sheet	-	-	-
A 124		GLY	0.89	-	Favored (51.54%) Glycine / -66.0,153.7	-	-	Favored (41.535%) beta sheet	-	-	-
A 125		ALA	0.83	-	Favored (31.65%) General / -149.8,150.2	-	0.04Å	Favored (59.437%) beta sheet	-	-	-
A 126		VAL	0.81	-	Favored (62.7%) Ile or Val / -110.2,121.2	Favored (94.4%) *t* chi angles: 175.8	0.08Å	Favored (58.007%) beta sheet	-	-	-
A 127		ALA	0.8	-	Favored (9.91%) General / -85.7,67.0	-	0.03Å	Favored (12.967%) beta sheet	-	-	-
A 128		LEU	0.81	-	Favored (48.92%) General / -125.1,147.7	Favored (81.3%) *mt* chi angles: 298.4,171.6	0.04Å	Favored (28.845%) beta sheet	-	-	-
A 129		ASP	0.82	-	Favored (25.22%) General / -107.6,112.5	Favored (82.1%) *m-30* chi angles: 295.1,345.4	0.02Å	Favored (25.846%) beta sheet	-	-	-
A 130		TYR	0.83	-	Favored (78.74%) Pre-Pro / -131.2,157.2	Favored (97%) *m-80* chi angles: 293,93.2	0.07Å	Favored (26.016%) beta sheet	-	-	-
A 131		PRO	0.81	-	Favored (52.47%) Trans-Pro / -58.1,152.4	Favored (77.6%) *Cg_exo* chi angles: 334.8,34.2,331.4	0.05Å	Favored (82.855%)	-	-	-
A 132		ALA	0.78	-	Favored (60.97%) General / -56.6,-28.8	-	0.04Å	Favored (38.327%)	-	-	-
A 133		GLY	0.74	-	Favored (38.64%) Glycine / -67.9,-5.3	-	-	Favored (42.126%)	-	-	-
A 134		THR	0.7	-	Favored (48.35%) General / -94.1,-5.0	Favored (75.8%) *p* chi angles: 61.2	0.06Å	Favored (21.886%)	-	-	-
A 135		SER	0.67	-	Favored (53.17%) General / -56.7,138.1	Favored (53.2%) *m* chi angles: 292.1	0.09Å	Favored (13.71%)	-	-	-
A 136		GLY	0.65	-	Favored (77.99%) Glycine / 92.4,-7.2	-	-	Favored (75.207%)	-	-	-
A 137		SER	0.64	-	Favored (85.6%) Pre-Pro / -58.5,136.2	Favored (65.3%) *m* chi angles: 297.2	0.07Å	Favored (32.007%)	-	-	-

A 138		PRO	0.65	-	Favored (64.54%) Trans-Pro / -70.4,150.8	Favored (65%) *Cg_endo* chi angles: 26.8,326.1,26.7	0.03Å	Favored (77.761%) beta sheet	-	-	-
A 139		ILE	0.67	-	Favored (67.82%) Ile or Val / -110.6,124.9	Favored (75.6%) *mt* chi angles: 300.9,169.3	0.06Å	Favored (66.779%) beta sheet	-	-	-
A 140		LEU	0.71	-	Favored (28.69%) General / -108.6,149.6	Favored (6.6%) *mp* chi angles: 282.6,63	0.04Å	Favored (45.976%)	-	-	-
**#**	**Alt**	**Res**	**High B**	**Clash > 0.4Å**	**Ramachandran**	**Rotamer**	**Cβ deviation**	**CaBLAM**	**Bond lengths**	**Bond angles**	**Cis Peptides**
			Avg: 1.07	Clashscore: 0.83	Outliers: 4 of 615	Poor rotamers: 0 of 510	Outliers: 0 of 564	Outliers: 16 of 613	Outliers: 5 of 617	Outliers: 10 of 617	Non-Trans: 1 of 616
A 141		ASP	0.74	-	Favored (6.05%) General / -84.4,-176.0	Favored (49.6%) *p0* chi angles: 68.3,13	0.05Å	Favored (37.245%)	-	-	-
A 142		LYS	0.77	-	Favored (56.33%) General / -62.0,-17.2	Favored (59.5%) *pttt* chi angles: 68.6,181.6,182.4,180.4	0.01Å	Favored (32.157%)	-	-	-
A 143		CYS	0.78	-	Favored (39.35%) General / -98.4,-2.3	Favored (73.6%) *m* chi angles: 297.4	0.05Å	Favored (44.309%)	-	-	-
A 144		GLY	0.77	-	Favored (65.8%) Glycine / 89.1,10.8	-	-	Favored (89.372%)	-	-	-
A 145		ARG	0.76	-	Favored (26.75%) General / -88.2,143.9	Favored (83.2%) *mtm180* chi angles: 298.6,180,297.7,177.2	0.07Å	Favored (34.568%)	-	-	-
A 146		VAL	0.74	-	Favored (39.52%) Ile or Val / -80.4,126.5	Favored (86.1%) *t* chi angles: 177.6	0.06Å	Favored (47.224%)	-	-	-
A 147		ILE	0.72	-	Favored (7.28%) Ile or Val / -110.2,17.8	Favored (39.1%) *pt* chi angles: 61.1,167.3	0.10Å	CaBLAM Disfavored (1.029%)	-	-	-
A 148		GLY	0.72	-	Favored (12.98%) Glycine / 129.0,171.5	-	-	Favored (15.332%)	-	-	-
A 149		LEU	0.75	-	Favored (26.66%) General / -108.3,150.9	Favored (59.8%) *mt* chi angles: 304.9,179.9	0.06Å	Favored (12.566%)	-	-	-
A 150		TYR	0.8	-	Favored (17.7%) General / -85.9,164.1	Favored (60.2%) *t80* chi angles: 188.5,82.5	0.11Å	CA Geom Outlier (0.419%)	-	-	-
A 151		GLY	0.88	-	Favored (5.22%) Glycine / 118.4,-32.5	-	-	CaBLAM Disfavored (4.339%)	-	-	-
A 152		ASN	0.99	-	Favored (22.53%) General / -82.7,117.3	Favored (30.6%) *t0* chi angles: 183.6,306.8	0.06Å	CaBLAM Outlier (0.72%)	-	-	-
A 153		GLY	1.13	-	Favored (31.5%) Glycine / -170.7,-167.8	-	-	Favored (33.431%)	-	-	-

#	Alt	Res	High B	Clash > 0.4Å	Ramachandran	Rotamer	Cβ deviation	CaBLAM	Bond lengths	Bond angles	Cis Peptides
A 154		VAL	1.3	-	Favored (28.78%) Ile or Val / -127.9,164.7	Favored (25.8%) *m* chi angles: 299.4	0.07Å	Favored (17.517%) beta sheet	-	-	-
A 155		VAL	1.46	-	Favored (13.83%) Ile or Val / -103.2,144.5	Favored (8.7%) *p* chi angles: 65.5	0.03Å	Favored (49.741%)	-	-	-
A 156		ILE	1.58	-	Favored (3.12%) Ile or Val / -115.7,174.6	Favored (43.5%) *pt* chi angles: 62,169.1	0.09Å	Favored (26.791%)	-	-	-
A 157		LYS	1.62	-	Favored (62.97%) General / -58.0,-26.9	Favored (97.5%) *mttt* chi angles: 290.3,179.2,180.2,178.3	0.01Å	Favored (38.268%)	-	-	-
A 158		ASN	1.57	-	Favored (37.66%) General / -89.0,6.8	Favored (54.1%) *p0* chi angles: 67.6,12.4	0.07Å	Favored (55.877%)	-	-	-
A 159		GLY	1.43	-	Favored (67.89%) Glycine / 93.3,-13.3	-	-	Favored (56.954%)	-	-	-
A 160		SER	1.25	-	Favored (9.99%) General / -83.7,175.0	Favored (50.3%) *m* chi angles: 291.4	0.06Å	Favored (30.436%)	-	-	-
**#**	**Alt**	**Res**	**High B**	**Clash > 0.4Å**	**Ramachandran**	**Rotamer**	**Cβ deviation**	**CaBLAM**	**Bond lengths**	**Bond angles**	**Cis Peptides**
			Avg: 1.07	Clashscore: 0.83	Outliers: 4 of 615	Poor rotamers: 0 of 510	Outliers: 0 of 564	Outliers: 16 of 613	Outliers: 5 of 617	Outliers: 10 of 617	Non-Trans: 1 of 616
A 161		TYR	1.07	-	Favored (35.15%) General / -117.0,152.2	Favored (77.1%) *m-80* chi angles: 294.1,81.1	0.04Å	Favored (49.113%) beta sheet	-	-	-
A 162		VAL	0.93	-	Favored (50.97%) Ile or Val / -124.4,117.6	Favored (76.8%) *t* chi angles: 178.2	0.06Å	Favored (55.681%) beta sheet	-	-	-
A 163		SER	0.84	-	Favored (35.94%) General / -101.3,117.9	Favored (27.7%) *t* chi angles: 172	0.05Å	Favored (55.943%) beta sheet	-	-	-
A 164		ALA	0.8	-	Favored (57.83%) General / -62.1,142.0	-	0.06Å	Favored (39.032%) beta sheet	-	-	-
A 165		ILE	0.82	-	Favored (31.6%) Ile or Val / -85.3,118.1	Favored (83.1%) *mt* chi angles: 299.6,171.4	0.03Å	Favored (51.758%) beta sheet	-	-	-
A 166		THR	0.89	-	Favored (9.42%) General / -104.3,99.7	Favored (36.4%) *p* chi angles: 54.1	0.09Å	Favored (25.282%) beta sheet	-	-	-
A 167		GLN	1.04	-	Favored (5.68%) General / -110.6,-36.6	Favored (18.3%) *tm-30* chi angles: 188.8,265.2,322.3	0.11Å	CaBLAM Outlier (0.025%)	-	-	-
A 168		GLY	1.27	-	Favored (48.81%) Glycine / 59.3,-129.2	-	-	Favored (10.275%)	-	-	-
A 169		LYS	1.6	-	Favored (13.15%) General / -155.4,135.6	Favored (88.2%) *tttt* chi angles: 184.2,175.9,179,179.9	0.01Å	CaBLAM Disfavored (1.193%)	-	-	-

A 170		ARG	2.03	-	Favored (50.24%)<br>General / -65.9,133.1	Favored (68.6%) *ttt180*<br>chi angles: 174.7,173.5,170.1,170.4	0.04Å	Favored (42.956%)	-	-	-
A 171		GLU	2.55	-	Favored (52.85%)<br>General / -127.8,138.8	Favored (49.8%) *mt-10*<br>chi angles: 298.7,185.1,118.3	0.00Å	Favored (57.318%)<br>beta sheet	-	-	-
A 172		GLU	3.09	-	Favored (7.49%)<br>General / -84.4,90.0	Favored (49.7%) *mm-30*<br>chi angles: 290.9,289.5,302.2	0.08Å	Favored (53.597%)<br>beta sheet	-	-	-
A 173		GLU	3.51	-	Favored (34.4%)<br>General / -56.8,-22.2	Favored (21.8%) *pt0*<br>chi angles: 72.3,183,6.7	0.04Å	Favored (6.974%)<br>beta sheet	-	-	-
A 174		THR	3.65	-	OUTLIER (0.04)%)<br>Pre-Pro / 67.3,132.8	Favored (91.6%) *m*<br>chi angles: 301.1	0.13Å	CaBLAM Disfavored (1.656%)<br>try beta sheet	-	-	-
A 175		PRO	3.43	-	Favored (71.62%)<br>Trans-Pro / -63.0,-20.5	Favored (37.8%) *Cg_endo*<br>chi angles: 22.9,325.6,31.2	0.01Å	CaBLAM Disfavored (1.124%)<br>try beta sheet	-	-	-
A 176		VAL	2.94	-	Allowed (0.15%)<br>Ile or Val / 61.2,-63.2	Favored (68%) *t*<br>chi angles: 171.9	0.12Å	Favored (51.54%)<br>beta sheet	-	-	-
A 177		GLU	2.36	-	Allowed (0.33%)<br>General / 65.5,-56.1	Favored (98.5%) *mt-10*<br>chi angles: 293.8,176.5,355.2	0.03Å	Favored (55.504%)<br>beta sheet	-	-	-
A 178		CYS	1.83	-	Favored (15.97%)<br>General / 49.1,51.6	Favored (74.6%) *m*<br>chi angles: 297.1	0.05Å	CaBLAM Disfavored (4.474%)<br>try beta sheet	-	-	-
A 179		PHE	1.42	-	Favored (23.13%)<br>General / -153.0,144.2	Favored (70.1%) *t80*<br>chi angles: 185.2,74.6	0.01Å	Favored (14.98%)<br>beta sheet	-	-	-
A 180		GLU	1.14	-	Favored (24.58%)<br>Pre-Pro / -133.8,135.4	Favored (34.2%) *tt0*<br>chi angles: 181.5,175.7,289.3	0.06Å	Favored (51.317%)	-	-	-
**#**	**Alt**	**Res**	**High B**	**Clash > 0.4Å**	**Ramachandran**	**Rotamer**	**Cβ deviation**	**CaBLAM**	**Bond lengths**	**Bond angles**	**Cis Peptides**
			Avg: 1.07	Clashscore: 0.83	Outliers: 4 of 615	Poor rotamers: 0 of 510	Outliers: 0 of 564	Outliers: 16 of 613	Outliers: 5 of 617	Outliers: 10 of 617	Non-Trans: 1 of 616
A 181		PRO	0.96	-	Favored (12.55%)<br>Trans-Pro / -48.8,-28.1	Favored (87.4%) *Cg_exo*<br>chi angles: 330.2,36.4,332.7	0.01Å	Favored (48.045%)	-	-	-
A 182		SER	0.84	-	Favored (63.09%)<br>General / -61.6,-20.2	Favored (90.3%) *p*<br>chi angles: 68.6	0.04Å	Favored (60.617%)	-	-	-
A 183		MET	0.77	-	Favored (42.42%)<br>General / -74.0,-5.6	Favored (85.3%) *mtp*<br>chi angles: 292.2,171.1,59.5	0.08Å	Favored (33.103%)<br>three-ten	-	-	-
A 184		LEU	0.73	-	Favored (36.97%)<br>General / -99.6,-1.9	Favored (81%) *mt*<br>chi angles: 302.2,177.6	0.07Å	Favored (55.657%)	-	-	-
A 185		LYS	0.7	-	Favored (33.72%)<br>General / -87.4,133.4	Favored (98.4%) *mttt*<br>chi angles: 293.4,181.5,179.7,180.7	0.03Å	Favored (27.204%)	-	-	-

A 186		LYS	0.68	-	Favored (47.8%) General / -59.3,143.9	Favored (98.1%) *mttt* chi angles: 292.6,181.9,179.7,180.2	0.01Å	Favored (13.706%)	-	-	-
A 187		LYS	0.68	-	Allowed (1.04%) General / 84.2,-6.8	Favored (11.2%) *tptm* chi angles: 184.4,68.3,174.3,292.8	0.06Å	CaBLAM Disfavored (3.695%)	-	-	-
A 188		GLN	0.69	-	Favored (39.03%) General / -95.6,134.0	Favored (50.6%) *tt0* chi angles: 183.4,171.1,315.3	0.02Å	Favored (24.857%)	-	-	-
A 189		LEU	0.71	-	Favored (29.2%) General / -120.2,117.9	Favored (5.3%) *tt* chi angles: 192.4,152.6	0.04Å	Favored (67.793%) beta sheet	-	-	-
A 190		THR	0.76	-	Favored (53.05%) General / -116.9,136.3	Favored (88.4%) *m* chi angles: 297	0.05Å	Favored (62.83%) beta sheet	-	-	-
A 191		VAL	0.82	-	Favored (69.11%) Ile or Val / -112.3,128.1	Favored (54.8%) *t* chi angles: 180.7	0.04Å	Favored (67.196%) beta sheet	-	-	-
A 192		LEU	0.91	-	Favored (23.04%) General / -101.7,111.0	Favored (83.4%) *mt* chi angles: 299.7,179.4	0.04Å	Favored (64.011%) beta sheet	-	-	-
A 193		ASP	1.02	-	Favored (6.64%) General / -82.5,87.6	Favored (51%) *m-30* chi angles: 295.2,356.5	0.01Å	Favored (52.461%) beta sheet	-	-	-
A 194		LEU	1.15	-	Favored (31.48%) General / -108.2,116.2	Favored (54%) *mt* chi angles: 305.6,176.2	0.07Å	Favored (46.482%) beta sheet	-	-	-
A 195		HIS	1.27	-	Favored (66.56%) Pre-Pro / -61.8,154.6	Favored (13.1%) *p90* chi angles: 76.6,78.2	0.08Å	Favored (45.594%)	-	OUTLIER(S) worst is CA-CB-CG: 4.6 σ	-
A 196		PRO	1.37	-	Favored (39.87%) Trans-Pro / -56.8,-21.4	Favored (65.8%) *Cg_exo* chi angles: 335.6,40,321.6	0.02Å	Favored (18.323%)	-	-	-
A 197		GLY	1.42	-	Favored (52.14%) Glycine / -104.2,12.7	-	-	Favored (63.786%)	-	-	-
A 198		ALA	1.43	-	Favored (44.01%) General / -78.0,-3.8	-	0.04Å	CaBLAM Disfavored (4.326%)	-	-	-
A 199		GLY	1.42	-	Favored (51.04%) Glycine / 76.7,32.3	-	-	Favored (68.378%)	-	-	-
A 200		LYS	1.37	-	Favored (51.61%) General / -56.1,-26.8	Favored (13.8%) *pttp* chi angles: 65.7,180,173,58.1	0.07Å	Favored (17.973%)	-	-	-
**#**	**Alt**	**Res**	**High B**	**Clash > 0.4Å**	**Ramachandran**	**Rotamer**	**Cβ deviation**	**CaBLAM**	**Bond lengths**	**Bond angles**	**Cis Peptides**
			Avg: 1.07	Clashscore: 0.83	Outliers: 4 of 615	Poor rotamers: 0 of 510	Outliers: 0 of 564	Outliers: 16 of 613	Outliers: 5 of 617	Outliers: 10 of 617	Non-Trans: 1 of 616
A 201		THR	1.31	-	Favored (9.06%) General / -93.8,-42.5	Favored (91%) *m* chi angles: 298.9	0.04Å	Favored (51.726%) alpha helix	-	-	-

A<br>202		ARG	1.23	-	Favored<br>(24.74%)<br>General /<br>-83.8,-34.3	Favored (75.9%)<br>*ttm-80*<br>chi angles:<br>191.3,184.6,293.8,275.2	0.10Å	Favored<br>(53.574%)<br>alpha helix	OUTLIER(S)<br>worst is NE--<br>CZ: 4.1 σ	-	-
A<br>203		ARG	1.15	-	Favored<br>(2.25%)<br>General /<br>-102.4,-56.5	Favored (43.5%)<br>*ttp-170*<br>chi angles:<br>180.9,163.4,57.6,179	0.06Å	Favored<br>(18.694%)<br>alpha helix	-	-	-
A<br>204		VAL	1.06	-	Favored<br>(41.31%)<br>Ile or Val /<br>-72.6,-49.4	Favored (88.6%) *t*<br>chi angles: 174	0.02Å	Favored<br>(67.188%)<br>alpha helix	-	-	-
A<br>205		LEU	0.98	0.49Å<br>HB3 with A<br>206 PRO<br>HD3	Favored<br>(50.14%)<br>Pre-Pro /<br>-52.8,-53.8	Favored (63.9%) *tp*<br>chi angles: 177.2,64.4	0.12Å	Favored<br>(83.197%)<br>alpha helix	-	-	-
A<br>206		PRO	0.91	0.49Å<br>HD3 with A<br>205 LEU<br>HB3	Favored<br>(51.69%)<br>Trans-Pro /<br>-50.8,-36.4	Favored (74.1%)<br>*Cg_exo*<br>chi angles:<br>328.9,37.8,331.3	0.01Å	Favored<br>(96.24%)<br>alpha helix	-	-	-
A<br>207		GLU	0.85	-	Favored<br>(70.9%)<br>General /<br>-71.5,-35.6	Favored (82.6%)<br>*mm-30*<br>chi angles:<br>290.3,299.5,307.8	0.07Å	Favored<br>(92.384%)<br>alpha helix	-	-	-
A<br>208		ILE	0.8	-	Favored<br>(91.39%)<br>Ile or Val /<br>-65.2,-46.2	Favored (98.1%) *mt*<br>chi angles: 292.7,168.6	0.16Å	Favored<br>(79.88%)<br>alpha helix	-	-	-
A<br>209		VAL	0.77	-	Favored<br>(91.12%)<br>Ile or Val /<br>-66.2,-42.5	Favored (94.8%) *t*<br>chi angles: 174.8	0.07Å	Favored<br>(81.761%)<br>alpha helix	-	-	-
A<br>210		ARG	0.75	-	Favored<br>(82.79%)<br>General /<br>-58.9,-39.9	Favored (91.8%)<br>*mtt180*<br>chi angles:<br>286.1,169.2,176.9,162.3	0.02Å	Favored<br>(79.265%)<br>alpha helix	-	-	-
A<br>211		GLU	0.74	-	Favored<br>(87.49%)<br>General /<br>-66.2,-38.3	Favored (68%) *mm-30*<br>chi angles:<br>291,291.8,309.5	0.05Å	Favored<br>(93.432%)<br>alpha helix	-	-	-
A<br>212		ALA	0.73	-	Favored<br>(76.04%)<br>General /<br>-61.9,-34.4	-	0.03Å	Favored<br>(84.398%)<br>alpha helix	-	-	-
A<br>213		ILE	0.74	-	Favored<br>(82.63%)<br>Ile or Val /<br>-68.6,-44.5	Favored (38.8%)<br>*mm*<br>chi angles: 298.2,303.9	0.14Å	Favored<br>(79.552%)<br>alpha helix	-	-	-
A<br>214		LYS	0.74	-	Favored<br>(74.38%)<br>General /<br>-60.5,-34.6	Favored (54.5%)<br>*mtmt*<br>chi angles:<br>288.5,190.3,286.8,182.8	0.06Å	Favored<br>(76.121%)<br>alpha helix	-	-	-
A<br>215		LYS	0.74	-	Favored<br>(45.64%)<br>General / -87.9,4.2	Favored (60.9%)<br>*mttm*<br>chi angles:<br>293.6,187.7,183,294.9	0.05Å	Favored<br>(50.94%)	-	-	-
A<br>216		ARG	0.74	-	Favored<br>(27.06%)<br>General / 55.7,43.9	Favored (14.2%)<br>*mpt180*<br>chi angles:<br>276.6,71.6,174.5,174.3	0.02Å	Favored<br>(29.679%)	-	-	-
A<br>217		LEU	0.73	-	Favored<br>(23.5%)<br>General /<br>-99.0,111.0	Favored (66%) *mt*<br>chi angles: 304.3,177.1	0.07Å	Favored<br>(44.166%)<br>beta sheet	-	-	-
A<br>218		ARG	0.71	-	Favored<br>(30.52%)<br>General /<br>-57.5,127.1	Favored (73.9%)<br>*ttm-80*<br>chi angles:<br>191.1,182.6,291.8,276.7	0.04Å	Favored<br>(33.363%)<br>beta sheet	-	-	-

A 219		THR	0.69	-	Favored (54.73%) General / -123.5,137.6	Favored (95.2%) *m* chi angles: 299.5	0.04Å	Favored (69.571%) beta sheet	-	-	-
A 220		VAL	0.68	-	Favored (58.96%) Ile or Val / -118.8,135.5	Favored (5.7%) *p* chi angles: 58.3	0.02Å	Favored (72.619%) beta sheet	-	-	-
**#**	**Alt**	**Res**	**High B**	**Clash > 0.4Å**	**Ramachandran**	**Rotamer**	**Cβ deviation**	**CaBLAM**	**Bond lengths**	**Bond angles**	**Cis Peptides**
			Avg: 1.07	Clashscore: 0.83	Outliers: 4 of 615	Poor rotamers: 0 of 510	Outliers: 0 of 564	Outliers: 16 of 613	Outliers: 5 of 617	Outliers: 10 of 617	Non-Trans: 1 of 616
A 221		ILE	0.68	-	Favored (72.64%) Ile or Val / -121.8,125.0	Favored (55.6%) *mt* chi angles: 305.5,170.3	0.08Å	Favored (72.193%) beta sheet	-	-	-
A 222		LEU	0.7	-	Favored (26.83%) General / -109.4,151.3	Favored (61.7%) *mt* chi angles: 304.4,176.2	0.07Å	Favored (46.828%) beta sheet	-	-	-
A 223		ALA	0.74	-	Favored (43.07%) Pre-Pro / -144.7,149.4	-	0.06Å	Favored (53.255%)	-	-	-
A 224		PRO	0.8	-	Favored (68.37%) Trans-Pro / -63.9,-23.3	Favored (40.4%) *Cg_endo* chi angles: 23.5,322.8,33.6	0.07Å	Favored (29.562%)	-	-	-
A 225		THR	0.85	-	Favored (48.11%) General / -136.0,156.9	Favored (60.8%) *p* chi angles: 63.9	0.03Å	Favored (24.13%) alpha helix	-	-	-
A 226		ARG	0.91	-	Favored (72.15%) General / -65.3,-31.1	Favored (87.2%) *mtt180* chi angles: 289.3,169.1,178,157.7	0.06Å	Favored (60.943%) alpha helix	-	-	-
A 227		VAL	0.95	-	Favored (85.2%) Ile or Val / -63.6,-48.8	Favored (64.2%) *t* chi angles: 171.4	0.07Å	Favored (74.604%) alpha helix	-	-	-
A 228		VAL	0.98	-	Favored (88.47%) Ile or Val / -65.3,-40.4	Favored (64%) *t* chi angles: 171.4	0.07Å	Favored (78.35%) alpha helix	-	-	-
A 229		ALA	1	-	Favored (75.49%) General / -58.0,-38.1	-	0.04Å	Favored (78.224%) alpha helix	-	-	-
A 230		ALA	1	-	Favored (85.42%) General / -60.9,-38.3	-	0.02Å	Favored (91.045%) alpha helix	-	-	-
A 231		GLU	1	-	Favored (80.88%) General / -64.9,-35.4	Favored (93.1%) *mt-10* chi angles: 292.4,187.6,350	0.09Å	Favored (88.686%) alpha helix	-	-	-
A 232		MET	1	-	Favored (85.51%) General / -67.4,-39.0	Favored (67.1%) *mtm* chi angles: 293.8,191.5,296.9	0.09Å	Favored (77.101%) alpha helix	-	OUTLIER(S) worst is CG-SD-CE: 4.1 σ	-
A 233		GLU	1	-	Favored (92.74%) General / -59.4,-45.0	Favored (46.4%) *tt0* chi angles: 184.9,177.9,56.4	0.05Å	Favored (78.555%) alpha helix	-	-	-
A 234		GLU	1	-	Favored (89.46%)	Favored (45.2%) *tt0* chi angles: 178.9,180.3,315.7	0.03Å	Favored (82.001%) alpha helix	-	-	-

					General / -63.2,-45.9						
A 235		ALA	0.99	-	Favored (70.95%) General / -58.6,-34.3	-	0.07Å	Favored (73.955%) alpha helix	-	-	-
A 236		LEU	0.98	-	Favored (18.8%) General / -101.0,18.8	Favored (88.5%) *mt* chi angles: 298.7,174.4	0.10Å	Favored (31.359%) alpha helix	-	-	-
A 237		ARG	0.95	-	Favored (46.3%) General / -50.4,-51.4	Favored (58.1%) *ttt-90* chi angles: 181,186.5,188.3,282	0.11Å	Favored (40.383%) alpha helix	-	-	-
A 238		GLY	0.92	-	Favored (3.86%) Glycine / -82.2,48.1	-	-	CaBLAM Outlier (0.18%)	-	-	-
A 239		LEU	0.88	-	Favored (72.24%) Pre-Pro / -138.7,157.3	Favored (81.5%) *mt* chi angles: 294.4,168.5	0.02Å	Favored (15.192%)	-	-	-
A 240		PRO	0.85	-	Favored (4.57%) Trans-Pro / -75.0,72.7	Favored (79.3%) *Cg_endo* chi angles: 30.2,323,28	0.04Å	Favored (6.942%) beta sheet	-	-	-
**#**	**Alt**	**Res**	**High B**	**Clash > 0.4Å**	**Ramachandran**	**Rotamer**	**Cβ deviation**	**CaBLAM**	**Bond lengths**	**Bond angles**	**Cis Peptides**
			Avg: 1.07	Clashscore: 0.83	Outliers: 4 of 615	Poor rotamers: 0 of 510	Outliers: 0 of 564	Outliers: 16 of 613	Outliers: 5 of 617	Outliers: 10 of 617	Non-Trans: 1 of 616
A 241		VAL	0.85	-	Favored (38.93%) Ile or Val / -116.7,140.5	Favored (10.5%) *p* chi angles: 62.5	0.04Å	Favored (24.955%) beta sheet	-	-	-
A 242		ARG	0.89	0.43Å NH1 with A 251 THR O	Favored (29.2%) General / -117.1,155.5	Favored (71%) *mtm180* chi angles: 299.8,186.7,293.7,182.8	0.12Å	Favored (49.603%) beta sheet	OUTLIER(S) worst is NE--CZ: 4.4 σ	-	-
A 243		TYR	1	-	Favored (52.67%) General / -120.4,127.8	Favored (63.7%) *t80* chi angles: 173.1,67.7	0.04Å	Favored (60.641%) beta sheet	-	-	-
A 244		MET	1.18	-	Favored (52.97%) General / -117.8,137.1	Favored (26.1%) *mmt* chi angles: 303,292,171.9	0.11Å	Favored (30.97%) beta sheet	-	-	-
A 245		THR	1.47	-	Favored (4.08%) General / -150.2,-172.1	Favored (10.8%) *t* chi angles: 188.4	0.15Å	Favored (17.698%)	-	-	-
A 246		THR	1.88	-	Favored (60.42%) General / -75.3,-16.5	Favored (69%) *p* chi angles: 62.6	0.05Å	Favored (42.066%)	-	-	-
A 247		ALA	2.41	-	Favored (57.86%) General / -87.4,-1.4	-	0.03Å	Favored (51.444%)	-	-	-
A 248		VAL	2.99	-	Favored (60.85%) Ile or Val / -111.7,119.8	Favored (79.5%) *t* chi angles: 178	0.06Å	Favored (29.3%)	-	-	-
A 249		ASN	3.5	-	Favored (9.16%) General / -84.9,65.4	Favored (87.3%) *m-40* chi angles: 294.3,318.4	0.02Å	CaBLAM Disfavored (3.277%)	-	-	-
A 250		VAL	3.81	-	OUTLIER (0)%)	Favored (81.2%) *t* chi angles: 176.6	0.08Å	CaBLAM Outlier	-	OUTLIER(S) worst is C-N-	-

#	Alt	Res	High B	Clash > 0.4Å	Ramachandran	Rotamer	Cβ deviation	CaBLAM	Bond lengths	Bond angles	Cis Peptides
					Ile or Val /<br>33.9,102.9			(0%)		CA: 4.2 σ	
A<br>251		THR	3.81	0.43Å<br>O with A 242<br>ARG NH1	Favored<br>(55.92%)<br>General /<br>-61.8,133.8	Favored (91.5%) *m*<br>chi angles: 299	0.05Å	Favored<br>(40.822%)	-	-	-
A<br>252		HIS	3.47	0.42Å<br>HB3 with A<br>253 SER HA	Favored<br>(14.01%)<br>General /<br>-123.4,167.3	Favored (84.6%) *m-70*<br>chi angles: 289.1,288.8	0.12Å	CA Geom<br>Outlier<br>(0.001%)	-	-	-
A<br>253		SER	2.91	0.42Å<br>HA with A<br>252 HIS HB3	Favored<br>(22.73%)<br>General /<br>-71.4,165.9	Favored (55.1%) *p*<br>chi angles: 73.8	0.16Å	CaBLAM<br>Disfavored<br>(4.263%)	-	OUTLIER(S)<br>worst is C-N-<br>CA: 4.5 σ	Cis<br>nonPRO<br>omega=<br>7.73
A<br>254		GLY	2.28	-	Favored<br>(50.74%)<br>Glycine /<br>-84.6,13.7	-	-	Favored<br>(10.623%)	-	-	-
A<br>255		THR	1.72	-	Favored<br>(54.64%)<br>General / -93.2,-2.9	Favored (58.1%) *p*<br>chi angles: 64.4	0.03Å	Favored<br>(12.558%)	-	-	-
A<br>256		GLU	1.3	-	Favored<br>(37.17%)<br>General /<br>-75.1,155.4	Favored (87.1%)<br>*mm-30*<br>chi angles:<br>299.1,292.4,342.1	0.04Å	Favored<br>(24.922%)	-	-	-
A<br>257		ILE	1.02	-	Favored<br>(5.14%)<br>Ile or Val /<br>-104.8,-39.1	Favored (92.6%) *mt*<br>chi angles: 296.5,171	0.10Å	Favored<br>(8.251%)	-	-	-
A<br>258		VAL	0.85	-	Favored<br>(66.05%)<br>Ile or Val /<br>-109.8,123.9	Favored (52.1%) *t*<br>chi angles: 181.1	0.09Å	Favored<br>(25.703%)	-	-	-
A<br>259		ASP	0.75	-	Favored<br>(41.5%)<br>General /<br>-109.7,120.1	Favored (95.3%) *m-30*<br>chi angles: 291.6,344.6	0.02Å	Favored<br>(70.559%)	-	-	-
A<br>260		LEU	0.7	-	Favored<br>(36.23%)<br>General /<br>-96.7,137.2	Favored (56.3%) *mt*<br>chi angles: 305.4,179.9	0.09Å	Favored<br>(39.863%)<br>beta sheet	-	-	-
**#**	**Alt**	**Res**	**High B**	**Clash > 0.4Å**	**Ramachandran**	**Rotamer**	**Cβ deviation**	**CaBLAM**	**Bond lengths**	**Bond angles**	**Cis Peptides**
			Avg:<br>1.07	Clashscore:<br>0.83	Outliers: 4 of<br>615	Poor rotamers: 0 of<br>510	Outliers:<br>0 of 564	Outliers:<br>16 of 613	Outliers: 5 of<br>617	Outliers: 10<br>of 617	Non-<br>Trans: 1<br>of 616
A<br>261		MET	0.7	-	Favored<br>(24.68%)<br>General /<br>-154.1,147.2	Favored (65.2%)<br>*ttm*<br>chi angles:<br>182.7,180.3,289.8	0.08Å	Favored<br>(38.541%)<br>beta sheet	-	-	-
A<br>262		CYS	0.7	-	Favored<br>(47.85%)<br>General /<br>-72.8,140.8	Favored (56.1%) *t*<br>chi angles: 181.8	0.07Å	Favored<br>(42.6%)	-	-	-
A<br>263		HIS	0.72	-	Favored<br>(61.61%)<br>General /<br>-52.4,-40.1	Favored (37.5%) *m-70*<br>chi angles: 289.1,312.3	0.10Å	Favored<br>(61.46%)	-	-	-
A<br>264		ALA	0.73	-	Favored<br>(81.86%)<br>General /<br>-60.9,-37.4	-	0.04Å	Favored<br>(77.783%)<br>alpha helix	-	-	-
A<br>265		THR	0.75	-	Favored<br>(64.73%)<br>General /<br>-72.2,-43.3	Favored (90%) *m*<br>chi angles: 298.2	0.03Å	Favored<br>(76.578%)<br>alpha helix	-	-	-
A<br>266		PHE	0.77	-	Favored<br>(66.08%)	Favored (93.4%)<br>*t80*	0.05Å	Favored<br>(84.075%)	-	-	-

#	Alt	Res	High B	Clash > 0.4Å	Ramachandran	Rotamer	Cβ deviation	CaBLAM	Bond lengths	Bond angles	Cis Peptides
					General / -57.8,-52.3	chi angles: 178.1,77.5		alpha helix			
A 267		THR	0.79	-	Favored (99.59%) General / -61.6,-43.0	Favored (92.4%) *m* chi angles: 297.7	0.05Å	Favored (84.397%) alpha helix	-	-	-
A 268		SER	0.81	-	Favored (94.28%) General / -60.1,-42.3	Favored (72.8%) *m* chi angles: 295.4	0.05Å	Favored (95.37%) alpha helix	-	-	-
A 269		ARG	0.87	-	Favored (84.66%) General / -64.3,-36.6	Favored (97.5%) *mtt-85* chi angles: 287.8,179.7,184.4,276.5	0.04Å	Favored (90.653%) alpha helix	-	-	-
A 270		LEU	0.97	-	Favored (81.72%) General / -67.1,-36.3	Favored (80.5%) *mt* chi angles: 291.5,176.5	0.02Å	Favored (90.238%) alpha helix	-	-	-
A 271		LEU	1.12	-	Favored (76.93%) General / -69.5,-37.1	Favored (94.6%) *mt* chi angles: 291.8,171.8	0.07Å	Favored (36.622%)	-	-	-
A 272		GLN	1.3	-	Favored (86.55%) Pre-Pro / -75.1,154.7	Favored (36.9%) *mt0* chi angles: 291.5,174.4,101.3	0.06Å	Favored (14.127%)	-	-	-
A 273		PRO	1.47	-	Favored (6.04%) Trans-Pro / -75.0,65.5	Favored (72.7%) *Cg_endo* chi angles: 31,322.6,27.8	0.03Å	Favored (7.512%)	-	-	-
A 274		ILE	1.58	-	Favored (74.1%) Ile or Val / -118.5,129.9	Favored (85.9%) *mt* chi angles: 298.9,170.8	0.05Å	Favored (23.967%)	-	-	-
A 275		ARG	1.58	-	Favored (34.89%) General / -62.3,128.2	Favored (83.7%) *ttt180* chi angles: 184.7,176.1,180.6,178.8	0.04Å	Favored (45.501%) beta sheet	-	-	-
A 276		VAL	1.45	-	Favored (57.45%) Pre-Pro / -93.5,124.4	Favored (78.2%) *t* chi angles: 178.1	0.06Å	Favored (40.823%) beta sheet	-	-	-
A 277		PRO	1.25	-	Favored (81.85%) Trans-Pro / -58.5,148.3	Favored (65.4%) *Cg_exo* chi angles: 335.6,34,330.8	0.04Å	Favored (82.357%) beta sheet	-	-	-
A 278		ASN	1.04	-	Favored (6.55%) General / -82.3,87.8	Favored (82.1%) *m-40* chi angles: 290.8,317.3	0.03Å	Favored (31.173%) beta sheet	-	-	-
A 279		TYR	0.86	-	Favored (36.19%) General / -79.8,141.6	Favored (81.6%) *m-80* chi angles: 293.7,105.5	0.04Å	Favored (19.982%)	-	-	-
A 280		ASN	0.74	-	Favored (16.55%) General / -97.3,-22.6	Favored (92%) *m-40* chi angles: 293.6,328.3	0.03Å	Favored (22.427%)	-	-	-
**#**	**Alt**	**Res**	**High B**	**Clash > 0.4Å**	**Ramachandran**	**Rotamer**	**Cβ deviation**	**CaBLAM**	**Bond lengths**	**Bond angles**	**Cis Peptides**
			Avg: 1.07	Clashscore: 0.83	Outliers: 4 of 615	Poor rotamers: 0 of 510	Outliers: 0 of 564	Outliers: 16 of 613	Outliers: 5 of 617	Outliers: 10 of 617	Non-Trans: 1 of 616
A 281		LEU	0.67	-	Favored (32.99%) General / -135.5,129.4	Favored (47.7%) *tp* chi angles: 176.5,66.9	0.04Å	Favored (35.863%)	-	-	-

A 282		ASN	0.64	-	Favored (17.79%)<br>General / -124.0,113.0	Favored (44.3%) *t0*<br>chi angles: 189.2,316.4	0.06Å	Favored (66.183%)	-	-	-
A 283		ILE	0.63	-	Favored (57.89%)<br>Ile or Val / -103.5,124.7	Favored (67.9%) *mt*<br>chi angles: 302.7,169.6	0.06Å	Favored (66.479%)<br>beta sheet	-	-	-
A 284		MET	0.64	-	Favored (27.14%)<br>General / -113.4,114.8	Favored (80.1%) *mtp*<br>chi angles: 300.5,185.1,74.3	0.03Å	Favored (66.865%)	-	-	-
A 285		ASP	0.67	-	Favored (35.26%)<br>General / -78.4,145.1	Favored (37%) *t70*<br>chi angles: 187.5,67.2	0.07Å	Favored (10.787%)	-	-	-
A 286		GLU	0.71	-	Favored (14.4%)<br>General / 49.1,52.4	Favored (50.5%) *mm-30*<br>chi angles: 296.5,291.7,3.9	0.03Å	Favored (13.905%)	-	-	-
A 287		ALA	0.75	-	Favored (47.67%)<br>General / -63.1,-14.4	-	0.03Å	Favored (12.09%)	-	-	-
A 288		HIS	0.77	-	Favored (59.3%)<br>General / -83.5,-8.8	Favored (60%) *p-80*<br>chi angles: 63.9,285.1	0.06Å	Favored (21.3%)	-	-	-
A 289		PHE	0.78	-	Favored (51.19%)<br>General / -60.9,131.6	Favored (20.7%) *t80*<br>chi angles: 189.1,53.4	0.07Å	Favored (34.407%)	-	-	-
A 290		THR	0.77	-	Favored (53.24%)<br>General / -90.0,3.5	Favored (66.4%) *p*<br>chi angles: 58.4	0.09Å	Favored (8.243%)<br>beta sheet	-	-	-
A 291		ASP	0.75	-	Favored (88.71%)<br>Pre-Pro / -59.4,135.5	Favored (15.6%) *t70*<br>chi angles: 184.4,96.6	0.06Å	Favored (40.696%)	-	-	-
A 292		PRO	0.73	-	Favored (12.25%)<br>Trans-Pro / -46.7,-32.5	Favored (77.3%) *Cg_exo*<br>chi angles: 329,37,332.6	0.08Å	Favored (69.212%)	-	-	-
A 293		SER	0.7	-	Favored (55.41%)<br>General / -76.8,-21.2	Favored (99.6%) *p*<br>chi angles: 65.4	0.06Å	Favored (73.493%)<br>alpha helix	-	-	-
A 294		SER	0.67	-	Favored (19.78%)<br>General / -86.2,-35.8	Favored (69.2%) *m*<br>chi angles: 296.6	0.05Å	Favored (67.605%)<br>alpha helix	-	-	-
A 295		ILE	0.65	-	Favored (80.91%)<br>Ile or Val / -67.5,-47.4	Favored (97.4%) *mt*<br>chi angles: 293.2,167.1	0.08Å	Favored (89.237%)<br>alpha helix	-	-	-
A 296		ALA	0.64	-	Favored (92.43%)<br>General / -61.4,-39.9	-	0.03Å	Favored (89.384%)<br>alpha helix	-	-	-
A 297		ALA	0.64	-	Favored (99.21%)<br>General / -61.8,-42.6	-	0.03Å	Favored (91.165%)<br>alpha helix	-	-	-
A 298		ARG	0.64	-	Favored (91.04%)<br>General / -62.1,-39.0	Favored (99.5%) *mtm-85*<br>chi angles: 286.2,192.2,294.4,275.5	0.03Å	Favored (87.819%)<br>alpha helix	-	-	-
A 299		GLY	0.65	-	Favored (45.6%)	-	-	Favored (90.714%)	-	-	-

#	Alt	Res	High B	Clash > 0.4Å	Ramachandran	Rotamer	Cβ deviation	CaBLAM	Bond lengths	Bond angles	Cis Peptides
					Glycine / -61.9,-53.1			alpha helix			
A 300		TYR	0.66	-	Favored (72.9%) General / -55.1,-49.0	Favored (90.8%) *t80* chi angles: 178.8,79.4	0.03Å	Favored (84.803%) alpha helix	-	-	-
#	Alt	Res	High B	Clash > 0.4Å	Ramachandran	Rotamer	Cβ deviation	CaBLAM	Bond lengths	Bond angles	Cis Peptides
			Avg: 1.07	Clashscore: 0.83	Outliers: 4 of 615	Poor rotamers: 0 of 510	Outliers: 0 of 564	Outliers: 16 of 613	Outliers: 5 of 617	Outliers: 10 of 617	Non-Trans: 1 of 616
A 301		ILE	0.67	-	Favored (96.44%) Ile or Val / -64.0,-45.6	Favored (90.9%) *mt* chi angles: 292.5,164.9	0.12Å	Favored (94.65%) alpha helix	-	-	-
A 302		SER	0.7	-	Favored (86.24%) General / -59.6,-40.1	Favored (72.6%) *m* chi angles: 295.7	0.06Å	Favored (95.284%) alpha helix	-	-	-
A 303		THR	0.72	-	Favored (89.68%) General / -63.1,-45.9	Favored (99.9%) *m* chi angles: 300.5	0.07Å	Favored (94.112%) alpha helix	-	-	-
A 304		ARG	0.74	-	Favored (71.64%) General / -61.5,-31.7	Favored (48.5%) *mmt180* chi angles: 291.4,288.1,184.6,186.2	0.02Å	Favored (75.351%) alpha helix	-	-	-
A 305		VAL	0.76	-	Favored (65.13%) Ile or Val / -71.0,-46.9	Favored (89.1%) *t* chi angles: 174.1	0.02Å	Favored (71.402%) alpha helix	-	-	-
A 306		GLU	0.77	-	Favored (69.64%) General / -61.8,-28.7	Favored (95.6%) *mt-10* chi angles: 290.3,179.4,5.3	0.05Å	Favored (72.916%) alpha helix	-	-	-
A 307		MET	0.77	-	Favored (58.84%) General / -76.0,-9.7	Favored (93.2%) *mtp* chi angles: 292.4,169.6,76.3	0.07Å	Favored (44.929%)	-	-	-
A 308		GLY	0.76	-	Favored (73.63%) Glycine / 80.3,17.5	-	-	Favored (80.661%)	-	-	-
A 309		GLU	0.73	-	Favored (17.53%) General / -92.2,-27.6	Favored (96.5%) *mt-10* chi angles: 295.3,179.1,346.4	0.03Å	Favored (9.356%)	-	-	-
A 310		ALA	0.7	-	Favored (34.38%) General / -148.6,164.4	-	0.04Å	Favored (13.747%)	-	-	-
A 311		ALA	0.68	-	Favored (19.11%) General / -102.1,154.3	-	0.05Å	Favored (53.722%)	-	-	-
A 312		ALA	0.66	-	Favored (35.43%) General / -142.0,146.3	-	0.05Å	Favored (70.532%) beta sheet	-	-	-
A 313		ILE	0.67	-	Favored (66.06%) Ile or Val / -125.3,123.9	Favored (72.9%) *mt* chi angles: 300.9,167.1	0.05Å	Favored (71.067%) beta sheet	-	-	-
A 314		PHE	0.7	-	Favored (54.5%) General / -106.8,130.3	Favored (19.4%) *m-10* chi angles: 291.9,350.4	0.05Å	Favored (68.976%) beta sheet	-	-	-
A 315		MET	0.76	-	Favored (52.31%)	Favored (62.4%) *mtt*	0.04Å	Favored (18.556%)	-	-	-

					General / -106.7,132.7	chi angles: 293.6,183.2,172.7		beta sheet				
A 316		THR	0.83	-	Favored (7.96%) General / -168.5,156.1	Favored (4.6%) *t* chi angles: 180	0.04Å	Favored (13.293%)	-	-	-
A 317		ALA	0.91	-	Favored (31.59%) General / -82.1,-29.5	-	0.06Å	Favored (20.378%)	-	-	-
A 318		THR	1	-	Favored (16.98%) Pre-Pro / -139.8,83.0	Favored (43.6%) *p* chi angles: 55.1	0.04Å	Favored (10.106%)	-	-	-
A 319		PRO	1.08	-	Favored (42.68%) Trans-Pro / -55.8,151.0	Favored (85.2%) *Cg_exo* chi angles: 333.9,35,331.6	0.09Å	Favored (20.885%)	-	-	-
A 320		PRO	1.14	-	Favored (63.79%) Trans-Pro / -53.2,137.7	Favored (96%) *Cg_exo* chi angles: 331.5,36.7,330.7	0.06Å	Favored (38.679%)	-	-	-
**#**	**Alt**	**Res**	**High B**	**Clash > 0.4Å**	**Ramachandran**	**Rotamer**	**Cβ deviation**	**CaBLAM**	**Bond lengths**	**Bond angles**	**Cis Peptides**
			Avg: 1.07	Clashscore: 0.83	Outliers: 4 of 615	Poor rotamers: 0 of 510	Outliers: 0 of 564	Outliers: 16 of 613	Outliers: 5 of 617	Outliers: 10 of 617	Non-Trans: 1 of 616
A 321		GLY	1.18	-	Favored (63.87%) Glycine / 96.9,-14.5	-	-	Favored (77.833%)	-	-	-
A 322		THR	1.19	-	Favored (19.47%) General / -82.8,165.8	Favored (68.6%) *p* chi angles: 62.6	0.05Å	Favored (32.35%)	-	-	-
A 323		ARG	1.19	-	Favored (7.21%) General / -125.9,23.6	Favored (77.5%) *mtt90* chi angles: 292.7,169.8,173.1,77	0.02Å	Favored (9.577%) beta sheet	-	-	-
A 324		ASP	1.19	-	Favored (9.37%) General / -88.1,93.0	Favored (66.4%) *t0* chi angles: 185.3,346.3	0.04Å	Favored (12.354%) beta sheet	-	-	-
A 325		ALA	1.2	-	Favored (57.38%) General / -82.0,-4.7	-	0.05Å	Favored (17.712%)	-	-	-
A 326		PHE	1.23	-	Favored (70.99%) Pre-Pro / -134.9,68.5	Favored (89.4%) *m-80* chi angles: 301.1,94.5	0.01Å	Favored (11.214%)	-	-	-
A 327		PRO	1.29	-	Favored (9.36%) Trans-Pro / -76.0,177.0	Favored (57.4%) *Cg_endo* chi angles: 32.2,320.8,30.6	0.05Å	CaBLAM Disfavored (2.778%)	-	-	-
A 328		ASP	1.37	-	Favored (10.13%) General / -59.2,157.7	Favored (91.4%) *m-30* chi angles: 290.1,350.2	0.01Å	Favored (5.111%)	-	-	-
A 329		SER	1.47	-	Favored (27.52%) General / -148.1,166.5	Favored (95.7%) *p* chi angles: 63.9	0.08Å	Favored (54.891%)	-	-	-
A 330		ASN	1.54	-	Favored (37.96%) General / -95.2,9.9	Favored (94.4%) *m-40* chi angles: 287,334.6	0.07Å	Favored (6.068%)	-	-	-
A 331		SER	1.55	-	Favored (11.25%)	Favored (38.5%) *t* chi angles: 181.1	0.09Å	CaBLAM Disfavored (3.327%)	-	-	-

					Pre-Pro / -157.0,140.4						
A 332		PRO	1.49	-	Favored (82.57%) Trans-Pro / -55.4,140.7	Favored (92.3%) *Cg_exo* chi angles: 333.1,35.7,330.7	0.04Å	Favored (62.252%)	-	-	-
A 333		ILE	1.38	-	Favored (69.15%) Ile or Val / -125.2,125.3	Favored (79.3%) *mt* chi angles: 300.7,172.3	0.08Å	Favored (60.592%) beta sheet	-	-	-
A 334		MET	1.25	-	Favored (31.82%) General / -84.3,140.1	Favored (70.8%) *mtm* chi angles: 294.9,176.3,285.4	0.05Å	Favored (46.288%) beta sheet	-	-	-
A 335		ASP	1.13	-	Favored (26.17%) General / -115.3,115.1	Favored (41.6%) *m-30* chi angles: 294.7,292.4	0.02Å	Favored (56.731%) beta sheet	-	-	-
A 336		THR	1.03	-	Favored (51.44%) General / -122.2,128.7	Favored (82.9%) *m* chi angles: 302.1	0.07Å	Favored (56.426%) beta sheet	-	-	-
A 337		GLU	0.96	-	Favored (29.95%) General / -87.2,121.6	Favored (90.3%) *tt0* chi angles: 183.5,177.7,353.8	0.03Å	Favored (42.376%) beta sheet	-	-	-
A 338		VAL	0.91	-	Favored (39.34%) Ile or Val / -135.4,161.4	Favored (23.2%) *m* chi angles: 301.4	0.08Å	Favored (37.772%) beta sheet	-	-	-
A 339		GLU	0.9	-	Favored (9.81%) General / -83.1,77.0	Favored (96.8%) *mt-10* chi angles: 296.5,180.3,353	0.01Å	Favored (16.452%) beta sheet	-	-	-
A 340		VAL	0.9	-	Favored (89.36%) Pre-Pro / -72.7,123.3	Favored (85.8%) *t* chi angles: 177.3	0.14Å	Favored (25.824%) beta sheet	-	-	-
**#**	**Alt**	**Res**	**High B**	**Clash > 0.4Å**	**Ramachandran**	**Rotamer**	**Cβ deviation**	**CaBLAM**	**Bond lengths**	**Bond angles**	**Cis Peptides**
			Avg: 1.07	Clashscore: 0.83	Outliers: 4 of 615	Poor rotamers: 0 of 510	Outliers: 0 of 564	Outliers: 16 of 613	Outliers: 5 of 617	Outliers: 10 of 617	Non-Trans: 1 of 616
A 341		PRO	0.93	-	Favored (90.07%) Trans-Pro / -60.3,148.4	Favored (58.1%) *Cg_exo* chi angles: 336.1,32.9,331.5	0.05Å	Favored (43.272%)	-	-	-
A 342		GLU	1	-	Favored (10.24%) General / -106.9,-28.2	Favored (76.4%) *mt-10* chi angles: 297.4,174.1,20	0.08Å	Favored (12.428%)	-	-	-
A 343		ARG	1.11	-	Favored (5.27%) General / -118.7,176.4	Favored (63.5%) *mmm-85* chi angles: 302.6,294.1,297.2,273.5	0.06Å	CaBLAM Disfavored (1.039%)	-	-	-
A 344		ALA	1.25	-	Favored (25.55%) General / -56.2,145.7	-	0.10Å	Favored (5.018%)	-	-	-
A 345		TRP	1.41	-	Favored (44.16%) General / -143.2,154.7	Favored (58.4%) *p-90* chi angles: 51,267	0.11Å	Favored (61.5%)	-	-	-
A 346		SER	1.53	-	Favored (13.04%) General / -110.1,-19.5	Favored (82.6%) *p* chi angles: 62.4	0.03Å	Favored (13.59%)	-	-	-

A 347		SER	1.59	-	Favored (26.32%) General / -162.7,164.7	Favored (83.8%) *p* chi angles: 67.5	0.02Å	CA Geom Outlier (0.036%)	-	-	-
A 348		GLY	1.55	-	Favored (13.05%) Glycine / 103.6,143.8	-	-	Favored (24.568%) three-ten	-	-	-
A 349		PHE	1.44	-	Favored (32.13%) General / 54.0,40.9	Favored (76.1%) *t80* chi angles: 182.1,71.4	0.02Å	Favored (5.497%)	-	-	-
A 350		ASP	1.3	-	Favored (18.16%) General / -57.3,-18.7	Favored (94.5%) *m-30* chi angles: 291.8,346.3	0.04Å	Favored (12.767%)	-	-	-
A 351		TRP	1.17	-	Favored (32.08%) General / -50.4,-36.2	Favored (25.6%) *m-10* chi angles: 285.7,329.5	0.10Å	Favored (44.921%)	-	-	-
A 352		VAL	1.07	-	Favored (47.94%) Ile or Val / -62.6,-29.9	Favored (5.5%) *p* chi angles: 69.5	0.14Å	Favored (59.463%) alpha helix	-	-	-
A 353		THR	1.02	-	Favored (16.2%) General / -103.2,-14.8	Favored (63.4%) *p* chi angles: 57.9	0.08Å	Favored (42.944%) alpha helix	-	-	-
A 354		ASP	0.99	-	Favored (58.82%) General / -82.6,-10.8	Favored (87.9%) *m-30* chi angles: 291.3,335.6	0.03Å	Favored (55.145%)	-	-	-
A 355		HIS	0.97	-	Favored (27.08%) General / -79.8,122.8	Favored (16.2%) *t-170* chi angles: 187.9,214.9	0.04Å	Favored (30.365%)	-	-	-
A 356		SER	0.95	-	Favored (59.21%) General / -81.6,-11.3	Favored (89.7%) *p* chi angles: 66.8	0.03Å	Favored (38.173%) beta sheet	-	-	-
A 357		GLY	0.92	-	Favored (37.1%) Glycine / -94.8,-170.3	-	-	Favored (30.377%) beta sheet	-	-	-
A 358		LYS	0.88	-	Favored (34.76%) General / -85.3,128.6	Favored (52.8%) *mttp* chi angles: 293.5,180.4,172.1,67.8	0.03Å	Favored (12.631%) beta sheet	-	-	-
A 359		THR	0.83	-	Favored (54.85%) General / -123.0,137.8	Favored (96.1%) *m* chi angles: 300.8	0.04Å	Favored (69.754%) beta sheet	-	-	-
A 360		VAL	0.79	-	Favored (58.53%) Ile or Val / -110.6,132.6	Favored (84.9%) *t* chi angles: 177.1	0.01Å	Favored (72.124%) beta sheet	-	-	-
**#**	**Alt**	**Res**	**High B**	**Clash > 0.4Å**	**Ramachandran**	**Rotamer**	**Cβ deviation**	**CaBLAM**	**Bond lengths**	**Bond angles**	**Cis Peptides**
			Avg: 1.07	Clashscore: 0.83	Outliers: 4 of 615	Poor rotamers: 0 of 510	Outliers: 0 of 564	Outliers: 16 of 613	Outliers: 5 of 617	Outliers: 10 of 617	Non-Trans: 1 of 616
A 361		TRP	0.78	-	Favored (19.07%) General / -123.0,113.4	Favored (6.1%) *t60* chi angles: 192.1,336.8	0.02Å	Favored (63.675%) beta sheet	-	-	-
A 362		PHE	0.79	-	Favored (20.03%) General / -85.6,111.4	Favored (5.6%) *t80* chi angles: 180.1,27.3	0.07Å	Favored (63.021%) beta sheet	-	-	-

A 363		VAL	0.82	-	Favored (20.83%) Pre-Pro / -110.9,135.1	Favored (65.6%) *t* chi angles: 179.3	0.12Å	Favored (51.085%)	-	-	-
A 364		PRO	0.87	-	Favored (20.51%) Trans-Pro / -52.3,-24.2	Favored (98.6%) *Cg_exo* chi angles: 332.2,36.1,331.7	0.06Å	Favored (6.96%)	-	-	-
A 365		SER	0.93	-	Favored (40.16%) General / -155.3,163.4	Favored (90.4%) *p* chi angles: 66.7	0.06Å	Favored (22.058%)	-	-	-
A 366		VAL	0.97	-	Favored (93.17%) Ile or Val / -60.4,-43.2	Favored (67.3%) *t* chi angles: 171.8	0.07Å	Favored (67.428%) alpha helix	-	-	-
A 367		ARG	0.98	-	Favored (99.7%) General / -62.7,-42.5	Favored (96.6%) *mtt-85* chi angles: 290.2,176.7,185.1,271.3	0.07Å	Favored (87.728%) alpha helix	-	-	-
A 368		ASN	0.98	-	Favored (75.35%) General / -67.9,-33.8	Favored (99.4%) *m-40* chi angles: 288.1,342.7	0.03Å	Favored (77.964%) alpha helix	-	-	-
A 369		GLY	0.95	-	Favored (56.46%) Glycine / -57.8,-51.7	-	-	Favored (91.953%) alpha helix	-	-	-
A 370		ASN	0.92	-	Favored (80.53%) General / -59.6,-38.2	Favored (98.7%) *m-40* chi angles: 287.3,339.7	0.04Å	Favored (78.957%) alpha helix	-	-	-
A 371		GLU	0.89	-	Favored (98.75%) General / -63.4,-41.6	Favored (80.3%) *mt-10* chi angles: 286.2,187.2,357.9	0.04Å	Favored (84.08%) alpha helix	-	-	-
A 372		ILE	0.87	-	Favored (83.52%) Ile or Val / -68.4,-41.8	Favored (94.9%) *mt* chi angles: 292,168.5	0.12Å	Favored (80.94%) alpha helix	-	-	-
A 373		ALA	0.85	-	Favored (87.3%) General / -58.8,-41.7	-	0.05Å	Favored (85.66%) alpha helix	-	-	-
A 374		ALA	0.85	-	Favored (93.02%) General / -62.2,-39.5	-	0.03Å	Favored (95.241%) alpha helix	-	-	-
A 375		CYS	0.86	-	Favored (86.37%) General / -65.7,-44.4	Favored (82.6%) *m* chi angles: 295	0.09Å	Favored (87.189%) alpha helix	-	-	-
A 376		LEU	0.88	-	Favored (79.98%) General / -67.3,-35.7	Favored (81.7%) *mt* chi angles: 289.2,170.1	0.09Å	Favored (84.457%) alpha helix	-	-	-
A 377		THR	0.89	-	Favored (93.69%) General / -63.3,-44.9	Favored (93.1%) *m* chi angles: 299.2	0.04Å	Favored (87.856%) alpha helix	-	-	-
A 378		LYS	0.89	-	Favored (69.04%) General / -63.2,-27.0	Favored (96.9%) *mttt* chi angles: 289.2,177,181.1,179.3	0.04Å	Favored (73.758%)	-	-	-
A 379		ALA	0.87	-	Favored (33.32%) General / -78.9,-1.0	-	0.01Å	Favored (50.111%)	-	-	-

#	Alt	Res	High B	Clash > 0.4Å	Ramachandran	Rotamer	Cβ deviation	CaBLAM	Bond lengths	Bond angles	Cis Peptides
A 380		GLY	0.83	-	Favored (56.29%) Glycine / 85.6,20.5	-	-	Favored (87.697%)	-	-	-
#	Alt	Res	High B	Clash > 0.4Å	Ramachandran	Rotamer	Cβ deviation	CaBLAM	Bond lengths	Bond angles	Cis Peptides
			Avg: 1.07	Clashscore: 0.83	Outliers: 4 of 615	Poor rotamers: 0 of 510	Outliers: 0 of 564	Outliers: 16 of 613	Outliers: 5 of 617	Outliers: 10 of 617	Non-Trans: 1 of 616
A 381		LYS	0.8	-	Favored (17.14%) General / -97.8,155.2	Favored (70.1%) *mmtt* chi angles: 307.5,297.4,185.4,182	0.07Å	Favored (35.41%)	-	-	-
A 382		ARG	0.77	-	Favored (22.37%) General / -87.3,112.7	Favored (98.6%) *mtm-85* chi angles: 296.3,186.7,294.2,274.8	0.04Å	Favored (42.422%) beta sheet	-	-	-
A 383		VAL	0.76	-	Favored (62.46%) Ile or Val / -123.1,135.6	Favored (42.6%) *t* chi angles: 182.9	0.08Å	Favored (58.389%) beta sheet	-	-	-
A 384		ILE	0.78	-	Favored (48.43%) Ile or Val / -123.2,139.9	Favored (18.6%) *tt* chi angles: 183.2,165.9	0.07Å	Favored (65.815%) beta sheet	-	-	-
A 385		GLN	0.84	-	Favored (35.25%) General / -115.5,119.2	Favored (68.8%) *tp40* chi angles: 175.7,69.8,58.4	0.04Å	Favored (68.581%) beta sheet	-	-	-
A 386		LEU	0.92	-	Favored (53.92%) General / -106.0,130.4	Favored (8.2%) *mp* chi angles: 278,58.9	0.06Å	Favored (38.567%) beta sheet	-	-	-
A 387		SER	1.03	-	Favored (26.26%) General / -145.8,166.3	Favored (92.6%) *p* chi angles: 64.5	0.06Å	Favored (25.889%)	-	-	-
A 388		ARG	1.14	-	Favored (20.95%) General / -49.9,-34.7	Favored (20.6%) *tpp-160* chi angles: 174,66.7,74.4,178.4	0.04Å	Favored (41.132%)	-	-	-
A 389		LYS	1.22	-	Favored (58.25%) General / -85.6,-8.5	Favored (72.3%) *mmtt* chi angles: 299.8,294.3,186.8,181.7	0.07Å	Favored (32.959%) alpha helix	-	-	-
A 390		THR	1.26	-	Favored (5.79%) General / -124.7,-17.9	Favored (64.9%) *p* chi angles: 63.2	0.04Å	Favored (12.555%) alpha helix	-	-	-
A 391		PHE	1.27	-	Favored (40.76%) General / -49.5,-41.6	Favored (68.2%) *t80* chi angles: 185.9,75.8	0.04Å	Favored (37.584%) alpha helix	-	-	-
A 392		GLU	1.25	-	Favored (65.76%) General / -60.7,-24.6	Favored (96.3%) *mt-10* chi angles: 293.3,176.7,5.8	0.05Å	Favored (36.863%) alpha helix	-	-	-
A 393		THR	1.23	-	Favored (3.78%) General / -106.5,-47.4	Favored (95.7%) *m* chi angles: 299.6	0.04Å	Favored (20.946%) alpha helix	-	-	-
A 394		GLU	1.21	-	Favored (77.67%) General / -66.5,-34.3	Favored (73.6%) *mm-30* chi angles: 295.6,296.5,307.9	0.04Å	Favored (74.017%) alpha helix	-	-	-
A 395		PHE	1.22	-	Favored (78.01%) General / -56.4,-47.9	Favored (87.8%) *t80* chi angles: 173.5,77	0.09Å	Favored (73.196%) alpha helix	-	OUTLIER(S) worst is CA-CB-CG: 5.2 σ	-

#	Alt	Res	High B	Clash > 0.4Å	Ramachandran	Rotamer	Cβ deviation	CaBLAM	Bond lengths	Bond angles	Cis Peptides
A 396		GLN	1.24	-	Favored (77.29%) General / -62.3,-34.8	Favored (95.8%) *mm-40* chi angles: 293.4,297.9,319.3	0.01Å	Favored (83.083%) alpha helix	-	-	-
A 397		LYS	1.28	-	Favored (78.33%) General / -65.6,-34.5	Favored (52.6%) *mtmt* chi angles: 289.2,190.4,286.6,188.6	0.08Å	Favored (83.563%) alpha helix	-	-	-
A 398		THR	1.31	-	Favored (68.3%) General / -67.7,-29.1	Favored (70.3%) *p* chi angles: 62.3	0.07Å	Favored (79.198%) alpha helix	-	-	-
A 399		LYS	1.3	-	Favored (33.59%) General / -72.3,-49.6	Favored (47.9%) *tttm* chi angles: 179.9,182.6,183.1,290.5	0.07Å	Favored (55.873%) alpha helix	-	-	-
A 400		ASN	1.24	-	Favored (54.37%) General / -94.2,-1.3	Favored (91.6%) *m-40* chi angles: 292.7,324.2	0.02Å	Favored (18.765%)	-	-	-
**#**	**Alt**	**Res**	**High B**	**Clash > 0.4Å**	**Ramachandran**	**Rotamer**	**Cβ deviation**	**CaBLAM**	**Bond lengths**	**Bond angles**	**Cis Peptides**
			Avg: 1.07	Clashscore: 0.83	Outliers: 4 of 615	Poor rotamers: 0 of 510	Outliers: 0 of 564	Outliers: 16 of 613	Outliers: 5 of 617	Outliers: 10 of 617	Non-Trans: 1 of 616
A 401		GLN	1.14	-	Favored (50.25%) General / -128.3,150.1	Favored (75%) *mt0* chi angles: 298.1,183,297.1	0.02Å	Favored (21.942%)	-	-	-
A 402		GLU	1.02	-	Favored (7.06%) General / -80.3,71.0	Favored (43.7%) *mm-30* chi angles: 295.2,289.8,300.1	0.00Å	Favored (25.631%)	-	-	-
A 403		TRP	0.9	-	Favored (40.7%) General / -75.2,147.0	Favored (96.1%) *m100* chi angles: 294.6,101.6	0.06Å	Favored (19.366%)	-	-	-
A 404		ASP	0.8	-	Favored (18.13%) General / -86.4,-37.7	Favored (54.8%) *m-30* chi angles: 295.3,300.8	0.07Å	Favored (14.948%)	-	-	-
A 405		PHE	0.73	-	Favored (48.36%) General / -133.2,141.9	Favored (86.4%) *m-80* chi angles: 299.7,87.1	0.07Å	Favored (33.884%)	-	-	-
A 406		VAL	0.7	-	Favored (62.16%) Ile or Val / -123.3,121.1	Favored (62%) *t* chi angles: 179.7	0.07Å	Favored (69.681%)	-	-	-
A 407		ILE	0.7	-	Favored (64.71%) Ile or Val / -108.9,128.4	Favored (80.8%) *mt* chi angles: 299.9,169.6	0.03Å	Favored (61.52%) beta sheet	-	-	-
A 408		THR	0.72	-	Favored (30.36%) General / -134.8,163.2	Favored (23.6%) *p* chi angles: 71.3	0.07Å	Favored (52.648%)	-	-	-
A 409		THR	0.76	-	Favored (2.1%) General / -98.9,-171.4	Favored (26.1%) *p* chi angles: 70.7	0.09Å	Favored (20.117%)	-	-	-
A 410		ASP	0.8	0.57Å OD2 with A 431 LYS NZ	Favored (6.3%) General / -72.9,2.7	Favored (54.2%) *p0* chi angles: 61.8,5.7	0.08Å	CaBLAM Disfavored (4.165%)	-	-	-
A 411		ILE	0.85	-	Favored (48.1%) Ile or Val / -62.3,-30.1	Favored (11.5%) *tp* chi angles: 193.1,64.9	0.03Å	Favored (48.325%)	-	-	-
A 412		SER	0.9	-	Favored (61.54%)	Favored (88.2%) *p* chi angles: 67	0.02Å	Favored (62.709%)	-	-	-

					General / -73.0,-15.1			three-ten			
A 413		GLU	0.96	-	Favored (69.41%) General / -64.9,-28.3	Favored (96.9%) *mt-10* chi angles: 293.3,178.7,5.5	0.03Å	Favored (31.075%)	-	-	-
A 414		MET	1.02	-	Favored (9.99%) General / -84.6,67.4	Favored (50.9%) *mmp* chi angles: 297,297.9,100.3	0.10Å	CA Geom Outlier (0.289%)	-	-	-
A 415		GLY	1.08	-	Favored (84.93%) Glycine / 79.0,11.7	-	-	Favored (50.829%)	-	-	-
A 416		ALA	1.13	0.40Å O with A 459 ARG NH1	Favored (4.61%) General / -78.1,78.8	-	0.05Å	Favored (24.557%)	-	-	-
A 417		ASN	1.16	-	Allowed (1.56%) General / -73.7,74.9	Favored (49.4%) *t0* chi angles: 182.8,7.7	0.08Å	Favored (58.453%) beta sheet	-	-	-
A 418		PHE	1.14	-	Favored (36.36%) General / -79.7,133.1	Favored (74.4%) *t80* chi angles: 180.1,69.8	0.04Å	Favored (26.322%) beta sheet	-	-	-
A 419		LYS	1.09	-	Favored (3.05%) General / -76.8,68.9	Favored (98.4%) *mttt* chi angles: 296.2,175.3,182,176.4	0.03Å	Favored (27.358%) beta sheet	-	-	-
A 420		ALA	1	-	Favored (31.58%) General / -80.1,147.1	-	0.06Å	Favored (16.617%)	-	-	-
**#**	**Alt**	**Res**	**High B**	**Clash > 0.4Å**	**Ramachandran**	**Rotamer**	**Cβ deviation**	**CaBLAM**	**Bond lengths**	**Bond angles**	**Cis Peptides**
			Avg: 1.07	Clashscore: 0.83	Outliers: 4 of 615	Poor rotamers: 0 of 510	Outliers: 0 of 564	Outliers: 16 of 613	Outliers: 5 of 617	Outliers: 10 of 617	Non-Trans: 1 of 616
A 421		ASP	0.91	-	Favored (15.63%) General / -95.5,-26.9	Favored (39.5%) *m-30* chi angles: 294,291.9	0.04Å	Favored (24.037%)	-	-	-
A 422		ARG	0.82	-	Favored (30.71%) General / -142.7,138.7	Favored (50.1%) *ttm170* chi angles: 173.5,174.6,286.7,164.2	0.07Å	Favored (39.718%)	-	-	-
A 423		VAL	0.75	-	Favored (74.03%) Ile or Val / -122.7,126.5	Favored (82.1%) *t* chi angles: 177.8	0.05Å	Favored (71.737%)	-	-	-
A 424		ILE	0.71	-	Favored (60.64%) Ile or Val / -108.2,121.0	Favored (82.2%) *mt* chi angles: 299.6,169.6	0.05Å	Favored (71.816%) beta sheet	-	-	-
A 425		ASP	0.68	-	Favored (16.88%) General / -121.7,111.2	Favored (61.5%) *t0* chi angles: 185.5,357.4	0.04Å	Favored (69.013%) beta sheet	-	-	-
A 426		SER	0.67	-	Favored (63.82%) General / -62.0,-20.4	Favored (91.4%) *p* chi angles: 66.6	0.12Å	Favored (7.053%) beta sheet	-	-	-
A 427		ARG	0.68	-	Favored (16.1%) General / 57.9,28.5	Favored (26.6%) *mmt90* chi angles: 294.8,288,190.2,82.2	0.09Å	Favored (7.556%)	-	-	-
A 428		ARG	0.7	-	Favored (49.16%)	Favored (97%) *mtt-85*	0.09Å	Favored (21.516%)	-	-	-

#	Alt	Res	High B	Clash > 0.4Å	Ramachandran	Rotamer	Cβ deviation	CaBLAM	Bond lengths	Bond angles	Cis Peptides
					General / -133.3,155.6	chi angles: 290.6,179.2,180.8,273.4					
A 429		CYS	0.73	-	Favored (31.32%) General / -153.8,152.9	Favored (21.6%) *p* chi angles: 58.7	0.10Å	Favored (50.722%)	-	-	-
A 430		LEU	0.77	-	Favored (18.49%) General / -102.3,107.5	Favored (4.4%) *mp* chi angles: 279.4,75	0.02Å	Favored (33.665%) beta sheet	-	-	-
A 431		LYS	0.81	0.57Å NZ with A 410 ASP OD2	Favored (41.87%) Pre-Pro / -111.2,150.4	Favored (71.1%) *mmtt* chi angles: 301.3,294.3,188.3,181.4	0.05Å	Favored (33.16%) beta sheet	-	-	-
A 432		PRO	0.87	-	Favored (32.23%) Trans-Pro / -68.1,134.7	Favored (43.6%) *Cg_endo* chi angles: 24.3,327.4,26.7	0.04Å	Favored (30.954%) beta sheet	-	-	-
A 433		VAL	0.95	-	Favored (55.11%) Ile or Val / -126.2,138.3	Favored (83.4%) *t* chi angles: 177.8	0.04Å	Favored (50.428%) beta sheet	-	-	-
A 434		ILE	1.05	-	Favored (38.24%) Ile or Val / -78.7,125.6	Favored (46.9%) *mm* chi angles: 305.7,301.7	0.09Å	Favored (46.273%)	-	-	-
A 435		LEU	1.15	-	Favored (47.36%) General / -106.0,123.0	Favored (57.8%) *tp* chi angles: 176.8,65.2	0.04Å	Favored (5.982%)	-	-	-
A 436		ASP	1.22	-	Allowed (0.57%) General / 54.2,-110.7	Favored (74.6%) *m-30* chi angles: 296.9,320.8	0.06Å	CaBLAM Disfavored (3.862%)	-	-	-
A 437		GLY	1.25	-	Favored (2.03%) Glycine / -101.3,-88.4	-	-	CaBLAM Outlier (0%)	-	-	-
A 438		GLU	1.22	-	Favored (33.3%) General / -90.5,-14.2	Favored (56.1%) *mt-10* chi angles: 293.9,179.6,293	0.02Å	Favored (7.492%)	-	-	-
A 439		ARG	1.14	-	Favored (29.44%) General / -91.3,140.8	Favored (81.7%) *ttt180* chi angles: 179.5,173.5,174.3,180.2	0.05Å	Favored (27.224%)	-	-	-
A 440		VAL	1.05	-	Favored (66.68%) Ile or Val / -116.0,132.0	Favored (80.6%) *t* chi angles: 176.5	0.10Å	Favored (67.638%)	-	-	-
**#**	**Alt**	**Res**	**High B**	**Clash > 0.4Å**	**Ramachandran**	**Rotamer**	**Cβ deviation**	**CaBLAM**	**Bond lengths**	**Bond angles**	**Cis Peptides**
			Avg: 1.07	Clashscore: 0.83	Outliers: 4 of 615	Poor rotamers: 0 of 510	Outliers: 0 of 564	Outliers: 16 of 613	Outliers: 5 of 617	Outliers: 10 of 617	Non-Trans: 1 of 616
A 441		ILE	0.96	-	Favored (16.1%) Ile or Val / -121.8,166.8	Favored (46.2%) *pt* chi angles: 61.8,171	0.06Å	Favored (33.606%) beta sheet	-	-	-
A 442		LEU	0.89	-	Favored (57.81%) General / -89.7,-4.3	Favored (74%) *mt* chi angles: 301.3,174.2	0.11Å	CaBLAM Disfavored (1.252%)	-	-	-
A 443		ALA	0.84	-	Favored (2.35%) General / 51.1,-128.5	-	0.05Å	CaBLAM Outlier (0.306%)	-	-	-
A 444		GLY	0.81	-	Favored (11.58%)	-	-	CaBLAM Disfavored	-	-	-

#	Alt	Res	High B	Clash > 0.4Å	Ramachandran	Rotamer	Cβ deviation	CaBLAM	Bond lengths	Bond angles	Cis Peptides
					Glycine / -130.7,-165.6			(1.234%)			
A 445		PRO	0.79	-	Favored (5.17%) Trans-Pro / -76.9,74.0	Favored (67.6%) *Cg_endo* chi angles: 31.3,325.1,23.8	0.07Å	CaBLAM Outlier (0.39%)	-	-	-
A 446		MET	0.78	-	Favored (95.13%) Pre-Pro / -70.5,147.9	Favored (66.8%) *mtm* chi angles: 291.7,174.6,288.4	0.08Å	Favored (14.857%)	-	-	-
A 447		PRO	0.78	-	Favored (81.89%) Trans-Pro / -55.4,139.5	Favored (99.4%) *Cg_exo* chi angles: 332.4,36.8,329.9	0.05Å	Favored (83.332%)	-	-	-
A 448		VAL	0.8	-	Favored (5.91%) Ile or Val / -76.0,155.7	Favored (27.5%) *m* chi angles: 298.8	0.09Å	Favored (47.837%)	-	-	-
A 449		THR	0.83	-	Favored (25.63%) General / -72.7,165.0	Favored (73%) *p* chi angles: 61.7	0.04Å	Favored (47.335%)	-	-	-
A 450		HIS	0.86	-	Favored (89.49%) General / -62.2,-38.5	Favored (80.1%) *m-70* chi angles: 289,294.1	0.08Å	Favored (65.361%)	-	-	-
A 451		ALA	0.88	-	Favored (99.17%) General / -61.0,-42.8	-	0.02Å	Favored (76.384%) alpha helix	-	-	-
A 452		SER	0.91	-	Favored (84.16%) General / -67.7,-40.6	Favored (24.6%) *m* chi angles: 288.3	0.08Å	Favored (84.081%) alpha helix	-	-	-
A 453		ALA	0.92	-	Favored (88.66%) General / -60.2,-40.0	-	0.04Å	Favored (94.827%) alpha helix	-	-	-
A 454		ALA	0.94	-	Favored (99.5%) General / -62.4,-42.5	-	0.10Å	Favored (97.528%) alpha helix	-	-	-
A 455		GLN	0.95	-	Favored (91.73%) General / -65.9,-41.2	Favored (33.1%) *tp40* chi angles: 187.5,65.2,11.9	0.10Å	Favored (97.679%) alpha helix	-	-	-
A 456		ARG	0.98	-	Favored (97.3%) General / -63.4,-43.5	Favored (14.9%) *tpp-160* chi angles: 182.1,62.5,58.1,168.7	0.05Å	Favored (86.342%) alpha helix	-	-	-
A 457		ARG	1.02	-	Favored (92.55%) General / -65.5,-42.0	Favored (71.9%) *ttt-90* chi angles: 181.5,174.8,176.5,272.4	0.04Å	Favored (61.7%) alpha helix	-	-	-
A 458		GLY	1.08	-	Favored (55.8%) Glycine / -55.0,-31.3	-	-	Favored (71.884%) three-ten	-	-	-
A 459		ARG	1.15	0.40Å NH1 with A 416 ALA O	Favored (25.63%) General / -47.4,-45.3	Favored (73.7%) *ttm-80* chi angles: 181.6,177,304.3,278.6	0.12Å	Favored (53.138%) three-ten	OUTLIER(S) worst is CD--NE: 4.1 σ	-	-
A 460		ILE	1.23	-	Favored (20.66%) Ile or Val / -74.5,-27.1	Favored (6.6%) *tt* chi angles: 204.3,169.8	0.08Å	Favored (72.84%) alpha helix	OUTLIER(S) worst is CB--CG1: 5.3 σ	-	-
#	Alt	Res	High B	Clash > 0.4Å	Ramachandran	Rotamer	Cβ deviation	CaBLAM	Bond lengths	Bond angles	Cis Peptides

			Avg: 1.07	Clashscore: 0.83	Outliers: 4 of 615	Poor rotamers: 0 of 510	Outliers: 0 of 564	Outliers: 16 of 613	Outliers: 5 of 617	Outliers: 10 of 617	Non-Trans: 1 of 616
A 461		GLY	1.32	-	Favored (63.24%) Glycine / -75.6,-0.4	-	-	Favored (53.254%) three-ten	-	-	-
A 462		ARG	1.42	-	Favored (55.88%) General / -90.2,1.9	Favored (94.6%) *mtt-85* chi angles: 295,178.7,186.7,268.4	0.10Å	Favored (50.02%)	-	-	-
A 463		ASN	1.53	-	Favored (47.64%) Pre-Pro / -122.1,87.4	Favored (56.4%) *m-40* chi angles: 301.6,286	0.07Å	Favored (5.059%)	-	-	-
A 464		PRO	1.63	-	Favored (68.75%) Trans-Pro / -57.1,148.5	Favored (84.9%) *Cg_exo* chi angles: 334,34.8,330.8	0.13Å	Favored (9.345%)	-	-	-
A 465		ASN	1.71	-	Favored (29.48%) General / 55.5,42.8	Favored (90.9%) *m-40* chi angles: 294,326.4	0.06Å	Favored (13.517%)	-	-	-
A 466		LYS	1.72	-	Favored (78.19%) Pre-Pro / -127.6,73.0	Favored (22.6%) *mtpp* chi angles: 289.2,169.3,63.2,66.1	0.06Å	CaBLAM Disfavored (2.892%)	-	-	-
A 467		PRO	1.66	-	Favored (61.95%) Trans-Pro / -63.8,-17.9	Favored (47.2%) *Cg_endo* chi angles: 24.9,325.6,29.1	0.01Å	Favored (44.126%)	-	-	-
A 468		GLY	1.53	-	Favored (90.72%) Glycine / -81.5,-3.1	-	-	Favored (45.046%)	-	-	-
A 469		ASP	1.36	-	Favored (51.23%) General / -60.2,144.0	Favored (86.3%) *m-30* chi angles: 283.5,353	0.05Å	Favored (27.745%)	-	-	-
A 470		GLU	1.18	-	Favored (37.19%) General / -137.6,136.0	Favored (88.4%) *tt0* chi angles: 187.6,172.9,4.3	0.03Å	Favored (71.782%) beta sheet	-	-	-
A 471		TYR	1.03	-	Favored (51.06%) General / -108.5,124.8	Favored (83.6%) *t80* chi angles: 174.2,74.4	0.03Å	Favored (72.193%) beta sheet	-	-	-
A 472		MET	0.92	-	Favored (43.98%) General / -112.2,143.0	Favored (42.1%) *mtt* chi angles: 286.6,182.3,163.9	0.04Å	Favored (58.361%) beta sheet	-	-	-
A 473		TYR	0.85	-	Favored (34.54%) General / -149.0,151.4	Favored (57.1%) *p90* chi angles: 61.2,92.4	0.03Å	Favored (52.114%) beta sheet	-	-	-
A 474		GLY	0.81	-	Favored (8.93%) Glycine / -120.9,-152.9	-	-	Favored (5.16%)	-	-	-
A 475		GLY	0.81	-	Favored (38.14%) Glycine / 53.1,-128.5	-	-	Favored (18.089%)	-	-	-
A 476		GLY	0.82	-	Favored (46%) Glycine / 174.5,-179.1	-	-	Favored (6.06%)	-	-	-
A 477		CYS	0.85	-	Favored (33.59%) General / -113.9,150.5	Favored (99.5%) *m* chi angles: 292.5	0.10Å	Favored (14.631%)	-	-	-

A<br>478		ALA	0.89	-	Favored<br>(24.51%)<br>General /<br>-94.8,145.9	-	0.06Å	Favored<br>(50.625%)<br>beta sheet	-	-	-
A<br>479		GLU	0.93	-	Favored<br>(13.66%)<br>General / -115.8,2.5	Favored (89.4%)<br>*mt-10*<br>chi angles:<br>298.5,185.1,0.9	0.04Å	Favored<br>(38.213%)	-	-	-
A<br>480		THR	0.96	-	Favored<br>(45.62%)<br>General /<br>-130.7,132.8	Favored (99.3%) *m*<br>chi angles: 300.4	0.02Å	Favored<br>(10.412%)	-	-	-
**#**	**Alt**	**Res**	**High B**	**Clash > 0.4Å**	**Ramachandran**	**Rotamer**	**Cβ deviation**	**CaBLAM**	**Bond lengths**	**Bond angles**	**Cis Peptides**
			Avg:<br>1.07	Clashscore:<br>0.83	Outliers: 4 of<br>615	Poor rotamers: 0 of<br>510	Outliers:<br>0 of 564	Outliers:<br>16 of 613	Outliers: 5 of<br>617	Outliers: 10<br>of 617	Non-<br>Trans: 1<br>of 616
A<br>481		ASP	0.95	-	Favored<br>(2.05%)<br>General /<br>-142.5,19.3	Favored (44.7%) *p0*<br>chi angles: 58.1,359.2	0.09Å	Favored<br>(6.015%)	-	-	-
A<br>482		GLU	0.92	-	Favored<br>(73.13%)<br>General /<br>-60.2,-34.0	Favored (99.6%)<br>*mt-10*<br>chi angles:<br>291.4,174.3,356	0.03Å	Favored<br>(42.66%)	-	-	-
A<br>483		GLY	0.87	-	Favored<br>(88.92%)<br>Glycine / -85.0,-0.7	-	-	Favored<br>(63.533%)	-	-	-
A<br>484		HIS	0.81	-	Favored<br>(44.77%)<br>General /<br>-74.5,141.9	Favored (44.3%)<br>*m170*<br>chi angles: 292.8,185.3	0.05Å	Favored<br>(40.835%)	-	-	-
A<br>485		ALA	0.75	-	Favored<br>(68.06%)<br>General /<br>-60.5,-28.0	-	0.05Å	Favored<br>(33.226%)	-	-	-
A<br>486		HIS	0.7	-	Favored<br>(63.02%)<br>General /<br>-56.5,-30.5	Favored (56%)<br>*m170*<br>chi angles: 289.2,170	0.09Å	Favored<br>(55.507%)	-	-	-
A<br>487		TRP	0.66	-	Favored<br>(54.25%)<br>General /<br>-77.1,-27.5	Favored (88.5%)<br>*m100*<br>chi angles: 298.9,107.8	0.10Å	Favored<br>(75.344%)<br>alpha helix	-	-	-
A<br>488		LEU	0.64	-	Favored<br>(21.58%)<br>General /<br>-85.6,-33.7	Favored (77.3%) *mt*<br>chi angles: 294.5,179.5	0.05Å	Favored<br>(70.936%)<br>alpha helix	-	-	-
A<br>489		GLU	0.62	-	Favored<br>(94.22%)<br>General /<br>-63.8,-39.4	Favored (57.8%)<br>*mt-10*<br>chi angles:<br>287.2,182.1,314.7	0.01Å	Favored<br>(89.413%)<br>alpha helix	-	-	-
A<br>490		ALA	0.6	-	Favored<br>(95.66%)<br>General /<br>-61.6,-40.7	-	0.07Å	Favored<br>(98.571%)<br>alpha helix	-	-	-
A<br>491		ARG	0.59	-	Favored<br>(87.39%)<br>General /<br>-66.8,-38.9	Favored (25.6%)<br>*tpp80*<br>chi angles:<br>182.3,60.7,65.3,84.2	0.03Å	Favored<br>(94.882%)<br>alpha helix	-	-	-
A<br>492		MET	0.59	-	Favored<br>(92.45%)<br>General /<br>-59.6,-45.2	Favored (61.4%)<br>*mtt*<br>chi angles:<br>289,174.4,172	0.05Å	Favored<br>(92.663%)<br>alpha helix	-	-	-
A<br>493		LEU	0.59	-	Favored<br>(99.37%)<br>General /<br>-62.8,-43.2	Favored (84.5%) *mt*<br>chi angles: 290.1,169.2	0.06Å	Favored<br>(96.608%)<br>alpha helix	-	-	-

A 494		LEU	0.6	-	Favored (98.04%) General / -63.8,-41.0	Favored (81%) *mt* chi angles: 289.1,171.1	0.05Å	Favored (88.514%) alpha helix	-	-	-
A 495		ASP	0.64	-	Favored (70.52%) General / -61.9,-29.9	Favored (96%) *m-30* chi angles: 286.6,348.9	0.04Å	Favored (73.671%) alpha helix	-	-	-
A 496		ASN	0.73	-	Favored (50.27%) General / -94.5,5.9	Favored (80.4%) *m-40* chi angles: 288.5,319.6	0.08Å	Favored (47.749%)	-	-	-
A 497		ILE	0.86	-	Favored (40.67%) Ile or Val / -88.2,128.3	Favored (94.7%) *mt* chi angles: 294.7,169.4	0.08Å	Favored (32.399%)	-	-	-
A 498		TYR	1.04	-	Favored (34.96%) General / -82.8,129.3	Favored (90%) *t80* chi angles: 179.8,77.4	0.03Å	Favored (50.648%) beta sheet	-	-	-
A 499		LEU	1.24	-	Favored (49.32%) General / -118.4,140.8	Favored (3.1%) *mp* chi angles: 284,82.3	0.05Å	Favored (56.613%)	-	-	-
A 500		GLN	1.38	0.47Å N with A 500 GLN OE1	Favored (64.38%) General / -58.5,-27.4	Favored (19.2%) *mp10* chi angles: 293.6,79.9,348.9	0.05Å	Favored (22.73%)	-	-	-
**#**	**Alt**	**Res**	**High B**	**Clash > 0.4Å**	**Ramachandran**	**Rotamer**	**Cβ deviation**	**CaBLAM**	**Bond lengths**	**Bond angles**	**Cis Peptides**
			Avg: 1.07	Clashscore: 0.83	Outliers: 4 of 615	Poor rotamers: 0 of 510	Outliers: 0 of 564	Outliers: 16 of 613	Outliers: 5 of 617	Outliers: 10 of 617	Non-Trans: 1 of 616
A 501		ASP	1.41	-	Favored (44.64%) General / -99.4,8.4	Favored (56%) *p0* chi angles: 63.7,357.4	0.02Å	Favored (51.979%)	-	-	-
A 502		GLY	1.32	-	Favored (83.31%) Glycine / 84.2,-4.5	-	-	Favored (68.714%)	-	-	-
A 503		LEU	1.16	-	Favored (47.6%) General / -63.4,131.2	Favored (40.1%) *tp* chi angles: 183,66.9	0.05Å	Favored (35.903%) beta sheet	-	-	-
A 504		ILE	0.99	-	Favored (41.41%) Ile or Val / -87.9,126.4	Favored (89.5%) *mt* chi angles: 298.1,170.7	0.04Å	Favored (38.776%) beta sheet	-	-	-
A 505		ALA	0.88	-	Favored (54.51%) General / -61.2,144.0	-	0.03Å	Favored (27.628%)	-	-	-
A 506		SER	0.82	-	Favored (17.57%) General / -121.9,164.4	Favored (86.1%) *p* chi angles: 67.8	0.03Å	Favored (28.682%)	-	-	-
A 507		LEU	0.83	-	Favored (49.79%) General / -70.1,147.5	Favored (94.7%) *mt* chi angles: 291.6,172.4	0.01Å	Favored (32.579%)	-	-	-
A 508		TYR	0.87	-	Favored (51.74%) General / -60.0,131.7	Favored (38.3%) *t80* chi angles: 178.7,57.5	0.06Å	Favored (31.033%)	-	-	-
A 509		ARG	0.93	-	OUTLIER (0.01)%) Pre-Pro / -14.4,-74.3	Favored (85%) *ttp80* chi angles: 179.7,184.8,65.5,80.7	0.16Å	Favored (21.609%)	-	-	-
A 510		PRO	0.98	-	Favored (66.73%)	Favored (41.6%) *Cg_endo*	0.04Å	Favored (18.291%)	-	-	-

					Trans-Pro / -61.9,-19.7	chi angles: 23.8,324.4,32.3		alpha helix			
A 511		GLU	1.01	-	Favored (15.66%) General / -91.0,-33.2	Favored (95%) *mt-10* chi angles: 297.6,175.9,352.1	0.07Å	Favored (43.22%) alpha helix	-	-	-
A 512		ALA	1	-	Favored (77.29%) General / -60.0,-36.5	-	0.02Å	Favored (58.344%) three-ten	-	-	-
A 513		ASP	0.97	-	Favored (61.35%) General / -60.1,-21.3	Favored (99.4%) *m-30* chi angles: 287.7,346.8	0.03Å	Favored (65.302%) three-ten	-	-	-
A 514		LYS	0.93	-	Favored (56.23%) General / -88.9,-7.3	Favored (98.7%) *mttt* chi angles: 295.7,178.2,177.8,178.5	0.02Å	Favored (63.882%)	-	-	-
A 515		VAL	0.88	-	Favored (65.32%) Ile or Val / -120.4,121.3	Favored (84.4%) *t* chi angles: 177.1	0.04Å	Favored (27.47%)	-	-	-
A 516		ALA	0.84	-	Favored (54.13%) General / -79.9,-17.0	-	0.03Å	Favored (13.889%)	-	-	-
A 517		ALA	0.79	-	Favored (54.11%) General / -63.9,146.4	-	0.03Å	Favored (35.243%)	-	-	-
A 518		ILE	0.75	-	Favored (24.57%) Ile or Val / -81.6,135.7	Favored (93.1%) *mt* chi angles: 296.2,169.2	0.05Å	Favored (23.517%)	-	-	-
A 519		GLU	0.71	-	Favored (52.6%) General / -56.6,138.2	Favored (56.4%) *mp0* chi angles: 295.2,88.8,358.8	0.00Å	Favored (29.538%)	-	-	-
A 520		GLY	0.68	-	Favored (72.67%) Glycine / 94.0,-9.7	-	-	Favored (82.442%)	-	-	-
**#**	**Alt**	**Res**	**High B**	**Clash > 0.4Å**	**Ramachandran**	**Rotamer**	**Cβ deviation**	**CaBLAM**	**Bond lengths**	**Bond angles**	**Cis Peptides**
			Avg: 1.07	Clashscore: 0.83	Outliers: 4 of 615	Poor rotamers: 0 of 510	Outliers: 0 of 564	Outliers: 16 of 613	Outliers: 5 of 617	Outliers: 10 of 617	Non-Trans: 1 of 616
A 521		GLU	0.66	-	Favored (67.72%) General / -59.5,-29.3	Favored (82.5%) *mt-10* chi angles: 286.4,179.6,12.8	0.11Å	Favored (34.166%)	-	-	-
A 522		PHE	0.65	-	Favored (43.02%) General / -101.2,6.7	Favored (85.2%) *m-80* chi angles: 295.8,105.3	0.08Å	Favored (17.807%)	-	OUTLIER(S) worst is CA-CB-CG: 4.6 σ	-
A 523		LYS	0.66	-	Favored (42.42%) General / -58.4,129.5	Favored (87.9%) *tttt* chi angles: 184.5,176.5,179.3,180.8	0.04Å	Favored (32.49%)	-	-	-
A 524		LEU	0.67	-	Favored (16.39%) General / -95.3,156.3	Favored (95.9%) *mt* chi angles: 297.3,175.6	0.01Å	Favored (36.837%)	-	-	-
A 525		ARG	0.68	-	Favored (22.71%) General / -76.4,166.9	Favored (99%) *mtt180* chi angles: 294.6,179,180.6,177.8	0.01Å	Favored (39.666%)	-	-	-
A 526		THR	0.69	-	Favored (68.94%)	Favored (95.8%) *m* chi angles: 299.6	0.01Å	Favored (46.999%)	-	-	-

					General / -53.5,-48.3						
A 527		GLU	0.69	-	Favored (81.86%) General / -65.2,-35.7	Favored (72.8%) *mm-30* chi angles: 290.7,294.7,308.1	0.01Å	Favored (82.877%) alpha helix	-	-	-
A 528		GLN	0.69	-	Favored (79.34%) General / -69.1,-39.1	Favored (97.9%) *mt0* chi angles: 292.6,175.5,317.3	0.03Å	Favored (95.932%) alpha helix	-	-	-
A 529		ARG	0.68	-	Favored (97.99%) General / -63.7,-41.7	Favored (30.5%) *mtp-110* chi angles: 286.8,178.5,63.9,251.1	0.03Å	Favored (97.382%) alpha helix	-	-	-
A 530		LYS	0.67	-	Favored (96.98%) General / -63.5,-40.4	Favored (54.7%) *mtmt* chi angles: 288.4,188.5,288.6,186.9	0.03Å	Favored (97.263%) alpha helix	-	-	-
A 531		THR	0.67	-	Favored (90.82%) General / -64.3,-44.7	Favored (89.1%) *m* chi angles: 298.3	0.04Å	Favored (86.987%) alpha helix	-	-	-
A 532		PHE	0.68	-	Favored (68.9%) General / -55.3,-50.7	Favored (49%) *t80* chi angles: 169.9,67	0.08Å	Favored (83.662%) alpha helix	-	OUTLIER(S) worst is CA-CB-CG: 6.4 σ	-
A 533		VAL	0.71	-	Favored (94.37%) Ile or Val / -64.8,-45.6	Favored (70.1%) *t* chi angles: 172.1	0.04Å	Favored (85.6%) alpha helix	-	-	-
A 534		GLU	0.75	-	Favored (87.73%) General / -59.8,-40.3	Favored (89.3%) *tt0* chi angles: 187.3,174.5,3.5	0.05Å	Favored (90.982%) alpha helix	-	-	-
A 535		LEU	0.8	-	Favored (99.09%) General / -61.8,-42.5	Favored (62.1%) *mt* chi angles: 286.8,165.6	0.07Å	Favored (91.995%) alpha helix	-	-	-
A 536		MET	0.86	-	Favored (75.11%) General / -69.3,-34.7	Favored (82.4%) *mtm* chi angles: 290.4,188.5,292.2	0.01Å	Favored (72.83%) alpha helix	-	-	-
A 537		LYS	0.91	-	Favored (21.72%) General / -84.7,-36.7	Favored (49.9%) *mtpt* chi angles: 290.3,173.7,73.8,182.3	0.09Å	Favored (59.397%) alpha helix	-	-	-
A 538		ARG	0.94	-	Favored (8.65%) General / -101.9,-36.5	Favored (84%) *mtp180* chi angles: 294.3,184.3,69.3,188.8	0.04Å	Favored (37.666%) alpha helix	-	-	-
A 539		GLY	0.94	-	Favored (66.02%) Glycine / -93.7,-1.3	-	-	Favored (33.641%)	-	-	-
A 540		ASP	0.91	-	Favored (30.79%) General / 53.8,43.6	Favored (24.8%) *t0* chi angles: 197.8,31.4	0.03Å	Favored (24.347%)	-	-	-
**#**	**Alt**	**Res**	**High B**	**Clash > 0.4Å**	**Ramachandran**	**Rotamer**	**Cβ deviation**	**CaBLAM**	**Bond lengths**	**Bond angles**	**Cis Peptides**
			Avg: 1.07	Clashscore: 0.83	Outliers: 4 of 615	Poor rotamers: 0 of 510	Outliers: 0 of 564	Outliers: 16 of 613	Outliers: 5 of 617	Outliers: 10 of 617	Non-Trans: 1 of 616
A 541		LEU	0.86	-	Favored (38.39%) Pre-Pro / -91.1,148.7	Favored (78.2%) *mt* chi angles: 302,176.5	0.07Å	Favored (21.965%) beta sheet	-	-	-
A 542		PRO	0.8	-	Favored (55.19%)	Favored (62.5%) *Cg_endo*	0.06Å	Favored (88.46%)	-	-	-

				Trans-Pro / -68.0,158.9	chi angles: 26.6,326,27.2					
A 543	VAL	0.75	-	Favored (91.84%) Ile or Val / -59.1,-46.7	Favored (70.8%) *t* chi angles: 172.2	0.11Å	Favored (67.517%)	-	-	-
A 544	TRP	0.71	-	Favored (62.04%) General / -54.9,-52.7	Favored (60.6%) *t-100* chi angles: 183.1,265.9	0.02Å	Favored (75.705%) alpha helix	-	-	-
A 545	LEU	0.68	-	Favored (77.15%) General / -66.4,-46.2	Favored (59.9%) *tp* chi angles: 181,59.5	0.02Å	Favored (74.634%) alpha helix	-	-	-
A 546	ALA	0.65	-	Favored (79.75%) General / -58.6,-39.2	-	0.06Å	Favored (89.369%) alpha helix	-	-	-
A 547	TYR	0.64	-	Favored (69.45%) General / -59.1,-51.6	Favored (83.7%) *t80* chi angles: 178.9,84.4	0.07Å	Favored (91.961%) alpha helix	OUTLIER(S) worst is CB--CG: 4.3 σ	-	-
A 548	GLN	0.63	-	Favored (98.1%) General / -63.4,-40.8	Favored (96%) *mt0* chi angles: 290.4,172.1,316.1	0.06Å	Favored (87.94%) alpha helix	-	-	-
A 549	VAL	0.62	-	Favored (92.63%) Ile or Val / -64.6,-46.4	Favored (73.5%) *t* chi angles: 172.5	0.10Å	Favored (86.788%) alpha helix	-	-	-
A 550	ALA	0.62	-	Favored (95.04%) General / -63.0,-39.5	-	0.04Å	Favored (82.434%) alpha helix	-	-	-
A 551	SER	0.62	-	Favored (65.79%) General / -67.2,-24.0	Favored (61.2%) *p* chi angles: 73	0.06Å	Favored (73.392%) alpha helix	-	-	-
A 552	ALA	0.63	-	Favored (56.97%) General / -82.3,-4.3	-	0.04Å	Favored (53.349%)	-	-	-
A 553	GLY	0.65	-	Favored (66.21%) Glycine / 79.2,22.1	-	-	Favored (86.178%)	-	-	-
A 554	ILE	0.68	-	Favored (43.05%) Ile or Val / -97.5,117.9	Favored (70.4%) *mt* chi angles: 302,169.2	0.08Å	Favored (30.416%)	-	-	-
A 555	THR	0.72	-	Favored (20.79%) General / -80.0,167.2	Favored (64.1%) *p* chi angles: 63.3	0.04Å	Favored (19.304%)	-	-	-
A 556	TYR	0.75	-	Favored (55.31%) General / -60.0,141.6	Favored (49.6%) *t80* chi angles: 190.5,78.8	0.05Å	Favored (28.344%)	-	-	-
A 557	THR	0.77	-	Allowed (1.3%) General / 81.7,-8.8	Favored (6.9%) *p* chi angles: 78	0.14Å	CaBLAM Disfavored (3.562%)	-	-	-
A 558	ASP	0.77	-	Favored (7.79%) General / -81.4,80.5	Favored (65.3%) *t0* chi angles: 185,344.6	0.05Å	Favored (15.375%)	-	-	-
A 559	ARG	0.75	-	Favored (5.33%) General / -59.1,-12.7	Favored (50.5%) *ptt-90* chi angles: 68.1,185.6,181.6,277.3	0.08Å	Favored (8.706%)	-	-	-

#	Alt	Res	High B	Clash > 0.4Å	Ramachandran	Rotamer	Cβ deviation	CaBLAM	Bond lengths	Bond angles	Cis Peptides
A 560		ARG	0.72	-	Favored (66.7%) General / -58.1,-31.0	Favored (87.7%) *mtm180* chi angles: 291,180.3,294.1,173.4	0.02Å	Favored (44.497%)	-	-	-
#	Alt	Res	High B	Clash > 0.4Å	Ramachandran	Rotamer	Cβ deviation	CaBLAM	Bond lengths	Bond angles	Cis Peptides
			Avg: 1.07	Clashscore: 0.83	Outliers: 4 of 615	Poor rotamers: 0 of 510	Outliers: 0 of 564	Outliers: 16 of 613	Outliers: 5 of 617	Outliers: 10 of 617	Non-Trans: 1 of 616
A 561		TRP	0.69	-	Favored (64.9%) General / -65.0,-18.0	Favored (71.9%) *p-90* chi angles: 64.3,265.2	0.11Å	Favored (66.803%) three-ten	-	-	-
A 562		CYS	0.67	-	Favored (50.21%) General / -85.2,-13.4	Favored (79.7%) *m* chi angles: 296.1	0.08Å	Favored (51.398%) alpha helix	-	-	-
A 563		PHE	0.67	-	Favored (5.12%) General / -120.7,-27.2	Favored (82.6%) *m-80* chi angles: 300.2,104.8	0.03Å	Favored (10.931%) alpha helix	-	-	-
A 564		ASP	0.69	-	Favored (6.85%) General / -80.1,74.5	Favored (40.8%) *t0* chi angles: 188.9,24	0.09Å	CaBLAM Disfavored (1.284%)	-	-	-
A 565		GLY	0.72	-	Favored (41.84%) Glycine / -168.9,179.2	-	-	Favored (24.085%)	-	-	-
A 566		THR	0.75	-	Favored (42.79%) General / -59.4,145.7	Favored (5.4%) *t* chi angles: 180.8	0.04Å	Favored (9.521%)	-	-	-
A 567		THR	0.78	-	Favored (62.23%) General / -55.2,-32.4	Favored (96.8%) *m* chi angles: 299.8	0.02Å	Favored (45.394%)	-	-	-
A 568		ASN	0.79	-	Favored (64.51%) General / -60.9,-22.6	Favored (99.6%) *m-40* chi angles: 287.6,340.9	0.02Å	Favored (59.124%)	-	-	-
A 569		ASN	0.8	-	Favored (42.19%) General / -97.5,9.5	Favored (98.4%) *m-40* chi angles: 290.7,340.3	0.05Å	Favored (29.116%)	-	-	-
A 570		THR	0.83	-	Favored (40.27%) General / -56.0,129.6	Favored (82.9%) *m* chi angles: 302.1	0.04Å	Favored (39.314%)	-	-	-
A 571		ILE	0.86	-	Favored (34.5%) Ile or Val / -87.1,131.6	Favored (92.1%) *mt* chi angles: 297.5,171.3	0.03Å	Favored (54.354%) beta sheet	-	-	-
A 572		MET	0.91	-	Favored (41.43%) General / -122.7,151.6	Favored (64.2%) *mtt* chi angles: 298.3,182.9,180.7	0.01Å	Favored (64.026%)	-	-	-
A 573		GLU	0.95	-	Favored (15.94%) General / -117.7,108.9	Favored (53.9%) *mt-10* chi angles: 294.4,179.9,109.5	0.02Å	Favored (15.964%)	-	-	-
A 574		ASP	0.96	-	Favored (32%) General / 53.1,43.1	Favored (71.2%) *m-30* chi angles: 298.6,327.8	0.01Å	Favored (37.312%)	-	-	-
A 575		SER	0.94	-	Favored (2.02%) General / 78.7,-5.6	Favored (5.1%) *p* chi angles: 85.1	0.07Å	CaBLAM Disfavored (2.898%)	-	-	-
A 576		VAL	0.88	-	Favored (40.77%)	Favored (77.7%) *t* chi angles: 178.1	0.07Å	Favored (25.632%)	-	-	-

					Pre-Pro / -96.0,127.5						
A 577		PRO	0.8	-	Favored (43.22%) Trans-Pro / -50.9,135.8	Favored (87.4%) *Cg_exo* chi angles: 330.2,37.3,331.5	0.05Å	Favored (90.448%) beta sheet	-	-	-
A 578		ALA	0.74	-	Favored (14.43%) General / -79.7,109.8	-	0.02Å	Favored (47.364%) beta sheet	-	-	-
A 579		GLU	0.72	-	Favored (30.08%) General / -94.7,140.3	Favored (95.1%) *mt-10* chi angles: 293.7,177.4,339.6	0.01Å	Favored (47.954%) beta sheet	-	-	-
A 580		VAL	0.73	-	Favored (73.59%) Ile or Val / -123.7,126.9	Favored (81.2%) *t* chi angles: 176.6	0.07Å	Favored (63.68%) beta sheet	-	-	-
**#**	**Alt**	**Res**	**High B**	**Clash > 0.4Å**	**Ramachandran**	**Rotamer**	**Cβ deviation**	**CaBLAM**	**Bond lengths**	**Bond angles**	**Cis Peptides**
			Avg: 1.07	Clashscore: 0.83	Outliers: 4 of 615	Poor rotamers: 0 of 510	Outliers: 0 of 564	Outliers: 16 of 613	Outliers: 5 of 617	Outliers: 10 of 617	Non-Trans: 1 of 616
A 581		TRP	0.78	-	Favored (31.34%) General / -84.0,124.7	Favored (97.5%) *m100* chi angles: 287.7,99.5	0.05Å	Favored (52.713%)	-	-	-
A 582		THR	0.83	-	Favored (13.17%) General / -88.7,167.7	Favored (70.3%) *p* chi angles: 62.3	0.07Å	Favored (41.735%)	-	-	-
A 583		LYS	0.87	-	Favored (27.68%) General / -59.5,-16.9	Favored (11.1%) *ptmt* chi angles: 66.8,186.7,286.9,185.7	0.02Å	Favored (26.624%)	-	-	-
A 584		TYR	0.87	-	Favored (43.24%) General / -101.0,8.0	Favored (85.1%) *m-80* chi angles: 293.2,103.3	0.04Å	Favored (57.385%)	-	-	-
A 585		GLY	0.84	-	Favored (79.44%) Glycine / 90.7,-8.7	-	-	Favored (73.29%)	-	-	-
A 586		GLU	0.78	-	Favored (34.12%) General / -83.1,137.4	Favored (90.9%) *tt0* chi angles: 186.3,178.7,4.1	0.01Å	Favored (31.251%)	-	-	-
A 587		LYS	0.71	-	Favored (44.04%) General / -97.9,127.7	Favored (34.8%) *ttpt* chi angles: 184.1,172.9,71.9,167.6	0.05Å	Favored (57.745%) beta sheet	-	-	-
A 588		ARG	0.65	-	Favored (51.97%) General / -131.8,151.0	Favored (95.2%) *mtt180* chi angles: 297.1,183.9,180.6,186.7	0.04Å	Favored (51.457%) beta sheet	-	-	-
A 589		VAL	0.61	-	Favored (39.48%) Ile or Val / -86.5,128.4	Favored (98%) *t* chi angles: 175.2	0.02Å	Favored (33.017%) beta sheet	-	-	-
A 590		LEU	0.6	-	Favored (29.23%) General / -67.8,126.4	Favored (87.1%) *mt* chi angles: 290.4,171.1	0.05Å	Favored (22.789%) beta sheet	-	-	-
A 591		LYS	0.6	-	Favored (12.59%) Pre-Pro / -146.7,71.3	Favored (97.8%) *mttt* chi angles: 296.3,179.1,180.2,174.6	0.07Å	CaBLAM Disfavored (4.396%) try beta sheet	-	-	-
A 592		PRO	0.61	-	Favored (81.71%)	Favored (51.1%) *Cg_exo*	0.03Å	Favored (32.392%)	-	-	-

					Trans-Pro / -61.0,150.9	chi angles: 337.7,36.3,325.5					
A 593		ARG	0.64	-	Favored (73.58%) General / -61.9,-33.1	Favored (97.4%) *mtt180* chi angles: 287.3,179.4,178.2,178.8	0.05Å	Favored (26.619%)	-	-	-
A 594		TRP	0.68	-	Favored (50.35%) General / -132.7,144.5	Favored (21.1%) *m-90* chi angles: 304.9,270.6	0.14Å	Favored (38.025%)	-	-	-
A 595		MET	0.72	-	Favored (40.06%) General / -134.3,132.9	Favored (14.3%) *tpt* chi angles: 182.2,65,193.7	0.04Å	Favored (68.823%)	-	-	-
A 596		ASP	0.78	-	Favored (12.69%) General / -117.1,105.8	Favored (51.5%) *t0* chi angles: 181.6,338.1	0.03Å	Favored (57.036%) beta sheet	-	-	-
A 597		ALA	0.84	-	Favored (21.5%) General / -57.5,-19.1	-	0.06Å	Favored (19.836%)	-	-	-
A 598		ARG	0.93	-	Favored (53.86%) General / -53.8,-32.7	Favored (83.9%) *ttt180* chi angles: 185.2,175.3,179.6,177.1	0.02Å	Favored (56.795%)	-	-	-
A 599		VAL	1.04	-	Favored (82.52%) Ile or Val / -61.5,-39.3	Favored (60.4%) *t* chi angles: 170.8	0.04Å	Favored (67.569%) alpha helix	-	-	-
A 600		CYS	1.16	-	Favored (26.83%) General / -94.5,-12.8	Favored (24.7%) *p* chi angles: 68.4	0.01Å	Favored (33.162%) alpha helix	-	-	-
**#**	**Alt**	**Res**	**High B**	**Clash > 0.4Å**	**Ramachandran**	**Rotamer**	**Cβ deviation**	**CaBLAM**	**Bond lengths**	**Bond angles**	**Cis Peptides**
			Avg: 1.07	Clashscore: 0.83	Outliers: 4 of 615	Poor rotamers: 0 of 510	Outliers: 0 of 564	Outliers: 16 of 613	Outliers: 5 of 617	Outliers: 10 of 617	Non-Trans: 1 of 616
A 601		SER	1.27	-	Favored (70.62%) General / -59.9,-51.2	Favored (36.1%) *t* chi angles: 181.3	0.08Å	Favored (33.742%) alpha helix	-	-	-
A 602		ASP	1.35	-	Favored (33.41%) General / -101.7,116.8	Favored (51%) *t0* chi angles: 185.8,332.3	0.07Å	Favored (26.125%) alpha helix	-	-	-
A 603		HIS	1.37	-	Favored (6.76%) General / -44.9,-41.0	Favored (92.2%) *t70* chi angles: 177,73.2	0.04Å	Favored (48.282%) alpha helix	-	-	-
A 604		ALA	1.33	-	Favored (83.31%) General / -61.1,-37.6	-	0.05Å	Favored (75.425%) alpha helix	-	-	-
A 605		ALA	1.23	-	Favored (83.22%) General / -67.8,-38.4	-	0.02Å	Favored (92.678%) alpha helix	-	-	-
A 606		LEU	1.1	-	Favored (82.26%) General / -67.7,-42.4	Favored (46%) *tp* chi angles: 182,55.8	0.07Å	Favored (93.47%) alpha helix	-	-	-
A 607		LYS	0.97	-	Favored (81.23%) General / -56.9,-47.2	Favored (53.5%) *tttm* chi angles: 181.8,178.1,185.7,293.9	0.01Å	Favored (92.35%) alpha helix	-	-	-

A 608		SER	0.86	-	Favored (97.21%) General / -61.6,-41.2	Favored (72.7%) *m* chi angles: 295.4	0.04Å	Favored (93.915%) alpha helix	-	-	-
A 609		PHE	0.78	-	Favored (87.08%) General / -59.3,-47.0	Favored (81.2%) *t80* chi angles: 183,83.2	0.03Å	Favored (96.875%) alpha helix	-	-	-
A 610		LYS	0.72	-	Favored (92.74%) General / -61.1,-40.2	Favored (49%) *mtpt* chi angles: 287.6,170.9,73.7,179.8	0.03Å	Favored (93.726%) alpha helix	-	-	-
A 611		GLU	0.68	-	Favored (94.02%) General / -64.7,-39.6	Favored (97.5%) *mt-10* chi angles: 290.4,179,0.4	0.03Å	Favored (97.518%) alpha helix	-	-	-
A 612		PHE	0.68	-	Favored (68.09%) General / -60.1,-51.8	Favored (80.1%) *t80* chi angles: 172.1,80	0.06Å	Favored (77.895%) alpha helix	-	-	-
A 613		ALA	0.72	-	Favored (77.84%) General / -59.8,-37.0	-	0.06Å	Favored (76.001%) alpha helix	-	-	-
A 614		ALA	0.82	-	Favored (68.25%) General / -62.2,-26.2	-	0.05Å	Favored (30.343%)	-	-	-
A 615		GLY	1	-	Favored (56.97%) Glycine / 85.2,20.6	-	-	Favored (74.548%)	-	-	-
A 616		LYS	1.25	-	Favored (54.95%) General / -77.2,-30.2	Favored (96.8%) *mttt* chi angles: 288.7,177.5,179.9,179.2	0.03Å	-	-	-	-
A 617		ARG	1.59	0.56Å HG3 with A 617 ARG OXT	-	Favored (37.9%) *ptt180* chi angles: 60.6,181.5,179.4,171.5	0.04Å	-	-	-	-

About MolProbity | Website for the Richardson Lab | Using ecloud x-H | Internal reference 4.5.2
