# Supplementary material for: Integrative proteome-wide structural analysis and high-throughput docking identify broad-spectrum antiviral scaffolds against Zika, Yellow Fever, West Nile, Saint Louis encephalitis, and Usutu viruses
Source: Front Cell Infect Microbiol. 2026 Apr 30;16:1723132. doi: 10.3389/fcimb.2026.1723132 (PMC13171538; doi:10.3389/fcimb.2026.1723132)
Supplement: Supplementary file 7 [file DataSheet7.zip › ZIKV/ZIKV_NS3/Mol_probity_Files/ZIKV_NS3_1FH-rama.pdf]

# MolProbity Ramachandran analysis

ZIKV_NS3_1FH.pdb, model 1

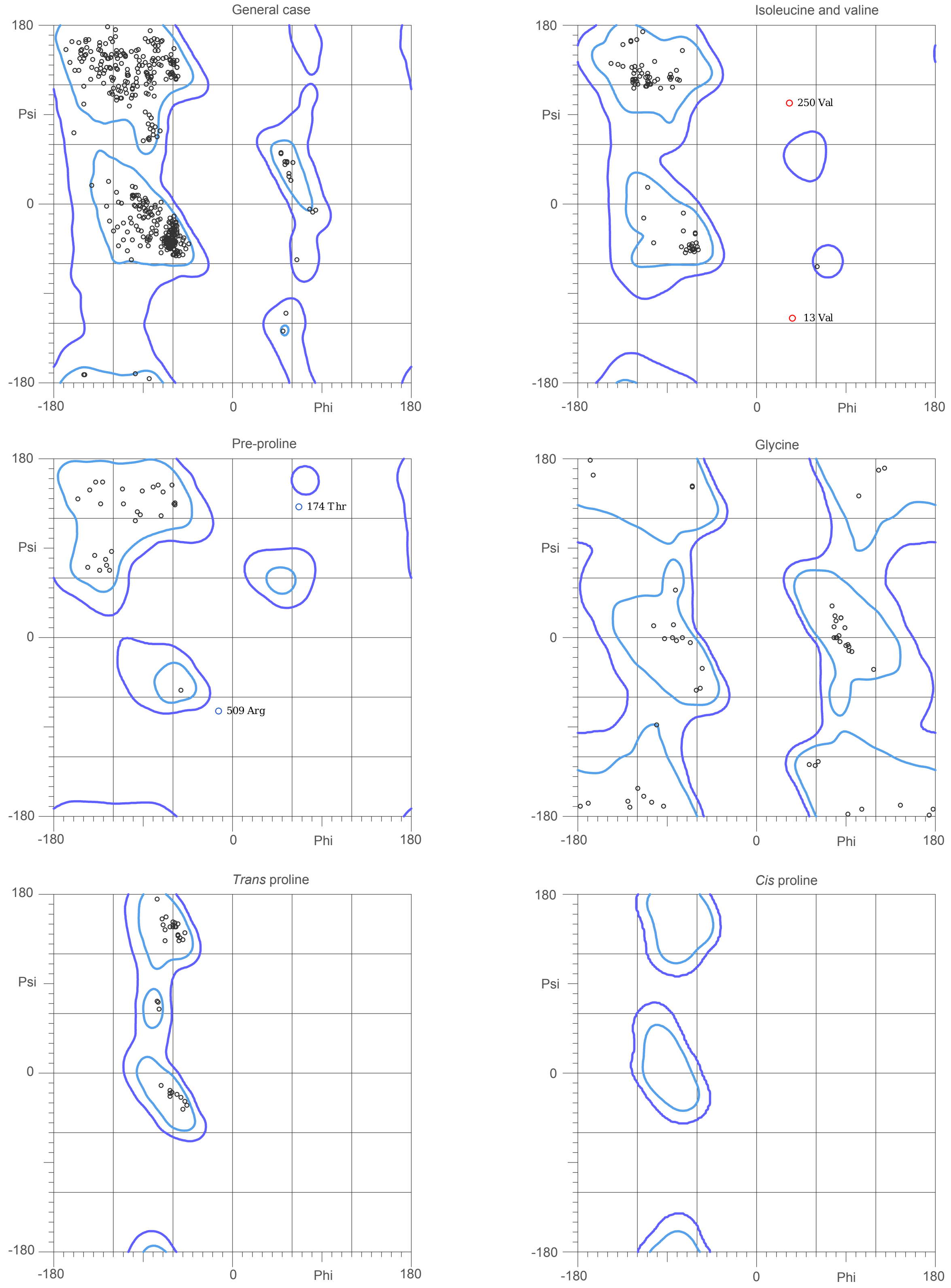


98.2% (604/615) of all residues were in favored (98%) regions.
99.3% (611/615) of all residues were in allowed (>99.8%) regions.

There were 4 outliers (phi, psi):
13 Val (36.7, -115.2)
174 Thr (67.3, 132.8)
250 Val (33.9, 102.9)
509 Arg (-14.4, -74.3)
