# Supplementary material for: Integrative proteome-wide structural analysis and high-throughput docking identify broad-spectrum antiviral scaffolds against Zika, Yellow Fever, West Nile, Saint Louis encephalitis, and Usutu viruses
Source: Front Cell Infect Microbiol. 2026 Apr 30;16:1723132. doi: 10.3389/fcimb.2026.1723132 (PMC13171538; doi:10.3389/fcimb.2026.1723132)
Supplement: Supplementary file 7 [file DataSheet7.zip › ZIKV/ZIKV_NS4a/Mol_probity_Files/ZIKV_NS4a_1FH-multi.table.pdf]

Viewing ZIKV_NS4a1FH-multi.table

Duke Biochemistry
Duke University School of Medicine

When finished, you should close this window.

Hint: Use File | Save As... to save a copy of this page.

All-Atom Contacts	Clashscore, all atoms:	102.28		0th percentile* (N=1784, all resolutions)
	Clashscore is the number of serious steric overlaps (> 0.4 Å) per 1000 atoms.			
Protein Geometry	Poor rotamers	2	2.00%	Goal: <0.3%
	Favored rotamers	95	95.00%	Goal: >98%
	Ramachandran outliers	3	2.40%	Goal: <0.05%
	Ramachandran favored	117	93.60%	Goal: >98%
	Rama distribution Z-score	1.45 ± 0.74		Goal: abs(Z score) < 2
	MolProbity score^	3.12		18th percentile* (N=27675, 0Å - 99Å)
	Cβ deviations >0.25Å	0	0.00%	Goal: 0
	Bad bonds:	77 / 973	7.91%	Goal: 0%
	Bad angles:	46 / 1317	3.49%	Goal: <0.1%
Peptide Omegas	Cis Prolines:	0 / 6	0.00%	Expected: ≤1 per chain, or ≤5%
	Twisted Peptides:	19 / 126	15.08%	Goal: 0
Low-resolution Criteria	CaBLAM outliers	13	10.6%	Goal: <1.0%
	CA Geometry outliers	7	5.69%	Goal: <0.5%
Additional validations	Chiral volume outliers	0/160		
	Waters with clashes	0/0	0.00%	See UnDowser table for details

In the two column results, the left column gives the raw count, right column gives the percentage.

* 100th percentile is the best among structures of comparable resolution; 0th percentile is the worst. For clashscore the comparative set of structures was selected in 2004, for MolProbity score in 2006.

^ MolProbity score combines the clashscore, rotamer, and Ramachandran evaluations into a single score, normalized to be on the same scale as X-ray resolution.

Key to table colors and cutoffs here:

#	Alt	Res	High B	Clash > 0.4Å	Ramachandran	Rotamer	Cβ deviation	CaBLAM	Bond lengths	Bond angles	Cis Peptides
			Avg: 6.09	Clashscore: 102.28	Outliers: 3 of 125	Poor rotamers: 2 of 100	Outliers: 0 of 114	Outliers: 16 of 123	Outliers: 66 of 127	Outliers: 37 of 127	Non-Trans: 19 of 126
1		GLY	14.18	-	-	-	-	-	-	-	-
2		ALA	10.23	0.58Å O with 6 VAL HG23	Favored (99.94%) General / -62.9,-42.9	-	0.05Å	-	OUTLIER(S) worst is C--N: 6.7 σ	-	Twisted nonPRO omega= 140.64
3		ALA	8.79	0.69Å HA with 68 ILE HD11	Favored (87.97%) General / -64.9,-45.0	-	0.06Å	Favored (75.036%)	OUTLIER(S) worst is C--N: 6.2 σ	OUTLIER(S) worst is C-N-CA: 5.0 σ	-
4		LEU	8.51	-	Favored (91.37%) General / -65.7,-39.6	Favored (92.4%) *mt* chi angles: 293.5,175.5	0.04Å	Favored (77.3%) alpha helix	OUTLIER(S) worst is CA--C: 4.8 σ	OUTLIER(S) worst is C-N-CA: 4.1 σ	-
5		GLY	7.38	-	Favored (98.43%) Glycine / -61.5,-44.1	-	-	Favored (97.46%) alpha helix	-	-	-
6		VAL	6.44	0.68Å HB with 68 ILE HD13	Favored (90.3%)	Favored (99.9%) *t* chi angles: 175.5	0.09Å	Favored (93.69%) alpha helix	-	-	-

					Ile or Val / -65.3,-41.0						
7		MET	7.15	0.73Å HG2 with 68 ILE HG23	Favored (98.84%) General / -61.2,-42.6	Favored (86.1%) *mtp* chi angles: 293.5,185.5,71	0.04Å	Favored (89.23%) alpha helix	OUTLIER(S) worst is C--N: 5.0 σ	-	-
8		GLU	7.09	-	Favored (90.45%) General / -65.9,-42.3	Favored (91.3%) *tt0* chi angles: 183.7,176.4,179.3	0.04Å	Favored (89.494%) alpha helix	OUTLIER(S) worst is C--N: 4.2 σ	-	-
9		ALA	5.96	0.67Å HB3 with 31 LEU HD11	Favored (98.26%) General / -62.2,-41.3	-	0.03Å	Favored (97.731%) alpha helix	OUTLIER(S) worst is CA--C: 4.5 σ	OUTLIER(S) worst is C-N-CA: 5.1 σ	-
10		LEU	6.59	0.90Å HD22 with 72 LEU HD22	Favored (94.37%) General / -64.9,-42.8	Favored (82.6%) *mt* chi angles: 289.5,169.5	0.01Å	Favored (96.702%) alpha helix	OUTLIER(S) worst is CA--C: 4.8 σ	-	-
11		GLY	7	-	Favored (97.5%) Glycine / -63.9,-39.6	-	-	Favored (95.793%) alpha helix	-	-	-
12		THR	6.46	-	Favored (96.5%) General / -60.8,-44.7	Favored (89.4%) *m* chi angles: 298.7	0.04Å	Favored (79.457%) alpha helix	-	-	-
13		LEU	6.63	1.09Å HD12 with 27 ASN HB2	Favored (55.92%) Pre-Pro / -66.7,-45.0	OUTLIER (0.1%) chi angles: 281.5,214.5	0.04Å	Favored (44.904%)	-	-	-
14		PRO	8.02	1.26Å CD with 14 PRO N	Allowed (0.18%) Trans-Pro / -82.2,-162.6	Favored (46.8%) *Cg_exo* chi angles: 327.5,357.5,29.4	0.10Å	CA Geom Outlier (0%)	OUTLIER(S) worst is N--CD: 19.8 σ	OUTLIER(S) worst is CA-N-CD: 13.3 σ	-
15		GLY	8.44	-	Favored (7.13%) Glycine / 113.5,-30.6	-	-	CA Geom Outlier (0.161%)	-	OUTLIER(S) worst is C-N-CA: 4.9 σ	Twisted nonPRO omega= -104.68
16		HIS	10.22	0.56Å H with 14 PRO HB3	Favored (34.28%) General / -149.6,164.7	Favored (97.1%) *m-70* chi angles: 298.2,282.2	0.01Å	CA Geom Outlier (0.002%)	OUTLIER(S) worst is C--N: 6.6 σ	-	Twisted nonPRO omega= -139.75
17		MET	10.63	1.06Å HE1 with 76 LYS HG2	Favored (2.76%) General / -96.8,-55.8	Favored (55.3%) *ttm* chi angles: 180.5,171.5,289.4	0.05Å	CaBLAM Outlier (0.286%) try alpha helix	OUTLIER(S) worst is C--N: 6.1 σ	OUTLIER(S) worst is C-N-CA: 4.7 σ	Twisted nonPRO omega= 142.14
18		THR	10.54	0.48Å HG23 with 124 GLU OE1	Favored (91.85%) General / -60.1,-45.8	Favored (88.5%) *m* chi angles: 298.6	0.03Å	Favored (58.538%) alpha helix	OUTLIER(S) worst is C--N: 6.3 σ	OUTLIER(S) worst is C-N-CA: 4.5 σ	-
19		GLU	10.5	0.49Å O with 22 GLN HB2	Favored (59.81%) General / -76.0,-35.0	Favored (70.7%) *tt0* chi angles: 179.5,178.5,156.6	0.08Å	Favored (70.29%) alpha helix	OUTLIER(S) worst is C--N: 5.4 σ	OUTLIER(S) worst is C-N-CA: 4.2 σ	-
20		ARG	11.47	1.31Å O with 24 ALA N	Favored (71.42%) General / -60.1,-51.0	Favored (79.8%) *ttp80* chi angles: 180.1,188.5,63.6,82.5	0.08Å	Favored (35.025%) alpha helix	OUTLIER(S) worst is CA--C: 4.2 σ	-	-
**#**	**Alt**	**Res**	**High B**	**Clash > 0.4Å**	**Ramachandran**	**Rotamer**	**Cβ deviation**	**CaBLAM**	**Bond lengths**	**Bond angles**	**Cis Peptides**
			Avg: 6.09	Clashscore: 102.28	Outliers: 3 of 125	Poor rotamers: 2 of 100	Outliers: 0 of 114	Outliers: 16 of 123	Outliers: 66 of 127	Outliers: 37 of 127	Non-Trans: 19 of 126
21		PHE	9.71	0.70Å CE1 with 120 ILE HD12	Favored (6.17%) General / -51.6,-25.0	Favored (28.5%) *t80* chi angles: 195.5,76.5	0.03Å	Favored (52.554%) alpha helix	-	-	-

22		GLN	9.73	0.49Å HB2 with 19 GLU O	Favored (6.76%) General / -78.9,-53.7	Favored (62.4%) *mt0* chi angles: 294.5,177.5,76.5	0.06Å	Favored (33.249%) alpha helix	OUTLIER(S) worst is C--N: 5.3 σ	OUTLIER(S) worst is C-N-CA: 5.2 σ	-
23		GLU	8.55	0.69Å HB2 with 20 ARG HA	Favored (66.52%) General / -65.9,-22.8	Favored (57.9%) *mt-10* chi angles: 294.1,178.5,117.8	0.04Å	Favored (36.682%) alpha helix	-	-	-
24		ALA	7.83	1.31Å N with 20 ARG O	Favored (97.33%) General / -63.7,-40.6	-	0.07Å	Favored (54.274%) alpha helix	-	OUTLIER(S) worst is C-N-CA: 4.3 σ	-
25		ILE	8.29	0.97Å HD11 with 116 LEU HD12	Favored (97.3%) Ile or Val / -60.6,-44.7	Favored (49.4%) *mm* chi angles: 302.5,299.5	0.03Å	Favored (83.311%) alpha helix	-	-	-
26		ASP	8.42	0.48Å O with 30 VAL HG13	Favored (99.23%) General / -62.5,-42.0	Favored (86.7%) *m-30* chi angles: 294.5,161.5	0.04Å	Favored (78.449%) alpha helix	OUTLIER(S) worst is CG--OD2: 14.3 σ	OUTLIER(S) worst is OD1-CG-OD2: 5.3 σ	-
27		ASN	7	1.09Å HB2 with 13 LEU HD12	Favored (97.09%) General / -61.9,-44.3	Favored (25.5%) *t0* chi angles: 189.5,296.6	0.01Å	Favored (89.363%) alpha helix	OUTLIER(S) worst is CA--C: 4.6 σ	-	-
28		LEU	6.92	-	Favored (87.9%) General / -65.0,-44.9	Favored (87.8%) *mt* chi angles: 291.5,174.5	0.03Å	Favored (85.818%) alpha helix	-	-	-
29		ALA	7.99	0.40Å O with 33 ARG HG2	Favored (66.27%) General / -56.2,-51.9	-	0.04Å	Favored (83.159%) alpha helix	OUTLIER(S) worst is C--N: 4.8 σ	-	-
30		VAL	7.91	0.48Å HG13 with 26 ASP O	Favored (80.98%) Ile or Val / -67.7,-38.8	Favored (32.5%) *m* chi angles: 297.5	0.03Å	Favored (73.072%) alpha helix	OUTLIER(S) worst is C--N: 6.9 σ	-	-
31		LEU	6.83	0.67Å HD11 with 9 ALA HB3	Favored (73.15%) General / -68.7,-44.8	Favored (59.2%) *mt* chi angles: 289.5,177.5	0.02Å	Favored (72.227%) alpha helix	-	-	-
32		MET	7.3	1.04Å HG2 with 61 LEU HD21	Favored (78.76%) General / -69.0,-40.8	Favored (71.2%) *mtt* chi angles: 291.5,176.5,179.5	0.01Å	Favored (65.278%)	-	-	-
33		ARG	9.27	0.44Å NH2 with 35 GLU HB2	Favored (5.19%) General / -92.5,-179.4	Favored (10.5%) *ptm-80* chi angles: 75.5,187.5,303.5,272.5	0.07Å	Favored (5.593%)	OUTLIER(S) worst is C--N: 4.6 σ	-	-
34		ALA	8.64	-	Favored (64.47%) General / -65.5,-17.3	-	0.02Å	Favored (6.125%)	-	-	-
35		GLU	9.12	0.72Å HG2 with 35 GLU O	Favored (47.65%) General / -92.3,6.2	Favored (91.4%) *tt0* chi angles: 182.5,179.5,5.5	0.07Å	CaBLAM Outlier (0.003%)	OUTLIER(S) worst is C--N: 4.4 σ	-	Twisted nonPRO omega= -136.73
36		THR	9.86	-	Favored (8.58%) General / -110.3,-28.4	Favored (39.3%) *p* chi angles: 54.5	0.01Å	CaBLAM Outlier (0.127%)	-	-	-
37		GLY	9.52	-	Favored (2.49%) Glycine / 96.7,108.2	-	-	CaBLAM Outlier (0.843%)	OUTLIER(S) worst is C--N: 4.8 σ	-	-
38		SER	7.84	0.45Å O with 41 TYR N	Favored (16.19%) General / -99.3,104.3	Favored (68.1%) *m* chi angles: 296.8	0.09Å	CA Geom Outlier (0.007%)	-	OUTLIER(S) worst is C-N-CA: 5.3 σ	Twisted nonPRO omega= 122.63

39		ARG	8.11	0.43Å O with 42 LYS HB3	Favored (17.54%) Pre-Pro / -62.1,-35.0	Favored (97.8%) *mtt-85* chi angles: 293.5,182.3,178.5,275.5	0.02Å	Favored (24.316%)	OUTLIER(S) worst is CA--C: 4.5 σ	-	-
40		PRO	7.38	0.83Å CD with 40 PRO N	Favored (56.35%) Trans-Pro / -65.2,-28.4	Favored (86.2%) *Cg_exo* chi angles: 330.5,316.5,60.3	0.02Å	Favored (63.134%) alpha helix	OUTLIER(S) worst is N--CD: 60.8 σ	OUTLIER(S) worst is N-CD-CG: 21.9 σ	-
**#**	**Alt**	**Res**	**High B**	**Clash > 0.4Å**	**Ramachandran**	**Rotamer**	**Cβ deviation**	**CaBLAM**	**Bond lengths**	**Bond angles**	**Cis Peptides**
			Avg: 6.09	Clashscore: 102.28	Outliers: 3 of 125	Poor rotamers: 2 of 100	Outliers: 0 of 114	Outliers: 16 of 123	Outliers: 66 of 127	Outliers: 37 of 127	Non-Trans: 19 of 126
41		TYR	5.84	0.45Å N with 38 SER O	Favored (79.29%) General / -59.3,-38.0	Favored (4.6%) *m-10* chi angles: 280.1,32.5	0.03Å	Favored (54.152%) alpha helix	OUTLIER(S) worst is CA--C: 4.5 σ	-	-
42		LYS	6.28	0.43Å HB3 with 39 ARG O	Favored (92.03%) General / -60.1,-45.7	Favored (86.2%) *tttt* chi angles: 181.4,177.5,179.1,180.5	0.04Å	Favored (83.485%) alpha helix	-	-	-
43		ALA	6.98	-	Favored (96.84%) General / -60.5,-43.5	-	0.02Å	Favored (91.79%) alpha helix	-	-	-
44		ALA	6.79	0.69Å O with 47 GLN O	Favored (93.27%) General / -64.7,-39.2	-	0.02Å	Favored (98.986%) alpha helix	-	-	-
45		ALA	6.65	0.62Å HA with 48 LEU HD13	Favored (89.23%) General / -66.4,-39.9	-	0.02Å	Favored (76.101%) alpha helix	OUTLIER(S) worst is C--N: 4.9 σ	-	-
46		ALA	7.29	-	Favored (82%) General / -60.0,-38.2	-	0.06Å	Favored (61.019%) alpha helix	-	OUTLIER(S) worst is C-N-CA: 4.6 σ	-
47		GLN	8.57	0.69Å O with 44 ALA O	Favored (21.11%) General / -110.0,110.3	Favored (44%) *tt0* chi angles: 176,183,326.1	0.02Å	Favored (20.959%) alpha helix	-	-	-
48		LEU	8.25	0.75Å HD23 with 100 ILE HG13	Favored (30.74%) Pre-Pro / -132.5,91.4	Favored (56.7%) *tp* chi angles: 182.1,59.5	0.01Å	Favored (6.02%) alpha helix	-	OUTLIER(S) worst is CA-C-N: 5.8 σ	-
49		PRO	8.35	0.58Å O with 53 GLU HB3	OUTLIER (0)%) Trans-Pro / -152.6,7.3	Favored (63.2%) *Cg_endo* chi angles: 31.5,324.5,25.6	0.08Å	CaBLAM Outlier (0%) try alpha helix	OUTLIER(S) worst is N--CD: 11.4 σ	OUTLIER(S) worst is CA-N-CD: 8.0 σ	Twisted PRO omega= 141.7
50		GLU	8.23	0.46Å O with 53 GLU O	Allowed (0.28%) General / -146.9,-65.4	Favored (92.3%) *tt0* chi angles: 181.5,180.5,175.5	0.07Å	Favored (48.982%) alpha helix	OUTLIER(S) worst is CA--C: 4.2 σ	-	Twisted nonPRO omega= -135.63
51		THR	7.71	-	Favored (73.84%) General / -67.8,-45.7	Favored (95.5%) *m* chi angles: 299.5	0.10Å	Favored (40.658%) alpha helix	OUTLIER(S) worst is CA--C: 4.1 σ	-	Twisted nonPRO omega= 147.11
52		LEU	6.42	0.68Å HD11 with 56 MET HE2	Favored (83.18%) General / -64.9,-36.1	Favored (44.7%) *tp* chi angles: 184.6,59.7	0.06Å	Favored (58.7%) alpha helix	-	-	-
53		GLU	6.1	0.58Å HB3 with 49 PRO O	Favored (25.12%) General / -88.0,147.2	Favored (87.9%) *tt0* chi angles: 180.5,174.9,171.5	0.06Å	CaBLAM Disfavored (1.284%) try alpha helix	OUTLIER(S) worst is CA--C: 4.9 σ	-	-

54		THR	6.58	-	OUTLIER (0.02)%) General / 90.4,-53.8	Favored (100%) *m* chi angles: 300.5	0.05Å	CaBLAM Outlier (0.752%) try alpha helix	-	OUTLIER(S) worst is C-N-CA: 4.4 σ	-
55		ILE	5.99	0.61Å HG23 with 60 LEU HD12	Favored (95.59%) Ile or Val / -61.2,-47.0	Favored (96.3%) *mt* chi angles: 292.1,168.2	0.01Å	Favored (52.381%) alpha helix	OUTLIER(S) worst is C--N: 5.8 σ	OUTLIER(S) worst is C-N-CA: 5.1 σ	-
56		MET	5.48	0.68Å HE2 with 52 LEU HD11	Favored (93.46%) General / -63.7,-44.5	Favored (27.2%) *mmt* chi angles: 290.5,302.5,180.5	0.07Å	Favored (67.555%) alpha helix	-	OUTLIER(S) worst is C-N-CA: 4.9 σ	-
57		LEU	5.42	0.80Å HD21 with 93 TRP CD1	Favored (51.29%) General / -77.9,-31.6	Favored (61.5%) *mt* chi angles: 288.5,175.5	0.02Å	Favored (59.808%)	OUTLIER(S) worst is C--N: 5.2 σ	OUTLIER(S) worst is C-N-CA: 5.1 σ	-
58		LEU	5.99	-	Favored (5.78%) General / -84.5,-52.9	Favored (89.9%) *mt* chi angles: 293.5,176.8	0.03Å	CaBLAM Outlier (0.057%)	OUTLIER(S) worst is C--N: 4.4 σ	OUTLIER(S) worst is C-N-CA: 4.6 σ	-
59		GLY	6.34	-	Favored (11.5%) Glycine / 138.4,169.1	-	-	CA Geom Outlier (0.201%)	-	OUTLIER(S) worst is O-C-N: 4.0 σ	Twisted nonPRO omega= -143.98
60		LEU	6.38	0.61Å HD12 with 55 ILE HG23	OUTLIER (0.03)%) General / 61.8,-92.1	Favored (84.2%) *mt* chi angles: 291.5,175.5	0.02Å	CaBLAM Disfavored (1.548%)	OUTLIER(S) worst is C--N: 4.3 σ	-	Twisted nonPRO omega= -97.08
**#**	**Alt**	**Res**	**High B**	**Clash > 0.4Å**	**Ramachandran**	**Rotamer**	**Cβ deviation**	**CaBLAM**	**Bond lengths**	**Bond angles**	**Cis Peptides**
			Avg: 6.09	Clashscore: 102.28	Outliers: 3 of 125	Poor rotamers: 2 of 100	Outliers: 0 of 114	Outliers: 16 of 123	Outliers: 66 of 127	Outliers: 37 of 127	Non-Trans: 19 of 126
61		LEU	5.06	1.04Å HD21 with 32 MET HG2	Favored (99.3%) General / -63.2,-42.1	Favored (58.2%) *tp* chi angles: 181.7,59.7	0.07Å	Favored (49.37%) alpha helix	OUTLIER(S) worst is CA--C: 4.7 σ	-	Twisted nonPRO omega= -145.21
62		GLY	4.47	0.50Å HA2 with 65 SER OG	Favored (97.27%) Glycine / -61.6,-45.0	-	-	Favored (90.208%) alpha helix	-	-	-
63		THR	4.65	-	Favored (81.96%) General / -57.8,-47.6	Favored (88.9%) *m* chi angles: 298.4	0.01Å	Favored (68.981%) alpha helix	OUTLIER(S) worst is CA--C: 4.3 σ	OUTLIER(S) worst is C-N-CA: 5.2 σ	-
64		VAL	4.59	0.47Å HG13 with 3 ALA HB2	Favored (99.54%) Ile or Val / -62.1,-44.9	Favored (66%) *t* chi angles: 171.6	0.06Å	Favored (72.23%) alpha helix	-	-	-
65		SER	4.57	0.89Å OG with 88 LEU HD13	Favored (76.17%) General / -69.8,-37.0	Favored (80.4%) *p* chi angles: 61.5	0.01Å	Favored (82.388%) alpha helix	OUTLIER(S) worst is C--N: 4.0 σ	-	-
66		LEU	3.63	0.49Å C with 66 LEU HD12	Favored (79.09%) General / -65.4,-34.8	Allowed (1.4%) *pp* chi angles: 75.8,89.5	0.02Å	Favored (78.981%) alpha helix	OUTLIER(S) worst is C--N: 6.3 σ	OUTLIER(S) worst is C-N-CA: 4.8 σ	-
67		GLY	3.39	-	Favored (51.31%) Glycine / -63.8,-51.7	-	-	Favored (73.882%) alpha helix	-	-	-
68		ILE	3.77	0.73Å HG23 with 7 MET HG2	Favored (96.33%) Ile or Val / -62.0,-46.5	Favored (95.7%) *mt* chi angles: 294.5,168.5	0.04Å	Favored (76.501%) alpha helix	-	-	-

69		PHE	3.36	0.41Å O with 73 MET HG2	Favored (22.53%) General / -85.3,-32.3	Favored (50%) *t80* chi angles: 190.5,86.5	0.04Å	Favored (57.52%) alpha helix	OUTLIER(S) worst is CA--C: 4.1 σ	-	-
70		PHE	3.54	-	Favored (63.58%) General / -51.6,-47.9	Favored (90.9%) *t80* chi angles: 177.8,81.1	0.03Å	Favored (65.316%) alpha helix	-	OUTLIER(S) worst is C-N-CA: 5.1 σ	Twisted nonPRO omega= 147
71		VAL	4.35	0.70Å O with 74 ARG HG2	Favored (98%) Ile or Val / -63.6,-43.0	Favored (92.6%) *t* chi angles: 174.5	0.01Å	Favored (89.865%) alpha helix	-	OUTLIER(S) worst is O-C-N: 4.1 σ	-
72		LEU	4.51	0.90Å HD22 with 10 LEU HD22	Favored (96.13%) General / -64.4,-42.3	Favored (58%) *tp* chi angles: 175.5,64.5	0.03Å	Favored (90.53%) alpha helix	OUTLIER(S) worst is CA--C: 4.0 σ	-	-
73		MET	3.7	0.41Å HG2 with 69 PHE O	Favored (73.42%) General / -63.4,-32.1	Favored (96.6%) *mmm* chi angles: 290.5,300.5,291.5	0.04Å	Favored (6%)	OUTLIER(S) worst is CA--C: 4.3 σ	-	-
74		ARG	4.73	0.77Å HG3 with 72 LEU O	Allowed (0.25%) General / 65.7,-152.8	Favored (86.8%) *mtt180* chi angles: 305.8,173.5,184.5,179.5	0.05Å	CaBLAM Disfavored (1.055%)	OUTLIER(S) worst is C--N: 4.8 σ	OUTLIER(S) worst is C-N-CA: 4.9 σ	-
75		ASN	5.29	-	Favored (3.33%) General / -86.9,54.9	Favored (98.6%) *m-40* chi angles: 290.5,339.5	0.06Å	CaBLAM Disfavored (1.085%)	OUTLIER(S) worst is C--N: 5.8 σ	OUTLIER(S) worst is C-N-CA: 5.0 σ	-
76		LYS	4.69	1.06Å HG2 with 17 MET HE1	Favored (34.23%) General / -84.3,135.7	Favored (51.8%) *tptt* chi angles: 184.5,62.6,178.5,180.5	0.03Å	Favored (31.317%)	-	-	-
77		GLY	4.95	0.41Å C with 76 LYS O	Favored (25.33%) Glycine / -67.0,-3.9	-	-	Favored (15.966%) beta sheet	OUTLIER(S) worst is C--N: 4.4 σ	-	-
78		ILE	4.62	0.78Å HG13 with 76 LYS HG3	Favored (93.02%) Ile or Val / -62.2,-41.9	Favored (89.9%) *mt* chi angles: 291.4,169.5	0.05Å	CaBLAM Disfavored (4.872%)	OUTLIER(S) worst is C--N: 4.5 σ	-	-
79		GLY	4.3	0.58Å HA3 with 83 PHE CE1	Allowed (0.61%) Glycine / 169.6,-115.7	-	-	Favored (17.074%)	-	-	-
80		LYS	4.06	1.12Å HD2 with 80 LYS O	Favored (21.34%) General / 54.8,47.9	OUTLIER (0%) chi angles: 65,64.5,185.5,61	0.04Å	CA Geom Outlier (0.142%)	OUTLIER(S) worst is N--CA: 4.1 σ	-	Twisted nonPRO omega= 122.42
**#**	**Alt**	**Res**	**High B**	**Clash > 0.4Å**	**Ramachandran**	**Rotamer**	**Cβ deviation**	**CaBLAM**	**Bond lengths**	**Bond angles**	**Cis Peptides**
			Avg: 6.09	Clashscore: 102.28	Outliers: 3 of 125	Poor rotamers: 2 of 100	Outliers: 0 of 114	Outliers: 16 of 123	Outliers: 66 of 127	Outliers: 37 of 127	Non-Trans: 19 of 126
81		MET	3.72	-	Favored (12.1%) General / -87.1,-43.7	Favored (53.8%) *ttm* chi angles: 184.5,171.5,288.6	0.08Å	CA Geom Outlier (0.269%)	-	OUTLIER(S) worst is C-N-CA: 5.3 σ	Twisted nonPRO omega= 135.41
82		GLY	3.27	-	Favored (97.78%) Glycine / -64.2,-42.8	-	-	Favored (48.144%) alpha helix	-	OUTLIER(S) worst is C-N-CA: 4.2 σ	-
83		PHE	2.38	0.60Å O with 87 THR HG23	Favored (80.18%) General / -60.0,-37.6	Favored (24.5%) *m-10* chi angles: 302.5,149.5	0.06Å	Favored (74.814%) alpha helix	-	-	-
84		GLY	2.57	-	Favored (86.75%) Glycine / -64.0,-47.3	-	-	Favored (95.209%) alpha helix	-	-	-

85		MET	3.37	0.89Å HA with 85 MET HE3	Favored (99.15%) General / -61.9,-43.3	Allowed (1.8%) *tpt* chi angles: 186.5,61.5,278.5	0.02Å	Favored (84.212%) alpha helix	-	-	-
86		VAL	2.95	0.56Å HG12 with 119 LEU HD11	Favored (95.05%) Ile or Val / -65.2,-42.8	Favored (92.6%) *t* chi angles: 174.5	0.02Å	Favored (90.606%) alpha helix	-	-	-
87		THR	2.02	0.81Å HG21 with 116 LEU HD23	Favored (91.3%) General / -65.8,-39.9	Favored (74.2%) *p* chi angles: 61.5	0.05Å	Favored (90.014%) alpha helix	-	-	-
88		LEU	2.79	0.89Å HD13 with 65 SER OG	Favored (97.9%) General / -63.7,-42.2	Favored (30.5%) *mt* chi angles: 293.5,160.5	0.01Å	Favored (91.156%) alpha helix	-	-	-
89		GLY	3.23	-	Favored (94.93%) Glycine / -58.3,-44.8	-	-	Favored (95.765%) alpha helix	OUTLIER(S) worst is C--N: 4.4 σ	-	-
90		ALA	2.65	0.44Å HB1 with 115 LEU HD13	Favored (85.83%) General / -62.7,-47.0	-	0.03Å	Favored (83.461%) alpha helix	OUTLIER(S) worst is C--N: 6.0 σ	-	-
91		SER	2.25	0.41Å O with 95 MET HG2	Favored (84.75%) General / -62.6,-47.2	Favored (73.5%) *m* chi angles: 295.5	0.02Å	Favored (76.206%) alpha helix	-	-	-
92		ALA	3.21	0.55Å HB1 with 56 MET SD	Favored (78.9%) General / -67.9,-43.6	-	0.02Å	Favored (84.752%) alpha helix	OUTLIER(S) worst is C--N: 4.9 σ	-	-
93		TRP	3.16	0.80Å CD1 with 57 LEU HD21	Favored (98.69%) General / -62.5,-41.3	Favored (90.3%) *t60* chi angles: 181.5,89.5	0.04Å	Favored (92.455%) alpha helix	OUTLIER(S) worst is C--N: 4.9 σ	-	-
94		LEU	2.55	1.52Å HB3 with 108 VAL CG1	Favored (74.34%) General / -70.0,-35.3	Favored (90.5%) *mt* chi angles: 291.3,170.5	0.03Å	Favored (76.854%) alpha helix	OUTLIER(S) worst is C--N: 4.2 σ	-	-
95		MET	3.1	0.93Å HA with 95 MET HE2	Favored (83.35%) General / -59.1,-39.8	Favored (6.8%) *mmp* chi angles: 288.5,311.5,78.5	0.02Å	Favored (74.738%) alpha helix	-	-	-
96		TRP	3.69	0.69Å CE3 with 100 ILE HD12	Favored (58%) General / -76.8,-34.5	Favored (68.7%) *p-90* chi angles: 69.4,267.3	0.05Å	Favored (90.407%) alpha helix	-	OUTLIER(S) worst is C-N-CA: 4.6 σ	-
97		LEU	3.55	0.46Å O with 100 ILE HB	Favored (83.4%) General / -58.3,-41.0	Favored (59.4%) *tp* chi angles: 181.6,60.7	0.03Å	Favored (77.978%) alpha helix	OUTLIER(S) worst is C--N: 5.8 σ	-	-
98		SER	3.96	0.49Å OG with 108 VAL HG21	Favored (83.53%) General / -57.6,-46.9	Favored (69.6%) *m* chi angles: 296.5	0.03Å	Favored (74.764%) alpha helix	-	-	-
99		GLU	6	0.43Å HG2 with 33 ARG HH11	Favored (94.64%) General / -63.2,-39.4	Favored (93.1%) *mt-10* chi angles: 289.5,176.5,157.5	0.01Å	Favored (68.808%)	-	-	-
100		ILE	5.49	0.75Å HG13 with 48 LEU HD23	Favored (83.17%) Ile or Val / -64.1,-38.7	Favored (94.1%) *mt* chi angles: 292.5,171.5	0.05Å	Favored (24.29%)	-	-	-
**#**	**Alt**	**Res**	**High B**	**Clash > 0.4Å**	**Ramachandran**	**Rotamer**	**Cβ deviation**	**CaBLAM**	**Bond lengths**	**Bond angles**	**Cis Peptides**
			Avg: 6.09	Clashscore: 102.28	Outliers: 3 of 125	Poor rotamers: 2 of 100	Outliers: 0 of 114	Outliers: 16 of 123	Outliers: 66 of 127	Outliers: 37 of 127	Non-Trans:

											19 of 126
101		GLU	4.63	0.50Å HB3 with 104 ARG HD2	Favored (23.23%) Pre-Pro / -137.7,84.1	Favored (91.8%) *tt0* chi angles: 185.5,179.5,175.5	0.09Å	CaBLAM Outlier (0.642%)	OUTLIER(S) worst is C--N: 4.5 σ	OUTLIER(S) worst is C-N-CA: 4.2 σ	-
102		PRO	4.71	1.37Å CD with 102 PRO N	Favored (90.06%) Trans-Pro / -64.7,148.1	Favored (91.1%) *Cg_exo* chi angles: 329.5,43.5,324.6	0.06Å	Favored (5.792%) alpha helix	OUTLIER(S) worst is N--CD: 17.9 σ	OUTLIER(S) worst is CA-N-CD: 4.2 σ	-
103		ALA	4.64	0.42Å C with 105 ILE N	Allowed (0.18%) General / 80.8,-42.1	-	0.05Å	CaBLAM Outlier (0.057%) try alpha helix	OUTLIER(S) worst is CA--C: 4.8 σ	-	Twisted nonPRO omega= -130.49
104		ARG	3.44	0.51Å O with 107 CYS HB2	Favored (56.89%) General / -58.0,-24.1	Favored (95.2%) *mtt-85* chi angles: 285.5,185.5,179.5,276.5	0.02Å	Favored (33.303%) three-ten	OUTLIER(S) worst is C--N: 5.2 σ	OUTLIER(S) worst is C-N-CA: 5.0 σ	-
105		ILE	3.33	0.42Å N with 103 ALA C	Favored (90.83%) Ile or Val / -66.4,-42.7	Favored (20.1%) *tt* chi angles: 191.7,170.6	0.11Å	Favored (73.201%) three-ten	-	-	-
106		ALA	4.12	-	Favored (89.1%) General / -59.0,-42.0	-	0.08Å	Favored (91.29%) alpha helix	OUTLIER(S) worst is C--N: 4.3 σ	-	-
107		CYS	3.98	0.51Å HB2 with 104 ARG O	Favored (82.34%) General / -66.4,-44.6	Favored (84.3%) *m* chi angles: 295.5	0.05Å	Favored (93.802%) alpha helix	-	OUTLIER(S) worst is C-N-CA: 4.9 σ	-
108		VAL	3.01	1.52Å CG1 with 94 LEU HB3	Favored (81.66%) Ile or Val / -57.0,-43.7	Favored (59.9%) *t* chi angles: 170.8	0.06Å	Favored (87.588%) alpha helix	OUTLIER(S) worst is C--N: 6.6 σ	-	-
109		LEU	2.96	0.52Å HD22 with 25 ILE HG22	Favored (88.41%) General / -65.8,-38.2	Favored (84.1%) *mt* chi angles: 294.5,178.5	0.02Å	Favored (94.538%) alpha helix	-	-	-
110		ILE	3.76	-	Favored (98.77%) Ile or Val / -62.3,-44.1	Favored (93.4%) *mt* chi angles: 295.5,170.5	0.01Å	Favored (94.629%) alpha helix	-	-	-
111		VAL	3.23	-	Favored (91.43%) Ile or Val / -64.2,-41.1	Favored (92.6%) *t* chi angles: 174.5	0.01Å	Favored (88.855%) alpha helix	OUTLIER(S) worst is C--N: 5.5 σ	-	-
112		VAL	2.84	0.76Å HG22 with 94 LEU HD12	Favored (92.79%) Ile or Val / -59.3,-45.9	Favored (65.4%) *t* chi angles: 171.6	0.03Å	Favored (94.974%) alpha helix	-	-	-
113		PHE	3.28	0.59Å O with 117 VAL HG23	Favored (92.16%) General / -64.6,-43.9	Favored (59.2%) *t80* chi angles: 187.5,89.5	0.01Å	Favored (89.092%) alpha helix	-	-	-
114		LEU	3.76	0.40Å O with 118 VAL HG23	Favored (93.59%) General / -63.3,-39.1	Favored (94.6%) *mt* chi angles: 295.5,174.5	0.02Å	Favored (95.654%) alpha helix	-	-	-
115		LEU	3.42	0.44Å HD13 with 90 ALA HB1	Favored (96.18%) General / -64.3,-42.8	Favored (86.6%) *mt* chi angles: 290.2,171.5	0.02Å	Favored (92.701%) alpha helix	-	-	-
116		LEU	3.36	0.97Å HD12 with 25 ILE HD11	Favored (83.46%) General / -59.8,-48.0	Favored (6.1%) *mt* chi angles: 271.5,164.5	0.05Å	Favored (81.493%) alpha helix	OUTLIER(S) worst is C--N: 5.7 σ	-	-
117		VAL	4.47	0.59Å HG23 with	Favored (78.07%)	Favored (68.9%) *t* chi angles: 172	0.01Å	Favored (76.509%)	OUTLIER(S) worst is C--N:	-	-

				113 PHE O	Ile or Val / -62.7,-37.1			alpha helix	6.3 σ		
118		VAL	6.09	0.40Å HG23 with 114 LEU O	Favored (87.74%) Ile or Val / -58.0,-46.9	Favored (84.1%) *t* chi angles: 173.5	0.02Å	Favored (70.936%) alpha helix	OUTLIER(S) worst is C--N: 5.3 σ	-	-
119		LEU	5.35	0.74Å HD12 with 87 THR HG22	Favored (7.38%) General / -73.3,-54.8	Favored (84.8%) *mt* chi angles: 291.1,174.5	0.04Å	Favored (77.94%) alpha helix	-	-	-
120		ILE	5.4	0.70Å HD12 with 21 PHE CE1	Favored (21.91%) Pre-Pro / -75.4,113.0	Favored (85.3%) *mt* chi angles: 298.9,170.1	0.04Å	Favored (28.72%)	-	-	-
**#**	**Alt**	**Res**	**High B**	**Clash > 0.4Å**	**Ramachandran**	**Rotamer**	**Cβ deviation**	**CaBLAM**	**Bond lengths**	**Bond angles**	**Cis Peptides**
			Avg: 6.09	Clashscore: 102.28	Outliers: 3 of 125	Poor rotamers: 2 of 100	Outliers: 0 of 114	Outliers: 16 of 123	Outliers: 66 of 127	Outliers: 37 of 127	Non-Trans: 19 of 126
121		PRO	8.35	1.34Å CD with 121 PRO N	Favored (98.35%) Trans-Pro / -58.8,-35.6	Favored (95.9%) *Cg_exo* chi angles: 331.5,43.5,323.4	0.07Å	CaBLAM Disfavored (3.067%)	OUTLIER(S) worst is N--CD: 19.5 σ	OUTLIER(S) worst is N-CD-CG: 4.0 σ	-
122		GLU	7.8	-	Favored (94.1%) Pre-Pro / -65.1,128.8	Favored (99%) *mt-10* chi angles: 292.5,179.5,0.5	0.06Å	Favored (11.24%) beta sheet	OUTLIER(S) worst is C--N: 6.9 σ	-	Twisted nonPRO omega= -148.65
123		PRO	8.4	1.02Å CD with 123 PRO O	Favored (87.29%) Trans-Pro / -64.5,145.2	Favored (54.5%) *Cg_endo* chi angles: 32.5,43.5,300.5	0.06Å	Favored (18.068%)	OUTLIER(S) worst is N--CD: 69.8 σ	OUTLIER(S) worst is CA-N-CD: 23.4 σ	-
124		GLU	10.4	0.48Å OE1 with 18 THR HG23	Favored (2.77%) General / -115.9,-47.8	Favored (83.3%) *tt0* chi angles: 179.5,175.5,167.5	0.07Å	CaBLAM Disfavored (2.314%)	-	-	Twisted nonPRO omega= 149.29
125		LYS	9.98	-	Favored (32.96%) General / -93.0,120.1	Favored (99.4%) *mttt* chi angles: 295,179.5,179.5,179.5	0.08Å	Favored (38.765%)	OUTLIER(S) worst is C--N: 5.6 σ	-	Twisted nonPRO omega= 137.41
126		GLN	10.84	0.48Å HG2 with 123 PRO HB3	Favored (98.85%) General / -62.3,-41.8	Favored (91.8%) *mt0* chi angles: 292.5,177.5,300.5	0.03Å	-	OUTLIER(S) worst is C--N: 4.3 σ	-	-
127		ARG	12.1	0.89Å HB3 with 127 ARG NH2	-	Allowed (1.3%) *ptp-110* chi angles: 59.5,194.5,66.5,280.5	0.06Å	-	-	-	-

About MolProbity | Website for the Richardson Lab | Using ecloud x-H | Internal reference 4.5.2
