# Supplementary material for: Integrative proteome-wide structural analysis and high-throughput docking identify broad-spectrum antiviral scaffolds against Zika, Yellow Fever, West Nile, Saint Louis encephalitis, and Usutu viruses
Source: Front Cell Infect Microbiol. 2026 Apr 30;16:1723132. doi: 10.3389/fcimb.2026.1723132 (PMC13171538; doi:10.3389/fcimb.2026.1723132)
Supplement: Supplementary file 7 [file DataSheet7.zip › ZIKV/ZIKV_NS4a/Mol_probity_Files/ZIKV_NS4a_1FH-rama.pdf]

MolProbity Ramachandran analysis

ZIKV_NS4a1FH.pdb, model 1

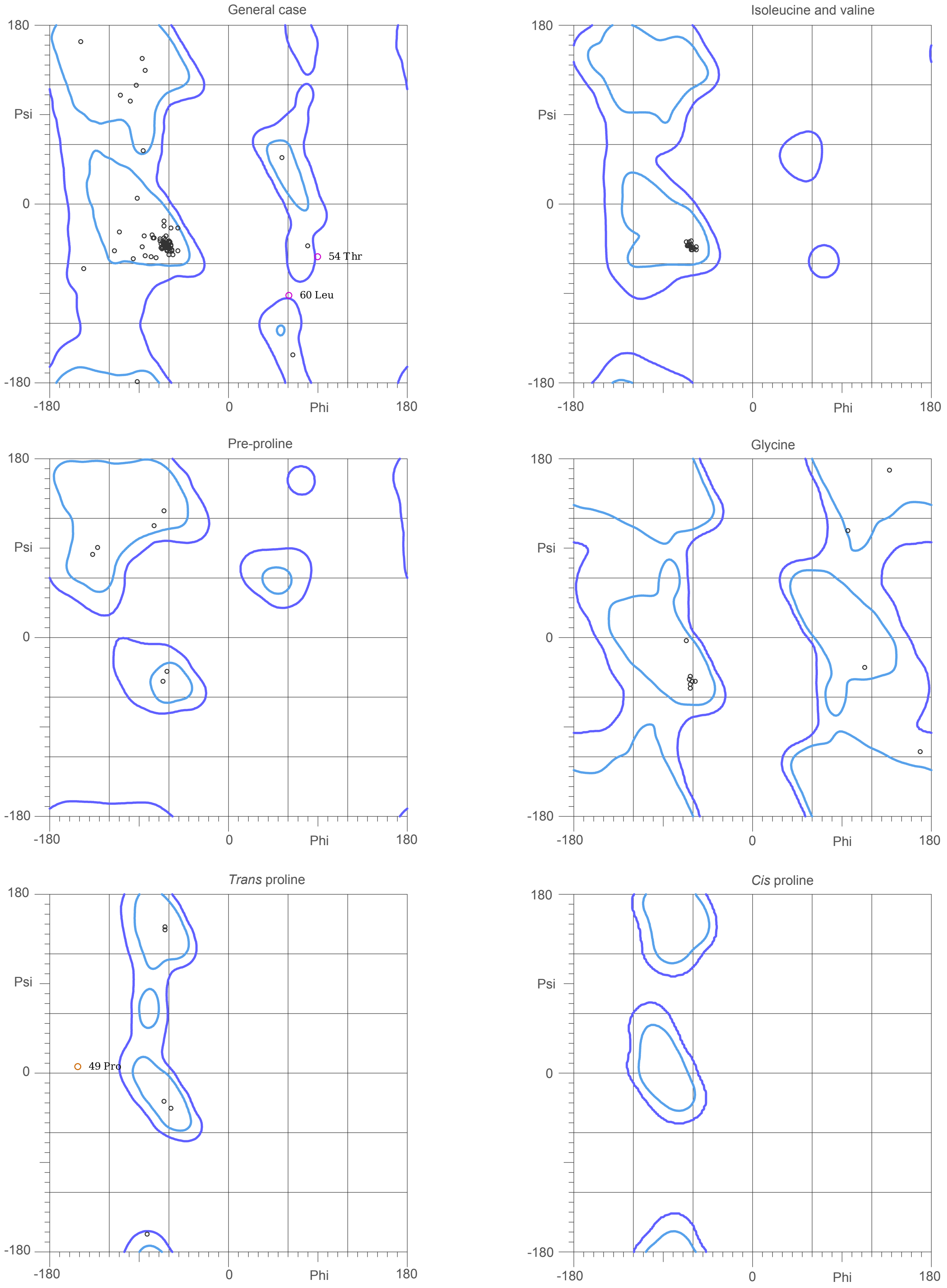

93.6% (117/125) of all residues were in favored (98%) regions.
97.6% (122/125) of all residues were in allowed (>99.8%) regions.

There were 3 outliers (phi, psi):
- 49 Pro (-152.6, 7.3)
- 54 Thr (90.4, -53.8)
- 60 Leu (61.8, -92.1)

http://kinemage.biochem.duke.edu

Lovell, Davis, et al. Proteins 50:437 (2003)
