# Supplementary material for: Integrative proteome-wide structural analysis and high-throughput docking identify broad-spectrum antiviral scaffolds against Zika, Yellow Fever, West Nile, Saint Louis encephalitis, and Usutu viruses
Source: Front Cell Infect Microbiol. 2026 Apr 30;16:1723132. doi: 10.3389/fcimb.2026.1723132 (PMC13171538; doi:10.3389/fcimb.2026.1723132)
Supplement: Supplementary file 7 [file DataSheet7.zip › ZIKV/ZIKV_NS4b/Mol_probity_Files/ZIKV_NS4b_1FH-multi.table.pdf]

Viewing ZIKV_NS4b1FH-multi.table

MolProbity

Duke Biochemistry
Duke University School of Medicine

When finished, you should close this window.

Hint: Use File | Save As... to save a copy of this page.

All-Atom Contacts	Clashscore, all atoms:	37.15		9th percentile* (N=1784, all resolutions)
	Clashscore is the number of serious steric overlaps (> 0.4 Å) per 1000 atoms.			
Protein Geometry	Poor rotamers	1	0.51%	Goal: <0.3%
	Favored rotamers	192	98.46%	Goal: >98%
	Ramachandran outliers	4	1.61%	Goal: <0.05%
	Ramachandran favored	236	94.78%	Goal: >98%
	Rama distribution Z-score	6.18 ± 0.49		Goal: abs(Z score) < 2
	MolProbity score^	2.41		52nd percentile* (N=27675, 0Å - 99Å)
	Cβ deviations >0.25Å	0	0.00%	Goal: 0
	Bad bonds:	93 / 1923	4.84%	Goal: 0%
	Bad angles:	52 / 2617	1.99%	Goal: <0.1%
Peptide Omegas	Cis Prolines:	0 / 9	0.00%	Expected: ≤1 per chain, or ≤5%
	Twisted Peptides:	12 / 250	4.80%	Goal: 0
Low-resolution Criteria	CaBLAM outliers	16	6.5%	Goal: <1.0%
	CA Geometry outliers	11	4.45%	Goal: <0.5%
Additional validations	Chiral volume outliers	0/312		
	Waters with clashes	0/0	0.00%	See UnDowser table for details

In the two column results, the left column gives the raw count, right column gives the percentage.

* 100th percentile is the best among structures of comparable resolution; 0th percentile is the worst. For clashscore the comparative set of structures was selected in 2004, for MolProbity score in 2006.

^ MolProbity score combines the clashscore, rotamer, and Ramachandran evaluations into a single score, normalized to be on the same scale as X-ray resolution.

Key to table colors and cutoffs here:

#	Alt	Res	High B	Clash > 0.4Å	Ramachandran	Rotamer	Cβ deviation	CaBLAM	Bond lengths	Bond angles	Cis Peptides
			Avg: 7.70	Clashscore: 37.15	Outliers: 4 of 249	Poor rotamers: 1 of 195	Outliers: 0 of 229	Outliers: 17 of 247	Outliers: 84 of 251	Outliers: 34 of 251	Non-Trans: 12 of 250
1		ASN	26.05	-	-	Favored (96.1%) *m-40* chi angles: 290.8,333.4	0.00Å	-	-	-	-
2		GLU	25.48	0.42Å O with 6 LEU HG	Favored (99.68%) General / -62.8,-43.1	Favored (92%) *tt0* chi angles: 183.4,178,178.4	0.01Å	-	OUTLIER(S) worst is C--N: 6.7 σ	-	-
3		LEU	25.78	-	Favored (94.59%) General / -60.2,-45.0	Favored (74.6%) *mt* chi angles: 290.2,175.5	0.06Å	Favored (75.21%)	-	-	-
4		GLY	25.42	-	Favored (93.43%) Glycine / -66.8,-38.9	-	-	Favored (91.463%) alpha helix	-	-	-
5		TRP	24.66	-	Favored (91.6%) General / -64.3,-38.6	Favored (89.3%) *t60* chi angles: 177.2,83.9	0.03Å	Favored (93.196%) alpha helix	-	-	-

6		LEU	24.24	0.52Å O with 10 LYS HG3	Favored (94.89%) General / -60.1,-44.2	Favored (70.6%) *mt* chi angles: 290.3,176.5	0.06Å	Favored (90.656%) alpha helix	OUTLIER(S) worst is C--N: 6.7 σ	-	-
7		GLU	24.31	-	Favored (97.19%) General / -63.9,-43.0	Favored (70%) *tp30* chi angles: 180.5,66.9,12.5	0.01Å	Favored (87.369%) alpha helix	-	-	-
8		ARG	24.02	-	Favored (97.55%) General / -63.9,-41.6	Favored (40%) *tpt170* chi angles: 172.5,62.6,178.5,170.4	0.02Å	Favored (90.506%) alpha helix	OUTLIER(S) worst is C--N: 6.0 σ	-	-
9		THR	23.29	-	Favored (99.51%) General / -63.1,-42.8	Favored (88.3%) *m* chi angles: 298.5	0.01Å	Favored (98.505%) alpha helix	-	-	-
10		LYS	18.29	0.73Å HD3 with 55 GLN HG2	Favored (95.91%) General / -63.9,-43.5	Favored (97.6%) *mttt* chi angles: 289.8,175.5,180.1,178.4	0.02Å	Favored (98.981%) alpha helix	OUTLIER(S) worst is C--N: 5.1 σ	-	-
11		ASN	23.38	-	Favored (91.6%) General / -63.3,-38.5	Favored (92.4%) *m-40* chi angles: 293.5,329.5	0.01Å	Favored (97.729%) alpha helix	-	-	-
12		ASP	23.05	-	Favored (93.52%) General / -64.8,-39.4	Favored (96.6%) *m-30* chi angles: 288.3,162	0.01Å	Favored (89.729%) alpha helix	OUTLIER(S) worst is CG--OD2: 14.2 σ	OUTLIER(S) worst is OD1-CG-OD2: 5.3 σ	-
13		ILE	22.96	-	Favored (82.33%) Ile or Val / -61.2,-39.4	Favored (97.1%) *mt* chi angles: 292.5,169.5	0.02Å	Favored (90.029%) alpha helix	-	-	-
14		ALA	23.03	0.49Å HA with 17 MET HE3	Favored (96.78%) General / -60.5,-42.7	-	0.03Å	Favored (84.558%) alpha helix	OUTLIER(S) worst is C--N: 5.6 σ	-	-
15		HIS	17.51	-	Favored (98.22%) General / -61.3,-42.2	Favored (57.2%) *m170* chi angles: 296.5,164	0.04Å	Favored (82.835%) alpha helix	OUTLIER(S) worst is C--N: 4.9 σ	-	-
16		LEU	17.21	-	Favored (90.16%) General / -60.7,-46.5	Favored (88.4%) *mt* chi angles: 296.5,171.5	0.03Å	Favored (80.269%) alpha helix	-	-	-
17		MET	17.42	0.49Å SD with 232 LEU HB3	Favored (85.95%) General / -61.5,-47.4	Favored (82.2%) *mtm* chi angles: 290.5,183.5,287.3	0.06Å	Favored (76.578%) alpha helix	OUTLIER(S) worst is C--N: 5.4 σ	-	-
18		GLY	18.02	0.56Å O with 22 GLU HB3	Favored (81.34%) Glycine / -68.8,-41.9	-	-	Favored (61.876%) alpha helix	-	-	-
19		ARG	17.76	0.61Å HA with 22 GLU O	Favored (4.72%) General / -70.1,175.1	Favored (87%) *mtm180* chi angles: 293,183.5,294.5,175.5	0.06Å	CaBLAM Outlier (0.092%) try alpha helix	OUTLIER(S) worst is C--N: 6.0 σ	OUTLIER(S) worst is C-N-CA: 4.5 σ	-
20		ARG	18.12	0.58Å HB2 with 19 ARG O	Allowed (0.33%) General / 79.9,-46.7	Favored (76.6%) *mtt90* chi angles: 297.1,171.2,180.5,81.1	0.02Å	CaBLAM Outlier (0.609%) try alpha helix	OUTLIER(S) worst is C--N: 6.5 σ	-	-
**#**	**Alt**	**Res**	**High B**	**Clash > 0.4Å**	**Ramachandran**	**Rotamer**	**Cβ deviation**	**CaBLAM**	**Bond lengths**	**Bond angles**	**Cis Peptides**
			Avg: 7.70	Clashscore: 37.15	Outliers: 4 of 249	Poor rotamers: 1 of 195	Outliers: 0 of 229	Outliers: 17 of 247	Outliers: 84 of 251	Outliers: 34 of 251	Non-Trans: 12 of 250

21		GLU	18.02	0.51Å N with 19 ARG O	Favored (97.11%) General / -64.1,-41.9	Favored (92%) *tt0* chi angles: 183.3,178,178.3	0.07Å	CaBLAM Disfavored (4.976%) try alpha helix	OUTLIER(S) worst is C--N: 5.4 σ	OUTLIER(S) worst is C-N-CA: 4.3 σ	Twisted nonPRO omega= 135.86
22		GLU	26.82	0.70Å HG2 with 23 GLY H	Favored (5.15%) General / -164.0,-177.6	Favored (37.9%) *tt0* chi angles: 185.5,180.5,123	0.01Å	CA Geom Outlier (0.001%)	OUTLIER(S) worst is C--N: 6.3 σ	-	Twisted nonPRO omega= -141.63
23		GLY	27.83	0.70Å H with 22 GLU HG2	Allowed (0.75%) Glycine / 110.6,-93.7	-	-	CaBLAM Outlier (0.117%)	-	-	Twisted nonPRO omega= -116.42
24		ALA	29.15	-	Favored (37.06%) General / -92.8,124.8	-	0.07Å	CA Geom Outlier (0.05%)	-	-	Twisted nonPRO omega= -126
25		THR	30.16	0.54Å HA with 28 PHE HB2	Favored (51.1%) General / -97.2,4.4	Favored (80.4%) *p* chi angles: 60.5	0.01Å	CA Geom Outlier (0.009%)	OUTLIER(S) worst is CA--C: 4.4 σ	-	Twisted nonPRO omega= 124.73
26		MET	30.95	-	Favored (89.79%) General / -66.3,-40.4	Favored (59.4%) *mtm* chi angles: 294.5,172.5,288.5	0.04Å	Favored (12.039%)	OUTLIER(S) worst is C--N: 6.7 σ	OUTLIER(S) worst is C-N-CA: 4.3 σ	-
27		GLY	31.63	-	Favored (45.53%) Glycine / -105.8,11.3	-	-	Favored (16.475%)	-	-	-
28		PHE	22.32	0.54Å HB2 with 25 THR HA	Favored (2.85%) General / -107.5,36.1	Favored (95.4%) *m-80* chi angles: 298.5,90.5	0.05Å	Favored (5.422%)	OUTLIER(S) worst is C--N: 5.2 σ	-	-
29		SER	21.78	-	Favored (78.47%) General / -61.8,-48.9	Favored (66%) *m* chi angles: 294.4	0.08Å	CaBLAM Disfavored (3.691%)	OUTLIER(S) worst is C--N: 6.5 σ	-	-
30		MET	15.47	-	Favored (82.02%) General / -66.7,-36.2	Favored (99.2%) *mtp* chi angles: 291.7,179.5,73.5	0.03Å	Favored (47.37%) alpha helix	OUTLIER(S) worst is C--N: 6.8 σ	-	-
31		ASP	13.91	0.62Å C with 31 ASP OD2	Allowed (1.95%) General / -112.3,-59.8	Favored (65.5%) *t0* chi angles: 185.5,172.5	0.03Å	Favored (38.702%) alpha helix	OUTLIER(S) worst is CG--OD2: 14.2 σ	OUTLIER(S) worst is OD1-CG-OD2: 5.3 σ	-
32		ILE	11.26	1.09Å HG12 with 247 LEU HD22	Allowed (1.58%) Ile or Val / -83.1,81.2	Favored (76%) *mt* chi angles: 301.1,170.7	0.01Å	Favored (24.244%)	OUTLIER(S) worst is C--N: 5.3 σ	-	-
33		ASP	11.71	0.55Å OD2 with 35 ARG HG3	Favored (7.98%) General / -84.4,83.5	Favored (60.9%) *t0* chi angles: 187.8,172.9	0.04Å	Favored (67.518%)	OUTLIER(S) worst is CG--OD2: 14.2 σ	OUTLIER(S) worst is OD1-CG-OD2: 5.3 σ	-
34		LEU	16.42	0.73Å HD11 with 39 ALA HB3	Favored (3.17%) General / -85.1,54.6	Favored (8.9%) *tp* chi angles: 200.7,62.3	0.03Å	Favored (24.712%)	OUTLIER(S) worst is CA--C: 4.2 σ	-	-
35		ARG	16.26	0.55Å HG3 with 33 ASP OD2	OUTLIER (0)%) Pre-Pro / -53.3,-92.3	Favored (83%) *mtm180* chi angles: 288.5,174.5,298.5,183.5	0.08Å	CaBLAM Disfavored (1.943%)	OUTLIER(S) worst is C--N: 4.2 σ	-	-
36		PRO	15.31	0.86Å CD with 36 PRO N	Favored (4.21%) Trans-Pro / -80.9,-178.1	Favored (91.1%) *Cg_exo* chi angles: 329.5,317.5,60.7	0.04Å	CA Geom Outlier (0.026%)	OUTLIER(S) worst is N--CD: 59.1 σ	OUTLIER(S) worst is N-CD-CG: 22.2 σ	-
37		ALA	9.46	-	Favored (56.53%) General / -113.3,128.7	-	0.07Å	CA Geom Outlier (0.309%)	OUTLIER(S) worst is C--N: 4.5 σ	OUTLIER(S) worst is C-N-CA: 5.0 σ	-

#	Alt	Res	High B	Clash > 0.4Å	Ramachandran	Rotamer	Cβ deviation	CaBLAM	Bond lengths	Bond angles	Cis Peptides
38		SER	7.2	0.43Å OG with 39 ALA N	Allowed (0.19%) General / -150.7,-62.3	Favored (80.3%) *p* chi angles: 61.5	0.08Å	CaBLAM Outlier (0.236%) try alpha helix	-	OUTLIER(S) worst is C-N-CA: 4.2 σ	-
39		ALA	6.9	0.73Å HB3 with 34 LEU HD11	Favored (95.53%) General / -62.6,-40.0	-	0.02Å	Favored (63.828%) alpha helix	OUTLIER(S) worst is C--N: 6.1 σ	-	-
40		TRP	7.57	0.40Å HE3 with 34 LEU HD23	Favored (89.8%) General / -64.1,-38.1	Favored (56.7%) *t-100* chi angles: 184.4,244.4	0.01Å	Favored (86.879%) alpha helix	OUTLIER(S) worst is C--N: 4.2 σ	-	-
**#**	**Alt**	**Res**	**High B**	**Clash > 0.4Å**	**Ramachandran**	**Rotamer**	**Cβ deviation**	**CaBLAM**	**Bond lengths**	**Bond angles**	**Cis Peptides**
			Avg: 7.70	Clashscore: 37.15	Outliers: 4 of 249	Poor rotamers: 1 of 195	Outliers: 0 of 229	Outliers: 17 of 247	Outliers: 84 of 251	Outliers: 34 of 251	Non-Trans: 12 of 250
41		ALA	6.91	-	Favored (98.47%) General / -62.4,-41.2	-	0.02Å	Favored (92.964%) alpha helix	-	-	-
42		ILE	6.28	0.69Å O with 46 LEU HD23	Favored (97.41%) Ile or Val / -61.2,-43.9	Favored (94.9%) *mt* chi angles: 293.5,170.5	0.04Å	Favored (93.745%) alpha helix	-	-	-
43		TYR	6.72	0.51Å HE1 with 22 GLU OE1	Favored (81.19%) General / -57.7,-47.9	Favored (59.5%) *t80* chi angles: 180.5,92.5	0.03Å	Favored (84.904%) alpha helix	OUTLIER(S) worst is C--N: 4.6 σ	-	-
44		ALA	6.83	-	Favored (82.79%) General / -65.7,-45.4	-	0.06Å	Favored (80.167%) alpha helix	-	-	-
45		ALA	6.18	-	Favored (94.59%) General / -62.9,-45.0	-	0.03Å	Favored (94.492%) alpha helix	OUTLIER(S) worst is CA--C: 4.5 σ	-	-
46		LEU	5.7	0.69Å HD23 with 42 ILE O	Favored (95.58%) General / -64.1,-40.2	Allowed (0.5%) *mm* chi angles: 271.1,308.9	0.03Å	Favored (94.659%) alpha helix	-	OUTLIER(S) worst is C-N-CA: 4.2 σ	-
47		THR	6.43	-	Favored (99.37%) General / -61.1,-42.9	Favored (89%) *m* chi angles: 298.6	0.01Å	Favored (97.812%) alpha helix	-	-	-
48		THR	6.46	0.87Å O with 52 PRO HG2	Favored (97.6%) General / -63.8,-42.6	Favored (89.2%) *m* chi angles: 298.6	0.06Å	Favored (97.969%) alpha helix	-	-	-
49		LEU	5.72	-	Favored (96.78%) General / -61.5,-41.1	Favored (85.8%) *mt* chi angles: 290.9,173.8	0.02Å	Favored (96.132%) alpha helix	-	-	-
50		ILE	5.38	0.48Å O with 54 VAL HG23	Favored (99.09%) Ile or Val / -62.1,-44.5	Favored (94.1%) *mt* chi angles: 291.9,169	0.03Å	Favored (82.289%) alpha helix	-	-	-
51		THR	6	-	Favored (90.18%) Pre-Pro / -53.7,-49.2	Favored (96.4%) *m* chi angles: 299.7	0.03Å	Favored (84.682%) alpha helix	-	-	-
52		PRO	5.92	0.92Å CD with 52 PRO N	Favored (22.3%) Trans-Pro / -65.5,-39.3	Favored (91.1%) *Cg_exo* chi angles: 329.5,321.5,59.6	0.01Å	Favored (97.817%) alpha helix	OUTLIER(S) worst is N--CD: 57.2 σ	OUTLIER(S) worst is CA-N-CD: 21.3 σ	-

53		ALA	4.97	-	Favored (99.39%) General / -62.5,-42.2	-	0.02Å	Favored (97.245%) alpha helix	-	-	-
54		VAL	5.22	1.04Å HG22 with 232 LEU CD1	Favored (97.22%) Ile or Val / -63.4,-42.7	Favored (72.9%) *t* chi angles: 178.5	0.02Å	Favored (97.586%) alpha helix	-	-	-
55		GLN	5.43	0.73Å HG2 with 10 LYS HD3	Favored (97.78%) General / -63.7,-42.9	Favored (84.4%) *mt0* chi angles: 290.5,175.7,1.5	0.01Å	Favored (97.574%) alpha helix	-	-	-
56		HIS	5.26	-	Favored (98.09%) General / -63.6,-42.6	Favored (90%) *t70* chi angles: 180.3,71.2	0.01Å	Favored (98.264%) alpha helix	-	-	-
57		ALA	4.58	0.62Å HB2 with 228 ARG HD2	Favored (99.79%) General / -62.8,-42.6	-	0.02Å	Favored (98.8%) alpha helix	-	-	-
58		VAL	5.09	0.55Å HG12 with 62 TYR CE1	Favored (99.58%) Ile or Val / -62.8,-44.5	Favored (80.8%) *t* chi angles: 176.5	0.03Å	Favored (89.481%) alpha helix	-	-	-
59		THR	5.1	-	Favored (85.17%) General / -67.5,-39.6	Favored (74.1%) *p* chi angles: 61.5	0.03Å	Favored (88.039%) alpha helix	-	-	-
60		THR	4.55	-	Favored (98.99%) General / -61.4,-42.6	Favored (95.2%) *m* chi angles: 299.5	0.02Å	Favored (94.666%) alpha helix	-	-	-
**#**	**Alt**	**Res**	**High B**	**Clash > 0.4Å**	**Ramachandran**	**Rotamer**	**Cβ deviation**	**CaBLAM**	**Bond lengths**	**Bond angles**	**Cis Peptides**
			Avg: 7.70	Clashscore: 37.15	Outliers: 4 of 249	Poor rotamers: 1 of 195	Outliers: 0 of 229	Outliers: 17 of 247	Outliers: 84 of 251	Outliers: 34 of 251	Non-Trans: 12 of 250
61		SER	4.75	-	Favored (96.47%) General / -63.7,-43.5	Favored (78%) *p* chi angles: 70.5	0.01Å	Favored (96.22%) alpha helix	-	-	-
62		TYR	4.58	0.55Å CE1 with 58 VAL HG12	Favored (94.35%) General / -64.3,-39.7	Favored (24.5%) *m-10* chi angles: 295.5,152	0.03Å	Favored (98.649%) alpha helix	OUTLIER(S) worst is C--N: 4.6 σ	-	-
63		ASN	4.78	-	Favored (90.82%) General / -64.1,-45.0	Favored (83.4%) *m-40* chi angles: 296.5,337.5	0.02Å	Favored (91.052%) alpha helix	-	-	-
64		ASN	3.43	0.44Å O with 68 MET HG2	Favored (96.54%) General / -63.5,-43.6	Favored (90.6%) *m-40* chi angles: 294.5,334.4	0.01Å	Favored (87.685%) alpha helix	-	-	-
65		TYR	4.21	-	Favored (95.17%) General / -63.7,-43.9	Favored (59.9%) *t80* chi angles: 185.5,91.5	0.02Å	Favored (97.12%) alpha helix	-	-	-
66		SER	4.11	-	Favored (93.82%) General / -64.5,-39.5	Favored (93.7%) *p* chi angles: 64.2	0.01Å	Favored (97.381%) alpha helix	-	-	-
67		LEU	3.85	-	Favored (87.45%) General / -67.0,-40.7	Favored (75.5%) *mt* chi angles: 291.3,177.5	0.02Å	Favored (97.618%) alpha helix	-	-	-

68		MET	3.05	1.17Å HA with 68 MET HE2	Favored (99.38%) General / -62.3,-42.4	Favored (2.4%) *mmp* chi angles: 293.2,296.7,71.5	0.01Å	Favored (98.237%) alpha helix	-	-	-
69		ALA	3.5	-	Favored (98.32%) General / -62.5,-43.6	-	0.02Å	Favored (97.39%) alpha helix	-	-	-
70		MET	4.03	-	Favored (86.39%) General / -65.6,-44.5	Favored (97.9%) *mtp* chi angles: 290.6,176.5,70.2	0.03Å	Favored (83.586%) alpha helix	OUTLIER(S) worst is N--CA: 4.1 σ	-	-
71		ALA	3.96	-	Favored (93.28%) General / -65.3,-42.4	-	0.02Å	Favored (85.57%) alpha helix	-	-	-
72		THR	3.62	-	Favored (97.88%) General / -63.0,-43.8	Favored (89.1%) *m* chi angles: 298.6	0.01Å	Favored (97.934%) alpha helix	OUTLIER(S) worst is C--N: 5.1 σ	-	-
73		GLN	4.59	-	Favored (95.37%) General / -62.2,-45.0	Favored (80.5%) *mt0* chi angles: 289.9,176,44.5	0.01Å	Favored (90.895%) alpha helix	OUTLIER(S) worst is C--N: 4.1 σ	-	-
74		ALA	3.83	-	Favored (99.22%) General / -62.0,-42.5	-	0.01Å	Favored (91.683%) alpha helix	-	-	-
75		GLY	3.07	-	Favored (91.33%) Glycine / -61.0,-47.4	-	-	Favored (95.441%) alpha helix	-	-	-
76		VAL	3.35	0.59Å CG2 with 210 SER HB3	Favored (39.88%) Ile or Val / -68.0,-28.1	Favored (80.6%) *t* chi angles: 176.5	0.06Å	Favored (59.634%) alpha helix	-	-	-
77		LEU	4.19	0.51Å O with 93 VAL HB	Favored (2.44%) General / -65.9,-60.9	Favored (94.4%) *mt* chi angles: 293.7,174.5	0.05Å	Favored (23.1%) alpha helix	-	-	-
78		PHE	4.12	0.53Å CZ with 80 MET HB3	Favored (40.95%) General / -70.7,-50.0	Favored (11.1%) *t80* chi angles: 180.5,38.6	0.07Å	Favored (5.413%)	-	-	-
79		GLY	4.96	-	Allowed (0.44%) Glycine / 66.3,-12.6	-	-	CaBLAM Disfavored (3.759%)	-	OUTLIER(S) worst is C-N-CA: 4.6 σ	-
80		MET	10.14	0.53Å HB3 with 78 PHE CZ	Favored (58.4%) General / -76.0,-30.1	Favored (61.9%) *ttm* chi angles: 183.4,182.6,287.5	0.01Å	CA Geom Outlier (0%)	OUTLIER(S) worst is C--N: 4.6 σ	-	Twisted nonPRO omega= 149.25
**#**	**Alt**	**Res**	**High B**	**Clash > 0.4Å**	**Ramachandran**	**Rotamer**	**Cβ deviation**	**CaBLAM**	**Bond lengths**	**Bond angles**	**Cis Peptides**
			Avg: 7.70	Clashscore: 37.15	Outliers: 4 of 249	Poor rotamers: 1 of 195	Outliers: 0 of 229	Outliers: 17 of 247	Outliers: 84 of 251	Outliers: 34 of 251	Non-Trans: 12 of 250
81		GLY	11.48	-	Favored (2.98%) Glycine / 102.9,-38.9	-	-	CaBLAM Disfavored (3.981%)	-	-	Twisted nonPRO omega= 123.37
82		LYS	13.65	-	Favored (94.13%) General / -64.0,-39.4	Favored (97.3%) *mttt* chi angles: 296.5,179.5,180.3,180.5	0.01Å	CaBLAM Disfavored (1.618%) try beta sheet	-	OUTLIER(S) worst is C-N-CA: 4.0 σ	-
83		GLY	13.26	-	Favored (4.23%)	-	-	CaBLAM Outlier	-	-	-

					Glycine / 110.5,121.2			(0.673%) try beta sheet			
84		MET	13.45	0.41Å SD with 86 PHE HD1	Favored (64.69%) Pre-Pro / -83.0,158.4	Favored (96.1%) *mtp* chi angles: 294.5,180.5,75	0.05Å	Favored (43.409%)	-	OUTLIER(S) worst is C-N-CA: 5.2 σ	Twisted nonPRO omega= 122.99
85		PRO	13.1	0.78Å CD with 85 PRO N	Favored (14.33%) Trans-Pro / -60.9,124.1	Favored (94.4%) *Cg_exo* chi angles: 331.3,316.5,59.8	0.08Å	Favored (6.623%)	OUTLIER(S) worst is N--CD: 66.5 σ	OUTLIER(S) worst is CA-N-CD: 22.6 σ	Twisted PRO omega= 136.32
86		PHE	12	1.07Å CD2 with 87 MET HB2	Allowed (0.58%) General / -135.8,-65.7	Favored (36.4%) *t80* chi angles: 193.6,78.5	0.03Å	CA Geom Outlier (0.332%)	OUTLIER(S) worst is C--N: 4.1 σ	OUTLIER(S) worst is C-N-CA: 4.8 σ	-
87		MET	10.6	1.07Å HB2 with 86 PHE CD2	Favored (11.94%) General / -163.3,149.4	Favored (6.7%) *ttt* chi angles: 203.5,174.5,196.5	0.03Å	Favored (6.847%)	OUTLIER(S) worst is C--N: 5.9 σ	-	Twisted nonPRO omega= -138.33
88		HIS	9.05	-	Favored (25.24%) General / -136.3,165.7	Favored (7.7%) *p90* chi angles: 62.9,60.5	0.07Å	Favored (52.64%)	OUTLIER(S) worst is C--N: 4.8 σ	-	Twisted nonPRO omega= 149.67
89		GLY	8.05	-	Favored (18.74%) Glycine / -129.6,176.1	-	-	Favored (31.205%)	-	-	-
90		ASP	9.16	-	Favored (98.3%) General / -63.5,-43.1	Favored (98.6%) *m-30* chi angles: 288.5,167.5	0.02Å	CaBLAM Disfavored (2.811%)	OUTLIER(S) worst is CG--OD2: 14.2 σ	OUTLIER(S) worst is OD1-CG-OD2: 5.3 σ	-
91		LEU	8.09	-	Favored (2.75%) General / -106.9,-52.5	Favored (72.6%) *tp* chi angles: 178.5,62.5	0.07Å	CA Geom Outlier (0.172%)	OUTLIER(S) worst is C--N: 5.7 σ	-	-
92		GLY	4.19	-	Favored (7.51%) Glycine / -168.3,142.5	-	-	Favored (9.342%)	-	-	-
93		VAL	3.64	0.65Å HG13 with 87 MET SD	Favored (59.92%) Pre-Pro / -49.6,-51.3	Favored (88.9%) *t* chi angles: 176.1	0.11Å	Favored (37.918%)	-	-	-
94		PRO	3.08	0.44Å HD3 with 77 LEU O	Favored (23.51%) Trans-Pro / -57.7,-46.9	Favored (34.1%) *Cg_endo* chi angles: 22.1,325.7,31.1	0.03Å	Favored (70.646%) alpha helix	OUTLIER(S) worst is N--CD: 4.2 σ	-	-
95		LEU	2.62	0.64Å HB3 with 199 ILE HG23	Favored (96.63%) General / -63.2,-43.9	Favored (20.5%) *mt* chi angles: 307.5,169.5	0.04Å	Favored (86.553%) alpha helix	OUTLIER(S) worst is C--N: 6.4 σ	-	-
96		LEU	3.28	-	Favored (89.76%) General / -58.6,-43.3	Favored (66.3%) *tp* chi angles: 175.5,61.8	0.01Å	Favored (90.799%) alpha helix	OUTLIER(S) worst is C--N: 6.6 σ	-	-
97		MET	3.28	0.63Å HE2 with 93 VAL HG12	Favored (92.42%) General / -65.5,-39.9	Favored (85.6%) *mtm* chi angles: 291.5,185.5,284.5	0.04Å	Favored (95.486%) alpha helix	OUTLIER(S) worst is C--N: 5.2 σ	-	-
98		MET	2.86	-	Favored (98.41%) General / -61.9,-41.9	Favored (80.8%) *mtm* chi angles: 288.7,185.5,287.5	0.01Å	Favored (96.883%) alpha helix	-	-	-
99		GLY	2.73	-	Favored (95.78%) Glycine / -64.0,-44.6	-	-	Favored (99.732%) alpha helix	-	-	-
100		CYS	3.44	-	Favored (95.67%)	Favored (99.1%) *m* chi angles: 289.5	0.02Å	Favored (98.65%) alpha helix	-	-	-

					General / -61.1,-41.0						

#	Alt	Res	High B	Clash > 0.4Å	Ramachandran	Rotamer	Cβ deviation	CaBLAM	Bond lengths	Bond angles	Cis Peptides
			Avg: 7.70	Clashscore: 37.15	Outliers: 4 of 249	Poor rotamers: 1 of 195	Outliers: 0 of 229	Outliers: 17 of 247	Outliers: 84 of 251	Outliers: 34 of 251	Non-Trans: 12 of 250
101		TYR	3.34	-	Favored (89.09%) General / -66.0,-43.0	Favored (62.9%) *t80* chi angles: 172.5,86.5	0.01Å	Favored (99.104%) alpha helix	-	-	-
102		SER	2.43	1.18Å O with 106 PRO HG2	Favored (91.08%) General / -64.7,-38.5	Favored (77.7%) *p* chi angles: 60.5	0.02Å	Favored (96.153%) alpha helix	OUTLIER(S) worst is C--N: 4.5 σ	-	-
103		GLN	3.25	0.46Å HE22 with 192 TRP CD1	Favored (98.12%) General / -63.7,-41.0	Favored (98.5%) *mm-40* chi angles: 291.9,300.5,314.5	0.01Å	Favored (93.149%) alpha helix	-	-	-
104		LEU	2.98	-	Favored (98.74%) General / -61.0,-42.7	Favored (94.6%) *mt* chi angles: 296.5,174.5	0.02Å	Favored (98.089%) alpha helix	-	-	-
105		THR	3.26	-	Favored (57.73%) Pre-Pro / -65.0,-41.9	Favored (96.2%) *m* chi angles: 299.7	0.01Å	Favored (96.79%) alpha helix	-	-	-
106		PRO	2.89	1.18Å HG2 with 102 SER O	Favored (13.45%) Trans-Pro / -74.5,-25.0	Favored (89.8%) *Cg_exo* chi angles: 329.5,315.5,61.6	0.01Å	Favored (93.086%) alpha helix	OUTLIER(S) worst is N--CD: 65.5 σ	OUTLIER(S) worst is CA-N-CD: 22.0 σ	-
107		LEU	3.69	0.48Å O with 111 VAL HG23	Favored (92.95%) General / -62.1,-39.5	Favored (96.3%) *mt* chi angles: 294.5,173	0.01Å	Favored (76.044%) alpha helix	-	-	-
108		THR	4.24	-	Favored (90.41%) General / -62.5,-46.0	Favored (88.4%) *m* chi angles: 298.5	0.04Å	Favored (97.046%) alpha helix	-	-	-
109		LEU	3.47	-	Favored (98.24%) General / -63.6,-42.8	Favored (58.2%) *tp* chi angles: 181.7,59.8	0.01Å	Favored (98.546%) alpha helix	-	-	-
110		ILE	3.32	-	Favored (98.46%) Ile or Val / -63.0,-42.9	Favored (95.3%) *mt* chi angles: 294.5,169	0.03Å	Favored (98.527%) alpha helix	-	-	-
111		VAL	4.26	0.48Å HG23 with 107 LEU O	Favored (98.17%) Ile or Val / -63.3,-42.9	Favored (72%) *t* chi angles: 178.5	0.03Å	Favored (99.052%) alpha helix	-	-	-
112		ALA	4.17	0.46Å O with 116 LEU HG	Favored (98.87%) General / -62.2,-42.0	-	0.02Å	Favored (98.686%) alpha helix	-	-	-
113		ILE	3.43	1.17Å HG21 with 184 VAL HG22	Favored (84.06%) Ile or Val / -64.4,-39.0	Favored (88.3%) *mt* chi angles: 291.9,172.5	0.09Å	Favored (96.989%) alpha helix	-	OUTLIER(S) worst is C-N-CA: 5.2 σ	-
114		ILE	4.19	-	Favored (90.13%) Ile or Val / -63.8,-40.7	Favored (96.9%) *mt* chi angles: 292.5,169.5	0.01Å	Favored (98.689%) alpha helix	-	-	-
115		LEU	4.45	-	Favored (98.16%) General / -63.6,-42.0	Favored (95%) *mt* chi angles: 296.4,175.5	0.01Å	Favored (97.719%) alpha helix	-	-	-

116		LEU	4.17	0.46Å HG with 112 ALA O	Favored (98.9%) General / -63.3,-41.0	Favored (94.2%) *mt* chi angles: 296.5,173.5	0.01Å	Favored (96.123%) alpha helix	-	-	-
117		VAL	3.5	1.00Å HG22 with 180 ILE HG21	Favored (91.3%) Ile or Val / -65.0,-41.2	Favored (92.9%) *t* chi angles: 174.5	0.03Å	Favored (96.999%) alpha helix	-	-	-
118		ALA	4.25	-	Favored (98.15%) General / -62.1,-41.4	-	0.02Å	Favored (97.578%) alpha helix	-	-	-
119		HIS	4.58	-	Favored (93.55%) General / -61.5,-40.2	Favored (82.6%) *t70* chi angles: 184.5,73.5	0.06Å	Favored (98.453%) alpha helix	-	-	-
120		TYR	3.91	0.57Å CE2 with 176 ILE HG21	Favored (90.85%) General / -62.9,-45.8	Favored (76.2%) *t80* chi angles: 180.5,87.5	0.01Å	Favored (96.811%) alpha helix	-	-	-
**#**	**Alt**	**Res**	**High B**	**Clash > 0.4Å**	**Ramachandran**	**Rotamer**	**Cβ deviation**	**CaBLAM**	**Bond lengths**	**Bond angles**	**Cis Peptides**
			Avg: 7.70	Clashscore: 37.15	Outliers: 4 of 249	Poor rotamers: 1 of 195	Outliers: 0 of 229	Outliers: 17 of 247	Outliers: 84 of 251	Outliers: 34 of 251	Non-Trans: 12 of 250
121		MET	3.98	0.50Å HE1 with 173 VAL CG1	Favored (96.19%) General / -63.0,-39.9	Favored (93.1%) *mmm* chi angles: 301.5,297.5,291.5	0.01Å	Favored (93.712%) alpha helix	-	-	-
122		TYR	4.86	0.84Å C with 125 PRO HD2	Favored (98.9%) General / -62.6,-41.4	Favored (55.7%) *t80* chi angles: 180.5,93.5	0.02Å	Favored (94.616%) alpha helix	OUTLIER(S) worst is N--CA: 4.0 σ	-	-
123		LEU	4.91	-	Favored (97.71%) General / -62.6,-43.9	Favored (80.6%) *mt* chi angles: 293.5,178.5	0.01Å	Favored (98.499%) alpha helix	-	-	-
124		ILE	4.3	0.73Å HG21 with 173 VAL HG22	Favored (42.65%) Pre-Pro / -65.0,-40.0	Favored (37.2%) *mm* chi angles: 295.4,299.9	0.04Å	Favored (93.689%) alpha helix	-	-	-
125		PRO	4.57	0.84Å HD2 with 122 TYR C	Favored (24.36%) Trans-Pro / -67.7,-34.7	Favored (84.5%) *Cg_exo* chi angles: 334.1,37.6,328.5	0.01Å	Favored (97.845%) alpha helix	OUTLIER(S) worst is N--CD: 4.8 σ	-	-
126		GLY	5.17	-	Favored (96.03%) Glycine / -59.9,-45.4	-	-	Favored (93.45%) alpha helix	-	-	-
127		LEU	5.21	-	Favored (97.39%) General / -64.0,-41.5	Favored (94.3%) *mt* chi angles: 296.4,173.5	0.01Å	Favored (90.196%) alpha helix	-	-	-
128		GLN	4.67	0.82Å HE21 with 169 LYS HB3	Favored (97.14%) General / -63.3,-43.6	Favored (91.8%) *mt0* chi angles: 289.5,175.7,307.5	0.01Å	Favored (99.26%) alpha helix	-	-	-
129		ALA	5.41	-	Favored (95.86%) General / -63.2,-44.3	-	0.02Å	Favored (97.822%) alpha helix	-	-	-
130		ALA	5.3	-	Favored (95.53%) General / -64.5,-40.4	-	0.02Å	Favored (96.275%) alpha helix	-	-	-
131		ALA	5.21	-	Favored (98.91%)	-	0.02Å	Favored (96.188%)	-	-	-

#	Alt	Res	High B	Clash > 0.4Å	Ramachandran	Rotamer	Cβ deviation	CaBLAM	Bond lengths	Bond angles	Cis Peptides
					General / -62.1,-43.4			alpha helix			
132		ALA	5.4	-	Favored (95.57%) General / -62.0,-45.0	-	0.04Å	Favored (86.217%) alpha helix	-	-	-
133		ARG	5.76	-	Favored (87.3%) General / -66.8,-38.8	Favored (98.9%) *mtt180* chi angles: 292.5,178.1,179.5,176.2	0.01Å	Favored (84.393%) alpha helix	-	-	-
134		ALA	5.54	-	Favored (90.39%) General / -59.1,-42.4	-	0.02Å	Favored (85.856%) alpha helix	OUTLIER(S) worst is C--N: 4.5 σ	-	-
135		ALA	5.38	0.53Å HB1 with 162 ILE HG12	Favored (96.36%) General / -61.5,-44.9	-	0.03Å	Favored (96.065%) alpha helix	-	-	-
136		GLN	5.89	0.50Å NE2 with 162 ILE HD13	Favored (98.16%) General / -61.9,-41.7	Favored (98.2%) *mm-40* chi angles: 291.9,298.5,314.5	0.01Å	Favored (97.903%) alpha helix	-	-	-
137		LYS	5.9	-	Favored (92.17%) General / -64.5,-38.8	Favored (96.5%) *mttt* chi angles: 289.3,179.2,180.8,180.5	0.01Å	Favored (92.512%) alpha helix	OUTLIER(S) worst is C--N: 4.1 σ	-	-
138		ARG	6	-	Favored (98.49%) General / -61.0,-43.7	Favored (84.2%) *ttp80* chi angles: 181.5,182.5,63,78.6	0.02Å	Favored (93.513%) alpha helix	-	-	-
139		THR	5.66	-	Favored (96.76%) General / -62.1,-40.7	Favored (95.4%) *m* chi angles: 299.5	0.02Å	Favored (96.999%) alpha helix	OUTLIER(S) worst is C--N: 4.0 σ	-	-
140		ALA	6.44	0.45Å O with 144 MET HG3	Favored (99.22%) General / -61.0,-42.8	-	0.07Å	Favored (97.042%) alpha helix	OUTLIER(S) worst is CA--C: 4.6 σ	-	-
**#**	**Alt**	**Res**	**High B**	**Clash > 0.4Å**	**Ramachandran**	**Rotamer**	**Cβ deviation**	**CaBLAM**	**Bond lengths**	**Bond angles**	**Cis Peptides**
			Avg: 7.70	Clashscore: 37.15	Outliers: 4 of 249	Poor rotamers: 1 of 195	Outliers: 0 of 229	Outliers: 17 of 247	Outliers: 84 of 251	Outliers: 34 of 251	Non-Trans: 12 of 250
141		ALA	7.41	-	Favored (83.81%) General / -61.8,-47.7	-	0.02Å	Favored (94.976%) alpha helix	-	-	-
142		GLY	14.74	-	Favored (99.87%) Glycine / -62.9,-41.4	-	-	Favored (91.336%) alpha helix	OUTLIER(S) worst is C--N: 4.6 σ	-	-
143		ILE	14.52	-	Favored (82.4%) Ile or Val / -68.9,-43.3	Favored (96.9%) *mt* chi angles: 292.7,169.5	0.02Å	Favored (85.831%) alpha helix	-	OUTLIER(S) worst is C-N-CA: 5.2 σ	-
144		MET	15.04	0.45Å HG3 with 140 ALA O	Favored (77.69%) General / -62.6,-34.8	Favored (85.1%) *mtm* chi angles: 291.5,185.5,286.5	0.02Å	Favored (44.771%)	OUTLIER(S) worst is C--N: 5.0 σ	-	-
145		LYS	6.6	0.48Å O with 147 PRO CD	Favored (19.1%) General / -77.0,169.0	Favored (98%) *mttt* chi angles: 292.4,179.8,179.5,181	0.02Å	CaBLAM Disfavored (1.575%)	-	-	-
146		ASN	23.31	0.43Å C with 146 ASN OD1	Allowed (1.95%) Pre-Pro / -67.5,107.6	Favored (54.6%) *t0* chi angles: 187.2,324.1	0.07Å	CA Geom Outlier (0.262%)	-	OUTLIER(S) worst is C-N-CA: 4.1 σ	-

147		PRO	21.18	0.97Å CD with 147 PRO N	OUTLIER (0)%) Trans-Pro / -150.8,-130.8	Favored (73.4%) *Cg_endo* chi angles: 29,35.5,303.1	0.06Å	CA Geom Outlier (0.007%)	OUTLIER(S) worst is N--CD: 53.9 σ	OUTLIER(S) worst is CA-N-CD: 20.8 σ	-
148		VAL	21.99	0.90Å HG23 with 147 PRO HD2	OUTLIER (0)%) Ile or Val / 84.7,158.0	Favored (89.8%) *t* chi angles: 176	0.02Å	Favored (14.493%)	-	OUTLIER(S) worst is C-N-CA: 4.8 σ	-
149		VAL	9.62	0.87Å O with 149 VAL HG12	Allowed (0.41%) Ile or Val / 51.0,49.4	Favored (72.3%) *t* chi angles: 178.5	0.09Å	CA Geom Outlier (0.004%)	-	-	-
150		ASP	5.7	0.62Å HB2 with 148 VAL O	OUTLIER (0.01)%) General / 134.4,-49.9	Favored (86.1%) *m-30* chi angles: 284.2,163.5	0.07Å	CaBLAM Outlier (0.689%) try alpha helix	OUTLIER(S) worst is CG--OD2: 14.2 σ	OUTLIER(S) worst is OD1-CG-OD2: 5.3 σ	Twisted nonPRO omega= -114.66
151		GLY	7.4	-	Favored (92.12%) Glycine / -64.3,-45.9	-	-	Favored (45.67%) alpha helix	OUTLIER(S) worst is C--N: 4.7 σ	-	-
152		ILE	4.53	-	Favored (96.66%) Ile or Val / -64.7,-43.6	Favored (91.7%) *mt* chi angles: 297.5,169.4	0.07Å	Favored (83.643%) alpha helix	-	OUTLIER(S) worst is C-N-CA: 4.9 σ	-
153		VAL	4.45	-	Favored (97.29%) Ile or Val / -61.9,-43.3	Favored (98.9%) *t* chi angles: 175.6	0.01Å	Favored (84.19%) alpha helix	-	-	-
154		VAL	6.93	0.40Å HG23 with 150 ASP O	Favored (87.43%) Ile or Val / -65.7,-40.3	Favored (73.1%) *t* chi angles: 172.5	0.01Å	Favored (95.504%) alpha helix	OUTLIER(S) worst is C--N: 4.7 σ	-	-
155		THR	6.35	-	Favored (99.43%) General / -62.6,-42.3	Favored (89.2%) *m* chi angles: 298.7	0.02Å	Favored (97.898%) alpha helix	-	-	-
156		ASP	6.44	-	Favored (97.58%) General / -63.9,-40.9	Favored (96.3%) *m-30* chi angles: 288.3,161.9	0.01Å	Favored (97.47%) alpha helix	OUTLIER(S) worst is CG--OD2: 14.2 σ	OUTLIER(S) worst is OD1-CG-OD2: 5.2 σ	-
157		ILE	4.11	-	Favored (99.09%) Ile or Val / -62.9,-43.6	Favored (94.2%) *mt* chi angles: 292,169	0.03Å	Favored (96.399%) alpha helix	-	-	-
158		ASP	4.55	-	Favored (94.17%) General / -64.7,-39.8	Favored (96.6%) *m-30* chi angles: 288.3,162	0.01Å	Favored (91.331%) alpha helix	OUTLIER(S) worst is CG--OD2: 14.2 σ	OUTLIER(S) worst is OD1-CG-OD2: 5.3 σ	-
159		THR	4.01	-	Favored (96.1%) General / -61.5,-45.0	Favored (96.2%) *m* chi angles: 299.7	0.02Å	Favored (94.519%) alpha helix	-	-	-
160		MET	4.1	1.09Å O with 164 PRO HG2	Favored (91.92%) General / -60.5,-46.0	Favored (98.4%) *mtp* chi angles: 293.5,174.1,69.5	0.01Å	Favored (87.617%) alpha helix	-	-	-
**#**	**Alt**	**Res**	**High B**	**Clash > 0.4Å**	**Ramachandran**	**Rotamer**	**Cβ deviation**	**CaBLAM**	**Bond lengths**	**Bond angles**	**Cis Peptides**
			Avg: 7.70	Clashscore: 37.15	Outliers: 4 of 249	Poor rotamers: 1 of 195	Outliers: 0 of 229	Outliers: 17 of 247	Outliers: 84 of 251	Outliers: 34 of 251	Non-Trans: 12 of 250
161		THR	4.25	0.43Å O with 165 GLN HG3	Favored (95.6%) General / -64.5,-42.8	Favored (95.9%) *m* chi angles: 299.6	0.03Å	Favored (86.559%) alpha helix	-	-	-

162		ILE	3.74	0.53Å HG12 with 135 ALA HB1	Favored (97.07%) Ile or Val / -63.2,-42.6	Favored (96%) *mt* chi angles: 293.5,169.5	0.01Å	Favored (99.184%) alpha helix	-	-	-
163		ASP	3.46	-	Favored (94.78%) Pre-Pro / -61.4,-46.6	Favored (95%) *m-30* chi angles: 291.5,161.5	0.03Å	Favored (96.102%) alpha helix	OUTLIER(S) worst is CG--OD2: 14.2 σ	OUTLIER(S) worst is OD1-CG-OD2: 5.3 σ	-
164		PRO	3.48	1.09Å HG2 with 160 MET O	Favored (56.77%) Trans-Pro / -63.5,-36.0	Favored (62%) *Cg_exo* chi angles: 328.5,316.5,61.5	0.04Å	Favored (99.092%) alpha helix	OUTLIER(S) worst is N--CD: 67.9 σ	OUTLIER(S) worst is CA-N-CD: 23.6 σ	-
165		GLN	4.21	0.43Å HG3 with 161 THR O	Favored (99.8%) General / -62.9,-42.6	Favored (93.4%) *mt0* chi angles: 295.5,176.5,314.4	0.01Å	Favored (98.657%) alpha helix	-	-	-
166		VAL	3.91	0.42Å O with 170 MET HG2	Favored (97.28%) Ile or Val / -62.0,-43.2	Favored (92.7%) *t* chi angles: 174.5	0.02Å	Favored (98.716%) alpha helix	-	-	-
167		GLU	4.48	-	Favored (97.45%) General / -63.9,-42.8	Favored (97.9%) *mt-10* chi angles: 292.5,181.5,167.5	0.01Å	Favored (97.762%) alpha helix	-	-	-
168		LYS	4.48	-	Favored (97.14%) General / -64.2,-41.4	Favored (96.5%) *mttt* chi angles: 289.1,179.2,180.7,180.4	0.01Å	Favored (90.824%) alpha helix	-	-	-
169		LYS	3.67	0.82Å HB3 with 128 GLN HE21	Favored (92.21%) General / -65.4,-42.6	Favored (62%) *mttm* chi angles: 290.3,182.5,181.5,295.8	0.01Å	Favored (88.174%) alpha helix	-	-	-
170		MET	3.8	0.42Å HG2 with 166 VAL O	Favored (96.85%) General / -63.6,-40.4	Favored (91.9%) *mmm* chi angles: 290,300.5,285.5	0.02Å	Favored (96.56%) alpha helix	-	-	-
171		GLY	4.17	-	Favored (97.47%) Glycine / -61.9,-44.7	-	-	Favored (95.509%) alpha helix	OUTLIER(S) worst is C--N: 4.6 σ	-	-
172		GLN	4.17	-	Favored (92.34%) General / -65.3,-43.0	Favored (71.3%) *mt0* chi angles: 294.5,175.7,62.5	0.02Å	Favored (88.986%) alpha helix	-	-	-
173		VAL	3.14	0.73Å HG22 with 124 ILE HG21	Favored (93.76%) Ile or Val / -64.3,-41.9	Favored (66.8%) *t* chi angles: 171.7	0.03Å	Favored (96.855%) alpha helix	-	-	-
174		LEU	3.61	-	Favored (92.83%) General / -65.5,-40.4	Favored (65.4%) *mt* chi angles: 290.2,177.5	0.02Å	Favored (95.732%) alpha helix	-	OUTLIER(S) worst is C-N-CA: 4.5 σ	-
175		LEU	4.11	-	Favored (94.03%) General / -64.4,-39.5	Favored (86.6%) *mt* chi angles: 291,173.8	0.02Å	Favored (97.376%) alpha helix	OUTLIER(S) worst is CA--C: 4.0 σ	-	-
176		ILE	3.32	0.57Å HG21 with 120 TYR CE2	Favored (96.4%) Ile or Val / -62.7,-42.5	Favored (94.5%) *mt* chi angles: 292,169	0.01Å	Favored (97.119%) alpha helix	OUTLIER(S) worst is C--N: 4.4 σ	-	-
177		ALA	2.7	-	Favored (94.94%) General / -60.2,-44.6	-	0.04Å	Favored (97.566%) alpha helix	-	-	-
178		VAL	3.63	-	Favored (98.3%) Ile or Val / -63.0,-42.9	Favored (92.5%) *t* chi angles: 174.5	0.03Å	Favored (98.292%) alpha helix	OUTLIER(S) worst is C--N: 5.2 σ	-	-

179		ALA	3.68	-	Favored (98.83%) General / -62.0,-43.4	-	0.02Å	Favored (98.037%) alpha helix	-	-	-
180		ILE	2.96	1.00Å HG21 with 117 VAL HG22	Favored (89.78%) Ile or Val / -65.7,-41.1	Favored (96.1%) *mt* chi angles: 293.5,169.4	0.06Å	Favored (96.907%) alpha helix	-	-	-
**#**	**Alt**	**Res**	**High B**	**Clash > 0.4Å**	**Ramachandran**	**Rotamer**	**Cβ deviation**	**CaBLAM**	**Bond lengths**	**Bond angles**	**Cis Peptides**
			Avg: 7.70	Clashscore: 37.15	Outliers: 4 of 249	Poor rotamers: 1 of 195	Outliers: 0 of 229	Outliers: 17 of 247	Outliers: 84 of 251	Outliers: 34 of 251	Non-Trans: 12 of 250
181		SER	3.06	-	Favored (90.71%) General / -64.1,-38.3	Favored (93.9%) *p* chi angles: 64.2	0.03Å	Favored (93.946%) alpha helix	-	-	-
182		SER	3.45	-	Favored (94.05%) General / -64.9,-39.8	Favored (77.6%) *p* chi angles: 70.6	0.02Å	Favored (96.133%) alpha helix	-	-	-
183		ALA	3.39	-	Favored (98.98%) General / -61.8,-42.4	-	0.02Å	Favored (97.791%) alpha helix	-	-	-
184		VAL	2.52	1.17Å HG22 with 113 ILE HG21	Favored (98.63%) Ile or Val / -62.8,-43.2	Favored (73.4%) *t* chi angles: 172.5	0.02Å	Favored (97.118%) alpha helix	OUTLIER(S) worst is CA--C: 4.7 σ	-	-
185		LEU	3.26	-	Favored (96.49%) General / -60.7,-44.4	Favored (93.6%) *mt* chi angles: 296.5,172.5	0.02Å	Favored (92.08%) alpha helix	-	-	-
186		LEU	4.05	-	Favored (93.82%) General / -65.0,-39.7	Favored (92.5%) *mt* chi angles: 293.5,175.5	0.02Å	Favored (93.636%) alpha helix	-	-	-
187		ARG	3.37	-	Favored (98.72%) General / -61.0,-42.6	Favored (35.5%) *tpt170* chi angles: 179.5,67.5,181.6,178.5	0.01Å	Favored (91.811%) alpha helix	-	-	-
188		THR	3.16	-	Favored (86.46%) General / -65.6,-44.5	Favored (96.2%) *m* chi angles: 299.7	0.04Å	Favored (88.613%) alpha helix	-	-	-
189		ALA	3.22	-	Favored (98.52%) General / -62.7,-40.9	-	0.02Å	Favored (96.358%) alpha helix	OUTLIER(S) worst is C--N: 6.2 σ	-	-
190		TRP	3.25	-	Favored (93.05%) General / -61.0,-45.9	Favored (86%) *t60* chi angles: 175.5,82.4	0.01Å	Favored (97.432%) alpha helix	OUTLIER(S) worst is C--N: 4.6 σ	-	-
191		GLY	2.41	-	Favored (99.69%) Glycine / -61.7,-42.0	-	-	Favored (99.568%) alpha helix	-	-	-
192		TRP	2.5	0.46Å CD1 with 103 GLN HE22	Favored (96.92%) General / -64.0,-42.9	Favored (52.6%) *m-10* chi angles: 291.8,355.5	0.02Å	Favored (97.885%) alpha helix	-	-	-
193		GLY	2.92	-	Favored (96.69%) Glycine / -64.6,-39.3	-	-	Favored (95.93%) alpha helix	-	-	-

194		GLU	3.06	-	Favored (99.12%) General / -62.4,-42.0	Favored (85%) *tt0* chi angles: 178.5,182.5,178.7	0.01Å	Favored (94.346%) alpha helix	-	-	-
195		ALA	2.07	-	Favored (96.22%) General / -62.4,-44.5	-	0.02Å	Favored (92.721%) alpha helix	-	-	-
196		GLY	2.58	-	Favored (97.69%) Glycine / -64.4,-42.2	-	-	Favored (98.063%) alpha helix	-	-	-
197		ALA	3.32	-	Favored (96.59%) General / -61.8,-44.6	-	0.02Å	Favored (97.573%) alpha helix	-	-	-
198		LEU	2.85	0.94Å C with 198 LEU HD23	Favored (94.41%) General / -65.2,-40.6	OUTLIER (0.2%) chi angles: 178.5,174.5	0.01Å	Favored (95.617%) alpha helix	-	-	-
199		ILE	2.32	0.64Å HG23 with 95 LEU HB3	Favored (91.55%) Ile or Val / -62.3,-41.4	Favored (93.6%) *mt* chi angles: 292,169.5	0.01Å	Favored (89.417%) alpha helix	-	-	-
200		THR	3.27	-	Favored (89.13%) General / -60.4,-46.7	Favored (88.8%) *m* chi angles: 298.4	0.01Å	Favored (81.638%) alpha helix	-	-	-
**#**	**Alt**	**Res**	**High B**	**Clash > 0.4Å**	**Ramachandran**	**Rotamer**	**Cβ deviation**	**CaBLAM**	**Bond lengths**	**Bond angles**	**Cis Peptides**
			Avg: 7.70	Clashscore: 37.15	Outliers: 4 of 249	Poor rotamers: 1 of 195	Outliers: 0 of 229	Outliers: 17 of 247	Outliers: 84 of 251	Outliers: 34 of 251	Non-Trans: 12 of 250
201		ALA	3.41	-	Favored (95.73%) General / -64.8,-41.5	-	0.03Å	Favored (81.737%) alpha helix	-	-	-
202		ALA	2.4	-	Favored (94.61%) General / -65.0,-40.3	-	0.02Å	Favored (92.945%) alpha helix	-	-	-
203		THR	2.62	0.46Å OG1 with 95 LEU HD13	Favored (86.6%) General / -58.6,-46.6	Favored (99.5%) *m* chi angles: 300.5	0.01Å	Favored (96.652%) alpha helix	-	-	-
204		SER	3.45	-	Favored (98.82%) General / -62.7,-43.4	Favored (66.5%) *m* chi angles: 294.4	0.02Å	Favored (94.238%) alpha helix	-	-	-
205		THR	3.22	-	Favored (94.33%) General / -65.2,-40.6	Favored (96.1%) *m* chi angles: 299.6	0.03Å	Favored (94.227%) alpha helix	OUTLIER(S) worst is C--N: 5.0 σ	-	-
206		LEU	2.49	-	Favored (94.34%) General / -64.4,-39.7	Favored (98.3%) *mt* chi angles: 293.5,172.4	0.02Å	Favored (97.278%) alpha helix	OUTLIER(S) worst is C--N: 5.6 σ	-	-
207		TRP	3.09	1.03Å O with 211 PRO HG2	Favored (91.7%) General / -64.8,-38.6	Favored (65.2%) *t-100* chi angles: 184.4,248.4	0.02Å	Favored (96.94%) alpha helix	-	-	-
208		GLU	4.23	-	Favored (97.91%) General / -62.0,-41.2	Favored (99.2%) *mt-10* chi angles: 292.5,178,178.3	0.02Å	Favored (98.515%) alpha helix	-	-	-
209		GLY	3.47	-	Favored (98.84%)	-	-	Favored (98.731%)	-	-	-

					Glycine / -62.3,-43.6			alpha helix			
210		SER	3.63	0.59Å HB3 with 76 VAL CG2	Favored (71.22%) Pre-Pro / -65.2,-44.8	Favored (93.9%) *p* chi angles: 64.2	0.01Å	Favored (98.315%) alpha helix	-	-	-
211		PRO	3.89	1.03Å HG2 with 207 TRP O	Favored (50.13%) Trans-Pro / -62.9,-38.2	Favored (46.7%) *Cg_exo* chi angles: 327.5,315.5,62.3	0.07Å	Favored (97.649%) alpha helix	OUTLIER(S) worst is N--CD: 73.9 σ	OUTLIER(S) worst is CA-N-CD: 25.8 σ	-
212		ASN	3.63	-	Favored (97.78%) General / -62.3,-43.9	Favored (90.8%) *m-40* chi angles: 294.5,334.4	0.01Å	Favored (94.056%) alpha helix	-	-	-
213		LYS	3.02	-	Favored (98.46%) General / -62.2,-43.6	Favored (97.8%) *mttt* chi angles: 291.5,179.1,180.7,179.5	0.01Å	Favored (94.494%) alpha helix	OUTLIER(S) worst is C--N: 4.9 σ	-	-
214		TYR	3.31	-	Favored (99.6%) General / -63.0,-41.5	Favored (78.2%) *t80* chi angles: 180.5,86.5	0.03Å	Favored (98.611%) alpha helix	-	-	-
215		TRP	5.07	-	Favored (96.05%) General / -64.7,-41.4	Favored (59.7%) *m-10* chi angles: 291.8,345.8	0.01Å	Favored (97.122%) alpha helix	-	-	-
216		ASN	4.64	-	Favored (97.38%) General / -61.2,-44.4	Favored (96.4%) *m-40* chi angles: 289.5,334.4	0.03Å	Favored (93.093%) alpha helix	-	-	-
217		SER	3.58	-	Favored (95.15%) General / -65.1,-41.4	Favored (65.5%) *m* chi angles: 294.3	0.01Å	Favored (93.714%) alpha helix	-	-	-
218		SER	3.71	-	Favored (94.31%) General / -64.6,-39.8	Favored (93.7%) *p* chi angles: 64.2	0.01Å	Favored (99.005%) alpha helix	-	-	-
219		THR	4.57	-	Favored (94.86%) General / -64.8,-42.7	Favored (89.6%) *m* chi angles: 298.7	0.02Å	Favored (99.083%) alpha helix	OUTLIER(S) worst is C--N: 5.0 σ	-	-
220		ALA	4.09	-	Favored (96.76%) General / -64.0,-40.6	-	0.02Å	Favored (97.507%) alpha helix	-	-	-
**#**	**Alt**	**Res**	**High B**	**Clash > 0.4Å**	**Ramachandran**	**Rotamer**	**Cβ deviation**	**CaBLAM**	**Bond lengths**	**Bond angles**	**Cis Peptides**
			Avg: 7.70	Clashscore: 37.15	Outliers: 4 of 249	Poor rotamers: 1 of 195	Outliers: 0 of 229	Outliers: 17 of 247	Outliers: 84 of 251	Outliers: 34 of 251	Non-Trans: 12 of 250
221		THR	3.72	-	Favored (98.58%) General / -63.5,-41.4	Favored (89.5%) *m* chi angles: 298.7	0.01Å	Favored (98.091%) alpha helix	OUTLIER(S) worst is C--N: 4.4 σ	-	-
222		SER	4.72	-	Favored (87.45%) General / -66.8,-38.9	Favored (80.4%) *p* chi angles: 61.5	0.02Å	Favored (98.157%) alpha helix	OUTLIER(S) worst is C--N: 4.5 σ	OUTLIER(S) worst is C-N-CA: 4.8 σ	-
223		LEU	4.89	-	Favored (99.72%) General / -62.9,-42.2	Favored (95.7%) *mt* chi angles: 293.5,173.8	0.02Å	Favored (98.042%) alpha helix	-	-	-
224		CYS	4.27	0.45Å O with 228 ARG HG2	Favored (98.3%) General / -63.3,-43.3	Favored (98.4%) *m* chi angles: 289.5	0.01Å	Favored (98.673%) alpha helix	-	-	-

#	Alt	Res	High B	Clash > 0.4Å	Ramachandran	Rotamer	Cβ deviation	CaBLAM	Bond lengths	Bond angles	Cis Peptides
225		ASN	4.59	-	Favored (97.83%) General / -63.9,-41.0	Favored (96.7%) *m-40* chi angles: 290.5,334.5	0.00Å	Favored (98.295%) alpha helix	-	-	-
226		ILE	5.3	-	Favored (98.92%) Ile or Val / -62.6,-43.9	Favored (93.8%) *mt* chi angles: 293.5,171.5	0.03Å	Favored (95.601%) alpha helix	OUTLIER(S) worst is C--N: 5.3 σ	-	-
227		PHE	5.14	-	Favored (95.56%) General / -64.0,-40.1	Favored (43.4%) *t80* chi angles: 175.5,93.5	0.01Å	Favored (90.734%) alpha helix	-	-	-
228		ARG	4.51	0.62Å HD2 with 57 ALA HB2	Favored (96.99%) General / -60.6,-42.5	Favored (48.8%) *mmt180* chi angles: 293.5,291.9,179.6,177.5	0.01Å	Favored (91.92%) alpha helix	OUTLIER(S) worst is C--N: 5.3 σ	-	-
229		GLY	6.81	-	Favored (97.45%) Glycine / -64.7,-40.4	-	-	Favored (98.875%) alpha helix	-	-	-
230		SER	7.23	-	Favored (97.86%) General / -62.8,-43.8	Favored (66.6%) *m* chi angles: 294.4	0.02Å	Favored (95.815%) alpha helix	-	-	-
231		TYR	5.95	-	Favored (98.19%) General / -61.0,-43.9	Favored (63.1%) *t80* chi angles: 180.5,91.5	0.01Å	Favored (99.524%) alpha helix	OUTLIER(S) worst is C--N: 4.5 σ	-	-
232		LEU	4.67	1.04Å CD1 with 54 VAL HG22	Favored (99.94%) General / -63.0,-42.8	Favored (94.2%) *mt* chi angles: 291.5,172.5	0.02Å	Favored (95.858%) alpha helix	-	-	-
233		ALA	5.66	-	Favored (95.62%) General / -64.8,-41.8	-	0.02Å	Favored (97.786%) alpha helix	-	-	-
234		GLY	5.76	-	Favored (99.38%) Glycine / -63.5,-41.0	-	-	Favored (98.893%) alpha helix	-	-	-
235		ALA	5.04	-	Favored (97.57%) General / -62.8,-43.9	-	0.02Å	Favored (98.003%) alpha helix	-	-	-
236		SER	5.38	0.56Å HA with 239 TYR CE2	Favored (99.49%) General / -63.0,-41.2	Favored (83.4%) *p* chi angles: 62.5	0.02Å	Favored (97.15%) alpha helix	-	-	-
237		LEU	6.46	-	Favored (95.82%) General / -64.6,-41.9	Favored (94.2%) *mt* chi angles: 292.5,174.5	0.02Å	Favored (84.851%) alpha helix	-	-	-
238		ILE	6.34	-	Favored (84.72%) Ile or Val / -61.2,-40.1	Favored (92.5%) *mt* chi angles: 293.6,172.5	0.03Å	Favored (77.505%) alpha helix	-	-	-
239		TYR	5.55	0.62Å C with 239 TYR CD1	Favored (54.81%) General / -77.1,-28.7	Allowed (1.4%) *p90* chi angles: 76,67.5	0.01Å	Favored (88.652%) alpha helix	OUTLIER(S) worst is C--N: 4.1 σ	OUTLIER(S) worst is C-N-CA: 4.3 σ	-
240		THR	6.18	0.53Å N with 239 TYR CD1	Favored (92.11%) General / -65.9,-40.9	Favored (96.3%) *m* chi angles: 299.7	0.01Å	Favored (94.616%) alpha helix	-	-	-
**#**	**Alt**	**Res**	**High B**	**Clash > 0.4Å**	**Ramachandran**	**Rotamer**	**Cβ deviation**	**CaBLAM**	**Bond lengths**	**Bond angles**	**Cis Peptides**
			Avg: 7.70	Clashscore: 37.15	Outliers: 4 of 249	Poor rotamers: 1 of 195	Outliers: 0 of 229	Outliers: 17 of 247	Outliers: 84 of 251	Outliers: 34 of 251	Non-Trans:

											12 of 250
241		VAL	7.17	-	Favored (91.27%) Ile or Val / -62.0,-41.5	Favored (66.3%) *t* chi angles: 171.7	0.05Å	Favored (98.34%) alpha helix	-	-	-
242		THR	6.58	-	Favored (99.71%) General / -63.0,-42.1	Favored (89.2%) *m* chi angles: 298.7	0.02Å	Favored (97.742%) alpha helix	-	-	-
243		ARG	6.5	0.47Å CD with 34 LEU HD22	Favored (99.23%) General / -62.2,-42.4	Favored (46.6%) *ttp-170* chi angles: 169.5,176.5,66.3,192.4	0.01Å	Favored (99.24%) alpha helix	-	-	-
244		ASN	7.29	0.48Å O with 247 LEU HB3	Favored (95.27%) General / -62.4,-44.9	Favored (93.9%) *m-40* chi angles: 293.5,334.5	0.01Å	Favored (86.442%) alpha helix	-	-	-
245		ALA	7.45	-	Favored (85.18%) General / -67.0,-42.5	-	0.03Å	Favored (77.444%) alpha helix	OUTLIER(S) worst is C--N: 4.9 σ	-	-
246		GLY	7.87	-	Favored (90.36%) Glycine / -57.2,-39.6	-	-	Favored (92.558%) alpha helix	-	-	-
247		LEU	8.41	1.09Å HD22 with 32 ILE HG12	Favored (75.22%) General / -57.2,-49.7	Favored (73.1%) *tp* chi angles: 178.1,62	0.06Å	Favored (81.48%) alpha helix	-	-	-
248		VAL	7.13	-	Favored (10.71%) Ile or Val / -89.2,-6.0	Favored (30.8%) *m* chi angles: 300.3	0.01Å	Favored (19.428%) alpha helix	OUTLIER(S) worst is C--N: 4.9 σ	-	-
249		LYS	7.95	-	Favored (81.03%) General / -62.8,-48.0	Favored (98.6%) *mttt* chi angles: 295.5,179.1,180.7,180.6	0.03Å	CaBLAM Disfavored (4.485%)	OUTLIER(S) worst is C--N: 6.2 σ	OUTLIER(S) worst is C-N-CA: 4.3 σ	-
250		ARG	8.32	-	Favored (69.56%) General / -69.9,-44.4	Favored (98.1%) *mtt-85* chi angles: 290.3,181.6,183.7,274.5	0.07Å	-	-	-	-
251		ARG	8.32	-	-	Favored (94.2%) *mtt-85* chi angles: 292.9,182.5,175.6,282.5	0.03Å	-	OUTLIER(S) worst is C--N: 6.1 σ	-	-

About MolProbity | Website for the Richardson Lab | Using ecloud x-H | Internal reference 4.5.2
