# Supplementary material for: Integrative proteome-wide structural analysis and high-throughput docking identify broad-spectrum antiviral scaffolds against Zika, Yellow Fever, West Nile, Saint Louis encephalitis, and Usutu viruses
Source: Front Cell Infect Microbiol. 2026 Apr 30;16:1723132. doi: 10.3389/fcimb.2026.1723132 (PMC13171538; doi:10.3389/fcimb.2026.1723132)
Supplement: Supplementary file 7 [file DataSheet7.zip › ZIKV/ZIKV_NS4b/Mol_probity_Files/ZIKV_NS4b_1FH-rama.pdf]

MolProbity Ramachandran analysis

ZIKV_NS4b1FH.pdb, model 1

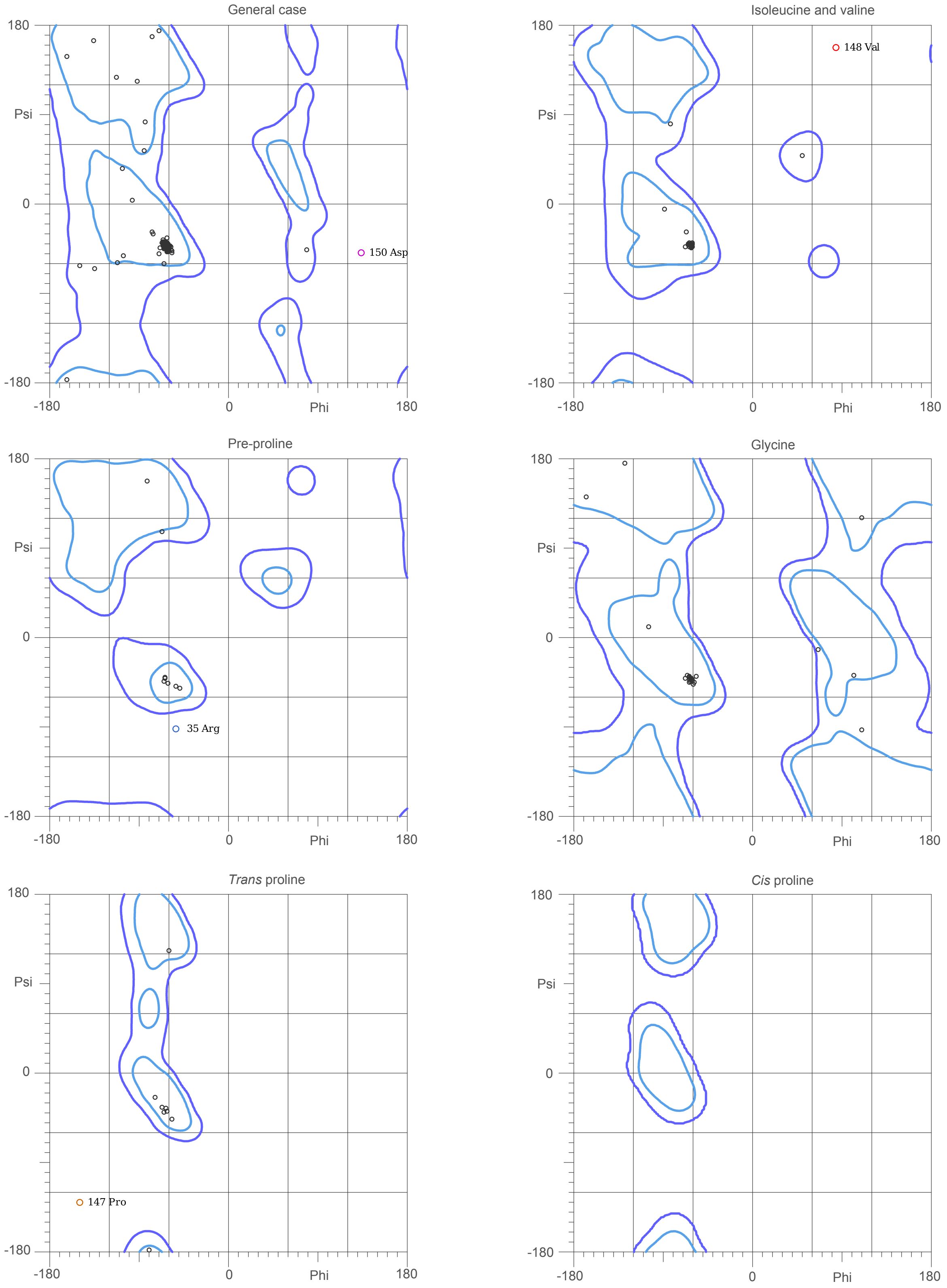

94.8% (236/249) of all residues were in favored (98%) regions.
98.4% (245/249) of all residues were in allowed (>99.8%) regions.

There were 4 outliers (phi, psi):
- 35 Arg (-53.3, -92.3)
- 147 Pro (-150.8, -130.8)
- 148 Val (84.7, 158.0)
- 150 Asp (134.4, -49.9)
