# Supplementary material for: Integrative proteome-wide structural analysis and high-throughput docking identify broad-spectrum antiviral scaffolds against Zika, Yellow Fever, West Nile, Saint Louis encephalitis, and Usutu viruses
Source: Front Cell Infect Microbiol. 2026 Apr 30;16:1723132. doi: 10.3389/fcimb.2026.1723132 (PMC13171538; doi:10.3389/fcimb.2026.1723132)
Supplement: Supplementary file 7 [file DataSheet7.zip › ZIKV/ZIKV_NS5/Mol_probity_Files/ZIKV_NS5_1FH-multi.table.pdf]

MolProbity

Viewing ZIKV_NS5_1FH-multi.table

Duke Biochemistry
Duke University School of Medicine

When finished, you should close this window.

Hint: Use File | Save As... to save a copy of this page.

All-Atom Contacts	Clashscore, all atoms:	2.02		99th percentile* (N=1784, all resolutions)
	Clashscore is the number of serious steric overlaps (> 0.4 Å) per 1000 atoms.			
Protein Geometry	Poor rotamers	0	0.00%	Goal: <0.3%
	Favored rotamers	761	99.22%	Goal: >98%
	Ramachandran outliers	3	0.33%	Goal: <0.05%
	Ramachandran favored	880	97.67%	Goal: >98%
	Rama distribution Z-score	0.05 ± 0.26		Goal: abs(Z score) < 2
	MolProbity score^	1.04		100th percentile* (N=27675, 0Å - 99Å)
	Cβ deviations >0.25Å	0	0.00%	Goal: 0
	Bad bonds:	11 / 7404	0.15%	Goal: 0%
	Bad angles:	18 / 10004	0.18%	Goal: <0.1%
Peptide Omegas	Cis Prolines:	0 / 33	0.00%	Expected: ≤1 per chain, or ≤5%
	Cis nonProlines:	1 / 869	0.12%	Goal: <0.05%
	Twisted Peptides:	1 / 902	0.11%	Goal: 0
Low-resolution Criteria	CaBLAM outliers	13	1.4%	Goal: <1.0%
	CA Geometry outliers	3	0.33%	Goal: <0.5%
Additional validations	Chiral volume outliers	0/1049		
	Waters with clashes	0/0	0.00%	See UnDowser table for details

In the two column results, the left column gives the raw count, right column gives the percentage.

* 100th percentile is the best among structures of comparable resolution; 0th percentile is the worst. For clashscore the comparative set of structures was selected in 2004, for MolProbity score in 2006.

^ MolProbity score combines the clashscore, rotamer, and Ramachandran evaluations into a single score, normalized to be on the same scale as X-ray resolution.

Key to table colors and cutoffs here:

#	Alt	Res	High B	Clash > 0.4Å	Ramachandran	Rotamer	Cβ deviation	CaBLAM	Bond lengths	Bond angles	Cis Peptides
			Avg: 0.95	Clashscore: 2.02	Outliers: 3 of 901	Poor rotamers: 0 of 767	Outliers: 0 of 820	Outliers: 14 of 899	Outliers: 10 of 903	Outliers: 16 of 903	Non-Trans: 2 of 902
A 1		GLY	7.47	-	-	-	-	-	-	-	-
A 2		GLY	6.44	-	Favored (10.15%) Glycine / -98.7,-138.2	-	-	-	-	-	-
A 3		GLY	5.12	-	Favored (55.83%) Glycine / -62.2,-17.4	-	-	Favored (5.577%)	-	-	-
A 4		THR	3.79	-	Favored (55.07%) General / -95.0,1.3	Favored (72.6%) *p* chi angles: 61.8	0.02Å	Favored (55.501%)	-	-	-
A 5		GLY	2.67	-	Allowed (1.85%) Glycine / -104.2,-85.4	-	-	Favored (28.171%)	-	-	-
A 6		GLU	1.88	-	OUTLIER (0)%) General / 98.7,-60.0	Favored (62.4%) *tp30* chi angles: 187.3,68.2,17.5	0.11Å	CA Geom Outlier (0.027%)	-	OUTLIER(S) worst is C-N-CA: 4.9 σ	-

A 7		THR	1.36	-	Favored (3.84%) General / -95.3,-176.6	Favored (11.7%) *t* chi angles: 189.9	0.04Å	CaBLAM Disfavored (2.276%)	-	OUTLIER(S) worst is C-N-CA: 5.6 σ	Cis nonPRO omega= -3.99
A 8		LEU	1.06	-	Favored (84.89%) General / -57.5,-44.5	Favored (60.9%) *tp* chi angles: 181.3,61.1	0.04Å	Favored (57.647%)	-	-	-
A 9		GLY	0.89	-	Favored (71.33%) Glycine / -60.8,-29.8	-	-	Favored (89.692%) alpha helix	-	-	-
A 10		GLU	0.81	-	Favored (62.18%) General / -74.2,-40.7	Favored (67%) *tp30* chi angles: 183.2,66,23.1	0.02Å	Favored (72.552%) alpha helix	-	-	-
A 11		LYS	0.78	-	Favored (82%) General / -57.1,-43.2	Favored (87.2%) *tttt* chi angles: 181.4,176.3,178.4,176.8	0.03Å	Favored (87.574%) alpha helix	-	-	-
A 12		TRP	0.78	-	Favored (80.93%) General / -61.6,-48.4	Favored (68.2%) *t60* chi angles: 181.4,78.2	0.06Å	Favored (97.361%) alpha helix	-	-	-
A 13		LYS	0.78	-	Favored (81.15%) General / -60.8,-37.2	Favored (20.4%) *mmmt* chi angles: 287.1,287.6,281.6,180.6	0.04Å	Favored (87.481%) alpha helix	-	-	-
A 14		ALA	0.79	-	Favored (98.39%) General / -61.4,-42.2	-	0.02Å	Favored (86.974%) alpha helix	-	-	-
A 15		ARG	0.8	-	Favored (97.27%) General / -61.6,-41.3	Favored (59%) *mmm-85* chi angles: 288.7,286,296.1,274.2	0.03Å	Favored (90.04%) alpha helix	-	-	-
A 16		LEU	0.82	-	Favored (90.1%) General / -61.8,-39.0	Favored (76.6%) *mt* chi angles: 288.1,171	0.02Å	Favored (79.979%) alpha helix	-	-	-
A 17		ASN	0.84	-	Favored (63.8%) General / -73.4,-31.3	Favored (91.7%) *m-40* chi angles: 286,335.4	0.09Å	Favored (83.812%) alpha helix	-	-	-
A 18		GLN	0.86	-	Favored (76.41%) General / -69.0,-35.2	Favored (97.8%) *mt0* chi angles: 290.7,171.6,341.6	0.01Å	Favored (40.435%)	-	-	-
A 19		MET	0.88	-	Favored (50.28%) General / -67.5,149.3	Favored (30.7%) *mmt* chi angles: 292.9,295.5,179.3	0.02Å	Favored (19.601%)	-	-	-
A 20		SER	0.9	-	Favored (22.4%) General / -70.2,165.3	Favored (98.7%) *p* chi angles: 65.3	0.07Å	Favored (43.914%)	-	-	-
**#**	**Alt**	**Res**	**High B**	**Clash > 0.4Å**	**Ramachandran**	**Rotamer**	**Cβ deviation**	**CaBLAM**	**Bond lengths**	**Bond angles**	**Cis Peptides**
			Avg: 0.95	Clashscore: 2.02	Outliers: 3 of 901	Poor rotamers: 0 of 767	Outliers: 0 of 820	Outliers: 14 of 899	Outliers: 10 of 903	Outliers: 16 of 903	Non-Trans: 2 of 902
A 21		ALA	0.91	-	Favored (77.25%) General / -58.1,-38.8	-	0.04Å	Favored (61.55%)	-	-	-

A 22		LEU	0.92	-	Favored (78.62%) General / -69.3,-39.0	Favored (92.7%) *mt* chi angles: 293.1,175.2	0.03Å	Favored (85.103%) alpha helix	-	-	-
A 23		GLU	0.91	-	Favored (82.3%) General / -68.2,-39.5	Favored (38.8%) *mt-10* chi angles: 289.2,166.5,310.7	0.04Å	Favored (79.041%) alpha helix	-	-	-
A 24		PHE	0.9	-	Favored (71.06%) General / -58.5,-51.2	Favored (89.1%) *t80* chi angles: 175.2,79.1	0.04Å	Favored (92.095%) alpha helix	-	-	-
A 25		TYR	0.88	-	Favored (95.01%) General / -61.5,-40.5	Favored (19.7%) *m-10* chi angles: 293,149	0.06Å	Favored (86.011%) alpha helix	-	-	-
A 26		SER	0.85	-	Favored (94.05%) General / -61.9,-40.0	Favored (72.2%) *m* chi angles: 295.8	0.02Å	Favored (74.776%) alpha helix	-	-	-
A 27		TYR	0.82	-	Favored (52.56%) General / -77.5,-36.2	Favored (46.4%) *m-80* chi angles: 290.5,117.4	0.03Å	Favored (56.539%) alpha helix	-	-	-
A 28		LYS	0.8	-	Favored (64.13%) General / -53.2,-39.8	Favored (86.4%) *tttt* chi angles: 180.6,176.7,176.9,181	0.05Å	Favored (66.869%) alpha helix	-	-	-
A 29		LYS	0.78	-	Favored (18.86%) General / -102.3,19.4	Favored (96.8%) *mttt* chi angles: 297.1,178.7,180.7,178.5	0.04Å	Favored (13.428%)	-	-	-
A 30		SER	0.77	-	Favored (32.29%) General / -121.3,119.5	Favored (40.1%) *t* chi angles: 178	0.03Å	CaBLAM Disfavored (1.005%)	-	-	-
A 31		GLY	0.75	-	Allowed (1.74%) Glycine / 90.2,56.9	-	-	Favored (7.822%)	-	-	-
A 32		ILE	0.74	-	Favored (6.35%) Ile or Val / -160.1,148.6	Favored (21.9%) *tt* chi angles: 191.2,166.1	0.04Å	Favored (31.57%)	-	-	Twisted nonPRO omega= -148.74
A 33		THR	0.73	-	Favored (51.59%) General / -107.1,133.5	Favored (96.1%) *m* chi angles: 299.7	0.05Å	Favored (51.723%) beta sheet	-	-	-
A 34		GLU	0.73	-	Favored (36.87%) General / -111.2,146.5	Favored (85.2%) *mt-10* chi angles: 289,184.2,339.4	0.10Å	Favored (49.967%) beta sheet	-	-	-
A 35		VAL	0.75	-	Favored (42.38%) Ile or Val / -89.8,127.5	Favored (62.6%) *t* chi angles: 179.6	0.09Å	Favored (41.862%) beta sheet	-	-	-
A 36		CYS	0.79	-	Favored (3.4%) General / -76.7,74.2	Favored (62.3%) *m* chi angles: 301	0.06Å	Favored (25.728%) beta sheet	-	-	-
A 37		ARG	0.85	-	Favored (50.11%) General / -83.6,-0.3	Favored (81.2%) *mtp180* chi angles: 293.9,179.8,69.5,197.4	0.10Å	Favored (8.946%)	-	-	-
A 38		GLU	0.92	-	Favored (73.03%) General / -54.4,-46.7	Favored (92.7%) *tt0* chi angles: 179.8,178.6,357	0.03Å	Favored (53.16%)	-	-	-
A 39		GLU	1.02	-	Favored (93.42%)	Favored (98.7%) *mt-10*	0.01Å	Favored (91.387%) alpha helix	-	-	-

					General / -63.7,-39.1	chi angles: 290.5,178.2,353.4					
A 40		ALA	1.14	-	Favored (96.7%) General / -64.3,-41.5	-	0.04Å	Favored (97.823%) alpha helix	-	-	-
**#**	**Alt**	**Res**	**High B**	**Clash > 0.4Å**	**Ramachandran**	**Rotamer**	**Cβ deviation**	**CaBLAM**	**Bond lengths**	**Bond angles**	**Cis Peptides**
			Avg: 0.95	Clashscore: 2.02	Outliers: 3 of 901	Poor rotamers: 0 of 767	Outliers: 0 of 820	Outliers: 14 of 899	Outliers: 10 of 903	Outliers: 16 of 903	Non-Trans: 2 of 902
A 41		ARG	1.26	-	Favored (95.83%) General / -64.6,-42.1	Favored (96.8%) *mtt180* chi angles: 288.4,173.3,177.9,167.4	0.04Å	Favored (97.842%) alpha helix	-	-	-
A 42		ARG	1.41	-	Favored (94.24%) General / -65.3,-41.5	Favored (97%) *mtt-85* chi angles: 288.1,180.1,181.5,276.2	0.01Å	Favored (94.4%) alpha helix	-	-	-
A 43		ALA	1.56	-	Favored (92.57%) General / -60.5,-40.7	-	0.05Å	Favored (85.294%) alpha helix	-	-	-
A 44		LEU	1.71	-	Favored (66.69%) General / -73.0,-38.6	Favored (91.6%) *mt* chi angles: 294.2,176.4	0.05Å	Favored (86.359%) alpha helix	-	-	-
A 45		LYS	1.83	-	Favored (77%) General / -62.3,-34.7	Favored (96.2%) *mttt* chi angles: 288.2,178.8,180.3,180	0.03Å	Favored (76.559%) alpha helix	-	-	-
A 46		ASP	1.91	-	Favored (56.14%) General / -89.2,1.1	Favored (84.7%) *m-30* chi angles: 289.5,334.8	0.02Å	Favored (54.741%)	-	-	-
A 47		GLY	1.92	-	Favored (79.32%) Glycine / 76.9,17.9	-	-	Favored (85.241%)	-	-	-
A 48		VAL	1.84	-	Allowed (1.27%) Ile or Val / -85.9,76.2	Favored (32.1%) *t* chi angles: 185.5	0.05Å	Favored (14.844%) beta sheet	-	-	-
A 49		ALA	1.69	-	Favored (67.04%) General / -59.8,-27.9	-	0.02Å	Favored (31.247%)	-	-	-
A 50		THR	1.49	-	Favored (37.9%) General / -97.3,11.0	Favored (78.2%) *p* chi angles: 60.3	0.02Å	Favored (42.188%)	-	-	-
A 51		GLY	1.29	-	Favored (49.78%) Glycine / -56.2,-26.4	-	-	Favored (5.041%)	-	-	-
A 52		GLY	1.1	-	Favored (2.13%) Glycine / 87.1,56.7	-	-	Favored (8.727%)	-	-	-
A 53		HIS	0.96	-	Favored (36.07%) General / -154.3,155.9	Favored (45.7%) *p90* chi angles: 63.8,84.6	0.10Å	Favored (14.285%)	-	OUTLIER(S) worst is CA-CB-CG: 4.3 σ	-
A 54		ALA	0.85	-	Favored (46.24%) General / -64.2,150.3	-	0.04Å	Favored (38.76%)	-	-	-
A 55		VAL	0.78	-	Favored (9.05%) Ile or Val / -101.0,-8.8	Favored (21.8%) *m* chi angles: 301.8	0.08Å	Favored (29.759%)	-	-	-

#	Alt	Res	High B	Clash > 0.4Å	Ramachandran	Rotamer	Cβ deviation	CaBLAM	Bond lengths	Bond angles	Cis Peptides
A 56		SER	0.73	-	Favored (28.89%) General / -153.5,150.6	Favored (41%) *t* chi angles: 176.4	0.04Å	Favored (22.68%)	-	-	-
A 57		ARG	0.7	-	Favored (54.78%) General / -53.0,-34.2	Favored (52.6%) *ttt90* chi angles: 189.7,182.5,185.5,92.3	0.08Å	Favored (54.227%) alpha helix	-	-	-
A 58		GLY	0.68	-	Favored (74.97%) Glycine / -59.2,-33.4	-	-	Favored (80.92%) alpha helix	-	-	-
A 59		SER	0.66	-	Favored (97.46%) General / -63.9,-42.5	Favored (71.8%) *m* chi angles: 295.2	0.12Å	Favored (78.062%) alpha helix	-	-	-
A 60		ALA	0.64	-	Favored (82.07%) General / -61.9,-36.7	-	0.03Å	Favored (81.133%) alpha helix	-	-	-
**#**	**Alt**	**Res**	**High B**	**Clash > 0.4Å**	**Ramachandran**	**Rotamer**	**Cβ deviation**	**CaBLAM**	**Bond lengths**	**Bond angles**	**Cis Peptides**
			Avg: 0.95	Clashscore: 2.02	Outliers: 3 of 901	Poor rotamers: 0 of 767	Outliers: 0 of 820	Outliers: 14 of 899	Outliers: 10 of 903	Outliers: 16 of 903	Non-Trans: 2 of 902
A 61		LYS	0.62	-	Favored (90.16%) General / -66.0,-42.0	Favored (91.3%) *mttt* chi angles: 290.9,179.4,188.5,176.7	0.01Å	Favored (85.434%) alpha helix	-	-	-
A 62		ILE	0.61	-	Favored (87.84%) Ile or Val / -66.0,-40.7	Favored (33.4%) *mm* chi angles: 294.1,299.1	0.06Å	Favored (81.939%) alpha helix	-	-	-
A 63		ARG	0.61	-	Favored (79.81%) General / -56.3,-46.5	Favored (4.2%) *tmm160* chi angles: 183.8,265,308.9,176.3	0.04Å	Favored (85.976%) alpha helix	-	-	-
A 64		TRP	0.62	-	Favored (94.32%) General / -60.5,-45.3	Favored (88%) *t60* chi angles: 184.7,89	0.02Å	Favored (98.869%) alpha helix	-	-	-
A 65		LEU	0.62	-	Favored (77.27%) General / -62.2,-34.9	Favored (82.6%) *mt* chi angles: 289.4,172	0.03Å	Favored (74.853%) alpha helix	-	-	-
A 66		GLU	0.63	-	Favored (50.89%) General / -73.4,-46.1	Favored (90%) *tt0* chi angles: 182.3,175.2,354.7	0.06Å	Favored (72.372%) alpha helix	-	-	-
A 67		GLU	0.65	-	Favored (73.68%) General / -60.9,-33.8	Favored (92.6%) *mt-10* chi angles: 286.8,178.3,342.2	0.04Å	Favored (76.104%) alpha helix	-	-	-
A 68		ARG	0.66	-	Favored (50.92%) General / -84.6,0.2	Favored (50.1%) *mmt180* chi angles: 295,288,180.2,181.4	0.03Å	Favored (57.006%)	-	-	-
A 69		GLY	0.67	-	Favored (76.29%) Glycine / 80.5,16.2	-	-	Favored (83.323%)	-	-	-
A 70		TYR	0.68	-	Favored (13.84%) General / -83.2,-45.5	Favored (89.6%) *m-80* chi angles: 292.8,100.6	0.03Å	CaBLAM Disfavored (4.614%)	-	-	-
A 71		LEU	0.68	-	Favored (53.72%) General / -115.6,135.8	Favored (39.4%) *tp* chi angles: 181.3,67.9	0.02Å	Favored (15.321%)	-	-	-

A 72		GLN	0.69	-	Favored (73.91%) Pre-Pro / -130.2,78.8	Favored (11.1%) *mm-40* chi angles: 296.2,287.1,264	0.04Å	Favored (10.958%)	-	-	-
A 73		PRO	0.7	-	Favored (97.75%) Trans-Pro / -58.4,142.4	Favored (53.7%) *Cg_exo* chi angles: 337.1,35.8,326.3	0.10Å	Favored (13.328%)	-	-	-
A 74		TYR	0.7	-	Favored (37.97%) General / -156.8,164.5	Favored (38.3%) *p90* chi angles: 68.4,99.8	0.05Å	Favored (7.153%)	-	-	-
A 75		GLY	0.69	-	Favored (42.88%) Glycine / 57.8,-126.7	-	-	Favored (41.822%)	-	-	-
A 76		LYS	0.68	-	Favored (35.73%) General / -93.0,122.8	Favored (37.5%) *ttpt* chi angles: 185,173.4,70.8,178.5	0.03Å	Favored (12.841%)	-	-	-
A 77		VAL	0.66	-	Favored (66.76%) Ile or Val / -113.2,130.7	Favored (59.4%) *t* chi angles: 180	0.08Å	Favored (68.427%)	-	-	-
A 78		VAL	0.65	-	Favored (70.12%) Ile or Val / -113.7,129.1	Favored (81.5%) *t* chi angles: 176.6	0.04Å	Favored (72.449%) beta sheet	-	-	-
A 79		ASP	0.64	-	Favored (12.3%) General / -114.3,104.7	Favored (61.5%) *t0* chi angles: 179.5,355	0.05Å	Favored (62.944%)	-	-	-
A 80		LEU	0.64	-	Favored (46.08%) General / -87.7,3.9	Favored (81.5%) *mt* chi angles: 296.2,179.9	0.06Å	Favored (5.92%)	-	-	-
**#**	**Alt**	**Res**	**High B**	**Clash > 0.4Å**	**Ramachandran**	**Rotamer**	**Cβ deviation**	**CaBLAM**	**Bond lengths**	**Bond angles**	**Cis Peptides**
			Avg: 0.95	Clashscore: 2.02	Outliers: 3 of 901	Poor rotamers: 0 of 767	Outliers: 0 of 820	Outliers: 14 of 899	Outliers: 10 of 903	Outliers: 16 of 903	Non-Trans: 2 of 902
A 81		GLY	0.65	-	Allowed (0.87%) Glycine / -140.7,47.3	-	-	Favored (15.284%)	-	-	-
A 82		CYS	0.65	-	Favored (69.62%) General / -65.3,-28.8	Favored (12.6%) *p* chi angles: 73.9	0.06Å	CaBLAM Outlier (0.236%)	-	-	-
A 83		GLY	0.66	-	Favored (36.37%) Glycine / 53.1,-127.8	-	-	Favored (34.07%)	-	-	-
A 84		ARG	0.66	-	Favored (51.23%) General / -54.1,-31.1	Favored (81.4%) *ttm-80* chi angles: 186.4,176.3,293,275.1	0.01Å	Favored (5.276%)	-	-	-
A 85		GLY	0.65	-	Favored (14.35%) Glycine / 113.6,10.7	-	-	Favored (72.153%)	-	-	-
A 86		GLY	0.64	-	Favored (11.69%) Glycine / -50.0,-56.6	-	-	Favored (21.58%)	-	-	-
A 87		TRP	0.63	-	Favored (69.31%) General / -70.5,-32.0	Favored (33.2%) *m-10* chi angles: 292,13.8	0.07Å	Favored (73.033%) alpha helix	-	-	-

#	Alt	Res	High B	Clash > 0.4Å	Ramachandran	Rotamer	Cβ deviation	CaBLAM	Bond lengths	Bond angles	Cis Peptides
A 88		SER	0.62	-	Favored (85.29%) General / -65.8,-44.6	Favored (54.4%) *m* chi angles: 292.5	0.05Å	Favored (86.493%) alpha helix	-	-	-
A 89		TYR	0.62	-	Favored (86.98%) General / -66.8,-38.8	Favored (30%) *m-80* chi angles: 287.1,125.2	0.02Å	Favored (94.369%) alpha helix	-	-	-
A 90		TYR	0.62	-	Favored (64.08%) General / -59.9,-52.8	Favored (90.9%) *t80* chi angles: 177.2,80.7	0.05Å	Favored (80.09%) alpha helix	-	-	-
A 91		ALA	0.64	-	Favored (73.63%) General / -58.4,-36.4	-	0.05Å	Favored (71.232%) alpha helix	-	-	-
A 92		ALA	0.66	-	Favored (66.01%) General / -60.8,-24.6	-	0.06Å	Favored (67.518%) alpha helix	-	-	-
A 93		THR	0.69	-	Favored (57.53%) General / -91.7,-0.1	Favored (61.7%) *p* chi angles: 63.7	0.02Å	Favored (56.714%)	-	-	-
A 94		ILE	0.71	-	Favored (39.56%) Ile or Val / -90.9,130.8	Favored (49.2%) *mm* chi angles: 303.7,302.8	0.08Å	Favored (27.557%)	-	-	-
A 95		ARG	0.74	-	Favored (35.32%) General / -58.2,146.1	Favored (98%) *mtt180* chi angles: 291.3,180,180.2,178.6	0.02Å	Favored (13.229%)	-	-	-
A 96		LYS	0.75	-	Favored (4.42%) General / 73.5,2.9	Favored (89.1%) *mttt* chi angles: 303.3,182.8,182.4,178	0.04Å	Favored (6.074%)	-	-	-
A 97		VAL	0.75	-	Favored (37.49%) Ile or Val / -87.5,130.3	Favored (97.5%) *t* chi angles: 175.2	0.05Å	Favored (14.555%)	-	-	-
A 98		GLN	0.74	-	Favored (7.11%) General / -106.6,-35.2	Favored (93.4%) *mm-40* chi angles: 295.6,292.2,307	0.06Å	Favored (27.83%)	-	-	-
A 99		GLU	0.71	-	Favored (16.67%) General / -152.2,136.4	Favored (91.7%) *tt0* chi angles: 181.3,175.6,358	0.01Å	Favored (33.589%)	-	-	-
A 100		VAL	0.69	-	Favored (73.83%) Ile or Val / -123.9,127.7	Favored (58%) *t* chi angles: 180.2	0.07Å	Favored (71.471%)	-	-	-
**#**	**Alt**	**Res**	**High B**	**Clash > 0.4Å**	**Ramachandran**	**Rotamer**	**Cβ deviation**	**CaBLAM**	**Bond lengths**	**Bond angles**	**Cis Peptides**
			Avg: 0.95	Clashscore: 2.02	Outliers: 3 of 901	Poor rotamers: 0 of 767	Outliers: 0 of 820	Outliers: 14 of 899	Outliers: 10 of 903	Outliers: 16 of 903	Non-Trans: 2 of 902
A 101		ARG	0.67	-	Favored (11.98%) General / -120.3,106.6	Favored (95.9%) *mtt180* chi angles: 298.1,181.1,177.9,184.3	0.03Å	Favored (64.411%) beta sheet	-	-	-
A 102		GLY	0.68	-	Favored (16.32%) Glycine / -101.4,144.0	-	-	Favored (56.951%) beta sheet	-	-	-
A 103		TYR	0.72	-	Favored (51.95%) General / -131.8,146.1	Favored (80.9%) *m-80* chi angles: 300.4,86.4	0.04Å	Favored (55.151%) beta sheet	-	-	-

A 104		THR	0.79	-	Favored (35.03%) General / -153.4,154.8	Favored (8.2%) *t* chi angles: 183.8	0.09Å	Favored (40.644%) beta sheet	-	-	-
A 105		LYS	0.9	-	Favored (44.44%) General / -78.9,-29.4	Favored (73.8%) *tttt* chi angles: 192.6,172.5,188,182.7	0.03Å	CaBLAM Disfavored (2.875%) try beta sheet	-	-	-
A 106		GLY	1.02	-	Favored (45.43%) Glycine / 59.9,-145.8	-	-	Favored (5.847%)	-	-	-
A 107		GLY	1.12	-	Favored (26.58%) Glycine / 103.0,177.9	-	-	Favored (9.211%)	-	-	-
A 108		PRO	1.18	-	Favored (4.2%) Trans-Pro / -74.1,72.0	Favored (64.8%) *Cg_endo* chi angles: 31.4,324.1,25	0.03Å	CaBLAM Outlier (0%)	-	-	-
A 109		GLY	1.19	-	Favored (3.02%) Glycine / 137.3,-1.4	-	-	Favored (12.653%)	-	-	-
A 110		HIS	1.16	-	Favored (11.74%) General / -99.2,165.4	Favored (99.2%) *m-70* chi angles: 299.7,290.4	0.10Å	Favored (31.199%)	-	-	-
A 111		GLU	1.11	-	Favored (92.79%) General / -61.9,-39.7	Favored (98.4%) *mt-10* chi angles: 290.2,179.7,355.4	0.04Å	CaBLAM Disfavored (1.034%)	-	-	-
A 112		GLU	1.06	-	OUTLIER (0.05)%) Pre-Pro / 63.8,140.5	Favored (39.8%) *mm-30* chi angles: 294.4,295.1,3.6	0.06Å	CaBLAM Disfavored (1.054%)	-	-	-
A 113		PRO	1.03	-	Favored (74.83%) Trans-Pro / -68.5,150.0	Favored (68.9%) *Cg_endo* chi angles: 27.2,327.2,25	0.02Å	Favored (48.913%)	-	-	-
A 114		MET	1.02	-	Favored (51.96%) General / -123.9,141.5	Favored (32.7%) *ttt* chi angles: 183,175.6,180.3	0.03Å	Favored (51.38%) beta sheet	-	-	-
A 115		LEU	1	-	Favored (20.37%) General / -93.4,108.8	Favored (72.9%) *mt* chi angles: 303.2,178.7	0.06Å	Favored (38.581%) beta sheet	-	-	-
A 116		VAL	0.98	-	Favored (50.4%) Ile or Val / -125.6,139.8	Favored (58.4%) *t* chi angles: 180.2	0.07Å	Favored (44.71%) beta sheet	-	-	-
A 117		GLN	0.95	0.46Å HE21 with A 121 TRP CD1	Favored (35.76%) General / -89.4,130.3	Favored (9.4%) *tm-30* chi angles: 204.5,278.1,315.8	0.05Å	Favored (16.098%) beta sheet	-	OUTLIER(S) worst is CG-CD-NE2: 5.0 σ	-
A 118		SER	0.92	-	Favored (31.5%) General / -154.1,166.9	Favored (90.1%) *p* chi angles: 66.8	0.03Å	Favored (13.945%)	-	-	-
A 119		TYR	0.88	-	Allowed (0.14%) General / -67.2,76.0	Favored (81.9%) *t80* chi angles: 176,72.3	0.06Å	CaBLAM Disfavored (2.103%)	-	-	-
A 120		GLY	0.85	-	Favored (2.88%) Glycine / 134.4,4.3	-	-	Favored (6.43%)	-	-	-

#	Alt	Res	High B	Clash > 0.4Å	Ramachandran	Rotamer	Cβ deviation	CaBLAM	Bond lengths	Bond angles	Cis Peptides
			Avg: 0.95	Clashscore: 2.02	Outliers: 3 of 901	Poor rotamers: 0 of 767	Outliers: 0 of 820	Outliers: 14 of 899	Outliers: 10 of 903	Outliers: 16 of 903	Non-Trans: 2 of 902
A 121		TRP	0.82	0.46Å CD1 with A 117 GLN HE21	Favored (52.65%) General / -55.9,-27.6	Favored (46.2%) *p-90* chi angles: 74.9,272.2	0.13Å	Favored (29.983%) alpha helix	-	-	-
A 122		ASN	0.8	-	Favored (47.5%) General / -71.3,-7.5	Favored (18.9%) *p0* chi angles: 61.6,298.3	0.05Å	Favored (40.145%) alpha helix	-	-	-
A 123		ILE	0.79	-	Favored (8.73%) Ile or Val / -110.3,11.8	Favored (34.9%) *pt* chi angles: 63.5,166.1	0.06Å	Favored (53.506%)	-	-	-
A 124		VAL	0.78	-	Favored (69.12%) Ile or Val / -111.7,126.5	Favored (69.3%) *t* chi angles: 178.8	0.09Å	Favored (30.219%)	-	-	-
A 125		ARG	0.78	-	Favored (49.61%) General / -116.8,124.5	Favored (51.8%) *ttm170* chi angles: 175.5,180.3,288.3,170.4	0.07Å	Favored (65.824%)	-	-	-
A 126		LEU	0.79	-	Favored (54.34%) General / -113.0,134.7	Favored (83%) *mt* chi angles: 301.2,177	0.04Å	Favored (70.449%) beta sheet	-	-	-
A 127		LYS	0.79	-	Favored (30.65%) General / -128.4,122.1	Favored (83.9%) *tttt* chi angles: 187.1,180,180.6,179.4	0.02Å	Favored (69.606%)	-	-	-
A 128		SER	0.8	-	Favored (16.5%) General / -104.3,158.4	Favored (76.6%) *p* chi angles: 60.2	0.04Å	Favored (7.311%)	-	-	-
A 129		GLY	0.8	-	Favored (57.7%) Glycine / 85.2,20.1	-	-	Favored (34.046%)	-	-	-
A 130		VAL	0.78	-	Favored (43.23%) Ile or Val / -101.0,115.8	Favored (64.7%) *t* chi angles: 179.4	0.12Å	Favored (28.277%)	-	-	-
A 131		ASP	0.77	-	Favored (7.89%) General / -84.1,92.5	Favored (61.4%) *t0* chi angles: 184.3,340.7	0.09Å	Favored (66.805%) beta sheet	-	-	-
A 132		VAL	0.76	-	Favored (11.99%) Ile or Val / -52.8,-27.3	Favored (52.6%) *t* chi angles: 169.7	0.07Å	Favored (34.172%)	-	-	-
A 133		PHE	0.75	-	Favored (64.31%) General / -68.6,-18.5	Favored (52.1%) *m-80* chi angles: 284.9,106.9	0.06Å	Favored (53.376%) alpha helix	-	-	-
A 134		HIS	0.75	-	Favored (21.7%) General / -111.1,15.2	Favored (99.8%) *m-70* chi angles: 297.6,287.2	0.03Å	Favored (39.4%)	-	-	-
A 135		MET	0.75	-	Favored (34.9%) General / -80.0,130.0	Favored (29.6%) *ttt* chi angles: 182.4,180.2,169.7	0.03Å	Favored (31.202%)	-	-	-
A 136		ALA	0.75	-	Favored (58.11%) General / -63.0,143.1	-	0.03Å	Favored (48.321%)	-	-	-

A 137		ALA	0.74	-	Favored (56.43%) General / -63.6,145.1	-	0.03Å	Favored (35.403%)	-	-	-
A 138		GLU	0.72	-	Favored (23.07%) Pre-Pro / -129.7,128.9	Favored (92.5%) *tt0* chi angles: 181.2,178.4,355.5	0.06Å	Favored (42.607%) beta sheet	-	-	-
A 139		PRO	0.69	-	Favored (81.2%) Trans-Pro / -55.4,138.8	Favored (84.1%) *Cg_exo* chi angles: 334.2,34.9,330.9	0.02Å	Favored (25.175%) beta sheet	-	-	-
A 140		CYS	0.67	-	Favored (14.39%) General / -154.9,175.0	Favored (28%) *p* chi angles: 65.2	0.05Å	Favored (38.243%)	-	-	-
**#**	**Alt**	**Res**	**High B**	**Clash > 0.4Å**	**Ramachandran**	**Rotamer**	**Cβ deviation**	**CaBLAM**	**Bond lengths**	**Bond angles**	**Cis Peptides**
			Avg: 0.95	Clashscore: 2.02	Outliers: 3 of 901	Poor rotamers: 0 of 767	Outliers: 0 of 820	Outliers: 14 of 899	Outliers: 10 of 903	Outliers: 16 of 903	Non-Trans: 2 of 902
A 141		ASP	0.64	-	Favored (14.55%) General / -94.6,-30.1	Favored (58.7%) *m-30* chi angles: 295.8,303.1	0.06Å	Favored (13.141%)	-	-	-
A 142		THR	0.62	-	Favored (28.83%) General / -139.9,132.6	Favored (64.1%) *m* chi angles: 303.3	0.06Å	Favored (33.676%)	-	-	-
A 143		LEU	0.61	-	Favored (32.74%) General / -116.3,118.4	Favored (58.8%) *tp* chi angles: 177.4,65.4	0.07Å	Favored (66.099%)	-	-	-
A 144		LEU	0.62	-	Favored (49.68%) General / -114.2,139.1	Favored (37.6%) *mt* chi angles: 308.6,177	0.09Å	Favored (52.457%) beta sheet	-	-	-
A 145		CYS	0.65	-	Favored (33.91%) General / -135.9,130.5	Favored (39.6%) *m* chi angles: 304.8	0.03Å	Favored (38.953%) beta sheet	-	-	-
A 146		ASP	0.69	-	Favored (2.65%) General / -136.9,33.8	Favored (32.5%) *t0* chi angles: 195.9,26.9	0.06Å	Favored (7.454%) beta sheet	-	-	-
A 147		ILE	0.74	0.51Å O with A 147 ILE HG23	Allowed (1.28%) Ile or Val / -88.5,76.1	Favored (18.9%) *tt* chi angles: 182.9,168.3	0.07Å	CaBLAM Disfavored (1.349%)	-	-	-
A 148		GLY	0.8	-	Favored (20.99%) Glycine / 177.2,158.7	-	-	CaBLAM Disfavored (2.959%)	-	-	-
A 149		GLU	0.87	-	Favored (49.88%) General / -135.2,149.3	Favored (91.6%) *mt-10* chi angles: 299.7,182,2	0.04Å	Favored (27.739%)	-	-	-
A 150		SER	0.92	-	Favored (32.9%) General / -68.8,159.2	Favored (61%) *m* chi angles: 298.7	0.04Å	Favored (11.245%) beta sheet	-	-	-
A 151		SER	0.95	-	Favored (28.88%) General / -155.4,152.3	Favored (38.3%) *t* chi angles: 177.6	0.07Å	Favored (48.759%)	-	-	-

A 152		SER	0.95	-	Favored (66.58%) General / -64.6,-21.0	Favored (89%) *p* chi angles: 66.9	0.02Å	Favored (49.148%)	-	-	-
A 153		SER	0.93	-	Favored (65.62%) Pre-Pro / -102.5,112.5	Favored (42.6%) *t* chi angles: 175.7	0.04Å	Favored (29.516%)	-	-	-
A 154		PRO	0.89	-	Favored (66.46%) Trans-Pro / -64.4,-21.6	Favored (44.2%) *Cg_endo* chi angles: 24.5,327,27.4	0.01Å	Favored (75.164%)	-	-	-
A 155		GLU	0.83	-	Favored (72.46%) General / -66.2,-31.6	Favored (99.5%) *mt-10* chi angles: 292,180.6,353.6	0.01Å	Favored (63.72%) alpha helix	-	-	-
A 156		VAL	0.78	-	Favored (24.15%) Ile or Val / -79.1,-43.5	Favored (92.1%) *t* chi angles: 174.4	0.04Å	Favored (71.927%) alpha helix	-	-	-
A 157		GLU	0.73	-	Favored (82.95%) General / -61.0,-37.5	Favored (93.2%) *mt-10* chi angles: 289.9,183.7,349.7	0.03Å	Favored (99.055%) alpha helix	-	-	-
A 158		GLU	0.7	-	Favored (70.22%) General / -64.0,-50.0	Favored (50.4%) *tt0* chi angles: 177.7,178.6,325.2	0.02Å	Favored (84.672%) alpha helix	-	-	-
A 159		THR	0.67	-	Favored (90.89%) General / -59.8,-45.9	Favored (89.9%) *m* chi angles: 298.7	0.05Å	Favored (90.151%) alpha helix	-	-	-
A 160		ARG	0.66	-	Favored (86.47%) General / -62.5,-46.9	Favored (96.7%) *mtt180* chi angles: 289.1,173.5,179.5,167.2	0.06Å	Favored (85.294%) alpha helix	-	-	-
**#**	**Alt**	**Res**	**High B**	**Clash > 0.4Å**	**Ramachandran**	**Rotamer**	**Cβ deviation**	**CaBLAM**	**Bond lengths**	**Bond angles**	**Cis Peptides**
			Avg: 0.95	Clashscore: 2.02	Outliers: 3 of 901	Poor rotamers: 0 of 767	Outliers: 0 of 820	Outliers: 14 of 899	Outliers: 10 of 903	Outliers: 16 of 903	Non-Trans: 2 of 902
A 161		THR	0.65	-	Favored (89.95%) General / -66.1,-41.8	Favored (93.8%) *m* chi angles: 299.3	0.04Å	Favored (83.048%) alpha helix	-	-	-
A 162		LEU	0.65	-	Favored (84.74%) General / -63.5,-36.7	Favored (98.3%) *mt* chi angles: 292.3,172.6	0.02Å	Favored (86.456%) alpha helix	-	-	-
A 163		ARG	0.66	-	Favored (91.39%) General / -62.7,-38.7	Favored (97.7%) *mtt180* chi angles: 288.2,173.8,178.6,170.9	0.02Å	Favored (94.883%) alpha helix	-	-	-
A 164		VAL	0.67	-	Favored (85.03%) Ile or Val / -67.2,-46.0	Favored (78.8%) *t* chi angles: 173	0.04Å	Favored (88.049%) alpha helix	-	-	-
A 165		LEU	0.68	-	Favored (85.18%) General / -62.3,-37.4	Favored (85.6%) *mt* chi angles: 290.1,172.8	0.03Å	Favored (90.361%) alpha helix	-	-	-
A 166		SER	0.7	-	Favored (90.91%) General / -65.1,-43.8	Favored (71.9%) *m* chi angles: 295.9	0.04Å	Favored (95.723%) alpha helix	-	-	-

A 167		MET	0.72	-	Favored (72.08%) General / -57.9,-50.8	Favored (48.2%) *ttp* chi angles: 177,190.7,70.4	0.06Å	Favored (90.741%) alpha helix	-	-	-
A 168		VAL	0.74	-	Favored (85.87%) Ile or Val / -59.5,-41.8	Favored (52.1%) *t* chi angles: 169.7	0.09Å	Favored (72.413%) alpha helix	-	-	-
A 169		GLY	0.76	-	Favored (13.85%) Glycine / -47.5,-52.7	-	-	Favored (88.364%) alpha helix	-	-	-
A 170		ASP	0.79	-	Favored (70.64%) General / -59.1,-33.3	Favored (91.6%) *m-30* chi angles: 284.8,347.2	0.08Å	Favored (73.146%) alpha helix	-	-	-
A 171		TRP	0.83	-	Favored (63.89%) General / -74.2,-37.4	Favored (35.5%) *m100* chi angles: 283,72.6	0.08Å	Favored (75.597%) alpha helix	-	-	-
A 172		LEU	0.88	-	Favored (67.1%) General / -68.0,-28.1	Favored (91.9%) *mt* chi angles: 293.3,175.7	0.05Å	Favored (66.315%) three-ten	-	-	-
A 173		GLU	0.94	-	Favored (60.79%) General / -62.8,-17.3	Favored (67.9%) *mm-30* chi angles: 288.4,293.7,311.9	0.10Å	Favored (65.291%)	-	-	-
A 174		LYS	1	-	Favored (51.75%) General / -85.1,0.4	Favored (73.7%) *mmtt* chi angles: 300.2,294.9,184.3,181.8	0.06Å	Favored (26.247%)	-	-	-
A 175		ARG	1.04	-	Favored (13.78%) Pre-Pro / 53.5,57.5	Favored (95.9%) *mtt180* chi angles: 298.4,183.3,180.2,178.2	0.03Å	Favored (18.511%)	-	-	-
A 176		PRO	1.03	-	Favored (4.64%) Trans-Pro / -77.9,54.0	Favored (47.6%) *Cg_endo* chi angles: 33.3,322.5,26.4	0.11Å	CaBLAM Outlier (0.074%)	-	-	-
A 177		GLY	0.98	-	Favored (30.61%) Glycine / 57.2,-122.9	-	-	Favored (25.528%)	-	-	-
A 178		ALA	0.89	-	Favored (19.23%) General / -86.8,159.1	-	0.01Å	CaBLAM Disfavored (4.029%)	-	-	-
A 179		PHE	0.8	-	Favored (42.31%) General / -153.9,160.9	Favored (41.8%) *p90* chi angles: 56.1,84.9	0.08Å	Favored (70.301%)	-	-	-
A 180		CYS	0.73	-	Favored (3.71%) General / -151.7,109.8	Favored (51.1%) *t* chi angles: 180.3	0.10Å	Favored (23.318%) beta sheet	-	-	-
**#**	**Alt**	**Res**	**High B**	**Clash > 0.4Å**	**Ramachandran**	**Rotamer**	**Cβ deviation**	**CaBLAM**	**Bond lengths**	**Bond angles**	**Cis Peptides**
			Avg: 0.95	Clashscore: 2.02	Outliers: 3 of 901	Poor rotamers: 0 of 767	Outliers: 0 of 820	Outliers: 14 of 899	Outliers: 10 of 903	Outliers: 16 of 903	Non-Trans: 2 of 902
A 181		ILE	0.68	-	Favored (75.18%) Ile or Val / -118.4,126.6	Favored (3.2%) *mp* chi angles: 299.4,96.9	0.07Å	Favored (55.44%) beta sheet	-	-	-

A 182		LYS	0.66	-	Favored (41.33%) General / -74.9,132.3	Favored (33.4%) *ttpt* chi angles: 186.2,170.6,73.7,185.5	0.04Å	Favored (46.159%) beta sheet	-	-	-
A 183		VAL	0.66	-	Favored (69.26%) Ile or Val / -115.3,122.4	Favored (70.6%) *t* chi angles: 178.7	0.14Å	Favored (72.518%)	-	-	-
A 184		LEU	0.68	-	Favored (31.9%) General / -81.7,-31.8	Favored (92.4%) *mt* chi angles: 295.1,171.7	0.05Å	Favored (23.249%)	-	-	-
A 185		CYS	0.71	-	Favored (9.77%) Pre-Pro / -145.1,63.2	Favored (35.2%) *p* chi angles: 63.2	0.04Å	Favored (5.916%)	-	-	-
A 186		PRO	0.75	-	Favored (61.26%) Trans-Pro / -61.2,-18.9	Favored (41.1%) *Cg_endo* chi angles: 23.6,326.6,29.1	0.06Å	Favored (18.12%)	-	-	-
A 187		TYR	0.79	-	Favored (65.21%) General / -68.2,-25.4	Favored (29.9%) *p90* chi angles: 69.7,83.6	0.03Å	Favored (33.582%)	-	-	-
A 188		THR	0.83	-	Favored (58.37%) General / -64.8,142.6	Favored (10.1%) *t* chi angles: 186.2	0.10Å	Favored (35.748%)	-	-	-
A 189		SER	0.86	-	Favored (77.46%) General / -58.0,-39.1	Favored (66.3%) *m* chi angles: 294.4	0.02Å	Favored (59.406%)	-	-	-
A 190		THR	0.88	-	Favored (97.44%) General / -62.3,-44.0	Favored (91.2%) *m* chi angles: 298.9	0.01Å	Favored (78.401%) alpha helix	-	-	-
A 191		MET	0.87	0.48Å HB2 with A 191 MET HE3	Favored (72.77%) General / -70.9,-39.2	Favored (32.7%) *ttm* chi angles: 192.9,172.1,300	0.04Å	Favored (75.309%) alpha helix	OUTLIER(S) worst is CG--SD: 4.3 σ	-	-
A 192		MET	0.86	-	Favored (77.5%) General / -57.8,-49.2	Favored (28.9%) *tmm* chi angles: 180.9,277.4,292.2	0.03Å	Favored (81.097%) alpha helix	-	-	-
A 193		GLU	0.84	-	Favored (98.42%) General / -60.8,-42.9	Favored (91.6%) *tt0* chi angles: 181.7,175.8,359.4	0.01Å	Favored (87.939%) alpha helix	-	-	-
A 194		THR	0.82	-	Favored (88.25%) General / -59.6,-46.7	Favored (88.6%) *m* chi angles: 298.6	0.04Å	Favored (90.783%) alpha helix	-	-	-
A 195		MET	0.81	-	Favored (86.45%) General / -67.0,-38.7	Favored (74.9%) *mtm* chi angles: 290.6,187.3,295.9	0.03Å	Favored (84.12%) alpha helix	-	-	-
A 196		GLU	0.8	-	Favored (98.62%) General / -61.1,-43.6	Favored (91.1%) *tt0* chi angles: 179.6,177.7,355	0.04Å	Favored (90.505%) alpha helix	-	-	-
A 197		ARG	0.8	-	Favored (98.86%) General / -63.4,-42.2	Favored (98.1%) *mtt-85* chi angles: 288.8,177.9,184.5,274.7	0.04Å	Favored (94.723%) alpha helix	-	-	-
A 198		LEU	0.81	-	Favored (90.32%) General / -65.1,-38.3	Favored (85.9%) *mt* chi angles: 290.1,171	0.02Å	Favored (93.62%) alpha helix	-	-	-

#	Alt	Res	High B	Clash > 0.4Å	Ramachandran	Rotamer	Cβ deviation	CaBLAM	Bond lengths	Bond angles	Cis Peptides
A 199		GLN	0.81	-	Favored (77.8%) General / -62.2,-48.9	Favored (54.5%) *tt0* chi angles: 177.2,177.9,55.9	0.04Å	Favored (88.856%) alpha helix	-	-	-
A 200		ARG	0.81	-	Favored (78.41%) General / -61.1,-35.9	Favored (97.3%) *mtt-85* chi angles: 288.3,179.7,181.2,275.6	0.03Å	Favored (74.045%) alpha helix	-	-	-
			Avg: 0.95	Clashscore: 2.02	Outliers: 3 of 901	Poor rotamers: 0 of 767	Outliers: 0 of 820	Outliers: 14 of 899	Outliers: 10 of 903	Outliers: 16 of 903	Non-Trans: 2 of 902
A 201		ARG	0.79	-	Favored (50.1%) General / -76.5,-40.4	Favored (43.1%) *tpt170* chi angles: 184.9,65.9,173.3,165.7	0.03Å	Favored (59.211%) alpha helix	-	-	-
A 202		HIS	0.77	-	Favored (13.49%) General / -113.5,-5.7	Favored (97%) *m-70* chi angles: 300.3,294.3	0.05Å	Favored (32.719%)	-	-	-
A 203		GLY	0.74	-	Favored (55.51%) Glycine / 77.6,29.4	-	-	Favored (78.153%)	-	-	-
A 204		GLY	0.71	-	Favored (17.97%) Glycine / -109.1,-157.2	-	-	Favored (28.33%)	-	-	-
A 205		GLY	0.68	-	Favored (43.66%) Glycine / 178.1,173.6	-	-	Favored (7.523%)	-	-	-
A 206		LEU	0.67	-	Favored (27.37%) General / -94.4,142.5	Favored (94.7%) *mt* chi angles: 295.8,173.7	0.02Å	Favored (10.64%)	-	-	-
A 207		VAL	0.66	-	Favored (33.1%) Ile or Val / -141.1,136.2	Favored (4.4%) *p* chi angles: 56.5	0.07Å	Favored (50.764%) beta sheet	-	-	-
A 208		ARG	0.67	-	Favored (42.35%) General / -95.3,129.7	Favored (29.7%) *tpt170* chi angles: 177.9,75.7,175.1,180.2	0.01Å	Favored (53.472%) beta sheet	-	-	-
A 209		VAL	0.68	-	Favored (26.54%) Pre-Pro / -96.5,132.4	Favored (71%) *t* chi angles: 178.6	0.09Å	Favored (46.801%)	-	-	-
A 210		PRO	0.7	-	Favored (22%) Trans-Pro / -55.5,-19.1	Favored (90.9%) *Cg_exo* chi angles: 333.2,34.8,331.8	0.06Å	Favored (55.516%)	-	-	-
A 211		LEU	0.71	-	Favored (58.51%) General / -86.5,-2.5	Favored (88.3%) *mt* chi angles: 298.4,173.5	0.08Å	Favored (44.292%)	-	-	-
A 212		CYS	0.73	-	Favored (39.08%) General / -75.9,147.3	Favored (82.3%) *m* chi angles: 294.9	0.04Å	Favored (35.956%)	-	-	-
A 213		ARG	0.73	-	Favored (34.14%) General / -77.4,153.0	Favored (51.5%) *mmt180* chi angles: 298.3,293,188.9,181.6	0.04Å	Favored (47.331%)	-	-	-
A 214		ASN	0.74	-	Favored (2.11%) General / -92.4,27.9	Favored (87.4%) *m-40* chi angles: 294.1,318.6	0.03Å	CaBLAM Outlier (0.828%)	-	-	-

A 215		SER	0.73	-	Favored (12.81%) General / -113.5,-13.4	Favored (82.4%) *p* chi angles: 62.3	0.06Å	Favored (17.636%) alpha helix	-	-	-
A 216		THR	0.72	-	Favored (49.03%) General / -126.6,129.7	Favored (84.6%) *m* chi angles: 301.8	0.05Å	Favored (22.08%)	-	-	-
A 217		HIS	0.7	-	Favored (9.21%) General / -86.1,65.7	Favored (43.6%) *t-90* chi angles: 196.5,296.1	0.10Å	Favored (14.947%)	-	-	-
A 218		GLU	0.68	-	Favored (25.06%) General / -99.4,147.6	Favored (13.1%) *pt0* chi angles: 61.7,177.4,32.5	0.04Å	Favored (12.393%) beta sheet	-	-	-
A 219		MET	0.66	-	Favored (43.69%) General / -142.2,158.5	Favored (81.5%) *mtp* chi angles: 299.3,184.1,77.7	0.02Å	Favored (47.425%) beta sheet	-	-	-
A 220		TYR	0.66	-	Favored (32.83%) General / -103.9,143.4	Favored (92.6%) *m-80* chi angles: 291.6,89.3	0.02Å	Favored (48.078%) beta sheet	-	-	-
**#**	**Alt**	**Res**	**High B**	**Clash > 0.4Å**	**Ramachandran**	**Rotamer**	**Cβ deviation**	**CaBLAM**	**Bond lengths**	**Bond angles**	**Cis Peptides**
			Avg: 0.95	Clashscore: 2.02	Outliers: 3 of 901	Poor rotamers: 0 of 767	Outliers: 0 of 820	Outliers: 14 of 899	Outliers: 10 of 903	Outliers: 16 of 903	Non-Trans: 2 of 902
A 221		TRP	0.66	-	Favored (24.14%) General / -104.9,111.4	Favored (21.9%) *t60* chi angles: 182.9,62.3	0.07Å	Favored (60.57%) beta sheet	-	-	-
A 222		VAL	0.68	-	Favored (64.4%) Ile or Val / -123.7,135.1	Favored (52.8%) *t* chi angles: 181	0.06Å	Favored (49.196%)	-	-	-
A 223		SER	0.71	-	Favored (56.76%) General / -63.4,144.7	Favored (80.8%) *p* chi angles: 61.6	0.09Å	Favored (22.274%)	-	-	-
A 224		GLY	0.75	-	Favored (35.64%) Glycine / 87.2,-18.6	-	-	Favored (54.067%)	-	-	-
A 225		ALA	0.79	-	Favored (6.13%) General / -86.2,60.3	-	0.06Å	Favored (7.643%)	-	-	-
A 226		LYS	0.84	-	Favored (50.65%) General / -61.0,131.4	Favored (98.1%) *mttt* chi angles: 292,181.1,180.8,178.2	0.04Å	CaBLAM Disfavored (1.274%) try beta sheet	-	-	-
A 227		SER	0.87	-	Favored (23.61%) General / -163.9,162.5	Favored (93.9%) *p* chi angles: 66.3	0.03Å	Favored (13.748%) beta sheet	-	-	-
A 228		ASN	0.89	-	Favored (21.41%) General / -80.4,117.7	Favored (27.6%) *t0* chi angles: 183.4,281.4	0.06Å	Favored (20.728%)	-	-	-
A 229		ILE	0.89	-	Favored (98.92%) Ile or Val / -61.8,-44.5	Favored (83.2%) *mt* chi angles: 290.2,168.6	0.10Å	Favored (49.952%)	-	-	-
A 230		ILE	0.88	-	Favored (99.53%)	Favored (92.4%) *mt* chi angles: 291.5,168.2	0.04Å	Favored (79.525%) alpha helix	-	-	-

					Ile or Val / -62.4,-44.8						
A 231		LYS	0.87	-	Favored (78.91%) General / -69.0,-37.6	Favored (97.2%) *mttt* chi angles: 290.1,179.4,180,179.4	0.02Å	Favored (79.488%) alpha helix	-	-	-
A 232		SER	0.85	-	Favored (96.31%) General / -64.3,-42.5	Favored (72.5%) *m* chi angles: 295.8	0.07Å	Favored (82.753%) alpha helix	-	-	-
A 233		VAL	0.83	-	Favored (76.77%) Ile or Val / -70.7,-44.0	Favored (83%) *t* chi angles: 173.4	0.07Å	Favored (80.539%) alpha helix	-	-	-
A 234		SER	0.81	-	Favored (99.04%) General / -62.6,-41.5	Favored (70.9%) *m* chi angles: 296.2	0.05Å	Favored (98.117%) alpha helix	-	-	-
A 235		THR	0.79	-	Favored (84.33%) General / -63.5,-46.8	Favored (92%) *m* chi angles: 299	0.04Å	Favored (88.829%) alpha helix	-	-	-
A 236		THR	0.78	-	Favored (93.78%) General / -59.8,-44.8	Favored (88.5%) *m* chi angles: 298.4	0.05Å	Favored (96.944%) alpha helix	-	-	-
A 237		SER	0.76	-	Favored (98.14%) General / -61.8,-41.8	Favored (63.3%) *m* chi angles: 297.7	0.08Å	Favored (93.142%) alpha helix	-	-	-
A 238		GLN	0.75	-	Favored (86.12%) General / -66.6,-38.0	Favored (94.8%) *tp40* chi angles: 187.7,63.4,54.4	0.02Å	Favored (98.62%) alpha helix	-	-	-
A 239		LEU	0.74	-	Favored (95.65%) General / -64.9,-41.4	Favored (91.4%) *mt* chi angles: 291.2,171.4	0.05Å	Favored (96.194%) alpha helix	-	-	-
A 240		LEU	0.74	-	Favored (84.44%) General / -66.2,-37.0	Favored (99%) *mt* chi angles: 292.4,172.4	0.03Å	Favored (91.712%) alpha helix	-	-	-
**#**	**Alt**	**Res**	**High B**	**Clash > 0.4Å**	**Ramachandran**	**Rotamer**	**Cβ deviation**	**CaBLAM**	**Bond lengths**	**Bond angles**	**Cis Peptides**
			Avg: 0.95	Clashscore: 2.02	Outliers: 3 of 901	Poor rotamers: 0 of 767	Outliers: 0 of 820	Outliers: 14 of 899	Outliers: 10 of 903	Outliers: 16 of 903	Non-Trans: 2 of 902
A 241		LEU	0.74	-	Favored (81.86%) General / -68.2,-40.9	Favored (28.1%) *tp* chi angles: 187.8,59.4	0.04Å	Favored (74.901%) alpha helix	-	-	-
A 242		GLY	0.76	-	Favored (70.12%) Glycine / -56.6,-36.1	-	-	Favored (90.379%) alpha helix	-	-	-
A 243		ARG	0.81	-	Favored (78.18%) General / -65.5,-34.4	Favored (96.5%) *mtt180* chi angles: 292.1,175.4,187.2,173.9	0.04Å	Favored (86.567%) alpha helix	-	-	-
A 244		MET	0.87	-	Favored (61.62%) General / -72.4,-25.7	Favored (99.1%) *mmm* chi angles: 293.6,301.2,291.9	0.07Å	Favored (68.268%) alpha helix	-	-	-
A 245		ASP	0.96	-	Favored (11.53%) General / -96.9,-33.6	Favored (61.9%) *m-30* chi angles: 295.3,306.6	0.06Å	Favored (28.476%)	-	-	-

A 246		GLY	1.04	-	Favored (49.28%) Glycine / -85.6,-172.9	-	-	Favored (22.23%)	-	-	-
A 247		PRO	1.09	-	Favored (82.56%) Trans-Pro / -58.7,148.5	Favored (59.7%) *Cg_exo* chi angles: 336,35.1,328.8	0.06Å	Favored (16.778%)	-	-	-
A 248		ARG	1.1	-	Favored (2.97%) General / -76.1,76.0	Favored (98.7%) *mtt180* chi angles: 294.2,177.1,179.2,182.1	0.01Å	Favored (29.512%)	-	-	-
A 249		ARG	1.05	-	Favored (85.62%) Pre-Pro / -58.5,133.4	Favored (84%) *ttt180* chi angles: 183.8,175.5,178.8,178.1	0.05Å	Favored (23.913%)	-	-	-
A 250		PRO	0.98	-	Favored (67.66%) Trans-Pro / -59.1,151.3	Favored (83.7%) *Cg_exo* chi angles: 334.3,33.7,332.6	0.07Å	Favored (71.296%)	-	-	-
A 251		VAL	0.91	-	Favored (30.72%) Ile or Val / -73.4,133.9	Favored (87.5%) *t* chi angles: 173.9	0.08Å	Favored (41.715%)	-	-	-
A 252		LYS	0.85	-	Favored (35.79%) General / -89.1,129.1	Favored (85.8%) *tttt* chi angles: 180.7,178.3,176.9,179.2	0.04Å	Favored (51.549%) beta sheet	-	-	-
A 253		TYR	0.81	-	Favored (30.94%) General / -101.9,144.1	Favored (77.4%) *m-80* chi angles: 288.1,90.1	0.09Å	Favored (59.665%) beta sheet	-	-	-
A 254		GLU	0.79	-	Favored (43.27%) General / -134.3,136.1	Favored (45%) *tt0* chi angles: 186.4,178.9,41.3	0.06Å	Favored (42.004%) beta sheet	-	-	-
A 255		GLU	0.79	-	Favored (55.07%) General / -57.5,138.5	Favored (89.8%) *tt0* chi angles: 186.7,175.1,1.8	0.03Å	Favored (50.817%)	-	-	-
A 256		ASP	0.79	-	Favored (7.67%) General / -65.4,168.1	Favored (20.7%) *t0* chi angles: 202.2,351.9	0.02Å	Favored (16.74%)	-	-	-
A 257		VAL	0.79	-	Favored (26.48%) Ile or Val / -77.4,119.7	Favored (85.3%) *t* chi angles: 177.2	0.04Å	Favored (17.125%)	-	-	-
A 258		ASN	0.8	-	Favored (10.26%) General / -85.5,74.9	Favored (87.7%) *m-40* chi angles: 292.9,319.5	0.06Å	Favored (63.118%) beta sheet	-	-	-
A 259		LEU	0.8	-	Favored (57.73%) General / -61.5,141.4	Favored (85.3%) *mt* chi angles: 297.3,180	0.07Å	Favored (19.877%) beta sheet	-	-	-
A 260		GLY	0.81	-	Favored (45.15%) Glycine / -76.9,-177.4	-	-	Favored (32.259%)	-	-	-
**#**	**Alt**	**Res**	**High B**	**Clash > 0.4Å**	**Ramachandran**	**Rotamer**	**Cβ deviation**	**CaBLAM**	**Bond lengths**	**Bond angles**	**Cis Peptides**
			Avg: 0.95	Clashscore: 2.02	Outliers: 3 of 901	Poor rotamers: 0 of 767	Outliers: 0 of 820	Outliers: 14 of 899	Outliers: 10 of 903	Outliers: 16 of 903	Non-Trans: 2 of 902
A 261		SER	0.82	-	Favored (7.5%) General / -115.0,171.7	Favored (63.1%) *m* chi angles: 294	0.07Å	CaBLAM Disfavored (4.73%)	-	-	-

A 262		GLY	0.85	-	Favored (20.91%) Glycine / 91.9,-151.6	-	-	Favored (24.11%)	-	-	-
A 263		THR	0.91	-	Favored (11.01%) General / -130.3,173.2	Favored (58.2%) *p* chi angles: 64.4	0.10Å	Favored (9.09%)	-	-	-
A 264		ARG	1.02	-	Favored (51.05%) General / -129.2,144.0	Favored (53.8%) *mtp180* chi angles: 287.3,183,61.8,212.3	0.10Å	Favored (35.892%) beta sheet	-	-	-
A 265		ALA	1.16	-	Favored (25.3%) General / -81.0,157.9	-	0.03Å	Favored (33.528%) beta sheet	-	-	-
A 266		VAL	1.31	-	Favored (18.81%) Ile or Val / -123.7,166.4	Favored (28.7%) *m* chi angles: 298.4	0.05Å	Favored (33.79%)	-	-	-
A 267		ALA	1.46	-	Favored (71.28%) General / -59.9,-33.0	-	0.04Å	Favored (6.134%)	-	-	-
A 268		SER	1.54	-	Favored (56.74%) General / -61.2,142.6	Favored (65%) *m* chi angles: 297.3	0.03Å	Favored (32.458%)	-	-	-
A 269		CYS	1.52	-	Favored (44.81%) General / -99.6,124.5	Favored (52%) *t* chi angles: 180.6	0.05Å	Favored (45.369%)	-	-	-
A 270		ALA	1.42	-	Favored (55.87%) General / -65.6,145.6	-	0.05Å	Favored (46.227%)	-	-	-
A 271		GLU	1.28	-	Favored (56.06%) General / -64.8,135.7	Favored (89.7%) *tt0* chi angles: 185.6,176,4.7	0.02Å	Favored (38.28%)	-	-	-
A 272		ALA	1.14	-	Favored (71.13%) Pre-Pro / -65.7,120.7	-	0.04Å	Favored (43.33%)	-	-	-
A 273		PRO	1.04	-	Favored (83.76%) Trans-Pro / -62.5,151.3	Favored (34.6%) *Cg_exo* chi angles: 339.4,33.7,327.6	0.08Å	Favored (69.777%)	-	-	-
A 274		ASN	0.97	-	Favored (6.64%) General / -80.1,97.0	Favored (58%) *t0* chi angles: 186.3,334.3	0.06Å	Favored (31.541%)	-	-	-
A 275		MET	0.93	-	Favored (63.25%) General / -64.0,-17.3	Favored (96.9%) *mmm* chi angles: 292.2,303.4,293.6	0.06Å	Favored (35.431%)	-	-	-
A 276		LYS	0.9	-	Favored (59.86%) General / -78.4,-13.4	Favored (98.9%) *mttt* chi angles: 293.8,179.5,179.6,178.9	0.01Å	Favored (61.96%) alpha helix	-	-	-
A 277		ILE	0.88	-	Favored (10.72%) Ile or Val / -98.3,-41.9	Favored (85.2%) *mt* chi angles: 295.3,173.8	0.07Å	Favored (35.334%) alpha helix	-	-	-
A 278		ILE	0.85	0.40Å HD13 with A 278 ILE HG21	Favored (11.09%) Ile or Val / -108.5,-10.6	Favored (38.7%) *pt* chi angles: 65.8,171.5	0.08Å	Favored (22.927%) alpha helix	-	-	-

#	Alt	Res	High B	Clash > 0.4Å	Ramachandran	Rotamer	Cβ deviation	CaBLAM	Bond lengths	Bond angles	Cis Peptides
A 279		GLY	0.83	-	Favored (77.12%) Glycine / -57.9,-35.8	-	-	Favored (59.187%) alpha helix	-	-	-
A 280		ARG	0.8	-	Favored (88.52%) General / -66.4,-38.9	Favored (43.3%) *tpt170* chi angles: 181.7,62.5,178,168.6	0.02Å	Favored (86.145%) alpha helix	-	-	-
#	Alt	Res	High B	Clash > 0.4Å	Ramachandran	Rotamer	Cβ deviation	CaBLAM	Bond lengths	Bond angles	Cis Peptides
			Avg: 0.95	Clashscore: 2.02	Outliers: 3 of 901	Poor rotamers: 0 of 767	Outliers: 0 of 820	Outliers: 14 of 899	Outliers: 10 of 903	Outliers: 16 of 903	Non-Trans: 2 of 902
A 281		ARG	0.79	-	Favored (84.4%) General / -67.0,-37.5	Favored (93.8%) *mtm-85* chi angles: 294.2,197.5,297.5,282.3	0.06Å	Favored (98.475%) alpha helix	OUTLIER(S) worst is CG--CD: 4.7 σ	-	-
A 282		ILE	0.79	-	Favored (97.79%) Ile or Val / -64.0,-44.2	Favored (91.5%) *mt* chi angles: 292.2,165.7	0.12Å	Favored (93.921%) alpha helix	-	-	-
A 283		GLU	0.79	-	Favored (87.87%) General / -60.3,-39.7	Favored (97.6%) *mt-10* chi angles: 288.7,173.7,355.2	0.05Å	Favored (86.356%) alpha helix	-	-	-
A 284		ARG	0.79	-	Favored (98.4%) General / -60.8,-43.2	Favored (20.6%) *tpt-90* chi angles: 177.5,72,187.3,276.4	0.06Å	Favored (87.841%) alpha helix	-	-	-
A 285		ILE	0.8	-	Favored (91.87%) Ile or Val / -64.9,-46.4	Favored (91.1%) *mt* chi angles: 295.2,166.5	0.08Å	Favored (89.655%) alpha helix	-	-	-
A 286		ARG	0.81	-	Favored (79.56%) General / -60.6,-36.8	Favored (97.6%) *mtt180* chi angles: 288.2,174.7,176.2,166.4	0.04Å	Favored (77.654%) alpha helix	-	-	-
A 287		ASN	0.82	-	Favored (62.59%) General / -72.5,-44.1	Favored (98.9%) *m-40* chi angles: 288.8,337.8	0.02Å	Favored (77.953%) alpha helix	-	-	-
A 288		GLU	0.83	-	Favored (78.69%) General / -60.7,-36.4	Favored (21.8%) *mm-30* chi angles: 288,281.3,310.4	0.08Å	Favored (59.047%) alpha helix	-	-	-
A 289		HIS	0.85	-	Favored (9.72%) General / -115.1,27.0	Favored (98.5%) *m-70* chi angles: 300.6,286	0.08Å	Favored (17.203%) alpha helix	-	-	-
A 290		ALA	0.89	-	Favored (63.67%) General / -54.6,-35.8	-	0.05Å	Favored (34.883%) alpha helix	-	-	-
A 291		GLU	0.95	-	Favored (66.13%) General / -63.3,-21.2	Favored (99.9%) *mt-10* chi angles: 292.1,179.4,355.9	0.00Å	Favored (52.379%) alpha helix	-	-	-
A 292		THR	1.04	-	Favored (13.24%) General / -116.6,5.3	Favored (75.5%) *p* chi angles: 60	0.07Å	Favored (25.484%)	-	-	-
A 293		TRP	1.14	-	Favored (41.84%) General / -74.6,132.5	Favored (21.9%) *t60* chi angles: 181.6,50.1	0.05Å	Favored (8.229%)	-	-	-
A 294		PHE	1.23	-	Favored (29.07%) General / -150.5,166.8	Favored (53%) *p90* chi angles: 66.3,95.2	0.01Å	Favored (34.675%) beta sheet	-	-	-

A 295		LEU	1.29	-	Favored (39.51%) General / -110.3,144.1	Favored (83.2%) *mt* chi angles: 301.3,177.9	0.02Å	Favored (50.769%) beta sheet	-	-	-
A 296		ASP	1.31	-	Favored (8.69%) General / -136.0,110.7	Favored (65.7%) *t0* chi angles: 186.6,345.1	0.04Å	Favored (49.298%) beta sheet	-	-	-
A 297		GLU	1.27	-	Favored (28.06%) General / -78.7,0.1	Favored (98.3%) *mt-10* chi angles: 293.9,179.5,359.3	0.01Å	Favored (17.146%)	-	-	-
A 298		ASN	1.19	-	Favored (25.18%) General / -104.4,16.9	Favored (67.7%) *m-40* chi angles: 294.5,281	0.03Å	Favored (6.67%)	-	-	-
A 299		HIS	1.1	-	Favored (21.34%) Pre-Pro / -50.1,136.5	Favored (70%) *t-90* chi angles: 184.6,275.9	0.11Å	Favored (20.594%)	OUTLIER(S) worst is CB--CG: 6.3 σ	-	-
A 300		PRO	1	-	Favored (30.81%) Trans-Pro / -71.4,-14.3	Favored (59%) *Cg_endo* chi angles: 26.3,324.9,28.8	0.15Å	Favored (9.465%) beta sheet	-	-	-
**#**	**Alt**	**Res**	**High B**	**Clash > 0.4Å**	**Ramachandran**	**Rotamer**	**Cβ deviation**	**CaBLAM**	**Bond lengths**	**Bond angles**	**Cis Peptides**
			Avg: 0.95	Clashscore: 2.02	Outliers: 3 of 901	Poor rotamers: 0 of 767	Outliers: 0 of 820	Outliers: 14 of 899	Outliers: 10 of 903	Outliers: 16 of 903	Non-Trans: 2 of 902
A 301		TYR	0.92	-	Favored (25.11%) General / -87.0,149.0	Favored (23.3%) *m-10* chi angles: 293.8,339.8	0.11Å	Favored (27.724%)	-	-	-
A 302		ARG	0.86	-	Favored (12.01%) General / -88.2,-42.9	Favored (63.7%) *ttt180* chi angles: 183.5,183,178.3,199.5	0.05Å	Favored (21.596%)	-	OUTLIER(S) worst is NE-CZ-NH2: 4.1 σ	-
A 303		THR	0.8	-	Favored (10.02%) General / -120.0,-5.8	Favored (78.2%) *p* chi angles: 60.9	0.08Å	Favored (13.343%)	-	-	-
A 304		TRP	0.76	-	Favored (26.9%) General / -90.9,143.3	Favored (34.2%) *m100* chi angles: 285.8,65.1	0.02Å	Favored (28.647%)	-	-	-
A 305		ALA	0.73	-	Favored (42.76%) General / -74.3,147.0	-	0.04Å	Favored (45.548%) beta sheet	-	-	-
A 306		TYR	0.71	-	Favored (54.07%) General / -110.3,132.6	Favored (63.3%) *t80* chi angles: 169.1,73.9	0.07Å	Favored (41.641%)	-	-	-
A 307		HIS	0.7	-	Favored (8.45%) General / -116.2,-23.3	Favored (96.3%) *m-70* chi angles: 303.7,291.4	0.07Å	CaBLAM Disfavored (3.412%)	-	-	-
A 308		GLY	0.7	-	Favored (26.48%) Glycine / 166.3,167.1	-	-	Favored (17.501%)	-	-	-
A 309		SER	0.72	-	Favored (44.69%) General / -134.4,158.1	Favored (51.3%) *m* chi angles: 300.9	0.10Å	Favored (57.259%)	-	-	-

#	Alt	Res	High B	Clash > 0.4Å	Ramachandran	Rotamer	Cβ deviation	CaBLAM	Bond lengths	Bond angles	Cis Peptides
A 310		TYR	0.76	-	Favored (42.76%) General / -150.6,157.2	Favored (45.6%) *p90* chi angles: 70.2,90.8	0.06Å	Favored (16.622%)	-	-	-
A 311		GLU	0.81	-	Favored (57.76%) General / -66.7,138.9	Favored (98.2%) *mt-10* chi angles: 291.1,181.1,354.4	0.09Å	Favored (21.893%)	-	-	-
A 312		ALA	0.86	-	Favored (31.32%) Pre-Pro / -153.2,154.9	-	0.07Å	Favored (28.247%)	-	-	-
A 313		PRO	0.93	-	Favored (87.01%) Trans-Pro / -65.5,150.3	Favored (36.8%) *Cg_endo* chi angles: 22.7,326,30.7	0.02Å	Favored (73.937%)	-	-	-
A 314		THR	1.01	-	Favored (41.93%) General / -74.6,132.6	Favored (87.2%) *m* chi angles: 301.4	0.04Å	Favored (38.308%)	-	-	-
A 315		GLN	1.12	-	Favored (52.34%) General / -132.6,147.6	Favored (66.9%) *mt0* chi angles: 299.4,175.1,64.4	0.08Å	Favored (13.073%)	-	-	-
A 316		GLY	1.23	-	Favored (31.71%) Glycine / 99.1,178.3	-	-	Favored (12.905%)	-	-	-
A 317		SER	1.34	-	Favored (22.39%) General / -157.1,148.8	Favored (46.8%) *t* chi angles: 180.2	0.05Å	Favored (29.538%)	-	-	-
A 318		ALA	1.4	-	Favored (31.3%) General / -106.2,7.1	-	0.03Å	Favored (38.326%)	-	-	-
A 319		SER	1.4	-	Favored (35.23%) General / -123.5,156.6	Favored (65.6%) *m* chi angles: 297.2	0.03Å	Favored (23.932%)	-	-	-
A 320		SER	1.33	-	Favored (39.41%) General / -148.9,162.7	Favored (88.3%) *p* chi angles: 62.9	0.03Å	Favored (46.857%)	-	-	-
**#**	**Alt**	**Res**	**High B**	**Clash > 0.4Å**	**Ramachandran**	**Rotamer**	**Cβ deviation**	**CaBLAM**	**Bond lengths**	**Bond angles**	**Cis Peptides**
			Avg: 0.95	Clashscore: 2.02	Outliers: 3 of 901	Poor rotamers: 0 of 767	Outliers: 0 of 820	Outliers: 14 of 899	Outliers: 10 of 903	Outliers: 16 of 903	Non-Trans: 2 of 902
A 321		LEU	1.2	-	Favored (36.06%) General / -93.3,133.9	Favored (7.1%) *tt* chi angles: 179.2,147.9	0.08Å	Favored (33.997%) beta sheet	-	-	-
A 322		VAL	1.05	-	Favored (38.1%) Ile or Val / -80.4,129.1	Favored (95.1%) *t* chi angles: 175.8	0.05Å	Favored (32.413%) beta sheet	-	-	-
A 323		ASN	0.91	-	Allowed (1.57%) General / -62.4,110.2	Favored (33.2%) *t0* chi angles: 183.2,311	0.07Å	Favored (40.683%)	-	-	-
A 324		GLY	0.81	-	Favored (49.68%) Glycine / -56.6,-52.5	-	-	Favored (38.656%)	-	-	-

A 325		VAL	0.75	-	Favored (93.7%) Ile or Val / -59.5,-44.9	Favored (55.9%) *t* chi angles: 170.2	0.04Å	Favored (78.933%) alpha helix	-	-	-
A 326		VAL	0.72	-	Favored (82.02%) Ile or Val / -68.5,-45.5	Favored (93.6%) *t* chi angles: 174.6	0.02Å	Favored (79.176%) alpha helix	-	-	-
A 327		ARG	0.71	-	Favored (79.01%) General / -56.1,-44.4	Favored (58.6%) *ttt180* chi angles: 181.3,179.8,185.8,199.9	0.07Å	Favored (89.903%) alpha helix	-	-	-
A 328		LEU	0.72	-	Favored (83.85%) General / -62.7,-36.7	Favored (84.2%) *mt* chi angles: 289.8,170.2	0.05Å	Favored (74.339%) alpha helix	-	-	-
A 329		LEU	0.74	-	Favored (56.16%) General / -91.7,-3.6	Favored (88.4%) *mt* chi angles: 299.1,175.5	0.07Å	Favored (43.646%)	-	-	-
A 330		SER	0.78	-	Favored (12.77%) General / -110.7,24.6	Favored (60.5%) *m* chi angles: 298.9	0.06Å	Favored (16.182%)	-	-	-
A 331		LYS	0.84	-	Allowed (1.65%) Pre-Pro / -36.1,-52.7	Favored (85.1%) *tttt* chi angles: 179,178.2,178.4,179.7	0.17Å	Favored (24.878%)	-	-	-
A 332		PRO	0.92	-	Favored (34.64%) Trans-Pro / -57.1,-19.8	Favored (83.2%) *Cg_exo* chi angles: 334.4,34.1,331.3	0.07Å	Favored (43.417%) alpha helix	-	-	-
A 333		TRP	1.01	-	Favored (44.35%) General / -99.9,3.1	Favored (43.7%) *m-90* chi angles: 294.2,268.8	0.07Å	Favored (53.412%) three-ten	-	-	-
A 334		ASP	1.11	-	Favored (49.58%) General / -86.1,1.7	Favored (6.3%) *t70* chi angles: 204.6,54.4	0.04Å	Favored (15.208%) three-ten	-	-	-
A 335		VAL	1.19	-	Favored (7.38%) Ile or Val / -116.6,10.4	Favored (31.6%) *m* chi angles: 297.1	0.07Å	Favored (53.321%)	-	-	-
A 336		VAL	1.23	-	Favored (63.99%) Ile or Val / -113.1,120.8	Favored (74.9%) *t* chi angles: 178.3	0.06Å	Favored (27.654%)	-	-	-
A 337		THR	1.23	-	Favored (87.67%) General / -59.3,-40.9	Favored (88.2%) *m* chi angles: 298.5	0.01Å	Favored (35.186%)	-	-	-
A 338		GLY	1.2	-	Favored (37.3%) Glycine / -55.8,-23.4	-	-	Favored (56.096%) alpha helix	-	-	-
A 339		VAL	1.15	-	Favored (11.02%) Ile or Val / -102.3,-47.8	Favored (92.9%) *t* chi angles: 174.5	0.06Å	Favored (30.706%) alpha helix	-	-	-
A 340		THR	1.09	-	Favored (61.19%) General / -73.8,-15.0	Favored (77.7%) *p* chi angles: 60.9	0.06Å	Favored (59.579%)	-	-	-
**#**	**Alt**	**Res**	**High B**	**Clash > 0.4Å**	**Ramachandran**	**Rotamer**	**Cβ deviation**	**CaBLAM**	**Bond lengths**	**Bond angles**	**Cis Peptides**
			Avg: 0.95	Clashscore: 2.02	Outliers: 3 of 901	Poor rotamers: 0 of 767	Outliers: 0 of 820	Outliers: 14 of 899	Outliers: 10 of 903	Outliers: 16 of 903	Non-Trans: 2 of 902
A 341		GLY	1.05	-	Favored (90.52%)	-	-	Favored (59.676%)	-	-	-

					Glycine / -82.0,-2.2						
A 342		ILE	1.02	-	Favored (36.13%) Ile or Val / -88.4,131.4	Favored (96.6%) *mt* chi angles: 294.4,167.6	0.16Å	Favored (8.064%)	-	-	-
A 343		ALA	1	-	Favored (40.13%) General / -152.5,163.2	-	0.04Å	Favored (18.036%)	-	-	-
A 344		MET	0.97	-	Favored (56.21%) General / -60.9,142.5	Favored (58.7%) *ttp* chi angles: 187.7,182.6,72.9	0.06Å	Favored (32.028%)	-	-	-
A 345		THR	0.95	-	Favored (17%) General / -70.2,167.9	Favored (68.4%) *p* chi angles: 58.9	0.05Å	Favored (21.212%)	-	-	-
A 346		ASP	0.92	-	Favored (14.52%) General / -80.2,109.2	Favored (64.2%) *t0* chi angles: 185.5,354.1	0.05Å	Favored (7.22%)	-	-	-
A 347		THR	0.89	-	Favored (25.68%) General / -109.4,9.0	Favored (55.1%) *p* chi angles: 56.8	0.02Å	Favored (14.114%) beta sheet	-	-	-
A 348		THR	0.87	-	Favored (47.43%) Pre-Pro / -74.7,166.2	Favored (63.2%) *p* chi angles: 63.4	0.10Å	Favored (29.601%)	-	-	-
A 349		PRO	0.85	-	Favored (44.78%) Trans-Pro / -50.4,-35.4	Favored (88.4%) *Cg_exo* chi angles: 330,38.3,330.1	0.09Å	Favored (88.364%)	-	-	-
A 350		TYR	0.85	-	Favored (69.12%) General / -62.0,-51.2	Favored (82.2%) *t80* chi angles: 180.9,84.4	0.06Å	Favored (75.419%) alpha helix	-	-	-
A 351		GLY	0.85	-	Favored (93.37%) Glycine / -59.7,-38.1	-	-	Favored (90.626%) alpha helix	-	-	-
A 352		GLN	0.86	-	Favored (88.47%) General / -66.6,-39.6	Favored (84.2%) *mt0* chi angles: 291.6,175.9,36.2	0.04Å	Favored (91.346%) alpha helix	-	-	-
A 353		GLN	0.87	-	Favored (71.32%) General / -70.8,-34.1	Favored (90.5%) *mm-40* chi angles: 290.4,293.9,306.4	0.03Å	Favored (75.576%) alpha helix	OUTLIER(S) worst is CG--CD: 4.9 σ	-	-
A 354		ARG	0.89	-	Favored (78.48%) General / -60.0,-49.2	Favored (80.2%) *mtp180* chi angles: 289.1,172.7,72.1,183	0.13Å	Favored (67.796%) alpha helix	-	-	-
A 355		VAL	0.91	-	Favored (41.72%) Ile or Val / -73.8,-34.9	Favored (6.3%) *p* chi angles: 69.1	0.08Å	Favored (68.791%) alpha helix	-	-	-
A 356		PHE	0.94	-	Favored (58.78%) General / -65.1,-51.9	Favored (21.2%) *t80* chi angles: 195,98.1	0.03Å	Favored (64.44%) alpha helix	-	-	-
A 357		LYS	0.96	-	Favored (67.57%) General / -60.4,-51.9	Favored (5.3%) *tmtt* chi angles: 191.9,267.1,180,177	0.03Å	Favored (68.458%) alpha helix	-	-	-
A 358		GLU	0.98	-	Favored (75.73%) General / -66.4,-33.4	Favored (66.9%) *mm-30* chi angles: 290,293,307.4	0.05Å	Favored (25.08%) alpha helix	-	-	-

#	Alt	Res	High B	Clash > 0.4Å	Ramachandran	Rotamer	Cβ deviation	CaBLAM	Bond lengths	Bond angles	Cis Peptides
A 359		LYS	0.99	-	Favored (2.89%) General / -113.2,-50.1	Favored (63.1%) *mttm* chi angles: 294.8,186.2,185.3,295	0.04Å	Favored (13.234%) alpha helix	-	-	-
A 360		VAL	1	-	Favored (73.01%) Ile or Val / -70.1,-37.8	Favored (84.4%) *t* chi angles: 177.1	0.12Å	Favored (80.627%) alpha helix	-	-	-
#	Alt	Res	High B	Clash > 0.4Å	Ramachandran	Rotamer	Cβ deviation	CaBLAM	Bond lengths	Bond angles	Cis Peptides
			Avg: 0.95	Clashscore: 2.02	Outliers: 3 of 901	Poor rotamers: 0 of 767	Outliers: 0 of 820	Outliers: 14 of 899	Outliers: 10 of 903	Outliers: 16 of 903	Non-Trans: 2 of 902
A 361		ASP	0.99	-	Favored (6.35%) General / -79.1,8.1	Favored (79.2%) *m-30* chi angles: 283.1,341.7	0.17Å	Favored (29.667%)	-	OUTLIER(S) worst is CA-CB-CG: 7.4 σ	-
A 362		THR	0.98	-	Favored (63.89%) General / -69.1,-17.7	Favored (60.5%) *p* chi angles: 57.4	0.06Å	CaBLAM Disfavored (3.508%)	-	-	-
A 363		ARG	0.94	-	Allowed (0.05%) General / 52.1,84.1	Favored (80.8%) *ttt180* chi angles: 183.4,173.4,181.4,174.4	0.11Å	CaBLAM Outlier (0.074%) try beta sheet	-	-	-
A 364		VAL	0.9	0.64Å O with A 364 VAL HG13	Favored (46.69%) Pre-Pro / -52.9,135.9	Favored (10.4%) *p* chi angles: 64.6	0.23Å	Favored (36.315%) beta sheet	-	-	-
A 365		PRO	0.84	-	Favored (48.86%) Trans-Pro / -73.8,154.4	Favored (72.3%) *Cg_endo* chi angles: 27.4,325,28	0.06Å	Favored (70.248%) beta sheet	-	-	-
A 366		ASP	0.78	-	Favored (70.53%) Pre-Pro / -86.2,124.2	Favored (38.6%) *m-30* chi angles: 288.2,308.1	0.11Å	Favored (26.258%)	-	-	-
A 367		PRO	0.72	-	Favored (35.7%) Trans-Pro / -66.9,162.9	Favored (42.5%) *Cg_endo* chi angles: 24,326.6,28.1	0.01Å	Favored (21.4%)	-	-	-
A 368		GLN	0.67	-	Favored (28.09%) General / -59.1,149.3	Favored (79.1%) *mt0* chi angles: 293.6,181,60.7	0.01Å	Favored (23.711%)	-	-	-
A 369		GLU	0.63	-	Favored (62.4%) General / -51.7,-42.6	Favored (61.5%) *tp30* chi angles: 182.7,69.3,24.5	0.01Å	Favored (61.022%)	-	-	-
A 370		GLY	0.61	-	Favored (49%) Glycine / -59.4,-53.1	-	-	Favored (91.156%) alpha helix	-	-	-
A 371		THR	0.6	-	Favored (73.43%) General / -62.1,-32.8	Favored (78.1%) *p* chi angles: 60.9	0.13Å	Favored (74.157%) alpha helix	-	-	-
A 372		ARG	0.59	-	Favored (81.31%) General / -61.2,-48.5	Favored (66.3%) *ttt-90* chi angles: 178.6,183.6,184.7,268.6	0.06Å	Favored (77.625%) alpha helix	-	-	-
A 373		GLN	0.59	-	Favored (86.51%) General / -66.3,-37.9	Favored (79.9%) *mt0* chi angles: 290.6,181.4,29.2	0.03Å	Favored (83.043%) alpha helix	-	-	-
A 374		VAL	0.59	-	Favored (96.85%) Ile or Val / -63.6,-45.7	Favored (68.7%) *t* chi angles: 172	0.07Å	Favored (93.114%) alpha helix	-	-	-

A 375		MET	0.6	-	Favored (87.66%) General / -58.5,-42.4	Favored (49.3%) *ttp* chi angles: 183.1,182.8,62.8	0.07Å	Favored (94.004%) alpha helix	-	-	-
A 376		ASN	0.6	-	Favored (94.75%) General / -63.3,-39.5	Favored (43.7%) *t0* chi angles: 189.3,63.4	0.03Å	Favored (95.59%) alpha helix	-	-	-
A 377		ILE	0.61	-	Favored (85.46%) Ile or Val / -66.5,-46.8	Favored (98%) *mt* chi angles: 293.3,167.4	0.02Å	Favored (77.189%) alpha helix	-	-	-
A 378		VAL	0.62	-	Favored (89.76%) Ile or Val / -66.8,-43.5	Favored (85.9%) *t* chi angles: 173.7	0.03Å	Favored (82.121%) alpha helix	-	-	-
A 379		SER	0.64	-	Favored (98.3%) General / -62.8,-43.6	Favored (70.4%) *m* chi angles: 296.3	0.09Å	Favored (98.408%) alpha helix	-	-	-
A 380		SER	0.66	-	Favored (99.67%) General / -62.7,-42.5	Favored (73.5%) *m* chi angles: 295.5	0.05Å	Favored (99.122%) alpha helix	-	-	-
**#**	**Alt**	**Res**	**High B**	**Clash > 0.4Å**	**Ramachandran**	**Rotamer**	**Cβ deviation**	**CaBLAM**	**Bond lengths**	**Bond angles**	**Cis Peptides**
			Avg: 0.95	Clashscore: 2.02	Outliers: 3 of 901	Poor rotamers: 0 of 767	Outliers: 0 of 820	Outliers: 14 of 899	Outliers: 10 of 903	Outliers: 16 of 903	Non-Trans: 2 of 902
A 381		TRP	0.68	-	Favored (85.01%) General / -60.8,-47.7	Favored (84.4%) *t60* chi angles: 174.9,85	0.03Å	Favored (91.632%) alpha helix	-	-	-
A 382		LEU	0.7	-	Favored (95.34%) General / -64.5,-40.3	Favored (40.5%) *tp* chi angles: 185.1,58.2	0.08Å	Favored (87.231%) alpha helix	-	-	-
A 383		TRP	0.74	-	Favored (90.37%) General / -59.7,-41.2	Favored (53.5%) *m100* chi angles: 283,118.9	0.09Å	Favored (85.902%) alpha helix	-	-	-
A 384		LYS	0.78	-	Favored (91.68%) General / -65.9,-41.4	Favored (96.6%) *mttt* chi angles: 288.8,178.1,181,179.4	0.01Å	Favored (96.841%) alpha helix	-	-	-
A 385		GLU	0.85	-	Favored (81.48%) General / -65.0,-35.5	Favored (76%) *mm-30* chi angles: 289.7,298,306.2	0.05Å	Favored (77.08%) alpha helix	-	-	-
A 386		LEU	0.93	-	Favored (62.23%) General / -73.3,-42.6	Favored (78.9%) *mt* chi angles: 295.4,169	0.09Å	Favored (71.174%) alpha helix	-	-	-
A 387		GLY	1.01	-	Favored (76.43%) Glycine / -60.5,-32.0	-	-	Favored (73.218%)	-	-	-
A 388		LYS	1.08	-	Favored (65.39%) General / -60.1,-25.5	Favored (97%) *mttt* chi angles: 286.6,174.9,180.3,175.6	0.02Å	Favored (29.295%)	-	-	-
A 389		ARG	1.12	-	Favored (3.66%) General / -115.4,-39.0	Favored (80%) *ttt180* chi angles: 181.2,174.6,175,172.3	0.03Å	Favored (22.744%)	-	-	-

#	Alt	Res	High B	Clash > 0.4Å	Ramachandran	Rotamer	Cβ deviation	CaBLAM	Bond lengths	Bond angles	Cis Peptides
A 390		LYS	1.12	-	Favored (3.21%) General / -125.7,90.4	Favored (97.9%) *mttt* chi angles: 295.6,184.1,177.9,184.9	0.03Å	Favored (18.817%)	-	-	-
A 391		ARG	1.09	-	Favored (66.72%) Pre-Pro / -78.9,145.7	Favored (63.9%) *mmm-85* chi angles: 292.6,289.7,295.8,276.5	0.02Å	Favored (18.612%)	-	-	-
A 392		PRO	1.03	-	Favored (58.57%) Trans-Pro / -53.5,142.5	Favored (84.5%) *Cg_exo* chi angles: 334.1,34.5,332	0.03Å	Favored (47.795%)	-	-	-
A 393		ARG	0.97	-	Favored (49.86%) General / -131.7,153.6	Favored (46.4%) *mtm180* chi angles: 299.1,192.4,292.3,156.6	0.07Å	Favored (49.063%) beta sheet	-	-	-
A 394		VAL	0.9	-	Favored (36.18%) Ile or Val / -86.4,130.7	Favored (82.4%) *t* chi angles: 176.8	0.04Å	Favored (38.91%) beta sheet	-	-	-
A 395		CYS	0.85	-	Favored (23.69%) General / -85.3,151.9	Favored (69.4%) *m* chi angles: 298.7	0.07Å	Favored (38.814%) beta sheet	-	-	-
A 396		THR	0.8	-	Favored (10.7%) General / -96.4,168.2	Favored (65.4%) *p* chi angles: 63.1	0.05Å	Favored (48.077%)	-	-	-
A 397		LYS	0.77	-	Favored (73.16%) General / -55.4,-49.2	Favored (35.9%) *ttpt* chi angles: 180.7,180.3,71.9,174.8	0.03Å	Favored (63.252%)	-	-	-
A 398		GLU	0.74	-	Favored (92.52%) General / -62.6,-39.1	Favored (98.8%) *mt-10* chi angles: 290.6,178.1,354	0.02Å	Favored (84.851%) alpha helix	-	-	-
A 399		GLU	0.73	-	Favored (89.34%) General / -63.6,-38.0	Favored (42.3%) *mt-10* chi angles: 288.2,167.8,311.8	0.07Å	Favored (94.143%) alpha helix	-	-	-
A 400		PHE	0.74	-	Favored (91.23%) General / -64.7,-44.2	Favored (69.4%) *t80* chi angles: 183.6,70.4	0.09Å	Favored (96.4%) alpha helix	-	-	-
**#**	**Alt**	**Res**	**High B**	**Clash > 0.4Å**	**Ramachandran**	**Rotamer**	**Cβ deviation**	**CaBLAM**	**Bond lengths**	**Bond angles**	**Cis Peptides**
			Avg: 0.95	Clashscore: 2.02	Outliers: 3 of 901	Poor rotamers: 0 of 767	Outliers: 0 of 820	Outliers: 14 of 899	Outliers: 10 of 903	Outliers: 16 of 903	Non-Trans: 2 of 902
A 401		ILE	0.77	-	Favored (91.97%) Ile or Val / -59.2,-46.5	Favored (97.8%) *mt* chi angles: 292.7,167	0.06Å	Favored (87.734%) alpha helix	-	-	-
A 402		ASN	0.84	-	Favored (91.66%) General / -64.2,-38.6	Favored (99.2%) *m-40* chi angles: 287.9,338.9	0.04Å	Favored (76.595%) alpha helix	-	-	-
A 403		LYS	0.97	-	Favored (63.07%) General / -74.2,-39.7	Favored (51.3%) *tptt* chi angles: 182.3,63.5,171,181.2	0.04Å	Favored (82.328%) alpha helix	-	-	-
A 404		VAL	1.16	-	Favored (51.43%) Ile or Val / -68.6,-31.5	Allowed (1.4%) *p* chi angles: 78.6	0.08Å	Favored (12.717%)	-	-	-

A 405		ARG	1.41	-	OUTLIER (0.03)%) General / 64.5,147.7	Favored (97.6%) *mtt-85* chi angles: 292.8,177.8,184.3,272.4	0.06Å	CaBLAM Disfavored (1.851%)	-	-	-
A 406		SER	1.68	-	Favored (63.33%) General / -62.3,-19.4	Favored (88.3%) *p* chi angles: 67	0.02Å	Favored (37.653%)	-	-	-
A 407		ASN	1.92	-	Favored (47.88%) General / -96.8,6.8	Favored (89.9%) *m-40* chi angles: 292.5,321.7	0.02Å	Favored (45.165%)	-	-	-
A 408		ALA	2.06	-	Favored (32.34%) General / -88.0,137.4	-	0.04Å	Favored (33.609%)	-	-	-
A 409		ALA	2.06	-	Favored (29.78%) General / -79.3,153.5	-	0.05Å	Favored (30.63%) beta sheet	-	-	-
A 410		LEU	1.92	-	Favored (41.74%) General / -115.6,146.4	Favored (88%) *mt* chi angles: 300.2,177.4	0.04Å	Favored (60.834%) beta sheet	-	-	-
A 411		GLY	1.71	-	Favored (41.73%) Glycine / -91.5,-169.4	-	-	Favored (28.872%) beta sheet	-	-	-
A 412		ALA	1.5	-	Favored (3.05%) General / -159.5,119.6	-	0.02Å	CaBLAM Disfavored (2.968%)	-	-	-
A 413		ILE	1.33	-	Allowed (1.78%) Ile or Val / -122.7,-27.4	Favored (42.9%) *pt* chi angles: 60.1,170.8	0.07Å	CaBLAM Disfavored (4.224%)	-	-	-
A 414		PHE	1.2	-	Favored (34.65%) General / -116.3,152.0	Favored (25.2%) *m-10* chi angles: 299.1,145.4	0.02Å	Favored (21.679%) alpha helix	-	-	-
A 415		GLU	1.12	-	Favored (70.1%) General / -61.3,-29.8	Favored (77.7%) *mm-30* chi angles: 292.4,296.8,306.6	0.03Å	Favored (59.814%) alpha helix	-	-	-
A 416		GLU	1.06	-	Favored (81%) General / -61.9,-36.3	Favored (87.8%) *mt-10* chi angles: 288.4,169,337	0.04Å	Favored (72.805%) alpha helix	-	-	-
A 417		GLU	1.03	-	Favored (49.46%) General / -78.2,-30.9	Favored (42.2%) *tt0* chi angles: 196.8,175.6,15.2	0.05Å	Favored (49.44%) alpha helix	-	-	-
A 418		LYS	1.01	-	Favored (25.87%) General / -59.8,-16.2	Favored (97.4%) *mttt* chi angles: 290.3,181.2,179.7,178.2	0.06Å	Favored (52.833%) three-ten	-	-	-
A 419		GLU	1	-	Favored (46.91%) General / -80.8,-2.4	Favored (96.3%) *mt-10* chi angles: 293.1,183.1,358.6	0.03Å	Favored (51.974%)	-	-	-
A 420		TRP	0.98	-	Favored (53.27%) General / -114.2,136.2	Favored (19.7%) *m-10* chi angles: 294,319.2	0.08Å	Favored (31.371%)	-	-	-
**#**	**Alt**	**Res**	**High B**	**Clash > 0.4Å**	**Ramachandran**	**Rotamer**	**Cβ deviation**	**CaBLAM**	**Bond lengths**	**Bond angles**	**Cis Peptides**
			Avg: 0.95	Clashscore: 2.02	Outliers: 3 of 901	Poor rotamers: 0 of 767	Outliers: 0 of 820	Outliers: 14 of 899	Outliers: 10 of 903	Outliers: 16 of 903	Non-Trans: 2 of 902

A 421		LYS	0.95	-	Favored (14.61%) General / -83.1,-44.9	Favored (87.7%) *tttt* chi angles: 183.9,176.5,179.5,180.2	0.01Å	Favored (23.041%)	-	-	-
A 422		THR	0.92	-	Favored (28.55%) General / -128.5,161.9	Favored (59.8%) *p* chi angles: 64.1	0.04Å	Favored (14.777%)	-	-	-
A 423		ALA	0.88	-	Favored (78.56%) General / -59.8,-37.2	-	0.05Å	Favored (67.025%) alpha helix	-	-	-
A 424		VAL	0.85	0.41Å HA with A 427 VAL HG22	Favored (89.66%) Ile or Val / -66.5,-42.1	Favored (58.4%) *t* chi angles: 170.6	0.15Å	Favored (89.324%) alpha helix	-	-	-
A 425		GLU	0.83	-	Favored (78.23%) General / -56.5,-42.3	Favored (47.3%) *tt0* chi angles: 181,174.7,56.7	0.04Å	Favored (96.11%) alpha helix	-	-	-
A 426		ALA	0.8	-	Favored (86.5%) General / -62.8,-46.8	-	0.08Å	Favored (97.484%) alpha helix	-	-	-
A 427		VAL	0.79	0.41Å HG22 with A 424 VAL HA	Favored (27.6%) Ile or Val / -68.7,-23.4	Favored (28.5%) *m* chi angles: 298.5	0.09Å	Favored (68.976%) alpha helix	-	-	-
A 428		ASN	0.79	-	Favored (55.68%) General / -89.0,1.4	Favored (87.7%) *m-40* chi angles: 288.3,326.9	0.04Å	Favored (53.502%)	-	-	-
A 429		ASP	0.78	-	Favored (63.14%) Pre-Pro / -89.6,115.2	Favored (41%) *t0* chi angles: 184.1,329.2	0.01Å	Favored (32.34%)	-	-	-
A 430		PRO	0.78	-	Favored (17.47%) Trans-Pro / -49.3,-29.5	Favored (86.1%) *Cg_exo* chi angles: 330.5,37.3,330.6	0.06Å	Favored (81.817%)	-	-	-
A 431		ARG	0.78	-	Favored (61.78%) General / -74.0,-29.1	Favored (92.3%) *mtm180* chi angles: 294,174.4,295.8,176.7	0.05Å	Favored (73.676%) alpha helix	-	-	-
A 432		PHE	0.78	-	Favored (74.03%) General / -60.6,-50.3	Favored (71.4%) *t80* chi angles: 170.6,80.2	0.11Å	Favored (76.452%) alpha helix	-	-	-
A 433		TRP	0.77	-	Favored (97.88%) General / -60.7,-43.3	Favored (53%) *t-100* chi angles: 189.5,246.8	0.07Å	Favored (93.505%) alpha helix	-	-	-
A 434		ALA	0.76	-	Favored (79.94%) General / -58.4,-39.5	-	0.04Å	Favored (91.302%) alpha helix	-	-	-
A 435		LEU	0.75	-	Favored (86.73%) General / -64.5,-37.2	Favored (90.3%) *mt* chi angles: 291,171.3	0.06Å	Favored (93.512%) alpha helix	-	-	-
A 436		VAL	0.74	-	Favored (96.18%) Ile or Val / -63.1,-46.4	Favored (54.7%) *t* chi angles: 170	0.05Å	Favored (92.62%) alpha helix	-	-	-
A 437		ASP	0.73	-	Favored (84.53%) General / -59.8,-39.3	Favored (86.6%) *m-30* chi angles: 284,344.8	0.06Å	Favored (92.224%) alpha helix	-	OUTLIER(S) worst is CA-CB-CG: 4.8 σ	-
A 438		ARG	0.73	-	Favored (83.75%)	Favored (98.9%) *mtt180*	0.03Å	Favored (88.338%)	-	-	-

#	Alt	Res	High B	Clash > 0.4Å	Ramachandran	Rotamer	Cβ deviation	CaBLAM	Bond lengths	Bond angles	Cis Peptides
					General / -65.8,-45.0	chi angles: 290.5,175.2,184.2,174.3		alpha helix			
A 439		GLU	0.72	-	Favored (75.88%) General / -67.7,-34.0	Favored (83.2%) *mm-30* chi angles: 290,299.3,312.1	0.10Å	Favored (78.467%) alpha helix	-	-	-
A 440		ARG	0.72	-	Favored (83.57%) General / -59.0,-47.9	Favored (49.9%) *ttm110* chi angles: 185.5,182.3,298.7,107.9	0.10Å	Favored (80.799%) alpha helix	-	-	-
**#**	**Alt**	**Res**	**High B**	**Clash > 0.4Å**	**Ramachandran**	**Rotamer**	**Cβ deviation**	**CaBLAM**	**Bond lengths**	**Bond angles**	**Cis Peptides**
			Avg: 0.95	Clashscore: 2.02	Outliers: 3 of 901	Poor rotamers: 0 of 767	Outliers: 0 of 820	Outliers: 14 of 899	Outliers: 10 of 903	Outliers: 16 of 903	Non-Trans: 2 of 902
A 441		GLU	0.73	-	Favored (91.69%) General / -59.7,-41.9	Favored (91.4%) *tt0* chi angles: 181.4,175.1,357.8	0.03Å	Favored (95.785%) alpha helix	-	-	-
A 442		HIS	0.76	-	Favored (99.31%) General / -63.1,-41.2	Favored (73.9%) *m-70* chi angles: 288.3,297.7	0.03Å	Favored (99.469%) alpha helix	-	-	-
A 443		HIS	0.79	-	Favored (98.51%) General / -63.1,-43.4	Favored (37.6%) *m170* chi angles: 286.7,192.1	0.08Å	Favored (98.114%) alpha helix	-	-	-
A 444		LEU	0.82	-	Favored (70.54%) General / -64.5,-29.5	Favored (96.8%) *mt* chi angles: 292.6,173.6	0.03Å	Favored (73.605%) alpha helix	-	-	-
A 445		ARG	0.85	-	Favored (53.67%) General / -87.2,1.2	Favored (97.7%) *mtt-85* chi angles: 290.9,182,181,275.6	0.05Å	Favored (46.364%)	-	-	-
A 446		GLY	0.88	-	Favored (18.8%) Glycine / 92.3,26.3	-	-	Favored (14.733%)	-	-	-
A 447		GLU	0.89	-	Favored (37.93%) General / -149.1,163.3	Favored (13.1%) *pt0* chi angles: 60.9,182,33.9	0.05Å	Favored (34.962%)	-	-	-
A 448		CYS	0.88	-	Favored (17.21%) General / -149.5,133.6	Favored (55.4%) *t* chi angles: 181.6	0.03Å	Favored (38.6%)	-	-	-
A 449		HIS	0.86	-	Favored (10.14%) General / -101.7,-32.8	Favored (19.9%) *m-70* chi angles: 296.8,242.9	0.06Å	Favored (19.42%)	-	-	-
A 450		SER	0.83	-	Favored (24.61%) General / -109.8,8.6	Favored (53.3%) *m* chi angles: 300.5	0.04Å	Favored (36.035%)	-	-	-
A 451		CYS	0.81	-	Favored (3.01%) General / -76.6,69.3	Favored (71.5%) *m* chi angles: 298	0.09Å	CaBLAM Disfavored (4.655%)	-	-	-
A 452		VAL	0.79	-	Favored (61.78%) Ile or Val / -123.0,135.8	Favored (97.6%) *t* chi angles: 175.2	0.06Å	Favored (28.322%)	-	-	-
A 453		TYR	0.79	0.58Å OH with A 605 GLN NE2	Favored (25.32%) General / -100.5,147.9	Favored (90.9%) *m-80* chi angles: 292.2,85.3	0.06Å	Favored (44.667%) beta sheet	-	-	-
A 454		ASN	0.81	-	Favored (15.52%)	Favored (41.6%) *t0* chi angles: 188.2,9.8	0.05Å	Favored (57.236%)	-	-	-

#	Alt	Res	High B	Clash > 0.4Å	Ramachandran	Rotamer	Cβ deviation	CaBLAM	Bond lengths	Bond angles	Cis Peptides
					General / -114.0,107.3			beta sheet			
A 455		MET	0.85	0.49Å C with A 455 MET SD	Favored (25.3%) General / -90.8,113.2	Allowed (1.6%) *tmt* chi angles: 176.2,275.2,170.2	0.05Å	Favored (47.162%) beta sheet	-	-	-
A 456		MET	0.91	-	Favored (23.47%) General / -122.9,161.8	Favored (76.3%) *mmm* chi angles: 306.9,295.5,289.3	0.02Å	Favored (24.439%) beta sheet	-	-	-
A 457		GLY	0.98	-	Favored (26.16%) Glycine / -81.7,147.0	-	-	Favored (25.907%) beta sheet	-	-	-
A 458		LYS	1.07	-	Favored (33.89%) General / -86.1,133.4	Favored (65.3%) *tttt* chi angles: 186,180.2,170.5,194.1	0.06Å	Favored (40.605%) beta sheet	-	-	-
A 459		ARG	1.16	-	Favored (5.34%) General / -79.1,70.0	Favored (94%) *mtt180* chi angles: 296,182.8,183.9,189.5	0.02Å	Favored (23.803%) beta sheet	-	-	-
A 460		GLU	1.26	-	Favored (24.43%) General / -81.7,157.8	Favored (69.2%) *mt-10* chi angles: 293.7,184.7,320.4	0.04Å	Favored (24.968%) beta sheet	-	-	-
#	Alt	Res	High B	Clash > 0.4Å	Ramachandran	Rotamer	Cβ deviation	CaBLAM	Bond lengths	Bond angles	Cis Peptides
			Avg: 0.95	Clashscore: 2.02	Outliers: 3 of 901	Poor rotamers: 0 of 767	Outliers: 0 of 820	Outliers: 14 of 899	Outliers: 10 of 903	Outliers: 16 of 903	Non-Trans: 2 of 902
A 461		LYS	1.37	-	Favored (27.06%) General / -111.8,114.2	Favored (11.1%) *mtmm* chi angles: 298.2,165.5,280.4,282.2	0.08Å	Favored (28.381%) beta sheet	-	-	-
A 462		LYS	1.5	-	Favored (43.21%) General / -131.3,157.5	Favored (98.6%) *mttt* chi angles: 294.5,185.2,180.5,178.9	0.05Å	Favored (37.861%) beta sheet	-	-	-
A 463		GLN	1.64	-	Favored (23.96%) General / -76.7,121.9	Favored (44.8%) *tt0* chi angles: 188.3,178.4,71.4	0.08Å	Favored (36.877%) beta sheet	-	-	-
A 464		GLY	1.78	-	Favored (86.87%) Glycine / -81.8,-8.3	-	-	CaBLAM Disfavored (4.059%)	-	-	-
A 465		GLU	1.9	-	Allowed (0.33%) General / 65.5,-55.8	Favored (97.6%) *mt-10* chi angles: 292.3,173.4,340	0.04Å	Favored (5.114%)	-	-	-
A 466		PHE	1.96	-	Favored (17.73%) General / -110.5,20.4	Favored (85.9%) *m-80* chi angles: 302.4,95	0.07Å	Favored (12.204%)	-	OUTLIER(S) worst is CA-CB-CG: 4.1 σ	-
A 467		GLY	1.93	-	Favored (88.47%) Glycine / 84.7,3.0	-	-	Favored (58.065%)	-	-	-
A 468		LYS	1.81	-	Favored (38.7%) General / -111.4,145.6	Favored (52.3%) *mttp* chi angles: 298.2,181.6,182.5,66.3	0.04Å	Favored (21.063%)	-	-	-
A 469		ALA	1.64	-	Favored (57.12%) General / -61.3,142.1	-	0.02Å	Favored (37.299%)	-	-	-
A 470		LYS	1.46	-	Favored (18.86%)	Favored (71.4%) *mmtt*	0.04Å	Favored (27.815%)	-	-	-

					General / -86.0,161.6	chi angles: 300.4,294.9,188.7,182.9					
A 471		GLY	1.29	-	Favored (44.6%) Glycine / -68.9,146.2	-	-	Favored (29.525%)	-	-	-
A 472		SER	1.17	-	Favored (43.23%) General / -71.4,152.3	Favored (66.2%) *m* chi angles: 294.4	0.12Å	Favored (51.254%) beta sheet	-	-	-
A 473		ARG	1.08	-	Favored (42.18%) General / -74.5,135.6	Favored (52.9%) *ttt180* chi angles: 182.8,169.6,177.1,158.5	0.05Å	Favored (28.647%) beta sheet	-	-	-
A 474		ALA	1.02	-	Favored (58.85%) General / -64.4,140.3	-	0.06Å	Favored (39.1%) beta sheet	-	-	-
A 475		ILE	0.98	-	Favored (54.4%) Ile or Val / -111.2,117.2	Favored (78.2%) *mt* chi angles: 300.7,172.9	0.13Å	Favored (67.907%) beta sheet	-	-	-
A 476		TRP	0.95	-	Favored (9.99%) General / -100.9,99.5	Favored (18%) *m100* chi angles: 299.6,61.9	0.08Å	Favored (71.674%) beta sheet	-	-	-
A 477		TYR	0.93	-	Favored (35.65%) General / -88.9,129.2	Favored (95.9%) *m-80* chi angles: 292.4,87.9	0.15Å	Favored (47.039%)	-	-	-
A 478		MET	0.91	0.41Å HE2 with A 453 TYR CZ	Favored (33.31%) General / -97.1,139.3	Favored (11.6%) *tpp* chi angles: 169.1,69.2,97.8	0.02Å	Favored (28.268%)	-	-	-
A 479		TRP	0.89	-	Favored (6.73%) General / -45.5,126.5	Favored (88.2%) *t60* chi angles: 180.4,84.2	0.11Å	Favored (46.815%)	-	-	-
A 480		LEU	0.87	-	Favored (44.49%) General / -50.9,-37.5	Favored (23%) *tp* chi angles: 189.9,64.8	0.05Å	Favored (32.657%)	-	-	-
**#**	**Alt**	**Res**	**High B**	**Clash > 0.4Å**	**Ramachandran**	**Rotamer**	**Cβ deviation**	**CaBLAM**	**Bond lengths**	**Bond angles**	**Cis Peptides**
			Avg: 0.95	Clashscore: 2.02	Outliers: 3 of 901	Poor rotamers: 0 of 767	Outliers: 0 of 820	Outliers: 14 of 899	Outliers: 10 of 903	Outliers: 16 of 903	Non-Trans: 2 of 902
A 481		GLY	0.84	-	Favored (82.78%) Glycine / -61.2,-33.0	-	-	Favored (90.315%) alpha helix	-	-	-
A 482		ALA	0.8	-	Favored (42.71%) General / -79.4,-31.4	-	0.07Å	Favored (74.973%) alpha helix	-	-	-
A 483		ARG	0.77	-	Favored (89.74%) General / -62.5,-38.4	Favored (78.3%) *mtp180* chi angles: 286.5,170.4,59.2,187.1	0.07Å	Favored (74.175%) alpha helix	-	-	-
A 484		PHE	0.74	0.45Å C with A 484 PHE CD2	Favored (77.45%) General / -58.0,-49.3	Favored (82.9%) *t80* chi angles: 173.6,81	0.13Å	Favored (89.589%) alpha helix	-	OUTLIER(S) worst is CA-CB-CG: 7.2 σ	-
A 485		LEU	0.71	-	Favored (79.97%) General / -64.6,-35.1	Favored (99.7%) *mt* chi angles: 292.6,172.6	0.08Å	Favored (81.814%) alpha helix	-	-	-

A 486		GLU	0.7	-	Favored (95.02%) General / -62.1,-40.2	Favored (98.1%) *mt-10* chi angles: 289.4,173.5,355.4	0.08Å	Favored (90.252%) alpha helix	-	-	-
A 487		PHE	0.69	-	Favored (66.74%) General / -63.9,-51.0	Favored (89.6%) *t80* chi angles: 179.7,79	0.05Å	Favored (79.057%) alpha helix	-	-	-
A 488		GLU	0.7	-	Favored (65.03%) General / -52.2,-45.2	Favored (93%) *tt0* chi angles: 180.1,177.8,357.3	0.04Å	Favored (66.8%) alpha helix	-	-	-
A 489		ALA	0.72	-	Favored (15.07%) General / -82.0,-45.6	-	0.08Å	Favored (31.6%) alpha helix	-	-	-
A 490		LEU	0.76	-	Favored (11.73%) General / -114.0,-18.3	Favored (84.5%) *mt* chi angles: 300.3,176.2	0.02Å	Favored (8.64%) three-ten	-	-	-
A 491		GLY	0.8	-	Favored (37.47%) Glycine / -55.6,-23.8	-	-	Favored (38.132%) three-ten	-	-	-
A 492		PHE	0.85	-	Favored (62.65%) General / -58.9,-24.6	Favored (39%) *p90* chi angles: 72.3,91.4	0.06Å	Favored (62.202%) three-ten	-	-	-
A 493		LEU	0.89	-	Favored (86.56%) General / -62.5,-37.6	Favored (95%) *mt* chi angles: 292.1,173.4	0.09Å	Favored (57.084%) three-ten	-	-	-
A 494		ASN	0.93	-	Favored (16.65%) General / -78.1,-48.2	Favored (98.3%) *m-40* chi angles: 290.9,340.2	0.07Å	Favored (56.602%) alpha helix	-	-	-
A 495		GLU	0.95	-	Favored (85.51%) General / -64.1,-36.9	Favored (72.8%) *mm-30* chi angles: 292.8,296.1,304.6	0.02Å	Favored (66.615%) alpha helix	-	-	-
A 496		ASP	0.96	-	Favored (57.37%) General / -92.3,-0.9	Favored (87.8%) *m-30* chi angles: 291.7,335.3	0.04Å	Favored (42.519%)	-	-	-
A 497		HIS	0.95	-	Favored (9.44%) General / 51.3,55.5	Favored (99.2%) *m-70* chi angles: 298.5,291.4	0.05Å	Favored (19.005%)	-	-	-
A 498		TRP	0.93	0.56Å CD1 with A 498 TRP N	Favored (64.98%) General / -60.8,-23.4	Allowed (1.3%) *p-90* chi angles: 63.6,316.3	0.04Å	Favored (42.98%)	-	-	-
A 499		MET	0.91	-	Favored (46.58%) General / -91.7,6.3	Favored (37.1%) *mmp* chi angles: 289.1,290.1,99.9	0.08Å	Favored (37.435%)	-	-	-
A 500		GLY	0.89	-	Favored (49.21%) Glycine / -64.3,152.8	-	-	Favored (53.477%)	-	-	-
**#**	**Alt**	**Res**	**High B**	**Clash > 0.4Å**	**Ramachandran**	**Rotamer**	**Cβ deviation**	**CaBLAM**	**Bond lengths**	**Bond angles**	**Cis Peptides**
			Avg: 0.95	Clashscore: 2.02	Outliers: 3 of 901	Poor rotamers: 0 of 767	Outliers: 0 of 820	Outliers: 14 of 899	Outliers: 10 of 903	Outliers: 16 of 903	Non-Trans: 2 of 902
A 501		ARG	0.87	-	Favored (71.44%) General / -58.6,-34.6	Favored (54.6%) *ttt90* chi angles: 187.7,170,179.2,79.2	0.04Å	Favored (54.609%)	-	-	-

A 502		GLU	0.85	-	Favored (62.44%) General / -59.4,-23.3	Favored (26.7%) *pt0* chi angles: 69,181.5,357.5	0.04Å	Favored (32.511%) alpha helix	-	-	-
A 503		ASN	0.82	-	Favored (3.35%) General / -111.4,-45.8	Favored (51.4%) *t0* chi angles: 185.7,53.1	0.03Å	Favored (26.689%) alpha helix	-	-	-
A 504		SER	0.79	-	Favored (48.2%) General / -87.6,-11.6	Favored (87.7%) *p* chi angles: 67	0.05Å	Favored (13.541%) alpha helix	-	-	-
A 505		GLY	0.77	-	Favored (4.89%) Glycine / 81.9,-59.1	-	-	Favored (5.007%) alpha helix	-	-	-
A 506		GLY	0.76	-	Favored (32.32%) Glycine / -102.6,-12.6	-	-	Favored (33.725%)	-	-	-
A 507		GLY	0.76	-	Favored (32.68%) Glycine / -81.5,152.8	-	-	Favored (17.061%)	-	-	-
A 508		VAL	0.78	-	Favored (5.87%) Ile or Val / -121.9,12.6	Favored (27.1%) *m* chi angles: 295.9	0.04Å	CaBLAM Disfavored (3.86%)	-	-	-
A 509		GLU	0.81	-	Favored (55.77%) General / -57.8,133.9	Favored (90%) *tt0* chi angles: 185.6,177.3,4.8	0.04Å	Favored (12.846%)	-	-	-
A 510		GLY	0.84	-	Favored (65.45%) Glycine / 97.2,-11.8	-	-	Favored (83.594%)	-	-	-
A 511		LEU	0.86	-	Favored (27.3%) General / -82.6,149.7	Favored (85.7%) *mt* chi angles: 297.6,180	0.05Å	Favored (41.717%)	-	-	-
A 512		GLY	0.87	-	Favored (46.2%) Glycine / -80.2,162.4	-	-	Favored (57.332%)	-	-	-
A 513		LEU	0.86	-	Favored (76.02%) General / -61.6,-34.6	Favored (94.2%) *mt* chi angles: 291.9,171.2	0.06Å	Favored (57.562%)	-	-	-
A 514		GLN	0.83	-	Favored (63.39%) General / -65.0,-16.4	Favored (22.5%) *pm20* chi angles: 67.4,269.9,28.9	0.02Å	Favored (45.319%) alpha helix	-	-	-
A 515		ARG	0.8	-	Favored (11.29%) General / -105.4,-27.7	Favored (92.1%) *mtt180* chi angles: 292.8,187,179.2,188.6	0.05Å	Favored (16.405%) alpha helix	-	-	-
A 516		LEU	0.77	-	Favored (74.9%) General / -60.5,-35.0	Favored (77.5%) *mt* chi angles: 291.2,176.7	0.07Å	Favored (64.575%) alpha helix	-	-	-
A 517		GLY	0.74	-	Favored (78.08%) Glycine / -58.9,-34.4	-	-	Favored (89.51%) alpha helix	-	-	-
A 518		TYR	0.72	-	Favored (43.05%) General / -79.1,-27.7	Favored (79.4%) *m-80* chi angles: 289.7,84.2	0.03Å	Favored (77.293%) alpha helix	-	-	-
A 519		ILE	0.7	-	Favored (85.4%)	Favored (37.9%) *mm*	0.03Å	Favored (76.986%)	-	-	-

#	Alt	Res	High B	Clash > 0.4Å	Ramachandran	Rotamer	Cβ deviation	CaBLAM	Bond lengths	Bond angles	Cis Peptides
					Ile or Val / -67.5,-41.0	chi angles: 297.9,304.1		alpha helix			
A 520		LEU	0.69	-	Favored (92.3%) General / -65.6,-40.1	Favored (5.9%) *mp* chi angles: 268,66	0.09Å	Favored (97.227%) alpha helix	-	-	-
**#**	**Alt**	**Res**	**High B**	**Clash > 0.4Å**	**Ramachandran**	**Rotamer**	**Cβ deviation**	**CaBLAM**	**Bond lengths**	**Bond angles**	**Cis Peptides**
			Avg: 0.95	Clashscore: 2.02	Outliers: 3 of 901	Poor rotamers: 0 of 767	Outliers: 0 of 820	Outliers: 14 of 899	Outliers: 10 of 903	Outliers: 16 of 903	Non-Trans: 2 of 902
A 521		GLU	0.7	-	Favored (98.34%) General / -61.5,-42.1	Favored (90.7%) *tt0* chi angles: 185.3,176.4,3.1	0.04Å	Favored (99.141%) alpha helix	-	-	-
A 522		GLU	0.71	-	Favored (74.52%) General / -59.7,-50.3	Favored (37.6%) *tt0* chi angles: 177.8,181.1,308.3	0.05Å	Favored (88.311%) alpha helix	-	-	-
A 523		MET	0.72	-	Favored (77.63%) General / -58.0,-39.2	Favored (20.3%) *ttp* chi angles: 173.7,171.6,49.4	0.08Å	Favored (73.503%) alpha helix	-	-	-
A 524		ASN	0.72	-	Favored (63.52%) General / -59.4,-24.5	Favored (30.6%) *t0* chi angles: 193.8,62.8	0.08Å	Favored (66.427%) alpha helix	-	-	-
A 525		ARG	0.73	-	Favored (58.49%) General / -89.5,-2.8	Favored (98.1%) *mtt-85* chi angles: 293.5,177.2,183.7,273.3	0.04Å	Favored (54.895%)	-	-	-
A 526		ALA	0.72	-	Favored (92.14%) Pre-Pro / -70.9,145.8	-	0.05Å	Favored (32.188%)	-	-	-
A 527		PRO	0.7	-	Favored (73.16%) Trans-Pro / -55.1,136.4	Favored (97.3%) *Cg_exo* chi angles: 331.8,35.2,333.2	0.05Å	CaBLAM Disfavored (2.835%)	-	-	-
A 528		GLY	0.68	-	Favored (31.93%) Glycine / 152.9,-170.2	-	-	Favored (41.284%)	-	-	-
A 529		GLY	0.66	-	Favored (25.18%) Glycine / -96.5,-149.1	-	-	CaBLAM Outlier (0.811%)	-	-	-
A 530		LYS	0.65	0.43Å NZ with A 674 ASP OD2	Favored (45.93%) General / -64.5,150.8	Favored (51.9%) *mttp* chi angles: 293.6,180.6,183.4,69.3	0.04Å	Favored (6.395%)	-	-	-
A 531		MET	0.64	-	Favored (32.67%) General / -90.8,137.0	Favored (62.2%) *ttm* chi angles: 183.9,177.5,288.5	0.06Å	Favored (46.766%)	-	-	-
A 532		TYR	0.65	-	Favored (30.95%) General / -118.0,155.0	Favored (85.3%) *m-80* chi angles: 295.5,83.2	0.05Å	Favored (58.603%) beta sheet	-	-	-
A 533		ALA	0.67	-	Favored (14.6%) General / -143.9,125.2	-	0.06Å	Favored (48.602%) beta sheet	-	-	-
A 534		ASP	0.71	-	Favored (26.36%) General / -113.4,154.8	Favored (93.9%) *m-30* chi angles: 292.6,341	0.15Å	Favored (34.172%) beta sheet	-	-	-

A 535		ASP	0.75	-	Favored (3.22%) General / -150.6,104.5	Favored (68.8%) *t0* chi angles: 182.9,351	0.13Å	Favored (11.137%) beta sheet	-	OUTLIER(S) worst is CA-CB-CG: 7.3 σ	-
A 536		THR	0.79	-	Favored (35.82%) General / -80.0,131.5	Favored (91.5%) *m* chi angles: 297.9	0.10Å	Favored (38.805%)	-	-	-
A 537		ALA	0.81	-	Favored (84.76%) General / -60.3,-38.8	-	0.06Å	Favored (17.449%)	-	-	-
A 538		GLY	0.83	0.42Å O with A 539 TRP C	Allowed (1.31%) Glycine / -139.2,41.8	-	-	CaBLAM Disfavored (2.625%)	-	-	-
A 539		TRP	0.82	0.42Å C with A 538 GLY O	Favored (8.37%) General / -44.4,-51.8	Favored (44.6%) *t-100* chi angles: 192.1,267.3	0.06Å	Favored (34.057%)	-	-	-
A 540		ASP	0.8	-	Favored (61.67%) General / -58.1,-25.8	Favored (97.2%) *m-30* chi angles: 289.1,343.3	0.06Å	Favored (53.607%) alpha helix	-	-	-
**#**	**Alt**	**Res**	**High B**	**Clash > 0.4Å**	**Ramachandran**	**Rotamer**	**Cβ deviation**	**CaBLAM**	**Bond lengths**	**Bond angles**	**Cis Peptides**
			Avg: 0.95	Clashscore: 2.02	Outliers: 3 of 901	Poor rotamers: 0 of 767	Outliers: 0 of 820	Outliers: 14 of 899	Outliers: 10 of 903	Outliers: 16 of 903	Non-Trans: 2 of 902
A 541		THR	0.78	-	Favored (21.47%) General / -98.8,-11.9	Favored (49.3%) *p* chi angles: 65.8	0.09Å	Favored (61.415%)	-	-	-
A 542		ARG	0.76	-	Favored (21.77%) General / -102.2,18.1	Favored (96.8%) *mtt-85* chi angles: 290.9,176.9,183.6,279.4	0.09Å	Favored (15.972%)	-	-	-
A 543		ILE	0.74	-	Favored (27.57%) Ile or Val / -89.7,114.4	Favored (49.3%) *mm* chi angles: 304.4,302.6	0.07Å	Favored (27.152%)	-	-	-
A 544		SER	0.74	-	Favored (17.18%) General / -79.9,169.9	Favored (96.1%) *p* chi angles: 66	0.05Å	Favored (21.719%)	-	-	-
A 545		LYS	0.74	-	Favored (79.69%) General / -61.9,-35.9	Favored (95.6%) *mttt* chi angles: 289.9,176.6,183.7,173.3	0.10Å	Favored (59.323%)	-	-	-
A 546		PHE	0.74	-	Favored (67.69%) General / -67.7,-28.5	Favored (24.1%) *m-10* chi angles: 295.4,342.5	0.07Å	Favored (73.97%) alpha helix	-	-	-
A 547		ASP	0.75	-	Favored (59.01%) General / -76.0,-37.0	Favored (29.6%) *m-30* chi angles: 289.5,294.5	0.10Å	Favored (84.544%) alpha helix	-	OUTLIER(S) worst is CA-CB-CG: 4.8 σ	-
A 548		LEU	0.77	-	Favored (88.84%) General / -66.0,-38.6	Favored (94.5%) *mt* chi angles: 291.8,172.9	0.01Å	Favored (94.691%) alpha helix	-	-	-
A 549		GLU	0.79	-	Favored (87.68%) General / -62.4,-38.0	Favored (97.6%) *mt-10* chi angles: 289.2,178,354.5	0.05Å	Favored (89.631%) alpha helix	-	-	-

A 550		ASN	0.82	-	Favored (69.87%) General / -71.4,-33.9	Favored (13.9%) *m-40* chi angles: 287.3,17.7	0.10Å	Favored (92.612%) alpha helix	-	-	-
A 551		GLU	0.85	-	Favored (77.11%) General / -64.4,-34.0	Favored (65.1%) *mm-30* chi angles: 288,295.1,343.6	0.07Å	Favored (67.511%) alpha helix	-	-	-
A 552		ALA	0.88	-	Favored (58.32%) General / -61.7,-18.1	-	0.03Å	Favored (56.77%) three-ten	-	-	-
A 553		LEU	0.9	-	Favored (66.32%) General / -61.6,-23.9	Favored (95.1%) *mt* chi angles: 291.9,171.6	0.03Å	Favored (55.293%) three-ten	-	-	-
A 554		ILE	0.93	-	Favored (32.76%) Ile or Val / -61.0,-24.4	Favored (15.9%) *tt* chi angles: 196,171.3	0.10Å	Favored (63.607%) three-ten	-	-	-
A 555		THR	0.97	-	Favored (61.87%) General / -72.0,-14.3	Favored (69%) *p* chi angles: 62.6	0.10Å	Favored (65.979%) three-ten	-	-	-
A 556		ASN	1	-	Favored (50.56%) General / -86.7,1.9	Favored (90.7%) *m-40* chi angles: 288.9,329	0.07Å	Favored (52.368%) three-ten	-	-	-
A 557		GLN	1.04	-	Favored (36.59%) General / -101.0,-0.4	Favored (92.2%) *mt0* chi angles: 296.8,180.3,324.8	0.03Å	Favored (55.68%)	-	-	-
A 558		MET	1.07	-	Favored (14.57%) General / -100.0,160.1	Favored (69.1%) *mtt* chi angles: 295.7,177.2,181.7	0.05Å	Favored (23.33%)	-	-	-
A 559		GLU	1.07	-	Favored (39.6%) General / -68.4,155.5	Favored (98.2%) *mt-10* chi angles: 294.3,180.5,356.3	0.05Å	Favored (47.115%)	-	-	-
A 560		GLU	1.05	-	Favored (74.83%) General / -58.4,-37.2	Favored (98.5%) *mt-10* chi angles: 290.1,176.6,352.6	0.01Å	Favored (54.595%)	-	-	-
**#**	**Alt**	**Res**	**High B**	**Clash > 0.4Å**	**Ramachandran**	**Rotamer**	**Cβ deviation**	**CaBLAM**	**Bond lengths**	**Bond angles**	**Cis Peptides**
			Avg: 0.95	Clashscore: 2.02	Outliers: 3 of 901	Poor rotamers: 0 of 767	Outliers: 0 of 820	Outliers: 14 of 899	Outliers: 10 of 903	Outliers: 16 of 903	Non-Trans: 2 of 902
A 561		GLY	0.99	-	Favored (38.22%) Glycine / -62.6,-53.9	-	-	Favored (81%) alpha helix	-	-	-
A 562		HIS	0.92	-	Favored (87.85%) General / -59.8,-46.9	Favored (17%) *t-170* chi angles: 182,183.4	0.05Å	Favored (75.175%) alpha helix	-	-	-
A 563		ARG	0.85	-	Favored (78.75%) General / -55.9,-45.0	Favored (73%) *ttt-90* chi angles: 180.6,176.1,183.1,273.6	0.06Å	Favored (79.765%) alpha helix	-	-	-
A 564		THR	0.79	-	Favored (93.31%) General / -61.6,-45.6	Favored (97.9%) *m* chi angles: 300	0.03Å	Favored (87.956%) alpha helix	-	-	-
A 565		LEU	0.75	-	Favored (84.9%) General / -65.0,-45.6	Favored (51.2%) *tp* chi angles: 183.7,61.7	0.04Å	Favored (85.603%) alpha helix	-	-	-

A 566		ALA	0.73	-	Favored (87.51%) General / -60.8,-39.0	-	0.03Å	Favored (83.583%) alpha helix	-	-	-
A 567		LEU	0.72	-	Favored (97.52%) General / -64.0,-41.4	Favored (78.4%) *mt* chi angles: 288.2,168.9	0.03Å	Favored (98.172%) alpha helix	-	-	-
A 568		ALA	0.71	-	Favored (97.84%) General / -63.0,-40.5	-	0.03Å	Favored (99.182%) alpha helix	-	-	-
A 569		VAL	0.72	-	Favored (95.51%) Ile or Val / -64.2,-45.7	Favored (59.1%) *t* chi angles: 170.7	0.04Å	Favored (84.473%) alpha helix	-	-	-
A 570		ILE	0.73	-	Favored (73.92%) Ile or Val / -65.5,-50.1	Favored (97.1%) *mt* chi angles: 293.8,168.2	0.09Å	Favored (73.213%) alpha helix	-	-	-
A 571		LYS	0.73	-	Favored (99.3%) General / -61.3,-43.2	Favored (86.3%) *tttt* chi angles: 180.5,173.9,175.5,178.9	0.05Å	Favored (60.778%) alpha helix	-	-	-
A 572		TYR	0.74	-	Favored (19.37%) General / -91.5,-25.1	Favored (61.3%) *m-80* chi angles: 294.5,78.4	0.08Å	Favored (47.547%) alpha helix	-	-	-
A 573		THR	0.75	-	Favored (7.06%) General / -111.9,-31.0	Favored (55.6%) *p* chi angles: 64.8	0.03Å	Favored (34.216%) alpha helix	-	-	-
A 574		TYR	0.75	-	Favored (12.76%) General / -95.4,-32.8	Favored (77.4%) *m-80* chi angles: 290.1,82.3	0.09Å	Favored (54.935%) alpha helix	-	-	-
A 575		GLN	0.74	-	Allowed (0.69%) General / -95.7,-70.9	Favored (56.9%) *tt0* chi angles: 182.2,179.5,330.8	0.01Å	Favored (15.22%) alpha helix	-	-	-
A 576		ASN	0.73	-	Favored (8.97%) General / -82.1,77.0	Favored (64.6%) *m-40* chi angles: 283.4,282.2	0.07Å	Favored (26.997%)	-	-	-
A 577		LYS	0.72	-	Favored (36.56%) General / -90.4,129.8	Allowed (0.4%) *ttmp* chi angles: 176.5,179.9,262.1,74.9	0.04Å	Favored (25.467%)	-	-	-
A 578		VAL	0.71	-	Favored (67.74%) Ile or Val / -110.9,124.5	Favored (71.1%) *t* chi angles: 178.6	0.06Å	Favored (68.445%) beta sheet	-	-	-
A 579		VAL	0.71	-	Favored (42.33%) Ile or Val / -131.9,142.7	Favored (9.8%) *p* chi angles: 62.1	0.03Å	Favored (62.089%) beta sheet	-	-	-
A 580		LYS	0.73	-	Favored (48.7%) General / -101.9,126.0	Favored (48.9%) *mtpt* chi angles: 298.6,175.4,69.8,180.8	0.10Å	Favored (66.899%) beta sheet	-	-	-
**#**	**Alt**	**Res**	**High B**	**Clash > 0.4Å**	**Ramachandran**	**Rotamer**	**Cβ deviation**	**CaBLAM**	**Bond lengths**	**Bond angles**	**Cis Peptides**
			Avg: 0.95	Clashscore: 2.02	Outliers: 3 of 901	Poor rotamers: 0 of 767	Outliers: 0 of 820	Outliers: 14 of 899	Outliers: 10 of 903	Outliers: 16 of 903	Non-Trans: 2 of 902

A 581		VAL	0.77	-	Favored (67.05%) Ile or Val / -126.8,134.1	Favored (24.7%) *t* chi angles: 186.2	0.05Å	Favored (58.632%) beta sheet	-	-	-
A 582		LEU	0.84	-	Favored (35.7%) General / -80.3,131.9	Favored (32.7%) *tp* chi angles: 186.1,65.8	0.04Å	Favored (43.087%) beta sheet	-	-	-
A 583		ARG	0.94	-	Favored (66.9%) Pre-Pro / -141.4,156.6	Favored (47.6%) *ptt90* chi angles: 68.9,175.3,171.8,93	0.13Å	Favored (41.614%) beta sheet	-	-	-
A 584		PRO	1.09	-	Favored (73.4%) Trans-Pro / -63.2,153.5	Favored (52.3%) *Cg_exo* chi angles: 336.8,34.5,329.1	0.10Å	Favored (60.018%)	-	-	-
A 585		ALA	1.24	-	Favored (38.27%) General / -146.4,162.4	-	0.04Å	Favored (11.399%)	-	-	-
A 586		GLU	1.37	-	Favored (36.99%) General / -54.5,129.7	Favored (21.1%) *tp30* chi angles: 182.7,66.5,49.7	0.06Å	Favored (9.153%)	-	-	-
A 587		GLY	1.43	-	Favored (28.75%) Glycine / 111.2,-12.2	-	-	Favored (19.577%)	-	-	-
A 588		GLY	1.4	-	Favored (69.07%) Glycine / 89.0,8.4	-	-	Favored (71.552%)	-	-	-
A 589		LYS	1.29	-	Favored (11.77%) General / -89.5,169.0	Favored (61%) *pttt* chi angles: 67.1,184.7,184,183.8	0.04Å	Favored (41.964%)	-	-	-
A 590		THR	1.14	-	Favored (44.12%) General / -118.9,147.3	Favored (42%) *p* chi angles: 54.9	0.07Å	Favored (48.873%) beta sheet	-	-	-
A 591		VAL	1	-	Favored (39.77%) Ile or Val / -135.2,161.5	Favored (20.1%) *m* chi angles: 302.4	0.10Å	Favored (49.057%) beta sheet	-	-	-
A 592		MET	0.9	-	Favored (33.72%) General / -101.3,141.7	Favored (95.8%) *mmm* chi angles: 299.6,297.1,292.6	0.12Å	Favored (49.172%) beta sheet	-	-	-
A 593		ASP	0.83	-	Favored (51.17%) General / -107.1,133.8	Favored (93.9%) *m-30* chi angles: 287.1,341.4	0.08Å	Favored (61.649%) beta sheet	-	-	-
A 594		ILE	0.8	0.41Å N with A 594 ILE HD12	Favored (51.09%) Ile or Val / -102.8,119.3	Allowed (1.8%) *mp* chi angles: 297.1,82.4	0.08Å	Favored (40.025%) beta sheet	-	-	-
A 595		ILE	0.8	-	Favored (23.44%) Ile or Val / -135.7,168.2	Favored (42.5%) *pt* chi angles: 61.2,174.4	0.08Å	Favored (25.985%) beta sheet	OUTLIER(S) worst is CB--CG1: 6.1 σ	-	-
A 596		SER	0.8	-	Favored (44.49%) General / -151.2,159.3	Favored (89.4%) *p* chi angles: 66.8	0.02Å	Favored (56.812%) beta sheet	-	-	-
A 597		ARG	0.81	-	Favored (17.5%) General / -161.0,151.1	Favored (18.4%) *ttp-170* chi angles: 207.9,175.1,73.4,200	0.07Å	Favored (41.675%)	-	-	-
A 598		GLN	0.83	-	Favored (39.17%)	Favored (68.7%) *mt0*	0.06Å	Favored (7.743%)	-	-	-

#	Alt	Res	High B	Clash > 0.4Å	Ramachandran	Rotamer	Cβ deviation	CaBLAM	Bond lengths	Bond angles	Cis Peptides
					General / -91.9,8.0	chi angles: 295.2,182.7,68.9					
A 599		ASP	0.85	0.45Å N with A 599 ASP OD1	Allowed (1.39%) General / -143.4,61.6	Favored (44.1%) *p0* chi angles: 58,356.2	0.12Å	CaBLAM Disfavored (4.797%)	-	-	-
A 600		GLN	0.87	-	Favored (11.77%) General / -150.3,128.2	Favored (49.6%) *tp40* chi angles: 174.6,75.5,52.6	0.05Å	Favored (7.092%)	-	-	-
**#**	**Alt**	**Res**	**High B**	**Clash > 0.4Å**	**Ramachandran**	**Rotamer**	**Cβ deviation**	**CaBLAM**	**Bond lengths**	**Bond angles**	**Cis Peptides**
			Avg: 0.95	Clashscore: 2.02	Outliers: 3 of 901	Poor rotamers: 0 of 767	Outliers: 0 of 820	Outliers: 14 of 899	Outliers: 10 of 903	Outliers: 16 of 903	Non-Trans: 2 of 902
A 601		ARG	0.89	-	Favored (25.53%) General / -94.5,144.5	Favored (89.5%) *mtt180* chi angles: 293.2,178.4,185.3,159.9	0.04Å	Favored (41.436%) beta sheet	-	-	-
A 602		GLY	0.91	-	Favored (13.62%) Glycine / -91.6,137.1	-	-	Favored (37.948%)	-	-	-
A 603		SER	0.93	-	Favored (65.86%) General / -66.5,-19.7	Favored (85.8%) *p* chi angles: 67.8	0.05Å	Favored (36.917%)	-	-	-
A 604		GLY	0.94	-	Favored (67.36%) Glycine / -95.7,6.4	-	-	Favored (61.311%)	-	-	-
A 605		GLN	0.94	0.58Å NE2 with A 453 TYR OH	Favored (41.72%) General / -74.0,148.8	Favored (47.4%) *mt0* chi angles: 292.4,184.4,269.3	0.09Å	Favored (41.033%)	-	-	-
A 606		VAL	0.93	-	Favored (52.04%) Ile or Val / -54.0,-41.9	Favored (73.6%) *t* chi angles: 172.5	0.02Å	Favored (14.816%)	-	-	-
A 607		VAL	0.91	-	Favored (6.21%) Ile or Val / -113.7,21.4	Favored (22.3%) *m* chi angles: 294.7	0.01Å	Favored (17.206%)	-	-	-
A 608		THR	0.89	-	Favored (90.7%) General / -59.6,-45.9	Favored (49.5%) *m* chi angles: 294.5	0.09Å	Favored (45.143%) alpha helix	-	-	-
A 609		TYR	0.86	-	Favored (89.73%) General / -62.3,-38.5	Favored (35.8%) *m-80* chi angles: 286.6,119.9	0.03Å	Favored (89.095%) alpha helix	-	-	-
A 610		ALA	0.82	0.42Å HB1 with A 498 TRP HE1	Favored (96.29%) General / -64.0,-43.2	-	0.06Å	Favored (93.365%) alpha helix	-	-	-
A 611		LEU	0.79	-	Favored (79.55%) General / -68.3,-36.6	Favored (93.1%) *mt* chi angles: 294.8,176	0.04Å	Favored (88.934%) alpha helix	-	-	-
A 612		ASN	0.76	-	Favored (94.1%) General / -63.0,-39.2	Favored (91.9%) *m-40* chi angles: 288.9,347.9	0.03Å	Favored (87.87%) alpha helix	-	-	-
A 613		THR	0.73	-	Favored (83.41%) General / -63.6,-46.9	Favored (82.6%) *m* chi angles: 302.2	0.07Å	Favored (95.798%) alpha helix	-	-	-
A 614		PHE	0.7	-	Favored (71.78%)	Favored (71.5%) *t80*	0.07Å	Favored (95.499%)	-	-	-

					General / -59.4,-51.0	chi angles: 178.1,88.7		alpha helix			
A 615		THR	0.68	-	Favored (88.5%) General / -59.2,-41.3	Favored (91%) *m* chi angles: 297.2	0.15Å	Favored (86.007%) alpha helix	-	-	-
A 616		ASN	0.67	-	Favored (78.98%) General / -63.3,-35.0	Favored (51.8%) *m-40* chi angles: 280.6,277	0.06Å	Favored (84.754%) alpha helix	-	OUTLIER(S) worst is CA-CB-CG: 4.7 σ	-
A 617		LEU	0.66	-	Favored (93.93%) General / -62.7,-39.4	Favored (80%) *mt* chi angles: 288.7,169.5	0.01Å	Favored (92.226%) alpha helix	-	-	-
A 618		VAL	0.64	-	Favored (93.31%) Ile or Val / -64.5,-46.2	Favored (64.9%) *t* chi angles: 171.5	0.05Å	Favored (91.37%) alpha helix	-	-	-
A 619		VAL	0.63	-	Favored (98.73%) Ile or Val / -62.6,-43.7	Favored (57.6%) *t* chi angles: 170.5	0.05Å	Favored (96.51%) alpha helix	-	-	-
A 620		GLN	0.63	-	Favored (79.75%) General / -67.1,-35.4	Favored (20.5%) *mm110* chi angles: 297.1,300.1,87.7	0.05Å	Favored (86.345%) alpha helix	-	-	-
**#**	**Alt**	**Res**	**High B**	**Clash > 0.4Å**	**Ramachandran**	**Rotamer**	**Cβ deviation**	**CaBLAM**	**Bond lengths**	**Bond angles**	**Cis Peptides**
			Avg: 0.95	Clashscore: 2.02	Outliers: 3 of 901	Poor rotamers: 0 of 767	Outliers: 0 of 820	Outliers: 14 of 899	Outliers: 10 of 903	Outliers: 16 of 903	Non-Trans: 2 of 902
A 621		LEU	0.62	-	Favored (88.25%) General / -62.4,-38.1	Favored (68.2%) *mt* chi angles: 286.7,170.1	0.03Å	Favored (89.468%) alpha helix	-	-	-
A 622		ILE	0.62	-	Favored (76.94%) Ile or Val / -71.0,-41.5	Favored (89.3%) *mt* chi angles: 296.6,166.7	0.11Å	Favored (91.014%) alpha helix	-	-	-
A 623		ARG	0.62	-	Favored (73.42%) General / -60.2,-34.3	Favored (84.1%) *mtp180* chi angles: 290.2,172.8,67.9,193	0.03Å	Favored (83.113%) alpha helix	-	OUTLIER(S) worst is NE-CZ-NH2: 4.1 σ	-
A 624		ASN	0.62	-	Favored (81.3%) General / -68.0,-37.3	Favored (60.6%) *m-40* chi angles: 283,278.1	0.06Å	Favored (88.26%) alpha helix	-	-	-
A 625		MET	0.62	-	Favored (77.99%) General / -65.1,-34.3	Favored (75.9%) *mtm* chi angles: 286.3,190.1,284.4	0.06Å	Favored (82.657%) alpha helix	-	-	-
A 626		GLU	0.63	-	Favored (76.75%) General / -63.9,-48.4	Favored (77.9%) *tt0* chi angles: 178.8,180.2,342.5	0.03Å	Favored (73.574%) alpha helix	-	-	-
A 627		ALA	0.63	-	Favored (75.12%) General / -59.5,-36.0	-	0.08Å	Favored (74.369%) alpha helix	-	-	-
A 628		GLU	0.63	-	Favored (34%) General / -89.7,8.0	Favored (55.8%) *mt-10* chi angles: 294.1,178.2,40.4	0.04Å	Favored (47.681%)	-	-	-
A 629		GLU	0.64	-	Favored (16.65%) General / 61.1,38.8	Favored (92.4%) *mt-10* chi angles: 299.5,183.2,356.5	0.05Å	Favored (16.143%)	-	-	-

A 630		VAL	0.65	-	Favored (14.38%) Ile or Val / -91.4,-47.2	Favored (82.1%) *t* chi angles: 176.7	0.07Å	CaBLAM Disfavored (1.109%)	-	-	-
A 631		LEU	0.66	-	Favored (5.97%) General / -118.6,97.9	Favored (82.8%) *mt* chi angles: 295.1,169.5	0.02Å	Favored (26.332%)	-	-	-
A 632		GLU	0.68	-	Favored (16.29%) General / -81.7,170.0	Favored (95.4%) *mt-10* chi angles: 295.5,182.1,349.2	0.02Å	Favored (16.347%)	-	-	-
A 633		MET	0.69	-	Favored (70.08%) General / -61.9,-29.3	Favored (95.1%) *mmm* chi angles: 290.8,303.8,292	0.04Å	Favored (57.47%)	-	-	-
A 634		GLN	0.7	-	Favored (82.69%) General / -64.3,-36.0	Favored (96.8%) *mt0* chi angles: 291.1,178.8,338.2	0.02Å	Favored (76.829%) alpha helix	-	-	-
A 635		ASP	0.71	-	Favored (46.16%) General / -78.7,-34.5	Favored (91.7%) *m-30* chi angles: 293.4,342.8	0.07Å	Favored (78.764%) alpha helix	-	-	-
A 636		LEU	0.71	-	Favored (70.63%) General / -65.8,-30.0	Favored (81.5%) *mt* chi angles: 291.3,175.7	0.10Å	Favored (75.806%) alpha helix	-	-	-
A 637		TRP	0.7	-	Favored (15.08%) General / -70.9,-53.4	Favored (18.7%) *t60* chi angles: 175.3,30.4	0.02Å	Favored (18.131%) alpha helix	-	-	-
A 638		LEU	0.69	-	Favored (11.99%) General / -110.4,-21.8	Favored (83.6%) *mt* chi angles: 301,176.9	0.06Å	CaBLAM Disfavored (4.754%)	-	-	-
A 639		LEU	0.69	0.46Å HG with A 639 LEU O	Favored (2.09%) General / 50.9,63.8	Favored (54.9%) *tp* chi angles: 182.5,58.9	0.14Å	Favored (8.715%)	-	-	-
A 640		ARG	0.68	-	Favored (64.64%) General / -68.3,-19.1	Favored (90.9%) *mtm180* chi angles: 295,175.1,297.3,178.2	0.09Å	Favored (25.007%)	-	-	-
**#**	**Alt**	**Res**	**High B**	**Clash > 0.4Å**	**Ramachandran**	**Rotamer**	**Cβ deviation**	**CaBLAM**	**Bond lengths**	**Bond angles**	**Cis Peptides**
			Avg: 0.95	Clashscore: 2.02	Outliers: 3 of 901	Poor rotamers: 0 of 767	Outliers: 0 of 820	Outliers: 14 of 899	Outliers: 10 of 903	Outliers: 16 of 903	Non-Trans: 2 of 902
A 641		LYS	0.67	-	Favored (82.39%) Pre-Pro / -130.6,70.2	Favored (72.3%) *mmtt* chi angles: 296.6,292.4,183.6,181.4	0.01Å	Favored (15.632%) alpha helix	-	-	-
A 642		PRO	0.65	-	Favored (71.07%) Trans-Pro / -63.5,-22.2	Favored (40.6%) *Cg_endo* chi angles: 23.5,326.7,28.9	0.03Å	Favored (50.717%) alpha helix	-	-	-
A 643		GLU	0.63	-	Favored (99.63%) General / -62.8,-42.2	Favored (92.1%) *tt0* chi angles: 181.6,176.7,0.1	0.02Å	Favored (58.461%) alpha helix	-	-	-
A 644		LYS	0.62	-	Favored (86.26%) General / -65.8,-37.5	Favored (48.6%) *mtpt* chi angles: 285.9,175.3,59.8,175.6	0.05Å	Favored (99.273%) alpha helix	-	-	-
A 645		VAL	0.6	-	Favored (85.59%) Ile or Val / -65.2,-47.9	Favored (82.7%) *t* chi angles: 173.4	0.08Å	Favored (83.062%) alpha helix	-	-	-

A 646		THR	0.59	-	Favored (81.01%) General / -62.4,-48.2	Favored (91%) *m* chi angles: 298.9	0.03Å	Favored (88.56%) alpha helix	-	-	-
A 647		ARG	0.59	-	Favored (93.05%) General / -63.3,-38.9	Favored (96.7%) *mtt-85* chi angles: 288.1,182.2,183.4,278.1	0.02Å	Favored (89.426%) alpha helix	-	-	-
A 648		TRP	0.6	-	Favored (91.48%) General / -61.9,-46.0	Favored (91.2%) *t60* chi angles: 183.5,87.9	0.04Å	Favored (94.194%) alpha helix	-	-	-
A 649		LEU	0.62	-	Favored (99.77%) General / -62.7,-42.6	Favored (80.3%) *mt* chi angles: 289.7,167.1	0.08Å	Favored (93.231%) alpha helix	-	-	-
A 650		GLN	0.63	-	Favored (91.62%) General / -63.9,-38.6	Favored (97.1%) *mt0* chi angles: 290.6,170.7,337	0.04Å	Favored (64.065%) alpha helix	-	-	-
A 651		SER	0.65	-	Favored (15.02%) General / -90.4,-35.7	Favored (66.9%) *m* chi angles: 297	0.04Å	Favored (34.445%) alpha helix	-	-	-
A 652		ASN	0.67	-	Favored (7.87%) General / -121.6,-15.8	Favored (69.1%) *m-40* chi angles: 295.2,283.2	0.05Å	Favored (10.108%) alpha helix	-	-	-
A 653		GLY	0.68	-	Favored (18.12%) Glycine / -49.8,-54.4	-	-	Favored (55.33%) alpha helix	-	-	-
A 654		TRP	0.68	-	Favored (92.78%) General / -59.4,-44.7	Favored (64.3%) *t-100* chi angles: 185.3,248.7	0.06Å	Favored (85.444%) alpha helix	-	-	-
A 655		ASP	0.68	-	Favored (71.71%) General / -62.2,-31.2	Favored (86.8%) *m-30* chi angles: 283.2,347.3	0.10Å	Favored (75.219%) alpha helix	-	-	-
A 656		ARG	0.68	-	Favored (63.38%) General / -74.3,-38.4	Favored (93.3%) *mmt-90* chi angles: 297,288.4,185.6,274.7	0.08Å	Favored (75.513%) alpha helix	-	-	-
A 657		LEU	0.67	-	Favored (89.24%) General / -61.7,-38.8	Favored (78.8%) *mt* chi angles: 288.5,170.2	0.03Å	Favored (77.234%) alpha helix	-	-	-
A 658		LYS	0.67	-	Favored (62.82%) General / -63.9,-17.1	Favored (56.9%) *mtmt* chi angles: 289.8,186.7,288,185.3	0.05Å	Favored (65.603%) three-ten	-	-	-
A 659		ARG	0.66	-	Favored (23.58%) General / -96.0,14.5	Favored (71.4%) *mtt90* chi angles: 287.6,165.7,171.6,74.8	0.04Å	Favored (26.53%)	-	-	-
A 660		MET	0.66	-	Favored (40.04%) General / -122.7,153.0	Favored (54.8%) *mtt* chi angles: 302.6,178.5,188.6	0.10Å	Favored (30.614%)	-	-	-
**#**	**Alt**	**Res**	**High B**	**Clash > 0.4Å**	**Ramachandran**	**Rotamer**	**Cβ deviation**	**CaBLAM**	**Bond lengths**	**Bond angles**	**Cis Peptides**
			Avg: 0.95	Clashscore: 2.02	Outliers: 3 of 901	Poor rotamers: 0 of 767	Outliers: 0 of 820	Outliers: 14 of 899	Outliers: 10 of 903	Outliers: 16 of 903	Non-Trans: 2 of 902

A 661		ALA	0.66	-	Favored (8.59%) General / -128.9,106.0	-	0.01Å	Favored (33.863%)	-	-	-
A 662		VAL	0.67	0.40Å HG22 with A 667 CYS SG	Favored (67.06%) Ile or Val / -121.0,133.6	Favored (54.1%) *t* chi angles: 180.8	0.01Å	Favored (33.035%)	-	-	-
A 663		SER	0.68	-	Favored (5.58%) General / -150.5,116.3	Favored (38.7%) *t* chi angles: 177.2	0.08Å	CaBLAM Disfavored (4.159%)	-	-	-
A 664		GLY	0.68	-	Favored (21.31%) Glycine / 59.7,-120.1	-	-	Favored (66.092%)	-	-	-
A 665		ASP	0.68	-	Favored (57.36%) General / -90.6,-3.7	Favored (45.9%) *m-30* chi angles: 302.3,338.4	0.13Å	Favored (12.683%)	-	-	-
A 666		ASP	0.68	-	Favored (24.7%) General / -107.2,152.0	Favored (65.5%) *m-30* chi angles: 301.2,317.1	0.09Å	Favored (17.822%)	-	-	-
A 667		CYS	0.67	0.40Å SG with A 662 VAL HG22	Favored (8.65%) General / -158.6,133.9	Favored (50.7%) *t* chi angles: 180.1	0.09Å	Favored (32.622%)	-	-	-
A 668		VAL	0.66	-	Favored (62.3%) Ile or Val / -111.6,131.7	Favored (85.6%) *t* chi angles: 177.6	0.03Å	Favored (68.54%) beta sheet	-	-	-
A 669		VAL	0.67	-	Favored (54.69%) Ile or Val / -130.8,138.0	Favored (48.4%) *t* chi angles: 181.8	0.04Å	Favored (67.336%) beta sheet	-	-	-
A 670		LYS	0.68	-	Favored (27.25%) Pre-Pro / -119.7,82.8	Favored (44.4%) *tttp* chi angles: 181.3,177.2,171.1,73.9	0.10Å	Favored (23.789%) beta sheet	-	-	-
A 671		PRO	0.7	-	Favored (55.1%) Trans-Pro / -70.7,158.6	Favored (70%) *Cg_endo* chi angles: 29.6,326.6,23.6	0.08Å	Favored (24.419%)	-	-	-
A 672		ILE	0.71	-	Favored (22.41%) Ile or Val / -62.1,-19.7	Favored (7.8%) *tp* chi angles: 199.8,66.5	0.05Å	Favored (12.744%)	-	-	-
A 673		ASP	0.72	-	Favored (26.63%) General / -150.1,145.9	Favored (10.2%) *t70* chi angles: 195.2,290.9	0.03Å	Favored (21.166%)	-	-	-
A 674		ASP	0.72	0.43Å OD2 with A 530 LYS NZ	Favored (43.37%) General / -60.7,-16.8	Favored (94.3%) *m-30* chi angles: 289.2,349.5	0.05Å	Favored (13.826%)	-	-	-
A 675		ARG	0.71	-	Favored (35.38%) General / -57.8,-20.4	Favored (99.6%) *mtm-85* chi angles: 286.4,190.4,294,274.6	0.02Å	Favored (26.427%)	-	-	-
A 676		PHE	0.7	-	Favored (35.68%) General / -52.8,-30.9	Favored (65.9%) *t80* chi angles: 186.7,77.5	0.03Å	Favored (50.885%) three-ten	-	-	-
A 677		ALA	0.69	-	Favored (63.27%) General / -56.9,-29.8	-	0.10Å	Favored (53.955%) three-ten	-	-	-
A 678		HIS	0.68	-	Favored (31.64%)	Favored (100%) *m-70*	0.08Å	Favored (48.248%)	-	-	-

#	Alt	Res	High B	Clash > 0.4Å	Ramachandran	Rotamer	Cβ deviation	CaBLAM	Bond lengths	Bond angles	Cis Peptides
					General / -100.8,-2.7	chi angles: 297.5,287.5					
A 679		ALA	0.68	-	Favored (5.11%) General / -84.9,58.6	-	0.06Å	CaBLAM Disfavored (4.817%)	-	-	-
A 680		LEU	0.69	-	Favored (16.31%) General / -103.8,-13.7	Favored (3.1%) *mm* chi angles: 275.6,290.1	0.04Å	Favored (7.873%)	-	-	-
**#**	**Alt**	**Res**	**High B**	**Clash > 0.4Å**	**Ramachandran**	**Rotamer**	**Cβ deviation**	**CaBLAM**	**Bond lengths**	**Bond angles**	**Cis Peptides**
			Avg: 0.95	Clashscore: 2.02	Outliers: 3 of 901	Poor rotamers: 0 of 767	Outliers: 0 of 820	Outliers: 14 of 899	Outliers: 10 of 903	Outliers: 16 of 903	Non-Trans: 2 of 902
A 681		ARG	0.69	-	Favored (95.69%) General / -64.5,-42.9	Favored (72.5%) *mtt90* chi angles: 294.9,178.2,189.1,90	0.05Å	Favored (46.68%)	-	-	-
A 682		PHE	0.69	-	Favored (72.86%) General / -58.5,-50.7	Favored (91%) *t80* chi angles: 176,78.6	0.05Å	Favored (77.432%) alpha helix	-	-	-
A 683		LEU	0.69	-	Favored (56.96%) General / -50.5,-46.4	Favored (38.1%) *tp* chi angles: 183.8,66.8	0.08Å	Favored (75.698%) alpha helix	-	-	-
A 684		ASN	0.7	-	Favored (68.36%) General / -72.4,-35.1	Favored (68.9%) *m-40* chi angles: 288.2,280.7	0.02Å	Favored (78.036%) alpha helix	-	-	-
A 685		ASP	0.7	-	Favored (72.14%) General / -63.1,-31.1	Favored (14.5%) *t70* chi angles: 195.7,66.9	0.09Å	Favored (76.272%) alpha helix	-	-	-
A 686		MET	0.71	-	Favored (20.13%) General / -83.0,6.0	Favored (85.5%) *mmm* chi angles: 301.3,305.9,286.1	0.01Å	Favored (42.249%)	-	-	-
A 687		GLY	0.73	-	Favored (84.02%) Glycine / 79.1,12.5	-	-	Favored (86.62%)	-	-	-
A 688		LYS	0.76	-	Favored (44.9%) General / -99.1,126.5	Favored (16.6%) *mmtm* chi angles: 310.4,294.2,185.3,272.2	0.07Å	Favored (22.31%)	-	-	-
A 689		VAL	0.81	-	Favored (69.14%) Ile or Val / -126.0,126.1	Favored (85.2%) *t* chi angles: 177.2	0.05Å	Favored (57.759%) beta sheet	-	-	-
A 690		ARG	0.88	-	Favored (35.81%) General / -70.1,128.3	Favored (26%) *tpt90* chi angles: 176,70,164.5,90.5	0.10Å	Favored (41.386%)	-	-	-
A 691		LYS	0.96	-	Favored (67.66%) General / -54.7,-39.0	Favored (89.4%) *mttt* chi angles: 287.1,175.5,181.1,169.3	0.10Å	Favored (26.971%)	-	-	-
A 692		ASP	1.03	-	Favored (39.55%) General / -102.5,5.8	Favored (70.9%) *m-30* chi angles: 297.5,311.2	0.07Å	Favored (19.13%) alpha helix	-	-	-
A 693		THR	1.08	-	Favored (6.17%) General / -129.2,14.8	Favored (78.9%) *p* chi angles: 60.3	0.02Å	Favored (14.219%)	-	-	-
A 694		GLN	1.08	-	Favored (2.31%)	Favored (20.5%) *mp10*	0.03Å	CaBLAM Outlier	-	-	-

					General / 52.9,-129.7	chi angles: 296.1,87.5,54.8		(0.548%)			
A 695		GLU	1.05	-	Favored (51.54%) General / -129.8,146.6	Favored (93.4%) *mt-10* chi angles: 299.2,180.9,1.6	0.04Å	CA Geom Outlier (0.136%)	-	-	-
A 696		TRP	0.98	-	Favored (20%) General / 59.6,30.4	Favored (67.8%) *m100* chi angles: 304.8,101.7	0.02Å	Favored (13.31%)	-	-	-
A 697		LYS	0.91	-	Favored (53.99%) Pre-Pro / -119.6,152.9	Favored (97.7%) *mttt* chi angles: 295.4,185.2,183.6,182.2	0.04Å	Favored (18.794%)	-	-	-
A 698		PRO	0.84	-	Favored (57.99%) Trans-Pro / -68.8,158.4	Favored (51.7%) *Cg_endo* chi angles: 25.4,325.4,28.9	0.02Å	Favored (68.701%) beta sheet	-	-	-
A 699		SER	0.79	-	Favored (22.41%) General / -92.9,148.0	Favored (39.7%) *t* chi angles: 176.9	0.04Å	Favored (33.204%) beta sheet	-	-	-
A 700		THR	0.76	-	Favored (36.52%) General / -77.4,130.0	Favored (89.5%) *m* chi angles: 298.7	0.02Å	Favored (40.765%) beta sheet	-	-	-
**#**	**Alt**	**Res**	**High B**	**Clash > 0.4Å**	**Ramachandran**	**Rotamer**	**Cβ deviation**	**CaBLAM**	**Bond lengths**	**Bond angles**	**Cis Peptides**
			Avg: 0.95	Clashscore: 2.02	Outliers: 3 of 901	Poor rotamers: 0 of 767	Outliers: 0 of 820	Outliers: 14 of 899	Outliers: 10 of 903	Outliers: 16 of 903	Non-Trans: 2 of 902
A 701		GLY	0.75	-	Favored (16.31%) Glycine / -108.7,155.0	-	-	Favored (64.092%) beta sheet	-	-	-
A 702		TRP	0.74	-	Favored (50.65%) General / -128.4,146.9	Favored (42.8%) *m-90* chi angles: 294.5,264.8	0.04Å	Favored (65.665%)	-	-	-
A 703		SER	0.74	-	Favored (58.8%) General / -88.3,-3.8	Favored (96.6%) *p* chi angles: 65.9	0.03Å	Favored (40.061%)	-	-	-
A 704		ASN	0.74	-	Favored (8.94%) General / -139.2,113.6	Favored (58.5%) *t0* chi angles: 184,351.9	0.06Å	Favored (10.997%)	-	-	-
A 705		TRP	0.73	-	Favored (10.27%) General / -51.2,-27.9	Favored (76.2%) *p-90* chi angles: 66.4,273	0.03Å	Favored (38.596%)	-	-	-
A 706		GLU	0.73	-	Favored (68.75%) General / -63.2,-26.3	Favored (60.6%) *mt-10* chi angles: 290.2,177,307.9	0.04Å	Favored (64.343%)	-	-	-
A 707		GLU	0.74	-	Favored (55.49%) General / -91.9,3.3	Favored (94.7%) *mt-10* chi angles: 297.1,181.4,0.1	0.01Å	Favored (51.687%)	-	-	-
A 708		VAL	0.76	-	Favored (60.4%) Pre-Pro / -97.3,120.8	Favored (65.4%) *t* chi angles: 179.3	0.10Å	Favored (32.992%)	-	-	-
A 709		PRO	0.79	-	Favored (47%) Trans-Pro / -74.2,155.1	Favored (74.7%) *Cg_endo* chi angles: 28.9,326.6,24.2	0.02Å	Favored (30.093%)	-	-	-
A 710		PHE	0.82	-	Allowed (1.21%)	Favored (13.2%) *t80* chi angles: 180.4,41.4	0.12Å	CaBLAM Disfavored (1.729%)	-	-	-

#	Alt	Res	High B	Clash > 0.4Å	Ramachandran	Rotamer	Cβ deviation	CaBLAM	Bond lengths	Bond angles	Cis Peptides
					General / -127.7,-59.3						
A 711		CYS	0.84	-	Allowed (1.62%) General / -138.8,77.4	Favored (56.8%) *m* chi angles: 302.3	0.07Å	CA Geom Outlier (0.416%)	-	-	-
A 712		SER	0.85	-	Favored (22.02%) General / 56.5,33.1	Favored (53.1%) *m* chi angles: 300.5	0.02Å	CaBLAM Outlier (0.331%)	-	-	-
A 713		HIS	0.83	-	Favored (26.66%) General / -162.3,160.3	Favored (40.2%) *p-80* chi angles: 65.6,269.5	0.04Å	Favored (15.905%)	OUTLIER(S) worst is CE1--NE2: 4.3 σ	-	-
A 714		HIS	0.8	-	Favored (12.92%) General / -118.4,166.2	Favored (58.4%) *m90* chi angles: 302.9,80	0.13Å	Favored (39.32%)	-	-	-
A 715		PHE	0.77	-	Favored (51.5%) General / -120.2,140.2	Favored (93.1%) *m-80* chi angles: 291.7,92.9	0.08Å	Favored (49.066%) beta sheet	-	-	-
A 716		ASN	0.75	-	Favored (40.08%) General / -111.1,144.5	Favored (82.7%) *m-40* chi angles: 292,315.4	0.04Å	Favored (53.605%) beta sheet	-	-	-
A 717		LYS	0.74	-	Favored (29.83%) General / -97.2,116.7	Favored (87.5%) *tttt* chi angles: 186,174.6,183.4,177.6	0.07Å	Favored (55.787%) beta sheet	-	-	-
A 718		LEU	0.76	0.44Å N with A 718 LEU HD22	Favored (21.38%) General / -108.8,154.9	Allowed (1.1%) *mm* chi angles: 290.3,302.1	0.09Å	Favored (32.769%) beta sheet	-	-	-
A 719		TYR	0.79	-	Favored (55.34%) General / -119.7,134.2	Favored (81.4%) *m-80* chi angles: 298,83.4	0.04Å	Favored (46.813%)	-	-	-
A 720		LEU	0.83	-	Favored (22.88%) General / -79.3,165.7	Favored (6.3%) *mp* chi angles: 277.6,70	0.01Å	Favored (40.139%)	-	-	-
**#**	**Alt**	**Res**	**High B**	**Clash > 0.4Å**	**Ramachandran**	**Rotamer**	**Cβ deviation**	**CaBLAM**	**Bond lengths**	**Bond angles**	**Cis Peptides**
			Avg: 0.95	Clashscore: 2.02	Outliers: 3 of 901	Poor rotamers: 0 of 767	Outliers: 0 of 820	Outliers: 14 of 899	Outliers: 10 of 903	Outliers: 16 of 903	Non-Trans: 2 of 902
A 721		LYS	0.86	-	Favored (5.07%) General / -46.7,-34.3	Favored (2.6%) *tmmt* chi angles: 181.2,261.9,292,176.2	0.03Å	Favored (49.531%)	-	-	-
A 722		ASP	0.87	-	Favored (56.96%) General / -89.8,0.5	Favored (74.6%) *m-30* chi angles: 297,319.6	0.07Å	Favored (57.406%)	-	-	-
A 723		GLY	0.85	-	Favored (62.55%) Glycine / 95.2,-15.8	-	-	Favored (60.055%)	-	-	-
A 724		ARG	0.83	-	Favored (54.52%) General / -66.2,146.5	Favored (48.8%) *ptt-90* chi angles: 65.9,188.1,179.9,279	0.09Å	Favored (42.656%)	-	-	-
A 725		SER	0.79	-	Favored (14.99%) General / -93.0,160.2	Favored (88.9%) *p* chi angles: 68.2	0.03Å	Favored (44.058%) beta sheet	-	-	-
A 726		ILE	0.76	-	Favored (19.83%)	Favored (21.8%) *tt* chi angles: 188.7,165.3	0.13Å	Favored (57.549%) beta sheet	-	-	-

#	Alt	Res	High B	Clash > 0.4Å	Ramachandran	Rotamer	Cβ deviation	CaBLAM	Bond lengths	Bond angles	Cis Peptides
					Ile or Val / -147.3,144.0						
A 727		VAL	0.73	-	Favored (59.02%) Ile or Val / -111.4,119.1	Favored (57.2%) *t* chi angles: 180.3	0.05Å	Favored (58.018%) beta sheet	-	-	-
A 728		VAL	0.72	0.40Å HG21 with A 769 PHE CZ	Favored (23.75%) Pre-Pro / -119.3,134.3	Favored (40.9%) *t* chi angles: 183.2	0.16Å	Favored (49.751%) beta sheet	-	-	-
A 729		PRO	0.72	-	Favored (84.77%) Trans-Pro / -66.0,151.1	Favored (40.8%) *Cg_endo* chi angles: 23.6,328,26.9	0.02Å	Favored (58.437%) beta sheet	-	-	-
A 730		CYS	0.73	-	Favored (26.09%) General / -143.5,134.9	Favored (31.8%) *t* chi angles: 188.6	0.03Å	Favored (61.105%)	-	-	-
A 731		ARG	0.75	-	Favored (47.18%) General / -136.4,147.1	Favored (80.8%) *ttt180* chi angles: 182.1,175.9,184.7,174.3	0.01Å	Favored (27.658%)	-	-	-
A 732		HIS	0.77	-	Favored (32.2%) General / -52.1,131.7	Favored (55.8%) *t-90* chi angles: 193.1,293.5	0.06Å	Favored (35.708%)	-	-	-
A 733		GLN	0.79	-	Favored (67.88%) General / -56.9,-34.3	Favored (60.2%) *tt0* chi angles: 180.9,177.1,52.5	0.04Å	Favored (52.187%)	-	-	-
A 734		ASP	0.8	-	Favored (73.37%) General / -62.6,-32.4	Favored (96%) *m-30* chi angles: 289.1,348.5	0.06Å	Favored (71.14%) alpha helix	-	-	-
A 735		GLU	0.8	-	Favored (52.69%) General / -76.9,-39.0	Favored (86.2%) *mt-10* chi angles: 295.2,180,16.8	0.03Å	Favored (84.349%) alpha helix	-	-	-
A 736		LEU	0.8	-	Favored (93.47%) General / -65.2,-39.8	Favored (84.2%) *mt* chi angles: 289.7,172.4	0.01Å	Favored (78.681%) alpha helix	-	-	-
A 737		ILE	0.79	-	Favored (28.91%) Ile or Val / -77.5,-45.6	Favored (93.1%) *mt* chi angles: 295.4,171.3	0.08Å	Favored (75.988%) alpha helix	OUTLIER(S) worst is CB--CG1: 4.3 σ	-	-
A 738		GLY	0.79	-	Favored (93.61%) Glycine / -59.4,-38.5	-	-	Favored (99.399%) alpha helix	-	-	-
A 739		ARG	0.8	-	Favored (72.89%) General / -57.3,-50.4	Favored (47.2%) *ttp-170* chi angles: 180,197.8,68.1,196.7	0.06Å	Favored (86.689%) alpha helix	-	-	-
A 740		ALA	0.83	-	Favored (68.34%) General / -59.0,-31.0	-	0.04Å	Favored (72.954%) alpha helix	-	-	-
#	Alt	Res	High B	Clash > 0.4Å	Ramachandran	Rotamer	Cβ deviation	CaBLAM	Bond lengths	Bond angles	Cis Peptides
			Avg: 0.95	Clashscore: 2.02	Outliers: 3 of 901	Poor rotamers: 0 of 767	Outliers: 0 of 820	Outliers: 14 of 899	Outliers: 10 of 903	Outliers: 16 of 903	Non-Trans: 2 of 902
A 741		ARG	0.9	-	Favored (34.74%) General / -82.4,2.0	Favored (27.2%) *mtp180* chi angles: 288,177.9,54.2,159.6	0.07Å	Favored (41.814%)	-	-	-

A 742		VAL	1.01	-	Favored (75.17%) Ile or Val / -119.6,129.6	Favored (81.1%) *t* chi angles: 176.6	0.03Å	Favored (32.4%)	-	-	-
A 743		SER	1.15	-	Favored (25.79%) Pre-Pro / -93.0,142.8	Favored (72.8%) *m* chi angles: 295.7	0.05Å	Favored (40.083%)	-	-	-
A 744		PRO	1.3	-	Favored (7.62%) Trans-Pro / -76.8,65.9	Favored (78.6%) *Cg_endo* chi angles: 30.7,324.6,25	0.02Å	CaBLAM Outlier (0.158%)	-	-	-
A 745		GLY	1.43	-	Favored (14.28%) Glycine / 152.4,166.5	-	-	CaBLAM Disfavored (3.649%)	-	-	-
A 746		ALA	1.48	-	Favored (19.68%) General / -93.6,-21.0	-	0.03Å	Favored (5.974%)	-	-	-
A 747		GLY	1.44	-	Favored (2.13%) Glycine / -90.4,71.0	-	-	CaBLAM Disfavored (1.609%)	-	-	-
A 748		TRP	1.31	-	Favored (50.72%) General / -128.6,147.3	Favored (66.8%) *m100* chi angles: 301.2,83.3	0.12Å	Favored (28.145%)	-	-	-
A 749		SER	1.15	-	Favored (15.53%) General / -87.9,165.0	Favored (93.5%) *p* chi angles: 64.3	0.06Å	Favored (41.062%)	-	-	-
A 750		ILE	1	-	Favored (48.18%) Ile or Val / -65.4,-29.8	Favored (10.1%) *tp* chi angles: 195.7,65.5	0.08Å	Favored (65.833%)	-	-	-
A 751		ARG	0.88	-	Favored (67.37%) General / -72.5,-39.7	Favored (97.7%) *mtt180* chi angles: 292.5,182.7,179.8,173.6	0.02Å	Favored (83.641%) alpha helix	-	-	-
A 752		GLU	0.79	-	Favored (86.51%) General / -66.7,-38.3	Favored (34.6%) *mt-10* chi angles: 290.3,165.2,308.5	0.03Å	Favored (83.015%) alpha helix	-	-	-
A 753		THR	0.73	-	Favored (92.88%) General / -60.5,-45.7	Favored (90.2%) *m* chi angles: 297.2	0.05Å	Favored (90.714%) alpha helix	-	-	-
A 754		ALA	0.69	-	Favored (88.23%) General / -60.4,-39.7	-	0.09Å	Favored (87.979%) alpha helix	-	-	-
A 755		CYS	0.66	-	Favored (96.35%) General / -64.4,-41.8	Favored (93%) *m* chi angles: 291.8	0.06Å	Favored (87.301%) alpha helix	-	-	-
A 756		LEU	0.64	-	Favored (83.52%) General / -66.8,-43.6	Favored (60.2%) *tp* chi angles: 181.2,60.2	0.01Å	Favored (80.019%) alpha helix	-	-	-
A 757		ALA	0.63	-	Favored (79.15%) General / -57.9,-40.0	-	0.07Å	Favored (78.751%) alpha helix	-	-	-
A 758		LYS	0.62	-	Favored (96.69%) General / -61.1,-44.9	Favored (54.1%) *tttp* chi angles: 185,173.4,175.6,65.8	0.07Å	Favored (84.71%) alpha helix	-	-	-

#	Alt	Res	High B	Clash > 0.4Å	Ramachandran	Rotamer	Cβ deviation	CaBLAM	Bond lengths	Bond angles	Cis Peptides
A 759		SER	0.62	-	Favored (75.44%) General / -63.1,-33.4	Favored (85.3%) *p* chi angles: 67.3	0.12Å	Favored (82.304%) alpha helix	-	-	-
A 760		TYR	0.63	-	Favored (78.42%) General / -69.0,-40.8	Favored (28.8%) *m-80* chi angles: 287.4,126.5	0.02Å	Favored (91.42%) alpha helix	-	-	-
**#**	**Alt**	**Res**	**High B**	**Clash > 0.4Å**	**Ramachandran**	**Rotamer**	**Cβ deviation**	**CaBLAM**	**Bond lengths**	**Bond angles**	**Cis Peptides**
			Avg: 0.95	Clashscore: 2.02	Outliers: 3 of 901	Poor rotamers: 0 of 767	Outliers: 0 of 820	Outliers: 14 of 899	Outliers: 10 of 903	Outliers: 16 of 903	Non-Trans: 2 of 902
A 761		ALA	0.64	-	Favored (98.65%) General / -63.5,-41.6	-	0.07Å	Favored (95.24%) alpha helix	-	-	-
A 762		GLN	0.65	-	Favored (92.12%) General / -65.3,-39.2	Favored (84.3%) *mt0* chi angles: 288.8,170.7,307.5	0.07Å	Favored (94.583%) alpha helix	-	-	-
A 763		MET	0.67	-	Favored (94.98%) General / -64.6,-40.2	Favored (98.8%) *mtp* chi angles: 291.5,177.9,75.9	0.04Å	Favored (92.593%) alpha helix	-	-	-
A 764		TRP	0.68	-	Favored (97.4%) General / -60.6,-42.8	Favored (21.3%) *m-10* chi angles: 282.1,340.7	0.03Å	Favored (93.937%) alpha helix	-	-	-
A 765		GLN	0.69	-	Favored (69.48%) General / -64.0,-28.0	Favored (95.6%) *mt0* chi angles: 288.5,176.2,327.9	0.05Å	Favored (60.714%) alpha helix	-	-	-
A 766		LEU	0.7	-	Favored (14.39%) General / -92.5,-33.8	Favored (96.3%) *mt* chi angles: 296.9,176.8	0.05Å	Favored (52.248%) alpha helix	-	-	-
A 767		LEU	0.69	-	Favored (8.16%) General / -104.2,-35.0	Favored (92%) *mt* chi angles: 298.4,175.7	0.03Å	Favored (37.835%) alpha helix	-	-	-
A 768		TYR	0.69	-	Favored (7.02%) General / -121.6,29.8	Favored (85.4%) *m-80* chi angles: 302.6,95.7	0.04Å	Favored (17.673%) alpha helix	-	-	-
A 769		PHE	0.67	0.40Å CZ with A 728 VAL HG21	Favored (22.28%) General / -57.8,-18.4	Favored (23.8%) *p90* chi angles: 68.5,80.4	0.06Å	Favored (11.402%) alpha helix	-	-	-
A 770		HIS	0.66	0.46Å H with A 770 HIS HD1	Favored (59.69%) General / -82.0,-8.7	Favored (2.1%) *p-80* chi angles: 66.9,319.4	0.07Å	Favored (47.769%)	-	-	-
A 771		ARG	0.65	-	Favored (36.9%) General / -92.0,126.0	Favored (34.7%) *ttm170* chi angles: 174.8,171.1,288.7,149.1	0.04Å	Favored (36.183%)	OUTLIER(S) worst is CZ--NH2: 6.0 σ	-	-
A 772		ARG	0.65	-	Favored (83.64%) General / -61.8,-37.3	Favored (96.9%) *mtt-85* chi angles: 288.6,180.4,183.7,278.1	0.03Å	Favored (49.359%)	-	-	-
A 773		ASP	0.64	-	Favored (63.23%) General / -73.8,-40.8	Favored (94.4%) *m-30* chi angles: 292.7,342.2	0.08Å	Favored (88.58%) alpha helix	-	-	-
A 774		LEU	0.64	-	Favored (64.21%) General / -72.9,-30.9	Favored (70.1%) *mt* chi angles: 292.1,179.1	0.09Å	Favored (77.59%) alpha helix	-	-	-

A 775		ARG	0.64	-	Favored (93.28%) General / -61.6,-40.0	Favored (43.1%) *mmm-85* chi angles: 284.2,280.7,298.2,280.1	0.03Å	Favored (78.247%) alpha helix	-	-	-
A 776		LEU	0.63	-	Favored (64.92%) General / -73.8,-36.2	Favored (2.2%) *mm* chi angles: 269,284.3	0.03Å	Favored (80.751%) alpha helix	-	-	-
A 777		MET	0.62	-	Favored (76.63%) General / -66.8,-46.0	Favored (99.7%) *mtp* chi angles: 292,176.3,73.5	0.07Å	Favored (81.989%) alpha helix	-	-	-
A 778		ALA	0.61	-	Favored (98.48%) General / -62.1,-41.7	-	0.06Å	Favored (90.436%) alpha helix	-	-	-
A 779		ASN	0.61	-	Favored (89.98%) General / -66.1,-39.6	Favored (98.3%) *m-40* chi angles: 290.8,340.7	0.05Å	Favored (98.664%) alpha helix	-	-	-
A 780		ALA	0.6	-	Favored (99.48%) General / -63.1,-41.8	-	0.04Å	Favored (90.545%) alpha helix	-	-	-
**#**	**Alt**	**Res**	**High B**	**Clash > 0.4Å**	**Ramachandran**	**Rotamer**	**Cβ deviation**	**CaBLAM**	**Bond lengths**	**Bond angles**	**Cis Peptides**
			Avg: 0.95	Clashscore: 2.02	Outliers: 3 of 901	Poor rotamers: 0 of 767	Outliers: 0 of 820	Outliers: 14 of 899	Outliers: 10 of 903	Outliers: 16 of 903	Non-Trans: 2 of 902
A 781		ILE	0.6	-	Favored (81.93%) Ile or Val / -68.4,-45.6	Favored (93.9%) *mt* chi angles: 293.1,166.1	0.02Å	Favored (86.768%) alpha helix	-	-	-
A 782		CYS	0.6	-	Favored (82.19%) General / -61.8,-36.8	Favored (93.1%) *m* chi angles: 291.9	0.04Å	Favored (89.128%) alpha helix	-	-	-
A 783		SER	0.61	-	Favored (81.77%) General / -68.3,-40.6	Favored (70.7%) *m* chi angles: 296.2	0.04Å	Favored (96.334%) alpha helix	-	-	-
A 784		ALA	0.62	-	Favored (82.28%) General / -65.6,-35.9	-	0.05Å	Favored (60.181%)	-	-	-
A 785		VAL	0.64	-	Favored (31.6%) Pre-Pro / -93.1,132.1	Favored (64.1%) *t* chi angles: 179.5	0.04Å	Favored (17.047%)	-	-	-
A 786		PRO	0.66	-	Favored (23.65%) Trans-Pro / -50.1,144.0	Favored (97.6%) *Cg_exo* chi angles: 332.7,36.6,329.8	0.04Å	Favored (42.34%)	-	-	-
A 787		VAL	0.68	-	Favored (34.6%) Ile or Val / -65.0,-24.0	Favored (29.7%) *m* chi angles: 300.7	0.03Å	Favored (36.485%)	-	-	-
A 788		ASP	0.71	-	Favored (57.53%) General / -91.9,-1.0	Favored (73.8%) *m-30* chi angles: 295.1,319.7	0.05Å	Favored (56.08%)	-	-	-
A 789		TRP	0.75	-	Favored (8.96%) General / -82.5,79.2	Favored (36.1%) *m-10* chi angles: 290.3,13.7	0.07Å	Favored (20.97%)	-	-	-
A 790		VAL	0.8	-	Favored (80.57%) Pre-Pro / -59.0,126.2	Favored (91.9%) *t* chi angles: 174.4	0.15Å	Favored (24.319%)	-	-	-

#	Alt	Res	High B	Clash > 0.4Å	Ramachandran	Rotamer	Cβ deviation	CaBLAM	Bond lengths	Bond angles	Cis Peptides
A 791		PRO	0.86	-	Favored (76.62%) Trans-Pro / -56.4,145.9	Favored (76.4%) *Cg_exo* chi angles: 334.9,34.1,331.6	0.02Å	Favored (47.897%)	-	-	-
A 792		THR	0.93	-	Favored (10.44%) General / -117.2,-15.4	Favored (65.4%) *p* chi angles: 63.1	0.04Å	Favored (13.031%) beta sheet	-	-	-
A 793		GLY	1.02	-	Favored (37.53%) Glycine / -94.1,-168.2	-	-	Favored (28.203%) beta sheet	-	-	-
A 794		ARG	1.13	-	Favored (27.95%) General / -116.8,156.0	Favored (94.1%) *mtt-85* chi angles: 292.1,177.7,178.5,268.4	0.03Å	Favored (33.312%)	-	-	-
A 795		THR	1.23	-	Allowed (0.65%) General / -118.6,-84.4	Favored (52.5%) *p* chi angles: 56.6	0.06Å	CaBLAM Disfavored (1.071%)	-	-	-
A 796		THR	1.33	-	Favored (19.34%) General / -95.5,-20.1	Favored (57.2%) *p* chi angles: 64.6	0.12Å	CaBLAM Disfavored (2.744%)	-	-	-
A 797		TRP	1.41	-	Favored (12.54%) General / 57.2,26.7	Favored (71.1%) *m100* chi angles: 299.7,82.4	0.04Å	Favored (10.471%)	-	-	-
A 798		SER	1.45	-	Favored (47.16%) General / -61.1,146.5	Favored (35.8%) *t* chi angles: 174.4	0.03Å	Favored (13.325%)	-	-	-
A 799		ILE	1.46	-	Favored (7.9%) Ile or Val / -95.0,5.6	Favored (41.3%) *pt* chi angles: 65,171.6	0.06Å	Favored (25.986%)	-	-	-
A 800		HIS	1.42	-	Favored (46.64%) General / -130.6,155.4	Favored (83.9%) *m-70* chi angles: 288.8,288.3	0.07Å	CaBLAM Disfavored (2.22%)	OUTLIER(S) worst is CB--CG: 6.6 σ	-	-
**#**	**Alt**	**Res**	**High B**	**Clash > 0.4Å**	**Ramachandran**	**Rotamer**	**Cβ deviation**	**CaBLAM**	**Bond lengths**	**Bond angles**	**Cis Peptides**
			Avg: 0.95	Clashscore: 2.02	Outliers: 3 of 901	Poor rotamers: 0 of 767	Outliers: 0 of 820	Outliers: 14 of 899	Outliers: 10 of 903	Outliers: 16 of 903	Non-Trans: 2 of 902
A 801		GLY	1.34	-	Favored (45.66%) Glycine / 89.7,173.8	-	-	Favored (31.009%)	-	-	-
A 802		LYS	1.22	-	Favored (11.65%) General / -118.2,167.3	Favored (94.9%) *mttt* chi angles: 293.8,187,177.5,183.7	0.04Å	Favored (15.185%)	-	-	-
A 803		GLY	1.11	-	Favored (13.79%) Glycine / -84.7,20.7	-	-	Favored (11.381%)	-	-	-
A 804		GLU	1	-	Favored (67.96%) General / -60.5,-28.0	Favored (59.2%) *mm-30* chi angles: 287.8,291.7,307.4	0.01Å	Favored (36.753%)	-	-	-
A 805		TRP	0.92	-	Favored (65.7%) General / -67.3,-21.0	Favored (60.1%) *p-90* chi angles: 51.3,264.8	0.02Å	Favored (52.03%) three-ten	-	-	-
A 806		MET	0.85	0.42Å HG2 with A 806 MET O	Favored (10.75%) General / -85.4,71.8	Favored (56.5%) *ttm* chi angles: 179.1,176.2,280.9	0.03Å	CaBLAM Disfavored (2.932%)	-	-	-

A 807		THR	0.81	-	Favored (4.64%) General / -150.4,-174.1	Favored (11.8%) *t* chi angles: 190.1	0.07Å	Favored (30.014%)	-	-	-
A 808		THR	0.79	-	Favored (5.96%) General / -127.1,-8.2	Favored (53%) *p* chi angles: 56.6	0.08Å	CaBLAM Outlier (0.829%)	-	-	-
A 809		GLU	0.78	0.45Å O with A 810 ASP C	Favored (57.56%) General / -58.5,136.4	Favored (82.8%) *tt0* chi angles: 186.4,177.9,12.3	0.03Å	Favored (21.002%) beta sheet	-	-	-
A 810		ASP	0.77	0.45Å C with A 809 GLU O	Favored (30.14%) General / -51.5,133.3	Favored (12.8%) *t70* chi angles: 191.4,276.7	0.09Å	Favored (37.306%)	-	-	-
A 811		MET	0.76	-	Favored (66.25%) General / -62.5,-22.5	Favored (96.6%) *mmm* chi angles: 292,302.2,295.3	0.14Å	Favored (27.745%)	-	-	-
A 812		LEU	0.75	-	Favored (72.54%) General / -68.9,-32.7	Favored (70.2%) *mt* chi angles: 288.5,173.9	0.06Å	Favored (72.564%) alpha helix	-	-	-
A 813		MET	0.74	-	Favored (72.32%) General / -71.1,-36.5	Favored (79.8%) *mtm* chi angles: 289.6,188.1,281.5	0.02Å	Favored (86.717%) alpha helix	-	-	-
A 814		VAL	0.72	-	Favored (80.72%) Ile or Val / -69.1,-44.7	Favored (84%) *t* chi angles: 177	0.05Å	Favored (83.589%) alpha helix	-	-	-
A 815		TRP	0.71	-	Favored (90.89%) General / -59.4,-45.6	Favored (61.4%) *t60* chi angles: 192.8,93	0.07Å	Favored (96.261%) alpha helix	-	-	-
A 816		ASN	0.7	-	Favored (95.44%) General / -60.5,-42.0	Favored (70.8%) *m-40* chi angles: 280.4,336.3	0.02Å	Favored (97.076%) alpha helix	-	-	-
A 817		ARG	0.71	-	Favored (76.77%) General / -61.0,-49.6	Favored (49.1%) *ttm110* chi angles: 184.2,174.1,302.2,111.7	0.03Å	Favored (74.414%) alpha helix	-	-	-
A 818		VAL	0.73	-	Favored (84.12%) Ile or Val / -65.7,-48.0	Favored (67.2%) *t* chi angles: 171.8	0.06Å	Favored (57.303%) alpha helix	-	-	-
A 819		TRP	0.76	-	Favored (16.27%) General / -97.4,-23.1	Favored (97.3%) *m100* chi angles: 290.8,102.7	0.05Å	Favored (29.056%) alpha helix	-	-	-
A 820		ILE	0.8	-	Favored (4.46%) Ile or Val / -110.6,-56.5	Favored (49.5%) *mm* chi angles: 302.1,300.1	0.03Å	Favored (17.011%) alpha helix	-	-	-
**#**	**Alt**	**Res**	**High B**	**Clash > 0.4Å**	**Ramachandran**	**Rotamer**	**Cβ deviation**	**CaBLAM**	**Bond lengths**	**Bond angles**	**Cis Peptides**
			Avg: 0.95	Clashscore: 2.02	Outliers: 3 of 901	Poor rotamers: 0 of 767	Outliers: 0 of 820	Outliers: 14 of 899	Outliers: 10 of 903	Outliers: 16 of 903	Non-Trans: 2 of 902
A 821		GLU	0.85	-	Favored (30.68%) General / -75.7,-46.6	Favored (98.3%) *mt-10* chi angles: 294.1,176.4,354.6	0.02Å	Favored (56.293%) alpha helix	-	-	-

A 822		GLU	0.89	-	Favored (50.65%)<br>General / -89.9,-9.0	Favored (94.1%) *mt-10*<br>chi angles: 294.8,184,359.2	0.05Å	Favored (31.379%)	-	-	-
A 823		ASN	0.94	-	Favored (7.61%)<br>General / -84.4,90.5	Favored (44.7%) *t0*<br>chi angles: 192.9,327.2	0.09Å	Favored (24.8%)	-	-	-
A 824		ASP	0.97	-	Favored (4.31%)<br>General / -59.1,-11.9	Favored (89.4%) *m-30*<br>chi angles: 284.9,344.2	0.06Å	Favored (14.179%)	-	-	-
A 825		HIS	0.98	-	Favored (55.73%)<br>General / -93.9,-0.4	Favored (98.7%) *m-70*<br>chi angles: 296.9,290.6	0.03Å	Favored (49.191%)	-	-	-
A 826		MET	0.97	-	Favored (31.96%)<br>General / -92.1,119.6	Favored (64.4%) *ttm*<br>chi angles: 182.4,178.9,283.6	0.01Å	Favored (37.053%)	-	-	-
A 827		GLU	0.94	-	Favored (89.27%)<br>General / -64.7,-37.9	Favored (99.3%) *mt-10*<br>chi angles: 291.2,177.1,353	0.02Å	Favored (45.862%)	-	-	-
A 828		ASP	0.89	-	Favored (2.47%)<br>General / -107.5,87.3	Favored (65%) *t0*<br>chi angles: 181,355.5	0.06Å	Favored (20.004%)	-	-	-
A 829		LYS	0.85	-	Favored (7.2%)<br>General / -83.2,63.5	Favored (98.9%) *mttt*<br>chi angles: 294.8,183.7,175.3,184.8	0.04Å	Favored (38.448%)	-	-	-
A 830		THR	0.81	-	Favored (54.99%)<br>Pre-Pro / -88.6,127.8	Favored (52%) *m*<br>chi angles: 294.8	0.03Å	Favored (32.807%)<br>beta sheet	-	-	-
A 831		PRO	0.78	-	Favored (63.09%)<br>Trans-Pro / -71.0,154.0	Favored (57.8%) *Cg_endo*<br>chi angles: 26.1,325.8,27.8	0.03Å	Favored (86.78%)<br>beta sheet	-	-	-
A 832		VAL	0.76	-	Favored (17.69%)<br>Ile or Val / -86.0,139.2	Favored (53.2%) *t*<br>chi angles: 169.8	0.08Å	Favored (47.579%)	-	-	-
A 833		THR	0.76	-	Favored (10.09%)<br>General / -110.8,-25.0	Favored (75.3%) *p*<br>chi angles: 61.3	0.07Å	Favored (21.865%)	-	-	-
A 834		LYS	0.76	-	Favored (50.66%)<br>General / -137.0,153.6	Favored (97.4%) *mttt*<br>chi angles: 296.4,183.2,180.1,179.8	0.04Å	Favored (23.58%)	-	-	-
A 835		TRP	0.76	-	Favored (66.25%)<br>General / -62.4,-22.6	Favored (69.5%) *m100*<br>chi angles: 278.7,108.6	0.07Å	Favored (47.551%)<br>alpha helix	-	-	-
A 836		THR	0.75	-	Favored (61.77%)<br>General / -69.9,-12.6	Favored (50.7%) *p*<br>chi angles: 65.6	0.06Å	Favored (59.921%)<br>three-ten	-	-	-
A 837		ASP	0.73	-	Favored (46.94%)<br>General / -84.6,-15.1	Favored (79.6%) *m-30*<br>chi angles: 296.3,335.5	0.15Å	Favored (46.253%)	-	OUTLIER(S)<br>worst is CA-CB-CG: 4.6 σ	-
A 838		ILE	0.71	-	Favored (71.88%)<br>Pre-Pro / -86.6,120.3	Favored (48.7%) *mm*<br>chi angles: 301.5,300	0.04Å	Favored (32.394%)	-	-	-
A 839		PRO	0.7	-	Favored (36.51%)	Favored (80.6%) *Cg_endo*	0.07Å	Favored (28.779%)	-	-	-

#	Alt	Res	High B	Clash > 0.4Å	Ramachandran	Rotamer	Cβ deviation	CaBLAM	Bond lengths	Bond angles	Cis Peptides
					Trans-Pro / -74.5,163.2	chi angles: 30.3,323.8,27.1		beta sheet			
A 840		TYR	0.69	-	Favored (39.64%) General / -121.7,152.2	Favored (82.6%) *m-80* chi angles: 300.9,87.6	0.06Å	Favored (34.692%)	-	-	-
**#**	**Alt**	**Res**	**High B**	**Clash > 0.4Å**	**Ramachandran**	**Rotamer**	**Cβ deviation**	**CaBLAM**	**Bond lengths**	**Bond angles**	**Cis Peptides**
			Avg: 0.95	Clashscore: 2.02	Outliers: 3 of 901	Poor rotamers: 0 of 767	Outliers: 0 of 820	Outliers: 14 of 899	Outliers: 10 of 903	Outliers: 16 of 903	Non-Trans: 2 of 902
A 841		LEU	0.69	-	Favored (53.83%) General / -68.7,137.1	Favored (33.8%) *tp* chi angles: 186.7,63.5	0.07Å	Favored (31.011%)	-	-	-
A 842		GLY	0.7	-	Favored (32.26%) Glycine / -56.6,147.6	-	-	Favored (48.532%)	-	-	-
A 843		LYS	0.7	-	Favored (60.69%) General / -51.6,-40.8	Favored (87.4%) *tttt* chi angles: 183.2,176.8,178.9,179.1	0.03Å	Favored (63.529%)	-	-	-
A 844		ARG	0.72	-	Favored (71.26%) General / -55.4,-40.4	Favored (60.4%) *ttp-170* chi angles: 182.2,176.2,63.6,196.5	0.02Å	Favored (72.492%) alpha helix	-	-	-
A 845		GLU	0.73	-	Favored (32.62%) General / -81.7,-30.6	Favored (13.1%) *mp0* chi angles: 286.8,83.5,36.9	0.16Å	Favored (81.745%) alpha helix	-	-	-
A 846		ASP	0.75	-	Favored (95.88%) General / -60.4,-42.4	Favored (45.5%) *m-30* chi angles: 276.9,343.2	0.09Å	Favored (76.396%) alpha helix	-	OUTLIER(S) worst is CA-CB-CG: 5.5 σ	-
A 847		LEU	0.77	-	Favored (83.6%) General / -66.7,-36.9	Favored (93.3%) *mt* chi angles: 291.7,171.3	0.07Å	Favored (83.612%) alpha helix	-	-	-
A 848		TRP	0.78	-	Favored (83.45%) General / -61.4,-48.0	Favored (83.2%) *t60* chi angles: 174.3,85	0.04Å	Favored (77.198%) alpha helix	-	-	-
A 849		CYS	0.78	-	Favored (87.97%) General / -64.4,-37.6	Favored (76.6%) *m* chi angles: 288.3	0.10Å	CaBLAM Disfavored (2.824%)	-	-	-
A 850		GLY	0.79	-	Favored (2.69%) Glycine / 121.1,119.8	-	-	CaBLAM Disfavored (1.179%)	-	-	-
A 851		SER	0.81	-	Favored (15.62%) General / -161.1,149.0	Favored (27.1%) *t* chi angles: 171.9	0.04Å	Favored (32.824%) beta sheet	-	-	-
A 852		LEU	0.84	-	Favored (30.25%) General / -87.8,7.4	Favored (45.1%) *mt* chi angles: 292.8,183	0.15Å	Favored (9.336%)	-	-	-
A 853		ILE	0.9	-	Favored (6.3%) Ile or Val / -52.0,-25.4	Favored (11.7%) *tp* chi angles: 192.3,65.2	0.03Å	Favored (31.865%)	-	-	-
A 854		GLY	0.95	-	Favored (82.78%) Glycine / -90.1,6.2	-	-	Favored (60.675%)	-	-	-
A 855		HIS	1.01	-	Favored (47.4%) General / -117.6,142.3	Favored (50.6%) *m-70* chi angles: 295.9,256	0.04Å	Favored (29.271%)	-	-	-

A 856		ARG	1.04	-	Favored (15.8%) Pre-Pro / -44.2,-52.7	Favored (81.6%) *ttt180* chi angles: 178.9,178.4,177.5,180.5	0.08Å	Favored (55.745%)	-	-	-
A 857		PRO	1.04	-	Favored (75.76%) Trans-Pro / -61.6,-23.0	Favored (27.1%) *Cg_endo* chi angles: 20.8,324.7,34.8	0.01Å	Favored (68.602%) alpha helix	-	-	-
A 858		ARG	1.01	-	Favored (70.97%) General / -69.5,-44.4	Favored (5.2%) *tmt170* chi angles: 187.2,275.1,185.8,192	0.07Å	Favored (68.088%) alpha helix	-	-	-
A 859		THR	0.96	-	Favored (93.24%) General / -61.9,-45.5	Favored (91%) *m* chi angles: 298	0.02Å	Favored (91.747%) alpha helix	-	-	-
A 860		THR	0.91	-	Favored (91.78%) General / -59.6,-45.5	Favored (92.6%) *m* chi angles: 299.1	0.03Å	Favored (97.088%) alpha helix	-	-	-
**#**	**Alt**	**Res**	**High B**	**Clash > 0.4Å**	**Ramachandran**	**Rotamer**	**Cβ deviation**	**CaBLAM**	**Bond lengths**	**Bond angles**	**Cis Peptides**
			Avg: 0.95	Clashscore: 2.02	Outliers: 3 of 901	Poor rotamers: 0 of 767	Outliers: 0 of 820	Outliers: 14 of 899	Outliers: 10 of 903	Outliers: 16 of 903	Non-Trans: 2 of 902
A 861		TRP	0.87	-	Favored (85.75%) General / -59.0,-47.3	Favored (52.2%) *t60* chi angles: 166.4,83.8	0.09Å	Favored (92.211%) alpha helix	-	-	-
A 862		ALA	0.85	-	Favored (83.28%) General / -59.2,-39.7	-	0.04Å	Favored (85.165%) alpha helix	-	-	-
A 863		GLU	0.83	-	Favored (81.59%) General / -63.3,-35.8	Favored (98.2%) *mt-10* chi angles: 290,179,354.9	0.03Å	Favored (75.65%) alpha helix	-	-	-
A 864		ASN	0.82	-	Favored (29.95%) General / -99.4,14.4	Favored (90.2%) *m-40* chi angles: 294,323.3	0.03Å	Favored (38.284%) alpha helix	-	-	-
A 865		ILE	0.8	-	Favored (70.96%) Ile or Val / -55.8,-42.6	Favored (34.6%) *mm* chi angles: 294.7,296.2	0.13Å	Favored (44.188%) alpha helix	OUTLIER(S) worst is CB--CG1: 4.5 σ	OUTLIER(S) worst is C-CA-CB: 4.0 σ	-
A 866		LYS	0.77	0.44Å NZ with A 895 GLU OE1	Favored (68.1%) General / -61.0,-27.2	Favored (53.3%) *mtpt* chi angles: 290.2,172.2,68.1,176.8	0.07Å	Favored (69.543%) alpha helix	-	-	-
A 867		ASP	0.75	-	Favored (31.32%) General / -81.5,-34.6	Favored (31.9%) *m-30* chi angles: 291.4,291.2	0.05Å	Favored (83.569%) alpha helix	-	-	-
A 868		THR	0.72	-	Favored (64.2%) General / -72.3,-29.9	Favored (68.3%) *p* chi angles: 62.7	0.04Å	Favored (92.423%) alpha helix	-	-	-
A 869		VAL	0.69	-	Favored (78.95%) Ile or Val / -69.0,-46.3	Favored (63.6%) *t* chi angles: 171.3	0.06Å	Favored (82.694%) alpha helix	-	-	-
A 870		ASN	0.67	-	Favored (85.5%) General / -60.8,-38.5	Favored (99.2%) *m-40* chi angles: 287.7,343.1	0.03Å	Favored (90.198%) alpha helix	-	-	-

#	Alt	Res	High B	Clash > 0.4Å	Ramachandran	Rotamer	Cβ deviation	CaBLAM	Bond lengths	Bond angles	Cis Peptides
A 871		MET	0.66	-	Favored (74.7%) General / -58.4,-50.2	Favored (45.3%) *ttp* chi angles: 175.5,189.1,66.1	0.06Å	Favored (85.848%) alpha helix	-	-	-
A 872		VAL	0.65	-	Favored (92.16%) Ile or Val / -63.7,-41.3	Favored (67.1%) *t* chi angles: 171.8	0.07Å	Favored (82.449%) alpha helix	-	-	-
A 873		ARG	0.65	-	Favored (87.68%) General / -60.1,-39.8	Favored (92.6%) *mtt180* chi angles: 288.6,179.2,172.2,165.5	0.06Å	Favored (89.337%) alpha helix	-	-	-
A 874		ARG	0.66	-	Favored (83.31%) General / -64.1,-36.2	Favored (97%) *mtt180* chi angles: 289,172.9,180.5,170.8	0.03Å	Favored (91.506%) alpha helix	-	-	-
A 875		ILE	0.68	-	Favored (86.87%) Ile or Val / -67.3,-45.1	Favored (98.8%) *mt* chi angles: 292.7,167.3	0.03Å	Favored (96.944%)	-	-	-
A 876		ILE	0.7	-	Favored (96.1%) Ile or Val / -64.2,-45.5	Favored (98%) *mt* chi angles: 292.8,168.6	0.07Å	Favored (5.832%)	-	-	-
A 877		GLY	0.72	-	Favored (8.99%) Glycine / 147.5,160.6	-	-	Favored (14.144%)	-	-	-
A 878		ASP	0.73	-	Favored (8.45%) General / -83.5,177.4	Favored (12%) *t0* chi angles: 204.3,336.8	0.02Å	Favored (13.233%)	-	-	-
A 879		GLU	0.74	-	Allowed (0.65%) General / 72.8,-46.5	Favored (98.9%) *mt-10* chi angles: 293.8,179.7,355.2	0.07Å	CaBLAM Outlier (0.911%)	-	-	-
A 880		GLU	0.74	-	Favored (46.66%) General / -59.6,144.8	Favored (64.2%) *mm-30* chi angles: 294.7,287,329.8	0.06Å	Favored (30.086%)	-	-	-
**#**	**Alt**	**Res**	**High B**	**Clash > 0.4Å**	**Ramachandran**	**Rotamer**	**Cβ deviation**	**CaBLAM**	**Bond lengths**	**Bond angles**	**Cis Peptides**
			Avg: 0.95	Clashscore: 2.02	Outliers: 3 of 901	Poor rotamers: 0 of 767	Outliers: 0 of 820	Outliers: 14 of 899	Outliers: 10 of 903	Outliers: 16 of 903	Non-Trans: 2 of 902
A 881		LYS	0.73	-	Favored (34.14%) General / -87.0,131.3	Favored (86.8%) *tttt* chi angles: 180.8,176.5,175.8,179.6	0.01Å	Favored (47.308%) beta sheet	-	-	-
A 882		TYR	0.73	-	Favored (37.39%) General / -120.5,152.9	Favored (73.6%) *m-80* chi angles: 301,85	0.07Å	Favored (53.558%) beta sheet	-	-	-
A 883		MET	0.72	-	Favored (55.62%) General / -113.3,132.3	Favored (51.5%) *tpp* chi angles: 176,68.4,71.5	0.02Å	Favored (46.965%) beta sheet	-	-	-
A 884		ASP	0.73	-	Favored (9.74%) General / -82.7,71.3	Favored (69.8%) *m-30* chi angles: 291.5,320.2	0.04Å	Favored (23.654%) beta sheet	-	-	-
A 885		TYR	0.74	-	Favored (66.04%) General / -66.5,-20.5	Favored (61.9%) *m-80* chi angles: 288.8,109.2	0.07Å	Favored (19.792%)	-	-	-

A 886		LEU	0.76	-	Favored (67.3%) General / -60.4,-27.2	Favored (86.6%) *mt* chi angles: 291.5,174.8	0.04Å	Favored (59.234%)	-	-	-
A 887		SER	0.78	-	Favored (60.05%) General / -68.4,-11.7	Favored (90.7%) *p* chi angles: 66.7	0.03Å	Favored (60.143%) three-ten	-	-	-
A 888		THR	0.81	-	Favored (56.41%) General / -89.9,-5.7	Favored (50.2%) *p* chi angles: 65.7	0.02Å	Favored (55.296%)	-	-	-
A 889		GLN	0.86	-	Favored (34.8%) General / -91.0,134.1	Favored (62.9%) *tp40* chi angles: 179.9,67.5,30.1	0.04Å	Favored (30.594%)	-	-	-
A 890		VAL	0.94	-	Favored (22.84%) Ile or Val / -58.4,-23.6	Favored (30.7%) *m* chi angles: 296.8	0.05Å	Favored (41.525%)	-	-	-
A 891		ARG	1.09	-	Favored (73%) General / -65.5,-31.8	Favored (59%) *ttt90* chi angles: 187.7,171.7,182.8,84.9	0.02Å	Favored (47.865%) alpha helix	-	-	-
A 892		TYR	1.36	-	Favored (11.01%) General / -117.6,-7.8	Favored (54.6%) *m-80* chi angles: 305.9,110.3	0.05Å	Favored (22.019%) alpha helix	-	-	-
A 893		LEU	1.79	-	Favored (64.52%) General / -54.4,-36.9	Favored (50.9%) *tp* chi angles: 183.7,61.7	0.05Å	Favored (42.184%) three-ten	-	-	-
A 894		GLY	2.46	-	Favored (56.6%) Glycine / -62.2,-17.8	-	-	Favored (73.856%) three-ten	-	-	-
A 895		GLU	3.44	0.44Å OE1 with A 866 LYS NZ	Favored (36.41%) General / -103.8,10.5	Favored (88.3%) *mt-10* chi angles: 298.8,185.4,1.9	0.01Å	Favored (51.458%) alpha helix	-	-	-
A 896		GLU	4.72	-	Favored (59.23%) General / -53.1,-35.0	Favored (92.2%) *tt0* chi angles: 183,178.7,3.2	0.02Å	Favored (42.463%) alpha helix	-	-	-
A 897		GLY	6.19	-	Favored (88.55%) Glycine / -81.6,0.0	-	-	Favored (60.698%)	-	-	-
A 898		SER	7.62	-	Favored (22.21%) General / -78.1,166.9	Favored (92.9%) *p* chi angles: 66.4	0.02Å	Favored (26.719%)	-	-	-
A 899		THR	8.83	-	Favored (78.85%) Pre-Pro / -82.2,124.0	Favored (94.3%) *m* chi angles: 299.4	0.04Å	Favored (25.873%)	-	-	-
A 900		PRO	9.76	-	Favored (37.58%) Trans-Pro / -52.6,-28.7	Favored (99.6%) *Cg_exo* chi angles: 332.4,35.1,332.5	0.05Å	Favored (78.698%)	-	-	-
**#**	**Alt**	**Res**	**High B**	**Clash > 0.4Å**	**Ramachandran**	**Rotamer**	**Cβ deviation**	**CaBLAM**	**Bond lengths**	**Bond angles**	**Cis Peptides**
			Avg: 0.95	Clashscore: 2.02	Outliers: 3 of 901	Poor rotamers: 0 of 767	Outliers: 0 of 820	Outliers: 14 of 899	Outliers: 10 of 903	Outliers: 16 of 903	Non-Trans: 2 of 902
A 901		GLY	10.46	-	Favored (84.71%) Glycine / -88.6,2.6	-	-	Favored (63.581%)	-	-	-
A 902		VAL	10.98	-	Favored (75.91%)	Favored (71.6%) *t* chi angles: 178.6	0.03Å	-	-	-	-

				Ile or Val / -120.0,127.9						
A 903		LEU	11.32	-	-	Favored (48.3%) *tp* chi angles: 181.6,65.6	0.03Å	-	-	-	-
