# Supplementary material for: Integrative proteome-wide structural analysis and high-throughput docking identify broad-spectrum antiviral scaffolds against Zika, Yellow Fever, West Nile, Saint Louis encephalitis, and Usutu viruses
Source: Front Cell Infect Microbiol. 2026 Apr 30;16:1723132. doi: 10.3389/fcimb.2026.1723132 (PMC13171538; doi:10.3389/fcimb.2026.1723132)
Supplement: Supplementary file 7 [file DataSheet7.zip › ZIKV/ZIKV_NS5/Mol_probity_Files/ZIKV_NS5_1FH-rama.pdf]

MolProbity Ramachandran analysis

ZIKV_NS5_1FH.pdb, model 1

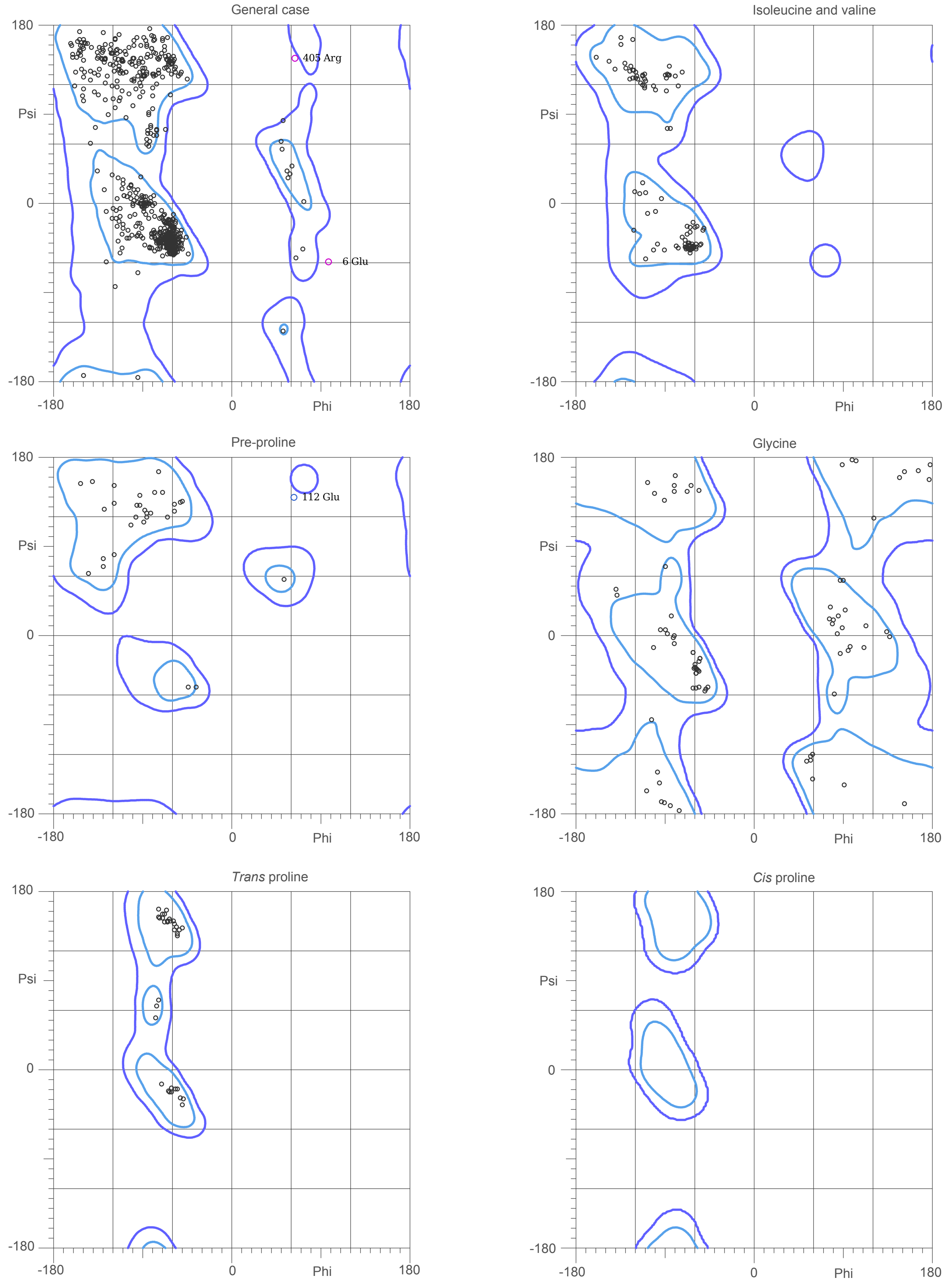

97.7% (880/901) of all residues were in favored (98%) regions.
99.7% (898/901) of all residues were in allowed (>99.8%) regions.

There were 3 outliers (phi, psi):
6 Glu (98.7, -60.0)
112 Glu (63.8, 140.5)
405 Arg (64.5, 147.7)
